# Characterization of *Campylobacter* spp. Strains Isolated From Wild Birds in Turkey

**DOI:** 10.3389/fmicb.2021.712106

**Published:** 2021-08-18

**Authors:** Cemil Kürekci, Fatih Sakin, Lennard Epping, Marie-Theres Knüver, Torsten Semmler, Kerstin Stingl

**Affiliations:** ^1^Department of Food Hygiene and Technology, Faculty of Veterinary Medicine, Hatay Mustafa Kemal University, Hatay, Turkey; ^2^Department of Pharmacology and Toxicology, Faculty of Veterinary Medicine, Hatay Mustafa Kemal University, Hatay, Turkey; ^3^Robert Koch Institute, Genome Sequencing and Genomic Epidemiology, Berlin, Germany; ^4^German Federal Institute for Risk Assessment, Department of Biological Safety, National Reference Laboratory for Campylobacter, Berlin, Germany

**Keywords:** *C. coli* clade, whole-genome sequencing, Eurasian coots, environmental Campylobacter, diagnostics

## Abstract

Turkey is an important stopover site for many migrating birds between Europe, Asia and Africa. *Campylobacter* spp. are frequently found in wildlife, in particular waterfowl, and distinct strains are disseminated within this reservoir. In this study, 183 wild birds of hunting areas in Turkey were collected and thermophilic *Campylobacter* spp. from cloacal swabs were isolated at a prevalence of 5.2% from song thrushes (6/116) and 93% from Eurasian coots (41/44). After PCR species differentiation and *flaA* restriction profiles determination, *C. jejuni* and *C. coli* strains were further investigated by whole genome sequencing. PCR target amplification of the *ceuE* gene, commonly used for *C. coli* species-identification was inefficient and even hampered in one isolate. A close look on the *ceuE* sequence revealed that various mismatches in the *ceuE* oligo annealing sites caused less efficient diagnostic detection. All *C. coli* isolates belonged to the environmental clade II and clade III, for which thirty-six novel MLST types were identified. Further single nucleotide polymorphism (SNP) analysis showed a high genomic divergence between the *C. coli* isolates. High variability was also implicated for putative plasmid-located genes detected in 51% of the *C. coli* isolates. Distinct gene variants in clades II and III *C. coli* were identified by a *k-mer* analysis. After substracting *k-mers* in common with *C. coli* clade I database, 11 and 35 distinct genes were identified in clades II and III isolates, mainly involved in surface structures and modifications as well as signal transduction, suggesting niche adaptation of *C. coli* strains in wild birds. All strains were susceptible against (fluoro-)quinolones, erythromycin, tetracycline, gentamicin and only one isolate was resistant against streptomycin, suggesting that the sensitive phenotype was due to absence of selective pressure and niche separation in wild birds in Turkey. We conclude that *Campylobacter* spp. isolates from wildlife and environmental sources are still scarce in the databases and that there is a need for more studies on thermophilic *Campylobacter* spp. from different places all over the world in order to complement our understanding on dissemination and adaptation to distinct niches of this global food-borne pathogen.

## Introduction

Thermophilic *Campylobacter*, in particular *C. jejuni* and *C. coli*, have become well-recognized as the commonest causative agent of acute gastroenteritis in humans in developed countries (Casey et al., 2017). In the developing world, campylobatecteriosis is much less investigated but it appears to primarily occur in children rather than adults and to be frequently associated with contaminated drinking water (Platts-Mills and Kosek, 2014). Among the several species known to potentially cause pathogenesis in humans, *C. jejuni* and *C. coli* were implicated in over 90% of reported human campylobacteriosis worldwide (Sheppard et al., 2009). The majority of cases with infection displayed watery or bloody diarrhea and abdominal cramps that could be accompanied by other symptoms including fever, vomiting and headaches (Pogreba-Brown et al., 2016). In addition, *Campylobacter* related infections may also result in extraintestinal manifestations such as bacteremia, meningitis, abortion and mycotic aneurysms and long-term complications like the Guillain-Barré syndrome and reactive arthritis (O’Brien, 2017). In fact, most people with campylobacteriosis completely recover within a week without medication, but in severe cases it could be life threatening and antibiotic therapy needs to be applied (Winn et al., 2006). It is quite difficult to make exact estimation of the number of people affected by *Campylobacter* spp., but globally, at least 96 million people every year suffer from campylobacteriosis (Kirk et al., 2015). Besides profound health impacts, *Campylobacter* associated infections have substantial economic costs worldwide. For example, it was estimated that losses due to these organisms reached up to $1.9 billion in the United States alone in 2013 (O’Brien, 2017).

*Campylobacter* spp. are ubiquitous in a wide range of wild and domestic animal species, particularly in poultry with asymptomatic carriage. Therefore, contaminated foodstuffs of poultry play the most significant role in the transmission of *Campylobacter* spp. to humans, although contaminated water and other foodstuff including milk, dairy products and red meats have been also recognized as potential source (Sheppard et al., 2009; Pogreba-Brown et al., 2016; Thépault et al., 2018). In addition, the presence of *Campylobacter* spp. in many wild bird species, particularly in waterbirds, has been documented previously (Waldenström et al., 2002; Cody et al., 2015). Evidence obtained from epidemiological studies using molecular characterization highlights the significance of wild animals as additional source of pathogenic *Campylobacter* to humans, attributing 2.1–3.5% of clinical cases to wild birds in England annually (Cody et al., 2015). Given the geographical location, Turkey is of great importance in terms of its avifauna and as well as having the significant stopover sites on the route of many migrating birds between the continents Europe, Asia and Africa. However, no studies to date have investigated the existence and prevalence of *Campylobacter* spp. in wild birds in Turkey so far. Thus, the present study was undertaken to determine; (i) the presence of *Campylobacter* spp. in wild bird population in Turkey, and (ii) to characterize the molecular epidemiology of wild bird-associated *Campylobacter* spp. isolates by whole genome sequencing (WGS).

## Materials and Methods

### Sampling Wild Birds and Isolation of *Campylobacter* spp.

**FIGURE 1:**
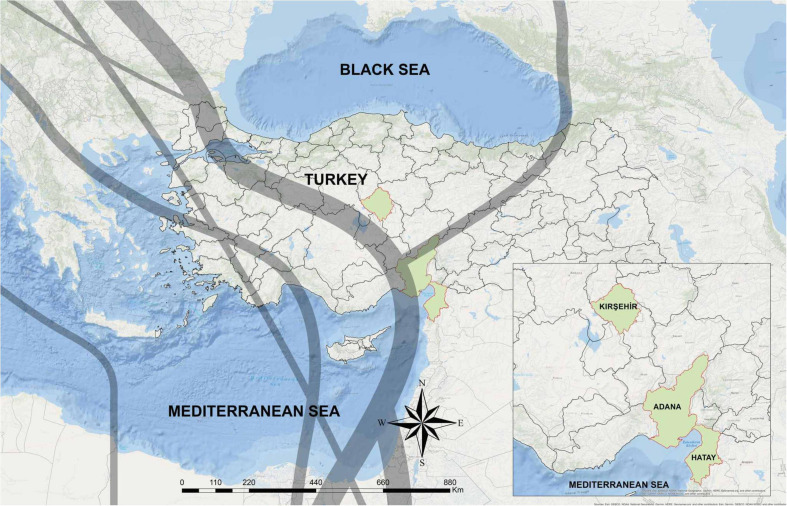
The geographic location of sampling sites in Turkey. The sampling areas are shown in green. Gray lines indicate two main bird migration routes on Turkey, adapted from Özkazanç and Özay (2019).

We sampled 183 birds belonging to five species: the European turtle dove (*Streptopelia turtur*; *n* = 3), the Eurasian coot (*Fulica atra*; *n* = 44), the song thrush (*Turdus philomelos*; *n* = 116), wild quails (*Coturnix coturix*; *n* = 15), and the red-crested pochard (*Netta fufina*; *n* = 5) during hunt activities from September 2017 to February 2018. Our sampling sites for coots and ducks included two lakes namely Kaman Lake (Kırsehir province; 39°.21′ N 33°.43′ E) and Adalı wetland (Adana province; 36°.30′N 34°.48′ E), while other bird samples were taken from Hatay province (36°.13′N 36°.10′ E) (Figure 1).

The cloacal swabs were directly streaked on mCCDA (Oxoid, Thermo Fisher Scientific Inc., Waltham, MA, United States) and incubated at 41.5°C under microaerobic atmosphere for about 48 h. All *Campylobacter* isolates were microscopically examined for phenotypical characteristics such as motility and typical morphology. In addition, absence of growth at 25°C on Columbia blood agar (ColbA; Merck & Co., Kenilworth, New Jersey, United States) containing 5% sheep blood (Oxoid) was checked. Subsequently, species identification was carried out using MALDI-TOF MS (Bruker Daltonics, Billerica, MA, United States) and real-time PCR (Mayr et al., 2010). The proteomic profiles were evaluated with the aid of manufacturer’s reference library (Bruker; version 6.0) and based on the score values as follows: a score of 2.300–3.000 indicates “highly probable species identification,” a score of 2.000–2.299 indicates “secure genus identification and probable species identification,” a score of 1.700–1.999 indicates “probable genus identification” and a score of ≤ 1.699 indicates “not reliable identification.” Strains were stored at −80°C by using the cryobank system (MAST Group Ltd., Bootle, United Kingdom).

### DNA Extraction and Molecular Identification of *Campylobacter* spp.

For DNA extraction, fresh bacterial culture (24 h growth on ColbA) was suspended in phosphate buffered saline to an OD_600nm_ of ∼2.0. This cell suspension was pelleted by centrifugation (∼14,000 rpm, 5 min) and then immediately frozen at −20°C before DNA extraction. Total genomic DNA was isolated from each strain using the PureLink genomic DNA mini kit (Thermo Fisher Scientific). Subsequently, the extracted genomic DNA was run on a 0.8% agarose gel by electrophoresis. Quality of the DNA was checked spectrophotometrically (NanoDrop, Thermo Fisher Scientific). In addition, the Qubit 3.0 fluorometer (Thermo Fisher Scientific) was used for nucleic acid quantification. Multiplex real time PCR was conducted on all isolates to identify species using the method described previously by Mayr et al. (2010). The PCR protocol consisted of 3 min at 95°C, followed by 45 cycles of 30 s at 95°C, 1 min at 60°C and 30 s at 72°C. *Campylobacter* spp. isolates were also characterized using RFLP-*flaA* typing as described previously (Nachamkin et al., 1993). After PCR amplification of the *fla*A gene, amplicons were digested with *Hpy*F3I (*Dde*I) restriction enzyme (Thermo Fisher Scientific) and electrophoresed on a 2.5% polyacrylamide gel. The phylogenetic relation of isolates was determined using the Dice correlation coefficient and the unweighted pair group mathematical average (UPGMA) clustering algorithm using BioNumerics software 7.2 (Applied-Maths, Belgium) (Supplementary Image 1).

### Determination of the Minimal Inhibitory Concentration of Antimicrobials by Microdilution

The frozen *C. jejuni* strains were recovered by streaking on ColbA containing 5% sheep blood and incubated under microaerobic conditions at 41.5°C for 24 h. The microdilution assay was performed according to M45-A (Clinical and Laboratory Standards Institute [CLSI], 2015) and VET06 (Clinical and Laboratory Standards Institute [CLSI], 2017) with the in-house validated modification of use of fetal calf serum instead of lysed horse blood in the culture medium for improved readability of *Campylobacter* growth. Cation-supplemented Mueller–Hinton broth (TREK Diagnostic Systems, United Kingdom) was supplemented with 5% fetal calf serum (PAN Biotech, Germany) and inoculated with 2–8 × 10^5^ colony forming units/mL using bacteria grown on ColbA. Minimum inhibitory concentrations (MICs) were determined using the European standardized microtiter plate format EUCAMP2 (TREK Diagnostic Systems). The minimum inhibitory concentration of six antibiotics, including ciprofloxacin (Cip), nalidixic acid (Nal), erythromycin (Ery), tetracycline (Tet), gentamicin (Gen), and streptomycin (Str) were determined after 2 days of incubation at 37°C under microaerobic conditions. Reference isolates (*C. jejuni* ATCC 33560, ATCC collection and *C. coli* 2012-70-443-2, DTU, Lyngby, Denmark) were used as quality control strains. The epidemiological cut-off values recommended by the European Committee on Antimicrobial Susceptibility Testing (EUCAST) were used to determine resistance.^1^

### Whole Genome Sequencing

In total, 39 *C. coli* were selected for WGS based on origin and the results of *flaA* typing analysis. In addition, *C. jejuni* (*n* = 3) isolates were included. Total genomic extraction protocol and subsequent quantification steps are aforementioned. The bacterial DNA was paired-end sequenced using the Illumina MiSeq sequencing platform and 301 × 2 cycles (Illumina Inc., San Diego, CA). DNA libraries were prepared using the Nextera XT DNA Library Prep kit or the Nextera DNA Flex Library Prep Kit according to the manufacturer’s instructions (Illumina, San Diego, United States). Quality of the libraries was assessed by gel analysis. The sequences were published within the BioProject No. PRJNA702752, BioSample No. SAMN17977770-SAMN17977811 at National Center for Bioechnoogy Information (NCBI) sequence read archive.^2^ New multilocus sequence typing (MLST) alleles and MLST-ST types were uploaded to PubMLST^3^.

### Sequence Analysis

Sequencing reads were treated for quality control, trimmed for adapters and *de novo* genomes were assembled using SPAdes 3.11.1 (Bankevich et al., 2012) with careful options (with mismatch correction) included in Ridom Seqsphere + v. 6.0.0 (2019-04) (Ridom GmbH, Germany) using default settings. Sequences were analyzed using the 7 housekeeping genes MLST scheme of PubMLST^3^. Quality trimming was performed in a window of 20 bp with Phred score 30. The obtained average coverage (processed, unassembled) was > 60-fold. Resistance genes and known gene mutations were detected with ResFinder 4.0 (Bortolaia et al., 2020; Center for Genomic Epidemiology, DTU, Denmark). Core genome alignments were calculated using Roary v.3.13.0 (Page et al., 2015) with a sequence identity of at least 80%. Phylogenetic trees for the core genome and for *ceuE* genes were built with RAxML-NG v.0.9.0 (Kozlov et al., 2019) (100 bootstraps). The phylogenetic trees were visualized with GrapTree v.1.5.0 (Zhou et al., 2018). SNP distances of the gene sequence alignment of the core genome alignment of clade II (1,220 orthologs genes) and clade III (1,274 orthologs genes) were calculated with snp-dists v.0.7.0.^4^ Clade specific genes were identified with a modified version of our in-house *k-mer* analysis workflow (Golz et al., 2020). *K-mers* of length 31 were obtained from each of the clade genomes and compared against a *C. coli* (clade I) specific *k-mer* database. *K-mers* that were absent in the database, were mapped back against each genome in order to identify clade II and clade III specific genes. Genes with a *k-mer* coverage of at least 80% within all strains per clade were further investigated and annotated with eggNOG v.5.0 (Huerta-Cepas et al., 2017, 2019). Putative plasmid sequences of the genomes were identified utilizing plasmidSPAdes v3.13.1 (Antipov et al., 2016). Homology of the putative plasmid genes were evaluated using Roary v.3.13.0 using a minimum sequence identity of 80%. Similarity of putative plasmid gene content carried by each strains was visualized with Phandango (Hadfield et al., 2018).

## Results

A total of 59 *Campylobacter* spp. strains were isolated from samples of coots and song thrush. *Campylobacter* was not detected in other tested bird species. Of the 59 *Campylobacter* spp. isolates, 36 were identified at species level (spectral scores ≥ 2.000) by MALDI-TOF MS. For the remaining 23 isolates, MALDI-TOF MS was able to identify only genus level (spectral scores between 1,700 and 1,999). In this study, 93% of coots (41/44) were colonized by thermophilic *Campylobacter* spp., of which all isolates (*n* = 53) except for one were identified as *C. coli* by species differentiating real-time PCR (Mayr et al., 2010). Song thrushes carried thermophilic *Campylobacter* spp. at a lower prevalence of 5.2% (6/116). Five of the six isolates were identified as *C. jejuni*. After initial identification, three isolates (two *C. jejuni* and one *Campylobacter* spp.) from song thrushes and one *C. coli* isolates from coot did not survive in the stock and were not analyzed further.

**FIGURE 2:**
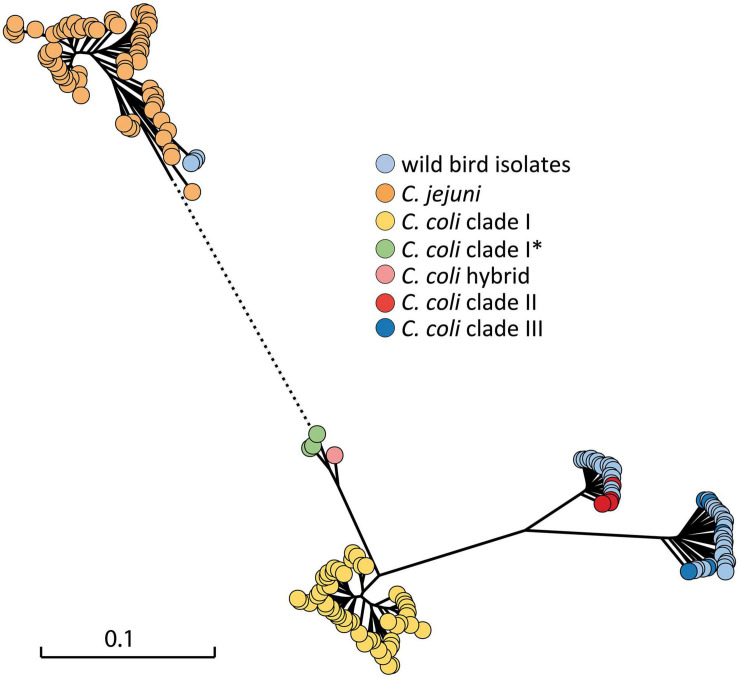
Maximum likelihood phylogenetic tree of 42 thermophilic *Campylobacter* spp. isolates from wild birds in the context of *C. jejuni* and *C. coli* complete genomes from NCBI (Supplementary Table 2) constructed on the basis of the core genome. The scale bar represents the distance between the sequences determined by the number of SNP differences in core genes. Dotted line indicates shortened distance to *C. jejuni* cluster, which is out of scale. *, subclade.

The *flaA* gene was successfully amplified and digested with *Hpy*F3I in 49 *C. coli* isolates. A high degree of variability was observed based on RFLP-*flaA* analysis, in which 31 different restriction fingerprinting profiles were determined when using a similarity cut-off value of 80% (Supplementary Image 1). Whole genome sequence data were obtained from 42 isolates. A phylogenetic analysis was performed based on 604 core genes, which were in common in the strains at identity of at least 80% (Figure 2). In order to determine how the wild bird *Campylobacter* spp. isolates (*n* = 42) attribute in broader phylogeny, a set of *C. jejuni* and *C. coli* covering different STs and clades from the NCBI database were included in the tree. One German isolate previously identified as *C. coli/C jejuni* hybrid (Golz et al., 2020) was also included. Twenty-two *C. coli* isolates appeared in clade III, and seventeen isolates clustered within clade II genomes, whereas the remaining three strains from song thrushes clustered with published *C. jejuni* genomes, as expected (Supplementary Table 1).

The MLST types of the *C. jejuni* isolates were determined as ST1259 (*n* = 2) and ST1356 (*n* = 1) based on the WGS data analysis (Supplementary Table 1). However, of the 39 *C. coli* isolates, only one was assigned to known MLST type (ST3310). The *C. coli* isolates carried one or more new alleles of the 7 housekeeping genes or an unknown ST. In total, 7 *aspA*, 10 *glnA*, 7 *gltA*, 11 *glyA*, 13 *pgm*, 9 *tkt*, and 9 *uncA* allele variants were identified and 36 new STs submitted to PubMLST. This showed that the isolated *C. coli* were not phylogenetically linked, except for four isolates (ST 10966; BfR-CA-15855, BfR-CA-15859 and ST 10970; BfR-CA-15860, BfR-CA-15861).

**FIGURE 3:**
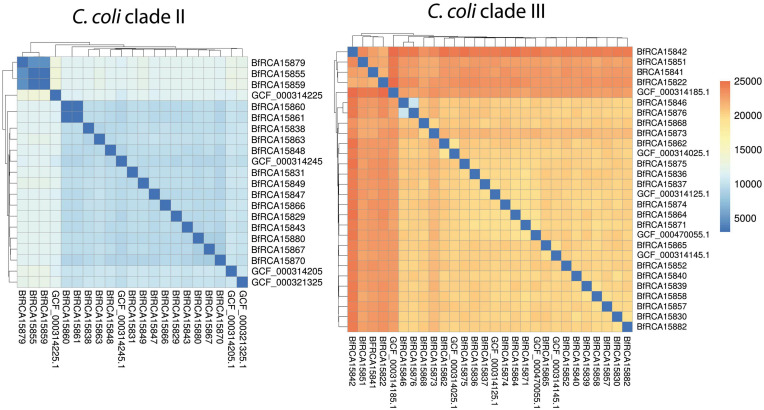
Pairwise SNP analysis show that *C. coli* clade II isolates were less diverse than *C. coli* clade III isolates. Heatmap color code of pairwise number of SNPs is shown on the right. NCBI closed genomes of *C. coli* clade II and clade III are included for comparison.

A further single nucleotide polymorphism (SNP) analysis was performed in order to gain more insight into the strain divergence of wild bird *C. coli* isolates (Figure 3). Clade II isolates differed from each other by around 4,500 to 10,000 SNPs within 1120 genes, except for two apparently clonal strain pairs mentioned above, which displayed either no detectable SNP (BfR-CA-15860 and BfR-CA-15861) or 2 SNPs (BfR-CA-15855 and BfR-CA-15859). Clade III wild bird isolates even displayed a higher genomic divergence with medial pairwise SNPs of more than 19,000 within 1274 genes.

All tested *Campylobacter* spp. strains were found to be susceptible to Cip, Nal, Ery, Tet, and Gen. The MIC values for Cip ranged between ≤ 0.12 and 0.25 μg/ml. The range of MICs against Nal was 2–8 μg/ml and for Ery and Tet between ≤ 1 and ≤ 0.5 μg/ml and for Gen between 0.25 and 1 μg/ml for all isolates, respectively. All isolates were also susceptible against Str with MIC values between 0.5 and 4 μg/ml, except one *C. coli* isolate (BfR-CA-15830), which showed resistance with a MIC value of 16 μg/ml. The following antimicrobial resistance (AMR) genes were observed in the isolates using ResFinder 4.0: *bla*_OXA–460_, *bla*_OXA–447_ and *aadE-Cc* genes. WGS showed that all *C. jejuni* isolates (*n* = 3) carried a *bla*_OXA–447_ gene, whereas two *C. coli* isolates had the *bla*_OXA–460_ gene (Supplementary Table 1). Correlating well with phenotypic sensitivity against the tested antimicrobial resistance results, there was no other known resistance genes or point mutations detected in the sequenced isolates. Interestingly, although the *aadE*-Cc gene was identified in five of the *C. coli* isolates, only one was phenotypically resistant against streptomycin (Supplementary Table 1). We confirmed that the AadE-Cc protein in four of the five isolates was 100% identical to each other, except for BfR-CA-15848, which harbored two non-synonymous mutation of A143V and T252A. Relative to the phenotypically resistant strain BfR-CA-15830, the genetic context, except for the 5′ flanking two genes (FHP99_01330 (NAD(P)H-dependent oxidoreductase and in some strains also FHP99_01325; N-methyl-L-tryptophan oxidase) was different.

**FIGURE 4:**
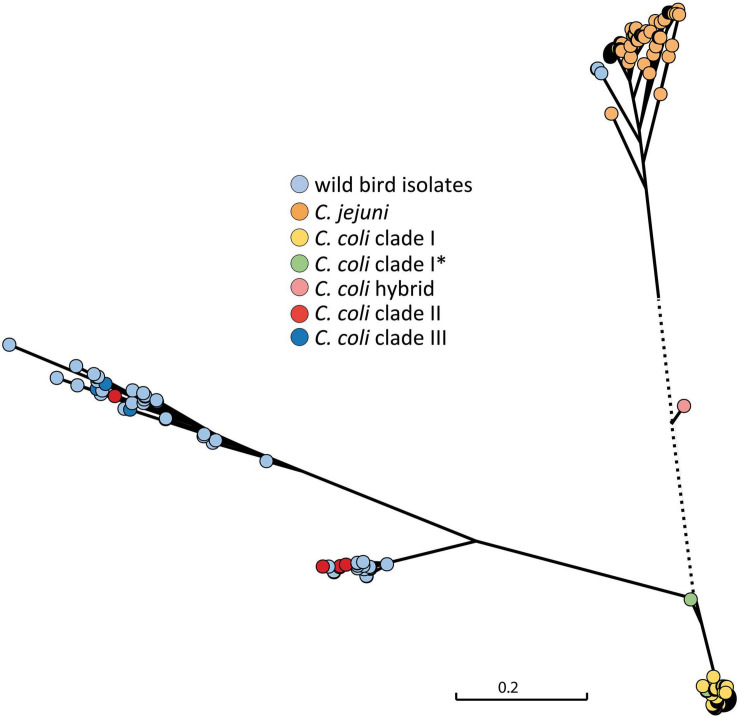
Phylogenetic tree of the *ceuE* target gene for *C. coli* species differentiation. *ceuE* of *C. coli* clade I, for which typing methods have been optimized, is significantly different to clade II and clade II *C. coli ceuE*. The *C. coli* hybrid isolate in between *C. jejuni* and *C. coli* clade I was originally detected by ambiguous real-time PCR results which were caused by *C. jejuni* introgression into the *C. coli* genome (Golz et al., 2020). The scale bar represents the distance between the sequences determined by the number of SNPs. Dotted line is out of scale. *, subclade.

From real-time PCR analysis of the *ceuE* target for *C. coli*, obtained Ct values were not always optimal and the *C. coli ceuE* target could not be detected in one isolate (BfR-CA-15822). Therefore, a comparison of the *ceuE* gene sequence of the isolates in this study with those of *C. coli* clade I and *C. jejuni* sequences accessible in the public database were further carried out. A phylogenetic SNP tree showed high variability of the *ceuE* gene of the wild bird isolates (Figure 4) and significant difference to the *C. coli* clade I of food-production chain origin, for which typing schemes have been optimized.

Mismatches in the annealing sites of the oligos and probe sequences of the real-time PCR (Mayr et al., 2010) were visualized by alignment of the 103 bp target fragment of *ceuE* (Supplementary Image 2). The wild bird *C. coli* isolates carried in total 16 distinct sequences within these 103 bp target fragment of *ceuE*, confirming phylogenetic divergence and hinting at independent evolutionary point mutations in *ceuE* in the individual *C. coli* isolates. We identified one to two point mutations in the *ceuE* reverse oligo and/or in the probe. The number of mismatches for the annealing site of the forward *ceuE* oligo was two to seven. While two point mutations were negligible for efficient amplification of the *ceuE* target, four to six point mutations led to less efficient amplification (Ct values > 30) and seven point mutations inhibited amplification in one isolate (see above).

In order to further reveal distinct gene variants in clade II and clade III *C. coli*, a *k-mer* analysis was performed with some modifications as previously reported (Golz et al., 2020). For this purpose, *k-mers* of 31 bases length were obtained from the genomes of either clade II or clade III isolates. After substracting *k-mers* in common with *C. coli* clade I database (Golz et al., 2020), the residual clade II or clade III specific *k-mers* were remapped to the original individual genome and the specific genes. Those genes, which were covered in length of at least 80% by *k-mers* within all strains per clade were annotated using eggNOG (Huerta-Cepas et al., 2017; Huerta-Cepas et al., 2019). Using this approach, 11 and 35 distinct genes were identified in clade II and clade III isolates, respectively (Supplementary Table 3). Among hypothetical proteins, the genes encode proteins involved in surface structures and modifications, like flagellar, capsule and protein glycosylation as well as signal transduction, suggesting niche adaptation of *C. coli* strains in wild birds.

**FIGURE 5:**
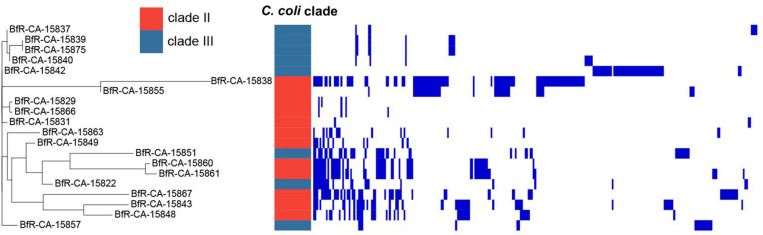
Phylogenetic tree of plasmid-located genes of wild bird isolates based on presence or absence. Strains are depicted on the left. Blue bars indicate presence of plasmid-located genes (Supplementary Table 4).

We also asked the questions whether the isolates harbor plasmids and which genes were putatively located on these epichromosomal elements. For this purpose, the plasmidSPAdes v3.13.1 (Antipov et al., 2016) tool was used, which identifies plasmids assuming independent replication from the chromosome and, thereby, differences in copy number of plasmids vs. chromosomes per bacterial cell. A gene copy number different from the chromosomal genes is reflected by different coverage depths of matching raw reads relative to the mean coverage depth of all genes. The stringency of this approach was increased by only considering assembly contigs, which matched to known *Campylobacter* plasmids with at least 30% of coverage lengths (Supplementary Table 4). Of the 42 isolates, 20 harbored genes with putative plasmid location (Figure 5). The number of plasmid-located genes varied between the strains, indicating the presence of different number of plasmids and/or different size, independent of the *C. coli* clade. In *C. jejuni* isolates no plasmid-located genes were identified with this approach.

BLAST analysis identified 275 genes, 96 of them with annotated putative function (Supplementary Table 4). Among these genes, typically plasmid-located genes encoding Trb, Tra, and VirB conjugative transfer genes, type VI secretion proteins, methyltransferases, replication initiation protein, plasmid partitioning protein and helix-turn-helix DNA regulatory proteins were present (Supplementary Tables 4, 5). A comparison of present gene homologs in the isolates showed that the strains were not only phylogenetically diverse, but also harbored diverse plasmids (Figure 5). None of the two resistance genes identified in the isolates was plasmid-located.

## Discussion

Several studies have previously investigated the occurrence of *Campylobacter* spp. in wild birds. To the best of our knowledge, this is, however, the first report of *Campylobacter* spp. in wild birds in Turkey. We herein document the high prevalence of *C. coli* in coots. *C. jejuni* was also detected in song thrushes, but the prevalence was lower. A similar isolation rate was reported in a Spanish study in which 78% of cloacal samples obtained from coots were found to be positive for *C. coli* (Antilles et al., 2015). The astonishing prevalence variability in wild birds has been attributed to several factors including but not limited to geographical location, seasonality and species (Antilles et al., 2015; Cody et al., 2015). Additionally, high *Campylobacter* prevalence in coots has been attributed to their life history of characteristics including coprophagic way of life (Alcaide et al., 2014; Antilles et al., 2015).

In the current study, phylogenetic analysis utilizing core genes and RFLP-*flaA* indicated considerable genetic diversity. MLST analysis revealed that three *C. jejuni* isolates displayed known MLST types (ST-1259 and ST-1356), previously reported in wild bird from Sweden sampled in 2000. On the contrary only one *C. coli* was identified with a known MLST type (ST-3310), which was previously identified in a duck from United Kingdom in 2007 (PubMLST). All the other *C. coli* isolates harbored new alleles and/or sequence types resulting in 36 different novel STs, reflecting the diversity of the genotypes, thus showing the presence of several different clones within the coot population despite their similar ecological niche. It has also been observed that some samples obtained from coots harbored at least two distinct *C. coli* strains with different sequence types, indicating polyclonal *C. coli* carriage. Coots are non-migratory birds, but many other bird species migrate across Europe, therefore are possibly exposed to and/or disseminate these genotypes during migratory behavior, which might explain the carriage of different genotypes.

Contrary to *C. jejuni* that presents many MLST-based clonal complexes, isolates of *C. coli* of clade I belong to two clonal complexes (Sheppard and Maiden, 2015). To date, *C. coli* clades have appeared to have strong host-genotype association, for instance clade I is being isolated from human clinical and livestock samples (Sheppard et al., 2010, 2013). In contrast, *C. coli* isolates within clades II and III are often associated with environmental samples (Sheppard et al., 2008; Sopwith et al., 2010). In fact, each clade appears to have distinct molecular characteristics, meaning that clade I has lower sequence diversity in MLST genes than those reported for clades II and III (Sheppard et al., 2010). Recently, Nilsson et al. (2018) also noted variations in genes, such as tricarballylate gene locus, involved in oxidative stress and some virulence factors. Therefore, it is not surprising to detect significant genetic divergence among clade II clade III genomes in the current study. In addition, it has been also noted that *C. coli* clade II and III presented difference in phenotypic colony morphology and in *in vitro* motility patterns (Nilsson et al., 2018).

It is anticipated that the proportion of antimicrobial resistance found in *Campylobacter* spp. isolates from wild animals are notably lower than in isolates obtained from livestock, which likely arises from antimicrobial use in animal production and as well as environmental barrier. Likewise, apart from one exception (one *C. coli* being Str resistant), none of the *Campylobacter* strains were resistant to the tested antibiotics in the current study. This finding is in accordance with results reported by Antilles et al. (2015) who also noted no resistance to antimicrobials among *Campylobacter* spp. from wild bird population in Spain. Additionally, the *aadE*-Cc gene conferring resistance to aminoglycosides was detected in five *C. coli* isolates, of which one was phenotypically resistant to Str. The comparative analysis of *aadE*-Cc gene sequences showed identical protein sequences in these strains (except for one of the sensitive strains), thus, it remains unclear why those genes were silent and awaits further research on the mechanism of deactivation of the *aadE*-Cc gene in *C. coli*. It might hint on the fact that *aadE* expression is not needed in the wild birds without selection pressure due to absence of Str in this environment and corroborates the usefulness of complementary information from genetic and phenotypic analysis for the estimation of antimicrobial resistance. This disparity between phenotype and genotype for Str was also noted by Painset et al. (2020) who detected Str susceptible strains of *C. jejuni* (ECOFF < 4 mg/L) despite having aminoglycoside resistance trait (*aadE*) in their genome. Though the phenotypic resistance was not determined against penicillin in the current study, some strains of *Campylobacter* appear to have *bla*_OXA_ gene variants, highlighting the potential role of the wild birds in the transmission of β-lactam resistance genes. Hull et al. (2021) recently showed the high prevalence of β-lactam antimicrobial resistance genes including *bla*_OXA_ variants among *C. coli* and *C. jejuni* strains from live food animals and retail meats.

Comparison of the sequences of the *ceuE* gene, which was shown to be involved in iron acquisition and is commonly used to identify *C. coli* (Mayr et al., 2010), revealed that some bases were substituted, resulting in late amplification signals in some and an ambiguous PCR results in one *C. coli* isolate. A similar phenomenon has recently been described in some *C. coli* strains predominantly isolated from eggshells, which was reasoned to the unfavorable environmental conditions (Golz et al., 2020). A high degree of horizontal gene exchange from *C. jejuni* to *C. coli* strains was identified in these strains by *k*mer analysis and subsequent recombination length characterization, resulting in novel hybrid strains that may influence the sensitivity of traditional diagnostic methods for identification such as PCR. Here, horizontal gene transfer does not seem to be the cause for the diagnostic problem but niche adaptation. As observed from the diversity of *ceuE* sequences of the isolates from wild birds, insertion and functional tolerance and/or improvements of spontaneous mutations seem to be the major driving force of *ceuE* diversification, in particular, in *C. coli* clade III isolates. It should be noted that diagnostics for thermotolerant *Campylobacter* spp. have been mainly adapted to isolates from food-producing animals, food and humans. Thus, it is not unexpected that environmental *Campylobacter* spp. might not completely be identified with traditional diagnostic tools, which may hint at the need for extension of the databases including more environmental and wildlife isolates.

## Conclusion

Wildlife, in particular waterfowl, is considered a significant reservoir for *Campylobacter* spp., although the implication in public health is still unclear. The presence of high occurrence of *C. coli* in coots might have ecological implications and impact on the dissemination of thermophilic *Campylobacter* spp. into public health relevant niches. There is a need for further analysis of these distinct and unusual *Campylobacter* strains and for improvements of diagnostic tools, including also wildlife and environmental isolates.

## Data Availability Statement

The datasets presented in this study can be found in online repositories. The names of the repository/repositories and accession number(s) can be found below: https://www.ncbi.nlm.nih.gov/bioproject/?term=(PRJNA702752)%20AND%20bioproject_sra[filter]%20NOT%20bioproject_gap[filter].

## Author Contributions

CK conceived and designed the experiments, performed laboratory work and analysis, and drafted the manuscript. FS collected the samples. LE analyzed the data. M-TK performed laboratory work. TS advised genome analysis, reviewed the manuscript, and provided suggestions. KS designed the experiments, analyzed the data, and drafted and reviewed the manuscript. All authors contributed to the article and approved the submitted version.

## Conflict of Interest

The authors declare that the research was conducted in the absence of any commercial or financial relationships that could be construed as a potential conflict of interest.

## Publisher’s Note

All claims expressed in this article are solely those of the authors and do not necessarily represent those of their affiliated organizations, or those of the publisher, the editors and the reviewers. Any product that may be evaluated in this article, or claim that may be made by its manufacturer, is not guaranteed or endorsed by the publisher.

## Footnotes

1
www.eucast.org

2
www.ncbi.nlm.nih.gov/sra

3
www.pubmlst.org

4
https://github.com/tseemann/snp-dists
