# Supplementary material for: Characterization of Campylobacter spp. Strains Isolated From Wild Birds in Turkey
Source: Front Microbiol. 2021 Aug 18;12:712106. doi: 10.3389/fmicb.2021.712106 (PMC8416542; doi:10.3389/fmicb.2021.712106)
Supplement: Supplementary file 1 [file Image_1.pdf]

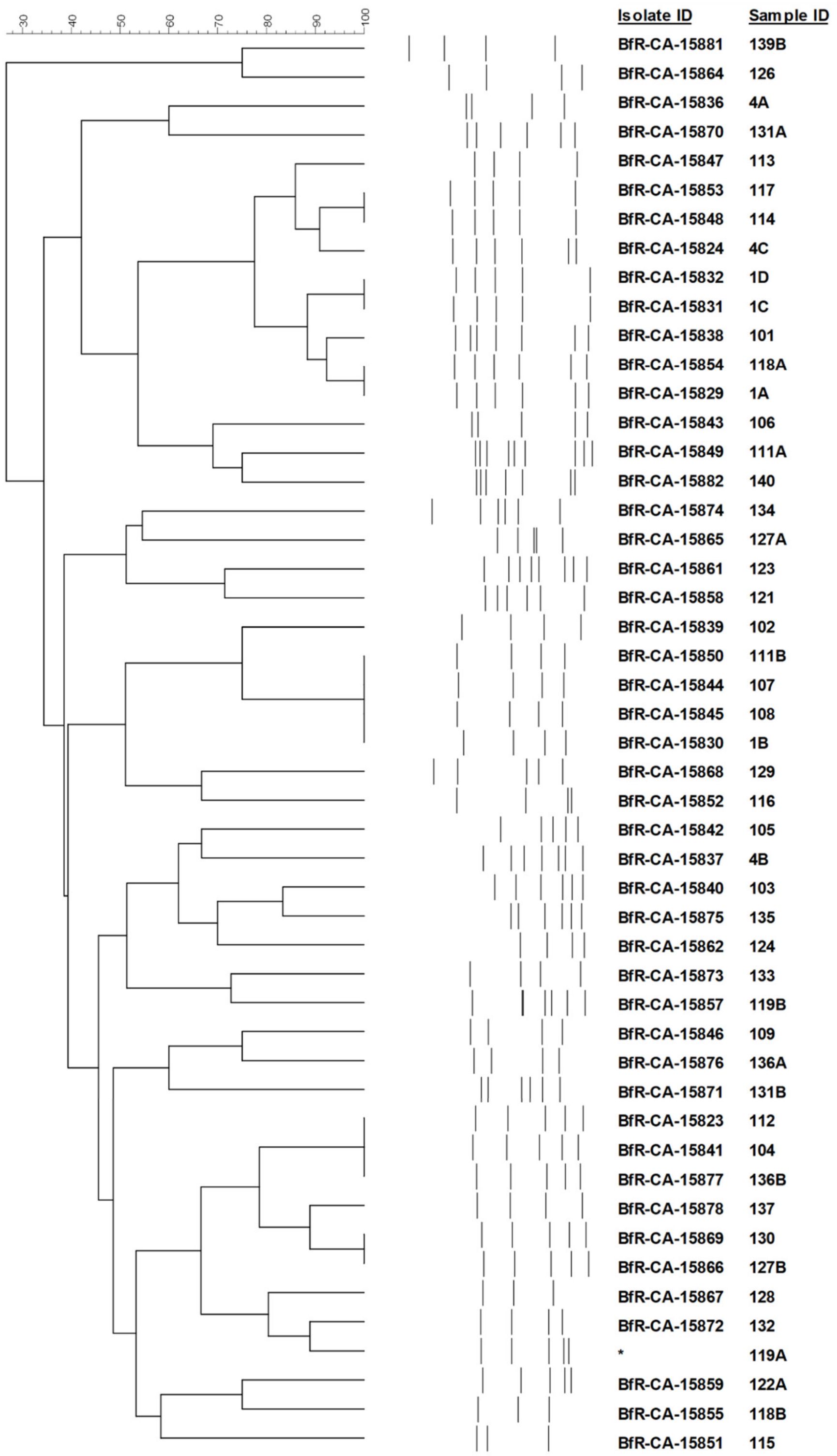

Image 1. Phylogenetic analysis of the *C. coli flaA* gene following HpyF3I digestion.

* This strain did not survive in the stock, thus there is no given isolate number for it.
