# Supplementary material for: Characterization of Campylobacter spp. Strains Isolated From Wild Birds in Turkey
Source: Front Microbiol. 2021 Aug 18;12:712106. doi: 10.3389/fmicb.2021.712106 (PMC8416542; doi:10.3389/fmicb.2021.712106)
Supplement: Supplementary file 8 [file Table_6.pdf]

```
LOCUS       DMACINML_1            261035 bp    DNA     linear       16-DEC-2020
DEFINITION  Campylobacter species strain strain.
ACCESSION
VERSION
KEYWORDS    .
SOURCE      Campylobacter species
  ORGANISM  Campylobacter species
            Unclassified.
COMMENT     Annotated using prokka 1.13 from
            https://github.com/tseemann/prokka.
FEATURES             Location/Qualifiers
     source          1..261035
                     /organism="Campylobacter species"
                     /mol_type="genomic DNA"
                     /strain="strain"
     gene            237..1007
                     /gene="hisJ"
                     /locus_tag="DMACINML_00001"
     CDS             237..1007
                     /gene="hisJ"
                     /locus_tag="DMACINML_00001"
                     /inference="ab initio prediction:Prodigal:2.6"
                     /inference="similar to AA sequence:RefSeq:CAL34871.1"
                     /codon_start=1
                     /transl_table=11
                     /product="histidine-binding protein precursor"
                     /protein_id="Prokka:DMACINML_00001"
                     /translation="MKKFLTAFLVAFTGLFLVACQNTKTENNASNEANTTLTLKVGTA
                     PNYKPFNYKQDSKLTGFDTDLVEEIAQKNGIEIVWVETNFDGLIPALKAGKIDMIASA
                     MSATDERRQSVDFTKPYYMSKNLYIKLKNNEALQTKTDLEGKKIGVQLGTLQENTAKT
                     IKDAQIQSNKDLNIAVLALKNNKIDAIVADEDTAKGFLAENSDLVSFYQETDGGEGFS
                     FAFDKDKQKEVIEIFNEGIDEAKASGFYDNLIKKYELE"
     sig_peptide     237..296
                     /gene="hisJ"
                     /locus_tag="DMACINML_00001"
                     /inference="ab initio prediction:SignalP:4.1"
                     /note="predicted cleavage at residue 20"
     gene            1394..2377
                     /gene="corA"
                     /locus_tag="DMACINML_00002"
     CDS             1394..2377
                     /gene="corA"
                     /locus_tag="DMACINML_00002"
                     /inference="ab initio prediction:Prodigal:2.6"
                     /inference="similar to AA sequence:RefSeq:CAL34863.1"
                     /codon_start=1
                     /transl_table=11
                     /product="magnesium and cobalt transport protein"
                     /protein_id="Prokka:DMACINML_00002"
                     /translation="MLYIYIKTQNALVQRINFNLDNQELPQNILWIDLLHPSAAEIAF
                     ISNEFNLEFPTKEEREEIELSAKYWEDNATITINAHFLIREFKNDNEEQNSIKLRTEI
                     VTFATAKNILFTIRYNEFSTFEEIQARILASPKNFEDGFDIIDKMFEVRVEKDADLLE
                     WIDKEARRLRTTVLEKKDEYSYDEMLKDISSLQELNMRVRDSLFDKRRAMTSLLKSDK
                     IDRDIKQNLTIVLKDLNSLVEFSVSQLNVLDNIQTILASQVNIEQNKIIKLFTVATVA
```

```
                     MMPPTLIGTIYGMNFKFMPELELHYAYPIVLAVMIISIIVPVIVFKKKGWL"
     gene            2396..2938
                     /locus_tag="DMACINML_00003"
     CDS             2396..2938
                     /locus_tag="DMACINML_00003"
                     /inference="ab initio prediction:Prodigal:2.6"
                     /inference="similar to AA sequence:RefSeq:CAL34862.1"
                     /codon_start=1
                     /transl_table=11
                     /product="molybdopterin biosynthesis protein"
                     /protein_id="Prokka:DMACINML_00003"
                     /translation="MDIIKIGILTLSDRASSGVYEDKATAEVERTLNSYIKNEIIYFK
                     ELIPDDYDLIIKKLLYLADEKECDLIITSGGTGPALRDVTPEATEAVCDKLMPGFGEL
                     MRLESLKFVPTAILSRQTAGIRNKSFIINLPGNPKAIAECLEPVFPAIPYCIDLIGGA
                     YIEGNDEVIQVFRPKKKCQN"
     gene            complement(2935..3120)
                     /locus_tag="DMACINML_00004"
     CDS             complement(2935..3120)
                     /locus_tag="DMACINML_00004"
                     /inference="ab initio prediction:Prodigal:2.6"
                     /inference="similar to AA sequence:RefSeq:CAL34861.1"
                     /note="hypothetical protein Cj0724"
                     /codon_start=1
                     /transl_table=11
                     /product="hypothetical protein"
                     /protein_id="Prokka:DMACINML_00004"
                     /translation="MFFKKKVPHKQNTRPKIPTNDIILDDRLYKFKNKIQKISKDEAS
                     VSLLARQLSRLIKANKF"
     gene            3250..4437
                     /locus_tag="DMACINML_00005"
     CDS             3250..4437
                     /locus_tag="DMACINML_00005"
                     /inference="ab initio prediction:Prodigal:2.6"
                     /inference="similar to AA sequence:RefSeq:CAL34860.1"
                     /codon_start=1
                     /transl_table=11
                     /product="putative integral membrane zinc-metalloprotease"
                     /protein_id="Prokka:DMACINML_00005"
                     /translation="MTLIAILCLYTALLSWISYNQIRFLEKEKDKQAQILSEEDYQNA
                     ADIAIENEKFKLFSNFYTLIVNIAWIGFGFLYLKELLISENSRLENTLFLLAFLIITS
                     ILNLPLSIYESFVKDKAHGFSNMTLKLFIKDTIKSLILTLIFGFLILYALLFCYDFFG
                     TFWWFAAFVFAFCVIVIINVIYPTLIAPIFNKMEKLEDENLLSKINDLMKRCGFNANG
                     VYVIDASKRDKRLNAYFGGLFKSKRVVLFDTLLKALSERELLAVLGHELGHFVHKDII
                     KALINGAITMFLLFFIFAHLPDFAYLESHLEGVNGGVFALLFIFANIFSFIISPLINA
                     LSRKNEFAADQHGSKLTSKEDMKNALIALARENKAFIKTSKIYTFFYLSHPSISDRIK
                     ALS"
     gene            4434..5234
                     /locus_tag="DMACINML_00006"
     CDS             4434..5234
                     /locus_tag="DMACINML_00006"
                     /inference="ab initio prediction:Prodigal:2.6"
                     /inference="similar to AA sequence:RefSeq:CAL34859.1"
                     /codon_start=1
                     /transl_table=11
```

```
                     /product="putative DNA methylase"
                     /protein_id="Prokka:DMACINML_00006"
                     /translation="MTIKNALIEAKSRLEKHENEALFILCEYLKKDRTWIFLNQDLEF
                     DSKPYFELIKRFQSGEPFEYIFEKADFWGLEFKVKKGVLIPRYDSEILLSQVLKICKN
                     NNFQNILEIGFGSGILSIVLAKELGIKITACDINPKALQLALENAKLHKVEHLIDFKL
                     YDFKQIKESYDFIFSNPPYIQNSYPIDLWVQNEPKEALFGGEKGYEILEEIITFSFER
                     KVKFLACEFGYDQKAILKQILYQKNFQAEFFQDEQGYDRAFVAQFAKK"
     gene            5276..5749
                     /locus_tag="DMACINML_00007"
     CDS             5276..5749
                     /locus_tag="DMACINML_00007"
                     /inference="ab initio prediction:Prodigal:2.6"
                     /inference="similar to AA sequence:RefSeq:AON66884.1"
                     /codon_start=1
                     /transl_table=11
                     /product="putative membrane protein"
                     /protein_id="Prokka:DMACINML_00007"
                     /translation="MKAINLFLLAAMIGIELILGIVVAPVIFYPANFIGEGVLSHFQS
                     GLMMTQIFIKMGYLLIFVSIFNLLFEIYSFIKEDMKFQIKFSKLILSILILILSLVFV
                     LYFTNTIVELQNLGEQATKSQEFLSIHNASEVVIKIILLMQVFLYFLSFKIAKKC"
     gene            5785..6534
                     /gene="flaC"
                     /locus_tag="DMACINML_00008"
     CDS             5785..6534
                     /gene="flaC"
                     /locus_tag="DMACINML_00008"
                     /inference="ab initio prediction:Prodigal:2.6"
                     /inference="similar to AA sequence:RefSeq:CAL34857.1"
                     /codon_start=1
                     /transl_table=11
                     /product="flagellin"
                     /protein_id="Prokka:DMACINML_00008"
                     /translation="MMISDASMMQQNYYLNNAQKASEKALENIAAVRAISGVDSANLA
                     IADSLRSQSSTIDQGIANAYDAIGVLQIADASLTNISQSADRLNELSVKMNNAALSDS
                     QKSMLRTEATRIQESINDSFNNATYNGKNVFQTMNFVVGSGTETTNLNPLATSGLSID
                     NQDSITSFMDQLGALRSEIGSGINAITSNINASVQNSINAKAAENNLLNNDMAKNVND
                     FNSNYLKENAAAFVAAQSNMQLQSKIASLLQ"
     gene            6571..7203
                     /locus_tag="DMACINML_00009"
     CDS             6571..7203
                     /locus_tag="DMACINML_00009"
                     /inference="ab initio prediction:Prodigal:2.6"
                     /inference="similar to AA sequence:RefSeq:AMK27605.1"
                     /codon_start=1
                     /transl_table=11
                     /product="YggS family pyridoxal phosphate enzyme"
                     /protein_id="Prokka:DMACINML_00009"
                     /translation="MTLEQILEKTKNIRLVAASKYVDANIIEKLFAQGITEFGENQVQ
                     ALIQKKEILDKKDLNIKWHFIGTLQSNKINLLIKQKPILWHSCNGLKIAKAMDKRLNY
                     KLDTLLEINSANELSKSGLNPDQAIEEYLQIQEECPNLNLCGVMSIGSHSEEQKEIIK
                     SFEITYKIYEKLQKHGAKICSMGMSNDFEIAIKCGSNMVRLGSILFKNLK"
     gene            complement(7220..10822)
                     /gene="dnaE"
                     /locus_tag="DMACINML_00010"
```

```
     CDS             complement(7220..10822)
                     /gene="dnaE"
                     /locus_tag="DMACINML_00010"
                     /EC_number="2.7.7.7"
                     /inference="ab initio prediction:Prodigal:2.6"
                     /inference="similar to AA sequence:RefSeq:CAL34855.1"
                     /codon_start=1
                     /transl_table=11
                     /product="DNA polymerase III, alpha chain"
                     /protein_id="Prokka:DMACINML_00010"
                     /translation="MSQFTHLHLHTEYSLLDGANKLKELAKTLKEQGATSVAMTDHGN
                     MFGAIDFYQTMRSQGLKPIIGLEAYLHNHEDISDKSSRQRFHLCLYAKNEVGYQNLMY
                     LSSQSYIKGLYYYPRINKKLLEEHSEGLICSSACLQGEVNWHLNIHSERNVRFGAKGY
                     EAAKEAALWYKKVFGDDFYFEIMRHGIGDQRAIDDDIIRLSKELNIKIIATNDTHYTF
                     KERAAAHEVFMCIAMGKKLNDPDRLRHSVHEFYVKSPAQMSELFADIPEAIENTQEIA
                     NKCNLELNLGNPTPPNFKFTREYAKEYNISLPEEGKEFSFDNDDVVFDELCKKGLEER
                     LKFVDISKHEEYKTRLEVEINIIKKMKFSGYMLIVHDFIKVAKDKGIPVGPGRGSAAG
                     SLVAYSLKITDLDPIPYSLLFERFLNPERVSMPDIDVDFCQDRRGEVIDYVIDKYGAD
                     KVAQVITFGKLLARGVIRDVARVCDMSIPDADELAKLIPEELKITLDAAYEKEPKIKE
                     FIERHPKGPEVWEYAKALEGLNRNAGMHAAGVVISNESLWKKTPLFRQSKNDERHLVT
                     QYSKDHLEDVDLIKFDFLGLKTLTLINNAIKLIKKRYNKDIVWEAIDVNDAKVYKTIQ
                     SGNTLGIFQIESGGMQNLNARLKPERFEDIIAVLALYRPGPMESGMLDDFIDRKHGLK
                     KIEYPFDTLESVLEPTYGVIVYQEQVMQIVQIIGGFSLGGADVVRRAMGKKDPEKMKK
                     LKSEFADGAEKQGYDRVKAEELWELIVKFAGYGFNKSHSAAYALITFQTAYLKTYYPS
                     EFMAALLTSEENNVDKIAVYIDEMKKMNIKLLPPSVNKAIREFSAVEQEDGKDAIVYG
                     LGAIKSVGIPAVENLLEARERGEFKNIDDFLSRIEPTKINKRTLESLIKAGAFDEFGY
                     TRKCLFDNMDNLTEGSKKMADARKNAASSLFGEDELTVGLQVNIVAKDEEFEVMEKLE
                     FEKEILGIYVSGHPLDRFYEKFKDIDYIKSIDFSTLKGNGEILSIGKIESFKSMMSKN
                     NKRYGRLEILDFYSTFEVTVFESNIEEIENILKNEASRNEAYGFLLSYKAEEGKLDSL
                     ALRSIKTLEELQEGEVKAVKKFSAKKNALENKDFVAEPKEFENNIIELDLSRLNRELV
                     YEIHEIARNAHNPNEKGNKKLVLKIVSTGSCLLYHTDFVISDSVSEKIINKYAG"
     gene            complement(11033..11362)
                     /locus_tag="DMACINML_00011"
     CDS             complement(11033..11362)
                     /locus_tag="DMACINML_00011"
                     /inference="ab initio prediction:Prodigal:2.6"
                     /inference="similar to AA sequence:RefSeq:CAL34854.1"
                     /codon_start=1
                     /transl_table=11
                     /product="putative ArsC family protein"
                     /protein_id="Prokka:DMACINML_00011"
                     /translation="MKVYGIKNCNSVKKALEFLNQKALEFDFIDIKKIDLNILENWLK
                     YKKFSEIINTAGTTSKKLGLNKDKIENLSELELKNIILENPSCIKRPVIERDNQIYIG
                     KEYEQIS"
     gene            complement(11359..12702)
                     /locus_tag="DMACINML_00012"
     CDS             complement(11359..12702)
                     /locus_tag="DMACINML_00012"
                     /EC_number="2.5.1.54"
                     /inference="ab initio prediction:Prodigal:2.6"
                     /inference="similar to AA sequence:RefSeq:CAL34853.1"
                     /codon_start=1
                     /transl_table=11
                     /product="putative phospho-2-dehydro-3-deoxyheptonate
```

```
                     aldolase"
                     /protein_id="Prokka:DMACINML_00012"
                     /translation="MTWTKNSWKNFSIKQQPIYSNQEELNKVLNKLEKLPPLVFAGEV
                     RELQNALARVCRKEAFLLQGGDCAESFANFGAVNIRDMFKILLQMAIVLTFAGGCPVV
                     KIGRMAGQFAKPRSSDYEEIDGVKLPSYRGDIINGFEFNEKARIPDPNRMLEAYYQSA
                     TTLNLLRGFAKGGLANLHEVHRWNLGFLKKSELHQQYTDISEKISQALDFMEACGINT
                     YNTPSLKEVDVYTSHEALLLPYEEALTRVDSLSGEIYDCSAHMLWIGERTRGLDEAHV
                     HFLSGVRNPLGVKIGPNASADDVIALANALNPNNEEGRLNIIIRMGAEKIATQLPKIF
                     SKLKKEGLNLVYSIDPMHGNTVKAGDFKTREFDKIMQEVRYFFEIAMSEGVYAGGVHL
                     EMTGQDVTECTGGASNVTAQSLEKRYETQCDPRLNADQALELAFLIADLVKKVRK"
     gene            complement(12727..13140)
                     /locus_tag="DMACINML_00013"
     CDS             complement(12727..13140)
                     /locus_tag="DMACINML_00013"
                     /inference="ab initio prediction:Prodigal:2.6"
                     /inference="similar to AA sequence:RefSeq:CAL34852.1"
                     /codon_start=1
                     /transl_table=11
                     /product="transthyretin-like periplasmic protein"
                     /protein_id="Prokka:DMACINML_00013"
                     /translation="MLSMKKILLALISIPIFLNAAEYQLSTHILDINSGQPASNVKVE
                     LYSLNKNQQWIKISEKFTEKNGRISDLLPYEKTENRPSGIYKLKFYTKDYYMSRKVDS
                     FYPFVEVSFELLKDQKHYHVPITLSPFGYSTYRGS"
     sig_peptide     complement(13078..13140)
                     /locus_tag="DMACINML_00013"
                     /inference="ab initio prediction:SignalP:4.1"
                     /note="predicted cleavage at residue 21"
     gene            complement(13293..13649)
                     /gene="rplS"
                     /locus_tag="DMACINML_00014"
     CDS             complement(13293..13649)
                     /gene="rplS"
                     /locus_tag="DMACINML_00014"
                     /inference="ab initio prediction:Prodigal:2.6"
                     /inference="similar to AA sequence:RefSeq:CAL34851.1"
                     /codon_start=1
                     /transl_table=11
                     /product="50S ribosomal protein L19"
                     /protein_id="Prokka:DMACINML_00014"
                     /translation="MKNKYIEQFEAKQIEGKNVPEFRAGDTLRLAIRIKEGDKTRIQN
                     FEGICIARRGNGVDETFIVRKMGANNVGVERIFPIYSESLESITVLRKGRVRRARLFY
                     LRDRRGKAARIKELKK"
     gene            complement(13660..14364)
                     /gene="trmD"
                     /locus_tag="DMACINML_00015"
     CDS             complement(13660..14364)
                     /gene="trmD"
                     /locus_tag="DMACINML_00015"
                     /EC_number="2.1.1.31"
                     /inference="ab initio prediction:Prodigal:2.6"
                     /inference="similar to AA sequence:RefSeq:CAL34850.1"
                     /codon_start=1
                     /transl_table=11
                     /product="tRNA (guanine-N1)-methyltransferase"
```

```
                     /protein_id="Prokka:DMACINML_00015"
                     /translation="MKFSFVSLFPNLLEFYFKDSILSRAIQKELFQLDFLNPRDFTDN
                     IYHKVDDYKIGGGAGLLMQVEPLYNTLKFIKDNEENPYFIFLNPSGKTFNQKDAKRLS
                     KKQNIVFVCGRYEGIDERVIEIFANEVFSIGDFILTGGELPALTLCDAIARNINGVLG
                     NACSLEEESFENGLLEAPSFAKPFIFEQNFKKFYTPSEFLKGNHAKIATLKTTLASCK
                     TKFFRPDLFLEHERKK"
gene                 complement(14361..14900)
                     /gene="rimM"
                     /locus_tag="DMACINML_00016"
CDS                  complement(14361..14900)
                     /gene="rimM"
                     /locus_tag="DMACINML_00016"
                     /inference="ab initio prediction:Prodigal:2.6"
                     /inference="similar to AA sequence:RefSeq:CAL34849.1"
                     /codon_start=1
                     /transl_table=11
                     /product="putative 16S rRNA processing protein"
                     /protein_id="Prokka:DMACINML_00016"
                     /translation="MSERNFIQVAKLGKTVGLKGYLKLYNLSDFIQQFKKGASFFTEN
                     GKSQLKIKHYNLANSSVLFEGYENVEAAKQLINLILYQSIEATRQTCILKKDEFFYFD
                     ILKCEVFDDERRLGKVVDILEVGDSYLFEIQSDQELIDKAYAKTFFIPYIDKYVNKID
                     IEKYSIFCTKDAFLILENS"
gene                 complement(14893..15135)
                     /locus_tag="DMACINML_00017"
CDS                  complement(14893..15135)
                     /locus_tag="DMACINML_00017"
                     /inference="ab initio prediction:Prodigal:2.6"
                     /inference="similar to AA sequence:RefSeq:CAL34848.1"
                     /note="hypothetical protein Cj0711"
                     /codon_start=1
                     /transl_table=11
                     /product="hypothetical protein"
                     /protein_id="Prokka:DMACINML_00017"
                     /translation="MVENFLREYAKLIADYPDKITTEKIKLDENFAEIILYADKVDTG
                     KLIGKNGKMINAIKTVVSACKSKDSMSYRVTVKALE"
gene                 complement(15140..15367)
                     /gene="rpsP"
                     /locus_tag="DMACINML_00018"
CDS                  complement(15140..15367)
                     /gene="rpsP"
                     /locus_tag="DMACINML_00018"
                     /inference="ab initio prediction:Prodigal:2.6"
                     /inference="similar to AA sequence:RefSeq:CAL34847.1"
                     /codon_start=1
                     /transl_table=11
                     /product="30S ribosomal protein S16"
                     /protein_id="Prokka:DMACINML_00018"
                     /translation="MTVIRLTRMGRTKRPFYRIVVTDSRKRRDGGWIESIGYYNPMVE
                     PEVVKFDSERLAYWKSVGAKLSDKVASITSK"
gene                 complement(15435..16772)
                     /gene="ffh"
                     /locus_tag="DMACINML_00019"
CDS                  complement(15435..16772)
                     /gene="ffh"
```

```
                     /locus_tag="DMACINML_00019"
                     /inference="ab initio prediction:Prodigal:2.6"
                     /inference="similar to AA sequence:RefSeq:CAL34846.1"
                     /codon_start=1
                     /transl_table=11
                     /product="signal recognition particle protein"
                     /protein_id="Prokka:DMACINML_00019"
                     /translation="MFELVSESFKSAINKLRFVDDEKALKNALDTLKKALLKADVHHK
                     VVKDLLSLIEEDVKKNGIGQKQFLEAIKANLENILNGDGKNQGFVFATKPPTVVLMVG
                     LQGGGKTTSTVKLANYLKLRNKKVLIAACDLQRLAAVEQLKQLCEANELELFYIENEK
                     DPIKVAKQALEKAKNSMADVLLVDTAGRLAIDEALMNELKAVKDTLNPDEIFYVADAM
                     SGQDGVKTASSFNELLQITGVILSKFDADTKGGIALGITKQIGIPLRFVGVGEKIADL
                     EIFIPERIVNRIMGEGDLATLAEKTATIIDEKEAKKLNQKIKKGEFNFNDFLTQMESV
                     KKLGSMKSIIGMIPGLSNVASSVKDLDLDNSKEIIHIKAMISSMTLKERENPDLLNNA
                     RKRRIAEGAGLSQVEVNRFLKQFANAAKLAKRFSGKKGMDSLMQMMNQARRQF"
     gene            complement(16839..17591)
                     /locus_tag="DMACINML_00020"
     CDS             complement(16839..17591)
                     /locus_tag="DMACINML_00020"
                     /EC_number="4.2.1.70"
                     /inference="ab initio prediction:Prodigal:2.6"
                     /inference="similar to AA sequence:RefSeq:CAL34845.1"
                     /codon_start=1
                     /transl_table=11
                     /product="putative ribosomal pseudouridine synthase"
                     /protein_id="Prokka:DMACINML_00020"
                     /translation="MQEKAYKLLALQENISNREAKDLIDKGCVFSHGKKVVVARALMS
                     DKTKFSIVKTSKPKVIFEDENIIAINKPYAYISEELEKKFNAKLLNRLDKETSGVILL
                     CKNEDFRKICIEEFKKQRVHKGYIAVLDGILAEEVEINEPILTIKTKNGALSKISKEG
                     LSAVSIFTPIMMQAKKTLAKIIIKTGRTHQIRVHAKFIKHGIIGDEKYAKISSDRMYL
                     HSYEIRILNYQFKAELDPSFAKLGFEIKNLDF"
     gene            complement(17572..18729)
                     /gene="kdtA"
                     /locus_tag="DMACINML_00021"
     CDS             complement(17572..18729)
                     /gene="kdtA"
                     /locus_tag="DMACINML_00021"
                     /inference="ab initio prediction:Prodigal:2.6"
                     /inference="similar to AA sequence:RefSeq:CAL34844.1"
                     /codon_start=1
                     /transl_table=11
                     /product="3-deoxy-D-manno-octulosonic-acid transferase"
                     /protein_id="Prokka:DMACINML_00021"
                     /translation="MIFLYYILVWIAFLLCAIPLFLLSLFKPKYKNSLKARFFLFRNI
                     SQKRSDVHFHVCSYGEAKSIKELVLKFDSRITTITQTGYDYAKEICNKVNYLAFENWI
                     PFWLKPSKVLVIFEAEYWLMFVFIAKLQKSKVILLNARISDNSYASYKKFRFFYKKIF
                     CYIDEVFAQSEADKIRLEDLGAKNVKIFPNIKSKLQICPAQKYFKPKQKLIIFASTHK
                     GEEELLLKHYKMDKQEKLIIAPRHPERFLEVEQLLHDKGLKFDKFSLLQNEDKKFNQD
                     ILLLDCLGELINFYAISDVVVLGGSFFEGIGGHNPIEVAHFNNVLISGVYIHNQKSLF
                     QEVDNVYFCEDLKELDGIIHNYNLKAKIAQNNDLSTIIQAIQEGIDARKSI"
     gene            complement(18726..19442)
                     /locus_tag="DMACINML_00022"
     CDS             complement(18726..19442)
                     /locus_tag="DMACINML_00022"
```

```
                     /inference="ab initio prediction:Prodigal:2.6"
                     /inference="similar to AA sequence:RefSeq:CAL34843.1"
                     /note="conserved hypothetical protein Cj0706"
                     /codon_start=1
                     /transl_table=11
                     /product="hypothetical protein"
                     /protein_id="Prokka:DMACINML_00022"
                     /translation="MNKYLEQLVLLSKIDQEIDSFEPKMESISKTLKDAENKIAKFNV
                     ELNNLENEIQDVENQKVQNNAHISEFSAKIKELSKKSGAVKTEKEANALKIEEDIAKE
                     QLDAANDEIVRLDKILENKELFKKELLEERAKEEQNLDEIRVSISSQMDGLEKERMNV
                     YAKKTKLVAEMNQKVLSFYEKIRKWAKNTAVVPVKKQACYGCFMKIYDKTYLSVIKGE
                     EIITCPHCGRILYKEQEDQN"
     gene            complement(19452..20177)
                     /locus_tag="DMACINML_00023"
     CDS             complement(19452..20177)
                     /locus_tag="DMACINML_00023"
                     /inference="ab initio prediction:Prodigal:2.6"
                     /inference="similar to AA sequence:RefSeq:CAL34842.1"
                     /note="conserved hypothetical protein Cj0705"
                     /codon_start=1
                     /transl_table=11
                     /product="hypothetical protein"
                     /protein_id="Prokka:DMACINML_00023"
                     /translation="MKLIEIYNFLDKLSPFETQEIWDNSGLLLGNFDDEIHNVYLSLD
                     IDEQLIQNAEENSLFITHHPLIFKGLKDLANSAYPRAFIKEMLRKNLSLISMHTNFDL
                     SHLNTYFIEEILGFEISFKDKFLAYVDVDMTFKELCTLVKKRLNLEILRTSFCGKEHL
                     KRLAVCTGSGGDLLNEVKADCYLSGDFKYHQALEAISNHLNLIDLGHFESERYFAQCL
                     VKHLKNLPLKVIITVSKNPFQYF"
     gene            complement(20164..21027)
                     /gene="glyQ"
                     /locus_tag="DMACINML_00024"
     CDS             complement(20164..21027)
                     /gene="glyQ"
                     /locus_tag="DMACINML_00024"
                     /EC_number="6.1.1.14"
                     /inference="ab initio prediction:Prodigal:2.6"
                     /inference="similar to AA sequence:RefSeq:CAL34841.1"
                     /codon_start=1
                     /transl_table=11
                     /product="glycyl-tRNA synthetase alpha chain"
                     /protein_id="Prokka:DMACINML_00024"
                     /translation="MTFSQMILNLQEFWQKQGCAIMQPYDMPAGAGTFHPATFLRSLG
                     KKPWATAYVAPSRRPTDGRYGENPNRLGAYYQFQVLMKPSPDNIQELYLKSLENLGFD
                     LKSHDVRFVEDNWESPSLGAWGLGWEVWLDGMEVTQFTYFQQVGGIAVDLVSAEITYG
                     LERIAMYLQNVDNVYDIVWSEFNGEKIKYADVHKRSEYEFSKYNFEISNVDFLNQQFE
                     NTYKECKSILEQGLALPAYDYCMLAAHTFNLLDARGAISVAQRQDYMLKIRELSKTCA
                     EIYKKNLDETD"
     gene            complement(21029..21583)
                     /locus_tag="DMACINML_00025"
     CDS             complement(21029..21583)
                     /locus_tag="DMACINML_00025"
                     /inference="ab initio prediction:Prodigal:2.6"
                     /inference="similar to AA sequence:RefSeq:CAL34840.1"
                     /note="hypothetical protein Cj0703"
```

```
                     /codon_start=1
                     /transl_table=11
                     /product="hypothetical protein"
                     /protein_id="Prokka:DMACINML_00025"
                     /translation="MQTYLELQEFCKLVHLNEDVVKGMMANGALNFKEEEGKIYIEAN
                     QGTFSVVPSASNSKPAMVNSMTLAGESFVEKTIGTILNLHEKVLDAKDETLDALKNEN
                     KFLKDALYSMQELYDEDRKTIETLNHELKHAREEIEFLKRKYKLMWSKTAEIFGARTE
                     PDLEMKKDENSSTTNEKLNENKEG"
     gene            complement(21594..22088)
                     /gene="purE"
                     /locus_tag="DMACINML_00026"
     CDS             complement(21594..22088)
                     /gene="purE"
                     /locus_tag="DMACINML_00026"
                     /EC_number="4.1.1.21"
                     /inference="ab initio prediction:Prodigal:2.6"
                     /inference="similar to AA sequence:RefSeq:CAL34839.1"
                     /codon_start=1
                     /transl_table=11
                     /product="phosphoribosylaminoimidazole carboxylase
                     catalytic subunit"
                     /protein_id="Prokka:DMACINML_00026"
                     /translation="MKFVSIVMGSKSDYEIMSECAKTLEKFGVKYELIISSAHRSPKR
                     TKEYIKEAETKGAKVFIAAAGMAAHLAGAVAAYTTKPVLGVPMPGSALASMDSLFSTV
                     QMPGGIPVGTLAVGKAGAKNAAYLAMQILALNDEALAEALKQDRKNQEENLISDSKSV
                     EVLL"
     gene            complement(22085..23341)
                     /locus_tag="DMACINML_00027"
     CDS             complement(22085..23341)
                     /locus_tag="DMACINML_00027"
                     /inference="ab initio prediction:Prodigal:2.6"
                     /inference="similar to AA sequence:RefSeq:CAL34838.1"
                     /codon_start=1
                     /transl_table=11
                     /product="putative protease"
                     /protein_id="Prokka:DMACINML_00027"
                     /translation="MIIPEIVAPAGNFTKLKIALAYGADAVYAGVNNFSLRSRTAREF
                     NYESFEEAIKYTHQRGKKVYVTLNGFHLGGQIESLKKHILKLKEMKPDAFIVASVGAM
                     ALVRELAPEIALHVSTQANVLNYLDARVYKDMGAKRVVIARELGLKDAKSLKENCDIE
                     LEAFVHGSMCFAYSGRCLISSVQSGRMSNRGSCANDCRFNYELYAKNPENGVLFRLEE
                     DENGTHVFNSKDLNLCSYIEKIMQEDCISAFKIEGRTKSEYYVALTTRTYKMAIQDAL
                     EGKFEASKYEKEIATLKNRGFTDGYLVSRPLEKADTQNHDTSIEEGTHQVHAIVEDGE
                     YFKCKGKIVLNKAYEILMPLGDKIESCDNELGKIYKTEDKYFVEFKKLISKNNKEFNE
                     IHSGNENEIKMPNKISSFSFLRKEIG"
     gene            complement(23338..24039)
                     /locus_tag="DMACINML_00028"
     CDS             complement(23338..24039)
                     /locus_tag="DMACINML_00028"
                     /inference="ab initio prediction:Prodigal:2.6"
                     /inference="similar to AA sequence:RefSeq:CAL34837.1"
                     /note="hypothetical protein Cj0700"
                     /codon_start=1
                     /transl_table=11
                     /product="hypothetical protein"
```

/protein_id="Prokka:DMACINML_00028"
/translation="MTQEELDALMNGDVNFDEDAESNNKSETESHLESEEGLMLEDVK
VSDYKPDPSVVWPPPPPNQEHKVVHQLDDVTKDSELKATEMMDKLESINSFFADSESL
FSEIDKALSKNIEIFSKLNEKFPNVVSFNEALQLNSKVKKASQEIIDKLQCGQDEVMM
AMDAMQYQDIHRQKIERVINVMRALSRYMSSLFEGRIDDKKRVSSAVHIEGDSTTDIV
SNDDIEALIASLGQK"
gene 24238..25668
/gene="glnA"
/locus_tag="DMACINML_00029"
CDS 24238..25668
/gene="glnA"
/locus_tag="DMACINML_00029"
/EC_number="6.3.1.2"
/inference="ab initio prediction:Prodigal:2.6"
/inference="similar to AA sequence:RefSeq:CAL34836.1"
/codon_start=1
/transl_table=11
/product="glutamine synthetase"
/protein_id="Prokka:DMACINML_00029"
/translation="MGKFVNNVDEFFNFCKQNEVLFVDFRFTDMIGTWHHITYNLHAI
SEETFEAGIPFDGSSIHGWQPIEKSDMILKPDAQSAFLDPFTADPTVIVFCDVYDIYK
GQMYEKCPRSIAKKAMEHLKNSGIADTAYFGPENEFFVFDSVKIVDTTHCSKYEVDTE
EGEWNDDKDFADSYNTGHRPRNKGGYFPVQPIDSLVDIRSEMVQTLEKVGLKTFVHHH
EVAQGQAEIGVNFGTLVEAADNVQIYKYIVKMVAHLNGKTATFMPKPLYGDNGNGMHV
HMSLWKDGVNLFYDKEGYGGLSQTAINYIGGVLKNARSVAAFTNPSSNSYKRIVPGFE
APCILTYSCQNRSASCRVPYGIGKNSARVEIRFPDSTANPYLAFVSLLMAGLDGIKNK
TIPVGPMDENLFDLTLDEIREKGIEQLPHTLRGSLEALIRYNAFLKPVMSDIFIDDYQ
HLKFETQVWPVEARPTAYEFKTCYSC"
gene complement(26038..26829)
/gene="flgG"
/locus_tag="DMACINML_00030"
CDS complement(26038..26829)
/gene="flgG"
/locus_tag="DMACINML_00030"
/inference="ab initio prediction:Prodigal:2.6"
/inference="similar to AA sequence:RefSeq:CAL34835.1"
/codon_start=1
/transl_table=11
/product="flagellar basal-body rod protein"
/protein_id="Prokka:DMACINML_00030"
/translation="MMRSLHTAATGMVAQQTQIDVTSNNIANVNTAGFKKGRAEFADL
MYQVMKYAGTSTSATTLSPSGIEVGIGVRPTAVTKVFTEGNLKATSTDGLDMAIAGNG
FFQIQLPDGTIAYTRNGQFTKDNEGNIVNADGYRLLPEMTIPEGATAINVATDGTVSV
MLPGEQQETQIGQVELVQFINPAGLHSMGDNLYLETGASGAPVAGIATQDGLGTIRHG
FIELSNVQLVEEMTDLITGQRAYEAGSKAITTSDDMLGIVNQLKR"
gene complement(26860..27672)
/gene="flgG2"
/locus_tag="DMACINML_00031"
CDS complement(26860..27672)
/gene="flgG2"
/locus_tag="DMACINML_00031"
/inference="ab initio prediction:Prodigal:2.6"
/inference="similar to AA sequence:RefSeq:CAL34834.1"
/codon_start=1

```
                     /transl_table=11
                     /product="flagellar basal-body rod protein"
                     /protein_id="Prokka:DMACINML_00031"
                     /translation="MQNGYYQATGGMVTQFNKLDVITNNLANINTSGYKRDDVVIADF
                     KRIFKETQDELPIENHTRDASRFVNTTIDGVPQVSQGYTDFSLGSLKATNNPLDLAMT
                     REDAFYLVQTKDGEIRLSKDGNFQLNEEGYLVNKQGYRILSSDYFNNPQNAGIRIPNG
                     AVYINVDRNGSIEVDGVQNARLFIAQVDDIRALQKDGDNVYRIDDLTRIRDLDNSNAV
                     RQGFSQGSNVNPVTEMVGLIEANRMVEMYQKVMTAHMDDLNQEAINKLAAVK"
gene                 complement(27813..28925)
                     /gene="ftsZ"
                     /locus_tag="DMACINML_00032"
CDS                  complement(27813..28925)
                     /gene="ftsZ"
                     /locus_tag="DMACINML_00032"
                     /inference="ab initio prediction:Prodigal:2.6"
                     /inference="similar to AA sequence:RefSeq:CAL34833.1"
                     /codon_start=1
                     /transl_table=11
                     /product="cell division protein FfsZ"
                     /protein_id="Prokka:DMACINML_00032"
                     /translation="MSEFLVEEMQHNKGAKIKVIGCGGGGGNMINHMVKMGLNDLDLI
                     AANTDAQAISNSLAKTKIQLGEKKTKGLGAGMLPEVGAESARESFEEIKASLSQSDIV
                     FIASGFGGGTGTGATPVIAQAAKEIGALTVSVVTMPFAFEGKQRKKLAENGLLELKKE
                     SDSILVIQNEKLLSIIDKKAGIKDAFKLVDDILARAVKGMVSILLDNGDINVDFADVR
                     TIMSHRGLALMGVGSASGENAIEEALSNAIESPLLDGMDIKGAKGVILHFKTSSNCSL
                     IEISAAANNIEEIVDENAKIIFGSTTDDSMEDRVEVTIIATGFEDRDSMAKKAAEEAE
                     APKKNPYLNLRKVSGGFDEEIMAQIETPTFLRRQMD"
gene                 complement(28942..30324)
                     /gene="ftsA"
                     /locus_tag="DMACINML_00033"
CDS                  complement(28942..30324)
                     /gene="ftsA"
                     /locus_tag="DMACINML_00033"
                     /inference="ab initio prediction:Prodigal:2.6"
                     /inference="similar to AA sequence:RefSeq:CAL34832.1"
                     /codon_start=1
                     /transl_table=11
                     /product="cell division protein FtsA"
                     /protein_id="Prokka:DMACINML_00033"
                     /translation="MNILGIDLGSTQTCAIVAQKDEEGLKIIGFAKAKTNGVKKGAIT
                     NIELASKSIEEAVRNAEMMSGVHYDKVVVSISGAYTKSVDSIGVVNIPNHEIGIKEIH
                     RAVSTAKHTANLPSGYEIIHVLPYNFKVNDLEHVDDPLGMSGNRLEVSTHIVISQESH
                     IKNLKKAVELADLRVDNIVLSGYASAIACLDDSEKELGAVLIDMGGAICDMVVHTGNS
                     IRYNDCLQIGSINITQDLSMALHTPLKEAEKIKLNYAALSQQPNALIQIPSMGDERKV
                     NEVSLDIISNVIYARAEETLMILAKILSDNRYANSIGGGVVLTGGMTKLAGIDELAPA
                     TFDNRSVRLATARKDLITGFSEIFNDPENTCAIGLCLYGAGYFTPYELDSNEKLRYKG
                     EIENYNRQIRQEIIPQKEVENETKSDFFGENLQENDTIILQEQLDFKEPKEKKPNVFS
                     NIWHKIMNQF"
gene                 complement(30327..31814)
                     /locus_tag="DMACINML_00034"
CDS                  complement(30327..31814)
                     /locus_tag="DMACINML_00034"
                     /inference="ab initio prediction:Prodigal:2.6"
                     /inference="similar to AA sequence:RefSeq:CAL34831.1"
```

```
                     /codon_start=1
                     /transl_table=11
                     /product="putative periplasmic protein"
                     /protein_id="Prokka:DMACINML_00034"
                     /translation="MLTWMQHHKKYLVITIWISTIAFVGAGFLGWGAYDFNLNRSSSV
                     AVVGDEKINYNEFNVRYNQIFNYYNQISNGGLNEESAKQLGIENAALSSLVEDKLLLN
                     FAKDLGLGSSENEVIQALAETRAFQDPSGDFNKTIYYELLNANNMTPKEYEKTLSNEI
                     IIGKLEQIFNLPQTDEELKMLGASYFMQDSLNIAKLEQDKENIQVNEEELKKLWNEHK
                     EDYKTKKIYEISTYYLPVDMQKIDDSKLEEFYNNENNKFKYKDFSGKIMSFEAAKKDV
                     AKDYALAQLKNTANAKFLELKEGKDNFQKDENITDSDVYYPLEALSRAKNGDVLRPSE
                     YQNGYIIIKLNKTNPIRTKTFDEAREELMPIYISEQVKKNLEEKAKQNLENFSGTNIG
                     FVSRDTVRNDAKISNILNEAEFSYFLMNVFNSDQNRSYVILNEDKAILYKINKQKLDM
                     NPEKFQQYRTMLNQNLQNLKANEMKQELIEELKKTYPIKIYYKGK"
     gene            31967..32902
                     /gene="mraW"
                     /locus_tag="DMACINML_00035"
     CDS             31967..32902
                     /gene="mraW"
                     /locus_tag="DMACINML_00035"
                     /EC_number="2.1.1.-"
                     /inference="ab initio prediction:Prodigal:2.6"
                     /inference="similar to AA sequence:RefSeq:CAL34830.1"
                     /codon_start=1
                     /transl_table=11
                     /product="S-adenosyl-methyltransferase"
                     /protein_id="Prokka:DMACINML_00035"
                     /translation="MKIPHIPVLLNEVNQAFKKLDSGYFLDCTVGFGGHSESLLKNHP
                     NLKLIACDQDKEALEFSKKRLHDFQDRTEFVLSNFSQILDQISHDKLNDLKGILADIG
                     VSSYQLDDNKRGFNLHSDFLDMRMNQDAKNSAFDIINNYTQEQLSDIFKKYGELNNGY
                     FIAQKICQERQKNKIKSAKELYEIIGKTKQNHRKISKATLVFQAIRIEVNQELEVLKT
                     FLEKLEKIKLKNCILAIICFHSLEDRIVKNYFKKWAKNCICDERAFKCECGNNHALGQ
                     IISKKAIVPSKEEIMINSRSSCAKMRIFHFKNMDK"
     gene            32902..33792
                     /locus_tag="DMACINML_00036"
     CDS             32902..33792
                     /locus_tag="DMACINML_00036"
                     /inference="ab initio prediction:Prodigal:2.6"
                     /inference="similar to AA sequence:RefSeq:AJK70823.1"
                     /codon_start=1
                     /transl_table=11
                     /product="membrane protein"
                     /protein_id="Prokka:DMACINML_00036"
                     /translation="MLDDDFLKEKQNIRQKMLKFSRAVNQGKPLDDDLKEEISSDDIL
                     RRRFKKRSNRFLDEVEEEYELEYTKKSNIYLKEDLINVKLEEKQSLAKKIFSKIKEKN
                     KNQEPKNKKTKKLFSFLKKKEIQTIQNKTTHNNTQEKYLHTDKQTEIKKLQNDKIQPT
                     QTDLKPDIRQSLENLQKKIQTQEIKAEIEKPEIQPIKTEYDEDAQKVKNVLLEGFSNA
                     TKEDKNLNFNHLLFAVLFVSFTLVLFAPQIYIRNQIYYLSREIATLRVEESVLDEENK
                     DLKRRLEIMRFQNQILDYLE"
     gene            complement(33780..34271)
                     /locus_tag="DMACINML_00037"
     CDS             complement(33780..34271)
                     /locus_tag="DMACINML_00037"
                     /inference="ab initio prediction:Prodigal:2.6"
                     /inference="similar to AA sequence:RefSeq:APA80970.1"
```

```
                     /codon_start=1
                     /transl_table=11
                     /product="membrane protein"
                     /protein_id="Prokka:DMACINML_00037"
                     /translation="MKKFKDFSCNFLFKISDQPVLLRDLLEANALFNDGMLVDPSKLN
                     FNFKILNSYIYFGIFCALILLPLLLITHYLFTRLDFHISIISAVAVTACVFISYDIFK
                     VYARKMISKKLIQKAWALHFPYFAYEKYSIMAGNFYKEALKEEIPKANLEQYVLEKII
                     HSK"
     gene            complement(34346..35536)
                     /locus_tag="DMACINML_00038"
     CDS             complement(34346..35536)
                     /locus_tag="DMACINML_00038"
                     /inference="ab initio prediction:Prodigal:2.6"
                     /inference="similar to AA sequence:RefSeq:AJK70820.1"
                     /codon_start=1
                     /transl_table=11
                     /product="acetate kinase"
                     /protein_id="Prokka:DMACINML_00038"
                     /translation="MKILVLNSGSSSIKFKFFDDKVVKASGLVEKIGEEKSKVILKNA
                     LNDEKFERELPIKNHEHGLQIVNELFKESGILEDLNALDGCGHRIVHGGRNLSEHCLV
                     DDYVLKEIDRVSIFAPLHNPAHLAGIKTMMKAAPNVKNTVIFDTAFHRTMPDFAYMYA
                     LPYDFYDKHNIRRYGFHGTSHAFVSARAAKLLGKDKEDLNAISAHLGNGASVCAIEGG
                     KSIDTSMGFTPLEGLVMGTRCGDLDPAILPFISNLKGLTIEEIDTLLNKKSGLYGICG
                     SNDFRDIGTKIEQGDDRARLAFDMFCYRLLKYIGAYFTVLPRTDAIIFTGGIGENAAS
                     VRQKVCERLSHLGVELDIGLNNEKSSCERMISTANSKVKIFIIPTDEELEIARITEEL
                     INKS"
     gene            complement(35550..37052)
                     /gene="pta"
                     /locus_tag="DMACINML_00039"
     CDS             complement(35550..37052)
                     /gene="pta"
                     /locus_tag="DMACINML_00039"
                     /EC_number="2.3.1.8"
                     /inference="ab initio prediction:Prodigal:2.6"
                     /inference="similar to AA sequence:RefSeq:EAQ72368.2"
                     /codon_start=1
                     /transl_table=11
                     /product="phosphate acetyltransferase"
                     /protein_id="Prokka:DMACINML_00039"
                     /translation="MANLYLMRSRSDELNTLITTKLLKNYSKIYKNIAIYCPVIYVHR
                     IPVLQGWLEEFDIKQTLKNAYSFTFREAMEEFSKDPHSFFNTILEEYEELNRKYDFVL
                     VNSFCEFGILDGFDLSIKLAKNLNTPIAAIINDEDKLIAQKYFDEALDGKDYALINEN
                     FNFEEVHKLQAYDFMTPHRFKYELIKKSVKNKKTVVLPESNDERILKAVEILLKSSVV
                     DLILLGDEQDIKQKADELALDLTAVKILNPLNSEYNEEFATLLYEARKSKGMSLEEAK
                     ELVKDRTYFGTLLIHSGKADAMVSGASTTTAETIRPALQIIKTQEGVDSVSGIFFMGL
                     DDKVLAFADCAVNPNPNAEQLATSAYVSAMTAKSFGIEPRIALLSYSSGDSGKGESVD
                     LVKEALKIAKEKYPELNIDGPMQFDCAYDPKTAAKKMPNSKIAGNVNVYVFPDLDAAN
                     ICYKAVQRTANALAIGPILQGLKKPVNDLSRGCLVDDIVDTVILSAIQAQ"
     gene            37157..37855
                     /gene="flgH"
                     /locus_tag="DMACINML_00040"
     CDS             37157..37855
                     /gene="flgH"
                     /locus_tag="DMACINML_00040"
```

```
                     /inference="ab initio prediction:Prodigal:2.6"
                     /inference="similar to AA sequence:RefSeq:CAL34824.1"
                     /codon_start=1
                     /transl_table=11
                     /product="putative flagellar L-ring protein precursor"
                     /protein_id="Prokka:DMACINML_00040"
                     /translation="MKKVLFYVLPFAFFGCSATVDPQISMKPPAYVEELAPKQSNNVE
                     TAPGSLFGKGDNPLFSDKKAMNVNDLVTVVIQESTTQSTQANKATSRTNTSNLGGGAL
                     TGGSGLLGTAIDKVNAYSNIGFQTNSTNNYQGTGAQSRNESFNTTISTRVIKILSNGN
                     YFIEGSRELLINGEKQIIQLSGVIRPYDIGQDNTIDSRYIADAKILYKTEGEVDRSTR
                     KPWGSKVIEAIWPF"
     gene            complement(37939..39012)
                     /gene="ispG"
                     /locus_tag="DMACINML_00041"
     CDS             complement(37939..39012)
                     /gene="ispG"
                     /locus_tag="DMACINML_00041"
                     /EC_number="1.17.7.1"
                     /inference="ab initio prediction:Prodigal:2.6"
                     /inference="similar to AA sequence:RefSeq:CAL34823.1"
                     /codon_start=1
                     /transl_table=11
                     /product="4-hydroxy-3-methylbut-2-en-1-yl diphosphate
                     synthase"
                     /protein_id="Prokka:DMACINML_00041"
                     /translation="MEYKRFKTRQIKVGDVLIGGDAPISVQSMLFTKTRDIEGSLEQL
                     NRLYFAGANIVRLACLDMADARALKEIKAKSPLPLIVDIHFNHNLAVFCAEFIDGVRI
                     NPGNIGSKENIKEVVKACKERGIPIRIGVNHGSIEKEFSDKYGYGVDAMLESALYNIK
                     LLEDLDFFDIKISMKTSDTQKTIEAYERLRPLCDYPFHLGVTEAGTKFHSTIKSSIAL
                     GNLLLKGIGDTMRVSMTGELEEEIKVARAILQDSGVQKSGVNIISCPTCGRIQSDLIS
                     AIKIVEEKTKHIKEPLNISVMGCVVNALGEAKGADVAIAFGKNQGLVIRHGEVVAKLK
                     ENELVDRFLAEVEDEVKSREIKE"
     gene            complement(39071..40909)
                     /gene="priA"
                     /locus_tag="DMACINML_00042"
     CDS             complement(39071..40909)
                     /gene="priA"
                     /locus_tag="DMACINML_00042"
                     /EC_number="3.6.1.-"
                     /inference="ab initio prediction:Prodigal:2.6"
                     /inference="similar to AA sequence:RefSeq:CAL34821.1"
                     /codon_start=1
                     /transl_table=11
                     /product="putative primosomal protein N'"
                     /protein_id="Prokka:DMACINML_00042"
                     /translation="MRYYELAVFGLYLQNLIFHSEEEIPALREVIIDLKNKKNLRALV
                     LKECQKPEFQTKEIKEITPFSLSCVQFELAKFISYYYTSKLGFVLGLFSTHQTYACDP
                     VKIENEPKLSQKQKEALEFVKTNPTSLLFADTGSGKTEIYICLIKQYLEQGKQVLLLM
                     PEISLTPQMQKRLEVYFKDKFFLWHSKISKKKKQENLERFCKGEALLVAGARSALFLP
                     FVNLALVIVDEEHDNSYKASNQPYINARDLALFLSSKFDIKVLLGSATPSLTSFYKQK
                     SFRLKGTFFESKKHFLYDESELGITPMLLGELKKSLENQKQAIIFLPTKANFRQIVCK
                     DCGQTIKCPFCSIAMSLHKKRNVLKCHYCNYTSLIEQNCPSCQGAMLEARKMGTAELQ
                     ELLQEALPLARIAKFDSDEITSIRKLNAILKDFNENNIDILIGTSMLAKGHDYHSVDL
                     SVILGLDEYLFRPNFKASEETLALAMQVAGRAGRKGEGRVLLQTKNRNFFERYIEDYD
```

```
                     SFLQDELENRKDLYPPFKRLLRIVIEDKEQKQAQNLCKKLVDQISHILKVEIIGYGAC
                     SIEMLKGQYRFYILLRSDFYKALIKIQEYALNFKNVSVDIDPIDFL"
     gene            complement(40906..41352)
                     /locus_tag="DMACINML_00043"
     CDS             complement(40906..41352)
                     /locus_tag="DMACINML_00043"
                     /inference="ab initio prediction:Prodigal:2.6"
                     /inference="similar to AA sequence:RefSeq:CAL34820.1"
                     /codon_start=1
                     /transl_table=11
                     /product="putative periplasmic protein"
                     /protein_id="Prokka:DMACINML_00043"
                     /translation="MKKAFTLLELIFVILILGILASLSFSFANQNKNNAKLLKLKIDY
                     EMLNSALALMRTQVELKQISAFSPNLDEAKINTQKEKLFYCQAGQNCTYSLLHTPIYS
                     NLNAWMKIAPNRYRFFLNAKEWVDFFYNADFAILECLSEKYCKELL"
     gene            complement(41349..41594)
                     /locus_tag="DMACINML_00044"
     CDS             complement(41349..41594)
                     /locus_tag="DMACINML_00044"
                     /inference="ab initio prediction:Prodigal:2.6"
                     /inference="similar to AA sequence:RefSeq:CAL34819.1"
                     /note="hypothetical protein Cj0682"
                     /codon_start=1
                     /transl_table=11
                     /product="hypothetical protein"
                     /protein_id="Prokka:DMACINML_00044"
                     /translation="MASDNLKQVIINVSAKDFIVKCSGEFANFLEDDIALISGGTKKI
                     ELKRFVDAFVKKSYENYILERELKKLIKAINEELPVK"
     gene            complement(41584..41814)
                     /locus_tag="DMACINML_00045"
     CDS             complement(41584..41814)
                     /locus_tag="DMACINML_00045"
                     /inference="ab initio prediction:Prodigal:2.6"
                     /inference="similar to AA sequence:RefSeq:CAL34818.1"
                     /note="hypothetical protein Cj0681"
                     /codon_start=1
                     /transl_table=11
                     /product="hypothetical protein"
                     /protein_id="Prokka:DMACINML_00045"
                     /translation="MFDEKIINTMSDKVNELIEKYNEVCEANENLRNELVSVKAQNEA
                     KSNQIMRLEEELKSRNIESEDIFKKIEAVLGK"
     gene            41969..43942
                     /gene="uvrB"
                     /locus_tag="DMACINML_00046"
     CDS             41969..43942
                     /gene="uvrB"
                     /locus_tag="DMACINML_00046"
                     /inference="ab initio prediction:Prodigal:2.6"
                     /inference="similar to AA sequence:RefSeq:CAL34817.1"
                     /codon_start=1
                     /transl_table=11
                     /product="excinuclease ABC subunit B"
                     /protein_id="Prokka:DMACINML_00046"
                     /translation="MLELTSDFKPSPDQKQAIQGIVKSIKKGNKYQTLLGVTGSGKTF
```

```
                     TMANVIKELNMPTLIMSHNKSLCAQLYSEFKGFFSNNHVEYFISYYDYYQPEAYIPRT
                     DVFIEKDSSTNEDLERLRLSATASLLSYEDVVCIASVSANYGLGNPNEYIGMVLIFEL
                     GMQISQKELLKKLVDMGYKRNDNFFDRADFRVQGDIIDIYPAYYEDEVVRLEFFGDEL
                     DAMYHYNVLENKKGKDLKRFILYPTSQFSVGETRLKQAIKDIKIELNERLAQFEHENK
                     LVEYQRLKQRVEFDLEMLTSTGMCKGVENYARHLTGLKEGDTPYTLFDYFAIKNRKFL
                     VIVDESHVSLPQFRGMFAGDRSRKQTLVDYGFRLPSALDNRPLMFDEFIHKDCQFLFV
                     SATPAPLELELSKENIFHQIMRPTGLLDPLIELKDSDNQVEILFDEAKKVIQRNERVL
                     VTVLTKKLAEELTRYYLELGIKVKYMHSDIDAIERNEIIRGLRSGAFDMLIGINLLRE
                     GLDLPEVSLIAIMDADKEGFLRSTTSLIQTMGRAARNVNGKVLLFCKKITKSMQEAMD
                     TTNERRKLQEAYNQKHNITPTSVKRHIEESLKNEENLGEIYRKGKKFEKMPANERAKI
                     IKELRKQMLEAAKALEFEKAAALRDEINKLKDL"
     gene            complement(43977..45563)
                     /gene="dcuB"
                     /locus_tag="DMACINML_00047"
     CDS             complement(43977..45563)
                     /gene="dcuB"
                     /locus_tag="DMACINML_00047"
                     /inference="ab initio prediction:Prodigal:2.6"
                     /inference="similar to AA sequence:RefSeq:CAL34811.1"
                     /codon_start=1
                     /transl_table=11
                     /product="anaerobic C4-dicarboxylate transporter"
                     /protein_id="Prokka:DMACINML_00047"
                     /translation="MDFLTSLDESIQFLIQIIIVLICLFYGAKKGGIALGLLGGIGIL
                     MLVFAFHIKPGKPAIDVMLTILAVVVASATLQASGGLDVMLQIAERVLRRNPKFLTIL
                     APFVTCFLTILCGTGHVVYTIMPIIYDIAIKNGIRPERPMAAASISSQMGIIASPVSV
                     AVVSLTALLLNAEHKLAGFDGYINLLQITIPSTLFGVLCVGIFSWFRGKDLDKDEVFQ
                     EKLKDPEFKKYVYGDSKTLLGVQLPKSNWIAMWIFLGAIALVALIGAFDSLKPNWGQK
                     MAYKAEINGTTLQWTPSGLNKGVVDLKQLGFDQTSIVEENGDKSSKFKEAINSGEIIA
                     IPQKDKLGNPSYDFISMVNVIQMFMLLAGALIIIFTKTDAKKIGSNEIFKSGMIALVA
                     VFGISWMADTMFAVHTPMMKKALEDVVTDYPWTYAIMLLLISKFVNSQAAAIAAFVPL
                     ALGIGVEPGIIVAFAAACYGYYILPTYPSDLATMQFDRSGTTHIGKFVINHSFILPGL
                     IGVISSCVAGYFLALIAGYL"
     gene            complement(45748..46998)
                     /gene="rpoN"
                     /locus_tag="DMACINML_00048"
     CDS             complement(45748..46998)
                     /gene="rpoN"
                     /locus_tag="DMACINML_00048"
                     /inference="ab initio prediction:Prodigal:2.6"
                     /inference="similar to AA sequence:RefSeq:CAL34810.1"
                     /codon_start=1
                     /transl_table=11
                     /product="RNA polymerase sigma-54 factor"
                     /protein_id="Prokka:DMACINML_00048"
                     /translation="MLRQKITQAPKTKISQTLRSWLPILQANIEDLKENLDKFAEENP
                     FLSVQEPIQTQEHNKNYFDSFYKRNVNSAFVDNKGFATKSVYELLSEQIIPPLFPTNK
                     SQELAGKIIECLNHEGYFEYDEEILKDYDINEIERIRARFKFLDPVGVGAKDYKEAFL
                     FALENIELDAELYDFCKMLIQDFENIQNYTKESLYKEALTIFKRFSIPPFLEYFEDSR
                     VIIPDIFVYRENNEIKVKINDDYYPEISIETDGLNHEYLNHYIKEAKNLVDALTMRKA
                     TLYKIGLMIVEYQYDFFMGKDIKPMTFKDLALDLERNASTISRAVANKYLSCDRGLIP
                     LRDFFAFALDEEGETSNVGVKDFVANLVKNENRLKPLSDSKILELIKKEFKVDIGRRT
                     ITKYRKHLNIPSSTDRKKLYELEG"
     gene            complement(46998..47726)
```

```
                     /locus_tag="DMACINML_00049"
     CDS             complement(46998..47726)
                     /locus_tag="DMACINML_00049"
                     /inference="ab initio prediction:Prodigal:2.6"
                     /inference="similar to AA sequence:RefSeq:ADT72413.1"
                     /codon_start=1
                     /transl_table=11
                     /product="ABC-transporter ATP-binding protein"
                     /protein_id="Prokka:DMACINML_00049"
                     /translation="MSKLEIVNLEKIIKKTKIIQGISLELNSGEVVGLLGPNGAGKTT
                     TFYMICGLIAPSSGKVLLDGLDITKEPLNKRARLGIGYLPQESSIFKDLSVEDNLLLA
                     AQIFYKDKKILHEKVEQMLELLSIEPIRLRKGLSLSGGERRRCEIARSLMCEPKFLLL
                     DEPFAGVDPIAVAEIQSLINELKKLDIGILITDHNVRETLAICDRAYVIRSGSLLASG
                     NAEEIANNKDVKKYYLGAEFKLLD"
     gene            complement(47719..48126)
                     /locus_tag="DMACINML_00050"
     CDS             complement(47719..48126)
                     /locus_tag="DMACINML_00050"
                     /inference="ab initio prediction:Prodigal:2.6"
                     /inference="similar to AA sequence:RefSeq:CAL34808.1"
                     /codon_start=1
                     /transl_table=11
                     /product="putative ATP/GTP-binding protein"
                     /protein_id="Prokka:DMACINML_00050"
                     /translation="MKEFVLAKDELKTMLQTLPKQGVVLLQGELASGKTSLVQAWVSF
                     LNLDEKVDSPTFSIMQKYENQDICVYHYDIYQEGLEGLLKNGLFENFFEEGLHLVEWG
                     DENLKKALLKLGIPTLEIKISVEDNKRKYVIHE"
     gene            complement(48123..48410)
                     /locus_tag="DMACINML_00051"
     CDS             complement(48123..48410)
                     /locus_tag="DMACINML_00051"
                     /inference="ab initio prediction:Prodigal:2.6"
                     /inference="similar to AA sequence:RefSeq:AHY39902.1"
                     /codon_start=1
                     /transl_table=11
                     /product="S4 domain-containing protein"
                     /protein_id="Prokka:DMACINML_00051"
                     /translation="MGFKNLIFLVKFCTMRVDKFLNAVNITKRRAISEDMCRSGVVGI
                     NGVIVKASKEVKVGDIIALHFTDYTQEYKVLALPTTKNIPKNAQSEYVVKL"
     gene            48510..49730
                     /gene="argG"
                     /locus_tag="DMACINML_00052"
     CDS             48510..49730
                     /gene="argG"
                     /locus_tag="DMACINML_00052"
                     /EC_number="6.3.4.5"
                     /inference="ab initio prediction:Prodigal:2.6"
                     /inference="similar to AA sequence:RefSeq:CAL34806.1"
                     /codon_start=1
                     /transl_table=11
                     /product="argininosuccinate synthase"
                     /protein_id="Prokka:DMACINML_00052"
                     /translation="MKNEVKKVVLAYSGGLDTSIILKWLQDEYKCEVVTFTADIGQGE
                     ELEPARKKALSLGIKEENVFIKDLREEFVKDYVFPMFRANTIYEGEYLLGTSIARPLI
```

```
                     AKTQAQIALQTGADAVSHGATGKGNDQVRFELGYLAFNPDLKIIAPWREWDLNSREKL
                     LAYAQKHGIDISKKKGKSPYSMDANLLHISYEGLVLEDPAHAPEEDMWRWSKSPKEAP
                     NESEIIELDFQKGDLVAINGEHLSPAGLLSKLNELGAKHGIGRLDIVENRYVGMKSRG
                     CYETPGGTILLKAHRAIESITLDREAAHLKDELMPKYASLIYNGYWFSPERMMLQALI
                     DESQKHANGRVKLELYKGNVMVIGRESANDSLFNAAYCTFEEDEVYNQKDAAGFIKLN
                     ALRFIIAGKNGRKF"
     gene            49745..50188
                     /gene="rplI"
                     /locus_tag="DMACINML_00053"
     CDS             49745..50188
                     /gene="rplI"
                     /locus_tag="DMACINML_00053"
                     /inference="ab initio prediction:Prodigal:2.6"
                     /inference="similar to AA sequence:RefSeq:CAL34805.1"
                     /codon_start=1
                     /transl_table=11
                     /product="50S ribosomal protein L9"
                     /protein_id="Prokka:DMACINML_00053"
                     /translation="MKVLLIKDVKALGKAGEIKEVKDGYGQNFLIAKGFAKAATNEVL
                     RKYESDKKKEAENLRFELANLEKLKEELSKVTLEISKPVGANGSLFGGVTKDEIAHAL
                     KEQTHIEIDKKSLECDTLKSLGTHEVSVKLGHAIHAKFNINIKAE"
     gene            50188..50730
                     /gene="hslV"
                     /locus_tag="DMACINML_00054"
     CDS             50188..50730
                     /gene="hslV"
                     /locus_tag="DMACINML_00054"
                     /EC_number="3.4.25.-"
                     /inference="ab initio prediction:Prodigal:2.6"
                     /inference="similar to AA sequence:RefSeq:CAL34804.1"
                     /codon_start=1
                     /transl_table=11
                     /product="ATP-dependent protease HslV"
                     /protein_id="Prokka:DMACINML_00054"
                     /translation="MFHATTILAYKGKNKSVIGGDGQVSFGNTVLKGNAVKIRKLNNG
                     KVLAGFAGSTADAFNLFDMFENLLQSSKGDLLKAAIDFSKEWRKDKYLRKLEAMMLVL
                     DRNHIFLLSGTGDVVEPEDGQIAAIGSGGNYALSAARALAKHANLDEEELVKSSLQIA
                     GEICIYTNTNIKTYVIEDEK"
     gene            50727..52046
                     /gene="hslU"
                     /locus_tag="DMACINML_00055"
     CDS             50727..52046
                     /gene="hslU"
                     /locus_tag="DMACINML_00055"
                     /inference="ab initio prediction:Prodigal:2.6"
                     /inference="similar to AA sequence:RefSeq:CAL34803.1"
                     /codon_start=1
                     /transl_table=11
                     /product="ATP-dependent Hsl protease ATP-binding subunit"
                     /protein_id="Prokka:DMACINML_00055"
                     /translation="MNLTPKEIVKFLDDYVIGQKKAKKIIAIALRNRYRRMQLSPELQ
                     DDIVPKNILMIGSTGVGKTEIARRLAKMMGFPFIKIEASKYTEVGFVGRDVESMVRDL
                     ANAALNLVKNEQREKNKDKIDEFIENKILEKLLPPLPKGISDEKQEEYKNSLEKMRTK
                     LRNGDLDESVIEIEISQSMFDTNPNLPPEMGAMQDIVKVIGVGSKKVKKEMKIKDAKN
```

ALKNEAGEKILDQESIKSEALKRAENEGIIFIDEIDKIAVSSGNSNRQDPSKEGVQRD
LLPIVEGSSVQTKIGTLKTDHILFIAAGAFHLSKPSDLIPELQGRFPLRVELDSLDDK
ALYEILTRPKNSLLKQYEQLLKTENLELEFDDEAIKEIAKIASKANEEMQDIGARRLH
TVIEKLLEDLSFEADEYAGKKFVVDKKMVEEKLGDIIENKDLARYIL"
gene 52043..52918
/locus_tag="DMACINML_00056"
CDS 52043..52918
/locus_tag="DMACINML_00056"
/inference="ab initio prediction:Prodigal:2.6"
/inference="similar to AA sequence:RefSeq:ALW55063.1"
/codon_start=1
/transl_table=11
/product="GTPase Era"
/protein_id="Prokka:DMACINML_00056"
/translation="MKSGFVSIIGRTNAGKSTLINSLLEEKIALVSHKQNATRRKIKA
IVMNGEDQIIFIDTPGLHESKATLNQFLIQSAIKSMGDCDVILFVASIFDSVKDYENF
LSLNPKVPHIIVLNKVDLADNGALLKKLNEYAKFSEYFKAILPYSCKKKSYQKPLLNE
LCKLLPEHEHFYDSEFLTPSSQKDIFRDFILESVYENLSEELPYSCEIMIKNTKETPN
LFIIDAQIITDTNSHKAMLIGKDGVTLKRIGKDARFKISKLIQNKVLLKLFVVVKKNW
QKDEVFLKKILNYEE"
gene 52908..53984
/locus_tag="DMACINML_00057"
CDS 52908..53984
/locus_tag="DMACINML_00057"
/inference="ab initio prediction:Prodigal:2.6"
/inference="similar to AA sequence:RefSeq:CAL34801.1"
/codon_start=1
/transl_table=11
/product="putative transmembrane protein"
/protein_id="Prokka:DMACINML_00057"
/translation="MKNKAHFIGFENLIYKQKNGNFEEDSLFKELTKECDLQNPFEYQ
LAFLKQDQIYHCFLTWAAKLPKTKFCFPEPLIFQSLFLENKIKEGNFCILEISSKKVF
LCFYEQGKFKTFKTLNFCDNVEEFINKSRILELLQHYESKILLSGKAHEIIDLISTKA
KLPLKIIQEDKITLSNHSIHHLDKNANFIKYYQKHLPWYFKFIFLFVLSFIISIGILS
LIDFTQYQSAKKTHIQNEISQNKIYEIQENQNQKLKANIEKLQLEIQTQDLLLEKYSE
QLSKITQNFKADKDTILILTKIITWLNDHSLKIANLMIDKTLITIKFSNEENFNKALQ
FTSPQFNLISQDKFLYEITLRALQ"
gene 53981..54538
/locus_tag="DMACINML_00058"
CDS 53981..54538
/locus_tag="DMACINML_00058"
/inference="ab initio prediction:Prodigal:2.6"
/inference="similar to AA sequence:RefSeq:CAL34800.1"
/codon_start=1
/transl_table=11
/product="putative periplasmic protein"
/protein_id="Prokka:DMACINML_00058"
/translation="MNQIEQFLENLSPREKILIYLTTILLAVLLSFHFYHSYLKSLFN
HEFFIQSDEVNKIIITNQDLNQQIKQLEEKLNSIQDKIKSYENHLSTFNKDYETYIKN
LQNLALKNNLQISNISRLQEENSYHRISVDILGDFNPILSFIQDVENSNLHYEIQKFE
IDNTSNLNLHLKLTLSFIALVKNKN"
gene 54628..56415
/locus_tag="DMACINML_00059"
CDS 54628..56415

```
                     /locus_tag="DMACINML_00059"
                     /inference="ab initio prediction:Prodigal:2.6"
                     /inference="similar to AA sequence:RefSeq:CAL34798.1"
                     /codon_start=1
                     /transl_table=11
                     /product="putative aminopeptidase"
                     /protein_id="Prokka:DMACINML_00059"
                     /translation="MNIYKTRVEKIRQFMIKEKLDAYLILSADPHLNEYLPSFYQSRA
                     FVSGFKGSAGSLIITLQDAFLWTDGRYWIQAKKELEGSDILLQKQDANNTFLKWLKEN
                     LNKEQNLGIDFSVLSLALQKEIQKNCKAELKNIDLISPIWENRPALPKNKVYEHELKY
                     CSYSRKEKLTLVREKMAKLQAQNHLISSLDDIAWITNLRGSDVNYNPVFLSHLLILEE
                     KALLFIDREKIDFELEKKLNLDGIWLKDYNEIQDELKKLQNTNLLIEPSKTTALLIEI
                     LDKSVEILEEINPSTHLKAIKTDKEIAHIQNAMIEDGVALCKFFAWLEENIENNTQIT
                     ELDIDTKVTEFRSQSPYYISNSFATIAGFNANAALPHYKAEKESFSYIQKNGLLLIDS
                     GGQYKNGTTDITRVVAIGELNKEQIHDYTLVLKAHIAISSTVFPKDITMPLLDAITRA
                     PLWQEQLDYAHGTGHGVGYFLNVHEGPQTLSYFSPVLEKTKAKEGMLTSIEPGIYRTG
                     KWGIRLENLVVNTKIKNPKNKDFGEFLYFKPLTLCPFEISCIDKTLLSIKEKAWINAY
                     HKEVYEKLSPKLHDNPKALKWLKERTETI"
     gene            complement(56465..58300)
                     /gene="pbpC"
                     /locus_tag="DMACINML_00060"
     CDS             complement(56465..58300)
                     /gene="pbpC"
                     /locus_tag="DMACINML_00060"
                     /inference="ab initio prediction:Prodigal:2.6"
                     /inference="similar to AA sequence:RefSeq:ABV52210.1"
                     /codon_start=1
                     /transl_table=11
                     /product="penicillin-binding protein 2"
                     /protein_id="Prokka:DMACINML_00060"
                     /translation="MRMRLVVGFILLFFIFLLSRVYYLSIKSNVYYEELAKQNAVKTE
                     FIAPTRGQISDRNGTPLAINDLGFSISIKPYLTLKRANRGVLEKELNQLQEFFPDLNV
                     TKLAELYKKNDSYYNQDFIKIIDFIPSQEMMKHYSELNLNENIRIDPAGQRKYPFGKL
                     ASHIIGYVGKADLQDIKENEIAKLTNFTGKSGIERYYNDVLQGEKGTKVYKVNALNQE
                     VEQLSYTSALSNDIELTIDIELQSFLTQLFEGNAGAAIIMDISDGSILAAGSFPEYDL
                     NPFVTGISYKEWDELSNNLDHPFTNKLINGYYPPGSVVKMGVGLSFLNSKSINPSTQF
                     FCNGSVELGGRFFRCWNRAGHGAVDLKHAIKASCDVYFYDGSLQVGIDQISRTLSRIG
                     FGAKTGVDLPNEFVGILPSREWKMQRYKQAWFQGDTLNTSIGQGNFLATPMQIARYTA
                     QIAKGGEVIPHFLKSVENNNSITEDKTQDSNEIFTLFEKSQLPYIRDAMYAVANEQGG
                     TSYHYLRNLEVKVAAKTGTAQVVGFSQADKNRVDEKQLKYYTRSHAWVTSYAPYSKPR
                     YVVTVLVEHSGRTISSGVATAKIYQKMIDLGYFKPESKVNSSKKN"
     gene            complement(58300..58794)
                     /locus_tag="DMACINML_00061"
     CDS             complement(58300..58794)
                     /locus_tag="DMACINML_00061"
                     /inference="ab initio prediction:Prodigal:2.6"
                     /inference="similar to AA sequence:RefSeq:CAL34796.1"
                     /codon_start=1
                     /transl_table=11
                     /product="putative integral membrane protein"
                     /protein_id="Prokka:DMACINML_00061"
                     /translation="MRKSISTYKVKLNGSYIFLGFLCILYQILGSIFFYMPLLYGVFF
                     CYMYFLLEERQKTFNKLDFRWYFSLFFLFFTDITYDFFLFSSWFAFAIFYYTCADWIK
                     TNFKIGKIIPVILVLCVYAFIFVLDAIFSYVGDQEFKMPSFGYMISIMFECLFAYMLF
```

```
                     RSKF"
     gene            complement(58796..59386)
                     /locus_tag="DMACINML_00062"
     CDS             complement(58796..59386)
                     /locus_tag="DMACINML_00062"
                     /inference="ab initio prediction:Prodigal:2.6"
                     /inference="similar to AA sequence:RefSeq:ABS44681.1"
                     /codon_start=1
                     /transl_table=11
                     /product="GTP-binding protein"
                     /protein_id="Prokka:DMACINML_00062"
                     /translation="MIISAKFISSLDKFDKNFASHFSEVAFLGRSNVGKSSLINSLCK
                     QKKLAKSSATPGKTQLINFFEVLCKKDEDKFSIHFIDLPGFGYAKVSKNLKEIWNKNL
                     DEFLKLRSSIKLFVHLIDSRHTNLDIDLNLDEYLKSFLKPDQRILKVFTKCDKLNQSE
                     KAKLKNNFKDAILISNLNKMGLDDLEHEIIKQTLGL"
     gene            complement(59370..59840)
                     /locus_tag="DMACINML_00063"
     CDS             complement(59370..59840)
                     /locus_tag="DMACINML_00063"
                     /inference="ab initio prediction:Prodigal:2.6"
                     /inference="similar to AA sequence:RefSeq:CAL34794.1"
                     /codon_start=1
                     /transl_table=11
                     /product="putative OstA family protein"
                     /protein_id="Prokka:DMACINML_00063"
                     /translation="MVRKIILFLMSISISLASQIEVKALNFYSDENKGESILSGNVEI
                     IRGDDVLTAEKVIIYTDKNRKPIRYEAVQNANFKIVLKGKTYKGSGDKFIYNVAKDIY
                     EIDGNAYINELESNQKLYGDRIVVDKRNNVYRVESKDKKPARFIFDLEQNDNKR"
     gene            complement(59825..60337)
                     /locus_tag="DMACINML_00064"
     CDS             complement(59825..60337)
                     /locus_tag="DMACINML_00064"
                     /inference="ab initio prediction:Prodigal:2.6"
                     /inference="similar to AA sequence:RefSeq:CAL34793.1"
                     /codon_start=1
                     /transl_table=11
                     /product="putative membrane protein"
                     /protein_id="Prokka:DMACINML_00064"
                     /translation="MAIRIFGILIALFTITFTILSLQDPYSLNIKSYALNFKNIEAFN
                     LNAYELNASNVKSYYKAQTWTRYVDKDEFDNFVNFNLDFNLSANKLELKGKEMNKVLF
                     EGNVTYIGSNNTKILADKVEYEPKNKILSTDTGFKALINGSIINGNTLNYDVEKKILQ
                     VQGVNAWLER"
     gene            complement(60328..60816)
                     /locus_tag="DMACINML_00065"
     CDS             complement(60328..60816)
                     /locus_tag="DMACINML_00065"
                     /inference="ab initio prediction:Prodigal:2.6"
                     /inference="similar to AA sequence:RefSeq:CAL34792.1"
                     /codon_start=1
                     /transl_table=11
                     /product="putative HAD-superfamily hydrolase"
                     /protein_id="Prokka:DMACINML_00065"
                     /translation="MIELIFLDVDGCLTDGKIIYTSNGSVIKEFDVKDGAAIEAWIKL
                     GKKIAIITGRNCPCVSARAKDLKIEIVHQGVKDKLTCAKKILEELNLDFSQCAAIGDY
```

```
                     FNDKNLLENVGLSFKPKDAHKDLKVDITLDKKGGKAAVAQMIEYIIKKNNMQEEWDKL
                     WL"
     gene            complement(60819..61658)
                     /locus_tag="DMACINML_00066"
     CDS             complement(60819..61658)
                     /locus_tag="DMACINML_00066"
                     /inference="ab initio prediction:Prodigal:2.6"
                     /inference="similar to AA sequence:RefSeq:CAL34791.1"
                     /codon_start=1
                     /transl_table=11
                     /product="putative lipoprotein"
                     /protein_id="Prokka:DMACINML_00066"
                     /translation="MQTIKQKLKKTLPKLLAASLVATFSSGCFGGLFSSVGSAQIYYP
                     SNDFHSSPSGSGSKGTMKPYTINGKTYYPTVVSVGETADGIASWYGPGFHGKKTSNGE
                     TYNQNGLTAAHKTLPMNTILKVTNLNNNRQVTVRVNDRGPFVNNRIIDLSKGAANQID
                     MIAAGTAPVRLEVIGFGSANSGNNVVHSNINYGTSGGIANNGQIYEGGNFMVQIGAFK
                     NPSGAQTIASRYKTYRTYSSTIRKSSVDGLSRVFLTGFRSEEEARDFAASGAFAGAFV
                     VRE"
     sig_peptide     complement(61566..61658)
                     /locus_tag="DMACINML_00066"
                     /inference="ab initio prediction:SignalP:4.1"
                     /note="predicted cleavage at residue 31"
     gene            complement(61603..62736)
                     /locus_tag="DMACINML_00067"
     CDS             complement(61603..62736)
                     /locus_tag="DMACINML_00067"
                     /EC_number="3.2.1.-"
                     /inference="ab initio prediction:Prodigal:2.6"
                     /inference="similar to AA sequence:RefSeq:CAL34790.1"
                     /codon_start=1
                     /transl_table=11
                     /product="putative secreted transglycosylase"
                     /protein_id="Prokka:DMACINML_00067"
                     /translation="MKKIILCFILFYNFLFAQTTTPEFYERQMSVLRNLDIDPSFISD
                     LAFVQSQQDLRSKHAPTLIDGIQNFSKVTPMIRKILAQQEVPEEILYLAMVESGLKAH
                     SVSNAKAVGVWQFMQPTARNLGLRIDAYVDERRDPVKSTYAAVHYLKSLKEEFGKWYL
                     ALLAYNCGNGKLRQAIKQAGSDDLRILLDSDKKYLSLETRNFIRKILTLAFLANDRDF
                     LLDKDGSLVNYALNNDFAKVDAPSSVALKDLAKNLNMDLATFKKYNPQFKHGFTPPGK
                     GYYMYIPLNKVAFFDKNFKVEKLAKVDTTIPMTKVYVVKSGDSLYKIAKTYNTSVEQI
                     RELNKIAKNHLSINQKLIIPIKENKNANYKTKAKENFTKVVSR"
     sig_peptide     complement(62683..62736)
                     /locus_tag="DMACINML_00067"
                     /inference="ab initio prediction:SignalP:4.1"
                     /note="predicted cleavage at residue 18"
     gene            complement(62739..63551)
                     /locus_tag="DMACINML_00068"
     CDS             complement(62739..63551)
                     /locus_tag="DMACINML_00068"
                     /inference="ab initio prediction:Prodigal:2.6"
                     /inference="similar to AA sequence:RefSeq:CAL34789.1"
                     /codon_start=1
                     /transl_table=11
                     /product="putative TatD-related deoxyribonuclease protein"
                     /protein_id="Prokka:DMACINML_00068"
```

```
                     /translation="MFLNLEFKDGLKIVDTHCHLDSEVFKEDLDELLKHSFDNGIEKI
                     IIPGADIKDLPYAADLASKYKNVFFAAGVHPYELEGFDEKILRAYLKDEKCVAVGECG
                     LDYFRFKSEDFKEREKEKEEQKRLFVAQLELAKEFKKPVIIHSREANNDTYEILHEHS
                     KDLVGGVLHCFNASEHLLRLSNDGFYFGIGGVLTFKNAKNLVNILPQIPKDRLLLETD
                     APYLTPEPYRGKRNEPLLTQLVANKMSEILDLPRQELLGICLENSYRLFFKG"
     gene            complement(63542..64786)
                     /gene="cbrR"
                     /locus_tag="DMACINML_00069"
     CDS             complement(63542..64786)
                     /gene="cbrR"
                     /locus_tag="DMACINML_00069"
                     /inference="ab initio prediction:Prodigal:2.6"
                     /inference="similar to AA sequence:RefSeq:CAL34788.1"
                     /codon_start=1
                     /transl_table=11
                     /product="two-component response regulator"
                     /protein_id="Prokka:DMACINML_00069"
                     /translation="MNKKILLIDDNKMLGKLLAKKIQTTLNREVDIVFSLAEVKALPD
                     DEYFLTFADLCLPDAPNGEVVDYLLAKNWPVIVLTASNDKATRDKFMDKDILDYIFKE
                     SDTCIDQIIDSIVKLEHYAKTKVILALSKLSERNEIKKLLTQRKFSVLAAAHGEEAMS
                     YLNDNNDIKLIIADANMPVISGSELLSEVRTRFSDSELGVIILGDKDDALEASLLVSG
                     ANEYLIKPLSKESLNCRLDKCLHYMANMQFLSIYNNLDPISGIKNSNALLNCVEDYLN
                     EIACKEEEFAFAFLDIDNLRNLNEEYGYEVGDKIVKICADEIINETKGRDIVGRYSAE
                     KICIVLKNISQERAIKILSRIRVNIKKAGILVNLDEVFFTASIGVVFAKSGDKFETLV
                     DKASKALSQAKANGKDRVEVCS"
     gene            complement(64875..66398)
                     /gene="recN"
                     /locus_tag="DMACINML_00070"
     CDS             complement(64875..66398)
                     /gene="recN"
                     /locus_tag="DMACINML_00070"
                     /inference="ab initio prediction:Prodigal:2.6"
                     /inference="similar to AA sequence:RefSeq:CAL34787.1"
                     /codon_start=1
                     /transl_table=11
                     /product="putative DNA repair protein"
                     /protein_id="Prokka:DMACINML_00070"
                     /translation="MINRVLMKENLGFKEVELEISQGLTVFTGLSGAGKSVLFKGILS
                     AFALSDSEAKLVEIALDDKIDLEEYGIESEDENVFKLLKDKNAKYFINNQSISKKSLI
                     HLSKKFIKYLSAKEINEFSNEKFLNLLDALECVKNPEFEDFLSHFKDDYKQWLQISEE
                     LTTILEEEKRIEELKELASTQIERISKINPKIGEYEELLNLKKKLSKKDKIEAAWEKA
                     NAIFELEKVVIDALNLSEKDTSFFSECLNELRIIAENEKIEDLDFDIEEVLNRIEDLS
                     SLIKRYESIENALEVLENKKNELAHYDNLSFEKKELEKKLEKIEKKINQSTQLLTQAR
                     MQNLKTLEDYLNNYLKNLYMKNVSLELVNISKITSLGKDEIKLSINAANLKNLSSGEL
                     NRLRLAFIATECKILNSGTGIIFLDEIDANLSGKEAMSIANVLNELAAFYQIFAISHL
                     PQLSSKAHNHFLVEKNASCSTVRKLKNEERIKELARMISGETITDEALEFAKTLFKA"
     gene            complement(66398..67258)
                     /locus_tag="DMACINML_00071"
     CDS             complement(66398..67258)
                     /locus_tag="DMACINML_00071"
                     /EC_number="2.7.1.23"
                     /inference="ab initio prediction:Prodigal:2.6"
                     /inference="similar to AA sequence:RefSeq:CAL34786.1"
                     /codon_start=1
```

```
                     /transl_table=11
                     /product="putative inorganic polyphosphate/ATP-NAD kinase"
                     /protein_id="Prokka:DMACINML_00071"
                     /translation="MQNKIDYKNIKKIGLAARPNSNLDKEILTLKEILEKKGVELLLY
                     KESSENIKLPKYDLDTLFELSDFVISLGGDGTLISLCRKACEHNKAVLGIHAGHLGFL
                     TDFKVDEAEVFFEAFFRGEFRVENPFLLSIILEANDGQITQKFAFNDVVISKDRKASM
                     AHIEVFRKAKKFNEYFGDGLIVATPAGSTAYNLSANGPIVYTLAQAFILTPVCSHSLT
                     QRPIVLPKGFDLEIGAKDCIFSIDGQENYKMNDFKSVKVGLSDKSVALIHPKNRDYFQ
                     ILREKLNWGH"
     gene            67351..69102
                     /gene="aspS"
                     /locus_tag="DMACINML_00072"
     CDS             67351..69102
                     /gene="aspS"
                     /locus_tag="DMACINML_00072"
                     /EC_number="6.1.1.12"
                     /inference="ab initio prediction:Prodigal:2.6"
                     /inference="similar to AA sequence:RefSeq:CAL34785.1"
                     /codon_start=1
                     /transl_table=11
                     /product="aspartyl-tRNA synthetase"
                     /protein_id="Prokka:DMACINML_00072"
                     /translation="MRSHYNTDLNSAHIGEVVKLCGWVNSYRDHGGVIFIDLRDRSGI
                     IQLVCDPNDSKEAHEIASRTRNEFVLIAEGKIRPRGEGLTNPKLKTGEIEVVVSKLII
                     ENESAVPPFGIGDESVNEELRLKYRFLDLRNPKLYENFALRSKACIAARNSLANMGFL
                     EVETPILTKATPEGARDYLVPSRVHQGEFYALPQSPQLFKQLLMCSGFDRYFQIAKCF
                     RDEDLRADRQPEFTQIDVEMSFCEQKDVIATAETFLKDIFKACGKEIQIPFRQMPYKE
                     AMENYGSDKPDLRFDLKLIDVIDIFAKSNNEIFANIAKDSKKNRVKALRVPKGDTIFS
                     KRQMQRFEEFVRKFGAQGLAFIQVKEDGLKGPLCKFFNEEDLNELTKRCELEVGDVVF
                     FGAGVKKTVLDYMGRFRIFLAGELNLINPDALEFLWVVDFPMFEQNDDGSYSAMHHPF
                     TMPKNIDETDLEEISSIAYDVVLNGVELGGGSIRIHKNDIQQKVFKLLNIDEEQQKEK
                     FGFLLDALSFGAPPHGGIAIGLDRLIMLVTGANSIREVIAFPKTQRAQCLMTDAPSVA
                     SNEAMRELGIKLRENVK"
     gene            69099..69674
                     /gene="adk"
                     /locus_tag="DMACINML_00073"
     CDS             69099..69674
                     /gene="adk"
                     /locus_tag="DMACINML_00073"
                     /EC_number="2.7.4.3"
                     /inference="ab initio prediction:Prodigal:2.6"
                     /inference="similar to AA sequence:RefSeq:CAL34784.1"
                     /codon_start=1
                     /transl_table=11
                     /product="adenylate kinase"
                     /protein_id="Prokka:DMACINML_00073"
                     /translation="MKELFLIIGAPGSGKTTDASLIAQADADVTHYSTGDLLRAEVAS
                     GSELGKTIDGFISKGNLVPLDVVVNTIVSALKTAPTKTVIIDGYPRSVEQMVEFDKVL
                     SEQNEICLKGVIEVRVSEQVAKDRVLGRSRGADDNEEVFYNRMKVYTEPLEEIISFYQ
                     KKKLHFIIDGERTIEPIVADMKELIKKIQSI"
     gene            69684..70202
                     /gene="ppa"
                     /locus_tag="DMACINML_00074"
     CDS             69684..70202
```

```
                     /gene="ppa"
                     /locus_tag="DMACINML_00074"
                     /EC_number="3.6.1.1"
                     /inference="ab initio prediction:Prodigal:2.6"
                     /inference="similar to AA sequence:RefSeq:CAL34783.1"
                     /codon_start=1
                     /transl_table=11
                     /product="inorganic pyrophosphatase"
                     /protein_id="Prokka:DMACINML_00074"
                     /translation="MDLSKIKIGDIPNKINAVIEIPYGSSIKYEIDKDSGAIMVDRVM
                     ASAMFYPANYGFIANTLADDGDPVDILVLNEYPIQAGAVIPCRLIGVLIMEDESGMDE
                     KLLAVPNSKIDARYDNIKTYTDLPQATLNKIKNFFETYKILEPNKWVKVQDFKDEKAA
                     IEILEKAIKNYK"
     gene            70250..70750
                     /gene="mrsA"
                     /locus_tag="DMACINML_00075"
     CDS             70250..70750
                     /gene="mrsA"
                     /locus_tag="DMACINML_00075"
                     /EC_number="1.8.4.11"
                     /inference="ab initio prediction:Prodigal:2.6"
                     /inference="similar to AA sequence:RefSeq:CAL34782.1"
                     /codon_start=1
                     /transl_table=11
                     /product="putative peptide methionine sulfoxide reductase"
                     /protein_id="Prokka:DMACINML_00075"
                     /translation="MKNIILGGGCFWCVEAAFENCEGIIHTEVGYTGGNDNPSYESVC
                     NGDGNIEVVKIIYNETKISLEQILSLFFKIHDPTSFDKQGADIGIQYRSAIFYESQSD
                     KNTIEDFIKKEQVNFPLPIVTQIYKLQRYYKAEDYHQHYFIHNPNQSYCRFVIAPKLE
                     KIKNYE"
     gene            complement(70975..71850)
                     /locus_tag="DMACINML_00076"
     CDS             complement(70975..71850)
                     /locus_tag="DMACINML_00076"
                     /inference="ab initio prediction:Prodigal:2.6"
                     /inference="similar to AA sequence:RefSeq:ABV52194.1"
                     /codon_start=1
                     /transl_table=11
                     /product="NOL1/NOP2/sun family protein"
                     /protein_id="Prokka:DMACINML_00076"
                     /translation="MEYNLASIYNDKELENLKNSFKLPKKICCFINRLKCDNLTLERE
                     FQDLNLEYEKLNEYCYLFKACDKSVLSSMQAFNEFKFYIQNYASYLCALNLDVKAGQS
                     VLDMCAAPGGKSINLANFMQNEGYLACNEASKDRFFTLQKNLKNYGVKARVFMKDGKN
                     IGNLCPLKFDKILLDAPCSTLAKMGLELEKSSKEIKNIAKLQKKLLHSALKALKVGGE
                     LVYSTCTFTKEENEEVIENALRSDFDIELLDINLENALAKNGQSREFEEISKCKRILP
                     CEEYDGFFIAKLRKN"
     gene            complement(71840..72220)
                     /locus_tag="DMACINML_00077"
     CDS             complement(71840..72220)
                     /locus_tag="DMACINML_00077"
                     /inference="ab initio prediction:Prodigal:2.6"
                     /inference="similar to AA sequence:RefSeq:ABS43581.1"
                     /note="conserved hypothetical protein TIGR00250"
                     /codon_start=1
```

```
                     /transl_table=11
                     /product="hypothetical protein"
                     /protein_id="Prokka:DMACINML_00077"
                     /translation="MRALALDVGLKRIGVALCIDKIAIPLEGIIRKNRNQAANEVKNL
                     IQIHDISLLIVGIPKGGSSEEEMTRRIKHFVSLLEFDKEIRFVDESGTSKEALGYGVA
                     NTRKKDAKLDSLAALIMIKDYFGI"
     gene            complement(72217..72987)
                     /locus_tag="DMACINML_00078"
     CDS             complement(72217..72987)
                     /locus_tag="DMACINML_00078"
                     /inference="ab initio prediction:Prodigal:2.6"
                     /inference="similar to AA sequence:RefSeq:AGQ95318.1"
                     /codon_start=1
                     /transl_table=11
                     /product="DNA processing protein DprA"
                     /protein_id="Prokka:DMACINML_00078"
                     /translation="MQNELPSNYLELFKELKNPPKKLYYKGNLKLLEQRKIAIIGSRR
                     MSTYTKNCVFELSSLLKNSKISVVSGGALGVDISASMAAMPSTIGVFANGLDEIYPRS
                     NEKIIKQIYENALALSENESHYKPKPYDFLLRNRLIIALSEAVVVAQADLQSGSMQSA
                     RLALELNKPLYVLPQRKNESDGTNLLLQERNANLLLDFKEFVSCFGIIEEEKDEFLDF
                     CRQGVSVDEATQIYGEKVYEYELEGKISIEGLFIRVLV"
     gene            complement(72977..74029)
                     /locus_tag="DMACINML_00079"
     CDS             complement(72977..74029)
                     /locus_tag="DMACINML_00079"
                     /inference="ab initio prediction:Prodigal:2.6"
                     /inference="similar to AA sequence:RefSeq:CAL34778.1"
                     /codon_start=1
                     /transl_table=11
                     /product="putative periplasmic protein"
                     /protein_id="Prokka:DMACINML_00079"
                     /translation="MSKKLIQIKRYLIIAILFLICVILFLGILIQYQDSQNALKFAQN
                     SSKNTTIEPKQENISNENHSGIFKEIPYKDENDNFQENNQSILEQQIKDLNLSSVIEQ
                     NLSKLDENLSLVPDQNLSKEENLIKDTNSSKIKQARLAIIIDDMANISQVKALQALKL
                     KLIPSFFPPDKNHIDTPKLALKFDFYMVHLPLAAMNYTKPELDTLNPSDSEKRIFKKI
                     KQIKKDFKDLKFINNHTGSLFTSDEKAMKKLYKAFEKEELIFVDSKTIASSKAPKVAK
                     ALGQIYIQRDVFLDNQDDVAYIKKQLMEAVNLAKKKGFAIAIGHPRKNTFKALEQSKD
                     LLESVELVYLSEIYAK"
     gene            complement(74032..75054)
                     /gene="ilvC"
                     /locus_tag="DMACINML_00080"
     CDS             complement(74032..75054)
                     /gene="ilvC"
                     /locus_tag="DMACINML_00080"
                     /EC_number="1.1.1.86"
                     /inference="ab initio prediction:Prodigal:2.6"
                     /inference="similar to AA sequence:RefSeq:CAL34777.1"
                     /codon_start=1
                     /transl_table=11
                     /product="ketol-acid reductoisomerase"
                     /protein_id="Prokka:DMACINML_00080"
                     /translation="MAVTVYYDKDCDLSLIKSKKVAIIGFGSQGHAHAMNLRDNGVNV
                     VIGLREDGSSFSKAKSAGFEVMSVSEASKVADVIMILAPDEIQADIFNAEIKPNLSEG
                     KAIAFAHGFNIHYGQIVAPKGIDVIMIAPKAPGHTVRNEFAIGGGTPCLIAIHQDESK
```

```
                     NAKNLALSYASAIGGGRTGIIETTFKAETETDLFGEQAVLCGGLSALIQAGFETLVEA
                     GYEPEMAYFECLHEIKLIVDLIYQGGIADMRYSISNTAEYGDYITGPKIITEETKKAM
                     KGVLKDIQDGNFAKDFILERRAGFARMHAERKNMNNSLIEQTGRNLRAMMPWISAKKL
                     IDKDKN"
     gene            75177..77111
                     /locus_tag="DMACINML_00081"
     CDS             75177..77111
                     /locus_tag="DMACINML_00081"
                     /inference="ab initio prediction:Prodigal:2.6"
                     /inference="similar to AA sequence:RefSeq:CAL34776.1"
                     /codon_start=1
                     /transl_table=11
                     /product="putative ribonuclease"
                     /protein_id="Prokka:DMACINML_00081"
                     /translation="MKEFLNNLNYGISAHEVSNEFKQILRELLANNIIKEHKNRYYLN
                     NGYVFGTLDISSKGTGFLQCFDESFKKDLLIENKNLKGANYKDIVAAKLLPLKKKRPS
                     AKVVLVLKRANETSLVITKRYGEAILGMNIKTGLSTALKASQKSLKALPLGTILKIEN
                     QDNNIIEVLGHIDDESIDEKISLALFNKNSEFSDACIKEALANGDSVDASMYENRLDL
                     RALPFCTIDPVHAKDFDDAIYFDTEKRELYVAIADVSEYVYAYSAIDKEARNRGFSIY
                     FPHIAIPMLPRPLSENICSLKPHLDRLAYCFKITLDLDCKVVKEELFETIINSKRRFN
                     YDEVDEILIQKPDLKELSWLYKLFEITKTLRKNRLKNAFEFRTEELRMNLDENLSLQS
                     TIFEKDTPSHNLIEDCMLLANKAAAKLIDVGVFRNHSSADMKKIDRLLNELLELGIDV
                     KLKPNLPELIRDIQALADELGLRAEVDKLIIKAQKKAEYSSENGGHFGLGFDKYSHFT
                     SPIRRYSDLILHRLLKAKQKKDEKLFNYLLLNIQSTCENLSVLEREADKVAYDFMDRK
                     FARWAAKNIGKKFKALVVQNDGICIAKLDDEIKGADIILYDTRVNLLESVEVQILEAD
                     IIMAKIYAKITQRLRKESNV"
     gene            77104..78069
                     /locus_tag="DMACINML_00082"
     CDS             77104..78069
                     /locus_tag="DMACINML_00082"
                     /inference="ab initio prediction:Prodigal:2.6"
                     /inference="similar to AA sequence:RefSeq:EAQ72985.1"
                     /note="conserved hypothetical protein"
                     /codon_start=1
                     /transl_table=11
                     /product="hypothetical protein"
                     /protein_id="Prokka:DMACINML_00082"
                     /translation="MYRKELQNLLSKNQIDNFFFLYGADNFQSELYADFIKEKYQADE
                     ILKLFFEEYSFTRASDFLSGGSLFSEKKLLEIKTSKKIPTKELKVLVELCKNNKDNFL
                     LLELYDESSKQSDIEKIFSPNFVRFFKANNAREGIELLSIKAKQLHIEITQNALFTLF
                     TSFDENLYLAASELNKFSGLRVDEKIIEQYCYSLNIGSFESFFEKILKRADFKNELEK
                     ILDNFNEIALINSLNSAFYRLFKIALYAKIYGKVDFKELLGYTPPPQVAQNLNEQAFS
                     LKIKHYKEIFNLLLKSEYELKTNSKLAKKEFLIATLLKLARIIKS"
     gene            complement(78703..79572)
                     /locus_tag="DMACINML_00083"
     CDS             complement(78703..79572)
                     /locus_tag="DMACINML_00083"
                     /inference="ab initio prediction:Prodigal:2.6"
                     /inference="similar to AA sequence:RefSeq:CKG88255.1"
                     /codon_start=1
                     /transl_table=11
                     /product="hypothetical protein"
                     /protein_id="Prokka:DMACINML_00083"
                     /translation="MLKISKRISTIVFIVLVFIAVVSSVYEFIHEALKFKEDNESKAR
```

```
                     ENLSILIKWSENEGKEELEYAKKLNKETYNQEKVTQMIIKNLKMIQTSIEDMQILTFY
                     GFADEDEELTRKASQIVLRLNMDIILYLLDNEKTFIGHQTYFLFDKERFKIFEDFLFF
                     LNARLEEDFLKKNDNDFEIIEIVTYINLLIGLDGAFANNMYLRELSIAPICDLNNPKT
                     IAILNGIEKIDIAVDRYINLINSKIKFIAYKDDYLKMKIENINNNYPKLKLGQKQINE
                     LKSIQSKLKECKQ"
     gene            complement(79767..80843)
                     /locus_tag="DMACINML_00084"
     CDS             complement(79767..80843)
                     /locus_tag="DMACINML_00084"
                     /inference="ab initio prediction:Prodigal:2.6"
                     /codon_start=1
                     /transl_table=11
                     /product="hypothetical protein"
                     /protein_id="Prokka:DMACINML_00084"
                     /translation="MRDKFLKRVLKLCFFTFFICNAAYALCTDELSDEDLKFIQNANL
                     NQVNFKCNINNQTYYYNNLTRKEYLQNLEELKAIYFNLNKEELKTLSYVIYLNEALFY
                     LKLDLNFFKNFFSVQKNDISNLENLRNSHYKQIENSLTLNQKEKLQNSFVLADQILQK
                     NAIAKDYTNISSKLIEQSKQENKEILKYIESSNKDIFSLKNINQLAFLYNANLLINEA
                     KNYPQRSALAYDKNEALKLLEILLKTMNKISNKEDKLVLLQGALITYNKAISSLDIDL
                     PLNEQKYKKIIKRKEYIILMGRFFQSDAFAYFNTNIKRKFDFKDDFIQIFPKRTSEIL
                     KLIAIYDQTKKTLMLTKEALKYKG"
     sig_peptide     complement(80766..80843)
                     /locus_tag="DMACINML_00084"
                     /inference="ab initio prediction:SignalP:4.1"
                     /note="predicted cleavage at residue 26"
     gene            complement(80833..81177)
                     /locus_tag="DMACINML_00085"
     CDS             complement(80833..81177)
                     /locus_tag="DMACINML_00085"
                     /inference="ab initio prediction:Prodigal:2.6"
                     /codon_start=1
                     /transl_table=11
                     /product="hypothetical protein"
                     /protein_id="Prokka:DMACINML_00085"
                     /translation="MPLYKECEVCKTKINKLQNIWNIFSGKLEIKCKNCKSKYGVSNK
                     IINWIYDTFGYLLLIPDNMLLIIIFFGGYFFVKYGLLVALYLLAIILLARKLIGIVLL
                     ILCKLKRIENER"
     gene            complement(81576..81851)
                     /locus_tag="DMACINML_00086"
     CDS             complement(81576..81851)
                     /locus_tag="DMACINML_00086"
                     /inference="ab initio prediction:Prodigal:2.6"
                     /codon_start=1
                     /transl_table=11
                     /product="hypothetical protein"
                     /protein_id="Prokka:DMACINML_00086"
                     /translation="MENLYETWEDEKEFKKYYNDFYLKTNQEEKHNNLHKKLLIYNQN
                     AKNDFLHMSLLGLDKIYLQALVDKNKIYFTNLSWEFALRIFDNKFLS"
     gene            complement(82051..82395)
                     /gene="hypA"
                     /locus_tag="DMACINML_00087"
     CDS             complement(82051..82395)
                     /gene="hypA"
                     /locus_tag="DMACINML_00087"
```

```
                     /inference="ab initio prediction:Prodigal:2.6"
                     /inference="similar to AA sequence:RefSeq:CAL34773.1"
                     /codon_start=1
                     /transl_table=11
                     /product="hydrogenase expression/formation protein"
                     /protein_id="Prokka:DMACINML_00087"
                     /translation="MHELSIVESLIELCEENAFAHKAKSVQEIYVKIGRLSGIEVELF
                     RRCFETFRENSELCKNAKLFIELAPLEILCLKCDQTSILEENVFKCPKCESIEYKISQ
                     GEDLHLMRLVME"
     gene            complement(82395..83369)
                     /gene="hypE"
                     /locus_tag="DMACINML_00088"
     CDS             complement(82395..83369)
                     /gene="hypE"
                     /locus_tag="DMACINML_00088"
                     /inference="ab initio prediction:Prodigal:2.6"
                     /inference="similar to AA sequence:RefSeq:CAL34772.1"
                     /codon_start=1
                     /transl_table=11
                     /product="hydrogenase isoenzymes formation protein"
                     /protein_id="Prokka:DMACINML_00088"
                     /translation="MKNISLAHGGGGEEMNELLTKLFKIFDNKILNESNDAAILGNLA
                     LSTDSFVLSPIFLDDEVNIGKLCVCGSVNDVLMVGAKPKYLSLGLILEEGFELEKLER
                     ILKSIKEECEKCEVELVCGDTKVVPKGKGDEIYINTTALGEIISKKESKNIKAGLSIL
                     VSGDVGRHGASVLIKRNELEADIKSDCKALNKEVLELLEKDIKVVAMRDATRGGLSAV
                     LNEWAKQSGNDLLIFEEKIKIQDEVLGLCELFGYEAFELANEGTFILCVEKEDELKAL
                     EILKKYNANASIIGEVLEEKKARVILENAYGAKRFLEAPKGELLPRIC"
     gene            complement(83366..84457)
                     /gene="hypD"
                     /locus_tag="DMACINML_00089"
     CDS             complement(83366..84457)
                     /gene="hypD"
                     /locus_tag="DMACINML_00089"
                     /inference="ab initio prediction:Prodigal:2.6"
                     /inference="similar to AA sequence:RefSeq:CAL34771.1"
                     /codon_start=1
                     /transl_table=11
                     /product="hydrogenase isoenzymes formation protein"
                     /protein_id="Prokka:DMACINML_00089"
                     /translation="MNFIDEFRDKDNILALKKLIEQEIKTPINIMEICGGHTHSIMKY
                     ALPSILPKEINFIHGPGCPVCVMPRVRIDTAIKLASMKDTIFCTLGDLLRVPGSEISL
                     LDLRARGADVRALYSPLEVLDIAKQNLNKTIIFFAIGFETTTPMSALLLEKVIEQNLK
                     NVFFHINHITVPAPVEAIMNDENVKINAFLGPSHVSVITGYGIYKPLAAKFKTPIAVS
                     GFEPVDIMESVLNIVRQINKGSFEVFNQYKRAVSEKGNEKAQNLVKKYFQVCDFEFRG
                     LGLIKEGGLELKEEFSAYDASKQFDCAVQSKSESKACICGQILRGLAKPYDCKVFGKA
                     CTPKSPIGSCMVSGEGACAAYYKYSKVNA"
     gene            complement(84441..84722)
                     /gene="hypC"
                     /locus_tag="DMACINML_00090"
     CDS             complement(84441..84722)
                     /gene="hypC"
                     /locus_tag="DMACINML_00090"
                     /inference="ab initio prediction:Prodigal:2.6"
                     /inference="similar to AA sequence:RefSeq:CAL34770.1"
```

```
                     /codon_start=1
                     /transl_table=11
                     /product="hydrogenase isoenzymes formation protein"
                     /protein_id="Prokka:DMACINML_00090"
                     /translation="MCLSIPSEILEIDELNNALVQTLGVKRKVNLDLIDEPLQKGDYV
                     LIHVGVAMEKIDKEAALESIKTYQEIVEKMNSGEIKSDEGDMGLNEFHR"
     gene            complement(84722..85471)
                     /gene="hypB"
                     /locus_tag="DMACINML_00091"
     CDS             complement(84722..85471)
                     /gene="hypB"
                     /locus_tag="DMACINML_00091"
                     /inference="ab initio prediction:Prodigal:2.6"
                     /inference="similar to AA sequence:RefSeq:AJP35105.1"
                     /codon_start=1
                     /transl_table=11
                     /product="Hydrogenase isoenzymes nickel incorporation
                     protein HypB"
                     /protein_id="Prokka:DMACINML_00091"
                     /translation="MCKDCGCSVNSAHTHEHSHHHDHHHENPSLKESKTIEVISKILS
                     KNDEEAKHNRAHFNEANTLCINLMSSPGSGKTTLLESTIKALKNELKIAVIEGDLETN
                     NDALRVKNAGALAYQITTGQSCHLDAFMVHEALHHLDIKGVDLLFIENVGNLVCPASY
                     DLGEHLNVVLLSVTEGSDKPQKYPVMFKKADIVLITKADLAHHFDFDIKEATKLIKEL
                     SPRADIITLDAKNGTNMELWYKLLKLKKELF"
     gene            complement(85548..87734)
                     /gene="hypF"
                     /locus_tag="DMACINML_00092"
     CDS             complement(85548..87734)
                     /gene="hypF"
                     /locus_tag="DMACINML_00092"
                     /EC_number="2.1.3.-"
                     /inference="ab initio prediction:Prodigal:2.6"
                     /inference="similar to AA sequence:RefSeq:CAL34768.1"
                     /codon_start=1
                     /transl_table=11
                     /product="carbamoyltransferase"
                     /protein_id="Prokka:DMACINML_00092"
                     /translation="MCRLGYKLQISGLVQGVGFRPLVFELACELGLCGEVKNDGFGVE
                     IILACTKEECEHFIYKLKEKLPPLARIDSIDITEQTTTNYTHFSIGTSLQNTKTTPML
                     SDFALCKECKKEFFDEKNPRFLYPFITCTHCGPRFSIIKNLPYDRCNTTMEALKMCEF
                     CKSEYEDPKNRRFHAQPISCPQCKIDVFLKNKKGEILAQDDEAFKACAKLLKQGKILA
                     IKGMGGFHLMCDAFNLEAIKELRLRKNRPKKPFALMCRDISDAKELCFVDKEEEKLLG
                     SILAPIVILKAKKAFSLIAPDVDKLGIMLAYTPLHLLLFRYFDGVLVATSANLSGESI
                     IKDEDNLLKKLGNVFDFYLDYAREIHNPSDDSIAQVVNGKTMFLRTSRGLNPTYLEIK
                     SDKKGVFLALGSELKNEFVIFYENKLLISPYIGDLKSVDVYERFFSLLDFFKQSYDLS
                     FDQILCDKHPNFSYTKEFYNTYKIQHHYAHFCALYFEYEKEFKKDEKALGFIFDGTGY
                     GDDGKIWGGEIFIGNLKQYERIVHFENFTLINSDIKNIQNLALSLIWHYDLEDEAKTF
                     LSKIPKIKLENLKKIHTHSTLQTSSLGRIIDAFGSIVFDIEKISYEAQIGLMCEAFYD
                     ENLDFSYELFSKDGEINFKELIEGALKDEKSKAITGMFNALANLIITFSKAYDLKVLL
                     GGGVFQNKTLLQILKAKNFDFLASLKYPCNDSSIALGQMVHFLKKD"
     gene            complement(87715..89232)
                     /locus_tag="DMACINML_00093"
     CDS             complement(87715..89232)
                     /locus_tag="DMACINML_00093"
```

```
                     /inference="ab initio prediction:Prodigal:2.6"
                     /inference="similar to AA sequence:RefSeq:CAL34767.1"
                     /note="hypothetical protein Cj0621"
                     /codon_start=1
                     /transl_table=11
                     /product="hypothetical protein"
                     /protein_id="Prokka:DMACINML_00093"
                     /translation="MTLEFDFVYSADNDIFEYLLRFYAKNYRYTLEKQEDKYHFSIDA
                     DEENLKAFCDSLNSMSHSIFLKKFDVKAGEFHQQEVKDKDFFPFTYLTYLNSKLYQEK
                     KELSKNEWGVFCECEFSSDLNEFQSLNEDNFDKNLNTAFDLLLKEKSIYLKDKKGIYE
                     ISLFKGDFECDFLMPCDIKAINSVFVCSNENLKLLASLEKPLMKLRLSAVFRKNHDLD
                     FNEFKLRLAQDLFSFALGLKLWQNEYKFLSVKKIKEFQKDFYITTLDNQIVVLEGFEF
                     INPRARELIFSKEDKNMARISYLLSRYGEKAFVLELSKEYEDLLLANKEINLLCLSLP
                     KNSKELYEEIQKDEIGARLLENFAKEFPLLNESFELKNNFYSLLCLVGRVLNLDENLH
                     KAGEKLLKIADESKMPRGVKIDYRLKEDKSFDYTRTLRSTMSFMLAGVDSANIAYGAV
                     ESLAYFLRDTYDDLREKKQSEMALISGSLFEHKALLRNTLKHLKNCQLSDVPLRI"
     gene            complement(89279..89926)
                     /locus_tag="DMACINML_00094"
     CDS             complement(89279..89926)
                     /locus_tag="DMACINML_00094"
                     /inference="ab initio prediction:Prodigal:2.6"
                     /inference="similar to AA sequence:RefSeq:AAW35768.1"
                     /note="conserved hypothetical protein"
                     /codon_start=1
                     /transl_table=11
                     /product="hypothetical protein"
                     /protein_id="Prokka:DMACINML_00094"
                     /translation="MARQSSSVKSFRYLNRDFFYQIKNIKTLRLKLNEKGEFHLSIPQ
                     FYPLEKAKEFLYKNEIWIQKTYLNFQSKKARISENEIYFLGKKYILNLDESFKKTRIS
                     KGEIQSASREDLDNFLKQNAKIIFSFYLKKWSKKTNLHYSHLSIKKMKTRWGSCNHKK
                     GYINLNLRLLEKNLRAIEYVILHELSHIKFPNHSKEFYDFIYSFMSDFRQREKEF"
     gene            complement(89902..91218)
                     /locus_tag="DMACINML_00095"
     CDS             complement(89902..91218)
                     /locus_tag="DMACINML_00095"
                     /inference="ab initio prediction:Prodigal:2.6"
                     /inference="similar to AA sequence:RefSeq:CAL34765.1"
                     /codon_start=1
                     /transl_table=11
                     /product="putative MATE family transport protein"
                     /protein_id="Prokka:DMACINML_00095"
                     /translation="MAKKQLSLTKLSVPIFWDLLSKYLTIIINTAMVSHYSNSLVGAM
                     GAGNLIADLFITIFSFLSVGCSVVIAQAIGARDLVLARKVIHQSLFLNALLGFVCAVF
                     IIWQGELLLRLANIPEEKLQDGIIYLRMLGICLFFDALGIVLAAIIRVYNMAYWVMFI
                     GFLMDIIVIIGNFYALHFTQSELLGVGLSNLVARFVAVAALLIILFYKLKIHLKIQEM
                     IRLEKAVVKKVLNIGGFSAGENLLWIVQYFIAFSFVAKLGSNNDSVQTIYFQISMLIM
                     LIGQAISIANEIIVGKLVGAKYQNIAYKHAWNALYLSVMASAFVAVLNFVFKDFTMDL
                     LDLKPELRELMLPLFTLSIFLEISRTFNIVMVNALRASGDAKFPFFSGLVFMMGVSLP
                     VGYVLCFHFGLGILGVWIGFCADEFLRGLVNAYRWKSKKWQGKALV"
     gene            complement(91603..93657)
                     /locus_tag="DMACINML_00096"
     CDS             complement(91603..93657)
                     /locus_tag="DMACINML_00096"
                     /inference="ab initio prediction:Prodigal:2.6"
```

```
                     /codon_start=1
                     /transl_table=11
                     /product="hypothetical protein"
                     /protein_id="Prokka:DMACINML_00096"
                     /translation="MSKKELYEKLIFAIEGQEVNGVIQAIRDGAEVDKEIVDIAYGFL
                     ESLEIEYADDKAFYAGCTAKNSDQDYIYRIVSRYAKGQKLDTIINTMFNRKTNPLKSK
                     GEIITPKNKRELIKLMNKKRQYLGDIDISNIKDFSELFTDVIRTDFGGIELWDTNHVV
                     NMNRMFEKFNFSKIKSDSPLFDWISNMDTSNVSDMGYMFSQSIGFNTDIGSWNTSNVL
                     NMSYMFLEAEDFNQNISAWDTSNVLCMVDMFHGAKRFKQNIDNWDVSSINKDYRATNA
                     KKYKFIDHENLCNYALYENCPTKPKWLMPCKKENGKYKPNTKLELILLAKDKNINLAD
                     VDISNINDLSLIFLHCERDFSGIESWNTSHVVNMSNMFAYSNMNQDISMWDTSKVAYM
                     DGMFQNTPFNQNINDWNISNVKDLSSMFYCAEDFNQPLDKWDTSRVESMHYMFYRALK
                     FNQDIGLWNTSKVKDMNHMFSNAESFNQNINNWNVSNVKNMNGMFFYTKKFNQPLDKW
                     DTGKVTNMASMFRSSKRFNQNISSWNVSHVKNFSYMFEETEDFNQPLNGWDITGTTSL
                     AYMFSEAKSFNSPLNEWDTSKIKDMTGMFEETEKFNQPLSDWDVSNVETMHAMFSESK
                     SFNQDISSWNIKSIEDLSYFLHEAKAYTYSLKSWRLNAIDVDNYYIVEGTNIEEPTWY
                     ES"
     gene            complement(93945..94685)
                     /locus_tag="DMACINML_00097"
     CDS             complement(93945..94685)
                     /locus_tag="DMACINML_00097"
                     /inference="ab initio prediction:Prodigal:2.6"
                     /inference="similar to AA sequence:RefSeq:APU77476.1"
                     /codon_start=1
                     /transl_table=11
                     /product="phosphate ABC transporter ATP-binding protein"
                     /protein_id="Prokka:DMACINML_00097"
                     /translation="MIAKTTNLNLFYGKKQALFDINMQIEQNKITALIGASGCGKSTF
                     LRCFNRMNDKIAKIDGLVEIEGKDVKNQDVVVLRKNVGMVFQQANVFVKSIYENISYA
                     PKLHGMIKSKDEEEALVVDCLQKVGLFEEVKDKLKQNALALSGGQQQRLCIARALAIK
                     PKLLLLDEPTSALDPISSGVIEELLKELSHNLSMIMVTHNMQQGKRVADYTAFFHLGE
                     LIEFGESKEFFENPKQEKTKAYLSGAFG"
     gene            complement(94682..95770)
                     /gene="pstA"
                     /locus_tag="DMACINML_00098"
     CDS             complement(94682..95770)
                     /gene="pstA"
                     /locus_tag="DMACINML_00098"
                     /inference="ab initio prediction:Prodigal:2.6"
                     /inference="similar to AA sequence:RefSeq:CAL34761.1"
                     /codon_start=1
                     /transl_table=11
                     /product="putative phosphate transport system permease
                     protein"
                     /protein_id="Prokka:DMACINML_00098"
                     /translation="MKKLFKKRQKASKSFKRLCKMGLYINLIFLCIFLGSVAYLGIGA
                     FKQTYIYVEADRNSPAYELLSRAEQRKIRTGQITEKSWLLANSEVDQYMKQNYNRLNE
                     EQRALVDNLVQKDEIALRFNTNFFLNGDSKSPENSGILSSAVGTLLVMLVCMAVSVPV
                     GVAAAIYLEEFAPQNLFTHFIEVCINNLASIPSILFGLLGLGVFINLFGMPRSSALVG
                     GLTLAIMSLPIIIVSTKAALKNVDINMKNAAYALGMTKVQMVKGIMLPLAMPMILTGS
                     ILTLAGAIGETAPLMIIGMIAFIPDVASSVLDATSVLPAQIYSWSAMPERAFLERTAA
                     GIIVLLGLLVVLNLSAVLLRKYFQGKLK"
     gene            complement(95767..96681)
                     /gene="pstC"
```

```
                     /locus_tag="DMACINML_00099"
     CDS             complement(95767..96681)
                     /gene="pstC"
                     /locus_tag="DMACINML_00099"
                     /inference="ab initio prediction:Prodigal:2.6"
                     /inference="similar to AA sequence:RefSeq:CAL34760.1"
                     /codon_start=1
                     /transl_table=11
                     /product="putative phosphate transport system permease
                     protein"
                     /protein_id="Prokka:DMACINML_00099"
                     /translation="MLKEKIIKFILFLCAFVSVVVSFAIMLTILIEALKFFQKESIST
                     FLFSSEWAADAAFMNADGTSKQGVFGAVSLFWGTFYISLIAMLTALPLGVMCAIYLGV
                     FAGKKSKNYLKPILEVIAGIPTVVFGFFAAIIVAPFIVWFFSLFGIQASFQSALGAGF
                     IMGIMIVPIVASLSQDCIEAVSEKRINGAYALGMTKKEVVFAVILPEAIPGIVAACLL
                     GLSRALGETMIVVMAASLRPNLTMNFLEDMTTVTVKIVEALSGDQAFDSSLALSAFSL
                     GLVLFIITLIINMFSVYLINRFHKRKNL"
     gene            complement(96691..97686)
                     /gene="pstS"
                     /locus_tag="DMACINML_00100"
     CDS             complement(96691..97686)
                     /gene="pstS"
                     /locus_tag="DMACINML_00100"
                     /inference="ab initio prediction:Prodigal:2.6"
                     /inference="similar to AA sequence:RefSeq:CAL34759.1"
                     /codon_start=1
                     /transl_table=11
                     /product="putative periplasmic phosphate binding protein"
                     /protein_id="Prokka:DMACINML_00100"
                     /translation="MKKILALSVASFALAGALSAADLKIAGSSTVYPFTSFVAEEYAA
                     IHNTKTPIVESLGTGGGFKVFCEGITDISNASRAMKFSEFETCKKAGVTDIVGIMVGY
                     DGIVLAQNKANTPLNITKKELFLALAKEVPQGGKLVPNPYTNWNQINKNLPNRKISVY
                     GPPSSSGTRDTVEELVMSDISKKIPEYKGEYKTIRQDGAYIPSGENDNLIVSKLTIDK
                     DAFGLFGYGFLVSNVDKINAADIDGIKADEKNIADGKYDLARSLFIYINAKKNPKEAF
                     EFAKIYMSDDLAKSGGELEKIGLVPLNNELLKASQKHIEDRKILTDELVKAGKVF"
     sig_peptide     complement(97624..97686)
                     /gene="pstS"
                     /locus_tag="DMACINML_00100"
                     /inference="ab initio prediction:SignalP:4.1"
                     /note="predicted cleavage at residue 21"
     gene            98023..98523
                     /gene="cft"
                     /locus_tag="DMACINML_00101"
     CDS             98023..98523
                     /gene="cft"
                     /locus_tag="DMACINML_00101"
                     /inference="ab initio prediction:Prodigal:2.6"
                     /inference="similar to AA sequence:RefSeq:CAL34758.1"
                     /codon_start=1
                     /transl_table=11
                     /product="ferritin"
                     /protein_id="Prokka:DMACINML_00101"
                     /translation="MLSKKVVDLLNEQINKEMYAANLYLSMSSWCYENSLDGAGLFLF
                     QHASEESEHARKLITYLNETDSHVELKEVKQPEQNFKSLLDVFEKTYEHEQSITKSIN
```

```
                     DLVEHMLANKDYSTFNFLQWYVSEQHEEEALFRGIVDKIKLISDNGNGLYLADQYIKN
                     LALSKK"
     gene            98573..99949
                     /locus_tag="DMACINML_00102"
     CDS             98573..99949
                     /locus_tag="DMACINML_00102"
                     /inference="ab initio prediction:Prodigal:2.6"
                     /inference="similar to AA sequence:RefSeq:CAL34757.1"
                     /codon_start=1
                     /transl_table=11
                     /product="putative acyltransferase family protein"
                     /protein_id="Prokka:DMACINML_00102"
                     /translation="MTYFSLEFSILMIVFFAIYWAFKDNYKIQNILILAFSYLIYILI
                     NPYFALILFIYTFFIHFFALLIFVRRKRYIFATCLTFIILNLCFFKYFPNIKGSLDQV
                     LSFLGLDFLNIELILPIGISFYTFASITYLVDVYKKHHLESFLNLATFLSFFPTLLAG
                     PIMRSAFFFEQAYQKREFKHANLIIILLIFGIVKKVLIANYLGIYAKNILDFPQSYNF
                     IQLLSAIYAYAVQIYCDFSGYVDLVCAFALMLGFTLPPNFNMPYLAKNLKEFWARWHI
                     SLSTFIRDYIYIPLGGNRKGMPRTIANILIAFILSGLWHGSTLAFGLWGLLHGLGIVF
                     IHLLALTKFSLQKIPALGRFLTFQFVCFTWIFFYYNKNLEDALEYFKACYNNFFQIPS
                     YNDIYMLVSFAVLFMIYPLFVNFREYCVKILNLTPFLLKPFVITFILLLVFAFMPNGI
                     PDFIYSSF"
     gene            99950..100930
                     /locus_tag="DMACINML_00103"
     CDS             99950..100930
                     /locus_tag="DMACINML_00103"
                     /inference="ab initio prediction:Prodigal:2.6"
                     /inference="similar to AA sequence:RefSeq:CAL34756.1"
                     /codon_start=1
                     /transl_table=11
                     /product="putative periplasmic protein"
                     /protein_id="Prokka:DMACINML_00103"
                     /translation="MKIIRFFFILIIVFILVCLVMNQSIFSYIEQKYHFAFYPKNDIL
                     QEANGFKNKLEQIRAILSNEPLPQNIEENIMVQEDMDELNASNDFIDKNTTIEDIQQT
                     EDENVSFIDNTKLEIHKGDEFLFIGDSLMQGVAIALNRDLIDLGLKANDLSKQNTGLS
                     YKSYFDWAKATKEAFAKNPNIKYLVVLVGANDPWDIKKGGVYHRFGSDSWIDIYTYRV
                     NEIINIAKQHNAKILWFEVPPVKKDELNEKIQILNKIYSEEILKNKQIFINTKLFFSV
                     NDEFSTYIKNENNKSVKMRTDDGIHFTPNGAKEMSKLLLEHITIKEENAN"
     gene            100920..102089
                     /locus_tag="DMACINML_00104"
     CDS             100920..102089
                     /locus_tag="DMACINML_00104"
                     /inference="ab initio prediction:Prodigal:2.6"
                     /inference="similar to AA sequence:RefSeq:CAL34755.1"
                     /codon_start=1
                     /transl_table=11
                     /product="putative periplasmic protein"
                     /protein_id="Prokka:DMACINML_00104"
                     /translation="MQIKQFKAILLLILIFSINLYANNTNNIIDEILNQTKNQSALSNY
                     ASKKDLKILEQKLKDQKNIGIRIYGDSHTAADFFPRVIRGYLIKSNSIGFAYPLQPKY
                     QQNLNLDYSYKNYEILNSRNTDTAGIDFPLGGIIAKAKEKGAKITLDTNLDKKKFKVG
                     FVFKAQNNTNAFSIKDAQNKSYELRSVANKWSYKEYELVFPLQISALQKNAQLGGYFI
                     TSKEDNAYLDTIAINGAKSDLWMSWNKKVSEQELKLLSNDLILLAYGSNDALFKGFEK
                     NKFKKNLKDWIRTLKTYNKNALIMLVSPPTVVKKQGKNYTLAPDFFAIRQALYEVAKE
                     EKTLLFDMHQFMQDSGGKNKWIEQKLSLQDVHLTIKGYELMAKKLLDDLKKYVHY"
```

```
     sig_peptide     100920..100985
                     /locus_tag="DMACINML_00104"
                     /inference="ab initio prediction:SignalP:4.1"
                     /note="predicted cleavage at residue 22"
     gene            complement(102159..103523)
                     /locus_tag="DMACINML_00105"
     CDS             complement(102159..103523)
                     /locus_tag="DMACINML_00105"
                     /inference="ab initio prediction:Prodigal:2.6"
                     /inference="similar to AA sequence:RefSeq:CAL34754.1"
                     /codon_start=1
                     /transl_table=11
                     /product="putative outer membrane efflux protein"
                     /protein_id="Prokka:DMACINML_00105"
                     /translation="MKNLLSILLAFFLSACGVKLSLPKEPTMSEESFKDLNITYDWYK
                     AYDNAKLNEFLNFVLLNNSDINTARSTLLAALARADLIDYDLYPTLSANLGLGGDKKL
                     NSGVQSKNFNNSLNLSYELDIYGKIRDSSSASEFSAKASAYDLENLKISMINTALNGV
                     FELAYFNDVDNLLKEYLANLEQMRELYAYKFELGKIEELDLLNIEQNLLRAKQNLLRN
                     EQNRNLLIKNLQDLIGKQEGFAYIDYFKALTLSDFKNLNPNFNIPLEALVYRPDVRSK
                     LNTLKSAFKDYASVQKSILPSISLNGALNGSDKQFDDSFKFEVLSGNIKISLPFLDYG
                     RVKQNIKISQFDYESTLITYEQSLQSAMNEFALNYKDYQSNTMLLQILKDTSFKQELI
                     TQAYWEKYNLGKSELKDYLDASNTLNSTKQSLLEARFNLLKTINSYYQITALSYDETH
                     LEMP"
     gene            complement(103525..105450)
                     /locus_tag="DMACINML_00106"
     CDS             complement(103525..105450)
                     /locus_tag="DMACINML_00106"
                     /inference="ab initio prediction:Prodigal:2.6"
                     /inference="similar to AA sequence:RefSeq:CAL34753.1"
                     /codon_start=1
                     /transl_table=11
                     /product="ABC-type transmembrane transport protein"
                     /protein_id="Prokka:DMACINML_00106"
                     /translation="MIFLNNICKNIGENAILKNVSLSIEKGEFVAIIGQSGSGKTSLL
                     NIIGTLDEPSNGSYIFDNYEVTQLNSDEKARLRREKIGFIFQRYNLLNLLSANDNVTL
                     PAVYAGKKVQERNFRAQELLGNLELEHKIESKPNELSGGQQQRVSIARALMNGGELIL
                     ADEPTGALDSKSGIMVLEILKKLNAQGHTIVLVTHDPKIAAQAKRVIEIKDGEILSDS
                     KKDKFAYQGQVKSMPKEKKTFTLFKNQAFECLKIAYSSILAHKLRSILTMLGIIIGIA
                     SVVCVVALGLGSQAKVLESIARLGTNTIEIRPGKGFGDLRSGKTRLNFSDLETLRSLD
                     YLEAVDAHSNTSGVATYTNISLSARAEGVGVNNFAIEGLKLQVGRILNNEDIETNANV
                     AVLDFNAKRNLFPHQKSEDVLGRVVIFNSQPFKIIGVLQKDTEKPIEDNVVRLYMPYT
                     TLMNKLTGDRNLREIIVKVKDDVSSTLAENAIIRILEIKRGQRDFFTFNSDTFKQAIT
                     ANKRTTTILTASVAVIALIVGGIGVMNIMLVSVSERTREIGIRMAIGARREDIMMQFL
                     IEAVMICSMGAILGVLLSIFVIFGFNTLSTDFPMVLNAYSVLLGLLSSVLIGVVFGFF
                     PARNAANLNPISALSKE"
     gene            complement(105450..106625)
                     /gene="macA"
                     /locus_tag="DMACINML_00107"
     CDS             complement(105450..106625)
                     /gene="macA"
                     /locus_tag="DMACINML_00107"
                     /inference="ab initio prediction:Prodigal:2.6"
                     /inference="similar to AA sequence:RefSeq:SQE24135.1"
                     /codon_start=1
```

```
                     /transl_table=11
                     /product="amidohydrolase"
                     /protein_id="Prokka:DMACINML_00107"
                     /translation="MKNKIVLIVVILALFGVGAYFVFFNNGEKINYLTQKVKKINISQ
                     TIEAVGKVYAKDQVDVGAQVSGQIIKLYVDVGSYVKQGDLIAQIDKDKQQNDLDITKA
                     QLESAKANLESKKVALEIASKQYQREQKLYAAKASSLENLETQKNNFYTLKANVAELN
                     AQVVQLEITLKNAQKDLGYTTITAPIDGVVINVAVDEGQTVNANQNTPTIVRIANLDQ
                     MEVRMEIAEADVGKIKIGTELDFSLLSDPQKTYQASIASIDPADTQISDSSTNSTSSS
                     SSSSSSSSSNNAIYYYAKFYVANKDDFLRIGMSIQNEIVVASVKDVIAVPTYTIKNDA
                     KGYYVEILQGQKAVKKYVKLGIKDSVNTQILEGLSEDELLIVSSSGDSAAPKLRLRF"
     gene            complement(106708..107898)
                     /locus_tag="DMACINML_00108"
     CDS             complement(106708..107898)
                     /locus_tag="DMACINML_00108"
                     /inference="ab initio prediction:Prodigal:2.6"
                     /inference="similar to AA sequence:RefSeq:CAL34751.1"
                     /codon_start=1
                     /transl_table=11
                     /product="putative amidohydrolase"
                     /protein_id="Prokka:DMACINML_00108"
                     /translation="MLERVKNLSDKYYEKIVNLRHQIHMHPELEFEEENTARLVCEIL
                     DEFGIKYEKNIAKTGILASIEGKKQSTKKPQCVLLRADMDALPVQEQTNLSYASKING
                     KMHACGHDGHTAGLLGAVLILNELRDEFSGTIKFMFQPAEEGSGGAKPMIEAGILENP
                     HVDAVFGCHLWGPLLENTAQIVSGEMMAGVDVFDLEFIGKGGHGAHPHTTIDPIVMAS
                     KFVSDVQCIISRRLRPVDAGVITIGKFHAGTTFNVIPQNAILQGTVRFLSDENQKLLQ
                     SSIENTAKAVALEFGGDFKLDYKREYPPLINDENAAKIARKAFSKVLGEQNIISVAKP
                     DMGAEDFAFLTRERMGAYVFVGISKDLKNPTLHHSSTFCWEDENLKVLMQGDSMMALE
                     FLNS"
     gene            complement(107903..108784)
                     /locus_tag="DMACINML_00109"
     CDS             complement(107903..108784)
                     /locus_tag="DMACINML_00109"
                     /inference="ab initio prediction:Prodigal:2.6"
                     /inference="similar to AA sequence:RefSeq:ADC28217.1"
                     /codon_start=1
                     /transl_table=11
                     /product="putative polyphosphate kinase"
                     /protein_id="Prokka:DMACINML_00109"
                     /translation="MQEKVPAKIQEDTKKDEIYVSIKKKKSTIEYEKDLKNLQIELLK
                     FQNYVKAKGLKVLILIEGRDAAGKGGAIKRLIEYLNPRGCRVVALEKPNDVEKTQWYF
                     QRYVTHLPSAGEIVIFDRSWYNRAGVEPVMGFCTPQQHKDFLREVPLFENMISNSNLM
                     FFKFYFSVSKEEQKRRFEKRRIDPLKQYKLSPVDQKSQELWDKYTLAKYSMLLASNTP
                     TCPWTIISSDDKKKARLNLLRFILSKVDYADKKIGDFSKIDQELVRSGEEEIRKMEAN
                     LEKIDNKKADEKIKDLD"
     gene            108879..110579
                     /gene="dsbD"
                     /locus_tag="DMACINML_00110"
     CDS             108879..110579
                     /gene="dsbD"
                     /locus_tag="DMACINML_00110"
                     /EC_number="1.8.1.8"
                     /inference="ab initio prediction:Prodigal:2.6"
                     /inference="similar to AA sequence:RefSeq:CAL34749.1"
                     /codon_start=1
```

```
                     /transl_table=11
                     /product="putative thiol:disulfide interchange protein"
                     /protein_id="Prokka:DMACINML_00110"
                     /translation="MRFIFTLLFSCYFLFASVLSLNEAFEVKSSSYDSAVSIDIKLGK
                     DIYLYSDKLKLYINQNDISHLINLPPSTQRLNEKVYYQDLNLALPSLLLERFAQSQNN
                     QIKLEFQGCSEQGLCYNPQTWYFDLKHKDKNFEISAPYKEKKKTLQVKSEESMIANFL
                     ATDKLFWILLSFLGYGLLLSLTPCILPMIPILSSLIVAKSETKFSKKHSFFLSFIYVF
                     SMSLAYAIAGVIASFLGASVQGLLQKPSVLIFFALIFVLLALAMFESFRFELPLKFQN
                     FLHKKSEKGKGVLGVALMGFLSALIVGPCVAAPLTGALIYIANTGDALLGASALFVMS
                     FGMGIPLLFIGLGLGFLKAGAWMQKVKIFFGFVMLAMAIWILSRIIETRYILIAFGIL
                     GVFFSVFMGIFEQAFSVITKIKKSLLILVLAYSLSIFLGGIFGAKNFLNPLNLNSNTL
                     LNSSKLNYNFINDLNALQKEIKTSTRPIMFNFTASWCENCKLLDELTFSDEKIQEKLK
                     NFHLIKIDLTQNSDKDLEIMKEFGVFGPPVLIFFENGKENLRITGFISADDLLQKLTK
                     "
     sig_peptide     108879..108929
                     /gene="dsbD"
                     /locus_tag="DMACINML_00110"
                     /inference="ab initio prediction:SignalP:4.1"
                     /note="predicted cleavage at residue 17"
     gene            110576..111244
                     /locus_tag="DMACINML_00111"
     CDS             110576..111244
                     /locus_tag="DMACINML_00111"
                     /inference="ab initio prediction:Prodigal:2.6"
                     /inference="similar to AA sequence:RefSeq:CAL34748.1"
                     /codon_start=1
                     /transl_table=11
                     /product="MOSC-domain containing protein"
                     /protein_id="Prokka:DMACINML_00111"
                     /translation="MKIKSLQIGHIKDYGGFKSAFIKNIYLKEVDIDFSGIINDNIAD
                     TKHHGGKNKALFANSYHNYPLWENFLGKKLECGMMGENLTLENLHEQNVCIGDIHRFE
                     DAILQVSEPRKPCVKISKIHHNPNFTQEIFKTGLSGWYYRVLEGRKIHAHSKIQLIER
                     NPINLSVLELNHLFYNPHQALKQNPTLLEKLSKLDTLISQNWHETIQKRLKNTYDLNY
                     MQNL"
     gene            111295..112638
                     /locus_tag="DMACINML_00112"
     CDS             111295..112638
                     /locus_tag="DMACINML_00112"
                     /inference="ab initio prediction:Prodigal:2.6"
                     /inference="similar to AA sequence:RefSeq:CAL34747.1"
                     /codon_start=1
                     /transl_table=11
                     /product="putative sodium-dependent transmembrane
                     transport protein"
                     /protein_id="Prokka:DMACINML_00112"
                     /translation="MQRQTWSSTLTYILTVAGATIGFGATWRFPYLVGENGGGAYVLV
                     FCIAMILIGIPVILAENVIGRKSMTNSVDAFEGKWKFIGYMGLLGSFGIMAYYMVLGG
                     WVFVYVFELIIGNFNLSHTVTKDFTEQFFNEKISFNPLGVGIFTTLFVLINYIILRRG
                     IIDGIEKSVKFLMPLLFICLILVVGRNLSLEGAMNGVKFYLTPDFDKILSRKLLIDVL
                     GQVFFALSLGFGVMITLSSHLSKNENMTKTAIYTGVLNTLIAVLAGFMIFPALFAAGL
                     EPDKGPSLVFQTLPIAFSHIHFGTIICILFFILLLIAALTTSLPIYQVIISVLEEKFK
                     LSKKTAINLTLGSIFILGNIPCILTYGPWRDITLVKGKNIFDSFDFISGNILFVLTAF
                     FCCIYVGWVLGKEQSIKELSNEGKLKSVGFKIWFYYIKFIIPLIILTIFVYGVTN"
     gene            112926..114188
```

```
                     /locus_tag="DMACINML_00113"
     CDS             112926..114188
                     /locus_tag="DMACINML_00113"
                     /inference="ab initio prediction:Prodigal:2.6"
                     /inference="similar to AA sequence:RefSeq:ADT73021.1"
                     /codon_start=1
                     /transl_table=11
                     /product="Major outer membrane protein"
                     /protein_id="Prokka:DMACINML_00113"
                     /translation="MKLVKLSLVAALAAGAFSVANAAPLEEAIKDIDVSGVLRYRYES
                     GRFGKDLNLVNGSSLNTSKQEHKYRAQLNFSGAIADNFKAFVQLDYNSKDGGYGVDSI
                     SNTSDTLNVRQLYLTYTDENVATSVILGKQQLNTIWTDNAIDGLVGTGVKVVNNSIDG
                     LTLAAFAVDSSNEAVHTTTNPKGDTDASPFLNWNKNIYGGAAVGSYEVFNGQLNPQLW
                     LAYMTDNAFLYAVDAAYSTTIFDGVNWTLEGAYLGNSLDNELKDAGFGNGNFFALNGG
                     IEVNGWDATLGGLYYGKKDKGTVTVIEDQGNIGSLLAGEEIFYTQGSQLNGDIGRNIF
                     GYVKAGYTFNETVRVGADFVYGGTKTDSTVTGHAGGGDKLEAVARVSYKYSPKLNFSA
                     WYSYVNVDNGDQGEGDHNTVRLQALYKF"
     sig_peptide     112926..112994
                     /locus_tag="DMACINML_00113"
                     /inference="ab initio prediction:SignalP:4.1"
                     /note="predicted cleavage at residue 23"
     gene            complement(114328..115452)
                     /gene="dnaJ-2"
                     /locus_tag="DMACINML_00114"
     CDS             complement(114328..115452)
                     /gene="dnaJ-2"
                     /locus_tag="DMACINML_00114"
                     /inference="ab initio prediction:Prodigal:2.6"
                     /inference="similar to AA sequence:RefSeq:EAQ72629.1"
                     /codon_start=1
                     /transl_table=11
                     /product="co-chaperone protein DnaJ"
                     /protein_id="Prokka:DMACINML_00114"
                     /translation="MELSYYEILEITQSADKDTIKKAYRKMALKYHPDRNQGDKEAED
                     KFKLVNEAYEVLSNEEKRAIYDRYGKDALKGGGFGSGGAGFGGFEDLGDIFSSFFGGG
                     FGGSSQRRKKSSDEKFQAELVVNLKLTFKEAVFGCKKNIDFTYKSSCKTCSGTGAKDG
                     KLQTCPKCKGRGQVGISQGFITFAQTCPDCKGSGESASEKCKDCKGRGYEELKDSVEV
                     NIPEGIDNGMNLRVAAKGNLSKSGNRGDLYVKVIVEEDDTFIRDDEDIYIEFPVFFTQ
                     AILGQSIKVPTIRGEATLNLPKGAKDGQRFVLEREGVKDVHSSRIGNQIVQIAIKFPN
                     SLNDEQKELLEKLSESFGIKDGMHHEQKGLFEKITHWFKS"
     gene            115598..116269
                     /gene="racR"
                     /locus_tag="DMACINML_00115"
     CDS             115598..116269
                     /gene="racR"
                     /locus_tag="DMACINML_00115"
                     /inference="ab initio prediction:Prodigal:2.6"
                     /inference="similar to AA sequence:RefSeq:CAL35376.1"
                     /codon_start=1
                     /transl_table=11
                     /product="two-component regulator"
                     /protein_id="Prokka:DMACINML_00115"
                     /translation="MINVLMIEDDPDFAQLLSEYLTQFNIKITNFENPKEALNVGIQG
                     YDCLILDLTLPGIDGLEVCREIRQKYNIPIIISSARGDLSDKVVGLQIGADDYLPKPY
```

```
                     DPKEMHARIMSLIRRTKRAENTSNENINSAFKIDERKHEITYQNKVLTLTPAEYEILE
                     YLIQQHGYSVSREQLVSRCKNLKDKDSKSLDVIIGRLRTKINDSSKSPKHIFSVRGIG
                     YKLIG"
     gene            116266..117516
                     /gene="racS"
                     /locus_tag="DMACINML_00116"
     CDS             116266..117516
                     /gene="racS"
                     /locus_tag="DMACINML_00116"
                     /inference="ab initio prediction:Prodigal:2.6"
                     /inference="similar to AA sequence:RefSeq:CAL35377.1"
                     /codon_start=1
                     /transl_table=11
                     /product="two-component sensor (histidine kinase)"
                     /protein_id="Prokka:DMACINML_00116"
                     /translation="MTKNYSIYTKLVILFVVTFFLVCVLFIVLLKIESNTYSEEEGFK
                     QESFIKNIINSYGNSSGVDIGAHLNNSGFVAIQNDYFAKIARIDGQNLFRAGNGENSC
                     TLSSLKYQNKLYLDVQCKDFNALFEEDTNNRVYNLLLIGFFFFSFLVVFMYFSVLKSL
                     EPLKKLRRQVAEVANGGKPDFENYQQDEVGKIAFEFQKAFKKNQELIQSRQLFLRTIM
                     HELKTPIGKGRIISEMLKEEKQKERLIDIFLRMDSLINEFAKIENLFSKNYNLQFKPV
                     HFSTILNEAKDYLMRDDFNKVVKLNLKHDALINVDMEIFPLILKNLIDNALKYSSNGT
                     CELYCCKECFVIKNPGNPLSEPIEHYFEAFTREPYQKVKGMGLGLYIVFEVCKLHNFD
                     IIYFYEEGKHCFKIFFGVKTNNDT"
     gene            117506..118081
                     /gene="recR"
                     /locus_tag="DMACINML_00117"
     CDS             117506..118081
                     /gene="recR"
                     /locus_tag="DMACINML_00117"
                     /inference="ab initio prediction:Prodigal:2.6"
                     /inference="similar to AA sequence:RefSeq:CAL35378.1"
                     /codon_start=1
                     /transl_table=11
                     /product="recombination protein"
                     /protein_id="Prokka:DMACINML_00117"
                     /translation="MTHKGVEKFNELVESFANLPTIGKKTAIRLAYHICTANQLEGMK
                     LAHNIENAIRFIKPCQQCGALSENELCEICTDEERDKNFLCIVQSPKDVLVIEESKSY
                     GGLYFVLDDLNDEKLAQLRQMILKLQSKELIFAFTHNLNSDAMIFYIEDKLKDLNLQF
                     SKIAQGIPSGVNLENVDFISLNKAINFRTKV"
     gene            complement(118083..118583)
                     /gene="hydD"
                     /locus_tag="DMACINML_00118"
     CDS             complement(118083..118583)
                     /gene="hydD"
                     /locus_tag="DMACINML_00118"
                     /inference="ab initio prediction:Prodigal:2.6"
                     /inference="similar to AA sequence:RefSeq:CAL35379.1"
                     /codon_start=1
                     /transl_table=11
                     /product="putative hydrogenase maturation protease"
                     /protein_id="Prokka:DMACINML_00118"
                     /translation="MFADEGIGVHLCKLLEKNYKFTHPNHSLNFVDGGTLALQLSYII
                     AEYDKMIVLDCIDADNSKIGDVFFFPYDAMPKKINWSGSAHEVEMLQTLQYMELMGDL
                     PPTQILACVPKRIKPMSFTLSQEIVSAGKIMEKTLLEYLAKEGFSYEKIANHSLQELA
```

```
                     DISYKN"
     gene            complement(118613..119305)
                     /gene="hydC"
                     /locus_tag="DMACINML_00119"
     CDS             complement(118613..119305)
                     /gene="hydC"
                     /locus_tag="DMACINML_00119"
                     /EC_number="1.12.5.1"
                     /inference="ab initio prediction:Prodigal:2.6"
                     /inference="similar to AA sequence:RefSeq:AAW35720.1"
                     /codon_start=1
                     /transl_table=11
                     /product="quinone-reactive Ni/Fe hydrogenase, cytochrome b
                     subunit"
                     /protein_id="Prokka:DMACINML_00119"
                     /translation="MQNKEEKLQRKAEYEFSIGLRLTHWVRAFAIVILVVTGYYLSYV
                     FQNPVSNGEPVNFMQAKYRMVHQAVGFVLIACVIFKVYLFFFDKISRKERISIADVLN
                     PKLWIEQIKFYLFLGKHPHLKGVYNPLQFVTYVFFYIVMVGIILTGLILYTHTYHEGL
                     GGLLYEVLRPLEAALGGLADVRTYHRILMWVILIFVPVHIYMAFFNAIKGKDGAIDAI
                     VSGYKFVKEEKN"
     gene            complement(119316..121031)
                     /gene="hydB"
                     /locus_tag="DMACINML_00120"
     CDS             complement(119316..121031)
                     /gene="hydB"
                     /locus_tag="DMACINML_00120"
                     /EC_number="1.12.5.1"
                     /inference="ab initio prediction:Prodigal:2.6"
                     /inference="similar to AA sequence:RefSeq:CAL35381.1"
                     /codon_start=1
                     /transl_table=11
                     /product="Ni/Fe-hydrogenase large subunit"
                     /protein_id="Prokka:DMACINML_00120"
                     /translation="MSQKIIVDPITRIEGHLRVEVVVDDNNVVKEAYAGSTLWRGIET
                     IVKGRDPRDAGFMTQRICGVCTFSHYKAGIVAVENALGITPPLNALLTRTLMNAALFL
                     HDHIVHFYQLHGLDWADVVSALSADVKKASDEAFKYTSMPYATGADKLLEVQQRLKTF
                     VDKGNLGPFANAYYGHPTYRLSPEQNLIVLSHYLECLRIQRIIAQCMAIFGAKNPHPQ
                     SLTVGGVTCVMDLLSPARMGEYMEKFKEVQDFVNRAYYPDLVMAGKAYANEPSVLNDI
                     GVNNLYTFQEFQLGRDEWLFESGIIKNGDLSKVYEVEEDKITEEATHSWYADNEPLHP
                     YDGKTNPNYTGLVEGESVDHHGNTVHSKVFDTKGKYSWIKAPRYEGNPMQVGPLANIV
                     VNYAKGNQNVVPVVDEFLKETGLPLNAVFSTLGRTAARCLEAKIVANNALKAFRNLVE
                     NLKVDESTCAPYVIDNSKEYKGRYMGHVPRGTLSHWCRIKNGVIENWQAVVPSTWNAS
                     PKDANGVGGSYEQCLIGLKIADVKQPLEIIRKIHSYDPCIACAVHVMDTKGNNLSEYK
                     INASL"
     gene            complement(121037..122176)
                     /gene="hydA"
                     /locus_tag="DMACINML_00121"
     CDS             complement(121037..122176)
                     /gene="hydA"
                     /locus_tag="DMACINML_00121"
                     /EC_number="1.12.5.1"
                     /inference="ab initio prediction:Prodigal:2.6"
                     /inference="similar to AA sequence:RefSeq:CAL35382.1"
                     /codon_start=1
```

```
                     /transl_table=11
                     /product="Ni/Fe-hydrogenase small chain"
                     /protein_id="Prokka:DMACINML_00121"
                     /translation="MIDYNQIESRLSALEKLPSLNPENSIPKALERSGFTRRDFMKWA
                     GAMTAFLALPASFTPMVAKAAELADRLPVIWLHMAECTGCSESLLRSDTPTIDSLIFD
                     YISLEYHETVMAAAGWQAEENLESAIEKYKNKYILMVEGGIPMGDTEKFLTIGPHGKS
                     GYELSKVASENALAIFAIGTCSSFGGIQAARPNPSNAQPLSKVTSKTVINVPGCPPSE
                     KNIVGNVLHYLLFGELPALDVYNRPKWAYGLRIHDLCERRGHFDAGEFVQNFGDEGAK
                     NGYCLYKVGCKGPYTFNNCSRERFNQHTSWPIQAGHGCIGCSEPNFWDTMGPFEEPMA
                     SRKFDTVFGLGADNVSDKIGIGVLTLTGVAIAAHAVIASMKKNEE"
     gene            complement(122308..124143)
                     /locus_tag="DMACINML_00122"
     CDS             complement(122308..124143)
                     /locus_tag="DMACINML_00122"
                     /inference="ab initio prediction:Prodigal:2.6"
                     /inference="similar to AA sequence:RefSeq:CAL35383.1"
                     /codon_start=1
                     /transl_table=11
                     /product="putative FAD dependent oxidoreductase"
                     /protein_id="Prokka:DMACINML_00122"
                     /translation="MKKANLIFKDNTPFSLDFDDFYFNSKDGLNESKFVYSEAFEFKE
                     NDTFIIAESGFGIGLNFFLTLKRFLQAKKRPKKLFYVSVEGFYIEKDKLREIYHKLGF
                     YEEFKEILEAFLKFYPKAKKGYYRFYFKDCFLDLIFDDIAVLSELDFNADVWFLDGFS
                     PSKNSAMFDENFIAQVARLSKIDTQICTFSASSILQKNLIKYGFEVQKTKGFRKREMI
                     KAFLRKENPILDKEAYFQRIPSLYKNKKVAIIGSGICGATLAYELSLRDFEVSVFEKN
                     DSLGCGASGNESGILSSLILKPDVALGEFSQLAFIEASRFYKQILNLNLNGVIEFAHT
                     PLMQERFISQKDNVLFKIDKNEAFLEDGGHIKPKEILKSLFEKSQAKMYFNHEYDFFQ
                     HQEDKFTLHFKNQKAIQDFDILIYAMGADTKDFLVYDGMLLSKVRGQVTHLKPFLDNA
                     FALSSKAYICPSDGDLQVIGASYDRLNSNPHPQKADDEENLQNIQEFLKGDEEIIIKG
                     SRVGFRSYSSDRFAIVGAAYDEAFYKQEYKALLWTKNKTQTLPQNIPNLYLNFAHGSR
                     AFSTSVLAARYLCALINEEPLGVFKKFIPCIHPARFLIRKLKKGI"
     gene            complement(124140..126110)
                     /gene="amiA"
                     /locus_tag="DMACINML_00123"
     CDS             complement(124140..126110)
                     /gene="amiA"
                     /locus_tag="DMACINML_00123"
                     /EC_number="3.5.1.28"
                     /inference="ab initio prediction:Prodigal:2.6"
                     /inference="similar to AA sequence:RefSeq:CAL35384.1"
                     /codon_start=1
                     /transl_table=11
                     /product="N-acetylmuramoyl-L-alanine amidase"
                     /protein_id="Prokka:DMACINML_00123"
                     /translation="MLRILVFCIALFSICFGVYENELANFDKNFIMSKNDAQVKFHHQ
                     LKSLYIQSVINEDEKTKIEILKRLIISSNALNLDDKSYANELEESGVSKASINALRKA
                     VIQDKQKLQSSSKNVELSSFDPPKSTPKPQQAKQEIVKTQNNKPQQTKKVDKVYVLKS
                     SKTSQGVVLDLNTNLEQEDIKSFALDEKGNYRYISDFEGVLEGGRKEYKFDDYRIVIS
                     QYNPKTIRIVAYAKEKIPIEFDFEDEKINISKKSQPVQKNISAKQETKTAVKKESDKV
                     VAKQEPKQVKTQEAKTNTKKEQADDEPLYVLDVDKSSNSVVLNLSDELDEKDITSFST
                     KDQKFFRQVVSFQGILEGSRKNFTFGQNAVTVTQYNPKTVRIVLSSPKEFKLFKELDG
                     KNLTLGFGGSVSKNTSVSSKSTSKTSNKNVLNSNFKSDKLIVLDAGHGGKDSGALSDK
                     KGTLKEKDIVLSTTLKLGNELKKRGYKVLYTRSSDKFINLRDRTKYANDKRADLFLSI
                     HANAAPSASRAKSSEGVETFFLSPARSERSKKAAEKENQGDFEEINYFSKQSILNFLN
```

```
                     REKIVASNKLAIDVQKNILTQTRKKYKIVDGGVREAPFWVLVGAQMPAILIEIGYITH
                     PSEGKRIANKSFQDTLVKGIADGVENYFYNNR"
     gene            complement(126110..127201)
                     /locus_tag="DMACINML_00124"
     CDS             complement(126110..127201)
                     /locus_tag="DMACINML_00124"
                     /inference="ab initio prediction:Prodigal:2.6"
                     /inference="similar to AA sequence:RefSeq:CAL35385.1"
                     /codon_start=1
                     /transl_table=11
                     /product="putative 2-nitropropane
                     dioxygenase,oxidoreductase protein"
                     /protein_id="Prokka:DMACINML_00124"
                     /translation="MSLKSLQIGKHTIKYPIFQGGMGLGISWDKLASAVSLNGGLGII
                     SSVGTGYYENRTHISKELNAKPYGSENFYSREGLQTLINNARKVCGDAPLGCNILCAS
                     NDYARIARDACEVGFNVIVSGAGLPTNLPEFTADFPDVALVPIISSPKALKIICKRWQ
                     SRYNRLPDAVVLEGPKSGGHQGFTYEQCLDPNYQLENLIAPVVEEAKNWGSFPVIAAG
                     GIWDKKDIENAISLGASGVQMGTRFIGTFECDASDEFKNVLLACKNEDIELIKSPVGY
                     PARGVRTNLLDLVDKRMGPKINCISNCVAPCGRGKEATKVGYCIADRLFDAWSGKKET
                     GLFFTGANGYRLEKLISVKELMDKLVNGE"
     gene            complement(127198..128403)
                     /gene="tyrS"
                     /locus_tag="DMACINML_00125"
     CDS             complement(127198..128403)
                     /gene="tyrS"
                     /locus_tag="DMACINML_00125"
                     /EC_number="6.1.1.1"
                     /inference="ab initio prediction:Prodigal:2.6"
                     /inference="similar to AA sequence:RefSeq:CAL35386.1"
                     /codon_start=1
                     /transl_table=11
                     /product="tyrosyl-tRNA synthetase"
                     /protein_id="Prokka:DMACINML_00125"
                     /translation="MDIKNILAEIKRGCAEIIDEERVENLIKNYYEKGENFFVKAGFD
                     PTAPDLHLGHSVVLTKMAYLQKHGAIVQFLIGDFTGQIGDPSGKSVTRKKLDKEQVLK
                     NAKTYEEQVFKILDPQKTQLKFNSQWLNELGAAGIVELTSTFSVARMLERDDFTKRFK
                     EQSPISICEFLYPLLQGYDSVALKCDIEMGGTDQKFNLLMGRTLQRIYNTGKEQAVMM
                     MPLLEGLDGVNKMSKSLGNYIGVTEKANDMYAKILSISDELMWRYYELLSTKSLEEIE
                     NMKKEISSGNLHPKLAKENLALEITARFHNEESAKAAKAEFDSVHSGNALPSDIKEFE
                     IQGEIWLAKALVECGLENSTSAARRSINANAVSVNSQKVSDEQMQLSAGEYILQVGKR
                     KFAKLKVNI"
     gene            complement(128419..130614)
                     /gene="spoT"
                     /locus_tag="DMACINML_00126"
     CDS             complement(128419..130614)
                     /gene="spoT"
                     /locus_tag="DMACINML_00126"
                     /EC_number="3.1.7.2"
                     /inference="ab initio prediction:Prodigal:2.6"
                     /inference="similar to AA sequence:RefSeq:CAL35387.1"
                     /codon_start=1
                     /transl_table=11
                     /product="putative guanosine-3',5'-bis(diphosphate)
                     3'-pyrophosphohydrolase"
```

```
                     /protein_id="Prokka:DMACINML_00126"
                     /translation="MKPVDEELLLEKLIEDIKNCRDLTRAKELLFSICQEDAIVEKAV
                     DYCIKSHEGQVRKSGEPYAVHPILVASLVAFLSENKATILAALLHDVIEDTQCTEEEL
                     RDQFGSEVLKLVLGLTKIVEIREDNLIPSKSKKSLTKSALTFRNMLLASIEDVGVLVV
                     KLCDRLHNMLTLDALREDKQKRISEETLVVYAPIAHRLGISSIKNYLEDLSFKYLMPD
                     EYKLIDNYINSNDQQMQLGLNEFISKIELLFLSNGFRQGSFQIQKRIKHSYSIYLKMQ
                     RKGVGIEEVLDLLGVRILVENVSDCYLALGVLHTNFNPLVSRFKDYIALPKQNGYQTI
                     HTTLFDTKNIIEAQIRTFDMHKIAEFGIAAHWKYKEDGSVVAPRLDWLADISMQSTNN
                     LENAEDYNAIELYEYAKDSLYVEDVAVYSPKGEIFTLPRGATVLDFAYEVHTKVGLHA
                     KSAYVNRVKVPLLTELKNGDIVRVVTSNDRFYRCSWVDSVKTGKAKASIREFCKQKMR
                     EINLLSAINMLSFIFSVDKNKIENWIEKENLGRRIRQVASDSAYLKDIVNGLKKYAKK
                     SYWFDKYEIKEQKIGNFLLYSNHKIANVDFDYCCHPKRGDAVLAFIENGNAIVHHKLC
                     DRADKMIDNNKDMVLIKWDSNIPKSYKLIFSLENKKGILAEFLAVLAKMQINLLTISL
                     SSDTNTVVDYFEITIEIPDNINPDNVRDRLKSYCKILDFTSLNDAYKEA"
     gene            complement(130598..130822)
                     /gene="rpoZ"
                     /locus_tag="DMACINML_00127"
     CDS             complement(130598..130822)
                     /gene="rpoZ"
                     /locus_tag="DMACINML_00127"
                     /EC_number="2.7.7.6"
                     /inference="ab initio prediction:Prodigal:2.6"
                     /inference="similar to AA sequence:RefSeq:CAL35388.1"
                     /codon_start=1
                     /transl_table=11
                     /product="putative DNA-directed RNA polymerase omega
                     chain"
                     /protein_id="Prokka:DMACINML_00127"
                     /translation="MENRIEEIAAKALERMGDDRYRLSLVVAKRAEQLADGAAPLVDI
                     DKSKLKFADIALYEIAENKITLEGLVETSR"
     gene            complement(130837..131553)
                     /gene="pyrH"
                     /locus_tag="DMACINML_00128"
     CDS             complement(130837..131553)
                     /gene="pyrH"
                     /locus_tag="DMACINML_00128"
                     /EC_number="2.7.4.-"
                     /inference="ab initio prediction:Prodigal:2.6"
                     /inference="similar to AA sequence:RefSeq:CAL35389.1"
                     /codon_start=1
                     /transl_table=11
                     /product="uridylate kinase"
                     /protein_id="Prokka:DMACINML_00128"
                     /translation="MQERKRVLVKFSGEALAGENGFGIENSILKFIANEIKELVKNNI
                     EVGIVIGGGNIIRGVSAAKGGLIKRTSGDHMGMLATVINAIAIQEALESCGLDVRVQS
                     AIQMEAFCETYIMRRAQRHLEKGRVVIFAAGTGNPYFTTDTTAILRAVEIDAQMVIKA
                     TKVNGVYDKDPNKFEDAVFLQTLSYDEAMQDNIKVMDDTAIALAKDNSLPIVVCNMFE
                     EGNLLKIIQGDNSLCSIVKN"
     gene            complement(131605..132807)
                     /locus_tag="DMACINML_00129"
     CDS             complement(131605..132807)
                     /locus_tag="DMACINML_00129"
                     /inference="ab initio prediction:Prodigal:2.6"
                     /inference="similar to AA sequence:RefSeq:CAL35390.1"
```

```
                     /codon_start=1
                     /transl_table=11
                     /product="putative peptidase M23 family protein"
                     /protein_id="Prokka:DMACINML_00129"
                     /translation="MRKILIFLCFFLLSNEIYANAINEKTKNLEENKRIQEQVNKKLE
                     DLANDILSGEKSLKELSAQIETLNSQTLKLEARAKAQNKDLNLLTSQNTDLLKNKANM
                     EGKLITLMAKDFAYDLPIPQGYVESKESFMAFEVLGSLDKILQDEIFKLSKDYEDISK
                     QIDSKQDQIKKINEDLKDYNTQLAKLQNLRQKQVSEINKQKTDRAIYAKKLENLQDQQ
                     DELRKTLNQLKIIESKQEDKKQTQKTASDSKITNNTQKIRQIGSSYQGSSTKRYTGKK
                     TIAPLESFTVKQKFGNYIDPVYNIKIFNENVVLRSKKADAVVKSVLDGKIVFAKDTSM
                     LARVVIVEHDNGIHTIYAHLDKIAPNIKVGKNVKKGAVVGRIKNDLTFEVTQKNFHIN
                     PLELISLN"
     sig_peptide     complement(132742..132807)
                     /locus_tag="DMACINML_00129"
                     /inference="ab initio prediction:SignalP:4.1"
                     /note="predicted cleavage at residue 22"
     gene            complement(132804..133610)
                     /locus_tag="DMACINML_00130"
     CDS             complement(132804..133610)
                     /locus_tag="DMACINML_00130"
                     /inference="ab initio prediction:Prodigal:2.6"
                     /inference="similar to AA sequence:RefSeq:CAL35391.1"
                     /codon_start=1
                     /transl_table=11
                     /product="putative integral membrane protein"
                     /protein_id="Prokka:DMACINML_00130"
                     /translation="MKFFKTHLSLILPLLFMMFAFEFILLTNATLRYYEELVNKDYNI
                     IVASSTELDKNVIKTKLSSFSDLELLDPKNLIDRLKNDISDKNLKILRDSLPRFYSIK
                     LNYLPTQNELNMIKNQLLSIPSISKVETFAKTHDKVYSLLVLMKFVFWLFLFIIILLS
                     FVLFLKQMKIWLFEHTERVEIMCLFGAPFWFRSFMLYKIVVLDCFIAFVILFIFFTQI
                     YNLSIVQDSLKAVDIILPPINFIVHLGVIFLVTLFICLLCVNSVMFRVKK"
     gene            complement(133597..134262)
                     /locus_tag="DMACINML_00131"
     CDS             complement(133597..134262)
                     /locus_tag="DMACINML_00131"
                     /inference="ab initio prediction:Prodigal:2.6"
                     /inference="similar to AA sequence:RefSeq:CAL35392.1"
                     /codon_start=1
                     /transl_table=11
                     /product="putative ABC transporter ATP-binding protein"
                     /protein_id="Prokka:DMACINML_00131"
                     /translation="MSNLIQAHKLSLGYDELVIKEASFTFKDDDFVFITGKSGSGKST
                     LLKSFYGDLELLSGQLEVCGSSMRKIGNLELLKLRQQIGIIFQDYRLIQEYSVEKNVM
                     LPLMIKGYSKKVCHDQASKLLKHVNLTFKADKLPNQLSGGEQQRVAMARALAHNPKLL
                     LCDEPTGNLDEYSSDIIWTLLKSARELLGTCVVVVTHRIPSNLRLNYRRFNIENGNMN
                     EIF"
     gene            complement(134311..135507)
                     /gene="trmB"
                     /locus_tag="DMACINML_00132"
     CDS             complement(134311..135507)
                     /gene="trmB"
                     /locus_tag="DMACINML_00132"
                     /EC_number="2.1.1.33"
                     /inference="ab initio prediction:Prodigal:2.6"
```

```
                     /inference="similar to AA sequence:RefSeq:CAL35393.1"
                     /codon_start=1
                     /transl_table=11
                     /product="putative tRNA (guanine-N(7)-)-methyltransferase"
                     /protein_id="Prokka:DMACINML_00132"
                     /translation="MPNFKARTLKEIILPFEKDGVEFVWMAYNDNVNLIYTKVEEESF
                     FLQVKKGENEFVVKVDKHTKPSKIAYLHKALYVFKQYFCEDIISEAFGIKNNALAEKT
                     PLIANDFEEVLARLEGRIYIEIGFGSGRHLLYQAKNNPHVLILGIEIYNPAITQVAKL
                     AKAQGIDNILLVQSDARLLLSVLQSHSVEKIFLHFPVPWDKKPHRRVVSKAFCKECAR
                     VLTAQGVFELRTDSYEYFDFTFKEFLDFSSPQFSIRKNENLEISSKYEDRWKRQEKDI
                     YDLWVWNLDKEENNPLSEEFDFSDIHLDKNDLMLLEKNFENFTLKKEDFFLHFENIYK
                     QGESLLLKIAFGAFNKPEHCYLHIGETISFVFKEPFKIQENLKAIKELKKSVKFHFNM
                     KHFLEN"
     gene            complement(135507..136739)
                     /locus_tag="DMACINML_00133"
     CDS             complement(135507..136739)
                     /locus_tag="DMACINML_00133"
                     /inference="ab initio prediction:Prodigal:2.6"
                     /inference="similar to AA sequence:RefSeq:CAL35394.1"
                     /codon_start=1
                     /transl_table=11
                     /product="putative fibronectin domain-containing
                     lipoprotein"
                     /protein_id="Prokka:DMACINML_00133"
                     /translation="MKKFHLSLCLGALTLLFSACSVSQLSNIGSKKELLVNESLPKID
                     NLKSLSDISNIAFEWDPLYDDNIKGFYLYRSNDANPEFKLVGTIKDKFQTHYVDAKLE
                     PNTKYYYMMRSFNNQGHISEDGKVVEVKTMPRLEAIAFAQAITNLPSRIKLIWRPHSD
                     PRVASYIIERAKADGQEFKKIAEVKNRLNAEFIDEDLKPEESFNYRIIAVGFDGVQSE
                     PSSVLNSTSKALPPQVEHLNASLDGANKIVLTWDAPKYKDFSYYKVYSTSSSLLPYTL
                     LAKTEQNSYEDIVEGAAKSRYYKVTMVDIDGLESSMPKNGVEGKTLGSPSSPSVILAQ
                     STSDGIELEWVDNDSRAVEYEVRRYGGGQNAIFKGIKEKRLKDIKALPGVEYSYEVIA
                     IDSVGLRSEPSKRVKAGQ"
     sig_peptide     complement(136674..136739)
                     /locus_tag="DMACINML_00133"
                     /inference="ab initio prediction:SignalP:4.1"
                     /note="predicted cleavage at residue 22"
     gene            complement(136687..137652)
                     /locus_tag="DMACINML_00134"
     CDS             complement(136687..137652)
                     /locus_tag="DMACINML_00134"
                     /EC_number="4.2.1.70"
                     /inference="ab initio prediction:Prodigal:2.6"
                     /inference="similar to AA sequence:RefSeq:CAL35395.1"
                     /codon_start=1
                     /transl_table=11
                     /product="putative ribosomal pseudouridine synthase"
                     /protein_id="Prokka:DMACINML_00134"
                     /translation="MQTFLVDESSRLDTFLAKNLQQSRNQIALMIEKNCVWVNDKIQN
                     KNSFKLKVGDRLVLNLPLVEEVKPKFDIDFDIEILYEDEDLLVLNKPSNLVVHGAKSV
                     KSATLVDWLIEKQYTLSTLGGEVRAGLVHRLDKETSGAILIAKNNFTHQKLSEQLSDK
                     TMGRIYLALIDLPLKEDKIIIDKALIRSSANAIKKIVTNSFVKGSKEAKSAFANLAKS
                     EDVSLIAAKLFTGRTHQIRAHLASINRHILGDELYGYRGKYECRIMLHAYFLYFIHPR
                     NGEKIFIQAPLMDDFKQILEKKFILGELNEKISLESLLGCFDSFI"
     gene            137730..138836
```

```
                     /gene="mrdB"
                     /locus_tag="DMACINML_00135"
     CDS             137730..138836
                     /gene="mrdB"
                     /locus_tag="DMACINML_00135"
                     /inference="ab initio prediction:Prodigal:2.6"
                     /inference="similar to AA sequence:RefSeq:CAL35396.1"
                     /codon_start=1
                     /transl_table=11
                     /product="RodA protein"
                     /protein_id="Prokka:DMACINML_00135"
                     /translation="MFIPDRRILTHFDYMQPILFIPIILISFFLIFEANAFLAEKQFV
                     YACVGIAAFSVFFLLPIRKLMWLIPIAYWVNILLLLSTDLFGVEKLGAKRWLEIPFTH
                     FTIQPSEIFKPSFILMLAYLIYQDPPPKNGYKLKQFLKLSFFIILPFLLIAQEPDLGS
                     AMVLLIVGFGVLFIMGVHYKIWLSIIIAIGISSPIIYTHFLKPYQKQRIHDFISEKPS
                     YQVAQSMIAIGNGGLIGKSQDEATQTHFNFLPISTSDFIFAYLIERFGFLGGFVLILL
                     YTLLIFHLLSLNQKLKEDYFARVAINCVALFIFIYAAVNISMTIGFAPVVGIPLPFFS
                     YGGSSFTIFMIFFGILQHLITFRYFWIDSKNKIK"
     gene            138883..138959
                     /locus_tag="DMACINML_00136"
     tRNA            138883..138959
                     /locus_tag="DMACINML_00136"
                     /product="tRNA-Met"
                     /inference="COORDINATES: profile:Aragorn:1.2"
                     /note="tRNA-Met(cat)"
     gene            139328..139591
                     /locus_tag="DMACINML_00137"
     CDS             139328..139591
                     /locus_tag="DMACINML_00137"
                     /inference="ab initio prediction:Prodigal:2.6"
                     /codon_start=1
                     /transl_table=11
                     /product="hypothetical protein"
                     /protein_id="Prokka:DMACINML_00137"
                     /translation="MKHFIFSGLIGLGALAFLAGCGDEAKTSDYYKAHLDEAKARVAE
                     CKKMEKMNETQQRDCSNANEAVLFQRDKNNKFYQSSPEAFEWK"
     gene            complement(140544..141023)
                     /locus_tag="DMACINML_00138"
     CDS             complement(140544..141023)
                     /locus_tag="DMACINML_00138"
                     /inference="ab initio prediction:Prodigal:2.6"
                     /inference="similar to AA sequence:RefSeq:AON64954.1"
                     /codon_start=1
                     /transl_table=11
                     /product="TPR repeat protein, Sel1 subfamily"
                     /protein_id="Prokka:DMACINML_00138"
                     /translation="MIVKNIKISNNIALKGKLMVMKYFIFVFTLIPVCVYSNDNYFDL
                     GKEQHKQGNYSKAKQYYEKACELNIKEACNGLGLLYFDGDGVKQDRTQAVSYFKKACE
                     LEYGEACGILGLLYLDGDGVKQDRTQAVNYFKKACELGYGETCDILGLLYLDGDGVK"
     repeat_region   141296..141925
                     /note="CRISPR with 10 repeat units"
                     /rpt_family="CRISPR"
                     /rpt_type=direct
     gene            complement(142072..142470)
```

```
                     /locus_tag="DMACINML_00139"
     CDS             complement(142072..142470)
                     /locus_tag="DMACINML_00139"
                     /inference="ab initio prediction:Prodigal:2.6"
                     /inference="similar to AA sequence:RefSeq:CAL35625.1"
                     /codon_start=1
                     /transl_table=11
                     /product="putative CRISPR-associated protein"
                     /protein_id="Prokka:DMACINML_00139"
                     /translation="MFDIPTKSKKEQKSANKFRVSLVKLGFFMMQFSVYVKICKGMTS
                     AKTAIKSVERILPPCGNIRVLTITEKQFDNMQILLGGVSFNEKMNDDKNLVLFEYDEK
                     TQDFKYSNDKIYKENKTKSNKIRQASLFEF"
     gene            complement(142490..142732)
                     /locus_tag="DMACINML_00140"
     CDS             complement(142490..142732)
                     /locus_tag="DMACINML_00140"
                     /inference="ab initio prediction:Prodigal:2.6"
                     /inference="similar to AA sequence:RefSeq:CAL35626.1"
                     /codon_start=1
                     /transl_table=11
                     /product="putative CRISPR-associated protein"
                     /protein_id="Prokka:DMACINML_00140"
                     /translation="MIEVFRPLVDLCVFDLKDIKESEFLDKEAKQRLIGILQEEIIIE
                     NKAYPLNRAINFYIQNFKNALLENDELMMVNLYDRR"
     gene            complement(142852..143244)
                     /locus_tag="DMACINML_00141"
     CDS             complement(142852..143244)
                     /locus_tag="DMACINML_00141"
                     /inference="ab initio prediction:Prodigal:2.6"
                     /inference="similar to AA sequence:RefSeq:ADT66761.1"
                     /codon_start=1
                     /transl_table=11
                     /product="CRISPR-associated protein Cas1"
                     /protein_id="Prokka:DMACINML_00141"
                     /translation="MAYDEAFKSILISNQAKLSLEFNHLVIKQDDKEAKFFLKDINFI
                     ILESLQASITSSLLSALAKYKIILLTCDESHHINGIFSPFLGHFISAKVAKKQIKVTL
                     QRKAILWQKIIKNKISNQAHILKITHHI"
     gene            complement(143234..146236)
                     /locus_tag="DMACINML_00142"
     CDS             complement(143234..146236)
                     /locus_tag="DMACINML_00142"
                     /inference="ab initio prediction:Prodigal:2.6"
                     /inference="similar to AA sequence:RefSeq:AFU43535.1"
                     /codon_start=1
                     /transl_table=11
                     /product="CRISPR-associated protein"
                     /protein_id="Prokka:DMACINML_00142"
                     /translation="MKILGFDIGINSIGWAFVEDNQLQDCGVRLFTKAEDPKTKESLA
                     LPRRNARSNRRRLGRRRSRLIALKHIISKELKLNYQDYIANDGELPKAYEGRLVSPYE
                     LRYKALNEKIEPKDLARVILHIAKHRGYMNKNEKKSSDNEKGKILSALKTNALKLEKY
                     QSVGEYFYKEFFQKYKENTKDFINIRNKEGSYENCVLASDLEKELKLILEKQKEWGYS
                     YNDNFIKEILKVAFFQRPLKDFSYLVGACTFFEDEKRACKNSYSAWEFVALTKIINEL
                     KSLEKESGELVSSQIINEILNHILDKGSITYKKFREYIKLHESMKFKSLKYDKDNAES
                     AKLIEFRKLVEFKKALGEHSLTREELDQIATYITLIKDNEKLKITLEKYSLNNEQIKN
```

```
                     LIEIDFNDHINLSFKALNLILPLMKEGKRYDEACKLLNLKTKSNNQKFDFLPAFCDSI
                     FAQELTNPIVNRAISEYRKVLNALLKKYGKMHKIHIELARDIGLSKKLRTKIEKEQKE
                     NYENNIWALNECENFGLKANAKNILKLKLWKEQKEFCIYSGKKISIEHLRDEKALEVD
                     HIYPYSRSFDDSFLNKVLVFTKENQEKLNKTPFEAFGANEERWSKIQALAQNLPYKKK
                     NKILDEAFKGKQQQDFISRNLNDTRYISTLIVKYTKEYLDFLPLDEKEDINLKSGEKG
                     SKIHVQTINGMLTSVLRHTWGFAQKDRNNHLHHALDATIVAYSTNAIIKAFSDFKKEQ
                     ELLKAKLYAKELTNDSYKHQAKFFEPFEGFREQILNQINKLFVSKPPRKRARGALHKE
                     TFYPKDEMIKKYNSQEGVEIALNCGKIRKIGTKYVENDTMVRIDIFKKQNKFYAIPIY
                     TMDFALGVLPNKIVIAGKDKKGNPKQWQEIDESYEFCFSLHKDDLVLIQKKDMQEPEF
                     AYYNSFDIGNSSICVEKHDNKFENLTDNQKLLFVNAKEGSVKAEKLGIQGLKIFEKYI
                     ITPLGEKIKADFKPREDIALKTSKKHGL"
     gene            complement(146318..147691)
                     /gene="dnaB"
                     /locus_tag="DMACINML_00143"
     CDS             complement(146318..147691)
                     /gene="dnaB"
                     /locus_tag="DMACINML_00143"
                     /EC_number="3.6.1.-"
                     /inference="ab initio prediction:Prodigal:2.6"
                     /inference="similar to AA sequence:RefSeq:ABS44015.1"
                     /codon_start=1
                     /transl_table=11
                     /product="replicative DNA helicase"
                     /protein_id="Prokka:DMACINML_00143"
                     /translation="MQQEHFDLDLERAILSSCIMSEDAYSSIAGDIEPKDFSLKAHQD
                     VFKAIIACVNAGEPISISFLKKHKKIDEQILTEIIATPSIIDLPAYVNELREKSIKRQ
                     LLSFAHLLPTRINDNRAVSEISDEIGKEIFNITNRVNTNDIKDIEIVLSELLEEFKKQ
                     KSLENKSVIGLDTGFEDLNTMTKGFKGGELIIIAARPGMGKTTLCLNFIEKVLRQDKG
                     VVMFSLEMPAAQIMQRMLSAKTSIPLQKILTADLNDNEWERIGDACNYYSKKKLFIYD
                     SGYATITDVRAILRRLKAQEESIGLCVIDYIGLMMSNSNFNDRHLQVSEISRGLKLLA
                     RELDMPIIALSQLNRSLEQRANKRPMMSDLRESGAIEQDADTILFVYRDEVYREQDEK
                     ERENKAKAEGKPYQRNFIPNPMQENAEILVGKNRNGPVGVVEVLFLKEKSCFVDKPRF
                     ESVEFQE"
     gene            147836..148765
                     /locus_tag="DMACINML_00144"
     CDS             147836..148765
                     /locus_tag="DMACINML_00144"
                     /inference="ab initio prediction:Prodigal:2.6"
                     /inference="similar to AA sequence:RefSeq:ADT65897.1"
                     /note="conserved hypothetical protein"
                     /codon_start=1
                     /transl_table=11
                     /product="hypothetical protein"
                     /protein_id="Prokka:DMACINML_00144"
                     /translation="MKKTLFSFALASLFVSQASSIEFQSGLSGNLSLGIGARDVKSNI
                     SPLANSNYLGGYNAKNSDTAAIPFIGLELYYGNLIDNDRVFLKNYNGRDLSGIALGYE
                     RAYLERFSTSFSFISSLREKAYANPYFIGNREETDVSKYGFRISQLYESDFGKFNASY
                     VFAKNKLDKESIEFDSLKREGNYHELELSYNYSLLNLGLIYDYNDADGKAQSYSRYGF
                     KIGTHLVFARNYILTPSLSLNQYKADGTDPIFNQKQDGSITKFNLKLVKNQFLGYDSL
                     YGFVNYGLEKRNSDIDFYDETYHILLTGVGYKF"
     sig_peptide     147836..147892
                     /locus_tag="DMACINML_00144"
                     /inference="ab initio prediction:SignalP:4.1"
                     /note="predicted cleavage at residue 19"
```

```
     gene            complement(148788..150119)
                     /locus_tag="DMACINML_00145"
     CDS             complement(148788..150119)
                     /locus_tag="DMACINML_00145"
                     /inference="ab initio prediction:Prodigal:2.6"
                     /inference="similar to AA sequence:RefSeq:CAL34706.1"
                     /codon_start=1
                     /transl_table=11
                     /product="putative MATE family transport protein"
                     /protein_id="Prokka:DMACINML_00145"
                     /translation="MSNIFSTLSPFRLFVKCAVPNVISMAFISFYYIVDGIFVGKYLG
                     NDALAALALIIPFIMISFALADMIAIGSAVQISMHLGLGKKHLARKIFSSSMLIIFLI
                     SCLVGILEYLLSPVLINFLDVSEDIKSMAKECMLVFALFAPFTMCSFALDNYLRICGK
                     TTYSMMMNVIIALSNIVLDYIFIVVLGWGLFSAALATCLGLMLGGIFGIFPFLFQNLE
                     LKFSSLYMNMKIFKNIVYNGSSEFFGNISGSLYGIFANLVLLKISGTQAVAAFSIVSY
                     IDNFIIMLIIAMGDAMQPALSYNYAKKDFSRIKDILKVMFFAGAILSLLSIVMIFVCG
                     KDLVGLFTQEGDQEFIIFAYMALMLFSFNYLFAWFNVLSGSFLTASNKASFSLILSLS
                     QNLFIPLCFLLILSSIMGLKGVWLSPFFAELCVLILACIFVRKVFKKLSFL"
     gene            complement(150345..151280)
                     /locus_tag="DMACINML_00146"
     CDS             complement(150345..151280)
                     /locus_tag="DMACINML_00146"
                     /inference="ab initio prediction:Prodigal:2.6"
                     /inference="similar to AA sequence:RefSeq:CAL34705.1"
                     /codon_start=1
                     /transl_table=11
                     /product="putative pyridine nucleotide-disulfide
                     oxidoreductase"
                     /protein_id="Prokka:DMACINML_00146"
                     /translation="MKKVDLIVVGAGPTGIGCAVEAKLKNKEVLVLEKSNNICQTLMQ
                     FYKDGKRVDMAYKGCEGTNHGHVPFQDGTKESTIETFQNALNEHNIEVEFGSEVESVK
                     NENGVFLVSTGKGVYECKNIIVAIGRMGKPNKPDYKLPGTLTKIINFNANSVLGDEKI
                     LVVGGGNSAAEYAVDLANSNKVSLCYRKKEFTRLNDINLKDIHEAGNSGKVELKLGID
                     IEELEDDNGKAKVKFNDGSSETYDRIIYAIGGSTPLDFLQKCGINVDDKGVPLMDENK
                     QSNVKGIFVAGDIATKNGASIVTGLNDAVKILAVI"
     gene            151351..152583
                     /gene="proA"
                     /locus_tag="DMACINML_00147"
     CDS             151351..152583
                     /gene="proA"
                     /locus_tag="DMACINML_00147"
                     /EC_number="1.2.1.41"
                     /inference="ab initio prediction:Prodigal:2.6"
                     /inference="similar to AA sequence:RefSeq:CAL34704.1"
                     /codon_start=1
                     /transl_table=11
                     /product="gamma-glutamyl phosphate reductase"
                     /protein_id="Prokka:DMACINML_00147"
                     /translation="MRNLLENIKKNSQKLLNLTPKDKENIILKLAHTLRKNFKTILES
                     NEKDITNFTKSGAMRDRLLLDEKRIFSLCDALEKIAYLEDPIGKISKGWVNYAGLKIE
                     KISIPIGLISVIYEARPSLSAEIIALMIKSSNACVLKGGSEAKFTNSAIFDLVYKVLE
                     EFNLQDCFAMFYERNEIMQILAFDDLIDVIIPRGSSNMIQEIASHTKIPLIKQDKGLC
                     HAFVDESANLNMALEIILNAKCQRVSVCNALETLLIHEKIAKDFIELLIPEFEKFKVK
                     IHAHENILEHFKNSKLEISKADESTFDTEWLDFAISVKSVKDCDEAIEHINQHSSLHS
```

```
                     ETIISNNALNINKFQRLLRSSCIYVNASTRFSDGGEFGFGGEVGISTSKLHARGPMGV
                     EDICTYKYLISGEGQIRK"
     gene            152580..153665
                     /locus_tag="DMACINML_00148"
     CDS             152580..153665
                     /locus_tag="DMACINML_00148"
                     /inference="ab initio prediction:Prodigal:2.6"
                     /inference="similar to AA sequence:RefSeq:CAL34703.1"
                     /codon_start=1
                     /transl_table=11
                     /product="putative integral membrane protein"
                     /protein_id="Prokka:DMACINML_00148"
                     /translation="MSKRKILKQQYKEIIQLNPTERVWQMPFFFALAVGFALSIAAYY
                     ERMDLGLIAMIGIMAFVYTTNTPMYHRMAVTMCCSFGLCLSFLIGLCTHFFPAFSPLI
                     IGLVAMASSILIRYYNIGAPGYFFFVFSCLLASFFPFPAKDYIFLVGLICIGGIIANI
                     AAFLYSVSVIYIFKNSPPKPVLQNGNLGFDIIFVDSIIIAFFVGFAVFLGAFLELDRS
                     YWVAVSTTVILQGANLKSVWIKQLQRILGTTVGILFAWWLLKIKFTPLEFVLLMMFLA
                     FIIEFLVVRNYALTVIFLTPYVTYLAEAASFGNLSVDMLIHARLQDIIIGSLLGLLGG
                     FVIHKPYLRKYFEYIAKYIFRVRLANR"
     gene            complement(153776..154345)
                     /gene="efp"
                     /locus_tag="DMACINML_00149"
     CDS             complement(153776..154345)
                     /gene="efp"
                     /locus_tag="DMACINML_00149"
                     /inference="ab initio prediction:Prodigal:2.6"
                     /inference="similar to AA sequence:RefSeq:CAL34697.1"
                     /codon_start=1
                     /transl_table=11
                     /product="elongation factor P"
                     /protein_id="Prokka:DMACINML_00149"
                     /translation="MASYSMGDLKKGLKIEIDGIPFKIVEYQHVKPGKGPAFVRIKIK
                     SFIDGKVLEKTFHAGDKCEAPNLEEKTMQYLYDDGENCQFMDTESYEQVAISDEDVGE
                     AKKWMLDGMMVDVLFHNGKAIGVEVPQVVELKIVETAPNFKGDTQGSNKKPATLETGA
                     VVQIPFHVLEGEVIRVDTVRGEYIERANK"
     gene            complement(154780..155052)
                     /locus_tag="DMACINML_00150"
     CDS             complement(154780..155052)
                     /locus_tag="DMACINML_00150"
                     /inference="ab initio prediction:Prodigal:2.6"
                     /inference="similar to AA sequence:RefSeq:CAL34696.1"
                     /note="hypothetical protein Cj0550"
                     /codon_start=1
                     /transl_table=11
                     /product="hypothetical protein"
                     /protein_id="Prokka:DMACINML_00150"
                     /translation="MKQWLSDFKLALIQEDVNKLEGLLNTLDLKKMLEDLARDFQSDE
                     LKDKLNDNLSQIKALLQEAVVLISEKKNSKACEIQKIQKALKYFKD"
     gene            complement(155036..155419)
                     /gene="fliS"
                     /locus_tag="DMACINML_00151"
     CDS             complement(155036..155419)
                     /gene="fliS"
                     /locus_tag="DMACINML_00151"
```

```
                     /inference="ab initio prediction:Prodigal:2.6"
                     /inference="similar to AA sequence:RefSeq:CAL34695.1"
                     /codon_start=1
                     /transl_table=11
                     /product="flagellar protein"
                     /protein_id="Prokka:DMACINML_00151"
                     /translation="MQNNLAYNAYSQNQAGIESPQKLIEMLYEGILRFCSRAKVAIRN
                     EDIEQRVHFVKRTTAIFIELINTLDYEKGGEVAHYLSGLYTREIQLLSLANLENNEAR
                     IDEVINVTKGLLEAWREVHSNEAMA"
     gene            complement(155429..157378)
                     /locus_tag="DMACINML_00152"
     CDS             complement(155429..157378)
                     /locus_tag="DMACINML_00152"
                     /inference="ab initio prediction:Prodigal:2.6"
                     /inference="similar to AA sequence:RefSeq:ALW55331.1"
                     /codon_start=1
                     /transl_table=11
                     /product="flagellar capping protein"
                     /protein_id="Prokka:DMACINML_00152"
                     /translation="MAFGSLSSLGFGSGVLTQETIDKLKEAEQQARVNPYTTKIEENT
                     TKQKDLTEIKTKLSTFQSAVSSLGDATAFAKRKVVASITDNPPASLTATSGVALQSMN
                     INVTQLAQKDVYQSKGLANDTGFINANLESSTNLTFFSKGKEYTVTIDKNTTYSDLVD
                     KINSATGGEIVAKMVNTGEKDTPYRLTLTSKETGEDSAISFYPGAKDSSGKYAVDENA
                     KSVFESLGWTLDDNALNAEGFDPATSKKGVGIIDDAENPLHIQQAQNANFTMDGIKMT
                     RSSNTITDIGVGITLTLNKTGEINFDIQQDSESITQAMQEMVDAYNDLVLNLNAATDY
                     NSETGTKGSLQGVTEVNSIRSSIVSILFQSQSVDGTVEDASGNKVSAKVMLSLQDYGL
                     SMTESGTLNFDSSAFESKVKEDTAMAESFFSGITQYKNINHTGELIKQGSLENYLNKV
                     EGDDNGISFDSGKFQIVSDFETYDLSKNADGTAFKLSGATEQEMLQNLADHINSKGIE
                     GLTVKVESYNQNGETGFKLNFKTDGNADFAIKGDNDFLKQFGLSQVSIAAEAVEGAGV
                     FAQLKTTLQGITGTDGSLTKYDESLTNDTKALTESKESAQKLIDTRYETMANQWLQYE
                     SILNKLNQQLTTVTNMINAANNSNN"
     gene            complement(157380..157748)
                     /gene="flaG"
                     /locus_tag="DMACINML_00153"
     CDS             complement(157380..157748)
                     /gene="flaG"
                     /locus_tag="DMACINML_00153"
                     /inference="ab initio prediction:Prodigal:2.6"
                     /inference="similar to AA sequence:RefSeq:CAL34693.1"
                     /codon_start=1
                     /transl_table=11
                     /product="flagellar protein"
                     /protein_id="Prokka:DMACINML_00153"
                     /translation="MEISKANGQLDAAFANLAQRASGAHQTNSDIQAGGNQSQGGDDK
                     QQGNLNNKLTDIVRKLNEQMDSLDTNVRFGYSDKIDSMYISVTEKSTGREIRQIPSEE
                     AMRLAEYFRDVIGMIFDKES"
     gene            complement(157910..159715)
                     /gene="ubiD"
                     /locus_tag="DMACINML_00154"
     CDS             complement(157910..159715)
                     /gene="ubiD"
                     /locus_tag="DMACINML_00154"
                     /EC_number="4.1.1.-"
                     /inference="ab initio prediction:Prodigal:2.6"
```

```
                     /inference="similar to AA sequence:RefSeq:CAL34692.1"
                     /codon_start=1
                     /transl_table=11
                     /product="putative 3-octaprenyl-4-hydroxybenzoate
                     carboxy-lyase"
                     /protein_id="Prokka:DMACINML_00154"
                     /translation="MKDFIKILKDNDLLKVYEEPVDIDLEIAHLAYIEAKKGENGKAL
                     LFKNPVDKRNNKQYKFPVLMNAFCNEKALNLAFGRDYKEVADEISKLTKLHIPTSFKA
                     KIDFFMNLLSLKNVPPKRLKADKALYDYELLNSLEELPILKTWEEDAGRFITMGQVYT
                     QNLDKTQNNLGMYRLQVSDKNELLMHWQIHKDGANFYHEYKNAGFKKMPVSIAIGGDP
                     LYIWCSQAPLPKGIFELLLYGFIKKTPAKLTPCENGIFVPYDSDIVIEGYVDLEEFKI
                     EGPFGDHTGFYTPAELFPVMKVEKIYTKKEAIYQATVVGKPPLEDKIMGLGTERIFLP
                     LLQTSVPDLIDYKMPENGVFHNLILAKIDAKYPAHAQQIMHAFWGVGQMSFVKHAIFV
                     DKKAPNLDDYDALIPYILNRFNANKILISEGICDQLDHASPNSCFGGKAGLDACEETI
                     VEELEILEDEKLLELFKTKIELVNLKQFYKESKSPIVCILLDKKEKIEQSFEKLLEFK
                     KHFRILVFLDTNNKLENPYMLVWRVVNNIDAKRDIFIKADRVGIDATAKGLVEGYERV
                     WPKQTDCTKSVIEDLILRNILENNSELFSKFEILG"
     gene            complement(159712..160638)
                     /gene="hemC"
                     /locus_tag="DMACINML_00155"
     CDS             complement(159712..160638)
                     /gene="hemC"
                     /locus_tag="DMACINML_00155"
                     /EC_number="2.5.1.61"
                     /inference="ab initio prediction:Prodigal:2.6"
                     /inference="similar to AA sequence:RefSeq:CAL34691.1"
                     /codon_start=1
                     /transl_table=11
                     /product="porphobilinogen deaminase"
                     /protein_id="Prokka:DMACINML_00155"
                     /translation="MKELVIATRKSQLALWQSEFIANFLSKTHGIAVRLEGFKTKGDV
                     ILDSPLAKIGGKGLFTKELEESMLRNEAHLAVHSLKDVPSFFPQGLVLAAISKREQSN
                     DAMLSQNYKDFLSLPKGARIGTTSLRRKMQLLLLRPDLEIISLRGNVNSRIEKLKNGD
                     FDAIILAMAGIKRLDLDKQVNFVYAFSKDELIPAASQGALGIESIDDEKILEFLKCLN
                     DDNALIETTIEREFIATLEGGCQVPIGINAELLNDEICVRAILGLPDGSEILKEKRMI
                     KKSEFKGFGESLAKEFIAKGAKEILKKAESMI"
     gene            complement(160635..161021)
                     /locus_tag="DMACINML_00156"
     CDS             complement(160635..161021)
                     /locus_tag="DMACINML_00156"
                     /inference="ab initio prediction:Prodigal:2.6"
                     /inference="similar to AA sequence:RefSeq:CAL34690.1"
                     /codon_start=1
                     /transl_table=11
                     /product="putative integral memnbrane protein"
                     /protein_id="Prokka:DMACINML_00156"
                     /translation="MNYKLSLSPLFLLELVASILFIVFFGLGNFLIFILASMIFGVIL
                     LGIFWKNMLEFRISDFKNMLTQFAFIMAAFLFIFPGIVTSIFGFFVLCFGLIFNFAKP
                     RYKRNYRQNNTSDEEIIDVEIIEEKK"
     gene            complement(161018..162724)
                     /gene="proS"
                     /locus_tag="DMACINML_00157"
     CDS             complement(161018..162724)
                     /gene="proS"
```

```
                     /locus_tag="DMACINML_00157"
                     /EC_number="6.1.1.15"
                     /inference="ab initio prediction:Prodigal:2.6"
                     /inference="similar to AA sequence:RefSeq:CAL34689.1"
                     /codon_start=1
                     /transl_table=11
                     /product="prolyl-tRNA synthetase"
                     /protein_id="Prokka:DMACINML_00157"
                     /translation="MKFSKLYAPSLKEAPKDATLPSHIFLTRAGFVEQIGSGLYNFLP
                     LGKKVLDKIRTIVKEEMDKAGAQEVSLSFVTPATLWQESGRYNVFGKELLRFKDRKEN
                     DFVLGPTHEEAMLSLVKNKITSYKQLPLHLYQIGLKFRDEARPRFGLLRCREFLMKDG
                     YSFHANEEDLTREFDLMHKTYSQILQRMGLEFRAVDADSGAIGGSGSKEFMVLAKNGE
                     DDILICENCDYAANVEAAIRANKTCDEERPEANYTSKFHTPDVKTIEALAQFFKINAF
                     YTIKAVVKKAIYENENKLVVFFIRGCDDLQETKAQNACNALELVDASEEELEKAGLVA
                     GFIGFVGLKNVDFYLDKELEGEKQMIMGANEKDYHLIGIDVVNLNKDRFKDLAEVKEN
                     DCCAKCGGKLRLSKGIEVGHIFKLGQKYSKAMNANFLDENGKSKPFYMGCYGIGVSRL
                     LAVAIEANHDEKGCIWNKTLAPFTLEIIVSNIKDEKSLEFATKLYEDLSNVGIEVLLD
                     DRNERFGVKMNDFELMGFPYALVVGKGLEKDELEFIDRKTLEKKILSSKEAFDFIKKS
                     VE"
     gene            complement(162714..164009)
                     /gene="hemA"
                     /locus_tag="DMACINML_00158"
     CDS             complement(162714..164009)
                     /gene="hemA"
                     /locus_tag="DMACINML_00158"
                     /EC_number="1.2.1.70"
                     /inference="ab initio prediction:Prodigal:2.6"
                     /inference="similar to AA sequence:RefSeq:CAL34688.1"
                     /codon_start=1
                     /transl_table=11
                     /product="glutamyl-tRNA reductase"
                     /protein_id="Prokka:DMACINML_00158"
                     /translation="MYYCISFTHKNTDISLRERLSFSDEAKKNEFLRLLSIHENIEEC
                     LVISTCNRVEIVAYVKEACAEYIIKSLALLCNVDKESLVQKADIFEDSGAIHHLFSVA
                     SSLDSLVVGETQIAGQLKDAFAFALKNNFCAVHLSRAIHSAFKCAAKVRNETQISKNP
                     ISVASVAVAKAKELLPLEGKSAIVIGAGEMGELAAKHLIAAGARVIILNRDIEKARIL
                     CERLGVLSECDSLNNLKKYLDEYELFFSATNASSAIITNSLIDEVSHKRYFFDIAVPR
                     DIDVSENEKVSVFAVDDLEVVVRNNLALREQEARMAYGIIGRETAEFFRYLNDLALTP
                     IIKAIRLQAKECANKQLQIALDKGYLKHSDEEEARKLIHQVFKAFLHTPTINLKHLQG
                     KMQSDTVINAMRYIFALENNLEGLNQYACEFNMENNDEI"
     gene            complement(164009..164902)
                     /locus_tag="DMACINML_00159"
     CDS             complement(164009..164902)
                     /locus_tag="DMACINML_00159"
                     /EC_number="2.5.1.-"
                     /inference="ab initio prediction:Prodigal:2.6"
                     /inference="similar to AA sequence:RefSeq:CAL34687.1"
                     /codon_start=1
                     /transl_table=11
                     /product="polyprenyl synthetase"
                     /protein_id="Prokka:DMACINML_00159"
                     /translation="MQPIDDLIKQYLQELDYEPVLTMLANTKSGKKLRSKLLLAIAGE
                     NANSYKICAAIELIHLASLLHDDIIDESKLRRGAKSVNAEFGTKNALMLGDILYSKAF
                     YELSQLDACFASIISDAVVKLAVGELMDVELSKSFNADKDKYLKMIYNKTAVLIEASA
```

```
                     RCGAILAKLDEKAFGEYGKNLGLAFQMIDDILDIKGDEKTLGKPAMNDFKEGKTTLPY
                     IYLCENLNDEDRTQLRNLFQKDLNNDEKTWMQIKFEETKALNKAISEAKEYAQQAALA
                     IKDFSNEQLQGIIQAMIDRDF"
     gene            complement(164919..165449)
                     /locus_tag="DMACINML_00160"
     CDS             complement(164919..165449)
                     /locus_tag="DMACINML_00160"
                     /inference="ab initio prediction:Prodigal:2.6"
                     /inference="similar to AA sequence:RefSeq:CAL34686.1"
                     /codon_start=1
                     /transl_table=11
                     /product="putative exporting protein"
                     /protein_id="Prokka:DMACINML_00160"
                     /translation="MSIRLLVFFALMANCIVLNAAPVFDYTYKFTLKKDERASVEIKE
                     LGYDNGVQNFDFYWTLFDNTNIVVHSKFRKYPRQFVMSLRRNLDWVTQTLIPDYKNPH
                     IDRARLILEFSEYKKGEAIFTVYIEDKDSRLEVKFLDPRKMQLTNPPQNNQVVPMIDF
                     NEPQVKPLIQRENTTN"
     sig_peptide     complement(165387..165449)
                     /locus_tag="DMACINML_00160"
                     /inference="ab initio prediction:SignalP:4.1"
                     /note="predicted cleavage at residue 21"
     gene            complement(165449..165712)
                     /locus_tag="DMACINML_00161"
     CDS             complement(165449..165712)
                     /locus_tag="DMACINML_00161"
                     /inference="ab initio prediction:Prodigal:2.6"
                     /inference="similar to AA sequence:RefSeq:CAL34685.1"
                     /note="hypothetical protein Cj0539"
                     /codon_start=1
                     /transl_table=11
                     /product="hypothetical protein"
                     /protein_id="Prokka:DMACINML_00161"
                     /translation="MDIFDEMFNKTPKEKFIEIIQNGNLGALEKVFENFFADHIAMIE
                     LLEKQGFNEMDVKSFILENGDFIQERQNDLFIELGAKILGHEG"
     gene            complement(165841..166398)
                     /gene="oorC"
                     /locus_tag="DMACINML_00162"
     CDS             complement(165841..166398)
                     /gene="oorC"
                     /locus_tag="DMACINML_00162"
                     /inference="ab initio prediction:Prodigal:2.6"
                     /inference="similar to AA sequence:RefSeq:CAL34684.1"
                     /codon_start=1
                     /transl_table=11
                     /product="OORC subunit of 2-oxoglutarate:acceptor
                     oxidoreductase"
                     /protein_id="Prokka:DMACINML_00162"
                     /translation="MKYQLRFGGEGGQGVITAGEILAEAAIKEGRQAFKASTYTSQVR
                     GGPTKVDIIIDDKEILFPYAVEGEVDFMLSTADKGYKGFRGGVKEGGIIVVEPNLVHP
                     ESEDYKKWQIFEIPIITIAKDEVGNVATQSVVALAIAAYMSKCIDLDILKETMLHMVP
                     AKTRDANSKAFDLGVKYAQETKAHS"
     gene            complement(166395..167240)
                     /gene="oorB"
                     /locus_tag="DMACINML_00163"
```

```
CDS             complement(166395..167240)
                /gene="oorB"
                /locus_tag="DMACINML_00163"
                /inference="ab initio prediction:Prodigal:2.6"
                /inference="similar to AA sequence:RefSeq:CAL34683.1"
                /codon_start=1
                /transl_table=11
                /product="OORB subunit of 2-oxoglutarate:acceptor
                oxidoreductase"
                /protein_id="Prokka:DMACINML_00163"
                /translation="MAFNYDEYLRTDKLPTQWCWGCGDGVVLKCIIRAIEKLGWNMDD
                VCLVSGIGCSGRMSSYVNCNTVHTTHGRAIAYATGIKLANPSKHVIVVSGDGDTLAIG
                GNHTIHGCRRNIDLTHIVINNFIYGLTNSQTSPTTPKGFYTVTAQFGNIDPNFDACEL
                TKAAGASFVARGNVIEANKLENLIYKALAHKGYSFVDVFSNCHINLGRKNKMGEAVAM
                LDWIKNRVVDKSKFESMDFEERKDKFPTGVLYEDSTQPEYCHAYEEVRRAAKEKRMVD
                LGALK"
gene            complement(167242..168366)
                /gene="oorA"
                /locus_tag="DMACINML_00164"
CDS             complement(167242..168366)
                /gene="oorA"
                /locus_tag="DMACINML_00164"
                /inference="ab initio prediction:Prodigal:2.6"
                /inference="similar to AA sequence:RefSeq:CAL34682.1"
                /codon_start=1
                /transl_table=11
                /product="OORA subunit of 2-oxoglutarate:acceptor
                oxidoreductase"
                /protein_id="Prokka:DMACINML_00164"
                /translation="MREVIATGNVLIAKAAIDCGCKFFGGYPITPSSEIAHELSHMLP
                ANDGTFIQMEDEISGVSVTIGAAMSGVKSMTASSGPGISLKAEQIGLAFIAEIPLVIV
                NVMRGGPSTGLPTRVAQGDLFQAKAPTHGDFASVAIAPASLEEAYTETIRAFNLAEKY
                MTPVFLLMDETVGHMNGKAVLPDLKDIEIINRKKFTGDKKDYKPYAAGENEPATLNPF
                FTGYRYHVTGLHHGDIGFPTEDGAIVKKNIERLIGKIKNNQDDICTYEEYMLDDAEFL
                IIAYGSVSRSAKEAINRLREEGIKVGLFRPITLYPVAEKKIAEVVSKFKKVMVSELNM
                GQYLEEIERVSSRRDFISLHRANGRPITPSEIIAKVKENI"
gene            complement(168375..168686)
                /gene="oorD"
                /locus_tag="DMACINML_00165"
CDS             complement(168375..168686)
                /gene="oorD"
                /locus_tag="DMACINML_00165"
                /inference="ab initio prediction:Prodigal:2.6"
                /inference="similar to AA sequence:RefSeq:CAL34681.1"
                /codon_start=1
                /transl_table=11
                /product="OORD subunit of 2-oxoglutarate:acceptor
                oxidoreductase"
                /protein_id="Prokka:DMACINML_00165"
                /translation="MSIAAPKDTPVWVDEHRCKACNICVSYCPAGVLAMRDDVHAVLG
                QMIEVVHPESCIGCTECETHCPDFAIMVAKRDEFKFAKLTPEAKDRAVAVKNNKYKKL
                A"
gene            complement(168701..169570)
                /gene="sucD"
```

```
                     /locus_tag="DMACINML_00166"
     CDS             complement(168701..169570)
                     /gene="sucD"
                     /locus_tag="DMACINML_00166"
                     /EC_number="6.2.1.5"
                     /inference="ab initio prediction:Prodigal:2.6"
                     /inference="similar to AA sequence:RefSeq:CAL34680.1"
                     /codon_start=1
                     /transl_table=11
                     /product="succinyl-coA synthetase alpha chain"
                     /protein_id="Prokka:DMACINML_00166"
                     /translation="MSILVNKNTKVIVQGFTGKEATFHAEQCIAYGTNIVGGVTPHKG
                     GQTHLGKPVFDTVADAVKATGADVSLIFVPAFAVGDSVIEAADAGIKLAVVITEHTPV
                     KDMMFAKQYANKKGMKIIGPNCPGIITSEECKLGIMPGFIFKKGCVGLISKSGTLTYE
                     AANQVVQGGYGISTAVGIGGDPIIGLAYKELLSEFEKDNETKAIVMIGEIGGSLEVEA
                     AKFIKENISKPVVAFIAGATAPKGKRMGHAGAIVGSADESAAAKKEALKSYGIHVVDS
                     PALIGEEIQKILG"
     gene            complement(169580..170743)
                     /gene="sucC"
                     /locus_tag="DMACINML_00167"
     CDS             complement(169580..170743)
                     /gene="sucC"
                     /locus_tag="DMACINML_00167"
                     /EC_number="6.2.1.5"
                     /inference="ab initio prediction:Prodigal:2.6"
                     /inference="similar to AA sequence:RefSeq:CAL34679.1"
                     /codon_start=1
                     /transl_table=11
                     /product="succinyl-coA synthetase beta chain"
                     /protein_id="Prokka:DMACINML_00167"
                     /translation="MNIHEYQAKAIFADNGIPTLKGKVAFSVEEAVENAKELGGSVWA
                     VKAQIHAGGRGLGGGVKIAKNLDEVKAYASQILGMNLVTHQTGPEGKLVQKLYIESGA
                     NIVKEYYLAILFNRMAEQITIIASSEGGMDIEKVAKESPEKIAKVGIDPQIGFKMFHG
                     LEVAKVLGLDKDESKKLISMIAKLYKLYMDKDMNMLEINPLIKTAEGDFYALDAKCSF
                     DDSALYRHPEIAELRDITEENPAEREAAEFGLSYVKLDGDVACMVNGAGLAMATMDII
                     NYSGAKPANFLDVGGGASAETVAKAFEIILRDKNVKVIFINIFGGIVRCDRIANGILE
                     ATKNVEVNIPIVVRLDGTNAAEAKAILDSSNLKNIKAATNLKNGAELVKSLVG"
     gene            complement(170740..171648)
                     /gene="mdh"
                     /locus_tag="DMACINML_00168"
     CDS             complement(170740..171648)
                     /gene="mdh"
                     /locus_tag="DMACINML_00168"
                     /EC_number="1.1.1.37"
                     /inference="ab initio prediction:Prodigal:2.6"
                     /inference="similar to AA sequence:RefSeq:CAL34678.1"
                     /codon_start=1
                     /transl_table=11
                     /product="malate dehydrogenase"
                     /protein_id="Prokka:DMACINML_00168"
                     /translation="MKITIIGAGNVGSSVAYALILRELANEIVLVDINEDLLIAKELE
                     LSQSIAALNLDIELICTKDYSCTKNSDIVLFSAGFARKDGQSREELLQLNANIMLDCA
                     KKIKEFNSDPLFIILTNPVDFLLNTLYESGIFSSKKIVAMAGVLDNARFKYEVAKKLK
                     AKISSVDTRLIGFHNDDMVLVKSYASVKNQKLGELLSEEEFEDLENEVKTGGAKVIKH
```

```
                     LKTSAYLAPASACVRMLESIRSGEFLPMSVILHGEFGVQNKALGTMARLGLEGVVEIM
                     KLELSDEEKDKVKKSLIKYQYIKEDK"
     gene            complement(171645..173849)
                     /gene="icd"
                     /locus_tag="DMACINML_00169"
     CDS             complement(171645..173849)
                     /gene="icd"
                     /locus_tag="DMACINML_00169"
                     /EC_number="1.1.1.42"
                     /inference="ab initio prediction:Prodigal:2.6"
                     /inference="similar to AA sequence:RefSeq:EAQ73167.2"
                     /codon_start=1
                     /transl_table=11
                     /product="isocitrate dehydrogenase, NADP-dependent"
                     /protein_id="Prokka:DMACINML_00169"
                     /translation="MQVTYTLTDESPALATYSFLPIVKAFLSKAHIGVKTSDISLSGR
                     ILATFSEYLKEDQRCEDALELLGELVKKSDANLIKTPNISASIPQLKAAIKELQEKGY
                     MLPNYPDEPKNDEEFQIKTKYQKVLGSAVNPVLRQGNSDRRSTKAVKDYAKNNPYRVV
                     EFNPNSKTRVSYMKEGDFFSNEKAVLIDEDCVASIEFVADSGKSEILKEGLKLEKNEI
                     LDATFMDVEKLQDFYAQEIKASKDNDLLFSLHLKATMMKVSDPILFGYAVKIFFKELF
                     DEFKDEFETLGINPNNGLSELLSKVETSSKKDEILKRYNEILNSRADISMVNSDKGIT
                     NLHVPSDVIVDASMPAMLKNGARLWDKEGKEKDTNAVIPDQTYATIYEAVIEDLHKNG
                     TLNPAKLGSVSNVGLMAKKAQEYGSHDKTFVAKEDGVFKIIAKGKVLLEHKVRKGDIY
                     RANQAKFDAVLNWIDLGIERADLTGAEAIFWLDSKRASNKIMIDLVQNRLKEKNKNIA
                     ILAPYEACLKSLELIRAGKDAISITGNVLRDYLTDLFPILELGTSAKMLSVVPMLNGG
                     AMFETGAGGSAPKQVEQLVEENHLRWDSLGEFLALQASLEFYANKCNNHKAKVLAECL
                     DEAIGEWLENNKAPSRKVKEDDNRTSHFYLAMYFANHLARQANDMELQSFFKDIALEF
                     SSNEEKIRAEFNDAQGVKVDLGGYYKFDDEKANKIMRPSATFNAIIEKIGQR"
     gene            complement(173966..176536)
                     /locus_tag="DMACINML_00170"
     CDS             complement(173966..176536)
                     /locus_tag="DMACINML_00170"
                     /inference="ab initio prediction:Prodigal:2.6"
                     /inference="similar to AA sequence:RefSeq:CAL34676.1"
                     /codon_start=1
                     /transl_table=11
                     /product="putative periplasmic protein"
                     /protein_id="Prokka:DMACINML_00170"
                     /translation="MKKKIVYVLLVLIVFISAIFLVLKNGILISNIQLSFLNLEQLYI
                     KLDKKLIVRAKNITFNEDTNASIQDDKNENSDFASKELLKITKNLKYLYTFVEEIDIQ
                     NLNIKDNHMRILFKNDEFFVDNDLLFLKLALHREGQEINADIKNLWLKDYNLSVDGNL
                     SINARSEFYNFKGQASGDLIDFKVNISYKNQNLAYKFEDINIRDITTIFNQAEKRITL
                     PEPLVLWVAHRAKGDFYHFDFIQGFVDLSKNNYYFDDISAWGYANNVKVRLDNQMNAI
                     NFPKLDLNLSNQKLNFTFNKASYNESDLSESKVFLYDLFDNKKHGIYLRIKSKNLKFD
                     EKLAKALKNYNLNLPFYQKSGKLGSDLELIIDFNEKGDLKYNGTLSLENAELTLANFN
                     VAKAFVKLNQNDLSIENASVKNEFLEANFNAKIDLANHKGIFDTQISRLYFDNGELFD
                     MRNQKTVINLDYKDDLQLSMPEWDLTLNFKEGLEVYANNSSVLIPHSPLLKNFGFMSA
                     KSIYYKSIDFNDFNAQIQDAHFKNNLLVDNKPYENDSFGIVRKNGVLDINTQSGLVSA
                     KIIDNNKTIHLKNLTYTYQKDKNASASSFDIAKNTQNIILNGENLALILADFNKTLKF
                     DKLEANLKSDILDARATRKNANFDLHYSPNDLKLFVNNINDEHLNEFLQKRAVQEGVF
                     NFSVVGSSTDYFEGEFNFKDTFIRDLKGVNQLISFIDTVPSLLMFKTPTFNEKGLSLH
                     DGRVVFNRKKDLLSFEAINLNGDSMDLYGLGSANLRLNTVDVDLELKTLKSASETISK
                     VPILNYVILGKNQEISTNIKVDGALDNPKFHTQILSDTLKTPFNLIKNIIQLPSNLFN
                     "
```

```
gene            176550..177455
                /locus_tag="DMACINML_00171"
CDS             176550..177455
                /locus_tag="DMACINML_00171"
                /inference="ab initio prediction:Prodigal:2.6"
                /inference="similar to AA sequence:RefSeq:EAQ73356.2"
                /note="conserved hypothetical protein"
                /codon_start=1
                /transl_table=11
                /product="hypothetical protein"
                /protein_id="Prokka:DMACINML_00171"
                /translation="MIFILGIFYYLTQPLKSNSVVFLPQGSIAQIITHLKQNKYEMNS
                IDKYILFFLGHPQSGWINIGTKELNRIEFLHKLTIAKAALETLTLIPGETSVIFLEQA
                AQQLELDKNTLLAEFEKQATYKEGVFLPETYKIPKGITEALLIQVLLKHAENTNRKTS
                EKIFGEYNAKKWHQYIITASVIQKEAANESEMPIVASVIYNRIKKGMKLQMDGTLNYG
                IYSHAKITPQRIRQDNSSYNTYKFEGLPKEAVCNVSLSAIRAAIFPAKTDYLYFVRDK
                ATGAHIFSTNLNDHNKAINLQKTNN"
gene            177527..177958
                /gene="flgB"
                /locus_tag="DMACINML_00172"
CDS             177527..177958
                /gene="flgB"
                /locus_tag="DMACINML_00172"
                /inference="ab initio prediction:Prodigal:2.6"
                /inference="similar to AA sequence:RefSeq:CAL34674.1"
                /codon_start=1
                /transl_table=11
                /product="flagellar basal-body rod protein"
                /protein_id="Prokka:DMACINML_00172"
                /translation="MINPFKSKELITSALAGRNLRNQLINANLANVDTPFYKARDIEF
                ETALVNRANEIFKKNDNKELQLAITEEGHQKPWKFPDPSKSTIYLRDGHLARNDANTV
                DLDVETTEMSKNTVMITALDGVLRRQSNIFSSILDASSKLS"
gene            177968..178462
                /gene="flgC"
                /locus_tag="DMACINML_00173"
CDS             177968..178462
                /gene="flgC"
                /locus_tag="DMACINML_00173"
                /inference="ab initio prediction:Prodigal:2.6"
                /inference="similar to AA sequence:RefSeq:CAL34673.1"
                /codon_start=1
                /transl_table=11
                /product="flagellar basal-body rod protein"
                /protein_id="Prokka:DMACINML_00173"
                /translation="MAYLSDFDISGYGLSAQRFRMNVISSNIANANTTRTAEGGPYRR
                REVIFKATDFDKLLNEQINKDNNFLKYENPLNDPSSPEEAKPAIQSVVVDKVVRDDKD
                FRMKYDPSHPDANAQGYVAYPNVNPVIEMADLIEATRAYQANVSAFTSAKTIAQSAID
                LLRG"
gene            178469..178768
                /gene="fliE"
                /locus_tag="DMACINML_00174"
CDS             178469..178768
                /gene="fliE"
                /locus_tag="DMACINML_00174"
```

```
                     /inference="ab initio prediction:Prodigal:2.6"
                     /inference="similar to AA sequence:RefSeq:CAL34672.1"
                     /codon_start=1
                     /transl_table=11
                     /product="flagellar hook-basal body complex protein"
                     /protein_id="Prokka:DMACINML_00174"
                     /translation="MNNINDLRLNNNISNTNKSQNSSDRIGDEFAKMLKNEIEDLDKT
                     QKTAEAAMTDIATGQVKDLHQAAIAITKAESSMKFMLEVRNKAISAYKEITRTQI"
     gene            178774..180579
                     /gene="pbpB"
                     /locus_tag="DMACINML_00175"
     CDS             178774..180579
                     /gene="pbpB"
                     /locus_tag="DMACINML_00175"
                     /inference="ab initio prediction:Prodigal:2.6"
                     /inference="similar to AA sequence:RefSeq:CAL34671.1"
                     /codon_start=1
                     /transl_table=11
                     /product="putative penicillin-binding protein"
                     /protein_id="Prokka:DMACINML_00175"
                     /translation="MQEHKKNRVSKVAFAYCMALFFMIIFLSSTFFLTSKRHIPNTEK
                     DQYSIALRGNIITKDNFTITSSRQIYRAEIDLRSINKDKFDLFLKLFQIYSGISDSEI
                     ADIKKRIQKQKKRSYHFILSQNLDSKQASYLKDLAKKLYIQGFFKAFTNNLGKAETRG
                     LSIIEHEEDRIYMSKDALTPAIGYTKMVLDPQSGILKNVGVKGLEKYYDACLNPLQNE
                     KIQGLKDIGGNIILNLNSLQQKKIDGCLLYLNISLKLQKSIEKAIDGRNEDLKANEII
                     VGVMDSKTGQILALASSRRYDPQNRGKDLSVLNASAIEYGYEAGSVIKPFIFTTALRL
                     GKLKTDEVINTYGGAYKLGRFTIRDDHKMDQMTMEEVIRYSSNIGMIQIAKRLSNLEI
                     IAGLKIFKFGEKSGIDLPYEQKGEIPNSKRLRDIEKSVLSYGYGLKTTFIQLLAAYNV
                     FNNNGVYITPHLAEKFYQDGRFVSLEEDIKKEVILTPQAAQTMQKILIDVIEKGTGKK
                     AITQGIIIGGKTGTARIAERQGYTSNRYNASFFGFANDAKHNYTIGVLVRNPTKPYSY
                     YAAQSALPMFKDVVDILINEDFLTPVIVENNQTLN"
     gene            complement(180572..182374)
                     /locus_tag="DMACINML_00176"
     CDS             complement(180572..182374)
                     /locus_tag="DMACINML_00176"
                     /inference="ab initio prediction:Prodigal:2.6"
                     /inference="similar to AA sequence:RefSeq:EAQ72995.1"
                     /codon_start=1
                     /transl_table=11
                     /product="Na/Pi-cotransporter, putative"
                     /protein_id="Prokka:DMACINML_00176"
                     /translation="MKSLEDRVQEDKNFLKYLKISFCGIFTLILIFALIRFSEFANLL
                     AGVAILLIGMTNLSLGFKSFSGGLLERILTRSTNTKFKSILFGAISTLIMQSSTLVSI
                     ISLSFLSAGLISLTAGVGIIFGANLGNTASSWLIVWLTRVNISILAIPLLVIGVLFFF
                     QKDNIIKSFGNIFIGIGFFFLGVDYIKNGFNEFQDAIDLSYFDFTGFKGVLIFVGLGA
                     LLTGIIQSSTATIAIIVAALLAGQISFENSLAATLGTSVGGVVTAVLASLSTNVEGKK
                     LAFANCIFNFTIAIIIIAIFPYFIDFLNGIAGILNIENLALKMALFHTLFNLLGIFVF
                     VLFIPQLVNFLNKSVKASKDKNKDRPLYLDSNLLKFSDTAIEALRKESEHLYDNAYAI
                     IAHAIGLSRKDIQSNKSFEEILQNKKWFSRNVDLDYLYQTRIKVLFEAIIDFSTKAQI
                     HIDDELKNHRIFTFKIAAKNLTEATKNLKLVQENIKKYSNCNNKDLALEYNKIRSNLG
                     ELLRSIQELRAVDDEKMYLIIKNLQKGKEILKEIDGLTLNNVEHLILVRKITTAEGIS
                     IINDTSFIAKIAKDLIDAVEIIFAREKVLWDDIN"
     gene            complement(182355..182789)
                     /locus_tag="DMACINML_00177"
```

```
     CDS             complement(182355..182789)
                     /locus_tag="DMACINML_00177"
                     /inference="ab initio prediction:Prodigal:2.6"
                     /inference="similar to AA sequence:RefSeq:CAL34667.1"
                     /codon_start=1
                     /transl_table=11
                     /product="putative membrane protein"
                     /protein_id="Prokka:DMACINML_00177"
                     /translation="MIGLYLLIAALSFLLLYFALKKLTLNIDEKALLEPIKSDIYPKF
                     CDIIDERIREFKDRIQNNSSALKDQDRKDELLEKLGDLSRELTFIQTMNLSNKSDSVW
                     QNELFDFLKELENLLLEYLENGEEEAENLRENLMSEFEKLRG"
     gene            complement(182786..183106)
                     /locus_tag="DMACINML_00178"
     CDS             complement(182786..183106)
                     /locus_tag="DMACINML_00178"
                     /inference="ab initio prediction:Prodigal:2.6"
                     /inference="similar to AA sequence:RefSeq:CAL34666.1"
                     /codon_start=1
                     /transl_table=11
                     /product="putative rhodanese-like domain protein"
                     /protein_id="Prokka:DMACINML_00178"
                     /translation="MITNLPASVWMKQDLSEYQIFDVRTPAEWEEGVLPHAECVALYD
                     NHGLLNANFLEEFQTRRDKNKKLAFICRGGHRSMMAANFIKNELGLESVNLDGGMLAL
                     KGWI"
     gene            complement(183103..184932)
                     /gene="htpG"
                     /locus_tag="DMACINML_00179"
     CDS             complement(183103..184932)
                     /gene="htpG"
                     /locus_tag="DMACINML_00179"
                     /inference="ab initio prediction:Prodigal:2.6"
                     /inference="similar to AA sequence:RefSeq:CAL34665.1"
                     /codon_start=1
                     /transl_table=11
                     /product="hsp90 family heat shock protein"
                     /protein_id="Prokka:DMACINML_00179"
                     /translation="MQFQTEVNQLLQLMIHSLYSNKEIFLRELVSNSSDALDKLNFLS
                     VSDDKYKSLKFEPRIDIKIGKDKKTLTISDNGIGMDKDDLINNLGTIAKSGTKSFLEN
                     LSGDAKKDSQLIGQFGVGFYSAFMVASKIEVLSKKALDDKAYLWISDANGYEIEDATK
                     EEQGTSITLYLKDDEFANSYKIESIIEKYSNHIQFPIFMEKEEFIPAKEGEEEGKTEL
                     KISQINKANALWRMQKSSLKAEDYERFYEQNFHDSNKPLLYLHTKSEGKLEYNSLFFI
                     PQNAPFDLYRVDYQSGLKLYVKRVFISDDDKELLPTYLRFVRGIIDVEDLPLNVSREI
                     LQENQILKGVKEASVKKILSELEKLKNKDKEKYLSFFKNFGKVLKEGLYGFGGEKDSL
                     LKLMLYKSTKGESLRSLEEYKNDIQGEQKEIFYIIGSNESLLRNSPLLEEYKQKNIEV
                     LLMDDEIDSLVTPMLGEYEGLKFVAINQVEDKNELSDEEKNEFASLVAKFKELLKDQV
                     EDVRLTSRLKDSPSCIVYDKNKPDFAMQQLLKQMGQEQNFKPILEINPKHAIFTGLKN
                     NETFSADIATLVLNMAKLSEGMGVDNPAEFNASLTKIITKAFS"
     gene            complement(185005..185373)
                     /gene="crcB"
                     /locus_tag="DMACINML_00180"
     CDS             complement(185005..185373)
                     /gene="crcB"
                     /locus_tag="DMACINML_00180"
                     /inference="ab initio prediction:Prodigal:2.6"
```

```
                     /inference="similar to AA sequence:RefSeq:CAL34664.1"
                     /codon_start=1
                     /transl_table=11
                     /product="CrcB protein"
                     /protein_id="Prokka:DMACINML_00180"
                     /translation="MLNTILVVGFGGFIGAVLRMLSINLINKILPHSISFGTLFVNIL
                     GSFMMGLLFSYAHHKGLSPAIKSFVSTGFLGAFTTFSTFSYENLLLLQSGNYFNFSLN
                     ILFNIILCLIAVWLGFIIFK"
     gene            complement(185367..186068)
                     /gene="plsC"
                     /locus_tag="DMACINML_00181"
     CDS             complement(185367..186068)
                     /gene="plsC"
                     /locus_tag="DMACINML_00181"
                     /EC_number="2.3.1.51"
                     /inference="ab initio prediction:Prodigal:2.6"
                     /inference="similar to AA sequence:RefSeq:CAL34663.1"
                     /codon_start=1
                     /transl_table=11
                     /product="putative 1-acyl-SN-glycerol-3-phosphate
                     acyltransferase"
                     /protein_id="Prokka:DMACINML_00181"
                     /translation="MKVVIYKKIKALYFWLFFIISVAFVVLCFCFIKSQDTLWKLRKS
                     WARFQKNILFCKYEVIGEFNLSANMILMNHQSTLDIIALEDLYPKNLCWIAKKELGEI
                     PLFKIAIKKPKLLCIDRKNPRDLVRVLKEAKQRLGEDRVLAIFPEGTRSRNEKLLKFQ
                     SGAKVLSDKLDLKVQPILIVDSAKILDTKSFSASSGVLKIICMDLVDTSDDKWLENTR
                     KKMQELLDQERAKLC"
     gene            complement(186040..187239)
                     /locus_tag="DMACINML_00182"
     CDS             complement(186040..187239)
                     /locus_tag="DMACINML_00182"
                     /inference="ab initio prediction:Prodigal:2.6"
                     /inference="similar to AA sequence:RefSeq:CAL34662.1"
                     /codon_start=1
                     /transl_table=11
                     /product="putative periplasmic protein"
                     /protein_id="Prokka:DMACINML_00182"
                     /translation="MRIILFLVFVCFSLFARSEISVFDGQDENMQKELQKLPKEEQQI
                     YQNIAPSDENSDFDSNIYDPFIPQSSLVLTTDDYVNKVYVGEAFPIILHAKTTEDTNF
                     DFNITLQKSKDLVFLNPDLKWEYIKGDYRAVLWFEAKTSNANLEQISVSLLRNGKVFQ
                     SASISIGAIQFENTPNNKNFSHLVASSLEVKKIKTSYFDDSNIITMIELSATNTNLKS
                     FFVNGVEKQGIENLKGDFNSSNAFYYAIFPSTKTDFEFSYFNKDTKKMENMSFKLKIS
                     DDEVSTQSDLNPTNRDFNIYKQYALWALTLILALWFVWKKNYFILAGALLCFILGFLV
                     DTNTENAILKAGSRAKILPTESSTYFYTANSNEKVEVLGKRLHYVKVLFQDGRIGWVE
                     SSDLQKN"
     sig_peptide     complement(187189..187239)
                     /locus_tag="DMACINML_00182"
                     /inference="ab initio prediction:SignalP:4.1"
                     /note="predicted cleavage at residue 17"
     gene            complement(187236..187880)
                     /gene="purQ"
                     /locus_tag="DMACINML_00183"
     CDS             complement(187236..187880)
                     /gene="purQ"
```

```
                     /locus_tag="DMACINML_00183"
                     /EC_number="6.3.5.3"
                     /inference="ab initio prediction:Prodigal:2.6"
                     /inference="similar to AA sequence:RefSeq:CAL34661.1"
                     /codon_start=1
                     /transl_table=11
                     /product="phosphoribosylformylglycinamidine synthase I"
                     /protein_id="Prokka:DMACINML_00183"
                     /translation="MKVAIIRFPGTNCEFDTQYAFEKIGVKAQVVWHEEKEINADLVV
                     LPGGFSYGDYLRCAAIAKIAPAMQGVIAHAKRGGYILGICNGFQILLESGLLEGAMKH
                     NNNLSFISKNQNLRVVSNNNVFLKNFKKDEIINLPIAHGEGNYYADEATLKKMQDKDL
                     ILLKYEPNPNGSVLDIAGICDENKKIFGLMPHPERACDKILGNDIGLRMLEGFM"
     gene            complement(187880..188125)
                     /gene="purS"
                     /locus_tag="DMACINML_00184"
     CDS             complement(187880..188125)
                     /gene="purS"
                     /locus_tag="DMACINML_00184"
                     /inference="ab initio prediction:Prodigal:2.6"
                     /inference="similar to AA sequence:RefSeq:CAL34660.1"
                     /codon_start=1
                     /transl_table=11
                     /product="phosphoribosylformylglycinamidine (FGAM)
                     synthase"
                     /protein_id="Prokka:DMACINML_00184"
                     /translation="MKIVVNVFLKNGVLDPQGKAIEKALHSLNFNEVKEARVAKQIIL
                     DLDESNEQKAKERVKSMCEELLVNSVIEDYELIVSEK"
     gene            complement(188134..188844)
                     /gene="purC"
                     /locus_tag="DMACINML_00185"
     CDS             complement(188134..188844)
                     /gene="purC"
                     /locus_tag="DMACINML_00185"
                     /EC_number="6.3.2.6"
                     /inference="ab initio prediction:Prodigal:2.6"
                     /inference="similar to AA sequence:RefSeq:CAL34659.1"
                     /codon_start=1
                     /transl_table=11
                     /product="phosphoribosylaminoimidazole-succinocarboxamide
                     synthase"
                     /protein_id="Prokka:DMACINML_00185"
                     /translation="MKKKELLYEGKGKKLYATEDENFLISEFKDDLTAFNAEKRGSES
                     GKGALNCKISTELFHLLEKNGIKTHLVETINDNEQVIKKCKIIPIEVIVRNVATGSLT
                     KRLGIKDGTVLPFVLVEFCLKNDELGDPFINDEHCLILNLVQNEAQIKEIKEMAKKIN
                     AILSPFFDAKNLRLIDFKIELGLTKDNELVLADEISPDSCRFWDKFSNEKLDKDRFRQ
                     DLGNVKMAYEEVLKRILN"
     gene            complement(188847..190169)
                     /locus_tag="DMACINML_00186"
     CDS             complement(188847..190169)
                     /locus_tag="DMACINML_00186"
                     /inference="ab initio prediction:Prodigal:2.6"
                     /inference="similar to AA sequence:RefSeq:CAL34658.1"
                     /codon_start=1
                     /transl_table=11
```

```
                     /product="putative secreted protease"
                     /protein_id="Prokka:DMACINML_00186"
                     /translation="MSSKRFLAAITGFTGSLALCVFLASNLQAKPKNQSDLMQQRLES
                     LDKLTKTLAIVEQYYVDDENITDLIDKSLSGLLSNLDAHSSFLNEKDFNDMKIQTSGE
                     FGGLGITVGMKDGALTVISPIEGTPADKAGIKSGDIILKINDEATLGINLNDAVDKMR
                     GKPKTQITLTIFRKGATKPFDVALTRDIIKVESVYAKLIENENILYLRVTNFDKNVVD
                     MTSKELKKHPDVKGVILDLRNNPGGLLNQATGLVNLFVDKGVIVSQKGRVTSENQEYK
                     ANPKNKISDSSLVVLVNGGSASASEIVSGALQDLKRAIIVGENTFGKGSVQQIIPINE
                     TEALRLTIARYYLPSGRTIQAVGVKPDIEVFPGKVNTQEDSFNIKESDLKQHLESELE
                     KLDKNEKKEDKKEQKNDKNIITQKQINDDAQLKSAIDTIKILNIKQGQ"
     sig_peptide     complement(190080..190169)
                     /locus_tag="DMACINML_00186"
                     /inference="ab initio prediction:SignalP:4.1"
                     /note="predicted cleavage at residue 30"
     gene            190328..190609
                     /locus_tag="DMACINML_00187"
     CDS             190328..190609
                     /locus_tag="DMACINML_00187"
                     /inference="ab initio prediction:Prodigal:2.6"
                     /inference="similar to AA sequence:RefSeq:CAL34657.1"
                     /note="conserved hypothetical protein Cj0510c"
                     /codon_start=1
                     /transl_table=11
                     /product="hypothetical protein"
                     /protein_id="Prokka:DMACINML_00187"
                     /translation="MKHTHTHSPKHIKAISNRLSRTIGHLEAIKKMVERDEDCSDILI
                     QLAAVKAAVNNTAKAILKEHLAHCMYHAVMEKDTQSIEDLNKAIDMFVK"
     gene            190746..193319
                     /gene="clpB"
                     /locus_tag="DMACINML_00188"
     CDS             190746..193319
                     /gene="clpB"
                     /locus_tag="DMACINML_00188"
                     /inference="ab initio prediction:Prodigal:2.6"
                     /inference="similar to AA sequence:RefSeq:ABS44657.1"
                     /codon_start=1
                     /transl_table=11
                     /product="ATP-dependent chaperone protein ClpB"
                     /protein_id="Prokka:DMACINML_00188"
                     /translation="MANIQEFLTDNMLSNLESAASLAIHSKNNEVVPLHLLWALIVDS
                     SSILNQICNKFNISKEALELEIKSKISNLATSSNVNRENMRFSNEFINSLESAKGLMS
                     ANGDNYLSVDTWLISESEKNPIREILAKFIDIKEFQKELQNLRAGRKIESKTSDETLD
                     SLNKFGIDLTAKASEGKLDPVIGREEEIERLMQILIRKTKNNPILLGEPGVGKTAIVE
                     ALAQRIIKKDVPKSLQNKKVIALDMSALIAGAKYRGEFEDRLKAVVNEVIKSENIILF
                     IDEIHTIVGAGASEGSMDAANILKPALARGELHTIGATTLKEYRKYFEKDAALQRRFQ
                     PVNVGEPSVNEALAMLRGIKEKLEIHHNVTINDSALVAAAKLSKRYIADRFLPDKAID
                     LIDEAAAELKMQIESEPSSLRKVRKDIETLEVENEALKMENDEKNQKRLDEISKELAN
                     LKEKQSALNSQFENEKAVFDSISAKKKEIDSLKNEAVFAKNKGEFQKAAELEYGKIPE
                     CEKEVANLEEKWKKMSENGVLLKNQVDEDLVAGILSKWTGISVQKMLTSEKQKFLEVE
                     KHLKESVIGQDKALSALARAIKRNKAGLNADNKPIGSFLFLGPTGVGKTQSAKALAKF
                     LFDDEKAMIRFDMSEFMEKHSVSRLLGAPPGYIGHEEGGELTEAVRRKPYSVLLFDEV
                     EKAHKDVFNVLLGILDDGRATDSKGVTVDFKNTIIILTSNIASSAIMNLSGKEQEDAV
                     KNELKNFFKPEFLNRLDDIITFNPLGKDEAYEIVKLLFKDLQKSLENKGIKASLSENA
                     ALLIAKDGFDPDFGARPLRRAIYDLIEDKLSDMILADKLDENDEIIIDAKNDEIVIEK
```

```
                     V"
     gene            complement(193364..195295)
                     /gene="pbpA_1"
                     /locus_tag="DMACINML_00189"
     CDS             complement(193364..195295)
                     /gene="pbpA_1"
                     /locus_tag="DMACINML_00189"
                     /inference="ab initio prediction:Prodigal:2.6"
                     /inference="similar to AA sequence:RefSeq:CAL34655.1"
                     /codon_start=1
                     /transl_table=11
                     /product="penicillin-binding protein"
                     /protein_id="Prokka:DMACINML_00189"
                     /translation="MKILKYIFSFFVMCFIAAFVYVAYLFTNADTQGYTFKEYKPPLT
                     TQIYDRNGKLVANIFEQHRFYANYEELPPRLIEALVAIEDTSFFEHDGVNIDAIFRAV
                     VKIIKSGGKTMEGASTLTQQFIKNTELTPERTITRKIREALLAYKMETILSKEQILER
                     YLNYIFFGHGYYGVKTAAQGYFHKNLNELSLKEIAMLVGMPKAPSSYDPTKHLDLSIA
                     RANNVVARMYTLGWISKAEYDEAMKEIPQIYDDTLTQNAAPYVVDEVIKQLSPSIKDL
                     KTGGYKITLNIDLDVQEMAQNALKFGYDEIVKRDKDANLSTLNGAMVVVNHQSGDVLA
                     LVGGVDYEKSNYNRATQSTRQPGSSFKPFVYQAAINLGYSPVSEIADISRIFEGGAGD
                     NKDWKPKNDGGKFLGLITLKEALTRSRNLATINLALNMGLDVLHSKLVEFGFRDIPAN
                     LSIVLGSFGISPLEYSKFYTMFGNYGVIKEPQIIRQVQDRNDQIIMEFNSNEYRVSDE
                     AQSFLVLDMMRNVVEHGTGRNARVEGIEIAGKTGTTNKNIDAWFCGLTPEIEALIWYG
                     NDNNKPMRYTEGGARTSAPVFREFLNKYLDKFPDTTRKFSIPNGVYRGSYKGKNAYYT
                     IKSPLPRANMKFNESEILF"
     gene            complement(195292..195843)
                     /gene="maf"
                     /locus_tag="DMACINML_00190"
     CDS             complement(195292..195843)
                     /gene="maf"
                     /locus_tag="DMACINML_00190"
                     /inference="ab initio prediction:Prodigal:2.6"
                     /inference="similar to AA sequence:RefSeq:CAL34654.1"
                     /codon_start=1
                     /transl_table=11
                     /product="Maf-like protein"
                     /protein_id="Prokka:DMACINML_00190"
                     /translation="MLILASSSVSRLNLLKEAKIEFQQISFDYDENLDKNLKPSVYVQ
                     KIVLEKERQFCTAYEEKFKDKTLLFADSIVCIGDKILTKANTKEEAYEMLDLQDGKCA
                     SILSAFLLKNSKKRVFSLSKTTLYFSKFDPHDLKDYVENGLYKGKAGAIMCEGFHKNY
                     IIKQEGNLNTALGLDTQNLKAYL"
     gene            complement(195847..198375)
                     /gene="alaS"
                     /locus_tag="DMACINML_00191"
     CDS             complement(195847..198375)
                     /gene="alaS"
                     /locus_tag="DMACINML_00191"
                     /EC_number="6.1.1.7"
                     /inference="ab initio prediction:Prodigal:2.6"
                     /inference="similar to AA sequence:RefSeq:CAL34653.1"
                     /codon_start=1
                     /transl_table=11
                     /product="alanyl-tRNA synthetase"
                     /protein_id="Prokka:DMACINML_00191"
```

```
                     /translation="MDVRSEYLNFFKSKGHEITPSSPLVPDDATLLFTNAGMVPFKSI
                     FIGEVPRPNPPRKTSCQTCIRAGGKHNDLDNVGYTARHHTFFEMLGNFSFGDYFKEQA
                     IAYAWEFVTEVLKLPKDRLYVTVHESDDEAFELWQKHIQKERIYRFGDKDNFWQMGDT
                     GPCGPCSEIFYDQGEEYFNGEEDYMGGDGDRFLEIWNLVFMQYERSADGKLTPLPKPS
                     IDTGMGLERVTAIKEGKFSNFDSSLFMPIINEIAKLCGKTYVYESGASFRVIADHIRS
                     SVFLLAQGTSFDKEGRGYVLRRILRRALRHGYLLGLKKPFMYNLVDVVCKLMGDHYTY
                     LNEKKDFIKEQIRLEEERFLSTIENGIEIFNEELKNTKEVFSGEVAFKLYDTYGFPLD
                     LTQDMLREKNLVVDEAKFEELMNEQKNRAKASWKGSGDKVASGDFKNLLEKFGENCFV
                     GYEKTECQATVLALLDEDFKEVSSLKGLGWVMLDNTPFYATSGGQIADTGYIGTSEVL
                     DTQKFFNLNLSLVKANEEIKLNDKIIAKIDTDKREQTARHHSATHLLHHALREILGSH
                     ISQAGSLVESNKLRFDFTHHKALSKEELESIEKRVNEMIISSSEAILENMPLEDAKKS
                     GAIALFNEKYQGNVRVLTLGESKELCGGTHVKNTAQIGSFYIVKESGVSAGVRRIEAV
                     VSRAALEFCKTFMNENLNLKEELKSNDLSLGVKKLKNEILKLKNELKNSNKSQLNSSN
                     INGVQICVECVESGDIKTMIDEFKNKFEKAAILLIQAKDDKITLAAGVKNCPLKAGNL
                     VKNIAQILGGNGGGRDDFATAGGKDISKIDEALKQALDLIQKGL"
     gene            198492..199571
                     /locus_tag="DMACINML_00192"
     CDS             198492..199571
                     /locus_tag="DMACINML_00192"
                     /inference="ab initio prediction:Prodigal:2.6"
                     /inference="similar to AA sequence:RefSeq:CAL34652.1"
                     /codon_start=1
                     /transl_table=11
                     /product="putative aminotransferase (degT family).
                     Functional classification-Misc"
                     /protein_id="Prokka:DMACINML_00192"
                     /translation="MNFINLQAQYLKYKEEIDSEIASVLSSSSFIGGAKLNEFETNLA
                     QYTHVQHAIGCSSGTSALYLALRALDIKQGDEVIVPSFTFIATAEMVALLGAKPVFVD
                     INLDNFNIDFELLKNAISPKTKAIIAVSMFGQMSNLQELNTFCESKNISLIEDGAQSF
                     GASFKGEKSCSIAKISCTSFFPSKPLGAYGDGGAIFCNDESIAKKLRILLNHGQTDRY
                     KHEFIGINARLDTLQAAILNAKLKHLDEEINKRQEIAKFYNENLTNCQIPKIDENAIS
                     AYAQYSVLVEDRARVIKAFEKANIPYAIHYPTPLHKQPCFSEFSHLKLEKSEFASEHI
                     LSLPFSAFLSQSEQESVIKIFKEGL"
     gene            199568..200680
                     /locus_tag="DMACINML_00193"
     CDS             199568..200680
                     /locus_tag="DMACINML_00193"
                     /inference="ab initio prediction:Prodigal:2.6"
                     /codon_start=1
                     /transl_table=11
                     /product="hypothetical protein"
                     /protein_id="Prokka:DMACINML_00193"
                     /translation="MKLDQVGSFLRPKILKEARENFALGKIDEEQLRSIEDSCIRDLL
                     EECDKAGIYYLSDGELRRSWWHLDFYWGFGGIKKIKKEKGYIFKGIETRAEGIEIESK
                     IECNNHPFLKDFERLMQIAKGLKIDTNRLKLTIPSPSMLLYMLFIRGGKNTEFNYYGT
                     DFTKLKNDILNAYENFYKEFAALGGVYLQLDDTSFGSLCDYEFCALNEINADDICEEY
                     VDFLNESLKTMPKNIMSAIHICRGNYRSRYVASGGYMKVAKKLFGELRVNKFFLEFDD
                     ERAGDFEPLKYINDQIAVLGILTSKTNESPSVEELKARVKIAAQFLSPKQIEISTQCG
                     FSSTEEGNEIFTHSQWEKIKLLKQVLSELENEGFFS"
     gene            200677..201531
                     /locus_tag="DMACINML_00194"
     CDS             200677..201531
                     /locus_tag="DMACINML_00194"
                     /inference="ab initio prediction:Prodigal:2.6"
```

```
                     /inference="similar to AA sequence:RefSeq:APU82387.1"
                     /codon_start=1
                     /transl_table=11
                     /product="oxidoreductase"
                     /protein_id="Prokka:DMACINML_00194"
                     /translation="MKIGIIGLGKMGQNHLNELGKNKNFKINALFDMVENKNLNAPFF
                     TNLDEFLNQDNDIIIIATPTNSHLEIARKVFCKCKCVLIEKPLALNLNEIDEISNLAK
                     DHDVKVGVGFCERFNPAVLALKKELQNEEIISINIQRFSTYPQRISDVGILQDLAVHD
                     LDLLHFLSGEKIINANILKSFNKDKQREDESIITCKLEKTIACVHQSWNSTQRLRKIT
                     LVSKNHFYEANLADFSLSKDGQSLELMTQTPLFGEHMALYDLFLNKENYLANIQNAYA
                     VQEILEEF"
     gene            201661..202572
                     /gene="hemH"
                     /locus_tag="DMACINML_00195"
     CDS             201661..202572
                     /gene="hemH"
                     /locus_tag="DMACINML_00195"
                     /EC_number="4.99.1.1"
                     /inference="ab initio prediction:Prodigal:2.6"
                     /inference="similar to AA sequence:RefSeq:EAQ73002.1"
                     /codon_start=1
                     /transl_table=11
                     /product="ferrochelatase"
                     /protein_id="Prokka:DMACINML_00195"
                     /translation="MKLVLFLNMGGATNLQDCETFLKNMFNDPYILGIKNKFIRRFVA
                     WIITKSRVKAMRENYKQMGGKSPLNELTQSLCEKLNAKTKDFKFDFVNLYVPPFANEV
                     LQKYTLNENDEIILFPLYPHHSSTTVTSSLEVLQNEISKQNIKAKVKTMDIFYENTLY
                     NEMIISHILAKKSEFDAKTLIFSAHSLPQSIIDKGDLYEKHVNHHVELLKERLKDHFD
                     EIILAYQSKLGPVKWLEPNTSDILANLDNKALIYPISFCIDCSETIFELGIEYKHLAK
                     CDYDLITCPNDSDEFAQFVLKYLSDFN"
     gene            complement(202645..203880)
                     /locus_tag="DMACINML_00196"
     CDS             complement(202645..203880)
                     /locus_tag="DMACINML_00196"
                     /inference="ab initio prediction:Prodigal:2.6"
                     /inference="similar to AA sequence:RefSeq:ADT65846.1"
                     /codon_start=1
                     /transl_table=11
                     /product="ammonium transporter, degenerate"
                     /protein_id="Prokka:DMACINML_00196"
                     /translation="MNAINSSFIVLCTLLVLLMTPALAMFYSGMVRSKNTLNTIMNCF
                     IVFGVITLQWVVLGFSLSFGKDEGLGLVGNFENFLLEGISGYNASGVPNILFVIFQMM
                     FALIASAIITGSLVGRIKLGVLVIFLLFWSTLVYDVLAHMIWSSEGFLLKRGSLDFAG
                     GGVVHISSGVAGLVGALMVGARKSDDEDKNAHSIPYAFLGAILLFIGWLGFNAGSAGE
                     MNDIAINAFIVSIISAACGFLSWVVLEWLIHKKPTILGGLSGLVAGLVGITPACGYVD
                     IYASLVIGALSSVFCYFGLSFIKYKLKWDDSLDAFSLHGIGGIWGGIATGLFASAKVN
                     PNVIAQNALGEGFFISGSLELLKEQIFAIVICVVLSALISFIIFKIISCFTDLRVKEE
                     VEQKGLDVSLHGEKAYTLA"
     gene            complement(204195..205193)
                     /locus_tag="DMACINML_00197"
     CDS             complement(204195..205193)
                     /locus_tag="DMACINML_00197"
                     /inference="ab initio prediction:Prodigal:2.6"
                     /inference="similar to AA sequence:RefSeq:AJK70653.1"
```

```
                     /codon_start=1
                     /transl_table=11
                     /product="tRNA 2-selenouridine synthase"
                     /protein_id="Prokka:DMACINML_00197"
                     /translation="MLNEVDFTEFQKENFSLLIDARSPREFLHSHLIGALNFYALNDE
                     EYQEIGTIYKKNQALAKARGASYICQNMSKHILELTQKFRIGDKIGIYCSRGGLRSKS
                     IAVILSELGYRVVRLKGGFKAYRSFLAHYFDNEINLDFFALCGNTGCGKTELLELLPH
                     HLNLEKMANHLGSSFGDILGAQPTQKAFEASLFHVLNSLKDFIFIESESRKIGEIVLP
                     LKLYEKMQKAFKIHCFCSLEKRIDRIQKIYQEKMTPLKFKECLAKISPYISVNFRQDL
                     LKSYEQREWQRLIALLLAYYDKSYKKPQKIDFELNTDDIFKAKEELLGYFKTQLLF"
     gene            complement(205183..205668)
                     /locus_tag="DMACINML_00198"
     CDS             complement(205183..205668)
                     /locus_tag="DMACINML_00198"
                     /inference="ab initio prediction:Prodigal:2.6"
                     /inference="similar to AA sequence:RefSeq:AJK70652.1"
                     /codon_start=1
                     /transl_table=11
                     /product="HIT family hydrolase"
                     /protein_id="Prokka:DMACINML_00198"
                     /translation="MQYLYAPWRSEYFEKEKSECPFCDCANKIKSDEELGVIFRAKHC
                     FGVMNRYPYSAGHFMIIPYLHEEHIENLSDEVWQEISKFVRLGVRILKSELHAGGVNI
                     GMNLSKDAGAGIAPHCHYHLVPRWAGDTNFITTIGETRVCGTNIEQVYQKLVLAFKNV
                     K"
     gene            complement(205679..206455)
                     /gene="trpC"
                     /locus_tag="DMACINML_00199"
     CDS             complement(205679..206455)
                     /gene="trpC"
                     /locus_tag="DMACINML_00199"
                     /EC_number="4.1.1.48"
                     /inference="ab initio prediction:Prodigal:2.6"
                     /inference="similar to AA sequence:RefSeq:CAL34646.1"
                     /codon_start=1
                     /transl_table=11
                     /product="indole-3-glycerol phosphate synthase"
                     /protein_id="Prokka:DMACINML_00199"
                     /translation="MILDKIFEKTKEDLKERKAQLPYDVLGRSLASNPFFPKDVKAAL
                     KRVENEVRIIAEVKKASPSKGVIRENFDPLDIALKYEKNGAAAISVLTEPHFFQGSLE
                     YLSMIRRYTRAPLLRKDFIFDEYQILEALVYGADFVLLIARMLSMKELKRLLEFTRHL
                     GLEALVEIHDKEDLSKAIFSGADIIGINHRNLDDFSMDMNLCEKLIPQIPNSKIIIAE
                     SGLENKDFLRHLQNLGVDAFLIGEYFMRQNDEGEALKALL"
     gene            complement(206452..207729)
                     /locus_tag="DMACINML_00200"
     CDS             complement(206452..207729)
                     /locus_tag="DMACINML_00200"
                     /inference="ab initio prediction:Prodigal:2.6"
                     /inference="similar to AA sequence:RefSeq:AVL47691.1"
                     /codon_start=1
                     /transl_table=11
                     /product="ATP-dependent nuclease subunit B"
                     /protein_id="Prokka:DMACINML_00200"
                     /translation="MYRNLLFVLIGFFLTACGASKTVIAYPNYEQQKSNEFDLRIMKA
                     YNYEYFNQYEEARDEFLALFRDYNITNFLENAFLLTLANNLDKKEEINNIAKPYLDKS
```

```
                     DNLKRLSALYALNSYNLNEAQKLIEELLKKKSDDPRNLELYGDVLMRQNNLKDAIKYY
                     QLAYNQIQNEEILFKLVGVYAILNDTSSIKNILETSKNINGCTLKTCILLAKIYNDEG
                     NDKALQEIYIELYNITKNQSFILALIELLNAQNKDKEALHFALKYNVDDDVKLYLYQR
                     LKLYGKAKDLSMQIYKHTQEKEYLLRAAIFEFEEANLEKKITPQVITLVSEKFSDAID
                     ENSDALYLNYYGYLLIDYDLDLEKGIKLVHLALKKEPQNLYYLDSLAWGYYKQGDCQK
                     AWEILKQTLEDKEFANSDESKAHAKAIKACIKP"
     gene            complement(207702..208076)
                     /locus_tag="DMACINML_00201"
     CDS             complement(207702..208076)
                     /locus_tag="DMACINML_00201"
                     /inference="ab initio prediction:Prodigal:2.6"
                     /inference="similar to AA sequence:RefSeq:CAL34644.1"
                     /note="conserved hypothetical protein Cj0496"
                     /codon_start=1
                     /transl_table=11
                     /product="hypothetical protein"
                     /protein_id="Prokka:DMACINML_00201"
                     /translation="MIFNKDFSYGFDENACQKCGGKCCTGESGNIFANKEELKALREH
                     LQLDEKEFALKYLKKVGFRMSFKEVEFEDGFACIFFDKEKRNCSIYDFRPMQCRTFPF
                     WEYFKTHQEELKKECIGICYLS"
     gene            complement(208066..208767)
                     /locus_tag="DMACINML_00202"
     CDS             complement(208066..208767)
                     /locus_tag="DMACINML_00202"
                     /inference="ab initio prediction:Prodigal:2.6"
                     /inference="similar to AA sequence:RefSeq:CAL34643.1"
                     /codon_start=1
                     /transl_table=11
                     /product="putative methyltransferase domain protein"
                     /protein_id="Prokka:DMACINML_00202"
                     /translation="MQDNIKLAQLNDGYRYNSDSLLLADFVLEMGVRNQVLEVGSGCG
                     IIGILLKKFVSNLDLSLLDIQQENIELIHRNLKQNNIQAEIFHEDFRNFQNNKKFDFI
                     VCNPPFYRDGAHESKNMHKNISKFQKYLTLDEFIARSNALLKPMGVLYFCYEALALDK
                     ICLALESKKLKLTKIRFVHTHQNTQARLVLVQARKSVKSPCEILPPFFVYKNENLSEQ
                     MQEIHSRFRLESYDI"
     gene            complement(208770..208925)
                     /locus_tag="DMACINML_00203"
     CDS             complement(208770..208925)
                     /locus_tag="DMACINML_00203"
                     /inference="ab initio prediction:Prodigal:2.6"
                     /inference="similar to AA sequence:RefSeq:CAL34642.1"
                     /codon_start=1
                     /transl_table=11
                     /product="putative exporting protein"
                     /protein_id="Prokka:DMACINML_00203"
                     /translation="MLKFFFVISLLSSLLFAQNFIDFASQNNVLKQEQNTSKENAPLK
                     RVKIPTH"
     sig_peptide     complement(208872..208925)
                     /locus_tag="DMACINML_00203"
                     /inference="ab initio prediction:SignalP:4.1"
                     /note="predicted cleavage at residue 18"
     gene            complement(209003..209079)
                     /locus_tag="DMACINML_00204"
     tRNA            complement(209003..209079)
```

```
                     /locus_tag="DMACINML_00204"
                     /product="tRNA-Arg"
                     /inference="COORDINATES: profile:Aragorn:1.2"
                     /note="tRNA-Arg(cct)"
     gene            complement(209107..211182)
                     /gene="fusA"
                     /locus_tag="DMACINML_00205"
     CDS             complement(209107..211182)
                     /gene="fusA"
                     /locus_tag="DMACINML_00205"
                     /inference="ab initio prediction:Prodigal:2.6"
                     /inference="similar to AA sequence:RefSeq:CAL34641.1"
                     /codon_start=1
                     /transl_table=11
                     /product="elongation factor G"
                     /protein_id="Prokka:DMACINML_00205"
                     /translation="MSRSTPLKRVRNIGIAAHIDAGKTTTSERILFFTGMSHKIGEVH
                     DGAATMDWMEQEKERGITITSAATTCFWKDHQINLIDTPGHVDFTIEVERSMRVLDGA
                     VAVFCSVGGVQPQSETVWRQANKYGVPRIVFVNKMDRIGANFYNVEDQIRNRLKANPV
                     PLQIPIGAEDNFKGVIDLVTMKALVWEDDTKPTDYVEKEIPAELKEKAEEYRTKMIEA
                     VSETSDELMEKYLGGEELSLEEIKTGIKAGCLSLSIVPMLCGTAFKNKGVQPLLDAVV
                     AYLPAPDEVANIKGEYEDGTEVSVKSTDDGEFAGLAFKIMTDPFVGQLTFVRVYRGCL
                     ESGSYAYNSTKDKKERIGRLLKMHSNKREEIKVLYAGEIGAVVGLKDTLTGDTLASEK
                     DKVILERMDFPDPVISVAVEPKTKADQEKMSIALNKLAQEDPSFRVSTDEESGQTIIS
                     GMGELHLEIIVDRMLREFKVEAEVGQPQVAYRETIRKTVEQEYKYAKQSGGRGQYGHV
                     FLRLEPLEPGSGYEFVNDIKGGVIPKEYIPAVDKGVQEALQNGVLAGYPVEDVKVTVY
                     DGSYHEVDSSEMAFKLAASMGFKEGARKAGAVILEPMMKVEVETPEDYMGDVIGDLNK
                     RRGQVNSMDERGGNKIITAFCPLAEMFGYSTDLRSQTQGRATYSMEFDHYDEVPKNVA
                     EEIIKKRNG"
     gene            complement(211195..211665)
                     /gene="rpsG"
                     /locus_tag="DMACINML_00206"
     CDS             complement(211195..211665)
                     /gene="rpsG"
                     /locus_tag="DMACINML_00206"
                     /inference="ab initio prediction:Prodigal:2.6"
                     /inference="similar to AA sequence:RefSeq:CAL34640.1"
                     /codon_start=1
                     /transl_table=11
                     /product="30S ribosomal protein S7"
                     /protein_id="Prokka:DMACINML_00206"
                     /translation="MRRRKAPVREVLPDPIYGNKVITKFINSLMYDGKKSTATTIMYG
                     ALELIDKKGGEKKGIDIFNDAIENIKPLLEVKSRRVGGATYQVPVEVRPARQQALAIR
                     WIISFARKRSERTMIDKLAAELLDAANSKGASFKKKEDTYKMAEANKAFAHYRW"
     gene            complement(211735..212118)
                     /gene="rpsL"
                     /locus_tag="DMACINML_00207"
     CDS             complement(211735..212118)
                     /gene="rpsL"
                     /locus_tag="DMACINML_00207"
                     /inference="ab initio prediction:Prodigal:2.6"
                     /inference="similar to AA sequence:RefSeq:CAL34639.1"
                     /codon_start=1
                     /transl_table=11
```

```
                     /product="30S ribosomal protein S12"
                     /protein_id="Prokka:DMACINML_00207"
                     /translation="MPTINQLVRKERKKVLEKSKSPALKNCPQRRGVCTRVYTTTPKK
                     PNSALRKVAKVRLTSGFEVISYIGGEGHNLQEHSIVLVRGGRVKDLPGVKYHIVRGAL
                     DTAGVAKRTVSRSKYGAKRPKASAK"
     gene            complement(212418..216968)
                     /gene="rpoC"
                     /locus_tag="DMACINML_00208"
     CDS             complement(212418..216968)
                     /gene="rpoC"
                     /locus_tag="DMACINML_00208"
                     /EC_number="2.7.7.6"
                     /inference="ab initio prediction:Prodigal:2.6"
                     /inference="similar to AA sequence:RefSeq:CAL34627.1"
                     /codon_start=1
                     /transl_table=11
                     /product="DNA-directed RNA polymerase beta' chain"
                     /protein_id="Prokka:DMACINML_00208"
                     /translation="MSKFKVIEIKEDARPRDFEAFQLRLASPEKIKSWSYGEVKKPET
                     INYRTLKPERDGLFCAKIFGPIRDYECLCGKYKKMRFKGVKCEKCGVEVANSKVRRSR
                     MGHIELVTPVAHIWYVNSLPSRIGTLLGVKMKDLERVLYYEAYIVENAGEAYYDNEST
                     KKVEYCDVLNEEQYQNLMQRYENSGFKARMGGEVVRDLLANLDLVALLNQLKEEMTAT
                     NSEAKKKTIIKRLKVVENFLNSNLNANTNNEDAVPNRPEWMMITNLPVLPPDLRPLVA
                     LDGGKFAVSDVNDLYRRVINRNTRLKKLMELDAPEIIIRNEKRMLQEAVDALFDNGRR
                     ANAVKGANKRPLKSLSEIIKGKQGRFRQNLLGKRVDFSGRSVIVVGPKLRMDQCGLPK
                     KMALELFKPHLLAKLEEKGYATTVKQAKKMIENKTNEVWECLEEVVKGHPVMLNRAPT
                     LHKLSIQAFHPVLVEGKAIQLHPLVCAAFNADFDGDQMAVHVPLSQEAIAECKVLMLS
                     SMNILLPASGKSVTVPSQDMVLGIYYLSLEKMGAKGSHKICTGIDEVMMALESKCLDI
                     HASIQTMVDGRKITTTAGRLIIKSILPDFVTENMWNKVLKKKDIAALVDYVYKQGGLQ
                     ITASFLDKLKNLGFEYATKAGVSISIADIIVPSDKQKAIDEAKKQVREIQNSYNLGLI
                     TSGERYNKIIDIWKTTNNTLSRDMMKLVEKDKEGFNSIYMMADSGARGSAAQISQLAA
                     MRGLMTKPDGSIIETPIISNFREGLNVLEYFISTHGARKGLADTALKTANAGYLTRKL
                     IDVAQNVKITVEDCGTHEGVEINEITADSSIIETLEERILGRVIAEDVIDPITNSVLF
                     AEGTLIDEEKARILGESGVKSVNIRTPITCKAKKGICAKCYGINLGEGKLVKPGEAVG
                     IISAQSIGEPGTQLTLRTFHSGGTASTDLQDRQVSAQKEGFIRFYNLKTYKNKEGKDI
                     VANRRNAAILLVEPKIKAPFKGIVNIENIHEDVIISIKDKKQEAKYILRKYDLAKPNE
                     LAGVSGSIDGKLYLPYQSGAEVEENESIVEMIKEGWNVPNRIPFASEILVKDGDPVVQ
                     NIKAGEKGILKFYILKGDGLDRVRNIKKGDIVKEKGFFVVVADENDREAKRHYIPRES
                     RIEFNDSEEVNFDSIIASAPKQEKSIIAEWDAYNNTIIAEIDGVVSFEDIEAGYSADE
                     QIDEATGKRSLVINEYLPSGVRPTLIIAGKGDKAVRYQLEPKTVIFVHDGDKISQADI
                     LAKTPKAAAKSKDITGGLPRVSELFEARKPKNAAVIAEIDGVVRFDKPLRSKERLIIQ
                     AEDGTSAEYLIDKSKHIQVREGEFIHAGEKLTDGVVSSHDVLKILGEKALHYYLISEI
                     QQVYRGQGVVISDKHIEVIVSQMLRQVKIIDSGHTKFIEGDLVSRRKFREENERILRL
                     GGEPAIAEPVLLGVTRAAIGSDSVISAASFQETTKVLTEASIAGKFDYLEDLKENVIL
                     GRMIPVGTGLYGEQNLKLKEQE"
     gene            complement(216961..221088)
                     /gene="rpoB"
                     /locus_tag="DMACINML_00209"
     CDS             complement(216961..221088)
                     /gene="rpoB"
                     /locus_tag="DMACINML_00209"
                     /EC_number="2.7.7.6"
                     /inference="ab initio prediction:Prodigal:2.6"
                     /inference="similar to AA sequence:RefSeq:CAL34626.1"
```

```
                     /codon_start=1
                     /transl_table=11
                     /product="DNA-directed RNA polymerase beta chain"
                     /protein_id="Prokka:DMACINML_00209"
                     /translation="MLDNKLRNRLRVDFSNISKQIEIPNLLQLQKASFDYFLNLENGE
                     SGIEKVFKSIFPIHDPQNRLSLEYVSSEIGKPKYTIRECMERGLTYSVNLKMKIRLTL
                     HEKDEKTGEKVGIKDIKEQEIYIREIPLMTDRVSFIINGVERVVVNQLHRSPGVIFKE
                     EESSTVVNKLVYTAQIIPDRGSWLYFEYDAKDVLYVRINKRRKVPVTMLFRALGYKKQ
                     DIIKLFYPIQTIHVKKDKFLTEFNPNDFMDRIEYDIKDEKGKIIHQAGKRLTKKKAEQ
                     LIKDGLKWIEYPVEILLNRYLANPIIDKESGEVLFDSLTLLDESKLAKIKEQKSFEIA
                     NDLANGVDAAIINSFAQDNETLKLLKQSENIDDENDLAAIRIYKVMRPGEPVVKDAAK
                     AFVNDLFFNPERYDLTKVGRMKMNHKLGLEVPEYVTVLTNEDIIKTAKYLIKVKNGKG
                     HIDDRDHLGNRRIRSIGELLANELHLGLAKMQKAIKDKFTSLNADIDKVMPYDLINPK
                     MITTTIIEFFTGGQLSQFMDQTNPLSEVTHKRRLSALGEGGLVKERAGFEVRDVHATH
                     YGRICPVETPEGQNIGLINTLSTYAKVNELGFVEAPYRKVENGRVTDQVVYLTATQEE
                     GLFIAPASTKVDAKGNIIEEFVEARQDGETILARREEVQLIDLCSGMVVGVAASLIPF
                     LEHDDANRALMGSNMQRQAVPLITSSAPIVGTGMENIIARDAWEAIKAKRGGVVEKVD
                     NKSIFILGEDEKGPFIDHYIMEKNLRTNQNTNFMQHPIVKKGDVVKAGQIIADGPSMD
                     QGELAIGKNALIAFMPWNGYNYEDAIVVSERIIREDTFTSVHIYEKEIEARELKDGIE
                     EITKDIPNVKEEDVAHLDESGIAKIGTHIKPGMILVGKVSPKGEVKPTPEERLLRAIF
                     GEKAGHVVNKSLYATASLEGVVVDVKIFTKKGYEKDDRALKSYDKEKMSLEKEHHDRL
                     LMMDREEMLRVCALLSKSALMSDQKIGDKNYTKGQKASLEDLEKINRFTLTTLIKAYS
                     KEVQKEYEELKNHFQNEKKKLKAEHDEKLEILEKDDILPSGVIKLVKVYIATKRKLKV
                     GDKMAGRHGNKGIVSTIVPEVDMPYLPNGKSVDIALNPLGVPSRMNIGQILESHLGLV
                     GLRLGDQIQEIFDRKQKDFIKELRAKMLEICSIPRLANEKEFIKNLSDEELLNYARDW
                     SKGVKFATPVFEGVNIEEFAKLFEIAKIDMDGKTELYDGRTGEKIAERVHVGCMYMLK
                     LHHLVDEKVHARSTGPYSLVTQQPVGGKALFGGQRFGEMEVWALEAYGAAHTLREMLT
                     IKSDDVEGRFSAYKALTKGENVPATGIPETFFVLTNELKSLALDVEIFDKDEDNE"
     gene            complement(221207..221584)
                     /gene="rplL"
                     /locus_tag="DMACINML_00210"
     CDS             complement(221207..221584)
                     /gene="rplL"
                     /locus_tag="DMACINML_00210"
                     /inference="ab initio prediction:Prodigal:2.6"
                     /inference="similar to AA sequence:RefSeq:CAL34625.1"
                     /codon_start=1
                     /transl_table=11
                     /product="50S ribosomal protein L7/L12"
                     /protein_id="Prokka:DMACINML_00210"
                     /translation="MAISKEDVLEFISNLSVLELSELVKEFEEKFGVSAAPVMVAGGA
                     AAGGAAAAAEEKTEFDIVLVDGGAKKIEVIKIVRALTGLGLKEAKDAVEQTPSTLKEG
                     VAKADAEEAKKQLEEAGAKVELK"
     gene            complement(221601..222080)
                     /gene="rplJ"
                     /locus_tag="DMACINML_00211"
     CDS             complement(221601..222080)
                     /gene="rplJ"
                     /locus_tag="DMACINML_00211"
                     /inference="ab initio prediction:Prodigal:2.6"
                     /inference="similar to AA sequence:RefSeq:CAL34624.1"
                     /codon_start=1
                     /transl_table=11
                     /product="50S ribosomal protein L10"
```

```
                     /protein_id="Prokka:DMACINML_00211"
                     /translation="MTRSEKVEIIAKLQEEFKISEAIVVCNYKGLSTKKLEELRNNAR
                     ENNVKVQIIKNTLASIALNNSGKTGLVLKDTNIYLWGEDQLSVSKVAAKFEENNDKFE
                     IKTAHIEGEVADVAKVRALAKMPSRNELLAMLLQVWNAPITNFTIGLNALKNKKESE"
     gene            complement(222230..222931)
                     /gene="rplA"
                     /locus_tag="DMACINML_00212"
     CDS             complement(222230..222931)
                     /gene="rplA"
                     /locus_tag="DMACINML_00212"
                     /inference="ab initio prediction:Prodigal:2.6"
                     /inference="similar to AA sequence:RefSeq:CAL34623.1"
                     /codon_start=1
                     /transl_table=11
                     /product="50S ribosomal protein L1"
                     /protein_id="Prokka:DMACINML_00212"
                     /translation="MAKITKRLKELSQKIDVSKEYALNEAIETVRNLKSAKFDETVEI
                     ALKLNVDPRHADQMVRGSVVLPAGTGKKVRVAVIAKDAKADEAKNAGADIVGSDDLIE
                     EIQKGNMNFDVLIATPNLMGLVGKVGRILGPKGLMPNPKTGTVTMDVAQAVNNAKSGQ
                     VNFRVDKQGNIHAGLGKVSFSKEQLWDNISTFVKAINKHKPAAAKGRYIKSAALSLTM
                     SPSVKLETQELLDMK"
     gene            complement(222987..223412)
                     /gene="rplK"
                     /locus_tag="DMACINML_00213"
     CDS             complement(222987..223412)
                     /gene="rplK"
                     /locus_tag="DMACINML_00213"
                     /inference="ab initio prediction:Prodigal:2.6"
                     /inference="similar to AA sequence:RefSeq:CAL34622.1"
                     /codon_start=1
                     /transl_table=11
                     /product="50S ribosomal protein L11"
                     /protein_id="Prokka:DMACINML_00213"
                     /translation="MAKKVVGEIKLQIAATKANPSPPVGPALGQQGVNIMEFCKAFNE
                     RTKDMAGFNIPVVITVYADKSFTFITKQPPATDLIKKAAGISKGTDNPLKNKVGKLTR
                     AQVLEIVDKKIADLNTKDRDQAAKIIAGSARSMGVEIVD"
     gene            complement(223439..223972)
                     /gene="nusG"
                     /locus_tag="DMACINML_00214"
     CDS             complement(223439..223972)
                     /gene="nusG"
                     /locus_tag="DMACINML_00214"
                     /inference="ab initio prediction:Prodigal:2.6"
                     /inference="similar to AA sequence:RefSeq:CAL34621.1"
                     /codon_start=1
                     /transl_table=11
                     /product="transcription antitermination protein"
                     /protein_id="Prokka:DMACINML_00214"
                     /translation="MSTHKWYAIQTYAGSEMAVKRAIENLVKDNGIEEQLKEIVVPTE
                     DVIEFKNGKEKISERSLYSGYVFAHLDLNTELWHRIQSLPKVGRFIGESKKPTPLTEK
                     DINLILEKVHNRAAPKPKISFEEGENVRITEGPFANFTAIVEEYDMVRGLLKLNVSIF
                     GRSTPVEILYSQVEKII"
     gene            complement(223985..224164)
                     /gene="secE"
```

```
                     /locus_tag="DMACINML_00215"
     CDS             complement(223985..224164)
                     /gene="secE"
                     /locus_tag="DMACINML_00215"
                     /inference="ab initio prediction:Prodigal:2.6"
                     /inference="similar to AA sequence:RefSeq:CAL34620.1"
                     /codon_start=1
                     /transl_table=11
                     /product="preprotein translocase SecE subunit"
                     /protein_id="Prokka:DMACINML_00215"
                     /translation="MEKLITYFKLSKAELRKVIFPLKEQVRNAYITVFVVVTVISLFL
                     ALVDWIMSSIVSAIV"
     gene            complement(224183..224258)
                     /locus_tag="DMACINML_00216"
     tRNA            complement(224183..224258)
                     /locus_tag="DMACINML_00216"
                     /product="tRNA-Trp"
                     /inference="COORDINATES: profile:Aragorn:1.2"
                     /note="tRNA-Trp(cca)"
     gene            complement(224270..224428)
                     /gene="rpmG"
                     /locus_tag="DMACINML_00217"
     CDS             complement(224270..224428)
                     /gene="rpmG"
                     /locus_tag="DMACINML_00217"
                     /inference="ab initio prediction:Prodigal:2.6"
                     /inference="similar to AA sequence:RefSeq:CAL34619.1"
                     /codon_start=1
                     /transl_table=11
                     /product="50S ribosomal protein L33"
                     /protein_id="Prokka:DMACINML_00217"
                     /translation="MRIKVGLKCEECGDINYSTYKNSKNTTEKLELKKYCPRLKKHTL
                     HKEVKLKS"
     gene            complement(224481..225680)
                     /gene="tuf"
                     /locus_tag="DMACINML_00218"
     CDS             complement(224481..225680)
                     /gene="tuf"
                     /locus_tag="DMACINML_00218"
                     /inference="ab initio prediction:Prodigal:2.6"
                     /inference="similar to AA sequence:RefSeq:CAL34618.1"
                     /codon_start=1
                     /transl_table=11
                     /product="elongation factor TU"
                     /protein_id="Prokka:DMACINML_00218"
                     /translation="MAKEKFSRNKPHVNIGTIGHVDHGKTTLTAAISAVLSRRGLAEL
                     KDYDNIDNAPEEKERGITIATSHIEYETDNRHYAHVDCPGHADYVKNMITGAAQMDGA
                     ILVVSAADGPMPQTREHILLSRQVGVPYIVVFMNKADMVDDAELLELVEMEIRELLSS
                     YDFPGDDTPIISGSALKALEEAKAGQDGEWSAKIMDLMAAVDSYIPTPTRDTEKDFLM
                     PIEDVFSISGRGTVVTGRIEKGIVKVGDTIEIVGIKDTQTTTVTGVEMFRKEMDQGEA
                     GDNVGVLLRGTKKEEVIRGMVLAKPKSITPHTDFEAEVYILNKDEGGRHTPFFNNYRP
                     QFYVRTTDVTGSIKLADGVEMVMPGENVRITVSLIAPVALEEGTRFAIREGGKTVGSG
                     VVSKIIK"
     gene            complement(225741..225815)
```

```
                     /locus_tag="DMACINML_00219"
     tRNA            complement(225741..225815)
                     /locus_tag="DMACINML_00219"
                     /product="tRNA-Thr"
                     /inference="COORDINATES: profile:Aragorn:1.2"
                     /note="tRNA-Thr(ggt)"
     gene            complement(225910..225986)
                     /locus_tag="DMACINML_00220"
     tRNA            complement(225910..225986)
                     /locus_tag="DMACINML_00220"
                     /product="tRNA-Gly"
                     /inference="COORDINATES: profile:Aragorn:1.2"
                     /note="tRNA-Gly(tcc)"
     gene            complement(225993..226078)
                     /locus_tag="DMACINML_00221"
     tRNA            complement(225993..226078)
                     /locus_tag="DMACINML_00221"
                     /product="tRNA-Tyr"
                     /inference="COORDINATES: profile:Aragorn:1.2"
                     /note="tRNA-Tyr(gta)"
     gene            complement(226140..226215)
                     /locus_tag="DMACINML_00222"
     tRNA            complement(226140..226215)
                     /locus_tag="DMACINML_00222"
                     /product="tRNA-Thr"
                     /inference="COORDINATES: profile:Aragorn:1.2"
                     /note="tRNA-Thr(tgt)"
     gene            complement(226349..227086)
                     /locus_tag="DMACINML_00223"
     CDS             complement(226349..227086)
                     /locus_tag="DMACINML_00223"
                     /inference="ab initio prediction:Prodigal:2.6"
                     /inference="similar to AA sequence:RefSeq:CAL34617.1"
                     /codon_start=1
                     /transl_table=11
                     /product="amino-acid ABC transporter ATP-binding protein"
                     /protein_id="Prokka:DMACINML_00223"
                     /translation="MSILKIKNLQKYYGSHHALKNINLEVKQKEVVVILGPSGCGKST
                     LLRCINGLEEIADGIIEIGQDKIDKNFKDWTRVRQKVGMVFQSYELFEHLSVKDNILL
                     GPLKVQKRAKEEVLQEAKIWLEKVGLAHKIDAYPRELSGGQKQRIAIVRSLCMNPELM
                     LFDEVTAALDPEIVREVLEVILNLAKDGMTMLIVTHEMGFARAVADRIIFMDNGEIIE
                     ENTPDDFFKNPKSERAKKFLNLFDYHK"
     gene            complement(227088..227744)
                     /locus_tag="DMACINML_00224"
     CDS             complement(227088..227744)
                     /locus_tag="DMACINML_00224"
                     /inference="ab initio prediction:Prodigal:2.6"
                     /inference="similar to AA sequence:RefSeq:CAL34616.1"
                     /codon_start=1
                     /transl_table=11
                     /product="amino-acid ABC transporter integral membrane
                     protein"
                     /protein_id="Prokka:DMACINML_00224"
                     /translation="MFDLLNPDTLSRLWKGLYITLEISIVSIIITSFGGLFLGILMSL
```

```
                     KNRYVYILCRFALEFVRVMPLLVWLFMVYFGLSRWLGINLSSVSSAIIVFSIWGSFEM
                     MDLVRTSLKSIPKHQYESAASLGLNKTQSYFYVIIPQAMRRLTPMSMNLLTRMIKSTT
                     FAYLIGAVELVKVGQQIIEFNNKNDLAPFIIYGLIFFIFFIICYPVTLYSRKLEKKWS
                     "
gene                 complement(227737..228438)
                     /locus_tag="DMACINML_00225"
CDS                  complement(227737..228438)
                     /locus_tag="DMACINML_00225"
                     /inference="ab initio prediction:Prodigal:2.6"
                     /inference="similar to AA sequence:RefSeq:CAL34615.1"
                     /codon_start=1
                     /transl_table=11
                     /product="amino-acid ABC transporter integral membrane
                     protein"
                     /protein_id="Prokka:DMACINML_00225"
                     /translation="MDFDFILANSNAFVAAAWLTLKLSFFGIIFSIIIGFFCILMSYF
                     KITIMEAFCKVYIEFSRNTPLLIQLFFLYYALPKFNIHLEQIPPLNLICLSVDEALRP
                     AFACAIVGLSFLGGSYMAESFRAGFEAIKKQQFEAGLSLGFTKLNNLRYVILPQALGV
                     CLPGISANIVFLIKETSVVSIIALPDLVTVMKGLNSLTYKTDELLLLLFLGYLCIILP
                     ISLFLFFLEKRFSNV"
gene                 complement(228449..229045)
                     /locus_tag="DMACINML_00226"
CDS                  complement(228449..229045)
                     /locus_tag="DMACINML_00226"
                     /inference="ab initio prediction:Prodigal:2.6"
                     /inference="similar to AA sequence:RefSeq:EAQ73447.2"
                     /codon_start=1
                     /transl_table=11
                     /product="transcriptional regulator, putative"
                     /protein_id="Prokka:DMACINML_00226"
                     /translation="MQEYLDFLKGIGKIRKFEKNNILFFEGEKALKFFILLKGRVRVY
                     KSTAGEKEITLHYFTPPNFIAEMPTFKRLRYPANAVCEEYCEILEIDFEDFEALCTQN
                     KEFNFLLISSLFEKIKILEKKLLENSLDLKSKLARYLLENEKNLAQISQRQIAVDLSV
                     RAESLSRILKEFKNSELIETKKGKIEILDKEGLKKGLW"
gene                 229127..229510
                     /gene="ctb"
                     /locus_tag="DMACINML_00227"
CDS                  229127..229510
                     /gene="ctb"
                     /locus_tag="DMACINML_00227"
                     /inference="ab initio prediction:Prodigal:2.6"
                     /inference="similar to AA sequence:RefSeq:CAL34613.1"
                     /codon_start=1
                     /transl_table=11
                     /product="group III truncated hemoglobin"
                     /protein_id="Prokka:DMACINML_00227"
                     /translation="MKYETINKESIAKLMELFYDKIRKDKDLGPIFNNAIGTSDEAWK
                     EHKAKIGNFWAGMLLGEGDYIGQPLKKHLDLPPFPQEFFGIWLGLFEESLDKIYNEEM
                     KNVILQRAQMIASHFQNMLYRFGGH"
gene                 complement(229513..231327)
                     /gene="recG"
                     /locus_tag="DMACINML_00228"
CDS                  complement(229513..231327)
                     /gene="recG"
```

/locus_tag="DMACINML_00228"
/EC_number="3.6.1.-"
/inference="ab initio prediction:Prodigal:2.6"
/inference="similar to AA sequence:RefSeq:CAL34612.1"
/codon_start=1
/transl_table=11
/product="ATP-dependent DNA helicase"
/protein_id="Prokka:DMACINML_00228"
/translation="MKIKENDFDFFKKLKIKSAIDLALILPKKIENLSPSKMPKENEL
CTQKIIIKSIHSKKGQLFGLGFCKEWNQDISFVFFHPRAWHFGVCKIGKELILHAKLT
RFGHIWQFNNPKILASFAGFAPKYQIQGLQDNKISEFIHQYLNYKNLKESGIEDKYIH
FLLNLHAYDEKSFLMFENLERFEKDLKYIEIYNFLKRLKAKKNHFKAYEIEIFNIANW
LKNLPFSPTRDQLNALKDIEQDLSSKEAKRRVIMGDVGCGKTLVLLGAALMVYPKQAI
LMAPTSILAHQLYEEAKKLLPEFMDILFIKGGKKEKDLEEKIKKTNLIIGTHALIHLQ
SHNAVLVMIDEQHRFGSAQRQQIYSLNEDELAPHFIQFSATPIPRTLSMIQSELLNFS
FIKEMPFKKDITTLCIQNEGFAKLSEKIKEEIAQNHQIIIIYPLVNPSDKIPYLSLEQ
ARKYWEDNYEKVFVTHGKDKQKDEILERFRDEGNILLSTTVVEVGISLPRLSTIVIVG
AERLGLATLHQLRGRVGRIGLKSTCYLYTKLKEIPNRLKEFASTLDGFKIAELDLKNR
LSGDLLDGFIQHGNEFKYFDFSKDEYILEKAKKDLMKI"

gene complement(231317..232537)
/locus_tag="DMACINML_00229"

CDS complement(231317..232537)
/locus_tag="DMACINML_00229"
/inference="ab initio prediction:Prodigal:2.6"
/inference="similar to AA sequence:RefSeq:CAL34611.1"
/codon_start=1
/transl_table=11
/product="zinc protease-like protein"
/protein_id="Prokka:DMACINML_00229"
/translation="MQNLEVNEIKIPFIFEENNDFPIVILKLVFRNCGRSYDEIAGLA
KMFARILNEGVDDNFFKELEFKAVNLEASSGFESLEINLSCLEENFEFALKHLENLLL
NPRFEEKILEKLKINALGELASKNSDFDYLAKNLLNSEIFECKEFQSPNDGDEKTIKQ
ISLSDLESFYKKNINLSNLVVILGGNLAQEKAKGLLDKLLAKLQKGSQNSQKTYEINS
KNKDIIQIRKESEQAYIYFAAPFFTKFNDKDFYLAKIALFILGQGGFGSRIMEEIRVK
RGLAYSAYAMLDMNASFTRVFGYLQTKNESAKEAKKIVKEVFKDFVKEGVSQNELVQA
KNFLIGSTPLRYESLSRRLSISFNEYYQGLPKGYYKEELKLIEKVELQELNAYIRKHN
EILDLNFASIANEN"

gene complement(232537..233583)
/locus_tag="DMACINML_00230"

CDS complement(232537..233583)
/locus_tag="DMACINML_00230"
/inference="ab initio prediction:Prodigal:2.6"
/inference="similar to AA sequence:RefSeq:CAL34610.1"
/codon_start=1
/transl_table=11
/product="putative radical SAM domain protein"
/protein_id="Prokka:DMACINML_00230"
/translation="MKRLDKEEALYLLRNANLVELGEMAYQRKLELHPEKITTFVVDR
NINYTNVCCIDCSFCAFYRHHKEDDAYILSFEEIGKKIEELEAIGGTQILFQGGVHPK
LKIEWYEELVSWIKEHYPNITVHGFSAVEIAYIAKASKISISEVLQRLQAKGLFSIPG
AGAEVLSDRVRDIIAPNKCDTATWLEVHRRAHEIGMKSTATMMFGTVESDEEIVDHFE
HLRKLQDETGGFRAFILWSFQSENTALIQKHPEIMKQSSNKYLRLLALARLYLDNFKN
LQSSWVTQGSLIGQLALKFGANDLGSTMMEENVVSAAGASYRMNQDEMIRLIKSLGEN
PAKRNTAYEILERF"

```
     gene            233656..234843
                     /locus_tag="DMACINML_00231"
     CDS             233656..234843
                     /locus_tag="DMACINML_00231"
                     /inference="ab initio prediction:Prodigal:2.6"
                     /inference="similar to AA sequence:RefSeq:CAL34609.1"
                     /codon_start=1
                     /transl_table=11
                     /product="putative MFS (Major Facilitator Superfamily)
                     transport protein"
                     /protein_id="Prokka:DMACINML_00231"
                     /translation="MNYIDLLKNNKNIRILTLVQFIVYFGAWFSQTGVFTLLVSLNAP
                     TWATATSAMLAFLPGVLLAPINGVIVEKNKPKKLLLSMISIELVSIFCLIFVTSLSML
                     WLLFILIFVRLCVASIYFQAEMSLLAKILNPEELKLANEMHSVIWAVSYTASMASAGI
                     FINFFGIKAAFLFDCCLIIIGILLLTRLHIPHFKQKIQNKFFTLIKEGFLYVLQNKII
                     LHLIILHAFIGFTAYETLVTLLAQHEYKEVLSAALVIGFLNAIRALALAFGPLFLSRI
                     TNNKNIFFLYLGQGLGIILWALTQFNFYISFIGLIAAGFCTSSLWAYTYTMIQKNCDK
                     AYYGRVIAYTDMVYLSFSALISQLMGLLFEQGLKLATITALLGMIFIFAAFYWKWFDK
                     KYL"
     gene            complement(234870..235958)
                     /gene="nusA"
                     /locus_tag="DMACINML_00232"
     CDS             complement(234870..235958)
                     /gene="nusA"
                     /locus_tag="DMACINML_00232"
                     /inference="ab initio prediction:Prodigal:2.6"
                     /inference="similar to AA sequence:RefSeq:CAL34608.1"
                     /codon_start=1
                     /transl_table=11
                     /product="transcription termination factor"
                     /protein_id="Prokka:DMACINML_00232"
                     /translation="MEKIADIIESIANEKNLDIESVKEKVITALINTAKRIYGQEYDF
                     FVDRKNLNLYQKITVVADNDERLNENKESYIEFSKAKKEAPDVEIGDELTYECSLENL
                     GRTAVNILHKELEYHIQKLLEQTIFEKYKNKVGQMVFGNVVRIDNEENTYIEIDELRA
                     FLPRKNRIKGEKFKVGDVVKAVIRRVYTDKGIKMELSRTSPKFLECLLEAEVPEIKDG
                     YVSVVKCARIPGERAKIILQANGANIDPVGATVGVKGVRINAVSKELHNENIDCIEFS
                     NEPEILIARALAPAIVSSVKIENEKAIVTLNSEQKSKAIGKSGINIRLAGMLSGYEIE
                     LNEVNTHTTNNTLSNEEALKNLQDLFKI"
     gene            236084..236326
                     /locus_tag="DMACINML_00233"
     CDS             236084..236326
                     /locus_tag="DMACINML_00233"
                     /inference="ab initio prediction:Prodigal:2.6"
                     /inference="similar to AA sequence:RefSeq:CAL34607.1"
                     /note="hypothetical protein Cj0459c"
                     /codon_start=1
                     /transl_table=11
                     /product="hypothetical protein"
                     /protein_id="Prokka:DMACINML_00233"
                     /translation="MDLKLARNLINEEPKNISLAKIEEAVNQEGQKFFYFDKENSHKD
                     LIALVEHFEKKGLSVYHRTIRYGLDSNDFMYEVHIL"
     gene            236323..237624
                     /gene="miaB"
                     /locus_tag="DMACINML_00234"
```

```
     CDS             236323..237624
                     /gene="miaB"
                     /locus_tag="DMACINML_00234"
                     /inference="ab initio prediction:Prodigal:2.6"
                     /inference="similar to AA sequence:RefSeq:CAL34606.1"
                     /codon_start=1
                     /transl_table=11
                     /product="putative tRNA 2-methylthioadenosine synthase"
                     /protein_id="Prokka:DMACINML_00234"
                     /translation="MSSKKLFIQTLGCAMNVRDSEHMIAELTQKDNYTLTEDIKEADL
                     ILINTCSVREKPVHKLFSEVGGFEKVKKSGAKIGVCGCTASHLGSEIFRRAPYVDFVL
                     GARNISKITEAVNTPKFMSVDIDYDESEFAFGDFRNSIYKSYINISIGCDKHCTYCIV
                     PHTRGDEISIPLNIIYKEAQKAVDKGAKEIFLLGQNVNNYGKRFRNEHKKMDFSDLLE
                     ELSTIEGLERIRFTSPHPLHMDDKFLEVFAKNPKVCKSMHMPLQSGSSQILKSMKRGY
                     TKEWYLDRALKLRELCPNVSISTDIIVAFPGESDKDFEETMEVLEKVRFEQMFSFKYS
                     KRPLTKAATMPNQIDEAIASSRLSTLQARHNEILDDIVKKQENKTFKVLFEELRAGNA
                     IAGRTDNNFLVQVEGSEELLGTFKEVKITNAKRMVLYGEII"
     gene            237587..238231
                     /locus_tag="DMACINML_00235"
     CDS             237587..238231
                     /locus_tag="DMACINML_00235"
                     /inference="ab initio prediction:Prodigal:2.6"
                     /inference="similar to AA sequence:RefSeq:AAW35094.1"
                     /note="conserved hypothetical protein"
                     /codon_start=1
                     /transl_table=11
                     /product="hypothetical protein"
                     /protein_id="Prokka:DMACINML_00235"
                     /translation="MQSVWFYMGKSFKIAFIARLVFILQWLIFITCKKTYKGDKVDQD
                     SYVILFWHGRLALMPFAFRHFGKKGKKAYVMISHHRDGEQIARIIKLFGLDTVRGSTS
                     KGASTALRSAFKILDENHDVVITPDGPRGPYHSISDGAVLLAQKKNVKIRILNYEARN
                     FWEFKSWDKMILPKPFTHLVYRLSEPLDISSLDKDQAKFFLLEHFKNIEQMDKF"
     gene            238240..239229
                     /locus_tag="DMACINML_00236"
     CDS             238240..239229
                     /locus_tag="DMACINML_00236"
                     /inference="ab initio prediction:Prodigal:2.6"
                     /inference="similar to AA sequence:RefSeq:CAL34604.1"
                     /note="hypothetical protein Cj0456c"
                     /codon_start=1
                     /transl_table=11
                     /product="hypothetical protein"
                     /protein_id="Prokka:DMACINML_00236"
                     /translation="MLFLLKKILPQLFTSIILEDKKNILKTSIYKNQKLISSNEKTFD
                     KSEKLLEYVKTLNSKFLFYHTALFLNVKEQGLIPSKNDKDFERFNVGKMSLKSVTLNN
                     ALIYTATEHIEYFNEFFEDYGGLDFLYSPFAILYHNILKEKPSNDKITLYAYRHEYIL
                     TLIICRGVEILYGDIIFFEEELGLEIENQEENLDSLGKDILSDTEVTLDNFNETLEDK
                     LNALDQFGDNDLEHNEDDSEFLDGKDKLDLEEMNQFGNDMELCRHIVMSIEKFYKDEK
                     YQSVFIDNIFILSTKELDQSAIEFLEEETLLEIQTKQINTLDSITELMQKELQ"
     gene            239226..239750
                     /locus_tag="DMACINML_00237"
     CDS             239226..239750
                     /locus_tag="DMACINML_00237"
                     /inference="ab initio prediction:Prodigal:2.6"
```

```
                     /inference="similar to AA sequence:RefSeq:AAW35092.1"
                     /note="conserved hypothetical protein"
                     /codon_start=1
                     /transl_table=11
                     /product="hypothetical protein"
                     /protein_id="Prokka:DMACINML_00237"
                     /translation="MTTYSFIKPRKKPIFTLFDKIWLGLFSFGIFFIFAIYFLYLTKI
                     ALTHNLIEEQKEQVLQFQNKTQENGQLYNILLEQSEIANTFYTQNQAFKNSLKNLFDI
                     IVKTDGITLESVEQEKNSLKLIGVTPTKEMFALLLETPLKSIFDQSFTTYYRLNNGWY
                     RFISISKQNSGVLQ"
     gene            239747..240265
                     /locus_tag="DMACINML_00238"
     CDS             239747..240265
                     /locus_tag="DMACINML_00238"
                     /inference="ab initio prediction:Prodigal:2.6"
                     /inference="similar to AA sequence:RefSeq:CAL34602.1"
                     /codon_start=1
                     /transl_table=11
                     /product="putative membrane protein"
                     /protein_id="Prokka:DMACINML_00238"
                     /translation="MKDKSLEELNILKIIVYVLSFITVCTALILFLLLPVLKDYKQTH
                     LRKNSQAAIFNAAKAKLDASENKLITLRTENNKSLEQFEQQFNLAHLQNFLEKYFESI
                     QITELELDKKEKYLTHRINIRALINNPRRLYDFVDALAQYNNLIKIDYPMTLKASDHG
                     IMINLNVQIYSN"
     gene            complement(240257..241549)
                     /gene="thiC"
                     /locus_tag="DMACINML_00239"
     CDS             complement(240257..241549)
                     /gene="thiC"
                     /locus_tag="DMACINML_00239"
                     /inference="ab initio prediction:Prodigal:2.6"
                     /inference="similar to AA sequence:RefSeq:CAL34601.1"
                     /codon_start=1
                     /transl_table=11
                     /product="thiamin biosynthesis protein ThiC"
                     /protein_id="Prokka:DMACINML_00239"
                     /translation="MKTQMLYAKEGIFTKEMEIVAQKEEVSKDFLLENIACGKIIIPA
                     NINHKSLDPNGIGLGLRTKVNVNLGVSNDCVDYSEEMKKVELAHKFGIEAIMDLSNYG
                     KTSRFRDELIAVSKAMIGTVPVYDAVGFLEKDLKQISARDFLDVVYHHAKSGVDFMTI
                     HAGINSRAARVFKESKRLTNIVSRGGSVLYAWMAMKEAENPFFEYYDDLLEICLKYDV
                     TLSLGDALRPGSTHDASDAAQISELIELSLLTQRAWEAGVQVMIEGPGHMAINEIEAN
                     MQIEKRICKGAPFYVLGPLVTDIGAGYDHISGAIGGAVAAAAGADILCYVTPAEHLRL
                     PNLNDVRDGIVATKIAAHAGDLAKLPKERKRDDEMSKARQDIDWEKMFKLAIDGEKAK
                     KMFNERRPDDLNSCSMCGKMCAMNTMNQVLKGENVSLV"
     gene            complement(241721..242482)
                     /gene="dnaQ"
                     /locus_tag="DMACINML_00240"
     CDS             complement(241721..242482)
                     /gene="dnaQ"
                     /locus_tag="DMACINML_00240"
                     /EC_number="2.7.7.7"
                     /inference="ab initio prediction:Prodigal:2.6"
                     /inference="similar to AA sequence:RefSeq:ADC28071.1"
                     /codon_start=1
```

```
                     /transl_table=11
                     /product="DNA polymerase III subunit epsilon"
                     /protein_id="Prokka:DMACINML_00240"
                     /translation="MSLQQIDQIISVLSKQSKPYEWVIKEFKKIEELKDYELDLETFE
                     LLGLGLTLDKNNIFTLQTRTKKIKEEIFCIVDIESTGGIKNGQILEIGAVKIKNLQEI
                     DRFSSLVQVKEIPENITELTGINLDMVKDAPSLTKVLNDFRLFLKDSIFVAHNVRFDY
                     GFISKALNECGFGILLNRRICTIEFAQCCIESPRYKLDVLKELLGIKNAHHRALNDAL
                     AAAEIFKHCLGKLPYHIKTTEELINFTKTSRIKQK"
gene                 complement(242479..243126)
                     /gene="rpe"
                     /locus_tag="DMACINML_00241"
CDS                  complement(242479..243126)
                     /gene="rpe"
                     /locus_tag="DMACINML_00241"
                     /inference="ab initio prediction:Prodigal:2.6"
                     /inference="similar to AA sequence:RefSeq:ADN90653.1"
                     /codon_start=1
                     /transl_table=11
                     /product="Ribulose-phosphate 3-epimerase"
                     /protein_id="Prokka:DMACINML_00241"
                     /translation="MYVAPSLLSANFLKLEEEIKAIDEAGADLLHIDVMDGHFVPNLT
                     FGPCVIEKISTITSIPLDVHLMVKDVGKFVDLFTPIKPKFISFHIESEPHPIRLCEYI
                     RSQGIHPAIVLNPHTPINSIKHMIDFVDMVLLMSVNPGFGGQKFLPLVYDKIKELRRL
                     IDERNAKVFIEVDGGINGLNASDLEAAGADILVAGSYIFSSNDYKTSISSLKLEF"
gene                 243264..243458
                     /gene="rpmB"
                     /locus_tag="DMACINML_00242"
CDS                  243264..243458
                     /gene="rpmB"
                     /locus_tag="DMACINML_00242"
                     /inference="ab initio prediction:Prodigal:2.6"
                     /inference="similar to AA sequence:RefSeq:ADN90652.1"
                     /codon_start=1
                     /transl_table=11
                     /product="50S ribosomal protein L28"
                     /protein_id="Prokka:DMACINML_00242"
                     /translation="MARICQITGKGPMVGNNVSHANNKTKRRFLPNLRTVRVTLEDGT
                     TRKMRIAASTLRTLKKQNSK"
gene                 243657..243872
                     /locus_tag="DMACINML_00243"
CDS                  243657..243872
                     /locus_tag="DMACINML_00243"
                     /inference="ab initio prediction:Prodigal:2.6"
                     /inference="similar to AA sequence:RefSeq:CAL34597.1"
                     /note="conserved hypothetical protein Cj0449c"
                     /codon_start=1
                     /transl_table=11
                     /product="hypothetical protein"
                     /protein_id="Prokka:DMACINML_00243"
                     /translation="MLHEYRELMSELKGKDAHFDKLFERHNELDDQIKDAEEGRALLS
                     DIEISNLKKEKLHIKDELNQYLSNYKK"
gene                 243882..244976
                     /locus_tag="DMACINML_00244"
CDS                  243882..244976
```

```
                     /locus_tag="DMACINML_00244"
                     /inference="ab initio prediction:Prodigal:2.6"
                     /inference="similar to AA sequence:RefSeq:CAL34596.1"
                     /codon_start=1
                     /transl_table=11
                     /product="putative MCP-type signal transduction protein"
                     /protein_id="Prokka:DMACINML_00244"
                     /translation="MFGSKINHTELQNIKNENQNLAHQLEKIKAENLELKDKISTLEQ
                     EALESKLKTDLLNVLLGGVLKNITIVQSDMLENVNKAEIISNYSKTSLAEMDELNHIA
                     NSINASLSDITESANKTRDVAGTLHRSVDEITNVINLIKDVSDQTNLLALNAAIEAAR
                     AGEHGRGFAVVADEVRKLAEKTQKATTEVEMNINLLKQNANEMYTQSEQVEKISINSN
                     AHIMSFSEKFTHLVNEAHSTNSNAVGIASEAFVSLAKLDHIAFKLNGYKEIFAKSGQE
                     LADHTSCRLGKWVASTGKERFGNSKSFTKINEPHQKVHENMNSAIRIASTEDASLGNT
                     QDVIIKKCENAENASLHLFDVFKEMLNETN"
     gene            complement(244991..245584)
                     /locus_tag="DMACINML_00245"
     CDS             complement(244991..245584)
                     /locus_tag="DMACINML_00245"
                     /inference="ab initio prediction:Prodigal:2.6"
                     /inference="similar to AA sequence:RefSeq:CAL34595.1"
                     /codon_start=1
                     /transl_table=11
                     /product="putative NUDIX hydrolase family protein"
                     /protein_id="Prokka:DMACINML_00245"
                     /translation="MIFKNLKELPFCESKYIKPKRFSYESNGKLCTWDFIESKDSVSI
                     LLYHKELKSFIFVRQFRIPLWYHQKQDKNYQKDENMGYTIELCSGLVDKDLPLEQIAK
                     EECIEEVGYAPKEFEKVGDFYTGFGSGASRQSFYFAIVDENDKVCEGGGVEDEQIEAV
                     FVKINEYEEFSKKIIHTPLLDFAYLWFLKEKKELCLK"
     gene            complement(245663..247729)
                     /locus_tag="DMACINML_00246"
     CDS             complement(245663..247729)
                     /locus_tag="DMACINML_00246"
                     /inference="ab initio prediction:Prodigal:2.6"
                     /inference="similar to AA sequence:RefSeq:EAQ73281.2"
                     /codon_start=1
                     /transl_table=11
                     /product="TonB-dependent receptor, putative, degenerate"
                     /protein_id="Prokka:DMACINML_00246"
                     /translation="MNAKKLKLSILCFCLIGLGEIKAEEQYQLDDVVVSASGFEQDLV
                     DAPASISVITKEELSKKPIKDIGEAISDIPGVDVTMNKTGTYDFSIRGFGSSYTLVLI
                     DGKRQSVANGFYDNGFSGSESGYLPPASMIERIEVIRGPASTLYGSDAVGGVINIITK
                     KNPNKTEASIEFNTLLQQHSNKYGNAGGFNAYVATPLIEDTLSISARIKYYDKAASDL
                     KWPTPVWNGGTQRPENYQIASHSPGYFTSLGFGSRLNWTVNDSNNVYLDVERYVNEIS
                     VNSTSGRAIKSERQLFKDNVVLNHDGNYEFGTSNAYLQYGSTKDKELHSQIWVGEGKV
                     VLPWNLDKYGNLVSTFGTRIDYETLKNDQESAGSEVRGKNLDQTTVALYGEGEYFISD
                     DLIFTTGLRYIYSDLFDSELTPRVYLVYHLNDNVAFKGGVAMGYKTPAAKQLTNGYYN
                     YSNDSASFGNPDLKPEESTNYELGVDFRIFDFAHYSITGFITDFKNQISSEELTGMQN
                     DIDCTNGVTCERSINLGKTQTKGVEFAFNTKPYNGFSVNSSYTFMDNRYKDGQTNWFG
                     GDRIENLPRHIAMLKLNYEQGKFSSYVKARARLDTIARSKGGGSGILPWQKYKPFYIV
                     DLGVNYKIDKQSSLSFTVQNLFDKDFFDPQVTRWDTSANPGGYANRYQDYTEGRSFWL
                     SYKYDF"
     gene            complement(247975..248808)
                     /gene="citE"
                     /locus_tag="DMACINML_00247"
```

```
     CDS             complement(247975..248808)
                     /gene="citE"
                     /locus_tag="DMACINML_00247"
                     /EC_number="4.1.-.-"
                     /inference="ab initio prediction:Prodigal:2.6"
                     /inference="similar to AA sequence:UniProtKB:P9WPE1"
                     /codon_start=1
                     /transl_table=11
                     /product="Citrate lyase subunit beta-like protein"
                     /protein_id="Prokka:DMACINML_00247"
                     /translation="MKAKSFLFVPSIYPERFIKALQSGANQIIIDLEDSVEEAKKEVG
                     RKNIADFSSQCAKNNDKFLIRINETQSAEFQKDLSLISTIKESNSIVGIVLPKAQNYE
                     DIDILSKFELPIIPIIESALGVENLDDIAKHPSVLALSFGSLDMTLDLNLQEGEGKNF
                     ILNSIRTQIVLKSVKYNLLSPINGVYPDIKNIDGLKEDLLFAKSMGFGGSLCIHPNQV
                     VPINEVFSPSAQQIAWAKEILSLRKNSNDIIFNHNGMMVDLPVIKKAEQILLDSNSNL
                     P"
     gene            complement(248819..249991)
                     /gene="yfdE"
                     /locus_tag="DMACINML_00248"
     CDS             complement(248819..249991)
                     /gene="yfdE"
                     /locus_tag="DMACINML_00248"
                     /EC_number="2.8.3.19"
                     /inference="ab initio prediction:Prodigal:2.6"
                     /inference="similar to AA sequence:UniProtKB:P76518"
                     /codon_start=1
                     /transl_table=11
                     /product="Acetyl-CoA:oxalate CoA-transferase"
                     /protein_id="Prokka:DMACINML_00248"
                     /translation="MLPLEGKLVLSIEQAIAAPFCTRQLADLGARVIKIERPEVGDLA
                     RGYDKRVDGIASHFVWVNRSKESLAIDLKDPDALEFIKTILPKVDIFVQNLAFGASKR
                     LGLDYENLKKYNPKIIVCDISGYGEGGNYGHKKAYDLLIQSESGLVSITGTQDECVKV
                     PISIADISAGMYAYSGILQALLYLEKTGKGSRVEISMLECMAEWMNYPLYYTYKNATP
                     PERKGAFHASITPYGPYKAKDGVVMFGLQNPREWESFCKIVLQDESLIDHEDFKNNTL
                     RTKNASKVKEIIEDAFKNLSAKEVINRLDEAKIANGNVNELNQVWEHPQLKARNRWRE
                     VDSEVGKIPALLPPATNNNYEARMDKIPALGEHTESILREFGFSDKINLFKEKKII"
     gene            complement(250004..250834)
                     /gene="meh"
                     /locus_tag="DMACINML_00249"
     CDS             complement(250004..250834)
                     /gene="meh"
                     /locus_tag="DMACINML_00249"
                     /EC_number="4.2.1.153"
                     /inference="ab initio prediction:Prodigal:2.6"
                     /inference="similar to AA sequence:UniProtKB:A9WC41"
                     /codon_start=1
                     /transl_table=11
                     /product="Mesaconyl-C(4)-CoA hydratase"
                     /protein_id="Prokka:DMACINML_00249"
                     /translation="MSYENFIGKQEKFTENINEKQVQKIVATLQSKQLKNGDALPYLW
                     QWCFFNEALTPDKLGRDGHPKLGLDKNSFLPSFGEVNRMWAGGRFKFITPLIIGEDAT
                     KITTIKKIQEKQGKSGKLTFVTLEHCYYQKEQLKIEEEQDIVYKQPAPPKLTSDVVAM
                     NADYSEKMQPDSTLLFRYSALTFNGHKIHYDFPYATQVEGYHGLVIHGPLLATYMINC
                     FVKNHPDKQIVNYSYRGMHALCVPDSFAVESKITDKNTAEVWINKNGFVAHSGTIHFK
```

```
                     "
     gene            complement(250845..252143)
                     /locus_tag="DMACINML_00250"
     CDS             complement(250845..252143)
                     /locus_tag="DMACINML_00250"
                     /inference="ab initio prediction:Prodigal:2.6"
                     /inference="similar to AA sequence:RefSeq:CAL34701.1"
                     /codon_start=1
                     /transl_table=11
                     /product="putative dicarboxylate carrier protein MatC"
                     /protein_id="Prokka:DMACINML_00250"
                     /translation="MMDTINIIILFSLIISIALGYVLKANIGVFAMLSAFIGAYLYGI
                     TPEKVINLWNGSLSLFFILLSITYFYGFAIANGTLNNLALKAVYSVRNRPWSIPIALY
                     ITVMVFVGIGPGHYAGFAFMSPLVLYIASKIKMNKILAAIIIYSGSCAGGFNPFTLGG
                     RVTFDLIEKLGYSAEASAIMQLNLGKNMFIAHTLIFIIGYFLLKGYKVKITSQITKPK
                     RLNAAQWKTIFIVCFVFAVIMIPSFFLALNPHDETLKKVTSIFNPVFVSFLGIVLAVI
                     FGIGSEKEAFKNVPWDLIFMIGSLSMLIELSKTTGIMENITTFMSAHGSSSSNWLYYL
                     LGILSSCMSAFASSMGVVMPTFFSIVPSIKALEPSLAFSIIAVFATFTGYSSFSTGGA
                     LVLAGAQDESEKRYLFIRLIILPLILVVFGFVLLFLGILN"
     gene            complement(252602..253540)
                     /gene="accA"
                     /locus_tag="DMACINML_00251"
     CDS             complement(252602..253540)
                     /gene="accA"
                     /locus_tag="DMACINML_00251"
                     /EC_number="6.4.1.2"
                     /inference="ab initio prediction:Prodigal:2.6"
                     /inference="similar to AA sequence:RefSeq:CAL34593.1"
                     /codon_start=1
                     /transl_table=11
                     /product="acetyl-coenzyme A carboxylase carboxyl
                     transferase subunit alpha"
                     /protein_id="Prokka:DMACINML_00251"
                     /translation="MASYLDFEKNIQQIDEDIINAQIKGDTEAVAILKKNLEKEIAKT
                     YKNLSDFGRLQLARHPDRPYALDYIDLILNDAYEIHGDRAFRDDPAIVCFMGYLGEKK
                     LIVIGEQKGRGTKEKIARNFGMPHPEGYRKALRVARLAEKFQIPILFLIDTPGAYPGL
                     GAEERGQSEAIATNLYELSDLKTPTIAVVIGEGGSGGALAIGVADKLAMMKNSVFSVI
                     SPEGCAAILWNDPSKSEAATKAMKVTADDLKSQGLIDDVIEEPINGAHRNKEAAAVAI
                     ADYVKKALDELEKIDPRELASNRMQKILQLGAFSES"
     gene            complement(253542..254756)
                     /gene="fabF"
                     /locus_tag="DMACINML_00252"
     CDS             complement(253542..254756)
                     /gene="fabF"
                     /locus_tag="DMACINML_00252"
                     /EC_number="2.3.1.41"
                     /inference="ab initio prediction:Prodigal:2.6"
                     /inference="similar to AA sequence:RefSeq:CAL34592.1"
                     /codon_start=1
                     /transl_table=11
                     /product="3-oxoacyl-[acyl-carrier-protein] synthase"
                     /protein_id="Prokka:DMACINML_00252"
                     /translation="MKRVVVTGIGMINALGLDKESSFKAICEGKSGVKKITLFDTTDF
                     PVQIAAEIENFDPLEVVDGKEVKKIDRFIQLGIKAAREAMQDANFDQNLDKEEFGVVS
```

```
                     AAGIGGLPNIEKNSVICSERGPRKISPFFIPSALVNMLGGLISIEHGLKGPNISCVTA
                     CAAGTHAIGEAYKSIALGNASKMLVIGAEAAICPVGIGGFASMKALSTRNDDPAHASR
                     PFDKERDGFVMGEGAGALVFEEYEEAKKRGAKIYAELVGFGESADAHHITSPTLDGPL
                     RAMKKALKMAGNPKVDYINAHGTSTPVNDKNETAAIKELFGSEIPAVSSTKGQTGHCL
                     GAAGAIEAVISIMALDNGIMPPTINQFVSDEECDLDYIPNEARKADLRVVMSNSFGFG
                     GTNGCVVFKKVD"
     gene            complement(254790..255023)
                     /gene="acpP"
                     /locus_tag="DMACINML_00253"
     CDS             complement(254790..255023)
                     /gene="acpP"
                     /locus_tag="DMACINML_00253"
                     /inference="ab initio prediction:Prodigal:2.6"
                     /inference="similar to AA sequence:RefSeq:CAL34591.1"
                     /codon_start=1
                     /transl_table=11
                     /product="acyl carrier protein"
                     /protein_id="Prokka:DMACINML_00253"
                     /translation="MATFDDVKAVVVEQLSIDADAVKMESKIIEDLGADSLDVVELIM
                     ALEEKFEVEIPDSDAEKLIKIEDVVNYIENLKK"
     gene            255138..255803
                     /locus_tag="DMACINML_00254"
     CDS             255138..255803
                     /locus_tag="DMACINML_00254"
                     /inference="ab initio prediction:Prodigal:2.6"
                     /inference="similar to AA sequence:RefSeq:CAL34590.1"
                     /codon_start=1
                     /transl_table=11
                     /product="putative transcriptional regulator"
                     /protein_id="Prokka:DMACINML_00254"
                     /translation="MLFSRLIETNSHIWDEYLHHEFVKKLENGSLKEENFLFYLKQDY
                     IYLIHYAKCYARLALNAKNAEELRFAMKFQNYIIEGEIELHKSILKLGINADKLNVKD
                     ESLTNIAYTRYMLSVGESGDFLDMLVALSACAIGYGYIGAEIYKRLGKEKLQNHPYKE
                     WILTYSSDEFQSEIKEFEDFLNSYTQKISQEKFENLSEIFYNVVRLENAFWEHALQMK
                     MDI"
     gene            complement(255823..256230)
                     /locus_tag="DMACINML_00255"
     CDS             complement(255823..256230)
                     /locus_tag="DMACINML_00255"
                     /EC_number="1.4.3.5"
                     /inference="ab initio prediction:Prodigal:2.6"
                     /inference="similar to AA sequence:RefSeq:CAL34586.1"
                     /codon_start=1
                     /transl_table=11
                     /product="putative pyridoxamine 5'-phosphate oxidase"
                     /protein_id="Prokka:DMACINML_00255"
                     /translation="MDLKEIAQFLDENTPAFLTTLGTCGNPRVRPMQSPLLVGDRIYF
                     CTSNNKGLFKHIQNYNGIEFCSCSREGVFLRLRANAVFESNAKVKELMFEKYPYLVSL
                     YETPQNSRFEVFYLDNLSAKMQFPNGDFKLYKA"
     gene            complement(256240..256983)
                     /gene="fabG"
                     /locus_tag="DMACINML_00256"
     CDS             complement(256240..256983)
                     /gene="fabG"
```

```
                     /locus_tag="DMACINML_00256"
                     /EC_number="1.1.1.100"
                     /inference="ab initio prediction:Prodigal:2.6"
                     /inference="similar to AA sequence:RefSeq:CAL34585.1"
                     /codon_start=1
                     /transl_table=11
                     /product="3-oxoacyl-[acyl-carrier protein] reductase"
                     /protein_id="Prokka:DMACINML_00256"
                     /translation="MKFSGKNVLITGASKGIGASIAKTLASFGLKVWINYRSKPELAD
                     ALKDEILAQGGNAAVIKFDASKEDEFENGIKTIVESDGELSYLVNNAGVTNDKLALRM
                     KLEDFTGVVDTNLSSAFLGCREALKTMSKKRFGAVVNIASIVGEMGNAGQTNYSASKG
                     GMIAMTKSFAKEGASRNLRFNCVTPGFIKSDMTEILSDEIKQAYQDNIPLKRFAEPEE
                     VANCVAFLLSDYASYVTGDVLKINGGLYM"
     gene            complement(257049..258527)
                     /gene="pgm"
                     /locus_tag="DMACINML_00257"
     CDS             complement(257049..258527)
                     /gene="pgm"
                     /locus_tag="DMACINML_00257"
                     /EC_number="5.4.2.1"
                     /inference="ab initio prediction:Prodigal:2.6"
                     /inference="similar to AA sequence:RefSeq:CAL34584.1"
                     /codon_start=1
                     /transl_table=11
                     /product="2,3-bisphosphoglycerate-independent
                     phosphoglycerate mutase"
                     /protein_id="Prokka:DMACINML_00257"
                     /translation="MRQKCILVITDGIGFNKEKDFNAFEAAKKPNYERLFKEVPNSLI
                     KTSGLAVGLPENQMGNSEVGHMCIGSGQIIYQNLVRINKAIESYELEKNANLKKLLQK
                     CKRVHVIGLYSDGGVHSMDTHFNALLEICAKNGNEVFAHAITDGRDVSPTSGLEFIKK
                     LKNHCEKIGVHFATLCGRFYAMDRDKRWERVKEYYDCLLGKSKGVPNLVDYVEECYQN
                     EITDEFIQAAKNENYKGMQEDDGLIFINFRNDRMKQLIEVLNSQNFKEFEREKVFENL
                     LTMSVYDDKFNIPVLFEKEEIKNTLSETISKAGLTQLHTAETEKYAHVTFFFNGGKEE
                     LLENETRVLIPSPKVKTYDEKPEMSAFEVCDAVRKGIEKGEDFIVVNFANGDMVGHTG
                     DFNAAVKAVEAVDACLGEVVECARKHDYAFIITSDHGNCEAMQDKKGNLLTNHTTFDV
                     FAFVEAKGVDKLKENMGLSNIAASVLKILNLEIPKEMNEALF"
     gene            258607..259668
                     /gene="mraY"
                     /locus_tag="DMACINML_00258"
     CDS             258607..259668
                     /gene="mraY"
                     /locus_tag="DMACINML_00258"
                     /EC_number="2.7.8.13"
                     /inference="ab initio prediction:Prodigal:2.6"
                     /inference="similar to AA sequence:RefSeq:CAL34583.1"
                     /codon_start=1
                     /transl_table=11
                     /product="phospho-N-acetylmuramoyl-pentapeptide-
                     transferase"
                     /protein_id="Prokka:DMACINML_00258"
                     /translation="MYYLSYFSDYAFFTYISVRAGFAFFIALCLSLFIMPKFITWAKN
                     KNANQPIYELAPQTHKVKSQTPTMGGLIFIACAVFTSLICVKLDNVFTITALFCLVLF
                     CLIGLVDDLGKILKKDNHSGLSPKAKLLAQISVALICILPLYFSPEFNTEFYLPFYKY
                     PLFDMKIFALAFWILVLISSSNAVNLTDGLDGLATVPSIFSLSTLGIFLYLSGNLNYS
```

```
                     EYLLLPKIQGLGEVVIICAALIGALMGFLWYNCYPAQVFMGDSGSLALGGFIGFLAII
                     SKNEILLLLVGFVFVLETISVILQVGSFKIFNKRVFKMAPIHHHFEKIGWVENKIIVR
                     FWMIALLSNLLALASIKLR"
     gene            259668..260876
                     /gene="murD"
                     /locus_tag="DMACINML_00259"
     CDS             259668..260876
                     /gene="murD"
                     /locus_tag="DMACINML_00259"
                     /EC_number="6.3.2.9"
                     /inference="ab initio prediction:Prodigal:2.6"
                     /inference="similar to AA sequence:RefSeq:CAL34582.1"
                     /codon_start=1
                     /transl_table=11
                     /product="UDP-N-acetylmuramoylalanine--D-glutamate ligase"
                     /protein_id="Prokka:DMACINML_00259"
                     /translation="MKISLFGYGKTTQAIAKNLVNKFGPFDIYDDHFQEENKDEWGNH
                     LLNPKEFDEKLSKLEIPSPGFPPNHILIKKAKNLQSEYDFFYDAMPKSIWISGTNGKT
                     TTTQMATHLLSHVGAIMGANVGFPLAELDLNAKIWILETSSFTLHYTHTAKPEIYALL
                     PISADHLSWHGSFENYVKDKLSVLERMNECDIAILPEIYAKTPTKAYIISYKDEFDLA
                     QKMDIDIKKISFKSPFLLDAVMALSIEKILLDTLSYELLNHFVMEKNKLEELQDNQGR
                     LWINDTKATNENAVMAALNRYKDKKIHLIIGGDDKGVDLSMLFNFMKDFDVELYAIGV
                     STQKMLDYALKANLKAHKCEFLENAVKEISKTLKNNEVALLSPACASLDQFSSYAQRG
                     EKFKEYVSKI"
ORIGIN
        1 aaacaagggt ttacaaaatc ttcatttagc tttataaact ttagtttata atttaaagat
       61 tttaaagtcc ttttttgaaa aaggaaatga aagtataact aattaagctt aaaggaggat
      121 taaataaatt atttttttaa ttaattttgg caaaaaatta aaaactagat ttttttgcca
      181 atcagctagc taactttgat ataatcatag agaaaatttt actataaagg aaaacaatga
      241 aaaagttttt aaccgctttc ttagttgcct tcacgggctt atttttagta gcttgccaaa
      301 acaccaaaac agaaaataat gcaagcaatg aagctaatac cacactcact ttaaaggttg
      361 ggactgctcc aaattacaaa ccttttaatt ataagcaaga ttctaaactt acgggttttg
      421 atactgattt ggttgaagaa attgctcaaa aaaatggtat tgaaattgtt tgggttgaaa
      481 ctaattttga tggattgatc cctgctttaa aggcgggcaa gatcgatatg atcgcttcag
      541 ctatgagtgc tacagatgaa agaagacaaa gtgtagattt tacaaaacct tattatatga
      601 gtaaaaatct ttatattaaa cttaaaaata atgaagctct tcaaacaaaa actgatctag
      661 aaggtaaaaa aataggagtt cagttaggaa ctttgcaaga aaatacagca aagactatca
      721 aagatgcaca aattcaaagt aataaggatt tgaatatagc tgttttagca ctaaaaaata
      781 ataaaatcga cgctattgtt gccgatgagg atactgctaa aggctttttg gctgaaaatt
      841 ctgatttagt aagcttttat caagaaacag atggtggcga aggttttagt tttgcttttg
      901 ataaagataa acaaaaagaa gttatagaaa tatttaatga aggtatagat gaagcaaagg
      961 ctagtggatt ttatgataac cttataaaaa aatacgaatt agaataagcg tgaaatttca
     1021 cgcttctatg atagaatttt aaatatcata ttttctctct tttgcaactt ttaaaagttt
     1081 ttattgttat tgcatacaaa aatcttgcta gtacactcca ataaagacaa aacaattata
     1141 aaattaccaa aaaagaaatt tgctactttt tataatcttt acttagataa tctttaagtc
     1201 aaaatttagc actaccacgc taatatctaa ataccatact aaatcaaaca attttcatct
     1261 ttagaataat aaagtcacga gttacaaatc taaaaaaaat tagaaattga attaagttct
     1321 agaaacattt tagatacaag ttgattttta actaatcaaa gctataattt caaagtcaaa
     1381 ataaggaacg actatgcttt acatttacat aaaaacacaa aatgccttag tgcaaagaat
     1441 taattttaat cttgataatc aagaattacc gcaaaatatc ttatggattg atttgcttca
     1501 tccaagcgcg gcagaaattg cctttatatc aaatgaattt aatcttgaat ttccaacaaa
     1561 ggaagagaga gaagaaatag aacttagcgc aaagtactgg gaggataatg ctactataac
     1621 tatcaatgct catttttga ttcgagaatt taagaatgat aacgaagagc aaaactcaat
     1681 taagcttcgc actgaaattg taacttttgc aacagctaaa aatattttat ttacaataag
```

1741 atataatgaa tttagcacct ttgaagaaat tcaagcaagg atattagcaa gtccaaagaa
1801 ttttgaagat ggttttgata ttatcgataa aatgtttgaa gtacgagttg aaaaagatgc
1861 ggatttgctc gagtggatag ataaagaagc aagacgctta agaacaactg ttttagagaa
1921 aaaagatgag tatagttatg atgagatgtt aaaagatatc tcaagtttac aagaacttaa
1981 tatgcgagtt agagattctt tatttgataa acgtcgtgct atgacttcct tactgaaaag
2041 tgataaaatc gatagggata taaaacaaaa tttaactata gttttaaaag acttgaattc
2101 tttagttgaa tttagtgttt cacaacttaa tgttctagat aatattcaaa ccattctagc
2161 aagtcaagtt aacatagagc aaaataaaat catcaaactt tttaccgttg caactgttgc
2221 catgatgccc cctactctca taggaacaat ttatggtatg aactttaaat ttatgcctga
2281 acttgagctt cactatgctt atcctatagt gcttgcggtg atgataattt ctatcattgt
2341 gcctgttatt gtttttaaga aaaagggttg gctataatac attaaaggag ataaaatgga
2401 tatcattaaa ataggaattt taaccttaag cgatagggca agtagtggag tatatgaaga
2461 taaagccact gctgaagttg aaagaactct aaattcatat attaaaaatg aaatcatcta
2521 ttttaaagaa cttataccag atgattatga tttgattatt aaaaaattac tttatctagc
2581 agatgagaaa gaatgcgact taataatcac tagcggcgga acaggaccgg cacttcgtga
2641 tgtaacacca gaagctacag aagcagtatg cgataaatta atgccaggtt ttggagaact
2701 catgcgttta gaaagcttaa aattcgtacc cacagctata ctttcaagac aaacagcagg
2761 aattcgcaat aaatctttta tcatcaatct acctggcaat cctaaagcca tagcagaatg
2821 cctagagcct gtatttcctg caataccttа ttgcatcgat ttaatagggg gagcttatat
2881 agaaggaaat gatgaagtga ttcaagtctt tagacctaag aaaaaatgcc aaaattaaaa
2941 cttattagct ttaataagcc tgcttaactg cctagcaagc aggcttactg atgcttcatc
3001 cttgcttatt ttttgtattt tatttttaaa tttataaagc ctatcgtcta aaattatatc
3061 attggttgga attttaggtc ttgtattttg tttatgcgga actttttttct tgaaaaacat
3121 ttttatcctt ttttgtaaat atacattatt ttaattgaaa aaaatattat ttttaatatt
3181 tatatcatta ttaactttat ttcatgattt taagttagaa ttcgcaaaaa aataaatcaa
3241 ggaaaataaa tgactttaat tgcaatttta tgtctttata cagcactttt atcttggatt
3301 tcttataatc aaattcgctt tttagaaaaa gaaaaagaca aacaagctca aattttaagc
3361 gaggaagatt atcaaaacgc agccgatatt gctatagaaa atgaaaaatt caagcttttt
3421 tcaaattttt atactctgat tgttaatatc gcttggatag gctttggatt tctctactta
3481 aaagaactat taatcagcga aaactctcgt cttgaaaata ctctattttt acttgctttt
3541 ttaattatca caagtatttt aaatttacct ttaagtattt atgagagttt tgttaaagac
3601 aaagctcatg gtttttcaaa tatgacttta aaacttttta ttaaagacac tataaaaagt
3661 cttatcttaa ctttgatttt tggattttta atcctttatg ccctactttt ttgttatgac
3721 tttttggca ctttttggtg gtttgcagct tttgtttttg ctttttgtgt gatagtgatt
3781 ataaatgtta tttatcctac tctaatcgct cctatattta acaaaatgga aaaattagaa
3841 gatgagaatt tgctaagtaa aatcaacgat ctcatgaagc gatgtggatt taatgcaaat
3901 ggggtttatg tgattgatgc tagcaaaaga gataagcgtt taaatgctta ttttggtgga
3961 ctttttaaaa gcaagagagt agtacttttt gacacacttt taaaagcctt aagcgagaga
4021 gaactcttgg cagttttagg acatgagctt ggacattttg tccacaaaga tattataaaa
4081 gcactgatta atggagcaat cactatgttt ttacttttct ttatttttgc tcatttgcct
4141 gattttgcat atctagaaag tcatctagaa ggagttaatg ggggtgtttt tgctctttta
4201 tttatatttg caaatatttt tagctttatt atctctcctt taataaatgc tttgagtaga
4261 aaaaatgaat ttgccgcgga tcagcacgga agtaagctta ctagcaagga agatatgaaa
4321 aatgcactca tagctttagc aagagaaaat aaagctttta ttaaaacaag taagatttat
4381 accttttttt accttagtca tccaagcatt agcgatagaa tcaaggctct ttcttgacta
4441 taaaaaacgc tcttatagaa gccaaatcta gacttgaaaa gcatgaaaac gaagccttgt
4501 ttatactttg tgaatatcta aaaaaagata ggacttggat ttttttaaat caagatctag
4561 aatttgactc taagccctat tttgaactta tcaagcgctt tcaatcaggg gagccttttg
4621 aatacatctt tgaaaaagca gatttttggg gcttggaatt taaagtaaaa aaaggggttt
4681 taatcccacg ttatgatagt gaaattttac tttcacaagt tcttaaaatt tgcaaaaata
4741 ataattttca aaatatatta gaaataggct ttggaagcgg aattttaagt atagttttag
4801 ctaaagaatt gggaataaaa attacagctt gtgatatcaa ccccaaagct ttacaactgg
4861 ccttagaaaa tgctaaatta cacaaagttg aacatttaat cgactttaaa ctttatgatt
4921 ttaaacaaat aaaagaaagt tatgatttta ttttttcaaa tcctccctat atccaaaatt

4981 catacccat agatctttgg gtacaaaacg aaccaaaaga agctctattt ggaggagaaa
5041 aaggatatga aattttagaa gaaattataa ctttctcctt tgaaagaaag gttaaatttt
5101 tagcatgcga atttggctat gatcaaaaag caattttaaa acaaatttta tatcaaaaaa
5161 attttcaagc tgaatttttt caagatgaac aaggatatga tcgtgctttt gtcgctcaat
5221 ttgctaaaaa ataagctttg tatgatatga taagttaaaa aattaaggag aaaaattgaa
5281 agcaatcaat ctatttttac ttgcagcaat gattggcata gaacttatac ttggcatagt
5341 tgttgctccg gtgatttttt acccagctaa ctttataggc gagggagttt taagtcattt
5401 tcaaagcggt ttaatgatga ctcaaatttt cattaaaatg ggatatttgt taatttttgt
5461 ttctattttc aacttattgt ttgaaatcta ttcttttata aaggaagata tgaaatttca
5521 gattaaattc tcaaaactaa tacttagcat actgatttta atcttaagct tagtatttgt
5581 tttatatttt acaaatacca ttgttgagct tcaaaattta ggcgagcaag ctacaaaaag
5641 tcaagaattt cttagcatac ataatgcaag tgaagtggtt ataaaaatta ttttattgat
5701 gcaagttttt ctatatttct taagttttaa aatagcaaaa aaatgctaga atattttata
5761 aaaatttaaa aggaggagca agctatgatg atctctgatg caagcatgat gcagcaaaat
5821 tattatctaa acaatgctca aaaagctagt gaaaaagctt tagaaaacat tgcagctgtc
5881 cgtgcaataa gtggagttga tagtgctaat ttagctattg ctgattcttt aagatctcaa
5941 tcaagcacta tagatcaagg cattgcaaac gcctatgatg ccatcggtgt tttacaaatt
6001 gcagatgcaa gccttacaaa tatttctcaa agtgctgata gacttaatga actctccgta
6061 aagatgaata atgcggcatt aagtgattct caaaaaagta tgttaagaac agaagcaaca
6121 cgcatacaag aatccatcaa tgattctttt aataatgcta cttataatgg aaaaaatgta
6181 tttcaaacta tgaattttgt agtaggaagt ggaactgaaa caactaattt aaatccattg
6241 gcaactagtg gtcttagcat agataatcaa gacagcatta caagttttat ggatcaactt
6301 ggtgctttaa gaagtgaaat aggttcaggt attaatgcta tcacatctaa tatcaatgct
6361 agcgtacaaa atagcatcaa tgcgaaagca gctgaaaata atttgctcaa taatgatatg
6421 gcaaaaaatg tcaatgattt taatagcaat tacctaaaag aaaatgcggc tgcttttgtt
6481 gctgcacaat ctaatatgca gcttcaaagc aaaattgcta gcttattgca atgataaaag
6541 ccctaatcta gggcttctct ttttataaaa atgactttag aacaaatttt agaaaaaacc
6601 aaaaatatcc gtcttgtagc agcaagcaaa tatgttgatg caaatattat cgaaaaactt
6661 tttgctcaag gaatcacaga atttggagaa aatcaagttc aagctttaat acaaaaaaaa
6721 gaaattcttg ataaaaaaga tttaaatata aaatggcatt ttataggaac gctacaaagc
6781 aataaaatca atcttttaat caagcaaaaa ccaatactct ggcattcttg taatggatta
6841 aaaatagcaa aagccatgga taaaagacta aattataaac tagatacctt actagaaatc
6901 aatagtgcca atgaactcag caaaagcggg cttaatccgg atcaagctat agaagaatat
6961 ttacaaattc aagaagaatg tcctaattta aatctttgcg gagttatgag cataggctca
7021 cacagcgaag aacaaaaaga aattataaaa agttttgaaa ttacctataa aatctatgaa
7081 aaacttcaaa aacacggagc taaaatttgc tccatgggaa tgagtaatga ttttgaaatc
7141 gctattaaat gcggatctaa tatggtaaga cttggaagta ttttatttaa aaatttaaaa
7201 taaaatccct tcgtatcttt tatcctgcat acttattgat aattttctca ctaacgctat
7261 cagaaataac aaaatcagta tgataaagca aacaagaacc cgtgctaaca attttcaaaa
7321 caagcttttt gttacctttt tcatttggat tatgcgcatt ccttgctatt tcgtgaattt
7381 cataaacaag ctctcgattc aatcttgaaa gatcaagctc tatgatattg ttttcaaatt
7441 ctttaggttc tgctacaaag tctttatttt ccaaggcgtt tttcttagcg ctaaattttt
7501 ttaccgcctt aacctctcct tcttgaagct cctctagagt tttaattgat cttaaagcta
7561 agctatctaa tttaccctcc tctgctttat agcttagtaa aaatccataa gcctcatttc
7621 tagaggcttc attttttaaa atattttcaa tctcttctat attagactca aaaaccgtca
7681 cttcaaaagt agaataaaaa tccaatatct caagcctacc ataacgcttg ttatttttac
7741 tcatcataga tttaaagctt tcaatcttcc ctatgcttaa aatttctcca ttgcctttaa
7801 gcgttgaaaa atctatgctt tttatataat ctatatcctt aaatttctca taaaaacgat
7861 caagcggatg tcctgaaaca taaattccca aaatctcttt ttcaaattca agcttttcca
7921 taacctcaaa ttcttcatcc ttggccacta tattaacttg taacccaaca gttaactcat
7981 cttctccaaa aagagaactt gctgcatttt tccttgcatc agccattttt ttagagcctt
8041 cggttaaatt gtccatatta tcaaacaagc actttctcgt atagccaaat tcatcaaaag
8101 cccccgcttt aataaggctt tctagggttc ttttgttgat ctttgtgggc tctatcctac
8161 ttaaaaaatc atcaatattt ttaaattcgc ctcgctctct tgcttcaagt aaattttcaa

```
 8221 ctgcagggat tcccacactt ttaatagctc caagtccata aacaatagca tctttaccat
 8281 cttcttgctc tactgcactg aattcgcgta tagctttatt aacagaaggc gggagaagtt
 8341 tgatattcat tttttttcatt tcatcgatat aaacagcgat tttatcgaca ttattttctt
 8401 cacttgttag caatgctgcc ataaattcac taggatagta ggtttttaag tacgctgttt
 8461 gaaaagtgat caaggcataa gccgcagagt gagatttgtt aaaaccatat cctgcaaatt
 8521 tgacaattaa ctcccaaagc tcttcggctt ttactctatc ataaccctgc ttttctgcac
 8581 catcagcaaa ttcacttttt aatttttca tttttcggg atctttttg cccatagcgc
 8641 ggcgcaccac atcagcacca cctagtgaaa atccacctat aatctgaaca atttgcataa
 8701 cttgctcttg ataaacaata accccatatg taggctccaa tacactttca agagtatcaa
 8761 atggatattc tatcttttta agcccatgtt ttctatctat aaaatcatca agcattcctg
 8821 attccatagg acctgggcga taaagagcaa gaaccgcaat aatatcttca aaacgctcag
 8881 gctttagcct tgcatttaag ttttgcattc cgcctgattc tatttgaaat atccctaaag
 8941 tatttccact ctgtatagtt ttataaactt tagcatcatt tacatcaata gcttcccaaa
 9001 ctatgtcttt attgtaacgc tttttaatga gcttgatagc attattgatt aaagttaggg
 9061 tttttaaacc caagaagtca aatttgataa ggtctacatc ctccaaatga tctttagagt
 9121 attgtgtaac caaatgtctt tcatcgtttt tgctttgcct aaaaagagga gtttttttcc
 9181 ataaactctc attagaaatc acaacacctg ctgcgtgcat acctgcattt ctattgagtc
 9241 cttcaagagc cttagcatac tcccaaactt caggaccctt tggatggcgt tctataaatt
 9301 ctttaatctt tggttctttt tcataagctg catcaagagt gattttaagc tcttcaggta
 9361 taagcttggc aagttcatct gcatcaggaa tactcatatc gcaaaccctt gccacatcac
 9421 gaatcacccc acgagctaaa agcttaccaa aagtaataac ctgcgcaacc ttatcagctc
 9481 catatttatc aatcacataa tcaatcactt ctccgcgtct atcttgacaa aaatccacat
 9541 caatatcggg cattgatacg cgttctggat ttaaaaaacg ctcaaaaagc aaactataag
 9601 ggatcggatc taaatctgta attttcaaag aataagcaac caaactaccc gcggctgaac
 9661 cccttccagg acctacagga attcctttat ctttggccac cttgataaaa tcatgaacga
 9721 ttagcatata gcctgaaaat ttcatttttt ttatgatatt gatttcaact tcaagccttg
 9781 ttttatattc ttcatgcttg cttatatcaa cgaattttaa cctttcctca agtccttttt
 9841 tacaaagctc atcaaaaacc acatcatcat tatcaaaact aaattcttta ccttcttcgg
 9901 gcaagcttat attatattct tttgcatatt ctcttgtaaa tttaaaattt ggtggggtag
 9961 gatttcctaa attgagctct aagttacatt tattagcaat ttcttgagta ttttcaatag
10021 cttcaggtat atcagcaaaa agctcactca tttgcgctgg agatttgaca taaaattcat
10081 gaacgctatg gcgtaaacga tcaggatcat tcagcttttt acccatagca atacacataa
10141 atacttcatg agcagccgct ctttccttaa aagtataatg ggtatcattt gtagcaataa
10201 ttttaatatt caattctttt gagagtctta taatatcatc gtctatagct ctttgatctc
10261 ctataccatg acgcataatt tcaaaataaa aatcatcgcc aaaaactttt ttataccata
10321 aagccgcttc tttagccgct tcataaccct tggctccaaa acgaacattt ctttcactat
10381 gaatatttaa atgccaattt acctcacctt gtaagcaagc tgaagaacag ataagtcctt
10441 cgctatgctc ttctaaaagc tttttattga tgcgggggata ataatacaaa cccttaatat
10501 agctttgaga acttaaatac attaaatttt gataaccaac ctcatttttt gcatataaac
10561 aaaggtgaaa tctttgacgc gagcttttat ctgaaatatc ttcatgattg tgcaaatatg
10621 cctcaagacc tataataggt tttaatcctt gagaacgcat agtttgataa aaatcaattg
10681 ctccaaacat attgccatga tctgtcatag ctacacttgt agcaccctgt tctttaagag
10741 ttttagctag ttctttaagt ttattagctc catcaagtaa agaatattct gtatgtaaat
10801 gaagatgggt aaattggctc atcgtcttcc ttgaaaaata taaatactaa agattttgat
10861 aaaaattata aatttttatt gctgaatttg aaagaattta tggctaaaaa ttacctgaaa
10921 tttatcatgg tgattttatt accatgagca aatatatttt aagaatattt aaaaaataaa
10981 atagtaaaag tttttaagat tgaaagaatg agttatcttt caagaaataa aattacgaaa
11041 tttgttcata ttctttgcca atataaattt gattatctct ttctataaca ggacgcttaa
11101 tacagcttgg attttctaaa atgatatttt tcaactcaag ctcacttaag ttctctatct
11161 tgtctttatt taaaccgagt tttttgagg tggtgcctgc agtattgata atttcgctaa
11221 atttttata tttaagccaa ttttcaagta tattcaaatc aatcttttta atatcgataa
11281 aatcaaactc tagagctttt tgatttaaaa attctagagc ttttttacg gaattacagt
11341 ttttaatccc ataaactttc attttctcac ctttttaacc aaatcagcaa ttaaaaacgc
11401 aagctcaaga gcttgatccg cattaagtct tgggtcacac tgtgtctcat agcgtttttc
```

11461 taggctttgc gcagtaacat tactagcacc acccgtacat tcagttacat cctgtcctgt
11521 catttcaaga tgaactccac ccgcatatac accttcactc atagctattt caaagaaata
11581 tcttacctct tgcatgattt tatcaaattc acgcgtttta aaatcacctg cttttacggt
11641 attgccatgc ataggatcta tactgtaaac taaatttaag ccctcttttt taagcttaga
11701 gaaaatttta ggcaattgag ttgctatttt ttcagcaccc attctgatga tgatatttaa
11761 tctaccctct tcattgttcg gatttaaagc attagctaaa gctatcacat catcagcact
11821 tgcatttgga cctattttca ctcccaaagg atttctaacc ccacttaaaa aatgcacatg
11881 agcctcgtca agacctctag ttctctcacc tatccaaagc atatgagccg aacagtcata
11941 aatttcaccg cttaaactat ctactctagt taaagcttcc tcataaggca aaagcaaagc
12001 ctcatgagag gtataaacat caacttcttt taaacttgga gtattatagg tattaatacc
12061 acaggcctcc ataaaatcta aagcttgaga gatcttctcg ctgatatcag tatattgttg
12121 atgcaattca ctttttttca agaagcctaa attccatcga tgcacttcgt gtaaattagc
12181 tagtcctcct ttagcaaaac cacgcaataa attcaaagta gtagcacttt gatagtaagc
12241 ttcaagcatt ctatttggat cgggaattct agctttttca ttaaattcaa agccattaat
12301 gatatcgcct cgataacttg gaagttttac tccatctatc tcttcataat cactacttcg
12361 tggcttagca aactgccctg ccatacgacc aattttaaca actggacaac cacccgcaaa
12421 agttaaaact atagccattt gaagtaaaat tttaaacata tctcgtatat ttactgctcc
12481 aaaatttgca aaactttctg cacaatctcc accttggagt aaaaaggcct cttttcggca
12541 tactcttgct aaagcatttt gaagctctct aacttcacct gcaaaaacca aaggaggaag
12601 tttttcaagt ttatttaaaa ctttattcaa ctcctcttga ttagaataaa taggctgttg
12661 tttaatagaa aaatttttcc aagaattttt agtccaagtc atcgttattt cctaaaagat
12721 ataatttcaa cttcccctat aggtcgagta accaaaagga gataaggtaa taggtacatg
12781 gtaatgtttt tgatctttta aaagttcaaa acttacttct acaaaggggt aaaagctatc
12841 aactttgcgt gacatataat aatctttggt ataaaatttg agtttataaa ttccagaagg
12901 acgattttct gtttttttcat aaggaagtaa atcgcttatt ctgccatttt tttctgtgaa
12961 tttttcactt atcttaatcc attgttgatt tttattcaaa ctataaagtt ctacttttac
13021 attggatgcg ggctggccag aattaatatc taaaatatgg gtgcttaact gatattcagc
13081 tgcgtttaga aatattggta tacttatgag agccaacaaa attttttttca tagaaagcat
13141 atttttctcc cttaataata ttttatatat ttaaatcata acaaaatatt attaacaata
13201 ataactaaat aataaatttt ataaattagt taaaaatata tttattcaaa aaacagaaag
13261 atactaaatc tgcaaaatta ttattttgca gattattttt taagttcttt aatacgtgct
13321 gctttaccgc gtctatctct aagatagaac aatctagcac gacgaacacg accttttctt
13381 aaaacagtaa tgctctctaa gctttcacta taaattggaa aaattctttc tacaccaacg
13441 ttattagcac ccatttttgcg aacaataaag gtttcatcta caccattacc tcttctagct
13501 atacaaattc cttcaaaatt ttgaatccta gttttatcgc cctctttgat gcgaattgca
13561 agtcttaaag tatcgccagc acgaaattct ggaacatttt ttccctcaat ttgttttgcc
13621 tcaaattgct ctatgtattt atttttcata aaaaattcct tattttttgc gttcatgctc
13681 taaaaataaa tctggacgaa aaaatttcgt tttgcaagac gctaaagtag tttttaaagt
13741 cgcaatttta gcgtgattac cctttaaaaa ctctgaagga gtgtaaaatt tcttgaaatt
13801 ttgttcaaaa ataaatggtt ttgcgaatga aggagcttcc aataaaccat tttcaaaact
13861 ttcctcttct aagctgcaag cattacctaa aactccattg atatttcttg cgatagcatc
13921 acaaagagta agcgctggaa gttctccgcc tgttaaaata aaatctccta tgctaaatac
13981 ttcattagca aaaatttcta ttactctttc atcaataccт tcatatctcc cacaaacaaa
14041 aacaatattt tgtttcttgc ttaatctctt tgcatctttt tgattaaaag tttttccact
14101 tggatttaga aaaataaaat aaggattttc ttcattatct ttgataaatt ttaaagtgtt
14161 ataaaggggt tcaacttgca tcaataagcc agctccaccg ccaattttat aatcatctac
14221 tttatgataa atattgtccg taaaatctct aggatttaaa aagtcaagct gaaagagttc
14281 tttttggatt gctcgagaaa gaatagaatc tttaaaataa aattcaagca aattaggaaa
14341 caaagaaaca aaactaaact tcatgaattt tctaagatta aaaaagcatc tttggtgcaa
14401 aaaatagaat atttttctat atcaatttta ttgacatatt tatcaatata gggaataaaa
14461 aaagttttag cataagcttt atcaattaac tcttgatcgc tttgaatttc aaataaataa
14521 gaatctccta cttcaagaat atccaccact ttccccaacc ttcgctcatc atcgaatact
14581 tcgcatttta aaatatcaaa ataaaaaaat tcatcctttt tcaatatgca agtttgtctt
14641 gtagcttcaa tactttgata aagaattaga ttaataagct gttttgcagc ttcaacattc

14701 tcataacctt caaacaaaac agaagagtta gctaaattgt agtgtttaat tttcaactga
14761 cttttgccat tttcagtgaa aaaagaagct cctttttttaa attgctggat aaagtcactt
14821 aaattataaa gttttaaata tcctttaagc ccaacagttt ttccaagttt ggcaacttga
14881 ataaaatttc tttcactcaa gtgctttaac cgttacacga tagctcattg aatctttgct
14941 cttgcaagct gaaacgactg tctttattgc attaatcatt tttccatttt taccgataag
15001 tttacccgtg tcaactttat cagcataaag tatgatttca gcaaaatttt catcaagctt
15061 tattttttct gttgtaattt tatctggata atctgctata agtttagcat actctcttaa
15121 aaaatttct accatgaaat tatttactag taatagaagc tactttatca ctaagttttg
15181 caccaacgct tttccaataa gctagacgct cagaatcaaa tttcaccact tcaggctcaa
15241 ccataggatt ataataccca atactttcta tccatccacc atcgcgacgt tttctactat
15301 ctgttactac tatacgataa aatggtcttt tagttcttcc cattcttgta agtctaatta
15361 ctgtcatata ctctccttaa aaataaaaat tttttaacta tagatactat gaaaagtatt
15421 tatagataaa aaatctaaaa ttgtcttcta gcttgattca tcatttgcat taaactatcc
15481 atgccctttt ttcctgaaaa tctcttggcg agtttagcag catttgcaaa ttgctttaaa
15541 aagcgattaa cctcaacttg agataagcca gcaccttctg caatacgacg ctttctagca
15601 ttattaagca aatctggatt ttctctttct tttagcgtca tagatgagat catagctttg
15661 atatgtatga tttctttaga attatctaga tcaagatctt ttacacttga agcaacatta
15721 gaaagtccag gtatcattcc tataatggat ttcatgctac ccaatttttt aacactctcc
15781 atttgagtta aaaaatcatt aaaattaaac tcgccttttt tgattttttg atttaatttc
15841 ttagcttctt tttcatcgat aatagtcgcg gtttttcag ctaaagtcgc caaatctccc
15901 tcacccatta tacgattaac tattctttcg ggaataaaaa tttctaaatc cgctatcttt
15961 tcacccacgc ctacaaatct caaaggaatt ccaatttgtt tagtgatacc taaagctata
16021 cctcctttgg tatctgcatc aaatttagat aaaattactc ctgtaatctg taaaagttca
16081 ttaaaacttg aagctgtttt aactccatct tgcccactca tagcatcagc aacataaaaa
16141 atttcatctg ggtttaaggt atcttttacc gcttttaatt cattcattaa agcttcatcg
16201 atagctaatc ttcctgcagt atcgactaat aatacatccg ccatagaatt ttttgctttt
16261 tctaaagctt gttttgcaac tttaatcgga tctttttcat tttcaatata aaaaagctct
16321 aactcattgg cttcgcaaag ctgtttgagt tgctctactg cagctaaacg ctgtaaatca
16381 caagcggcta tcaaaacttt tttattgcga agttttaagt agttagcaag cttaacagta
16441 cttgtagttt tacctccacc ttgcaaacct accattaaaa caacagttgg aggcttagta
16501 gcaaaaaacaa aaccttgatt ttttccatcg ccatttagaa tattttctaa atttgcttta
16561 atagcttcta aaaattgctt ttgccctatg ccattttttt tcacatcttc ttcaataaga
16621 gaaagtaaat ctttaaccac cttatggtgt acatctgctt ttaatagagc ttttttttaag
16681 gtatctaaag catttttaag cgctttttca tcatcaacaa aacgaagttt attaattgct
16741 gatttaaaag attcgctgac tagttcaaac actttattac ctttctatta ttttcaaatt
16801 taagttgagc attatacaaa atcttttctt aaatggcttt aaaaatctaa attttttatc
16861 tcaaaaccaa gctttgcaaa actaggatct aattctgcct taaactgata atttaaaatt
16921 cgaatttcat aactatgcaa atacatccta tcgcttgaaa tcttagcata tttttcatct
16981 cctatgattc catgctttat aaatttagca tgcactctta tctgatgagt tctacctgtt
17041 ttaataataa ttttagctaa agttttttta gcttgcatca taataggagt aaaaatacta
17101 accgcgctta aaccttcttt agaaatttta cttaaagctc catttttagt ttttatagtt
17161 aatataggct cattaatctc aacttcttct gctaaaattc catctaaaac agctatataa
17221 cctttatgaa ctctttgttt tttaaactct tcaatgcata tctttcgaaa atcttcattt
17281 ttacaaagca atatgacacc actagtttct ttatcaagac gattaagcaa cttcgcattg
17341 aatttttttt caagctcctc gctgatatag gcatagggtt tatttatagc aatgatattt
17401 tcatcttcaa aaattacttt aggtttgctc gttttcacaa tactaaattt cgtcttatca
17461 ctcatcaaag ctcttgccac aaccaccttt ttaccatgag aaaaaacgca acctttatct
17521 atcaaatctt ttgcttcgcg atttgaaata ttttcttgca aagctaaaag tttatatgct
17581 ttttcttgca tcgatacctt cctgtatagc ttggattatt gttgataaat cattattttg
17641 tgctattttt gcttttaaat tataattatg aattatacca tcaagttctt ttaaatcttc
17701 gcaaaaatag acattatcta cttcttgaaa taagcttttt tgattatgta tataaactcc
17761 acttatcaat acattgttaa aatgagctac ttctatggga ttatgccctc ctatcccttc
17821 aaaaaaagaa ccacctaata caaccacatc tgaaatggca taaaaattta taagctcacc
17881 taaacaatcc aataataaaa tatcttgatt aaatttttta tcttcatttt gcaataaact

17941 aaatttatca aatttcaaac ctttatcatg caacaattgc tccacttcca aaaaacgttc
18001 aggatgcctt ggggcgatga ttaatttttc ttgtttatcc atcttataat gcttgagtaa
18061 aagttcttct tcgcccttat gagtgcttgc aaaaataatc aatttttgct taggtttaaa
18121 gtatttttgt gcgggacata tttgcaattt ggatttaata tttggaaaaa ttttaacatt
18181 tttagcacct aaatcttcca atcttatttt atccgcttca ctttgtgcaa aaacttcatc
18241 aatatagcaa aaaatttttt tataaaaaaa tctaaatttt ttatagcttg catatgaatt
18301 atctgaaatt ctagcattta gtaaaataac cttgcttttt tgaagctttg ctataaaaac
18361 gaacattaac caatactcag cttcaaaaat cactaaaacc ttgctaggtt tgagccaaaa
18421 tggaatccaa ttttcaaaag caagataatt tactttattg caaatttctt tagcatagtc
18481 ataacccgtc tgcgtaatag tcgtaattct tgaatcaaat ttcaaaacaa gctctttaat
18541 gctttttgcc tctccatatg agcaaacatg aaaatgcaca tccgatcttt tttgagatat
18601 attacgaaag agaaaaaatc tagcctttaa gctattttta tattttggct taaataaaga
18661 aagtaaaaaa agtgggatag cgcagagtaa aaaagctatc cacactaaaa tataataaag
18721 aaaaatcaat tttgatcttc ttgctcttta tataaaattc ttccacaatg agggcaagtt
18781 ataatctctt cacctttaat cactgacaaa taagttttat cataaatttt catgaagcag
18841 ccataacaag cttgtttttt aacgggaact acggctgtat ttttagccca ttttctaatc
18901 ttctcataaa agcttaaaac tttttgattc atctctgcaa ctaatttagt ctttttagca
18961 taaacattca ttctttcttt ctccaagcca tccatttgag aactaatgct tactcttatt
19021 tcatctaaat tttgctcctc ttttgctctt tcttccaaaa gttctttttt gaaaagctct
19081 ttatttttcta aaatttttatc caaacgaaca atctcatcat tagccgcatc aagctgctct
19141 ttagcgatat cttcttcgat ttttaaagca tttgcttctt tttctgtctt aacagctcca
19201 ctctttttag aaagctcttt gattttagct gaaaactctg aaatatgagc gttattttga
19261 actttttgat ttttctacatc ttgaatttca ttttctaaat tatttaattc aacattaaat
19321 ttagcaatct tattttcagc atcttttaaa gtcttgctaa tgctttccat cttcggttca
19381 aagctgtcaa tttcttgatc tatttttgat aaaagcacta attgttcgag atatttattc
19441 atttattttc cttaaaaata ttgaaatgga tttttttgaaa ctgttattat aacttttagc
19501 ggcaaatttt tcaagtgttt taccaaacat tgagcaaaat agcgttcgct ctcaaaatgt
19561 cctaaatcaa tcaaatttag atgattgctt atggcttcta aggcttgatg gtatttaaaa
19621 tcgccactta aatagcaatc tgccttaact tcatttaata aatctccccc gctccctgtg
19681 caaacagcca agcgttttaa atgctccttg ccacaaaaac ttgttcttag aatttctaaa
19741 tttaagcgct ttttaaccaa ggtgcaaagc tctttaaaag tcatatccac atctacataa
19801 gctaaaaatt tatctttaaa agaaatttca aatcccaaaa tttcttctat aaaataagta
19861 tttaaatgac tgagatcgaa attagtatgc atagaaatta aagaaagatt ttttctaagc
19921 atctccttaa taaaagctct aggataagct gaatttgcca aatccttcaa acctttaaaa
19981 atcaaaggat gatgtgtgat aaacaaggaa ttttcttctg cgttttgaat caattgctca
20041 tctatatcaa gacttaaata aacattatga atttcatcgt caaaatttcc aagtaaaaga
20101 ccactattat cccaaatttc ttgagtttca aaaggactta gcttatctaa aaaattataa
20161 atttcaatca gtttcatcta aatttttctt ataaatttca gcacaagttt tcgaaagctc
20221 gcgaattttt agcatataat cttgtctttg cgcaacagaa atagcacccc ttgcatcaag
20281 caagttaaaa gtatgagccg caagcataca ataatcataa gcaggcaaag ctaaaccttg
20341 ctctaaaata cttttacatt ctttataagt gttctcaaat tgctgattta aaaaatctac
20401 attactgatt tcaaaattat atttactgaa ttcatattca ctgcgtttat gtacatctgc
20461 gtatttaatt ttttctccat taaattcgct ccaaacaata tcataaacat tatccacatt
20521 ttgaagatac atggcaattc tctcaagtcc ataagtgatt tcagcactta ctaaatctac
20581 cgcgattccg ccaacttgct gaaaataggt aaattgagtc acttccatac catcgagcca
20641 aacttcccaa ccaagacccc aagcgccaag gcttggactt tcccaattgt cctcaacaaa
20701 acgcacatca tgacttttaa gatcaaagcc caaattctca aggcttttta aataaagctc
20761 ttgaatatta tcaggacttg gcttcattaa aacttgaaat tgataataag ctcccaggcg
20821 attaggattt tctccatatc ttccatctgt tggacggcgc gaaggagcta cataggcagt
20881 agcccaaggt tttttaccta aacttcttaa aaaagttgcc ggatgaaagg ttccagcccc
20941 tgctggcata tcataaggct gcataatagc acaaccttgt ttttgccaaa attcttgtaa
21001 atttaatatc atttgcgaaa aagtcatttt aaccttcttt attttcattt aatttttcat
21061 ttgttgtgct agaattttca tctttttca tttccaaatc aggctcagtt cttgccccaa
21121 aaatttcagc tgtcttactc cacataagct tatatttgcg ttttaaaaat tctatttctt

21181 ctcgggcatg ttttaattca tggtttaaag tttcaatagt ttttctatct tcatcataaa
21241 gctcttgcat tgaataaagt gcatctttta aaaatttatt ttcatttttt aaagcatcaa
21301 gggtttcatc ctttgcatct aaaaccttct catgcaaatt taaaatcgtc cctatggttt
21361 tttctacaaa gctttctcct gctaaagtca tagaatttac catggcaggc tttgaattag
21421 aagcgcttgg aacaacgcta aaagttcctt gatttgcctc gatataaatt tttccctctt
21481 cttctttaaa attcaaagcg ccatttgcca tcattccttt tacaacatct tcatttaaat
21541 gcacaagttt gcaaaattct tggagctcta aataagtttg cattttcatc cttttataat
21601 aaaacttcta cgctcttaga atcgcttatt aaattttctt cttgattttt tctatcttgt
21661 tttaaagcct ctgccaaagc ttcatcattt aaagcaagaa tttgcatagc caaataagct
21721 gcatttttag cccctgcttt acccactgct aaagttccta cgggaattcc acctggcatt
21781 tgtacagttg aaaataaaga atccatactc gctaaagcac ttcctggcat aggaactcct
21841 aaaacaggtt tagtcgtata agccgcaact gctcctgcta aatgtgctgc cattccagca
21901 gctgcgataa aaactttagc tccttttgtt tctgcttctt taatgtattc tttagttcgc
21961 tttggactac ggtgcgcaga actgataata agttcatatt ttacaccaaa tttttcgagt
22021 gttttagcac attcactcat gatttcataa tcactcttgc ttcccatcac aattgataca
22081 aatttcatcc aatttccttt cttaaaaagc taaaactgct gattttattt ggcattttaa
22141 tttcgttttc atttccgcta tgaatttcat taaattcttt attattttta gaaataagtt
22201 ttttaaattc tacaaaatat ttatcttctg ttttataaat tttacccaat tcattatcac
22261 aagactcgat tttatctcct aaaggcatta aaatttcata ggctttattt aaaacaatct
22321 tgcccttgca tttaaaatat tcaccatcct caacgatagc atgcacttga tgagtgccct
22381 cttctatact tgtatcgtga ttttgcgtat ctgctttttc caaaggacgc gagacaagat
22441 aaccatctgt aaagccacga tttttaagtg tggcaatttc tttttcatac ttacttgctt
22501 caaatttgcc ttctaaagca tcttgtatag ccattttata agttctagtt gtaagtgcga
22561 cataatactc acttttagta cgcccttcta ttttgaatgc acttatacaa tcttcttgca
22621 taattttttc tatataagaa cacaaattta aatctttaga attaaataca tgagtaccat
22681 tttcatcttc ttccaaacga aaaagtactc cattttcagg attttttgca taaagctcat
22741 aattaaatct acaatcattc gcgcaagagc cacgattact cattcttcca ctttggacag
22801 aactaatcaa acagcgaccc gagtaggcaa aacacatcga tccatgcaca aaagcttcga
22861 gctcaatatc acaattttct tttaagcttt tagcatcttt aagacccaat tctcttgcga
22921 taacaactcg ttttgcaccc atatctttat aaactcttgc atcaagatag tttaaaacat
22981 tagcttgagt agacacatgc aaagctattt caggagctaa ctctcgaacc aaggccatcg
23041 cacccactga agctactata aaagcatcgg gcttcatttc tttaagtttt aaaatatgtt
23101 tttttaaaga ttctatttgt ccgcccaaat gaaagccatt taaagtaaca taaacctttt
23161 tgcctctttg atgagtatat ttaatagctt cttcaaaact ctcataatta aattccctag
23221 ctgtgcgaga acgcaaagaa aaattattaa cccctgcata aactgcatcg gctccataag
23281 ctaaggcgat ttttaattta gtaaaatttc ccgcaggagc tacaatctct ggaataatca
23341 tttttgacct aaactcgcta tgagtgcttc tatatcatca ttacttacaa tatcagtagt
23401 agaatcacct tcaatatgaa ccgcagaact cacacgtttt ttatcatcaa ttctaccttc
23461 aaataaagaa ctcatataac gactcaaggc gcgcattaca ttgataacgc gttcaatttt
23521 ttgacggtga atatcttggt attgcatagc atccattgcc atcattactt catcttgtcc
23581 gcattgtaat ttatcaataa tttcttggct tgcttttttc acttttgaat ttaattgtaa
23641 tgcctcatta aaactaacaa catttgggaa tttctcgttc agttttgaaa atatttcaat
23701 attcttactc aatgccttat caatttcact aaacaaactt tcagaatctg caaagaaact
23761 attaatactt tctaatttat ccatcatctc ggtagctttt aattcgctat ctttagtaac
23821 atcatcgagc tgatgaacca ctttatgctc ttggtttggc gggggtggtg gccatacaac
23881 actaggatct ggtttataat cagacacctt tacatcttct aacatcaatc cctcttcaga
23941 ttctaaatga gactctgttt cgcttttatt gttagactct gcatcctcat caaaatttac
24001 atcaccattc atcaaggcgt caagttcttc ttgagtcatc tttttccttt tctaaaccta
24061 gattttattt tggtactacg attataatca aatttatcta aaatgaaaat ataaatttaa
24121 attttcatag ttttgtaagt aaatttcaaa aaatgattaa aaaaatatat taattaaacc
24181 tgaattaaaa aaaatatgat acaattttca taaattatct taaagaagga gtaaaagatg
24241 ggcaaatttg ttaacaatgt tgatgaattc tttaattttt gcaaacaaaa cgaagttctt
24301 tttgtagact ttcgctttac tgatatgata ggaacttggc atcatatcac ttataatctt
24361 catgcaatta gtgaagaaac ttttgaagca ggaattccat ttgatggtag ctcgattcat

24421 ggatggcagc ctattgaaaa atcagatatg attttaaaac ctgatgcgca aagtgcattt
24481 ttagatcctt ttacagcgga tcctacggtt atagtttttt gtgatgttta tgatatatat
24541 aaagggcaaa tgtatgaaaa atgtccaaga agtatagcaa aaaaagcaat ggagcacctt
24601 aaaaatagcg gtatagctga tactgcttat tttggtccag aaaatgaatt ttttgttttt
24661 gatagtgtaa aaattgtaga tacaacccat tgttctaaat acgaagtgga tacagaagag
24721 ggagagtgga atgatgataa agatttcgca gatagctaca acacaggcca tcgtccacga
24781 aataaaggtg gatattttcc tgttcaacct atagattctt tagtagatat tcgttctgaa
24841 atggttcaaa ctcttgaaaa agtaggactt aaaacctttg ttcatcatca tgaagttgca
24901 caagggcaag cagaaattgg ggtaaatttt ggaacacttg tagaagctgc ggataatgta
24961 caaatctaca aatatatcgt taaaatggta gctcatttaa atggaaaaac agcaactttt
25021 atgccaaaac ctctttatgg cgacaatgga aatggtatgc atgttcatat gagtctatgg
25081 aaagacggtg taaatttatt ctatgacaaa gaaggctatg gaggacttag tcaaactgct
25141 attaactata taggcggggt tttgaaaaat gctcgttctg ttgcagcttt taccaatcca
25201 agctctaatt catacaaaag aatagttcca ggttttgaag ctccttgtat tttaacctat
25261 tcttgccaaa atagaagtgc aagttgtcgc gtgccttatg gaataggtaa aaattcagct
25321 cgtgttgaaa ttcgtttccc tgatagcact gctaatcctt atctagcttt tgttagtctt
25381 ttaatggcag ggcttgatgg aattaaaaac aaaaccattc ctgtaggacc tatggatgaa
25441 aacttatttg atctcacctt agatgaaatt cgtgaaaaag gcatagagca actaccacat
25501 accctaagag gaagtcttga agctttaatt cgttacaacg ctttcttaaa acctgtaatg
25561 agcgatatct ttatagatga ttatcaacat ttaaaatttg aaacccaagt ttggcctgtt
25621 gaagcacgtc caactgcata cgaatttaaa acttgctact cttgctaaat aaaaagcaaa
25681 gcgaaatttt aaacgctttg ctttattttt attgataact tcttaaattc ttacttttca
25741 atattgtata aattcacttt caaaaagaat caaaaattat tattgttaat attgcatcag
25801 aatttctaat ccctaatttt atatatttta gaaattgatc gattatactt catttgaatt
25861 gtttttttcat tattttaaac ttgaataata ttttagattt aatgtttgat aattttatac
25921 cattgttgag aaatataatc ggataatgta attataaata tgtaaacatt ttagccaatt
25981 aataattgga aaaatttatg gcataaaaat accataaatt tatatgatta atttaaatta
26041 tctttttaat tgattgacaa ttccaagcat atcatcactt gtcgtgatag cctttgaacc
26101 tgcttcgtaa gctctttgcc ctgtgataag atcagtcatt tcttcaacaa gctgcacatt
26161 acttagctct ataaaaccat gtcttattgt tccaagtcca tcttgagttg ctatacctgc
26221 cacaggtgca ccgcttgctc ctgtttcaag ataaagatta tctcccatag aatgaagtcc
26281 agctgggttg ataaattgta caagttcaac ttgaccgatt tgtgtttctt gttgttctcc
26341 tggaagcatt acagaaactg tgccatcagt agccacatta atcgcagttg ctccttctgg
26401 tatagtcatt tcaggcaaaa gcctataacc atctgcatta acaatattcc cctcattatc
26461 ttttgtaaat tgcccatttc ttgtataagc tatcgtgcca tcagggagtt gaatttgaaa
26521 aaaaccattt ccagcaattg ccatatcaag accatctgtg cttgtagcct ttaaattacc
26581 ttcagtaaaa acctttgtca cagctgtcgg gcgtacacca atacctactt ctatgcccga
26641 tggagaaagt gttgttgctg aagttgaagt tcctgcatat ttcataactt ggtacattag
26701 atcagcaaat tctgcacgac ccttttttaaa acctgctgtg ttgacatttg caatattgtt
26761 tgaagtaaca tcaatttgtg tttgctgtgc taccattccc gtagcagcag tatgaagtga
26821 tctcatcatt ttctatcctt taatctattt tatattattt tatttaacag ctgcaagttt
26881 attgatagct tcttggttta aatcatccat atgagctgtc ataacttttt gatacatttc
26941 taccattcta tttgcctcta tgagccctac catttcggtt actggattga cattagagcc
27001 ttgagaaaaa ccttggcgga ctgcattgga attatccaaa tctctaatac gcgttaaatc
27061 atctatccta taaacattat ccccatcttt ttgcaaagct cttatatcat ctacttgagc
27121 gataaataat cttgcatttt gaacaccatc tacttcaatg cttccatttc tgtccacatt
27181 gatataaacc gcgccatttg ggatacgaat tcctgcattt tgaggattat taaaataatc
27241 actgcttaaa attctatacc cttgcttatt gaccaaataa ccctcttcat taagctgaaa
27301 atttccatct ttgcttaatc ttatttcacc atctttagtt tgaaccaaat aaaaagcatc
27361 ttccctagtc atagccaaat ctaaaggatt gttagtcgct tttaaagagc ctaagctaaa
27421 atctgtatat ccttgtgaaa cttgaggaac accatctata gtagtattta caaatcttga
27481 tgcatctctt gtatgatttt ctataggtaa ctcatcctga gtttctttaa aaatcctttt
27541 aaaatctgca attacaacat catctctttt gtaaccactc gtgttaatat tggcaagatt
27601 atttgtgata acatcaagtt tattaaattg agttaccatc ccgcccgttg cttgataata

27661 tccattttgc atatttttca cctataaatt ttaatatttt aatttataaa gcaaagagtg
27721 ttccaacttt ttaaaagaat gaattttagg ggattagatg aataaaaata aaaagcaaga
27781 tttataaaaa tcttgctaaa aaatatttcg ttttagtcca tttgacggcg taagaaagta
27841 ggagtttcaa tttgagccat tatctcttca tcaaaaccac cacttacttt tctaaggttt
27901 aaataaggat ttttttagg agcttcagcc tcttctgcag cttttttagc catactgtct
27961 ctatcttcaa atcctgtagc aataatagta acttctaccc tatcttccat gctatcatca
28021 gtagtagaac caaaaataat ctttgcattc tcatcaacaa tttcttcaat attatttgca
28081 gcagcagaaa tttcaatcaa agagcaatta gaacttgttt taaaatgcaa aataacccct
28141 ttagcccctt taatatccat accatctaat aatggagatt ctatggcatt agaaagagcc
28201 tcttcgatag cattttctcc actagcacta cctacaccca taagtgctaa accacgatga
28261 ctcattatgg ttctaacatc agcaaagtca acattgatat cgccattatc taaaagtata
28321 gaaaccatac ccttaacagc acgagctaaa atatcatcaa ccaatttaaa agcatcctta
28381 atccctgctt ttttatcaat aatacttaag agtttttcat tttgaatcac aaggatagaa
28441 tcactttctt ttttgagttc aaggagtcca ttttcagcaa gtttttttct ttgttttcct
28501 tcaaaagcaa aaggcatagt tacaacagaa acagttaaag cacctatttc tttagctgct
28561 tgtgcaatga ctggagtagc gcctgttcct gtgccaccac caaaacctga agcgataaaa
28621 acgatatcac tttgactaag actagcttta atctcttcaa agctttctct agcactctct
28681 gccccaacct ctggcaacat acctgcgcct agtccctttg tttttttctc gccaagttga
28741 attttggttt ttgctaatga atttgaaata gcttgtgcat cagtattagc cgcgattaaa
28801 tcaaggtcat ttaagcccat cttgaccata tgattaatca tatttccacc gccaccaccg
28861 cagcctataa ctttaatttt agcgccttta ttatgttgca tctcttctac taaaaattcg
28921 ctcataaaaa ctttctcctt gttaaaattg attcattatt ttatgccaaa tatttgaaaa
28981 aacattaggt tttttctctt ttggctcttt aaaatctaat tgttcttgta atattattgt
29041 atcattttct tgcaaatttt cgccaaaaaa atcagatttt gtttcatttt ccacttcttt
29101 ttgtggaatt atttcttgtc taatttgacg gttgtaattt tctatctccc ctttatatct
29161 taactttca tttgaatcaa gttcataagg cgtaaaataa cctgcaccat ataaacaaag
29221 tcctatagca caagtatttt caggatcgtt aaaaatttca ctaaatccag taatcaaatc
29281 ttttctagca gtagcaagtc taacagaacg gttatcaaaa gttgcaggag ccaactcgtc
29341 aatacctgct aatttagtca ttccacccgt caatacaaca ccaccaccta tactatttgc
29401 ataacgatta tcacttaaaa ttttagctaa aatcattaaa gtttcttcag ctcttgcgta
29461 aattacattt gaaatgatat ctaaagaaac ttcatttact tttctttcat ctcccataga
29521 aggaatttgt atcaatgcat taggttgttg agaaagagct gcataattta atttaatttt
29581 ctcagcttcc tttaaagggg tatgcaaagc catcgataaa tcttgggtaa tattaatcga
29641 tccaatttgc aaacaatcat tatagcgaat tgaatttcca gtatgaacta ccatatcgca
29701 gatggctcca cccatatcaa tcaaaacagc acctagttct ttttcactat catctaaaca
29761 agctatagct gaagcataac cagaaaggac tatattatca actcttaaat cagccaactc
29821 aacagctttt tttaaatttt taatatgaga ttcttgagaa ataactatat gagttgaaac
29881 ctccaaacga ttaccgctca tacccaaagg atcatctaca tgttctaaat cattcacctt
29941 aaaattataa ggtaaaacat ggataatttc ataaccgctt ggaagatttg cagtgtgctt
30001 agctgtactt accgcacgat gaatttcttt gatgcctatc tcatgatttg gtatatttac
30061 cacacctatg ctgtcaacac ttttagtata ggcacctgaa atagaaacca ccactttatc
30121 ataatgaaca ccgctcatca tttctgcatt tctaactgct tcttcaatcg attttgaggc
30181 aagttcgata ttagtaatag cgcctttttt tacaccatta gtttttgctt ttgcaaaacc
30241 aataattttt aaaccttcct catctttttg agcgacaatg gcacaagttt gtgtcgaacc
30301 taagtcaatt cctaatatat tcaaaattat ttacctttat aataaatttt tattggatat
30361 gtttttttaa gctcttcgat caattcctgt ttcatttcat tagcttttaa gttttgtaaa
30421 ttctgattaa gcattgtgcg atattgctga aatttttcag gattcatatc aagcttttgc
30481 ttattaattt tataaagtat agccttatct tcatttaaaa taacataaga acggttttga
30541 tcagaattaa aaacattcat taaaaaataa ctaaattccg cttcatttaa tatattacta
30601 atcttagcat catttcttac agtatctctg cttacaaaac ctatattggt tccgctaaaa
30661 ttttctaaat tttgcttagc tttttcttct aaattctttt ttacctgttc gctgatataa
30721 ataggcataa gctcttccct agcctcatca aaagtcttag ttcttatagg attggttttg
30781 ttaagtttga tgataatata accatttga tattcactcg gtcttaacac atctccattt
30841 ttcgctctag ataaagcttc aagtgggtaa taaacatctg agtctgtgat attttcatct

30901 ttttggaaat tatctttacc ttcttttaat tctaaaaatt tagcattagc cgtattttta
30961 agctgcgcta aagcataatc tttggcaaca tcctttttag ccgcttcaaa actcatgatt
31021 tttccgctaa aatctttata tttaaattta ttattttcat tattataaaa ttcttctagc
31081 ttgctatcat cgattttttg catatccact ggtagataat aagtggaaat ctcatatatt
31141 ttttttgttt tataatcttc tttgtgttca ttccaaagtt ttttaagctc ttcttcatta
31201 acttgtatat tttctttatc ttgctctagt tttgctatat ttaaagaatc ttgcataaaa
31261 tagctagctc ctagcatttt caactcttca tctgtttgag gaagattaaa tatttgctct
31321 agtttaccga taataatttc atttgaaaga gtttttttcat attcttttgg agtcatattg
31381 tttgcattta aaagttcgta gtaaattgtt ttattgaaat cgcctgaagg atcttgaaag
31441 gctctagttt cagcaagtgc ttgaataact tcattttcgc ttgaacctaa tcccaaatct
31501 ttagcaaaat ttaacaaaag tttatcttca actaaagagc ttaatgcggc attttcgatt
31561 cctaattgct tagcgctttc ttcatttaaa ccaccattgc taatttggtt gtaataatta
31621 aaaatctgat tatatcttac attaaattca ttataattga ttttttcatc tccaactaca
31681 gcaacactcg aacttctatt aagattaaaa tcataagcac cccaacctaa aaacccggct
31741 ccaacaaaag ctatggtgct aatccatata gtaataacaa gatatttttt gtgatgttgc
31801 atccaagtaa gcatatcatt ccttgataaa aattaaacta catattttat cgcagaagtg
31861 tttaattaga gtttaattag tagtttttttg ttaaatattt tcattttgat tttagttaaa
31921 atttcgttat aattatagct tataaaaatt ttataggata taggatttga aaattccaca
31981 cattcccgtt ttactcaatg aagtaaatca agcatttaaa aaactagact cggggtattt
32041 tttagattgt actgtgggtt ttggtggtca tagcgaatcc ttacttaaaa atcatccaaa
32101 ccttaaactt atagcatgcg atcaagataa agaagcgctt gaattttcaa aaaagcgtct
32161 tcatgatttt caagatagaa cagaatttgt tctgagtaat tttagtcaaa ttttagacca
32221 aatttcacac gataaactga acgatctcaa aggcatttta gctgatattg gagtatcttc
32281 ttatcaactt gatgataata aacgcggatt taatcttcat tctgattttt tagatatgag
32341 aatgaatcaa gatgctaaaa attcagcttt tgatattatt aataattata cccaagagca
32401 attgagtgat atttttaaaa aatatggaga attaaataat ggatacttta tcgctcaaaa
32461 aatttgtcaa gaaagacaaa aaaataaaat aaaaagcgct aaagaacttt atgaaatcat
32521 aggaaaaaca aaacaaaatc atagaaaaat ttctaaagca actctagttt tccaagctat
32581 cagaatagaa gtcaatcagg aacttgaagt tttaaaaact tttttagaaa aattagaaaa
32641 aattaaatta aaaaattgta tcctagctat tatttgtttt cattctttag aagataggat
32701 agtaaaaaac tattttaaaa aatgggctaa aaattgcatc tgtgatgaaa gagcctttaa
32761 atgtgaatgt ggaaataatc atgctttagg acaaattata agtaaaaaag ccatagttcc
32821 aagcaaagag gaaattatga ttaactctcg ttcaagctgt gcaaaaatgc ggatatttca
32881 ttttaaaaat atggataaat aatgctagat gatgatttct taaaagaaaa acaaaatata
32941 cgccaaaaaa tgcttaaatt ttctcgtgcc gtcaatcaag gtaaaccttt ggatgatgat
33001 ttaaaagaag aaatctctag tgatgatatt ttaagacgcc gctttaaaaa acgttcaaat
33061 cgtttcttag atgaagtaga agaagagtat gagttagaat atacaaaaaa atcaaatata
33121 tatttaaaag aagacttgat aaatgttaaa ttagaagaaa aacaatcttt ggctaaaaaa
33181 atttttttcca aaataaaaga aaaaaataaa aaccaagaac caaaaaataa aaaaacaaaa
33241 aaactttttt cttttctgaa gaaaaaagaa attcaaacta tacaaaataa aactacgcac
33301 aataacactc aagaaaaata tttgcacaca gacaagcaaa cagaaattaa aaaattacaa
33361 aatgataaga tccaacccac acaaactgac ttaaaacctg atataagaca aagcctagaa
33421 aatttacaaa aaaaaataca aacacaagaa ataaaagcag agattgaaaa acccgaaatt
33481 cagcccatta aaacagaata cgatgaagat gcgcaaaagg ttaaaaatgt attactagaa
33541 ggatttagca atgctactaa agaagataaa aatctcaatt tcaatcattt gctttttgct
33601 gtattatttg tcagttttac tcttgtttta tttgcaccac aaatttatat aagaaatcaa
33661 atttactatc ttagtcgaga aattgctact ttaagagtag aagaaagtgt acttgatgaa
33721 gaaaataaag atttaaaaag acgcttggaa atcatgcgct ttcaaaatca aattttagat
33781 tatttagaat gaatgatttt ttctaaaaca tattgttcta aatttgcttt tggaatttct
33841 tcctttaagg cttctttata aaaattgcca gccataatag aatattttttc atacgcaaaa
33901 taaggaaaat gcaaagccca cgctttttgt attaattttt ttgaaatcat tttgcgagca
33961 taaaccttaa aaatatcgta agaaataaaa acacatgcgg taacagctac ggcactgatt
34021 atactgatat gaaaatcaag acgagtaaaa agataatgcg taatgagtaa taatggtaat
34081 aaaatcaaag cgcagaaaat tccaaaatat atataagaat ttaaaatttt aaaattaaaa

34141 ttaagtttgg aaggatccac aagcattcca tcattaaata aagcattagc ttcaagtaaa
34201 tcgcgcaaga gaacaggctg gtcggatatc ttaaaaagaa aattacaact aaaatcttta
34261 aatttttca tatttttac acttttaaat ttttagttta aaatactatc taaagaaaat
34321 taaatttggg ttaaaacaaa agcttttagc ttttgtttat caactcttct gtgattctag
34381 caatctcaag ttcttcatct gtaggaatga taaaaatctt tactttagaa ttagcggtgc
34441 taatcattct ttcacagctt gatttttcat tatttaatcc tatatcaagt tcaacaccaa
34501 gatgagataa tctttcacaa actttttggc gaaccgaagc ggcattttca cctataccac
34561 ctgtaaatat aatagcatcc gttctaggta aaaccgtaaa ataagcccct atatatttta
34621 aaaggcggta acaaaacata tcaaaagcca aacgcgctct atcatcaccc tgctctattt
34681 tcgttcctat atctctaaaa tcattagaac cgcaaattcc ataaagcccg ctttttttat
34741 taagcaaagt gtcgatttcc tctattgtta agcccttaag attactaata aatggtaaaa
34801 ttgctggatc taaatcacca cagcgagttc ccataacaag accttcaaga ggagtaaaac
34861 ccatagaagt atctatactt ttacctcctt caatagcaca aacacttgca ccatttccaa
34921 gatgtgctga aattgcattt aaatcttctt tgtcttttcc aagcaattta gcagctctag
34981 cacttacaaa agcatgagaa gtaccatgaa aaccatatct tcttatattg tgcttatcat
35041 aaaaatcata aggtaaagca tacatataag caaaatcagg catagtacga tgaaatgctg
35101 tatcaaaaat cactgtattt ttaacattag gcgcagcttt catcatcgtt ttaatgcccg
35161 ctaaatgcgc aggattatgc aaaggcgcaa aaatactaac tctatcaatt tcttttaata
35221 cataatcatc aaccaaacaa tgctcgctta aatttcttcc cccatgcact atacgatgac
35281 cacagccatc tagcgcattt aaatcttcta aaattccaga ttctttaaaa agctcattga
35341 caatttgtaa accatgttcg tgatttttta taggaagttc tctttcaaat ttttcatcgt
35401 ttaaagcatt ttttagtata acttttgatt tttcttctcc aattttttcc accaaaccac
35461 tagctttaac caccttatca tcaaaaaatt taaatttaat agacgaggag cctgaattta
35521 aaactaaaat cttcatttat tttcctttct tactgtgctt gtatggcact taaaataact
35581 gtatccacta tatcatcaac caagcatccg cgacttaaat catttacagg ttttttaagt
35641 ccttgcaaaa taggaccgat tgctaaagca tttgctgttc tttgtacagc cttatagcaa
35701 atgtttgcgg catcaagatc aggaaaaaca tatacattta cattgcccgc tatcttagaa
35761 tttggcattt ttttagcagc agttttaggg tcataagcac aatcaaattg cataggacca
35821 tcaatattta attctggata tttttccttg gcaattttca aagcttcttt aacaagatct
35881 acgctctcgc ctttgccgct gtctccgcta gaataagaaa gcaaggctat ccttggctca
35941 ataccaaaag acttcgcagt catggcactc acataagcac tggttgcaag ttgttcagca
36001 tttggatttg gatttacagc gcagtctgca aaagctaaga ctttatcatc aagccccata
36061 aagaaaattc ccgaaacaga atcaacaccc tcttgagttt taataatctg aagtgcagga
36121 cgaatagttt cagctgtagt tgtagatgct ccacttacca tagcatcagc ttttccactg
36181 tgtattaaca aggttccaaa ataagtccta tctttaacta attctttagc ctcttcaagg
36241 ctcattccct tagattttct tgcttcatat aacaaagtag caaattcttc attgtattct
36301 gaattaagcg gattgagtat ttttactgcg gttaaatcaa gcgctaattc atcagctttt
36361 tgtttgatat cttgctcatc acccaacaaa ataagatcaa ccacactgct ttttaacaaa
36421 atttcaactg cttttaaaat tctttcatca ttactttctg gcaaaacaac agttttttta
36481 tttttaacac ttttttttaat gagttcatat ttaaaacgat gtggagtcat aaaatcataa
36541 gcttgcaact tatgcacttc ttcaaaatta aaattttcat taatcaaggc gtaatctttg
36601 ccatctaaag cttcatcaaa atacttttga gctattaact tatcttcatc attgataata
36661 gccgctatag gagtgttaag atttttcgcc aattttatgc ttagatcaaa accatcaagt
36721 attccaaatt cacaaaaact attaacaaga acaaaatcat atttacgatt taattcttca
36781 tattcttcta agatggtatt gaaaaaacta tgcggatctt tgctaaattc ttccatagct
36841 tctctaaaag taaaactata agcatttttt aatgtttgct tgatatcaaa ttcctcaagc
36901 catccttgca aaacaggaat acgatgtaca taaatcacag gacaataaat agctatattt
36961 ttatatatct tgctatagtt tttcaataat ttcgtggtga ttaatgtatt taactcatca
37021 ctcctagatc tcatcaaata aagatttgcc attattttct ccttggtttt aaaaagtgga
37081 acactctttg ctttatactt acttgaaaaa ctattttaac aaataaaaat gaaatttcat
37141 catttaaagg acgaaaatga aaaaagtttt attctatgtg ttaccttttg catttttttgg
37201 atgttctgca acagtagatc cacaaatttc tatgaagccg cctgcttatg tagaagagct
37261 tgcacctaaa caaagcaaca atgtagaaac tgcacccgga tcactttttg gaaaaggaga
37321 taatccttta ttctctgata aaaaggcgat gaatgtaaat gatttagtta ctgtggttat

37381 ccaagaaagc accactcaaa gcactcaagc caataaggcc acaagcagaa ccaatacttc
37441 caatttagga ggaggagctt taacaggtgg aagtggcttg cttggaactg caattgataa
37501 agtaaatgct tattctaata taggctttca aaccaatagt actaataact accaaggaac
37561 aggtgctcaa agtcgtaatg aaagttttaa tactacaatt tcaacacgcg tgattaaaat
37621 tttatctaat ggcaattatt ttatcgaagg ttcaagagag cttttgatta atggagaaaa
37681 acaaatcata caacttagcg gtgtgattcg tccttatgat atcggtcaag ataatactat
37741 agatagtaga tatatagcag atgcaaaaat tctttacaaa actgaaggtg aagtggatag
37801 aagtactaga aaaccttggg gaagcaaagt catagaagct atatggcctt tttaaataaa
37861 cttaaaattt aagtccttaa aattaggact taaattttat tgcgatttta ttttaaacat
37921 ctatacttaa aataaaatct attcttttat ctctctactc ttaacctcat cttctacttc
37981 agctaaaaat ctatccacaa gttcattttc tttgagtttt gctacaactt ctccatgtct
38041 tataacaagt ccttgatttt ttccaaaagc tatagctaca tcagcaccct tagcctcgcc
38101 taaagcatta acaacacagc ccataacact gatatttaga ggctctttga tatgtttagt
38161 tttttcttct acgattttaa tagcgctaat taaatcactt tgaattcttc cacaagttgg
38221 acatgagatg atatttactc cactttttttg cactccgcta tcttgcaaaa ttgctcttgc
38281 gactttgatt tcttcttcga gctcaccggt cattgaaact ctcatagtat cacctatacc
38341 tttaagaagc aaattgccca aagcaataga actttttatc gtgctgtgaa atttagtccc
38401 tgcttctgtt actcctaaat gaaaaggata atcacaaagc ggtcttaatc tttcataagc
38461 ttctatggtc ttttgagtat cagaagtttt catcgaaatt tttatatcaa aaaaatccaa
38521 atcctctaaa agcttaatat tatacaatgc actttctagc attgcgtcta cgccatatcc
38581 atatttatcg ctgaattctt tttctatgga gccatgattg acacctatac gtataggaat
38641 acctcgctcc ttacaagctt ttaccacttc tttaatattt tcttttgagc ctatattgcc
38701 tggatttata cgaactccat caataaattc agcacaaaat acagctaaat tgtgattaaa
38761 atgtatatct acaatcaaag gcaaaggact ttttgcctta atctctttca aagctcttgc
38821 atccgccata tccaaacatg ccaaacgcac gatattagct cctgcaaaat aaagccgatt
38881 aagttgctct aaagaacctt caatatctct tgttttagta aagagcatag attgcaccga
38941 aataggcgca tctccaccta ttaaaacatc acccacttta atttgtcttg tcttaaatct
39001 tttatattcc attttcagct tctttttgta aataattttt aaaattttag ctattttttg
39061 ctttaattcc tcataaaaaa tcaataggat ctatatctac gctaacattt ttaaaattta
39121 aagcatattc ttgtatttta attaaagctt tataaaaatc gctacgaagt aatatataaa
39181 aacgatattg ccctttttaac atttctatgc tacaagctcc atatcctata atttcaactt
39241 ttaaaatgtg actgatttga tcaactaatt ttttacacaa attttgagcc tgcttttgct
39301 ctttatcttc gatcacaatt ctcaaaagac gcttaaaagg aggataaaga tcttttctgt
39361 tttctaattc atcttgcaaa aagctatcat aatcctctat atatcgttca aaaaaattcc
39421 tattttttgt ttgtaaaagc acccttcctt cgcctttacg ccctgctctt cctgctactt
39481 gcatagccaa agctaaagtt tcttcacttg ctttaaaatt agggcgaaac aaatactcat
39541 caagtcctaa aataacgctt aaatctacac tatgataatc atgtccttta gcaagcatag
39601 aagttcctat aagtatatca atattatttt cgttaaaatc tttcaaaatg gcattgagtt
39661 ttcgaatgct tgtaatctca tcgctgtcaa atttagctat ccttgccaaa ggtaaagctt
39721 cttgcaaaag ttcttgtaat tctgctgtac ccatcttcct agcctctagc attgcacctt
39781 gacaactagg acaattttgc tctatcaaag aagtataatt acaataatga cactttaaaa
39841 catttctttt tttatgtaaa ctcatagcaa tagaacaaaa aggacatttt atggtttgcc
39901 cacaatcttt gcaaacaatt tgcctaaaat tagccttcgt gggtaaaaaa atgatagctt
39961 gcttttgatt ttccaaactt tttttcaact cacctaaaag catagggggtt atacccagtt
40021 cgctttcatc gtataaaaaa tgtttcttac tttcaaaaaa agtaccctta agcctgaaac
40081 tcttttgctt gtaaaaactc gttaaagaag gagtagcaga gcctaaaagc actttgatat
40141 caaattttga actcaaaaaa agcgctaaat ccctagcatt aatatagggt tgattggacg
40201 ctttgtaaga attatcatgt tcttcatcca ctatgacaag tgctaaattt acaaaaggta
40261 aaaataaagc cgatcttgct cctgcaacca aaagcgcttc acccttgcaa aaacgctcta
40321 aattttcttg ctttttttttc tttgaaattt tagaatgcca tagaaaaaat ttatctttaa
40381 aatatacctc taaacgcttt tgcatctgtg gagtaagaga aatttctggc attaaaagca
40441 aaacttgttt tccttgttct aaatattgct ttattaaaca aatataaatt tcagttttcc
40501 cactacctgt atctgcaaaa agcaaagaag tgggattagt ttttacaaat tctaaagctt
40561 ctttttgctt ttggcttaat ttgggttcat tttctatttt aaccggatca caagcatagg

40621 tttgatgagt agaaaaaagt cctaatacaa agcctagttt tgaagtataa taatacgaaa
40681 taaatttcgc caattcaaat tgcacacagc ttaacgaaaa aggtgtaatt tctttaatct
40741 cttttgtttg aaattcaggc ttttggcatt cttttaatac taaagctcta agattttttt
40801 tatttttaa atcaataata acctccctta aagccggaat ttcttcctcg ctatgaaaga
40861 ttaaattttg caaataaagt ccaaaaactg ctaattcata atatctcata ataattcttt
40921 acaatatttt tcgctaagac attctaaaat agcaaaatca gcattataaa aaaaatcaac
40981 ccattctttt gcatttaaaa aaaatctata tcgattaggt gctattttca tccaagcatt
41041 taaattagag taaataggcg tatggagtaa agaataagtg caattttgac cagcttgaca
41101 ataaaataac ttttcttttt gagtattgat ttttgcttca tcaagattag gagaaaaagc
41161 actgatttgt ttgagttcaa cttgggtacg cataagagct aaggctgaat ttagcatttc
41221 ataatctatt ttcaatttta ataatttcgc attattttta ttttgattgg caaaagagaa
41281 gcttaaactt gccaatatcc ctaaaatcaa tatcacaaaa ataagctcta aaagagtaaa
41341 agccttttttc attttacagg gagttcttca ttgatcgctt ttataagttt tttaagttct
41401 ctttctaaga tataattctc atagcttttt tttacaaaag catcgacgaa acgctttaat
41461 tctatctttt tagtgccacc agaaataaga gcgatatcat cttccaaaaa attcgcaaat
41521 tctccactgc atttaacaat gaaatccttt gcagaaacat tgataataac ttgttttaaa
41581 ttatcacttg ccaagtacag cctctatttt tttgaaaata tcttcacttt ctatattgcg
41641 agacttaagc tcttcttcta aacgcatgat ttgattgctc tttgcttcat tttgcgcttt
41701 tacgcttaca agttcgtttc ttaaattttc atttgcttcg cacacttcat tgtatttttc
41761 aatcaactca ttgactttat ctgacattgt gttaataatt ttttcatcga acataggttt
41821 gccttctgtt atagaaattt aaaataaaaa tattttttttg taattgtagc agaaaaaaat
41881 ttaaatttag gtttttttata gtcaattaat ttttaattaa agcattttct tttaaaattt
41941 ttctaaaaaa ctttcaaaaa gtaggaaaat gttagaactt acaagtgatt ttaaaccaag
42001 tcctgatcaa aaacaagcaa tacaaggcat agtaaaaagt ataaaaaaag gtaataaata
42061 tcaaacctta ctcggcgtaa caggaagtgg aaaaactttt accatggcaa atgtcataaa
42121 agaactcaat atgccaactc ttatcatgag ccacaataaa agcctttgtg cacaacttta
42181 cagcgaattt aaaggatttt tttctaacaa tcatgtggaa tattttattt cttattatga
42241 ttattatcag cctgaagctt atataccacg taccgatgtt tttatagaaa aagacagctc
42301 tacaaatgaa gatctagagc gtttaagact tagtgcaacc gcttcgcttt taagttacga
42361 agatgtggtt tgcatcgcta gtgtttcagc taattatggg ttgggaaatc caaatgaata
42421 cattggaatg gtattgattt ttgaactggg catgcaaatt tcacaaaaag aacttttgaa
42481 aaaacttgta gatatgggct ataaaagaaa tgataatttc tttgatcgtg cagattttcg
42541 cgtacaaggt gatattatag atatttatcc tgcttattat gaagatgaag tagtaaggct
42601 tgaatttttc ggtgatgaac tggatgcaat gtatcattat aatgttttag aaaacaaaaa
42661 aggcaaagat ttaaaacgct ttatacttta tcctacaagc caatttagcg taggagaaac
42721 tagactaaag caagctatta aagatataaa gatagaactt aatgagcgtt tggcgcaatt
42781 tgaacatgaa aacaagcttg tagaatacca acgccttaaa caacgcgttg aatttgatct
42841 tgaaatgctt acaagtacag gaatgtgcaa aggggttgaa aactatgcta gacatttaac
42901 aggattaaaa gaaggagata caccttatac actttttgat tattttgcta taaaaaatag
42961 aaaaatttctt gttattgtag atgaaagtca tgtttctttg ccacaatttc gcggtatgtt
43021 tgcaggagat agaagcagga aacaaacttt agtagattat ggttttcgtc ttccatcagc
43081 tcttgataat cgccctttaa tgtttgatga atttatccat aaagattgtc agtttctttt
43141 tgtttcagct acccctgccc cacttgagct tgaacttagt aaagaaaata tctttcatca
43201 aatcatgcgt ccaacgggac tacttgatcc tttgatagaa cttaaagata gcgataatca
43261 agttgaaatt ttatttgatg aagccaaaaa agttatacaa agaaatgaac gcgttttggt
43321 tactgtgctg actaaaaaac tagctgaaga acttacaaga tattatttag aacttggcat
43381 taaagtaaaa tacatgcatt cagatattga tgcaatagag cgcaatgaaa tcataagagg
43441 cttaagaagt ggtgcttttg atatgttaat aggcataaat ttgcttagag aaggacttga
43501 tttgcctgag gtttcgctta ttgccattat ggatgcggat aaggaaggtt ttttaagaag
43561 cactacaagt ttaattcaaa ccatgggaag agccgctaga aatgtcaatg gtaaggtttt
43621 actcttttgt aaaaagatta caaaatctat gcaagaagct atggatacca caaatgagcg
43681 tcgcaaactt caagaagctt ataatcaaaa acataatatt actccaactt cagtcaagcg
43741 acatattgaa gaaagtttaa aaaatgaaga gaatttagga gaaatttatc gcaaaggtaa
43801 aaaatttgaa aaaatgcctg ctaatgaaag agctaaaatc ataaaagaac ttcgtaaaca

43861 aatgcttgaa gctgctaaag cactcgaatt tgaaaaagca gcggctttaa gagatgagat
43921 caataaatta aaagatttat aatattttta ttttgcagga aaaatcctgc aaaatattaa
43981 agatatcctg ctattaaagc taagaaatat cccgcaacac aagaactaat cacacctata
44041 agacctggta aaataaaact atgattgata acaaatttgc ctatatgagt tgtacctgat
44101 ctatcaaatt gcatagtagc taagtcgctt ggataagttg gtaaaatata atacccatag
44161 caagctgctg caaaagccac gataatacct ggctcgacac ctattcctaa ggctaaagga
44221 acaaaagccg caatagctgc tgcttgagag tttacaaatt ttgaaataag taaaagcatg
44281 attgcataag tccaaggata atctgtaacc acatcttcaa gagctttttt catcatagga
44341 gtatgaacag caaacatagt atcagccatc caagaaattc caaaaactgc aacaagagct
44401 atcataccag atttaaaaat ctcatttgaa ccgatttttt tcgcatctgt tttagtaaag
44461 ataataatca aagctccagc taaaagcata aacatttgta ttacatttac catagagatg
44521 aaatcataac taggattgcc taatttatct ttttgaggga ttgcaataat ctcgcctgaa
44581 tttatcgctt ctttaaattt gctactctta tctccatttt cttctactat tgaagtttga
44641 tcaaaaccta attgcttaag atctacaaca cctttattta atccacttgg agtccattgt
44701 aatgtagtgc cattaatctc cgctttataa gccatttttt gaccccaatt tggtttaaga
44761 gaatcaaatg caccaattaa ggcaaccaat gcaatagctc ctaagaaaat ccacattgca
44821 atccaattgc tttttggaag ctgaacacct aaaagtgttt tagaatcgcc ataaacatat
44881 tttttaaatt caggatcttt aagtttttct tgaaaaactt catctttatc caaatccttt
44941 cccctaaacc aagaaaaaat tcccacacat aaaacaccaa aaagcgtact aggtatagta
45001 atttgaagaa gattgatata gccatcaaaa cctgctaatt tatgctctgc atttaaaagc
45061 aaagctgtta agcttacaac tgcaacagaa acaggacttg caataatacc catttgcgaa
45121 gatatagaag ctgctgccat aggacgctct ggacgaattc catttttaat cgctatgtca
45181 taaataatag gcatgatagt ataaaccaca tgtcctgtcc cacaaagtat ggttaaaaag
45241 caagttacaa aaggagctaa aatggttaag aatttaggat tgcgtcttaa aactctttca
45301 gctatttgaa gcatcacatc aagaccgcca cttgcttgta aagtcgcact tgcaacaacc
45361 acagctaaga tagtaagcat tacatctata gcaggctttc caggctttat atgaaaagca
45421 aaaacaagca ttaaaatacc tatacctcca agtaagccaa gggctatacc accttttttc
45481 gcaccgtaaa aaagacaaat taaaacaatg atgatttgta ttaaaaactg aatactctca
45541 tctagactgg ttaaaaagtc cattttaact ccttttatgg aataaattta aaaattgttg
45601 tgattatagc acaattttaa ataaaaaaac atataattaa aataatatac taatgaatat
45661 taaaatatac attaaaatat cattaataca aattcagtgt atttttatat ttttgatttc
45721 aagattaaaa aagtgataaa ttgagtatta tccttcaagt tcgtataatt tttttctatc
45781 tgttgaactt ggtatattta agtgtttacg atatttagta atagtgcgtc gtcctatatc
45841 gactttaaat tcttttttga tcaattctag aattttacta tcgcttaaag gttttaaacg
45901 attttcattt ttaactaaat ttgcgacaaa gtccttaaca ccgacatttg aagtctcgcc
45961 ctcttcatca agcgcaaaag caaaaaaatc acgcaaaggg ataagtcccc tatcacaact
46021 taagtattta tttgccactg cccttgaaat ggttgaagca ttacgctcta aatcaagagc
46081 tagatcttta aaagtcatag gtttaatatc tttacccatg aaaaaatcat attgatattc
46141 tactatcata agtcctattt tataaagcgt tgcttttctc atcgttaaag catcgactaa
46201 attttttgct tcttttatgt agtgatttaa atattcatga tttaaaccat cagtctcaat
46261 agaaatttca ggataataat catcattgat ttttaccttg atttcattat tttccctata
46321 aacaaagata tcaggtataa taacgcgaga atcttcaaaa tactccaaaa aaggcggtat
46381 actaaaacgc ttaaaaatcg ttaatgcttc tttatacaag gactctttag tataattttg
46441 tatattttca aaatcttgaa taagcatttt acaaaaatca taaagctctg catcaagttc
46501 tatattttct aatgcaaata aaaatgcttc tttataatct tttgccccca ctcccacagg
46561 atcaagaaat ttaaatcttg ctcttatgcg ctctatttca tttatatcat aatcttttaa
46621 aatttcttca tcgtattcaa aatatccctc atgatttaag cattctatga ttttacctgc
46681 aagttcttgg gatttatttg taggaaaaag aggaggtata atctgctcgc ttaaaagttc
46741 atatacactt tttgtggcaa aacctttatt gtccacaaat gctgaattta cattgcgttt
46801 ataaaaagaa tcaaaataat tcttattatg ctcttgggtt tgaatgggtt cttgaacact
46861 caaaaaagga ttttcctcgg caaatttatc taaattttct tttaaatctt ctatatttgc
46921 ttgtaatata ggcaaccatg aacgcagagt ttgagaaatc ttggttttag gagcttgggt
46981 aatcttttgc cttaacatca atcaaggagt ttaaactccg ctcctaaata gtatttttta
47041 acatctttat tatttgctat ttcttcagca tttccgcttg cgagcaaaga gcctgatctt

47101 atcacataag ctctatcaca aattgccaaa gtttcacgaa cattatgatc ggtaattaaa
47161 attccaatat caagcttttt aagctcattg ataagacttt gaatttcagc taccgcgata
47221 ggatccactc cagcaaaagg ctcatcaaga agcaaaaatt taggctcaca catcaaagat
47281 cttgcgattt cacaccgcct tctttccccg ccacttaagc tcaaaccttt tcttaaacga
47341 ataggttcta tacttaaaag ttcgagcatt tgctccactt tttcatgtaa aattttctta
47401 tctttataaa aaatttgagc agctaaaagc aaattatctt caacgcttaa atccttaaaa
47461 atgctgcttt cttgaggtaa gtaacctatg cccagccttg ctcgtttatt taaaggctct
47521 ttagtaatat ccaaaccatc taaaagcact tttccgctac ttggagctat aagtccacat
47581 atcatataaa atgtagttgt ttttccggct ccattaggtc ctaaaagtcc aacaacttca
47641 ccactattta gttctaagga tataccttgg ataatctttg tttttttaat aatttttttct
47701 aaatttacaa tttctaattt actcatgaat tacatacttt cttttattat cttctacact
47761 aatttttatt tccaaggtag gaattccaag ttttagcaaa gctttttttta aattctcatc
47821 tccccattca accaaatgca agccctcttc aaaaaaattc tcaaacaaac catttttttaa
47881 aagtccttcc aaaccctctt gatatatatc ataatgataa acacatatat cttgattttc
47941 atatttttgc atgatagaaa aggtgggaga atctactttc tcatctaaat ttaaaaagct
48001 cacccaagct tgcactaggc ttgttttacc gctggctaat tctccttgca ataaaacaac
48061 accttgtttt ggtaaagttt ggagcattgt ttttaattca tctttggcta aaacaaattc
48121 tttcataatt tcaccacata ctcgctttgt gcatttttttg gaatattttt ggtagtaggt
48181 aaagccaaaa ccttatattc ttgcgtataa tcagtaaaat gcaaagcgat aatatctcct
48241 actttaacct ctttgctcgc tttcactatc actccattaa tccccactac accacttcta
48301 cacatatcct cagaaatagc tcttctttta gtaatattaa ccgcatttaa aaatttatct
48361 actctcatag tacagaattt taccaaaaaa attaaatttt taaaacccat tttatccaaa
48421 ctagcctttc ttcatcaatt taaaaacttt tctttaatga attttggata caatttaaca
48481 attaaatcaa tattaacaaa ggatactcta tgaaaaatga agtaaaaaaa gtcgttttgg
48541 catattctgg cggacttgat acaagtatta ttttaaaatg gcttcaagat gaatacaaat
48601 gcgaagtagt aactttttaca gcagatattg gacaaggtga agagcttgaa cctgctagaa
48661 aaaaagctct ttcattggga attaaagaag aaaatgtttt cattaaagat ttaagagaag
48721 aatttgttaa agattatgta tttcctatgt ttagagccaa taccatttat gaaggtgaat
48781 acctacttgg aactagcata gcaagacctt tgattgcaaa aactcaagca caaattgctc
48841 tacaaacagg tgctgatgcg gtaagtcatg gagctacagg taaaggaaat gatcaagtgc
48901 gttttgaatt aggctatcta gcttttaatc ctgatttaaa aatcatcgct ccttggcgtg
48961 aatgggattt aaacagccgt gaaaaactct tagcttatgc acaaaaacat ggcattgata
49021 tttctaagaa aaaaggaaaa tccccttatt ctatggatgc aaatttactt catatttctt
49081 acgagggact tgttttagaa gatcctgcgc acgcgcctga agaggatatg tggagatgga
49141 gcaaaagtcc aaaagaagcg ccaaatgaaa gcgaaattat agaacttgat tttcaaaagg
49201 gagatttagt tgcgatcaat ggagagcatt taagtcctgc tgggctttta agcaaactta
49261 acgagcttgg tgcaaaacat ggcataggac gccttgatat agtagaaaat cgctatgtgg
49321 gaatgaaaag tcgtggctgc tatgaaacac caggaggaac tatactctta aaagcacacc
49381 gtgctattga aagtattaca ctagatagag aagctgcgca tttaaaagat gaacttatgc
49441 caaaatatgc gagtttgatt tataatggct attggttctc acctgaaaga atgatgcttc
49501 aagctctaat cgatgaatct caaaaacatg ccaatggtag agtaaaactt gaactttata
49561 aaggcaatgt aatggttata ggccgagaaa gcgctaatga tagcttattt aatgcagctt
49621 attgcacctt tgaagaagat gaagtataca atcaaaaaga cgcagcaggt tttatcaaac
49681 tgaatgcttt gcgttttatt atcgcgggaa aaaacggaag aaaattttaa acaaggatta
49741 aaaaatgaaa gtattattaa tcaaagatgt taaagctctt ggaaaagcag gtgaaatcaa
49801 agaagtaaaa gatggttatg gtcaaaattt tctcattgct aaaggctttg ctaaggcagc
49861 aacaaatgag gttttaagaa aatacgaaag cgacaagaaa aaagaagcgg agaatttacg
49921 ctttgagctt gcaaacttag aaaaactaaa agaagagctt agtaaagtta cgcttgaaat
49981 ttcaaagcct gtaggagcta atggaagttt atttggcggg gtaactaaag atgaaattgc
50041 ccatgcttta aaagagcaaa cccatataga aatcgataaa aaaagcttgg agtgcgatac
50101 cctaaaaagc cttggcactc atgaagtcag cgttaaatta ggtcatgcta tccatgcaaa
50161 atttaatata aacataaagg ctgaataatg tttcatgcaa caactatttt agcttacaaa
50221 ggcaaaaaca aatccgtaat cggcggagat ggtcaagtaa gctttggcaa tactgtttta
50281 aaaggtaatg cagtaaaaat aagaaaactc aataatggca aagttttagc aggttttgca

50341 ggatcaacag ccgatgcttt taatcttttt gatatgtttg aaaatctact tcaaagctct
50401 aagggtgatc ttttaaaagc agcaattgat ttttctaaag aatggcgaaa ggataaatat
50461 ctaagaaaac tcgaagctat gatgctagta cttgatagaa atcatatttt cttactttcg
50521 ggcacaggcg atgtagtcga gcctgaagat ggacaaatcg ctgccatagg aagtggtgga
50581 aattacgcgc tttcagcagc tagagctttg gcaaaacatg caaatttaga tgaagaagaa
50641 ctagttaaat caagccttca aatcgcgggt gaaatttgca tttataccaa taccaatata
50701 aaaacttatg taattgagga tgaaaaatga atttaactcc taaagaaatc gttaaatttt
50761 tagatgatta tgtcatagga caaaaaaaag ctaaaaaaat tatagcaata gctctaagaa
50821 accgctatcg tagaatgcaa ctcagccctg aactccaaga tgatatagtg ccaaaaaata
50881 tccttatgat aggatcaaca ggtgtgggca aaactgaaat tgcaaggcgt ttggctaaga
50941 tgatgggctt tccttttatc aaaatcgaag cgagtaaata cacagaagta ggctttgtag
51001 gtcgcgatgt agaaagtatg gtaagagatc ttgcaaatgc agctttaaat ttggtaaaaa
51061 atgaacaaag agaaaaaaat aaagataaaa tcgatgaatt tatagaaaat aaaattttag
51121 aaaaacttct tcccctttg cctaaaggta tcagtgatga aaaacaagaa gagtataaaa
51181 atagccttga aaaaatgaga acaaaactta gaaatgggga tttagatgaa agcgtgatag
51241 agatagaaat ttcgcaaagc atgtttgata ccaatccaaa tttgccgcct gaaatgggag
51301 caatgcaaga tatagtcaaa gttataggcg ttggcagcaa aaaagtcaaa aaagaaatga
51361 aaattaaaga tgccaaaaat gctcttaaaa atgaagctgg agagaaaatt ttagatcaag
51421 aaagtatcaa aagcgaagct ttaaaaagag cagaaaatga aggaattatt tttatagatg
51481 agattgataa aattgcagtt tcaagtggaa attcaaatcg tcaagatcca agcaaagagg
51541 gtgtgcaaag agatttgctt cctattgttg aaggctcgag cgtgcaaaca aaaatcggca
51601 ctttaaaaac cgatcatatc ctttttatag cagcaggagc tttccatctt agcaaaccaa
51661 gcgatttaat ccctgaactt caaggtcgtt tccctttaag ggtggagcta gatagtcttg
51721 atgataaagc actttatgaa attttaacac gccctaaaaa ttcactttta aaacaatacg
51781 aacaactttt aaaaactgaa aatttagaac ttgaatttga tgacgaggcg atcaaagaga
51841 ttgcaaaaat tgcctctaag gccaatgaag aaatgcaaga tataggcgct agacgcttgc
51901 atacggttat agaaaaatta cttgaagatc tcagctttga agcagatgaa tatgcgggta
51961 aaaaattcgt agttgataaa aaaatggtag aagaaaaact cggagatatt atagaaaata
52021 aagatcttgc aaggtatatt ttgtgaaaag tggctttgtt agcatcatag ggcgcaccaa
52081 tgcaggaaaa agcactctta ttaattcctt actagaagaa aaaatcgccc tagtttctca
52141 caaacaaaac gctacaaggc gtaaaattaa agctatagtc atgaacggtg aagatcagat
52201 catctttata gatacacctg gtcttcatga aagcaaagca acgcttaatc aatttcttat
52261 ccaaagtgct ataaaatcta tgggtgattg cgatgttatc ttgtttgttg ctagtatttt
52321 tgacagtgta aaagattatg aaaatttcct aagcttaaat cctaaagttc ctcatatcat
52381 cgttcttaac aaagtagatc tagctgataa tggcgcactt ttaaaaaaac tcaacgaata
52441 tgcaaaattc agtgagtatt ttaaggctat cttgccttat tcttgcaaga aaaagagtta
52501 tcaaaaacca ctgctaaatg agctatgtaa gctcttgcct gaacatgagc atttttacga
52561 tagcgaattt ttaactccta gttctcaaaa ggatattttt agagatttca tacttgaaag
52621 tgtttatgaa aatttaagcg aagaattgcc ttattcttgc gaaattatga taaaaaatac
52681 caaagaaaca cccaatcttt ttatcatcga tgcacaaatc atcacagata caaattccca
52741 taaagctatg cttataggaa aagatggagt aacacttaaa agaataggca aagatgcgag
52801 atttaaaatc tcaaaactca ttcaaaataa agtgctttta aagcttttg tcgttgtaaa
52861 gaaaaattgg caaaaagatg aagtttttct taaaaaaatt ctaaattatg aagaataaag
52921 ctcattttat aggttttgaa aatcttatct ataaacaaaa aaatggtaat ttcgaagaag
52981 atagcctttt taaagagcta acaaaagaat gtgatcttca aaatcctttt gaatatcaat
53041 tggcttttt aaagcaagat caaatctatc attgcttttt aacttgggct gcaaaactac
53101 ccaaaaccaa attttgcttc cccgaacctc tcatctttca aagcttattt ttagaaaata
53161 aaatcaaaga agggaatttt tgcatcttag aaataagctc aaaaaaggta tttttgtgtt
53221 tttatgagca aggtaaattt aaaacctta aaacactcaa tttttgtgac aatgtagaag
53281 aatttataaa taaaagccgt attttagagc ttttacaaca ttatgaaagc aaaatactgc
53341 taagcggaaa agctcatgaa attatcgatc ttataagcac gaaggcaaaa cttcctttga
53401 aaataattca agaagataaa atcacacttt caaatcacag cattcatcat cttgataaaa
53461 atgccaattt catcaagtat tatcaaaaac atcttccttg gtatttcaaa tttattttt
53521 tatttgttct aagttttata atcagcattg gaattttatc attgattgat tttacacaat

53581 accaaagtgc taaaaaaaca catatacaaa atgaaatttc acaaaataaa atctatgaaa
53641 ttcaagaaaa tcaaaatcaa aaacttaagg ccaatataga aaaacttcaa ttagaaattc
53701 aaactcaaga tcttttacta gaaaaatact ctgagcaatt gtctaaaatt acgcaaaatt
53761 ttaaagctga taaagataca attttgattt taacaaagat tattacatgg ttaaatgatc
53821 attctttaaa aattgctaat ctcatgatag ataaaaccct tatcaccata aaatttagca
53881 acgaagagaa ttttaataaa gctcttcaat ttacaagtcc tcaatttaac ctcatctcgc
53941 aggataaatt cttgtacgaa atcaccttaa gagcattaca atgaatcaaa ttgagcaatt
54001 tttagaaaat ctaagtccta gagaaaaaat tttaatatat cttacaacca tacttctagc
54061 agtattgtta tcctttcatt tttaccattc ttacttaaaa agtttgttta atcatgaatt
54121 ttttatccaa agcgatgaag taaataaaat tatcataaca aatcaagatt taaaccaaca
54181 aataaaacag cttgaagaaa aactaaattc tatccaagat aaaataaaaa gctatgaaaa
54241 tcatcttagt actttcaata aagactacga aacatatatt aaaaatttac aaaatctagc
54301 cctaaaaaac aatcttcaaa tctctaatat atcaagatta caagaagaaa attcttatca
54361 tagaatttca gtcgatattt tgggtgattt taatcctata ctttctttta tacaagatgt
54421 tgaaaattca aatttacact atgaaattca aaaatttgaa attgacaata catcaaattt
54481 aaatctccat ttaaaactaa cgctttcttt tattgcccta gtaaaaaata aaaactaaaa
54541 caaatactat agctttttat ctataaaagc tataatttta ttttgataga aaaatttata
54601 aaataacgca aatttaaaag gaaaaaaatg aatatttaca aaacaagagt tgaaaaaatc
54661 agacaattta tgataaaaga aaaacttgac gcttatttga tactaagtgc tgatccacat
54721 ttaaatgagt atttgcctag tttttatcaa agtagagcat ttgtgagcgg ttttaaaggc
54781 tctgctggaa gcttgatcat cactcttcaa gatgcttttt tatggacaga tggaagatac
54841 tggattcagg caaaaaagga attagaaggt agtgacatac ttttacaaaa acaagatgca
54901 aataatacct ttttaaaatg gctaaaagaa aatttaaaca aggaacaaaa tttaggtata
54961 gatttttcag ttttatcctt agctttgcaa aaagaaatac aaaaaaactg caaagccgaa
55021 ctaaaaaata tcgatttaat tagccctatt tgggaaaatc gtcctgcact acccaaaaat
55081 aaagtttatg aacacgaatt aaaatattgc tcctattcta gaaaagaaaa actaacatta
55141 gtacgtgaaa aaatggctaa actacaagca caaaaccatc ttatttccag tcttgatgat
55201 atagcttgga ttactaattt aagaggaagc gatgtaaact acaatcctgt atttttaagt
55261 catttgctaa tcttggaaga aaaggctttg ctttttatag atagggaaaa aattgatttt
55321 gagcttgaaa aaaaattaaa tctcgatgga atttggctta aggattataa tgaaattcaa
55381 gatgaactta aaaaactcca aaatacaaat ttactcattg agccttcaaa aacaactgct
55441 ttactcattg aaattttaga taaaagcgtt gaaattttag aagaaattaa tcctagcacc
55501 catttaaaag ctatcaaaac agataaagaa atcgctcaca tacaaaatgc catgatagaa
55561 gatggggtag ctttatgtaa attctttgct tggcttgaag aaaatatcga aaataataca
55621 caaatcactg aacttgacat cgataccaaa gtcaccgaat ttagatctca aagtccttat
55681 tatattagca atagttttgc cactatagca ggatttaacg ctaatgcagc tttgccgcat
55741 tataaagctg aaaaagaaag cttttcttat attcaaaaaa atggattatt gcttatagat
55801 tcgggtgggc aatacaaaaa tggcaccacg gacattacta gagttgtagc tataggtgaa
55861 ttaaacaaag aacaaattca tgattatacc ctagttttaa aagcacacat agccatatca
55921 agcacggtct ttccaaaaga tattaccatg cctttgcttg atgctatcac aagggctcct
55981 ttatggcaag aacaacttga ttatgcacac ggcactggtc atggagtggg ttattttta
56041 aatgtccatg aaggtccgca aactctttct tattttagcc ctgtgcttga aaaaacaaag
56101 gctaaagaag gtatgctaac ttctatagaa cctggaattt ataggacggg aaaatggggc
56161 attaggcttg aaaatttagt tgttaataca aaaataaaaa atcccaaaaa caaagatttt
56221 ggtgaatttt tatattttaa acccttaaca ctttgtcctt ttgaaataag ttgtattgac
56281 aaaaccttat tgagtatcaa agaaaaagcg tggattaatg cttatcataa ggaagtttac
56341 gaaaaactct cccctaagct tcatgacaat ccaaaggctt taaaatggct aaaagaaaga
56401 actgagacga tttaactaaa aattaaaatc ttagaaaaat taatattgta taaaattaat
56461 ttttctagtt ctttttcgag ctattaactt tgctttctgg cttaaaatat ccaagatcta
56521 tcattttttg ataaatcttt gccgtagcaa cacctgagct tatagttctt ccgctgtgtt
56581 ctaccaaaac agtaaccaca taacgcggtt tagaataagg cgcataagaa gttacccaag
56641 catgcgatct tgtataatac ttaagttgct tttcatcgac tctattttta tcagcttgag
56701 aaaaacctac cacttgtgca gttcctgttt tagcagcaac ttttacttct aaattacgca
56761 aataatgata agaagtacca ccttgctcat tagcaactgc atacatcgca tctcttatat

56821 agggaagttg gctttttca aaaagagtga aaatttcatt actatcttgt gttttatctt
56881 ctgtgatgct attattattt tctacacttt ttagaaaatg gggaataact tcaccgcctt
56941 ttgcaatttg agctgtatac ctagcaatct gcataggtgt agccaagaaa tttccttgcc
57001 ctatggaagt atttaaggta tcgccttgaa accaagcttg cttatatctt tgcattttcc
57061 attccctact tggcaagatc cccacaaatt cattaggtaa atccacacca gttttagcac
57121 caaaacctat gcgtgataaa gtacgactta tttgatctat acccacttgc aaacttccat
57181 cgtaaaaata aacatcacag cttgctttta tcgcatgttt taaatctaca gccccatgcc
57241 ctgctctatt ccaacaacga aaaaatctcc caccaagctc tacgctacca ttacaaaaaa
57301 attgggttga gggattaata ctttttgaat ttaaaaaaga caaaccaacg cccatcttca
57361 caacagaacc tgggggataa tatccgttaa taagcttatt tgtaaaagga tgatcaagat
57421 tattagaaag ctcgtcccat tctttataag aaattccagt tacaaaagga tttaaatcat
57481 actcaggaaa acttcctgct gccaaaatag aaccatcact aatatccata atgatagcag
57541 cacccgcatt accttcaaaa agttgtgtta aaaagctttg gagttctata tctatggtaa
57601 gttctatatc attagaaagt gccgaagtat aggaaagctg ctccacttct tgatttaaag
57661 cattaacctt ataaacttta gttccttttt ctccttgtaa aacatcatta taataacgct
57721 ctattccgct ctttcctgta aaattagtca gctttgcaat ttcattttct ttaatatctt
57781 gtaagtctgc cttacctaca tagcctataa tatgagaagc taattttcca aaaggatatt
57841 ttctttgccc tgcaggatct atgcgtatat tttcatttaa attgagttct gaataatgct
57901 tcatcatttc ttgtgaaggt ataaaatcta taattttaat aaaatcttga ttataataag
57961 agtcattttt cttataaagt tcagcaagct tggtaacatt taaatcagga aaaaattctt
58021 ggagttggtt taattctttt tctaatacac ctctatttgc tctttttaaa gttaaataag
58081 gcttaataga aatactaaaa cccaaatcat taatcgccaa aggagtacca ttacgatctg
58141 aaatttgacc acgagtagga gctataaatt cagtttttac agcgttttgc ttggcgagtt
58201 cttcataata cacatttgat tttatactta gataatatac acggcttaac aaaaatataa
58261 aaaacagcag tataaagcca acaaccaaac gcattctcat taaaacttac tcctaaatag
58321 catataagca aacaaacact caaacataat cgatatcata tagccaaaac ttggcatttt
58381 aaactcttga tctcccacat aagaaaaaat agcatccaaa acaaaaataa aagcataaac
58441 acaaagaacg agtataacgg ggataatttt tcctatttta aaatttgttt taatccaatc
58501 cgcgcaagta taataaaaaa tagcaaaagc aaaccaagaa gaaaatagaa aaaaatcgta
58561 agttatatct gtaaaaaaca aaaaaaacaa agaaaaatac catctaaaat caagcttatt
58621 aaatgttttt tgtctttctt ctaaaagaaa atacatatag caaaaaaata caccataaag
58681 caaaggcata taaaaaaaaa tagaacctaa aatttgatat aaaatacaca aaaagcctaa
58741 aaaaatataa cttccgttca gttttacttt atatgtgctg atactttttc tcattttata
58801 aacctaaagt ttgcttaata atttcatgtt ctaaatcatc aagtcccatt ttatttaaat
58861 tagatatcaa aatagcatct ttaaaattat ttttaagttt agctttttcg ctttgattaa
58921 gcttatcaca ttttgtaaaa acctttaata ttctctgatc aggctttaaa aaactttttta
58981 aatactcatc taaatttaaa tcaatatcta aattcgtatg tcttgaatca atcaaatgca
59041 caaaaagttt aatcgaactt ctcaatttta aaaactcatc caaattttta ttccatattt
59101 cttttagatt ttttgaaacc ttcgcataac caaagccagg caagtcaata aaatgaatac
59161 tgaatttatc ctcatctttt ttacataaaa cttcaaaaaa attaatcaat tgcgtttttc
59221 caggagtagc agaactcttg gcgagttttt tttgcttaca taaggaatta atcaaagaac
59281 ttttccccac atttgatctt cctaaaaatg ccacttcgct aaaatgagat gcaaaatttt
59341 tatcgaattt atccaaagag ctgataaatt tagcgcttat tatcattttg ttctaaatca
59401 aatataaatc gagcaggctt tttatccttg ctttcaacac gataaacatt atttcgttta
59461 tctactacta tcctatctcc ataaagtttt tgattacttt caagctcgtt gatataggca
59521 tttccgtcaa tttcataaat atctttagct acattataaa taaatttatc cccacttccc
59581 ttataggttt ttccctttaa aacgatttta aaattagcat tttgcacagc ttcataacga
59641 ataggtttgc gatttttatc tgtatatata atcacttttt cagcagttaa aacatcatcg
59701 ccccttatga tttcaacatt accgctgagt atgctttcgc ccttgttttc atctgaataa
59761 aaatttaagg ccttaacctc tatctggcta gctaaagata tgcttatact catcaaaaat
59821 aaaattatct ttctaaccac gcattcactc cttgaacttg taaaattttc ttttccacat
59881 cgtagtttag agtatttccg ttaataatac ttccatttat caaagcttta aatcctgtat
59941 ccgtgcttaa aattttattc ttaggctcat attcaacttt atcagcaagt attttagtat
60001 tattagagcc tatataagtt acatttccct caaacaaaac cttattcatt tccttgcctt

60061 taagttctag tttatttgca cttaaattaa aatccaaatt gaaattaaca aaattatcaa
60121 actcatcttt atctacataa cgcgtccaag tttgtgcttt gtaatatgat tttacattac
60181 ttgcattaag ctcataagca tttaaattga aagcttcgat atttttaaaa tttaaagcat
60241 aggatttaat atttaaagaa taaggatctt gcaaacttaa aattgtaaaa gtaatagtaa
60301 aaagcgcaat caaaattcca aatattctta tagccaaagc ttatcccatt cctcttgcat
60361 attatttttc tttataatat attctatcat ttgagccaca gctgctttac cacctttttt
60421 atcaagtgtg atatcgactt ttaaatcttt atgtgcatcc ttaggtttaa aactaagacc
60481 tacattttca agtaaatttt tatcattaaa ataatcccct atggccgcac attgagaaaa
60541 atccaaattc agctcttcta aaattttttt agcacaagtt aatttatctt taacaccttg
60601 atgcacaatt tctatcttca aatccttagc acgagcactc acgcaaggac agtttcttcc
60661 agtaataata gcaatttttt tacccaattt aatccaagct tcgatagccg caccatcttt
60721 gacatcaaat tctttaatca cacttccatt agaagtgtag atgattttac catctgtaag
60781 acaaccatct acatctaaaa atatcaattc tatcattttt actcacgcac aacaaaagca
60841 ccagcaaatg caccacttgc cgcaaaatct cttgcttcct cttcacttct aaatcctgtc
60901 aagaaaacac gactcaaacc atcaactgaa cttttgcgta tagttgaaga ataagttcta
60961 taagttttat atctagatgc tatagtttga gctcctgatg ggtttttaaa cgctccaatt
61021 tgcaccataa aatttccacc ctcatagatt tgaccgttat tggcaattcc accgctagtt
61081 ccataattta tatttgaatg aacgacatta ttacctgaat tagccgaacc aaaacctata
61141 acctcaagtc ttacgggtgc tgttcctgca gcaatcatat cgatttgatt tgcagcgccc
61201 ttggaaagat ctataattct attatttaca aaaggacccc tatcgttaac acgcacggta
61261 acttggcggt tattatttaa gttggttacc tttaaaatgg tattcatagg caaagtttta
61321 tgagctgcgg ttaaaccatt ttgattataa gtttccccat tagaagtttt tttaccatga
61381 aagcctggcc cataccaact tgctatacca tctgctgttt cacctactga tacaacagtt
61441 ggataataag tcttgccatt tatggtataa ggtttcatag tccccttgct tccagaacct
61501 gagggactac tatgaaaatc gttgcttgga taatatattt gtgctgaacc tacgctagaa
61561 aaaagacctc caaagcatcc gctagaaaaa gttgcaacta aactagcggc taacaacttt
61621 ggtaaagttt tctttagctt ttgttttata gtttgcattt ttattctcct taataggtat
61681 gattaatttt tgattaatgc ttaagtgatt tttagcgatt ttattcaatt ctcgaatttg
61741 ctctacgcta gtattgtaag ttttggcgat tttatataaa gaatcacctg attttacaac
61801 ataaacttta gtcataggta tagttgtatc cacttttgca agcttttcaa ctttaaaatt
61861 tttatcaaaa aaagctactt tatttaaagg aatatacata taatatccct tacctggagg
61921 agtgaaacca tgtttaaatt gtggattata ttttttaaaa gttgctaagt ccatatttaa
61981 atttttagct aaatctttta atgcaactga agaaggagca tcaacttttg caaaatcatt
62041 atttaaagcg taattaacca aagagccatc tttatcaagt aaaaaatctc tgtcgttagc
62101 caagaaagct agagttaaaa ttttacgtat aaaatttcta gtttccaaag aaagatattt
62161 tttatcagaa tcaagcaaaa ttcttaaatc atcacttcct gcttgcttta ttgcttgacg
62221 caattttcca tttccacaat tataagctaa aagtgctaaa taccatttgc caaactcttc
62281 ttttaaactt tttaaataat gaaccgcagc atatgtggat tttacaggat cgcgtctttc
62341 atcaacataa gcatcaattc taagtcctaa atttcttgct gttggttgca taaattgcca
62401 cacgcctaca gccttagcat tagaaacact atgtgctttt aatcccgatt ctaccattgc
62461 tagatataat atttcttctg gaacttcttg ctgcgctaga atttttctta tcataggagt
62521 aacttttgag aaatttgta taccatcaat caaagtcggt gcatgcttcg atcttaaatc
62581 ctgttgactt tgcacaaaag ccaaatcact tataaaactt ggatcaatat ctaaatttct
62641 taaaacactc atttgtcttt cataaaattc aggagtagta gtttgcgcaa aaagaaagtt
62701 gtaaaataaa ataaaacata atataatttt tttcatattt aacctttaaa aaataatcta
62761 taggaatttt ccaaacaaat ccctaaaagc tcttgtcttg gtaaatcaag aatttcactc
62821 attttattgg ccaccaattg agtcaataaa ggctcattcc ttttaccgcg ataaggctct
62881 ggagtaagat agggcgcatc tgtttctagc aacaatctat cttttggaat ttgtggtaaa
62941 atatttacta aatttttagc atttttaaat gtcaaaacac caccgattcc aaaataaaat
63001 ccatcattgc taaggcgcaa taaatgttcg cttgcattaa aacaatgcaa aacccctcca
63061 acaagatctt tagaatgttc atgtaaaatt tcataagtat cgttatttgc ttcgcgagag
63121 tggatgataa caggcttttt gaattcttta gctagttcaa gctgggctac aaaaagtctt
63181 ttttgctctt ctttttcttt ttctctttct ttaaaatctt cacttttaaa acgaaaataa
63241 tcaagcccgc attcacctac agccacgcat ttttcatctt ttaaatacgc tcttagaatt

63301 ttctcatcaa aaccttctag ctcataagga tgtacgcctg cggcaaaaaa tacattttta
63361 tatttactag caagatctgc cgcataaggc aaatctttaa tatctgcgcc tggtatgatg
63421 atttttttcta tgccattatc aaacgaatgt tttaaaagct cgtctaaatc ctctttaaac
63481 acttcactat ctaaatgaca atgagtatcg acgatcttta aaccatcttt aaattcaaga
63541 tttaagaaca tacttctact ctatctttac cattggcctt agcttgcgaa agtgcttttg
63601 aagctttatc aactaaggtt tcaaatttat ctccactctt agcaaaaacc actcctatag
63661 aagcggtaaa aaagacttca tccaaattga ctaaaattcc agcttttttt atattgactc
63721 gaatgcgaga aagaatttta atcgctcttt cttgtgaaat attctttaat actatgcaaa
63781 ttttttcagc actatatcta ccaactatat cacgtccttt ggtttcgtta ataatctcat
63841 cagcacaaat tttgacgatt ttatccccta cttcatagcc atattcttca tttaaatttc
63901 ttaaattgtc tatatccaag aacgcaaaag caaattcttc ttctttgcat gcaatttcat
63961 ttaaataatc ctcaacacaa ttcaacaaag cattggaatt ttttatccca gaaataggat
64021 caagattgtt ataaatactt aaaaattgca tattggccat ataatgaaga catttatcta
64081 agcgacaatt taaactttct ttacttaaag gttttatcaa atactcatta gccccactta
64141 caagcaaact agcctctaaa gcatcatctt tgtcgcctaa aataattaca cctaattcgc
64201 tatcactaaa acgagttctt acctcactta aaagctcgct tccactgata acaggcatat
64261 ttgcatcagc aataataagc tttatatcat tattatcatt taaatagctc attgcttcct
64321 cgccatgagc agcagccaaa acactaaatt ttctttgtgt gagaagtttt tttatttcat
64381 tacgctcaga aagtttactt agggctaaga taaccttggt tttagcatag tgttcaagct
64441 taacaataga atctatgatc tgatctatac aggtatcgct ttctttaaag atataatcta
64501 aaatatcttt atccataaat ttatctcttg tagctttatc attacttgca gtcaatacaa
64561 taacaggcca attttttgcc aagagataat ccaccacttc tccattaggt gcatcaggca
64621 agcataaatc tgcaaaagtt aaaaaatatt catcatcggg caatgcttta acttcagcaa
64681 ggctaaaaac aatatcaact tcacgattta aagtagtttg tatcttttta gctaaaagct
64741 ttccaagcat tttattatca tcaataagta aaattttttt attcataatc tataaaaata
64801 cctttttttaa aactaaatct ttataataaa attaaaaaaa tcccaattct aacacaaaca
64861 aataaaaatt ccgcttaagc tttaaacaaa gttttagcaa attccaaagc ttcatctgta
64921 atagtctcgc cacttatcat tcttgctaat tctttaattc tctcttcatt cttaagtttt
64981 ctaaccgtgc tacaagaagc attttttttca accaaaaaat gattatgagc ttttgaagaa
65041 agctgtggca agtgagatat agcaaaaatt tgataaaaag cagcaagctc atttaaaaca
65101 ttggctatac tcatcgcttc cttaccgctt aaattagcat caatctcatc taaaaatatt
65161 atccctgtgc ctgaatttaa aattttacac tcagtagcaa taaaagcaag tcttaagcga
65221 ttaagttcac ctgagcttaa attttttaaa tttgcagcat tgatacttaa cttgatctca
65281 tcttttccca aagaggtgat ttttgaaata tttactaatt ctaaactcac atttttcata
65341 taaagatttt ttaaataatt atttaaataa tcctctaaag tttttaaatt ttgcatgcga
65401 gcttgagtta ataactgggt actttgattt attttctttt cgattttttc tagttttttt
65461 tcaagttctt tttttcaaa gcttaaatta tcataatgag cgagttcatt ttttttattt
65521 tctagcactt ctaaagcatt ttctatactc tcataacgct tgatcaaaga ggataaatcc
65581 tcgattctat tgagtacttc ttctatatca aaatccaaat cttcgatttt ttcattttcg
65641 gctatgatac gaagttcatt taaacattcg ctaaaaaaac tcgtatcttt ttcactgaga
65701 tttaaagcat caatgactac tttttctaat tcaaaaatag catttgcctt ttcccaagct
65761 gcttctattt tgtctttttt tgataatttc tttttaagat ttaaaagctc ttcgtattct
65821 ccgattttag gattgatttt agaaattctt tcaatctgtg tgcttgctaa ttctttaagc
65881 tcttcaatcc tttttttcctc ttctaaaatt gtagtcaatt cttctgaaat ttgaagccat
65941 tgtttataat cgtctttaaa atgagataaa aaatcttcaa attcaggatt tttcacacat
66001 tctaaagcat ctaagagatt taaaaatttc tcattactaa attcattgat ttctttggcg
66061 cttaggtatt taataaattt tttacttaga tgaatcaaac ttttttttaga aatgctttga
66121 ttattgataa aatatttcgc attcttatcc tttaaaagtt taaaaacatt ttcatcttca
66181 ctttctatgc cgtattcttc taaatctatc ttatcatcaa gtgcaatctc aacaagcttt
66241 gcctcactat cgcttaatgc aaaagcagac aatataccct taaaaagtac agactttccc
66301 gctccgctta aacccgtaaa gactgtaagt ccttgagaaa tttcaagctc aacttcttta
66361 aagccaagat tttctttcat taaaactcta tttatcatca atgtccccaa ttcaattttt
66421 ctcttaatat ctgaaaataa tctcgatttt taggatgtat aagtgccaca cttttatcgc
66481 taagccccac cttaacactt ttaaaatcat tcattttgta attttcttgt ccatctatac

66541 taaaaataca atctttcgcg cctatttcaa gatcaaaacc cttaggcaat actataggac
66601 gttgagttaa agaatgcgaa caaacaggtg tgagtataaa agcttgtgct aaagtataaa
66661 ctataggtcc atttgcactt aaattgtatg ctgtagagcc tgctggagta gctactataa
66721 gaccatctcc aaaatattca ttaaattttt tagccttgcg aaatacttct atatgtgcca
66781 tagaagcttt tctatcctta ctgatcacta catcattaaa agcaaatttt tgcgtgattt
66841 gaccatcatt agcttctaaa ataatactca ataaaaaagg attttctacc ctaaattctc
66901 ccctaaaaaa agcttcaaaa aaaacttcag cttcatccac cttaaaatcc gttaaaaatc
66961 ctaaatgacc tgcatgaatt cctaaaaccg ctttattgtg ctcacaagct tttcggcata
67021 aagaaatcaa agttccatca ccccctaagg aaattacaaa atccgaaagc tcaaacaaag
67081 tatcaaggtc gtattttggc aattttatat tttcagaact ttccttataa agtaaaagtt
67141 ctacaccctt tttttctaaa atttctttta gagttaaaat ttccttgtct aaatttgaat
67201 ttggacgcgc cgcaagccct attttttaa tgtttttata atctatttta ttttgcataa
67261 aaaattataa caaatcttag ctaagcaaaa gaataaaaat attataattt taacttatat
67321 gttaaaaaaa ttaattttca aggaaaaatt ttgcgaagtc attacaacac agacttaaat
67381 tctgcccaca taggcgaagt agttaaactt tgcggttggg tcaatagcta ccgagatcat
67441 ggcggcgtaa tttttattga tttaagagat cgtagcggta ttattcaact tgtatgcgat
67501 ccaaatgata gcaaagaagc tcacgaaata gcctctcgca caagaaatga atttgtttta
67561 atagccgaag gaaaaattcg accccgagga gaaggactta caaatcctaa attaaaaacc
67621 ggtgaaattg aagtagtagt aagcaagctc attatcgaaa atgaaagcgc tgtccctcct
67681 tttggcatag gcgatgaaag tgtaaatgaa gaattgcgtc ttaaatatcg ctttttagat
67741 ttaagaaatc ctaagcttta tgaaaatttt gcactacgct ctaaagcttg tatagccgct
67801 agaaattctt tggcaaatat gggattttta gaagttgaaa cccctatcct aaccaaagct
67861 actccagaag gagcaaggga ttatcttgtg ccatcacgcg tgcatcaagg agaattttat
67921 gctcttcctc aaagtccaca acttttcaag caacttttaa tgtgtagtgg ctttgatcgt
67981 tattttcaaa tcgctaaatg ttttagagat gaagatttaa gagcagatcg tcagcctgaa
68041 tttacacaaa ttgatgttga aatgagtttt tgcgagcaaa aagatgtgat tgccacagct
68101 gaaacctttt taaaagatat cttcaaagct tgcggtaaag aaatccaaat tcctttttaga
68161 caaatgcctt ataaagaggc tatggaaaac tacggaagcg acaagcctga tttgcgtttt
68221 gatttgaaac tgatcgatgt tatagatatc tttgcgaagt caaataatga aatttttgca
68281 aatattgcca aagacagtaa aaaaaatcgt gttaaagctt tacgcgttcc aaagggtgat
68341 actatctttt caaaaagaca aatgcaaaga tttgaagaat ttgtacgcaa atttggggct
68401 caaggcttag cctttataca agttaaagaa gatggtctta aaggccctct ttgcaaattt
68461 ttcaatgaag aggatttaaa cgagcttacc aagcgttgtg aacttgaagt aggcgatgtg
68521 gtatttttttg gtgcaggagt taagaaaact gtgcttgatt atatgggaag gtttagaatt
68581 ttcctagctg gagaacttaa tttaatcaac cctgatgctt tagaattctt atgggtagtt
68641 gattttccta tgtttgaaca aaatgatgat ggaagctatt ctgcaatgca tcatcctttc
68701 actatgccaa aaaatatcga tgagactgac ttagaagaaa tctcatctat cgcttatgat
68761 gttgtactca acggcgttga actaggtggc ggtagtataa gaattcataa aaacgatatc
68821 caacaaaaag tctttaaatt gcttaacatc gatgaagaac aacaaaaaga aaaatttgga
68881 tttttacttg atgcactaag ttttggtgct ccaccacacg gaggtatcgc cataggactt
68941 gacagactta tcatgctagt aacaggggca aatagcataa gagaagttat agcatttcca
69001 aaaactcaaa gagcacaatg cttgatgaca gatgcgcctt cagttgctag caatgaagcc
69061 atgagagaat taggaatcaa attaagggag aatgtaaaat gaaagaatta tttttaatta
69121 taggtgcgcc aggaagtgga aaaactacag atgctagttt gattgcacaa gctgatgcgg
69181 atgttactca ttattctaca ggagatcttt taagagcgga agtagcaagt ggtagcgagc
69241 ttggtaaaac catagatggt tttatctcta aaggaaattt agttccgcta gatgtagtgg
69301 taaataccat agtttcagca ctaaaaactg ctcctacaaa aacagttatt atcgatggtt
69361 atccaagaag tgtagaacaa atggtagaat ttgataaggt tttaagcgag caaaatgaaa
69421 tttgtcttaa gggtgtaatc gaagtaagag ttagcgagca agtagctaaa gatcgcgttt
69481 taggaagaag tcgtggtgca gatgataacg aagaagtatt ttacaataga atgaaagtat
69541 ataccgagcc gctagaagaa atcatttctt tttatcaaaa gaaaaaactt catttcatca
69601 tcgatggaga acgcactatc gaacctatcg ttgctgatat gaaagaactg attaaaaaaa
69661 ttcaaagtat ataaggataa aaaatggact taagcaaaat caaaatcgga gatattccaa
69721 acaaaatcaa tgctgtaatt gaaattccat acggctcaag cattaaatac gaaatcgata

69781 aagatagcgg tgcgatcatg gtggatcgcg ttatggctag cgcgatgttt tatccagcaa
69841 attatggttt tatcgcaaac acactagctg atgatggcga tcctgtggat atcttagtct
69901 taaatgaata ccctatccaa gcaggcgcag ttattccatg tcgtttaatc ggtgttttaa
69961 tcatggaaga tgaaagtgga atggatgaaa aacttcttgc tgtgcctaat tctaaaatcg
70021 atgcaagata tgacaatatc aaaacctata ctgatttacc acaagcaact ctaaataaaa
70081 tcaaaaattt ctttgaaact tataaaatct tagaacctaa caaatgggta aaagtgcaag
70141 atttcaaaga tgaaaaagct gctattgaaa ttttagaaaa agcgattaaa aactacaaat
70201 aaattcttta tttagccttt tataggctaa atctttaagg aaaatcccta tgaaaaatat
70261 cattttaggt ggtggatgtt tttggtgcgt agaagctgct tttgaaaatt gcgaaggcat
70321 tatacacact gaagtgggct acacaggtgg aaacgataac cctagctatg aaagcgtttg
70381 caatggcgat ggtaatatag aagtagttaa aatcatttat aatgaaacca aaatttcctt
70441 agaacaaatc ttaagcttat tttttaaaat tcatgatcca actagctttg acaagcaagg
70501 agctgatata ggaattcaat atcgttcggc gattttttat gaaagccaaa gcgataaaaa
70561 cactatagaa gattttataa aaaaagaaca agtcaatttc ccacttccaa ttgtcacaca
70621 aatttacaaa cttcaaagat attacaaagc agaagattat catcaacatt attttataca
70681 caatcccaac caaagctatt gtcgttttgt tatagcgcct aagctagaaa aaatcaaaaa
70741 ttacgagtga aattatcttt ttaagttttg aaaattttac tttttattca caggcttttc
70801 acgatgtgat tttcttttca aagtacctaa aaataggact ttaagtttta aattttccga
70861 aaaaacattt attgtttttt aagttttttt gtgaatttat ttgaagatgt gaataatttt
70921 aatatataat aaaattttag cttctatacg aacttaatct taaagaattg aaaatcaatt
70981 tttccttaat ttagcgatga aaaacccgtc atattcctca cagggcaaaa tgcgtttgca
71041 tttagaaatc tcctcaaatt ctctactttg cccattttta gccaaagcat tttctaaatt
71101 tatatccaaa agttctatat caaaatcact tcttaaagca ttttctatca cttcttcatt
71161 ttcctctttg gtaaaagtac aagtgctata aacaagctct ccacctactt tcaaagcctt
71221 gagtgcagaa tgtaaaagtt ttttttgaag tttggctatg tttttaatct ctttagaact
71281 tttttctaat tctaaaccca tttttgctaa agtagaacaa ggtgcatcaa gtaaaatttt
71341 atcaaatttt aagggacata aatttcctat atttttacca tctttcataa aaactcgagc
71401 ttttactcca taatttttca aattcttttg caaagtaaaa aacctgtcct tacttgcctc
71461 attgcaagct agatagcctt cattttgcat aaaattagca agatttatac tttttccacc
71521 aggcgctgca cacatatcta aaacgctttg ccctgctttt acatctaaat ttaaagcaca
71581 caaataagat gcataatttt gtatataaaa tttaaattca ttaaaagctt gcatagaact
71641 taaaacactt ttatcacaag ccttaaaaag atagcagtac tcgttaagct tttcatactc
71701 taaattcaaa tcttgaaatt ctctttcaag tgttaaatta tcacatttta ggcgatttat
71761 aaaacaacat atttttttag gcaatttaaa agaattttta agattttcaa gctctttatc
71821 attataaata ctagctagat tatattccaa aataatcctt tatcatgatt aaagctgcta
71881 aagagtctaa tttagcatct tttttgcgtg tattagctac gccatacccct aaagcttctt
71941 tgcttgttcc actttcatct acaaagcgga tttctttatc aaattcaagt aaagatacaa
72001 aatgcttaat gcgtcttgtc atttcttctt cgcttgaacc tcctttggga atacctacga
72061 ttaacaaaga aatatcatga atttggatta aattttttaac ttcatttgca gcttggttgc
72121 ggtttttttct gattatacct tctaaaggaa tggcgatttt atctatacaa agcgccacgc
72181 ctatacgctt taaacccaca tctaaggcca aagctctcat accaaaaccc ttataaaaag
72241 accttctatg gaaatttttc cttcaagctc gtactcatag actttttctc cataaatttg
72301 agttgcctca tccacgctta cgccctgtct acaaaagtct aaaaattcat ccttttcttc
72361 ttctataata ccaaaacaac ttacaaattc tttaaaatct aaaagtaaat ttgcatttct
72421 ttcttgtaaa agtaaatttg tcccatcgct ttcatttttg cgttggggta aaacatataa
72481 aggtttatta agctccaaag caagccttgc gctttgcata gaaccacttt gcaaatctgc
72541 ttgcgcaaca acaacagctt cactcaaagc gataatcagt ctatttctaa gtaaaaaatc
72601 ataaggttta ggcttataat gggattcatt ttcacttaaa gccaaagcat tttcataaat
72661 ttgttttata atttttttcgt tacttctagg ataaatttca tcaagaccgt tagcaaaaac
72721 ccctatggtg cttggcatag ccgccatact tgcgcttata tcaacaccta aagcacctcc
72781 gcttacaaca cttatttttg aattcttaag caagcttgaa agttcaaaaa cacaattttt
72841 agtgtaagta ctcatacgcc tagagcctat aatggctatc tttctttgtt ctaagagttt
72901 taaatttcct ttataataaa gttttttagg aggatttttc aactctttaa aaagttctag
72961 ataattgctt ggaagttcat tttgcataaa tttcacttaa atacactaac tcaacacttt

73021 caagtaaatc tttgctttgc tctaaggctt taaaggtatt ttttctaggg tgtcctatag
73081 ctatcgcaaa gccttttttc ttagcaagat ttaccgcctc catgagttgt tttttaatat
73141 aagcaacatc gtcttgattg tctaaaaata catctctttg tatataaatt tgacctaagg
73201 ctttagctac tttaggtgct ttagaacttg ctatggtttt agaatctaca aagatcaatt
73261 cttctttttc aaaagcctta tagagttttt tcattgcttt ttcatcactt gtaaataagc
73321 ttcctgtgtg attattgata aattttaaat ctttaaaatc tttttttatt tgttttattt
73381 ttttaaaaat acgcttttca ctatcgcttg gatttaaggt atcaagctca ggcttagtat
73441 aattcatcgc cgctaaaggc aagtgcacca tataaaaatc aaattttaaa gcaagctttg
73501 gagtatcgat atgattttta tcaggcggaa aaaatgaagg gataagcttg agttttaaag
73561 cctgtaaagc cttaacttga cttatatttg ccatatcatc tataataata gcaagacgag
73621 cttgtttaat tttgcttgaa tttgtatctt ttataagatt ttcttccttg cttaaatttt
73681 gatctggcac aaggcttaaa ttttcatcta gtttgcttaa attttgttct ataacactgc
73741 ttaaatttaa atctttgatt tgttgttcta agatactttg attgttttct tggaaattat
73801 cattttcatc tttgtaagga atttctttaa aaatacccga atgattttca ttagatatat
73861 tttcttgctt aggttctatt gttgtatttt tactagagtt ttgagcgaat tttaatgcat
73921 tttgagaatc ttgatattga atcaaaattc ccaaaaataa aatcacgcat attaaaaaaa
73981 gtatagcgat gattaagtaa cgcttgattt gaatcagttt ttttgacaat tttagttctt
74041 atctttatca attaattttt tagcactgat ccaaggcatc attgcgcgta aattacggcc
74101 tgtttgctcg attaaagaat tattcatatt tttgcgttct gcatgcattc ttgcaaagcc
74161 tgcacggcgt tctaaaatga aatctttagc aaagtttcca tcttgtatat cttttaaaac
74221 acctttcatg gctttttttag tttcttcagt gataattttt ggccctgtaa tataatcacc
74281 gtattctgca gtgtttgaaa tagaataacg catatctgca attccacctt gatagatcaa
74341 atctacgata agttttatct cgtgcaaaca ctcaaaataa gccatttcag gctcatatcc
74401 tgcttcaacc aaagtttcaa atcctgcttg aatcaaagca ctaagcccac cacaaagtac
74461 agcttgctca ccaaaaagat ctgtttctgt ttcagcttta aaagttgttt ctatgatacc
74521 tgtacgacct ccaccaatag cacttgcata gcttaaagct aaatttttag catttttgct
74581 ctcatcttga tgaatcgcta tcaagcaagg agttcctcca cctatagcaa attcatttct
74641 tactgtatga ccaggtgctt ttggagctat catgattaca tctataccct taggagctac
74701 gatttgacca taatgaatat taaaaccatg tgcaaaagct atagccttac cttcgcttaa
74761 atttggcttt atttccgcat taaaaatatc agcttgaatt tcatccggag ctaaaatcat
74821 aatcacatct gctacttttg aagcttcgct cacgctcatc acctcaaaac ctgcactttt
74881 tgctttagaa aaacttgatc catcctcacg aagcccgatg acaacattaa ctccattgtc
74941 ccttaaattc atagcatgag catgtccttg agatccaaag cctataattg ccactttttt
75001 tgatttaatc aaacttaaat cacagtcttt gtcataataa actgtaacag ccattatttt
75061 actccttaat aaattcaaaa ttaaagagct tattataaca ataaagtttt aaatttcata
75121 tttttttat aaactttatg caagaattct caagaaaatt tacataaagt aaagcagtga
75181 aagaattttt aaacaatctt aattatggca taagtgctca tgaagttagt aatgaattta
75241 aacaaattct aagagagctt ctagctaata acatcatcaa agaacacaaa aatagatatt
75301 atcttaataa cggctatgtt tttggaactc ttgatatttc aagtaaaggc acaggatttt
75361 tacagtgttt tgatgagagt tttaaaaaag atttgcttat agaaaataaa aatttaaaag
75421 gagcaaatta caaagatatc gttgctgcta aattacttcc cttaaagaaa aaacgcccta
75481 gtgctaaagt agttttggtt ttaaaaagag ccaacgaaac ctcacttgtc atcaccaaaa
75541 gatacggcga agcaattctt gggatgaata ttaaaacagg attaagcacc gccttaaaag
75601 cctcgcaaaa atccctaaaa gcactacctt tggggacaat tttaaaaata gaaaatcaag
75661 ataacaacat catagaagtt ttagggcata tcgatgatga aagcatagat gaaaaaattt
75721 ctcttgcact ttttaacaaa aacagtgaat ttagcgatgc ttgtatcaaa gaagctttag
75781 ccaatggaga tagcgttgat gcaagtatgt atgaaaatcg cttagatcta agagctttgc
75841 ctttttgcac tatagatcct gtgcatgcaa aagattttga tgatgcaatt tattttgata
75901 cagaaaaacg cgaactctat gtcgcaatcg ccgatgtaag cgagtatgtt tatgcttata
75961 gcgctattga taaagaagct agaaatcgtg gattttctat atattttcca catattgcca
76021 tacctatgtt gcctcgccct ttgagtgaaa acatctgttc tttaaagcct catttagatc
76081 gcttggcgta ttgttttaaa atcacgcttg atttagactg caaagtcgta aaagaagaac
76141 tttttgaaac catcatcaac tcaaaacgcc gttttaatta tgatgaagtg gatgaaattc
76201 tgattcaaaa gccagactta aaagaacttt cttggcttta taagcttttt gaaatcacaa

76261 aaactttacg caaaaatcgc cttaaaaacg cctttgaatt ccgcactgaa gaactaagga
76321 tgaatttgga tgaaaattta agtcttcaaa gcacgatttt tgaaaaagac acaccttcac
76381 ataatttaat cgaagattgt atgcttttag ccaacaaagc cgccgcaaaa ctcatcgatg
76441 taggtgtttt tagaaatcac tccagtgcgg acatgaaaaa gatagataga cttttaaatg
76501 agcttttaga gcttggaatc gatgtgaaat taaaacccaa cctgcctgag cttatacgcg
76561 atatacaagc tttagccgat gagcttggct taagggcaga agtggataaa ctcatcatta
76621 aagctcaaaa aaaggcggaa tattctagcg aaaacggagg gcattttggc ttgggttttg
76681 acaaatactc acattttaca agccctatac gcagatattc tgatcttatc ttgcatagac
76741 ttttaaaagc caaacaaaaa aaagatgaaa aactttttaa ttacctactt ttaaatatac
76801 aaagcacttg cgaaaacctt agtgtgcttg aaagagaggc cgataaagtc gcttatgatt
76861 ttatggatag gaaatttgca agatgggcag ctaaaaatat aggcaaaaaa tttaaagcct
76921 tagtagtgca aaatgatgga atttgtatag caaaacttga tgatgagatc aaaggagctg
76981 acatcatcct ttatgataca cgggtaaatt tgctagaaag tgtagaagtg caaattttag
77041 aagctgacat catcatggca aaaatttatg caaaaatcac ccaaagactt agaaaggaaa
77101 gtaatgtata gaaaagaact tcaaaattta ctttccaaaa atcaaattga taattttttc
77161 tttctttatg gagctgataa ttttcaaagt gagctttatg ctgattttat caaggaaaaa
77221 taccaagcag atgaaatttt aaaattattt tttgaagaat acagctttac tcgtgcaagt
77281 gatttctaa gcggaggttc gctttttagc gaaaaaaaac ttttagaaat caaaacctct
77341 aaaaaaattc ccacaaaaga acttaaagtt ttagttgagc tttgcaaaaa taataaagac
77401 aatttcttac ttttagaact ttacgatgaa agctctaaac aaagcgatat agaaaaaatc
77461 ttttctccta attttgtgcg ttttttcaag gccaataacg caagagaagg gatagagctt
77521 ttaagcataa aagcaaaaca actccatata gaaatcactc aaaatgcttt atttaccctt
77581 tttacaagct ttgatgagaa tttgtatctt gctgctagtg agcttaataa attcagcggc
77641 ttaagagtag atgaaaaaat catagagcag tattgttata gtttaaacat aggaagtttt
77701 gaaagcttct ttgaaaaaat tttaaaaaga gctgatttta aaaatgagct tgagaagatt
77761 ttagacaatt ttaatgaaat cgcactgatc aactctttaa attctgcctt ttaccgcctt
77821 tttaagatcg ctctttatgc taaaatttat gggaaggtgg attttaaaga actcttaggc
77881 tatactcccc cgcctcaagt agcacaaaat ttaaacgagc aagctttcag tctaaaaatc
77941 aaacactata aagaaatttt caacctttta cttaaaagtg aatatgagct taaaactaat
78001 tccaaactcg ctaaaaaaga atttctaatc gccactttgc taaagcttgc aaggattata
78061 aaaagttaga ttattgacaa tctcatttct tatttttgct ctttaaattt attgtaaaat
78121 ttctctgctt aaatgagatt attatttaaa aaaatcaatt tatacttcac tccatcacaa
78181 gtcgcatgag gtgcaaatcc tcaccttgtg agattttata ctctatactt tcgcatttag
78241 gacacttaaa aacattttct tctaaaatgc ttgtttggtc acattttaaa cacaaaattt
78301 caagcagtgt aagttctgta aattttacat tcaaatcttt aaaatcttct agttttgatt
78361 gataatcatc tacaatttta ccctatcctt agggcttggg tttaaagcca tttttttcaat
78421 gattctaaat tcttgttctt tttcaaaacg atgatttgct tcccaatgaa tcgtatataa
78481 aggagaataa agtaattttg caagcacagc aaatattata ataattatta tacaaggtat
78541 ataaatcctt taagtttgta aaaaggttta ttcatttata ctcctaaaaa tctcagctat
78601 tttgcaataa ttattgataa tttaatcctt gcattaatga acatatttca tatattcaaa
78661 tctcctaaag attttaatta ttttcttttt gtaattcttc attcattgtt tacactcttt
78721 tagtttagat tgtatggatt taagttcatt gatttgtttt tgaccgagtt tgagttttgg
78781 ataattattg tttatatttt ctattttcat tttaagataa tcatctttat aggcaataaa
78841 tttaattttt gaatttataa gattgatata tctatcaact gcaatatcaa ttttttctat
78901 gccatttaga attgctatgg ttttgggatt atttaaatca caaataggag ctatggaaag
78961 ttcccttagg tacatattgt tagcaaacgc accatctaag ccaatcaata gattaatata
79021 tgttacaatt tctattattt caaaatcatt atcatttttt ttcaaaaaat cctcttctaa
79081 acgagcattt aaaaaaaata aaaaatcttc aaaaatttta aatctttcct tatcaaataa
79141 aaaataagtt tgatgtccta taaaagtttt ttcattatct aaaagataaa gtataatatc
79201 catatttaat cttaaaacaa tttgactagc ttttcttgta agctcttcat cttcatctgc
79261 aaatccataa aaagttagaa tttgcatatc ttcaatactt gtttgtatca tcttaagatt
79321 tttaataatc atttgcgtaa ctttttcttg gttataagtt tctttgttta atttttttagc
79381 atactctaat tcctctttgc cttcattttc actccacttg attaaaatac taagattttc
79441 tcttgctttg ctttcattat cttctttaaa ctttagagct tcatggataa actcataaac

79501 actactaaca actgcaataa aaaccaatac aataaaaact atagtagaaa ttctttttga
79561 tattttaagc aagtttttta cctttaaaaa taatattatt ttgtattata atcttatcaa
79621 atcaaagatt aaagtcttgt tcttttgtgt tgcttgtatt ttgatttata cttatatctt
79681 tttgttttaa atttaaagaa ggattatttt tagaatttgg cgagtttgtt tttataggga
79741 tgaagatagt taaaaatcaa atatatttat cctttatatt ttaaagcttc ctttgtaagc
79801 attaaagttt tttttgtttg atcgtaaatt gcaatgagtt ttaaaatttc actcgttctt
79861 tttggaaaaa tttgaataaa atcatcttta aaatcaaatt ttctttttat attggtatta
79921 aaataagcaa acgcatcact ttgaaaaaat cgtcccatta aaataatata ttcttttctt
79981 ttgattattt ttttatattt ttgttcattt agtggcaaat caatatctaa gctcgatatt
80041 gctttattat aagtaatcaa agcaccttgt aaaagcacaa gcttatcttc tttatttgaa
80101 attttattca ttgtttttaa aagtatttct aaaagtttta atgcttcatt tttatcataa
80161 gcaagagctg atctttgtgg ataattttta gcctcattta taagtaaatt tgcattataa
80221 agaaaggcta attgattgat atttttaaga gagaagatat ctttgtttga actttcaata
80281 tactttaaaa tttctttatt ttcttgtttg ctctgctcta taagcttaga ggaaatatta
80341 gtataatcct ttgcaattgc gtttttttgt aaaatttgat ctgctaaaac aaaagagttt
80401 tgtaattttt ctttctgatt taatgtcaga ctattttcta tttgcttata atgagaattt
80461 cttaagtttt caaggtttga aatatcgttt ttttgcacag aaaaaaagtt tttaaaaaaa
80521 ttcaaatcaa gttttagata aaaaagtgct tcattaagat agatgacata gcttagtgtt
80581 tttaattctt ctttgttcag attaaaataa atcgccttaa gttcttctaa gttttgtaaa
80641 tattcttttc gggtaaggtt gttatagtag tatgtttgat tgtttatgtt gcatttaaaa
80701 ttaacttgat ttaaatttgc attttgtata aattttaaat cctcatcgct caactcatca
80761 gtacacaaag cataagcagc gttgcaaata aaaaatgtaa aaaaacataa ttttaatact
80821 ctttttaaaa atttatctct cattttctat ccttttttaat ttgcaaagaa taagcaaaac
80881 aataccaatt aatttcctag cgagaaggat aattgccaat aagtaaagtg ctactaataa
80941 tccatatttg acaaaaaaat atccaccaaa aaatattata attaaaagca tattatctgg
81001 aataagcaat aaatatccaa aagtatcata tatccagttt ataattttat tagaaacacc
81061 atatttactt ttgcaatttt tgcattttat ttctaatttg cccgagaata tattccaaat
81121 attttgcaat ttattgattt ttgttttaca aacttcacat tctttataaa ggggcatttt
81181 atttatccta tttttttctt tattattttc tatgactttt tatctttatc ttaaggcttc
81241 ttggttaatt tcatttgcat tcatcagcta tttccttgat tttatttaag aaattattac
81301 caagttcaaa taaatctggt attaaatatt tttcaaattc gctttctttg tattaaagaa
81361 tgattttttc tgaatttgtg gcaatatcta atagtttttt ttgtgtctaa tttacaaggt
81421 tctttatttg ttatcaagaa attacataca aaagtcgata ccatagaatg aaattccacc
81481 ctagcttcaa aataaagatt tttatctaag gtatttatcc attctagcat atcattataa
81541 tgctttaaaa attcaatata ttgttctatt tgtttttaac ttaaaaattt attatcaaaa
81601 attctaagtg caaactccca agaaagattg gtaaaataaa ttttattttt atcaactaat
81661 gcttgcagat atatcttatc taaacctaat aaagacatat gtaaaaaatc attcttagca
81721 ttttgattat agattaataa ttttttatgt aagttattgt gcttttcttc ttgatttgtt
81781 ttaagataaa aatcattgta gtattttttta aattcttttt catcttccca agtttcatat
81841 aaattttcca caaacaattc tttttctttc ttttgataat acctatccca aaaaataagc
81901 tctaaaggtc gttgtaaaaa atgataagct aagcttgtaa aaataattca aactataagt
81961 acaccgcaag gtatataaaa tattttaagt ttataaaaag gtttttttcat aaaataataa
82021 tccaagattt tttaagaatt tagacaaaat ttactccatc acaagtcgca tgaggtgcaa
82081 atcctcacct tgtgagattt tatactctat actttcacat ttagggcact taaaaacatt
82141 ttcttctaaa atacttgttt gatcgcattt taaacacaaa atttcaagcg gtgcaagctc
82201 tataaaaagc tttgcatttt tgcaaagctc tgaattttcc ctaaaagttt caaaacaacg
82261 cctaaaaagc tctacttcta taccgcttaa acgacctatt tttacataaa tctcttgcac
82321 gcttttagct ttatgagcaa aagcattttc ttcgcaaagt tcgattaaag actctactat
82381 actaagctcg tgcattagca aattctcggt aaaagttcgc ccttaggagc ttctaaaaag
82441 cgtttagctc cataagcatt ttctaaaatc acacgagctt ttttctcttc taaaacttct
82501 cctatgatac tagcatttgc attgtatttt tttaaaattt ccaaagcctt tagttcatcc
82561 tctttttcaa cgcaaagtat aaaagtgcct tcatttgcaa gttcaaaagc ctcatagcca
82621 aaaagctcac aaagtcctaa aacctcatct tgtattttta tcttttcttc aaaaatcaaa
82681 agatcattcc cgctttgctt tgcccattcg tttaaaactg cactaagtcc cccacgcgtt

82741 gcatcacgca tagctacgac ttttatatct ttttccaaaa gctccaaaac ttccttattt
82801 aaagccttgc agtcgctttt tatatctgct tctaattcat ttcttttgat caaaacacta
82861 gccccgtgcc tgcccacatc acctgaaaca agtatgctaa gtcctgcttt gatattttta
82921 ctctcttttt tagagatgat ttcacctaaa gcagtggtgt tgatataaat ttcatccccc
82981 ttgccttttg gcaccacttt agtatcgcca caaaccaact caacctcgca tttttcacac
83041 tcttctttaa tgctttttaa aatgcgttca agtttttcta gctcaaagcc ctcttctaaa
83101 ataagtccta agctaaggta ttttggtttt gcccctacca ttaaaacatc attaacagaa
83161 ccgcaaacac aaagcttacc tatatttacc tcatcatcta aaaagatagg acttaagaca
83221 aaagaatccg tgcttaaagc taaatttccc aaaatcgccg catcattgct ttcatttaaa
83281 attttattat caaagatctt aaaaagctta gttaaaagct cgttcatctc ttctccgcca
83341 cccccatgag ctaaagaaat atttttcatg cattcacctt tgagtattta taatacgccg
83401 cacaagctcc ctcgcctgaa accatacagc ttcctatagg gctttttgga gtacaggctt
83461 taccaaaaac cttacaatca taaggctttg ccaaacctct taaaatttgt ccgcaaatac
83521 aagccttact ttcactttta ctttgcactg cgcagtcaaa ttgcttgctc gcatcataag
83581 cgctaaattc ttcttttaat tccaaaccac cctcttttat aagccctaaa ccacggaatt
83641 caaaatcaca aacttgaaag tattttttaa ccaaattttg tgctttttca ttgccttttt
83701 cactcaccgc tcttttgtat tgattaaaaa cttcgaaact gcctttattg atttgtctta
83761 ctatatttaa aacgctttcc atgatatcca caggctcaaa accactcact gctatagggg
83821 ttttaaactt agccgctaaa ggtttataaa tcccataacc tgtgatcaca ctcacatgag
83881 aaggccctaa aaaggcattg atctttacat tttcatcatt cataatcgct tctacaggtg
83941 ctggaactgt gatatggttg atatgaaaaa agacattttt taaattttgc tctattactt
84001 tttcaagaag taaagcactc ataggcgtag tagtttcaaa acctatggca aaaaagatga
84061 tggttttgtt taaattttgc ttagcaatat ccaaaacttc aagcggagaa taaagcgctc
84121 ttacatccgc tccccttgct cttaaatcaa gcaaagaaat ttcacttcct ggaaccctta
84181 aaagatctcc taaggtgcaa aaaatcgtat ctttcataga agcaagtttg atcgcagtat
84241 caatgcgaac ccttggcatc acacaaacag gacagcctgg cccatggatg aaattgatct
84301 ctttaggcaa aatagaaggc aaagcgtatt tcataatact atgcgtatgc ccaccacaaa
84361 tttccatgat attaatcggt gttttaattt cttgttctat aagctttttt aaggctaaaa
84421 tattgtcttt atctctaaat tcatcgatga aattcattta aacccatatc cccttcatcg
84481 cttttgatct cgccactgtt catcttttct actatttctt gataggtttt tatactttct
84541 aaagcggctt ctttgtcgat tttttccatg gcaacaccta catggattaa aacataatca
84601 cccttttgca aaggctcatc gatcaaatcc aaattcactt ttcttttaac acctagagtt
84661 tgcaccaagg cattgttaag ctcatcgatt tctaaaattt cagaaggtat agataaacac
84721 attaaaaaag ctccttttttg agtttaagta gtttatacca aagctccata ttggttccat
84781 ttttagcatc aagagtgatg atatcagctc ttggactgag ttcttttatg agttttgtag
84841 cttctttgat atcaaaatca aaatgatgag ccaaatccgc cttagtaatc aaaacaatat
84901 ctgctttttt aaacatcaca ggatattttt gtggcttatc actgccttca gtcacagaaa
84961 gtaagactac atttaaatgc tcgcccaaat catagctcgc aggacaaact aaattcccca
85021 cattttctat aaaaagtaaa tccactcctt tgatatctaa atgatgcaaa gcctcatgca
85081 ccataaaagc atctaaatga cagctttgtc ctgtagtgat ttgatacgct aaagctccag
85141 cattttttac tcttaaagcg tcgttattgg tttctaaatc cccttcaatc actgcgattt
85201 taagttcgtt tttcaaagcc ttgatcgtgc tttctaaaag cgtagttttt ccgcttcctg
85261 gagagctcat gagattgata caaagtgtat ttgcttcatt aaaatgcgcc ctattgtgct
85321 tggcttcttc gtcattttta cttaagattt tagaaattac ttctatggtt ttgctttctt
85381 ttaggcttgg attttcatga tgatgatcat gatgatgaga atgctcatga gtatgagccg
85441 aatttactga acagccacaa tccttacaca tattatgttc cttttgaaag tttttctttg
85501 attttatctt taaaagtatt aaattttaat gagttttaaa gatattatca gtcttttttt
85561 aaaaaatgca ccatttgtcc tagggctatg gagctatcat tgcaagggta ttttaaagat
85621 gcgagaaagt caaaattttt agcttttaaa atttgaagta aggttttatt ttgaaaaact
85681 ccacccccta aaagcacttt taaatcataa gccttgctaa aagttataat caaatttgcc
85741 aaagcattaa acatacccgt aatagccttg ctttttttcat cctttaaagc accctcaatg
85801 agttctttaa agttgatttc tccatcctta gaaaaaagct cgtaagaaaa gtctaaattt
85861 tcatcataaa atgcttcgca catcagccca atttgtgctt cgtaagaaat tttttctata
85921 tcaaaaacta tactcccaaa agcatcgata atgcgtccta aagaacttgt ttgaagtgta

85981 gagtgggtgt gaatttttttt aagattttca agttttattt ttggaatttt agataaaaat
86041 gtcttagcct catcttctaa atcataatgc caaatcaagc tcaaagctaa attttgtata
86101 ttttttatat cgctattaat aagggtgaaa ttttcaaaat gcacgattct ttcgtattgt
86161 tttaaattcc ctataaaaat ctcgccaccc caaatttttc catcatcgcc ataacccgtg
86221 ccatcaaaga taaaacccaa agctttttca tcttttttaa attcttttttc gtattcaaaa
86281 taaagtgcgc aaaaatgtgc gtaatggtgc tgaattttat aagtattgta aaattctttt
86341 gtatagctaa aattaggatg tttatcgcaa agtatttgat caaaacttaa atcataactt
86401 tgtttaaaaa aatcaagcaa agaaaaaaat ctctcataca catcaacact ttttaaatcc
86461 cctatataag gagaaatcaa aagtttattt tcataaaaaa tcacaaattc gtttttttaat
86521 tcggagccca aagctaaaaa aactcctttt ttatcgcttt taatttccaa ataagtcgga
86581 tttaaacccc ttgaagtgcg taaaaacata gtttttccat ttacaacttg agcaatgcta
86641 tcatcgcttg gattgtgaat ttctcttgca tagtctagat aaaaatcaaa aacattgcca
86701 agttttttaa gcaaattatc ctcatctttg ataatgcttt caccacttaa atttgcactt
86761 gttgctacta aaaccccatc aaaatatcta aaaagtaaaa gatgcagtgg agtgtaagct
86821 aacataatgc cgagtttatc tacatcaggg gcgattaaag aaaaggcttt tttagctttt
86881 aaaatcacaa taggagctaa aatagaacca agtagttttt cttcctcttt atccacaaaa
86941 caaagctctt tagcatcgct tatatctcta cacattaagg caaagggttt tttggggcgg
87001 ttttttctaa gtcttaattc ttttatggct tcaagattaa aggcatcaca catcaaatga
87061 aatcccccca tgcctttgat agctaaaatt tttccttgct ttaaaagctt agcacaggct
87121 ttaaaagcct catcatcttg ggctagaatt tcaccctttt tattttttaa aaaaacatcg
87181 attttacact gagggcaact tataggctgg gcatgaaaac gcctattttt aggatcttca
87241 tattcacttt tgcaaaactc acacattttc aatgcttcca ttgtggtatt gcacctatca
87301 taagggagat ttttaatgat gctaaatctt ggcccacaat gcgtacaagt gataaaagga
87361 tataaaaaac gcggattttt ttcatcaaaa aattcttttt tgcactcttt acaaagtgca
87421 aaatcactaa gcattggagt agtttttgta ttttgcaaag aagtacctat gctaaaatgg
87481 gtataatttg tagtcgtttg ctctgtgata tcaatgctat ctatccttgc taaaggcggg
87541 agcttctctt tgagtttata gatgaaatgc tcgcattctt ctttggtgca agcaaggatg
87601 atttctaccc caaaaccatc atttttttact tctccgcaaa gtcctaattc acaggcaagc
87661 tcaaagacaa gaggacgaaa gcctacgcct tgaacaagtc ctgaaatttg aagtttatat
87721 cctaagcggc acatcgctta actgacaatt tttaagatgt tttaaagtat ttctaagcaa
87781 agctttatgc tcaaacaaag atccactaat caacgccatt tcactctgtt ttttttctct
87841 taagtcatca taagtatcgc gtaaaaaata agccaagctt tccacagctc cataagcgat
87901 atttgcacta tctacccctg ctagcataaa gctcatagta gatcttaagg tcctagtata
87961 atcaaaactt ttatcttctt tcaaacgata atcaatcttc actccgcgag gcattttaga
88021 ttcgtctgcg atttttaaaa gtttttctcc tgccttgtgt aaattttcat ccaaatttaa
88081 caccctaccc accaagcaaa gtaaagaata aaaattgttt ttaagttcaa aactctcatt
88141 taaaagcgga aattctttgg caaaattttc taaaagtctt gcgccgattt catctttttg
88201 tatctcttca taaagctctt tagaattttt aggtaaagaa aggcaaagca aattgatctc
88261 tttatttgcg agcaataaat cttcatactc cttactaagc tctaacacaa aagccttttc
88321 tccataacga cttaaaagat aagaaattct tgccatattt ttatcttctt tagaaaagat
88381 aagctctctt gctcttgggt tgataaattc aaaaccctct aatactacta tttgattatc
88441 taaagtcgtg atataaaaat ctttttgaaa ttcttttatc tttttaacgc ttaaaaattt
88501 atactcattt tgccaaagct ttaatcctaa agcaaaagaa aacaaatcct gcgctagcct
88561 taacttaaat tcattaaaat caagatcgtg attttttcta aatacagcgc ttaatcttag
88621 cttcatcaat ggtttttcca aactcgcaag aagttttaga ttttcatttg aacaaacaaa
88681 gacagaattt atagctttga tatcgcaagg cattaaaaaa tcgcactcaa aatccccttt
88741 aaataaagaa atttcataaa tgcccttttt atcctttaaa taaatacttt tttcttttag
88801 caacaaatca aaagcagtgt ttaaattttt atcaaaatta tcttcattaa gactttgaaa
88861 ttcgtttaaa tcacttgaaa attcacactc gcaaaataca ccccattcat ttttacttaa
88921 ttcttttttt tcttgataga gctttgaatt gagataagta agataagtga aagggaaaaa
88981 atctttatcc tttacttctt gctgatgaaa ttctcctgcc ttaacatcaa attttttcaa
89041 aaaaatacta tgactcatag aatttaaaga atcacaaaaa gcctttaaat tttcttcgtc
89101 cgcatcaatg ctaaaatgat atttatcctc ttgtttttct aaagtataac gataattttt
89161 cgcataaaaa cgcagcaaat attcaaatat atcattatcc gcactataaa caaaatcaaa

89221 ttccaaagtc atattgtgcc ttttttgaaa aataaactat aaatatagca ttttttactt
89281 aaaattcttt ttctctttgc ctaaaatcgc tcataaaaga atagataaaa tcataaaatt
89341 ctttgctgtg gtttgggaat ttaatgtggc ttaattcatg taaaatcaca tattctatag
89401 ctcttaagtt tttttctaaa agtcttaaat ttaagttgat atagcctttt ttatgattgc
89461 aagatcccca acgcgttttc attttttaa tactcaaatg agaataatgc aaatttgttt
89521 ttttactcca tttttaaga taaaaagaaa aaatgatttt tgcattttgt tttaaaaaat
89581 tatccaaatc ttctcttgaa gcactttgaa tttcaccctt tgaaattcgc gtttttttaa
89641 agctttcatc taaatttaaa atatattttt tacctaaaaa atatatttca ttttcgctaa
89701 tccttgcttt tttgctttga aaattaagat aagtttttg tatccaaatt tcatttttat
89761 ataaaaattc ttttgctttt tctagcggat aaaattgagg gatacttaaa tgaaattcgc
89821 ccttttcatt gagctttaaa cgcaaggttt taatgttttt gatttgatag aaaaaatccc
89881 tatttaagta acgaaaactt tttacactag agctttgcct tgccatttct tactcttcca
89941 tctataagca tttacaagcc ctcttaagaa ctcatctgca caaaaaccta tccatacccc
90001 caaaattcca agcccaaaat gaaaacaaag cacatagcct actggcaaag aaacacccat
90061 cataaaaaca agtccgctaa aaaatgggaa tttcgcatct ccgcttgctc ttagggcatt
90121 gaccatgact atattaaaag tgcgtgaaat ttcaagaaaa atagaaagcg taaataaagg
90181 aagcataagc tctctaagct caggttttaa atcaagcaag tccatggtaa aatctttgaa
90241 aacaaaattt aaaaccgcca caaaagcact ggccatcacg ctcaaataca aggcattcca
90301 agcgtgcttg taggcgatat tttggtattt tgcacccact aatttaccca cgatgatttc
90361 atttgctatg ctgattgctt gtccaataag catgataagc attgaaattt gaaaataaat
90421 cgtttgcacg ctatcattgt ttgatccaag ttttgccaca aaagaaaaag cgataaaata
90481 ctgcactatc caaagcaaat tttctcccgc tgaaaaaccg cctatgttta aaactttttt
90541 caccacagct ttttcaagcc ttatcatttc ttgaatttta agatgaattt ttaatttata
90601 aaaaagtatg ataagcaaag cagctactgc tacaaaacgc gcaacaagat tgctaagccc
90661 tactcctaaa agctcgcttt gagtaaaatg cagggcgtaa aaattgccta taatgactat
90721 gatatccatc aaaaagccta taaacataac ccaataagcc atattataaa ccctgataat
90781 agcagccaat acaatcccca aagcatcaaa aaacaaacaa attcctagca ttcttagata
90841 gataatgcca tcttgtaatt tttcttcagg gatattagca agcctaagca aaagctctcc
90901 ttgccatatg ataaacaccg cgcaaacaaa acccaagagc gcatttaaaa acaagctttg
90961 atgtatcact tttcttgcta agactaaatc ccttgctcct atggcttgag cgatcacaac
91021 actacaaccc acacttaaaa aactaaaaat cgtgataaac aaatcagcga ttaaattccc
91081 cgctcccata gcacccacaa gggaatttga ataatgcgaa accatagcag tattgatgat
91141 gatagtaagg tattttgaaa gcaaatccca aaaaatagga acactgagtt ttgttaggga
91201 aagttgtttt tttgccatga ttattctttt aaaaatatta aaatctaaga ttataacaaa
91261 attaacctaa tttagacaaa ttttatactc aaagatgagc taaaatttaa aaagaaaaaa
91321 ggcattgttg tatcttttct ggcttaaaaa tttattttta tagtgatgat tttataataa
91381 ttttttaaga attttatgaa tgattttttt aaacgctatg ataataatgt caacattttt
91441 gaaattttgt atatagtcca tagataaatt tacgaatatt tatcttattt acacttgaaa
91501 cacctcgatc ttgacattgt tttagaataa gtaaagactg ccttaaccta tatattaaga
91561 caatctttat tttataatat tcttaatata aatattacat gtttatgatt cataccatgt
91621 tggctcttct atatttgtac cttctacaat atagtaatta tcaacatcta ttgcattaag
91681 tcgccagctt ttaaggctgt atgtataagc ttttgcttca tgtaaaaaat agcttaaatc
91741 ttctatactt tttatattcc agctgcttat atcttgatta aagcttttac tttcagaaaa
91801 catagcatgc atagtttcta catttgatac atcccagtca ctaagtggtt ggttaaattt
91861 ctcagtttct tcaaacatgc cagtcatatc ttttatttta gatgtatccc attcattaag
91921 tggtgaatta aaagattttg cctcactaaa catatatgct aatgatgttg tgcctgttat
91981 atcccaccca ttaagaggct ggttaaaatc ttctgtttct tcaaacatat atgaaaaatt
92041 tttaacatgt gatacattcc atgagctgat attttgatta aatcttttgc ttgatctaaa
92101 cattgacgcc atatttgtaa ctttgcctgt atcccattta tcaagaggct ggttaaattt
92161 ttttgtataa aaaaacatac cattcatatt tttcacatta gatacattcc agttattaat
92221 attttgatta aaactttctg cattagaaaa catatgattc atatctttta cttttgatgt
92281 gttccataag ccaatatctt gattaaattt taaagctcta taaaacatat aatgcatact
92341 ttctacacgg cttgtatccc acttatcaag aggctgatta aaatcttctg cacaataaaa
92401 catagagctc aaatctttta cattagatat attccagtca tttatatttt ggttaaaagg

92461 tgtattttgg aacataccgt ccatatatgc tacttttgat gtatcccaca tactaatatc
92521 ctggttcata ttgctatatg caaacatatt gctcatattt accacatggc ttgtattcca
92581 tgattctatg ccactgaaat ctctttcaca gtgtagaaat attaatgata aatcattaat
92641 attagatata tcaacatcag caagatttat atttttatct tttgcaagta atattaattc
92701 cagttttgta ttaggcttat acttaccatt ttctttttta catggcatga gccatttagg
92761 cttagtaggg cagttttcat ataaagcata gttacataaa ttttcatgat ctatgaactt
92821 atattttttt gcattagtag ccctgtaatc tttatttatg ctgcttacat cccagttatc
92881 tatattttgt ttaaatcttt ttgccccatg aaacatatcc accatacata atacattaga
92941 tgtatcccat gcacttatgt tctgattaaa atcttctgct tccaaaaaca tatagctcat
93001 gtttaataca tttgatgtat tccagctgcc tatatcagta ttaaagccaa ttgattggga
93061 aaacatataa cccatatcac ttacattgga agtatccata ttgcttatcc agtcaaataa
93121 agggctgtcg cttttaattt tactaaaatt aaacttttca aacattctgt tcatatttac
93181 cacatggttt gtatcccaaa gttctatgcc accaaaatct gttcttatta catctgtaaa
93241 taattctgaa aaatctttaa tatttgaaat atcaatatca cctaaatact gccttttctt
93301 attcattagc tttataagtt ctcttttatt tttaggtgta attatttcac ctttactttt
93361 aagaggatta gtttttctat taaacatggt atttataata gtatcaagct tttgcccctt
93421 agcatatctt gaaacaattc tataaatata atcctgatct gaatttttag cagtgcagcc
93481 agcataaaaa gctttatcat cagcatattc tatttctaaa ctttctaaaa atccataagc
93541 tatatcaact atttccttat ctacttctgc cccatctcta atggcttgga taactccatt
93601 aacctcttga ccttcaatag caaatattag cttttcatac aattcttttt tactcatttt
93661 gtttctccaa atatttaaaa aatttccgag taacaaaatt atatactata aataataatt
93721 ttaatacaat aaaaataaac actattataa acacatcaaa acaataaatt tttaaatttc
93781 aaattttaag tttattttat ttttcaaaaa atagggattt aaatttatat tattttatct
93841 ttagaaaaag gaaagagaaa tttatcttac tctctcctat tttataaaaa agtctttgtc
93901 aaataagatt tatagatggg ataaaaaatt taatattaaa aatctcaccc aaaagcacca
93961 cttaaataag ctttggtttt ttcttgcttt ggattttcaa aaaattcctt actttcccca
94021 aattctataa gctctccaag gtgaaaaaat gctgtataat cagccacgcg tttgccttgt
94081 tgcatattgt gcgtaaccat gatcatagaa agattatggc ttaattcttt taaaagctct
94141 tcaatcacgc ctgaacttat aggatcaagt gcagaagttg gctcatctaa aagcaaaagt
94201 ttaggtttga tcgctaaagc tcttgcgata caaaggcgtt gttgttgtcc gcctgaaaga
94261 gctagagcat tttgcttgag cttgtcttta acttcttcaa aaagccctac tttttgcaaa
94321 caatccacca ccaaagcttc ttcctcatcc ttgcttttaa tcataccatg aagttttgga
94381 gcataagaga tattttcata aatgcttttt acaaaaacat tagcttgttg aaaaaccata
94441 ccgacatttt tacgcaaaac tactacatct tgattttta catcttttcc ttctatttct
94501 actaaaccat cgatttttgc gattttgtca ttcattctgt taaaacaacg caaaaaggta
94561 gattttccac atcctgaagc accgattaat gcagtgattt tattttgctc gatttgcata
94621 ttgatatcaa ataaagcttg tttttttcca taaaataaat ttaaatttgt tgtttttgct
94681 atcactttaa ttttccttga aaatattttc ttaataaaac cgcacttaaa tttagcacca
94741 ccaaaagccc taaaagcacg atgatacccg ctgcagttct ttctaaaaac gcacgctctg
94801 gcatagccga ccatgaataa atttgagcag gtaaaacgct agttgcatca agcactgaac
94861 ttgctacatc agggataaaa gctatcatac cgataatcat caaaggtgca gtctcaccta
94921 tggctcctgc taaagtaagg atagaacctg ttaaaatcat aggcatagcc aaaggaagca
94981 tgatgccctt taccatttgc acctttgtca tacccaaagc ataggcagca tttttcatgt
95041 tgatatccac gtttttaaa gctgctttgg ttgaaactat gatgataggc aagctcatga
95101 tcgctaaagt aagcccgcct accaaagcac ttgaacgcgg cattccaaaa agattgataa
95161 acacgccaag tcctaaaagt ccaaataaaa tactaggaat gcttgcaaga ttgttaatac
95221 acacttctat aaaatgtgta aataaatttt gcggtgcaaa ttcttccaag taaatcgctg
95281 ctgcaactcc tactggaaca cttactgcca tacacacaag cataacaagc aaagttccta
95341 cagccgaaga taaaattcct gaattttcag gcgattttga atccccattt aaaaagaaat
95401 tcgtattaaa acgaagtgct atttcatctt tttgtacaag attatcaacc aaagctcttt
95461 gctcttcatt taagcggttg taattttgtt tcatatactg atccacttct gaatttgcta
95521 aaagccatga tttttcagtg atttgtcctg ttcttatctt tctttgctct gcacgcgata
95581 aaagctcata agcaggcgaa tttctatccg cttccacata aatataagtt tgcttaaacg
95641 ccccaattcc aagatatgcc acacttccta aaaagataca aagaaaaatg agattgatat

95701 aaagtcccat tttacaaagt cttttaaaag actttgaggc tttttgtcgt tttttgaaaa
95761 gctttttcat aaatttttcc ttttgtgaaa gcggttgata agataaacgc taaacatatt
95821 gatgattaaa gtgatgatga aaagcaccaa acctaaagaa aaggcactta aagctaaaga
95881 actatcaaaa gcttgatccc cacttaatgc ttctacgatc ttaaccgtaa ccgttgtcat
95941 atcttctaag aaattcattg tcaaatttgg acgcaaagaa gctgccatta ccactatcat
96001 ggtttcaccc aaagcccttg aaagccctaa aagacaagct gctactatac ctggaatagc
96061 ctcaggtaaa atcacagcaa aaaccacttc tttttttgtc atccccaaag cataagctcc
96121 gttaatcctt ttttcactca cagcttcgat acaatcttgc gacaaagaag ccactatagg
96181 cactatcatg atccccatga taaatcccgc acctaaagca ctttgaaaac tcgcctgtat
96241 accaaacaaa gaaaaaaacc atactataaa aggtgctacg attatagctg caaaaaatcc
96301 aaaaacaaca gtaggaatcc ctgcgataac ttctaggata ggcttgaggt agtttttaga
96361 ttttttaccg gcaaaaacac ccaaatatat agcacacatt acccccaaag gtagagctgt
96421 tagcatggcg attaaagaga tataaaaagt tccccaaaaa agcgacactg caccaaaaac
96481 cccttgcttg ctcgtgccat ctgcattcat aaaagctgca tcagccgccc attcgcttga
96541 aaataaaaaa gtacttatgc tttctttttg aaagaatttt aaagcttcaa ttaaaatcgt
96601 tagcatgatg gcaaagctta ccaccacact aacaaaagcg cataagaaaa gtataaattt
96661 aattattttt tcttttagca aagtttttcc ttaaaatact tttccagctt ttacaagctc
96721 atcggttaag atttttctat cttctatatg tttttgagaa gcttttaaaa gctcattatt
96781 taaaggaaca agccctattt tctcaagctc tcctccactt tttgctaaat catcactcat
96841 ataaattttt gcaaattcaa aggcttcttt agggtttttc tttgcattaa tatagataaa
96901 caaacttctt gcaagatcgt attttccatc agcaatattt ttttcatctg cttttatacc
96961 atcgatatct gctgcattga ttttatccac attgcttact aaaaatccat aaccaaaaag
97021 tccaaaagca tctttatcga tagtaagttt tgaaacgatt agattatcat tttcaccact
97081 tgggatataa gctccatctt ggcgtatggt tttatactcg cctttgtatt caggaatttt
97141 tttagaaata tcactcatta caagctcttc tacggtatct cttgtacctg agcttgatgg
97201 aggaccataa acgctgattt tacgattagg taaattttta ttgatttgat tccagtttgt
97261 ataaggattt gggacaagtt tgccaccttg cggaacctct ttagctagag ctaggaaaag
97321 ttctttttta gtgatgttta aaggtgtgtt ggctttattt tgcgctaaaa ctataccatc
97381 ataacctacc ataatcccta ctatatcagt aactcccgct tttttgcaag tttcaaattc
97441 actaaatttc atcgctcttg aagcattaga aatatcagtt atgccctcgc aaaatacttt
97501 aaatccaccc cctgttccta aactctctac gataggagtt tttgtattgt gaatcgctgc
97561 atactcttca gctacaaaag aagtaaatgg atacacagtt gatgaacctg cgatttttaa
97621 atcagccgca cttaaagcac ccgctaaagc aaaacttgca acacttaaag ctagaatttt
97681 tttcattttt tctccttact tttaaaaaag taacggaagt ttaggaaaag tgggaaacat
97741 tttggaaaca tttttttac ttcgcaacgg aaataaattc cgtcttgcga caaaggactt
97801 agtccttttg aaacctctaa aggcacgccc acctaaaaat ctttgcagat tttcagcctt
97861 tttaaaattc tatctcataa caaaatataa attatttcat ttagaaacat tttattttta
97921 ttttatatat tttgatttca atcctttaaa aatttatttt gttttaaaaa aatcaatata
97981 tcattttaaa aacattctaa aaaataaaaa caaggagata gaatgctttc aaaaaaagta
98041 gttgatttat taaacgagca aatcaataaa gaaatgtatg cagctaattt atatctaagt
98101 atgagttctt ggtgctatga aaatagtctt gatggagctg gactctttct ttttcaacac
98161 gcaagcgaag aaagtgaaca cgcaagaaaa cttataactt atcttaatga aacagattca
98221 catgtagagc ttaaagaagt taagcaacct gaacaaaatt tcaaatcttt acttgatgtt
98281 tttgaaaaaa cttacgagca tgaacaaagc attacaaaat ctattaatga tcttgtagag
98341 cacatgttag ccaataaaga ttattctact tttaattttc ttcaatggta tgtgagcgaa
98401 caacacgaag aagaagcact tttccgtggt atagtagaca aaatcaaact catcagtgat
98461 aatggaaacg gcttgtatct agctgatcaa tatatcaaaa atttagcctt gtctaaaaaa
98521 taatattttt gccttgaaaa aggcaaaatt acaatttcat cttaggtagc gtatgactta
98581 tttttcttta gaatttagca ttttaatgat tgtatttttt gccatatatt gggcatttaa
98641 agataattat aaaatacaaa atattttaat cttagctttt tcatatctta tttatatact
98701 gattaatcct tattttgctt taattttatt tatttatact ttttttatac attttttcgc
98761 cttacttatc tttgtgcgtc gtaaacgata tatctttgct acttgtctaa cttttataat
98821 attaaattta tgttttttca aatattttcc aaacatcaaa ggaagtcttg atcaagtgct
98881 aagctttttg gggcttgatt ttttaaatat tgaacttatc ttacctatag gtattagttt

98941 ttatactttt gcttctatta cttatcttgt agatgtatat aaaaaacacc atttagaaag
99001 ctttttaaat ttagcaacct ttttatcatt tttccctacc ctacttgcag gaccgattat
99061 gcgtagtgcg tttttttttcg aacaagctta tcaaaaacgc gaatttaaac atgcaaattt
99121 gatcatcatt ttgcttattt ttggtatagt taaaaaagtt cttattgcta attacttagg
99181 aatttatgct aaaaatattt tagattttcc tcaatcttat aatttcatac aacttttaag
99241 tgcaatttat gcttatgcgg tgcaaatata ttgcgatttt agtggatatg tagatctagt
99301 ttgcgctttt gctttaatgc taggttttac tctaccaccg aattttaata tgccttattt
99361 agcaaaaaat cttaaagaat tttgggctag atggcatatc agcctttcaa ctttcataag
99421 ggattatatc tatattcctt taggaggcaa tagaaaagga atgcctcgca ccatagccaa
99481 tatactcatc gcttttatcc tttcgggctt atggcatgga agtactttag cttttggact
99541 ttggggatta ttgcatggat taggtattgt atttatccac ttgcttgcct taaccaaatt
99601 cagccttcaa aaaattccag ctttaggaag atttttaacc tttcaatttg tatgttttac
99661 ttggattttc ttttattata ataaaaattt agaagatgct ttagaatact tcaaagcttg
99721 ctataataac tttttccaaa ttcctagcta taatgatatc tatatgcttg taagttttgc
99781 tgtgcttttt atgatttatc ctttatttgt caattttaga gaatattgtg taaagatttt
99841 aaatcttacg ccgtttttat taaagccgtt tgtaatcact tttattttgc ttttggtgtt
99901 tgcttttatg ccaaatggta ttcctgattt tatttattcg agtttttaaa tgaaaattat
99961 tagatttttc tttattttaa ttatagtgtt tatacttgtt tgccttgtga tgaatcaaag
100021 catttttttct tatatcgagc aaaaatatca ctttgcattt tatcctaaaa acgatatttt
100081 acaagaggcc aatggtttta aaaataaact agaacaaatc cgtgcaatac taagcaatga
100141 gcccctgcct caaaatatag aagaaaatat catggttcaa gaagatatgg atgaacttaa
100201 tgcaagtaat gattttatcg ataaaaacac caccattgaa gatatacagc aaacagaaga
100261 tgaaaatgtc agttttattg acaataccaa gctagaaatt cacaaaggtg atgaattttt
100321 atttataggt gattctttaa tgcaaggtgt ggcaatcgca cttaatcgcg atttaattga
100381 tctaggttta aaagctaatg atcttagcaa gcaaaataca ggactttctt ataaatctta
100441 ttttgactgg gcaaaagcga ccaaagaagc ttttgcaaaa aatcctaaca ttaaatatct
100501 cgtcgtccta gttggagcaa atgatccttg ggatattaaa aaaggtgggg tttatcatcg
100561 ctttggttcg gatagctgga ttgatatcta cacttataga gtaaatgaaa tcatcaatat
100621 agccaaacaa cataatgcaa aaattctttg gtttgaagtc cctcctgtta aaaaagatga
100681 gctcaatgaa aaaatacaaa ttctaaataa aatttacagc gaagaaattc taaaaaacaa
100741 gcaaattttt atcaatacaa aattattttt tagcgtcaat gatgaatttt caacctatat
100801 caaaaacgaa aacaacaaaa gtgtaaaaat gagaacagat gatggaattc attttacccc
100861 aaatggcgca aaagagatgt caaaactctt gctcgaacat ataacaatca aggaagaaaa
100921 tgcaaattaa gcaatttaaa gctatattat taatccttat attttcaatc aatttatacg
100981 caaacaacac aaacaatatc atagatgaaa ttttaaatca aaccaaaaat caaagcgctt
101041 tgagtaatta tgcttctaaa aaagatttaa agattttaga acaaaaactc aaagatcaaa
101101 aaaatatagg cattagaatt tatggagatt ctcacacggc agcagatttt tttccgcgtg
101161 ttattagagg atatttaatc aaatcaaatt ccataggctt tgcttatcct ttacaaccta
101221 aatatcaaca aaacttaaat cttgattatt cttataaaaa ttatgaaatt ttaaactcaa
101281 gaaatacaga caccgcaggt attgattttc ctttaggtgg gataatagcc aaagctaaag
101341 aaaaaggtgc aaaaatcact cttgatacca atttagacaa aaaaaaattt aaagtaggtt
101401 ttgtctttaa ggctcaaaat aacaccaatg cctttagtat taaagatgct caaaacaaaa
101461 gttatgagtt aagatcagtg gctaataaat ggtcatataa agaatacgaa cttgtttttc
101521 ctttgcaaat ttctgctcta caaaaaaacg ctcaattggg aggatatttt atcaccagca
101581 aagaagataa tgcctattta gataccatag cgatcaatgg ggcaaaaagt gatctttgga
101641 tgtcgtggaa taaaaaagta agcgagcaag aattaaaact cctatctaat gatttaatct
101701 tgcttgctta tggttctaat gacgcacttt ttaagggctt tgaaaaaaat aaatttaaaa
101761 agaatttaaa agattggatt cgcacactca aaacttataa caaaaacgcc cttatcatgc
101821 ttgtttctcc tcctactgtg gtaaaaaaac aaggtaaaaa ctatacccta gctcctgatt
101881 tttttgctat aaggcaagca ctttatgagg tagccaagga agaaaaaact cttctttttg
101941 acatgcacca attcatgcaa gatagtggtg gaaaaaacaa atggatagaa caaaagctct
102001 ccttgcaaga tgtacattta accattaaag gctatgaatt aatggctaaa aaattgcttg
102061 atgatttaaa aaaatatgtg cattattgat tttaatttta tcaatataat ttaaaaaagc
102121 cttatttgct tgatattttt agcagaagcg atataaaatt aaggcatttc caaatgggtt

102181 tcatcatagc ttaaagctgt gatttgataa taagaattga tggttttaag aagattaaac
102241 ctagcttcta acaaactttg tttagtggaa tttaaagtat tagaagcatc taaataatct
102301 tttaattcac ttttacccaa attatatttt tcccaataag cttgagtaat cagctcttgt
102361 ttaaagctag tatcttttaa aatttgaagc aacatagtat tgctttgata gtctttataa
102421 tttaaagcaa attcattcat agcactttgc aaactctgct cataagtaat aagtgtactc
102481 tcataatcaa actgcgaaat tttgatattt tgctttactc tgccatagtc taagaaaggc
102541 aaagaaattt ttatattacc actaagaact tcaaatttaa aactatcatc aaattgttta
102601 tcactaccat ttaaagcccc atttagagaa atgcttggca agattgattt ttgcaccgaa
102661 gcatagtctt taaaagcgga tttaagggta ttgagcttag aacgcacatc aggacgatac
102721 acaagagctt ctaaagggat gttaaaatta ggatttaaat tcttaaaatc agaaagagtt
102781 aaagctttaa aataatcaat ataagcaaaa ccctcttgct tgcctatcaa atcttgtaga
102841 ttttttataa gtaaattgcg attttgctca ttgcgtaata aattctgttt tgctcttaat
102901 aaattttgct cgatattaag caaatcaagt tcttcgattt ttccaagctc aaatttataa
102961 gcatagagct ctctcatttg ttctaaatta gctagatact ctttaagtaa attatccaca
103021 tcattaaaat aagcaagttc aaaaacacca tttaaagctg tattaatcat actgattttt
103081 aaattttcta gatcataagc actagccttt gcactgaatt cactcgcact tgagctatcc
103141 cttatctttc cataaatgtc aagctcatag cttaaattta agctattatt aaaattctta
103201 ctttggactc cggaatttaa cttcttatct ccaccaagcc ccaaatttgc acttaaagtc
103261 ggatacaaat cataatctat caaatcagcc cttgctaaag ctgcaagcaa agtacttcta
103321 gctgtattta tatcgctatt gtttaacaaa acaaaattta aaaattcatt caatttagca
103381 ttatcgtaag ccttgtacca atcataagtg atatttaagt ctttaaagct ctcctcgctc
103441 attgttggct ctttaggtaa gctaagtttt actccacagg cgcttaaaaa aaatgctagt
103501 aaaatactca ataaattttt cattttattc ctttgataag gcgctgatag gatttaaatt
103561 ggctgcattt cttgcaggga aaaatccaaa aacaaccccg attaaaacag aactcaatag
103621 ccccaaaagc acagaatacg catttaaaac cataggaaaa tcagtgctta aggtattaaa
103681 accaaaaatc acaaaaatag aaagcaaaac tcctaaaatc gcacccatag agcatatcat
103741 caccgcttcg atcaaaaact gcatcatgat atcttctctt ctagcgccta ttgccatacg
103801 aattcctatt tctcttgttc tttcactcac tgaaacaagc atgatattca tcacgcctat
103861 acctccaaca atcaaagcaa tcaccgccac cgaagcagta agtatagttg tcgtgcgttt
103921 atttgccgtg atagcttgct tgaaagtatc agaattaaaa gtaaaaaaat ccctctgtcc
103981 gcgtttgatc tctaaaattc taataatggc attttcagcc aaggtagagc tcacatcgtc
104041 ttttactttt actataattt cacgcaaatt tctatcaccc gtaagcttat tcatcagtgt
104101 agtataaggc atataaaggc gtactacatt atcttctatg ggtttttctg tgtctttttg
104161 taaaactcct atgattttaa agggttgaga attaaaaatc accacacgcc ctaaaacatc
104221 ttcgctcttt tgatgaggaa aaagatttct cttagcatta aagtcaagca ccgctacatt
104281 agcattagtt tctatatctt cattatttaa aattctccca acttgaagct ttaaaccctc
104341 tatggcaaaa ttattcaccc ctactccctc agctctagcg cttaaagaaa tgttagtata
104401 agttgctacg cctgaagtat tagagtgcgc atctaccgct tctaaataat ctaaacttct
104461 aagtgtttct aagtcgctaa aattaagcct tgttttgcct gaacgcaaat ccccaaaacc
104521 tttaccaggg cggatttcaa tggtattggt tccaagtctt gcgatggatt ctaaaacctt
104581 agcttgagag ccaagcccta aggccaccac gcacacaacc gaagctatac ctatgatgat
104641 ccctagcatg gttaaaatag aacgaagttt atgtgcaagt atggaagaat aagctatttt
104701 taaacattca aaggcttgat ttttaaacaa ggtaaaagtt tttttctctt ttggcatact
104761 tttaacctgt ccttgatagg caaacttatc ttttttgcta tcacttaaaa tctctccatc
104821 tttgatttct atcactcttt tagcctgtgc tgctatcttt ggatcgtgcg ttacaagtac
104881 tatggtatgc ccttgtgcgt ttaatttttt taaaatttca agcaccataa taccgctttt
104941 actatctaaa gctcctgttg gctcatcggc taaaataagc tctccaccat tcatcaaagc
105001 tctagctata gaaacacgct gttgctgtcc tccacttagc tcattaggct ttgattctat
105061 cttgtgctca agctctaaat ttcctaaaag ttcttgagct ctaaaatttc tttcttgcac
105121 tttttttaccc gcataaacag caggtaatgt gacattgtca tttgcgctta gaagatttaa
105181 aagattgtag cgttgaaaaa taaagcctat tttttcgcgt cttaatctcg ctttttcgtc
105241 gctattgagc tgggtaactt cataattatc aaaaatataa ctcccattgc ttggctcatc
105301 taaggttcct atgatattta aaagcgaagt ttttccactc ccactttgcc ctatgatagc
105361 gacaaattcg ccttttttcta tacttaaact tacatttttt aatatggcat tctcgcctat

105421 atttttacaa atattgttta aaaaaatcat caaaatctca atcttaactt aggagcagca
105481 ctatcgccac ttgaacttac tatcagtaat tcatcctcgc ttaaaccttc taaaatttga
105541 gtatttacag aatctttaat gcctaattta acatattttt ttactgcttt ttgaccttgc
105601 aaaatttcaa cataataccc ttttgcatca tttttaatcg tataagtagg cacagcaata
105661 acatctttaa cacttgctac aacaatttca ttttgtatac tcataccaat acgcaaaaaa
105721 tcatctttat ttgccacata aaatttagca taataatata tagcattgtt agaacttgaa
105781 cttgaagaac tagagcttga agaagaagta gaattggtac ttgaatcgct tatttgagta
105841 tctgcaggat ctatacttgc tatacttgct tgataagttt tttgaggatc gctaagcaaa
105901 gaaaaatcaa gttctgtgcc gatttttatc ttaccaacat ccgcttcggc tatctccatt
105961 cttacttcca tttgatctaa attagctatg cggactatgg ttggggtgtt ttgattggca
106021 tttacagttt gtccttcatc gactgctaca ttgatcacaa ctccatcaat aggcgcagtg
106081 atggttgtat atcctaaatc tttttgagca tttttaagag tgatttcaag ctgaactact
106141 tgagcattaa gctctgcaac atttgccttt aaagtgtaaa aattattttt ttgagtttct
106201 aaattttcaa gagaacttgc cttagcagca taaagttttt gttctctttg gtattgttta
106261 cttgcgattt caagggcgac ttttttactt tctaaattgg ccttagcact ttcaagctgc
106321 gcctttgtga tatctaaatc attttgctgt ttatctttat caatttgtgc gatcaaatca
106381 ccttgtttta catacgaacc cacatccaca taaagcttga tgatttgccc gctcacttga
106441 gcacccacat caacttgatc tttagcataa actttcccta ccgcttctat ggtttgagaa
106501 atatttattt ttttaacctt ttgggtgaga taattgattt tttcaccatt attgaaaaat
106561 acaaaataag cacccactcc aaaaagcgct aaaatcacta caatcaaaac gatcttattt
106621 ttcatcctaa gccttaaacc aaaaaaaata aatcttagaa ttctacttgg gcaaagtgaa
106681 tttttttgtga atttttttaat tcattttttta agaatttaaa aattctaaag ccatcatact
106741 atcaccttgc attaagactt ttaaattttc atcttcccag caaaaagtag agctatggtg
106801 caaggtaggg tttttcaaat cttttgaaat acccacaaaa acataagcgc ccattctctc
106861 acgcgttaaa aaagcaaaat cctcagcccc catatcaggc ttagctacgc taatgatatt
106921 ttgctcgcct aataccttgc taaaagcttt tctagctatt tttgcagcat tttcatcatt
106981 gatcaaagga ggatattctc ttttataatc aagcttaaaa tcacccccaa attccaaagc
107041 aacagctttg gctgtatttt ctatggagct ttgtaaaagt ttttgatttt catcgcttaa
107101 aaaaacgcact gttccttgta agatagcatt ttgtgggatc acattaaaag tcgttcctgc
107161 atgaaatttt cctatggtga taacccccgc atccacaggc cttaaacgcc ttgaaatgat
107221 acattgcaca tcgctgacaa atttgctcgc catgactata ggatcgatag ttgtatgagg
107281 gtgtgctcca tgtccgcctt ttcctataaa ttcaagatca aaaacatcaa cccccgccat
107341 catctcaccg cttacaattt gagcagtatt ttcaagcaaa ggcccccata aatggcatcc
107401 aaaaaccgca tctacatggg gattttcaag tatgcccgct tctatcatag gcttagcacc
107461 cccacttcct tcttcagcag gctgaaacat aaatttaatc gttccgctaa attcatctct
107521 tagctcattt aagatcaaaa ccgctcccaa aagccctgct gtgtgtccat cgtgaccaca
107581 tgcgtgcatt tttccattga tttttgaagc ataactaaga tttgtttgct cttgcacagg
107641 caaagcgtcc atatccgctc ttaaaagcac gcattgaggt tttttagtgc tttgtttttt
107701 tccttctatg cttgctaaaa ttccggtttt agctatattt ttttcatatt taataccaaa
107761 ttcatctaaa atttcgcaca ccaatcttgc tgtattttcc tcttcaaatt caagctcagg
107821 gtgcatatga atttgatgtc ttaaattgac aattttttca tagtatttat cacttaaatt
107881 tttaacgcgc tctaacatag ttttagtcca aatcctttat tttttcatct gctttttttat
107941 tgtctatttt ttctaaattt gcttccattt tgcgaatttc ttcttcgccg cttcttacta
108001 attcttgatc aatttttgaa aaatctccaa tttttttatc cgcataatcc actttttgaca
108061 atataaaacg caacaaatta agccttgctt ttttcttatc atctgaagaa atgatagtcc
108121 atggacaggt tggggtatta gatgctaaaa gcatagaata ctttgccaag gtatatttat
108181 cccataattc ttgagatttt tgatccacag gagaaagttt gtattgcttt aaagggtcaa
108241 tgcggcgttt ttcaaagcgt cttttttgct cctctttaga aacagaaaaa taaaatttaa
108301 aaaacatcaa attgctattt gaaatcatat tttcaaacaa aggcacctct cttaaaaagt
108361 ctttatgctg ctgtggggta caaaaaccca tcacaggctc tactcctgcg cggttatacc
108421 atgatctatc aaagatcaca atctcacccg ctgaaggcaa atgcgtcaca tagcgttgaa
108481 aataccattg tgtttttttca acatcattag gcttttcaag tgctaccaca cgacatcccc
108541 taggatttag atactctatc aaacgcttga tcgcaccacc cttgcctgct gcatcgcgtc
108601 cttctataag tataagcact tttaaaccct ttgcttttac ataattttga aattttaaaa

108661 gctcgatttg caaatttttt aaatcttttt catattctat agtgcttttt ttcttcttga
108721 tagaaacata aatttcatct ttttttgtat cctcttgaat ttttgccgga actttttctt
108781 gcataagcaa cctttataaa atatttaaag caaatttacc ataacaaact ataattcaca
108841 ttaaaatcat aaattttaca cttgagttta ggataataat gcgttttatt tttacacttt
108901 tatttagctg ttattttttta tttgcttctg ttttatcttt aaatgaagcc tttgaagtaa
108961 aatcgagttc ttatgatagc gctgtatcaa tcgatattaa attgggaaaa gatatttatc
109021 tttattctga taaactcaaa ctctatatta atcaaaacga tatcagccat ttaatcaatc
109081 ttccgcctag cactcaaaga ctcaacgaaa aagtgtatta tcaagattta aatttagccc
109141 tgcctagcct actgcttgag cgttttgcac aaagtcaaaa caatcaaatc aaactcgaat
109201 ttcaaggctg ctccgaacaa ggcttgtgtt acaacccccca aacttggtat tttgatctaa
109261 agcacaaaga taaaaatttt gaaatttcag cccccctataa agaaaagaaa aagactcttc
109321 aagtaaaatc cgaagaaagt atgatagcaa atttcttagc cacagataag ctttttttgga
109381 tcttactaag ctttttaggc tatggacttt tactttcact tactccttgt attttgccaa
109441 tgattcctat attatcttca cttatcgtag ccaaaagtga aacaaaattt tctaaaaaac
109501 acagcttttt tttatctttt atttatgtat tttctatgtc tttagcttat gctatcgccg
109561 gagttatagc aagtttttta ggagcaagcg ttcaaggact tttgcaaaaa cctagtgttt
109621 tgatcttttt tgcattgatt tttgtattgc ttgctttagc aatgtttgaa agttttcgct
109681 ttgaactacc cttaaaattt caaaattttc tccacaaaaa aagtgaaaaa ggcaaaggag
109741 tgctaggtgt agccctcatg ggctttttat ccgctcttat cgtaggtcct tgcgtagccg
109801 ctcctctaac tggtgctttg atctacatag ccaatacagg cgatgcgctt ttaggagcta
109861 gcgctctttt tgtcatgagt tttggcatgg gaattccttt acttttcatt ggtctaggtc
109921 ttggcttttt aaaggcggga gcttggatgc aaaaagtaaa aattttcttt ggttttgtta
109981 tgctagctat ggctatttgg atactttctc gtatcattga aacaagatat attttgatcg
110041 cttttggaat tttgggcgta ttttttagtg tatttatggg aattttcgaa caagctttta
110101 gcgtgataac taagattaaa aaaagtctat tgattttggt tttggcttat tctttaagta
110161 tatttttagg tgggatattt ggcgcaaaaa atttcttaaa tcctttgaat ttaaattcaa
110221 atacactctt aaattcttca aaattaaatt ataactttat aaacgatctt aatgctcttc
110281 aaaaagagat taaaaccagc acccgtccta tcatgtttaa ctttacagca tcttggtgtg
110341 aaaattgcaa actcttagat gagcttactt ttagtgatga aaaaattcaa gaaaaactca
110401 aaaattttca tttaatcaaa atcgatctca ctcaaaacag cgacaaggac ttagaaatca
110461 tgaaagaatt tggtgttttt ggcccaccag ttttgatatt ttttgaaaat ggtaaagaaa
110521 atcttagaat cactggtttt atcagtgcag atgatttatt acaaaaattg acaaaatgaa
110581 aattaaaagc ttgcaaatag gacatatcaa ggattatggc ggctttaaaa gcgcctttat
110641 aaaaaatatt tatttaaaag aagtggatat tgatttttcg ggcattatta acgacaatat
110701 agcagataca aaacatcatg gcggaaaaaa taaagccttg tttgcaaatt cttatcataa
110761 ttacccccctt tgggaaaatt ttcttggaaa aaaacttgag tgtggtatga tgggagagaa
110821 tttaaccctt gaaaatttac acgaacaaaa tgtttgtata ggcgatatac atcgatttga
110881 agatgctatc ttgcaagtaa gcgaaccgcg caaaccttgt gtgaaaattt caaaaattca
110941 ccacaatcca aatttcacac aagaaatttt taaaacagga ttaagcgggt ggtattatcg
111001 ggttttagag ggtagaaaaa ttcatgcaca ttctaaaatt cagcttatag aaagaaatcc
111061 cataaattta agcgtcctag aactcaatca tttattttac aatcctcatc aagcactcaa
111121 acaaaatcca actttgcttg aaaaattaag caaactcgat acgctcattt cacaaaattg
111181 gcacgaaact atacaaaaac gcttaaaaaa tacctacgat ttaaactata tgcaaaattt
111241 ataatttttt aaagtattct taggctacaa tttaattttt ttgaaaggat aagcatgcaa
111301 agacaaacat ggagtagcac tcttacttat atcttaaccg tcgcaggagc aaccataggc
111361 tttggtgcaa cttggcgttt tccttatctt gtaggagaaa atgggggtgg tgcttatgtt
111421 ttagtatttt gtatagcaat gatactcatt ggtatccctg ttatcttagc tgaaaatgtc
111481 atcggtagaa aatccatgac aaacagcgtc gatgcttttg aaggtaaatg gaaatttata
111541 ggctatatgg gacttttggg tagttttggt atcatggctt attatatggt cttaggtggc
111601 tgggtttttg tctatgtttt tgagttaatc ataggcaatt tcaatctctc tcatactgta
111661 accaaagatt ttaccgagca attttttaat gaaaaaattt ctttcaatcc tttaggtgta
111721 ggaattttta ctacactttt tgtccttatc aattatatca ttttaaggcg tggcattata
111781 gatggtatag aaaaatcagt taaattcctc atgcctttgc ttttcatttg tcttatactt
111841 gttgtaggac gcaacttaag ccttgaaggt gccatgaatg gtgtaaaatt ttatcttacg

111901 cctgattttg acaaaattct atcccgaaaa ctactcatcg atgttctagg acaagtcttt
111961 tttgcgcttt ctctgggttt tggcgtgatg ataactcttt catctcatct tagcaaaaat
112021 gaaaacatga caaaaactgc catttataca ggagttttaa acactcttat tgctgtgcta
112081 gcaggcttta tgatctttcc tgcgcttttt gcagcaggac ttgagcccga taaaggacct
112141 tctttagtgt ttcaaaccct gcctattgcg ttttctcata tccacttcgg gactatcatt
112201 tgcattttat tttttatact tttactcatc gctgcattaa cgacaagttt gcctatatat
112261 caagttatca tcagtgtttt agaagaaaaa ttcaaacttt ctaaaaaaac agctattaat
112321 ttaaccctag gttcgatttt catactagga aacattcctt gtatactcac ttatgggcct
112381 tggagagata ttactcttgt taaaggaaaa aatattttcg atagttttga ttttatcagc
112441 ggaaatatct tatttgtttt aacagccttt ttttgctgta tctatgtagg ttgggttctt
112501 ggcaaagagc aaagcataaa agagcttagt aatgaaggta aattaaaaag cgtgggattt
112561 aaaatttggt tttattatat taagttcatc ataccttttaa tcattcttac aattttcgtt
112621 tatggagtta caaattaatt ttttataaat gtataaacac aagcgatttg aatcgcttta
112681 tctcttatgt ttgattactt ctcatttact tttaaggctt atacttccaa atgaaccgaa
112741 aacattcata agaaaaactc cttaaataat gggcctagta aataccaagc taggtctatt
112801 tttattatat tttctactct aaaaatttag aatttttatt attttaagtg ctttttaaga
112861 aaaaactata gatttatgtg ctacaattac agcgttttac ttaattttaa acaaggagaa
112921 ttctcatgaa actagttaaa cttagtttag tagcagctct tgctgcgggt gctttctcag
112981 tggcaaatgc tgctccactt gaagaagcga tcaaagatat cgatgtatca ggtgttttaa
113041 gatacagata cgaatcaggt agatttggaa aagatcttaa cttggtaaat ggtagtagct
113101 taaatacaag caagcaagag cataaataca gagctcaact taacttcagc ggtgctatag
113161 cggacaactt caaagctttc gttcaacttg actacaattc aaaagatggt ggttatggag
113221 tagatagcat tagcaataca agcgatactt taaatgttcg ccaattatac ttaacttaca
113281 cagatgaaaa tgtagctaca agtgtaatcc taggtaaaca acaactcaac accatctgga
113341 ctgataatgc aatcgatggt ttagtaggaa caggtgttaa agtagtaaat aacagcatcg
113401 atggtttaac cctagctgct tttgctgttg atagttctaa tgaagcagta catactacta
113461 ccaatcctaa aggtgatact gatgcaagcc catttttaaa ttggaataaa aatatttatg
113521 gtggtgcagc tgtcggttct tatgaagtat ttaatggtca attaaaccca caattatggt
113581 tagcttatat gactgacaat gctttcttat atgctgtaga tgcagcttat agcactacta
113641 tctttgatgg agtaaattgg actcttgaag gtgcttattt aggaaacagc cttgacaacg
113701 aactcaaaga tgctggcttc ggaaatggta acttctttgc tttaaatggt ggtattgaag
113761 taaatggctg ggatgcaact cttggtggtt tatactacgg taaaaaagat aaaggtactg
113821 taactgtaat cgaagatcaa ggaaatatcg gatctttact tgcaggtgaa gaaatttctt
113881 acactcaagg ttctcaactt aatggagata taggtagaaa tatcttcgga tatgttaaag
113941 caggctatac tttcaacgaa acagttcgtg taggtgctga cttcgtatac ggtggaacta
114001 aaacagattc tacggttaca ggacacgcag gtggtggaga taaacttgaa gcagttgcaa
114061 gagtaagcta caaatactct ccaaaactta acttctcagc atggtattct tatgtaaatg
114121 ttgataatgg tgatcaagga gaaggtgatc acaatactgt aagacttcaa gctctttaca
114181 aattctaaga agctctaaag tctaacttca aggtggagtt ttactccacc tttttttata
114241 tcttcttttt aaagcttaaa tttatataaa atatctttct aacttaagtt caaagaaatt
114301 ttcccaagct aaaaagcttg aagaaaatta agatttaaac caatgggtta tttttttcaaa
114361 caatcctttt tgctcatggt gcataccatc ttttatccca aaactttcac tgagtttttc
114421 taaaagttct ttttgctcat cattgagtga atttggaaat ttgatagcaa tttgcacgat
114481 ttgattgcct atgcgagaac tatgcacatc ttttactccc tctctttcaa gcacaaatct
114541 ttgcccatct tttgcaccct ttggcaaatt taaagtcgcc tcccctcgta tggtaggcac
114601 ctttatactt tgtcctaata tggcttgagt aaaaaatact ggaaattcta tataaatatc
114661 ctcatcatct cttataaaag tatcatcctc ttcaacgata actttaacat acaaatcccc
114721 tctattgcca cttttactaa gattgccttt agctgcaact cttaaattca tgccattgtc
114781 tataccttca gggatgttta cttctacact atccttaagc tcttcatagc ccctaccctt
114841 acaatcttta catttctcac ttgcactttc tccactcccc ttacaatcag gacaagtttg
114901 cgcaaaagta ataaaacctt gagaaattcc tacttgtccc ctacccttac attttggaca
114961 agtttgaagc tttccatcct tagctcctgt tccactacaa gttttacatg aagatttgta
115021 agtaaaatct atattttttt tacagccaaa caccgcttct ttaaaagtaa gttttaaatt
115081 caccacaagt tcagcttgaa atttttcatc acttgatttt ttgcggcgtt gcgatgatcc

115141 accaaagcca ccaccaaaaa aactagaaaa aatatcaccc aaatcttcaa agccaccaaa
115201 acctgctccc cctgatccaa aaccgccacc ctttaaagca tctttaccat atctatcata
115261 aatagcgcgt ttttcttcat tgcttaaaac ttcataagct tcatttacaa gcttgaattt
115321 atcttcagct tctttatctc cttgatttct atcggggtgg tattttaaag ccatttttct
115381 ataagctttt ttaatggtat ctttatcagc actctgagta atttctaaaa tttcatagta
115441 gcttaactcc atatttttcc tttaaataat aaaatattta taaaatttgt aaaaataaaa
115501 aatataattt tagcaaaaat aaactcaata atttatcaaa atttaaataa aattatgtaa
115561 atatataaat cttacataaa taataaagga taacaaaatg attaatgtgt tgatgataga
115621 agatgatcct gattttgccc aattattgtc agaatattta actcaattta atatcaaaat
115681 cacaaatttt gaaaatccta aagaagcttt aaatgtaggt atacaaggct atgattgttt
115741 aatcttagat ttaactctac ctggaataga cggacttgaa gtttgtagag aaatcagaca
115801 aaaatacaat attcccatca tcatttcatc tgctagagga gatttgagtg ataaagtagt
115861 aggacttcaa atcggtgcag atgattattt accaaaacct tatgatccaa aagaaatgca
115921 cgcaagaatc atgagtctta ttcgtcgcac aaaaagagct gaaaatacta gcaatgaaaa
115981 tatcaattca gcttttaaaa tcgatgaaag aaaacacgaa atcacttatc aaaacaaggt
116041 cttaactttta actcctgccg aatatgaaat tttagaatac ctcatacagc aacacggata
116101 tagcgtatca agagaacaac ttgtaagtcg ctgtaaaaat cttaaagata aagactctaa
116161 aagccttgat gttatcatcg gtcgtttaag aactaagata aatgatagtt ctaaatctcc
116221 aaaacatata ttttcagtta gaggtatagg atataaactt ataggatgac aaaaaattat
116281 tctatttata ccaaacttgt tattttattt gttgtaacct tctttttagt ttgtgtttta
116341 tttattgtac ttttaaaaat agaaagcaat acttacagcg aagaagaagg ctttaaacaa
116401 gaaagtttta taaaaaatat catcaattct tatggcaatt cttctggggt tgatatagga
116461 gctcatctta ataatagtgg atttgttgcc atacaaaatg actattttgc aaaaatcgca
116521 aggatagatg gacaaaacct ttttcgtgca ggaaatggag aaaattcctg tactctctct
116581 tctttaaaat atcaaaacaa actctatctt gatgtccaat gcaaagattt caacgcttta
116641 tttgaagagg atactaacaa tagagtgtat aatcttttat taataggctt tttctttttt
116701 tctttcttag tagtttttat gtatttttca gtattaaaat ccctcgaacc gctcaaaaaa
116761 ttaagaagac aagtcgcaga agtagctaat ggtggaaaac ctgattttga aaactaccaa
116821 caagatgaag taggtaaaat agcctttgaa tttcaaaaag cttttaaaaa aaatcaagaa
116881 cttatacaat caagacaact ttttctacgc accatcatgc atgagcttaa aactcctatc
116941 ggaaaaggta ggattatatc tgaaatgcta aaagaagaga agcaaaaaga aagattgatt
117001 gatatttttt taagaatgga ttccttgatt aatgaatttg caaaaataga aaatcttttt
117061 tcaaaaaact acaatcttca gtttaaacct gtacatttta gcactatact taatgaagct
117121 aaagattatt taatgcgtga tgatttcaat aaagttgtca agcttaactt aaaacacgat
117181 gccttgatta atgtagatat ggaaattttt cctttgattt taaaaaatct cattgataat
117241 gctttaaaat attcaagcaa tgggacttgt gagctttatt gttgtaaaga atgttttgtg
117301 ataaaaaatc ctggtaatcc attaagtgag cctatagaac attattttga ggcttttacg
117361 cgtgaaccat atcagaaagt taaggggatg ggtttaggac tttatatcgt ttttgaagtg
117421 tgtaagctac ataattttga tattatttat ttttacgaag aaggcaaaca ttgctttaaa
117481 attttttcg gagtaaaaac aaacaatgac acataagggt gtagaaaaat ttaacgaact
117541 tgtagaaagt tttgcaaatt tgcccactat cggtaaaaaa actgctattc gccttgctta
117601 tcatatctgc acagcaaatc aacttgaagg tatgaaactc gcacacaata tagaaaatgc
117661 catacgcttt ataaagcctt gtcagcaatg cggagcttta agcgaaaacg aactttgcga
117721 aatttgcacc gatgaagaaa gggataagaa tttcttatgt atagtgcaaa gtcctaaaga
117781 tgttttggtt atagaagaaa gtaaaagcta tgggggtttg tattttgtct tagatgattt
117841 aaatgatgaa aaactcgccc aactaagaca aatgatacta aaattacaaa gcaaagaact
117901 catttttgct tttactcaca atttaaattc cgatgcaatg attttttaca tagaagataa
117961 acttaaggat ttaaatttgc aatttagcaa aatcgcgcag ggtattccaa gtggagttaa
118021 tttggaaaat gttgacttta tatctctaaa taaagccatt aattttcgca ctaaggttta
118081 aatcagttct tataagaaat gtcagctaat tcctgcaaag aatgatttgc tattttttca
118141 tagctaaagc cctctttagc taaatactca agcaaagttt tctccatgat tttacccgca
118201 ctaacaatct cttgcgatag agtaaaactc ataggcttga tacgctttgg aacgcaagct
118261 aaaatttgcg tagggggcaa atctcccatt aattccatat attgcagggt ttgaagcatt
118321 tctacttcgt gtgcactacc gctccaattg atctttttag gcattgcatc ataaggaaag

118381 aaaaatacat cgccgatttt tgaattatct gcatctatac aatcaagcac tatcatcttg
118441 tcatattctg cgataatata gctaagttgc aaggctaagg tgcctccatc gacgaaattt
118501 aaactgtgat ttggatgggt aaatttataa tttttctcca aaagtttgca aagatgaacg
118561 cctatgcctt catctgcaaa cataatattt cctataccaa ggataagaaa tttcaatttt
118621 tctcttcttt aacaaattta taaccgctaa ctatagcatc gatcgcacca tctttacctt
118681 taatcgcatt gaaaaatgcc atatagatat gaacgggcac aaagattaaa ataacccaca
118741 ttaaaattct atggtaagtt ctaacatcag ctaaacctcc aagagcggcc tctaaaggtc
118801 ttaacacttc atacaacaat cctcccaagc cctcatgata agtatgagta taaagaatca
118861 gtcctgttag aataatcccc accataacaa tataaaaaaa tacataagtt acaaattgca
118921 aaggattata aacacctttt aaatgtggat gttttcccaa aaatagataa aatttaatct
118981 gctctatcca taattttgga ttaagcacat ctgctatact gatgcgctct ttgcgactta
119041 tcttatcgaa aaagaaaaga taaactttaa aaatcacaca agcaatcaaa acaaaaccga
119101 cagcttgatg caccattctg tattttgctt gcatgaaatt aaccggctcg ccattgctta
119161 caggattttg aaaaacatag gaaagataat atcctgttac aaccaaaatc actatagcaa
119221 aagctctcac ccaatgtgtt aaacgcaaac ctatgctaaa ttcatattca gcctttcttt
119281 gcaatttttc ttctttattt tgcataaaag cccctttata aactcgcgtt tattttgtat
119341 tcgcttagat tatttccctt agtatccatt acatgcaccg cacaagcaat acaaggatca
119401 taagagtgaa tttttctaat gatttcaagt ggttgtttta catcagcgat ttttaatcct
119461 atcaaacatt gctcatagct tccgcccacg ccattagcat cttttggact tgcattccaa
119521 gtagaaggaa caactgcttg ccaatttttct atcactccat ttttaatcct gcaccagtga
119581 cttagcgttc cgcgtggcac atgccccata tagcggcctt tgtactcttt agaattatcg
119641 atcacataag gcgcacaagt gctctcatct acttttaaat tttctactaa attacggaaa
119701 gcttttaaag cattattagc cacaattttt gcttccaaac aacgcgcagc agttcttcca
119761 agagtactaa atacagcatt taaaggaagt cctgtttctt ttaaaaactc atctaccaca
119821 ggaacaacat tttgattacc cttagcataa tttacaacga tattagccaa aggtcctact
119881 tgcataggat ttccttcata gcgtggagct ttaatccagc tatatttacc cttagtatca
119941 aatactttag aatgcactgt atttccatga tgatctacac tttcaccctc tacaagtcct
120001 gtataatttg gatttgtttt accatcataa ggatgtaaag gctcattatc tgcataccaa
120061 gaatgagtcg cttcttcagt gatcttatct tcttctactt catatacctt gcttaaatcg
120121 ccatttttaa taattccact ttcaaacaac cactcatctc taccaagctg gaattcttgg
120181 aaagtataaa gattatttac cccaatatcg tttagcacac taggttcgtt tgcataagct
120241 tttcctgcca taacaagatc aggataataa gcacggttaa caaagtcttg tacttctttg
120301 aatttttcca tatactcacc cattctagca gggcttagca aatccataac gcaagttaca
120361 ccacccacgg ttaagctttg cggatgagga tttttagcac cgaagatagc catacattga
120421 gctatgattc tttgaattct taaacactct agataatgtg ataaaacgat caaattttgt
120481 tctggactta agcgataagt aggatgacca taataagcat ttgcaaaagg tcctagattt
120541 cctttatcta caaatgtttt aagtctttgt tgcacttcaa gaagtttatc cgctcctgtt
120601 gcataaggca ttgaagtata tttaaaagcc tcatcacttg ctttttttaac atcagcactt
120661 aaagcactta ctacatctgc ccaatcaagt ccatgaagtt gataaaaatg cacgatatga
120721 tcgtgtaaga aaagtgcggc attcattaaa gttcttgtta aaagcgcatt taaaggcgga
120781 gtaataccca aagcattttc aaccgcaact atacccgctt tatagtgaga aaaagtacaa
120841 acaccgcaaa ttctttgggt cataaagccc gcatctcttg gatcgcgtcc tttaacaatg
120901 gtttcaatac cacgccataa agtagaaccc gcataagctt ctttaaccac attgttatca
120961 tcaacaacaa cttcaactct taagtgtcct tctattcttg taattggatc tactattatc
121021 ttttgactca tgtattttat tcctcatttt ttttcataga tgcgatcacc gcatgtgctg
121081 caatagcaac gcctgtaagc gttagaacac ctatacctat tttatctgat acattatcag
121141 ctcctagccc aaaaacagta tcaaatttac gacttgccat aggctcttca aaaggcccca
121201 tagtatccca gaaatttggt tcagaacaac ctatacagcc atgtcctgct tggataggcc
121261 aagaagtgtg ctgattaaat ctttctctag agcagttatt gaaagtataa ggacccttac
121321 aacccacttt ataaagacaa taaccatttt ttgcaccctc atcgccaaag ttttgcacaa
121381 attcacccgc atcaaaatgt ccgcgtcttt cgcaaagatc gtgaattctc aaaccataag
121441 cccattttgg tctattataa acatcaagcg caggcaactc gccaaaaagt aagtaatgta
121501 aaacatttcc tactatattt ttttcacttg gcggacaacc tgggacatta ataaccgttt
121561 tgcttgttac cttgcttaga ggttgagcat tgcttgggtt tggtcttgca gcttgaattc

121621 caccaaagct cgaacaggtt cctatcgcaa aaatagccaa agcattttca cttgccactt
121681 tagaaagctc atagccactc tttccatgtg gtcctatggt taaaaatttc tctgtatctc
121741 ccatagggat accgccttca accattaaaa tatatttgtt tttatatttt tcaatggcac
121801 tttctaaatt ttcttcagcc tgccaacccg ctgcagccat aacggtttca tggtattcta
121861 aagaaatata atcaaatatt aaactatcaa tagttggagt atcgcttctt aataaactct
121921 cgctacaacc cgtacattca gccatatgaa gccaaatcac aggaagtctg tctgcaagtt
121981 ctgcagcttt tgccaccata ggggtaaaac tcgctggcaa tgctaaaaat gctgtcatcg
122041 cccctgccca tttcataaaa tctcttctag taaagccgga tctttctaaa gcttttggta
122101 tagaattttc aggatttaaa gaaggaagtt tctcaagtgc acttaaacgg gactctattt
122161 gattataatc aatcattgaa attccttaat taatgtaatt tgttctactt aataataaaa
122221 caaagcaaat taaaaatgta ttaaaattta aatttcgata gaaaaatata aacaaaactt
122281 aaagatatat taataaaaac taaaaattta tatccctttc tttagcttgc gaatcaaaaa
122341 tcttgcaggg tgtatacaag gaataaattt tttaaatacc cccaaaggct cttcattaat
122401 caaagcacaa agatatcttg cagctaaaac agaggtgcta aaagctcttg aaccatgagc
122461 aaaattaaga tataaattgg ggatattttg aggcaaagtc tgtgtcttgt tttttgtcca
122521 taataaggct ttatattctt gcttataaaa agcttcatca taagctgccc ctacaatagc
122581 aaacctatcg ctagaataag atctaaaacc cactctagag cctttgatga taatttcctc
122641 atcacctttt aaaaattctt gtatattttg caaattttct tcatcatcag ctttttgtgg
122701 atgtgggttt gaattaagcc tatcataact tgcaccaata acttgcaaat ccccatcact
122761 aggacaaata taagcttttg aagaaagggc aaatgcatta tctaaaaaag gttttagatg
122821 agtaacctgt cctctaacct tacttaaaag cattccatca taaactaaaa aatccttagt
122881 atcagcaccc atagcataga ttaaaatatc aaaatcttgg attgcttttt ggtttttaaa
122941 atgtaaagta aatttgtctt cttggtgttg aaaaaaatca tattcgtgat taaaatacat
123001 ttttgcctga ctttttttcaa aaagcgattt taaaatttct ttaggcttga tgtgtccgcc
123061 atcttccaaa aaagcttcat tcttgtcaat cttaaataaa acattatctt tttggcttat
123121 aaaacgctct tgcattaaag gagtatgagc aaattctatc actccgttta gatttaaatt
123181 taaaatttgc ttataaaaac gactcgcctc gataaaagca agttgagaaa attcgcccaa
123241 agcaacatca ggttttaaaa tcaaagagct taaaattcca ctttcattgc cacttgcacc
123301 acatcctaaa ctatcatttt tttcaaaaac actgacttca aaatcacgca aagaaagctc
123361 ataagccaaa gtcgcaccac atatacccga gcctataatg gctacttttt tgtttttata
123421 taaacttgga attctttgaa aataagcttc tttatctagg attggatttt ctttgcgtaa
123481 aaaagctttg atcatctctc gctttctaaa acctttagtt ttttgaactt caaagccata
123541 ttttataaga tttttttgta aaatactcga agcagaaaag gtgcaaattt gggtatcaat
123601 ctttgaaagt cgagcaactt gagctatgaa attttcatca aacatagctg aatttttact
123661 cggagaaaaa ccatctaaaa accaaacatc agcattaaaa tcaagctcac taagaacagc
123721 aatatcatca aagattaaat ccaaaaagca gtctttgaaa taaaagcgat aatatccttt
123781 tttagcctta ggataaaact ttaaaaaagc ttctaaaatt tctttaaatt cttcataaaa
123841 tccaagctta tgataaattt ccctaagctt gtctttttcg atataaaaac cctcaacact
123901 gacataaaat aattttttag gacgcttttt tgcttgaaga aatcttttaa gagttaaaaa
123961 gaaattaagt cctataccaa agccactttc agctatgata aatgtatcat tttctttaaa
124021 ttcaaaagcc tcgctataaa caaatttact ttcatttaaa ccatcttttg agttaaaata
124081 aaaatcatca aaatccaaag aaaatggagt attatcttta aagattaaat tagcttttttt
124141 catctattgt tataaaaata attctccaca ccatcagcta taccсttaac caaagtgtct
124201 tgaaaacttt tatttgcgat tctttttcct tcacttggat gagtaatgta tccgatttct
124261 atcaaaatag caggcatttg agccccaaca agaacccaaa aaggagcttc tcttactcca
124321 ccatcaacaa ttttatattt cttgcgagtt tgggttaaga tatttttttg cacatcgata
124381 gcaagtttgt ttgaagccac aatcttttct ctgtttaaga aatttaaaat gctttgtttt
124441 gaaaaataat taatctcttc aaaatcacct tgattttctt tttcggcagc ctttttactt
124501 ctttcacttc tagcaggact taggaaaaaa gtctccacac cctcgctgct ttttgctctt
124561 gatgcactag gcgctgcatt cgcatggata gataaaaaca aatccgccct tttatcatta
124621 gcatactttg ttctatctct aagattgata aatttatctg aacttcttgt gtaaagtact
124681 ttataaccgc gtttttttaag ctcatttcca agttttaaag tcgtgcttaa aactatatct
124741 ttttctttta aagtgccctt tttatcactt aaagcaccgc tatccttacc cccatgtccc
124801 gcatcaagca ctatgagctt atcggatttg aaattagaat ttaaaacatt tttattgcta

124861 gtttttgaag tgcttttact tgaaacagaa gtgtttttac ttacactacc accaaagcct
124921 aaagtcaaat ttttgccatc taattcttta aaaagtttaa attccttagg tgaactaagt
124981 acaatgcgaa ctgtttttgg attatactga gtaactgtaa cagcattttg cccaaaagta
125041 aaattcttgc gactaccttc taaaattcct tgaaagctca ctacttgcct aaagaatttt
125101 tgatcttttg tgctaaaact tgtaatatct ttttcatcaa gctcatcact taaatttaaa
125161 acaacagaat tagaactttt atctacatct aaaacatata aaggttcatc atctgcttgc
125221 tctttttag tatttgtttt agcttcttga gttttaactt gcttaggctc ttgttttgca
125281 actactttat cactttcttt ttttactgct gtctttgttt cttgtttagc agaaatattt
125341 ttttgtacag gttgtgattt tttagaaata tttatttttt catcttcaaa atcaaactct
125401 ataggaattt tttccttagc ataagcaaca atacgaatag tttttggatt gtattgcgaa
125461 atcactatgc gataatcatc aaatttatat tcttttcttc caccttctaa aacaccttca
125521 aaatcactga tataacgata atttcccttt tcatccaatg caaaactttt tatatcttct
125581 tgctctaaat tggtatttaa atcaagcacc actccttgac ttgtcttact tgattttaaa
125641 acataaacct tatctacttt ttttgtctgc tggggtttat tgttttgcgt ttttacaatt
125701 tcttgtttag cttgttgggg ttttggagtg cttttagggg gatcaaaact agaaagctcg
125761 acatttttag aactagactg aagtttttgt ttatcctgaa taactgcctt tcttaaagca
125821 ttgatactag ctttgctaac tccgctttct tctagttcat tcgcataaga tttatcatct
125881 aaatttaaag cattggaact tataatcaat ctttttaaaa tttcgatttt agtttttttca
125941 tcttcattaa ttacactttg tatataaaga ctttttagct ggtgatgaaa tttaacttga
126001 gcatcatttt ttgacataat gaaattctta tcaaagttag caagctcatt ttcatacacc
126061 ccaaaacaaa tactaaaaag cgcgatacaa aaaactaaaa ttctaagcat tattcaccat
126121 tgacaagttt atccataagc tctttaacac tgattaattt ttctaaacga tagccattag
126181 ctcctgtaaa aaataatcct gtttcttttt ttcccgacca tgcgtcaaaa agtctatctg
126241 ctatacaata acccaccttt gttgcctctt tacctcggcc acatggcgct acacaattac
126301 tgatacaatt gatctttggt cccattcttt tatcgactaa atcaagcaaa ttagtcctta
126361 ctcctcttgc aggatatcct actggggatt tgataagttc tatatcttca tttttacatg
126421 caagcaatac atttttaaac tcatcacttg catcacattc aaaagtgcct ataaaacgag
126481 ttcccatttg aaccccacta gcacctaaag aaatggcatt ttctatatct tttttatccc
126541 aaattccacc cgctgcaatc actggaaaag aaccccaatt tttagcctct tcaaccacag
126601 gtgcgattaa attctcaagt tgataattag gatctaaaca ttgctcatag gtaaaacctt
126661 gatgtccacc gctcttaggt ccttcaagca caacagcatc aggcagacgg ttatatctgc
126721 tttgccatct tttacagatg atttttaaag ccttaggcga agagattata ggaactaaag
126781 caacatcagg gaaatccgct gtaaattcag gtaaattagt aggaagtcca gcgcctgaaa
126841 ctataacatt aaaacccact tcacaagcat cacgagcaat tcttgcataa tcattacttg
126901 cacataaaat attacaaccc aaaggcgcat caccgcatac ttttcttgca ttatttatca
126961 aagtttgcaa gccttctcgt gaataaaaat tttcactacc ataaggctta gcattcagtt
127021 ctttgctaat atgggtgcga ttttcataat atcctgttcc aacagatgag ataataccaa
127081 gaccgccatt taatgacact gccgaagcta atttatccca gcttatacca agccccattc
127141 cgccttgaaa aatgggatat tttatagtgt gttttcctat ttgaagggat tttaaactca
127201 tatattcacc tttaattttg caaattttct cttacctact tgtaaaatat actcccctgc
127261 gctaagttgc atttgctcat cgcttacttt ttgtgaattt acactcactg catttgcatt
127321 aatgcttctt cttgctgcag aggtagaatt ttccaaaccg cattctacta aagctttagc
127381 aagccaaatt tcgccttgta tctcaaattc ttttatatcg cttggcaaag catttccact
127441 atgcacgcta tcaaattcgg cttttgcagc cttagcactt tcttcattgt gaaaacgcgc
127501 agtaatctct agtgccaaat tttccttagc aagtttagga tgtaaattgc cactagaaat
127561 ttcttttttc atattttcaa tttcttctaa actttagta cttaaaagct cgtaataacg
127621 ccacattaac tcatcactaa tacttagaat tttagcatac atatcattag ctttttctgt
127681 aacgccgata taatttccca agcttttact cattttattt acaccatcta aaccctctaa
127741 aagaggcatc atcatcacgg cttgttcttt tccggtatta taaattcttt gcagggttct
127801 tcccattaag aggttaaatt tttgatccgt tccacccatt tctatatcac attttaaggc
127861 tacgctatcg tatccttgga gtaaaggata taaaaattca catatagaaa taggactttg
127921 ttctttaaaa cgctttgtaa aatcatcgcg ctctagcatt cttgccacac taaaggttga
127981 agtaagctct actattccag cagctcctag ttcattgagc cattgagaat taaattttaa
128041 ttgcgtcttt tgaggatcta aaatcttaaa tacctgctct tcataagttt tggcattttt

128101 taatacttgt tctttatcaa gctttttgcg tgtaacgctt tttccactag gatcgccaat
128161 ttgccctgta aaatcaccga ttaaaaattg aactatagct ccgtgttttt gcaaatatgc
128221 cattttggtt aaaaccacgc tatggcctaa atgtaaatca ggtgcagtag gatcaaatcc
128281 cgcttttacg aaaaaatttt caccctttc gtaataattt ttaatcaaat tttcaactct
128341 ttcttcatcg atgatttctg cacaccctcg tttgatttct gcaagtatat tttttatatc
128401 cattttccac cttttagttt aagcttcttt ataagcgtca tttaatgaag taaaatctaa
128461 aattttacaa taagacttca atctatctcg gacattatca ggatttatat tgtcaggaat
128521 ttctatcgtg atttcaaaat aatccaccac tgtattagta tcacttgaca aactaatcgt
128581 taataaatta atctgcattt tagccaaaac tgccaaaaac tctgctaaaa ttcctttttt
128641 attttctaaa gaaaaaatta atttgtatga tttaggtata ttagaatccc atttaatcaa
128701 aaccatgtct ttattattat ctatcatttt gtctgcccta tcacagagtt tatgatgaac
128761 tatagcgttt ccattctcga taaatgcaag cacagcatca cctctttttg gatggcaaca
128821 gtaatcaaaa tccacattag caattttatg attagaatat aataaaaaat ttccaatttt
128881 ttgctctttg atttcatatt tatcaaacca ataagacttc ttagcatatt ttttaagccc
128941 attgactata tcttttaaat aagcactatc gctagctact tgtcgtatgc gcctacctaa
129001 attctctttt tcgatccaat tttctatctt attcttatct acgctaaaaa taaaacttaa
129061 catattaatc gcgcttaaaa gattgatttc tctcattttt tgcttgcaaa attctcttat
129121 actcgctttt gcttttcctg tttttacact atcaacccaa gagcaacgat aaaatctatc
129181 attggaagtt acaacccgta ctatatctcc atttttaagc tcggttaaaa gggggacttt
129241 tacgcgatta acataagcgc tttttgcgtg aagccctact ttagtatgta cttcataagc
129301 aaaatcaagc acggtagccc ctcgcggtaa ggtaaaaatt tcaccctttg gagaataaac
129361 agccacatcc tctacataaa gactatcttt tgcatactca taaagctcaa tagcattata
129421 atcttctgca ttttcaagat tgttcgtgct ttgcattgaa atatcagcaa gccaatcaag
129481 tcgcggagcc acaacgcttc catcttcttt atatttccaa tgcgcagcta taccaaattc
129541 agcgatttta tgcatatcaa aagtacgaat ttgtgcttcg ataatatttt tcgtatcaaa
129601 aagtgttgtg tgtatggttt gatagccatt ttgttttggt aaggctatgt agtctttaaa
129661 acgcgaaacc aagggattaa aatttgtatg taaaacccct aaagccaaat aacaatcgct
129721 cacattttca accaaaatcc tcacgccaag tagatccaag acctcttcta tacccacacc
129781 ctttctttgc attttaagat agatagaata actgtgcttg atacgttttt gaatttgaaa
129841 acttccttgc ctaaagccat tgctaagaaa taaaagttcg atttttgaaa taaattcatt
129901 aaggcctagt tgcatttgct gatcgtttga attgatataa ttatcaataa gtttatactc
129961 atcaggcatt aaatatttaa aacttaaatc ttctaaataa tttttaatac tagaaattcc
130021 aagtctatgt gctatgggtg cataaacgac taaagtttct tcgctgatac gcttttgctt
130081 atcttctctt aaagcatcta aggttagcat attgtgaagt ctatcgcaaa gttttaccac
130141 caaaacccca acatcttcaa tacttgctaa aagcatattt ctaaatgtaa gagcagattt
130201 ggttaaactt ttctttgatt ttgaaggaat aagattatct tctctgattt ctacgatttt
130261 tgtaaggcct aaaacaagct ttaaaacttc agatccaaac tgatctctaa gttcttcttc
130321 agtacattgt gtatcttcga tcacatcatg taaaagcgcg gctaaaattg tggctttatt
130381 ttcacttaaa aaagcaacta agcttgcaac caagatggga tgaactgcat aaggttctcc
130441 actttttctt acctgacctt cgtgagactt tatgcaataa tccacagctt tttctacaat
130501 agcatcttct tgacaaatgc taaaaagtag ttcttttgct cttgttaaat ctctacaatt
130561 tttaatatct tcaatgagtt tttcgagcaa taattcctca tcgactggtt tcaactagac
130621 cctctaaagt gattttattt tctgctatct catataaagc aatatctgcg aattttaatt
130681 tacttttatc aatatcaacc aaaggtgctg caccatcagc taactgctct gctcttttttg
130741 caacaaccaa agaaagacgg tagcgatcat ctcccattct ttctagtgct ttagctgcta
130801 tttcttctat tctattctcc atttatttct ccttgattag tttttaacta tagaacaaag
130861 actgttatcg ccttgtataa ttttcaataa atttccttct tcaaacatat tgcatacaac
130921 tataggtaaa gaattgtcct tagctagagc gattgcagtg tcatccataa ctttaatgtt
130981 atcttgcatt gcctcatcat agcttagggt ttgtaaaaat actgcatctt caaatttatt
131041 aggatcttta tcataaactc cgtttacttt tgttgcttta ataaccattt gtgcatcaat
131101 ttcaaccgct cttaagatag ctgttgtatc tgttgtaaaa taaggatttc ctgtgcctgc
131161 agcaaagatt acaaccctac ctttttctaa atgtctttga gcccttctca tgatataagt
131221 ttcgcaaaaa gcttccattt gaatcgcact ttgcactctc acatcaagtc cgcaactttc
131281 taaagcttct tggatagcaa tagcattaat aacagtcgca agcattccca tgtgatctcc

131341 gcttgtgcgt ttgataagac cacctttgc cgctgaaaca ccgcgaatga tatttccacc
131401 acctattact atacctactt ctatattgtt tttaactagt tctttgatct catttgctat
131461 gaattttaaa atagaatttt caataccaaa accattttct ccggctaaag cttctcctga
131521 aaattttact aaaactcttt ttctctcttg catttttct ccttaatcag cttgaaatta
131581 tattgaaaat tgcattaaaa ttagctaatt taaactaata agttctaaag gattgatatg
131641 aaaattcttt tgtgtcactt caaaagttaa atcattttt atcctaccta ctaccgcacc
131701 tttttttaca tttttaccga ctttaatatt gggcgctatc ttgtctaaat gtgcataaat
131761 agtatgaatt ccattatcat gttcgactat cacaactctt gctagcatac tcgtatcttt
131821 agcaaaaact atctttccat ctaaaacact ttttaccaca gcatcagctt ttttacttct
131881 taaaacaaca ttttcattga aaattttaat attataaaca ggatcaatat aatttccaaa
131941 tttttgcttt acagtaaaag actctaaagg agctatggtt tttttacctg tataacgctt
132001 agtagagctt ccttgataac tagagccaat ttgacgaatt ttttgggtat tattagtgat
132061 tttactatca ctagcagttt tttgagtttg ctttttgtct tcttgcttgc tttctataat
132121 ttttaactga tttaaggttt ttcttaactc atcttgctga tcttgcaaat tttcaagttt
132181 tttagcgtaa attgcccgat cggtttttg tttgttaatc tcgctgactt gcttttgtct
132241 taaattttga agttttgcca attgggtatt ataatctttt aaatcctcat tgatcttttt
132301 gatttgatct tgcttgctgt cgatttgctt gcttatatct tcataatctt tagagagttt
132361 aaaaatttca tcttgtaaaa ttttatccaa gctacctaaa acctcaaaag ccataaaact
132421 ttccttactt tcaacataac cttgaggaat aggcaaatca taagcaaaat ctttagccat
132481 taaagttata agcttgccct ccatattggc tttatttttc aaaagatccg tattttgact
132541 cgtgagtaaa tttaagtctt tattttgtgc tttagctcga gcttcaagct taagggtttg
132601 agaattaaga gtttcaattt gagcgcttaa ttcttttagg ctcttttctc cgcttaatat
132661 atcatttgcc aaatcttcaa gctttttatt tacctgctct tgaatgcgtt tgttttcttc
132721 taaattttta gtttttcat tgattgcatt agcataaatt tcattgctta gtaaaaaaaa
132781 acaaagaaaa attaaaattt ttctcatttt ttcaccctaa acatcaccga atttacacac
132841 aataaacata taaataaagt cactaaaaaa atcacgccca aatgcacgat aaaattaata
132901 ggaggcaaga taatatcaac cgcttttaga ctatcttgaa ctatagaaag attataaatt
132961 tgtgtaaaaa atataaataa aataacaaaa gcaataaaac aatccaaaac cacaatctta
133021 tacaacataa aagatctaaa ccaaaaaggt gcaccaaaca agcacataat ctctacacgc
133081 tcagtatgct caaaaagcca aattttcatt tgttttaaaa ataaaacaaa tgaaagcaaa
133141 ataatgataa ataaaaataa ccaaaataca aatttcatca aaacaagtaa ggaataaacc
133201 ttatcatgtg ttttagcaaa agtctcaacc ttgctaatgc ttggaatgct taaaagttga
133261 tttttaatca tattaagctc attttgcgta ggtaaataat taagcttaat gctataaaat
133321 ctcggcaaag aatctcgtaa aatttttaaa tttttatcgg aaatatcatt ttttaagcga
133381 tctatcaaat ttttaggatc taaaagttct aagtcagaaa aggaagaaag tttagttttt
133441 ataacattct tgtcaagttc agtagagctt gcgacgatga tattataatc tttattgaca
133501 agttcttcgt aatatcttaa ggtagcattt gtaagcaaaa taaactcaaa agcaaacatc
133561 ataaataata agggtaaaat caaagataag tgggttttaa aaaatttcat tcatatttcc
133621 attttctata ttaaaacgac gataattcag tcttaaatta ctcggaattc tatgtgtaac
133681 caccacaaca caagtaccca aaagctctct ggctgatttt aaaagcgtcc aaataatatc
133741 tgaagaatac tcgtctaaat ttcccgtagg ctcatcacaa agcaaaagct taggattgtg
133801 tgctaaagct ctagccatag ctactctttg ctgttctcct ccacttagtt gattgggcaa
133861 tttatctgct ttaaatgtca aatttacatg ctttaaaagc ttgcttgctt gatcatgaca
133921 aacttttta ctataacctt taatcataag aggaagcata acattttttt ctacgctata
133981 ttcttgtata agtctataat cttggaatat aataccaatt tgctgacgca attttaaaag
134041 ctctaaattt ccaattttc gcatagaaga tccgcacact tcaagttgac ccgaaagaag
134101 ctccaaatcc ccataaaaag attttaacag agtgcttttt ccacttccgc ttttacctgt
134161 gataaaaaca aaatcatcat ctttaaaagt gaaactagct tctttaataa ccaattcatc
134221 ataacctaaa gaaagtttat gtgcttgaat taaatttgac atcccatcct ctttgaattt
134281 caaaattcgt aaattatgct ttattttgc ttaattttca agaaaatgtt tcatattaaa
134341 gtgaaattta acacttttt taagctcttt tatagctttt aaattttctt gtattttaaa
134401 aggctcctta aaaacaaagc taatcgtctc tcctatatgc aaataacaat gctcaggctt
134461 attaaacgca ccaaaggcta tctttaacaa taaactttcc ccttgcttgt aaatattttc
134521 aaaatgcaag aagaaatcct ctttctttaa agtaaaattt tcaaaatttt tctctaaaag

134581 cattaaatca tttttatcta aatgtatatc cgaaaaatca aattcttctg ataaaggatt
134641 attttcttct ttatctaaat tccaaaccca aagatcataa atatcttttt cctgtctttt
134701 ccatctgtct tcgtatttgc tagaaatttc taaattttca ttttttctaa tgctaaattg
134761 tggcgatgaa aaatctaaaa attccttaaa agtaaagtcg aaatactcat aactatccgt
134821 tctaagttca aacacccctt gggctgtaag cacccttgcg cattctttgc aaaatgcttt
134881 agaaacaaca cgacgatgag gctttttatc ccaaggaact ggaaagtgta agaaaatttt
134941 ttctacagaa tgagactgaa gaacgcttaa taaaagccta gcatcacttt gaaccaataa
135001 aatattatca ataccctgtg ctttcgcaag cttagccacc tgcgtgatgg ctggattata
135061 aatttctatc ccaaggatta aaacatgggg attattttta gcttggtaaa gcaaatgcct
135121 tcctgaacca aagccgattt caatgtaaat tctcccctca agtcttgcca atacttcctc
135181 aaaatcatta gcaatcaaag gagtttttc agccaaagca ttgtttttta taccaaaagc
135241 ctcgctgata atatcctcgc aaaaatattg cttaaaaaca tataaagcct tatgtaaata
135301 ggctatttta gaaggcttag tgtgtttatc taccttaact acaaattcat tttctccttt
135361 tttaacctgt aaaaaaaagc tttcttcttc gacctttgtg tagattaaat tcacattatc
135421 attatatgcc atccaaacaa attctacccc atctttttca aagggtagaa tgatttcttt
135481 aagtgttcta gctttaaaat ttggcactat tgccctgcct taactctctt tgaaggctct
135541 gaacgaaggc caacagaatc aatcgctatc acttcataac tatactctac acccggtaaa
135601 gctttgatat ctttcaatct tttctctttt atgcctttaa aaatagcatt ttgcccaccg
135661 ccatagcgtc taacttcgta ttcaactgct cttgagtcat tatccaccca ttctaattca
135721 ataccatcac tggtgctttg agccaaaatc acactaggag aagatggact ccctaaagtt
135781 ttgccctcta ctccattttt aggcatagaa ctttcaagcc catctatatc taccatagtt
135841 accttatagt atctactctt tgcagcacct tcaactatat cctcataaga attttgctcg
135901 gttttcgcca aaagagtata aggaagcaaa cttgaactcg tagaataaac tttataatac
135961 gaaaaatctt tatatttagg agcatcccat gttaaaacta ttttattggc tccatctaaa
136021 ctcgcattta aatgttcaac ttgaggtggc aaagccttgc ttgtagaatt taaaacagaa
136081 cttggctcac tttgcactcc atcaaaccca accgctataa ttctataatt aaaactctcc
136141 tctggcttta aatcctcatc tataaattca gcatttaaac gatttttttac ctcagcaatt
136201 tttttaaatt cttgtccatc tgccttagct ctttctatga tataagaagc tactcttgga
136261 tctgaatgcg gacgccaaat caatttaatt cggcttggta gattggtaat agcttgtgca
136321 aaagcaatag cctctaatct tggcatagtt tttacttcaa ccacctttcc atcttctgaa
136381 atatgtcctt gattattaaa acttctcatc atatagtaat atttcgtatt tggctcaagc
136441 ttagcatcta cataatgggt ttgaaattta tccttaatcg ttcctacaag tttaaattca
136501 ggatttgcat catttgaacg atataaataa aatcctttga tattatcatc atataaagga
136561 tcccattcaa acgctatatt actaatatcg ctaaggcttt taagattgtc aatctttggc
136621 aagctttcat tgactaaaag ttctttcttt gaacctatat tggaaagttg cgaaacactg
136681 caagcactaa ataaaagagt caaagcaccc aagcaaagac tcaagtgaaa tttttttcatt
136741 aagctctcct aaaataaatt ttttttctaa aatttgttta aaatcgtcca ttaatggagc
136801 ttgaataaat atttttttctc catttctagg atgaataaaa tacaaaaaat acgcatgtag
136861 cataatccta cactcgtatt ttcccctata accatatagt tcatctccta aaatatgacg
136921 atttatacta gctaaatgtg ctctgatttg atgcgttcta cctgtaaaaa gcttagctgc
136981 aataaggctc acatcctcgc tttttgccaa gttagcaaaa gcacttttag cctccttaga
137041 acccttaaca aagctatttg taactatttt tttaatagca ttagcacttg atcgaatcaa
137101 agctttatct atgataattt tatcctcttt tagtggcaaa tcaattaacg caaggtaaat
137161 tcttcccata gttttatcgc taagttgctc gctaagcttt tggtgggtaa aattattttt
137221 cgctatcaat atagctccgc ttgtttcttt atcaagccta tgaacaagcc ctgcacgcac
137281 ttctccaccc aaggtagaaa gggtgtattg tttttctata agccaatcca ccaaagtcgc
137341 acttttaaca ctctttgcac catgaactac caaatttgag ggtttgttta aaaccaacaa
137401 atcctcatct tcgtataaaa tttctatatc aaaatctata tcaaatttag gtttaacttc
137461 ctccacaagc ggtaaattta aaacaagcct atctccgact tttagcttaa atgaattttt
137521 attttgaatt ttatcattaa cccaaacgca gtttttttct atcatcaagg ctatttgatt
137581 gcgactttgc tgaagatttt tagctaaaaa agtatcgagc cttgaagatt catcaactaa
137641 aaaagtttgc atttttttcct tataatttta tataatttag agttttattt tataattata
137701 ctttaaatac tacaaattga aggcaaattt tgtttatacc cgatagaaga attttaacac
137761 attttgatta tatgcaacct attttattta tacctattat tttgatttca ttttttttga

137821 tatttgaggc caatgctttt ttagctgaaa aacaatttgt ctatgcctgt gtaggaattg
137881 ctgcatttag cgtgttcttt ttgttgccta tccgcaaact gatgtggctt atacctatag
137941 cttattgggt aaatattctt ttacttttaa gcacagatct ttttggtgtt gaaaagctag
138001 gcgctaaaag atggcttgaa attcctttta cacactttac cattcaacct tctgaaattt
138061 ttaaacctag ttttatttta atgcttgctt atttgatcta tcaagatcca ccgcctaaaa
138121 atggctataa attaaaacaa tttctaaaac ttagtttttt tatcattttg ccttttttac
138181 tgatcgctca agagcctgat ttaggaagtg ctatggtgct tttaatcgta ggctttggcg
138241 tgctttttat catgggtgtg cattataaaa tttggcttag tatcatcatc gccatcggta
138301 taagttctcc tatcatttat acgcattttt taaaacccta tcaaaaacaa agaattcatg
138361 attttatctc agaaaaaccg agttatcaag tcgcacaatc catgatagct ataggcaatg
138421 gaggccttat aggaaaatct caagatgaag ctacgcaaac acattttaat tttttgccta
138481 tctccacaag tgattttatc tttgcgtatc ttatagagag gtttggtttt ttggggggat
138541 ttgtattgat tttgctctat actctactca tttttcattt gctaagctta aatcaaaagc
138601 ttaaggaaga ttatttttgct agggttgcta taaactgcgt agcattgttt atttttattt
138661 atgctgcggt aaacatttcg atgactatag gatttgcgcc tgttgttggt atcccgctgc
138721 cttttttttag ctatggagga agttctttta cgatttttat gattttcttt ggaattttac
138781 aacatttgat tacttttaga tatttttgga tagatagtaa aaataaaata aaataatttt
138841 aagcaaaaat aagatataat tttatcttta aaaaattaag gcggatttat agctcagttg
138901 gttagagcaa ccggctcata accggttggt cgcaggttcg agtcctgcta aatccaccat
138961 tatattaatt acacttatac aatctaaaaa aacttatatt tttttacccg cttagtaaat
139021 caaaactaga ttaaaaattt ttagtgtatt ttaactgcat tatattaaat taagtaaaac
139081 ttaagcaaaa ttccaatgga aatttacagg ctatgaaaac tgcttaaaaa acaaaatcag
139141 ttaattgcga atcaaagcaa tatgtcaatc acaaaataat aaagatattt taaaacaagt
139201 aaaaataaaa aactttttca aaaagataaa aaataataat ctttatcaat caagtcctaa
139261 tatttttaaa tagaaataaa atgatgttaa aatattttgt ttctatatta aatcaaggag
139321 aaaaacaatg aaacatttta tttttagcgg attaattggt ttaggtgctt tggctttttt
139381 agcaggttgt ggcgatgagg caaaaacaag cgattattat aaagcgcact tagatgaggc
139441 aaaagctaga gttgcagaat gtaaaaaaat ggagaaaatg aatgaaactc aacaaaggga
139501 ttgctctaat gcaaatgaag cggtattatt tcaaagagac aaaaataata aattttatca
139561 atcaagtcct gaagcttttg aatggaagta aattagcttt agttttattt ttatgctttt
139621 tgattccaaa tttagctttt ggggctgaaa gtcccgaagg ggcgttaaca ctaatcaaaa
139681 atggactctt atcttggaca ctcctcatta aaaccgcttg tttgtagata ttttgaattt
139741 ttgttttggt ctctttttaa atttctttat aataaaatca aatgcgtatg tgcattaata
139801 taaaaaataa aaaaatatgc atgtagtatt tatacgtagt agttaatatt aatattatat
139861 ttcttaggtt tttaaaaaat agtaatttta atacaaagaa aaaaaataag agaagtccta
139921 taaaatagta ttttgattga atatttgttt gatttttaag gttgaaataa gaacattaaa
139981 acttaaagca atgtaaaaat atcaatttga agatattgat tatttaaaaa cttgagtaga
140041 gtttgtgagt cttatttgat attaattcag tttaaaactt ttagagtaaa gtaaaacttg
140101 ttttattatt tttatcttat ttattatatt atttacagtc taatttttac gtattaaaga
140161 aatagtagaa tttatatatg tccatagctt attatggttt attttaagct ataataaatt
140221 gttaagatgt ttttccaaga taaattgaca atgagttaaa ggtattttgt actcaaggct
140281 taacccattg ttttcataat ttattataag caagatgaaa ttaaaaatta gttgttaacg
140341 aataccttttt tcatttaata ttttatattc attgcaacct ttttgaaatc ctaaatcaca
140401 agctttgccg tagtattctt ttgcagaaga taaatttttt ctaacacctt gaccgtcatg
140461 atacaaattt cctagatttg tacaaacatc tccataccct aattcacaag ctttttttaaa
140521 gtaacttact gcttgagtgg atcttactta acaccatcgc catcaagata taaaagtcca
140581 agaatatcac aagtttctcc ataccctaat tcacaagctt ttttaaagta atttactgct
140641 tgagtgcgat cttgtttaac accatcgcca tcaagatata aaagtccaag aataccacaa
140701 gcttctccat actctaattc acaagctttt ttaaagtaac ttactgcttg agtgcgatct
140761 tgtttaacac catcgccatc aaaatataaa agtccaagac cattacaagc ttcttttata
140821 tttaattcac aagctttttc atagtattgt ttagctttag aataattacc ttgtttatgt
140881 tgttcttttc caagatcaaa ataattatca tttgaataaa cgcaaacagg tattaaagta
140941 aatacaaata tgaaatattt cattaccatc aattttcctt ttaaagctat attatttgaa
141001 atttttatat tctttacaat catattcatc tcctaattca caagattttt cataattaat

141061 tcttgctgta tggcaatctc tttcgatgta cttacacttc tttaataaaa taaccatcgt
141121 tcgaaaatac atttgttgtt aataaaatta agaactaata tatttaattt tatttgttgc
141181 cttttataat taattgttaa ttatagcata aattataatt tacaattgat aatatgttat
141241 gtaaaaatta aagtttgaaa aaatttattt tttcaaattc caaaagcgac atattgtttt
141301 agtctctttt taaatttctt tatggtaaaa taaagagctt gttaaaaacg atatatacac
141361 tgttttagtc tctttttaaa tttctttatg gtaaaattcg tttgaagtgc tccattcata
141421 gtattcggtt ttagtctctt tttaaatttc tttatggtaa aataaaacgc ttttattgtt
141481 tatacctttc atagttttag tctcttttta aatttcttta tggtaaaatt agagagatta
141541 gcaaatcatc ttcacatagg ttttagtctc tttttaaatt tctttatggt aaaatccatc
141601 atcaatcatt gaaagtaaaa ttgcgtttta gtctcttttt aaatttcttt atggtaaaat
141661 ggtaaagagt taaaagagca attagaattg gttttagtct ctttttaaat ttctttatgg
141721 taaaattttt agcctaaagc tatcagttaa aaagccgttt tagtctcttt ttaaatttct
141781 ttatggtaaa attaagagtt ttaaaaaggc ttaaaggtat aaagttttag tctcttttta
141841 aatttcttta tggtaaaatt ttctatatga aaagagttta gaagaggcgg ttttagtctc
141901 tttttaaatt tctttatggt aaaatggact ttgaacaaat aaagaaaaat agagcttttt
141961 gtatacaaaa atgtataaaa atatgctatc attttaaaag agaaatttaa aaagagacta
142021 aaataagtgg ttttcggtta gccacgcagg gttacaatcc ctttaaaacc gttaaaattc
142081 aaacaaacta gcttgtctaa ttttattaga cttagttttg ttttctttgt atatcttgtc
142141 attgctgtat ttaaaatctt gtgtttttc atcgtattca aaaagaacta aatttttatc
142201 atcattcatt ttttcattaa aagaaactcc gcctaaaagt atttgcatat tgtcaaattg
142261 tttttcagtt atggttaata ctcttatatt gccacaaggg ggtaaaattc tttctacgga
142321 tttgattgct gttttagccg aagtcattcc tttgcaaatt ttaacatata cgctaaattg
142381 catcataaaa aagccaagtt ttacaagcga aactctaaat ttatttgcac tttttttgctc
142441 tttcttactt ttagtgggta tatcaaacat caataacact ctcataaatt tatcttctat
142501 catataaatt taccatcata agttcatcat tttccaataa agcattttta aaattttgaa
142561 tataaaaatt tatagcccta ttcaaaggat aagctttatt ttcaataatg atttcttctt
142621 gcaaaatacc aattaatctt tgctttgctt ccttatctaa aaattcactc tcttttatat
142681 cttttaaatc aaatacgcac aagtcaacca aaggcctaaa tacttctatc aaatcatcac
142741 aaagattaaa atcattatag atattatcgt gctttatacc ttgccatggt aaaagtccac
142801 ttatgcaaac cgctctaact ataagtgatc ttatgacagc atagccgtaa tttagatatg
142861 atgggtaatt tttaaaatat gggcttgatt tgaaattttg tttttaatga ttttttgcca
142921 caaaatcgct tttctttgta gtgttacttt aatttgcttt ttagctactt ttgcactgat
142981 aaaatgacct aaaaatggag aaaaaattcc attgatatga tgagattcat cgcaagttaa
143041 aagtatgatt ttatacttgg ctagagcgct aagcaagctt gaagtgatac ttgcttgcaa
143101 agattctagt atgataaaat tgatatcttt gagaaaaaac ttggcttctt tatcgtcttg
143161 ttttatcaca agatgattaa actcaaggct tagttttgct tgattggata tcaatatgct
143221 tttaaaagct tcatcatagg ccatgttttt tgctcgtctt taaggcaata tcttcccttg
143281 gtttaaaatc agccttaatt ttttctccaa gcggggttat aatgtatttt tcaaaaattt
143341 tcaatccctg aattcctaat ttttcagcct ttacacttcc ttcttttgca tttacaaaca
143401 atagcttttg attgtctgtt aagttttcaa atttattatc atgtttttca acacaaatac
143461 ttgagttacc gatatcaaaa ctattataat aagcaaattc gggttcttgc atatcttttt
143521 tttgtatcaa aaccaaatca tctttatgca aagaaaaaca aaactcatag ctttcatcta
143581 tttcttgcca ttgctttgga ttgccctttt tatctttacc tgcaattact attttatttg
143641 gtaaaactcc taaagcaaaa tccatagtat aaataggtat agcataaaat ttattttgtt
143701 ttttaaaaat atcaatcctt accatggtat cattttctac atattttgta cctatttttc
143761 ttatttttcc acaatttagt gctatttcta caccttcttg tgagttgtat tttttaatca
143821 tttcatcttt agggtaaaat gtttccttat gcaaagctcc tcttgctctt tttctaggag
143881 gttttgaaac aaaaagcttg tttatttgat ttaaaatttg ctctctaaac ccctcaaaag
143941 gctcaaagaa cttagcttga tgtttataac tatcatttgt aagttcttta gcataaagtt
144001 ttgcttttaa aagctcttgt tctttttaa aatccgaaaa agcttttata atggcatttg
144061 tgctataagc gactattgtg gcatcaagcg cgtgatgaag atggttgttt ctatcttttt
144121 gagcaaatcc ccaagtatgc cttaaaactg atgtaagcat tccattgatt gtttgtacat
144181 gtattttact tcctttttct ccacttttta gatttatatc ttctttttca tctaaaggta
144241 aaaaatctaa atattctttt gtgtatttaa caataagagt tgaaatatat cttgtatcat

144301 ttaaattcct actgataaaa tcttgctgtt gcttgccttt aaaagcttcg tctaaaattt
144361 tatttttctt tttgtaggga agattttgag ctaaagcttg aattttactc catctttctt
144421 catttgctcc aaaggcttca aaaggtgttt tattgagttt ttcttgattt tctttagtaa
144481 aaaccaaaac cttatttaaa aaagaatcat caaaacttcg tgaataagga tatatatgat
144541 caacctctaa agctttttca tctcttaagt gttctatgga tatttttttа ccactataaa
144601 tacaaaattc tttttgttct ttccaaagtt ttaattttaa gatattttta gcatttgcct
144661 taagcccaaa attttcacac tcatttaaag cccaaatatt attctcatag ttttcttttt
144721 gctctttttc aatcttagtc ctaagctttt tactcagccc tatatctctt gctaattcta
144781 tatgaatttt atgcattttt ccatattttt taagcaaagc atttaaaact tttctatact
144841 cgcttatagc acgatttacg ataggattgg taagctcttg tgcaaaaata ctatcgcaaa
144901 aggcaggtaa aaaatcgaat ttttgattgt ttgattttgt tttcaaattt aaaagcttgc
144961 aagcctcatc gtatcttttt ccctctttca tcaaaggcaa gattagatta agtgctttaa
145021 aactaagatt tatatgatca ttgaaatcaa tttctattaa attcttgatt tgctcgttat
145081 ttaaactata cttttctaaa gtaattttta atttctcatt atctttaatt agagtgatat
145141 aagtcgctat ttgatctagt tcttccctag ttaaggaatg ctctcctagg gcttttttaa
145201 attcgactaa ttttctaaat tcgatcagct ttgcactttc tgcattgtct ttatcatact
145261 ttaggctttt aaatttcata ctttcatgaa gtttaatgta ttctctaaat tttttataag
145321 tgatacttcc tttatctaag atatgattta aaatttcatt aataatctga ctcgatacaa
145381 gctctccgct ctctttctct aagcttttta gctcattgat gatcttggtt aacgctacga
145441 attcccaagc actatatgaa tttttacaag ctcttttttc atcttcaaaa aaagtgcaag
145501 ctcctactaa atatgaaaaa tctttcaaag gtctttgaaa aaatgcaact tttagaattt
145561 cctttataaa attatcatta taagaatatc cccattcttt ttgtttttct aaaatcagtt
145621 ttaattcttt ttctaaatca cttgctagaa cgcaattttc ataactgcct tctttattgc
145681 gtatatttat aaaatctttt gtattttctt tatatttttg gaaaaattct ttataaaaat
145741 actctcctac actttgatat ttttctaatt ttaaagcatt tgtttttaaa gcgcttaaaa
145801 ttttaccctt ttcattgtca cttgattttt tttcattttt attcatatag ccacgatgtt
145861 ttgctatgtg taaaatcacc cttgctaaat ctttgggttc tatttttttca ttaagtgctt
145921 tataccttag ctcataagga cttacaagcc ttccttcata agctttaggc aattctccat
145981 cgtttgctat ataatcttga taatttaatt ttaattcttt tgaaataata tgctttaaag
146041 caatcagtct tgatcttctt cttcctagtc gtcttctatt gcttcttgca ttgcgtctag
146101 gtaaagctaa agattctttt gttttaggat cttcagcctt agtaaataat ctcacaccac
146161 aatcttgcaa ttgattgtct tcaacaaaag cccaacctat agagttaatc cctatatcaa
146221 aacctaaaat tttcattcgt tctcactttt atatagtatt tatgattgat tttaaataac
146281 attgctttca tatcaaagca cctctaacaa tttaacccta ctcctgaaat tcaacactct
146341 caaatcttgg tttatccaca aagcaagatt tttcttttaa aaataaaacc tcgacaaccc
146401 ctacagggcc atttctgttt ttgccgacta aaatttcagc attttcttgc ataggattag
146461 gaatgaaatt tctttgataa ggtttgcctt cggctttagc cttgttttca cgttcttttt
146521 catcttgttc cctataaacc tcatcgcgat acacaaataa aatcgtatcc gcatcctgct
146581 ctatagcccc actttcacgc aaatcactca tcataggacg cttatttgca cgctgttcta
146641 aagaacgatt gagctgcgaa agtgcgatga taggcatatc aagctctctt gctaaaagct
146701 taagccctct tgaaatttcg ctgacttgta aatgtctatc attgaaatta gaattactca
146761 tcataagccc tatataatcg atcacacaaa gccctatact ttcttcttga gcctttaaac
146821 gccttaaaat cgctcttaca tctgtgatag tcgcataacc actatcatag ataaataatt
146881 tctttttaga ataataattg caagcatcgc caatgcgttc ccattcgtta tcatttaaat
146941 ccgcagtcaa gattttttgc aaaggaatgg aagtttttgc cgaaagcatt ctttgcatga
147001 tttgagcagc aggcatttca agagaaaaca tcaccactcc cttatcctgt cttaaaacct
147061 tttctataaa attcaagcac aaagttgttt ttcccatacc tgggcgcgct gcgatgatga
147121 taagctcccc gcctttaaaa cccttagtca tagtatttag atcttcaaat cctgtatcaa
147181 gtcctatcac ggatttgttt tcaaggcttt tttgtttttt aaattcttcc aaaagctcgc
147241 ttaagactat ttctatatct ttgatatcgt tggtgtttac acgattagtg atgttaaaaa
147301 tttccttgcc gatttcatca gaaatttcac tgactgcgcg attatcattg atccttgttg
147361 gcaaaagatg ggcaaaactt aaaagctgtc ttttaatcga cttttcgcga agttcattga
147421 cataagcggg aagatctatt atcgaaggag ttgcgataat ctcggttaaa atttgctcgt
147481 ctattttttt atgctttttt aaaaagctaa tcgatatagg ctctccagca ttcacacaag

147541 caataatagc tttaaaaaca tcctgatgag ctttaagact aaaatcttta ggttctatat
147601 ctcctgctat gctagaataa gcgtcctcgc tcataataca actacttaaa attgcgcgtt
147661 ctaaatccaa atcaaaatgc tcttgctgca cctattttcc ttttttgagt aaaatttatt
147721 gaaagatttt atctaaaaga atctaaaaat tcacataagc tttttataaa aaattaaagt
147781 aatattagtt acaataaaat tataatttta atcaaaaact atcaaggagt ttatcttgaa
147841 aaaaacctta ttttcttttg ctttggcctc tttatttgta agtcaagctt catccataga
147901 atttcaaagt ggtcttagcg gaaatttaag cttagggata ggagcaagag atgttaaaag
147961 caatatctca cctctagcaa atagcaacta tctaggtggg tataacgcta aaaattccga
148021 tactgctgcc ataccttttta taggtcttga gctttattat ggcaatttaa tcgataatga
148081 tagagtattt cttaaaaact ataacggaag agatttaagc ggtattgctt tgggttatga
148141 aagagcttat ttagaacgct ttagcacttc tttttctttc atttcatctt taagagaaaa
148201 agcttatgct aatccttatt ttataggtaa tcgtgaagaa actgatgtga gtaaatacgg
148261 ctttagaatt tcacaacttt acgaaagtga ttttggaaaa ttcaatgctt cttatgtttt
148321 tgcaaaaaac aaactagata aagaaagtat agaatttgat tctcttaaac gagagggaaa
148381 ttatcatgag ttagaattaa gctataatta ttctttatta aacttaggct taatctatga
148441 ttataatgac gctgatggaa aggctcaaag ctactcaaga tatggcttta aaataggcac
148501 acatctagtt tttgcaagaa attatatcct aactccaagc ctaagcttaa atcaatacaa
148561 agcagatggc acagatccta tttttaacca aaaacaagat ggtagtatca caaaatttaa
148621 tctcaagctc gtaaaaaatc aattcttagg ctatgactca ctttatggct ttgtaaatta
148681 tggcctagaa aaaagaaata gtgatataga tttttacgat gaaacatacc acatcttgct
148741 tacaggtgta ggatataaat tctaaaatca atccaaagaa actatcatca taaaaacgat
148801 agtttcttaa agacttttct aacaaaaata caagctaaaa tcaaaacaca aagttcagca
148861 aaaaatggac taagccatac tccttttaaa cccatgatag aagataaaat caaaagaaaa
148921 cacaaaggaa taaaaagatt ttgactcaag cttaaaatca aagaaaaact cgctttattg
148981 ctcgctgtta aaaaagaacc acttaaaaca ttaaaccacg caaaaagata attaaaagaa
149041 aacaacatca aagccatata agcaaaaatg ataaattctt gatcaccttc ttgagtaaac
149101 aatcctacca aatccttacc gcaaacaaat atcatcacga tagacagcaa agacaaaatc
149161 gcacccgcaa aaaacattac ctttaaaata tctttaatcc ttgaaaaatc ctttttagca
149221 taattataac ttaacgcagg ctgcatcgca tcacccatag cgataataag cataatgata
149281 aaattatcaa tatacgaaac gatagaaaaa gcagcaaccg cttgagtgcc tgaaattttt
149341 aataaaacta aattggcaaa tatgccatat aaagaacctg agatattgcc aaaaaattca
149401 ctgcttccat tataaactat atttttaaaa atcttcatat tcatatacaa acttgagaat
149461 ttaagctcta aattttgaaa taaaaaaggg aaaattccaa aaataccgcc tagcataagt
149521 cccaaacaag tcgcaagagc tgctgaaaac aagccccaac ctaacacaac gatgaaaata
149581 taatcaagca ctatattact taatgctata atcacattca tcatcatact ataagttgtt
149641 tttccacaaa ttctaagata attatccaaa gcaaaagagc acatggtaaa gggtgcaaac
149701 aaagcaaaca caagcataca ctcttttgcc attgatttga tatcttcact tacatctaaa
149761 aaattaatca aaacaggact taaaagatat tctaaaatgc ccaccaaaca tgaaataaga
149821 aagataatta acatactaga gctaaagatt tttctagcta aatgtttctt cccaagtcct
149881 aaatgcatag aaatttgcac agctgaacca atggctatca tatcagccaa agcaaaagat
149941 atcataataa aaggaataat caaggccaaa gccgctaaag cgtcatttcc tagatatttt
150001 ccaacaaaaa ttccatcaac aatataataa aaagaaataa atgccatgct tattacatta
150061 ggcacagcac atttaacaaa aagcctaaaa gggcttaagg tgctaaaaat atttgacata
150121 aaaaatcctt taaaatattt aacaatacaa atatcttaaa aggagctaat agagtgtttg
150181 aagtcctagt atgtagaaag ctaagcgata ggtttctatc ggtatgattt ttttattgtt
150241 tttgttttgt gtttgcataa taatatttaa aaattaaaat ataataaaga aattataata
150301 ttgaaagttt aataagtcaa atttagccca agggccaaat ttgattaaat tacggcaagt
150361 atctttacag cgtcatttaa gcctgttact atgcttgcgc catttttagt tgcaatatct
150421 cctgctacaa aaatgccttt tacattgctt tgtttatttt catccattaa aggaactcct
150481 ttatcatcga cattgattcc gcatttttgt aaaaaatcaa gaggagtaga accaccgata
150541 gcataaatga ttctatcata agtctcactg ctgccatcgt taaatttcac tttagcttta
150601 ccattatcat cctcaagctc ttctatatct ataccgagtt ttaattctac tttgcctgaa
150661 tttccagctt catgaatatc tttaagattg atatcattta gccttgtaaa ttctttcttg
150721 cgataacaaa ggcttacttt atttgagtta gctaaatcca ccgcatattc tgcagctgaa

150781 tttccgcctc ctacaacaag aattttttca tcacctaaaa cagaattagc attgaaatta
150841 ataatctttg ttaaagttcc aggaagttta taatcaggct tattaggctt acccattcta
150901 cctatagcaa cgatgatatt tttacactca taaacaccct tgcctgtgct tactaaaaat
150961 actccatttt catttttaac actctctact tcagagccaa attcaacttc tatattatgc
151021 tcatttaaag cgttttgaaa agtttctatg gtgctttctt tggttccatc ttgaaaaggg
151081 acatgtccgt gatttgtccc ttcacaaccc ttataagcca tatcaactct tttaccatct
151141 ttgtaaaact gcatcaaagt ttgacaaata ttgtttgatt tctccaatac caaaacttct
151201 ttatttttaa gcttggcttc aaccgcacaa ccaattcctg tcggccctgc gccaactaca
151261 attaaatcta ctttttttcat tcaaatttac ctttcaattc taaatttttt tgtataattt
151321 atcaagtttt ttgtaaataa aggattaaaa atgcgaaatt tacttgaaaa tattaagaaa
151381 aattctcaaa aattgctcaa tttaacaccc aaagataaag aaaatattat attgaaacta
151441 gctcacactt taagaaagaa tttcaaaacc atattagaat ccaatgaaaa agatataaca
151501 aatttcacca aaagtggagc aatgcgagat aggcttttac tagatgaaaa acgcattttt
151561 tctctttgtg atgctttgga aaaaattgct tatttagaag atcctatagg taaaatttct
151621 aaaggttggg ttaattatgc gggcttaaag atagaaaaaa tcagcattcc cataggacta
151681 atcagtgtaa tttatgaggc aagaccgagt ctaagtgctg aaatcattgc tttaatgata
151741 aaaagctcca atgcttgcgt gcttaaagga ggcagtgaag caaaatttac aaattcggca
151801 atatttgatc ttgtttataa agtactagaa gagtttaatt tgcaagattg ttttgctatg
151861 ttttatgaaa gaaatgagat catgcaaatt ttagcttttg atgatttaat cgatgtgatt
151921 atccctcgtg gaagttcaaa tatgatacaa gaaatcgcta gtcatacaaa aattccactc
151981 atcaaacaag acaaaggttt atgccatgct tttgtagatg agagtgcaaa tttaaatatg
152041 gctttagaaa tcatccttaa tgcaaaatgc caaagagtaa gtgtttgtaa tgccctagaa
152101 accctttttaa tccatgaaaa aattgctaaa gattttatag aacttttaat ccctgaattt
152161 gaaaaattta aggtaaaaat tcacgcacat gaaaatatct tagaacattt taaaaactca
152221 aaattagaaa tttcaaaggc tgatgaaagc acttttgata ctgaatggct tgattttgct
152281 atcagtgtaa aatcagtaaa agattgcgat gaggctatag agcatatcaa ccaacacagc
152341 tctttacact ctgaaaccat aatctcaaac aatgctttaa atataaacaa atttcaacgc
152401 cttctacgct catcttgtat ttatgtcaat gcctcgactc gttttagtga tggggggtgaa
152461 tttggctttg gtggggaagt gggaatttcc acaagcaaac ttcatgcaag aggtccaatg
152521 ggtgttgaag atatttgcac ttataaatac ctaatcagcg gagaaggaca aataagaaaa
152581 tgagtaaaag aaaaatttta aaacaacaat ataaagaaat catacaactt aatccaactg
152641 aaagagtttg gcaaatgcct tttttctttg ccttagcagt tggatttgca cttagtattg
152701 ctgcatatta tgagcgtatg gacttgggac taatcgctat gataggaatc atggcttttg
152761 tttatacaac caatacacct atgtatcata gaatggctgt tacaatgtgc tgttcttttg
152821 gtttgtgctt atcttttttta ataggacttt gcacccattt ttttcctgcg ttttctccgc
152881 ttataattgg tctagtggct atggctagtt ctattttgat acgctattat aacataggcg
152941 ctccggggta ttttttcttt gttttttcct gtttattggc ttcatttttt ccttttccag
153001 caaaagatta tatttttcta gtgggattga tttgtatagg tggcatcatt gccaacatag
153061 ctgctttttt atactctgta tctgtcattt atatttttaa aaattcccca cccaagcctg
153121 ttttgcaaaa tgggaattta ggctttgata ttatctttgt agattctata attattgctt
153181 tttttgtcgg atttgctgta tttctaggag catttttaga acttgataga agttattggg
153241 ttgcagttag cacgactgtt atcttacaag gagctaattt aaaatcagtt tggataaaac
153301 agcttcaacg cattcttggg acaacggttg gaattttatt tgcatggtgg cttttaaaaa
153361 tcaaatttac tcctttagaa tttgtcttgt tgatgatgtt tttggccttt ataatagagt
153421 ttttggtagt tagaaactat gccttaaccg taatttttct taccccttat gtgacttatt
153481 tagctgaagc agcttctttt ggaaatttaa gtgttgatat gctgatacac gctcgcttgc
153541 aagatattat cataggtagt cttttggggc ttctaggggg ttttgttatc cataaacctt
153601 atttaagaaa atattttgaa tacattgcaa aatacatttt tagagtaaga ttggcaaata
153661 gataattctt ttaaaattca gcttttaagc ctatgcgcat taaaagctga aataatatat
153721 tttaaaaatt cctgtataaa tatcttaatt tataaaagct tacaaaattt gatatttatt
153781 tattagctct ttcaatatac tccccgcgca ctgtatcaac gcgtataact tcaccttcta
153841 aaacatgaaa aggaatttgt acaactgcac ctgtttcaag tgtagcaggt tttttgtttg
153901 aaccttgagt atcgccttta aaatttggag ctgtttctac aatcttaagt tctacaactt
153961 gcggaacttc cacgccgata gctttgccgt tgtgaaaaag tacatctacc atcataccat

154021 caagcatcca tttttttgct tcccctacat cttcatcact gattgcaact tgctcgtaag
154081 attctgtatc cataaattgg caattttcac catcatcata aagatactgc atggtttttt
154141 cttccaaatt tggagcttca catttatctc ctgcgtgaaa agtttttttct aaaaccttac
154201 catcaataaa agatttgatt tttatacgca caaaagcagg acctttttcct ggttttacat
154261 gttgatattc aactatttta aaaggaattc catcgatttc gattttaagc cctttttttta
154321 aatcacccat tgaataagat gccataaatt ttccttttta tattaaaaat aaattataat
154381 tttaacaaaa attatttgat atcacaatat aattgataat aaaataagca tttttttgtt
154441 agaatttagc tttaaagcat agtaaatgct cgcttcttta aaaagaagag gaaagtccga
154501 gctgcgaaag acaaacattc catctaacgg atggctaggg cgacctaagg gacagtgcaa
154561 cagaaagaaa actaccacga aagtggaaaa ggtgaaacgg tgaggtaaaa gcttaccagc
154621 gattttggca acaatttcgg ctatgtaaac ccaatgtgca gcaagaaggg atggttaagg
154681 tctttatttt aacccttcgc ttgattttgt ttgcgaaaac aaaactagat aaatgagcat
154741 tctagacaga actcggctta tcgctatgct ttaaaaattt taatctttga aatattttaa
154801 agctttttga atttttttgaa tttcacaagc tttagaattc tttttttcac ttattaaaac
154861 aaccgcttct tgtaaaagtg ctttaatttg acttaaatta tcatttagtt tatcttttaa
154921 ctcatcactt tgaaaatctc ttgcaagatc ttcaagcatt ttttttaaat caagtgtatt
154981 tagcaagcct tcaagcttat tgacatcctc ttgtatgagc gcgagtttaa aatcactaag
155041 ccattgcttc attgctatgc acttctctcc aagcttctaa caagccttta gttacattga
155101 taacctcatc aatccttgct tcattatttt ctaaatttgc caaagataaa agttgaatct
155161 ctcttgtata aagaccgcta agataatgag ccacttcgcc acctttttca taatctaaag
155221 tattgataag ctctataaaa atagccgtag ttctttttac aaaatgcact ctttgttcta
155281 tatcttcgtt tcttatcgct actttagccc ttgagcaaaa acgcaaaatt ccttcataaa
155341 gcatttcgat aagtttttgc ggagattcta tccccgcttg attttgagaa taagcattgt
155401 aggctaaatt attttgcatg atttatcctt aattatttga attatttgct gcattaatca
155461 tatttgtaac agtagtgagt tgttgattaa gcttatttag aatactttca tattgtaacc
155521 attgatttgc catagtttca tacctagtat ctatcagttt ttgagctgat tcttttgact
155581 cagtaagagc ttttgtatca ttggttaaac tttcatcata ttttgttaaa gaaccatcag
155641 ttcctgttat accttgtaaa gtcgttttaa gctgtgcaaa aacacctgca ccctcaacag
155701 cttcagctgc tatagaaact tggctcaagc caaattgctt aagaaaatca ttatcccctt
155761 taatcgcaaa gtcagcattc ccatctgttt taaaattaag cttaaaacct gtttctccat
155821 tttgattata agattcaacc tttacagtta aaccctcaat ccctttgcta ttgatatggt
155881 cggctaaatt ctgaagcatt tcttgttctg ttgcaccact taacttaaaa gctgttccat
155941 cggcattttt agacaaatca taggtttcaa aatcacttac aatttgaaat ttgcccgaat
156001 caaagcttat gccattatca tcgccttcta ctttatttaa ataattttca agactgccct
156061 gtttaatcaa ttcacccgta tgatttatat ttttatattg agtaataccc gaaaaaaagc
156121 tctcggccat agctgtatct tcttttacct tgctttcaaa ggcagaagaa tcaaaattta
156181 aagttcctga ttctgtcata cttaaaccat aatcttgcaa agaaagcata accttagcgc
156241 ttactttatt gccgctagca tcttctactg ttccatccac tgattgagat tgaaaaagta
156301 tcgaaacaat acttgaacgt atagaattta cctcagttac accctgtaaa gatcctttag
156361 ttccagtttc gctgttataa tcggttgctg cgttaagatt taaaactaaa tcattataag
156421 catcaaccat ttcttgcata gcttgagtga tagactcgct atcttgctga atatcaaaat
156481 taatttcacc cgttttattt aaagtcagag taattcctac accaatatcg gtaatagtat
156541 tgctcgatct tgtcatctta atgccatcca tggtaaaatt agcattttga gcttgttgga
156601 tatgtaaagg attttcagca tcatctataa tacctacgcc ttttttactt gtagcaggat
156661 caaacccttc tgcatttaaa gcattatcat caagtgtcca acccaaactt tcaaaaacac
156721 tttttgcatt ttcatctact gcatattttc cgctactatc tttcgcacct ggataaaaac
156781 ttatggcact atcttcgcct gtttccttgc ttgtcaaagt taaacgatat ggagtgtctt
156841 tttcgcctgt atttaccatt ttagctacta tttctccacc tgtagcacta ttgattttat
156901 ctactaaatc gctataagtt gtatttttat ctatggttac agtgtattct ttacctttag
156961 aaaaaaaggt taaattggtt gaactttcca aatttgcatt gataaaccct gtatcatttg
157021 ctaagccttt gctttgataa acatcttttt gtgcaagttg agtaacattg atattcatac
157081 tttgcaaggc cacacccgaa gtagctgtaa gacttgcagg aggattatct gtaatacttg
157141 ccacaacctt tctcttagca aaagcagtag catctcccaa agaagaaacc gcgctttgaa
157201 aagtagaaag tttagtttta atctctgtta agtcttttttg ttttgttgta ttttcttcaa

157261 tctttgtagt ataaggatta acacgagctt gttgctctgc ttcttttaat ttatctatag
157321 tttcttgtgt taaaacccct gatccaaatc ccaaacttga taaactacca aatgccattt
157381 taactctcct tatcaaaaat cataccgatt acatctctaa agtattcagc caatctcata
157441 gcttcttcgc taggaatttg acgaatttct cttccggtac ttttctctgt cacgcttata
157501 tacattgaat caattttatc gctatatcca aatctgacat tagtatctag cgaatccatt
157561 tgctcattta attttcgcac aatatctgtc aatttattgt tcaaatttcc ttgctgttta
157621 tcatcgcctc cttggctttg atttcctcca gcttgaatat ctgaattagt ttgatgcgct
157681 ccacttgctc tttgagctaa attcgcaaaa gctgcatcta actgcccatt tgcctttgat
157741 atttccattt taaaatcctt tttaaaattt aatattgctg tttaaaggct atatcgacaa
157801 aattataaat ttatgaatac tttataaaga aaaatctatt tttcattaca aaaaaataac
157861 aagcaaaaag tgtgccaaaa gttatcaaaa agctgaattt aagatagaat tagcctagaa
157921 tttcaaattt tgaaaaaagt tctgagttat tttccaaaat atttcttaaa atcaaatcct
157981 caatcacgct tttagtacaa tcagtttgtt taggccaaac tctttcataa ccctccacta
158041 agcctttagc agtagcatca atccctactc tatctgcttt aataaaaata tctcttttag
158101 cgtctatatt gtttaccacg cgccatacaa gcatataagg attttcgagt ttgttattgg
158161 tatctaaaaa tactaaaatt ctaaaatgct ttttaaattc caaaagcttt tcaaagcttt
158221 gctctatttt ttcttttta tctagcaaaa tacaaactat aggactttta ctttctttgt
158281 aaaattgctt taaattaaca agctctattt tggttttaaa aagctctaat aatttttcat
158341 cttctaaaat ttcaagctct tctactatgg tttcttcaca agcatcaagc cctgcctttc
158401 ctccaaagca agaattaggc gaagcatgat ctaattgatc acaaattcct tcagagatta
158461 aaattttatt tgcattaaaa cgatttaaaa tataaggtat caatgcatca taatcatcta
158521 aatttggggc tttcttatct acaaaaatag catgcttaac aaagctcatt tgtcccactc
158581 cccaaaaagc atgcataatc tgctgtgcat gagcaggata ttttgcatca atttttgcca
158641 agataagatt atgaaaaacc ccattttcag gcattttata atcgattaaa tcaggaactg
158701 aagtttgcaa aaggggtaaa aatattcttt cggttccaag ccccatgatc ttatcttcta
158761 aaggaggttt tcccacaaca gttgcttgat aaatagcctc tttttggta taaattttt
158821 ctaccttcat cacaggaaaa agctctgcag gagtataaaa acccgtatga tcgccaaaag
158881 gtccttcgat tttaaattct tctaaatcca cataaccctc gatcactata tcactatcat
158941 aaggtacaaa aataccattt tcacaaggtg tcagtttggc tggagttttt ttaataaaac
159001 cataaagtaa aagttcaaaa attcctttag gcaaaggagc ttgagaacac caaatataaa
159061 gaggatcccc gcctatggct atacttacag gcattttttt aaagccagca tttttatact
159121 cgtgataaaa atttgcccca tctttatgaa tttgccagtg cattaaaagc tcatttttat
159181 cgctaacttg taaacgatac atgccaagat tattttgagt tttatccaaa ttttgtgtat
159241 aaacttgccc catagtaata aatctacccg catcttcttc ccaagtctta agtataggaa
159301 gttcttcaag agaatttaaa agctcataat catacaaagc tttatctgct tttaatcttt
159361 taggtgggac atttttgaga cttaataaat tcataaaaaa atctattttt gctttaaaac
159421 tagtaggaat atgaagcttg gttagctttg aaatttcatc agccacctct ttataatctc
159481 gtccgaaagc caaatttaat gctttttcat tacaaaaagc attcatcaaa acaggaaatt
159541 tatattgttt attatttcgt ttatctacag gatttttaaa aagcaaagcc ttaccatttt
159601 cacctttttt agcttctata taagctaaat gggcgatttc aagatctata tctacaggtt
159661 cttcatacac ttttagcaaa tcattgtctt ttagaatttt aataaaatct ttcatatcat
159721 actttcagct tttttcagaa tttctttagc tcccttggcg ataaattcct ttgctaaact
159781 ctcaccaaag cctttaaatt cgcttttttt aatcattctt ttttctttta aaatttcact
159841 accatcaggc aaaccaagta tggcgcgcac acaaatctca tcatttagaa gttctgcatt
159901 aataccaata ggcacttgac atcctccttc taaagtagcg ataaattctc tttctatggt
159961 agtttctatc aaagcattgt catcatttaa acactttaaa aattctaaaa tttttttcatc
160021 atctatactc tctataccca aagccccttg agatgctgct gggattagct catctttgct
160081 aaatgcataa acaaaattca cttgtttatc taaatccaaa cgcttaatac ctgccatagc
160141 taagataatc gcatcaaaat ccccattttt taatttttct atacgagaat tcacattgcc
160201 acgcaaggaa ataatttcta aatcaggtct taataataaa agttgcattt tgcgtctaag
160261 gcttgtagtg ccaatccttg caccttttgg caaagaaaga aaatctttat aattttgact
160321 taacatcgca tcattgcttt gttctctttt gctgatcgca gccaaaacca aaccttgagg
160381 aaaaaaactt ggtacatctt ttaagctatg cacagccaaa tgtgcttcat ttcttaacat
160441 actttcttct agctccttgg taaaaagccc tttacccccct atttttgcca agggactatc

160501 taagatcaca tctcctttag ttttaaaacc ttctaaacga actgctatgc catgagtttt
160561 gcttaaaaaa tttgctataa attcactttg ccaaagagct aattggcttt ttcttgtagc
160621 tatgacaagt tctttcattt tttctcctct atgatttcaa catcgataat ttcttcatca
160681 gaagtattat tttgcctata gtttcttttg tatctaggtt tggcaaagtt aaaaatcaag
160741 ccaaaacata aaacaaaaaa tccaaaaatg ctcgttacta ttcctggaaa gataaacaaa
160801 aaagccgcca ttataaaagc aaactgggta agcatatttt taaaatcact tatacgaaac
160861 tcaagcatat ttttccaaaa aattccaagc aaaattactc caaaaatcat actagccaaa
160921 ataaaaatta aaaaatttcc caaaccaaaa aatacgataa ataaaatact tgcaacaagt
160981 tctaataaaa aaagcggact aaggctgagc ttgtaattca ttctacactc tttttaataa
161041 aatcaaaagc ttctttactt gatagaattt ttttctctag tgtttttcta tctataaatt
161101 ctagctcatc cttttctaaa cccttaccca caaccaaagc ataaggaaag cccataagct
161161 caaaatcatt cattttgacc ccaaaacgct catttctatc atcaagtaaa acctcaatac
161221 ccacattgct taaatcctca taaagtttgg tagcaaattc taaagacttc tcgtctttga
161281 tgtttgaaac tataatttca agtgtaaaag gagctaaggt tttattccaa atacaaccct
161341 tctcatcatg atttgcctca atcgctactg caagtaagcg tgaaactcct atgccatagc
161401 atcccatata aaaaggcttg cttttgccat tttcatctaa gaaatttgca ttcatagcct
161461 tagaatattt ttgtcctagt ttaaaaatat gccctacttc tatacccttg cttaatctta
161521 attttccacc acatttagca caacaatcat tttctttaac ttctgccaaa tccttaaaac
161581 gatccttgtt taaattaacc acatcaatgc ctatcaaatg ataatctttt tcatttgctc
161641 ccattatcat ttgtttttcg ccttcaagct ctttatctaa ataaaaatca acatttttca
161701 aacccacaaa gcctataaaa cctgcaacca atcccgcttt ttctaattct tcttcgctcg
161761 catctacaag ctcaagagca ttgcaagcgt tttgtgcttt agtttcttgt aaatcatcgc
161821 aaccgcgtat aaaaaataca actaatttat tttcattttc gtaaatagct ttttttacta
161881 cagctttgat agtataaaaa gcattgattt tgaaaaattg tgctaaagct tctatggttt
161941 taacatctgg agtgtgaaat ttacttgtat aatttgcctc aggacgctct tcatcgcaag
162001 ttttattggc tctaattgca gcttcaacat ttgcagcata atcacaattt tcacaaatta
162061 aaatatcgtc ctcgccattt tttgccaaaa ccataaattc tttagaacca cttccgccaa
162121 tcgctccact atcagcatca acagctctaa attctaaacc cattctttgt aagatttgag
162181 aataagtctt atgcatcaaa tcaaattcac gcgttaaatc ctcttcgttt gcatgaaaac
162241 tatagccatc tttcattaaa aattcacgac atcttaaaag cccaaatctt ggtcttgctt
162301 catcgcgaaa ttttagccct atttgataca aatgcaaggg aagttgttta taagatgtga
162361 ttttattttt taccaaagaa agcatagcct cttcatgcgt tggacctaga acaaaatcat
162421 tttcttttct atctttaaaa cgcaaaagct ctttaccaaa aacattataa cgcccacttt
162481 cttgccataa agttgctgga gtcacaaagc tcaagcttac ttcctgcgct cctgctttat
162541 ccatttcttc tttaacaatg gtgcgaattt tatccaatac tttttttccc aaaggtaaaa
162601 aattataaag cccgctaccg atttgctcaa caaaaccagc acgtgttaaa aaaatatgac
162661 tcggtaaagt cgcatctttt ggagcctctt taagacttgg tgcatataat ttgctaaatt
162721 tcatcattat tctccatatt aaattcacat gcgtattgat ttaaaccttc aagattgttt
162781 tctaaagcaa aaatataacg catagcatta atcactgtat cactttgcat tttcccttgt
162841 agatgcttta aatttatagt tggagtatgc aaaaaagcct taaatacttg atggattaat
162901 tttcttgcct cttcttcatc agaatgtttt aaatatcctt tatctaaagc gatctgaagc
162961 tgtttgttag cacattcttt tgcttgcaag cgaatcgctt taataatagg agttaaagct
163021 aaatcattta aatatctaaa aaattctgcc gtctcacgcc cgatgatacc atacgccata
163081 cgcgcttctt gttctcttaa agccaaattg tttcgcacga caacttcaag atcatcaact
163141 gcaaaaaacag atactttttc attttcgctg acatcaatat ctcgcggtac agcaatatca
163201 aaaaaatatc tcttatgaga aacctcatcg attaaagaat ttgtaataat agcacttgaa
163261 gcattggtag ctgagaaaaa aagctcgtat tcatcaagat attttttttaa attatttaaa
163321 ctatcgcact cacttaaaac acccaatctc tcacaaagaa tacgggcttt ttctatatct
163381 cggtttaata ttataaccct agctccagca gcgattaaat gcttggctgc aagctctccc
163441 atctcacctg cacctataac tatagcactt tttccctcta aaggcaaaag ttctttagct
163501 ttggctactg ccacgctagc aacggaaata ggattttttg aaatttgagt ttcattacga
163561 accttagccg cacatttaaa tgcactgtga attgctcttg aaagatgtac tgcacaaaaa
163621 ttattttttta aagcaaaagc aaaagcatct tttaattgtc ctgcaatttg tgtttctcca
163681 acaaccaagc tatcaagcga acttgcaact gaaaaaagat gatgaatagc tccgctatct

163741 tcgaaaatat cggctttttg aaccaagctt tctttgtcta cattgcacaa taaagctaag
163801 gatttgatga tatattctgc acaagcttct tttacgtaag ctactatttc tacacgattg
163861 caagtgctta taaccaagca ttcttcaata ttttcatgaa tgcttagcaa tctcaaaaat
163921 tcattttttt tcgcctcatc agaaaaactc aatctctctc ttaaagagat atctgtattc
163981 ttatgggtaa aagatataca ataatacatt aaaaatccct atctatcata gcttgaatta
164041 taccttggag ttgttcatta gaaaaatctt ttatggctaa tgctgcttgc tgggcatatt
164101 ctttagcttc cgatatagct ttatttaaag cttttgtttc ttcaaatttt atttgcatcc
164161 atgttttttc atcattgttt aaatcttttt gaaacaaatt ccttagctga gtcctatcct
164221 catcatttaa attttcacaa agataaatat aaggcaaagt ggttttaccc tctttgaaat
164281 cattcatagc aggtttacca agggtttttt catcaccctt gatatctaaa atatcatcaa
164341 tcatttgaaa tgcaaggcct aaatttttgc catattcccc aaaagctttt tcatctaatt
164401 ttgccaaaat agccccgcac cttgcacttg cttcgattaa aactgcggtt ttattataaa
164461 tcatctttag atatttatct ttatctgcat taaaactttt gctaagctca acatccataa
164521 gctcacccac agcaagctta acaaccgcat ctgaaataat acttgcaaaa caagcatcta
164581 gttggctcaa ttcataaaaa gcttttgaat ataaaatatc tccaagcata agggcgtttt
164641 ttgtaccaaa ttcagcatta acagactttg caccgcgtct taatttgctt tcatctatga
164701 tatcatcatg caacaaactt gctaaatgta tgagttctat tgcggcacaa attttataag
164761 aatttgcatt ttctcctgca attgctaaaa gtaactttga acgcaatttt ttgcctgatt
164821 tggtattagc aagcatagtt aaaacaggct cataatcaag ctcttgcaaa tattgtttta
164881 tcaaatcatc tattggttgc accctatatc cttcaaattt agtttgtggt attttctcgt
164941 tgtattaatg gttttacttg aggctcatta aaatctatca taggcactac ttgattattt
165001 tgaggtggat ttgttaattg cattttttctt ggatcaagaa atttcacttc gagtctagaa
165061 tccttatctt ctatatacac agtaaaaatt gcctcgcctt ttttgtattc actaaattct
165121 aaaataagtc ttgctctatc aatatgtgga tttttataat caggaattaa agtttgagtt
165181 acccagtcca aatttcttct taaactcatc acaaattgac gcggatattt gcgaaattta
165241 gaatgaacga ctatattggt attatcaaac aaagtccaat aaaaatcgaa attttgaacc
165301 ccattatcat aaccaagctc tttaatctcc acgctagctc tctcatcttt tttaagtgta
165361 aatttatatg tataatcaaa aacaggagcg gcatttaaaa caatacaatt tgccatcaaa
165421 gcaaaaaaaa ccaaaagcct aatgctcatt agccctcatg tcctaaaatt ttagcaccta
165481 attctataaa aaggtcattt tgcctttctt gaataaaatc tccattttca agtataaagc
165541 ttttcacatc catttcattg aaaccttgtt tttctaaaag ctcgatcata gcaatatgat
165601 cagcaaaaaa gttttcaaat actttttcta aggctcctaa atttccattt tgtataattt
165661 ctataaattt ttccttagga gttttattaa acatttcgtc aaaaatatcc attgaatttc
165721 cttaataaat atgtttttga ttataaaatt ttaatcgtta aaagtagcta aattaaaaaa
165781 tgaaaaagtt taaaaattta ttttaagaag caataaattt gggaataaac ccaaatttat
165841 ttaagaatga gctttagttt cttgtgcgta tttaacacct aaatcaaaag ctttagaatt
165901 tgcatctctt gttttagctg gaaccatatg aagcatagtt tctttaagaa tatccaaatc
165961 aatacattta ctcatataag cggcaatagc caatgcaaca actgattgag tagccacatt
166021 acctacttca tctttagcta tagtaatgat aggaatttca aaaatttgcc atttttttata
166081 atcttcgctt tcaggatgaa ccaaattcgg ctctacaact atgataccac cctcttttac
166141 tccaccgcga aagcctttgt aacccttatc tgcagttgaa agcataaagt ctacttcgcc
166201 ctctactgca taaggaaaaa gaatttcttt atcatcaatg ataatatcaa ccttagttgg
166261 acctccacgc acttgggaag tataagttga agctttaaaa gcttgacgac cttctttgat
166321 cgctgcttca gctaaaattt ctcctgcggt gataacacct tgaccaccct caccaccaaa
166381 tcttaattga tatttcattt taaggctcct aaatcaacca ttcttttttc tttggctgca
166441 cgacgcactt cttcataagc atgacaatat tctggctgcg tgctatcttc atataaaaca
166501 cctgttggga atttgtcttt tctttcttca aaatccatac tttcaaattt agacttatcc
166561 acaacacgat ttttaatcca atcaagcata gccactgctt cacccatttt attttttcta
166621 cctaggttaa tatggcagtt tgagaaaaca tcaacaaagc tataaccttt gtgtgctaaa
166681 gctttataaa ttaaattttc taatttattt gcctcaataa catttcctct tgctacaaaa
166741 gaagctccag cagctttagt taattcacaa gcatcaaaat ttggatcaat atttccaaat
166801 tgcgcagtta ctgtataaaa acctttttgga gtagttggag aagtttgaga attagtaagc
166861 ccataaataa agttattaat tacaatatga gttaaatcta tatttctacg acatccatgt
166921 atagtatgat ttccaccaat tgccaaagta tcgccatcac cacttactac aataacatgt

166981 ttgctaggat tggctagttt tatgcctgtc gcataagcaa tagctctacc atgagtagtg
167041 tgcacggtat tgcaatttac ataagagctc attctaccgc tacatcctat acctgaaaca
167101 agacaaacat cgtccatatt ccaacccagc ttttctatcg cacggataat acattttaaa
167161 actacaccat caccacaacc ccaacaccat tgagtaggaa gtttatcagt tcttaaatat
167221 tcatcataat taaatgccat tttatatatt ctcctttact ttagcaatga tttcgctagg
167281 tgttatagga cggccatttg cacgatgtaa gctaataaaa tcacgacgag aacttactct
167341 ttcaatctcc tcaagatatt gtcccatatt taattcactt accattactt ttttaaattt
167401 gcttaccact tcagcaattt tcttttcagc tacaggataa agcgtgatag ggcggaaaag
167461 tcctacctta ataccttctt ctctaagtct attgatagct tctttagcag aacgacttac
167521 gcttccataa gctataatta aaaactcagc atcatcaagc atatactctt cataagtaca
167581 aatatcatct tggttatttt taattttacc tataagtctt tctatgtttt tcttaactat
167641 agcgccatct tctgttggaa aacctatatc tccatgatga agtccagtta catgatagcg
167701 gtagccagta aaaaatggat tgagtgttgc aggttcgttt tcgcccgccg cataaggttt
167761 ataatctttt ttatcgcctg tgaatttttt acggttaata atttctatat cttttaaatc
167821 aggtaatacc gctttaccat tcatatgccc tacagtttca tccataagta aaaacacagg
167881 agtcatgtat ttttcagcta aattaaacgc tcttatggtt tctgtataag cttcttctag
167941 gctcgctggt gcgatagcta cactggcaaa atccccatga gtaggtgctt ttgcttgaaa
168001 caaatctcct tgagccaccc ttgttggaag acctgttgaa ggaccaccac gcataacatt
168061 tacgataaca agtggaattt cagcgataaa cgcaagaccg atttgctctg cttttagtga
168121 aattccagga ccactacttg cagtcattga ttttacaccg ctcatagcag caccgatagt
168181 cacactcaca cctgaaattt catcttccat ttgtataaaa gttccatcat ttgcaggaag
168241 catatggctt aattcatgtg caatttcaga acttggtgta ataggataac ccccaaaaaa
168301 tttacatcca caatcaattg ctgcttttgc aattaaaaca ttacctgttg ctataacttc
168361 tctcattgtc tttcctatgc taattttta tatttattat tttttactgc tacggctcta
168421 tctttagcct ctggagtcag tttggcaaat ttaaattcat ctctttttgc caccataata
168481 gcaaaatctg gacaatgtgt ttcgcactca gtacaaccta tacaggactc agggtgtaca
168541 acctctatca tttgtcccaa aaccgcatga acatcatcgc gcatagctaa aacccctgca
168601 gggcaatagc taacgcagat attgcaagct ttacatctat gctcatctac ccaaacaggg
168661 gtatcttttg gagctgctat actcatatct ttttcctttt ttagcctaat atttttttgta
168721 tttcttcacc gattaaagct ggagaatcca ctacatgaat tccataagac tttaacgctt
168781 ctttttttagc tgcagcactt tcatcagcac tacctactat agctcctgcg tgtcccattc
168841 ttttaccttt tggcgcagtc gctcccgcaa tgaatgctac cacaggtttg ctaatatttt
168901 ctttaataaa ttttgctgct tctacttcta agctaccgcc gatttcccct atcataacaa
168961 tagcttttgt ttcgttatct ttttcaaatt cacttaaaag ttctttataa gcaagtccta
169021 tgataggatc gccaccaatt cctactgctg ttgaaattcc atacccgcct tgaactactt
169081 gattagctgc ttcataagtt aaagttccac tttttgaaat aagccctacg caaccctttt
169141 tgaaaataaa gcctggcata atgcctaatt tgcattcttc agaagtgatg attcctgggc
169201 aatttggtcc tataattttc ataccttttt tatttgcata ctgcttggca aacatcatat
169261 ctttaaccgg agtatgttct gtaattacaa cagcaagctt aatacccgca tccgctgcct
169321 cgattacact atcacccaca gcaaaagcag gaacaaaaat caaacttaca tcagctcccg
169381 tagccttaac tgcatccgca actgtatcaa aaacaggctt gccaagatga gtttgaccac
169441 ccttatgagg agttactcct cctactatat ttgtaccata agctatgcat tgctctgcat
169501 gaaaagttgc ttctttacct gtaaaacctt gaactattac ttttgtattt ttattaacca
169561 atatactcat tttttatcct tatcctacta aactttttac taattctgca ccatttttaa
169621 gattggttgc tgctttgata tttttaagat ttgagctatc taaaatcgct tttgcttcag
169681 ctgcatttgt accatcaaga cgcactacga taggaatatt aacctcaaca tttttagttg
169741 cttctaaaat accatttgca attctatcgc aacgaacaat accaccaaaa atattgataa
169801 atattacttt aacattttta tctcttaaaa taatctcaaa agcttttgcg actgtttcag
169861 cagacgcacc accacccaca tctaagaaat ttgcaggttt tgcaccgcta taattgataa
169921 tatccattgt tgccatagca agtcctgcgc catttaccat acaagcaaca tcaccgtcta
169981 attttacata acttaaacca aattcagccg cttctctttc agcaggattt tcctctgtaa
170041 tatctctaag ttcagcaatc tctggatggc gataaagcgc actgtcatca aaactacatt
170101 ttgcatcaag agcataaaaa tctccttctg cggttttaat taaaggattg atttcaagca
170161 tattcatatc tttatccatg taaagcttat aaagttttgc tatcatagaa ataagttttt

170221 tgctttcatc tttatctagt cccaaaacct ttgctacttc aagaccatgg aacattttaa
170281 agccaatttg tggatcaatg cctacttttg caattttttc agggctttct ttagctactt
170341 tttctatatc cattcctcct tcgcttgagg caatgattgt aatttgctct gccattctgt
170401 taaataaaat agctaaataa tattctttta ctatatttgc accactttct atgtaaagtt
170461 tttgcaccaa tttaccctca ggtcctgttt gatgagtaac caaattcata cctaaaattt
170521 gacttgcata ggcctttacc tcatctaaat ttttagcgat tttaacacca ccgccaagac
170581 cacgaccacc tgcatggatc tgagccttaa cagcccaaac actaccgcct aactctttag
170641 cattttctac agcttcttct acgctaaatg caacctttcc ctttaaagta gggataccat
170701 tgtctgcaaa aatcgctttt gcttgatatt catgtatatt catttatctt cctttatata
170761 ttgatatttt attaatgatt tttttacctt gtctttttct tcgtcgctaa gctcaagctt
170821 cataatctca acaacaccct ctaaacccaa tcttgccata gttcctaagg ctttattttg
170881 cactccaaat tccccatgca aaatcacact cataggcaaa aattctccac ttcttatgga
170941 ttcgagcatt cttacacaag cgcttgcagg tgccaaatat gctgaagtct taagatgttt
171001 aatcaccttt gcaccaccgg ttttaacttc attttccaaa tcttcaaatt cctcttcgct
171061 taaaagctct ccgagttttt gatttttaac acttgcataa gattttacta aaaccatatc
171121 atcattatga aaaccaatta atcttgtatc cacgcttgaa attttggctt ttaacttttt
171181 agctacctca tacttaaatc ttgcattgtc taaaacccct gccatagcaa caattttttt
171241 agaagaaaaa attccgctct cgtaaagagt attgagcaaa aagtccacag gatttgtcaa
171301 aatgataaaa agtggatcag aattaaattc tttaattttt ttagcacaat caagcataat
171361 gtttgcattg agttgcaaaa gctcttctct gctttgacca tcttttcttg caaatcctgc
171421 actaaaaagc acaatatcag aattttttgt acaagaataa tccttggtgc aaattaattc
171481 tatatccaaa tttaaagcag ctatgctttg ggataattct aattcctttg caatcaataa
171541 atcttcattg atatcaacta aaacaatctc attcgccaac tcacgcaaaa ttaaagcata
171601 ggctacactt gaacctacat tccctgcacc gatgattgtg attttcatct ttgtcctatt
171661 ttttctatga tagcattaaa agttgcactt ggacgcataa ttttatttgc tttttcatca
171721 tcaaatttat aataaccgcc taaatcaacc ttcacgcctt gagcgtcatt aaattcagct
171781 ctaatttttt cttcattaga ggaaaattct aaagcaatat ctttaaaaaa gctttgaagc
171841 tccatatcat ttgcttgtct tgctaaatga tttgcaaaat acattgctaa ataaaaatgg
171901 cttgtacgat tatcatcttc ttttactttt cttgatggag ctttattatt ttcaagccat
171961 tctcctatag cttcatcaag acattctgct aagactttag ctttatgatt attgcattta
172021 ttagcataaa attctaagct tgcttgtagg gctaaaaatt cacccaagct atcccaacgc
172081 aaatggtttt cttctactaa ttgttctact tgctttggtg ctgaacccccc tgctccggtt
172141 tcaaacatag ctccaccatt taacattggt acaactgaaa gcatttttgc acttgttcca
172201 agctctaaga taggaaaaag atcggttaaa taatctctta aaacatttcc cgtaatagaa
172261 atggcatctt ttccagcgcg aatcaattct aagcttttta agcaagcttc ataaggagca
172321 aggatagcta tatttttatt tttttcctta agacggtttt gaactaaatc tatcataatt
172381 ttattgctag ctcttttgct atctaaccaa aaaatcgcct cagctcctgt taaatcagct
172441 ctttctatgc ccaagtcaat ccaatttaaa acagcatcaa atttagcttg attagctcta
172501 taaatgtcac cttttctaac tttatgctct aataaaacct taccccttagc aatgatttta
172561 aacactccat cttctttagc aacaaaagtt ttatcatgtg agccgtattc ttgagctttt
172621 ttagccatca agcctacatt agatacgcta cctaattttg caggatttaa agtgccattt
172681 ttatgcaaat cttcaataac tgcttcatag atagtcgcat aagtttgatc agggataact
172741 gcatttgtat ctttttcttt gccttcttta tcccataatc ttgccccatt tttaagcata
172801 gcaggcatag aagcatcaac aataacatcg cttggaacat gcaaattagt aatgccctta
172861 tctgaattta ccatagaaat atcagcacga ctatttaaaa tttcattata tctttttaaa
172921 atctcatctt ttttacttga agtttctacc ttgcttaaaa gctcgcttaa tccattgttt
172981 ggattaattc caagtgtttc aaattcatct ttaaattcat caaataattc tttaaaaaag
173041 atttttaccg cataaccaaa aagtatagga tcgcttacct tcatcattgt agcttttaaa
173101 tgaagagaaa aaagcaaatc attatccttg cttgctttga tttcttgagc ataaaaatct
173161 tgcaattttt ctacatccat aaaggttgca tctaaaattt cattttttttc aagctttaag
173221 ccttctttta aaatttcact tttgccacta tctgcgacaa attcaatact tgctacacaa
173281 tcctcatcga ttaaaaccgc tttttcattt gaaaagaaat caccttcttt catataagat
173341 acacgagttt tagaattagg attaaactca accacgcgat aaggattatt cttcgcataa
173401 tctttaaccg ctttagtaga acggcgatct gaattgcctt gtctaagcac aggattaacc

173461 gctgaaccta aaacctttttg atattttgtt tttatttgaa attcttcatc gttttttggt
173521 tcgtctgggt agtttggtaa catataaccc ttttcctgca attctttaat cgcagctttc
173581 agctgtggaa tagaagctga aatatttgga gtcttaatta aatttgcatc tgattttttt
173641 acgagctcac ccaaaagctc caaagcatct tcgcatcttt gatcttcttt tagatactca
173701 ctaaaagtag ctaaaattct tccagataac gaaatatcag aagtttttac tcctatatgc
173761 gccttgctta aaaaagcttt tactataggt aaaaacgaat aggtcgcaag cgctggagac
173821 tcgtctgtca aagtgtaagt aacttgcatt ttctgtcctt ttaaacacta attttatttt
173881 tcaatctaaa gtatattaca attaaaaaaa aatgcaagag taaatttaaa gtttgtttta
173941 gttttaaaac aaactttaaa tacttttaat taaatagatt agaaggaagt tgtatgatat
174001 ttttaatcaa gttaaaagga gtttttaaag tatcacttaa aatttgggta tgaaattttg
174061 gattatccaa agcgccatcg acttttatat tagtacttat ttcttgattt ttccctaaaa
174121 taacataatt taaaatcgga acctttgaaa tagtttcaga agctgatttt aaagttttta
174181 attccaaatc tacatcaacg gtatttaatc ttaagtttgc gctacctaaa ccatacaaat
174241 ccatactatc tccatttaaa ttaatcgctt caaaactaag taaatctttc ttacgattaa
174301 aaacaaccct tccatcatgc aaacttaaac ctttttcatt aaaagtagga gttttaaaca
174361 tcaaaagact tggaaccgta tcgataaaag atataagctg attaacccct ttaaggtccc
174421 ttataaaagt atctttaaaa ttaaattccc cctcaaaata atctgtgctg ctgcctacca
174481 cactaaaatt aaaaacaccc tcctgcacgg ctcgcttttg taaaaattca ttaagatgct
174541 catcgtttat attgttgaca aataatttca aatcattagg actataatga agatcaaaat
174601 ttgcattctt gcgagtagcc ctagcatcta agatatcgct ctttaaattt gcctctagtt
174661 tatcaaattt taaagtttta ttaaaatctg ctaaaatcaa agcaagattt tctccattga
174721 gtataatatt ttgagtattc tttgctatat caaacgaaga agcggaagca tttttatctt
174781 tttgataagt ataagtcaaa tttttaagat gtatggtttt attattatca ataatcttgg
174841 cactgaccaa accactttgt gtatttatgt ctaaaacacc atttttacgg acaataccaa
174901 agctatcatt ttcataaggt ttattatcga ctaacaagtt gtttttaaaa tgagcatcct
174961 gtatttgtgc attaaagtca ttaaaatcta tacttttata ataaatactt tttgcactca
175021 taaaaccgaa attttttaat aaaggcgaat gaggtattaa aacactagaa ttattggcat
175081 atacttcaag tccttcttta aaatttaaag tcaaatccca ttctggcata ctaagctgca
175141 aatcatcttt atagtctaaa tttatcactg tttttttgatt tctcatatca aaaagctcac
175201 cattgtcaaa atacaatctt gaaatttgag tatcaaaaat acccttgtgg ttggctagat
175261 cgattttagc attaaaattt gcttccaaaa actcattttt tacacttgca ttttctatgc
175321 tcaagtcatt ttgattcagt ttaacaaaag ctttagccac attaaaattt gccaatgtaa
175381 gctccgcatt ttctaaagac aaagtgccat tgtattttaa atcacctttt tcattaaaat
175441 cgattataag ctctaaatca cttccaagct ttccactttt ttgataaaaa ggtaaattta
175501 aattataatt ttttaatgcc ttggcaagct tttcatcaaa ttttaaattt ttagatttga
175561 tacgcaaata aattccatgt tttttattgt caaataaatc atataaaaac accttacttt
175621 cacttaagtc tgattcatta taacttgcct tattgaaagt aaaatttaat ttttgattgc
175681 ttaaatttaa atcaagtttt ggaaaattaa tagcattcat ttgattgtct aaacgaactt
175741 ttacattatt tgcataaccc catgcactga tatcatcaaa ataataatta tttttagata
175801 aatcaacaaa accttgaata aagtcaaaat gatagaagtc ccctttagct ctatgtgcaa
175861 cccaaagaac taaaggttca ggaagtgtta ttcttttttc tgcttgattg aaaatagtag
175921 taatatctct aatgttaata tcttcgaatt tataagctaa gttttgattt ttataagaaa
175981 tatttacttt aaaatcaatt aaatcgccac ttgcttggcc tttaaaatta taaaattcac
176041 ttcttgcatt aatacttaaa tttccatcta cacttaaatt ataatcttta agccaaagat
176101 ttttgatgtc agcattgatt tcttgcccct ctctgtgtaa ggcaagtttt aaaaacaata
176161 aatcattatc tacaaaaaat tcatcatttt taaacaatat acgcatatga ttatctttaa
176221 tattgagatt ttgtatgtct atttcctcaa caaaagtata aagatatttt aaattcttag
176281 taatctttaa caactcctta gaagcaaaat cagaattttc attcttatca tcttgaatgc
176341 ttgcattagt atcttcatta aatgtaatat tttttgctcg cacaatgagt tttttatcta
176401 atttaatata taattgctcc aaatttaaaa aacttaattg gatattagat atgagaatac
176461 cattcttaag aactaaaaat atagccgaga taaatacaat taaaacaagt aatacatata
176521 caattttctt tttcattaga aacttctttt tgatatttat tttaggaatt ttttattatt
176581 taacacaacc tttaaaaagc aattctgttg tttttttgcc tcaaggttct attgctcaaa
176641 ttataacaca tctaaagcaa aataaatacg aaatgaactc gattgataaa tatatcttgt

176701 ttttcttagg ccatcctcaa tcaggttgga taaatatagg cacaaaagaa ctcaatagga
176761 tagaattttt acacaaactc actatagcca aagcagcatt agaaactctt actctaatcc
176821 ctggagaaac cagtgtaatt tttttagaac aagctgcaca acaattagaa cttgataaaa
176881 acacgctttt agctgaattt gaaaaacaag ccacatataa agaaggagtt tttcttcctg
176941 aaacctacaa aattccaaaa ggaatcacag aagcactttt aattcaagtt ttactcaaac
177001 atgcagaaaa tacaaataga aaaacttcgg aaaaaatttt tggcgaatac aatgctaaaa
177061 aatggcacca atatatcata acagcgtcag tcattcaaaa agaagcggcc aatgaaagcg
177121 aaatgcctat agttgcaagc gttatttata atcgtatcaa aaaaggtatg aaacttcaaa
177181 tggatgggac tttaaactat ggaatttatt ctcatgcaaa aatcactcct caaagaataa
177241 gacaagataa tagttcctat aatacttata aatttgaagg tttgcctaaa gaagcggttt
177301 gcaatgtttc tctatctgca attcgtgctg ctatctttcc tgcaaaaact gattatttat
177361 attttgtaag agataaagcc acaggagcgc atatttttag caccaatctt aacgatcaca
177421 acaaggctat taatttgcaa aaaacaaata attgaaattg taaaaattta tgcaagttgg
177481 aacagtattt gcttgttaat ttttgaaatt ataaaaagga taaaaaatga ttaacccttt
177541 caaatcaaaa gaacttatca caagtgcttt agcgggtaga aatttaagaa atcaactcat
177601 caatgcaaac cttgcaaatg ttgacactcc tttttataaa gcaagagata ttgaatttga
177661 aactgctttg gtaaatcgtg caaatgaaat ctttaaaaaa aatgacaata aagaattgca
177721 attagcaata accgaagaag gacatcaaaa accttggaaa tttccagatc ctagcaaatc
177781 tactatttat ctaagagatg gacatttagc aagaaatgat gctaacactg tagatttaga
177841 tgttgagact actgagatga gtaaaaatac agtgatgatt acagctcttg atggggtctt
177901 aagaagacaa agtaatattt ttagctctat acttgatgca agttctaaat taagttaaag
177961 gtaaattatg gcatacttaa gtgattttga tattagcgga tatggtttaa gtgctcaacg
178021 ctttagaatg aatgttatta gctccaatat agctaatgca aatacaacaa gaacagctga
178081 aggcggacct tatagaagac gcgaagtgat ctttaaagca acagattttg acaagctttt
178141 aaatgagcaa atcaataaag acaataactt tttaaaatat gaaaatcctt taaatgatcc
178201 aagctcgcct gaagaggcaa agcctgctat acaaagtgtt gttgtagata aagtcgtaag
178261 agatgataaa gattttcgta tgaaatacga tccctctcat cctgatgcta atgcgcaagg
178321 ctatgtagca tatccaaatg taaatcctgt catcgaaatg gcagatttaa ttgaagcaac
178381 aagagcttat caagctaatg ttagtgcttt tacaagtgca aagaccatag ctcaaagcgc
178441 gattgattta ttaagaggat aataaaatat gaataatatt aatgatttaa gattaaataa
178501 caacatttca aatacaaaca aaagccaaaa ctctagtgat agaatcggtg atgaatttgc
178561 caaaatgctt aaaaatgaaa ttgaagattt agataaaact caaaaaactg cagaagctgc
178621 gatgacggat attgcaacag gacaagtaaa agatttacac caagcagcta ttgctattac
178681 aaaagctgaa agcagtatga aatttatgct agaagtccga aataaagcta tcagtgctta
178741 taaagaaatc acaagaacac aaatttaact tatatgcaag agcataagaa aaatcgcgtt
178801 tcaaaagtcg cctttgctta ctgcatggca cttttttta tgattatttt tcttagctct
178861 accttttttc tcacttcaaa acgccatatc cctaataccg aaaaagatca atactctatt
178921 gccttgcgcg gaaacatcat cactaaagat aattttacta tcacaagctc gagacaaatt
178981 tatcgcgcag aaattgattt aagaagtatc aataaagata aatttgattt atttttaaaa
179041 ctttttcaaa tatatagcgg tattagcgat agtgaaattg cagatattaa aaaaagaatc
179101 caaaaacaaa aaaaacgatc ctatcatttt atcctttctc aaaatttaga ttctaaacaa
179161 gcaagttatc ttaaagatct tgcaaaaaag ctttatatac aaggattttt taaagcattt
179221 accaataact taggaaaagc cgaaacaagg ggcttgagta tcatagaaca cgaagaagat
179281 agaatttata tgtccaaaga tgccttaact ccagccatag gttatacaaa aatggtttta
179341 gatcctcaaa gtggaatttt aaaaaatgtt ggagtaaaag ggcttgaaaa atactacgat
179401 gcttgtctaa atccgctcca aaacgagaaa atccaaggtt taaaagatat aggtggaaat
179461 ataatcttaa atttaaattc attacagcaa aaaaagattg atggttgtct tttatatctt
179521 aatatatcct taaaacttca aaaaagcatt gaaaaagcga ttgatggaag aaatgaagat
179581 ttaaaagcta atgaaatcat agtcggagtc atggatagta aaacaggtca aattttagct
179641 cttgcaagct ctagacgtta tgatccgcaa aatcgcggca aagatttatc tgttttaaat
179701 gcaagcgcca tagagtatgg atacgaagca ggatcggtta tcaagccttt tatttttaca
179761 actgctctaa gacttggcaa actcaagacg gatgaagtga ttaataccta tggcggagcc
179821 tataaactgg gtcgttttac cattagagat gatcataaaa tggatcaaat gactatggaa
179881 gaggtaatta ggtattcttc taatataggg atgatacaaa ttgccaaaag attgagcaat

179941 cttgaaatca ttgctgggct taaaattttt aaatttggtg aaaagagtgg gattgatttg
180001 ccttacgaac aaaaaggaga aattcctaat tccaagcgtt taagagatat agaaaagtcg
180061 gtattaagtt atggatatgg tttaaaaaca acctttatac aacttcttgc tgcctataat
180121 gtttttaata acaatggggt ttatataact ccacatttgg ctgaaaaatt ctatcaagat
180181 ggacgctttg taagtcttga agaggatatc aaaaaagaag ttatcttaac cccgcaagca
180241 gcacaaacca tgcaaaaaat tcttattgat gttatagaaa aaggtacagg aaaaaaagcc
180301 atcactcaag gcataatcat aggaggcaaa acaggaaccg cgcgtattgc agaaaggcaa
180361 ggctatactt ctaatcgcta caatgcttct tttttcggtt ttgctaatga tgcaaaacat
180421 aattatacca taggagtttt agtaagaaat cctactaaac cttatagtta ttatgctgcg
180481 caaagtgctt tgcctatgtt taaagatgtt gtagatattt taatcaatga ggatttttta
180541 acccctgtca ttgtagaaaa taaccaaact cttaattaat atcatcccaa agcacctttt
180601 ctcttgcaaa aatgatttca acagcatcta tgagatcttt agcgatctta gcgataaaac
180661 ttgtatcatt gattatggat atgccctctg cggtagtgat ttttctaacc aaaataagat
180721 gttcgacatt attaagagtt aaaccatcaa tttcttttaa aatttcctta cctttttgaa
180781 gatttttaat aattaaatac attttttcat catcaacagc cctaagctct tgaatacttc
180841 ttaataattc tcctaaatta gatcttattt tattgtattc taaggccaaa tctttattgt
180901 tgcaattaga gtattttttg atattttctt gaactaattt taaattttta gttgcctctg
180961 taagattttt agcagcgatt ttaaaagtaa aaattctatg atttttaagc tcatcatcta
181021 tatgaatttg tgctttggtt gaaaaatcga tgatcgcttc aaaaagcact ttgattctag
181081 tttgatacaa ataatccaaa tccacatttc tgctgaacca tttttatttt tgtaaaattt
181141 cttcaaaact tttattgctt tgtatatcct tgcgacttaa acctatagca tgagcgataa
181201 tcgcatacgc attatcataa agatgctcgc tttcttttcg caaggcttcg atagctgtat
181261 cgctaaattt caataaatta gaatcaagat ataaaggtcg atccttgttt ttatccttag
181321 acgctttaac gctcttgttt aaaaaattta ccaattgcgg gataaacaat acaaaaacaa
181381 atattcccaa aagattaaac aaggtatgaa ataaagccat tttcaaagct aaattttcta
181441 tattcaaaat tcctgctatc ccatttaaaa aatcaataaa ataaggaaaa atagctatga
181501 ttataattgc aatagtaaaa ttaaaaatac aatttgcaaa agcaagcttc ttaccttcaa
181561 cattagtgct taaagaagct agcacagctg ttaccacacc tcctacgcta gtgcctaaag
181621 tcgctgctaa agaattttcg aaactgattt gtcctgccaa taaggctgcg actataatgg
181681 ctattgtcgc tgtgcttgat tgaataatac ctgtaagcaa agccccaagt cctacaaaaa
181741 tcagcactcc cttaaaacct gtaaaatcaa aataagacaa atctatagca tcttgaaatt
181801 cattaaaacc atttttata taatccactc ccaaaaagaa aaaacctata cctataaaaa
181861 tattcccaaa actttttata atattgtctt tttgaaagaa aaataaaact ccaatcacaa
181921 gcaagggtat agccaagata gaaatattaa cccttgtaag ccaaacaatc agccaagaac
181981 tcgcagtatt tcctaaattt gcaccaaaaa taatccccac accagcggtc aaagatataa
182041 gtcctgcact taaaaaagaa agggaaataa ttgaaactaa agtcgaagat tgcatgataa
182101 gcgtactaat agcaccaaaa agtatgcttt taaatttcgt atttgttgag cgagtaagga
182161 ttctttcaag taaaccgcca ctaaaagatt taaaccctaa acttaaattt gtcattccta
182221 taagcaagat tgcaactcca gctaataagt tggcaaattc actaaagcga attaaagcaa
182281 agatcaaaat caaggtaaaa ataccgcaaa aacttatctt gagatatttt aaaaagtttt
182341 tgtcttcttg caccctatcc tctaagcttt tcaaattcac tcattaaatt ttctcttaaa
182401 ttttcagcct cttcttcgcc attttccaaa tactcaagca ataaattttc aagctctttt
182461 aaaaaatcaa aaagctcatt ttgccataca ctatcacttt tattacttaa attcattgtt
182521 tgtataaagg taagctctct gcttaaatcc cctaattttt ccaataattc atcttttcta
182581 tcttgatctt ttaaggcaga actattattt tgaattctat ccttaaattc tctaattctt
182641 tcatcaataa tatcacaaaa tttaggataa atatctgatt ttataggctc aagcagtgct
182701 ttttcatcta tatttaaagt aagtttttta agtgcaaaat aaaggagtaa aaaagataat
182761 gctgctatta acaaataaag tcctatcata tccaaccttt taatgccaac atcccaccat
182821 ctaaatttac actttctaaa ccaagttcat ttttaataaa attggcagcc atcatacttc
182881 tatgacctcc acgacaaata aaagcaagtt ttttattttt atcccttctt gtttgaaatt
182941 cttctaaaaa attagcattt aaaagtccgt gattatcata taaagctaca cattcagcat
183001 ggggtaaaac accctcttcc cattcagctg gagtgcgaac atcaaaaatt tgatattcgc
183061 ttaaatcctg cttcatccaa acacttgctg ggagatttgt aatcatgaga aagccttagt
183121 aatgattttt gtaagacttg cattaaattc agccggatta tccacgccca taccttcgct

183181 aagttttgcc atgtttaaaa ccaaagttgc tatatcggca ctaaaagttt cattattttt
183241 aaggcctgta aaaattgcgt gctttggatt gatttccaaa ataggtttaa agttttgctc
183301 ttgccccatt tgttttaaaa gttgctgcat tgcaaaatca ggtttatttt tatcataaac
183361 aatacaactt ggactatctt ttaaacgact tgtcagtctc acatcttcaa cttgatcttt
183421 taaaagctct ttaaatttag ccactaatga agcaaattca tttttttctt catcacttaa
183481 ttcattttta tcttcaactt gattaatagc tacaaatttc aagccctcat attctccaag
183541 cataggagta accaaagaat cgatctcatc atccatgagt aatacttcta tatttttttg
183601 cttatattct tcaagcaaag gtgaatttct aagcaagctt tcattgcttc caatgatata
183661 aaaaatttct ttttgttcgc cttgtatatc gtttttgtat tcttctaaag agcgtaagct
183721 ttcgcctttg gtgcttttat aaagcatgag ttttaaaaga ctgtcttttt cgccaccaaa
183781 accataaagt ccttctttta ataccttgcc aaaattctta aaaaagctta aatatttttc
183841 tttatcttta tttttaagtt tttcaagctc gctaagtatt tttttaacac tggcttcttt
183901 tacacctttt aaaatttgat tttcttgtaa aatttcacgg cttacattaa gtggtaaatc
183961 ctccacatcg ataattccgc gtacaaaacg caaataagtc ggtaaaagct ctttatcgtc
184021 atcactgata aatacacgct ttacataaag ttttaaaccg ctttgataat ccacacgata
184081 aagatcaaaa ggtgcatttt gcgggataaa aaataaagaa ttgtattcta attttccttc
184141 gcttttggtg tgaagataaa gcaagggttt attagaatcg tgaaaatttt gctcataaaa
184201 tctttcataa tcctctgctt taagacttga tttttgcatt ctccatagag cattagcttt
184261 gttaatctga gaaattttaa gctcagtttt tccttcttcc tcgccctctt tagcagggat
184321 aaattcttct ttttccataa aaattggaaa ttgaatatga tttgaatatt tttctataat
184381 gctttcaatt ttataagaat ttgcaaattc gtcatctttt aaatataaag tgatacttgt
184441 cccttgctct tctttagtcg catcttcaat ctcatagcca tttgcatcag aaatccaaag
184501 ataggcttta tcatctaaag ctttttttgct taaaacttcg attttgctag ctaccataaa
184561 tgcagaataa aaacctacgc caaattgtcc tataagttga gaatcttttt ttgcatcacc
184621 acttaaattc tctaaaaagc tttttgtacc actttttgct atggttccaa gattgttaat
184681 aaggtcatct ttatccatac ctataccatt atcactaata gtaagggttt ttttatcttt
184741 accgatttta atatctattc taggttcaaa ttttaaactt ttatatttat catcactcac
184801 gcttaaaaaa ttaagtttat ctaaagcatc acttgaattt gatacaagtt ctcttaaaaa
184861 tatttccttg tttgagtata aggaatgaat catcaattgt aaaagttgat ttacttcagt
184921 ttgaaactgc atttttttctc ctttataaat tttgaaacag atattttagc aaaaaataca
184981 aactctaagg taaatttcaa gagcttactt aaaaattata aaaccaagcc aaactgctat
185041 gagacaaaga attatattga ataaaatatt taaagaaaaa ttaaaataat ttccactttg
185101 taaaagcagt aaattttcat aagaaaaagt agaaaaagtc gtaaaagccc ctaaaaatcc
185161 tgtacttaca aagcttttaa tggcgggaga taaaccctta tgatgcgcat aagaaaataa
185221 aagccccatc ataaaagatc ctaagatatt aacaaaaaga gtaccaaagc ttatagaatg
185281 aggtaaaatt ttatttatca aattgataga aagcattctt aaaactgccc ctataaaacc
185341 cccaaaacca acaactaaaa tagtatttag caaagcttag ctctttcttg atctagcaat
185401 tcttgcattt tttttctagt attttcaagc catttatcat cactcgtatc cactaaatcc
185461 atgcagatga ttttcaacac tccagaacta gcactaaaac ttttggtatc taaaattttc
185521 gcagaatcga caattaaaat aggctgcacc tttaagtcaa gcttatcgct taaaacctta
185581 gcaccacttt gaaatttaag caatttttcg tttctagaac gcgttccctc aggaaaaatt
185641 gccaaaactc tatcttcacc caatctttgc ttagcttctt ttaaaacacg caccaaatcg
185701 cgtggatttt ttctatctat acaaagaagc ttgggttttt taatggcgat tttaaaaaga
185761 ggaatttcac ccaattcttt tttagcaatc cagcataaat ttttaggata caaatcctct
185821 aaagctataa tgtctaaagt gctttgatga ttcattaaaa tcatatttgc actaagatta
185881 aattctccta tgacttcata tttgcaaaaa agaatatttt tttgaaatct agcccaagat
185941 tttcgaagtt tccaaagagt atcttgggac tttataaaac aaaagcataa cacaacaaaa
186001 gctacgctta taataaaaaa taaccaaaaa tataaagctt taattttttt gtaaatcact
186061 actttcaacc catcctatcc taccatcttg aaaaagcact ttgacataat gaagtctttt
186121 gcctaacacc tcaacctttt cgttagaatt tgctgtataa aaataagttg atgattcagt
186181 tggtaaaatt ttagctctag aacccgcttt taaaattgca ttttctgtgt tagtatctac
186241 aagaaaacca agtataaaac ataaaagcgc tcctgctaaa ataaaataat ttttcttcca
186301 aacaaaccat aatgccaaaa taagagttaa agcccaaaga gcatattgtt tataaatatt
186361 aaaatcccta tttgtaggat ttaaatcgct ttgagtgctt acctcatcat cgctgatttt

186421 aagcttaaag ctcatatttt ccattttttt agtgtctttg ttaaaataag aaaattcaaa
186481 atctgtttta gtcgatggaa aaatagcata ataaaaagca ttagaagaat taaaatcacc
186541 ctttaaattt tcaataccct gtttctcgac tccatttaca aaaaaactct taagatttgt
186601 atttgtagca cttaattcaa tcattgtaat aatatttgaa tcatcaaaat aactggtttt
186661 aattttttta acttccaaag aacttgccac aagatgtgaa aagtttttat tattgggcgt
186721 attttcaaac tggatagcac ctatactaat actagcactt tgaaaaactt ttccatttct
186781 taaaagactg actgaaatct gctccaaatt tgcatttgaa gttttagcct caaaccacaa
186841 aacagcccta taatcgcctt taatgtactc ccattttaaa tcaggattta aaaaaaccaa
186901 gtccttgctt ttttgcaaag tgatattaaa atcaaaattt gtatcttctg tagttttggc
186961 atgaaggatt atgggaaacg cctctccaac atatactttg ttgacataat catcagtagt
187021 aagaactaaa gagctttggg gtataaaagg atcatatata ttactatcaa aatcggaatt
187081 ttcatcactt ggagcgatat tttgataaat ttgctgctcc tcttttggaa gtttttgtag
187141 ttcttttttgc atattttcat cttgaccatc aaaaacagaa atttcacttc gagcaaaaag
187201 actaaaacaa acaaaaacta gaaaaagtat tattctcaca taaagccttc aagcattctt
187261 aagcctatat cattacctaa aattttatcg caagcacgct ctggatgagg cataagacca
187321 aaaatttttt tattttcatc acaaattcct gcgatatcta acacagagcc attaggattt
187381 ggttcatatt ttaaaaggat taaatcctta tcttgcattt ttttcaaagt ggcttcatct
187441 gcataataat ttccttcacc atgggcaata ggaagattga tgatttcatc tttcttaaaa
187501 tttttttaaga aaacattatt attcgaaaca actcttaaat tttgattttt tgaaataaaa
187561 cttaaattat tattatgctt catggcacct tctaaaagtc cgctttcaag caaaatttga
187621 aaaccattgc aaattcctaa aatatatcca cctcttttcg catgtgcaat aacgccttgc
187681 atagcaggag caattttagc aattgctgca catcttaaat aatctccata agaaaatcct
187741 ccaggtaaaa ccactaagtc tgcattaatt tctttttctt cgtgccaaac gacttgcgct
187801 tttacgccga ttttttcaaa agcatattgg gtatcgaatt cacagtttgt tccaggaaat
187861 ctaataatag caactttcat catttctcac ttacaataag ctcataatct tcgattacac
187921 tatttaccaa aagctcttca cacatgcttt ttacgcgctc ttttgctttt tgttcattac
187981 tctcatctaa atcaagtata atttgtttag ctactcttgc ttcttttact tcgttaaaat
188041 ttaaagaatg taaagccttt tctatagctt ttccttgggg atctaaaaca ccgtttttca
188101 aaaaaacatt aacaactatt ttcattatat tccttagttt aaaattcttt ttaaaacctc
188161 ttcataggcc attttaacat ttcctaaatc ttgacgaaat ctgtctttat ctagcttttc
188221 attgctaaat ttatcccaaa atctacagct atcaggactg atttcatctg ctaaaacgag
188281 ctcgttatct ttagttaaac ccaactcaat tttaaaatca ataagtctta aatttttagc
188341 atcaaaaaaa ggacttaaga tcgcattaat ctttttagcc atttctttaa tttctttaat
188401 ctgtgcctca ttttgtacta aatttaaaat caaacaatgc tcatcattaa taaaaggatc
188461 tcctagctca tcatttttaa gacaaaattc caccaaaaca aaaggtaaaa ccgtgccatc
188521 tttaataccc aatctttttg taagcgatcc tgttgcaaca ttgcgtacta taacctcaat
188581 aggtataatt ttacattttt ttatcacttg ctcattgtcg ttaatggttt caaccaaatg
188641 ggtttttatg ccattttttt caagtaaatg aaaaagctcg gtgctgattt tgcaatttaa
188701 tgcgcccttt ccgctttcac ttcctctttt ttcggcatta aaagctgtta aatcatcttt
188761 aaattcactt ataagaaaat tttcatcttc ggtagcatag agtttttttac ccttgccttc
188821 ataaagtaat tcttttttttt tcataattat tgtccttgtt taatatttaa gattttgata
188881 gtatctatgg cagattttaa ctgcgcatca tcattaattt gcttttgagt aataatattc
188941 ttatcatttt tttgttcttt cttgtcttct tttttctcat ttttatcaag tttttctagc
189001 tcactctcta aatgctgctt taaatcgctt tctttgatat tgaaactatc ttcttgagta
189061 ttgacttttc ctggaaacac ttcaatatcg ggttttacac ccactgcttg aatagtgcgt
189121 ccacttggca aataatatct tgcaatagta agtcttaaag cttcagtttc attaatcgga
189181 ataatttgct gaacactgcc ttttccaaaa gtattttctc ctacaattat agctcgctta
189241 agatcttgca aagccccgct aacaatctca ctagcactcg cacttcctcc atttacaagc
189301 accactaaag aactatcaga aattttattt tttggatttg ctttatactc ttgattttca
189361 cttgtaacgc gtcctttttg agaaacaatc actcctttat ctacgaacaa attaacaagt
189421 ccagtggctt gatttaaaag gccgccagga ttatttctta aatccaaaat tacacccttt
189481 acatcaggat gtttttttaag ctctttgctt gtcatatcta caacattttt atcaaaattt
189541 gtaactctta aataaagtat attttcattt tcaataagct ttgcataaac actttctact
189601 ttgataatat cacgcgttaa agcaacatca aaaggcttag ttgctccttt tctaaatata

189661 gtaagagtga tttgtgtctt tggttttcca cgcattttat ccactgcatc atttaagttt
189721 attcccaaag tagcttcatc attgattttt aatataatat cacctgattt aatccctgct
189781 ttatccgctg gagttccttc tataggagag ataacagtca aggcgccatc tttcatacct
189841 acagtgatcc caagcccacc aaattcacca cttgtttgaa ttttcatatc gttaaaatct
189901 ttttcattta aaaaagagga atgtgcatca agattactca gaagcccaga aagtgactta
189961 tcaatcaaat ctgtaatatt ttcatcatct acataatact gctcaactat agctaaagtt
190021 ttagtcagtt tatccaaaga ctctaagcgt tgttgcatta aatcagattg attctttggt
190081 tttgcttgta ggttgcttgc taaaaaaaca caaagagcca aagagcctgt aaagcctgta
190141 atagcggcta gaaatctttt actactcaaa atgaactcca aaattaaata ttttaagccc
190201 aaattttaat aactttttgct taaaaattcg taaataatac taaatttgtt ataagtttaa
190261 tggatttaaa aaaattaatg ctaaaatatt ttatattttt taattttaaa gcaagaaaga
190321 atatataatg aaacacactc atacacattc tccaaaacat atcaaagcta taagtaatcg
190381 cctaagtcgt accataggac atttagaagc tattaaaaaa atggttgaaa gagatgagga
190441 ttgtagtgat attcttatcc aacttgctgc ggtcaaagca gcggttaaca ataccgccaa
190501 agccatactt aaagaacatt tagcccattg tatgtatcat gcagtgatgg aaaaagatac
190561 acaaagtata gaagatttaa acaaagctat tgatatgttt gtcaagtagc cttttttttaa
190621 tactctgaat ttaatacttt caaataaata cttaatttta ttgcaaaaat attataaaat
190681 aacttgacat tatttgacta aagttatata atgctatata tataaaactt gaaaaaggat
190741 atacaatggc aaatatacaa gaatttttaa ccgacaatat gctttcaaat cttgaaagcg
190801 cggcatcttt agcaattcat tctaaaaata atgaagttgt ccctttgcat cttttatggg
190861 ctttaattgt agacagttca agtattttaa atcaaatttg taataaattt aatatttcaa
190921 aagaagcttt agagcttgaa atcaaaagca aaatttcaaa tcttgcaaca agctctaatg
190981 taaatcgtga aaatatgcgt ttttctaatg aatttatcaa ttctttagaa agtgctaaag
191041 gactcatgag tgccaatggt gataactatt taagtgtaga tacttggctt ataagtgaaa
191101 gtgaaaaaaa ccctattaga gaaattttag ctaaatttat agatattaaa gaatttcaaa
191161 aagaactcca aaatctaaga gcgggacgca aaatcgaaag caaaacaagt gatgaaacct
191221 tagatagctt aaataaattt ggtatagatt taaccgcaaa agcaagtgaa ggaaagcttg
191281 atcctgttat aggcagagaa gaagaaatcg aacgcttgat gcaaattctt atacgcaaaa
191341 cgaaaaacaa tcctatactt ttaggtgaac caggagtagg gaaaacagca atcgtagaag
191401 ctttagcaca aagaattatc aaaaaagatg taccaaaatc tttacaaaat aaaaaagtca
191461 tagcacttga tatgagtgca ttgatcgcag gggcaaaata tcgtggagaa tttgaagata
191521 gattaaaagc ggtggtaaat gaggttatta aaagtgaaaa tatcatactt tttatcgatg
191581 aaattcacac catagtaggc gcaggagcaa gcgaaggaag tatggatgcg gcaaatattt
191641 taaagccagc acttgcaaga ggcgaactac acaccatagg agcaaccact cttaaagaat
191701 atcgcaaata ctttgaaaaa gatgcagcct tgcaacgccg ttttcaaccc gtaaatgtag
191761 gcgaacctag tgtaaatgaa gctttagcaa tgctaagagg tattaaagaa aaacttgaaa
191821 ttcatcacaa tgtcaccata aatgatagcg ctttagtagc ggcagctaag ctttcaaaac
191881 gctatatagc cgatcgtttt ttacccgata aagcaattga tttaatcgat gaagctgcgg
191941 ctgaacttaa aatgcaaatt gaaagcgaac caagctcttt aagaaaagtg cgcaaagaca
192001 tagaaacctt agaagtagaa aatgaagctt taaaaatgga aaatgatgaa aaaaatcaaa
192061 aaagactaga tgaaatttcc aaggagctag caaatttaaa agaaaaacaa agtgccttaa
192121 actctcaatt tgaaaatgaa aaagcagtct ttgatagcat aagtgcaaag aaaaaagaaa
192181 ttgatagtct aaaaaatgaa gccgtttttg caaaaaataa aggtgaattc caaaaggctg
192241 cagaattaga atatggcaaa attccagaat gcgaaaaaga agtggcaaat ttggaagaaa
192301 aatggaaaaa aatgagtgaa aatggagtct tacttaaaaa tcaagttgat gaagatttgg
192361 ttgctggaat tttaagtaaa tggacaggaa ttagcgtgca aaaaatgctg acttcagaaa
192421 aacaaaaatt tttagaagta gaaaaacacc taaaagaaag cgtaattgga caagataaag
192481 ctctaagtgc tttagcaagg gctattaaac gcaataaagc aggattaaat gcagacaaca
192541 agcctatagg aagcttttta tttttaggac ctacaggagt aggaaaaaca caatctgcta
192601 aggctttggc gaaattttta tttgatgatg aaaaagcaat gattcgcttt gatatgagtg
192661 aatttatgga aaaacatagt gtttcaagac tcttaggagc acctcctggc tatataggac
192721 atgaagaggg tggcgagctt actgaagcgg tgcgtagaaa gccttatagc gtgcttttat
192781 ttgatgaagt cgaaaaagct cataaagatg tatttaatgt gcttttagga attttagatg
192841 atggtagagc aaccgatagc aaaggtgtaa ccgtggattt taaaaatacc attatcattt

192901 taacttccaa tatcgcctca agtgctatca tgaatttaag tggaaaagaa caagaagatg
192961 cggtaaaaaa tgagttaaaa aatttcttca agcctgaatt tttaaatcgc ttagacgata
193021 tcatcacctt taatccacta ggaaaagatg aggcatatga gatagttaag cttttattta
193081 aagacttgca aaaaagtttg gaaaataaag gcataaaagc aagtcttagc gaaaatgctg
193141 cacttttaat cgctaaggac ggatttgatc ctgactttgg tgctagacct ttaagaaggg
193201 ctatttatga tttaatcgaa gacaaactaa gcgatatgat acttgcagat aagcttgatg
193261 aaaacgatga gatcatcatc gatgcaaaaa atgatgagat tgttatcgaa aaagtttgac
193321 tctttttaga agaaatagac ttttctattt cttcgcatac tttttaaaat aaaatctcac
193381 tttcgttaaa tttcatattt gctcttggta gaggcgattt gatagtataa taagcatttt
193441 tccccttgta gcttcctcta tataccccat ttggaatact gaattttctt gtagtatcag
193501 gaaatttatc taagtattta tttaaaaatt ctctaaatac aggagcagag gtcctagccc
193561 caccctctgt ataacgcata ggcttattat tgtcatttcc ataccaaatc aaagcttcaa
193621 tctcaggcgt taagccacaa aaccaagcat cgatattttt gtttgtggtt cccgtttttc
193681 ctgctatttc tataccttca acccttgcat tgcgccctgt gccatgctct acaacatttc
193741 tcatcatatc taaaaccaaa aagctttgtg cttcatcgct cactctgtat tcatttgagt
193801 taaattccat aataatttga tcatttctat cttgcacttg cctaataatt tgtggctctt
193861 ttataactcc gtaatttcca aacatagtat aaaatttaga atactccaaa ggagaaattc
193921 caaaacttcc taaaacaatc gataaattag ccggaatatc tctaaagcca aattctacaa
193981 gttttgaatg aagtacatcc agtcccatat tgagtgcgag attgatagta gctagatttc
194041 ttgagcgtgt taaggcttct tttagggtaa tcaagccaag gaatttacct ccatcgtttt
194101 tgggtttcca gtctttatta tcccctgctc caccttcaaa aattctagaa atatccgcaa
194161 tctcactcac aggcgaataa ccaagattaa tagctgcttg atatacaaaa ggcttaaagg
194221 agctacccgg ttgtcgcgtg ctttgagtgg ctcggttgta attgcttttt tcataatcaa
194281 cccctcctac caaagctaaa acatctccgc tttgatgatt aacaaccacc atagctccat
194341 ttagcgtgct taagtttgca tctttatcgc gttttactat ttcatcataa ccaaatttta
194401 aggcgttttg agccatttct tgtacatcta aatctatatt taaagtgatt ttatagccac
194461 ctgttttaag atctttaatg ctaggactta attgcttaat cacctcatct acgacataag
194521 gagctgcatt ttgcgttaaa gtatcatcat aaatttgagg aatttctttc atagcctcat
194581 cgtattctgc tttagaaatc caacctaatg tatacattct tgctacaaca ttatttgctc
194641 ttgcgataga aagatctaaa tgttttgtag gatcataact acttggtgct tttggcatac
194701 ccacaagcat agcaatctct ttaagactta actcgtttag attcttatga aaatatcctt
194761 gcgcagctgt tttaacccca taatacccat gcccaaaaaa gatataattt aaatatcttt
194821 ccaaaatttg ctctttgctt aaaatcgttt ccattttgta ggcgagcaag gcttcacgaa
194881 ttttacgagt gatggttctt tcaggagtta attctgtgtt tttgataaat tgctgggtaa
194941 gcgttgaagc cccctccatg gtttttccac cactttttat gattttaaca acagctctaa
195001 aaatagcatc aatattcacc ccatcatgct caaaaaagct agtatcttct atagctacaa
195061 gagcttctat aaggcgtgga ggtaattctt cataatttgc ataaaaacga tgttgttcga
195121 agatattagc tactaatttt ccatttctat cgtaaatttg cgttgtaagt ggaggtttgt
195181 attctttaaa agtatatcct tgtgtatctg catttgtaaa aagataagct acataaacaa
195241 atgcagcgat gaaacacatc acaaaaaatg aaaatatata ttttaaaatt ttcataaata
195301 agcctttaaa ttttgagtat caagccctaa agcagtattt aaatttcctt cttgtttgat
195361 aatatagttt ttatgaaaac cctcacacat aatcgctcct gccttaccct tataaagtcc
195421 attttctaca taatctttca aatcatgagg gtcaaatttt gaaaaataaa gcgttgtttt
195481 tgaaagagag aaaactcttt tttttgaatt ctttaacaaa aaagcactta aaatgctagc
195541 acattttcca tcttgtaaat caagcatttc ataagcttct tctttggtat ttgccttagt
195601 aagaatttta tctcctatgc aaactatgct gtctgcaaag agtaaagttt tatctttaaa
195661 tttttcttca taagctgtgc aaaaattgtct ttctttttct aggacaattt tttgcacata
195721 aacactaggc tttaaatttt tgtctaaatt ttcatcatag tcaaagctta tttgttgaaa
195781 ttcaatcttt gcctctttga gtaaattcaa tctagaaaca gaacttgaag caagaattaa
195841 cattgcttaa agtccttttt ggatcaaatc taaagcctgt tttaaagctt catctatctt
195901 gcttatatct ttaccacctg ccgtagcaaa atcatctctt ccgccgccat tgccacctaa
195961 aatttgagca atatttttaa ccaaatttcc agcttttaaa ggacaatttt tcacacctgc
196021 cgcaagggtt atcttatcat cttttgcttg tattagcaaa atagcagctt tttcgaattt
196081 gtttttaaat tcatcaatca tagtttttat atcaccactt tctacgcatt caacgcaaat

196141 ttgaactccg ttgatattgc ttgagttaag ctgtgattta ttggaatttt taagctcatt
196201 ttttaatttt agaatttcat ttttaagttt ttttacccca agacttaaat cattgctttt
196261 aagctcttct ttaagattta aattttcatt cataaaagtc ttgcaaaatt ctaaagctgc
196321 tcttgaaaca accgcttcta tacgccttac acctgcactt acaccacttt ctttaactat
196381 gtaaaaactt cctatttgag ctgtattttt tacatgagtt cctccgcaaa gctctttact
196441 ttcacctaag gttaaaacgc gtacattgcc ttgatatttt tcattaaaca aagcaatggc
196501 tccacttttt ttagcatctt ctaaaggcat attttctaaa attgcttcgc ttgagctaat
196561 gatcatttca ttaactcttt tttcaatact ttctaattct tccttgctta aagctttgtg
196621 atgagtaaaa tcaaatctta atttattgct ttcaaccaaa gaacctgctt gactgatatg
196681 agagcccaaa atttctctta aagcgtggtg caaaagatgg gtagccgaat gatggcgtgc
196741 agtttgttct cttttatcgg tatcaatttt tgctatgatc ttatcattaa gcttgatttc
196801 ttcattagct tttacaagac taagattgag attgaaaaac ttttgagtat ctagcacttc
196861 gctagttcct atatatcctg tatcagcaat ttgcccccca cttgttgcat aaaaaggagt
196921 attatcaagc ataacccagc ctaaaccttt aagacttgaa acttctttaa aatcttcatc
196981 taaaagagct aaaacggtgg cttgacattc tgtttttca taacccacaa agcaattttc
197041 tccaaatttt tcaagtaaat ttttaaaatc tccacttgct accttatcgc cgcttccttt
197101 ccaagaagct tttgcacgat ttttttgctc attcatcaat tcttcaaatt tagcctcatc
197161 taccacaaga tttttttctc ttaacatatc ttgcgttaaa tcaagtggaa aaccataagt
197221 atcataaagt ttaaatgcaa cctctccgct aaaaacttct ttggtgtttt taagctcttc
197281 attaaaaatt tcaataccgt tttctatagt gcttaaaaat ctctcttctt ctaagcggat
197341 ttgctctttt ataaaatctt tcttttcatt aaggtaggta taatgatcac ccataagctt
197401 gcaaaccaca tcaactaaat tatacataaa aggtttttta agccccaata aatacccatg
197461 tcttaaagca cggcgtaaaa tacggcgtaa aacatagccc ctaccctctt tatcaaaact
197521 cgttccttga gcaagcaaaa atacactcga tcttatatga tcagctataa ctctaaagct
197581 agctccactt tcataaacat aagtttttcc acaaagtttt gcaatttcat tgataatagg
197641 cataaaaagc gagctgtcaa aattgctaaa tttgccctct ttgatagctg taaccctctc
197701 aagccccatt cctgtatcaa tacttggttt tggcaaaggt gtaagtttac catcagcact
197761 tctttcatac tgcatgaaaa caaggttcca aatttctaag aacctatctc catctccacc
197821 catataatct tcttcgccat taaaatactc ttcaccttga tcataaaaaa tctcactaca
197881 aggaccacaa ggacctgtat cgcccatttg ccaaaaatta tccttatcac caaatctata
197941 aattctttct ttttggatat gtttttgcca caattcaaac gcttcatcat cgctttcatg
198001 tacggttaca taaagtctat ctttaggaag ttttaatacc tcagttacaa attcccaagc
198061 ataagcgata gcttgttctt taaaataatc cccaaaactg aaatttccta acatttcaaa
198121 aaaagtatga tggcgagcgg tatagcctac attatctaaa tcattgtgct ttccccctgc
198181 tcttatacaa gtttggcaac ttgttttgcg tggtgggttt ggacgaggca cttctcctat
198241 aaatatactt ttaaaaggca ccattcctgc atttgtaaaa agcaaagttg catcatcagg
198301 aactaaagga cttgaagggg tgatttcatg acccttagac ttaaaaaaat ttaaatattc
198361 acttctaaca tccataagat tttcctaaga aatttttata aaagcatatc ataccaaaat
198421 tactttaaat taagtaaaac taaaatacaa taagcctttt taattaattt ctttaatttt
198481 caaggttttt tatgaatttt atcaatcttc aagctcaata tcttaaatat aaagaagaaa
198541 tcgattctga aatcgcaagt gttttatcaa gctcctcttt tatagggggt gcaaaattaa
198601 atgaatttga aacaaattta gcccaataca ctcatgtgca acacgccata ggatgctcaa
198661 gtggcacaag tgctttatat cttgctttaa gggctttaga tatcaaacaa ggagatgaag
198721 tcatagttcc tagctttact tttatagcca ccgctgaaat ggtagcacta ctgggagcta
198781 agcctgtatt tgtagatata aatttagata attttaatat cgattttgaa ctcttaaaaa
198841 atgccatcag ccccaaaaca aaagcaatta ttgctgtgag tatgtttggt caaatgagta
198901 atttacaaga attaaacacc ttttgcgaaa gtaaaaatat aagcttgatt gaagatggcg
198961 cacaaagttt tggagcaagc tttaagggtg aaaaatcttg ctctattgcc aaaatttcat
199021 gcactagctt ttttccctct aagcccttag gagcttatgg cgatggtgga gcgatttttt
199081 gcaatgatga gagcatagct aaaaaattaa gaattttact caaccacgga caaactgatc
199141 gttataaaca tgaatttata gggataaatg ctcgtcttga tactcttcaa gcggccattt
199201 taaatgcaaa actaaagcac ctagatgaag aaattaacaa aagacaagaa atagcaaaat
199261 tctacaacga aaatttgaca aattgtcaaa ttccaaagat tgatgaaaac gccatcagtg
199321 cttatgcaca atatagcgtt ttagtggaag atagagctag agttataaaa gcttttgaaa

199381 aagcaaacat tccttatgct atccactatc ctacgccact tcataaacaa ccttgcttca
199441 gtgaattttc gcatttaaaa ttagaaaaat cagaatttgc gagtgagcat attttatcct
199501 tgccattttc agcattttta agtcaaagtg agcaagaaag tgtaattaaa atatttaagg
199561 aaggactatg aaattagatc aagtgggctc tttttaaga cccaaaattt taaaagaagc
199621 tagagaaaat tttgccttgg gaaaaatcga tgaagaacaa cttcgcagca ttgaagattc
199681 ttgtataaga gatcttttag aagagtgtga taaggcggga atttattatt taagcgatgg
199741 agaattacgc cgttcttggt ggcatcttga tttttactgg ggttttggtg gcattaaaaa
199801 aatcaaaaaa gaaaagggtt atatctttaa agggatagaa acaagagccg aaggtattga
199861 aattgaaagt aaaatcgagt gcaataatca tccttttta aaagattttg aaagattaat
199921 gcaaatcgca aaaggtttaa aaatagatac aaatcgctta aaacttacca ttccaagccc
199981 ttcaatgctt ttatatatgc tttttatccg tggaggcaaa aacacagaat ttaattacta
200041 tggtacagat tttacaaaac ttaaaaatga tattttaaat gcttatgaaa atttttacaa
200101 agaatttgca gctcttggtg gagtatattt gcaacttgat gacacaagct ttggttcttt
200161 atgtgattat gaattttgtg ctttaaatga aattaacgca gatgatattt gcgaagaata
200221 tgtagatttt ttaaatgaga gtttaaaaac tatgccaaaa aacataatga gcgcgattca
200281 tatttgtcgt ggaaactacc gcagtcgcta tgtggcaagc ggaggctata tgaaagtcgc
200341 taagaagctt tttggcgaac ttagagtcaa taaattcttc ttagaatttg atgatgaaag
200401 ggctggagat tttgaacctt taaaatatat aaatgatcaa attgcggttt taggaattct
200461 tactagcaaa accaatgaaa gtccgagcgt agaagagtta aaagcaagag tgaaaatagc
200521 agctcaattt ttaagtccaa aacaaattga aatcagcaca caatgtggct ttagctccac
200581 tgaagaagga aatgaaatct tcactcattc tcaatgggaa aaaattaaac ttttaaaaca
200641 agttttaagt gagcttgaaa atgaaggatt tttctcttga aaataggaat tataggcctt
200701 ggaaaaaatgg ggcaaaacca tttaaacgag cttggtaaaa ataaaaattt taaaatcaat
200761 gctttgtttg atatggttga aaataaaaat ttaaatgcac cctttttac caatttagat
200821 gaatttttaa atcaagataa tgatatcatc atcattgcaa caccgacaaa ttcacattta
200881 gaaatagcta gaaaggtttt ttgtaagtgc aaatgcgtat tgattgaaaa acctttagcg
200941 ctgaatttaa atgaaataga tgaaatttca aatttagcta aagaccatga tgtaaaagtg
201001 ggtgtgggat tttgcgagag atttaatcct gcggttttag ctctaaaaaa agagcttcaa
201061 aacgaggaaa ttatcagcat caatatacaa agattttcca cttaccctca aagaatcagt
201121 gatgtaggaa ttttacaaga tttggctgtg catgaccttg acttgcttca ttttttaagt
201181 ggagagaaaa ttataaacgc taacatttta aaaagcttca ataaagacaa acaaagagaa
201241 gatgagagca ttataacttg caagctagaa aaaacgatcg cttgtgtgca tcaaagctgg
201301 aacagtacgc aaagactaag aaaaattaca cttgtaagta aaaatcattt ttacgaggca
201361 aatttagcag acttttcctt aagcaaagac ggacaaagct tggagctgat gacacaaaca
201421 ccgctttttg gggagcatat ggctttatat gatttgtttt taaataagga aaattaccta
201481 gcaaatatac aaaatgctta cgctgtgcaa gaaattttag aagaatttta atttttctaa
201541 aataatgtaa catcatccaa ataacttgag cttcaataaa atcttcaaat acttaagata
201601 aaaaaattta ttgattttaa atccatactg aagtaaaata aaactaaaaa aggtttttaa
201661 atgaaattgg ttttattttt aaatatgggt ggagccacaa atttacaaga ttgtgaaact
201721 tttttaaaaa atatgtttaa cgatccttat attttaggta ttaaaaataa atttataaga
201781 agatttgtag cgtggatcat tacaaaatct cgcgtaaaag ctatgagaga aaattataaa
201841 caaatgggtg gaaaatctcc acttaatgaa cttactcaaa gtttgtgcga aaaacttaat
201901 gcaaaaaacaa aagactttaa atttgacttt gtcaatcttt atgtgccacc ttttgcaaat
201961 gaagttttgc aaaaatacac tttaaacgaa aatgatgaaa tcattctttt tcctctttat
202021 ccacatcact caagcaccac agttacttca tcgcttgaag ttttacaaaa tgaaatttca
202081 aaacaaaaca tcaaagcaaa agtaaagacc atggacattt tttatgaaaa tacgctttat
202141 aatgaaatga ttatttctca cattttggct aaaaaaagtg agtttgatgc taaaactttg
202201 attttttcag cacattcttt accgcaaagc attattgaca agggcgattt atatgaaaaa
202261 catgtcaatc atcatgtaga gcttttaaaa gaaagattaa aagatcattt tgatgagatt
202321 atcttagcct accaatcaaa actaggtcca gtaaaatggc ttgaaccaaa tacaagcgat
202381 attttggcaa atttagataa caaagctttg atttatccta tttctttttg catagattgt
202441 tctgaaacga tttttgagct aggtatagag tataaacatt tagcaaagtg tgattatgat
202501 ttaatcactt gtccaaatga tagcgatgaa tttgctcaat ttgttttaaa gtatttatct
202561 gattttaact gagaatgaat tttgaagtcc ttgtctatca ttttttaaag attaacaagg

202621 actttttggt aaaaatttag ccaactaagc caaagtataa gctttttccc cgtgcaagct
202681 tacatctaag ccttttgct ctacttcttc ttttaccctt aagtctgtaa aacaagaaat
202741 aatcttaaaa ataataaagc ttataagagc tgacaaaacc acgcaaatca caatagcaaa
202801 aatttgctct tttaaaagct ctaaacttcc actgataaaa aatccctcgc caagtgcatt
202861 ttgggcaatc acattaggat ttactttagc acttgcaaaa agccctgtgg caattcctcc
202921 ccaaatacct ccgattccat gcaaagaaaa agcatctaaa gaatcatccc atttgagttt
202981 gtatttgata aaactcaaac caaaataaca aaatacagaa gacaaagccc ctataacaag
203041 agaagcgtaa atatctacat aaccacaagc tggagtaatc cccacaagcc ctgctacaag
203101 cccactaagc ccacctaaaa tagtcggttt tttatgaata agccactcaa gtactaccca
203161 agataaaaag ccacaagccg ctgatataat acttactata aaagcattga tagcaatatc
203221 attcatttcg ccagcacttc cagcattaaa acctaaccat cctataaaaa gcaaaatcgc
203281 acccaagaaa gcataaggaa tagaatgcgc gtttttatcc tcatcatcac tttttctagc
203341 gcctaccatc aaagctccta caagtccggc aaccccagag ctaatatgca caacaccgcc
203401 cccagcaaaa tcaagacttc ctcttttaag caaaaatccc tcgctactcc aaatcatatg
203461 cgctaacaca tcataaacta aagtactcca aaaaagtaga aatatcacca agacacccag
203521 tttaattctt cccaccaaag agcctgttat aatagcagat gcaataagtg caaacatcat
203581 ttgaaaaatc acaaaaagaa tattcggaac gcctgaagcg ttgtatccgc taataccctc
203641 taagaggaaa ttctcgaaat ttcctacaag cccaagtcct tcatctttac caaaagaaag
203701 actaaatcca agcactaccc attgtaaagt aatgacacca aaaacgataa agcaattcat
203761 tatggtatta agagtatttt tacttcttac catacctgaa taaaacattg ccaaagctgg
203821 agtcataagc aaaactagca aagtacacaa cacaataaaa cttgaattta tcgcattcat
203881 ttacctatcc tttcttacaa aatttaattg cgagataatt atatttaaaa tggtttaaaa
203941 aaatataaat ttaagaaata ttttatatat tttttttatc aaagtttatt tttttatctg
204001 aaattaccat aaatagggac tttaaaaaaa tatttcaatt ctctataact caaatttact
204061 agcattaaga taaaaaattg gagctaaact gaaaaaattt aacaaaatta aaaaagaatt
204121 cttgcaagat ggtaaagaaa aattttgatg atttggtaaa agttttttaa attaaaagta
204181 gaaattatct agtattaaaa aagcaattgc gttttaaaat agcccaaaag ctcttctttg
204241 gctttgaaga tatcatcagt gtttagctca aaatctattt tttgtggctt tttataactc
204301 ttatcataat aagccaaaag caaggcaata agcctttgcc attctctttg ctcataactt
204361 tttagcaaat cctgacgaaa attgacactg atataaggac taatcttagc aagacattct
204421 ttaaatttta aaggggtcat tttttcttga tagatctttt gtatcctgtc tatgcgcttt
204481 tctaaagaac aaaaacaatg aattttaaat gctttttgca ttttttcata aagctttaaa
204541 ggcaaaacta tttcacctat tttacgactt tcgctctcta taaagataaa atcttttaaa
204601 gaatttaaaa catgaaaaag gcttgcttca aaagcttttt gcgtaggctg agcacctaaa
204661 atatcaccaa aagaagatcc tagatgattt gccatttttt ctaaatttaa atgatggggc
204721 aaaagctcca aaagctctgt ttttccacag cctgtattac cacaaagagc aaaaaaaatct
204781 aaatttattt cattatcaaa ataatgcgcc aaaaaacttc tataagcttt aaagccaccc
204841 tttaaacgca ccactctata gcctaattcg ctcaaaatca ctgcaataga ttttgaacgc
204901 aaacccccac gagagcagta aattccgatc ttatctccta tacggaattt ttgagtaagc
204961 tctaagatat gctttgacat attttgacaa atataactcg cgcccctagc cttagccaaa
205021 gcttgatttt ttttataaat tgtccctatt tcttgatatt cttcatcatt taaagcataa
205081 aaattcaacg cccctatcaa atgagaatgt aaaaattccc taggacttct tgcatcgatt
205141 aaaagagaga aattttcttt ttgaaattcc gtaaaatcta cttcatttaa catttttaaa
205201 tgccaaaaca agtttttgat aaacttgttc tatattggta ccacaaaccc tagtctctcc
205261 tatagtggtg ataaaattag tatctcctgc ccaacgcggc actagatgat aatgacaatg
205321 aggagctatc ccagcacccg catctttact caaattcata ccgatattta ctccgcctgc
205381 atggagttcg ctttttaaaa ttcttacccc tagacgcaca aatttactta tctcttgcca
205441 aacttcatca cttaaatttt ctatatgctc ttcatgcaaa taaggtatta tcataaaatg
205501 ccctgcagaa taaggatagc gattcatcac gccaaaacaa tgcttagctc taaaaatcac
205561 ccctaattct tcatcgcttt tgattttatt agcacaatca caaaaagggc actcgctctt
205621 ttctttttca aaatactcac ttcgccatgg tgcatacaaa tactgcattt ttatcctttt
205681 aaagcaaggc tttcaaagcc tctccttcat cattttgacg cataaaatat tctccaatta
205741 aaaaagcatc aacacctaaa ttttgcaaat gtcttaaaaa atctttattc tctaaaccac
205801 tttctgcgat aataattttt gaatttggaa tttgtggaat aagcttttca cataaattca

205861 tatccataga aaaatcatcc aaattgcgat gatttatacc tataatatca gctcctgaaa
205921 aaatcgcttt actcaaatct tctttatcgt gaatttccac caaagcttca agtcccaaat
205981 gccttgtaaa ttcaagtaat cttttaagct ctttcatact taacattcta gcgataagca
206041 aaacaaaatc cgccccataa accaaagctt ctaaaatttg atactcatca aaaataaaat
206101 cctttcgcaa caaaggagca cgcgtataac ggcgtatcat actaaggtat tctaaactcc
206161 cttgaaaaaa atgcggttct gttaaaactg aaatagcagc agcgccattt ttttcgtatt
206221 ttaaagctat atccaaagga tcgaaatttt cccttataac ccccttgctt ggactggctt
206281 ttttaacctc tgcgataatt cttacttcat tttctactct ttttaaagca gctttaacat
206341 ctttaggaaa aaaaggattt gatgctaaag atcgtcccaa cacatcataa ggcagttgcg
206401 ccttacgctc tttcaaatct tctttagttt tttcaaaaat tttatctaaa atcatggttt
206461 aatacacgcc tttatcgctt ttgcatgagc tttgctctca tcagaatttg caaattcttt
206521 atcctctaaa gtttgcttca aaatttccca agccttttga caatcaccct gtttataata
206581 cccccacgct aaagaatcta aataatataa attttgaggc tctttttttca aagcaagatg
206641 aactaatttt ataccctttt caagatctaa atcataatct atcaacaaat atccataata
206701 atttaaatac aaggcatcac tattttcatc aattgcatcg gaaaattttt cactcacaag
206761 agtaataact tgtggggtga tttttttttc caaatttgct tcttcaaatt caaaaattgc
206821 tgcacgcaaa agatattctt tctcttgagt gtgtttataa atttgcatac taagatcttt
206881 tgccttgccg tatagtttta atctttgata caaataaagc tttacatcat catctacatt
206941 atattttaag gcaaaatgca atgcttcttt atctttattt tgagcattta aaagctcgat
207001 aagagctaga ataaaacttt gatttttgt aatattataa agctctatat aaatttcttg
207061 taaagctttg tcatttccct cgtcattata aatctttgct agtaaaatac aagttttaag
207121 agtacaacca ttaatatttt ttgaagtttc aagtatattt ttaatgcttg aagtatcgtt
207181 taatatcgca taaacaccca caagcttaaa caagatctcc tcgttttgaa tttgattata
207241 agctagttgg taatacttta tggcatcttt taaattgttt tggcgcatta aaacatctcc
207301 ataaagctct aaatttcttg gatcatcact tttttctttt aaaagctctt cgataagctt
207361 ttgagcctcg ttaagattgt aagaatttaa ggcataaagt gcactaagac gcttaagatt
207421 atcactctta tcaagataag gtttagctat attatttatt tcttcttttt tgtcaagatt
207481 atttgctaga gtcagcaaaa aagcattttc taaaaaatta gtaatattat aatccctaaa
207541 aagtgccaaa aactcatctc tagcttcttc gtattgatta aaatattcat aattataagc
207601 tttcatgatt cttaaatcaa attcattact cttttgttgc tcataattag gataagctat
207661 aaccgttttt gaagccccgc acgctgttaa aaaaaagcct attaagacaa ataacaaatt
207721 cctatacatt ctttttttag ctcctcttga tgtgttttaa aatattccca aaaaggaaag
207781 gtacggcatt gcatcggacg aaaatcgtag atagagcagt ttcttttctc cttatcaaaa
207841 aaaatacatg caaatccatc ttcaaattca acctccttaa agctcattct aaagcctacc
207901 tttttttaaat attttaaagc aaattctttt tcatctaatt gcaaatgttc tcgcaaagcc
207961 tttagctctt ctttatttgc aaaaatattt ccactttctc ctgtgcagca ttttccacca
208021 catttctgac aagcattttc atcaaatccg taagaaaaat ctttattaaa tatcataact
208081 ctccaaccta aatctagagt gaatctcttg catttgttca cttaaattct catttttata
208141 aacaaaaaaa ggaggtaaaa tctcacatgg tgactttaca ctttttctag cttgcaccaa
208201 tacaagcctt gcttgagtgt tttgatgagt atgcacaaaa cgaattttag ttaattttaa
208261 ctttttactc tctaaagcaa ggcaaatttt atccaaagct aaagcctcgt aacaaaaata
208321 caataccccc ataggcttta acaaagcatt agatctagca ataaattcat ctaatgttaa
208381 atatttttga aatttactta tatttttatg catattttta ctctcatgag ctccatctct
208441 ataaaaaggt ggattgcaaa ctataaagtc aaatttttta ttgttttgaa aattcctaaa
208501 atcttcgtgg aatatctcag cttgtatatt gttttgtttt aaattacgat gaattaattc
208561 tatattttct tgctgtatat caagcaaaga taaatctaaa ttagaaacaa attttttaag
208621 taaaattcct ataataccac atcctgaacc tacttctagg acttgatttc ttacacccat
208681 ttctaaaaca aaatcagcca aaagcaaaga atcgctattg taacgataac catcattcaa
208741 ttgagctaat ttaatattat cttgcatttt caatgagttg gaattttaac tcttttcaaa
208801 ggagcatttt ctttgcttgt gttttgttct tgttttaaaa cattattttg acttgcgaaa
208861 tctataaaat tttgagcaaa aagtaaagag gataataacg atattacgaa aaaaaatttc
208921 aacataattt tccttgaaat ttgaattcta ttttgagttt atttaaaaaa cttagcttgg
208981 caaaataaat acaaacttta aatggtgtcc acggcgagat tcgaactcac ggcctcaaaa
209041 ttaggaattt tgcgctctat cctgctgagc tacgaggaca aaagcgggaa aatcccgctt

209101 taatacttaa ccatttctct ttttaatgat ttcctcagca acatttttag gaacttcatc
209161 ataatgatca aattccatag aataagtcgc acgaccttga gtttgacttc taagatcagt
209221 tgagtatcca aacatttcag ccaaaggaca aaatgctgtg atgattttat ttccacctct
209281 ttcatccata ctatttactt gaccgcgacg cttattaaga tcgccaatta catcacccat
209341 ataatcttca ggagtttcaa cttcaacctt catcataggc tcaaggatta cagcacctgc
209401 ttttctagca ccctctttaa agcccataga agcagcaagt ttaaatgcca tctctgatga
209461 atccacctca tggtagcttc catcataaac agttacttta acatcttcta ctggataacc
209521 tgctaaaaca ccatttgta atgcttcttg aacaccctta tctactgcag gaatatattc
209581 ttttggaatt accccacctt tgatatcatt aacaaactca tatccactac ctggctcaag
209641 tggctcaaga cgtaagaata catgtccata ttgaccgcgt ccacctgatt gtttagcgta
209701 tttgtattct tgctcaacag tttttctaat agtttcacga taagcaactt gaggttgtcc
209761 cacttcagct tctactttaa attcacgaag catacgatca acgataattt caagatgcaa
209821 ttcacccata cctgaaatga tagtttgacc actttcttca tctgtagaaa ctctaaagct
209881 tggatcttct tgagctagtt tatttaatgc aatagacatt ttttcttgat ctgctttggt
209941 ttttggctct actgcaacag aaataacagg atctgggaaa tccattcttt caagaattac
210001 tttatctttt tcactcgcaa gagtatctcc tgttaaagta tctttaagtc ctacaactgc
210061 accgatctct cctgcgtaaa gaactttaat ctcttctctt ttattagagt gcatttttaa
210121 caaacgaccg attctctctt ttttatcttt ggttgagtta taagcataag aaccgctttc
210181 taaacatcca cgataaacac gcacgaaagt taattgtcct acaaatgggt cagtcatgat
210241 tttaaatgca agtcctgcaa attcaccatc atcagttgat tttacagaaa cttctgtacc
210301 atcttcatat tctcctttga tgtttgcaac ttcatctgga gctggcaaat aagctacaac
210361 agcatcaagt aaaggttgaa caccttatt tttaaatgca gtcccacaaa gcataggaac
210421 aatagaaaga cttaaacatc ctgctttgat acctgtttta atttcttcaa ggcttaattc
210481 ttcaccgcct agatattttt ccatcaactc atctgaagtt tctgaaactg cttctatcat
210541 tttagtgcga tattcttccg ccttttcttt aagttcggca ggaatttctt tttctacata
210601 atctgttggc ttagtatcat cttcccaaac taaagctttc atagttacaa gatcgataac
210661 acctttgaaa ttatcttcag caccgattgg aatttgaagt ggaactggat tagcttttaa
210721 gcgattgcga atttgatcct ctacattata gaaatttgca ccgattctgt ccattttatt
210781 tacaaaaact attcttggaa caccgtattt atttgcttgt ctccaaacag tttcgctttg
210841 aggttgcacc ccacctactg aacaaaatac tgcaacagca ccatcaagga cacgcataga
210901 tctttcaact tctatggtga aatccacgtg gcccggagtg tctataagat tgatttgatg
210961 atctttccaa aaacaagtcg tcgcagcaga ggtaatagta atacctcttt ctttttcttg
211021 ttccatccag tccatagtcg cagcaccatc atgcacttcg cctattttat ggctcatacc
211081 tgtaaagaaa agaattctct cacttgtagt tgtttttccc gcatcaatat gtgcagcaat
211141 accgatattt ctaactcttt ttaaaggagt acttctagac atttttttctc cttcttacca
211201 gcgatagtga gcaaatgctt tatttgcttc agccatttta taagtatctt ctttttttctt
211261 aaatgaagca cctttgctat ttgccgcatc taaaagttca gctgcaagtt tatctatcat
211321 agttctttca cttctttttc tagcaaaaga aataatccaa cgaattgcca aagcttgttg
211381 tctagcagga cgaacttcta ctggaacttg ataagtcgct ccacctacgc ggcgagattt
211441 tacttctagc aaaggtttaa tattttcaat cgcatcatta aagatatcaa taccttttt
211501 ttcgccacct tttttatcga taagttctaa agcgccatac ataatagtag tagctgtgct
211561 ttttttacca tcatacatta aagaattaat aaattttgtg attactttgt taccataaat
211621 cggatcaggc aagacttctc ttaccggagc ttttcttctt ctcattattt ttccttcaaa
211681 ttttaaattt tactcaaaca aaatcaaatc taacggattc gtctgtctaa aaaattactt
211741 agcacttgct ttagggcgtt ttgcaccata tttagaacga gaaactgttc tttttgcaac
211801 acccgcagta tcaagcgcac cacgcacaat atgatatttc acacctggta agtcttttac
211861 cctaccgcca cgcactaaaa caatgctgtg ttcttgtagg ttgtgacctt caccaccgat
211921 atagctaatc acttcaaagc cactagtaag tcttactttg gcaacttttc ttaacgctga
211981 gtttggtttt ttaggagttg ttgtataaac cctagtgcaa actccccttc tttgtgggca
212041 atttttaagc gctggagatt tagatttttc taaaactttt ttgcgctctt ttctaaccaa
212101 ttgatttatg gtaggcacaa taattccttt ctttaaaaaa tataataaac tttggatgat
212161 atctaaattt tacttataag attataaact ataaataatt taaatctttt tgcaaatttt
212221 ctaaaaataa atttacaaag ataaatgttt gattggtaga aaatgaaaat actttaccat
212281 tttaatattt gttttgtatt tcagttttat aaatatctta aattttataa tataaaaata

212341 aagaagagaa aataatcaaa ttgagctttt caaatcaaga taaaataaaa aaatagccta
212401 aaaataggct attttcatta ctcttgttct ttgagtttta aattttgttc tccgtaaagt
212461 cctgttccaa caggaatcat tctacccaaa ataacatttt cttttaagtc ttctaagtag
212521 tcaaatttac ctgcgatact tgcttcagtg agtacttttg ttgtttcttg gaatgaagct
212581 gcagaaatca cactatcact tcctatcgct gctcttgtaa cacctaaaag tacaggctca
212641 gcaatagctg gctctccacc caatctaaga attctctcat tttcttcgcg gaatttgcgt
212701 cttgaaacca aatctccttc gataaattta gtatgtccac tatcgataat tttaacctgt
212761 cttaacattt gagaaactat aacctcaata tgcttatcag aaataacaac cccttgacct
212821 cggtatactt gttgaatttc agaaatcaag taataatgca aggctttttc gcctaaaatt
212881 ttaagcacat cgtggcttga aactactcca tcagttagct tctcgcccgc gtgaataaat
212941 tcaccttctc ttacttgaat atgttttgat ttatcgatca aatactctgc gcttgtgcca
213001 tcttctgctt ggataatcaa tctttctttt gagcgtaaag gcttatcaaa acgaaccaca
213061 ccatcaatct ctgcaattac tgctgcattc tttggttttc ttgcctcaaa aagctctgaa
213121 actcttggaa gaccccctgt gatatctttt gatttcgccg ctgcttttgg agtttttgcc
213181 aaaatatctg cttgagaaat tttatcacca tcatgaacga aaataaccgt ttttggctcc
213241 aattgatagc gcactgcttt atcgcctttg cctgcgatta ttaaggttgg tcttaccccg
213301 cttggtaaat attcattaat cactagcgag cgtttgcctg ttgcttcatc gatttgctca
213361 tccgcactat aacctgcttc tatatcttca aaacttacca caccatcaat ctctgcaatg
213421 atagtgttat tataagcatc ccattctgcg ataatagatt tttcttgttt tggtgcacta
213481 gcaattatag aatcaaaatt aacctcttcg ctatcattaa attctattct tgactccctt
213541 ggaatatagt gtctttttgc ctctctatca ttttcatcag caactacaac aaaaaatcct
213601 ttttctttta ctatatcacc ttttttgata tttctaaccc tatcaagtcc atcaccttta
213661 agaatataga attttagaat ccccttttcc cctgctttga tattttgtac aacaggatct
213721 ccatctttta caagaatttc acttgcaaat ggaatacggt ttggaacatt ccaaccctct
213781 ttaatcatct caacaatact ttcattttct tcaacttcag caccactttg atatggtaaa
213841 taaagttttc cgtcaatgct tccgctaacg cctgctaatt catttggttt agcaagatcg
213901 tattttctaa gaatatattt tgcttcttgt tttttatctt ttatagaaat aattacatct
213961 tcgtgaatat tttctatatt gacaatacct ttaaatggtg ccttaatttt tggctccaca
214021 agtaaaatag ccgcatttct acgattagct actatatctt taccttcttt gtttttataa
214081 gttttaaggt tataaaatct tataaaacct tctttttggg cacttacttg cctatcttgt
214141 aaatccgtac tagcagttcc cccgctatgg aaagttctaa gtgtaagttg agttcctggc
214201 tcaccaatag attgagcaga aatgataccc actgcctcac ctggcttaac aagcttgcct
214261 tcgccaagat taattccata acattttgca caaattcctt ttttagcttt acaagtgatt
214321 ggagtgcgaa tatttacact ttttacacca ctttctccta atattctagc tttttcttca
214381 tctatcaaag tgccttctgc aaaaagcaca gaatttgtaa taggatctat tacatcttca
214441 gctataaccc tacctaaaat tctctcttct aaagtttcta taatagaact atctgcggtg
214501 atctcattaa tttcaacacc ttcgtgcgtt ccgcaatctt caacagtgat ttttacattt
214561 tgtgccacat cgataagttt tcttgtcaaa taacccgcgt ttgctgtttt aagagcagta
214621 tctgccaaac cttttctggc tccgtgagtt gaaataaaat actcaagaac attcaaacct
214681 tcacggaaat ttgaaataat aggtgtttca ataatcgatc catcaggttt agtcataagc
214741 cctctcatag cagcaagctg agaaatttga gctgcactac ccctagcacc tgaatctgcc
214801 atcatataaa tagaattaaa gccttcctta tctttttcca caagcttcat catatctctt
214861 gaaagagtat tattggtagt tttccaaata tcaataattt tgttatatct ttcacctgaa
214921 gtgattaaac caagattata agaattttga atttctctta cttgcttttt agcctcatca
214981 atagcctttt gtttatcact aggaacgata atatcagcaa tagatataga aacgcctgct
215041 ttagttgcat attcaaaacc taaattttta agtttatcta aaaaacttgc agtaatttgt
215101 aaaccaccct gtttgtatac ataatccaca agcgcagcaa tatctttctt ttttaaaacc
215161 ttattccaca tattctcagt aacaaaatca ggcaaaatag atttaataat aagtcttcct
215221 gccgttgtag taatcttcct gccatcaacc atagtttgaa tactagcatg aatatcaagg
215281 catttacttt caagagccat cataacttca tcaatgcctg tgcatatttt atgtgagcct
215341 tttgctccca ttttttccaa agaaagataa taaattccca aaaccatatc ttgagaaggt
215401 acagttacag actttccact tgcaggcaag agaatattca tggaagaaag cattaaaacc
215461 ttacactctg caatcgcttc ttgagaaagc ggcacatgta ctgccatttg atccccgtca
215521 aagtctgcgt taaatgccgc acaaactaaa ggatgaagct gtattgcttt tccttcaacc

215581 aaaacaggat gaaaagcttg gatagaaagc ttatgtagag tcggagcacg gtttagcata
215641 acaggatgcc cttttaccac ttcttctaaa cattcccaaa cttcattggt tttgttttct
215701 atcatttttt tagcttgttt tactgtagta gcataacctt tttcttcaag tttagctaat
215761 aaatgtggtt taaaaagctc taaagccatt tttttaggca agccacattg atccattcta
215821 agttttggcc ctacaacaat aacgctacga cctgaaaaat ccaccctttt acccagcaag
215881 ttttgtctaa aacgaccttg cttacctttg ataatttcac tcaaagattt taatggacgc
215941 ttatttgctc ctttaactgc attagcacga cgaccattat caaaaagcgc atctacagct
216001 tcttgaagca ttcttttttc atttcttatg ataatttcag gcgcatcaag ctccataagt
216061 tttttaagac gtgtatttct attgataacg cggcgatata aatcatttac atcagaaact
216121 gcgaatttac ctccatctag agcaaccaaa ggacgcaaat caggtggtaa tacaggtaaa
216181 tttgtaatca tcatccattc aggacgattt ggtactgcat cttcattgtt tgtatttgcg
216241 tttaaatttg aatttaaaaa gttttctaca acttttaaac gttttataat ggtttttttc
216301 ttagcctctg aatttgtagc agtcatttct tctttaagct ggttcaaaag tgccacaaga
216361 tctaaatttg ctagcaagtc gcgaactacc tcgccaccca ttcttgcttt aaaaccactg
216421 ttttcataac gctgcattaa attttgatat tgctcttcgt ttaatacgtc gcagtattct
216481 actttttttg tgctttcatt atcatagtat gcctctcctg cattttctac aatataagct
216541 tcataataaa gtacgcgctc aagatctttc atcttaacgc caagaagtgt tcctatacga
216601 cttggaagag aattaacata ccaaatatga gctactggag ttacaagttc aatgtgtccc
216661 attctagaac gacgaacttt tgaatttgca acttcaaccc cacatttttc gcatttaaca
216721 cccttaaaac gcattttttt gtatttacca caaagacatt cataatctct aataggacca
216781 aaaatttttcg cacaaaaaag tccatctctt tctggtttta aggttctata attaatagtt
216841 tctggctttt ttacttcgcc ataagaccaa gatttaattt tttcaggact cgcaagtcta
216901 agttgaaatg cttcaaagtc cctaggtctt gcatcttctt tgatttctat tactttaaat
216961 ttactcattg tcttcatcct tgtcaaaaat ctcaacatct aaagctagag atttaagttc
217021 attagtcaat acaaaaaatg tctccggaat tcctgttgct gggacatttt ctcctttggt
217081 aagcgcttta taggcactga atctaccttc aacatcatca gattttatag taagcatctc
217141 tcttaaagta tgagctgccc cataagcttc aagtgcccaa acctccattt caccaaatct
217201 ttgtccacca aagagtgctt taccacccac aggctgctga gtaaccaaac tataaggtcc
217261 agtacttcta gcatgaactt tttcatcaac caaatggtga agttttagca tatacataca
217321 tcccacatga actctctcag cgattttttc tccagtacga ccatcgtaaa gctctgtttt
217381 accatccatg tcaatctttg ctatttcaaa aagttttgca aactcttcta tatttactcc
217441 ctcaaaaaca ggggttgcaa atttaacacc cttgctccaa tctcttgcat aatttaaaag
217501 ctcctcatca cttaaatttt taatgaattc tttttcattt gcaagtcttg ggatagaaca
217561 tatttcaagc atttttgctc ttaattcttt aatgaaatct ttttgttttc tgtcaaaaat
217621 ttcttgaatt tgatcaccca gtctaagccc tacaagacct aaatggcttt ctaaaatttg
217681 tcctatattc atacggcttg gaaccccaag tggattaagt gcgatatcta cactttttcc
217741 atttggtaaa taaggcatat ccacttcagg tactatagta gaaactatac ctttgtttcc
217801 gtggcgtcct gccattttat cacccacttt aagcttgcgt tttgttgcga tatacacctt
217861 aacaagctta attactccac ttggtaagat atcatctttt tctaaaattt caagcttctc
217921 atcgtgctca gctttaagct ttttcttctc attttggaaa tgattttta actcttcgta
217981 ctctttttga acttctttag aataagcttt aattaaagta gtcaaggtaa aacgattgat
218041 tttttccaaa tcttctaggc ttgctttttg tccttttgta tagtttttat cacctatttt
218101 ttgatcactc atcaaagcag atttagaaag caacgcgcaa acgcgtagca tctcttctct
218161 atccatcatt aaaagtctat catgatgctc tttttctaag gacatttttt ctttatcata
218221 cgattttaaa gctctatcgt ctttttcata tccttttttg gtgaaaattt tcacatcaac
218281 aactacacct tctaaagaag cagtagcata aagggattta ttcaccacat ggccggcttt
218341 ttcaccaaaa atcgctctta aaagtctttc ttcaggcgtt ggcttaactt caccttttgg
218401 agaaacctta cctactaaaa tcatacctgg ttttatatga gtaccaattt ttgctatacc
218461 gctttcatcc aaatgcgcaa catcttcttc tttaacattt ggtatatctt tagtaatctc
218521 ttctatacca tcttttaatt ctctagcttc tatttctttt tcgtaaatat gaacactagt
218581 aaaagtatct tcgcgtataa ttctttcact tactactata gcatcttcgt agttatatcc
218641 attccaaggc ataaaagcaa tcaaagcatt ttttccgata gcaagttcac cttgatccat
218701 agaaggacca tcagctataa tttgccctgc tttaaccaca tctccctttt taacaatagg
218761 atgttgcata aaatttgtat tttggtttgt acggagattt ttttccataa tataatgatc

218821 tataaatgga ccttttttcat cttcaccaag gataaatatg cttttgttat ctactttttc
218881 aacaacccca cctcttttag cctttatagc ttcccaagca tcacgagcaa tgatattttc
218941 catacctgtt ccaacaatag gtgctgaaga agttataagc ggaactgctt gacgttgcat
219001 gtttgagccc atcaatgccc tatttgcatc atcatgctct aaaaatggga tcaaagaagc
219061 agcaacaccc acaaccatac ccgagcaaag atctataagc tgtacttctt cacgtcttgc
219121 aagtatagtt tctccatctt gtcttgcttc gacaaattct tctattatat tgcctttagc
219181 atctactttt gtagaagctg gagcaataaa caatccttct tcttgagtag ctgttaaata
219241 cacaacctga tccgttactc ttccattttc tacttttcta taaggcgctt caacaaatcc
219301 gagctcattt actttagcat aagtagataa ggtattgatc aaaccgatat tttgcccttc
219361 tggagtttca acaggacaaa ttctaccata atgcgtcgca tgaacatccc gtacttcaaa
219421 acccgctctt tctttaacaa gtccaccttc tcctaaagct gacaaacgac gtttatgcgt
219481 gacttcgctt aaaggattgg tttgatccat aaattgtgag agttgtccac ctgtgaaaaa
219541 ttcaataatt gtcgttgtaa tcattttagg attgatcaaa tcataaggca ttactttgtc
219601 aatatctgca ttcaaagagg taaatttatc tttgatagct ttttgcattt tagcaagacc
219661 caaatgaagt tcattcgcta aaagttctcc gatagaacga atacgacgat ttcctaagtg
219721 atccctatca tcaatatgac ccttaccatt ttttacttta attaaatatt ttgcagtttt
219781 gataatatct tcattggtta gaactgttac atattctgga acctcaagcc ctaatttgtg
219841 attcattttc atacgaccta ctttagtaag atcatatctt tcagggttaa aaaataaatc
219901 attaacaaag gctttagctg catcttttac aacaggctca cctggacgca ttactttata
219961 aattctaata gctgccagat cattttcatc atcaatattt tcactttgtt ttaataattt
220021 taaagtttca ttatcctgtg caaacgagtt aataatagct gcatccactc catttgccaa
220081 atcgttagct atttcaaaac ttttttgctc tttaatttta gcaagtttgc tttcatcaag
220141 taaggttagg gaatcaaata aaacttcgcc actttcttta tcaataatag gattagccag
220201 ataacgattt aataaaattt ctactggata ttctatccat tttaaaccat ctttaataag
220261 ctgctcagct ttcttcttag ttagtctttt accagcttga tggataattt ttcctttttc
220321 atccttgata tcatattcta ttctatccat aaaatcattt ggattaaatt ccgttaagaa
220381 tttatctttt ttaacatgaa ttgtctgaat aggataaaat aatttaataa tatcttgttt
220441 tttatacccc aaagctctaa aaagcattgt tactggtact tttcttcttt tattaatcct
220501 tacatacaat acatctttgg catcgtattc aaaataaagc caagaaccgc gatcaggaat
220561 aatttgagca gtgtatacaa gcttattaac aacagtcgag ctttcttctt ctttgaaaat
220621 tacacccgga cttctatgaa gctgattaac cacaactctt tcaactccat tgataataaa
220681 agaaactcta tctgtcatca atggaatttc tctgatataa atttcttgtt ctttgatatc
220741 ttttatacca actttttcac ctgttttctc atctttctca tgtaaagtaa gacggatttt
220801 catttttaaa tttacagagt aagttaaacc tctttccata cactcacgaa ttgtgtattt
220861 tggttttcca atttcgctgc taacatactc aagacttaac ctattttgtg gatcatggat
220921 aggaaagatt gatttaaaaa ctttttctat tccactttca ccgttttcaa gatttaaaaa
220981 ataatcaaaa cttgcttttt gaagttgaag aagatttgga atttctattt gctttgaaat
221041 attggagaaa tctactctta aacgatttct taatttattg tctaacatat tgcatatacc
221101 tcgtggtaaa ttttgtaaat ttaaaaaaga agacctaata tctaggtaaa aaataaaaaa
221161 gaagacttac taaaaaagta aatcttctta aacttttaaa gcttaattac ttaagttcta
221221 ctttagcacc agcttcttca agttgttttt tagcttcttc tgcatcagct ttagccacgc
221281 cttcttttaa agttgaaggt gtttgctcta ctgcatcttt tgcttcttta agtccaaggc
221341 cagttaaagc acgaacgatt ttaattactt cgatcttttt agcaccacca tcaactaaaa
221401 cgatatcaaa ttcagttttt tcttcagctg cagctgctgc accacctgct gctgcaccac
221461 ctgctaccat tacaggagca gcagacacgc cgaatttttc ttcaaattct tttacaagct
221521 cagaaagctc aagaacactt aaatttgaaa taaattctaa tacatcttct ttagaaattg
221581 ccattttttta tcctttatta ttattcagat tcttttttat ttttaagcgc atttaaacca
221641 atagtgaaat tggtgattgg cgcattccaa acttgcaaaa gcatagcaag caactcgttg
221701 cgagaaggca ttttcgctaa agctctaacc ttagcaacat ctgcaacttc gccttcaata
221761 tgggcagttt taatttcgaa tttatcatta ttttcttcga atttagctgc tacttttgaa
221821 acgctaagtt ggtcctcgcc ccaaagataa atattagtat ctttaagaac cagacctgtt
221881 ttaccggaat tattaagagc aatgctagcc aaagtgtttt taataatttg aacttttaca
221941 ttgttttctc tcgcattatt tctaagttct tcaagctttt tagtgctcaa gcctttgtaa
222001 ttacaaacta caatcgcttc gctaatttta aattcttctt gaagtttagc aataatctca

222061 actttttcgc ttctagtcac attttctcct ttccgattga gatttcaagc agagtggttt
222121 tacccaaatt aagctttcgc ctcgactatc tccaatctaa aataaacatc tcaaaaaact
222181 aaaaattctt tcaaatgttt atttaaaaag agttgcatta tagcaactct tacttcatat
222241 caagtaattc ttgtgtttca agttttacag aaggactcat tgttaaagat aaagccgcac
222301 ttttaatata tctaccttta gctgctgctg gtttatgttt attaattgct ttaacaaaag
222361 tagaaatatt atcccaaagt tgctctttag aaaaacttac ctttccaaga cctgcgtgaa
222421 tatttccttg tttatctaca cggaaattta cctgtccact tttagcattg ttaactgctt
222481 gagctacatc catagtaact gttccagtct ttggatttgg catcaaacct ttaggaccta
222541 agattctacc tactttacct acaagaccca ttaaattagg agttgcgatt aatacatcaa
222601 aattcatatt gccttttga atttcttcta tcaaatcatc acttcctaca atatctgctc
222661 cagcattttt tgcttcatct gccttagcat cttttgcaat aacagccaca cgaacttttt
222721 tacctgttcc cgcaggaagc acaacagaac ctctaaccat ttgatcggca tgtcttggat
222781 caacattaag tttaagtgcg atttcaacag tttcatcaaa ttttgctgac tttaaatttc
222841 ttactgtttc aattgcttca tttaaagcat actctttgct tacatcaatt ttttgtgata
222901 attcttttaa tcttttagtt attttagcca tatatttctc cgcattttta aagtgctacc
222961 gctttttacc ttagcggtaa aaggttttaa tctacaattt caacacccat agagcgagct
223021 gaacctgcaa taattttcgc agcttgatct ctatccttag tattcaaatc tgcaattttt
223081 ttatcaacaa tttctaaaac ttgggcacga gttaatttac ctaccttatt tttaagtgga
223141 ttgtctgtac ctttagaaat tccagctgct tttttgatta aatctgtagc aggtggttgt
223201 tttgtaataa aagtaaagct tttatcagca taaacagtaa taacaacagg gatattaaat
223261 cctgccatat cctttgttct ttcattaaaa gctttacaaa attccataat atttacacct
223321 tgttgaccta aagcagggcc tacaggtggc gatggattag cttttgtagc tgcaatttgc
223381 aatttaattt cgcctacgac tttttttagcc ataacaaatc tccttacttg atataaaatt
223441 aaataatttt ctcaacttga gaatacaaga tctctaccgg agtagaacgt ccaaaaatag
223501 aaacattaag ttttagtaat ccgcgaacca tatcgtattc ttctactata gcagtaaagt
223561 ttgcaaatgg accttctgta attcttacat tttccccttc ttcgaaagaa attttaggct
223621 ttggagcagc acggttatgt acctttttcca aaattaaatt aatatctttt tctgtcaaag
223681 gagtaggttt ttttgattct ccaataaaac gaccaacttt tggaagagat tggatacgat
223741 gccaaagttc tgtatttaaa tcaagatgtg caaaaacata accagaatac aaacttcttt
223801 cgcttatctt ctcttttcca tttttaaatt caataacatc ttcagttgga acaacaattt
223861 cttttagttg ctcttcgatt ccattatctt ttactaaatt ttcaatggct cttttaaccg
223921 ccatttcgct accagcataa gtttggattg cataccattt atgagtactc aaaatttgtt
223981 ctccttatac aatcgcagaa acaatagagg acataatcca atccactagc gccaaaaaca
224041 aagaaataac cgtaactact acaaaaactg taatataagc atttcttacc tgttctttta
224101 aaggaaaaat tactttcctt aattcggctt ttgataattt aaaataagtt attaattttt
224161 ccatttattt aaaccttaat catggcaggg caagagggat tcgaaccccc aaccatcgga
224221 tttggaatcc ggcgctctac cgttggagct attgccctag ggcataagct taacttttta
224281 gcttaacttc tttgtgaagt gtgtgttttt ttaatcttgg gcaatatttt tttaattcca
224341 atttttctgt agtatttttg ctatttttat aggtgctata attaatatca ccacattctt
224401 cacatttcaa accaacttta attctcattt atattttcct taaaaaattt aaagccaagt
224461 actaaacttg gcttttaaaa ttacttaata attttagaaa caacacctga accaacagtt
224521 ttaccacctt cacggatagc aaaacgagta ccttcttcaa gagctactgg tgcaatcaag
224581 cttacagtga ttcttacatt ttcacctggc ataaccattt caacgccatc agctaattta
224641 atagaacctg ttacatctgt tgttcttaca tagaattgtg gtctatagtt attaaagaat
224701 ggagtatgtc taccaccttc atctttattt aagatataaa cttcagcttc gaaatcagta
224761 tgtggagtaa ttgattttgg tttagcaaga accataccgc ggataacttc ttcttttttt
224821 gtaccacgaa gaagaacacc aacattatct cctgcttcac cttgatccat ttcttttcta
224881 aacatttcaa cgccagttac agttgttgtt tgagtatctt taataccaac gatttctata
224941 gtatcaccaa cttttacaat accttttttca attctacctg taacaacggt accacgacct
225001 gaaattgaga aaacatcttc aattggcatt aagaaatctt tttcagtatc acgagttgga
225061 gttggaatat agctatcaac agcagccata agatccataa ttttcgctga ccattcacca
225121 tcttgtccag cttttgcttc ttcaagagct tttaaagcag aacctgaaat aataggtgta
225181 tcatcacctg ggaaatcata agaacttaat aattctctaa tttccatttc aaccaattct
225241 aaaagttcag catcatcaac catatctgct ttattcataa aaacaacaat atatggaaca

225301 cctacttggc gtgaaagaag aatgtgctct ctagtttgtg gcataggacc atccgctgca
225361 gaaacaacaa gaatagcacc gtccatttga gcagcacccg taatcatgtt tttaacgtag
225421 tcagcgtgac ctggacaatc aacgtgtgcg tagtggcgat tatctgtttc atattcgata
225481 tgagaagttg caatagtgat acctctttct ttttcttcag gagcgttatc aatattatca
225541 taatctttaa gttctgccaa acctcttctt gaaagaacag cagaaatagc agctgtcaaa
225601 gtagttttac cgtggtcaac gtgaccgata gtaccaatgt ttacatgtgg cttattacgt
225661 gaaaattttt ctttagccat tttttcctcc atataaaaat ttaacaaggc aagtctatat
225721 ttataatttt ttcaatttta tggagctcat agcgggaatt gaacccgcga cctctccctt
225781 accaagggag tgctctacct ctgagccata cgagcgctca gaaaaattat ataaaaacat
225841 agacaactaa aaaataattt taggggattt actgtaagtt agaaaaaaat acagctccca
225901 gtcaaacttt ggagcgggaa acgggactcg aacccgcgac cctcagcttg gaaggctgat
225961 gctctagcca actgagctac tcccgcaaag catggtggtg agtcgtggat tcgaaccacg
226021 gaaggcgaaa gccagcagat ttacagtctg ccctcgttgg ccacttgagt aactcaccat
226081 aaataaaata ttacatctaa cttttatcgt aatattctgg tcaaacactg ctaaaataat
226141 ggagctggtt aagggacttg aaccccсgac ctgctgctta caaggcagct gctctaccaa
226201 ctgagctaaa ccagcaaatt aatttaaaga cgatattata tcttaaaaaa aatagtttgt
226261 caagattaag cactataaat tatttaaaaa acattctaat aattaaaatt tctcatcaaa
226321 aatccaaaaa ctaggatttt cattaaaatt atttatgata atcaaataaa ttaagaaatt
226381 tcttagctct ttcactttta ggatttttaa aaaagtcatc gggagtattt tcttcaataa
226441 tttctccatt atccataaag attattctat cagctactgc tcttgcaaat cccatctcgt
226501 gagtaactat aagcatagtc attccatctt ttgccaaatt taaaatcact tctaaaacct
226561 cacgaacgat ttcaggatca agtgctgctg taacctcatc aaaaagcata agctctggat
226621 tcatacaaag actacgcact atggctattc tttgtttttg ccccccactt agctctcttg
226681 gataagcatc aattttatgc gccaaaccta ctttttcaag ccaaatttta gcttcttgta
226741 aaacttcttc tttagccctt ttttgtactt ttaaaggacc taaaagtatg ttatctttca
226801 cgcttaaatg ttcaaataat tcataggatt gaaataccat gcccactttt tgacgcaccc
226861 ttgtccaatc tttaaaattt ttatcgatct tatcttgtcc tatttctata atcccatctg
226921 caatttcctc aagtccatta atgcaacgca aaagagtgga ttttccacaa ccacttggac
226981 ctaaaattac caccacttct ttttgtttta cttctaaatt aatattcttt aaagcatgat
227041 gagagccata atatttttgt aaattttta tctttaaaat actcatttta actccatttt
227101 ttttccaatt ttctagaata taaagtaaca ggatagcaaa ttataaaaaa gataaaaaag
227161 attaaaccat atataataaa tggtgctaaa tcatttttgt tattaaattc tatgatttgc
227221 tgtcctactt ttacaagctc taccgcaccg attaaataag caaaagttgt gcttttaatc
227281 atgcgtgtta acaaattcat actcataggc gttaaacgac gcatagcttg tgggattatg
227341 acataaaaat aactttgagt tttatttaaa ccaagacttg ctgcactttc gtattggtgc
227401 ttaggaatgc tttttaaaga agttctcacc aaatccatca tttcaaaact tccccaaata
227461 ctaaaaacga taatagccga acttacactg cttaaattta tacctaacca tctagaaagt
227521 ccaaaatata ccataaaaag ccatactaac aacggcataa cacgaacaaa ttccaaagca
227581 aaacggcata gaatataaac ataacgattt ttaaggctca tcaaaatacc taaaaaaagc
227641 cctccaaaag aagttataat aatgcttacg atagaaattt caagcgtaat atacaaaccc
227701 ttccaaagcc ggcttagggt atctggattt aataaatcaa acatttgaaa accttttttc
227761 caaaaagaac aaaaacaaag aaataggtag aatgatacac aaataсссса aaaacaaaag
227821 caataaaagc tcatctgttt tataagtaag agaatttaaa cccttcataa cagtgactaa
227881 atccggcaaa gctataatac tcacaacaga ggtttcttta ataagaaaaa caatattcgc
227941 acttattcct ggcaagcaaa ctcctaaagc ttgaggtaat ataacataac gcaaattatt
228001 caatttagta aaacctaaag aaagccctgc ttcaaattgc tgctttttaa tagcctcaaa
228061 accagcccta aaactctctg ccatataaga accacccaag aaactaagtc ctacaatagc
228121 acatgcaaac gctggacgca aagcctcatc tacgcttaag cagatcaaat tcaaaggtgg
228181 aatttgttct aaatgaatat taaatttagg caaagcataa tataagaaaa aaagctgtat
228241 taacaaagga gtgttacgag aaaattctat ataaaccttg caaaaagctt ccataatcgt
228301 tattttaaaa taactcatta aaatacaaaa aaagcctata attatagaaa aaataatacc
228361 aaagaaacta agttttaaag taagccaagc agctgcaacg aaagcatttg aattagccag
228421 tataaaatca aaatccattt tattcctatt accaaagtcc tttttttaaa ccctctttat
228481 ctagtatttc aattttaccc tttttgtct caattagctc tgaattttta aattctttta

228541 aaattcttga caaagattca gcacgaacgc ttaaatccac tgcgatttgt ctttgcgaaa
228601 tttgagctaa attcttttca ttttctaaaa gatatcttgc aagtttgctt ttaagatcta
228661 aagaattttc taataatttt ttttctaaaa ttttaatttt ttcaaaaaga gatgaaataa
228721 gtaagaaatt aaattcttta ttttgagtac acagggcttc aaaatcctca aaatcaattt
228781 ctaaaatttc acaatattct tcacaaacag catttgctgg atatctcaaa cgcttaaatg
228841 taggcatttc ggctatgaaa ttgggtggtg taaaataatg caaagtaatc tccttttcac
228901 ctgctgtact cttatataca cgcaccctac cctttaaaag aataaaaaat tttaatgcct
228961 tttcgccctc aaaaaataaa atattatttt tttcaaattt tcttattttg cctatgcctt
229021 ttaaaaaatc caaatactct tgcattgtaa tatttaacct atgttaattt tatctcattt
229081 tgtttgtgct atattaacga taaattttta tccaaaggca aacataatga aatatgaaac
229141 cataaacaaa gaaagtatag ctaaacttat ggagcttttc tatgacaaaa taagaaaaga
229201 taaagattta ggacctattt tcaacaacgc cataggaact agtgatgaag catggaaaga
229261 acataaggca aaaataggaa atttttgggc tggaatgcta ctaggagagg gtgattatat
229321 cgggcaacct ctgaaaaaac atcttgactt acctcctttt ccacaagaat tttttggaat
229381 ttggctagga ctttttgaag aaagtttaga taaaatttac aatgaagaaa tgaaaaatgt
229441 tattttacag cgcgcacaaa tgatagcctc gcattttcaa aatatgcttt atcgttttgg
229501 cggacattaa gattatattt tcatcagatc tttttttgct ttttccaaaa tgtattcatc
229561 tttggaaaaa tcaaaatatt taaattcgtt tccatgctgg ataaatccat caagcaaatc
229621 tccactcaaa cgatttttaa gatcaagctc tgcgatttta aaaccatcta aagtactagc
229681 aaattctttt aatcgattcg gaatttcttt gagttttgta taaagataac aagtgctttt
229741 aagtccaata cgacctacgc gtccacggag ttgatgcaag gtagcaagac ctaagcgttc
229801 tgccccccacg ataacgatag tgctaagcct tggcaatgaa attcctactt ctacaaccgt
229861 agtgcttaac aaaatatttc cctcatcgcg aaaccgctcc aaaatttcat ctttttgctt
229921 gtctttccca tgcgtaacaa aaactttttc ataattatcc tcccaatatt tccttgcttg
229981 ctctaaagaa agataaggaa ttttatcgct tggattgact aaggggtaga tgatgatgat
230041 ttgatgattt tgcgcaattt cctctttgat tttttcacta agttttgcaa aaccctcatt
230101 ttgtatacaa agagtggtaa tatccttttt aaaaggcatt tccttgataa aactaaaatt
230161 taaaagctct gattgtatca tgcttaaagt tcttggaatg ggcgttgcgg aaaattgtat
230221 aaaatgtggg gctaattcat cttcatttaa agagtaaatt tgctgacgct gcgcagaacc
230281 gaagcgatgt tgctcatcta tcattacaag cacggcatta tgactttgaa ggtgaatgag
230341 tgcatgagtt cctataatca aattggtttt tttgattttt tcttccaaat ctttttcttt
230401 tttgccccct ttgataaata aaatatccat aaattcaggt aaaagttttt ttgcctcttc
230461 ataaagctga tgggcaagta tgctagtagg agccattaag atagcttgtt ttggatacac
230521 cattaaagca gcacctagca aaaccaaagt cttaccacag cccacatctc ccataatcac
230581 tcgtctttta gcctctttag agcttaaatc ttgctctata tcctttaaag catttaattg
230641 atcgcgagtg ggcgaaaaag gtaaattttt tagccaattg gctatattaa aaatttcaat
230701 ttcataagct ttaaaatggt ttttttttagc ttttaagcgt tttaaaaaat tataaatttc
230761 aatatatttt aaatctttct caaaacgctc caaattctca aacattaaaa aactttttc
230821 atcataagca tgcaaattta aaagaaaatg gatgtattta tcttcaatcc cgctttcttt
230881 taaattttta taattaagat attggtgtat aaattcagaa attttgttat cttgtaagcc
230941 ttgaatttga tattttggag caaagcctgc aaaagaagct aaaattttag gattattaaa
231001 ctgccaaata tgaccaaaac gcgtaagttt tgcatgtaaa ataagctctt tgcctatttt
231061 gcaaactcca aagtgccaag ccctaggatg aaaaaacaca aaagaaatgt cttgattcca
231121 ttccttgcaa aatcccaaac caaaaagctg gcctttttc gaatgaatgc ttttaatgat
231181 tattttttga gtacacagct cattttcttt cggcatttta ctaggactca aattttcgat
231241 tttttaggt aagataagtg ctaaatcgat cgcgctttta atttttaatt ttttaaaaaa
231301 atcaaaatca ttttctttaa ttttcatttg caatacttgc aaaatttaaa tctaaaattt
231361 cattatgctt tcgtatgtaa gcatttaact cttgaagttc tactttttct atgagcttta
231421 attcttcttt ataatatcct tttggcaatc cctgataata ctcgttaaag cttatcgaca
231481 aacgcctact aagactttca taacgcaaag gagttgaacc tatgagaaaa tttttagctt
231541 gtacaagctc attttgactc actccttctt ttacaaaatc tttaaatact tctttaacta
231601 tctttttagc ttctttagca ctttcatttt tagtttgcaa atagccaaaa actctagtaa
231661 aagatgcatt catatcaagc atagcataag cagaatacgc aagccctctt ttaacacgaa
231721 tttcttccat tatccttgat ccaaaaccgc cttgacctaa aataaatagt gcgatttttg

231781 caagataaaa atccttatca ttaaattttg taaaaaaggg tgcagcaaag taaatatagg
231841 cttgttcgct ttcttttctg atttggatta tgtctttatt ttttgagtta atttcataag
231901 ttttttgtga attttgactg cctttttgaa gttttgctaa taatttatct aataagcctt
231961 tagccttttc ttgagctaaa tttccaccta aaatcactac aagattgctt aaattaatat
232021 ttttcttata aaaactttcc aagtcgctta aagaaatttg cttgatagtt ttttcatcac
232081 catcatttgg actttgaaat tctttgcatt caaaaatttc gctattaagc aaattttttg
232141 ctaaataatc aaaatcacta tttttactag caagctcacc tagggcatta atttttagtt
232201 tttctaaaat tttttcttca aatctaggat ttaaaagtaa attttctaag tgttttaaag
232261 caaattcaaa attttcttcc aagcaagaaa gattgatttc caaactttca aaaccactac
232321 ttgcttccaa atttacagct ttaaattcca attctttaaa aaagttatcg tctacaccct
232381 cgtttaaaat tcttgcaaac atctttgcaa gtcctgctat ctcatcataa cttcttccac
232441 agtttctaaa aacaagcttt aaaataacaa taggaaaatc attattttcc tcaaaaataa
232501 aaggaatttt tatctcatta acttctaaat tctgcattaa aacctttcta aaatttcata
232561 agctgtattg cgcttagcgg gattttcgcc taaactttta atcaatctta tcatctcatc
232621 ttgattcatt ctataactcg cacccgcagc gcttacaaca ttttcctcca tcatagttga
232681 acctaaatca ttggccccaa attttaaagc aagttgccca atcaatgaac cttgtgttac
232741 ccaagaactt tgcaaatttt taaaattatc caaataaagt ctagctaaag ctaagagtct
232801 taaatatttg tttgaacttt gcttcataat ttcaggatgt ttttggatta atgctgtatt
232861 ttcgctttga aagctccata agatgaaggc cctaaaacct cctgtttcat cttgtaattt
232921 tcttaaatgt tcaaaatgat cgacaatctc ctcatcactt tcaaccgttc caaacatcat
232981 agtagcagta cttttcatac caatttcatg agctcttcta tgcacttcta accaagtagc
233041 agtatcgcat ttattgggcg caataatatc tcgcactcta tcacttaata cctcagcacc
233101 cgctccaggt attgaaaaca aacctttagc ttgaagtctt tgtaaaactt cagaaattga
233161 aattttagaa gccttagcaa tataagcaat ctcaactgca gaaaagccat gcacggtaat
233221 atttggataa tgctctttta tccatgaaac aagctcttca taccattcta tttttagttt
233281 aggatgcacc ccaccttgaa aaagaatttg tgtccctccg atagcttcga gctcttcaat
233341 ttttttaccg atttcttcaa agcttaaaat ataagcatca tcttctttat gatggcgata
233401 aaaagcacaa aaagaacaat ctatacaaca aacattagta taatttatat ttctatccac
233461 aacaaaggta gtgatttttt caggatgaag ttctaatttt ctttggtaag ccatttctcc
233521 aagctctact aaatttgcat tccttaacaa ataaagagct tcttctttat ctaatctttt
233581 caagttattt ccttaaaatc catttttagc tttattatag catttaaaaa cataaattat
233641 gctatagttt cgcttatgaa ttatattgac ttattgaaaa ataataaaaa tattagaata
233701 ctaaccttag tgcaatttat cgtatatttt ggtgcgtggt tttcccaaac aggtgttttt
233761 actcttttag taagccttaa cgctcccact tgggcaacag ctacaagcgc tatgcttgct
233821 tttttaccag gggttttgct tgctcctatt aatggagtta tagtagaaaa aaataagcct
233881 aaaaagcttt tattgagtat gattagcata gagcttgttt ccatttttctg tcttatcttt
233941 gtaacaagtc ttagtatgct ttggctttta tttattttaa tttttgttag actttgcgtg
234001 gcttcgattt atttccaagc tgaaatgagt ttgctagcta aaattttaaa tccagaagaa
234061 ctcaaacttg ccaatgaaat gcatagtgta atttgggcag tttcttatac agcaagtatg
234121 gcaagtgcag gaatttttat caattttttt ggtataaaag cagcattttt gtttgattgt
234181 tgccttataa ttattggaat tttactttta actagacttc atattcctca ttttaaacaa
234241 aaaattcaaa ataaattttt cactttgatt aaagaaggtt ttttatatgt tttgcaaaac
234301 aaaatcatcc ttcatcttat catattgcat gcttttatag gatttactgc ttatgaaaca
234361 ctcgtaactt tattagctca acacgaatat aaagaagttt tatctgctgc tttagttata
234421 ggatttttaa atgctataag agctttagct ctagcctttg gtcctctttt tctaagtaga
234481 attacaaaca ataaaaatat attttttcta tatctaggtc aaggtttggg aattatactt
234541 tgggctttaa ctcagtttaa tttttatatt tcatttatag gtcttattgc tgcgggattt
234601 tgcacctcat ctctttgggc ttatacttat acaatgattc agaaaaactg cgataaagct
234661 tactatggaa gagtgattgc ttatacagat atggtttatc ttagctttag tgctcttatt
234721 tctcaactca tgggtttatt attcgaacaa ggtcttaaac tcgctacgat tacagctttg
234781 cttggaatga tatttatatt tgcagctttt tattggaaat ggtttgataa gaaatatttg
234841 tgaattttat aaattcacaa attaaaagat taaattttaa ataaatcttg caagtttttt
234901 agagcttctt cattgcttag agtattgttt gtggtatgag tattaacctc attaagttcg
234961 atttcataac cgctaagcat acccgcaaga cgaatattaa taccactttt tccaatagct

235021 ttgctttttt gctcgctgtt taaagttaca attgcttttt cattttcaat ctttacagaa
235081 ctcactatag caggggctaa ggctcttgca attaaaattt caggctcatt gctaaattca
235141 atacaatcta tattttcatt gtgtaattct ttgcttactg cattgatcct aaccccttttt
235201 acacctacag tagctcccac aggatcaata tttgctccat ttgcttgaag tataattttt
235261 gctctttctc caggaattct agcacattta actacgctta catatccgtc tttaatttca
235321 ggcacctccg cttcaagcaa gcattctaga aatttagggc tcgtgcgact tagctccatt
235381 ttaatgccct tatctgtata aacgcgacgt atcacagcct taacaacatc gcccacttta
235441 aatttttcac ccttaatgcg atttttcctt ggcaaaaaag ctcttagctc atctatctct
235501 atataagtat tttcttcatt atcaattcgc acaacatttc caaaaaccat ttggccgact
235561 ttatttttat atttttcaaa aatagtttgc tcaagcagct tttgtatgtg gtattctaat
235621 tctttgtgta aaatatttac agctgttctt cctaaatttt ctaaagagca ttcataggtg
235681 agctcatcac cgatttcaac atcaggtgct tcttttttgg ctttgctaaa ttcaatgtag
235741 ctttctttat tttcattcaa tctctcatca ttgtcagcca caactgtaat tttttgataa
235801 agatttaaat tttttctatc tacaaaaaag tcatattctt gcccataaat tctttttgct
235861 gtatttatca aagctgtaat aactttttct tttacgcttt ctatatccaa atttttctca
235921 ttcgcaatgc tttctataat gtctgcgatt ttttccataa ttatcgatac cttttctttt
235981 gggatttttt taagaaagta gattaaaagt atatctttaa taagtttaat taaaagtaaa
236041 atttgataaa attatagatt taaaagacgc taaggataca attatggatt taaaattggc
236101 aagaaattta atcaatgaag agcctaaaaa tattagtcta gctaaaattg aagaggctgt
236161 aaatcaagaa ggacaaaagt ttttttattt tgataaagaa aattcacaca aagatcttat
236221 tgccttggtt gagcactttg aaaaaaaagg cttaagtgtt tatcatagaa ctatcagata
236281 tggtttagat agtaatgatt ttatgtatga ggttcacatt ctttgagctc gaaaaaactt
236341 tttattcaaa ccttaggctg tgcaatgaat gtcagagatt ctgaacatat gatagcagaa
236401 ctgacacaaa aggataatta tactttaaca gaagatatca aagaagcaga tttgatactt
236461 atcaatacct gctctgtgcg tgaaaaacct gtacataagc ttttttcaga agtaggtgga
236521 tttgaaaagg ttaaaaaatc gggtgcaaaa ataggagttt gtggttgcac agcttcgcat
236581 ttaggaagtg aaatctttcg tcgtgcccct tatgtggatt ttgtattagg agctaggaat
236641 atttctaaaa ttaccgaagc tgttaacact cctaaattta tgagcgttga tatagattat
236701 gatgagagtg aatttgcttt tggtgatttt agaaatagca tttacaaatc atatattaat
236761 atctctatag gttgtgataa acattgtact tattgtatag ttccgcacac aagaggagat
236821 gaaatttcta tccctttaaa tattatctac aaagaagcac aaaaagcagt agacaaaggc
236881 gctaaggaaa ttttcctact aggacaaaat gttaataatt acggcaaaag atttagaaat
236941 gaacataaaa aaatggattt ttcagatctt ttagaagaac ttagcaccat agaaggttta
237001 gagcgtatcc gcttcacaag tcctcatcct ctacatatgg atgataaatt tttagaagta
237061 tttgccaaaa atccaaaagt ttgtaaatca atgcatatgc ctttacaaag cggctcaagt
237121 caaattttaa agtctatgaa gcgtggctac actaaagagt ggtatctaga tagggcatta
237181 aagttaagag aactttgccc aaatgttagc atatcaacag atattattgt tgcatttcca
237241 ggagaaagcg ataaagattt tgaagaaact atggaggttt tagaaaaagt acgctttgaa
237301 caaatgtttt cttttaaata ctccaagcgt cctttaacca aagctgctac tatgccaaat
237361 caaatcgatg aagcaatagc ttcaagtcga cttagtactc tacaagctcg tcataatgaa
237421 attttagacg atattgtcaa aaaacaagaa aataaaactt ttaaagttct ttttgaagaa
237481 ttaagagcgg gcaatgctat tgcgggtaga acagataaca attttttggt gcaagttgaa
237541 ggcagtgaag aactcttagg aacttttaaa gaagtaaaaa ttactaatgc aaagcgtatg
237601 gttttatatg gggaaatcat ttaaaatagc ttttatagca cgcttagttt tcattctaca
237661 atggctgatt tttattactt gtaaaaaaac ttataaaggc gataaagttg atcaggattc
237721 ttatgtgata ctttttttggc atgggcgtct tgcccttatg ccttttgcct ttaggcattt
237781 tggtaaaaaa ggaaaaaaag cttatgttat gatctctcat catagagatg gagagcaaat
237841 tgctagaatt attaaattat ttggactaga tacagtgcga ggaagtactt caaagggagc
237901 cagcacagct cttcgaagtg cttttaaaat tctagacgaa aatcacgatg tagtcatcac
237961 tcctgatgga ccacgcggac cttatcatag tatttcagat ggagcagtat tgcttgcgca
238021 gaaaaaaaat gttaaaataa gaattttaaa ctatgaggcg agaaattttt gggaatttaa
238081 aagttgggat aaaatgattt tgcctaagcc ttttactcat ttagtctata ggcttagcga
238141 gcccttggat atttcaagtt tagataaaga tcaagctaaa ttttttttac ttgaacattt
238201 taaaaatatt gaacaaatgg ataaatttta aggaataaaa tgttattttt acttaaaaaa

238261 atattaccac aacttttcac aagtattatt ttagaagaca aaaaaaacat ccttaaaact
238321 tctatatata aaaaccaaaa acttataagt agcaatgaaa aaactttcga caaaagtgaa
238381 aaattattag aatatgtaaa aacactcaat agcaaatttt tattttacca tacagcatta
238441 tttttaaatg ttaaagagca aggtttgatt cctagtaaaa atgataagga ttttgaacga
238501 tttaatgtag gcaaaatgag cttaaaatcc gtcacattaa ataatgcttt aatatacaca
238561 gcgacagaac acatagaata ttttaatgaa ttttttgagg attatggtgg tttggatttt
238621 ttatattctc cttttgctat actttatcat aatattttaa aagaaaaacc aagtaatgac
238681 aaaataactc tttatgctta tagacatgag tacattttaa cccttataat ttgccgaggt
238741 gttgaaattt tatatggaga tatcattttc tttgaagaag aattgggctt agaaattgaa
238801 aatcaagaag agaatttaga tagcctaggc aaagatatat taagcgatac tgaagtaacg
238861 cttgataatt ttaatgaaac tctagaagat aagctcaacg ctcttgatca atttggtgat
238921 aatgatctag aacataacga agacgattca gagttcttag atggtaagga taaactcgat
238981 cttgaagaga tgaatcaatt tggcaacgac atggagcttt gtcgccatat agtcatgagt
239041 attgaaaaat tttacaagga tgaaaaatat cagagtgttt ttattgataa tatttttatc
239101 cttagcacaa aagaactcga tcaaagcgct attgaatttt tggaagaaga aactttatta
239161 gaaattcaaa ccaaacaaat caatacttta gattcaatta cagaactcat gcaaaaggag
239221 cttcagtgac aacctatagc ttcataaagc ctagaaaaaa gcctatcttt acactctttg
239281 ataaaatatg gctgggactt tttagttttg gtatattttt tatatttgca atatattttt
239341 tataccttac caagattgca ttaacacata atctaataga agaacaaaaa gaacaagttt
239401 tacaatttca aaataaaaca caagaaaatg gacaattata taatattctc ttagaacaaa
239461 gtgaaattgc aaataccttt tatacccaaa accaagcttt taaaaatagc ttaaaaaatc
239521 tttttgacat aatagtcaag accgatggaa tcactcttga aagcgtagag caagaaaaaa
239581 attctcttaa gcttataggg gttacaccta caaaagaaat gtttgcctta cttttagaaa
239641 ctccattgaa aagtattttt gatcagagtt ttacaactta ttatcgctta aacaatggtt
239701 ggtatcgctt cattagtata agtaaacaaa attcaggagt tttacaatga aagataaaag
239761 tcttgaagaa ttaaatattt taaagattat tgtttatgtt ttaagcttta tcactgtatg
239821 tactgctttg attttatttt tattattgcc tgtgctaaaa gattacaagc aaacccattt
239881 aagaaaaaat tcacaagcag ctatatttaa tgcagcgaaa gctaagttag atgccagtga
239941 aaataaatta atcacactaa gaacagaaaa caataaaagc ttagaacaat ttgaacaaca
240001 attcaatcta gcacatcttc aaaatttttt agaaaaatac tttgaaagta tacagataac
240061 agagcttgaa ttagataaaa aagaaaaata tcttacccat agaatcaaca ttcgagcatt
240121 gataaataat cctagacgct tatatgactt tgtagatgcc ttagctcaat ataacaatct
240181 tataaagatt gattatccca tgaccttaaa ggctagtgat catgggataa tgataaatct
240241 taatgtgcaa atttactcaa actaaactta cattctcgcc ttttaaaacc tgattcattg
240301 tattcatagc acacattttt ccacacatag agcaagaatt taaatcatca gggcgtcttt
240361 cgttaaacat ttttttagct ttttcgccat caatagcaag tttaaacatt ttctcccagt
240421 caatatcttg tcttgcctta ctcatttcat catctctttt tctttctttt ggaagtttag
240481 ctaaatcccc tgcatgagct gcaatcttag tagctactat accatctcta acatcattta
240541 aattaggcaa tcttaaatgc tcagcaggag ttacataaca aagtatatca gcacccgcag
240601 cagcagccac agcaccaccg atagctccgc ttatatgatc atatccagca ccaatatccg
240661 taaccaaagg ccctagaaca taaaaaggag cacctttgca aatgcgtttt tcaatttgca
240721 tattggcttc aatctcatta attgccatat gcccaggtcc ttctatcatc acttgaacac
240781 ctgcttccca tgctctttga gtgagtaaag ataattcgat aagttcagaa atttgcgctg
240841 catcacttgc atcatgtgta gatcctggac gcaaagcatc gcctaaagat agggtcacat
240901 cgtattttaa acaaatttca agcaaatcat catagtattc aaaaaaagga ttttcagctt
240961 ctttcatagc catccacgca taaagcaccg atcctcctct agaaactata ttagtaagtc
241021 ttttactttc tttaaaaact ctagccgcac gcgaatttat acccgcatgt atagtcataa
241081 aatcaactcc gctttttgca tgatgataaa ccacatctaa aaaatcccta gcgctgattt
241141 gttttagatc tttttctaaa aatcctaccg catcataaac tggaactgtt cctatcatgg
241201 cttttgaaac tgcgataagc tcgtctctaa agcgacttgt tttgccataa ttacttaaat
241261 ccataatagc ttcaattcca aatttgtgag caagctctac ttttttcatc tcctcactat
241321 aatcaacaca atcatttgaa acacctaaat tcacattcac tttagtacga agtccaagtc
241381 ctatgccatt tggatcaaga cttttatgat tgatattcgc cggaatgata atcttaccac
241441 aagctatatt ttcaagcaaa aaatctttgc ttacttcttc tttttgagca acaatctcca

241501 tttctttggt aaaaatacct tctttagcat aaagcatttg agttttcata tttatccttt
241561 aaaaaatttg gataaatgag ggtgcaagta aattgtagaa agtataaatc ttcccgacgc
241621 tagcattatc tagttccggt tcggtctaag cttttagctt actctcagcc tgtatcacaa
241681 gctcccgtca tttatgtttt aattatactc tttaaatatt ttatttttgc tttatacgag
241741 aagtcttagt aaaatttata agttcttcag tggttttgat atgataagga agtttgccaa
241801 ggcaatgttt aaaaatttca gccgcagcca aagcatcatt taaagctctg tgatgagcat
241861 tttttatccc taaaagttct tttaaaacat ctagtttata tcttgggctt tctatacagc
241921 attgcgcaaa ttctatagtg cagatacgac gattaagcaa aattccaaaa ccgcattcat
241981 ttaaggcctt ggaaataaaa ccataatcaa aacgcacatt atgtgcgacg aaaatactat
242041 cttttaaaaa aagtctaaaa tcatttaaaa ccttagtcaa actaggcgca tctttgacca
242101 tatctaaatt aattcctgta agttctgtaa tattttcagg aatttctttt acctgcacca
242161 aggagctaaa acgatcaatt tcttgcaaat tttttatctt aactgcccct atttctaaaa
242221 tttgtccgtt tttaatccca cctgtacttt ctatatctac aatacaaaaa atttcttctt
242281 taatcttttt tgtacgcgtt tgtaaggtaa aaatattatt tttatctaaa gttaaaccca
242341 aacccaaaag ctcaaaagtt tccaaatcaa gctcataatc ttttaattct tcaatcttct
242401 taaattcctt gattacccac tcatagggct tactttgctt acttaaaaca gaaataatct
242461 gatcaatttg ctgcaaactc aaaattcaag ctttaaagat gaaattgaag ttttataatc
242521 atttgaagaa aaaatataac ttccagcgac taaaatatct gcacccgcgg cttctaaatc
242581 gcttgcattt aaaccattga ttccgccatc aacctctata aaaaccttag catttctttc
242641 atcgatcagt cttcttaatt ctttgatttt atcatatact aaaggtaaaa atttttgtcc
242701 gccaaagcca ggattaacac tcattaaaag caccatatct acaaagtcta tcatatgttt
242761 tatagaattt ataggggtat gtggatttaa aacaatagca gggtgaattc cttgacttct
242821 tatatactcg caaagcctta taggatgagg ctcgctttct atatgaaaag aaataaattt
242881 aggtttgata ggggtaaaaa gatctacaaa tttacctaca tcttttacca tcaaatgaac
242941 atctaaagga attgaagtaa tagttgaaat tttttctatc acacagggtc caaaggttaa
243001 atttggaaca aaatgtccat ccataacatc aatatgcaaa agatcagctc ccgcctcatc
243061 gattgcttta atctcttctt ctagttttaa aaaattggcc gataataaac tcggagctac
243121 atacataaaa ttagcctcat aaagtaaaaa attttaaaga aaaacatttt atctatttat
243181 aaattaaaaa tagtataaat atttgaatat tttaagctta aatttgatag aatcttgatt
243241 taaaatttcc aaaggatgca aatatggcaa gaatatgtca aatcacaggt aagggaccaa
243301 tggtgggtaa taatgtcagc catgctaaca ataaaacaaa aagacgcttt ttacctaatc
243361 ttagaacagt tcgcgtaacc ctagaagatg gtactacaag aaaaatgaga attgctgctt
243421 ctactttaag aactttaaaa aaacaaaact ctaaataaac tcttgaggat tcgtcctcaa
243481 tgaccttaaa attcttcaat tatcattacc ttatttaata aattgctaat ttgtaacata
243541 tttaagattt attgaattgt tcagtatttt ttaagccaaa aatatcattt aattagctat
243601 aatgtaacat atcatttaat attttttaaa ttttatcaat ttttaaggag aattttatgc
243661 tacatgaata tcgcgaactt atgtcagagt taaaaggaaa agatgcgcat tttgataagt
243721 tatttgagcg ccataatgaa cttgacgatc aaattaaaga cgcagaagaa ggaagagccc
243781 tattaagtga tattgaaatt tcaaatctta aaaaagaaaa attacatatt aaagatgagt
243841 tgaaccaata tctatcaaac tacaaaaaat agggtacaaa tatgtttgga agtaaaataa
243901 atcatacaga acttcaaaat atcaaaaatg aaaaccaaaa tcttgctcat cagcttgaga
243961 aaatcaaggc tgaaaattta gagttaaaag ataaaatttc aaccttagag caagaagctt
244021 tagagtcaaa attaaaaacc gatcttttaa atgtattatt aggcggtgtt ttgaaaaata
244081 ttaccatagt tcaaagcgat atgcttgaaa atgttaataa agctgaaatt atttctaatt
244141 attctaaaac ttcactagca gaaatggatg aattaaatca tatagctaat tctataaatg
244201 cttctttaag cgatattaca gaatcagcta ataaaacacg cgatgttgca ggaaccttac
244261 atcgtagtgt tgatgagatt actaatgtaa tcaatcttat caaagatgta tccgatcaaa
244321 ccaatcttct agcattaaat gctgccatag aagctgctag agcaggagaa catggtagag
244381 gttttgctgt tgtggcagat gaagttagaa aattagctga aaaaactcaa aaagctacaa
244441 cagaagtgga aatgaatatc aatcttctaa aacaaaatgc aaatgaaatg tatactcaaa
244501 gtgagcaagt ggaaaaaatt tctattaatt ctaatgcaca tattatgagt ttttcagaaa
244561 aatttaccca cttagtcaat gaagcacact ctacaaattc aaatgccgta ggcattgctt
244621 cagaagcatt tgtatctttg gcaaagcttg atcatatagc atttaaactc aatggatata
244681 aagaaatttt tgcaaaatct ggacaagaat tagccgatca tacaagttgt cgtttaggca

244741 aatgggtagc aagtactgga aaagaaagat ttggaaacag caaaagcttt acaaaaatca
244801 atgagccaca tcaaaaagta catgaaaata tgaatagtgc tatccgtatc gcgagtacag
244861 aagatgcaag cttaggcaat actcaagatg taattatcaa aaaatgtgaa aatgctgaaa
244921 atgcatcttt acatttattt gatgtattta aagaaatgct taatgaaaca aattaatcaa
244981 attctttgcc ttatttaagg caaagctctt tcttttcttt taaaaaccac aaataagcaa
245041 aatcaagcaa gggtgtatgg attatctttt tagaaaattc ttcatactcg ttaatcttta
245101 caaaaacagc ctctatttgc tcatcctcaa cgccacctcc ttcacaaacc ttatcattct
245161 catctacaat tgcaaaataa aaactttgtc tgctagctcc tgaaccaaag cctgtataaa
245221 aatcacccac tttttcaaat tctttaggag cataacctac ttcttcgata cattcttctt
245281 tagctatttg ttccaagggt aaatctttat ccacaagtcc tgaacaaagt tctatggtat
245341 agcccatatt ttcatctttt tgatagtttt tatcttgctt ttggtgatac caaagcggta
245401 tgcgaaattg acgaacgaaa ataaagcttt tcaattcttt atggtataaa agaattgaaa
245461 cgctatcttt ggattctata aaatcccaag tgcaaagctt gccattactt tcatagctaa
245521 atcttttagg tttgatatac ttagactcac aaaaaggaag ctcttttaaa tttttaaata
245581 tcataaaaca aagcctttat tttttcttca ttatactttg tttttacttg ttttaagtta
245641 agatgctctc cttttttca ttttaaaaat catatttgta acttaaccaa aaacttcttc
245701 cttctgtata atcttgatag cgatttgcat aacctcctgg attagcacta gtatcccatc
245761 ttgtaacttg tggatcaaag aaatctttgt cgaaaagatt ttgtactgta aaacttaaac
245821 ttgattgttt atcgatttta taatttactc ccaaatccac tatataaaaa ggcttatatt
245881 tttgccatgg taaaattcca ctaccgccac cttttgatct ggctatagta tcaagtcttg
245941 cacgtgcttt aacataagag ctgaatttac cttgctcgta atttaatttt agcatagcaa
246001 tatgtcttgg caaattttct attctatctc caccaaacca atttgtctgt ccatccttat
246061 aacgattatc catgaaagta taacttgaat tgacactaaa accattataa ggttttgtat
246121 tgaaagcaaa ttcaacaccc ttggtttgcg ttttgccaag attgatagaa cgctcacatg
246181 taacaccatt ggtacaatca atatcatttt gcattcctgt taattcttca ctactgattt
246241 gatttttaaa atcagtaata aatcctgtaa tagaataatg agcaaaatca aatatcctaa
246301 aatcaactcc caactcataa tttgtacttt cttcaggttt taaatcagga tttccaaaag
246361 aagcactgtc attgctataa ttataataac cattagtaag ttgttttgct gctggagttt
246421 tatagcccat agctacacct cctttaaaag caacattatc atttaaatga taaacaagat
246481 atactctagg agttagttca gaatcaaaca aatcgctata aatataacgc aagcctgtag
246541 taaaaatcaa atcatcgcta ataaaatatt caccctcacc ataaagcgca accgtagttt
246601 gatcaagatt tttacccctt acttcactac ctgcactttc ttgatcgttt ttaagcgtct
246661 catagtctat tcttgttcca aaagtgctta ccaaatttcc atatttatcc aaattccaag
246721 gcaagacaac ctttccttcc cctacccaaa tttgactatg taattcttta tctttggtgc
246781 tgccatactg taaatacgca ttacttgttc caaattcata atttccatca tgatttaaaa
246841 caacattatc tttaaacagc tgcctttcgc ttttgatagc cctaccgctt gtagaattta
246901 ctgaaatttc attgacataa cgctcaacat ctaaataaac attattacta tcatttacag
246961 tccaattcag tcttgatcca aaacccaaac tcgtaaaata tccaggacta tgacttgcga
247021 tttgataatt ttcaggtctt tgagtaccac cattccaaac cggcgtaggc cattttaaat
247081 cagaagctgc cttatcataa tacttaatcc tagcagaaat tgaaagcgta tcttcaatca
247141 aaggtgtagc cacataagca ttaaaaccac ccgcatttcc atatttatta gaatgctgct
247201 gtaaaagggt gttaaattct atactcgctt cggttttatt aggatttttt ttagtaatga
247261 tatttattac cccgcctacc gcatcactac cataaagcgt agaagcaggt ccgcgtatca
247321 cttcaatacg ctctatcata gatgcgggag gtaaatatcc tgattcgcta ccactaaaac
247381 cattatcata aaaaccatta gcaacgcttt gacgctttcc atcaatcaaa accaaagtat
247441 aagaactccc aaaacctcgt atgctaaaat cataagtacc ggttttattc atagtaacat
247501 caacacctgg aatatctgaa attgcctctc ctatatcctt aatgggtttt ttactaagtt
247561 cttccttggt aataacagaa atactagcag gcgcatccac taagtcttgc tcaaaaccac
247621 ttgcgctcac taccacatca tcaagttggt attgttcttc tgctttaatt tcacctaatc
247681 ctatcaaaca aaaacacaaa atgcttaatt taagcttttt agcattcatc gtcctttttc
247741 ctttcttgta ttaggctatt atgatagtta aaatcaattt tgaagtatag ataaaaatga
247801 ctttaaattc acttaacttt gataataatt ataaaaacaa ttttaaagct taaaatttgc
247861 acaaaacata gctttttaga aatattataa gttattttaa aagacaagta agaggataaa
247921 aatatcttaa aaaaaagtat tatatttata cacaaataat ttattcaaat ctccttatgg

247981 gagatttgaa ttgctatcta acagaatttg ttctgctttc ttaataacgg gtagatccac
248041 catcattcca ttatgattaa aaatgatatc attgctgttt tttctaagcg ataaaatttc
248101 ttttgcccat gcaatttgtt gagctgatgg gctaaaaacc tcattgattg gtacaacttg
248161 atttggatga atacacaaag atcctccaaa gcccattgat tttgcaaata gcaaatcttc
248221 ttttaatcca tcaatatttt ttatatcagg ataaactcca ttgattggcg ataaaagatt
248281 gtattttacg ctttttagaa caatctgcgt tctaatgctg tttaaaataa aattcttccc
248341 ctccccctct tgcaaattta aatccaaagt catatctaaa cttccaaaac ttaacgctaa
248401 aacactcgga tgcttagcaa tatcatctaa attttccact cccaatgcac tttcgatgat
248461 aggtattata ggaagttcaa atttgctaag tatatctata tcctcataat tttgcgcctt
248521 tggcaataca atgccaacaa tactattaga ttctttaata gttgaaatta aggacaaatc
248581 tttttgaaat tccgcgcttt gtgtttcatt aatccttata agaaatttat cgttattttt
248641 agcacattga cttgaaaaat ctgcaatatt ttttcttcca acttcttttt ttgcctcttc
248701 aacagaatct tccaaatcaa taataatttg attagcaccg ctttgtaagg cttttataaa
248761 cctttctgga taaattgatg gaacaaataa aaaacttttta gctttcatag aaaaatcctt
248821 aaataatctt tttttctttg aacaaattta tcttatcaga aaaaccaaat tctctaagaa
248881 ttgattctgt gtgttctcca agggctggaa ttttgtccat tctagcctca taattgttat
248941 ttgttgccgg cggtaaaagt gcaggtatct tcccaacctc gctatcaact tccctccaac
249001 gatttctagc ttttagctgt ggatgttccc atacttgatt gagttcgttg acatttccat
249061 ttgcgatttt tgcttcatca agacgattta taacttcttt tgcgctaagg tttttaaaag
249121 cgtcttcgat tatttctttt acttttgatg catttttggt tcttaaggta ttgtttttaa
249181 aatcttcgtg atcaattaat gattcatctt gcaatacaat tttacaaaag ctttcccatt
249241 ctcttggatt ttgcaagcca aacattacaa ctccatcttt tgccttgtaa ggaccatatg
249301 gagtaatact tgcatgaaat gcaccttttc tctctggagg agtcgcattt ttataggtgt
249361 aataaagagg ataattcatc cattcagcca tacattctag catagaaatt tctaccctag
249421 aaccctttcc agtttttttct aagtataata atgcctgcaa aataccgcta taagcataca
249481 ttcctgcgga aatatctgcg atactgattg gaacttttac acattcatct tgcgtacccg
249541 ttatggaaac taagccactt tctgattgta ttaaaagatc atatgctttt ttatgtccat
249601 aatttccacc ttcaccatat ccagaaatat cgcaaactat aatctttggg ttgtattttt
249661 ttagattttc ataatccaat cccaatcttt tagatgcacc aaatgctaaa ttttgcacaa
249721 aaatatccac ttttggtaaa atcgtcttga taaattcaag cgcgtcagga tcttttaaat
249781 caatagccaa actttcctta ctgcgattta cccagacaaa atgactagct ataccatcta
249841 ctcttttatc atatcctctt gccaaatctc ccacttcagg tctttctatt ttaataactc
249901 tagcccctaa atccgccaat tgtcttgtac aaaaaggtgc tgctattgct tgttctatac
249961 ttaaaaccag ttttccttct aaaggtaaca tctcttctcc tttttattta aaatgaatag
250021 tgccagaatg cgctacaaat ccatttttat ttatccatac ttcagcagta tttttatcag
250081 taatttttga ctccacagca aaactatcag gaacacaaag tgcatgcata cctctataac
250141 tataattaac aatttgttta tcgggatgat tttttacaaa gcaattaatc atataagttg
250201 ccaataaagg accatgtata actaaaccat gataaccctc aacttgcgtt gcataaggaa
250261 aatcatagtg gatcttatgg ccattaaagg ttaaagcaga atagcgaaac aaaagcgtgg
250321 aatcaggttg catttttca gaataatctg cattcatagc aactacatcg ctcgtaagct
250381 ttggaggtgc tggttgttta taaacaatat cctgttcttc ctcgattttt aactgctctt
250441 tttgataata gcagtgctct aaggtaacaa aagtaagttt tccacttttt ccttgttttt
250501 cttgaatctt ttttattgtg gttatttttg ttgcatcttc tccaataatc aaaggggtaa
250561 taaatttaaa tctacctcct gcccacatac gattgacttc accaaagctt ggcaaaaagc
250621 tatttttatc tagtcctagt ttaggatggc catctctgcc taatttatct ggagttagtg
250681 cttcattaaa aaagcaccat tgccaaagat atggaagggc atcgccattt tttaattgct
250741 tagattgtag cgttgcaaca attttttgca cctgtttttc attgatgttt tctgtaaatt
250801 tttcttgttt tccaataaaa ttttcataac tcattttttt ctccttaatt taaaatacct
250861 aaaaataata atacaaaacc aaatactacc aaaattaaag gtaatattat caatcttata
250921 aaaagatagc gtttttcact ttcatcttgt gcaccagcca aaaccaaggc tccgcctgtt
250981 gaaaaacttg aatatccagt aaatgttgca aatactgcga ttatagaaaa tgcaagtgat
251041 ggctcaagcg ctttgattga tggtacgata gagaaaaatg ttggcataac cacccccatg
251101 cttgaggcaa acgcactcat acaagaagat aaaattccaa gcaaataata aagccaatta
251161 ctgctacttg aaccgtgcgc actcataaat gttgtgatat tttccattat gcctgttgtt

251221 ttactgagct caataagcat agaaagtgat ccaatcatga aaatcaaatc ccaaggcaca
251281 ttcttaaatg cttctttttc actaccaatt ccaaatatta ctgctaaaac gattccaaga
251341 aatgaaacaa aaacagggtt aaaaatggaa gttacttttt ttagtgtttc atcatgagga
251401 tttaaagcaa gaaaaaaaga cgggatcata ataaccgcaa aaacaaagca cacaataaaa
251461 atcgtcttcc attgcgcagc atttaatctt tttggttttg taatttgcga agtaattttc
251521 accttataac cttttagcaa aaaatatccg attataaaaa tcaaagtatg tgcaataaac
251581 atatttttac ctaaatttaa ttgcattatt gcagaagctt ctgctgaata accaagtttt
251641 tcgataagat caaaagttac tctgccacca agggtaaagg gattaaatcc acctgcacag
251701 gaaccagaat aaataataat agctgctaaa atcttattca ttttgatttt gctagcaata
251761 taaagaacca aaggactcat aaaagcaaag cctgcataat gtcctggtcc aattcctaca
251821 aataccataa ctgtaatata aagtgcaata ggaatactcc aaggtctgtt cctaacagaa
251881 tacacagctt ttaatgcaag attgtttagc gtaccatttg caatcgcaaa accataaaaa
251941 taagtaatag aaagaagtat aaaaaaataaa cttaaacttc cattccaaag attaatcacc
252001 ttttctggtg ttataccgta aaggtatgcc ccaataaagg ccgaaagcat cgcaaagact
252061 ccgatatttg ctttaagaac atagcccaaa gcaattgata ttatgaggga aaataaaata
252121 ataatattaa tagtatccat catttagctc cttatcttga gtaattttaa gataattttc
252181 tactaacttc caataaaaaa cacttaaata ctatcaacaa atttttgaca aataaaaatt
252241 ttatttaata atgttaccaa tattctcata ttttaaattt attgttactt aattaagcaa
252301 atttaaaaaa attatatatt tcatttttat atatttttaa tatgaaagtg atattttaaa
252361 tattaataaa tttttattac aatattaccc atttgtcaaa ttattttta tccattaaaa
252421 agtataaatg cttctttaca tcttctatat tgttttattt gcaatataga ggatataaag
252481 tattcattat ttatctctta tatttattaa actttttttg taggttttgc tttattgtca
252541 aaattttaat tttctagaat aatttgaaaa ttttaaagaa tatttttatt aagtagagtt
252601 tttagctttc gctaaaagct ccaagttgaa gaatttttttg catgcgattt gaagcaagtt
252661 ctcgtggatc tattttttct aattcatcca aggctttttt aacataatca gctatagcaa
252721 cagcagcagc ttctttattg cgatgggctc cgttgatagg ctcttcaatc acatcgtcaa
252781 ttaagccttg actttttaaa tcatctgcag taactttcat agcttttgtt gctgcttcac
252841 ttttagaagg atcattccaa agtatagccg cacaaccctc aggagaaatg acagaaaaaa
252901 ctgaattttt catcattgca agcttatcag ccacacctat agccaaagct ccaccacttc
252961 ctccttctcc tataaccaca gctatagtag gagtttttaa atcgctaagt tcatacaaat
253021 ttgtagcaat agcttcactt tgaccacgtt cttctgctcc caaacctgga taagcacccg
253081 gggtatctat caaaaacaaa ataggaattt gaaatttctc tgcaagtctt gccactctta
253141 aagcttttct ataaccttca ggatggggca tgccaaaatt tctagcgatc ttttcttttg
253201 tgccacgtcc cttttgttct cctataacta taagtttttt ttctcctaaa tatcccataa
253261 aacaaacaat agcaggatca tctctaaaag cacgatctcc gtgaatttca taagcatcat
253321 ttaaaataag atctatataa tcaagtgcat aagggcgatc tggatgacga gcaagttgca
253381 aacgtccaaa atcgcttaaa tttttataag ttttagcaat ttcttttttcg agattttttt
253441 taagtatagc aacagcctcg gtatcaccct taatttgagc attgataata tcttcatcaa
253501 tttgctgaat atttttttca aaatctaaat acgaagccat atcaatctac tttttttaaat
253561 accacacaac catttgtgcc gccaaagcca aaagaattac tcatcacaac tcttaaatcc
253621 gcttttctgg cctcatttgg tatataatct aaatcacatt cttcatcgct aacaaattga
253681 ttaatagtag gtggcataat tccattgtct aatgccatga tactaataac cgcttcaata
253741 gctcctgcag cacccaaaca atgccctgtt tgtccttttg ttgagctaac cgctggaatt
253801 tcacttccaa aaagctcttt aattgcagct gtttcatttt tgtcatttac tggcgttgaa
253861 gttccatgtg cattaatata atctaccttt ggatttcctg ccatttttaa ggcttttttc
253921 atcgctctta aaggtccatc taatgtaggt gaggtaatgt gatgcgcatc agcactttct
253981 ccaaaaccaa ccaattcagc ataaatctta gctccacgct ttttagcctc ttcatactct
254041 tcaaaaacta aagcacctgc accctctccc attacaaaac catctctttc tttatcaaaa
254101 ggtcttgaag cgtgagcagg atcatcattt ctagtagaaa gggctttcat cgaagcaaaa
254161 ccaccgattc ctacaggaca aatagcagct tctgcgccaa ttactaacat tttgctagca
254221 ttacccaaag ctatactttt ataagcttct cctatggcat gagttcctgc tgcacaagcc
254281 gttacacaag aaatattagg tcctttaagt ccgtgttcta tagaaattaa accgcctagc
254341 atatttacta aagctgaagg tataaaaaat ggagaaattt tacgaggacc acgttcagaa
254401 caaataacag aattcttttc tatatttggc agacctccaa ttcccgcagc tgaaacaact

254461 ccaaattctt ctttgtctaa attttgatca aaattagcat cttgcattgc ttcacgagca
254521 gctttaatac ctagctgaat aaaacgatcg attttttttaa cttctttacc atctactact
254581 tctagaggat cgaaattttc aatttctgct gcaatttgaa ctggaaagtc tgtagtatca
254641 aaaagagtga ttttttttcac accactctta ccttcacaaa tcgccttaaa agaactttct
254701 ttatctaaac ccaaggcatt gatcatacca atacctgtta ctacaactct tttcaagaac
254761 aagctccttg taaagtgaat tttctaaaat tattttttaa gattttcgat ataatttaca
254821 acatcctcaa ttttaattag tttttcagca tcactatctg ggatttctac ttcaaatttt
254881 tcttctaatg ccataataag ctctacaaca tccaaagaat ctgcacctaa atcttcaatg
254941 atttttgatt ccatcttaac agcatccgca tcaatgctta attgctctac caccactgct
255001 tttacatcat caaatgttgc catttatata tctccttgat aaaaattaaa ataaagcgta
255061 ttttacaata aaaaacttaa aatatttata gtttttgact atactttata aaaaccattg
255121 aattttagga gtaagaaatg cttttttctc gacttattga aacaaattcg cacatttggg
255181 atgaatattt acatcatgaa tttgtaaaaa aattagaaaa tggaagttta aaagaagaaa
255241 attttctttt ttatctcaaa caagattata tctatctcat ccattatgct aagtgctatg
255301 cacgacttgc cttaaatgca aaaaatgccg aagaactacg ctttgcaatg aaatttcaaa
255361 actacattat agaaggagaa attgaacttc ataaaagtat tttaaaactt ggcatcaatg
255421 ctgataaatt aaatgttaaa gatgaaagtt tgaccaatat agcttacaca cgctatatgt
255481 taagcgtggg tgaaagtgga gatttttttag atatgttagt ggctttaagt gcttgtgcca
255541 taggttatgg ttatatagga gctgaaattt ataaaagact cggcaaggaa aaacttcaaa
255601 atcaccctta taaagaatgg attttaacct atagttcaga tgaatttcaa agtgaaatta
255661 aagaatttga agattttttta aattcttata ctcaaaaaat atctcaagaa aaatttgaaa
255721 atcttagcga aattttctat aatgtagtgc gtttagaaaa tgctttttgg gaacatgctt
255781 tgcaaatgaa aatggatatt taaagcaagg aaaatccttg ctttaagctt tgtaaagttt
255841 aaaatctccg tttggaaact gcattttagc acttaaattg tcaagataaa aaacttcaaa
255901 tctagaattt tgtggagttt cataaaggct tacaagataa ggatattttt caaacattaa
255961 ctcttttact tttgcgttag attcaaaaac tgcatttgcc ctaagtctta aaaaaacacc
256021 ctctctagag caagaacaaa attcaatacc attataattt tgtatatgtt taaaaagtcc
256081 tttattatta gaagtgcaaa aataaattct atcccctaca agcaaagggc tttgcatagg
256141 acgaactcta ggatttccac aagttcctaa agttgtcaaa aatgcaggag tattttcatc
256201 taaaaattgt gcgatttctt ttaaatccat atgctttcct tacatatata atccgccatt
256261 gattttaagc acatcacctg tcacataaga agcataatca cttaataaaa acgctacaca
256321 atttgcaact tcttcaggtt ctgcaaagcg ttttaaaggg atattatctt gataagcttg
256381 tttgatttca tcgcttaaaa tctctgtcat atcactttta ataaagcctg gagtaacaca
256441 attaaagcgt aaatttctac tcgctccctc tttagcaaaa gacttagtca tagcaatcat
256501 tccaccttta ctagcagaat aattagtctg acctgcattc cccatttcac cgacaattga
256561 agcaatattt acaaccgcgc caaaacgctt tttactcatt gtttttaaag cttctctaca
256621 acctaaaaac gctgaactca aatttgtatc tactacacca gtaaaatcct ctagtttcat
256681 tcttaaggca agtttatcgt ttgtaacgcc tgcattattt acaagataac taagttctcc
256741 atcgctttct actatagttt ttataccatt ttcaaactca tcttctttgc ttgcatcaaa
256801 tttaatcaca gccgcattgc caccttgtgc aagaatttca tcttttaaag catctgcaag
256861 ttctggtttt gaacgataat taatccaaac ctttaatcca aaacttgcca aagtttttgc
256921 aatagaagca ccaataccct tgcttgcgcc tgtaatcaaa acattttttc cactaaattt
256981 catttctatc cttaaaaaaa atataaaaat taaattttaa caaaaatcta tatcaaaagg
257041 caaacagatc aaaataaagc ctcattcatt tcttttggaa tttctaaatt taatatttta
257101 agcacactcg cagcgatatt actaagcccc atattttcct tcagtttatc cactcccttg
257161 gcttctacaa aagcaaaaac atcaaaagtt gtatgattgg tcaataaatt tcctttttta
257221 tcttgcatag cttcacaatt tccatgatca cttgtaataa taaaggcata atcatgcttt
257281 cttgcgcatt caaccacttc acccaaacaa gcatctactg cctctacagc cttaacagca
257341 gcgttaaaat ctcctgtatg acctaccata tcaccatttg caaaattcac cactataaaa
257401 tcttcaccct tttctatccc ttttctaacc gcatcacata cttcaaaagc actcatttca
257461 ggcttttcat cgtaagtttt tacctttggg cttgggatta aaacccttgt ttcattttct
257521 aaaagctctt cttttcctcc attgaaaaag aaagtgacat gggcatattt ttcagtttct
257581 gcagtgtgaa gttgagtaag tcctgcttta gagatggttt cagaaagtgt gtttttaatt
257641 tcttcttttt caaaaagtac tggaatatta aatttatcat cataaacact catagtaagt

257701 aaattttcaa acactttctc tctctcaaat tccttgaaat tttgagaatt taaaacttcg
257761 atcagttgtt tcattctatc atttctaaaa ttaataaaaa tcaatccatc atcttcttgc
257821 atacctttat aattctcatt tttagccgct tggataaatt catcagttat ttcattttga
257881 taacattctt ccacataatc aaccaaattt ggcacaccct tacttttacc caataaacaa
257941 tcataatatt ccttgactct ttcccaacgc ttatctctat ccatagcgta aaaacgcccg
258001 caaagcgtag caaaatgcac acctattttt tcacaatgat ttttaagctt ttttataaat
258061 tctagtccgc ttgtagggct tacatcgcgt ccatcggtaa ttgcatgtgc aaaaacttca
258121 tttccatttt tagcacaaat ttcaagcaaa gcattaaaat gcgtatccat agaatgaacc
258181 ccaccatcac tatataaacc gataacatgc accctttttgc atttttgtaa aagttttttt
258241 aaatttgcgt ttttctcaag ttcgtaactc tctatagctt tattaatgcg caccagattt
258301 tgatagataa tttgcccact acctatgcac atatgaccca cttcactatt tcccatttga
258361 ttttcaggca aacctactgc taaaccacta gttttgatta aagaatttgg tacttcttta
258421 aaaagcctct cgtaatttgg ctttttagca gcctcaaaag cattaaaatc tttttccttg
258481 ttaaaaccta tcccgtctgt aataaccaaa atacattttt gtctcatttt ttactctttt
258541 ataagaaatt ttttggctag attttactaa aattaagctt ttaaaaacca aaaagaaggc
258601 tagcttttgt attatctttc ttattttagc gattatgctt tttttactta tatcagtgtg
258661 cgcgcgggct ttgcgttttt tattgcttta tgtttatctt tattcattat gcctaaattt
258721 attacttggg ctaaaaataa aaatgcaaat caacccattt acgaattagc acctcaaact
258781 cacaaagtca aatctcaaac gcctacaatg ggtgggctta ttttcatagc ttgtgcagtc
258841 tttacaagcc ttatttgtgt caaattggat aatgtattta caatcacagc cttattttgt
258901 cttgtgcttt tttgtcttat cggacttgtg gatgatttgg gtaaaatttt aaaaaaagac
258961 aaccactcag gacttagtcc aaaagcaaaa cttttagccc aaataagcgt ggcattaatc
259021 tgtattttgc ctttatattt tagtcctgaa tttaatacgg aattttatct tcctttttat
259081 aaatatccgc tttttgatat gaaaattttt gctcttgctt tttggatttt ggtcttaatc
259141 tcaagctcta atgctgtcaa tttaaccgat gggcttgatg gacttgcaac tgttcctagt
259201 atttttttctt tatctacctt gggtatattt ttatatttga gtggaaattt aaactatagc
259261 gaatatttac ttttacctaa aattcaaggc ctaggtgaag tagtgattat ctgtgcagca
259321 ttgattggag ctcttatggg atttttatgg tacaactgct accctgcaca agtttttatg
259381 ggagatagtg gaagcttggc tttgggtgga tttataggct ttttagctat tataagtaaa
259441 aatgaaatct tacttttact cgtaggcttt gtctttgtac ttgaaaccat ttctgtcatt
259501 ttacaagtag gaagctttaa aatttttaac aaaagagtat ttaaaatggc gcctatacac
259561 catcattttg aaaaaatagg ctgggtagaa aataaaatca tagtacgttt ttggatgata
259621 gccttgctat ctaatttact tgcacttgca tcgataaagt taagataatg aaaatttcgc
259681 tttttggata tggaaaaacc acacaggcaa tagccaaaaa tttagttaat aaattcggtc
259741 cttttgacat ctatgatgat cactttcaag aagaaaataa agatgaatgg ggcaatcatc
259801 ttttaaatcc taaagaattt gatgaaaaac taagcaagct tgaaattcca agccctggat
259861 ttccacccaa tcatattctt ataaaaaaag caaaaaattt acaaagcgaa tatgattttt
259921 tttatgatgc tatgccaaaa tctatttgga tcagtggcac aaatggcaaa accacaacta
259981 cacaaatggc tacacacctg ctttcgcatg taggagcaat aatgggagca aatgttggtt
260041 ttcctctagc cgaacttgat ttaaatgcca aaatttggat acttgaaact tcatctttta
260101 cacttcacta tacccatact gcaaaacctg aaatttacgc actcttaccc ataagtgcgg
260161 atcatctttc atggcatgga agttttgaaa attacgtaaa agataaattg agtgttttgg
260221 aaagaatgaa tgagtgtgat attgcgattt tgcctgaaat ttatgcaaaa acacccacaa
260281 aagcttatat tatctcctat aaagatgaat ttgatcttgc tcaaaaaatg gatattgata
260341 taaaaaaaat atcttttaaa agtccttttt tgcttgatgc tgttatggct ttaagtatag
260401 aaaaaatttt acttgatacc ttaagctatg aacttttaaa tcattttgtt atggaaaaaa
260461 ataaacttga agaattgcaa gataatcaag gtagactttg gatcaatgat actaaagcga
260521 ccaatgaaaa cgctgtaatg gccgctctta accgctataa agataaaaaa attcatctca
260581 ttataggcgg tgatgataag ggtgtagatt taagcatgtt atttaatttt atgaaagatt
260641 ttgatgtaga actctatgcc ataggggtta gtactcaaaa aatgttagat tatgctttaa
260701 aagcaaattt aaaagctcat aagtgtgaat ttttagaaaa tgctgtcaaa gaaatttcca
260761 aaacgttaaa aaataatgag gtagctttat taagccctgc ttgtgcaagc ttagatcaat
260821 ttagctctta tgctcaacgt ggagaaaaat ttaaagaata tgtgagtaaa atttaacaac
260881 cccaactcac ttctctcacc ccaaacacat tcaaacaata aaaaaaccca aacaaattta

```
   260941 ccagcctagc aaatcaatat cttaacacca cccaaaaaac catacaatga aattataaac
   261001 caaacacaaa gacaacacaa ttaagctaaa aatta
//
LOCUS       DMACINML_2            253157 bp    DNA     linear       16-DEC-2020
DEFINITION  Campylobacter species strain strain.
ACCESSION
VERSION
KEYWORDS    .
SOURCE      Campylobacter species
  ORGANISM  Campylobacter species
            Unclassified.
COMMENT     Annotated using prokka 1.13 from
            https://github.com/tseemann/prokka.
FEATURES             Location/Qualifiers
     source          1..253157
                     /organism="Campylobacter species"
                     /mol_type="genomic DNA"
                     /strain="strain"
     gene            complement(246..1586)
                     /gene="radA"
                     /locus_tag="DMACINML_00260"
     CDS             complement(246..1586)
                     /gene="radA"
                     /locus_tag="DMACINML_00260"
                     /inference="ab initio prediction:Prodigal:2.6"
                     /inference="similar to AA sequence:RefSeq:CAL35320.1"
                     /codon_start=1
                     /transl_table=11
                     /product="putative DNA repair protein RadA (ATP-dependent
                     protease)"
                     /protein_id="Prokka:DMACINML_00260"
                     /translation="MAKNKILFECQACGNQQSKWLGKCPECGSWDSFIELKSEQIKVL
                     KEIAQASTKASEAICIEDVELEHFSRCSTDDDELDLVLGGGLVEGSLVLIGGSPGVGK
                     STLLLKIASNLAKKNKKVLYVSGEESKAQIKLRADRLEANTPNLFLLTELCLENILEE
                     LHKKDYEILIIDSIQTLYSNKIASAAGSITQVREITFELMRVSKTYNISTFIIGHITK
                     EGAIAGPRVLEHMVDVVLYFEGDATKEIRLLRGFKNRFGGTSEVGIFEMTPKGLISAK
                     DLANRFFTRGKATSGSALGVVMEGSRALVLEVQALVCESSYPKRSATGYEKNRLDMLL
                     ALLERKLEIPLGHYDVFINISGGVRVNETAADLAVVAAIISSFKNRPLSKDCVFIGEL
                     SLNGEIREVFSLDTRLKEAKMQKFKNAIIPSKPLEDVGLKCFVAKELAQVLEWM"
     gene            1720..2571
                     /locus_tag="DMACINML_00261"
     CDS             1720..2571
                     /locus_tag="DMACINML_00261"
                     /inference="ab initio prediction:Prodigal:2.6"
                     /inference="similar to AA sequence:RefSeq:ABS43588.1"
                     /codon_start=1
                     /transl_table=11
                     /product="carbon-nitrogen hydrolase"
                     /protein_id="Prokka:DMACINML_00261"
                     /translation="MQKLLKVALIQFAPKSYEVQNNLDKALKLAHLALEQGAKLIVLP
                     ELYDSAYCVEDQDEKFSLNLHQAHPNLDALQKLAKNYQAYIVACSIEKDTQLYDSAYI
                     ISHKGLLGVYRKIYLWGNEKERFARGDKYPIFELEFENFKLKLGLQICYEIGFSEGSR
                     FLALQGAEIICFPSAFGKARTYVWDLASRARALENGVFVLAANRSGSEISKINNETLT
                     FAGKSKIINPKGEILKEILEEEGFIIAELDLEEVQIQRKNLPYLKDLNLKLNQEILTN
```

```
                     IHLNTKE"
     gene            2574..3455
                     /gene="pdaA"
                     /locus_tag="DMACINML_00262"
     CDS             2574..3455
                     /gene="pdaA"
                     /locus_tag="DMACINML_00262"
                     /EC_number="3.-.-.-"
                     /inference="ab initio prediction:Prodigal:2.6"
                     /inference="similar to AA sequence:RefSeq:SQE24513.1"
                     /codon_start=1
                     /transl_table=11
                     /product="polysaccharide deacetylase family protein"
                     /protein_id="Prokka:DMACINML_00262"
                     /translation="MAKEILVAYGVDIDAVAGWLGSYGGEDSPDDISRGLFAGEVGIP
                     RLLKLFKKYNIPATWFAPGHSIETFPEQMKMIVDAGHEIGAHGYSHENPIAMSAKQEE
                     DVLLKSIELIEKISGKKPSGYVAPWWEFSNITNELLLKHGIKYDHSLMHNDFTPYYVR
                     VGDKWTKIDYSKDAKEWMKPLVRGQETDLIEIPANWYLDDLPPMMFIKKSPNSFGFVS
                     PRDIGQMWIDQFDWVYREMDYAIFPMTIHPDVSARPQVLLMHERIIEHINKHEGVKWV
                     NLNDMADDFSKRFPRKK"
     gene            3497..3832
                     /locus_tag="DMACINML_00263"
     CDS             3497..3832
                     /locus_tag="DMACINML_00263"
                     /inference="ab initio prediction:Prodigal:2.6"
                     /inference="similar to AA sequence:RefSeq:ABS44315.1"
                     /note="conserved hypothetical protein"
                     /codon_start=1
                     /transl_table=11
                     /product="hypothetical protein"
                     /protein_id="Prokka:DMACINML_00263"
                     /translation="MCILCGEMISTLHWSELNFKEEKHELSVGEEQKERLKIRLKKVK
                     ILNEILEFYGLKLKEWQNSKYILSNKKGRDIIVNDLGDLWIKASELEKKSFDVLDENL
                     LHFLRAKHG"
     gene            3825..4769
                     /locus_tag="DMACINML_00264"
     CDS             3825..4769
                     /locus_tag="DMACINML_00264"
                     /inference="ab initio prediction:Prodigal:2.6"
                     /inference="similar to AA sequence:RefSeq:ABS43326.1"
                     /note="conserved ATP/GTP binding protein"
                     /codon_start=1
                     /transl_table=11
                     /product="hypothetical protein"
                     /protein_id="Prokka:DMACINML_00264"
                     /translation="MAKIPVHVVTGFLGSGKTTFLKEILSNQTLNDIALVINELGEVS
                     LDHKLIQTDFIEEQTLFLNAGCACCNKREDLQKKFKELLDSFENKGKRPQRVILETSG
                     LANPAPIIFTFQSDVFLANHFELANIITCIDAFEGLNHLQNEEACNQVLSSDFLILTK
                     KDLNPNTQSLEEKINTLYPNIQIISKENFNFDMLSSHHKIQKEIKNIEIKSHKDDISS
                     ITLIFDKAINWNIFSIWLSMLLHEHGSKILRVKGLINTEESYLTNINGVGHLIYHPTH
                     TKISSLNHPSQLVFIAKNLKLDKIKESLENFLKLEQLN"
     gene            complement(4766..5632)
                     /gene="ftsY"
                     /locus_tag="DMACINML_00265"
```

```
     CDS             complement(4766..5632)
                     /gene="ftsY"
                     /locus_tag="DMACINML_00265"
                     /inference="ab initio prediction:Prodigal:2.6"
                     /inference="similar to AA sequence:RefSeq:CAL35321.1"
                     /codon_start=1
                     /transl_table=11
                     /product="putative signal recognition particle protein"
                     /protein_id="Prokka:DMACINML_00265"
                     /translation="MFNFFKKGLAKTLESIVGVKGENKKITKDLLEEILLEADVAYEI
                     VEEIIYYLPPSDEVKKEDLKRVMGSYFLYENKEFLDEKPFVELILGVNGAGKTTSIAK
                     LANLYKSQGQKVILGACDTFRAGAIEQLRLWSEKVGVDIVLTQQGHDPSAVAFDTISK
                     ARAKDFDRVIIDTAGRLQNQKNLANELEKIVRISAKAMPNAPHRKILVLDGTQGNAGI
                     LQAKAFNELVQLDGVIITKLDGTAKGGALFSIARELELPILYIGVGEKMQDLIEFNAQ
                     NFLDTLLEGVFD"
     gene            complement(5632..6189)
                     /locus_tag="DMACINML_00266"
     CDS             complement(5632..6189)
                     /locus_tag="DMACINML_00266"
                     /inference="ab initio prediction:Prodigal:2.6"
                     /inference="similar to AA sequence:RefSeq:CAL35322.1"
                     /codon_start=1
                     /transl_table=11
                     /product="putative lipoprotein thioredoxin"
                     /protein_id="Prokka:DMACINML_00266"
                     /translation="MKIKKVFTLAILLSFFVACSNDKEKDQSDMNASVQSSLSQSDSL
                     RFNLNLIDGNSVFVKKDNENLNFADGDKATLFVFFTTWCSPCIAEIPHLNKLQEKYEE
                     QFNIVGVLLEDKSDEELKAFVEENQILYKIANGENNYLLAKILGGVNGIPTMFLYDKN
                     SQLVNQYLGLIPEEMLEIDIQKAIL"
     sig_peptide     complement(6127..6189)
                     /locus_tag="DMACINML_00266"
                     /inference="ab initio prediction:SignalP:4.1"
                     /note="predicted cleavage at residue 21"
     gene            6816..8369
                     /locus_tag="DMACINML_00267"
     CDS             6816..8369
                     /locus_tag="DMACINML_00267"
                     /inference="ab initio prediction:Prodigal:2.6"
                     /inference="similar to AA sequence:RefSeq:CAL35324.1"
                     /codon_start=1
                     /transl_table=11
                     /product="HD/KH domain containing protein"
                     /protein_id="Prokka:DMACINML_00267"
                     /translation="MIESLIALITAIVGLGIGYLVAKKINDAKYEIFVEQAKAKAKAI
                     EYEAELILKDAKNSILNAELEVKKKYEDKTHKIQKEFNQKFDELSKKEQKLQLQEESL
                     KENQEELLRSKKQMLDLQSDADKLKNQYQAKLNDVLNILEHSAGLTQDEAKNIILEKV
                     EENSRNEIAHIVRRYEEEARNEAKRRANYIIAQATSRFAGEFAAERLINVVNIKNDEL
                     KGRIIGKEGRNVKTLEMVLGVDIIIDDTPGAIIVSCFNLYRRAIATKVIELLVEDGRI
                     QPARIEEIHEKVCKEFDNAILEEGETILMDLGLSKVHPEITKLIGKLKYRASYGQNAL
                     AHSLEVAHLAGIIAAECGGDENLARRAGILHDIGKALTHDFEGSHVDLGAELCKRYKE
                     HPVVINAIYAHHGHEEATSIESAAVCAADTLSAARPGARREVLEAFLKRVSELEEIAK
                     SKEGIKNAYAINAGREIRVIANAKLVNDDESVLLAKEIAAEIQEKMQYPGEIKVNVIR
                     ELRAIEYAK"
     gene            8379..8936
```

```
                     /locus_tag="DMACINML_00268"
     CDS             8379..8936
                     /locus_tag="DMACINML_00268"
                     /inference="ab initio prediction:Prodigal:2.6"
                     /inference="similar to AA sequence:RefSeq:CAL35325.1"
                     /codon_start=1
                     /transl_table=11
                     /product="putative integral membrane protein"
                     /protein_id="Prokka:DMACINML_00268"
                     /translation="MQDMIDTLLKYGYIVLFFYSLGGGMVGILAAGVLSSQGKMDLTL
                     CISIAFVANTLGSTLLFILGKYYKKDIMPYFKNHRRKLALAMMKTKQHGVVLLITQKF
                     VYGLKTFIPIAAGIAKYQFIKFFIINTLASLLWAVLLGYSAYAFGFVIEAIFNKLSSY
                     PYIAPLFLIVLVGIIWFYLAKFSKK"
     gene            8936..10195
                     /locus_tag="DMACINML_00269"
     CDS             8936..10195
                     /locus_tag="DMACINML_00269"
                     /inference="ab initio prediction:Prodigal:2.6"
                     /inference="similar to AA sequence:RefSeq:CAL35326.1"
                     /codon_start=1
                     /transl_table=11
                     /product="putative competence family protein"
                     /protein_id="Prokka:DMACINML_00269"
                     /translation="MSFWNSLYLNLKEFRYLFLFGLIVFIFNIFLEYNHFLTLKEKKH
                     YLVENALLLHYYPKYNKNNKKYWVLKLKTKDFILYSTSFKDLNLSKNQLLNLKIITNN
                     ISFRDYLRKSFYAPSYGFEASGYFKNNVIISYFLNQHTNEKIQEFYGALFFALPISSE
                     LRKDVNYYGVAHLIAISGYHIGLLFALVFFILTPLYSFFQKRYFPYRSLRFDLSVFIF
                     ILLFAYAYLIGFVPSYIRSLIMALWGFYLLCKNIKILSFFTLFISIYLCIALYPRLLL
                     SVGFLFSCLGVFYIFLYLHHFSKFFHNFLNIVLLNIWTFFCMIIPVLYFFPLISYQQF
                     LAIILSGIFIVFYPLVLCLHFIAHGNLLDTVLIDFFNIKFYAIDIKIPTWILISYLTL
                     SFIAMRYKILALLCVFANFIPFIMIMI"
     gene            complement(10139..10945)
                     /locus_tag="DMACINML_00270"
     CDS             complement(10139..10945)
                     /locus_tag="DMACINML_00270"
                     /inference="ab initio prediction:Prodigal:2.6"
                     /inference="similar to AA sequence:RefSeq:ABS43802.1"
                     /codon_start=1
                     /transl_table=11
                     /product="YihY family protein"
                     /protein_id="Prokka:DMACINML_00270"
                     /translation="MKRIFAILSNLRDKEILNYAAALSFYTILSLIPILFVCFSVFTQ
                     ISSFKVYYEKAKQIIFAFLIPAQQDIVAAYLDTFLNNSVNLGIVGLIAMTFTSLAFFS
                     GYDFVINRITKNEPKGLWQSISSYWTLLTLVPLGLGLSFYISGLIQQALDDYKIGFNF
                     FEILPFVIIWGLFFISYSSSIHKGTLKSLILVSFAAGAIWYIGKNLFVYYIVYNKTYA
                     SVYGSFSTILFFFIWIYISWIVYLFGIKIYYFLNHNHDKGDKIRKNTKKG"
     gene            complement(10957..12339)
                     /gene="glcD_1"
                     /locus_tag="DMACINML_00271"
     CDS             complement(10957..12339)
                     /gene="glcD_1"
                     /locus_tag="DMACINML_00271"
                     /inference="ab initio prediction:Prodigal:2.6"
                     /inference="similar to AA sequence:RefSeq:CAL35328.1"
```

```
                     /codon_start=1
                     /transl_table=11
                     /product="putative glycolate oxidase subunit D"
                     /protein_id="Prokka:DMACINML_00271"
                     /translation="MKKEFEQYFIKLLGSENAYFDDIHKRAYSYDATKKHYLPDGVLF
                     PRNEEDISQILKFCNENNIIIIPRGAGSGFTGGALAVNGGLILSFEKHMNKILEIDLE
                     NLVAVVQPGVINIHLQREVAKHGLFYPPDPASMEYSSLGGNVSENAGGMRAAKYGITK
                     DYVMALRAVLPNGEIIRAGKRTIKDVAGYNLAGILIASEGSLAVLSELTLKLIALPKF
                     KKTAFGIFPSVKSAMNAVYKSLASGVNPVSMEFLDNLSIRAVESKFNKGLPVDAGAIL
                     IADVDGNVKEAIDEDLKILGEHFLDAGASQFKIAKDEQEAADIWFARRNCSQSIAMYG
                     TLKLNEDITVPRSKLPALLEGIDEISKKYGFKIPCFGHTGDGNVHTNVMVPDKNDEEQ
                     VKKGYEAVEEVFKLTVELGGTLSGEHGIGLSKAPFMKLAFSDAEMNLMRNIKKAFDPN
                     NILNPFKMGL"
gene                 complement(12344..13063)
                     /locus_tag="DMACINML_00272"
CDS                  complement(12344..13063)
                     /locus_tag="DMACINML_00272"
                     /inference="ab initio prediction:Prodigal:2.6"
                     /inference="similar to AA sequence:RefSeq:CAL35329.1"
                     /codon_start=1
                     /transl_table=11
                     /product="putative exporting protein"
                     /protein_id="Prokka:DMACINML_00272"
                     /translation="MLKFLAFILSLSISLFAVDLNLKPVKMELLKVEDIYGYIEDSPD
                     IKLNSSGVVIQRFQTSKSIIARASVIAKEKGLAKLKFSVFADLEQDALPLPNVVPQKG
                     DEVVLNFLYDRGLVIAPDEQTYNELVSSFPEIYFTHIDILGAQLIRSSSLAPKRSDFR
                     KFCADNAVGILIFALEDKAKIVDCQDFSELYELAIPKPSSIQVPFYSRIGGYKSNFFD
                     FNSQEVGNYYRYYDALINILR"
gene                 13187..14347
                     /locus_tag="DMACINML_00273"
CDS                  13187..14347
                     /locus_tag="DMACINML_00273"
                     /inference="ab initio prediction:Prodigal:2.6"
                     /inference="similar to AA sequence:RefSeq:CAL35330.1"
                     /codon_start=1
                     /transl_table=11
                     /product="putative peptidase M23 family protein"
                     /protein_id="Prokka:DMACINML_00273"
                     /translation="MKKLLLLFTFVIQSFAALSVEELTWDNGDTLLKFLQRNSIPISL
                     YYGLDREDQELASDIAYKVKYQVLKDENNNIEQVLIPISDDLQIHIYKDKNNQYTLTF
                     TPVSYQKEDRILHLTIESSAYQDVYEESGSSTLARAMVRSFRGSVNFRNIQKGDEVTL
                     YYEQKRRMGRLWGDIAVRMATVEINKNAQEVFAFNDIFYDRDGKEVEAFLLTKPVNYT
                     RISSTFSTARYHPILKRYRAHLGIDYAAPTGTPVKSAGKGTITFVGTKGGYGNVIQVK
                     HDSGYMTLYAHLSRFAKIKKGQKVNQGQLIGYVGSTGMSTGPHLHFGVYLNNKAINPA
                     SVVKIAKSELSGKAKEEFKNTIVKYKGIVNEALAANKPNPPKEEDFENYIEF"
sig_peptide          13187..13246
                     /locus_tag="DMACINML_00273"
                     /inference="ab initio prediction:SignalP:4.1"
                     /note="predicted cleavage at residue 20"
gene                 complement(14344..14550)
                     /locus_tag="DMACINML_00274"
CDS                  complement(14344..14550)
                     /locus_tag="DMACINML_00274"
                     /inference="ab initio prediction:Prodigal:2.6"
```

```
                     /inference="similar to AA sequence:RefSeq:CAL35331.1"
                     /note="hypothetical protein Cj1216c"
                     /codon_start=1
                     /transl_table=11
                     /product="hypothetical protein"
                     /protein_id="Prokka:DMACINML_00274"
                     /translation="MYEKKDLRVLKIIQKAREFGDADLLNEALVNQLVNAEFKEMESK
                     EREGLIILLNSLIEAKDKALLSNP"
     gene            complement(14553..15233)
                     /locus_tag="DMACINML_00275"
     CDS             complement(14553..15233)
                     /locus_tag="DMACINML_00275"
                     /inference="ab initio prediction:Prodigal:2.6"
                     /inference="similar to AA sequence:RefSeq:CAL35332.1"
                     /note="conserved hypothetical protein Cj1217c"
                     /codon_start=1
                     /transl_table=11
                     /product="hypothetical protein"
                     /protein_id="Prokka:DMACINML_00275"
                     /translation="MGRSGENYLSLLDNQKVSVFCAYDKDYNILRAKVHKENLFSHLG
                     FKDIEKCVFMDQIHSSKVEIYDESLENLSCDGLISIEKNTALCVLSADCLPLILWHES
                     GVIAALHSGRKGSFENILKECVDKIYTKNPNLDMQKFHLFILPSICAKNYEIDGEILE
                     FAKVEFKDFLYERRLDLKALVKFQAQNLGIKNIVDSNICSFDDKTFYSYRRDKTSKRF
                     VSVVYLKG"
     gene            complement(15197..15808)
                     /gene="ribA_1"
                     /locus_tag="DMACINML_00276"
     CDS             complement(15197..15808)
                     /gene="ribA_1"
                     /locus_tag="DMACINML_00276"
                     /EC_number="2.5.1.9"
                     /inference="ab initio prediction:Prodigal:2.6"
                     /inference="similar to AA sequence:RefSeq:CAL35333.1"
                     /codon_start=1
                     /transl_table=11
                     /product="putative riboflavin synthase alpha chain"
                     /protein_id="Prokka:DMACINML_00276"
                     /translation="MFNGLIREIAKVKSYQNNVLNLKANYRPNLGDSVAVNGACLSVT
                     KLHSDGFELELSHESRKHIAVQNLKDKVHIEPALRYGDRIDGHLMQGHIDFIGKLEKI
                     EKDENGIDFYISLPKEAMKFMARKGSIGIDGVSLTINEIVENGVRLTIIPITFKETLF
                     KEYKIGREINIESDLLARYIYAQMQDKNKGLSWEEVERITYLY"
     gene            complement(15818..18358)
                     /locus_tag="DMACINML_00277"
     CDS             complement(15818..18358)
                     /locus_tag="DMACINML_00277"
                     /inference="ab initio prediction:Prodigal:2.6"
                     /inference="similar to AA sequence:RefSeq:CAL35334.1"
                     /codon_start=1
                     /transl_table=11
                     /product="putative periplasmic protein"
                     /protein_id="Prokka:DMACINML_00277"
                     /translation="MKKILYGIVAFIVVLLIAFYTLLFTSFGNNIVANFIQDKIKQST
                     GLDANITQFTLRFSSLDIEANLANMADLKLEGNLSLFKLGFDLDYIINLDKNYAKNLG
                     LNLNQNLAFSGKINGKSSDFMINGKGYLFGSNVLLDARIYEYSPIALNLSANDLQISE
```

```
                     LLALFGRGNLAKGTIDIASKISAKDLKPDGNAIIKLDNTYINYAEIKKEFNIDLPENS
                     NLKAEILANIKDDTVYTVSKIYNSYLTLQTQKTLYNLIQNTLNTDFDLSVPNLAKLEK
                     LTKTKLNGSLEVKGDASVANNALSSLNANVIGLGGEVKANLKNNQLNLNASNASLEKI
                     LALIGYGGIVSGNLNANLVSEGLDFANFNADVKINNAKLNSAEIKKIAKLELPNTSFS
                     LDAKAKAKNGNISYNALLASNLLNIKKLNGTYNLKNAELNTDLNAFIDDLSQFNAIAG
                     QNLQGKAELNAKAHIIGAQVQNLNADANIAGGLIKANSNGKKLDLDINKLDLSKVFTI
                     VGMPNYASGIVNAKARLDDINFNNLNGKVNIDAKGILNAAVLSQLMDKKFPNNTNYDL
                     NVKADLKNNVADFEGVLNSSLANLSNFKGKFDINKMILNSNYALDIADFSKLGFLLDR
                     KLKGKASFNGNIGFDKTLNFTALSKNLFEGKLDSTLKNNILNAKLEGVNLSTLASGLD
                     FIDIYQGKANLEVKYNLISEQGEVNADMKEGKLKPNAITAALKLITLKDITTDVFHTA
                     NAKALINKENIKLDLNMQADRSYVLISSGMLNSKSGSLNLPFDIKIDRANFKGNITGT
                     TQKPQVKLEAGSVVNSIKNIVGDKAQDGVQKAGEKVDEGINKLLNKIF"
     gene            18522..18782
                     /gene="groES"
                     /locus_tag="DMACINML_00278"
     CDS             18522..18782
                     /gene="groES"
                     /locus_tag="DMACINML_00278"
                     /inference="ab initio prediction:Prodigal:2.6"
                     /inference="similar to AA sequence:RefSeq:CAL35335.1"
                     /codon_start=1
                     /transl_table=11
                     /product="10 kD chaperonin (cpn10)"
                     /protein_id="Prokka:DMACINML_00278"
                     /translation="MNFQPLGKRVLVKRVEETKTTASGIIIPDNAKEKPLTGEVVAVS
                     KEITDIANGDKIVFAKYGGTEIKLDNSEYLVLNLDDILGILK"
     gene            18804..20441
                     /gene="groEL"
                     /locus_tag="DMACINML_00279"
     CDS             18804..20441
                     /gene="groEL"
                     /locus_tag="DMACINML_00279"
                     /inference="ab initio prediction:Prodigal:2.6"
                     /inference="similar to AA sequence:RefSeq:CAL35336.1"
                     /codon_start=1
                     /transl_table=11
                     /product="60 kD chaperonin (cpn60)"
                     /protein_id="Prokka:DMACINML_00279"
                     /translation="MAKEIIFSDEARNKLYEGVKKLNDAVKVTMGPRGRNVLIQKSFG
                     APTITKDGVSVAKEVELKDSLENMGASLVREVASKTADQAGDGTTTATVLAHAIFKEG
                     LRNITAGANPIEVKRGMDKACEAIVAELKKLSREVKDKKEIAQVATISANSDEKIGNL
                     IADAMEKVGKDGVITVEEAKSINDELNVVEGMQFDRGYLSPYFITNAEKMTVELSNPY
                     ILLFDKKIANLKDLLPVLEQIQKTGKPLLIIAEDIEGEALATLVVNKLRGVLNISAVK
                     APGFGDRRKAMLEDIAILTGGEVISEELGRTLESATVQDLGQASSVIIDKDNTTIVNG
                     AGEKANIDARVNQIKAQIAETSSDYDREKLQERLAKLSGGVAVIKVGAATETEMKEKK
                     DRVDDALSATKAAVEEGIVIGGGAALIKAKAKINLDLKGDEAIGAAIVERALRAPLRQ
                     IAENAGFDAGVVVNSVENAKDENTGFDAAKGEYVNMLESGIIDPVKVERVALLNAVSV
                     ASMLLTTEATISEIKEDKPAMPDMSGMGGMGGMGGMM"
     gene            complement(20946..22151)
                     /locus_tag="DMACINML_00280"
     CDS             complement(20946..22151)
                     /locus_tag="DMACINML_00280"
                     /inference="ab initio prediction:Prodigal:2.6"
                     /inference="similar to AA sequence:RefSeq:AVS37715.1"
```

```
                     /codon_start=1
                     /transl_table=11
                     /product="sensor histidine kinase"
                     /protein_id="Prokka:DMACINML_00280"
                     /translation="MAKKVIRQILLIYLTTTGIFLAIFFTLWYQKLYEELVVVKGTSL
                     RENHRNIIINILNSRFVPMNESASNIAQSTGLKFAIFDKKQAIFDNLDFDFRKARVEL
                     KGRGIYDGKVFFLDRMNTDNYFLKHANEDNIHTESGLKILIQGEDVKKDLVWIRVKVF
                     SFAIMAFCVLGLIAYILVKIALKPLEDKIITLNRFIKDSTHELNTPLSVILMSIEQLE
                     NQKLEHNTKFTRIKLAAKSLSQVYSDLVFYNFPNTLELNKQELDLKALIEERLEYFKI
                     FFEQKKISLKLNLHQASIFASKNQISKLIDNLLSNAIKYNKKGGEILVELKTGFLSIA
                     DTGCGISKTNLKHIFDRYARFNTDQGGFGIGLSLVKKICDDNDIKIICESVENEGSVF
                     KLSWDTKKI"
     gene            complement(22144..22809)
                     /gene="dccR"
                     /locus_tag="DMACINML_00281"
     CDS             complement(22144..22809)
                     /gene="dccR"
                     /locus_tag="DMACINML_00281"
                     /inference="ab initio prediction:Prodigal:2.6"
                     /inference="similar to AA sequence:RefSeq:CAL35338.1"
                     /codon_start=1
                     /transl_table=11
                     /product="two-component regulator"
                     /protein_id="Prokka:DMACINML_00281"
                     /translation="MAARILLLEDDLSLSEIIEEFLIDEGYEVYLCTNAKEALELAYE
                     KHFDVWILDVKVPLGDGFSLLKELRDCGKQTPAIFMTSLNTTEDLKEGYNAGCDDYIR
                     KPFELAELSIRIQALLKRAFSHKNEDYEDLGNGFKFSFTSQILYHFDKALTLPSKEIK
                     LLSLLLKNKNNFLSTERIFEELWEYDEEPSELSLRAYIKNLRKILGKEKIINQRGRGY
                     CYG"
     gene            22944..23543
                     /locus_tag="DMACINML_00282"
     CDS             22944..23543
                     /locus_tag="DMACINML_00282"
                     /inference="ab initio prediction:Prodigal:2.6"
                     /inference="similar to AA sequence:RefSeq:CAL35339.1"
                     /codon_start=1
                     /transl_table=11
                     /product="putative iron-binding protein"
                     /protein_id="Prokka:DMACINML_00282"
                     /translation="MLPKWDSSFSVHNAKIDDQHKKLFELAAKVEIVSDRSVSKNEVK
                     ELLAEFFNYMKDHFNDEEKYMQLIAYPAFEEHKKIHKEIIQTMINLIKDIKSTNDLKE
                     KLYIIAKKWLLEHILYEDMKVEKWRSSSLATDEGKDVSFEAADDSDDDKPQFYLYKCN
                     CPGKVHDVPYGIHQKIELQGHKFTCKTCKQAITFFQKHS"
     gene            23557..23922
                     /locus_tag="DMACINML_00283"
     CDS             23557..23922
                     /locus_tag="DMACINML_00283"
                     /inference="ab initio prediction:Prodigal:2.6"
                     /inference="similar to AA sequence:RefSeq:ADT66500.1"
                     /codon_start=1
                     /transl_table=11
                     /product="Ribbon-helix-helix protein, copG family"
                     /protein_id="Prokka:DMACINML_00283"
                     /translation="MKQETRNYSLRMPLELKERLDNLSKNLSKPKSSIVREALEAYLN
```

```
                     EIEDFSFAVNALEELKDGDYSKASKKIDKVVENLQKESDDLGDFDFAIEALEELKDGD
                     YSKASKKIDKIVKNLKKPE"
     gene            complement(24006..24080)
                     /locus_tag="DMACINML_00284"
     tRNA            complement(24006..24080)
                     /locus_tag="DMACINML_00284"
                     /product="tRNA-Asn"
                     /inference="COORDINATES: profile:Aragorn:1.2"
                     /note="tRNA-Asn(gtt)"
     gene            complement(24160..25410)
                     /locus_tag="DMACINML_00285"
     CDS             complement(24160..25410)
                     /locus_tag="DMACINML_00285"
                     /inference="ab initio prediction:Prodigal:2.6"
                     /inference="similar to AA sequence:RefSeq:CAL35341.1"
                     /codon_start=1
                     /transl_table=11
                     /product="putative two-component sensor (histidine
                     kinase)"
                     /protein_id="Prokka:DMACINML_00285"
                     /translation="MNRSSIFYTITFIFIFAGVSVILGFLWLIEYDQQNYTRELNTKY
                     SLIANARLLNFAGIISEKEFEEQTKNYNKMESVVEVRQIRRILFGGEVLARVEVNNGL
                     IEIISYDRQVYLNIIFDGKVNLYKDQDYQTYRYFIIKAIAVAVICILVLLYIYIFKKL
                     KPLKALKRQIDKFAQGKLNDIEDVSTGVDEISQVSEAFYQAIVQIRKLNQSRQFFLRN
                     IMHELKTPITKGLITLEMLEDNKYKERLGSIFNRLEILINEFAAIEQITSGAAFINRK
                     KYNILDVLDEAKEIAMRDDNNIRIFMDESFFVNVDFKLFTTAIKNMIDNGIKHSENGF
                     IQIDIMDDYICFKNRGPELNNTLEYYTQAFTQGGSKQKSSFGLGLYIVNTILESHGMK
                     LDYDYEEGVNLFYFRNLKSIIVKE"
     gene            complement(25407..26081)
                     /locus_tag="DMACINML_00286"
     CDS             complement(25407..26081)
                     /locus_tag="DMACINML_00286"
                     /inference="ab initio prediction:Prodigal:2.6"
                     /inference="similar to AA sequence:RefSeq:CAL35342.1"
                     /codon_start=1
                     /transl_table=11
                     /product="putative two-component regulator"
                     /protein_id="Prokka:DMACINML_00286"
                     /translation="MTNILMIEDDLELAEITAEYLEKFDMKVDIAHEPYIGLSKLALK
                     EYQLIILDLSLPGLDGLEVCEEIRKKYDTPIIISSARHDITDKVNALELGADDYLPKP
                     YNPKELQARIKSHLRRISNTKSAIAKSVKDLVYDQYKHIITMKGQELTLTNAEFDILS
                     YLIKKEGGVVSREELVYNCSSISEDSSNKSIDVIISRIRQKMGDDPKTPKYIHSIRGI
                     GYKLTQ"
     gene            complement(26164..27582)
                     /gene="htrA"
                     /locus_tag="DMACINML_00287"
     CDS             complement(26164..27582)
                     /gene="htrA"
                     /locus_tag="DMACINML_00287"
                     /EC_number="3.4.21.-"
                     /inference="ab initio prediction:Prodigal:2.6"
                     /inference="similar to AA sequence:RefSeq:CAL35343.1"
                     /codon_start=1
                     /transl_table=11
```

```
                     /product="serine protease (protease DO)"
                     /protein_id="Prokka:DMACINML_00287"
                     /translation="MKKLILSLSLASALFAANINFNETSITTNRVNPAAGDTILSYHD
                     SIKDAKKSVVNISTSKTITRVNRPSPLDDFFNDPYFKQFFDFDFPQRRGGNDKEVVSS
                     LGSGVIISQDGYIVTNNHVVDDADTISVSLPGSDTEYKAKLIGKDPKTDLAVIKIEAN
                     NLWAITFTNSDDLMEGDVVFALGNPFGVGFSVTSGIISALNKDNIGLNQYENFIQTDA
                     SINPGNSGGALVDSRGFLVGINSAILSRGGGNNGIGFAIPSNMVKDIAKKLIEKGRID
                     RGFLGVTISALQGDTKKAYKNQDGALITDVQKGSSADEAGLKRGDLVTKVNDKVIKSP
                     SDLKNYIGTLEIGQKIILDYERDGENKRVDFTLKGEKENPKAVQNDLIEGMTLRNLDP
                     RFKERLQIPKDVNGVFVESVKDKSRAKDSGFQEGDIIIGVGQNEIKNLKDLEQALKQV
                     KKKEFTKIWVYRNGFATLLILK"
     sig_peptide     complement(27532..27582)
                     /gene="htrA"
                     /locus_tag="DMACINML_00287"
                     /inference="ab initio prediction:SignalP:4.1"
                     /note="predicted cleavage at residue 17"
     gene            27791..28690
                     /gene="cbpA"
                     /locus_tag="DMACINML_00288"
     CDS             27791..28690
                     /gene="cbpA"
                     /locus_tag="DMACINML_00288"
                     /inference="ab initio prediction:Prodigal:2.6"
                     /inference="similar to AA sequence:RefSeq:CAL35344.1"
                     /codon_start=1
                     /transl_table=11
                     /product="putative curved-DNA binding protein"
                     /protein_id="Prokka:DMACINML_00288"
                     /translation="MNSLYETLGVSKNASADEIKKAYRRLARQYHPDINKEKGAEEKF
                     KEINAAYEILSDEKKRAQYDQYGDSMFGGQSFHDFSRNAGGVNLDDILKDLFGGGFGG
                     GSSKGSRFNGFSSKGFNAGFGGFGGFEEEDLDSTIELSIPFEKAVTGGEYSFSLQGET
                     IKFKIPHGIKQGEKLRIRNKGKKGRNGTRGDLIVVVKLGESEIYQREDDDLYQKVDIS
                     LKTALFGGKVTVNTLKESKKEATITIPANSKNGQKIRLKGYGVQNRKSDIYGDMYLVL
                     NVVLPNTETLDEKLIEMLKEKLS"
     gene            28706..29080
                     /gene="hspR"
                     /locus_tag="DMACINML_00289"
     CDS             28706..29080
                     /gene="hspR"
                     /locus_tag="DMACINML_00289"
                     /inference="ab initio prediction:Prodigal:2.6"
                     /inference="similar to AA sequence:RefSeq:CAL35345.1"
                     /codon_start=1
                     /transl_table=11
                     /product="heat shock transcriptional regulator"
                     /protein_id="Prokka:DMACINML_00289"
                     /translation="MEHHYDEPVYLISVVAKVLSIHPQTLRQYEREGLIEPSRTDGKI
                     RLYSQRDIDRIKLILRLTRDMGVNLAGVDVILKLKNQLHEFENLIDELRLELSKQQNP
                     NASSKAVVKHKNSFDLIFYEKK"
     gene            29081..30703
                     /gene="kefB"
                     /locus_tag="DMACINML_00290"
     CDS             29081..30703
                     /gene="kefB"
```

```
                     /locus_tag="DMACINML_00290"
                     /inference="ab initio prediction:Prodigal:2.6"
                     /inference="similar to AA sequence:RefSeq:CAL35346.1"
                     /codon_start=1
                     /transl_table=11
                     /product="putative glutathione-regulated potassium-efflux
                     system protein"
                     /protein_id="Prokka:DMACINML_00290"
                     /translation="MGNFLEIFLITAAIAIVLNVIFKKFEIPTIIGYIAAGEIISEIY
                     HLSGKGEIAHIAEFGIVFLMFTIGLEFSFKHLMAMKQEVFLNGSLQMLTCGFVFMLLA
                     IGILGLGDKSATIVGFALALSSTAVVLKILNDNGDINEQYGRKALGILLFQDIAVIPL
                     LLLVDFFSSNNQNIGQLLLTTLLSAAILIALLFFIGKYLVDRIFRLIIRASSQEIFIS
                     TILFMVIGASFLAHYFGFSYSLGAFIAGALIAETKYKHKIEADLIPFRDLLLGLFFIT
                     VGMQIQPSIVAQNWFIICVLTLLIMGLKFGIVCGFLFLYTKKRVALKTAFSIAQVGEF
                     ALAIFSLLQAKSMLDVETAQILIVVSIITMIITPFVLNNIRKIANVVEDMTLNTNVVQ
                     QSTHSTLKNHLVIFGYGRLGQEIVQKIKNTGVPYLVLESDLNLVELGISRGENVVFAN
                     AAQEETLKIANITECAVAIVTITNEAKLEMICQVLASYPKPIDTIIHVNGSLEKMLFS
                     SIDENIRIVRSEKVIARNLVQEALECRIHKNT"
     gene            30713..31045
                     /locus_tag="DMACINML_00291"
     CDS             30713..31045
                     /locus_tag="DMACINML_00291"
                     /inference="ab initio prediction:Prodigal:2.6"
                     /inference="similar to AA sequence:RefSeq:CAL35347.1"
                     /note="hypothetical protein Cj1232"
                     /codon_start=1
                     /transl_table=11
                     /product="hypothetical protein"
                     /protein_id="Prokka:DMACINML_00291"
                     /translation="MAKIDEKTLRVKYLRALEKFANAAISALKKENFDKEKFQERMEK
                     NKKIVEKCEAVNLNSSYNKTLENFVNNCLDFSKQKEELLSLANALDKLKKGEKKEKHK
                     TYLKDYYD"
     gene            31038..31658
                     /locus_tag="DMACINML_00292"
     CDS             31038..31658
                     /locus_tag="DMACINML_00292"
                     /inference="ab initio prediction:Prodigal:2.6"
                     /inference="similar to AA sequence:RefSeq:CAL35348.1"
                     /codon_start=1
                     /transl_table=11
                     /product="putative HAD-superfamily hydrolase"
                     /protein_id="Prokka:DMACINML_00292"
                     /translation="MINVFFDMDGTLIDSANAISCAVNEIRNELNLSPLSREKIMQTI
                     NTPNVDWAKELYSIDNFHHSSFKDGFEKYFIKHYEQSVVLFEGIKDILEFLKEQNCFL
                     AIATNAPQSSLSSILKKHEIIPYFDKILGVSLGIEPKPHPMMLDLLKSEAPHKTSIFI
                     GDSQKDRECAKNANMPYFHAKWYQKDLQENEFSNASELKNFLERYL"
     gene            31712..33706
                     /gene="glyS"
                     /locus_tag="DMACINML_00293"
     CDS             31712..33706
                     /gene="glyS"
                     /locus_tag="DMACINML_00293"
                     /EC_number="6.1.1.14"
                     /inference="ab initio prediction:Prodigal:2.6"
```

/inference="similar to AA sequence:RefSeq:CAL35349.1"
/codon_start=1
/transl_table=11
/product="glycyl-tRNA synthetase beta chain"
/protein_id="Prokka:DMACINML_00293"
/translation="MSELLIEIGTEELPAIPLLKELANIEKKWKDVLEDYRLVSDFKF
YYTPRRLVIFHENFASKQEDSFAEFIGAPKNVAYKEGQLSAAGQSFLQKAGISESELS
FKEIKGKEVLYHQKAIKGLQSQEVLGEMIHQFLKSLNFGKSMRWGANSFEFIRAIRSI
VCILDDNLVEFESYGVKSAKKTFVHRSVSYDLQEFNNAKEYFSLLEKNHIILDPLQRK
ERILKQFKLLESQNNIQIGEDEELLAEVIAITEYPNALLGSFEEEYLQIPSEVIITSM
RENQRYFAVFNEKGLSNHFIVVSNAVCEDYSKIIHGNERVLRARLSDAMFFYQNDLQS
GLNPEKLAKMTYLEGLGTMQDKSLREIKIAEVLCQMLNNDKIANISTAIKYAKADLAT
QMVYEFTDLQGIMGSYYAQKMGLDYEICLAIKEQYLPNSEQAPLPSTEFSSIVALANK
LDTLIGLFSIGKIPSGTKDPYALRRAANGIIKIALNLNKEFDIQALLEKLASHYKNFD
MQILKDFIFERLYTFYSVNASFIKAVLSSKNTDLIHINQSVKALIELSKKANFQENFT
TFKRLANIANKTSHKVDENLFIEEAENKLYKAFKEKIQAHSLQEKLENLFSLKPFIDE
FFDKVMINAEDEKLKNNRQALIYEIYEEFLKIADLKELSL"

gene 33703..34524
/locus_tag="DMACINML_00294"

CDS 33703..34524
/locus_tag="DMACINML_00294"
/inference="ab initio prediction:Prodigal:2.6"
/inference="similar to AA sequence:RefSeq:CAL35350.1"
/codon_start=1
/transl_table=11
/product="putative peptidase M23 family protein"
/protein_id="Prokka:DMACINML_00294"
/translation="MKAFWLFLSLALWLFGAQNFELIKGQALFLELDKKDFISLKNED
KILATFPHPKNQEKILAIFSLPYKNPPQNTKLIALYKNKKEEIFIKTLEGNYKSEKLS
VENKKIFPPKAVKERIAKEFKEANAIYSSYTPKALFDGSFNTPLDSFITSDFGKARTF
NEKVASYHSGTDFRAAVGTPIYAANSGIVKIAKDRYFAGKSVVIDHGFGIYSQYYHLS
KIEVKVGQRIKKGDFLGLSGATGRVSGPHLHFGILAGGKQVDPLDFISKFNTLFQ"

gene 34521..35471
/locus_tag="DMACINML_00295"

CDS 34521..35471
/locus_tag="DMACINML_00295"
/inference="ab initio prediction:Prodigal:2.6"
/inference="similar to AA sequence:RefSeq:CAL35351.1"
/note="conserved hypothetical protein Cj1236"
/codon_start=1
/transl_table=11
/product="hypothetical protein"
/protein_id="Prokka:DMACINML_00295"
/translation="MKFSEFFHTWLHESYYKNAVSIGKNGDFFTAVSVGNLFGTLLAK
HFLDLIDKKVLKFPLELVEIGANEGYLSRDFLAALLEFRPEIFSKLSYFIIEPHEKLR
NLQKKTLEGVEFTHKRSLKECHFENAFFFCNELFDSFACELIDDDKMAFVQDFKLVFE
PMKAKLKEKCEILNLKKGELSLELEEFFKDLDKASKKFIFASFDYGVFNPEQFSIRIY
QKHEVFNPFEITLKDFFGKSDLTYNVNFTQIQQLIKEHDFKLLALKKQNQALIDFGFE
ELLKYTKDKNLKTYENFLSQSKILFFNFDEKFHFFEFAKF"

gene complement(35543..36508)
/locus_tag="DMACINML_00296"

CDS complement(35543..36508)
/locus_tag="DMACINML_00296"
/inference="ab initio prediction:Prodigal:2.6"

```
                     /inference="similar to AA sequence:RefSeq:CAL35352.1"
                     /codon_start=1
                     /transl_table=11
                     /product="putative phosphatase"
                     /protein_id="Prokka:DMACINML_00296"
                     /translation="MLGIDLGSNTLRAVFINEKFEKLDEYEFIIGSAKNLDKTGKISD
                     EAIEKLRNALQEIAKKYDLSQAKAAATAAFRKASNTSEIFTRLKQEFQIDFKVIDAKS
                     EAKISVLGMKMGLLNLGLNLDCGYCDLGGASCEFSFRENFQSFDFGIISFYEKCKIKR
                     SVNSFSFKKILQKYPDFPYKFKDKKLKIHFLIHDNFLKTMAFEAFKQSNELKKMLKQS
                     KMKNIVLNSGVPTALAALKKGLNYEKYNAKKINGSRLNVNDFLNFGQRLWHMDEKRAS
                     FWVGNTRKNYLVAGCFLLFSIFEREKLIVIDEGLREGLCMADLNF"
     gene            36564..37337
                     /gene="pdxJ"
                     /locus_tag="DMACINML_00297"
     CDS             36564..37337
                     /gene="pdxJ"
                     /locus_tag="DMACINML_00297"
                     /inference="ab initio prediction:Prodigal:2.6"
                     /inference="similar to AA sequence:RefSeq:EAQ72683.1"
                     /codon_start=1
                     /transl_table=11
                     /product="pyridoxal phosphate biosynthetic protein PdxJ"
                     /protein_id="Prokka:DMACINML_00297"
                     /translation="MLLGVNIDHIAVLRQARMVNDPDLLEAAFIAAKFGDQITLHVRE
                     DRRHAQDFDLENIIRFCKSPINLECALNDEILNLALKLKPHRITLVPEKREELTTEGG
                     LNLNHDKIKESIEKLQNAEIEVSLFINPSLEDIQKSYELKADFIELHTGHYANLHNAL
                     FSNISHTTFALKEFDLSKKILQNQFEEELSNIEICAKKGVELGLKVAAGHGLNYKNVS
                     QIVNIKEICELNIGQSIVARSVFVGLQNAILEMKALLQR"
     gene            37334..38434
                     /gene="pdxA"
                     /locus_tag="DMACINML_00298"
     CDS             37334..38434
                     /gene="pdxA"
                     /locus_tag="DMACINML_00298"
                     /EC_number="1.1.1.262"
                     /inference="ab initio prediction:Prodigal:2.6"
                     /inference="similar to AA sequence:RefSeq:ABS43783.1"
                     /codon_start=1
                     /transl_table=11
                     /product="4-hydroxythreonine-4-phosphate dehydrogenase"
                     /protein_id="Prokka:DMACINML_00298"
                     /translation="MKKLAVSIGDINGIGLEILARSHEKLSQICTPYYFIHESLLQKA
                     LKLLNLELLNAKIVAFKDAKNYEFTLLKKHNSLEIYSFGLPLNLKVDENFDIKAGEID
                     AKSGLYSFLSFKAASYFVYKNHAHALLTLPIHKKAWENAGLEFKGHTDALRFFFKKNA
                     IMMLGCKELFVGLFSEHIPLAKVSKKITFQNLSIFLKDFYQETRFKKIGLLGFNPHAG
                     DYGVIGGKEEELMQKAIAFVNAFLNSKKDEKFFKKALKDENLQKELLLNFNTKGVYLP
                     YPLVADTAFTKANLKACNRLIAMYHDLGLAPLKALYFEKSINVSLNLPIIRVSVDHGT
                     AFDKAYKNAKISTKSYFEAAKFALELPSKASI"
     gene            complement(38418..39023)
                     /locus_tag="DMACINML_00299"
     CDS             complement(38418..39023)
                     /locus_tag="DMACINML_00299"
                     /inference="ab initio prediction:Prodigal:2.6"
                     /inference="similar to AA sequence:RefSeq:ASI87753.1"
```

```
                     /codon_start=1
                     /transl_table=11
                     /product="Hypothetical protein"
                     /protein_id="Prokka:DMACINML_00299"
                     /translation="MKIILLTILSFLSLWGLSFDELIYKNEVKPSFDCFKVKDNGKND
                     DELIICNKIGAINEFENKKLALMDNIFISLYQELLKNANENIKEDFKLVVKKMFRERK
                     NCTKATSNDYIQASQDPQSPFVYKTDCIEKTYARAFLELMQKAKKDTQSKERLKQIFN
                     NQVDRYENLIQMSIQDPIDLEIFIDNLAQEGLIDDNAKLKL"
     gene            39271..39582
                     /locus_tag="DMACINML_00300"
     CDS             39271..39582
                     /locus_tag="DMACINML_00300"
                     /inference="ab initio prediction:Prodigal:2.6"
                     /inference="similar to AA sequence:RefSeq:CAL35357.1"
                     /note="hypothetical protein Cj1242"
                     /codon_start=1
                     /transl_table=11
                     /product="hypothetical protein"
                     /protein_id="Prokka:DMACINML_00300"
                     /translation="MVVKDIQTTSSSMFSATTKVKEKSTSADEFQATLNEVKNKEDKE
                     DKKTSSSEFTNEDLDLSAVKEDFKSYAWQKMREDQYKKNEEDLLNKLFATIDAGNTAK
                     I"
     gene            39736..40758
                     /gene="hemE"
                     /locus_tag="DMACINML_00301"
     CDS             39736..40758
                     /gene="hemE"
                     /locus_tag="DMACINML_00301"
                     /EC_number="4.1.1.37"
                     /inference="ab initio prediction:Prodigal:2.6"
                     /inference="similar to AA sequence:RefSeq:CKG95321.1"
                     /codon_start=1
                     /transl_table=11
                     /product="uroporphyrinogen decarboxylase"
                     /protein_id="Prokka:DMACINML_00301"
                     /translation="MIFIDACFKKPTPYTPIWMMRQAGRYLPEYMKVRQEAGDFLSLC
                     KDYKRASEVSLQPVDILGVDAAIIFSDILVVPLEMGMNLRFEKGEGPVFQTPISTLED
                     LNKLDDQNASKKLNYVYDSLKLTREKLPLDKALIGFCGSPWTIATYMIEGSGSKNYAK
                     CKKMLYQNPELLHEILNKLTQVLKSYLEEQIKAGANAVQIFDSWASALECDKFFDFSF
                     KYMLEISDFIKNKYPHIPVILFPKGISGYLDKIDGNFDVFGVDWSTPLDLARDKLSHQ
                     YTLQGNMEPCRLYDKKAIKQGVENILEIMQNKAHIFNLGHGILPDIPVENAKYFIQLV
                     QESSAK"
     gene            40755..41657
                     /locus_tag="DMACINML_00302"
     CDS             40755..41657
                     /locus_tag="DMACINML_00302"
                     /inference="ab initio prediction:Prodigal:2.6"
                     /inference="similar to AA sequence:RefSeq:CAL35359.1"
                     /codon_start=1
                     /transl_table=11
                     /product="putative radical SAM domain protein"
                     /protein_id="Prokka:DMACINML_00302"
                     /translation="MKILFGPINSRRFGRSLGIDLSPSKKQCNFDCVYCELEAQKPQA
                     KQDEIVSVEEIILEVQSALEKNILFDFLTLTANGEPSLYPHLDKLISSLRKIAKDKKL
```

```
                     LILSNGTAVLDQDKFNTLLKLDVVKFSLDSAISKTFYRIDRALKNIDLEKMIKKMAEF
                     KTQFKGDLVMEILVVKDLNDNEEEFIALNQALAKIAPLRVDLSTIDRPPAYAVKKISE
                     ERLLELSKLITSTPVLLPKRHYEGEKLNFGEEELLKMLHLRSQSEIDIENKLDKASQV
                     LLDKLIKEEKVKILNLAGVKFYKA"
     gene            complement(41654..42847)
                     /locus_tag="DMACINML_00303"
     CDS             complement(41654..42847)
                     /locus_tag="DMACINML_00303"
                     /inference="ab initio prediction:Prodigal:2.6"
                     /inference="similar to AA sequence:RefSeq:CAL35360.1"
                     /codon_start=1
                     /transl_table=11
                     /product="putative membrane protein"
                     /protein_id="Prokka:DMACINML_00303"
                     /translation="MLLVWIIFIFNIILAFLSIDFARIHESFEQIKLAFFASIFILSL
                     TLIFFLFWQKRALAKKDQKILLIKNEFIHSKEIDIETSLDLIREKIAHLNQDLFKISH
                     EKETYAKLLEDSLYQIGNIEGVVRCLDEEEASREIKSLVLNALKHQKEQIRLGLNQAL
                     EYHKSIGVAQGNVLQFEASAAIVSDDELESFLLANLLSYFGIENTIFKSLSFDINDFH
                     VVFIKDKILEQSSKKADDFIVFGRSKNLYYEYFLSLPFEKKELETILQRKLDKICVLK
                     FQTPYQNNVLLFKQNEFDATLFFNIIEKQCSQNMRVNSFSELKKELKKEAYRLVLLDY
                     ELIKFDLEQMRSILSAYKKQHPQSHIIFFSKERVRDFDCVSEVLNDISRNDLIALIRK
                     YLPKN"
     gene            complement(42849..44663)
                     /gene="uvrC"
                     /locus_tag="DMACINML_00304"
     CDS             complement(42849..44663)
                     /gene="uvrC"
                     /locus_tag="DMACINML_00304"
                     /inference="ab initio prediction:Prodigal:2.6"
                     /inference="similar to AA sequence:RefSeq:CAL35361.1"
                     /codon_start=1
                     /transl_table=11
                     /product="excinuclease ABC subunit C"
                     /protein_id="Prokka:DMACINML_00304"
                     /translation="MTEILLKNTLKEELQTLPSNAGVYQYFNAEGKLLYVGKAKNLKN
                     RVRSYFAFTPNLHANPKNSLRIQKMIEEAVHLEFITTNSEADALILENSFIKQLHPKY
                     NILLRDDKTYPYIYVDFNEEFPRFEITRKLVKKSKIKYFGPFFKGARELLDALYLYYP
                     LKQKASCKSPCIFYQISRCLAPCDKKITQEEYRLILDKAINALLSPSILVKNLEKQMF
                     HLAQNENYEEAAKIRDQIATIRDLEIKVEIDIAKLEDFEIFALACESSMLSTLRFVVQ
                     NGKIISANSKITPLKNNTDFDKNEIYKQLILENFSIDIPLVTNTIYVYEEFEDRELLE
                     EILSQRFDKKISIKVPKIGEKRRICDLAFQNACLNIQKEQKNSDFNIQKELKNYFELE
                     NLPNYIEVFDNSHMQGVANVGAMVAYKMGAWDKSNYRKFHLKYKNDYEQMREVLTRRA
                     LDFDKMPAPDLWLIDGGKALLDLAKEIILSSGANVDILAISKEKIDAKAHRAKGGAKD
                     KIHSLKGEFALSINDKKLQFLQKLRDEAHRFAISFHQNTKKKQDLSSSKLAKLGLSSG
                     IIQKLLAYYGSFEAIYEANFEELRQLVGKKSAQKIKED"
     gene            complement(44656..45132)
                     /locus_tag="DMACINML_00305"
     CDS             complement(44656..45132)
                     /locus_tag="DMACINML_00305"
                     /inference="ab initio prediction:Prodigal:2.6"
                     /inference="similar to AA sequence:RefSeq:CAL35362.1"
                     /note="hypothetical protein Cj1247c"
                     /codon_start=1
                     /transl_table=11
```

```
                     /product="hypothetical protein"
                     /protein_id="Prokka:DMACINML_00305"
                     /translation="MEKFQSPKIIKNTSAILNDLMSKSHYRPLKTLFFCKDFLNSFPP
                     AKQRLIARIYIKNHILNIVTLNSAAYQELNHDNSKIYIKVLIKKYGKMYPLSGFSDIK
                     DLKIFTQKYNNTIKNNENFTAKKPYIELSLGKFENKIENKILAKKFEEIRQMIKND"
     gene            45269..46804
                     /gene="guaA"
                     /locus_tag="DMACINML_00306"
     CDS             45269..46804
                     /gene="guaA"
                     /locus_tag="DMACINML_00306"
                     /EC_number="6.3.5.2"
                     /inference="ab initio prediction:Prodigal:2.6"
                     /inference="similar to AA sequence:RefSeq:CAL35363.1"
                     /codon_start=1
                     /transl_table=11
                     /product="GMP synthase (glutamine-hydrolyzing)"
                     /protein_id="Prokka:DMACINML_00306"
                     /translation="MKKADILVLDFGSQYTQLIARRLREQGVYAEILPFNVSLADIKA
                     KEPKGIILSGGPASVYATDAYFCDKEVFNLGLPILGICYGMQLMAHHYKANVAPAGHK
                     EYGKASIEIQKDSDLFKNLPKKQIVWMSHSDKVENLPQGFEVLATSENSPYCVFANEE
                     KKFFALQFHPEVQHSEFGKSILKNFAKYACNCESIWNMGSFAKTQAEKIREEVGNDKV
                     LCAVSGGVDSSVVAALLANAIKDQVIVVFVDNGLLRSGEKEQVEFMFKNTLGINLISI
                     DASKIFLDRLAGVTDPEQKRKIIGNTFIEVFEEEAKKHKDVKFLAQGTLYTDIIESSV
                     IGASKTIKSHHNVGGLPEKMNLKLVEPLKEIFKDEVRALGIELGLSKEIVYRHPFPGP
                     GLAIRIMGEVNRPSLELLRKADVILLEELRSTGWYDKTWQAFCVLLNVKSVGVMGDNR
                     TYDNTVCVRVVDASDGMTATFSHIPYEILENISRRIINEVEGINRVVYDISSKPPATI
                     EWE"
     gene            46986..47594
                     /gene="cfa_1"
                     /locus_tag="DMACINML_00307"
     CDS             46986..47594
                     /gene="cfa_1"
                     /locus_tag="DMACINML_00307"
                     /EC_number="2.1.1.79"
                     /inference="ab initio prediction:Prodigal:2.6"
                     /inference="similar to AA sequence:RefSeq:CAL35298.1"
                     /codon_start=1
                     /transl_table=11
                     /product="cyclopropane-fatty-acyl-phospholipid synthase"
                     /protein_id="Prokka:DMACINML_00307"
                     /translation="MENSIKDSYNKICKKWSDFRKSTSTNKCIIDFANDLKSNSRVLD
                     IGCGTGYPIASYLSKQGFQVTGIDISEEMIKQAQKLNLHNATFLVENILNFKTDKKYD
                     AIIAFDSIWHISHDKQECIYQIISSLLTPGGLFLFTHGKNDGEIISTMWGESFYHSAL
                     DLEKVHKLLKQNGFDILSSIEDYIEETTGDRELLIIAKKKEL"
     gene            complement(47740..48297)
                     /gene="rsmA"
                     /locus_tag="DMACINML_00308"
     CDS             complement(47740..48297)
                     /gene="rsmA"
                     /locus_tag="DMACINML_00308"
                     /EC_number="2.1.1.-"
                     /inference="ab initio prediction:Prodigal:2.6"
                     /inference="protein motif:HAMAP:MF_00607"
```

```
                     /codon_start=1
                     /transl_table=11
                     /product="Ribosomal RNA small subunit methyltransferase A"
                     /protein_id="Prokka:DMACINML_00308"
                     /translation="MFFYEYLKNPKQIGAFCSSSQKLGFVMTQNINLRQADYIVEIGP
                     GTGVFTENILKYKNKKAQFFAIEINKNMAEKLKRKIANLDIEIGSAENLPYFLRQRSI
                     ANADVIVSGIPWSLLKDHEQEKLLKIIHKSLKPNGYFSTFAYVLPTFSSLKFRQKIFS
                     FFSEVRISRVVWQNFPPAVVYYCKK"
gene                 48431..49891
                     /locus_tag="DMACINML_00309"
CDS                  48431..49891
                     /locus_tag="DMACINML_00309"
                     /inference="ab initio prediction:Prodigal:2.6"
                     /inference="similar to AA sequence:RefSeq:CAL35364.1"
                     /note="hypothetical protein Cj1249"
                     /codon_start=1
                     /transl_table=11
                     /product="hypothetical protein"
                     /protein_id="Prokka:DMACINML_00309"
                     /translation="MHGKIAIYMDSTGRGTVTNSANTFFDFSRQIWNDKKSMPSVGML
                     VEFRTLSSDKKAENGAPAPVSKTITGIKPSKFQEFKEGDFITEHDFWKTDSDDELEDL
                     QNSRRSAYITELYRSIDFDSIEKIPLSFTIPQAIQKYFAHEILSVEALQANLQDEKDI
                     PCILDYLILKRFLFKAYDTLIFMDNSIDQTQFSALKSIMMHLENSYKQMIADKKPNIT
                     KIFNETFLSLQCHYQALIATIDTRKNRLASLEAQMKALQSEIQLKSNAANTDPEKLKA
                     RQEKLAKLQKEAEYYRTTLKRLGAIQEDFYKKNYNIFENAFKLSREKLFKKIITGLNL
                     CATIMDVKIWHLSLKSSGVKNSYFTMSNIENSFCSLSFAEHYLSRLNKAALNPFDQKL
                     LVYIQKITKEQRKKFLVVTSDLDLLCKLKIETFAQNPYFIVKYAPKKVNYQSLMRDNS
                     FDIVFIDEKHVWESVADIILEGKHFDKIGKTKFKLI"
gene                 50132..51382
                     /gene="purD"
                     /locus_tag="DMACINML_00310"
CDS                  50132..51382
                     /gene="purD"
                     /locus_tag="DMACINML_00310"
                     /EC_number="6.3.4.13"
                     /inference="ab initio prediction:Prodigal:2.6"
                     /inference="similar to AA sequence:RefSeq:CAL35365.1"
                     /codon_start=1
                     /transl_table=11
                     /product="phosphoribosylamine--glycine ligase"
                     /protein_id="Prokka:DMACINML_00310"
                     /translation="MKIMILGSGAREYSIALALKRVDENLEFYFAPGNGATESLGTNL
                     NFKDPVVLATYAKEKDFDLCIVGSESFLAEGIVDIFKQHNIPIFGPTKAAAMLETSKS
                     FMKSFLKKYRIKTAKFLNTNDIEKAKNFILSLTPPIVVKADGLCAGKGVIIAKTHEEA
                     IEEASKMLSGESFGEAGKLIVIEEFLDGFELSIFAVCDGNDFVLLPAAQDHKKLLDGD
                     EGPNTGGMGAYAPSSLANESLLRKIQKDIIIPTLAGMKKEDSEFCGVLFIGIMVVGNK
                     PYVLEFNVRFGDPECEVLMPLIENPLELILATTQKRLRHTKIKIKKDYAVGVVCASEN
                     YPYKSSPKSEISITNIPENTHISYAGVSLEDGKLMADGGRVLVCVGTGSSIEEAQKKA
                     YELCENVHFKGKQFRKDIAHQVIQ"
gene                 51379..51831
                     /locus_tag="DMACINML_00311"
CDS                  51379..51831
                     /locus_tag="DMACINML_00311"
                     /inference="ab initio prediction:Prodigal:2.6"
```

/inference="similar to AA sequence:RefSeq:CAL35366.1"
/note="conserved hypothetical protein Cj1251"
/codon_start=1
/transl_table=11
/product="hypothetical protein"
/protein_id="Prokka:DMACINML_00311"
/translation="MKEDLLTRLERENLTIASFNKRILAYFIDNLVLSLIVFVIFYDK
LLNAKDLFDTAQILGDFYLGFLLLHITYQTIFTYLYGASLGKMVCKIIILDEDLLDKP
NLNQSCIRSIVRQISDMAFMLGFAWALSNDLRKTWEDYLARTIVVNVA"

gene 51821..53869
/locus_tag="DMACINML_00312"

CDS 51821..53869
/locus_tag="DMACINML_00312"
/inference="ab initio prediction:Prodigal:2.6"
/inference="similar to AA sequence:RefSeq:ADT66527.1"
/codon_start=1
/transl_table=11
/product="organic solvent tolerance protein, putative"
/protein_id="Prokka:DMACINML_00312"
/translation="MWRKILFLSASLTLLDAAQVDIYALDAKKQGDILTANEDVVIFS
DFYFITANKAIYNEKTGDLELFGDVNILRGQNERSHSNYAKINLNSNKANFNNFFFSN
NNLEVWFQSKTSYLDENIFKSEISAVSSCNVEDPDWEIRFSEGWLNRENNFVHLYNAK
LYVKDFPVFYLPYFGFSADTHRQSGLLIPKIVLKNSEGLYYEQPIYIAPYENWDLELN
PQIRTNRGFGLYSTLRFVDSPYSMGEVNLGAFRENSSYYHDENLKNQSHYGAELKYAR
NDLIKTLLGDNFQEGLWVDATYLNDIDYLNLGSRDYRDLNSLVTSKINYFLADENNYY
GAYAKYYIDTSALNNDNTLQEYPSFQYHRFLNNFFDERLRYSFDASFHNFYRPVGPYA
NQLNLNLPISYHNAFFDDFLHFTFTERFYASFLNYSHYPEKEHEHYFRNTHDFNLYTD
LSKAYDNFFHTLNFGLGYILPGAKSGSISEDYLEESNKEEEHFGLYTTQYFYNNEGQK
KIKHRLNIDYLNKQGSFDEISNLLSYYYNNNISFNSEIVYSDLQDRFTNIISQMEIKP
NSKFNWSFSHAYQNDEYGKYSFIGTRANYLASANYHLFGGIWFDTQRAHANMWELGYT
FQRKCWNYSLMYRERIDPQLTSAGITAKNQSGIYFIFNFYPLGGVKYDFSLAETESKI
"

gene 53886..56102
/locus_tag="DMACINML_00313"

CDS 53886..56102
/locus_tag="DMACINML_00313"
/inference="ab initio prediction:Prodigal:2.6"
/inference="similar to AA sequence:RefSeq:AGQ94461.1"
/codon_start=1
/transl_table=11
/product="polynucleotide phosphorylase"
/protein_id="Prokka:DMACINML_00313"
/translation="MLNIFKSLTNLLYGKKGQIMQYSIEINGNLEVFDIEKVAKQAAG
AVLMRQGKSVVLATVAREEKPVEEDFLPLTVQYIEKAYAAGKIPGGYVKRETKPSDAE
TLTARIIDRSLRPLFPKGYAYPTQIVVMVLSADPQVDLQVMSLNAASVALYLSDIPMK
APVCGVRIGRIEEKFVLNPNNEELKNSTLDLYVAGVKDELLMIEMRALPNQKEDEIFI
EAPYADVLTQTTSQSMNELSEDEILEALSLAQKAILNGSNAYEEAFLKHKKSSDLNFK
DEIENFELLAFIENNFHDQIAQAINQMAKSERASELNKIAEKILNFESATKWDEESIL
NALAKVKHKIVRSQILNTKKRADGRNLNEVRPITIETNILPNAHGSCLFTRGQTQALV
VATLGSENDAQMVDVLTEKNPINERFMVNYNFPGFSVGEASPIKAPGRRELGHGNLAK
RALYPSIDENYPYVIRLVSEILESNGSSSMATVCGGSLALRAAGVPSLKLVAGVAMGL
IFEGDQYAILTDIMGLEDHDGDMDFKVAGSKDGITALQMDIKLGGIDQKILKEALYQA
KEGRIHILNIMEEALKEIVVNEEVLPKLELFNVDPSKIVDIIGQAGKTIKEIIEKFGV
SIDLDREKGEVKIAGNENEQIKAAKDYIINITSSQKNNKRGFKEKDLSDFEIGEEFEG

                     KVKKITNFGAFIELKNGIDGLLHNSKSKNLNLSENQILKVKVSEIKNGKISVDLCE"
gene                 56099..56581
                     /locus_tag="DMACINML_00314"
CDS                  56099..56581
                     /locus_tag="DMACINML_00314"
                     /inference="ab initio prediction:Prodigal:2.6"
                     /inference="similar to AA sequence:RefSeq:CAL35369.1"
                     /note="hypothetical protein Cj1254"
                     /codon_start=1
                     /transl_table=11
                     /product="hypothetical protein"
                     /protein_id="Prokka:DMACINML_00314"
                     /translation="MSEFLTHPFKPFFDKDSKILILGSFPSVKSREDGFYYQHPRNRF
                     WPILETLFDTRLENITKQQAFLKEKNIALWDVLQSCKIKNSDDKTISYAKANDLNLIL
                     SQAQIQAIFTTGKSAYQFFTKFHPHLKAIALPSSSPANLNFSFEQLLQSYKIIKNFIK
                     "
gene                 complement(56519..56788)
                     /locus_tag="DMACINML_00315"
CDS                  complement(56519..56788)
                     /locus_tag="DMACINML_00315"
                     /inference="ab initio prediction:Prodigal:2.6"
                     /codon_start=1
                     /transl_table=11
                     /product="hypothetical protein"
                     /protein_id="Prokka:DMACINML_00315"
                     /translation="MFDFFINYMIFCAKDLSKLFLGTLALFLFVFFGIMFFICEGVFL
                     YVLLFCEIILFLYMLLGIFYIRNQKLFYKIFNYFIALQKLFKRKI"
gene                 57179..57388
                     /locus_tag="DMACINML_00316"
CDS                  57179..57388
                     /locus_tag="DMACINML_00316"
                     /inference="ab initio prediction:Prodigal:2.6"
                     /inference="similar to AA sequence:RefSeq:CAL35370.1"
                     /codon_start=1
                     /transl_table=11
                     /product="putative isomerase"
                     /protein_id="Prokka:DMACINML_00316"
                     /translation="MPFVNIRITKENGEPTNEQKQELIAGVTDLLARVLNKNKASTVV
                     IIDEIDTDNYGLGGKSITQVRKEKS"
gene                 complement(57385..58041)
                     /locus_tag="DMACINML_00317"
CDS                  complement(57385..58041)
                     /locus_tag="DMACINML_00317"
                     /inference="ab initio prediction:Prodigal:2.6"
                     /inference="similar to AA sequence:RefSeq:CAL35371.1"
                     /codon_start=1
                     /transl_table=11
                     /product="putative membrane protein"
                     /protein_id="Prokka:DMACINML_00317"
                     /translation="MELILLSLIVILLVFVIIKQFKNSSKTEEKDIHISNDITQLKSI
                     GELSVFQVFSKEIVTKKDSAFNGIWKSILGWSLSERQIALIFEFEITFLYDLRDKNFE
                     IVVLDNDHYKIIMPECKYKHSIIDMKFYDEKNAKFLPFLLPDSINSTGLSFSENDKNK
                     LIREAKEEVKELSLNLIRNLESKIHKSARDTLEAIAKGFGAKKVEFEFKDNTQKLDIN
                     "

```
gene            complement(58059..59243)
                /locus_tag="DMACINML_00318"
CDS             complement(58059..59243)
                /locus_tag="DMACINML_00318"
                /inference="ab initio prediction:Prodigal:2.6"
                /inference="similar to AA sequence:RefSeq:CAL35372.1"
                /codon_start=1
                /transl_table=11
                /product="putative efflux pump"
                /protein_id="Prokka:DMACINML_00318"
                /translation="MENFNRTLWVCWFGVFTTSMGLSQIAPILPLYIKELGHTNTSEI
                AFYSGLAFGITPLGMAIFSPLWAYLGAKYGYKNMLLRASLGMSALTLWLSFAHDAMEV
                VLIRGLTGIVSGFTAAAVVFIAVIAPKEKVAYALGTLSTASISGSLLGPLFGGVVAEI
                FSISAVFDLVAFLLACSFVTIFLFIRERKIQKEAKKNTQEFKENKKLIVIIFITTFII
                QFGTFGVMPILSLYVEQIYQGGNLALWAGVVVAASGISNLFFAPKLGKIADKIGPSKI
                IFGALIFCGICFYLQSLATNVYALIFVRLLIGIGLGGLLPCVNALLKKGVSAKNLSLV
                FGFNQTCQFLGNFCGAFGGGLITAHFSVELVFAFICCIFILNAIIFLIFERNYIFSNQ
                GL"
gene            59297..59752
                /locus_tag="DMACINML_00319"
CDS             59297..59752
                /locus_tag="DMACINML_00319"
                /EC_number="3.1.3.48"
                /inference="ab initio prediction:Prodigal:2.6"
                /inference="similar to AA sequence:RefSeq:CAL35373.1"
                /codon_start=1
                /transl_table=11
                /product="putative phosphotyrosine protein phosphatase"
                /protein_id="Prokka:DMACINML_00319"
                /translation="MKKIVFVCLGNICRSPMAEFVMKDLIKKANLEKEFFVTSAGTSG
                EHDGENMHYGTQNKLKSLDIKYQNFTSKKLTQVLCDESDFLITMDNSNFKNVQKNYKN
                IQNKLLKITDFASHLGYDEVPDPWYSGNFDETYQIISLACKNLLNHLQK"
gene            complement(59754..60623)
                /locus_tag="DMACINML_00320"
CDS             complement(59754..60623)
                /locus_tag="DMACINML_00320"
                /inference="ab initio prediction:Prodigal:2.6"
                /inference="similar to AA sequence:RefSeq:CAL34746.1"
                /note="hypothetical protein Cj0600"
                /codon_start=1
                /transl_table=11
                /product="hypothetical protein"
                /protein_id="Prokka:DMACINML_00320"
                /translation="MLVSPIEKIQFQGFEFYIKRDDLLGEINGNKARKLAFYLSQNYD
                QGQRFISYGGIQGNALVALSIFATKRNLILVYACEKIPTYLRNSPCGNYKLALENNVY
                FIENHSNKDLKTFALSLCEKDDIFIEQGVANLNAELGYIQLAKEIESQSKTLGLDFDI
                FLPSGTGTSAAFLAKNSKFKVFTCACVGDEYYLKTQILNLIKNYDFSNLEILKSDKKY
                HFGKPYLEFYQLYKDLKQECLVEFDLLYDMLGLSIALKREWKNPLLYIHQGGISGNSS
                MLERYIFKYCKDK"
gene            complement(60633..61583)
                /locus_tag="DMACINML_00321"
CDS             complement(60633..61583)
                /locus_tag="DMACINML_00321"
                /inference="ab initio prediction:Prodigal:2.6"
```

```
                     /inference="similar to AA sequence:RefSeq:CAL34745.1"
                     /codon_start=1
                     /transl_table=11
                     /product="putative OmpA family membrane protein"
                     /protein_id="Prokka:DMACINML_00321"
                     /translation="MKNEQEEGNFWIAYADLMAGLLFVFILLIGAIVVKYVLTQSDLR
                     EIKDNLNKQEARLEESKEELRNKEAIVFKLSSDLNNASSALNLINSQKAELEANITNY
                     EQLSKELNSSIDSKDKQILILLGQLEKKDEEIRNLQESFDKAKEKVQNLSLIRENLSK
                     ELQAKLDSNITIDQKTGSISLPSEVLFDKDSYTLKNEAKASLRKILSEYFNAIINDPK
                     ILSNIENIIIEGHTDSDGSYIYNLDLSQKRAYEVMNFIYTFYKDTRLQKLLMASGRSF
                     SDPVLVNGVEDKDKSRRIEIKFSIKNDNALKDVEKFFEFR"
     gene            complement(61580..63034)
                     /locus_tag="DMACINML_00322"
     CDS             complement(61580..63034)
                     /locus_tag="DMACINML_00322"
                     /inference="ab initio prediction:Prodigal:2.6"
                     /inference="similar to AA sequence:RefSeq:SQE24126.1"
                     /codon_start=1
                     /transl_table=11
                     /product="membrane protein"
                     /protein_id="Prokka:DMACINML_00322"
                     /translation="MDIRSDEISELILPESKEKKGYLVYLKIIFIPALLYVFILLGYY
                     NIINFKVELHTVVMIGFIFFTALVFARHSSEYAYSIFEQQKDEFKQALKRHIMKHFLT
                     IGKDTKSNASFDDFAYAYVKGSRNENFASIGAAIFPMMGILGTFISIALSMPNFSSSD
                     TAALEQEIALLLSGVGTAFYVSIYGIFLALWWMFFEKFGKSKIERLLNRQKNSTSGFF
                     WTKEELDQRYLSESLQHFEKIGAIFKQVSNQDFFAELDHTIDRKFGIFQDMLNVEEKA
                     IRLSGEHIKQTMGELSRAQRDQRDLGKMYGEMLNGIGMLNQNLKEINMRMSEQYNRLL
                     DISNEKIQHFDKTLLAFDEKVDRFGKNFELYEKAILESQEKVFEGFKTSLFEGMHKFK
                     EVYEEEKSIDAKIKMMDKLKQEIKNLNEETNEVMAKIGKTPSLTQEEQDNIENEILSS
                     EKITENTEQTKEIQNDSDTLDSNKNFNKEEKEDK"
     gene            complement(63133..64197)
                     /locus_tag="DMACINML_00323"
     CDS             complement(63133..64197)
                     /locus_tag="DMACINML_00323"
                     /EC_number="4.1.2.13"
                     /inference="ab initio prediction:Prodigal:2.6"
                     /inference="similar to AA sequence:RefSeq:APA79036.1"
                     /codon_start=1
                     /transl_table=11
                     /product="Fructose-bisphosphate aldolase class II"
                     /protein_id="Prokka:DMACINML_00323"
                     /translation="MGLLDIVKAGVVSGDELNKVYEYAKTQGFAIPAVNVVGTDSINA
                     VLEAAKKVNSPVIIQFSNGGAKFYAGKNCPQGDVLGAISGAKHVHLLAKAYGIPVILH
                     TDHAARKLLPWIDGLIEANAEYKKTHGQALFSSHMLDLSEEDLEDNLSTCETYLKKLD
                     SLGISLELELGCTGGEEDGVDNTGIDNSKLYTQPEDVALAYERLGKISDKFSIAASFG
                     NVHGVYKPGNVSLQPEILKNSQKFVKEKFGLEADKPINFVFHGGSGSELKDIKDAVSY
                     GVVKMNIDTDTQWAFWDGVREYEAKNRAYLQGQIGNPEGDDKPNKKYYDPRVWLRSGE
                     ESMIKRLELAFEDLNCIGRN"
     gene            complement(64197..65018)
                     /gene="peb4-cbf2"
                     /locus_tag="DMACINML_00324"
     CDS             complement(64197..65018)
                     /gene="peb4-cbf2"
                     /locus_tag="DMACINML_00324"
```

```
                     /inference="ab initio prediction:Prodigal:2.6"
                     /inference="similar to AA sequence:RefSeq:CAL34742.1"
                     /codon_start=1
                     /transl_table=11
                     /product="major antigenic peptide PEB-cell binding factor"
                     /protein_id="Prokka:DMACINML_00324"
                     /translation="MRKVSLIAAALIAGVALNLNAATIATVNGKNISDTEVSEFFAPM
                     LRGEDFKSLPDHQKNALLQQYIMQDLILQDAKKQNLEKDPLYKKELERAKDSILINVY
                     QEKILNSIKIDSSKVKAFYEQNKEKYIKPAAVQAKHILVSSEEEAKDIIKELKGLKGK
                     ALDDKFSQLAKEKSIDPGSKNQGGELGWFDQSTMVKPFTDAAFALKNGTITTTPVKTN
                     FGYHVILKENSRAKEQIKFDDVKKGIENGLKFEEFKKVINQKGQDLLNNAKVEYK"
     sig_peptide     complement(64953..65018)
                     /gene="peb4-cbf2"
                     /locus_tag="DMACINML_00324"
                     /inference="ab initio prediction:SignalP:4.1"
                     /note="predicted cleavage at residue 22"
     gene            65101..65727
                     /gene="nth"
                     /locus_tag="DMACINML_00325"
     CDS             65101..65727
                     /gene="nth"
                     /locus_tag="DMACINML_00325"
                     /EC_number="4.2.99.18"
                     /inference="ab initio prediction:Prodigal:2.6"
                     /inference="similar to AA sequence:RefSeq:CAL34741.1"
                     /codon_start=1
                     /transl_table=11
                     /product="endonuclease III"
                     /protein_id="Prokka:DMACINML_00325"
                     /translation="MKRNSEIKELFLKHFDKPTTELKFSNLYELLVCVMLSAQCTDKR
                     VNLITPELFKAYPDITSLANANLSSLKTYIQSCSFYNNKAQNLIKMAQSVCENFNAQI
                     PLEEEKLKSLAGVGQKTAHVVLIEWCGANCMAVDTHVFRVSHRLGLSKAKTPEATEED
                     LTRIFKDNLNYLHQAMVLFGRYVCKAKKPLCKECFLNHLCKSKDKELE"
     gene            65724..66374
                     /locus_tag="DMACINML_00326"
     CDS             65724..66374
                     /locus_tag="DMACINML_00326"
                     /EC_number="3.1.30.-"
                     /inference="ab initio prediction:Prodigal:2.6"
                     /inference="similar to AA sequence:RefSeq:CAL34740.1"
                     /codon_start=1
                     /transl_table=11
                     /product="putative DNA/RNA non-specific endonuclease"
                     /protein_id="Prokka:DMACINML_00326"
                     /translation="MKFIIFLLAFTLHVFALEAYKPDKDFASYFNPLNCSVVLDKFFY
                     INCYDYKLKGTKAVAYKVEASNLKEKQIKKRARFEDDTNIPKKYRSTWSDYKNSGYTR
                     GHIASNASFRFSKAAQTSVFLMSNITPQNAQVNATVWNEIEQRERSLAFKFQSVEVLN
                     LILYGKNPKFIKNGIAIPSSYIKIIKTPRFKECYEVPNNDIQSEEINDYKISCNKF"
     sig_peptide     65724..65774
                     /locus_tag="DMACINML_00326"
                     /inference="ab initio prediction:SignalP:4.1"
                     /note="predicted cleavage at residue 17"
     gene            66384..67016
                     /locus_tag="DMACINML_00327"
```

```
     CDS             66384..67016
                     /locus_tag="DMACINML_00327"
                     /inference="ab initio prediction:Prodigal:2.6"
                     /inference="similar to AA sequence:RefSeq:CAL34739.1"
                     /codon_start=1
                     /transl_table=11
                     /product="putative integral membrane protein"
                     /protein_id="Prokka:DMACINML_00327"
                     /translation="MEISALTITILYIIGISAEGMTGALAAGRHKMDLFGVIFIALVT
                     AIGGGSIRDVLLGHYPLTWVKHPEYIVLICLCALVATKIPRIVSRLESLFLTLDAIGL
                     VVFSILGAQIAIEQNHGFIIAISAAVITGVFGGILRDILCMRIPLVFQKEIYAGIAII
                     AGAIYYALMVYLQLHALICTLVTLFVGVTARLLAIKYQWSLPIFSYNEEK"
     gene            67094..67312
                     /locus_tag="DMACINML_00328"
     CDS             67094..67312
                     /locus_tag="DMACINML_00328"
                     /inference="ab initio prediction:Prodigal:2.6"
                     /inference="similar to AA sequence:RefSeq:CAL34737.1"
                     /codon_start=1
                     /transl_table=11
                     /product="putative lipoprotein"
                     /protein_id="Prokka:DMACINML_00328"
                     /translation="MNKAILLTLIGLFFTACEQTKSVEYYKAHPEEAKKRSDECRLKS
                     IISQDCVNAHSVAIPKEDWDEGHDTQTR"
     gene            complement(67316..68020)
                     /locus_tag="DMACINML_00329"
     CDS             complement(67316..68020)
                     /locus_tag="DMACINML_00329"
                     /inference="ab initio prediction:Prodigal:2.6"
                     /inference="similar to AA sequence:RefSeq:CAL34736.1"
                     /codon_start=1
                     /transl_table=11
                     /product="putative SAM-dependent methyltransferase"
                     /protein_id="Prokka:DMACINML_00329"
                     /translation="MKDELFKEKLTKQFEFDKSVASVFDDMINRSVPFYKENLELCAN
                     LIKKLTKEGVKVCDLGCSSANFLIFLASLRKDLKLFGVDNAPSMLEIAKSKAQAYGLN
                     IEFFEANLCDFVFFQSDVFVANYTLQFIRPPKRQELVNEIYKNLNKGGIFIMSEKILY
                     EDAFLSKNMIELYADYKERQGYSKIEIAAKREALENVLIPYSEKENLDMLKNAGFEKI
                     ESVFKWVNFETFIAFK"
     gene            complement(68024..68863)
                     /gene="ribF"
                     /locus_tag="DMACINML_00330"
     CDS             complement(68024..68863)
                     /gene="ribF"
                     /locus_tag="DMACINML_00330"
                     /EC_number="2.7.1.26"
                     /inference="ab initio prediction:Prodigal:2.6"
                     /inference="similar to AA sequence:RefSeq:CAL34735.1"
                     /codon_start=1
                     /transl_table=11
                     /product="putative riboflavin kinase/FMN
                     adenylyltransferase"
                     /protein_id="Prokka:DMACINML_00330"
                     /translation="MLNIFTTTLKNNIKTLAIGCFDGVHLGHKKLIECLDDSGALLII
```

```
                     DKFKGKKLCTNQDKAFLAQKEIIELDFESIKALDGKDFLQALKEEFVNLERIVVGFDF
                     SFGKNRSYKAKDIEALSGIKTTIIDEFRLGGVGVHASLIKEYLTRGDIKKANCFLGRN
                     YAIKGKLIKGQGLGSKELFATLNLECEEYFLPKNGVYASIVSFHDKRYKSVSFVGVRS
                     SDMQFAIESHIIEKFDESLAIGEVLELEFVEFLRENQKFNDLAQLKEQISKDINQAKA
                     CLG"
gene                 complement(68829..69578)
                     /gene="tlyA"
                     /locus_tag="DMACINML_00331"
CDS                  complement(68829..69578)
                     /gene="tlyA"
                     /locus_tag="DMACINML_00331"
                     /inference="ab initio prediction:Prodigal:2.6"
                     /inference="similar to AA sequence:RefSeq:CAL34734.1"
                     /codon_start=1
                     /transl_table=11
                     /product="putative hemolysin"
                     /protein_id="Prokka:DMACINML_00331"
                     /translation="MRYDIFVAKRLNLSRNKALELIENEEILLNSKPYKASFDVQNLL
                     KNKIADQEELLNSKELNLELLGELYVSRAAFKLKYFLENHNINIANKTCLDIGSSTGG
                     FVQILLEHKALKVSALDVGDNQLHPSLRDDKRIEIIENTDLRTFKSEQKFDFITCDVS
                     FISLTHLLSYIDNLALKDIVLLFKPQFEVGKQAKRDKKGVLKDEKAVFKAMREFENAC
                     ARLGWILQCCEESKIKGKEGNVEYFYYYTKK"
gene                 complement(69575..70579)
                     /locus_tag="DMACINML_00332"
CDS                  complement(69575..70579)
                     /locus_tag="DMACINML_00332"
                     /inference="ab initio prediction:Prodigal:2.6"
                     /inference="similar to AA sequence:RefSeq:ALW79391.1"
                     /codon_start=1
                     /transl_table=11
                     /product="membrane protein"
                     /protein_id="Prokka:DMACINML_00332"
                     /translation="MSLLKNNIQLFVLFSIFAFLFFWQKFAWVSLFLIPIFLAFFLEF
                     FYFLRLRKNIIKEATMIKNSLIYRVSAGDFYIYSLSFFMALFALASLFLNLISFEKQD
                     GFFLFILLPLFLFFFKQKLQLQFLDNAYNDFRIIILSSLILALLYAIFNGVVNPIQSF
                     NLEDFNQSIIHYKNSKFFVFDLISQILTLINALKEYFLYSLGLFWFRVLNFIFDFINF
                     FIFCSFVAYLYNFAFKAKKKTYVFVFSFFVALASFFIVEDKNQNPKAYQKELVLMMNN
                     LSFLKEQNLSMLQNDKDRLVKNLKQVQELLDKNAFEIGIWWFSKDKEELQKSLNESLQ
                     "
gene                 complement(70560..72503)
                     /gene="ligA"
                     /locus_tag="DMACINML_00333"
CDS                  complement(70560..72503)
                     /gene="ligA"
                     /locus_tag="DMACINML_00333"
                     /EC_number="6.5.1.2"
                     /inference="ab initio prediction:Prodigal:2.6"
                     /inference="similar to AA sequence:RefSeq:CAL34732.1"
                     /codon_start=1
                     /transl_table=11
                     /product="DNA ligase"
                     /protein_id="Prokka:DMACINML_00333"
                     /translation="MTKKEYLEKVALAASWMRAYYEKDEPLASDAEYDTLIRELRAFE
                     EQNENEISPDSPTQKIAPTIQSEFKKLSHLSRMWSMEDVFDESELRAWAKRARCEENF
```

```
                     FIEPKFDGASLNLLYDNGRLISGATRGDGEIGEDITLNVLEIDNIPKTINYKEKIEIR
                     GEVVILKDDFEKINEKRARLNQSLFANPRNAASGSLRQLDTSITRERNLKFYPWGVGQ
                     NSLEFSKHSEIMSFIRELGFLKDDFVKLCVNLDEVLKAYNELLSLRDKKPMMMDGMVV
                     RVDDLALCKELGHTVKFPKFMAAFKFPALEKTTRLIGVNLQVGRSGVITPVAVLEPVN
                     LDGVIVKSATLHNFDEIARLDAKINDFVSVIRSGDVIPKITKVFKERRDGLELDIARP
                     QFCPTCKSELLDEGTLIKCQNIDCEDRLVNSIIHFVSKKCLNIDGLGESIVELLFKEK
                     KITTLESIFHLKFSDFEGLEGFKEKKINNILNAIEQARDCELFRFITALGIEHIGEVA
                     AKKLALSFGKEWHKQSLEAYANLEGFGEQMALSLCEFSRVNSARIDEFYKLLNLKEQK
                     VQIQEDSVIFGKTFVITGTLSRPREEFKNLIESLGGKVSGSVSKKTDYVLFGQEAGSK
                     LDKAQELGVKCIDENEFNELIEK"
     gene            complement(72504..73646)
                     /gene="folP"
                     /locus_tag="DMACINML_00334"
     CDS             complement(72504..73646)
                     /gene="folP"
                     /locus_tag="DMACINML_00334"
                     /EC_number="2.5.1.15"
                     /inference="ab initio prediction:Prodigal:2.6"
                     /inference="similar to AA sequence:RefSeq:AOH51600.1"
                     /codon_start=1
                     /transl_table=11
                     /product="dihydropteroate synthase"
                     /protein_id="Prokka:DMACINML_00334"
                     /translation="MKFFKINPNTDFSLLCSFINPHKMGQKIMSEKTQIYFILIKDIT
                     TPAANILKQDALRVGAELITHKEVITAKITHSNALLMATKEQIHKLINKEKLQDFGLK
                     NLARFLENDFSKPKQAELMAVINVNEDSFNADSRVSYKDFEERLNEILALNPEYIDIG
                     AVSSRPKSVYCGKEEEFRRLKNVLDLIYDKNYYKKAIFSLDSFDEYCLEYALNKGFKF
                     INDISSLRNLNLAKLASKYNAKYCLMHMQNDPLTMQDDPRYDDLLDEMSSFFKEKLEI
                     LDALGVKESILDVGIGFGKSAEHNMILIKHLEHFLQFKKPLLVGASRKSVINAYYESE
                     VKDRLAGTLYLHLKAFENGASIIRVHDLYEHKQLFAMANAMQSIGV"
     gene            complement(73643..74254)
                     /locus_tag="DMACINML_00335"
     CDS             complement(73643..74254)
                     /locus_tag="DMACINML_00335"
                     /inference="ab initio prediction:Prodigal:2.6"
                     /inference="similar to AA sequence:RefSeq:CAL34730.1"
                     /note="hypothetical protein Cj0584"
                     /codon_start=1
                     /transl_table=11
                     /product="hypothetical protein"
                     /protein_id="Prokka:DMACINML_00335"
                     /translation="MFVSKIIITDDFDGIRAELLKQFHPNSLRFIPKEVASEFLIDDA
                     KAVEKESYIAETSEKIIVLMANSFRIEAQNFLLKLLEEPPKNIKFLIVVPSKNLLLPT
                     IKSRLICEKRNKIKAKNTLNLELNKMDLKTLFEFLQENENLDKAELMEKIALIAEESV
                     KFKDFNADELEFFYESYELAKLNSKSSLILATLLLNYYTKNEK"
     gene            complement(74254..74787)
                     /locus_tag="DMACINML_00336"
     CDS             complement(74254..74787)
                     /locus_tag="DMACINML_00336"
                     /inference="ab initio prediction:Prodigal:2.6"
                     /inference="similar to AA sequence:RefSeq:CAL34729.1"
                     /note="hypothetical protein Cj0583"
                     /codon_start=1
                     /transl_table=11
```

```
                     /product="hypothetical protein"
                     /protein_id="Prokka:DMACINML_00336"
                     /translation="MSDFLSFTLENIRNGGTFMAWMESRRLEWAPLMAARLKYLLEGR
                     TFVLMCDEQRAWYEEYFLANINSKTTRPMLPFVSLKSLCKKKIQNIEDIALLNDLLDI
                     SFPNGFIYFYIGSASDKKSLIAKSRDDSLLWLFDEQLQDSFYLNSKDKDLDVKLITLY
                     KLFDTSLDAILFSKVSL"
     gene            complement(74788..75990)
                     /gene="lysC"
                     /locus_tag="DMACINML_00337"
     CDS             complement(74788..75990)
                     /gene="lysC"
                     /locus_tag="DMACINML_00337"
                     /EC_number="2.7.2.4"
                     /inference="ab initio prediction:Prodigal:2.6"
                     /inference="similar to AA sequence:RefSeq:CAL34728.1"
                     /codon_start=1
                     /transl_table=11
                     /product="aspartokinase, alpha and beta subunits"
                     /protein_id="Prokka:DMACINML_00337"
                     /translation="MLIVQKYGGTSVGTLERIDAVAERVIKSAKEGHQLVVVVSAMSG
                     VTNTLIEQAEYFSKNPNGKDMDMLLSSGERVTSALLSIALNERGYPSLAFSGRKAGIV
                     TDSVSTKARISYIDTQEITKALNEGKIVVVAGFQGVDKEGDVTTLGRGGSDLSAVAVA
                     GALKADLCEIYTDVDGVYTTDPRIEPKAKKLDKISYEEMLELASLGAKVLQNRSVELA
                     KKLNVKLVTRSSFNDNEGTMITKEDGMEQALVSGIALDKNQARVTLRNIEDKPGIAAE
                     IFSVLADENINVDMIIQNVGVDGATNLGFTVPQNELDLAKNAMQKILGSKTTIESDSA
                     VVKVSIVGVGMKSHSGVASKAFKALADEGINIGMISTSEIKISMIVHEKYGELAVRAL
                     HECYGLDK"
     gene            complement(75990..76460)
                     /gene="nudH"
                     /locus_tag="DMACINML_00338"
     CDS             complement(75990..76460)
                     /gene="nudH"
                     /locus_tag="DMACINML_00338"
                     /EC_number="3.6.1.-"
                     /inference="ab initio prediction:Prodigal:2.6"
                     /inference="similar to AA sequence:RefSeq:CAL34727.1"
                     /codon_start=1
                     /transl_table=11
                     /product="putative NUDIX hydrolase family protein"
                     /protein_id="Prokka:DMACINML_00338"
                     /translation="MEKEKKYRPNVAAIVLSSAYPFECKLFIAKRSDMDNIWQFPQGG
                     IDEGEDPKSAVLRELKEEIGTNEVEIIAEHPEWLSYDFPEKIAKKMYPYDGQSQKYFL
                     VRLKHGAKININTKHPEFDAYQFVGVKQVFETINHFKRNIYVKVIKYFEEKGYI"
     gene            76555..77622
                     /locus_tag="DMACINML_00339"
     CDS             76555..77622
                     /locus_tag="DMACINML_00339"
                     /EC_number="1.3.98.3"
                     /inference="ab initio prediction:Prodigal:2.6"
                     /inference="similar to AA sequence:RefSeq:AHW91665.1"
                     /codon_start=1
                     /transl_table=11
                     /product="coproporphyrinogen III oxidase"
                     /protein_id="Prokka:DMACINML_00339"
```

```
                     /translation="MHLYIHIPFCESKCHYCAFTSLKKHDYENKYFEALYKDISFHLQ
                     KLNIKKNSIKTLFIGGGTPSAVSAKNFEMILTLIEPFLSKNIEFSSEANPNSADIDWL
                     KTMKNLGLNRISFGAQSFHPKKLEFLGRIHDQKMIFKALENANKLGFKKINLDLIYDT
                     KLDDKKMLQFELEQLKQIKNLITHLSAYNLTIEPLSTFAKKDHFKKNAPYLMKFFIQE
                     LQKLGFLQYEISNFAKNKAQICKHNLAYWKGLDYLGCGLSAVSCYKNERFYTAKNLKD
                     YLQNPVFRDVEKLSQEDLNLEHLFLGLRSIVGIEECKLSEIQKEKANLLVREKKLKFE
                     KGRYYNLNFLISDELALYLSS"
gene                 77686..78090
                     /gene="tatB"
                     /locus_tag="DMACINML_00340"
CDS                  77686..78090
                     /gene="tatB"
                     /locus_tag="DMACINML_00340"
                     /inference="ab initio prediction:Prodigal:2.6"
                     /inference="similar to AA sequence:RefSeq:CAL34725.1"
                     /codon_start=1
                     /transl_table=11
                     /product="sec-independent protein translocase"
                     /protein_id="Prokka:DMACINML_00340"
                     /translation="MSFGEIIVILIVAILVLGPDKLPEAIVQIAKILKALKRNIDDAK
                     SSIEKEIRINDLKEEAKKYKDEFSSANENIRKKLSFEEFDDLKRDILDKTKVDLTFDS
                     RENSTPNPNEQNPNENQKPKLNPFENQEKVEK"
gene                 78091..78837
                     /gene="tatC"
                     /locus_tag="DMACINML_00341"
CDS                  78091..78837
                     /gene="tatC"
                     /locus_tag="DMACINML_00341"
                     /inference="ab initio prediction:Prodigal:2.6"
                     /inference="similar to AA sequence:RefSeq:CAL34724.1"
                     /codon_start=1
                     /transl_table=11
                     /product="sec-independent protein translocase"
                     /protein_id="Prokka:DMACINML_00341"
                     /translation="MFEELRPHLIELRKRLFISVACVIVMFIVCFAFRSYILDVLKAP
                     LIQALPEVAKHVNVIEVQEALFTAMKVSFFAAFIFSLPVIFWQFWKFVAPGLYDNEKR
                     LVVPFVSFASIMFALGACFCYFIVVPLAFKFLINFGLSEDFNPVITIGTYVDFFTKVV
                     VAFGLAFEMPVIAFFFAKIGLIDDSFLKRHFRIAVLAIFVFSAFMTPPDVLSQFLMAG
                     PLCGLYGLSILIVQKVNPAPKDEEKEESDK"
gene                 78834..79859
                     /gene="queA"
                     /locus_tag="DMACINML_00342"
CDS                  78834..79859
                     /gene="queA"
                     /locus_tag="DMACINML_00342"
                     /EC_number="5.-.-.-"
                     /inference="ab initio prediction:Prodigal:2.6"
                     /inference="similar to AA sequence:RefSeq:ABS44030.1"
                     /codon_start=1
                     /transl_table=11
                     /product="S-adenosylmethionine:tRNA
                     ribosyltransferase-isomerase"
                     /protein_id="Prokka:DMACINML_00342"
                     /translation="MKDLLLSSYDYTLPNELIATYPVYPKEDAKLLVFERKNNQISHT
```

```
                     TFKNLQDFLPDCTLFFNDTKVIKARIYGNKSSGGKIELFLHQPCLGLQDSLFLAQIKG
                     RVKKDEILSFEQNLKAQVVELLDDGLRKVQFFQNNEILNTFKLYELLDVIGHVPLPPY
                     IKRSDEKSDLEDYQSIFAKNLGAVAAPTASLHFSENMLENLKKRHEIYHLTLHVGAGT
                     FKSVECENLENHKMHSEFFDIPKKACEVIDSQKKILGIGTTVTRTIEYYARTKQSQGF
                     CDLFLHPHNPPLRQDYLLTNFHLPKSTLIMLVSAFIGRQNCLKLYDIAIKEKYRFYSY
                     GDAMLIL"
     gene            complement(79862..80815)
                     /gene="lpxD"
                     /locus_tag="DMACINML_00343"
     CDS             complement(79862..80815)
                     /gene="lpxD"
                     /locus_tag="DMACINML_00343"
                     /EC_number="2.3.1.-"
                     /inference="ab initio prediction:Prodigal:2.6"
                     /inference="similar to AA sequence:RefSeq:CAL34722.1"
                     /codon_start=1
                     /transl_table=11
                     /product="UDP-3-O-[3-hydroxymyristoyl] glucosamine
                     N-acyltransferase"
                     /protein_id="Prokka:DMACINML_00343"
                     /translation="MRLKEIAEFLSLEYEGEDIEITALNSLLRANFTELTYCDGERNI
                     KDIPNTGAAAILVAKEYENLVPKDTKALITQSPHLCFAFLSKIFAKPLFSNAKEKVQN
                     IAKSAKIMPNVYIGNNVNIGENVVIMAGAYIGDNVSIGEESIIHPNVVIYNDSKIGKK
                     CHLLANCVIGSDGFGYAHNKNGEHYKIYHNGNVILEDFVEIGACTTIDRAVFDSTIIK
                     AGTKVDNLVQIGHNCDIGQNCIIVAQTGISGSSELGRNVVMGGQSATSGHLRIGDFST
                     IAARGGVSKNLEGGRVYGGFPIMLQKDWLKLQAKIAMKFKE"
     gene            complement(80812..81276)
                     /gene="ilvH"
                     /locus_tag="DMACINML_00344"
     CDS             complement(80812..81276)
                     /gene="ilvH"
                     /locus_tag="DMACINML_00344"
                     /EC_number="2.2.1.6"
                     /inference="ab initio prediction:Prodigal:2.6"
                     /inference="similar to AA sequence:RefSeq:CAL34721.1"
                     /codon_start=1
                     /transl_table=11
                     /product="acetolactate synthase small subunit"
                     /protein_id="Prokka:DMACINML_00344"
                     /translation="MRRVLSVIVLNEHGVLSRIVGLFSGRGYNIDSLTVAPLPGGEFS
                     RINIVTFGDERVFEQIVKQLHKLIPTYKVIERDEFIEKEMALVKIPLSENLGGLDAVL
                     KAYNGTIANSNENFLFLLVADDTNRIENFLKTIKKYNPSDIVRSGSVLMELK"
     gene            complement(81273..82973)
                     /gene="ilvI"
                     /locus_tag="DMACINML_00345"
     CDS             complement(81273..82973)
                     /gene="ilvI"
                     /locus_tag="DMACINML_00345"
                     /EC_number="2.2.1.6"
                     /inference="ab initio prediction:Prodigal:2.6"
                     /inference="similar to AA sequence:RefSeq:CAL34720.1"
                     /codon_start=1
                     /transl_table=11
                     /product="acetolactate synthase large subunit"
```

```
                     /protein_id="Prokka:DMACINML_00345"
                     /translation="MRKISGSAMICEALKEENVKIVFGYPGGAALNIYDEIYLQKYFK
                     HILVRHEQAAVHAADAYARMSGEVGVAVVTSGPGFTNTITGLATAYSDSIPLVLISGQ
                     VANSLIGTDAFQEIDAVGISRPCVKHNYLVTCIEEFPRILKEAFYIAKSGRKGPVHID
                     VPKDVSAALGIWDYPKEISMKTYKPIYKGNSKQIKKLAENLEEAKRPLFYLGGGCIAS
                     NAADEIRELVKITQIPAVETLMALGTLRSDDELNLKMAGMHGSYTANMALSECDLLVA
                     IGARFDDRITGKTSEFAKHSKIVHIDIDPSSISKIINAHFPIVGDIKEVVKELLEELK
                     KNQFKSNIHEWRQTLKRYDELYPLAYEDSDEVLKPQWVIQECAKLAPDARIITDVGQH
                     QMWVAQFYPFNYPRQLATSGGQGTMGYSLPAALGAKLAVGEEVVINFVGDGSVLMNIQ
                     ELMTAYENGIKTINIILNNSFLGMVRQWQSMFYEEHFSATDLSLQADFVKIAEGFGCE
                     GYNIKTKDEFHTAFKQALESNKTSLLNVFIDRFEDVLPMVPAGGAIYNMILPKLKDKQ
                     "
     gene            complement(83051..83494)
                     /locus_tag="DMACINML_00346"
     CDS             complement(83051..83494)
                     /locus_tag="DMACINML_00346"
                     /inference="ab initio prediction:Prodigal:2.6"
                     /inference="similar to AA sequence:RefSeq:CAL34719.1"
                     /codon_start=1
                     /transl_table=11
                     /product="putative GatB/Yqey family protein"
                     /protein_id="Prokka:DMACINML_00346"
                     /translation="MTLKEQILNDIKEAMKQKDDFKRDTLRTLNAAFKQVEVDERIEL
                     DDERILKIITSEIKKRKDAIELYSKGGREDLAQKEQKEIALFESYLPQQLSDEELQTA
                     LKELIANLGVSSLKDQGLVMKEAKAKFGARVDGKRLNVTLRELLN"
     gene            complement(83511..84530)
                     /gene="ribB"
                     /locus_tag="DMACINML_00347"
     CDS             complement(83511..84530)
                     /gene="ribB"
                     /locus_tag="DMACINML_00347"
                     /inference="ab initio prediction:Prodigal:2.6"
                     /inference="similar to AA sequence:RefSeq:EAQ73046.2"
                     /codon_start=1
                     /transl_table=11
                     /product="3,4-dihydroxy-2-butanone 4-phosphate synthase"
                     /protein_id="Prokka:DMACINML_00347"
                     /translation="MKFVSIEQAIKDLQEGKMLVMVDAEDRENEGDLIFPAQFSTKEK
                     VNFMIKEARGVVCVALAEELARKFELPLMVPKNTSNHETAFTITVDAKDATTGVSAYE
                     RDMTIKIFADDNAKASDFVCPGHINPLIAKKGGVLERTGHTEGTVDLCQLAGLKQACV
                     ICEIVKDDGDMARRSDLEEFCAKHNLNMIAVSDIIEYRLKNESLIKLQEKSDAILAGF
                     KAQKFIFTDHNNAQHIAFAFKEIQKCENVKFHISGSDFELLTSDKFSKLLDQICFLSE
                     NGGVIVFMQGEKSSATQFKNYGIGAQILRYFGIEEIKLMSQSCDKDYIGLKGFGLNLK
                     ICNFN"
     gene            84637..84711
                     /locus_tag="DMACINML_00348"
     tRNA            84637..84711
                     /locus_tag="DMACINML_00348"
                     /product="tRNA-Gln"
                     /inference="COORDINATES: profile:Aragorn:1.2"
                     /note="tRNA-Gln(ttg)"
     gene            84743..84818
                     /locus_tag="DMACINML_00349"
     tRNA            84743..84818
```

```
                     /locus_tag="DMACINML_00349"
                     /product="tRNA-Met"
                     /inference="COORDINATES: profile:Aragorn:1.2"
                     /note="tRNA-Met(cat)"
     gene            complement(85033..85108)
                     /locus_tag="DMACINML_00350"
     tRNA            complement(85033..85108)
                     /locus_tag="DMACINML_00350"
                     /product="tRNA-Phe"
                     /inference="COORDINATES: profile:Aragorn:1.2"
                     /note="tRNA-Phe(gaa)"
     gene            85250..86593
                     /gene="ktrB"
                     /locus_tag="DMACINML_00351"
     CDS             85250..86593
                     /gene="ktrB"
                     /locus_tag="DMACINML_00351"
                     /inference="ab initio prediction:Prodigal:2.6"
                     /inference="similar to AA sequence:RefSeq:CAL35397.1"
                     /codon_start=1
                     /transl_table=11
                     /product="putative K+ uptake protein"
                     /protein_id="Prokka:DMACINML_00351"
                     /translation="MKQFGLDRRTFKILLAGYIVIALLGAILLHSSWAHTSPISFIDA
                     FFTSTSAVSMTGLIVKNTAVDFTLAGQIIILILVQIGGLGYMSIGLFVYLIIRKKVGF
                     NGRNLLKESLFYPSMEGLFKFFKKVLLFIFTIELIGTILLTMRFALEMNFGKALWFGF
                     FHSISAFNNSGFTIFENGLLAYKHDVAINLIFTSLIIIGGLGYFVLVELYFFQRKRLQ
                     NLSLHTKVVIMATLFLIVSSTLIIFLLEYANPKSIGSFSLFDKILSSYFIAINYRTAG
                     FNTLDMSGLHDASLFFGSLFMVIGGAPGGTAGGMKITTVVVLLFYAYWSIRNGRVRIF
                     NHEIPQEIISRAFIIAVGSAVYIVIAVIFLSLLESEFNFIHLLFETSSAFATVGISVG
                     DGGDLSLCALFSNPSKIIIIIMMLSGRIGVFAFLLSVFQQDKAMHIKFPKGKIYL"
     gene            86606..87256
                     /gene="ktrA"
                     /locus_tag="DMACINML_00352"
     CDS             86606..87256
                     /gene="ktrA"
                     /locus_tag="DMACINML_00352"
                     /inference="ab initio prediction:Prodigal:2.6"
                     /inference="similar to AA sequence:RefSeq:CAL35398.1"
                     /codon_start=1
                     /transl_table=11
                     /product="putative K+ uptake protein"
                     /protein_id="Prokka:DMACINML_00352"
                     /translation="MKKLSYGVIGLGKFGSVVADELIAGGHTVIIADKDEEALKSIQN
                     SPSYAYILDSTNISALKEAGFHDVEVVIVSIGENVEKSILTLMALKDIGVNNIIAKAT
                     SNIHGQILSKLGATKVIYPEKESAKKLVKDFLTKDADYEVFDLSANTIRAIKITIDDK
                     LAGNSLKHIAQNMKVISYKKLNTDWEILPDLETTTVYGGDVVILLGTVKELREFEH"
     gene            complement(87257..87964)
                     /locus_tag="DMACINML_00353"
     CDS             complement(87257..87964)
                     /locus_tag="DMACINML_00353"
                     /inference="ab initio prediction:Prodigal:2.6"
                     /inference="similar to AA sequence:RefSeq:CAL35399.1"
                     /note="conserved hypothetical protein Cj1285c"
```

```
                     /codon_start=1
                     /transl_table=11
                     /product="hypothetical protein"
                     /protein_id="Prokka:DMACINML_00353"
                     /translation="MREIGFLIPWIKMIFGKIDYINLLPLHIYLKKYPLPNGYKASME
                     YKKGVPSKLNKDLFYRRVDAAIVSSIESARKKYKNLDLGICANKRVLSVLVERNTLNA
                     KDPSSATSNALAKVLKQEGRVIIGDKALKLYLQDPSKYIDLCAKWHEKTGLPFVFARF
                     SCVQKKALYKQILKKFPKTKIKIPYYILQNYATTRNLDIKDMRYYLDEIIYYKISTKE
                     KAALKRFIKACKALNPT"
     gene            complement(87930..88559)
                     /gene="upp"
                     /locus_tag="DMACINML_00354"
     CDS             complement(87930..88559)
                     /gene="upp"
                     /locus_tag="DMACINML_00354"
                     /EC_number="2.4.2.9"
                     /inference="ab initio prediction:Prodigal:2.6"
                     /inference="similar to AA sequence:RefSeq:CAL35400.1"
                     /codon_start=1
                     /transl_table=11
                     /product="uracil phosphoribosyltransferase"
                     /protein_id="Prokka:DMACINML_00354"
                     /translation="MKNIHHICHPLIEHKLGFLRDKETKPFHFRMLIDEISTFLLFEA
                     TKDLCLKEAQIQTPVANAKVKRLDEKIMICPILRAALGMLDSIFRLIPDASVGFLGFV
                     RNEKTLKADFYFQKLPKDAKERTAIVIDPMFATGGTAIDACNFLKDQGVKKIKFISIL
                     AAPQGLENFAKIHSDVELYVASIDESLNEKGYIVPGLGDAGDRVFNTLD"
     gene            complement(88556..89791)
                     /locus_tag="DMACINML_00355"
     CDS             complement(88556..89791)
                     /locus_tag="DMACINML_00355"
                     /EC_number="1.1.1.38"
                     /inference="ab initio prediction:Prodigal:2.6"
                     /inference="similar to AA sequence:RefSeq:CAL35401.1"
                     /codon_start=1
                     /transl_table=11
                     /product="malate oxidoreductase"
                     /protein_id="Prokka:DMACINML_00355"
                     /translation="MNLKEEALRYHLGGKIDIKPSKPMNSSHDLSLAYSPGVAEPCIE
                     IASNNELAYTYTNKANLVAIVSDGSAVLGLGNIGAQASKPVMEGKACLFKKFADVNAY
                     DLEIEAHSIEEIVAFCKAIAPTFGGINLEDISAPKCFEIEAALQNLGIPVMHDDQHGT
                     AIISTAGLMNAMEISGKKFEDIKVVVSGAGAAGIASARMYRNLGVKNIILVDSKGVVN
                     KKRTDLNQYKLEFVSDTQADTLKEAMKDADVFLGLSAPKILDDEMILSMAKDPVIFAL
                     ANPIPEVMPEDVARLRKDAIVGTGRSDYPNQINNVLGFPFIFRGALDVRATKITENMK
                     VAAARALADLAKLPVSDAVKNAYNISHLEFGKDYVIPKPFDERVKAVVSTAVAAAAVK
                     DGVALLKEFDEKAYFESLK"
     gene            complement(89791..91182)
                     /gene="gltX2"
                     /locus_tag="DMACINML_00356"
     CDS             complement(89791..91182)
                     /gene="gltX2"
                     /locus_tag="DMACINML_00356"
                     /EC_number="6.1.1.17"
                     /inference="ab initio prediction:Prodigal:2.6"
                     /inference="similar to AA sequence:RefSeq:CAL35402.1"
```

```
                     /codon_start=1
                     /transl_table=11
                     /product="glutamyl-tRNA synthetase"
                     /protein_id="Prokka:DMACINML_00356"
                     /translation="MTGKLTTRFAPSPTGYLHIGGLRTALYSYLYARKNKGNFLLRIE
                     DTDLKRNSKEATQAIIEAFKWCGLDYDGEVTYQSERFDLYKKYIQKLLDEGKAYYCYM
                     SKEELDELRAKQEAAKERPRYDGRYRDFTGTPPANIEPVVRIKAPQEGEICFVDGVKG
                     EVKFKVEDILDDFIIARSDGSPTYNFTVVIDDALMGVSDVIRGDDHLSNTPKQIVLYE
                     ALGFEIPKFYHVAMIHGEDGKKLSKRHGATDVMEYKAMGILPQALLNFLVRLGWSHGD
                     DEVFSLEDLKKLFDPNHINKSASCYNFKKLEWLNAHYIKTLPFEEINRQLKDLGFDLS
                     AYAKAGFLLDLLRERAKTLLEIISGAKSIIEAPQNYDENAINKFINENNLLLLQNYAN
                     ELNEEKSAKDFEEFTNEFLQKNEVKLKDLAQPIRIALTGSAVSPSIFEVLEFLGVNEC
                     KKRIEKFLDFKGK"
     gene            91247..92062
                     /locus_tag="DMACINML_00357"
     CDS             91247..92062
                     /locus_tag="DMACINML_00357"
                     /inference="ab initio prediction:Prodigal:2.6"
                     /inference="similar to AA sequence:RefSeq:CAL35403.1"
                     /codon_start=1
                     /transl_table=11
                     /product="putative periplasmic protein"
                     /protein_id="Prokka:DMACINML_00357"
                     /translation="MKKILLSFIFFISLTHANTINAIAVVVDKEPITTYDIEQTMKAL
                     NIPKNQALGILINEKMELSQIKQFGIYTSELELDNAIDKMLMQNKISLEDFKKDLQVK
                     GQNYEQFRSNFKKDFEKRKLYEKIASTAKTDFSDEGAKNFFEQNKNKFTFFTEINVNI
                     YRSNNAQDLENIRAAIKTPLKAEKAILTPNNSDPRLLGLLSPIPLGGFSPVLNSQKGY
                     EIYQVNDKSKGQTPEYYQIKNEVLNAYVSEQRQNYIQDYFDKLRSKINIEYLR"
     sig_peptide     91247..91300
                     /locus_tag="DMACINML_00357"
                     /inference="ab initio prediction:SignalP:4.1"
                     /note="predicted cleavage at residue 18"
     gene            complement(92090..93421)
                     /gene="accC"
                     /locus_tag="DMACINML_00358"
     CDS             complement(92090..93421)
                     /gene="accC"
                     /locus_tag="DMACINML_00358"
                     /EC_number="6.3.4.14"
                     /inference="ab initio prediction:Prodigal:2.6"
                     /inference="similar to AA sequence:RefSeq:CAL35404.1"
                     /codon_start=1
                     /transl_table=11
                     /product="biotin carboxylase"
                     /protein_id="Prokka:DMACINML_00358"
                     /translation="MEIKSILIANRGEIALRALRTIKEMGKKAICVYSEADKDALYLK
                     YADASICIGKARSSESYLNIPAIISAAEIAEADAIFPGYGFLSENQTFVEICAKHNIK
                     FIGPSVEAMNLMSDKSKAKQVMQRAGVPVIPGSDGALAGAEAAKKLAKEIGYPVILKA
                     AAGGGGRGMRVVEHEKDLEKAYWSAESEAMTAFGDGTMYMEKYIQNPRHIEVQIIGDS
                     FGNVIHIGERDCSMQRRHQKLIEESPAILLDEKTRARLHETAVKAAKAISYEGAGTFE
                     FLVDKNLDFYFIEMNTRLQVEHCVSEMVSGIDIIEQMIKVAEGYALPSQENVRLKGHS
                     IECRITAEDPKTFLPSPGKIIKYVPPAGRNVRMESHCYQDYSVPPYYDSMIGKLVVWA
                     EDRNKAISKMKVALDELIIGGIKTTKDFHLAMMDNPDFINNNYDTNYLARH"
     gene            complement(93423..93878)
```

```
                     /gene="accB"
                     /locus_tag="DMACINML_00359"
CDS                  complement(93423..93878)
                     /gene="accB"
                     /locus_tag="DMACINML_00359"
                     /EC_number="6.4.1.2"
                     /inference="ab initio prediction:Prodigal:2.6"
                     /inference="similar to AA sequence:RefSeq:CAL35405.1"
                     /codon_start=1
                     /transl_table=11
                     /product="putative biotin carboxyl carrier protein of
                     acetyl-CoA carboxylase"
                     /protein_id="Prokka:DMACINML_00359"
                     /translation="MTKEEIKELVNLFAEANISKIKIKEQDGFEIELERDMCCDVPAP
                     LACPPVPAPQPINVNVVNEAQPSSAKSNKPSINSPMVGTFYQAPSPGAAPFVKVGSSV
                     KKGDTIAIIEAMKIMNEIEAEFDCRIVEILVADGQPVEFGMPLFTVEKL"
gene                 94037..94597
                     /gene="dcd"
                     /locus_tag="DMACINML_00360"
CDS                  94037..94597
                     /gene="dcd"
                     /locus_tag="DMACINML_00360"
                     /EC_number="3.5.4.13"
                     /inference="ab initio prediction:Prodigal:2.6"
                     /inference="similar to AA sequence:RefSeq:CAL35406.1"
                     /codon_start=1
                     /transl_table=11
                     /product="putative deoxycytidine triphosphate deaminase"
                     /protein_id="Prokka:DMACINML_00360"
                     /translation="MGLKADNWIRKMALEHQMIEPFCEANIGKGVVSYGLSSYGYDIR
                     VGREFKIFTNVNSTVVDPKNFLEENVVDFEGDVCIVPANSFALARTIEYFKMPDDVLA
                     ICLGKSTYARCGIIVNVTPFEPGFEGHITIEISNTTPLPAKIYANEGIAQVLFLQGDE
                     KCDTTYKDKKGKYQAQTGITLPRILK"
gene                 94651..95655
                     /gene="pseB"
                     /locus_tag="DMACINML_00361"
CDS                  94651..95655
                     /gene="pseB"
                     /locus_tag="DMACINML_00361"
                     /inference="ab initio prediction:Prodigal:2.6"
                     /inference="similar to AA sequence:RefSeq:CAL35407.1"
                     /codon_start=1
                     /transl_table=11
                     /product="UDP-GlcNAc-specific C4,6 dehydratase/C5
                     epimerase"
                     /protein_id="Prokka:DMACINML_00361"
                     /translation="MFNGKNILITGGTGSFGKTYTKVLLENYKPNKIIIYSRDELKQF
                     EMSSIFNANCMRYFIGDVRDKERLNVAMRDVDFVIHAAAMKHVPVAEYNPMECIKTNI
                     HGAQNVIDACFENGVKKCIALSTDKACNPVNLYGATKLASDKLFVAANNIAGNKQTRF
                     SVTRYGNVVGSRGSVVPFFKKLIAQGAKELPITDTRMTRFWISLEDGVKFVLSNFERM
                     HGGEIFIPKIPSMKITDLAHALAPNLSHKIIGIRAGEKLHEIMISSDDSHLTYEFENY
                     YAISPSIKLVDQESDFSINALGEKGQKVKDGFSYSSDNNPQWASEKELLDIINHTEGF
                     "
gene                 95656..96786
```

```
                     /gene="pseC"
                     /locus_tag="DMACINML_00362"
     CDS             95656..96786
                     /gene="pseC"
                     /locus_tag="DMACINML_00362"
                     /inference="ab initio prediction:Prodigal:2.6"
                     /inference="similar to AA sequence:RefSeq:CAL35408.1"
                     /codon_start=1
                     /transl_table=11
                     /product="C4 aminotransferase specific for PseB product"
                     /protein_id="Prokka:DMACINML_00362"
                     /translation="MITYSHQNIDQSDIEAVLNALKDEILTGGKKVDEFEEALCEYIG
                     IKHACVLNSATSALHLAYTALNVKDKIILTTPLTFAATANAALMAGAKVEFIDIKNDG
                     NIDEKKLEARLVKNSTDIGAISVVDFGGNSVEMDEISNLAKKYNIPLIDDASHALGSE
                     YKGKKVGSMADLSIFSFHPVKPITTFEGGAVVSNNEELISKIKLLRSHGIVKKRLWDS
                     DMMELGYNYRLSDVACALGINQLKKLDHNLEKREEIASFYDKEFDKNPYFSTIKIKDY
                     KKSSRHLYPILLFPEFYCPKEEIFENLLKAGLGVQVHYKPTYEFSFYKKLLGEIRLEN
                     ADNFYKAELSIPCHQEMSLKDAKFVRDTLFSTLEKVKKDYCG"
     gene            96779..98095
                     /locus_tag="DMACINML_00363"
     CDS             96779..98095
                     /locus_tag="DMACINML_00363"
                     /inference="ab initio prediction:Prodigal:2.6"
                     /inference="similar to AA sequence:RefSeq:ALJ17966.1"
                     /codon_start=1
                     /transl_table=11
                     /product="aminopeptidase"
                     /protein_id="Prokka:DMACINML_00363"
                     /translation="MDKLDFRHKDFEQTGKAMYELAKELFPIPRSITGQGFRASLEIL
                     NKTLGENILKFHSIKSGTRVFDWIVPDEWNAKEAYIITPEGKKICDFKKHNLHLLNYS
                     EAIDKEIELEELEKHLYSIEEMPDAIPYVTSYYKRRWGFCITHNERKNLKKGLYKVFI
                     DAKHDENGVLNYADFIIPSTQNSKDEILLSSYLCHPSMANNELSGPIVAIFLAKWLLG
                     LKERKYNYRFVIIPETIGSIIYLNKHLDHLKKHVKAGFVLSCLGDDNAYSLIHTPKEN
                     TLSDKVAMHTLKNKENFKEFSFLERGSDERQYNAPLVNLNIVGICRTKYGEYKEYHTS
                     KDDLNFISVKGLMGGLKAMQEIILNLEINAIYENTTICEPNLGKRGLYHTINNGIKQK
                     PISVDFLAYCDGQNDIIDIANILNIQAYELKELLEKILEYKLVRKI"
     gene            98092..98877
                     /locus_tag="DMACINML_00364"
     CDS             98092..98877
                     /locus_tag="DMACINML_00364"
                     /inference="ab initio prediction:Prodigal:2.6"
                     /inference="similar to AA sequence:RefSeq:ADN91469.1"
                     /codon_start=1
                     /transl_table=11
                     /product="aminoglycoside N3'-acetyltransferase"
                     /protein_id="Prokka:DMACINML_00364"
                     /translation="MKKLFQYNQKIYTQKDLVNTLKNIGIKNGDIICVHTEIFHFQTP
                     LLSKNEFLNSFLEALFKAIGKEGTLIMPTFTYSFCKNEVYDKLNSKCTVGILNEFFRK
                     QEGVKRTNDPIFSFAVKGAKEDLFLYKEQKSCFDEGCIYNILKNNNGKIVLLGTQLLG
                     YTFTHFIEEQARVPYRYFKPFKGDIIYENSNKKTYKINYYVRDLHKKSEVSILKQIAL
                     LKNSNNFKLEKIGNAEIVSIDCQAYFDETMKALKQDPYCLLKD"
     gene            98879..100387
                     /locus_tag="DMACINML_00365"
     CDS             98879..100387
```

```
                     /locus_tag="DMACINML_00365"
                     /EC_number="6.3.2.-"
                     /inference="ab initio prediction:Prodigal:2.6"
                     /inference="similar to AA sequence:RefSeq:CAL35421.1"
                     /codon_start=1
                     /transl_table=11
                     /product="putative amino acid activating enzyme"
                     /protein_id="Prokka:DMACINML_00365"
                     /translation="MTTHIYNFLEKSLANFSDKTAFVEPFAKERKEITYKNFDLFSKK
                     VASEILKKLGNDNPTQSPILIILPKGIDCLISFFGVALSGNFYTLLDEKSPKERVEKV
                     IEILKPKLFITSKELKFDLALPTLYTEDFKSFDTDENLIQVAKEKHIDTNLLYVLFTS
                     GSTGIPKGVSIAHKSVIDYTFWVCETFKFDENEILANQAPFYFDNSILDIFSSVKAGA
                     TLHLLPNHLFAFPNKILECLEKEKVSATFWVPSVLIYFANTEALDNFNLINLKKILFA
                     GEIMPNKQLNIWRKHLPDTLFANLYGPTEITVDCSFYIVDREFKDEELLPIGKACKNT
                     ELLVFDESMNLISSKQVGVKGELYVRGTSLSLGYYNDKEKTQKAFIQNPLHDNYLDLL
                     YKTGDIVAYNERGELLCYGRADNQIKYMGHRIELGEIESVINSHPKVKNSACIFKDKI
                     ICFYESDEELDLKTFCKERLVSYMIPNKLIKLDKFQFNQNGKIDRKVLNEFI"
     gene            100374..101117
                     /locus_tag="DMACINML_00366"
     CDS             100374..101117
                     /locus_tag="DMACINML_00366"
                     /inference="ab initio prediction:Prodigal:2.6"
                     /inference="similar to AA sequence:RefSeq:AAW35938.1"
                     /codon_start=1
                     /transl_table=11
                     /product="acetyltransferase, GNAT family"
                     /protein_id="Prokka:DMACINML_00366"
                     /translation="MNLFEEFEKNYFKGFKLSNWNESISILKEKFHKKKLSLESYDSN
                     FFFYENNSHLLYYFINDTNKKNKLKPSFIKISIKHEKYLDDHLNFIHLHSFNVELECQ
                     EMCLKNKNIILHDFPFITQANIQDYEQIYNFFLKHFSKDFLFYFSKNDIKNKINQILI
                     YKENGIIRGALIYTLTIKSAFLDFIAVDKNLHYKNVAFALLNTYFKKNFNTHLFKLFV
                     SVHNLKALKFYQRAGFTLNTAKILYYKNF"
     gene            101144..101380
                     /gene="acpP4"
                     /locus_tag="DMACINML_00367"
     CDS             101144..101380
                     /gene="acpP4"
                     /locus_tag="DMACINML_00367"
                     /inference="ab initio prediction:Prodigal:2.6"
                     /inference="similar to AA sequence:RefSeq:ADN91472.1"
                     /codon_start=1
                     /transl_table=11
                     /product="Putative acyl carrier protein"
                     /protein_id="Prokka:DMACINML_00367"
                     /translation="MQNTLELIKQFFINIDRSDIDENMENLLSEDIIDSMDIMSLVAE
                     IEKYYKKPLKAEFIKAENFESFQTIKNMLEQAMK"
     gene            101382..102533
                     /locus_tag="DMACINML_00368"
     CDS             101382..102533
                     /locus_tag="DMACINML_00368"
                     /inference="ab initio prediction:Prodigal:2.6"
                     /inference="similar to AA sequence:RefSeq:AVS37640.1"
                     /codon_start=1
                     /transl_table=11
```

```
                     /product="FkbM family methyltransferase"
                     /protein_id="Prokka:DMACINML_00368"
                     /translation="MNFINKKNQNNITTIYILRFPLFEKIKNIHAHIYGGIEYISYTF
                     KIFSFKFYTTKKTLELSDPEFLSKNLENLLNQSNQKQWNQSFYMQDIIGKYLVENNTH
                     EKILKLCKNLDNESKALIFRILTRLKNFQTNPNYQIKYTKEEKQDLEKLQNEFFSNIL
                     PISKGIFGWNGYFLPIKQFEISVFWHKHAMHTFSNQTLEKIKNKNIIDVGGFIGDSAI
                     IFEKEFTNQKVYSFEAVSKNYDLMLKTLELNNSKRIIPVKNALGSKEEKLQISISGYS
                     SSLKHGESNSNYEEVEVITLDSYIEKNPMEIGFIKVDIEGFEQEFLKGAINTIKAQKP
                     AMLISIYHNPNDFFDIKPLIESWNLGYKFKIYRPIDFYVSLETALYCEI"
     gene            complement(102530..103738)
                     /locus_tag="DMACINML_00369"
     CDS             complement(102530..103738)
                     /locus_tag="DMACINML_00369"
                     /inference="ab initio prediction:Prodigal:2.6"
                     /inference="similar to AA sequence:RefSeq:ADN91473.1"
                     /codon_start=1
                     /transl_table=11
                     /product="hypothetical protein"
                     /protein_id="Prokka:DMACINML_00369"
                     /translation="MLINQTFKIDSCDDVELGIKRTSKLEYRISYDDEKEMRAIVFII
                     GGIGANANISFLDFDREYIAKNFDVVVVHVFYHCFCARRSIDEEYNALTIFTKEDLPK
                     LAQALFGMGINMSVSLENAHQCYELLNQYITALKSQGKLAQNYQARLTSTFIPPNGDY
                     QNFGIMAAIDHINVLKDLVKKFPNFKNLPKIYGGGSYGGYLSLLIAKIAPWYVDGVID
                     NSGSVLPLLNYIIGRNLNRSEFVFNNPNTFVEMFVKTYWTREDENSPYFFANENYMIR
                     ALLNSFHLQIQAKKNNNIIFIGYHSAKDEFDTSKDKETLFESYKALGYDSTLYLIKDE
                     SQIDGRFIKDLKHGMRITDKALFKKELPLMLEKLQGRKSLMQENSISYPCGDKIFTFK
                     DDKMSFALHC"
     gene            103828..104526
                     /gene="pseF"
                     /locus_tag="DMACINML_00370"
     CDS             103828..104526
                     /gene="pseF"
                     /locus_tag="DMACINML_00370"
                     /EC_number="2.7.7.43"
                     /inference="ab initio prediction:Prodigal:2.6"
                     /inference="similar to AA sequence:RefSeq:CAL35425.1"
                     /codon_start=1
                     /transl_table=11
                     /product="putative acylneuraminate cytidylyltransferase"
                     /protein_id="Prokka:DMACINML_00370"
                     /translation="MKNLCIIPARGGSKRIPKKNIIDFLGKPLIAYSIESALNSGIFD
                     EVVISSDDEEIIKIACQFGAKAPFVRDEKLSDDYSSSTAVIQNAIEILAKKGQVYDNV
                     CALYATAPLISGEILKKAYEKFLENEGKFLFSATEFDYPIQRAFYLNEKNQVHMFDES
                     FYNSRSQDLTKAYHDAGAFYFGKSKAWLENDFLFKPHSSVYLLPRNLVCDIDTLEDLE
                     FAKILYKVNYESSL"
     gene            104510..105331
                     /locus_tag="DMACINML_00371"
     CDS             104510..105331
                     /locus_tag="DMACINML_00371"
                     /inference="ab initio prediction:Prodigal:2.6"
                     /inference="similar to AA sequence:RefSeq:APU78225.1"
                     /codon_start=1
                     /transl_table=11
                     /product="nucleotidase"
```

```
                     /protein_id="Prokka:DMACINML_00371"
                     /translation="MKVLFRSDSSSQIGFGHIKRDLVLAKQYSDVSFACLALEGSLID
                     EIPYPVYELESSSIYELIDLIKEQKFKLLIIDHYDISCDDEKLIKLETGVKILSFDDE
                     IKAHHCDILLNVNAYSKASDYKGLVPFRCELRCGFSYALIRDEFYKESKIKREKIWDY
                     FICIGGTDIKNLSMQIASDLPKNKKIAIATSSSNKNLKKLEKFTQDHENVKLFIDEEI
                     SKLMNESTKLIISASSLVNEALLLKANFKAICVAKNQEKLANWLVQKGYEVEYRL"
gene                 105328..105789
                     /locus_tag="DMACINML_00372"
CDS                  105328..105789
                     /locus_tag="DMACINML_00372"
                     /inference="ab initio prediction:Prodigal:2.6"
                     /inference="similar to AA sequence:RefSeq:APU78226.1"
                     /codon_start=1
                     /transl_table=11
                     /product="acetyltransferase"
                     /protein_id="Prokka:DMACINML_00372"
                     /translation="MIYLKDFAHLDKQEILLVWQWRNDEKISQFMKIKYIDFQEHLNF
                     ITSLKKDQSKKYFLVFKDNEAIGVIDFINIAKDSCEFGLYAKPDLKGMGQILMQEIKK
                     YTFEKLKIKKLKACVFKQNKRALDLYLKNSFCIKNEDENMFYVEFMGGGNS"
gene                 105829..106464
                     /locus_tag="DMACINML_00373"
CDS                  105829..106464
                     /locus_tag="DMACINML_00373"
                     /inference="ab initio prediction:Prodigal:2.6"
                     /inference="similar to AA sequence:RefSeq:AHW92334.1"
                     /codon_start=1
                     /transl_table=11
                     /product="formyltransferase"
                     /protein_id="Prokka:DMACINML_00373"
                     /translation="MKIAILTSPNQWFIPYAKELNQQIENSKLFYNHTEIFNFDIVFI
                     LSYHKIIDKNFLKHNKHNIVIHASNLPQGKGWAPLFHQVIEGKNEITFSLFEADDKAD
                     NGDIYLQKNLKLNGLELYEELREKQARFTLNMCLEFLKLYPNIQAKKQVGKESFYPKR
                     SPKDSELDINKSLNEQFNLLRIASNEDFPAFFYKDGKKFILKIYLDSTGGG"
gene                 106649..106942
                     /locus_tag="DMACINML_00374"
CDS                  106649..106942
                     /locus_tag="DMACINML_00374"
                     /inference="ab initio prediction:Prodigal:2.6"
                     /inference="similar to AA sequence:RefSeq:ADN91477.1"
                     /codon_start=1
                     /transl_table=11
                     /product="hypothetical protein"
                     /protein_id="Prokka:DMACINML_00374"
                     /translation="MSLNHIILGSINLDKLNKTTASFGFYSNPFSHLSGLGRILEQSS
                     IYLACNFLKIKVLKLEIFQENLQVINLHKKFGFAITKETIKHDKKIFIMQLVF"
gene                 106988..108019
                     /gene="pseI"
                     /locus_tag="DMACINML_00375"
CDS                  106988..108019
                     /gene="pseI"
                     /locus_tag="DMACINML_00375"
                     /EC_number="4.1.3.-"
                     /inference="ab initio prediction:Prodigal:2.6"
                     /inference="similar to AA sequence:RefSeq:CAL35431.1"
```

```
                     /codon_start=1
                     /transl_table=11
                     /product="Pse synthetase"
                     /protein_id="Prokka:DMACINML_00375"
                     /translation="MQIGNFNTDEKVFIIAELSANHSGSLEMALKSIKAAKEAGADAI
                     KIQTYTPDSLTLDCDKDDFIIKGGLWDQRKFYELYESAKTPYEWHSQIFEAAQDAGIL
                     CFSSPFAKEDVEFLKRFDPIAYKIASFEANDENFVRMIAKENKPTIVSTGIASEEELF
                     KICEIFKEEKNPNLIFLKCTSAYPAQISDMNLKGIISLKEKFGVEVGLSDHSFGHLAP
                     VMAVALGARVIEKHFMLDKNIKSEDSKFSLDFDEFKAMVEAVREAESALGDGSLALDE
                     KSLKNRVFARSLYASKDIKKGEIFSEENVKSVRPSFGLHPKFYKELLGKTATKDIEFG
                     TALKQGDFQ"
     gene            108016..108984
                     /gene="rmd_1"
                     /locus_tag="DMACINML_00376"
     CDS             108016..108984
                     /gene="rmd_1"
                     /locus_tag="DMACINML_00376"
                     /EC_number="1.1.1.281"
                     /inference="ab initio prediction:Prodigal:2.6"
                     /inference="similar to AA sequence:RefSeq:AJA55542.1"
                     /codon_start=1
                     /transl_table=11
                     /product="GDP-6-deoxy-D-mannose reductase"
                     /protein_id="Prokka:DMACINML_00376"
                     /translation="MKILVTGADGFIGSHLCESLVQKGYKVRALSQYNSFNFWGHLEK
                     SPYLKDMEVVSGDLRDSFFCEKITQGMDAIFHLGALIAIPYSYEAPQSYVDTNVKGTL
                     NMLEAAKKNEISHFIHTSTSEVYGTARYVPIDEKHPLQPQSPYSASKIAADMMALSYY
                     NAFNLNVNIARPFNTYGPRQSARAIIPTIITQILSGVKELKLGDLSPTRDLNFVLDTC
                     EGFISLLKLNHFGEVYNIGSGTEYSMQEVLDLIQKILNQNVKIIQDEKRLRPKNSEVF
                     RLKCDSSKLQNATHWQSKISLEEGLKLSIEYFKENLASYKSEIYNV"
     gene            108977..110128
                     /locus_tag="DMACINML_00377"
     CDS             108977..110128
                     /locus_tag="DMACINML_00377"
                     /inference="ab initio prediction:Prodigal:2.6"
                     /inference="similar to AA sequence:RefSeq:CDH63148.1"
                     /codon_start=1
                     /transl_table=11
                     /product="Bacillosamine/Legionaminic acid biosynthesis
                     aminotransferase PglE;
                     4-keto-6-deoxy-N-Acetyl-D-hexosaminyl-(Lipid carrier)
                     aminotransferase"
                     /protein_id="Prokka:DMACINML_00377"
                     /translation="MFENEITFIKKLFNKEKIALHEPCFIGNEKKYLLECIDSGFVSS
                     VGEFVTRFEEALKQRTKARFVIATNTGTAALHIALLANGIDENCEVITQSISFVATAN
                     AIAYTGARAIFLDIDENTLSLSPKALQNFLENHSYQKDGFTYNKITHKRIKACVVMHT
                     FGLSANIEKLCEICKNYNIFLIEDAAEALGSTYKNKALGTFGKCGILSFNGNKIITGG
                     SGGAILCDDENLANLAKHLSTTAKIPHPYEYDHDIIGYNYRLCNINAAILLAGLEKLD
                     FFLENKRELAQIYKEFFAHHKACKFIDERKNSKSNFWLNALCFKDENLRNIFLQECLK
                     NNIFARPVWKSLPSLKPFQTWQKDELLNTQKLEKCLVNLPSSVRLDKEK"
     gene            110128..110847
                     /locus_tag="DMACINML_00378"
     CDS             110128..110847
                     /locus_tag="DMACINML_00378"
```

```
                     /inference="ab initio prediction:Prodigal:2.6"
                     /inference="similar to AA sequence:RefSeq:CDH63149.1"
                     /codon_start=1
                     /transl_table=11
                     /product="FIG00471635: hypothetical protein"
                     /protein_id="Prokka:DMACINML_00378"
                     /translation="MKKFINNEEISNNIGTNEEIWENIFSNKEWGKYPSENLIRFIAR
                     NFYNVKNRNEIRILELGLGTGANLWFCAREGFRVSGIEWSKTGVERFQKRLSDENLSS
                     QIDEIKIGDYEQKLDEFKDESFDAWIDSYSLAYNDFKKTKTIIEKAISKLKSGGKFFS
                     ITPSFNNEGFEKNENLGYHECKPTLGSDAFTGVIRYCDEEDLKELYNGKNYQITDIKI
                     ARFASLEKNLNELYIIEGQKL"
     gene            110851..111555
                     /locus_tag="DMACINML_00379"
     CDS             110851..111555
                     /locus_tag="DMACINML_00379"
                     /inference="ab initio prediction:Prodigal:2.6"
                     /codon_start=1
                     /transl_table=11
                     /product="hypothetical protein"
                     /protein_id="Prokka:DMACINML_00379"
                     /translation="MQNILLKQFLEERKKKYKNGIYHYTQVNFSYNSNHIEGSTLSEE
                     QTRYIYDRNSFLANENQIINTNDILETKNHFLAFDFILDSVFDEINEAYIKKLHSIVK
                     NNCSDIEIIGEFKQRQNYVGDLKTSAPHLVKEHLEKLFQGYKVHNIEDIIDFHFHFEK
                     IHPFEDGNGRVGRLLMFKECLKNNITPFIVDEEHKLFYYRGLKNYENQKGFLIDTYLS
                     CQDKYKEILNYFQIVC"
     gene            111609..112328
                     /locus_tag="DMACINML_00380"
     CDS             111609..112328
                     /locus_tag="DMACINML_00380"
                     /inference="ab initio prediction:Prodigal:2.6"
                     /inference="similar to AA sequence:RefSeq:ABS43939.1"
                     /codon_start=1
                     /transl_table=11
                     /product="methyltransferase domain family protein"
                     /protein_id="Prokka:DMACINML_00380"
                     /translation="MNNAAFWENIFSNKEWGKYPSENLIRFIARNFYNVKNRNEIRIL
                     ELGLGTGANLWFCAREGFRVSGIEWSKTGVERFRKRLNDENLSSQIDEIKIGDYEQKL
                     DEFKDESFDCIIDSCSLCCNNFEKTKHIFDKAILKLKPNGKFFSSTIAKGIVGYDKNK
                     EDFQLPNDGIYTHVGMIRFSNKEDIQKLYKNDNFKQTSLKVQSIEDNGALIDKLFIIE
                     GEKNIMGGGNSKKLLQGVENE"
     gene            112321..113286
                     /locus_tag="DMACINML_00381"
     CDS             112321..113286
                     /locus_tag="DMACINML_00381"
                     /inference="ab initio prediction:Prodigal:2.6"
                     /inference="similar to AA sequence:RefSeq:SQE24790.1"
                     /codon_start=1
                     /transl_table=11
                     /product="putative protein involved in methicillin
                     resistance"
                     /protein_id="Prokka:DMACINML_00381"
                     /translation="MSRIFLEDFLKNHKNYSYLLDDIYLNKKYASLYGEVFEFSYQKE
                     VKLFKMIAIKEKIPNTKFYDLQSPYGYGGIYCNVNDKNFIKEALENLKLKAVEQNIIA
                     FFIRFHLFDENLKNYHYLLPFFTKNKETIIVNTNENIENIRTNYSPRIRSYIKKARKE
```

```
                     INIEFAQKEDYKDFFKLYTHTMQRNNADKFYFFNERYFEKLFNFKESIILKASLDNKI
                     LAFASFFLCGDFSYYHLSANSLKSNANAALLDFFFEYANQNACKFCFLGGGVQNEDTL
                     FKFKEKFSTLRANFYVGGMIFNKEEFDFLNKNFTNKYFLKYRFDD"
     gene            113703..114707
                     /gene="neuB2"
                     /locus_tag="DMACINML_00382"
     CDS             113703..114707
                     /gene="neuB2"
                     /locus_tag="DMACINML_00382"
                     /EC_number="4.1.3.-"
                     /inference="ab initio prediction:Prodigal:2.6"
                     /inference="similar to AA sequence:RefSeq:CAL35440.1"
                     /codon_start=1
                     /transl_table=11
                     /product="N-acetylneuraminic acid synthetase"
                     /protein_id="Prokka:DMACINML_00382"
                     /translation="MKKALIIAEAGVNHNGDLNLAKKLIEVAAKSGADFVKFQSFKAE
                     LCVSKNAKKATYQLKTTAKDESQLEMIKKLELDFNAHQLLISHAKQCGIAFLSTPFDL
                     ESIELLDDLGLEVFKIPSGEITNLPYLKKIAKLNKKIILSTGMSNLGEIEAALEVLCK
                     EGTQRANITLLHCTTEYPAPFDEVNLKAMQTLKNAFNLDVGYSDHTKGIHISLAAIAL
                     GASVIEKHFTLDKNMSGPDHKASLEPDELQELCTKIREIESALGDGIKQASKSERKNI
                     EIARKSLVAKKKIKKGEIFSEENLTTKRPASGISAMRYDEYLGKKASKDYEEDELIHE
                     "
     gene            114700..115857
                     /locus_tag="DMACINML_00383"
     CDS             114700..115857
                     /locus_tag="DMACINML_00383"
                     /EC_number="5.1.3.14"
                     /inference="ab initio prediction:Prodigal:2.6"
                     /inference="similar to AA sequence:RefSeq:ABS43139.1"
                     /codon_start=1
                     /transl_table=11
                     /product="UDP-N-acetylglucosamine 2-epimerase"
                     /protein_id="Prokka:DMACINML_00383"
                     /translation="MSKRKICIVSATRAEWYLLKNLCLEIQNDEDLELQIIATGAHLS
                     PEFGLTYKEIEKEFKIAKKIPILLASDDKISLCKAMALANIGFSEAFESLKPDIVVIL
                     GDRYEMLSVASVCLMMNLPIAHLCGGELTLGAIDDSIRHSISKMAHLHFVSTQTYKNR
                     LLQLGEEENRVFNVGSLASPIIKNINFLDKNELAKVLEFDLEQNIYLITYHPVTLNLQ
                     NSKKEIDILLKTLDKLNNASLIFTKANADENGLLINEILKDYCEKNPKKAKLFDNLGS
                     QKYLSIMKIAKAMIGNSSSGISESPFFKTPCINIGSRQKGRLRTQNIIDCEFENLDQA
                     FEKLESKDFKQALYHFKNPYENDKNPNIIIKTCLKNANLDTILQKKFIDLR"
     gene            115862..116893
                     /locus_tag="DMACINML_00384"
     CDS             115862..116893
                     /locus_tag="DMACINML_00384"
                     /inference="ab initio prediction:Prodigal:2.6"
                     /inference="similar to AA sequence:RefSeq:CAL35442.1"
                     /codon_start=1
                     /transl_table=11
                     /product="putative sugar-phosphate nucleotide transferase"
                     /protein_id="Prokka:DMACINML_00384"
                     /translation="MDINKLKLTPDSSIEEALKVVGQERVRLGIIVDKKDNFLGVISD
                     SNIRKALIKGKNLQSKIKDIYTKNPITIKENTSKNELLKLSAKTDIYDFPVLNDKKEV
                     ISIKSISSLLNRQVNSNFVIIMAGGLGTRLKELTKDTPKPMLKVGKKPILESIVQRLK
```

```
                     EQNFENFIFCVNYKKQIIEDYFKKGKDFGIKISYIKERKKLGTAGALSLIKQDFKESF
                     IVMNADILTELDFNELLKAHKDSKALMSVCVREFEQQIPFGVIEQNKGFITNITEKPV
                     QKFLVSAGIYVCEPEILELLNKNEYLDMPELIKLAMQKGRVNTYLIHDYWIDIGRLDE
                     FVKANEEFK"
     gene            116890..117795
                     /locus_tag="DMACINML_00385"
     CDS             116890..117795
                     /locus_tag="DMACINML_00385"
                     /inference="ab initio prediction:Prodigal:2.6"
                     /inference="similar to AA sequence:RefSeq:CAL35443.1"
                     /note="hypothetical protein Cj1330"
                     /codon_start=1
                     /transl_table=11
                     /product="hypothetical protein"
                     /protein_id="Prokka:DMACINML_00385"
                     /translation="MKVLIIGFGSIGKKHFLALNELGFLVDVLSKSYEFSHFEGKNFR
                     LFKDLEELNLQDYDLFIIANITTSHFKTLEFLDQNVKEKIILVEKPLFQKDMEFIPSN
                     NNQIYIAYLLRFHPMIQDLKQLIDEKEVYFAKFICNSYLPNWRKLDYRQNYSAKKELG
                     GGVMLDLSHEIDLAFYFFKELSLKFSQNLKISELEITSDDFTFLALSAKNTSIHIELD
                     YFSKLTQRKILFHTKENSFEADLVHNKLNIYDKTNNFKSKTYENDTFKTLQNMYKAIL
                     EKDQNLCTLEEARRVLRLCDEARNG"
     gene            117788..118495
                     /gene="ptmB"
                     /locus_tag="DMACINML_00386"
     CDS             117788..118495
                     /gene="ptmB"
                     /locus_tag="DMACINML_00386"
                     /EC_number="2.7.7.43"
                     /inference="ab initio prediction:Prodigal:2.6"
                     /inference="similar to AA sequence:RefSeq:CAL35444.1"
                     /codon_start=1
                     /transl_table=11
                     /product="acylneuraminate cytidylyltransferase (flagellin
                     modification)"
                     /protein_id="Prokka:DMACINML_00386"
                     /translation="MGEILCTICARGGSKGVKNKNIRKINDLEMIAYSIIQAKNSKLF
                     KHIVISTDSEEIAKIALKYGGEVFFKREAHLASDTAAKIPVMRDALLRSEEYFKCQFD
                     ALIDLDASAPLRSSSDIIKAFETFCQNQNDNLITAVPARRNPYFNLIEVQDGKVVKSK
                     SGNFTTRQSAPKCYDMNASIYIFKRDFLLQNDSVFGENTGLFIMDESTAFDVDSELDF
                     KIVEFLIKEKNLQAKDF"
     gene            118495..119265
                     /gene="ptmA"
                     /locus_tag="DMACINML_00387"
     CDS             118495..119265
                     /gene="ptmA"
                     /locus_tag="DMACINML_00387"
                     /inference="ab initio prediction:Prodigal:2.6"
                     /inference="similar to AA sequence:RefSeq:CAL35445.1"
                     /codon_start=1
                     /transl_table=11
                     /product="putative oxidoreductase (flagellin
                     modification)"
                     /protein_id="Prokka:DMACINML_00387"
                     /translation="MLENKIIFVAGACGRIGKALCKKILQNKGIAILADINENHLSIL
```

KTELENEFKKELLSLRLDITSKESLNYAIDQAFKKYSKIDGFVNSSYPVGKDWGKIAY
YEASYEQICESLNLHLGGFILASQEFVKFFKKQSYGNIINLSSIMGVFAPKFENYENT
AMQSSLEYSVIKAGINHLGAWLAKELFNTNIRVNTLASGGILDNQANIFLEKYRKCCA
SKGMLDAEDICGTLVFLLSDESKFITGQTLVVADGWGL"
gene 119265..121118
/gene="maf-2"
/locus_tag="DMACINML_00388"
CDS 119265..121118
/gene="maf-2"
/locus_tag="DMACINML_00388"
/inference="ab initio prediction:Prodigal:2.6"
/inference="similar to AA sequence:RefSeq:SQE24803.1"
/codon_start=1
/transl_table=11
/product="maf-2"
/protein_id="Prokka:DMACINML_00388"
/translation="MNNELFLKNTQALFEVDQILAYELRRIKHCFNFALQGDNLENLN
IIHLSSKESIYKNPYEELSNNLTFFKQEYSKYPTLFFYGLGSGAFYKALLTNQNHKQL
IVFESELELFYLAFHLFDFSKDIRNERLILFYTPNLNTTQLNALLNHKKIKFSIKTYD
FHIHSEFYLSHYKDIIQNLNQSLIEIIKFIVLAYGNDPKDSHIGIEHTLKNLNTLVYG
SVFQHFLKYNRAKSKNAIIVSTGPSLTKQLSLLKEYASKATIFCADSSYPILARAGIK
PDYVLSLERVAATSEFFNNDFKEFDKDILFLITSVTHENTLKYLEKNHRNYMLVLRPL
LFSSHLKLDDFGYLGVGMSVANMAYELAVALRHENIILIGQDLAYDDLGHSHPKDHIY
GEQGDKFQDKLSVRAYGGKGLVKTQLSWQLFRQSFEKDIPLVKEKLQITTYNCTEGGA
RIEGSIEKPFKELCESILDTNLEKPFKNDFKLEPKEAQKILEKTKKHLLNQVKISKNL
IEEIQNKLKTLPNILNEKDINLDFKKLKKLELDFQSLATKMKSYGLMSELLQALVFHQ
ECDLLKLKVQPCKNQEEEQKQILLCLKYHHEKFSELILYMQIQNELIEKYI"
gene 121130..123085
/locus_tag="DMACINML_00389"
CDS 121130..123085
/locus_tag="DMACINML_00389"
/inference="ab initio prediction:Prodigal:2.6"
/inference="similar to AA sequence:RefSeq:ATM02447.1"
/codon_start=1
/transl_table=11
/product="PseD protein"
/protein_id="Prokka:DMACINML_00389"
/translation="MTFTEEQKNRYKKNFNALSDNLILKKDLAKLSKSKKFKILQGQD
PLDINLLNTFTHKKIYNKPLEELNALLASYNEKYILYPVLYFYGLGNGILYKALCQNP
KLQHIIIFENDIELIFTSLHLIDLSQEIKEGKIHILSSKKNKEQFLSIFNVQPFLNFL
RVYFLELHSNYYESEHDEIIKLNQDIHHAIKEAVCVRGNNPKDALQGIEQFIFNLPNM
LTHPNAKELINTRKGLDKNAIIVSTGPSLTKQLSLLKEYASKATIFCADSSYPILARA
GIKPDYVMMLERTDMTAEFFNHDFGDFDKGIIFCIAALVHPKAIEYLEKQNRTYILIE
KSLHFESYMKLHSFGYLNLVSSVAHMAYELAMALKFENIIFIGQDLAYAKDGCSHPKD
YQNSPTYETNMYEHLSTKAYGGKGEVKTHFVWNMFRTILEKHIINTPKTIKVYNCTEG
GARIEGSIEKPFKELCENLLTNDLNKPFPSLNLPSQDKQNERMLKAFYKIQKSIQNCD
KLELFVLEKQQSILEKINQEEFKNLSYDDKELAQGIIEDIDTLKNEIEKIHTFYEDLG
EILQPLYCQFEFNLAKIYVLNPLTKEDWVNKTLLWIKEHLDWIAYILIHLKAQKQALQ
SSILPLENELLQRNFKHLLDKIKRKTK"
gene 123082..124992
/locus_tag="DMACINML_00390"
CDS 123082..124992
/locus_tag="DMACINML_00390"
/inference="ab initio prediction:Prodigal:2.6"

```
                     /inference="similar to AA sequence:RefSeq:AIW10510.1"
                     /codon_start=1
                     /transl_table=11
                     /product="PseE protein"
                     /protein_id="Prokka:DMACINML_00390"
                     /translation="MKTTDIFEKNLLSLSGKEYENLIKKLKKIKESKKFSPLFDAKDP
                     LNLNIIENSTKEKIYQNPLKELESKIDLIQKKYLKYPVLFFYGIGNGFLYKALLQNPH
                     FKRIIIFEKEIELIFLALNLADFTQELKKGRLIIILSSEMNDTKANSLFSLPEIKLLF
                     KSYFLDLHSDFYEKYSKDIIKINNINKNMIIFFSLRQGNDPKDALQGVDQFVRHIPHM
                     ITHPSLSELIKKRYKKAKNAIIVATGPSLQKQIPLLKKYHKKASIFCLDASYSILHEN
                     DIKPDYVLSLERVVATSEFFNNDFKEFDKDILFILLTLTHPQTIKYLEANKRSYILTP
                     RDLPFSQSIKHLKEFSSLNGMSVAHMALVLAYHLGHENVILIGQDLAFGKDGSSHSKG
                     YGRGEELAKKETQALPQTKVKAYGGKGLVKTTTIWELFIKVFENITTIINAQKNCTIY
                     NATEGGARIGGTIEKPFKELCENLLDKELKKPLTKVNKLSRKEQNLLLLKSYKQIKTF
                     IKRSEKIAKELRKTGKQIRNLTQEIKQKYKLEQISKNIDEIKTKLASSNYAYLAEVLG
                     PSVYHAESSLGELYLQNISNESDKQNKLIAWIYAHEAWIDEVHNLIILQNSVLKNAII
                     PLRDMVEKRKLV"
     gene            complement(125014..126738)
                     /gene="flaB"
                     /locus_tag="DMACINML_00391"
     CDS             complement(125014..126738)
                     /gene="flaB"
                     /locus_tag="DMACINML_00391"
                     /inference="ab initio prediction:Prodigal:2.6"
                     /inference="similar to AA sequence:RefSeq:ADC28911.1"
                     /codon_start=1
                     /transl_table=11
                     /product="flagellin"
                     /protein_id="Prokka:DMACINML_00391"
                     /translation="MGFRINTNVAALNAKANSDLNSKALDQSLARLSSGLRINSAADD
                     ASGMAIADSLRTQASTLGQAINNGNDAASILQTADKAMDEQLKILDTIKTKATQAAQD
                     GQSLKTRTMLQADINRLMEELDNIANTTSFNGKQLLSGSFINQEFQIGAQSNQTIKAS
                     IGATQSSKIGVTRFETGTQISDSGTAQITIKNYNGIEDFKFQSVVISTSVGTGLGALA
                     EEINKYADKTGVRASFNVQTVGAMAITKGNTTDDFTINGVKIGKIEFEAGDKNGSLVA
                     SINAVKDTTGVEASLSDDGRLVLTSREGRGIKVSGFTPSMGILAEQAENYGRLSLVKN
                     DGRDIAISGTGLSAAGFGEGQMVSQSSVSLRETKGQISAQIADAMGFNNYEGGGRFLA
                     DYSSISSYMSAAGSGMSAGSGFSVGSGKNMSVMLSANVGFVGNAQSMLSNFYNVSEGS
                     GFSAGSGQSQFAQMKATALGSTDKTAGVTTLKGAMAVMDIAETATINLDQIRADIGSV
                     QNQLQVTINNITVTQVNVKSAESTIRDVDFAAESANFSKYNILAQSGSYAMSQANAVQ
                     QNVLKLLQ"
     gene            complement(126834..128681)
                     /locus_tag="DMACINML_00392"
     CDS             complement(126834..128681)
                     /locus_tag="DMACINML_00392"
                     /inference="ab initio prediction:Prodigal:2.6"
                     /inference="similar to AA sequence:RefSeq:APU79916.1"
                     /codon_start=1
                     /transl_table=11
                     /product="motility accessory factor"
                     /protein_id="Prokka:DMACINML_00392"
                     /translation="MSILEKNIQALLSGVNEPLGNKLLNFIQTKTCSRFSMDGNLNIY
                     DKNNDIFMYENLEELNYFYQNILEKTLRYPFICIYGIGNALLIKNLAKHYRHLFVFES
                     EIELFILALSALDLSEELCSGKIYLVDVCEPKMELQLSILFDQRDIFEWLNLYEMFIN
                     CNFYKLYYQDTISKTDKKCIDNIFLVIRNFDPNDQLTTICYKQFLQNIPHLLQNIPFQ
```

RLLSERKAKFDTAVVVSAGPSLHKQLSLLRQYQENVVIFCADGALNMLENEGIVADYV
LNLDIKDLTVKFFNCSKPLDYSKTIAVLAANTHPNVVKHVQTKLPCCVALRDKYLYEQ
FHLDDFGYIDTGTHVSHFSYTLALALGFKNIVMIGQDLAFDEKGNSHSKDFVFGEKFD
HTLNMPTLKVLAYEGKGEVLTHIAWNDYRIKLEYLIARNNDKAVFYNATEGGAYINFA
KNISFKECCEKFFTQKKPSLNIPKPFTYNRSLKFLSKIFLHLKNDFQSAQDILRDAHL
LNNALINVLNVKKTLPLDFLQNIYQSITDFDFILDNNTFIQDDVLKGTFGYRGNFLSE
VIKKRFEDETHYFLSLIHAYQKWLEKFIEKLEMKSFIIRDAMLEYEKLI"

gene complement(128691..129935)
/locus_tag="DMACINML_00393"

CDS complement(128691..129935)
/locus_tag="DMACINML_00393"
/inference="ab initio prediction:Prodigal:2.6"
/inference="similar to AA sequence:RefSeq:ALJ18007.1"
/codon_start=1
/transl_table=11
/product="carbonic anyhydrase"
/protein_id="Prokka:DMACINML_00393"
/translation="MIVNKTYEIDSCDDVELGIKRESKLEFKLCFDDEKEMKALVFIV
PGLGGDANENYREHLAEFVANEFSVAVVSVNYHCIGNRPQTGSTFYLDDIDKLILQTS
CETLGIKINPDKLNSLEDLNFTLKKVDTILEERKTQRKIVSNFKLSIHLSLQPAKNEY
QNFGVMQAQDLLNAALYLKAHAPFNTMGGGMPVIMIGSSHGGYLAHLAAKFAPWLVDG
VIDNSSYAKFLWRLIGFGKEIDFTKYYCFGTAIFFKHIHIYCSDKTFWTSNSSSNNFF
SIPRRMIRYILEPNHIENQANYPKPYYTSYHSFYDKEIAPPDEKLELYKYLKQFDFDA
ELHMIKNPDQIDGKFIKNLDHGMGMSIKTLISKELPCMLEKILSNSRKEWKNKSISYP
CDGLIYHFSEKDNKINLDITKT"

gene complement(129922..130449)
/locus_tag="DMACINML_00394"

CDS complement(129922..130449)
/locus_tag="DMACINML_00394"
/inference="ab initio prediction:Prodigal:2.6"
/inference="similar to AA sequence:RefSeq:CAL35455.1"
/codon_start=1
/transl_table=11
/product="putative periplasmic protein"
/protein_id="Prokka:DMACINML_00394"
/translation="MKRAFTILELVFVIVILGILAAIALPKFSSSRDEAEVSKSLNNL
KTLINDISIYALKNSDLSTMNFMSNVSGVENVDLKNFTGIKEVNFRVGEDKECIKLIF
IDKNDFVLMGISSNEASKNAIINIANNPKQEFQNLDFTSSSKNKACVALSKNENFKNL
ASQTYILIGGFDDSK"

gene complement(130452..131459)
/locus_tag="DMACINML_00395"

CDS complement(130452..131459)
/locus_tag="DMACINML_00395"
/inference="ab initio prediction:Prodigal:2.6"
/inference="similar to AA sequence:RefSeq:CAL35456.1"
/codon_start=1
/transl_table=11
/product="putative glycoprotease"
/protein_id="Prokka:DMACINML_00395"
/translation="MKNLILAIESSCDDSSIAVMDKDTLECKFHKKISQEMEHNVYGG
VVPELAARLHSEALPRILEQCKGYFDKLCAIAVTNEPGLSVSLLSGISMAKTLALSLN
LPLMAINHLKGHIYSLFLEESICLDMGILLVSGGHTMVLYLGKDGKIEILARTNDDSF
GESFDKVAKMMNLGYPGGVLIENLAQKAKLKNIHFNIPLKHSKELAYSFSGLKNAVRL
EILKHENLDEDIKAEIAYGFENAACEHIMDKLEKIFNLYKFKTFGVVGGASANLNLRS

```
                     RLQNLCEKYQSKLKLAPLKYCSDNALMIARAGVDTYKRKEFVELEEEILSPKNKNFLR
                     I"
     gene            complement(131456..132850)
                     /locus_tag="DMACINML_00396"
     CDS             complement(131456..132850)
                     /locus_tag="DMACINML_00396"
                     /inference="ab initio prediction:Prodigal:2.6"
                     /inference="similar to AA sequence:RefSeq:CAL35457.1"
                     /codon_start=1
                     /transl_table=11
                     /product="putative periplasmic protein"
                     /protein_id="Prokka:DMACINML_00396"
                     /translation="MKFFISIIFFISGLFALDLEFSVGENGKSLDDNNTVLIFGGIQG
                     DEPGGFHAASLLLSDYNITKGKIIVAPNLAFDSIIKRSRGNNGDLNRKFASLSPKDPD
                     YQTVQRIKELILLPEVSMVINLHDGWGFYKPTYIDAMQNPKRWGNSSVIDTSEINASK
                     YPNLEGIATQTVNSVNASLADPKHAYHLKNTKTQELGDTEMLKALTYFVISNHKAAFA
                     NEASKNLPVNLRAYYHLLAIENYLKTAGIEFTRTFELTPQGVDKAINQELEVKLFDDK
                     ILLSLKNPRQVINYVPFPINKELNYNTSNELTAVIAENNSFYIQYGNRFQTRLYPEYL
                     EFSNPFDKVTLQVDGNETIVNFGTKLQVKENFIVPRIKGVRVNIIGFDHGRDESNMLV
                     SKKNMQKPYSLDMAGKIYRVEFYELRGANLQQFLEDSVESKLIKNAKILDLSTLKTAR
                     SKDKFIGSILVEFE"
     gene            complement(132847..133923)
                     /locus_tag="DMACINML_00397"
     CDS             complement(132847..133923)
                     /locus_tag="DMACINML_00397"
                     /EC_number="1.1.1.267"
                     /inference="ab initio prediction:Prodigal:2.6"
                     /inference="similar to AA sequence:RefSeq:CAL35458.1"
                     /codon_start=1
                     /transl_table=11
                     /product="1-deoxy-D-xylulose 5-phosphate reductoisomerase"
                     /protein_id="Prokka:DMACINML_00397"
                     /translation="MIVFGSTGSIGLNALKLATLKNIKISALACGENISLLNEQIEQF
                     KPEFVAIKNSKDKHLVKHDKVFVGQEGLEEILSLCEDDLLLNAIVGFAGLKSTLKAKE
                     LGKKIALANKESLVVAGKFLKGAKFLPVDSEHSALKFLLEGKKDIAKLYITASGGAFY
                     KHKIKDLSHVSVKDALKHPNWNMGSKITIDSATMANKLFEIIEAYHLYDFKNIDALIE
                     PKSLVHAMCEFKNGASTAYFSRADMKLSISEAIFSKHDSKILEPVDFVKLSSLKFYKI
                     STKKYPIFKLKNELLNNPDLGVIINAANEIGVQNFLDNKIQFLDIAHVIFKALDHFGV
                     PKISSIDEVFEYDYKTREYLRGMK"
     gene            complement(133920..134648)
                     /gene="cdsA"
                     /locus_tag="DMACINML_00398"
     CDS             complement(133920..134648)
                     /gene="cdsA"
                     /locus_tag="DMACINML_00398"
                     /EC_number="2.7.7.41"
                     /inference="ab initio prediction:Prodigal:2.6"
                     /inference="similar to AA sequence:RefSeq:CAL35459.1"
                     /codon_start=1
                     /transl_table=11
                     /product="phosphatidate cytidylyltransferase"
                     /protein_id="Prokka:DMACINML_00398"
                     /translation="MFNAARIIWGVVMAIAVILIALIDQFIINFIVFAFILYLAFNEA
                     KKLFNLEDVSIIPLVLAFILGTISEIPLAFGALAALLVLGYLVYKKAPSLKAVWIYLY
```

PSLPVLALWQVYLDDGMFALFWLIAIVAICDSAAYFIGKLLGKTPFSQTSPNKTLEGV
IGGLVCASILGTLIGIFVYSFWLSLLCSFFVAFFAVIGDLLESYFKREAGVKDSGDLI
PGHGGILDRIDAVIIAAFVMVALL"
gene complement(134657..134995)
/locus_tag="DMACINML_00399"
CDS complement(134657..134995)
/locus_tag="DMACINML_00399"
/inference="ab initio prediction:Prodigal:2.6"
/inference="similar to AA sequence:RefSeq:CAL35460.1"
/codon_start=1
/transl_table=11
/product="putative coiled-coil protein"
/protein_id="Prokka:DMACINML_00399"
/translation="MPVSPIGNMNFVNQNMAYPATQASNELAKEGFAASLNMAAFNEK
EKALNKLEKVNETQEIKEEIKEKAEQEEKKKKQKQEAKKDDKDENLEEQTEEASFKNA
QSIHHIDISI"
gene complement(134998..136305)
/locus_tag="DMACINML_00400"
CDS complement(134998..136305)
/locus_tag="DMACINML_00400"
/inference="ab initio prediction:Prodigal:2.6"
/inference="similar to AA sequence:RefSeq:CAL35461.1"
/codon_start=1
/transl_table=11
/product="putative fibronectin/fibrinogen-binding protein"
/protein_id="Prokka:DMACINML_00400"
/translation="MKYTELMQLQKFFSQFKKIDFIKRVNDNILELSFDKQRFIFDLT
RGMSAIYTAKLMSKNYNAPFDFMLKKYFNNAFIKEVKLLQGNRILCFSVRVDKAYKSY
ESKIYFEFTGKNTNVIITDEKDLIIEALRHIDKSYRVVKPNVVLEALKPYKMDEKFEE
IKNFNDYFTQKFEILHANKIKQIQTLKLAQIDKKIENLEELYLALDKEELLLDQALNL
RKQADILFANLSILKEYEREFELDDFEGKKVKFKLDLSPKESANLFYKNAKKLEQKAR
NLNLQRENLKEKLDFAKGLKEMLSKAKNEFELEILLPKKSTKKNQENKQDSGIANFYF
NEFKICVGKNEKGNENLLKSAKKDDLWLHVRDIPSSHVLIISNKQKISEEVIEFSARL
CVNFSGLKKGSYWVDYTLKNFVKVQQKAFVKYTNFKSINITKD"
gene 136368..136943
/gene="mobA"
/locus_tag="DMACINML_00401"
CDS 136368..136943
/gene="mobA"
/locus_tag="DMACINML_00401"
/inference="ab initio prediction:Prodigal:2.6"
/inference="similar to AA sequence:RefSeq:CAL35462.1"
/codon_start=1
/transl_table=11
/product="putative molybdopterin-guanine dinucleotide
biosynthesis protein"
/protein_id="Prokka:DMACINML_00401"
/translation="MKLENLNCVILCGGKSSRMGEDKSKLIFKNQTLTQFQVEKFSKI
FKNVYYVSAKEDKFGAKFKLIKDCPEFEIYSPMLALYSILSNFKDEFVFILSVDSPNLS
DKELLKMLAFLEQDYQIIIAKTPSHKHPLCGFYHSSVAYLCKELLEKNEQKIALLFSK
AKTHFVEFEDETPFLNLNFYQEYEQFKSEYE"
gene 136940..137935
/gene="pldA"
/locus_tag="DMACINML_00402"

```
CDS             136940..137935
                /gene="pldA"
                /locus_tag="DMACINML_00402"
                /EC_number="3.1.1.32"
                /inference="ab initio prediction:Prodigal:2.6"
                /inference="similar to AA sequence:RefSeq:AOH52353.1"
                /codon_start=1
                /transl_table=11
                /product="phospholipase A1"
                /protein_id="Prokka:DMACINML_00402"
                /translation="MKKILFLIFFYSLVFCDDFEQALEFEKKGQYEKAMQIYKKLALQ
                NSSKQEFQENNASLNSNTKHEMQATQKAASTKEDFSRIALENYLGEDSSFNPLGISSY
                KMNYFLPFAYSFGSLGGENRKTEMKFQLSIKKRLFEDLLGLGEKYYVGYTQTSWWQNY
                KHSSPFRETNYQPEFFVDIPLHFEEYKFLNNLRVGILHESNGKGDENLESRSWNRIYA
                SSVFLYQRFLFVPRIWYRIPENSEDDDNPEITHYMGNFDINMGYLGNDYFINLMLRNN
                LDFHDNKAAVQVDIGYDIFDNGIYWYLQYFNGYGESLIDYNKRLQRLSTGFLISY"
gene            138068..139036
                /gene="ceuB"
                /locus_tag="DMACINML_00403"
CDS             138068..139036
                /gene="ceuB"
                /locus_tag="DMACINML_00403"
                /inference="ab initio prediction:Prodigal:2.6"
                /inference="similar to AA sequence:RefSeq:CAL35464.1"
                /codon_start=1
                /transl_table=11
                /product="enterochelin uptake permease"
                /protein_id="Prokka:DMACINML_00403"
                /translation="MLFKHLFSLNILLILLVIFGILSLFIGVIRINLDDIFSLSATQL
                EIILLTRIPRLIAILLTGMSLSICGLIMQQLTQNKFVSPTTAGTMDCAKFGILISLIF
                FAGASFFTQALIASIFALLGSFIFIQILRKIKLKDVIFVPLIGLMFGGIINAITTFFA
                YALNYIQNIQGWLQGSMANVMQGNYELLYISLPLFILAYFLAHKITIAGMGEDLALNL
                GVSYNTILFLGLIIVSIITSVVIVSVGVIPFLGLIIPNLVAIYRGDNLKKNLIYIALC
                GALFLLICDIISRLVIFPFEMPLSITAGVLGSLIFIFLLLKRKTYA"
gene            139029..139967
                /gene="ceuC"
                /locus_tag="DMACINML_00404"
CDS             139029..139967
                /gene="ceuC"
                /locus_tag="DMACINML_00404"
                /inference="ab initio prediction:Prodigal:2.6"
                /inference="similar to AA sequence:RefSeq:CAL35465.1"
                /codon_start=1
                /transl_table=11
                /product="enterochelin uptake permease"
                /protein_id="Prokka:DMACINML_00404"
                /translation="MRKKILILSFITLIMALVFIFAGLKDFDEYALKNRSLQIAAIVI
                VAICIAVSTIIFQTLCNNKILTPAIIGLDSLYMLLQSALVFSLGSANLSVYKNDINFL
                ITLACMVVFSLGLYKILFSSDKSIYLIMLLGLIFGTLFSTLSSFFEILIDPDEFMVIQ
                GRMFASFDNVAFDVLALAYIVTLLSFVWIFRYMKFLDPLSLGKDLSINLGINYQKISK
                HLMIIIAILTSISTALVGPITFLGLLVVNITYELFKTSKHSILLLACVLISILALLGG
                VFFVSRVFDYNTTISVIINFLGGIYFIYLVLKGNKL"
gene            139964..140719
                /gene="ceuD"
```

```
                     /locus_tag="DMACINML_00405"
     CDS             139964..140719
                     /gene="ceuD"
                     /locus_tag="DMACINML_00405"
                     /inference="ab initio prediction:Prodigal:2.6"
                     /inference="similar to AA sequence:RefSeq:CAL35466.1"
                     /codon_start=1
                     /transl_table=11
                     /product="enterochelin uptake ATP-binding protein"
                     /protein_id="Prokka:DMACINML_00405"
                     /translation="MIRLQNITKCYDKKAIISDLSIDFHKGKITSIIGANGAGKSTLL
                     ALASRLIKPDSGRIYIDGMDLTLYKEQDLAQKISILKQQNHINLRLKVEELVAFGRFP
                     HSQGRLNANDKIKIDEALEYMGLNSLRNEFLDSLSGGQKQRAFIAMIIAQDTEFIMFD
                     EPLNNLDMKHSVQIMQLMKNLVKDFNKSIAVVLHDINFASIYSDEIIALKNGKLLKQG
                     LKDEIIHEDVLRQIYDMDIPVSQIDGKKICIYF"
     gene            140734..141723
                     /locus_tag="DMACINML_00406"
     CDS             140734..141723
                     /locus_tag="DMACINML_00406"
                     /inference="ab initio prediction:Prodigal:2.6"
                     /inference="similar to AA sequence:RefSeq:APA81643.1"
                     /codon_start=1
                     /transl_table=11
                     /product="Enterochelin uptake periplasmic binding protein"
                     /protein_id="Prokka:DMACINML_00406"
                     /translation="MKKSLVFAFFAFFLSLILTACDSKSNENNASAATKTSTTVKVIP
                     ISMSDEGDSFLVKDSLGENKIPKNPSKVVILDLGILDTFHALRLNDKVAGVPAKNLPK
                     YLQQFKDKPSVGGVQQVDFEAINALKPDLIIISGRQSKFYEKLKEIAPTMFVGLDNAN
                     FLSSFENNVLSVAKLYGLEKEASEKIADIKNEIEQAKSMVDEDKRALIVLTNSNKISA
                     FGPQSRFGIIHDVLGINAVDENVKVGTHGKSINSEFILEKNPDYLFVVDRNIIVGNKE
                     RAQGILDNALVAKTNAATNNKIIYLDPEYWYLASGNGLESLKTMILEVTNAIK"
     sig_peptide     140734..140796
                     /locus_tag="DMACINML_00406"
                     /inference="ab initio prediction:SignalP:4.1"
                     /note="predicted cleavage at residue 21"
     gene            141888..142442
                     /locus_tag="DMACINML_00407"
     CDS             141888..142442
                     /locus_tag="DMACINML_00407"
                     /inference="ab initio prediction:Prodigal:2.6"
                     /inference="similar to AA sequence:RefSeq:SQE24824.1"
                     /codon_start=1
                     /transl_table=11
                     /product="integral membrane protein"
                     /protein_id="Prokka:DMACINML_00407"
                     /translation="MINKNKLFRQIHIYVSLFFLPCAVLFALTGIVYIFGADQDTGLK
                     VENYKLVKNIEVGKEKDALIQYLQENNLKIPSNTEVIESKDKGITIGGAHYSANIALV
                     AQNEYAITLKTRSLLGDMIMLHKDKGAWYFSVLSIGFGIALFLLYISGLIITLFASKK
                     DRQKQLATLGAGVVITIFLAYLSL"
     gene            142510..142600
                     /locus_tag="DMACINML_00408"
     tRNA            142510..142600
                     /locus_tag="DMACINML_00408"
                     /product="tRNA-Ser"
```

```
                     /inference="COORDINATES: profile:Aragorn:1.2"
                     /note="tRNA-Ser(gct)"
     gene            complement(143077..144909)
                     /gene="nrfA"
                     /locus_tag="DMACINML_00409"
     CDS             complement(143077..144909)
                     /gene="nrfA"
                     /locus_tag="DMACINML_00409"
                     /EC_number="1.7.2.2"
                     /inference="ab initio prediction:Prodigal:2.6"
                     /inference="similar to AA sequence:RefSeq:CAL35469.1"
                     /codon_start=1
                     /transl_table=11
                     /product="putative periplasmic cytochrome C"
                     /protein_id="Prokka:DMACINML_00409"
                     /translation="MKKNFLRLAIVVLVLLIVGVLWLNNDINQKKEDEANKNAIAANA
                     DFSLLSDDNPNFERWGKVFPEQLKMYLTVEEEEPKATEFGGNLSYSKLIRFPQLTILW
                     AGYPFSLDFNEERGHFWVQVDQMKTARNNKDFLNAHGLKAFKGQPAACMNCHSGWTPW
                     LINNIAKGDFVAFNSTNYWTMIKNVPAVDGIVENSPEHAGPHGGKRMGVTCADCHNPN
                     DMSLRLTRPAAINALVARGYEKDPVQGVKATREEMRTLVCSQCHVEYYFKPTGEKVKV
                     MGETIVDDSSKKWWDGTQKNYDEYEFWRDGNKPKEIEANGIMLTFPWSEWKKGQPFRI
                     EMLDDYYEKVRNVFGADFTHKLTGAPIIKIQHPESELYSGGVHAANGVSCADCHMPYV
                     REGAKKISQHNITSPLRDINSACKTCHKQSEDYLRAQVRDIQNSVAHDQRTAEYAIVS
                     LIMDTKKLRDELGNMEKFQTDGKADASKISEELKEVLELHRKSQMRADFVNAENSTGF
                     HNPREASRMLLQAVDMAKTGQIKLIEIANANGINDFKTSNLGFEDIQKFNPGELYYKV
                     DVNGHKAGERYYGDEIEVNGNPPKELLEHDKNLAPYNYQVIDKK"
     gene            complement(144925..145416)
                     /gene="nrfH"
                     /locus_tag="DMACINML_00410"
     CDS             complement(144925..145416)
                     /gene="nrfH"
                     /locus_tag="DMACINML_00410"
                     /inference="ab initio prediction:Prodigal:2.6"
                     /inference="similar to AA sequence:RefSeq:CAL35470.1"
                     /codon_start=1
                     /transl_table=11
                     /product="putative periplasmic cytochrome C"
                     /protein_id="Prokka:DMACINML_00410"
                     /translation="MKKSLNLFGIFLVLLFVFFAVGFYTFYNAKGTSYLSNASESCNN
                     CHIMNEVYNEYMAGPHAQKVKGEARATCVDCHLPHGFLAKWIAKAESGLNHAYAFTFK
                     LDELPTNLSATEKSRKMIQDNCVRCHADFAQTAINATTNPHADKSLKCASCHQDVGHK
                     HGI"
     gene            145600..147684
                     /gene="ppk"
                     /locus_tag="DMACINML_00411"
     CDS             145600..147684
                     /gene="ppk"
                     /locus_tag="DMACINML_00411"
                     /EC_number="2.7.4.1"
                     /inference="ab initio prediction:Prodigal:2.6"
                     /inference="similar to AA sequence:RefSeq:CAL35471.1"
                     /codon_start=1
                     /transl_table=11
                     /product="polyphosphate kinase"
```

```
                     /protein_id="Prokka:DMACINML_00411"
                     /translation="MQTNPNMFLNRELSWLRFNSRVLDQCSRPLPLLERLKFVAIYCT
                     NLDEFYMIRVAGLKQLFSAGVNISSSDEMSPLQQLKAIRKYLHKEKDLLEHYFNEIIS
                     DLEKENLFIKSYENLDNNLKQKCDEYFFSTIFPVIVPIAVDATHPFPHLNNLSFSLAV
                     KICDKTHPELIKFGMIRIPRVLPRFYEVSANVYVPIESIVEKHTEEIFPGYKLLASAA
                     FRVTRNADMVIEEEEADDFMMILEQGLKLRRKGAFVRLQIQKGADEQIVEFLNTHMKI
                     FHKDVYEYSILLNLPSLWQIAGNKTFTHLLSPLYAPKTLPPFDENLSIFDAIDKEDIL
                     IIQPFESFDPVYKFIKEASKDPEVISIRMTLYRVEKNSNIVQALIDAASDGIQVTVMV
                     ELKARFDEENNLHWAKALENAGAHVIYGITGFKVHAKVSQVIRKKGDKLKFYMHLSTG
                     NYNASSAKIYTDVSYFTSKVEFARDTTSFFHILSGFSKNRRLQTLSMSPNQIKEKILE
                     MIALEASKGSEGVIIAKMNSLVDSDIIKALYGASIKGTQIDLIVRGICCLKPNEEFSK
                     NIRVRSIIGKYLEHARVFYFKHSEPNYFISSADWMPRNLERRLELMTPIYDERSKAKL
                     AQFLRLQLSDNLLAYELQNDGEYTKVASNEKVIDSQQILEEYVSKIYKTLKKDADQSR
                     ATYLASKLFKEN"
     gene            complement(147817..148704)
                     /locus_tag="DMACINML_00412"
     CDS             complement(147817..148704)
                     /locus_tag="DMACINML_00412"
                     /inference="ab initio prediction:Prodigal:2.6"
                     /inference="similar to AA sequence:RefSeq:CDH62331.1"
                     /codon_start=1
                     /transl_table=11
                     /product="FIG00470172: hypothetical protein"
                     /protein_id="Prokka:DMACINML_00412"
                     /translation="MALPSMGHTAPATENVKLKQSVYETIIKIGATETPILNKIGTSK
                     VTNPLTHSWLTDTFEEPKKNANLELSQFVGETKNTAQKTTNATQIFITEVMVSKALLK
                     ANQYGGNEMEYQIGKKTKEHKMDMEYALFGLGRDNDVKKSVFKDYIQAQEATSGEMAG
                     IFHYVAKGNSRFADGKRGNVLAFDEAGDWSGTATELTEDKLNQILQNIWNAGVTPKDV
                     FLGADLKGAINKFATRILGNETKLVGQVVSLETDFGTVNFHMHRLLSPKYGLGDVLIA
                     GDFQYMKHGLYIPTTFNRS"
     gene            complement(148783..149442)
                     /locus_tag="DMACINML_00413"
     CDS             complement(148783..149442)
                     /locus_tag="DMACINML_00413"
                     /inference="ab initio prediction:Prodigal:2.6"
                     /codon_start=1
                     /transl_table=11
                     /product="hypothetical protein"
                     /protein_id="Prokka:DMACINML_00413"
                     /translation="MQCFLIDELNITESEYDRFLDKHDRESSNNYNYGAIKIFSDNNT
                     IDGDKLQEYFFGTEKKYDVFISHFSQEKDKAIQLKKFLEEECKVNAFVDSYVWGNYRK
                     ILSYIKEELKADNEEYLLANLHIMLQSSLRNIIESARYFIFINSKEHCNNNTIYSPWV
                     YYELQEANRKHSNETNLNMENLCESFDISIKRDVYSFLKKFTIVDDMLYFKRHINKFF
                     I"
     gene            149529..149996
                     /locus_tag="DMACINML_00414"
     CDS             149529..149996
                     /locus_tag="DMACINML_00414"
                     /inference="ab initio prediction:Prodigal:2.6"
                     /codon_start=1
                     /transl_table=11
                     /product="hypothetical protein"
                     /protein_id="Prokka:DMACINML_00414"
                     /translation="MEKEKILIEELKMIQDIIKRMADNSFKLKKWCLTVVVVTMLFQE
```

```
                     GVNNFIPIISLFGFWFLDSYYLYIEREFRNLYNTKARQFNNNANLKQSMQSHFIFEID
                     GKFTLKKLFIVGFSKTILYFYGVILILILLVKYKKNVFNFVDNLIISIGAINT"
     gene            complement(150195..150710)
                     /locus_tag="DMACINML_00415"
     CDS             complement(150195..150710)
                     /locus_tag="DMACINML_00415"
                     /inference="ab initio prediction:Prodigal:2.6"
                     /inference="similar to AA sequence:RefSeq:ABS44288.1"
                     /note="conserved hypothetical protein"
                     /codon_start=1
                     /transl_table=11
                     /product="hypothetical protein"
                     /protein_id="Prokka:DMACINML_00415"
                     /translation="MNLNTVEYNLTRQMIINAKESTDKGIKKDIPSALRGKIEQELNI
                     QPLKEFGENYTEYYHDGKGALQKLLIEKQGQVAGAFHRKDLGDIDLVWGEVIDKVKHK
                     GYGLSHIIDEHPELDLNIIPEIIEKGEIAQRKNREKSFNIVFKDYILGLNKGFNKNGE
                     NQWIVTAFEKK"
     gene            complement(151298..151656)
                     /gene="ssrA"
                     /locus_tag="DMACINML_00416"
     tmRNA           complement(151298..151656)
                     /gene="ssrA"
                     /locus_tag="DMACINML_00416"
                     /product="transfer-messenger RNA, SsrA"
                     /inference="COORDINATES: profile:Aragorn:1.2"
     gene            complement(151657..152067)
                     /locus_tag="DMACINML_00417"
     CDS             complement(151657..152067)
                     /locus_tag="DMACINML_00417"
                     /inference="ab initio prediction:Prodigal:2.6"
                     /inference="similar to AA sequence:RefSeq:CAL35473.1"
                     /note="hypothetical protein Cj1361c"
                     /codon_start=1
                     /transl_table=11
                     /product="hypothetical protein"
                     /protein_id="Prokka:DMACINML_00417"
                     /translation="MGILTKLELDYEIDDIEKFLQFFRTMCDRFEPLIIQLGSDSVRY
                     KEAIKELETLAHNTAWAARRLNLEEVTDFCVFCEEMMAQANRFNGPASDEFTDWMLLM
                     SDQFEKYCRSYENDDSVLAVFNPLIVNVPNIISK"
     gene            152158..153168
                     /gene="ruvB"
                     /locus_tag="DMACINML_00418"
     CDS             152158..153168
                     /gene="ruvB"
                     /locus_tag="DMACINML_00418"
                     /EC_number="3.6.1.-"
                     /inference="ab initio prediction:Prodigal:2.6"
                     /inference="similar to AA sequence:RefSeq:CAL35474.1"
                     /codon_start=1
                     /transl_table=11
                     /product="holliday junction DNA helicase"
                     /protein_id="Prokka:DMACINML_00418"
                     /translation="MDRIVEIEKYSFDETYETSLRPSNFDGYIGQENIKKNLNVFISA
                     AKKRNECLDHILFSGPAGLGKTTLANIISYEMGANIKTTAAPMIEKSGDLAAILTNLS
```

```
                     EGDVLFIDEIHRLSPAIEEVLYPAMEDYRLDIIIGSGPAAQTIKIDLPKFTLIGATTR
                     AGMLSNPLRDRFGMQFRLEFYKDEELAIILQKAALKLNKTCENEAALEIAKRSRSTPR
                     IALRLLKRVRDFADVNDEETITKERAKEALNSLGVNELGFDAMDLRYLELLTEAKRKP
                     IGLSSIAAALSEDENTIEDVIEPYLLANGYIERTAKGRIASTKSFSVLKLNYEQTLFD
                     EN"
gene                 153178..154221
                     /gene="amaA"
                     /locus_tag="DMACINML_00419"
CDS                  153178..154221
                     /gene="amaA"
                     /locus_tag="DMACINML_00419"
                     /inference="ab initio prediction:Prodigal:2.6"
                     /inference="similar to AA sequence:RefSeq:CAL35475.1"
                     /codon_start=1
                     /transl_table=11
                     /product="acid membrane antigen A"
                     /protein_id="Prokka:DMACINML_00419"
                     /translation="MQTGKIFLICFTLVVLSLLLYLFKDFLLVIIIASLMAVATSNAN
                     AKFLSLTNGRKFLASMLTTACMVLLFFAPFVYAMIELAKALKNFDINLVTQTLNYVKN
                     YQFTLPESLSFLEPKIKEFLASIDLNSISKQILNYASSFTKSGAKFLIDMVLICIFYF
                     FANLYGTELVIYIKSVIPIDKQELDDILSEVGNVMAVVLYSMIIIAILQGALFSIIAM
                     FYGYDAILMGVIFAVSSLIPALGGALVYIPISLYEFASSGLGSALVIFIYSVVVISFI
                     ADTLVKPLIIKWINEKLVKTPTKINELLIFLAMIAGISTFGFWGIILGPAILTFFIST
                     IRLYIILKDKNLI"
gene                 complement(154268..155659)
                     /gene="fumC"
                     /locus_tag="DMACINML_00420"
CDS                  complement(154268..155659)
                     /gene="fumC"
                     /locus_tag="DMACINML_00420"
                     /EC_number="4.2.1.2"
                     /inference="ab initio prediction:Prodigal:2.6"
                     /inference="similar to AA sequence:RefSeq:CAL35476.1"
                     /codon_start=1
                     /transl_table=11
                     /product="fumarate hydratase"
                     /protein_id="Prokka:DMACINML_00420"
                     /translation="MEYRIEHDTMGEIKVPNDKYWGAQTERSFENFKIGCEKMPKVLI
                     YAFANLKKSLALVNNKLGKLDDAKKSAIVQACDEIIAGKFDDNFPLAIWQTGSGTQSN
                     MNMNEVIANRATEIMGGDFRKEKLVHPNDHVNMSQSSNDTFPTAMSIVAVEQVEKKLI
                     PALDELIATFEKKVKEFEGIIKIGRTHLQDATPLTLAQEFSGYLSMLLHSKEQIITSL
                     PTLRELAIGGTAVGTGLNAHPELSEKVSEELSKLIGTKFVSSPNKFHALTSHDAINFT
                     HGAMKGLAANLMKIANDIRWLASGPRCGLGELNIPENEPGSSIMPGKVNPTQCEAITM
                     VAVQVMGNDAAIGFAASQGNFELNVFKPVIIYNFLQSLDLLADSMHSFNIHCAVGIEP
                     NREKIEYNLHNSLMLVTALNPHIGYENAAKVAKNAHKKGISLKESAMELGLVSEEDFA
                     KFVDPAKMIGPKA"
gene                 complement(155794..157590)
                     /gene="glmS"
                     /locus_tag="DMACINML_00421"
CDS                  complement(155794..157590)
                     /gene="glmS"
                     /locus_tag="DMACINML_00421"
                     /EC_number="2.6.1.16"
                     /inference="ab initio prediction:Prodigal:2.6"
```

```
                     /inference="similar to AA sequence:RefSeq:CAL35478.1"
                     /codon_start=1
                     /transl_table=11
                     /product="glucosamine--fructose-6-phosphate
                     aminotransferase (isomerizing)"
                     /protein_id="Prokka:DMACINML_00421"
                     /translation="MCGIVGYIGKNEKKHTILSGLKELEYRGYDSAGLAVMKNGELDF
                     FKAVGKLENLANKCANFTSEEFGFAIGHTRWATHGKPTEINAHPHLGQYSCVIHNGII
                     ENYKEIKNKLESQGVSFLSQTDTEVIVQLFEFYAEKMEVFEAWKKTIEELRGAFATLL
                     VSKKDPTRVFFAKNAAPLIVGKSKNREFYFASADTPLIGLCDEVIYLEDLSLGYASEN
                     ELVIYENNQLKQNSFTKLSGDKAYAKKDGFRFFMEKEIYEQSRVMSEVLMGRINGDEV
                     VFDELENENLSTVDEITLCACGTSYHAAMASAYLFERLAKVKAKVEIASEFRYREAIV
                     KPNSLFIVISQSGETADTLEALKIAKEQGAKTFAICNVDNSNIVRLAHLSLLTRAGIE
                     KGVASTKAFATQVLTLWMLAIFIAQKKKIDMSAEIKALLHTPNCVAVSTKLHEKIHRL
                     SKRYLDGHGFFFIGRDVFYPLALEGALKLKELSYLHAEGYPAGEMKHGPIALADSKLY
                     TIALMPKTLLYEKTKSNVEELIARDSTVLCVSPLDFDLSDDFIKTNEQDHYMCEFFEM
                     MVITQLLAMEISIRLGNDVDMPRNLAKSVTVE"
     gene            complement(157594..159942)
                     /locus_tag="DMACINML_00422"
     CDS             complement(157594..159942)
                     /locus_tag="DMACINML_00422"
                     /inference="ab initio prediction:Prodigal:2.6"
                     /inference="similar to AA sequence:RefSeq:CAL35479.1"
                     /codon_start=1
                     /transl_table=11
                     /product="putative nucleotidyltransferase"
                     /protein_id="Prokka:DMACINML_00422"
                     /translation="MLNKELELFKKNLNTYAQNYRKFFLKQGSFSLYHSKNMDNFLKQ
                     TYDIVLKECFENFLPASDNIPFCVLASKGYAKNNLCANESISLIFVYKDIKAYHLKPM
                     IKAFIEILNDVHLQIDYVVLELNGLYNASNELKTTIIGARFICGSKILFKSVKEKFDS
                     ILRANKNEFAQILLANFKDFNLPFIKQEFNIKKDFGGLDHLRALESLLILFKNSPKNY
                     ALNFIDEKNLSELRLAADFLLSLKSAINLQSNKDQDEFLFSNIHEIAELMYRKGKKNL
                     DAEKILVQKALQSMHTIGFYTHFLAYKIQNELQNLTQKQYQFKNLAEALTFLLDLEDQ
                     DIVFDLDLVFALKNLSYGKKDLEKALALFEKIFFKRHSFCILKLLLDSGILKELCKPF
                     GMVRFLSDEEGDYSFDEQAFLLLKEFEKYEDELESLKNLNADEKMILKLVILLSAINN
                     ENEISLASIYRAYCVKFNLKNDIFELGLRIFKNHNALKELAEKEDVYNPIIICALLSK
                     VENLKTLELLHTLTWLKAKALNRNPFFYKVIDRILENAKQGFDDENLLDETARRVKKE
                     LTLKRTKLFLDQNAILQDKITHIKSNLFIIKNTFEDIVEIARFAKENDFKFWFSNSTN
                     LSLQIVAFKDFKIEIVLTALANLNLVFMNLFELFDDKIYLRFEYDNIITDEQKIKLCE
                     LLNSNLSGFSSKKIKKPIIKKDELKLDLNYSKIYAKLSLNTKDQQGLMAYMMNIFNEL
                     GLVLSAAKIQTIRQRTRNTFIFQKNDNLEKNEEKLLNSLISE"
     gene            160085..161149
                     /locus_tag="DMACINML_00423"
     CDS             160085..161149
                     /locus_tag="DMACINML_00423"
                     /inference="ab initio prediction:Prodigal:2.6"
                     /inference="similar to AA sequence:RefSeq:CAL35480.1"
                     /codon_start=1
                     /transl_table=11
                     /product="putative radical SAM domain protein"
                     /protein_id="Prokka:DMACINML_00423"
                     /translation="MKNLLEKLENEERLNENEANALWDLDLFTLAKFAHKKRTKLHGK
                     KVYFNINRHINPTNICADTCKFCAFSAHRKNPNPYLMSHEEIMKIVDDTITRGTKEVH
                     IVSAHNKDTSWQWYLEIFKMIKEKYPQMHIKAMTAAEIDFLHRRFKMSYEEVIEKMLE
```

```
                     YGVDSMPGGGAEIFDEEIRKKICHGKVSSENWLKIHKLWHDKGRQSNATMLFGHIEER
                     HHRIDHMLRLRALQDESGGFNAFIPLVWQRDNSYIQTDKIMDSEEILKTIAISRLVLD
                     NIKNIKAYWATMTLNLAMVAQEFGANDLDGTIEKESIQSAGGAKSAKGTNLKTFIEMI
                     KTANLTPVERDSLYNELQTY"
     gene            161160..162479
                     /locus_tag="DMACINML_00424"
     CDS             161160..162479
                     /locus_tag="DMACINML_00424"
                     /inference="ab initio prediction:Prodigal:2.6"
                     /inference="similar to AA sequence:RefSeq:CAL35481.1"
                     /codon_start=1
                     /transl_table=11
                     /product="putative permease"
                     /protein_id="Prokka:DMACINML_00424"
                     /translation="MDFFKLKENGTNVRTEVVAGLTTFLAMVYIIPVNSSIVGNTGMP
                     IEALITATALITIVATTFNAFFANTPVAMSVGMGLNAYFTFAVCLGQKIPWQSALGAV
                     FISSVIFLILSFTSFRLWVIRNIPKDLRLAICAGIGCFIAFLGLSQMGIISSNKDTLV
                     SIGNFKNPHVLFGIFTLILIIFFWAIKLRGAFILGVLISSIIAWIFGIDNASFPEQIF
                     SLPNFSNENGLGAIFLELDIKSALNIAMIPIILTFFITQLFDSIGTITGIGERGKIFD
                     DAKNGEKKLGKTLMADATGSALGALGGTSTVTAFVESTTGVESGGRTGFTALVVAICF
                     AFTLFLLPLFKAIPANAIYPVLVMVGILMFMEVKNIDFKDSAIAVASFFTIIMMPFTY
                     SITTGFAFGFLSYLLVRIFKREWDKINLGIIVLSLISLGNFLLIALQ"
     gene            162497..162940
                     /locus_tag="DMACINML_00425"
     CDS             162497..162940
                     /locus_tag="DMACINML_00425"
                     /inference="ab initio prediction:Prodigal:2.6"
                     /inference="similar to AA sequence:RefSeq:CAL35482.1"
                     /codon_start=1
                     /transl_table=11
                     /product="putative nucleotide phosphoribosyltransferase"
                     /protein_id="Prokka:DMACINML_00425"
                     /translation="MIFYSYEEFKEDVKILAKEIKEDFNPDALLAIARGGMSLGHSLA
                     VALQTRNLFALNSIHYDDTKKLDTIEIFNIPDLSKHKKILLIDDIVDSGESLAEIKKV
                     LLEKFPHIELKIATIFYKKTALLEPDFKIKEAKEWVEFYWDIHID"
     gene            162982..163680
                     /locus_tag="DMACINML_00426"
     CDS             162982..163680
                     /locus_tag="DMACINML_00426"
                     /inference="ab initio prediction:Prodigal:2.6"
                     /inference="similar to AA sequence:RefSeq:ABS44654.1"
                     /codon_start=1
                     /transl_table=11
                     /product="lipoprotein, VacJ family"
                     /protein_id="Prokka:DMACINML_00426"
                     /translation="MRLKLISCILLFSTIFAFANELDLNDFEEEYQSYKVNDPLSGYN
                     KAMTSFNVTLYDYGFRPILKGYNAITPEFIRTGVRNFFDNLFSPLRFVGNVLQFKFEE
                     AGEEFKRFSANTIMGFGGIMDPASKMGLKKHPADLGTVLAHWGVGSGFHIVLPVLGPS
                     NLRDALTLPATWYASPTAYIDPTWASIAISAYGFGNELSFRLDEIDEIYYNTPNLYPF
                     IRDAYEQRRNELSK"
     sig_peptide     162982..163041
                     /locus_tag="DMACINML_00426"
                     /inference="ab initio prediction:SignalP:4.1"
                     /note="predicted cleavage at residue 20"
```

```
gene            163690..164259
                /locus_tag="DMACINML_00427"
CDS             163690..164259
                /locus_tag="DMACINML_00427"
                /inference="ab initio prediction:Prodigal:2.6"
                /inference="similar to AA sequence:RefSeq:CAL35484.1"
                /codon_start=1
                /transl_table=11
                /product="putative periplasmic protein"
                /protein_id="Prokka:DMACINML_00427"
                /translation="MKKIFLILTLSLNIFALQLDEISGTMQKNINKSLQLLQNNKEDK
                KQAADGIFLIFDHIIDYKLMAQLSLSKDYSKLNPKEQEKFASAFEASLKKSFTDKLNL
                YKDQVLMVKNGELKNAKRYFLTSSMIVDGEEKNIIFKFYNNNNDWLIYDIDVLGVSIV
                QTYRSQFGDILANQDFDTLLQKLENITIE"
gene            164259..166721
                /locus_tag="DMACINML_00428"
CDS             164259..166721
                /locus_tag="DMACINML_00428"
                /inference="ab initio prediction:Prodigal:2.6"
                /inference="similar to AA sequence:RefSeq:CAL35485.1"
                /codon_start=1
                /transl_table=11
                /product="putative ntegral membrane protein"
                /protein_id="Prokka:DMACINML_00428"
                /translation="MFAKFLKIFIYHPKSTFFTTLFICLFLSFFASKLSVDASAESLL
                LEDDKDLKIFRELSKHYKSDNFLLLAFKPEDENPFSNKNLNKLKELHQELEQVSLVER
                VFSIINAPLLQSSENTDLKELLKNIPNIESKDINLTKAQNEILNSPFYKNNIISKDGK
                TTGLIIYLKPDTIYNELIEKRDLSTDDNEKEQIRLAIKKHQEQQKVITKQGLDTIKNI
                IKKYEKDEDRFYLGGVSMIADDMITYIKSDLMLYGISLVFLLACALYCFFRSWRFVFL
                PLFICFISLSAASGVFALLNFQITVISSNYVALVLIITLSVVIHLITHFIEISKLYPK
                AKIQRLVLQTLLAKANPSFYAIITTMIGFFSLMLSNIEPIIKLGIMMSIGIGLALIFS
                YLFLASALVLLKPKKYYKKEFKFNFLAFCAKTSLDPKKRPIIYILSIVTIILALVGIS
                KLRVENSFVNYFKDGSEIKKGLLVIDKILGGTLPLDIIIRFPNNENNSNLNDVFDSFE
                SEFDALATKDTYWFNSQKTRIAKKVHEFLENKEFIGSVLSLNSLLSLGKNINDGKDLD
                DFTLAFLNENLPLELKQELLSPFVNIEENELRFSIRIIDSDPKLRRNEFLTNLKQGLE
                TLLKNDGVEIQITGIMLLYNNMLQSLFSSQFDTLIFVILVIFVLFIIIFRSLKFSIIA
                LLVNIIPLSMVFALMGILGIPLDMMSITIAAIAIGIGVDDTIHYIYRFKEEIKTKSVE
                EAILASHLSIGSALYYTTIGIVLGFSVMMSSNFIPTIYFGVLTVFVMILLLSGSLFLL
                PSLLITIYTKKTKALNPYKVHK"
gene            complement(166755..167363)
                /locus_tag="DMACINML_00429"
CDS             complement(166755..167363)
                /locus_tag="DMACINML_00429"
                /inference="ab initio prediction:Prodigal:2.6"
                /inference="similar to AA sequence:RefSeq:CAL35486.1"
                /note="conserved hypothetical protein Cj1374c"
                /codon_start=1
                /transl_table=11
                /product="hypothetical protein"
                /protein_id="Prokka:DMACINML_00429"
                /translation="MKILLATSNKHKVIELKELLKEFEIYAFDEILTAFEIEENGNSF
                KENALIKARAVFKALSDKQKNDFIVLSDDSGICVDVLGGKPGIYSARFSGKGDDKSNR
                DELVKQMGALGFKTSKAHYVAAIALVSLGGEWTTHGSMHGDVIDIERGENGFGYDSLF
                IPKGFDKTLAELSNNEKNQLSHRFKALELARIILKVLNKGKE"
```

```
gene            complement(167335..167790)
                /locus_tag="DMACINML_00430"
CDS             complement(167335..167790)
                /locus_tag="DMACINML_00430"
                /inference="ab initio prediction:Prodigal:2.6"
                /inference="similar to AA sequence:RefSeq:ADN90629.1"
                /codon_start=1
                /transl_table=11
                /product="Integral membrane protein"
                /protein_id="Prokka:DMACINML_00430"
                /translation="MNRFQTFSLMTEGKVNSELLMDYKDKLETLSDEAMFYFCSLELK
                NPIIGLILGVTPAFILSGLTFDRFYKGDMGLGFVKMAMWAFIFIGLLIAGFFDSSSML
                VVWIFNIIALFIWNILDFFLVWQGIKNDNLAKIIQFLEQDNENFISNKQ"
gene            167933..169228
                /locus_tag="DMACINML_00431"
CDS             167933..169228
                /locus_tag="DMACINML_00431"
                /inference="ab initio prediction:Prodigal:2.6"
                /inference="similar to AA sequence:RefSeq:AAW36002.1"
                /codon_start=1
                /transl_table=11
                /product="multidrug resistance efflux transporter,
                putative"
                /protein_id="Prokka:DMACINML_00431"
                /translation="MLNNVLPLSFIVGTRFFGLFIVLPVLSFYALELQGANEFLVGLL
                VGVYALTQMALQMPFGILSDKIGRKKTMLIGLIIFIIGSLICSWTDNIYTMLVGRMLQ
                GAGAIGAVATAMISDFITEENRGKAMAIMGSFIGLSFAASMVISPLMSAKWGLSSLFD
                LSAALSLLCIILLYTVVPKENKITHENTKTPFLHLIKQKNLALMNFTNFMQKMLMSIA
                FLSIPIILVKHLGFDEHKLWIVYTASMIAGFIAMGFSGSLGEKRGLAKQILLLGIAFF
                ILSYIFFVFSNSITFFIVAVVIFFIGFNLHEPIMQSCASKFCKVHEKGAALGLFNAFG
                YGGSFIGGVIGGIFLHLNKLELLAIILVALSIVWFIALLYLKNPSEFKNIYLPLETTL
                NFSEFNKNLGVVDIYKNSKNLVIKFDSKLTNKEELESKI"
gene            complement(169230..169757)
                /locus_tag="DMACINML_00432"
CDS             complement(169230..169757)
                /locus_tag="DMACINML_00432"
                /inference="ab initio prediction:Prodigal:2.6"
                /inference="similar to AA sequence:RefSeq:EAQ72915.1"
                /codon_start=1
                /transl_table=11
                /product="lipoprotein, putative"
                /protein_id="Prokka:DMACINML_00432"
                /translation="MRKIHLISALFLALFLSACAKSAFVQSSIQTSNEGIFLKAKQGQ
                SFSITLQNPSKIQSALKDELALRLKNLGLKEVSLNADYEILINLVDFKKHSYAQRITS
                SGRFFYDFDPFENSGEWYIENYYTMQVNIQISSKNTPQKTSLFARTAYLSDKKRCQLS
                LENKIINQISSFFYF"
sig_peptide     complement(169695..169757)
                /locus_tag="DMACINML_00432"
                /inference="ab initio prediction:SignalP:4.1"
                /note="predicted cleavage at residue 21"
gene            complement(169757..170467)
                /locus_tag="DMACINML_00433"
CDS             complement(169757..170467)
                /locus_tag="DMACINML_00433"
```

```
                     /inference="ab initio prediction:Prodigal:2.6"
                     /inference="similar to AA sequence:RefSeq:CAL35492.1"
                     /codon_start=1
                     /transl_table=11
                     /product="putative periplasmic protein"
                     /protein_id="Prokka:DMACINML_00433"
                     /translation="MKKISLILACSMSLFAASNSEISDFYSKSIKAQFPNATVSIGNR
                     QKVGNTGFESVIVTITLEGQKQEQILFTKDNLIVPDIIDLKTKTSYAQEYEMKKFQEA
                     RANFTKNVKPVVQEETMLIALGDKSKPAIYVFSDPECPYCRDHLAQIKDELKNYQVNY
                     ILTPVHGKSAFEKSALIYKESKKAKSDDEKIAILNKYYDANIKSYPKVSDSELKEVFS
                     LYEKYRSLGLSATPTIIK"
     sig_peptide     complement(170417..170467)
                     /locus_tag="DMACINML_00433"
                     /inference="ab initio prediction:SignalP:4.1"
                     /note="predicted cleavage at residue 17"
     gene            complement(170516..172315)
                     /gene="selB"
                     /locus_tag="DMACINML_00434"
     CDS             complement(170516..172315)
                     /gene="selB"
                     /locus_tag="DMACINML_00434"
                     /inference="ab initio prediction:Prodigal:2.6"
                     /inference="similar to AA sequence:RefSeq:CAL35491.1"
                     /codon_start=1
                     /transl_table=11
                     /product="putative selenocysteine-specific elongation
                     factor"
                     /protein_id="Prokka:DMACINML_00434"
                     /translation="MNSLLIGTAGHIDHGKTSLIKAINGFEGDSLKEEKERQITINLS
                     FSNLNYKQKHLSFIDVPGHKDLVKTMISGAFGFSACLFVVDINEGLKEQSIEHLEVLE
                     LLGVKDIILVLSKCDLCENPKEQEQKILNSLEFTPMKVFHTSIKDQESIERLKNYLLN
                     LECKNANENLVFRYYIDRVFSLKGIGTIVTGSLNEGKISLNEKIICLDNQKELVVKNI
                     QNHESNFDEISAFNRVALSLNCDYKELKKGYVLSKKGFFKGFKECDVLIKAKRLKNEK
                     MLFCVGTKSIECKISILKELENNEFFAHLEFEKNLFLCFNEKFILLQNNRVVGGGEVL
                     NPVSEPLKKEQKNKFLMLLKNKELKKAFEFLKNTHKYGFGLLSSYQRFKLSHEEALNL
                     AKELSGVFIDEKNLNIYNLLALDELKKFISFILEKNPYAMLSATSLALRLPWASAEFC
                     EFGLQNLSNLLEFEKGVYFKKGIDYEKLRERNNDELYNILKKQGIKPQAPYNLYEFLE
                     LDRKSGDEILKKLTKQNRVIRLSHNLFVEKNALDKLMQECLEILKSQSLDIQSMKERF
                     NLSRKYAIAYLEYLDKDERVQKKDEKRFLKTNI"
     gene            complement(172312..173634)
                     /gene="selA"
                     /locus_tag="DMACINML_00435"
     CDS             complement(172312..173634)
                     /gene="selA"
                     /locus_tag="DMACINML_00435"
                     /EC_number="2.9.1.1"
                     /inference="ab initio prediction:Prodigal:2.6"
                     /inference="similar to AA sequence:RefSeq:CAL35490.1"
                     /codon_start=1
                     /transl_table=11
                     /product="L-seryl-tRNA(SeC) selenium transferase"
                     /protein_id="Prokka:DMACINML_00435"
                     /translation="MNKFRTFPQIGSLIDDESLKEYPFYLRSHFCKSVVAKLKANITE
                     NSWDKNTILEKIQQEIQKHLLKDLQSVINATGVVIHTNLGRSVIDESIFEKSKDVLCN
```

```
                     YANIEFDLENGKRGSRYALVLEKLKMLFECEDALVVNNNAAAVFLVLHSLCFDKELIT
                     SRGELVEIGGSFRVSEVIKAAGVKLCEVGTSNKTHLKDYEKAINENTKMLLKTHKSNF
                     ALIGFHSEVGIKDLGNLAKEKGLLSYYDLGSGWCENLNPKLIKNEPKVKDVIKHCDIL
                     SFSGDKLFGSVQAGIILGKKELIDKIKENQLLRMLRVDKITLAFLNESLKAYLEKDYE
                     KITSLKLLNDEITYIKEKALRVQNEIQVKSELKMSKSLVGGGSMPDKTLETCILAFDG
                     NALKLQEKFREKNIIGRIENEAFVLDFRTIREKDLSKLIQIINTMENL"
gene                 173704..175365
                     /locus_tag="DMACINML_00436"
CDS                  173704..175365
                     /locus_tag="DMACINML_00436"
                     /inference="ab initio prediction:Prodigal:2.6"
                     /inference="similar to AA sequence:RefSeq:AAW36003.1"
                     /codon_start=1
                     /transl_table=11
                     /product="iron-sulfur cluster-binding domain protein"
                     /protein_id="Prokka:DMACINML_00436"
                     /translation="MKDFVFIQNENLIPLPDEINIWGKCKDEEVLISNEKAQKAQIYA
                     PEINFYLKNSQDEILERSKNILKLYEARANVYDLGLDLEQNKEVKNRIILVDSDDAMA
                     DFFKKQNFKIISLKNEEILAVFGSIGELCAVILNQGEEVEIDFDILLFNTNSNEIRKD
                     FTRQSGCYNISNFKDQESLLEFLQDKSPKYTYKTYITYDSSVCQYHERRSEHCAKCAE
                     ICPTVAILKDDESKHLEFSQVDCLGCGGCISVCPSGSLDYAPMPRESFFELCEFYKEK
                     KILIIPKKMSLENLNIKLPKDVLPFMIEGEKWLSSMHFLTLLQSSGANLVFYTDFISR
                     GSAEAIDLLNTFFERKFQKEAIFTAKDEKELQNALEKQEFIENLSFDFHNNTLTTREN
                     FAKRMQNLIQNDDFGVKESGEWLRYGKIEINANTCTLCLSCVGACNVGALIADTKENA
                     LKFNASLCTTCGYCEVSCAEKDTLKLTRSGMEFNPSYFEYQTMAKDELFACIECGKEF
                     ATKKAVEKIANLMKPKFGNDESKIKTLYCCADCKAKVMILAMQKR"
gene                 complement(175528..176019)
                     /gene="fldA"
                     /locus_tag="DMACINML_00437"
CDS                  complement(175528..176019)
                     /gene="fldA"
                     /locus_tag="DMACINML_00437"
                     /inference="ab initio prediction:Prodigal:2.6"
                     /inference="similar to AA sequence:RefSeq:CAL35494.1"
                     /codon_start=1
                     /transl_table=11
                     /product="flavodoxin"
                     /protein_id="Prokka:DMACINML_00437"
                     /translation="MSVAVIYGSAMGNTEGAANTIASKLGISDVLNVADIDAAKINSY
                     DKLICGTSTWGSGDLQDDWDGFDFSGLSLGGKTVAVFGMGDSESYSDTFCGGMGKLAE
                     NLKNAGANLVGEVSTDGYTFESSDAVVDGKFVGLALDNDNQEDQTEARIDAWVEQIKA
                     HFA"
gene                 complement(176019..176714)
                     /locus_tag="DMACINML_00438"
CDS                  complement(176019..176714)
                     /locus_tag="DMACINML_00438"
                     /inference="ab initio prediction:Prodigal:2.6"
                     /inference="similar to AA sequence:RefSeq:CAL35495.1"
                     /note="hypothetical protein Cj1383c"
                     /codon_start=1
                     /transl_table=11
                     /product="hypothetical protein"
                     /protein_id="Prokka:DMACINML_00438"
                     /translation="MLFGFEDKREFIPKVYSSLCKQELVKTFLIQYNASVDSVLRIPL
```

```
                     SYARSAKNLKKPFQNFLQDAIHMPFGKIKNIDKNLTLNISYFQKRKGLIFKTKIFQNV
                     DILRLLRAYFRGICFDSQVLFDFYVYDKISHQNQNRSIVQNDNLIIIDNKIAVLPLCK
                     EVDLQNLNIDSEIQKISKFIYQNQFEQICIVCPRNKNFTHFVQIKHFLCDLNKTMLKL
                     VPYSITNKLIRRK"
     gene            complement(176714..177031)
                     /locus_tag="DMACINML_00439"
     CDS             complement(176714..177031)
                     /locus_tag="DMACINML_00439"
                     /inference="ab initio prediction:Prodigal:2.6"
                     /inference="similar to AA sequence:RefSeq:CAL35496.1"
                     /note="hypothetical protein Cj1384c"
                     /codon_start=1
                     /transl_table=11
                     /product="hypothetical protein"
                     /protein_id="Prokka:DMACINML_00439"
                     /translation="MSVLVIGADEITPIKAVLYDLGAKKIEHWDARNENRVNRKPIPC
                     DTECIVMLTSFLNHNTMKKIKNEAKKRKIPLVCAKRSVSCVYCEYCKIFNLDKEFSCS
                     KAE"
     gene            177222..178646
                     /gene="katA"
                     /locus_tag="DMACINML_00440"
     CDS             177222..178646
                     /gene="katA"
                     /locus_tag="DMACINML_00440"
                     /EC_number="1.11.1.6"
                     /inference="ab initio prediction:Prodigal:2.6"
                     /inference="similar to AA sequence:RefSeq:CAL35497.1"
                     /codon_start=1
                     /transl_table=11
                     /product="catalase"
                     /protein_id="Prokka:DMACINML_00440"
                     /translation="MKKLTNDFGNIIADNQNSLSAGAKGPLLMQDYLLLEKLAHQNRE
                     RIPERTVHAKGSAAYGEIKITADLSAYTKAKIFQKGEVTPLFLRFSTVAGEAGAADAE
                     RDVRGFAIKFYTKEGNWDLVGNNTPTFFIRDAYKFPDFIHTQKRDPRTHLRSNNAAWD
                     FWSLCPESLHQVTILMSDRGIPASYRHMHGFGSHTYSFINDKNERFWVKFHFKTLQGI
                     KNLSNKEAAELIAKDRESHQRDLYNAIENKDFPKWKVQVQILAEKDADKLGFNPFDLT
                     KIWPHSIVPLMDIGEMILNQNPQNYFNEVEQAAFSPSNIVPGIGFSPDKMLQARIFSY
                     PDAQRYRIGTNYHLLPVNRARSEVNTYNVAGAMNFDSYKNGAAYYEPNSYEESPKEDK
                     SYLEPDLVLEGNAQRYAPLDNDFYTQPRALFNIMNQNQKEQLFENIAASMEGVEEKII
                     ARALGHFEKISPDYAQGIKKALGK"
     gene            178704..179171
                     /locus_tag="DMACINML_00441"
     CDS             178704..179171
                     /locus_tag="DMACINML_00441"
                     /inference="ab initio prediction:Prodigal:2.6"
                     /inference="similar to AA sequence:RefSeq:CAL35498.1"
                     /codon_start=1
                     /transl_table=11
                     /product="ankyrin-repeat containing protein"
                     /protein_id="Prokka:DMACINML_00441"
                     /translation="MITLSLEEEKRFAELCKMAFNFARNNEYENLKIMIEAGLNVNLK
                     THKGDTLLMLAAYNNSYECAKMLLEKGALVDEKNDRGQTPLAGVCFKGYLPMCELLVK
                     HGANIDENNGLGMTPYSFAIMFGRKDVAEFLLKSSKKSLSKRLSLGILKLFKK"
     gene            complement(179175..179843)
```

```
                     /locus_tag="DMACINML_00442"
     CDS             complement(179175..179843)
                     /locus_tag="DMACINML_00442"
                     /inference="ab initio prediction:Prodigal:2.6"
                     /inference="similar to AA sequence:RefSeq:CAL35499.1"
                     /codon_start=1
                     /transl_table=11
                     /product="helix-turn-helix containing protein"
                     /protein_id="Prokka:DMACINML_00442"
                     /translation="MDEKQIEQFVKLTNFLGQVLGKQYEVVFHVLSKEGAYIAAIANS
                     HVSGRTLESPLSAFASELVQNKAYLKEDFLCDYKALIGKSKLVRGSTFFIKNSDELVG
                     ILCINHDTSMMRDLVCKIIDIEKLDDMGEFLNQNKENNTSVDSLGNIETLSHSIEEIL
                     AQSIDMNYLNSDYQLSIAQKEEITKTLYQKGIFNIKGSVPIVARLLKISEPSVYRYLK
                     NFKS"
     gene            180017..180379
                     /locus_tag="DMACINML_00443"
     CDS             180017..180379
                     /locus_tag="DMACINML_00443"
                     /inference="ab initio prediction:Prodigal:2.6"
                     /inference="similar to AA sequence:RefSeq:CAL35500.1"
                     /codon_start=1
                     /transl_table=11
                     /product="putative endoribonuclease L-PSP"
                     /protein_id="Prokka:DMACINML_00443"
                     /translation="MSNYPKAIGPYSAYREANGFLFISGQIPINPASGEIESSDIKEQ
                     TKQSLKNIGAILEENGISYDQVIKTTCLLADISDFAAFNEVYAQFFKSPYPARSAFAV
                     KDIPKGAKVEIEVIAKKG"
     gene            180383..181909
                     /locus_tag="DMACINML_00444"
     CDS             180383..181909
                     /locus_tag="DMACINML_00444"
                     /inference="ab initio prediction:Prodigal:2.6"
                     /inference="similar to AA sequence:RefSeq:EAQ72574.1"
                     /codon_start=1
                     /transl_table=11
                     /product="cryptic C4-dicarboxylate transporter DcuD,
                     authentic frameshift"
                     /protein_id="Prokka:DMACINML_00444"
                     /translation="MLGLAFSLFSVFLLVFMLYKKINAHMALLLSGLLLLSITSLANF
                     LIHTFPENYVLLNYPNIYAFLNALDFYQLKHAILEKDSLNLGFFDIFQVFNQTMSNTL
                     AGLGLTLMAIAGFSAYMDHVGASYALFKVFEKPLKMVKSPYILLLVAYFIVQFLVLFI
                     PSHAGLGLLLMVTMYPILVRSGVSKLSALSVIGICQYIDHGPGSGNVIMASEKAEIDP
                     AIYFVHYQLPTTLPIIVAVGIAIYFCNKFFDKKDNFVFNAQEVEKELSENKDKENELK
                     KPPKIYAILPVIPLVLILGFSSVLDSILVLMGISNEEEVKAAASSAIKMNVPVAMVIS
                     TFVAIAFEMIRYKSVVETLNSIMIFFKGMGHLFVITVSLIVCGQVFASGLLSVGFVDT
                     LIDFCKNAGFGVLAIIIAVSILLAVCAFLMGSGNAAFFSFAPLIPDIAKRFGIETITM
                     IAPIQIMTGFGRCVSPIAPAILAISAIAKVSPFAVVKRTAIPMLVAAIVNIIMTYIYL
                     "
     gene            181921..183084
                     /gene="metC"
                     /locus_tag="DMACINML_00445"
     CDS             181921..183084
                     /gene="metC"
                     /locus_tag="DMACINML_00445"
```

```
                     /EC_number="4.4.1.8"
                     /inference="ab initio prediction:Prodigal:2.6"
                     /inference="similar to AA sequence:RefSeq:AAW36015.1"
                     /codon_start=1
                     /transl_table=11
                     /product="cystathionine beta-lyase"
                     /protein_id="Prokka:DMACINML_00445"
                     /translation="MNSKTKLIHCGRGDQNVEVRSVNPTLMRASTILFKDHATWQKYR
                     ELRKTDRVLSYGARGTTTNFELEKLICTLEGGYRAQLFPTGLAALAMVLLNYASKDAH
                     FLITDAIYGPVRTICELFLEKMGVEIDFLKADASDVEEKIKPNTKLILCESPGSILYE
                     IIDLPKLCKIAHSHNIPVAIDNTYSSGYFLNPLELGVDISVIAATKYLSGHSDVTMGV
                     VVINEKEWKNFDKLPEALGFTTSPDDAYLVLRGMRTLDVRMKAHEKSADEIVEFLQTR
                     KEIKTIFYPKLKTHPNHEIFMRDHKGANGMVTIEFAEGYSKEDAIKFVDELEYFSIGA
                     SWGGYESLATVTTPPRTATDWSKRGPFVRFHIGLEDTKDLIADLTKAFDSIKK"
     gene            183094..184461
                     /locus_tag="DMACINML_00446"
     CDS             183094..184461
                     /locus_tag="DMACINML_00446"
                     /inference="ab initio prediction:Prodigal:2.6"
                     /inference="similar to AA sequence:RefSeq:CAL35504.1"
                     /codon_start=1
                     /transl_table=11
                     /product="putative fumarate lyase"
                     /protein_id="Prokka:DMACINML_00446"
                     /translation="MTGISVFDHKLLADSWSTQDMRAVFCEENRIQKWLDVEAALAKA
                     QAKLNIIPQEAADEIAKKAYYKFMDMDFIFNEFKKTKHPLVPTVRGLEKACDNNLGEY
                     VHFGVTTQDIIDTGLVLQFKEAMTLVKSELKAIAKALAKLAKTHKNTAMMGRTLALQA
                     LPITFGHKVAIWLSELDRHFERILELEKRLYVGSIVGAVGTKASLSDECNEVEKLTLE
                     NLGLEVPNISWQPARDRFIELGFVLGNINATFNKIAHQLLILSHNEIDEVAEPFGKGQ
                     VGSSTMPHKRNPAVSENAVTVSNAFKANLAILSDIERHEHERDGQVWKMEWKLLPEMF
                     LMLSVVLANMKFALSDLEVKKDKMLKNLDTLKGFVLAERVMFALSDHYGKQHAHEIVY
                     ENAMLGIEQQKTFKEVLLADTRVSQVLKEKDINALLDATSYVGYAPKLVDEFLEKIQN
                     HAILQ"
     gene            184470..185810
                     /locus_tag="DMACINML_00447"
     CDS             184470..185810
                     /locus_tag="DMACINML_00447"
                     /inference="ab initio prediction:Prodigal:2.6"
                     /inference="similar to AA sequence:RefSeq:AAW36017.1"
                     /codon_start=1
                     /transl_table=11
                     /product="MmgE/PrpD family protein"
                     /protein_id="Prokka:DMACINML_00447"
                     /translation="MLYSEILADFIFNLQYENIPDAVVQRAKELMLDSLGTAIAASKE
                     ECVLNAFKAFENLSTEKNTPVWVNDKKLDPIYAAMLNGIASHALDFDDTHTEAILHAS
                     AILTPLCLNYGFHISKNAKKIIKAFIVGWEVAARVGIASKGTFHKRGFHTTAIAGIFG
                     SASASAVLLDLNKQQIINALGLAGSFASGINEFLSNGSNSKVLHIANAIKNGILVANF
                     AKNNMSGPLSIFEGRDNIFKCFGIEQECDKTELDKGLGEIWQSMQVSIKPYPSCHFAH
                     GLIDCAIALKNDGLKADEIKSIRCFVDEVPISFICDPLEAKYTPNSAYEAKFSMPFLM
                     ALGFFDGKITLKSYQNLKRKEVLEFAQKITYEKRKSQGFPKYFPGHMEAILQDGKVIC
                     KDVFINKGNFDNPLSFEELKDKFINNAKENLTMDKIHDILEQIKHLEKQTDLSL"
     gene            complement(185980..186144)
                     /locus_tag="DMACINML_00448"
     CDS             complement(185980..186144)
```

```
                     /locus_tag="DMACINML_00448"
                     /inference="ab initio prediction:Prodigal:2.6"
                     /inference="similar to AA sequence:RefSeq:ALW35763.1"
                     /codon_start=1
                     /transl_table=11
                     /product="mobilization protein"
                     /protein_id="Prokka:DMACINML_00448"
                     /translation="MPKIATAHCNPKKQPALNHNDRTNDNAKTITKELTHLNEYSCTS
                     DEVRKNLKII"
     gene            complement(186110..186382)
                     /locus_tag="DMACINML_00449"
     CDS             complement(186110..186382)
                     /locus_tag="DMACINML_00449"
                     /inference="ab initio prediction:Prodigal:2.6"
                     /codon_start=1
                     /transl_table=11
                     /product="hypothetical protein"
                     /protein_id="Prokka:DMACINML_00449"
                     /translation="MQKQYPEVYSLEESLAILKKYKDDVSKKDYEEIKSTICGHAIED
                     MFANEEDIIMLVKMSAYNLSSDEIIAEYKEKGFVEYAKNCHRSLQS"
     gene            complement(186559..187149)
                     /locus_tag="DMACINML_00450"
     CDS             complement(186559..187149)
                     /locus_tag="DMACINML_00450"
                     /inference="ab initio prediction:Prodigal:2.6"
                     /inference="similar to AA sequence:RefSeq:APB40452.1"
                     /codon_start=1
                     /transl_table=11
                     /product="replication initiation protein"
                     /protein_id="Prokka:DMACINML_00450"
                     /translation="MSLNGKYTKTLYRLLKQFRTTGKARFEWEDFCRIMDIPQDYRQS
                     EIDKWILKPAIKELSKERDLFDQIRVPFKNLAYEKEKAKGRGRGGKVSGITFTFKPEN
                     IQMQKLENESQKIMSDEQKYLKILNNMKLNQVRFVYNDKLWQFNDFDFNEFKIIAVEL
                     IRDEYENLNFGNHMHFNAKNQEQFFKMIDTFRNGIR"
     gene            187802..187894
                     /locus_tag="DMACINML_00451"
     CDS             187802..187894
                     /locus_tag="DMACINML_00451"
                     /inference="ab initio prediction:Prodigal:2.6"
                     /codon_start=1
                     /transl_table=11
                     /product="hypothetical protein"
                     /protein_id="Prokka:DMACINML_00451"
                     /translation="MKKIILGLAVIASLSSLALAKITVSQQPIV"
     gene            188003..188209
                     /locus_tag="DMACINML_00452"
     CDS             188003..188209
                     /locus_tag="DMACINML_00452"
                     /inference="ab initio prediction:Prodigal:2.6"
                     /inference="similar to AA sequence:RefSeq:ASI88145.1"
                     /codon_start=1
                     /transl_table=11
                     /product="Hypothetical protein"
                     /protein_id="Prokka:DMACINML_00452"
```

```
                     /translation="MILVTPCGVIDTTNFGKSEVNRQTIDFSLKFQEKKEKVEAEAKK
                     EEQSNSTNTDKADNKKDQLLDISV"
     gene            complement(188587..188859)
                     /locus_tag="DMACINML_00453"
     CDS             complement(188587..188859)
                     /locus_tag="DMACINML_00453"
                     /inference="ab initio prediction:Prodigal:2.6"
                     /codon_start=1
                     /transl_table=11
                     /product="hypothetical protein"
                     /protein_id="Prokka:DMACINML_00453"
                     /translation="MQKQYPEVHSLEESLAILKKYKDDVNKEDYEGIKSVISNHAIES
                     IYMNELDIIAMIKQSALKLSADEIIAEYKEKGFVEYAKNCHRSLQS"
     gene            complement(189007..189129)
                     /locus_tag="DMACINML_00454"
     CDS             complement(189007..189129)
                     /locus_tag="DMACINML_00454"
                     /inference="ab initio prediction:Prodigal:2.6"
                     /inference="similar to AA sequence:RefSeq:ASI88145.1"
                     /codon_start=1
                     /transl_table=11
                     /product="Hypothetical protein"
                     /protein_id="Prokka:DMACINML_00454"
                     /translation="MILVTPCGVMDIVNFAKSKINKQIFRYICLILKICYNVIF"
     gene            189394..189618
                     /locus_tag="DMACINML_00455"
     CDS             189394..189618
                     /locus_tag="DMACINML_00455"
                     /inference="ab initio prediction:Prodigal:2.6"
                     /inference="similar to AA sequence:RefSeq:CAL35506.1"
                     /codon_start=1
                     /transl_table=11
                     /product="putative ferrous iron transport protein"
                     /protein_id="Prokka:DMACINML_00455"
                     /translation="MTLNELKDGQSAIIKEIKAYKELHSRLLSFGFMKNKTLKKVHSS
                     LKNATIMVEFESTCVILRSNEAETIQVEPL"
     gene            189615..191456
                     /locus_tag="DMACINML_00456"
     CDS             189615..191456
                     /locus_tag="DMACINML_00456"
                     /inference="ab initio prediction:Prodigal:2.6"
                     /inference="similar to AA sequence:RefSeq:ALW62441.1"
                     /codon_start=1
                     /transl_table=11
                     /product="iron transporter FeoB"
                     /protein_id="Prokka:DMACINML_00456"
                     /translation="MKKITIALAGQPNVGKSLLINTLCKANMKVGNFSGVTVEKAVAK
                     TRYKGYELEFIDLPGTYSLDGYSEEEKIARHFLESKNYDIIVNVLDATNLERNLILSA
                     ELLSLNKKMLLALNMCDEAKKEGIELDTSILSKEFQINVVEISAKTKQNLELLLEKII
                     ILFENKFTPKSRFSTPLCEKSLEKEDILTLVNELSRKVITYKKEERNLTKKIDALLIH
                     KFFGLPIFLFLMWLLFQLTFTLGQIPMDYIESGFNALGEWVKNNIHNTFIASALADGA
                     IAGVGAVVLFLPNIIILFLGIALLETTGYMSRVAFLLDGILHKFGLHGKSFIPLITGF
                     GCSVPAFMATRTLKNKRDRLLTLFVINFMSCGARLPVYVLFIGAFFPSEEAGNYLFGI
                     YLLGAILGLIAAKILRTTAFRGIDEPFVMEMPKYRMPNWHLVWFMVFNKAKMYLKKAG
```

```
                     TFILVASLLIWFASNFPNNESNLDDFSAQERAVEQSYLGQFGKTIEPIFSPLELDWKL
                     SVSLVSGLAAKEVMISTMGVLYSLGKEVDETSSDLKGIIAKNIPLPSAVAYILFVMIY
                     NPCFAATIVFSKESGKIKYTFFLFLFTCISAYIVAFIGLNITKFVIN"
     gene            complement(191453..192955)
                     /gene="hydA2"
                     /locus_tag="DMACINML_00457"
     CDS             complement(191453..192955)
                     /gene="hydA2"
                     /locus_tag="DMACINML_00457"
                     /inference="ab initio prediction:Prodigal:2.6"
                     /inference="similar to AA sequence:RefSeq:CAL35508.1"
                     /codon_start=1
                     /transl_table=11
                     /product="putative Ni/Fe-hydrogenase small subunit"
                     /protein_id="Prokka:DMACINML_00457"
                     /translation="MAKLSNEELKDILIKRIEKIENSDLVDKKTINEESVKALAKHLS
                     LGNEIPALAQKFFELAPKTKVVWLHLCECTGCSESLLRADLPSFDELVFDFFSLEYHE
                     TLMAANGTKAEELLEHVLKEDFVLAVEGGVAAIDTFFLTIGAEGESGYEILEKLAARA
                     KAIFAVGTCSSYGGIQAAYPNPSKTCGISEVLTQKVVNIPGCPPSDVNIIVTLTYFAL
                     FGILPELDEQNRPVWAYGKCLHDLCERKAKFESGIFAEHFDDEKAKSGACLFKIGCKG
                     PYTYNNCPKVKFNAKTSWPVAAGHGCIACSEKNFWDEFGNYEKPMANPFSYAKLINQE
                     FSTEFALEEQIQILSLMDFEFESNLKLILQNIAKNKLGALLVENYKTSFEKNFIFIEQ
                     NFDENPMPSSDIWKYFEINFILAKGEFLQDKNDFLKAAQNYSFKHASPYDFKLTLNEQ
                     KSKLDVSKSFRMPLIYLCGGLDFEALAYSVLKAFEKNIKSVIDFNKQKAG"
     gene            complement(192958..193782)
                     /gene="fabI"
                     /locus_tag="DMACINML_00458"
     CDS             complement(192958..193782)
                     /gene="fabI"
                     /locus_tag="DMACINML_00458"
                     /EC_number="1.3.1.9"
                     /inference="ab initio prediction:Prodigal:2.6"
                     /inference="similar to AA sequence:RefSeq:CAL35509.1"
                     /codon_start=1
                     /transl_table=11
                     /product="enoyl-[acyl-carrier-protein] reductase [NADH]"
                     /protein_id="Prokka:DMACINML_00458"
                     /translation="MIMKGKKGLIVGVANNKSIAYGIAKSCYEQGASLAFTFLNDALK
                     KRVEPIAEEFGSNFVYELDVNNNEHLDSIAEKIKKDLGEIDFIVHAVAYAPKEALENS
                     FLETSKEAFDIAMGTSVYSLLSLTRAVLPVLKENGAILTLSYLGGVKYVPHYNVMGVA
                     KAALESSVRYLARDLGIKGIRVNAISAGPIKTLAASGIGDFRMILKYNEINAPLKRNV
                     SIEDVGNSAMYLLSDLARGVTGEVHYVDAGYNIMGMGDVTKDENGNTVLCWDNQKG"
     gene            complement(193786..194457)
                     /gene="tpiA"
                     /locus_tag="DMACINML_00459"
     CDS             complement(193786..194457)
                     /gene="tpiA"
                     /locus_tag="DMACINML_00459"
                     /EC_number="5.3.1.1"
                     /inference="ab initio prediction:Prodigal:2.6"
                     /inference="similar to AA sequence:RefSeq:CAL35510.1"
                     /codon_start=1
                     /transl_table=11
                     /product="putative triosephosphate isomerase"
```

```
                     /protein_id="Prokka:DMACINML_00459"
                     /translation="MIFAANLKCNHTRSSFKLYANILNQYLEAKNDDIIVFPPSIAFL
                     ENELNFIQGAQNFYPCINGAFTGELGKEHLDEFGIKCVLIGHSERRALGDENLIKAKF
                     NFAKEHAYKIIFCIGEDLELKNSNQTLDFLKKQLEVIDLDYKNLIIAYEPIYSIGSGV
                     SAKKEDIAKVLEFLSSLTQAPLLYGGSVNEDNIKDILNIKHCSGVLIGSAALKAENFI
                     KLIKG"
     gene            complement(194454..195656)
                     /gene="pgk"
                     /locus_tag="DMACINML_00460"
     CDS             complement(194454..195656)
                     /gene="pgk"
                     /locus_tag="DMACINML_00460"
                     /EC_number="2.7.2.3"
                     /inference="ab initio prediction:Prodigal:2.6"
                     /inference="similar to AA sequence:RefSeq:CAL35511.1"
                     /codon_start=1
                     /transl_table=11
                     /product="phosphoglycerate kinase"
                     /protein_id="Prokka:DMACINML_00460"
                     /translation="MSDIISIKDIDLSKKKVFIRCDFNVPQDDFLNITDDRRIRSAIP
                     TIRYCLDNGCSVILASHLGRPKEISSKYSLEPVAKRLARLLDKEVIMAKDVIGEDAKS
                     KAANLKASEILMLENLRFEKGETKNDENLAKELASMAEVYINDAFGVCHRAHASVEAI
                     TKFFDENHKGAGFLLQKEIEFAENLIKRPARPFVAVVGGSKVSGKLQALTNLLPKVDK
                     LIIGGGMAFTFLKAQGFDIGNSLLEEELLEEANKILTKGKNLGVKIYLPVDVVAAPAC
                     SQESPMKFVPSQEIPSGWMGLDIGPASVRLFKEVISDAQTIWWNGPMGVFEIDKFSKG
                     SIKMSHAISDGHATTVVGGGDTADVVARAGDADEMTFISTGGGASLELIEGKELPGVK
                     VLRSGGAQ"
     gene            complement(195659..196657)
                     /gene="gapA"
                     /locus_tag="DMACINML_00461"
     CDS             complement(195659..196657)
                     /gene="gapA"
                     /locus_tag="DMACINML_00461"
                     /inference="ab initio prediction:Prodigal:2.6"
                     /inference="similar to AA sequence:RefSeq:CAL35512.1"
                     /codon_start=1
                     /transl_table=11
                     /product="glyceraldehyde 3-phosphate dehydrogenase"
                     /protein_id="Prokka:DMACINML_00461"
                     /translation="MAVKVAINGFGRIGRCVARIISKRDDIELVAINDTTDIELTKYL
                     FKYDTVHGEFEGSVENEGDDLIINGKKIKVFKSRDIKELDFAKYGAQIVLECTGAHLT
                     IEKCQGFLDMGVQKVIMSAPAKDNTPTYVLGVNSQNYKGENIISNASCTTNCLGPVCR
                     VLQDNFGIEKGLMTTIHAYTNGQSIIDAKTKDKRRSRAAAQNIIPTSTGAAKAMKLVM
                     PELNGKLHGQSMRVPVIDVSSVDLTAQLSKKVSKEELNEAFRKAAATNLKGILMVDDD
                     ERVSSDFITCSYGAVVVSDLTQVIADDFVKVVAWYDNEWGYSSRLVDMAVYIANKA"
     gene            196719..197264
                     /gene="nadD"
                     /locus_tag="DMACINML_00462"
     CDS             196719..197264
                     /gene="nadD"
                     /locus_tag="DMACINML_00462"
                     /EC_number="2.7.7.18"
                     /inference="ab initio prediction:Prodigal:2.6"
                     /inference="similar to AA sequence:RefSeq:CAL35513.1"
```

```
                     /codon_start=1
                     /transl_table=11
                     /product="putative nicotinate-nucleotide
                     adenylyltransferase"
                     /protein_id="Prokka:DMACINML_00462"
                     /translation="MKIALFGGSFDPPHKGHDAIVKEALAKLDIDKLIIMPTFINPFK
                     KGFFADEKQRFAWVNKLWGKLEKVEICDFETKQKRPVPSIESVEYLYKIYHPSKFYLL
                     IGADHLEKLHLWHDFKKLNSLVEFIIANRNDIEIPKNFKDLKTDIKIASSFIRSTLDT
                     HEVCDEIKDEVKNYYEKLQKN"
     gene            197293..197619
                     /locus_tag="DMACINML_00463"
     CDS             197293..197619
                     /locus_tag="DMACINML_00463"
                     /inference="ab initio prediction:Prodigal:2.6"
                     /inference="similar to AA sequence:RefSeq:CAL35514.1"
                     /note="conserved hypothetical protein Cj1405"
                     /codon_start=1
                     /transl_table=11
                     /product="hypothetical protein"
                     /protein_id="Prokka:DMACINML_00463"
                     /translation="MQERVDLIVKILDEKKAEDIKTFDMSEQDYFVKYVIIAATLGEK
                     HALSLIDELKTKLKAKGEEFLNIESSEEWSVIDLGDILIHLLTPEHRGIYNIEELLES
                     LKKIKV"
     gene            complement(198257..199621)
                     /locus_tag="DMACINML_00464"
     CDS             complement(198257..199621)
                     /locus_tag="DMACINML_00464"
                     /EC_number="5.4.2.-"
                     /inference="ab initio prediction:Prodigal:2.6"
                     /inference="similar to AA sequence:RefSeq:CAL35516.1"
                     /codon_start=1
                     /transl_table=11
                     /product="putative phospho-sugar mutase"
                     /protein_id="Prokka:DMACINML_00464"
                     /translation="MLDVIFREYDIRGLYGKELNEKSVKAIGFCLGQTMLAKGCKNVS
                     VGYDARYSADELYKYLVSGLNKAGIQVYNIGLVPTPLGYFSLYEGMKFDANIMITGSH
                     NPKDYNGFKITIGKESFFGAELKEFSKEVYKHLDDEIEENLEAQKYDILSLYVNFMCE
                     QFSFLKDFNYKFAIDCSNGAAGVVIEPLVKALNLKAHVMFSNPDGQFPNHEPDPTEEK
                     NLHAIKKFLNENQEYLLAFAFDGDADRMVALSKTHIFCGDELCYLFAKNIPNPRILGE
                     VKCSKNLFDEVAKFGTIFMGKTGHSNIKKMMKEKDIDLAAEVSGHIFFKHRYFGYDDG
                     IYAFLRALELVYKGFDLESMISALPKLYTTPEIKIPVSEEEKFKLVDEFKKAIERGAL
                     EGVKSLCEIDGARIDFGYGWALLRASNTSPYLITRFEASSLEQAKELETKVFKLFNDI
                     KESV"
     gene            199734..200270
                     /gene="fliL_1"
                     /locus_tag="DMACINML_00465"
     CDS             199734..200270
                     /gene="fliL_1"
                     /locus_tag="DMACINML_00465"
                     /inference="ab initio prediction:Prodigal:2.6"
                     /inference="similar to AA sequence:RefSeq:CAL35517.1"
                     /codon_start=1
                     /transl_table=11
                     /product="putative flagellar protein"
```

```
                     /protein_id="Prokka:DMACINML_00465"
                     /translation="MDEELENNEEKKKKGGSLVIIIVILLFVLLLSIMGVIAWLISSS
                     SSDESEVKAAPKEEVKVDKAKVTAPTQRGSDFANIGPMYPLDPFTLNLLSDSGSRYVK
                     CTIELEQNTELLKPELDKKVAVIRDIIIHALTAKTFEEVSTQKGKERLKDELVGKINE
                     ILTDGFIKNVYFTDFVVS"
gene                 200270..200617
                     /gene="acpS"
                     /locus_tag="DMACINML_00466"
CDS                  200270..200617
                     /gene="acpS"
                     /locus_tag="DMACINML_00466"
                     /EC_number="2.7.8.7"
                     /inference="ab initio prediction:Prodigal:2.6"
                     /inference="similar to AA sequence:RefSeq:CAL35518.1"
                     /codon_start=1
                     /transl_table=11
                     /product="holo-[acyl-carrier protein] synthase"
                     /protein_id="Prokka:DMACINML_00466"
                     /translation="MRIGCDIVAISRIDTIYQKHGNAFLDKFLNQNEQKLIKSSSTLA
                     GFWAAKEAASKALGVGISKECGFLDIEILKDSKNAPHLKLASHVLEKFKITSTSLSIS
                     HDNGFAIAVVALD"
gene                 complement(200620..201042)
                     /locus_tag="DMACINML_00467"
CDS                  complement(200620..201042)
                     /locus_tag="DMACINML_00467"
                     /inference="ab initio prediction:Prodigal:2.6"
                     /inference="similar to AA sequence:RefSeq:CAL35519.1"
                     /codon_start=1
                     /transl_table=11
                     /product="putative membrane protein"
                     /protein_id="Prokka:DMACINML_00467"
                     /translation="MKRVKARIRANFRNRIKRNLKGSLKEKLAGTILLCAIVPLAICG
                     YLFIVIVGTFFNTARARQGVRALDHFVNASLFNGYAWESVSSHAWRERNRKKWAKVVI
                     KITDFFQKDHCKRANKREQPVVDFILSRNLDKQTIGKR"
gene                 complement(201039..202409)
                     /locus_tag="DMACINML_00468"
CDS                  complement(201039..202409)
                     /locus_tag="DMACINML_00468"
                     /inference="ab initio prediction:Prodigal:2.6"
                     /inference="similar to AA sequence:RefSeq:CAL35520.1"
                     /codon_start=1
                     /transl_table=11
                     /product="putative cytochrome P450"
                     /protein_id="Prokka:DMACINML_00468"
                     /translation="MSQCPFFPKPYKNKASTLLTFLLKRRSWLDGLYERSYKMQTGYV
                     KMPNFDLYVINDTKEVKRMMVDEVKEFPKSAFLHELLSPLLGESIFTTNGEVWKKQRE
                     LLRPSFEMTRISKVFNLMSEAVSDMMKRFEKYPNASIIEVDEAMTFVTADVIFRTIMS
                     SKLDEEQGKKILDAFVTFQEQSVHTAMRRMFRFPKWLSYVLGDRKRAKAGDVIRQALS
                     DIIKPRYDAVSSGKVEDFEDILGSLLLVVDAQTNQRFSFEEILDQVAMLFLAGHETTA
                     SSLTWTLYLLSLYPDEQEKAYKEIIQVLQGEDIQISHLKQFKYLTNIFKESLRLYPPV
                     GFFAREAKKDTKVRDKMIKKGSGVVIAPWLIHRHEGFWTNPHEFKPSRFEGEYKKDAY
                     LPFGVGERICIGQGFAMQEAILILANILKKYKLELEEGFVPDVVGRLTIRSANGMRIK
                     FSKRES"
gene                 complement(202406..203527)
```

```
                     /locus_tag="DMACINML_00469"
     CDS             complement(202406..203527)
                     /locus_tag="DMACINML_00469"
                     /inference="ab initio prediction:Prodigal:2.6"
                     /inference="similar to AA sequence:RefSeq:CAL35521.1"
                     /codon_start=1
                     /transl_table=11
                     /product="putative integral membrane protein"
                     /protein_id="Prokka:DMACINML_00469"
                     /translation="MDREVFYIAGYDPKSYRFYYDLFKKNLKDYSHRFDLKAKMGGIE
                     KNGNFPFFKINCENTQTRYHFLTWNDIVKKNWSQSYKDALMDCYSFFRIYTITGLFLK
                     FGKESIYQLVTGYYPFFYVIFSLLLSLGLALGSFVFLQDYIPSFLAIVIGIVLGFLLN
                     RFLFKLGRKLAVFWIARICAFCATWKEKRLGAMEQRIKLFADEILKFLKQNENKQDYE
                     LILVAHSVGTIVCIEVLEHILKQNLDKKVLDKLKILTLGECIPLTSYQKNSDGFRKKL
                     EFVSAFDLKWYDYTSIIDGACFPQVDFFRTSGVKANFTPPFLSAKFHTLYEKNEYKKI
                     KRDKNKAHFLYLYSPHIKGSYDFFAFIVAPKFLEEKVKI"
     gene            complement(203594..204778)
                     /locus_tag="DMACINML_00470"
     CDS             complement(203594..204778)
                     /locus_tag="DMACINML_00470"
                     /inference="ab initio prediction:Prodigal:2.6"
                     /inference="similar to AA sequence:RefSeq:ABS43455.1"
                     /codon_start=1
                     /transl_table=11
                     /product="capsule polysaccharide export protein KpsS"
                     /protein_id="Prokka:DMACINML_00470"
                     /translation="MKFRDKIKKEFSGKNVLLLQGPVGTFFHRLAIKMKKNKTKVFKL
                     NFNGGDFLFYPSGKRCKCDEKDLENFYENFFKEKKIDAIVMYNDCRLIHAKAIKVAKE
                     LGIGIWIFEEGYLRPYCITFEKDGVNANSSLPRDKNFYLSCNISTKESIKEIPGGFKF
                     MAFSAFLYWLFSFLLAPFFNNKLHHRTLYPFEFLFWFSSLYRKYLYKFTEKKLNQKIY
                     SLEKKYFLAILQVYNDTQIKHHYKKNIEEFIEETILSFANHARAKSYLVFKHHPMDRG
                     YRNYSKLINELSQKYHVEGRILYVHDTYLPTLLKRALGCITINSTVGLSAILEGCPTK
                     VCGNAFYDFEGLAYPKKLQFFWREAHAYKPNPNLVLNFKNYLLNTNQFNGNFYKNSFL
                     SR"
     gene            complement(204775..206802)
                     /locus_tag="DMACINML_00471"
     CDS             complement(204775..206802)
                     /locus_tag="DMACINML_00471"
                     /inference="ab initio prediction:Prodigal:2.6"
                     /inference="similar to AA sequence:RefSeq:ABS43950.1"
                     /codon_start=1
                     /transl_table=11
                     /product="capsule polysaccharide export protein KpsC"
                     /protein_id="Prokka:DMACINML_00471"
                     /translation="MKFHTTSKKLIKNVKDFYKIVLYKAYKSIDKEDVFVGWGRKNSG
                     LKAIALAKKHHAKFMLLEDGFLRSLNLGIENSPSFSIVKDDVGIYYDASTPSRLENIL
                     NTYEFSPKELAWAKKAIELIKKEKLSKYNNNLCIPQELFSANEERVLIITQVANDASL
                     RFGLASDFSTQDIINDALKENPNAKIYIKIHPDVLSGKKQSDFSMQDLPSRCVILKEN
                     YNPIELLSHFKKVYTKTSGMGFEALMLGCECVCYGMPFYAGWGLTQDKLECKRRVKKR
                     SLEEVFCAAYILYSEYFNPYLNQKSDIFDTIFTLARYKKIEQVNSHTLYFLGFTLWKR
                     WFMKPFFKAKNNKIIFLNSLNELYKANLNSEDKIFIWGKKYDKALLTKDFKNEIFLVE
                     DGFLRSVFLGSDLTRPFSLIVDSKGLYVDPNHPSDLEEVLQNHEFDDTLRQRAKKLII
                     TITQNKFSKYNGLKHEKLDFNTNKKIILIPAQVEDDASMILGGAGFDTLKLLQSVRAA
                     NKDAFIVFKPHPDVLSGNRKGLKDKDIILKYCDEIIENVSIDSAINACDEVHTITSTS
```

```
                     GFDALLRGKKVVVYGKPFYAGWGLTQDLHHISRRTRVLSLEELVAGVLILYPRYIHPK
                     SKNLCEVELALDIMLALQKDYFSKRWLRILIDFRILMLRKIRRICEVFIKR"
     gene            complement(206838..208529)
                     /locus_tag="DMACINML_00472"
     CDS             complement(206838..208529)
                     /locus_tag="DMACINML_00472"
                     /inference="ab initio prediction:Prodigal:2.6"
                     /inference="similar to AA sequence:RefSeq:EAQ72594.1"
                     /codon_start=1
                     /transl_table=11
                     /product="galactosyltransferase"
                     /protein_id="Prokka:DMACINML_00472"
                     /translation="MKTVGVVIPIYNVEKYLRECLDSVINQSYTNLEIILVNDGSTDE
                     NSLNIAKEYTLKDKRITLFDKKNGGQSTARNVGIEYFSGEYKLKNKTQSIKENSLIEF
                     NIEGNNPYEIYTVYKSYKAFNDEKDLTKFTYPIIDYIIFLDSDDYWELNCIEECVPRM
                     DGVEVVWFDYKFLYNEFKPDMPYKSMFEYAYISKECIINSSEWKDIILNSQAGLFWFG
                     WHGMIRFPYLKEIKLKFINGIIHEDHHFGIVLFALSKLIYIYYRKVYIYRVRSNSTCN
                     HSGSNEPCIPSYLSVLLNCNLNKEQIFKYNKYYSYCLIYKELDLFFNMYKKDIFFEIL
                     RKRFLPIYKAVCLNIFDLYCDPLNIFPKLPSDFFITFLKKNEKKIENLNAIIQEKNKM
                     NEQIQIQIQNLNQIIKNKEEVIQIQIQNLNQIIKNKEEVIQIQNNQIIFQAKYGMAKS
                     RIQNQLSYKLGQAMIINSKSLLGYLIMPMILLNIIISHKQAQKAYKLKIKKNPNLALP
                     PLESYPDYKEALKEKECLTYKLGEALIKASNNWYGGGYIKLLLEIRKLKKEFKKK"
     gene            complement(208559..208993)
                     /gene="lacA"
                     /locus_tag="DMACINML_00473"
     CDS             complement(208559..208993)
                     /gene="lacA"
                     /locus_tag="DMACINML_00473"
                     /EC_number="2.3.1.18"
                     /inference="ab initio prediction:Prodigal:2.6"
                     /inference="similar to AA sequence:RefSeq:SQE24952.1"
                     /codon_start=1
                     /transl_table=11
                     /product="putative sugar transferase"
                     /protein_id="Prokka:DMACINML_00473"
                     /translation="MSDKCKIVIGDNCFFNGGNISLLCGEMNTHIDIGNDCLFASDIT
                     LATSDNHSIFNKETKKRLNYAGNIVIGKHCWICDDVKILNHSYIGDECVIATKALVVS
                     FPKKLFSFFYQKPPNNCIIGGIPARILKKNIVWHREIKEFLE"
     gene            complement(209083..209277)
                     /locus_tag="DMACINML_00474"
     CDS             complement(209083..209277)
                     /locus_tag="DMACINML_00474"
                     /inference="ab initio prediction:Prodigal:2.6"
                     /codon_start=1
                     /transl_table=11
                     /product="hypothetical protein"
                     /protein_id="Prokka:DMACINML_00474"
                     /translation="MVIKNNDERLQNIKICGVNNEIYGIDYVPSICNLNISIFGNNNK
                     VYIPKDLKINKNKGGGYLLI"
     gene            complement(209349..210830)
                     /gene="mshA"
                     /locus_tag="DMACINML_00475"
     CDS             complement(209349..210830)
                     /gene="mshA"
```

```
                     /locus_tag="DMACINML_00475"
                     /EC_number="2.4.1.250"
                     /inference="ab initio prediction:Prodigal:2.6"
                     /inference="protein motif:HAMAP:MF_01695"
                     /codon_start=1
                     /transl_table=11
                     /product="D-inositol-3-phosphate glycosyltransferase"
                     /protein_id="Prokka:DMACINML_00475"
                     /translation="MLKHYIHCHQAFDLKKGGSVGYISSLCEAFRRYNDSFLTDKKTK
                     CSFLFPDIPTDQYIPNDILEIIVEQRFKYTQNYHIKTVSKNLLQKRQDWFREIIPFSE
                     YVKIDPKNIASIHIHGAYNFLPIYNSLIRLGVDSRVVKILTTHNPYQPTMEDMYLTCR
                     NMKWRNDDKEMFYYYFNERDKWAFKLADCLFFPTEESLEGYYKLWPEFASLIKNKPIY
                     FCPTGTAINKPADDKKFLKESLNIPKDAKIFLYLGRFIDVRGYDILIDAAKQIISKND
                     KIYFIVVGEKHVVPINSKQWIQIPFTDKPQEYINIADACLCPNRGSLFDLSMIEFLAF
                     GKPIICSYVGGYRWLENKTSGVLYSSVEDIDKFADNILLFSQFSREKINKMQNDNLNL
                     YKKCLSLNSFQENYCNTIDNIYRDFGISKKMYSISYCDKASVFSLNADNKKEFFTKGE
                     PVIKSLEIAKKVESKQEIFNRKFKKLMRNPYVFFKDFLIKRLG"
     gene            complement(210834..211562)
                     /locus_tag="DMACINML_00476"
     CDS             complement(210834..211562)
                     /locus_tag="DMACINML_00476"
                     /inference="ab initio prediction:Prodigal:2.6"
                     /codon_start=1
                     /transl_table=11
                     /product="hypothetical protein"
                     /protein_id="Prokka:DMACINML_00476"
                     /translation="MLNHYIILPSDELHYIKLKDLQVLKKNINVKVIYTQDYVFHSLS
                     PIGIQFYDKEIIDISSIVDLKKGDALPISLKQLMLIDFKNTSSIHLIGNNKQLFFLLQ
                     ILEILHATSIKIFWTRLGDFQVKRINNQQSEISIIEENFLNLNEIKIKNYIMQICKEE
                     IKIPLKTIKKYNFSSNATLSQHNGNYEVVFSVQDKILLRSFISFQKQIVNTVDNINPI
                     KRKIKKLIKNPKRFFKDFIIKKIR"
     gene            complement(211556..213550)
                     /locus_tag="DMACINML_00477"
     CDS             complement(211556..213550)
                     /locus_tag="DMACINML_00477"
                     /inference="ab initio prediction:Prodigal:2.6"
                     /inference="similar to AA sequence:RefSeq:CAL35547.1"
                     /codon_start=1
                     /transl_table=11
                     /product="putative sugar transferase"
                     /protein_id="Prokka:DMACINML_00477"
                     /translation="MNQIGVSIIVPCYNVADYVGKCLQSLCKQTLEEIEIIIINDGST
                     DGTHLVIEDFIKKNKISSIQYYCKENGGYGSAVNLGIYHAKGEYIAICEPDDYVDEMY
                     YEILYKIAKDENMDVVLYNGYIENRPYFGHLAITTYTSVKLNCVLSTEELKNRFLSCG
                     TAIYAAIYRKIFLLDNQITLPETCKVYQDVPFVAKVFSICKKIRYVMGAKYYYLRGRA
                     EQSVANPSRFVDIIPAIKDLVEFLISNENKLLLDKAYVLGFAVAHLIGRYQISIACNA
                     LDTAEKIKNYIETVLCDDVILQLYSKNFLKNNGFNVDKVLIREVDLNPVNYYSLPTIQ
                     NFSLQASYNEIISYMGLKLLFAQNENFSFNILKTLEWELSYFINIPMADPGEQIKEVC
                     LKLLNSYNANILINKFPKLVAYISVLLNNSIEELTIPVPRLREDGAQILDWFYNRSAI
                     KANLNTQTDLCKEIKDYFNQSEERFLSYIENKTICVVGNSPCELNKNKGCVIDNYDIV
                     IRFNNYKTNGFEKDYGSKTNVWAVSPALETITTKDLSQFDFMISNDSSFYTSPFERSS
                     IYKIMLLDLPFFQIQTLEFIKRTQLRTLSHGIVMLCYLLKHRERLKDFAYYGFSLEEQ
                     SQGISHYYNGDPSSGKILPFHNWNKEAKILQQINKELKQC"
     gene            complement(213554..214831)
```

```
                     /locus_tag="DMACINML_00478"
     CDS             complement(213554..214831)
                     /locus_tag="DMACINML_00478"
                     /inference="ab initio prediction:Prodigal:2.6"
                     /inference="similar to AA sequence:RefSeq:AJK71602.1"
                     /codon_start=1
                     /transl_table=11
                     /product="glycosyl transferase family 2"
                     /protein_id="Prokka:DMACINML_00478"
                     /translation="MKNFKISIIIPSLNSITYICECVDSVLNQTLKDIEIICVDAGST
                     DGTLEVLREYEKNDERLKVIVSDKKSYGYQMNLGIKEAKGEYLGIVESDDYIKENMYE
                     NLYNIAIEKKCDIVKGDMLNFWDKEERVYEYRLLNWTEVLYNKILNFKTDKRILTESN
                     IINPSGIYKLDFIKKYNILCNETPGSAFQDTSFWFQLQVLASSIFLVKEAYYYYRQDN
                     INSSCNSLDKIYCICEEYDFIEQFVLKNKFYDILRIVHYLRYRGYRWNLYRIHSSYRL
                     EFLYKFQKDFKRLKTISENSFSLFKKWEIEQLQEIMSNPKKFYNEVARYKKGAIEFME
                     NTPEYKIGKQAIICKSRKDFVELFRLINTNTTKPNGNLEKKYCDYYEGQSYKEHLSYK
                     IGKILLGLKNNYFLALTIPFQIVFCYIKFKLKG"
     gene            complement(214839..215930)
                     /locus_tag="DMACINML_00479"
     CDS             complement(214839..215930)
                     /locus_tag="DMACINML_00479"
                     /inference="ab initio prediction:Prodigal:2.6"
                     /inference="similar to AA sequence:RefSeq:AJK71603.1"
                     /codon_start=1
                     /transl_table=11
                     /product="UDP-galactopyranose mutase"
                     /protein_id="Prokka:DMACINML_00479"
                     /translation="MYDYLVVGTGLFGSIFAYEASKKGYKCLVLEKRSHIGGNCYTEN
                     IENINVHKYGAHIFRTSDENIWKYMQQFCRFNHFVNSPIANFKGEIYNLPFNMNTFSK
                     LWGIFSPKEAREIITKQSQEIQGIPRNLEEHVIKFVGRDIYEKFVKGYTEKQWGRDCK
                     DLPASIIRRIPVRYIYDNNYFNDPYQGIPIGGYTAIFEKMLENCEVLLNIDFLKHKKE
                     FQNKAKKIIFTGTIDSFYNYEYGPLEYRSLRFEHKILNTDNYQGVAVVNYTDKETPYT
                     RTIEHKHFEFGKQEKTVVSEEYPLEWTKDIEPYYPINDDKNQKLYEKYLELAKKETNV
                     YFGGRLGEYKYYDMQDSVKSALEFSKKEL"
     gene            complement(215976..217364)
                     /locus_tag="DMACINML_00480"
     CDS             complement(215976..217364)
                     /locus_tag="DMACINML_00480"
                     /inference="ab initio prediction:Prodigal:2.6"
                     /inference="similar to AA sequence:RefSeq:CAL35255.1"
                     /codon_start=1
                     /transl_table=11
                     /product="putative glycosyltransferase"
                     /protein_id="Prokka:DMACINML_00480"
                     /translation="MQTKTVGVVIPIYNVEKYLRECLDSVINQSYTNLEIILVNDGST
                     DENSLNIAKEYTLKDKRITLFDKKNGGQSTARNVGIEYFSGEYKLKNKTQSIKENSLI
                     EFNIEGNNPYEIYTVYKSYKAFNDEKDLTKFTYPIIDYIIFLDSDDYWELNCIEECVP
                     RMDGVEVVWFDYRAVYDKVKRKHLSQMTHFDYKNEVIITSKEWLDRAKERRLFYFWFA
                     WQGMVNFTFLKNIKLKFIKGIFAEDCHFGVLLFALSKNIYIFPKQIYIYRLRESSSMN
                     FTRKKWAIHPDSHLKKIDIFENSNETRLYYETTSWMQIALEFIKFIHSKHHLSDEIKQ
                     HFLPVVCNKALTLQKFDKDPLYLKKYAKNLKIYIQNQPLGAVSRVKEYLSYKIAKEIS
                     RKKSIMKLTLAFSVVKVSVQHQKEVRRYKKDIKRDVLNKRLPLEFYRDYQKAIKLKNF
                     FKKFLIQSIFYK"
     gene            complement(217354..219000)
```

```
                     /locus_tag="DMACINML_00481"
     CDS             complement(217354..219000)
                     /locus_tag="DMACINML_00481"
                     /inference="ab initio prediction:Prodigal:2.6"
                     /inference="similar to AA sequence:RefSeq:CAL35551.1"
                     /codon_start=1
                     /transl_table=11
                     /product="putative sugar transferase"
                     /protein_id="Prokka:DMACINML_00481"
                     /translation="MPLLSVIIPFGLSKERSYIQERVYEKAQYFKTDEKIEFIFVEGY
                     SSQEHNLKTYILKNNHIYLKDESQKEYFSQGKCRNFGASYANASVLLFLDLDCFISFD
                     SLEKILKLIKIKNILNNPNALLVLPVVYLTQEASEILKEYPMDLWDVLIQEDLISGKN
                     FLVKFFAPSSTSSLIINRHKFLEIGGNNENFIGHGYEDFDLFARVLNSCLKFEKVPLN
                     LNYDSRNWNFYSFKGFRSWFSLLGYELCFYGIYMYHLWHIEPNQNGYMDKKHKNHKLF
                     YKHLKKIKDYSIDPLQISSVKNTKILVIANKKYNFRALSVYLGKFVYKSLDELAYQNK
                     KEIEIYLQEEKFDKILLDKNIYNEDLHEIFKNYLCVYFELGILPDSWLFYKDTNELYD
                     RRYWDIDLDKLQIKEMQAYITSLKFEEKKKILTFIQKLGFSKDDDLYKIVFYLKNELY
                     SFAKNDGFYQIIFDKKKMLMLGNYDKEYYSLKSFVYKPYLYEIKRVRFFSKILEFLGL
                     NFLLSLISQTKFYRLARKLFFNPRDFFKDSKFYKRHNNAN"
     gene            complement(219006..219953)
                     /gene="kpsF"
                     /locus_tag="DMACINML_00482"
     CDS             complement(219006..219953)
                     /gene="kpsF"
                     /locus_tag="DMACINML_00482"
                     /inference="ab initio prediction:Prodigal:2.6"
                     /inference="similar to AA sequence:RefSeq:CAL35552.1"
                     /codon_start=1
                     /transl_table=11
                     /product="D-arabinose 5-phosphate isomerase"
                     /protein_id="Prokka:DMACINML_00482"
                     /translation="MDALKTAKEVFATEAKAIEDLALNLDENFSKAIELMLHTKGRCI
                     VSGMGKSGHIGAKIAATLASTGTPSFFIHPGEALHGDLGMLTPDDVLIAISNSGETEE
                     ILKIIPAIKKRKIPLIAMCGKKNSTLVKQGDIFLNISVKEEACPLQLAPMSSTTATLV
                     MGDALAAALMKARNFRPDDFALFHPGGSLGRKLLTRVSDLMVSKNLPIVHPDTEFNDL
                     VDVMTSGKLGLCLVLENEKLVGIITDGDLRRALKANDKPRFDFKAKEIMSINPKVVDA
                     DAMASEAEEIMLKYKIKEIVVSKEDKVVGIIQLYAIGNV"
     gene            complement(219963..221594)
                     /gene="kpsD"
                     /locus_tag="DMACINML_00483"
     CDS             complement(219963..221594)
                     /gene="kpsD"
                     /locus_tag="DMACINML_00483"
                     /inference="ab initio prediction:Prodigal:2.6"
                     /inference="similar to AA sequence:RefSeq:CAL35553.1"
                     /codon_start=1
                     /transl_table=11
                     /product="capsule polysaccharide export system periplasmic
                     protein"
                     /protein_id="Prokka:DMACINML_00483"
                     /translation="MKKILILFLSCIFCFGAIDVSQISAGGNSSTTSSSQNIENNISK
                     SAPTQIPVFGAELFNGNFKNYTQRVYNPDYKIAVGDQISLKIWGAVEFEQILVVDSQG
                     NIFIPKVGAVNLLGVKNSALVSVIKAQVNKIYKNNVFVYADMNAYQNVSVFVTGSVNA
                     PGLYQGLSSDSVIQYLDKAGGINLEYGSFRDIQILRNNAVIKKIDLYDFLLKGQMDLF
```

```
                 PFRSGDVVLVGNVQSYVFVNGDVQRPFRFELANDIKTLFDLARVAGAKPIVTNAILRS
                 YGNDHKLEVSAYNKMQFSKVLLKTGDEVQFNPEYISQNISITVNGEHSGLKTLVVRKG
                 TTLEDVSRLIVANGQSDMNALQVFRKSVAKTQKDLINAQLKELETLALTSPSVTSQGA
                 AIKAEQAKLILEFIQRARELEPKGQIVIDKPKSYGEVILEEGDTINVPSKNNLIIVQG
                 EVTLPGAFVYNKGEDLKYYINLAGGYGERADTSKVLVIRNNGKAQRYSGSVDMMPGDS
                 VLVLPKVESENLQIFSMLTQILYQIAVATNVVLNI"
sig_peptide      complement(221517..221594)
                 /gene="kpsD"
                 /locus_tag="DMACINML_00483"
                 /inference="ab initio prediction:SignalP:4.1"
                 /note="predicted cleavage at residue 26"
gene             complement(221596..222711)
                 /gene="kpsE"
                 /locus_tag="DMACINML_00484"
CDS              complement(221596..222711)
                 /gene="kpsE"
                 /locus_tag="DMACINML_00484"
                 /inference="ab initio prediction:Prodigal:2.6"
                 /inference="similar to AA sequence:RefSeq:CAL35554.1"
                 /codon_start=1
                 /transl_table=11
                 /product="capsule polysaccharide export system inner
                 membrane protein"
                 /protein_id="Prokka:DMACINML_00484"
                 /translation="MTKINTFFNKIKDLSIFDSFKIVWILMIFVIIYYILIAADRYVS
                 TITMSVKSTTGSTQASGVLSLLTATSNTSEDIKFLQGYIESLDMLKILDEKIHLKKLY
                 NEQYIDLFFSLSSSSSIESYLKYYQSRVKVHVDDKTGLLNVEVEGFTPESAHLIAKTI
                 MQESEKFINEISHKAAREQMSFAEEELVKYKERYQKAQNDLIAFQNKYGVFDPLKQAE
                 AKAGLVTQLESDIAQREAKLLTMQSYMNDHAPEIVTLKAEIAALKKQLVKERSKISAD
                 NSSQKLNDLAAKFQDLTIEAGFAQSAYEAALKAYESARIEALRKIKQLVIVQTPDVPQ
                 SAKYPEKIYNILTAFIVLSLIFGVIKFVKMIIEEHKY"
gene             complement(222711..223373)
                 /gene="kpsT"
                 /locus_tag="DMACINML_00485"
CDS              complement(222711..223373)
                 /gene="kpsT"
                 /locus_tag="DMACINML_00485"
                 /inference="ab initio prediction:Prodigal:2.6"
                 /inference="similar to AA sequence:RefSeq:CAL35555.1"
                 /codon_start=1
                 /transl_table=11
                 /product="capsule polysaccharide export ATP-binding
                 protein"
                 /protein_id="Prokka:DMACINML_00485"
                 /translation="MIKVLNLTKSYPLFNGGRHYVFKDFTFEFPENCSIGLMGRNGAG
                 KSTLMKLLSGSELPDRGKIITNKKFSWPLGLSGAFQGSLTARDNAKFVARVYGYKGEE
                 LHEKVKFVEDFAELGKFFDEPMNTYSSGMSARIAFGLSMAFDFDYYLIDEAGAVGDPK
                 FREKSHKLYKERLSQSKVIMVSHNVAEIKEWCDKIIFMENGKATVYDDVDEGIAVYQG
                 KK"
gene             complement(223370..224152)
                 /gene="kpsM"
                 /locus_tag="DMACINML_00486"
CDS              complement(223370..224152)
                 /gene="kpsM"
```

```
                     /locus_tag="DMACINML_00486"
                     /inference="ab initio prediction:Prodigal:2.6"
                     /inference="similar to AA sequence:RefSeq:ABS44297.1"
                     /codon_start=1
                     /transl_table=11
                     /product="capsular polysaccharide ABC transporter,
                     permease protein"
                     /protein_id="Prokka:DMACINML_00486"
                     /translation="MLNVIHALFFRELKTRFGKNKYLGYFWVIGEPMSVILMLTIIVT
                     IIREYHHQLMPEGISIFMFLISGIVPYFMFRSIVTQLMNGIAANLPLFAYKPVKPIHV
                     FIARTLLEFCIYFVIFVAVLFIVGWFFRLEVIPRHFLSVMICIFLLMCSGFALGMVFA
                     IIGYFLEPLKTLLNYFSIVFYWGSGVIFPTWLMPKPILDIFYYNPLLHVMELLRFNFF
                     ENYPLQDDYTYTYPVFCIICTSFVGLFLYYYNRQALTAVRRT"
     gene            complement(224211..224627)
                     /locus_tag="DMACINML_00487"
     CDS             complement(224211..224627)
                     /locus_tag="DMACINML_00487"
                     /inference="ab initio prediction:Prodigal:2.6"
                     /inference="similar to AA sequence:RefSeq:CAL35557.1"
                     /note="hypothetical protein Cj1449c"
                     /codon_start=1
                     /transl_table=11
                     /product="hypothetical protein"
                     /protein_id="Prokka:DMACINML_00487"
                     /translation="MDERILEFIKNEQLLSWAMSDEKGVYIANAFYAFDEKNLAFIIA
                     SHEDTKHIKLASENPSIALNIAKESKIAFLKGIQAKAKFKSASKEQIKIYFSKFPFAK
                     LDKSAKIYALELDWLKFTNNALGLGKKLEFYKDKIL"
     gene            224704..225267
                     /locus_tag="DMACINML_00488"
     CDS             224704..225267
                     /locus_tag="DMACINML_00488"
                     /inference="ab initio prediction:Prodigal:2.6"
                     /inference="similar to AA sequence:RefSeq:AMK28270.1"
                     /codon_start=1
                     /transl_table=11
                     /product="ATP-binding protein"
                     /protein_id="Prokka:DMACINML_00488"
                     /translation="MQIDASKNQSFSMDYTTKSGKHLALSMYDNQSLSYSNDEEGKSL
                     NLKRQYGFSFTFEGSKLTQADLDEIKNAMKEVEPMIKDFLANSKVGELKPKEIIESAM
                     QMANVLPTPNDENHQNAIMNNFTNKFSDLLKQNQSNDKDINASMLEDSKKLLDEVLEQ
                     MKKQLEKQQEKAKENQNNAEKGLNLYA"
     gene            225307..225996
                     /gene="dut"
                     /locus_tag="DMACINML_00489"
     CDS             225307..225996
                     /gene="dut"
                     /locus_tag="DMACINML_00489"
                     /EC_number="3.6.1.23"
                     /inference="ab initio prediction:Prodigal:2.6"
                     /inference="similar to AA sequence:RefSeq:CAL35559.1"
                     /codon_start=1
                     /transl_table=11
                     /product="dUTPase"
                     /protein_id="Prokka:DMACINML_00489"
```

```
                     /translation="MKNVEILENMLKLQQKLNDETNGLNWEEGYTKEGKLISWRRCIY
                     MECAELIDSFAWKHWKNISSPTNWENVRMEIVDIWHFILSLLLEEYTNKKDKNFQAIA
                     NEIISVSVFQDFCKEEGNPSENDLYSILNDIELIIHKCSGFGFSLGELLSNYFILAIK
                     CGLNLEILYKTYIGKNILNIFRQNNGYKQGTYKKDWNGKEDNEVLAEILEQELDFDTI
                     YKKLEEYYKKV"
gene                 225998..226708
                     /locus_tag="DMACINML_00490"
CDS                  225998..226708
                     /locus_tag="DMACINML_00490"
                     /inference="ab initio prediction:Prodigal:2.6"
                     /inference="similar to AA sequence:RefSeq:CAL35560.1"
                     /codon_start=1
                     /transl_table=11
                     /product="putative integral membrane protein"
                     /protein_id="Prokka:DMACINML_00490"
                     /translation="MKILRLAIKDFFTLQFLKFALIPLTFSFILMIFLAIFGFSFLLD
                     YFNSLFSVGEDSFWAWFYALHFVQILITLISVLFSGFVIIFASVFLALFITSFLTPLI
                     VKQINNKYYHHEVQNISNMYIIFEIFKIFLKFIGIFLLCTLALFLPFINLFVYYLAFY
                     YLFHKLLMLDITSSVLDKENFKIFNAQASTFEFKFSTLCFYLLSSIPLLGIFLQVFFV
                     IFLTHLAYQRILKLEVKG"
gene                 complement(226691..227656)
                     /gene="tilS"
                     /locus_tag="DMACINML_00491"
CDS                  complement(226691..227656)
                     /gene="tilS"
                     /locus_tag="DMACINML_00491"
                     /EC_number="6.3.4.-"
                     /inference="ab initio prediction:Prodigal:2.6"
                     /inference="similar to AA sequence:RefSeq:CAL35561.1"
                     /codon_start=1
                     /transl_table=11
                     /product="putative tRNA(Ile)-lysidine synthase"
                     /protein_id="Prokka:DMACINML_00491"
                     /translation="MQIKDEILSLLKKGRNLLAFSYGSDSSALFHFLVQKKIDFDLVM
                     INYKTRKNSDLEEQKAKELALEFHKKIFIKIAPVIKGNFEKEARDFRYDFFEKICLEQ
                     NYDKLILAHHLNDQLEWFLMQLSRGAGLAEILGMQECEKRQNYTLLRPLLFTSKDEIL
                     SYLKENDIFYFQDESNENEKYFRNYIRKNFSNAFVSKFHQGLKRSFFYLDEDRKKLYD
                     LESIKEIQGLMICPKNESLIAKVVKMKGLLLSAAQRKEFLKGDCVLGGKIGIVYKDEK
                     AIVFKYEICQKLPKEFKEACRIAKIPRLLRAYLYNHKIDISALNF"
gene                 complement(227646..228965)
                     /gene="yliG"
                     /locus_tag="DMACINML_00492"
CDS                  complement(227646..228965)
                     /gene="yliG"
                     /locus_tag="DMACINML_00492"
                     /inference="ab initio prediction:Prodigal:2.6"
                     /inference="similar to AA sequence:RefSeq:ABS43656.1"
                     /codon_start=1
                     /transl_table=11
                     /product="MiaB-like tRNA modifying enzyme YliG_"
                     /protein_id="Prokka:DMACINML_00492"
                     /translation="MSKLYLMSLGCNKNLVDSEIMLGRLSAYELCDEPSKADVLIVNT
                     CGFIDSAKKESINAILDLHEQRKKDSLLVVTGCLMQRYREELMKELPEVDLFTGVGDY
                     ERIDEMILKKTNLFSNSTYLQGENSKRIITGSNSHAFIKIAEGCNQKCSFCAIPSFKG
```

```
                     KLKSREISSIIAELKDLVARGYKDFSFIAQDTSSYLFDKGEKDGLIRLIDEVEKIEGI
                     KAARILYLYPTSASEALIKRIIASKIFVNYFDMPLQHISDNMLKIMKRGANSTRLKEM
                     LNLMKSAPDSFLRTGFIVGHPGESDADFEELCEFIKDFGFDRVSVFAYSKEEDTAAFD
                     MEQVPFKVINKRLKIIEKIVDEVIEKSFEKEVGKKRLVVCTGESSEGEFFIAAKDLRW
                     DREIDGEILINESECGNLEMGQIYECEIIQNVDKKLIAKALRKIDAN"
gene                 229039..230136
                     /locus_tag="DMACINML_00493"
CDS                  229039..230136
                     /locus_tag="DMACINML_00493"
                     /inference="ab initio prediction:Prodigal:2.6"
                     /inference="similar to AA sequence:RefSeq:AGQ94588.1"
                     /codon_start=1
                     /transl_table=11
                     /product="peptide chain release factor 2"
                     /protein_id="Prokka:DMACINML_00493"
                     /translation="MDNYEFSELLKTLKNKVGNIASIIKPENIQTRLKKIEELENSPS
                     FWNDVKQAGIIGKEKTKITNLLKNYENAFNALNDASELFDLANSENDTETLEALFNDA
                     PKLEDTITSLEISMLLSGENDGKNAIVSIHPGAGGTESNDWASILYRMYLRFCEREGF
                     KVETLDFQEGEEAGLKDVSFLVKGENAYGYLKAENGIHRLVRTSPFDSAGRRHTSFSS
                     VMVSPELDDDIEIEIEEKDIRIDYYRASGAGGQHVNKTESAVRITHFPTGIVVQCQND
                     RSQHKNKATAFKMLKSRLYELELMKQQDSANAGEKSEIGWGHQIRSYVLFPYQQVKDN
                     RSGEAFSQVDNILDGDIKKMIEGVLIALKSE"
gene                 complement(230219..230611)
                     /locus_tag="DMACINML_00494"
CDS                  complement(230219..230611)
                     /locus_tag="DMACINML_00494"
                     /inference="ab initio prediction:Prodigal:2.6"
                     /inference="similar to AA sequence:RefSeq:ADC29315.1"
                     /codon_start=1
                     /transl_table=11
                     /product="putative lipoprotein"
                     /protein_id="Prokka:DMACINML_00494"
                     /translation="MKKILTSALALGVMTLIIGCGDGDLVELKNNGNDQAMFDLGNQE
                     IMKEYPNYKLYDYTSVRAISDFKDPEGKIKEMKRDLEIDKYPYSNYVFLLNPNDKKDY
                     KVVYVYCDSDLKKRIYCPVVDAYRIYAR"
gene                 complement(230732..231850)
                     /gene="truD"
                     /locus_tag="DMACINML_00495"
CDS                  complement(230732..231850)
                     /gene="truD"
                     /locus_tag="DMACINML_00495"
                     /EC_number="5.4.99.-"
                     /inference="ab initio prediction:Prodigal:2.6"
                     /inference="similar to AA sequence:RefSeq:CAL35565.1"
                     /codon_start=1
                     /transl_table=11
                     /product="tRNA pseudouridine synthase D"
                     /protein_id="Prokka:DMACINML_00495"
                     /translation="MNLAEENTIFKPLYSLKHSPINAYFSKNSDDFVVRERPLYEFSG
                     KGEHLILHINKKDLSTNEALRILSELSGVKMRDFGYAGLKDKQGSTFQYLSMPKKFES
                     FLANFSHPKLKILETFIHENKLRIGHLKGNSFFIRLKKVLPSDALKLEQALINLDKQG
                     FANYFGYQRFGKFGDNYKEGLEILRGKKMKNVKMKEFLISAFQSELFNRYLSKRVELS
                     HFANDFTEKELAQIYSISKEEAKELKKQEQFFKLLKGEVLGHYPFGKCFLCEDLSAEL
                     ERFKARDISAMGLLIGAKAYEASQGLALNLENEIFKDALEFKAKMQGSRRFMWSYLED
```

```
                     VKWRYDEQKAHFCMEFFLQKGSYATVVLEEILHKNLFE"
gene                 complement(231819..232640)
                     /gene="thiL"
                     /locus_tag="DMACINML_00496"
CDS                  complement(231819..232640)
                     /gene="thiL"
                     /locus_tag="DMACINML_00496"
                     /EC_number="2.7.4.16"
                     /inference="ab initio prediction:Prodigal:2.6"
                     /inference="similar to AA sequence:RefSeq:CAL35566.1"
                     /codon_start=1
                     /transl_table=11
                     /product="putative thiamin-monophosphate kinase"
                     /protein_id="Prokka:DMACINML_00496"
                     /translation="MNKEDFIIKAFLNEKNGDDGAIVDKWCLSKDLFFENVHFKREWL
                     TLEQIATKAMLVNISDAIVMNAKPKYALLGLALPNSLGEDEIKALQKGFLKTAKKFNI
                     KIIGGDTISNDKIDISITIISKVRNKAIFRKGLKKGHLLAYTGVLGKSLRGLEILQNG
                     GTLNSNHVFIKPKLRPDFFYEIAPFISCAMDISDGLSKDLSRLLAQNKCGISWFKTLD
                     DYTLYSGEEYEILFAFDEKEYPKIEKIAKKHGVKINIFGKAVKGKYEFSGREHHF"
gene                 232722..233795
                     /locus_tag="DMACINML_00497"
CDS                  232722..233795
                     /locus_tag="DMACINML_00497"
                     /inference="ab initio prediction:Prodigal:2.6"
                     /inference="similar to AA sequence:RefSeq:CAL35567.1"
                     /note="hypothetical protein Cj1459"
                     /codon_start=1
                     /transl_table=11
                     /product="hypothetical protein"
                     /protein_id="Prokka:DMACINML_00497"
                     /translation="MKNLELRIFRFDKQKDYEAYYKPYVYDNYENFATLYDLLLQVQD
                     DDIYFDFEKNDKSYIVVNKEFLPLDTALDILVKKYDFNLTIEPLSTKRSVKDLIINKD
                     DFLEKFKYLAPFANEEDKKLYEKYDYLYYSSEILDFLPEYMGDAVFYLASKMIEKYPN
                     KKIKILKTICDTQKGIFYHLPSKNEDLENIIKNLQKEIIDLKLINEAALEFDLPKINA
                     FDNEIKELGEVKYDFDNFNIACYGFKIKDDIKSKIKAHFISYENSDKNNGFSLLHLNP
                     ELSYKMAANIILDAYDSGADFIVVNQAKDFYMFDTCSKKLMQSSGREFKDFYVLSYFE
                     FLSLIQGIKNPSLQNHDLKVSLI"
gene                 233792..234175
                     /locus_tag="DMACINML_00498"
CDS                  233792..234175
                     /locus_tag="DMACINML_00498"
                     /inference="ab initio prediction:Prodigal:2.6"
                     /inference="similar to AA sequence:RefSeq:CAL35568.1"
                     /note="hypothetical protein Cj1460"
                     /codon_start=1
                     /transl_table=11
                     /product="hypothetical protein"
                     /protein_id="Prokka:DMACINML_00498"
                     /translation="MKISLECKDLIIEKALELFLKEHLVMKKDCDFIISDEKISTQKP
                     LFIIAKNSPFLSVPFSKETLINSLNEFDSALKATAQKLADERRRVLEARIDEIANEFK
                     KDYQSKIDLAISELKDKLVKALMYE"
gene                 234168..234857
                     /locus_tag="DMACINML_00499"
CDS                  234168..234857
```

```
                     /locus_tag="DMACINML_00499"
                     /EC_number="2.1.1.171"
                     /inference="ab initio prediction:Prodigal:2.6"
                     /inference="similar to AA sequence:RefSeq:ANS24505.1"
                     /codon_start=1
                     /transl_table=11
                     /product="methyltransferase, putative"
                     /protein_id="Prokka:DMACINML_00499"
                     /translation="MNNEFVSVKDFLQGKKNIHFNDSTQPNKKEKTKKTLPKLYTSIE
                     SGIYKGKKLLLPSLQTTRSTKSIVKKCVFNVLRLDLRNKIFIEAFGGSALMAAEALSN
                     YALKAYAIELDLNAYKIALENAKNIDTNLKVIHANTFEMLPKLIKDSKEDIILYLDPP
                     FDIREGFSGIYEKIYNFLEKLKLDALFCIILEHNSKIKTPDNIANFTKTKEKKFGSTS
                     LSFYQKNILEE"
     gene            234920..235966
                     /gene="flgI"
                     /locus_tag="DMACINML_00500"
     CDS             234920..235966
                     /gene="flgI"
                     /locus_tag="DMACINML_00500"
                     /inference="ab initio prediction:Prodigal:2.6"
                     /inference="similar to AA sequence:RefSeq:CAL35570.1"
                     /codon_start=1
                     /transl_table=11
                     /product="flagellar P-ring protein"
                     /protein_id="Prokka:DMACINML_00500"
                     /translation="MRVLTFFLLLMTSVFATQIKDIANTVGVRDNQLIGYGLVVGLNG
                     SGDGTSSKFTLQSISNLLQGMNIKVDPNDIKSKNTAAVMVTAKLPAFAKSGDKLDITV
                     SSMGDAKSLQGGTLLLTALRGIDGEIYAIAQGSISTGGLTARPGGTGSHSTAATVMGG
                     ANVEREIPQNFSQIDDLTLSLKVADFKTASDIERILNTIFDEEVARAIDSRTVKLKKP
                     EDLSNVDFMARVLEQDIAYTPQSKVIIDERTGTVIAGVDIEVEPVLITHKDITIKIDP
                     NNNAAAAQNEIDMKDGGFVDPNSNTLRINNAKSTVANIARMLNKLGATPNDIIAIMEN
                     LKRAGAINADLEII"
     sig_peptide     234920..234970
                     /gene="flgI"
                     /locus_tag="DMACINML_00500"
                     /inference="ab initio prediction:SignalP:4.1"
                     /note="predicted cleavage at residue 17"
     gene            235966..236307
                     /locus_tag="DMACINML_00501"
     CDS             235966..236307
                     /locus_tag="DMACINML_00501"
                     /inference="ab initio prediction:Prodigal:2.6"
                     /inference="similar to AA sequence:RefSeq:CAL35571.1"
                     /note="hypothetical protein Cj1463"
                     /codon_start=1
                     /transl_table=11
                     /product="hypothetical protein"
                     /protein_id="Prokka:DMACINML_00501"
                     /translation="MKVDNFLSTYSMNNALLERAAKARNLESDFKINNNEIITKTKED
                     EALKEQTDAFEAFFLKQVLDVSLKTQNSLFGKDASDEIYSSMYNDTMSKALSGGMGFS
                     KLLYDFLKERG"
     gene            236371..236568
                     /locus_tag="DMACINML_00502"
     CDS             236371..236568
```

```
                     /locus_tag="DMACINML_00502"
                     /inference="ab initio prediction:Prodigal:2.6"
                     /inference="similar to AA sequence:RefSeq:CAL35572.1"
                     /note="hypothetical protein Cj1464"
                     /codon_start=1
                     /transl_table=11
                     /product="hypothetical protein"
                     /protein_id="Prokka:DMACINML_00502"
                     /translation="MINPIQQSYVGNATLNTTKTDKETKTNDTQKTENDKAARIAEQI
                     KNGSYKIDTKATAQAIAESLI"
gene                 236607..237041
                     /locus_tag="DMACINML_00503"
CDS                  236607..237041
                     /locus_tag="DMACINML_00503"
                     /inference="ab initio prediction:Prodigal:2.6"
                     /inference="similar to AA sequence:RefSeq:CAL35573.1"
                     /note="hypothetical protein Cj1465"
                     /codon_start=1
                     /transl_table=11
                     /product="hypothetical protein"
                     /protein_id="Prokka:DMACINML_00503"
                     /translation="MLKQRLDEVNAILTKLIELTQEDIENIKIAKHDTVASSVEEKNK
                     LIAEFSAAKKRLDSALVELNNSSTKGLSELLDEEDKQKLDLLKKNLQTLHTTNKEYAK
                     FVLIVKDFLDGLVNKMFDMNDGTNNAYGDKKTTPESIFKINV"
gene                 237051..238877
                     /gene="flgK"
                     /locus_tag="DMACINML_00504"
CDS                  237051..238877
                     /gene="flgK"
                     /locus_tag="DMACINML_00504"
                     /inference="ab initio prediction:Prodigal:2.6"
                     /inference="similar to AA sequence:RefSeq:CAL35574.1"
                     /codon_start=1
                     /transl_table=11
                     /product="putative flagellar hook-associated protein"
                     /protein_id="Prokka:DMACINML_00504"
                     /translation="MGIFGTLYTGVTGLKASEVQIATTSNNISNANATFYTRQRVVQT
                     TNGFVTTGGVQVGTGTAIESIVRLHDEYSYYKLKGASNQLEYTKYMASTLEEIAQRFP
                     DLQSTGILQDLENYNKAWNDFASNPNENATKIALVKASQTLTESVNNTFATLDKIQKK
                     VNDDIKNTVDEINRIGEEIATINKQIYGQEALPTDHANELRDRRDELELTLSKLVNAV
                     ASKNEINQDNRLDTTIIDPGHQYNLSIEGFSIVDGVNFHPLKLDYDDKNKAYSIYYET
                     PDEKVRDLTAKINGGQLGAQLDLRGRNYSKSEGKYSDGIIQGYMDSLNTFAKTMINET
                     NNLYASSAKSSATSDYLPGLTEDIPLVNYDRTIQPGSFDIVIYDDKGDKKLTKTITVD
                     VNTTMEDIMRQITANTDDNGNNNANDDVDDHINASFSYDAKTGDSLFQINAKSGFKVA
                     IEDKGTNFAGAFSIGGFFSGTNASDIKVKDSILNDPSTVRASLNGVDSGNDMANKIIQ
                     LQYSKVNFYNEDGTIDNLTMEEYYRKLTGKIGSDGENNNVVNASNQTLYNSVYSEYQS
                     KSGVNTNEELAALIQWQSSYGAAAKIVTTVDQMLDTLLGLKS"
gene                 238894..239658
                     /locus_tag="DMACINML_00505"
CDS                  238894..239658
                     /locus_tag="DMACINML_00505"
                     /inference="ab initio prediction:Prodigal:2.6"
                     /inference="similar to AA sequence:RefSeq:CAL35575.1"
                     /note="hypothetical protein Cj1467"
```

```
                     /codon_start=1
                     /transl_table=11
                     /product="hypothetical protein"
                     /protein_id="Prokka:DMACINML_00505"
                     /translation="MKNFIQLKTKLDSLVKEKNTNKALFESPDPLQIAKFHNDEFIAL
                     LCALFAYGNAKNIVNFLKKLDFSLLDLSQKQIQKELQNLKYRFQNERDIQEIFLTFSR
                     LKNEISLHELFSTAYEKRENTTDAILAFLQKIKTLNSYSSYGYDFFFGKIWQNTPTSP
                     LKRYNMYLRWMVRKDELDLGLFTKIHTKDLLIPLDTHTHKISLTLGLLKRKIYDYKSV
                     LELTRNLKKLDANDPIKYDFALYRLGQNKALSKLKE"
     gene            239662..240426
                     /locus_tag="DMACINML_00506"
     CDS             239662..240426
                     /locus_tag="DMACINML_00506"
                     /inference="ab initio prediction:Prodigal:2.6"
                     /inference="similar to AA sequence:RefSeq:CAL35576.1"
                     /codon_start=1
                     /transl_table=11
                     /product="putative integral membrane protein"
                     /protein_id="Prokka:DMACINML_00506"
                     /translation="MGFEHLDTIYYFVLFFVALFAGFIDSIVGGGGLITLPALIACGI
                     PAHLSLATNKLQSVFGSFTATLTYFKSTTLPHLAYGIFFTALGAAIGSYSVLFVKDEH
                     LKLIILIFLTLTFLYTALRPSLGKHESEAKIKNIKFFHLICGLTLGFYDGFLGPGTGS
                     FWIFACVMLLGFNMKKASINTKILNFTSNVVALFIFLWQYELLWIVGLLMGVGQILGA
                     YLGSKLVLKTNGKFIKTLFLIVVGATIIKVAWDYFS"
     gene            complement(240423..241601)
                     /gene="ctsF"
                     /locus_tag="DMACINML_00507"
     CDS             complement(240423..241601)
                     /gene="ctsF"
                     /locus_tag="DMACINML_00507"
                     /inference="ab initio prediction:Prodigal:2.6"
                     /inference="similar to AA sequence:RefSeq:AAW36076.1"
                     /codon_start=1
                     /transl_table=11
                     /product="general secretory pathway protein F"
                     /protein_id="Prokka:DMACINML_00507"
                     /translation="MKLYELEILKDKTKQKKHLRARNLNEAQAKAIAKKWEILNIKEM
                     QKAGFKRLDNEKFILFFQELSLLCEVGISIKDALNELNKIYSHKMIAKLCDNLNLGQN
                     LSLAFENANFGLTHAELALIKTSQNTGKLSEIFMQISRLRQNNVENEQRVKKALRYPL
                     IVFLSVCAAFVLLMLFVVPNFKDLFEDLNLTLPFITELIIYIYNFLDKYILLVLCLII
                     VVLFLALFLYRKYYFFSYFCDSLLVKIPLLSKFIIYQQNYSFFMIFSLLLKSGISLLD
                     AFRLATLALNNKKILLCYQEVKTFMDSGLEISYAFKKVDLFDPLVFSMLSVAMKSSKL
                     EFLSEEIAKYYKNKSENLMDKFLSFLEPIMTLWVAILVLFLALGIFLPIWELSSGVNF
                     "
     gene            complement(241588..243150)
                     /gene="ctsE"
                     /locus_tag="DMACINML_00508"
     CDS             complement(241588..243150)
                     /gene="ctsE"
                     /locus_tag="DMACINML_00508"
                     /inference="ab initio prediction:Prodigal:2.6"
                     /inference="similar to AA sequence:RefSeq:CAL35578.1"
                     /codon_start=1
                     /transl_table=11
```

```
                     /product="putative type II protein secretion system E
                     protein"
                     /protein_id="Prokka:DMACINML_00508"
                     /translation="MIERFLFQDYINDKITLEQIYHEHKIDSESFLKSLASDENISYS
                     TLEELDLSLGEKFPCSFLLKFKILPILSKENTLYIASSKPCSLELLDEIRIFYEAKDI
                     EVLIANEFKIMKFLYKLQVKEKLQNLSANLRLEWKENKYQNEQSYISQIFDFILDEIL
                     EFNPSDIHIEARENDALIRFRVDGILREFACLEKDIYEALVFHIKFLSQLNVAESRKA
                     QDGNFELKIKENRYDFRISSLPLQNGESIVIRILKHNEEILDLENLNLGEKNLTILKK
                     ALYNPYGMILLTGPTGSGKSTSLYACLNELKSIEKKIISVEDPIEYKMPLVQQILLNP
                     KAGLEFNNALRAILRQDPDIIMIGEIRDEESLDIAIKASLTGHLLLSTLHTNNAISTI
                     DRLIDMNAKPYLIASALRIIIAQRLVRKLCPKCKQKSTKIYEIKGDFFEAKGCEHCHH
                     SGYQGRELISECLYIDEEIAQVIREGGYKNKILELAKAKGFQSMFEQGLQKAKEGITS
                     IDELVRVLNEAL"
     gene            complement(243143..243739)
                     /locus_tag="DMACINML_00509"
     CDS             complement(243143..243739)
                     /locus_tag="DMACINML_00509"
                     /inference="ab initio prediction:Prodigal:2.6"
                     /inference="similar to AA sequence:RefSeq:CAL35579.1"
                     /codon_start=1
                     /transl_table=11
                     /product="putative membrane protein"
                     /protein_id="Prokka:DMACINML_00509"
                     /translation="MNIEERVRELEDKYKRYLLKKYFKFLFIFAALVVCMVISFFVIQ
                     NFYKQKALALEALEYKKTLNHQIAQAQIANEKNKILQERLSKDENKDELKEELKTQID
                     IYSKLLNISDLKRNFYQNPSYEKAMNLAARYYENKDYEKSIFWSLKANDIDRKNSSSW
                     ILFSKAKQALGKDEEAKQALDTYVSYYGLIEFDKEIDD"
     gene            complement(243736..244332)
                     /gene="ctsP"
                     /locus_tag="DMACINML_00510"
     CDS             complement(243736..244332)
                     /gene="ctsP"
                     /locus_tag="DMACINML_00510"
                     /inference="ab initio prediction:Prodigal:2.6"
                     /inference="similar to AA sequence:RefSeq:CAL35580.1"
                     /codon_start=1
                     /transl_table=11
                     /product="putative ATP/GTP-binding protein"
                     /protein_id="Prokka:DMACINML_00510"
                     /translation="MNNIIAFRQECFNQINALLKNKALILLWGKSGSGKSVLLQRLAL
                     HHNEDFTNQIFTDEKDFHKFIQDKKRKIIILDEVGMYSPDILELIRIYSDQMSFVLSS
                     HKKINLFEKEYFKSRFSAMFWLQKPNINELSSYIKMKFDQELSQKSLKFLMKIYQGNL
                     RYLDKILQSFCELNAYLNNNKNQDYILRLSALEQGFLK"
     gene            complement(244325..245743)
                     /gene="ctsD"
                     /locus_tag="DMACINML_00511"
     CDS             complement(244325..245743)
                     /gene="ctsD"
                     /locus_tag="DMACINML_00511"
                     /inference="ab initio prediction:Prodigal:2.6"
                     /inference="similar to AA sequence:RefSeq:CAL35581.1"
                     /codon_start=1
                     /transl_table=11
                     /product="putative type II protein secretion system D
```

protein"
/protein_id="Prokka:DMACINML_00511"
/translation="MIRLIFIKFLFFTNLIYAIECQKRLFDISINEDLSIEESLRELA
SYCSFSVVVKDTIAKEKLKTVQSSINIHQMSLDEIFKLFIEEQDLAYKFDGKVLKISA
LQTKIFKINYITSVREGQSITKASVDSKPRQSEYYSAFEDNEDNMIKSMEKFDFWQNI
EKEIMILLSSSHENYDIKAPIVNPNAGLVIVTGTHSQLQRVKAYLQKLESRLKKQVII
DVSILAVNLNKNHSSGINWQNFNIGLNSQTSDNTNSFMQIQNGQGFVKNLGLRANLNF
DSIVNFLSQSGKTSVLSNPKLMALNNQQAIISIGDTVNYQVKESSKGTENGTTVSESY
SNYSIFIGILLNILPEISDDGKIMLRINPSLSDFKYPEDNKRQKEPRTIAPDTIQKKL
SSVVQVENNQTLILGGLISHNRSQNNNSVNFLSKIPILGSLFRSEDENSNITEIVFII
TPSIVDNTHIPSLRELGFKYNE"

gene complement(245721..246017)
/locus_tag="DMACINML_00512"

CDS complement(245721..246017)
/locus_tag="DMACINML_00512"
/inference="ab initio prediction:Prodigal:2.6"
/inference="similar to AA sequence:RefSeq:CAL35582.1"
/note="hypothetical protein Cj1475c"
/codon_start=1
/transl_table=11
/product="hypothetical protein"
/protein_id="Prokka:DMACINML_00512"
/translation="MLSYEDFEEYFKILNTKQSVELGNFQNPFFNPTFEKIKEIKITA
IMLDRAKINNKWYKKGDKIGEAVITDITTKEIILRYDTLDFKIAFKNNDKINIY"

gene complement(246056..249616)
/locus_tag="DMACINML_00513"

CDS complement(246056..249616)
/locus_tag="DMACINML_00513"
/inference="ab initio prediction:Prodigal:2.6"
/inference="similar to AA sequence:RefSeq:CAL35583.1"
/codon_start=1
/transl_table=11
/product="pyruvate-flavodoxin oxidoreductase"
/protein_id="Prokka:DMACINML_00513"
/translation="MGKIMKTMDGNEAAAYAAYAFTEVAGIYPITPSSPMADYTDMWA
AAGKKNLFGVPVKIVEMQSEAGAAGTVHGSLQTGALTTTYTASQGLLLKIPNMYKIAG
QLLPCVIHVAARSLAAQALSIFGDHQDIYAARQIGFAMLCSHSVQETMDLAGVAHLCA
IKGRVPFLHFFDGFRTSHEIQKVEVMDYAHFDRLLDRKALLEFRNSALNPENPKTRGT
AQNDDIYFQTREVSNRFYDALPDVVNEYMQEISKITGREYKPFTYYGHKEPERIVIAM
GSVTQTLEEVVDHLNSKGEKVGIVKVYLYRPFSLKYFFDVMPKSVKKIAVLDRTKEPG
SLGEPLYLDIKSAFYGKENAPVIVGGRYGLSSKDVDPAQMIAVFENLKLDNPKDGFTV
GIIDDVTHTSLQTGEKISLGDESAIECLFYGLGADGTVGANKNSIKIIGDKTDFYAQA
YFAYDSKKSGGYTRSHLRFSKKPIRSTYLVSTPHFIACSVAAYLEIYDVLAGIRKGGT
FLLNSIWNAEETIRQLPDAVKKTLAEKEVNFYIINATKLARDIGLGNRTNTIMQSAFF
KLAKIIPYEDAQKYMKELAYKSYSKKGDAIVEMNYKAIDVGADGLVKVEVDPSWKNLE
IKEKEQTNAYKGTEFVEKIVKPMNAAKGDELPVSTFLGYEDGSFEHGTTEYEKRGVGV
MVPRWIEANCIQCNQCASVCPHAVIRPFLINDEEMAKAPRGVKDHALEAKGTKGEKLS
FKIQVSPLDCTGCELCVHECPTKEKSLVMVPLQEEMDFGEQENADYLFKEITYKDDIL
NKESTKGAQFAQPLFEFHGACPGCGETPYITLITRLFGERMIIANATGCSSIYGGSAP
STPYRKSVKNGHGPAWGNSLFEDNAEFGLGMKIATENTRYRIENIMNENMQKVPNALA
ALFKEWIANKDKGAESVEIKDKMIPILEQNTDIKAVQDILNLKHFLSKKSHWIFGGDG
WAYDIGYGGLDHVLASGENVNILVLDTEVYSNTGGQSSKSSRTGAVAQFAAAGKPIQK
KDLGQIAMTYGYIFVAQVNSTANYSHLIKAIMAAEAYDGPSLIICYSPCIAHGIKGGL
GYSGEQGELATKCGYWPLYTFDPRLEEQGKNPLTMVGKEPDWDLYESFLMNEVRYNSL

```
                     KKSNPEHAKELFERNKKDAQRRYRQLKRIAMADFSNEVES"
     gene            complement(249719..250357)
                     /locus_tag="DMACINML_00514"
     CDS             complement(249719..250357)
                     /locus_tag="DMACINML_00514"
                     /inference="ab initio prediction:Prodigal:2.6"
                     /inference="similar to AA sequence:RefSeq:CAL35584.1"
                     /codon_start=1
                     /transl_table=11
                     /product="putative hydrolase"
                     /protein_id="Prokka:DMACINML_00514"
                     /translation="MKKTILFDLDGTLIDSTDAILNSFQGAFKILGLTPKNNEEIKNL
                     IGHPLEHMFKFLYQDKANLSQEFVLAYREIYSQIYLEQTTLLPKAKEALELAFEIGDL
                     GIVTTKGGKFTPLLLEHLGVKKFFKTLITLEDVTNPKPHAEPILLALEKMQKTKENAY
                     MIGDTILDIQAAKAANITPLALSCGYGDEDELKRYSKVFSSAYKSVLYIKNN"
     gene            complement(250357..251355)
                     /locus_tag="DMACINML_00515"
     CDS             complement(250357..251355)
                     /locus_tag="DMACINML_00515"
                     /inference="ab initio prediction:Prodigal:2.6"
                     /inference="similar to AA sequence:RefSeq:AGQ94609.1"
                     /codon_start=1
                     /transl_table=11
                     /product="outer membrane fibronectin-binding protein"
                     /protein_id="Prokka:DMACINML_00515"
                     /translation="MRKLLLCLGLASVLFGADNNVKFEITPTLNYNYFEGNLDMDNRY
                     APGIRLGYHFDDFWLDQLELGLEHYSDVKYTNSTLTTDITRTYLSAIKGIDLGEKFYF
                     YGLAGGGYEDFSHGAFDNKSGGFGHYGAGLKFRLSDSLALRLETRDQISFHDADHSWV
                     STLGVSFGFGAKQEKVVVEQTKEVVSKPQVVTPAPAPVVSQSKCPAEPREGALLDENG
                     CEKTIYLEGHFDFDKVNINPAFEEKIKEIAKILDENVRYDTILEGHTDNTGSRSYNQK
                     LSERRAESVAKELEKFGVDKSRIQTVGYGQDKPRSSNDTKEGRADNRRVEAKFILN"
     gene            complement(251477..251866)
                     /gene="rpsI"
                     /locus_tag="DMACINML_00516"
     CDS             complement(251477..251866)
                     /gene="rpsI"
                     /locus_tag="DMACINML_00516"
                     /inference="ab initio prediction:Prodigal:2.6"
                     /inference="similar to AA sequence:RefSeq:CAL35586.1"
                     /codon_start=1
                     /transl_table=11
                     /product="30S ribosomal protein S9"
                     /protein_id="Prokka:DMACINML_00516"
                     /translation="MATTYATGKRKTAIAKVWVKPGSGKISVNGIDLNTWLGGHEAIK
                     LKVVQPLLVTKQETSMDIKATTLGGGYSAQAEALRHGISRALAAMDADFRALLKPKGL
                     LTRDSRTVERKKYGRRKARRSPQFSKR"
     gene            complement(251869..252294)
                     /gene="rplM"
                     /locus_tag="DMACINML_00517"
     CDS             complement(251869..252294)
                     /gene="rplM"
                     /locus_tag="DMACINML_00517"
                     /inference="ab initio prediction:Prodigal:2.6"
                     /inference="similar to AA sequence:RefSeq:EAQ72516.2"
```

```
                     /codon_start=1
                     /transl_table=11
                     /product="ribosomal protein L13"
                     /protein_id="Prokka:DMACINML_00517"
                     /translation="MTKITKPNEVKREWIVLDAEGKRFGRLLTEVATILRGKNKPCFT
                     PNVDCGDYVIIINASKAVFTGANKAEDKLYHRHSGYFGSVKSEKFGDLLEKNPAKLYK
                     LAVRGMLPKTNLGRAMLKKLKIYAGSEHPHTAQIAKEGK"
     gene            complement(252587..252850)
                     /locus_tag="DMACINML_00518"
     CDS             complement(252587..252850)
                     /locus_tag="DMACINML_00518"
                     /inference="ab initio prediction:Prodigal:2.6"
                     /codon_start=1
                     /transl_table=11
                     /product="hypothetical protein"
                     /protein_id="Prokka:DMACINML_00518"
                     /translation="MQKTGQIFTQYGLKTEEFITYGVFDDLLFTFEHYLEEDNDCISF
                     SVSFRVKSMKSDEILCKIIGLDQPNLDFKILNQYGVRSFPFSL"
     gene            complement(252963..253094)
                     /locus_tag="DMACINML_00519"
     CDS             complement(252963..253094)
                     /locus_tag="DMACINML_00519"
                     /inference="ab initio prediction:Prodigal:2.6"
                     /inference="similar to AA sequence:RefSeq:CKG87616.1"
                     /note="Domain of uncharacterised function (DUF1910)"
                     /codon_start=1
                     /transl_table=11
                     /product="hypothetical protein"
                     /protein_id="Prokka:DMACINML_00519"
                     /translation="MKKHTRLRDPLDTWDLEGAAIVKLMNLDKEEFKSYKYFPYDLI"
ORIGIN
        1 aggaatgata aaaataccca cgctacacac aaaccaagca atttgcccac ttgatatatt
       61 gtctattcct ttgctttcta gttttgcttt gagtttagaa taaataggaa tttgcaaatt
      121 tactccttag tttaaaaggt aaatatattt aagtatcact caatgttttt tatcttttttg
      181 cctttattta acccatatac aaacccttgc tagatgatag tgactagttt acttgcaaaa
      241 tagcatcaca tccattctaa aacttgggca agctctttgg ctacaaagca tttaagtcct
      301 acatcttcta aaggtttaga tggaatgata gcatttttaa atttttgcat tttggcttct
      361 tttaaacgag tatcaagact aaaaacttct cttatttctc catttaaact caattcccct
      421 ataaaaacac aatccttact caaaggacga tttttaaaac tagaaataat agccgcaacc
      481 acagccaaat cagccgcagt ttcattcact cttactccac cgctaatatt tataaagaca
      541 tcataatgcc caagtggaat ttctaatttt ctttcaagta aagctaaaag catgtcaagg
      601 cgattttttt catagcctgt ggcactgcgt ttaggataag aactctcgca aactaaagct
      661 tgcacttcta aaaccaaagc acgagaaccc tccattacaa cacctaaagc actgcctgaa
      721 gtagccttac ctcgcgtaaa aaaacgattg gctaaatctt ttgcactgat caaaccttta
      781 ggagtcattt caaaaattcc cacctcactt gtgccgccaa agcgattttt aaaacccctt
      841 aaaagtctaa tttctttagt tgcatcccct tcaaaataaa gcaccacatc aaccatatgt
      901 tctaaaactc tagggcctgc tatggctcct tctttggtaa tatgccctat tataaaagtg
      961 ctgatattat aagttttgct tactcgcata agttcaaaag tgatctcacg cacttgagtg
     1021 atacttccag ccgctgaagc aattttgttt gaatataaag tttgtatgga atcaataatc
     1081 agaatttcat aatctttctt atgcaattct tccaaaatat tttcaagaca aagttctgta
     1141 agtaaaaata aattaggagt attagcctca agtcgatccg ctcttaattt gatttgcgct
     1201 ttgctttctt ctccgcttac ataaagaact tttttatttt ttttagccaa attagaagca
     1261 atctttagca aaagtgtaga tttacctact ccaggactac cacctatgag caccaaagaa
     1321 ccctcaacaa gccctccgcc taaaaccaaa tccagctcat catcatcagt actacatctt
```

1381 gaaaaatgct caagttctac atcttctata cagatagcct cgcttgcctt tgtgcttgct
1441 tgtgcgattt cttttaaaac tttaatttgt tcagatttta attctataaa actatcccaa
1501 gaaccacact caggacattt tccaagccat ttgctttgct gatttccgca agcttggcat
1561 tcaaaaagaa ttttattttt agccatgata cttctttaaa gaatatttat tgcaataatt
1621 tttcacatta taaccaaaat taaattaaaa tttaacaaaa agtcaaataa aattaagttt
1681 tatatgatta taattaaata aaataaaaaa ggctagagca tgcaaaaatt actaaaagtc
1741 gccctaatcc aatttgctcc aaaatcttac gaggttcaaa acaatctcga taaagcctta
1801 aagcttgccc atcttgcatt ggagcaaggt gctaagctga tagttttacc tgaactttac
1861 gatagtgctt attgtgtaga agatcaagat gaaaaattta gcttaaattt acatcaagca
1921 catcctaatc ttgatgcatt acaaaaatta gctaaaaact atcaagctta tatcgtagca
1981 tgctctatag aaaaagatac ccaactttat gatagtgctt acatcattag tcacaaaggt
2041 cttttaggtg tttatagaaa aatttatctt tggggtaatg aaaaagaaag atttgcaaga
2101 ggagacaaat atcctatttt tgaactagaa tttgaaaatt ttaaactcaa attaggtttg
2161 caaatttgtt atgaaatagg ctttagcgaa ggatctagat ttttagcact tcaaggagct
2221 gaaatcattt gttttccttc agcttttggt aaggcaagga cttatgtttg ggatttagca
2281 agtagggcta gagctttaga aaatggtgtt tttgtcttag ctgctaaccg atctggaagt
2341 gaaatttcta agataaataa tgaaacttta acctttgctg gaaaatcaaa aattatcaat
2401 cctaaaggag aaattttaaa agaaatccta gaagaagaag gttttatcat agctgaactt
2461 gatcttgaag aagttcaaat tcaaagaaaa aatttacctt atcttaaaga tttaaatctc
2521 aaattaaatc aagaaatatt aacaaacatc catcttaata caaaggagta aaaatggcaa
2581 aagaaattct agttgcttat ggcgtggata ttgatgcggt agcaggatgg ctaggaagct
2641 atggaggtga ggattctcct gatgatattt caagaggtct ttttgctggt gaagttggta
2701 ttcctaggct tttaaaactc tttaaaaaat ataatattcc tgcaacttgg tttgctccag
2761 gacattccat agaaaccttt cctgaacaaa tgaaaatgat agtggatgca ggacatgaaa
2821 taggtgccca tggatattca catgaaaatc ctattgcaat gagtgctaaa caagaagaag
2881 atgttttact taaaagtata gaacttatag aaaaaatcag cggaaaaaaa ccaagtggat
2941 atgtagcacc ttggtgggaa ttttcaaata tcaccaatga gctcttgctt aaacacggca
3001 ttaaatacga ccattcttta atgcataatg acttcacacc ttattatgtg cgtgttggag
3061 acaaatggac taaaattgat tatagtaaag atgccaaaga atggatgaag cctttggtac
3121 gcggacaaga aacagatttg attgaaattc ctgcaaattg gtatttagac gatcttcctc
3181 ctatgatgtt tataaaaaaa tctccaaata gctttggttt tgtttcccca agagatatag
3241 gtcaaatgtg gatagatcag tttgattggg tttatagaga aatggattat gctatttttc
3301 ctatgactat acatcctgat gtaagtgcta gacctcaagt tttactcatg catgaaagaa
3361 ttattgaaca tatcaacaaa cacgaaggtg taaaatgggt aaatttaaac gatatggcgg
3421 atgatttttc aaaacgcttt ccaagaaaaa aataaattcg caaggctttt agccttgtga
3481 attcaaaagg ataaaaatgt gtattttatg tggcgaaatg ataagtactt tgcattggag
3541 cgagttaaat tttaaagaag aaaaacacga acttagtgta ggtgaagaac aaaaagaaag
3601 attaaaaata aggcttaaaa aagtcaaaat tttaaatgaa attttagaat tttatggatt
3661 aaaacttaaa gaatggcaaa attcaaaata catattaagc aataaaaaag gtcgcgatat
3721 tatcgttaat gatttaggcg atttatggat taaagctagc gagcttgaaa aaaaatcatt
3781 cgatgtgttg gatgaaaatc tacttcattt tttaagagct aaacatggct aaaattcccg
3841 ttcatgttgt taccggtttt ttaggaagtg gaaaaacaac ctttttaaaa gaaatattaa
3901 gcaatcaaac cctgaatgat attgctcttg taatcaacga actaggagag gtttccctag
3961 atcataaact tatacaaaca gattttatcg aggaacaaac tttattttta aatgcaggtt
4021 gtgcttgctg caataaaaga gaagatttgc aaaagaaatt taaagaatta cttgacagtt
4081 ttgaaaacaa gggtaaaaga cctcaaagag ttatattaga aacctcaggc ttagcaaatc
4141 ctgcacctat cattttcact tttcaaagcg atgtgttttt agctaaccat tttgaattag
4201 ccaatattat cacttgcatt gatgcttttg aaggacttaa tcacttacaa aatgaagaag
4261 cttgcaatca agtattaagc tctgattttt taatacttac aaaaaaagat cttaatccca
4321 atacccaaag cttagaggaa aagataaaca ctttatatcc caatatacaa atcataagca
4381 aagaaaattt taatttcgat atgctttcta gccatcataa aattcaaaaa gaaattaaaa
4441 atatagaaat aaaaagtcat aaagatgata taagttctat aactcttatt tttgacaagg
4501 ctataaattg gaatattttt agcatttggc ttagcatgct tttgcatgaa cacggatcaa
4561 aaattctaag agtaaaagga cttatcaata cagaagaatc ttatctaacc aacattaatg

4621 gagtaggaca tctgatttat catcctacgc atactaaaat ttcgagccta aaccatcctt
4681 cgcagttagt atttatagca aaaaatttaa aattagacaa gattaaagaa tccttagaaa
4741 attttttaaa actcgagcaa ttaaattaat caaaaacacc ctctaagagt gtgtctaaga
4801 aattttgtgc attaaattct atcaaatctt gcattttttc acccacccct atatacaaaa
4861 taggaagttc taattcacgc gctatactaa aaagtgctcc accctttgct gtgccatcaa
4921 gtttagtaat aataactcca tcaagctgaa caagctcatt aaaagcttta gcttgtaaaa
4981 tgccggcatt accttgagtg ccatcaagta ctaaaatttt tctatgtggg gcattaggca
5041 tagctttagc tgaaattcta acgatttttt ctagttcgtt ggctaaattt ttttgatttt
5101 gcaaacgacc cgctgtgtca atgataaccc tatcaaaatc cttagctcta gccttgctaa
5161 tagtatcaaa agcgactgcc gaagggtcgt gaccttgttg agttaaaact atatccaccc
5221 ctactttttc agaccaaagc cttaattgtt ctatcgcccc tgccctaaaa gtatcacaag
5281 cacctaaaat cactttttga ccttggcttt tgtataaatt agcaagctta gctatgcttg
5341 tggttttgcc tgcaccattt actcctaaaa taagttctac aaagggtttt tcatctagga
5401 attctttatt ttcataaaga aaataagaac ccattacacg ctttaaatct tctttcttaa
5461 cctcatcact aggtggcaaa taataaataa tttcttctac aatctcatat gctacatctg
5521 cttcaagtaa aatttcctct agtaaatctt tggtaatttt tttattttca cctttaacac
5581 ccactatact ttcaagtgtt tttgcaagtc ctttttttaaa aaaattaaac attaaagtat
5641 cgccttttgt atatcaattt ccaacatttc ttctggaatc aatcctagat attggttgac
5701 aagctgggaa tttttatcat ataaaaacat agtaggaatt ccatttaccc cgcctaaaat
5761 tttagccaaa agataattat tttcaccatt ggctatttta tataaaattt gattttcttc
5821 aacaaaagcc ttaagctcct catcactctt atcttcaagt aaaacgccta ctatattaaa
5881 ctgctcttca tatttttcct gaagcttgtt aagatgagga atttctgcaa tgcaaggaga
5941 gcaccaagtg gtaaaaaaca caaataaagt cgccttatct ccatcggcaa aatttaaatt
6001 ttcattgtct ttttttacaa aaactgaatt tccgtctatt aaattgagat taaatctcaa
6061 gctatcactt tggcttaagc ttgattgaac gcttgcattc atatcacttt gatctttttc
6121 tttatcatta ctacatgcaa caaaaaaact caagagtata gctagtgtaa atactttttt
6181 aattttcatt ctatattttc cttgtatctt tgtaaaaaat gttaaaatta tatcatagtt
6241 aagataataa tttttaaaaa ggcacacgat gcaaaaaaca tcttttagga ctctgcaaaa
6301 aaaccgcctc gcacaacaca aaaaacttaa atttaaacaa gattttatag tatttaagga
6361 atgttttaac ttgattaaaa aaacaaaagc aaaaaatatt cttattttta ttcctctggg
6421 gtatgagcca aatttactca aatttagaca tatttttagt aaaaatcata aactttttgt
6481 cccatttatg caagataaaa gtttaaaaat tgtaaaatta agattacctt ttgttaaaaa
6541 aaggtttggg gtattagaac ccatggactc ctttttgaaa actaaaattg atatagctat
6601 cattccggtt ataggagtag atagagagtt aaaaagaata ggacatgggc aaggttttta
6661 tgatagattt tttgaaaatt taaactataa acctcttgta atttttactc aaagtatcaa
6721 tgctataagt gaaaaaaaat taacccaaga gcatgatatt gcgggtgaat tttatatcaa
6781 cccttataaa aaatattaca agaaagacaa caaatatgat agaatcactt atcgcactta
6841 taaccgctat agtcggtctt ggaataggat atttagttgc aaaaaaaatc aatgatgcca
6901 aatatgaaat ttttgtcgag caagcaaaag cgaaggcaaa agccatagaa tatgaagctg
6961 aattgatttt aaaagatgcc aaaaattcaa tccttaatgc cgaactcgaa gtgaaaaaaa
7021 aatatgagga taaaactcac aaaattcaaa aagaattcaa tcaaaaattc gatgaattat
7081 ccaaaaaaga acaaaaactt caacttcaag aagaaagctt aaaagagaat caagaagaat
7141 tactacgctc caaaaaaacaa atgcttgatt tacaatcaga tgctgacaaa ctcaaaaatc
7201 aataccaagc aaaactcaat gatgttttaa atattttaga acattcagca gggctcactc
7261 aagatgaagc aaaaaatatc attcttgaaa aagtagaaga aaactcacgc aatgaaattg
7321 cccatattgt taggagatac gaggaggaag ctagaaatga ggctaaaaga agggcaaatt
7381 atatcatagc acaagcaacc tctcgttttg caggagaatt cgctgctgaa agacttatta
7441 atgttgtcaa tatcaaaaac gatgagttaa aagggcgcat tataggcaaa gaaggacgca
7501 atgttaaaac tttagaaatg gttttaggag tagatattat cattgatgat acacctggag
7561 ctattatagt aagttgtttt aatctttatc gccgtgctat tgctactaaa gtgattgaac
7621 ttttggtaga agatggaaga attcaacctg cgcgcataga agaaattcat gaaaaagtat
7681 gtaaagaatt tgataatgct attttagaag aaggtgaaac tatactgatg gacctaggac
7741 tttctaaggt ccacccagaa atcactaaac ttataggaaa gcttaaatac cgcgcaagct
7801 atggacaaaa tgctttagca cactctttag aagtagctca tttagcagga atcatcgctg

7861 ctgagtgtgg aggggatgaa aatttagcac gacgcgcggg aattttacac gatataggca
7921 aggctttaac acatgatttt gaaggctctc atgtggattt gggagcagag ctttgcaaac
7981 gctataaaga gcatcctgtg gttatcaatg ctatttatgc tcaccatgga cacgaagaag
8041 ctacaagtat agaatcagca gctgtttgcg cagccgatac gctcagcgct gcaagaccgg
8101 gtgctagacg cgaagtttta gaagctttct taaaaagagt aagcgaactt gaagaaattg
8161 ccaaaagcaa agagggtatt aaaaacgctt atgcgattaa tgcaggaaga gaaattcgtg
8221 tcattgcaaa tgcaaaactt gtaaatgatg atgaaagcgt gcttttagcc aaagaaatag
8281 ccgctgaaat tcaagaaaaa atgcaatatc ctggagaaat caaggtaaat gtcatacgcg
8341 aactaagagc catagaatac gctaaataag ggatcagtat gcaagatatg atagatacct
8401 tattaaaata tggttatata gttttatttt tctattcttt aggtggtgga atggtaggta
8461 ttttggctgc tggagtttta agttctcaag gaaaaatgga cttaacttta tgtataagca
8521 tagcttttgt agccaatacc ctaggatcca ccttactttt tatacttgga aaatactata
8581 aaaaagatat tatgccttat tttaaaaatc atcgccgaaa acttgccctt gctatgatga
8641 aaaccaaaca acatggagtc gttttgctta taactcaaaa atttgtctat ggcttaaaaa
8701 cttttatccc catagctgca ggtatagcaa aatatcaatt tattaaattt ttcatcatca
8761 atactcttgc tagcttgctt tgggcagtac ttttaggata tagcgcttat gcttttggtt
8821 ttgttataga agcaattttt aacaaattaa gctcttatcc ttatatcgca cctttatttt
8881 taatcgtatt ggttggaatt atttggtttt atttggctaa attttctaag aaataatgtc
8941 tttttggaat tctttatatt taaaccttaa agaattccgc tatttattcc tatttggcct
9001 tatcgttttt atttttaata tatttttaga atacaatcat tttttaaccc tcaaagaaaa
9061 gaaacattat ctagtagaaa atgccttgct tttacattat tatccaaaat acaataaaaa
9121 taataaaaaa tactgggttt taaaacttaa aacaaaggat tttatccttt atagtacaag
9181 ttttaaggat ttaaatttga gtaaaaatca gcttttaaat ttaaaaataa ttacaaacaa
9241 tatttctttt agggattatc ttagaaaaag cttttatgct cctagctacg gttttgaagc
9301 atcaggatat tttaaaaata atgttatcat ttcttatttt ttaaaccaac atacaaatga
9361 aaaaatacaa gaattttatg gagctttatt ttttgctttg cccatctctt cggagcttag
9421 aaaagatgtt aattattatg gtgttgctca tttaatagca atcagtggtt atcatatagg
9481 tttgcttttt gctttggtgt tttttatact cacgcccttg tatagtttct ttcaaaaaag
9541 atatttccct tatagaagct tacgctttga tttaagtgtt tttattttta tcttgctttt
9601 tgcctatgct tatcttatag gttttgtgcc ttcttatatt cgctctttaa tcatggctct
9661 ttggggtttt tatctacttt gtaaaaatat aaaaattcta agctttttta ccctctttat
9721 aagcatatat ctttgtatag ctctttatcc taggctttta ttgagcgtgg gatttttatt
9781 ttcttgtttg ggagtttttt atattttttt gtatttacac catttttcaa aattctttca
9841 taatttctta aatattgttt tactgaacat atggacattt ttttgtatga ttatccctgt
9901 gctttatttt tttcctctta ttagctatca acaattctta gctattattt tgagcggaat
9961 ttttatagtc ttttatcctt tggttttatg tttgcatttt atagcacatg ggaatctttt
10021 agatacggta ttgattgatt tttttaatat taaattttat gctatagata ttaaaattcc
10081 aacttggatt ttaatatctt atcttacact ctcttttata gctatgcgtt ataaaatttt
10141 agcccttctt tgtgtttttg cgaattttat ccccttttatc atgattatga tttaaaaaat
10201 aataaatttt aatcccaaaa agataaacaa tccaagaaat ataaatccaa ataaagaaaa
10261 aaagtatagt agaaaaagaa ccataaacac tggcataggt tttattataa actatataat
10321 acacaaataa attctttcct atataccaaa tagctcccgc tgcaaaggaa actagtatca
10381 aactcttaag agtaccttta tgaatagaac ttgaataaga tataaaaaac aaaccccaaa
10441 taatcacaaa aggcaaaatt tcaaaaaaat taaaacctat tttataatca tctaaagctt
10501 gctgaatcaa acccgatata taaaaactaa gtcctaagcc tagtgggaca agagttaaaa
10561 gcgtccagta agaactgatg ctttgccaaa gtccttttgg ctcatttttt gtaatgcgat
10621 tgattacaaa atcgtagcct gaaaaaaaag ctaaagaagt aaaagtcatc gctataaggc
10681 ccactatacc caaatttaca ctattgttta aaaatgtgtc aagataagca gcaactatat
10741 cttgttgtgc aggtattaaa aaagcaaaaa tgatttgttt ggctttttca taataaacct
10801 taaagcttga aatttgcgta aaaacagaaa aacacacaaa caaaatcggt attaaagata
10861 aaatagtata aaaactaagt gctgcggcat aatttaaaat ttctttatcc cgcaaattag
10921 acaaaatagc aaaaattctc ttcaatcttt tccttttttaa agtcccattt taaaaggatt
10981 aagaatatta tttggatcaa aggctttttt aatatttctc atcaaattca tttcagcatc
11041 agaaaaagct aatttcataa aaggagcctt agaaagccct attccatgtt caccacttaa

11101 ggttcctcca agctccacag taagtttaaa tacttcttct actgcctcat aacctttttt
11161 cacctgttct tcatcatttt tatcaggaac cataacatta gtatgcacat ttccatcgcc
11221 agtatgacca aaacaaggga ttttaaaacc atattttttta gaaatttcat ctatgccttc
11281 aagcaaagca ggaagttttg atcgcggcac agtaatatcc tcatttaatt ttaaagtccc
11341 atacatagct atactttgag agcaatttct tctagcaaac caaatatctg ctgcctcttg
11401 ttcatcttta gctattttaa attgacttgc tcctgcgtct aaaaaatgct ctcctaaaat
11461 ttttaaatct tcatcaatag cttcttttac attgccatct acatcagcaa tcaaaatagc
11521 ccctgcatcc acaggcaaac ctttgttaaa tttactttct accgctctaa tgctaagatt
11581 atctaaaaat tccatagata caggattaac ccccacttgca agactcttat aaactgcatt
11641 catagcactt tttacacttg gaaaaattcc aaaagctgtt ttcttaaatt taggcaaagc
11701 gataagtttt aaagtaagct cacttaaaac cgctaaagaa ccctcactcg cgattaaaat
11761 acctgcaaga ttatacccctg caacatcttt gatggtacgc ttacccgcac gaataatttc
11821 tccattgggt aaaacagccc ttaaagccat aacataatct ttagtaatgc catactttgc
11881 agctctcata cctccagcat tttcgcttac attgcccccct aagctagaat actccatact
11941 agcaggatca ggcggataaa aaagcccatg tttggccact tctctttgta aatgtatatt
12001 aatcacacct ggttgcacta ctgcgactaa attttcaaga tcgatttcta aaattttatt
12061 catatgcttt tcaaaactta aaatcaatcc cccattgaca gccaaagcac cccctgtaaa
12121 acccgaacct gctccgcgag gaatgatgat aatattattt tcattgcaaa attttaatat
12181 ttgagaaata tcctcttcgt ttcttggaaa aagtactcca tcgggcaaat aatgcttttt
12241 tgtagcatca taactataag cacgcttatg aatatcatca aaataggcat tttcactgcc
12301 tagaagtttt ataaaatatt gttcaaattc tttttttcatt gatctacctt agaatattaa
12361 ttaaagcatc ataatatcta taataattac ccacttcttg agaattaaaa tcaaaaaaat
12421 tacttttata gccacctata cgagaataaa aaggaacttg tatactactt ggcttaggta
12481 tagctaattc ataaagctca ctaaaatctt gacaatcaac tatctttgcc ttatcctcta
12541 aagcaaaaat caaaattccc acagcattgt ctgcgcaaaa ttttctaaaa tcactacgct
12601 ttggagcaag agatgaagag cggatgagtt gtgctcctaa gatatcaata tgggtaaaat
12661 aaatttctgg aaagctagat acaagttcat tataagtttg ttcatctgga gcgatcacca
12721 agcctctatc ataaagaaaa tttaaaacca cctcatcacc cttttgcggc acaacattgg
12781 gtaatggtaa ggcatcttgt tcaagatcgg caaaaacact gaattttaat tttgccaaac
12841 cctttttcttt agctataaca ctagctcttg ctataatgct ttttgaagtt tgaaaacgct
12901 gtataacaac tccgcttgag tttaatttaa tatcaggact atcttcaata taaccataaa
12961 tgtcttcaac cttcaaaagt tccattttta caggctttaa atttaaatct actgcaaaaa
13021 gactgatgct taagcttaaa ataaatgcta gaaatttaag caaactaact ccttaaaaaa
13081 tcaatttaat gtattataat cctttttttgt taagcaattg taagaattat aaagtaaaat
13141 atagcttttg aaaatttaaa aatcctcgaa aaaataaggt ctttatatga aaaaactctt
13201 actcttattt acatttgtta ttcaaagttt tgctgcgctt agtgttgagg agcttacttg
13261 ggataatggc gatactttac ttaaattctt acaaagaaat tctatcccta tatctcttta
13321 ttatgggctt gatagagaag atcaagaact tgcttctgat atagcctata aagtaaaata
13381 ccaagtttta aaagatgaaa ataacaatat agaacaagtt ttgattccaa tcagtgatga
13441 tttacaaatt catatttata aagataaaaa caatcaatac acccttacct ttactcctgt
13501 ttcttatcag aaagaagata gaattttaca tttaacaatc gaaagttcag cctatcaaga
13561 tgtttatgaa gaaagtggta gtagtacttt agctcgtgct atggtgcgct cttttcgagg
13621 aagtgttaac tttcgcaata tccaaaaagg cgatgaagtt accctttatt acgagcaaaa
13681 aagacgcatg ggaagacttt ggggagatat cgctgtaaga atggctacgg tagaaattaa
13741 taaaaatgct caagaagtat ttgcttttaa tgatattttt tacgatcgtg acggaaaaga
13801 agtggaggct tttttattaa ctaagcctgt aaattacaca agaatatcat caacttttag
13861 caccgcaaga tatcatccta tactcaaacg ctaccgtgcg caccttggta tagactatgc
13921 agcacctact ggaacacctg taaaaagtgc aggcaaagga acgatcacct ttgtaggaac
13981 taaaggagga tatggaaatg tcatacaagt aaagcatgat tcaggctata tgactttata
14041 tgctcattta agtcgttttg caaaaattaa aaaaggtcaa aaagtaaatc aaggtcaact
14101 cattggctat gtaggatcaa cgggtatgag tacaggtcct catttgcatt ttggtgttta
14161 tctaaacaac aaagccatca atcctgcttc agtggttaaa attgctaaat cagagctaag
14221 tggtaaagcc aaggaagaat ttaaaaacac catagtaaaa tataaaggaa ttgtaaacga
14281 agctttagct gctaataagc caaatcctcc taaagaagaa gattttgaaa attatataga

14341 attttaagga ttgctaagca aagccttatc tttagcttca atcaaggaat taagaagaat
14401 tataagccct tctctttcct tgctttccat ctctttaaat tcagcattaa caagctgatt
14461 tactaaagct tcattaagta aatccgcatc cccaaactct ctagcttttt gaatgatttt
14521 taatactctt aaatcctttt tttcatacat tcttaaccct ttaaataaac aacgctcaca
14581 aagcgttttg aagtcttatc acgacgataa gaataaaacg ttttatcatc aaaactacaa
14641 atattactat ctacgatatt tttaatacct aaattttgag cctgaaattt tactaaagct
14701 tttaaatcaa gtcttctttc atataaaaaa tctttaaatt ctacttttgc aaattctaaa
14761 atttctccgt ctatttcata gtttttagca caaatactag gcaggataaa aagatgaaat
14821 ttttgcatat ctaaatttgg attttttgta taaattttat ccacacactc ttttaaaata
14881 ttttcaaaac tcccttttct tcctgaatga agcgctgcta taacgccact ttcatgccat
14941 aaaatcaaag gcaagcaatc cgcacttaaa acacaaagtg cggtattttt ctctatgctt
15001 atcaaaccat cacaacttaa atttctaaa ctttcatcat aaatttcaac tttactggaa
15061 tgaatttgat ccataaaaac acatttttca atatccttaa aacctagatg agaaaataaa
15121 ttttctttat gaactttagc tctaaggata ttatagtctt tatcataagc gcaaaaaaca
15181 ctgacttttt gattatctag taaagataag taattctctc cacttcttcc catgataatc
15241 ctttattttt atcttgcatt tgtgcataaa tataacgagc aagcaaatcg ctttctatat
15301 tgatttctcg gcctatttta tactctttaa aaagtgtttc cttaaaagta ataggtataa
15361 tggttaatct aacgccattt tctacaattt cattaatagt aagactaact ccatctatac
15421 ctatactgcc ttttcttgcc ataaatttca ttgcttcttt aggtaagctt atataaaaat
15481 caattccatt ttcatctttt tcaatctttt caagcttccc gataaaatcg atatgtcctt
15541 gcatcaaatg tccatctatt ctatctccat accttaaagc aggttctata tgcactttat
15601 cttttaaatt ttgtactgct atgtgcttac gactctcgtg tgaaagttcg agttcaaaac
15661 catcgctatg aagtttagtc acgctcaaac aagctccatt aacagctaca ctatcgccca
15721 aattagggcg ataatttgcc tttaaattca aaacattatt ttgataagat ttaaccttag
15781 caatctctct tataagtccg ttaaacatta tttaacctta aaaaatttta ttcaataatt
15841 tattgatacc ctcatccact ttttctcctg ctttttgaac tccatcttgg gctttatcgc
15901 ctacaatatt tttaatagaa tttactacac tacccgcttc aagtttaact tggggctttt
15961 gtgttgttcc tgttatattg cctttaaaat tggctctatc tatcttaata tcaaaaggca
16021 aattcaagct tccgctttta gaattcagca tacctgaaga aataaggaca taagaacgat
16081 ccgcttgcat atttaaatca agcttgatat tttctttgtt aataagtgct ttagcattgg
16141 cagtatgaaa aacatcagta gtgatatctt ttaaggtaat aagcttaaga gctgcagtga
16201 tagcattagg tttaagctta ccctctttca tatctgcatt aacctctcct tgctcgctta
16261 taagattata cttaacttct aaatttgcct tgccttgata tatatctata aaatcaagtc
16321 cacttgccaa agtgcttaaa tttacaccct ctaactttgc atttaaaata ttatttttca
16381 aagtgctatc aagttttcct tcaaataaat ttttactcaa tgcagtaaaa tttaaagttt
16441 tatcaaagcc gatatttcca ttaaagcttg cttttccctt gagctttcta tcgagtaaga
16501 aacccaactt tgaaaaatca gcaatatcca aagcataatt cgaatttaaa atcattttat
16561 taatgtcaaa ttttccttta aaattactaa gattagccaa agaagaattt aacacgccct
16621 caaaatcagc aacattgttt tttaaatccg ctttaacatt taagtcgtaa tttgtattgt
16681 ttgggaattt tttatccatt aactgactta aaactgcagc atttaaaata cccttagcat
16741 ctatatttac cttgccatta agattgttaa aatttatatc atctaaacgc gcttttgcat
16801 taactatccc acttgcataa tttggcatac ccacaatagt aaaaacctta gaaagatcaa
16861 gcttgtttat atctaagtct agtttttttc cattagaatt tgctttgatt aaaccacctg
16921 ctatatttgc atcagcgttt aaattctgaa cttgagcacc tataatatgc gcttttgcat
16981 taagttctgc cttgccttgt aaattttgcc ctgctatagc attaaattga gacaaatcat
17041 caataaaagc atttaaatct gtgtttaatt ctgcattttt aagattgtag gttccattaa
17101 gtttttttgat attgagcaaa ttggacgcta agagcgcatt ataagatata ttgccatttt
17161 tagccttggc tttagcatca agactaaagc tcgtatttgg aagttcaagt ttggctattt
17221 ttttaatctc agcactattt aattttgcat tattgatttt tacatctgca ttaaaatttg
17281 caaagtccaa tccctcgctc acaagatttg catttaaatt cccagaaacg ataccaccat
17341 aacctataag agctagtatt ttttctaaac tggcattact tgcattaaga tttaattgat
17401 tgttttttaa atttgccttc acttctccgc caagccctat aacattagca tttaaactag
17461 ataaagcatt attagccaca ctcgcatcgc cttttacttc caaagatcca tttagctttg
17521 ttttggtaag tttttcaagc tttgcaagat taggaaccga tagatcaaaa tctgtattta

17581 gggtattttg aataagatta taaagagttt tttgagtttg taaagttaaa taactattat
17641 aaattttact cactgtatag actgtatcat ctttgatatt tgctaaaatt tcagctttaa
17701 gattagaatt ctcaggtaaa tctatattaa attctttttt gatttctgca taatttatat
17761 aagtattatc aagtttaatg atagcatttc catcaggttt taaatcttta gcagaaattt
17821 tacttgctat atctatggtt cctttagcca aatttcctcg tccaaaaagt gctagtaatt
17881 cagagatttg aagatcgtta gcgcttaaat ttaaagcgat aggagaatat tcataaattc
17941 tagcatccaa aagtacatta gagccaaaca aatatccctt gccattgatc ataaaatcgc
18001 tcgattttcc attaatcttc cctgaaaaag ccaaattttg attcaaattt aaacctaaat
18061 ttttagcata atttttatct aaattgatga tataatcaag atcaaaaccg agcttaaaaa
18121 gactcaaatt tccttctagt tttaaatcag ccatattagc caaatttgcc tcaatatcaa
18181 gacttgaaaa acgcagagta aactgagtga tattggcatc aagacctgta ctttgcttga
18241 ttttatcttg gataaaattt gcgactatat tattaccaaa gctagtaaaa agcagagtat
18301 aaaatgctat caaaagcact acaataaaag ccactattcc ataaagaatt ttcttcattt
18361 aaacatcctt tagttttttt aattaattat ctttagtctt attaactaaa gttttataaa
18421 tatttttact caaagtattg acaaaattca tttttatag tatgatatta tcactcaaaa
18481 ttaaagagtg ctaaaaatca atatttttaa ggatggacaa aatgaatttt caacctttag
18541 gaaagcgtgt tttagtaaaa cgcgtagaag aaaccaaaac aacagcttca ggtataatta
18601 taccagataa tgctaaagaa aaaccactca caggtgaagt tgttgcagta agcaaagaaa
18661 ttactgatat tgcaaatgga gataaaatcg tatttgctaa atacggtgga acagaaatca
18721 aacttgataa tagcgaatat ttagttttaa atttagatga tattttagga attttaaaat
18781 aatttacaaa aaaggataaa caaatggcaa aagaaattat tttttcagat gaagcaagaa
18841 acaaacttta cgaaggtgtt aaaaaattaa acgatgcagt aaaagttact atgggaccaa
18901 gaggacgtaa tgttttaatc caaaaaagct ttggtgcacc tactattact aaagatggtg
18961 taagcgtagc caaagaagta gaacttaaag acagtcttga aaatatgggt gcttcacttg
19021 ttagagaagt agcaagcaaa acagcagatc aagcaggtga tggtacaaca actgcaactg
19081 ttttagcaca cgctatcttt aaagaaggtt taagaaatat cacagctggg gcaaatccta
19141 tcgaagttaa acgcggtatg gacaaagctt gcgaagcaat cgtagccgaa cttaaaaaac
19201 tttctcgtga agtaaaagat aaaaaagaaa ttgctcaagt tgctacaatt tcagcaaatt
19261 cagatgaaaa aatcggaaat ttaatagcag atgctatgga aaaagtaggc aaagatggtg
19321 ttatcactgt tgaagaagca aaatcaatca atgatgaatt aaatgtagtt gagggtatgc
19381 aatttgacag aggttatcta agtccttatt ttataaccaa tgcagaaaaa atgactgtag
19441 aactttcaaa tccttatatc ttgctttttg ataaaaaaat tgcaaattta aaagatttat
19501 taccggtttt agaacaaatt caaaaaacag gaaaaccact tttaatcatt gctgaagata
19561 tcgaaggtga agcacttgca actttagttg taaacaaact acgcggtgta ttaaatatct
19621 ctgctgtaaa agctccaggc tttggagata gaagaaaagc tatgcttgaa gatatagcga
19681 ttttaacagg cggtgaagta atctctgaag aacttggaag aactcttgaa agtgctacag
19741 tacaagatct tggacaagct tcaagtgtaa tcatcgataa agataataca actatcgtaa
19801 atggcgcagg cgaaaaagca aatattgacg caagagtaaa tcaaatcaaa gctcaaatag
19861 ctgaaacaag ctctgattat gatagagaaa aattacaaga aagacttgct aaattaagcg
19921 gtggtgttgc agttattaaa gtaggtgcag caactgaaac tgaaatgaaa gagaaaaaag
19981 atcgcgttga tgatgcttta agtgcaacta aagcagcagt tgaagaaggt atagtaatag
20041 gcggtggcgc agctttaatc aaagctaaag ctaaaatcaa tcttgattta aaaggcgatg
20101 aagctatcgg cgcggctata gttgaaagag ctttaagagc acctttaaga caaatcgctg
20161 aaaatgcagg atttgatgca ggtgtagttg taaacagcgt tgaaaatgct aaggatgaaa
20221 atacaggctt tgatgctgca aaaggcgaat atgtaaatat gcttgaaagt ggaattatcg
20281 atccggttaa agtagaaaga gtagctttac ttaatgcagt ttctgtagct agcatgcttt
20341 taacaacaga agcaacaatc agcgaaatca aagaagataa acctgcaatg cctgatatga
20401 gtggcatggg cggaatgggt ggcatgggcg gaatgatgta agccctacta ccaataatca
20461 gcaaattttt gctgattatt tctttttaaa ctgccaaatt ttgtaagata caaaaaataa
20521 aaaaataaat acgcattaaa aatctgccaa gatacaataa aaaacaatat catgatcacg
20581 ccataaaaat ttgcaaagcg agtcatttat cagaaagtct tcacatttat caaaatataa
20641 tctaaacata aaaacgaaat taaaatgcaa agattgctta aatcatcttg ggtcaatgat
20701 ctcgcaagaa atgatcttcc tgtgtatgtt tacccatacc aagccttatt aagtagcaac
20761 aggcacagct atcctctttg ctctttggca tatttacgaa cagcttacat aactgtataa

20821 gataaatcag ctttttttaat aaatttattt tatagaaaaa taaattttta ttgataataa
20881 ctataaaata agaaagtgtt tatagagctt caagcaagtg aatacaaaaa ttttattatc
20941 ctttttcaaa tcttctttgt atcccaactt aacttaaaaa cactgccttc attttccact
21001 gattcgcaaa tgattttaat atcattatca tcgcaaattt tttttaccaa agaaagccct
21061 atgccaaagc ctccttgatc agtattaaat ctcgcatacc tatcaaaaat gtgcttaaga
21121 tttgtctttg aaattccaca acctgtgtct gcaatgctta aaaaacctgt ttttagctcc
21181 accaaaatct ctccaccttt tttgttgtat ttgattgcat tacttaaaag attgtctatg
21241 agtttggaaa tttggttttt agaagcgaaa atacttgctt gatgtaaatt aagctttaga
21301 ctgattttct tttgctcaaa aaatatttta aaatattcta gtctttcttc aatcaatgcc
21361 tttaagtcaa gttcttgttt attaagctct aaggtattag gaaaattata aaaaacaaga
21421 tctgaataca cttgagacaa actttttgca gctaatttta tccttgtaaa tttggtatta
21481 tgctctagtt tttgattttc aagttgttct atactcatca aaatcacgct taaaggagtg
21541 ttaagctcat gggttgaatc tttgataaat cgattaagag tgatgatctt atcttctaat
21601 ggttttagag caatttttac taaaatataa gctatcaagc ccaatacaca aaaagccatg
21661 atggcaaaag aaaaaacttt tactctaatc caaacaagat cctttttaac gtcctcgcct
21721 tggattaaaa ttttcaatcc gctttcagta tgtatattat cttcatttgc atgctttaaa
21781 aaataattat ccgtattcat acgatcaaga aaaaatactt taccatcata aattccccta
21841 cccttaagct caacccttgc ttttctaaaa tcaaaatcca gattatcaaa aatagcctgt
21901 tttttatcaa aaatagcaaa tttaagtcca gtgctttggg cgatattgct agcactttca
21961 ttcatgggaa caaaacgaga atttaggata tttataatga tatttctatg attttccctt
22021 aaagaagtac cttttacaac tactaattct tcatataatt tttgatacca taaagtaaaa
22081 aagatcgcta aaaaaatacc tgtggtagtc aaataaatca ataaaatttg cctaatgact
22141 tttttagcca tagcaatatc ctctgcccct ttgattgata atttttttctt ttcctaatat
22201 tttacgcaaa tttttaatat acgctcttaa actaagttca ctaggctctt catcatactc
22261 ccaaagttct tcaaaaattc tttcagtgct taaaaaatta tttttatttt ttaaaagtaa
22321 agataataat ttaatctctt tacttggcaa agttaaagct ttatcaaaat ggtaaagaat
22381 ttgcgaggta aagctaaatt taaacccatt tcctaaatct tcataatctt catttttatg
22441 agaaaaagcc ctctttaaca aagcttgaat gcgaattgaa agttctgcaa gctcaaaggg
22501 cttgcgtata taatcatcac aaccggcatt ataaccctct tttaaatctt ctgttgtatt
22561 taaagaagtc ataaaaatag caggagtttg ttttccacaa tctcttaatt cttttaataa
22621 agaaaatcca tcgcccaaag gaaccttaac atctaatatc catacatcaa aatgtttttc
22681 ataagcgagc tctaaagcct ctttagcatt ggtacataaa taaacttcat agccctcatc
22741 gattaaaaat tcttctataa tctcacttaa acttaaatca tcttctaaga gcagaattct
22801 agctgccaca agataccttt ctttgatatt tcttgaaatt gtatcaaaaa atattgaaaa
22861 aatggtttaa tacaaattaa tttatattta acttattttt atgttataat ctttaatgca
22921 aataaatttt tcaaggagtc aaaatgcttc caaaatggga cagcagtttt agtgtgcaca
22981 atgccaaaat tgatgatcag cataaaaaac tttttgagct tgcagcaaag gttgaaattg
23041 tttctgatag atcagtaagc aaaaatgaag taaaagagct tttagctgaa tttttcaact
23101 acatgaaaga tcattttaat gatgaagaga aatatatgca actcattgct tatccagcat
23161 ttgaagaaca taaaaaaatt cataaagaaa ttatacaaac catgattaat cttattaaag
23221 atatcaaatc aacaaatgat cttaaagaaa agctttatat tatagcaaaa aaatggctct
23281 tagagcacat actctatgaa gacatgaaag tagaaaaatg gagaagctct tctttagcaa
23341 cagacgaagg taaagatgtc agctttgaag cagcggatga tagcgacgat gacaagccgc
23401 aattttatct ttataaatgc aattgcccag gtaaagtaca tgatgtccct tatggcatcc
23461 atcaaaaaat tgaattacaa ggacacaaat tcacttgcaa aacctgtaag caagcaatta
23521 catttttttca aaaacattcc tagaaaggtt aagttaatga aacaagaaac tagaaattat
23581 tcccttagaa tgcctttaga acttaaagaa cgcttagata atttgagtaa aaatctttcc
23641 aagcctaaat caagtatagt tagagaggcc ttagaagctt atcttaatga aattgaagat
23701 ttttcttttg cagtcaatgc tttagaagaa ttaaaagacg gagattattc aaaagcgagt
23761 aagaagatag ataaggttgt agaaaatctt caaaaggaat cagatgattt aggtgatttt
23821 gattttgcta tcgaagcctt agaagaacta aaagacggag attattcaaa agcgagtaag
23881 aagatagata aaatcgttaa aaatctcaaa aaaccagaat aaacattata aatactttat
23941 cacattgtga taaagtttat taagttatat caattaaaaa atatgatttt atacagagtt
24001 agaagtggct ccggatgtag gattcgaacc tacgaccaat cggttaacag ccgactactc

24061 taccgctgag ctaatccgga atatcttaaa aagtaaaatg taattatatt tgatttttaa
24121 aactttgtca agtatttttt caaaaaaatg caaaaaattt cattctttaa caataatact
24181 ttttaaattt ctaaaataaa acaaattcac accctcttca taatcataat caagcttcat
24241 tccatgagat tctaaaatgg tattaactat ataaagtcca agtccaaagc ttgatttttg
24301 ctttgagcct ccttgagtaa aagcttgagt gtaatactct aaagtattat ttaattcagg
24361 acctctattt ttaaaacaaa tataatcatc cattatatca atttgtataa agccattttc
24421 agaatgctta ataccattat caatcatatt tttaatcgca gtggtaaaaa gcttgaaatc
24481 tacattcaca aaaaagctct catccataaa aatgcgtatg ttattatcat ctcgcatagc
24541 gatttcttta gcctcatcta aaacatctaa aatattatat tttttgcggt tgataaatgc
24601 tgctccggaa gtaatttgtt ctatggctgc aaattcgttg ataagaattt caagacgatt
24661 aaatatgctt cctaatcttt ctttgtattt gttatcctct aacatttcaa gagtgataag
24721 tcctttagta atcggggttt taagctcgtg cataatattt cttaagaaaa attgacgcga
24781 ttgattgagc ttacgaattt gaacgattgc ttgataaaaa gcttcactta cttgagaaat
24841 ttcatccact cccgtgctta catcttctat atcattaagt tttccttgag caaatttatc
24901 aatttgcctc tttaaagctt ttagaggttt taattttta aatatataaa tataaagcaa
24961 tacaagtata caaataacag ctacggctat ggctttaatg ataaaatatc tataagtttg
25021 ataatcttga tctttataaa gatttacctt gccatcaaaa ataatattta aatacacttg
25081 tctatcataa gagatgattt caatcaagcc attattgacc tcgactctag ctaacacttc
25141 tccgccaaat agaattctac gaatttgcct aacctctaca acagactcca ttttgttata
25201 gttttttgtt tgctcttcaa attcttttc gctaattatc cctgcaaaat ttaaaagcct
25261 tgcgttagct attaaagaat acttggtgtt taattctcta gtgtaatttt gctgatcata
25321 ttctatgagc cataaaaagc caagaataac actaacgcca gcaaaaataa aaataaaagt
25381 tatggtataa aaaatagatg aacgattcat tgagtaagct tatagcctat acctctgata
25441 gaatggatat attttggagt ttttggatca tcacccattt tttgacgaat gcggctaata
25501 attacatcga tacttttatt gctcgaatct tcactgattg aagaacaatt ataaacaagc
25561 tcttcgcgac tgacaacacc gccttctttt ttgatcaaat agcttaaaat gtcaaattct
25621 gcattggtta aagtaagctc ttgacccttc attgtgataa tgtgcttgta ttgatcgtaa
25681 acaagatctt taacactttt tgctatggca ctttttgtat ttgaaatgcg tcttaagtga
25741 ctcttgatgc gtgcttgaag ttctttagga ttgtaaggct ttggcaaata atcatccgcg
25801 cccaattcta aagcattaac cttatctgta atatcatgtc tagcacttga gataataata
25861 ggcgtatcat actttttacg aatttcttca cacacttcaa gcccatcaag tcctggtaaa
25921 gaaagatcta aaattataag ttgatattct tttaaggcta gctttgaaag acctatataa
25981 ggctcatgtg cgatatcaac tttcatatca aatttttcta aatattcagc cgtaatttca
26041 gcaagttcta aatcatcttc tatcataagg atatttgtca ttgcattcct tttactatat
26101 ttaatctcgg tttttaaatt ttaaactaaa atatataatg aaagggaaac cctttcgtaa
26161 aaactattta agtatcaaca aagttgcaaa accattgcga taaacccaaa ttttagtaaa
26221 ttcttttttc tttacttgct ttaaagcttg ttctaaatct tttaaatttt taatttcatt
26281 ttgtcctaca cctatgatga tatcaccttc ttgaaatcct gaatccttag ccctactctt
26341 atctttaaca ctttctacaa aaaccccatt tacatctttt ggaatttgca aacgctcttt
26401 aaatcttgga tccaaattcc ttaaagtcat accctcaatc aaatcatttt gaacagcctt
26461 aggatttttct ttttctcctt tgagggtaaa atcaactctt ttattttccc catctctttc
26521 ataatcaagt ataatttttt gacctatctc taaagtacct atatagtttt taagatcact
26581 tggactttta attactttat cattcacctt agtcaccaaa tctccgcgct taagtcctgc
26641 ttcatcggca cttgagcctt tttgcacatc agtaattaaa gcaccatctt gatttttata
26701 agctttttta gtatcaccct gtaaagcaga aatagttaca cccaaaaatc ctctatcaat
26761 cctacctttt tcaatgagct ttttagctat atcttttacc atatttgaag gtattgcaaa
26821 tccaatacca ttatttccac ccccacgaga gagaatggct gaatttatcc ctactaaaaa
26881 tccacgacta tccactaagg caccgcctga atttccagga ttaattgaag catctgtttg
26941 aataaagttt tcatattggt ttaaacctat attgtcttta tttaaagcag aaattatccc
27001 acttgtaaca ctaaatccaa caccaaaagg atttcctaaa gcaaaaacaa catctccttc
27061 cattaaatca tctgaatttg tgaaagtaat agcccaaaga ttatttgcct ctattttaat
27121 caccgctaaa tctgtctttg gatccttgcc tataagcttt gctttatatt ctgtatcact
27181 gcctggtaaa cttacgctaa tggtatctgc atcatctact acatggttat ttgtaacaat
27241 ataaccatct tgcgaaataa tcacgcctga acccaaagaa ctgactactt ctttatcatt

27301 ccctcctctt ctttgtggaa aatcaaaatc aaaaaattgt ttaaaataag gatcattaaa
27361 aaaatcatcc aaaggacttg gacgattgac ccttgtgata gtttttgaag tagagatatt
27421 tacaacagat tttttagcat cttttatcga atcatgataa gaaagaattg tatctccagc
27481 cgcaggatta acacgattgg ttgttatgct tgtttcatta aaattgatat ttgcagcaaa
27541 taaagcactt gctaaactca atgataaaat aagctttttc attttgcttc cttttttgtt
27601 tatattattc ttaaaaaaca tatgatttgt aaaatatttt acgcatttca taagtataaa
27661 accgaaaatt tacaaaattg taaatgaaag cttgagtgat aaaaatttat aaaacttgat
27721 tgacttaggc taaagttttt gatataattt ctacttaaat aaaacaatta tatatttttt
27781 aaggaataac atgaatagct tatatgaaac acttggagtg tctaaaaatg ctagtgcaga
27841 tgagataaaa aaagcctatc gtcgtttagc aagacaatat catcctgata tcaataaaga
27901 aaaaggtgcc gaagaaaaat ttaaagaaat caatgctgct tatgaaattt taagcgatga
27961 aaaaaaacgc gcacaatatg atcaatacgg agactccatg tttggagggc aaagtttcca
28021 tgacttctca agaaatgcag gtggagtcaa tttagatgat atcttaaaag atctctttgg
28081 cggaggtttt ggtggaggct cttctaaagg aagtcgtttt aatggttttt catcaaaagg
28141 ctttaatgct ggatttggag gttttggtgg atttgaagaa gaagatttgg attctactat
28201 agaacttagc attcctttg aaaaagcagt aacaggtgga gaatatagct ttagccttca
28261 aggagaaacc atcaaattta aaattccaca cggaatcaaa caaggagaaa agcttagaat
28321 tcgcaacaaa ggcaaaaagg ggcgtaacgg aacacgcggg gatttaattg ttgtagtcaa
28381 attaggagaa agtgaaatat atcaaagaga agatgatgac ttatatcaaa aagttgacat
28441 ctctttaaaa acggctctat ttggtggaaa agttaccgtt aatacccta aagaaagtaa
28501 aaaagaagcc accataacca tacctgcaaa ttctaaaaat ggacaaaaaa ttcgtcttaa
28561 aggctatgga gtacaaaatc gcaaaagtga tatctatgga gatatgtact tggtattaaa
28621 tgttgtttta ccaaacactg aaaccctaga tgagaaatta atcgaaatgt taaaagaaaa
28681 actatcataa aaaaggagta aagtcatgga acaccattat gatgaacccg tatatctaat
28741 aagcgtcgta gcaaaagtgt taagtataca tccacaaacc ctaagacaat acgaaagaga
28801 aggcttgata gagcctagta gaactgatgg aaaaataaga ctctattctc aaagagatat
28861 cgatcgtata aaacttatct tgcgtttaac gcgtgatatg gggtaaatc tagcaggagt
28921 agatgtaatc ttaaagctta aaaatcaact ccacgaattt gaaaatttaa ttgatgaatt
28981 acgcttagag cttagcaagc aacaaaatcc aaacgcaagc tcgaaagcgg ttgtaaaaca
29041 taaaaatagc tttgatttaa tttttatga aaaaaaatag atgggtaatt ttttagaaat
29101 ttttttaatt acagctgcca tagctatcgt tcttaatgtt atttttaaga aatttgaaat
29161 tcctactatt atcggctaca ttgccgcagg agagattatt tctgaaattt atcatctaag
29221 cggcaagggt gagattgcac atattgctga atttggtata gtatttttaa tgtttaccat
29281 agggcttgaa ttttctttta agcatttaat ggctatgaaa caggaagtat ttttaaatgg
29341 ttccttgcaa atgcttacct gtggctttgt ttttatgttg ctagctatag gaattttagg
29401 acttggtgac aaaagtgcaa ccatagtagg ttttgctcta gctctttctt caaccgctgt
29461 ggtgcttaaa attttaaatg ataatggcga tatcaatgag caatatggaa gaaaagcttt
29521 aggaatttta ctttttcaag atatagctgt gattccttta ttattgcttg tagatttctt
29581 ctcttcaaac aatcaaaaca taggacaatt attactcaca acccttcttt cagcggccat
29641 tttaattgct ctgcttttct ttataggtaa atacttagtc gataggatat ttaggctcat
29701 catacgcgcc tcctcacaag aaatttttat cagcactata ctttttatgg tgattggagc
29761 aagctttttg gcgcattatt ttggtttttc ttattcttta ggagcgttta ttgcaggagc
29821 cttgatagca gaaactaaat acaaacacaa aatcgaagca gatttaatcc cttttagaga
29881 cttgctttta ggtttatttt ttataaccgt aggaatgcaa attcaaccta gcatcgtagc
29941 gcaaaattgg tttattatct gtgttttaac tctgctcata atgggtttaa aattcggcat
30001 agtgtgtggt tttttatttt tatataccaa aaaaagagtg gcattaaaaa ctgccttttc
30061 cattgcccaa gttggagaat ttgccttagc gatcttttca ctcttgcaag caaaaagcat
30121 gttagatgtt gaaactgctc aaattttgat tgtcgtttct attataacta tgattatcac
30181 tccttttgta ttaaacaata taagaaaaat agccaatgta gtagaagata tgactttaaa
30241 taccaatgtc gttcagcaaa gcactcacag cactctaaaa aatcatcttg taatctttgg
30301 atatggacgc ttagggcaag agatagtgca aaaaatcaaa aatactggtg ttccttattt
30361 ggttttagaa agtgatttaa atttggtaga gcttggtata agtcggggcg aaaatgtcgt
30421 ttttgctaat gctgctcaag aagaaacact caaaatagcc aacataacag aatgtgcagt
30481 agcaatcgta accataacca acgaagctaa actcgaaatg atttgtcaag tcttagcttc

30541 ttatccaaag cctattgaca caattatcca tgtcaatggt agtcttgaaa aaatgctttt
30601 ttcaagcatt gatgagaata ttcgcattgt aagatctgaa aaagtcatcg caagaaattt
30661 ggtccaagaa gctctagaat gcagaattca taaaaacact taaaggataa tattggcaaa
30721 aattgatgaa aaaactctaa gagttaaata tttaagagct ttagaaaaat ttgcaaatgc
30781 agctatttca gctttaaaaa aagaaaattt tgataaagaa aaatttcaag aaagaatgga
30841 aaaaaataaa aaaatagttg aaaaatgcga ggcagtaaat ttaaattcaa gctataataa
30901 aaccttagaa aattttgtaa ataattgcct tgacttctca aaacaaaaag aagagcttct
30961 aagccttgca aatgctttgg ataaactcaa aaaaggcgaa aaaaaagaaa aacataaaac
31021 ttatttgaag gattattatg attaatgtat tttttgatat ggacggcacc ttaatcgaca
31081 gcgcaaacgc tatatcttgt gctgttaatg agataagaaa tgaactaaac ttaagcccac
31141 tttctaggga aaaaattatg caaaccatca ataccccaaa tgtagactgg gcgaaagagc
31201 tttatagtat cgataatttc catcattcta gctttaaaga tggttttgag aaatatttca
31261 tcaaacacta tgagcaaagc gtagttttat ttgaagggat taaagatata ttggaatttt
31321 taaaagaaca aaattgtttt ttagctatcg ctactaatgc accccaaagc tcactttcta
31381 gtatacttaa aaaacatgaa atcattcctt attttgataa aattttaggc gtaagtcttg
31441 gtatagaacc aaaacccat cctatgatgc ttgatctttt aaagtctgaa gcaccccata
31501 aaacaagtat ttttataggc gatagtcaaa aagatagaga atgtgcaaaa aatgcgaata
31561 tgccttattt tcacgctaaa tggtatcaaa aggatttgca agaaaatgaa tttagcaatg
31621 caagcgaact taaaaacttt ttagagagat atttataagt tttttaatga aaattgagct
31681 aaaataaaat aaatttttaa aagagtaata tatgagtgaa ttattaatag aaatcggaac
31741 agaagagcta cctgctattc ctttactaaa agaattagcc aatatagaaa aaaaatggaa
31801 agatgtttta gaagattatc gcttggtgag tgattttaaa ttttactata ctcctcgtcg
31861 tttagtgatt tttcatgaaa atttcgcaag caaacaagaa gatagctttg ctgaatttat
31921 aggtgcgcct aaaaatgtcg cttataaaga ggggcaatta tcagccgcag ggcaaagctt
31981 tttacaaaaa gcaggtatta gcgaaagtga gctttctttt aaagagatca agggcaagga
32041 agtcttatat catcaaaaag ccattaaagg tttgcaaagt caagaagttt taggagagat
32101 gattcatcaa ttcttaaaaa gccttaactt tggcaaaagt atgcgctggg gtgctaattc
32161 ttttgaattt atccgtgcaa tccgctctat agtttgtatt ctagatgata atttagtcga
32221 atttgaaagc tatggcgtta agagtgctaa aaagactttt gtacatagaa gtgtaagcta
32281 tgacttgcaa gaatttaata acgcaaaaga gtattttagc cttttagaga aaaatcatat
32341 catcttagat ccgctccaaa gaaaagaaag aatactcaag caatttaaac tcctagaaag
32401 ccaaaacaat atccaaatag gcgaagatga agagcttttg gctgaagtta ttgctatcac
32461 agaatatcct aatgcacttt taggaagttt tgaagaagaa tatcttcaaa ttcctagcga
32521 agttatcatt acttccatga gagaaaatca acgctatttt gcagtcttta atgaaaaagg
32581 tctaagtaat cattttatcg tggtaagtaa cgcggtgtgc gaggattact caaaaatcat
32641 tcatggcaat gaaagggttt tacgcgcaag acttagtgat gcaatgtttt tctatcaaaa
32701 cgacttgcaa agcggcttaa atcctgaaaa acttgctaaa atgacttatc ttgaaggcct
32761 aggaacaatg caagataaaa gcctaagaga gataaaaatc gctgaagttt tatgccaaat
32821 gctaaacaat gataaaatcg caaacataag tactgcgatc aaatacgcaa aagctgactt
32881 agcaacgcaa atggtttatg aatttacaga tttgcaaggg attatgggaa gctattatgc
32941 acaaaaaatg ggcttagatt atgaaatttg tcttgctata aaggagcaat acctgccaaa
33001 ttccgaacaa gctccacttc ctagcactga attttcaagc atagtcgccc ttgcgaacaa
33061 gcttgatact ctcataggac tctttagtat aggcaaaatt ccaagtggca caaaagatcc
33121 ttacgcctta aggcgcgctg ctaatgggat aatcaaaatt gccctaaatt taaacaaaga
33181 atttgacatt caagcactac tagaaaaact agcaagccac tataaaaact ttgatatgca
33241 aattttaaaa gattttattt ttgaaagact ttataccttt tatagcgtta acgcttcttt
33301 tatcaaagcg gttttaagtt ctaaaaatac agatcttatc cacatcaatc aaagcgtaaa
33361 agcccttata gaattaagca aaaaagctaa ttttcaagaa aatttcacta cctttaaaag
33421 actggccaat atcgctaata aaacttcaca caaagtcgat gaaaacctct ttattgaaga
33481 agcagaaaat aaactttata aagcctttaa ggaaaaaatt caagcacact ctttacaaga
33541 aaaacttgaa aatctctttt cgcttaaacc ttttatagat gaatttttcg ataaagtaat
33601 gatcaatgct gaagatgaaa aacttaaaaa taatcgccaa gcacttatct atgaaattta
33661 cgaagaattc ctaaaaatcg ctgatcttaa agagttaagc ctatgaaagc tttttggctt
33721 tttctaagcc ttgctctttg gctttttgga gctcaaaatt tcgagcttat caaaggacaa

33781 gctttatttt tggaactgga taaaaaagat tttatttctt taaaaaatga ggataaaatt
33841 ttagccacct ttcctcatcc taaaaatcaa gaaaaaattt tagctatatt ttcactgcct
33901 tataaaaatc cacctcaaaa tacaaaactg attgctcttt ataaaaacaa aaaagaagaa
33961 atttttataa aaactttaga aggcaattat aaaagtgaaa aattaagcgt agaaaataaa
34021 aaaatttttc cacctaaagc cgttaaagaa cgcattgcta aagaatttaa agaagctaat
34081 gcaatttata gctcttacac tccaaaagct ttatttgatg gctcttttaa tactccttta
34141 gattctttta tcacaagtga ttttggcaaa gcaagaacct ttaatgaaaa agttgcaagc
34201 tatcacagcg gaacagactt tagagctgct gtgggaactc ctatctatgc ggctaattca
34261 ggcatagtaa aaatagcaaa agatcgttat tttgcaggaa aatccgtagt cattgatcat
34321 ggttttggaa tttactcgca gtattatcat ctttctaaaa tcgaagtcaa ggtaggacaa
34381 aggattaaaa aaggtgattt tttaggactg agtggagcta caggaagggt tagtggacca
34441 cacttgcatt ttggtatttt agctggaggc aagcaggtag atcctttgga ttttatctct
34501 aaattcaata cactttttca atgaaattca gtgaattttt tcatacttgg ctgcatgaaa
34561 gttattataa aaacgctgtt agtataggta aaaatggaga tttttttacc gcagtaagcg
34621 tgggaaatct ttttggcact cttttagcaa agcatttttt agatcttata gataaaaaag
34681 tcttaaaatt tcccttagaa cttgtagaaa taggagccaa tgagggctat ttaagcaggg
34741 atttttttagc tgctttactt gaatttaggc ctgaaatttt ttccaagctt tcttatttta
34801 tcatagaacc tcatgaaaaa ttaagaaatt tacagaaaaa aactttagaa ggcgtggaat
34861 ttacacataa gagaagctta aaagaatgtc attttgaaaa tgccttttc ttttgcaatg
34921 agctttttga tagttttgct tgtgaattaa tagatgatga taaaatggcc tttgtgcaag
34981 attttaaact tgtttttgaa cccatgaagg ctaaattaaa agaaaaatgc gaaattttaa
35041 atttaaaaaa aggcgaatta agcctagaat tggaagaatt ttttaaagat ttggataaag
35101 cttctaaaaa atttatcttt gcaagttttg attatggcgt gtttaatccc gaacaattta
35161 gcattagaat ttatcaaaaa catgaagttt ttaatccttt tgaaatcact ttaaaagatt
35221 tttttggtaa aagcgattta acctataatg ttaattttac tcaaattcaa cagcttataa
35281 aagaacatga ttttaaactc ttagctctaa aaaaacaaaa ccaagccttg attgattttg
35341 gttttgaaga attattaaaa tacaccaaag ataaaaatct taagacttat gaaaattttc
35401 tttcacaaag taaaatttta ttttttaatt ttgatgaaaa atttcacttc tttgaatttg
35461 caaaattcta gcattaacac ttctcataaa ataaagcgtt gaaagtttat ctttataatt
35521 ttttacataa aaacaagaat gattaaaaat ttaaatcagc catacaaagc ccctctctta
35581 aaccctcatc aatcactata agcttttctc tctcaaaaat gctaaaaagt aaaaaacatc
35641 ccgcgaccaa ataattttta cgcgtatttc ccacccaaaa acttgctctt ttttcatcca
35701 tatgccaaag tctttgccca aaatttaaaa aatcattgac atttaatctt gagccattga
35761 ttttcttcgc attatatttt tcataattta atcctttttt taaagccgct agagccgtag
35821 gaacgcctga atttaaaacg atatttttca tcttactttg tttaagcatt ttttttaatt
35881 cattgctttg tttaaaagcc tcaaaagcca tggtttttaa aaaattatca tggattaaaa
35941 aatggatttt tagtttttta tctttaaatt tataaggaaa atcaggatat ttttgaagta
36001 tttttttaaa agaaaaagaa tttacgcttc tttttatctt gcatttttca taaaaactga
36061 taataccaaa atcaaaactt tgaaaattct ctctaaaaga aaattcacaa gatgcacctc
36121 ctaaatcaca atatccacaa tccaaattta agcctaaatt taaaagcccc attttcatac
36181 ccaaaacact gattttagct tcacttttgg catctataac cttaaaatca atttgaaatt
36241 cttgctttaa acgagtaaaa atttcacttg tattgcttgc ctttctaaaa gctgcagttg
36301 cggctgcttt tgcttggctt agatcgtatt ttttagctat ttcttgaagt gcgttgcgaa
36361 gtttttctat ggcttcatcg cttatctttc ccgttttatc taaattttta gccgagccta
36421 tgataaattc atattcatct aacttttcaa atttttcatt tataaaaacc gctcttaaag
36481 tatttgagcc aagatctata cctaacatcg ctttccttat ttaaatttat agtataatag
36541 caaaaaagtt ctaaaggaaa aatatgcttt taggtgtaaa tatcgatcac attgcagtat
36601 taagacaagc tagaatggta aatgatcctg atcttttaga agctgctttt atagcagcca
36661 aatttgggga tcaaatcact ttacatgtaa gagaagatcg ccgtcatgct caagattttg
36721 acttagaaaa tatcatacgc ttttgtaaaa gtcctattaa tttagaatgt gctttaaatg
36781 atgagattct aaacttagcg cttaaattaa aacctcatcg catcacctta gtgcctgaaa
36841 aaagagaaga gcttaccaca gaaggtggac taaacttaaa tcatgataaa attaaagaaa
36901 gtatagaaaa acttcaaaat gcagaaattg aggtttcgct ttttatcaat cctagcttag
36961 aagacataca aaaatcatat gagttaaaag ctgattttat agaacttcac acagggcatt

37021 atgcaaattt acataacgca ctttttagca atatctctca caccaccttt gccttaaagg
37081 aatttgattt aagtaaaaaa atattgcaaa atcaatttga agaagaatta agcaatatag
37141 aaatttgtgc taaaaaagga gtagagcttg gattaaaagt cgctgctggg catggactaa
37201 actataaaaa tgtaagccaa atagtaaata tcaaagaaat ttgcgagctt aatatcggcc
37261 aaagtattgt agcaagatcg gtttttgtag ggctacaaaa tgcgatttta gaaatgaaag
37321 cgctgcttca aagatgaaaa aattagccgt cagtataggc gatattaatg gcatagggct
37381 tgaaattcta gcacgctcac atgaaaaatt aagccaaatt tgcactcctt attattttat
37441 ccatgaaagc ttgctccaaa aagctttaaa acttttaaat ttagagcttt taaatgcaaa
37501 aatcgtagct tttaaggatg ctaaaaatta tgaatttaca ctacttaaaa agcataattc
37561 tcttgaaatt tattcttttg gcttgccttt aaatttaaaa gtggatgaaa actttgacat
37621 taaagcagga gagatagatg caaaaagtgg actttatagc tttttaagct ttaaggcggc
37681 gagctatttt gtttataaaa atcacgcaca cgccctactt actctaccta tacataaaaa
37741 agcttgggag aatgcggggc ttgaatttaa aggacatact gacgcactaa gattcttttt
37801 taaaaaaaat gccatcatga tgttaggatg caaagaactt tttgtaggac tttttagcga
37861 gcatattccc ttagcaaaag taagcaaaaa aatcactttt caaaatttaa gtattttttt
37921 aaaagacttt tatcaagaaa ctcgctttaa aaaaatcgga cttttgggtt ttaacccgca
37981 tgcaggagat tatggggtaa taggtggcaa agaagaagaa cttatgcaaa aagctatcgc
38041 tttcgttaat gctttttaa attctaaaaa agatgaaaaa ttctttaaaa aagctctaaa
38101 agatgagaat ttacaaaaag aattgctttt aaattttaat acaaaaggtg tttatttgcc
38161 ctacccttta gtcgcagata cagctttcac aaaagcaaat ttaaaagcat gcaatcgctt
38221 gatagctatg tatcacgatc ttggcttagc acctttaaag gctttgtatt ttgaaaaaag
38281 tatcaatgtg agtttaaatt tacccattat acgcgtaagt gttgatcatg gcaccgcatt
38341 tgataaggcc tataaaaatg ctaaaatcag tactaaaagc tattttgaag cagctaaatt
38401 tgctctagaa ttgccctcta aagcttcaat ttagcgttat catcgatcaa gccctcttgg
38461 gctagattat ctataaaaat ttccaaatct ataggatctt gaatgctcat ttggattaaa
38521 ttttcatacc tatctacttg attattaaaa atttgcttta atctttcttt gctttgagta
38581 tcttttttag ctttttgcat aagctctaaa aacgctctag cataagtttt ttctatacaa
38641 tcagtcttat aaacaaaagg cgattgagga tcttgacttg cttggatata atcatttgat
38701 gtggctttag tgcaattttt tctttctcta aacatttttt taacaaccaa tttaaaatct
38761 tcttttatat tttcatttgc attttttaaa agctcttgat ataaacttat aaagatatta
38821 tccatcaaag caagtttttt attttcaaat tcattaatag cacctatttt attgcatata
38881 attaattcat catcattttt gccattatct ttaaccttaa agcaatcaaa gcttggtttg
38941 acttcatttt tatatataag ctcatcaaaa ctcaagcccc ataagctaag aaaacttaaa
39001 attgtcaata aaattatctt cattgcctct ccttgttttt tgttttattg taacaaaaat
39061 atttttaatt tttttaaaaa aaatcaaaat acaaaatttt taaccatcac aatacggatt
39121 tattttaaga aaaattaaca aagccataaa aactcttttt atcttaattt ttgataagtt
39181 attcaaaatt tgcaaaaaaa tcttttaaaa attctaaatt ggaataaaaa ttgcttatct
39241 aatttaaaat caacaacaaa ggaggagaaa atggtggtaa aagatatcca aacaacttca
39301 agctctatgt ttagtgctac aaccaaagtt aaggaaaaaa gcacttcagc agatgaattt
39361 caagctactc taaatgaagt taaaaacaaa gaagataagg aagataaaaa gacaagttca
39421 agcgaattta ctaatgaaga tcttgatctt agtgctgtta aagaagattt taaatcttac
39481 gcttggcaaa aaatgagaga agatcaatac aagaaaaatg aagaagattt actcaataaa
39541 ctttttgcaa ccatcgatgc gggaaatact gctaaaatat aaatttaaac ttaaaatttc
39601 attgcgagaa tttttcaatt ctcgtataat tttataaaat accaaaataa gacaatcata
39661 aatctttcac acaataaaac acatttattt ttatcaattt ctgttataat tttatcaaaa
39721 aaacaaagga aaactatgat ttttatcgac gcttgcttta aaaaacccac accttataca
39781 cccatttgga tgatgcgtca agcaggaaga tatttgcctg aatatatgaa agttagacaa
39841 gaggcgggag attttttatc tttatgtaaa gactacaaaa gagcttcgga ggtttcttta
39901 caacctgttg atattttagg ggttgatgca gcgattatct tttcagacat tttagtagtc
39961 cctcttgaaa tggggatgaa tttacgcttt gaaaaaggcg aaggaccggt atttcaaaca
40021 cctatttcaa ccctagaaga tcttaacaag ctagatgatc aaaatgcttc taaaaaacta
40081 aactatgttt atgattcttt aaaactaaca cgcgaaaaat tgcctcttga taaagctttg
40141 ataggatttt gtggcagccc ttggaccata gcaacttata tgatagaagg aagtgggagt
40201 aaaaattatg caaaatgcaa aaaaatgctt tatcaaaatc cagaactttt gcatgaaatt

40261 ttaaataaac tcacccaagt tttaaaatct tacctagaag aacaaatcaa agcaggtgcg
40321 aatgcagtgc aaatttttga cagctgggcg agtgctttag aatgtgataa atttttttgat
40381 ttttcattta aatacatgct tgaaatttct gattttatca aaaataaata ccctcacata
40441 cctgtaattt tatttcctaa aggtattagt ggttatttgg ataaaatcga tggaaatttt
40501 gatgtttttg gggttgattg gagcactcct cttgatctag cacgcgataa attatcccac
40561 caatacaccc tacaaggcaa tatggaacct tgcagacttt atgataaaaa agctatcaaa
40621 caaggcgtag aaaatattct tgaaattatg caaaataaag cccatatttt caatctagga
40681 catggaatct tacctgatat tcctgttgaa aatgcaaaat actttatcca actcgtgcaa
40741 gaaagttcag ccaaatgaaa attctctttg gtcccatcaa ttcaagacgc tttggaagat
40801 ctttaggcat tgacctaagt cctagtaaga aacaatgcaa ttttgattgc gtatattgcg
40861 aacttgaagc tcaaaaacct caagcaaagc aagatgagat tgtaagcgta gaagaaatta
40921 ttttagaagt tcaaagtgct cttgaaaaaa atatcttatt tgactttctt accttaacag
40981 ccaatggaga accaagcctt tatccgcatt tagataaatt aatctcatcc ttacgcaaga
41041 tcgccaaaga taaaaaattg ctcattttaa gcaatggcac tgcagtttta gatcaagata
41101 aattcaatac cttgttaaag ctagatgtgg ttaaattcag cctagatagt gctatttcta
41161 aaacctttta ccgtatcgat agagctttaa aaaatatcga tttggaaaaa atgataaaaa
41221 aaatggcaga atttaaaacc caatttaaag gcgatcttgt aatggaaatc cttgtcgtaa
41281 aagatcttaa tgacaacgaa gaagaattta tcgctttaaa tcaagcttta gctaaaatag
41341 cgcccttaag agttgattta agcactatcg atagacctcc agcttatgcg gttaaaaaaa
41401 taagcgaaga aagactttta gaactttcta aactcataac ttccactcct gtgcttttac
41461 ctaaaaggca ttatgagggc gaaaagttaa attttggtga agaagaatta ttaaaaatgc
41521 ttcatctacg ctcgcaaagt gagatagata tagaaaataa attagataaa gcttcccaag
41581 ttcttttaga taagcttata aaagaagaaa aagtgaagat tcttaatcta gcaggagtta
41641 aattctataa agcctaattt ttaggcaaat attttctaat caaagctata agatcatttc
41701 tactgatatc atttaaaact tcacttacac aatcaaaatc cctcactctt tctttagaaa
41761 aaaatataat atgactttga gggtgttgct ttttataagc gctaagaata gaacgcatct
41821 gctcaagatc aaatttaatc aattcataat caagcaatac taaacgataa gcttcctttt
41881 ttagttcttt ttttaattcg ctaaaagaat ttacacgcat attttgagag cattgttttt
41941 ctatgatatt gaaaaacaaa gttgcatcaa actcattttg tttaaaaagc aaaacattat
42001 tttgataagg agtttgaaat tttaaaacac aaattttatc aagttttctt tgcaaaatag
42061 tttcaagctc tttttttttca aaaggcaagc ttaaaaaata ctcataatat aagtttttac
42121 tacgcccaaa cacgataaaa tcatcggcct ttttagaact ttgctctaaa attttatcct
42181 taatgaaaac cacatgaaaa tcattaatat caaaactcaa gctcttaaaa atagtatttt
42241 ctataccaaa ataagacaat aaattggcta gcaaaaatga ctcaagctca tcatcgctta
42301 ctattgcagc acttgcttca aattgcaaaa cattaccctg tgctactcct atgcttttat
42361 ggtattctaa ggcttgatta agtccaagtc taatttgttc tttttgatgt tttaatgcat
42421 ttaaaacgag tgatttgatt tctctacttg cttcttcctc atctaaacat cttacaacac
42481 cctcgatatt gccaatttga tataaactat cttctagcaa ttttgcgtaa gtttcttttt
42541 catgagaaat cttgaacaag tcttgattta aatgtgctat tttttcccga atcaaatcca
42601 aactcgtttc gatatctatc tctttagaat gtataaattc atttttaatt aataaaattt
42661 tttgatcttt cttagccaaa gctcgctttt gccaaaaaag gaaaaatatt aaagttaaag
42721 ataaaataaa aatactagca aaaaaagcaa gtttaatttg ctcaaaactt tcatgaattc
42781 ttgcaaagtc tatacttaaa aaagccaaaa taatattaaa aataaaaata atccaaacca
42841 ataacaaatc aatcttcctt tattttttgc gctgattttt ttcctactaa ttggcgcaat
42901 tcttcaaaat ttgcctcata aatagcttca aaacttccat aatacgccaa aagtttttgt
42961 ataattcccg aactaagccc taattttgca agcttagagc ttgataaatc ttgtttttttc
43021 ttagtatttt gatgaaaact tatggcaaaa cgatgtgctt catctcttaa tttctgtaag
43081 aattgtaatt ttttatcgtt aatacttagg gcaaattctc cttttaagga atgaattttg
43141 tctttagccc ctcctttagc acgatgagct ttagcatcga ttttttcttt tgagattgct
43201 aaaatatcaa catttgcccc gctacttaag atgatttcct tagctaaatc aagcaaagcc
43261 tttcccccgt ctataagcca caaatcagga gcaggcattt tatcaaaatc taatgcccta
43321 cgcgttaaaa cttcgcgcat ttgctcatag tcatttttat attttaaatg aaattttcta
43381 taatttgact tatcccaagc acccatttta taagctacca tagctcctac attcgccaca
43441 ccttgcatat gagaattatc aaaaacctct atataatttg gcaaattttc aagctcaaaa

43501 taatttttaa gttctttttg tatattaaaa tcactatttt tttgctcttt ttgtatattt
43561 aaacacgcat tttgaaatgc caaatcgcaa attcttcttt tttctcctat tttaggaacc
43621 ttaatactga tttttttatc aaatctttgg cttaaaattt cttctaaaag ctccctatct
43681 tcaaattctt cataaacata aatagtattt gtaaccaagg gaatgtctat gctaaaattt
43741 tctaaaataa gttgtttata aatctcattt ttatcaaaat ctgtattatt tttaagtggg
43801 gtgattttag aattggcact gatgattttt ccattttgca cgacaaaacg caaagttgaa
43861 agcatagagc tttcacaagc taaagcaaaa atttcaaagt cttctaattt agcgatatca
43921 atctcaactt taatttccaa atcccttatc gtagcaatct gatctcttat ttttgccgct
43981 tcttcataat tctcattttg agctaaatga aacatttgtt tttccaaatt tttcactaaa
44041 atactagggc ttaaaagggc attaatggct ttatctaaaa tcaaacgata ttcttcttga
44101 gtgatttttt tatcacaagg tgcaagacag cgagaaattt ggtaaaaaat acaaggggat
44161 ttacaacttg ctttttgttt taatggataa tacaaataaa gtgcatctaa aagctctcta
44221 gctcctttaa aaaagggacc aaaatattta attttactct tttttactaa ttttctagtg
44281 atttcaaaac gcgggaattc ttcattaaaa tctacataaa tataaggata agtcttatca
44341 tctcttagta aaatattgta ttttggatga agttgcttga taaaagaatt ttccaaaatc
44401 aaagcatcag cttctgaatt tgtcgtaata aattccaaat gcactgcttc ttctatcatt
44461 ttttgaattc ttaaagaatt ttttgggtta gcatgcaaat tcggagtaaa agcaaaataa
44521 gatcttacgc ggtttttaag atttttagcc ttgcctacat ataaaagttt tccttcggcg
44581 ttaaaatact gatagactcc agcatttgaa ggtagagttt gaagctcttc tttgagagtg
44641 tttttaagca aaatttcagt catttttat catttgcctt atttcttcaa atttctttgc
44701 taaaatttta ttttcaattt tgttttcaaa ttttccaagg cttaattcta tgtagggttt
44761 tttagccgtg aaattttcat tattttttat tgtattgtta tatttttgtg taaaaatttt
44821 taaatctttt atatctgaaa atccactcaa aggatacatt tttccgtatt tttttatgag
44881 aactttaatg taaattttgc tattatcgtg atttaattct tggtaagcgg ctgaatttaa
44941 agtcactata tttaaaatat gatttttat ataaattcta gcgattaaac gctgtttggc
45001 aggcggaaaa gaatttaaaa aatctttaca aaaaaataag gtttttaagg gtcgataatg
45061 agacttactc atcaagtcat taagaatggc gctagtattt ttaattatct ttggactttg
45121 aaatttttcc ataagatgat tttagcattt tttttcttaa ttttttttgtg tgcttgtggc
45181 tataagggta acccacaata cactagctac gaacaaaacg gttcagtaaa aaacattaaa
45241 aaatacgaac aattaaatag gtggtagaat gaaaaaagca gatattttag ttttggattt
45301 tggatcgcaa tatactcagc ttatagcaag aagattgaga gagcaaggcg tttatgctga
45361 gattttgcct tttaatgtaa gtttagcaga tataaaagca aaagaaccta agggtattat
45421 tttaagtggt ggccctgcaa gtgtttatgc aacagatgcg tacttttgtg ataaagaagt
45481 ttttaatcta ggcttaccga tacttggaat ttgctatggt atgcagctta tggctcatca
45541 ctataaagcc aatgttgctc cagctggaca caaagaatac ggcaaagcaa gtatagaaat
45601 tcaaaaagat agtgatttgt ttaaaaatct acctaaaaaa caaatcgttt ggatgagtca
45661 ttctgataag gttgaaaatt taccacaagg ttttgaagtt ttagcaacga gtgaaaatag
45721 cccgtattgt gtttttgcaa atgaagagaa aaaattcttt gctttacaat tccaccctga
45781 agtgcagcac agcgaatttg gtaaaagtat cttgaaaaat tttgcaaaat atgcttgcaa
45841 ttgcgaaagt atttggaata tgggatcttt tgcaaaaact caagctgaaa aaattcgcga
45901 agaagtgggc aatgataaag ttctttgtgc tgtaagtgga ggggtagata gcagtgtcgt
45961 agcagccttg ctagcaaatg cgatcaaaga tcaagttata gtagtttttg tagataatgg
46021 acttttaaga agtggtgaaa aagaacaagt tgaatttatg tttaaaaata ccttaggaat
46081 taatcttata agtattgatg cgagcaagat tttcctagat cgcttagcag gagtaactga
46141 ccctgagcaa aaacgcaaaa tcataggcaa taccttcata gaagtttttg aagaagaggc
46201 taaaaagcat aaagatgtga aattttagc ccaaggcacg ctttatactg atatcataga
46261 aagctcggtt ataggtgcaa gtaaaactat caaaagtcac cacaatgtgg gcggattgcc
46321 tgaaaagatg aatcttaagc ttgttgaacc attaaaagaa atttttaaag atgaagtgcg
46381 tgccttaggt atcgagcttg gattaagcaa agaaatagtt tatcgccatc ctttcccagg
46441 ccctggtctt gctatacgca tcatgggaga agtcaatcgc ccaagccttg aattattacg
46501 taaagcggat gttatcttgc ttgaagaatt aagaagtaca ggttggtatg ataaaacttg
46561 gcaagctttt tgtgtattgt taaatgtaaa aagtgttggc gttatgggag ataaccgaac
46621 ttatgataat actgtttgcg ttcgcgttgt tgatgcaagc gatggaatga cagcgacttt
46681 ttcacatata ccttatgaaa ttttagaaaa tatctcgcgt cgtattatca atgaggttga

46741 aggcattaac cgcgtagttt atgatatctc aagtaaacca cctgcaacca tagaatggga
46801 ataaagtaaa aaatctgaaa acctgataat gatacacagg ttttcaagaa tttaaatctt
46861 ctttagcgaa tttttaatac ttaaaaaagt caacatgcaa agcatgaaaa aaggctatat
46921 caagcgtatt ggttttattt tataaaaaaa atcaaaaaat gttataagtt ttgagggata
46981 tacagatgga aaatagtata aaagattcgt acaataaaat ttgtaaaaag tggagtgatt
47041 ttcgaaaaag cacttcaacc aataaatgca ttattgattt tgcgaatgat ttgaagtcaa
47101 attcaagagt gctagatatt ggttgtggaa caggatatcc tattgcttcg tatttatcaa
47161 aacaaggttt tcaagttact gggatagata tttcagaaga aatgataaaa caagcacaaa
47221 aacttaactt acataatgct actttttag tagagaatat tttaaatttc aaaacagata
47281 aaaaatatga tgcaatcatc gcatttgatt ctatttggca tattagtcac gataagcaag
47341 aatgtatcta tcaaatcatt tcttctttgt tgacacctgg tggattgttt ttatttaccc
47401 atggaaaaaa tgatggcgaa attatcagca caatgtgggg agaatcattt taccatagcg
47461 ctctagattt agaaaaagta cataaattat taaagcaaaa cggatttgat atcttatctt
47521 ccatagaaga ttatatagaa gaaactacgg gtgatcgaga attgctcatt attgctaaaa
47581 agaaagaatt atgatatagc tttacttaaa agaagtaatc tagagcaatt accaagctta
47641 gcccaaattt tacaaaatcc aaatgcaatg tatgcgttct aggataatct catatcataa
47701 agcgttaaat tattttttag caaaaaatgg gtgtaaaatt tatttcttac aataatacac
47761 cacagcaggt gggaaatttt gccaaacaac tcttgaaatt cttacctctg aaaaaaaaga
47821 aaatatcttt tgacggaatt ttaaacttga aaaagtaggc aatacataag caaaagtact
47881 aaaataacca ttgggtttta agcttttatg aatgattttt aaaagctttt cttgctcatg
47941 atctttaagc aaactccacg gaattccact aacaatcaca tctgcattag ctatacttct
48001 ttgccttaaa aaataaggta aattttcagc acttcctatt tctatatcca aatttgcaat
48061 ttttctttta agtttctctg ccatattttt attgatttct atagcaaaaa attgcgcttt
48121 tttgttttta tattttaaaa tattttccgt aaaaacacct gtgccaggtc caatttctac
48181 gatatagtct gcttgacgta aattaatatt ttgagtcata acaaaaccta gtttttggga
48241 gctagagcaa aaggcaccga tttgttttgg atttttgaga tattcataaa aaaacattca
48301 atttttccta tagtaaaaaa tataactaaa aaaattttta aaatcaaaca tgcgatttta
48361 tccttttgct tataaaattt ttataaaaat aaagctaaaa ttatctttta aaaactttca
48421 aggatgaaat atgcatggaa aaatagctat atatatggat tccacagggc gtggaactgt
48481 aacaaattcg gcaaatactt tttttgactt tagtcgtcaa atttggaatg ataagaaaag
48541 tatgcccagt gttgggatgc ttgttgaatt tcgcacgctc tctagtgata aaaaagccga
48601 aaatggcgct cctgcacctg taagcaaaac tatcacaggg ataaaacctt ccaaatttca
48661 agaatttaaa gagggtgatt ttatcactga acatgatttt tggaaaaccg atagcgatga
48721 tgagcttgaa gatttacaaa attctcgaag aagtgcatat attacagaac tttatagaag
48781 cattgatttt gattctatag aaaaaattcc attatctttt accattcctc aagctataca
48841 aaaatatttt gcccatgaaa ttttatctgt tgaagctttg caagctaatt tacaagatga
48901 aaaagatatt ccttgtattt tggattatct tatccttaaa agattttat ttaaagctta
48961 tgatacttta atttttatgg ataatagcat agatcaaacc caatttagcg ccttaaaaag
49021 cattatgatg cacttagaaa attcttacaa acaaatgata gcagataaaa aacccaatat
49081 cactaaaata tttaatgaaa ctttcttatc tttacaatgt cattaccaag ctttaatagc
49141 taccatagat acaagaaaaa accgccttgc ttcacttgag gcacaaatga aagctttaca
49201 atctgaaatt caactcaaaa gcaatgcggc caatacagat cctgaaaaac tcaaagcaag
49261 acaagaaaaa ttagcaaaac ttcaaaaaga agcggagtat tatagaacaa cccttaaaag
49321 actgggtgct atacaagagg atttttataa gaaaaattat aatattttg aaaatgcttt
49381 caaactttca cgcgaaaaac tctttaaaaa aataattaca ggtcttaatt tatgtgctac
49441 gataatggat gttaaaattt ggcatttatc cttaaaatca tcaggggtta aaaattcata
49501 ttttacaatg agcaatatag aaaattcctt ttgttctctt tcatttgcag aacattatct
49561 aagtcgttta aacaaagcag cactcaatcc ttttgatcaa aaacttttag tttatatcca
49621 aaaaattacc aaagaacaaa gaaaaaaatt tttagtcgta acttcggatt tggatttgct
49681 ttgcaaatta aaaatagaaa cctttgcaca aaatccctat ttcatagtaa aatacgctcc
49741 taaaaaagtc aattatcaat ccttaatgcg tgataatagt tttgatatag tttttattga
49801 tgaaaagcat gtatgggaaa gtgtagcgga tattatttta gaaggaaaac attttgataa
49861 aatagggaaa accaaattta aactgattta aacatagaat tttaagctaa ataaagctat
49921 aatttacttg cctaaacagt tcgtgatatt taaagtgatc caacatattt ttattaactg

49981 tgattgtgcg atttaccgtg ttctgtggcg tcgtttgagt ttcgaaagaa gcgagaagtt
50041 gcagccttta aaaattgcct agcggttttc tttgactttt tggggtcaat ctttattaag
50101 cggctgtttg ggtttgatac aaggagaata aatgaaaata atgattttgg gtagcggcgc
50161 tcgtgaatat tctatcgctt tagctctaaa gcgtgtggat gaaaatttag aattttattt
50221 tgctcctggt aatggtgcta ccgaaagtct tggaaccaat cttaacttta aagatcctgt
50281 agttttagct acttatgcaa aagagaaaga ttttgatcta tgcatagtag gtagtgaaag
50341 ttttttagct gaaggcattg tagatatttt taaacaacac aatattccta tttttggccc
50401 tacaaaagct gcggctatgc ttgaaacttc taagtctttt atgaaaagtt ttttaaaaaa
50461 ataccgtatc aaaacggcaa aatttttaaa tacaaacgat atagaaaaag caaaaaactt
50521 tatattaagc ttaacgcctc ctattgtcgt taaagccgat gggctttgtg ctggcaaagg
50581 ggtaatcatt gcaaaaaccc atgaagaagc tatagaagaa gcatctaaaa tgcttagtgg
50641 agaaagtttt ggtgaagcag gcaagcttat tgtgatagaa gaatttttag atggttttga
50701 gcttagtatt tttgcagtat gcgatggtaa tgactttgtg cttttacctg ctgcacaaga
50761 tcataaaaaa cttttagatg gagacgaagg accaaataca ggtggtatgg gggcatacgc
50821 tccaagctcc ttagcaaatg aaagcttgct tagaaaaata caaaaagata tcattatccc
50881 aactcttgca ggtatgaaaa aagaagatag tgaattttgc ggggtattgt ttataggtat
50941 tatggttgta ggtaacaaac cttatgtttt ggaatttaat gtgcgttttg gagatcctga
51001 atgcgaagtt ttaatgcctt tgattgaaaa tcctctagag cttattttag ccactacgca
51061 aaaacgatta agacacacta aaataaaaat caaaaaagac tatgcagtag gtgtagtatg
51121 tgcgagcgag aattaccctt acaaaagctc tccaaaaagt gaaatttcta taacaaatat
51181 tcctgaaaat acacatattt cttatgctgg agtaagctta gaagatggca aacttatggc
51241 agatggtggg cgtgtattag tttgcgttgg tacaggttca agcatagaag aagcacagaa
51301 aaaagcttac gagctttgcg agaatgttca ttttaaagga aaacaattta gaaaagatat
51361 tgctcatcag gttattcaat gaaagaagat ttgcttacgc gtcttgagcg agaaaatctt
51421 accattgcta gctttaacaa aaggatattg gcgtacttta ttgataatct tgtactttcg
51481 ctgattgtat ttgttatttt ttacgacaaa ttgcttaatg ctaaagattt gtttgataca
51541 gctcaaattt taggtgattt ttacttagga tttttattgc ttcatattac ctaccaaaca
51601 atttttactt atttatatgg agcaagttta ggaaaaatgg tatgcaaaat cattatttta
51661 gatgaagatt tattagacaa gcccaattta aaccaaagct gcatacgctc aatcgtaaga
51721 caaataagcg atatggcatt tatgttaggc tttgcttggg ctttgagcaa tgatttacgc
51781 aaaacttggg aagattatct agcaaggaca atagtagtta atgtggcgta aaattttatt
51841 tttaagcgct agccttacct tactcgatgc cgcccaagtg gatatttatg cacttgatgc
51901 taaaaaacaa ggagatatac tcactgcaaa tgaagatgta gttatttttt cagactttta
51961 ttttatcact gccaataaag ctatttacaa tgaaaaaacc ggagatttag aactttttgg
52021 tgatgtaaat atattaagag gtcaaaatga acgctctcat tctaactacg ctaaaatcaa
52081 tttaaattca aacaaggcca attttaacaa tttcttcttt tctaataata atctagaagt
52141 atggtttcaa agcaaaacaa gctatttaga tgaaaatatt ttcaaaagcg aaatttcagc
52201 tgtttcaagt tgcaatgttg aagatcctga ttgggaaatt cgcttttctg aaggatggct
52261 caatagggaa aataattttg tccatttata taatgccaag ctatatgtta aagattttcc
52321 tgtattttat ttgccttatt ttggctttag tgcggatact catcgccaaa gtggactttt
52381 aatcccaaag atagtcttaa aaaacagcga gggtttatat tatgagcagc ctatttatat
52441 agcgccttat gaaaattggg atctagaatt aaatcctcaa atcagaacca atcgtggctt
52501 tggactttac tccaccttaa gatttgttga ttctccttat tccatgggag aggtaaattt
52561 aggagccttt agggaaaatt catcctatta tcatgatgaa aatttaaaaa atcaaagtca
52621 ttatggtgca gaattaaaat atgctagaaa tgatttaatt aaaactttac tgggtgataa
52681 ttttcaagaa ggcttatggg tagatgctac ctatcttaat gatatagatt atcttaatct
52741 tggcagtagg gattatcgtg atttaaattc attggttact tcaaaaatca attattttct
52801 agcagatgaa aataattatt atggggctta tgcaaagtat tatatcgata cttctgcatt
52861 aaataacgat aataccctac aagaataccc aagctttcaa tatcatcgct ttttaaataa
52921 ctttttttgat gaacgcttgc gttattcttt tgatgcatct tttcataatt tttatcgtcc
52981 cgtaggccct tatgcaaatc aactgaattt aaatttgcct atttcttacc ataatgcttt
53041 ttttgatgat ttcttacatt ttacttttac tgaacgcttt tatgcttcat ttttaaatta
53101 tagtcattat cctgaaaaag agcatgaaca ttattttaga aatacacatg attttaatct
53161 ttatactgat ttatctaaag catatgataa tttttttcac actcttaatt ttggtttagg

53221 gtatattctt ccaggagcca aatcaggaag tatttcggag gattatcttg aagaatccaa
53281 caaagaagaa gaacactttg gcctttatac tactcaatac ttttacaata atgaaggcca
53341 aaaaaaaatc aaacatagac ttaatattga ttatttaaac aagcaaggca gttttgatga
53401 aataagcaat ttattaagtt attattataa taacaacatc agttttaata gtgagattgt
53461 ttactctgat cttcaagatc gttttacaaa tatcatcagt caaatggaaa tcaaacctaa
53521 ttctaaattt aattggagct tttcgcacgc ctatcaaaat gatgagtatg gaaaatatag
53581 ctttataggt actcgtgcga attaccttgc tagtgcaaat tatcatttat ttggaggaat
53641 ttggtttgat acccaaagag cacatgctaa tatgtgggaa cttggatata cctttcaacg
53701 caaatgttgg aattattctt taatgtatag agaaagaatt gatccgcaac ttacaagtgc
53761 gggaattact gctaaaaatc aaagcggtat ttattttatt tttaacttct atcctttagg
53821 tggagttaaa tacgattttt ctttagcaga aacggagagt aaaatttaaa attaaggaat
53881 gagatttgct aaatattttt aaaagtttga caaatttatt atatggaaaa aaaggacaaa
53941 ttatgcaata cagtatagaa attaatggta atttagaagt atttgatata gaaaaagtag
54001 ctaaacaagc tgcaggtgct gtacttatgc gacaaggaaa aagtgtcgtt ttagctacag
54061 ttgcaagaga agaaaagcct gttgaagaag actttttacc tctaactgtt cagtatatag
54121 aaaaagcgta tgcagcagga aaaatcccag gtggttatgt taaaagagaa actaaaccaa
54181 gcgatgcaga aaccttaact gcacgcatca tagacagaag cttgcgtccg ctttttccaa
54241 aaggatatgc ttacccaact caaattgtcg taatggttct ttctgctgat ccacaagttg
54301 atctacaagt tatgagtcta aatgcagcaa gcgtagcact ttatcttagc gatattccta
54361 tgaaagctcc tgtttgtgga gttcgcatag gacgcataga agaaaaattt gtcctaaatc
54421 caaacaatga agagcttaaa aatagcaccc ttgatcttta tgtagcaggg gttaaagatg
54481 aacttttaat gatagaaatg cgtgctttac ctaatcaaaa agaagatgaa atctttatcg
54541 aagcacctta tgcagatgtt ttaactcaaa caacttcaca aagcatgaat gaacttagcg
54601 aagatgaaat tttagaagca ctaagcctag ctcaaaaagc tatattaaat ggctctaatg
54661 cttacgaaga agctttttttg aaacataaaa aatcaagcga tttaaatttc aaagatgaaa
54721 tagaaaattt cgaacttctc gcttttatag aaaataattt tcacgatcaa atcgcacaag
54781 ctatcaatca aatggcaaaa agtgaaagag cgagcgaatt aaataaaatt gctgaaaaaa
54841 tattaaattt tgaaagcgca acaaaatggg atgaagaaag cattttaaat gctttagcaa
54901 aagtcaaaca taaaatcgtt cgtagccaaa ttttaaatac aaaaaaaaga gcggatggta
54961 gaaatttaaa tgaagtgcgt cctatcacca tagaaacaaa tattttacct aatgcacatg
55021 gctcttgcct ttttacacgc ggacaaaccc aagctttagt tgtagcgact ttaggcagtg
55081 aaaatgatgc gcaaatggtt gatgtgttaa ccgaaaaaaa tcctatcaat gaacgcttta
55141 tggttaatta taattttcca ggcttttcag taggcgaagc aagtcctatc aaagcacctg
55201 gaagaagaga attaggacat ggaaatttag ccaaaagagc cctttatcca agcatagatg
55261 aaaattatcc ttatgttatc cgtttggtaa gtgaaatttt agaaagcaat ggctctagct
55321 ctatggcaac ggtttgtggt ggatctttag ctttaagagc agcaggagtg cctagcttaa
55381 aacttgtagc aggggtagct atggggctta tttttgaagg cgatcaatat gctattttaa
55441 ctgatatcat gggcttagaa gatcatgatg gggatatgga ttttaaagta gcaggtagta
55501 aagacggtat cacagcttta caaatggata tcaaattggg cggtattgat caaaaaatac
55561 tcaaagaagc cttatatcaa gccaaagaag gtagaatcca tatcttaaac atcatggaag
55621 aagcacttaa agaaattgtt gttaatgaag aggtgttacc aaaactcgag cttttcaatg
55681 ttgatccttc aaaaatagtc gatatcatag gccaagctgg aaaaactatc aaagaaatta
55741 ttgaaaaatt tggtgtttct atagatttag atcgtgaaaa gggtgaagta aaaattgcgg
55801 gcaatgaaaa cgaacaaatc aaagctgcaa aagattatat tatcaatatc acaagctctc
55861 aaaaaaataa caaaagaggc tttaaagaaa aagatctatc agactttgaa ataggcgaag
55921 aatttgaagg aaaagtcaaa aaaatcacga attttggagc ctttatagaa cttaaaaatg
55981 gaattgatgg cttgcttcat aattctaaaa gtaaaaatct caatttaagc gaaaatcaaa
56041 ttttaaaagt aaaagtaagt gagattaaaa atggtaaaat ctctgtggat ttatgcgagt
56101 gagtgagttt ttaactcatc cttttaaacc ctttttgat aaggattcta aaattttgat
56161 tttaggatct tttccttcag ttaaatcaag agaagatgga ttttactatc agcatccaag
56221 aaatcgtttt tggcctatat tagaaacact ttttgataca agattagaaa atataacaaa
56281 acaacaagcg tttttaaaag aaaaaaatat cgccctttgg gatgtgcttc aaagctgtaa
56341 gattaaaaat tcagatgata aaaccataag ttacgcaaag gctaatgatt taaatttaat
56401 cctttctcaa gcacagatcc aagctatttt taccacagga aaaagtgctt accaattttt

56461 cactaaattt catcctcatt taaaagccat agccctaccc tcaagctctc ctgcaaattt
56521 aaatttttct tttgaacaac ttttgcaaag ctataaaata attaaaaatt ttataaaata
56581 atttttgatt acgtatgtaa aaaataccca aaagcatata taaaaataaa attatttcac
56641 aaaaaagtaa tacatataaa aacacacctt cgcatataaa aaacataatt ccaaaaaaga
56701 caaataaaaa taaagctaaa gttcctaaaa acaatttaga aagatcttta gcacaaaaaa
56761 tcatataatt tataaaaaaa tcaaacattt taagatttat gatattcttt gcaaagtcta
56821 tttttgctag cttctattaa taattcattt ttactagcac aagttttatc tccaactcct
56881 ggtgcatttg ctataccaag tcccattcca aacatccaag caaaaggtac acctctttct
56941 tgtttttgct ccaaatattc gcaaaaaaag cattagcact accatacaac aacatcgttc
57001 caaaaaaaac atgcaataat tttatacata taagcctctt tttaaggaat gttttgtatc
57061 aaattataat cttgtaatat taattatcaa aataattttt ataaaaaatg agcttttcta
57121 ataagcttgt attaatgttt tctatttaaa atgattaaaa aatataaagg agttttaaat
57181 gccttttgta aatatacgca ttactaaaga aaacggcgag cctacaaacg aacaaaaaca
57241 agaacttatc gcaggagtaa cggatttact cgctagggtt ttaaataaaa acaaagcctc
57301 aaccgtagta atcattgatg aaatcgatac agacaattac ggcttaggtg gaaaaagtat
57361 cacccaagta agaaaagaaa aaagctagtt tatatctagc ttttgagtat tgtctttaaa
57421 ttcaaactct accttttttg ctccaaaacc tttggctata gcttctaaag tatctctagc
57481 gcttttgtga attttacttt ctaaatttct aataagattt aatgacaatt ctttaacctc
57541 ttctttagct tctcttatga gcttattttt gtcattttca ctaaagctaa gtcctgtgct
57601 attaatagaa tcaggcagca aaaaaggcaa aaatttagca tttttttcat cataaaattt
57661 catatctata atgctgtgct tgtatttaca ttcaggcata atgatcttat aatgatcatt
57721 atctaaaact acaatttcga aatttttatc tcttaaatcg tacaaaaatg taatctcaaa
57781 ttcaaaaatc aaagcaatct gtctttcgct taaagaccaa cccaaaatac ttttccaaat
57841 tccgttaaaa gcactatctt ttttagtaac aatttcttta ctaaatactt gaaaaacact
57901 gagttctcct atggatttaa gctgggtaat atcattgcta atatgaatat ctttctcttc
57961 tgttttagaa ctatttttaa actgctttat gatgacaaat acaagcaaaa taacaatcaa
58021 actaagtaaa atgagttcca taaaatcacc tttctaaatt ataaaccttg attagaaaaa
58081 atataatttc tttcaaatat caaaaaaatt atagcattta aaataaaaat acagcaaata
58141 aaagcaaaaa caagttctac actaaaatgc gctgttatca atcctcctcc aaaagcaccg
58201 caaaaatttc ctaaaaactg acaagtttga ttaaaaccaa atacaaggct taaattttta
58261 gcactaacac ctttttttaag caaagcattc acacaaggca aaagccctcc aaggcctatt
58321 cctataagca atcttacaaa aataagtgca taaacatttg tagctaagga ttgtaaataa
58381 aaacaaattc cacaaaatat caaagcacca aaaataatct tactagggcc gattttatct
58441 gctatttttc caagtttggg cgcaaagaaa agattactaa tcccgcttgc agccacaacc
58501 acacccgccc aaagagctaa atttccacct tgataaattt gttctacata aagacttaaa
58561 ataggcatta ccccaaaagt tccaaactga ataataaaag ttgtgatgaa aataataaca
58621 ataagttttt tattttcctt aaattcttga gtattctttt ttgcttcttt ttgaattttt
58681 ctctcacgaa taaaaagaaa aattgtcaca aaagaacaag caagcaaaaa ggctaccaag
58741 tcaaaaacag cactaatact aaaaatttct gccacaactc cgccaaataa aggacccaat
58801 aaactcccac tgatagatgc agtagaaagc gtacctaagg cgtaagctac tttttctttg
58861 ggtgctatta cagcaataaa aacaacagct gctgcggtaa atcctgatac aatgcctgtt
58921 aaacctcgta tcaaaaccac ttccatagca tcatgggcaa aactaagcca cagcgttaaa
58981 gctgacattc ccaagcttgc tcgaagtagc atattttat agccgtattt tgcacccaaa
59041 tatgcccaca agggcgaaaa aatagccatt ccaagcggtg taattccaaa agctaatcct
59101 gagtaaaatg cgatttcgct agtatttgta tgtcctaatt ctttgatata taaaggcaag
59161 attggagcga tttggctaag tcccatactt gtagtaaata cgccaaacca acatacccat
59221 aaagtcctat tgaaattttc cataaaatta ttgtatgata ttttttataa ttttcaatta
59281 aatttcaagg atgaaaatga aaaagatagt ttttgtatgt cttggaaata tttgtcgttc
59341 ccccatggct gaatttgtta tgaaagatct cattaaaaag gcaaacttag aaaaagaatt
59401 ttttgtaacc agtgcaggaa cctcaggaga gcatgatgga gaaaatatgc actatggcac
59461 tcaaaataaa ctcaaaagct tggatataaa atatcaaaat tttacaagca aaaaactcac
59521 tcaagtactt tgtgatgaaa gtgatttttt aattactatg gataattcca atttcaagaa
59581 tgttcaaaaa aattataaaa atattcaaaa taaactttta aaaatcactg attttgcttc
59641 tcatttagga tatgatgaag ttcctgatcc atggtatagt gggaattttg atgaaactta

59701 tcaaatcatt tctttagcgt gtaaaaatct tttaaatcat ctacaaaaat aaattattta
59761 tctttgcagt atttaaatat atatctttct aacatagagg aatttccaga aattcctcct
59821 tgatggatat aaagcaaggg atttttccat tctcttttta aggctatact caatcctagc
59881 atatcataaa gtaaatcaaa ttctactaga cattcttgtt ttaaatcttt ataaagctga
59941 taaaattcta aataaggttt accaaaatga tattttttat cactttttag aatttctaaa
60001 ttcgaaaaat cataattttt aataagattt aaaatttgag ttttaaggta gtactcatct
60061 ccaacgcaag cacaagtaaa aactttaaat ttggaatttt tagctaaaaa tgcagccgaa
60121 gttcccgtgc ctgagggtag aaaaatatca aaatctaaac ctaaagtttt actttgactt
60181 tctatttcct tggccagttg aatataacct aattcggcat ttaaattagc aactccttgc
60241 tctataaaaa tatcatcttt ttcacacaaa gaaagcgcaa atgtttttaa gtctttattg
60301 ctgtgatttt cgataaaata aacattgttt tctaaggcta atttataatt tccacaagga
60361 ctatttctta gataggtagg aatcttttca caagcataaa caagaatcaa atttctttta
60421 gtcgcaaata tactaagtgc taccaaagca ttaccttgaa tgccaccata acttataaag
60481 cgttgacctt gatcatagtt ttgagaaaga taaaaagcaa gtttctagc tttgtttcca
60541 ttgatttctc ctagcaaatc atcacgcttg atataaaatt caaatccttg aaattgaatt
60601 ttttctatgg gagaaactaa catttaaaat tcttaacgaa attcaaaaaa cttttcaaca
60661 tcttttaaag cattgtcatt tttaatactg aatttaatct ctatgcggcg gcttttatct
60721 ttatcctcta ccccattaac aagaacagga tcagaaaacg atctaccact tgccatcaaa
60781 agtttttgca accttgtatc cttataaaaa gtatagataa aattcattac ctcataagcg
60841 cgtttttgag ataaatcaag attataaata taagatccat cactatctgt atgtccttcg
60901 atgatgatat tttctatgtt agataaaatc tttggatcat tgatgatagc attaaaatac
60961 tcacttaaaa tttttcttaa actcgccttg gcttcatttt taagcgtgta agaatcttta
61021 tcaaataata cctcagaagg tagagaaatg gatcctgttt tttgatctat ggtaatattg
61081 ctatcaagtt tagcttgcaa ttctttgctt aaattctcgc gaatcaaact taaattttga
61141 accttttctt ttgccttgtc aaagctttct tgtaaatttc taatttcttc atctttcttt
61201 tctagctgac ctaacaaaat caaaatttgc ttatctttac tatcgataga tgaattaagc
61261 tctttactta attgttcata attagtgata tttgcttcaa gttctgcttt ttgagaattt
61321 ataagattta aagcacttga agcattattt aaatcagagc ttaatttaaa aacaatagct
61381 tctttatttc taagctcttc tttactttct tctaagcgtg cttcttgttt gttaagatta
61441 tctttgattt ctcttagatc actttgagtt aaaacgtatt taactacaat agcacctatt
61501 aacaggataa aaacaaaaag tagcccagcc atcaagtcag cataggctat ccaaaaatta
61561 ccttcttctt gctcattttt catttatctt ctttttcttc tttattaaaa tttttattag
61621 aatctaaagt atcgctatcg ttttgtattt ctttagtttg ttctgtattt tcagttatct
61681 tttcgcttga aagaatttca ttttctatat tatcttgctc ttcttgagta agactaggag
61741 ttttacctat cttagccatc acttcatttg tttcttcatt taaattcttg atttcttgct
61801 ttaatttatc catcattttg attttcgcat cgatactttt ttcttcctca taaacttctt
61861 taaatttatg catacccctcg aaaagacttg tcttaaagcc ttcaaaaacc ttttcttgac
61921 tttctaaaat agctttttca tataattcaa aatttttacc aaaacgatcc accttttcat
61981 caaaagcaag caaagtctta tcaaaatgct gaattttttc attgctgata tcaagaagac
62041 ggttatattg ctcagacatt cgcatattga tttcttttaa attttgatta agcattccta
62101 taccatttag catttcacca tacatttttc caagatctct ttgatctctt tgtgccctgc
62161 ttaactcgcc catagtttgc ttaatatgct ccccactcaa tcttatagct ttttcttcta
62221 cattgagcat atcttgaaaa attccaaatt ttctatctat agtatgatca agttctgcaa
62281 aaaaatcttg attgcttact tgtttaaaaa tagcaccgat tttttcaaaa tgttgcaagc
62341 tttcactcaa atatctttga tcaagttctt cttttgtcca aaaaaatccg cttgtagaat
62401 ttttttggcg atttaacaaa cgctcgatct tactctttcc aaatttttca aaaaacatcc
62461 accaaagagc tagaaaaata ccataaattg aaacataaaa agcagtccct acaccactta
62521 gtaaaagagc gatctcttgc tctaaagccg ctgtatcact agagctaaaa ttaggcatag
62581 ataatgctat actgataaag gttcctaaaa ttcccatcat aggaaaaata gccgcaccaa
62641 tactagcaaa attttcattc cttgaaccct taacataagc ataagcaaaa tcatcaaaac
62701 tcgcattgga ttttgtatct ttacctatgg taagaaaatg tttcataata tgtcttttta
62761 aagcttgttt aaattcatct ttttgttgtt caaaaatact atatgcatac tcagaactat
62821 gccttgcaaa cacaagggct gtgaaaaaaa taaagcctat cataacaaca gtatgcaatt
62881 cgactttgaa atttataata ttataataac ccaataaaat aaaaacataa agtaaagcag

62941 gtataaaaat aatttttaaa taaactaaat atcctttttt ctctttactt tctggcagaa
63001 tgagttctga aatttcatcc gatcttatat ccataacact aataccccat tagctaaatt
63061 ctattttaaa gattattatc aagaaattca aaaaatcttt aaaataaaat ttaggaagat
63121 taagccaaag gcttaatttc ttcctataca atttaaatct tcaaaagcaa gctctaaacg
63181 ttttatcata ctttcctcac cgcttcttag ccaaacacgc ggatcgtagt atttttttatt
63241 aggcttatca tcgccttctg gattaccaat ttgaccttgt aaatatgcac gattctttgc
63301 ttcatactca cgaactccat cccaaaatgc ccattgagta tctgtatcga tattcatttt
63361 taccacacca taactcactg catctttgat atcttttagt tcacttccgc ttccaccatg
63421 aaaaacaaaa ttgatcggct tatcagcttc taggccaaat ttttctttta caaatttttg
63481 tgaattttta agaatttcag gctgtaagct tacgttgcct ggtttataaa ctccatgcac
63541 atttccaaag ctggctgcta ttgaaaattt atcgctgatt tttccaagtc tttcatatgc
63601 aagtgctaca tcttcaggct gggtataaag tttagaatta tcaatacctg tattatccac
63661 tccatcttct tctccacccg tgcagccaag ttcaagttcg agtgaaattc ccaaagaatc
63721 aagcttttta agatatgttt cgcaagtgct tagattgtct tctaaatcct cttcgctaag
63781 atcaagcata tgagagctaa ataaagcctg tccatgggtt tttttatatt cagcatttgc
63841 ttctataagc ccatctatcc aaggcaaaag ttttctagca gcatgatctg tatgcaaaat
63901 cactgggata ccataagcct tagcaagcaa atgcacatgc ttagctccac taatcgctcc
63961 taatacatca ccttgtggac aattttttcc tgcataaaat ttcgcccctc cattagaaaa
64021 ttggatgata acaggagaat ttactttttt agcagcttct aaaacagcat tgatcgaatc
64081 agttcccact acattaacag cagggatagc aaaaccctgt gttttagcgt attcataaac
64141 tttattaagc tcatctccgc taacaacccc tgcttttacg atgtctaaca agcccattat
64201 ttgtattcca ctttagcatt gtttaacaaa tcttgacctt tttgattgat cacttttttta
64261 aattcttcaa atttcaaacc attttcaatg ccttttttaa catcatcaaa tttgatttgt
64321 tcttttgcac gagaattttc ttttaagatt acatggtaac caaaatttgt ttttacagga
64381 gtagttgtaa tagtaccatt tttaagtgca aatgctgcgt cagtaaaagg ttttaccatt
64441 gttgattgat caaaccaacc caattctcca ccttgatttt ttgaacctgg atcgattgat
64501 ttttctttag caagttgact gaatttatca tctaaagctt tgccttttaa acctttgagt
64561 tctttaataa tatctttagc ctcttcctcg ctagatacta aaatgtgctt agcttgaact
64621 gcagcaggtt tgatatattt ttctttattt tgttcataaa atgctttaac tttagaagaa
64681 tcaattttaa tagaattaag aatttttctt tgataaacat taataagtat gctatctttt
64741 gcgcgttcta attctttttt ataaagagga tctttttcca aattttgttt tttagcatct
64801 tgtagtatca aatcttgcat gatgtattgt tgtaaaagag cattttttttg atgatcaggc
64861 aagcttttaa aatcctctcc tctaagcatc ggtgcaaaaa attcactcac ttcagtatcg
64921 cttatatttt taccatttac agtagctata gtagctgcat ttaaatttaa agctacacct
64981 gcaattaaag ctgcggcaat taaagaaact tttctcattt tagacttcct tttttaataa
65041 aatagattaa aatcaattat atctcaactt ggtttaaagc taattaatta aaataaaaaa
65101 atgaaaagaa attcagaaat caaagaactt tttttaaaac attttgacaa gcccaccaca
65161 gaattaaagt tttcaaatct ttatgaactt ttagtctgtg tgatgctttc tgcgcaatgc
65221 actgacaaaa gggttaatct tatcacccct gagcttttta aagcttatcc tgatatcaca
65281 agtcttgcaa atgcaaattt atcgagcctt aaaacttata tacaaagctg ttctttttac
65341 aataacaaag ctcaaaatct catcaaaatg gctcaatcag tctgtgaaaa tttcaatgcc
65401 caaattccat tagaggaaga aaaattaaaa tccttagctg gagtaggaca aaaaacagcc
65461 catgtggttt tgattgaatg gtgcggtgca aactgcatgg cagtagatac tcatgttttt
65521 cgtgtttctc atcgcttagg acttagcaag gccaaaactc ccgaagctac cgaagaggat
65581 ttaacgcgca tatttaaaga taatcttaac tatctccatc aagctatggt gctttttggg
65641 cgctatgttt gcaaggctaa aaaaccccta tgcaaagaat gttttctaaa ccatctttgc
65701 aaaagtaaag acaaggagct tgaatgaaat ttattatctt tttactagca tttacacttc
65761 atgtttttgc tttagaggcc tataaacccg ataaagattt tgcatcttat tttaatcctc
65821 taaattgctc cgttgttttg gataaatttt tttatatcaa ttgttatgat tataaactca
65881 aaggcaccaa agccgtagct tataaggtag aagcgtctaa tctgaaagaa aaacagatca
65941 aaaaacgagc tcgctttgaa gatgatacaa atatccctaa aaaataccgt agcacttgga
66001 gtgattataa aaatagtggc tatacccgag ggcacatagc ttcaaatgct tcttttcgct
66061 tcagtaaagc agcacaaaca tcggtttttt taatgagcaa tatcactcca caaaatgcac
66121 aagttaacgc aacggtttgg aatgaaatcg aacaaagaga aagaagtctt gcttttaaat

66181 ttcaaagtgt tgaagtgcta aatctaatac tttatggtaa aaatcctaaa ttcattaaaa
66241 atggtatcgc tataccgagt tcttatatta agatcatcaa aactcctcga tttaaagaat
66301 gctatgaggt gccaaataac gatatccaaa gtgaagaaat caatgattat aaaatttctt
66361 gcaataaatt ctaagggcta tcaatggaaa ttagtgcttt aactataact atactttata
66421 ttataggaat ttcagcagag ggcatgacag gtgctttggc tgcaggcaga cataaaatgg
66481 atctttttgg agtaattttc atagctttag taactgccat aggaggcgga agcatacgcg
66541 atgtgctttt aggtcattat ccgcttactt gggttaaaca ccctgaatat attgttttaa
66601 tttgcctttg tgctcttgtt gctactaaaa ttccacgcat tgttagtaga ttagaaagtt
66661 tatttttaac acttgatgcc atagggcttg tagtctttag catacttggt gcacaaattg
66721 caatagaaca aaaccatggc tttatcatcg caatctctgc agcagtgatc acaggtgttt
66781 ttggtggaat tttaagagat attttatgta tgagaattcc tttagttttc caaaaagaaa
66841 tttatgcagg aattgcaatc atagcagggg cgatttatta cgctttaatg gtttatcttc
66901 aactgcatgc tttaatttgc acgcttgtaa cgctatttgt aggagttaca gcaagacttt
66961 tagcaatcaa atatcaatgg tctttgccta ttttttccta taatgaggaa aaataatgaa
67021 ttgtatttat aaaaatttgc taagataaaa agaagcgtga taaaagttta attatgcttt
67081 ttaaggaaat actatgaata aagcaatact acttaccctt attggtttat tttttacagc
67141 ttgtgaacaa accaaaagtg tagaatatta caaagctcat cctgaagaag ccaaaaaaag
67201 aagtgatgaa tgtcgcttga aatctatcat cagtcaagat tgtgtaaatg ctcatagcgt
67261 tgctatccca aaagaagatt gggatgaagg ccatgataca caaacaagat aatttttatt
67321 taaaggctat aaaggtttca aaattaaccc acttaaaaac actttctatt ttttcaaatc
67381 ctgcattttt aagcatatct aaattttctt tttcactata agggataaga acattttcca
67441 aagcctccct tttggctgca atttcgattt tagaataacc ttgtctttct ttataatctg
67501 cataaagttc tatcatattt ttagacaaaa aagcatcttc ataaagaatt ttttcactca
67561 tgataaaaat accacctttg tttaaattct tataaatttc attaacaagt tcttgtcttt
67621 taggcggacg aatgaattgc aaagtataat tagccacaaa tacatcactt tgaaaaaata
67681 caaaatcaca caaattcgct tcaaaaaatt cgatatttaa gccataagct tgtgcttttg
67741 atttggcgat ttcaagcata gaaggtgcat tatccacacc aaaaagctta agatcttttc
67801 ttaagctggc taaaaaaatt aaaaaattcg cactagaaca acccaaatca caaaccttta
67861 ctccctcttt agtcaatttt tttatcaaat ttgcacaaag ttctaaattt tctttataaa
67921 aaggcacaga gcggttaatc atatcatcaa aaacacttgc aacactctta tcaaattcaa
67981 attgtttggt taattttttct ttaaaaagtt catctttcat tctctatcct aaacacgctt
68041 tagcttgatt tatatctttt gaaatttgtt ctttaagttg ggcaagatcg ttaaattttt
68101 gattttctct tagaaattct acaaattcaa gctctaaaac ttcccctata gccaagcttt
68161 catcaaattt ttctataata tgactttcta tggcaaattg catatcgcta gaacgcacgc
68221 ctacaaaact tacgctctta tagcgtttat catgaaaact cacaatgcta gcataaactc
68281 catttttagg taaaaaatat tcctcgcatt ctaaatttaa agttgcaaaa agctcttttg
68341 agccaagtcc ttgacccttt ataagcttac ctttgattgc ataatttcgt cccaaaaagc
68401 aatttgcctt tttaatatca ccccgtgtca aatactcttt aatcaagctt gcatgaacac
68461 ccactccacc taatctaaat tcatctataa tcgttgtttt aatcccgcta agtgcttcta
68521 tatcttttgc tttatagctt ctattttttc caaaagaaaa atcaaaacct acaactatac
68581 gctccaaatt tacaaattcc tcttttaaag cttgtaaaaa atccttacca tctaaggcct
68641 tgatgctttc aaaatcaagt tctataatct ctttttgagc taggaatgct ttgtcttgat
68701 tggtgcaaag ctttttaccc ttaaatttat cgataatcaa aagagccccc gaatcatcta
68761 ggcactctat aagcttttta tgccctaaat gaactccatc aaaacatcct atggctaagg
68821 ttttaatatt atttttttagt gtagtagtaa aaatattcaa cattaccctc ttttcctttg
68881 attttagact cttcacaaca ttgcaagatc caacctaagc gcgcacaagc attttcaaat
68941 tctctcatag ctttaaacac agccttttca tcttttaaaa caccctttttt gtctcttttt
69001 gcttgcttac caacttcaaa ttggggttta aaaagcaaaa ctatatcctt taaagctaaa
69061 ttatcaatat acgaaagcaa atgagttaaa gaaataaaac tgacatcaca agtgataaaa
69121 tcaaattttt gttcgctttt aaaagttctt aaatccgtat tttcaataat ttcaatcctt
69181 ttatcatcgc gtaaactagg atggagttga ttatcgccca catctaaagc gcttactttc
69241 aaagccttgt gttctaataa aatttgcaca aaaccgcccg tgcttgaacc tatatcaaga
69301 caagttttat ttgctatatt gatattatga ttttctaaaa aatatttaag cttaaaagct
69361 gcacgagaaa cataaagctc acccaaaagc tctaaattta attctttgga atttaaaagt

69421 tcttcttgat ctgctatctt gtttttaagc agattttgca catcaaaaga agctttataa
69481 ggcttggagt taagcaaaat ttcttcattt tctatcaatt ctaaagcttt atttctgcta
69541 agatttaaac gctttgctac aaaaatatca tatctcattg aagactttca tttaaggatt
69601 tttgtaattc ttctttatct ttagaaaacc accaaattcc tatttcgaag gcatttttat
69661 ccaaaagttc ttgcacttgt tttaagtttt taactagtct atctttatca ttttgaagca
69721 tacttaaatt ttgctctttt aaaaaagaaa gattattcat catcaaaaca agctcttttt
69781 gataagcttt tggattttgg tttttatcct ctacgataaa aaatgaagct agagctacaa
69841 aaaatgaaaa gacaaaaaca taagttttct tttttgcctt aaaagcaaaa ttataaagat
69901 aggccacaaa ggagcaaaaa ataaaaaaat taataaaatc aaaaataaaa tttaaaaccc
69961 taaaccaaaa aagacccaaa gaatataaaa aatactcttt taaagcattt ataagtgtta
70021 aaatttgaga aattaaatca aaaacaaaaa actttgagtt tttataatga ataatacttt
70081 gattaaaatc ttcaagatta aaactttgta taggatttac cactccatta aaaatcgcat
70141 ataacaaagc taaaatcaaa cttgataaaa tgattattct aaaatcatta taggcattat
70201 ctaaaaactg caactgaagt ttttgcttga aaaaaaataa aaataaaggc aagagtataa
70261 acaaaaaaaa tccatcttgt ttttcaaaac tgatcaaatt taaaaacaaa gaagcaagcg
70321 caaacaaagc cataaaaaaa ctcaagctat agatataaaa atctcccgca ctcacacgat
70381 aaatcaaact atttttaatc atcgttgctt cttttatgat attttttcta agccttaaaa
70441 aataaaaaaa ttccaaaaaa aatgctaaaa aaatagggat taaaaaaagc gaaacccaag
70501 caaatttttg ccaaaaaaat aaaaatgcaa aaatactaaa taaaacaaat agctgaatat
70561 tatttttcaa taagctcatt aaattcgttc tcatcaatac attttacacc cagctcttga
70621 gccttgtcga gtttagaacc tgcttcttga ccaaaaagca cataatcagt ctttttagaa
70681 accgatccgc taaccttgcc tccaaggctt tctattagat ttttaaactc ttctctaggg
70741 cgcgaaagcg tgcctgtgat cacaaaagtc ttgccaaaaa taacactatc ttcttgaatt
70801 tgtacttttt gctcttttaa atttaaaagc ttataaaact catcaattct tgcgctattt
70861 acacgagaaa actcacacaa agaaagagcc atttgctcac caaagccttc taaatttgca
70921 taagcttcca aactttgctt gtgccattct ttaccaaaac ttaaagctag ctttttagca
70981 gctacttctc caatatgctc gatccccaaa gcagtgataa aacgaaaaag ctcacaatcc
71041 cttgcttgct caatagcatt taaaatatta ttgatctttt tttctttaaa accttctagg
71101 ccttcaaaat cgctaaattt aagatgaaaa atgctctcga gagtggttat tttttttctct
71161 ttaaataaaa gttcaactat actttctcca agtccgtcaa tatttaaaca tttttttagaa
71221 acaaaatgaa tgatagaatt tacaagccta tcttcacaat caatattttg acatttgatc
71281 aaagttccct catctaaaag ttcgcttttg caagtaggac aaaactgagg tctagctata
71341 tcaagttcta agccatctct acgctcttta aaaactttag ttatttttgg tatcacatca
71401 ccacttctta tcacgcttac aaaatcattg atttttgcat caagtcttgc aatttcatca
71461 aaattatgaa gtgtggcaga ttttacaata actccatcta aatttacagg ctctaaaacc
71521 gccacaggag tgataacccc gcttcttcca acctgcaaat ttacccctat taaacgcgta
71581 gtttttttcca aggctggaaa tttaaaagct gccataaatt ttggaaattt cacagtatga
71641 cctaactctt tacagagtgc aagatcatct actcttacca ccattccatc catcatcata
71701 ggctttttat ctcttaggct taaaagctca ttataagctt ttaaaacctc atctaaattt
71761 acacaaagct ttacaaagtc atcttttaaa aagccaagct ctcttataaa actcataatc
71821 tcgctgtgtt tactaaattc aagtgagttt tgccctaccc cccaaggata gaatttcaaa
71881 tttctttctc ttgtgatgct tgtgtcaagt tgtcttaaac tcccacttgc ggcattgcgt
71941 ggattggcaa acaagctttg gtttaaacgg gctctttttt cattgatttt ttcaaaatca
72001 tcttttaaaa tcaccacttc gccgcgaatt tctatctttt ctttatagtt tatagttttt
72061 gggatattgt cgatttcaag cacattaagt gtgatatcct cacctatttc cccatctcct
72121 cttgtcgcac cgcttattag tctaccatta tcataaagca aatttaagct tgctccatca
72181 aatttaggct ctataaaaaa attctcttca catcttgcac gctttgccca tgctctaagc
72241 tcactctcat caaaaacatc ttccatcgac cacatacgac ttaaatgaga aagtttttta
72301 aattcgcttt gtatagtagg ggctattttt tgcgtaggag aatcaggaga aatttcattt
72361 tcattttgct cttcaaaagc tcttaattct ctaatcaaag tatcatattc tgcatcactt
72421 gctaaaggct catccttttc ataatacgcc ctcatccaag aagcagccaa ggcaactttt
72481 tctaagtact cttttttttgt catttaaact cctatgcttt gcatcgcatt tgccattgca
72541 aaaagctgtt tatgctcata taaatcatgc actcttataa tacttgcgcc attttcaaaa
72601 gcctttaaat gcaaatataa agtccccgct aagcgatctt taacttcact ttcataatac

72661 gcattgatca cgcttttacg acttgctcct acaagcaagg gctttttaaa ttgcaagaaa
72721 tgttctaaat gcttgattaa aatcatatta tgttctgcac ttttaccaaa gccaattccc
72781 acatctaaaa tactttcttt cactcctaaa gcatctaaaa tttcaagttt ttctttaaaa
72841 aaactactca tttcatcaag caaatcatca tatcttggat catcttgcat ggttaaaggg
72901 tcattttgca tatgcataag gcagtatttt gcattgtatt tacttgcaag tttagctaaa
72961 ttaagatttc ttaagctact gatatcattg ataaatttaa agcccttatt taaagcgtat
73021 tctaagcaat attcatcaaa actatccaaa ctaaaaatgg ctttttata ataattttta
73081 tcataaatta aatccagcac atttttcagt cttctaaact cttcttcctt gccacaatac
73141 acgcttttg gcctagagct taccgctcct atatcaatat actcaggatt taaagctaaa
73201 atttcattta atctttcttc aaaatcttta taactcaccc tagaatcagc attaaaacta
73261 tcttcattga catttataac tgccataagc tctgcttgct ttggttttga aaaatcattt
73321 tctaaaaatc tagctaaatt tttaagccca aaatcttgaa gcttttcttt gtttataagt
73381 ttgtgaattt gttcttttgt agccattaaa agcgcattag aatgtgtgat tttagcagtg
73441 ataacttcct tgtgtgttat aagctcagct cccactctta aggcatcttg ttttaaaata
73501 tttgccgcag gagtagttat atctttaatc aaaataaaat aaatttgagt tttttcactc
73561 ataattttt gccccatttt atgaggattt ataaaagagc aaagtaaact aaaatcagtg
73621 tttggattga ttttaaaaaa tttcattttt cattctttgt ataataattt aataaaagcg
73681 tagccagtat caaagagctt ttagaattta actttgcaag ctcatagctt tcataaaaaa
73741 attcaagctc atcggcatta aaatctttaa atttaacact ttcttctgca atcagtgcga
73801 ttttttccat taattctgct ttatctaaat tttcattttc ttgtaaaaac tcaaacaaag
73861 tctttaaatc cattttatta agttctaaat ttaaagtatt tttagccttt attttgttgc
73921 gtttttcaca aataagacgc gattttatgg taggtaaaag taaattttta gaaggaacta
73981 cgataagaaa tttaatattt tttggcggct cttcaagaag cttgagtaaa aaattttgag
74041 cctctatcct aaaagaattt gccattaaaa caatgatttt ttcacttgtt tctgcgatat
74101 agctttcctt ttcaaccgct ttagcatcat cgattaaaaa ctcacttgca acttctttag
74161 gtataaaacg caaagaattg ggatgaaatt gctttaaaag ttcagcccta ataccatcaa
74221 aatcatcggt gatgataatt ttacttacaa acactaaaga ctcactttgg aaaataaaat
74281 cgcatccaaa ctcgtatcaa aaagtttata aagagtgatg agtttgacat ctaaatcttt
74341 atctttggaa ttcaaataaa aactatcttg caattgctca tcaaaaagcc ataaaagact
74401 atcgtcgcgt gatttagcga ttaatgattt cttatcgctt gcactcccta tataaaaata
74461 aataaagcca ttaggaaacg aaatatcaag taaatcatta agcaaggcta tatcttcgat
74521 attttgtatc tttttttac ataaagattt taaagataca aaagggagca tgggacgagt
74581 agttttggaa tttatatttg ccaaaaagta ttcttcatac caagcccttt gttcatcaca
74641 catcaaaaca aaggttcgcc cctctaaaag gtattttaat cttgcagcca ttaaaggagc
74701 ccattctaaa cgacgagact ccatccaagc cataaatgtt ccgccattgc ggatattttc
74761 aagtgtaaaa cttaaaaaat cgctcattta tttatcaagt ccatagcact catgcaaggc
74821 tcttaccgca agctcgccat atttttcatg cacaatcatt gaaattttaa tctcgcttgt
74881 agaaatcatg cctatattaa taccctcatc tgctaaagct ttaaaggctt tgcttgctac
74941 acctgaatgt gatttcatac ctacgcccac tatagaaact ttaaccacag cactatcgct
75001 ttctatggtt gtcttagaac ctaaaatttt ttgcatagca ttttttgcca aatcaagctc
75061 attttgaggc acagtaaatc ctaaatttgt agctccatct actccaacat tttgtataat
75121 catatctaca ttgatatttt catctgctaa aacagaaaaa atttcagccg cgattcctgg
75181 tttatcttct atatttctta aagttactct tgcttgattt ttatctaaag caataccact
75241 aactaaagct tgttccattc catcttcttt tgttatcatt gtaccctcat tatcattaaa
75301 actacttcta gtaactaatt ttacatttaa tttttttgca agttcaaccg agcgattttg
75361 caaaacctta gctcccaaac tagcaagctc aagcatttct tcataagaaa ttttatcaag
75421 tttttttgcc tttggttcaa tccttggatc tgtagtataa actccatcca catcggtata
75481 aatttcacac aaatccgcct taagagctcc tgcaacagca accgcactca aatcacttcc
75541 gccacgccct aaagttgtaa catcaccctc tttatcaact ccttgaaagc ccgctacaac
75601 gactattttt ccttcattta aagccttggt aatttcttga gtgtcaatat aagaaattct
75661 agcctttgta gaaacgctat ctgtaacaat tcctgctttt ctccctgaaa aagcaagaga
75721 aggatatcct ctttcattga gtgcgataga aagcaaagca ctcgttacgc gctctccact
75781 acttaaaagc atatccatat ctttgccatt aggatttttg ctaaagtatt ctgcttgctc
75841 tattaaagta tttgtaactc cactcatcgc tgaaactaca accacaagct gatgtccctc

75901 ttttgcactt tttataacac gctctgctac cgcatcaatg cgttctaaag tgccaacgct
75961 tgttccgcca tatttttgaa ctataagcat taaatataac ctttctcttc aaaatattta
76021 ataactttaa cataaatatt tctcttgaaa tgatttatgg tttcaaacac ttgttttact
76081 ccgacaaatt gataagcatc aaattcagga tgttttgtat taatattaat tttagcaccg
76141 tgttttaatc gcactaaaaa atatttttgg ctttgcccat cataaggata catttttta
76201 gcaatctttt ctggaaagtc ataactcaac cattcaggat gctcagctat gatttccact
76261 tcattcgtgc caatctcttc cttaagctct cttaatacag cacttttagg gtcttctccc
76321 tcatcgatac caccttgtgg aaactgccaa atattatcca tatcgcttct tttggctata
76381 aaaagcttac actcaaaagg ataagcactt gataaaacaa tagcagcgac attgggtcta
76441 tacttttttt ctttttccac ttttttcctt caattttaag cttataattt taacctaaat
76501 gaaatcaaaa tttcattaaa ataagaaaat aacattataa aatttaaggt ttttatgcat
76561 ttatatattc atatcccttt ttgcgaaagc aaatgccatt attgtgcttt tacaagccta
76621 aaaaaacatg attatgaaaa caagtatttt gaggctttat ataaagatat ttcctttcat
76681 ttgcaaaaat taaatattaa aaaaaatagt attaaaaccc tctttatagg aggaggtacc
76741 ccaagtgctg ttagtgcaaa aaattttgaa atgatcttaa cacttataga accttttta
76801 agtaaaaata tagaattttc aagcgaggct aatcccaatt cagccgatat agactggctt
76861 aaaacaatga aaaatttagg ccttaatcgt atctcttttg gagcgcaaag ctttcatcct
76921 aaaaaactag aatttttagg aagaattcat gatcaaaaaa tgatttttaa agcattggaa
76981 aatgcaaata aactgggatt taaaaagatt aatttagatc ttatttatga tacaaaatta
77041 gatgataaaa aaatgcttca atttgaactt gaacaattaa aacaaattaa aaatttaatc
77101 actcatctaa gcgcttataa tcttaccata gagccactga gtacttttgc aaaaaaggat
77161 cattttaaaa aaaacgcccc ctatttgatg aaatttttca tccaagaact tcaaaagctt
77221 ggatttttac aatacgaaat cagtaatttt gccaaaaata aagcccaaat ttgcaaacac
77281 aatttagcct actggaaagg tttggattat ctaggctgtg gtttgagcgc ggtgagttgt
77341 tataaaaacg agcgttttta tacggctaaa aatttaaaag attatcttca aaatcctgtc
77401 tttagggatg ttgaaaaact aagtcaagaa gatttaaatt tagaacatct atttttagga
77461 ctaagaagta tagtgggtat tgaagaatgc aaattaagcg aaattcaaaa agaaaaagcc
77521 aatttacttg taagagaaaa aaagcttaaa tttgagaaag ggcgttacta taatcttaac
77581 tttttaatta gcgatgagct tgccttatat ctctcatctt aaattcaact tttttaagta
77641 aaatatttct tttataaaat ttcaaatatt ttaagagtat tgtttatgag ttttggtgaa
77701 attattgtaa ttttaatagt agcaatttta gttttagggc ctgataaact tcctgaagcc
77761 atagtgcaaa tagcaaaaat tttaaaggct ttaaaacgca atatcgatga tgcaaaatca
77821 agcatagaaa aagaaattcg tatcaatgat ttaaaggaag aagctaaaaa atacaaagat
77881 gaattttcaa gtgcaaatga aaacatccgt aaaaaactta gttttgaaga atttgatgat
77941 ttaaaaagag atattttgga taaaactaaa gtggatttga cttttgatag cagagaaaat
78001 tccaccccta acccaaacga acaaaatcca aatgaaaatc aaaaacccaa actcaatcca
78061 tttgaaaatc aagaaaaagt agaaaaataa atgtttgaag aattaagacc ccatttaata
78121 gaacttagaa agcgtctttt tattagcgtt gcttgtgtga ttgtaatgtt tatagtatgt
78181 tttgcttttc gtagctatat cttagatgtg ttaaaagcac ctcttattca ggctttacct
78241 gaagtagcta aacatgtcaa tgttatcgaa gttcaagagg ctttatttac agctatgaag
78301 gtaagttttt ttgctgcttt tatctttttct ttgcctgtta ttttttggca attttggaaa
78361 ttcgtagccc caggccttta cgataatgaa aaacgccttg tggtaccttt tgtaagcttt
78421 gcaagcatta tgtttgctct aggggcttgt ttttgctatt ttattgtagt acctttagct
78481 tttaaatttt taatcaattt tggtttaagt gaagatttta atcctgtaat caccatagga
78541 acttatgtgg actttttttac aaaagtagtt gttgcttttg gcttggcttt tgaaatgcct
78601 gttatagcat ttttctttgc taaaattgga cttattgatg atagtttctt aaaacgccat
78661 tttcgcatag ctgttttagc tatctttgta ttttcagcct ttatgactcc acctgatgtc
78721 ttatctcaat ttttaatggc tgggccactt tgcggacttt atggactttc tattttaatc
78781 gttcaaaagg taaatcctgc tccaaaagat gaagaaaaag aagaaagcga taaatgaaag
78841 atttacttct ttcaagctat gattatacct tacccaacga acttatagct acttatcctg
78901 tgtatcctaa agaagatgcc aaactcttag tttttgagcg aaaaaataat caaatttctc
78961 atactacttt taaaaattta caagatttct tacccgattg caccctcttt tttaatgata
79021 ctaaagtcat taaagcaaga atttatggga ataaatcaag cggtggaaaa atagaacttt
79081 ttctacatca accctgttta ggtttgcaag attcactttt tttagcacaa attaaagggc

79141 gcgttaaaaa agatgaaatt ttatcttttg aacaaaattt aaaagctcaa gttgtagaac
79201 ttttagatga tggcttacgc aaagtacaat tttttcaaaa taatgaaatt ttaaatactt
79261 tcaaacttta tgaactcctt gatgtaatag gacatgtacc cttacctcct tacatcaaaa
79321 gaagtgatga aaaaagtgat ttagaagatt atcaaagtat ttttgctaaa aatttaggag
79381 cagtagcggc tcctacggct agtttacatt ttagcgaaaa tatgcttgag aatttaaaaa
79441 agaggcatga aatttatcat ttgactttgc acgttggcgc aggcactttt aaaagtgtag
79501 aatgtgaaaa tttagaaaat cataaaatgc acagtgaatt ttttgatatt cctaaaaaag
79561 catgtgaagt gattgattct caaaaaaaga ttttaggtat aggcactaca gtaacaagga
79621 ctatagaata ctacgcaaga actaaacaaa gccaaggctt ttgtgatctt tttttacatc
79681 ctcacaaccc accccctaaga caagactatc ttttaactaa ttttcatcta ccaaaatcaa
79741 ctctgattat gttagtttca gcatttatag gaaggcaaaa ttgccttaaa ctttatgata
79801 ttgccataaa agaaaaatat cgtttttact cttatggcga tgctatgctt attttataaa
79861 tttattcttt aaatttcata gcaatttttg cttgaagttt aagccaatct ttttggagca
79921 ttataggaaa acctccataa actctgccac cttcaagatt tttactcaca ccaccacgag
79981 ccgcaatggt actaaaatcc cctattctta aatgtccact cgttgcactt tgtccgccca
80041 taaccacatt gcgtccaagc tcacttgagc ctgaaattcc agtttgtgca acgatgatac
80101 aattttgccc tatatcgcaa ttgtgtccta tttggacaag attatctact tttgttcctg
80161 ctttgatgat agtgctatca aacactgctc tatctattgt tgtgcaagct cctatttcaa
80221 caaaatcttc taaaataaca ttaccattgt gatagatttt ataatgctct ccatttttat
80281 tgtgtgcata accaaaccca tcacttccta ttacgcaatt tgcaagcagg tggcattttt
80341 tgccaatttt actatcatta taaatcacca cattaggatg gataatactc tcttcgccta
80401 tgctgacatt atctcctata taagctcctg ccataataac aacattttcg cctatattga
80461 cattattgcc tatataaaca tttggcatga tttttgcact ttttgctata ttttgaactt
80521 tttcttttgc attgctaaat aaaggcttag caaaaatttt actcaaaaaa gcaaagcata
80581 aatgcggact ttgggtaata agagcctttg tatctttagg cactaaattt tcatattctt
80641 tagcgaccaa aatcgctgct gctcctgtgt tgggtatatc tttgatattt ctttcgccat
80701 cacaataagt aagctcagta aaatttgctc ttaataaaga atttaaagcc gttatttcaa
80761 tatcttcgcc ttcgtattct aaacttaaaa attccgctat ttcttttaat ctcatttaag
80821 ctccattaaa accgatccac tgcgaacaat atcgctagga ttgtattttt taattgtttt
80881 tagaaaattt tctatgcggt ttgtatcatc agctacaagt aagaaaagaa aattttcatt
80941 actatttgca atagtaccat tataagcttt caaaacggca tcaagcccac ccaaattttc
81001 actgagtgga atttttacaa gtgccatttc tttttctata aattcatctc tttctattac
81061 tttataagta ggtataagtt tatgcaactg ctttactatt tgctcaaaaa cacgctcatc
81121 tccaaaagtt actatattga tacgagaaaa ttctccacct ggtaaaggag cgacggttaa
81181 gctatcgata ttatatcccc tacctgaaaa aagtcctaca atacgagaaa gcacaccatg
81241 ttcatttaaa actataaccg ataaaacacg tctcattgct tatcctttaa ttttggtaaa
81301 atcatattat aaatagcccc gcccgctggc accataggaa gtacatcttc aaaacgatct
81361 ataaatacat taagcaagct tgttttattg gactctaagg cttgcttaaa tgctgtgtga
81421 aattcatctt ttgtttttat attatatcct tcacatccaa aaccttcagc tatttttaca
81481 aaatctgctt gcaagcttaa atctgtagcc gaaaaatgct cttcataaaa catactttgc
81541 cactggcgaa ccattcctaa gaatgaattg tttaaaataa tatttatggt tttaatacca
81601 ttttcataag ctgtcattaa ttcttgaata ttcattaaaa cagaaccatc gcccacaaaa
81661 tttataacaa cttcttctcc tactgcaagc tttgcgccca aagcagcagg taaagaatat
81721 cccattgttc cttgcccacc actggttgca agctgtcttg gataattaaa aggataaaat
81781 tgcgcaaccc acatttgatg ctgtcccaca tcagtaatta tccttgcatc tggagcaagt
81841 tttgcgcact cttgtattac ccattgaggt tttaaaacct catcactatc ttcataagct
81901 aaaggataaa gctcatcata gcgtttaaga gtttgtctcc actcatggat atttgactta
81961 aattgatttt ttttcaattc ttccaaaagc tctttaacaa cttctttgat atctccaact
82021 ataggaaaat gcgcattgat aatcttagaa atagaacttg gatctatatc aatatgcact
82081 attttagaat gcttagcaaa ttcacttgtt ttacctgtaa tcctatcatc aaaacgcgcg
82141 cctattgcta ctaataaatc acattcactt aaagccatat tagccgtata gcttccatgc
82201 attcctgcca ttttaagatt tagctcatca tcgcttctta aggttccaag agccattagg
82261 gtttcaacgg ctggaatttg agtaatctta acaagctccc tgatctcatc agccgcattt
82321 gacgctatac atccaccccc aagataaaaa agaggacgct tggcttcttc taaattttca

82381 gccaatttct taatttgttt tgaatttcct ttataaatag gcttataagt tttcattgaa
82441 atttcttttg gataatccca aatgccaagt gctgcgctta catcttttgg tacatcaatg
82501 tgcacaggac cttttcttcc actttttgct atgtaaaaag cttcttttag tattcttgga
82561 aattcttcta tacaagtaac taaataatta tgttttacac agggacgaga aattcctaca
82621 gcatcaatct cttgaaaagc atcggtgcct attaatgaat ttgcaacttg acctgaaatc
82681 aaaaccaaag gtatagaatc actataagct gtcgcaagtc ctgtgatagt attggtaaaa
82741 ccaggaccac ttgtaacaac agctacaccc acttcaccgc tcattctcgc ataagcatca
82801 gctgcatgca cagcagcttg ctcatgacgc accaaaatat gcttaaaata cttttgtaaa
82861 taaatttcat cataaatatt taaggctgct ccaccaggat aaccaaaaac tatcttaaca
82921 ttctcttctt ttaaagcttc gcaaatcatt gccgaaccgc ttattttttct cataaggcct
82981 cctttgaaaa tcaaattata acaataattc aatcttttat ttagataaaa tataaaaaag
83041 tttgaaaatt ttaatttaaa agctctctaa gtgttacatt taatcttttt ccatccactc
83101 tggctccaaa tttagccttt gcttccttca ttaccaaacc ttgatctttt aaagaactaa
83161 cccctaaatt tgcaatcaac tcttttaagg ctgtttgtaa ttcctcatcg cttaattgtt
83221 gaggtaaata actttcaaaa agtgctattt ctttttgttc tttttgcgcc aaatcttctc
83281 ttccaccttt ggaataaagc tctatagcat ctttgcgctt tttaatctca cttgtgataa
83341 tttttaaaat tctttcatca tctaattcta ttctttcatc tacttcaacc tgtttaaacg
83401 cagcatttag cgttcttaaa gtatcgcgtt taaaatcatc tttttgcttc attgcttctt
83461 taatatcatt taatatctgt tcttttaaag tcattgtata tccttttatt ttagttaaaa
83521 ttacaaattt taagatttaa tccaaaacct tttaagccta tataatcctt atcgcaactt
83581 tgcgacataa gcttaatctc ttctatacca aaataccttta gaatttgcgc acctatgcca
83641 taatttttaa attgagttgc gcttgatttt tcaccctgca taaaaactat cactcctcca
83701 ttttcactta aaaaacaaat ttgatctaat aatttagaaa atttatcaga agttaaaagc
83761 tcaaaatcac ttccactaat atgaaattta acattttcac atttttgaat ttctttgaat
83821 gcaaatgcta tatgttgagc attattatga tctgtaaaaa taaatttttg cgctttaaaa
83881 cctgctaaaa tagcatcact tttttcttgt aatttaataa ggctttcatt ctttaaacga
83941 tattctataa tatcagaaac ggctatcata tttagattat gcttggcgca aaattcctcc
84001 aaatcacttc ttcttgccat atctccatca tctttaacaa tctcacaaat cacacaagct
84061 tgtttaagcc ctgctaattg acaaagatca accgttcctt ctgtgtgacc tgtgcgttct
84121 aaaacaccac ctttttttagc tatcaaagga tttatatgtc cagggcatac aaaatcactt
84181 gcttttgcat tatcatctgc aaaaattttt atcgtcatat ctctttcata ggcactcaca
84241 cctgtggttg catcttttgc atccactgtg atcgtaaaag ctgtttcatg atttgaagta
84301 tttttgggta ccattaaagg aagttcaaat tttcttgcaa gctcttctgc gagtgctaca
84361 catacaactc ctctagcttc ttttatcata aaatttacct tttctttagt gctaaattga
84421 gctggaaaaa tcaaatctcc ttcattttct ctatcttctg catctaccat tacaagcatt
84481 tttccttctt gtaaatcctt aatcgcttgt tcaatgctta caaacttcat taataatcct
84541 taaatattat tttttttaaa attataactt tatttgcttg acaaataaat taaaattgat
84601 tataatctcg tttttacttt ttgaaaatta aatcattggg gtatcgccaa gcggtaaggc
84661 aacaggtttt ggtcctgtca ttcaggggtt cgaatccctt taccccatcc acaaatcaaa
84721 cttcaaacac aaattcagtt gtcgcgaagt agagcagtgg ttagctcgtc gggctcataa
84781 cccgaaggtc gggagttcaa atctcccctt cgcaaccaaa atctatctac aactttaaat
84841 cctaaatata caaaaattat ttattttaat tttttagaat gtattttttta atcaaaaaag
84901 tcgcattata aaataataaa aaataaatta tagctaacat gttaagatat aatatgagat
84961 aaatttttag ataaaattta aatgaataac ttaggaagaa attaaagtca tttttaataa
85021 aaaataaaat gatggtggtt agaggcagaa tcgaactgcc gacacgcaga ttttcagtct
85081 gctgctctac cgactgagct atccaaccaa atcatcttta aaaaataaag tgtaattata
85141 cttttttttc tttaatttaa gtttaaatat aaaaataaaa ttttatttgc cttaaagttt
85201 taatccgata gaatagtcga tttaaaatca aacaaccaaa gaggattttt tgaaacaatt
85261 tggcttagac agacgaactt ttaaaatttt gctagcagga tatattgtga ttgctttact
85321 tggtgctatt cttttgcatt caagctgggc tcatacaagt cctattagtt ttatcgatgc
85381 tttttttact agtacttcag ccgttagtat gactggactt attgttaaaa atacagcagt
85441 tgattttacc ttggcaggac aaattattat tttaatttta gttcaaattg gtggcttagg
85501 ttatatgagt atagggcttt ttgtttactt aatcatacgc aaaaaagtag gttttaatgg
85561 tagaaatttg cttaaagaat ctttatttta tccttcaatg gagggacttt ttaaattctt

85621 taaaaaagtt ttactattta ttttcacaat agaacttata ggcaccatac ttcttactat
85681 gcgttttgca cttgaaatga attttggcaa ggccttatgg tttggatttt ttcactcaat
85741 cagcgctttt aataattcag gctttactat ttttgaaaat ggacttcttg cttacaaaca
85801 cgatgtagct attaatctta tctttacttc tcttattatc attggtgggc ttgggtattt
85861 tgtcttagtt gaattgtatt tttttcaaag aaaaagactg caaaatttaa gcttgcatac
85921 taaagttgtt attatggcga cccttttttct tattgtttct tcaactctta ttatttttct
85981 tttagaatat gctaatccaa aatccatagg tagcttttct ctttttgata aaattttaag
86041 ttcttatttt attgcgatca attaccgcac agcagggttt aatacactcg atatgagtgg
86101 tttgcatgat gcgagtttgt tttttggctc tttgtttatg gtgattggag gagcaccggg
86161 tgggacggca ggcggaatga aaatcacaac tgttgtagtg cttttgtttt atgcttactg
86221 gagcattcgc aatggaagag taagaatttt caaccacgaa attccacaag agattatttc
86281 aagagcattt attatagcag tgggttcggc tgtttatata gttatagcgg ttattttcct
86341 ttctttgcta gaatcagaat ttaattttat tcatctgctt tttgaaacct catcagcatt
86401 tgcaacagta ggaatttcag taggagatgg aggagatctt tcgctttgtg ctttatttag
86461 caatccaagc aaaattatta ttattattat gatgttaagc ggaaggattg gtgtttttgc
86521 atttttactt tctgtctttc aacaagataa agcaatgcat atcaaatttc ctaaaggaaa
86581 aatctattta taaaggttaa atataatgaa aaagcttagt tacggggtta taggacttgg
86641 aaaatttgga tccgtagtcg cagatgagct tattgctgga gggcatactg taatcatagc
86701 cgataaagat gaagaagctt taaaaagcat acaaaattca ccaagctatg cttatatctt
86761 agattctact aatatttcag cacttaaaga agcaggtttt catgatgtag aagtcgtgat
86821 tgtaagcata ggcgaaaatg ttgaaaaatc catacttacc ctaatggctc ttaaggacat
86881 aggggttaat aatattatcg caaaagctac ttctaatatt catgggcaaa ttctatccaa
86941 actaggagct acaaaagtaa tttatcccga aaaagaaagt gctaaaaaac ttgtaaaaga
87001 ctttttaact aaagatgcag attatgaagt ttttgatcta tcggctaata ccatccgcgc
87061 tattaaaatc accatagatg ataaattagc gggaaattca ttaaaacaca ttgcgcaaaa
87121 tatgaaagtt atcagctata aaaagctcaa tacagactgg gaaatactgc ctgatttaga
87181 aacaactact gtttatggtg gagatgttgt aatcttactt ggaacagtta aagaacttag
87241 agaatttgaa cattaatcaa gtaggattta aagccttgca agcttttata aaacgcttta
87301 aggctgcttt ttctttggtg gaaattttat aatatataat ttcatctaga taatatctca
87361 tatcttttat atccaaattt ctcgttgtcg cataatttg aagtatataa taaggaattt
87421 taatctttgt ttttggaaat ttttttaaga tttgtttata taaagctttt ttttgcacac
87481 aagaaaatct agcaaaaaca aaaggtaatc ctgtttttc atgccatttt gcacacaaat
87541 caatatactt agaaggatct tgcagataaa gctttaaagc cttatcacct ataatcaccc
87601 tcccctcttg ttttaaaacc ttagctaaag cattagaagt tgccgagctt ggatctttag
87661 cattgagcgt atttctttcc actaaaacac tcaaaactct tttattggca caaatgccca
87721 aatctaaatt tttatatttt tttctagcac tttctatgct tgaaacaata gcagcatcaa
87781 ctcttctata aaacaaatcc ttatttagct tacttggcac acctttttttg tattccatgc
87841 ttgctttata accattgggt agtggatact ttttaagata aatatgcaaa ggtaaaagat
87901 taatataatc aatttttcca aaaatcattt taatccaagg tattaaaaac cctatctccc
87961 gcatccccaa gtcctggaac tatataacct ttttcattta agctttcatc aattgaggct
88021 acataaagtt caacatcgct gtgaattttt gcaaaatttt caagaccttg aggggctgcg
88081 agtatagaaa tgaatttaat ctttttttacc ccttgatctt ttaaaaaatt gcaagcatct
88141 atggctgtac cacctgttgc gaacataggg tctataacaa tagcagtacg ttcttttgca
88201 tctttaggaa gtttttgaaa ataaaaatcc gccttgagcg ttttttcatt tctaacaaaa
88261 cccaaaaatc ctacactagc atcaggaata agtctaaaaa tactatcaag catacccaaa
88321 gccgccctta aaatagggca tatcataatc ttttcatcta atctttttttac cttggcatta
88381 gcaacagggg tttgaatttg tgcttctttt aaacataaat ctttagtcgc ttcaaaaagc
88441 aagaaagtag aaatctcatc aatgagcatt ctaaaatgaa agggtttggt ttctttatcg
88501 cgcaaaaatc ctaatttatg ctctatcaaa ggatggcata tatgatgaat atttttcatt
88561 ttaaactctc aaaatatgct ttttcatcaa attctttaag caaagctacc ccatctttta
88621 cagctgcagc agccactgca gtacttacaa cagccttaac tctctcatca aaaggttttg
88681 ggataacata atcttttcca aattctaagt ggcttatatt gtaagcattt ttcaccgcat
88741 cgcttacagg tagctttgct aaatccgcca aagctcttgc tgcagctact ttcatatttt
88801 cagtgatttt tgtcgcacgc acatctaaag ctccacggaa aataaaagga aagcctaaaa

88861 cattattgat ttgattagga taatcactcc ttcctgttcc tacaatagca tcttttctaa
88921 gtcttgctac atcttcaggc atcacctctg gaataggatt tgccaaagca aaaataacag
88981 gatcttttgc catagacaaa atcatttcat catctagtat ctttggcgca ctcagtccca
89041 aaaatacatc cgcatctttc atagcctctt ttaaagtatc tgcttgtgta tcacttacaa
89101 attcaagttt gtattgattt aaatccgttc ttttttttatt gaccacaccc ttgctatcta
89161 caagaatgat atttttaaca cctaaatttc tatacattct agcactagca atacccgcag
89221 ctcctgctcc gcttacaaca actttaatgt cttcaaattt tttaccgctt atctccatag
89281 cattcataag ccctgcggtt gagataatag cagttccatg ctgatcatcg tgcattacag
89341 gaattcctaa attttgcaaa gccgcttcga tttcaaaaca tttaggcgct gaaatatctt
89401 ctaaattgat tccaccaaaa gtcggtgcta tggctttgca aaaagcaaca atttcttcaa
89461 tactgtgtgc ttcgatttct aaatcatacg catttacatc agcaaatttt ttaaacaagc
89521 aagccttacc ttccataaca ggtttagaag cttgcgcacc gatatttcca agacctaaaa
89581 ccgcagaacc atcactaacg atagctacta aattcgcttt gtttgtataa gtataagcaa
89641 gctcattgtt agaagcaatt tcaatgcaag gttctgcaac acctgggcta taagccaaag
89701 ataaatcatg gcttgaattc ataggttttg atggttttat atctatctta ccccctaagt
89761 ggtatcttaa agcctcttct tttaaattca ttattttcct ttaaaatcta aaaatttttc
89821 tattcttttt ttacattcat tgacccctaa aaactctaaa acttcaaaaa tgctaggact
89881 aaccgcactt cctgttagag ctatgcgtat aggttgagcc aaatctttta attttacctc
89941 atttttttgt aagaattcat tagtaaattc ttcaaaatct tttgcacttt tctcttcatt
90001 taactcattt gcatagtttt gaagcagtaa aagattattt tcatttataa atttatttat
90061 agcattttca tcataatttt gtggcgcttc aatgatactt ttagctccgc taataatttc
90121 aagtaaagtt tttgcgcgtt ctcttaataa atccagcaaa aatcctgctt ttgcataagc
90181 gcttaaatca aagcctagat cttttaattg gcgattgatt tcttcaaaag gtaaagtctt
90241 gatataatga gcattgagcc attcaagttt tttaaaatta taacaagaag cacttttgtt
90301 aatatgattt ggatcaaaaa gctttttaag atcttctaaa gaaaaaacct catcatcacc
90361 gtgactccaa cccaagcgca ctaaaaagtt taataaggct tgaggtaaaa ttcccatagc
90421 cttgtattcc atcacatcgg ttgctccatg tcgttttgat agctttttgc catcttctcc
90481 atgtatcata gctacatggt aaaatttagg aatttcaaat cctaaagcct cataaagcac
90541 gatttgctta ggagtatttg aaaggtgatc atctcctcta atcacatcac ttactcccat
90601 taaagcatca tcaattacaa ctgtaaaatt ataagtaggg cttccatcgc ttcttgctat
90661 gataaaaatca tctaaaatat cttctacttt aaatttcacc tctcctttaa caccatctac
90721 aaaacaaatt tcaccctctt gaggagcttt tatacgcact acaggttcta tatttgctgg
90781 aggagtgcct gtaaaatctc tatatctgcc atcatatctt ggacgttctt tagctgcttc
90841 ttgtttagca cggagctcat caagttcttc tttactcata taacaataat aagccttgcc
90901 ctcatctaaa agtttttgta tatatttttt ataaagatca aagcgttcag attggtaagt
90961 tacctcgcca tcataatcca aaccgcacca tttaaaggct tctatgatag cttgcgtagc
91021 ttctttagaa tttcttttca aatccgtatc ttcaatacga agtaaaaaat tacccttgtt
91081 ttttcttgca tacaaatagc tataaagagc agttcttaaa ccgcctatat gtaaatatcc
91141 tgtagggctt ggagcgaaac gagtcgtgag ttttcctgtc atttttttgcc ttttcttaaa
91201 ttttaattga tataatgcga tatttacact taaataaagg tttgaaatga agaaaatttt
91261 actaagtttt atattttttta taagcttaac tcatgcaaat actataaatg ctattgccgt
91321 agtggtagat aaagagccta ttacaaccta tgatatagag caaaccatga aagctttaaa
91381 tatacctaaa aatcaagctt tgggaatttt gattaatgaa aaaatggaac tttcacaaat
91441 aaaacaattt ggaatttaca caagcgaact tgagcttgat aatgctattg ataaaatgtt
91501 aatgcaaaat aaaatttcgc ttgaagactt taaaaaagat ttgcaagtca aggggcaaaa
91561 ttacgaacaa tttcgtagta attttaaaaa agatttcgaa aaaagaaaac tctatgaaaa
91621 aattgccagc accgctaaaa ccgattttag cgatgaagga gctaaaaatt ttttcgaaca
91681 aaataaaaac aaatttacat tttttaccga aattaatgta aatatctatc gttctaataa
91741 cgctcaagat ctagaaaata taagagctgc tataaaaact ccacttaaag cagaaaaagc
91801 tatcctaact cctaacaatt cagatccaag actcctagga cttttatcac ctatcccttt
91861 aggaggcttt tctccggttt taaattcaca aaagggttat gaaatttatc aagtaaatga
91921 taaatctaaa gggcaaactc cagaatatta tcaaattaaa aatgaagtgc tcaatgccta
91981 tgtaagcgag caaagacaaa attatataca agattatttt gataaattac gctcaaaaat
92041 caatatagaa tatttaagat aaaatcaaga tggaaaaaat ccatcttgat caatgtctag

92101 caagatagtt agtatcgtaa ttattattaa taaaatcagg attatccatc attgcaagat
92161 gaaaatcttt agtggtttta atcccaccta taatcagctc atctaaagct actttcattt
92221 ttgaaatagc tttgttgcga tcttctgccc aaaccacaag tttccctatc atagagtcat
92281 aatacggtgg cacgctatag tcttgatagc aatggctttc cattctgaca ttgcgtcctg
92341 caggtggaac atacttgata attttacctg gacttggcaa gaaagtctta ggatcttcag
92401 ctgtgattct acactctata gaatgtcctt ttaatcttac attttcttga gatggtaaag
92461 catagccttc agctactttg atcatttgtt caataatatc aatcccactt accatttcac
92521 tcacacaatg ctcaacttgc aaacgcgtat tcatctctat aaaataaaaa tccaaatttt
92581 tatccactaa aaattcaaaa gttcctgcac cctcatagct tatagcttta gcagctttta
92641 ctgctgtttc atgaagtctt gcacgggttt tttcatcgag taaaattgct ggagattctt
92701 ctataagttt ttggtggcgt ctttgcatag agcaatctct ctcgccaata tggatcacat
92761 ttccaaaact atctccgatg atttgaactt ctatatggcg tggattttgt atatattttt
92821 ccatatacat agtcccatca ccaaaagctg tcatcgcttc actttcagct gaccaatatg
92881 ctttttcaag atctttttca tgctctacta cacgcattcc acgacctccg cctccagctg
92941 ctgcttttaa aatcacagga taaccgatct ctttggcaag ttttttagct gcttcagcgc
93001 ctgctaaagc cccatcactt cctggtataa caggaactcc tgctctttgc atcacttgct
93061 tggcttttga tttatcgctc atcaaattca tagcttcaac cgaaggtcct ataaatttga
93121 tattgtgttt agcacaaatt tcaacaaaag tttgattttc acttaaaaat ccataacctg
93181 gaaaaatcgc atccgcttct gcaatttcag ctgctgaaat aatagcaggt atattgagat
93241 agctttcaga acttcttgct tttcctatac aaatacttgc atctgcatat tttaaataca
93301 aagcatcttt atctgcttca gaataaacgc aaattgcttt ttttcccatt tctttaattg
93361 ttcttaaagc tcgaagtgct atttctccac gattagcaat taaaatgctt ttaatttcca
93421 ttttataatt tctccaccgt aaataaaggc ataccaaatt ctacaggctg accatctgca
93481 actaaaattt ctactattct acaatcaaat tcagcttcga tctcattcat gattttcatc
93541 gcttcgataa tagcaatagt atcgcctttt ttaactgaac tacccacttt tacaaaagga
93601 gctgcacctg ggcttggagc ctgatagaaa gtacccacca tagggctatt tatacttggt
93661 ttgtttgact ttgcacttga aggctgtgct tcattaacta cattaacatt tataggttgt
93721 ggagctggaa cgggtggaca agccaaagga gctggaacat cacaacacat atctctttca
93781 agttcgattt caaatccatc ttgctctttg atttttattt tgctgatatt agcttcagca
93841 aataaattta ctaattcttt aatctcttct tttgtcatat tttcatctcc tgattttaag
93901 aacttaatct agtattatat taaaaaactt gaaatttaac tttaaaaaat aaaatttcac
93961 gcttaaaagt ttcataaaat ataaaaaata agctataatc caaaaaaatt atgtttaaaa
94021 aataaaggta tattaaatgg gtttaaaagc agacaattgg ataagaaaaa tggcactcga
94081 gcatcaaatg atagagcctt tttgcgaagc aaatataggc aagggcgttg taagctacgg
94141 actttcaagc tatggttatg atatacgcgt ggggcgcgaa tttaagattt ttaccaatgt
94201 aaattctacc gtagtggatc caaaaaattt tttagaagaa aatgtggtag attttgaagg
94261 tgatgtatgt atagtccctg ctaattcttt tgcacttgct agaactatag agtattttaa
94321 aatgcctgat gatgttttag ctatttgtct tggaaaaagc acttatgcaa gatgtggcat
94381 aatcgtaaat gtaactcctt ttgaaccagg ctttgaaggg catataacca tagaaatttc
94441 aaacactacc cccttgcctg caaaaattta tgccaacgag ggcattgcac aggtattgtt
94501 tttacaaggt gatgaaaaat gcgatacaac atacaaagat aaaaaaggca aatatcaagc
94561 tcaaacaggc ataaccctgc caagaatttt aaaataggaa cgcttattgc tttaagtttt
94621 ttaagtgata atttggcaaa ggaaggcaaa atgtttaatg gaaaaaatat cttaattact
94681 ggtggtacag gctcttttgg aaaaacctat actaaggttt tattagaaaa ttataagcct
94741 aataaaatca tcatttattc tcgcgatgag cttaaacaat ttgaaatgtc aagcattttt
94801 aatgctaatt gcatgcgtta ttttataggc gatgtaagag ataaagaacg cttaaatgtt
94861 gctatgcgcg atgtagattt tgttatacat gctgctgcga tgaaacatgt tcctgttgcc
94921 gaatataatc ccatggaatg tatcaaaacc aatatccatg gcgctcaaaa tgtaatcgat
94981 gcttgttttg aaaatggagt aaaaaaatgc attgctctaa gcacagataa agcttgcaat
95041 cctgtaaatt tatatggtgc taccaaactt gcaagcgata aactttttgt tgctgcaaac
95101 aatatagcag gcaataaaca aacacgattt agcgttacaa gatatggaaa tgtagtaggc
95161 tcaagaggct cggtagtgcc tttctttaaa aagcttatcg cgcaaggtgc taaagaactt
95221 cctatcacag atactagaat gacacgcttt tggatttctc ttgaagatgg ggtaaaattt
95281 gtattgagta attttgaaag aatgcacgga ggagaaattt ttatccctaa aattccttcg

95341 atgaaaatta cagatctagc ccacgcttta gctccaaatt taagccataa aatcataggc
95401 ataagagcgg gagaaaaact tcatgaaatc atgatttcaa gtgatgatag ccatttaacc
95461 tatgaatttg aaaactacta tgccataagt cctagtatca agcttgtaga tcaagagagt
95521 gattttagca tcaatgcctt aggtgaaaaa ggacaaaaag taaaagatgg attttcttat
95581 agttcagata acaatccaca atgggcgagc gaaaaagagc ttttagatat catcaatcat
95641 actgagggtt tttaaatgat tacttattct catcaaaata tcgatcaaag cgatatagaa
95701 gcggttttaa acgccttaaa ggatgaaatt ttaaccggtg gcaaaaaagt cgatgaattt
95761 gaagaagcac tttgcgaata cataggaatt aagcatgctt gtgtcttaaa ttcagccact
95821 tccgctcttc atcttgccta tacagcttta aatgtcaagg ataaaataat cttaaccact
95881 cctttaactt ttgcggctac agctaatgca gctttaatgg caggagctaa agtagaattt
95941 atcgatatta aaaatgatgg caatatagat gagaaaaagc tcgaagcaag gcttgtaaaa
96001 aatagcaccg atataggagc tattagcgtg gtggattttg gaggcaatag cgttgaaatg
96061 gatgagattt caaatcttgc taaaaaatac aatatccctt taatcgatga tgcaagccat
96121 gctttaggta gcgagtataa gggtaaaaaa gtgggttcta tggcggattt aagtattttt
96181 tcttttcacc ctgttaagcc tattaccact tttgagggtg gcgctgtagt aagcaataat
96241 gaagaattaa tctccaaaat aaaactttta agaagccatg gtatcgttaa aaaaagactt
96301 tgggatagcg atatgatgga acttggctat aattatcgct tgagtgatgt tgcttgtgct
96361 ttagggataa atcaacttaa aaaactagat cacaatcttg aaaaacgcga agaaattgca
96421 agtttttatg ataaagaatt tgacaaaaat ccttattttt ccactataaa aatcaaagat
96481 tataaaaaaa gttcaagaca tttgtatcct attttactct ttccagaatt ttattgcccc
96541 aaagaagaaa tttttgaaaa tttactcaaa gctggacttg gagtacaggt gcattataaa
96601 ccaacttatg agtttagttt ttataaaaag ctcttaggag aaataagact tgaaaatgcg
96661 gataattttt acaaagccga acttagcata ccttgtcatc aagaaatgag cttaaaagat
96721 gcaaaatttg taagagatac tttatttagc acacttgaaa aagttaaaaa ggactattgt
96781 ggataagcta gactttagac acaaagactt tgaacaaacg ggaaaggcta tgtatgaact
96841 tgccaaggag ctttttccta tccctcgaag tatcacagga caaggtttta gagctagctt
96901 agaaatttta aataaaactc tgggagaaaa tatactcaaa tttcactcta tcaaaagtgg
96961 cacaagggtt tttgactgga tagtccctga tgaatggaac gctaaagaag cctatatcat
97021 cacaccagaa ggaaagaaaa tttgtgattt taaaaagcac aatctacatc ttttaaacta
97081 cagcgaagcc atagataaag aaatagaact tgaagaactt gagaagcatc tttactccat
97141 agaagaaatg cctgatgcca tcccttatgt gacaagttat tataaaaggc gttggggatt
97201 ttgcatcacg cataatgaaa gaaaaaattt aaaaaaagga ctatacaaag tctttatcga
97261 tgcaaaacac gacgaaaatg gagttttaaa ttatgctgat tttataatcc ctagcactca
97321 aaattcaaaa gatgaaattt tactctcaag ctatctttgt catccttcta tggcaaataa
97381 cgaacttagt ggccctatag tagccatttt cttagccaaa tggcttttag gtttaaaaga
97441 aagaaaatac aattaccgct ttgttatcat tcctgaaacc ataggctcta taatttatct
97501 taataagcat ttagatcact taaaaaaaca tgttaaagca ggttttgtgc tttcttgctt
97561 aggagatgat aatgcttact cgcttataca tactcctaaa gaaaatactt taagcgataa
97621 agttgccatg cacacactca aaaataagga aaattttaaa gaatttagct tcttggagcg
97681 tgggagcgat gaaaggcaat acaatgctcc gcttgtgaat ttaaacatag taggaatatg
97741 ccgcacaaaa tatggtgaat ataaagaata ccatacatca aaagatgact taaatttcat
97801 ttcagtcaag ggtttaatgg gcggacttaa ggctatgcaa gaaataatct taaatctaga
97861 aatcaatgcc atatacgaga acaccacaat atgcgaaccc aatcttggca aaagaggact
97921 ttatcatacg ataaataacg gaatcaaaca aaaacctatc agtgttgatt tcctcgccta
97981 ctgcgatggt caaaatgata tcattgatat agctaacatc ttaaatatac aagcttatga
98041 gttaaaagag cttttagaaa aaattttaga atataaacta gtgagaaaga tatgaaaaaa
98101 ctttttcaat ataatcaaaa aatatacact caaaaagatc ttgttaatac tttaaaaaat
98161 ataggtatta aaaatggtga tataatttgt gtccatacag aaatctttca ttttcaaact
98221 ccgctactct ctaaaaatga atttttaaat tcatttttag aagctctttt taaagccata
98281 ggaaaagaag gaacccttat catgccaact tttacttata gcttttgcaa aaatgaagtt
98341 tatgataaac ttaattccaa gtgtacagta ggaatattaa atgaattttt tagaaagcaa
98401 gaaggtgtaa aagaacgaa tgaccctata ttttcctttg ctgtaaaagg tgccaaagaa
98461 gatttgtttc tatataaaga acaaaaatcg tgttttgatg aaggttgtat ttataatata
98521 cttaagaata ataatggtaa aattgtcctt ttagggacgc aacttttagg ctacactttt

98581 actcatttta tagaagagca agcacgagtt ccttatagat attttaagcc atttaaaggt
98641 gatattatct atgaaaatag taataaaaaa acatataaaa tcaattacta tgttcgagac
98701 cttcacaaaa aatcggaagt aagtatttta aaacaaattg ctcttttaaa aaatagcaat
98761 aattttaagt tggaaaaaat aggcaatgcg gaaatcgtat ctatagactg tcaagcttat
98821 tttgatgaaa ctatgaaagc tttaaaacaa gatccatatt gtttattaaa ggattaaaat
98881 gacaactcat atctacaact ttttagaaaa aagccttgct aactttagcg acaaaactgc
98941 ttttgtagag ccttttgcca aagaaagaaa agaaatcact tataaaaatt ttgatctttt
99001 ttccaaaaaa gtagccagtg aaattttaaa aaaattagga aatgacaatc ctactcaaag
99061 ccctattttg ataatactgc ctaaaggaat tgattgtttg atttcatttt ttggagtggc
99121 actcagtgga aatttttata cacttttaga tgaaaaaagt cctaaagaaa gagtagaaaa
99181 agtcatagaa attttaaagc ctaagctttt tatcacttca aaagaattaa aatttgattt
99241 agctttacct acgctttaca cagaagattt taaaagcttt gatacagatg aaaacttgat
99301 ccaagttgcc aaagaaaaac atatcgatac taatttactc tatgttcttt ttacaagtgg
99361 aagtacagga attccaaaag gagtaagcat agcacataaa agtgtgattg attatacttt
99421 ttgggtgtgt gaaactttta aattcgatga aaatgagatt ttagccaatc aagcaccatt
99481 ttattttgac aatagtattt tagatatttt ttcaagtgta aaagcagggg caactttaca
99541 ccttttaccc aatcatcttt ttgcttttcc aaataaaatt ttagaatgcc tagaaaaaga
99601 aaaggttagc gctacatttt gggtaccttc agtactgatt tactttgcaa ataccgaggc
99661 tttagataat tttaatttaa taaatttaaa aaaaattctc tttgctgggg aaatcatgcc
99721 taataaacaa ttaaacattt ggcgcaaaca tttacctgat acactttttg caaatcttta
99781 tggacctact gaaatcactg ttgattgttc tttttatata gtagatcgtg aatttaaaga
99841 tgaagaactt ttacccatag gcaaagcttg taaaaataca gagcttttag tatttgatga
99901 aagtatgaat ttgataagtt ctaaacaagt gggcgtaaaa ggtgaacttt atgtgagagg
99961 aacttcttta tctttagggt attataacga caaagaaaag acacaaaaag cttttataca
100021 aaatcctttg cacgataact acttagatct tttatataaa acaggagata tagtggctta
100081 taatgaacga ggcgagcttt tatgctatgg acgcgcggat aatcagatca aatacatggg
100141 acaccgcatt gaacttggag aaatagaaag cgtgatcaac tctcatccaa aagtcaaaaa
100201 tagtgcttgt atttttaaag acaaaattat ttgtttttat gaaagtgatg aagagcttga
100261 tctaaaaact ttttgcaaag aaaggttagt ttcttatatg atacctaata aacttatcaa
100321 attagataaa tttcaattca atcaaaatgg taaaattgat aggaaagttt taaatgaatt
100381 tatttgaaga atttgaaaaa aactatttta aaggatttaa actaagtaat tggaatgaaa
100441 gtatatcaat attgaaagaa aaatttcata aaaaaaaatt atctctcgaa agttatgatt
100501 caaatttctt tttttatgaa aataattctc atcttttata ttacttcatc aacgacacaa
100561 ataaaaaaaa taaattgaaa cctagtttta ttaaaatttc aataaaacat gaaaagtact
100621 tagatgacca tttaaatttt atacatctac attcttttaa tgtggaattg gaatgtcaag
100681 aaatgtgttt aaaaaataaa aatattattt tgcatgattt tccttttatt actcaagcta
100741 atattcaaga ctatgagcaa atatataatt ttttttgaa gcatttttca aaagatttct
100801 tattttattt ttcaaaaaac gatataaaaa ataaaataaa tcagattctt atctataaag
100861 aaaacggcat aattcggggt gcgttaattt ataccctaac aattaagtcc gctttcttgg
100921 attttattgc agttgataaa aacttacatt ataaaaatgt agcctttgct cttcttaata
100981 cttattttaa gaaaaatttc aacacccatt tatttaaact ctttgtcagt gtccataatt
101041 taaaagcttt aaaattttat cagcgagcag gatttacttt aaatacagct aaaatattgt
101101 attataaaaa cttttaacaa atttaaaatc acaaaggaat aaaatgcaaa acacattaga
101161 attaattaag cagtttttta tcaatataga tagatctgat atagatgaaa atatggaaaa
101221 tcttttaagc gaagatatta tcgatagtat ggatataatg tctttagttg ccgagataga
101281 aaaatattat aaaaaacctt taaaagctga atttattaaa gcggaaaatt ttgaaagttt
101341 tcaaactatc aaaaatatgt tagaacaagc gatgaaataa tatgaatttc attaataaga
101401 aaaatcaaaa taatattaca acaatctata tactaagatt tcctttattt gaaaaaataa
101461 aaaatatcca tgcacatatt tatggtggta tagaatatat ttcctatact tttaaaattt
101521 ttagctttaa attctatact acaaaaaaaa ctttagaatt atccgatcct gaatttttat
101581 caaaaaattt agaaaatttg ctcaatcaat caaatcaaaa gcaatggaat caaagttttt
101641 atatgcaaga tattataggt aagtatttag tagaaaataa tacccatgaa aaaatcctta
101701 agctctgcaa aaatttggat aacgaaagta aagccttaat ttttagaatt ctaacaagac
101761 taaaaaattt tcaaacgaat ccaaattatc aaatcaaata cacaaaagaa gaaaaacaag

101821 acctagaaaa acttcaaaat gaatttttttt caaatatcct accgatctct aaaggaattt
101881 ttggatggaa tggatatttt ttacccatca aacaatttga aatttctgtt ttttggcaca
101941 aacatgctat gcataccttt agcaaccaaa ccctagaaaa gataaaaaat aaaaatatta
102001 ttgatgtggg cggttttata ggtgatagtg ccattatctt tgagaaagaa tttaccaacc
102061 aaaaagtgta tagttttgaa gcagttagta aaaattacga tttaatgcta aaaacactag
102121 agcttaataa tagcaaaaga atcattccag tcaaaaatgc cttaggttct aaagaagaaa
102181 aattgcaaat cagtatttca ggctatagct cgagcttgaa acacggtgaa tcaaattcta
102241 attacgaaga agtggaggtt ataactctag attcttatat cgagaaaaat ccaatggaga
102301 taggctttat aaaggttgat atagaaggat ttgaacaaga atttttaaaa ggtgctatta
102361 atacaataaa agctcaaaaa cctgcaatgc ttattagtat ttatcacaat cctaatgatt
102421 tttttgatat taaaccttta atagagtctt ggaacttggg atataaattt aaaatttata
102481 ggcctataga tttttatgtt agtttagaaa ctgctttgta ttgtgaaatt tagcaatgca
102541 aggcaaaact cattttatca tccttaaagg taaaaatttt atctccacaa ggataagaga
102601 tagaattttc ttgcattaaa gattttcttc cttgtaattt ttcaagcatt aaaggaagct
102661 ctttttttaaa caaagcttta tcggttattc tcataccatg ttttaaatct ttaataaacc
102721 taccatctat ttgactttca tcttttatca aatacaaagt cgaatcataa cctaacgctt
102781 tataactttc aaacagcgtt tctttatctt tagatgtgtc aaattcatct tttgcgctat
102841 gatatcctat gaaaataata ttattatttt ttttagcctg aatttgcaaa tggaaagaat
102901 tgagtaaagc gcggatcatg taattttcat tggcaaaaaa ataaggtgaa ttttcgtctt
102961 ctcttgtcca ataagtttta acaaacattt ctacgaatgt attaggatta ttaaaaacaa
103021 attcagatct attgagattt cttcctatta tataatttag aagaggcaaa accgagcctg
103081 aattatcaat caccccatcc acataccaag gagctatttt agctataagt aaagaaagat
103141 atcctccata tgaccctccc ccataaattt ttggaagatt tttaaaattt ggaaattttt
103201 tcacaagatc ttttaaaaca ttgatatgat caatagctgc cataatgcca aaattttgat
103261 aatctccatt aggtggaata aaagttgaag taagtctagc ttgataattt tgcgctaatt
103321 tcccttgtga ttttaaagct gtaatatact gattaagtaa ttcataacat tgatgtgcat
103381 tttcaagact aacactcata ttaataccca taccaaataa agcctgagct aatttaggta
103441 gatcttcttt tgtaaatatt gtcaatgcat tatattcttc atcaatactt cttctagcac
103501 aaaaacaatg ataaaaaaca tgcaccacta caacatcaaa atttttagca atatactctc
103561 tatcaaaatc taaaaaagaa atattagcat tagcgccaat ccctcctatg ataaaaacta
103621 tagctctcat ttctttttca tcgtcataac ttatacgata ttcaagtttt gaagttcttt
103681 taatacccaa ttctacatca tcacaagagt ctattttaaa ggtttgattt ataagcatta
103741 ttttcctatc tgtattcttt tatgtgtatt taaagacaat tatacttgat ttttgataaa
103801 ataattccca aagaattaag gaaaaaaatg aaaaatcttt gcataatacc tgctcgcggg
103861 ggttcaaaga gaattcctaa aaaaaatatc attgattttt taggaaaacc tttgatcgct
103921 tatagtatag aaagtgcttt aaattcgggt atttttgatg aagttgttat ttcaagtgat
103981 gatgaggaaa ttataaaaat agcttgtcaa tttggcgcta aagctccttt tgtccgtgat
104041 gaaaaattaa gcgatgatta tagttctagc acagcagtga ttcaaaatgc gattgaaatt
104101 ttagctaaaa aagggcaagt ttatgacaat gtttgtgctc tttatgccac tgctccttta
104161 attagcggtg aaattttaaa aaaagcttat gaaaaatttc tagaaaatga aggtaagttt
104221 ttattttcag ctaccgaatt tgactatcct atacaaaggg ctttttatct aaatgaaaaa
104281 aatcaagttc atatgtttga tgaaagtttt tacaactctc gctcacagga tttaaccaaa
104341 gcttatcatg acgcaggagc tttttacttt ggcaaaagca aggcttggct agaaaatgat
104401 tttttattta aaccccattc tagtgtgtat ttactaccac gaaatttagt ttgcgatatc
104461 gatactttag aagatttaga atttgcaaaa atactttata aggtaaatta tgaaagttct
104521 ctttagaagt gatagttcta gtcaaatagg ctttggtcat atcaaacgcg atcttgtctt
104581 agctaaacaa tacagcgatg taagttttgc ctgcttggca ttagaaggct ctttgatcga
104641 tgaaattcct taccctgttt atgagcttga aagttcaagc atttatgaac tcatcgatct
104701 tatcaaagag caaaaattca aacttttaat cattgatcat tatgatatca gttgtgatga
104761 tgaaaagctt ataaaacttg aaacaggagt taaaatctta agttttgatg atgagataaa
104821 agctcatcat tgcgatattt tgctcaatgt caatgcctac tcaaaagcaa gtgattataa
104881 aggtttggtg ccttttcgtt gcgagttaag atgtgggttt tcttatgctt taattagaga
104941 tgaattttat aaagagagca agataaaaag agagaaaatt tgggattatt tcatttgcat
105001 aggtggcaca gatatcaaaa atctttctat gcaaattgcc tctgatttac ccaaaaacaa

105061 aaaaattgca atcgctacaa gctcgtccaa taaaaatctc aaaaaacttg aaaaattcac
105121 tcaagatcat gaaaatgtaa agctctttat agatgaagaa atttcaaaat taatgaatga
105181 aagcacaaag cttatcatca gtgctagttc tttagtcaat gaggccttgc ttttaaaggc
105241 taattttaaa gccatttgtg tcgctaaaaa tcaagaaaaa ctagccaatt ggctagttca
105301 aaaaggctat gaagtggagt ataggctttg atatatttaa aagattttgc tcatttagat
105361 aaacaagaaa ttttactcgt atggcagtgg cgtaatgatg aaaaaataag ccagtttatg
105421 aagataaaat atatcgattt tcaagagcat ttaaatttca ttactagctt gaaaaaagat
105481 caaagtaaaa aatatttttt ggtttttaag gataatgaag ctattggcgt aattgatttt
105541 atcaatatcg cgaaagattc ttgtgaattt gggctttatg caaaacctga tttaaaaggt
105601 atggggcaaa ttttaatgca agaaatcaaa aaatacacct ttgaaaaatt aaaaataaaa
105661 aaattaaaag cttgtgtttt taagcaaaac aaaagagctt tggatttata tttaaaaaat
105721 agtttttgta ttaaaaatga ggatgaaaat atgttttatg tagaatttat gggggggggg
105781 aatagttaaa tttcaaccta aatttcttat tatacaaagg ctagcggtat gaaaatagcc
105841 atactcacct cgcctaatca atggtttatc ccttatgcta aagaacttaa ccaacaaata
105901 gaaaattcca agctttttta taatcataca gagattttta attttgatat agtatttata
105961 ctttcttatc ataagataat agataaaaat tttctaaaac acaataaaca caatatcgtc
106021 atccatgctt caaatttgcc ccaaggaaaa ggctgggctc cactttttca tcaagtcata
106081 gagggcaaaa atgaaatcac cttttcgctt tttgaagcag atgataaagc tgataatggt
106141 gacatttatc tgcaaaaaaa ccttaaatta aatggcttag aattgtatga agaactaaga
106201 gaaaagcaag ctcggtttac tctaaatatg tgtttagaat ttttaaaact atatcccaat
106261 atacaagcaa aaaaacaagt aggtaaagaa agtttttatc ctaaaagaag tccaaaagat
106321 agtgaacttg atataaataa aagtttaaac gaacaattta atctcttacg catagctagc
106381 aatgaagatt ttccagcttt tttttacaaa gatggtaaaa aatttatact caaaatttat
106441 ctagattcta cagggggggg gtaaagcctt aattttaaat cttgaaaact ttaaaacctt
106501 aaataaaaca aaaaaagaga aaattagact ttttagaaat cacccaaata ctaaaaaata
106561 tctatataat cagtatttta tctccaaaag agaacatttt aattttattc aaaaattaaa
106621 aaagaattct aaaaaaactt acttttgttt gagtttaaat catataattt taggaagtat
106681 caatcttgac aaattaaaca aaactacagc aagctttggt ttttactcca atcctttttc
106741 acatcttagt ggcttaggaa gaattttaga gcaaagctct atatatttag cttgtaattt
106801 tctaaaaata aaagtcttaa aattagaaat ttttcaagaa aacttacagg ttatcaattt
106861 gcacaaaaaa tttggttttg ccattaccaa agaaacaatc aaacatgata aaaaaatttt
106921 tataatgcaa ttagtttttt aaaaaaagtc gatataatgc aaaacaagaa ccaaaaaagg
106981 caaaatcatg caaataggaa attttaatac cgatgaaaaa gtttttatca tagcagagct
107041 atccgcaaat cactcaggaa gtcttgaaat ggcgcttaaa agcataaaag cagccaaaga
107101 agcaggagca gatgcgataa aaattcaaac ctacactcca gatagtctta cgctagattg
107161 tgataaagat gattttatca tcaaaggggg gctttgggat cagcgcaaat tttatgaact
107221 ttacgaaagt gccaaaaccc cttatgagtg gcattctcaa atttttgaag ctgcacagga
107281 tgcaggcata ctttgttttt caagcccttt tgcaaaagag gatgtagagt ttttaaaacg
107341 ctttgatccc atcgcttata aaattgcctc ctttgaagca aatgatgaaa atttcgtgcg
107401 tatgattgcc aaagaaaaca agcctaccat tgtttcaact ggtattgcaa gcgaagaaga
107461 gctttttaaa atatgcgaaa ttttcaaaga agaaaaaaat cctaatctga tttttttaaa
107521 atgcacctca gcttatccag cacaaatttc tgatatgaat ttaaaaggca ttataagttt
107581 aaaagaaaaa tttggcgttg aggtagggct tagcgatcat agctttggac acttagctcc
107641 tgttatggca gttgcacttg gagctagagt gatagaaaaa cattttatgc ttgataaaaa
107701 tataaaaagc gaagatagca aattcagcct tgattttgat gaatttaaag ccatggtaga
107761 agcagtaaga gaggctgaaa gcgctttggg cgatggctct ttagccttag atgaaaaaag
107821 tcttaaaaac cgcgtttttg ctagaagttt atatgcaagc aaagatatca aaaaaggtga
107881 aatttttagc gaagaaaatg taaaatcagt gcgtccttct tttggacttc atcctaaatt
107941 ttacaaagag cttttaggaa aaactgccac taaagatata gaatttggca cagctttaaa
108001 acaaggagat tttcaatgaa aatcctagta acaggtgcag atggttttat aggctctcat
108061 ctttgtgaga gtttggttca aaaaggctat aaagtaagag ctttaagtca atataattct
108121 tttaattttt ggggacattt ggaaaaaagt ccgtatttaa aggatatgga agttgtaagt
108181 ggggatttaa gagatagctt tttttgtgaa aaaatcactc aaggaatgga tgccattttt
108241 catcttggag ctttaattgc cattccttat tcttacgagg caccgcaaag ttatgtagat

108301 actaatgtaa aaggcacttt aaatatgcta gaagctgcga aaaaaaatga aatttctcat
108361 tttatacaca cttctacaag cgaagtttat ggcactgcaa gatatgtgcc tatcgatgaa
108421 aagcaccctt tacaacctca aagcccctat tctgctagta aaatcgcagc ggatatgatg
108481 gcgcttagtt attataatgc ttttaattta aatgtaaata ttgctcgtcc tttcaatacc
108541 tatggaccaa gacaaagtgc aagagccatt atacccacta tcataacaca aattttaagt
108601 ggggtaaaag aattaaaact tggggatttg agtccgacaa gggatttaaa ctttgtcctt
108661 gatacttgcg aaggctttat ctcgcttttg aaattaaatc attttggaga agtgtataac
108721 ataggctctg ggactgaata ctccatgcaa gaagtgcttg atcttataca aaaaattcta
108781 aatcaaaatg taaaaatcat ccaagatgaa aaaagattac gcccaaaaaa tagtgaggtt
108841 tttagactaa aatgcgattc aagcaagctt caaaatgcaa cccattggca aagcaaaata
108901 agtcttgaag aaggcttaaa actaagcata gaatatttta aagaaaattt agcaagttat
108961 aaaagtgaaa tttacaatgt ttgaaaatga aatcactttt atcaaaaaac ttttcaataa
109021 agaaaaaatc gctttacatg aaccttgctt tatcggaaat gaaaaaaaat atctcttaga
109081 atgcattgat agcggttttg tttcaagcgt gggtgagttt gtaacgcgtt ttgaagaagc
109141 tttaaaacaa agaacaaaag ctcgatttgt catcgctaca aatacaggca ccgccgcact
109201 tcatatcgct ttacttgcaa atggtattga tgaaaattgt gaggtgatta cccaaagcat
109261 tagctttgtt gcaacggcaa atgccattgc ttacacagga gctagggcta tttttttaga
109321 tattgatgaa aatactttga gtttaagtcc taaagcttta caaaattttt tagaaaatca
109381 cagctaccaa aaagatggct ttacttacaa taaaatcaca cataagcgca tcaaagcttg
109441 cgttgtaatg catacttttg gtcttagtgc taacatagaa aagctttgcg aaatttgtaa
109501 aaattataat attttttttaa tcgaagatgc cgccgaagct ttaggaagca cttataaaaa
109561 caaggcttta ggtacttttg gaaaatgtgg aattttaagt tttaatggaa acaaaatcat
109621 cacaggtggg agtggtggag cgattttatg cgatgatgaa aatttggcaa atcttgcaaa
109681 gcatttaagt actactgcta aaatacctca tccttatgaa tacgatcatg atatcatagg
109741 ctataattat agactgtgca atataaatgc cgcaatttta cttgcgggac ttgaaaagct
109801 agatttcttc ctagaaaaca aaagagaatt ggcacaaatt tataaggaat tttttgcaca
109861 tcataaagct tgtaaattta tagatgaaag aaaaaattca aaaagtaatt tttggctcaa
109921 tgctttatgt tttaaagatg aaaatttaag aaatatattt ttacaagaat gtttaaaaaa
109981 caatatcttt gcgcgtcctg tatggaaaag cttgcctagc cttaaacctt ttcaaacttg
110041 gcaaaaagat gagcttttaa atactcaaaa attagaaaaa tgtttggtaa atttacctag
110101 cagtgtaaga ttagataagg aaaaataatg aaaaaattta taaataatga agaaatttcg
110161 aacaatattg ggactaatga agaaatttgg gaaaatattt tttccaataa agaatgggga
110221 aaatatccta gtgaaaactt gataagattt atagctagaa atttttataa tgtaaaaaat
110281 agaaatgaaa ttcgtattct agaattagga cttggtacgg gagcaaattt atggttttgt
110341 gcaagagaag gttttagagt aagtggtatt gaatggagta aaacaggagt agaaagattt
110401 caaaagcgtt taagtgatga aaatttaagt tctcaaatag atgagataaa aattggagat
110461 tatgagcaaa aattggatga atttaaagat gagagctttg atgcatggat agatagctat
110521 tctttggctt ataatgattt taaaaaaaca aagactatta tagaaaaagc catctcaaaa
110581 ctaaaaagtg gagggaaatt tttttccata actcctagtt ttaataacga aggctttgaa
110641 aaaaatgaaa atttgggtta tcatgaatgc aaaccaactc ttggaagtga cgcatttaca
110701 ggcgttataa gatattgtga tgaagaagat ttaaaagaac tctataatgg caaaaattat
110761 caaattaccg atattaaaat tgctcgtttt gctagcctag aaaaaaatct taacgagctt
110821 tatattattg aaggacaaaa attataaatt atgcaaaaca tacttttaaa acaattttta
110881 gaagaaagaa agaaaaagta taaaaatgga atttatcatt atactcaagt taatttttcg
110941 tataattcaa accatatcga aggaagcaca ttaagcgagg aacaaacaag atatatttat
111001 gatagaaact ctttttttagc caatgaaaat caaattatca atactaatga tattttagaa
111061 actaaaaatc attttttagc ctttgatttt atcttggata gtgtttttga tgaaattaat
111121 gaagcatata ttaaaaagct tcatagtatt gttaagaata attgttctga tattgaaatt
111181 attggagaat ttaaacaaag gcaaaattac gtgggcgatt taaaaacttc tgctccgcat
111241 cttgtaaagg agcaccttga aaaactattt cagggttata aagtacacaa tatagaggat
111301 attatagatt ttcattttca ttttgaaaaa atacatcctt ttgaagatgg caatggcaga
111361 gtgggaagac ttttaatgtt taaagaatgt ttaaaaaata atatcactcc ctttattgtc
111421 gatgaagaac acaagttgtt ttattatagg gggcttaaaa attatgaaaa ccaaaaaggt
111481 tttttaatcg atacttactt atcttgtcag gataaatata aagaaatctt aaattatttt

111541 caaatcgtct gctaaaaaag caagtgaaaa atcaaatttt aaaaaacaag gaggaggtaa
111601 aataaaatat gaataatgct gcgttttggg aaaatatttt ttccaataaa gaatggggaa
111661 aatatcctag tgaaaacttg ataagattta tagctagaaa tttttataat gtaaaaaata
111721 gaaatgaaat tcgtattcta gaattaggac ttggtacggg agcaaattta tggttttgtg
111781 caagagaagg ttttagagta agtggtattg aatggagtaa aacaggagta gaaagatttc
111841 gaaagcgttt aaatgatgaa aatttaagtt ctcaaataga tgagataaaa attggagatt
111901 atgagcaaaa attggatgaa tttaaagatg agagctttga ctgcataatc gatagttgct
111961 cgctttgttg caataacttt gaaaaaacta aacacatttt tgataaagcg attttaaaat
112021 taaagccaaa tggaaaattc ttttcaagta ccatagcaaa aggtattgta ggatatgata
112081 agaacaaaga agattttcaa cttccaaatg atggaattta cactcatgtg ggaatgatac
112141 gcttttcaaa caaggaagat attcaaaagc tttataaaaa tgataacttt aaacaaacca
112201 gtcttaaagt tcaaagcatt gaagataatg gcgccttaat cgataaactt ttcatcatag
112261 aaggagaaaa aaatataatg gggggggga actctaaaaa actcctacaa ggagtggaaa
112321 atgagtagaa tttttttaga agatttttta aaaaatcaca aaaactactc ttatctttta
112381 gatgatattt atttaaataa aaaatatgca agtctttatg gagaagtatt tgaattttct
112441 taccaaaaag aagtaaaatt atttaaaatg attgctatta aggaaaaaat tccaaataca
112501 aaattttacg atttgcaaag cccttatgga tatggtggga tttattgcaa tgttaatgat
112561 aaaaatttta taaaagaagc tcttgaaaat ttaaaactta aagctgtgga gcaaaatatt
112621 atagcttttt ttatccgttt tcatcttttt gatgaaaatt taaaaaacta tcattatctt
112681 ttgccttttt ttactaaaaa taaagaaact attatcgtaa atacaaatga aaatatagaa
112741 aatattcgta caaattatag ccctaggata agatcctata ttaaaaaagc aagaaaagaa
112801 ataaatatag aatttgccca aaaggaagat tataaagact ttttcaaact ctatacacac
112861 actatgcaaa gaaataatgc ggataaattt tattttttca atgaaagata ttttgaaaaa
112921 ttattcaatt tcaaagaaag cataatttta aaagccagtt tagataataa aattttagct
112981 tttgcttcat tttttctttg tggagatttt tcttattatc acctaagcgc caattcgtta
113041 aaatctaacg ccaatgcagc tttgcttgat tttttctttg aatatgccaa ccaaaatgct
113101 tgcaaatttt gtttttttggg cggaggagtg caaaatgaag atacactttt taaatttaag
113161 gaaaaatttt ccacactaag ggcaaatttt tatgtgggtg gcatgatttt taataaagaa
113221 gaattcgatt ttttaaataa aaacttcaca aataaatact ttttaaaata ccgctttgac
113281 gattaaacat catgttatac agctagttgt tatccttaat ttttactaaa ctataaagta
113341 tatttaattt ttgttatata ttaacaaaaa ttaaataata aataaattta taagtaaaat
113401 atcagagata aaaaacctga agcaaaaacg atccctagaa acagagttgt aaataatcta
113461 aacataagta aacttatcta taacaaaata aacaaacttt tcaacaaaat agatattcat
113521 ctttataaat caaaaccttt tactgcacaa ggaaatacaa tttttatgca aaacatcaaa
113581 aaaatattat tatttaaaat aaaacagaat actttttgct atctttgtta taatgacaag
113641 taaaaaatac tatgtttcgt aataaaaata gtatttttta cctccatata aaaggcaaaa
113701 aaatgaaaaa agctttaatc atagcagaag caggggttaa tcacaatggt gatttaaatt
113761 tggccaaaaa gctgatcgaa gttgctgcca aaagtggagc tgattttgtt aaatttcaaa
113821 gtttcaaagc cgaactttgc gtgagtaaaa atgctaaaaa agcaacttat cagttaaaaa
113881 ccacagccaa agatgaaagt caattagaaa tgattaaaaa actagagctt gactttaacg
113941 cacaccagct tttaatctct catgctaaac aatgcggtat tgcttttctt tcaacgcctt
114001 ttgatttaga aagcattgaa cttttagatg atttaggact tgaagttttt aaaattccaa
114061 gtggggaaat cacaaattta ccttatctaa aaaaaattgc aaaattaaat aaaaaaatca
114121 tactctccac aggaatgtca aatttaggcg agatagaagc ggctttagaa gtactttgca
114181 aagaaggcac ccaaagagca aatatcaccc tacttcactg cactaccgaa taccccgcac
114241 cctttgatga ggttaattta aaagctatgc aaaccttaaa aaatgctttt aatcttgatg
114301 taggctattc agatcacacc aaaggtatac atatttcttt ggccgctatc gccttagggg
114361 caagcgtgat agaaaagcat tttaccttag ataaaaatat gtcaggacct gatcataaag
114421 cctctttaga acccgatgaa ctccaagaac tttgcactaa aatcagagaa atcgaaagtg
114481 ctttaggcga tggcataaaa caagcaagca aaagtgaaag gaaaaatatc gaaattgcaa
114541 gaaaaagcct agtagctaaa aagaaaatta aaaaaggaga aatttttagc gaagaaaacc
114601 ttaccacaaa acgccctgct agtggtataa gcgcgatgcg ttatgatgaa tacttgggca
114661 aaaaagccag taaagattat gaagaagatg agctaatcca tgagtaaaag aaaaatttgc
114721 atagtcagtg caacaagagc tgagtggtat cttttaaaaa atttatgcct tgaaatacaa

114781 aatgatgaag acttagaact tcaaattatc gccacaggag cacatttaag ccccgaattt
114841 ggactaacct ataaagaaat agaaaaagaa tttaaaatcg ctaaaaaaat tcctatttta
114901 ctcgcaagtg atgataaaat aagcctttgt aaagctatgg ctttggcaaa tattggcttt
114961 agcgaagcat ttgaaagctt aaaacctgat atcgtagtaa ttttaggcga tcgttatgaa
115021 atgttaagcg tggctagtgt gtgcttgatg atgaatcttc ctatagccca tctttgcggt
115081 ggagagctta ccttaggagc cattgatgat agcataagac atagcatttc taaaatggca
115141 catttgcatt ttgtaagcac ccaaacttat aaaaatcgtc ttttgcaact tggagaagaa
115201 gaaaatcgtg tatttaatgt cggatctttg gcaagtccta ttataaaaaa tatcaatttt
115261 ttggataaaa atgagcttgc aaaagtcttg gaatttgact tagagcaaaa tatttattta
115321 attacttatc atcctgtaac tttaaattta caaaattcta aaaaagaaat cgatatttta
115381 ttaaaaactt tagataaact taacaatgca agtttgattt ttaccaaagc caatgcagat
115441 gaaaatgggc ttttaatcaa tgaaatttta aaagattact gcgaaaaaaa tcctaaaaaa
115501 gctaaacttt ttgacaatct tggctcacaa aaatatctta gcattatgaa aatagccaaa
115561 gccatgatag gaaatagctc aagcggtata agcgaaagtc ctttctttaa aacccccttgt
115621 atcaatatag gatcaagaca aaaagggcgt ttaagaactc aaaatatcat tgattgtgaa
115681 tttgaaaatt tagatcaagc ttttgaaaaa ctagaaagca aagattttaa acaagcattg
115741 tatcatttta aaaaccctta cgaaaatgac aaaaatccaa atataatcat aaaaacttgc
115801 ctaaaaaacg cgaatttgga tacaatttta caaaaaaaat tcattgattt aaggtaaaac
115861 cttggatata aataaactca aactcacacc tgattctagc attgaagaag ccttaaaagt
115921 agtaggacaa gaacgcgttc gccttggaat cattgttgat aaaaaggata atttcttagg
115981 tgtgatcagt gattcaaaca tcagaaaagc tttgataaag ggtaaaaatt tacaaagcaa
116041 aatcaaagac atctatacta aaaatcctat taccatcaaa gaaaatacaa gcaaaaatga
116101 acttttaaaa ctaagcgcaa agactgatat ttatgatttt cctgtgctaa atgacaaaaa
116161 agaagtcatt tctataaaat ccatttcttc cttactaaac cgccaagtaa attcaaattt
116221 tgtcatcata atggcaggtg gacttggaac ccgtttaaaa gaactgacta aagatactcc
116281 aaaacctatg ctaaaggttg gaaaaaagcc tattttagaa agtatagtgc aaagattaaa
116341 agagcaaaat tttgaaaatt tcatcttttg tgtaaattat aaaaagcaaa ttatcgagga
116401 ttattttaaa aaaggtaaag atttggtat aaaaatttct tatatcaaag agcgcaaaaa
116461 actaggtaca gcaggggctt taagtcttat caagcaagat tttaaagaaa gctttatcgt
116521 aatgaatgca gatattttaa ccgagctcga ttttaacgag cttttaaaag cgcataaaga
116581 ttctaaagct ttaatgagcg tatgcgtaag agaatttgag cagcaaattc cttttggagt
116641 gatagaacaa aataaaggct ttatcacaaa tatcacagaa aagccggtgc aaaaattttt
116701 agtgagtgca ggaatttatg tatgtgagcc tgaaatttta gaacttttaa ataaaaacga
116761 ataccttgat atgccagagc ttattaaact agctatgcaa aaaggcaggg taaatactta
116821 tttaatccat gattattgga tcgatatagg aagacttgat gagtttgtca aggcaaatga
116881 ggaatttaaa tgaaagtttt aatcataggc tttggaagca taggaaaaaa acattttttta
116941 gccctaaatg aacttggatt tttagtagat gtgctttcaa aaagctatga attttcgcat
117001 tttgaaggta aaaactttag actttttaaa gatttagaag aattaaattt acaagattat
117061 gacttattta tcatcgcaaa tatcactaca agtcatttta aaactcttga atttttagat
117121 caaaatgtaa aagaaaaaat catacttgtg gaaaaacctt tatttcaaaa ggatatggag
117181 tttattccta gcaacaataa tcaaatttat attgcttatc ttttaagatt tcatcctatg
117241 attcaagatt taaaacaact cattgatgaa aaagaagttt attttgctaa atttatttgc
117301 aattcttatt tgcctaattg gcgaaaactt gattataggc aaaattatag cgccaaaaaa
117361 gagctaggtg gaggggtaat gcttgattta tcccatgaga ttgatttggc tttttatttt
117421 tttaaagaac tttctttaaa attttctcaa aatttaaaaa tttcagagct tgaaataaca
117481 agtgatgatt ttactttttt ggcattgagt gcgaaaaata cttcaattca tatcgagctt
117541 gattattttt caaaattaac tcaaagaaaa attctttttc ataccaaaga aaatagcttt
117601 gaagccgatt tagttcataa taaattaaat atttatgata aaactaacaa ttttaaaagt
117661 aaaacttatg aaaatgatac ttttaaaacc ttgcaaaata tgtataaagc gatcctagaa
117721 aaggatcaaa atttatgcac tttagaagaa gcaaggaggg ttttaagatt gtgtgatgag
117781 gcaagaaatg ggtgaaattt tatgcacaat ttgtgcaaga ggtggaagca aaggcgttaa
117841 aaataaaaac atacgcaaga ttaacgatct tgagatgata gcttatagca ttatccaagc
117901 taaaaattca aagcttttta aacatatcgt tataagcaca gatagcgagg aaatagccaa
117961 aatagcgcta aaatatggcg gcgaagtttt tttcaaaaga gaagctcatc tagcaagcga

118021 tacagctgct aaaattcctg taatgcgtga tgctttatta agaagtgaag agtattttaa
118081 atgccaattt gacgccctaa tcgatcttga cgcttcagca ccgcttagat caagtagtga
118141 tatcataaaa gcttttgaaa cattttgtca aaatcaaaat gataatctaa tcaccgctgt
118201 tcctgctaga cgcaatcctt attttaatct aatcgaagtg caagatggaa aagttgtaaa
118261 atccaaatca ggaaatttta ccacacgcca aagcgcacct aaatgttatg atatgaatgc
118321 aagtatttat atatttaaaa gagatttttt actgcaaaat gacagcgttt ttggtgaaaa
118381 tacagggctt ttcatcatgg atgaaagcac tgcttttgat gtagatagcg aacttgattt
118441 taaaatagta gaatttttga tcaaagaaaa aaacttacaa gcaaaggatt tttaatgcta
118501 gaaaataaaa ttatctttgt agcaggagct tgtggacgca taggcaaagc actttgtaaa
118561 aagatattgc aaaacaaagg catagccatt ttagccgata tcaatgaaaa tcatttatcc
118621 atattaaaaa cagaacttga aaatgagttt aaaaaagagc ttttaagcct taggcttgac
118681 attacttcta aggaaagttt aaattatgct atcgatcaag cttttaaaaa atactctaaa
118741 atcgatggct ttgtgaattc tagctatcct gtaggaaaag attggggcaa gatagcttat
118801 tatgaagctt catatgagca aatttgcgaa agtttaaatt tacacttggg gggatttatc
118861 ttagcttcgc aagaatttgt gaaatttttt aaaaaacaaa gctatggcaa tatcatcaat
118921 cttagctcta ttatgggggt ttttgcgccc aaatttgaaa attatgaaaa taccgccatg
118981 caaagttctt tagaatacag cgtgatcaaa gcagggatta atcatcttgg tgcttggctc
119041 gcaaaagagc tttttaatac aaacattcgt gtaaatacct tagcaagtgg tggaatttta
119101 gacaatcaag ccaatatttt tttagaaaaa taccgcaaat gctgcgcaag caagggtatg
119161 ttagatgcag aagatatttg cggaaccttg gtatttttac tcagtgatga gagtaaattt
119221 atcacgggac aaaccttagt tgtagccgat ggatggggac tttaatgaat aatgaacttt
119281 tcttaaaaaa tactcaagca ctttttgaag tggatcaaat tttagcttat gagttaagaa
119341 gaattaagca ttgttttaat ttcgcattac aaggcgataa tctagaaaat cttaatataa
119401 tccatctatc tagcaaagaa agtatttata aaaatcctta tgaagaatta agcaacaatt
119461 taactttttt caagcaagaa tactccaaat atcctacttt atttttttat ggtttgggta
119521 gcggtgcgtt ttataaagcc ttactcacaa accaaaatca caaacaactc atcgtttttg
119581 aaagcgagct tgagcttttt tacttggctt ttcatctatt tgattttagc aaagatatta
119641 gaaatgaaag attgatttg ttttatacac ccaacttaaa taccacccag cttaatgctt
119701 tgctaaatca taaaaaaatt aaattcagca ttaaaactta tgattttcat attcatagcg
119761 aattttatct ttctcattat aaagacatca ttcaaaatct taatcaaagt cttattgaaa
119821 tcatcaaatt tatagtccta gcttatggta atgatcctaa agattcgcat ataggcattg
119881 aacatacttt aaaaaaccta aataccttag tttatgggag tgtttttcaa cactttttaa
119941 aatataaccg tgcaaaatcc aaaaatgcca tcatcgtttc aaccggcccc tcacttacaa
120001 aacaactctc tcttttaaaa gaatacgcct caaaagcaac gatcttttgt gctgattctt
120061 cttatcctat acttgcaaga gcgggtataa agcctgatta tgtcttaagt cttgaaaggg
120121 tagcggcaac aagtgagttt tttaataatg attttaaaga atttgataaa gatattttat
120181 ttctaatcac atcagtaacc catgaaaata ccctaaaata tcttgagaaa aatcatagaa
120241 attatatgct tgtattaaga cctttgcttt tttcttcaca tttaaaatta gatgattttg
120301 gttatcttgg tgtaggaatg agcgttgcca atatggctta tgaattggct gttgctttaa
120361 gacatgaaaa tatcatttta atcggacaag atttagctta tgatgattta ggacattctc
120421 acccaaaaga tcatatttat ggagaacaag gtgataaatt tcaagataaa cttagtgtta
120481 gagcctatgg gggcaaagga cttgtaaaaa ctcaactttc ttggcaactt ttcagacaaa
120541 gttttgaaaa agatatacct cttgttaaag aaaagctaca aattactaca tataattgca
120601 ccgaaggtgg agcaaggata gaaggaagta tagaaaaacc ttttaaagaa ctttgtgaaa
120661 gtatacttga tacaaattta gaaaaacctt ttaagaatga ttttaagtta gaacccaaag
120721 aagcgcaaaa aattttagaa aaaacaaaaa aacaccttct aaatcaagta aaaataagta
120781 aaaatttaat agaagaaatt caaaataaac tcaagacttt gccaaatatt ttaaacgaaa
120841 aagatatcaa tttggatttt aaaaaactta agaaattaga gcttgatttt caaagcttgg
120901 ctactaaaat gaaaagctat ggccttatga gcgagctttt acaagcttta gtttttcatc
120961 aagagtgtga tcttttaaaa ctaaaagtac agccttgcaa aaaccaagaa gaagaacaaa
121021 aacaaatttt gctttgtttg aaatatcatc atgaaaaatt cagtgaattg attttatata
121081 tgcaaattca aaacgaactc atagaaaaat acatctaaag gagagaaata tgacttttac
121141 agaagagcaa aaaaatcgtt ataaaaaaaa cttcaacgcc ctatctgata accttatcct
121201 taaaaaagat cttgcaaaac tttctaaaag taaaaaattt aaaatcctac aagggcaaga

121261 tccacttgat ataaatcttt taaacacctt tactcacaaa aaaatttaca acaaaccctt
121321 agaagagcta aatgctttgc ttgcaagcta taatgaaaaa tacattctat accctgtgct
121381 ttatttttat ggtttaggca atggcatact ttataaggct ctttgtcaaa accctaaatt
121441 acaacacatc atcatttttg aaaatgatat agagcttatc tttacaagct tgcatcttat
121501 tgacttatct caagaaatca aagaaggaaa aatccacatt ctctcttcta aaaagaacaa
121561 agagcaattt ctttctattt ttaatgtcca gccttttttta aattttttaa gggtgtattt
121621 tttagaactt catagcaatt attatgaaag tgaacatgat gaaatcatca agctcaatca
121681 agacatacat cacgccatca aagaagcagt atgcgtgcgc ggaaataatc caaaggatgc
121741 cttgcaaggc atagaacaat ttattttttaa cttgcctaac atgcttaccc atcctaacgc
121801 taaagaatta atcaatacaa gaaaaggcct tgataaaaat gccatcatcg tttcaaccgg
121861 cccctcactt acaaaacaac tctctctttt aaaagaatat gcctcaaaag caacgatctt
121921 ttgtgctgat tcttcctatc ctatacttgc aagagcgggt ataaagcctg attatgtgat
121981 gatgcttgaa agaacagata tgacagctga atttttttaat cacgattttg gtgactttga
122041 taagggcatc atcttttgta ttgctgcttt ggtacatcct aaggctattg agtatttaga
122101 aaagcaaaat cgaacctata ttttaattga aaaatcactt cattttgaaa gctatatgaa
122161 gctacattct tttggttatt taaacttagt ttcaagtgtg gcacatatgg cttatgaact
122221 agctatggct ttaaaatttg aaaatatcat ttttatagga caggatctag cctatgcaaa
122281 agatggttgc tctcatccaa aagactatca aaactcacct acttatgaaa caaatatgta
122341 tgaacatctc tcaaccaagg cttatggtgg aaagggcgag gtaaaaaactc attttgtatg
122401 gaatatgttt cgcaccattt tagaaaagca tatcatcaac actccaaaaa ctatcaaggt
122461 ttataattgc accgaaggtg gagcaaggat agaaggaagt atagaaaaac cttttaaaga
122521 actttgtgaa aatttattga ctaatgattt aaataaacct tttccaagtc ttaatcttcc
122581 aagtcaagat aagcaaaatg aaagaatgtt aaaggccttt tacaaaattc aaaaaagcat
122641 tcaaaattgc gataaattag aactttttgt cctagaaaaa caacaaagca ttctagaaaa
122701 aatcaaccaa gaagaattta aaaacctaag ctatgatgat aaagaattag ctcaaggtat
122761 cattgaagat atcgatactt taaaaaatga aatagaaaaa atccacactt tttatgaaga
122821 tctgggtgaa attttacaac ccctatactg tcaatttgag tttaatttgg caaaaattta
122881 tgttttaaat ccactgacaa aagaagattg ggtaaataaa actttattgt ggattaaaga
122941 gcatttggat tggattgctt atattttaat acatcttaaa gctcaaaaac aggctttaca
123001 aagcagtatt ttgcctttag aaaatgaact tttacaaaga aattttaagc acttacttga
123061 taaaatcaaa aggaaaacaa aatgaaaaca acagatatat ttgaaaaaaa tcttctttct
123121 ctttcaggaa aagaatacga gaatttgatt aaaaaactta aaaaaatcaa agaaagtaaa
123181 aaattttctc cccttttttga tgctaaagat ccgcttaatt taaacatcat agaaaactcc
123241 actaaagaaa aaatttatca aaatcctttg aaagaattag aaagcaaaat cgatctcatt
123301 caaaaaaaat accttaaata tcccgtttta tttttttatg gcataggcaa tggcttttta
123361 tataaagcct tattacaaaa cccacatttt aaacgcatta tcatttttga aaaagaaatt
123421 gaactcatat ttcttgcttt aaatttagcc gattttaccc aagagcttaa aaagggaaga
123481 ttgattatca ttttaagcag tgaaatgaat gatactaagg cgaattcttt attttctttg
123541 cctgaaataa aattattgtt taaatcctat tttttggatt tgcattcaga tttttatgaa
123601 aaatactcaa aagatattat caaaatcaac aatatcaata aaaatatgat tatcttcttc
123661 tctctaagac aaggcaatga tcctaaagat gccttgcaag gtgtggatca atttgtaagg
123721 catatacctc atatgatcac tcatccaagt cttagtgagc tgattaaaaa aagatataaa
123781 aaggctaaaa atgccatcat tgtagctact ggtccaagcc ttcaaaaaca aattccactt
123841 ttaaaaaaat accacaaaaa agctagcatt ttttgtcttg atgcttctta ctcgatcttg
123901 catgaaaatg atatcaaacc tgattatgtt ttaagccttg aaagagtggt ggcaacaagt
123961 gagtttttta acaatgactt taaagaattt gataaagata tattgtttat ccttttaacg
124021 cttacccatc cacaaaccat caaatatcta gaagctaata aaagatctta tatacttaca
124081 ccaagagact tgcctttttc acaaagcatt aaacatttaa aagaattctc atcgcttaat
124141 ggaatgagtg tggcacatat ggctttagtt ttagcttatc atttagggca tgaaaatgtg
124201 attttaatcg gacaagatct agcctttgga aaagatggat cctcacactc aaaaggctat
124261 ggcagaggtg aagagctagc taaaaaagaa acacaggctc tacctcaaac taaagtaaag
124321 gcttatgggg gtaaaggttt agttaaaaca acgacgattt gggagctttt tattaaggtt
124381 tttgaaaaca taactactat catcaatgca caaaaaaatt gcactattta taatgctaca
124441 gaagggggtg caagaatagg tggaaccata gaaaaacctt ttaaagaact ttgtgaaaat

124501 ttactggata aagagcttaa aaaacctctc acaaaggtca ataaattaag tagaaaagag
124561 caaaatcttt tacttttaaa atcctataaa caaatcaaaa ctttcatcaa aagaagtgaa
124621 aaaatcgcaa aagaacttag aaaaactgga aagcagatta gaaatttaac ccaagagatc
124681 aagcaaaaat acaaattaga gcaaatcagt aaaaatatag atgagattaa aacaaaactt
124741 gcaagtagca attatgctta tttggctgaa gttttaggac caagtgttta tcatgcagaa
124801 agctctttgg gtgagcttta tttgcaaaat attagcaatg aaagcgataa acaaaacaaa
124861 ctcattgctt ggatttatgc acatgaagct tggattgatg aagtgcataa tttaatcatc
124921 ttgcaaaata gcgttttaaa aaatgccatt ataccttttaa gagatatggt agaaaaaaga
124981 aagcttgtgt aatcgctcta aaaagagcga tttttattgt aaaagtttta aaacattttg
125041 ttgtacagca ttggcctgac tcatcgcata agatccactt tgagctagga tattgtattt
125101 agaaaaattt gcactctctg cagcaaagtc tacatctctg atggttgatt cagctgattt
125161 aacatttact tgagttacag taatgttgtt gatagtaact tgaagttgat tttgcaccga
125221 gcctatatct gctcttattt ggtcaagatt gattgtagca gtttcagcta tatccatcac
125281 agccatagca ccttttagag ttgtaacccc tgcagtttta tccgttgaac ctagcgcagt
125341 agctttcatt tgagcaaatt gagattgtcc tgatccagca gaaaaaccac tgccttcaga
125401 tacattataa aaattactta gcatagattg agcatttcca acaaaaccaa cattagcact
125461 tagcattaca gacatatttt taccactacc tacagaaaaa ccagaaccag cactcatacc
125521 agaaccagct gcactcatat atgaactgat agaagaataa tccgctaaga atcttccgcc
125581 accttcatag ttgttaaatc ccatagcatc agcaatttga gctgaaattt gtcccttagt
125641 ttctcttaaa gatactgaag attgagaaac catttgaccc tctccaaaac ctgctgctga
125701 aagtccagtt cctgaaatcg ctatatctct accatcgttt ttaacaagag aaagacggcc
125761 ataattttct gcttgttctg ctaaaatacc catacttgga gtaaaaccac tgactttaat
125821 acctctaccc tctcttgaag taagaaccaa tcttccatca tcactcaaag aagcctcaac
125881 cccagtagta tcttttacag cattgataga agctactaaa gatccatttt tatcacctgc
125941 ttcaaattct attttaccga ttttcactcc attgattgta aaatcatctg tagtgtttcc
126001 ttttgtaatg gccattgcac caacagtttg cacattaaaa ctagctctaa cgcctgtttt
126061 atcagcatat ttattgatct cttcagccaa agctccaagt cctgttccta ctgaagtaga
126121 aataacaacg ctttgaaatt taaagtcttc tataccatta tagtttttaa tagtaatttg
126181 cgcagtacca ctgtctgaaa tctgtgttcc cgtttcaaat cttgttacac cgattttaga
126241 agactgagtc gctccgattg aagctttgat ggtttgattt gactgtgcac cgatttggaa
126301 ttcttgattg ataaaactac cacttaaaag ttgtttaccg ttaaatgaag ttgtatttgc
126361 gatattgtca agttcttcca tcaaacgatt gatatctgct tgaagcatag ttcttgtttt
126421 taaactttgt ccatcttgag ctgcttgagt cgctttagtc ttgatagtat ctaagatttt
126481 aagttgctca tccatagcct tatctgcagt ttgcaagata cttgctgcat cgttaccatt
126541 gtttatagct tgacctaagg tagaagcttg agttcttaag ctatctgcta tcgccatccc
126601 tgaagcatca tctgctgcag agttaattct aagacctgaa ctaagtcttg caagcgattg
126661 atccaatgct ttgctattta gatctgagtt tgctttagca tttaatgctg caacgtttgt
126721 gttaatacga aatcccattt taaatccttt taaatgaatt caaactcatc catgagcttg
126781 ttactcacta tatcgtttag aaaaaaaaat actttatagt tttttgtaaa aatttatatc
126841 aatttctcat attctaacat tgcatccctt ataataaagg atttcatctc taatttctcg
126901 ataaactttt caagccattt ttgataagca tgaatcaaac ttaaaaaata atgtgtttca
126961 tcctcaaatc ttttttttat aacttcagat aaaaaattcc cacgatagcc aaatgttcct
127021 tttaaaacat catcttgtat aaaagtgtta ttatctaaaa taaaatcaaa atcagtaatg
127081 ctttgataaa tattttgtaa aaaatctaaa ggtaaagttt ttttcacatt taatacattt
127141 atcaaagcat tatttaacaa atgagcatct cgtaaaatat cttgagcact ttgaaaatcg
127201 tttttttaaat gtaaaaaaat cttagacaaa aacttaagcg atcgattata ggtaaagggt
127261 ttgggtatat taagagaagg tttttttctgg gtaaaaaatt tttcacagca ttctttaaaa
127321 cttatatttt ttgcaaaatt aatataagca ccccccttctg tagcattata aaatactgct
127381 ttatcattat ttcttgcaat aagatattca agctttatgc ggtaatcatt ccaagcaata
127441 tgagttaaaa cctcaccttt tccttcataa gctagaactt ttagggttgg catatttaga
127501 gtatgatcaa acttttctcc aaaaacaaaa tcttttgaat gtgaatttcc tttttcatca
127561 aaggctaaat cttgtcctat cataacgata tttttaaaac ctagagctag agccaaagta
127621 taagaaaaat gacttacatg ggttcctgta tctatatagc caaaatcatc caaatgaaac
127681 tgttcataca aatatttatc tcttaatgca acgcagcaag gtagttttgt ttgcacgtgt

127741 tttacaacat taggatgtgt atttgctgct aaaaccgcta tggttttaga ataatctaaa
127801 ggttttgaac aattaaaaaa tttcactgtc aaatctttta tatccaaatt caatacatag
127861 tctgcaacaa taccctcatt ttcaagcata tttaaagcac catctgcaca aaaaataact
127921 acattttctt gatattgtct taacaaagaa agttgtttat gaagactagg accagctgaa
127981 actacaacgg ctgtatcaaa tttggctttg cgttcgctta aaagtctttg aaagggtata
128041 ttttgtaata aatggggtat attttgcaaa aattgcttgt aacaaatagt tgttagttga
128101 tcattgggat caaaatttct aataactaaa aaaatattat ctatacactt tttatcagtt
128161 tttgatatag tatcttgata atacaattta taaaaattac aatttataaa catctcataa
128221 agatttaacc attcaaatat atctctttga tcaaataata tactaagctg aagctccatt
128281 tttggctcac aaacatccac aagataaatt ttcccactac aaagctcttc actcaaatct
128341 agtgcgctta aagctaagat aaaaagttct atctcacttt caaatacaaa aagatgtcta
128401 taatgcttag ctaagttttt gataagcaag gcattgccta taccatagat acatataaaa
128461 ggatatctta aagttttttc taaaatgttt tgataaaaat aatttaattc ttctaagttt
128521 tcatacatga aaatatcatt gtttttatca tagatattta aatttccatc catactaaaa
128581 cgagagcaag tcttagtttg tataaaattt aaaagtttat ttcctaaggg ttcattgact
128641 ccgcttaata aagcttgtat gtttttttct aggatactca tggaatttcc ttaagttttt
128701 gtgatatcta gattgatttt attgtctttt tcgctaaaat gatatatcaa accatcacaa
128761 gggtagctta tgcttttgtt tttccattct tttctagaat tagataaaat tttttcaagc
128821 atacaaggaa gttctttaga aatcaaagtt tttatagaca ttcccatacc atgatctaag
128881 tttttgataa atttaccatc aatttgatct ggattttttta tcatatgaag ctcggcatca
128941 aaatcaaatt gctttaaata tttataaagt tctaatttct catcgggcgg agctatttct
129001 ttatcataaa aagaatgata gcttgtataa tagggttttg gataatttgc ttgattttct
129061 atatgatttg gctctagtat gtatcttatc attcttcttg gtatagaaaa aaagttattt
129121 gagcttgaat tacttgtcca aaaagtttta tccgaacaat aaatatggat atgtttaaaa
129181 aaaatagccg ttccaaaaca atagtatttt gtaaaatcaa tttccttacc aaaacctata
129241 agacgccaaa gaaacttagc ataactagaa ttatctatca cgccatctac aagccaaggt
129301 gcaaatttag ctgctaaatg tgctaaatat cctccatgag aactacctat catgataacg
129361 ggcatacccc cccccatagt gttaaaaggt gcatgagcct taagataaag agctgcattt
129421 aataaatctt gcgcttgcat tactccaaaa ttttgatact catttttagc aggctgcaaa
129481 cttaaatgaa tgcttagttt aaaatttgaa acaatttttc tttgagtttt acgctcttct
129541 aggattgtgt ctactttttt aagtgtaaaa tttaaatctt ctaaagaatt taacttatct
129601 ggattgattt ttatgcctaa agtttcacaa ctcgtttgca aaatcaactt atctatatca
129661 tctaaataaa aagtagaacc cgtttgggga cgatttccta tacaatgata atttacgcta
129721 acaacagcta cactaaattc atttgctaca aattcagcta aatgctcacg ataattttca
129781 tttgcatccc cgccaagtcc agggactata aagaccaaag ctttcatttc tttttcatca
129841 tcaaaacaaa gtttaaattc aagctttgat tctcttttta taccaagttc tacatcatca
129901 caagaatcaa tttcataagt tttatttact atcatcaaat ccccctatca aaatataagt
129961 ttgactcgct aaattcttaa aattctcatt tttacttaag gccacgcaag ctttattttt
130021 tgagctgctt gtaaagtcta agttttgaaa ttcttgtttt gggttattgg ctatgttgat
130081 tatggcattt ttacttgctt cattgctaga aattcccatc aatacaaaat catttttatc
130141 aatgaaaatc agctttatac actccttatc ctctcctacc ctaaaattta cttcttttat
130201 acctgtaaaa tttttaagat caacattttc cactccactt acattgctca taaaattcat
130261 agtgcttaaa tcactatttt ttaaggcata gatacttata tcgttgatta aagttttaag
130321 attgtttaag gatttactca cctcagcttc atctctacta gaactgaatt ttggcaaagc
130381 gattgctgct aaaattccta aaatcacgat aacaaaaaca agttccaata tagtaaaagc
130441 tcttttcact tttatatcct taaaaaattt ttattttttg gacttaaaat ctcctcttca
130501 agttccacaa attcttttct tttataagta tcaaccccag ctcttgcaat cattaaagca
130561 ttatcagagc agtattttaa aggagctagt tttaatttgc tttgatactt ttcacataaa
130621 ttttgcaaac gagaacgcaa atttaaattc gcacttgctc ctcctacaac accaaaggtt
130681 ttaaacttgt aaagattaaa aatcttttct aatttatcca tgatatgttc acatgctgca
130741 ttttcaaatc cataagcaat ttctgctttt atatcttcat ctaaattttc atgttttaaa
130801 atttccaaac gcaccgcatt tttaagccct gaaaaactat aagcaagctc cttggagtgt
130861 tttaaaggga tattaaagtg gatatttttt aatttggctt tttgggctaa attttctata
130921 agtactccgc caggatagcc caaattcatc atcttagcta ccttatcaaa actttcgcca

130981 aagctatcat cgttagttct tgctaaaatt tctatttttc catctttccc aagataaaga
131041 accatggtat gcccgccact tacaagcaaa attcccatat caagacaaat tgactcctct
131101 aaaaaaagac tataaatatg gcctttaaga tgattgatag ccattaaagg caaattaaga
131161 ctaagcgcta aagtctttgc catacttatg ccactaagca aagaaacact aagtccaggt
131221 tcatttgtca cagctatggc acagagttta tcaaaatacc ccttgcattg ctcaagtatc
131281 cttggcaagg cttcactatg aagtcttgct gcaagctcgg gcactactcc gccataaaca
131341 ttgtgctcca tttcttgaga aattttttta tgaaatttac actctaaagt atctttatcc
131401 atcactgcta tagagctatc atcacaagaa ctttctatgg ctaaaataag atttttcatt
131461 caaattccac taaaatagaa ccgataaatt tatctttaga tcttgcggtt tttagagtag
131521 aaagatcaag aattttagca tttttgataa gtttgctttc gacactatct tctaaaaatt
131581 gttgcaaatt agcccctctt aattcataaa attctacacg ataaattttt cctgccatat
131641 ctaaagaata aggcttttgc atattttttt tagaaactaa catattgctt tcatctctgc
131701 catgatcaaa acctatgata tttacacgca cacctttaat ccttggcaca ataaaatttt
131761 ctttaacttg aagttttgtt ccaaaattaa ctatagtttc attgccatct acttgtaaag
131821 ttactttatc aaaagggttg ctaaattcaa gatattcagg ataaagtctt gtttgaaatc
131881 ggttgccata ttggatataa aaagaattat tttcagctat aactgcagta agctcattgc
131941 tagtgttata atttaattct ttgttgatag gaaaaggtac ataattgata acttgtcttg
132001 gatttttaa agaaagtaaa attttatcat caaaaagctt gacttcaagc tcttgattga
132061 tagctttatc aacaccttgt ggggtaagct caaaagttct agtaaattca attcctgctg
132121 tttttagata gttttcgatg gctaaaagat ggtaataagc ccttaaattt acaggcaaat
132181 ttttacttgc ttcattcgca aaagcggctt tgtgattgga tataacaaaa tatgttaaag
132241 cttttagcat ttcagtatcg ccaagctctt gagttttggt atttttaaga tgataagcat
132301 gttttggatc ggctaaggaa gcattgacac tatttacagt ttgagtggct ataccttcaa
132361 gattagggta tttactagca ttgatttcac ttgtatcaat cacgcttgaa tttccccaac
132421 gcttagggtt ttgcatagca tcaatatatg tgggcttata aaaaccccaa ccatcgtgaa
132481 gattgatcac catactaact tcaggaagca agataagctc tttaatgcgt tgaactgttt
132541 gataatcagg atcttttgga cttagacttg cgaatttgcg gtttaaatct ccattatttc
132601 cacgcgagcg cttgataatg ctatcaaaag ccaagttagg agcaactatg attttacctt
132661 tagtgatatt ataatcactc aaaagcaaac ttgctgcgtg aaatccacca ggctcatcac
132721 cttgaattcc cccaaaaatc aaaactgtat tattatcatc taagcttttt ccattttctc
132781 ctacactaaa ttccaaatcc aaagcaaaaa gtccacttat aaaaaaaata atcgatataa
132841 aaaatttcat ttcattcccc ttaaatactc tcttgtttta taatcatact caaaaacttc
132901 atcaatactt gaaattttag gcacgccaaa atgatccaat gctttaaaaa taacatgagc
132961 aatatctaaa aattgtattt tattatctaa aaaattttgt acccctattt cattagcagc
133021 attgataatc acgcctaaat caggattgtt taaaagctcg tttttgagtt taaaaatagg
133081 atattttttt gtgctgattt tataaaattt taaactagaa agtttaacaa agtcaacagg
133141 ctctaaaatt ttactatcat gcttagaaaa aatagcctct gatatggata atttcatatc
133201 tgctcttgaa aaatacgccg tgcttgcgcc atttttaaac tcgcacatag catgaactaa
133261 agattttggt tcgattaaag catcgatatt tttaaaatca tacaaatgat aagcttcgat
133321 gatttcaaaa agcttatttg ccattgtcgc actatctatc gtgatctttg aacccatatt
133381 ccaatttggg tgctttagag catctttaac actcacatgg cttaaatctt taattttatg
133441 cttataaaaa gctccaccgc ttgcagtaat ataaagcttt gctatatctt ttttaccctc
133501 aagcaaaaat ttcaaagccg aatgctcact atctacgggt aaaaacttcg ccccttttag
133561 aaattttcca gcaactacca agctttcttt attagctaaa gctatttttt taccaagctc
133621 ctttgctttt aaggtgcttt tcaatcctgc aaaacccaca atagcattca gtagcaaatc
133681 atcttcgcac aaacttaaaa tttcttctaa gccctcttgt cctacaaaaa ctttatcgtg
133741 ttttactaga tgcttatctt tagagttttt aatcgctaca aattcgggtt taaattgttc
133801 tatttgctca tttaaaagac ttatattttc cccgcaagca agagcagaaa tcttgatatt
133861 ttttaaagtg gcaagtttta gagcattaag ccctatactt cctgtacttc caaaaactat
133921 cataaaagag caaccatcac aaaagcagcg ataatcaccg catcaattct atctaaaatt
133981 ccaccatgtc ctggtataag atctccgcta tcctttacgc ccgcttctct tttaaaataa
134041 ctctcaagca aatcaccaat aactgcaaaa aatgctacaa aaaacgagca aagcaaagaa
134101 agccaaaaac tataaacaaa aattcctatc aaggtaccca aaatactcgc acaaaccagt
134161 ccacctatca cgccttctaa ggttttatta ggactggttt gtgaaaaggg agtttttccc

134221 aaaagcttgc ctataaaata ggctgcgcta tcgcaaattg caacaatagc aatcaaccaa
134281 aacaaagcaa acattccatc atctaaatac acctgccaaa gcgctaatac aggcaagctc
134341 ggataaagat aaatccaaac agctttttaag ctaggagctt ttttatacac aagataaccc
134401 aacaccaaaa gtgctgctaa agcaccaaaa gctaaaggaa tttcactaat ggttccaagt
134461 ataaaggcta aaaccaaagg gataatgctt acatcttcta aattaaataa ttttttttgct
134521 tcattaaaag ctagatacaa tataaacgca aaaacaataa aattgataat aaactgatcg
134581 attaaagcaa tgagtatcac tgctatagcc atcactacgc cccaaataat tcttgctgca
134641 ttaaacattt ttttccttat atgctgatat ctatatgatg aatactttgt gcatttttaa
134701 agcttgcttc ttctgtttgc tcttctaagt tttcatcctt atcatctttt tttgcttctt
134761 gtttttgttt tttctttttt tcctcttgtt cggctttttc tttaatctct tctttgattt
134821 cttgagtttc attgactttt tcaagcttgt ttaaggcttt ttctttttca ttaaaagctg
134881 ccatatttaa tgaagctgca aaaccttcct ttgcaagctc attacttgct tgagtagcag
134941 gataagccat attttgatta acaaaattca tatttcctat tgggctaact ggcattttta
135001 atcctttgtg atattgatac ttttaaaatt tgtatatttt acaaacgcct tttgctgaac
135061 cttaacaaaa ttctttaaag tataatcgac ccaatatgaa cctttcttta atcctgaaaa
135121 attcacgcaa agcctcgcac taaattctat cacttcttcg ctgatttttt gcttgtttga
135181 aataatcaaa acatgagagc ttggaatatc ccttacatgc aaccacaaat catctttttt
135241 tgcactcttt agcaaatttt cattgccttt ttcattttta cctacacaaa ttttaaactc
135301 attaaaataa aaattagcta tgccactatc ttgcttgttt tcttgatttt tcttagtgct
135361 ttttttaggc aataaaattt caagctcaaa ttcattttta gctttactta gcatttcttt
135421 taaaccctta gcaaagtcta gtttttcttt taaattttct ctttgtaaat ttaaatttct
135481 agccttttgt tctaattttt tagcattttt ataaaataaa ttagcacttt cttttggact
135541 taaatcaagt ttaaatttta cctttttttcc ttcaaaatca tcaagttcaa attctctttc
135601 atattctttt aaaatgctta aattagcaaa taaaatatcc gcttgctttc ttaaatttaa
135661 agcctgatcc aacaaaagct cttctttatc caaagcaaga taaagctctt ctaaattttc
135721 tattttttta tcaatttgag cgagtttaag tgtttgaatt tgtttgattt tatttgcatg
135781 taaaatttca aatttttgag taaaataatc attaaaattt tttatttctt caaatttttc
135841 atccatttta taaggcttta aagcctctaa aaccacatta ggcttaacaa cgcggtaact
135901 tttatcaata tgccttaaag cctctataat caaatctttt tcatctgtga taattacatt
135961 agtattttta cccgtaaatt caaaataaat cttactttca tagctcttat aagccttatc
136021 aaccctaaca ctaaaacata aaatacgatt gccttgaagc aatttcactt ctttgataaa
136081 agcattgtta aagtattttt ttagcatgaa atcaaaagga gcgttataat ttttactcat
136141 caattttgca gtataaatag cactcattcc acgagttaaa tcaaaaataa atctttgttt
136201 atcaaagctt agctctaaaa tattatcatt aacacgcttg ataaaatcga ttttttttaaa
136261 ctgggaaaaa aatttttgta attgcataag ttctgtgtat ttcataaaag agattataag
136321 atatttttga tgaatttatg atagaattga aataaaaaag gaaatttttg aaacttgaaa
136381 atttaaattg tgtgatttta tgtgggggta aatcaagtcg catgggagag gataaaagca
136441 aattgatttt taaaaatcaa accttaaccc aatttcaagt agaaaaattt tcaaaaatat
136501 ttaaaaatgt ttatgtgagt gctaaagagg acaaatttgg agcaaaattt aaactcataa
136561 aagattgtcc tgaatttgaa atttactccc ccatgcttgc gctttattct atactttcaa
136621 attttaaaga tgaatttgtt tttattttaa gcgtagatag tcctaatcta agcgataaag
136681 aacttttaaa aatgctagcc tttttagagc aagattatca aatcatcatt gccaaaaccc
136741 cctcacacaa acatccttta tgcggatttt atcacagctc tgtagcttat ctttgtaaag
136801 aattattaga aaaaaatgaa caaaaaatcg ctttattgtt ttctaaagcg aaaacccact
136861 ttgtggaatt tgaggatgaa actcctttct taaatcttaa tttttatcaa gaatatgagc
136921 aatttaaaag cgagtacgag tgaaaaaaat tctttttttg atattttttt atagtttagt
136981 attttgtgat gattttgaac aagctttaga atttgagaaa aaagggcaat atgaaaaagc
137041 tatgcaaatt tataaaaaac tagccttgca gaattcttct aaacaagaat tccaagaaaa
137101 taatgcttct ttaaattcaa acacaaagca cgaaatgcaa gctacccaaa aagcagctag
137161 cacaaaagag gatttttcgc gtatcgcttt ggaaaattat ttaggagagg attcaagctt
137221 taatcccctt ggaatcagct catataagat gaattatttt ctaccctttg cttatagctt
137281 tggctcatta ggcggggaaa atagaaaaac tgaaatgaaa tttcaactca gtattaaaaa
137341 aagattgttt gaagatttgt taggattggg tgaaaaatat tatgtaggct atactcaaac
137401 atcttggtgg caaaattata aacattcctc accctttaga gaaacaaatt atcagcctga

137461 attttttgta gatattcctt tgcattttga agagtataaa tttcttaaca atctaagagt
137521 gggaattttg catgagagca atggcaaagg agatgagaat ttagaatcac gctcttggaa
137581 tagaatttat gcttcaagcg tatttttata tcagcgtttt ttatttgttc ctagaatttg
137641 gtatagaatt cctgaaaaca gcgaagatga tgataatcct gaaatcacac attatatggg
137701 aaattttgat atcaatatgg gatatttagg aaatgattat tttatcaatc ttatgctaag
137761 aaataatctt gattttcatg ataataaagc tgcggtgcaa gtagatatag gatatgatat
137821 ttttgataat ggaatttatt ggtatttaca atacttcaat ggctatggag agagcctcat
137881 agactacaac aagcgcttac aaagactttc tacgggattt ttaatttcat attaaattta
137941 attttctttt ttataaatta aattaaaaca attgattgag attttacaaa tttttatagt
138001 tttttaaatt attttatttt aaaatacgat tttatttttg ataattatta ttactataag
138061 gaaaaaattg ctttttaaac acttgtttag tttaaatatt cttttaatct tacttgtcat
138121 ttttggaata ttaagcttat ttataggggt tattcgtatc aatttagacg atattttttc
138181 actcagcgct acccagcttg aaatcatttt gcttacaaga attccacgac ttatcgcaat
138241 tttactcaca ggtatgagtc ttagtatatg tgggctcatc atgcaacaac tcacacaaaa
138301 taaatttgtt tccccaacaa ctgcagggac tatggattgt gctaaatttg gtatattgat
138361 ttctttaata ttttttgcag gagcctcttt ttttacccaa gccctcatcg catctatttt
138421 cgcactttta ggctctttta tatttatcca aattttaaga aaaatcaaac tcaaagatgt
138481 gattttcgtg cctttgatag gcttaatgtt tggcgggatt ataaatgcta taaccacttt
138541 ttttgcctat gctttaaatt atatacaaaa tatccaaggt tggctacaag gcagcatggc
138601 aaatgttatg cagggaaatt atgaattgct ttatatttct ttaccgcttt ttatacttgc
138661 ttattttcta gctcataaaa tcactatagc aggtatggga gaagatcttg ctttaaatct
138721 tggggtttct tataatacaa tcttattttt aggtttaata atagtttcta ttatcactag
138781 tgtggtgatt gtaagtgttg gagttatacc ttttttaggt cttattatcc ctaatcttgt
138841 tgctatttat cgcggggata atcttaagaa aaatctcatc tatatagcat tatgtggggc
138901 tttgtttttg cttatttgcg atattatctc aaggcttgtg atttttcctt ttgaaatgcc
138961 attaagcata actgcgggtg ttttaggatc tttgatcttt atttttcttt tactcaaaag
139021 gaaaacttat gcgtaaaaaa atcttaatct tgagttttat aactcttata atggcacttg
139081 tttttatatt tgcgggttta aaagattttg atgaatacgc attaaaaaat cgctctttgc
139141 aaatcgcagc tattgttata gtggctattt gtatagctgt atcaactata atttttcaaa
139201 ctctttgcaa taacaaaatt ttaacccctg ctatcatagg cttagattct ttatatatgc
139261 ttcttcaaag cgcccttgtc ttttctttgg gttctgcaaa tttgagcgta tataaaaatg
139321 atattaattt tttaatcact ctagcttgta tggttgtatt ttctttaggg ctttataaaa
139381 ttctttttag tagcgacaaa agcatttatc ttatcatgct tttaggcttg atttttggaa
139441 cgctttttag caccttaagc tcttttttttg aaattcttat cgatcctgat gaatttatgg
139501 ttatccaagg aagaatgttt gcaagctttg ataatgtagc ctttgatgtt ttagctcttg
139561 cttatatcgt tactttactt agttttgttt ggatttttcg ttatatgaaa ttcttagatc
139621 ctttaagctt aggcaaggat ttatctatca atctagggat aaactatcaa aaaatttcaa
139681 aacaccttat gataataatt gctattttaa cctctattag cacagcgctt gtgggcccta
139741 tcaccttttt aggactttta gtagtaaata tcacttacga acttttcaaa acctctaaac
139801 atagtatttt actacttgct tgcgtgctca ttagcatttt agccttgctt gggggagtgt
139861 tttttgtctc tagggttttt gattacaata caactataag cgttattatc aattttctag
139921 gtggaatata ttttatctat cttgtattaa agggcaataa attatgataa gattacaaaa
139981 tatcactaaa tgctacgata aaaaagctat tatttcagat cttagcatcg attttcacaa
140041 aggaaaaatc acttctatta taggagcaaa tggagcagga aaatctactc ttttagccct
140101 tgcaagccgt ttaatcaaac ctgatagcgg gcgaatctat atcgatggta tggatttaac
140161 actatataaa gaacaagatc ttgcgcaaaa aatttccatt cttaaacaac aaaaccatat
140221 caatttaaga ttaaaagttg aagaattagt cgcatttgga cgctttccac actctcaagg
140281 aaggctcaat gcaaacgata aaataaaaat cgatgaagcg cttgagtata tgggacttaa
140341 ttctctaaga aatgaatttt tagacagctt aagcgggggg caaaaacaaa gagcttttat
140401 agcgatgatt atcgctcaag atactgaatt tatcatgttt gatgaacctc ttaacaatct
140461 tgatatgaaa cacagtgtgc aaatcatgca acttatgaaa aatttagtaa aagactttaa
140521 taaaagcata gctgttgtgc ttcacgatat caattttgct tcgatttatt cagatgaaat
140581 tatagcccta aaaaatggaa aacttttaaa acaggggtta aaagatgaaa ttatccacga
140641 agatgtttta agacaaattt atgatatgga tatccctgta agtcagattg atgggaaaaa

140701 aatttgtatt tatttctaaa taaaggagaa tatatgaaaa aatctttagt ttttgcattt
140761 tttgcatttt ttttaagtct aattttaacg gcttgcgatt caaaatctaa tgaaaataac
140821 gcaagcgccg caacaaaaac tagcactacg gtaaaagtta tacctattag catgagtgat
140881 gagggcgata gctttttggt aaaagatagt ctaggtgaaa acaaaattcc taaaaatcca
140941 tctaaggttg tgattttaga tcttggaatt ttagatactt ttcatgctct aagacttaac
141001 gataaggttg caggagttcc agctaaaaac ttgccaaaat acttacaaca atttaaagat
141061 aaacctagcg taggtggggt tcagcaagtt gattttgaag ctattaacgc tttaaaacct
141121 gatcttatta tcatttctgg acgccaaagc aaattctatg aaaaattaaa agaaatcgct
141181 ccaactatgt ttgtagggct tgacaatgcg aatttcttaa gctcttttga aaataatgtt
141241 ttaagcgttg caaaacttta tggcttagaa aaagaagctt ctgaaaaaat tgcagatatt
141301 aaaaatgaga tagaacaagc aaaaagcatg gtagatgaag acaaaagagc tcttattgtt
141361 ctaacaaatt ctaacaaaat ttctgctttt ggacctcaat ctcgctttgg aatcattcac
141421 gatgttttag gtatcaatgc tgtggatgaa aatgtaaaag tgggtacaca tggaaaaagc
141481 attaattctg aatttatact agaaaaaaat cctgattatc tatttgtagt cgatagaaat
141541 atcatcgtag gcaacaaaga acgcgcacaa ggtatacttg ataatgcact tgtagctaaa
141601 actaacgctg ctacaaataa taaaatcatt tatcttgatc ctgaatactg gtatctagca
141661 agtggcaatg gattagaatc actaaaaacc atgattttag aagtaacaaa cgccattaaa
141721 taaactattt gtaagaggat aaacttctct tacaccctaa ctaaatccaa ccaacaataa
141781 caaatattaa aataaattta agtttatatc tactataaaa aattcacttt cgcttcacat
141841 tcttttgtca aaataccata caaattgata atcaaaaagg agagaaaatg attaacaaaa
141901 acaaattatt tagacaaatt cacatttatg tgagtttatt tttcttaccc tgtgctgtac
141961 tttttgcact tacagggatt gtttatatct ttggagccga tcaagataca ggcttaaagg
142021 tagaaaatta taaacttgta aaaaatatag aagttggtaa agaaaaagat gcactcatcc
142081 aatacctaca agaaaacaat ttaaaaattc catctaatac tgaagttatt gaaagcaaag
142141 ataagggtat aaccataggc ggagcacatt atagtgcaaa tatagctctt gttgctcaaa
142201 acgagtatgc aataactctt aaaactagat ctttactagg cgatatgatc atgcttcata
142261 aagataaagg tgcttggtat ttttctgtgc ttagcatagg ttttggtatt gctttatttt
142321 tgctctatat ttcaggtctt ataattactt tatttgctag caaaaaagat agacaaaaac
142381 agctcgcaac gcttggagct ggggttgtta tcactatatt tttagcttat cttagtcttt
142441 aatttttttа tctagctttt ttaagctagg tattatctaa ttttttatat aatcacactt
142501 taatttattg gacagatggg tgagcggctg aaaccacacc cctgctaagg gtgcagatct
142561 taatcgggtc tcgagggttc aaatccctct ctgtccgcca ctgactttca aaaatttata
142621 atcccatgat ttaaacacag tttcaaatca atgatcttaa tattttaagc gatattttta
142681 atctttaata ttactttaca taaaaatatc caaaaaatta aattttatcc ctactttaat
142741 cagttaaaca atatagcatt ttaaaattat aaaaactctt tatttgagcg tatttaattt
142801 tatattttac aagattaagc tatttatttc tgattttaaa atgctaaaaa atacattaaa
142861 aatataagct taaattatct attttacata ctcttatctt ttttataaag cttatacatg
142921 ataatgcttt ataatctaaa gccaaattgt agattaaaat tttactttta taaatatcgc
142981 atcacttaaa cacagcgcat aaaaaaatat aaaaataaaa aatatagaat ttatttgaag
143041 gatataaaaa gccctaaaat atagggcttt taagaattat tttttatcaa taacttgata
143101 attatacgga gcaaggtttt tatcgtgttc taaaagttct tttggaggat tgccatttac
143161 ttctatttca tcaccataat aacgctcccc tgctttatgt ccattaacat ctactttata
143221 ataaagctct ccaggattga atttttgtat atcttcaaaa cctaaatttg aagttttaaa
143281 atcattaatg ccattagcat tagcaatttc tataagctta atttgtcctg ttttagccat
143341 atctacagct tgtaaaagca ttcttgaagc ctcacgagga ttgtggaaac ctgttgaatt
143401 ttcagcatta acaaaatcag ctctcatttg agatttgcgg tgaagctcta aaacttcttt
143461 aagttcttca ctgatcttgc ttgcatcagc tttaccatcg gtttggaatt tctccatatt
143521 tcctagctca tcgcgtaatt ttttagtatc catgattaag ctaacgatag catattcagc
143581 tgttctttga tcgtgtgcta cgctattttg tatatcacga acttgagctc ttaaataatc
143641 ctcgctttgt ttatggcaag ttttacaagc agaattaata tctcttaatg gagaagtgat
143701 attgtgttga gaaattttct tagcaccttc tctaacataa ggcatatgac aatcagcaca
143761 acttactcca ttagcagcat gtacaccacc actataaagc tcactttcag ggtgttgaat
143821 tttaatgatt ggagcacctg ttaatttatg agtgaaatct gcaccaaata cattgcgaac
143881 tttttcataa taatcatcta gcatttcaat tctaaaaggc tgaccttttt tccattcact

143941 ccatgggaat gttagcatga taccattagc ttcaatttct tttggcttgt tgccatctct
144001 ccaaaattca tattcatcat aattcttttg agtaccatcc caccattttt tagaactatc
144061 atcaacaatg gtttcaccca ttacttttac tttttctcct gttggtttaa aataatactc
144121 aacatggcat tgtgaacaaa ctaaagttct catctcttct cttgtagctt ttacaccttg
144181 tactggatct ttttcatagc ctctagcaac taaagcatta attgcagcag gacgagtaag
144241 tcttaaactc atatcatttg ggttatgaca atctgcacaa gtaacaccca ttcttttacc
144301 accatgcggt cctgcatgct ctggagaatt ttctactata ccatcaacag ctggaacatt
144361 tttaatcata gtccaataat ttgtagagtt aaatgcgaca aaatctccct ttgcaatatt
144421 attaataagc catggagtcc atccactatg acaattcata caagctgctg gctgaccctt
144481 aaatgcttta agtccgtgag cattaagaaa atctttatta tttcttgcgg ttttcatttg
144541 atctacttga acccaaaaat gccctctttc ttcgttaaaa tcaagactga aaggatatcc
144601 ggcccaaagt atagttaatt gaggaaaacg aataagtttt gaataagata aattaccccc
144661 aaattcagta gccttaggtt cttcttcttc aactgttaaa tacattttaa gttgttctgg
144721 aaaaactttc ccccatcttt caaaatttgg attatcatcg ctaagtaaag aaaaatccgc
144781 atttgcagca atggcatttt tatttgcctc gtcttctttt ttttgattta tatcattatt
144841 gagccataaa actcccacta taagcaaaac taaaacaacg atagctaagc gtaaaaaatt
144901 ttttttcatt caatttctcc ttaattaaat tccatgtttg tgacccacat cttgatgaca
144961 agaagcacat tttaaagatt tgtctgcgtg tggatttgtt gtagcattga tggctgtttg
145021 agcaaaatca gcatgacagc gaacacaatt atcttgtatc atttttctgc ttttctcagt
145081 cgcgctcaaa tttgtaggaa gctcatcgag tttaaaagta aaagcataag catgatttaa
145141 accactctct gctttagcta tccacttagc caaaaaacca tggggcaagt gacaatcaac
145201 acaagttgcc ctagcctctc cttttacctt ttgtgcatgc ggtcctgcca tatattcatt
145261 ataaacctca ttcataatat ggcaattatt acaagattca cttgcattac ttaagtaaga
145321 tgtacctttt gcattataaa aggtgtaaaa tcctaccgca aagaacacaa aaagcaaaac
145381 aagaaaaata ccaaaaagat tcaaagattt tttcaaaatt ttcctcctta aactttcaga
145441 ataaatgaaa ttgtgatttt aacatatagc tttttaagat aaatttaaag atatatttac
145501 tcttaattaa ccagaataaa aattttgaat tttatttcta taaaaaactc attattttca
145561 aaaataaatg ttaaaataac accaaaaaaa ggagttttaa tgcaaacaaa tccgaatatg
145621 tttttaaata gagaactttc ttggctacgc tttaactcac gggtactaga tcaatgttcg
145681 cgtccattgc ctttattaga acgccttaaa tttgtagcta tatattgtac taatttggat
145741 gaattttata tgatacgcgt tgcaggatta aaacaacttt tctcagctgg agttaatata
145801 agcagcagtg atgagatgtc tcctttacaa caattaaaag ctatacgcaa atatcttcac
145861 aaagaaaaag atctgttaga gcattatttt aatgaaatta taagtgatct tgaaaaagaa
145921 aatcttttta ttaaaagcta tgaaaatcta gacaacaacc taaaacaaaa atgtgatgaa
145981 tatttttttct ctactatctt tcctgttatc gttccaatcg ctgtagatgc aactcatcct
146041 tttccgcatt taaacaatct ttcattttct ttagcggtta aaatttgcga taaaacccat
146101 cctgagctta tcaaatttgg aatgattcga attccaagag ttttacctcg tttttatgaa
146161 gtgagtgcaa atgtttatgt tccaattgaa agcatagtgg aaaaacacac agaagaaatt
146221 tttcctggct ataaactcct tgcttcagct gcttttagag taactagaaa tgcggatatg
146281 gtgatagaag aggaagaagc tgatgatttt atgatgatac tagagcaagg cttaaaactt
146341 cgtagaaagg gtgcttttgt aagattgcaa attcaaaagg gcgcagatga acaaatcgta
146401 gaatttctta atacccatat gaaaattttc cacaaggatg tttatgagta ttctatctta
146461 ctaaatcttc ctagcctttg gcaaattgct ggcaataaaa cctttactca tcttttaagc
146521 ccgctttatg cacccaaaac cttgccacct tttgatgaaa atttatccat ctttgatgcg
146581 atagataaag aagatatact catcattcaa ccttttgaaa gctttgatcc tgtttataaa
146641 ttcatcaaag aagcaagcaa ggatcctgaa gttatttcca taagaatgac tctttataga
146701 gtagagaaaa actctaatat agtccaagct ttaatcgacg cagcgagcga tgggatacaa
146761 gttacagtta tggtagaact taaagcccgc tttgatgagg aaaacaatct tcactgggca
146821 aaagctttag aaaatgctgg agctcatgtg atttatggaa tcactggctt taaagttcat
146881 gccaaagttt ctcaagttat acgcaaaaaa ggcgataagc ttaaatttta tatgcattta
146941 agcacgggaa attataatgc aagcagtgct aaaatctaca ccgatgtaag ttattttacg
147001 agcaaagttg aatttgcaag ggatacaaca agctttttc acatactttc aggatttagt
147061 aaaaatcgtc gccttcaaac cctctctatg agtccaaatc aaatcaagga aaaaattcta
147121 gaaatgatag ctttagaggc aagtaagggg agcgaaggag ttatcattgc aaaaatgaat

147181 tctcttgtag atagcgatat cataaaagct ctttatggag ctagcataaa aggaactcaa
147241 atcgatctta tcgttcgtgg aatttgctgt ttgaaaccta atgaagaatt cagcaaaaac
147301 atacgcgtta gaagtattat aggaaaatat ttagaacatg ctagagtgtt ttattttaaa
147361 cacagcgagc caaattattt tatatcaagt gcggattgga tgccaagaaa tttagagcgt
147421 cgtttagaac ttatgactcc tatatatgat gaaagaagca aggcaaaatt agcacaattc
147481 ttacgcttgc aactcagcga taatttactt gcttacgagc ttcaaaatga tggagaatat
147541 acaaaagttg ctagcaatga aaaagtgatt gactcacaac aaattttaga agaatatgtc
147601 agtaaaatct ataaaactct taaaaaagat gctgatcaaa gtcgcgcaac ttatctagcc
147661 tctaagcttt ttaaagaaaa ctgataaaat ggtggaagcg aggggaattg aacccctgtc
147721 caaaaataac cccacaaaga cttctacatg tttagcaaag gtgaaaactt catctagcta
147781 aacccacctt ccaaaatttc aggctagact aagactttaa cttcgattaa aagtagtagg
147841 aatataaagt ccgtgtttca tatattgaaa atcacctgct attaaaacat cgcccagacc
147901 atatttagga cttaataatc tatgcatgtg aaaatttacc gtcccaaaat ctgtctcaag
147961 acttactact tgacctacta attttgtttc attacctaaa attcttgtag caaatttatt
148021 aatagctcct tttagatctg ctcctaaaaa tacatcttta ggcgtaactc ctgcattcca
148081 aatattttgt aagatttgat tgagtttatc ttctgtaagt tcagttgcag ttccgctcca
148141 gtctcccgct tcatcaaaag ctagtacatt tccacgcttt ccatctgcaa atctactatt
148201 ccctttggct acatagtgaa aaatccctgc catttctcca cttgttgctt cttgtgcttg
148261 aatataatct ttaaaaaccg atttttttac atcattatct ctgcccaaac caaataaagc
148321 atattccata tccattttat gctctttggt ttttttgccg atttgatatt ccatctcatt
148381 gccgccatac tgattggctt ttagtaaggc ttttgacacc ataacttctg tgataaaaat
148441 ttgagtagca ttggtggttt tttgagcagt gtttttttgtt tcccctacaa attggctcaa
148501 ttctaaattt gcgttctttt ttggttcttc aaaagtgtct gtaagccaac tatgggttaa
148561 aggatttgta acttttgaag tgcctatttt atttaaaatt ggtgtttcag tagctccaat
148621 tttaataatc gtttcatata ctgattgttt taatttaaca ttttccgttg cgggtgctgt
148681 atgtcccatt gaaggtaaag ccattttga attctcctta gtttattttt aaggattttt
148741 ccaaaaatgg ctatttcaaa aatagtgtgt tttgaaatga ttttaaataa aaaatttatt
148801 tatatgacgt ttaaaataga gcatatcatc aactattgta aattttttaa gaaaagagta
148861 aacatctctc tttatagata tatcaaagct ttcacagagg ttttccatat ttaaatttgt
148921 ttcattgcta tgttttctat ttgcttcttg tagctcataa taaacccatg gagaatatat
148981 ggtattgtta ttgcaatgtt ctttgctatt aataaatata aaatacctag cagattctat
149041 aatatttctt agcgaggatt gtaacatgat atgtaaatta gcaagtaaat attcttcatt
149101 atcggcttta agttcttctt taatatagct taatattttc ctataattac cccaaacata
149161 agaatctaca aaagcattaa ctttgcattc ttcctccaaa aatttcttaa gttgtattgc
149221 cttgtctttt tcttgactaa aatgtgatat aaaaacatca tattttttt ctgttccaaa
149281 aaaatattct tgtagtttat cgccatcaat agtgttatta tcgctaaata tttttattgc
149341 tccatagtta taattatttg aactttcgcg gtcgtgttta tctaaaaatc gatcgtattc
149401 agattctgta atgtttagtt catcaatcaa aaaacattgc atattttact ccttatttta
149461 atgcctgatt atattataat actaattaat attaagtaaa atataaaaaa tattacaagg
149521 atagatttat ggaaaaagaa aaaatactta ttgaagagct aaaaatgata caagatatca
149581 ttaaaagaat ggctgataat tcttttaagc ttaaaaaatg gtgcttaacg gtagtagtag
149641 ttactatgct ttttcaagaa ggggttaata attttatacc tataatctca ctttttggtt
149701 tttggtttct agattcatat tatttatata tagagagaga atttagaaac ttgtataata
149761 caaaagcaag acaatttaac aataatgcca atttgaaaca atctatgcag agtcatttta
149821 tttttgagat tgatggaaag tttacattga aaaaattatt tattgttgga ttttctaaga
149881 ctatattata tttttatgga gtgattttaa ttttgatatt acttgttaag tataaaaaaa
149941 atgtttttaa ttttgttgat aatttaatca tttccattgg tgctattaac acttaaagtg
150001 tcagcattaa acatagtttc agtgcgtatc agctgagcta ggggcgataa tattaataaa
150061 aattaatcac ctacctttta tcatgcttaa ttaaaatatt acatggattt atgctgcaat
150121 taataaaata gttaaaaggg agacaatcat cccttttttg taaaatcacc agcgtatgct
150181 gtcttagtga ttttctattt cttttcaaaa gcagttacta tccactgatt ttccccattt
150241 ttattaaatc ctttattaag ccctaaaata taatctttga aaactatatt gaaagatttt
150301 tctctatttt ttcgttgtgc tatctctccc ttctcaataa tctcaggtat tatatttaaa
150361 tccaattcag gatgctcatc gataatatga gataaaccat aacctttatg ttttacctta

150421 tctattactt ctccccaaac caaatcaata tctcctaaat ctttcctatg aaaggcacct
150481 gctacctgtc cttgtttttc aataagtaat ttttgtaaag cacctttttcc atcatgataa
150541 tactctgtat aattttcacc aaattctttt aaaggctgta tatttaattc ttgttcgatt
150601 ttacctctta aagcacttgg aatatctttt tttatccctt tatcggtgct ttctttagca
150661 ttgattatca tctgtctagt taggttgtat tcaacagtat ttaaattcat cttatctaga
150721 aaatcaagct tgttattttt atttctttt agaaaatcat catatctttt tagaatatct
150781 tcactagctt ttttatcatc ttgtattttt tcatcaagtt tttgctttat actttttgca
150841 ttttaaattg gccttgttca ttaaaaagta tcttctttga tcaaatcttt aactttagaa
150901 cttttctaac ttcaactccc tgttttattt tatctattaa attgatttta aattttgctt
150961 gaatggaatt ttaaagaata atatttgctc tatctttgaa gatatctaac tataagactt
151021 tatttgatgc tatttgattt atttgcgaat tggtattttc tatacctaat aaattatgat
151081 tttttatcct tataaaagta gtttatatca tttttaatct ttatttatat taaatcactt
151141 tttttgattt ttatttcata tttcaattta aaccattgtt acttatttgt aaaataggaa
151201 atttaatata ttaaaatcac caccaattga tatcttatag ccatttatgt cttcgccaaa
151261 aattaaaaaa tatcaaaaaa attaaaaagc aatgtaatgg tggaagcgag gggaattgaa
151321 cccctgtcca aaaataaccc cacaaagact tctacatgtt tagcaaaggt gaaaacttca
151381 tctagctaaa ctcaccttcc aaaatttcag gctagactaa gactttaact tcgattaaaa
151441 gttgtcaatt ttcaatctac actatcaaag atgaccaaaa ttcaagctag atagtatcac
151501 ttgagtttcg ggctcaactg aacttacgca gctttagcgt aagcaggagc aaatttaacg
151561 ttatttgcgt ttaattttaa tttggacttt ttacgctttg tccaaagcga catgccatct
151621 aagcagactt actcctgtcg aagccaagtc gctcccttat ttagaaataa tatttggaac
151681 attaacaatt aaaggattaa aaacggctaa aacagaatca tcattttcat acgaacggca
151741 atatttttca aactgatcgc tcatcaaaag catccagtct gtaaattcat cgctagctgg
151801 cccattaaaa cgatttgctt gagccatcat ctcttcacaa aatacacaaa aatcagttac
151861 ttcttcaaga tttaaacgcc ttgctgccca agcagtatta tgcgccaaag tttcgagttc
151921 ttttatagct tctttataac gcacgctatc gcttccaagc tgtatgatta aaggttcaaa
151981 tctatcgcac attgttctaa aaaattgtaa aaattttttct atgtcgtcga tttcgtaatc
152041 taattctaat tttgttaaaa ttcccatttt taaaccgtat tgctaaagtt taatgcaaat
152101 tgtagcaaat tttagctaaa atcttatttt aattttccaa agtgtaagaa tgaaaatatg
152161 gacagaatag tagaaataga aaaatattct tttgatgaaa cttatgaaac ttctttgcgt
152221 ccttcaaatt tcgatggcta catagggcaa gaaaatatta aaaagaattt aaatgttttt
152281 attagcgctg caaaaaaaag aaatgaatgt ttagatcata tactttttag tgggcctgcg
152341 ggacttggca aaaccacatt ggctaatatt atttcttatg aaatgggtgc aaatatcaaa
152401 acaaccgccg cacctatgat agaaaaaagc ggagatttgg ctgcaatttt aaccaattta
152461 agcgaaggag atgtgctttt tatcgatgaa attcatcgct taagccctgc tattgaagaa
152521 gtactttacc ctgcaatgga agattatagg ctagatatca tcatcggtag tggccctgcg
152581 gcacaaacta taaaaatcga tttgcctaaa tttacactta ttggtgctac aacgcgtgca
152641 ggaatgctta gtaatccttt aagagatcgt tttggaatgc aatttagact tgaattttat
152701 aaagatgaag agcttgctat catacttcaa aaagctgcac taaagctcaa taaaacctgc
152761 gaaaatgaag ctgcgcttga aattgctaaa agaagtcgct ctacccctag aatcgcactt
152821 agacttttaa aaagagtgag ggattttgca gatgttaatg acgaagaaac gatcaccaaa
152881 gaaagagcca aagaagctct aaattcctta ggtgtaaatg agcttggttt tgatgcaatg
152941 gatttaaggt atttagaact tctaacagaa gctaaaagaa aacctatagg gctttctagc
153001 atagcagcag ctttgagcga agatgaaaat actattgaag atgtaataga gccttatttg
153061 cttgcaaatg gctacataga acgcactgcc aaaggtcgta tagcaagcac aaaaagcttt
153121 agcgtgctca aacttaatta tgaacaaact ttatttgatg aaaattgaag gaaaaaaatg
153181 caaactggaa aaatttttct catctgtttt actttagtag ttttatctct tttgctttat
153241 ctttttaaag atttcttact cgtcattatt atcgcaagct tgatggcagt tgctacctca
153301 aatgcaaacg ctaaattctt aagtctgaca aatggacgca aatttttagc atctatgcta
153361 acaactgcat gtatggtttt gcttttcttc gctccctttg tctatgcaat gatcgaacta
153421 gctaaggctt taaaaaactt tgatatcaat ctcgtcacac aaacgctcaa ttatgttaaa
153481 aattatcaat ttaccctacc tgaaagtctt agctttttag aacctaaaat aaaagaattt
153541 ctagcttcta tagacttaaa tagtatctct aagcaaattt taaattatgc ttcaagtttt
153601 acaaaatcag gagctaaatt tcttatagat atggtgctaa tctgtatatt ttatttttt

153661 gccaatctat atggaacaga actggtaatc tatataaaat ctgtcattcc tattgacaaa
153721 caagagcttg atgatatttt aagcgaagtg ggtaatgtta tggctgtggt gctttattct
153781 atgatcatta tcgccatact tcaaggagca ctttttagta tcattgctat gttttacgga
153841 tacgatgcta tcttaatggg tgtaattttt gccgtaagct ctctcatacc agctctaggt
153901 ggagctttgg tatatatccc tataagtctt tatgaatttg cttcaagtgg tcttggttca
153961 gcacttgtta tttttattta ttctgttgtt gttatctctt ttatcgctga tactctagtt
154021 aaacctttaa tcattaaatg gattaatgaa aaactcgtaa aaacaccgac taaaatcaat
154081 gagcttttga ttttcttagc gatgatagct ggaatttcaa catttggttt ttggggtatt
154141 attttagggc ctgcaatttt aactttttttt atttcaacaa taagacttta tataatttta
154201 aaagataaga atttaattta aaataaaaaa tgtttctctc acacaagaga gaaacatcaa
154261 gctagactta agcttttggt cctatcattt tagcaggatc aacaaattta gcaaagtctt
154321 cttcactaac caaacctaac tccatagcac tttctttcaa agaaatacct tttttatgtg
154381 catttttagc taccttagca gcattttcgt atccaatatg tggatttaat gcagtaacta
154441 acattaaaga attatgtaga ttgtattcta ttttttcacg atttggttct atgcctacag
154501 cacaatgaat attgaatgaa tgcatagaat cagcaagcaa atcaagactt tgtaagaagt
154561 tatagataat cacgggctta aatacattga gctcgaaatt tccttgactt gcagcaaaac
154621 ctatagcagc atcatttccc ataacttgaa ccgctaccat agtaatagct tcacattgtg
154681 taggattaac cttacctggc atgatggagc ttcctggttc attttcaggg atattaagct
154741 caccaagtcc gcatctaggg cctgaagcta accatctgat atcatttgct attttcatta
154801 aatttgcggc caagcccttc atcgctccat gggtaaaatt gatagcgtca tggcttgtta
154861 aagcgtggaa tttgtttgga cttgaaacaa attttgtacc tataagcttg ctaagctcct
154921 cactcacttt ttcgctaagc tctggatgtg cattcagtcc tgttcctacc gctgttccgc
154981 ctattgccaa ttctcttaaa gttggtaaag aagtgataat ttgctctttt gagtgtaaaa
155041 gcatagaaag atagccactg aattcttgtg ctaaagtaag cggtgtagca tcttgtaaat
155101 gtgtgcgtcc tattttgata attccttcaa attcttttac cttttttttca aaagtagcga
155161 taagttcatc aagcgcagga ataagctttt tctctacttg ctctactgca actatactca
155221 tagcagttgg gaaagtatcg ttagaacttt gagacatatt tacatgatcg tttggatgta
155281 caagtttttc tttacgaaaa tctccaccca taatttcagt tgcacgattt gcaatcactt
155341 cattcatatt catattgctt tgtgtacctg atcctgtttg ccaaattgcg agtgggaagt
155401 tatcatcaaa tttacctgcg ataatttcat cgcaagcttg aactatagca cttttttttag
155461 catcatctag cttgccaagc ttgttattta ccaaagccaa agattttttta agatttgcaa
155521 aagcatagat tagcaccttt ggcatctttt cgcaaccaat tttgaaattt tcaaaacttc
155581 tctctgtttg agcaccccaa tatttgtcat ttggaacttt aatttcgccc atagtatcat
155641 gttctattct gtattccatt aggtttcctt tctagaaaat tgttatgcaa ttatttaaac
155701 aaaaataagc ataaaattag cttataattt aaaatttatt tattttttta gaatttatga
155761 gagtttatgt gcagttttat aactgcacgg ggtttattca acggttacac tttttgctaa
155821 atttcttggc atatctacat cattgccaag tcttatagaa atttccattg ctagaagttg
155881 agtgattacc atcatttcaa aaaattcaca catataatga tcttgctcat tggtttttat
155941 aaaatcatcg cttaaatcaa aatccaaagg agaaacacaa agaacagtag aatctctagc
156001 tatcaactct tcaacatttg atttggtttt ttcataaagc agggttttag gcattaaagc
156061 tatggtataa agtttagaat cagccaaagc tataggtccg tgcttcattt ctcctgcagg
156121 ataaccctca gcatgcaaat aagaaagttc ttttaatttt aaagctcctt ctaaagccaa
156181 aggatagaaa acatctctac ctataaagaa aaatccatgg ccatctaaat aacgcttaga
156241 taaacgatga atttttttcat gcaatttagt acttactgca acacaattag gagtgtgcaa
156301 aagagctttg atttcggcac tcatatcgat ttttttcttt tgagcaataa aaattgccaa
156361 catccaaagt gttaaaacct gagttgcaaa agccttggtt gaagctacac ctttttcaat
156421 gcctgcacgc gttaaaaggc ttaaatgtgc caaacggact atattagaat tatccacatt
156481 gcagatggca aatgttttag ctccttgctc ttttgcaatt tttaaagctt ctaaagtatc
156541 tgcagtttcc ccgctttgag aaattacgat aaataaagaa ttaggcttta caatcgcttc
156601 tcggtagcga aattcactag caatttctac cttagcttta accttagcta aacgctcaaa
156661 caaatacgcg cttgccatag cagcatgata gctagttcca caagcacaaa gcgtgatttc
156721 atcaactgta ctcaaattct cattttcaag ctcatcaaaa accacttcat cgccattaat
156781 gcgtcccatc aaaacctcgc tcattacgcg gctttgttca taaatttctt tttccataaa
156841 aaatcgaaag ccatcttttt ttgcataagc tttatcgcct gaaagcttgg taaaggaatt

156901 ttgttttagt tgattgtttt cataaataac aagttcattt tcactagcat aacccaaact
156961 taaatcctca agataaatca cctcatcaca aagtcctatc aagggtgtat cagcgcttgc
157021 aaaataaaat tcacgatttt tagattttcc tacgattaaa ggagcggcat ttttagcaaa
157081 aaatacacga gtaggatctt ttttactcac taaaagtgtg gcaaaagcac ctctaagctc
157141 ttctatcgtt tttttccaag cttcaaaaac ttccattttt tctgcataaa attcaaaaag
157201 ttgaactata acttctgtat ctgtttgact tagaaaactc actccttgac tttcaagctt
157261 atttttaatc tctttataat tttctataat accattatga atcacacaag aatactgacc
157321 taaatgagga tgagcattga tttcagtagg ttttccatgg gtcgcccagc gtgtatgccc
157381 tatagcaaaa ccaaattctt cacttgtaaa atttgcacat ttattggcca aattttcaag
157441 cttacctacg gctttgaaaa aatcaagctc tccatttttc atcaccgcta aacccgcact
157501 atcataaccg cgatattcaa gttctttaag cccacttaaa atagtatgtt ttttttcatt
157561 ttttcctata taacctacaa ttccgcacat tgtttactca cttattaaag aatttaatag
157621 tttttcttca tttttctcta aattgtcatt tttttgaaaa ataaaggtat tacgcgttct
157681 ttggcgtatg gtttgaattt ttgctgcaga taaaaccaaa cccaactcgt taaaaatatt
157741 catcatatat gccattaaac cttgttgatc ttttgtattc aagctaagtt ttgcatagat
157801 tttagaataa tttaaatcca attttaactc atcttttttta atgattggct ttttgatctt
157861 ttttgaagaa aaaccgctta aatttgaatt taaaagttcg caaagtttga tttttttgctc
157921 gtctgtaatg atattatcat actcaaatct taaataaatt ttatcatcaa ataactcaaa
157981 taaattcata aaaaccaaat ttaaatttgc taaagcagtt aaaacaattt ctattttaaa
158041 atccttaaaa gcaacgattt gaaggcttaa attggtacta ttgctaaacc aaaatttaaa
158101 atcattttcc tttgcaaatc gtgcaatttc aacaatatct tcaaaagtat ttttaatgat
158161 aaaaagattg gattttatat gagtgatttt atcctgtaaa atggcatttt gatctaagaa
158221 aagctttgtt ctcttaagtg ttaattcttt ttttacgcgc cttgcagttt catcgagtaa
158281 attttcatca tcaaaacctt gtttggcatt ttctaaaatt ctatctatca ccttataaaa
158341 aaatggatta cgatttaaag cttttgcttt taaccaagta agagtatgca agagctctaa
158401 ggtttttaaa ttttcaacct tagaaagcaa agcacatata atgataggat tataaacatc
158461 ttctttttca gcaagctctt ttaaggcatt gtgattttta aaaattctaa gcccaagttc
158521 aaaaatatca ttttttaaat taaatttaac acaataagct ctatatatac tcgctaaaga
158581 aatttcattt tcattattaa tagcacttaa aagtataact aactttaaaa tcattttctc
158641 atctgcattt aaatttttca aactctcaag ctcgtcttcg tatttttcaa attcttttaa
158701 aagcaaaaat gcttgctcat caaaactata atccccctcc tcatcgctta aaaaacgcac
158761 cataccaaaa ggcttacata attcttttaa aattccacta tcaagcaaaa gttttaagat
158821 gcaaaaacta tgtcttttaa aaaatatttt ttcaaaaagg gctaaagctt tctctaaatc
158881 ctttttttcca taacttaaat ttttaagagc aaaaaccaaa tctaaatcaa aaacaatatc
158941 ttgatcttct aaatccaaca aaaaagtcaa tgcttctgct aaatttttaa attggtattg
159001 tttttgcgta agattttgca attcattttg aattttataa gctaaaaaat gagtataaaa
159061 acctatagta tgcatacttt gcaaggcttt ttgcactaag attttttcag catctagatt
159121 tttcttgccc tttctataca tcaattctgc aatctcgtga atattgctaa ataaaaattc
159181 atcttgatct ttattgcttt gcaaatttat agcagatttt aaggacaata aaaaatcagc
159241 cgctaagcgc aactcgctta aattttttttc atctataaaa tttaaagcat aatttttagg
159301 ggaattttta aaaagaatca acaaactttc caaggctcgt aagtgatcta agcctccaaa
159361 atcttttttt atgttaaatt cttgcttgat aaaaggaagg ttaaaatctt taaaatttgc
159421 aagtaaaatt tgagcaaatt cgtttttatt tgcacgaagt atgctatcaa atttttcctt
159481 gactgattta aataaaattt tagatccgca tataaatcga gcacctatta tggttgtttt
159541 aagttcatta ctggcattat aaagcccatt tagctctaaa accacataat caatttgcaa
159601 atgcacatca tttaatattt ctataaaagc tttaatcata ggttttaaat gataagcttt
159661 gatatcctta taaacaaaaa ttaaagaaat gctttcattt gcacaaagat tatttttttgc
159721 atatccttta cttgccaaaa cacaaaaagg tatattatcg cttgcaggca aaaaattttc
159781 aaaacattct tttaatacta tatcataagt ttgctttaaa aaattatcca tatttttcga
159841 atgataaaga ctaaatgatc cttgttttaa aaaaaatttt ctatagtttt gtgcataggt
159901 attgagattt ttcttaaaaa gttctaattc tttatttaac aaatctttac tctcttttaa
159961 aattttattt ataagatttt aacaaaaata tcttttattt tggctttgct ataaactctt
160021 tttcagtaag caaaatttat aatgtttttt taaaaattct aaaattttat agaagtgaaa
160081 atcaatgaaa aatctacttg aaaaactaga aaatgaagaa agattaaacg aaaatgaggc

160141 aaatgctcta tgggatttag atctttttac cttagctaaa tttgctcaca aaaaacgcac
160201 taagcttcat ggaaaaaagg tgtattttaa tataaatcgt catataaatc ctacaaatat
160261 ttgtgcggat acatgcaaat tttgcgcctt ttcggcacac cgaaaaaatc caaatcctta
160321 tcttatgagt catgaagaaa ttatgaaaat agttgatgat actatcactc gtggcactaa
160381 agaagtgcat attgtttcag cacacaataa agacacaagt tggcagtggt atttagaaat
160441 ttttaaaatg attaaagaaa aatacccca aatgcatata aaagccatga cagcagcaga
160501 aattgacttt ttacatcgcc gttttaaaat gagctatgaa gaagtgatag aaaaaatgct
160561 tgaatatggc gtggattcta tgcctggagg tggagcagaa atttttgatg aagaaatacg
160621 caaaaaaatc tgtcatggaa aagtaagcag tgaaaattgg ctcaaaattc acaaactttg
160681 gcatgataaa ggcagacaaa gcaatgctac tatgcttttt ggacatatcg aagaaaggca
160741 ccataggatc gatcatatgt taagacttcg tgctttacaa gatgaaagtg gaggttttaa
160801 cgcatttatt cctttggttt ggcaaagaga taatagctat atacaaacag ataagattat
160861 ggacagtgaa gaaattttaa aaaccatagc tatttcccgt ttggtgcttg ataatatcaa
160921 aaatattaaa gcttactggg cgaccatgac tttaaattta gctatggttg cacaagaatt
160981 tggcgcaaac gatctagatg gcaccataga aaaagaaagt atacaaagcg caggaggagc
161041 aaaatctgct aagggtacta acctaaaaac ctttatcgaa atgataaaaa cagcaaattt
161101 aactcctgta gaacgcgata gtttatataa tgaactacaa acctactaag gattaaaaaa
161161 tggatttttt taaactcaaa gaaaatggca ctaatgttcg aactgaagtt gtagcggggc
161221 taacaacctt tttagctatg gtttatatca ttcctgtaaa ttcaagtata gtaggtaata
161281 caggcatgcc tatagaagct ttaattaccg caactgcttt aatcaccatt gtcgcgacta
161341 cttttaatgc ctttttttgct aatactccag tagccatgag tgttggaatg ggtttaaatg
161401 cttattttac ttttgcagtt tgcttgggac aaaaaatacc atggcaaagc gccttaggag
161461 cagtatttat ctcaagtgtt atttttctaa ttctttcttt tacatccttt cgtctttggg
161521 ttatacgcaa tatacctaaa gatttacgct tagccatttg cgcagggatt ggatgtttta
161581 tcgctttttt aggtttaagc caaatgggca ttataagctc taataaagat actttagtca
161641 gtataggaaa ttttaaaaat ccccatgtac tttttggaat ttttactcta attttaatta
161701 tattttttttg ggctataaaa ctaagaggag cttttatttt aggtgtttta ataagctcta
161761 ttatagcttg gatttttggg attgataatg caagctttcc tgagcaaatc ttttccttgc
161821 ctaattttag taatgaaaat ggcctaggag ctattttttt agaacttgat atcaaaagtg
161881 ctttaaatat cgctatgata cctattatcc ttactttttt tatcactcaa ctttttgata
161941 gcataggcac tattacaggc ataggcgaaa gaggtaaaat ttttgatgat gcaaaaaatg
162001 gagagaaaaa attaggcaaa actctcatgg cagatgctac aggatcagcc ctaggtgcct
162061 taggcggaac ttcaactgtg actgcttttg tagaaagcac aacaggggtt gaaagtggag
162121 gtagaacagg atttactgct ttagttgtag ctatttgctt tgcttttaca cttttttttgc
162181 ttccactttt taaagccatt cctgcaaatg ccatataccc tgtgcttgta atggtgggaa
162241 ttttaatgtt tatggaagtt aaaaatatag attttaaaga cagtgctatt gcagtagcta
162301 gtttttttac catcattatg atgcctttta cttattcgat taccacgggt tttgcttttg
162361 gttttctttc ttatttattg gttagaattt ttaagagaga atgggataag attaatcttg
162421 gcattatagt tttaagctta atttccttag gaaatttctt actcattgct ttacaataaa
162481 aaaaaggggg acaaaaatga ttttttattc ttatgaagaa tttaaagaag atgttaaaat
162541 tttagctaaa gaaatcaaag aagattttaa tcctgatgca cttttagcca tagcaagagg
162601 agggatgagc ttgggacatt ctttagcagt tgctcttcaa acacgcaatc tttttgctct
162661 aaattccatc cactatgatg atacaaaaaa acttgatact attgagattt ttaacattcc
162721 tgatcttagc aaacataaaa aaattctact cattgatgat attgtggata gcggggaaag
162781 tttagctgaa attaaaaaag ttttgcttga aaaatttcca catatcgaac ttaaaattgc
162841 tacgattttt tataaaaaaa ccgctctttt agagcctgat tttaagatta aagaggctaa
162901 agaatgggtt gaattttact gggatattca tattgattaa aatctgctat aatagcggct
162961 tttaattttt gggagtttta tttgagatta aaattaatat cttgtatttt actctttagc
163021 acgatctttg cttttgcaaa tgagcttgat ttaaatgatt ttgaagaaga atatcaaagc
163081 tataaagtaa atgacccgct ttctggctat aataaagcaa tgacaagctt taatgtaacc
163141 ttgtatgatt atggctttcg ccctattctt aaaggctaca atgccatcac tcctgaattt
163201 attcgcacgg gcgtaagaaa tttttttgat aatttatttt cacccctacg ctttgtaggc
163261 aatgtcttgc aatttaaatt tgaagaagca ggagaagaat ttaaaagatt tagtgctaat
163321 accattatgg gctttggtgg gattatggat cctgcaagca aaatgggact taaaaaacat

163381 cctgctgatt taggaactgt tttggcacat tggggagtag gaagtggttt tcatatcgtt
163441 ttacccgtac ttggacctag caatctaaga gatgcattga ccttacctgc aacttggtat
163501 gcaagcccaa cagcttatat cgatcctact tgggcaagca tagctataag cgcctatggt
163561 tttggaaacg aacttagttt taggcttgat gagattgacg aaatttatta taatactcct
163621 aatttatatc catttatacg cgatgcttac gagcaaagac gcaatgaact tagcaaataa
163681 aggaaaaaaa tgaaaaaaat atttttaatt ttaactttat ctttaaatat ttttgcctta
163741 caattagatg aaatttcagg cactatgcaa aaaaatatca ataaaagctt gcaacttcta
163801 caaaacaata aagaagataa aaaacaagct gctgatggaa tttttctaat ttttgatcat
163861 ataattgatt ataaactcat ggctcaactg agtctttcta aggattattc taaattaaat
163921 cctaaagaac aagaaaaatt tgctagtgct tttgaagcaa gccttaaaaa aagctttact
163981 gataaactca atctttataa agatcaagtt ttaatggtaa aaaatggaga attaaaaaat
164041 gcaaaacgat atttttttaac ctcttcgatg atagtagatg gagaggaaaa aaatatcatc
164101 tttaaatttt ataacaacaa taatgactgg cttatttatg atatcgatgt tttgggtgtg
164161 agtatagttc aaacctatag atctcaattt ggagatattt tagcaaacca agattttgat
164221 accctgcttc aaaaacttga gaatataact atagaataat gtttgcgaaa ttcttaaaaa
164281 tttttattta tcatccaaaa agtacttttt ttaccactct ttttatatgc ttgtttttaa
164341 gctttttttgc ttctaaatta agcgtagatg caagtgctga gagcttgctt ttggaggatg
164401 ataaggattt aaaaattttt agagaacttt ctaaacatta caaaagtgat aattttttac
164461 ttttagcttt taaacccgaa gatgaaaatc ctttttcaaa taaaaattta aacaaattaa
164521 aagagcttca tcaagaatta gaacaagttt cattagttga gcgtgttttt agcatcatta
164581 atgctccttt gctccaaagc tctgaaaata cggacttaaa agaactttta aaaaatatcc
164641 ccaatataga aagcaaggat atcaacctca ccaaagccca aaatgaaatt ttaaacagtc
164701 ctttttataa aaacaatatc atttcaaagg atggtaaaac aacagggctt atcatctatc
164761 ttaaacccga tacaatctat aacgaactca tagaaaaacg cgatcttagc acagatgata
164821 atgaaaaaga acaaattcgc ctagcgatta aaaaacatca agaacaacaa aaagtcatta
164881 caaaacaagg cctagatacg atcaaaaata tcatcaaaaa atacgaaaaa gatgaggata
164941 gattctatct tggtggggta agtatgatcg ctgatgatat gataacttat ataaaatccg
165001 atctcatgct ctatggaatt tctcttgtgt ttttattggc ttgtgcactt tattgttttt
165061 ttcgctcatg gcgttttgtc tttttacctc tctttatttg ttttataagc ttaagtgcag
165121 caagcggtgt ttttgctctt ttaaattttc aaatcactgt tatttcttca aactatgtcg
165181 ccttagtcct aatcatcact ctaagcgtag ttatccacct catcacacat tttatagaaa
165241 ttagcaagct ttatccaaaa gctaaaattc aacgccttgt acttcaaacc ctacttgcaa
165301 aagcaaatcc tagtttttat gccattatta ccaccatgat aggctttttc tctctcatgc
165361 tttcaaatat agaacctatt ataaaacttg gtattatgat gagtataggc atagggcttg
165421 cacttatttt tagctatttg tttttagcaa gtgctttggt acttcttaaa ccaaaaaaat
165481 actataaaaa agaatttaaa tttaatttct tagctttttg tgccaagaca agtctagatc
165541 ctaaaaaacg ccctattata tacatactct ctattgttac cattatctta gctctagtgg
165601 gtatttctaa actaagggtt gaaaattctt ttgtaaatta ttttaaagat gggagtgaga
165661 tcaaaaaagg cttattggtt attgataaaa ttttaggcgg aaccttgcct ttagatatca
165721 ttattcgttt tcccaacaat gaaaacaact ctaatttaaa tgatgtattt gatagctttg
165781 aaagcgaatt tgatgctcta gcgacaaagg atacttattg gtttaattct caaaaaacgc
165841 gtattgcaaa aaaagtacat gaatttttgg aaaataaaga atttataggc tcggttctaa
165901 gtcttaatag ccttttaagc ttaggaaaaa acatcaatga tggcaaagat ttagatgatt
165961 ttactcttgc gtttttaaac gaaaatttac ccctagaact caaacaagaa cttctttcac
166021 cctttgttaa tatagaagaa aatgagttaa gatttagcat acgtattata gattctgatc
166081 caaaactaag gcgtaatgag tttttaacaa atctcaagca agggctcgaa acacttttaa
166141 aaaatgatgg tgtagaaatt caaatcacag gcattatgct actttataac aatatgcttc
166201 aaagcctttt ttcatcacaa tttgatacgc ttatttttgt tatcttggtt atctttgtat
166261 tgtttatcat tatttttaga agtttgaaat tttcaattat cgccctactg gttaatatca
166321 ttcccttaag tatggtcttt gctcttatgg gaattttagg catacccttta gatatgatga
166381 gtattaccat agctgctata gctataggca taggagtgga tgataccata cattatatct
166441 atcgttttaa agaagagatt aaaaccaaaa gcgtagaaga agcgatttta gcctcacatc
166501 ttagcatagg atcggcactt tattatacaa ccattggtat agttttaggc tttagcgtta
166561 tgatgagtag caattttatc cctacgattt attttggagt tttaactgta tttgtaatga

166621 ttttgctttt aagcggaagt ttgtttttac tcccaagtct tttaattaca atttatacta
166681 agaaaaccaa agctttaaac ccgtataaag tacataaata aagacaacta ctaaaatata
166741 tctgatgaaa tttctcactc ttttccttta tttaaaactt ttaaaataat ccttgcaagt
166801 tccaaagctt taaagcgatg tgagagttga tttttttcat tattgctaag ttctgccaag
166861 gttttatcaa agcctttagg gataaaaagg ctatcatagc caaaaccatt ttcacccctt
166921 tctatatcga tcacatctcc atgcatactg ccatgtgttg tccattcgcc acctaagctt
166981 accaaagcaa tggcagccac ataatgggcc ttgcttgttt taaaacccaa agcccccatc
167041 tgttttacaa gctcatcacg attgctctta tcatctcctt tgccactaaa acgagcagaa
167101 taaattcctg gcttaccacc caaaacatca acacaaattc cactatcatc acttaaaaca
167161 atgaaatcat ttttttgttt atcactcaaa gccttaaaaa cagccctcgc ttttataaga
167221 gcattttcct taaaactatt gccattttct tctatttcaa aggcggttaa aatttcatca
167281 aaggcataaa tttcaaattc ttttaaaagt tcttttaatt ctataacctt gtgtttattg
167341 cttgttgcta ataaaatttt cattatcttg ctccaaaaat tgaattattt tcgctaaatt
167401 atcattttta atcccttgcc aaactaagaa aaaatcaagt atattccata taaaaagagc
167461 gatgatatta aaaatccata ctacaagcat agaagatgaa tcgaaaaaac ctgcgataag
167521 caaacctata aaaataaaag cccacattgc catttttaca aaaccaagcc ccatatcccc
167581 tttataaaaa cgatcaaaag taagccccga taaaataaaa gcaggtgtta cgcctaaaat
167641 aagtcctata atgggatttt tcaattccaa agaacaaaaa taaaacattg cctcatcgct
167701 taaggtttcc aatttatcct tataatccat caataactcc gaatttactt taccttctgt
167761 cattaaagaa aaagtttgaa aacgattcat aaatctatcc ttttaaaaaa ttaaaatctt
167821 acttttttaa gccttttaaa aagataaaat aaaaaactta acaatcctta atataattca
167881 tagtaaaatt atcttttttt gtaaaattta agatttttag gaaaccaaaa taatgttaaa
167941 taatgtctta cccctatctt ttatcgttgg aacaagattt tttggacttt ttatagtttt
168001 gcctgtgctt agcttttacg ctcttgaact ccaaggcgca aatgaatttt tagtaggact
168061 tctagtaggt gtttatgctc taacccaaat ggctttacaa atgccttttg gaatactaag
168121 cgataaaatt gggcgtaaaa aaactatgct tatagggctg attatcttta ttatagggtc
168181 tttaatctgc tcttggactg ataatattta tacaatgcta gtaggtagaa tgcttcaagg
168241 tgcaggtgct attggcgcag tagctacggc aatgattagc gattttatca ccgaagaaaa
168301 tcgcggtaaa gctatggcga taatgggatc ttttatagga ctatcttttg cagcttctat
168361 ggtaatctcg cctttaatga gtgctaaatg gggggctttct agtcttttttg atcttagtgc
168421 tgctctatcg cttttatgta tcatcttgct ttatactgtc gttccaaaag aaaataaaat
168481 cactcatgaa aataccaaaa ctcctttttt acatcttatc aaacaaaaaa atttagccct
168541 aatgaatttt accaatttca tgcaaaaaat gcttatgagt atagcatttt taagtattcc
168601 tatcatttta gtaaaacatt taggctttga tgagcataag ctttggattg tctatacagc
168661 atctatgata gcaggtttta tcgccatggg attttcggga agcttgggcg aaaaaagagg
168721 cttagcaaag caaattttac ttttagggat agcttttttt atcctttctt atatcttttt
168781 tgttttttct aattccatta cttttttttat cgttgcagtt gtgatatttt ttatagggtt
168841 taacttgcac gaacccatta tgcaaagctg tgcttctaaa ttctgtaaag tacatgaaaa
168901 aggtgctgct ttaggacttt ttaatgcttt tggatatggt ggaagtttta taggaggtgt
168961 aataggaggt atatttttac atcttaacaa attagaattg ctggctatta ttttagtagc
169021 tttgtctatt gtttggttta tagccctact atatttaaaa aatccttctg aatttaaaaa
169081 tatttatttg cctttagaaa caactttaaa ttttagcgaa tttaataaaa atttaggtgt
169141 tgtagatatt tataaaaact caaaaaacct agtcattaaa tttgatagca agctgacaaa
169201 caaagaagaa ttagaaagta aaatttaaat caaaaataaa aaaagcttga aatttgattt
169261 ataattttat tttctaagga aagctgacaa cgctttttat cactaagata agccgtgcgg
169321 gcaaataagc ttgtttttttg tggggtattt ttagagctaa tttgtatatt tacttgcata
169381 gtatagtaat tttcaatata ccattcccca ctattttcaa aaggatcaaa atcataaaaa
169441 aatcttcccg aacttgtaat tctttgggca taagaatgct ttttaaaatc tacaagattg
169501 atcaaaattt cataatcagc attcaaagat acttctttta atcctaaatt tttaagcctt
169561 aaagctaatt catcttttag agcactttga attttacttg gattttgcaa agtgatacta
169621 aaactttgcc cttgtttggc ctttaaaaaa atgccttcat tgcttgtttg tatagagctt
169681 tgaacaaagg cagactttgc acaagcactc aaaaagagtg ctagaaataa agctgaaatt
169741 agatgaattt ttctcattat ttgatgatgg ttggagtagc actaaggcct aaagaacgat
169801 atttttcata aagagaaaat acttctttta actcgctatc gctcaccttt ggataagatt

169861 tgatatttgc gtcataatat ttatttaaaa tagctatttt ctcatcatca ctcttagctt
169921 ttttactctc tttgtaaatc aatgctgatt tttcaaaagc tgatttacca tgcactggag
169981 ttaagatata attgacttga taatttttaa gctcatcttt aatctgtgct aaatgatctc
170041 tacaataagg acattctgga tctgaaaata cataaatagc aggtttgctt ttatcgccta
170101 aagctataag cattgtttct tcttgaacta cgggtttaac atttttagtg aaatttgccc
170161 tagcctcttg gaatttttc atttcatatt cttgagcata agaagtttta gtttttaaat
170221 caataatatc aggaacgata agattatctt ttgtaaaaag aatttgctct tgcttttgtc
170281 cttcaagcgt tatcgtaaca atcacgcttt caaagcctgt atttccaacc ttttggcgat
170341 tacctatact cactgttgca tttggaaatt gtgctttaat gcttttagaa taaaaatcac
170401 tgatttcaga attgctagca gcaaataaag acattgagca agcaaggatt aagcttattt
170461 ttttcattta ttttccttaa aatcaaatat taatgtttta ttttataatg atattctaaa
170521 tatttgtttt taaaaatctt ttttcgtcct ttttttgcac tctctcatct ttatcaaggt
170581 attctaaata agctatggca tattttcttg aaagattaaa acgctctttc atactctgta
170641 tatcaaggct ttgactcttt aaaatttcta ggcattcttg cataagttta tctaaggcat
170701 ttttttctac aaaaagattg tgagaaagtc ttataacacg attttgcttg gtaagttttt
170761 ttaaaatttc atctccactt tttctgtcta gttctaaaaa ttcatataaa ttataagggg
170821 cttgaggttt tatgccttgt tttttaagta tattgtatag ctcatcattg tttctttctc
170881 taagcttttc ataatcaatt cctttttaa aatacacccc cttttcaaat tcaagtaaat
170941 ttgatagatt ttgaagtcca aactcgcaaa attccgcact ggcccaagga agtcttaggg
171001 ccaaagaagt tgctgaaagc atggcataag gatttttttc aagtataaaa gaaatgaatt
171061 ttttaagctc atctaaggct aagagattat aaatattaag attttttca tctataaaaa
171121 ccccacttaa ttctttggct aaatttaaag cttcttcgtg acttaactta aatctttggt
171181 aacttgagag caagccaaag ccatatttat gcgtgttttt tagaaattca aaagcctttt
171241 ttaattcttt atttttcaaa agcattaaaa atttattttt ttgttccttt ttcaaaggct
171301 cgctcacagg atttaaaacc tctcctccgc ccacaacacg attattttgc aataaaataa
171361 atttttcatt aaaacataaa aaaagatttt tttcaaattc aagatgagca aaaaattcat
171421 tattttccaa ctcttttaaa atacttatct tacactctat gctttttgta cccacacaaa
171481 aaagcatttt ttcatttttt aatcttttag ctttaatcaa aacatcacat tctttaaagc
171541 ctttaaaaaa accttttttg cttaaaacat aacctttttt taattcttta taatcacaat
171601 ttaagctcaa agccacacgg ttaaaagctg aaatttcatc aaaattactt tcgtgatttt
171661 gtatattttt aactacaagt tctttttgat tgtcaagaca aatgattttt tcatttaggg
171721 aaattttacc ttcattgagg cttccagtaa ctatggttcc tatgcctttt aaagaaaaaa
171781 ccctatcgat ataataacga aaaacaagat tttcattagc attcttgcat tctaaattta
171841 aaagataatt ttttaagcgc tcaatgcttt cttgatcttt tatcgaagta tgaaacactt
171901 tcataggtgt aaactcaagt gagtttagaa ttttttgctc ttgctccttg ggattttcac
171961 ataaatcgca tttactaagc actaagataa tatctttcac acctaaaagt tcaagtactt
172021 ctaaatgttc tatgctttgc tcttttaaac cctcattaat atcgactaca aataaacaag
172081 cactaaaacc aaaagctcca cttatcatcg ttttaaccaa gtctttatgg cctggcacat
172141 cgataaaaga aaggtgtttt tgcttataat ttaaattaga aaaacttaaa ttaatcgtga
172201 tttgtctttc tttttcctct tttaaactat ccccttcaaa accatttata gccttaatca
172261 aagaagtttt tccatgatca atatgccctg cagtaccaat tagtagtgaa ttcataaatt
172321 ttccatagta ttgataattt gaattaattt tgataaatct ttttctctaa ttgtcctaaa
172381 atctagcaca aaagcttcat tttctatacg ccctatgata tttttttctc taaatttttc
172441 ttgcaatttt aaagcattgc catcaaaagc taaaatacaa gtttctaaag ttttatcagg
172501 catagaacca ccccctacca aactttttact cattttcaat tcacttttaa cctgaatttc
172561 attttgcacc cttaaagctt tttctttaat ataagtgatt tcgtcattta aaagctttaa
172621 acttgtgatt ttttcgtagt ctttttcaag ataagcctta agactttcat ttaaaaaagc
172681 aagtgtgatt ttatccactc ttaacattct tagcagttga ttttctttaa ttttatcaat
172741 caattctttt ttaccaagta taattccagc ttgcacactg ccaaaaagct tatccccact
172801 aaagctcaaa atatcacaat gctttatcac atctttgact ttaggctcgt tttttatcag
172861 ttttggattt aaattctcac accaacccga acctaaatca taataactca aaagtccttt
172921 ttcttttgcc aaatttccta aatccttgat gccaacctca ctatgaaaac caatcaaagc
172981 gaaattagat ttatgggttt ttaaaagcat tttagtattt tcatttatag ctttttcata
173041 atcttttaaa tgtgttttat tgctcgtgcc tacttcacaa agctttactc ctgcagcttt

173101 aatcacttca ctcacgcgaa aacttccgcc aatttcaaca agctcgcctc ttgaagtgat
173161 gagttcttta tcaaaacaaa gagaatgaag caccaaaaaa actgctgcag cattgttgtt
173221 tacaaccaaa gcatcttcgc attcaaaaag cattttaagc ttttcaagca ctaaagcata
173281 gcgactccct cttttaccgt tttctaaatc aaattcaata ttagcataat tgcaaagcac
173341 atctttggac ttttcaaaaa tactctcatc aataacactg cgccctaaat ttgtatgaat
173401 aaccacaccc gtagcattaa tgacactttg taaatctttg agtaaatgtt tttgaatttc
173461 ttgctgtatt ttttctaaaa tagtattttt atcccatgaa ttttcagtta tattcgcctt
173521 gagtttagca acaacacttt tgcaaaaatg agatcttaaa taaaaaggat attcttttaa
173581 actctcatca tcaatcaagc ttccaatttg cggaaaagtt ctaaatttgt tcatcttaag
173641 ccttaagaaa atatttggtt attttaccat agaatttgac aaattgacaa tcaaggaaaa
173701 tcaatgaaag actttgtttt catacaaaat gaaaatctta ttcccttgcc tgatgagata
173761 aatatttggg gaaaatgcaa ggatgaagaa gttttaattt ctaatgaaaa agcacaaaaa
173821 gcacaaattt atgctccaga aattaatttt tatcttaaaa attcacaaga tgaaatttta
173881 gaaagaagta aaaacatatt aaaactttat gaagctaggg caaatgtcta tgatttagga
173941 cttgatttag aacaaaacaa agaagtaaaa aaccgcatta ttttggtaga ttctgatgat
174001 gctatggctg atttttttaa aaaacaaaat tttaaaatca taagcttaaa aaatgaagaa
174061 attctagcgg tatttggcag tataggcgag ctttgcgctg tgattttaaa tcaaggagaa
174121 gaagtagaaa ttgattttga tattttactt tttaatacaa attctaacga aattcgcaag
174181 gattttacaa gacaaagtgg ttgttataat attagcaatt ttaaagatca agaaagttta
174241 cttgaatttt tacaagataa aagtccaaaa tacacatata aaacctatat cacctatgat
174301 tctagtgtgt gccaatacca cgaaagacgc agtgagcatt gcgcaaaatg tgctgaaatt
174361 tgtcctacgg tagccatttt aaaagatgat gaaagcaagc atttagaatt ttctcaagta
174421 gattgtctag gctgtggagg ctgtatcagc gtatgtccta gtggctcgct tgattatgca
174481 cccatgccta gggaaagttt ttttgaactt tgtgaatttt ataaagaaaa aaagatttta
174541 atcataccta aaaaaatgtc tttagaaaat ttaaacataa aactacccaa agatgttcta
174601 ccttttatga tagagggtga aaaatggctt tcttccatgc attttctaac tcttttgcaa
174661 agcagtggtg caaatttggt tttttatacg gattttattt cgcgaggaag tgctgaagcg
174721 atagatcttt taaacacttt ttttgaaaga aaatttcaaa aagaagctat ttttacagcc
174781 aaagatgaaa aagaattgca aaacgcttta gaaaagcaag aatttataga aaatttaagt
174841 tttgattttc acaataacac tcttaccact agagaaaatt ttgccaaaag aatgcaaaat
174901 ttaatccaaa atgatgattt tggcgtaaaa gaaagtggtg aatggttaag atatggaaaa
174961 atagaaatca atgcaaacac ctgtactctc tgtctttctt gtgtaggagc ctgtaatgta
175021 ggagctttga ttgccgatac taaagaaaat gcactcaaat ttaatgcttc attatgcacc
175081 acctgtggat actgcgaagt aagttgtgcc gaaaaagata ctctaaaact tacgcgtagt
175141 ggaatggaat ttaatccaag ttattttgaa taccaaacca tggcaaaaga tgaacttttt
175201 gcttgtatag aatgcggaaa agaatttgct accaaaaaag cggtagaaaa aattgcaaat
175261 ttaatgaaac ctaaatttgg taatgacgag agcaagataa aaactcttta ttgctgtgca
175321 gattgtaaag caaaggtgat gatacttgcc atgcaaaaaa gataaaatcg atttttgagt
175381 gcgaatttaa tattttaatg gtgtataaat ttcaaacctc ttctatataa aaagcagtct
175441 tttatctttc taagcgggtt ttgaaaaaaa gtagaataat taaaaatata ctgaaatatc
175501 ggcaaaggaa gccgatattt ttaaaaatta agcaaaatga gctttgattt gctctaccca
175561 agcgtcaatt cttgcttcag tttgatcttc ttgattgtca ttatcaagtg caagacctac
175621 aaattttcca tcaacaactg catcgcttga ttcaaatgta taaccatcag ttgaaacttc
175681 acctactaaa tttgcgcctg catttttttaa attttctgca agttttccca taccgccgca
175741 gaaagtgtct gaatagcttt cgctatcacc cattccaaat acagctacag tttttccacc
175801 aaggctaaga cctgaaaaat caaaaccatc ccaatcatct tgtaaatcgc cacttcccca
175861 agtagaagtt ccacaaatta atttatcata agaattaatt ttagctgcat caatatctgc
175921 aacatttaaa acatcactaa ttcctaattt tgaagcaatt gtatttgctg ctccttctgt
175981 atttcccata gcacttccat aaattactgc tactgacatt attttctcct tataagttta
176041 tttgttattg aatacgggac taattttaac atagttttat ttaaatcgca taaaaaaatgt
176101 tttatttgaa caaaatgtgt gaagtttttg ttacgcggac aaactatgca aatttgttca
176161 aattgatttt gatatataaa tttagatatt ttttgaattt cactatcgat atttaaattt
176221 tgaagatcta cttctttgca aagaggcaaa accgcaattt tattatctat gataatcaaa
176281 ttatcatttt gaacaatgct tcgattttga ttttgatgag aaattttatc atatacataa

176341 aaatcaaata aaacttgtga atcaaaacaa atacctctaa aataagccct taaaagtctt
176401 aaaatatcga cattttgaaa tattttagtc ttgaaaatca aaccctttct tttttgaaaa
176461 taagaaatgt tcaaagtaag gtttttgtcg atattttta ttttaccaaa aggcatatgt
176521 atcgcatctt gcaagaagtt ttgaaaaggc ttttttaaat tttttgcact tcttgcatat
176581 gaaagcggga ttcttaaaac actatcaaca ctagcattgt attgaatcaa aaaagttttg
176641 acaagctctt gcttacataa agaactataa actttaggga taaattctct tttatcttca
176701 aatccaaata gcattattct gccttagagc aagaaaattc tttatctaaa ttaaatatct
176761 tacaatactc acaatacaca caacttacac ttctttttgc acaaactaat ggaatttttc
176821 gttttttagc ctcattttta atttttttca tagtattgtg atttaaaaaa cttgtaagca
176881 tcactataca ttctgtatca caaggaatgg gtttgcgatt gactctattt tcattgcgcg
176941 catcccaatg ctctattttc tttgcaccta aatcatacaa aacagcctta ataggtgtta
177001 tttcatctgc accaataacc aaaactgaca taagctctcc tttgtgattt ttattttaaa
177061 ttattttgat aattattatt ataatataat caaactaaat ttattttgaa atttattatc
177121 atttatatta tttatcgatt tattgagttt aattcagtta actttaattt ttaatagata
177181 taatttctca attaataaaa attattattt aggagaaaat gatgaaaaaa ttaactaacg
177241 acttcggaaa tattatagcc gataatcaaa actctttaag tgcaggtgca aaaggccctt
177301 tacttatgca agattatctt ttacttgaaa aacttgctca tcaaaataga gaaagaattc
177361 cagaaagaac agtgcatgca aaaggaagtg cggcttatgg cgaaataaaa attacagcgg
177421 atttaagtgc ttataccaaa gctaaaattt ttcaaaaagg tgaagttact ccattgtttt
177481 tacgcttttc aacagtagca ggtgaagcag gtgcagcaga tgccgaacgc gatgttagag
177541 gttttgctat taaattttac accaaagaag gaaactggga cttagtagga aataacactc
177601 cgactttctt cattcgtgat gcttataaat tccctgattt tatccatact caaaaaagag
177661 atccaagaac acatttaaga agtaataatg ctgcttggga tttttggagc ttatgtcctg
177721 aaagcttaca ccaagtaacc attcttatga gcgatagagg aattccagca agttatcgtc
177781 atatgcacgg ttttggaagc catacttata gctttatcaa tgacaaaaat gaaagatttt
177841 gggtaaaatt tcattttaaa accctacaag gcattaaaaa tcttagcaat aaagaagccg
177901 ctgaacttat cgctaaagat agagaaagtc accaaagaga tctttacaat gctatagaaa
177961 ataaagattt cccaaaatgg aaagttcaag ttcaaattct tgctgaaaaa gatgctgata
178021 aactaggctt taatcctttt gatttaacta aaatttggcc gcatagcata gtgcctttaa
178081 tggatatagg tgaaatgatc ttaaatcaaa atccacaaaa ttattttaat gaagtagaac
178141 aagcagcttt tagcccaagc aatatagtac ctggtatagg ttttagtcct gataaaatgc
178201 tacaagctag aattttctca tatcctgatg cacaaagata tagaatagga actaattatc
178261 accttttacc tgtaaatcgt gctagaagtg aagtaaatac ttacaatgtt gcaggtgcta
178321 tgaattttga tagctataaa aatggtgctg cgtattatga gccaaatagc tatgaagaaa
178381 gcccaaaaga agataaaagc tatcttgaac ctgatttagt tttagaaggc aatgcacaaa
178441 gatacgcacc tttagataat gatttttaca ctcaaccaag agcgttgttt aacatcatga
178501 atcaaaatca aaaagaacaa ctttttgaaa atatagcagc ttctatggaa ggtgtagaag
178561 aaaaaatcat tgctagagct ttaggacatt ttgaaaaaat ttcacctgat tatgcacaag
178621 gtatcaaaaa agctttagga aaataatttt tacttgccac tttttaaggt ggcaaactaa
178681 agattataaa taaaggaaaa atgatgataa cacttagctt agaagaagaa aaaagatttg
178741 ccgaactttg caaaatggct tttaattttg ctagaaacaa tgaatatgaa aatcttaaaa
178801 tcatgataga agcaggattg aatgtgaatt taaaaacaca taaaggagat accctgctta
178861 tgcttgcagc ttataataat tcttacgaat gtgcaaaaat gcttttagaa aagggtgcac
178921 ttgttgatga aaaaaatgat cgtgggcaaa cacctttggc aggggtttgt tttaagggct
178981 atttgcctat gtgcgagctt ttggtaaaac acggagcaaa tatagatgaa aataacggcc
179041 taggtatgac gccttatagc tttgctatca tgtttggccg taaagatgtg gctgagtttt
179101 tactcaaaag ctctaaaaaa agcttaagta aacgcctttc tttaggaatt ttaaaattat
179161 ttaaaaaata aattctatga tttaaaattc tttaaatacc tataaacact aggctctgaa
179221 atttttaaga gtctagccac tataggcaca gaacccttga tattaaaaat accctttttga
179281 tataaagttt ttgtaatctc ttctttttgc gctatactga gttgatagtc tgaatttaaa
179341 taattcatat caatactttg ggctaaaatt tcttctatag aatgacttag ggtttctata
179401 tttcctaaac tatccacact ggtattattt tctttatttt gatttaaaaa ttcacccata
179461 tcgtctaatt tttctatatc tataatctta caaaccaaat ctctcatcat actagtatcg
179521 tgattgatac ataaaattcc cactaattca tcagaatttt ttataaaaaa tgttgaacct

179581 ctaacaagtt ttgattttcc gattaaagcc ttataatcac acaaaaaatc ttcttttaaa
179641 taagctttat tttgcacaag ctcactcgca aaagcactca aaggagactc aagagttcgg
179701 ccacttacat ggctgtttgc aatagctgct atataagcac cctctttaga aagcacatga
179761 aaaactactt catactgctt gcctaaaact tgtcctaaaa aattcgtaag ctttacaaat
179821 tgctcaattt gtttttcgtc catgaaaata ttcctaaaaa ataaatttag aatccaaagg
179881 ttaaatttta actttaattt tttaaaaaca ttcttaaaaa taatatttta ttattgtgat
179941 aataaaatat tgacaaatta ttattatcgt gataaaatac atttgtttaa tttacattaa
180001 caaaaaagga gaaaaaatgt caaattatcc aaaggctatc ggcccttatt cagcttatag
180061 agaagcaaat ggctttcttt ttatctcggg acaaatccct atcaatccag catcaggaga
180121 gatagaaagc tctgatatca aagagcaaac caaacaatct ctaaaaaata taggagctat
180181 attagaagaa aatggaattt cttacgatca agttatcaag acaacttgcc ttttagcaga
180241 tatttcagat tttgctgcct ttaatgaagt gtacgcacaa tttttcaaat ctccatatcc
180301 tgcaagaagt gcatttgcag taaaagatat accaaaagga gctaaagtag aaatagaagt
180361 tattgctaaa aaaggataaa acatgttagg tttagctttt tctttattta gcgttttttt
180421 gcttgtattt atgctttata aaaaaatcaa tgcccatatg gctttgcttt taagtggact
180481 tttactactc agtataacaa gcttggctaa ttttttaata catacattcc ctgaaaacta
180541 tgtattgtta aattatccta atatttacgc ttttttaaat gctttagatt tttaccaatt
180601 aaagcatgca attcttgaaa aagattcttt aaatttaggt ttttttgata tctttcaagt
180661 ctttaaccaa accatgtcaa atactctagc aggtttagga ctaacactaa tggctatagc
180721 aggtttttct gcttatatgg atcatgttgg ggcttcttac gcactcttta aagtttttga
180781 aaaacccttta aaaatggtaa aatcccctta tattttactt ttagttgcat attttatcgt
180841 gcaatttttg gttcttttta tcccttctca tgcaggttta gggcttttgc ttatggttac
180901 tatgtatcct atattggttc gctctggagt ttcaaaactt tcagccctaa gcgtaattgg
180961 tatatgtcaa tatatcgatc atggtcctgg aagcggcaat gtaatcatgg cctcagaaaa
181021 agctgaaatt gatcctgcta tttattttgt tcattatcaa ctccctacaa ccctacctat
181081 cattgtagct gtaggtattg ctatatattt ttgcaataaa ttctttgata aaaaagataa
181141 ttttgttttt aatgctcaag aagtagaaaa agaactcagt gaaaataaag ataaagaaaa
181201 tgaacttaaa aaacctccaa aaatttatgc tatcttgcct gttatacctt tagtattaat
181261 acttggattt agctctgtgc tcgatagcat tttggtttta atgggaatta gtaacgaaga
181321 agaagtaaaa gctgcagcta gttcagctat taagatgaat gttcctgtgg ctatggtgat
181381 ttcaactttt gttgcgattg cttttgaaat gatacgctat aaaagcgtag tagaaacact
181441 taattctatt atgattttct ttaaaggaat gggacattta tttgtcatca ctgtttcttt
181501 gatagtgtgc ggtcaagtgt ttgcaagtgg gcttttatct gttggctttg tagatacttt
181561 aatagatttt tgtaaaaatg caggatttgg agtacttgct atcatcatcg ctgtttcaat
181621 tttactcgca gtttgtgctt tcttaatggg ttcaggaaat gcagcatttt ttagctttgc
181681 cccacttatc ccagatatag caaaacgctt tggtattgaa actattacca tgatagcgcc
181741 tattcaaatc atgacaggtt ttggaagatg tgttagccct atagcacctg ctattttagc
181801 tatctcagct attgcaaaag taagtccttt tgctgtggtt aaaagaacag ctattcctat
181861 gcttgtagcg gctattgtaa atattattat gacttatatt tatctttaaa aggagaaaaa
181921 atgaactcaa aaactaaact tattcattgc ggtagaggcg atcaaaatgt tgaagtaaga
181981 tcagttaatc ctacgcttat gcgtgcatca accatacttt ttaaagatca tgccacttgg
182041 caaaaatata gagaattaag aaaaacagat cgtgtattaa gctatggcgc tagaggaacg
182101 acaaccaact tcgaactcga aaaacttatc tgcactttag aaggtggcta cagagctcag
182161 cttttcccaa caggacttgc agcacttgcc atggtgcttt taaactatgc tagcaaagac
182221 gcacattttt taattactga tgcaatttat gggcctgtga gaacaatttg cgaattattt
182281 ttagaaaaaa tgggtgttga gattgatttt ttaaaagccg atgcaagcga tgtagaagaa
182341 aaaatcaagc ctaatactaa gctcattcta tgcgaaagcc ctggttctat actttatgaa
182401 atcatagatt taccaaagct ttgcaaaatt gcacactctc ataatattcc tgtagctatt
182461 gataatactt attcaagcgg atacttccta aatccacttg agctaggcgt agatatttcg
182521 gttatagctg cgacaaaata tcttagcggt cactctgatg ttactatggg tgtagtagtt
182581 atcaatgaaa aagaatggaa aaattttgac aaacttcctg aagctttagg ctttacaaca
182641 agtcctgatg atgcttattt ggttcttcgt ggtatgagaa ctttagatgt aagaatgaaa
182701 gctcatgaaa aaagtgcaga tgaaatcgta gaatttttac aaacaagaaa ggaaattaaa
182761 actattttct atccaaaact caaaacccat ccaaaccatg aaatttttat gcgcgatcat

182821 aaaggtgcta atggtatggt tactatcgaa tttgcagagg gttatagtaa agaagatgct
182881 attaaatttg tagatgagtt agaatatttt tccataggtg ctagctgggg tggctatgaa
182941 agtttagcaa ctgtaactac tccaccaaga actgcaacag attggagcaa aagaggtcca
183001 tttgtaagat ttcatatagg acttgaagat actaaagact tgattgctga tttaactaag
183061 gcttttgata gcattaaaaa ataaggaaat aaaatgactg gaattagtgt atttgatcat
183121 aaattattag cagattcatg gagcacgcaa gatatgcgtg ctgtttttg tgaagaaaat
183181 cgcatacaaa aatggctcga tgttgaagca gcattagcaa aagcacaagc aaaacttaat
183241 atcatccctc aagaagcagc agatgaaatc gcaaaaaaag cttattataa atttatggat
183301 atggatttta tctttaatga atttaaaaaa accaaacatc ccttagttcc tactgttagg
183361 ggcttagaaa aagcttgcga taataatttg ggtgaatatg tacattttgg ggtaaccact
183421 caagatatca ttgacacagg acttgttttg caatttaaag aagctatgac tcttgtaaaa
183481 agtgaattaa aagccattgc aaaagcttta gcaaaacttg ctaaaacaca caaaaatact
183541 gctatgatgg gaagaactct tgctttacaa gctttaccta tcacctttgg gcataaggta
183601 gccatctggc ttagcgagct tgatcgtcat tttgaaagaa tacttgaact tgaaaaaaga
183661 ctttatgtag gcagcatagt aggagctgtt ggaactaaag caagtttaag cgatgaatgc
183721 aatgaagtag aaaagctaac tttggaaaat ttaggacttg aagtacctaa tatctcttgg
183781 caacctgcac gcgatcgttt tatagagctt ggttttgtgc taggcaatat caatgcaact
183841 tttaacaaaa tcgctcatca attactcata ctctcacaca atgaaatcga tgaagtagca
183901 gaaccttttg gcaaagggca agtaggatct agcactatgc cacacaaaag aaatcctgcg
183961 gtaagtgaaa atgctgttac tgtaagcaat gcctttaaag caaatttagc gatcttaagc
184021 gacatagaaa gacacgagca tgaaagagat gggcaagttt ggaaaatgga atggaagctt
184081 ttacctgaaa tgtttttaat gctttctgtg gttttagcaa atatgaaatt tgcccttagt
184141 gatctagaag ttaaaaaaga taaaatgctt aaaaatcttg acacccttaa aggctttgta
184201 ttagcagaac gcgttatgtt tgcgctaagt gatcattacg gcaaacaaca cgcccatgaa
184261 attgtttatg aaaatgctat gctaggtatt gagcaacaaa aactttcaa agaagtattg
184321 cttgcagata caagagtatc tcaagtctta aaagaaaaag atatcaacgc tttacttgat
184381 gcaacaagct atgtaggata tgcgcctaaa ctggtagatg aatttttaga aaaaattcaa
184441 aatcatgcta ttttacaata ggaaaaacga tgttatatag cgaaattttg gcggatttta
184501 ttttcaatct tcagtatgaa aatatccctg atgcagtcgt tcaaagggct aaagagctta
184561 tgcttgatag ccttggaacg gccatagcag caagtaagga agaatgcgtt ttaaacgcct
184621 ttaaagcttt tgaaaattta agcacagaaa aaaatacccc tgtatgggta aatgataaaa
184681 aactagatcc tatttatgcg gccatgctaa atggcattgc ttctcacgca cttgattttg
184741 acgatacgca tacagaagcg attttacatg caagtgcgat tttaactcct ctttgtttaa
184801 attatggttt tcatatcagt aaaaatgcta aaaaaattat aaaagctttt attgttggct
184861 gggaagttgc tgctagagtg ggcattgcaa gcaaagggac tttccacaag cgtggttttc
184921 ataccacagc tatcgctgga atttttggaa gcgcgagtgc gagtgcggtt ttacttgatt
184981 taaacaagca acaaatcatc aatgctttag gacttgctgg aagttttgca agcggaatta
185041 atgagttctt atctaatggt tctaattcaa aagtcttgca tatagcaaat gctattaaaa
185101 atggaatttt agtcgctaac tttgctaaaa ataacatgag tggaccttta agcatttttg
185161 aaggcaggga taatatcttt aaatgttttg gcatagaaca agaatgcgat aaaacagaac
185221 ttgataaagg cttgggtgaa atttggcaaa gtatgcaagt ttctataaag ccttatccaa
185281 gttgtcattt tgctcatggt cttattgatt gtgccatagc tttaaaaaat gatggcttga
185341 aagcagatga aatcaaaagc attcgttgtt ttgtagatga agttcctatt tcttttattt
185401 gcgatcctttt agaagcaaaa tatactccaa atagtgctta tgaagctaaa ttttccatgc
185461 ctttttttgat ggctttaggt ttttttgatg gtaagattac tcttaaatcc tatcagaatt
185521 taaaacgaaa agaagtactt gaatttgcac aaaaaatcac ttatgaaaaa agaaaatctc
185581 aaggttttcc aaaatatttt cctggacata tggaagctat tttgcaagat ggaaaagtga
185641 tttgcaaaga tgtttttatc aataaaggta attttgacaa tcctttaagc tttgaagaat
185701 taaaagataa attcatcaac aacgcaaaag aaaacctcac catggataaa attcacgata
185761 ttttagaaca aataaagcat ttagaaaagc aaactgatct ttcattgtaa gtcctaaaaa
185821 gacttacaat aactccttac ctatttcttt tagctaagtt ttaatctatc gttttctctc
185881 ttgctttcat ttgtgataat taaaaaagca ttcttaagtt agcccataat cttgctttga
185941 cattgtgttt ttcaaagctt agaatgtttt ttcataggct tagatgattt tcaaattctt
186001 acgcacttca tcactagtgc aagagtattc atttaaatgc gtaagttctt tagtgatggt

186061 tttagcatta tcgttggttc tatcattgtg atttaatgca ggttgttttt taggattgca
186121 atgagcggtg gcaatttttg gcatattcta caaatccctt ttctttgtat tctgctatta
186181 tctcatcact acttagatta tatgcactca tttttactag cataataata tcttcttcat
186241 ttgcaaacat atcttcaata gcgtgtccgc aaatggtaga tttaatttct tcatagtctt
186301 ttttgcttac atcatcttta tatttttttaa gtattgcaag gctttcttct aagctataca
186361 cttcgggata ttgtttttgc atttttaaat tttttccttt tttttggtgt tttaaaagct
186421 aataaaatca aaaaatcatt tattgatttt agttgatttt attagcgggg ttaaggggtt
186481 gcacccctta caagctcgac acttggcaca agtggcagat agcttgtatc cttcaataag
186541 ataattatag caaatttttt acctaatccc attcctaaaa gtatcaatca ttttaaaaaa
186601 ctgctcttgg tttttagcat taaagtgcat atgatttcca aagtttaaat tctcatactc
186661 atctcttata agctctactg caataatttt aaattcatta aaatcaaaat cgttaaattg
186721 ccaaagctta tcattataaa caaagcggac ttgattaagt ttcatattgt ttaaaatttt
186781 taaatatttt tgctcatcgc tcattatttt ttggctttca ttttctagct tttgcatttg
186841 gatattttca ggtttgaaag taaaggtaat gcctgaaacc ttgccaccac gcccccctccc
186901 ttttgctttt tctttttcat aggcaagatt tttaaaaggc actctaattt gatcgaaaag
186961 atcgcgttct ttagaaagct cttttatggc aggttttaaa atccatttat cgatttcact
187021 ttgcctataa tcttgcggta tatccataat cctgcaaaaa tcttcccact caaatcgtgc
187081 tttgcctgtg gttctaaatt gctttaaaag tctataaaga gttttagtat atttaccatt
187141 aaggctcaca aactcgctaa gttcaaaggc tgtaaaatta gcgcttagtt gatttaaaat
187201 gtattcaaat tgcggattaa cttgtatttc tgtactctct aaatcttttg agcttgcata
187261 aacataaatt gcaaattctg taaaaaggtt tattgttctg tgtgttgtta cttcttttttc
187321 tcctctagtt gtttttttcga ttattgtaaa atcagcttta aaaaactttt ctcttaaggg
187381 taatgaaaat tctaataaac tctcattggt ataatcactt ttaagaatgc ttcttaaatc
187441 actaggataa aatgttactt tatttttctt ctttaagttt taccattaaa ccacataata
187501 aatcctgttc tatctcatta aagctagata taggtattgt caatattatt caccctctta
187561 atagtttaga ttccccgctt tttttaatag catctctatc ccctataata taagcgtttg
187621 aaagttccct aaaaatataa aaaataaaaa atcgcgtacg cgcgagagaa aacaaaaaag
187681 ttgagtaatt tttttcaatg tttaagcaaa tttttgtttt tttctgttat ttgatattaa
187741 attaagtttt tacttttata attttttata tatcagaatt ttaatcatag aaaggattaa
187801 aatgaaaaaa attatcttag gtttagcagt tattgcttca ttatctagcc ttgccttagc
187861 aaaaattact gtatcacaac agcctatagt ttaaccacat tatgaaggtt gtattttagt
187921 ttatcaatgg gagagattat agcaaatgca ataatcaaga taatattaat tatgaacttt
187981 gcaaactaag aggagagtat aaatgatact tgtaacaccc tgtggtgtta tagacaccac
188041 taattttggc aaaagtgaag taaataggca aactatagat ttttctttaa aatttcaaga
188101 aaaaaaagag aaagtagaag ctgaagctaa aaaagaagaa caaagtaatt ctacaaatac
188161 agataaagca gacaataaaa aagatcagct tttagatata tccgtttaat tcttagcatt
188221 agggtgagag cttgtcgttc gctcgtttta aacgagcttc gtgcccctga ctctacttat
188281 aacaaatcat agcacctatt taggtgcaat ttgctaaagc taattaaata gtgttattct
188341 taaaaaaggg taaaatatcg attttagggc tatttttata ataaatcatc ttcttatatt
188401 tttcattata ggttatttta gctaaagtta gttttattac aatagaatta cctaaattta
188461 tctataatga cttggagtag taaggcagga attgctatac aaatgcaaag ttttatcgct
188521 aaagctctaa aataaggacg ctaaaaaata acttgcaaac aagaaaatat ttatgcaggt
188581 tgctttttag gattgcaatg agcggtggca atttttggca tattctacaa atcccttttc
188641 tttatattcg gcaatgattt catcagcact taactttaaa gcactttgtt ttatcatggc
188701 gataatatct aattcattca tgtaaatact ctctatcgca tgattactta taacagattt
188761 tataccttca taatcttcct tatttacatc atctttatat tttttaagta ttgcaaggct
188821 ttcttctaag ctatgcactt ctggatattg tttttgcatt tttgaatttt tcctttttttg
188881 gtgtttcaaa agctaataaa atcaaaaaat catttattga ttttagttga tttttattag
188941 cggggttaag ggttgcaccc cttacaagct cgatacttgg cacaagtggc ggataacttg
189001 tatcctttaa aaaataacat tgtagcatat ttttaagatt aagcagatat atctaaaaat
189061 ttgtttattt attttacttt tagcaaaatt tacgatgtcc ataacaccac aaggcgttac
189121 aagtatcatt tatactctcc tattgataaa ctaaaacaca accttcgtaa catagttgaa
189181 ttatagtaag tttcatattg tttaaaaaag gcggtaaaaa gttttaacaa gatataaatt
189241 cttcaagctc aaaaactgtg aaattaacac caaatagata aaacaaggtt ttcttttata

189301 tagtatattt ccattgtttc aaacaaattt gcaagacttc taaacatttt tttagtgtat
189361 attttataca attgcatcaa aggaaatata tcaatgactc tcaatgaatt aaaagatgga
189421 caaagtgcga ttataaaaga aattaaagct tataaagaac ttcattctag gcttttaagt
189481 tttggtttta tgaaaaataa aacattaaaa aaagtacact cttctttaaa aaatgcaacc
189541 attatggtgg aatttgaaag cacttgcgtg attttaagat caaatgaagc tgaaactata
189601 caagtggaac ccttatgaaa aaaattacta ttgcactagc aggacaaccc aatgttggca
189661 aaagcctact tatcaatacg ctttgcaagg ctaatatgaa agtaggaaat tttagcggag
189721 ttaccgtaga aaaagccgta gcgaaaacac gctataaagg ttatgaactt gaatttatag
189781 acttgcctgg gacttattct ttagatggtt atagcgaaga agaaaaaatc gctcgtcatt
189841 ttctagaaag taaaaattac gatatcatag tcaatgtttt agatgctaca aatttagaac
189901 gcaatcttat tcttagtgca gaacttttaa gtcttaataa aaaaatgctt cttgctctaa
189961 atatgtgcga tgaggctaaa aaagaaggca tagagcttga tacttcaatt ttaagcaaag
190021 aatttcaaat caatgttgtt gaaatttcag ctaaaacaaa acaaaattta gaactcttac
190081 ttgaaaaaat cattattttg ttcgaaaata aatttacacc caaatcaaga ttttccactc
190141 cgctttgcga aaaaagctta gaaaaagaag atatactaac cctagtgaat gagctttcaa
190201 gaaaagttat tacttacaaa aaagaagaaa gaaatcttac taaaaaaatt gatgcattac
190261 tcattcataa gtttttggc ttacctatat ttttattttt aatgtggctt ttatttcagc
190321 tcacttttac cttaggacaa attcctatgg attacataga atcaggcttt aatgccttag
190381 gtgagtgggt taaaaacaat atccataaca ctttcatcgc gtcagcttta gctgatggag
190441 ctattgcagg tgttggagct gtagtgcttt ttttgcctaa tatcattatt ttattttag
190501 gcattgctct tttagaaact acaggctata tgtcaagagt ggctttctta ttagatggaa
190561 ttttgcataa atttggttta catggaaaaa gcttcattcc gctcatcact ggttttggct
190621 gctctgtgcc tgcttttatg gctacacgca cgcttaaaaa taaaagagat agacttttaa
190681 cactttttgt catcaacttt atgagttgtg gggcaagact tcctgtttat gtattattta
190741 ttggtgcatt ttttccaagc gaagaagcgg gaaattatct ttttggaatt tatcttctag
190801 gtgctatttt aggacttatt gcagccaaaa tcttaagaac aactgcattt cgtggcattg
190861 atgaaccttt tgtaatggaa atgccaaaat accgtatgcc aaattggcat ttagtatggt
190921 ttatggtatt taataaagct aaaatgtatc ttaaaaaagc agggaccttt atacttgtag
190981 cttccttgct tatatggttt gcgagcaatt ttccaaacaa tgaaagcaat ttagatgatt
191041 ttagtgctca agaaagagct gttgagcaaa gttatctagg gcaatttggc aaaacaattg
191101 agcctatttt ttctccgctt gagcttgatt ggaaattaag cgtatcctta gtcagtggtt
191161 tggccgcaaa agaagtaatg atttctacca tgggggtgct ttattctctt ggaaaagaag
191221 tggatgaaac aagcagtgat ttaaaaggca ttatcgctaa gaatatccct ttgcctagtg
191281 cggtggctta tattttattt gtaatgattt acaatccttg ctttgctgca accatagtct
191341 tttcaaaaga aagcggaaaa ataaaatata ctttttttcct ttttcttttt acttgtatca
191401 gcgcttatat agtagctttt atagggttaa atattacaaa atttgtaatc aattaacccg
191461 ctttttgttt attaaagtct attacacttt tgatattttt ctcaaaagcc ttcaaaacac
191521 tataagcaag ggcttcaaaa tcaagccctc cgcatagata aatcaaaggc atacgaaaag
191581 atttactcac atcaagtttt gatttttgct cattaagagt aagtttaaaa tcataaggac
191641 ttgcatgctt aaagctgtaa ttttgcgccg cttttaaaaa gtcgttttta tcttgtaaaa
191701 attcaccctt tgccaaaata aaattgatct caaaatactt ccaaatatca ctagaaggca
191761 taggattttc atcaaaattt tgctctataa aaataaagtt tttttcaaaa gatgttttat
191821 agttttctac aagcaaagcg cctaatttat ttttagcgat attttgcaag ataagcttta
191881 aattgctttc aaattcaaaa tccatcaaag acaagatttg gatttgctct tctagagcaa
191941 attctgtgct aaattcctga tttataagct tagcataaga aaaaggattt gccataggtt
192001 tttcataatt tccaaattca tcccaaaaat tcttttcgct acaagctata cagccatgtc
192061 ctgcagctac aggccaagaa gttttagcgt taaattttac tttagggcaa ttattataag
192121 tataaggacc tttacagcct attttaaata aacaagctcc acttttggct ttttcatcat
192181 caaaatgttc tgcaaaaatt ccgctctcaa atttagcctt tctttcacac aaatcatgca
192241 aacacttacc ataagcccaa acgggacgat tttgctcatc aagctcaggc aaaattccaa
192301 ataaagcgaa ataagtcaaa gttacgatga tatttacatc acttggagga caacctggga
192361 tattaactac tttttgggtt aaaacttctg aaataccaca agttttactt gggtttggat
192421 aagcagcttg aattccccca taagaagaac aggttcccac agcaaaaata gccttagctc
192481 tagctgctaa tttttccaaa atttcataac cactctcacc ctctgcacct atggtaagaa

192541 aaaatgtatc aatagcagca acaccgcctt caaccgccaa aacaaaatct tctttcaaaa
192601 catgctctaa aagttcttca gcttttgtcc cattggctgc cattaaagtt tcatggtatt
192661 ctaaagaaaa aaagtcaaaa accaactcat caaaacttgg caaatctgca cgcaataagc
192721 tctcactaca acctgtacac tcacaaagat gtagccaaac aaccttggtc ttaggagcaa
192781 gttcgaaaaa cttttgcgca agagctggaa tttcatttcc caaagataaa tgtttagcaa
192841 gagcttttac gctttcttca ttgatagttt ttttatctac caagtctgaa ttttcgattt
192901 tttcaatgcg ttttataagt atatctttta actcttcatt gctgagtttt gccattatta
192961 tcctttttga ttatcccaac aaagcacagt atttccattt tcgtcttttg tgacatctcc
193021 cattcccatg atattatatc ctgcatcaac ataatgcacc tcgcccgtaa caccacgagc
193081 taaatcactg agtaagtaca tcgctgaatt tcccacatct tcaatgctta cattgcgttt
193141 taacggagca ttgatttcat tgtattttaa tatcattcta aaatcaccta ttccacttgc
193201 tgctaaagtt ttaataggcc ctgccgaaat cgcatttaca cgaattcctt tgatacctaa
193261 atctcttgcc aaataacgca cagagctttc aagagctgct tttgctacac ccataacatt
193321 ataatgaggc acatatttta ccccgccaag ataacttaaa gttaaaattg caccattttc
193381 ttttaataca ggtaatactg cacgcgttaa agaaagcaaa gaatacacag aagttcccat
193441 agctatatca aaagcctctt ttgaagtctc taagaatgaa ttttctaaag cttcttttgg
193501 tgcataagca acagcatgca ctataaaatc aatctctcct agatcttttt taattttttc
193561 ggcaatagaa tctaaatgtt cgttgttatt tacatcaagc tcataaacaa agtttgatcc
193621 aaattcttct gctataggct caacgcgctt ttttaatgca tcatttaaaa aagtaaaagc
193681 taaacttgct ccttgttcat agcaagactt tgctatacca taagctatag atttattatt
193741 ggctacgcct actataaggc ctttttttgcc tttcattatc atcatttatc ctttaattag
193801 ttttataaaa ttttcagctt ttaacgcagc agatcctatt aaaacaccgc tgcaatgttt
193861 tatatttaaa atatctttga tattatcctc atttacactt ccgccataaa gcaaaggagc
193921 ttgcgtaagc gaagaaagaa attcaagcac tttagcaata tcttcttttt ttgcacttac
193981 gccacttcct atagagtaaa taggctcata agcaataatg agatttttat aatctaagtc
194041 aataacttca agttgttttt ttaaaaaatc taaggtttga tttgaatttt taagctccaa
194101 atcctcgcct atacaaaaaa tgattttata agcatgctct ttagcaaaat taaatttagc
194161 tttgattaga ttttcatctc ctaaagctct tctttcagaa tgtcctatca aaacacattt
194221 gattccaaat tcatccaaat gctcttttcc aagctcccct gtaaaagcac cattgataca
194281 aggataaaaa ttttgtgccc cttgtataaa atttaattca ttttctaaaa aagctatact
194341 aggcggaaaa acgatgatat catcgttttt tgcttctaaa tactgattta aaatatttgc
194401 ataaagttta aaacttgatc ttgtgtgatt gcattttaaa tttgcagcaa atatcattgt
194461 gctcccccac ttcttaaaac ttttacacct ggcaattctt taccttcgat aagctcaagc
194521 gaagcacctc cgcctgtaga aataaaagtc atttcatctg catctccagc acgcgcgaca
194581 acatcagccg tatcgccacc ccctacaaca gtagtagcat gtccatcgct gatagcatgg
194641 ctcattttta tgctgccttt tgaaaattta tcgatctcaa aaacacccat aggcccattc
194701 caccaaatag tctgtgcatc agaaataacc tctttaaaaa gtctcacact tgcaggacct
194761 atatctaagc ccatccagcc acttggaatt tcttgcgaag gtacaaattt cattggtgat
194821 tcttgagagc aagcaggagc agcaactaca tccacaggta agtaaatttt aacgcctaaa
194881 ttctttcctt tggtaagaat tttattggct tcttctaaaa gctcttcttc taaaagtgaa
194941 tttcctatat caaaaccttg agcctttaaa aaggtaaatg ccataccgcc accgatgata
195001 agtttatcaa cttttggaag taaatttgta agagcttgaa gctttccgct tacttttgat
195061 ccacccacaa cagcaacaaa aggacgcgca ggacgcttga taagattttc agcaaattca
195121 atctcttttt gcagtaaaaa tcccgcaccc ttatgatttt catcaaaaaa tttagtaata
195181 gcctctacgc tagcatgcgc tctatgacat accccaaaag catcattgat ataaacctcg
195241 gccatagaag caagttcttt agctaaattt tcatcatttt tagtttcacc cttttcaaaa
195301 cgcaaatttt caagcattaa aatttcactt gcttttaaat ttgctgcttt gctttttgca
195361 tcttcaccta taacatcttt agccataatc acttctttat ctaaaagcct tgctaaacgc
195421 ttagctacag gctctaaaga atatttagaa ctaatctctt ttgggcgacc taaatgcgaa
195481 gctaaaatca cactgcagcc attatctaaa caatatctta tcgtagggat agccgatctt
195541 atgcgtctat catcagtaat gtttaaaaaa tcatcttgag ggacattaaa atcacatctt
195601 ataaaaactt tttttttgct tagatcaata tcttttatag aaataatatc actcattttt
195661 aagccttatt tgcaatatat actgccatat ctactaggcg gcttgagtat ccccactcat
195721 tgtcatacca agcaacaact ttaacaaaat catctgctat aacttgagtc aaatcactta

195781 ctacaactgc cccataagaa caagtgataa aatcgctcga tactctttca tcatcatcaa
195841 ccattaaaat gccttttaaa tttgtagctg cagcttttct aaaggcttca ttaagctctt
195901 ctttacttac ttttttacta agctgagcgg ttaaatccac acttgaaaca tcaataacag
195961 gcacacgcat actttgtcca tgaagtttac cattaagttc aggcattaca agcttcatcg
196021 cttttgctgc acctgtagaa gttggaatga tattttgagc agcagcacgc gagcggcgtt
196081 tatctttagt ctttgcatca ataatgcttt gtccatttgt ataagcatgt atggttgtca
196141 taagtccttt ttctatacca aaattatctt gtaaaacacg acaaacagga cctaagcagt
196201 tagtcgtaca acttgcatta gagatgatat tttcaccttt ataattttgt gaatttacac
196261 ctaaaacata agtaggagta ttatcttttg caggagcact catgatcact ttttgcactc
196321 ccatatctaa aaacccttgg catttttcta ttgttaaatg agctcctgta cactctagaa
196381 caatttgtgc accatatttt gcaaaatcaa gctcttttat atcacgactt ttaaatactt
196441 tgattttttt accattgata attaaatcat ctccttcatt ttccacactg ccttcaaatt
196501 ctccatgcac tgtgtcatat ttaaaaagat attttgtaag ttcaatatct gtagtatcat
196561 ttatcgctac aagttcgata tcatctcgtt ttgaaataat tcttgctaca caccttccta
196621 tgcgaccaaa accatttata gcaactttta cagccatttt gtttcctttt gcgaagtttt
196681 ttatgctaat attttactaa aaaaaggtta aaattttaat gaaaattgca ctttttggtg
196741 gcagttttga cccaccgcat aaaggacatg acgctattgt caaagaagct ttagcaaaac
196801 ttgatatcga taaactcatt attatgccta cttttatcaa tccttttaag aaaggatttt
196861 ttgctgatga aaaacaaaga tttgcttggg taaataaact ttgggggaaa ctagaaaaag
196921 tagaaatttg cgactttgaa accaagcaaa aacgccctgt accaagcatt gaaagtgtag
196981 aatatcttta taaaatttac catcctagca aattttatct tctaatcggt gcagatcatt
197041 tagaaaaact tcatctttgg catgatttta aaaaacttaa ttccttagta gaatttatca
197101 tcgcaaatcg caatgatata gaaattccaa aaaattttaa agatttaaaa accgatataa
197161 aaatcgcttc atcttttata aggagtaccc tagatacaca tgaagtttgc gatgaaatta
197221 aagatgaagt gaaaaattac tatgaaaaat tacaaaaaaa ctgatacaat ttcaaaaatt
197281 taaaggaaaa aaatgcaaga aagagtagat ttaatcgtta aaattttaga tgagaaaaaa
197341 gctgaagaca taaaaacctt tgatatgagc gaacaagatt attttgtaaa atacgttatc
197401 atcgcagcta ccttgggtga aaaacatgct ttatctttga tcgatgagct taagaccaaa
197461 ctcaaagcta agggtgaaga atttttaaat atagaaagca gtgaagaatg gagtgtgatc
197521 gatctaggtg atatacttat ccatctttta acacctgaac accgaggaat ttataatata
197581 gaagaattat tagaaagtct taaaaaaatc aaggtataag tttaaaatgc aaaaaatgca
197641 ctaaaaattt caaaaaataa ataaatttta tgattttttt gagctttcta atgagctttt
197701 tgtccccatt ataaagcaaa atatcgttta aatttcatag caaacttaat ggataattta
197761 ttccgcccat gttttgagcg gtcatctctt ctttttttggt ttaattatct tatttggttt
197821 attttgggaa ttttttaaat ttttccttca aataggaatg attttaatcc cacttagagt
197881 agtctttatc atccttgtgc cctttcattt agaaaatttt tttaggcaaa actttttatt
197941 ttttagaaaa tattttttgt ttggcatttg caattttact aatcgttgca attattatgc
198001 catttttaga acgaatttcc aaaatatttt tctaaatatc ctatgcgttt ttcaaggctt
198061 ggatgtgtgg caaaaatacc atcaaaggct ttcaagaaaa acaagtacat gcaattttgt
198121 ttaagttttt tatcataaaa gtggatcttt ggtgtatatt tttgctacac tcgcacaaac
198181 aatgcgttat ataaatgaga aaaagctctt gttttcgtgc ttttctctaa atttacaaat
198241 aaacctaaaa tacttattaa actgactctt taatatcgtt aaaaagttta aatactttag
198301 tttcaagctc ttttgcttgc tctaaagagc ttgcttcaaa acgcgtgata agataaggac
198361 tggtattgga tgcacgaagt aaagcccatc cgtaaccaaa atcaatccta gccccatcga
198421 tttcacaaag actcttcacc ccttctagcg caccccttttc tatcgctttt ttaaattcat
198481 ctacaagctt gaatttttct tcttcgctca ctggaatttt aatctcaggt gtagtataaa
198541 gttttggtaa ggcgctaatc atactctcta aatcaaagcc cttataaaca agctctaaag
198601 ctcttaaaaa ggcataaatt ccatcgtcat atccaaaata tctgtgctta aagaaaatat
198661 ggccgctcac ttcagctgct aaatcaatat ctttttcttt catcatcttt ttgatatttg
198721 aatgccctgt ttttcccata aaaatagtac caaattttgc cacctcatca aaaagatttt
198781 tagagcattt tacctcacct aaaattctag gattaggaat atttttcgca aacaaataac
198841 aaagctcatc tccacaaaaa atatgagttt tacttaaagc caccattcta tctgcatcac
198901 catcaaaagc aaatgctaaa agatattctt gattttcatt taaaaatttc tttatagcgt
198961 gtaaattttt ttcttctgtg ggatcgggtt catggtttgg aaattgtcca tcaggatttg

199021 aaaacataac atgagctttt aaatttaaag cttttaccaa aggctctatc acaaccccag
199081 cagctccatt ggagcagtcg attgcaaatt tataattaaa atcctttaaa aaactaaatt
199141 gctcacacat aaaattcaca taaagactta aaatatcgta tttttgagct tctaaatttt
199201 cttcaatctc atcgtctaaa tgcttataaa cttcttttga aaattcttta agttcagccc
199261 caaaaaaact ttctttacct atagtgattt taaaaccatt gtaatccttt ggattatgcg
199321 aacctgtgat cataatattt gcatcaaatt tcataccctc ataaagacta aaatatccaa
199381 gtggagtggg gacaagtcct atgttataaa cttgtatacc tgctttatta agcccgctga
199441 ctagatattt ataaagctca tcagcactat atcttgcatc ataacccaca cttacatttt
199501 tacaaccttt tgcaagcatg gtttgtccca aacaaaaacc tatagcttta acactttttt
199561 cgttaagttc cttaccataa agtccgcgta tatcatactc cctaaaaatc acatctagca
199621 tatttttttcc ttaagtcttt ttaataaatt tcttttttat aatacaaaat ttggatagaa
199681 aagaaaattt tgctacaatt ttacattaat ttcactaaat aaaggaaaaa ttaatggatg
199741 aagaattaga aaataacgaa gaaaaaaaga aaaaaggcgg ctcgcttgtt atcattatag
199801 taattttact ttttgtctta cttcttagca tcatgggtgt aattgcatgg cttatctcaa
199861 gtagctcaag cgatgaaagc gaagtcaaag cagctccaaa agaagaggta aaagtcgata
199921 aagctaaagt tacagcaccg acacaacgcg ggagtgattt tgcaaatata gggcctatgt
199981 atcctcttga tccttttact ttaaatttac ttagtgatag tggctcaaga tatgtaaaat
200041 gcactataga actagagcaa aatacagagc ttttaaaacc cgaacttgat aaaaaagtag
200101 cagtaattcg cgatatcatt atccatgctc taactgcaaa aacctttgag gaagttagca
200161 cacaaaaagg caaagaaaga ctcaaagatg aacttgttgg aaaaatcaat gaaattctca
200221 ccgatggctt catcaaaaat gtttatttta ctgattttgt agtctcttaa tgcgtatagg
200281 ttgcgatata gttgccattt ctaggataga tacaatctat caaaaacatg gcaacgcctt
200341 tcttgataaa tttctaaacc aaaatgagca aaaattgata aaatctagct caactttagc
200401 aggtttttgg gctgcaaaag aagcagcaag caaagctttg ggtgtaggaa tttcaaaaga
200461 atgtggcttt ttggatatag aaattttaaa agatagcaaa aatgcccctc atttaaaact
200521 cgcttctcat gtattggaaa aatttaagat cacttccact tcccttagta tctcacatga
200581 taatggtttt gctattgctg tagttgcttt ggattgattt tatcttttgc ctatagtttg
200641 cttatctaaa tttcttgaaa gtataaaatc aacaacaggt tgctctcttt tattggctct
200701 tttgcaatga tctttttgaa agaaatctgt gattttaatc actactttg cccatttttt
200761 tctatttctt tcgcgccacg catgcgaaga tacgctttcc caagcatagc cattaaacaa
200821 acttgcattt acaaaatgat ccaaggctct aacaccttgt cttgctctag ctgtattaaa
200881 aaaagttcct actatcacta tgaacaaata tccacaaatt gctaaaggca ctatggcaca
200941 aagcaagatt gtccctgcta atttttcttt taaactgcct tttaaattac gcttaatgcg
201001 gttgcgaaaa ttggcacgaa ttcttgcttt gactcttttc atgactctct tttgctaaat
201061 ttgatcctca ttccatttgc agagcgtatg gttaagcgac ctacaacatc tggaacgaaa
201121 ccttcttcaa gttctaattt atattttttc aaaatattag ccaaaattaa aatagcctct
201181 tgcatcgcaa aaccttgacc tatgcaaatt ctttccccta ccccaaaagg taaataagca
201241 tcctttttat actcaccttc aaaacgagag ggtttaaatt cgtgtggatt agtccaaaat
201301 ccttcatgtc tatgtatcag ccaaggtgct ataacaactc ctgaaccttt tttaatcatc
201361 ttatctctta ctttagtatc ttttttgct tctcttgcaa aaaagcctac aggcggataa
201421 agtcttaaag actctttaaa aatattggtt aaatatttaa attgttttaa atgtgaaatt
201481 tgtatatctt caccttgtaa aacttgaata atttctttat aagcttttc ttgttcatca
201541 ggataaagac tgagtagata aagcgtccat gttaaagaac ttgctgtcgt ttcatggcct
201601 gctaagaaaa gcatagctac ttgatctaga atttcttcaa aagaaaaacg ctgattagtc
201661 tgcgcatcca ccaccaaaag caaagaaccc aaaatatctt caaaatcctc tacttttcca
201721 ctacttaccg catcatatct tggctttata atatcactta aagcttgcct gatcacatca
201781 cctgctttag cacgcttacg gtcacccaac acataagaaa gccatttagg gaaacgaaac
201841 atacgacgca tagctgtatg tacactttgc tcttgaaaag tcacaaaagc atctaggatt
201901 ttttttcctt gctcttcatc taatttagaa gacatgatgg tgcgaaaaat aacatcagca
201961 gtaacaaaag tcatagcctc atccacttcg ataatagaag cattaggata tttttcaaaa
202021 cgcttcatca tatcgcttac tgcttcactc atgagattga aaacctttga aatacgagtc
202081 atttcaaaac ttgggcgtaa aagttcgcgt tgctttttcc aaacttcacc atttgttgta
202141 aatatgctct ctcccaaaag agggcttaaa agttcgtgca aaaatgcact ttttggaaat
202201 tctttaacct catcaaccat cattcttttg acttctttag tatcgttgat cacataaaga

202261 tcaaaatttg gcatttttac ataacctgtt tgcattttat agctacgctc ataaagacca
202321 tcaagccatg atctgcgttt tagtaaaaag gttaaaagtg ttgaagcttt atttttataa
202381 ggtttgggaa aaaacggaca ttgactcata tttttacctt ttcttctaaa aatttaggtg
202441 ctacaataaa agcaaaaaaa tcataacttc ctttaatatg aggactataa agatataaaa
202501 aatgtgcttt atttttatct cttttaattt ttttatattc attttttca tacaaggtat
202561 gaaatttagc acttaaaaat ggcggagtaa agttagcttt taccccgctt gtacggaaaa
202621 aatccacttg tggaaaacac gctccatcga ttattgaagt ataatcatac cattttaaat
202681 caaatgcaga aacaaattca agtttttttc taaaaccatc cgaatttttt tgatagcttg
202741 ttaaaggaat gcattctcct aatgtaagaa tttttaattt atccaatact tttttatcta
202801 aattttgttt taaaatatgc tctaaaactt ctatacaaac tatagttcca acactatgag
202861 cgaccaaaat gagttcgtaa tcttgcttgt tttcattttg ctttaaaaat tttaaaattt
202921 catctgcaaa aagctttatt ctttgctcca tagcacccaa tcttttttct ttccaagtag
202981 cacaaaaagc acatatcctt gctatccaaa atacggccaa ttttctacct agcttgaata
203041 aaaaacgatt gagtaaaaat cctagaacta tacctataac aatagccaaa aaagaaggga
203101 tataatcttg caaaaaaaca aaacttccca aagctaagcc taaagacaat aacaaagaaa
203161 aaataacata aaaaaaagga taataacccg taacaagctg ataaatcgat tctttaccaa
203221 atttaagaaa aaggcctgtg atggtataaa ttctaaaaaa actataacaa tccatcaaag
203281 catctttgta gctttgagac cagttttttt taactatatc attccaagtt aaaaaatgat
203341 agcgcgtttg agtgttttcg caatttatct taaaaaatgg aaagtttcca tttttttcaa
203401 taccacccat ctttgcttta agatcaaatc tatgagagta atcttttaaa ttcttcttaa
203461 ataaatcata ataaaatcta tagctcttag gatcatagcc cgctatataa aatacttctc
203521 tatccataaa aatacatttt cttttttca aggattataa cattttgttt taaaagctgt
203581 cttaatcgga gttttatcga cttaaaaaag aatttttgta aaaattgcca ttgaattgat
203641 ttgtatttaa aagatagttt ttgaaattta aaaccaagtt aggattgggt ttgtaagcat
203701 gagcttcgcg ccaaaaaaat tggagctttt taggataagc taagccttca aaatcataaa
203761 aagcattgcc acaaacctta gtaggacagc cctccaaaat agcgcttaaa cccacagtgg
203821 aattaatcgt gatacaacct aaagcccttt ttaaaagagt aggcaagtaa gtatcatgca
203881 catataaaat tcttccttct acatggtatt tttgacttag ctcatttata agtttagaat
203941 aatttctata tcccctatcc ataggatgat gtttaaaaac aagataagac tttgctcttg
204001 cgtgatttgc aaaagaaagg atggtttctt ctataaattc ttctatgttt tttttataat
204061 ggtgcttaat ttgcgtatca ttataaactt gtaaaatagc caaaaaatac tttttttcaa
204121 ggctataaat tttttgattg agtttttttt ctgtgaattt ataaaggtat tttctgtata
204181 aagaactaaa ccaaaataaa aattcaaaag gatataaggt gcgatgatga agcttgttgt
204241 taaaaaaagg agctagcaga aaagaaaaaa gccaatacaa aaaagcacta aaggccataa
204301 atttaaaacc acctggaatt tcttttatgc tttcttttgt agaaatattg caagaaagat
204361 agaaattttt atcgcgaggc aaggaggagt ttgcattgac cccatctttt tcaaaagtga
204421 tacaataagg ccttaaatac ccctcttcaa aaatccaaat accaatgcca agctccttag
204481 ctactttgat agcttttgca tggatcaaac ggcaatcatt atacatcaca atagcatcta
204541 tcttttttc tttaaaaaag ttctcataaa aattttcaag atccttttcg tcgcacttgc
204601 acctttttcc acttggatag aaaagaaaat cccctccgtt aaaattaagc ttaaaaactt
204661 tggttttatt ttttttcatc tttatagcaa ggcggtgaaa aaaagtaccc acaggtcctt
204721 gtaaaagcaa gacattttta ccgctaaatt cttttttaat cttatctcta aatttcatct
204781 ttttataaaa acttcacata ttcttcttat ctttctaagc attaaaattc taaaatcaat
204841 taaaattctt agccatcttt ttgaaaaata atctttttgc aaagctaaca ttatatccaa
204901 agcaagctca acttcacata aatttttact ctttggatga atatatcttg gataaaggat
204961 caaaactcct gccacaagct cttctaaact aagcactctt gtgcgtcttg agatatgatg
205021 taaatcttga gtcaaacccc agcccgcata aaaaggcttt ccataaacca ctactttctt
205081 acctcgtaaa agtgcatcaa aaccgctagt agaagtgatg gtatgtactt catcgcatgc
205141 atttatggcg ctgtctatgc tgacattttc tatgatctcg tcgcagtatt ttaaaatgat
205201 atccttatct tttaaaccct tgcgatttcc acttaaaaca tcaggatgag gcttaaaaac
205261 gataaaggcg tctttatttg cagctcttac gctttgtaaa agctttaagg tgtcaaaacc
205321 tgctccacct aagatcatcg aagcatcatc ttctacttga gcggggatga gaatgatttt
205381 tttatttgta ttaaagtcaa gtttttcatg ctttaggccg ttgtactttg aaaatttgtt
205441 ttgtgttata gtgatgatga gctttttggc tctttgtctt aaagtatcat caaattcatg

205501 attttgcaaa acctcttcta aatcgctagg gtgattagga tctacataca aacccttgct
205561 atctactatc aaagaaaaag ggcgtgtaag gtctgaacct aaaaaaaccg agcgcaaaaa
205621 gccatcttct actaaaaaaa tttcattttt aaaatcttta gtcagcaaag ctttatcata
205681 ctttttaccc caaatgaaga ttttatcttc ggaatttaaa ttcgctttgt aaagttcatt
205741 aagtgagttt aaaaaaataa ttttattgtt tttggccttg aaaaatggct tcataaacca
205801 acgcttccat aaagtaaagc ctaaaaaata taaagtatga gaatttactt gctctatctt
205861 tttatatctt gctagggtaa aaatggtatc aaaaatatca ctttttttgat tcaaataagg
205921 attaaaatac tcgctgtata ggatataagc agcacaaaaa acctcttcta aagatctttt
205981 tttaaccctt cttttacact ctagcttatc ttgagttagc ccccatcctg cataaaaagg
206041 cataccataa catacacatt cacatcctag catcaaagct tcaaaaccca tgcccgaagt
206101 cttagtataa actttttttaa aatggcttaa taattctatg ggattataat tttcttttaa
206161 aatcacgcat ctactgggta aatcttgcat gctaaaatca ctttgttttt taccacttag
206221 cacatcagga tggattttga tatatatttt agcatttgga ttttctttta gggcgtcatt
206281 tatgatatct tgggtcgaaa aatcacttgc taaaccaaat cgaagtgaag cgtcatttgc
206341 cacttgagtg ataattaaaa cgcgctcttc gttggcgctg aaaagctctt ggggtatgca
206401 aagattgtta ttatacttgc tgagtttttc tttttgata agctctatgg cttttttttgc
206461 ccaagctagc tctttggggc taaactcata ggtatttaaa atattttcaa gccttgaagg
206521 agtgctcgca tcataataaa tccccacatc atctttaact atagaaaaac tcggactatt
206581 ttctatgcct aaatttaatg agcgtaaaaa accatcttct aaaagcataa atttagcatg
206641 atgtttttttg gctagagcta tagctttcaa gcctgaattt ttcctgcccc agcctacaaa
206701 aacatcttct ttatctatgc ttttataggc cttatacaag actatcttgt aaaaatcttt
206761 aacatttttt ataagttttt ttgaagtggt gtgaaatttc ataaatctct ctttaaatta
206821 aaaagagatt atatcattta tttctttttta aattccttct ttagcttcct aatctcaagc
206881 aataatttga tatacccccc cccataccaa ttattacttg cttttataag ggcctctcct
206941 agcttatagg tcaaacactc tttttctttt aaagcttctt tataatcagg gtaagattct
207001 aaagggggta aagctaaatt tggattttttc tttattttta atttataagc tttttgggct
207061 tgtttgtgcg aaattattat atttaataat atcatgggca ttattaaata acctagtaaa
207121 gattttgaat ttattatcat ggcttgacct agtttataag agagttggtt ttgaatgcgg
207181 gatttggcca ttccatattt agcttggaaa ataatttgat tattttgaat ttggataact
207241 tcttctttat tttttattat ttgatttaga ttttgaattt gaatttggat aacttcttct
207301 ttatttttta ttatttgatt tagattttga atttgaattt gaatttgttc attcatttta
207361 ttttttttctt gaatgatggc gtttagattt tctatcttct tttcattttt ctttagaaat
207421 gtaataaaaa aatcagaagg tagcttagga aaaatattta aaggatcaca atataaatca
207481 aaaatattta aacacaccgc tttatatata ggcaaaaatc tttttcttaa aatttcaaaa
207541 aaaatatctt ttttatacat attaaaaaac aagtcgagtt ctttataaat gagacaataa
207601 ctataatatt tattatattt aaaaatttgt tctttattta aattgcaatt taataaaaca
207661 ctcaagtaac taggaataca cggttcattt gaaccggaat gattacaagt actattactg
207721 cgcactctat atatataaac tttcctataa tatatatata ttagctttga caaagcaaat
207781 aagacaatgc caaaatggtg atcttcgtgt ataattccat tgataaattt taattttatt
207841 tcctttagat atggaaatct tatcatacca tgccaaccaa accaaaacaa tcctgcttga
207901 gaatttaaaa taatatcttt ccactcactt gagttaatta tacattcttt actaatatat
207961 gcatattcaa acatagattt atatggcata tctggcttaa actcattata taaaaattta
208021 taatcaaacc aaaccacctc taccccatcc attctaggta cacattcttc tatgcagttt
208081 aattcccagt aatcatcaga atctaaaaag ataatataat caataatagg ataagtaaat
208141 tttgttaaat ctttttcatc attaaaagct ttatagcttt tatatacagt gtatatttca
208201 taaggattat taccttctat gttaaattct attaaagaat tttctttgat gctttgggtt
208261 ttatttttga gtttgtattc tccactaaag tattctatgc ccacatttct agctgtactt
208321 tgaccaccat tttttttatc aaaaagagta attcttttat cttttaaagt atattctttt
208381 gctatgttta atgagttttc atctgtgcta ccatcattta caagtatgat ttctaagttt
208441 gtataacttt gattgataac actatctaag cattctctta aatattttc tacattatag
208501 atagggatta ctacaccgac ggttttcata tattacctct aggtttattt cttaaaattc
208561 attctaaaaa ttcttttatt tctctatgcc aaacaatatt tttttttaat attctagcag
208621 gaattcctcc tataatacaa ttatttgggg gcttttggta aaaaaatgaa aataatttct
208681 taggaaaaga aacaactaga gccttagtag ctattacaca ttcatcgcct atataagaat

208741 gatttaatat ttttacatca tcacatatcc aacaatgttt acctataact atatttccag
208801 cgtaatttaa tctttttttt gtttctttgt taaaaatact atgattgtct gaagttgcaa
208861 gtgttatatc tgatgcaaac aagcaatcat tgccaatatc aatatgcgta ttcatttctc
208921 cacacaataa cgaaatattt ccaccattaa aaaaacaatt atcacctata actattttac
208981 atttatctga caaagctcca ataccacctt tgaatattat acaattgagt gttcgattta
209041 catatacatt ttttccaatt ttaatttcat tattatttcc atttatatta ataaataccc
209101 ccccccctta tttttattta tttttaagtc ttttggaata taaactttat tattgtttcc
209161 aaaaatagag atatttaaat tgcatattga tggtacatag tctatgccat atatttcatt
209221 attgacacca caaattttaa tattttgcaa tctttcgtca ttattttttta tcaccataat
209281 caacttcctt attagaaaaa tttttagaat aagcaatatt ttattaaata attttataat
209341 ttcaattatt atcctaatct ctttattaaa aaatctttaa aaaatacata tggatttctc
209401 attagctttt tgaactttcg attgaaaatc tcttgcttac tctcaacttt tttagcaatt
209461 tccaaagatt ttatcacagg ctctcctttt gtgaaaaatt ctttttttatt atcagcattt
209521 aacgaaaata cagatgcttt atcacagtag ctaatgctat acattttttt acttatgcca
209581 aaatctctat atatattatc tatagtattg cagtaattct cctgaaaact atttaaagag
209641 agacattttt tatataaatt aagattatca ttttgcattt tatttatttt ctctctcgaa
209701 aactgagaaa acaataaaat attatcagca aatttatcaa tatcttcaac agaggaataa
209761 agcactccac tagttttatt ctccaaccaa cgatatccac ctacataaga gcatattatt
209821 ggttttccaa atgctaaaaa ttcaatcata cttaaatcaa ataaactacc tctatttgga
209881 cataaacaag catcggctat attgatatat tcttgtggtt tatcagtaaa tggtatttgt
209941 atccattgtt ttgaattaat tggcacaaca tgcttctcgc caacaactat aaagtaaatt
210001 ttatcatttt tgctaataat ttgttttgca gcatcaatta aaatatcata acctcttaca
210061 tctataaacc ttccaaggta aagaaatatc ttagcatctt ttggaatatt taaactttct
210121 tttaaaaatt tcttatcatc tgcgggtttg tttatagctg tacctgtagg acaaaaataa
210181 ataggtttat tttttatcaa agaagcaaac tctggccata atttataata tccttctaaa
210241 ctttcctcgg taggaaaaaa caagcaatct gcaagcttaa aagcccattt atctctttcg
210301 ttaaaataat aataaaacat ttctttatca tcatttcgcc atttcatatt tctgcaagtg
210361 agatacatat cttccatagt tggctgatat ggattatgtg ttgtaagaat ttttactact
210421 cttgaatcca cgcctaatct aattaaagaa ttatatattg gcaaaaaatt ataagctcca
210481 tggatatgta tagaagctat attttttgga tctattttga catattctga aaatgggatg
210541 atttctctaa accaatcttg tcttttttgt aataaatttt ttgaaacagt tttaatatga
210601 tagttttgtg tatacttaaa tctttgctca actatgattt ccaatatatc atttgggata
210661 tattggtcag taggaatatc aggaaacaga aaagaacatt ttgtcttttt gtctgtcaaa
210721 aaagaatcat tatatcttct aaatgcttca cataaggaag atatatatcc aacacttccg
210781 cctttttttta aatcaaatgc ttgatggcaa tgaatgtagt gctttaacat gttttacctt
210841 attttttttta taataaaatc tttaaagaac ctttttggat ttttaatgag tttttttaatt
210901 tttcttttta taggatttat attatccacg gtatttacaa tttgcttctg aaaactaata
210961 aatgaacgca ataatatttt atcttgcaca gaaaaaacaa cttcataatt accattgtgc
211021 tgacttaatg ttgcattgga actgaagtta tattttttta ttgtttttaa aggtatttta
211081 atttcttcct tacaaatttg cattatatag tttttttattt taatctcatt taaatttaaa
211141 aaattttctt ctataatact gatttcagat tgttgattat ttatcctttt aacctgaaaa
211201 tcaccaagtc tagtccaaaa tattttaata gaagtggcat gtaaaatttc taaaatctgc
211261 aataaaaaaa acagttgctt attattgcct attaaatgta ttgatgatgt atttttaaaa
211321 tcaattaaca ttaactgttt taaagaaata ggtaaagcat cgcctttttt caaatcaact
211381 atactagaaa tatctataat ttctttatca taaaattgaa ttcctatggg acttaaagag
211441 tggaaaacat aatcttgcgt ataaataacc ttaacattta tattttttttt taaaacttgt
211501 agatctttta acttaatata atgcaattca tcggatggaa gtataatata atgatttaac
211561 attgttttaa ttccttatta atttgttgaa gtatttttgc ttctttattc cagttgtgaa
211621 atggtaatat ttttccactg cttggatctc cattataata atgagaaatt ccttgagatt
211681 gttcttctaa agaaaatcca tagtaagcaa aatcttttaa tctttctctg tgctttaaaa
211741 gataacaaag catcacaata ccatgagata aagttcttag ttgcgttctc ttaataaatt
211801 ctaatgtctg tatttgaaaa aaaggtaaat ccaaaagcat tatcttataa atactacttc
211861 tttcaaatgg agaagtataa aaacttgaat cattggatat cataaaatca aattgggata
211921 aatcttttgt tgttattgtt tcaagagccg gagataccgc ccatacattg gttttggaac

211981 cataatcttt ttcaaaacca tttgttttat aattattaaa acgtataaca atatcataat
212041 tatctataac gcaacccctta tttttattta actcacaagg agagtttcca acaacacaaa
212101 tagttttatt ctctatgtaa ctcaaaaatc tttcttcaga ttgattaaaa taatctttaa
212161 tttctttaca taaatccgtt tgagtattta aattagcttt gattgcactt ctattataaa
212221 accaatcaag aatttgagca ccatcttctc ttaatcgtgg aactgggatt gttaattctt
212281 caatactgtt gtttaacaat actgaaatat atgctactaa cttaggaaat ttattaatca
212341 aaatatttgc attataacta tttaatagct tcaaacatac ttctttgatt tgttctcctg
212401 ggtctgccat aggaatattt ataaaataac ttaattccca ttctaaagtc tttaaaatat
212461 taaaagaaaa attctcattt tgagcaaata ataattttaa gcccatatat gaaataattt
212521 cattataact tgcttggagt gaaaaattct gtattgtagg tagtgaataa taattaactg
212581 gatttaaatc tacttcacga atcaatactt tatcaacatt aaaaccatta tttttttaaaa
212641 aatttttcga ataaagttgc aatatcacat catcacataa aacagtctct atataatttt
212701 taattttttc agcggtatct aaagcattac aagcaataga aatttgatac ctacctatta
212761 gatgagcaac agcaaaacct aaaacatatg ctttatcaag taaaagtttg ttttcattag
212821 aaattaaaaa ttctacaaga tcttttatag caggtataat atcaacaaat ctacttggat
212881 tagctactga ttgctctgct cttcctctta aataataata ctttgctccc ataacataac
212941 gaattttttt acatatagaa aaaacctttg ctacaaaagg aacatcttga taaactttac
213001 aagtttctgg taaagtaatt tggttatcca ataaaaaaat ttttctataa atagctgcat
213061 aaatagcagt tccacaactt aaaaagcgat tttttaattc ctcggtactt aaaacacaat
213121 ttaattttac actagtatat gtagttattg ctaaatgacc aaaataaggg cgattttcga
213181 tatatccatt gtataagaca acatccatat tctcatcttt tgcaatttta tataaaattt
213241 cataatacat ttcatctaca taatcatcag gttcgcatat agctatatat tctcctttag
213301 catgataaat tcctaaattt acagcactac catatccacc attttccttg caatagtact
213361 gaatgctact gattttattt ttttttataa aatcttctat tacaagatgc gtaccatctg
213421 tagaaccatc attaataata atgatctcta tctcttctaa tgtttgtttg cataaagact
213481 gcaaacactt gcccacataa tctgctacat tataacaagg aactataatc gaaacgccta
213541 tttgattcat attctaacct tttagtttaa atttaatata acaaaacact atttggaatg
213601 gtatagttaa tgctaaaaaa taattatttt tcaatcccaa taaaattttt ccgattttat
213661 agcttaaatg ttctttgtac gattgacctt cataatagtc gcaatatttt ttttccagat
213721 tgccatttgg tttagtggta ttagtattga tcaatctaaa taattctaca aaatccttac
213781 ggctcttgca aataatagcc tgtttaccta ttttatattc tggtgtattt tccataaact
213841 caatagcacc ttttttatat ctagctactt cattataaaa ttttttagga ttagacataa
213901 tctcttgaag ttgttctatc tcccattttt taaataaaga gaaactattt tcactaatag
213961 tttttaatct tttaaaatct ttctgaaatt tatacaaaaa ttccaaccta taactcgagt
214021 gaattcgata aagattccaa cgatatccac gatatcttaa ataatgaact atacgcaaaa
214081 tatcataaaa tttatttttc aatacaaatt gttctataaa atcgtattct tcgcaaatac
214141 aatatatttt atctagacta ttacaagaag aattgatatt atcttgtcta taataataat
214201 atgcttcttt cactaaaaaa atactgctag ctaaaacttg caattgaaac caaaagctag
214261 tatcttgaaa agcagaacct ggagtttcat tgcacaatat attgtatttt tttataaaat
214321 ccaatttata aattccacta ggatttataa tgtttgactc agttaaaatt ctcttatctg
214381 ttttaaaatt taatatttta ttatatagaa cttctgtcca atttaacaac ctatattcat
214441 aaactctttc ttctttgtcc caaaaattta acatatctcc tttaactata tcgcattttt
214501 tttctatagc tatattatat aaattttcat acatattttc ttttatataa tcatcacttt
214561 caactatgcc taaatactca ccttttgctt ctttgatgcc caaattcatc tgataaccat
214621 agcttttttt atccgaaact atgactttta gtctttcatc gtttttctca tactctctaa
214681 gcacttcaag agtaccatca gtggatccag catcaacgca aattatttcg atatctttta
214741 aggtttgatt taatacacta tcaacacact cacaaatata tgttatagaa tttaaagatg
214801 gtataattat agatatttta aaatttttca tatctaagtc ataattcttt tttagaaaat
214861 tctaatgcgg atttaacact atcctgcata tcatagtatt tatactcacc tagtcttcca
214921 ccaaaataca cattggtttc ttttttggct agttctaaat atttttcata taatttttga
214981 tttttatcat cattgatagg ataatacggt tctatatctt tagtccactc tagtggatat
215041 tcttcactta ctactgtttt ttcttgcttt ccaaattcaa aatgtttatg ctcaatcgtt
215101 cttgtatatg gagtttcttt atctgtgtaa tttaccacag ctacaccttg ataattatct
215161 gtatttaaaa tcttatgctc aaatcttaag cttctatatt ctaaaggacc atactcataa

215221 ttataaaaag aatcaatagt tccagtaaaa atgatttttt ttgctttatt ctgaaattct
215281 tttttatgtt ttaaaaaatc aatattcaag agcacttcac aattttctaa cattttctca
215341 aaaatagccg tgtatccccc tataggtata ccttgatatg ggtcattaaa ataattatta
215401 tcataaatat atcttactgg gattcttcta ataatcgatg caggtaaatc cttacaatcc
215461 cttccccact gcttttcagt atagccttta acaaattttt catatatatc tcttccaaca
215521 aattttataa catgctcttc aagattccta gggatacctt gtatttcttg gctttgtttt
215581 gttataattt ctctagcttc ctttggactg aaaatacccc ataatttact aaaggtattc
215641 atattaaaag gtaaattata aatctctcct ttaaaattag ctatgggcga atttacaaaa
215701 tgattaaatc tgcaaaattg ttgcatatat ttccatatat tttcatcaga tgttctgaaa
215761 atatgcgcac cgtatttatg aacattaata ttttcgatat tttcagtgta gcagtttcca
215821 cctatatgag atcttttttc taacactaga catttatagc ccttcttgct tgcttcataa
215881 gcaaaaatac taccaaacaa tccggttcca actactaaat aatcatacat acaaaaagcc
215941 tttttaattt ttaaaattaa aagtatagca aataattatt tataaaatat actttgaatt
216001 aaaaattttt taaaaaaatt ttttaatttt attgcctttt gataatctct ataaaattcc
216061 aaaggcaatc ttttatttaa aacatctctt ttaatatctt ttttatatct tctcacttct
216121 ttttgatgtt gtacagatac cttaacaaca ctaaaagcta atgttaattt cattatactt
216181 ttttttctag aaatttcttt tgcaatttta taagaaagat attctttcac tctgctaact
216241 gcacctaagg gttgattttg tatatatatt tttaaatttt tggcatattt ttttagatac
216301 aatggatctt tatcaaattt ttgtaaagtt aaagctttat tgcaaactac tggtaaaaaa
216361 tgctgtttaa tttcatcgct taaatgatgc ttagaatgga taaattttat aaattctagt
216421 gcgatttgca tccagcttgt agtctcataa tacaatcttg tctcattcga attttcaaaa
216481 atatcaatct ttttcaaatg cgaatcagga tgtatcgccc attttttacg ggtaaaattc
216541 atagagcttg attctcgtaa acgataaatg tatatttgct ttggaaatat ataaatattt
216601 ttactcaaag caaacaataa caccccaaaa tgacaatctt ccgcaaatat ccccttgata
216661 aattttagtt ttatgttttt aagaaaagta aaatttacca taccttgcca agcaaaccaa
216721 aaataaaaaa gtcttctttc cttagctcga tcaagccatt cttttgaagt gataatcact
216781 tcatttttat aatcaaagtg agtcatttgg ctaagatgtt ttcttttgac tttatcatac
216841 accgctctat aatcaaacca aaccacctct accccatcca ttctaggtac acattcttct
216901 atgcagttta attcccagta atcatcagaa tctaaaaaga taatataatc aataatagga
216961 taagtaaatt ttgttaaatc tttttcatca ttaaaagctt tatagctttt atatacagtg
217021 tatatttcat aaggattatt accttctatg ttaaattcta ttaaagaatt ttctttgatg
217081 ctttgggttt tatttttgag tttgtattct ccactaaagt attctatgcc cacatttcta
217141 gctgtacttt gaccaccatt ttttttatca aaaagagtaa ttcttttatc ttttaaagta
217201 tattcttttg ctatgtttaa tgagttttca tctgtgctac catcatttac aagtatgatt
217261 tctaagtttg tataactttg attgataaca ctatctaagc attctcttaa atatttttct
217321 acattgtaga tggggattac tacaccgacg gttttagttt gcattgttat gtcttttata
217381 aaatttagaa tctttaaaaa aatccctagg attaaaaaat aattttcttg ctaaacgata
217441 aaatttagtc tgcgaaatca aacttaataa gaaatttaaa cccaaaaatt ctaaaatttt
217501 ggaaaaaaat ctcacccttt ttatctcata caaataaggc ttataaacaa aagattttaa
217561 agaataatac tctttgtcat aatttcccaa cataagcatt tttttcttat caaaaataat
217621 ttgataaaaa ccatcatttt tagcaaaact ataaagctca ttttttaaat aaaaaacaat
217681 tttatacaaa tcatcatctt tactaaatcc taatttctgg ataaaagtaa gaattttctt
217741 tttttcttca aatttcaaag aagtgatgta agcttgcatt tctttaattt gtaatttatc
217801 taaatcaata tcccaatacc ttctatcata aagctcattg gtatctttat aaaacaacca
217861 agaatcaggt aaaattccaa gctcaaaata cacacaaaga tagtttttaa aaatttcgtg
217921 caaatcttcg ttgtatatat ttttatctag taaaatttta tcgaattttt cttcttgtaa
217981 ataaatttca atttcttttt tattttgata agcaagctca tctaagcttt tataaacgaa
218041 cttgcctaaa taaacgctca aagctctaaa attatatttt ttatttgcaa taactaaaat
218101 tttagtattt tttacacttg aaatttgcaa aggatctata gaataatctt ttattttttt
218161 caagtgtttg taaaataact tatgattttt atgcttttta tccatataac cattttgatt
218221 tggctctata tgccaaagat ggtacatata aattccataa aaacatagct cataaccaag
218281 taaagaaaac caagaacgaa aacctttaaa gctataaaaa ttccaatttc tagaatcata
218341 atttaaattt aaaggcacct tctcaaattt taaacaagag tttaaaactc tagcaaaaag
218401 atcaaaatct tcatagccat gacctataaa attttcatta tttccgccaa tttctaaaaa

218461 tttatgtcga ttgataatca aactcgaagt cgaacttgga gcaaaaaact taacaagaaa
218521 atttttttcca ctgatcaaat cttcttgaat taatacatcc cataaatcca ttgggtattc
218581 ttttaaaatt tcactagcct cttgcgttaa atatactacg ggcaatacta aaagagcatt
218641 gggattgttt aagatatttt tgatttttat aagttttaaa attttttcaa gactatcaaa
218701 agaaataaaa caatcaagat ctaaaaataa aagtacacta gcattagcat aactagcacc
218761 aaaatttcga catttgcctt gagaaaaata ctctttttga ctctcatctt tgagataaat
218821 gtgattgttt tttagaatat atgtcttaag gttatgctct tgcgaagaat aaccctcgac
218881 aaatataaat tctatctttt catcggtttt aaaatactgt gccttttcat aaactctttc
218941 ctgtatataa gagcgttctt tactcaaacc aaaaggtatt atcacagata ataagggcat
219001 aaatactaca cattccctat cgcataaagc tgtatgatac ctactacttt atcttcttta
219061 gaaacgacaa tttctttgat tttatacttt agcatgatct cttctgcctc acttgccata
219121 gcatcagcat cgactacttt aggatttata ctcatgatct ctttagcttt aaaatcaaat
219181 cttggtttat cattagcctt taaagcgcgg cgcaaatccc catcggttat aatgcctact
219241 aacttttcat tttcaagcac caaacaaagt cctaatttcc cacttgtcat cacatcaaca
219301 agatcattaa attcagtatc aggatgaact ataggaagat ttttagatac catcagatca
219361 cttaccctag ttaaaagctt tctacccaaa cttccacctg gatgaaaaag agcaaaatca
219421 tcaggtctaa aatttctagc cttcatcaaa gcagctgcta aagcatcgcc cattaccaaa
219481 gtagcagttg tcgaagacat cggagcaagt tgcaaagggc atgcttcttc ttttacagat
219541 atatttaaaa aaatatcccc ttgttttaca agagtagagt ttttctttcc acacatggct
219601 ataagaggga ttttacgttt tttaatcgca gggatgattt ttaaaatctc ttctgtttca
219661 cctgaatttg aaatagctat caaaacatca tcaggagtaa gcatcccaag atccccatgc
219721 aaagcctctc caggatggat aaaaaagctt ggagttcctg tgcttgctaa agtcgcagct
219781 atcttagctc ctatatggcc tgatttaccc atgccactga ctatacaacg cccctttgta
219841 tggagcataa gttctatggc tttagaaaaa ttttcatcca aatttaaagc caaatcctct
219901 atagccttag cctctgtcgc aaaaacttct ttagctgtct ttaaagcatc cataatcaca
219961 ccttagatat ttaatactac attcgttgca acggcaattt gatataaaat ttgcgttagc
220021 atagaaaaaa tttgtaaatt ctcactttcg accttaggca aaactaagac agaatcacct
220081 ggcatcatat caacacttcc actatatctt tgagccttgc cattgttgcg tataacaagt
220141 actttggagg tatctgctct ttctccataa cctcctgcaa gattgatata gtattttaaa
220201 tcttcaccct tattataaac aaaagctcct ggtaaagtaa cctcaccttg gactatgata
220261 agattgtttt tactagggac attgatagta tcaccctctt ctaatataac ctctccataa
220321 gattttggtt tatcgataac aatttgcccc ttaggctcta attctcttgc tctttggata
220381 aactcaagta tcaattttgc ttgttctgct ttgatagctg caccctgaga agtcactgaa
220441 ggactagtta aagcaagggt ttcaagctct ttaagctgag cattgataag atctttttga
220501 gttttagcaa cactttttct aaaaacttgc aaggcattca tgtccgattg cccatttgcc
220561 acgatcagtc ttgaaacatc ctctaacgtc gttccttttc gaaccaccaa agttttaagc
220621 ccgctatgct cgccattgac tgttatactg atattttgcg aaatgtattc gggattaaac
220681 tgcacctcat cacccgtttt taataaaact ttagaaaatt gcattttatt ataagcactt
220741 acttcaagct tatgatcatt tccataactt cttagtatcg cattagttac tataggtttt
220801 gcacctgcta cgcgggctaa atcaaataaa gttttaatat catttgcaag ctcaaagcga
220861 aacggtcttt gcacatctcc atttacaaaa acataacttt ggacattgcc caccaaaacc
220921 acatcgccac ttctaaaagg aaaaagatcc atttgaccct taagtaaaaa atcatacaaa
220981 tctatctttt taatcacagc attattgcgt aaaatttgaa tatctctaaa acttccgtat
221041 tctaaattta tcccacctgc cttatcaaga tactggatca cagaatccga gctaagacct
221101 tgataaagcc ctggagcatt aacactgcct gtgacaaaaa cgctaacatt ttgataagca
221161 ttcatatccg cataaacaaa aacattattt ttataaattt tatttacctg agctttaatc
221221 acacttacaa gagcactatt ttttacccct agcaaattta ccgctcctac cttagggata
221281 aaaatatttc cttgagaatc cacaactaaa atttgttcaa attcaaccgc accccaaatt
221341 ttaagactga tttgatcgcc cactgcgatt ttatagtcag gattataaac cctttgagtg
221401 taatttttga aatttccatt aaataattct gcgccaaata caggaatttg agtaggggca
221461 ctcttgctga tattgttttc tatattttga gaagagctag ttgtgctaga attcccgcct
221521 gcgctaatct gtgaaacatc tatagctcca aaacaaaaaa tacaacttaa aaataaaatt
221581 aatatttttt tcattttaat acttgtgctc ctctataatc attttaacaa atttaatcac
221641 cccaaaaatc aaagataata caataaatgc tgttaagata ttataaattt tttcagggta

221701 ttttgcactt tggggtacat caggggtttg aacgataact agttgtttaa tctttcttaa
221761 agcttctatc cttgcacttt cataagcttt caaagccgcc tcataagcac tttgagcaaa
221821 gcctgcttct atggttagat cttgaaattt agcagctaga tcattgagtt tttgagaaga
221881 attatcggct gaaattttag atctttcttt aactaattgt ttttttaaag cagcgatttc
221941 agcctttagg gttacaattt caggtgcgtg atcattcata tagctttgca tggttaaaag
222001 cttagcttct ctttgagcga tatctgattc aagttgagtt actaagccgg ctttagcttc
222061 tgcttgtttt agaggatcaa aaactccgta tttattttga aaggcaatca aatcattttg
222121 cgctttttga tagcgttctt tatattttac caattcttct tccgcaaagc tcatttgctc
222181 tcttgctgcc ttgtgtgaaa tttcattgat aaatttttca ctttcttgca taatggtttt
222241 ggcgattaaa tgcgcagatt ctggagtaaa tccttcaacc tcgacattta aaagtcctgt
222301 tttatcatcc acatgcactt taacgcggct ttgataatat tttaaataac tttctataga
222361 gctagaactt gataagctaa aaaataaatc gatatattgc tcattgtaaa gtttttttag
222421 atgaattttt tcatctagaa tctttaacat atcaagcgat tctatatagc cttgtaaaaa
222481 cttgatatct tcgctagtat tagaagttgc ggttaaaagc gataaaaccc cgcttgcttg
222541 agtgcttcca gtagttgatt ttacactcat ggtgatagta cttacataac gatcagcggc
222601 aattaaaata taataaatga tcacaaaaat catcaagatc catactatct tgaaagaatc
222661 aaaaatactt agatctttaa ttttattaaa aaatgtattt atttttgtca ttattttttt
222721 ccttgataga cggctatgcc ctcatctaca tcatcataaa cagtagcttt tccattttcc
222781 ataaagataa ttttatcaca ccattcttta atttcagcca cattgtgtga aaccatgatc
222841 actttagatt ggctcaatct ttctttatat aatttatggc ttttttctct aaatttagga
222901 tctcctacag ctcctgcctc atcgattaga taataatcaa aatcaaaagc catgctaagc
222961 ccaaaagcta tcctagcact catacccgaa gaataagtat tcataggctc atcaaaaaat
223021 ttgccaagct ctgcaaaatc ttctacaaat ttgacttttt catgaagctc ttcaccctta
223081 tagccataaa ccctagctac aaatttagca ttatctctag ctgttaagga tccttgaaaa
223141 gctccactta gtcccaaggg ccaagaaaat tttttattgg tgatgatttt tcctctatcg
223201 ggaagctcag agccacttaa aagcttcatt aaagttgatt ttcctgcacc atttctaccc
223261 attaaaccta tactacaatt ttcaggaaat tcaaaagtaa aatctttaaa aacataatgc
223321 cttccgccat taaacaaagg ataagatttg gttaaattta aaacttttat catgttcttc
223381 tcactgctgt taaagcttgt ctattgtaat aatataaaaa aagtcccaca aaagaagtgc
223441 atatgatgca aaatacagga taagtataag tataatcatc ttgcaaagga taattttcaa
223501 aaaaattaaa tcttaaaagc tccataacat gaagcaaggg gttgtaataa aatatatcaa
223561 gaataggttt tggcataagc caagtaggaa agataacacc tgaaccccaa taaaatacta
223621 tactaaaata attaagcaaa gtctttaagg gctctaaaaa atatccaatg atagcaaaaa
223681 ccatacccaa agcaaaaccc gaacacataa gtaaaaatat acaaatcatc acgcttaaaa
223741 aatgcctagg gataacttct aatctaaaaa accaacctac tataaacagt actgctacaa
223801 atataacaaa ataaatacaa aactcaagca aagtcctagc gataaaaaca tgtatgggtt
223861 ttacaggctt gtaggcaaac aagggcaaat ttgctgctat gccattcata agctgagtga
223921 ctatacttct aaacataaaa taaggaacta tgcctgaaat taaaaacata aaaatagaaa
223981 tcccttcagg catgagctgg tgatgatatt ctctaataat agtaactata atggtaagca
224041 ttaagattac actcataggc tcgccaataa cccaaaaata acccagatat ttatttttac
224101 caaatctagt ctttaattct ctaaaaaaca aagcgtgaat tacatttaac atttatttat
224161 atacctaatt tttgcataag ctataatcat aactcatttt tttaaacaaa tcataaaatt
224221 ttatctttat aaaattcaag ctttttgcca agtcctaaag cattgtttgt aaatttcagc
224281 caatcaagct ctaaggcata aatttttgca ctcttatcca attttgcaaa aggaaatttt
224341 gaaaaataaa tttttatttg ctccttgctt gcgcttttaa atttggcctt agcttggata
224401 ccttttaaaa aagctatctt gctttctttg gctatattta gtgctatact aggattttcg
224461 ctagctaatt ttatatgctt cgtatcttca tgtgaggcga tgataaaagc taaatttttt
224521 tcatcaaaag cataaaaagc attggctata taaacacctt tttcatcact catagcccaa
224581 gataaaagct gctcattttt tataaattct aaaattcttt catccattgg gtaaaattat
224641 actaaatttt tttaaaaaat aaccgatttt tcattttaag catatcaaca caaggggagc
224701 aaaatgcaaa ttgatgcgag caaaaatcaa agtttttcaa tggactatac aacaaaaagt
224761 ggcaaacacc tagccttgtc tatgtatgat aatcaaagct taagttatag caatgatgaa
224821 gaaggtaaaa gtcttaatct taaacgccaa tatggtttta gcttcacttt tgagggctct
224881 aaactcaccc aagctgatct tgatgagata aaaaatgcaa tgaaagaagt cgaacccatg

224941 ataaaagact ttttggcaaa ttctaaagta ggagaattaa aaccaaaaga aatcatagaa
225001 tcggcaatgc aaatggcaaa tgtattgcca acgccaaatg atgaaaacca tcaaaatgct
225061 atcatgaata attttacaaa taaatttagt gatttactca aacaaaatca aagcaatgat
225121 aaagatatca atgcatccat gcttgaagat agcaaaaaac tactagatga agttttagag
225181 caaatgaaaa agcagcttga aaaacagcaa gaaaaagcta aagaaaatca aaataatgca
225241 gaaaaaggtt taaatcttta cgcttaaatt ttataataaa tcataaaaaa ttaaaaaagg
225301 cacaagatga aaaatgttga aattttagaa aatatgctca agctccaaca aaaactcaat
225361 gatgaaacta atggtttaaa ttgggaagaa ggttatacca aggaaggaaa gctcatcagc
225421 tggagacgtt gtatttatat ggaatgtgcc gaacttatag attcttttgc ttggaaacat
225481 tggaaaaaca tttcaagccc tacaaattgg gaaaatgttc gtatggaaat cgtagatatt
225541 tggcatttta tcctcagtct tttacttgaa gaatatacaa ataaaaagga taaaaatttc
225601 caagccatag cgaatgaaat catctcagtt agcgtttttc aagatttttg taaagaagag
225661 ggcaatccta gcgaaaatga tctttatagc attttaaacg acatagagct tatcattcat
225721 aaatgcagtg gctttggatt tagccttgga gagctacttt ctaattattt tatcctagcg
225781 attaaatgtg gacttaatct tgaaattctt tataaaacct acataggaaa aaatatttta
225841 aatattttta gacaaaacaa tggctacaag caaggtactt ataagaaaga ttggaacgga
225901 aaagaagata atgaagtatt ggctgaaatt ttagagcaag agcttgattt tgatactatt
225961 tataaaaaat tagaagaata ttataaaaag gtgtaatttg aaaattttaa gacttgctat
226021 taaggatttt ttcacacttc aatttttgaa atttgcctta attccactta cttttagttt
226081 tattttgatg atatttttgg caatttttgg tttttctttt ttgcttgatt attttaattc
226141 tttatttagt gttggagaag actcgttttg ggcatggttt tacgctcttc attttgttca
226201 aattttaatt acgcttataa gtgttttatt ttcaggcttt gttataattt ttgcttcagt
226261 tttcctagcc ctttttatca cttcttttct tactcccctc atcgtaaaac aaatcaataa
226321 caaatactat catcacgaag tacaaaacat ctcaaatatg tatataattt ttgaaatttt
226381 caaaatattt ttaaaattta taggaatttt tttactttgc accttggctt tgtttttgcc
226441 ttttatcaat ctttttgtgt attatcttgc tttttattat ctttttcata agcttttaat
226501 gcttgatata acaagttcgg ttttagataa agaaaatttt aaaattttta acgctcaagc
226561 atctaccttt gaatttaaat tcagcacttt atgtttttac ttgctctcat ctatcccgct
226621 tttaggaatt tttttgcaag tattttttgt gatttttta acacatttag cttatcaaag
226681 aattttaaaa ttagaagtta agggctgaaa tatcgatttt gtgattgtat aaataagctc
226741 ttaaaagtct tggtatttta gcgattctgc aagcttcttt aaattctttt ggcaattttt
226801 gacaaatttc atatttaaaa acgatagcct tttcatcctt atacacgatg cctatttttc
226861 cacctaaaac acaatcgccc ttcaaaaact cttttctttg tgctgcactt aaaagcaaac
226921 ctttcatttt tacaacctta gcaatcaaac tttcattttt aggacaaatc ataagacctt
226981 gaatttcttt tatagactcc aaatcataga gtttttttct atcttcatca agataaaaaa
227041 aacttctttt taagccttga tgaaatttac tcacaaaggc attggaaaaa ttttttctta
227101 tataatttct aaaatatttt tcattctcat tgctctcatc ttgaaaataa aaaatatcat
227161 tctcttttaa ataacttaaa atttcatcct tactcgtaaa aagcaaaggg cgtaaaaggg
227221 tgtaattttg gcgcttttca cactcttgca tccctaaaat ttcggccaaa cccgctccgc
227281 gagaaagctg cattaaaaac cactccaatt gatcattgag atgatgggct aagataagct
227341 tgtcataatt ttgctcaaga catatttttt caaaaaaatc ataacgaaaa tccctagctt
227401 ctttttcaaa attccccttt atcacaggag cgatttttat aaaaattttt ttatgaaatt
227461 ccaaggcaag ctccttggct ttttgctctt ctaaatcgct atttttctc gttttataat
227521 taatcatcac aagatcaaaa tcaatctttt tttgcactaa aaaatgaaaa agcgcactag
227581 aatcacttcc ataagaaaaa gcgagtaaat ttctaccctt ttttaacaag gataaaattt
227641 catctttaat ttgcatctat ttttcttaaa gccttggcta tgagcttttt atcaacattt
227701 tgaatgattt cgcattcata aatttgtccc atttccaaat tcccacattc gctttcattg
227761 ataagaattt ctccatcaat ctctctatcc catcttaaat ctttagctgc gataaaaaac
227821 tcaccctcgc tactctcacc tgtacaaaca actaagcgct tttttcccac ttctttttca
227881 aaactttttt ctatgacttc atctacaatt ttttctatga ttttaagtct tttattgatc
227941 actttaaaag gcacttgctc catatcaaaa gcagctgtat cttcttcttt agaatacgca
228001 aaaacactca ctctatcaaa accaaaatct ttgataaatt cacaaagctc ctcaaaatcc
228061 gcatcgcttt cacctgggtg ccctacgata aaacccgtgc gcaaaaagct atcaggtgca
228121 cttttcatca aatttaacat ttctttcaat cttgtgctat ttgctccacg cttcataatc

228181 ttaagcatat tgtcgctaat gtgttgcaaa ggcatatcaa aataatttac aaaaatttta
228241 gaagctataa tgcgcttgat caaagcctca ctcgcacttg taggataaag atataaaatt
228301 ctagctgctt taataccctc gatcttttct acttcatcaa tcaagcgtat aagcccatct
228361 ttttcacctt tatcaaacaa ataagaactc gtatcttgtg cgataaaaga aaaatcctta
228421 taacccctag ccaccaaatc tttaagctcg gctataatgc tagaaatttc acgcgatttt
228481 aacttaccct taaaacttgg gatcgcacaa aagaacatt tttgattgca accttcagcg
228541 atttttataa aagcatgtga attagatcct gtgatgatgc gtttggagtt ttcaccctgc
228601 aaataagtcg aatttgaaaa taaattggtt tttttaagta tcatttcatc gattctttca
228661 taatcaccca caccagtaaa aagatcaact tcaggaagtt ctttcataag ctcttcacgg
228721 taacgctgca tcaaacaacc cgtcacaacc aaaagcgaat cttttttttct ttgctcatgc
228781 aaatctaaaa tcgcattgat actttctttt tttgcactat ctataaagcc acaggtattt
228841 acgattaaaa catcagcctt acttggctca tcgcaaagct cataagcgct aagacgccct
228901 agcataattt cactatcgac taaattttta ttacagccta aagacataag ataaagtttt
228961 gacatttttt tcctatatta aagttttatt ttgatataat ctttgatttt aaaaatataa
229021 ttatagcaaa gaaattttat ggataattac gaatttagcg aacttttaaa aactttaaaa
229081 aacaaagtcg gcaacatagc ctctatcatc aagcctgaaa acatccaaac aaggcttaaa
229141 aagatcgaag agcttgaaaa ttctccttct ttttggaatg atgtcaagca agcaggaatc
229201 ataggcaaag aaaaaaccaa gattaccaat ctactaaaaa actacgaaaa tgctttcaat
229261 gctctaaatg acgcaagcga acttttcgat ctagccaaca gcgaaaacga tacagaaacg
229321 cttgaagctt tatttaatga tgcgccaaaa cttgaagata ccatcacaag tcttgaaatt
229381 tccatgcttt taagcggaga aaatgatggc aaaaatgcta ttgtttctat tcatcctggt
229441 gcaggtggaa cagagagcaa tgactgggca agcatacttt atagaatgta tttaagattt
229501 tgcgaaagag agggtttcaa agtcgaaact ttagactttc aagagggcga ggaagcagga
229561 cttaaggatg tgagcttttt ggttaaaggc gaaaatgctt atgggtattt aaaagctgaa
229621 aatggaattc accgcttggt aagaacctct ccttttgata gcgcaggacg ccgtcacaca
229681 agcttttcaa gcgttatggt aagccctgaa cttgatgatg atatagaaat agaaatcgaa
229741 gaaaaagata taagaataga ttattataga gcaagtggag cgggtggaca acatgtcaac
229801 aaaacagaat cagccgtaag aatcacacat tttccaaccg gtatagtcgt acaatgtcaa
229861 aatgatagaa gccaacacaa aaacaaagcc accgctttta aaatgctaaa atctcgcctt
229921 tatgagcttg aactcatgaa acaacaagat agtgctaatg caggtgaaaa aagtgagata
229981 ggctgggggc atcaaatccg ctcttatgtg cttttccctt atcaacaagt caaagacaat
230041 cgaagtggcg aggctttctc tcaagtagat aatatccttg atggagatat taaaaaaatg
230101 atagagggtg ttttaatcgc tttaaaatca gagtaacagg cttaaaaata agtaatttcc
230161 gcagaatgtc ggtataaaaa agaattagaa agatagcggg attttcccgc ttgttttatt
230221 atcttgcata aattctataa gcatcaacca caggacaata aattcttttt ttaagatcac
230281 tatcgcaata aacataaaca actttatagt ctttttttatc gtttggattt aataagaaaa
230341 catagtttga gtatggatat ttatctattt ctaaatcacg cttcatttct ttgattttac
230401 cttctggatc tttaaaatca ctaattgctc tgactgaagt ataatcataa agcttataat
230461 ttggatattc tttcataatc tcttgattac ctaaatcaaa catagcttga tcgttgccat
230521 tgttttttaa ctcgactaaa tcgccatcgc cacaacctat aatcaaagtc ataactccca
230581 aagccaaagc acttgttaaa atttttttca tttttgctcc tattttaaaa attaaattaa
230641 tataattgta cctaaattta aaaatttaaa tatcattttt gtaataaatt tttaaagtaa
230701 aaatcacatt aaaatttaac tcgcaatcat ttcattcaaa caaattttta tgtaaaatct
230761 cttctaaaac cacggtagca taagagcctt tttgcaagaa aaactccatg caaaaatgag
230821 ctttttgctc atcataacgc catttaacat cttctaaata cgaccacata aagcgtcttg
230881 agccttgcat tttggcttta aattccaaag cgtctttaaa aatttcattt tccaaattta
230941 aagccagtcc ttgacttgct tcgtaggctt tagcaccgat taaaagtccc atagcactga
231001 tatctcttgc tttaaagcgt tcaagttcgg cacttaaatc ctcacataaa aaacattttc
231061 caaaaggata atgccccaaa acctcgcctt ttaaaagttt aaaaaactgc tcttgctttt
231121 taagttcttt agcttcttct ttggaaatac tataaatttg tgctaattct ttttcagtaa
231181 aatcatttgc aaaatgcgat aattccactc ttttactcaa ataacgatta aaaagctcgc
231241 tttgaaaagc agagattaaa aactccttca ttttgacatt tttcatcttt ttaccacgta
231301 aaatttcaag cccttctttg taattgtccc caaattttcc aaagcgttga taaccaaaat
231361 aatttgcaaa accctgttta tccaaattga ttaaggcctg ctctaatttt aaagcatcgc

231421 taggcaaaac ttttttttaag cgtataaaaa aagaatttcc ttttaaatgg cctattctta
231481 atttattttc atgaataaaa gtttctaaaa ttttaagctt aggatgagaa aaattagcca
231541 aaaaactctc gaatttttta ggcatagaaa gatattgaaa agtagagcct tgcttgtctt
231601 taagcccagc atagccaaaa tcacgcatct taacccccact taattcgctt aaaattctta
231661 aagcttcatt agtgcttaaa tctttttttat tgatatgcaa gataagatgt tcgcccttgc
231721 cactaaattc atacaaaggc ctttcacgca ccacaaaatc atcactattt ttactaaaat
231781 acgcattaat aggactgtgt tttaagctat ataaaggctt aaaaatggtg ttctcttccg
231841 ctaaattcat actttccttt cactgctttt ccaaaaatat ttatttttac gccatgtttt
231901 tttgcaattt tttctatctt tggatactct ttttcatcaa atgcaaataa aatttcatat
231961 tcttccccac tataaagcgt ataatcatca agtgttttaa accaagaaat accgcattta
232021 ttttgtgcta aaagtcttga taaatcttta ctcaaaccat ctgaaatatc catagcgcaa
232081 gatataaaag gtgcgatttc ataaaaaaaa tcaggcctta attttggttt tataaagaca
232141 tgatttgaat ttaaagtgcc gccattttgc aaaatttcaa gccctcttaa acttttaccc
232201 aaaacccctg tataagcaag caaatgtcct tttttagtc ctttctaaa aatagcctta
232261 tttctcactt tagaaatgat agtaatgcta atatcaatct tatcattact aatcgtatca
232321 ccgcctataa ttttgatatt aaatttttta gccgttttta aaaaaccttt ttgcaaggcc
232381 ttaatctcat cttcacctag actatttggc aaggcaagcc ctaaaagtgc gtattttggc
232441 ttagcattca tcacaatagc gtctgaaata tttaccagca ttgcctttgt agcaatttgc
232501 tctaaagtca gccattctct tttaaaatgc acattttcaa aaaacaaatc cttactcaag
232561 caccatttat cgacgatagc accatcatcg ccgttttttt catttaaaaa agctttgatt
232621 ataaaatctt ccttattcat gaaaagattt tataaaattt ccttttaaat tctagtgaat
232681 tttaaaaaaa ttttatataa tacttatcaa aaggaaaaag catgaaaaat ttagaactta
232741 gaatttttag atttgataaa caaaaagatt atgaggccta ttataaaccc tatgtttatg
232801 ataattatga aaattttgcc actctttatg acttgctttt gcaagtgcaa gatgatgata
232861 tttattttga ttttgaaaaa aatgataaaa gctatatcgt agtcaataaa gaatttttac
232921 cattagacac agctttggat atattagtca aaaagtatga ttttaacctt accatagagc
232981 cactaagcac taaaagatca gtaaaagatc ttatcatcaa taaagatgat tttttagaaa
233041 aatttaaata ccttgctcca tttgcaaatg aagaagataa aaagctttat gaaaaatatg
233101 attatttgta ttattcaagt gaaattttag actttttacc tgaatatatg ggcgatgcag
233161 tgttttatct ggcttcaaaa atgatagaaa aatatcctaa caaaaaaata aaaattttaa
233221 agacaatttg tgacacgcaa aaaggtatct tttatcatct gccaagtaaa aatgaagatt
233281 tggaaaatat catcaaaaat ttacaaaaag aaatcataga tttaaaactt atcaatgagg
233341 ctgctttaga atttgattta ccaaaaatca atgcttttga taatgaaatc aaagagcttg
233401 gtgaagtaaa atatgatttt gataatttta atatcgcttg ttatggtttt aagatcaaag
233461 atgatataaa atcaaaaatc aaagcccatt ttatctctta tgaaaatagc gataaaaaca
233521 atggattttc attacttcat ctaaatcctg aacttagcta taaaatggca gcaaatatta
233581 tccttgatgc ttacgatagc ggtgctgatt ttatagtggt aaatcaagca aaagattttt
233641 atatgtttga cacttgctcg aaaaaactta tgcaaagtag cggaagagaa tttaaggatt
233701 tttatgtttt gagctatttt gaattccttt ctttaatcca agggattaaa aatccttctt
233761 tgcaaaatca tgacttgaaa gtaagtttga tatgaaaatt tctctagaat gtaaagattt
233821 gatcatcgaa aaagccttag agcttttttt aaaagagcat ttagtgatga aaaaagattg
233881 tgattttatt ataagtgatg aaaaaatctc tactcaaaag cctttattta ttatcgccaa
233941 aaactcaccc tttttaagtg tgccttttag caaagaaact ctcataaatt ccttaaatga
234001 attcgatagc gctctaaaag caacggctca aaagctggct gatgaaagaa gaagggtact
234061 agaagcaagg atagatgaaa tcgcaaatga atttaaaaaa gactatcaaa gcaagataga
234121 tcttgctatc agtgagctta aagataaatt agttaaagct ttaatgtatg aataatgaat
234181 ttgtaagtgt taaggatttt ttacagggta aaaaaaatat ccattttaat gactctaccc
234241 agcccaacaa aaaagaaaaa accaaaaaaa ctttgcctaa attatataca agcatagaaa
234301 gtggcatata taaaggcaaa aaactgcttt tacctagtct tcaaaccaca agaagcacta
234361 aaagtattgt aaaaaaatgt gtttttaatg tcttaagatt ggatttaaga aataaaattt
234421 ttatcgaagc ttttggagga agtgctttaa tggctgctga agctttgagt aattacgctc
234481 ttaaggctta tgccatagag cttgatttaa acgcttataa aattgcttta gaaaatgcta
234541 aaaatataga tacaaattta aaagtaattc atgctaatac ttttgaaatg ttacccaaac
234601 ttataaaaga ttctaaagaa gatattattt tatatcttga tccgcctttt gatataagag

234661 aaggttttag tggaatttat gaaaaaattt ataatttctt agaaaaatta aaattagatg
234721 ctttattttg tatcatttta gagcataatt ccaaaataaa aactcctgat aatatagcga
234781 attttaccaa aacaaaagaa aaaaaattcg gatcaactag tcttagtttt tatcaaaaaa
234841 atattctaga agaataaatt ttaatcttgg aacacttatt gcttctttta ttctaactat
234901 cagattaaag gaagaatcga tgagggtttt aacatttttt ttacttttga tgacgagtgt
234961 ttttgcaaca caaataaaag atatagctaa taccgtaggc gtaagagata atcaacttat
235021 aggctatggt cttgttgtag ggctaaatgg tagcggagat ggaacaagct cgaaatttac
235081 actacaatcc atatccaatc ttttgcaggg tatgaatatc aaagtagatc ctaacgatat
235141 caaatcaaaa aatacagcag cggttatggt aacagccaaa cttccagcct ttgcaaaaag
235201 tggagataaa cttgatatca cagtttcatc tatgggcgat gcaaaatctt tacaaggtgg
235261 aacactcttg cttaccgcac ttcgtgggat cgatggggaa atttatgcta tcgcacaagg
235321 ttctatctca acaggaggat taactgctag accaggagga acaggctctc actctactgc
235381 tgctactgtg atgggtgggg ctaatgtaga aagagaaatt ccacaaaatt ttagccaaat
235441 tgatgattta accctaagct taaaagtagc ggattttaaa acagcaagtg atatagaaag
235501 aattttaaat accatatttg atgaagaagt agctagagct attgattctc gcacagtaaa
235561 acttaaaaaa cctgaagatc tttctaatgt tgattttatg gcaagagttt tagagcaaga
235621 tattgcttat actcctcaaa gtaaggtaat tatcgatgaa agaacaggaa cagtgattgc
235681 gggagtggat attgaggttg agcctgtttt aatcacccat aaagacatta ccataaaaat
235741 tgatccaaac aataatgcag cagcggctca aaatgaaatc gatatgaaag atggtggctt
235801 tgtagatcct aattcaaaca ccctaagaat caacaatgcc aaaagtacag ttgcaaacat
235861 tgctagaatg ctaaacaaac ttggagcaac gccaaatgat atcatcgcta ttatggaaaa
235921 tttaaaaaga gcgggcgcta tcaatgcaga tttggagatc atctaatgaa agtagataat
235981 tttttaagca cttatagtat gaataatgcc ctgcttgaac gtgcagctaa agcaagaaat
236041 ttagaatctg attttaaaat caataacaat gagataataa caaaaaccaa agaagatgaa
236101 gccttaaaag agcaaactga tgcttttgaa gctttttttc taaagcaagt tttagatgta
236161 tctttaaaaa ctcaaaattc actttttggg aaagatgcaa gtgatgaaat ttattcatct
236221 atgtataatg ataccatgag taaggcctta agtggcggta tgggattttc aaaattatta
236281 tacgattttt taaaagaaag gggttaagtt ttttaatttt tggccgatat agtttgtaga
236341 ggatttatat aaagatcaaa ggaggcagaa atgatcaatc ctatacaaca aagttatgtg
236401 gggaatgcca ctttaaatac aacaaaaaca gataaagaaa ctaaaacaaa cgatactcaa
236461 aaaacagaga atgataaagc tgcgagaatt gcagagcaaa tcaaaaatgg tagctataaa
236521 atagacacaa aagctacagc tcaagcaata gcggaatctt taatttgatt tcattctcct
236581 ttttttgagt taaaaaggag aatgaaatgc ttaaacaaag attagatgaa gtcaatgcta
236641 tcttaacaaa actcatagaa ttaacacaag aagatataga aaatataaaa attgcaaagc
236701 atgatacggt tgcttcaagt gttgaagaaa aaaacaagct tattgctgaa tttagcgctg
236761 ctaaaaagcg acttgattct gctttagttg aacttaataa tagctctaca aaaggactaa
236821 gcgaattgct tgatgaggaa gataagcaaa aattagatct acttaaaaaa aatttacaaa
236881 cccttcatac cacaaataaa gaatatgcga aattcgttct tattgtcaaa gattttcttg
236941 atggcttagt aaataaaatg tttgatatga atgatggaac aaataatgct tatggagata
237001 aaaagactac tccagagtca atttttaaaa tcaatgttta aggattaaag atgggtattt
237061 ttggaacttt atatacagga gttaccggtt taaaagcaag cgaggtgcaa attgcaacta
237121 caagtaataa catctccaat gcaaatgcaa ccttttatac tcgtcaaaga gtagtacaaa
237181 ctacaaatgg ctttgttaca acaggtggag tacaagttgg aacaggaaca gccatagaaa
237241 gcatagtccg cttgcatgat gagtattcat attataagct taaaggtgct tcaaatcagt
237301 tagaatatac aaaatatatg gcctcaactc tagaagaaat tgcccaacgc ttccctgatt
237361 tgcaaagtac tggaatttta caggacttag aaaattataa taaagcatgg aatgactttg
237421 cttcaaatcc aaatgaaaat gctactaaaa tagcccttgt aaaagcttct caaaccctaa
237481 cagaaagtgt aaataacacc tttgccacct tagataaaat ccaaaaaaaa gtaaatgatg
237541 atatcaaaaa cactgtggat gaaatcaata gaatcggtga agaaatcgct actatcaata
237601 aacaaattta tggtcaagaa gctctaccaa cagatcatgc aaatgaactt agggatagaa
237661 gagatgagct tgagcttact ctttcaaaat tggtcaacgc agtagcaagc aaaaatgaaa
237721 tcaatcaaga caatagactt gatactacaa tcatcgatcc aggacatcaa tacaatctca
237781 gcatagaagg ttttagcata gtagatggtg tgaattttca tcctttaaaa cttgattatg
237841 atgataaaaa taaagcctat agtatttatt atgaaactcc agatgaaaaa gttcgcgatc

237901 tgactgcaaa aatcaatggc ggacaactag gagctcaact tgacttacgc ggaagaaatt
237961 atagcaagag tgaaggaaaa tatagcgatg gaatcatcca aggctatatg gactctttaa
238021 atacctttgc aaaaaccatg atcaatgaaa ccaataatct ttatgcaagc tcggcaaaaa
238081 gctcggcaac ttctgattat cttccaggac ttacagagga tattcctttg gtaaattatg
238141 acagaactat acaaccagga agctttgata tagttattta cgatgataag ggtgataaaa
238201 aacttacaaa aactattacc gttgatgtaa ataccaccat ggaagatatc atgcgtcaaa
238261 tcactgctaa cacagatgat aatggcaaca ataatgcaaa tgacgatgtt gatgatcata
238321 tcaatgcaag ttttagctat gatgcaaaaa caggagacag tttatttcaa atcaatgcaa
238381 aatcaggctt taaagtagcc atagaagata agggaaccaa ttttgctggt gcatttagca
238441 taggaggatt ttttagcgga actaatgcta gcgatatcaa agtaaaagat tctatcttaa
238501 atgatccaag cacggtaaga gctagcttaa atggtgttga tagtggaaat gatatggcaa
238561 ataaaatcat acaacttcaa tacagtaaag tgaattttta caatgaagat ggaacaattg
238621 ataatcttac tatggaagag tattaccgca aacttacggg taaaataggt tctgatggtg
238681 agaataacaa tgtcgtaaat gcaagcaatc aaacgcttta taactccgtt tatagcgaat
238741 atcaatctaa aagtggagtc aatacaaacg aagaattagc cgctctcata caatggcaat
238801 caagctatgg cgctgcagca aaaatcgtaa ccacagtaga tcaaatgcta gataccttgt
238861 tagggcttaa atcctaagct ctagcggtat caagtgaaaa attttatcca gcttaaaacc
238921 aaactcgatt ctttagtcaa agaaaaaaat accaacaagg ctctttttga aagtcctgac
238981 cctttgcaga tcgctaaatt tcataatgat gaatttattg ctttactttg tgctttattt
239041 gcttatggaa atgctaaaaa tatagtcaat tttttaaaaa agcttgattt ttctttgctt
239101 gatttaagcc aaaaacaaat tcaaaaagaa cttcaaaatt taaaataccg ctttcaaaat
239161 gaaagagata tacaagaaat ttttctcacc ttttcaagac ttaaaaatga aatttcactt
239221 catgagcttt ttagcacagc ctatgaaaaa agagaaaata ccaccgatgc gatcttagct
239281 tttttgcaaa aaatcaagac tttaaattcg tattcaagtt atggttatga ttttttcttt
239341 ggtaaaattt ggcaaaatac tcctacttca cctcttaagc gttataatat gtatctaaga
239401 tggatggtta gaaaagatga acttgatctt ggccttttta ccaaaattca cacaaaagat
239461 cttttaatcc cacttgatac acatacacat aaaatttcct tgactttagg tcttctaaag
239521 cgtaaaatat acgactataa aagcgtttta gaactcacac gtaatttaaa aaaattggat
239581 gcaaatgatc ctataaaata tgattttgca ctttatcgtt tggggcaaaa taaagcactt
239641 agtaaattaa aggagtaaaa aatgggcttt gaacatctag atacgattta ttattttgta
239701 ttgttttttg tggccctttt tgcaggattt attgattcta tcgtgggtgg aggtggactt
239761 atcacacttc ctgcgcttat agcttgcgga attcctgcac atctttcttt agcaaccaat
239821 aaactccaaa gtgtctttgg ctcttttaca gcgactttaa cttattttaa atcaaccacc
239881 ctacctcatc ttgcttatgg tatttttttc acagccttag gcgcagccat aggaagttac
239941 agcgtgcttt ttgtaaaaga tgagcattta aaacttatca ttttgatttt cttaacttta
240001 acttttttat atactgcttt gcgtcctagc ttgggcaaac acgagagtga agcaaaaata
240061 aaaaatatca aattttttca tttaatttgt ggattaactt tgggttttta tgatggcttt
240121 ttggggcctg ggacaggttc attttggatt tttgcttgtg taatgctact aggttttaac
240181 atgaaaaaag caagtatcaa tacaaaaatt ctaaatttca ctagcaatgt cgttgccctt
240241 ttcatctttt tgtggcaata tgaactttta tggatagtgg gtttacttat gggtgtagga
240301 caaattttag gagcctatct tggatcaaaa ttagtactta aaaccaatgg taaatttata
240361 aaaaccctat ttttaatcgt agtgggtgct actatcatca aagttgcttg ggattatttt
240421 tcttaaaaat ttactcctga gctaagttcc catatcggca aaaaaattcc aagcgctaaa
240481 aacaatacta aaatcgcaac ccacaaggtc attataggct ctaaaaagct taaaaactta
240541 tccattaaat tttcactttt atttttgtaa tactttgcaa tctcctcact taaaaactca
240601 agcttagaac tcttcatagc tacactcaac atagaaaaaa ccaaaggatc aaaaagatct
240661 actttttttaa aagcataact tatttcaagg ccagaatcca taaatgtttt aacttcttga
240721 taacataata atatcttttt attatttaga gccaaagtgg ctagcctaaa agcatctaaa
240781 agagaaattc cgctttttaa aagcaaggaa aaaatcataa aaaaagaata attttgctga
240841 taaatgataa atttagacaa taaaggaatt tttacaagta agctatcgca aaaatatgaa
240901 aagaaataat attttctata taaaaataaa gcaagaaata aaacaactat gattaaacaa
240961 agcacaagca aaatatattt gtctaaaaaa ttataaatat aaattataag ctcagttata
241021 aaaggtaggg ttaaatttaa atcctcaaat aaatctttaa aattaggcac aacaaaaagc
241081 attaacaaaa caaaagctgc gcatacgctt aaaaaaacaa tcaaaggata tcttaaagct

241141 tttttgactc tttgttcatt ttctacatta ttttgcctta gtcttgaaat ttgcataaaa
241201 atttcactta attttcctgt gttttgggaa gttttaataa gggctaattc cgcatgagta
241261 agcccaaaat ttgcattttc aaaggcaagg cttaaatttt gtcctaaatt taaattatca
241321 caaagcttag caatcatttt gtgagaatat attttattaa gctcatttaa agcatctttt
241381 atgctgattc ctacttcgca aagcagggat aattcttgaa agaaaaggat aaatttctca
241441 ttatccaatc tcttaaaacc cgctttttgc atctctttga tatttaaaat ttcccatttt
241501 ttagcgatag cttttgcttg agcttcattt aaatttctag ctcttaaatg ctttttctgt
241561 tttgttttgt ctttaagaat ttcaagctca taaagcttca ttaagcaccc ttacaagctc
241621 atctatgcta gttatgccct cttttgcttt ttgcaaaccc tgctcaaaca tactttgaaa
241681 accctttgct ttggcaagct ctaaaatttt atttttataa ccaccctctc taataacttg
241741 agctatttcc tcatcgatat ataaacactc gctaataagc tccctacctt gatatccact
241801 atgatgacaa tgctcacaac ctttagcctc aaagaaatca ccttttattt cataaatctt
241861 agtgcttttt tgcttgcatt taggacaaag tttgcgcact aaacgctggg caataatgat
241921 tcttaaagca gaagcgatca aataaggctt tgcattcata tcaatgagtc tatctatggt
241981 agaaatggca ttatttgtat gcaaggtact taaaagcaag tgtcctgtta aggaagcctt
242041 aatcgctata tcaagactct cctcatctct gatttctcct atcatgataa tatcaggatc
242101 ttgtcttaaa atagctctta gggcattatt aaattcaagc cctgcttttg gatttagcaa
242161 aatttgctga actagaggca ttttatattc tatgggatct tccacagaaa ttattttctt
242221 ttctatactt tttagctcat ttaaacaagc atacaaactt gtactttttc cacttcctgt
242281 aggacctgtt agtaaaatca ttccataagg gttgtatagg gcttttttta aaattgtaag
242341 atttttttcg cccaaattta aattttctaa atccaaaatt tcttcgttgt gttttaaaat
242401 tcttatcaca atgctttcgc cattttgcaa aggcaaagat gaaatcctaa aatcatatct
242461 attttctttg attttaagct caaaatttcc atcttgtgcc tttcttgatt ctgcaacatt
242521 aagctgtgat aaaaatttaa tatgaaaaac caaagcctca taaatatctt tttcaagaca
242581 agcaaattcg cgtaaaattc catccactct aaatcttatc aaagcatcat tttctcttgc
242641 ttctatgtgt atatcactgg gattaaattc aagaatttca tccagtataa aatcaaaaat
242701 ttgactgata taactttgct cattttggta tttattttct ttccattcta acctaagatt
242761 ggcacttaaa ttttgaagct tttccttaac ttgaagttta tataaaaatt tcataatctt
242821 aaattcattt gcgatcaaaa cttctatatc tttagcttcg taaaaaattc taatttcatc
242881 taaaagctct aaggaacagg gtttagaact tgctatataa agtgtatttt ccttactcaa
242941 aatgggtaga attttaaatt tcaaaagaaa actacatgga aatttttcac ccaaactaag
243001 atcaagttct tctaaagtag aataagaaat attttcatcg ctagctaaag attttaaaaa
243061 actttcacta tcgattttat gctcgtgata aatttgctca agggtgattt tatcattgat
243121 ataatcttga aataaaaatc gttcaatcat ctatttcctt atcaaattca atcaaaccat
243181 aataactcac ataagtatcc aaggcttgct tagcctcctc atcttttcct aaagcttgtt
243241 tggccttgga aaataaaatc catgaagatg aatttttttct atcgatatca ttagccttta
243301 aagaccaaaa aatagacttt tcataatctt tattttcata atatcgagca gctaaattca
243361 tagccttttc ataacttgga ttttggtaaa aatttctttt taaatcactt atatttaaaa
243421 gtttagaata aatatcgatt tgagttttta actcctcttt taactcatct ttgttttcat
243481 ccttggataa tctttcttgc aaaattttat ttttttcatt tgcaatttgt gcttgtgcga
243541 tttgatgatt gagtgttttt ttatattcta aagcctccaa agctaaggcc ttttgcttgt
243601 aaaaattttg tatcacaaaa aagcttataa ccatacacac caccaaagca gcaaagataa
243661 ataaaaattt gaaatatttt ttcaataaat atcttttgta tttatcctca agctcacgca
243721 ctcgctcttc tatattcatt ttaaaaaccc ctgctctaac gcgcttagtc tcaaaatata
243781 atcttgattt ttgttgttgt ttaaataagc gtttaactcg caaaaacttt gaagtatttt
243841 gtctaaatat cttaaattgc cttgataaat tttcataaga aattttaaag actttttgaga
243901 aagctcttga tcaaatttca ttttaatata actgcttaat tcatttatat taggcttttg
243961 aagccaaaac atagcactaa aacgactttt aaaatactcc ttttcaaaaa gattgatttt
244021 tttatgtgag ctaagtacaa aactcatttg atcactataa atccgaatta attctaatat
244081 atcagggcta tacatcccca cttcatctag tatgataatt tttcgttttt tatcttgtat
244141 aaatttatga aaatcttttt catctgtaaa aatttgatta gtaaaatcct cattatgatg
244201 caacgcaagt ctttgcaata aaacactttt cccactaccg cttttacccc aaagtaaaat
244261 caaagctttg ttctttaata aggcattgat ttgattaaaa cattcttgtc taaaagcaat
244321 tatattattc attgtattta aatcctaact ccctaagact aggtatatgt gtattgtcta

244381 ctatacttgg ggtgatgata aaaacaattt ctgtgatatt ggaattttca tcttcacttc
244441 taaataaaga acccaaaata ggaattttag ataaaaaatt cacagaatta ttattttgtg
244501 agcgattatg agagatgagt ccgcccaaaa ttaaagtttg attattttct acttgcacca
244561 cacttgaaag cttttttga attgtatcag gtgcgatagt gcggggttct ttctgtcttt
244621 tattgtcctc tggatactta aaatcactca aacttggatt gattctaagc atgatttttc
244681 catcatcaga aatttcgggc aaaatattaa gcaaaattcc tatgaaaata gaataattac
244741 tataactttc acttactgtt gtaccatttt cagtgccttt agagctttct tttacttgat
244801 aattcacagt atcgcctatg gaaataatgg cttgttgatt gtttaaagcc attaatttag
244861 gattagacaa aacactggtt tttccacttt gggataaaaa attaacaata gaatcaaaat
244921 tcaaattcgc tcttaaaccc aaatttttaa caaaaccttg accattttgt atttgcataa
244981 aagaatttgt attatcactt gtttgagaat ttagtcctat attgaaattt tgccaattga
245041 tcccactaga atgattttta ttaagattta cagcaagtat gctcacatct ataataactt
245101 gtttcttaag acggctttca agcttttgca aataagcttt tacgcgttga agctgagaat
245161 gcgttccagt aacgataaca agtccagcat taggattgac tatgggtgct tttatgtcgt
245221 agttttcatg cgaagagcta agtaaaatca taatttcttt ttctatattt tgccaaaagt
245281 caaatttttc catactctta atcatattat cttcattatc ttcaaaagca ctataatact
245341 cactttgcct tggtttagaa tccactgaag ctttagttat gctttgtcct tctcttactg
245401 aagtgatata gttgatttta aaaatttag tttgtaaggc agaaattttt aacaccttgc
245461 catcaaattt ataagctaaa tcttgttctt ctataaaaag tttaaaaatt tcatctaaac
245521 tcatttggtg gatattgata ctgctttgaa cagtttttaa tttttcttta gcaatcgtat
245581 ctttaaccac cacactaaaa gaacaataac tagcaagctc ccttaagctc tcttctatac
245641 tcaaatcttc atttatgctt atatcaaata agcgcttttg gcattctatg gcataaatga
245701 gatttgtaaa aaatagaaat ttaataaata ttaatcttat cattgttttt aaatgctatt
245761 ttaaaatcca aagtatcgta tcttaaaata atttcttttg tagtaatatc agtaatcacg
245821 gcctccccta tcttatcacc ctttttatac catttattat tgatctttgc tctatcaagc
245881 ataatagcag taatttttat ttctttgatt ttttcaaaag taggattgaa gaatggattt
245941 tgaaaatttc caagttctac actctgcttt gtatttaaaa tcttgaaata ttcttcaaaa
246001 tcctcatatg ataacaaagt gcaaaagcac aatgttatac ttaaaaaata ttttatcagc
246061 tctcaacctc attgctaaaa tcagccatag caattctttt aagttgtcta tagcgacgct
246121 gtgcatcttt tttattgcgt tcaaataatt cttttgcatg ctctggattt gattttttca
246181 aagaattata gcgcacctca ttcattaaaa agctctcata aagatcccaa tcaggctctt
246241 ttcctaccat agttaaagga tttttaccct gctcttctaa tcttggatca aaagtataaa
246301 gaggccaata accacatttt gtagcaagtt cgccttgctc gcctgaatat ccaagtccgc
246361 ccttaatgcc atgagcgata caaggagaat agcatataat caaagaagga ccatcataag
246421 cttcagctgc cataatagcc ttaattaaat ggctataatt tgcagtagaa ttaacttgcg
246481 ctacaaaaat ataaccataa gtcatagcaa tctgacccaa atctttttc tgtataggct
246541 ttcctgctgc tgcaaattgt gcaactgcac ctgttcttga agattttgaa ctttgacccc
246601 ctgtatttga atacacttct gtatcaagta ctaaaatatt tacattttca ccgcttgcta
246661 aaacatgatc aagtccgcca taacctatat cataagccca gccatcgcca ccaaaaatcc
246721 agtgagattt tttacttaga aaatgtttta aatttaaaat atcctgaaca gctttaatat
246781 cagtgttttg ttctaaaata ggtatcatct tatctttgat ttctactgat tctgcgcctt
246841 tgtctttatt agcaatccat tctttaaaca atgctgctaa agcgttaggt actttttgca
246901 tattttcatt cataatattt tcaatacgat atcttgtatt ttcagttgca attttcatac
246961 ccaagccaaa ttcagcatta tcttcaaaca atgaatttcc ccaagcagga ccgtgtccat
247021 ttttcacgct ttttctataa ggagtagaag gagccgaacc tccataaatc gaactacatc
247081 ccgtagcatt agcgataatc attctttcgc cgaataatct tgtaattaaa gtgatataag
247141 gagtttcacc acaacctgga catgctccat ggaattcaaa taaaggctga gcaaactgcg
247201 cccctttagt gctttcttta tttaagatat catctttgta agtaatttct ttaaataaat
247261 aatctgcatt ttcttgctca ccaaaatcca tttcttcttg aagaggaacc ataaccaaag
247321 atttttcttt agtaggacac tcatgcacac aaagctcaca gcctgtacaa tcaagcggag
247381 aaacctgaat tttgaaactt aatttttcac ctttagtgcc cttagcttct aaagcgtgat
247441 cttttactcc ccttggagct ttagccattt cttcatcatt gatcaaaaat ggtctaataa
247501 ccgcatgtgg acatacagaa gcacattgat tacattggat acaattagcc tctatccatc
247561 ttggcaccat taccccaacg ccgcgttttt catattcagt cgttccatgc tcaaagcttc

247621 catcttcata acccaagaaa gtagatacag gtaattcatc ccctttggct gcattcatag
247681 gtttaacgat cttttcaaca aattcggtac ccttgtaagc attggtttgt tctttttctt
247741 tgatttctaa atttttccag cttggatcaa cttctacttt tacaagtcca tctgctccta
247801 catcaatagc tttgtaattc atctctacaa tagcatcgcc ttttttgcta taagacttat
247861 aagctagctc tttcatatat ttttgagcat cttcataagg aatgattttt gctagtttga
247921 aaaaggcaga ttgcatgata gtatttgtac gatttccaag tcctatatca cgagcaagtt
247981 ttgtagcatt gatgatataa aaattcactt ctttttcagc caaagtcttc ttaactgcat
248041 ctggaagttg tcttatggtt tcttctgcat tccaaatact atttaaaagg aaagtccctc
248101 cttttcgaat tcctgctaaa acatcataaa tttcaagata agctgctacc gaacaagcga
248161 tgaaatgtgg agttgaaaca agataggttg agcggatcgg ctttttagaa aatcttaaat
248221 ggcttcttgt atagccccct gatttttttag aatcataagc aaaatacgct tgtgcataaa
248281 aatctgtttt atctccgatg attttaatag aatttttatt agcacccaca gtaccgtctg
248341 cacctagtcc ataaaataaa cattctatag cactttcatc ccctaatgaa atcttttcgc
248401 ctgtttgcaa agaagtgtga gtaacatcat cgataatccc aactgtaaag ccatcttttg
248461 gattatcaag cttaagattt tcaaacaccg ctatcatttg cgcaggatca acatccttag
248521 aagaaagtcc atatcttcct cctacgataa caggagcatt ttctttgcca tagaaagctg
248581 atttgatatc aagatataaa ggctctccaa gacttcctgg ctctttagtt ctatctaaaa
248641 cagctatttt tttcacagat tttggcataa catcaaagaa gtattttaaa ctgaatggac
248701 gatataaata aactttttaca atccctactt tttcgccctt agaattaaga tgatcaacaa
248761 cctcttcaag agtttgagtc acagaaccca tagctataac aattctttca ggctctttgt
248821 gtccataata agtaaatggt ttatattctc tacctgtgat ttttgaaatt tcttgcatat
248881 attcatttac aacatctggt aaagcatcat aaaaacggtt gcttacctct cttgtttgaa
248941 aataaatatc atcattttga gccgttccgc gtgttttagg attttcagga tttagagcac
249001 tatttctaaa ttcaagtaaa gctttacgat ctaataatct atcaaaatgt gcataatcca
249061 ttacttcaac tttttgaatt tcatgtgaag ttctaaaacc atcaaagaag tgcaaaaacg
249121 gcactcttcc tttaatcgca cataagtgtg ccactccggc taagtccata gtctcttgta
249181 cagaatgtga acaaagcata gcaaaaccaa tctgtcttgc agcataaata tcttgatgat
249241 caccaaatat agataaagct tgcgctgcta aagaacgcgc tgctacatgg atcacacagg
249301 gaagtagttg acctgcgatt ttgtacatat ttggaatttt tagtaataaa ccttgagaag
249361 ctgtataagt tgtggttaaa gctcctgttt gtagtgatcc gtgcactgta cctgctgcac
249421 ctgcctcact ttgcatttca acgattttta ctggaacacc aaaaagattt tttttacccg
249481 cagctgccca catatcagtg taatcggcca tgggagagct gggtgtaata ggataaatac
249541 cggctacttc agtaaaggcg tatgcagcat atgcagcggc ctcatttcca tccatagttt
249601 tcataatttt acccatagaa accttccttt attattaatt gataattttt ataatttttc
249661 ggccaccaat ttactataaa ttctcttaaa aggcgtttaa caaaaacttg aagttagttt
249721 aattattttt tatatataaa actgatttat aggcacttga aaataccttta gaatagcgtt
249781 ttaactcatc ttcatctcca taaccacaac tcaaagccaa aggggtaata tttgccgcct
249841 ttgctgcttg gatatctaag atcgtatcgc ctatcatata ggcattttct ttagtttttt
249901 gcatttttttc caaggctaaa agtatgggtt ctgcatgagg ttttggattt gttacatctt
249961 caagtgttat taaagtttta aagaattttt tcactcctaa atgctctaac aacaatggag
250021 taaattttcc acccttagtt gtaactatac ccaaatctcc gatctcaaaa gctaattcta
250081 aagcttcttt tgctttaggt aaaagagtgg tttgttctaa ataaatctgt gaataaattt
250141 cacgataagc tagaacaaat tcttgactta aattcgcctt atcttgataa aggaatttaa
250201 acatatgctc taaaggatga cctataagat ttttaatctc ttcgttattt tttggagtta
250261 gtccaaggat tttaaaagct ccttggaaag aatttaagat ggcatcagta gaatcaatca
250321 aagtgccatc taagtcaaat aaaatagttt ttttcattag tttaaaataa atttagcctc
250381 tactcttcta ttatccgctc taccttcttt ggtatcatta cttgagcgag gtttatcttg
250441 accatagcca accgtttgga tacgactttt atctacgcca aatttttcaa gctcttttgc
250501 aacactttcg gcgcgtcttt ctgaaagttt ttgattgtat gatcttgaac ctgtattatc
250561 agtatgaccc tctaaaatag tatcatatct tacattttca tctaaaattt tagcaatttc
250621 tttgattttc tcttcaaagg ctggattgat atttacttta tcaaaatcaa aatgcccttc
250681 caaataaatt gtttttcgc aaccattttc atctaacaaa gcaccctctc ttggttctgc
250741 aggacatttt gattgtgaaa ctacaggagc tggagcggga gttacaactt gaggtttgct
250801 aaccacttct tttgtttgct ccactacaac tttttcttgc ttagcaccaa aaccaaaact

```
   250861 aacacccaaa gttgaaaccc aactatgatc cgcatcatgg aaagaaattt gatctcttgt
   250921 ttcaagtctt aaagctaaag aatcactaag gcgaaatttt aaacctgcgc cataatgacc
   250981 aaatcctcca cttttattat caaaggcacc atgagaaaaa tcctcgtatc ccccaccagc
   251041 taaaccataa aaataaaatt tctcacctaa atcaatgcct ttaatagcac tcaaataagt
   251101 tctagtaata tcggtggtaa gagtagaatt tgtatatttt acatccgagt aatgttctaa
   251161 acctagttct aattgatcaa gccaaaaatc atcaaaatga tatcctaatc taatccctgg
   251221 tgcatagcga ttatccatat ctaaattacc ttcaaaataa ttgtaattca aagtaggagt
   251281 aatttcaaat tttacattgt tatctgcacc aaataaaacg cttgccaacc ctaaacatag
   251341 taataacttt ctcatcaaaa gcctttcata gataaattca cttaagtagt gcgaatttca
   251401 attatatcat aaaatataaa aaaaataaat agtaaataga gataaaatat aaaaagcggg
   251461 aaaaccccgc tgaagattaa cgtttagaga attgtgggct tcttcttgct ttgcggcgtc
   251521 cgtatttttt acgctcaaca gttctgctat ctctagtaag aagtccttta ggttttaata
   251581 aagctctaaa atcagcatcc atagccgcta aagctcttga aattccgtgt cttaaagcct
   251641 cagcttgagc actataacca ccacctaaag tcgttgcttt aatatccatt gaagtttctt
   251701 gtttagttac aagtaaaggc tgaactactt taagtttgat tgcttcatgt ccaccaagcc
   251761 aagtatttaa atcgatacca ttaacgctga ttttaccgct acctggtttt acccatactt
   251821 ttgctatagc agtttttctt ttacctgttg catatgttgt tgccatgatt attttccttc
   251881 tttagcaatt tgcgcagtgt gtgggtgctc actacccgca taaattttta atttttttaag
   251941 catcgctcta cccaaatttg ttttaggaag catacctcta actgctaatt tgtataattt
   252001 tgcaggattt ttttcaagta aatctccgaa tttttcgctt tttacgcttc caaaatatcc
   252061 tgaatgtctg tgataaagct tatcttctgc tttatttgca cctgtaaata cagcttttga
   252121 agcattgatg ataatcacat aatcaccaca atcaacatta ggagtaaagc aaggtttgtt
   252181 tttacctctt aaaattgttg ctacttcagt taaaagacga ccaaagcgtt tgccttctgc
   252241 gtctaaaaca atccattctc gtttaacttc gtttggcttt gttatctttg tcataattct
   252301 ttacctttgc caaaaatttt aaaatgtgat tgtataaaaa acaatattaa tttttactta
   252361 atttaagttt aaataaagaa ataattttta tttttaata tatttaaaaa ttttattata
   252421 ctgatgatta tgcactataa acttttaata atcaaattaa tttttcatca ccaaccaaag
   252481 gcgatattaa ggaataaatt tttaatattt caaatcattt acttcgaagt atttttaaat
   252541 tctttttttg aatttaccca tataactcat ctaataccca aagcttttat aaagaaaatg
   252601 gaaaagatct aacgccatat tgatttaaaa ttttaaaatc caaattaggc tgatcaagtc
   252661 ctattatctt acataaaatt tcatcacttt tcatagattt tactctaaaa gaaacactaa
   252721 aacttatgca atcattatct tcttctaaat aatgttcaaa agtaaaaagt aagtcgtcaa
   252781 aaacaccata agtgataaat tcttctgttt ttaaaccata ttgtgtgaaa atttgtccag
   252841 tttttttgcaa aattttagaa caatatcatc taagatttct tgtcgtattg ttgaaattcc
   252901 tttgtagaat tattttacta ttttttactt tgtttaattt gaaaattaaa aacccactaa
   252961 gattaaatta aatcataagg aaaatattta tagcttttaa attcttcttt atctaaattc
   253021 attagcttta caattgctgc accttcaaga tcccaagtat ctagaggatc tcttaatctt
   253081 gtatgttttt tcatatattg atgtaaaaac tcaatatcct tagtagctac aaattcttta
   253141 ataagctcgt gttcttt
//
LOCUS       DMACINML_3            185032 bp    DNA     linear       16-DEC-2020
DEFINITION  Campylobacter species strain strain.
ACCESSION
VERSION
KEYWORDS    .
SOURCE      Campylobacter species
  ORGANISM  Campylobacter species
            Unclassified.
COMMENT     Annotated using prokka 1.13 from
            https://github.com/tseemann/prokka.
FEATURES             Location/Qualifiers
     source          1..185032
                     /organism="Campylobacter species"
                     /mol_type="genomic DNA"
```

```
                     /strain="strain"
     gene            complement(234..1229)
                     /gene="ansA"
                     /locus_tag="DMACINML_00520"
     CDS             complement(234..1229)
                     /gene="ansA"
                     /locus_tag="DMACINML_00520"
                     /EC_number="3.5.1.1"
                     /inference="ab initio prediction:Prodigal:2.6"
                     /inference="similar to AA sequence:RefSeq:EAQ71964.1"
                     /codon_start=1
                     /transl_table=11
                     /product="L-asparaginase"
                     /protein_id="Prokka:DMACINML_00520"
                     /translation="MAEAKPKIAILATGGTIAGSIDSAVATTGYTAGVVGVDVLIKAV
                     PQIQDLANISGEQIANIDSSNMRDEIWLKLAKKINKLFDEGVDGVVITHGTDTMEETA
                     YFLNLTVKSDKPVVLVGAMRPSTAISADGPKNLYNAVALAADKEAKNKGVMVAMNDKI
                     LSARGVVKTHSLNVDAFSSPNFGDVGYIVDGKVFFYNNISKAHTKNSPFDVSKLTSLP
                     KVDILYTYSNDGSAIAAKALFDNGTKGIVVAGSGAGSIHEDQKNALKELIKQGLDVVV
                     SSRVAAGRVAVSDTDKKLGFISAEDLNPQKARVLLMLALTKTSDPKKIQEYFLKY"
     gene            complement(1367..2938)
                     /gene="recJ"
                     /locus_tag="DMACINML_00521"
     CDS             complement(1367..2938)
                     /gene="recJ"
                     /locus_tag="DMACINML_00521"
                     /EC_number="3.1.-.-"
                     /inference="ab initio prediction:Prodigal:2.6"
                     /inference="similar to AA sequence:RefSeq:CAL34209.1"
                     /codon_start=1
                     /transl_table=11
                     /product="putative single-stranded-DNA-specific
                     exonuclease"
                     /protein_id="Prokka:DMACINML_00521"
                     /translation="MKTINKSEIKKILASRFEKDLHTKLCDLPLPCCLKDAYKAANRI
                     KEAIDKNEKIAIVGDYDVDGIISCVIMAEFFDDIGFDYTVRIPNRFKDGYGLNAEIIN
                     ELDVNLIITVDNGIAAFEAAKLCKEKNIDLIITDHHMPQDILPDAFAIINPKQKDCDF
                     PDIEICGAQVAWYLIAALKEVCKLKYDMGKFLELLAIAIVADMMELRDLNRALVRRGI
                     ECINKSKRVAFKAIKQYYQKDKFALDNIGFLIAPLINSAGRMDDASISYEFLHTKDLD
                     KASAYLEQIISFNESRKDEEKQLFEDSLSQIDENDSCIVVSGLNWHEGVLGIVASRLA
                     KHFNKPAFVFSQNDQRLKGSARSVGKIDILALISKANSILSNYGGHKGAAGLSLDLDS
                     FEKFKTIIKKECMEISQSEFLDTDEILGILDPNEIDFEMLEILESFEPFGHKNPRPFF
                     ILENLYVKNKKFLGKDEKHLKLILTKDNKTIEALFFNFDKEPQLCENINLLGSISKNE
                     FRGLATPQFIIKEIL"
     gene            complement(2925..4559)
                     /gene="pyrG"
                     /locus_tag="DMACINML_00522"
     CDS             complement(2925..4559)
                     /gene="pyrG"
                     /locus_tag="DMACINML_00522"
                     /EC_number="6.3.4.2"
                     /inference="ab initio prediction:Prodigal:2.6"
                     /inference="similar to AA sequence:RefSeq:CAL34208.1"
                     /codon_start=1
```

```
                     /transl_table=11
                     /product="CTP synthase"
                     /protein_id="Prokka:DMACINML_00522"
                     /translation="MSKQTKYIFVTGGVLSSLGKGIAAASIATLLKNSGLKVSILKAD
                     PYINVDPGTMSPFEHGEVFVTDDGAETDLDLGHYERFLDESLSQDNNFTTGRVYQSVI
                     EKERRGEYLGKTIQVIPHIVGEIKERIKKAGEGKDILIVEIGGTVGDIEGLPFLEAIR
                     ALRLEVGKNNAMNIHLTLVPFIKAAGELKTKPTQHSVGELRRIGISPDMIICRSEKAL
                     DRDLKDKIAISCGVEKNCVIESVDAASIYQIPLNFLKQDILNPIASILDLKNLKPNME
                     NWDILVKRVIAPSNEVKIAFVGKYVDLKESYKSLTEAIIHAGAALDTRVELQWVDSEK
                     LENLESAEAFKNVSGILVAGGFGYRGVEGKIKAIHYARENKIPFLGICLGMQLALVEF
                     ARHVLKIEDANSSEFDDKCSNPIVYLIDEFMDASGKKQIRTAKTPLGGTMRLGSYECK
                     VKPNSLLAKVYNNAKNIKERHRHRYEANPKYRKEFESKGLIVSGESEGLIEAVELNNH
                     PFFLAVQFHPEFTSRLERVNPVICGFIKAAISYEDN"
gene                 4672..5295
                     /gene="thyX"
                     /locus_tag="DMACINML_00523"
CDS                  4672..5295
                     /gene="thyX"
                     /locus_tag="DMACINML_00523"
                     /EC_number="2.1.1.148"
                     /inference="ab initio prediction:Prodigal:2.6"
                     /inference="similar to AA sequence:RefSeq:CAL34207.1"
                     /codon_start=1
                     /transl_table=11
                     /product="thymidylate synthase"
                     /protein_id="Prokka:DMACINML_00523"
                     /translation="MQITLLFHTPLSVCSHATRTCWQSFDKGDCGGEKDKELIDRVGN
                     KFKHASTLEHLNYTFYIQGISRACLQEVARHRHTSPSVKSTRYTLKELRNESEFKIGD
                     FENASRYLVLCGNEEVDNASIQALENLRLILQKSISLDIAKYCLPESYKTELTLTINA
                     RSLQNFISLRSSKSALWEIRNLANALFEALPQDHQFIFEHCLHKDIH"
gene                 5414..6799
                     /locus_tag="DMACINML_00524"
CDS                  5414..6799
                     /locus_tag="DMACINML_00524"
                     /inference="ab initio prediction:Prodigal:2.6"
                     /inference="similar to AA sequence:RefSeq:CAL34206.1"
                     /codon_start=1
                     /transl_table=11
                     /product="putative sodium:dicarboxylate family
                     transmembrane symporter"
                     /protein_id="Prokka:DMACINML_00524"
                     /translation="MDKQFFQMFLMFSQIQTVAIIAVLFVIFYILKKMRDKKVNFSLR
                     MLFALFIGLGLGFALQYLAQFPDSKEASTIIWYNEAKHWFGFVSSVFVAFIKMLVIPL
                     VGICIIKVIIEIDKNIKISSLLGISLFWILFSTAIAASLGILLAYNFQLGDNFTAYEG
                     ARQIREIQTFSSIILGLIPSNIISVMNKENIIAIVIFAFFVGICAKKVSKKEEYTQAF
                     QTFENFVLVFYNIMMNMTATVIRFMPYAVVCMMANVLLSNGFEAIKTAGLFIILTYIA
                     MFIMFGVHFLLLASQGLNPIKYAKKAFPVWLFAFSSRSSLGTLPMTTSTLQNKFGVNP
                     AVANFVASIGTTTGLNGCAGYFPAMAAVFVAFATHTPIDFTFALMIVLVAVIGSLGIA
                     GVPGSATMAASIMLAGIGFGDNFVMLSLILAIDPIIDMARTASNVSGAMTSALCTAKN
                     LKAIDKEIYNS"
gene                 complement(6831..9200)
                     /gene="nrdA"
                     /locus_tag="DMACINML_00525"
CDS                  complement(6831..9200)
```

```
                     /gene="nrdA"
                     /locus_tag="DMACINML_00525"
                     /EC_number="1.17.4.1"
                     /inference="ab initio prediction:Prodigal:2.6"
                     /inference="similar to AA sequence:RefSeq:CAL34205.1"
                     /codon_start=1
                     /transl_table=11
                     /product="ribonucleoside-diphosphate reductase alpha
                     chain"
                     /protein_id="Prokka:DMACINML_00525"
                     /translation="MKVIKRNGRTEELDVSKIKKCTFDAIKDLEGVNLSELELDAKIQ
                     FRDGISTEEIQKTLIKTAVDKIDIDCPNWTFVAARLFLFDLYKKVNGMNRYNHLREYF
                     EKGEKEGRILLGLKEKYDLDDLNDYIMPERDMQFTYLGIKTLYDRYLIKDSKGMPIEL
                     PQQMFMAIAMFLAQNEFNPQEWAKKFYDLISKFELMLATPTLSNARTTRHQLSSCYIG
                     STPDNIEGIFDSYQEMALLSKFGGGIGWDWSKVRAMGGSIDGHKNAAGGIIPFLKITN
                     DIAVAVDQLGTRKGAIAVYIEPWHMDVSDFIDLRKNSGEERRRAHELFPALWINDLFM
                     KRVRANDKWTLFDPADTQDLCDLYGEAFEKRYEEYEKDESIVKEIVEAKELWKKILLN
                     YFETGLPFLCFKDNANKANPNAHVGIIRSSNLCTEIFQNTEPNYYKIKVIFENGDEKH
                     FDEEERVVIDGGYEKPAKKISTLDSFNGNKVYIVEKYKSDGKTAVCNLASINLSKVYT
                     KEDIERVVPTAIRMLDNVIDLNFYPHRKVKDTNLKSRAIGLGVMGEAQMLAEARIHWG
                     SDEHLNKIDEIMEKISYEAINASSNLAIEKGSYPDFEGSNWSKGIFPIDVASEKAKAL
                     TLREGLFNQSECDWDKLREKVKADGMRNGYLMAIAPTSSISILVGTTQTIEPVYKRKW
                     FEQNLSGMIPVVVPNLSLETWQYYTPAYELDQRILVKAAAVRGKWIDQGQSLNIFLSL
                     DKASGGYLNDIYQLAWELGVKSTYYLRSESPDSEKINVADRSIECEGCQ"
     gene            complement(9215..10543)
                     /gene="purB"
                     /locus_tag="DMACINML_00526"
     CDS             complement(9215..10543)
                     /gene="purB"
                     /locus_tag="DMACINML_00526"
                     /EC_number="4.3.2.2"
                     /inference="ab initio prediction:Prodigal:2.6"
                     /inference="similar to AA sequence:RefSeq:CAL34204.1"
                     /codon_start=1
                     /transl_table=11
                     /product="adenylosuccinate lyase"
                     /protein_id="Prokka:DMACINML_00526"
                     /translation="MVERYSREIMAKKWDMQAKYDAWLKVELAAVKAWNKLGLINDTD
                     CEKILKNAKFDIARIDEIEKTTKHDVIAFLTSVSESLGEESRFVHYAMTSSDCIDTAV
                     ALQIKESLDLILEDVSLLLEVIKKRALEHKNTLMVGRSHGIHGEPITFGLVLAIWYDE
                     ILHAKELLEHAREVISYGKISGAMGNFAHAPLKFEEEVCKNLDLKPAPVSNQVIQRDR
                     YAQVISAIAILASSCEQIAVAIRHFQRTEVYEAEEYFSAGQKGSSAMPHKRNPVLSEN
                     ITGLCRVLRSFVTPALENVALWHERDISHSSVERFILPDAFVTADFMLMRLTNLIDKL
                     LVYPENMMKNLNLTGGLVFSQRVLLELPFKGISREEAYKIVQRNAMKVWADLQNGKAA
                     INENNESLFLLALLADEDLRKSLNEEDIRKCFDYNYYTKNVDAIFARTFK"
     gene            10584..11519
                     /locus_tag="DMACINML_00527"
     CDS             10584..11519
                     /locus_tag="DMACINML_00527"
                     /inference="ab initio prediction:Prodigal:2.6"
                     /inference="similar to AA sequence:RefSeq:ADT65449.1"
                     /codon_start=1
                     /transl_table=11
                     /product="RNA pseudouridylate synthase family protein"
```

```
                     /protein_id="Prokka:DMACINML_00527"
                     /translation="MEFYLKKFRERLSLAYTKIKLSNNGKKAFQVLIDNLKININEAQ
                     KLIDKKRLFCNGILVEEKNKILQGNVELIVYENNPKGVEIAFENEDFAVLEKESGILS
                     HPNGRHCKYSLSDEIWHLWGKEACVAHRLDKETSGLILVAKHKKAQIELKGLFEKKQI
                     QKEYLALASGEIKANFIVDQAMDLTKDYDDVKTRMQICENGKKAITEFEVLSFYPKIN
                     ATLLLCKPLTGRQHQIRVHLFYKNHKILGDPLYGLEKKDIEKILDEKMDIEERIKLSG
                     AKRLCLHSYRLKFNYDGENFDIFSKKGIEKILTAF"
gene                 11647..12513
                     /locus_tag="DMACINML_00528"
CDS                  11647..12513
                     /locus_tag="DMACINML_00528"
                     /inference="ab initio prediction:Prodigal:2.6"
                     /inference="similar to AA sequence:RefSeq:ADT65448.1"
                     /codon_start=1
                     /transl_table=11
                     /product="fumarylacetoacetate hydrolase family protein"
                     /protein_id="Prokka:DMACINML_00528"
                     /translation="MKFVNFKLNQKATLGVLNSSGKVINLADLDINVRDMNELIINFD
                     NFKHKFKDLDSKTAYEIPKEDYLAPIIEPRQDIICLGINFLDHAKESAQFKGEKFEER
                     EYPVYFGKRCNQATAPFGSISLHADVTSQLDYECELAFILSKDAYKIKAEDAKDYIFG
                     YTIINEISARDLQKRHKQFYRAKSLEGSTIMGPYITSVDEIAYPPELNLQSYVNKELR
                     QNSNTKFFIFDIGYVLEELSSGMLLKAGTIISMGTPSGVGMGLNPPVFLKKGDEVRCV
                     IENLGEICNVIT"
gene                 12571..12738
                     /gene="dba"
                     /locus_tag="DMACINML_00529"
CDS                  12571..12738
                     /gene="dba"
                     /locus_tag="DMACINML_00529"
                     /inference="ab initio prediction:Prodigal:2.6"
                     /inference="similar to AA sequence:RefSeq:CAL34199.1"
                     /codon_start=1
                     /transl_table=11
                     /product="disulfide bond formation protein"
                     /protein_id="Prokka:DMACINML_00529"
                     /translation="MEFLELLLVLIALILIIKKPEKENLAFGLVIVAWLLMVFFYIGH
                     KTGALLTIMNL"
gene                 12753..14270
                     /gene="dsbI"
                     /locus_tag="DMACINML_00530"
CDS                  12753..14270
                     /gene="dsbI"
                     /locus_tag="DMACINML_00530"
                     /inference="ab initio prediction:Prodigal:2.6"
                     /inference="similar to AA sequence:RefSeq:CAL34198.1"
                     /codon_start=1
                     /transl_table=11
                     /product="disulfide bond formation protein"
                     /protein_id="Prokka:DMACINML_00530"
                     /translation="MNEINKTKNFYTLMCLAGFLIILLPVGIANFVFGYMWGDSPCTL
                     CWGQREAMIFIGVMALFIVRYGMKGKYLAALLIMTAVGLYQSFAHYGNHAHRDLDQGF
                     GLAVFGIHTYFWAEVVFWAVVLLLGVMFAFAPKFGSFDKELNGEKFRKFTKFSFAAVL
                     ISTLIVASNVFQAFVSTGIPPYVGQGDPVRFSLNPKYIIWSTGGWNGLWQNISFLGKR
                     DVKAPDYAFAPASEKLGIKFDNDTNNSPFAEIDDELKIIDEQTINFDKAINTLDYIND
```

```
                     EFVASSKWDVAFLDNNFSTKEGFELDPYFSATIDPIIGIIPYKENKFLLMGSNKSFLR
                     FAKNPNADEALQYADFVKGNDQFEGQGKDLGRGRLDTVRAKFNHVASMTTDDHYLYLA
                     TVPNNKDSKTFVISKISLKDLVLSAEFTPKAELKEGKTLGDLYITSMAFKDDEIYALS
                     KNHNVIAIINPTKEEVVKTIAFPSSITNARSIFFKDGKIHILSYQDGANKLYTLK"
     gene            complement(14290..14721)
                     /gene="yjaB"
                     /locus_tag="DMACINML_00531"
     CDS             complement(14290..14721)
                     /gene="yjaB"
                     /locus_tag="DMACINML_00531"
                     /EC_number="2.3.1.-"
                     /inference="ab initio prediction:Prodigal:2.6"
                     /inference="similar to AA sequence:UniProtKB:P09163"
                     /codon_start=1
                     /transl_table=11
                     /product="putative N-acetyltransferase YjaB"
                     /protein_id="Prokka:DMACINML_00531"
                     /translation="MIKHYEKLNQEQAKGLLEIWEQSVKATHNFLDEKNFRTIKEYLQ
                     NADFKDLNFLFAYKNDEFVGFLGADKECIQMLFVKPEYFRKKIGSKLLQKALDDFKIK
                     KVEVNLDNPNALKFYQKFGFEKVGEYKDDFGFILIKMEIKS"
     gene            complement(14724..15410)
                     /locus_tag="DMACINML_00532"
     CDS             complement(14724..15410)
                     /locus_tag="DMACINML_00532"
                     /inference="ab initio prediction:Prodigal:2.6"
                     /inference="similar to AA sequence:RefSeq:CAL34197.1"
                     /codon_start=1
                     /transl_table=11
                     /product="putative transcriptional regulatory protein"
                     /protein_id="Prokka:DMACINML_00532"
                     /translation="MSKKALCIISGGMDSTLCAYLAKKEGYEIIALHFDYQQRTQEKE
                     KECFKQICKALNVEKSYIFDVSFIANIGGNALTDQSIHIPKNELSISDTPPITYVPFR
                     NGIFLSIAGSLAEKENCESIFIGVVEEDGSGYPDCTEKFIQKAQEFINEGTSKHFITS
                     LKTPLVNLNKTQIVELALKENVPLELTWSCYESEDEACGECDSCLLRLRGFERAGFKD
                     KIEYKKYKGL"
     gene            15810..17036
                     /locus_tag="DMACINML_00533"
     CDS             15810..17036
                     /locus_tag="DMACINML_00533"
                     /inference="ab initio prediction:Prodigal:2.6"
                     /inference="similar to AA sequence:RefSeq:CAL34196.1"
                     /note="hypothetical protein Cj0015c"
                     /codon_start=1
                     /transl_table=11
                     /product="hypothetical protein"
                     /protein_id="Prokka:DMACINML_00533"
                     /translation="MINIKLIEHIFKAASISRWNDYPRMANLVELDKQAHKFIIAYFI
                     AKMEKDVDMKIIIEGGIFEFLSRVVVTDIRPDVYHEIVRQKKAEVNAWVLSKIEPMIE
                     DIEDGEFLKRFEAYLNGNTYAKERLILKAASYFATRWEFNIVYQTSAFLNDIDEIKNK
                     VDEELEDYYELIGARKIALNQKIAKIIDLSGRLRFQKRWAQTPRIPETAVLGHMLVVA
                     ILGYFYSLKIKACDKRLENNFYCALFHDLPESLTRDIISPVKYGIDGLHTIINDYEMK
                     LINDRILPFVPENLRAEFSYILGIREGRSNEANFVKNEFENRTYKNAKIELCSGSLSS
                     FNQNEFGAIDGKALKYCDKIAAFIEAGLSISYGVKSKELESGFEDMFKFFNESPTIDG
                     VNFLEICREFRDYFKI"
```

```
gene            complement(17082..18758)
                /gene="ilvD"
                /locus_tag="DMACINML_00534"
CDS             complement(17082..18758)
                /gene="ilvD"
                /locus_tag="DMACINML_00534"
                /EC_number="4.2.1.9"
                /inference="ab initio prediction:Prodigal:2.6"
                /inference="similar to AA sequence:RefSeq:CAL34194.1"
                /codon_start=1
                /transl_table=11
                /product="dihydroxy-acid dehydratase"
                /protein_id="Prokka:DMACINML_00534"
                /translation="MRSDAIKKGHLKAPNRSLLRACGLKDEDFDKPFIGVANSYIDII
                PGHYFLNEYAKIIKDEIRKNGCIPFEFNTIGVDDGIAMGHEGMLYSLPSREIIANSVE
                SVMNAHQLDALICIPNCDKITPGMLMGALRVNVPTIFVSGGPMRSGVTKKGEKISLSS
                VFEAVGAYEANKISEEEFKDIECSACPSGGSCSGMFTANSMNTLCEAMGIALEGNGTI
                LALSKEREELLRKAARRICEIALDERFKIKNIITQKAIRNAMVVDMAMGGSTNTILHM
                LAISREAGVALDIKDLNFISSKVAHIAKIAPSLNTVYMDDIHKAGGVSAVMAEISSRE
                GHILELDALTITGENLQERLKNAKIKDETIIRKVDNAYSKVGGLAVLFGNLAEQGCVV
                KTAGITGERKFKGKAVCFNSQDEAIKGIIKGKVQKGDVCVIRYEGPKGGPGMQEMLSP
                TSLIMGMGLGADVALITDGRFSGATRGLSIGHVSPEAAEGGLIGLLKDGDEIEIDVDA
                YTINANLSDEEIAKRKNEFVMPQKEVKSRWLRMYQKLVTNASKGAVLDME"
gene            18909..19556
                /locus_tag="DMACINML_00535"
CDS             18909..19556
                /locus_tag="DMACINML_00535"
                /inference="ab initio prediction:Prodigal:2.6"
                /inference="similar to AA sequence:RefSeq:AMP62754.1"
                /codon_start=1
                /transl_table=11
                /product="rubrerythrin family protein"
                /protein_id="Prokka:DMACINML_00535"
                /translation="MRQYESYKCPKCGNEVEVQNVGGGKLSCCGVEMECITQDLTAVN
                LMKAFAGESMARNKYDLFADVAEEEGWHAVARHFREAAENEKWHARAEFKAYHEIVDG
                KPLEVTTKNLVAAAEGENYEHTTMYPNFAKIAEDEGKKAIARLFTAIGKVEIEHEREY
                LALKKMLEEEEFFNSEVEELWVCEVCGHIHRGKKAPAACPLCKAPKEYFKREFLG"
gene            19561..20169
                /gene="rnhB"
                /locus_tag="DMACINML_00536"
CDS             19561..20169
                /gene="rnhB"
                /locus_tag="DMACINML_00536"
                /EC_number="3.1.26.4"
                /inference="ab initio prediction:Prodigal:2.6"
                /inference="similar to AA sequence:RefSeq:CAL34191.1"
                /codon_start=1
                /transl_table=11
                /product="ribonuclease HII"
                /protein_id="Prokka:DMACINML_00536"
                /translation="MGDFSPFLRENLKTLFEENELLTPFDIDLVGIDEAGRGALAGPM
                MMAACKLHKDLEGLCDSKKLSEKRREELYEIILLHSDYLILAFSSEKIDEIGLSACLK
                MGLTLIKKHFKTENNFLYDGNTNLGISGIKTQIKADANIKQVSAASILAKVSKDRVMN
                FLAKDFPCYEFEKNKAYGTKAHKELIAKFGICKLHRKSFKLL"
```

```
     gene            complement(20164..21615)
                     /gene="gltD"
                     /locus_tag="DMACINML_00537"
     CDS             complement(20164..21615)
                     /gene="gltD"
                     /locus_tag="DMACINML_00537"
                     /EC_number="1.4.1.13"
                     /inference="ab initio prediction:Prodigal:2.6"
                     /inference="similar to AA sequence:RefSeq:CAL34190.1"
                     /codon_start=1
                     /transl_table=11
                     /product="glutamate synthase (NADPH) small subunit"
                     /protein_id="Prokka:DMACINML_00537"
                     /translation="MGNARGFLDFKRVDFKKVAPKERVLNFKEFTTLLDKKEQEIQGG
                     RCMDCGVAFCHTGVMSEGKDVGCPLNNLIPEWNDLIYRSLWKEAYERLDLTNPFPEFT
                     GRVCPAPCEDSCVCAINGKSVSIKNNELAIIENAFKENLVSPNKPKQYNGKKIAIIGS
                     GPAGLACANTLNSLGYKVSVFERSDKVGGLLMYGIPDMKLDKSIVDRRVDLLKESGIE
                     FKVNENIDSKDKVSKLLKEFDALVLCTGASKPIDLDIEGRKLKGVEFALDFLTQNTKT
                     LLKTGKGADTAKGKNVLVIGSGDTSVDCIAVATRQGAKSIVRFERSPKRPLQRSQNNP
                     WPLKADIFTTDYGLEEAIAVYEKDPREYQKMTKKFLGKDKVESVEACDLKREFKEGKA
                     INVELPNSNQNYKADLVLLAMGFSGSEDAIASNFGVSLDEKNNVKTKKFQTTHEKIFA
                     CGDARKGQSLVVWAIKDGIECALSIHENLAKQS"
     gene            complement(21625..26115)
                     /gene="gltB"
                     /locus_tag="DMACINML_00538"
     CDS             complement(21625..26115)
                     /gene="gltB"
                     /locus_tag="DMACINML_00538"
                     /EC_number="1.4.1.13"
                     /inference="ab initio prediction:Prodigal:2.6"
                     /inference="similar to AA sequence:RefSeq:CAL34188.1"
                     /codon_start=1
                     /transl_table=11
                     /product="glutamate synthase (NADPH) large subunit"
                     /protein_id="Prokka:DMACINML_00538"
                     /translation="MDLENILENNQSVGLYHPKNEHDACGIAAVANIRGIASYKVICD
                     ALEILMNLEHRGGAGAEENSGDGAGILIQIPHDFFMTQELGFELPQKGDYAVAQMFLS
                     PNADAKEEAKEIFLQGLKDKNLEFLGFREVPFNPSDIGASALKAMPYFLQAFVKKPST
                     VNAGLEFERVLYACRRLIEKRALHVPKFYFSSFSSRTIVYKGMLLSTQLSDFYLDFKD
                     VNMKSAIALVHSRFSTNTFPSWERAHPNRYMVHNGEINTIRGNVDSIRAREGLMESEY
                     FENLDEIFPIIAKPSSDSAMFDNTLEFLALNGRTLEEAFMMMVPEPWHKNENMGSKKR
                     AFYEYHSLLMEPWDGPAAIVFTDGVIMGASLDRNGFRPSRYYLTKDDMLILSSETGAL
                     KLDENNIKAKKRLEPGKLLLVDTARGRVIADNEIKEHYANAKPYKQWLKNLVELEKQH
                     SGVYKHKFLKEDEVLKLQKAFGWSYDELKMSVASMAQNGKEALAAMGVDTPLAILSKT
                     YQPLYNYFKQLFAQVTNPPLDAIREEIVTSTRIYLGSEGNLLKPDENNAKRVKISLPI
                     ISNEELFEVKALNNFQVKEFSILYEYSKVTLEQALDNLCVKIEEEIKKGVSIIILSDK
                     GVDDKNAYIPALLAVSGVHNYLVRKNLRTHTSIIIESGEPREIHHFACLLGYGATVIN
                     PYLVYESIQKLIENKDLNVSYDKAVENFIKASSSGIVKIASKMGVSTLQSYNGSALFE
                     CLGLSSKVIDKYFTSTTSRIEGMDLEDFEKELIALHKHAFNDTHKALDSKGIHSFRSA
                     KEEHLIDPLVIFNLQQACRNKDYKSFKKYSALVDEKQVNLRSLMEFDFSEAISIDKVE
                     SAESIVKRFRTGAMSYGSISKEAHECLAEAMNKIGGKSNSGEGGEDEERYEIKDGVNK
                     NSAIKQVASGRFGVDLNYLTHAKELQIKVAQGAKPGEGGQLMGFKVYPWIAKARHSTA
                     GVTLISPPPHHDIYSIEDLAQLIYDLKNANKDAKISVKLVSENGIGTVAAGVAKAGAN
                     LILVSGYDGGTGASPRTSIPHAGIPWELGLAETHQTLILNKLRDRVRLETDGKLMNGR
```

DLAIAALLGAEEFGFATAPLIVMGCTMMRVCHLNTCPFGIATQDKELRDRFKGKVDDV
VNFMYFIAEELREYMARLGFERLDDMIGRVDKLRQKPMQGKAGKVNLDKILKSLPTYN
RTAVHFKDYKDNKLEKTIDYRILLPLCKNAVEKKEPIKLSLEVGNQSRTFATMLSSEI
LKTYGKDALSEDSIQIKAIGNAGNSFGAFLLKGIKLEIVGDSNDYLGKGLSGGKIIAK
ISNEATFSPEENIIAGNACLYGATEGEVYLDGIAGERFCVRNSGAKAVVLGTGVHGCE
YMTGGLVVVLGDVGANFAAGMSGGVVYIFGRHNEAHVNTELVDIKDLSAKDEKELKTM
IENHIAYTNSKKAKDILEKFDKKDFFKVMPRDYEKMLEMIELCKDEEDPSLAAFLKIT
QK"

```
gene            complement(26249..27550)
                /locus_tag="DMACINML_00539"
CDS             complement(26249..27550)
                /locus_tag="DMACINML_00539"
                /inference="ab initio prediction:Prodigal:2.6"
                /inference="similar to AA sequence:RefSeq:CAL34187.1"
                /codon_start=1
                /transl_table=11
                /product="putative Na+/H+ antiporter family protein"
                /protein_id="Prokka:DMACINML_00539"
                /translation="MTLLTNPIIISVVLMTLLCLFRFNVLLSLLISALVAGIFSHIEI
                MKTMSILIDGMKGNLKTALSYILLGAIAAAISRTNLTAYLIKVVSRFISHKKYLLLLS
                IALISCFSQNLIPIHVAFIPLLIPPLLSLFNKLKIDRRAVACALTFGLTTPYMVLPIG
                FGLNFQDLLRENLEKNGVNVSLVDVTNAMYYAAICMVLGLFLALFVFYRKPREYQEIE
                IQKIDFDNIKMGRKEWGVLVGLILTLVLQIFTMNLPLSGLLGFISMVILGGVEYKSVN
                DIFDDGLKLMGFIAFVMLVAAGYGEVLKQSGAVNELVNSVVPWMQENKFLAVFLMLLI
                GLIITMGIGTSFGTIPIIATLFCPLCLELGFSAALIIFILGVAGALGDAGSPASETTM
                GTSVGLSVDKQHDHIKDTCIPTFIFYNGPLLILGSIIAMFL"
gene            27793..28152
                /locus_tag="DMACINML_00540"
CDS             27793..28152
                /locus_tag="DMACINML_00540"
                /inference="ab initio prediction:Prodigal:2.6"
                /inference="similar to AA sequence:RefSeq:ABS43397.1"
                /codon_start=1
                /transl_table=11
                /product="hypothetical protein"
                /protein_id="Prokka:DMACINML_00540"
                /translation="MNLNAEQKNAVLRFKKFVSFRNKISLNLSLIVLICYYIFVLGIG
                LMPEILGYKLGPSSITLGIMVGIGLILLCIISTGIYTFIANYFLDKEQEEVIKNLENE
                GLLDALKDGKINYKELV"
gene            28149..29795
                /locus_tag="DMACINML_00541"
CDS             28149..29795
                /locus_tag="DMACINML_00541"
                /inference="ab initio prediction:Prodigal:2.6"
                /inference="similar to AA sequence:RefSeq:ABS44294.1"
                /codon_start=1
                /transl_table=11
                /product="cation/acetate symporter ActP"
                /protein_id="Prokka:DMACINML_00541"
                /translation="MKVFIFLSLIFNLALSAGFDLGEVHQKPINITAVSMFLVFVLAT
                LFITYYSNKKSKSKSGFYTAGGNITGMQNGVAIAGDYMSAASFLGITALVFTNGFDGL
                IYSIGFLVGWPIILFLIAEKFRNLGKFTFADITAYRLEAKPIRVISAISALSVIIFYL
                IAQMVGAGQLIQLLFGLPYTFAVVLVGILMICYVTFGGMHATTWVQIIKAILLLAGAT
                FMAVMILYLTKFDLKYYFDLAISHHPKGENIMKPGTFLPDTISALSLGLALMFGTAGL
```

```
                     PHILMRFFTVKDAKEARKSVFYATGFIGYFYILTFIIGFGAIALLLGNPQFTNADGSF
                     NGASNMIAVTLADVLGGDVFLGFISAVAFATILAVVAGLAISGAGAISHDLYVNVIKK
                     GDVKHEDEMRVTKIATIAIGIFAILLGIVFEKQNVAFTVGLAFAIAASVNFPILLLCI
                     YWKNLTTKGAFWGGLIGLIVVLALVILSPSIWVKSFGFSEAIFPYDHPALFSMPLSFI
                     LIYIISKLDNSKRAKKDKEGFEAQDFRAQSGVGVSEAVAH"
     gene            complement(29917..30351)
                     /gene="petJ"
                     /locus_tag="DMACINML_00542"
     CDS             complement(29917..30351)
                     /gene="petJ"
                     /locus_tag="DMACINML_00542"
                     /inference="ab initio prediction:Prodigal:2.6"
                     /inference="protein motif:HAMAP:MF_00594"
                     /codon_start=1
                     /transl_table=11
                     /product="Cytochrome c6"
                     /protein_id="Prokka:DMACINML_00542"
                     /translation="MKKIALFFVLFVCLLNLDAKDKEWLPKGESISVYEYIPNNPRSP
                     AAFSSVDAKKLNANQRKGQQVYSKWCIACHGQGMPGTNALSALYKDQGIPALLEDRTD
                     LSPDLVMIFVRYGKHSMPFFRKTEINDKELQYLGEYLGRNYK"
     gene            complement(30348..32018)
                     /gene="glcD_2"
                     /locus_tag="DMACINML_00543"
     CDS             complement(30348..32018)
                     /gene="glcD_2"
                     /locus_tag="DMACINML_00543"
                     /inference="ab initio prediction:Prodigal:2.6"
                     /inference="similar to AA sequence:RefSeq:CAL35328.1"
                     /codon_start=1
                     /transl_table=11
                     /product="putative glycolate oxidase subunit D"
                     /protein_id="Prokka:DMACINML_00543"
                     /translation="MILPKNVSQSDFTAAVAKFEKALGKEWVFKTQEDLDLYRDAYSP
                     QWDDDDEPIPSLALAPKNVEEVQAIVKIANEFKIPLFPISTGKNLGYGSSAPQQRGQV
                     VVDLKRMNKIIEVDDKRNFCIVEPGVSYFDLYEYVEKNNLNVFLDIPDPGWGSPLGNA
                     LDHGWGYSYGMYRDHFGSHCGMEVVLANGEILRTGMGALPKAKTFAENKYGYGPYVDG
                     LFSQSNFGIVTKMGFWMMPKPEHYMLLSIKMKKREDLIPAVEILNYLEDSFIVGWPQY
                     FSPLNPPYGKPMNAELKGYLTSKNGLPDMDKIQNYALKNKIPYWNIDVSVYGCKEVCY
                     ANLEYIKQRFKIIEGVDISIVQEFSLPLNLEQKKQLKHKVTLGIPNMEIFWLSTRGEA
                     LEPSDGHVWFSPIIPRDGKELLKCQEVYMELFREFGEESPITPFSHPRSWMYRAFCFM
                     LAFNNSRTDKAHNLKVRKMYRQMVQTAAEHGWGDYRAAPTFQDDVMGAYSYNNHILRR
                     FNEQLKDCIDPNGILAPGRGGIWPKNLRDERFVNNKRDALRMKEGKEK"
     gene            complement(32015..32392)
                     /locus_tag="DMACINML_00544"
     CDS             complement(32015..32392)
                     /locus_tag="DMACINML_00544"
                     /inference="ab initio prediction:Prodigal:2.6"
                     /codon_start=1
                     /transl_table=11
                     /product="hypothetical protein"
                     /protein_id="Prokka:DMACINML_00544"
                     /translation="MLEVSRRNFLKATALSAVCLSGLNASFLEFSQAKQVKYFIKDSE
                     IPYQENFINADEILLVEKDISNIYSKLKEAFENKALIGSVSSGTTFFVIRTMASDYGM
                     RVAYEKKQGDFYTWILAPKGVKL"
```

```
gene            complement(32564..34876)
                /gene="gyrB"
                /locus_tag="DMACINML_00545"
CDS             complement(32564..34876)
                /gene="gyrB"
                /locus_tag="DMACINML_00545"
                /EC_number="5.99.1.3"
                /inference="ab initio prediction:Prodigal:2.6"
                /inference="similar to AA sequence:RefSeq:CAL34184.1"
                /codon_start=1
                /transl_table=11
                /product="DNA gyrase subunit B"
                /protein_id="Prokka:DMACINML_00545"
                /translation="MQENYGASNIKVLKGLEAVRKRPGMYIGDTNIGGLHHMIYEVVD
                NSIDEAMAGHCDTIDIEITTEGSCIVSDNGRGIPVDMHPTENMPTLTVVLTVLHAGGK
                FDKDTYKVSGGLHGVGVSVVNALSKKLVATVERNGEIYRQEFSEGKVISEFGVIGKSK
                KTGTTIEFWPDDTIFEVTEFDYDILAKRFRELAYLNPKITINFKDNRVGKSESFHFEG
                GISQFVSDLNKKEALTKPIFFGVDEEDVNVEVALLYNDTYSENLLSFVNNIKTPDGGT
                HEAGFRMGLTRVISNYIEANASAREKDSKITGDDVREGLIAIVSVKVPEPQFEGQTKG
                KLGSSYVRPIVSKASFEYLSKYFEENPIEAKAIMNKALMAARGREAAKKARELTRKKE
                SLSVGTLPGKLADCQSKDPSESEIYLVEGDSAGGSAKQGRERSFQAILPLRGKILNVE
                KARLDKILKSEQIQNMITAFGCGIGEDFDLSKLRYHKIIIMTDADVDGSHIQTLLLTF
                FFRFMNELVANGHIYLAQPPLYRYKKGQKKEIYLKDEKALNDYLIETGIESSNYEGIG
                LNDLKDFLKIVAAYRSVLNELKKRFNVISVIRYLIENPDFIKENNEELFKIVKAFLEK
                EGHNILNHYINENEIRIYVQTENGLEELIINDDLFSHPLYEEANYIFNKIKDRDLSFE
                KDILDILDEVEKNAKKGAYIQRYKGLGEMNPEQLWETTMDPSVRRLLKITIEDAQSAN
                DTFNLFMGDEVEPRRDYIQTHAKDVKHLDV"
gene            complement(34914..35981)
                /locus_tag="DMACINML_00546"
CDS             complement(34914..35981)
                /locus_tag="DMACINML_00546"
                /inference="ab initio prediction:Prodigal:2.6"
                /inference="similar to AA sequence:RefSeq:ADT65433.1"
                /codon_start=1
                /transl_table=11
                /product="DNA polymerase III subunit beta"
                /protein_id="Prokka:DMACINML_00546"
                /translation="MKLSINKNTLESAVLLCNAYVEKKDSSTITSHLLFEADEDKLII
                RASDFEIGINYKIRKIRVESGGFATANAKSIADVIKSLNNEEVVLETIDNFLFIRQKS
                TKYKLPMFNHEDFPNFPKTEGKNQFDIDSSDLSRSLKKILPSIDTNNPKYSLNGAFLD
                IKTDRINFVGTDTRRLAVYTLEKANNQEFHISIPKKAIMEMQKLFYEKIEIYYDENML
                IAKNDNFEFFTKLINDKFPDYEKVIPKTFKQELKFSTEDFIDSLKKISVVTEKMKLHF
                NKDKIIFEGISLDNMEAKTELEIETGVSEEFNLNIKIKYLLDFLTSIEEEQFTLSVNE
                PNLAFVVNSQGLSMVIMPMIL"
gene            complement(36146..37468)
                /gene="dnaA"
                /locus_tag="DMACINML_00547"
CDS             complement(36146..37468)
                /gene="dnaA"
                /locus_tag="DMACINML_00547"
                /inference="ab initio prediction:Prodigal:2.6"
                /inference="similar to AA sequence:RefSeq:CAL34182.1"
                /codon_start=1
                /transl_table=11
```

```
                     /product="chromosomal replication initiator protein"
                     /protein_id="Prokka:DMACINML_00547"
                     /translation="MNPNQILENLKKELNENEYENYIAILKFNEKQSKADFLVFNAPN
                     ELLAKFIQTKYGKKISHFYEVQSGNKASVLIQAQSQKHSGKSTKIDIAHIKAQSTILN
                     PSFTFESFVVGDSNKYAYGACKAISQKDKLGKLYNPIFIYGPTGLGKTHLLQAVGNAS
                     LEMGKKVIYATSENFINDFTSNLKNGSLDKFHEKYRNCDVLLIDDVQFLGKTDKIQEE
                     FFFIFNEIKNNDGQIIMTSDNPPNMLKGITERLKSRFAHGIIADITPPQLDTKIAIIR
                     KKCEFNDINLSNDIINYIATSLGDNIREIEGIIISLNAYANILGQEITLELAKSVMKD
                     HIKEKKENISIEDILSLICKEFNIKPSDVKSSKKTQNVVTARRIAIYLARELTSLTFS
                     QLANFFVMKDHTAISHSVKKIKELMEDDEQIKTKIEELKNKILTKSQS"
gene                 37570..38046
                     /gene="ruvC"
                     /locus_tag="DMACINML_00548"
CDS                  37570..38046
                     /gene="ruvC"
                     /locus_tag="DMACINML_00548"
                     /inference="ab initio prediction:Prodigal:2.6"
                     /inference="similar to AA sequence:RefSeq:ADN91856.1"
                     /codon_start=1
                     /transl_table=11
                     /product="Holliday junction resolvase"
                     /protein_id="Prokka:DMACINML_00548"
                     /translation="MKILGIDPGSRNCGYAIIESNKGKNTLIEAGLIKIQPSTLQYQI
                     TELCEGLDLIFKNHSFDEVAIEDIFFAYNPKTVLKLAQFRGALSLKILQIHGDFAEYT
                     PLQVKKAVTGKAKATKEQVAFMVKRLLGLSKDIKPLDITDAIAVALTHAANLRVRV"
gene                 38240..40801
                     /locus_tag="DMACINML_00549"
CDS                  38240..40801
                     /locus_tag="DMACINML_00549"
                     /inference="ab initio prediction:Prodigal:2.6"
                     /inference="similar to AA sequence:RefSeq:AJK83707.1"
                     /codon_start=1
                     /transl_table=11
                     /product="flagellar hook protein FlgE"
                     /protein_id="Prokka:DMACINML_00549"
                     /translation="MMRSLWSGVSGLQAHQVAMDVEGNNISNVNTNGFKYSRADFGTM
                     FSQTVKIATTPTDGRGGQNPLQIGLGVSVSSTTRIHSQGSVQTTNSNTDVAINGDGFF
                     MVSDDGGLTNYLTRDGAFKLDAYGNFVNNSGFIVQGWNINWDDQTIDSSRSPQNIFID
                     PGMHIPAAKSTEVAIKANLNSGLNIGTSSRNLYALDSVHGYNNKTQRAEDENDTGTTQ
                     FYTTSKNSVEVTEKGVDAGSLFNASGTGLNLRDGQGIWVSYADAKYSTNKVGASAFDA
                     NLQANQTAAFWGSADQRVNLDITLNGVKIQNAEIQNIDDAIAYINTFTAPTDTRDGTG
                     VRAVKSADGSGIEFVNDNSDGTTDNMKNINLIVSDANTAGELWNATWNAGTSSFTFDQ
                     NNINKNNTSLWTADTMAPNPPNGNVTLTGTDRVEVITAHKYIYSSNPVDIGPMTNPDG
                     GPAFQPGANETTRPTDPASAAYWDAMNGGLLNTNARTFRTTEDLRELLQRDARYGVDY
                     DGSGSFAAADVNQAVKVVVTANGNFAISNAKEDSSVPPNAINGVADATTTTPKAMSFN
                     ITAYSNKAGTVSTNDAFTAIFKAWDGPLTVGGSIKESEQLKLSAFSAGLEIFDSLGSK
                     HTLEVQFVKQNTTQDGGNEWQMIIRVPEPAEINTTGEGPNNIIVGTARFNNDGSLASY
                     TPRTINFSPNNGAAPNQQIKLSFGTSGSNDGLVSSNSASTLTGQATDGYASGNLKPDA
                     IRVDEKGNLLGEFTNGKTFAVAKIAMASVANNSGLEEIGGNLFKVTANSGNIVVGEAG
                     TGGRGEMKTSALEMSNVDLSRALTELIIIQRGYQANSKTISTSDQMLQTLIQLKQ"
gene                 41227..42498
                     /gene="metB"
                     /locus_tag="DMACINML_00550"
CDS                  41227..42498
```

```
                     /gene="metB"
                     /locus_tag="DMACINML_00550"
                     /EC_number="2.5.1.49"
                     /inference="ab initio prediction:Prodigal:2.6"
                     /inference="similar to AA sequence:RefSeq:CAL35821.1"
                     /codon_start=1
                     /transl_table=11
                     /product="putative O-acetylhomoserine (thiol)-lyase"
                     /protein_id="Prokka:DMACINML_00550"
                     /translation="MNFTKETLALHGAYNFDTQRSISVPIYQNTAYNFENLDQAAARF
                     NLQELGNIYSRIGNPTSDVLGQRLANVEGGAFGIPVSSGMAACFYALVNLASSGDNVA
                     YSNKIYGGTQTLISHTLKNFGIEAREFDIDDLDSLEKVIDQNTKAIFFESLSNPQIAI
                     ADIEKITQIAKKHKIATICDNTVATPFLLQPFKYGVDIIVHSLSKYVSGQGSALGGAL
                     IERKDLNDLLKNNDRYKAFNTPDPSYHGLNLNTLDLPIFSIRIIITWLRDLGASLAPQ
                     NAWLLLQGLETLAVRIEKHSQNAEKVANFLNSHPDIKGVNYPTLTSNAYHNLFKKYFD
                     KNFASGLLSFEARDYEHARRICDKTQLFLLAANLGDSKSLIIHPASTTHSQLSEEELQ
                     KAGIKKTTVRLSIGLENSDDLIADLKQAIES"
     gene            42510..43544
                     /locus_tag="DMACINML_00551"
     CDS             42510..43544
                     /locus_tag="DMACINML_00551"
                     /inference="ab initio prediction:Prodigal:2.6"
                     /inference="similar to AA sequence:RefSeq:ABS44543.1"
                     /codon_start=1
                     /transl_table=11
                     /product="inner membrane protein YeeE"
                     /protein_id="Prokka:DMACINML_00551"
                     /translation="MLSGIIVGLLLGFVLQRGRFCVVGAYRDVFLSKKFTIFIALFIV
                     VALQSIGVWALHSLGYISIKPQEFYWLSTIIGGIIFGFGMVIAGGCATGTWYRAGEGL
                     IGSIAALLFYAFSASAVKYGVLLPLQDNLNTFKISDGTFYQSLGISPWILVLVLSLVV
                     GFFVIKELRKPRLKVARLKPRKQGISHILFEKSWHPFVTAVLVALIAILAWPLSSLSG
                     RNYGLGITTPSANLVQYLSTGDIQFVDWGVFLVLGIALGSFLGAKLSGEFRFRLPDKK
                     TLAYASIGGILMGVGASLAGGCTIGNGLVETSLFSYKGWVATVFFLIGAYIATFYTII
                     LPTRKATQKI"
     gene            43554..43775
                     /locus_tag="DMACINML_00552"
     CDS             43554..43775
                     /locus_tag="DMACINML_00552"
                     /inference="ab initio prediction:Prodigal:2.6"
                     /inference="similar to AA sequence:RefSeq:ABS43826.1"
                     /note="conserved domain protein"
                     /codon_start=1
                     /transl_table=11
                     /product="hypothetical protein"
                     /protein_id="Prokka:DMACINML_00552"
                     /translation="MVTLDTRGKVCPFPLVEAKNLVQTLKSGEELEILFDCTQATETI
                     PQWAAEEGHEIVDFELLGDAEWRIKLIKK"
     gene            43786..44667
                     /gene="metA"
                     /locus_tag="DMACINML_00553"
     CDS             43786..44667
                     /gene="metA"
                     /locus_tag="DMACINML_00553"
                     /inference="ab initio prediction:Prodigal:2.6"
```

```
                     /inference="similar to AA sequence:RefSeq:AVL46551.1"
                     /codon_start=1
                     /transl_table=11
                     /product="homoserine O-succinyltransferase"
                     /protein_id="Prokka:DMACINML_00553"
                     /translation="MPLIIPENIPAYELLKEHAFIMGSRRAKHQDIRPQEILIINLMP
                     KKIETENQILSLLANSPLQVNITLLATTSYVGKNTPFTHLEKFYKGLEEVKKRKFDGA
                     IVTGAPVEQMDFEKVAYWEELLEIFDFLQQNVTSSMYICWGAMAALKHFYGVDKIALD
                     KKIFGIYKHDKVSPDLLLTNLDEKVLMPHSRHSSMDEEQISALQKQGKLKVLLRNKKI
                     GSALLRDEKNIFILGHLEYFKDTLHQEYVRDNFIQKAKNYYDKKGNIKYNWRSNANTI
                     FANWLNYDVYQSTPFVL"
     gene            complement(44672..45271)
                     /locus_tag="DMACINML_00554"
     CDS             complement(44672..45271)
                     /locus_tag="DMACINML_00554"
                     /inference="ab initio prediction:Prodigal:2.6"
                     /inference="similar to AA sequence:RefSeq:CAL35819.1"
                     /codon_start=1
                     /transl_table=11
                     /product="putative periplasmic protein"
                     /protein_id="Prokka:DMACINML_00554"
                     /translation="MKKSIVIIGGSVAGLSAALFFASAKNDELDFDITIFDDDKADLK
                     AAAIYNVPFFPKGAKADEIYTHIKAQIASMLEVKYIDSKVVSISGEKGDFTVSDEQGL
                     GIKADYIIVATGATKSEIKGLEDFVIPHELMPKPNKFCFKHHGRQMIKEGIYAAGLAS
                     GVTTMVACAMGSANEAACAILSDIKGVVSVYHDTPTTRN"
     sig_peptide     complement(45089..45271)
                     /locus_tag="DMACINML_00554"
                     /inference="ab initio prediction:SignalP:4.1"
                     /note="predicted cleavage at residue 61"
     gene            45430..45813
                     /locus_tag="DMACINML_00555"
     CDS             45430..45813
                     /locus_tag="DMACINML_00555"
                     /EC_number="3.5.4.16"
                     /inference="ab initio prediction:Prodigal:2.6"
                     /inference="similar to AA sequence:RefSeq:CAL35818.1"
                     /codon_start=1
                     /transl_table=11
                     /product="putative GTP cyclohydrolase I"
                     /protein_id="Prokka:DMACINML_00555"
                     /translation="MRYGEKEIKEFDVENMEIWPNDAKNDYIIKITLPEFMCCCPRSG
                     YPDFATIYLEYIPDQFVVELKAIKLYINTFMHRNVSHEASINEIYNTLKDKLKPKWIK
                     VVGDFNPRGNVHTVIECRSDMVVPE"
     gene            45810..46607
                     /gene="hindIIIM"
                     /locus_tag="DMACINML_00556"
     CDS             45810..46607
                     /gene="hindIIIM"
                     /locus_tag="DMACINML_00556"
                     /EC_number="2.1.1.72"
                     /inference="ab initio prediction:Prodigal:2.6"
                     /inference="similar to AA sequence:RefSeq:CKH09182.1"
                     /codon_start=1
                     /transl_table=11
```

```
                     /product="Modification methylase HindIII"
                     /protein_id="Prokka:DMACINML_00556"
                     /translation="MKFNDLDLNNWKSCDINTDSLWLISSRDKSGKHRNIYHGNFIPQ
                     IPNQLIRRYTKKDDLIIDPFLGSGTTLYECEKLNRKCIGLDINESILEFVLDNFGINF
                     DNKRYFLGNCDNTDAKKVDEFLENALNKLESKKAQFIFLHPPYMDIVKFSEKKEDLSQ
                     ISDLKKFKDKFLFTCQNMLKYLEKSRYFALVIADLYRNSEIVPLGFELMNLIKQNFKV
                     KLKGIIVKNIEGNRGKLGINAIWRYRALRSDYYIFKHEYIFVFKKEF"
gene                 46608..47687
                     /locus_tag="DMACINML_00557"
CDS                  46608..47687
                     /locus_tag="DMACINML_00557"
                     /inference="ab initio prediction:Prodigal:2.6"
                     /inference="similar to AA sequence:RefSeq:ATM02703.1"
                     /note="DUF262 domain-containing protein"
                     /codon_start=1
                     /transl_table=11
                     /product="hypothetical protein"
                     /protein_id="Prokka:DMACINML_00557"
                     /translation="MDLEIQMEDKRNLLRADRLDMSFGEIISNYEEDNLIIQPSYQRS
                     FRWNDYQKTRFIESVLLGIPIPPIFVAEDKDNGKWELIDGLQRVSTILSFFGLLKENG
                     KIDDRLNKFKLLEGEILTELKDKNIDSFPLKLQLTIKRFVCRVEILRWDTNSEIKYQL
                     FSRLNTGGEALKEQEIRNCIYRGDLNDLINNLAENKDFKDVIGATERQEKEMYLSELV
                     LRFMAFYYRHNDLIFKTSGSIQGFLSDFMRSNMEKNQDDFYQVKQVFLNVISYLNENK
                     LSDLFRDNRARQFNANHYDTIMYILAKNLNRFQDSQEFRQIIQNLLKNKEYIDKSGSS
                     TYATKRMQEKIKFAEEFIKNEFTRN"
gene                 47668..48303
                     /locus_tag="DMACINML_00558"
CDS                  47668..48303
                     /locus_tag="DMACINML_00558"
                     /inference="ab initio prediction:Prodigal:2.6"
                     /codon_start=1
                     /transl_table=11
                     /product="hypothetical protein"
                     /protein_id="Prokka:DMACINML_00558"
                     /translation="MSLQEINEELLQEFRWRCDEMILLRDIVDSCEKDEYQKFILKMS
                     IPIIYAHLEGFIVSACKIVLQYLNTLKLSSNDCSEKFIMMAFDKDIKSLSDSQHLEKK
                     IKHFCRIQEKLLCQLKFETKVNVKSNLTFEVLENLCLKFDISSVKLKDYKDILDQLVN
                     IRNAIAHGENSYNFQNFGDIEKYIDLFEALCLDFYQVLFNILEDEKYKKEK"
gene                 48306..48980
                     /locus_tag="DMACINML_00559"
CDS                  48306..48980
                     /locus_tag="DMACINML_00559"
                     /inference="ab initio prediction:Prodigal:2.6"
                     /inference="similar to AA sequence:RefSeq:CKH09127.1"
                     /codon_start=1
                     /transl_table=11
                     /product="hypothetical protein"
                     /protein_id="Prokka:DMACINML_00559"
                     /translation="MDIPFKEGTANYYDFTIMSDLQWHCTKCELKAAQAKTWQTWRHQ
                     GIQLDTDEKGNFYKMQLCCKCNMNTVHRKLKSLEILEDTKTRSGISVSLARRIKNCLR
                     NEEAIYLREMSPNELEIDHKFPQIRWKTDESKNDGSMSEEEIKNKFILLTRSNNLLKS
                     RYCEKCFKNGTRGYFPGIKFWYKGNEKWQGEDDNDEKGCEGCFWYDPYKWREELNRIS
                     QIQINL"
gene                 48977..49984
```

```
                     /gene="fokIM_2"
                     /locus_tag="DMACINML_00560"
     CDS             48977..49984
                     /gene="fokIM_2"
                     /locus_tag="DMACINML_00560"
                     /EC_number="2.1.1.72"
                     /inference="ab initio prediction:Prodigal:2.6"
                     /inference="similar to AA sequence:RefSeq:CKH09081.1"
                     /codon_start=1
                     /transl_table=11
                     /product="D12-class N6-adenine-specific DNA
                     methyltransferase"
                     /protein_id="Prokka:DMACINML_00560"
                     /translation="MNYIGSKFKLSSFLFKSIDETLKAKNKALEDCIFCDIFAGTSAV
                     GKLFKTKVKQVIANDKEFYSFILAKNYIQNSNLLTRANELIDILNYQIPLIKNKIYKY
                     YAIGGGENRQYFSDLNAMKIDSIRVQIEIWKEQNFINKEEYYFLLASLLESADKVANT
                     ASVYGAFLKHLKKSAQKKMVLFPAEFQQSHNKHQVFNKNANELIREIKGDILYLDPPY
                     NNREYGANYHVLNTIAIYDDFIPQGKTGLRAYEKSAWCKKNLVFNALEDLIKNANFKF
                     IFLSYNDEGLLSLEQIKQIFKKYGKYDLKNQIYKRFKADSSRICKQDQTIEYLHILEK
                     K"
     gene            50063..50707
                     /locus_tag="DMACINML_00561"
     CDS             50063..50707
                     /locus_tag="DMACINML_00561"
                     /inference="ab initio prediction:Prodigal:2.6"
                     /inference="similar to AA sequence:RefSeq:CAL35815.1"
                     /codon_start=1
                     /transl_table=11
                     /product="putative outer membrane protein"
                     /protein_id="Prokka:DMACINML_00561"
                     /translation="MNKKLLSIIAGVALFSSSALAAEESGFFIGADAAYVQTKVKGDL
                     KHNKTGAVFNGDLSSSIPILGLRMGYRFNDLHRLYAAYNYSDEFSDIIRTPNLRIEGD
                     FNTHKFLLGYDFTPEIFKRVRAVAGGYLGYAKTSLDLKTSLLSLSQDFDGFVYGAKIG
                     AIFELGTSNELEVGFKAEQIEYNTRNYYQETIGSNFYDPRQTNYGLYLGYTYKF"
     sig_peptide     50063..50128
                     /locus_tag="DMACINML_00561"
                     /inference="ab initio prediction:SignalP:4.1"
                     /note="predicted cleavage at residue 22"
     gene            complement(50731..51360)
                     /locus_tag="DMACINML_00562"
     CDS             complement(50731..51360)
                     /locus_tag="DMACINML_00562"
                     /inference="ab initio prediction:Prodigal:2.6"
                     /inference="similar to AA sequence:RefSeq:CAL35814.1"
                     /note="hypothetical protein Cj1720"
                     /codon_start=1
                     /transl_table=11
                     /product="hypothetical protein"
                     /protein_id="Prokka:DMACINML_00562"
                     /translation="MLKELLEIKKEMEPVIHEANVKLNVLAREVIVRRKEYEIYGPMV
                     DRVYLDNAIYVKVMSSGRDVKTDNVPIKTGFYMVFVAPEKESTKDIKNKLKVAYEGLD
                     NTFIATLIRSCQRFKEIISKTQATLSKASKMNVLIKTNLGEASSAIKFNITIEYTKEN
                     QKLTRDNSKSAGSFRDTRTYINLVVDKNTDSQICEKLLDDVERYFIGGG"
     gene            51697..53232
```

```
                     /locus_tag="DMACINML_00563"
     CDS             51697..53232
                     /locus_tag="DMACINML_00563"
                     /inference="ab initio prediction:Prodigal:2.6"
                     /inference="similar to AA sequence:RefSeq:APA51564.1"
                     /codon_start=1
                     /transl_table=11
                     /product="2-isopropylmalate synthase"
                     /protein_id="Prokka:DMACINML_00563"
                     /translation="MKNNKIIIFDTTLRDGEQALGSSLNINQKLQIALALENLGVDVI
                     EAGFPVSSQGDFEAVKNIASKVKNSTICALSRALDKDIDTAYEALKVADSFRIHTFIA
                     TSTLHIQDKLKKDFDEILAMAQRAIARARNYTDDVEFSCEDAGRTPIDNLCFMVENAI
                     KAGARTINIPDTVGYTLPSEFANIIKILFNKVPNIDKAVISVHCHNDLGMATGNSLSA
                     ILQGARQIECTMNGLGERAGNCALEEVVMAIKTRSDYLKGFYTDIKCENISKTSKLVS
                     AITNESIPSHKAIVGSNAFSHSSGIHQDGVLKNRQTYEIISPSSIGLHENRMLMTARS
                     GRAMIKTCLDNLGYDEKTYNLDEVYESFLRLADKKGQVYDYDLEALMFLSYENDEENE
                     LVLEKLSVISGTIPTACVCVRIKEELKTEACTGNGPIEAVFNCISRITGLNPVLKAYS
                     INAKSSGVDAQGQVDVDLEFKGRKFHGKGLSTDVIEASAQAFISAYNAIYRSLKVEER
                     KAV"
     gene            53229..54305
                     /gene="leuB"
                     /locus_tag="DMACINML_00564"
     CDS             53229..54305
                     /gene="leuB"
                     /locus_tag="DMACINML_00564"
                     /EC_number="1.1.1.85"
                     /inference="ab initio prediction:Prodigal:2.6"
                     /inference="similar to AA sequence:RefSeq:CAL35812.1"
                     /codon_start=1
                     /transl_table=11
                     /product="3-isopropylmalate dehydrogenase"
                     /protein_id="Prokka:DMACINML_00564"
                     /translation="MNTYKIAVLPGDGIGPSVMKEALKILNFVAQKHGFKLETKEAKI
                     GGASIDAYGVALSDETLKLCEQSDAILFGSVGGPKWDNLPIDQRPERASLLPLRKHFN
                     LFANLRPCKIYESLRHASPLKDEIIQKGVDILCVRELTGGIYFGKQELGKESAYDTEI
                     YTKAEIERITRLAFESARIRNKKVHLIDKANVLASSILWRDVVGQIAKEYEDIKLEYM
                     YVDNAAMQIIKNPSTFDVMLCSNLFGDILSDELAAISGSLGLLCSASLNDKGFGLYEP
                     AGGSAPDIAHLNIANPIAQILSAALMLKYSFKEEKAAQDIENAIAIALERGKMTKDLN
                     ANSYLSTDEMGDCIVEILKENNNG"
     gene            54298..55704
                     /gene="leuC"
                     /locus_tag="DMACINML_00565"
     CDS             54298..55704
                     /gene="leuC"
                     /locus_tag="DMACINML_00565"
                     /EC_number="4.2.1.33"
                     /inference="ab initio prediction:Prodigal:2.6"
                     /inference="similar to AA sequence:RefSeq:CAL35811.1"
                     /codon_start=1
                     /transl_table=11
                     /product="3-isopropylmalate dehydratase large subunit"
                     /protein_id="Prokka:DMACINML_00565"
                     /translation="MAKTLYEKVFDSHVVYQKENELPILYIDRHLIHEVTSPQAFSGL
                     KMANRKMARSDLTLATIDHDVSTKSTDLDACSGMAKEQITTLMQNTKEFGVRLLGLGD
```

```
                     KNQGIVHIVSPELGFTLPGVTLVCGDSHTATHGAFGALAFGIGTSEVEHVMATQTLKQ
                     AKLKTMKIECKGQFQKGVYAKDLILYLIAKYGTAKGTGYAIEFCGELIESLSMEARMT
                     LCNMAIEFGAKVGMIAPDEITFEYIKGKEFAPKGEEFEKHCEYWRSLKSDEGAQYDES
                     IVLDVSQIQPQISYGTNPSQVIAINERIPKVSDFSNPSDQKSLLDALDYVNLEQDQSL
                     EGVKIDIVFIGSCTNGRLEDLKIAADILKGRKIHKDVKALIVPGSMQVRKEAENLGLD
                     KIFIEAGCEWRYAGCSMCLGMNDDKASAGQRVASTSNRNFVGRQGKGSITHLMSPASA
                     AACAIEGFICDNRKYLGV"
gene                 55706..56308
                     /gene="leuD"
                     /locus_tag="DMACINML_00566"
CDS                  55706..56308
                     /gene="leuD"
                     /locus_tag="DMACINML_00566"
                     /EC_number="4.2.1.33"
                     /inference="ab initio prediction:Prodigal:2.6"
                     /inference="similar to AA sequence:RefSeq:CAL35810.1"
                     /codon_start=1
                     /transl_table=11
                     /product="3-isopropylmalate dehydratase small subunit"
                     /protein_id="Prokka:DMACINML_00566"
                     /translation="MQKFTTHKGIACPLEYANIDTDQIIPKQFLLAVSKQGFGKHLFH
                     DLRYLDDKESVLNMDFNLNKKEYQNASILVSFENFGSGSSREHAPWALVDYGIRAIIA
                     PSFADIFKNNALGNGLLTIELAKDEVVSIIKQLKESEDKNIEISLFEKKVFFKDQVFS
                     FDLDEFRRTCLLEGLDNIALTLKYEEQIKAYEKNSKSFLV"
gene                 complement(56288..56818)
                     /locus_tag="DMACINML_00567"
CDS                  complement(56288..56818)
                     /locus_tag="DMACINML_00567"
                     /inference="ab initio prediction:Prodigal:2.6"
                     /inference="similar to AA sequence:RefSeq:ADN91846.1"
                     /codon_start=1
                     /transl_table=11
                     /product="Acetyltransferase, GNAT family"
                     /protein_id="Prokka:DMACINML_00567"
                     /translation="MQDFVIEKATQKDLKSILNIAKDALNSMKAMNFNQWDENYPNEE
                     IFKEDIKTQELYLCKEKDEILGFICINEKFEPEFYEQINFKKSYDNQAFYLHRLAVKE
                     NAKGKGVAQKLLNFCETYAKNEGKYSLRADTHSKNIPMNSLFKKLNFEFCGAFHIPCY
                     EDEFLAYEKILNQKAF"
gene                 complement(57045..57120)
                     /locus_tag="DMACINML_00568"
tRNA                 complement(57045..57120)
                     /locus_tag="DMACINML_00568"
                     /product="tRNA-Val"
                     /inference="COORDINATES: profile:Aragorn:1.2"
                     /note="tRNA-Val(gac)"
gene                 complement(57122..57197)
                     /locus_tag="DMACINML_00569"
tRNA                 complement(57122..57197)
                     /locus_tag="DMACINML_00569"
                     /product="tRNA-Ala"
                     /inference="COORDINATES: profile:Aragorn:1.2"
                     /note="tRNA-Ala(ggc)"
gene                 complement(57331..58296)
                     /locus_tag="DMACINML_00570"
```

```
CDS             complement(57331..58296)
                /locus_tag="DMACINML_00570"
                /inference="ab initio prediction:Prodigal:2.6"
                /inference="similar to AA sequence:RefSeq:ADT73490.1"
                /codon_start=1
                /transl_table=11
                /product="lipopolysaccharide core biosynthesis protein
                LpsA"
                /protein_id="Prokka:DMACINML_00570"
                /translation="MLQSRLFMNLKGIGISFISRYFFQTNLHKIFNKILKYNVKELEE
                IQTRVKYYNQINDFFTLTEKEKIGKFPFKNTSYAFDAYEISKYFNNDFLWNKKFGDIK
                YTFKEPTICKSRPLENNANNILLKLDKNRHFCFLKDNINYENKRNIAIFRGAVYQKHR
                KEFFHSYFNKPFCDLGDTSKQLSQWKKNFLSKEEQMKYKFIISLEGNDVASNLKWAMN
                SNSLVLAPKITCETWFMEGTLKPNYHFALIDNDNLATVIEYFNNHPKDALEIINNAHQ
                YIKKFVDKKKEFYIGILVLTKYFYYSGQLDLNKDECEREILELIK"
gene            complement(58407..58494)
                /locus_tag="DMACINML_00571"
tRNA            complement(58407..58494)
                /locus_tag="DMACINML_00571"
                /product="tRNA-Ser"
                /inference="COORDINATES: profile:Aragorn:1.2"
                /note="tRNA-Ser(gga)"
gene            58599..58675
                /locus_tag="DMACINML_00572"
tRNA            58599..58675
                /locus_tag="DMACINML_00572"
                /product="tRNA-Met"
                /inference="COORDINATES: profile:Aragorn:1.2"
                /note="tRNA-Met(cat)"
gene            complement(59116..59220)
                /locus_tag="DMACINML_00573"
CDS             complement(59116..59220)
                /locus_tag="DMACINML_00573"
                /inference="ab initio prediction:Prodigal:2.6"
                /inference="similar to AA sequence:RefSeq:SQE25200.1"
                /codon_start=1
                /transl_table=11
                /product="small hydrophobic protein"
                /protein_id="Prokka:DMACINML_00573"
                /translation="MTALDIFQIIFIFVVVLIGFVGMIWVISKDKSNR"
gene            complement(59217..60287)
                /locus_tag="DMACINML_00574"
CDS             complement(59217..60287)
                /locus_tag="DMACINML_00574"
                /inference="ab initio prediction:Prodigal:2.6"
                /inference="similar to AA sequence:RefSeq:CAL35807.1"
                /codon_start=1
                /transl_table=11
                /product="putative radical SAM domain protein"
                /protein_id="Prokka:DMACINML_00574"
                /translation="MKELINILDFLPEELEEKIKPMFRVKQIYQWIYQKYANNFSDMS
                SLPKDLRLELAQTYHFAPLKCVKNEQSKDGSIKYLFELIDGLRIESVLLPMKEEQFDD
                EGKRISHTRYTICVSSQVGCKSGCSFCLTAKGGLKRNLSAGEIVGQILWIKKQNNIPY
                ERRVNIVYMGMGEPLDNLKNVSKAVKILSQNDGLAISPRRQTISTSGLAKQIKELGEM
```

NLGVLLAISLHAVNDELRTELMPINKAYNIAAIMDAVRAFPIDQRKRVMFEYLLIDGI
NDKLEHAKELVKLLNGIKAKVNLILFNPHEGSIYHRPKLENAIKFQDLLSAKGVTCTI
RESKGLDISAACGQLKERAKEL"

```
gene            complement(60307..60843)
                /locus_tag="DMACINML_00575"
CDS             complement(60307..60843)
                /locus_tag="DMACINML_00575"
                /inference="ab initio prediction:Prodigal:2.6"
                /inference="similar to AA sequence:RefSeq:CAL35806.1"
                /note="hypothetical protein Cj1712"
                /codon_start=1
                /transl_table=11
                /product="hypothetical protein"
                /protein_id="Prokka:DMACINML_00575"
                /translation="MIVCAGGNENFSFAKAIGIGLIESTFHLTQLCLKEKPSKLVFIG
                TCGLYDKGEILGIYKSSHACNIEFSYISDQFYTPANIEIKLNNQNVSRETIKVNSSNY
                ICQNSQAAKEFAKLGFFAENMEAFSVLSVAKNLDIDAECILCATNFCNENAHEDFIKN
                HQKAKEKLEEYLKINHYI"
gene            60929..61723
                /gene="ksgA"
                /locus_tag="DMACINML_00576"
CDS             60929..61723
                /gene="ksgA"
                /locus_tag="DMACINML_00576"
                /EC_number="2.1.1.-"
                /inference="ab initio prediction:Prodigal:2.6"
                /inference="similar to AA sequence:RefSeq:CAL35805.1"
                /codon_start=1
                /transl_table=11
                /product="putative dimethyladenosine transferase (16S rRNA
                dimethylase)"
                /protein_id="Prokka:DMACINML_00576"
                /translation="MMKAKKHYGQNFLNDKSILSKIIQAIPKDTENIVEIGPGLGDLT
                QELLKVSHVKAYEIDDDLIPILRKKFQKELECGKLNLLHQDASKLAYFDEKKYFLVAN
                LPYYVASHLILQALEDKNCSGLIVMVQKEMALKFCAHSGESDFSALGVLSAMICEREI
                LFDVEPECFNPPPKVVSAVMKMIKFRAYQDICEIHAFKAFLKDCFKAPRKQLLGNLKN
                YKAELAEVFQELKLNLNLRPHELCVDSYLKIYEKVKEEYERKQQRK"
gene            61698..63728
                /locus_tag="DMACINML_00577"
CDS             61698..63728
                /locus_tag="DMACINML_00577"
                /inference="ab initio prediction:Prodigal:2.6"
                /inference="similar to AA sequence:RefSeq:CAL35804.1"
                /codon_start=1
                /transl_table=11
                /product="putative metallo-beta-lactamase family protein"
                /protein_id="Prokka:DMACINML_00577"
                /translation="MNENNKGNSENTNIPNSNSKNNKRYKYRNRRKKLADSISENSQN
                STHEFSNNEKINTKKNSTNTQPLESENIEKPSEKKKKRNRNLPSKLTGNEDWQIALAE
                CIEANRVSHENRLHPLKYNNSSEHKIRITPLGGLGEIGGNISVFETNNDAIIIDIGMS
                FPDGTMHGVDIIIPDFDYVRKIKDKIRGIVITHAHEDHIGAVPYFFKEFQFPIYATPL
                ALGMISNKFEEHGLKAERKWFRPVEKRKVYEIGEFDIEWIHITHSIIDASALAIKTKA
                GTIIHTGDFKIDQTPIDGYPTDLGRLAHYGEEGVLCLLSDSTNSYKEGYTKSESSVGP
                TFDQIFARTKGRVIMSTFSSNIHRVYQAITYGLKYGRKVCVIGRSMERNLYTTMELGY
```

```
                     IKLDRKIFIDADEVSKYKDNEVLIVTTGSQGETMSALYRMATDEHKFIKIKPSDQIII
                     SAKAIPGNEASVSAVLDYLLKAGAKVAYQEFSEIHVSGHASIEEQKLMLTLVKPKFFL
                     PVHGEYNHINKHKETAIKCGIPEKNIYLMSDGDQVELCQKYIKRVKTVKTGKVFVDNQ
                     INKQIADDVVIDRQKLADSGIVVIIAQLDKASKTLINKPRVFSYGLVADKQDHAFSKE
                     MAEVLGQFFVNVKDEVLNDPRFLENQIRQVLRKHIFRKIKKYPTIVPTIFVM"
gene                 63770..64531
                     /locus_tag="DMACINML_00578"
CDS                  63770..64531
                     /locus_tag="DMACINML_00578"
                     /EC_number="4.2.1.70"
                     /inference="ab initio prediction:Prodigal:2.6"
                     /inference="similar to AA sequence:RefSeq:CAL35803.1"
                     /codon_start=1
                     /transl_table=11
                     /product="putative ribosomal pseudouridine synthase"
                     /protein_id="Prokka:DMACINML_00578"
                     /translation="MRINKFISHNSPYSRREADELIKQGLVKINNKIALLSDEVKTND
                     KVFVKGKALRKKTQFSVIIYHKQKGEIVSKRDDRGRKTIYDTLPKQFNTWLSVGRLDF
                     ASEGLLLLTDSPVIAHALMNSDLEREYYLKLKGVINKQVIEAMQNGLEIKNEKKGAHA
                     KTKITSMSFAPFLDFEIFGTSGGYTKLRVIINEGKNRELRRFFGHFDLEVMDLKRVAF
                     GALDLGMLKAGKHRYLENGEYEKLRDFLKVNNIYY"
gene                 64786..65097
                     /gene="rpsJ"
                     /locus_tag="DMACINML_00579"
CDS                  64786..65097
                     /gene="rpsJ"
                     /locus_tag="DMACINML_00579"
                     /inference="ab initio prediction:Prodigal:2.6"
                     /inference="similar to AA sequence:RefSeq:CAL35802.1"
                     /codon_start=1
                     /transl_table=11
                     /product="30S ribosomal protein S10"
                     /protein_id="Prokka:DMACINML_00579"
                     /translation="MERIRLKLKAYDHRVLDRTVAAIVEAVKRTGADIRGPIPMPTKI
                     KRYTVLKSPHINKDSREQFEIRIHARMLDIVAATPDTVDSLTKLDLAPEVSVEVRAMG
                     K"
gene                 65107..65682
                     /gene="rplC"
                     /locus_tag="DMACINML_00580"
CDS                  65107..65682
                     /gene="rplC"
                     /locus_tag="DMACINML_00580"
                     /inference="ab initio prediction:Prodigal:2.6"
                     /inference="similar to AA sequence:RefSeq:CAL35801.1"
                     /codon_start=1
                     /transl_table=11
                     /product="50S ribosomal protein L3"
                     /protein_id="Prokka:DMACINML_00580"
                     /translation="MEYIVEKIGMSRTIANPSIAVTLLRVVNAKVCEVDNGKAIVAYS
                     KGKVNNKCVAGQQKKYNLSAEFNRFATLEVANTEAGDLDETPLNEAKVLKVSFNTKGR
                     GYSGVMKRHNFAGGPASHGSRFHRRHGSIGNREWPGRVQPGMKMAGHYGNTKVTVKNE
                     VVSYDAENKILVVKGAVPGYNGAMGKIRIAK"
gene                 65679..66293
                     /gene="rplD"
```

/locus_tag="DMACINML_00581"
CDS 65679..66293
/gene="rplD"
/locus_tag="DMACINML_00581"
/inference="ab initio prediction:Prodigal:2.6"
/inference="similar to AA sequence:RefSeq:CAL35800.1"
/codon_start=1
/transl_table=11
/product="50S ribosomal protein L4"
/protein_id="Prokka:DMACINML_00581"
/translation="MSKVVVLNDKLEKAGELELPAKYAEVNPHNLYLYVKSYLASLRA
NTAHTKGRSDVSGGGKKPWRQKGRGGARAGSTRTNVWVGGAVAFGPTNERNYYQKVNK
KQKRLALERALADKAAKGALFTADSLAIESGKTKDANAVIKKLGVKDALIVKDLLDEK
TLLAYRNLANCYVVDISEVNAYLISVFNAVIIEKSALESITKEG"
gene 66296..66577
/gene="rplW"
/locus_tag="DMACINML_00582"
CDS 66296..66577
/gene="rplW"
/locus_tag="DMACINML_00582"
/inference="ab initio prediction:Prodigal:2.6"
/inference="similar to AA sequence:RefSeq:CAL35799.1"
/codon_start=1
/transl_table=11
/product="50S ribosomal protein L23"
/protein_id="Prokka:DMACINML_00582"
/translation="MADITDIKTILYTEKSLNLQEQGVVVIQTSPKMTKTGLKAVLKE
YFGVTPQSINSLRMDGKVKRFRGRLGQRNDYKKFYVKLPEGVSLESMEA"
gene 66579..67409
/gene="rplB"
/locus_tag="DMACINML_00583"
CDS 66579..67409
/gene="rplB"
/locus_tag="DMACINML_00583"
/inference="ab initio prediction:Prodigal:2.6"
/inference="similar to AA sequence:RefSeq:CAL35798.1"
/codon_start=1
/transl_table=11
/product="50S ribosomal protein L2"
/protein_id="Prokka:DMACINML_00583"
/translation="MAIKTYKPYTPSRRYITGLSSEDITAKPSVRSLLVKLPAHAGRN
SYGRITSRHKEAGAKKLYRIIDFKRRKFGIEGKVEAIEYDPYRNCRIALISYKDGEKR
YILQPRGLSVGDIVAAAESGLDIKPGNAMKLKNIPVGTIVHNVELKPGKGGQMIRSAG
AYAQLMGKEEKYVILRLASGEMRQVLAECMASIGEVGNEEWANVTIGKAGRNRHRGIR
PQTRGSAMNPVDHPHGGGEGKKNSGRHPVTPWGKPTKGAKTRRKKASDKLIISRRKGK
"
gene 67411..67692
/gene="rpsS"
/locus_tag="DMACINML_00584"
CDS 67411..67692
/gene="rpsS"
/locus_tag="DMACINML_00584"
/inference="ab initio prediction:Prodigal:2.6"
/inference="similar to AA sequence:RefSeq:CAL35797.1"

```
                     /codon_start=1
                     /transl_table=11
                     /product="30S ribosomal protein S19"
                     /protein_id="Prokka:DMACINML_00584"
                     /translation="MARSLKKGPFVDDHVMKKVIAAKKANDNKPIKTWSRRSTIIPDM
                     IGLTFNVHNGKSFIPVYITENHIGYKLGEFAPTRTFKGHKGSVQKKIGK"
     gene            67703..68110
                     /gene="rplV"
                     /locus_tag="DMACINML_00585"
     CDS             67703..68110
                     /gene="rplV"
                     /locus_tag="DMACINML_00585"
                     /inference="ab initio prediction:Prodigal:2.6"
                     /inference="similar to AA sequence:RefSeq:EAQ72756.1"
                     /codon_start=1
                     /transl_table=11
                     /product="ribosomal protein L22"
                     /protein_id="Prokka:DMACINML_00585"
                     /translation="MSKALIKFIRLSPTKARLIAREVQGMNAELAMASLKFMPNKGAK
                     YIANAISSAVANGGFEANEVVVKSCRVDAGAVLKRFRPRARGSASRIRKPTSHILVEV
                     AKMEVKTEAKKAVAKKTPVKKTSTKKVTAEKES"
     gene            68110..68811
                     /gene="rpsC"
                     /locus_tag="DMACINML_00586"
     CDS             68110..68811
                     /gene="rpsC"
                     /locus_tag="DMACINML_00586"
                     /inference="ab initio prediction:Prodigal:2.6"
                     /inference="similar to AA sequence:RefSeq:CAL35795.1"
                     /codon_start=1
                     /transl_table=11
                     /product="30S ribosomal protein S3"
                     /protein_id="Prokka:DMACINML_00586"
                     /translation="MGQKVNPIGLRLGINRNWESRWFPTKANLVENIGEDYKIRAFLK
                     RKLYYAGISQILVERTAKKLRVTVVAARPGIIIGKKGSDVDNLRKELQDLIGKDVNIN
                     IKEERKAGASAQLAAESVATQLEKRIAFRRAMKKVIQGAQKAGAKGIKVSVSGRLGGA
                     EMARTEWYLEGRVPLHTLRAKIDYGFAEARTTYGNIGVKVWIFKGEVLQKGLQAEKTE
                     ESAPAKKQRRTRRSK"
     gene            68814..69239
                     /gene="rplP"
                     /locus_tag="DMACINML_00587"
     CDS             68814..69239
                     /gene="rplP"
                     /locus_tag="DMACINML_00587"
                     /inference="ab initio prediction:Prodigal:2.6"
                     /inference="similar to AA sequence:RefSeq:CAL35794.1"
                     /codon_start=1
                     /transl_table=11
                     /product="50S ribosomal protein L16"
                     /protein_id="Prokka:DMACINML_00587"
                     /translation="MLMPKRTKYRKMMKGRNRGYANRGTEFTFGEFALKATEAGRINS
                     RQIEAARIALTRFVKRQGKTWIRVFPDKPLTKKPLETRMGKGKGAVEEWVMNIKPGRI
                     IYEMAGVSEEMAREALTLAMHKLPFKTKFVTRESQNEIY"
     gene            69226..69411
```

```
                     /gene="rpmC"
                     /locus_tag="DMACINML_00588"
     CDS             69226..69411
                     /gene="rpmC"
                     /locus_tag="DMACINML_00588"
                     /inference="ab initio prediction:Prodigal:2.6"
                     /inference="similar to AA sequence:RefSeq:ADN91829.1"
                     /codon_start=1
                     /transl_table=11
                     /product="50S ribosomal protein L29"
                     /protein_id="Prokka:DMACINML_00588"
                     /translation="MKYTEIKDKTAAELATMLKEKKVLLFTLKQKLKTMQLTNPKEIS
                     EVRKDIARINTAINALK"
     gene            69421..69672
                     /gene="rpsQ"
                     /locus_tag="DMACINML_00589"
     CDS             69421..69672
                     /gene="rpsQ"
                     /locus_tag="DMACINML_00589"
                     /inference="ab initio prediction:Prodigal:2.6"
                     /inference="similar to AA sequence:RefSeq:CAL35792.1"
                     /codon_start=1
                     /transl_table=11
                     /product="30S ribosomal protein S17"
                     /protein_id="Prokka:DMACINML_00589"
                     /translation="MAFKREIQGVVVKIAGEKTASILVERKVVHPRYRKIVKRFKKYL
                     IHDERNEVKVGDTVVAVECRPLSKRKSFRLKSVLATGVE"
     gene            69672..70040
                     /gene="rplN"
                     /locus_tag="DMACINML_00590"
     CDS             69672..70040
                     /gene="rplN"
                     /locus_tag="DMACINML_00590"
                     /inference="ab initio prediction:Prodigal:2.6"
                     /inference="similar to AA sequence:RefSeq:CAL35791.1"
                     /codon_start=1
                     /transl_table=11
                     /product="50S ribosomal protein L14"
                     /protein_id="Prokka:DMACINML_00590"
                     /translation="MIQSFTRLAVADNSGAKELMCIKVLGGSKRRYATVGDVIVASIK
                     KALPNGKVKKGQVVKAVIVRTKKEIHRDNGSLIRFDENAAVILDNKREPIGTRIFGPV
                     GREVRYGGFMKIVSLAPEVL"
     gene            70040..70273
                     /gene="rplX"
                     /locus_tag="DMACINML_00591"
     CDS             70040..70273
                     /gene="rplX"
                     /locus_tag="DMACINML_00591"
                     /inference="ab initio prediction:Prodigal:2.6"
                     /inference="similar to AA sequence:RefSeq:CAL35790.1"
                     /codon_start=1
                     /transl_table=11
                     /product="50S ribosomal protein L24"
                     /protein_id="Prokka:DMACINML_00591"
```

/translation="MAVKLKIKKGDSVKVITGDDKGKTGKVLAVYPKTLKVVVEGCKI
AKKAIKPSEKNPNGGFINKEMPMDISNVAKVQE"
gene 70280..70822
/gene="rplE"
/locus_tag="DMACINML_00592"
CDS 70280..70822
/gene="rplE"
/locus_tag="DMACINML_00592"
/inference="ab initio prediction:Prodigal:2.6"
/inference="similar to AA sequence:RefSeq:CAL35789.1"
/codon_start=1
/transl_table=11
/product="50S ribosomal protein L5"
/protein_id="Prokka:DMACINML_00592"
/translation="MRLKEKYDQSIKPALVKEFDIKNPMLIPAIEKIVISVGAGELAK
DQKVLQNVADTISLIAGQKAVITKAKKSVAGFKVREGFPVGVMVTLRKENMFAFLDKL
ISIALPRVKDFRGLSRDGFDGRGNYNFGLDEQLMFPEVEYDKILRTHGMNISIVTTAQ
NDKEAQKLLELIGVPFTKGK"
gene 70824..71009
/gene="rpsZ"
/locus_tag="DMACINML_00593"
CDS 70824..71009
/gene="rpsZ"
/locus_tag="DMACINML_00593"
/inference="ab initio prediction:Prodigal:2.6"
/inference="similar to AA sequence:RefSeq:ADN91824.1"
/codon_start=1
/transl_table=11
/product="30S ribosomal protein S14 type Z"
/protein_id="Prokka:DMACINML_00593"
/translation="MAKKSMIAKAARKPKFKVRGYTRCQICGRPHSVYRDFGICRVCL
RKMGNEGLIPGLKKASW"
gene 71019..71414
/gene="rpsH"
/locus_tag="DMACINML_00594"
CDS 71019..71414
/gene="rpsH"
/locus_tag="DMACINML_00594"
/inference="ab initio prediction:Prodigal:2.6"
/inference="similar to AA sequence:RefSeq:CAL35787.1"
/codon_start=1
/transl_table=11
/product="30S ribosomal protein S8"
/protein_id="Prokka:DMACINML_00594"
/translation="MINDIISDSLTRIRNAGMRKLETTKLLHSKVVEALVGIFQAKGY
IESFNVIEEDKKKFINVVLKYDEKGKSVINELKRVSKPGRRVYKGKEEIKRFKNGYGT
IVVSTSRGVLANDEAYKAGVGGEILCTIW"
gene 71492..72028
/gene="rplF"
/locus_tag="DMACINML_00595"
CDS 71492..72028
/gene="rplF"
/locus_tag="DMACINML_00595"
/inference="ab initio prediction:Prodigal:2.6"

```
                     /inference="similar to AA sequence:RefSeq:CAL35786.1"
                     /codon_start=1
                     /transl_table=11
                     /product="50S ribosomal protein L6"
                     /protein_id="Prokka:DMACINML_00595"
                     /translation="MSRIGKQPIAIPSGVEVKLEGNLLKFKKGNLAKELDTKANVNVE
                     IKDNNILFSPKGEDRQSRAYWGTYRALAYNIVVGLTQGFSKTLEINGVGYKAALKGKV
                     LELSLGFSHPINYDIPEGIEITVDKNNVIIKGSDKQVVGQVAAQIREFRPPEPYKGKG
                     VKYSDERIIRKAGKTSKK"
     gene            72038..72394
                     /gene="rplR"
                     /locus_tag="DMACINML_00596"
     CDS             72038..72394
                     /gene="rplR"
                     /locus_tag="DMACINML_00596"
                     /inference="ab initio prediction:Prodigal:2.6"
                     /inference="similar to AA sequence:RefSeq:CAL35785.1"
                     /codon_start=1
                     /transl_table=11
                     /product="50S ribosomal protein L18"
                     /protein_id="Prokka:DMACINML_00596"
                     /translation="MRANVLKRKLILRIKRKKRIRAKISGCESFPRISVFKSNRTLYI
                     QAIDDVKAVTLAAVDGRKLGVKANKEGAKKIAAEFAKVLKAKQIEQAVFDRNGYVVYHG
                     VIAALAESLRENGIRL"
     gene            72406..72849
                     /gene="rpsE"
                     /locus_tag="DMACINML_00597"
     CDS             72406..72849
                     /gene="rpsE"
                     /locus_tag="DMACINML_00597"
                     /inference="ab initio prediction:Prodigal:2.6"
                     /inference="similar to AA sequence:RefSeq:CAL35784.1"
                     /codon_start=1
                     /transl_table=11
                     /product="30S ribosomal protein S5"
                     /protein_id="Prokka:DMACINML_00597"
                     /translation="MEKYNREEFEEVIVDIGRVTKVVKGGRRFRFTALVIVGNRKGLV
                     GVGYGKAKEVPDAIRKAVDDAFKNIVEVKTKGSTIAHDVEVKYNASRILLKPASEGTG
                     VIAGGSTRPIVELAGIKDILTKSLGSNNSANVVRATIKALTMLKG"
     gene            72854..73246
                     /gene="rp10"
                     /locus_tag="DMACINML_00598"
     CDS             72854..73246
                     /gene="rp10"
                     /locus_tag="DMACINML_00598"
                     /inference="ab initio prediction:Prodigal:2.6"
                     /inference="similar to AA sequence:RefSeq:CAL35783.1"
                     /codon_start=1
                     /transl_table=11
                     /product="50S ribosomal protein L15"
                     /protein_id="Prokka:DMACINML_00598"
                     /translation="MNLTKAAGSTHKTKRIGRGQGSGMGKTATKGGKGQTARKGYNEK
                     RGFEGGQQPLQRRLPKVGFTSKIAKPYVINVEKITAVKELNEITIESIKSVHKISKSV
                     TKIKLIGASAKDLASKIKDENISVTGSK"
```

```
gene            73246..74511
                /gene="secY"
                /locus_tag="DMACINML_00599"
CDS             73246..74511
                /gene="secY"
                /locus_tag="DMACINML_00599"
                /inference="ab initio prediction:Prodigal:2.6"
                /inference="similar to AA sequence:RefSeq:CAL35782.1"
                /codon_start=1
                /transl_table=11
                /product="preprotein translocase subunit"
                /protein_id="Prokka:DMACINML_00599"
                /translation="MNRALTNKILITLAFLFAYRVLAYVPVPGVNADVIAEFFNDNQN
                NALGLFNVFSGGAAERFSIISLGIMPYITASIIMELLAATFPNIGKMKKERDGMQKYM
                QIIRYATIVITLVQSIGVAIGLQSLHGRGGAGAIMIENLNMFIALCAISMLAGTMLLM
                WIGEQITQRGIGNGISLIIFAGIVSGIPRAISGTVGQINSGEMNFLTAFVILVLILIT
                IGVIIYVELGERRIPISYSRKVVMQNQNKRIMNYIPIKINLSGVIPPIFASAILMFPT
                TILQTSTNPYLQAINDFLNPNGYLFHVLTFLFVIFFAYFYASIVFNAKDIAENLKKQG
                GFIPGIRPGEGTANYLNEVASRLTLSGSIYLGLVATLPWVLVKFMGVPFNFGGTSVLI
                VVQVALDTMRKIEAQIYMNKYQTLNAIGL"
gene            complement(74568..75827)
                /locus_tag="DMACINML_00600"
CDS             complement(74568..75827)
                /locus_tag="DMACINML_00600"
                /inference="ab initio prediction:Prodigal:2.6"
                /inference="similar to AA sequence:RefSeq:CAL35781.1"
                /codon_start=1
                /transl_table=11
                /product="putative efflux protein"
                /protein_id="Prokka:DMACINML_00600"
                /translation="MKKKSYKFHPNDTKLDKKMVEITEKSGFKIAVFSMAAMTMLGSV
                LISSALPAINRHFEDILIQSGASVETNFTLAHLDILVRLVLTLPAIFVVILSPFAGIL
                MDKLGKLKFVFPAMVVWTLAGVSGFFLNNIYAILTSRAIFGMATAFIMTGASALLGDY
                YSRGGFNRRENALSLQGFFCAVGGAIFISIAGFVSSYSWRYPFLVYGLGILITLVAMI
                YLFEPRKFKFYNHTKIEEKTNYWKFFPIYFIGFFIMVVYYISPTQLPYYIEEHLGLDP
                KFIGISMSISALCYGIFSLSYKYIMRFLSIKTIYVATLFIVGCSFLILFLIDDFIAVL
                FALALLGMGGGIMLVNNTAYLFSICPENARARAYGILASCIFLGQFLSPIISQPIVRQ
                LGLVDAFLIWAILNFVVCIVFLFLKQR"
gene            76070..78172
                /gene="topA"
                /locus_tag="DMACINML_00601"
CDS             76070..78172
                /gene="topA"
                /locus_tag="DMACINML_00601"
                /EC_number="5.99.1.2"
                /inference="ab initio prediction:Prodigal:2.6"
                /inference="similar to AA sequence:RefSeq:CAL35780.1"
                /codon_start=1
                /transl_table=11
                /product="DNA topoisomerase I"
                /protein_id="Prokka:DMACINML_00601"
                /translation="MKKNLIIVESPAKAKTIGNFLGKEYEVIASKGHIRDLPKSSFGI
                KIENDEFIPEYRITSDHSALVKELKEKAKGAKEIYLATDEDREGEAIAYHIAKAIGKD
                ENVLPRIVFHEITKNAIENALKNPRKLDMNSVNAQQTRRLLDRIVGYKLSPLLGQKIQ
```

RGLSAGRVQSAALKIIVDREKEIRAFVPLEYFSIDMTFQKDLDAELVEFDKAKIEKLT
LTNKDRAKLVFEACKNENYAINDIESKERKIAPPPPFMTSTLQQSASNRLGFNPKKTM
MIAQKLYEGVNTHEGVMGVITYMRTDSLNLAKEAVENARKFIQTNFGKDYLPSKANAY
TTKAKGAQEAHEAIRPTNLNFTPEIAAKFLDKDELKLYTLIYNRFLACQMNPAISQTQ
NVFVKNDRAVFKISGRKILFDGYYKVYGDMDKDKILPNLKIGENLKVQNLEMNSHFTE
PPSRYSEAGLVKKLESLGIGRPSTYAPTISILTSRDYVKIDKKQLIPSEVAFSVTEVL
EKNFSDIVDSKFTSNLENTLDEIAEDKANWQETLKEFYYPFMRKVEEGKTKIASQKTV
TKLGENCPDCGGELAIRKGRFGEFVACLNFPKCKYSRNLKNESKNEAEVSASKAKAKG
IGITCPSCKEGEIVERLSKRGKFYGCSAYPKCNFISKYKPSDEKCEECGENLVIKELK
KGTFLECLKCKIKKEIKD"

gene 78175..79011
/gene="bioB"
/locus_tag="DMACINML_00602"

CDS 78175..79011
/gene="bioB"
/locus_tag="DMACINML_00602"
/EC_number="2.8.1.6"
/inference="ab initio prediction:Prodigal:2.6"
/inference="similar to AA sequence:RefSeq:CAL35779.1"
/codon_start=1
/transl_table=11
/product="putative biotin synthase"
/protein_id="Prokka:DMACINML_00602"
/translation="MKVMLCAISNIASGNCSEDCKYCTQSAYVKTNIQKYRQKEISQI
VLEAKIAKKNEALGFCLVTAGLGLDDEKLEYVCKAAHAVQKEVPNLLLIACNGMASVE
QLKELKKAGIFSYNHNLEASKEFFPQICTTHTWESRFQTNLNAKEAGLMLCCGGIYGM
GESEEDRISLRKSLQELQPFSTPINFFIANENLKLQVPKLSADEALKIISDTKEALPD
TVVMVAGGREVVLQERQYEIFKAGAGAIVIGDYLTTKGEEPSRDIVKLKEMGFTFASE
CH"

gene 78992..80167
/locus_tag="DMACINML_00603"

CDS 78992..80167
/locus_tag="DMACINML_00603"
/inference="ab initio prediction:Prodigal:2.6"
/inference="similar to AA sequence:RefSeq:CAL35778.1"
/codon_start=1
/transl_table=11
/product="putative transmembrane transport protein"
/protein_id="Prokka:DMACINML_00603"
/translation="MHPSVIDSQGLIDLQILIVIALCLLFSPHIAKILRLPISATEII
LGALIAYFGFIGKSENFTILANVGFYYLMFIAGMEVNLRAFFNMDKEVAKKSFFYIIL
LYSFSSAIVWIFELSFVFVLIIPVMSVGLLSLLYKDFGKECYWLNTAMIVATLAEVVS
IVLLTIAGAFLREGTNIIDVVQSILYLNIFLGLCLLSFKILGVLFWWYPQLKVVLMPW
EDKNEKDIRFCMAIFILIIVAMIITKLEIVLGSFIAGSFIATFFDHKKDLEHKLSSFG
HGFLIPIFFIHIGTTFDLKMILEYEIVLDAILLMFVMVGLRILCASVFWKKIGFQNMI
LFGLSHSMPLTLLIATATLGYSGKVISEELYSALILTALFEAIVVMSAIKFISNIKK"

gene 80297..81565
/locus_tag="DMACINML_00604"

CDS 80297..81565
/locus_tag="DMACINML_00604"
/inference="ab initio prediction:Prodigal:2.6"
/inference="similar to AA sequence:RefSeq:ADN91805.1"
/codon_start=1
/transl_table=11

```
                     /product="Citrate synthase"
                     /protein_id="Prokka:DMACINML_00604"
                     /translation="MSNSVTVTDNRNGKSYEFPIYDGTTGPSVVDMGSFYKQTGMFSY
                     DEGLTSTATCKSKITYIDGEEGVLMHRGYPIEWLAENKLFLDVVHLLLYKELPSSERL
                     EAFRYELKKRSYIHEGMHRLFDSFPDNAHPMAVLQGAVSSLSAFYPDHLNMNVREEYM
                     EMAARVVAKIPTIVAAAYRYKNGFPMAYPNLDRGFTENFLYMLRTYPYDHVELKPIEV
                     KALDTVFMLHADHEQNASTSTVRAVGSTHAHPYACISAGIGALWGHAHGGANEGVIRM
                     LEEIATVDRVDEFIKRAKDKNDPFRLMGFGHRVYKNFDPRAKVLKKLRDQLIDELGID
                     TNLIKVATRIEEIALQDDYFVQRNLYPNVDFHSGLILKALGIPNEMFATLFVIGRTPG
                     WIAQWIEQKEQETLKIVRPRQLYLGETSKI"
     gene            81639..82418
                     /gene="cysQ"
                     /locus_tag="DMACINML_00605"
     CDS             81639..82418
                     /gene="cysQ"
                     /locus_tag="DMACINML_00605"
                     /inference="ab initio prediction:Prodigal:2.6"
                     /inference="similar to AA sequence:RefSeq:ADN91804.1"
                     /codon_start=1
                     /transl_table=11
                     /product="CysQ protein"
                     /protein_id="Prokka:DMACINML_00605"
                     /translation="MFKLDNLLKTAIDASNEASKAILKERKNLQIWQKQDKTPLTSAD
                     LASNEILNDILGKTDIKILSEEGLLNQEESKNLQSYWLIDPLDGTSGFLKGSDEFCIM
                     ISLIYEARPVLALIKNPSKGDIFYAHQNTRVYKNDKILDTNEQDFINNQSKALLSVNH
                     LSKEDENFAKKYHLEPINIGSGLKFCAILEGKAGIYKRFEKLNSWDISAGDFLVNQNG
                     GFMGTFSKEMILYSPLNYKCEPFICVSQKSFLNIYIHKNSI"
     gene            82443..83204
                     /locus_tag="DMACINML_00606"
     CDS             82443..83204
                     /locus_tag="DMACINML_00606"
                     /inference="ab initio prediction:Prodigal:2.6"
                     /inference="similar to AA sequence:RefSeq:CAL35775.1"
                     /codon_start=1
                     /transl_table=11
                     /product="putative periplasmic protein"
                     /protein_id="Prokka:DMACINML_00606"
                     /translation="MKIFCILLIGILLNSSLFANNFSQKKIIKLEQQESFEIIDLQQN
                     VAKQNLDQQKGIFDSSSLVGKKESIRKDNDVDFAIVMSFRQNFGYTLDGFSINAKEFS
                     TLFSKNLVDNLKLNFVNSAANGVYQDSRTLARFSPKDSKLLNVSPFLKKEKDKSQIYA
                     KFTDYLVVINLDDFYVNITNYFVAITKNAVANINFKVVSTSNGKILTSKNVRLNFALH
                     AQDPRINYQELVNQMPNMLAEVINKELGKLNLIVQ"
     sig_peptide     82443..82502
                     /locus_tag="DMACINML_00606"
                     /inference="ab initio prediction:SignalP:4.1"
                     /note="predicted cleavage at residue 20"
     gene            complement(83211..83384)
                     /locus_tag="DMACINML_00607"
     CDS             complement(83211..83384)
                     /locus_tag="DMACINML_00607"
                     /inference="ab initio prediction:Prodigal:2.6"
                     /codon_start=1
                     /transl_table=11
                     /product="hypothetical protein"
```

```
                     /protein_id="Prokka:DMACINML_00607"
                     /translation="MQININLEEMTKWQITYELKRILSSLDPITAHEIAKELRQEFLS
                     IEELQDRIRQIND"
     gene            complement(83498..84274)
                     /gene="murB"
                     /locus_tag="DMACINML_00608"
     CDS             complement(83498..84274)
                     /gene="murB"
                     /locus_tag="DMACINML_00608"
                     /EC_number="1.3.1.98"
                     /inference="ab initio prediction:Prodigal:2.6"
                     /inference="similar to AA sequence:RefSeq:CAL35772.1"
                     /codon_start=1
                     /transl_table=11
                     /product="UDP-N-acetylenolpyruvoylglucosamine reductase"
                     /protein_id="Prokka:DMACINML_00608"
                     /translation="MIIDFKKYSSVKIGGEFEVEVLDQIREFDGFLIGGANNLLVSPK
                     PKNIGILSDNFNFIEILEQNKDFLHLRIGCKTKASQMYRFSKENNLYGFEYLSKIPGT
                     LGGLLKMNAGLKDECISQNLVKIATSKGEILRENIDFSYRFCPLNMPFFWAEFKLGFG
                     FDKIKDENLKNARNNQPSGASFGSIFKNPKGDFAGRLIEAVGLKGFSKGGAMLSDKHA
                     NFLINKKNASFEDAIFLIELAKKKVFEEFGINLEEEVIIV"
     gene            complement(84271..84540)
                     /gene="fliQ"
                     /locus_tag="DMACINML_00609"
     CDS             complement(84271..84540)
                     /gene="fliQ"
                     /locus_tag="DMACINML_00609"
                     /inference="ab initio prediction:Prodigal:2.6"
                     /inference="similar to AA sequence:RefSeq:CAL35771.1"
                     /codon_start=1
                     /transl_table=11
                     /product="flagellar biosynthetic protein"
                     /protein_id="Prokka:DMACINML_00609"
                     /translation="MDESTLVALGVQTFKITLLLSLPMLLAGLIAGLIISIFQATTQI
                     NEMTLSFVPKIILVVIVIIFLMPWMTTTMIDFTENILNQIPTFIK"
     gene            complement(84552..85415)
                     /locus_tag="DMACINML_00610"
     CDS             complement(84552..85415)
                     /locus_tag="DMACINML_00610"
                     /inference="ab initio prediction:Prodigal:2.6"
                     /inference="similar to AA sequence:RefSeq:CAL35770.1"
                     /note="conserved hypothetical protein Cj1674"
                     /codon_start=1
                     /transl_table=11
                     /product="hypothetical protein"
                     /protein_id="Prokka:DMACINML_00610"
                     /translation="MKKTITIAHSPDADDIFMYMAIKLGWIGNDFDYKNTALDIQTLN
                     DLALRNEFDATAISFGLYPLIAQEYALLRTAVSFGEGYGPKLIKKQNTHLKRNFKIAL
                     SGANTTNALIFRMKYPEARIIYKNFLEIENAVLSGEVDAGVLIHESILEFDSSLCVEA
                     EIWDIWLELAQENLPLPLGGMALRRSLPLSDAIKIERDLTNAVKIADSNRRILAPMLM
                     ERNLIRVDEKKLDTYLNLYANKNSICMNEMQFKAVDVLFKLGYDYKFYDKIIHAKDYL
                     IPSEYEEFRNS"
     gene            85525..86556
                     /gene="recA"
```

```
                     /locus_tag="DMACINML_00611"
     CDS             85525..86556
                     /gene="recA"
                     /locus_tag="DMACINML_00611"
                     /inference="ab initio prediction:Prodigal:2.6"
                     /inference="similar to AA sequence:RefSeq:CAL35769.1"
                     /codon_start=1
                     /transl_table=11
                     /product="recA protein"
                     /protein_id="Prokka:DMACINML_00611"
                     /translation="MDDNKKKSLDAALKSLDKAFGKGTILRLGDKEVEQIDSIGTGSV
                     GLDLALGIGGIPKGRIIEIYGPESSGKTTLTLHIIAECQKAGGVCAFIDAEHALDVKY
                     AKNLGVDTDNLYISQPDFGEQALEIVETIARSGAIDLIVVDSVAALTPKAEIEGDMGD
                     QHVGLQARLMSQALRKLTGIVHKMNTTVIFINQIRMKIGAMGYGTPETTTGGNALKFY
                     ASVRLDVRKVATLKQNEEPIGNRVKVKVVKNKVAPPFRQAEFDVMFGEGLSREGELID
                     YGVKLDIVDKSGAWFSYKDKKLGQGRENSKAFLKENPEIADEITKEIQNSMGIEGMIS
                     GSEDEEGEE"
     gene            86556..87800
                     /gene="eno"
                     /locus_tag="DMACINML_00612"
     CDS             86556..87800
                     /gene="eno"
                     /locus_tag="DMACINML_00612"
                     /EC_number="4.2.1.11"
                     /inference="ab initio prediction:Prodigal:2.6"
                     /inference="similar to AA sequence:RefSeq:CAL35768.1"
                     /codon_start=1
                     /transl_table=11
                     /product="enolase"
                     /protein_id="Prokka:DMACINML_00612"
                     /translation="MLVIEDVRAYEVLDSRGNPTVKAEVVLSDGSMGAAIVPSGASTG
                     SKEALELRDNDERFGGKGVLKAVSNVNETIADEILGLDALNQAQLDDTLRELDGTNNY
                     SKLGANATLGVSMATARAAANALGVPLYRYLGGANASILPVPMCNIINGGAHASNNID
                     FQEFMIMPFGFTSFKEALRSVCEIYAILKKELVNLGHSAALGDEGGFAPNLANNTEPI
                     DLLMTCIKKAGYENRVKIALDVASTEFFKEGKYHMEGKAFSSEDLIERYVELCAKYPI
                     CSIEDGLAENDFEGWIKLTEKLGNKIQLVGDDLFVTNEDILREGIIKKMANAVLIKPN
                     QIGTITQTMRTVRLAQRNNYKCVMSHRSGESEDAFIADFAVALNTGQIKTGALARGER
                     TAKYNRLLEIELESDEFLGEKL"
     gene            87866..88069
                     /locus_tag="DMACINML_00613"
     CDS             87866..88069
                     /locus_tag="DMACINML_00613"
                     /inference="ab initio prediction:Prodigal:2.6"
                     /inference="similar to AA sequence:RefSeq:ADN91797.1"
                     /codon_start=1
                     /transl_table=11
                     /product="hypothetical protein"
                     /protein_id="Prokka:DMACINML_00613"
                     /translation="MVFWAFLVIVGAIYFGNMLFGQYSLDTLLSLEATKEELNKKISF
                     LKEQNAKAQKDYFELQGLYPNEN"
     gene            88059..88721
                     /gene="cgpA"
                     /locus_tag="DMACINML_00614"
     CDS             88059..88721
```

/gene="cgpA"
/locus_tag="DMACINML_00614"
/inference="ab initio prediction:Prodigal:2.6"
/inference="similar to AA sequence:RefSeq:CAL35766.1"
/codon_start=1
/transl_table=11
/product="putative periplasmic protein"
/protein_id="Prokka:DMACINML_00614"
/translation="MKTKILGIFLIFISLLNAEENPFKSEQNMSMIVPPDFQKEDIKF
NSNARVLKSVSFNYINLDGSEEVLNIDINKSIDWHDTYTLVRSKSPDPSKVFDVSVTI
PERNDSKKESNNTASIEIPLQVDKIYDFISYAVYKNKIKLNTNDELISDFSIGNPSKI
VVDFKSDLVNPTKNIRISNSLFKRIDFGSHKGYYRLVIYLDGNYNYNIQKDATGYMIN
LL"

gene 88721..89560
/locus_tag="DMACINML_00615"

CDS 88721..89560
/locus_tag="DMACINML_00615"
/EC_number="6.5.1.1"
/inference="ab initio prediction:Prodigal:2.6"
/inference="similar to AA sequence:RefSeq:CAL35765.1"
/codon_start=1
/transl_table=11
/product="putative ATP-dependent DNA ligase"
/protein_id="Prokka:DMACINML_00615"
/translation="MRIFIFLWCACFAFASDILLLSKISEQEIQNKNFNGYLMSEKLD
GIRGIWEAGKFKTRQDNPIHTPSYFTYNFPSFKLDGELWIARDKFDEVSALIRSDNLD
SSLWRSVTYNVFDVPNACEEFKLTSCTLSNRLKVLEQYLQQNPNPYIKIIKQIPIKDQ
EHLKEFYKDIILNKGEGVVIRKDLAPYEKGRSKNAFKLKPYEDAECKVIGYTEGKGKF
QGKIGALLCQMPNDRVIRIGSGLKDKDRENPPKIGSIVTYKFNGLTKNSLPRFPVFLH
IRD"

gene 89607..89684
/locus_tag="DMACINML_00616"

tRNA 89607..89684
/locus_tag="DMACINML_00616"
/product="tRNA-Pro"
/inference="COORDINATES: profile:Aragorn:1.2"
/note="tRNA-Pro(tgg)"

gene 89709..89785
/locus_tag="DMACINML_00617"

tRNA 89709..89785
/locus_tag="DMACINML_00617"
/product="tRNA-His"
/inference="COORDINATES: profile:Aragorn:1.2"
/note="tRNA-His(gtg)"

gene 89790..89866
/locus_tag="DMACINML_00618"

tRNA 89790..89866
/locus_tag="DMACINML_00618"
/product="tRNA-Arg"
/inference="COORDINATES: profile:Aragorn:1.2"
/note="tRNA-Arg(tcg)"

gene 89881..89957
/locus_tag="DMACINML_00619"

tRNA 89881..89957

```
                     /locus_tag="DMACINML_00619"
                     /product="tRNA-Arg"
                     /inference="COORDINATES: profile:Aragorn:1.2"
                     /note="tRNA-Arg(tct)"
     gene            89963..90047
                     /locus_tag="DMACINML_00620"
     tRNA            89963..90047
                     /locus_tag="DMACINML_00620"
                     /product="tRNA-Leu"
                     /inference="COORDINATES: profile:Aragorn:1.2"
                     /note="tRNA-Leu(tag)"
     gene            90248..91003
                     /gene="proB"
                     /locus_tag="DMACINML_00621"
     CDS             90248..91003
                     /gene="proB"
                     /locus_tag="DMACINML_00621"
                     /EC_number="2.7.2.11"
                     /inference="ab initio prediction:Prodigal:2.6"
                     /inference="similar to AA sequence:RefSeq:EAQ71967.2"
                     /codon_start=1
                     /transl_table=11
                     /product="glutamate 5-kinase"
                     /protein_id="Prokka:DMACINML_00621"
                     /translation="MQRIVVKVGSHVISDENLLNLKRFENLVAFLAKLMQKYEVILVT
                     SAAISAGCTKLHIDRKNLINKQVLAAIGQPFLISVYNEFLAKFGKMGGQILLTGKDFD
                     SRKATKHAKNAIDAMIDLGILPIINENDATAIEEIVFGDNDSLSAYATHFFEADLLVI
                     LSDIDGFYDKNPSEFSDAKRLEKVEFIKEEWLNVSVKTGSEHGTGGIVTKLKAAKFLL
                     ENDKKMFLASGFDLSVAKAFLLENKQIGGTLFE"
     gene            91083..91286
                     /locus_tag="DMACINML_00622"
     CDS             91083..91286
                     /locus_tag="DMACINML_00622"
                     /inference="ab initio prediction:Prodigal:2.6"
                     /codon_start=1
                     /transl_table=11
                     /product="hypothetical protein"
                     /protein_id="Prokka:DMACINML_00622"
                     /translation="MKKLARIFLLVVAFALSAFAYDPYACSLQNLINLGSCNPNLVPN
                     DEYACWALPNVRVNGELVCAEYY"
     sig_peptide     91083..91145
                     /locus_tag="DMACINML_00622"
                     /inference="ab initio prediction:SignalP:4.1"
                     /note="predicted cleavage at residue 21"
     gene            91286..92323
                     /locus_tag="DMACINML_00623"
     CDS             91286..92323
                     /locus_tag="DMACINML_00623"
                     /inference="ab initio prediction:Prodigal:2.6"
                     /inference="similar to AA sequence:RefSeq:EAQ72322.1"
                     /note="conserved hypothetical protein"
                     /codon_start=1
                     /transl_table=11
                     /product="hypothetical protein"
```

```
                     /protein_id="Prokka:DMACINML_00623"
                     /translation="MSILVNAVDTFYSTKNEAVFSASKSENIKRNVADFNTDNNTKNS
                     VYFFSDDKEISLNLSNESMELLTNHFSANDFLKLSNGNIGLSGKAAAFVEGWYKDIMI
                     NRNFLNADRDKNGRIENNEYLSLKNSVEDVAKNQDLSKNFGAVVLRMAQSTTGYVENT
                     ISDKSLSIEALMDESIKSDRNFDGIITRLEYSAKGGTDMQGYTNWAIDALNDILDDSD
                     KAKKIHLVVDPTSEIWHSYAGKTLNEVKAYYDNPFRATSGTSASGFLNIVIEEPRILP
                     GNDLENEKEQLLNEFPEFRALIQNNPNITKEKLLKLKNQQRITHNYQDHQKQDLQKEF
                     TQNFFKTDLKA"
     gene            92455..93375
                     /gene="fmt"
                     /locus_tag="DMACINML_00624"
     CDS             92455..93375
                     /gene="fmt"
                     /locus_tag="DMACINML_00624"
                     /EC_number="2.1.2.9"
                     /inference="ab initio prediction:Prodigal:2.6"
                     /inference="similar to AA sequence:RefSeq:CAL34269.1"
                     /codon_start=1
                     /transl_table=11
                     /product="methionyl-tRNA formyltransferase"
                     /protein_id="Prokka:DMACINML_00624"
                     /translation="MMKKIIFMGTPSYATCILKALLEDENFELLALFTQPDKAVGRKQ
                     ILTPSDTKAFLLQKAPQVPIFTPNSLKDESVIEQIRALKPDFIVVAAYGKILPKAILD
                     IAPCINLHASLLPKYRGASPIQSAILNADEKSGVCTMLMEEGLDTGAVLESVECDIRD
                     KNVNEVFEILASLAAKLILSTLLNYEKLLPKKQDESLATHCKKIKKEDGLVSLDNARE
                     IYQKYLAFTPWPGIFLENGLKFIELELVDELKQNAKMGEILELEKESFLLACKQGILR
                     IKKLQESGKKALDGRTYLNGKRLKSADSLC"
     gene            93338..93991
                     /gene="birA"
                     /locus_tag="DMACINML_00625"
     CDS             93338..93991
                     /gene="birA"
                     /locus_tag="DMACINML_00625"
                     /EC_number="6.3.4.15"
                     /inference="ab initio prediction:Prodigal:2.6"
                     /inference="similar to AA sequence:RefSeq:CAL34270.1"
                     /codon_start=1
                     /transl_table=11
                     /product="putative biotin--[acetyl-CoA-carboxylase]
                     synthetase"
                     /protein_id="Prokka:DMACINML_00625"
                     /translation="MEKGLKVQIACVESIDSTHLFLCEQIRSGKINENFALYALNQTK
                     GVGSRENLWQSSSGNLHLSFCIKESDLPSDLPLASVSIYFAYLLKEILQEKGSRIWLK
                     WPNDLYLDDKKVGGIISTKISDFIVGGMGLNLKFAPHDAALCDVEISLEDLIEKFLQR
                     LEKKFLWKHIFSKYMLEFEKSRQFSVHYEGKVFSLENAFLYEDGSILLGDKRVYSLR"
     gene            93988..94773
                     /locus_tag="DMACINML_00626"
     CDS             93988..94773
                     /locus_tag="DMACINML_00626"
                     /inference="ab initio prediction:Prodigal:2.6"
                     /inference="similar to AA sequence:RefSeq:CAL34271.1"
                     /codon_start=1
                     /transl_table=11
                     /product="parA family protein"
```

/protein_id="Prokka:DMACINML_00626"
/translation="MSEIITIANQKGGVGKTTTAVNLAASLAVAEKKVLLIDVDPQAN
ATTGLGFNRNNYEYNIYHVFIGRKKLSDIILKTELPQLHLAPSNIGLVGIEQELAKGE
NNEKKMILKNQIQEVLDEYDFIIIDSPPALGSITINAFVASDSVIIPIQCEFYALEGV
AMVLNTIKIIKKTINPKLKVRGFLPTMYSSQNNLSKDVVEDLKQNFKKQLFTINGNED
DFIVIPRNVKLAESPSFGKPIILYDIKSPGSLAYQNLAYSILG"

gene 94778..95614
/locus_tag="DMACINML_00627"

CDS 94778..95614
/locus_tag="DMACINML_00627"
/inference="ab initio prediction:Prodigal:2.6"
/inference="similar to AA sequence:RefSeq:CAL34272.1"
/codon_start=1
/transl_table=11
/product="parB family protein"
/protein_id="Prokka:DMACINML_00627"
/translation="MGLNKNRGLSTLMGDVGEVYSRELGLDKNQVSLIEISKITPNPF
QPRKTFDEVALNELANSIKEHGLIQPIIVLKKNDSFILVAGERRLRATQILGEENILA
FVSDSDESKLRELALIENIQRENLNPIELANSYKDLIEVYNITQENLAELIHKSRTQI
TNTLRLLNLDSKTQELIASGKISQGHAKVLVGLDKEDEKVLVDSILGQKLSVRDTERL
VQKVKNKENIEDEEFENSMQNLKQILNKIGFMCKNNKKELIIRLDNIDKIKKLTEILN
KI"

gene 95673..96098
/gene="atpF_1"
/locus_tag="DMACINML_00628"

CDS 95673..96098
/gene="atpF_1"
/locus_tag="DMACINML_00628"
/EC_number="3.6.3.14"
/inference="ab initio prediction:Prodigal:2.6"
/inference="similar to AA sequence:RefSeq:CAL34273.1"
/codon_start=1
/transl_table=11
/product="ATP synthase F0 sector B' subunit"
/protein_id="Prokka:DMACINML_00628"
/translation="MFEDMHPSIMLATMAIFLAMIVILNTMLYKPLLKFMDERNDSIK
NDENKVKENSQEMLGANDEVEAIHVSTREEIHKIKQNAINAAKEEAQQAIKAKKEELE
RKMANFYTELISQKKELQDHLSAHLPDLKQALQNNIKQH"

gene 96110..96622
/gene="atpF_2"
/locus_tag="DMACINML_00629"

CDS 96110..96622
/gene="atpF_2"
/locus_tag="DMACINML_00629"
/EC_number="3.6.3.14"
/inference="ab initio prediction:Prodigal:2.6"
/inference="similar to AA sequence:RefSeq:CAL34274.1"
/codon_start=1
/transl_table=11
/product="ATP synthase F0 sector B subunit"
/protein_id="Prokka:DMACINML_00629"
/translation="MKKLFYFIFLLPLCAFGASNGSGEYDIVPRTINFLIFIAILYYF
VANPFKAFYKNRILRISLKLDEIQKKLLESKAKKLDTMKKLEEAKANAAAALVVAKKE
AEVLAQKIKEETQNELALLEKHFEEQKSYEFRKMEKEVVSLTLKEIFEDSNIALKQNE

```
                     IIDIMMKKVS"
gene                 96622..97143
                     /gene="atpH"
                     /locus_tag="DMACINML_00630"
CDS                  96622..97143
                     /gene="atpH"
                     /locus_tag="DMACINML_00630"
                     /EC_number="3.6.3.14"
                     /inference="ab initio prediction:Prodigal:2.6"
                     /inference="similar to AA sequence:RefSeq:CAL34275.1"
                     /codon_start=1
                     /transl_table=11
                     /product="ATP synthase F1 sector delta subunit"
                     /protein_id="Prokka:DMACINML_00630"
                     /translation="MNDLIAKRYAKAITLRADISEFYDNLSILSSAFALPKFKNIIES
                     NQIQKEKKLEFVISLIEKASPSFNNFLKLLAENSRLSCIPQIAKELERQKSFKENIFL
                     GTVYSKEVLSEDSIKELESRLGAKFGVKIKLQNKAGLNEGVKISLEELGYEISFSMKA
                     LQNKMSEYILKII"
gene                 97156..98661
                     /gene="atpA"
                     /locus_tag="DMACINML_00631"
CDS                  97156..98661
                     /gene="atpA"
                     /locus_tag="DMACINML_00631"
                     /EC_number="3.6.3.14"
                     /inference="ab initio prediction:Prodigal:2.6"
                     /inference="similar to AA sequence:RefSeq:CAL34276.1"
                     /codon_start=1
                     /transl_table=11
                     /product="ATP synthase F1 sector alpha subunit"
                     /protein_id="Prokka:DMACINML_00631"
                     /translation="MKFKADEISSIIKERIENFDLNLEIEETGKIISVADGVAKVYGL
                     KNIMAGEMVEFDNGDKGMALNLEESSVGIVILGKGEGLKEGTSVKRLKKLLKVPVGEA
                     LIGRVVNALGEPIDAKGVINSNEFRFVEEKAKGIMARKSVHEPLHTGIKAIDALVPIG
                     RGQRELIIGDRQTGKTTVAVDTIISQKGQGVVCIYVAIGQKQSTVAQVVKRLEEHGAM
                     DYTIVVNAGASDPAALQYLAPYTGVTMGEFFRDNAKHALIVYDDLSKHAVAYREMSLI
                     LRRPPGREAYPGDVFYLHSRLLERASKLNDELGAGSLTALPIIETQAGDVSAYIPTNV
                     ISITDGQIFLETDLFNSGIRPAINVGLSVSRVGGAAQIKATKQVSGTLRLDLAQYREL
                     QAFAQFASDLDEASRKQLERGQRMVELLKQPPYSPLSVEKQVVLIFAGTKGYLDDVAV
                     SKIREFEDGIYPFIEAKYPDLFEQIRSKKALDAELEEKLAKAINEFKANHL"
gene                 98671..99555
                     /gene="atpG"
                     /locus_tag="DMACINML_00632"
CDS                  98671..99555
                     /gene="atpG"
                     /locus_tag="DMACINML_00632"
                     /EC_number="3.6.3.14"
                     /inference="ab initio prediction:Prodigal:2.6"
                     /inference="similar to AA sequence:RefSeq:CAL34277.1"
                     /codon_start=1
                     /transl_table=11
                     /product="ATP synthase F1 sector gamma subunit"
                     /protein_id="Prokka:DMACINML_00632"
                     /translation="MSNLKEIKRKIKSVHNTQKTTNAMKLVSTAKLKKAEEAAKRSRI
```

```
                     YAQKIDEILSEISFQVNKIVHNEEDSRFVLFHKKNQIKNVDLIFITADKGLCGGFNIK
                     TLKAVSELLKEYEEKNINVRLRAIGKTGIEYFNFQKIELLEKYLGLSSSPDYDKACSV
                     IQAAVDDYLNGITDEVVLVHNGYKNMITQELKISHLIPVEPKQIEGNSNSLLELEPED
                     TDLLGDLMKTYFEYNMYYALIDSLAAEHSARMQAMDNATNNAKARVKQLNLAYNKARQ
                     ESITTELIEIISGVESMK"
     gene            99581..100978
                     /gene="atpD"
                     /locus_tag="DMACINML_00633"
     CDS             99581..100978
                     /gene="atpD"
                     /locus_tag="DMACINML_00633"
                     /EC_number="3.6.3.14"
                     /inference="ab initio prediction:Prodigal:2.6"
                     /inference="similar to AA sequence:RefSeq:ABS43225.1"
                     /codon_start=1
                     /transl_table=11
                     /product="ATP synthase F1, beta subunit"
                     /protein_id="Prokka:DMACINML_00633"
                     /translation="MQGFISQVLGPVVDVDFNDYLPQINEAIVVNFESEGKKQKLVLE
                     VAAHLGDNRVRTIAMDMTDGLVRGLKAEALGAPISVPVGEKVLGRIFNVTGDLIDEGE
                     EVAFDKKWAIHRDPPAFEDQSTKSEIFETGIKVVDLLAPYAKGGKVGLFGGAGVGKTV
                     IIMELIHNVAFKHSGYSVFAGVGERTREGNDLYNEMKESNVLDKVALCYGQMNEPPGA
                     RNRIALTGLTMAEYFRDEMGLDVLMFIDNIFRFSQSGSEMSALLGRIPSAVGYQPTLA
                     SEMGKFQERITSTKKGSITSVQAVYVPADDLTDPAPATVFAHLDATTVLNRSIAEKGI
                     YPAVDPLDSTSRMLDPNIIGEEHYKVARGVQSVLQKYKDLQDIIAILGMDELSEEDKL
                     IVERARKIEKFLSQPFFVAEVFTGSPGKYISLEETIAGFKGILEGKYDHLPENAFYMV
                     GNIDEAIAKADKLKG"
     gene            100982..101371
                     /gene="atpC"
                     /locus_tag="DMACINML_00634"
     CDS             100982..101371
                     /gene="atpC"
                     /locus_tag="DMACINML_00634"
                     /EC_number="3.6.3.14"
                     /inference="ab initio prediction:Prodigal:2.6"
                     /inference="similar to AA sequence:RefSeq:CAL34279.1"
                     /codon_start=1
                     /transl_table=11
                     /product="ATP synthase F1 sector epsilon subunit"
                     /protein_id="Prokka:DMACINML_00634"
                     /translation="MDNLISFEIVTPMGVIYQGEVKSVTLPGSEGEFGVLKGHAALVS
                     SLKSGVIDIEKADLNHELIAIDAGHAKVDEDKISVLAKGAVWISGSSESEIEKNLESA
                     KELIKSMSSDNTALAATFSKLDNAKVR"
     gene            101372..101926
                     /gene="exbB3"
                     /locus_tag="DMACINML_00635"
     CDS             101372..101926
                     /gene="exbB3"
                     /locus_tag="DMACINML_00635"
                     /inference="ab initio prediction:Prodigal:2.6"
                     /inference="similar to AA sequence:RefSeq:CAL34280.1"
                     /codon_start=1
                     /transl_table=11
                     /product="putative MotA/TolQ/ExbB proton channel family
```

```
                     protein"
                     /protein_id="Prokka:DMACINML_00635"
                     /translation="MNFEAIFQFFGSSSIITYIVLAWLSLYFILSFFILFSRMTYLAA
                     WKNKEKESLESLLLGEKDLSRTRSILRKCTDTSLSHLEIYKNLASRRASNGLTWLSIV
                     ASTSPFIGLFGTVISILETFGGLGTQNSLSIIAPKISEALVATGCGILVAIPAYTFHL
                     IIKRKAFELLSIIDSEIKVISSNK"
gene                 101937..102326
                     /gene="exbD3"
                     /locus_tag="DMACINML_00636"
CDS                  101937..102326
                     /gene="exbD3"
                     /locus_tag="DMACINML_00636"
                     /inference="ab initio prediction:Prodigal:2.6"
                     /inference="similar to AA sequence:RefSeq:CAL34281.1"
                     /codon_start=1
                     /transl_table=11
                     /product="putative exbD/tolR family transport protein"
                     /protein_id="Prokka:DMACINML_00636"
                     /translation="MPFDDEKPELNITPLVDIMLVLLAILMVTAPSITYEEKINLPQG
                     SQKSTSAPSVKSLIVSINAKKEIFLNQEKYSFVSFADNLAQKKAQFNTDEPVFIRADK
                     SLKYDDVIFVLRSIKNLGFSKVALQTE"
gene                 102331..103107
                     /locus_tag="DMACINML_00637"
CDS                  102331..103107
                     /locus_tag="DMACINML_00637"
                     /inference="ab initio prediction:Prodigal:2.6"
                     /inference="similar to AA sequence:RefSeq:CAL34282.1"
                     /codon_start=1
                     /transl_table=11
                     /product="putative periplasmic protein"
                     /protein_id="Prokka:DMACINML_00637"
                     /translation="MKEYGLGKINSFLLAVAVYIFIIILIFFRLVTHMEPAIQYTDIQ
                     DSFIDIELMEPSKKVVNEQSTPKDIQKPTQELDIEKLFAQTTNKAVKTEDVDQKASNF
                     NELFGSIKEIQEEKTTKIQSSAKSETRSSSKTQASELVKQLNDSLLEEESINQGESIK
                     NQKTGIYDEFLGKVVRTITQRWRQYHPNSENISVKVKIFIDENGNFGYTSVEKSGNSL
                     YDAKVAEFLENQKGKFIAYPPQNKSISITMNLKDEAQIQK"
gene                 103121..104332
                     /gene="tolB"
                     /locus_tag="DMACINML_00638"
CDS                  103121..104332
                     /gene="tolB"
                     /locus_tag="DMACINML_00638"
                     /inference="ab initio prediction:Prodigal:2.6"
                     /inference="similar to AA sequence:RefSeq:CAL34283.1"
                     /codon_start=1
                     /transl_table=11
                     /product="putative TolB precursor protein"
                     /protein_id="Prokka:DMACINML_00638"
                     /translation="MKKIVTVFLIFLGILWAEDPVIDVVNSGVVLPKIIVKDNSNLSD
                     INLKRSFYNIIVNDLKVSSNFEVVSDASSEASNYTFEYDLSKNGNALNLHVKIKAGGL
                     EKSSQTYALNGVEQYPFLAHKSVKASVNALGLAPVEWMDHKILIARNSSSKKSQIIMA
                     DYTLTYQKVIIDGGLNLFPKWGNKEQSIFYYTAYDHDRPTLYRYDLRTNKASKILSSG
                     GMVVASDVSLDGSKLLVTMAPKDQPDIYLYDLKSKNLTQLTNYSGIDVNGNFIGVDDS
                     KFVFVSDRLGYPNIFIQNVNNGSAEQAVFHGKNNSAVSTYKDFLVYSSREPNQVGVFN
```

```
                IYLMSINSTGIRQFTANGKNLFPRFSSDGGSIVFIKYLGAQSALGVIRVNANKTFYFP
                LKVGKIQSIDW"
gene            104409..104906
                /gene="pal"
                /locus_tag="DMACINML_00639"
CDS             104409..104906
                /gene="pal"
                /locus_tag="DMACINML_00639"
                /inference="ab initio prediction:Prodigal:2.6"
                /inference="similar to AA sequence:RefSeq:CAL34284.1"
                /codon_start=1
                /transl_table=11
                /product="peptidoglycan associated lipoprotein (omp18)"
                /protein_id="Prokka:DMACINML_00639"
                /translation="MKKILFSSIAAFALVISGCSTKSTSVSGDTSVDSNRGTGGSDGW
                DIDSKISQLNDTLGKVYFDFDKFNIRPDMQNVVNTNANIFNNEVSGVSITVEGNCDEW
                GTDEYNQALGLKRAKAVKEALIAQGVNSDRIAVKSYGETNPVCTEKTKACDAQNRRAE
                FKLSR"
sig_peptide     104409..104477
                /gene="pal"
                /locus_tag="DMACINML_00639"
                /inference="ab initio prediction:SignalP:4.1"
                /note="predicted cleavage at residue 23"
gene            104910..105863
                /locus_tag="DMACINML_00640"
CDS             104910..105863
                /locus_tag="DMACINML_00640"
                /inference="ab initio prediction:Prodigal:2.6"
                /inference="similar to AA sequence:RefSeq:CAL34285.1"
                /codon_start=1
                /transl_table=11
                /product="putative periplasmic protein"
                /protein_id="Prokka:DMACINML_00640"
                /translation="MKKIFSVALIGATLLYAESSAFGAGDLTSNSPYGLTSSEKLLKE
                KLDTLSNNNAQANSRINEIEQRVEGLQSTLEGINSQYAKSNSRLTQLETNFEAIENNL
                STELQNLKAYVEESRKIQDANNKQIKKILTELSSLVDSINANYVSKDDLNSTNLSIKN
                TVVVPLSTDDKNITSSNENNISVASTQEKEVKQIDDSWKKKKNNEILDLAIKDLNNNS
                YENSKIKLNYLIQKQYKPARANFWLGEIEYKQKKYNNAIVYYKKSSALSTKGDYFPKL
                LYHTAISLDKIGDPKTANGFYKALKTNYPNSPEAKASPNRK"
sig_peptide     104910..104981
                /locus_tag="DMACINML_00640"
                /inference="ab initio prediction:SignalP:4.1"
                /note="predicted cleavage at residue 24"
gene            105879..106445
                /gene="slyD"
                /locus_tag="DMACINML_00641"
CDS             105879..106445
                /gene="slyD"
                /locus_tag="DMACINML_00641"
                /EC_number="5.2.1.8"
                /inference="ab initio prediction:Prodigal:2.6"
                /inference="similar to AA sequence:RefSeq:EAQ73020.1"
                /codon_start=1
                /transl_table=11
```

```
                     /product="FKBP-type peptidyl-prolyl cis-trans isomerase
                     SlyD"
                     /protein_id="Prokka:DMACINML_00641"
                     /translation="MAIEKNSVVSMFYELKDANTNEVLESNLYAQPISFILGKGQILE
                     ALEEEVMKLDCPSNADVVVKKEKGLGEYDENAVQVLPKEQFAGIDLKIGMELFGEGEN
                     GETVRVTVKEIGDNDVTIDYNHPYAGRDLLFSLNIVDARVASEDELLTGIIAGSHSCG
                     CGSGHGHDHHGHGGGGCCGGGGGGCGCH"
     gene            106402..107367
                     /gene="fabD"
                     /locus_tag="DMACINML_00642"
     CDS             106402..107367
                     /gene="fabD"
                     /locus_tag="DMACINML_00642"
                     /inference="ab initio prediction:Prodigal:2.6"
                     /inference="similar to AA sequence:RefSeq:AVS36599.1"
                     /codon_start=1
                     /transl_table=11
                     /product="[acyl-carrier-protein] S-malonyltransferase"
                     /protein_id="Prokka:DMACINML_00642"
                     /translation="MDAAVAVAADVVATKMNAVFIFPGQGSQSLGMGKDFYENSKTAK
                     ELLENASDFCKIDFKNLLFNENEDLNKSEFTQPAILLNSLMAYSALSELKPDIKAKFS
                     LGHSLGEFSALAINGAFSFLEATMLVHKRGLFMQEDCAKVEAGMMVILGLDDKKVEEL
                     CRKARDEDKKIFAANYNCDGQIVVAGLKPDLASYESVFKEAGAKRAMLLNMSVASHCP
                     LLENASNKLCIELERILEPNFKAVISNATAKAYTSKQEALELLKTQLVAPVLYKQSIK
                     ACENEADYFIEFGASVLKGLNKKITSKETYALTNMNDIEEFLKVI"
     gene            107368..108054
                     /gene="pfs"
                     /locus_tag="DMACINML_00643"
     CDS             107368..108054
                     /gene="pfs"
                     /locus_tag="DMACINML_00643"
                     /EC_number="3.2.2.16"
                     /inference="ab initio prediction:Prodigal:2.6"
                     /inference="similar to AA sequence:RefSeq:CAL34288.1"
                     /codon_start=1
                     /transl_table=11
                     /product="5'-methylthioadenosine/S-adenosylhomocysteine
                     nucleosidase"
                     /protein_id="Prokka:DMACINML_00643"
                     /translation="MKIAILGAMSEEITPLLEILKDYTKLEYANNTYYFANYKNHELI
                     LAYSKIGKVNSTLSASVMIEKFGAQVLLFTGVAGAFNPELEIGDLLYATKLAQYDLDI
                     TAFGHPLGFVPGNEIFIKTDDKLNNLALEVAKELNIKLRAGIIATGDEFICDEAKKAK
                     IREIFNADACEMEGASVALVCDALKVPCFILRAMSDKAGEKAEFDFDEFVINSAKISA
                     NFVLKMCEKL"
     gene            108051..108806
                     /locus_tag="DMACINML_00644"
     CDS             108051..108806
                     /locus_tag="DMACINML_00644"
                     /inference="ab initio prediction:Prodigal:2.6"
                     /inference="similar to AA sequence:RefSeq:ABS43221.1"
                     /codon_start=1
                     /transl_table=11
                     /product="PP-loop family protein"
                     /protein_id="Prokka:DMACINML_00644"
```

```
                     /translation="MINLSKKLIRQVAQANAKFGLIKDGDRVLLGLSGGKDSLALAHL
                     LKRMQAHAPFKFELEAATLSYGMGEDYSGLHAHCEEHGIKHSVIDSNIYEVSGDTIRK
                     NSSFCSYFSRMRRGALYTYALEKGFNKLAIAHHLDDAAESFFMNFIHNGALRTLAPIY
                     QSKRGITVIRPLIFVRERQLRDNATQNELQVIGNEFCPGMKLSEKNVKFPHAREEAKQ
                     LLANLEKEHPKLFTSLKTAFSNLHTESFWLEKA"
gene                 108811..109332
                     /locus_tag="DMACINML_00645"
CDS                  108811..109332
                     /locus_tag="DMACINML_00645"
                     /inference="ab initio prediction:Prodigal:2.6"
                     /inference="similar to AA sequence:RefSeq:CAL34290.1"
                     /codon_start=1
                     /transl_table=11
                     /product="putative hydrolase"
                     /protein_id="Prokka:DMACINML_00645"
                     /translation="MKKAFVLVDYQNDFIDGSLGFEKALKIKENILNTLNQIDFNTTH
                     LLLTYDTHDEDYLRSKEGLNLPIEHCIKDSLGWQMPKEFTPFLQKAHKVFHKNTFGSL
                     EFANFIAKSEYEEIHFAGLVSHICVFCNIILAFSAKPNSRLILHQNLSASFDENLEKS
                     AFDLLRAYGIEIL"
gene                 109332..109946
                     /locus_tag="DMACINML_00646"
CDS                  109332..109946
                     /locus_tag="DMACINML_00646"
                     /inference="ab initio prediction:Prodigal:2.6"
                     /inference="similar to AA sequence:RefSeq:CAL34291.1"
                     /note="hypothetical protein Cj0120"
                     /codon_start=1
                     /transl_table=11
                     /product="hypothetical protein"
                     /protein_id="Prokka:DMACINML_00646"
                     /translation="MQGFILHTQKVKDEDLIVYILSPKELVKAYRFYGLRHSSILNGY
                     KIDFALDENPSFLPRLKDVLHLGFVWIMDREKMLIWQEFIRLLYHHLKDAQVLESFYF
                     ELLDECVKRFEKQNPKRVIVDAYLKILEFEGRLHQEFRCFACDESIENPITLIRAFLP
                     SHHTCALGYAFEKQKLKNFYESKNCAIFGDEEIGNLYQLIKEGL"
gene                 109943..110350
                     /locus_tag="DMACINML_00647"
CDS                  109943..110350
                     /locus_tag="DMACINML_00647"
                     /inference="ab initio prediction:Prodigal:2.6"
                     /inference="similar to AA sequence:RefSeq:CDH61952.1"
                     /codon_start=1
                     /transl_table=11
                     /product="Metal-dependent hydrolase YbeY, involved in rRNA
                     and/or ribosome maturation and assembly"
                     /protein_id="Prokka:DMACINML_00647"
                     /translation="MILSDEKCEFLEPIASFLSSKDVELVFVESKEMQEINLEQRKQD
                     KTTDVLSFPLENIDESLPLGSVVINIDLAKQKAKELGHSFEEEVSLLFIHAMLHLLGF
                     DHEVDNGEMREKEKELIEHFNLPKSLIIRTLED"
gene                 110482..111684
                     /locus_tag="DMACINML_00648"
CDS                  110482..111684
                     /locus_tag="DMACINML_00648"
                     /inference="ab initio prediction:Prodigal:2.6"
                     /inference="similar to AA sequence:RefSeq:CDH61953.1"
```

```
                     /codon_start=1
                     /transl_table=11
                     /product="FIG00470444: hypothetical protein"
                     /protein_id="Prokka:DMACINML_00648"
                     /translation="MINGINSYSSYNYTNTFSNNTSNTKSSNVTNDNSNFVSDKSRAV
                     DKILGYGVDEYGFFTSDFNEAAGLPKDYKIFAEGLNNFVNSFLKRQPFYSQIDIAETI
                     GNTYNSFSSFVKDNGLGENLKEQDLQNLPQKDGINYWQELQSGNAFIWKLVDENVADL
                     GSNKYQIQNGEISKGGALVAFFTNNLSFVEGKTTLLGKLAGLNSNTDNDKIKEFLSFM
                     NANPLKYSYEQEGWGNPMGDSLSIWNYMNRAKNLGNDKLTKTIENLGKEYNQLINADM
                     SLEEFKTKYLDFKQRHDEFVKSLEEAEKAKGIDYNNPTKSTNNKETSEEDKEKPFKPI
                     QAESKSETYKDDNKMNELLKKLLETKFGTSDELEILFGMKFSDDNAGEFNKILSLNSA
                     PKIIDIKA"
gene                 111720..111956
                     /locus_tag="DMACINML_00649"
CDS                  111720..111956
                     /locus_tag="DMACINML_00649"
                     /inference="ab initio prediction:Prodigal:2.6"
                     /inference="similar to AA sequence:RefSeq:CKG68105.1"
                     /codon_start=1
                     /transl_table=11
                     /product="FIG00470444: hypothetical protein"
                     /protein_id="Prokka:DMACINML_00649"
                     /translation="MIGINSTFNNSFVNLNIKNNNNSSLNSNTNVKNLQDTQDTTKTS
                     NKVLGYEVDKDGFFTSDFNKAAGLPILHNFHPLK"
gene                 112352..112999
                     /locus_tag="DMACINML_00650"
CDS                  112352..112999
                     /locus_tag="DMACINML_00650"
                     /inference="ab initio prediction:Prodigal:2.6"
                     /inference="similar to AA sequence:RefSeq:EAQ72008.1"
                     /note="conserved hypothetical protein"
                     /codon_start=1
                     /transl_table=11
                     /product="hypothetical protein"
                     /protein_id="Prokka:DMACINML_00650"
                     /translation="MLKKISSALILGSLLVSSAFARDFLAKVSNGALSDNSQGVKTLN
                     FDEMKQVKGGYRVSFKLVANDELLAFAKTTPSEIGYYTDNNGDTYYWNSQGLRQFGGM
                     CGIEVTNCMVNNKHTYANQKRLWETMSIVGDDPYHYYLGYTVKRNIGVSNLGQRFVYF
                     SYGVAVINEQLGSWYRVNSSAVLNNYQVIKEISSKYKNDMEMALSGNYSRYRNKL"
sig_peptide          112352..112417
                     /locus_tag="DMACINML_00650"
                     /inference="ab initio prediction:SignalP:4.1"
                     /note="predicted cleavage at residue 22"
gene                 112996..113784
                     /locus_tag="DMACINML_00651"
CDS                  112996..113784
                     /locus_tag="DMACINML_00651"
                     /inference="ab initio prediction:Prodigal:2.6"
                     /inference="similar to AA sequence:RefSeq:CAL34229.1"
                     /note="conserved hypothetical protein Cj0055c"
                     /codon_start=1
                     /transl_table=11
                     /product="hypothetical protein"
                     /protein_id="Prokka:DMACINML_00651"
```

```
                     /translation="MINSINSNLNYNYNTSNFKQDKNIDFKINKHGVAVVTGEGALPY
                     RGKKFEYNPKIFGLDENISKKDMQEFNNFMKSNALKDPNKIEKSSLKNLGKDDNWDIK
                     DEDIKLMNFSSYSQMFNLGSDIAIKASNFRNEFNKLLQEDLTLEEFKTKYLDFKQRHD
                     EFVKEFETAMGDKMLLANNNDISMQEDKEKPFKPIQAESKNKETYKDDSKMNELLKKL
                     LEGKFSTSKELELLFGMKFSDDDAGEFNKILSLNSTPKSIDIKA"
gene                 113820..114809
                     /locus_tag="DMACINML_00652"
CDS                  113820..114809
                     /locus_tag="DMACINML_00652"
                     /inference="ab initio prediction:Prodigal:2.6"
                     /inference="similar to AA sequence:RefSeq:CDH61953.1"
                     /codon_start=1
                     /transl_table=11
                     /product="FIG00470444: hypothetical protein"
                     /protein_id="Prokka:DMACINML_00652"
                     /translation="MIGINSTFNNSFVNLNIKNNNNSSLNSNTSNNSVKNLQDTQDTT
                     KTNNKVLGYGVDKDGFFTSDFNEKAGIPKDIKIHSSTMESLVKISTNEKNPFRAFESI
                     DIAKTVGNAYKILSQVVGEDVLNSKDSFSLEEIANFPQGYEFNRQTMQVTKIYNSATD
                     YLNADIDFDYENTTDTKINKLFFNTTENLMKGNSATDIFNNNGGEENINTGVFFDTTP
                     SKYTNKDGTITKGGLLVAIINENIYTREGEATRQGKTEGFDKSMSGEEVKKAWNDRNY
                     NLKLNPNNIVQGAGIVDTNNENIEFTPLIEIMQKEQKELIKYLQRKQKIDITI"
gene                 114829..115425
                     /locus_tag="DMACINML_00653"
CDS                  114829..115425
                     /locus_tag="DMACINML_00653"
                     /inference="ab initio prediction:Prodigal:2.6"
                     /inference="similar to AA sequence:RefSeq:CKG56548.1"
                     /codon_start=1
                     /transl_table=11
                     /product="bacteriocin-type signal sequence
                     domain-containing protein"
                     /protein_id="Prokka:DMACINML_00653"
                     /translation="MLKKISSALILGSLLVSSAFAGDFLAKVSNGALSDNSQGVKALN
                     LDEMKQVKGGYLVGFKFFKDRPNLISEVYAIADFSEYELSNLNKGLCGAGDEKCSNPS
                     RDRLISWLQASANSPAEYRPVYKVKRQIKYSNLGQPYVLFTYGVGVYNVKNGQIYQYN
                     SSPMLNNNTIMREIAYKYQNSIQDAMGGWNPRYFNKIS"
sig_peptide          114829..114894
                     /locus_tag="DMACINML_00653"
                     /inference="ab initio prediction:SignalP:4.1"
                     /note="predicted cleavage at residue 22"
gene                 115442..116137
                     /locus_tag="DMACINML_00654"
CDS                  115442..116137
                     /locus_tag="DMACINML_00654"
                     /inference="ab initio prediction:Prodigal:2.6"
                     /inference="similar to AA sequence:RefSeq:CKG56531.1"
                     /codon_start=1
                     /transl_table=11
                     /product="FIG00470444: hypothetical protein"
                     /protein_id="Prokka:DMACINML_00654"
                     /translation="MVNFDTQYTNINYLSNNTNNKKSVLKNDDDYFNSVIKKFNFSEK
                     TKEEQGYNGSPDVKEVENSLYKRYMEDKLSKDEIKILNKFGTDYLTEGVDLSALHKRK
                     VDLLKTLTSMNEFEKIWGAERAQREKQFFNELGKPLTQEDIDKFLANKNNQKEDKEKP
                     FKPIQAESKNKETYKDDNKMNELLKKLLEGKFSTSKELELLFGMKFSDDDTGEFNKIL
```

```
                     SQNTSAKIIDIKA"
gene                 116173..117399
                     /locus_tag="DMACINML_00655"
CDS                  116173..117399
                     /locus_tag="DMACINML_00655"
                     /inference="ab initio prediction:Prodigal:2.6"
                     /inference="similar to AA sequence:RefSeq:CDH61953.1"
                     /codon_start=1
                     /transl_table=11
                     /product="FIG00470444: hypothetical protein"
                     /protein_id="Prokka:DMACINML_00655"
                     /translation="MIGINSTFNNSFVNLNIKNNNNSSLNSNTSNNSVKNLQDTQDTT
                     KTNNKVLGYEVDKDGFFTSDFNEAAGIPKDYKIYAKGAENFVNYITNLNFKSFTNIDI
                     AKSLGNAYKVFSQLIDEPSGNFTTEDLSKIPLGFSYDKKSFQVENIYQTQKEFDSALS
                     ANNNLQIYQSPKQLALSFPSWNENSPNDYTKKNSDIFAPSSHIDIGASFYKNDNGTIS
                     KGGVLMAFFAGMSGQNPLIEGETTIAGKLNGYDKNISQSQVEDLNEFIKQNPIKYGIT
                     GDIGTDFINVLKLKNNISDIDEFKKQWLEMKAKSDEMGEVYKAKQKEVISSQETSEED
                     KEKPFKPIQAESKSETYKDDNKMNELLKKLLETKFGTSDELEILFGMKFSDDNAGEFN
                     KILSLNSTPKSIDIKA"
gene                 complement(117420..118346)
                     /locus_tag="DMACINML_00656"
CDS                  complement(117420..118346)
                     /locus_tag="DMACINML_00656"
                     /inference="ab initio prediction:Prodigal:2.6"
                     /inference="similar to AA sequence:RefSeq:CAL34294.1"
                     /codon_start=1
                     /transl_table=11
                     /product="putative tRNA-dihydrouridine synthase"
                     /protein_id="Prokka:DMACINML_00656"
                     /translation="MIDFSKKPLFLAPMAGFSDLPFRNVVKKFGADITISEMISSNAL
                     VYESSKTLHMLERAELENPYIVQIAGGDKEVLKKAVQMLNEMDFVDGIDFNCGCPVNK
                     VVKQCAGSALLENLELFKSLVGVIKENNKKSLTSVKFRLGFNEKYPEKMAKICESLGV
                     DFVSIHGRTRKQLYSGKADYDSIASAKASVKIPVIANGDINAQNAKEVYEITKCDGLM
                     IGRASVGNPWIFYEIKSGKNVDEKLKKEIILTHFDEMIKHYKDQGVSIFRKHLHEYSK
                     GHKDASAFRDSVNRIDNVEEMRKKIEEFFSLT"
gene                 complement(118343..119329)
                     /locus_tag="DMACINML_00657"
CDS                  complement(118343..119329)
                     /locus_tag="DMACINML_00657"
                     /inference="ab initio prediction:Prodigal:2.6"
                     /inference="similar to AA sequence:RefSeq:CAL34295.1"
                     /codon_start=1
                     /transl_table=11
                     /product="putative membrane protein"
                     /protein_id="Prokka:DMACINML_00657"
                     /translation="MRIRLFFIASFIYIALIFALAWYLELGNYTLNFNTYSFELPIMI
                     WLILPLIPLVFFAIIHMGFYYFLLTLKFKHFFKDSTKFENFTRDLLLEKESNISFQTK
                     EFRQVAQLAKTLKTHEKIPNANKINEILDLIDGIKKEEYFNLNKFKLENNNILFLENE
                     KNHIKNDINYAYSKIKNLSQTQDEFQDLAFESLIKKGTYEQIKNIKITKKPCHILTLI
                     KRFKEGNLELNTAEYEVLLSHNILGEKDYLDIAKLSTKLLNPDALIGIFNKIKNEKGE
                     ALRAYLYLLAEFGLLDELREQIHNDDKKFNDFKAFLTLREKNIKIDLNQLIQ"
gene                 complement(119326..119679)
                     /locus_tag="DMACINML_00658"
CDS                  complement(119326..119679)
```

```
                     /locus_tag="DMACINML_00658"
                     /inference="ab initio prediction:Prodigal:2.6"
                     /inference="similar to AA sequence:RefSeq:CAL34296.1"
                     /note="hypothetical protein Cj0125c"
                     /codon_start=1
                     /transl_table=11
                     /product="hypothetical protein"
                     /protein_id="Prokka:DMACINML_00658"
                     /translation="MKKNEIQVFKTILENRKKTILENLQSNSKEIEALHNSVPSDSVD
                     FSVIETGSQIDFAISANLKQELEEIEDSLEKIKDGTYGICESCDEEIAIERLKIKPHA
                     KYCIVCRENLEKGEL"
     gene            complement(119691..120140)
                     /locus_tag="DMACINML_00659"
     CDS             complement(119691..120140)
                     /locus_tag="DMACINML_00659"
                     /inference="ab initio prediction:Prodigal:2.6"
                     /inference="similar to AA sequence:RefSeq:CAL34297.1"
                     /note="conserved hypothetical protein Cj0126c"
                     /codon_start=1
                     /transl_table=11
                     /product="hypothetical protein"
                     /protein_id="Prokka:DMACINML_00659"
                     /translation="MQVNIFHIQKNDEFKIWGEKYAKLISKFADLKEHNLFNKKIAAA
                     QNLNAQAAKLSYEEAFAPYTKGFCIVLDERGKELTSVEFSKLIADKNNLNFFIGGAYG
                     LRDEFSQSLDFRLSLSKLTLAHQFVKILLLEQIYRAFCINSNHPYHK"
     gene            complement(120142..120984)
                     /gene="accD"
                     /locus_tag="DMACINML_00660"
     CDS             complement(120142..120984)
                     /gene="accD"
                     /locus_tag="DMACINML_00660"
                     /EC_number="6.4.1.2"
                     /inference="ab initio prediction:Prodigal:2.6"
                     /inference="similar to AA sequence:RefSeq:CAL34298.1"
                     /codon_start=1
                     /transl_table=11
                     /product="acetyl-coenzyme A carboxylase carboxyl
                     transferase subunit beta"
                     /protein_id="Prokka:DMACINML_00660"
                     /translation="MNFADIFSKIRRQQPSTKEAPNHWVKCQSCHALMYYKEIESCFN
                     VCPKCSYHMRISPMDRIKLLSDENTFVEYDANLEAIDPLKFVDSKSYKKRLSEGESKT
                     GRKSAVISGECLIDGLKTQLVVFDFSFMGGSLGSVEGEKIVRAIQRAITDKTPVVIVS
                     ASGGARMQESTYSLMQMSKTSAALKLLSKEKLPYISVLTDPTMGGVSASFAWLGDLII
                     AEPGALVGFAGARVIKQTIGADLPEGFQKAEFLLEHGLIDAIVERSDMKKYLSDTLKF
                     FCGK"
     gene            complement(120999..121715)
                     /locus_tag="DMACINML_00661"
     CDS             complement(120999..121715)
                     /locus_tag="DMACINML_00661"
                     /inference="ab initio prediction:Prodigal:2.6"
                     /inference="similar to AA sequence:RefSeq:CAL34299.1"
                     /codon_start=1
                     /transl_table=11
                     /product="putative inositol monophosphatase family
```

```
                     protein"
                     /protein_id="Prokka:DMACINML_00661"
                     /translation="MKQFLDACLKANLQISKYLNNICESDLEFSPDLGFDNNQSYKLD
                     LKCEKIFTEYLSDLGQIFSEESGLIGENSPYKIILDPLDGSSNFVSKIPFYGTSAALF
                     KDEEAQSAFICNLANNEILIFDDNKNLKSNLFNPNYSPFLPNRFSQIGIVEKITLCPN
                     LINYLAKNKLKFRSLGATALSIAYAPYFSFVLVLGKTRVFDTAGALMLCKNLYIENNE
                     NFLLISKDKKIFDIILEFLK"
     gene            complement(121715..123931)
                     /locus_tag="DMACINML_00662"
     CDS             complement(121715..123931)
                     /locus_tag="DMACINML_00662"
                     /inference="ab initio prediction:Prodigal:2.6"
                     /inference="similar to AA sequence:RefSeq:AON66352.1"
                     /codon_start=1
                     /transl_table=11
                     /product="outer membrane protein assembly complex, YaeT
                     protein"
                     /protein_id="Prokka:DMACINML_00662"
                     /translation="MKKIISICALVALSNAAVIKEIKFSGLNHLSNASALNLTGLKIG
                     ETINLDKINTAILNLYKQNYFENIAVEEDNGVLNIIVTEKPSIAKISITGIASNDRKQ
                     VESILGIKRGTLFDEASAKEASERIKAYYEAKSYFDTIVEYRKKTLENTEGLELEFIV
                     NRGENIIINNVNLSGAEKFSYSDIEPAIVNKEKEFMGWMWGRNDGKLKIFELSNDSAR
                     ISDEYMKKGYLDVQVSSPYLKTYTDTYQANLTYFIKEGKPYKIQSISIENPLFSEEEN
                     KQNLENLRSTQGKLINIEDIRKDVKTIETQSADLGYAFAEVYPDIQKNDQTQEATVVF
                     KVIPHDKVYIRNVIISGNSRTVDRVVRRELYITEGNLYNRTDLSESTNALRRTSYFDD
                     VEIKEEKVDDTHIDLIVNVKEASTGAISGGIGYGSSDGLLLNASLSDTNIFGSGIKSS
                     VSVDKSDDTLSGRISLINPRILDSQYSLGGTLYSNDYEWDNYSEKNYGFDITLGRQFA
                     RYYNVSLTYNLEQSDIYHLSPTLLRTGYELGKTIKSSVTPAITFNNTDDYYLPRKGII
                     ASTSLEYAGLGGDQEFLSSSTKFNFYQGLEDYIGYDLIYRYKASFYKVWDEGYLPINQ
                     RIYLGGIRSLRGFESRTVSPKNEWGDEVGGTIAFANSVELSFPLIDRIKLRGSVFFDY
                     GMIGNKNLDEIQRMSTGLGIEWITPIGPLQLVFAKPLNDKKGDDTNTFEFNLGTRF"
     gene            124007..124834
                     /gene="tyrA"
                     /locus_tag="DMACINML_00663"
     CDS             124007..124834
                     /gene="tyrA"
                     /locus_tag="DMACINML_00663"
                     /EC_number="1.3.1.12"
                     /inference="ab initio prediction:Prodigal:2.6"
                     /inference="similar to AA sequence:RefSeq:CAL34301.1"
                     /codon_start=1
                     /transl_table=11
                     /product="putative prephenate dehydrogenase"
                     /protein_id="Prokka:DMACINML_00663"
                     /translation="MKVAIIGLGLIGGSLGLCLKENKLISCVYGLDSNKENEQDALKL
                     GLVHELIEFENLHLCDMIFIATPVNAIIEILQKLVDLPSNITIIELGSTKRKIIEKLP
                     AKLTKQTLFAHPMAGTENSGPKAAFKELYKDAVCVLCDSEVADDLHQKRAVEIFSHLG
                     MRIVFMDSTAHDHHAAIISHLPHVISFSLANFVMKEEDKRNIVHLAGGSFKGMSRIAK
                     SSPIMWGSIFEQNKDNVLSSIEFFQKELEFCKKMIEEDKNEELKSWMQKANTLREIL"
     gene            124931..126304
                     /locus_tag="DMACINML_00664"
     CDS             124931..126304
                     /locus_tag="DMACINML_00664"
                     /inference="ab initio prediction:Prodigal:2.6"
```

```
                     /inference="similar to AA sequence:RefSeq:CAL34302.1"
                     /codon_start=1
                     /transl_table=11
                     /product="putative peptidase M23 family protein"
                     /protein_id="Prokka:DMACINML_00664"
                     /translation="MARKKGKFYLYLLILVLIGALAFFASKMNGFETTPPKILMPDII
                     YSDLKKPISVHVKDDEGFIESVQIFLQKEGNGTGMLIVDEKIQNSNDITLQVALPKLA
                     YKEKVKSFIMEIKARDNSFWNFFNGNEASKKVLVMIDDSAPKVNILSNSYQIEQGGAA
                     SVVFSASDANLDKVYIETNTGKIFKATPYLKEGYYAALIAWDARDEEFRAYIIATDKA
                     GNITKERIRYYFVNRKYRVSNINLTDRFLDGKIEDLAQEYAPKDNNFTRFEKFKFVNE
                     TLRDNNEILIHKITSEVPESKIDNFKIDLFLPLRNGMKVADFADHRYYSYNGQFVSDS
                     YHMGIDLASVAEAPIISNNPGKVVFAEENGIYGLNLIVYHGFGVYSLYGHCSSKNVEI
                     DEILSKKSVIGRTGVSGLALGDHLHFGVLVQGVETRPEQWQDKKWIENNIYNVLNEGK
                     KIILGKN"
     gene            126304..127188
                     /gene="lpxC"
                     /locus_tag="DMACINML_00665"
     CDS             126304..127188
                     /gene="lpxC"
                     /locus_tag="DMACINML_00665"
                     /EC_number="3.5.1.-"
                     /inference="ab initio prediction:Prodigal:2.6"
                     /inference="similar to AA sequence:RefSeq:CAL34303.1"
                     /codon_start=1
                     /transl_table=11
                     /product="UDP-3-O-[3-hydroxymyristoyl] n-acetylglucosamine
                     deacetylase"
                     /protein_id="Prokka:DMACINML_00665"
                     /translation="MKQLTLAKAVKGVGIGLHKGEPIEITLEPLEANSGIVFFRSDLN
                     ATYKASPENVINTQMATVLGDERGFISTIEHLMSAINAYGIDNVRIVLNANEAPVMDG
                     SSISFCMMLDEAGVKELDAPKKIMVIKKPIEVRDGDKYVRLTPTKEPRINYTIKFDNA
                     VIGEQSYNFEFSKKNYIENIARARTFGFLKDVQALRDMNLALGGSLENTIVVDDNRIL
                     NPEGLRFKDEFVRHKILDAIGDLTLLGYRVFGDYISYAGSHHLNHLLTKEVLKDKDAY
                     EIVTLEKANEKAYEKVFA"
     gene            127227..127628
                     /locus_tag="DMACINML_00666"
     CDS             127227..127628
                     /locus_tag="DMACINML_00666"
                     /inference="ab initio prediction:Prodigal:2.6"
                     /inference="similar to AA sequence:RefSeq:ADN90360.1"
                     /codon_start=1
                     /transl_table=11
                     /product="hypothetical protein"
                     /protein_id="Prokka:DMACINML_00666"
                     /translation="MIGVYQDDKLVKSIESEEKASEFVPKILKILLKEFSFDELIYAN
                     GPGSYMGIKISYVSLSTLSIVKNIPLFAISAFELNSNQPIAAHKNMCFVKKEDEIILE
                     ENTAGEFFLPPNLSKLNKKDDNLPFYFLSAI"
     gene            127646..128524
                     /gene="thrB"
                     /locus_tag="DMACINML_00667"
     CDS             127646..128524
                     /gene="thrB"
                     /locus_tag="DMACINML_00667"
                     /EC_number="2.7.1.39"
```

```
                     /inference="ab initio prediction:Prodigal:2.6"
                     /inference="similar to AA sequence:RefSeq:CAL34305.1"
                     /codon_start=1
                     /transl_table=11
                     /product="putative homoserine kinase"
                     /protein_id="Prokka:DMACINML_00667"
                     /translation="MKILVPATSANLGPGFDCLGLSLALFNHTIIEKSNFFSISVNGE
                     GSENLSLKKNNIFVSIFNEIYQRLSGRKDHFRFIFDNTIPLSRGLGSSSAVIIGAIAS
                     AYEMAGFKADKEKILNEALKYENHPDNIAPAALGGFVVSVVENEKVFSIKKDLDENLN
                     AVVVIPNVAMSTEQSRNALPSNLSLKDSVFNLCHSSFLTACFLEKKYDLLALASKDLL
                     HQTRRMQNLPELFEVQKFALENKALMSTLSGSGSSFFSLAYKDDAKHLAQKMEQKFGH
                     FKVLCLEFDNKGFKIC"
gene                 128518..128811
                     /locus_tag="DMACINML_00668"
CDS                  128518..128811
                     /locus_tag="DMACINML_00668"
                     /inference="ab initio prediction:Prodigal:2.6"
                     /inference="similar to AA sequence:RefSeq:CAL34306.1"
                     /note="conserved hypothetical protein Cj0135"
                     /codon_start=1
                     /transl_table=11
                     /product="hypothetical protein"
                     /protein_id="Prokka:DMACINML_00668"
                     /translation="MLKKEKKRYNFTKDKHEPIRMCIVCKNRYLQKSMHRFKHFQGEL
                     YKDFDFGRSVYLCETCILSEGKVLQKAFFRSCKNLNTKITQQNLKEIVLNGKN"
gene                 128798..131368
                     /locus_tag="DMACINML_00669"
CDS                  128798..131368
                     /locus_tag="DMACINML_00669"
                     /inference="ab initio prediction:Prodigal:2.6"
                     /inference="similar to AA sequence:RefSeq:AJK82078.1"
                     /codon_start=1
                     /transl_table=11
                     /product="translation initiation factor IF-2"
                     /protein_id="Prokka:DMACINML_00669"
                     /translation="MAKIRIHEIAKELGYDSKEIIEKANELGLNIKTASNAVEPEVAA
                     GIYEYIQTKIIPEAFKKNLKQDPIKKAKAAPKEEKKEEKPSKEVEVKAPAKVVEEAKP
                     EPKSPEGPKENIQESLASATLAKRRGLVIVKKKKDEEEAAKKEEPKATTPSNEERLSL
                     KTMFSNADENLKKKKKEKKPFVATKKESAEKMDFLEGHDFADISLEDEDVVVLPDFSV
                     KEQEKPQNTMKKPPNFLRQSVGNSANLGLEGGIQRRSRKKPPKKVEKKENEAVSSVEI
                     SKEIRVYEFADKIGKSVSEVISKLFMLGMMTTKNDFLDEDAIEILAAEFGIEINIINE
                     ADEFDYVKDYEEEVDEKDLVSRAPVITIMGHVDHGKTSLLDYIRKSRVASGEAGGITQ
                     HVGAYMVEKNGRKITFIDTPGHEAFTAMRARGASITDIVIIVVAADDGVKPQTKEAIN
                     HAKAAGVPIIIAINKMDKEAANPDMVKTQLAEMEIMPVEWGGSYEFVGVSAKTGMGIE
                     DLLEIVLLQADILELKANPKSFAKASVIESSIQKGRGAVATIIVQNGTLKVGSTIVAG
                     EAYGKVRAMSDDQGKALKEIKPGECGVIVGLSEVADAGEILIAVKSDKEAREYANKRH
                     EYNRQKELSKSTKVSIDELGAKIKEGNLKALPVILKADVQGSLEALKASLEKLRNDEI
                     KVNIIHSGVGGITQSDIELASASENSIVLGFNIRPTGEIKERAKDKGVEIKTYNVIYN
                     LLDDVKALLGGMMSPIISEEQLGQAEIRQVINVPKIGQIAGCMVTEGVINRGAKIRLI
                     RDGVVVYEGNVSSLKRFKDDAKEVAKGYECGVGIEGCDDMRVGDYIESYKEVEEQASL
                     "
gene                 131365..131724
                     /gene="rbfA"
                     /locus_tag="DMACINML_00670"
```

```
CDS             131365..131724
                /gene="rbfA"
                /locus_tag="DMACINML_00670"
                /inference="ab initio prediction:Prodigal:2.6"
                /inference="similar to AA sequence:RefSeq:CAL34308.1"
                /codon_start=1
                /transl_table=11
                /product="putative ribosome-binding factor A"
                /protein_id="Prokka:DMACINML_00670"
                /translation="MNPSEIKKLRTESILKELIPEALANLDDENLKNLCVVDVECKKG
                RYDAFVYLDKMFFNTHEQEKILNSLKKASRALQNYCMSEQGWYRCPNFHFKFDDRLEY
                QNHMDALFEKIKKDKNE"
gene            131717..132604
                /gene="ctkA"
                /locus_tag="DMACINML_00671"
CDS             131717..132604
                /gene="ctkA"
                /locus_tag="DMACINML_00671"
                /EC_number="2.7.11.1"
                /inference="ab initio prediction:Prodigal:2.6"
                /inference="similar to AA sequence:UniProtKB:Q9ZKJ5"
                /codon_start=1
                /transl_table=11
                /product="Serine/threonine-protein kinase CtkA"
                /protein_id="Prokka:DMACINML_00671"
                /translation="MNNFYEILQFNSKQFIEPLGTKEKFWIQDKKYLFKIGRENTLEN
                CSEKIACEIAKLINLPCAEYELAVYKNGNISYGVASRNFLNSTSNLVLGNELLSETLQ
                EYDKNAKYKLKKYTLDNVLSLHVFLDNPSILENMIGCLVFDVFIGNQDRHHENWGIVF
                DEKTREINLAPSFDHAAGIASKISEEAAKIKLQTKDKNQQVLAYCLKSNSAFCDESGR
                RLKNLDVLKNILNKREQNISTFAKQWIDKIVSVDQSACSNIIDKIPNEFIGEFQELFI
                LEMLNINKKLICDLVRENI"
gene            132601..133314
                /locus_tag="DMACINML_00672"
CDS             132601..133314
                /locus_tag="DMACINML_00672"
                /inference="ab initio prediction:Prodigal:2.6"
                /codon_start=1
                /transl_table=11
                /product="hypothetical protein"
                /protein_id="Prokka:DMACINML_00672"
                /translation="MILKVCWRSSKAKKWFFIGELSYENNRYKFVYDENIYEAQEQGF
                ELFTNLSDVKLNYYSYELFPVFANRLLSKSRSEYKKYQEWLDLKDDKNPLEELSKNNG
                FRVVDFIALYLIPKVQKKYVVEFFLHNVDCIFEYQKCLNQIKQGDNLYLAKDIQNKFD
                KNAILVRTKDPMSILGYIPRIYNEDIASILAYDENATLSIKKINKDAPVQFQILCRFE
                AKWPKNFIAFDTRKDKNES"
gene            133304..133747
                /locus_tag="DMACINML_00673"
CDS             133304..133747
                /locus_tag="DMACINML_00673"
                /inference="ab initio prediction:Prodigal:2.6"
                /inference="similar to AA sequence:RefSeq:AGV48750.1"
                /codon_start=1
                /transl_table=11
                /product="ribosome maturation protein RimP"
```

```
                     /protein_id="Prokka:DMACINML_00673"
                     /translation="MNLEALCKEVNLELYDSELTSENGKKIYRVYVMKPLDEKGERKG
                     VNLDDCARLSEILSPIFDVEPPVSGEYFLEVSSAGLERKLSKIEHFAKSINELVKITT
                     NEKEKIEAKIISVEDENITLENLENQEKTTLKFSDIKKARTFVEW"
gene                 complement(133757..134743)
                     /locus_tag="DMACINML_00674"
CDS                  complement(133757..134743)
                     /locus_tag="DMACINML_00674"
                     /inference="ab initio prediction:Prodigal:2.6"
                     /codon_start=1
                     /transl_table=11
                     /product="hypothetical protein"
                     /protein_id="Prokka:DMACINML_00674"
                     /translation="MLIHDKKFDINLKFYFFMKYLVKINFSDTQITNILYHISKNETS
                     LYKLKQHINLNTKNGISQWNYLEQIIYEVRNNIDFNVFIFALKIYSIKNFLLELAKIR
                     QRNNIIRLSKIDPLSLEYDKITIYNPYSTRVNGALLALTFFNHIDNNQFFHDNIDKEY
                     FASICQLSQELKKHGLEPNQIFMLIFNESINQSIISNSGSNYESRILEKLESIGINQE
                     AIIKKHDENDSSTEFDFFFNLNGKSYGIGAKRTLRERYKQFIKTVQMSTIDVMIEITL
                     GLDLGEEKAKAIRNHGVYLIVSDEIYEQNKFLQNIDGVFGSSQFTLELLENL"
gene                 complement(134730..135695)
                     /locus_tag="DMACINML_00675"
CDS                  complement(134730..135695)
                     /locus_tag="DMACINML_00675"
                     /inference="ab initio prediction:Prodigal:2.6"
                     /inference="similar to AA sequence:RefSeq:ASE91810.1"
                     /codon_start=1
                     /transl_table=11
                     /product="DNA (cytosine-5-)-methyltransferase"
                     /protein_id="Prokka:DMACINML_00675"
                     /translation="MINNTDKLKAIDLFAGIGGIRIGFKNIFQEKLEFVFSSEIDKFA
                     CQTYFCNFNELPHGDITQINENDIPKHNILLAGFPCQAFSIAGHRKGFNDIRGTLFFD
                     VARIAKKHKPEIIFLENVKGFKNHDKGKTFTVVKNTLEDMGYDVFSEVLNARNFGIPQ
                     NRERIYIVAFLKNKFKNISFSFNELKNIKIKSKLGDILEKNIDEKYTISDKLWAGHQR
                     RKLEHAKKGNGFGYSLFNHDSEYTSTISARYYKDGSEILIEQKNKNPRKLTPREAARL
                     QGFPNSFKITTSDNQSYKQFGNSVAIPVIENIAKLIHKELYNANT"
gene                 135914..136225
                     /locus_tag="DMACINML_00676"
CDS                  135914..136225
                     /locus_tag="DMACINML_00676"
                     /inference="ab initio prediction:Prodigal:2.6"
                     /inference="similar to AA sequence:RefSeq:APA48481.1"
                     /codon_start=1
                     /transl_table=11
                     /product="restriction endonuclease"
                     /protein_id="Prokka:DMACINML_00676"
                     /translation="MLKPDLYIENKMILDAKWKIPDSNEDKKHGIAQSDLYQMFAYAC
                     KFKIHDIKLIYPLCERTMNLQNTIKELEFNANEHLAFLNDNSLLKTNIKVQVIFAPLP
                     F"
gene                 complement(136214..137020)
                     /locus_tag="DMACINML_00677"
CDS                  complement(136214..137020)
                     /locus_tag="DMACINML_00677"
                     /inference="ab initio prediction:Prodigal:2.6"
                     /inference="similar to AA sequence:RefSeq:CAL34312.1"
```

```
                     /codon_start=1
                     /transl_table=11
                     /product="putative ABC transporter integral membrane
                     protein"
                     /protein_id="Prokka:DMACINML_00677"
                     /translation="MFEILNFTFFQNALLAAILVSIACGVVGTLVMINRLFSIAGGIT
                     HGAFGGIGIAFYFSLPILISTGIFTLFLAFLVAFLSKRYEHRSDSIVAVIWAFGMAFG
                     IILIDLSPGYSTDLMAYLFGNILAVGMQDLWLMAIVDGVVIVLMLLFYRQFEALSFDV
                     EFAKVRGINTSFFHYLLIALMAFCIVISIRLVGIILIMALLSIPSFIAENFTKKLGLI
                     MILASVLSMIFCVLGLIISYYFNLSSGACIIAVACFGFLIHLLAKFLLKR"
     gene            complement(137013..137867)
                     /locus_tag="DMACINML_00678"
     CDS             complement(137013..137867)
                     /locus_tag="DMACINML_00678"
                     /inference="ab initio prediction:Prodigal:2.6"
                     /inference="similar to AA sequence:RefSeq:CAL34313.1"
                     /codon_start=1
                     /transl_table=11
                     /product="putative ABC transporter ATP-binding protein"
                     /protein_id="Prokka:DMACINML_00678"
                     /translation="MLFFEISNLNYAYNNETILKNINLSYDSKDFLSIIGPNGAGKST
                     LVKLILGLLKTKKTINFHGLKKNEIGYVPQHTLANPNFCPRVIEIVLMGLVNKKIFGF
                     YSKKDKNKALEALASVGMQDFWDRKINELSGGQKQRVFIARALVDECKMLILDEPTAS
                     VDSKSAIQIFELLSSLHEKGIGILLICHDINLVLAYSDKIAYLNKELFLHTNTKEKDK
                     SVFLKHLYENHSHFCDVEMSLNSCFCDEENCEKKKICEQEFARRNLKKSNFKKENFCL
                     KFSKESHV"
     gene            complement(137881..138735)
                     /locus_tag="DMACINML_00679"
     CDS             complement(137881..138735)
                     /locus_tag="DMACINML_00679"
                     /inference="ab initio prediction:Prodigal:2.6"
                     /inference="similar to AA sequence:RefSeq:CAL34314.1"
                     /codon_start=1
                     /transl_table=11
                     /product="putative periplasmic solute binding protein for
                     ABC transport system"
                     /protein_id="Prokka:DMACINML_00679"
                     /translation="MKKIFLFFIFLGLFKSFLYAKTLQPSENPNLPLISVSIAPQAFF
                     VKKIADDTLNINILSTNTHKPKSKSNPMKKLEKSELYFTIGLEFEKNLTDKLKQKFPK
                     LEIIDMQKDIPLAKTSSSNSKEILDPFTWLDPILVQNIALNTYNALVQKYPQNKSLYK
                     RNLDKFLTELDVLNLQISSKLQKVKNKEFITYHPAWSYFAKRYNLVQNHVEFLGKKLK
                     NEDLKNLGALIKDKNIKVIFVQTHFPENTAKTLEKEYNVKIYKIDYLSYDWENELLKT
                     ANAFYENL"
     gene            complement(138906..139844)
                     /gene="trxB_1"
                     /locus_tag="DMACINML_00680"
     CDS             complement(138906..139844)
                     /gene="trxB_1"
                     /locus_tag="DMACINML_00680"
                     /EC_number="1.8.1.9"
                     /inference="ab initio prediction:Prodigal:2.6"
                     /inference="similar to AA sequence:RefSeq:CAL34317.1"
                     /codon_start=1
                     /transl_table=11
```

```
                     /product="thioredoxin reductase"
                     /protein_id="Prokka:DMACINML_00680"
                     /translation="MLDVAIIGGGPAGLSAGLYTTRGGLKNVVMFEKGMPGGQITSSS
                     EIENYPGVAQVMDGISFMAPWSEQCMRFGLKHEMVGVEQISKNEDGSFTIKLEGGKTE
                     LAKAVIVCTGSAPKRAGFKGEDEFFGKGVSTCATCDGFFYKNKEVAVLGGGDTALEEA
                     LYLANICSKVYLIHRRDEFRAAPSTVEKVKKNEKIELITSASIDEVYGDKMGVTGVKV
                     KLKDGSIRDLNVPGIFTFVGLNVRNEILKQDDGKFLCDMEEGGQVSVNLKMQTSVPGL
                     FAAGDLRKDAPKQVICAAGDGAVAALSAMAYIESLH"
     gene            complement(139979..140293)
                     /gene="trxA"
                     /locus_tag="DMACINML_00681"
     CDS             complement(139979..140293)
                     /gene="trxA"
                     /locus_tag="DMACINML_00681"
                     /inference="ab initio prediction:Prodigal:2.6"
                     /inference="similar to AA sequence:RefSeq:CAL34318.1"
                     /codon_start=1
                     /transl_table=11
                     /product="thioredoxin"
                     /protein_id="Prokka:DMACINML_00681"
                     /translation="MGKYIELTSDNFAQAKEGVALVDFWAPWCGPCRMLAPVIDELAN
                     DFDGKAKICKVNTDEQGDLAAEFGVRSIPTLIFFKNGEVVDQLVGAQSKQAISDKLNS
                     LL"
     gene            complement(140348..140686)
                     /locus_tag="DMACINML_00682"
     CDS             complement(140348..140686)
                     /locus_tag="DMACINML_00682"
                     /inference="ab initio prediction:Prodigal:2.6"
                     /inference="similar to AA sequence:RefSeq:CAL34319.1"
                     /note="conserved hypothetical protein Cj0148c"
                     /codon_start=1
                     /transl_table=11
                     /product="hypothetical protein"
                     /protein_id="Prokka:DMACINML_00682"
                     /translation="MGFASYLSGILGEDKACEFLKKQKFEILKRNFRSKFGEIDIIAR
                     KDGILHFIEVKFTQNNYEVSERLNIKKYERILKTIEFYELKHGILNDFQIDLICISDD
                     MIQFFENISF"
     gene            complement(140686..141936)
                     /gene="hom"
                     /locus_tag="DMACINML_00683"
     CDS             complement(140686..141936)
                     /gene="hom"
                     /locus_tag="DMACINML_00683"
                     /EC_number="1.1.1.3"
                     /inference="ab initio prediction:Prodigal:2.6"
                     /inference="similar to AA sequence:RefSeq:CAL34320.1"
                     /codon_start=1
                     /transl_table=11
                     /product="homoserine dehydrogenase"
                     /protein_id="Prokka:DMACINML_00683"
                     /translation="MKVAILGYGVVGSAVARVLIQNKEIIRARCGQDIIPVIALAKSQ
                     KENPLIPIVTNVDEILQRDDIDVFVELMGGVETPFKIISEILKKNKPVVTANKAMLAY
                     HRYELEELAKNTTFGYEASVAGGIPIIKALKEGLSANNILSIEGILNGTSNYILSAMN
                     SKGLNFDDVLKKAQELGYAEADPTFDIEGQDAAHKLLILASIAYGLRAKPEDILIEGI
```

```
                     SKISAEDMYFAKEFDFTIKLLGIAKAQNSTVELRVHPTMINKDKMIAKVDGVMNAISI
                     VSDILGESLYYGAGAGGDATASAVIADLMDIARNEPKGPMMGFENNLNFKLLNKDEIY
                     TKYYLRLKVEDKTGVLSKITQLMSENNISIDSFLQKPKQGEIYSTLFFTTHHTFEKSI
                     QNLLSILEKQDFIKAKPFMMRIEE"
     gene            complement(141938..143143)
                     /locus_tag="DMACINML_00684"
     CDS             complement(141938..143143)
                     /locus_tag="DMACINML_00684"
                     /EC_number="2.6.1.1"
                     /inference="ab initio prediction:Prodigal:2.6"
                     /inference="similar to AA sequence:RefSeq:CAL34321.1"
                     /codon_start=1
                     /transl_table=11
                     /product="aminotransferase"
                     /protein_id="Prokka:DMACINML_00684"
                     /translation="MFDEIRFNTIERLPNYVFAEVNAIKMAARRAGKDIIDFSMGNPD
                     GKTPQHIIDKLCESANKDKTSGYSTSMGIYKLRLAICNWYKRKYGVNLDPENEVVATM
                     GSKEGFVNLARAIINPGDVAIVPAPAYPIHTQAFIIAGGNVAKMPLKYNEKFELDENK
                     FFEDLDKTLHESIPRPKYVVVNFPHNPTTVTCEKSFYERLVATAKKERFYIISDIAYA
                     DLTYDDYKTPSILEVEGAKDVAVETYTLSKSYNMAGWRVGFTVGNKRLVAALKKIKSW
                     FDYGMYTPIQVAATVALDGDQSCVDEIRATYDKRMHILLEAFENAGWKLHKPRASMFV
                     WAKLPESKMHLKSLEFSKQLLQHAEVAVSPGIGFGEAGDEYVRIALIENENRIRQAAR
                     NIKKYLKEE"
     gene            complement(143156..143962)
                     /locus_tag="DMACINML_00685"
     CDS             complement(143156..143962)
                     /locus_tag="DMACINML_00685"
                     /inference="ab initio prediction:Prodigal:2.6"
                     /inference="similar to AA sequence:RefSeq:CAL34322.1"
                     /codon_start=1
                     /transl_table=11
                     /product="putative periplasmic protein"
                     /protein_id="Prokka:DMACINML_00685"
                     /translation="MVKKIFIFLFLANSVFAVSSLELAKNLVANPSKDSQLRLLFPDS
                     SYIDNKGNPDIAKISRILKTNSLINLTLSDPQTLRLNFKAKADSVVFFKILTDALTNL
                     GYIYFIPTDMILRDGNIDYTIQVESQYILDPGALYSLLKDNSVYIDNIKRIGTYDYEY
                     DLNFENAQLNTNIDLKLNSTQTLERPLKDYVFTLQGASNLIIDAHDADFWFPKVLFLD
                     KNLNLIKAIKSQAQNNHFSELIPSGAVYAIVSDMYNLDNIRRGLKITLKK"
     sig_peptide     complement(143888..143962)
                     /locus_tag="DMACINML_00685"
                     /inference="ab initio prediction:SignalP:4.1"
                     /note="predicted cleavage at residue 25"
     gene            complement(143956..144900)
                     /locus_tag="DMACINML_00686"
     CDS             complement(143956..144900)
                     /locus_tag="DMACINML_00686"
                     /inference="ab initio prediction:Prodigal:2.6"
                     /inference="similar to AA sequence:RefSeq:CAL34323.1"
                     /codon_start=1
                     /transl_table=11
                     /product="putative membrane protein"
                     /protein_id="Prokka:DMACINML_00686"
                     /translation="MLNWKKIEDLNLKEVAARTKIELDFLEALVKKDFVTLNRFNVRG
                     FVKILSREYELDFTDFNEEFETYLNENNLNDTAKPKIITPKLDAYTQKSTGFVSFIVI
```

```
                     VIILALVGFGIYYFDYIKSFFQNDQNNSSAAVVDIIGQAQSNLKALENNVVVIDNQAE
                     ENETEANLTQIQNTELNNTKEEIKQISTTQNNTNAKEENLSTNDSLATTNQEVTELTK
                     APNKEAHFKASGKIWIGLIDLSNYRKTGLVKENDFNLSLEVNRLVLTGAAALSVFDEN
                     GKEQKFPAGNSKRFLIKDGKITSISAAEFMKLNKGKEW"
     gene            complement(144893..145576)
                     /locus_tag="DMACINML_00687"
     CDS             complement(144893..145576)
                     /locus_tag="DMACINML_00687"
                     /inference="ab initio prediction:Prodigal:2.6"
                     /inference="similar to AA sequence:RefSeq:CAL34324.1"
                     /codon_start=1
                     /transl_table=11
                     /product="putative rRNA methylase"
                     /protein_id="Prokka:DMACINML_00687"
                     /translation="MIVYGKQIFFYILERHKELINELYLAKECDKETFKKIANSGFKI
                     KKLDFKTAQAYAKGGNHQGFLLDIKEYEFKDLHTLKKGEFIAILYGISDVGNIGAIVR
                     TAYALGVDGLIFIGERLAMEGVIRTSSGAALDLEIALNNDIFTVLNELKQVGFKLFAS
                     ASGGKQIHHYKVDKGKKALILGSEGLGLSAKIIKKCDECVGIEMKNNFDSLNVSVAFA
                     ILCDRMINA"
     gene            complement(145589..146413)
                     /locus_tag="DMACINML_00688"
     CDS             complement(145589..146413)
                     /locus_tag="DMACINML_00688"
                     /inference="ab initio prediction:Prodigal:2.6"
                     /inference="similar to AA sequence:RefSeq:ALF91223.1"
                     /codon_start=1
                     /transl_table=11
                     /product="tetrapyrrole methylase family protein"
                     /protein_id="Prokka:DMACINML_00688"
                     /translation="MLYFIPTPIGNLSDISFRALELLKTCNVFFCEDTRVSKSLLSLL
                     SSKFEIDFGDKQFISFHTHNEKKVLENLDLEFFDQDIAFLSDAGMPGISDPGQFLIDF
                     ALKNDIQFEVLPGANAALVALVSSALCEKEFIFMGFLANKGKERQRDIQKILNHTYPS
                     IIYESPKRILSLIEQIAILDSQREIFAIKEISKKFETKFKAKAIDLFDILKQTNLKGE
                     WVVVLAKNDKDSIGNSLCENDILELEIPLKTKSKLLAKMNGKNPKEIYQKLLLSQS"
     gene            complement(146416..146616)
                     /gene="rpmE"
                     /locus_tag="DMACINML_00689"
     CDS             complement(146416..146616)
                     /gene="rpmE"
                     /locus_tag="DMACINML_00689"
                     /inference="ab initio prediction:Prodigal:2.6"
                     /inference="similar to AA sequence:RefSeq:CAL34326.1"
                     /codon_start=1
                     /transl_table=11
                     /product="50S ribosomal protein L31"
                     /protein_id="Prokka:DMACINML_00689"
                     /translation="MKKEIHPEYVECKVSCACGNTFVTKSNKSELRVDICSSCHPFFT
                     GSEKIVDAAGRVEKFKKKYAMQ"
     gene            complement(146695..147348)
                     /locus_tag="DMACINML_00690"
     CDS             complement(146695..147348)
                     /locus_tag="DMACINML_00690"
                     /inference="ab initio prediction:Prodigal:2.6"
                     /inference="similar to AA sequence:RefSeq:CAL34327.1"
```

```
                     /note="conserved hypothetical protein Cj0156c"
                     /codon_start=1
                     /transl_table=11
                     /product="hypothetical protein"
                     /protein_id="Prokka:DMACINML_00690"
                     /translation="MQFLYHKQAGEEILKLKGEEFAHLKARRIKENEILSLRNLEDDF
                     IYDYKISNLDRNSCLLYFLNKHFKPHSQSELNLALAVIDTKILEKTLPFLNELGVANL
                     HLVFTEFSQRNFKLDFERLEKIIISSCEQCGRSRKMQIQSYKNIQEFSKSFPDAVLVD
                     FEGEIKEFDKTRLYFIGPEGGFSPKEKQMFKEKICLKVPNILRSQSAVVAVAAKILL"
     gene            complement(147349..147756)
                     /locus_tag="DMACINML_00691"
     CDS             complement(147349..147756)
                     /locus_tag="DMACINML_00691"
                     /inference="ab initio prediction:Prodigal:2.6"
                     /inference="similar to AA sequence:RefSeq:CAL34328.1"
                     /codon_start=1
                     /transl_table=11
                     /product="putative integral membrane protein"
                     /protein_id="Prokka:DMACINML_00691"
                     /translation="MDKSYEFFLALHLYSLYASGFLMLFYLVLTQGNFKTEFIFIRRI
                     RLFLPIYYVFLALMLFTGFLLLAIKQFQMHLNLWLMIFSWIFIFALAIFHFTQFKKAR
                     RLRRYITFRRISFMILFCELVLLILPFLTGKYL"
     gene            complement(147760..148179)
                     /locus_tag="DMACINML_00692"
     CDS             complement(147760..148179)
                     /locus_tag="DMACINML_00692"
                     /inference="ab initio prediction:Prodigal:2.6"
                     /inference="similar to AA sequence:RefSeq:CAL34329.1"
                     /codon_start=1
                     /transl_table=11
                     /product="putative heme-binding lipoprotein"
                     /protein_id="Prokka:DMACINML_00692"
                     /translation="MGKNLSILISLFVILFLNACSNDKEFNHKNQDTTEEIVQIEQND
                     EKTEFSDTNLPLPVDDEANIGEEYDINPSVINSLYKQKCATCHGEKGEIKTKKGIAIK
                     NLSSEKFIQRLQNLKDEAHNFLTQEQRENLAKYIGKE"
     sig_peptide     complement(148120..148179)
                     /locus_tag="DMACINML_00692"
                     /inference="ab initio prediction:SignalP:4.1"
                     /note="predicted cleavage at residue 20"
     gene            complement(148179..148760)
                     /locus_tag="DMACINML_00693"
     CDS             complement(148179..148760)
                     /locus_tag="DMACINML_00693"
                     /EC_number="4.2.3.12"
                     /inference="ab initio prediction:Prodigal:2.6"
                     /inference="similar to AA sequence:RefSeq:CAL34330.1"
                     /codon_start=1
                     /transl_table=11
                     /product="putative 6-pyruvoyl tetrahydropterin synthase"
                     /protein_id="Prokka:DMACINML_00693"
                     /translation="MIIRKLFEFENAHIVRFCSSSRCKSSIHGHSYKVEVLLESKYLD
                     NAGMVYDFGLLKTHMRQIIDSFDHAITLFNQDDESYLNEMKKYSQRWVSLPVNVSAEN
                     FCRVFFILIDALLKQTKMINGEQGVNLQSIIVHETRTGYAQGFKEDAYSPTMPKIALE
                     NIEFSPAIKAEWSDIEFYDKLKNLHIFTNPKEV"
```

```
gene            complement(148757..149500)
                /locus_tag="DMACINML_00694"
CDS             complement(148757..149500)
                /locus_tag="DMACINML_00694"
                /inference="ab initio prediction:Prodigal:2.6"
                /inference="similar to AA sequence:RefSeq:CAL34331.1"
                /codon_start=1
                /transl_table=11
                /product="putative radical SAM domain protein"
                /protein_id="Prokka:DMACINML_00694"
                /translation="MQLVESFLSIQGEGKYSGKLAIFMRFAGCNFNCLGFGVKLIKNG
                KTLKGCDTIRAVFTKEFNEEYENLNANELFKRVLDLKKDFDPIVVITGGEPLIHHENP
                EFIHFIQTLLENNFKVHFESNGSIELDFEKYPFYKECIFALSVKLQNSGISKEKRLNF
                NALKGFKHYAKDSFYKFVLNSSQLESSALEILEILEKAPNEIFCMPMGENEQNLKQNA
                LKIAEFCIKNGYNYSDRIHIRLWNDKEGV"
gene            complement(149503..150465)
                /gene="moaA"
                /locus_tag="DMACINML_00695"
CDS             complement(149503..150465)
                /gene="moaA"
                /locus_tag="DMACINML_00695"
                /inference="ab initio prediction:Prodigal:2.6"
                /inference="similar to AA sequence:RefSeq:CAL34332.1"
                /codon_start=1
                /transl_table=11
                /product="putative molybdenum cofactor biosynthesis
                protein A"
                /protein_id="Prokka:DMACINML_00695"
                /translation="MLIDQFGRKINYLRISVTQRCNFRCLYCMPKIPFNHQPKENLLS
                FEELFLFVKVAIDEGIEKIRITGGEPLLRKDLSIFIKMINDYKKDLDLAITTNGFLLK
                DFAKDLKDAGLKRLNISLDTLESKKAKTLAQKDVLDSVLAGIDEALNVGLKVKLNTVV
                LKGLNDDELISLLEFAKSKNIQIRFIEFMENIHAYGKLQGLKRDEIIQILSQKYQIKL
                IKKAEKAPVSIYSADDYEFGIIDPHSHEFCDSCNRIRLSAEGLLIPCLYFDEALSIKE
                AVRKGDINAAAKILQEVLRNKPEKNRWSVVDNETSSRAFYQTGG"
gene            complement(150481..150999)
                /locus_tag="DMACINML_00696"
CDS             complement(150481..150999)
                /locus_tag="DMACINML_00696"
                /inference="ab initio prediction:Prodigal:2.6"
                /inference="similar to AA sequence:RefSeq:CAL34333.1"
                /codon_start=1
                /transl_table=11
                /product="putative periplasmic protein"
                /protein_id="Prokka:DMACINML_00696"
                /translation="MSNDVLYLVFMIVLLVAILAYMNIKDKESSAKINKLQGVVEDIT
                KELHYLRKELGVKDEGDQEEEDYKITLLKEEIQITLEKQISAKITPVLRTLKTMEHII
                EDFQNEQQNRILNLEQKAQSMTKLTPNYDTEEQKIVDLFKEGKSIEQIAKDLRMGTGN
                VELVLKFKQLIK"
gene            complement(150992..151492)
                /locus_tag="DMACINML_00697"
CDS             complement(150992..151492)
                /locus_tag="DMACINML_00697"
                /inference="ab initio prediction:Prodigal:2.6"
                /inference="similar to AA sequence:RefSeq:CAL34334.1"
```

```
                     /note="hypothetical protein Cj0163c"
                     /codon_start=1
                     /transl_table=11
                     /product="hypothetical protein"
                     /protein_id="Prokka:DMACINML_00697"
                     /translation="MGISLFKAGFEASLECCDIENNHFLQEYLKLGAISQLSKWKKLA
                     LRVDFDEGEKIIFDLLLSLKEDILRLEDSLDKNKDLLTLKQKGVVESLNFEYLNFLDE
                     ILEEGKEYYIRFDLNNQKMAIFARAQNKNLAQIIKIKPEDRIAFDAFVVQLQRDMIRN
                     KKDKNE"
     gene            complement(151483..152367)
                     /gene="ubiA"
                     /locus_tag="DMACINML_00698"
     CDS             complement(151483..152367)
                     /gene="ubiA"
                     /locus_tag="DMACINML_00698"
                     /EC_number="2.5.1.-"
                     /inference="ab initio prediction:Prodigal:2.6"
                     /inference="similar to AA sequence:RefSeq:CAL34335.1"
                     /codon_start=1
                     /transl_table=11
                     /product="putative 4-hydroxybenzoate
                     octaprenyltransferase"
                     /protein_id="Prokka:DMACINML_00698"
                     /translation="MNIIWNKFKDILELVVFKHSIFALPFLFSSMIVASKLVNDSAWF
                     GFKALILGIICAVSARNFAMATNRLMDEDIDKDNPRCANRPNVSGKIGRKSVWIFIIL
                     NAIIFIVCSYFINTLAFYLSFPVLFVLAIYSAFKRFSSLAHLVLGFCLGLAPIAGSVI
                     VMGEIHIYSVILCLGVTFWTAGFDLLYSLQDMEYDKKVGLHSIPAKFGSKATLFISAF
                     CHVLAVLFWLLFVWQVWGVALGNIALLGVIISGIILAFEHKIVHKNFAHIDKAFFTLN
                     GYLSIIFFIFIWVDLLWG"
     gene            152438..153307
                     /gene="miaA"
                     /locus_tag="DMACINML_00699"
     CDS             152438..153307
                     /gene="miaA"
                     /locus_tag="DMACINML_00699"
                     /EC_number="2.5.1.75"
                     /inference="ab initio prediction:Prodigal:2.6"
                     /inference="similar to AA sequence:RefSeq:CAL34336.1"
                     /codon_start=1
                     /transl_table=11
                     /product="tRNA delta(2)-isopentenylpyrophosphate
                     transferase"
                     /protein_id="Prokka:DMACINML_00699"
                     /translation="MFFQIALIGTTASGKTYIANALAKEFNAVVLSLDSLCVYKEINI
                     ANAKPSKDELMNLNYFGVNLLSVDEHFNVELFIREYQKAKEFALMNDLPLIIVGGTGF
                     YLKTMIDGLSQKIQDTTSILSNDEIYTLLLKIDPAYKIEKNDTYRLRKWLSIYENTKE
                     IPSDFLKRTQKDGVLKDIEIYELVWDREILRKRIEARTKEMIDSGLIEEARMLFSNFD
                     NNLKALNSIGLKECKEYLENKISLKELENLITIHTSQLAKRQRTFNKKFQSTPLEFSK
                     ALTLLRNKFLAKK"
     gene            complement(153293..153856)
                     /locus_tag="DMACINML_00700"
     CDS             complement(153293..153856)
                     /locus_tag="DMACINML_00700"
                     /inference="ab initio prediction:Prodigal:2.6"
```

```
                     /inference="similar to AA sequence:RefSeq:AHW91179.1"
                     /codon_start=1
                     /transl_table=11
                     /product="integral membrane protein"
                     /protein_id="Prokka:DMACINML_00700"
                     /translation="MDLYSLILLSCALAMDAFAVSLCKGFSVKKLNLRHYVITGAYFG
                     GFQALMPAIGYVIGITFASFIASIDHWIAFILLSLIGLKMIKESFENENCDTNSNQFG
                     FKIMFALAVATSVDALAIGVSFAFLDVNLFLALFLIGSITFILCAIALEIGNKFGTRF
                     KNKAEFLGGAVLIILGIKILIEHLFFS"
     gene            154029..154103
                     /locus_tag="DMACINML_00701"
     tRNA            154029..154103
                     /locus_tag="DMACINML_00701"
                     /product="tRNA-Glu"
                     /inference="COORDINATES: profile:Aragorn:1.2"
                     /note="tRNA-Glu(ttc)"
     gene            154265..154672
                     /locus_tag="DMACINML_00702"
     CDS             154265..154672
                     /locus_tag="DMACINML_00702"
                     /inference="ab initio prediction:Prodigal:2.6"
                     /inference="similar to AA sequence:RefSeq:AHY39679.1"
                     /codon_start=1
                     /transl_table=11
                     /product="integral membrane protein"
                     /protein_id="Prokka:DMACINML_00702"
                     /translation="MSYDTNFFLLYFKDKVPKDSLIFLKESLEKASEEQKEKLLFTKL
                     KNPLYGLLFAFLFPGLDRIYNGNIILGILKIISVILVSMGLVIAEDKNDESTMLIFIN
                     LFFILSVWVILDYFLVWKDICQNNLKKIFEVLQ"
     gene            154669..155097
                     /locus_tag="DMACINML_00703"
     CDS             154669..155097
                     /locus_tag="DMACINML_00703"
                     /inference="ab initio prediction:Prodigal:2.6"
                     /inference="similar to AA sequence:RefSeq:AHY39679.1"
                     /codon_start=1
                     /transl_table=11
                     /product="integral membrane protein"
                     /protein_id="Prokka:DMACINML_00703"
                     /translation="MKQDKTILLIFKDKIPQERLFILGEELKKSQKDLSALYAISLKN
                     PLVGLVLSITVGLFGVDRFYKGDILLACIKLAFFIIPLFATFAAFIALLNKSHSIFID
                     YFAIFALMFVVASIWKLVDIYLVFVGIKKDNFYKILNFLS"
     gene            155205..155843
                     /gene="sodB"
                     /locus_tag="DMACINML_00704"
     CDS             155205..155843
                     /gene="sodB"
                     /locus_tag="DMACINML_00704"
                     /EC_number="1.15.1.1"
                     /inference="ab initio prediction:Prodigal:2.6"
                     /inference="similar to AA sequence:RefSeq:CAL34339.1"
                     /codon_start=1
                     /transl_table=11
                     /product="superoxide dismutase (Fe)"
```

```
                     /protein_id="Prokka:DMACINML_00704"
                     /translation="MFELRKLPYDTNAFGDFLSAETFSYHHGKHHNTYVTNLNNLIKD
                     TEFATKDLVSIIKSSSGGVFNNAAQVYNHDFYFDCITPNACEVKGDIKAALEKEFGSL
                     ENFKAEFIKGATGVFGSGWFWLVYNTKNQKLEFVGTSNAATPITEDKVPLLVVDVWEH
                     AYYVDHRNARPAYLEKFYAHINWEFVAKAYEWALKEGMGSVSFYANELHPVK"
gene                 complement(155871..157076)
                     /locus_tag="DMACINML_00705"
CDS                  complement(155871..157076)
                     /locus_tag="DMACINML_00705"
                     /EC_number="1.5.1.7"
                     /inference="ab initio prediction:Prodigal:2.6"
                     /inference="similar to AA sequence:RefSeq:CAL34341.1"
                     /codon_start=1
                     /transl_table=11
                     /product="putative saccharopine dehydrogenase"
                     /protein_id="Prokka:DMACINML_00705"
                     /translation="MKNLLIIGAGGVSRVATVKCAMNSDTFSKITLASRTKSKCDEIA
                     SFIKERLGVEIQTAQIDADDSDAVVELIKKTGAQILLNVALPYQDLSLMDACIKAGID
                     YVDTANYEHPDLAKFEYKEQWARNDKFKEAGILGLLGSGFDPGVTNVFCAYAQQNLFD
                     EISYIDILDCNAGDHGYAFATNFNPEINLREVSAKGRYWEKGEWIETEPMEIKMEWDY
                     PEVGVKDSYLLYHEELESLVKNIKGLKRIRFFMTFGQSYLTHMKCLENVGMLGIKPVM
                     HQGKEIIPIEFLKTLLPDPASLGPRTKGYTNIGCVIRGVKDGKDRQVYIYNVCNHEEC
                     YKETGAQAVSYTTGVPAMIGTKLIAKGIWQGKGVFNMEEFDAKPFMDELNSQGLPWKI
                     IEMTPSLGE"
gene                 complement(157112..158017)
                     /gene="cfbpC"
                     /locus_tag="DMACINML_00706"
CDS                  complement(157112..158017)
                     /gene="cfbpC"
                     /locus_tag="DMACINML_00706"
                     /inference="ab initio prediction:Prodigal:2.6"
                     /inference="similar to AA sequence:RefSeq:CAL34342.1"
                     /codon_start=1
                     /transl_table=11
                     /product="putative iron-uptake ABC transport system
                     ATP-binding protein"
                     /protein_id="Prokka:DMACINML_00706"
                     /translation="MLEIKNLSKNFGKIQALKNINLHVKEGEFLSILGGSGSGKSTLL
                     RIIAQLEQASSYEFLKCKGEVAMMFQNYALFPHLNVEKNILFALYDKKDKKQILEDLL
                     KTFEIENLRYKKIDEISGGQAQRVAFARAVARGCKLLLLDEPFSNLDQNLKQDLRREL
                     KKLIQKQKITAIMVTHDIEDAYCMSDKIAFLDKGEILAHADPKELYFKPNHKSARILP
                     DLNIIEEELDLEDEFFQWIASKNYTFGYAELKIGNRFEATILQKEFLGAFYRLKVRYK
                     NIEFFMLLSSNYPLKEKINFDIINF"
gene                 complement(158004..159620)
                     /gene="cfbpB"
                     /locus_tag="DMACINML_00707"
CDS                  complement(158004..159620)
                     /gene="cfbpB"
                     /locus_tag="DMACINML_00707"
                     /inference="ab initio prediction:Prodigal:2.6"
                     /inference="similar to AA sequence:RefSeq:CAL34343.1"
                     /codon_start=1
                     /transl_table=11
                     /product="putative iron-uptake ABC transport system
```

```
                     permease protein"
                     /protein_id="Prokka:DMACINML_00707"
                     /translation="MLKTLKYYKIGAILLALFLTLPIFGIFAELFYILFQNFNTSDLA
                     EISSIKENLSHFFDYLFLKFIKDTLIVSIGVLLLSLILGVSSAYLMANYNFYFCRILE
                     KLLILPLAIPAYILAFVYVGIMDFQGFFHEIFGFRIDFFNHYGVIFVLGISLYPYIYL
                     FAKTAFKSEAIEAYEVAKIMGYSEFRIFSRVMLLSARPAIFSGALLVLMETLSDYGAS
                     AYLGVDTFSAGIFKLWYDLNDSYSSSILSGILMIFVFLIMYIDYYYKNKHHYSFNQNL
                     TLFIKKRKLSHIKQILACFYCFIIALLGFILPFIWLVYWGLKDFKLFETEFYIISFQT
                     IILAFITALITTLLAYFLMFSSRIIKNNFFSLCILKLSSLGYSIPAAALGISMIVLFV
                     FLDKIFHLNLLGTSLIILVFAYIIRFLASAIYSLEGGYNKIHLNTDEASLNLRPSYLI
                     LFFKIHTPLMKHFLFLAFIIVFIDTIKELPLSRILAPFGFETLSVKAFWFASDERIYD
                     AALPSLLIVLLSLIVVIWMDKITRKDDVRN"
gene                 complement(159620..160624)
                     /gene="futA1"
                     /locus_tag="DMACINML_00708"
CDS                  complement(159620..160624)
                     /gene="futA1"
                     /locus_tag="DMACINML_00708"
                     /inference="ab initio prediction:Prodigal:2.6"
                     /inference="similar to AA sequence:RefSeq:SQE23619.1"
                     /codon_start=1
                     /transl_table=11
                     /product="iron ABC transporter periplasmic iron-binding
                     protein"
                     /protein_id="Prokka:DMACINML_00708"
                     /translation="MKKIFSLCLLGFSLLGAAELNIYSARHYDADFQIIKKFEEKTGI
                     KVNHTQAKASELIKRLSLEGSNSPADIFITADISNLTEAKNSGLLSPVKSKYLEDTIP
                     AHLRDKDGEWFAITKRARIIAYNKNANTDISKMKNYEDLAKPEFKGQIVMRSATAPYS
                     KTLLASIIANDGDENAKAWAKGVLDNLATKPKGGDRDQARQVFAGEAKFAVMNTYYIG
                     LLKNSKNPKDVEVGNSLGIIFPNQDNRGTHINISGIAMTKSSKNQEAAKKFMEFMLTP
                     EIQKILTDSNYEFPIRNDVELSQTVKDFGTFKEDQIPVSQIAEKVKEAVKIYDQVGFR
                     "
sig_peptide          complement(160571..160624)
                     /gene="futA1"
                     /locus_tag="DMACINML_00708"
                     /inference="ab initio prediction:SignalP:4.1"
                     /note="predicted cleavage at residue 18"
gene                 complement(160625..160747)
                     /locus_tag="DMACINML_00709"
CDS                  complement(160625..160747)
                     /locus_tag="DMACINML_00709"
                     /inference="ab initio prediction:Prodigal:2.6"
                     /inference="similar to AA sequence:RefSeq:CAL34345.1"
                     /codon_start=1
                     /transl_table=11
                     /product="putative lipoprotein"
                     /protein_id="Prokka:DMACINML_00709"
                     /translation="MKKLVLFLFCLGFAFACSEHSNADFKDHDKTKYNSQYKDK"
sig_peptide          complement(160676..160747)
                     /locus_tag="DMACINML_00709"
                     /inference="ab initio prediction:SignalP:4.1"
                     /note="predicted cleavage at residue 24"
gene                 160989..161840
                     /locus_tag="DMACINML_00710"
```

```
     CDS             160989..161840
                     /locus_tag="DMACINML_00710"
                     /inference="ab initio prediction:Prodigal:2.6"
                     /inference="similar to AA sequence:RefSeq:CAL34346.1"
                     /codon_start=1
                     /transl_table=11
                     /product="putative iron transport protein"
                     /protein_id="Prokka:DMACINML_00710"
                     /translation="MKFYTMLSTLFISFMLSACAVLQKPSPLQENKDFYILNTHTQEK
                     ISFEDMILELLKADVILLGEKHDEAKHKISQVMIFNALEGNLSSQNISFDVALEMLAS
                     TEQNHLDKAFKNKKNIKVNELANALNWDKGWKWKDYDQFVNAVFYSRAKILGANLSRS
                     EITSIYNGVQPLKGYVSTTNEVKKQIFDIISLSHKLNPQENKELLDKLVEIQQFKDRR
                     MADVLVHHTNKVLLLAGNYHTSKKIGVPLHIQDFKSDKKIVVVNLNYGKVDLKDSDYI
                     FIYKGGE"
     sig_peptide     160989..161051
                     /locus_tag="DMACINML_00710"
                     /inference="ab initio prediction:SignalP:4.1"
                     /note="predicted cleavage at residue 21"
     gene            161837..164113
                     /locus_tag="DMACINML_00711"
     CDS             161837..164113
                     /locus_tag="DMACINML_00711"
                     /inference="ab initio prediction:Prodigal:2.6"
                     /inference="similar to AA sequence:RefSeq:AON66399.1"
                     /codon_start=1
                     /transl_table=11
                     /product="Putative outer membrane siderophore receptor"
                     /protein_id="Prokka:DMACINML_00711"
                     /translation="MKKLSLICVMGLCFANFAFSEELEFDSLEISGSRIKNDEKPFVT
                     PGATSTRDNLNSSTQSIDSIVRSVPGAYTQVDQAQGGVSVNIRGMTGLGRVNTQIDGV
                     TQTFFGSASDDSFHGQIGTSSFTAPIDKNFLVALDVNRGTFSGANGANALMGSANFRT
                     IGVDDIVQEGRVFGLLGKYSYGSNAIGPDFMGAIAGKTMLENNTSLGVLFAYSGQNVS
                     QNYKIGGGGYIGDQKPPIDTDDDGIPDDNLAAPINPNNLYNQPRSQLFKVEYKSDISE
                     AILNYRAYKNNLAGRKITNDTYQIDYRLNPDSNLLDLKFLFAYNDGKQKYNQGSTWGY
                     HDMSGVKTKNRAVTFDLSNTMNKEFSQDSNLYFTYGVNILNNHYSNDFPQDRVLLPYI
                     LTSFYPKGKQDIKTLYLDTSFTQGIFTLNSNVNWTNAQLSGYKGICSMANPYCQPKNA
                     TNLEKDYNNFNYSLMLSADIHPLFNPFISYSKSHRIPNVQEYFFTHDASFEHNMNTFL
                     KAESADTYQIGFNSFTHEILNDSDTLGFKMLYYDTKVKNYIYNRRYWQKADDAVFLMQ
                     LNDDEKAKFHGVELEFKYDTGFFYSILSYTYQKSKHKFSDTESLEFGGAQSGQSQFAQ
                     LPEHYANLDMGVRLFEEKLTLGALAKYTGKAKRIVPVGSLDDDPSNPDAMAPLKTTDE
                     LPKIPTIVDLYANYKILKNFTIKAEVQNLFDKNYMDALYSYNTGENQNAGGLFDPIYI
                     YNNSARGRTFIVSFEYKY"
     sig_peptide     161837..161899
                     /locus_tag="DMACINML_00711"
                     /inference="ab initio prediction:SignalP:4.1"
                     /note="predicted cleavage at residue 21"
     gene            164126..164887
                     /gene="exbB1"
                     /locus_tag="DMACINML_00712"
     CDS             164126..164887
                     /gene="exbB1"
                     /locus_tag="DMACINML_00712"
                     /inference="ab initio prediction:Prodigal:2.6"
                     /inference="similar to AA sequence:RefSeq:CAL34348.1"
```

```
                     /codon_start=1
                     /transl_table=11
                     /product="biopolymer transport protein"
                     /protein_id="Prokka:DMACINML_00712"
                     /translation="MLKKIFLFCFLLLLNLNAEVNVTDTNLSFDSPKIEAEVPKIELSL
                     SSLYQNADEVVKGVIYILVLFSVLAWAVFISKLMQFYFMKKNLDFSIHKIKELKNLNE
                     LDQTKGFAGVLSLEIQDELEKSEYKNDHIKERIELRLQNTISKQITASKNGLSLLASI
                     GASAPFIGLFGTVWGIMNAFIGIANLGNASLAVVAPGIAEALFATAFGLVAAIPAVLF
                     YNYLTRKNLKLMHHLDELANFVYILFHRSYFNDKN"
     sig_peptide     164126..164185
                     /gene="exbB1"
                     /locus_tag="DMACINML_00712"
                     /inference="ab initio prediction:SignalP:4.1"
                     /note="predicted cleavage at residue 20"
     gene            164874..165284
                     /gene="exbD1"
                     /locus_tag="DMACINML_00713"
     CDS             164874..165284
                     /gene="exbD1"
                     /locus_tag="DMACINML_00713"
                     /inference="ab initio prediction:Prodigal:2.6"
                     /inference="similar to AA sequence:RefSeq:CAL34349.1"
                     /codon_start=1
                     /transl_table=11
                     /product="biopolymer transport protein"
                     /protein_id="Prokka:DMACINML_00713"
                     /translation="MIKTEIAHKEEELSEINITPFIDIMLVLLIVFMAVTPLITSSIK
                     IELPKSSQQAEDKLKNPIILYLNTDNTLAINDDKLSLENLSSALDIKTKGNKEEIIYF
                     HIDKSVKYEDIMQVMQKLKENGYGKIALSSKKVD"
     gene            165286..166077
                     /gene="tonB1"
                     /locus_tag="DMACINML_00714"
     CDS             165286..166077
                     /gene="tonB1"
                     /locus_tag="DMACINML_00714"
                     /inference="ab initio prediction:Prodigal:2.6"
                     /inference="similar to AA sequence:RefSeq:CAL34350.1"
                     /codon_start=1
                     /transl_table=11
                     /product="TonB transport protein"
                     /protein_id="Prokka:DMACINML_00714"
                     /translation="MKSSVIFGFVSSLVLHALVLMFFLFSFYTQEKSSGVDFKQGVEF
                     TSIMMVSELPIGELKEVSIDQKKSNSQDKNKKQDEKMSLNSQDKNKKQDEKMSLNSQD
                     KNAVLKAQKKIEKQDENQAQKEIANASENSKFKNETLSAPLQSNEDKTQTIVSGNAKE
                     QIKSYQALLMAHLAKFKKYPQEAIMQKQEGVVRIRVSIDESGNVLSKELKKSCPYAVL
                     NDEVLSLFKRASPLPKPPKEMLKDGEKISFVMPIDYNIKDYLGKK"
     gene            166133..167353
                     /locus_tag="DMACINML_00715"
     CDS             166133..167353
                     /locus_tag="DMACINML_00715"
                     /inference="ab initio prediction:Prodigal:2.6"
                     /inference="similar to AA sequence:RefSeq:CAL34351.1"
                     /codon_start=1
                     /transl_table=11
```

/product="putative transmembrane transport protein"
/protein_id="Prokka:DMACINML_00715"
/translation="MFSSFFASKKWALWAYLGLFLLLFFLYMQTSLNVAINSWYSDFY
NVLQKPKIELLDSNSTQKAQENFENNASLIQEANQKAQENFQKANFINKGALYYYQSL
LEYFFNSRAMIEKESYSASDFYALILVFLAIAIPYVLIATINIYFASVYAFKWREAMT
FSYLKFWKNKDDNIEGSSQRIQEDTYNFSKIVESLGLSFIRALMTLVAFIPILWTLSD
VVSKALFANLSENSSFYFLKNIDGLLVYVALLISLGGLIVSWFVGIKLPGLEYNNQKA
EAAFRKELVYAEDNRKEYAKNETMIELFTGLKFNYKRLFLHYGYFNIWLILFEQMIVI
VPFLIMAPGLFAGAIGLGIVMQINNAFDQVRSSFSVFITNWTTITQLRSIYKRLKEFE
KNIEYAKNKNKNHL"
gene complement(167344..168477)
/gene="ynfM"
/locus_tag="DMACINML_00716"
CDS complement(167344..168477)
/gene="ynfM"
/locus_tag="DMACINML_00716"
/inference="ab initio prediction:Prodigal:2.6"
/inference="similar to AA sequence:UniProtKB:P43531"
/codon_start=1
/transl_table=11
/product="Inner membrane transport protein YnfM"
/protein_id="Prokka:DMACINML_00716"
/translation="MKMLKILIIFCATLTLSIVYAPQPIAPLLSNYFHVNLHQISWII
SVTLIPLAIAPLVYGYLLEIFSLKKILVVSLFLCSIFGIIGNLNDNFYFFLFLRFLQA
LCIPAILTALLTLLSRLESDNIQKNIAIYVGATTFGGFVGRVFGSFLSDLFSWHFALN
FFSILMMVCAFLFLFFKEHPISLNTQTSLRDFLPFIKELRFMVILFCVFTIFFSFQSI
VSFLPFHLKESIKDITQTQIGLVYLGFLTGVISSLLIGKTIEILGSKIRTAIFGFLVF
ITGCFCMIVGNFYFAFFAMFVFCSGMFISHCIFSGILNSIADKKGLANGIYLTFYYSG
GALGSVLPSFYYESFGWEFLCIFTAFLLFIALIIFGKFRHFYK"
gene 168589..169950
/locus_tag="DMACINML_00717"
CDS 168589..169950
/locus_tag="DMACINML_00717"
/inference="ab initio prediction:Prodigal:2.6"
/inference="similar to AA sequence:RefSeq:CAL34352.1"
/codon_start=1
/transl_table=11
/product="putative integral membrane protein with
hemolysin domain protein"
/protein_id="Prokka:DMACINML_00717"
/translation="MDPSQVLDLNQTLPTASFDAGYSILMVVIALALVLLNGFFVLSE
FSIVKVRRSKLEEMVKEKKVGAKKALEVTSRLDTYLSACQLGITLSSLALGWIGEPAI
AKMLEIPLANLGFNGVIIHTIAFIIAFSIITLLHVVLGELVPKSIAIAIADKAVLWVA
RPLHWFWILFLPCIKIFDFLAAMTLKLFGIKPAKESELTHSEEEIKIIASESQKGGVL
DEFETEIIRNAVDFSDTVAKEVMTPRKDMICLNKQKTYAENMQIICDHKHTRFPYIDG
SKDTILGMIHIRDIVQNELSDKSENLDTFVKPLILVPENISISKVLVMMNKERSHTAL
VIDEYGGTAGILTMEDIMEEIIGEIKSENEEDSYKKLAENIYEFQGRCDIETVEEMLL
ISYDEDLEQVTIGGYVFNLLGRLPVVGDRIEDELCYYEVKKMDGNSIERVKVVKKTNK
DEE"
gene complement(169963..171147)
/locus_tag="DMACINML_00718"
CDS complement(169963..171147)
/locus_tag="DMACINML_00718"
/inference="ab initio prediction:Prodigal:2.6"

```
                     /inference="similar to AA sequence:RefSeq:AAW34772.1"
                     /codon_start=1
                     /transl_table=11
                     /product="Ser/Thr protein phosphatase family protein"
                     /protein_id="Prokka:DMACINML_00718"
                     /translation="MRILLILRGNYYAGQEEFIKENRLKDYTLDLNSLRLLSGSVSKI
                     VSKYKNLNLKNDEELYKILLQFLEMRMQKGEFCIINAYNETLKVYKELAKQYRYNVYV
                     IDFKSSLEECLEKNEQKAKERGYIVSQSLLKKTHHLLERIPKKYTILNPNDWKKCLYQ
                     MPNLSKYRKIHHIGDIQGCFSVLKEYLQELKDDECYIFLGDYIDRGIENGKVLKFLLK
                     ICERENVYLLEGNHERHLIKWANGELSSSKEFNENTLKDFRKEKLTPRDARKFYPYLK
                     ECLYYKFHEKQVFCSHGGVNFLPSKAYQISFMSSYDFIYGVGNYEESQKVASQFCEFT
                     KDNVYQIFGHRNRTKLPVQIEKRVFLCEGKVDDGGHLRIVTLDKKGFECIELKNKVYR
                     KK"
     gene            complement(171156..171365)
                     /locus_tag="DMACINML_00719"
     CDS             complement(171156..171365)
                     /locus_tag="DMACINML_00719"
                     /inference="ab initio prediction:Prodigal:2.6"
                     /inference="similar to AA sequence:RefSeq:CAL34354.1"
                     /codon_start=1
                     /transl_table=11
                     /product="putative phnA domain protein"
                     /protein_id="Prokka:DMACINML_00719"
                     /translation="MPKDANGTELNAGDSVSVIKDLKVKGASTTLKRGTTIKNIKLTS
                     KEGEIEAKVDKFGVIVLKTEFLKKI"
     gene            complement(171415..172155)
                     /locus_tag="DMACINML_00720"
     CDS             complement(171415..172155)
                     /locus_tag="DMACINML_00720"
                     /inference="ab initio prediction:Prodigal:2.6"
                     /inference="similar to AA sequence:RefSeq:ABS44192.1"
                     /codon_start=1
                     /transl_table=11
                     /product="integral membrane protein, TerC family"
                     /protein_id="Prokka:DMACINML_00720"
                     /translation="MFEWVFSAEMWIALATLIGLEIVLGIDNIIFIAILVGRLPKEKR
                     QRARIFGLSLAMITRLLLLLSLFWIMKLTTPLFSVFSQEISGRDIILILGGLFLIAKS
                     TLEIHHDIDNAGEKSDEEVLKEGAKRGFFSILIQIAVLDIVFSLDSVITAVGMVNNIE
                     IMMIAVIVAVFVMMLASKSISNFVDDNPTIKILALAFLILVGVTLVAEGLDFHISKTY
                     VYFAMAFSLGVESINIYIKKKRLAQEKS"
     gene            172263..173249
                     /locus_tag="DMACINML_00721"
     CDS             172263..173249
                     /locus_tag="DMACINML_00721"
                     /inference="ab initio prediction:Prodigal:2.6"
                     /inference="similar to AA sequence:RefSeq:AOH52015.1"
                     /codon_start=1
                     /transl_table=11
                     /product="ankyrin domain protein"
                     /protein_id="Prokka:DMACINML_00721"
                     /translation="MKKLEDIKAMNYKQKDELEDLVLEAIRDNNLAKVKEILKDYPVK
                     ISCYELHFKNDNEEYPLFDPYYLIMRAAYACEENNNDFSILDYLFDEYGLSLKDPKYN
                     FCFTDMKYIKEANDKYILIKKAKESNIICRNALIYYYILNADNPNSHIIKYLVNRGAK
                     FEVHDEGSFGWTPMHFWARRNNYELLELAIKGGANVNMQTLLDPESEYNETLLFEAVK
```

EAETYRVTQLLIELGANVNFATPRTPLDDAKGSRNKKLLKDAGAMTSNEIRKKFNLPA
YDSSHCEIDGKTDFDLLGKYRDECSKLLNDAIKKAKENGEIKNIDAKIIKNL"

```
gene            173287..174039
                /locus_tag="DMACINML_00722"
CDS             173287..174039
                /locus_tag="DMACINML_00722"
                /inference="ab initio prediction:Prodigal:2.6"
                /codon_start=1
                /transl_table=11
                /product="hypothetical protein"
                /protein_id="Prokka:DMACINML_00722"
                /translation="MGNKDFGYFLNALENNDIKALEILLFDEELSNMQDENGNTLLHH
                AARLGSAGGVAMLIDCNPFIRNNDGKTAFNIAYDNDDVYIAKMIDNVKDRWQRKHGRY
                EEKLDESEMDIPFYLTDDKESILKQFKEENITTESLLDTSYGDGMVVLQSVLNAWNLS
                FINEKMPPVVEILIDQANGDVEALIDGFDEHMEQWENEVWDEPAVYAGKTEDGEDDYR
                YETDPDEWIREIIPFDKWDELKKQMRLYAVKK"
gene            174254..174469
                /locus_tag="DMACINML_00723"
CDS             174254..174469
                /locus_tag="DMACINML_00723"
                /inference="ab initio prediction:Prodigal:2.6"
                /codon_start=1
                /transl_table=11
                /product="hypothetical protein"
                /protein_id="Prokka:DMACINML_00723"
                /translation="MYKFSKYLIHFCRGDEMLKYSQFIFYNLNKDLKQIHMNFTLNNK
                NNYKLITEKLAITWAKLNFLHPFKERQ"
gene            174679..175611
                /locus_tag="DMACINML_00724"
CDS             174679..175611
                /locus_tag="DMACINML_00724"
                /inference="ab initio prediction:Prodigal:2.6"
                /inference="similar to AA sequence:RefSeq:AOH52011.1"
                /codon_start=1
                /transl_table=11
                /product="ankyrin domain protein"
                /protein_id="Prokka:DMACINML_00724"
                /translation="MKTLEDIKKLNFKIKMEIQKQLFDFISNNDLENVKNLLKDYPVK
                ESFYEAHFTYHHNNEDYELSLFDPAASLLKAAFTCGNYNNDFSILDYLFDEYGLSLKD
                SKYNFAFPDMKHIKEANDKYILMKKVEDDPCIYQKALIYAYILGAKNPNSQIIKYLVN
                RGAKFEVHDEGYSGRTPMHFWARRNNYELLELAIKGGANVDMQTRLIQESEYNETLLF
                EAVKEAETYRVTQLLIELGANVNFATPSTPLDIAKGSRNKKLLKDAGAMTSEQIRKKF
                NIYWDDKECKKDKNYIEKHSKLLNDAIKKAKESE"
gene            175657..176130
                /locus_tag="DMACINML_00725"
CDS             175657..176130
                /locus_tag="DMACINML_00725"
                /inference="ab initio prediction:Prodigal:2.6"
                /inference="similar to AA sequence:RefSeq:CKG87488.1"
                /codon_start=1
                /transl_table=11
                /product="hypothetical protein"
                /protein_id="Prokka:DMACINML_00725"
                /translation="MKNKNLLDKDTQEVFKYYLKNDSYLKGALDGLKKYERRAFISSI
```

```
                     LSYEYKNLVENGNLKVDEKYKAILPPNLIAILEYDKSEFNGANLRNFINDLEFELEHI
                     EMELYDDPADFEGGDYYNEDSECDEDYTVEDWVEDQVSDIRDFLYALKDFKSKYR"
     gene            complement(176182..176757)
                     /gene="purN"
                     /locus_tag="DMACINML_00726"
     CDS             complement(176182..176757)
                     /gene="purN"
                     /locus_tag="DMACINML_00726"
                     /EC_number="2.1.2.2"
                     /inference="ab initio prediction:Prodigal:2.6"
                     /inference="similar to AA sequence:RefSeq:CAL34356.1"
                     /codon_start=1
                     /transl_table=11
                     /product="phosphoribosylglycinamide formyltransferase"
                     /protein_id="Prokka:DMACINML_00726"
                     /translation="MLVKLAVLFSGNGSNLQNILEKLHKKTIGKNTYEVVLCLCNKKD
                     AYGIQRAKNFDLESVIIEHKDYKNREEFDEILVKKIKESGADLTILAGFMRILSPVFT
                     KNIKAINLHPSLLPLFKGAHAIKESYESDMKVAGVSVHWVNEELDGGKIIAQKAFEKQ
                     NLTFEEFEAKIHALEHEILPLSVVEIFENCH"
     gene            complement(176748..178118)
                     /locus_tag="DMACINML_00727"
     CDS             complement(176748..178118)
                     /locus_tag="DMACINML_00727"
                     /inference="ab initio prediction:Prodigal:2.6"
                     /inference="similar to AA sequence:RefSeq:CAL34357.1"
                     /codon_start=1
                     /transl_table=11
                     /product="putative kinase"
                     /protein_id="Prokka:DMACINML_00727"
                     /translation="MKTITDDIKTLEQNAINKGLDELILMENAGLNLAKLIKKEAKKI
                     RKQYKIKKVKILFLLGGGNNAADGLVALRNLKHAKAYKLGFKENEIFKKQEQILKNFG
                     FKFAKKEPKFKKFHIVVDCILGTGTNREADEKTKEILEKINQSKALKIACDIPTNLGF
                     TPCFKADITQCMGALKEILLEDFAKEFVGKIKLANLGVNAKKFSLDSKAFLLEEKDLK
                     TIERNTSSNKGNFGHIYIIASASAGTLAGLGALNFGSGLVSLVAKKSFSPLLMLKEKI
                     ENNASAIALGMGLENLDILKDEILQNIPLVLDANCFLSEAVLWYLSRKDVVITPHPKE
                     FIRLYKMCFDEDLSIEDLQRNRFFYAKKFAQNYDCVLVLKGANPIIIQKEKIFVVNLG
                     NEALAKGGSGDVLSGMIAALLGVKFSALEAAKNACLAHALVAKKYKFNKNSFDAIKLI
                     KGLKCL"
     gene            complement(178102..178800)
                     /locus_tag="DMACINML_00728"
     CDS             complement(178102..178800)
                     /locus_tag="DMACINML_00728"
                     /inference="ab initio prediction:Prodigal:2.6"
                     /inference="similar to AA sequence:RefSeq:CAL34358.1"
                     /note="conserved hypothetical protein Cj0189c"
                     /codon_start=1
                     /transl_table=11
                     /product="hypothetical protein"
                     /protein_id="Prokka:DMACINML_00728"
                     /translation="MENKSIAIFIDAENIPAKYAKSIFDIASDYGEIIIKRIYGDWTQ
                     KNIQAWKEQIAEYSLIAMQQFNFAANKNSSDMYLITEIMSIFYEKDIDIFVIVSSDSD
                     YTSLIQKLRENKKQVIGMGLEKSIKSYVNAFSEFFYLDQDENKKEDILSKDYLRALIN
                     ITEQLIDEKGRAEYAQIRTNMNRKYSDFNPQNYGFKNFRALIQKFLPKMKKFEEEREK
                     NIYFLVKKDYENYY"
```

```
gene            complement(178802..180307)
                /locus_tag="DMACINML_00729"
CDS             complement(178802..180307)
                /locus_tag="DMACINML_00729"
                /inference="ab initio prediction:Prodigal:2.6"
                /inference="similar to AA sequence:RefSeq:CAL34359.1"
                /note="conserved hypothetical protein Cj0190c"
                /codon_start=1
                /transl_table=11
                /product="hypothetical protein"
                /protein_id="Prokka:DMACINML_00729"
                /translation="MKKLKCVSFDENLDIIDVESTFTRGLPNLSIVGLANIAIKESIE
                RIKATLLTCNFSFPAKKITINLSPSGIPKKGSHFDLVIATLILLQNEDFDDFFVVGEL
                GLDGSIKSTNELFSLLLFLSTKVKSAKVVVPKNIALKASMIPNLEIYGLEHLNEVIEF
                FKEKNYEKFKFTQSHPLFANPLIIENEIFIKNHHFELDFKDIKGQEKAKRACLIAALG
                MHNILFEGSPGSGKSMCAKRLVEIMPPQSLNEVLMQNAYMSLNSKDCEFTQKRALRHP
                HHTSTRASIFGGGAKNAKIGEIALANGGVLFFDEFPHFNKQIIESLREPLEDHKIHIS
                RVNSKTTYETKFSFVAAQNPCPCGNLFSKNLSCVCSENEIKRYKNHISAPIMDRIDLY
                VAMDEISKDDKSSTNSKEMSERILKAFIFGKKRGQKEFNGKLSDVELQKFCILEKDAK
                DTLDLAISRYNLSQRALNKILKVSRSIADFEEKELLNKNHILEALSFRIRS"
gene            complement(180304..180831)
                /gene="def"
                /locus_tag="DMACINML_00730"
CDS             complement(180304..180831)
                /gene="def"
                /locus_tag="DMACINML_00730"
                /EC_number="3.5.1.88"
                /inference="ab initio prediction:Prodigal:2.6"
                /inference="similar to AA sequence:RefSeq:CAL34360.1"
                /codon_start=1
                /transl_table=11
                /product="polypeptide deformylase"
                /protein_id="Prokka:DMACINML_00730"
                /translation="MVRKIITYPNSRLFLNSEPVKQFDQELHTLLDDMYDTMIASQGV
                GLAAIQVDIPLRALIVNILDENEEQKKEDLLEIINPQIIPLDEEKITCTEGCLSVPDF
                FEEVERYNHILLKYQDRFGNFKELEAQGFLAVAIQHENDHLNGHLFIEKISFLKRQKF
                DKEFKKKLKNQKKSK"
gene            complement(180859..181443)
                /locus_tag="DMACINML_00731"
CDS             complement(180859..181443)
                /locus_tag="DMACINML_00731"
                /inference="ab initio prediction:Prodigal:2.6"
                /inference="similar to AA sequence:RefSeq:AIW09470.1"
                /codon_start=1
                /transl_table=11
                /product="ATP-dependent Clp protease proteolytic subunit"
                /protein_id="Prokka:DMACINML_00731"
                /translation="MYIPYVIEKTSRGERSYDIYSRLLKDRIIMLSGEIHDELAASIV
                AQLLFLEAEDPQKDIYLYINSPGGVITSGFSIYDTMNYIKPDVCTICIGQAASMGAFL
                LSCGTEGKRFALPNSRIMIHQPLGGARGQATDIEIQAKEILRLKAILNDILAKNTKQK
                VTKIVKDTERDFFMSAQEAKEYGLIDKVLEKSFK"
gene            complement(181443..182768)
                /gene="tig"
                /locus_tag="DMACINML_00732"
```

```
     CDS             complement(181443..182768)
                     /gene="tig"
                     /locus_tag="DMACINML_00732"
                     /EC_number="5.2.1.8"
                     /inference="ab initio prediction:Prodigal:2.6"
                     /inference="similar to AA sequence:RefSeq:ANS23308.1"
                     /codon_start=1
                     /transl_table=11
                     /product="trigger factor (peptidyl-prolyl cis /trans
                     isomerase, chaperone)"
                     /protein_id="Prokka:DMACINML_00732"
                     /translation="MEVKAKQLDSVNATASVKIPSGMIKSEVENLAKKASKNVKMDGF
                     RPGKVPVAAVLKRYQKELTQDAEQNLFKSAVDSALKELKKEVKELVGEPYFEKFDRQD
                     GEIVAELALSFKPEFKLDGYEELIPEYQTPKVTKKEIDEKKEELLKRFATPEAIKTKR
                     ALKEGDFAKFDFEGFVDEKAFDGGKAENYVLEIGSKQFIPGFEEGMVGMKSGEEKDIK
                     VTFPKEYGAAHLAGKDAVFKVKLHEIQELKLPELDEETLKKLLPNEEKPTVELLDEKL
                     KEQIKNEKLFKLVNDELKAKFADALIEKYNFDLPRGIVEQETDMQFRNAFNTFSEKEI
                     EELKTSKEKYQEKRDSFKEEAQKSVKLTFIIDELAKLRKIEVNDQELIQAIYFEAYRY
                     GMNPKEHLENYKKQGALPAVKMALIEEKLFSDIFMPKADKASKKEKEDK"
     gene            182889..183461
                     /locus_tag="DMACINML_00733"
     CDS             182889..183461
                     /locus_tag="DMACINML_00733"
                     /EC_number="3.5.4.16"
                     /inference="ab initio prediction:Prodigal:2.6"
                     /inference="similar to AA sequence:RefSeq:CDH62018.1"
                     /codon_start=1
                     /transl_table=11
                     /product="GTP cyclohydrolase I type 1"
                     /protein_id="Prokka:DMACINML_00733"
                     /translation="MQEKFENCVKTMLEIIGENPTREGLVKTPNRVFKAYEFLTSGYK
                     KNVKEILNNALFESSNNEMVLVRDIEFYSLCEHHLLPFFGRVHVAYIPDQKVVGLSKI
                     PRLVEVYARRLQIQEQLTEQIAEALMENVGAKGVGVVIEARHMCVEMRGVQKANSTTT
                     TSALRGLFLKNEKTREEFFSLINSPKQVRF"
     gene            183461..184846
                     /gene="fliI"
                     /locus_tag="DMACINML_00734"
     CDS             183461..184846
                     /gene="fliI"
                     /locus_tag="DMACINML_00734"
                     /EC_number="3.6.3.14"
                     /inference="ab initio prediction:Prodigal:2.6"
                     /inference="similar to AA sequence:RefSeq:CAL34364.1"
                     /codon_start=1
                     /transl_table=11
                     /product="flagellum-specific ATP synthase"
                     /protein_id="Prokka:DMACINML_00734"
                     /translation="MNLEKLRSKLGKENLSAVFGEITKISATSIEIRGLKTGVGDIIK
                     LVSNENENLNTLAMVVEIKEQFSYLSPFSFIEGFKIGDRAFISDAGMQIGVSDELLGR
                     VVDPFMRPKDGKGAIEATKYMPIMRAPIDAMKRGLIEEVFPVGVKTIDALLTCGVGQK
                     LGIFAGSGVGKSTLMGMIVKNSKAPIKVVALIGERGREIPEFIQKNLGGKLDDTVIIV
                     ATSDDSALMRKYGAFCAMSVAEYFKEQGKDVLFIMDSVTRFAMAQREIGLALGEPPTT
                     KGYPPSVLSLLPQLMERTGKEEGKGTITAFFTVLVDGDDMSDPIADQSRSILDGHIVL
                     SRELTDFGIYPPINIQNSASRVMGDIISPEHKLWARKFKRLNSLLKENEVLLRIGAYQ
```

KGSDKELDEAIAKKEFMQKFLGQNPEESFEFEETIRLLSQIDANVAPSAVQQNINMGS
SSATLPNPNLK"
ORIGIN
1 aaacaagggt ttacaaaatc ttcatttagc tttataaact ttagtttata atttaaagat
61 tttaaagtcc ttttttgaaa aaggaaatga aagtataact aattaagctt aaaggaggat
121 taaataaggc tagaaattta gccttatttt cgagatggtt gaaatttgag agtttaaaca
181 aactaattta gagaagataa aaaccttccc taagctttaa aaattcctaa atttcagtat
241 tttaaaaagt attcttggat ttttttagga tcgcttgttt ttgtaagagc taacataagc
301 aaaactcttg ctttttgcgg atttaaatct tcagcactaa taaaacccaa tttttttatct
361 gtatcactca ccgcaacgcg tcctgctgca acccttgagc ttacaacaac atcaagccct
421 tgtttgatga gttcttttaa agcatttttt tgatcttcat gtatgcttcc tgcaccactc
481 cctgctacaa caatgccttt agttccatta tcaaataaag cttttgcagc aatggcacta
541 ccatcatttg aataagtata gagtatatct acctttggca aacttgtgag cttacttaca
601 tcaaaaggac tattttttgt gtgcgcttta cttatgttat tatagaaaaa aacttttcca
661 tctactatat aacctacatc tccaaaatta ggagaagaaa aagcatcaac atttaaacta
721 tgagttttta ccacgcccct agcacttaaa attttatcgt tcatagcgac cataacgcct
781 ttatttttgg cttctttatc agcagccaat gctacagcat tgtaaagatt ttttgggcca
841 tctgcgctta tagcagttga tggacgcata gcgccaacta aaactacagg cttatcgctt
901 ttaaccgtta aatttagaaa ataagcggtt tcttccatgg tatcagtacc gtgagtaatg
961 actacaccat ctactccctc atcaaaaagt ttattgattt ttttagcaag ctttaaccaa
1021 atttcatcac gcatattaga gctatcgata tttgctattt gctctccgct aatattagcc
1081 aaatcttgaa tttgtggaac tgctttgatt aaaacatcta cgcccacaac ccctgctgta
1141 taacccgttg tggccacagc actatcgata gaacctgcta tagttccacc cgttgctaaa
1201 atagcaattt ttggtttggc ttctgccata catactccta tagataaaaa tacccatacg
1261 cacaatagta actttttcac aaagcccccct tatttattaa atttaagttt cgtaatgaaa
1321 catatatata atattaataa ataataaata aaaattttat aaatatttac aaaatttctt
1381 taataataaa ttgcggggtt gctaatcctc taaattcatt cttagaaatg cttccaagta
1441 aattaatatt ttcgcacaat tgcggttctt tgtcaaaatt aaaaaacaaa gcttctatag
1501 ttttattatc ttttgtgagt ataagcttta gatgtttttc atcttttcct aagaattttt
1561 tatttttaac ataaagattt tctaggataa aaaaaggacg cggattttta tgtccaaaag
1621 gctcaaaaga ttctaaaatt tcaagcattt caaaatcgat ttcattagga tctaaaatac
1681 ctaaaatttc atctgtatct aaaaattcac tttgagaaat ttccatacat tctttttta
1741 ttatggtttt aaatttttca aaagaatcaa gatctaagct aaggcctgca gcacccttgt
1801 gtccaccata attgcttaat atagaatttg ctttagaaat caaagctaaa atatcgattt
1861 tcccaacact tcttgcactt ccttttaagc gttgatcgtt ttgtgaaaat acaaaagcag
1921 gtttattaaa atgttttgct aaacgacttg ctactatccc taaaactccc tcatgccaat
1981 ttaaacccga tacaacaata caagaatcat tttcatcgat ttgactcaag ctatcttcaa
2041 aaagctgttt ttcttcatct ttacgacttt cattaaaact gatgatttgc tctaagtaag
2101 ctgaagcttt atccaagtcc tttgtatgca aaaattcata agaaatgctt gcatcatcca
2161 ttcttccagc actattgata agaggagcga ttaaaaatcc tatattatca agtgcaaatt
2221 tatctttttg ataatattgc ttaatcgcct taaaagcaac tcttttagat ttgttaatac
2281 actctatccc tcgcctaact aatgccctat ttaaatctct taactccatc atatctgcaa
2341 caatggcaat ggctagaagt tctaaaaatt tgcccatatc gtattttaat ttacaaacct
2401 cttttaaagc agctattaaa taccaagcta cttgcgctcc acaaatttcg atatctggaa
2461 aatcacaatc tttttgctta ggattaataa tcgcaaaagc atcaggcaaa atatcttggg
2521 gcatatgatg atcagttata attaaatcaa tattttttttc cttacaaagc ttagctgctt
2581 caaaggctgc tataccatta tccactgtaa taatcaaatt tacatcaagc tcattgataa
2641 tttcagcatt taaaccatat ccatctttga agcgattagg aattcttact gtataatcaa
2701 aacctatatc atcaaaaaat tctgccataa tgacacaaga gataatccca tcaacatcat
2761 aatcgcccac tatagcaatt ttttcatttt tatctatagc ttctttaatg cgatttgctg
2821 ctttataagc atcttttaaa caacaaggca aaggtaaatc gcaaagcttg gtatgtaaat
2881 ctttttcaaa acgcgaagct agaatttttt taatttcact cttattaatt gtcttcatag
2941 cttatagccg ccttgataaa accacaaatc acaggattaa cgcgctctaa tcttgaagtg
3001 aattcagggt gaaattggac agctaaaaag aaaggatgat tgtttagctc gacagcctct

3061 ataagtcctt cgctctctcc acttactatt aatcctttgc tttcaaattc tttgcgatat
3121 tttggattag cctcgtagcg gtggcggtgg cgttctttaa tattttttgc attattataa
3181 accttagcca aaagggagtt aggcttgact ttacactcat aagatcccaa acgcatagtt
3241 ccgcccaaag gtgttttagc agtacgaatt tgttttttac cgctcgcatc cataaattca
3301 tcgatgagat aaacgatagg attagaacat ttatcatcaa attcacttga atttgcatct
3361 tcgattttta acacatgtct agcaaattct accaatgcaa gctgcatacc caaacaaatt
3421 cccaaaaatg gaattttgtt ttctcttgcg taatgaatag cctttatttt accctctact
3481 ccgcgataac caaagccccc tgcaaccaaa attccactca catttttaaa cgcttcagcg
3541 ctttctaaat tttcaagctt ttcgctatct acccattgaa gttctactct agtatcaagc
3601 gctgcacctg catgaatgat ggcttctgta agactcttat aactttcttt tagatccaca
3661 tatttaccca caaaagcgat tttgacttca ttgctcggag ctataactct ttttactaga
3721 atatcccaat tttccatatt tggttttaaa ttttttaaat ccaaaataga agcgatagga
3781 tttaaaatat cttgttttaa gaaattcagt ggaatttgat aaatacttgc tgcatcaaca
3841 ctttctataa cgcaattttt ttcaacacca caagaaatag caattttatc ttttaaatct
3901 ctatccagcg ctttttcact acgacaaata atcatatcag gactaatacc tatgcgtctt
3961 agttcgccta cgctgtgttg ggtaggtttg gtttttagct cccctgcagc cttgataaaa
4021 ggcactaggg ttaaatggat attcatagca ttgtttttac ccacttccaa acgcaaagca
4081 cgaatagctt ctaaaaaagg caaaccttcg atatctccga ctgttccacc tatctctacg
4141 attaagatat ccttgccctc tcctgctttt ttaatacgct ctttaatctc tcctacgata
4201 tgtgggataa cttgaatagt tttaccaaga tattctccac gtctttcttt ttcgatcacg
4261 ctttgataaa cacgccctgt ggtgaaatta ttatcttgtg ataaactctc atctaaaaaa
4321 cgctcataat gccctaaatc aagatcagtt tccgctccat catctgtaac aaaaacttct
4381 ccgtgttcaa atggactcat agtaccagga tcaacattga tataaggatc tgcttttaaa
4441 atgcttactt taagtcctga atttttcaat aaagtcgcga tacttgcagc tgctattccc
4501 ttgcccaagg agcttaaaac tcctcctgta acaaaaatat atttagtttg tttactcatt
4561 atcgataagc cttaattttt tgtctaatta taacgcgata aaacttttta tttcattaga
4621 attttaagta aaaaatgtaa gaattatgaa aaaatttatc aaggaaaatt tatgcaaatc
4681 actttacttt ttcatacccc tctttctgta tgctcacatg caacaagaac ttgctggcaa
4741 agttttgata aaggtgattg tggcggagaa aaagataaag aactcatcga tcgcgtagga
4801 aataaattta aacatgcctc tactttagaa catttaaatt atacttttta tattcaagga
4861 atttcaaggg cttgcttaca agaagttgct agacatcgtc atacaagccc tagtgttaaa
4921 agcacgcgct atactctaaa agaacttcgc aatgaaagtg aatttaagat aggagatttt
4981 gaaaacgcaa gccgatatct tgttctttgc ggtaacgaag aagttgataa tgctagcatt
5041 caagctttag aaaacttacg ccttatttta caaaaaagca taagcttgga tatagccaaa
5101 tactgcttgc ctgagagtta taaaaccgaa cttactttaa caatcaatgc tagaagtttg
5161 caaaatttta tttctttgcg tagctcaaaa tctgctcttt gggagattcg aaatttggcc
5221 aatgctttat ttgaagcctt accacaagat catcaattta ttttcgagca ttgtttgcat
5281 aaagatattc attaaatcat acttatttta tcttatttat aatattttta tggtattatc
5341 cataagatca aggttttatg gatatacttt aaatatattt catacttgat atagtttctt
5401 acaaggactt tttatggata aacaattttt tcaaatgttt ttaatgttct cgcaaataca
5461 aactgtagct attattgctg tgctttttgt aatttttttac attcttaaaa aaatgcgaga
5521 taaaaaggta aatttctccc ttcgtatgct ttttgccctt tttataggtt taggtttagg
5581 ttttgcttta caatatctag ctcaatttcc agatagtaaa gaagcaagta ctatcatttg
5641 gtacaatgaa gcaaaacatt ggtttggttt tgtaagctca gtttttgtag cttttataaa
5701 aatgttagtt attccgcttg tgggtatttg tatcattaaa gtgattatag aaattgataa
5761 aaatataaaa atttcttcct tgcttggtat aagcctcttt tggatacttt ttagtactgc
5821 tatagctgct tctttaggta tattgctagc ttataatttt caattgggtg ataattttac
5881 tgcttatgaa ggagcaagac aaattcgtga aattcaaact ttttcgagta ttattttagg
5941 tttaattcct agcaatatca tttctgttat gaataaagaa aatatcatag ccattgtaat
6001 ttttgctttt tttgtaggaa tttgtgcaaa aaaagtttca aaaaaagaag aatatacaca
6061 agcttttcaa acttttgaaa attttgtctt agttttttat aatattatga tgaatatgac
6121 agctacggtg attcgattta tgccttatgc tgtagtttgc atgatggcaa atgttttatt
6181 aagcaatggt tttgaagcaa tcaaaactgc aggacttttt atcattctta cctatatagc
6241 tatgtttata atgtttggag tgcatttttt acttcttgct tctcaaggat taaaccctat

6301 aaaatacgcg aaaaaagctt ttcctgtatg gttgtttgca ttcagttcaa gatcttcttt
6361 agggactttg cctatgacga cttcaacttt gcaaaacaaa tttggagtaa atcctgccgt
6421 ggctaatttt gttgcctcta taggaacaac aacgggactt aatggttgtg cgggttattt
6481 tccggctatg gcagcagttt ttgtggcatt tgctacgcac acgcctatag attttacttt
6541 tgctttgatg atagtattag tagctgttat cggatcttta ggtattgctg gagttcctgg
6601 aagtgcgact atggcagctt ctattatgct cgcagggatt ggatttgggg ataattttgt
6661 tatgcttagc cttattttag ccattgatcc tatcattgat atggcaagaa ctgctagcaa
6721 tgtttcagga gctatgactt cagcgctttg tactgcaaaa aatttaaaag ccatagataa
6781 agaaatttac aattcttaaa aagatttaaa cctaatatag gtttaaatcc tcattggcaa
6841 ccttcacatt ctatgctacg atcagcaaca tttatctttt cgctatcagg actttcgctt
6901 cttagatagt aagttgattt tactcctagc tcccaagcta gttgatatat atcatttaag
6961 tagcctccac ttgctttatc aagtgataaa aagatattta agctttgtcc ttgatcaatc
7021 cattttccac gcactgctgc agctttaaca agaattcttt gatcaagttc gtaagctggg
7081 gtataatact gccaagtttc aagacttaaa ttaggcacta caacaggtat catgccgctt
7141 aaattttgct caaaccactt acgcttatag acaggttcga tagtttgtgt tgttcctacc
7201 aaaatagaaa ttgaagaagt aggagcaata gccatcaaat aaccatttct catgccatct
7261 gcttttactt tttctctaag tttatcccaa tcgcattcgc tttgattaaa caatccttct
7321 cttagagtta aagctttggc tttttcactt gctacatcaa taggaaaaat ccccttactc
7381 caatttgatc cttcaaaatc aggataagaa ccttttttcta tcgctaaatt tgagcttgcg
7441 ttaatagcct catagctaat tttctccata atttcatcga ttttgtttaa atgctcatca
7501 ctgccccaat gaattcttgc ttcagcaagc atttgtgcct cgcccataac gccaagtcct
7561 atggctcgag attttaaatt tgtatccttt acttttctat gagggtaaaa atttaaatca
7621 ataacattat caagcatacg aatagcagtt ggcaccacgc gctctatatc ttctttggta
7681 tataccttgc taagatttat gcttgctaga ttacaaactg cggtttttcc atcgctttta
7741 tatttttcta cgatatagac tttgtttcca ttaaagctat caagagttga aattttctta
7801 gcaggctttt catatccccc atcgataaca acgcgttctt cttcatcaaa atgtttttca
7861 tcaccatttt caaatataac tttaatttta taataatttg gctctgtatt ttgaaaaatt
7921 tctgtacata aatttgaact tctgatgatc cctacatgtg catttggatt ggctttattt
7981 gcattatctt taaaacataa aaaaggcaaa cctgtttcaa aataattaag caaaatcttt
8041 ttccaaagct ccttagcttc tactatttct ttaactatac tttcatcttt ttcgtactct
8101 tcataacgct tttcaaaagc ttccccgtat aaatcacaca aatcttgtgt atccgcagga
8161 tcaaaaagag tccatttatc atttgctcta actcttttca taaaaagatc gttaatccac
8221 aaagcaggga aaagctcatg tgcgcggcgt ctttcttcgc ctgaattttt acgcaaatcg
8281 ataaaatcac ttacatccat atgccaaggt tcgatataaa cagcgatcgc acccttttctt
8341 gtgccaagct gatccacagc cacagctata tcattagtaa tttttaaaaa aggaataata
8401 ccccctgcag cgtttttatg cccatctata cttccaccca ttgcacgaac tttagaccaa
8461 tcccagccta tacctccgcc aaatttagac aataatgcca tttcttgata agaatcaaaa
8521 attccctcga tattatcagg agttgagcct atgtaacaag agctaagctg gtggcgtgtt
8581 gttcttgcgt ttgataaagt aggagttgca agcatgagtt caaatttaga gataagatca
8641 taaaattttt ttgcccattc ttgaggattg aattcattct gtgctaaaaa catagcaata
8701 gccataaaca tttgctgagg aagttctata ggcataccct tgctatcttt aatcaaatat
8761 ctatcgtata gagttttaat gccaagatag gtaaattgca tatcgcgttc aggcataata
8821 taatcattta aatcatctaa atcgtatttt tctttaagcc cgagtaaaat tctaccttcc
8881 ttttcgccct tttcaaaata ctcgcgtaaa tgattataac gattcattcc attcactttt
8941 ttatacaagt caaacaaaaa aagtctagcc gcaacaaaag tccagtttgg acaatcaata
9001 tcgattttat ccacagcagt tttaatcaat gtttttgaa tttcttcagt tgaaattcca
9061 tctcgaaatt gaattttagc atcaagctcg agctcgctta gattaacacc ttctaaatct
9121 tttatagcat caaaagtgca ttttttgatc ttagaaacgt ctaattcttc tgtgcgacca
9181 tttcttttaa taactttcat tgtattcctt gtccttattt aaaagttctt gcaaagattg
9241 catcaacatt ttttgtgtaa taattataat caaagcactt tcttatatct tcttcattca
9301 agctttttct caaatcctca tcagctaaaa gcgcaagtaa aaataagctt tcattgtttt
9361 cattgatagc agctttacca ttttgtaaat cagcccaaac tttcatcgca tttctttgga
9421 ctattttata agcttcttca cggctaatgc ccttaaatgg aagctccaat aaaacgcgtt
9481 gtgaaaatac aagtccgcct gtaagattta gattttcat catattttca ggataaacca

9541 aaagcttatc aatcaaattt gtaagacgca tgagcataaa atcagctgta acaaaagcat
9601 caggcaagat aaatctttca acacttgaat gcgatatatc tctttcgtgc cataaagcta
9661 cattttccaa agcaggagtt acaaaagatc taagcacacg acaaagtcct gtgatatttt
9721 cacttagaac tggatttctt ttatgtggca tagccgagct tcctttttgt cctgcactaa
9781 aatactcttc agcctcatac acttctgttc tttgaaaatg tcttatcgca acagcaattt
9841 gctcgcacga tgaagccaaa atcgcaatag ctgaaatcac ttgcgcataa cgatctcttt
9901 ggataacttg atttgaaaca ggcgcgggtt taagatctaa atttttacat acttcttctt
9961 caaattttaa aggagcatgg gcaaaatttc ccattgcacc gctaattttt ccatagctta
10021 tcacttctct tgcatgctct aaaagctctt ttgcatgcaa aatttcatca taccaaatcg
10081 caagtaccaa accaaaagtg ataggctctc catgaattcc atggcttcta cctaccatta
10141 aagtattttt atgttccaaa gcgcgttttt taatcacttc taaaagcaaa gaaacatctt
10201 ctaaaatcaa atccaagctt tcttttatct gtaaagctac agcagtgtcg atacaatcag
10261 aacttgtcat tgcataatgt acaaaacgac tttcctctcc taaactttca cttacacttg
10321 taagaaaagc gatcacatca tgcttagtgg tttttctat ctcatcaatt ctagctatat
10381 caaatttagc atttttaaga attttttcac aatctgtatc atttataagg cctagtttat
10441 tccaagcttt tacagcagct agctctactt ttagccatgc atcatatttt gcttgcatat
10501 cccatttttt ggccatgatt tctctgctat atctttcaac catctttgaa acctttttag
10561 ctataatttt aaaaataatg aaaatggaat tttatctaaa aaaatttaga gaaaggttaa
10621 gtttggctta cacaaaaata aaactgtcaa ataatggaaa aaaggcgttt caagtgctta
10681 tagataattt gaaaatcaat ataaatgaag cccaaaaact catagataaa aaaaggcttt
10741 tttgcaatgg aattttagta gaagaaaaga ataaaatctt acaaggtaat gtagaattaa
10801 tagtctatga aaacaacccc aaaggagtag aaatcgcttt tgaaaatgaa gattttgcag
10861 tacttgaaaa agaaagtgga attttaagcc accctaatgg tagacactgc aaatacagcc
10921 ttagtgatga aatttggcac ctttggggta aggaagcgtg tgtagctcat aggcttgata
10981 aagaaacaag cggactcata ctcgtagcca aacacaaaaa agcacaaatt gagcttaaag
11041 gcctgtttga aaaaaaacaa attcaaaaag aatatcttgc ccttgcaagt ggggaaatca
11101 aagcaaattt catagtcgat caagcgatgg atttgacaaa ggattatgat gacgttaaaa
11161 ccagaatgca aatttgcgag aatggaaaaa aagcgatcac agaatttgaa gttttgagct
11221 tttatcctaa aatcaatgct acgcttttgc tttgcaaacc actcacaggc agacaacatc
11281 aaatcagagt acatttattt tataaaaatc acaaaatcct aggcgatcca ctctatggtc
11341 ttgaaaaaaa agatatagaa aaaattttag atgaaaaaat ggacatagaa gaacgcataa
11401 agctaagcgg tgccaaacgc ttgtgtttgc actcttatcg tttaaaattt aactatgatg
11461 gagaaaattt tgatattttt tcaaaaaagg gtatagaaaa aattttaaca gcattttaaa
11521 acaaaactga tattttattt catatctaac aagctatcat tttttataaa aaaattttaa
11581 aataagaatt tactttttgt taaaaattta gtataatcac aaaataaaaa tactaggatt
11641 ttataaatga aatttgttaa ttttaaactc aatcaaaaag ccactctagg ggtgcttaac
11701 tctagtggaa aagttataaa tttagccgat cttgatatca atgttcgcga tatgaatgaa
11761 ttgattataa attttgataa ttttaaacat aaatttaaag atttagattc taaaacagct
11821 tatgaaattc caaaagaaga ttatctagcc cctatcatag aaccgcgtca agatattata
11881 tgtcttggga ttaatttttt agatcatgcc aaagaatctg cacaatttaa aggcgaaaaa
11941 ttcgaagaaa gagaatatcc tgtgtatttt ggaaaacgct gcaatcaagc cacagctcct
12001 tttggcagca tatctttgca tgctgatgtc acttctcaac tagactatga atgcgagctt
12061 gctttcattt taagtaaaga tgcttataaa atcaaagcag aagatgcgaa agactatatt
12121 tttggctata ccattatcaa tgaaatttca gcacgagatc tccaaaaacg ccacaagcaa
12181 ttttaccgcg caaaaagcct tgagggaagc actataatgg gaccttatat tacaagtgtt
12241 gatgagattg cttatccgcc cgagcttaat ttacaaagtt atgtcaataa agaattgcga
12301 caaaattcca atacaaaatt ttttattttt gacatcggat atgtcttaga agaactaagc
12361 tcaggaatgc ttttaaaggc tggtactatc atatcaatgg gaactccaag tggcgtagga
12421 atgggcttaa atccacccgt tttttcttaaa aaaggtgatg aagtacgctg tgtgatagaa
12481 aatttaggag aaatttgcaa tgtaataaca taaaatctca ttaaaaatca tagaaatgtt
12541 ttttacgaaa tttaataaaa aggagatatc atggagtttt tagagctttt attagtttta
12601 atcgccttaa tactaatcat caaaaagcca gaaaaagaaa atttggcttt tggcttagtg
12661 atagtagctt ggcttttaat ggttttttc tatatcggcc acaaaacagg tgcgctttta
12721 acgataatga atctatgaag aaaggctgaa gaatgaacga aataaataaa acaaaaaatt

12781 tctacactct tatgtgtttg gcaggctttt tgattattct tttgcctgtg gggattgcaa
12841 attttgtatt tggttatatg tggggtgata gcccatgcac tctttgttgg ggacaaagag
12901 aagcgatgat ttttatcggg gttatggcac tttttatcgt gcgttatggt atgaaaggca
12961 aataccttgc cgctcttttg atcatgactg cagtaggtct ttatcaatct tttgcacatt
13021 atggcaatca cgctcatagg gatttagatc aaggttttgg tttggcggtt tttggtatcc
13081 atacttattt ttgggctgaa gtagtctttt gggctgtagt cttgctttta ggtgtgatgt
13141 ttgcttttgc tcctaaattt ggatcttttg acaaagaatt aaacggcgaa aaatttagaa
13201 aatttactaa atttagcttc gcagcagttt taatcagcac ccttatcgtt gcatcaaatg
13261 tctttcaagc ttttgtaagc acaggaattc ctccttatgt ggggcaaggt gatcctgtaa
13321 gatttagtct taatccaaaa tatatcattt ggagcacagg gggttggaat ggtttatggc
13381 aaaacatttc tttcttagga aaacgcgatg taaaggctcc tgattatgct tttgcacctg
13441 caagtgaaaa attaggtata aaatttgata atgatacaaa taattctcca tttgcagaaa
13501 tcgacgatga gcttaaaatt atcgatgaac aaaccataaa ttttgacaaa gcgatcaata
13561 ctcttgatta tattaatgat gaatttgtag caagttctaa atgggatgta gcgtttttgg
13621 ataacaattt tagcacaaaa gaaggttttg agcttgatcc ttatttttca gctactatcg
13681 atcctattat aggtatcatt ccttataaag aaaataaatt tcttctcatg ggatccaata
13741 aatctttcct aagatttgca aaaaatccaa atgcagatga agctttacaa tatgcagatt
13801 ttgttaaagg aaatgaccaa tttgaaggtc aaggaaaaga tttgggtcgt ggaagacttg
13861 atactgttag agcaaaattc aaccatgtag caagtatgac aacagatgat cattatcttt
13921 atcttgcaac cgtgcctaac aacaaagatt ctaaaacctt tgtaatttct aaaatttctt
13981 taaaagatct tgttctttca gcagaattta ctccaaaagc agaacttaaa gagggtaaaa
14041 ctttgggcga tctttatatc acttctatgg cttttaaaga tgatgaaatt tatgcactca
14101 gtaaaaatca caatgtaatt gccataatca atccaaccaa agaagaagtg gtaaaaacca
14161 tagcttttcc aagctctatc acaaatgcaa gaagtatttt ctttaaagat ggaaaaattc
14221 acatcctttc ttatcaagat ggagcaaata agctttatac tttaaaataa atttgaagtc
14281 ctagtgattc taggacttaa tttccatttt gattaaaata aaaccaaaat catctttata
14341 ttctcccact ttttcaaatc caaatttttg ataaaatttt aaagcatttg gattatcaag
14401 attaacttct actttcttga tcttaaaatc atctaaagct ttttgtaaaa gcttggagcc
14461 aattttttta cgaaaatatt ctggttttac aaaaagcatt tgaatacatt ctttatcagc
14521 tcctaaaaat cccacaaatt catcattttt gtaggcaaat aaaaaattta aatctttaaa
14581 atcagcattt tgtaaatatt ctttaatagt tcgaaaattt ttttcatcta aaaaattatg
14641 cgttgcctta acgctttgct cccaaatttc taaaagtcct ttagcttgtt cttgattcaa
14701 tttttcataa tgttttatca ttttcacaat cctttatatt ttttatattc gattttatct
14761 ttaaatcctg ccctttcaaa acctcttaaa cgaagcaaac aactatcaca ttctccacaa
14821 gcttcatctt cgctttcata acaagaccaa gtaagctcta aaggcacatt ttcttttaaa
14881 gcaagctcta caatttgagt tttgttaaga tttacgagag gggtttttaa agaagttata
14941 aaatgcttac ttgtgccttc attgataaat tcttgagctt tttgtataaa tttttcagta
15001 caatcaggat aaccgctacc atcttcttct actacaccta taaaaatact ttcacaattt
15061 tccttttccg ctaaagatcc tgctatgctt aaaaaaattc cattgcgaaa aggcacataa
15121 gtaataggcg gagtatcgct tatgctcagc tcatttttag ggatatggat actttgatct
15181 gttagagcat tacctcctat atttgctata aaactcacat caaaaatata agatttttct
15241 acatttaaag ccttgcaaat ttgcttaaaa cactcttttt ctttttcttg tgtgcgttgt
15301 tgataatcaa aatgcaaggc aataatctca tatccttctt ttttggccaa ataagcacac
15361 aaagtgctat ccattccacc gctaatgata caaagtgctt ttttactcat ttttatatcc
15421 ttaaatttgc tgtttttgca attctagcaa atattttta aaatttacac ttagtgtaaa
15481 gtcccatttt aacactatgt aataatggct aacaatctat gtttcatttc atttgactta
15541 aattcaaatt agtgatatga taatggtatt taaaaagggc tggggagcac caaatcatta
15601 attctatttt tatggatgat tttaggattt gcgaagtatt ttaaaaaaat actaatctta
15661 aaatttcatc cataaaagca gattctacaa atcaccaaaa actacactca atcattcaaa
15721 tttcatatcg attcaatctc ttcttcaatt tatttattat tttaaaaaat aaaatgataa
15781 aatttgaaaa aaattaaaag gcgatttaaa tgataaatat aaaacttata gaacatattt
15841 tcaaagcagc ttccatcagt cgttggaatg attatccaag aatggcaaat ttagtcgagc
15901 ttgataaaca agcgcataaa tttatcatag cttatttcat tgcaaaaatg gaaaaagatg
15961 tggatatgaa aattatcata gagggtggaa tttttgaatt tttaagccgc gttgtagtta

16021 ctgatatacg ccctgatgta tatcatgaaa tcgtacgtca aaaaaaagcc gaagttaatg
16081 cttgggtttt aagtaaaatc gaacccatga tagaagatat agaagatgga gagtttttaa
16141 agcgttttga agcgtatcta aatggcaata cctatgccaa agaaaggctt atcttaaaag
16201 ctgcatcgta ttttgctaca agatgggaat ttaacatagt ctatcaaacc tcagcttttt
16261 taaatgatat cgatgagatt aaaaacaaag tcgatgaaga attggaagat tattatgaac
16321 tcataggagc tagaaaaatc gctcttaatc aaaaaattgc aaaaatcatc gatttaagcg
16381 gaagacttcg ttttcaaaag cgttgggctc aaaccccacg catacctgaa actgctgttt
16441 taggacatat gcttgtagtg gcgattttgg gatattttta ttcacttaaa atcaaagctt
16501 gcgataaaag gcttgaaaac aatttttatt gtgctttatt tcatgattta cctgaatctt
16561 taacacgcga tatcatcagc cctgtaaaat atggcataga tggacttcac actattatca
16621 atgattatga aatgaaactg atcaatgaca gaattttacc cttcgtgcct gaaaatttaa
16681 gggctgaatt ttcttatatc ttaggcatta gagagggcag aagcaatgaa gcaaattttg
16741 tcaaaaatga atttgaaaat cgcacctata aaaatgccaa aatcgagctt tgtagtggaa
16801 gcttaagctc ttttaatcaa aatgaatttg gagcgattga tggtaaggct ttaaaatact
16861 gcgacaaaat cgctgctttt atcgaagcag gacttagtat aagctatggg gtaaaatcta
16921 aagaacttga aagcggtttt gaagatatgt ttaaattttt taacgaaagc cctacgattg
16981 atggggtaaa tttccttgaa atttgtagag aatttaggga ttattttaaa atttaatctt
17041 attttgatta taaactccaa gagcggaatg aaagaactct ttcattccat atctaaaact
17101 gcacccttac tagcatttgt tacaagtttt tgatacattc taagccatct ggatttgact
17161 tctttttgag gcattacaaa ttcatttttg cgttttgcaa tttcctcatc gcttaaatta
17221 gcattgatag tataagcatc tacatcgatt tctatctcat ctccatcttt taaaagccct
17281 ataagcccgc cctcagcagc ttcaggagaa acatgaccta tgcttaaacc ccttgttgca
17341 ccgctaaatc ttccatcggt aatcaaagcc acatccgcac caagccccat acccatgata
17401 agtgaagttg gacttaacat ctcttgcata cctggaccac cctttggccc ttcataacgg
17461 atcacgcaaa catcgccttt ttgtacttta cctttaatga tacctttaat agcctcatct
17521 tgagaattaa aacaaacagc tttgcccttg aattttctct ctcctgtaat acctgcagtt
17581 tttacaacac agccttgctc agccaaattt ccaaataaaa ccgcaagccc ccctacttta
17641 gaataagcat tatctacctt acgaatgatg gtttcatctt tgattttggc attttttaaa
17701 cgctcttgta aattttctcc tgtgatggtt aaagcatcaa gttctaaaat atgcccttct
17761 ctgcttgaaa tttcagccat tacagcactt acaccccctg ctttatggat atcatccata
17821 taaacagtat taagtgatgg agcgattttt gcaatatgcg caaccttgct tgaaataaaa
17881 tttaaatcct tgatatcaag cgcaactcct gcttctcttg aaatagctag catatgtaag
17941 attgtatttg tgcttccacc cattgccata tctactacca tagcatttct tatagctttt
18001 tgagtgataa tatttttaat tttaaatctt tcatctaaag cgatttcaca aattcttctt
18061 gcagcctttc ttaaaagctc ttctctttct ttgctaagcg ctaaaatcgt accatttccc
18121 tccaaagcta tacccatagc ttcacataaa gtattcatag aatttgcagt aaacataccc
18181 gagcaagatc ctccgcttgg acaagcagaa cattctatat ctttaaattc ttcttcgctg
18241 attttattgg cctcatatgc tcctacagcc tcaaaaactg aactaagact tattttttca
18301 cccttttttag tcacacccga acgcatagga ccaccgctta caaaaatagt cggtacattt
18361 acccttaaag cacccattag catacctggg gtgattttat cgcaatttgg aatacaaatt
18421 aaagcatcta gctgatgtgc attcataacg ctttcaaccg aatttgcgat gatttcacgg
18481 ctcggtaaag aataaagcat accctcatga cccatagcta taccatcatc cacacctata
18541 gtattaaatt caaaaggaat gcaaccattt tttcgaattt catcttttat aatttttgca
18601 tattcattta aaaaataatg ccctggaata atatcaatat agctatttgc cacgcctatg
18661 aaaggtttat caaaatcctc atcttttaaa ccacatgctc taagtaaaga gcggtttggt
18721 gcttttaaat gcccttttt aatcgcatca cttctcacta aaaactcctt aatattttt
18781 taatattata acttaatttt cataaaattt acttgcaaga aacaagcttt ctatacatat
18841 ttttcatcaa ttgacaaaaa tatttttta taatacaatg ctttttgatt atcaaaaagg
18901 agtgcaaaat gagacaatat gaatcctata aatgtccaaa atgcggtaac gaagtagaag
18961 ttcaaaatgt aggcggtgga aaacttagct gttgtggtgt tgagatggag tgtatcactc
19021 aagatttaac tgcggtaaat ttgatgaaag cctttgctgg agaatcaatg gctagaaata
19081 aatacgattt atttgcagat gtagctgaag aagaaggatg gcacgcggtt gcaagacatt
19141 ttagagaagc agcagaaaat gaaaaatggc atgcaagagc tgaatttaaa gcttatcacg
19201 aaatcgttga tggcaaacct ttagaagtaa caactaaaaa cctagttgca gcggctgagg

19261 gtgaaaacta cgaacatacc accatgtatc caaattttgc taaaatcgct gaagatgaag
19321 gtaaaaaagc tatagcaaga ttatttactg cgattggaaa agtagaaata gaacacgaaa
19381 gagaatacct agcccttaaa aaaatgcttg aagaggaaga attctttaat agcgaagtag
19441 aagaactatg ggtttgtgaa gtttgtggac atatccatcg tggcaaaaaa gctcctgcag
19501 cctgtccttt atgtaaagca cctaaagaat actttaagcg tgaattttta ggctaaatcc
19561 ttgggcgatt tttcgccctt tcttagagaa aatttgaaaa ctttatttga agaaaacgaa
19621 cttttaactc catttgatat tgatttagtt ggcattgatg aagcaggacg aggtgcctta
19681 gcaggtccta tgatgatggc agcttgtaaa ctacacaaag atcttgaagg gctttgtgat
19741 tctaaaaaat tgagcgaaaa aagacgcgaa gaactttatg aaatcatctt gcttcattca
19801 gattatctca tccttgcttt ttcttcagaa aaaattgatg aaatagggct tagcgcttgt
19861 ttaaaaatgg gtttaacgct cataaaaaaa cactttaaaa cagaaaataa tttcttatac
19921 gatggcaata caaatttagg cattagcggt ataaaaactc aaatcaaagc cgatgccaat
19981 atcaaacaag taagtgcagc aagtatactt gcaaaggtga gtaaagatag ggtgatgaat
20041 tttttagcta aagattttcc ttgctatgag tttgaaaaaa acaaggctta tgggacaaaa
20101 gcccataagg aattgatagc aaaatttgga atttgcaagc ttcatcgcaa aagttttaaa
20161 cttttatgat tgctttgcta aattctcatg tatgcttaaa gcacactcta taccatcttt
20221 tatagcccaa acgactaaag attgtccttt tctagcatct ccacaagcaa agattttttc
20281 atgtgtggtt tggaatttct ttgttttgac attgtttttc tcatccaaac ttactccaaa
20341 attactagcg atagcatctt cacttccgct aaaacccata gccaaaagca ctaaatccgc
20401 tttatagttt tgattagaat ttggaagttc gacatttata gccttgccct ctttgaattc
20461 gcgttttaaa tcacatgctt ctacgctttc tactttatct ttacctaaga atttttttgt
20521 catttttttga tattctctag gatcttttc atatacagca atggcttctt ctaaaccata
20581 atctgtcgta aagatatcag cttttaaagg ccaaggattg ttttggcttc tttgtaaagg
20641 tctttttgga cttctttcaa aacgcactat ggacttagct ccttgtcttg tagctacagc
20701 gatacaatca acgcttgtat ccccacttcc tataaccaag acatttttac cctttgctgt
20761 gtcagcaccc ttgcctgttt ttagcaaggt ttttgtattt tgcgttaaga aatcaagagc
20821 aaattcaaca ccctttagtt ttctaccctc tatatcaaga tcgataggtt tgctcgctcc
20881 tgtgcaaagc actaaagcat caaattcttt taaaagttta gaaaccttat ctttgctgtc
20941 tatattttca tttaccttaa attctatacc actttctttg agtaaatcaa cgcgtctatc
21001 gactatactt ttatcaagct tcatatcagg aattccatac attaaaagcc cgcccacttt
21061 atcgcttctt tcaaaaacac ttaccttgta gccaagtgaa tttagggtat tagcacaagc
21121 aagtccagca ggcccacttc ctatgatggc gattttttta ccattgtatt gtttaggttt
21181 gtttggactt actaaatttt ctttaaaagc attttctatg atggctagct cgttattttt
21241 gatacttacg ctttttccat taatcgcaca cacacaagaa tcctcacaag gagccggaca
21301 aacacggccg gtaaattcag gaaaaggatt ggtaagatca agtctttcat aagcttcttt
21361 ccataaagaa cgataaataa gatcattcca ttcagggatg agattgttta aaggacagcc
21421 tacatccttg ccttcactca tcacacccgt atggcaaaat gctaccccac aatccataca
21481 tcgtccgccc tgaatttctt gttctttctt gtctaaaagc gttgtaaatt ctttgaaatt
21541 taaaactctt tctttaggag caactttttt aaaatctacc cttttaaaat ctaaaaaacc
21601 tcttgcattt cccattttta ctccttattt ttgtgtgatt ttcaaaaatg ctgccaaact
21661 tggatcttct tcatctttgc aaagctcgat catctctaac attttttcat aatctcttgg
21721 cataacttta aaaaagtctt ttttgtcaaa tttttcgagt atatccttag ccttttttaga
21781 attagtataa gcgatatgat tttctatcat cgtttttaat tctttttcat ctttagcgct
21841 aagatcttta atatccacaa gctcggtatt tacatgagct tcattgtgtc ttccaaagat
21901 ataaaccaca cccccgctca tacctgcagc aaagttcgca cccacatcgc caagcactac
21961 gacaagcccc cctgtcatat actcacaacc atgcacaccg gttcctaaaa ctaccgcttt
22021 agctcctgaa tttcttacac aaaatctttc tcctgctata ccatctaaat acacttcacc
22081 ctcggttgct ccatacaaac aagcatttcc tgcgatgata ttttcttcag gtgagaaagt
22141 agcttcattt gaaattttag cgatgatctt acctccgctt aaacccttac ctagataatc
22201 attgctatca cctacgatct caagcttaat accctttaac aaaaatgctc caaagctatt
22261 acctgcattt cctatggctt tgatttgtat gctatcttca cttaaagcgt ctttgccata
22321 agtttttaga atttcacttg aaagcatagt agcaaaagta cgactttgat ttcctacttc
22381 taaagagagt ttgataggct ctttttctc cacagcattt ttacaaagtg ggagtaaaat
22441 tctataatca atcgtttttt caagtttatt gtctttatag tctttaaaat gcacagcggt

22501 tctgttatag gtaggtaagg attttaaaat tttatccaaa ttcactttac ctgctttacc
22561 ttgcataggt ttttgacgga gtttatcgac tcttcctatc atatcatcaa ggcgttcaaa
22621 accaagtctt gccatatatt ctctaagctc ttcagctata aaatacatga aattaaccac
22681 atcatctact ttgcctttaa aacgatctct aagttcttta tcttgagtgg caataccaaa
22741 aggacaagta ttaagatgac aaactctcat catcgtacag cccataacaa tcaaaggcgc
22801 agtagcaaag ccaaattctt cagctcctaa aagtgcagct atagctaaat cgcgtccatt
22861 catcagcttt ccatcggttt ctaatcttac tctatctcta agcttgttta agatcaaagt
22921 ttgatgcgtt tcagctaagc ctaactccca aggaattccc gcatgtggta tagaagttct
22981 agggcttgct cctgttcctc catcataacc tgaaacaagg attaaattcg ctcctgcttt
23041 tgccacacct gcagcaactg ttcctatacc gttttcactt acaagtttta ctgaaatttt
23101 ggcgtctttg ttcgcatttt ttaaatcata aatgagttga gccaaatcct caatagaata
23161 aatatcatga tgaggtggtg gagaaatcag cgtcacaccc gctgtagaat gtctagcctt
23221 agctatccaa ggataaactt taaagcccat taattgtccg ccctcacctg gttttgcacc
23281 ttgagcgact ttgatttgta attcttttgc gtgggttaaa taatttaaat ccaccccaaa
23341 acgcccactt gctacttgtt tgatagcgga atttttattg actccatctt tgatttcata
23401 gcgttcctca tcttcaccgc cctcgcctga atttgatttt ccgcctattt tattcatcgc
23461 ttctgctaag cactcatgag cttctttaga aatagaacca taactcatag cacctgttct
23521 aaaacgctta actatacttt cagcactttc gaccttgtca atactgatag cctcactaaa
23581 atcaaattcc attaaagaac gcaaattcac ttgtttttca tcaactaagg cagagtattt
23641 tttaaagctt ttatagtctt tattgcgaca agcttgctga agattaaaaa tcacaagtgg
23701 atcgattaag tgttcttctt tagcacttct aaagctgtga attcctttag aatctaaagc
23761 cttatgtgta tcattaaaag cgtgtttatg taaagcgata agttcttttt caaaatcttc
23821 taaatccata ccctcaatgc gtgaagttgt agaagtaaag tatttatcaa tcactttaga
23881 acttaagccc aaacactcaa aaagagcaga gccattataa ctttgtaaag tagaaactcc
23941 cattttagaa gcaattttta ctataccact tgaacttgct ttgatgaaat tttctaccgc
24001 tttatcataa cttacattta agtctttatt ttctataagt ttttgtatgc tttcataaac
24061 caaataagga tttataactg ttgcaccata gcctaaaaga caagcaaaat gatgaatttc
24121 tcttggctca ccactttcta tgataatgct tgtgtgagtt cttaaattct ttcttaccaa
24181 ataattatgc acgcccgaaa ccgcaagtaa agcagggata taagcatttt tatcatccac
24241 ccctttatcg cttaagatga tgatagaaac accttttta atctcttctt ctatttttac
24301 acaaagatta tctaaagctt gttctaaagt aactttagaa tactcataaa gtatagaaaa
24361 ttctttaact tggaaattgt ttaaagcctt aacctcgaaa agctcttcat tgcttataat
24421 aggcaaagaa attttaacac gctttgcatt attttcatcc ggttttagca aattcccttc
24481 acttcctaaa tatatccttg tagaagtaac aatctcttct cttatcgcat ctaaaggtgg
24541 attagtcact tgagcaaaaa gttgtttaaa ataattatac aaaggctgat aagtcttaga
24601 aagaatagcc aaaggagtat ccacacccat agccgctaaa gcttctttgc cattttgtgc
24661 catgcttgct acactcattt taagctcatc atagctccat ccaaaagctt tttgaagttt
24721 taaaacctca tcttctttta aaaatttatg tttataaacc ccgctgtgtt gtttttcaag
24781 ctctactaaa tttttaagcc attgtttata aggcttcgca ttagcataat gctctttgat
24841 ttcattatcg gctattaccc tacctcttgc agtatctaca agtaaaagtt ttccaggttc
24901 taagcgtttt ttagctttga tattgttttc atcaagtttt aaagctcctg tttcgcttga
24961 aagtatgagc atatcatctt ttgtaagata atatcttgaa ggacgaaaac catttctatc
25021 taaactcgcc cccatgataa ccccatcagt aaaaactata gcagcaggtc catcccaagg
25081 ctccatcaat aaagaatgat actcataaaa agcacgcttt ttacttccca tattttcatt
25141 tttatgccat ggttctggca ccatcatcat aaaagcttct tctaaagtgc gtccatttaa
25201 agctaaaaat tctaaagtat tatcaaacat agcagaatcg ctacttggtt ttgctatgat
25261 aggaaaaatt tcatctaaat tttcaaaata ttcgctctcc atcaaacctt ctctagctct
25321 tatgctatcg acatttcctc gtatggtgtt aatctctcca ttgtgtacca tatagcgatt
25381 tggatgagct ctttcccagc ttgggaaggt attagtcgaa aagcgagaat gcaccaaagc
25441 aatggcggat ttcatattga catctttaaa atcaagataa aaatcgctca attgagtaga
25501 aagaagcatt cctttataaa ctatagtgcg tgaagaaaag cttgaaaaat aaaatttagg
25561 cacatgcaaa gctctttttt ctataagacg acgacaagca taaagcactc tttcaaattc
25621 aagtcctgca ttaaccgtgc ttggtttttt tacaaaagct tgtaaaaaat aaggcatagc
25681 ctttaaagca cttgctccta tatcactagg attaaaaggc acctctctaa aacctagaaa

25741 ttctaaattt ttatctttta aaccttgtaa aaatatttct tttgcttctt ctttggcatc
25801 tgcattaggt gataaaaaca tttgcgctac agcataatcg cctttttgag gaagctcaaa
25861 accaagctct tgagtcataa aaaaatcatg aggaatctga atcaaaattc ccgctccatc
25921 gcctgaattt tcttcagctc ccgcaccgcc tctgtgttca agattcatca aaatttctaa
25981 agcgtcacaa ataaccttat aagaagcaat accgcgtata ttggcaactg cggcaatacc
26041 acaggcatcg tgttcatttt tagggtgata taaacctacg ctttgattgt tttctaagat
26101 attttccaaa tccattttat tctccttata agattgattt taaatttgat agcttgttaa
26161 ggattataac atatattttt ttaatatcga gatattttt atcttaaaaa taaataaatt
26221 aaaaaatatt tcaatggatt tataaaaatt ataaaaacat cgctatgata cttcctaaaa
26281 tcagcaaagg cccattataa aaaataaaag tagggataca agtatctttt atatgatcgt
26341 gttgcttatc tacacttaat cccacactcg ttcccatcgt cgtttcactt gctggacttc
26401 ctgcatctcc taaagctcct gcaacccta agataaatat gataagagct gcgctaaaac
26461 caagctctaa gcaaagagga caaaaaagtg tagctatgat aggaatagtc ccaaaagaag
26521 tacctatacc catagtaata ataagaccta ttaaaagcat taaaaataca gccaaaaatt
26581 tattttcttg catccaaggg actacggaat ttacaagctc atttacagca ccactttgtt
26641 ttaaaacctc tccataacct gccgctacaa gcataacaaa agcgataaaa cccattagtt
26701 tgagtccatc atcaaaaata tcattgacac ttttatattc aactccaccc aaaatcacca
26761 tagaaataaa acccaaaagc cctgataaag gcaaattcat cgtaaaaatt tgtaaaacta
26821 aggtcaaaat aagccctact aaaacacccc attctttacg tcccattttg atattgtcaa
26881 aatcaatttt ttgaatttct atttcttgat attctcttgg ttttctataa aatacaaaaa
26941 gtgctaaaaa aagccccaat accatacaaa tagcagcata atacatcgca tttgttacat
27001 ctactaagct gacattaacc ccatttttct ctaaattttc tcttaaaaga tcttggaaat
27061 taagtccaaa acctatgggt aaaaccatat aaggtgttgt aagtccaaag gttaatgcac
27121 aagcaacagc cctacgatcg atttttaatt tattaaaaag acttaaaagt ggtgggatta
27181 acaaaggaat aaacgccaca tggataggaa tcaaattctg cgaaaaacaa gaaatcaaag
27241 ctatagataa aagcaaaaga tattttttgt gagagataaa acgactgact accttaatca
27301 aataagctgt taaatttgtc cttgaaatcg cagcagcaat agcacctaaa agtatataac
27361 ttaaagcggt ttttaaattt cccttcattc catcgattaa aatactcata gttttcataa
27421 tctcaatatg agaaaaaatt ccagcaacta aagcagaaat caaaagactc aaaagtacat
27481 taaaacgaaa taaacaaagt aaagtcatca aaactacact gatgatgata ggatttgtaa
27541 gcagggtcaa aatttttcct tgtgaattta tatgtgaatt tataaaagct taattctaaa
27601 gaaaattttt ttaaaatgag ataaaaatta tcaatgaatt aatatgtaaa ttttagtaac
27661 attaataaat atatgttact ttttttcata tttaagataa aaattctatt attacacaaa
27721 tttttaaaat aaaaagccta aatttattga aatttttgtt atattgaatc ataaacattg
27781 aaaggagtac ccatgaattt aaatgcggag caaaaaaatg ctgttttgag atttaaaaaa
27841 tttgtttctt ttagaaacaa aatatctctt aacctatctt tgatagtgct tatttgttac
27901 tatatctttg ttctaggtat aggacttatg cctgaaattt tagggtataa actgggtcct
27961 agttccatca cgcttggtat tatggtaggc attggcctga ttttattgtg tattatttcc
28021 acaggaattt acacttttat cgcaaattat tttcttgata aagagcaaga agaagtgatt
28081 aaaaacttag aaaatgaggg attacttgat gcgttaaaag atggaaaaat caattataag
28141 gagctagtat gaaagtcttt atattttaa gtttaatctt caatttagct ttaagcgccg
28201 gttttgattt aggagaagtt catcaaaaac ctataaatat aactgctgtt agtatgtttt
28261 tagtctttgt tttagctact ttgtttatca cctattattc taacaaaaaa tccaaatcca
28321 aaagtggatt tttataccgcc ggaggaaata tcacaggaat gcaaaatggt gtagctatag
28381 cgggagatta tatgagtgca gcaagctttc ttggaatcac cgctttagtt tttactaatg
28441 gctttgatgg acttatttat tctataggat ttttagtagg ttggcctatt attttatttt
28501 taattgcaga aaaatttaga aatcttggca aatttacctt tgcagatatt accgcttatc
28561 gtttagaagc taagcctata cgcgttatat cggctatttc tgctttaagt gttattatct
28621 tttatcttat tgctcaaatg gtaggagcag ggcaactcat acaattgctt tttggtttgc
28681 cttatacctt tgctgttgtt ttggttggaa ttttaatgat ttgttatgta acttttggag
28741 gaatgcatgc aaccacttgg gtgcaaatca tcaaagccat tttacttcta gcaggggcta
28801 cttttatggc tgttatgatt ttatatctta cgaaatttga tttaaaatat tattttgatt
28861 tagccatttc tcatcatcca aaaggtgaaa atataatgaa accagggact tttttacctg
28921 ataccatatc agctctttct ttaggtttgg ctcttatgtt tggaactgca ggtttacctc

28981 atatacttat gcgatttttt actgtaaaag atgctaaaga agctagaaag tcagtatttt
29041 atgcaacagg atttataggg tatttttata tacttacttt cattataggc tttggtgcca
29101 tagcactctt acttggcaat cctcaattta ccaatgccga tggaagtttt aatggggcta
29161 gcaatatgat agcagtgacc ttagctgatg ttttgggtgg agatgtcttt ttaggattta
29221 tttcagcagt ggcttttgct actattttgg cagtagttgc aggacttgcg atttcaggtg
29281 caggagctat aagtcatgat ttatatgtca atgttatcaa aaagggtgat gtcaaacatg
29341 aagatgaaat gagagttaca aaaattgcaa ctatagctat aggaattttt gctattttgc
29401 taggcattgt ctttgaaaaa caaaatgttg cttttaccgt aggacttgct tttgctattg
29461 ctgcgagtgt aaatttttcct atactttttac tctgtattta ttggaaaaat cttaccacta
29521 aaggtgcttt ttggggaggg ctaataggac ttatagtcgt tcttgctctt gtgattttaa
29581 gccctagtat atgggtaaaa agttttggtt ttagtgaggc tattttttcct tatgatcatc
29641 ctgctttatt ttcaatgcct ttaagtttta tactcattta tatcatttca aaattagata
29701 attctaaaag agctaaaaaa gataaagaag gttttgaagc gcaagatttt agagctcaaa
29761 gcggtgtggg agtgagtgaa gctgtggcgc attaaacaaa aaaagcttct gtgcaattta
29821 gcaaagaagc tttttaaat ttaaaccagt aaatattctc atttttaaaa gtattttagg
29881 actttactta acataaagtc ctttagagtt catcttttat ttataatttc tacctaaata
29941 ctcacctaaa tactgcaatt ctttatcatt gatttcagtt ttgcggaaaa atggcatcga
30001 atgtttacca taacgcacaa aaatcattac caaatcaggg ctcaaatccg ttctatcttc
30061 taaaagtgca ggaattcctt gatctttata aagagctgaa agtgcatttg tcccaggcat
30121 tccttgacca tgacatgcta tacaccactt actataaact tgctgtcctt ttctttgatt
30181 tgcatttaat tttttagcat caacgcttga aaacgctgca ggggatcttg gattgtttgg
30241 aatatattca taaacactta tagattcacc ctttggcaac cattctttat ccttagcatc
30301 taaattcagc aagcaaacaa ataaaacaaa aaataaagcg atttttttca tttttcttta
30361 ccttctttca ttcttaatgc gtcgcgtttg ttattgacaa aacgctcatc tcttaaattt
30421 ttaggccaaa tgcctccacg accaggtgct aaaattccat ttggatcgat acaatcttta
30481 agctgctcgt taaagcggcg taaaatatga ttgttataag aataagcacc cattacatca
30541 tcttggaaag tcggagccgc acgataatct ccccatccat gttctgcagc agtttgaacc
30601 atttgacgat acattttacg cacttttaga ttgtgagctt tatccgttct tgagttatta
30661 aaggctaaca taaagcaaaa tgcacgatac atccaacttc taggatgtga aaaaggtgtt
30721 ataggagatt cttccccaaa ttctctaaat aattccatat acacttcttg gcattttaaa
30781 agctctttac catcgcgcgg aataataggt gagaaccata catgcccatc acttggctct
30841 aaagcctcac ctcttgtgct aagccagaaa atttccatat ttggaattcc aagagtaacc
30901 ttatgtttaa gctgcttttt ttgctccaaa ttcaaaggta agctaaattc ttgaacgata
30961 gaaatatcta caccctcaat gatcttaaat ctttgtttga tatattctaa attcgcatag
31021 cacacttctt tacagccata aactgaaaca tcgatattcc aataagggat tttattttttt
31081 aaagcataat tttgaatttt atccatatca ggtaagccat ttttacttgt aagatagcct
31141 tttaactccg cattcatcgg ttttccataa ggtggattta atggactaaa atactgcggc
31201 catcccacaa taaaactatc ctcaagataa tttaaaattt caaccgctgg gatcaaatcc
31261 tcgcgttttt tcatttttat agataaaagc atataatgtt ctggttttgg catcatccaa
31321 aagcccattt ttgtaacgat accgaagtta gattgtgaaa aaagtccatc tacataagga
31381 ccatagccgt atttattttc tgcaaaagtc ttagcttttg gtaaagcacc catacccgtg
31441 cgtaaaattt ctccattagc taaaacaact tccattccac aatgtgagcc aaagtgatca
31501 cgatacattc catagctgta tccccaacca tgatctaaag cgtttccaag tggagaaccc
31561 caacctggat caggaatatc taaaaaaaca ttgagattat ttttttctac atactcataa
31621 agatcaaaat aactcactcc aggttcaacg atacagaaat ttcttttatc atctacttcg
31681 atgattttat tcattctttt taaatctaca acaacctgcc ctctttgctg tggtgcagaa
31741 gaaccatagc ctaaattctt acctgtggaa ataggaaaaa gaggaatttt aaattcattt
31801 gctattttta ctatagcttg aacttcttct acatttttag gtgcaagtgc taaagaaggg
31861 ataggttcgt catcatcatc ccattgcggg ctatacgcat ctctataaag atctaaatcc
31921 tcttgggttt taaataccca ttctttaccc aaagcttttt caaattttgc tactgcagct
31981 gtaaaatcac tttgcgaaac attttttggt aaaatcataa tttaactcct tttggtgcta
32041 aaatccaagt ataaaaatca ccttgttttt tttcatacgc aacgcgcata ccataatccg
32101 acgccatagt gcgaatcaca aaaaaagttg tcccactact cacacttcca ataagtgctt
32161 tattttcaaa agcttcttta agttttgagt aaatattgct gatatctttt tcaacaagta

32221 aaatttcatc tgcgttaata aaattttctt gataaggaat ttcactatcc ttgataaaat
32281 atttcacttg tttagcttga gaaaattcca aaaaagaagc attcaaacca gaaagacaaa
32341 cagcacttaa agccgtagct tttaaaaaat ttctacgcga aacttctaac atacatcaat
32401 cctttttaagt atttagtatt acaaattata aatctaaaaa cttaaaaaat atttaaaaaa
32461 taatttgagt aaaaataaaa tatgaattat tgtaataaag tgtgaaattt ttaaacaaaa
32521 agatagaatt tagtgtcaaa tttaaatctg acactaaaaa attttacaca tctaaatgtt
32581 ttacatcttt agcatgtgtt tggatataat cgcgtcttgg ttcaacctca tcgcccatga
32641 agagattaaa ggtatcatta gcactttgtg catcttcgat agtgattttt aaaagtcttc
32701 ttacgcttgg atccatagtt gtttcccaaa gttgttcagg attcatttca cccaaacctt
32761 tatagcgttg gatataagca cctttttag cattttttc tacttcatct aagatatcta
32821 agatatcttt ttcaaagctc aaatcacgat cttttatttt attaaagata taatttgctt
32881 cttcataaag agggtgtgaa aataaatcat cattgatgat aagttcttcc aaaccatttt
32941 cagtttgcac ataaatgcga atttcgtttt cattgatata atgatttaaa atattatgcc
33001 cttcttttttc caaaaaagct ttaacgattt taaaaagttc ttcattattt tctttgataa
33061 agtcaggatt ttcgatcaag tatctgatca cagaaatcac attgaaacgc tttttaagct
33121 catttaatac cgagcgataa gcggcaacga tttttagaaa atcttttaaa tcatttaagc
33181 caattccttc ataattagaa ctttcaattc ctgtttcgat tagataatca tttaaagctt
33241 tttcatcttt gagataaatt tctttctttt gaccttttt atagcggtaa agcggtggtt
33301 gggcaagata aatgtgtcca ttagccacaa gttcattcat aaaacggaag aagaaagtta
33361 aaagtagagt ttgtatgtga gaaccatcta catcggcatc tgtcatgata atgattttat
33421 gatatcttag ttttgaaaga tcaaaatcct cacctatacc acaaccaaaa gcagtaatca
33481 tattttgaat ttgttcggat tttaaaattt tatcaagtct tgctttttca acatttagaa
33541 ttttaccacg caaaggcaaa atcgcttgaa aagatctttc tcttccttgt tttgcagaac
33601 cgcccgcaga atccccttcc acaagataaa tttcactctc acttggatct ttactttgac
33661 aatcagctaa tttcccaggc aaagttccta cactcaagct ttctttttta cgcgttaatt
33721 ctcttgcttt ttttgctgct tctcttcctc tagctgccat taaagcttta ttcataatag
33781 cttttgcttc gataggattt tcttcaaaat atttactaag atattcaaaa cttgcttttg
33841 aaactatagg acgcacataa gaagagccaa gttttccttt agtttgtcct tcaaattgag
33901 gttcaggcac ttttacactc actatagcga taagtccttc acgcacatca tcacctgtga
33961 ttttagagtc tttttctcta gcactagcat ttgcttctat atagttgcta atcactcttg
34021 ttaaacccat tctaaatcca gcttcatgcg ttccgccatc aggggttttg atattgttta
34081 caaaagaaag taagttttcg ctataagtat cattataaag taaagccact tctacattta
34141 catcttcttc atctacgcca aaaaatatag gtttagttaa agcttctttt ttatttaaat
34201 cgctaacaaa ttgtgaaatt ccaccttcaa aatgaaaact ttcactttta cctacacgat
34261 tatctttaaa atttatagtg atttttggat ttaaatatgc aagttcacga aatcttttag
34321 ccaaaatatc ataatcaaat tctgttactt caaaaattgt atcatcaggc caaaattcta
34381 tggtagttcc tgttttttttg ctctttccta taacaccaaa ttcactgatg actttacctt
34441 ctgaaaattc ttggcgataa atttctccat ttctttcaac cgtagctaca agttttttag
34501 aaagtgcatt tacgactgaa acgcccacac catgcaaacc ccctgaaact ttataagtat
34561 ctttatcgaa ttttcccccт gcatgaagaa cagttaaaac aacagttaag gttggcatat
34621 tttcagttgg gtgcatatcc acaggaattc cacgaccatt atcactaacg atacagcttc
34681 cttcggtagt gatttctata tctatagtat cacaatgtcc tgccattgct tcatcgatag
34741 aattatctac tacttcataa atcatatgat gaagtccgcc tatattggta tctcctatat
34801 acatacctgg gcgttttcta acagcttcta agccttttaa aactttaata ttactcgcac
34861 cataattttc ttgcataatt ttcctttgtc ttttgtctaa agattttaaa tttttataaa
34921 atcataggca tgataaccat agataagcct tgagaattta ccacaaaggc taagttaggt
34981 tcatttacac ttaaagtgaa ttgttcttct tcgattgaag ttaagaaatc aagtaggtat
35041 tttatcttga tattaagatt aaattcttca cttactcctg tttcgatttc aagttcagtt
35101 tttgcttcca tattgtctaa gcttatacct tcaaagatga ttttatcttt gttgaaatga
35161 agtttcattt tttcagttac aacactgatt tttttaagac tatctataaa atcttcagtt
35221 gaaaatttaa gttcttgttt gaaagttttt gggataactt tttcataatc agggaattta
35281 tcattgataa gttttgtaaa gaattcaaaa ttatcatttt tagcgataag catattttca
35341 tcataataaa tttcgatttt ctcataaaaa agtttttgca tttccatgat agcttttttta
35401 ggaatgctga tgtgaaattc ttgattattt gctttttcaa gagtataaac tgcaaggcgt

35461 cttgtatcgg ttccaacgaa atttattcta tctgttttta tgtctaaaaa tgcaccattt
35521 agagaatatt tagggttgtt agtatcaatg cttggtagaa tttttttaag agatctactt
35581 aaatcacttg aatcaatatc aaattgattt ttgccttctg ttttagggaa atttggaaaa
35641 tcttcatgat taaacatagg aagtttgtat tttgtacttt tttgtcttat aaataaaaaa
35701 ttatctatgg tttctaatac cacttcttca ttgtttaaac tttttatcac atcggctatg
35761 cttttcgcat ttgcagtagc aaaacctccg ctttctacac gaatttttct tattttatag
35821 tttatcccta tttcaaaatc actcgctctg atgataagtt tatcttcatc agcttcaaaa
35881 agtaaatgtg aagtgatagt gctagaatct ttttttcta cataggcatt acataaaagt
35941 accgctgatt caagggtatt tttattgata cttagtttca ttttttctcc ttggctttat
36001 tatatataat ttaaattcat tgtaataata agactattga aattgtgaaa ttctttagca
36061 aaaaattatt aaatttgtat tttaccatat tttgcttaat tatattttttt cacattttgc
36121 ttttcttttt ttcacatttt ttcacttaac tttgactttt tgtaagaatt ttgtttttta
36181 attcttcgat ttttgttttt atttgttcgt catcttccat gagttctttt atttttttca
36241 cactatgtga aatagctgtg tgatctttca ttacaaagaa attagcaagt tgtgaaaaag
36301 taaggcttgt aagctccctt gctaggtaga ttgctatgcg tcttgcagta acgacatttt
36361 gagttttttt gctagatttc acatcgcttg gcttgatgtt aaattcttta cagatcaaag
36421 ataaaatatc ttctatgctt atattttctt tcttttcttt gatatgatct ttcatcacgc
36481 tttttgcaag ttcaagagtg atttcttgtc cgagtatgtt agcataggca tttaagctta
36541 tgatgatacc ttctatttct cttatattat cccctaaaga agtggcgata taattgatga
36601 tatcattaga aagattgata tcgttaaatt cacatttttt gcgtatgata gctatttttg
36661 tatcgagttg aggcggagtg atatctgcta tgataccatg agcaaaacga ctttttaagc
36721 gttcggtgat acctttttagc atattgggtg gattatcgct tgtcatgatg atttgcccgt
36781 cattgttttt aatctcatta aatataaaga aaaattcttc ttgaattttg tctgtttttc
36841 ctaaaaattg cacatcatct atgagtaaaa catcacaatt tctatatttt tcgtgaaatt
36901 tatctaaaga gccatttttt aaatttgaag taaaatcatt gataaaattt tcactcgtag
36961 cataaatcac tttttttccc atttccaaac ttgcatttcc cacagcttga agcaaatgtg
37021 ttttgccaag tcctgtaggc ccatagataa agataggatt gtaaagtttt ccaagtttgt
37081 ctttttgtga gatagcttta caagctccat aagcgtattt gttagaatcc cccactacaa
37141 aactttcaaa agtaaaagaa ggatttaaaa tcgtgctttg tgctttgata tgagcgatat
37201 cgattttagt gcttttgcca ctatgctttt ggctttgtgc ttgtatcaag acgcttgctt
37261 tatttccact ttgcacttca taaaaatgtg aaattttttt accatatttt gtttgtatga
37321 atttggctaa aagctcatta ggagcgttaa agactagaaa atctgctttg ctttgttttt
37381 cgttaaattt taagatagcg atataatttt cgtattcgtt ttcattgagt tcttttttta
37441 aattttcaag tatttgattt ggattcataa tctctcttta gtctttgtaa gcttataaat
37501 tttatcttaa attttataaa aaaaggttaa aatttatttt ttgcatttca ttaaatacaa
37561 aggaaaaatt tgaaaatttt aggtatagat cctggttcaa gaaattgtgg ttatgctatc
37621 atagagtcaa ataagggtaa aaatacttta atcgaagcag gacttataaa gatacagcct
37681 agcactttac aataccaaat cacagagctt tgcgagggtt tggatttgat ttttaaaaac
37741 cattcttttg atgaggtagc catagaggat atcttttttg cttataatcc aaaaaccgtt
37801 ttaaaactcg cccaatttcg tggagcccta tccttaaaaa ttcttcaaat tcacggagat
37861 tttgcagaat acacccccctt acaagtaaaa aaagccgtta caggcaaggc aaaagcgact
37921 aaagaacaag tagcctttat ggtaaaaaga cttttaggac tgagtaaaga tatcaagcct
37981 ttagatatca ctgatgccat agcagttgct ttaacgcacg cagcaaattt aagagttagg
38041 gtatgatttt attacacaaa agccccaaac tccataaatt ctcaaaatac ctttaaattt
38101 tttctatatt tttaaaaaat ttcataaaaa atttctaaag ttggaacaga acttgcttat
38161 aaactctaca taaacgcaaa aagtttttcg aaacttcaag cgtcatttta aaaaagcaat
38221 attttataaa ggatttaaaa tgatgagatc actttggtct ggcgtaagcg gacttcaagc
38281 acaccaagtt gcaatggatg ttgagggtaa taacatttca aatgttaata ctaacggttt
38341 taaatattct cgtgcagatt ttggaacaat gttttctcaa accgttaaaa tcgctactac
38401 tccaactgat ggaagaggag ggcaaaaccc acttcaaatc ggacttggag tttcggtgag
38461 ttctacaaca agaattcatt ctcaaggttc ggttcaaact accaatagta atactgatgt
38521 agcgatcaat ggcgatggct tttttatggt aagtgatgat ggcggactta caaattatct
38581 tactcgcgat ggagctttca aactcgatgc ttatggtaac tttgtaaata actcaggctt
38641 tatcgttcaa gggtggaata tcaattggga tgatcaaacc atagacagct caagaagccc

38701 acaaaatatc tttatcgatc caggtatgca tatccctgca gcaaaatcca ctgaagtagc
38761 tatcaaagca aatttaaata gtggtttaaa tataggaact tcaagtagaa atctttatgc
38821 attggattca gttcatggtt acaataataa aactcaaaga gctgaagatg aaaacgatac
38881 aggaacaact caattttata ccacttctaa aaattctgtt gaagttacag aaaaaggtgt
38941 ggatgcaggc tcacttttca atgcaagcgg aacagggctt aatctaagag atggacaagg
39001 aatttgggtt tcttatgcgg atgcgaagta ttcgaccaat aaagtaggtg ctagtgcttt
39061 tgatgcaaat ttacaagcaa atcaaactgc agctttttgg ggaagtgcag atcaaagagt
39121 aaatttagat ataactttaa atggtgtaaa aattcaaaat gctgaaattc aaaacataga
39181 tgatgctatc gcttatatca ataccttcac cgctccaacc gacacaagag atggaacagg
39241 tgtaagagca gttaaaagtg cagatggctc gggtatagaa tttgtcaatg ataattcaga
39301 tggcactaca gataatatga aaaatattaa tcttatagtt tcagatgcaa atacagcagg
39361 ggagctttgg aatgctactt ggaatgcagg tacaagcagt tttacatttg atcaaaataa
39421 tattaataaa aataatactt ctttgtggac agctgataca atggcaccta atccaccaaa
39481 tggcaatgta acattaacag gaacagatag ggtagaagtt atcactgctc ataaatatat
39541 ttatagttca aatcctgtag atataggccc tatgacaaat cctgatggag gcccagcatt
39601 tcaacctggt gctaatgaaa ctacaagacc aaccgatcct gcttcagcag cttattggga
39661 tgctatgaat ggcggacttt taaataccaa tgcaagaacc tttagaacca cagaggattt
39721 aagagaactt ttacaaagag atgctagata tggggtggat tatgatggaa gtggatcttt
39781 tgcagcagca gatgtcaatc aagctgtaaa ggttgtcgta acagctaatg gaaattttgc
39841 tatttctaac gctaaagaag attcatcagt accgccaaat gctataaatg gtgtagctga
39901 tgctactaca acaactccaa aagcaatgag ttttaatatc actgcttatt ctaataaagc
39961 tggcacagta agtactaacg atgcttttac agctatcttt aaagcttggg atggtccttt
40021 aacagtgggt ggttcgatca aagaaagtga gcaacttaag ctttctgctt tttcagcagg
40081 acttgaaatt tttgactctt taggctctaa acatacttta gaagtgcaat ttgtcaagca
40141 aaataccact caagatgggg gtaatgaatg gcaaatgatc atccgcgttc cagagcctgc
40201 agagatcaac accacaggcg aaggcccaaa taatatcatc gtaggaacag caagatttaa
40261 caatgatggt tctttggcaa gctatacacc aagaacgatt aatttctcgc caaataacgg
40321 tgctgcgcca aatcaacaaa tcaagctttc ttttggaaca agtggaagta atgacgggct
40381 tgtaagctca aattcagctt caactctaac aggacaagca actgatggtt atgcttcagg
40441 aaacttaaag cctgatgcta tacgcgtaga tgaaaaagga aatcttttag gtgaatttac
40501 caatggtaaa acctttgcag tagctaaaat cgcaatggct tcagtagcca ataactcagg
40561 tcttgaagaa ataggtggaa atcttttcaa agttactgca aatagtggta atatagtagt
40621 aggcgaagca ggaacagggg gtcgtggtga gatgaaaacc tcagcgcttg aaatgtcaaa
40681 tgtggattta agtcgtgctt taacagagct tatcattata caaagaggtt atcaagcaaa
40741 ctcaaaaacc atttcaacaa gcgatcaaat gcttcaaacc ttaatccagc ttaagcaata
40801 attatcttaa gctttttaga ttctttataa aatttcccgc cttgtgcggg atacaacctt
40861 gctttacaag cctatcttgg tagccttgta atactaggtt gggcttgaac tgccttcaat
40921 ttattgctaa ctcgctcttt ttaagagtga gttgcaataa aattttaagc aatgtgatat
40981 aaatttttta aaatttaatt ttatttttat tttttaaaga taaaataatc aataattttt
41041 tagaaaggag aaaaaatgtc ttatattagt tttaatttaa tgctttcttc tttctgtatg
41101 cgtgcttggc acgcttaatc tactcgtata tactcatatt ctataaattt acatttccaa
41161 acaatctagt ttaaagccga taaaaggctt tttagtattt ttttaattaa caaaaaagga
41221 taaaaaatga attttactaa agaaacttta gcattacacg gagcttataa ttttgacact
41281 caaagaagta ttagtgtgcc tatatatcaa aacactgcat ataattttga aaatttagat
41341 caagctgcag cgagatttaa tcttcaagaa cttggcaata tttactcaag gataggtaat
41401 cctacaagcg atgttttagg acaaagactt gctaatgtcg agggtggggc ttttggaatt
41461 cctgtttcta gtggtatggc agcttgtttt tacgcacttg tgaatttggc aagttcgggg
41521 gataatgtcg cgtattcaaa caaaatttat ggtggaactc aaaccttgat ttctcataca
41581 cttaaaaatt tcggtataga agctagggaa tttgatatag atgatttgga tagcttagaa
41641 aaagttatag atcaaaacac aaaagcgatt tttttcgaaa gtctttcaaa tcctcaaatt
41701 gccatagctg atatagaaaa aataacccaa atagcaaaaa aacataaaat cgctaccatt
41761 tgtgataata ccgttgctac tcctttttta ctccaacctt ttaaatatgg tgtagatata
41821 atcgtgcata gtttaagtaa atatgtaagc ggtcaaggca gtgctttagg tggcgcactc
41881 atagaaagaa aagatttaaa tgacttgctt aaaaataacg atagatataa agcttttaac

41941 actcctgatc caagttatca tggactgaat ttaaatacac ttgatttgcc aatttttagc
42001 attagaatca tcatcacttg gcttagagat ttaggagcta gcttagcgcc tcaaaatgct
42061 tggttgcttt tacaaggact tgaaacttta gcagtgcgta tagaaaaaca cagccaaaat
42121 gctgaaaaag ttgcgaattt tttaaattct catcctgata tcaagggtgt aaattatcct
42181 actttaacaa gcaatgctta tcataattta tttaaaaaat attttgataa aaattttgct
42241 agtggacttt taagctttga agctagagat tatgagcatg ctagaagaat ttgtgataaa
42301 actcaacttt tcttacttgc tgcaaattta ggtgatagta aatctttgat catccatcct
42361 gcttctacta ctcattcgca actaagcgaa gaagaacttc aaaaagcagg cattaagaaa
42421 accactgtgc gtttaagcat aggacttgaa aatagcgatg atttgatagc ggatttaaaa
42481 caagctatag aaagttaaag agtgtattta tgttaagtgg aattatagtt ggtttgttat
42541 taggctttgt tttgcaaagg ggacgatttt gcgttgtagg agcttataga gatgtatttt
42601 taagtaaaaa atttacaatt tttatagcac tttttatcgt cgttgcattg caaagcattg
42661 gtgtttgggc tcttcattct ttaggatata tttcgattaa gcctcaagaa ttttattggc
42721 tttcaacgat tattggtggg attatttttg gttttggtat ggtaattgca ggtggctgtg
42781 ccacaggaac ttggtataga gcaggagagg gcttgatcgg ttctatcgcg gctttattgt
42841 tttatgcttt tagtgcaagt gctgtaaaat atggagttct tttgcctttg caggataatt
42901 taaatacttt taaaatttct gatggaactt tttatcaaag cttgggaatt tctccttgga
42961 ttttggtgct tgtattgagt ttggttgtcg gctttttgt gattaaagaa ttacgcaaac
43021 cgcgccttaa agtcgcaaga ttaaagccta gaaagcaagg aatttcacat atattgtttg
43081 aaaaatcgtg gcaccctttt gttacggctg ttttggttgc attgatcgcc attttagctt
43141 ggcctctttc aagcttaagt ggaagaaatt atggtttagg gattacaact ccttcggcaa
43201 atcttgtaca ataccttagc acaggtgata ttcagtttgt tgattgggga gttttttag
43261 ttcttggaat cgcacttggg tcttttttag gtgctaaatt atcaggagaa tttagatttc
43321 gtcttcctga taaaaaaacc ctagcttatg cgagcatagg aggaatttta atgggggttg
43381 gagcttctct tgctggaggc tgtaccatag gtaatggatt agtagaaaca tcactttttt
43441 cgtataaagg ttgggttgct acggtatttt tcttaatagg tgcttatata gctactttt
43501 atacaattat attgcctacg cgtaaggcaa cacaaaagat ataaggataa gatatggtaa
43561 ctttagatac tagaggaaag gtttgtccct ttccactcgt tgaagcaaag aatttggttc
43621 aaactttaaa aagcggtgaa gaattagaaa ttcttttga ttgtacacaa gctactgaaa
43681 caatcccaca atgggctgct gaagaagggc atgagatcgt tgattttgaa cttttaggcg
43741 atgcagaatg gagaataaag cttattaaaa aataaaggag aaaaaatgcc acttattatt
43801 ccagaaaata tacccgctta tgagctttta aaagagcatg cttttatcat gggatcaagg
43861 agggcaaaac atcaagatat ccgtccgcag gagattttaa tcatcaatct tatgccaaaa
43921 aaaattgaaa cagaaaatca aattttaagc ttacttgcaa attctccttt gcaagtaaat
43981 atcacacttt tagctactac tagctatgtg ggtaaaaaca ctccttttac tcatttggaa
44041 aaattttaca aaggtcttga agaggttaaa aaacgcaaat ttgatggtgc tattgtaaca
44101 ggagctcctg tggaacaaat ggattttgaa aaggttgctt actgggaaga attgctagaa
44161 atttttgatt ttttacagca aaatgtaaca agttctatgt atatttgttg gggtgctatg
44221 gctgctttaa aacattttta tggcgtagat aaaatcgctt tagataagaa aattttttgga
44281 atttataagc atgataaagt ttcaccggat ttacttttaa cgaatttaga cgaaaaagtt
44341 ttaatgcctc attcaagaca ttctagtatg gatgaagagc aaatttcagc actacaaaaa
44401 caaggaaaat tgaaagtttt gcttagaaat aaaaaaattg gttcggcttt gttaagagat
44461 gaaaagaata tttttatttt aggccatttg gagtatttta aagatacttt acaccaagaa
44521 tatgtaagag ataatttcat acaaaaagct aaaaattatt atgataaaaa aggcaatata
44581 aaatacaatt ggcgttcaaa tgcaaatact atttttgcaa attggctcaa ttatgatgtg
44641 tatcaaagca caccttttgt gctttgatga attaatttct tgtagtaggg gtatcatgat
44701 atacgcttac tacgcctttg atatcgctca aaatcgcgca agctgcttca ttggcacttc
44761 ccatagcgca agctaccatg gtggtaacac ctgaagcaag ccctgctgca taaattcctt
44821 ctttgatcat ttgtcttcca tgatgtttga aacaaaattt gtttggtttt ggcataagtt
44881 catgaggtat gacaaagtct tcaagccctt tgatttcact tttagttgct cctgttgcta
44941 cgatgatata atctgctttt atacccaaac cttgctcatc actaaccgta aaatcgcctt
45001 tttctccact aatgcttact acttttgaat cgatatattt tacctcaagc atagaagcaa
45061 tttgtgcttt tatgtgagtg taaatttcat ctgcttttgc acctttttgga aaaaaaggaa
45121 cattataaat tgctgctgct tttaaatccg ctttatcatc atcaaaaata gtgatatcaa

45181 aatcaagctc atcattttta gctgaagcaa aaaataaagc tgcgctaagt cctgctacac
45241 tgccacctat aattacaatt gatttttca tttttttcct tttttaaaga tttgtcttat
45301 tctagtataa aaagctttat ttaatctaat atctatattt tttatgtaat ttgctaaaaa
45361 tatttatttt tcaattgctc taaaagtaat tttttatata atttttaaaa tttttaataa
45421 ggaaaattca tgcgttatgg tgaaaaagaa attaaagaat ttgatgtaga aaatatggaa
45481 atttggccta atgatgcgaa gaatgattat attataaaaa tcactttgcc tgaatttatg
45541 tgttgttgtc cgcgttcggg ttaccctgac tttgcaacga tttatcttga gtatatacct
45601 gatcaatttg tggttgaact taaagcaata aaactttata tcaatacttt tatgcataga
45661 aatgtttcac atgaagcaag tatcaatgaa atttacaata ccttaaaaga caaactcaag
45721 ccaaaatgga ttaaggttgt aggggatttt aatccacgcg gaaatgtaca cacagtgata
45781 gaatgtcgta gcgatatggt ggtgccagag tgaaatttaa tgatcttgat ttgaacaact
45841 ggaaaagttg tgatataaat acagattctt tatggcttat ttctagtcgt gataaaagtg
45901 gaaagcatag aaatatttat catggtaatt ttataccaca aattcctaat cagctcatcc
45961 gtcgttatac aaaaaaagat gatttaatta tagatccttt tttgggaagt ggaacaacgc
46021 tctatgaatg tgaaaaatta aatagaaaat gtatagggtt agatattaat gagagtattt
46081 tggaatttgt attagataat ttcggaataa attttgataa taagcgttat tttttaggaa
46141 attgtgataa tactgatgcc aaaaaagttg atgagttttt agaaaatgct ttgaataagc
46201 ttgaaagtaa aaaagctcag tttatatttt tacatccgcc ttatatggat atagttaaat
46261 ttagtgagaa aaaagaagac ttaagtcaaa tttcggattt aaaaaaattt aaagataaat
46321 ttttatttac ttgtcaaaat atgttaaaat acttagaaaa aagtcgttat tttgctttag
46381 tgatagcaga tttatatag aatagcgaaa ttgtcccttt gggttttgag cttatgaatt
46441 tgataaaaca aaattttaaa gttaaactaa aaggaattat tgttaaaaat atagaaggaa
46501 atcgtggaaa attaggtatt aatgccatat ggagatatag agcattaagg agtgattatt
46561 atatttttaa gcatgagtat atttttgtat ttaaaaagga gttttagatg gatttagaaa
46621 tccaaatgga agataaaaga aatttattaa gggcagatag gctcgatatg tcttttggag
46681 aaattattag caactatgaa gaggataatt taattattca accttcttat caaagatctt
46741 ttaggtggaa tgattatcaa aaaacaagat ttatcgaatc agttttactc ggtattccca
46801 ttcctcctat ttttgttgca gaggataaag ataatggtaa atgggaactt atagatgggc
46861 ttcagcgtgt ttctactatt ttatcatttt ttggtctttt aaaagaaaat ggaaaaattg
46921 acgatagatt aaataaattt aaattacttg aaggagaaat tttaacagaa ttaaaagata
46981 aaaatattga ttctttccct ttaaagttgc agcttactat taaaagattt gtttgtagag
47041 ttgaaatttt gcgatgggat acaaattctg agataaaata tcaacttttt agccgtttaa
47101 atacaggagg ggaagcatta aaagagcaag aaattcgcaa ttgtatttat agaggagacc
47161 taaatgattt aattaataat ttagcagaaa ataaagattt taaagatgta attggtgcta
47221 cagaaagaca agaaaaagaa atgtatctta gtgagcttgt tttacgcttt atggcttttt
47281 attatagaca taatgattta atttttaaaa ctagtggaag tatacaaggt ttcttatcgg
47341 atttcatgcg ttctaatatg gaaaaaaacc aagatgattt ttatcaagtc aagcaagttt
47401 ttttaaatgt aataagttat ttgaatgaaa ataagctttc tgatttattt agagataata
47461 gggctagaca gtttaatgct aatcattatg atacaataat gtatattttg gcaaaaaatc
47521 taaatagatt ccaagattca caagaatttc gacaaataat acaaaattta ttaaaaaata
47581 aggaatatat tgataaatcc ggttcaagta cttatgccac aaaaagaatg caagaaaaaa
47641 tcaaatttgc agaagaattt ataaaaaatg agtttacaag aaattaatga agaactcttg
47701 caagagtttc ggtggcgttg cgatgaaatg atattgctga gggatatagt agactcatgt
47761 gaaaaagatg aatatcagaa atttatttta aagatgagca ttccaataat ctatgcgcat
47821 ttggaaggat ttattgtttc tgcttgtaaa attgtattgc aatatttaaa tactttgaag
47881 ctttctagta atgattgttc tgaaaaattt atcatgatgg catttgacaa agatataaaa
47941 tcactttcag actctcaaca tctagagaaa aagattaaac atttttgtcg tattcaagag
48001 aagttattat gtcaattaaa atttgagaca aaggttaatg taaagtcaaa tttaacattt
48061 gaagttttgg aaaatctttg tttaaaattt gatattagta gtgtaaaatt gaaagactat
48121 aaagatatac ttgatcaact tgtaaatatt aggaatgcta ttgcacatgg tgaaaattct
48181 tataatttcc aaaattttgg cgatattgag aagtatattg atttgtttga ggctttatgt
48241 ttagattttt atcaagtttt atttaatata cttgaagatg aaaaatataa aaaggagaag
48301 taaatatgga tataccctt aaagaaggaa cagcaaatta ttatgatttt accataatga
48361 gtgatttgca atggcattgc acaaaatgtg aattaaaagc agcacaagct aaaacttggc

48421 aaacttggag acatcaaggt attcagctag atactgatga aaaagggaat ttttacaaaa
48481 tgcaattatg ctgtaaatgt aatatgaata ccgttcatag aaaactaaaa agcttagaaa
48541 ttttggaaga tactaagaca agaagcggta tctctgttag tttagctaga agaattaaga
48601 attgtcttag aaatgaagaa gctatttatc taagagaaat gtctcctaat gaattggaaa
48661 ttgatcataa atttccacag attagatgga aaacagatga gagtaaaaat gatggttcta
48721 tgagtgaaga agaaataaaa aataaattta tattattaac tagaagtaat aatttattaa
48781 aatcgcgtta ttgcgaaaaa tgttttaaaa atggcactag aggatatttt cctgggatta
48841 aattttggta taaaggtaat gaaaaatggc aaggtgaaga tgataacgat gaaaaagggt
48901 gcgaaggctg tttttggtat gatccatata aatggagaga agaattaaat agaatttcgc
48961 aaatacaaat aaatctatga attatattgg gtcaaaattc aagctttcat ctttttttatt
49021 taaaagtatt gatgagactt taaaagcaaa aaataaagca ctagaagact gtatattttg
49081 tgatattttt gcaggaacat cagcagtagg aaagcttttt aagacaaaag taaaacaagt
49141 cattgctaat gataaggaat tttatagttt tattttggcg aaaaactata tacaaaactc
49201 aaatttatta acgcgtgcta atgaacttat agatatttta aactatcaaa tccctttaat
49261 aaagaataaa atttacaaat attatgcaat aggtggtgga gaaaataggc agtattttag
49321 tgatcttaat gcaatgaaaa ttgatagcat aagagtacaa attgaaattt ggaaagaaca
49381 aaattttata aataaagaag aatattattt tttgttggca agcttacttg aaagtgcaga
49441 taaagtggca aatactgctt ctgtttatgg ggcttttttta aaacatctta aaaaatcagc
49501 acaaaaaaaa atggtgttat ttcctgcaga atttcagcaa tcacataata aacatcaagt
49561 ttttaataaa aatgcaaatg aattgataag agagattaaa ggtgatatac tttatcttga
49621 tccaccgtat aataacagag aatatggtgc aaactatcat gtattaaata ctattgcaat
49681 atatgatgat tttattcctc aaggcaagac agggcttaga gcttatgaaa aatctgcttg
49741 gtgtaagaaa aatttggttt ttaatgcttt ggaagatttg attaaaaatg ccaattttaa
49801 gtttattttt ttaagttata atgacgaagg gttgctttct ttagaacaaa tcaaacaaat
49861 ctttaaaaaa tatggaaaat atgatttaaa aaaccaaatt tataagcgtt tcaaggctga
49921 ttcttctcga atttgcaaac aagatcaaac catagaatac ttacatatct tagaaaaaaa
49981 atgaaaaagt aacaaaaata atactatatt aattatcaat ataatataat gcgttttgca
50041 aatccaattt aaaggaaaga ttatgaataa aaagctttta agtataattg cgggtgttgc
50101 gttattttca agttcagctt tagctgcgga agaatcaggt ttttttatag gtgcggatgc
50161 agcttatgtt caaactaaag ttaaaggaga tttaaaacat aataaaaccg gtgctgtttt
50221 taatggtgat ttatctagta gcattcctat tttaggttta agaatgggtt atcgttttaa
50281 tgatttacat agattgtatg ctgcttataa ttactctgat gaattcagtg atattataag
50341 aactcctaat cttagaatag agggtgattt taacacacat aaattcttat taggttatga
50401 ttttactcca gaaattttta aaagagtaag agctgttgcg ggtggatatt taggctatgc
50461 aaaaacgagt cttgatttaa aaacatcttt attgtcttta tctcaagatt ttgatggttt
50521 tgtttacgga gcaaaaatag gtgctatatt tgaattaggt acttctaatg agctcgaagt
50581 aggttttaaa gcagaacaaa tcgaatacaa tactagaaat tattaccaag agaccatagg
50641 atcgaatttt tatgatccaa gacaaacaaa ttatggctta tatttaggtt atacttacaa
50701 attttaatga agtattttat cggtaggatc tcaacccccca ccgataaaat atctttctac
50761 atcatctaac aatttttcac aaatttgaga atcggtattt ttatccacta caaggttgat
50821 ataagttcta gtatctctaa aacttcctgc tgattttgaa ttgtcgcgtg ttaatttttg
50881 attttcttta gtgtattcta tggtgatgtt aaatttaata gctgaacttg cttctcctag
50941 attggttttt attaaaacat tcattttcga agctttagag agagtagctt gggttttaga
51001 tatgatttct ttaaatcttt gacaagagcg gatgagtgtc gcaatgaatg tattatcaag
51061 tccttcataa gctactttaa gtttattttt gatgtctttt gtactttctt tttcaggagc
51121 aacaaaaacc atgtaaaaac ctgttttgat tggaacatta tccgttttta catcacgccc
51181 acttgacata actttaacat aaattgcatt atcaagatag acacgatcca ccataggtcc
51241 gtaaatttcg tactcttttc tacgaactat tacttcacga gctaatacat ttaattttac
51301 attagcttca tggataacag gctccatttc tttttttgatc tctaaaagct cttttaacat
51361 caacatcctt tttgttactt aaaattacaa ttataacaat aaatttctta atttgtaaat
51421 ttttattttg tgtattaact aaaaatatgc taaattcaaa actatataat tttatgaagg
51481 aggcgacaat gacacaggtt tttaaaaatt caattcaagt caaagagcaa aaaaataaag
51541 aaaatcactt taacctgatg ctgcaactct cactcaccct aaactaaaat ttttattttt
51601 aatcattttc taaaattttt atttactat tgtttttaag tttttgaatt tttaataaaa

51661 ttcattcagt ttatttcatt ttttaaggat taaaatatga agaataataa aattatcatc
51721 tttgatacaa ccttacgcga tggtgaacaa gccttaggca gttctttaaa tataaatcaa
51781 aaattacaaa ttgccttagc gcttgaaaat ttaggtgttg atgtgataga agcaggtttt
51841 cctgtatctt cacaaggaga ttttgaagca gtaaaaaaca tagcctcaaa agtaaaaaat
51901 tcaaccattt gtgcgctttc tagagcttta gataaagata tagatacagc ttatgaggct
51961 ttaaaagtgg cggattcttt tagaattcat acttttatag ccacttcgac tttgcatata
52021 caagataaat taaaaaaaga ttttgatgaa attttggcta tggcacaaag agcgatcgca
52081 agagctagaa actatactga tgatgtggaa ttttcgtgcg aggatgcagg aagaactcct
52141 attgataatc tttgttttat ggtagaaaat gctatcaaag caggggctag aactatcaat
52201 atacctgata ctgtgggtta taccttacct agtgaatttg ctaatatcat caaaatttta
52261 tttaacaaag tgccaaatat agacaaagct gtcatttctg tgcattgtca caatgactta
52321 ggtatggcaa caggcaatag cttaagtgct attttacaag gcgcaagaca aatagaatgt
52381 accatgaatg gacttggcga aagggctgga aattgtgctt tagaagaagt tgtaatggcg
52441 ataaaaacaa gaagcgatta tttaaaaggt ttttatacag atatcaagtg cgaaaatatt
52501 tctaaaactt caaaattagt ttcagccatc actaatgaaa gtataccttc gcataaagcc
52561 atagtaggaa gtaacgcttt ttctcatagt tctggaattc atcaagatgg agtgcttaaa
52621 aatagacaaa cttatgaaat tataagccct agcagtatag gacttcatga aaatcgtatg
52681 cttatgactg caagaagcgg tagggctatg attaaaactt gtcttgataa tttaggatat
52741 gatgaaaaaa cttataattt agacgaagtt tatgagagtt ttttacgcct tgcggataaa
52801 aaaggacagg tttatgatta tgacttagag gctttaatgt ttttaagtta tgaaaatgat
52861 gaagagaatg aattagtact tgaaaaatta agtgtaatca gtggcactat cccaacagct
52921 tgtgtttgtg tgcgtataaa agaagagtta aaaactgaag cttgtaccgg aaatgggcct
52981 atagaagcgg tgtttaattg catctctaga atcacaggct taaaccctgt tttaaaagca
53041 tatagcatta atgcaaaaag ttcaggtgtg gatgcacaag ggcaagtgga tgtggattta
53101 gaatttaaag ggagaaaatt ccatggtaaa gggctttcaa cagatgttat agaagcatca
53161 gcacaagcct ttatcagtgc ttacaatgca atttatcgat ctttaaaagt tgaagaaagg
53221 aaagcagtat gaatacttat aaaatagcag ttttgccagg agatggcata gggccttcag
53281 tgatgaaaga ggctttaaaa attttaaatt ttgttgccca aaaacacggc tttaaattag
53341 aaacaaaaga agcaaagata ggtggggcaa gtatagatgc atatggggta gcattaagcg
53401 atgagacttt aaagctttgt gagcaaagtg atgctatttt gtttggttca gtaggagggc
53461 ctaagtggga taatttaccc atagatcaaa gacctgaaag agcttcgctt ttacctttaa
53521 gaaagcattt taatttattt gcaaatttgc gtccttgtaa aatttatgaa agcttaagac
53581 atgcttcgcc cttaaaagat gaaattatcc aaaaaggtgt agatatctta tgtgttagag
53641 agcttacagg tgggatttat tttggtaagc aagaattagg caaagaaagt gcttatgata
53701 ctgaaattta cactaaagca gagatagaaa ggatcacacg ccttgctttt gaaagtgcaa
53761 gaataagaaa taaaaaagtg catctaatcg acaaagcaaa tgttttagca agttctattt
53821 tatggcgtga tgtagtagga caaattgcaa aagaatacga agatatcaag ctagaataca
53881 tgtatgtaga taatgcagct atgcaaatta ttaaaaatcc tagcactttt gatgtaatgc
53941 tttgttctaa tctttttggc gatattttaa gtgatgaatt agcagctata agcggatctt
54001 tgggcttgct ttgttctgcg agtttaaatg ataaaggttt tgggctttat gagccagcag
54061 gtggttcagc tcctgatata gctcatttaa atatagccaa tcctatagcc caaattttaa
54121 gtgctgcttt gatgttaaaa tatagtttta aagaagaaaa agctgcacaa gatatagaaa
54181 atgccatagc catagcttta gagcgtggaa aaatgactaa ggatttaaat gcgaattctt
54241 atttaagcac cgatgaaatg gggggattgta tagtagaaat tttaaaggag aataacaatg
54301 gctaaaactt tatatgaaaa agtatttgat tctcatgtgg tttatcaaaa ggaaaatgaa
54361 ctccctatac tttatatcga tagacattta atccacgaag taacaagccc tcaagctttt
54421 tcaggtctta aaatggcaaa tcgtaaaatg gcaagatcgg atttaacttt agcgactatt
54481 gatcatgatg tatcgactaa aagcacagat ttggatgctt gttcaggtat ggcaaaagag
54541 cagattacga ctttgatgca aaatactaaa gaatttggag tcaggctttt aggcttaggc
54601 gataaaaatc aaggtatagt gcatatagta agtcctgagc ttggttttac cttaccaggt
54661 gtaactttgg tttgtgggga ttctcacact gcaacacatg ggcttttgg agctttggct
54721 tttggtatag gtacaagtga ggtcgagcat gtaatggcta cgcaaacttt aaaacaagca
54781 aagctcaaaa ccatgaaaat agaatgcaag ggtcagtttc aaaagggtgt ttatgctaag
54841 gatttgattt tatatcttat agcaaaatat ggcaccgcaa aaggcacagg ctatgccata

54901 gaattttgtg gagagttgat agaaagcttg agtatggaag ctagaatgac gctttgcaat
54961 atggctatag aatttggtgc aaaagtcggt atgatagctc ctgatgagat tacttttgaa
55021 tatataaaag ggaaagaatt tgctccaaag ggagaagaat ttgaaaagca ttgtgagtat
55081 tggagaagtt taaaaagcga tgagggcgcg caatatgatg aaagcatagt ccttgatgta
55141 agccaaattc aaccacaaat cagctatgga actaatcctt ctcaagttat tgctataaat
55201 gaaagaattc caaaagtcag tgattttagc aatccaagcg atcaaaaatc cttactcgat
55261 gccttagatt atgtgaattt agagcaagat caaagcttag aaggggtaaa aatcgatatt
55321 gttttcatag gttcttgtac aaatgggcgt ttagaagatc ttaagatagc tgcggatatt
55381 ttaaaagggc gtaaaattca taaggatgtt aaagctttaa tcgtacctgg ttctatgcaa
55441 gtgagaaaag aagctgaaaa tttaggactt gataaaattt tcattgaagc agggtgcgag
55501 tggagatatg caggatgttc aatgtgtctt ggtatgaatg atgataaagc aagcgcggga
55561 caaagagtcg cttcaacttc aaatcgtaat tttgtaggca gacagggtaa gggttctatc
55621 acccacttaa tgagtcctgc aagcgcagca gcctgtgcca tagaaggctt tatttgtgat
55681 aatagaaaat atttaggagt ttaaaatgca aaaatttact actcataaag gtatagcttg
55741 tcctttagaa tatgccaata tcgacacaga tcaaatcatc cctaagcaat ttttacttgc
55801 tgtttcaaag cagggctttg gtaagcattt gtttcatgat ttgcgttatt tagacgataa
55861 agaaagcgtt ttaaatatgg attttaatct caataaaaaa gaataccaaa acgcttctat
55921 tttagtaagt tttgaaaatt ttggaagcgg ttcttcaagg gagcacgcac cttgggcttt
55981 ggtggattat ggtataagag ctattatcgc gccttctttt gcagatattt ttaaaaataa
56041 tgctttagga aatggtttgc ttactataga gcttgctaaa gatgaggttg taagcattat
56101 aaagcaactg aaagaatctg aagataaaaa tatagaaatt tctttatttg agaaaaaggt
56161 gtttttttaaa gatcaagttt tttcttttga tttggatgaa tttcgtagaa cctgtttgct
56221 agaagggctt gataatatag ctttaacttt aaaatatgaa gaacaaatca aggcttatga
56281 gaaaaactca aaaagctttt tggtttagga ttttttcata agctaaaaac tcatcttcat
56341 agcaagggat atgaaaagcc ccgcaaaact caaaatttaa ctttttaaag agcgaattca
56401 tagggatatt tttactatga gtatccgctc ttaagctata ttttccttca tttttagcat
56461 aagtttcaca aaaatttaaa agcttttgtg ctacgccttt gcctttagca ttttctttta
56521 cagccaaacg gtgcagataa aaagcttgat tatcgtagga ttttttaaag ttgatttgct
56581 cgtaaaattc gggttcgaat ttttcattga tgcaaataaa acctagaatt tcatcctttt
56641 ctttacaaag ataaagctct tgtgttttga tatcttcttt aaaaatttct tcattaggat
56701 aattttcatc ccattgatta aaattcatag ctttcatgga atttagagcg tcttttgcta
56761 tgttaagtat tgattttaaa tctttttgtg tagctttttc tataacgaaa tcttgcataa
56821 aaaatccttt ttttataaat gtaaaattag tgtttgattt taatatcttt ggtaagtttt
56881 aaagaatcct attgggtttt aaaaatccca aaaagaaccc taattaatta aaaatcatta
56941 taacacaaaa gcaacaaatt gaaatcaaaa atcctttagt ttgtttgtat atagcgcttt
57001 taaggtattt tttgaaagct gaagaaattc aaagaaataa aaaatggtgg ctccgattgg
57061 acttgaacca acgaccacta ccatgtcaag gtagtgctct accaactgag ctacggaacc
57121 atggtggagc atagcgggtt cgaaccgctg accccaacgc tgccagcgtt gtgctctccc
57181 agctgagcta atgcccctta gagttgtgtt aaaattctag cgcaatatta ctttttttttg
57241 gctgaaaacc aaagttaaaa tcacgattta gaagcaaaga actataagaa tttaagacat
57301 gaagataaga atcggttaaa agaatttaaa ctatttgatt aattctaaaa tttctctttc
57361 gcattcatct ttgttaagat cgagttgccc agaatagtaa aaatatttag tcaaaactaa
57421 aatccctata taaaattctt ttttcttatc tacgaatttc ttgatatatt gatgagcatt
57481 attaataatt tctagcgcat cttttggatg gttgttaaaa tattcgatta ccgtagctag
57541 gttatcgtta tcaattaaag caaaatgata atttggtttt aaagtccctt ccatgaacca
57601 agtttcgcaa gttatttttg gggctaagac taaagaattg gaattcattg cccattttaa
57661 attgcttgct acatcgttgc cttctaaaga gattataaat ttatatttca tttgttcttc
57721 tttacttaaa aagtttttttt tccattgact caattgcttt gaagtatctc ctaagtcgca
57781 aaatggttta ttaaaataag aatgaaaaaa ttcttttcta tgtttttgat atacagcgcc
57841 tctaaaaatt gctatgtttc ttttattttc ataatttata ttgtctttta aaaaacaaaa
57901 atgcctattt ttatcaagtt ttaataaaat attgtttgca ttattctcta gaggtcttga
57961 tttacaaatg gttggctctt taaaagtata ttttatatca ccaaattttt tattccataa
58021 aaaatcattg ttaaagtatt tgctaatttc ataagcatca aaagcataag aggtattttt
58081 aaaaggaaat tttcctattt tttccttttc tgttaatgta aaaaaatcat ttatttgatt

58141 gtaatactta acccgagttt gtatttcttc taattctttg acattgtatt ttaaaatttt
58201 attgaaaatt ttatgtaaat ttgtttgaaa aaaatatctt gatataaaag atattccaat
58261 gccttttaaa ttcataaata atcttgattg taacattatg agatccataa ataaagaatt
58321 tataatattt taccaaaaat attaatgatt attaagcagt taagaaattt taataaaata
58381 ataatattta atattaaatt taataatggc agacaggaag ggattcgaac cctcgaaagc
58441 ttgcacttta cacgcgttcc aggcgtgctc cttcaaccgc tcggacacct gtctaattaa
58501 aaattaaatt atatctaaaa aagctttaaa taaaatttta gtaaaaaaga gtataattac
58561 attttcattt tgtaaaatga tctttaaaga tttattatgt cagggtagct cagctggtta
58621 gagcgctggt ctcataagcc ggaggtcggg agttcaagtc tccccttga caccatttcc
58681 aaaacttctt ttagaaaaat cctcaaaaat tataaatttt agctcaatgg ttttaaatct
58741 atgaatttgt tttagaattt aatctctatt ctaaatttta gcatttaaat ttgtgtatct
58801 tttatatggc ttgaaaaata aaatttgttc gaagagatgg ttttttgaga taaggtatat
58861 tttataaaag ataggaattt gattattgta ttttaaagat taatgtttaa aaatttatct
58921 cgaaataatc aaaaaaataa tcaaatttaa agacacaagg ggaatttgtt attagaaaat
58981 taacttaaaa tagacttttg ataagagtat agtatcataa aattataatt tgattgatat
59041 ttaaatttat caaagcgcac atcgttttat ttataaattt aaatatctta aaatttcaat
59101 aagagattta ttcaattagc gattgctttt atctttagaa atcacccaaa tcatacccac
59161 aaaacctatc aaaacaacga caaatataaa aatgatttga aaaatatcta aagctgtcat
59221 aattctttcg ccctttcttt gagttgtcca caagctgctg aaatatcaag tcccttgctt
59281 tctcttatgg tgcaagttac tcctttagcg cttaataaat cttgaaattt gattgcattt
59341 tcaagtttag gacgatgata aatgctaccc tcgtgaggat tgaaaagtat caaattcact
59401 ttggctttta taccatttaa aagcttgaca agttcttttg catgttcaag tttgtcgttg
59461 atgccatcga tgagtaaata ttcaaacatt actctttttc tttgatctat aggaaaagcg
59521 cgcacagcat ccataatcgc agctatatta taagctttat ttataggcat gagttcggtt
59581 cggagttcgt cattgactgc gtgaagggag atagcaagta aaacgcccaa attcatttcg
59641 cctagttctt tgatttgttt tgctaagccg ctagtactta tggtttgtct gcgaggactg
59701 atagcaagtc catcattttg agagagaatt ttaaccgcct tggaaacatt tttaagattg
59761 tcaagaggtt ctcccatacc catatagact atattgacac gacgctcata ggggatatta
59821 ttttgttttt tgatccataa aatttgtccg actatctctc ccgcgctcaa atttcttttt
59881 aagcctcctt tggcggtaag acaaaaacta cagccacttt tacagcctac ttgagaggaa
59941 acgcaaatgg tatatcttgt atgtgaaatt cttttacctt catcgtcaaa ttgttcttct
60001 ttcataggca aaagtacact ttctatcctt aagccatcga taagttcaaa aagatatttg
60061 atacttccat ctttgctttg ttcattttta acgcatttaa gaggagcgaa atggtaagtt
60121 tgcgcaagtt ctaaacgcaa atcttttggt agacttgaca tatcggaaaa attatttgca
60181 tatttttgat atatccattg ataaatttgt ttcacgcgaa acataggctt gatttttct
60241 tccaattctt caggtaaaaa gtctaagata ttaatgagtt ctttcaagat tgctttcctt
60301 tgattgctaa atataatgat taattttaa atattcttct agtttttctt ttgccttttg
60361 atgatttttt ataaaatctt catgagcatt ttcattgcaa aaattagtcg cacataaaat
60421 acattctgca tctatatcta aatttttagc cacgcttaaa actgaaaaag cttccatgtt
60481 ttcagcaaaa aaacctagtt ttgcaaattc ttttgctgct tgtgaatttt gacaaatata
60541 attagaagaa ttgactttga ttgtttcacg agaaacattt tgattgttga gtttgatttc
60601 aatgtttgca ggagtgtaga attgatcaga aatataagaa aattcaatat tgcaagcatg
60661 agagctttta taaattccta gaatttctcc tttatcgtaa agtccacatg ttcctataaa
60721 tacaagtttt gaaggttttt cttttaaaca aagctgtgtt aaatgaaagg ttgattctat
60781 caagcctata cctatggctt tggcaaaaga aaaattttca tttccacctg cgcaaactat
60841 catttacaat atcctaaaaa taatttagca agaattttag ctaaaaaggc ttaaaattaa
60901 gaatttttag ctaaaataaa aaactattat gatgaaagca aaaaaacatt acggacaaaa
60961 tttttaaac gataaaagta ttttatcaaa aatcatccaa gccataccca aagatactga
61021 aaatatagta gagattgggc ctggcttagg tgatttgacg caagagcttt taaaagtttc
61081 tcatgtaaag gcttatgaga tagatgatga tcttatacct attttgcgta agaaatttca
61141 aaaagaactt gaatgtggaa aattaaattt gcttcatcaa gatgcaagta aacttgcata
61201 ttttgatgaa aagaagtatt ttttggtcgc taatttgcct tactatgttg caagccattt
61261 gattttgcaa gccttagaag acaaaaattg ctcgggtttg attgtaatgg ttcaaaaaga
61321 aatggcattg aaattctgtg ctcatagcgg agaaagtgat ttttcagctc taggagtttt

61381 aagcgctatg atttgtgagc gagaaatact atttgatgta gaacctgaat gttttaatcc
61441 gccgccaaaa gtagtatcag cagtaatgaa aatgattaaa ttcagagctt atcaggatat
61501 ctgtgaaatt catgctttta aagctttttt aaaagattgt tttaaagcac caagaaagca
61561 actattaggg aatttaaaaa attataaagc tgaattagcg gaggtttttc aagagttaaa
61621 actcaattta aatttacgcc cacatgaact ttgcgtcgat tcatacctta aaatttacga
61681 aaaagtaaag gaagaatatg aacgaaaaca acaaaggaaa tagtgaaaac acaaacattc
61741 ctaactcaaa ttcaaaaaac aacaagcgct ataaatacag aaatcgtaga aaaaaattag
61801 ccgattctat cagtgaaaat tctcaaaatt caactcatga attttctaac aacgaaaaga
61861 taaataccaa gaaaaattct acaaataccc aacctttaga gagtgaaaat atagaaaaac
61921 caagcgaaaa aaagaaaaaa agaaatcgca atctcccttc taagctcact ggaaatgaag
61981 attggcaaat cgcgcttgca gagtgtatag aagcaaaccg tgtttcacat gaaaatcgct
62041 tacatccttt aaaatacaat aattctagcg agcataaaat tcgcatcact ccactaggcg
62101 gcttagggga aatcggtgga aatatcagtg tttttgaaac caataatgat gcgattatca
62161 tcgatatcgg tatgagcttt ccagatggaa ctatgcacgg ggtggatatc atcatccctg
62221 attttgatta tgtaagaaag attaaagata agattcgtgg tatagttatc actcatgcac
62281 atgaagatca tatcggtgcg gtgccttact tctttaaaga atttcaattc cctatttatg
62341 ctacgccttt agctttggga atgatttcaa ataaatttga agagcatggc ttaaaagctg
62401 aacgaaaatg gtttcgtcct gtagaaaaac gcaaagttta tgagatcggt gaatttgata
62461 tagagtggat acacatcaca cactccatca tcgatgcttc tgctttagct atcaaaacta
62521 aagcagggac tatcatccat acaggagatt ttaagatcga tcaaactcca atcgatggtt
62581 atccaacaga tttggggcgt ttagcgcatt atggcgaaga aggagttttg tgtcttttaa
62641 gcgatagtac taattcatac aaagaaggct atacgaaaag cgaaagttct gtaggaccga
62701 cttttgatca aatttttgca cgcactaagg gtagggtgat catgagtact tttagctcaa
62761 acatccaccg tgtttatcaa gctattactt atggtttaaa atacggcaga aaagtttgtg
62821 ttattggtcg ttctatggag agaaatcttt ataccactat ggaattaggt tatattaaac
62881 tcgatagaaa aattttcatt gatgcagatg aggttagtaa gtataaagac aatgaagtgc
62941 ttatcgtaac cacaggaagt caaggtgaaa ctatgagtgc gctttataga atggcaacgg
63001 atgagcataa atttataaaa ataaaaccaa gcgatcaaat catcatttca gctaaagcta
63061 tcccaggcaa tgaagcgagt gtttcagcgg tgcttgatta tcttctaaaa gcgggtgcta
63121 aagtagcgta tcaagaattt agcgaaattc atgtaagcgg gcatgcaagt atagaagagc
63181 aaaaattaat gcttactcta gttaagccta aattcttctt gccagtacat ggagaataca
63241 atcatatcaa taaacacaaa gaaacagcta taaaatgcgg aattcctgaa aaaaatattt
63301 atcttatgag cgatggcgat caagtagagc tttgtcaaaa atatatcaaa cgcgtgaaaa
63361 cagttaaaac aggcaaggtt tttgtggata atcaaatcaa taaacaaatc gctgatgatg
63421 tggtaattga tcgtcaaaaa cttgcagata gtggtatagt tgtcattatc gcacagcttg
63481 ataaagcaag taaaacattg attaataagc caagagtatt tagctatgga cttgtagcag
63541 ataagcaaga tcatgctttt tctaaagaaa tggcagaggt tttaggacaa tttttttgtca
63601 atgttaaaga tgaagtactt aatgatccaa gatttttgga aaatcaaatt cgccaagtat
63661 tgagaaaaca tatttttaga aagattaaaa aatatccaac catagttcca actatttttg
63721 taatgtaaaa tggggcgttt ttcgccctta aattttcaca ggaaatcaaa tgagaatcaa
63781 taaattcata tcgcataata gcccttattc acgccgcgaa gctgatgagc ttattaagca
63841 aggtttggta aaaataaaca acaaaatagc ccttttaagc gatgaagtaa agacaaatga
63901 taaagtcttt gttaagggta aggctttgcg taaaaaaacg caatttagcg tgattattta
63961 tcacaagcaa aagggtgaaa ttgttagcaa aagagatgat cgcggaagaa aaactatcta
64021 tgataccctg ccaaagcagt ttaatacttg gctaagtgta ggaaggcttg actttgctag
64081 tgaaggtttg cttttgctta cagattctcc tgtgatcgct catgctttga tgaatagtga
64141 tttagaaaga gaatattatc tcaaactcaa aggtgtgatc aataagcaag ttatagaagc
64201 gatgcaaaat ggactagaaa ttaaaaatga aaaaaaaggc gcacacgcaa aaacaaagat
64261 tacttctatg agttttgctc ctttttttgga ttttgaaatt tttggaacaa gtggcggata
64321 tacaaagcta agagtgatta taaatgaggg taaaaataga gagcttagac gtttttttgg
64381 gcattttgac ttagaagtaa tggatcttaa acgcgttgct tttggcgcct tagatcttgg
64441 aatgctaaaa gcaggaaaac atcgctatct tgaaaatggc gaatatgaaa aattaagaga
64501 tttttttaaaa gtcaataata tatattatta attatacttt tatgtatgat tttatttaaa
64561 tacattatta aaaattatag ggttgaaatt caaatataaa taccaactag tagtttttaa

64621 atttaatttc catttcataa tcaaaatata attatcatcc ttttaaaaag gaaaataatt
64681 atattttttt aaaatttcct tgacatttaa aagccgaatt tactatactc acatttctat
64741 ttttggttag ttttaaacta acgagttctt ttaaaaggaa aatttatgga aagaatcagg
64801 cttaagctaa aagcttatga ccatcgagta ctagacagaa cagttgcagc aattgtagaa
64861 gctgtcaaaa gaacaggtgc ggatatcaga ggtccgatac caatgcctac taaaatcaaa
64921 cgctatacag ttttaaaatc tccacatatt aacaaagatt ctcgtgagca gtttgagata
64981 agaattcacg cgcgtatgct agatatagta gcagcgactc cagatacagt tgattcttta
65041 actaagcttg atttagcgcc agaagttagc gttgaagtaa gagctatggg taaataagga
65101 taaaatatgg aatatattgt agaaaaaata ggaatgagta gaacaattgc taatccaagt
65161 atcgctgtca ctttactaag agttgtgaat gcaaaagtat gtgaagttga taatggtaaa
65221 gctatcgttg catactcaaa aggaaaagta aacaataaat gtgttgcagg acaacaaaaa
65281 aaatataatc tttctgcaga atttaacaga tttgcgactt tagaagtagc aaacactgaa
65341 gcaggagatt tagatgaaac tccattaaat gaagcaaaag ttttaaaagt aagcttcaat
65401 accaaaggta gaggttatag cggtgttatg aaaagacata acttcgcagg gggtccagca
65461 agccacggtt caagatttca tagacgccat ggatctatcg gtaatagaga atggccaggt
65521 cgcgttcaac caggtatgaa aatggcagga cattatggaa atactaaagt aactgttaaa
65581 aacgaagttg tatcttatga tgctgaaaac aaaattttag tagttaaggg tgcagttcca
65641 ggttataacg gtgctatggg taaaataagg attgcaaaat gagtaaagta gttgttttaa
65701 atgataaatt agaaaaagca ggcgaacttg aattacctgc aaaatatgca gaagtaaatc
65761 cacacaatct ttatttatat gtaaaatctt atctcgcaag ccttagagca aatacagctc
65821 atactaaagg tagaagtgat gtaagcggtg gtggtaaaaa accttggaga caaaaaggtc
65881 gtggtggcgc tagagcgggt tctactagaa cgaatgtttg ggtaggcggt gcggttgctt
65941 ttggtccaac aaatgaaaga aattattatc aaaaagtaaa taaaaagcaa aaaagattag
66001 cgcttgaaag agctttagct gataaagcag ctaagggtgc actttttaca gcagattctt
66061 tagctattga aagcggtaaa accaaagatg caaatgcagt gattaaaaaa cttggtgtta
66121 aagatgcttt gattgttaaa gatttactag atgaaaaaac acttctagct tatagaaatt
66181 tagcaaactg ttatgtagtt gatataagcg aagttaatgc ttatttgata tccgtattta
66241 atgctgtaat cattgaaaaa tcagcgttag aatctattac aaaagaggga taattatggc
66301 agatattact gatattaaaa caatacttta tactgaaaaa agtttaaatt tacaagaaca
66361 aggtgtcgta gttatccaaa cttcaccaaa aatgactaaa acaggcttaa aagcagtttt
66421 aaaagaatat tttggtgtaa ctccacaaag catcaattct ttaagaatgg atggaaaagt
66481 aaaacgcttt agaggtcgtt tgggacaaag aaatgactat aaaaaattct atgttaagct
66541 accagaaggt gttagcttag aaagtatgga ggcttaagat ggcaattaaa acttataaac
66601 catatactcc aagtagaaga tatattacag gtttaagttc tgaagatatt acagcaaaac
66661 caagtgtacg ctctttactt gtgaaacttc cagcacacgc aggacgtaat agctacggaa
66721 gaattacaag ccgtcataaa gaagcaggag ctaaaaaact ttacagaatt atcgatttta
66781 aacgccgcaa atttggcata gaaggaaaag tagaagcgat cgaatacgat ccatatagaa
66841 attgtagaat tgctttgatt tcttacaaag atggtgaaaa aagatatatt cttcaaccac
66901 gcggactaag tgttggtgat attgttgcag ctgctgaaag cggacttgat ataaaaccag
66961 gtaatgcaat gaaacttaaa aacattcctg tgggtactat tgttcataat gttgagttaa
67021 agccaggcaa aggtggtcaa atgatccgct cagcaggagc ttatgcgcaa cttatgggta
67081 aagaagaaaa atatgttatc ttaagacttg caagcggtga aatgagacaa gttctagctg
67141 aatgtatggc aagtataggt gaagtaggta acgaagaatg ggcaaatgtg actatcggta
67201 aagcaggtag aaatcgccac agaggtattc gcccacaaac tcgtggttct gcaatgaacc
67261 cagtagatca cccgcacggt gggggtgaag gtaagaaaaa ctcaggccgt catccagtaa
67321 ctccatgggg taaaccaacc aaaggtgcga aaacgcgtcg taaaaaagct agcgataagc
67381 taataatttc aagaagaaaa ggaaagtaag atggctagat cactaaaaaa aggtcctttt
67441 gttgatgatc atgtaatgaa aaaagtcatc gctgctaaaa aggctaatga taataaacct
67501 attaaaactt ggtctagaag aagtactatc attcctgata tgataggttt aactttcaat
67561 gttcacaatg gcaaaagttt tattcccgtt tatatcactg aaaatcatat cggttataaa
67621 cttggagaat ttgctccaac acgcacattt aaaggccata aaggttctgt gcaaaagaaa
67681 attggtaagt aagggataag atatgagtaa agcattaatt aaattcataa gattatctcc
67741 aactaaagca agattgattg ctagagaagt tcaggggatg aatgcagaat tagctatggc
67801 tagtttaaaa tttatgccaa acaagggtgc taaatatata gctaatgcaa tttcaagtgc

67861 agtagcaaat ggcggttttg aagcaaatga agtagtggta aaaagttgcc gtgtagatgc
67921 tggtgctgta ttaaaaagat ttagaccgcg tgctagagga agtgcaagcc gtatcagaaa
67981 acctacttca cacatcttag tagaagtagc aaaaatggaa gttaaaactg aagcaaaaaa
68041 agcagtagct aaaaaaactc ctgttaaaaa aacaagtact aaaaaagtaa cggcagaaaa
68101 ggaaagctaa tgggacaaaa agtaaatcca atcggtctaa gactaggaat taatagaaac
68161 tgggaatcaa gatggtttcc aactaaagca aatttagtgg aaaatatcgg tgaagattac
68221 aaaatcagag ctttcttaaa aagaaaactt tattatgcgg gaatcagcca aattttagta
68281 gaaagaactg ctaaaaagct tcgcgtaact gttgtagctg caagaccagg tattatcatt
68341 ggtaaaaaag gtagtgatgt tgataattta agaaaagaac tacaagattt aatcggcaaa
68401 gatgtaaata tcaacatcaa agaagaaaga aaagcgggtg cttcagctca acttgctgct
68461 gaaagcgttg caactcaact tgaaaaaaga atcgctttta gaagagcaat gaaaaaagta
68521 attcaaggtg cgcaaaaagc aggtgctaag ggattaaag tttcagtttc tggtcgttta
68581 ggcggtgctg aaatggcaag aactgaatgg tatcttgaag gtcgtgtgcc acttcatact
68641 ttaagagcaa aaatcgatta cggttttgca gaggctagaa ctacttatgg aaacataggt
68701 gttaaagttt ggatcttcaa aggtgaagta ttgcaaaaag gcttacaagc tgaaaaaaca
68761 gaagaaagtg ctccagctaa aaagcaaaga cgcacaagaa ggagtaaata atcatgttaa
68821 tgccaaaaag aactaaatat cgcaaaatga tgaaagggcg taacagaggt tatgctaatc
68881 gtggaactga atttactttt ggtgaatttg cattaaaagc aactgaagca ggacgcatta
68941 attcacgcca aattgaagca gctcgtatcg cattaactcg ttttgttaaa agacaaggta
69001 aaacttggat tagagttttt ccagataaac ctttaactaa aaaacccttta gaaactcgta
69061 tgggtaaagg taaaggtgca gttgaggaat gggtaatgaa tatcaagcca ggtcgtatta
69121 tttatgaaat ggcaggtgtg agcgaagaaa tggctagaga agcattgact ttggctatgc
69181 ataaactacc atttaaaact aagtttgtta caagagagag ccaaaatgaa atatactgag
69241 attaaagaca aaacagcagc tgagcttgca acaatgctaa aagaaaaaaa ggtgctttta
69301 ttcactttaa aacaaaagct aaaaacaatg caacttacta atccaaaaga gattagcgaa
69361 gttagaaaag acattgctag aatcaataca gcaattaacg ctttaaaata aggataaaaa
69421 atggcattta aaagagaaat acaaggcgtt gttgtgaaaa tcgcaggtga gaaaacagcg
69481 agtattttgg ttgaaagaaa agtggttcat ccaagatata gaaaaattgt taaacgcttt
69541 aaaaaatatc taattcatga tgagagaaat gaagttaaag tgggagatac cgttgtggcg
69601 gttgaatgca gaccactttc taaaagaaaa tcatttcgct taaaatctgt attagctaca
69661 ggagttgagt aatgattcaa agttttacaa gacttgcagt tgctgataat agcggtgcaa
69721 aagaattaat gtgtattaaa gttttaggtg gtagtaaaag aagatatgct actgtaggcg
69781 atgtaattgt tgcatctata aaaaaagctt taccaaatgg taaggtaaaa aaaggtcaag
69841 tagtaaaagc agttattgtt agaactaaaa aagaaattca tagagataat ggttctttaa
69901 ttcgttttga tgaaaatgct gcggttattc ttgataacaa aagagagcct atcggaactc
69961 gtatctttgg gccagtgggt agagaagtta gatatggtgg ctttatgaaa attgtttcac
70021 tagcaccgga ggtattataa tggcggttaa attaaagatt aaaaaaggtg atagcgttaa
70081 ggttatcaca ggtgatgaca aaggcaagac aggtaaagtg ttagcagtat atccaaaaac
70141 acttaaagtg gttgttgagg gatgtaagat tgctaaaaaa gctataaaac ctagtgaaaa
70201 aaatccaaat ggtggtttta tcaataaaga aatgccaatg gatatttcta atgtggcaaa
70261 agttcaggag taagcgacga tgagattgaa agaaaaatac gatcaaagca tcaaacctgc
70321 tttggtaaaa gaatttgata ttaaaaatcc tatgcttatc cctgcgatag aaaaaatcgt
70381 tattagcgta ggtgcgggtg aacttgctaa agatcaaaaa gtattgcaaa atgttgcaga
70441 tactatttct ttgatagcag gacaaaaagc tgtgatcacc aaagctaaaa aatcagttgc
70501 aggatttaaa gtgcgtgaag gttttcctgt aggtgtaatg gtaactttaa gaaaagaaaa
70561 tatgtttgct tttcttgata aattaatttc tattgcttta ccaagagtga aagactttag
70621 aggtttaagt agagatggtt ttgatggaag aggaaattac aatttcggac ttgatgagca
70681 gttgatgttc ccagaagttg agtatgataa aattttaaga actcatggta tgaacatttc
70741 tatagttaca acagcacaaa acgataaaga ggcacaaaag ttattagaac ttatcggtgt
70801 gccatttaca aaaggaaagt aagatggcta aaaaatcaat gattgcaaaa gcggcacgca
70861 aacctaaatt taaagtaaga ggctatacaa gatgccaaat ttgtgggcgt ccgcattcgg
70921 tttatagaga ttttggaatt tgtagagttt gcctaagaaa aatgggcaat gaaggcttga
70981 ttccaggtct taaaaaagca agctggtaag gaaataaaat gataaatgat ataatttcag
71041 attcactaac aagaattcgc aatgccggaa tgagaaagct tgaaacaacc aagcttttgc

71101 attctaaagt tgttgaagct ttagttggaa tttttcaagc taaaggatat attgaaagtt
71161 tcaatgttat cgaagaagat aaaaagaaat ttatcaatgt agttttaaaa tatgacgaaa
71221 aaggtaaaag cgttatcaat gaacttaaaa gagtttctaa accaggtcgt cgtgtttata
71281 agggtaaaga agaaattaaa agatttaaaa acggttatgg tactatcgta gttagtacaa
71341 gtcgtggcgt tttagcaaac gatgaagctt ataaagcggg tgttggcggc gaaattttat
71401 gtaccatttg gtaaaaatct gctttttatg gcattgggta tccattcttt taaaaaagta
71461 gaaatatcct agacaaataa aaaggaaaaa tatgtctcgt ataggtaaac aaccaatcgc
71521 tatccctagt ggagtagagg ttaaattaga aggaaatttg cttaaattta aaaaaggaaa
71581 tttagcaaaa gagcttgata caaaagcgaa tgtaaatgtt gagattaaag ataataacat
71641 tctttttttct cctaaaggtg aagacagaca aagtagagca tattggggaa cttatagagc
71701 tttagcttac aatattgttg taggcttaac tcaaggtttt tcaaaaactc ttgaaatcaa
71761 cggagtgggt tataaagctg ctttaaaagg taaagtttta gaattaagcc tcggtttttc
71821 acatcctatc aactatgata ttccagaagg aattgaaatc actgtagata aaaacaatgt
71881 tatcattaaa ggaagtgata agcaagtagt aggacaagtt gctgctcaaa ttcgtgaatt
71941 tagaccacct gagccatata aaggaaaagg cgttaagtat tctgatgaac gcattatccg
72001 caaagctggt aagacatcta aaaagtaagg ataggcaatg agagcaaatg tactaaaaag
72061 aaaactaatt ttaagaatta aaagaaaaaa aagaattaga gcaaaaattt caggatgtga
72121 aagcttccca agaatttctg tttttaaatc aaatagaact ctttatatcc aagcgattga
72181 tgatgttaaa gctgtaactt tagcagcagt agatggacgc aaacttggcg ttaaagcaaa
72241 caaagaaggt gctaaaaaaa tcgctgctga atttgctaaa gtgttaaaag ctaaacaaat
72301 agaacaagct gtgtttgata gaaatggtta tgtatatcac ggagtaattg cagcattagc
72361 tgaatcttta agagaaaatg gtattaggct ataaggggta aatcgatgga aaaatataat
72421 agagaagaat ttgaagaagt aatcgtcgat atcggcagag ttactaaggt tgttaaaggt
72481 ggtagaagat ttagatttac tgctttagtt attgttggaa accgcaaagg tttagtaggt
72541 gttggttatg gcaaggctaa agaggttcct gatgctattc gcaaagcagt tgatgatgca
72601 tttaaaaata tcgttgaagt gaaaactaaa ggatcaacta ttgctcatga tgttgaagta
72661 aaatacaacg caagtagaat tttacttaaa ccagctagcg aaggtacagg agttatcgcg
72721 ggcggttcta ctcgtcctat cgtagagctt gcgggtatta aagatatctt aactaagtct
72781 ttaggttcaa ataactcagc taacgtggtt cgtgctacaa tcaaagcttt aacaatgcta
72841 aaaggataag aagatgaatt tgacaaaagc agcgggatca acgcataaaa ccaaaagaat
72901 aggccgtggc caaggaagtg gaatgggtaa aaccgccact aaagggggca aaggtcaaac
72961 cgctagaaag ggttataatg aaaaaagagg ttttgaagga ggtcagcaac ctcttcaaag
73021 aagattacca aaagttggtt ttacttctaa aattgcaaaa ccttatgtga ttaatgttga
73081 aaaaatcaca gccgtaaaag aacttaatga aattacaatc gaaagcatca aaagcgttca
73141 taaaatttca aagtctgtta ctaaaattaa attaattggt gcaagcgcaa aagacttagc
73201 atcaaaaatt aaagacgaga atatcagcgt caccggatca aaataatgaa tagggcattg
73261 acgaataaga ttttaatcac cttagctttc ttgtttgctt atagggttct ggcttatgtg
73321 ccagttcctg gcgtcaatgc tgatgtgatt gcagaatttt ttaacgataa tcaaaacaat
73381 gctttgggtt tatttaatgt ttttagtggt ggggcagcag aacgcttttc tatcatctct
73441 ttaggtatta tgccttatat tactgcttcg attatcatgg agcttttagc agctactttc
73501 ccaaatatcg gtaaaatgaa aaaagaacgc gatggtatgc aaaaatatat gcaaattatc
73561 cgttatgcaa ctatagtaat cactttagtt caaagtatcg gtgtggctat agggcttcaa
73621 agcttacacg gaagaggtgg agcaggcgct ataatgatag aaaatttaaa tatgtttatt
73681 gcgctttgtg caatttctat gcttgcagga actatgcttt tgatgtggat aggggaacaa
73741 atcacacaaa gaggtatagg aaatggtatt tctttgatta tttttgcagg tattgtttca
73801 ggaattccaa gagctatttc gggcacagta gggcagatta attcaggcga aatgaatttc
73861 ttaaccgctt ttgttatcct tgttttgatt ttaatcacta tcggtgtgat tatatatgta
73921 gagcttggag aaagaagaat tcctatttct tactcgcgta aagtagtaat gcaaaatcaa
73981 aataagcgta ttatgaatta tattccaatt aaaatcaatt taagtggagt tattccgcca
74041 atttttgcaa gtgcaatttt aatgtttcct acgactattt tgcaaacaag tacaaatccg
74101 tatttgcaag ctatcaatga ctttttgaat ccaaatggtt atctatttca tgttttgact
74161 ttcttgtttg tgattttctt tgcgtatttt tatgcttcta tagtatttaa tgctaaggat
74221 atagcggaaa atcttaaaaa acaaggaggt tttatccctg gaattcgtcc aggcgaaggg
74281 acagcaaatt atcttaatga agtagcttct cgtcttactt tatcgggttc tatttattta

74341 ggacttgtag caaccttgcc ttgggttttg gttaagttta tgggtgtgcc ttttaatttt
74401 ggaggtactt cagtattgat tgtagttcaa gttgcattgg acacaatgag aaaaattgaa
74461 gctcaaattt atatgaacaa ataccaaacc ttaaatgcaa taggtttgta aatctttaac
74521 acaaggagat agctccttgt gttttactct tactataaaa acattttta tctttgtttt
74581 aaaaataaaa atacgataca aactacaaaa tttaaaatag cccatatcaa aaaagcatca
74641 acaagtccta attgtcttac tataggctga gagataatcg gactcaaaaa ttgccctaaa
74701 aatatacagc tagctaaaat tccataagct ctagctcgtg cattttcagg acagattgaa
74761 aaaagataag cagtgttatt tacaagcatg atgcctccac ccattccaag caaggctaag
74821 gcaaataaaa cagcgataaa atcatcaatc aaaaagagta ttaaaaaaga acaacccacg
74881 ataaataaag tagcaacata aatcgtttta atgcttaaaa aacgcatgat atatttataa
74941 cttagagaaa aaataccata acaaagcgca gagatcgaca ttgaaattcc tataaatttg
75001 ggatctaagc ctagatgctc ttctatataa taaggaagtt gtgtaggaga aatataataa
75061 accaccatga taaaaaatcc tataaaataa ataggaaaaa atttccagta attagttttt
75121 tcttctatct ttgtatgatt ataaaattta aattttcttg gctcaaaaag ataaatcatc
75181 gctactaggg tgattaaaat tccaagccca taaaccaaaa aaggatagcg ccaagaataa
75241 ctcgaaacaa aacccgctat agagataaat attgcaccac ctaccgcaca aaaaaagcct
75301 tgaagactta aggcattttc tctacggtta aagccaccac gactgtaata atcccccagc
75361 aaagccgatg cacctgtcat tataaaagcc gttgccattc caaaaatagc tcttgaagta
75421 agtatggcat agatattatt taagaaaaaa ccacttactc cagcaagtgt ccaaacaacc
75481 attgcaggaa aaacaaattt taatttaccc aatttatcca ttaaaattcc cgcaaaagga
75541 gataaaatca caacgaaaat agcaggtagg gttaatacta aacgcactaa aatatcaaga
75601 tgtgccaagg taaaatttgt ttctacgcta gcacctgatt ggattaaaat atcttcaaaa
75661 tgtctattga ttgcaggtaa tgctgatgag attaacacac ttcctagcat agtcattgcg
75721 gccattgaaa ataccgcgat tttaaagcct gatttttccg tgatctcaac cattttttta
75781 tctaattttg tatcattggg gtgaaattta taagattttt tcttcatatg tggacctttt
75841 ttggttttat tatactccaa aaattttctt ttgtatttaa agttacttct atttgtaatt
75901 tataccttcc atttttcatc tgtatgggta aaatatcttt cactataagt agaatttcaa
75961 aaatttttag ctaaattcag cttttaattt tttataggaa ttatttatct ttaattattg
76021 atagtatttt tagataacaa gaagatgaaa tttataaagg tagtgataaa tgaaaaaaaa
76081 tttgattata gtcgaatctc ctgcaaaagc aaaaactata ggcaattttc taggtaaaga
76141 atatgaggtc atagcttcta aaggacatat aagggatttg cctaaatcaa gttttggtat
76201 aaaaatagaa aacgatgaat ttatccccga gtatagaatt acaagtgatc atagcgcttt
76261 ggttaaagag cttaaggaaa aagcaaaggg agcaaaagag atttatttag caaccgatga
76321 ggatagagag ggtgaggcta tagcttatca tatcgcaaaa gctataggta aagatgaaaa
76381 tgttttaccg cgtattgttt ttcatgagat tactaaaaat gctatcgaaa atgctttaaa
76441 aaatcctaga aaactggata tgaattctgt caatgctcag caaactaggc gtttgttaga
76501 tcgtattgta gggtataaat taagtccttt actaggacaa aaaattcaaa gaggcttaag
76561 cgcgggtcgt gttcaaagtg cagctttaaa gattatcgtt gatcgtgaga aagaaattcg
76621 tgcttttgtt cctttagaat attttagtat agatatgact tttcaaaaag atttagatgc
76681 agagcttgtc gaatttgata aagcaaagat agaaaagctt actcttacaa ataaagatcg
76741 tgcaaagctt gtttttgaag cgtgtaaaaa tgaaaattat gctataaatg atatagaaag
76801 caaagaaaga aaaatcgctc cacctccacc ttttatgact tcaaccctac aacaaagcgc
76861 aagcaatcgt ttgggattta accctaaaaa aactatgatg atagctcaaa agctttatga
76921 gggagtaaat acccatgagg gtgtgatggg ggttattacc tatatgagaa cagatagttt
76981 gaatttagcc aaagaggcag ttgaaaatgc aagaaaattc atacagacaa attttggcaa
77041 agactatctt ccaagcaaag caaatgctta tactacaaaa gctaaaggcg cacaagaagc
77101 tcacgaagct atacgcccta ctaatttaaa ttttactcca gaaatcgcag ctaaattttt
77161 agataaagac gaattaaagc tctatacgct tatctataat cgtttttttag cttgtcagat
77221 gaatcctgct atctctcaaa ctcaaaatgt ttttgtaaaa aatgacagag cagtgtttaa
77281 gataagcggt aggaaaattc tttttgatgg gtattataaa gtttatggcg atatggataa
77341 ggataaaatt ttgccaaatc ttaaaatcgg tgaaaattta aaagtgcaaa atttagaaat
77401 gaattcacat tttacagaac ctccttcaag atattctgaa gctggacttg tgaaaaaact
77461 tgaaagctta ggtataggtc gtccttcaac ctatgcaccg actatttcta tacttacaag
77521 tcgtgattat gtaaaaatcg ataaaaaaca actcattcct agtgaagttg cttttagcgt

77581 aactgaagtg cttgaaaaaa attttagcga tatagtagat agcaaattta cttcaaatct
77641 tgaaaatact ttagatgaga tcgcagaaga taaagccaat tggcaagaga ctttaaaaga
77701 attttactat ccttttatga gaaaggttga agaaggaaaa acaaaaattg caagccaaaa
77761 aacggtgact aaattgggtg aaaattgccc tgattgtggc ggtgagcttg cgattagaaa
77821 agggcgtttt ggagaatttg tagcttgttt aaattttcca aaatgcaagt attcaagaaa
77881 tttaaaaaat gaaagcaaga atgaagctga agtaagtgct agcaaggcta aagcaaaggg
77941 tataggcatt acttgtccaa gttgtaaaga gggtgagata gtagagcgcc tttcaaaacg
78001 cggtaaattt tatggttgta gcgcttatcc aaaatgtaat tttataagca aatataaacc
78061 aagcgatgaa aaatgtgaag agtgtggaga gaatttggta ataaaagagt taaaaaaggg
78121 tacattctta gaatgtttaa aatgtaaaat taaaaaagaa ataaaggatt gattatgaaa
78181 gttatgcttt gtgctatttc aaatatagca agtggaaatt gttctgaaga ttgtaaatac
78241 tgcacccaaa gtgcttatgt aaaaacaaat atacaaaaat atcgtcaaaa agaaatttca
78301 caaattgttt tagaggctaa gatagctaag aaaaatgaag ctttgggttt ttgtttggtt
78361 acagcagggc ttggtcttga tgatgaaaaa ctcgaatatg tttgcaaagc agcacatgca
78421 gtgcaaaaag aagtacccaa tttgcttctt atagcctgta atggtatggc aagtgtggag
78481 caattaaaag agcttaaaaa agcaggaatt ttctcttata atcacaatct tgaagcttca
78541 aaagaatttt ttccacaaat ttgcaccact catacttggg aaagtagatt tcaaaccaat
78601 ttaaacgcta aagaagcagg gcttatgctt tgttgtggtg gaatttatgg tatgggggaa
78661 agcgaagagg ataggataag cttgagaaaa tcattgcaag aattgcaacc tttttcaacg
78721 cctattaatt ttttcattgc aaatgaaaat ttaaaattgc aagtcccaaa attaagtgct
78781 gatgaggctt taaaaattat aagcgatact aaagaagctt tacctgatac tgtagttatg
78841 gtagcaggag gtcgtgaggt ggtattgcaa gaaagacaat atgaaatttt taaagcaggc
78901 gcgggtgcta ttgtaatagg agattatctt actacaaaag gcgaagagcc aagtcgtgat
78961 atagtaaaac ttaaagagat gggatttact tttgcatccg agtgtcattg attcacaggg
79021 tttaatcgat ttacaaattc ttattgttat agctttatgt ttactatttt ctccacatat
79081 agctaaaatt ttgcgcttgc cgatttcagc tactgaaatt attttagggg cacttatagc
79141 gtattttggt ttcataggca agagtgaaaa ttttactatt ttagctaatg tgggttttta
79201 ttatcttatg tttatagcgg gtatggaagt caatttacgc gcttttttta atatggacaa
79261 agaagttgcc aaaaaaagct ttttttatat cattttgctt tattcttttt catctgctat
79321 agtatggatt tttgaacttt ctttcgtgtt tgttttgatt attccggtta tgagtgtggg
79381 gcttttatcc ttgctttata aagactttgg taaagagtgt tactggctta atacagctat
79441 gatagtagca acattagctg aggttgtttc catagtgctt ttaactatag ctggagcttt
79501 tttacgcgag ggaacgaata ttattgatgt agtacaaagc attttatatc ttaatatatt
79561 tttgggtctt tgtttgctta gttttaaaat tttgggcgta ctttttttggt ggtatcccca
79621 acttaaagtc gtactgatgc cttgggaaga caaaaatgaa aaagatataa gattttgtat
79681 ggcaattttt attttgatta ttgtggctat gattattaca aaacttgaaa ttgttttagg
79741 ttcttttatc gcaggctcat ttatcgcaac ctttttttgat cataaaaagg atttagagca
79801 taaactttct agttttggac acggcttttt aatcccaatt tttttcatcc atataggaac
79861 tacttttgat cttaaaatga ttttggaata cgaaatcgta cttgatgcaa ttttacttat
79921 gtttgtaatg gtgggtttga gaattttatg tgctagtgtt ttttggaaaa aaattggttt
79981 tcaaaatatg attttatttg gactaagtca ttctatgcct ttaactcttc ttattgctac
80041 agctacgcta ggatactcag gaaaagttat cagtgaagaa ctttattcgg cactaatttt
80101 aacggcttta tttgaagcta ttgttgtgat gagtgcgatt aaatttattt caaatattaa
80161 aaagtaatct aaatatcaca ggttactttt ttactcaaaa aggttaatga atttttaaat
80221 tcttgataac gaaaaagttg aaaatcgcct tttttagcta gaattagtaa aagtaaagat
80281 taaaaaagga gcaaaaatgt caaattctgt tactgtaact gacaatagaa atggtaaaag
80341 ctatgaattc cctatatatg atggtactac tggtccaagt gttgtggata tgggtagttt
80401 ttataaacaa acaggaatgt tttcttatga tgagggttta acttctacag ctacttgtaa
80461 atcaaaaatt acttatattg atggtgaaga gggtgtttta atgcaccgtg gttatcctat
80521 agagtggctt gctgaaaata aacttttttt agatgtagtc catcttttac tttataaaga
80581 acttccaagt agcgaaagac tcgaagcttt tagatatgag cttaaaaaac gctcttatat
80641 acatgagggt atgcatcgtc tttttgattc tttccctgat aatgctcatc ctatggcagt
80701 tttacaaggc gctgtttcat cacttagtgc tttttatcct gatcatttaa atatgaatgt
80761 tagagaagaa tacatggaaa tggcagctag agttgtggca aaaattccta ccatagtggc

80821 tgctgcttat cgttataaaa atggttttcc tatggcttac ccaaatttgg atcgtggatt
80881 tactgagaac tttttatata tgttaagaac ttatccttat gatcatgttg aacttaaacc
80941 tatagaagta aaagcactcg atactgtatt tatgcttcat gctgatcatg agcaaaatgc
81001 ctctacttct actgttcgtg ctgtaggttc tactcatgcg catccttatg catgtatttc
81061 agcaggtatt ggagcgcttt ggggacatgc tcacggaggc gcaaatgaag gtgttattag
81121 aatgcttgaa gaaatagcta ctgttgatag agtagatgaa tttattaaaa gagcaaaaga
81181 taaaaacgat ccattccgct tgatgggctt tggacatagg gtttataaaa attttgatcc
81241 acgcgccaaa gtgcttaaaa agcttcgtga tcaactaatt gatgaattgg gtattgatac
81301 gaatttgatt aaggtggcaa ctcgtataga agagattgct ttgcaagatg attattttgt
81361 acaaagaaat ctttatccaa atgtagattt tcatagcggc ttaattttaa aagctctagg
81421 aattccaaat gaaatgtttg caacgctttt cgtgatcgga cgtacaccag gctggatagc
81481 acaatggata gagcaaaaag aacaagaaac actaaaaatt gttcgcccaa gacagcttta
81541 tcttggagaa acaagtaaaa tttaaatgat tgtgtgaaat taactttttc acacaaactt
81601 cttatataat ttcaaaagaa tattttgaaa gtcttacaat gtttaaactc gataatttac
81661 taaaaaccgc catagatgct agtaacgaag cttctaaggc tattttaaaa gaaagaaaaa
81721 acctacaaat ttggcaaaag caagataaaa ccccccttaac atcagccgat cttgcttcaa
81781 atgaaatttt aaatgatata ttgggaaaaa cggatattaa aattttatct gaagaaggat
81841 tgttaaatca agaagaaagt aaaaatttgc aaagttactg gcttattgat ccgctcgatg
81901 gtacaagtgg atttttaaaa ggtagtgatg aattttgtat tatgatcagt ttgatttatg
81961 aagctcgtcc tgttttagct cttataaaaa atccttcaaa aggcgatatt ttttacgctc
82021 atcaaaatac tagagtttat aaaaatgata aaattttaga tacaaatgag caagatttta
82081 taaacaatca atccaaagct cttttaagcg tcaatcatct tagcaaagaa gatgaaaatt
82141 ttgccaaaaa atatcactta gaacccataa acataggatc agggcttaaa ttttgcgcta
82201 ttttagaagg taaagcggga atttataagc gttttgaaaa gttaaatagt tgggatattt
82261 cagctggaga ttttttagtt aatcaaaatg gtggttttat gggaactttt tctaaggaaa
82321 tgattttata ttctccctta aattataagt gcgaaccttt tatttgcgtt tctcaaaaaa
82381 gtttttttaaa tatatatata cataaaaaca gcatataatt ttaaaattat taaaggataa
82441 aaatgaaaat tttttgtatt ttgcttatag gtattttatt aaattctagt ctttttgcaa
82501 ataatttttc gcaaaaaaaa attatcaaac tagaacagca agaaagtttt gaaatcatag
82561 atcttcaaca aaatgttgca aaacaaaatt tagatcagca aaaaggaatt tttgatagct
82621 ctagcttggt gggtaaaaaa gaaagtattc gtaaagataa tgatgttgat tttgctatag
82681 ttatgagttt tagacaaaat tttggctata ctttggatgg ttttagtata aatgctaaag
82741 agttttctac tctttttttct aagaatttag tagataattt aaaattaaat tttgtaaatt
82801 cagccgcaaa tggtgtttat caagattcaa gaaccttagc ccgctttagc cctaaagact
82861 ctaaattact taatgtatct cctttttttaa aaaaagaaaa ggataaaagt caaatttatg
82921 ccaaatttac agactatctt gtggtgataa atttagatga tttttatgta aatatcacaa
82981 attattttgt cgcaataacc aaaaatgcag tagctaatat taacttcaaa gttgtttcta
83041 caagcaatgg caaaatactt acatcaaaaa atgtaagatt aaatttttgca cttcatgcac
83101 aagatcctag gataaattat caagaacttg tgaatcaaat gccaaatatg cttgctgaag
83161 ttataaacaa agagttagga aaattaaatt taatagtgca ataaaaaagc ttaatcgttg
83221 atttgcctaa tgcgatcttg tagttcttct atgcttaaaa attcttgtct taattctttt
83281 gcgatttcat gcgcggttat aggatcaaga cttgacaaaa ttcttttaag ctcgtaagtg
83341 atttgccatt tagtcatttc ttctaggttg atatttattt gcatgaatta tccttttatc
83401 gtaagaaaaa attgtttgat tgtagcgtga atttgattaa aaaatgctat tttttctaaa
83461 atcatacata attaaaaaag aagtataaat tgagttttta aacgataatc acttcttctt
83521 ctaaatttat accaaattct tcaaaaacct tttttttagc aagctcgata agaaaaatag
83581 catcttcaaa actcgcattt tttttattga ttaaaaaatt cgcatgctta tcgcttaaca
83641 tcgcacctcc tttactaaag cccttaagtc ctacagcttc aatgagtctt cctgcaaaat
83701 ctccttttgg atttttaaag atagagccaa agcttgctcc gctaggttga ttgtttcttg
83761 catttttttaa attctcatct tttatcttat caaaaccaaa accgagttta aattcagccc
83821 aaaaaaaaagg catatttaaa ggacaaaagc ggtagctaaa atcaatattt tcgcgcaaaa
83881 tttcaccttt tgaagtggca attttgacta aattctgact gatgcactca tctttaagtc
83941 ctgcattcat ttttaaaagt ccgccaagtg taccaggaat tttgctaagg tattcaaaac
84001 cataaagatt attttcttta gaaaaacgat acatttgact tgcttttgtt ttacaaccta

84061 tgcgaagatg taaaaagtct ttgttttgct ctaaaatttc aataaaatta aaattatcgc
84121 ttaaaattcc tatattttta ggcttaggac ttacaaggag attgtttgca ccgcctatta
84181 aaaagccatc aaattcacga atttgatcca aaacttcaac ctcaaattcg cctccaatct
84241 ttacagatga atatttttta aaatcgataa tcatttgata aaagttggaa tttgatttaa
84301 aatattttca gtaaaatcaa tcattgtcgt agtcatccaa ggcattaaaa agatgatgac
84361 tattaccacc aagataattt taggtacaaa agaaagcgtc atttcattaa tctgtgttgt
84421 agcctgaaaa atactgataa taagccccgc gataagccct gctaaaagca tgggcaaaga
84481 aagcaaaagt gtgattttaa aggtttgcac tccaagagca accaaggtgc tttcatccat
84541 tttaatcctt tttaagaatt tctaaattct tcatactcgc tcgggataag ataatcctta
84601 gcatgtatga ttttatcata aaatttataa tcatatccca gtttaaaaag cacatctact
84661 gctttaaatt gcatttcgtt catacagatg gagtttttat tggcatataa attcaaataa
84721 gtatcaagct ttttctcatc aacgcggatt aaatttcttt ccatcagcat aggggctaaa
84781 atgcggcgat tagagtctgc tatttttaca gcatttgtaa gatctctttc tatcttgata
84841 gcatcactaa gcggcaaaga acgacgcaaa gccattccgc ccaaaggtag gggtaaattt
84901 tcttgcgcaa gttctagcca tatatcccaa atttcagctt ctacgcaaag actactgtca
84961 aattctaaaa tgctttcatg gattaaaacc cctgcatcta cttcgccact taaaaccgca
85021 ttttcaatct ctaagaaatt tttataaatg attctagctt ctggatattt catacgaaaa
85081 attagagcat tagtagtatt tgcaccactt aatgcaattt taaaatttct ttttaaatgt
85141 gtattttgtt ttttgataag ttttggtcca taaccctcac caaaacttac tgcagtgcgt
85201 aaaagagcgt attcttgagc aatcaaagga taaagtccaa acgagattgc cgtagcatca
85261 aattcatttc ttaaagccaa atcatttaaa gtttgtatgt ctaaagctgt atttttatag
85321 tcaaaatcat tccctatcca tcctagttta attgccatat acataaaaat atcatcagca
85381 tcgggcgaat gtgcgattgt aattgttttt ttcataactt tcctaatttt taatgaaata
85441 atttttattc tatcaaaata aactattatt taattttatt ttgttaaaat tctagctaaa
85501 aatttgcaat ttttaaggaa aataatggac gataataaga aaaaatcgct tgatgctgcc
85561 ttaaaatcgc tcgataaagc ttttggcaag ggcactattt taaggcttgg agataaggaa
85621 gtagagcaga ttgatagcat aggaacgggt tctgtaggac ttgatcttgc gcttggtata
85681 ggcggaattc caaaaggaag aattattgaa atttatggac ctgaaagctc gggtaaaaca
85741 acgctaactt tacatattat agcagagtgt caaaaagcgg gtggggtttg tgcctttatc
85801 gatgctgagc atgctttgga tgtaaaatat gctaaaaatt tgggcgtaga tacggataat
85861 ctttatattt ctcaacctga ttttggtgaa caagctttag agatcgttga aaccatagca
85921 cgaagtgggg ctatagattt gatagttgta gatagtgttg cagcacttac tccaaaagct
85981 gaaatcgaag gcgatatggg agatcagcat gtagggcttc aagcaaggct tatgtctcaa
86041 gcattaagaa agcttacagg aattgtccat aaaatgaata caaccgtgat ttttatcaac
86101 caaattcgta tgaaaattgg tgctatgggt tatggaactc ctgaaacaac aacaggagga
86161 aatgcgctta aattctacgc ttcagtgcgt ttagatgtta gaaaagtggc gactttgaaa
86221 caaaatgaag agcctattgg taaccgtgtt aaagttaaag tagtaaaaaa taaagtagca
86281 cctccattta gacaagccga atttgatgta atgtttggag aaggtttaag ccgcgagggt
86341 gaattgatcg attatggtgt aaaacttgat atagtagata aaagcggtgc ttggtttttct
86401 tacaaagata aaaaattggg tcaaggtaga gaaaattcaa aagctttctt aaaagaaaat
86461 ccagaaatag ccgatgaaat taccaaagaa attcaaaatt ctatgggtat agagggtatg
86521 attagcggta gtgaagatga agaaggagaa gaataatgtt agtaattgaa gatgttagag
86581 catatgaagt gcttgatagt agaggaaatc caaccgtaaa agcagaagtt gtacttagtg
86641 atggaagcat gggtgcagct attgttccaa gtggtgcaag tacaggttct aaggaggctt
86701 tagaacttcg tgataacgat gaaagatttg gtggaaaagg tgttttaaaa gcagtttcta
86761 atgtaaatga aactatagct gatgaaattt taggactaga tgctttaaat caagctcagc
86821 ttgatgatac tttacgcgag cttgatggta caaacaatta ctcaaaatta ggtgcaaatg
86881 cgactttggg agtatctatg gcaacagcgc gtgcagcagc aaatgcttta ggagtgcctt
86941 tatatcgtta tttagggggt gcaaatgcaa gtattttgcc tgtaccaatg tgcaatatca
87001 tcaatggtgg tgctcatgca agcaataata tagactttca agaatttatg atcatgcctt
87061 ttggtttcac aagctttaaa gaagctttgc gttcagtttg tgaaatttat gcgatcttga
87121 aaaaagagct tgtaaattta ggacattctg cagctttagg tgatgaagga ggttttgctc
87181 caaatttagc taacaataca gagcctattg atcttttaat gacttgtatt aaaaaagcag
87241 gttatgaaaa tcgtgttaaa atcgctcttg atgtagcaag cactgaattt tttaaagaag

87301 gcaaatacca catggagggc aaagcttttt caagtgaaga tttgattgaa cgttatgtag
87361 agctttgtgc aaaatatcca atttgcagca ttgaagatgg tttggctgaa aatgattttg
87421 agggctggat caaacttact gaaaaattag gcaataaaat tcagcttgta ggcgatgatt
87481 tatttgtaac aaatgaagat atcttaagag aaggtatcat taaaaaaatg gcaaatgctg
87541 tgcttatcaa gccaaatcaa attggaacta ttactcaaac tatgagaact gttcgtttag
87601 cacaaagaaa caattataaa tgtgtgatga gtcacagaag cggtgagagt gaagatgctt
87661 ttatagcaga ttttgctgta gctttaaaca cagggcaaat caaaacagga gctttagcaa
87721 gaggtgaaag aactgcaaaa tataaccgct tgcttgaaat cgagcttgaa agtgacgaat
87781 ttttaggaga aaaactctga gtgatttact caaagaatat gacgaaagca ccaagaaaaa
87841 gagcttttat gcccatctta taaagatggt tttttgggct tttctcgtca ttgtaggagc
87901 tatttatttt ggaaatatgc tttttgggca gtattcttta gataccсttc tttcgcttga
87961 agcaaccaaa gaagaattaa ataaaaaaat atcttttctc aaagagcaaa acgccaaagc
88021 acaaaaagat tattttgaat tacaaggatt ataccccaat gaaaactaaa attttaggaa
88081 tatttttgat ttttatttct ttattaaatg ctgaggaaaa tcctttcaaa tcagagcaaa
88141 acatgagtat gattgtgccg cctgattttc aaaaagagga tattaaattt aattctaatg
88201 caagagtttt aaaaagcgta agttttaatt atatcaatct tgatggaagc gaagaggtct
88261 taaatattga cattaacaaa agcatagatt ggcatgatac ttataccctt gtgcgttcta
88321 aaagtccaga tccttcaaag gtttttgatg tttcagtaac catacctgag cgtaatgatt
88381 ccaaaaaaga atccaacaat acagcaagta tagaaatacc attacaggtt gataaaatct
88441 atgattttat atcctatgcg gtgtataaaa ataaaattaa actcaatacc aatgatgagc
88501 ttattagcga tttttccata ggtaatccta gtaaaattgt ggtagatttt aaatcagatc
88561 tagtaaatcc tactaaaaat atacgcatta gcaattccct ttttaagcgc atagattttg
88621 gctctcataa gggatattat aggcttgtga tatatttgga tggaaattat aattacaata
88681 tccaaaaaga tgccacagga tatatgatca atctattgtg atgagaattt ttatttttct
88741 ttggtgtgcc tgctttgctt ttgctagtga tattttgctt ttaagcaaga taagcgaaca
88801 agaaattcaa aataagaatt ttaatggtta tttgatgagt gaaaagcttg atggaatcag
88861 aggaatttgg gaagcaggaa aatttaaaac tcgtcaagat aatccaatcc atactccttc
88921 ttatttcact tataattttc ctagctttaa acttgatgga gagctttgga tagctaggga
88981 taaatttgat gaggtgtcag ctttaattcg tagcgataac ttagactcaa gtctttggag
89041 aagtgtcaca tataatgttt ttgatgtgcc caatgcttgt gaagaattta aacttacttc
89101 ttgcacttta agcaaccgtc ttaaggtttt agagcagtat ttgcaacaaa atcccaatcc
89161 ttatataaaa atcataaagc aaattcctat aaaagatcaa gagcatttga aagaatttta
89221 taaagatatt atcttaaata agggtgaagg tgtagtcata cgcaaggatc ttgctcctta
89281 tgaaaaaggt agaagtaaaa atgcttttaa gttaaaaccc tatgaagatg ctgaatgtaa
89341 agtcataggt tataccgaag gcaagggcaa attccaaggt aaaattggtg ccttgctttg
89401 tcaaatgcca aatgataggg taattagaat aggtagtggt ttaaaagata aagatagaga
89461 aaatccacct aaaataggct ctattgtcac ttataaattc aatggactga ctaaaaattc
89521 tttaccgcgt tttccagttt ttttacatat aagagattaa aattcttgac tttgttttct
89581 ttttctgata taatacctac ttttttcggg gtgtagcgca gtctggttag cgcacttggt
89641 ttgggaccaa ggggccgaag gttcgaatcc tttcaccccg accatttttt ataaatttat
89701 acaaaatggt gagtgtagct cagtcggtta gagcatcaga ttgtggtcct gagggtcgtg
89761 ggttcaattc ccatcactca ccccactaag cgttcgtagc tcaactggat agagcaacag
89821 acttcggatc tgtaggttat gggttcaatt cctatcgggc gcaccaccaa atttttatat
89881 gcgttcatag ctcagctgga tagagcaacg gccttctaag ccgtaggtca gaggttcgaa
89941 tcctcttgag cgtaccatac tagcggatgt ggtgaaattg gcagacacgc cagacttagg
90001 atctggtgca gcaatgcgtg aaggttcaag tcctttcatc cgcaccacaa tttaaaatta
90061 tatctccctt aaattagtgc aatcgtcttt ttctttacaa aataaaaatt tcattttatt
90121 ttaaatttta tttcgatttt tggaaaatta atcattttta ttaaaaaatt tgataaaatt
90181 ttttaagttt ataaaatgaa tttttgattt aaaaatttca ataccatata aaattaaaga
90241 gaaaagtatg caaagaatag tcgtaaaagt aggctctcat gtgatcagcg atgagaatct
90301 tttaaattta aagcgctttg agaatttggt ggcttttttg gccaaattaa tgcaaaaata
90361 tgaagttatt ttagttactt cagctgctat ttcagctgga tgtaccaagc ttcatataga
90421 tagaaaaaat ttaattaata aacaagtttt agctgcgata gggcaacctt ttttaatcag
90481 tgtttataat gaatttttag ccaaatttgg caaaatgggt gggcaaattt tgcttacagg

90541 aaaagatttt gattcaagaa aggcgactaa gcatgctaaa aatgctattg atgcgatgat
90601 tgatttagga attttgccta ttatcaatga aaatgatgcc acggctattg aagagattgt
90661 ttttggggat aacgatagct taagtgctta tgcgacgcat ttttttgaag ctgatttgct
90721 tgtgattttg agtgatattg atggttttta tgacaaaaat cctagtgaat ttagtgatgc
90781 aaagcgtttg gaaaaagttg aatttatcaa agaagaatgg cttaatgtaa gtgttaaaac
90841 aggaagcgaa cacggaacag gcggtatagt tactaagcta aaagcagcta aatttttatt
90901 ggaaaatgat aaaaaaatgt ttttagcaag tggttttgat ttaagcgtgg ctaaggcatt
90961 tttgctagaa aataaacaaa taggcggaac tttatttgag tgattataaa tttttatttt
91021 tccttaagtt ttgaagagta aaataaatta tgtgcataat tcataatact aaggagaaat
91081 caatgaaaaa attagcaaga atttttcttt tagttgttgc ttttgcgctc agtgcttttg
91141 catacgatcc ttatgcttgc tctttgcaaa atttaattaa tctaggtagt tgcaatccta
91201 atttagtgcc taatgatgag tatgcttgtt gggctttacc taatgttaga gtaaatggcg
91261 agcttgtttg tgcggagtat tattaatgtc tattttagta aatgctgtag atacttttta
91321 ttcgaccaaa aatgaagctg tattttcagc ttctaaaagc gaaaatataa aaagaaatgt
91381 cgcggatttt aatactgata ataatacaaa aaattccgtg tattttttta gtgatgataa
91441 agaaatttct ttgaatttaa gcaatgaaag catggaactt ttgacaaatc attttagcgc
91501 aaatgatttt ttaaaacttt ctaatggaaa tataggctta agcggaaaag cagccgcttt
91561 tgtagaaggc tggtataaag atattatgat aaatagaaat tttttaaatg ccgataggga
91621 taaaaatggc agaatagaaa ataacgagta tttatcttta aaaaattcag tagaagatgt
91681 tgcaaaaaat caagatttaa gtaagaattt tggggcagtt gttttaagaa tggctcaaag
91741 tacaacaggc tatgttgaaa atacaattag cgataaaagc ttaagtatag aagcacttat
91801 ggatgaaagt ataaaaagtg atagaaattt tgatgggatt attacgaggc ttgagtattc
91861 tgctaaaggt ggtacagata tgcaaggcta tacgaattgg gctatagatg ctttaaatga
91921 tattttagat gatagcgata aagccaaaaa aattcattta gttgtcgatc ctacaagcga
91981 aatttggcat tcgtatgcag gcaaaacatt aaatgaggtt aaggcttatt atgataatcc
92041 ttttagagcg acgagtggaa cttctgcttc tggtttttta aatatagtga ttgaagaacc
92101 aagaatttta ccaggcaatg atttagaaaa tgaaaaagag caattgctta atgaatttcc
92161 tgaatttaga gctttgatac aaaacaatcc caatattaca aaagaaaaac ttttaaaact
92221 taagaatcag caaagaatca cacacaatta ccaagatcat caaaaacaag atttgcaaaa
92281 agaatttaca caaaatttct ttaaaacaga tcttaaggcc taaattccct taaaaaattc
92341 ggcaatttcc aaaccttaca aatttttata aagatgctcc attttagact ggattgagtt
92401 agctttaaat tcaaatttgc tataatcaaa accttgaatt ttacacaaag cgaaatgatg
92461 aaaaaaatta tttttatggg cacaccaagc tatgcaactt gtattttaaa agctctttta
92521 gaagatgaaa attttgagct tttagcgctt tttactcagc ctgataaagc tgtgggtaga
92581 aagcagattt taacaccaag cgatactaaa gcatttttgc ttcaaaaagc tccacaagtt
92641 cctattttta cgccaaattc tttaaaagat gagagtgtta tagagcaaat tcgtgcttta
92701 aagcccgatt ttatagtggt tgctgcttat ggaaaaattt tacctaaggc tattttagat
92761 atagctcctt gtatcaatct acacgcttcg cttttgccta aataccgagg cgctagtccg
92821 atccaaagtg cgattttaaa tgccgatgaa aaaagcggag tttgcacaat gcttatggaa
92881 gaagggttgg atacaggagc tgtgcttgag agtgtggagt gcgatataag agataaaaat
92941 gtaaatgaag tttttgaaat tttagcaagc ttagcagcaa aacttatcct ttcgactctt
93001 ttaaattacg aaaaactcct tccaaaaaaa caagatgaaa gcttggctac acattgtaaa
93061 aaaattaaaa aagaagatgg gcttgtgagt ttagataatg caagagaaat ttatcaaaaa
93121 tacctagctt ttacaccttg gcctggaatt tttttagaaa atggtttgaa atttatagaa
93181 cttgagcttg tagatgagtt gaagcaaaat gctaaaatgg gtgaaatttt agaactcgaa
93241 aaagaaagtt ttttgcttgc ttgcaaacaa ggaattttac gcattaaaaa gcttcaagaa
93301 agcggaaaaa aggctcttga tggtaggact tatttaaatg gaaaaaggct taaaagtgca
93361 gatagcttgt gttgaaagta ttgattctac ccatcttttt ttatgcgaac aaattcgaag
93421 tggtaaaatc aatgaaaatt ttgcccttta tgccttaaat caaacaaaag gtgtaggcag
93481 tagagaaaat ttatggcaaa gctctagtgg aaatttgcat ctttcttttt gcatcaaaga
93541 aagcgattta cctagcgatt tgcctttggc ttctgtgagt atttattttg cttatttgtt
93601 aaaggaaatt ttacaagaaa aaggttctag aatttggctt aaatggccca atgatttata
93661 cttagatgat aaaaaagtag gagggataat aagcacaaaa atttcagatt ttatagtagg
93721 tggtatgggt ttaaatttaa aatttgctcc tcatgatgct gctttgtgcg atgttgaaat

93781 ttcacttgaa gatttgatag agaagttttt gcaaagacta gagaaaaaat ttttatggaa
93841 gcatattttt agcaagtata tgttagaatt tgaaaaatca agacaattta gtgtccatta
93901 tgaaggtaag gtattttcac ttgaaaatgc tttttatat gaggatggat caattttact
93961 aggcgataaa agggtgtata gtttaagatg agtgagatta ttactatagc aaatcaaaaa
94021 ggtggagtag gtaaaaccac aacagctgtg aatttggcag catctttagc agtggccgaa
94081 aaaaaagttc ttttaattga tgtggatcca caagcaaatg ctacaacagg tttgggtttt
94141 aatcgtaaca attatgaata caatatttac catgttttta taggtagaaa aaaactttcc
94201 gatattattt taaaaacaga attgcctcaa cttcatttag ctccttcaaa tataggtctt
94261 gtaggtatcg aacaagaact tgccaagggt gaaaacaacg aaaaaaaaat gatacttaaa
94321 aaccaaattc aagaagtgct tgatgagtat gattttatca tcatcgattc tccgcctgca
94381 cttggaagta tcactatcaa tgcttttgtt gctagcgata gtgttatcat acctatacaa
94441 tgtgagtttt atgcgcttga aggtgtggcg atggttttaa atacgattaa aattattaaa
94501 aaaactatca atcctaaatt aaaagtgcga ggatttttgc cgactatgta tagctcgcaa
94561 aataatcttt ctaaagatgt tgtggaggat ttaaagcaga attttaaaaa gcagcttttt
94621 actattaatg gcaatgaaga tgattttatc gttatacctc gcaatgtaaa attagctgaa
94681 agcccaagct ttggaaagcc tatcatactt tatgatataa aatctcctgg ttctttggct
94741 tatcaaaatt tagcatattc gattttagga taaaacaatg ggtcttaata aaaatagagg
94801 cttaagcacc ttaatgggag atgtaggaga ggtttatagt agagaattag gtcttgataa
94861 aaatcaagtg agtcttatag aaatttcaaa aatcactcca aatccttttc aaccaagaaa
94921 aacttttgac gaagtggctt taaatgaact tgcaaattct ataaaagagc acggattaat
94981 acaacccata attgtgctta aaaaaaacga tagttttatc ttggtagctg gcgaaagaag
95041 gcttagagct actcaaattt taggagagga aaatatacta gcctttgtat cggatagtga
95101 tgagagtaag cttagggaat tagcactcat agaaaatatc caaagggaaa atttaaatcc
95161 tatcgaactt gcaaattctt ataaggattt aatagaagtt tataatatta ctcaagaaaa
95221 tttagccgaa ctcattcata aaagcagaac gcaaatcaca aatactttaa ggcttttaaa
95281 tttagattct aaaacacaag agcttatagc aagcggtaag atttcacaag gtcatgctaa
95341 ggttttagta ggtttagata aagaagatga aaaagtgctt gtagatagca ttttgggaca
95401 aaaacttagc gtaagagata cagaaagact tgttcaaaaa gttaaaaata aagaaaatat
95461 agaagatgaa gaatttgaaa attctatgca aaatttaaaa caaattttaa ataaaatcgg
95521 ttttatgtgc aaaaataata aaaaagaact cattattcgt ttagataata ttgataaaat
95581 taaaaaactg actgaaattc ttaacaaaat ttaacaaagt cttagttttt atttgttaca
95641 ataaaagact tataatatcc caaaggagat ctatgtttga agatatgcat ccttccataa
95701 tgcttgctac tatggctatt tttttagcta tgatagtgat tttaaacact atgctatata
95761 agccccttttt gaaatttatg gatgaaagaa atgattctat aaaaaatgac gaaaataaag
95821 tcaaagaaaa ttcacaagaa atgctaggag ccaacgatga ggttgaagca attcatgtga
95881 gtacaagaga agaaatccat aaaatcaaac aaaatgccat caatgcagct aaagaggaag
95941 ctcaacaagc tataaaagct aaaaaggaag aactagagcg taaaatggct aacttctata
96001 ccgaattaat atctcaaaaa aaagaacttc aagaccattt gtcagcacat ttgcctgacc
96061 ttaaacaagc tttgcaaaat aatattaaac agcactaata aggagaaaga tgaaaaagct
96121 attttacttt attttttac tgcctttatg tgcctttggt gcatctaatg gtagtggaga
96181 gtatgatatt gttccaagaa caataaattt cttaattttt atagccatat tgtattattt
96241 tgtagcaaat ccttttaaag ctttttataa aaatcgaatt ttaagaattt ctttaaaatt
96301 agatgaaatt caaaagaagc ttttggaaag taaagcaaaa aaacttgaca caatgaaaaa
96361 gcttgaagaa gctaaagcaa atgcagcagc agctttagtt gtagcaaaaa aagaagcaga
96421 ggtgcttgct caaaaaatta aagaagaaac tcaaaatgag ctcgctttac ttgaaaaaca
96481 ttttgaagaa caaaaaagct atgaatttag aaaaatggaa aaagaagttg tgtctttaac
96541 tcttaaagag atttttgaag attcaaatat tgctttaaaa cagaatgaaa tcattgatat
96601 tatgatgaag aaggtgtctt aatgaatgat ttaatagcca aaagatacgc aaaagctata
96661 actttaagag cagatatttc agaattttat gataatttgt ctattttaag ctcagctttt
96721 gccctaccta aatttaaaaa tatcatagaa tcaaatcaaa ttcaaaaaga aaaaaaatta
96781 gaatttgtta tttctttaat agaaaaagct agtcctagtt ttaataattt tttaaaatta
96841 ttagcagaaa attcaagact ctcttgcatt cctcaaattg ctaaagagct cgaaagacag
96901 aaatctttta aagaaaatat ttttttagga accgtttatt ctaaagaagt attaagcgaa
96961 gatagtatca aagagcttga aagtagactt ggtgctaagt ttggtgtcaa aattaaattg

97021 caaaataaag caggtttaaa tgaaggtgtg aaaatttcac tagaagaatt aggttatgaa
97081 atttcttttt caatgaaagc tcttcaaaac aagatgagcg aatatatact caaaattatt
97141 taaggagaat gtgtaatgaa atttaaagct gatgaaatca gttctatcat aaaagaaaga
97201 atcgaaaact ttgatttaaa tttagagatc gaagaaacag gaaaaattat ttcagtagct
97261 gatggtgtag ctaaggttta tggtcttaaa aatattatgg ccggtgaaat ggtagaattt
97321 gacaatggcg ataaaggtat ggcattaaac cttgaagaaa gtagcgtggg tatagttata
97381 cttggaaaag gagagggctt aaaagaaggt acttctgtaa aacgccttaa aaaacttctt
97441 aaagttccag taggagaagc tcttattggt cgcgtggtga atgcgcttgg tgaaccaatt
97501 gatgccaaag gggttatcaa tagcaatgaa tttagatttg tagaagaaaa agctaaaggt
97561 attatggcta gaaaaagtgt gcatgaacct ttacatacag ggattaaagc tatagatgca
97621 cttgtgccga ttggtcgcgg tcaaagagag cttatcatag gtgatagaca aacaggtaaa
97681 accactgtag ctgtagatac tattatttct caaaaaggac aaggggtagt ttgtatttat
97741 gtagctatag gacaaaaaca aagtacagtg gctcaagttg taaaaagact tgaagagcat
97801 ggagctatgg attatactat agttgtaaat gcgggcgctt cagatccagc agcacttcaa
97861 tacctagcac cttacacagg tgtaactatg ggtgagtttt ttagagataa tgcaaaacac
97921 gctttaattg tttatgatga tttgagtaag catgctgtgg cttatcgtga aatgtctttg
97981 attttacgcc gtcctccagg tcgcgaggct tatcctggtg atgttttcta tcttcactca
98041 agacttttag aaagagctag taaattaaat gatgagttag gtgcaggttc tttaacagct
98101 ttgccaatta tcgaaactca agcaggagat gtttcagctt atattccaac taatgttatt
98161 tcgattaccg atggacaaat tttcttagaa actgatttat ttaactcagg aattcgtcca
98221 gctatcaatg tgggtctatc tgtttctcgt gtgggtggtg cggctcaaat taaagcaact
98281 aaacaagttt caggaacttt aagacttgat cttgcacaat atagagaact tcaagctttt
98341 gctcaatttg caagtgattt agatgaagca agtagaaaac aactagagcg cggacaaaga
98401 atggttgaat tgttaaaaca acctccttat tctccactta gtgttgaaaa acaagtagtt
98461 ttaatttttg caggaacaaa gggttatttg gatgatgttg ctgtttcaaa aatcagagaa
98521 tttgaagatg gaatttatcc atttatagaa gcaaaatatc cagatctttt tgaacaaatt
98581 cgttcgaaaa aagctttaga tgcagagcta gaagagaaat tagctaaagc aataaacgag
98641 tttaaagcaa atcatttata aggttctttt atgtctaatt taaaagaaat taaaagaaaa
98701 ataaaaagcg tccacaatac gcaaaaaaca accaatgcaa tgaagcttgt ttctactgcc
98761 aaattaaaaa aggcagaaga agcagcaaaa agatctagga tttatgcgca aaaaattgat
98821 gagattttaa gtgaaatttc atttcaagtc aataagattg ttcataatga agaggattca
98881 agatttgttt tatttcataa gaaaaatcaa atcaaaaatg tggatctaat cttcataaca
98941 gcagataaag ggctttgtgg tggttttaat atcaagaccc taaaggctgt aagtgaactt
99001 ttaaaggaat atgaagaaaa aaatatcaat gttcgtttaa gagctattgg aaaaacagga
99061 attgagtatt ttaattttca aaaaatagag cttttggaaa aatatcttgg cttaagctca
99121 agtcctgatt atgataaagc ttgctctgtt attcaagctg ctgtagatga ttatttaaat
99181 ggaataacag atgaagttgt tttagtgcat aatggatata aaaatatgat cacgcaagag
99241 ttaaaaattt ctcatctaat cccagtagag ccaaaacaaa tcgaaggaaa ctcaaattca
99301 cttttagaac ttgaacctga agatacagat cttttgggag atttgatgaa aacatatttt
99361 gaatacaata tgtattatgc attaattgat tccttggctg cagaacatag tgcaagaatg
99421 caggctatgg acaatgccac caataacgca aaagctagag ttaaacaact taatttagct
99481 tacaataaag caagacaaga atctatcact actgaactta tcgaaattat cagtggtgtt
99541 gagtcaatga aatagtaaat ttttaaggag agtataaata atgcaaggat ttatttcaca
99601 agtattaggt ccagttgttg atgtagattt taacgactat ttgcctcaaa ttaatgaagc
99661 cattgttgta aattttgaaa gtgaaggcaa aaaacaaaaa cttgttttag aagtagcagc
99721 acacttgggc gataatagag ttagaactat tgctatggat atgacagatg gcttagtaag
99781 aggacttaaa gcagaagctt taggcgctcc tattagtgtt cctgtgggtg aaaaagtttt
99841 aggaagaatt tttaatgtta cgggagattt gatcgacgaa ggcgaagaag ttgcttttga
99901 taaaaaatgg gcaattcata gagatccacc tgcatttgaa gatcaaagca caaaaagcga
99961 aatttttgaa acaggtatta aggttgtgga tttgcttgct ccttatgcaa aaggtggtaa
100021 agtaggactt tttggtggtg ctggcgttgg taaaacggtt attattatgg agcttattca
100081 caatgttgcg tttaaacata gcggttattc ggtatttgcg ggtgttggtg agagaactcg
100141 tgaggggaat gatctttata atgagatgaa agaaagtaat gttttggata aagttgcctt
100201 atgttatgga caaatgaatg aaccaccagg ggcaagaaac cgtattgctt taacaggtct

100261 tactatggct gagtatttta gagatgaaat gggtcttgat gttttgatgt ttatcgataa
100321 tatctttaga ttttcacaat caggctctga aatgtctgcg cttttaggaa gaattccttc
100381 agctgtgggt tatcaaccga ctttggcaag tgaaatgggt aaattccaag aaagaattac
100441 ttcaactaaa aaaggttcaa ttacttcagt tcaagcggtt tatgttccag cagatgactt
100501 aacagaccca gctcctgcaa ctgtttttgc tcacttagat gcaacaacgg ttttaaatag
100561 atctattgct gaaaagggta tttatcctgc agttgatcca cttgattcaa cttcaagaat
100621 gcttgatcca aatatcatcg gtgaagagca ctataaagtt gcacgcggtg ttcaatctgt
100681 acttcaaaaa tataaagatt tgcaagatat cattgcaatt ttaggtatgg atgaattaag
100741 tgaagaagat aaattgatcg ttgaaagagc aagaaaaata gagaaattct tatcacaacc
100801 attcttcgtt gcagaagtat ttacaggtag ccctggaaaa tatatttctc ttgaggaaac
100861 tattgccggc tttaagggaa ttttagaagg taaatacgat cacttgccag aaaatgcttt
100921 ctatatggtg ggtaatatcg acgaagctat tgcaaaagca gataaactga aaggttaatt
100981 tatggacaat ttaatcagtt ttgaaatagt tacacctatg ggtgtaattt atcaagggga
101041 ggtaaaatca gttaccttgc caggaagcga aggagaattt ggagtactta aaggacatgc
101101 tgctttggtt tcatctttaa aatctggtgt aattgatata gaaaaagctg atttaaatca
101161 tgagttaatc gcaattgatg caggtcatgc taaggtagat gaagataaga tcagcgtttt
101221 ggctaagggc gcggtttgga tttcgggttc tagtgaaagt gaaatagaga aaaatcttga
101281 aagcgctaaa gaattaatca aatctatgag ttcagataat acagctttag ctgctacctt
101341 ttcaaagctt gacaatgcaa aggttaggta gatgaatttt gaagcgatat ttcagttttt
101401 tggaagctca agcataatca cttatatagt cttagcttgg ctttccttat actttatact
101461 ttcttttttt atactttttt caagaatgac ttatttagcg gcttggaaaa ataaagaaaa
101521 agaaagtcta gaatctctac tcttgggcga aaaagatttg agtagaacgc gctctatttt
101581 aagaaagtgt acagatacta gcttatcaca tcttgagatt tataaaaatt tagcaagtcg
101641 tcgtgcatct aatgggctta cttggctcag tatagtagct tcaacctctc catttatagg
101701 actttttgga acagttattt ctatacttga gacttttggt ggtttaggga cacaaaattc
101761 tttaagcatt atcgcaccta agatcagtga agctttggtt gctacaggct gtggtatttt
101821 agttgctatt cctgcttata cttttcatct tattattaaa agaaaggctt ttgaactttt
101881 aagtattatt gatagtgaga ttaaagttat tagttctaat aaataggatt taagatatgc
101941 catttgatga tgagaaacca gagcttaata taactccttt ggttgatatc atgcttgttt
102001 tattggctat tttaatggta acagctccaa gcattactta tgaagaaaaa atcaatcttc
102061 ctcaaggaag tcaaaaatcc acaagtgctc ctagtgttaa aagcttgatt gtcagtatca
102121 atgccaaaaa agaaatattt ttaaatcaag aaaaatatag ttttgtaagt tttgcggata
102181 atcttgcaca aaagaaagca caatttaata ccgatgagcc agtgtttatt agagccgata
102241 agagtttaaa gtatgatgat gttatttttg tcctaagaag tattaaaaat ttaggcttta
102301 gtaaagtcgc cttacaaaca gaataagctc atgaaagaat atggtttagg caagataaat
102361 tcatttttgc ttgctgttgc ggtttatatt tttattataa ttcttatttt ttttagactt
102421 gtaactcaca tggaacctgc aatccaatac actgatatcc aagatagttt tatcgatata
102481 gagcttatgg agccttcaaa gaaagtcgta aacgaacaaa gtacccctaa ggatattcaa
102541 aaacctactc aagagcttga tattgaaaaa ctttttgcac aaactacaaa taaagcagta
102601 aaaactgaag atgtggatca aaaagctagt aattttaatg agctttttgg tagtatcaaa
102661 gagatacaag aggaaaaaac taccaaaata caatcaagcg ccaaatcgga aacaagaagc
102721 agctcaaaaa ctcaagcctc agaacttgta aagcaattaa acgatagttt acttgaagaa
102781 gaaagtatca atcaaggtga aagtattaaa aatcaaaaga caggaattta tgatgagttt
102841 ttaggaaagg tagtgcgcac cattacacaa agatggaggc aatatcatcc taatagtgaa
102901 aatatttcag ttaaggtaaa aatttttatt gatgaaaatg gaaattttgg ttatacttcg
102961 gtcgaaaaaa gtggaaattc tttatatgat gcaaaggtag ctgaattttt ggaaaaccaa
103021 aaaggtaaat ttatagctta tccacctcaa aataaaagca taagtattac tatgaattta
103081 aaagatgagg cacaaattca aaaataattt aggagtaaaa atgaaaaaaa ttgtaacagt
103141 atttttaatt tttttaggaa tactttgggc agaagatcct gtgattgatg ttgtaaattc
103201 gggtgtagtc ttaccaaaaa tcattgttaa agacaattcg aatttaagtg atattaattt
103261 aaagcgcagt ttttataata tcattgtaaa tgacttgaag gtaagttcaa attttgaagt
103321 tgtaagtgat gcaagttcag aagcttcaaa ttatactttt gagtatgatt taagtaaaaa
103381 tggtaatgct ttaaatttgc atgtcaagat taaagcaggg ggcttggaaa aatcaagcca
103441 aacctatgct ttaaatggtg tagaacagta tcctttttta gctcacaaaa gcgtaaaggc

103501 ttctgtaaat gcattaggac ttgcgcctgt tgaatggatg gatcataaaa ttttgattgc
103561 tagaaattca agctctaaaa aaagtcaaat tatcatggca gattatactt taacatatca
103621 aaaagtaatc attgatggag gattgaattt atttcctaaa tggggaaata aagagcaaag
103681 catttttat tataccgcat atgatcatga tagaccaacg ctttatcgtt atgacttaag
103741 aaccaataaa gcaagtaaaa ttttatcaag tggaggtatg gttgtagcaa gtgatgtgag
103801 cttagatggc agtaaattac ttgtgactat ggctccaaaa gatcagcctg atatttattt
103861 atatgattta aagtctaaaa atttaactca acttacaaat tattcgggta tagatgtaaa
103921 tgggaatttt ataggcgtag atgatagtaa atttgttttt gttagcgatc gcttaggtta
103981 tcctaatatt tttatacaaa atgtcaataa tggatctgct gagcaagcgg tttttcatgg
104041 taaaaataat tccgcagttt caacttataa agattttta gtatactcta gtcgtgagcc
104101 aaatcaagta ggtgttttta atatttattt aatgtcaatt aatagcacag gtattcgtca
104161 gtttaccgca aatggaaaaa atttatttcc tagattttca agtgatggtg ggagtatagt
104221 ctttattaaa tatcttggag cacagagtgc tttaggtgtt attcgtgtta atgcgaacaa
104281 gactttctat tttccattaa aagttgggaa aattcagtct attgattggt aaaaatttgt
104341 aatttttggt taaatttaat taattttaga tataatgcta acaatttatt aactttgaaa
104401 ggtcataaat gaaaaaaatt ctttttagtt ctattgcagc atttgcattg gttatcagtg
104461 gttgtagcac aaaaagcact agcgtaagtg gtgatacaag tgtagattcg aatcgtggaa
104521 caggtggtag tgatggctgg gatattgatt caaaaatttc tcaacttaat gatactttag
104581 gaaaagtgta ttttgatttt gataaattta atatccgccc agatatgcaa aatgttgtta
104641 atacaaatgc taatatcttc aacaatgaag taagcggtgt aagtatcact gttgaaggta
104701 actgcgatga gtggggaaca gatgagtata atcaagcttt aggtttaaaa agagcaaaag
104761 cagttaaaga agctctaatc gctcaaggtg taaattctga cagaatcgct gttaaaagct
104821 atggcgaaac aaatccagta tgtacagaaa aaacaaaagc ttgcgatgct caaaatcgtc
104881 gtgcagaatt taaactatca agataattga tgaaaaaaat attctcagta gctcttatcg
104941 gagctacttt actttatgca gaaagctctg cttttggtgc aggagattta accagtaatt
105001 cccccctatgg tttaacatca agtgaaaaat tactcaagga aaaattagat actttaagca
105061 ataataacgc acaagcaaat tctagaatta atgagattga acaaagagtt gaaggattgc
105121 aaagtacctt agaaggtata aattcgcagt atgctaaatc caattctaga ctaacacaac
105181 ttgaaactaa ttttgaagct atagaaaata atttaagcac agaacttcaa aatttaaaag
105241 cttatgttga agaaagtaga aaaattcaag atgctaacaa taagcaaatt aagaaaattt
105301 taaccgaatt aagttctttg gtcgattcta ttaacgcaaa ttatgtatct aaagatgact
105361 taaatagtac aaatttaagt atcaaaaata cagttgtcgt gcctctatcg actgatgata
105421 aaaacataac ttctagcaat gaaaataaca tcagtgttgc ttcaacgcaa gaaaaagaag
105481 ttaaacaaat agatgattct tggaaaaaga aaaaaaataa tgaaatttta gacttggcta
105541 tcaaagattt aaataataat tcttatgaga atagtaaaat taagttaaat tatctcatac
105601 aaaaacaata caaacccgca agagcaaatt tttggttagg agaaattgag tataagcaaa
105661 aaaagtataa taatgctatt gtttattata aaaaaagctc tgctttgagc actaaagggg
105721 attattttcc aaagctctta tatcacacag cgatttctct tgataagata ggagatccaa
105781 aaacagctaa tggattttat aaggctttaa agacaaatta tcctaactca ccggaagcaa
105841 aagcttcccc aaatagaaaa taatatcaag gagaaaaaat ggctatagaa aaaaatagcg
105901 tagtttcaat gttttatgaa ctaaaagatg caaatacaaa tgaagtttta gaatcaaatt
105961 tatatgctca acctatttct tttattttag gcaaaggtca aattttagaa gctttagaag
106021 aagaagtgat gaaacttgat tgccctagta atgctgatgt tgtagttaag aaagaaaaag
106081 gtttaggcga atacgatgaa aatgcggtgc aagttttacc aaaagaacaa tttgcaggta
106141 ttgatttaaa aataggtatg gagcttttg gtgagggcga aaatggtgaa accgttcgcg
106201 ttactgttaa agaaattggc gataatgatg tgactattga ttataatcat ccttatgcag
106261 gtcgtgattt gctttttct ttaaatatag tagatgctag agtagcaagc gaagatgagc
106321 ttcttacagg cattatcgca ggaagccata gttgtggttg tggaagtgga cacggacacg
106381 atcatcacgg acatggcggt ggtggatgct gcggtggcgg tggcggcgga tgtggttgcc
106441 actaagatga acgctgtatt tatttttcca ggtcaaggct ctcaaagtct tggcatgggt
106501 aaagattttt atgaaaactc caaaaccgca aaagaacttc tagaaaacgc cagcgatttt
106561 tgtaaaattg attttaaaaa tttattattt aatgaaaatg aagatttaaa caaaagtgaa
106621 tttacacaac ctgccatact cttaaattct ttaatggcgt atagtgcttt gagcgaatta
106681 aaacctgata ttaaggctaa attttcttta ggccattctt tgggtgaatt ttcagctttg

106741 gcgatcaatg gtgcttttag ctttttagaa gcaactatgc ttgtgcataa aagagggctt
106801 tttatgcaag aggattgtgc aaaggttgaa gcaggtatga tggtaatttt agggcttgat
106861 gataagaaag tggaagaact ttgtcgaaaa gctagagatg aagataaaaa gatttttgca
106921 gctaattata actgcgatgg gcaaatcgtt gttgcaggtt taaaacctga tttagcaagt
106981 tatgaaagtg tatttaagga agcaggtgct aaaagggcta tgcttttaaa tatgagtgta
107041 gcaagccatt gtccgctttt agaaaacgcg tctaataagc tttgcataga gcttgaaaga
107101 atactagagc ctaattttaa agccgtgatt tccaatgcta cagctaaagc ttatacgagc
107161 aaacaagaag ctcttgagct tttaaaaacc caacttgtag ctcctgtgct ttataaacaa
107221 agcattaaag cttgtgaaaa tgaagcagat tattttatag aatttggcgc tagcgtgctt
107281 aaaggtttaa ataaaaaaat cacttcaaaa gaaacctatg ctttaacaaa tatgaatgat
107341 atagaagaat ttttaaaggt aatataaatg aaaatagcaa ttttaggtgc aatgagtgaa
107401 gaaatcactc ctttgcttga aatattaaaa gattatacaa aattagaata tgcaaataat
107461 acttattatt ttgcaaacta taaaaaccat gaacttattc ttgcttattc taagataggc
107521 aaggtaaatt ctaccctaag tgctagtgta atgatagaaa aatttggagc gcaagttttg
107581 ctttttacag gtgttgcagg agcgtttaat cctgagcttg agattggcga cttgctttat
107641 gcgactaaat tagcacaata tgatcttgat atcactgctt ttgggcatcc attgggtttt
107701 gttccaggaa atgaaatttt tataaaaaca gatgataagt taaacaatct tgccttagaa
107761 gtcgcaaaag aattaaatat caaacttcgt gcaggtatta ttgcaacggg tgatgaattt
107821 atctgcgatg aggcaaagaa agcaaaaatc agagagattt ttaatgccga tgcttgcgaa
107881 atggaaggag caagtgtggc tttagtgtgt gatgctttaa aagtgccttg ttttatctta
107941 agggcgatga gtgataaagc cggcgaaaag gcagaatttg attttgatga atttgtgatc
108001 aattcagcta aaatttcggc taattttgtg cttaaaatgt gtgaaaaatt atgataaatc
108061 ttagcaaaaa attaatacgc caagttgcac aagctaatgc taaattcgga ctcattaaag
108121 atggagatag ggttctttta gggcttagcg gcggaaaaga ttctttagct ttagcacatc
108181 ttttaaaaag aatgcaagct cacgcacctt ttaaattcga gcttgaagca gcaactttaa
108241 gctatggtat gggtgaggat tactcagggc ttcatgcgca ttgcgaggag cacgggatta
108301 aacacagcgt gattgattca aatatctacg aggtttcagg cgatacaata agaaaaaatt
108361 caagcttttg tagttatttc tcaagaatgc gtcgaggtgc tttgtatact tatgctcttg
108421 agaaaggttt taacaaatta gctatcgcgc accatttaga tgatgcagct gagagctttt
108481 ttatgaattt tatccataat ggagctttaa gaactttagc gcctatttat caaagcaaac
108541 gaggtattac ggttatacgc cctttgattt ttgtgcgcga aagacaactt agagataatg
108601 ctacgcaaaa tgaacttcag gttataggaa atgaattttg ccctggtatg aaactaagcg
108661 aaaaaaatgt aaaatttcca catgcaagag aagaagcaaa gcaactttta gctaatttag
108721 aaaaagaaca tcctaagctt tttacgagtt taaaaactgc tttttcgaat ttgcacacag
108781 aaagtttttg gctagaaaag gcataatttg atgaaaaaag cttttgtttt ggtagattat
108841 caaaatgatt ttatcgatgg aagtttgggc tttgaaaaag ctttaaaaat caaagaaaat
108901 atccttaata ctctcaatca aatcgatttt aacaccacac atttgcttct tacttatgat
108961 actcatgatg aggattattt aaggagtaaa gaagggttaa atttgcctat tgagcattgt
109021 ataaaagaca gtttgggttg gcaaatgcct aaagaattta caccctttt gcaaaaagct
109081 cataaggttt ttcataaaaa tacttttgga agtttagaat ttgcaaattt tatcgcaaaa
109141 agtgaatacg aagaaattca ttttgcagga cttgtttccc atatttgcgt cttttgtaat
109201 atcattttag cttttagcgc gaagccaaat tcaagactta tactccatca aaatttaagt
109261 gcaagttttg atgaaaattt agaaaaatcc gcttttgatc tacttagagc ttatggcatt
109321 gaaatacttt aatgcaaggc tttatacttc atacacaaaa agtaaaagat gaggatttga
109381 ttgtttatat tttaagccct aaagagcttg ttaaggcgta tcgtttttat gggcttagac
109441 attctagtat tttaaatggt tataaaattg attttgcttt agatgaaaat cctagttttt
109501 tgccaagact taaggatgtt ttgcatttgg gttttgtatg gattatggat cgtgaaaaaa
109561 tgctgatttg gcaggaattt atccgtcttt tgtatcatca tttaaaagat gctcaagtgc
109621 ttgaaagttt ttattttgag cttttagatg aatgtgtaaa acgttttgaa aagcaaaatc
109681 ccaaacgcgt aattgtagat gcttatctta aaattttaga atttgaggga aggctccatc
109741 aagaatttag atgttttgct tgtgatgaaa gcatagaaaa tcccatcact ttaatccgtg
109801 ctttttttacc ttcgcatcac acttgtgctt taggttatgc atttgaaaag cagaaattaa
109861 aaaattttta tgaaagtaaa aattgtgcga tttttggtga tgaagaaata gggaatttgt
109921 atcaattgat caaggagggt ttatgatatt aagtgatgaa aaatgcgaat ttttagagcc

109981 tatagcaagt tttttaagct ctaaggatgt ggaacttgtt tttgtagaaa gtaaggaaat
110041 gcaagagatt aacttagagc aaagaaagca agataaaacc accgatgtgc tttcttttcc
110101 acttgaaaat atcgatgaga gcttgccttt gggttctgtt gtgattaata tagatttggc
110161 aaaacagaaa gctaaagagt taggacatag ttttgaagag gaagtaagct tgctttttat
110221 ccatgccatg cttcatttgc ttggatttga tcatgaggta gataatggag aaatgagaga
110281 aaaagaaaaa gagcttatag aacactttaa tctgcccaaa agtttgatta taaggacttt
110341 ggaagattaa gtaagatttt tttctttaga cactagaaga cttgtgcttt atattttaaa
110401 tatatgagta aaaatactta agaaaaaatc atttttaccg atttactttg taaatgaaat
110461 tttatttaca aggtggcaaa gatgattaat ggtataaatt cttattcaag ctacaattac
110521 acaaatactt ttagcaataa tacttcaaat acaaaatcat ctaatgttac aaatgataac
110581 tcaaactttg taagcgataa atcacgagca gttgataaaa ttttaggcta tggcgtagat
110641 gaatatggct tcttcacgag tgattttaac gaagctgcag gattgccaaa agattataag
110701 atttttgcag agggtttaaa taattttgta aatagctttc ttaaaagaca accttttac
110761 tcgcaaatag acattgccga aactatagga aatacttata attcttttc atctttcgta
110821 aaagataatg gattaggaga aaatcttaaa gaacaagatt tacaaaattt accacaaaaa
110881 gatggaataa attattggca agagctacaa agtggtaatg ctttcatttg gaaacttgtt
110941 gatgaaaatg tagctgactt aggttcaaac aaatatcaaa tacaaaatgg agaaatatcc
111001 aaaggtggtg ccttagttgc ttttttaca aataatctta gctttgttga aggaaaaact
111061 actttattag gtaaattagc tggacttaat agcaatacag acaatgataa aataaaagaa
111121 tttttatcct ttatgaatgc aaatccttta aaatactctt atgaacaaga aggttggggc
111181 aatcctatgg gagatagttt atctatatgg aattatatga atagagccaa gaatttagga
111241 aatgataaac tcactaaaac tattgaaaat ttaggaaaag aatataatca attaatcaat
111301 gctgatatga gtttagaaga atttaaaact aaatatcttg atttcaaaca aagacacgat
111361 gagtttgtaa aatcactaga agaagcagaa aaggcaaaag gtatagatta taataatcct
111421 acaaaaagca caaataacaa agaaacttca gaagaagaca aagaaaagcc attcaagcct
111481 atacaagcag aaagtaaaag cgaaacttac aaagatgata ataaaatgaa tgagctttta
111541 aaaaagctac tagaaactaa atttggcaca agtgatgagc tagaaattct ttttggtatg
111601 aaatttagtg atgataatgc aggggaattt aataaaattc tttctttaaa ttctgcacca
111661 aaaattatag atattaaggc ttaaaaatat aaaaatttaa attataaaaa ggattaaaaa
111721 tgataggtat caactcaact ttcaataact cctttgtcaa tcttaatatt aaaaacaata
111781 acaactcttc cttaaattca aatactaatg taaaaaattt acaagatacc caagacacta
111841 ctaaaacaag caataaagtc ttaggttatg aagtagataa agatggcttt tttacaagcg
111901 attttaataa agctgcaggc ttaccaatct tgcataactt ccaccccctg aaataagcat
111961 gtcaaactta ggtgttaggg cgtaaatgaa agtttgagtt aaaaacactt gctttgtatc
112021 attccaaatt gtaggatctc cgcctattgt tatattaggt taaagagaat acgaatttgt
112081 atttcttgtg ctaagtaaag aaaagcttgt aatacttctt aagcctattt gctttttaaa
112141 caaactgtct atatttagtt catcactagc aaaaaacaca actgactaaa accataaatg
112201 ttaaataaat ctttttttatt attgcctttc taaatatttt taaaaatttt tattcaaaat
112261 agtaaatatt tcaaaataaa aatccgattt taatatataa tttatgatat tattcaaata
112321 aagagtaaaa tagttaagaa aaggagaagt tatgctaaag aaaatatcaa gcgctttgat
112381 tttaggttct ttattggtaa gttctgcttt tgctagggat tttttagcta aggttagtaa
112441 tggtgcttta agtgataatt ctcaaggggt taagactctt aattttgatg agatgaaaca
112501 agttaagggt gggtatagag ttagttttaa gctagttgca aatgatgagc ttttagcttt
112561 tgccaaaact acaccgagtg aaataggata ctatacagat aacaatggcg atacatatta
112621 ttggaatagt cagggattaa ggcaatttgg tggaatgtgt ggtatagaag tcacaaattg
112681 tatggtaaat aataaacata catatgctaa ccaaaaaaga ctttgggaaa ctatgagcat
112741 agtaggcgat gatccatacc actattatct tggttataca gtaaaaagaa atataggtgt
112801 atcaaattta ggacaaagat ttgtatattt ttcctatggc gtagctgtta ttaatgagca
112861 attaggttct tggtataggg tgaattcaag tgctgtttta aataattatc aagttattaa
112921 agaaattagt tccaaatata aaaatgatat ggaaatggct cttagtggta attattcgag
112981 atatagaaat aagctatgat aaatagtata aattcaaatt taaactataa ctataatact
113041 tcaaatttta agcaagataa aaatattgac tttaaaataa acaagcatgg agtagctgtt
113101 gtaacaggag aaggtgcttt gccttatcgt ggaaaaaaat ttgagtataa tcctaaaata
113161 ttcggacttg atgaaaatat tagcaaaaaa gatatgcaag aatttaataa ctttatgaag

113221 tcaaatgctt taaaagatcc aaataaaata gaaaagtctt cacttaaaaa tcttggtaaa
113281 gatgataatt gggatataaa agatgaagat attaaattga tgaacttttc gtcttactct
113341 caaatgttta atcttggatc tgatattgct ataaaagcat ctaattttag gaatgaattt
113401 aataaacttt tacaagaaga tttaacccta gaagaattta aaactaaata tcttgacttc
113461 aagcaaagac acgatgagtt tgtcaaagaa tttgaaacag ctatgggtga taaaatgcta
113521 cttgctaata ataatgatat tagtatgcaa gaagacaaag aaaagccatt taaacctata
113581 caagcagaaa gtaaaaacaa agaaacttac aaagatgata gtaaaatgaa tgaactttta
113641 aaaaaactat tagaaggtaa atttagcact agtaaagaat tagaacttct ttttggtatg
113701 aaatttagcg atgatgatgc aggagaattt aataaaattc tttctttaaa ttctacacca
113761 aaaagtatag atattaaggc ttaaaaatat aaaaatttaa attataaaaa ggattaaaaa
113821 tgataggtat caactcaact ttcaataact cctttgtcaa tcttaatatt aaaaacaata
113881 acaactcttc cttaaattca aatacttcaa ataatagtgt aaaaaatcta caagataccc
113941 aagacactac taaaacaaat aataaagttt taggctatgg cgtagataaa gatggctttt
114001 ttacaagtga ttttaacgag aaagcaggca ttccaaaaga tataaaaatt cattctagca
114061 ctatggaaag cttagtaaaa atatctacaa atgaaaaaaa tccatttaga gcttttgaga
114121 gtatagacat tgctaaaacc gtaggaaatg cctataaaat tttaagtcaa gttgtaggcg
114181 aagatgtgct aaattctaaa gatagcttta gcttagaaga gatagccaac ttcccacaag
114241 gctatgaatt taatcgccaa actatgcaag taacgaaaat ttataattct gctacggatt
114301 atttaaatgc tgatatagat tttgattatg aaaatacaac tgatacaaaa ataaacaaat
114361 tgttttttaa tactactgaa aatcttatga aaggaaattc tgccacagat atttttaata
114421 ataatggcgg tgaagaaaat attaatacag gcgtattttt tgatacgaca ccaagcaaat
114481 acactaataa agatggcact attactaaag gtgggctttt agtagctatt ataaatgaga
114541 atatctatac tagagaagga gaggctacgc gtcaaggaaa aaccgaaggc tttgataagt
114601 ctatgagtgg tgaagaagtc aaaaaagctt ggaatgatag aaactataat ttaaagttaa
114661 atccaaataa tatagttcaa ggtgctggta ttgtcgatac aaataatgaa aatatagaat
114721 ttacaccttt aattgaaata atgcaaaaag agcaaaaaga attaataaaa tacttacaga
114781 gaaaacaaaa aattgatata acaatttaaa tcacattaag gagaaagtat gctaaagaaa
114841 atatcaagcg ctttgatttt aggttcttta ttggtaagtt ctgcttttgc tggggatttt
114901 ttagctaaag ttagcaatgg tgctttaagt gataattctc aaggggttaa agctcttaat
114961 ttagatgaga tgaaacaagt taagggtggg tatttagtcg gttttaaatt ttttaaagat
115021 cgtccaaatt taatttcaga agtttatgca attgcagatt tttcagaata cgaacttagc
115081 aatcttaaca agggtctatg tggtgcagga gatgaaaaat gctcaaaccc tagtagagat
115141 agattaattt cttggttgca agcgagtgct aattcccctg ctgaatatcg tcctgtatat
115201 aaggtaaaaa gacaaataaa atactcaaat ttaggacagc cttatgtttt atttacttat
115261 ggagtcggtg tttataatgt aaagaatgga caaatatatc aatataattc tagtcctatg
115321 ttaaataata atacaatcat gagagaaatt gcttataaat atcaaaattc tatacaagat
115381 gctatgggtg gttggaatcc aagatatttt aataaaatat cataaaaaaa aggggttttc
115441 tatggtaaat tttgatactc aatatacaaa tattaattat ctaagcaata acacgaacaa
115501 taaaaaatct gtgcttaaaa acgatgatga ttattttaat tccgtgataa aaaaatttaa
115561 tttctctgaa aaaaccaaag aagaacaagg atataatgga tctcctgatg taaaagaagt
115621 agaaaactct ttatataaaa gatatatgga agataaacta agcaaggatg aaataaaaat
115681 acttaataaa tttggcacag actatcttac agaaggcgta gatttgtccg cacttcataa
115741 acgaaaagtt gatcttttaa aaactcttac aagtatgaat gaatttgaga aaatatgggg
115801 tgctgaaagg gcgcaaagag aaaaacaatt ttttaatgaa cttggtaagc cgcttactca
115861 agaagatata gataagttct tagctaataa aaacaatcaa aaagaagaca aagaaaagcc
115921 atttaagcct atacaagcag aaagtaaaaa caaagaaact tacaaagatg ataataaaat
115981 gaatgaactt ttaaaaaaac tattagaagg taaatttagc actagtaaag aattagaact
116041 tctttttggt atgaaattta gcgatgatga tacaggggaa tttaataaaa ttctttctca
116101 aaacacttcg gcaaaaatta tagatattaa ggcttaaaaa tataaaaatt taaattataa
116161 aaaggattaa aaatgatagg tatcaactca actttcaata actcctttgt caatcttaat
116221 attaaaaaca ataacaactc ttccttaaat tcaaacactt caaataatag tgtaaaaaat
116281 ctacaagata cccaagacac tactaaaaca aacaataaag ttttaggtta tgaagtagat
116341 aaagatggct tttttacaag cgattttaac gaagcagcgg gaattccaaa agattataag
116401 atttatgcta aaggtgctga aaattttgtt aattatatca ctaaccttaa ttttaaatct

116461 tttaccaata tagatattgc caaaagttta ggtaatgctt ataaggtttt ttcacagctt
116521 attgatgaac cgagtggaaa ttttactact gaagatttaa gcaaaatccc tttaggattt
116581 tcttatgata aaaaaagctt tcaggtagaa aatatctatc aaacccaaaa agagtttgat
116641 agtgctttaa gtgcaaacaa taatttacaa atttatcaaa gtcctaaaca attggcgtta
116701 agttttccaa gttggaatga aaattctcct aacgattaca ctaaaaagaa tagcgatata
116761 tttgctccaa gttcccacat agatattgga gcatcatttt ataaaaatga taatggcact
116821 ataagcaaag gtggtgtttt aatggcattt tttgcaggta tgtcaggaca aaatcctctt
116881 atagaaggtg aaacaaccat agcaggaaaa cttaatggct atgataaaaa tataagccaa
116941 agtcaagtag aagatttaaa tgaatttata aaacaaaatc ctatcaaata cggcataaca
117001 ggagatatag gaacagattt tataaatgtt ttaaaactta aaaacaatat tagcgatata
117061 gatgaattta aaaaacaatg gctagaaatg aaagccaaaa gtgatgaaat gggagaagta
117121 tataaagcaa agcaaaaaga agttatttca tcgcaagaaa cttcagaaga agacaaagaa
117181 aagcctttta aacctataca agcagaaagt aaaagcgaaa cctacaaaga tgataataaa
117241 atgaatgagc ttttaaaaaa gctactagaa actaaatttg gcacaagcga tgagctagaa
117301 attcttttg gtatgaaatt tagtgatgat aatgcagggg aatttaataa aattctttct
117361 ttaaattcta caccaaaaag catagatatt aaggcttaag attttttaaa ttataaaaat
117421 caagttaaac taaaaaattc ttctattttt tttcgcattt cttcgacatt gtcgatgcga
117481 ttaacactat ctctaaaggc tgaagcatct ttatggcctt tggaatactc gtgtaaatgt
117541 ttacgaaata tactcacacc ttgatcttta tagtgtttta tcatttcgtc aaaatgagtg
117601 agtatgattt ctttttgag tttttcatct acatttttac cacttttgat ttcataaaat
117661 atccaaggat ttcccacgct tgcacgccct atcataagtc catcacattt tgtgatctca
117721 taaacctctt tagcattttg agcattgata tcaccatttg cgatcacagg aattttcacg
117781 cttgctttag cactagcaat agagtcataa tccgcttttc cactataaag ctgctttctt
117841 gtgcgtccgt gaatgcttac aaagtccacg cccaaacttt cgcaaatttt agccattttt
117901 tcgggatatt tttcattaaa gcctaagcga aatttaacac ttgtaaggct tttttattg
117961 ttttctttaa tcactcccac aaggctttta aaaagctcta aattttctag cagagcactt
118021 cctgcacatt gtttgacgac tttatttaca ggacaaccac aattaaagtc aatgccatct
118081 acaaaatcca tttcattaag catttgaact gctttttaa gcacttcttt atcacccct
118141 gcaatttgga ctatataagg gttttcaagt tcggctcttt ctagcatatg taaggttttg
118201 gagctttcat aaaccaaagc attggagctt atcatttcac ttatcgtgat atctgcacca
118261 aattttttta ccacattgcg aaaaggcagg tccgaaaaac ccgccatagg agctaaaaat
118321 aaaggctttt tgctaaaatc tatcattgta taagctgatt taaatcgatt ttgatatttt
118381 tttcacgcag ggttaaaaag gctttaaaat cattgaattt tttgtcatca ttatgaattt
118441 gctctctgag ttcatctaaa agtccaaatt cagccaaaag atataaatac gctcttaaag
118501 cttcaccttt ttcatttttg attttgttaa aaattcctat aagtgcatca ggatttaaaa
118561 gcttagtact tagttttgct atatcaagat agtcttttc gccaaggata ttgtgagaaa
118621 gtaaaacttc atattcggca gtattaagct ctaaatttcc ttctttaaaa cgcttgatga
118681 gtgtaaggat atgacaaggt tttttgtga ttttgatatt tttaatttgc tcataagttc
118741 ctttttttat caaactttca aaagccagat cttggaattc gtcttgagtt tgactaaggt
118801 ttttgatttt ggaataagca taatttatat catttttgat atgatttttt tcattttcta
118861 gaaataagat attattattt tcgagtttaa atttgttaag attgaagtat tcttcttttt
118921 taatgccgtc tataagatct aaaatttcat tgattttgtt tgcattaggg atttttcat
118981 gtgtttttag ggttttagct agctgtgcta cttgtctaaa ttcctttgtt tgaaagctga
119041 tatttgattc tttttccaaa agaaggtcgc gggtaaaatt ttcaaatttg gtagaatctt
119101 tgaaaaaatg cttaaatttt aaagtgagta aaaaataata aaaacccata tgaatgatgg
119161 caaaaaacac taaaggtatc aaaggtaaaa taagccatat cattataggt agctcgaagc
119221 tatatgtatt gaaatttaaa gtataatttc ctaattctaa ataccaagca agagcaaata
119281 taagagctat ataaataaag cttgctataa aaaataatct aattctcata actctccttt
119341 ttctaaattt tcgcggcata cgatgcagta ttttgcatga ggtttgattt taagtctttc
119401 tatggcgatt tcttcatcgc aagattcgca tattccataa gtgccatctt tgattttttc
119461 taaagaatct tctatttcct ctaattcttg ttttaaatta gcactgatag caaaatcaat
119521 ttgcgatcct gtttcaataa cagaaaaatc cacactatca cttgggacgc tattatgtaa
119581 ggcttcaatt tctttagaat tgctttgcaa attttccaat atagtttttt ttctattttc
119641 taagatagtt ttaaaaactt gtatttcatt ctttttcatt gtttaacctt ttatttgtga

119701 taaggatgat ttgagttgat acaaaaagca cgataaattt gttctaaaag taaaattttt
119761 acaaattgat gtgctaaggt gagtttgctt aatgaaagtc taaaatctaa actctggctg
119821 aattcatccc ttaggccata agcaccacct atgaaaaaat ttaaattatt tttatctgct
119881 atgagttttg aaaattctac gcttgtaagc tccttgcctc tttcatcaag aactatgcaa
119941 aagccttttg tataaggagc aaatgcctct tcatagctta atttagcagc ttgtgcattt
120001 aaattttgcg cagcagctat ttttttatta aagagattgt gttctttaag atctgcaaat
120061 ttggagataa gttttgcgta cttctcaccc caaattttaa attcatcgtt tttttgtatg
120121 tgaaaaatat taacttgcaa gttattttcc acaaaagaat ttaagagtat cgctaaggta
120181 ttttttcata tctgaacgct ctactatcgc atctatgagt ccatgttcga gtagaaattc
120241 agctttttgg aagccttcgg gtaaatctgc acctatggtt tgtttaatta ctcttgctcc
120301 tgcaaaaccc accaaagcgc ctggttcagc gatgattaaa tccccaagcc aagcgaaaga
120361 agcgctcacg ccacccatag taggatcggt taatacacta atataaggga gtttttcttt
120421 gcttaaaagt tttaaagcag cacttgtttt actcatttgc attaaagaat aagtactttc
120481 ttgcattcta gctccaccgc ttgcactcac aataacaaca ggagttttat ctgtgatggc
120541 tctttggatt gccctaacga ttttttcgcc ttctacagag cctaaagatc ctcccatgaa
120601 agaaaaaatca aatactacaa gttgagtttt aaggccatct ataaggcatt ctccactaat
120661 gaccgcagat tttctaccgg ttttgctttc gccctcgctc aagcgttttt tataggactt
120721 gctgtcaaca aatttgagcg gatctatggc ttctaaattt gcatcatatt caacaaaggt
120781 attttcatca cttaggagtt ttatcctatc cataggagaa attctcatat gataagaaca
120841 tttagggcat acattaaaac aagattctat ttctttataa tacattaaag catgacaact
120901 ctggcatttt acccaatgat ttggagcttc tttggtgctg ggttgttgtc ttctgatttt
120961 tgaaaaaata tctgcaaaat tcataataat tcacctaatt attttaaaaa ttccaagatt
121021 atatcaaaaa ttttcttatc tttacttatg agaaggaaat tttcattgtt ttctatatat
121081 aaatttttac acaacattaa agctcctgca gtatcaaaga ctcgagtttt tcctaaaact
121141 aaaacaaaac taaaatacgg agcataagca atgcttaaag ctgtagcacc caaagagcga
121201 aatttaagct tatttttcgc gaggtaattg ataagatttg ggcaaagtgt tattttttct
121261 acgatcccaa tttgagaaaa acgattaggc aaaaaaggag aataattagg attgaaaaga
121321 tttgatttta aatttttatt atcatcaaaa attaaaattt cattattggc tagattgcaa
121381 ataaaagcac tttgagcttc ttcatcttta aaaagtgctg cagatgtccc ataaaagggt
121441 attttagaga caaagtttga acttccatct aagggatcaa gaatgatttt ataaggacta
121501 ttttctccga tcaatccact ttcttcggaa aaaatttggc ctaaatcact aagatattct
121561 gtaaagattt tttcacattt tagatcaagc ttatagcttt gattattatc aaagccaaga
121621 tcaggagaaa actcaagatc gctttcacaa atattattaa gatacttgct aatttgtaaa
121681 tttgctttta ggcaagcatc tagaaattgt ttcattaaaa gcgtgttcct aggttaaatt
121741 caaaagtatt ggtatcatcg ccttttttat cattgagcgg tttagcaaat acaagttgta
121801 atggcccaat aggagtgatc cattctatac ctaaacctgt actcattctt tgaatttcat
121861 ctagattttt attgcctatc ataccataat caaaaaatac actaccgcga agtttaattc
121921 tatcgattaa agggaagcta agttctacgg aatttgcaaa agctatggtt ccacccactt
121981 catctcccca ctcattttta ggacttacag ttctactttc aaaaccgcga agagagcgaa
122041 taccacctag ataaattctt tgattgattg gaagatagcc ctcatcccaa actttataaa
122101 aacttgcttt ataacgataa ataagatcat agcctatata gtcttctaaa ccttgataaa
122161 agttaaattt tgtacttgat gaaaggaatt cttgatctcc tccaagtcct gcatattcta
122221 gacttgttga agcgataata ccttttcttg ggagataata atcatcagtg ttgttaaaag
122281 taatagcagg agttacagag ctttttatag ttttgccaag ctcataacct gttcttaaaa
122341 gtgttgggct tagatgatag atatcacttt gctcaagatt gtaagttaaa cttacattat
122401 aatatcttgc aaactggcgt cctagggtga tatcaaaacc ataatttttt tcagagtaat
122461 tatcccattc ataatcatta gaataaagcg ttccgcctaa gctgtattga ctatcaagaa
122521 tgcgtgggtt tataagactg attcttcctg ataaggtatc atcactttta tctacgctaa
122581 cagagctttt aatacctgaa ccaaaaatat tggtatctga taatgatgca ttgagcaata
122641 agccatcgct tgaaccatag ccaatacctc ctgaaatagc acccgtagaa gcttctttaa
122701 cattgacaat taagtctata tgtgtatcat ctactttttc ttctttgatt tctacatcat
122761 caaaataaga cgttcttctt aaagcatttg tagattcgct taaatcggtg cgattgtata
122821 aatttccttc agtgatataa agttcgcggc gaactactct gtcaactgtg cgagaatttc
122881 ccgaaatgat aacatttctt atatagactt tatcatgagg aatgacttta aacacaacgg

122941 tagcttcttg ggtttgatca ttttttgaa tatcaggata aacttctgca aaagcatagc
123001 ctaaatcagc actttgtgtt tctatggttt ttacatcttt acgaatatct tctatattaa
123061 tgagtttgcc ttgggttgag cgtagatttt ctaaattttg cttattttcc tcttcgctaa
123121 ataaaggatt ttctatactt atgctttgaa ttttataagg cttaccttct ttgataaaat
123181 aagttagatt agcttgataa gtatcagtat aggttttaag atagggcgag gatacttgca
123241 catcaagata tccttttttc atatattcat cactgattct tgcactatca ttgcttagtt
123301 caaaaatttt aagttttcca tcatttcttc cccacatcca gcccataaat tctttttctt
123361 tattgactat agcaggttca atatctgaat acgaaaattt ctctgctccg cttaaattga
123421 cattattaat gataatattt tctccgcgat tgactataaa ttcaagctct aatccctcgg
123481 tattttctag agtttttttt ctgtattcta ctatggtatc aaaatagctt ttagcttcgt
123541 aataagcttt gatgcgttcg cttgcttctt tggcacttgc ttcatcaaac aaggttcctc
123601 tttttatacc caaaatactt tctacttgct ttctatcatt agatgcaatt cctgtaatgc
123661 ttattttagc tatgcttggt ttttctgtga cgatgatatt taaaacacca ttatcttctt
123721 ctacggcaat attttcgaaa taattttgtt tataaaggtt aagtatggcg gtatttattt
123781 tatcaagatt tattgtttcg ccgattttca atcctgtgag atttaaagca cttgcatttg
123841 aaagatgatt taatccgcta aatttaattt ctttaattac tgccgcatta ctcagtgcaa
123901 ccaaggcaca aatgctgatg atttttttca tttattttta cctaaagctt tattaaaaat
123961 ttgtataata gcagaaaaaa tttaacaaag ggattgagat aatattatga aagtagcgat
124021 tatagggctt ggattaatag gggttcttt aggactttgc ttaaaagaaa ataaattaat
124081 ttcttgtgtg tatggtttag actcaaataa agaaaatgag caagatgctt taaaattagg
124141 actcgttcat gagcttatag aatttgaaaa tttgcattta tgtgatatga tttttattgc
124201 aacacctgta aatgccatta tagagatttt acaaaaatta gtggatctac cttcaaatat
124261 aactattata gagcttggaa gtacaaaaag aaaaatcata gaaaaattac ccgcaaaact
124321 taccaaacaa actcttttg cccatcctat ggcaggaact gaaaatagtg gtccaaaagc
124381 agcttttaaa gagctttata aggatgctgt ttgtgtgttg tgcgatagtg aagtggctga
124441 tgatttgcac caaaaaagag cagtggaaat tttttcgcat ttgggtatga ggattgtttt
124501 tatggatagc acggcacatg atcatcatgc tgctattatt tcacatttac cgcatgtgat
124561 tagtttttct ttagcaaatt ttgtgatgaa agaggaggat aaaagaaata tcgttcattt
124621 agcgggcggt tcttttaagg gtatgagtag gattgctaaa tcatctccta tcatgtgggg
124681 gagtattttt gaacaaaaca aagataatgt tttaagctcc attgaatttt ttcaaaaaga
124741 attagaattt tgtaaaaaga tgattgaaga ggataaaaat gaagaattaa agtcttggat
124801 gcaaaaagcc aatactttaa gagaaatttt gtgaaatttg cttgatattt actgaaatta
124861 taataaaatt tttttaagtt ttttaagaaa aaataaattt tgttaaaata aaaatatatt
124921 aggaaaaaat atggcaagaa aaaagggaaa attttatcta tatcttttaa ttttggtttt
124981 aattggtgct ttagcgtttt ttgcttcaaa aatgaatggt tttgaaacga cacctcctaa
125041 aatccttatg ccagatatca tttatagtga tcttaaaaaa cctatttcgg ttcatgtaaa
125101 agatgatgaa ggttttattg aaagtgttca aatcttcttg caaaaagaag gcaatggaac
125161 aggtatgctt atagtagatg aaaaaattca aaattcaaat gatattacct tacaagttgc
125221 tctaccaaaa cttgcttata aagaaaaagt aaaatctttt attatggaga ttaaagctag
125281 ggataatagt ttttggaatt ttttcaatgg taatgaagcc agtaaaaaag tccttgttat
125341 gatcgatgat agtgctccaa aggtaaatat attgagcaat tcttatcaga ttgagcaagg
125401 gggtgccgct agcgttgtat tctcggctag tgatgcgaat ttggataagg tttatataga
125461 aacaaataca ggaaaaatct ttaaggctac tccttattta aaagaaggat attacgcagc
125521 tcttatagct tgggatgcaa gagatgaaga atttcgtgct tatattattg ccacagacaa
125581 ggctggcaat atcactaaag agcgtataag gtattatttt gtcaatcgca aataccgcgt
125641 ttcaaatatc aatcttacag atagattttt agatggcaag atagaagatt tagcacaaga
125701 gtatgcacca aaagataata attttacgcg ttttgagaaa tttaaatttg tgaatgaaac
125761 cttaagagac aataatgaaa ttttgataca taagatcact tcagaagtcc ctgaaagtaa
125821 aatcgataat tttaaaatcg atttattttt accacttcga aatggtatga aggtagcaga
125881 ttttgctgat cataggtatt attcttacaa tggacaattt gtaagcgatt cttatcatat
125941 ggggattgat ttagcaagtg tagcagaagc tcctattata agcaataatc caggtaaagt
126001 cgtatttgca gaagaaaatg gaatttatgg acttaattta atcgtctatc atggttttgg
126061 agtttatagt ctttatgggc attgttcttc taaaaatgtt gaaattgatg agattttaag
126121 caaaaaaagt gtaataggta gaacaggtgt aagtggattg gctttgggag atcatttgca

126181 ctttggtgtt ttggttcaag gtgttgaaac acgccctgag caatggcaag ataaaaaatg
126241 gatagaaaat aatatttaca atgttttaaa cgaaggtaaa aaaataattt taggaaaaaa
126301 ttaatgaaac aattaacttt agcaaaagct gtaaaaggcg tgggtatagg gcttcataaa
126361 ggagagccta tcgagattac cctagagcct ttagaagcaa atagtgggat agtttttttt
126421 agaagcgatt taaatgctac ttataaggca agtcctgaaa atgttatcaa tacccaaatg
126481 gcaacggttt tgggcgatga aagagggttt atttccacca tagagcattt aatgagcgca
126541 atcaatgctt atgggatcga taatgttcgt atcgttttaa atgccaatga agctccagtg
126601 atggatggtt caagtattag tttttgtatg atgcttgatg aagcaggggt taaagagctt
126661 gatgcaccta aaaaaattat ggtgattaaa aagcctatag aggtaagaga tggtgataaa
126721 tatgtgcgtt taactccgac taaagaaccg cgtattaatt atactatcaa gtttgataat
126781 gctgttatag gagaacaaag ttataatttt gaattcagta agaaaaacta tatagaaaat
126841 atagcaagag ctaggacttt tggtttttta aaagatgttc aagctcttag agatatgaat
126901 ttagcattgg gcggaagctt ggaaaatact atagttgtag atgataatcg tattttaaat
126961 cctgaaggtt tgcgctttaa ggatgaattt gtaagacata aaattttaga cgcgattggg
127021 gatttaactt tgcttgggta ccgtgttttt ggagattata tttcatatgc tggaagccat
127081 catttaaatc atttactcac taaggaagtg ttaaaagata aagatgctta tgagatagta
127141 actcttgaaa aagcaaatga aaaagcttat gaaaaggttt ttgcataaaa aattcattat
127201 tgcttaatgc tttatctaaa cctttgatga tcggtgttta tcaagatgat aaacttgtaa
127261 aaagtataga gagcgaagaa aaggcaagtg agtttgtgcc aaaaatttta aagattttgc
127321 taaaagaatt tagctttgat gagttaattt atgctaatgg tccaggctct tatatgggga
127381 ttaaaatttc ttatgtaagt cttagcacgc tgagtatagt gaaaaatatc cctcttttg
127441 ctatcagtgc ttttgagctt aacagcaatc aacccattgc cgctcataaa aatatgtgtt
127501 ttgtgaaaaa agaagatgag atcatcttgg aggaaaatac agctggagag tttttttac
127561 ctccaaattt aagcaagctt aataaaaaag atgataatct acctttttac tttttatctg
127621 caatttaaag aatttaggaa aaaaattgaa aattttagta cctgctacaa gtgcgaattt
127681 aggccctggg tttgattgtt tgggattgag tttagcactt tttaatcata ctattatcga
127741 gaagtcaaat ttttttagca tcagtgtcaa tggcgaagga agtgagaatt taagtcttaa
127801 aaaaaataat atttttgtaa gtatttttaa tgaaatttat caaagattaa gcggtaggaa
127861 agatcatttt agatttattt ttgataatac cattccttta tctcgcggac ttggtagctc
127921 ttcggcagtg attatcggcg ctattgcgag tgcttatgag atggcgggtt ttaaagcaga
127981 taaagagaaa attttaaatg aagcgttaaa atacgagaat catcctgaca atatagctcc
128041 tgcagcgctt ggcggctttg tagtatctgt ggtagaaaat gaaaaagttt tcagcataaa
128101 aaaagattta gatgaaaatt taaatgctgt cgtggttata cctaatgtag cgatgagtac
128161 agaacaaagt cgtaatgcct tgccttcgaa tttgagtttg aaagatagtg tttttaatct
128221 ttgccattct tcttttctta cagcttgttt tttggaaaaa aagtatgatt tacttgctct
128281 tgctagcaag gatttattgc accaaacgcg tagaatgcaa aatttacccg agctttttga
128341 ggtgcaaaaa tttgctcttg aaaataaggc tttaatgagt acgctttcag gttcaggctc
128401 aagctttttt agtttagcat acaaagatga tgcaaagcat ttagctcaaa aaatggagca
128461 gaaatttgga cattttaaag ttctttgttt ggaatttgac aataaaggct ttaaaatttg
128521 ctaaaaaaag aaaaaaaaag atataatttt acaaaagata aacatgagcc tattcgaatg
128581 tgtatagtct gtaaaaatcg atatttgcaa aaaagtatgc atcgatttaa acattttcaa
128641 ggcgagcttt ataaagactt tgattttgga cgcagtgttt atttgtgtga gacttgcatt
128701 ttgagtgaag gcaaagtctt gcaaaaagca tttttagat cttgcaaaaa tttaaataca
128761 aaaatcactc agcagaattt aaaggagata gttttaaatg gcaaaaatta gaattcatga
128821 aattgcgaaa gaattaggct atgatagcaa agaaattatc gagaaagcaa atgaattagg
128881 attaaatatc aagaccgcat ctaatgctgt agaacccgaa gttgcagcag gaatttatga
128941 gtatatacaa actaaaatca ttcccgaggc tttcaagaaa aatttaaaac aagatcctat
129001 aaaaaaagct aaagctgcac ctaaagaaga aaaaaaagaa gaaaaaccat ctaaagaagt
129061 agaagtaaaa gctccagcta aagttgtaga agaagcaaag cctgaaccaa aaagtcctga
129121 agggcctaaa gaaaacattc aagagagctt agcgagtgcg actttagcta aaagaagagg
129181 gcttgtgatc gttaaaaaga aaaaagatga agaagaagcc gcgaaaaaag aagagccaaa
129241 ggctactact ccttcaaatg aagaacgcct tagtttaaaa actatgtttt caaatgcgga
129301 tgaaaattta aagaaaaaga aaaaagagaa aaagcctttt gtcgcgacta aaaaagaaag
129361 tgctgaaaaa atggactttt tagaaggtca tgattttgct gatatttctt tagaagatga

129421 agatgtggtg gttttacctg attttagcgt aaaagaacaa gaaaaaccac aaaataccat
129481 gaaaaaacca cctaactttt tgcgtcaatc agttggaaat tcagcaaatc ttggacttga
129541 gggcggaatt caaagaagaa gtcgtaaaaa accacctaaa aaagttgaga aaaaagaaaa
129601 tgaagcggtt tcgagcgtag aaatttcaaa agaaattcgt gtttatgaat ttgcagataa
129661 gataggcaaa agcgtaagtg aagtgatttc aaaacttttc atgctgggta tgatgacaac
129721 taaaaatgat tttttggatg aagatgcgat tgaaatttta gctgctgaat ttggcataga
129781 gatcaatatc atcaacgaag cagatgagtt tgattatgta aaagactatg aagaagaagt
129841 tgatgagaaa gatttggtaa gtagagcacc ggtgattact atcatggggc acgttgatca
129901 tggtaaaact tctttgcttg attatatcag aaaatctcgc gttgctagcg gcgaagcagg
129961 ggggattact caacatgtgg gcgcttatat ggtggaaaaa aatgggcgta aaatcacttt
130021 tatagatact ccaggtcacg aagcttttac tgctatgcgt gcaagaggtg caagcataac
130081 agatatcgtt atcatcgttg tagcagcaga tgatggggta aaaccacaaa ctaaagaagc
130141 gattaatcac gctaaagcag caggtgtgcc tatcatcata gcgattaaca aaatggataa
130201 agaagctgca aatcctgaca tggtaaaaac tcaacttgcc gaaatggaaa tcatgccagt
130261 agaatggggc ggatcatatg aatttgtagg agtttctgct aaaacaggaa tgggtattga
130321 agatttgctt gaaatcgtgc ttttacaagc tgatatttta gagctcaaag ccaatccaaa
130381 aagctttgca aaagcaagtg ttatagagag ttctatccaa aaaggacgcg gtgcggtagc
130441 tactatcatc gtgcaaaatg gaacgcttaa agtaggaagt actatcgttg cgggcgaagc
130501 ttatggaaaa gtgcgcgcaa tgagcgatga tcaaggcaaa gctttaaaag agatcaaacc
130561 gggtgaatgc ggtgttatcg tagggcttag cgaagtagct gatgcaggcg aaattttaat
130621 cgcagtgaag agcgataaag aagcaagaga gtatgctaac aagcgccacg aatacaatcg
130681 ccaaaaagaa ctcagcaaat cgactaaagt aagtatagat gagcttggag cgaagatcaa
130741 agaaggaaac ttaaaagccc ttcctgtgat tttaaaagct gatgtgcaag gatcgttaga
130801 agccttaaaa gcaagcttag aaaagcttag aaatgatgaa attaaagtca atatcataca
130861 cagcggagta ggggggatca cgcaaagtga tatagagctt gcaagtgcaa gtgaaaactc
130921 tatcgtacta ggctttaaca tacgcccaac cggagagatc aaagagcgtg ctaaagataa
130981 aggcgtggaa ataaaaactt ataatgtcat ttataatcta cttgatgatg taaaagcctt
131041 gctaggtggt atgatgagtc ctatcatctc tgaagaacaa cttggacaag cagagattag
131101 acaagtgatc aatgtaccaa aaatcggtca aattgcaggt tgtatggtaa ctgaaggcgt
131161 gattaaccgc ggggctaaaa ttcgccttat ccgtgatgga gttgtggttt atgaaggaaa
131221 tgtaagctca cttaaacgct ttaaagatga tgctaaagaa gtggcaaaag gctatgaatg
131281 tggcgtaggc atagaagggt gtgatgatat gagagtgggt gattatatag aaagctataa
131341 agaagtagag gaacaagcaa gtctatgaat ccaagtgaga ttaaaaaact tcgcaccgaa
131401 agtatcttaa aagaacttat ccctgaagct ttggcaaatt tagatgatga gaatttgaaa
131461 aatctttgcg tggtggatgt agagtgtaaa aaaggcagat acgatgcctt tgtatatctt
131521 gataaaatgt tttttaatac tcacgagcaa gagaagattt taaattcttt aaaaaaagcg
131581 agcagagctt tgcagaatta ttgtatgagc gaacaaggct ggtatagatg tccaaatttt
131641 cactttaaat ttgatgatag acttgagtat caaaaccata tggacgcact ttttgaaaag
131701 ataaaaaaag ataaaaatga ataattttta tgaaatatta caatttaact caaaacaatt
131761 tattgagcca ttggggacaa aggaaaaatt ttggattcaa gataaaaaat atctatttaa
131821 aattggtcga gaaaatactt tagaaaattg tagcgaaaag attgcgtgtg agatcgcaaa
131881 acttataaat ttaccttgtg ctgaatatga attagctgtt tataaaaatg gaaatatttc
131941 atatggagtg gctagtagaa attttctaaa ttctacttct aatttggtct taggcaatga
132001 gcttttaagt gaaacattac aagaatatga caaaaatgca aaatataaat taaaaaaata
132061 taccttagac aatgttttgt ctttacatgt ttttcttgat aatccatcca ttttagaaaa
132121 tatgataggg tgtttggtat ttgatgtttt tatcggtaat caagacagac atcatgaaaa
132181 ttgggggatt gtttttgatg agaaaacaag agagattaat ttagccccaa gttttgatca
132241 tgcggcaggt atagcttcaa aaataagtga ggaagcagct aaaataaaac ttcaaaccaa
132301 ggataaaaac caacaagttt tagcgtattg tcttaaatca aatagtgctt tttgtgatga
132361 gagtggcagg cgtttaaaaa atttagatgt attgaaaaat atcttaaata aaagagaaca
132421 aaatataagt acttttgcaa aacaatggat tgacaaaatt gtttcagttg atcaaagtgc
132481 ttgtagtaat ataattgata aaatcccaaa tgagtttata ggggaatttc aagaattatt
132541 tattttagaa atgctcaata taaataaaaa acttatttgt gatttagtaa gggaaaatat
132601 atgattttaa aagtgtgttg gagatcatca aaagctaaaa aatggttttt tataggagag

132661 ctttcttatg aaaacaatcg gtataagttc gtttatgatg aaaatatcta tgaagcacaa
132721 gaacaaggat ttgaattatt tacaaattta agtgatgtaa agttaaatta ttatagttat
132781 gaattatttc cagtatttgc caatagattg ctttcaaaat caagatcgga atataaaaaa
132841 tatcaagaat ggttagattt aaaagatgat aaaaatccat tagaagaatt atctaaaaac
132901 aatggtttta gagttgttga ttttatagct ttgtatttga tacccaaagt acaaaaaaaa
132961 tatgtagtag agttttttttt acataacgtt gattgcattt ttgaatatca aaaatgctta
133021 aatcaaataa aacaaggcga taatctttat ttggctaagg atattcaaaa taaattcgat
133081 aaaaatgcta tattggtacg aactaaagat cctatgagta ttttaggcta tataccaaga
133141 atttataatg aagatatagc atcgatttta gcatacgatg aaaatgcgac tttaagtatc
133201 aagaaaatca ataaagatgc accagttcag tttcaaattt tgtgtcgatt tgaagcaaaa
133261 tggcctaaaa attttatagc atttgataca cgaaaggata aaaatgaatc ttgaagcttt
133321 atgcaaagaa gtgaatttag agctttatga tagcgaatta actagcgaaa atggtaagaa
133381 aatttaccgc gtttatgtga tgaagccctt agatgaaaag ggtgaaagaa aaggtgtaaa
133441 tctggatgat tgtgcaaggc ttagtgaaat tttatcaccc attttcgatg tggagccacc
133501 tgtaagcggg gagtattttt tagaagtatc ttcagcagga cttgagagaa aacttagcaa
133561 gatagagcat tttgcaaaaa gtatcaacga gcttgtaaaa atcacaacca atgaaaaaga
133621 aaaaatcgaa gcaaaaatca tcagcgtaga ggatgaaaat atcactttag aaaatttaga
133681 aaatcaagaa aaaactactt taaaatttag cgatatcaaa aaagctagaa cttttgtaga
133741 atggtaaagt taaaaattat aaattttcta gcaattctaa tgtaaattgc gatgaaccaa
133801 ataccccatc tatgttttgt agaaatttgt tttgttcata tatttcatct gatacaatta
133861 aatatactcc gtgattgcga atagcttttg ctttttcttc gcctaaatcc aatcctaaag
133921 ttatttcaat cataacatct attgtactca tttgaaccgt ttttataaat tgtttatatc
133981 tttctcttaa ggttcttttt gctcctattc cataggattt tccattcaaa ttaaaaaaga
134041 aatcaaattc tgtagaacta tcgttttcat catgtttttt gattattgct tcttggttta
134101 ttccaataga ttctaatttc tctaatattc tactttcata gttgctacca ctgttgctta
134161 taatagattg attaatactt tcattgaaaa taagcataaa aatttgattt ggttctaagc
134221 catgtttttt taactcttga gaaagttggc aaatacttgc aaaatattct ttatctatat
134281 tgtcatgaaa gaattgatta ttgtctatat gattaaaaaa tgttaatgct aataaagctc
134341 cattaaccct agttgaatat ggattatata tagttatctt gtcatactct aaagatagcg
134401 gatctatttt tgacaatcta atgatattat tcctttgtct tatctttgct aattctaata
134461 aaaaattttt aattgagtaa atttttaaag caaatataaa tacattaaaa tctatattat
134521 ttctaacttc gtaaataatt tgttctaaat aattccattg gcttatacca tttttagtat
134581 ttaaattaat atgttgtttt agtttatata aggaagtttc attttttgat atatgatata
134641 aaatgtttgt tatttgcgta tctgaaaaat taattttaac taaatatttc ataaaaaagt
134701 aaaatttcaa attgatatca aatttttat catgtattag cattatataa ctccttatga
134761 attaatttag caatattttc aattacggga atggctacac tattgccaaa ttgtttatag
134821 ctttgattat cgcttgttgt tattttaaaa ctattaggaa agccttgcaa tctagcagct
134881 tctcttgggg tgagttttct aggattttta tttttttgtt caattagtat ttcggagcca
134941 tctttataat atctagcgct aatagtgctg gtatattctg aatcatgatt aaatagagaa
135001 tagccaaatc catttccttt tttggcgtgt tctagttttc tacgctggtg tccagcccat
135061 aatttatctg aaatagtata tttttcatct atattttttt ctaaaatatc tcctaattta
135121 gattttattt tgatattttt taactcatta aaactaaatg atatattttt aaatttattt
135181 tttaaaaaag ctacaatata tattctttct ctattttgcg gtattccaaa gtttctagca
135241 tttaatactt ctgaaaaaac atcatagccc atgtcttcta aagtattttt tacaacagta
135301 aaagtctttc ctttatcatg atttttaaat cctttaacat tttctaaaaa aataatctct
135361 ggtttgtgtt tctttgctat gcgtgcaaca tcaaaaaata atgttcctct aatatcatta
135421 aagcctttcc tatgcccagc aatagaaaaa gcttgacatg gaaatcctgc taataatata
135481 ttgtgttttg gaatatcatt ttcatttatt tgtgttatat ctccatgtgg tagttcgtta
135541 aaattgcaaa aatatgtttg acaagcaaat ttatctatct ctgaagaaaa aacaaattct
135601 agcttttctt gaaatatatt tttaaagcct atgcgtattc caccaattcc ggcaaataaa
135661 tcaatagctt ttagtttgtc tgtgttatta atcatgttat ccttgtatat tcgcaataat
135721 aattagggta tattatacta tgttaaagct agaaaaaaat taattaagat atcaattgcc
135781 ttgctttttc ctatggaaaa actttttgaa gattatgtag ctcatatgct aaaaaaagca
135841 aatccgactc aaaatatcaa aacccaaagt agtgaaaaat acctgatctc aaaaaaaagg

135901 tgaaaaatgc tttatgttaa agcctgattt gtatatagaa aataaaatga ttttagatgc
135961 taagtggaaa attccagata gcaatgaaga taaaaagcat ggcatagcac aaagtgattt
136021 gtatcaaatg tttgcttatg cttgcaaatt taaaattcac gatattaaac tgatctatcc
136081 tttatgtgaa agaacgatga atttgcaaaa tacgataaaa gaattagaat tcaatgcaaa
136141 tgagcatttg gcatttttaa atgataatag ccttttgaaa acaaatataa aagtgcaagt
136201 gatttttgca cctttacctt tttagtaaaa actttgctag caagtgtatc aaaaagccaa
136261 agcaagctac agctatgata caagcaccgc ttgagagatt aaaataatag cttattataa
136321 gccctaaaac gcaaaaaatc atactcaata cacttgctaa aatcattatc aaacctagct
136381 tttttgtaaa attttcagct ataaaactag gtatgctaag caaagccatg atcaaaataa
136441 tccctacaag gcgtatagaa atcactatgc aaaatgccat aagtgcgatt aaaagatagt
136501 gaaaaaagct tgtatttatc cctcgcactt ttgcaaattc aacatcaaag cttaaagctt
136561 caaattgtct ataaaataaa agcattaaaa caatcacaac cccatctact atcgccatca
136621 gccatagatc ttgcattcct actgctaaaa tatttccaaa aagataagcc atcaaatccg
136681 tgctataacc cggacttaaa tctataagta tgatcccaaa agccatccca aaagcccaaa
136741 tcacagcgac tatactatcg cttctgtgct cataacgctt cgataaaaat gctaccaaaa
136801 aggctaaaaa gagcgtgaaa atacctgtgc tgatcaatat aggcaaagaa aaataaaacg
136861 ctataccaat gcccccaaaa gctccatgtg taatgcctcc tgctatggaa aagagtcggt
136921 ttatcatcac aagtgtacct acgactccac aagcaatgct gactaaaatc gcagccaata
136981 aagcattttg aaaaaaggtg aaatttaaaa tttcaaacat ggctttcctt tgaaaatttc
137041 aaacaaaaat tttctttttt gaaatttgat ttttttaaat ttcttcttgc aaattcttgt
137101 tcgcaaattt ttttcttttc gcaattttct tcatcgcaaa agcaagaatt taaactcatt
137161 tctacatcgc aaaaatgcga atggttttca taaaggtgtt ttaaaaatac gcttttgtct
137221 ttttctttgg tattggtatg caaaaagagt tctttgttta aatacgcgat tttatcacta
137281 taagcaagca cgagattgat atcgtggcaa atgagtaaaa ttcctatacc cttttcatgt
137341 aaagagctta ggagttcaaa aatttgaatt gcggatttgc tatcaacgct tgctgtaggc
137401 tcatctaaga tcagcatttt gcattcatca accaaagctc tagcgataaa aacgcgttgt
137461 ttttgacctc cgcttaattc attgattttt ctatcccaaa aatcctgcat gcctacactt
137521 gctagagctt ctaaggcttt gtttttatct tttttgctat aaaaaccaaa aattttttta
137581 ttgacaagcc ccattaaaac aatttctata acccttgggc aaaagttagg atttgctaaa
137641 gtgtgttgag ggacatagcc gatttcattt tttttaagcc catgaaaatt gatagttttt
137701 ttagtcttta aaagtcctag gatgagtttt acaagtgtgg attttccagc accattaggg
137761 ccgatgatgg aaagaaaatc tttactatca tagcttaagt tgatattttt taagattgtt
137821 tcgttattgt aagcgtaatt aagatttgat atttcaaaaa aaagcattaa aaaaacctaa
137881 ctataaattt tcataaaaag cgtttgctgt tttcaaaagt tcattttccc aatcataaga
137941 taaataatct atcttataaa tttttacatt atattctttt tctaaagttt ttgctgtatt
138001 ttcaggaaaa tgagtctgta caaaaataac tttaatattt ttatctttaa tcaacgcacc
138061 caaatttttt aaatcttcat tttttagttt ttttcctaaa aattctacat ggttttgcac
138121 aagattataa cgcttagcaa agtatgacca agcgggatga tatgttataa attctttatt
138181 ttttactttt tgtaattttg aagagatttg taaatttaag acatctaact ctgttaaaaa
138241 tttatctaaa ttacgcttgt ataagctttt gttttgaggg tatttttgaa ctagagcatt
138301 gtaggtattt aaggctatgt tttgaactaa aataggatca agccaagtaa aaggatctaa
138361 aatttctttg gagttgctag agctagtttt agctagagga atatcttttt gcatgtctat
138421 gatttcaagt tttggaaatt tttgttttag tttgtctgtc aaattctttt caaattctaa
138481 gcctatggta aagtaaagct cacttttttc aagctttttc atagggtttg atttggattt
138541 tggcttgtgt gtatttgtag ataaaatgtt gatatttaga gtatcatccg ctattttttt
138601 aacaaaaaaa gcttgtgggg ctatgcttac gctgattaaa ggtagatttg gattttcact
138661 tggttgtaaa gttttagcat aaagaaagga tttaaaaagt cccaaaaata taaaaaaaag
138721 gaatattttt ttcattattt tataccctaa agagaatgaa atttaaaaat tataacaata
138781 ttaagtttat aaatagaatt tctagttttt atatctatgt taaaaaaagt attttagatt
138841 gtttttttatg gaaattttga tattttatct aaaatcaaga ctaaaatcta ggataaaatc
138901 ctagattaat gcaagctttc tatataagcc atagcgctaa gtgctgctac agctccatcg
138961 cccgctgcac aaataacttg ttttggagca tcttttctaa gatcacctgc tgcaaaaagc
139021 ccaggaacgc tagtttgcat tttaagattg acacttactt gcccaccttc ttccatatcg
139081 cataggaatt ttccatcatc ttgctttaaa atttcatttc ttacattcag ccccacaaag

139141 gtaaaaattc ctggcacatt taaatctcta atgctaccat cttttaattt tacttttacg
139201 cctgtaacac ccattttatc accatatact tcatctatgc ttgcactagt gataagttct
139261 atttttttcat ttttctttac tttttctacc gtagaaggtg cagctctaaa ttcatctctg
139321 cggtgaatta gataaacttt agaacaaata tttgctagat ataatgcttc ctctaaagca
139381 gtatcaccac cgcctaaaac cgctacttct ttatttttat agaaaaaacc atcgcaagtt
139441 gcacaagtgc tcacaccttt gccaaaaaat tcatcctcac ctttaaaacc tgctcttttt
139501 ggggcagaac cagtacaaac tatcaccgct tttgcaagct ctgttttgcc accttctagt
139561 ttaatagtaa aacttccatc ttcatttttt gaaatttgtt ctacacctac catttcatgc
139621 tttaagccaa aacgcataca ctgttcgctc caaggtgcca taaaagaaat tccatccata
139681 acttgtgcaa cacccggata gttttcaatt tcagaactag aagtaatttg ccctcctggc
139741 atcccttttt caaacattac tacattttta agaccacctc tagttgtgta aagtccagcg
139801 ctaagtcctg caggtcctcc acctataatt gctacatcta acattatttt tcctttgtat
139861 ttgaatttta ttgataataa tatacaaatt attcttaata gtagttaata aaaattatta
139921 tttagtataa atttgaaatt tttggatgaa aggctagtaa aactagcctt gaagattttt
139981 atagtaaaga atttaattta tcgctgattg cttgttttga ttgagcacca actaattgat
140041 ctactacttc accattttta aagaagataa gagtagggat agagcgaaca ccaaattcag
140101 ctgccaaatc accttgctcg tctgtattta ctttacaaat ttttgcttta ccatcaaaat
140161 catttgcaag ttcatcaatt actggagcaa gcattctgca aggtccacac catggagccc
140221 aaaaatcaac taaagcaaca ccttcttttg cttgtgcaaa attatcactt gttaattcaa
140281 tatattttcc cataattttt cctttcacta atttctgtaa ttgtatttta ttttttttaaa
140341 tattagctta aaaactgata ttttcaaaaa attgtatcat atcatcacta atgcagataa
140401 gatcgatttg aaaatcattc aaaatcccgt gttttaattc ataaaattct atggttttta
140461 aaattctttc atattttttta atatttaaac gctcagaaac ttcataattg ttttgagtaa
140521 atttaacttc gataaaatgt aaaattccat cttttctcgc aataatatca atctcgccga
140581 atttagaacg aaaattacgc tttaaaattt caaatttttg cttttttaaa aattcgcaag
140641 ccttatcttc gcctaaaatt ccactaagat atgatgcaaa tcccattatt cttctatacg
140701 catcataaaa ggcttagcct tgataaaatc ttgtttttcc aagatagaaa gtaaattttg
140761 aatacttttt tcaaaggtat gatgagtagt aaaaaataaa gtgctataaa tttcaccctg
140821 ttttggtttt tgcaaaaagc tgtctatcga aatattattt tcactcataa gttgagtaat
140881 tttagataaa acccctgttt tatcttctac ttttaatctt aaataatatt ttgtataaat
140941 ttcatccttg tttaaaagtt taaaatttaa attattttca aagcccatca taggaccttt
141001 gggttcattt cttgctatat ccataagatc agcgataaca gcacttgctg tagcatctcc
141061 tcctgcaccc gctccataat aaagactttc accaagtata tcactgacta tgctgatagc
141121 attcattact ccatcgactt ttgctatcat tttatcttta ttgatcatag taggatgcac
141181 tcttaattct actgtggaat tttgagcttt ggctatacct aaaagcttga tagtaaaatc
141241 aaattctttt gcaaaataca tatcttcagc agaaattttg ctgatacctt cgattaaaat
141301 atcttcaggt ttggcacgca aaccataagc aatacttgct aaaatcaaaa gtttatgtgc
141361 agcatcttgt ccttctatat caaaggttgg atctgcttct gcataaccaa gctcttgtgc
141421 tttttttagc acatcatcaa aatttaaacc cttactattc atagcgctta aaatataatt
141481 gctcgtgcca ttgagtatgc cttcgatact taagatattg ttcgcactaa gcccttcttt
141541 taaggctttg ataatgggaa ttccacccgc aacacttgct tcataaccaa aagttgtatt
141601 tttagccaac tcttcaagct catagcgatg ataagcaagc atagctttat ttgcagtaac
141661 tacaggtttg tttttttttaa gaatttcgct gatgatttta aaaggtgttt caaccccacc
141721 cattaactcc acaaaaacat ctatatcatc tcgttgcaaa atttcatcta catttgtaac
141781 gatagggatt aggggatttt ctttttggct tttggcaaga gcaataacag gaatgatatc
141841 ttgtccacat cttgcacgaa tgatttcttt gttttgaatt aaaacacgag caacagcact
141901 ccctacaacc ccatatccta aaatagcaac tttcatttta ttcttctttc aagtattttt
141961 taatattgcg cgcagcctga cgaatgcggt tttcattttc tattaaagca attcttacat
142021 attcatctcc tgcttcacca aagccgatcc ccggactcac tgctacttca gcgtgctgta
142081 aaagttgttt tgaaaattct aagcttttta gatgcatttt gctttcagga agttttgccc
142141 aaacaaacat gctagctctt ggtttatgaa gtttccaacc cgcattttca aatgcttcta
142201 gcaagatatg cattctttta tcgtaagtag cacgaatttc atctacacaa ctttgatccc
142261 catctaaagc tacggtagca gctacttgta taggagtata catgccataa tcaaaccatg
142321 attttatctt tttaagagca gctactaagc gcttatttcc tactgtaaat cctacacgcc

142381 agcctgccat attgtaagat tttgaaagag tgtaggtttc tacggcaaca tcttttgcac
142441 cctctacttc taaaatagaa ggggttttat aatcatcata ggttaaatcc gcataagcaa
142501 tatcagaaat gatataaaat cgctcttttt tcgcagttgc aactaatctt tcataaaagc
142561 ttttttcgca agttaccgtt gttggattgt gtggaaaatt taccactaca tattttgggc
142621 gcgggatact ttcatgcagg gttttatcta aatcttcaaa gaatttattt tcatcaagtt
142681 caaatttttc attatatttg agaggcattt tagccacatt tcctcctgct atgataaaag
142741 cttgagtatg gataggataa gctggagctg gtactatggc aacatctcct ggattgataa
142801 tcgctcttgc taaattcaca aaaccctctt tagaacccat agtcgctact acttcatttt
142861 caggatctaa atttactccg tattttcttt tataccaatt gcaaatagct aggcgaagct
142921 tataaatccc catagaagta gaataacctg aagttttgtc tttattggcg ctttcgcaaa
142981 gtttatcaat gatatgttga ggagttttac catcgggatt tcccatagaa aaatcgatga
143041 tatcctttcc agcacgtctt gctgccattt tgatcgcatt tacttctgca aaaacataat
143101 ttggaagcct ttctatggta ttgaaacgaa tttcatcaaa cattttttctt cctttttatt
143161 tttttagggt gatttttaaa cctcttcgga tattgtctaa attatacata tcgcttacaa
143221 ttgcataaac tgcaccgctt gggataagct ctgaaaaatg attattttga gcttgactct
143281 tgattgcttt aatgagattt aaatttttat ctaaaaatag tactttagga aaccaaaaat
143341 cagcatcgtg cgcatcaata atcaaattcg aagccccttg gagagtgaac acataatctt
143401 taagcggtct ttctaaagtt tgtgtagaat taagttttaa atcgatattg gtattaagct
143461 gagcattttc aaaatttaaa tcatactcat aatcataagt acctatgcgc ttaatattgt
143521 ctatataaac tgaattatct tttaaaagac tatataaagc acctggatct aaaatgtatt
143581 gagattctac ttgtatggtg tagtctatat ttccatcgcg taaaatcata tcagtaggga
143641 taaaatatat atatccaaga tttgtaagtg cgtcagttaa gatcttaaaa aatacaacag
143701 aatccgcttt ggctttgaaa ttgagcctta aagtttgagg gtctgatagg gttaggttga
143761 ttaaggaatt ggtttttaaa attcttgaaa ttttagctat gtctggattg cctttattgt
143821 caatatagct agaatcagga aataaaagac gcaattgaga atctttagaa ggattagcaa
143881 ctaaattttt agctaattct aaactagaaa cagcaaaaac agaattagct aaaaataaaa
143941 aaataaagat tttttttacc attctttacc tttgtttaac ttcataaatt cagctgccga
144001 aatacttgta atttttccat ctttaatcaa aaagcgcttt gaatttcccg caggaaattt
144061 ttgttctttg ccattttcat caaaaacact taaagccgca gctcctgtta aaaccaaacg
144121 atttacttct aaagaaagat taaaatcatt ttcttttaca agaccagttt ttctataatt
144181 tgataaatca atcaagccta tccaaatttt acccgaagct ttaaaatgtg cctctttatt
144241 gggagctttt gtaagttctg tgacttcttg atttgtcgtt gctaaagaat catttgtgct
144301 taaattttct tctttggcat ttgtattgtt ttgcgtggta gaaatttgtt ttatttcttc
144361 ttttgtattg ttaagttctg tattttgtat ttgagttaaa ttggcttctg tttcgttttc
144421 ttcagcttga ttatcaatca caacgacatt attttctaaa gcttttaaat tagattgtgc
144481 ttgtccgata atatctacaa cggccgcact tgagttattt tggtcatttt ggaaaaaaga
144541 tttaatataa tcaaaataat aaattccaaa acccacaagt gctaaaatta taactataac
144601 tataaaagat acgaaacctg ttgacttttg agtataagca tctaatttag gggttattat
144661 cttaggtttg gctgtatcgt taagattgtt ttcattgaga taggtttcaa attcttcatt
144721 aaaatctgta aaatcaagtt cgtattcacg acttaaaatt tttacgaaac cgcggacatt
144781 aaaacgattt aaagtaacaa aatctttttt aactaaagct tctaaaaaat ctaattcgat
144841 ttttgttcta gcagcaactt cttttaaatt taaatcttca attttttttcc aattaagcat
144901 ttatcatcct atcacacagt attgcaaaag ctacactaac attaaggcta tcgaaattat
144961 ttttcatttc tatgcctaca cattcatcgc atttttttat tattttagca cttaatccca
145021 aaccttcact tcctaaaatt aaagcttttt tgcctttatc gactttataa tgatgaattt
145081 gtttgcctcc gctcgcactt gcaaaaagtt taaatcctac ttgcttaagc tcatttaaaa
145141 cggtaaaaat atcattattt aaggcgattt caagatctag tgccgctccg ctacttgtgc
145201 ggataacgcc ttccatcgcg agtctttctc cgataaaaat aagcccatcc actcctaaag
145261 cataagctgt tcttacgata gctcctatat ttccaacatc actaatacca taaagaatag
145321 ctataaattc acccttttta agagtgtgta aatctttaaa ttcatactct ttaatatcta
145381 gcaaaaagcc ttgatggttt ccacctttag cataagcttg ggctgtttta aaatcaagtt
145441 ttttgatctt aaagcctgaa ttagcaatct ttttaaaagt ttctttgtcg cactctttag
145501 ctaaataaag ctcatttata agctctttat gtcgttctaa aatataaaaa aatatctgtt
145561 ttccataaac tatcattcta aattttacct aactttgact taaaagcagt ttttgataaa

145621 tttcttttgg attttttcca ttcatttttg ccaaaagttt gctttttgtt tttaaaggta
145681 tttcaagctc taaaatatcg ttttcacata aagaattacc aatgctatct ttatcgtttt
145741 tagctagcac gacaacccac tccccccttta aatttgtctg ttttaatata tcaaaaagat
145801 ctatagcttt ggctttaaat ttggtttcaa attttttaga aatttcttta atggcaaaaa
145861 tttctctttg actatctaaa atagctattt gctcgattaa agacaagatg cgtttagggg
145921 attcatagat aatgctagga taggtatgat tgagtatctt ttggatatct ctttggcgtt
145981 ctttgccttt attggctaaa aaacccataa aaataaattc tttttcgcat aaagccgagc
146041 ttacaagtgc caccaaggct gcattagcac caggtaaaac ttcaaattgg atatcatttt
146101 ttaaagcaaa atcaattaaa aattgcccag gatcactaat tcctggcata cccgcatcac
146161 ttaaaaaagc aatatcttga tcaaaaaatt ccaaatctaa attttctaaa actttttttt
146221 cattatgagt atgaaaggat atgaattgtt tatcgccaaa atcgatttca aatttagatg
146281 aaagtaaaga aagtaaagac ttgctcaccc ttgtatcttc gcaaaaaaaa acattgcaag
146341 tctttaaaag ctctaaagca cgaaaagaaa tatcgcttaa atttcctatg ggcgtaggaa
146401 taaaataaag cataattatt gcattgcgta tttttctta aatttctcta cacgacctgc
146461 tgcatctacg atttttttcac tacctgtgaa gaaaggatgg cagcttgaac aaatatctac
146521 tcttaactca cttttgtttg atttggtaac aaaagtattg ccacaagcgc agctaacttt
146581 gcattctaca tattctggat gaatttcttt tttcatagct ttttccttaa aaataaaaca
146641 ataagcggtg attatattaa atttaatata aaaaaaacat taatttaagt aaaattaaag
146701 caatattttt gcagccactg caacgacagc actttggctt cttaaaatat taggcacttt
146761 aaggcaaatt ttttctttaa acatttgttt ttctttgggg ctaaatccac cctctggccc
146821 tataaaataa agccgagttt tatcaaattc ctttatttca ccttcaaaat caaccaaaac
146881 agcatcagga aaagatttgc taaattcttg tatattttta tagctttgaa tttgcatttt
146941 tcgacttctt ccgcattgtt cacaagatga aatgataatt ttttctaatc tttcaaaatc
147001 aagtttaaaa tttctttgag aaaattcagt aaaaacaagg tgtaaatttg caactccaag
147061 ttcatttaaa aaagggagtg ttttttctaa aatttttgta tcgataactg ccaaagctaa
147121 attaagctcg ctttgtgaat ggggcttaaa atgtttattt aaaaaataaa gcaaacaaga
147181 attacggtcc aaatttgaaa ttttataatc ataaataaaa tcatcttcta aatttcttaa
147241 ggataaaatt tcattttctt taattcttct tgcttttaaa tgggcaaatt cttcgccctt
147301 aagttttaaa atttcctctc ctgcttgttt gtggtataaa aattgcattt ataaatattt
147361 gcctgttaaa aaaggtaaaa ttaaaaggac aagttcgcaa aataaaatca taaaactaat
147421 gcgtctaaaa gttatatagc gtcttaatct acgagctttt ttaaattgtg tgaaatgaaa
147481 aatagctaaa gcaaaaataa aaatccaaga aaaaatcatc aaccaaaggt ttaaatgcat
147541 ttgaaattgt ttaatggcta aaagcaaaaa gcccgtaaag agcatcaagg ctaaaaaaac
147601 atagtatata ggtaaaaaca aacgaattct acggataaag ataaattcgg ttttaaaatt
147661 tccttgagtt aagacaagat aaaaaagcat taaaaatcca ctagcgtaaa gactataaag
147721 gtgtagggct aaaaaaaatt cataactttt gtccatttat tactccttgc cgatatattt
147781 tgctaaattt tccctttgtt cttgggttaa aaaattatga gcttcatctt ttaaattttg
147841 aagtctttga ataaattttt cactgcttaa atttttaatt gctatgcctt ttttggtttt
147901 aatttctcct ttttctccat ggcaagtggc acatttttgc ttgtataaag aattaataac
147961 acttggattt atgtcgtatt cttcaccaat attagcctca tcatcaactg gtaagggtaa
148021 attagtatcg ctaaattctg tttttttcatc attttgttca atttgcacta tctcttcagt
148081 agtatcttgg tttttatgat tgaattcttt atcattacta caagcgttta aaaataaaat
148141 tacaaataag cttattaaaa tacttaagtt tttacccatt aaacctcctt aggatttgtg
148201 aaaatgtgca aattttttaa tttatcgtaa aattctatat cgctccattc tgcttttata
148261 gcaggtgaaa attctatatt ttcaagtgcg atttttggca tggtaggact ataagcatct
148321 tctttaaatc cttgtgcata gcctgttctt gtttcatgta ctataatgct ttgcaaattt
148381 acaccttgtt cgccatttat catcttagtt tgctttaata aagcatcgat gagtataaaa
148441 aatactcggc aaaaattttc agcactgaca ttaacaggca aactcaccca gcgttgtgag
148501 tattttttca tttcgtttaa ataagattca tcatcttgat taaaaagcgt aatagcatga
148561 tcaaagctat cgataatttg gcgcatatgg gtttttaaaa gcccaaagtc ataaaccata
148621 cccgcattat ctaaatactt gctttcaagc aaaacttcga ccttgtaaga atgcccatga
148681 atactacttt tacaacggct tgaagagcaa aatctaacaa tatgagcatt ttcaaattca
148741 aataattttc ttataatcat acaccctctt tatcattcca aagacgaata tggattctat
148801 cagaataatt ataaccattt ttaatacaaa actcggctat ttttaaagca ttttgtttta

148861 aattttgctc attttctccc atgggcatac aaaaaatttc attaggcgct ttttctaaaa
148921 tttctaaaat ctctaatgcc gaactttcta attgactaga atttaaaaca aatttataaa
148981 aactatcttt ggcataatgc ttaaagcctt taagagcgtt aaaatttaat ctttttttctt
149041 tacttatccc actattttga agcttgacac ttagagcaaa aatacattct ttataaaaag
149101 gatatttttc aaaatcaagt tctatacttc cattgctttc aaaatgcact ttgaaattat
149161 tttctaacaa agtttggata aaatggataa attctggatt ttcatgatgg attaaaggtt
149221 cgcctccagt aatcaccaca ataggatcaa aatctttttt taaatcaagc acgcgtttaa
149281 aaagctcatt agcatttaaa ttttcatact cttcgttaaa ttctttagta aaaactgctc
149341 ttatggtatc gcaaccttta agagtttttc catttttaat aagttttacc ccaaagccta
149401 aacaattaaa attacaccct gcaaaacgca taaaaatagc taatttcccg ctgtatttgc
149461 cttctccttg tatgcttaaa aaactttcaa caagttgcaa atttaacctc cggtttgata
149521 aaaggcacga gatgaggttt cattatctac tacgctccat ctgttttttt caggtttatt
149581 gcgcaatact tcttgtaaaa tttttgcagc ggcattaata tcaccttttc tcactgcttc
149641 tttaatgctt aaagcttcat caaaataaag acaaggtata agtaagcctt cagcgcttaa
149701 acgaatacgg ttacaagaat cacaaaattc atggctgtgt ggatctataa tcccaaattc
149761 ataatcatct gcactataaa tacttacagg agctttttca gctttttttaa tgagttttat
149821 ttgatatttt tgacttagaa tttgtatgat ttcatctctt tttaagcctt gtaattttcc
149881 ataagcgtgt atattttcca taaattctat aaagcgaatt tgaatatttt tagattttgc
149941 aaattccaaa agagatatca attcatcatc atttaaaccc tttagcacaa cagtatttag
150001 tttgactttt aagcctacat ttaaggcctc atcaatccct gctaagacac tatctaaaac
150061 atctttttgt gcgagagttt ttgctttttt gctttctagg gtatcaagag agatatttaa
150121 acgcttcagt ccagcatctt taagatcttt agcaaaatct tttaataaaa aaccattggt
150181 tgtaattgct aaatcaagat ctttttttata atcatttatc atttttataa atatacttaa
150241 gtctttacgc aataaaggct ctcctccagt gatacgaatt ttttcaattc cttcatcaat
150301 agccacttta acaaacaaaa aaagttcttc aaagctcaat aaattttctt taggttgatg
150361 attaaaagga atttttggca tacaataaag acaacgaaaa ttacatcttt gagttactga
150421 tattctaaga taattaatct ttctgccaaa ttgatcaata agcattacta cccctagtga
150481 ttatttgata agttgtttaa attttaaaac aagctcgaca tttccagtgc ccatacgcaa
150541 atcttttgca atttgctcta tgctttttcc ctctttaaac aaatccacaa ttttttgttc
150601 ttcggtatca taatttggtg ttaattttgt catactttgt gctttttgtt ctaaattcaa
150661 aatacgattt tgttgttcat tttgaaaatc ttctataatg tgctccatag tttttaaagt
150721 tcttaaaaca ggggtaattt ttgcactgat ttgtttttca agtgtgattt gaatttcttc
150781 ttttaataga gtgattttat aatcctcttc ctcttgatca ccctcatctt ttacgcctaa
150841 ttctttgcgt aaataatgaa gctcttttgt aatatcttca actactcctt ggagtttgtt
150901 tatttttgca ctactttctt tatctttgat attcatataa gctaatattg caactaaaag
150961 tacgatcata aaaactaaat aaagcacatc gttactcatt tttgtccttt ttatttctta
151021 tcatatctct ttgcaattga accacaaaag catcaaaagc tattctgtct tcgggtttta
151081 ttttgataat ttgagctaaa tttttatttt gagctctagc aaaaatagcc atttttttgat
151141 tattaagatc aaatcttatg taatattctt ttccttcttc taaaatttca tctaaaaaat
151201 ttaaatactc aaaatttaaa gattcaacta cgccttttttg tttgagtgtt aaaagatctt
151261 tattttttatc caaagaatcc tcaagtctta aaatatcttc ttttaagctc aaaagtaaat
151321 caaaaatgat tttctcgccc tcatcaaaat ctacacgcaa ggcgagtttt ttccattttg
151381 aaagttgaga aatggcacct aattttaagt attcttgtaa aaagtggtta ttttcaatat
151441 cacaacactc taaagaagct tcaaatcctg ccttaaaaag acttatcccc atagcaaatc
151501 aacccaaata aaaataaaaa atattatact taaatatcca tttaaagtaa aaaacgcttt
151561 atcgatgtga gcaaaatttt tatgtacaat tttatgttca aaagccaaaa taataccgct
151621 gataatcacc ccaagtaatg cgatatttcc aagtgctact ccccaaacct gccaaacaaa
151681 aagtagccaa aacaatactg ctaatacatg acaaaaagcc gagataaata atgtcgcttt
151741 agagccaaat ttagcaggga tagaatgaag ccccactttt ttatcatact ccatatcttg
151801 taaagaataa agcaagtcaa atccagccgt ccaaaaagtc acccctaaac ataaaatcac
151861 gctataaatg tgaatttcac ccataactat cacactacct gcaataggtg caagtcctaa
151921 acaaaatcct aaaaccaagt gcgctaaaga actaaagcgt ttaaaagcag agtaaattgc
151981 taatacaaaa agcacaggaa aagagagata aaaagccaaa gtattgataa aataagaaca
152041 tactataaaa ataattgcat taaggatgat aaaaatccaa acgcttttgc gtcctatttt

152101 tccactgaca ttagggcgat ttgcacagcg aggattgtct ttatcgatat cttcatccat
152161 taaacgattt gttgccatag caaaatttct tgcacttaca gcacaaataa tacctaaaat
152221 caaagcctta aaaccaaacc aagcgctatc attaacgagt tttgatgcca ctatcataga
152281 tgaaaataaa aagggcaagg caaaaataga atgcttaaat accacaagtt ctaaaatatc
152341 tttaaattta ttccaaatta tattcatgtt tccactttaa aatatttttt gctatgattt
152401 tactaaaatt ttcataaatt ttaggataaa ttatcacatg ttttttcaaa tcgcacttat
152461 aggaaccaca gccagcggaa agacttatat agcaaatgcc ttagctaaag aattcaatgc
152521 agtggtttta agccttgata gtctttgtgt ttataaagaa atcaatatag ctaatgctaa
152581 gccatctaaa gatgagctta tgaatttaaa ttactttgga gtaaatttat taagcgttga
152641 tgagcatttt aatgtagaat tgtttataag agaataccaa aaagcaaaag aatttgctct
152701 tatgaatgat ctacccttga ttatagtggg tggaacaggc ttttatctaa aaacgatgat
152761 agatggttta agtcaaaaaa ttcaagatac aactagcatt ctaagtaatg atgaaattta
152821 cactcttttg ttaaaaattg atccggctta taaaatagaa aaaaatgata cttatcgtct
152881 tagaaaatgg ctaagcattt atgaaaatac aaaggaaatt cctagtgatt ttttaaaaag
152941 aacacaaaag gatggagtgc ttaaggatat tgaaatttac gagcttgtat gggataggga
153001 aattctaaga aagcgcatag aagcaagaac aaaagaaatg attgatagtg ggcttataga
153061 agaagcaaga atgctttttt caaattttga taacaatctt aaagcattaa attctatagg
153121 gcttaaggaa tgtaaagagt atttagaaaa taaaatttct ttaaaagaac ttgaaaattt
153181 aatcaccata catacatcac agcttgctaa gcgtcaaaga acttttaata aaaaattcca
153241 aagcactcct ttggaatttt ctaaagcttt aacactactt agaaataaat ttttagctaa
153301 aaaataaatg ctcaattaaa atttttatcc ccaagattat caaaacagct cccccctaaaa
153361 attcagcttt atttttaaag cgagtgccaa atttattgcc tatttctaaa gcaatagcac
153421 ataagataaa agtaatagaa cctattaaaa atagtgctaa aaataaatta acatctaaaa
153481 aagcaaaact aactcctata gctaaggcat caacgcttgt agctacagct aaagcaaaca
153541 taattttaaa gccgaattga tttgaattag tgtcgcaatt ttcattctca aaagattctt
153601 taatcatttt tagaccgatt aaagagagta aaataaaggc tatccaatga tcaatacttg
153661 cgataaagga tgcaaaagtt atgcctataa catatccaat ggctggcatt aaagcttgaa
153721 aaccgccaaa ataagcccct gtaataacat agtgtcttaa atttagtttt ttcacactaa
153781 atcctttaca caaagataca gcaaaagcat ccatggctaa tgcacaggat aaaagaataa
153841 gactgtaaag atccatgatg actcctatga aagatgtaaa agttgagaat tataataaaa
153901 atttcttaat taattttttt gatgaaacta tgattaaaaa tttatagcag cggattgtat
153961 ttaattaaac tttaagaata aaaaatatat aattcaactt catttttatt tttactcctt
154021 ttaaaagtgg cccattcgtc tagcggttag gacatcgccc tttcacggcg gtaacacgag
154081 ttcgagtctc gtatgggtca ccacttaaat cttttttat taatatttcc aaactttatt
154141 tacagatagt ttttaaattc tatttaatgt ttattatcgc ttttgtaaaa aatagattta
154201 taaatttgta agaattttgt ttgtagaatt ttattttatt aaaatgatga ggattgataa
154261 ataaatgagt tatgatacaa attttttctt attatatttt aaagataagg ttccaaagga
154321 ttccttgatt tttcttaagg aaagtttaga aaaagcaagt gaagaacaaa aagaaaaact
154381 tttatttact aaactgaaaa atcccctata tggactttta tttgcttttt tgtttcctgg
154441 tttggataga atttataatg gcaatataat tttaggtata cttaaaatca tatcagtaat
154501 acttgtttcc atgggtttgg taatagcaga agataaaaac gatgaaagca ctatgctgat
154561 ttttataaat ttatttttca tactaagtgt ttgggtgatt ttggattatt ttttggtatg
154621 gaaagatatt tgtcaaaata atcttaaaaa aatttttgaa gttttacaat gaagcaagat
154681 aaaacgatat tgctcatatt taaagataag attcctcaag aaaggctatt tatcttaggg
154741 gaagaattaa aaaaaagcca aaaagatcta agtgctcttt atgctatctc tcttaaaaat
154801 cctttggttg gccttgtatt aagtataact gtgggattat ttggggttga tagattttat
154861 aagggcgata tcttgttagc ttgtattaaa ttggcttttt ttataatacc tttatttgct
154921 acttttgctg cttttatagc attgttaaat aagagccatt cgatttttat agattatttt
154981 gctatttttg ctttaatgtt tgttgttgct agcatttgga agcttgtgga tatttatctt
155041 gtttttgtgg gtattaaaaa agataatttt tataaaattc ttaactttct ttcttaaaaa
155101 atatcttgaa aatttaaaga agtagttaaa gactttctta gtctttttat ataattttta
155161 tgctacaata ttttgtcaaa cttactaaat taaaggagaa aattatgttt gaattaagaa
155221 aattacctta tgataccaat gcttttggtg atttttttaag cgctgaaact tttagctatc
155281 atcatggaaa acatcacaat acctatgtaa caaatcttaa taatcttatc aaagatactg

155341 aatttgcaac caaagatctt gtaagtatta tcaaaagttc aagtggtgga gtatttaaca
155401 atgctgctca agtttataat catgattttt attttgattg cattacgcca aatgcctgtg
155461 aggtaaaagg cgatattaaa gcagctttag aaaaagaatt tggctcttta gagaatttca
155521 aagctgaatt tatcaaagga gcaacaggag tttttggttc gggttggttc tggttagttt
155581 ataatactaa aaatcaaaaa ttagaatttg tgggtacttc aaatgcagct acacctatca
155641 ctgaagataa agttccttta cttgttgtgg atgtttggga gcatgcttat tatgtagatc
155701 atcgcaatgc gcgtcctgcg tatttggaaa aattctatgc tcatattaac tgggaatttg
155761 tagcaaaagc ttatgaatgg gctttaaaag aaggtatggg atcagttagc ttttatgcaa
155821 acgaacttca ccctgtaaaa taatacaagg catcttaaaa gatgcctttt ttactctcct
155881 aaacttggag tcatttcgat aatcttccat ggaagtcctt gagaattaag ctcatccata
155941 aaaggttttg cgtcaaattc ttccatatta aatacacctt ttccttgcca aattccttta
156001 gcgattagtt ttgtacctat catcgcagga actcctgtag tgtagcttac tgcttgcgcg
156061 ccggtttctt tgtaacattc ttcgtgattg caaacattat aaatataaac ttgtctgtct
156121 ttaccatctt ttacgccacg gattacacaa cctatattgg tataaccttt tgtgcgagga
156181 cctaggcttg caggatcagg aagtagggtt tttaaaaatt ctataggaat gatttctttg
156241 ccttggtgca taacaggttt tatacctagc attccaacat tttcaaggca tttcatatga
156301 gttaaataac tttgcccaaa agtcataaag aagcgaattc ttttcaaacc tttgatattt
156361 tttactaagc tttcaagctc ttcgtgataa agcaaataac tatcttttac gcctacttca
156421 ggataatccc attccatttt tatttccata ggttcggttt ctatccactc gccttttttcc
156481 cagtagcgtc ccttagcaga aacttctcta agattgattt caggattaaa attagttgca
156541 aaagcatagc catgatcgcc cgcgttacaa tctaaaatat ctatgtaaga aatttcatca
156601 aataaatttt gctgcgcata agcacaaaaa acatttgtaa caccaggatc aaacccactt
156661 cctaaaagtc ctaaaattcc tgcttcttta aatttatcat ttcttgccca ttgttcttta
156721 tactcaaatt tagctagatc tggatgctca tagtttgctg tatcaacata atcaattcct
156781 gcttttatac aagcatccat caagcttaaa tcctgataag gcaaagccac attaagtaaa
156841 atttgcgccc ctgttttttt aataagctct acaaccgcat cgctatcatc tgcatcgatt
156901 tgcgcagttt gaatttctac tcctagacgc tctttgataa agctagcaat ttcatcgcat
156961 ttacttttgg ttctacttgc tagagtgatt ttactaaagg tatctgaatt cattgcgcat
157021 tttacggtag ctacgcgact tactccgcct gcgcctatga ttaaaagatt tttcattgat
157081 tgtgtcctta aagaaatttc aggaatttta gctaaaaatt gataatatca aaatttattt
157141 tttcttttag cgggtaattt gagctgagca acataaaaaa ctctatattt ttatatctta
157201 ctttgagtct ataaaaagct cctaaaaatt ctttttgtaa gattgtagct tcaaaacgat
157261 ttcctatttt aagttctgca tagccaaaag tataattttt tgaagcgatc cactgaaaaa
157321 attcatcttc taaatcaagt tcttcttcta tgatatttaa atcaggtaaa attctagcac
157381 ttttatgatt aggcttaaaa taaagctctt taggatctgc atgagctaga atttctcctt
157441 tatcaagaaa ggcgatttta tcagacatgc aataagcatc ctcgatatcg tgagttacca
157501 taatggctgt aattttttgt ttttgtatga gtttttttag ctctcttctt aagtcttgtt
157561 ttaaattctg atccaaatta gaaaaaggtt catctaaaag taaaagctta cagcctcttg
157621 ctactgccct agcaaaggct actctttgag cttgtccgcc tgaaatttca tcaatttttt
157681 tatatcttaa attctcaatt tcaaaggttt ttaacaaatc ctctaaaatt tgttttttat
157741 ctttttttatc atagagtgca aaaagtatat ttttttcaac atttaaatga ggaaataaag
157801 cataattttg aaacatcatc gccacttcac ccttgcattt taaaaattca taagagcttg
157861 cttgttctag ttgggcaatg attcttaaaa gtgtactttt accacttcca ctccctccta
157921 aaatgcttaa aaattctcct tctttaacat gaagattgat attttttaaa gcttgtattt
157981 tgccaaaatt ttttgataag tttttaattt ctaacatcat ctttccttgt gattttatcc
158041 atccaaataa cgactattaa agaaagtaaa acaatgagta gtgaaggtaa agcagcatca
158101 taaattctct catcacttgc aaaccaaaaa gcttttacac ttagagtttc aaaaccaaaa
158161 ggagctaaaa ttctactcaa aggaagttct tttatagtat cgataaaaac aatgataaaa
158221 gccaaaaata aaaaatgttt cattaaggga gtatgaattt taaaaaataa gatcaaatag
158281 ctaggtctta aatttaaact cgcttcatct gtatttagat gaattttatt ataacccccct
158341 tccaaagaat aaatcgcact cgctagaaaa cgaataatat aagcaaaaac taaaatgatc
158401 aatgaagtgc ctaataaatt aagatgaaaa attttatcta aaaacacaaa aaggacaatc
158461 atgctaattc ctaaggcagc tgcgggtata gaataaccca aagaacttag cttaagaata
158521 cataggctga aaaagttatt ttttatgatt cttgagctaa acattaaaaa ataagccagt

158581 aaagtcgtaa tcaaagctgt gatgaaagcc aatataatgg tttgaaaact aatgatataa
158641 aattctgttt caaaaagttt aaaatctttt aacccccaat aaacaagcca tataaaaggc
158701 aagataaatc ctaataaagc gatgataaaa caataaaaac aggctaaaat ttgctttata
158761 tgacttaatt ttcttttttt gataaaaaga gttaaatttt gattaaaact ataatgatgc
158821 ttgtttttat aataataatc tatatacatg attaaaaaaa caaaaatcat caaaattcct
158881 gataaaatac tcgatgaata agaatcattt aaatcatacc atagtttaaa aatccctgcc
158941 gaaaaggtat ctactcccaa ataagccgaa gctccatagt cgcttaaagt ttccatcaat
159001 acaagcaaag ctccagaaaa aatagcaggc cttgcgctta aaagcatgac gcgtgaaaaa
159061 attctaaatt cagaataccc catgatttta gccacttcat aagcttctat ggcttcactt
159121 ttaaaagcgg ttttagcaaa taagtaaata taaggatata aagaaattcc taagacaaaa
159181 ataaccccat aatggttgaa aaaatctatc ctaaaaccaa aaatttcatg aaaaaagcct
159241 tgaaaatcca taatcccaac atatacaaaa gctaaaatat aagcaggtat tgccaagggt
159301 aaaataagta atttttctaa aatcctgcaa aagtaaaaat tataatttgc catcaaataa
159361 gcgctactta cacctaaaat caagcttagc aatagcactc ctatgctaac tatcaaagta
159421 tctttgatga attttaaaaa caaataatca aaaaaatgac tcaaattttc tttaatagat
159481 gaaatttcag ccaaatcgct tgtattgaaa ttttgaaaaa gaatataaaa aagctcagca
159541 aagataccaa aaataggcag ggtaagaaaa agggcaagta aaattgcccc tatcttgtaa
159601 tactttaggg ttttaagcat tatctaaaac cgacttgatc ataaatttta accgcttctt
159661 taacctttc tgctatttga ctcacaggta tttgatcttc tttaaaagtt ccaaaatctt
159721 ttactgtttg gcttaattct acatcatttc ttataggaaa ttcataattg ctatcagtga
159781 gaattttttg tatttctgga gttagcataa attccataaa cttcttagct gcttcttgat
159841 ttttgcttga ttttgtcata gcaataccgc tgatatttat atgagttcct ctattgtctt
159901 gatttggaaa aataatacct aaagaatttc caacttctac atcttttgga tttttagaat
159961 tttttaaaag tcctatataa taagtattca taacagcaaa tttagcctca cctgcaaaaa
160021 cttgtctagc ttgatcccta tctccaccct ttggtttggt tgcaagattg tcaagcacac
160081 ccttagccca tgctttagca ttttcatctc catcattagc tataatagaa gctaaaagag
160141 ttttgctata aggagctgta gcacttctca taacgatttg ccctttaaat tcaggcttag
160201 ccaaatcttc atagtttttc attttgctaa tgtcagtgtt tgcattttta ttgtaagcaa
160261 taattcttgc tctttttgta atcgcaaacc attccccatc tttatctctt aaatgagcag
160321 gtatagtatc ttctaaatat tttgatttaa cagggcttaa aagtccagaa ttttttgctt
160381 ctgtaagatt ggaaatatct gctgtgatga aaatatcagc aggcgaattg cttccttcaa
160441 gcgaaagtct tttgatgagt tctgaagctt tagcttgagt atgattaact ttaatgcctg
160501 ttttctcttc aaattttttt ataatctgaa aatcagcatc ataatgtctt gccgagtaaa
160561 tgttaagttc tgcagcacct agtaaagaaa aacctaataa acataaagaa aagatttttt
160621 tcatttattt gtccttatat tgagaattat atttagtttt atcatgatct ttaaaatcag
160681 cattagagtg ttcagagcac gcaaatgcaa aacctaagca aaataaaaat aaaactaatt
160741 ttttcattat ttctcccctt ttttgtgatt aaaattagac taatttttga taagggatat
160801 taatattaaa aagtaaatat aaagtaattt aatgattttt ctaatgataa gtattttcat
160861 tttattataa ttaattttga tattaattat aaataattaa atttaatata aatatttaag
160921 agtattttta tatataaaat attaatattc aatatcaaaa ataaaccaca aagaaagtag
160981 aaataataat gaaattttat accatgttaa gcacattatt tataagcttt atgttaagcg
161041 cttgtgctgt tttgcaaaaa ccatctccac ttcaagaaaa caaagatttt tacatactta
161101 atacccacac tcaagaaaaa atttcttttg aagatatgat tttagaatta ttaaaagcag
161161 atgtgatact gcttggcgaa aaacacgatg aagcaaagca caagataagc caagttatga
161221 tttttaatgc cttagaggga aatttaagtt ctcaaaatat aagttttgat gtagctttag
161281 aaatgctagc aagcacagag caaaatcact tagataaagc tttcaaaaat aagaaaaaca
161341 ttaaggttaa tgaacttgcc aatgcactaa attgggataa gggttggaaa tggaaagatt
161401 atgatcaatt tgttaatgca gtttttttatt caagagcaaa aatattaggt gcaaatcttt
161461 cacgctcgga aattacaagt atttataatg gagttcagcc tttaaaaggt tatgtttcaa
161521 ccacaaatga agtaaaaaaa caaattttg acattatatc cttaagccat aagttaaacc
161581 ctcaagaaaa taaagaattg ctagataaat tagtagaaat tcaacaattc aaagacaggc
161641 gtatggcaga tgtattagtg catcatacaa ataaagtttt attgctagct ggaaattatc
161701 atactagcaa gaagatagga gttcctttgc atatacaaga ctttaagagt gataaaaaaa
161761 tagtcgtggt gaatttaaat tatgggaagg tagatttaaa agatagtgat tatattttta

161821 tttataaggg aggagaatga aaaaattaag tttaatttgt gttatgggtt tatgttttgc
161881 taattttgct ttttctgaag aattagaatt tgatagcctt gaaatttcag gctcaagaat
161941 caaaaacgat gaaaagcctt ttgtaactcc tggagcaaca agcacaaggg ataatttaaa
162001 ttcaagtacc caaagtatag attctatagt gcgtagtgtt cctggtgcat acactcaagt
162061 agatcaagca caaggtggag tttcggtaaa tattcgcggt atgacaggac ttggaagagt
162121 aaatacccaa attgatggtg taactcagac ttttttggc tctgcaagcg atgatagctt
162181 ccatggacaa attggaactt caagttttac agccccaata gataaaaatt ttttagtcgc
162241 tttagatgtc aatcgtggaa cttttagtgg ggcaaatgga gctaatgctt taatgggttc
162301 agctaatttt agaactatag gtgtagatga tatagtgcaa gaaggaagag tttttggcct
162361 tttaggaaaa tactcttatg gtagcaatgc tataggacct gattttatgg gcgctatagc
162421 aggaaaaact atgttagaaa ataatactag cttgggcgtt ttatttgctt atagtgggca
162481 aaatgtttct caaaattata aaattggagg cggaggttat ataggcgatc aaaaacctcc
162541 tatcgacacc gatgatgatg gcattccaga cgataattta gcagcaccta taaatccaaa
162601 taatctttat aatcagccta gaagtcaact ctttaaagtg gaatacaaat ccgatattag
162661 cgaggcaatt ttaaattata gagcttacaa gaacaatctt gcgggtagaa agattacaaa
162721 tgatacctat caaattgatt atcgtttaaa tcctgattct aatcttttag atctaaagtt
162781 tttatttgct tacaatgatg gaaaacaaaa atataatcaa ggcagcactt ggggttatca
162841 cgatatgagt ggagttaaga ctaaaaatcg agcggtaact tttgatctta gtaataccat
162901 gaataaagaa ttttcacaag attcaaattt atattttact tatggggtta atattttaaa
162961 caatcattat agtaatgatt ttccacaaga tagagtgctt ttgccttata ttttaaccag
163021 tttttatcct aaaggaaaac aagatatcaa aaccctttat ttggatacga gttttacaca
163081 aggaattttt actttaaatt ctaatgtcaa ttggactaat gcgcaattaa gtggatataa
163141 aggaatttgt agcatggcta atccttattg ccagccaaaa aatgcgacaa atttagaaaa
163201 agattataat aattttaatt attctttgat gctttcagca gatatccatc cgctttttaa
163261 tcctttttatt tcttattcta aaagccatag aattccaaat gtccaagagt atttttttcac
163321 ccatgatgca agttttgagc ataatatgaa tacattttta aaggctgaaa gtgcagatac
163381 ttatcaaatc ggttttaatt cttttacaca tgaaatttta aatgatagcg atactttagg
163441 ttttaaaatg ctttattatg atactaaggt aaaaaattat atttacaata gaagatattg
163501 gcaaaaagct gacgatgctg tttttcttat gcagttaaat gatgatgaaa aagctaaatt
163561 tcacggggtt gaacttgaat ttaaatatga tacgggattt ttttattcta tactatctta
163621 tacttatcaa aaatccaagc ataaattttc agatacagaa tctttagaat ttggaggtgc
163681 gcaatcaggc caaagccaat ttgcccaact tccagagcat tatgctaatc ttgatatggg
163741 tgtaagatta tttgaagaaa agctaactct aggagctctt gctaaataca caggaaaagc
163801 aaaaagaata gttcctgtag gtagcttaga tgatgatcca agcaatcctg atgctatggc
163861 tccgcttaaa actacagatg aattgccgaa aataccaact attgttgatc tttatgcaaa
163921 ttataaaatt ttgaaaaact ttacaatcaa agcagaagta caaaatttat ttgacaaaaa
163981 ctatatggat gcactttact cttacaacac aggggaaaac caaaatgctg gaggattatt
164041 tgatcctatt tatatttata ataactctgc tcgcggtcgc acctttatag taagttttga
164101 atataaatat taaggaaata cataaatgct aaaaaaaata tttttatttt gtttttttatt
164161 gttaaattta aatgctgaag tgaatgtaac agatacaaat ttatcttttg attctcctaa
164221 aatagaagcc gaagtaccaa agatagagct ttccctttct agtctttatc aaaatgctga
164281 tgaggtggta aaaggcgtta tttacatatt ggttttattt tctgttttag cgtgggcagt
164341 ttttatttct aagcttatgc aattttattt tatgaagaaa aatttagact tctctataca
164401 caagataaaa gaattaaaaa atctcaatga attagatcag acaaaaggtt ttgcaggtgt
164461 tttatcttta gaaattcaag atgagcttga aaaaagtgaa tataaaaatg atcatataaa
164521 agagcgtata gaattgcgct tgcaaaatac catctcaaaa caaatcacag caagcaaaaa
164581 tggcttgagc ttgcttgcga gtataggtgc tagtgcacct tttatagggc tttttggaac
164641 agtttggggg attatgaatg catttatagg tatagcaaat cttggcaatg cttctttagc
164701 agttgttgca cctggtatag ctgaagcctt gtttgctaca gcttttggct tggtagctgc
164761 tattcctgcg gtgctttttt ataattatct tacaagaaaa aatttaaaac ttatgcacca
164821 tttagatgag cttgcgaatt ttgtctatat actttttcat aggagttatt ttaatgataa
164881 aaactgaaat agctcacaaa gaagaggagt taagtgagat taatataact ccttttatag
164941 atattatgct tgtattattg atcgttttta tggctgttac tcctcttatc acaagttcta
165001 tcaagataga actacctaag agttcacagc aagcagagga taagcttaaa aatcccatca

165061 tactttattt aaataccgat aatactttgg ccattaatga tgataaatta agcctagaaa
165121 atctttcaag tgctttggat ataaaaacca aaggtaataa agaagaaatt atttattttc
165181 atatcgataa aagtgtaaag tatgaagata ttatgcaggt aatgcaaaaa ttaaaagaga
165241 atggctatgg aaaaatagct ctttcaagca aaaaggtgga ttaatatgaa gtctagtgtg
165301 atttttgggt ttgtgtcgag tttggtattg catgctttgg tattaatgtt ttttcttttt
165361 tctttttata ctcaagaaaa atcaagcgga gttgatttta aacaaggagt ggaatttact
165421 tccattatga tggtatcaga gcttcctatt ggagaattaa aagaagtttc aatcgatcaa
165481 aaaaagagca attctcaaga taaaaacaaa aaacaagatg aaaaaatgag tcttaactcc
165541 caagataaaa acaaaaaaca agatgaaaaa atgagtctta actcccaaga taaaaatgct
165601 gtattaaaag ctcaaaaaaa gatagaaaag caagatgaaa atcaagccca aaaagaaata
165661 gctaatgcaa gtgaaaattc aaaatttaaa aatgaaacac ttagtgcccc actacaaagc
165721 aatgaagata aaacacaaac tatagtaagt ggcaatgcta aagaacaaat taaaagttat
165781 caagccttgc ttatggctca tttggctaaa tttaaaaaat atcctcaaga agcgataatg
165841 caaaagcaag aaggtgttgt aagaattcgc gtgagtatcg atgaaagtgg aaatgtccta
165901 agtaaggagc ttaaaaaatc ttgcccctat gcagtgctta atgatgaagt tttaagttta
165961 tttaaaagag cttctccatt gccaaaacca cctaaagaaa tgttgaaaga tggcgaaaaa
166021 attagttttg ttatgcctat tgattacaat attaaagact atttaggtaa aaaataaact
166081 cttgattaaa taaaattgat tagaattttc gttttgagat taaggcggtg gtatgttttc
166141 ttcgtttttt gcaagtaaaa aatgggcttt atgggcttat ttggggcttt ttttgctatt
166201 gtttttcttg tatatgcaaa caagtttaaa tgttgcgatc aattcatggt atagtgattt
166261 ttataatgtt ttacaaaaac caaaaataga acttttagat tctaattcaa cacaaaaagc
166321 acaagaaaat ttcgaaaata atgcaagttt gatccaagag gccaatcaaa aagcacaaga
166381 aaatttccaa aaagctaatt ttatcaataa aggcgcactt tattattatc aaagtttatt
166441 ggaatatttt tttaattcta gggctatgat tgaaaaagaa agctattctg cgagtgattt
166501 ttatgctttg attttagtat ttttagctat tgctattcct tatgttttaa tcgcaacgat
166561 taatatttat tttgctagtg tttacgcctt taagtggcgt gaagctatga ctttttctta
166621 tttgaaattt tggaaaaata aggatgataa catagaagga agttcgcaaa gaattcaaga
166681 agatacttat aatttttcta aaattgtaga aagcttagga cttagtttta taagagcttt
166741 aatgactctt gtagctttta taccgatttt atggacttta agcgatgttg taagcaaggc
166801 tttatttgca aatttaagtg agaattctag cttttatttt ttaaaaaaca tcgatggttt
166861 gcttgtttat gttgcgcttt tgatttcttt aggaggatta atcgtatcgt ggtttgtagg
166921 aattaaactt ccaggacttg aatacaacaa tcaaaaagcc gaagcagctt ttagaaaaga
166981 gcttgtttat gctgaagata accgcaaaga atacgctaaa aatgaaacca tgatagagct
167041 ttttacggga cttaaattca attataaaag acttttttg cactatggat actttaatat
167101 ttggttgatt ttatttgagc aaatgatagt tatcgttcca tttttaatca tggctccagg
167161 actttttgcg ggagctatag gacttggtat tgtaatgcaa attaataatg cttttgatca
167221 agtaagaagt tcttttagtg tttttatcac aaattggaca actatcaccc aacttagaag
167281 tatatataag cgtttaaaag aatttgaaaa aaatatcgaa tacgcgaaaa ataaaaacaa
167341 aaatcattta taaaaatgtc taaatttgcc aaaaattatc aaagcgatga aaagtaaaaa
167401 ggctgtgaaa atacataaaa attcccagcc aaaactttca taataaaaac ttggcaatac
167461 agaacccaaa gcgcccccac tataataaaa tgttaaataa attccattgg ctaagccttt
167521 tttatccgcg atcgaattta aaatccctga aaatatgcaa tgacttataa acattccgct
167581 acaaaataca aacatagcaa aaaaggcaaa ataaaaattc cctactatca tacaaaaaca
167641 acccgttata aaaactaaaa atccaaatat agctgtgcga atttttgagc ctaaaatttc
167701 tatggtttta cctataagta gtgagcttat tactccggtt aaaaatccca aataaacaag
167761 tccaatttgt gtttgagtga tatcttttat actttcttta agatgaaaag gtaaaaaact
167821 tactatgctt tgaaaagaga aaaagatagt aaaaacacaa aataatatca ccataaatct
167881 aagttctttg ataaagggta aaaaatccct caaggaagtt tgagtgttta agcttatagg
167941 atgctcttta aaaaacaaaa ataaaaaagc acaaaccatc attaaaatac taaaaaaatt
168001 taaggcaaaa tgccaagaaa aaagatcgct caaaaaactt ccaaaaaccc tcccaacaaa
168061 accaccaaaa gttgtagcac ctacataaat ggctatgttt ttttgtatat tgtcgctttc
168121 taaacgacta agcaaggtaa gaagtgctgt cagtatagca ggtatacaaa gagcttgtaa
168181 aaatctaaga aatagaaaaa aataaaaatt atcgtttaag tttccaataa taccaaaaat
168241 agaacataaa aacaaagaaa caacaagaat tttctttaag gaaaaaattt caagtaaata

168301 accataaacc aaaggtgcga ttgctaaggg aataagcgta acactgatta tccaagaaat
168361 ttggtgcaaa tttacatgaa aataattaga caataagggt gctataggtt gtggagcata
168421 gacaatagat agggttaatg tagcacaaaa aataatcaga attttaagca ttttcatgct
168481 ttaaattaaa ctccaatgca aattaatttt agcttaaaaa taatttttaa gctaaaattt
168541 tacttttaat tgtgtataat taagtatttt atttataagg agcttttatt ggaccccagt
168601 caggttttag atttaaacca aactttacct acagcgtctt ttgatgcagg atattctata
168661 cttatggttg tcattgcact agctttagtg cttttaaatg gctttttcgt tttatctgaa
168721 tttagtattg ttaaagtgcg tcgttcaaaa cttgaagaaa tggttaagga aaaaaaagta
168781 ggtgctaaaa aagcactaga agttacttca agacttgata cctatcttag tgcttgtcag
168841 ctaggaatta ctttaagttc tctagccctt ggttggatag gtgagcctgc tatagcaaaa
168901 atgttggaaa ttcctcttgc aaatcttgga tttaatggag ttattatcca tactatagct
168961 tttattatcg cctttagtat tatcactctt ttgcatgtgg ttttaggcga gcttgttcca
169021 aaaagcattg caattgctat tgcagataaa gctgtgcttt gggtagcaag acctcttcat
169081 tggttttgga tacttttctt gccttgtatt aaaattttcg atttcttgc ggctatgact
169141 ttgaagcttt ttggtatcaa gcctgccaaa gaaagcgagt taactcatag tgaagaagag
169201 attaaaatca tagcaagcga aagccaaaaa ggcggtgttt tagatgaatt tgaaactgaa
169261 attatccgca atgcggttga tttttcagat actgttgcaa aagaagtgat gacacctcgt
169321 aaagatatga tttgtctaaa taaacaaaaa acttatgctg aaaatatgca aatcatttgc
169381 gatcataagc atacgcgttt tccatatata gatggttcta aagatactat tttaggtatg
169441 atacatatac gcgatatagt acaaaatgag cttagtgata agagtgaaaa tttagatact
169501 tttgttaagc ctttgatttt agttcctgaa aatattagca tttcaaaagt tcttgtgatg
169561 atgaataaag agcgttctca cactgcactt gtgattgatg aatacggtgg aacagctgga
169621 attttaacca tggaagatat catggaagaa attatcggtg agattaaaag tgaaaatgag
169681 gaagacagct ataaaaaact tgctgaaaat atctatgaat tccaaggtcg ttgcgatata
169741 gaaaccgttg aagaaatgct tttgataagc tatgatgagg atttagagca agtaacgata
169801 ggtggttatg tgtttaatct tctaggacgc ttgcccgtag tgggtgatcg cattgaagat
169861 gagctttgtt actatgaagt taaaaaaatg gatggtaatt ctatagagcg tgttaaggtg
169921 gttaaaaaaa ccaacaaaga tgaagaataa gccttttttg gcttatttct ttctataaac
169981 tttatttta agttctatac actcaaaacc ttttttgtct aaagttacta tacgcaaatg
170041 tcccccatca tctactttac cttcgcacaa aaatactctt ttttctattt gcacaggcaa
170101 ttttgtgcgg tttctatgtc caaagatttg ataaacattg tcttttgtaa attcacaaaa
170161 ctgacttgct actttttgac tttcttcata atttcctacg ccataaataa aatcataact
170221 tgacataaag cttatttgat aagccttaga aggtaaaaaa tttaccccgc catgcgagca
170281 aaaaacttgt ttttcatgaa atttataata cagacattct tttaagtaag gatagaattt
170341 tctagcatcc ctaggcgtga gtttttcttt tctaaaatct ttgagtgtat tttcgttaaa
170401 ttctttagag cttgatagtt ctccattggc ccattttatc aaatgtcttt cgtgatttcc
170461 ttcaagtaaa tatacattct ctctctcgca aattttaagc aagaatttta agaccttgcc
170521 atttctatg cctctatcga tataatcccc taaaaagata tagcattcat catctttgag
170581 ctcttgtaga tattctttta agacgctaaa gcagccttga atatcaccta tgtggtgaat
170641 tttcctatac ttactaagat taggcatttg ataaaggcat tttttccaat cattaggatt
170701 taaaatcgta tattttttag gaattctttc aagcaggtga tgggtttttt ttaaaagtga
170761 ttgtgagact atatagccac gctctttggc tttttgttca ttttttttcaa gacattcttc
170821 taaagagctt ttaaaatcta taacataaac attatagcga tattgtttag ctagctcttt
170881 gtaaacctta agggtttcat tataagcatt aatgatgcaa aattcacctt tttgcatacg
170941 catttctaaa aattgaagta aaattttata taattcttca tcattttta gatttaaatt
171001 tttatattta cttacgattt tacttacact tcctgaaagc aaacgcaaag aatttagatc
171061 aagagtgtaa tcttttaagc gatttctttt gataaattct tcttgtcctg cgtagtaatt
171121 ccccccttaaa ataagtaaaa tacgcaagtt tttgcctaaa ttttctttaa aaattcagtc
171181 tttagcacaa taacgccaaa tttatctact ttagcttcaa tttcgccttc tttagaagta
171241 agtttgatat tttttatcgt tgttccgcgt tttaaagtag tgctagcacc tttaactttt
171301 aaatctttta tcacacttac gctatctcca gcatttaatt ctgttccatt tgcatcttta
171361 ggcatttttt tcctttttaa atttttatat cattgtatat tttatcatca ttttctaaga
171421 tttttcttga gctaagcgct tctttttgat atagatattg atggattcta cacccaaaga
171481 aaaagccata gcaaaatata cataagtttt tgaaatatga aaatccaaac cttcggcaac

171541 taaagttaca ccaactaaaa tcaaaaatgc tagagctaaa atcttaatcg ttggattatc
171601 atcaacgaaa tttgagatac ttttgcttgc aagcatcatt acaaaaacag ctacaatcac
171661 tgcaatcatc ataatttcta tattattaac catacccaca gcagtaataa ccgaatcaag
171721 agagaaaacg atgtctaaaa cagcaatttg tatcaatatg ctaaaaaatc ctcttttagc
171781 tccctctttt aaaacctctt catcactttt ttctccagca ttatcaatat catgatgaat
171841 ttcaagtgta gattttgcta tcaaaaataa tccacctaag atcaaaataa tatctcttcc
171901 tgaaatttct tgtgaaaata cgctaaaaag cggggttgta agtttcataa tccaaaataa
171961 agaaaggagc aataacaaac gcgtaatcat cgccaagctc aaaccaaaaa ttcttgccct
172021 ttgtcttttt tctttaggga gtcttcctac caaaatagcg ataaaaatga tattatctat
172081 ccccaaaaca atctctaagc caatcaaagt tgctagagcg atccacattt cagcactaaa
172141 aacccactcg aacataactt tccttaaaat tttgaaattt tttaaagtgt aattttataa
172201 cttcaaagca aatattaaat attttgatat agtatataat taaattttta taaggaaaaa
172261 taatgaaaaa actagaagat atcaaagcta tgaattacaa acaaaaagat gagttagaag
172321 atttagttct tgaggctata agagataata atttagctaa agtaaaagag attttaaaag
172381 attatcctgt aaaaatttct tgttatgagc ttcattttaa aaatgataac gaagaatatc
172441 ctttatttga tccttattat ttgataatga gagctgcata tgcttgtgaa gaaaataata
172501 atgatttttc tattttagat tatttatttg atgagtatgg cttaagtcta aaagatccta
172561 aatataattt ttgctttaca gatatgaaat atatcaaaga agctaatgat aaatatattt
172621 taataaaaaa agcaaaagaa agcaacatta tttgtcgaaa cgctttaatt tattattata
172681 tactcaatgc tgataatcca aactctcata tcattaagta tctagtcaat cgtggggcta
172741 aatttgaagt gcatgatgaa ggatcatttg gttggactcc tatgcatttt tgggctagac
172801 gtaataatta tgaattatta gaactagcca ttaaaggagg agctaatgta aatatgcaaa
172861 ccctacttga tccagagagt gaatacaatg aaaccctttt atttgaagct gtaaaagaag
172921 cagaaaccta tagggttaca cagcttttaa tcgaacttgg agccaatgta aattttgcta
172981 ccccaagaac ccctttagat gatgcaaaag gatctagaaa taaaaaactt ttaaaagatg
173041 caggagcaat gacttcaaat gaaattagaa agaaatttaa tttaccagca tatgattctt
173101 ctcattgtga aattgatgga aaaactgatt ttgacttatt gggtaaatat cgtgatgaat
173161 gttccaaact tttaaacgac gctataaaaa aggccaagga gaatggagaa attaagaata
173221 tagatgctaa aataataaag aatttataac aataaattta aataaaattt acttttaagg
173281 aaaaacatgg gaaataaaga ctttggctat tttttaaatg ctttagaaaa caatgatata
173341 aaagcacttg aaattttact ctttgatgaa gagttatcaa atatgcaaga tgagaatggc
173401 aatacactac tccaccatgc agcaagacta ggtagcgcag gaggtgtggc aatgctaatt
173461 gattgcaatc cttttatccg taataatgat ggtaaaacag cttttaatat agcttacgat
173521 aatgatgatg tctatatagc caaaatgatt gataatgtta aagataggtg gcaaagaaag
173581 catggtaggt atgaagaaaa attagatgaa agcgaaatgg atattccgtt ttatttaact
173641 gatgataaag aaagtatttt aaaacaattc aaggaagaaa atatcacaac tgaaagttta
173701 ttagatactt cctatggaga tggaatggtt gtattgcaat ctgttttaaa tgcatggaat
173761 ttaagtttta ttaatgaaaa aatgcctcct gttgttgaaa ttttaataga tcaagccaat
173821 ggggatgttg aagctttaat agatggattt gatgaacata tggagcaatg ggaaaatgag
173881 gtttgggatg aacctgctgt atatgctgga aaaactgaag atggtgaaga tgattatcgc
173941 tatgaaaccg atcctgatga atggataaga gaaattatac cttttgataa atgggatgaa
174001 ttaaaaaaac aaatgcgttt atatgcagtt aaaaaatgat tttgatagac taataaccca
174061 taataagctc gggttattag gtaaaactta gagataaaaa tgcgtaaaat aacgcaagtt
174121 agatccttta aattaaaact gcaatttagt ttttgggatt ttgcacattt taaccatatt
174181 caatacaaac aaatatattt tttgcaagat atttttaaat ggaccggtaa agatagaaat
174241 gatttaagct ttagtgtata aatttagtaa atatcttatt catttttgtc gtggcgatga
174301 aatgttaaaa tactctcaat ttatatttta taatttaaat aaagatttaa aacaaataca
174361 catgaatttt actctaaata ataaaaataa ttataaattg attacagaaa aattagcaat
174421 cacttgggct aaacttaatt ttttacatcc atttaaagaa agacaatgaa aaacacaaag
174481 gatcaagaag tggttttatc taccacagac atgctcacag aagtataaaa acaatattta
174541 aagaaatgta tttacatcat atcaaaggag tagcacctaa taaaatcatg atagaaagat
174601 taaatttaaa taatcctgct aatatagaag aagaattaaa caaaaacaaa caaatacaaa
174661 aaaactaaag gagcaacaat gaaaacatta gaagatatca aaaaattaaa ttttaaaata
174721 aaaatggaaa ttcaaaaaca attatttgat tttattagta ataatgactt agaaaatgtt

174781 aaaaatcttt taaaagatta tcctgtaaaa gaaagctttt atgaggcaca ttttacatat
174841 catcataata atgaagatta tgagttatct ttatttgatc cagcagcaag cttactaaaa
174901 gctgcattta cttgtggaaa ttataataat gatttttcta ttttagatta tttatttgat
174961 gagtatggtt taagtttaaa agattctaaa tataattttg cttttcctga catgaaacat
175021 atcaaagaag ctaatgataa atatatttta atgaaaaaag tagaagatga tccttgtatt
175081 tatcaaaaag ctttaattta tgcttatata ttaggcgcta aaaatccaaa ctctcaaatc
175141 attaagtatc tagtcaatcg tggggctaaa tttgaagtgc atgatgaagg atattccggt
175201 aggactccta tgcatttttg ggctagacgc aataattatg aattattaga actagccatt
175261 aaaggaggag ctaatgtaga tatgcaaacc cgacttattc aagagagtga atataatgaa
175321 acccttttat ttgaagctgt aaaagaagca gaaacctata gggttacaca acttttaatt
175381 gaacttggag ccaatgtaaa ttttgctacc ccaagcaccc ctttagatat tgcaaaagga
175441 tctagaaata aaaagctttt aaaagatgca ggagcaatga cttcagaaca aatcagaaag
175501 aagtttaata tatattggga tgataaagag tgtaaaaaag ataaaaacta tatagaaaaa
175561 cactccaaac ttttaaacga tgctataaaa aaggctaaag aaagtgaatg agggaataga
175621 aattaacttt gctaaaataa actataagga aagataatga aaaataaaaa tttattggat
175681 aaagatacac aagaagtatt taagtactat cttaaaaatg atagttattt aaaaggtgct
175741 ttagatggtt taaaaaaata cgagcgaaga gcttttataa gtagcatctt aagctatgaa
175801 tataaaaatc tagtggaaaa tggcaattta aaagtcgatg aaaaatataa agctatttta
175861 ccacctaact taatcgccat tttagaatat gataaaagtg aatttaatgg tgcaaatctt
175921 cgaaatttta taaatgattt ggaatttgaa ttagaacata tagaaatgga actctatgat
175981 gatcctgctg actttgaagg gggagattat tataacgaag atagcgaatg tgatgaggat
176041 tatacagtag aggattgggt agaagatcaa gtaagcgata ttagagattt tttatatgct
176101 ttaaaagatt ttaaaagtaa ataccgataa aacactcaag taaatttaat tttctaaaat
176161 ttagcgataa aatatcttaa cttaatgaca attttcaaaa atttctacta cacttaaggg
176221 taaaatttca tgctctaggg cgtgaatttt tgcttcaaat tcttcaaagg ttaaattttg
176281 tttttcaaaa gccttttgag cgatgatttt ccctccatct aattcttcat ttacccaatg
176341 cacactcact ccagctactt tcatatcgct ttcgtagctt tctttgatag catgagctcc
176401 tttaaaaagg ggtagtaaag atggatgaag attaattgct ttgatatttt ttgtaaaaac
176461 agggcttaaa attcgcataa atccagccaa gatagtaaga tctgctccgc tttctttgat
176521 ttttttttacc aaaatttcgt caaattcttc tctgttttta taatctttgt gttcgatgat
176581 cacgctttca aggtcaaagt ttttagctct ttgtatccca taagcatctt ttttattgca
176641 aagacaaagc acgacttcat aggtgttttt acctatagtt tttttatgaa gtttttctaa
176701 gatattttgt aaattacttc catttccact aaaaagtacc gctaatttta caagcatttt
176761 aatcctttta taagttttat cgcatcaaag ctgtttttgt taaatttata tttttttagcc
176821 acaagagcgt gtgctaaaca agcattttta gccgcttcca aagcgctgaa ttttacccct
176881 aaaagcgcag ctatcatacc gcttaaaaca tcgccacttc cgccttttgc taaagcttca
176941 tttccaagat ttacaacaaa gatttttttct ttttgtataa tgataggatt tgcacctttt
177001 aaaacaagaa cacaatcata attttgtgca aattttttag cataaaaaaa gcgatttctt
177061 tgcaaatctt ctatacttaa atcctcatca aaacacattt tataaagtct tataaattct
177121 tttggatgcg gggtgatgac tacatctttt ctgcttagat accaaagcac agcttcactt
177181 aaaaagcaat ttgcatccaa aaccaaagga atattttgca aaatctcatc ttttaaaata
177241 tctaaatttt caagtcccat acctaaggca atggcacttg cattgttttc aatcttttct
177301 tttaacatta aaagaggtga aaaacttttt tttgcgacta aggatacaag cccactgcca
177361 aaatttaaag ctccaagtcc tgctagggtt cctgcacttg cactagctat tatatagata
177421 tgaccgaaat ttcctttatt ggagcttgta tttctttcta tggtttttaa atctttttct
177481 tcaagtaaaa aagctttgga atcaagacta aattttttag cattgacacc aaggtttgcg
177541 agtttgattt tgcctacaaa ttctttagca aaatcctcaa gtaaaatttc ttttaaagct
177601 cccatacact gcgtaatatc ggctttaaag caaggagtaa aacccaaatt tgtaggtatg
177661 tcacaagcga tttttaaagc tttgctttga tttatttttt ctaaaatttc ttttgttttt
177721 tcatctgctt cgcggtttgt tcctgtgcct aagatacaat caacaacaat atgaaatttt
177781 ttaaatttag gctctttttt tgcaaattta aaaccaaaat tttttaaaat ttgctcttgt
177841 tttttaaaaa tttcattttc tttaaagcct agtttataag cttttgcatg ctttaaattt
177901 cttaaagcta ctaggccatc agcagcgtta ttgcctccgc ccaataaaaa aagtattttt
177961 actttcttta tcttgtattg tttgcgaatt ttttttgctt ctttttttgat aagcttagct

178021 aaatttaatc ctgcgttttc cattaaaata agctcatcta agcctttatt tatagcattt
178081 tgttctaaag ttttaatatc atcagtaata gttttcataa tctttttca ccagaaaata
178141 aatatttttt tctcgctctt cttcaaattt tttcatttta ggtaaaaatt tctgaattaa
178201 agcacgaaaa tttttaaagc cataattttg cggattaaaa tcagaatatt tgcggttcat
178261 attggtgcga atttgtgcgt attcggcgcg tcctttttca tcgatgagtt gttctgtgat
178321 attgatcaaa gctcttaaat aatccttgct taaaatatct tctttttgt tttcatcttg
178381 atcaaggtaa aaaaactcac taaaagcgtt tacataagat tttatagact tttcaagacc
178441 cattcctata acttgctttt tgttttctct tagcttttga atgagcgagg tataatcact
178501 atcgctagaa acaatgacaa aaatatcaat atctttctca taaaatatac tcataatttc
178561 agtgattaaa tacatatcac ttgaattttt atttgctgcg aaattaaact gctgcatagc
178621 gattaaagaa tactcggcaa tttgctcttt ccaagcttga atattttttt gagtccaatc
178681 tccataaata cgcttgataa ttatttcacc ataatctgaa gcgatatcaa aaatactttt
178741 tgcgtatttt gcgggtatat tttccgcatc aataaaaata gctatgcttt tattttccaa
178801 tttagctcct tatcctaaaa ctcaaagctt ctaaaatatg atttttatta agaagttctt
178861 tttcttcaaa atcggcaata gatcttgaaa cttttaaaat tttatttaaa gctctttgcg
178921 aaagattata gcgcgaaata gctaaatcca aagtatcctt ggcatctttt tctaaaatac
178981 aaaatttttg taattctaca tcgcttaatt tgccattgaa ttctttttgg ccgcgttttt
179041 tgccaaagat aaaagctttt aaaattcttt cgctcatttc tttcgaattt gtgcttgatt
179101 tatcatcttt actgatctca tccatagcta cataaagatc aattctatcc ataatagggg
179161 ctgaaatgtg atttttatat cttttaatct cattttcact gcaaacgcaa gacagatttt
179221 tagaaaataa atttccacaa ggacaaggat tttgagcggc aacaaaactg aatttagttt
179281 cataagtggt ttttgaattg actcttgaaa tatgaatttt atgatcttct agaggttctc
179341 ttagactttc gataatttgt ttgttaaaat gaggaaattc atcaaaaaat aaaactccac
179401 cattagctag agcgatttca cctatcttcg catttttgc tccaccacca aaaatactag
179461 ctcttgtaga ggtatgatga gggtgtctta aggctctttt ttgcgtaaat tcgcaatctt
179521 tagaatttaa agacatataa gcattttgca ttaaaacctc atttaaactt tgtggaggca
179581 tgatttcaac aaggcgtttt gcacacatac ttttgccact tcctggactt ccttcaaaaa
179641 gtatattatg catgcctagt gctgctatca aacaagctct tttagctttt tcttgtcctt
179701 tgatatcttt aaaatcaagt tcaaaatgat gatttttaat aaaaatttca ttttctataa
179761 tcaaaggatt tgcaaataaa ggatggcttt gggtgaattt gaatttctca tagttttttt
179821 ctttaaaaaa ttcaatcact tcatttaaat gctcaagtcc gtaaatttct agattgggta
179881 tcatagaagc ttttagagct atattttttg ggactacaac ttttgcactt tttacctttg
179941 tggataaaaa taaaagcaaa gaaaaaagct cattggtgct tttgatactt ccatcaagac
180001 caagctcgcc taccacaaaa aaatcatcaa agtcttcatt ttgcaacaat ataagcgtag
180061 cgataactaa atcaaaatga gaaccttttt ttggaattcc tgaagggcta agattgatgg
180121 tgattttttt agcaggaaat gaaaaattgc aagttaaaag tgtggctttg atacgctcga
180181 tactttcttt gattgcaata tttgcaagtc cgactatgct taaattaggc aagcctcttg
180241 taaatgtaga ttctacatcg atgatatcta aattctcatc aaagcttaca cattttaatt
180301 ttttcattta gatttttttt gatttttaag tttctttttg aattctttat caaatttttg
180361 tctttttaaa aaagaaatct tttctataaa taaatgacca ttaagatgat cattttcatg
180421 ctggatggcc acagctaaaa atccttgagc ttctaattct ttaaaatttc caaagcgatc
180481 ttgatatttg agtaagatat gattataacg ctccacttct tcaaagaagt caggcacact
180541 taagcaaccc tcggtgcagg tgattttttc ttcatctaaa ggaatgattt gcggatttat
180601 aatttctagt aaatcttctt ttttctgctc ttcattttca tctaaaatat ttacgatcaa
180661 tgcacgaagt gggatatcta cttgtatagc agccaaaccc actccttgac tagcaatcat
180721 agtatcatac atatcatcaa gtaaagtatg aagttcttga tcaaattgct ttacaggttc
180781 agaatttaaa aaaagtctag aattaggata agtgataatt tttctaacca taaacaacct
180841 ttatttaaat gattttaatc atttaaaact tttttctaaa accttatcga tcaaaccata
180901 ctctttagct tcttgtgctg acatgaaaaa atctctttct gtgtctttta ctattttagt
180961 gactttttgc ttggtatttt ttgccaagat atcattaagt atagctttta atcttaaaat
181021 ttctttagct tgaatttcta tatccgtagc ctgtcctctt gcgccaccta aaggctgatg
181081 tatcatgatg cgtgaattag gcaaagcaaa acgcttgccc tctgttccgc aactcaataa
181141 aaatgcgccc atagatgcag cttgacctat gcagatagta caaacatcag gcttgatata
181201 attcatagta tcatagatac taaaaccgct tgtgatcacg ccacctggag aattgatata

181261 aagatatata tctttttgtg gatcttcagc ttctaaaaaa agaagctgtg ctacgataga
181321 agcggcaagt tcatcgtgaa tttcgccact tagcataata attctatctt ttaaaagacg
181381 cgaataaata tcataacttc tttcaccgcg acttgttttt tcgattacat aaggtatgta
181441 cattatttat cttctttctc ttttttactt gctttatcag cttttggcat aaaaatatcg
181501 ctgaaaagtt tttcttcgat caaagccatt ttaactgcag gtaaagcccc ttgtttttttg
181561 taattttcta aatgttcttt tggattcatt ccatagcgat aagcttcaaa ataaattgct
181621 tggatcaatt cttgatcatt tacttcaatt ttgcgaagtt ttgcaagctc atcaatgata
181681 aaagtaagtt ttacactttt ttgtgcttct tctttgaaag aatcgcgttt ttcttgatat
181741 ttttctttgc ttgttttaag ttcttcaatt tctttttcgc tgaatgtgtt aaatgcgttg
181801 cggaattgca tatcagtttc ttgctctact ataccgcgtg gtaagtcaaa attgtatttt
181861 tcgattaaag catcagcaaa tttagctttt agttcatcat ttacaagctt gaaaagcttt
181921 tcgtttttga tttgttcttt aagtttttca tctagaagtt caactgttgg tttttcttca
181981 tttggtaaaa gtttttttgag tgtttcttca tcaagttcag gtaattttaa ctcttgaatt
182041 tcgtgcaatt ttactttaaa tactgcatct tttcctgcta aatgtgctgc gccgtattct
182101 ttaggaaagg ttacttttat atctttttct tccccacttt tcattcctac catacccttct
182161 tcaaagcctg gtatgaattg tttagagcct atttctaaaa cataattttc agctttaccc
182221 ccatcaaaag ctttctcatc tacaaaaccc tcaaaatcaa attttgcaaa atcaccctct
182281 tttaaagctc ttttagtttt aatagcttca ggagtagcaa agcgttttaa taattcttct
182341 tttttttcat caatttcttt tttagttact tttggagttt gatactcagg aatcaattct
182401 tcatagccat cgagtttaaa ttcaggttta aaagaaagag ctaattcagc aacaatttct
182461 ccatcttgac gatcaaattt ttcaaaatac ggttcaccta caagttcttt tacttctttt
182521 tttaattctt ttaaagcact atcaacggca gatttaaaaa gattttgttc tgcatcttga
182581 gtcaattctt tttgatatct tttaagcacg gcagcaacag gaacttttcc tggtctaaag
182641 ccatccattt ttacattttt agaagctttt ttagctaaat tttctacttc gcttttaatc
182701 atccctgaag gaatttttac actagcagtc gcattaacag agtctaattg ctttgctttt
182761 acttccataa cattttttcct tataaaataa aatttgcttc aatcatagca aatttttcct
182821 tattagttgc ataaaatttt aaagcatttt atgttaaaat gagctaagaa aaaaatacgg
182881 agaataattt gcaagaaaaa tttgaaaatt gcgtaaaaac tatgcttgaa atcatagggg
182941 aaaatccaac tagagaagga cttgtaaaga cgcctaatcg tgtttttaaa gcttatgagt
183001 ttttaacaag tggatataaa aaaaatgtca aagaaatatt aaataatgct ttatttgaaa
183061 gctcaaataa cgaaatggtt ctagtgcgcg atatagagtt ttatagtctt tgtgagcatc
183121 accttttacc ttttttttggt cgcgtacatg tggcttatat ccctgatcaa aaagtggttg
183181 ggcttagcaa aataccgcgt cttgtagaag tatatgcgag aagacttcaa attcaagaac
183241 aacttaccga acaaattgct gaagctttga tggaaaatgt tggcgcaaaa ggcgttggtg
183301 tggttataga agcaagacat atgtgtgttg aaatgcgtgg ggtgcaaaaa gcaaattcta
183361 ctactaccac ttcggctctt cgcggacttt ttttaaaaaa tgaaaaaaca cgcgaagaat
183421 ttttttcact cattaattct cctaagcaag ttcgtttttg atgaatttag aaaaacttcg
183481 ttctaaactt ggcaaagaaa atttaagtgc tgtttttgga gagatcacta aaatttctgc
183541 tacaagtata gaaattcgag gacttaaaac aggtgttggc gatatcatta aacttgtttc
183601 aaatgaaaat gaaaatttaa acaccttagc tatggttgtt gaaatcaaag agcaatttag
183661 ctatctaagc cctttttctt tcatagaagg ttttaaaata ggcgaccgtg cttttataag
183721 tgatgcagga atgcaaatag gcgttagtga tgagctttta ggaagggtag tggatccttt
183781 tatgcgccct aaagatggca agggtgctat agaagcaact aaatatatgc ctatcatgcg
183841 tgcacctatt gatgcgatga aaagaggact tatagaagaa gtttttcctg tgggagttaa
183901 aaccatagat gcgcttttaa cttgtggtgt ggggcaaaaa ttaggcattt ttgcaggaag
183961 tggtgtagga aaatctaccc ttatgggtat gatagttaaa aattcaaaag cccctatcaa
184021 ggtagtcgca cttataggtg aaagggggcg cgaaattcct gaatttatac aaaaaaattt
184081 aggtggaaag cttgatgata cagtaattat cgttgcaaca agtgatgata gtgctttaat
184141 gcgaaaatat ggagcatttt gtgctatgag cgtggctgaa tactttaaag aacaaggcaa
184201 agatgtactt tttatcatgg atagcgtaac gcggtttgcc atggcacaaa gagagatagg
184261 acttgctctt ggtgagcctc ctactacaaa aggttatcct ccaagtgttt taagcctctt
184321 gcctcaactt atggaaagaa caggaaaaga agagggtaaa ggaactataa cggcattttt
184381 tactgttttg gttgatggag atgatatgag tgatcctatt gccgatcaaa gccgttctat
184441 cctagatggg catattgttt taagtcgtga gcttacggat tttggaattt atcctcctat

```
   184501 taatatacaa aattcagcct caagggtaat gggcgatatc ataagtcctg aacacaaact
   184561 ttgggctaga aaatttaaac gcttaaattc gcttttaaaa gaaaatgaag tattgcttcg
   184621 tatcggtgct tatcaaaagg gtagtgataa ggagcttgat gaagccatag ctaaaaagga
   184681 atttatgcaa aaatttttag gacaaaatcc tgaggaaagc tttgaatttg aagaaactat
   184741 aagactttta agtcaaattg atgctaatgt tgcccctagt gcagtgcagc aaaatattaa
   184801 tatgggaagt tctagtgcaa cgctacctaa tcctaatctt aaataatctt attttgtatt
   184861 aaaataattt cataaatata gaaatgtaaa tttcatcaac aaattaaatc tttagattga
   184921 tactcgcata tcaatctaaa ttttatatat cagtctctaa ttaaaacctt ttctttttca
   184981 catcttcaag tatattatta gctatatcac ttacagtatt agaaataata gc
//
LOCUS       DMACINML_4            166796 bp    DNA     linear       16-DEC-2020
DEFINITION  Campylobacter species strain strain.
ACCESSION
VERSION
KEYWORDS    .
SOURCE      Campylobacter species
  ORGANISM  Campylobacter species
            Unclassified.
COMMENT     Annotated using prokka 1.13 from
            https://github.com/tseemann/prokka.
FEATURES             Location/Qualifiers
     source          1..166796
                     /organism="Campylobacter species"
                     /mol_type="genomic DNA"
                     /strain="strain"
     gene            complement(641..3007)
                     /gene="pflA"
                     /locus_tag="DMACINML_00735"
     CDS             complement(641..3007)
                     /gene="pflA"
                     /locus_tag="DMACINML_00735"
                     /inference="ab initio prediction:Prodigal:2.6"
                     /inference="similar to AA sequence:RefSeq:CAL35662.1"
                     /codon_start=1
                     /transl_table=11
                     /product="paralysed flagellum protein"
                     /protein_id="Prokka:DMACINML_00735"
                     /translation="MKIIFLVLLSICTLFSFELALNTGRENNQAFAVLHASNDLDFTC
                     QKIIIEDKMHFECEIMGIVDNKLSDQSFTAFDLKFIKEPQKIKMIIIPKISARMFDLS
                     QNIYADKELNSSSMHKSKSFTFIFTPELEHVKDYDGLDFNINFPHESLPYVGALDLNS
                     DPVIIPQSADINTYLRIKNEYDKANYTQVITDAQNAINRYRGSIFMNEFILYKLRAQS
                     QIYTQDPSMRDQQVLEKMIDEAKNWNRTFTSDKNFPEVLHIMLRTYIALSQRADIEYI
                     MSILNNEQPNSYFTQLARLDYADYIYPLNEKERAIDIYEDIYFNTKNLDLAARAAMSL
                     IKDYLANNQIDKAVQYVNTILKANPEYFPKDMFRSLELAKLFNQHKQYDISASIYEDV
                     FLKMPKIDSRYEQVLKDLALTLAMTSRSSDAKKYLDLYMDNYLDGKYLDEIRKANDEV
                     FFALADNNATFLHQRYANLMKEYAQKDENIANKALDEDVALYYKEGNLSAVLAYKDQI
                     ENKKLTNSTKLLEQAATQLLNNDLKADNCINAVNIFTQFNSYEIGQKIENKKQMLACL
                     IRTSNMQQAMDYIDKNHNEDSIFYGLQKASILYDNKQYPLALNIAREIANSRILKSDE
                     ENFKAYYLQFLSYLKMDDYNMAIKILQILESFPMNFTMVEAYDNLLIYANDHNMQTTI
                     LNYAPKAIDYQNLKGINLFSPNLEFIYLDTLAKTNQNEESLAVLIDLLKLKLSDEDRA
                     RALYIQSMTYERMQNTQAQKESLKQCLELKTTSNWQNLCRDKNQILAQ"
     sig_peptide     complement(2945..3007)
                     /gene="pflA"
                     /locus_tag="DMACINML_00735"
```

                     /inference="ab initio prediction:SignalP:4.1"
                     /note="predicted cleavage at residue 21"
     gene            complement(3020..4393)
                     /gene="nuoN"
                     /locus_tag="DMACINML_00736"
     CDS             complement(3020..4393)
                     /gene="nuoN"
                     /locus_tag="DMACINML_00736"
                     /EC_number="1.6.5.3"
                     /inference="ab initio prediction:Prodigal:2.6"
                     /inference="similar to AA sequence:RefSeq:CAL35663.1"
                     /codon_start=1
                     /transl_table=11
                     /product="NADH dehydrogenase I chain N"
                     /protein_id="Prokka:DMACINML_00736"
                     /translation="MLGNIDLSSLNIPLSYPFAFLIAVAIVLLLCSGFWKFHRTFYMA
                     TSSLSLIVSVFLILNNIAAQGLEAKGFLGTLNNDIVSLYASLVILCFSFLYLLMQKEE
                     NQGEFYSLFLFMVAGLLLMVSSSNLILIFIGLESSSLALYTLIAMRGSNNAISSAIKY
                     FSVAAVGSGFFVMASALIYIRTGTLDLGLKLALDKDPMLLGAGVMIFVLCAIKLSLAP
                     FHFWLKDVYSSAHVNLVAFISVVPKIAMFVVVIRLFSFLNHLGFENILNVLAVFSMLI
                     AALAALSQKDVKKMFAYSSIVHSSFVLAACIPLLNAADDVLNSIFVYWVLFGFANYGV
                     FLILSTFKGSSFEEFNALLLKKPLIALSLSFCVLSLAGIPPFGAFWGKVMVLKTLIVA
                     DYWYLALFMALASLLMLYAYLKLIIHALFIKTQAKVSEHLNFIQSFILALCTLISVFA
                     LFLLLKI"
     gene            complement(4383..5873)
                     /gene="nuoM"
                     /locus_tag="DMACINML_00737"
     CDS             complement(4383..5873)
                     /gene="nuoM"
                     /locus_tag="DMACINML_00737"
                     /EC_number="1.6.5.3"
                     /inference="ab initio prediction:Prodigal:2.6"
                     /inference="similar to AA sequence:RefSeq:CAL35664.1"
                     /codon_start=1
                     /transl_table=11
                     /product="NADH dehydrogenase I chain M"
                     /protein_id="Prokka:DMACINML_00737"
                     /translation="MLNYLIFFPLIAAFVTLVLNRGGIKVFSVIASLMVLGLNIKIWQ
                     DYLNGINLEYQLPFKVVNFMSYHIGADSIALILMLLSSLMIFLSFLFLKIEQKAMVSC
                     IFFLEFAIMGLFSSLDGLLFYVFWEFSLLPLLYIMGVYGKDYRAGIKFFVYAFAGSIL
                     MLLALIYQAYATYKLLNIFTFDIEIWKNNASAMSFNEQILLFGAFFVAFAIKAPLFPF
                     HTWAPKVYANSPILVSMMLVAFKMAPFGFLRFCLPLFPDASVYFMPLVVALCIVSIIY
                     NALIAYRASNLKELIAYSSISHLGVMILGIFSFNALGLSGAVFYMFAHGIVTGALFLM
                     AELLYKKYGTLEISYYHSLASKAPLFTIFFTLILLASISLPLTLSFIGEFLILLGVAK
                     LNLLYALLAGLVIILGAIYMLYVFRKMFFMQKESQLETCSLHAREILSLIPIVVLIFY
                     LGIAPKIFLDPLNKDADFIVEKMKERAIDDQTIRFLNHLEGENNVR"
     gene            complement(5875..7665)
                     /gene="nuoL"
                     /locus_tag="DMACINML_00738"
     CDS             complement(5875..7665)
                     /gene="nuoL"
                     /locus_tag="DMACINML_00738"
                     /EC_number="1.6.5.3"
                     /inference="ab initio prediction:Prodigal:2.6"

```
                     /inference="similar to AA sequence:RefSeq:CAL35665.1"
                     /codon_start=1
                     /transl_table=11
                     /product="NADH dehydrogenase I chain L"
                     /protein_id="Prokka:DMACINML_00738"
                     /translation="MQNLALISLFSPFVAFLFASCFALSERKIFVAYICSLLIGISAF
                     CSLYLLFNNQAFNVSLFEWFIGLSFGFNIDAISLTMMSVVGVVATCVHFYSIFYMAHD
                     KDFNKFFAYLGLFVFSMLFLVMSDNFLGLFVGWEGVGLCSWLLIGFWYKNNTYSFAAN
                     EAFIMNRIADLGMLLGIFWLYIQAGTLKYNEVFAIAQNLDHNALVLIASCLFIGAMGK
                     SAQFPLHTWLADAMAGPTPVSALIHAATMVTAGVYLVIRAGEIYSLVPEVSYFIALLG
                     AFVAIFAASMALVARDLKRIIAYSTLSQLGYMFVAAGLGAYGIALFHLVTHAFFKSLL
                     FLGAGNVMHAMDDQLDIKKMGGLFKSLKITAIFMTIGSLALAGIYPLAGFFSKDLILG
                     YSFIAHYHGIFLILLISAFLTAFYSFRLLMLVFFTPSRHDKHPHEASKIALLAMSPLV
                     VLAIIAGLFEHSFFEYLSTQLIFIDAQNKLVMICASVAAVLGVVLAIIAYKASWFKDS
                     IEQNKIHKLLSNDYFIPKFYHEFIVSKYESLCAVLKHCDTYIFDAIVEKIAYYCNLIS
                     QKIIMTNSLNLMLRFLVAGFVILLILVWVV"
     gene            complement(7668..7961)
                     /gene="nuoK"
                     /locus_tag="DMACINML_00739"
     CDS             complement(7668..7961)
                     /gene="nuoK"
                     /locus_tag="DMACINML_00739"
                     /EC_number="1.6.5.3"
                     /inference="ab initio prediction:Prodigal:2.6"
                     /inference="similar to AA sequence:RefSeq:CAL35666.1"
                     /codon_start=1
                     /transl_table=11
                     /product="NADH dehydrogenase I chain K"
                     /protein_id="Prokka:DMACINML_00739"
                     /translation="MVEKYFFIAIAMFIIGLIGILKRQNLIMLFISSEILLNAANLAL
                     VAASKMHQDLNGQVFALFVMGVAACEVAVGVAFCVLWYRRKGTLELKSLKEEV"
     gene            complement(7961..8476)
                     /gene="nuoJ"
                     /locus_tag="DMACINML_00740"
     CDS             complement(7961..8476)
                     /gene="nuoJ"
                     /locus_tag="DMACINML_00740"
                     /EC_number="1.6.5.3"
                     /inference="ab initio prediction:Prodigal:2.6"
                     /inference="similar to AA sequence:RefSeq:CAL35667.1"
                     /codon_start=1
                     /transl_table=11
                     /product="NADH dehydrogenase I chain J"
                     /protein_id="Prokka:DMACINML_00740"
                     /translation="MIEILAFTFFSVVVLGFFLVAVLSKTMLYSLSALAGGMVFLSGF
                     YFLLDAEFLGVIQIIVYSGAVLGLYSFAMMFFDNSKEFKEKLRAKKSFFLLVILSAVL
                     LLFMLLGFKHQNIASDLALSDPNLFDYNKQLAFAIFSKYLLAFEFIAILLLIALICAI
                     VLTHKELTKER"
     gene            complement(8473..9138)
                     /gene="nuoI"
                     /locus_tag="DMACINML_00741"
     CDS             complement(8473..9138)
                     /gene="nuoI"
                     /locus_tag="DMACINML_00741"
```

```
                     /EC_number="1.6.5.3"
                     /inference="ab initio prediction:Prodigal:2.6"
                     /inference="similar to AA sequence:RefSeq:CAL35668.1"
                     /codon_start=1
                     /transl_table=11
                     /product="NADH dehydrogenase I chain I"
                     /protein_id="Prokka:DMACINML_00741"
                     /translation="MKNYYLIDEKRKTPTSTWQKISQALKRSVKLELFVGLWVVMREM
                     LKRNNSATIKYPFEKVKLDNRYRAVHRLMRFIESENERCIGCGLCEKICISNCIRMET
                     SLDENARKKVGNYSINLGRCIYCGFCAEVCPELAIVHGTEYENAAEQRSYFGYKQDFL
                     TPIDKLKNQVEFEGAGSLRKDADLLVKKTPNYYEVLQEREKNLCTDEECALPNLAPQG
                     EVK"
     gene            complement(9148..10146)
                     /gene="nuoH"
                     /locus_tag="DMACINML_00742"
     CDS             complement(9148..10146)
                     /gene="nuoH"
                     /locus_tag="DMACINML_00742"
                     /EC_number="1.6.5.3"
                     /inference="ab initio prediction:Prodigal:2.6"
                     /inference="similar to AA sequence:RefSeq:CAL35669.1"
                     /codon_start=1
                     /transl_table=11
                     /product="NADH dehydrogenase I chain H"
                     /protein_id="Prokka:DMACINML_00742"
                     /translation="MSDFAFFALEALIKCIIVIAIFATLAGLATYAERKVLAYFQRRI
                     GPDMVGPFGLIQLVADMIKLFTKEDIIPSNSQKLIFAIAPLIAAICAFVSLAAIPMLP
                     EFTLFGRTIQPLIADINVALLFVIGTSGLCFYAIFLGGLASNNKWSIIGAARGLVAII
                     SYESVGALALIAIVMLVGSFSLIDINNYQSDGFFSWLIFKQPLAFALFVIALFIETNR
                     TPLCLTENEADIVAGYGTEYSGLRWGMFFIGEYASMIAGAILITLLFLGGFNDFYFIP
                     GWIMMIVKSSFIFFWYFWARAAFPQLRPDQVMRMCYLILIPLAVLNLLITAFAVLI"
     gene            complement(10139..12601)
                     /gene="nuoG"
                     /locus_tag="DMACINML_00743"
     CDS             complement(10139..12601)
                     /gene="nuoG"
                     /locus_tag="DMACINML_00743"
                     /EC_number="1.6.5.3"
                     /inference="ab initio prediction:Prodigal:2.6"
                     /inference="similar to AA sequence:RefSeq:CAL35670.1"
                     /codon_start=1
                     /transl_table=11
                     /product="putative NADH dehydrogenase I chain G"
                     /protein_id="Prokka:DMACINML_00743"
                     /translation="MTIKINGIDCAFEEGEYILNIARRNDIFIPAICYLSGCSPTLAC
                     RMCMVEADGKKVYSCNTKAKEGMVVESDLQNLWDERNEIMQAYCINHPLECGVCDKSG
                     ECELQNFTHKSRVNIQRHWIKDTHKPHKHWGMINYDPALCIVCERCITVCKDKIGESA
                     LKTVPRGGDSVDNSFKESMGKDAYAIWTKFQKSLIGPANGDTLDCSFCGECTSVCPTG
                     ALIGSKFQYTSNIWELKRIPASNPHSSDCELMYYDIKQSGISNQKPKIYRVSNDFSFA
                     SLNKAARFAFDTQNEASKDEKAFKELIELFEKNEIKNIKFNSFITNEEALILQNFKKK
                     FGLNLINNEALKFKDFLEEFISNSGEFYNATTQDITKSDFLVVAGTLLRYDAPTLSYK
                     INNALVMNKGSGLYFHPIEDAGIAKYSKNFISHTHKSGDEEQILYFLLQKFSQDENIK
                     ANLAEFFINENKEIEESINEEIIEQVVEKDEEGNEISKEVKKVVPKKVKKTIEVKRSV
                     FAKNLGIDEDKLEDLLLKKANFTLIIGSDFYFHKNAKKLAKLLALIQNTTPFKVFLNP
```

```
                     THTNTLGVAMICDLDESMQEGKTLGYNERGDFSFSYEEHANLASSSLNQQEGTFLNYD
                     KKVVPTNAALEFDGYFLNDLANALGFDEEYTINYTKRLPINKGFSPLEFDSLDNFYTN
                     GGECKRGYDLNLECCKKSAKNEFVAPHLQNLTLQEDEILLYSANPSYQFGRFSNRASA
                     INEVIFLGVSQNLAKEKNLQDKDLVKLKIKDKELSLSVRIDKDIKNGAFLPYFDEKID
                     TLSFFDERFIVANLEKLGASHE"
gene                 complement(12598..13290)
                     /locus_tag="DMACINML_00744"
CDS                  complement(12598..13290)
                     /locus_tag="DMACINML_00744"
                     /inference="ab initio prediction:Prodigal:2.6"
                     /inference="similar to AA sequence:RefSeq:CAL35671.1"
                     /note="hypothetical protein Cj1574c"
                     /codon_start=1
                     /transl_table=11
                     /product="hypothetical protein"
                     /protein_id="Prokka:DMACINML_00744"
                     /translation="MSYERALCFVDMLSLKNKILCEKIQGENIYISCLEDKNLNAKFY
                     KCEIASLSYVLALLCKMSEMDNFQDLDEGYLSAESCFGEEEASEILDFLENAKYLIVD
                     ENINFYKDSENIKYFLNFLSQKYKLKILDSSEKECDFKNTKLSTLQELDSYDGLVLFR
                     ANLKDDNLHCSKQFLQIAKCKDKSEVEVLAKDFSFKTTLCLDENLQGTIAFLNYDHQG
                     FDFTPIRIKEAK"
gene                 complement(13287..13514)
                     /locus_tag="DMACINML_00745"
CDS                  complement(13287..13514)
                     /locus_tag="DMACINML_00745"
                     /inference="ab initio prediction:Prodigal:2.6"
                     /inference="similar to AA sequence:RefSeq:CAL35672.1"
                     /note="hypothetical protein Cj1575c"
                     /codon_start=1
                     /transl_table=11
                     /product="hypothetical protein"
                     /protein_id="Prokka:DMACINML_00745"
                     /translation="MRRVDLRKSKELFEDLAQIIKEAQKGEVLVVLFEIGDFSPVEKS
                     FSFVKEQGCELLNSLKFNQVDWTIVIKKESV"
gene                 complement(13511..14737)
                     /gene="nuoD"
                     /locus_tag="DMACINML_00746"
CDS                  complement(13511..14737)
                     /gene="nuoD"
                     /locus_tag="DMACINML_00746"
                     /EC_number="1.6.5.3"
                     /inference="ab initio prediction:Prodigal:2.6"
                     /inference="similar to AA sequence:RefSeq:CAL35673.1"
                     /codon_start=1
                     /transl_table=11
                     /product="NADH dehydrogenase I chain D"
                     /protein_id="Prokka:DMACINML_00746"
                     /translation="MQIPSKLKPYYENIAFEQEDSKMIINLGPQHPSAHGNLRLILEL
                     DGEQVVKARPTIGYMHRGMEKMAENMIYQEFIPTTDRMDYIAASANNYAYCAAVEKLC
                     GLEIPRRAAVIRMILLELNRIASHLLWLATHALDIGAMSVFLYCFREREYVLDLIEKY
                     CGARLTHSSMRIGGVMLDLPEGFLNELLAFCDKFPKDIADYEALLDDNRIWRLRTENV
                     GVVSKEQAMNWGCTGVMLRGSGVRYDIRKEEPYLLYNEIDFGVPYATQGDSYARYKVY
                     MQEFRESLKILRQCAALYKDTPPEILANHPEYVSSSKEQILTQNYSLMQHFVLITQGL
                     KPPKGEVYVPTESPKGELGFFIHSDGTGRPYRLKARTPSYWHCAFFEEMLVGTYLADV
```

```
                     VAIMGNVNIVLGEIDR"
     gene            complement(14739..15530)
                     /gene="nuoC"
                     /locus_tag="DMACINML_00747"
     CDS             complement(14739..15530)
                     /gene="nuoC"
                     /locus_tag="DMACINML_00747"
                     /EC_number="1.6.5.3"
                     /inference="ab initio prediction:Prodigal:2.6"
                     /inference="similar to AA sequence:RefSeq:CAL35674.1"
                     /codon_start=1
                     /transl_table=11
                     /product="NADH dehydrogenase I chain C"
                     /protein_id="Prokka:DMACINML_00747"
                     /translation="MRKYSDKKNAQLQNYYKDRFYHAPHTQKLDVNESVFKQDYEVLK
                     TEVDIVNSFIELDFWVIEIKKEDNVKTLQMLQTLGYLNFTEASAIDFVADKNGFEVFY
                     QLLNLEKRLRVRVRTFVGVKERLQSVAHIFKGANWSEREIYDMFGIFIINHPNLKRIL
                     MPDDWYGHPLLKTYPLKGDEFAQWYEIDKIFGKEYREIVGAEQRDPGFADDKDTLNFA
                     RLYHEVPKGGSRKEVSFKQEYQEDGGVAFVKKVKRDEAKILDKRR"
     gene            complement(15530..16033)
                     /gene="nuoB"
                     /locus_tag="DMACINML_00748"
     CDS             complement(15530..16033)
                     /gene="nuoB"
                     /locus_tag="DMACINML_00748"
                     /EC_number="1.6.5.3"
                     /inference="ab initio prediction:Prodigal:2.6"
                     /inference="similar to AA sequence:RefSeq:CAL35675.1"
                     /codon_start=1
                     /transl_table=11
                     /product="NADH dehydrogenase I chain B"
                     /protein_id="Prokka:DMACINML_00748"
                     /translation="MAEHQVNYASGLPVVLTSVDKLVQWGRSNSLWALSYGLACCAIE
                     MMAAGGSRYDFDRFGTIFRASPRHSEVMIIAGTLCKKHAEFTRRLYDQMPDPKWVISM
                     GSCANTGGMFNTYSTVQGVDRIIPVDIYVPGCAPRPESFQFALMILQKKIRKEKASRK
                     MAPKRLV"
     gene            complement(16015..16404)
                     /gene="nuoA"
                     /locus_tag="DMACINML_00749"
     CDS             complement(16015..16404)
                     /gene="nuoA"
                     /locus_tag="DMACINML_00749"
                     /EC_number="1.6.5.3"
                     /inference="ab initio prediction:Prodigal:2.6"
                     /inference="similar to AA sequence:RefSeq:CAL35676.1"
                     /codon_start=1
                     /transl_table=11
                     /product="NADH dehydrogenase I chain A"
                     /protein_id="Prokka:DMACINML_00749"
                     /translation="MSHIDASHPYFGIFLMLVLASVIFFGLVFLASKIGNNFAAKNRK
                     KLGLGIYECGPIPVKQANKINSQFFIVALIFILLDIEVVFLFPWALIFKDLGWFGLLE
                     VFVFILLLGVGFLYAYKKGAFAWQSIK"
     gene            complement(16520..17185)
                     /locus_tag="DMACINML_00750"
```

```
CDS             complement(16520..17185)
                /locus_tag="DMACINML_00750"
                /inference="ab initio prediction:Prodigal:2.6"
                /inference="similar to AA sequence:RefSeq:CAL35677.1"
                /codon_start=1
                /transl_table=11
                /product="putative peptide ABC-transport system
                ATP-binding protein"
                /protein_id="Prokka:DMACINML_00750"
                /translation="MLEVKNLGKFYELKKHWYLKKEKHLIFENVNFSLKENENLMILG
                QSGAGKSTLARILCFLENPSFGEVIYKNLNPHKLDKTKQRLLRKEIQYCFQDQKAALN
                PYKKIKNLIQDGLENFNIKKEQEKILKFFDCFNLKKQILEQKPYELSGGEAARVGLIR
                ALILEPRLLILDEITSSLDIKNSKEILNFLHHYQQENLISYIFITHQDEFFVNFKCKK
                MKL"
gene            complement(17178..17888)
                /locus_tag="DMACINML_00751"
CDS             complement(17178..17888)
                /locus_tag="DMACINML_00751"
                /inference="ab initio prediction:Prodigal:2.6"
                /inference="similar to AA sequence:RefSeq:ADC29128.1"
                /codon_start=1
                /transl_table=11
                /product="putative peptide ABC-transport system
                ATP-binding protein"
                /protein_id="Prokka:DMACINML_00751"
                /translation="MKVRNLSLKLNQKKIFENISFDIKIAKSLMIVGESGVGKSLLGK
                SLIRLLDPKFEINADEWSFNEVSVLNLNQNELRAFRSKVGLVLQDAELSLYPYLDIGN
                LFHLILKTHTKLNQKDYKRYAFDLLEKLGFDDIDLLWHSYANELSLGMARRVSLALAL
                LSKPQILICDEITASLDKENMQKIIQILKELKNTLGLVCITHDLNLVNSLADEVLFLE
                KNQAQLFKADEFLRIYHA"
gene            complement(17885..18640)
                /locus_tag="DMACINML_00752"
CDS             complement(17885..18640)
                /locus_tag="DMACINML_00752"
                /inference="ab initio prediction:Prodigal:2.6"
                /inference="similar to AA sequence:RefSeq:CAL35679.1"
                /codon_start=1
                /transl_table=11
                /product="putative peptide ABC-transport system permease
                protein"
                /protein_id="Prokka:DMACINML_00752"
                /translation="MFLILGALFAPLLAPFDVLESHLNHLHQAPNLTYYLGADFLGRD
                VFSRLLFALRISLFIGICSSFLSIFFALFYLFLARCFFYTFWMRILDLFLALPAFLLM
                MFFQSFVGVNIFLMIFLIALVHWPFIARIIESELKRLENLDFYKASMALGRTKFRAFF
                KDLLPALKSLIFVLFVFNIIHAIATEATLSFFGLGLGFNIPTLGTLLNESSKAIFIGA
                WWMILFPLLALMFLFLPLLWLGNYLQKIGGVRS"
gene            complement(18666..19610)
                /locus_tag="DMACINML_00753"
CDS             complement(18666..19610)
                /locus_tag="DMACINML_00753"
                /inference="ab initio prediction:Prodigal:2.6"
                /inference="similar to AA sequence:RefSeq:CAL35680.1"
                /codon_start=1
                /transl_table=11
```

/product="putative peptide ABC-transport system permease protein"
/protein_id="Prokka:DMACINML_00753"
/translation="MLKRLVFSVFIAFFSTFLCFVLLYFSKGSVAYANKVNSQSKELV
QRIEANLGLDKPLLEQYQIWLFKALKGDLGVSFLSGESVLKLIKERMANTLILSLSAL
VLLFLLSVFLALLGHYYKESFIDKLITFLAFNFFALPPFVLALFFVLIFGIFWQVLPI
TGTSDIGFEDDFFNRLEHLILPVLVLVLSHLALFLRIARNCINETFSQIFVQNLYARA
LREKDIYFLVLRYSLSPIIAYFGGSALSFMMGTYVVESVFAYEGLGSLLFESILFKDF
PMALALIFFSVLLAAFFTFLSDVFAWILNPRLKRLKFV"

gene complement(19610..21148)
/locus_tag="DMACINML_00754"

CDS complement(19610..21148)
/locus_tag="DMACINML_00754"
/inference="ab initio prediction:Prodigal:2.6"
/inference="similar to AA sequence:RefSeq:AHY40756.1"
/codon_start=1
/transl_table=11
/product="peptide ABC transporter, periplasmic peptide-binding protein"
/protein_id="Prokka:DMACINML_00754"
/translation="MLRWFVLLFLLFFNLEAKIPKDTLIVAVENEISRINPAYSEDHD
AVINLVFSGLTRFDEHMSLKPDLATSWNVSKDGLSYDIFLREDVLWHDGVKFSADDVK
FSLEAFKNPKNNSSVYVNFEDIKSIEILNPYHLKITLSKPFPAFLDALSIGMLPKHLL
SDKDLNTASFNQNPIGTGPYKFVKWKKGEYVEFKANENFYLAKVKTPRLIIKHIFDPS
VASVELKNGKIDAALIDVSLLNIFKKDENFKILRERSADYRALMFNLDNEFLKDIQIR
KALNYAVDKQSIVKNLLHDYGFVANHPLERSWADSKAFKTYEYNPKKAQDLILALGFK
KNDEGIFEKDGNILEFEIWTMSNDPLRVSLAGILQSEFKKIGVIAKVVAKPAGSFDYS
KVDSFLVGWGSPLDPDFHTFRVFESSQDSALNNEGWNFGHYHDKKVDIALQKARNTLN
LEERKKYYKEFIDALYENPPFIFLTYLDFALVYNAKLEGIKARNLGHHGVGFTWNIYE
WSKK"

gene complement(21259..24030)
/locus_tag="DMACINML_00755"

CDS complement(21259..24030)
/locus_tag="DMACINML_00755"
/inference="ab initio prediction:Prodigal:2.6"
/inference="similar to AA sequence:RefSeq:AHY40757.1"
/codon_start=1
/transl_table=11
/product="putative D-lactate dehydrogenase, Fe-S protein, FAD/FMN-containing protein"
/protein_id="Prokka:DMACINML_00755"
/translation="MANFKAFYNEAKYIFKDRIFNDYARCYAYGIDASCYFYIPKIVI
IAKNEDEIKQVIQLAHTYKTPISFRAAGTSLSGQSSCDGVLVVIKFAFKKIKINKDAS
EITLGCGVVGIHANESLAFLKKKIGPDPATINSALIGGIINNNSSGMCCGTKDNSYKT
LRSIRVILANGSILDTSDALSVAQFKNENKKLINELREIKEEINANKELKDLIIKKFK
IKNTTGYSLNAFVDYDDEIDILAHLLVGSEGTLGFVSEVKLAVLDDLEFKACALLFFD
NINNAANTIKEFAKVDFISSAEIMDYASLKAASSYDELRDILADIKEGDTCVLIQSEH
SNELKLDENINKIKEISKLAYKSYFSKNKAEYDLWWKIRKALLPIAASLRKAGSTVIT
EDVCFNIEDLADGIKSIQELFYKYGFSDNGIIFGHALAGNIHFIITPDLNNKLEFDNF
SNLVKEMSNIVASYGGSIKAEHGTGRMVAPFVEVEWGKQAYLINKKIKSIFDKENLFN
PDVIISDDKDIYKKNIKQASLIDEKLNTCMECGFCERFCPSNEYTITPRQRIAILREI
KRLESLNDDESKAKLKDIKKYYNHLVDSSCAACGVCSFSCPLGINFADFSLKYRKNNI
GFMSKILGNLAYKNHEKTLKIAKFSLSIANKFDNLSLDNKLEKASNFLSAIPRTRAYL
PKVNDYELKSRKRAYNVVYFTSCLNKSFKPNEKMYDKRSLQEVFESLCEKANIGIIYA

PNDLCCGKAYENFQDIQDKNIQKINDFLSNIDSPIVLDHSACSAKLISDHLKYEIYDL
SEYLLKFIAPKLRIDKINEDVGLYIMCAARKLGLNENIIKLAKLCTNGKVLIDNDTYC
CGFAGYKGFFNPKLNINATKGFKKFYAKTNIKRGFSTSSTCEIGLSDATGISWQHIAY
LLDECSEAI"

```
gene            24194..24616
                /gene="cgb"
                /locus_tag="DMACINML_00756"
CDS             24194..24616
                /gene="cgb"
                /locus_tag="DMACINML_00756"
                /inference="ab initio prediction:Prodigal:2.6"
                /inference="similar to AA sequence:RefSeq:CAL35683.1"
                /codon_start=1
                /transl_table=11
                /product="single domain hemoglobin"
                /protein_id="Prokka:DMACINML_00756"
                /translation="MTQEQIQIIKDCVPILQKNGEDLTKEFYKVMFNDYPEVKPMFNM
                EKQASGEQPKALAMAILMAAKNIENLENMRSFVDKVAITHVNLGVKEEHYPIVGACLL
                KAIKNLLNPDEATLKAWEVAYGKIAEFYIDIEKKLYEK"
gene            complement(24637..26268)
                /locus_tag="DMACINML_00757"
CDS             complement(24637..26268)
                /locus_tag="DMACINML_00757"
                /inference="ab initio prediction:Prodigal:2.6"
                /inference="similar to AA sequence:RefSeq:CAL35684.1"
                /codon_start=1
                /transl_table=11
                /product="putative ABC transporter"
                /protein_id="Prokka:DMACINML_00757"
                /translation="MPFILELLKQNKFKFISFLLFSFISSAVGVLTLIFINDYLLKNT
                QNIPILYFIALLIVFFLSSTIVELGLSVFGQNFIFKMQRRVVKQILDTPLLKVAKVGK
                ARILASLGSDVRNVSFGLLRLPDFLQSSILILCTSVYLCYLSPQIFTLCAIWIIVVFT
                INNFLMMKVYSYFRKARENDDALQNNYQNILDGHKELLINRYRAKLYYEDEFENNARS
                KKKNNTLGNLFNNLSSNFTNVALLALVGVEFYLALEFKWASVAEATTIALSILFLRTP
                LVSMIGSFPTLLLAKIALDKIAKLELDDYKEHFEKTNFIKDWRQISFKNTSFSYDDNF
                HLNPVNLELKKGELIFLIGKNGSGKSTFCMLLTGLFKPSEGEIFVDDMKIDDDNLDIY
                RSLVSAVFSDFHLFTKTLAKEKFADEEKIASWLEFLELKGKTRVEDHELVLTKLSTGQ
                KKRLAMLIALLEERDILVLDEWAADQDPVFRRFFYKKLLPLLKEQGKTIFAITHDDAY
                FDSADRIFLAEEGNISELKGENIKELARNLVEKFD"
gene            complement(26313..27620)
                /locus_tag="DMACINML_00758"
CDS             complement(26313..27620)
                /locus_tag="DMACINML_00758"
                /inference="ab initio prediction:Prodigal:2.6"
                /inference="similar to AA sequence:RefSeq:AHY40760.1"
                /codon_start=1
                /transl_table=11
                /product="L-Proline/Glycine betaine transporter ProP"
                /protein_id="Prokka:DMACINML_00758"
                /translation="MKKSKLGTKEFKILGLSSLGGTLEFYDFIIFVFFAEYIAKVFFP
                KDMSEFWTLLNTYGAFAAGYLARPLGGIVMAHFGDKFGRKNMFMLSILLMVLPTFTLA
                FIPGYETLGFLAPFLLILIRIFQGIAIGGELPGAWVFVREHCKESQKAFSLSCLNSAM
                ALGILLGSMVFLLINAFFSTEEIAAYAWRIAFFVGGIFGIISIYLRRFLQETPIFKQM
                QKERSLSSFPLKDLFKEENIFKNTLASIFITWVLTGCVVVLILLMPKFMPSILGLSAI
```

```
                     EGSYLQVLGILGITLGGVFMGYLVDKLGLFKICIFFSLVFAFFSCLYFYALYDLKNLV
                     LTCILYTSVCFLGGINVFAPILMSEVFRAKIRFSGISFSYNIAYAIAGGVTPQLVLWL
                     NILATRSKNPFLYGMSMYMIFLALLAICAVFLVKDKINFDNRS"
     gene            27924..28142
                     /locus_tag="DMACINML_00759"
     CDS             27924..28142
                     /locus_tag="DMACINML_00759"
                     /inference="ab initio prediction:Prodigal:2.6"
                     /codon_start=1
                     /transl_table=11
                     /product="hypothetical protein"
                     /protein_id="Prokka:DMACINML_00759"
                     /translation="MMIIVGILGLIISTAVLLACGVFWVLFGIVFWGVILAFMLLDLS
                     GSYGVVALYILFIVLNLILIYKITRRKK"
     gene            28139..28573
                     /locus_tag="DMACINML_00760"
     CDS             28139..28573
                     /locus_tag="DMACINML_00760"
                     /inference="ab initio prediction:Prodigal:2.6"
                     /codon_start=1
                     /transl_table=11
                     /product="hypothetical protein"
                     /protein_id="Prokka:DMACINML_00760"
                     /translation="MKKLALALGGIALGACGGKIYQGIKNTWDKLIFKDLITLRKVIK
                     QYEEIEQIYPSYLNDLKFLEFRKTHKYDTETIYNIKDHLFSDLKKEKLIQIGTTMEAF
                     MEYSDKYLQDDITALKISWKFCAMKLKFDGALLQLQDVNKMV"
     gene            28583..28993
                     /locus_tag="DMACINML_00761"
     CDS             28583..28993
                     /locus_tag="DMACINML_00761"
                     /inference="ab initio prediction:Prodigal:2.6"
                     /inference="similar to AA sequence:RefSeq:CKH05500.1"
                     /codon_start=1
                     /transl_table=11
                     /product="hypothetical protein"
                     /protein_id="Prokka:DMACINML_00761"
                     /translation="MQENLKEIYEEFEFLNTQFASYFNDEEFANFKKDFEAKNALNYR
                     DFEEFKLEETDKKRLKDIQECLLAYINFCSSKDIKEKRVEVISKTFMPMFLISRLYFA
                     LKEEKLDMQIRKILKEKDELEEEKSLWISKVLPF"
     gene            29074..30198
                     /locus_tag="DMACINML_00762"
     CDS             29074..30198
                     /locus_tag="DMACINML_00762"
                     /inference="ab initio prediction:Prodigal:2.6"
                     /inference="similar to AA sequence:RefSeq:AJK70711.1"
                     /codon_start=1
                     /transl_table=11
                     /product="GTP-binding protein"
                     /protein_id="Prokka:DMACINML_00762"
                     /translation="MENKSQENTVDLNAIMSELLQQGQEMYNSSKKQDKLDKLNILII
                     GGTGVGKSTLINTVFGKEVAKTGDGAPVTQAIQKCEKKGLCIYDSKGLEKNDPSIVED
                     LKNLLDKQKQKEVSEQIHIAWLCICEATRRVEPLEKELYDLLKQYNFPTIVAITKAMQ
                     NINDKGEKFDEIVKNELKATEVIRIRAIETKLDDDEENILKPMGIKDLLNKSYNLLPE
                     AQQNALARKQIYDRDMKKIQCKKDATSKLNYYSTTAGAVAATPIPFSDIALILPTQIA
```

```
                     MIVHISSIYNLDFDKESAKKVTLALVGVCGAGFGIRLGVGAALKLIPGLGSLAGGAIN
                     ATVATTATKAMGELYIKYLDDHFDDIENNKSLSFNFEDYK"
     gene            30235..30651
                     /locus_tag="DMACINML_00763"
     CDS             30235..30651
                     /locus_tag="DMACINML_00763"
                     /inference="ab initio prediction:Prodigal:2.6"
                     /codon_start=1
                     /transl_table=11
                     /product="hypothetical protein"
                     /protein_id="Prokka:DMACINML_00763"
                     /translation="MQVKTEEELAKAVNNNENRIEIEGDLAEKVIKIKASGNVAWAVA
                     IGSIGVAVIAIISLFKNSESKNSQSPKVAFVASGIGATTAAGAVGVLGLATTISAIKI
                     AVSGGGKDVLKKLRNYKMEKISDTKIVLKNELLSKG"
     gene            30654..30893
                     /locus_tag="DMACINML_00764"
     CDS             30654..30893
                     /locus_tag="DMACINML_00764"
                     /inference="ab initio prediction:Prodigal:2.6"
                     /inference="similar to AA sequence:RefSeq:AAW35817.1"
                     /note="conserved hypothetical protein"
                     /codon_start=1
                     /transl_table=11
                     /product="hypothetical protein"
                     /protein_id="Prokka:DMACINML_00764"
                     /translation="MFCKLLKAFISNPRSWFLFYRLRKLGFCIKENFDKETKYSAVCK
                     SLDELVKYKNKHPNDFDNFFKIFKEILNDYEKSKK"
     gene            30905..31207
                     /locus_tag="DMACINML_00765"
     CDS             30905..31207
                     /locus_tag="DMACINML_00765"
                     /inference="ab initio prediction:Prodigal:2.6"
                     /inference="similar to AA sequence:RefSeq:AAW35817.1"
                     /note="conserved hypothetical protein"
                     /codon_start=1
                     /transl_table=11
                     /product="hypothetical protein"
                     /protein_id="Prokka:DMACINML_00765"
                     /translation="MYYNKIFSALSLANSFKSTIFKRISSPAQSARMIDFALEIKSIF
                     SHNSKDEIPIKILDKLVEYKNKYPNDFDEMFEILKEILDEYEKNPSEVKKNLKELF"
     gene            31209..32306
                     /locus_tag="DMACINML_00766"
     CDS             31209..32306
                     /locus_tag="DMACINML_00766"
                     /inference="ab initio prediction:Prodigal:2.6"
                     /inference="similar to AA sequence:RefSeq:CAL34716.1"
                     /codon_start=1
                     /transl_table=11
                     /product="putative ATP/GTP binding protein"
                     /protein_id="Prokka:DMACINML_00766"
                     /translation="MQNLLILYNPYYQQDVIKLHLEILLDKGQVAFGKIKSKLKEPLS
                     YEMDENKISHNQKLSEIYKNTSADNPLQLFLSDYANLFVAKVVKISKEVDENIIPKYY
                     QEKNLEVEDYFLITDLRELVRENFTLIRDKFLANFTTENNHTYAIYGNNYSYPLVVKQ
                     KEEINYFYDEINKYYLSVYKNQEYLKMQENFIQFIFGKKFFYMLHPDSINNLILAELE
```

```
                     LQQNLENPLYDFTSIIVKYSKTIEYEVYDFAKKIFTKLINQNSHLSSINYSVQGKEFN
                     FKQFFINKPNIGTIKFLFKNKEIQELLDKDIKGFINYEFNRTLDEFQTIRNHAVHAKS
                     PTLEQTLKIRNLILGIENTSILKRVLEYKFK"
gene                 32318..32488
                     /locus_tag="DMACINML_00767"
CDS                  32318..32488
                     /locus_tag="DMACINML_00767"
                     /inference="ab initio prediction:Prodigal:2.6"
                     /inference="similar to AA sequence:RefSeq:CKG72518.1"
                     /codon_start=1
                     /transl_table=11
                     /product="hypothetical protein"
                     /protein_id="Prokka:DMACINML_00767"
                     /translation="MIKPKPFLKKCPKCGKMFLCTPKSDCLNPSDFFCDKCSGKKDTN
                     LISKIISAIKKV"
gene                 32490..33350
                     /locus_tag="DMACINML_00768"
CDS                  32490..33350
                     /locus_tag="DMACINML_00768"
                     /inference="ab initio prediction:Prodigal:2.6"
                     /inference="similar to AA sequence:RefSeq:CAL34717.1"
                     /codon_start=1
                     /transl_table=11
                     /product="putative transcriptional regulator"
                     /protein_id="Prokka:DMACINML_00768"
                     /translation="MAHDKLAMRLAHILMRFNNGGRFSLEELASEFNVDIRTIQRDLN
                     QRLSFMPIQKENGKYFLESFALGKLSVKDIQNFAMLSGVQELYPKLDEDFITDLLNSK
                     VNSIFMIKNNGFEKVDYENFEIISVAILKHNILNFNYKEKSREVKPYKLLNYQGIWYL
                     IADEKDKLKHFTFSKIKNIHKNNEIFTPKEEFLDQILHDKNIWLDDSKEAIVKLDKNA
                     KEYFFRKNILNNFELIDEDEQSYTLKVYFSYDDELLNVVKQWIPYIKIEKPFELKEKL
                     DSILKDYLEN"
gene                 33416..33634
                     /gene="infA"
                     /locus_tag="DMACINML_00769"
CDS                  33416..33634
                     /gene="infA"
                     /locus_tag="DMACINML_00769"
                     /inference="ab initio prediction:Prodigal:2.6"
                     /inference="similar to AA sequence:RefSeq:CAL35687.1"
                     /codon_start=1
                     /transl_table=11
                     /product="translation initiation factor IF-1"
                     /protein_id="Prokka:DMACINML_00769"
                     /translation="MAKDDVIEIDGTVLEALPNANFKVELDNKHVILCHIAGKMRMHY
                     IRIMPGDKVKVELTPYSLDKGRITFRYK"
gene                 33921..34286
                     /gene="rpsM"
                     /locus_tag="DMACINML_00770"
CDS                  33921..34286
                     /gene="rpsM"
                     /locus_tag="DMACINML_00770"
                     /inference="ab initio prediction:Prodigal:2.6"
                     /inference="similar to AA sequence:RefSeq:CAL35689.1"
                     /codon_start=1
```

```
                     /transl_table=11
                     /product="30S ribosomal protein S13"
                     /protein_id="Prokka:DMACINML_00770"
                     /translation="MARIAGVDLPKKKRIEYGLTYIYGIGLFTSRKILDKTGISYDKR
                     VHELSEDEAAAIRKEIQENYMVEGDLRKQVAMDIKALMDLGSFRGLRHRKGLPVRGQK
                     TKTNARTRKGKRKTVGAKS"
     gene            34296..34688
                     /gene="rpsK"
                     /locus_tag="DMACINML_00771"
     CDS             34296..34688
                     /gene="rpsK"
                     /locus_tag="DMACINML_00771"
                     /inference="ab initio prediction:Prodigal:2.6"
                     /inference="similar to AA sequence:RefSeq:CAL35690.1"
                     /codon_start=1
                     /transl_table=11
                     /product="30S ribosomal protein S11"
                     /protein_id="Prokka:DMACINML_00771"
                     /translation="MAKRKIVKKKVVKKNIAKGIVYISATFNNTMVTVTDEMGNAIAW
                     SSAGGLGFKGSKKSTPYAAQQAVEDALNKAKEHGIKEVGIKVQGPGSGRETAVKSVGA
                     MEGIKVTFLKDITPLAHNGCRPPKRRRV"
     gene            34718..35344
                     /gene="rpsD"
                     /locus_tag="DMACINML_00772"
     CDS             34718..35344
                     /gene="rpsD"
                     /locus_tag="DMACINML_00772"
                     /inference="ab initio prediction:Prodigal:2.6"
                     /inference="similar to AA sequence:RefSeq:CAL35691.1"
                     /codon_start=1
                     /transl_table=11
                     /product="30S ribosomal protein S4"
                     /protein_id="Prokka:DMACINML_00772"
                     /translation="MARYRGPVEKLERRFGVSLALKGERRLAGKSALDKRPYAPGQHG
                     ARKGKISEYGLQLREKQKAKFMYGVSEKQFRRLFAEAARRDGNTGVLLIQLLEQRLDN
                     VVYRMGFATTRRFARQLVTHGHILVNGKRVDIPSFRVEAGAKIEVIEKSKNNPQITRA
                     IELTAQTGIVAWVDVEKDKRFGIFTRKPEREEVIIPVEERFIVELYSK"
     gene            35358..36371
                     /gene="rpoA"
                     /locus_tag="DMACINML_00773"
     CDS             35358..36371
                     /gene="rpoA"
                     /locus_tag="DMACINML_00773"
                     /EC_number="2.7.7.6"
                     /inference="ab initio prediction:Prodigal:2.6"
                     /inference="similar to AA sequence:RefSeq:CAL35692.1"
                     /codon_start=1
                     /transl_table=11
                     /product="DNA-directed RNA polymerase alpha chain"
                     /protein_id="Prokka:DMACINML_00773"
                     /translation="MRNITTSAYTPTEFTIENISDTVAKISAWPFEIGYGITLAHPLR
                     RLLYTSTIGYAPTAIHIDGVAHEFDSMRGMLEDVALFIINLKKLRFKLKSDSNKEIVE
                     FSFKGPKEIYGKDLNNDQVEVVNTDAYLATINEDAELKFTLIIEKGIGYVPSEEIKEL
                     INDPKFIALDAFFTPVREATYDIEKVLFEDNPDYEKVVLTVTTDGQITPNEAFQNALE
```

AMYKQLSVFDKITNVRSIIKSQATSNELENTKLLQNITDLNLSARSYNCLEKAGIVYI
GELALMSVNELAGLKNLGKKSLDEIKSIMESIGFPVGTSKLSDNKDLLKKKIDELKAQ
NEG"

```
gene            36375..36728
                /gene="rplQ"
                /locus_tag="DMACINML_00774"
CDS             36375..36728
                /gene="rplQ"
                /locus_tag="DMACINML_00774"
                /inference="ab initio prediction:Prodigal:2.6"
                /inference="similar to AA sequence:RefSeq:CAL35693.1"
                /codon_start=1
                /transl_table=11
                /product="50S ribosomal protein L17"
                /protein_id="Prokka:DMACINML_00774"
                /translation="MRHKHGYRKLGRTSSHRAALLKNLTIALVNSGKIETTLPKAKEL
                RGYVERLITRARLGDFNAHRAVFAALQDKNATNKLVTEIAPNFKERNGGYTRIIKTRI
                RRGDAAEMAFIEFVA"
gene            36948..37847
                /gene="hisG"
                /locus_tag="DMACINML_00775"
CDS             36948..37847
                /gene="hisG"
                /locus_tag="DMACINML_00775"
                /EC_number="2.4.2.17"
                /inference="ab initio prediction:Prodigal:2.6"
                /inference="similar to AA sequence:RefSeq:CAL35694.1"
                /codon_start=1
                /transl_table=11
                /product="ATP phosphoribosyltransferase"
                /protein_id="Prokka:DMACINML_00775"
                /translation="MQENSRLRIAIQKSGRLSKESVELLNKCGIKMHIHEQSLIAFST
                NLPIDILRVRDDDIPGLIFDGVVDLGIIGENVLEENELERKSLGEEANYKLLTKLDFG
                YCRLSLALPQEKKFDNLKDFENLRIATSYPQLLKRFMKENNIHYKNCMLTGSVEVAPR
                ANLADAICDLVSSGATLQANNLKEVKVIYQSRACLIQKQNTLSSEKQALVDKIMLRVA
                GVMQARESKYIMLHAPKEKLDKIQSLLPGVEKPTILPLAHDEKNVALHMVSKENLFWE
                TMEALKEEGASSILVLPIEKMLK"
gene            37855..39141
                /gene="hisD"
                /locus_tag="DMACINML_00776"
CDS             37855..39141
                /gene="hisD"
                /locus_tag="DMACINML_00776"
                /EC_number="1.1.1.23"
                /inference="ab initio prediction:Prodigal:2.6"
                /inference="similar to AA sequence:RefSeq:CAL35695.1"
                /codon_start=1
                /transl_table=11
                /product="histidinol dehydrogenase"
                /protein_id="Prokka:DMACINML_00776"
                /translation="MQIINFNELDEKQKQEVLKRPAIAAKDEISQIVSSIIKEVREKG
                DEALIEQALKFDKAQISSIKVSEEELENASSRLDKDLKEAILIAYENIKKFHEAQIPK
                EVAIETTKGVKCEVLTRPIEKVGLYIPGGLAPLFSTVLMLAIPAKIAQCEKIVLASPA
                KINDATLFCAKLCGVDEIYQMGGAGAIAALAYGTKSVLKVDKIFGPGNAFVTEAKRQV
```

```
                     SSDINGAAIDMQAGPSEVLVIADEKANVKFVASDLLSQAEHGADSQVILVCLSKEFAQ
                     SASDEVSRQLENLPRKELASKSIANSKIIIAKDLDEALLISNLYAPEHLIIQTQNPRE
                     LLDKVKHAGSVFLGELSPESMGDYASGTNHVLPTYGLTKTHSSLGLADFSKRMSVQEL
                     SKEGFLNLGKSVEILAANEHLDAHKNAVTFRLESLK"
     gene            39138..40199
                     /gene="hisB"
                     /locus_tag="DMACINML_00777"
     CDS             39138..40199
                     /gene="hisB"
                     /locus_tag="DMACINML_00777"
                     /EC_number="3.1.3.15"
                     /inference="ab initio prediction:Prodigal:2.6"
                     /inference="similar to AA sequence:RefSeq:CAL35696.1"
                     /codon_start=1
                     /transl_table=11
                     /product="imidazoleglycerol-phosphate
                     dehydratase/histidinol-phosphatase"
                     /protein_id="Prokka:DMACINML_00777"
                     /translation="MSEKILFIDRDGTIIEEPKSDFQIDSLEKLRFEKGAIPTLLKLK
                     NFGFKFVLVSNQDGLGTQSFPKEDFEIAHQKMLDILQSCGIEFKDIFICPHFENENCE
                     CRKPKTALLDEYIKHNLYNKEQSFVIGDRDTDMILASNLGVRGLKYSENLTWKEIEEE
                     ILNSFRTASISRITKETNIRVKVCLNGRKIAINTGVPFFDHMLEQIAVHGGIGLEISC
                     KGDLEIDEHHSVEDVALALGSAIKQALGDKIGITRYGFVLPMDECLASCAIDFCNRPH
                     LVYKAKFKKEKLGELSTEMIEHFFYSLSYSMGASLHLKVKGKNDHHKAEGLFKAFAKA
                     LKMAVKIESENLASSKGVI"
     gene            40196..40783
                     /gene="hisH"
                     /locus_tag="DMACINML_00778"
     CDS             40196..40783
                     /gene="hisH"
                     /locus_tag="DMACINML_00778"
                     /EC_number="2.4.2.-"
                     /inference="ab initio prediction:Prodigal:2.6"
                     /inference="similar to AA sequence:RefSeq:AHW92663.1"
                     /codon_start=1
                     /transl_table=11
                     /product="imidazole glycerol phosphate synthase subunit
                     HisH"
                     /protein_id="Prokka:DMACINML_00778"
                     /translation="MKLIIIDTACANLASLKFCLDRLGFNAKISRDLKDLESADKLFL
                     PGVGTAAKAMSNLESFNLTNFIKNTKKPLLGICLGMQILGDFSEELDQETLGIMPFKT
                     QKFELPKEYSLPHMGWNEVASSHPLFKGLNGAYFYFVHSYHVVLNDYTIATSDYGVKF
                     SASLAKDNFYGVQFHPERSGEAGEILISNFIKDIT"
     gene            40780..41508
                     /locus_tag="DMACINML_00779"
     CDS             40780..41508
                     /locus_tag="DMACINML_00779"
                     /EC_number="5.3.1.16"
                     /inference="ab initio prediction:Prodigal:2.6"
                     /inference="similar to AA sequence:RefSeq:ABS43305.1"
                     /codon_start=1
                     /transl_table=11
                     /product="1-(5-phosphoribosyl)-5-[(5-
                     phosphoribosylamino)methylideneamino]imidazole-4-
```

```
                     carboxamide isomerase"
                     /protein_id="Prokka:DMACINML_00779"
                     /translation="MIIPALDLIDGQVVRLVKGDYEQKKVYKYDPLEKFREYENAGAT
                     WLHLVDLSGAKDPNKRQLTLIEKLANEVSVNLQVGGGIRTKEEIQALLDCGVKRVVIG
                     SLAITNPTLCVEILQHFGSEAIVLALDTILKEDYMIAINAWQDTSDKRLLEVLEFYNI
                     KGLKHILCTDISRDGTMQGSNAELYELIHKNFPKIHTQASGGVASLEDLKKLKGICSG
                     VIVGKALLDGVFSVEEGIKCLQNA"
     gene            41490..42257
                     /gene="hisF"
                     /locus_tag="DMACINML_00780"
     CDS             41490..42257
                     /gene="hisF"
                     /locus_tag="DMACINML_00780"
                     /EC_number="4.1.3.-"
                     /inference="ab initio prediction:Prodigal:2.6"
                     /inference="similar to AA sequence:RefSeq:CAL35700.1"
                     /codon_start=1
                     /transl_table=11
                     /product="imidazole glycerol phosphate synthase subunit"
                     /protein_id="Prokka:DMACINML_00780"
                     /translation="MLTKRIIACLDVKDGRVVKGTQFKNHKDMGDIVELAKRYSQSGI
                     DELVFYDIAASARKERISREWVSEVAKNINIPFCVAGGIKSEEDAAELLANGADKISI
                     NSPALNDPDLITRLAKSFGVQCVVVGIDSFKDENGNLKVFKYTGDENTSKHSGKNTIE
                     WIKEVQDLGAGEIVLNMMNQDGVKNGYDLEQLSLAKKECKVPLIASGGAGKMEHFLEA
                     FKIGVDGALAASVFHQQIIDIKELKNYLKTQGVSIRI"
     gene            42260..42883
                     /gene="hisI"
                     /locus_tag="DMACINML_00781"
     CDS             42260..42883
                     /gene="hisI"
                     /locus_tag="DMACINML_00781"
                     /EC_number="3.6.1.31"
                     /inference="ab initio prediction:Prodigal:2.6"
                     /inference="similar to AA sequence:RefSeq:CAL35701.1"
                     /codon_start=1
                     /transl_table=11
                     /product="phosphoribosyl-AMP cyclohydrolase/
                     phosphoribosyl-ATP pyrophosphohydrolase"
                     /protein_id="Prokka:DMACINML_00781"
                     /translation="MQNFQNLNEKINWQKVNGLIPVIIQDAKSCEVLMLGFMDEKALE
                     KSLESKKVVFFSRTKNRLWMKGEESGNFLNIVDLSLDCDNDTLLILVDPIGATCHIGN
                     ISCFENVSKSADFVFLARLEKLINSRKNADENTSYTAKLFKKGTKRIAQKVGEEGVET
                     ALAATVKDKDELICEAADLMYHLSVLLADANLSFSDVIAKLKERHKE"
     gene            complement(43500..44663)
                     /gene="dapD"
                     /locus_tag="DMACINML_00782"
     CDS             complement(43500..44663)
                     /gene="dapD"
                     /locus_tag="DMACINML_00782"
                     /EC_number="2.3.1.117"
                     /inference="ab initio prediction:Prodigal:2.6"
                     /inference="similar to AA sequence:RefSeq:CAL35702.1"
                     /codon_start=1
                     /transl_table=11
```

```
                     /product="putative
                     2,3,4,5-tetrahydropyridine-2-carboxylate
                     N-succinyltransferase"
                     /protein_id="Prokka:DMACINML_00782"
                     /translation="MIINTKEDFLLLIKQIEQKNGYRKPKAFGIARLDRGQLNKNKIL
                     QASFALVNYEQNFGSAAIMLEAFMQRGVEIDFEASEFVQILKLEDIDYALACFKPFLE
                     EEGHKNIDILKIIKDKFKDDEFAFVCLFEDKEPLSVESVYLKLYLLSTKKVPLRSLNL
                     NGAFGLLSNVAWSDDKPIELEYLRENEMRLKMSNQYPKIDFVDKFPRFLAHIIPEDNT
                     RILESSKVRMGASLAAGTTIMPGASYVNFNAGTTGACMVEGRISSSAIVGEGSDVGGG
                     ASILGVLSGTSGNAISVGKACLLGANSVTGIPLGDNCIVDAGIAVLEGTKFLLKDREE
                     LAKLNPYFDFSKEIYKGIELKGLNGLHFRQDSISGAMIVCLNKKAIKLNEALH"
     gene            complement(44681..45787)
                     /gene="mrp"
                     /locus_tag="DMACINML_00783"
     CDS             complement(44681..45787)
                     /gene="mrp"
                     /locus_tag="DMACINML_00783"
                     /inference="ab initio prediction:Prodigal:2.6"
                     /inference="similar to AA sequence:RefSeq:CAL35703.1"
                     /codon_start=1
                     /transl_table=11
                     /product="putative ATP/GTP-binding protein (Mrp protein)"
                     /protein_id="Prokka:DMACINML_00783"
                     /translation="MKEQILEKLKSVKYPGFDKDIVSFNFVKDIKIENENVNVELEIV
                     SSNPQVSEELRKNIDEALASLNLKNLQIHIITPKIPEEKSNSRSGKNIAPQVKNFIMV
                     SSGKGGVGKSTTTVNLAISMAKMGKRVGILDADIYGPNIPRMLGETKTQPEVVGQRLK
                     PILSHGVYMMSMGVLIEEGQGLMWRGAMIMKAIEQLLADVIWPELDVLFLDMPPGTGD
                     AQITSAQSIPITAGVCVSTPQTVSLDDSKRALDMFNKLHIPIAGVIENMSGFLCPDNG
                     KEYDIFGKGTAEEMAKAYKSEVLAQIPIEMIVREGGDEGKPVSFYHPESVSAKRYLMA
                     AEKIWQFIEKINNEGGADNSAIQPVMNGKSACSH"
     gene            45907..47022
                     /gene="ispDF"
                     /locus_tag="DMACINML_00784"
     CDS             45907..47022
                     /gene="ispDF"
                     /locus_tag="DMACINML_00784"
                     /EC_number="2.7.7.60"
                     /inference="ab initio prediction:Prodigal:2.6"
                     /inference="similar to AA sequence:RefSeq:CAL35704.1"
                     /codon_start=1
                     /transl_table=11
                     /product="2-C-methyl-D-erythritol 4-phosphate
                     cytidylyltransferase/ 2-C-methyl-D-erythritol
                     2,4-cyclodiphosphate synthase"
                     /protein_id="Prokka:DMACINML_00784"
                     /translation="MANLSLIMLAAGNSTRFDMGVKKQFLRLGDDPLWLYATRNLSTF
                     YPFKKIVVTSSNTSYMKKFARNYEFVEGGATRAESLKKALEAIDSEFVMVSDVARVLV
                     SKSLFDRLIENLDKADCITPALKVADTTLFENEALQREKIKLIQTPQISRTHLLKQAL
                     NQNLDFTDDSTAISAMGGKIWFVEGEENTRKITFKEDLKKLDLPRPSNEIFCGNGFDV
                     HEFGEERPLLLGGVEIHPTMGLKAHSDGDVLAHSLTDAILGAACLGDIGELFPDTDEK
                     FKNANSMNLLKEAYKRVKELGFELVNVDICVMAQTPKLKDFKEAIAQNIANTLNIDEF
                     RVNIKATTTEKLGFIGRKEGMAVLASANLKYFDWTKI"
     gene            47019..47906
                     /locus_tag="DMACINML_00785"
```

```
CDS             47019..47906
                /locus_tag="DMACINML_00785"
                /inference="ab initio prediction:Prodigal:2.6"
                /inference="similar to AA sequence:RefSeq:CAL35705.1"
                /codon_start=1
                /transl_table=11
                /product="putative two-component regulator"
                /protein_id="Prokka:DMACINML_00785"
                /translation="MKVLIIENEIYLAQSISIKLSDVGYSCEIINSFDERNPEKYYDI
                ILLSTNTSNFLKAVTEFKHSIIILLISYISTDTVSNPLKLGACDYIQKPFMIEELIRK
                IKHYQDFKKLSILNKAYKSYIKSRLETVKIPEYNYKKLKLPVVLKSNKQSSADAFVFN
                YINEYDITLSYVDLSVSNSVEKAMKLPTENNLLFLSNFQVLKPLEREKLLEFIKNKSV
                ILHTNSNIEDLELPCINLNDNEKNIDSNQILTIDEYVKYIILNYQNVFPDTDLSKKLG
                ISRKSLWEKRKKYEISKKK"
gene            47884..49044
                /locus_tag="DMACINML_00786"
CDS             47884..49044
                /locus_tag="DMACINML_00786"
                /EC_number="2.7.7.4"
                /inference="ab initio prediction:Prodigal:2.6"
                /inference="similar to AA sequence:RefSeq:CAL35706.1"
                /codon_start=1
                /transl_table=11
                /product="putative sulfate adenylyltransferase"
                /protein_id="Prokka:DMACINML_00786"
                /translation="MKSAKKNKSIVIDKNELGILSLIKEGLLGSCTHLMDEHETKQIL
                KTGKSDKESFPYPLSFAPKISEEFIKDIRIGSKIDLILNDETVGNITLKSKFKNDKNF
                SNIFSPHTCSLDNMGEICIGGEIEIYNSTIQKIKEEFHKTKENLNAHKITAIVSSFDP
                LHRAHERMFRWTIDKADLVVVFLVESFDANGFDFDLKQNYLKKFIQNYLPPDRIFIFP
                LKDINLFHAHLNPSLESILAKSLGCTKLVVGQNHTGLGMFYDDNQPKTILDDFSKDYD
                IEVIVLPEFVFCDACRMIVSTRSCPHGCHHHLHYNSQSLKELLRAGIIPPAVFMRKEV
                SSIILSSIFPNRFKNLQKIYNDLFATDGILEYKSDEEFYQKLLETHQMSYMV"
gene            49054..49554
                /gene="pgpA"
                /locus_tag="DMACINML_00787"
CDS             49054..49554
                /gene="pgpA"
                /locus_tag="DMACINML_00787"
                /EC_number="3.1.3.27"
                /inference="ab initio prediction:Prodigal:2.6"
                /inference="similar to AA sequence:RefSeq:CAL35707.1"
                /codon_start=1
                /transl_table=11
                /product="putative phosphatidylglycerophosphatase"
                /protein_id="Prokka:DMACINML_00787"
                /translation="MQKLFLTFFYSGCAKKAPGTFGTLAALIPAFFILKYLGITTLFL
                LSILIFIASIRVIDDYERKTGIHDDKHIVIDEVAGVFLACAIAASGANSLLNFFMAFV
                FFRIFDITKPSIIGKIDKKTKGGLGVMLDDMLAGLFAGLLSAVIYGFLLKLDLVFWDK
                DLINLF"
gene            49631..49894
                /gene="rpsT"
                /locus_tag="DMACINML_00788"
CDS             49631..49894
                /gene="rpsT"
```

```
                     /locus_tag="DMACINML_00788"
                     /inference="ab initio prediction:Prodigal:2.6"
                     /inference="similar to AA sequence:RefSeq:CAL35708.1"
                     /codon_start=1
                     /transl_table=11
                     /product="30S ribosomal protein S20"
                     /protein_id="Prokka:DMACINML_00788"
                     /translation="MANHKSAEKRARQTIKKTERNRFYRTRLKNITKAVREAAAKNDK
                     NAANEALKIANKSIHAMVSRGFLKKQTASRRVSRLALLVNKIA"
     gene            49911..50978
                     /gene="prfA"
                     /locus_tag="DMACINML_00789"
     CDS             49911..50978
                     /gene="prfA"
                     /locus_tag="DMACINML_00789"
                     /inference="ab initio prediction:Prodigal:2.6"
                     /inference="similar to AA sequence:RefSeq:CAL35709.1"
                     /codon_start=1
                     /transl_table=11
                     /product="peptide chain release factor 1"
                     /protein_id="Prokka:DMACINML_00789"
                     /translation="MLASKLDPFLKRFEELNSLLSSSDIISDISKMTALSKEQKNLEP
                     IVLKAKEYLKTLDSIEENKTLLNDAELGELAKEELKNLEELKPKLEEELKILLLPKDP
                     NDERNIFLEIRAGTGGDEASLFVGDLVKAYARYAENRGYKLEIVSSSEGSVGGFKEII
                     MLVKGAGAFSRLKYEGGTHRVQRVPQTESQGRIHTSAITIAVMPEVDDIEIEINPNDL
                     KIDVMRSSGHGGQSVNTTDSAVRITHLPTGIVVVNQDGKSQHKNKESAMKVLKARLYE
                     MQESERLAQESEARKSQVGSGDRSERIRTYNFPQNRISDHRINLTLYRLDAILQDGLF
                     DEVIEPLIAHYQAESLQEQNL"
     gene            complement(51009..51764)
                     /locus_tag="DMACINML_00790"
     CDS             complement(51009..51764)
                     /locus_tag="DMACINML_00790"
                     /inference="ab initio prediction:Prodigal:2.6"
                     /inference="similar to AA sequence:RefSeq:CAL35710.1"
                     /codon_start=1
                     /transl_table=11
                     /product="putative pyridoxamine 5'-phosphate oxidase"
                     /protein_id="Prokka:DMACINML_00790"
                     /translation="MSFESIISHMNDHHQSNLIDLCKKFGAVKDPKEVRLVGVDFEGL
                     DIAYNDNENLRIEFPKKANEETLKDAIIALCMSAKTKKDLSGVAKEAKEFMLSFNSVS
                     LATLNADKEVVCSYAPFVNTSWGNYIYISEVSEHFNNIKENPNNIEIMFLEDESKAAS
                     VILRKRLRYRVKASFIERGEMFDKIYDEFEKQTGGEGGIKTIRNMLDFHLVKLEFQKG
                     RFVKGFGQAYDIENENVTHVGANSSPHKFPHKH"
     gene            51969..54074
                     /locus_tag="DMACINML_00791"
     CDS             51969..54074
                     /locus_tag="DMACINML_00791"
                     /inference="ab initio prediction:Prodigal:2.6"
                     /inference="similar to AA sequence:RefSeq:AHY40787.1"
                     /codon_start=1
                     /transl_table=11
                     /product="TonB-dependent heme receptor"
                     /protein_id="Prokka:DMACINML_00791"
                     /translation="MNPSKRVLKLSLLVFLLNTSINAQELNDNIQMQKVVVSATGFEQ
```

```
                     DADNNLRNVIVIDGKDLEKRGFNTLEQALERISGISFVNFGLGRNIDMRGQGSKSNIA
                     VKVMVNGNAINVLDNSHGVTPLESINLNNIERIEIIPGGGSVLYGNGTRGGVINIITK
                     KEKSDAFALNLKGSSYDHGNLGGNLGVYGAKKINDYLTFGFDIQGFNRDGYQEGYNQK
                     GYFANTKAYLNTSDNSDLTLNYNYFESKNTSSGYLTKAQVQSDPTQKGNSDNITQINR
                     PEIALDYHHYFSDLYEIQLGAFWQNQKIRYLKDEIPMMGTIAYQTGSGFEDTLTGINL
                     KNKINYKDDSYFIFGYEFANHDAKRTSIVHYNVPVIQMSHTMTTLMDMDKQSHSIFAL
                     DSHQFNDIFSISGGARYEYSLYDTHRNYTSKMIMGGRPLPSNPELFDTDDTSNNFALE
                     ITPNFKYSDTGNIYAKYERGFISPTPAQLVNKDQANQKYYTANLDPEIFDTFELGIND
                     FWWDFYGFNLTFFYTLSKDEISYLGNPHATGGAFWKYYNIDQTRRLGAELNLSQFLFS
                     DSLILKESLSYLDAKISKGVNDGLRIPYVSKIKATAGVEYAWNKNFSSYIDLSYFSRA
                     KDGGVVDENTGKMSRNSWIKDYFLTDIGVNYNYRNLQILAGIRNLFDKQYYTYQDSIN
                     DEYLAGNGRNYYVEFKYAF"
     sig_peptide     51969..52040
                     /locus_tag="DMACINML_00791"
                     /inference="ab initio prediction:SignalP:4.1"
                     /note="predicted cleavage at residue 24"
     gene            54034..55035
                     /gene="chuB_1"
                     /locus_tag="DMACINML_00792"
     CDS             54034..55035
                     /gene="chuB_1"
                     /locus_tag="DMACINML_00792"
                     /inference="ab initio prediction:Prodigal:2.6"
                     /inference="similar to AA sequence:RefSeq:ABV53113.1"
                     /codon_start=1
                     /transl_table=11
                     /product="putative hemin transport system permease
                     protein"
                     /protein_id="Prokka:DMACINML_00792"
                     /translation="MAEITMLNLNMRSKNLVIIGLFLAYIITCIIALCLGDENLSPKE
                     LLSYAFGENEILRQIIIDGRLPRIIMAILIGMLLASSGAVTQNVFSNPIADPYIIGIA
                     SAATFGSVLAYLLKLPDYYYGILGFVCSAIFSLAIFKVSSKASIATLLIIGIAASSFL
                     GAFTSFFTYLIGEDSFKIVAWLMGNIGSASWFRIGILILPLIFCLLYFYAHKNELNII
                     LSGDDEARNLGVDAQKLKINLLIVSSLAVSFAVAFTGLIAFVGLIIPHTVRLLLRNYD
                     NALVIPFCTAFGGLFLLICDTLARTLIAPVQIPIGVLTAFFGAPVFLYLALSARRFL"
     gene            55032..55811
                     /gene="chuC"
                     /locus_tag="DMACINML_00793"
     CDS             55032..55811
                     /gene="chuC"
                     /locus_tag="DMACINML_00793"
                     /inference="ab initio prediction:Prodigal:2.6"
                     /inference="similar to AA sequence:RefSeq:CAL35713.1"
                     /codon_start=1
                     /transl_table=11
                     /product="putative hemin uptake system ATP-binding
                     protein"
                     /protein_id="Prokka:DMACINML_00793"
                     /translation="MILKIQNLSFSYHKKELLKNINLELQNKAFIGILGPNGSGKSTL
                     LKLILKNLNISKGEISLFNTDIKNFSLKEFAQLCGFVPQNSGLNTPLKVIDVLLMSKF
                     TNLKHAFCDYSKEDILEVENFAKTLKLENFLERNILSLSGGEFQRVLLARALLKKPKI
                     LFLDEPTSALDLNHAIELLSLCERLIKQNNIAVVAILHDLNLASMFCDKVLFLKEGEI
                     KYFGSTKELFTKEILKEIYNLNCEIVYKDLKPYILALKEKI"
     gene            55808..56614
```

```
                     /gene="chuD"
                     /locus_tag="DMACINML_00794"
     CDS             55808..56614
                     /gene="chuD"
                     /locus_tag="DMACINML_00794"
                     /inference="ab initio prediction:Prodigal:2.6"
                     /inference="similar to AA sequence:RefSeq:CAL35714.1"
                     /codon_start=1
                     /transl_table=11
                     /product="putative hemin uptake system periplasmic
                     hemin-binding protein"
                     /protein_id="Prokka:DMACINML_00794"
                     /translation="MKKILIIMSLFLTLLNAKERIVVLDPASIETLFMLNAQDQIVGI
                     ANLQHSNIYPEEKTSKITSVGTFSHPSLEKIVALKPSLVILSSYSLNLEEGLKNFGIK
                     SINLKAQRLDEIKNNIQILAELTNKEEQGKKLLEEFEQGLQELQKNPLNKSGIYLYSS
                     NPLMAFNDNSLIADILRLIGIKNLSPQSEIARPIISAEYILKQNPDLLILGINADGNI
                     LDTNALLKNTKAAQMGQIYHNKNTHILLRLSPKIIDRIKEFKTRLENQNF"
     gene            complement(56611..57468)
                     /locus_tag="DMACINML_00795"
     CDS             complement(56611..57468)
                     /locus_tag="DMACINML_00795"
                     /inference="ab initio prediction:Prodigal:2.6"
                     /inference="similar to AA sequence:RefSeq:CAL35715.1"
                     /codon_start=1
                     /transl_table=11
                     /product="putative radical SAM domain protein"
                     /protein_id="Prokka:DMACINML_00795"
                     /translation="MKFKKLYIELSDICGLKCDFCPSKKALRGVMSVENFSKLAKQIW
                     DKSEIFTFHLLGDPLLLNNLEEYLQIAKNHQMKLEITTSGFYFSPKNSKLLLKYDNIH
                     QINISLMAFLSQNKLSLEQYFKPILEFCKEHLEYKKPSFINLRLWNLDTDFKAPSENL
                     PIYEFLSKEFGVQILTHLAKNRLQRHILLHQNKLFKWPSLKDKPLYTQGKCHALKEQI
                     GILSDGTLVPCCLDTQGDIPLGNVFNAEFKSLLDSKRIQEIKKAFEENKRIEKLCQSC
                     EFFKTRLSD"
     gene            57638..58900
                     /gene="kgtP_1"
                     /locus_tag="DMACINML_00796"
     CDS             57638..58900
                     /gene="kgtP_1"
                     /locus_tag="DMACINML_00796"
                     /inference="ab initio prediction:Prodigal:2.6"
                     /inference="similar to AA sequence:RefSeq:CAL35716.1"
                     /codon_start=1
                     /transl_table=11
                     /product="alpha-ketoglutarate permease"
                     /protein_id="Prokka:DMACINML_00796"
                     /translation="MQELTRAKKIRSIVAASSGNLVEWFDFYIYAFTATYFAHTFSTS
                     DNPIVQQINAFGVFAAGFFMRPIGSWLFGSLADKVGRKKSMVVSVVLMALGSFMIAAL
                     PSKDVVGDFAIILLLLARLIQGLSVGGEYGIAATYLSELATEGKRGFYSSFQYVTLIG
                     GQLLAVASISIMLFFFSIDEMKDYAWRILFVVGGILALGSLLVRKVMNESATQIHKHD
                     DRGTLKALFTSSWKQFLMVLGITAGGSLAFYTITTYTKTFMENSGMDKTLVNNLFLGA
                     LFILMIIQPLFGYIGDKIGHKRSLIIFCILAFVGIYPIFNLISSNAQSNSSLVFILVV
                     LLFIILSFYTSVAGIFKAKLFPEHVRALGTGLGYAISNAIFGGSAPWVALQFKNAGME
                     NGFFVYIAVLTLLMFIATLCLPKKSELN"
     gene            complement(58886..59905)
```

```
                     /locus_tag="DMACINML_00797"
     CDS             complement(58886..59905)
                     /locus_tag="DMACINML_00797"
                     /inference="ab initio prediction:Prodigal:2.6"
                     /inference="similar to AA sequence:RefSeq:AHW92684.1"
                     /codon_start=1
                     /transl_table=11
                     /product="A/G-specific adenine glycosylase"
                     /protein_id="Prokka:DMACINML_00797"
                     /translation="MQKQMIQDLQVKLLLWYDKNGRKNLPWRNLQSKDCDERLKDIDR
                     AYGVYISEIMLQQTQVKSVLERFYFPFLQKFPTLQSLAKANEDELLKAWQGLGYYTRA
                     RNLKKSALECVDKFEGKLPKKLDELKKLSGIGAYTAGAIACFAYDQKVSFVDGNIRRV
                     LSRLFALENPKMNELERKAKELLNLEDAFNHNQALLDIGALICVSKNAKCGICPLYDF
                     CQGKFNTELYPKAKKISYENASLNLFIFEWGKKFAIQKSQDKLYKGMYNFPFFKEEEG
                     EISKNMKFLGEFKHNYTKYKLNIKVYHQILKEENKNYQFKSLKELESTALSILSLKAL
                     RLIKL"
     gene            60230..60865
                     /locus_tag="DMACINML_00798"
     CDS             60230..60865
                     /locus_tag="DMACINML_00798"
                     /inference="ab initio prediction:Prodigal:2.6"
                     /inference="similar to AA sequence:RefSeq:AII23819.1"
                     /codon_start=1
                     /transl_table=11
                     /product="hemerythrin family protein"
                     /protein_id="Prokka:DMACINML_00798"
                     /translation="MDHHKLIPLWDERYNINHQKIDTQHQRLFELAAQVENAVFKFVK
                     RDELKEILTELFTYMKEHFHDEEEFMQEIRYPRLYEHKQMHKNIVHEMSQLIQNIKST
                     NDLKEKLYSIMSNWLSRHILSHDVMIIAWVNENLKKDENKTLELNDLPPKQILTQEEN
                     FEFIYSCPCKIDHRLSFKEHESIANEGKIMRCKKCQELLFFIRSQEKTYKG"
     gene            60869..61465
                     /locus_tag="DMACINML_00799"
     CDS             60869..61465
                     /locus_tag="DMACINML_00799"
                     /inference="ab initio prediction:Prodigal:2.6"
                     /inference="similar to AA sequence:RefSeq:CAL35339.1"
                     /codon_start=1
                     /transl_table=11
                     /product="putative iron-binding protein"
                     /protein_id="Prokka:DMACINML_00799"
                     /translation="MLPKWDKIYSVHNGKIDNQHKKLFELAAKVEHIFDKPVHRDEIK
                     ALLTDFFNYMKYHFDDEEKYMQFIRYPDFERHRQIHKEIIQSMIKIIQNIKTTNDLKE
                     KLYIMVKKWLLEHILYEDMRVEQWRIGSLSSENGADITFEEIKDDEEEAVIYFYACEC
                     IGQIHDVPSGIHKKIQNEGAKFKCKKCKFPLEFFKKGY"
     gene            61518..62276
                     /locus_tag="DMACINML_00800"
     CDS             61518..62276
                     /locus_tag="DMACINML_00800"
                     /inference="ab initio prediction:Prodigal:2.6"
                     /inference="similar to AA sequence:RefSeq:CAL35718.1"
                     /codon_start=1
                     /transl_table=11
                     /product="putative periplasmic protein"
                     /protein_id="Prokka:DMACINML_00800"
```

```
                     /translation="MLAFFKGLGIGFLCLVLFIAGVIFNVEFLNKNKTTQTLNFSRSI
                     ETSMEAKPDIFRANINFWANENLSTQTFLSNEDKIQISNTFNQILERSKKENFCSGGS
                     FSLEPNFSYKDGVQTPKGQRLHANLECEFKADKLEDFNKLLNDLNSIIAKSEFIGIST
                     PALEPKFSKQTLKDNKEKLYSELLKKAYSYENIYSLDLNKTCILKNLNIESIPNFYPR
                     ALNSMKSADNMELSLPIIKDEEQSISGTALFICK"
gene                 62335..62409
                     /locus_tag="DMACINML_00801"
tRNA                 62335..62409
                     /locus_tag="DMACINML_00801"
                     /product="tRNA-Gly"
                     /inference="COORDINATES: profile:Aragorn:1.2"
                     /note="tRNA-Gly(gcc)"
gene                 62420..62508
                     /locus_tag="DMACINML_00802"
tRNA                 62420..62508
                     /locus_tag="DMACINML_00802"
                     /product="tRNA-Leu"
                     /inference="COORDINATES: profile:Aragorn:1.2"
                     /note="tRNA-Leu(taa)"
gene                 62530..62603
                     /locus_tag="DMACINML_00803"
tRNA                 62530..62603
                     /locus_tag="DMACINML_00803"
                     /product="tRNA-Cys"
                     /inference="COORDINATES: profile:Aragorn:1.2"
                     /note="tRNA-Cys(gca)"
gene                 62635..62722
                     /locus_tag="DMACINML_00804"
tRNA                 62635..62722
                     /locus_tag="DMACINML_00804"
                     /product="tRNA-Ser"
                     /inference="COORDINATES: profile:Aragorn:1.2"
                     /note="tRNA-Ser(tga)"
gene                 62800..63129
                     /locus_tag="DMACINML_00805"
CDS                  62800..63129
                     /locus_tag="DMACINML_00805"
                     /inference="ab initio prediction:Prodigal:2.6"
                     /codon_start=1
                     /transl_table=11
                     /product="hypothetical protein"
                     /protein_id="Prokka:DMACINML_00805"
                     /translation="MKKFILISSILSSFFVFTQASELPGDEIQPEVKAIKEHFKDKVE
                     KVEFEAWAKGMGLNFSTQKYLNNQNYKKYAKTMAKLIRKTRGVKGKVEICYEGTPQKR
                     VHKCNKF"
sig_peptide          62800..62862
                     /locus_tag="DMACINML_00805"
                     /inference="ab initio prediction:SignalP:4.1"
                     /note="predicted cleavage at residue 21"
gene                 63223..64311
                     /gene="trxB_2"
                     /locus_tag="DMACINML_00806"
CDS                  63223..64311
                     /gene="trxB_2"
```

```
                     /locus_tag="DMACINML_00806"
                     /inference="ab initio prediction:Prodigal:2.6"
                     /inference="similar to AA sequence:RefSeq:CKG86610.1"
                     /codon_start=1
                     /transl_table=11
                     /product="thioredoxin reductase"
                     /protein_id="Prokka:DMACINML_00806"
                     /translation="MFKIVLLISIIFGLNACKGEDMNNNEDTNKDIKTVYAPNAVGID
                     LNTKTNEFLFNQRKKPTGKYTKEYTKALLEAVNIVDNSPYKKFYEPKYLDPEFHTGQK
                     STLIEFKEWQSIYLKDPVKGSLAPWTKAEKAYYNSLDGEGRYNYLVKRSGLVCTPIDL
                     KDSALIRPKRPKEKRFINAYKEGMKDYKEAKRLDYKGYDLFQKAIKNLSYAYEEGKDY
                     KAGLALAELGYSKDYFRAIIGKLDQDENNEVLLDKLINEFLNANYRSIRIYEELIEKY
                     DLGDAYWGLYVYSRKIEDTVFDDRFYFAQLKDSSEELYKNAFEHGAYGAFGAKANTIY
                     SDLIAGEYQLCLGILGNKKAFYEASCIS"
     gene            64289..64981
                     /gene="trxB_3"
                     /locus_tag="DMACINML_00807"
     CDS             64289..64981
                     /gene="trxB_3"
                     /locus_tag="DMACINML_00807"
                     /inference="ab initio prediction:Prodigal:2.6"
                     /inference="similar to AA sequence:RefSeq:CKG86610.1"
                     /codon_start=1
                     /transl_table=11
                     /product="thioredoxin reductase"
                     /protein_id="Prokka:DMACINML_00807"
                     /translation="MKQAVYLSDSGLKSRAFQALWLGVQLGDEKCLEGLYHPLYGIHE
                     NPLKRQLMKDFAKNPPYDKYGMLPFLDELISTEWIIDPNEYNFIYDINNRVVTAMLNG
                     IDKGKYKDPRDVDSTPESRWEFDQEIDAYKEGSVRSYSYDLPNHWSEADVEIYLEELY
                     LHAKLAALTPPQGYPNAPRYYSPERLEFIYEKHKLDAKLDPRIPAIYRANFPEELRAK
                     IRAFAKEHGIEE"
     gene            64983..65963
                     /gene="ribD"
                     /locus_tag="DMACINML_00808"
     CDS             64983..65963
                     /gene="ribD"
                     /locus_tag="DMACINML_00808"
                     /EC_number="3.5.4.-"
                     /inference="ab initio prediction:Prodigal:2.6"
                     /inference="similar to AA sequence:RefSeq:CAL35719.1"
                     /codon_start=1
                     /transl_table=11
                     /product="riboflavin-specific deaminase/reductase"
                     /protein_id="Prokka:DMACINML_00808"
                     /translation="MKNFYMNLALNEAWKYQFLTYPNPAVGCVILDKNEKILAIKAHE
                     KAGCAHAELNAIAHAFTSLNPEISLPQEANAMHEFICKNHRGIFKDSIAFVTLEPCSH
                     QGKTPPCAKLFSELGFKKVFISVKDENEIASKGADFLKKQGVEIEFGVLEDEGKKLLK
                     PFLKWQKGQFKLFKLALSMNGSPFGKIVSNELSRTYAHKIRSVIDLLVLGGETIRKDR
                     PILDARLCKGKAPNLCILSHHNLENFDKSIPLFNVPHRQIYTHIPNKAKFLMYEGGEN
                     FLKNFKDEMDMFLIFQSSSLNDEKNVTMSLNFKPLYRNFLGSDTYGIYEL"
     gene            65944..66465
                     /locus_tag="DMACINML_00809"
     CDS             65944..66465
                     /locus_tag="DMACINML_00809"
```

```
                     /inference="ab initio prediction:Prodigal:2.6"
                     /inference="similar to AA sequence:RefSeq:CAL35720.1"
                     /codon_start=1
                     /transl_table=11
                     /product="putative membrane protein"
                     /protein_id="Prokka:DMACINML_00809"
                     /translation="MAFMSFSGFFYARNDLRLFKIEKKNEIKSFFYKDYTLASFKDEL
                     NLNNEIFFYQSLKENLFKENDEILISNLGKKIILFRNFTQNSDNFAEAKLKQVLLLTF
                     LFLASVFFASLAAINEFGAIDLVFLMICLLLLVMGIINLGLLFKQIRILKSFSKEEMK
                     EFLTQRMKKYTKK"
     gene            complement(66524..67888)
                     /gene="tdcG"
                     /locus_tag="DMACINML_00810"
     CDS             complement(66524..67888)
                     /gene="tdcG"
                     /locus_tag="DMACINML_00810"
                     /EC_number="4.3.1.17"
                     /inference="ab initio prediction:Prodigal:2.6"
                     /inference="similar to AA sequence:RefSeq:AJA55834.1"
                     /codon_start=1
                     /transl_table=11
                     /product="L-serine dehydratase TdcG"
                     /protein_id="Prokka:DMACINML_00810"
                     /translation="MSNLNIFKIGVGPSSSHTLGPMLAGNLFCKKIAKKLEKIEKIQI
                     ILYGSLSLTGKGHLSDKAVIWGLNALEAKNLSAAIQEEVNKNATDKGKINLCGEKELR
                     FDYEKDLIFSKDFLPLHENGMQIQAFNASGEIVDQETYYSIGGGFVLTAAELEKRNKG
                     ISQGRKKKLDIELNNAKQALELCEKRDWDLAELSYRYELQFHTKEEIRSYCLEIWEVM
                     QEVYYNGTHPNEDYLPGKLHLKRRAKGLKERVTMTADPMGIIDFISLYAIAIAEENAS
                     GAKVVTAPTNGACAVIPAVMLYLKNHTIGFNDEKAIEFLLVAMLIGSFYKKNASISGA
                     EAGCQAEIGSASSMAAGAMATVLGANAFKACNAAEMAMEHHLGLTCDPVGGLVQIPCI
                     ERNAFGAIKAISAARMAMTRKSIPIVSLDEVIETMYETGKDMNYKYKETSLGGLATNL
                     KTVC"
     gene            complement(67899..69221)
                     /locus_tag="DMACINML_00811"
     CDS             complement(67899..69221)
                     /locus_tag="DMACINML_00811"
                     /inference="ab initio prediction:Prodigal:2.6"
                     /inference="similar to AA sequence:RefSeq:ABS43832.1"
                     /codon_start=1
                     /transl_table=11
                     /product="serine transporter"
                     /protein_id="Prokka:DMACINML_00811"
                     /translation="MNTPKWTRYDTRWVLSLFGTAVGAGILFLPIKAGVGGFWPVIAM
                     AVIIFPMVYLSHRALSRFVSQANGNDKDITHAAEEYFGRGVSIFISVLYFFAIFPICL
                     AYCVGITNTFESFIYHQFLPLLNSEGVFADIIRAMYEVSADDKGKVVASLFPFYRLLL
                     AFILVSIFMLIMLFSEELITKVCEWLVYPLCAILFIFSLYLIPQWSFESFSAIPGVKE
                     FITIVWLTLPVLVFSFNHSPAISTFSLSVKRQYKEHAVEKADQILFRTSTMLLFFVMF
                     FVISCVLSLSPAELNEARAQNIPVLSYFANKLDNPFISYGGPLIAFLAISSSFFGHYF
                     GAREGAYGIVRKCCKLAGSENPDLKKIAICSTLVMYIIMLITAYINPSILVFIESLGG
                     PIIAAILFLMPIIAIYTVSKMKKFQNKALDAFVFVTGVLTIITVIYTF"
     gene            complement(69491..69907)
                     /locus_tag="DMACINML_00812"
     CDS             complement(69491..69907)
                     /locus_tag="DMACINML_00812"
```

```
                     /inference="ab initio prediction:Prodigal:2.6"
                     /inference="similar to AA sequence:RefSeq:CAL35723.1"
                     /codon_start=1
                     /transl_table=11
                     /product="putative periplasmic protein"
                     /protein_id="Prokka:DMACINML_00812"
                     /translation="MKLKLGLAALLGATVLFARDIIVPASELPRNAQDFISKNFKTAQ
                     IALVKKDIDSYDVTLNDGTEIDFIITGEWKDVDGKYKALPNSILPNIMPKISASSNAQ
                     IIEVSKEINGYKFELSNQLKIYTDAQGNILGQKFDY"
     sig_peptide     complement(69851..69907)
                     /locus_tag="DMACINML_00812"
                     /inference="ab initio prediction:SignalP:4.1"
                     /note="predicted cleavage at residue 19"
     gene            complement(69976..70713)
                     /locus_tag="DMACINML_00813"
     CDS             complement(69976..70713)
                     /locus_tag="DMACINML_00813"
                     /inference="ab initio prediction:Prodigal:2.6"
                     /inference="similar to AA sequence:RefSeq:CAL35724.1"
                     /note="hypothetical protein Cj1627c"
                     /codon_start=1
                     /transl_table=11
                     /product="hypothetical protein"
                     /protein_id="Prokka:DMACINML_00813"
                     /translation="MQENHNFISFVLLDEAKFDFLKLKKILEDDFDIWIADENFTQDG
                     VIFSFKNMLITLNLIKNPIPNNEAEYYANFNFMWKDALEQTQKHKAHLLISVLSQSET
                     KLDQAKLFTQISSVCLQDKHALGFYTGGVVLEPKFYIENAKMLEDNRLPVYNWIYVSV
                     YPGDNGNNAYTYGLRNFDKLELEVCDANIEEKELFFTLYDIILHILTYDLTLKDNDTL
                     KFEDGRILSFKKSQGIGVENESLKIIF"
     gene            70880..71305
                     /gene="exbB-2"
                     /locus_tag="DMACINML_00814"
     CDS             70880..71305
                     /gene="exbB-2"
                     /locus_tag="DMACINML_00814"
                     /inference="ab initio prediction:Prodigal:2.6"
                     /inference="similar to AA sequence:RefSeq:EAQ72747.2"
                     /codon_start=1
                     /transl_table=11
                     /product="TonB system transport protein ExbB"
                     /protein_id="Prokka:DMACINML_00814"
                     /translation="MEFLKDYIDLIIFIILGVMAFIAFWCVIERVLFFRKIDFKNYEN
                     QEQFDDAISENLTTIYIIYSNAPYIGLLGTVVGIMITFYEMGIAGNIDVKSIVVGLSL
                     ALKATALGLLVAIPALMAYNALLRKVSLLSNAYKVSKNA"
     gene            71298..71687
                     /gene="exbD"
                     /locus_tag="DMACINML_00815"
     CDS             71298..71687
                     /gene="exbD"
                     /locus_tag="DMACINML_00815"
                     /inference="ab initio prediction:Prodigal:2.6"
                     /inference="similar to AA sequence:RefSeq:AAW36224.1"
                     /codon_start=1
                     /transl_table=11
```

```
                     /product="biopolymer transport exbD protein"
                     /protein_id="Prokka:DMACINML_00815"
                     /translation="MLKLPKNEGLNIVPFIDIMLVLLAIILSISTFIAHGKIEINLPV
                     SENALSLNNDEKKLSISIDENNIFYLDDKTTDLKGLSDAISSLHKKTIVELKSDKNAK
                     FESFVQIIDLLKAKEHENFQIITEKNR"
     gene            71684..72367
                     /gene="tonB2"
                     /locus_tag="DMACINML_00816"
     CDS             71684..72367
                     /gene="tonB2"
                     /locus_tag="DMACINML_00816"
                     /inference="ab initio prediction:Prodigal:2.6"
                     /inference="similar to AA sequence:RefSeq:CAL35727.1"
                     /codon_start=1
                     /transl_table=11
                     /product="putative TonB transport protein"
                     /protein_id="Prokka:DMACINML_00816"
                     /translation="MKNALSNHKSQAFYISFLFFLPLAFLLFNSANFFKIEFKSEDSF
                     TLAMKQFTQNQPTQTPPIPTNSEPIQHHKPKPIKQPIKKTKKAAENQVKPIQAINPIQ
                     KSTPFIAQTHTTHNQALKNSDAKPTQLTYGKDNHPALEKIQKAIMEQARKNYPRQARK
                     MRMQGVVEVEFLWKENKSLAELKIIQSSGHNLLDKSALESIRKASAFFPYYQSDLRIV
                     LPIVYNLKA"
     gene            complement(72413..73297)
                     /locus_tag="DMACINML_00817"
     CDS             complement(72413..73297)
                     /locus_tag="DMACINML_00817"
                     /inference="ab initio prediction:Prodigal:2.6"
                     /inference="similar to AA sequence:RefSeq:SQE25111.1"
                     /codon_start=1
                     /transl_table=11
                     /product="hypothetical protein"
                     /protein_id="Prokka:DMACINML_00817"
                     /translation="MDKLTINDFNVSLPSSQNITNRKEILELKKEEQEKVKVNDISDN
                     QISGFKVVFENKQGELMALKISDENYKNLQSHFGSFTNYIARDDGTIRLNGEANEFIA
                     NWFEKISSEFFNPNSLNNKEESNHINVKFHKNTLFESMQNLAFKGEDELSLSANLQTR
                     LNFAIEKDVNFDGNINEFDVKKESLTDILRDIKNASGGSADATKITDPQKSDENDEVK
                     IKKKKDEKDVKDLLEIAKEKGLSALSADEQAKLKASNPQIFEELQNKSLGNLEQNLKK
                     DLMVQIGNNEAIFVDKRV"
     gene            complement(73337..73537)
                     /locus_tag="DMACINML_00818"
     CDS             complement(73337..73537)
                     /locus_tag="DMACINML_00818"
                     /inference="ab initio prediction:Prodigal:2.6"
                     /inference="similar to AA sequence:RefSeq:CKH06754.1"
                     /codon_start=1
                     /transl_table=11
                     /product="periplasmic protein"
                     /protein_id="Prokka:DMACINML_00818"
                     /translation="MRKLLFIALVACFVNSFAYGKTENNNKNSQVRNSCVDYEIRLKQ
                     CNSLPTQHKRQACRLGLFAACK"
     sig_peptide     complement(73481..73537)
                     /locus_tag="DMACINML_00818"
                     /inference="ab initio prediction:SignalP:4.1"
                     /note="predicted cleavage at residue 19"
```

```
gene            73670..74653
                /locus_tag="DMACINML_00819"
CDS             73670..74653
                /locus_tag="DMACINML_00819"
                /inference="ab initio prediction:Prodigal:2.6"
                /inference="similar to AA sequence:RefSeq:ABS44159.1"
                /codon_start=1
                /transl_table=11
                /product="thiamine biosynthesis protein:ExsB"
                /protein_id="Prokka:DMACINML_00819"
                /translation="MKALALFSGGLDSMLAMKLITSQGIEVKALNINIGFGSTSDKSE
                IMKKRAAMVGADFEMIDVRNSYLQKVLFNPQYGYGKHFNPCIDCHAFMFKTALSMLKE
                ENASFIITGEVLGQRPMSQRGDAMAKVKKLALDEEDLILRPMCAKNLPLTKPEREGWV
                DREKLEGISGRSRKRQLELAAKFGLEDFESPGGGCLLTLDSFAKKIRDFIEFDKDMQV
                NDAQLLKYGRHLRLPNGAKMIIGRNELENELLRNLKTPKYEVIELGDLIGAYSLLSAN
                ADEKDLELALKIALTYTKHEANKSYELRFKNQNYKSIAFEDKKEINPFFIS"
gene            complement(74656..76308)
                /gene="fhaC"
                /locus_tag="DMACINML_00820"
CDS             complement(74656..76308)
                /gene="fhaC"
                /locus_tag="DMACINML_00820"
                /inference="ab initio prediction:Prodigal:2.6"
                /inference="similar to AA sequence:UniProtKB:P35077"
                /codon_start=1
                /transl_table=11
                /product="Filamentous hemagglutinin transporter protein
                FhaC"
                /protein_id="Prokka:DMACINML_00820"
                /translation="MRFLKIIFLLMSFICLSQADDSAKTLNLLDKRQQQLHIQKEFDE
                LEKKQQQEKDVYLENQDSEDKIYIFKQIQFKKNDTLTMQTNNILKKYINQPLSVSDIY
                NMIKELTNFIVSKGYSTSSVTIDGINQERDILFLDLKYGFVGEVYLNGDNNTTRLDFG
                MPLKKGDKFNIYDLDTGIENLNNGARDVKIAVKASENYGYSDIFINLQSKPPDLILDF
                DNSGYKAKGEYKASAYLSLSNIFNLNDSIRFGFIKGLLKDMSQERENVYVANYTLPIQ
                SYQLSYSMQYSDNINMIEGYDNSFIKNTDTSLRHKIMLKKILHRTSKDKFSIYANLGI
                KDDVNEIDNFRLESSSGRYSSIASGVEYSTLAFGGFLFLNLEYEKGIPFLGSKKDSKD
                SLYKIEFNKVNFNLSYQKSFYANDGLAFLYQNSSGASYSNESLLYADKFLIGDEYTVR
                GFKESSAALDYGAYSNNTIYLQFLNAPKYLRALEPFVGLDMGYGRDYLLPNKDFLAGV
                AFGFRYNLNHLSLNLTASKALHKSSNMPSETIPIYLRASVFF"
sig_peptide     complement(76249..76308)
                /gene="fhaC"
                /locus_tag="DMACINML_00820"
                /inference="ab initio prediction:SignalP:4.1"
                /note="predicted cleavage at residue 20"
gene            complement(76309..81933)
                /gene="fhaB"
                /locus_tag="DMACINML_00821"
CDS             complement(76309..81933)
                /gene="fhaB"
                /locus_tag="DMACINML_00821"
                /inference="ab initio prediction:Prodigal:2.6"
                /inference="similar to AA sequence:UniProtKB:P12255"
                /codon_start=1
                /transl_table=11
```

/product="Filamentous hemagglutinin"
/protein_id="Prokka:DMACINML_00821"
/translation="MEFNKKKIVRLSLNLTLLGALFPCVLFSADLRQRNGNVIVSRTA
DLTQSSNKTDVINIKNPNQNGVSHNQFDEFSVQDGVIFNNSLKDGNSQIGGWVGKNPN
LKQNANTIINEISSKQASSINGAVEVFGKSANLIFANENGFSVNGAAFLNTKGVTLST
GKFNGDFTEVTSNGRVAIGEKGVMVDGDYFNVISRGIDLAGSIAHYQSGKNLSDINFI
AGLNKVDLSTPNAPKIVASKQKDEKIDYGIDGKYLGSMYANTVTLISTEEGVGVRHSG
VIRGIEDVIVKVKDKAEFQAIGVNSNGSVKIDAKDIKTSLINADNIDLKASGKVTNEG
LYKGKQIQINANNFENAKQVNFSQETKDIFDINEENSKIYADNLALNTLDKTSNFGFI
NALNDIKVDTNSFDNQGEISANKDISLTLNDDAFVNNGKILSQKDIQIQANKDLTLNH
GNLYAQNLLHIKSLNDLNINSKLENASSIDLDAKNIYVKNLVASGKELNLHADENLVN
DAYLFSNGDFRAQATTLTNNSTFNSLNNLYLNAHTIHNNALIFADKDLNIEANILNNN
ANLTGNLEILTEYAQGFGQVGYGDTAFKRWNMEVGVTGIVKYNNLLDSKQASIQAGKD
ININTQSQDNARQINNSGKILAQGNIVSFGNIDNKSLSKEISVREVLEQIRVNGFFAE
EYLGSWNTNSTYYFVDRESLLDALKYFSTTQAKDHQRESAWNALKDAAAKNSALNQYF
SLLFGSDYASKRFVPDQKEWNLDAKIVFKAKSEAAIASNGKLDLNAKEYNQDLVSSLD
KNFALVDSDLKKATNSQDLILNSIPDLINSGLFTLENKTNNNITYEYTTNTSIIDESE
YFGFLNILNQFKNKNFDEFIVIGDPFFENQLINAMYANALQSNTKLSEEEIKKLLENG
AKYANENGLNLGQGLAKNQAVDQDMILYVTIKQKGKNIMAPMLYLSKETIANNEIKSN
LTGKTGIDINTQSFNSLLSGITSEGQININAGNEINLQSSNLNAQNINLSANNVNIDT
VLGIDEKGSYNSIQKGEIKANNVVNIDSVNDLNIKNSDISTSANDSKIVLISQKGDVN
IKNDYSKSSSFEQINTDSQKSNTTTSIDGVLSTNIKGANVGIVSNQDVNIIASNINAD
SKIDINANNVNIKDAHELSKTKTDGVYLKPVEISYESANLETSKSISSTLNSKGDINI
NTNGNIAITGSTLQADKSANLNGENITIENGENKISSSSHSSTSSIAGYKQSSVNTDA
SLVSSSQIQTDNLNLNAQKNVSIKGSELNGGEIDIQADKVDFIAAQNKIHTESDSLAF
GVFANANLELTGNGVAVNYSFVQDKANANTTNSSNSITQGGVRSDLLGTAEVGLEFSK
TEKISDELSYVSNTIKGSNININANNALDIGGANFEASKDINLRAGEIASTKYEDTKT
QSTTGFGLYIKERIEATSPIASAINQSAQNAAAKNENLGVNYGIVASQALANTANIFS
NNLINTTSNQTLGFKFNQDKSQSSSENISKINAGGNLNLESTKGSIVLNGVEAKANSI
NIEAKDNVILNAAKSKNSSSSSSLEIAATNKQTAGYHAVDGGTVGEGIEGFVSASHSK
TNETKFTNTNLNANNVNINTGKDFALNGANVKASNDAILNVGGNLEVNSLADSLNSQK
YSALLNVSGTGGLSSNHLVTGSVAGTLGIGYAKTDKTSVTTQSGISAANEINGHVNGN
LNLQGGILNSDSQKGNLLVDGKVTNSKVSVYEKSDGATIRLSGGTNQSFGGVVDIDDH
IDKTGGVNSAVNINININNNKNHGISTDTQNTTDTQDHSWAGGTINLNSSISKIKNGISK
VTGSNSTSNQPSNQPSKSDGPYINESTQTTRL"
gene complement(82188..83276)
/gene="aroC"
/locus_tag="DMACINML_00822"
CDS complement(82188..83276)
/gene="aroC"
/locus_tag="DMACINML_00822"
/EC_number="4.2.3.5"
/inference="ab initio prediction:Prodigal:2.6"
/inference="similar to AA sequence:RefSeq:CAL35731.1"
/codon_start=1
/transl_table=11
/product="chorismate synthase"
/protein_id="Prokka:DMACINML_00822"
/translation="MNTFGTRLKFTSFGESHGAAVGCVIDGMPAGVKFDEDFLQSELD
KRKGGSKFATPRKEGDKVQVLSGVFEGYTTGHPIAMVVFNENAHSKDYDNLKDLFRPA
HADFTYFHKYGIRDHRGGGRASARESVARVAAGAVAAMLLREFDICVQSGVFGVGRFV
SNLKEEELDFEFAKQSEIFCLDPNLEDEFKNEILGARNSKDSVGASVYTRAKGMLVGL
GEVLYDKLDSKLAHALMGINAVKAVEIGEGINASKMRGSQNNDALKGGEFLSNHSGGI
LGGISNGQDLILKTYFKPTPSIFLKQSSMDKFGNDLEFELKGRHDPCVGVRGSVVVSA

```
                     MVRLVLADCLLLHASANLNNLKNAYGVK"
     gene            complement(83276..83950)
                     /gene="rnc"
                     /locus_tag="DMACINML_00823"
     CDS             complement(83276..83950)
                     /gene="rnc"
                     /locus_tag="DMACINML_00823"
                     /EC_number="3.1.26.3"
                     /inference="ab initio prediction:Prodigal:2.6"
                     /inference="similar to AA sequence:RefSeq:EAQ72743.2"
                     /codon_start=1
                     /transl_table=11
                     /product="ribonuclease III"
                     /protein_id="Prokka:DMACINML_00823"
                     /translation="MKNIELLEQKLAYNFKDKDLLIHALTHKSFKKPYNNERLEFLGD
                     AVLDLVVGEYLFHKFAKDAEGDLSKLRAALVNEKSFAKIANSLNLGDFIFMSAAEENN
                     GGKEKPSILSDALEAIIGAIHLEAGFDFAKNIALKLIEKNFPQIDAKILIKDYKTKLQ
                     EITQAKLGQTPQYETVRAFGPDHLKQFEIALILNGQELARAIAGNKKEAQQMAAKITL
                     EKLGAL"
     gene            complement(83937..84377)
                     /gene="rnhA"
                     /locus_tag="DMACINML_00824"
     CDS             complement(83937..84377)
                     /gene="rnhA"
                     /locus_tag="DMACINML_00824"
                     /EC_number="3.1.26.-"
                     /inference="ab initio prediction:Prodigal:2.6"
                     /inference="similar to AA sequence:RefSeq:CAL35733.1"
                     /codon_start=1
                     /transl_table=11
                     /product="ribonuclease HI"
                     /protein_id="Prokka:DMACINML_00824"
                     /translation="MRHIEIYTDGSCLKNPGFGGWAYILRYNQHQKEGFGAEANTTNN
                     RMELKAIIEALKALKEPCEISLFTDSNLMVQSINEWLEAWIKKDFKGKKNVDLWKEYI
                     NMAKPHKIKAYWVKAHNGHLENERCDTLAREAALKIARENDEKY"
     gene            complement(84364..85347)
                     /locus_tag="DMACINML_00825"
     CDS             complement(84364..85347)
                     /locus_tag="DMACINML_00825"
                     /inference="ab initio prediction:Prodigal:2.6"
                     /inference="similar to AA sequence:RefSeq:CAL35734.1"
                     /codon_start=1
                     /transl_table=11
                     /product="putative periplasmic protein"
                     /protein_id="Prokka:DMACINML_00825"
                     /translation="MDFFFVEYRDPLVGLIILTVLIFVVAVANYIWKVFASKDEEQKL
                     EKFIKKFEMDNIHKDLLRNEGLSFGNLSFLAEIFTKSGEFEKATQIYLIALEKSKDKQ
                     EREFIFFALAKVYFKAGFLERAKEVLLQALKIRPRNIQTLKLLKIVYLKLRKHKENLE
                     LLGCLFELGENVKEEKEFLKALDFLASSLSDEEKKEHILKLQIDNNSMLGRLVFEKYH
                     IFLNQDFSSICDLLYKENKAFNLQNKEYFEFFYALGLIEDEKPKDIVFKNSNFKMLKI
                     LKDNSFKARLEFSYRCTECKSVMPLFFYHCPVCYEFNTCQIIYEVKNNETY"
     gene            85418..87244
                     /gene="dnaG"
                     /locus_tag="DMACINML_00826"
```

```
     CDS             85418..87244
                     /gene="dnaG"
                     /locus_tag="DMACINML_00826"
                     /EC_number="2.7.7.-"
                     /inference="ab initio prediction:Prodigal:2.6"
                     /inference="similar to AA sequence:RefSeq:EAQ72538.2"
                     /codon_start=1
                     /transl_table=11
                     /product="DNA primase"
                     /protein_id="Prokka:DMACINML_00826"
                     /translation="MITKESIENLSQRLNIVDIIENYIEVKKQGSSFVCVCPFHADKN
                     PSMHINPTKGFYHCFACKAGGDAFKFVMDYEKLSFADAVEKVANLCNFTLSYTKEKNE
                     NKKELRTILPSLNAYFKSNLKHHKEALDYLYKRMLNDQDIAKFELGFAGSSEDSIRLF
                     HNEKIPLEDAMSVGALKKDENNEFYASFIWRITFPIYDHKDLLVGFGGRTLNPNVAAK
                     YVNSPQNILFDKSRIFYAFNIAKENIAKKKEMIVCEGYMDAIAFHKAGFNNAVAVLGT
                     ALTENHLPLIRRYEAKVILCFDNDEAGLNAATRSAFLLSTHKIDGKVALLEGGKDPAE
                     LVANNQSAKLHTLLEKGMDLGEFYIRRILSSFSLNSALDKQKALENIQKYTFSLEPLV
                     ASSYVSLVSKLLGVDEKLIVLSSNQKSNKTIDFNASFQQTQNHNPKSHITELELLNYL
                     KNNPNTHELFIKIGDIRCFKHRVLLEKILEHKSYEDSDIREFESKNFSKDLSQIEFLW
                     GICKVNLAFLNHINITTPYLALKKQIFTLIEKNFTLLSKNLNDYELIGFLKENLFLLK
                     NEKDEEKLEALFKNLHRFFSAKNLDIKILKNNSEIDYNEEPF"
     gene            87342..87611
                     /locus_tag="DMACINML_00827"
     CDS             87342..87611
                     /locus_tag="DMACINML_00827"
                     /inference="ab initio prediction:Prodigal:2.6"
                     /inference="similar to AA sequence:RefSeq:CAL35736.1"
                     /codon_start=1
                     /transl_table=11
                     /product="NifU protein"
                     /protein_id="Prokka:DMACINML_00827"
                     /translation="MPFSDEELINPVKASLDKSMPMLERDGGGLEFLGIKNGVVYVHL
                     IGACKGCASSGTTLKYGLERQLKIDIHPEITIVNLNGGAEDFAKL"
     gene            87598..88155
                     /locus_tag="DMACINML_00828"
     CDS             87598..88155
                     /locus_tag="DMACINML_00828"
                     /inference="ab initio prediction:Prodigal:2.6"
                     /inference="similar to AA sequence:RefSeq:APA51474.1"
                     /codon_start=1
                     /transl_table=11
                     /product="histidine kinase"
                     /protein_id="Prokka:DMACINML_00828"
                     /translation="MQNYKKLGIEHFLRKDFKTAKIYFSLAYEKRKNKRLFHFITLCD
                     LALKSPKEAFLLFEFYLEHYQIKNIDKDLEEILHTVELKNEKNADEAENEIQEAHALN
                     YKDFLKSEEQIGFKKSFENIIHSTKLVIDNRDDFLDFLEKLLDNGYKDITLNYIESVM
                     PHFWANERFIKLQEKLIGFKSEIKA"
     gene            88139..89422
                     /gene="murE"
                     /locus_tag="DMACINML_00829"
     CDS             88139..89422
                     /gene="murE"
                     /locus_tag="DMACINML_00829"
                     /EC_number="6.3.2.13"
```

```
                     /inference="ab initio prediction:Prodigal:2.6"
                     /inference="similar to AA sequence:RefSeq:CAL35738.1"
                     /codon_start=1
                     /transl_table=11
                     /product="UDP-N-acetylmuramoylalanyl-D-glutamate--2,
                     6-diaminopimelate ligase"
                     /protein_id="Prokka:DMACINML_00829"
                     /translation="MKLKLDHSFITDNTLECEKDCFFLKTAQNMNFSSKALEKGAKII
                     DVEECKKLLKIDENIKIIGITGTNGKTTTAAAIYSILLDLGFKCGLCGTRGAFINDEQ
                     IDEKSLTTSPILKTLEYLQLATQKKCDFFIMEVSSHALVQNRIEGLNFAAKIFTNITQ
                     DHLDFHGNFENYKAAKELFFTDESLKFINKDALMIKFNVRNAFTYGIENPSLYQVKAY
                     SLEDGINAIVALKDQSFHIDSPLLGLFNLYNLLAASACVNELVKPDLKDLEKAISGFG
                     GVCGRVEQVANGVIVDFAHTPDGIEKVLDTLKNKKLIVVFGAGGDRDKTKRPLMGKIV
                     EHFAKIAIITSDNPRSEEPKDIMNEILSGFQNPDKALMIEDRKEAINKALKLKEKDDL
                     VVILGKGDENTQEIKGVKYPFSDKAIVNEILKNQG"
     gene            89426..89731
                     /locus_tag="DMACINML_00830"
     CDS             89426..89731
                     /locus_tag="DMACINML_00830"
                     /inference="ab initio prediction:Prodigal:2.6"
                     /inference="similar to AA sequence:RefSeq:CAL35739.1"
                     /note="conserved hypothetical protein Cj1642"
                     /codon_start=1
                     /transl_table=11
                     /product="hypothetical protein"
                     /protein_id="Prokka:DMACINML_00830"
                     /translation="MFENMDFSKMGELLNQVQEKAKNIELELANREFSAKSGAGLVKV
                     SANGKGEIIDVSIDDSLLEDKESLQILLISAINDVLAMVAQNRTSLASDVLGGFGKL"
     gene            89728..90819
                     /locus_tag="DMACINML_00831"
     CDS             89728..90819
                     /locus_tag="DMACINML_00831"
                     /inference="ab initio prediction:Prodigal:2.6"
                     /inference="similar to AA sequence:RefSeq:CAL35740.1"
                     /codon_start=1
                     /transl_table=11
                     /product="putative periplasmic protein"
                     /protein_id="Prokka:DMACINML_00831"
                     /translation="MKFLFFLVAFLVSLSAIERPKFEDFAAGYERNKASMLNYEGMPA
                     FALSENLLAVLKQPNAKLNKYVKYDPFLNLYLVRTDFSLIPTPMGDEEKLTRNDWIGI
                     WDPNRAYIGHIKYLAQNIDEKDQLDFSTKVGLLGTPCCEMMGIALNNSSFIGNRYLKH
                     FMKYNDVYWGDIGVDFAVRENKIYVNNVRKSGQFLINDEIISVDGTPFKDIRKLNEKI
                     LFADRGSTLYFQVLRDNVDLNISTEVFAKDLSKFNLPSNKPKPKPTSFRSNIGLSVNS
                     SLVVTRVDAGSKAALAGFMVGDKILRANNVILKDFKELQTLLGTGNDFNILIERKSDK
                     LPLSGLNNDLGGVHSGGDGKFQFFIRLVK"
     gene            90816..91655
                     /gene="ispA"
                     /locus_tag="DMACINML_00832"
     CDS             90816..91655
                     /gene="ispA"
                     /locus_tag="DMACINML_00832"
                     /EC_number="2.5.1.10"
                     /inference="ab initio prediction:Prodigal:2.6"
                     /inference="similar to AA sequence:RefSeq:CAL35741.1"
```

```
                     /codon_start=1
                     /transl_table=11
                     /product="geranyltranstransferase"
                     /protein_id="Prokka:DMACINML_00832"
                     /translation="MKELFIKHLENHLPKVQSFHPFFNEALAVMLRAGGKHFRAQLLL
                     GIVEAKMPSLIPNALNAALALEFIHTYSLIHDDLPAMDNADFRRGTPTLHKTYDETTA
                     ILVGDALNTEAFLLLSNLNLKESIKINLIRTLAFNAGLNGMILGQAIDCYFEDKKLNL
                     EELEFLHIHKTARLIAAALKMGCEICELDEKESEKIYQIGLKLGLIFQINDDIIDATM
                     SEEESGKPTKHDEHKNSFVNLLGLKNAIQSKEDLINECKNDLNDLDANLAKMIKSLMI
                     KYL"
     gene            91665..93563
                     /gene="tkt"
                     /locus_tag="DMACINML_00833"
     CDS             91665..93563
                     /gene="tkt"
                     /locus_tag="DMACINML_00833"
                     /EC_number="2.2.1.1"
                     /inference="ab initio prediction:Prodigal:2.6"
                     /inference="similar to AA sequence:RefSeq:CAL35742.1"
                     /codon_start=1
                     /transl_table=11
                     /product="transketolase"
                     /protein_id="Prokka:DMACINML_00833"
                     /translation="MDLSILQEQANTLRFLSADMVQKANSGHPGAPLGMADILTALSY
                     HLSHNPKNPTWLNRDRLIFSGGHASALLYSFLHLSGYDLSLEDLKNFRQLHSKTPGHP
                     EISTQGVEIATGPLGQGIANAVGFAMAAKKAQNLLGKELINHKVYCLCGDGDLQEGIS
                     YEACSLAGLHKLDNLIIIYDSNEISIEGDVSLAFNEDVKMRFESQGFEVLSINGHDYE
                     EIDKALKQAKEANKPCLIIAKTTIAKGAGELEGSHKSHGAPLGEEVIKKAKEKAGFDP
                     NLSFHIPKAAKIRFESAVELGDLEEAKWKDKLEKSEKKELLEKLLNPDFTKINYPDFK
                     GKDLATRDSNGEILNALAKDLEGFLGGSADLAPSNKTELYGMGDFVEGKNIHFGIREH
                     AMAAINNAFARYGIFLPFSATFFIFSEYLKPAARIAALMKIKHFFIFTHDSIGVGEDG
                     PTHQPIEQLSTFRAMPNFLTFRPADGVENVKAWQIALNADIPSAFVLSRQKLKALNEP
                     VFGDVKNGAYLLKESKDAKFTLLASGSEVSLCLESANELEKQGFACNVISMPCFELFE
                     KQDKAYKDRLLQGEVIGVEAAHSNELYKFCDRVYGIESFGESGKDKDVFKHFGFSTPQ
                     ILNFILDK"
     gene            93566..94681
                     /gene="iamB"
                     /locus_tag="DMACINML_00834"
     CDS             93566..94681
                     /gene="iamB"
                     /locus_tag="DMACINML_00834"
                     /inference="ab initio prediction:Prodigal:2.6"
                     /inference="similar to AA sequence:RefSeq:CAL35743.1"
                     /codon_start=1
                     /transl_table=11
                     /product="putative ABC transport system permease"
                     /protein_id="Prokka:DMACINML_00834"
                     /translation="MEANFKFQKEENKVFIFGIWDKTSVSKIKIKDLLSQISQTNCIF
                     DFSQLNEIDMAGVRFFLALEHDLKKNNILILRQGLNTRFKSLFELCEKNYQRIDEKQE
                     KKFNFTDFFISLGILSFDLLKILQKFISFTGAFFTSLFLCVKNPKNFRFISFLYHIEN
                     SAFKALPIVILTALLVGVVLAYQAAYQLAQFGANIFIVDLVGISATRELAPLIAAIVI
                     AGRSASSYTAQIGVMKITDEINAMNTMGFRSFEFIIIPRVMALVIAMPLIVALSDAIS
                     ILGGMVIAKINLDISFGEFLRRFREAVAMKHIIIGLVKAPIFGFLIGLIACFRGFEVK
                     NTTQSIGIYTTKSVVNAIFWVIAFDALFSVVLTIMEV"
```

```
gene            94682..95404
                /gene="iamA"
                /locus_tag="DMACINML_00835"
CDS             94682..95404
                /gene="iamA"
                /locus_tag="DMACINML_00835"
                /inference="ab initio prediction:Prodigal:2.6"
                /inference="similar to AA sequence:RefSeq:CAL35744.1"
                /codon_start=1
                /transl_table=11
                /product="putative ABC transport system ATP-binding
                protein"
                /protein_id="Prokka:DMACINML_00835"
                /translation="MIIKAQNIITRFGNKVVHDGVSFEINKNEIFGILGGSGSGKSVL
                LKQMLMLEHFDEGEYEILGFKLKNISEEDALALRKKWGVVFQFSALFSFFSVYENIAI
                PLKEYTNLDPIAIKELVLMKLKMVGLNESVLKQLPSELSGGMQKRVAIARALALDSKL
                LFLDEPTSGLDPHSSREFDDLILELKQSFDLNIVLVTHDKESMKNLLERFIILEDKKV
                AFCGNYEELKAQNERLFKRFME"
gene            95407..96300
                /locus_tag="DMACINML_00836"
CDS             95407..96300
                /locus_tag="DMACINML_00836"
                /inference="ab initio prediction:Prodigal:2.6"
                /inference="similar to AA sequence:RefSeq:CAL35745.1"
                /codon_start=1
                /transl_table=11
                /product="putative ABC transport system periplasmic
                substrate-binding protein"
                /protein_id="Prokka:DMACINML_00836"
                /translation="MENKANYFFVGLFVFGVFFASIGFMLWLGGYSKEESFEYYEIHT
                QESVAGLGIKAPVRLLGVEIGNVEDISIYDKENLGVNILIKIKKGTPIKEDTFATLQL
                QGITGLKFVQLQGGSKNSPKLQAKDKEEFPVIQFKESFFAAIDRQSEHIFSLIKRADD
                KSKELFSDKNLKNLEILLNNLAQLSATLNENSKTLNHNIANTSLKLGEMAENVSLSAK
                GFNASLKDIQEGALALSSFAKKANAKLDSYDDLRLSLDENLELLKKVLLESNILIQNL
                QKSPSDLIFKETKPKLGPGEK"
gene            96300..96902
                /locus_tag="DMACINML_00837"
CDS             96300..96902
                /locus_tag="DMACINML_00837"
                /inference="ab initio prediction:Prodigal:2.6"
                /inference="similar to AA sequence:RefSeq:CAL35746.1"
                /codon_start=1
                /transl_table=11
                /product="putative lipoprotein"
                /protein_id="Prokka:DMACINML_00837"
                /translation="MKKFILIAILALIFNACSIPTSTLDKTQSFILKNDGIPLQANFI
                KSDKILKIQNASLPLYLHSRSIIYIQKGVSGAYAYHYWADLPNNLYQSLLLSKFEQSQ
                IFKTLLSQGSSIGSDYILESRIDSFEQVINENENYISISISVNFIDAKESKIIAHRLF
                SVKEELDKIDIFNVYQSFEKALNFIGNEIVLWINTTLDEK"
sig_peptide     96300..96356
                /locus_tag="DMACINML_00837"
                /inference="ab initio prediction:SignalP:4.1"
                /note="predicted cleavage at residue 19"
gene            96954..97454
```

```
                     /locus_tag="DMACINML_00838"
     CDS             96954..97454
                     /locus_tag="DMACINML_00838"
                     /inference="ab initio prediction:Prodigal:2.6"
                     /inference="similar to AA sequence:RefSeq:CAL35747.1"
                     /note="hypothetical protein Cj1650"
                     /codon_start=1
                     /transl_table=11
                     /product="hypothetical protein"
                     /protein_id="Prokka:DMACINML_00838"
                     /translation="MKNELDVFKKHLGEIQGVSEFKATQICSQINDANDFIGALQILD
                     LTLKKIEKSITERLDEQNDDLQKRTLDATSSQLIQNCSFMGTALFGNIFNVYVGQKLF
                     EFEISNPLLVLEKAGYEGVLAYVQDKRDEIKDILCELSSAITMGIGMDNLNLSNSTTD
                     FKNLFR"
     gene            complement(97786..98544)
                     /gene="map"
                     /locus_tag="DMACINML_00839"
     CDS             complement(97786..98544)
                     /gene="map"
                     /locus_tag="DMACINML_00839"
                     /EC_number="3.4.11.18"
                     /inference="ab initio prediction:Prodigal:2.6"
                     /inference="similar to AA sequence:RefSeq:CAL35748.1"
                     /codon_start=1
                     /transl_table=11
                     /product="methionine aminopeptidase"
                     /protein_id="Prokka:DMACINML_00839"
                     /translation="MIELKKPAEIEKLRIANQIVAKTLDFLENEIKAGMSLKQIDKMA
                     EDFILSLGAKPSFKGLYGFPGAICTSLNQVCIHGIPDEKIIKEGDILGLDVGSLIDGY
                     YGDAARTIAIGKISSTDEALISCAKDALYHAIDIIREGMRFKELSAALGEFIDSRGFV
                     PLRGYCGHGIGRKPHGEPEILNYLEKGANAKSGPKIKNGMVFCIEPMICQKDGTPKHF
                     NGKWDAGSVDELNSAHYEHCVAIINGRAEILSTL"
     gene            complement(98554..99297)
                     /gene="murI"
                     /locus_tag="DMACINML_00840"
     CDS             complement(98554..99297)
                     /gene="murI"
                     /locus_tag="DMACINML_00840"
                     /EC_number="5.1.1.3"
                     /inference="ab initio prediction:Prodigal:2.6"
                     /inference="similar to AA sequence:RefSeq:CAL35749.1"
                     /codon_start=1
                     /transl_table=11
                     /product="glutamate racemase"
                     /protein_id="Prokka:DMACINML_00840"
                     /translation="MKIGVFDSGVGGLSVLKSLYEARLFDEIIYYGDTARVPYGVKDK
                     DTIVKFCLEALDFFSKFNIDMLIIACNTASAYALDALRSRADFPIYGVIDAGVEATKK
                     ALLDKNKKILVIATKATIHSKEYQNRLQEQGFTNVDALATGLFVPMVEEGIFEGEFLQ
                     SAMRHYFKGIETPNALILACTHFPLLANSLSAYFGSKTKLIHSGDAIVEFLKQRQKLL
                     LKEKKAKLHFYASSDVKSLENTAKIWLNL"
     gene            complement(99299..99760)
                     /gene="nlpC"
                     /locus_tag="DMACINML_00841"
     CDS             complement(99299..99760)
```

```
                     /gene="nlpC"
                     /locus_tag="DMACINML_00841"
                     /inference="ab initio prediction:Prodigal:2.6"
                     /inference="similar to AA sequence:RefSeq:EAQ72731.1"
                     /codon_start=1
                     /transl_table=11
                     /product="lipoprotein NlpC"
                     /protein_id="Prokka:DMACINML_00841"
                     /translation="MRFIFFVIMVAFCIGGCSLSQNTSHTKINYNVDKETRLRAIANE
                     WKKTPYVLGGSTKRGADCSGFAQSTLAQFNTHIPRTTTKQLRSGIKVSKSKLKTGDLV
                     FFKTGRGPNGMHVGIYMSKGKFIHLSTKGGVREVDLNSSYWKNRYIGARRY"
     sig_peptide     complement(99692..99760)
                     /gene="nlpC"
                     /locus_tag="DMACINML_00841"
                     /inference="ab initio prediction:SignalP:4.1"
                     /note="predicted cleavage at residue 23"
     gene            complement(99769..100938)
                     /gene="nhaA2"
                     /locus_tag="DMACINML_00842"
     CDS             complement(99769..100938)
                     /gene="nhaA2"
                     /locus_tag="DMACINML_00842"
                     /inference="ab initio prediction:Prodigal:2.6"
                     /inference="similar to AA sequence:RefSeq:CAL35751.1"
                     /codon_start=1
                     /transl_table=11
                     /product="Na(+)/H(+) antiporter"
                     /protein_id="Prokka:DMACINML_00842"
                     /translation="MNKVLLKLKALVLNEAFGGFLLIICTILALLVQNGSLSEHYREL
                     LNLKVGVSVGEFELNKPFLLWINDGLISIFFFAIGLELKKEFLHGDFKNPKNIVLPFM
                     GALGGILIPALIFAIVNIGDAYTLKGWAIPTATDTAFALAILMMCGKHIPSSLKLFLL
                     SLAIFDDVGAILIIAIFYTTKLSIFAFIVAGIAIFMMLILNILGITRKSFYFICSIIL
                     WVSVLKSGVHATLAGIITAFFIPMQTKNGEDFLEGIYESLKFWIAFVILPLFAFANAG
                     VNLSNIDLNAIFSGVSIGIFLGLFIGKQLGVFLFSYFAIKFKLASLPQNANLKQLYGV
                     CILTGIGFTMSLFIDGLAYEVSDIFNYADNLAILIASFCSGIWGFIYLKFFTARS"
     gene            complement(100935..102083)
                     /gene="nhaA1"
                     /locus_tag="DMACINML_00843"
     CDS             complement(100935..102083)
                     /gene="nhaA1"
                     /locus_tag="DMACINML_00843"
                     /inference="ab initio prediction:Prodigal:2.6"
                     /inference="similar to AA sequence:RefSeq:CAL35752.1"
                     /codon_start=1
                     /transl_table=11
                     /product="Na(+)/H(+) antiporter"
                     /protein_id="Prokka:DMACINML_00843"
                     /translation="MQAVKKIILSETFPGILLIFFTLLALLFKNSSLSVIYTDFFHAN
                     FTIGFDHFQIAKSLDLWINDGLIAIFFLCIGLELKYEILRGQLKNIRSVSLPIFGALG
                     GMIVPALIFSAINYSHEFAMKGWAIPTATDIAFAVGILMLLGSKIPSSLKLFLLSLAI
                     FDDLGAIIIIALFYTDQLSTLAIIVCLLCVFILFVLNYFHVTHLPLYVLVGVVLWIAM
                     LKSGVHATLAGVIIALFIPLDTKNKKPYLHEVHKDLNPWVVYFILPLFAFANAGIDLR
                     DMHFESIVSPVSMGIMLGLFLGKQLGVFGFCYLAIKLKLAKLPENIKYKKFYGICILT
                     GIGFTMSLFIDGLAYKNSNIFEHADKLAILIASFLSAIVGFIYLKIVK"
```

```
     gene            complement(102207..102389)
                     /locus_tag="DMACINML_00844"
     CDS             complement(102207..102389)
                     /locus_tag="DMACINML_00844"
                     /inference="ab initio prediction:Prodigal:2.6"
                     /inference="similar to AA sequence:RefSeq:CAL35753.1"
                     /note="hypothetical protein Cj1656c"
                     /codon_start=1
                     /transl_table=11
                     /product="hypothetical protein"
                     /protein_id="Prokka:DMACINML_00844"
                     /translation="MISDATMSNANLLTAVNTSVLKKSMDTNEVLMNELIKGMESASG
                     ASAPQASSSSGLDIYA"
     gene            102562..104634
                     /locus_tag="DMACINML_00845"
     CDS             102562..104634
                     /locus_tag="DMACINML_00845"
                     /inference="ab initio prediction:Prodigal:2.6"
                     /inference="similar to AA sequence:RefSeq:CAL35754.1"
                     /codon_start=1
                     /transl_table=11
                     /product="putative iron permease"
                     /protein_id="Prokka:DMACINML_00845"
                     /translation="MKFFKILFVLSMLFLANSAFARVDDYIAEANTIKNILEESIKSY
                     KKGDNLNAKKLSEDAYFQHFENMEGSIGRNIGKKAITMERKFTNLRRLYKDKAPIEQI
                     DALIESLYYDLDEVAPILQNGYRLKAEASDTNYDKAAAEKSSMQANAKREAEAEALIA
                     QMMGIDKNDLAKTNPSSFDTQNETIDDSVSSDLQAAAAMDARLQFILDNISTKFSQAA
                     NAFKNKDYQASKDFLNDALFNDYRNTKVEVLINKFTKAGNDQKIQQAIRTLIRQINNT
                     NIDEKNLRNDLDSIEEQLFDIFLQIPNSELSNLKIEGFNDETKGKDYTKVSDDIKLAL
                     DEILKNYEGFSASIVDDLQAIYLDIFEASGMENKIGAVDSALKLKIESLFSKSVALIK
                     ASADKKELEATFNDLEQLIASSVDKIQDSSPYSLFIWALGIILREGLEALIIVVAIVS
                     YLVQSGNKNRLNIAYSALFTGIILSFITAFGLSWLFKENAGQSRELIEGITMLIAVFL
                     LFYVGFWLLSNAQNKKWVSFIKQGAIDAISNNSAKTLWFTVFLAVYREGAETVLFYQA
                     LFFDAKTSTDLSAIFGGLGLGILVLIVLYFLLKAGAIRIPIKQFFYICSYIIFYMIFV
                     FTGKGIAELIEGKLIVPSLISMNFEPILWLGIYPYYETLIPQFIVLILLIIGILLTKQ
                     ISKKGVKS"
     sig_peptide     102562..102627
                     /locus_tag="DMACINML_00845"
                     /inference="ab initio prediction:SignalP:4.1"
                     /note="predicted cleavage at residue 22"
     gene            104631..105170
                     /gene="p19"
                     /locus_tag="DMACINML_00846"
     CDS             104631..105170
                     /gene="p19"
                     /locus_tag="DMACINML_00846"
                     /inference="ab initio prediction:Prodigal:2.6"
                     /inference="similar to AA sequence:RefSeq:CAL35755.1"
                     /codon_start=1
                     /transl_table=11
                     /product="periplasmic protein p19"
                     /protein_id="Prokka:DMACINML_00846"
                     /translation="MIKKFLSVVAAAAAISTNLFAGEVPIGDPKELNGMEIAAVYLQP
                     IEMEPRGIDLAASLADIHLEADIHALKNNPNGFPEGFWMPYLTIAYELKNTDTGAIKR
```

```
                     GTLMPMVADDGPHYGANIAMEKDKKGGFGVGNYELTFYISNPEKQGFGRHVDEETGVG
                     KWFEPFKVDYKFKYTGTPK"
     sig_peptide     104631..104696
                     /gene="p19"
                     /locus_tag="DMACINML_00846"
                     /inference="ab initio prediction:SignalP:4.1"
                     /note="predicted cleavage at residue 22"
     gene            105252..106655
                     /locus_tag="DMACINML_00847"
     CDS             105252..106655
                     /locus_tag="DMACINML_00847"
                     /inference="ab initio prediction:Prodigal:2.6"
                     /inference="similar to AA sequence:RefSeq:CAL35756.1"
                     /codon_start=1
                     /transl_table=11
                     /product="putative integral membrane protein"
                     /protein_id="Prokka:DMACINML_00847"
                     /translation="MSIYFVHFLSSILPLSILMAFITPNKKYIFKSFLAVFLGFLFGY
                     FAFFIAAQFLKTENLIFNFNFIFIACLLLSFVLYFWQKIEILSFILLGILSFCIALHY
                     YNLTQDFPIFNGVLIDSEAISSLGFIALALLICILIFFFLKWQKNINKKASFVLFLIL
                     IFIEGDKVLANILLTLMRNSFIETHTFLVSFVGKSNYFGVFGIYVYLFFIIFLAFLSL
                     QIRKKNIVKKQILDIAYRKNEAKNTLINRYFSSVFISCILSFCIILYFFMVSSKPLSI
                     DEPTELLPDKNGKFIFDVALLRDNKLHRFAYISEQGKVIRFFLINKREDRDSPVAVFD
                     ACAICGDMGYIKKEGELICISCNVRIFLPSVGKTGGCNPIPLKYDYDGEKITIDVKDV
                     VAGSNYFSQIKDIQVQDPVSKDKIINTQAPFSYSYKGITYYFSNEKNYEEFKKDPMKY
                     VEDTEALFLIQRRNNAS"
     gene            106645..107940
                     /locus_tag="DMACINML_00848"
     CDS             106645..107940
                     /locus_tag="DMACINML_00848"
                     /inference="ab initio prediction:Prodigal:2.6"
                     /inference="similar to AA sequence:RefSeq:CAL35757.1"
                     /codon_start=1
                     /transl_table=11
                     /product="putative ABC transport system permease"
                     /protein_id="Prokka:DMACINML_00848"
                     /translation="MLVKMITHSIFRNKIQKFLAFLTCFLATLLLCTMLNITLSIGDE
                     VTKQLKSYGSNILVLPKGSSLSIEIGNELYEPLKNKNYLEEKNLYMLKDIYWRNNITA
                     FAPFLEGKVEIKSNDSSKKALLYGTYFQKAIEIKDDDDFITGIKSLYPYLKVQGEWAK
                     DDSNEIMLGEDFAKNNDLKINDTIKLIGENKISKEVKVVGILLHANPKMSNKIIAPLN
                     LAQELLEKKGLYASAEVRAFTIPESALSEKVRRMGEEKLDQIEYDKWYCSAYVGSIAS
                     QISDGLPGADAKALNAISDAQSLVVKKIQSLMAITCIICLIVASIAISSLMSSEIHRR
                     KKEIGLLKVLGANTFQIYLLFAGENLIIALVSAVFGFIFGIGLSELISLGIFGYFIDI
                     AFIALPLSLVFAGLIAILGCLLPIKNIANLSPAGVLYGR"
     gene            107930..109048
                     /locus_tag="DMACINML_00849"
     CDS             107930..109048
                     /locus_tag="DMACINML_00849"
                     /inference="ab initio prediction:Prodigal:2.6"
                     /inference="similar to AA sequence:RefSeq:CAL35758.1"
                     /codon_start=1
                     /transl_table=11
                     /product="putative integral membrane protein"
                     /protein_id="Prokka:DMACINML_00849"
```

```
                     /translation="MVDKFFLNELFKSIAFSYQRLFIIVLSVFIGALTCSAFLNIYFD
                     IDTKLSKELKAYGANVIIEPKNDKELILNQDYEEAKKNLKARALTPFLYTFLNLGSTS
                     GVVLGTDFRALKITKPFIEVKEGSFSLSDFDENSAFLGINLSKQLGLKAGNELQIYNP
                     ENGKTTKLTIKGILLSNDELDSIVLAPLSVVQNLSDTFGIQYANAVVYGNFDEINTKT
                     KNISNESINAKPISSVSLSEGLVLDKIKALMFLIILVVLIIVTTSVNTTLSSIIFSRK
                     KEIALRLALGAKKSEIFKLFACEYFIISLFASIIGAFCGIFLANIFGYLIFNASIDFR
                     FKAVFMALIISLFFAFLAAFFPIKKALKINVCENLKGE"
     gene            109048..109695
                     /locus_tag="DMACINML_00850"
     CDS             109048..109695
                     /locus_tag="DMACINML_00850"
                     /inference="ab initio prediction:Prodigal:2.6"
                     /inference="similar to AA sequence:RefSeq:CAL35759.1"
                     /codon_start=1
                     /transl_table=11
                     /product="putative ABC transport system ATP-binding
                     protein"
                     /protein_id="Prokka:DMACINML_00850"
                     /translation="MKELIQIKNLSKEFGKVKALDNINLNIYKGEWLAIMGPSGSGKS
                     TLLNILSLMDNPSSGKYILDNEDLEQINEEQKITLRREKIGLIFQQFHLIPYLSALEN
                     VMLSQYYHSSVDEEDAKAVLEKVGLSHRLSHLPSQLSGGEQQRVCIARALINNPEILL
                     ADEPTGNLDEANEKIVLETLQKLKNEGKTIVLITHNPELAKFADRTLILQHGVLK"
     gene            109692..110180
                     /locus_tag="DMACINML_00851"
     CDS             109692..110180
                     /locus_tag="DMACINML_00851"
                     /inference="ab initio prediction:Prodigal:2.6"
                     /inference="similar to AA sequence:RefSeq:CAL35760.1"
                     /codon_start=1
                     /transl_table=11
                     /product="putative periplasmic thioredoxin"
                     /protein_id="Prokka:DMACINML_00851"
                     /translation="MKKVFLFFISLFLLNACSFDTNQNQGKIGQIGAEISAKDTSGKA
                     VKLADDKSDIKVLVFFQNGCPSCLQELPSLDKFMQEHPNKISVYAINSVDKAEVVQVL
                     AEQLNFKNIKVLTDDLKITNDRYAVFSTPTTIILKDGIIKERILGEKPWQTFESKLIS
                     LL"
     sig_peptide     109692..109748
                     /locus_tag="DMACINML_00851"
                     /inference="ab initio prediction:SignalP:4.1"
                     /note="predicted cleavage at residue 19"
     gene            110141..110644
                     /locus_tag="DMACINML_00852"
     CDS             110141..110644
                     /locus_tag="DMACINML_00852"
                     /inference="ab initio prediction:Prodigal:2.6"
                     /inference="similar to AA sequence:RefSeq:APA81931.1"
                     /codon_start=1
                     /transl_table=11
                     /product="putative lipoprotein thioredoxin"
                     /protein_id="Prokka:DMACINML_00852"
                     /translation="MANFRIQTYLFALITALFFIACDSGENFKALNSDKIYNFSYNGF
                     EKSLKLDEQTQNFALVFFTKDCGVCKEQIPILQDLAKNYDFNIFVVLGDAKDAKDAKA
                     WADEKGLSHLALFYERKAAKYLSGAVGEIYGVPVLSFFKESKMDEKFIGLTPYNVLEN
                     EIKKLKI"
```

```
gene            complement(110746..111792)
                /locus_tag="DMACINML_00853"
CDS             complement(110746..111792)
                /locus_tag="DMACINML_00853"
                /inference="ab initio prediction:Prodigal:2.6"
                /inference="similar to AA sequence:RefSeq:EAQ71968.1"
                /codon_start=1
                /transl_table=11
                /product="GTP-binding protein, GTP1/Obg family"
                /protein_id="Prokka:DMACINML_00853"
                /translation="MFIDSVKITLASGDGGKGAVSFRREKHVPLGGPDGGDGGNGGDI
                IFVCDNNTHTLVNFKGKRELRAQNGAGGMGRNKNGKKGENLELIVPEGTQVIDAQTNE
                VLLDLTKEGQRELFLKGGKGGLGNTHFKHATNQRPDYAQPGIKGESRLVRLELKLIAD
                VGLVGFPNVGKSTLISVVSNAKPEIANYEFTTLTPKLGLVDVDEYNSFVMADIPGIIE
                GASGGKGLGLAFLKHIERTSFLLFVLDPMREMPLKEQFIVLRNELEKFSNELFGREFG
                IMISKSDSVNLGEDFAEQITQNIANLEEYLKSIDNPQSFLIKVSSLEKTGLKELKFML
                LEEIKKLREKPEKR"
gene            complement(111894..112148)
                /gene="rpmA"
                /locus_tag="DMACINML_00854"
CDS             complement(111894..112148)
                /gene="rpmA"
                /locus_tag="DMACINML_00854"
                /inference="ab initio prediction:Prodigal:2.6"
                /inference="similar to AA sequence:RefSeq:AJA54214.1"
                /codon_start=1
                /transl_table=11
                /product="50S ribosomal protein L27"
                /protein_id="Prokka:DMACINML_00854"
                /translation="MAHKKGQGSTQNNRDSIGRRLGVKKFGGEFVRAGNIIIRQRGTA
                THAGNNVGIGKDHTIFALIDGFVKFERKDKDRKKVSVYPA"
gene            complement(112159..112467)
                /gene="rplU"
                /locus_tag="DMACINML_00855"
CDS             complement(112159..112467)
                /gene="rplU"
                /locus_tag="DMACINML_00855"
                /inference="ab initio prediction:Prodigal:2.6"
                /inference="similar to AA sequence:RefSeq:CAL34265.1"
                /codon_start=1
                /transl_table=11
                /product="50S ribosomal protein L21"
                /protein_id="Prokka:DMACINML_00855"
                /translation="MYAIIKHSGKQYKVSVGDELKLDHFEAESKANIEVSEVLAINDK
                ELKVGAPFVAGAKVVLEVINHGKDKKVVIYKKRRRKDSKLKRGFRRQFTRVVVKDIKA
                "
gene            complement(112625..113611)
                /locus_tag="DMACINML_00856"
CDS             complement(112625..113611)
                /locus_tag="DMACINML_00856"
                /inference="ab initio prediction:Prodigal:2.6"
                /inference="similar to AA sequence:RefSeq:CAL34264.1"
                /codon_start=1
                /transl_table=11
```

```
                     /product="putative periplasmic protein"
                     /protein_id="Prokka:DMACINML_00856"
                     /translation="MKKIFLFLTILSFMWAQDDVIIYDYVPLEDVNLSSLDPVQSLKT
                     LPHQRNKFQKVVQGNGTGESKEEALNNALIDALSQFKGVSSVNLRQEIQSVDLNFSHL
                     GQISQKSKSILQNVSKGRIDTYKVDKIDQNGTLWQVEISAFKSLYQNDNKPKLAILND
                     KNKEETYLKRALYDAFSQDENFTLLEREHDFKDENKIIKSEDSSSEESYKLGNVLGAD
                     YILEFEILDITQAKQSKISYQNNTKASINIAYKLIFYPTRELVFSKTFNTTFTLSGDS
                     KKEQKNWENIALFMQREMEKSLFFDTLEEDNKQEGKENTYKLHENGGVNLGF"
     gene            complement(113611..114234)
                     /locus_tag="DMACINML_00857"
     CDS             complement(113611..114234)
                     /locus_tag="DMACINML_00857"
                     /inference="ab initio prediction:Prodigal:2.6"
                     /inference="similar to AA sequence:RefSeq:CAL34262.1"
                     /codon_start=1
                     /transl_table=11
                     /product="putative lipoprotein"
                     /protein_id="Prokka:DMACINML_00857"
                     /translation="MKKNIFIIFSAFFAVFFAACGAPVYTDGYASQKKQGDALTLGLD
                     REDFEKTAEIMIDSMLNDPAFANIQPGQRKVIAIGKIINDTPQRIDTDKLISKITIAL
                     RKSGKFVLTTAVAAGGARDEMSEAVRELRDNDEFNQNTISSKGTLVAPDFSISGKIRQ
                     DNVKLKNGDIQAEYFFYLSVTDLNSGLAYWEDERTIDKTGSSKSVTW"
     sig_peptide     complement(114169..114234)
                     /locus_tag="DMACINML_00857"
                     /inference="ab initio prediction:SignalP:4.1"
                     /note="predicted cleavage at residue 22"
     gene            complement(114266..115618)
                     /locus_tag="DMACINML_00858"
     CDS             complement(114266..115618)
                     /locus_tag="DMACINML_00858"
                     /inference="ab initio prediction:Prodigal:2.6"
                     /inference="similar to AA sequence:RefSeq:CAL34260.1"
                     /codon_start=1
                     /transl_table=11
                     /product="putative lipoprotein"
                     /protein_id="Prokka:DMACINML_00858"
                     /translation="MRYKTIVFSILAMVFSACSTHQEQNVFYQQNLFSQTCNKQFFQE
                     QQEKINQNKDVIFIGLNTGYIARDCKDFNLSNSIFDKVEDSYKYDVDLQSLSKKSTRT
                     LASTLINEGVNDYQGAWYERTMLNVYKGLNFMSLGDFVNARVEFNRALMRQEKAKEYF
                     ASEIKKAYQEASKEEKLRQNIDNNIKIVSKQYENLFKDFNAQKEYTNPYVTYISSIFF
                     FMDYDYKKAADLLKEVYVLNLNEEIEKEFKIFDQYSSSLNPKKLKKYIFIIYENGLSP
                     ALDEFSLTLPFIFDDYITTVSIALPILTKRNASYDYLNIANNHQNFTTSNTFDFDQIV
                     ASEFKANLTPIIIKSLISSTLKTSLNMAVAKNDESGILSLATNIFSIVTTRSDLRFWN
                     FLPKNIQIMMIENDGLVQIYDNKNQKIYSSEVDIDKNVLIVVRSFASQFPTRVYKIEN
                     "
     sig_peptide     complement(115559..115618)
                     /locus_tag="DMACINML_00858"
                     /inference="ab initio prediction:SignalP:4.1"
                     /note="predicted cleavage at residue 20"
     gene            complement(115687..117393)
                     /locus_tag="DMACINML_00859"
     CDS             complement(115687..117393)
                     /locus_tag="DMACINML_00859"
                     /inference="ab initio prediction:Prodigal:2.6"
```

```
                     /inference="similar to AA sequence:RefSeq:AHY39361.1"
                     /codon_start=1
                     /transl_table=11
                     /product="anaerobic C4-dicarboxylate transporter"
                     /protein_id="Prokka:DMACINML_00859"
                     /translation="MDTIMIILQIIVLLGAIFIGIRLGGIAIGYAGGLGVVVLGLVLG
                     MKPGNIPWDVILIIAAAIAAISAMQQAGGLDYMVRVTEKILRSKPRFINYLAPACGWL
                     LTILAGTGNAVFSLMPVVVDVAKSQNIKPSAPLSLMVVSSQIGITASPVSAAVVYMSG
                     VLEPFGWSYPALIGIWIGTTFIACMITAFIVSLITPMDLSKDPVYQERLKAGLVKDAG
                     AILHSEDKPGAKLSVAIFLITVLAVVFYATTISDVVRNPIVNYVNSKIYPAYDQIMET
                     KQDLLGNLGENNLSKSINMQFAIIEKTLVSDANTSVFTQAIGDSKKSILSTLNNDVLV
                     KELSQTGFNIQNFKVKLEDHFSKLASLNSSRVQGVAFFEIVRSYIDPVKVSRDQAIMG
                     FLLAAAALITWLCKVEPSKILDTSVFKSGMTACICVFGVAWLGNTFVAGHEASIKEVA
                     GDWVKQSPAMLAVAFFFASMLLYSQAATAKAIVPVIVTALGISATNPQDSYMLVACFA
                     AVSALFVLPTYPTLLGAVQMDDTGTTRIGKFIFNHSFFIPGVLAIAIAVALGFVVAPM
                     LM"
     gene            complement(117409..118815)
                     /gene="aspA"
                     /locus_tag="DMACINML_00860"
     CDS             complement(117409..118815)
                     /gene="aspA"
                     /locus_tag="DMACINML_00860"
                     /EC_number="4.3.1.1"
                     /inference="ab initio prediction:Prodigal:2.6"
                     /inference="similar to AA sequence:RefSeq:CAL34258.1"
                     /codon_start=1
                     /transl_table=11
                     /product="aspartate ammonia-lyase"
                     /protein_id="Prokka:DMACINML_00860"
                     /translation="MGTRKEHDFIGELEISDEVYYGVQTFRAVENFNISHERLKDFPR
                     FVRALARVKKAAAMANYELGLLDKDIQDAIIKACDKILEGGYYDQFVVDMIQGGAGTS
                     TNMNANEVIANIALELMGHKKGEYQYLHPNDHVNLSQSTNDAYPTALHLALHDYLSDL
                     AKAMEHLKKAYERKAEEFKDVLKMGRTQLQDAVPMTLGREFKTFAVMMGEDIQRVLEA
                     RKLILEINLGGTAIGTGINSHPNYPKVVERKIREVTGFEYTVAEDLIEATQDTGAYVQ
                     ISGVLKRVATKLSKVCNDLRLLSSGPKCGLNEINLPKMQPGSSIMPGKVNPVIPEVVN
                     QVCYAVIGADVTVTFASEGGQLQLNVFEPVIAYSLFNSIVMLEKAMHTLADKCIDGIS
                     ANEKICSDFVYNSVGIVTALNPYIGYENSASIAKEAMSTGKKVADIALERGLLSKEQI
                     DEILTPANMLNPHMEAKK"
     gene            119078..119773
                     /gene="ung"
                     /locus_tag="DMACINML_00861"
     CDS             119078..119773
                     /gene="ung"
                     /locus_tag="DMACINML_00861"
                     /EC_number="3.2.2.-"
                     /inference="ab initio prediction:Prodigal:2.6"
                     /inference="similar to AA sequence:RefSeq:CAL34257.1"
                     /codon_start=1
                     /transl_table=11
                     /product="uracil-DNA glycosylase"
                     /protein_id="Prokka:DMACINML_00861"
                     /translation="MKEITINIDDIKINNDWKEFLKEEFSKSYFLEIKKHYIQALNNK
                     AIIYPPSNLTFNAFNLTPLNELKIVLLGQDPYHQPNQAMGLSFSVPYGIKIPPSLLNI
                     YKELKADLDIEPSKSGDLSSWAKQGVLLLNSILSVEAGKPASHSSWGWQEFSDAVISK
```

```
                LSLEKSGLIFMLWGNYAKSKKALIDTKKHFILEAAHPSPLARTGFLGCKHFSKANEIL
                KNLGKKPINWQLA"
gene            119874..120566
                /locus_tag="DMACINML_00862"
CDS             119874..120566
                /locus_tag="DMACINML_00862"
                /inference="ab initio prediction:Prodigal:2.6"
                /inference="similar to AA sequence:RefSeq:CAL34256.1"
                /codon_start=1
                /transl_table=11
                /product="putative amino acid recemase"
                /protein_id="Prokka:DMACINML_00862"
                /translation="MKTIGIIGGMSFESTITYYKTINEAINNQLGNLNSAKILLYSVN
                FEEIANLQKNGEWEKAAQILGECAKKLELGGADFILIATNTMHKIFHQIQKNTKIPLV
                HIAQSTAKILKQQNIHTIGLLGTQYTMMEDFYKNVLKEENIKTITPNQNDMQELSDII
                FNELCRGKIKENSKEKYLKIIQKLKDEGAQGIVLGCTEIGLLISQKDTSIPIFDTALI
                HALDAVNLALKD"
gene            complement(120563..120763)
                /locus_tag="DMACINML_00863"
CDS             complement(120563..120763)
                /locus_tag="DMACINML_00863"
                /inference="ab initio prediction:Prodigal:2.6"
                /codon_start=1
                /transl_table=11
                /product="hypothetical protein"
                /protein_id="Prokka:DMACINML_00863"
                /translation="MGKLERTIRIIILAMVIFSIGVYYASWWALIGFIPLLTGIFGVCP
                IRVWTGKQACPLGVCPISKKKD"
gene            120894..121517
                /locus_tag="DMACINML_00864"
CDS             120894..121517
                /locus_tag="DMACINML_00864"
                /inference="ab initio prediction:Prodigal:2.6"
                /inference="similar to AA sequence:RefSeq:ADT65502.1"
                /codon_start=1
                /transl_table=11
                /product="transcriptional regulator, Crp/Fnr family
                protein"
                /protein_id="Prokka:DMACINML_00864"
                /translation="MDKEKILKEYFKNYNLENKDFEAMVEKSYFKEFDKNTILDDCLG
                FVIVLKGGFRAFILGQNAKEITVFKLKQNEECVICSHCIFETISYNLTLESFDDTQIL
                VVPVKIYSQLKDKYPLIANYTLNLIAKRFNSLINILEQALFTPLHHRVKMFLKENAKE
                GKITFTHEEIALHLGSTREVISRILKTMQKEGFIQQNRKEITLLKAL"
gene            complement(121598..121702)
                /locus_tag="DMACINML_00865"
CDS             complement(121598..121702)
                /locus_tag="DMACINML_00865"
                /inference="ab initio prediction:Prodigal:2.6"
                /inference="similar to AA sequence:RefSeq:ADN90311.1"
                /codon_start=1
                /transl_table=11
                /product="hypothetical protein"
                /protein_id="Prokka:DMACINML_00865"
                /translation="MLHALFLLVWPLVIYVSYKFIMLNINQLEKDNKL"
```

```
gene            complement(121716..122840)
                /gene="cydB"
                /locus_tag="DMACINML_00866"
CDS             complement(121716..122840)
                /gene="cydB"
                /locus_tag="DMACINML_00866"
                /EC_number="1.10.3.-"
                /inference="ab initio prediction:Prodigal:2.6"
                /inference="similar to AA sequence:RefSeq:CAL34255.1"
                /codon_start=1
                /transl_table=11
                /product="cytochrome bd oxidase subunit II"
                /protein_id="Prokka:DMACINML_00866"
                /translation="MFFGLELGGLQIYWWVILSLLGGLLVFMFFVQGGQTLIDELSSD
                ELEKTMLVNSLGRKWELGFTTLVLFGGAAFAAFPLFYSTSFGGAYWAWLCILFCFILQ
                AVAYEYRKKENNVYGSKTYEVFLKINGYLGVFLIGVAISSFFSGSHFVLNEHNFVSWQ
                NPLRGLELLFNPFNYLLGIALVFLSRILGAAYFMNNIKDENIKAKAVKKLSINSILFL
                PFFLGFLAWIFIKEGFGVDEKGIVSMAENVYLYNFLDNILFAILFIIGVVFVLLGMIQ
                GSKGCSKTIFTLGFGSVLTVFALFLSIGLGSSAFYPSLSDLQSSLTIHNASSSYYTLS
                VMAYVSLLVPFVLAYIVYVWNAMDRVKITREEMKDSEHLY"
gene            complement(122840..124402)
                /gene="cydA"
                /locus_tag="DMACINML_00867"
CDS             complement(122840..124402)
                /gene="cydA"
                /locus_tag="DMACINML_00867"
                /EC_number="1.10.3.-"
                /inference="ab initio prediction:Prodigal:2.6"
                /inference="similar to AA sequence:RefSeq:CAL34254.1"
                /codon_start=1
                /transl_table=11
                /product="cytochrome bd oxidase subunit I"
                /protein_id="Prokka:DMACINML_00867"
                /translation="MSELSSVDWSRAQFALTALYHFLFVPLTLGLSFIIAIMETIYVK
                TGNERWKKITKFWLSLFAINFAIGVATGIIMEFEFGTNWANYSWFVGDIFGAPLAVEG
                IMAFFLEATFFAVMFFGWDKVSKKFHLVSTWCVAIGSNLSAFWILVANGWMQYPVGMQ
                FNPDTARNEMQSFFEVAFSPVAISKFLHTIGSGYVISALFVMGISAWFILKGRHIIEA
                KKSLVVGASFGLVCSVFLFFSGDESAYRVTQTQPMKLAAMEGVYQGEHRAGLVPFGIL
                NPKKTIDNNESVFLFDITIPYALSILGNRDPNSFVPGIEDLIHGNEDRGIEPMQERID
                KGKIAIQALKDYRLAKDNNDTMAMAIHKSILEANFKDFGYGYLEKPTDAIPPVALTFY
                SFHIMVALGSLFFLLFIATLYLTMANDIEKFRKILWLCLLCIPLGYIAAEAGWIVAEV
                GRQPWAIQDLMPVHVAATELGKVNVQISFWIFAVLFTALLIAEIKIMLTQIKKGFDAH
                AGHSTLMGKGEK"
gene            complement(124402..124632)
                /locus_tag="DMACINML_00868"
CDS             complement(124402..124632)
                /locus_tag="DMACINML_00868"
                /inference="ab initio prediction:Prodigal:2.6"
                /inference="similar to AA sequence:RefSeq:CAL34253.1"
                /codon_start=1
                /transl_table=11
                /product="putative membrane protein. Functional
                classification-Membranes, lipoproteins and porins"
                /protein_id="Prokka:DMACINML_00868"
```

```
                /translation="MYLKCFYFFFMRIFNFYKEGFKNLTLGKTLWKIIFIKLFIMLVI
                LKLFVFDVNFNSIFKNDNEKSNFVIENLIKEK"
gene            125119..126786
                /gene="lctP"
                /locus_tag="DMACINML_00869"
CDS             125119..126786
                /gene="lctP"
                /locus_tag="DMACINML_00869"
                /inference="ab initio prediction:Prodigal:2.6"
                /inference="similar to AA sequence:RefSeq:AAW34667.1"
                /codon_start=1
                /transl_table=11
                /product="L-lactate permease"
                /protein_id="Prokka:DMACINML_00869"
                /translation="MWQQIYDPFNSIWLSALFAFLPILCFLACLVFFKLKGYQAGFLT
                VVLASVIALFVYDMPWNLVGASFVQGFTNGMWPIAWIIIAAIFLYKLSIKSGSFEIIK
                KSVMSITPDHRIQVILIGFCFGSFLEGAIGFGGPVAITAALLVGLGLRPLQAAGLCLI
                ANTAPVAFGAVGIPIIAMANLVGVEAHSVSAMVGRMLVPLSLTIPFFIVFLMDGLKGV
                KETFPAILVAALSFTATQFWSSNHLGAELPDIISAIVSLAVTTIFLKFWKPKNIFRLD
                GETHFTEDNTLNFGDVLKAWTPFILLIICIIIWTQPWFKAIFDKDGFLSYTSITLQFS
                NLTGGILSPSITGIGEAKPLSLGLGIDLINGKTVAQAGTAILLAAFLTIAVLRIKAED
                AAECFWATLKEMAIPCLTIGLVVAFAFISKNSGMSTTLGLAFAHTGDAFAFFSPIIGW
                IGVFLTGSDTSANLLFGTLQQVSAQKLGVSEALFLAANSVGGVVGKMISPQSIAIACA
                AVGLVGKESDLFKFTLKYSIAFIILIGIWTCIIAFFLPGIIPEVVAK"
gene            126939..127676
                /locus_tag="DMACINML_00870"
CDS             126939..127676
                /locus_tag="DMACINML_00870"
                /inference="ab initio prediction:Prodigal:2.6"
                /inference="similar to AA sequence:RefSeq:CAL34248.1"
                /codon_start=1
                /transl_table=11
                /product="putative oxidoreductase iron-sulfur subunit"
                /protein_id="Prokka:DMACINML_00870"
                /translation="MKKVYFYATCLGTAAMQESVLNGIKLLRREGVEVIFKKNQTCCA
                QPSFNSGYFNESREIALYNVDLFDKDYPIVVPSGSCAGMMSHDYKELFKDRPEFSRVK
                DFSSRVIELSQYLDEVLNVNYEDKGEPIKVTWHSNCHALRVQKSIQASKNLIRKLKNV
                ELVELQYEEECCGFGGTFSVKEPEISNAMVRNKIKDIQNTGVKYLISGDGGCLLNIDG
                TMRRMGLDVKGIHLYEFLFKRLEGERL"
gene            127673..129112
                /locus_tag="DMACINML_00871"
CDS             127673..129112
                /locus_tag="DMACINML_00871"
                /inference="ab initio prediction:Prodigal:2.6"
                /inference="similar to AA sequence:RefSeq:CAL34247.1"
                /codon_start=1
                /transl_table=11
                /product="putative iron-sulfur protein"
                /protein_id="Prokka:DMACINML_00871"
                /translation="MSQKIPHEEIVHIKLNDIQMRENLMTAMHTLQKNRLGVIDARFQ
                DWQGLRAKAKQAKNNALMSLKDRLLEFEKNATKNGIQVHWASSDEDACEIVYEIMREK
                NITKLLKGKSMASEEIGLNHYLEKKGLKAIETDLGELILQLNEEPPLHIVVPAIHRNR
                HEIGQIFKDKLGAKLENDEPESLNAIARDHLRKDFEGLKLGLSGVNFAMSREGAFWLI
                ENEGNGRMCTTAPDIHIALCGVEKVMESFEDAATMVSLLTPSATGQFIPTYNNIITGP
```

```
                     RKNGDLDGPKEVHVILFDHNRTKMLAHKDYYEALRCIRCGACMNFCPVYDQIGGHAYQ
                     TTYPGPIGEVISPNIFGIDHTGDILNFCSLCGRCSEVCPVQIPLADLIRKLRCDKIGQ
                     GENPPMGAKNIHHNAMEAFAFKQFKKAATKGERWRFALSKAHYFNWFVQNLSGALPVI
                     KKWYAFKELPQIKKDLYKEIQAIEGVIYE"
     gene            129105..129761
                     /locus_tag="DMACINML_00872"
     CDS             129105..129761
                     /locus_tag="DMACINML_00872"
                     /inference="ab initio prediction:Prodigal:2.6"
                     /inference="similar to AA sequence:RefSeq:CAL34246.1"
                     /note="conserved hypothetical protein Cj0073c"
                     /codon_start=1
                     /transl_table=11
                     /product="hypothetical protein"
                     /protein_id="Prokka:DMACINML_00872"
                     /translation="MSKINEISNKSKEKILSALNEAYEAKDFTYQASIDPVEHIQTKE
                     DALAEMKQKMSDNKYIVENATKETLEEKINEIITKYNYKKMIYGTDLGLDLNKIQAEE
                     KICFDKEIENLRYEVFHSDFSIVHARAGVSSHGVALIPSSKGQPRMLSLAPKLCIVLL
                     KKEDIVKSLSEALNLVKTENEILPTNILFIAGPSRTADIELITVFGVHGPQIAHIVIY
                     "
     gene            129948..130670
                     /locus_tag="DMACINML_00873"
     CDS             129948..130670
                     /locus_tag="DMACINML_00873"
                     /inference="ab initio prediction:Prodigal:2.6"
                     /inference="similar to AA sequence:RefSeq:AII23819.1"
                     /codon_start=1
                     /transl_table=11
                     /product="hemerythrin family protein"
                     /protein_id="Prokka:DMACINML_00873"
                     /translation="MQIIEIYLEKTLDKLIPAWDEKYSINDEKIDAQHQKLFELAAKI
                     ENAVYKFVQRDELKEILTELFNYMKEHFSNEEDYMQEISYPYLNEHKIMHKNIIHDMS
                     HLIQNIKTTNDLKEKLYTIMSDWLLEHILHHDVMIGNWLSEQLLAKQEEELEKLQQED
                     LQQEEKQYENNPNPIPQVKTEYDFIYSCPCRTNHFLSYIEHLDIICHKKQLRCKKCQK
                     NLFYVRSEIKTEKRNNDVSKVG"
     gene            130651..131262
                     /locus_tag="DMACINML_00874"
     CDS             130651..131262
                     /locus_tag="DMACINML_00874"
                     /inference="ab initio prediction:Prodigal:2.6"
                     /inference="similar to AA sequence:RefSeq:ADN90300.1"
                     /codon_start=1
                     /transl_table=11
                     /product="Non-heme iron protein, hemerythrin family"
                     /protein_id="Prokka:DMACINML_00874"
                     /translation="MFPKWDDNYSVHNAKIDEQHKKLFELAGKVEFMFDRPVYKDDIK
                     ALLADFFNYMKDHFNDEEKYMKLIGYPDLEEHKKIHKEIIQSMIDLIKNIKSTNDLKE
                     KLYTVSKKWLLEHILYEDMKVEQYRRSSLSSEDDSEVSFEEVKEEKDDETALYFYTCE
                     CVGQIHDVPFGIHQKIQLKGAKFKCKKCKMAIQFYKKHSESFE"
     gene            complement(131491..132522)
                     /locus_tag="DMACINML_00875"
     CDS             complement(131491..132522)
                     /locus_tag="DMACINML_00875"
                     /inference="ab initio prediction:Prodigal:2.6"
```

```
                     /inference="similar to AA sequence:RefSeq:CAL34243.1"
                     /note="hypothetical protein Cj0069"
                     /codon_start=1
                     /transl_table=11
                     /product="hypothetical protein"
                     /protein_id="Prokka:DMACINML_00875"
                     /translation="MKKNIVFFEVRGGSDKGEDGYRKDTMPMVNALKAKGWNAEVIFF
                     EVGKKDEIYNYVKENFDGYVSRINPGNLKEENEYFDMLRKLCQEGLVGMPHPDAMIGY
                     GAKDALTKLADTDLVPSDTYAYYDIKTFKENFPKSLAKGERVLKQNRGSTGEGIWRVS
                     VEGEAKGEVLPLDTKIKCTEAKDNHVEHRELGEFMDFCEQYIIGDNGMLVDMTFLPRI
                     KEGEIRLLMLYNTPVNVVHKKPAEDADAFSATLFSGAQYRYDKPEDWQNLVDMFLGEL
                     PKVRAKLGNYDLPLIWTADFILDNDEKGNDKYVLGEINCSCVGFTSHLELADEVAQNI
                     INIVSKAKN"
     gene            complement(132636..133532)
                     /gene="pspA"
                     /locus_tag="DMACINML_00876"
     CDS             complement(132636..133532)
                     /gene="pspA"
                     /locus_tag="DMACINML_00876"
                     /inference="ab initio prediction:Prodigal:2.6"
                     /inference="similar to AA sequence:RefSeq:CAL34242.1"
                     /codon_start=1
                     /transl_table=11
                     /product="protease"
                     /protein_id="Prokka:DMACINML_00876"
                     /translation="MQILKSFFKGLGSVIKFINTYFKTFVLLLIVVWLFTPSSNSNSS
                     YANLERIDLKGEILDSSEVLEKIINAKNNDNIKGVLFVIDSPGGAFAPSMELALAIKD
                     LKAKKPVIAYASGTMASGSYLAGVGANKILANPASFIGSIGVIMQGADLSELAKKIGI
                     KEQTIQAGEFKSAGTLTRAWSEKEREFLQNLINESYDLFSEFVAKERNLELDKRNDWA
                     NARVFLAQNAKDLGLIDELSNYENAKKELQELAKVLNPIWKEEGRIDRFLNRLESQSS
                     NFLSKVMGDVILRLNANALELK"
     gene            complement(133520..134749)
                     /locus_tag="DMACINML_00877"
     CDS             complement(133520..134749)
                     /locus_tag="DMACINML_00877"
                     /inference="ab initio prediction:Prodigal:2.6"
                     /inference="similar to AA sequence:RefSeq:CAL34241.1"
                     /codon_start=1
                     /transl_table=11
                     /product="putative amidohydrolase family protein"
                     /protein_id="Prokka:DMACINML_00877"
                     /translation="MYIISAKYIFLCDEQFSILENKAFVFEDKILELGELEELKKRYP
                     KAKLIKTSPNCVILPAFINPHTHLEFSANSTTLHFGEFLTWLKSVIKSRSMLNEQAKE
                     ELILTNIQKMQKSGIGTIGEISSFGSDLNPCVKASQNGMRVIFFNEILGINEAQIQDK
                     KQEFLTRFENSLKFKDEFFMPAISVHSAYSTHPELAKFATNLAKKQKLLISTHFLESK
                     AENAWLKEAKGGFKEWFKNFTPNPKPLYKVSEFIQLFKGIRTLFTHCVYLKEMDLLDK
                     NLHSITHCAFSNRLLSQKTFDLKKALKSGLNIHLGTDGLSSNISLSILDEMRANLLVH
                     TNFDLLKLAPKLLQMATLYPAKALNLNLGEIKQGKIADFSVFELGECDKEQAPLQFIL
                     NAKEVQKLFIKGKECKF"
     gene            134842..135330
                     /gene="aroQ"
                     /locus_tag="DMACINML_00878"
     CDS             134842..135330
                     /gene="aroQ"
```

```
                     /locus_tag="DMACINML_00878"
                     /EC_number="4.2.1.10"
                     /inference="ab initio prediction:Prodigal:2.6"
                     /inference="similar to AA sequence:RefSeq:CAL34240.1"
                     /codon_start=1
                     /transl_table=11
                     /product="3-dehydroquinate dehydratase"
                     /protein_id="Prokka:DMACINML_00878"
                     /translation="MKIMVIQGPNINMLGVREVGIYGAMKMEDIHTQMKMAASQNNVE
                     LDFFQSNFEGEIVDKIQECLGTVDGIIINAAGYTHTSVAIRDAISAVALPTIEVHISN
                     VYRREEFRQKSLIAPVCSGSIVGFGPFGYHLALMGVIQICEQVKNLRAAQAQQMQAGA
                     QN"
gene                 135333..135815
                     /gene="folK"
                     /locus_tag="DMACINML_00879"
CDS                  135333..135815
                     /gene="folK"
                     /locus_tag="DMACINML_00879"
                     /EC_number="2.7.6.3"
                     /inference="ab initio prediction:Prodigal:2.6"
                     /inference="similar to AA sequence:RefSeq:CAL34239.1"
                     /codon_start=1
                     /transl_table=11
                     /product="putative
                     2-amino-4-hydroxy-6-hydroxymethyldihydropteridine
                     pyrophosphokinase"
                     /protein_id="Prokka:DMACINML_00879"
                     /translation="MLKIKGARRLETSRFFPYFSQKKKGFKYLALVGLGSNIEPEKTR
                     FNKLFRVMMEDKRFKILATSPFLINEAFGFKAQKDFTNATMLMQTNLHARAFLKVLLF
                     YELKFKRKRTFKNAPRTLDLDLLYFSQKVKRDEGCMVPHTGANQRISVILPLGLTKGL
                     "
gene                 135816..137246
                     /gene="flhF"
                     /locus_tag="DMACINML_00880"
CDS                  135816..137246
                     /gene="flhF"
                     /locus_tag="DMACINML_00880"
                     /inference="ab initio prediction:Prodigal:2.6"
                     /inference="similar to AA sequence:RefSeq:CAL34238.1"
                     /codon_start=1
                     /transl_table=11
                     /product="flagellar biosynthesis protein"
                     /protein_id="Prokka:DMACINML_00880"
                     /translation="MGQLIHTFTVEDTEQIIPKVKEDYGDKALIITNKQIRPKTLNRA
                     ALYEVMVAIEESDYEEHLKKMGKSLPPKKTIAKTLEKQNLEEKVNASIPQNNEDEDVI
                     LDFSNTRANLAKTKTNDTNYQSFPQSKINPNPTMGFNDFKERLSEVSNEISKVTNTPL
                     VDNYAPNPNYNKKIENFEKQINKLNDKIDLLADMMWDEKAEARNNLIIPPEFATIYKK
                     AKESGMLEKHLEAIMKATIENMPAAMKTNKDAVQRYFYSLLRNILPCRVESEIKKQKI
                     MMLVGPTGVGKTTTLAKLAFRYAYGDKRYKTGIITLDTYRIGAVEQLFQYAKMMKLPI
                     IDSIEPKDLDEAIKSLNNCEVILVDTIGNSQYDQNKLAKTKEFLMHSNAEIDVSLVVS
                     ANTKHEDLMEIYKNFSFLNIDTLIITKFDETKVFGNIFSLIYETNIPLSFFSTGQEVP
                     DDIEVANSDFLVRCILEGFNKGKDNE"
gene                 137239..138105
                     /locus_tag="DMACINML_00881"
```

```
CDS             137239..138105
                /locus_tag="DMACINML_00881"
                /inference="ab initio prediction:Prodigal:2.6"
                /inference="similar to AA sequence:RefSeq:CAL34237.1"
                /codon_start=1
                /transl_table=11
                /product="putative ATP-binding protein"
                /protein_id="Prokka:DMACINML_00881"
                /translation="MNNQANKLRSLMNQNGGKKSQNTHFIAITSGKGGVGKSTISANL
                ANVLANNGYKVGLFDADIGLANLDVILNVRIQKNLLHVLRGECSLEDILIEVKPNLWL
                IPGESGDEILKYNDKNIYERFLNQASILDELDFLIIDTGAGIGGNILNFLEMSDEVVV
                VTVPDPAAITDAYATIKTTSKTKENLLMLFNVVKNENEALKVFENIKKVADANIKNHL
                KLEFLGHLSASKDVSGSIKKRTLFTDENSASSDELKALASKLLYRLERKVLDNVTSRS
                FSNFFRKIIERF"
gene            138125..138481
                /locus_tag="DMACINML_00882"
CDS             138125..138481
                /locus_tag="DMACINML_00882"
                /inference="ab initio prediction:Prodigal:2.6"
                /inference="similar to AA sequence:RefSeq:CAL34236.1"
                /codon_start=1
                /transl_table=11
                /product="putative integral membrane protein"
                /protein_id="Prokka:DMACINML_00882"
                /translation="MRPENYVAFFTVCGFFIGLAFSVISIEEAFDILIFTCFITFMFY
                VFIHIAIMNFIDIKKISGRIFNKHDYEKTSNNIINDLVIREKKMDIILEKLNEEREEL
                KKNESKERRKNAKRAA"
gene            138462..139154
                /gene="fliA"
                /locus_tag="DMACINML_00883"
CDS             138462..139154
                /gene="fliA"
                /locus_tag="DMACINML_00883"
                /inference="ab initio prediction:Prodigal:2.6"
                /inference="similar to AA sequence:RefSeq:CAL34235.1"
                /codon_start=1
                /transl_table=11
                /product="RNA polymerase sigma factor for flagellar
                operon"
                /protein_id="Prokka:DMACINML_00883"
                /translation="MQKEPPKAYAQMIKDEQDQLVLSYMPALRAMAFRLKERLPSSID
                VNDLISIGVEEMIKLSRRYDKEQNDSFWGFARKRVNGAMLDFLRSLDVMSRNNRKIIK
                DIDILIDEYFREHEEEPDDEYLAQKLGLDIEKIKEVRTAHAISYTLSIDDQLELYNED
                DTLEKLEKEQLLEKIHEVLEDLKERDQLIIQLYYYEELSLKEISEILEISESRISQIH
                KKLLKKLRERLV"
gene            139154..140233
                /gene="fliM"
                /locus_tag="DMACINML_00884"
CDS             139154..140233
                /gene="fliM"
                /locus_tag="DMACINML_00884"
                /inference="ab initio prediction:Prodigal:2.6"
                /inference="similar to AA sequence:RefSeq:CAL34234.1"
                /codon_start=1
```

```
                     /transl_table=11
                     /product="flagellar motor switch protein"
                     /protein_id="Prokka:DMACINML_00884"
                     /translation="MAEILSQEEIDALLEVVDDNTDTPTASNSKDGKDERNIVVYDFK
                     RPNRVSKEQLRTIKGIHDKLARNLASQISSMMRSIVETKLHSVDQMTYGEFLMSLPSP
                     TSFNVFSIKPLDGNCVLEINPSIAFPMIDRLLGGQGDSYEASRELTDIELNLLDSILR
                     IIMQRLKESWATVTEIYPSIEAKESSPNVVQIVSQNEIVIMVVMEIIIGNSSGMVNIC
                     YPVVHLESILSRLANRDIMMGETSAKKSRNKELKTLIGRAEVVYEAILGKTLINVHEF
                     LELKQGDILRLDREADDKAIVSIDKKDVFLAQIGLHRFRKSIKILELIRTDKDEIKEI
                     LERYEEERKAKASVYDEPEEEEEEI"
     gene            140230..141066
                     /gene="fliY"
                     /locus_tag="DMACINML_00885"
     CDS             140230..141066
                     /gene="fliY"
                     /locus_tag="DMACINML_00885"
                     /inference="ab initio prediction:Prodigal:2.6"
                     /inference="similar to AA sequence:RefSeq:CAL34233.1"
                     /codon_start=1
                     /transl_table=11
                     /product="putative flagellar motor switch protein"
                     /protein_id="Prokka:DMACINML_00885"
                     /translation="MINEFLKLFTNECTSTIEGLTGKSAEFSEYKEFDVSSQDTLKPP
                     LVYAVFNIKEGGKIGILAGAVLMSAIGEWMMGEEEISKNDQLGPDEMDAAKEAIQNII
                     SAFSTTLGAQKDIPKMDFEISSCEFVAENVDFKDFTKLYFFDVKIEDLEEQISLIVDQ
                     TLHNILSGKAADDGHAHSSSNSENKGFEISEELKNINLIMDVRLPVRVRIGNKKMLLK
                     DVLTMDIGSVVELNQLANDPLEILIGDKRIAYGEVVIVDGNFGVQITEIGSKKERLEQ
                     LR"
     gene            141134..142279
                     /locus_tag="DMACINML_00886"
     CDS             141134..142279
                     /locus_tag="DMACINML_00886"
                     /inference="ab initio prediction:Prodigal:2.6"
                     /inference="similar to AA sequence:RefSeq:CDH61953.1"
                     /codon_start=1
                     /transl_table=11
                     /product="FIG00470444: hypothetical protein"
                     /protein_id="Prokka:DMACINML_00886"
                     /translation="MINGINSYSSYNYTSTFNNTFNTKSSNIINDNSNLVSDKSGAVS
                     KILGYGVDKDGFFTSDFNEAAGLPKDYKIYAKDIENFVNIQTKSLLSSYINIDIAKSI
                     GNAYKVFSQLLNDELNNKINFTKEDLVKIPQGFEFDQNTLEINKKYNLNDKEYKDFMS
                     NYQNYSPTSHNIKIMTFFSLDDKSDIFKYSLIDPTPYSNNDKTITKSGIFMTFFTGFQ
                     AKNYFIEGQTSILGKMQGFDKTMSQSEIDNLNKFISENSMNFKGNIFKLFDLLNSDMS
                     IDDFKQKILEQKENLNNPTKSTNNKETSEEDKKKPFKPIQAQSSSETYKDDSKMNELL
                     KKLLETKFGTSDELEILFGMKFSDDDTGEFNKILSLNSAPKSIDIKA"
     gene            142315..143385
                     /locus_tag="DMACINML_00887"
     CDS             142315..143385
                     /locus_tag="DMACINML_00887"
                     /inference="ab initio prediction:Prodigal:2.6"
                     /inference="similar to AA sequence:RefSeq:CDH61953.1"
                     /codon_start=1
                     /transl_table=11
                     /product="FIG00470444: hypothetical protein"
```

```
                     /protein_id="Prokka:DMACINML_00887"
                     /translation="MIGINSTFNNSFVNLNIKNNNNSSLNSNTNVKNLQDTKQYSKTS
                     NKVLDYEIDNEGYFTSDFNEKAGIPKDIKIHSSTMENFIKAQNNGSLKSYANIDIAKT
                     VGNAYKIVSQLVEKTPQLKRKSSFSKEDLANYFPQNYLIDKNTLEVKQTFSYEEMKDI
                     IAKGGFKNINENEKLSPSFFNFDNTDEKSELLPFNPDILHSAKNTALSWDTSADKYTN
                     NDGSITMGGLLIGFLSNNVNTGVTAQPLIAGETTVWGKLQGLDNSLSENEIKELNNKL
                     SNTYFSNLSDAFALLNGTSDIKEFENKLNSLNQNNKTNSSNFLEWVQQITKEHQKSLF
                     EILKQPDNINKIYLLINIDIRA"
gene                 143502..144107
                     /locus_tag="DMACINML_00888"
CDS                  143502..144107
                     /locus_tag="DMACINML_00888"
                     /inference="ab initio prediction:Prodigal:2.6"
                     /inference="similar to AA sequence:RefSeq:ABS44176.1"
                     /codon_start=1
                     /transl_table=11
                     /product="lysine decarboxylase family protein"
                     /protein_id="Prokka:DMACINML_00888"
                     /translation="MDTKISQDLEKFANIPNLKNAITFFGSARLKEDSFYYQEAKKLA
                     NLCVENGFCVVSGGGGGIMRAANAGAAKCKNDANFIKSVGFNILLPFEQKLNDFVEYN
                     ITFESLAIRKMALIEKSLAFVIFPGGFGTLDELCEVLTLKQLGFKNNVPIILFGSEFW
                     QGFDEFVRNSLLKLEVISKGDELKYEITDDLDYIINTLKEL"
gene                 144107..145123
                     /gene="trmU"
                     /locus_tag="DMACINML_00889"
CDS                  144107..145123
                     /gene="trmU"
                     /locus_tag="DMACINML_00889"
                     /EC_number="2.1.1.61"
                     /inference="ab initio prediction:Prodigal:2.6"
                     /inference="similar to AA sequence:RefSeq:CAL34227.1"
                     /codon_start=1
                     /transl_table=11
                     /product="tRNA
                     (5-methylaminomethyl-2-thiouridylate)-methyltransferase"
                     /protein_id="Prokka:DMACINML_00889"
                     /translation="MKILVAMSGGVDSTVTAYKLKNAGHEVIGCYMKLHGKPNYHEEN
                     IKKVEKVANFLQIPYHVLDLQEDFKNKVYMPFVDTYKEGKTPNPCALCNRFIKLGKLL
                     EFAKSLGCEKLATGHYARLENNLIRTAVDESKDQSYFLASADKEALKYLIFPLGEMKK
                     EDVKKFASTIEVLKSFATQKESSEICFVEDTYVQVLDQFMDTKIPGEVLDSSGKVVGK
                     HEGYMHYTIGKRRGFEVRGAHEPHFVLKINPKQNQIIVGTKEELKINEFKLKNINLFI
                     DAKELDCEVKIRYRSKSTPCKVIISDDKSASISLKESVYGLASGQMAVFYDDDKVIAS
                     GFIE"
gene                 complement(145158..146966)
                     /gene="yflS"
                     /locus_tag="DMACINML_00890"
CDS                  complement(145158..146966)
                     /gene="yflS"
                     /locus_tag="DMACINML_00890"
                     /inference="ab initio prediction:Prodigal:2.6"
                     /inference="similar to AA sequence:RefSeq:AJP34568.1"
                     /codon_start=1
                     /transl_table=11
                     /product="Putative malate transporter YflS"
```

/protein_id="Prokka:DMACINML_00890"
/translation="MEKKKIPMLILCILIALAFWIIPTPIGLEENSWHFLGLFIAVIM
AVILQVMPLGAVCMIAIAIVALSGITTTQSSVRATHIKTLEAIVLKDNSNIYKVAMDK
KSQASVLENLIKANVAKNLEPKVNEILKSNSNIEYQIDSLSKLYAKATSKVATQKLVD
ESKIAALNSLALEFMSQKDIDEKVNTAISSLKSKTGIKDALSGFSNSLIWLIVVSIIV
ARGVIKTGLGERLAYYFISIFGKKTLGIAYSIAFCETILAPVTPSNTARAGAIINPIV
QAIARSFKSTPEDGTQNKIGTYLSLVNFQANPISSAMFITATAPNPLVVDLVAQATNL
EVHLTWGQWALGMFLPGIAAMLLMPLVIYFLSPPEIKSTPNAKIFAKGKLEELGAMKG
SEKIMLGVFVLLLLLWAGALGFLFGISLDATSVALLGLSLVLVSGVLTFGEVLAEKAA
WNTLVWFSALVMMATLLGKLGVTQFLAEALGEFASAMGLGEISIMIFLSLAFLYTHYF
FASTTAHISAMFFVFYSAGLALGAPPLLYAFIMIASGNVMMALTHYATGTAPVIFGTG
YVSLKKWWSVGFVISVIDITVMILVGLVWWKFLGFY"
gene complement(147482..149119)
/gene="flgE"
/locus_tag="DMACINML_00891"
CDS complement(147482..149119)
/gene="flgE"
/locus_tag="DMACINML_00891"
/inference="ab initio prediction:Prodigal:2.6"
/inference="similar to AA sequence:RefSeq:CAL34223.1"
/codon_start=1
/transl_table=11
/product="flagellar hook protein"
/protein_id="Prokka:DMACINML_00891"
/translation="MMTSFYNGLSGVKTNSFDIDVAANNIANVNTMGFKYSTTQFKDI
FYSTITSQSTNPTQGGYGTGAASSQIVFEQGSPVASEGEFDVALQGKGFFGVLGADGE
AYYTRNGAFRRDAAGNLVDSYGNFVLGTMNPAFAGVSYSDRVAGLMGDYLNTGTPVNS
GFTVNSNDVFNIGTTASQGVIKVPVNMYLPPQVTQNVKWSGSLNTNATTEVVKVDLDP
AKFNVQKTEDGKYIVSGSVSKEDVFSAKAGDRILLNFIDDNGVKTSFEATLDENLNFT
SNELDLKGLDPNSIKLETAQISTEQQKANKDILESSIYNADGSKSILRLTLERILPQE
GENIQYKAIAQIFNSDGEAVGNPTEGSMVFDKNGALLQNNITSIANPDGGTINIDLGT
PYDANKPGSGYSGIYIKEGVAKNIVTQQDGVAEGFFDQYSISDDGSIVAQFSNGKAAI
VGKLALYNFINEQGLAAMGDNIFAATANSGDASFIMKDGQVINTAEFKGGFLEQSNVD
LSVQLSNLIATQKSFDASSKSITTSDQMIQKAINMKR"
gene complement(149123..149998)
/gene="flgD"
/locus_tag="DMACINML_00892"
CDS complement(149123..149998)
/gene="flgD"
/locus_tag="DMACINML_00892"
/inference="ab initio prediction:Prodigal:2.6"
/inference="similar to AA sequence:RefSeq:CKG56285.1"
/codon_start=1
/transl_table=11
/product="flagellar basal body rod modification protein"
/protein_id="Prokka:DMACINML_00892"
/translation="MLTSSDWTMTNTNTNTNTNTATATTTSTNTTSDTSSTSSSGLAS
NPNATLDKDAFLKLLLIELQHQDPTDPMDSDKMLTQTSQLSALEMQQNTNTTMQKMVE
TMQLLSNSFSTSMSTSAIGAIGKMATVSDNKIKLTGSDEIIALKMYLPEDSDENGVTL
EVYDSNNKLVFSEKSGEQSVSQGLFTMEWPGRNNDGVYAGDGEYTVKMVYNNKNGEKI
TANYGTYPIEGIIFKEGLAYAKMAGQEVPFDAITEITDYKLSSSSSTGSGSSGSDSSG
GSSGTTEDSESEERA"
gene complement(150055..152127)
/locus_tag="DMACINML_00893"

```
     CDS             complement(150055..152127)
                     /locus_tag="DMACINML_00893"
                     /inference="ab initio prediction:Prodigal:2.6"
                     /inference="similar to AA sequence:RefSeq:ALW54855.1"
                     /codon_start=1
                     /transl_table=11
                     /product="flagellar hook-length control protein"
                     /protein_id="Prokka:DMACINML_00893"
                     /translation="MSNLTPSNDILNLTPSKTNTTHSSSSKTKEQDLKKSDNTTQDDT
                     ESFLNSLLNSIEETNEFLPDHMKISQKEVVNEAMDKLQNGVFSESDKISIFESASFMQ
                     ILSLLDKLKADTADIKLANLSTQLSQLVKTEANFNALKSASNLSELLNIAKDLGLNVK
                     NIKVDRLLDLKATFPNLDKANFFKGAVDNVFKEIINNKLAHISKNLNQNVQNLNHTSH
                     ANLNQNSKNIKSKDDTSLLSQTLKNLDAIISSKNEKKDKAEIKNDNVKIQTDGETTEL
                     KNTSKNLNHSESLKELDQENLTNNDKKLPNQEVQNKEHTKELKNDKNTLLNEPLKKDD
                     KELKNTNKANAQDLTPSKNNTTQDKLPMQEKVSTQEKTMPQEKVSTQEKTILQDNKAS
                     VQEKPLNNKEGLNQETNNIKNTKDLNQALDKNNNLSENPKEQVKNENTNFNKTSNKEN
                     IRDSQNLSLNLNPKDLNLNKDNKSSVKDNTLKDIKQNFTEQKVSFENLNKTQFVSNKD
                     NTNFTHYTNKENYTQEQGKIQSENTEKNPLDDLNSLVKDLNKISQNNTKNITPKETLQ
                     HFSQDLKAAMEQYKAPITKLSITLNPHNLGEVEVTLVQRGNNLHINFNSNTNAMNLFI
                     QNQAEFKNSLVNMGFTGLEMNFSDQGKREQNQNQGKNRSGYGFKDALEGKTENEKVNL
                     ELVLAKYF"
     gene            complement(152141..152464)
                     /locus_tag="DMACINML_00894"
     CDS             complement(152141..152464)
                     /locus_tag="DMACINML_00894"
                     /inference="ab initio prediction:Prodigal:2.6"
                     /inference="similar to AA sequence:RefSeq:CAL34220.1"
                     /note="hypothetical protein Cj0040"
                     /codon_start=1
                     /transl_table=11
                     /product="hypothetical protein"
                     /protein_id="Prokka:DMACINML_00894"
                     /translation="MSKPLNTEFFIELDTQLINRKSEVLLQVLDLLETLKSNQNEKMA
                     RISSELTELLENEENLEKITNAQNLEELLDILVALNEDKMIKVYENAYLKEKFPHLAS
                     SQFLK"
     gene            152593..154401
                     /gene="typA"
                     /locus_tag="DMACINML_00895"
     CDS             152593..154401
                     /gene="typA"
                     /locus_tag="DMACINML_00895"
                     /inference="ab initio prediction:Prodigal:2.6"
                     /inference="similar to AA sequence:RefSeq:CAL34219.1"
                     /codon_start=1
                     /transl_table=11
                     /product="GTP-binding protein TypA"
                     /protein_id="Prokka:DMACINML_00895"
                     /translation="MENIRNIAVIAHVDHGKTTMVDELLKQSGTFSEREQISERVMDS
                     NDIEKERGITILSKNTAINYKGTKINIIDTPGHADFGGEVERVLKMIDGVLLLVDAQE
                     GVMPQTKFVVKKALSLGLKPIVVINKIDKPAADPERVINEIFDLFVALDANDEQLDFA
                     IVYAAAKNGYAKLDLNDESDNMEPLFKTILERVPAPSGTNENPLQLQVFTLGYDNFVG
                     KIGIARIFNGVVKKNQSVMLAKADGTKVNGRISKLIGFMGLEKMDIEEAGSGDIVAIA
                     GFEALDVGDSVVDPNNPMPLDPLHIEEPTLSIVFSVNDGPLAGTEGKHVTSNKIAERL
                     EAEMKTNIAMKYESTGEGKFKVSGRGELQITILAENMRREGFEFCMGRPEVIVKVEDG
```

VKTEPFEHLVIDVPEEFSGAVIEKLGKRKAEMKTMAPTGDGQTRLEFEIPARGLIGFR
SQFLTDTKGEGVMNHSFLEFRPFSGAVEKRNNGALISMENGVALGYSLFNLQERGVLF
IEPQTKVYTGMIIGEHSRPNDLDVNPIKGKNLTNVRASGSDDAIKLVPPRKLSLERAL
EWIEEDELVEVTPQNVRVRKRYLDPTQRKRMEKAKS"

```
gene            154401..155051
                /locus_tag="DMACINML_00896"
CDS             154401..155051
                /locus_tag="DMACINML_00896"
                /EC_number="2.7.7.-"
                /inference="ab initio prediction:Prodigal:2.6"
                /inference="similar to AA sequence:RefSeq:CAL34218.1"
                /codon_start=1
                /transl_table=11
                /product="putative poly(A) polymerase family protein"
                /protein_id="Prokka:DMACINML_00896"
                /translation="MDLKSLENRRLYILKRLGILKFLSIIEALLVGFLAFVFTKDILI
                AIILAVFVGIFFFRFTAKKLKLAKKELEFDALNLFLRRFGAKFRKESLSQKDFLKLEL
                SDNLKDFKSQNCFEFKEFKIYDIHFIDENKRFFCGILLEILKPSKNPSFEDEEKIYVK
                LQDKNFTLNHIFSKDNHYLIATLKNPFFIDLKESLEKNFKNLENNLKLIEEKIIKI"
gene            complement(155048..156397)
                /locus_tag="DMACINML_00897"
CDS             complement(155048..156397)
                /locus_tag="DMACINML_00897"
                /inference="ab initio prediction:Prodigal:2.6"
                /inference="similar to AA sequence:RefSeq:SQE23472.1"
                /note="Uncharacterized protein conserved in bacteria"
                /codon_start=1
                /transl_table=11
                /product="hypothetical protein"
                /protein_id="Prokka:DMACINML_00897"
                /translation="MPNFNQNEQIKCPSCGSFIDISTALYAQILDKAKQEMLKQKKEF
                EDEVNAKRAEYIKALNDLKAQKLEQEKLINEQVSQKLLLEKQKFEQELLIQRQNFQKE
                FSEKFNKEHENEMKIMQEELEKKSKELTEFLSVKAENERLKREQKENEERLKFQAKEE
                AFKEFKEQESKNLEFERDKMRLEFQKSTQEQELKYKELETNFKSVAQKLEDAQRRIEQ
                GSQQLQGEAAELLIEEYIQNEYIGDEVKEVPKGVNGADCLHIVKDSFGNICGSILYES
                KRTKEFNKEWIDKLKLDSIAAKSDIAVLITKTMPKEKQKTHFKEGILICTFAEFKGVL
                AVLRESIVNAYKLKNALQNKDEKNHILYEYLNSKEFNTQITFILKTYQNMKEELEAEK
                RALQNIWKKRERAIENLSFNSTAIVSSLNAIFSDLQGGNLIGEEGLKSLENLAKDED"
gene            156503..157699
                /locus_tag="DMACINML_00898"
CDS             156503..157699
                /locus_tag="DMACINML_00898"
                /inference="ab initio prediction:Prodigal:2.6"
                /inference="similar to AA sequence:RefSeq:EAQ71913.1"
                /codon_start=1
                /transl_table=11
                /product="drug resistance transporter, Bcr/CflA family"
                /protein_id="Prokka:DMACINML_00898"
                /translation="MQKHTKIHGFSKLKLIIILALMSSIAPLSTDMYLPALSHVEQSF
                HTSKALTQLSIASFFLAFALGQLIYGPLSDIFGRKIPALVGIFFFIVSSLFCVIIDNI
                YAFIALRFFEALGGCAGVVIARAIVNDLFEIKEAAGIFALMMVFSSLAPMLSPTFGSF
                LLEYFSWHSIFTTLFALGILLFLMILFGLKESAPHLKNKKFSHEEAMKSYKFVLKDKR
                FLTYVFCAALALAAMFAYITGSSFVFTQFFGLSEQQFGILFGVNALGFVICANINARL
                VRKFESEKILSKALIIMFVSTLILLINAFLYPNLFLFEASIFTSIAMLGFIAPNTTTL
```

```
                     AMARFKDHSGTASAVLGFIQFALAGLISSVVSALDANTPIILACVMCACVLIANGVYF
                     QAKRKA"
     gene            complement(157696..159174)
                     /locus_tag="DMACINML_00899"
     CDS             complement(157696..159174)
                     /locus_tag="DMACINML_00899"
                     /inference="ab initio prediction:Prodigal:2.6"
                     /inference="similar to AA sequence:RefSeq:ASI86604.1"
                     /codon_start=1
                     /transl_table=11
                     /product="membrane protein"
                     /protein_id="Prokka:DMACINML_00899"
                     /translation="MCKKTLNKDVRISLIRQVRKEVLSEKLLENEEKKGLKKNIYKRI
                     IGYWENASKDINEDLLKTEVKKISYEMISRKILSKELQIEEKNIFVNSDTIYIRPRAH
                     YSVGEDMKDAIKRQLSERLPIKKVNIGLFTKFSLQFKQCDFKCRIGEEYYREPKHITV
                     IEVMYLEYKNLAFVNCNFFEEVFFTWQKKFKLLTMENCHFYKAVYFSGVFNENTFFNS
                     SYFRDYINFYTCEFEKTACFYGVTFDKTPNFPQAIFKGNINVVNTNLNFTFDDLQERI
                     KQEYKDFNKAKEKKPLDKIANDFRDSFRNFKSTLIKDNNLLDASNFHKYELYCKELEL
                     KNKKNKTLKDKIDKWQLSFYHKLCDHHTDLLLNLRWLVVLIGSFALLYFTSRVIQDIN
                     LLKVLNQYGVCLSIVGVFNLLCLYWFGCIKKFDFFVYFNLIAVLWVICYKPKIIFGIV
                     NLIGDNSYNGFENVLITIYTILLALVLFSLQKTARKNSIVPS"
     gene            complement(159252..161162)
                     /locus_tag="DMACINML_00900"
     CDS             complement(159252..161162)
                     /locus_tag="DMACINML_00900"
                     /inference="ab initio prediction:Prodigal:2.6"
                     /inference="similar to AA sequence:RefSeq:CDH61872.1"
                     /codon_start=1
                     /transl_table=11
                     /product="Putative integral membrane protein"
                     /protein_id="Prokka:DMACINML_00900"
                     /translation="MMRKAFEKILNTLEKCCEETHQNDKSEYEIKSEYEIRRQLFNIL
                     SKNNPNNIKEYDISIPSDEKTKETIKEIKGKEDFRQGDELFSDDEKWYLIRKATLDLS
                     SLCDNMNNIQYNAIAQCNIIFSECNISLSDKQSENNRIVKTNLYFYNCTFEKALIKLE
                     NIIFEKTLTFNQCAFYNDLEIYQNQFLDHLVFINCSNEQNDKKIISLDLQDNEFKGYL
                     FIKNCTIKNISLWKNKFKNRCYFVDSKFGYKDKDNQVKLNFSNAHFEDEVYFNNSEFY
                     NYADFHECKFDNIACFYGVKFYKAPNFSQVVLKGNLNALNITSNFVFDDLEKQIDQEY
                     EDFNKDKKEQDKKPLYKFSNDFRDSFRTFKNALIKDNNLLDASNFHKCELYCKELELK
                     NKKATTFKDIVDRYQLFFYRKLCDHHTDLLKAFHNLLIVIMLFGMFSFVFDKFKQPSH
                     IENDIKYNIVKVDSNENYIFKEHNKTTYNLLSLNIEKEIQDLDISKFCFTFTLGLLFA
                     VVILSNANCLWFLFLLLLEVIVFYIGLPKSIFSHLLIILSFVCLFVFILVLDDEFERF
                     LFVSISYAVCIFILLAKPSLLLPIFGSFLDKDSNTTYPLLFSLSVVYFILVVLVLFSL
                     QKTARKNSIVPS"
     gene            complement(161180..162841)
                     /locus_tag="DMACINML_00901"
     CDS             complement(161180..162841)
                     /locus_tag="DMACINML_00901"
                     /inference="ab initio prediction:Prodigal:2.6"
                     /inference="similar to AA sequence:RefSeq:AFU42170.1"
                     /codon_start=1
                     /transl_table=11
                     /product="Putative integral membrane protein"
                     /protein_id="Prokka:DMACINML_00901"
                     /translation="MDSEILTLSQDEIENIKKELCRILGYRCKDKIEVINEVNINNFG
```

FAHDKKKHKNLFIISNKEKNLLDLEIDLKKMSSLADMDYQIKFEGYTIKFTDSFSKEK
IKIGQNLYFNNCDFKVIELKNICFKNFEIDGCRYESEQKKPIVFRACEFKELSLNNSI
FSESIKFEEETKIKYLDINLSEFKKYFFMQNCIIENISLWKNKFKDRCYFVDSKFGYK
DKVNKAELNFSNAHFENNAYFNNSEFYNYADFHECEFEKIACFYGVKFDKTPNFSQAI
FKGNLNVVNTNLNFTFDNLEEKIKQEYGSYKSQRIAKEVGVIPNLYQEKSLDKFANDF
RDSFRNFKSTLIKDNNLLDASNFHKYELYCKELELKNKKDKTLKDRIDKWQLSFYRKL
CDHHTDILQSLNSLILVIGIFVISSVVMVASFNYRLGYKPILEHWYFSLDFYNHHINS
IIQDNYLFVVKVNFAMLFGYLGLVGFALCLKYIREFFIIISYGVTLSVLAVSPKILIP
AMGIFTDKRSMLDPLSTIGGIYTIVFGFVVFSFIKTIRKNSIVPS"

gene complement(162881..164551)
/locus_tag="DMACINML_00902"

CDS complement(162881..164551)
/locus_tag="DMACINML_00902"
/inference="ab initio prediction:Prodigal:2.6"
/inference="similar to AA sequence:RefSeq:CKG55942.1"
/codon_start=1
/transl_table=11
/product="KAP family protein"
/protein_id="Prokka:DMACINML_00902"
/translation="MEYKQQIKNFLEDEENYSLLISGSWGVGKTYLWKEIENEINPKI
IHKENLWKKFKSNINFLNHLIILFSIIKQCVGITLISITEYFNLCNYIKNPNKQVKNI
VYVSLFGKEHYKEVLEEITLKAYKHNKILYFIRNITFWKLSVGTFLQLFITKNFKNII
VCLDDLERKSDKLDIKDILGLINQLKEEKCKVILISNKDELSNNKEKDKEIFDTYKEK
CIDLNIHIRSKTEVVSQILKEKNPEISKEIIPDSIYQIENLRNLNKIIKALEFFNKEL
QLADRLEQNKEYKAFFIEISKKIILEYENMDSYNLNEEKQQGIPLPRIFLKTINSIIK
DYTNNGLIYSIPNDKKEDFEKELKEYRFYSGLFALRKLLEDSIQYPNGTNKNKLEIFF
NSVDEKQLDDFVYELGFINFIALDRVFHFDKNERNLKMKKYCLELAKDCKYDEKFINI
ICNGDEALKQYYEGLEIKEITIKQNNFSKKIQKIQDKYKYIKSYSDFYAHLEKINKSS
EEEIRDFLNNEMIFSYCDIYFSIKEANKSNYPRLYSVYKSFEDEKKSN"

gene complement(164551..164667)
/locus_tag="DMACINML_00903"

CDS complement(164551..164667)
/locus_tag="DMACINML_00903"
/inference="ab initio prediction:Prodigal:2.6"
/inference="similar to AA sequence:RefSeq:ADT71802.1"
/codon_start=1
/transl_table=11
/product="type II restriction-modification enzyme"
/protein_id="Prokka:DMACINML_00903"
/translation="MKIYSKTLKAKDNENKINSLVYKLYNLTEDEIKIIESK"

gene complement(164686..165789)
/locus_tag="DMACINML_00904"

CDS complement(164686..165789)
/locus_tag="DMACINML_00904"
/inference="ab initio prediction:Prodigal:2.6"
/inference="similar to AA sequence:RefSeq:AFU42170.1"
/codon_start=1
/transl_table=11
/product="Putative integral membrane protein"
/protein_id="Prokka:DMACINML_00904"
/translation="MAFDSLVLKARISFLFSIFKDEALFFNINPNNCIFHNVEFNKIN
FIHNDDINVESYAESYVFQNAIFKDTLSFEGMEFERLRFDNVLFNGVVTFSNTKLNTK
PQFINCTFSNQFNIEHQYIKYSDKDIENKINNIQDKNDKFRALLNLRDLFRKLKSNRI
AHHNLIDASELRTQELYARELELKYKEKKSLREKIERWQLSFYHKLCDHHTDILQSLN

SLILVIGIFVLSSVAMVAIFNYRLGYKPILEHWYFSLDFYNHHINSIIQDNYLFVVKV
NFAMLFGYLILVGFALCLKYIREFFIIISYAITLLVLVVSPKILIPAMGFFTDKRAML
DPLSTMGGIYTIVFGFVVFSFIKTIRKNSIVPS"

ORIGIN

```
        1 gctattattt ctaatactgt aagtgatata gctaataata tacttgaaga tgtgaaaaag
       61 aaaaggtttt aattagagac tgatatataa aatttagatt gatatgcgag tatcaatcta
      121 aagattttga tttgttggta cttttagttg attactcgtt gatatctttt aattcattat
      181 tcattaacac ctaggtagat tgggttggtt tttatatagt tctttgtttg atgatttatt
      241 ctatattttt tgatattatt gcttttatgg attagttgcg tatctattga atttatttct
      301 catttagctt aaaataaatc gccttttttg attaaattcc ttgcttaact tcatttgtct
      361 actctaaaaa ttactcactt tataaattac ttaaacagct aacatacact atataacttt
      421 ttgcttgttt gtaaatttat gcatatttat acttaaaatt ttaaactaac atagaaagga
      481 tctatataag aaataaaaag gtttttacaa aaaaatatag tggaattttt aaaaaataaa
      541 atatgatttt ggtttttatt gcgatgattt aaaaaattca agccatacta tacaattaaa
      601 acaaaaacta tttcaataca tatagaaaat caaattaagc ttattgggct aaaatttgat
      661 ttttatctcg acataaattt tgccagttag aagtagtttt aagctctaaa cactgcttta
      721 gactttcttt ttgcgcctga gtattttgca ttctttcata agtcatactt tgtatatata
      781 aagctctagc cctatcttca tctgaaagct ttaattttaa caaatctatt aatactgcta
      841 aagattcttc attttgattg gttttggcta aggtatcaag atagataaac tctaaatttg
      901 gactaaaaag attgattcct tttaggtttt gatagtcaat agccttaggt gcataattta
      961 aaatcgttgt ttgcatattg tgatcgtttg cgtaaattaa taaattatca taagcttcaa
     1021 ccattgtaaa attcatagga aagctctcta aaatttgtaa aattttgata gccatattat
     1081 aatcatccat tttcaaataa gacaaaaatt gcaaataata agctttaaaa ttctcctcat
     1141 cactctttaa aattctagaa tttgctattt ctcgagctat atttaaagct aatggatatt
     1201 gtttattatc ataaagtata gatgcttttt gcaaaccata aaaaatagaa tcctcattat
     1261 gatttttatc gatataatcc attgcttgtt gcatatttga agttcttata agacaagcta
     1321 gcatttgttt tttattttct atcttttgtc caatttcata ggaattaaat tgtgtaaata
     1381 tattaacagc attgatacaa ttatcggctt ttaagtcatt atttagaagc tgagtagctg
     1441 cttgctctaa caactttgta gaatttgtta atttcttatt ttctatctga tctttatagg
     1501 ctaacactgc gcttaaatta ccctctttat aataaagcgc gacatcctca tctaaggctt
     1561 tatttgctat attttcatct ttttgagcat attctttcat taaatttgca taacgctgat
     1621 gcaaaaatgt agcattatta tccgctaaag caaagaaaac ctcatcattt gcttttctaa
     1681 tttcatctaa atattttcca tctaaataat tatccatata caaatcaaga tattttttag
     1741 catcacttga tcttgaagtc atagctaatg ttaaagcaag atcctttaaa acttgctcat
     1801 atctactatc aattttaggc atttttaaaa aaacatcttc gtaaatactt gcacttatgt
     1861 catattgttt gtgctgatta aaaagtttag caagctccaa agatctaaac atatcctttg
     1921 gaaaatattc aggattagcc tttaaaattg tattgacata ttgcacagct ttatcgattt
     1981 gattgttagc aagataatct ttgattaaac tcatcgctgc tcttgctgct aaatctaaat
     2041 ttttagtatt aaaataaata tcctcataaa tatctatagc tctttctttc tcatttaaag
     2101 gatagatata atcagcataa tcaagtcttg ctagctgagt aaaataacta ttgggttgtt
     2161 cgttatttaa aatcgacata atatattcta tatcagcacg ctgcgataag gcgatatagg
     2221 ttcttagcat aatatgcaag acttctggaa aattcttatc acttgtaaaa gtacgattcc
     2281 aatttttagc ctcatcaatc atttttttcca aaacttgctg atcacgcata cttggatctt
     2341 gagtgtaaat ttgactttgt gctctaagct tataaagtat aaactcattc ataaaaatac
     2401 taccacgata acgattgatg gcattttgag catctgtaat cacttgagta tagttggcct
     2461 tatcatactc atttttttata cgcaaataag tattgatatc ggcactttga gggataatta
     2521 caggatctga atttaaatcc aaagctccaa cataaggcaa gctttcatgc gggaaattga
     2581 tattaaaatc aagtccatca taatctttta catgctctaa ttctggagta aaaataaaag
     2641 taaaactctt acttttatgc atacttgaag aatttagctc tttatccgca taaatatttt
     2701 gcgatagatc aaacattctt gcgcttattt taggtataat aatcatttta atcttttgag
     2761 gctctttaat aaattttaaa tcaaaagcag taaagctttg atcacttagt ttattatcca
     2821 ctatacccat aatctcgcat tcaaaatgca ttttatcttc aataataatt ttttgacaag
     2881 taaaatctaa gtcattacta gcatgtaaga ccgcaaaagc ttgattgttc tcgcggcctg
     2941 tatttagggc taattcaaaa gaaaaaagag tgcaaatact cagcaatacc aaaaatataa
```

3001 ttttcataca ataattatat tatattttaa gtaacaaaaa taaagcaaac acactaatca
3061 aagtgcaaag agctaaaata aaactctgaa taaaatttaa atgctctgaa actttagctt
3121 gagtttttat aaataaagca tggataatta acttcaaata agcataaagc ataagaagtg
3181 atgccaaagc cataaaaaga gctaaatacc aataatcagc gacaattaaa gtttttaaca
3241 ccatcacctt gccccaaaaa gcaccaaatg gaggaattcc agctaatgaa agcacacaaa
3301 aactaagcga taatgcaatc aaaggctttt taagcaataa agcattaaat tcttcaaatg
3361 agcttccctt aaaggtacta agaatcaaaa atacaccata attagcaaag ccaaaaagca
3421 cccaataaac aaatatagaa tttaaaacat catcagccgc atttaacaaa ggaatacatg
3481 ctgctaaaac aaaactagaa tgcactatag aactataagc aaacattttt ttgacatctt
3541 tttggctcaa tgcggctaaa gctgcaataa gcattgaaaa tactgccaat acatttaaaa
3601 tattttcaaa tcctaaatga tttaaaaagc taaaaagtct gataactaca acaaacattg
3661 ctatttttgg gacaacagaa ataaaagcaa ctaaatttac atgcgctgaa gaatacacat
3721 cttttaacca aaaatgaaaa ggagctaaag aaagtttgat ggcacataaa acaaaaatca
3781 taactcctgc tcctaaaagc ataggatctt tatcaagagc caattttaag cccaaatcca
3841 aagtacctgt tctaatataa atcaaagctg atgccataac aaaaaagccc gaacccactg
3901 ccgcaacgct aaagtattta atagcacttg aaatagcatt attgcttcca cgcatagcaa
3961 taagtgtata aagagccaag gaagagcttt caagtcctat gaaaatcaag atcaaatttg
4021 agcttgaaac cataagcaaa agccccgcta ccataaataa aaacagacta taaaattcgc
4081 cttgattttc ttctttttgc atcaaaagat acaaaaatga aaagcacaaa ataaccaaag
4141 aagcatacaa agaaacaata tcattattta gagttcctaa aaatcctttt gcttctaagc
4201 cttgcgcagc gatattattt aaaattaaaa aaacactcac aatcaaagat aaagagctag
4261 tagccatata aaaagttcga tgaaatttcc aaaaacccga acaaagtaaa agcacgatag
4321 ccacagcaat caaaaatgca aaaggatagg ataaagggat atttaagctg cttaaatcga
4381 tattacctaa cattattttc cccttctaaa tgatttaaaa atcgaatggt ttgatcatca
4441 atagctcttt ctttcatttt ttcaacaatg aaatctgcat ctttatttaa aggatctaaa
4501 aaaatctttg gtgctatacc caaataaaaa atcaaaacca ctataggtat caaagaaaga
4561 atttctcttg catgcaagga gcaagtttca agctgacttt ctttttgcat aaaaaacatt
4621 tttctaaata cataaagcat ataaatagca cctaaaatga tcacaagtcc tgccaaaaga
4681 gcataaagta aattaagttt tgcaacacct aaaagaatta aaaactctcc aataaaagat
4741 agtgtaagtg gcaaagatat acttgcaagc aaaatcaaag taaagaaaat agtaaataaa
4801 ggtgctttac tagctaaaga atgataataa gaaatttcaa gtgtgccata tttttttatat
4861 aaaagctcag ccattaaaaa caaagctccc gtaacaatac catgtgcaaa catataaaat
4921 accgctccac taagccccaa agcattaaaa gaaaaaatac ctaaaatcat aaccccaagg
4981 tgagaaatcg aactataagc gatcaattct tttaaatttg aagcgcggta agcgattaag
5041 gcattataaa ttatacttac tatacacaaa gctacaacta aaggcataaa atacacactc
5101 gcatcaggga aaagtggcaa gcaaaagcgt aaaaatccaa aaggagccat tttaaatgct
5161 acaagcatca tagaaactaa aataggtgaa tttgcataaa cttttggcgc ccaagtatga
5221 aaagggaaaa gtggtgcttt aatagcaaag gcaacaaaaa atgctccaaa aagtaaaatt
5281 tgttcgttaa aactcatagc cgaagcgtta tttttccaaa tttctatatc aaaagtgaag
5341 atatttaaaa gtttgtaggt cgcataggct tgataaatta aagccaaaag cattaaaata
5401 gaacccgcaa aggcataaac aaaaaattta atccccgctc tataatcttt accatatacc
5461 cccataatat aaagcaaagg taaaagtgaa aattcccaaa atacataaaa aagtaaacca
5521 tcaagcgaag aaaataatcc cataatcgca aattctaaaa agaatatgca agacaccatt
5581 gctttttgtt caatctttaa aaataaaaaa cttaaaaata tcatcaaaga gcttaaaagc
5641 attaaaatca aagctatact atcagcgcct atatgataac tcatgaaatt tactacctta
5701 aaaggaagtt gatattcaag gtttattcca tttaaataat cttgccaaat cttgatattt
5761 aaacccagca ccattaagct cgctatgacg ctaaaaactt ttataccacc acgatttaaa
5821 acaagagtta caaaagcggc aattaaagga aaaaagatta gataatttaa cattttacac
5881 cacccatacc aaaataagca aaatcacaaa tcctgccacc aaaaatctaa gcatcaaatt
5941 aagactattt gtcataatta ttttttgcga aatcaaatta caataataag caattttctc
6001 cactatagca tcaaaaatat aagtatcaca atgttttaaa accgcacaca agctttcgta
6061 ttttgaaact ataaattcat gataaaactt aggaataaaa taatcattgc tcaaaagctt
6121 atggatttta ttttgctcta tgctatcttt aaaccaagaa gccttataag ctataatagc
6181 taaaactaca cccaaaacag ctgctacact cgcacaaatc attacaagtt tgttttgagc

6241 atcgataaaa ataagttgag tgctaagata ttcaaaaaaa ctatgctcaa acaagcccgc
6301 aataatagct aaaaccacca aaggactcat agccaaaaga gctattttac ttgcttcatg
6361 tggatgttta tcatgtctag aaggagtgaa aaataccaac attaaaagtc taaaactata
6421 aaaagcagtt aaaaaggctg agattaaaag aattaagaaa attccatgat aatgagcgat
6481 aaaagaataa cccaaaatca aatcttttga gaaaaagcct gctaaaggat aaattcctgc
6541 taaagccaaa gaacctatag tcataaaaat agcagtgatt tttaaagatt taaaaagccc
6601 acccattttt ttaatatcta gttgatcatc cattgcatgc ataacatttc cagcacctaa
6661 aaagagtaaa gatttgaaaa aagcatgggt tactaagtga aataaagcta taccataagc
6721 tccaagtcct gcagccacaa acatatatcc aagctgcgat aaagtagaat aagcaataat
6781 gcgttttaaa tccctagcca ccaaagccat ggaagctgca aaaatagcca caaatgctcc
6841 cagcaaagca ataaaataag aaacctcagg cactaaagaa taaatttctc ctgcacgaat
6901 aaccaaataa acccccgcag ttaccatggt agcagcatgg attaaggctg aaacaggtgt
6961 aggtcctgcc attgcatctg caagccaagt atgaagagga aattgtgcgg attttcccat
7021 agctccgata aataaacaac tcgcaatcaa aaccaaagca ttatgatcta aattttgcgc
7081 tatggcaaat acttcattgt attttaaagt acctgcttgg atataaagcc aaaaaattcc
7141 taaaagcata cccaaatccg caatacgatt catgataaaa gcttcattag ctgcaaaaga
7201 ataagtatta tttttatacc aaaaaccgat taaaagccaa gagcaaagcc ctactccttc
7261 ccagcctaca aaaagtccta aaaaattatc actcattact aaaaaaagca ttgaaaatac
7321 aaaaagacct aaataagcga aaaatttatt aaaatcttta tcgtgagcca tataaaaaat
7381 actataaaaa tgcacacaag ttgctacaac acccacaaca ctcatcatag taagagaaat
7441 agcgtctatg ttaaatccaa agcttaaacc tataaaccac tcaaataaac ttacattaaa
7501 agcttgattg ttaaaaagta gatacaaaga acaaaatgca cttataccga tcaataaaga
7561 acatatataa gcgacaaaaa tttttctttc acttaaagca aaacaacttg caaataaaaa
7621 agctacaaaa gggctaaaaa gagaaatcaa agctaaattt tgcattatca aacctcctct
7681 tttaggctct ttaattctaa agtgcctttt cttctatacc aaagcacaca aaatgccaca
7741 cccacagcca cttcacaagc agccacaccc atcacaaaaa gagcaaaaac ttgtccattt
7801 aaatcctgat gcatcttaga agctgcgaca agagccaaat ttgctgcatt gagtaaaatt
7861 tcacttgaaa taaagagcat gatgagattt tgtcttttta aaataccgat taatcctata
7921 ataaacattg caatagctat aaaaaaatac ttttctacca ttatctttcc tttgtaagct
7981 ctttatgtgt aagcacgatc gcacaaatta aagcgattaa aagcaaaata gctataaatt
8041 caaaagctaa aagatatttt gaaaaaatag caaaagcaag ttgcttatta taatcgaata
8101 aatttggatc acttaaagcc aaatcactcg cgatattttg gtgcttaaag cccaaaagca
8161 taaataaaag taaaacggca cttaaaatta ccaaaagaaa aaaacttttc ttagctctta
8221 atttttcttt gaattctttg gaattgtcaa aaacatcat agcaaagcta taaagcccaa
8281 gcacagcccc gctataaact ataatttgta tcactcctaa aaactctgca tcaagtaaaa
8341 aataaaaacc cgataaaaaa accattccac ccgctaaagc agaaagagaa taaagcatag
8401 ttttactcaa aactgctacc aagaaaaagc ctaaaaccac tacactaaaa aatgtaaaag
8461 ctaaaatttc tatcatttaa cttctccttg tggagctaaa tttggcaatg cacattcttc
8521 atcggtgcat aaattttttt ctctttcttg taaaacttca taataatttg gagttttttt
8581 cacaagtaaa tccgcatcct ttctaagact acccgcaccc tcaaattcaa cttgattttt
8641 aagcttatca ataggggtta aaaaatcttg cttatagcca aaataagatc tttgttcagc
8701 tgcattttca tattctgtac catggactat agcaagttct ggacaaactt cagcacaaaa
8761 accacaataa atacaacgcc ccaaattaat actataattt cccacctttt tacgagcatt
8821 ttcatctaaa gaagtttcca tcctaataca attactgata caaatctttt cacaaagccc
8881 gcatcctata catctttcat tttcactttc tataaaacgc ataagacggt gtacggcacg
8941 gtaacggttg tcaagcttta ccttttcaaa gggatatttt atggttgcac tattatttct
9001 ttttagcatt tcacgcataa ccacccacaa tcccacaaaa agctctaatt tcacacttct
9061 ttttaaagct tgtgaaatct tctgccaagt gctagtagga gtctttcttt tttcatcgat
9121 taaataataa tttttcatca caaactccta tataagtact gcaaaggcag taattaaaag
9181 atttaaaacc gccaaaggaa ttaaaattaa ataacacatt ctcatcactt gatcagggcg
9241 aagttgtgga aaagctgctc tagcccaaaa ataccaaaag aagataaaac ttgattttac
9301 tatcatcata atccagccag gtataaaata aaagtcatta aaaccgccta aaaataaaag
9361 cgtgatcaaa atcgctcctg ctatcataga ggcatattct cctataaaaa acatacccca
9421 tcttaaaccc gaatactccg taccataacc cgcaacaatg tcagcttcat tttcagttaa

9481 acaaagtgga gttctattgg tttcgataaa taaagcaatc acaaaaagcg caaaagctaa
9541 aggctgttta aaaataagcc atgaaaaaaa tccatcactt tgataattat taatatctat
9601 caaagaaaaa gatcctacaa gcataacaat agctattaaa gcaagagctc ctacactctc
9661 ataagaaatg atggctacaa gccctcttgc agcacctatg attgaccatt tgttattgct
9721 tgcaagccca cctaaaaata tagcataaaa acaaagcccc gaagttccta taacaaaaag
9781 caaagccaca ttaatatcgg caatcaaagg ctgtatagtg cgaccaaata atgtaaattc
9841 aggaagcata ggaatagccg ctaaagatac aaaagcacaa atggccgcga tcaaaggcgc
9901 tatagcaaaa atcaattttt gagaatttga aggtatgata tcttctttag taaaaagttt
9961 tatcatatct gctacaagct gaataagtcc aaaaggtcct accatatcag gtccgatgcg
10021 tctttgaaaa taagccaaaa cctttctttc tgcataagtt gcaagtcctg ctaaggtagc
10081 aaaaattgca atcactataa tacacttaat taaagcctct aaagcaaaaa aagcaaaatc
10141 actcatgact cgctcctaat ttttctaaat ttgccacaat aaatctttca tcaaaaaaac
10201 ttagcgtgtc gatttttttca tcaaaataag gtaaaaaagc cccatttttt atgtctttat
10261 ctatacgcac actaagactt aattccttat ctttgatctt aagttttaca agatctttat
10321 cttgtaagtt tttttctttt gccaaattct gactaacccc taaaaaaatc acttcgttga
10381 ttgcacttgc acgatttgaa aatctaccaa attgataact aggatttgca ctgtaaagca
10441 aaatttcatc ttcttgtaag gttaaatttt gcaaatgagg agctacaaat tcattcttag
10501 cgcttttttt acagcattct aaatttaaat catacccctct tttgcattct ccaccgttgg
10561 tataaaaatt atcaagacta tcaaattcca aaggagaaaa acctttgtta ataggaagtc
10621 ttttggtgta attgatcgta tactcctcat caaaacctaa agcattagct aaatcattta
10681 aaaagtatcc atcaaattct aaagctgcat tggttggaac tacttttttta tcataattta
10741 aaaaagtacc ctcttgctga tttaaacttg agctagctaa attagcatgt tcttcataag
10801 aaaagctaaa atctcctctt tcattataac ccaaagtctt accttcttgc atactctcat
10861 ctaaatcaca aatcatagca acgcctaagg tatttgtatg tgtaggattt aaaaagactt
10921 taaaaggagt tgtattttgg atcaaagcta gtaatttggc taattttttta gcattcttgt
10981 gaaaataaaa atcacttcct ataatcaaag taaaattagc ctttttaagc aataaatctt
11041 ctaatttatc ctcatctata cccaaatttt tagcaaaaac cgagcgtttt acttctatgg
11101 tttttttcac ttttttaggc acaacttttt ttacttcttt agaaatttca ttgccttctt
11161 catctttttc tacaacttgc tctataattt cttcattaat actttcttct atttctttat
11221 tttcatttat aaaaaattca gccaaatttg cttttatatt ttcatcttga ctgaattttt
11281 gaagcaagaa atataaaatt tgttcctcat ctccgctttt gtgagtatgt gaaatgaaat
11341 ttttagaata ctttgcaatg cctgcatctt ctatagggtg aaaataaagt cctgagcctt
11401 tattcatcac taaggcattg ttgattttat agctaagcgt aggcgcatcg tatcttaaaa
11461 gcgttcctgc aacgactaag aaatcgcttt ttgtgatatc ttgagttgta gcattataaa
11521 actcaccgct attgcttata aattcttcca aaaagtcttt aaattttaaa gcttcgttat
11581 tgataagatt taagccaaat ttcttttttga aattttgtaa aatcaaagct tcttcgttgg
11641 taataaagct attaaattta atattttttaa tctcattttt ttcaaaaagt tctataagct
11701 ctttaaaagc tttttcatcc ttacttgcct cattttgtgt atcaaaagca aatcttgcag
11761 ccttatttaa actcgcaaaa gaaaaatcat tgcttacgcg gtaaatttta ggtttttgat
11821 tgctaatgcc gctttgtttg atatcataat acataagctc acaatcactt gaatgtggat
11881 ttgaagcagg aattctttta agctcccaaa tattagaagt atattgaaat ttagagccta
11941 tcaaagctcc tgttggacat acactagtac actctccaca aaaagaacaa tcaagcgtat
12001 ctccgtttgc agggcctatc aaactttttt gaaatttcgt ccaaatcgca taagcgtctt
12061 tacccatact ttctttaaaa ctattatcta cactatcgcc tccacgaggt actgttttta
12121 aagcactctc gccgatttta tccttgcaaa ctgtgataca acgctcacat actatacaaa
12181 gtgcaggatc gtaattgatc attccccaat gtttatgcgg tttatgcgta tctttaatcc
12241 aatgtctttg gatatttaca cggcttttgt gggtgaaatt ttgcaattca cattcgcctg
12301 atttatcaca aaccccacat tctaaaggat ggttaataca ataagcttgc atgatttcat
12361 ttctttcatc ccaaagattt tgcaaatcac tttctacaac catacccttct tttgctttag
12421 tattgcaaga atacactttt ttaccatcag cctctaccat gcacatacga caagccaaag
12481 tcggactaca gcctgaaaga tagcaaattg ctgggataaa aatatcattt cttcttgcta
12541 tatttaaaat atattcgcct tcttcaaagg cgcaatctat gccatttatt ttaattgtca
12601 ttttgcttcc tttatgcgaa ttggagtaaa atcaaaacct tgatgatcgt aatttaaaaa
12661 ggctatagtg ccttgcaaat tttcatccaa acaaagtgtg gttttaaagc taaaatcttt

12721 agccaaaact tcaacttcac ttttatcttt acattttgca atttgcaaaa attgcttaga
12781 acaatgtaaa ttatcatctt ttaaatttgc tctaaaaagc accaagccat cataactatc
12841 aagctcttgt aaggtactca attttgtatt tttaaaatca cattcttttt cactactatc
12901 taaaatttta agtttgtatt tttggcttag aaaatttaga aaatatttga tattttcact
12961 gtctttgtaa aaattaatat tttcatctac aattaagtat tttgcatttt ctaaaaaatc
13021 taaaatttca ctcgcctctt cttcgccaaa acaagattct gcactcaaat atccctcatc
13081 taagtcttga aaattatcca tttcgctcat cttgcaaagc aaagctaaaa cataactcaa
13141 actcgcaatt tcacatttat aaaatttcgc atttaaattc ttatcttcaa ggcaagaaat
13201 ataaatattt tcaccttgaa ttttttcaca taaaatttta tttttaagac ttagcatatc
13261 cacaaaacaa agcgctcttt cgtaactcat acgctttcct ttttaatcac tatagtccaa
13321 tccacttgat taaatttcaa ggaatttaaa agctcgcagc cttgctcttt aacaaaagag
13381 aagcttttct ctacgggtga aaagtcaccg atttcaaaaa gcaccaccaa aacttctcct
13441 ttttgagctt cttttatgat ttgtgctaaa tcttcaaaaa gctctttact ctttcttaaa
13501 tccactcttc tcatcgatct atctcaccta aaactatatt tacatttccc ataatggcca
13561 caacatcagc caaataagtc cccacaagca tttcttcaaa aaatgcacaa tgccaataac
13621 taggcgttct agcctttaaa cgataaggcc ttcccgtacc atcactatgg ataaaaaatc
13681 caagctcgcc cttaggactt tcagttggca cataaacttc accctttgga ggttttaaac
13741 cttgagttat aagcacaaaa tgctgcatta aagaataatt ttgcgttaaa atttgctctt
13801 ttgaactgct gacatattca ggatgattgg ccaaaatttc aggcggagta tctttgtaaa
13861 gcgcggcaca ttgtctaagt attttaagac tttcgcgaaa ttcttgcata taaactttat
13921 atctagcata agaatctcct tgagttgcat aaggcacgcc aaagtctatt tcattataaa
13981 gtaaataagg ctcttccttg cgtatatcat atcttacacc acttcctctt aacatcaccc
14041 ctgtacagcc ccaattcatc gcctgttctt tgcttacaac acctacattt tcagttctta
14101 ggcgccaaat tctattatca tctaataaag cttcataatc agcgatatct ttaggaaatt
14161 tatcgcaaaa tgccaaaagc tcgtttaaaa aaccttcagg caaatcaagc ataaccccgc
14221 ctattctcat agaagaatgt gtaagtcttg ccccacaata tttttctatc aaatccaaaa
14281 catactcgcg ttctctaaag caatacaaaa atacactcat cgccccaata tcaagcgcgt
14341 gagttgcaag ccaaagcaaa tgcgatgcaa tgcgattaag ctctaaaagt atcattctta
14401 tcactgctgc acggcgtgga atttctaaac cacaaagctt ttcaacggct gcgcaataag
14461 cataattatt agccgaagca gcgatataat ccatcctatc agttgtcggg ataaactctt
14521 gatatatcat attttcagcc attttttcca taccacgatg catatagcct atggtagggc
14581 gcgcttttac aacctgctct ccatcaagtt ctaaaataag gcgtaaattt ccatgcgctg
14641 aagggtgttg tggccctaaa ttgatgatca tttttgagtc ttcttgctca aaagctatat
14701 tttcataata aggttttaac ttgctaggaa tttgcatctt aacgcctttt gtctaaaatt
14761 tttgcttcgt cgcgtttgac ttttttttaca aaagcaacgc ctccatcttc ttggtattct
14821 tgtttaaaag atacctcttt tctagatccg ccttttggta cttcatgata caatcttgca
14881 aaattgagtg tatctttatc atctgcaaaa cctggatctc tttgctcagc tcctacgatt
14941 tcgcggtatt ctttaccaaa aattttatcg atttcatacc actgcgcgaa ttcatctcct
15001 ttcaaaggat aggtttttaa aagaggatgg ccataccaat catcaggcat taaaatacgc
15061 tttaaattag gatgatttat gataaaaatt ccaaacatat cataaatttc tctttcgctc
15121 caatttgcac ccttaaaaat atgcgctacg ctttgtaagc gttcttttac acccacaaaa
15181 gttctaacac gtacccttaa tcttttttct aaatttaaaa gctggtaaaa tacttcaaaa
15241 ccatttttat cggctacaaa atcaatagca cttgcttctg taaaatttaa ataccctaag
15301 gtttgaagca tttgtagggt tttaacattg tcttcttttt taatttctat cacccaaaaa
15361 tcaagttcta taaaagaatt tacaatatca acttctgttt ttaaaacctc ataatcttgc
15421 ttaaatacac tctcattaac atctaatttt tgagtatggg gtgcatgata aaatctgtct
15481 ttataataat tttgcagttg ggcatttttt ttatcgctat attttctcat cacaccaacc
15541 tttttggagc cattttgcga ctggcttttt cttttcggat ttttttctgc aaaatcatta
15601 aagcaaactg aaaactttca gggcgcggag cacagcctgg cacataaata tcaactggaa
15661 tgatcctgtc tacaccttga acagtagaat aagtattaaa catacccccct gtatttgcac
15721 agcttcccat agaaatcacc catttaggat caggcatttg atcgtatagc cttcttgtaa
15781 attcagcgtg tttcttacat aaagtccctg cgatgatcat cacttcacta tgacgcggag
15841 aagctctaaa aatcgttcca aatctatcaa aatcatatct tgatcccccct gctgccatca
15901 tctcaatcgc gcaacaggcg agtccgtagg ataaagccca caaagaatta cttctccccc

15961 attgaacaag cttatctacg cttgttaaaa ccactggcaa accactggca taatttactt
16021 gatgctctgc catgcaaacg ctcctttttt ataagcatat aaaaatccaa cccctaaaag
16081 taaaataaaa acaaatactt ctaaaagccc aaaccatcct aaatctttaa aaatcaaagc
16141 ccaaggaaat aaaaacacca cttctatatc taaaagaata aaaatcaaag ctacgatgaa
16201 gaattgggaa ttaatcttat tagcttgttt tactggaatt ggaccacatt cataaattcc
16261 aagtcctagt tttttacgat tttttgcagc aaaattatta ccgattttag aagctaaaaa
16321 aaccaatcca aaaaaaataa cactagctag cactaacatt aaaaatatac cgaaataggg
16381 atgcgaagca tcaatgtggg acattttttc tccttttttt aaacactttg aatcacttca
16441 caacaaaaat taaaatttac actctgttct attttactct aaactttcta tattgaaact
16501 taataagaat aaatattgtt taaagtttca tttttttaca cttaaagtta acaaaaaact
16561 catcttgatg agtgataaaa atataagaaa tcaaattttc ttgctgataa tgatgtaaaa
16621 aatttaagat ttcttttgaa tttttatat caagactgga agtaatttca tctaaaataa
16681 gcaatcttgg ctctaagatc aaagcacgga taagtcctac ccgagcagcc tctcctccac
16741 ttaactcata gggtttttgt tctaaaattt gtttttttaa attaaagcaa tcaaaaaatt
16801 ttaaaatctt ttcttgctct tttttatat tgaaattttc tagaccatct tggataagat
16861 ttttgatttt tttataggg tttaaggctg ctttttgatc ctgaaaacaa tactgaattt
16921 cttttcttaa aagtctttgt tttgttttat cgagcttatg gggatttaaa tttttatata
16981 taacttcacc aaaacttggg ttttctaaaa aacaaagtat cctagctaaa gtgctttttc
17041 ctgcgccact ttgccccaaa atcatcaaat tttcattctc ttttaaagaa aaattgacat
17101 tttcaaaaat caaatgtttt tcttttttta aataccaatg ttttttaagc tcataaaatt
17161 tacctaaatt tttaacttca agcatgataa atccttaaaa attcatctgc tttaaaaagc
17221 tgtgcttgat ttttttctaa aaaaagcacc tcatctgcta aagaattgac taaatttaaa
17281 tcatgcgtga tacaaactaa gcctaaagta ttttttaact cttttaatat ttgaatgatt
17341 ttttgcatat tttctttatc taaagaagct gtaatctcat cacaaattaa aatttgaggc
17401 ttgctcaata aagctagagc caaactcact cttcttgcca tacccaaact taactcattt
17461 gcataagaat gccacaataa atctatatca tcaaagccta atttttcaag taagtcaaaa
17521 gcatatcttt tatagtcttt ttgatttaat ttcgtatgag tttttaaaat aagatgaaat
17581 aaatttccta tatccaaata cggataaaga ctaagctctg catcttgtaa aacaagtccg
17641 actttagaac gaaatgcacg aagctcgttt tgatttaaat ttagaacaga aacttcatta
17701 aagctccatt catctgcgtt gatttcaaat ttaggatcaa gcagtctgat taaggatttt
17761 cccaaaagac tttttccaac cccactctca cccacaatca tcaaactctt agctatttta
17821 atatcaaaac ttatattttc aaagatcttt ttttgattta attttaagct taaatttctt
17881 accttcaaga tcttactcca ccgatttttt gcagataatt tccaagccat aaaaggggta
17941 agaataaaaa cataagagct aaaagtggaa acaatatcat ccaccaagca cctataaaaa
18001 tagcttttga agattcgttt aacaaggttc ctaaagtcgg tatattaaaa ccaagtccta
18061 agccaaaaaa actcaaagtc gcttcagtag ctatggcatg aatgatatta aatacaaaaa
18121 gaacaaatat taaagatttt aaagctggca ataaatcctt aaaaaaagct ctaaatttag
18181 tccttcctag cgccatactt gctttataaa agtctaaatt ttctaatctt tttagctcgc
18241 tttctataat ccttgctata aaaggccaat gcactaaagc gattaaaaaa atcattaaaa
18301 atatatttac acccacaaaa ctttgaaaaa acatcatcaa taaaaaagca ggcaaagcta
18361 aaaaaagatc taaaattcgc atccaaaaag tataaaaaaa acatcttgct aaaaataaat
18421 aaaaaagagc aaaaaatata gacaaaaaag aactacaaat tcctataaat aaagagattc
18481 tcaaagcaaa aagcaaacgc gaaaatacat ctctgcctaa aaaatcagcc cctagataat
18541 aagtcaaatt aggagcttgg tgcaaatgat ttaaatgact ttctaaaaca tcaaaaggag
18601 ccaataaagg cgcaaacaaa gcacctaaaa tcaaaaacat taaaggtgta agaaagagta
18661 ttatcttaaa caaatttcaa ccttttttaat cttggattta aaatccaagc aaaaacatca
18721 cttaaaaatg taaaaaaagc agcaagtaaa acactaaaaa atatcaaagc caaagccata
18781 ggaaaatctt taaaaagaat gctttcaaaa agcaaactcc caagaccctc ataagcaaaa
18841 acactctcaa ccacataagt tcccatcata aagcttaaag cactgcctcc aaaataagct
18901 ataatagggc ttaaagaata tcttaaaacc aaaaaataaa tatctttttc ccttaaagct
18961 ctagcgtata aattttgaac aaaaatttgc gaaaaagttt cattgataca atttctagct
19021 atgcgcaaaa acaaagctaa atgagataaa acaagcacta aaacaggcaa aataagatgt
19081 tctaaacggt taaaaaaatc atcttcaaaa cctatatcac tagtccctgt tataggcaaa
19141 acctgccaaa aaattccaaa aatcaaaaca aaaaataaag ccaaaacaaa aggtggtaaa

19201 gcaaaaaaat taaaggctaa aaaagtgatt aatttatcta taaaactttc tttataataa
19261 tgccccaaaa gagcaagaaa aacactgagt aaaaacaaaa gcactaaggc actcaggctt
19321 aaaatcaagg tatttgccat cctttcttta atgagcttta aaacactttc cccgcttaaa
19381 aaagaaacgc ccaaatcccc ttttaaagcc ttaaaaagcc aaatttgata ctgctctagc
19441 aagggcttat ccagccctaa atttgcttct attctttgaa ccaattcctt gctttgagaa
19501 ttgactttat ttgcatacgc tacactgcct ttgctaaaat aaagcaaaac aaagcataaa
19561 aaagtactaa aaaatgcgat aaaaacacta aaaactaagc gttttagcac tatttttac
19621 tccactcata aatattccaa gtaaagccta ctccatgatg gcctaaattt cttgctttaa
19681 taccctcaag cttagcatta taaaccaagg caaaatcaag ataagttaga aaaataaaag
19741 gaggattttc atacaaagca tcaataaatt ctttataata ctttttacgc tcttccaaat
19801 ttaaagtatt tctagctttt tgcaaggcta tatctacctt tttatcatga taatgtccaa
19861 aattccaacc ctcattattt aaggcactat cttgcgaact ttcaaacact ctaaaggtgt
19921 gaaaatcagg atctaaagga cttccccaac ccactaaaaa actatctact ttagaataat
19981 caaaactgcc cgcaggcttt gccacaacct tagctataac ccctattttt ttaaactcac
20041 tttgcaaaat tcctgccaaa ctcaccctta aaggatcatt actcatagtc caaatttcaa
20101 attctaaaat attgccatct ttttcaaaaa taccctcatc atttttctta aaacctaaag
20161 ccaaaatcaa atcttgggct tttttaggat tgtattcata agttttaaaa gctttagaat
20221 ccgcccacga acgctctaaa ggatgattgg ctacaaaacc ataatcatgc aaaagatttt
20281 taactatact ttgcttatca acagcataat ttaaagcctt tcttatttga atatctttta
20341 aaaattcatt atctaaatta aacattaaag ctctataatc agcagatctt tcacgcaaaa
20401 tcttaaaatt ttcatccttt ttaaaaatat tcaataaaga aacatcaatc aatgccgcat
20461 caattttgcc atttttaagc tctacgcttg caactgaagg atcaaaaata tgtttgatga
20521 taagccttgg agtttttact tttgcaagat aaaaattctc attggcttta aactctacat
20581 attcaccctt tttccatttt acaaatttat aaggacctgt gcctatggga ttttgattaa
20641 aagaagcagt gtttaagtct ttatcactaa gtaaatgctt aggaagcatt cctatactta
20701 aagcgtctaa aaatgcagga aaaggtttag aaagcgtgat tttaagatga taaggattta
20761 aaatttctat acttttaatg tcttcaaaat tcacataaac tgaggaatta tttttaggat
20821 ttttaaaagc ttcaagactg aatttaacat catcagcact aaatttcact ccatcgtgcc
20881 acaaaacatc ttcacgcaaa aagatatcat agctaagtcc atccttgctt acattccacg
20941 acgtagccaa atcgggcttt aagctcatat gctcatcaaa acgcgtaagt cctgaaaata
21001 caagattaat caccgcatca tgatcctcgc tatatgcagg atttatcctt gaaatttcat
21061 tttccacagc tacgattaaa gtatcttttg gaattttagc ctcaagattg aaaaacaata
21121 aaaacagcaa aacaaaccat ctaagcactt gcaactcctt gaaatttaaa gattaaaaaa
21181 ttataaaata aaaaacaaga agccacaagc cctatagggg gataatttgt ttaaattgtg
21241 gggtttattt attctatgtt aaattgcttc actgcattca tccaataaat acgctatatg
21301 ctgccaagat atacccgtag catcacttaa tcctatctcg caagtgcttg atgtactaaa
21361 tccgcgcttg atattcgttt tggcataaaa ttttttaaag ccctttgtag cattaatatt
21421 taatttagga ttgaaaaaac ccttatatcc agcaaatccg caacaatagg tatcattatc
21481 tattaatact tttccatttg tgcatagctt tgcaagcttg attatatttt catttaagcc
21541 aagttttcta gcagcacaca tgatatacaa tcctacatct tcattgattt tatcaatgcg
21601 taacttaggg gcaataaatt ttaacaaata ctcacttaaa tcataaattt catactttaa
21661 atgatcacta attaatttag cactacaagc actatgatct agcactatgg gtgagtctat
21721 attgcttaaa aaatcattga ttttttgtat atttttatct tggatatctt gaaaattctc
21781 ataagcctta ccacaacata aatcgttggg cgcataaata atacctatat ttgctttttc
21841 gcacaagctc tcaaatactt cttgcaagct tcttttgtca tacatttttt catttggctt
21901 aaagctttta ttaagacagc ttgtaaaata aaccacatta taagctcttt tacgactttt
21961 taactcataa tcattcacct taggcaaata cgccctagtt cttggtatgg cgctaagaaa
22021 attgcttgct ttttcaagtt tattatcaag acttaaatta tcaaatttat ttgcgatact
22081 taatgaaaat ttagcaattt ttagagtttt ttcatggttt ttatatgcta gatttcctaa
22141 aattttagac ataaatccta tattattttt tctatatttt aatgaaaaat cagcaaaatt
22201 aatccccaaa gggcacgaaa aagagcacac cccacaagca gcacaagagc tatctactaa
22261 atgattgtaa tattttttaa tatcctttaa ttttgctttt gactcatcat catttaaact
22321 ttcaagacgt tttatctctc ttaaaatagc aattctttgt cttggagtaa ttgtatattc
22381 attcgatgga caaaatcttt cgcaaaatcc acactccata caggtattta atttctcatc

22441 aattaaactc gcttgcttga tatttttctt ataaatatct ttatcatcac taatgattac
22501 atcaggatta aataaattct ccttatcaaa gatacttttg attttttttgt tgattagata
22561 agcttgctta ccccactcta cctccacaaa aggcgctacc atgcgccctg tgccatgttc
22621 agcttttata cttccaccat acgaagctac tatattagac atttctttaa ctagatttga
22681 aaaattatca aattcaagct tattatttaa atccggcgta atgataaaat gaatattgcc
22741 cgctaatgca tggccaaaaa tgattccatt atcactaaaa ccatatttat aaaacaattc
22801 ttgaatgctt ttaattccat cagctaaatc ctcgatatta aaacacacat cttcagtaat
22861 caccgttgaa cctgcctttc taaggctagc tgctataggc aaaagcgctt ttctaatctt
22921 ccaccataaa tcatattcgg ccttattctt gctaaaatat gacttataag ctaattttga
22981 aatttctttt attttattta tattttcatc aagcttaagc tcattactat gctcgctttg
23041 gatcaataca caagtatcac cctctttaat atcagctaaa atatctctta gctcatcata
23101 gcttgatgca gcttttaagc ttgcatagtc catgatttca gcagagctta taaaatcaac
23161 tttagcaaat tcttttatag tgtttgcagc attattaata ttatcaaaaa acaataaagc
23221 acaggcctta aattccaaat catctaaaac agcaagctta acctcactta caaatcccaa
23281 agtcccttca gagccaacaa gcaaatgtgc taaaatgtct atttcatcat cataatcaac
23341 aaaggcatta aggctatagc ctgtggtatt tttgatttta aattttttga ttattaaatc
23401 ttttaattct ttattggcat taatctcttc tttgatctct ctaagctcat taattaattt
23461 tttattctca tttttaaatt gagccacact taaagcatcg ctagtatcaa gtatgctacc
23521 atttgccaaa atcaccctaa tgcttcttaa ggttttatag ctattatcct tagttccaca
23581 gcacattccg cttgagttgt tattgataat ccctccaatt aaagctgaat ttatagtagc
23641 tggatcaggt cctatttttt tctttaaaaa agcaagactt tcattagcat gaatacctac
23701 tacaccacat cccaaagtga tttcgctagc atctttgtta atttttattt ttttaaaggc
23761 aaatttaatc accacaagta ccccatcaca agagctttgc ccacttaaac ttgtgcccgc
23821 tgccctaaat gatatgggag ttttataagt atgtgctaat tgaataactt gcttaatttc
23881 atcttcgttt ttagcaataa tcactatttt tggaatataa aaataacacg acgcatcaat
23941 gccataagca taacatctag cataatcatt aaagattcta tccttaaaaa tatacttagc
24001 ctcattataa aaagctttaa aatttgccat gatccctctt tttaatttat tatcaattat
24061 ataacttaat aatttaaaga caattttaaa tttatatttt tgagcataaa tttaaccgaa
24121 gtcaattttt ttatctggat taaaagttag aataccctcc attttaaaaa tttaaagaac
24181 taaaaggaga actatgacac aagaacaaat tcaaattatc aaagattgcg taccaatttt
24241 acaaaaaaac ggggaagatt taacaaaaga gttttacaaa gtaatgttta atgactatcc
24301 tgaagtaaaa cctatgttta atatggaaaa acaagcatct ggggagcaac cgaaagcctt
24361 agctatggct attttaatgg cagctaaaaa tatagaaaat ttagaaaata tgagatcatt
24421 tgtggataaa gttgccataa ctcatgtaaa tttaggggtt aaagaagagc attatcctat
24481 agtaggagct tgtcttttaa aagctatcaa aaatctttta aatcctgacg aagcaacact
24541 taaagcttgg gaagttgctt atggaaaaat cgctgaattt tatatagata tagaaaaaaa
24601 gctatatgaa aaataataaa agcccatttt tgggctttaa tcaaatttct ctaccaaatt
24661 tctagccaat tctttgatat tttcaccctt taattcgctg atatttcctt cttcagccaa
24721 aaaaattcta tctgcactat caaaatacgc atcatcatga gtaattgcaa aaatcgtctt
24781 accctgttct tttaataaag gtaaaagctt tttgtaaaaa aatcttctaa aaaccggatc
24841 ttgatctgct gcccattcat caagcactaa aatatctctt tcttcaagca aggctataag
24901 cattgccaaa cgcttttttt gtcccgtaga aagcttggtt aatacaagct cgtgatcctc
24961 cactctagtt ttacccttaa gctctaaaaa ttcaagccaa gaagcaattt tttcttcatc
25021 tgcaaatttc tctttcgcta aagtcttagt aaagagatga aaatcactaa acacagccga
25081 tactaaagac ctataaatat ctaaattatc atcatcaatt ttcatatcat ctacaaaaat
25141 ttcaccctca cttggcttaa aaagccctgt taaaagcata caaaaagtag attttccgct
25201 tccgtttttt cctattaaaa atatcaattc tcctttttta agctccaaat ttacaggatt
25261 tagatgaaaa ttatcatcat aagaaaaact cgtattttta aaagaaattt gacgccaatc
25321 cttgataaaa ttggtttttt caaaatgctc tttataatca tcaagttcta gttttgctat
25381 cttatctaga gcgatttttg ctaaaagcaa ggtaggaaaa gatcctatca tcgaaaccaa
25441 aggggttctt aaaaataaaa ttgacaatgc tatcgttgtt gcttcggcta cactcgccca
25501 cttaaattcc aaggctaaat aaaattccac ccctactaaa gctagaagtg ctacatttgt
25561 aaaatttgat gaaagattgt taaaaagatt gcctagggtg ttatttttct ttttagatct
25621 tgcattgttt tcaaattcat cttcataata aagcttagcg cggtagcgat tgattaaaag

25681 ctctttatgg ccatctaata tattttgata attattttgc aaagcatcat catttttctct
25741 tgctttgcga aaatacgaat aaactttcat catcaaaaaa ttattaatag taaaaaccac
25801 aataatccaa atcgcacaaa gggtaaaaat ttgaggtgaa agatagcaca aataaacact
25861 agtgcaaagt atcaatatac ttgattgtaa aaaatcaggc aatctcaaaa gcccaaaaga
25921 tacatttctt acatcactgc ctaaagaagc taaaattctt gccttaccta cttttgcgac
25981 ttttaataaa ggagtatcta ggatctgctt tactacgcgt ctttgcatct tgaaaatgaa
26041 attttgacca aaaacgctta atcctagctc tactatagta gagctaagaa aaaacactat
26101 aagcaaagcg ataaagtata aaatagggat attttgtgta tttttaagca aataatcatt
26161 gataaaaatt agggttaaaa cccctacagc acttgagata aaagaaaaaa gcaaaaaaga
26221 gatgaattta aatttatttt gtttaagcaa ttctaaaata aaaggcattg ataaaaactc
26281 cgaaagtata attaaaaatt aattatagca ttttaagagc gattatcaaa atttatttta
26341 tcctttacaa ggaaaacagc acaaatagcc aataaagcta gaaaaatcat atacatactc
26401 attccatata aaaatggatt tttacttcta gtagcaagga tattgagcca caaaacaagt
26461 tgaggagtaa ctcctcctgc gatagcataa gcgatattat acgaaaaaga aattcctgaa
26521 aatcttattt tagccctaaa aacttcactc attaaaatag gagcaaagac attaatccca
26581 cctaaaaaac aaacacttgt atataaaata caggttaaga caagattttt aagatcatat
26641 aaagcataaa aatacaagca agaaaaaaat gcaaaaacta aagaaaagaa aatacaaatt
26701 ttaaacaagc ctaatttatc cacaagatag cccataaaca ctccgcctag tgttatacct
26761 aaaattccta gaacttgtaa ataacttcct tcaatcgcac tcaagcccaa aatacttggc
26821 ataaatttag gcattaataa gataagcact acaacacaac ctgttaaaac ccaagttata
26881 aagatagacg cgagcgtatt tttgaaaata ttttcttctt taaataagtc cttaagcgga
26941 aaagaactca agcttcgttc tttttgcatt tgtttaaaaa taggagtttc ttgtaaaaat
27001 ctacgcaaat aaatagaaat aatcccaaaa attccaccca caaaaaaagc aatacgccat
27061 gcataagctg cgatttcttc ggtgctaaaa aaagcattaa tcagtaaaaa taccatactt
27121 cctagtaaaa ttcccaaagc catagccgaa tttaaacaac tcaaagagaa agccttttgg
27181 ctttctttgc aatgttccct cacaaaaacc caagcaccag gtaattcccc gcctatagca
27241 ataccttgaa aaattcttat aagtataagc aaaaacggcg ctaaaaaacc aagagtttca
27301 taaccaggta taaaagccaa tgtaaaagta ggaagcacca taagcaaaat acttaacata
27361 aacatatttt tacgcccaaa tttatcacca aaatgcgcca tcactatacc acctaaaggg
27421 cgcgctaaat aacctgcagc aaaagctcca taagtattta acaaagtcca aaattcactc
27481 atatctttag gaaaaaatac ttttgcgata tattcagcaa aaaatacaaa gatgataaaa
27541 tcataaaatt ctaaagttcc acctagtgac gaaagtccta aaattttaaa ttcttttgtg
27601 cctagttttg attttttcat tattttatac cttaaagaat tttaaaaata accattatac
27661 tacaaatatt tataaaacaa acaatcattt tcaatttaat tttaatgata aaaatcataa
27721 aaatcagctt aagcaaaatt tttaataatt tttcaaaaaa acgacacaag ctgtcataag
27781 aatactttat acttttttg aaattaaaat caaggagaaa aaatgcaaac ccaagcttca
27841 aaaaccctaa atacttttta caagataaaa agaactatat ttttatctat agcgcaaaat
27901 aacaatattt tagcaaggta aaaatgatga ttattgtagg gattttagga cttatcatta
27961 gcacagcggt tttgttagct tgtggggtgt tttgggtttt atttgggatt gtattttggg
28021 gagtgatttt agcttttatg cttttggatt taagtggaag ttatggtgtg gtggcacttt
28081 atatactttt tatagtgctt aatttaattt taatttataa aattacaagg agaaagaaat
28141 gaaaaaatta gctttagctt taggcggaat cgcactaggt gcttgtggtg gaaaaatcta
28201 tcaaggtatt aaaaatactt gggataaatt gatttttaaa gatctcatca cccttagaaa
28261 agtcatcaaa caatacgaag aaatagagca aatttatcct agctatttaa acgatcttaa
28321 atttttagaa tttagaaaaa cccacaaata cgatacagaa actatttata atataaaaga
28381 tcatttattt agcgatctaa aaaaagaaaa gcttatccaa ataggcacta caatggaagc
28441 ttttatggaa tatagtgata aatatcttca agatgatata acagcattaa aaatcagctg
28501 gaaattttgc gctatgaaat taaaatttga tggtgctttg ttgcagttac aagatgtaaa
28561 caagatggta taaagagtta aaatgcaaga aaatttaaaa gaaatttatg aagaatttga
28621 atttttaaat actcaatttg caagctattt taatgatgaa gaatttgcaa attttaaaaa
28681 agattttgaa gcaaaaaatg ctttaaatta ccgcgatttc gaagaattta aactagaaga
28741 aacagataaa aaaaggctta aagatataca agaatgttta ttggcttata tcaacttttg
28801 tagcagtaaa gatataaaag aaaagagagt agaggtgatt tctaaaacct ttatgcctat
28861 gtttcttatt tctaggttgt attttgcctt aaaagaagaa aaactagata tgcaaattcg

28921 caaaatactt aaggaaaaag atgagttgga agaggaaaaa tctttatgga tttccaaagt
28981 cctgcctttt taagaatctt taatgaaatc ttaaaatata ttgattttta ataaacaaaa
29041 tgttataatt tataaaattt ttaaggagat aaaatggaaa ataaatcgca agaaaacaca
29101 gttgatttaa atgcaataat gagtgagttg ctgcaacaag gacaggaaat gtataactca
29161 tctaaaaaac aagataagtt ggataaatta aatatcttaa ttattggcgg aacaggcgtt
29221 ggcaaaagca cacttattaa cactgtgttt ggtaaagaag tagcaaaaac aggtgatggc
29281 gcaccagtaa cacaagcgat acaaaaatgt gaaaaaaagg gtttgtgtat ttacgatagt
29341 aaaggacttg agaaaaatga tcctagcata gtagaagatt taaaaaattt actagacaaa
29401 caaaagcaaa aagaagtaag cgaacaaatt catatagctt ggctttgtat ttgtgaggca
29461 actaggagag ttgagccatt agaaaaagaa ctttatgatt tgctcaaaca atacaatttt
29521 cctactatag tggcaatcac caaagcaatg caaaatataa atgacaaagg tgaaaaattt
29581 gatgaaattg taaaaaatga gctaaaggct acagaagtga ttcgcattag agctatagaa
29641 acaaaattag atgatgatga agaaaatatt ttaaaaccaa tggggataaa agatttatta
29701 aacaaatctt ataacctttt gcctgaagca caacaaaatg ctttagcaag aaagcaaatc
29761 tatgatagag atatgaaaaa aatacaatgt aaaaaagatg ctacttctaa gctaaactac
29821 tactctacaa cagctggtgc tgtagcagct acgcctatac cattttcaga tatagcactt
29881 attttaccaa cacaaatagc catgatagtg catattagta gtatatataa tttggatttt
29941 gacaaagaaa gtgcaaaaaa ggtaacttta gcattggtag gggtttgcgg agctggtttt
30001 ggcataaggc ttggagttgg agctgcatta aaattgatac ctggtcttgg aagcttagca
30061 ggtggcgcta tcaatgctac agttgcaaca actgccacaa aagcaatggg agagctttat
30121 ataaaatatc ttgatgatca ttttgatgat attgaaaaca ataaatcttt atcatttaat
30181 tttgaagatt ataaataagc attagaattt ctaatgctta gacaaggagt ttttatgcaa
30241 gttaaaaccg aagaagaatt agctaaggct gttaataata atgaaaatcg tatagagata
30301 gagggtgatt tggcggaaaa agtaattaaa atcaaagctt ctggaaatgt cgcttgggcg
30361 gtagctatag gtagtatagg tgttgctgta attgcaataa tttctctttt taaaaattct
30421 gaatctaaaa attctcaatc tcctaaagta gcttttgtgg ctagtggaat aggtgctaca
30481 acggcagcgg gtgctgttgg agtcttagga ttagcaacaa caataagtgc aataaaaatt
30541 gctgtatctg gtggtggaaa ggatgtttta aaaaaactaa gaaactataa aatggaaaaa
30601 atttctgaca caaaaatcgt attaaaaaac gaattattat ctaaaggata aaaatgtttt
30661 gtaagctttt aaaggctttt attagcaatc caagaagttg gtttttgttt tatcgtttgc
30721 gaaaacttgg tttttgtata aaagaaaatt tcgataaaga aacaaaatat tcagcagtat
30781 gcaaaagtct cgacgagcta gttaaatata aaaataaaca tccaaatgat tttgataatt
30841 tttttaagat ttttaaagaa attttgaatg attatgaaaa aagtaaaaaa tgaggtgtaa
30901 taaaatgtat tataataaaa tcttttcggc tttgtcttta gcgaatagct ttaaatctac
30961 gattttcaaa cgcatatcaa gtccggcaca aagtgctaga atgatcgatt ttgctttaga
31021 aatcaaaagc atattttctc acaattcaaa agatgaaata cctattaaaa tattagacaa
31081 attagttgaa tataaaaata aatatccaaa tgattttgat gaaatgtttg aaattttaaa
31141 agagatttta gatgagtatg aaaaaaatcc aagcgaagtt aaaaaaaatt taaaagaact
31201 tttctaaaat gcaaaatctc ttaattctct ataatcctta ctatcaacaa gatgttataa
31261 aattgcattt agaaatttta ttagacaaag gtcaggtagc atttggtaaa ataaaatcta
31321 aactaaaaga gccactttct tatgaaatgg atgaaaataa aatctcacac aatcaaaaac
31381 tatcagaaat ttacaaaaat acctctgctg ataatccttt acagcttttt ttgagtgatt
31441 atgcaaattt atttgtagca aaagtagtaa aaataagtaa agaagtagat gaaaatatca
31501 ttccaaaata ttatcaagag aaaaatttag aggtggagga ttatttctta atcacagatt
31561 taagagaatt ggtaagagaa aatttcacac tcataagaga taaattttta gctaacttca
31621 caacagaaaa taaccacact tacgcaattt atggcaataa ctattcttat cctttagtag
31681 tcaaacaaaa agaagaaatt aattattttt atgatgagat aaacaaatac tacctaagcg
31741 tttataaaaa tcaagagtat ttaaaaatgc aagaaaattt tatacaattt atctttggta
31801 aaaaattttt ttatatgcta catcctgata gtatcaataa ccttatccta gcagagctag
31861 aattgcaaca aaacttagaa aatccacttt atgacttcac ttcaataata gtaaaatact
31921 ctaaaactat agaatatgaa gtatatgact ttgccaaaaa gatatttaca aaactcatca
31981 accaaaattc ccatctttca tccataaact atagcgtaca aggaaaagaa tttaacttta
32041 aacagttttt catcaacaaa cctaatatcg gaacaattaa gtttttattt aaaaataaag
32101 aaattcaaga gcttttggac aaggatataa aaggctttat caattatgaa tttaatagaa

32161 ccttagatga atttcaaaca atacgaaatc atgcagtaca tgcaaaatca ccgactttag
32221 aacagacatt aaaaattcga aatttaattt taggtataga aaatactagc attttaaaac
32281 gcgtattaga gtataaattc aaataaaaag gataaaaatg ataaaaccaa agccttttct
32341 taaaaaatgt ccaaagtgtg gaaaaatgtt tttatgtact cctaagagtg attgtttaaa
32401 tccaagtgat tttttctgcg ataaatgtag cggcaaaaaa gatacaaact tgataagcaa
32461 aattatttct gcaattaaaa aggtttaata tggcgcacga taaacttgca atgcgtttag
32521 ctcatatctt aatgcgtttt aataatggag gaaggtttag cctagaagaa ttagcaagtg
32581 aatttaatgt agatattaga accatacaaa gagatttaaa ccaaaggctt tcttttatgc
32641 ctatccaaaa agaaaatgga aagtattttt tagaatcttt tgcactaggc aagcttagcg
32701 ttaaagatat acaaaatttt gctatgttaa gcggggttca agagctttat ccgaagctag
32761 atgaggattt tataacagat ttgctaaact ctaaagtaaa tagcattttt atgataaaaa
32821 acaatggctt tgaaaaagtt gattatgaaa attttgaaat cattagtgtt gcgattttaa
32881 aacacaatat tttaaatttt aactataaag aaaaaagtcg cgaagtcaaa ccttataagc
32941 ttttaaacta tcaaggaatt tggtatttaa tagctgacga aaaggacaaa ttaaagcatt
33001 ttacttttag caaaattaaa aatattcaca aaaataatga aatttttacc cctaaagaag
33061 aatttttaga ccaaattcta catgataaaa atatttggct agatgatagc aaggaagcta
33121 tcgtaaaact tgataaaaat gctaaagagt atttttttag aaaaaatatt ttaaacaact
33181 ttgaactcat cgatgaagac gagcaaagct atactctaaa agtatatttt tcctatgatg
33241 atgagcttct aaatgtagta aaacaatgga taccctatat aaagatagaa aaacctttttg
33301 agctgaaaga aaaactagat agcattttaa aagattactt ggaaaactaa tttcttaata
33361 aattcaaata ttttttgcta taatcacagt tttattttaa tgagaggaga aaattttggc
33421 aaaagacgat gtgattgaaa tcgatggcac agtgcttgaa gcattaccta atgcaaattt
33481 taaagttgaa cttgacaaca agcatgtgat tttatgtcat atagcaggaa aaatgcgtat
33541 gcactatata agaatcatgc caggggataa agttaaagtt gaacttacac cttatagcct
33601 tgataaaggg cgtatcactt ttagatataa ataatataag ttatttaaaa gctaatttta
33661 gataaaatta acatttttgc gacaaaatgt atcgaactat atgaagaggt atttttaaaa
33721 ttcaccactt attttaaaaa tagttggtta ttcataaaac ctgatgcagt cgtaaatgaa
33781 gtggaattca tttaaggaga actcatgaaa gtgagaccat cagttaaaaa gatgtgcgac
33841 aagtgcaaag tagttcgccg taaaggcgta gttcgcatta tttgcgaaaa tccaaaacat
33901 aaacaaagac aaggataaac atggctcgta tcgcaggtgt ggatttacca aagaaaaaaa
33961 gaattgagta tggacttacc tatatttatg gtataggact ttttacttca agaaaaatct
34021 tagataaaac aggcatttct tatgacaaaa gagtgcatga actaagcgaa gatgaagcag
34081 cagctatcag aaaagaaatc caagaaaact atatggttga aggggattta agaaaacaag
34141 ttgctatgga tataaaagca cttatggatt taggaagctt tagaggctta agacacagaa
34201 aaggcttacc agttcgcgga caaaaaacaa aaacaaacgc aagaactaga aaaggtaaga
34261 gaaaaactgt tggtgcaaaa tcataaggat aaaagatggc aaaaagaaaa atcgtaaaga
34321 aaaaagtagt taaaaaaaat atagcaaaag gtattgttta tatcagtgca acttttaaca
34381 ataccatggt aactgttaca gatgaaatgg gaaatgctat cgcttggagc agtgcgggcg
34441 gtttaggatt taaaggttct aaaaaatcaa ctccttatgc ggcacaacaa gcagtagaag
34501 acgctttaaa taaagcaaaa gaacacggaa tcaaagaagt aggcattaaa gtacaaggac
34561 caggaagcgg tagagaaact gctgttaaaa gtgtaggtgc tatggaagga atcaaagtaa
34621 ctttcttaaa agatattact ccattagctc ataatggttg cagaccgcct aagcgtcgtc
34681 gtgtctaaga tataagaaaa aattaggaga attaattatg gcaagatata gaggaccagt
34741 agaaaaatta gaaagacgct ttggtgttag cttagcatta aaaggagaaa gaagactagc
34801 cggaaaaagc gcattagata aacgcccata tgcaccagga caacacgggg caagaaaagg
34861 taaaatcagc gaatacggac tccaattaag agaaaaacaa aaagctaaat ttatgtatgg
34921 agtaagtgaa aaacaattca gaagactttt tgcagaagct gctagaagag atggcaatac
34981 aggggtttta cttatccaac ttttagaaca aagacttgac aatgtagttt atagaatggg
35041 ctttgctaca actcgtcgtt ttgcaagaca acttgtaact catggacaca ttttagtaaa
35101 tggaaaaaga gtagatatcc ctagctttag agtagaagca ggagcaaaaa tcgaagtaat
35161 agaaaaaagc aaaaataatc ctcaaatcac aagagcgatc gagcttactg ctcaaacagg
35221 tatcgttgct tgggtagatg tggaaaaaga taaaagattt ggaattttca ctagaaaacc
35281 tgaaagagaa gaagttatca ttccagtaga ggaaagattc atcgttgagc tttactctaa
35341 gtaatagggg ctaaatcatg agaaacatta caacatctgc ttatacgcca acagaattca

35401 ctatagaaaa tattagtgat actgtggcta aaatcagtgc ttggcctttt gagatcggct
35461 atggtattac cctagcccat cctttacgcc gtttacttta tactagcact ataggctatg
35521 ctccaaccgc tattcatatc gatggcgtgg cgcatgaatt tgatagtatg cgtggtatgc
35581 ttgaagatgt agcacttttt atcatcaatc ttaaaaaatt acgcttcaaa cttaaaagcg
35641 attctaataa agaaatcgtt gagtttagtt tcaaaggccc taaagaaatt tatggtaaag
35701 atttaaacaa cgatcaagtt gaagtcgtaa atacagatgc atatttagca acaatcaatg
35761 aagatgctga acttaaattt actcttatca tagaaaaagg cattggttat gtaccaagtg
35821 aagaaatcaa agaacttatc aatgatccaa aattcatagc tcttgatgct tttttcactc
35881 ctgtaagaga ggcgacttat gatattgaga aagttttatt tgaagacaat cctgactatg
35941 aaaaagtagt tttaacagta actactgatg gacaaatcac tccaaatgaa gcttttcaaa
36001 atgctttaga agctatgtat aagcaattat cagtatttga taaaattaca aatgttagaa
36061 gtatcataaa aagtcaagct acaagcaatg aattagaaaa tactaaatta ttgcaaaata
36121 ttactgatct caatttaagt gcaagaagtt acaattgcct tgaaaaagca ggaattgttt
36181 atatcggaga gctcgcttta atgagtgtta atgaacttgc tggacttaaa aatttaggta
36241 aaaaatcttt agatgaaatt aaaagcatta tggaaagcat aggcttccct gtaggtactt
36301 ctaagcttag tgataataaa gatttgctta agaaaaaaat cgatgaatta aaagcacaaa
36361 acgaaggata aagaatgaga cataaacacg gatatagaaa acttggcaga acttcatctc
36421 atcgtgctgc cttgttaaaa aatttaacta tagctttagt taatagcggt aaaatcgaaa
36481 caactctacc aaaagctaaa gaattaagag gctatgtaga aagattgatt actagagcaa
36541 gacttggaga ttttaacgct cacagagcag tttttgctgc tttacaagat aaaaatgcta
36601 caaacaaact tgttacagaa atagctccaa atttcaaaga aagaaatggt ggctatacaa
36661 gaattatcaa aacaagaatt cgccgcggtg acgcagctga aatggctttt attgaatttg
36721 tagcctaaaa attccagctt aagctggaat tttaaaattt tctccttata attttttaac
36781 ctactaattt tttacaaatc caaaaattca aaatcatatt tttaaatcta atttctaaaa
36841 aataataaaa attcttaaat gattatttaa aaaataattt ttagccaaaa ttttaactac
36901 ttatgctaat ataatttttt atttttataa atgcaaggat ataaaatatg caagaaaatt
36961 ctcgtttgcg tatagctata caaaaatcag gcagactctc taaagaatcc gtagaacttt
37021 taaacaaatg cggaattaaa atgcatatac atgagcaaag tctcatagct ttttcaacca
37081 acctacccat agatatctta agagttagag atgatgatat acctggtctt atctttgatg
37141 gagttgtaga tcttggcatt ataggcgaaa atgttttaga agaaaatgaa ctcgaaagaa
37201 agagcttggg cgaagaggca aattacaagc ttttgacaaa gcttgatttt ggttattgta
37261 gactttcact tgccttgcct caagagaaaa aatttgataa tttgaaagat tttgaaaatt
37321 taagaatcgc cacttcttat ccacaacttt taaaacgctt tatgaaagaa aacaatattc
37381 attataaaaa ttgtatgctt acaggttcgg ttgaagtagc tccaagagca aatttagccg
37441 atgcgatttg tgatcttgtt tcaagcggcg caacactaca agcaaacaat cttaaagaag
37501 ttaaagttat ttaccaatct cgtgcttgtt tgatacaaaa acaaaatact ctaagtagtg
37561 aaaaacaggc tttggttgat aaaatcatgc ttcgcgttgc aggggtaatg caagcaagag
37621 aaagcaaata catcatgctt catgctccaa aagaaaaact tgacaaaatc caatccctac
37681 ttccaggtgt agaaaagcct actatcttac ctttagcaca tgatgaaaaa aatgtagcct
37741 tgcatatggt aagtaaagaa aatctttttt gggaaactat ggaagcttta aaagaagaag
37801 gtgcgagttc gattttagtt ttacctattg aaaaaatgtt aaagtgagta aaaaatgcaa
37861 ataataaatt ttaacgagtt agacgaaaaa caaaaacaag aagtattaaa acgccctgca
37921 atagcagcaa aagatgaaat ttcacagata gtaagttcta tcattaagga agtaagagaa
37981 aaaggcgatg aagctttgat agaacaggct ttaaaatttg acaaagccca aatttcaagc
38041 atcaaagtaa gcgaagaaga gcttgaaaac gcaagctcac gcttggataa agacttaaaa
38101 gaagcgattt taatagctta tgaaaatatc aaaaaattcc atgaggcgca aattccaaaa
38161 gaagtagcca ttgaaaccac caagggtgta aaatgcgaag ttttaacacg ccctattgaa
38221 aaagtaggac tttatatacc agggggtttg gcgcctttat tttcaacggt tttaatgcta
38281 gcaattcctg caaaaatagc tcaatgtgaa aaaattgttt tggcaagccc tgcaaaaata
38341 aacgatgcta cactttttg cgctaagctt tgcggagtag atgaaattta tcaaatgggt
38401 ggtgcaggag ctatagcagc actagcttat ggaaccaaaa gcgttttaaa agtagataaa
38461 atctttgggc caggtaatgc ctttgttacc gaagctaaac gccaagtaag cagtgatata
38521 aatggagctg ctatagatat gcaagcagga cctagtgaag ttttagtcat agcagatgag
38581 aaagcaaatg ttaaatttgt agcaagcgac ttgctttcgc aagctgaaca cggtgcagac

38641 tctcaagtga ttttggtatg cttaagcaaa gaatttgcac aaagcgcgag tgatgaagta
38701 agccgtcagc ttgaaaattt accaagaaaa gaattagcgt ctaaaagcat tgcaaattct
38761 aaaatcatca ttgcaaaaga cttagatgaa gctcttttga tctcaaatct ttacgcacct
38821 gagcacttaa tcatacaaac acaaaatcct cgtgagcttt tagataaagt caaacacgct
38881 ggatctgtat ttttaggtga gttaagccca gaatccatgg gggattatgc aagtgggaca
38941 aatcatgtct tgccaactta tggacttacc aaaacgcatt ctagcttggg cttggctgat
39001 tttagcaaaa gaatgagcgt gcaagaactt agcaaagaag gctttttaaa tttaggaaaa
39061 agtgttgaaa ttttagctgc aaatgaacat ttagacgcac ataagaatgc agtaactttt
39121 cgcttagaaa gtctaaaatg agtgaaaaaa tccttttat cgatagagat ggcaccatca
39181 tagaagaacc aaagagcgat tttcaaatcg atagccttga aaaacttcgt tttgaaaaag
39241 gtgctatccc tactctttta aaacttaaaa attttggctt taaatttgtg cttgtttcta
39301 atcaagatgg ccttgggact caaagctttc ctaaagaaga ttttgaaata gcgcatcaaa
39361 aaatgttaga tattttacaa agctgtggta tagaatttaa agatattttc atttgtccac
39421 attttgaaaa tgaaaattgc gagtgtagaa agccaaaaac tgccctttta gatgagtata
39481 tcaagcacaa tctttacaat aaagaacaaa gctttgtgat cggcgatcga gatactgata
39541 tgatcctagc ttcaaattta ggtgttcgtg ggttaaaata cagcgagaat ttgacttgga
39601 aagagataga agaagaaatt ctaaattcct ttcgcaccgc aagcatttcg cgtatcacta
39661 aagaaaccaa tatccgtgta aaagtatgtt taaatggcag aaagattgct attaatacag
39721 gagttccttt tttcgatcat atgcttgagc aaatcgcagt acatgggggt ataggacttg
39781 aaatttcttg caagggtgat ttagaaattg atgagcatca tagcgttgaa gatgttgctt
39841 tagctttagg aagtgccata aaacaagctt taggagataa gataggcatc acaagatatg
39901 gctttgtctt acctatggat gagtgtttgg caagttgtgc gatcgatttt tgcaaccgtc
39961 cgcatttagt ttataaagcc aaatttaaaa aggaaaaatt aggcgaatta agtactgaaa
40021 tgatagaaca ctttttttac tcgctaagct actcaatggg agcgagttta cacttaaagg
40081 ttaaaggtaa aaacgatcat cacaaagccg aagggctttt taaagctttt gcaaaagcct
40141 tgaaaatggc tgtaaaaatc gaaagtgaaa atttagccag ctctaaaggg gtaatatgaa
40201 actgatcatc atcgatacag catgtgcaaa tttagcgtct ttgaaatttt gtcttgatag
40261 actaggcttt aatgctaaaa tcagtagaga tttaaaagat ttagaaagtg cggataagct
40321 ttttttacca ggggtcggaa cagcagcaaa agcaatgagc aatttagaaa gctttaatct
40381 aacaaatttt atcaaaaata ccaaaaaacc cttacttgga atttgcctag gaatgcaaat
40441 tttaggggat ttttcagaag aactagatca agaaactcta ggcattatgc cttttaaaac
40501 tcaaaaattt gaacttccaa aagagtattc tttaccgcac atggggtgga atgaagttgc
40561 aagctcacac ccacttttta agggtttaaa tggagcttat ttttattttg tacacagtta
40621 tcatgtagta ttaaatgact ataccatagc tacaagtgat tatggggtaa aatttagcgc
40681 aagtttagca aaagataatt tttatggcgt acaatttcac cctgaaagaa gtggagaagc
40741 gggggaaatt ttaatatcaa atttcattaa ggatataaca tgataatccc tgcacttgat
40801 ttgatcgatg ggcaagtagt acgccttgtt aaaggcgatt atgaacaaaa aaaagtatac
40861 aaatacgatc ctttagaaaa atttagagaa tacgaaaacg caggagcaac ttggcttcat
40921 ttagtggatt taagcggagc taaagatcca aataaaagac aacttaccct gatagaaaaa
40981 ttagcaaatg aagtcagtgt caatttgcaa gtgggtggtg gaattcgcac caaagaagag
41041 atacaagctt tgcttgattg tggagtaaag cgtgtagtga tcggctcttt ggcgattaca
41101 aatcctacgc tttgtgttga aattttacaa cattttggaa gcgaagctat cgttttggca
41161 cttgatacga ttttaaaaga agattatatg atagccatta acgcttggca ggatacgagc
41221 gataaaagac ttttagaggt tttagaattt tataatatca agggtctaaa acacatactt
41281 tgcactgata tttcaagaga tggcaccatg caaggaagca atgctgaact ttatgaactc
41341 atccataaaa actttcctaa aattcacact caagcaagtg gggcgtagc gagcttagaa
41401 gatcttaaaa aactcaaggg tatttgtagt ggggttatcg taggaaaagc tttattagat
41461 ggagttttta gcgtagaaga agggataaaa tgcttacaaa acgcataatt gcatgtttag
41521 atgtaaaaga tggtagagta gtaaagggaa cacagtttaa aaatcataaa gatatgggag
41581 atatcgtaga acttgcaaag cgttattctc aaagcgggat cgatgagctt gtgttttatg
41641 atattgccgc aagcgctaga aaagaacgta tttcaagaga atgggtaagc gaagtggcta
41701 aaaatatcaa tatccctttt tgcgtggcag gcggtattaa aagcgaagaa gatgcagccg
41761 aacttttagc caatggagcg gataaaattt ctatcaactc ccctgcttta aacgatccag
41821 atcttatcac tcgtttagcc aaaagttttg gagtgcaatg cgttgtagtg ggcattgata

41881 gctttaaaga tgaaaatgga aatttaaagg ttttcaaata cacaggtgat gaaaacacta
41941 gcaaacatag cgggaaaaat acgatcgagt ggattaagga agtgcaggat ttgggtgctg
42001 gagagatagt tctaaatatg atgaatcaag atggtgttaa aaatggatat gatttagaac
42061 aattaagcct tgcaaaaaaa gaatgcaaag tacctttgat cgcaagcggt ggagcaggaa
42121 agatggagca ctttttagaa gcctttaaaa tcggagtaga tggggcttta gcagcttctg
42181 tttttcatca acaaatcatt gatattaaag aattaaaaaa ctacttaaaa acccaaggtg
42241 taagtataag gatataaaaa tgcaaaattt ccaaaattta aatgaaaaaa tcaattggca
42301 aaaagtaaat gggcttatcc ctgtcataat acaagatgca aaaagctgtg aagttttaat
42361 gctaggtttt atggatgaga aagctttaga aaaaagtttg gaaagcaaaa aggtcgtatt
42421 tttttcgcgt actaaaaatc gtctttggat gaagggtgaa gagagtggaa atttttttaaa
42481 tatcgttgat ttaagccttg attgcgataa tgatacactt ttaattttag ttgatcccat
42541 aggagctact tgccatatag gcaatatttc ttgctttgaa aatgtatcta aaagtgcaga
42601 ttttgtcttt ttagcaagac tggaaaaact tataaattca cgcaaaaatg cggatgaaaa
42661 tacctcctat actgcaaagc tttttaaaaa aggaacaaag cgtattgcac aaaaagtcgg
42721 cgaagaaggt gtggaaactg ccttggcagc aactgttaaa gataaagatg agcttatttg
42781 cgaggcggct gatttgatgt atcatttaag tgttttactt gcggatgcaa atttaagttt
42841 ttcagatgtg attgccaaac ttaaagaaag acataaagaa taaaattgaa atatcatttt
42901 aggctttctg atcttttttta tctaccctgc attaatgatg tttttcatct taatgcatag
42961 aaaatatata tctgaagtac caatgttagg ttttcataca aacatggaaa tttttaaacg
43021 agcgagtatt tattattttt atttagcaat tatatctctt tatttttagt gtggaaaaaa
43081 gttatttcaa agatataaag tttagatttt ataaatactt tttattttgg ggttgctttt
43141 gtctttgtgt gtttttcttg cttaccttgc ctttagatgg gcatttaagc tttagtgcct
43201 ttgctgtgtg ttgtttagtt ttgtctttgt ttttaaattt atttattttt gctttaagaa
43261 aatttcttat tttacttgga aaaaacttat ttttataata actacattac taggtatttg
43321 tgtaggtttg gcttatatct ttttttgcct ttattatcaa ctttatattt ttgacaatta
43381 cgatttttta ggtaaatttt ataatatccc ttcttataaa agacctttga attttatatt
43441 ttaatgaaaa ataaacaaaa aagcctaaaa ttaggctttt gctgataatt tagagcgaat
43501 taatgcaaag cttcgttaag cttgatcgct tttttattta agcacactat catcgctccg
43561 ctgatagagt cttgacggaa atgaagtcca tttaagccct taagctcgat acccttataa
43621 atctctttac taaaatcaaa ataaggatta agtttagcaa gctcttcacg atctttaagt
43681 aaaaattttg ttccttcaag cactgcaatt cccgcatcta cgatacaatt atcacccaaa
43741 ggaattcctg ttactgaatt tgctcctaaa agacacgctt tgcctacact tatagcattt
43801 ccacttgttc cgcttaaaac acctaaaatc gatgcaccac cgcccacatc agagccttca
43861 cctacgatag cactcgaact tatacgccct tcaaccatac aagcccctgt tgttcctgcg
43921 ttaaaattca catagcttgc acctggcatg attgtagtac ctgcagccaa agatgcaccc
43981 attcttacct tagagctttc taaaattctt gtattatctt cagggatgat atgtgctaaa
44041 aatcttggga atttatccac aaaatcgatt tttggatact gattgctcat ttttaaacgc
44101 atttcgtttt ctcttaaata ctcaagctcg ataggcttat catcactcca agctacattg
44161 cttaaaagcc caaaagctcc atttaaattt aaacttctta aaggaacttt tttggtagaa
44221 agcaaataaa gtttcaaata aacactttcc acactcaaag gctctttatc ttcaaaaaga
44281 caaacaaaag caaattcatc gtctttaaat ttatctttga taatctttaa aatatcgata
44341 tttttatgtc cctcttcttc taaaaaaggt ttaaagcaag caagcgcata atcaatatcc
44401 tcaagcttaa gaatttgtac aaattcactt gcttcaaaat caatctcaac accccttttgc
44461 ataaaagctt caagcataat cgccgcagaa ccaaaatttt gctcataatt taccaaagca
44521 aaactggctt gtaaaatttt atttttatta agctgaccac gatcaagtct tgctatacca
44581 aaagctttag gttttctata tccatttttt tgttcaattt gcttaattaa aagtaaaaaa
44641 tcttctttag tgttaataat catttttttct ccttaaaatt ttaatgcgaa caagcacttt
44701 ttccattcat cacaggttgg attgcagaat tatctgcgcc gccttcatta ttgatttttt
44761 ctataaattg ccaaattttt tcagctgcca ttaaataacg ctttgcactt acactttcag
44821 gatgataaaa acttacaggc tttccttcat ctccgccttc tcttactatc atctctatgg
44881 gaatttgtgc taaaacttca ctcttgtaag cttttgccat ttcctctgca gtgcctttac
44941 caaaaatatc atactctttg ccattatcag gacataaaaa gccactcata ttttctatca
45001 caccggcgat tggaatgtga agtttgttaa acatatctaa agctctttta ctatcatcta
45061 aagaaacggt ttgtggggtg cttacacaaa cgccagcagt aattggaata ctttgtgccg

45121 aagtaatttg cgcatctcct gttcctggag gcatatctaa aaataataca tcaagttcag
45181 gccaaatcac atctgcaagc aattgctcga tagctttcat gatcatagct ccacgccaca
45241 taagcccttg accttcttcg attaaaaccc ccatactcat catataaaca ccatggctta
45301 aaataggctt taatctttgc cctaccactt caggctgagt tttagtttcg cctaacatcc
45361 ttgggatatt tggcccataa atatccgcat ctaaaatccc aactcttttt cccattttag
45421 ccatagaaat agccaaattt acagtggttg tagacttacc tactccaccc tttccacttg
45481 aaaccatgat gaaatttttt acttgagggg cgatattttt accacttctt gaattgcttt
45541 tttcttcagg aatttttgga gtaatgatat gaatttgtaa attcttcaaa tttaaagaag
45601 ctaaagcttc atcaatattt ttacgtaatt cttcgcttac ttgcggattt gatgaaacaa
45661 tttcaagctc aacattaaca ttttcatttt cgatttttat atcttttaca aaattaaaac
45721 tcactatatc cttatcaaaa ccaggatatt ttacactttt taatttctct aaaatttgct
45781 ctttcatctt ttttccttaa cttttaaaaa atcaatttta ctaataaaat gataaaattt
45841 agctttttat aagtgtataa tatggtaaca ttatatataa attaatattt ataacaaagg
45901 gaaaaaatgg ctaatcttag cttgattatg ctagcagctg gaaattctac tcgctttgat
45961 atgggagtaa aaaaacaatt tctacgcctt ggcgatgatc ctttatggct ttatgctacg
46021 cggaatttaa gcacttttta tccttttaaa aaaatagttg ttacttctag taatacttct
46081 tatatgaaaa aatttgctag aaattatgaa tttgttgaag gtggtgccac tagagcagaa
46141 tccttaaaaa aagccctaga agccatagat agcgaatttg taatggtaag tgatgtagca
46201 agagttttag taagcaaaag cttgtttgat cgtttgattg aaaatttaga taaagcagat
46261 tgtatcactc cggctttaaa agtcgcagat actacgcttt ttgaaaatga agccttgcaa
46321 agagaaaaaa tcaaactcat tcaaactcct caaatttcac gcactcactt gctcaaacaa
46381 gccttaaatc aaaatttaga tttcacagat gatagcacag ctatatctgc aatgggcgga
46441 aaaatttggt ttgttgaagg tgaagaaaat actagaaaaa taacctttaa agaagatctt
46501 aaaaagcttg atttgcctcg accaagcaat gaaatctttt gtggaaatgg ttttgatgtt
46561 catgaatttg gagaagaacg ccccttgctt ttaggtggag ttgaaattca tcctaccatg
46621 ggcttaaaag cacacagcga tggagatgtt ttagctcact ctttaaccga tgcgattttg
46681 ggtgcagctt gtttaggaga tataggcgaa ctttttcctg atacagatga aaaatttaaa
46741 aatgcaaatt ctatgaatct tttaaaagag gcatataaaa gagttaagga gttgggcttt
46801 gaacttgtaa atgtagacat ttgcgtgatg gcacaaactc caaaacttaa agattttaaa
46861 gaagccatag cccaaaatat agcaaatact ctcaatatcg atgaatttag agtaaatata
46921 aaagctacta ctactgaaaa gttaggtttt atagggcgta aagaaggtat ggctgtttta
46981 gcaagtgcga atttaaaata ttttgattgg acgaaaatat gaaagtttta attatagaaa
47041 atgaaattta tctagctcaa agcataagta ttaaacttag tgatgtgggt tatagttgcg
47101 agatcatcaa ttcctttgat gagcgtaacc ctgaaaaata ttatgatatt attttacttt
47161 ctaccaatac tagcaatttt ttaaaagcag ttacagaatt taaacatagt attattattt
47221 tacttatttc ttatatcagc acagacacgg tttcaaatcc tttaaaactt ggtgcgtgcg
47281 attatataca aaaacctttt atgatagaag aattgatacg aaaaatcaaa cattatcaag
47341 actttaaaaa gctttctata ttgaataaag cttataaaag ctatatcaaa tcaagacttg
47401 aaacagttaa aattccagaa tacaactata aaaaattaaa actccccgta gttttaaaat
47461 ctaacaaaca aagttcagct gatgcctttg tgtttaacta tatcaacgaa tacgacatca
47521 ctctttctta tgttgatcta tctgtgagta attcagtaga aaaagcgatg aaacttccaa
47581 cagaaaacaa tcttttattc ttgagcaatt ttcaagtttt aaaaccgcta gagcgtgaaa
47641 aattacttga atttatcaaa aataaatccg ttattttgca tactaattca aatatagaag
47701 atttagaact cccgtgtatc aatcttaatg ataatgaaaa aaatatcgat agtaatcaaa
47761 ttttaaccat agatgaatat gttaaatata tcattttaaa ttatcaaaat gtctttcctg
47821 atacagatct ttcaaagaaa ttaggaattt ctcgtaagtc tttatgggag aaaaggaaaa
47881 aatatgaaat cagcaaaaaa aaataaaagt atcgttatag ataaaaatga actaggcatt
47941 ctttctctta tcaaagaggg tttgcttgga agttgcaccc atttaatgga tgaacatgaa
48001 acaaaacaaa ttcttaaaac gggtaaatcc gataaagaaa gttttcctta tcctttgtcc
48061 tttgctccaa aaatttcaga agaattcatt aaagacattc gcataggaag caaaatcgat
48121 cttatcctta atgatgaaac ggtgggaaat atcacactta aaagcaaatt taaaaatgat
48181 aaaaattttt ccaatatctt tagcccacat acttgctctt tagataatat gggtgaaatt
48241 tgcatcggtg gagaaataga aatttataat tctacaattc aaaaaatcaa agaagaattt
48301 cataaaacca aagaaaatct taatgcccat aaaattactg cgatagtttc aagctttgat

48361 cctttgcata gagcacatga aagaatgttt cgctggacta tagataaggc ggatttggtt
48421 gttgtttttc ttgtagaatc ttttgacgca aatggctttg attttgattt gaaacaaaat
48481 tatttgaaaa aatttattca aaattatttg ccaccggata gaattttcat cttcccgctt
48541 aaagatatca atctttttca cgctcattta aatcctagtc tagaaagcat tttagccaag
48601 agtcttggat gtactaaact tgtcgtaggg caaaatcaca caggacttgg aatgttttat
48661 gatgataatc aacctaaaac catactagat gatttttcta aagattatga tatagaagtg
48721 attgttttac ctgaatttgt attttgcgat gcgtgtcgta tgatagtaag tactcgatct
48781 tgccctcatg gatgccatca tcatttacat tataattctc aatccttaaa agaattgtta
48841 agagcaggta ttatacccc tgctgttttt atgcgaaaag aagtttcaag tattatctta
48901 tcttctattt ttcctaatcg atttaaaaat ttgcaaaaaa tctataatga tcttttcgca
48961 acagatggaa ttttagaata taaaagcgat gaagaatttt atcaaaagct tttagaaact
49021 catcaaatgt cttatatggt ttgaaaggaa acaatgcaaa aacttttttt aacttttttt
49081 tactcaggat gtgcaaaaaa agcaccagga acttttggaa ctttagcggc tttgattcct
49141 gcttttttta tactaaaata cttaggaatc actaccttat ttttactgag tattttgatt
49201 tttattgcca gtataagagt gatcgatgat tatgaaagaa aaacagggat tcacgatgat
49261 aaacatatag taattgatga agttgcagga gtttttctag cgtgtgctat cgctgcaagt
49321 ggcgctaatt ctttactgaa ttttttttatg gcctttgtgt ttttccgtat ttttgatatc
49381 acaaaacctt ctatcatagg aaaaatcgat aaaaaaacca aaggcggact tggtgttatg
49441 ctagatgata tgctagcagg attgtttgca ggacttttaa gtgcagtgat ttatggtttt
49501 ttacttaaat tagatttagt cttttgggat aaagacttga taaatttatt ttgaaatttt
49561 gcttaaatta atatattttt aaattttatt cttatataat ttagtcttta atttcaaaaa
49621 aggaaaataa atggcaaacc ataaatctgc tgaaaaaaga gcaagacaaa ctatcaaaaa
49681 aacagaaaga aatagatttt atagaacaag acttaaaaat atcactaaag ctgtaagaga
49741 agctgctgct aaaaacgata aaaacgctgc taacgaagct ttaaaaatag caaacaaaag
49801 cattcacgct atggtaagtc gtggatttct taaaaaacaa acagcttctc gtcgtgtaag
49861 tcgcctagct cttctagtaa ataaaattgc ataatctttc ataattttta atgttagcta
49921 gtaaactaga tccttttttta aaacgctttg aagagttaaa ttctcttctt agtagttctg
49981 atatcatcag cgatatcagt aaaatgactg cactttctaa agaacaaaag aatttagaac
50041 ctatcgtctt aaaagctaaa gaatacctaa aaactctaga tagcatagaa gaaaataaaa
50101 ctctacttaa tgatgctgaa cttggagagc tcgcaaaaga agagttaaaa aatttagaag
50161 agttaaagcc taagctcgaa gaagaactta aaattttgtt actacccaaa gatccgaatg
50221 atgaaagaaa tatttttctt gaaattcgtg caggaacggg tggagatgag gcttctttat
50281 ttgtaggcga tcttgttaaa gcctatgcaa gatatgctga aaatcgtggc tataaacttg
50341 aaatcgttag ctctagcgaa ggaagtgtag gtggttttaa agaaattatc atgctagtta
50401 aaggtgcggg agctttctca agactgaagt atgaaggtgg aacacatcgc gttcaacgcg
50461 ttccacaaac cgaatctcaa ggacgcatac acacttctgc tataactata gcggttatgc
50521 ctgaagtaga tgatatagag atagaaatca accctaatga tcttaagata gatgttatgc
50581 gctcttcagg acacggagga caaagtgtaa atactactga ttctgctgtt agaatcactc
50641 accttcctac aggtatagtt gtagtcaatc aagatggaaa aagtcaacac aaaaataaag
50701 aaagtgctat gaaagtctta aaagcaagac tttacgaaat gcaagaaagc gaacgcttgg
50761 cacaagaaag cgaagcaaga aaatcccaag taggaagtgg agatagaagc gaacgcatac
50821 gcacttataa ttttccacaa aatcgcatca gcgaccatcg catcaatctt accctatatc
50881 gtttggatgc tattttacaa gatggacttt ttgatgaagt tatcgaacct ttaatagcac
50941 actatcaagc agaatcatta caagagcaga atttataatt tcattagtct tgaaaacaag
51001 actaatgatt agtgtttatg cgggaattta tgcgggctac tatttgcacc cacatgggtt
51061 acattttcat tttctatatc ataagcttgt ccaaaacctt ttacaaaacg acccttttga
51121 aattcaagtt ttacaagatg aaaatcaagc atattgcgta tagtttttat tccaccttct
51181 ccacctgttt gtttttcaaa ctcatcataa attttatcaa acatttcccc acgctcgata
51241 aaactagcct tgaccctata acgcaatctt ttacgaagta taacagaagc tgccttgctc
51301 tcatcctcta aaaacattat ttctatattg tttggatttt ctttgatatt attaaaatgc
51361 tcacttactt cactgatata aatataattt ccccatgaag tatttacaaa tggcgcataa
51421 gaacaaacca cttccttatc cgcatttaaa gtcgctaaag aaaccgaatt aaagctcaac
51481 ataaactctt ttgcttcttt tgctacccca ctaagatcct ttttagtctt tgcactcata
51541 caaagtgcaa taatagcatc tttaagagtt tcttcatttg ctttttttagg aaattctatg

51601 cgtaaatttt cattatcatt ataagctata tctaagccct caaaatcaac tccgaccaat
51661 cttacttctt taggatcttt cactgctcca aattttttac aaagatcgat caaattagat
51721 tgatggtgat cattcatatg agaaataata ctttcaaaac tcattttta ccctttcttt
51781 aatttttata gaataaaata tcaagctaaa aatggtatca attatcaaat taaaaaggac
51841 ttaaaaagac taaaatcatt taaatttcaa aaatttattc taatttaagt tgataaagga
51901 taaattttct cttcaaatat tatgatatat attatcatta tttattttat ttaacaagga
51961 gtgaaaattt gaatccgagc aagagagttt tgaagctatc tttgctagta tttttattaa
52021 atactagtat caatgcacaa gaattaaatg ataatattca aatgcaaaaa gttgttgtaa
52081 gtgcaacagg atttgaacaa gatgcggata ataacttacg caatgtgatt gttattgatg
52141 gcaaagactt agaaaaaaga ggatttaata ccttagaaca agctctagaa agaatttcag
52201 gcattagttt tgtaaatttt ggactaggac gcaatatcga tatgcgtgga caaggaagca
52261 aatccaatat agctgtgaaa gttatggtaa atggaaatgc tatcaatgtc cttgataatt
52321 ctcatggtgt aacccccttta gaaagtatca atctaaacaa tatagaacgc atagaaatca
52381 taccaggggg aggctctgta ctttatggaa atggcactag aggcggtgtg attaatatca
52441 tcacaaaaaa agaaaaatcc gatgcttttg ctctaaatct taaaggcagt agttacgatc
52501 atggaaattt aggaggaaat ttaggggttt atggggctaa aaaaatcaat gattatctaa
52561 catttggttt tgatatacaa ggctttaacc gcgatggcta tcaagaaggc tacaatcaaa
52621 aaggctattt tgccaataca aaagcctatc taaacactag tgataatagc gatttaaccc
52681 taaattataa ttattttgaa agcaaaaata caagcagtgg ttatcttaca aaagcacaag
52741 ttcaaagcga cccaacccaa aaaggaaata gtgataatat cactcaaatc aatagacctg
52801 aaatcgcttt agattatcat cactatttta gtgatttata tgaaattcaa ctcggagcat
52861 tttggcaaaa tcaaaaaata agatatctaa aagatgaaat tccaatgatg ggcaccatag
52921 cttatcaaac tggtagtggt tttgaagata cacttacagg tatcaattta aaaaataaaa
52981 tcaattataa agatgattct tattttatct ttggttatga gtttgcaaat catgatgcca
53041 aaagaacaag catagttcat tataatgttc cagtaattca aatgagtcat accatgacaa
53101 ccctaatgga tatggataag caaagccatt ctatttttgc tctagactct catcaattta
53161 atgatatttt tagtatttca ggtggggcaa gatacgaata cagtctttat gatacccata
53221 gaaattacac aagtaaaatg ataatgggtg gtagaccact accaagcaat ccagagcttt
53281 ttgacactga tgatacaagc aataactttg ctcttgaaat cactccaaat ttcaaatatt
53341 ctgatactgg aaatatatat gctaaatacg aaagaggttt catctctcca actcctgcac
53401 aacttgtcaa taaagatcaa gcaaatcaaa aatattacac tgccaattta gatcctgaaa
53461 tttttgatac ctttgaactt gggattaatg atttttggtg ggatttttat ggttttaatt
53521 tgactttctt ttacactcta agcaaggatg aaatttctta tcttggaaat ccacatgcta
53581 caggaggggc attttggaaa tactacaata tcgaccaaac gcgtcgttta ggggcggagt
53641 taaaccttag tcaattttta ttcagtgata gtttaatcct caaagaaagc ctaagctatc
53701 ttgatgctaa aatttctaaa ggtgtcaatg atggacttag aattccttat gtttcaaaaa
53761 taaaagccac agccggagta gaatacgctt ggaataaaaa tttctcaagc tatatagatc
53821 taagctattt ttcaagagca aaagatggcg gagttgttga tgaaaataca ggaaagatgt
53881 ctagaaattc ttggattaag gattattttt taacggatat tggtgtaaat tataattata
53941 gaaatttaca aattttagca ggcattcgca atctttttga caagcaatat tacacttatc
54001 aagatagtat aaatgatgag tatctagctg gaaatggcag aaattactat gttgaattta
54061 aatatgcgtt ctaaaaattt agtgattata gggctttttt tagcctatat aatcacctgt
54121 atcatcgcac tttgtttagg agatgaaaat ttaagcccta aagaacttct ctcctatgct
54181 tttggcgaaa atgaaatttt gcgtcaaatc atcatagatg ggcgtttgcc tcgtattata
54241 atggctattt taatcggtat gcttttagct agcagtggag cagtaacgca aaatgtattt
54301 tcaaacccca tagcagatcc ttatattata ggcatagctt cagcggcaac ctttggatct
54361 gttttagcgt atttgttaaa actgcctgat tattattatg gaattttagg ctttgtttgt
54421 tctgccattt tttcattagc gatctttaaa gtatcttcaa aagcttccat agctacttta
54481 ctcatcatag gcatcgcagc ttcatctttt ctaggagctt ttacctcatt ttttacctat
54541 cttatagggg aagattcttt taaaatagtc gcttggctta tgggaaatat aggttcagct
54601 tcttggtttc gtataggaat tttgattttg cctttgattt tttgtctttt gtatttttac
54661 gcacataaaa atgaattaaa tatcatttta agtggagatg atgaggcaag aaatttagga
54721 gttgatgccc aaaaattaaa aatcaatctt ttgatagtct cttctttagc ggtttctttt
54781 gcagtagctt ttacaggact tatagccttt gtaggactta taataccaca tactgtaaga

54841 ctcttgctta gaaattatga caatgctttg gttattcctt tttgtaccgc ttttggaggg
54901 ctttttttac tcatttgcga tactctagca agaactctaa tagcccctgt gcaaattccc
54961 ataggagttt taaccgcatt ctttggagct cctgtatttt tatacttggc tttaagtgct
55021 aggagatttt tatgatatta aagatacaaa atttaagttt tagctatcat aaaaaagaat
55081 tgttaaaaaa tatcaattta gagcttcaaa ataaagcttt tataggtatt ttagggccca
55141 atggttcagg taaaagcacg cttttaaagc ttatacttaa gaatttaaat ataagcaagg
55201 gcgaaatatc gctttttaat accgatataa aaaacttttc ccttaaagaa tttgcacaac
55261 tttgcggttt tgtacctcaa aattcgggtt taaacacacc cttaaaagtt attgatgtgc
55321 ttttgatgag caaatttaca aatttaaaac atgctttttg tgattattct aaagaagata
55381 ttttagaagt tgaaaatttt gcaaaaactt taaaattgga aaacttttta gaaagaaata
55441 tactttcttt aagtggtgga gaatttcaaa gagtgctttt agcaagagct ttgcttaaaa
55501 aacccaaaat tctttttttta gatgaaccca cttcagccct tgatttaaac catgctatcg
55561 agcttttaag cctttgcgaa agattgataa aacaaaacaa tatcgcagtt gtagcgattt
55621 tgcatgattt aaatttagcc tctatgtttt gcgataaggt attgttttta aaagaaggag
55681 aaataaagta ttttggaagc acaaaagagc tttttaccaa agaaattcta aaagaaattt
55741 acaatttaaa ttgcgagatt gtctataaag acttaaaacc ttatatctta gccttaaagg
55801 aaaaaatatg aaaaaaattt taatcattat gagtttattt ttaaccctac tcaatgccaa
55861 agaacgcatt gtagtgcttg atcctgcaag catagaaaca ctttttatgc taaatgcaca
55921 agatcaaatc gtaggtattg caaatttgca acactcaaat atctaccctg aagaaaaaac
55981 ttcaaaaata actagcgtag gaactttttc acacccttct cttgaaaaaa tagttgccct
56041 aaagccaagc ttagtgatat taagctcgta ttctttaaat ttagaagaag ggcttaaaaa
56101 ctttggaatc aaaagtatca atttaaaagc ccaacgcctt gatgaaatca aaaacaatat
56161 acaaattcta gcagaactca caaacaaaga agagcaaggc aagaaacttt tagaagaatt
56221 tgaacaagga ttgcaagaat tgcaaaaaaa tcccttaaat aaaagtggaa tttaccttta
56281 ttctagcaac ccactcatgg cctttaatga taattcttta atagcagata tattaagact
56341 cataggcatt aaaaacctaa gccctcaaag cgaaatagca cgccctataa tatcagcaga
56401 atacattcta aaacaaaatc ctgatttatt gatcttaggc attaatgcag atggcaacat
56461 tttagatacc aatgctttgt taaaaaatac aaaagcagcc caaatgggac aaatttatca
56521 caataaaaac acccatatat tgttacgctt aagcccaaaa atcatagata gaattaaaga
56581 atttaaaaca agattagaaa atcaaaattt ttaatcactc aatcttgttt taaaaaattc
56641 acagctttga caaagctttt ctatacgctt attttcttca aaggcctttt tgatttcttg
56701 aatgcgttta gaatcaagaa ggctcttaaa ttcagcattg aaaacattac ccaaaggtat
56761 atcaccctga gtatccaaac aacaaggcac gagcgtgccg tcacttaaaa tgcctatttg
56821 ctcttttaaa gcatggcatt ttccttgagt ataaaggggt ttatctttta aacttggcca
56881 tttaaaaagc ttgttttgat gaagtaaaat atgcctttga agacgatttt tagccaaatg
56941 tgtaagaatt tgaaccccaa attctttact taaaaattca taaataggca aattttcact
57001 aggagcttta aaatcagtat ctaaattcca aagtctaaga ttgataaagc ttggtttttt
57061 atactctaaa tgctctttgc aaaactctaa aataggctta aaatattgtt ccaaagaaag
57121 cttattttgg cttaaaaaag ccattaaaga gatattgatt tgatggatat tgtcatattt
57181 taataataat ttagaatttt taggactaaa ataaaagcca cttgtagtaa tttctaattt
57241 catttgatga tttttagcaa tttgcaaata ttcttctaaa ttatttaaaa gcaaaggatc
57301 gcccaaaaga tgaaaagtaa aaatttcgct tttatcccaa atttgctttg cgagtttaga
57361 aaaattttct acactcatca ctccacgcaa ggcttttttt gaaggacaaa aatcacattt
57421 taaaccacaa atatcgctta attctatgta taatttttta aatttcatca tatatccaag
57481 agtatttttt tagcaatatt taaaaaatag attaaaataa gtaacatttt atacatattt
57541 ttatatattt tttaattatt ttatatactt tcaaaacttt taaataaaat ccacattata
57601 cttgaaaaaa ttaaaacttt aaaatgaagg aaatttcatg caagaactta ctcgagcgaa
57661 aaaaattcgt tccattgtag ctgcaagcag cggaaattta gtagaatggt tcgattttta
57721 tatttatgcc tttacagcaa cttattttgc tcatactttt tcaacttctg ataatcctat
57781 cgtgcaacaa atcaatgctt ttggagtctt tgctgcagga tttttcatgc gtcctatagg
57841 atcttggctt tttggttctt tagctgataa ggtgggtcgt aaaaaatcca tggttgtatc
57901 tgtggtttta atggctttgg gctcttttat gatagcagct ttacctagta aagatgtggt
57961 gggggatttt gctattatct tgcttttact tgcaagactt atacaaggat tgagtgtagg
58021 cggagaatac ggcatagcag cgacctatct ttccgaactt gccaccgaag gcaagcgggg

58081 tttttactct tctttccaat acgtaaccct cataggcgga cagcttttag cagtagctag
58141 tattagtata atgcttttct tttttagtat tgatgaaatg aaagattatg catggagaat
58201 actttttgtt gtgggtggaa ttttagctct tggctctctt cttgtgcgta aagtgatgaa
58261 tgaaagcgcg acacaaattc acaaacatga tgatagaggg acgcttaaag ctttatttac
58321 aagctcttgg aaacaatttt taatggtgct tggtatcacc gcaggtggat ctttggcatt
58381 ttatactatc actacctata ccaaaacttt tatggaaaat tcaggtatgg ataaaacact
58441 ggttaataat cttttttttag gtgctttatt tatacttatg attatacaac cgctttttgg
58501 atatatcggc gataagatag gacataagcg atccttgatc atcttttgta ttttagcctt
58561 tgtaggaatt tatcctattt tcaatcttat atcaagcaat gctcaaagca attcttcttt
58621 ggtatttatc ttagtagtgc ttttatttat tattctaagt ttttacactt cagtggctgg
58681 aatttttaaa gccaaacttt ttcctgaaca tgtaagagcc ttaggcacgg gcttgggcta
58741 tgcgatatct aatgctatct ttggaggatc tgcaccttgg gtggccttac aatttaaaaa
58801 tgcaggaatg gaaaatggct tttttgtcta tattgctgtg cttactctac ttatgtttat
58861 agcaactttg tgcctgccta aaaaatcaga gcttaattag ccttaaagcc tttaaagaaa
58921 gtatcgaaag tgcggtactt tctaattctt ttaagctttt aaattgataa tttttatttt
58981 cctcttttaa aatttgatga taaactttaa tgtttaattt atactttgta taattatgtt
59041 taaattctcc caaaaatttc atatttttag aaatttcacc ctcctcttct ttaaaaaaag
59101 gaaaattata catacccttta taaagtttat cttgactttt ttgtatggca aattttttgc
59161 cccattcaaa tataaaaaga tttaagcttg cattctcgta agatattttt ttagccttag
59221 gataaagctc tgtgttaaat ttaccttggc aaaaatcata caagggacaa attccacatt
59281 ttgcattttt gctcacacat attaaagcac ctatatcaag taaggcttgg ttatgattaa
59341 aagcatcttc caaatttaaa agctccttag cctttctttc aagctcgttc atttttggat
59401 tttccaaggc aaaaaggcgt gaaagcacgc ggcgtatatt gccatcaaca aaagaaactt
59461 tttgatcgta agcaaaacaa gcaatggcac ctgcagtata agctcctata ccacttaatt
59521 tttttaattc atcaagtttt ttaggcaatt taccttcaaa tttatctaca cattctaagg
59581 ctgacttttt taaatttcta gcacgagtat aatacccaag cccctgccaa gcttttaaaa
59641 gctcgtcttc atttgcctta gccaaacttt gcaaggtagg aaatttttgt aaaaaaggaa
59701 aataaaacct ctctaaaact gattttactt gagtttgctg gagcataatt tcactgatat
59761 aaaccccata agctctatca atatctttta atctttcatc acaatctttg ctttgcaaat
59821 tacgccatgg caaatttttg cgtccatttt tatcatacca aagaagtaat ttaacttgta
59881 agtcttgaat catttgtttt tgcatgagtt agagtataac aaaaattgca taaaaattta
59941 caatgtatta tagtgatatt ttaataatta aaatcaaaaa tcattttata aatttatata
60001 aaaaatccca ttttacatac aatacaaagc tataaataat aaaaatataa aaataaatat
60061 aacttaaata taaattcaaa taaaaaataa aaaagcaaga caatatcatt attttaaaag
60121 ctttaattta gaataaaata tttaaaatac atttttaatt ttcaaaatta tattctttgt
60181 tataattatt taaatcagta tttttaaacg catttaaagg gctaaatcaa tggatcatca
60241 caagctaatt ccccctttggg atgagcgcta taatatcaac catcaaaaaa tcgataccca
60301 gcaccaaaga ctctttgaac ttgcggcaca agttgaaaat gcggttttta aatttgttaa
60361 acgcgatgaa ttaaaagaaa ttttaaccga actttttacc tacatgaaag agcattttca
60421 tgatgaggaa gaatttatgc aagaaattcg atatccacgc ctttatgagc ataagcaaat
60481 gcataaaaat atcgtgcatg aaatgtctca attgatacaa aatataaaat ccacaaatga
60541 tttaaaagaa aaactatact ctatcatgtc caattggctc tctaggcata ttttaagcca
60601 tgatgttatg attattgctt gggtaaatga gaatttaaaa aaagatgaaa ataaaacact
60661 tgagttaaat gatttgccac caaaacaaat tctaacacag gaggaaaatt ttgaatttat
60721 ctattcttgt ccttgcaaga tagatcatag gttaagtttt aaagagcatg aaagtattgc
60781 taatgaaggc aagattatga gatgtaaaaa atgtcaagag cttttatttt ttattagaag
60841 ccaagaaaaa acttataaag gataaagctt gcttccaaaa tgggataaaa tctatagcgt
60901 tcataatggc aaaatagaca atcaacacaa aaagcttttt gaacttgcag ccaaggttga
60961 acatatattt gataagcccg ttcatagaga tgaaattaaa gctttactta cagatttttt
61021 caactatatg aaataccatt ttgatgatga agaaaaatat atgcaattca ttcgttatcc
61081 ggactttgaa aggcatcgcc aaatccacaa agaaatcatc caatccatga taaaaattat
61141 tcaaaatata aaaaccacca atgacctaaa agaaaaactt tatattatgg taaaaaaatg
61201 gcttttagaa cacatacttt atgaagatat gagagtagaa caatggagaa tagggtcttt
61261 aagctctgaa aatggagctg atatcacttt tgaagagatt aaagatgatg aagaagaggc

61321 tgtaatttat ttttatgctt gtgagtgcat aggacaaatt catgatgtgc cttcaggaat
61381 tcacaaaaaa atacaaaatg aaggtgcaaa atttaagtgt aaaaaatgca aatttccttt
61441 agaatttttc aaaaaaggat attagttttt atatttattt tgctacaatt atttaatttt
61501 tattaaagga taaagaaatg ttagcatttt ttaaaggctt agggatagga tttttatgct
61561 tggtgctttt tatagctgga gtgatttttta atgtagaatt tttaaataaa aacaaaacta
61621 ctcaaacttt aaatttttca agaagtatag aaacaagtat ggaagctaaa cctgatattt
61681 ttcgtgcaaa tatcaacttt tgggcaaatg aaaatttaag cacccaaacc tttttaagca
61741 atgaagacaa aattcaaatt tccaatactt ttaatcaaat tttagaacgc tccaaaaaag
61801 agaatttttg cagtggtgga agtttctcgc ttgaacctaa tttttcttat aaagacggag
61861 tccaaactcc taaaggacaa agacttcatg caaatttaga atgtgagttt aaagcggaca
61921 agctagagga ttttaacaaa cttttaaatg atttaaactc aatcattgca aaaagtgaat
61981 ttataggtat ttctacacct gctttagagc ctaaattctc aaaacaaacc ctaaaagaca
62041 ataaagaaaa actttatagc gaacttctaa aaaaagctta ttcttatgaa aacatttatt
62101 ctttagatct taacaaaact tgtatattaa aaaatctaaa tattgaatca attccaaatt
62161 tttatccaag ggctttaaat agtatgaaaa gtgctgataa tatggagctt tccttaccta
62221 taataaaaga tgaagagcaa agcattagtg gcactgcttt atttatttgc aagtaatcaa
62281 agcaaaataa actcatttta agtttatttt gctagaattt catctttagc tttcgcggga
62341 atagctcagg ggtagagcac aaccttgcca aggttggggt cgcgagttcg aatctcgttt
62401 cccgctccat taatcacccg cccgggtggt ggaattggta gacacaaggg acttaaaatc
62461 cctcggaatt tttcttccgt gccggttcaa gtccggcctc gggcaccact ttttctaaaa
62521 aattatcagg gcgacatagc caagcggtaa ggcatgggcc tgcaaagcct tgatctccgg
62581 ttcgaatccg gatgtcgcct ccaaaaaagg caattaattt ttacaacttc aattcgggag
62641 atggctgagt ggtcgaaagc ggcggtcttg aaaaccgttg agggtcacac ctccaggggt
62701 tcgaatccct ttctcccggc cactttttga aatttttcat tagtttaaca aactctgtta
62761 tgataaattg atttgatcta aaattaagga aatcaattta tgaaaaaatt catcttaatc
62821 tcatctatac tttcatcttt ttttgtattt acccaagcaa gcgaactgcc aggcgatgaa
62881 atccaacccg aagtaaaagc tatcaaagaa cattttaagg acaaagtaga aaaggtagaa
62941 tttgaagcat gggcaaaagg tatgggttta aacttctcaa ctcaaaaata tcttaacaat
63001 caaaactata aaaaatatgc taaaactatg gcaaaactca ttagaaaaac aagaggagtt
63061 aagggaaagg ttgaaatttg ttatgaagga acgcctcaaa aacgagttca taaatgcaat
63121 aaattttaat tcctgactta aattaatatc ataaaaattt ttaagttttt ttcactataa
63181 aaaagctaca atttcttaat tatatatcat taaggaaaag gcatgtttaa aattgttttg
63241 ctcatatcaa taatctttgg cttaaatgct tgcaaaggag aggatatgaa taacaatgaa
63301 gataccaata aagatattaa aacagtctat gcgcctaatg ctgtgggcat agatcttaac
63361 acaaaaaacca atgaattttt atttaatcaa cgaaaaaaac ccacaggcaa atacactaaa
63421 gaatacacca aagccctttt agaagctgtc aatatagtcg ataattcccc ttataaaaaa
63481 ttttatgagc ctaaatattt agatcctgaa tttcacacag ggcaaaaatc cactctaata
63541 gaatttaaag aatggcaaag catatattta aaagatcctg ttaaaggaag cttagctcct
63601 tggaccaaag cagaaaaagc ttattataat tctttagatg gagaaggaag gtataattat
63661 ttagtaaaaa gaagtggctt agtatgtact cctatagatc taaaagatag tgccttaata
63721 agacctaaaa gacctaaaga aaaaagattt ataaatgctt ataaagaagg tatgaaagat
63781 tataaagaag ctaagagact agattataaa ggatatgatc tttttcaaaa agccattaag
63841 aatttaagct atgcttatga agagggtaaa gattataaag caggacttgc tttagcagaa
63901 cttggatatt caaaagatta ttttagagcc attataggta aattagacca agatgaaaac
63961 aatgaagttt tattggataa actaatcaat gaatttttaa atgctaatta tagaagcata
64021 aggatttatg aagagcttat agaaaagtat gacttagggg atgcttattg gggtttatat
64081 gtttattctc gtaaaataga agatacagta tttgatgata gattttattt tgctcagctt
64141 aaagatagca gtgaagagct ttataaaaat gcttttgaac atggagctta tggagctttt
64201 ggtgctaagg caaatactat atattctgat ttaattgcag gagaatatca actttgctta
64261 ggtatattgg gcaataaaaa ggctttttat gaagcaagct gtatatctta gtgattctgg
64321 tttaaaatca agagcctttc aagctctttg gcttggagtg caattaggag atgaaaagtg
64381 tttagaagga ttgtatcatc ctttatatgg aatacatgaa aaccctttaa aaagacaact
64441 catgaaagac tttgctaaaa atcctcctta tgataaatac ggaatgcttc ctttcttaga
64501 tgagttaatc tctacagaat ggattataga tccaaatgaa tataatttta tttatgatat

64561 taataataga gtagtgacgg caatgctaaa tggaatcgat aagggtaaat ataaagatcc
64621 aagagatgtg gattctactc ctgaaagtag atgggaattt gaccaagaaa tagatgctta
64681 taaagaaggt tctgtaagaa gttatagtta tgatttgccc aatcactgga gcgaagcaga
64741 tgtagaaatt tatttagaag agctttattt acatgctaaa ctcgcagccc tcactcctcc
64801 tcaaggctat cctaatgcgc caagatacta tagccctgaa aggttagaat ttatctatga
64861 aaaacataaa ctagacgcta aacttgatcc tagaatccct gctatataca gagctaattt
64921 tcctgaagag cttagagcta agataagagc ttttgctaaa gagcatggaa tagaggaatg
64981 aaatgaaaaa tttttatatg aatttagcat taaatgaagc ttggaaatat cagtttttaa
65041 cctaccctaa tcctgctgta ggttgcgtta tactagataa aaatgaaaaa attttagcca
65101 taaaagccca tgaaaaagca ggatgtgctc atgcagaatt aaatgctata gcccatgctt
65161 ttacaagttt aaaccctgaa atttcattac ctcaagaagc aaatgcgatg catgaattta
65221 tttgcaagaa tcatagaggt atttttaaag atagcatagc ctttgttacg cttgaacctt
65281 gctctcatca aggaaaaaca cctccttgtg ctaaactttt tagcgagctt ggctttaaga
65341 aagtttttat cagtgttaaa gatgagaatg agattgcaag caaaggtgca gattttttaa
65401 aaaaacaagg agtggagatt gaatttggcg tgcttgaaga tgaaggaaaa aaacttttaa
65461 aaccctttt aaaatggcaa aaagggcaat ttaaactttt caaacttgcc ctttctatga
65521 atggctcgcc ttttggaaaa atcgtaagta atgagctaag tcgtacttat gcgcataaaa
65581 ttcgttccgt gattgattta cttgtgttag gaggagaaac gataagaaaa gatcgtccta
65641 tacttgatgc aagactttgc aaaggcaagg cgcccaatct ttgtatctta agtcatcaca
65701 atttagaaaa ttttgacaaa agcattcctt tatttaatgt acctcatcgc caaatttata
65761 ctcatattcc taataaagct aaatttttaa tgtatgaagg tggggaaaat tttctaaaaa
65821 atttcaaaga tgaaatggat atgtttttga tttttcaaag ttcaagttta aatgatgaaa
65881 aaaatgttac aatgtcttta aattttaagc cactttatag aaattttta gggagtgaca
65941 cttatggcat ttatgagctt tagtggattt ttttatgcaa gaaatgattt aagattattt
66001 aaaatagaaa agaaaaacga aatcaaatct ttttttata aagattatac gctagcaagc
66061 ttcaaagatg agctaaattt aaacaacgaa atcttttttt atcaaagttt gaaagaaaat
66121 ttatttaaag aaaatgatga aattttaatt tcaaatttag gcaaaaaaat catacttttt
66181 agaaatttca ctcaaaatag tgataatttt gctgaagcaa aattaaaaca agtcttgctt
66241 ttaacttttt tatttttagc gagtgtattt tttgcaagct tggctgccat caatgaattt
66301 ggtgccatcg atcttgtttt tttgatgatt tgcttgttgc ttttagttat gggtatcatt
66361 aaccttggct tactttttaa acaaattcgt atcttaaaaa gttttagcaa agaagaaatg
66421 aaggaatttt taacccaaag aatgaaaaaa tacacaaaga aataaacaaa ataccttaaa
66481 ttttctaaaa aatttatcta ttaaaagtgt gcgaagtaaa atcttagcac actgttttta
66541 aatttgtagc aagtccgcct aaagaagttt ctttatattt ataattcata tccttgccgg
66601 tttcatacat agtttctatc acctcatcaa gactgactat aggaatggat ttgcgtgtca
66661 ttgccattct agctgcactg atggctttta tcgctccaaa agcgtttctt tcaatacatg
66721 gaatttgaac caaacccct acgggatcac aagtaagtcc taaatgatgc tccattgcca
66781 tttcagcagc attacaagcc ttaaaagcgt tagctcctaa aaccgtagcc attgctcctg
66841 ctgccataga acttgcactg cctatttcag cctgacaacc tgcctctgcc ccgctaatac
66901 ttgcattttt cttataaaaa gaacctataa gcatagctac taataaaaat tcgattgcct
66961 tttcatcatt aaaacctata gtatgatttt taagataaag cataacagca ggaattactg
67021 cacaagctcc attagtagga gcagttacaa cctttgctcc acttgcattc tcttcagcaa
67081 tagcaatagc gtataaagag ataaaatcaa taatccccat aggatcagca gtcattgtaa
67141 ccctttcttt aagccccttt gctctacgct ttaagtgaag ttttcccggt aaataatcct
67201 cgtttggatg tgtaccattg taatacactt cttgcatcac ttcccaaatt tctagacaat
67261 acgatcttat ttcttctttc gtatgaaatt gcaactcata tctataagaa agttcggcca
67321 aatcccaatc tctttttca caaagttcta aggcttgctt tgcattatta agctctatat
67381 ctagtttttt ctttctgcct tgggatatcc ctttatttct cttttccaat tccgcagccg
67441 ttaaaacaaa accaccgcct atagaatagt aagtttcttg atccacgatc tcaccgctag
67501 cattaaaagc ttgaatttgc ataccatttt catgcaaagg caaaaaatct ttagaaaaaa
67561 ttaaatcttt ttcataatca aatcgaagct ctttttcccc gcatagattg atcttcccct
67621 tgtcggtagc atttttattg acttcttctt gtatagctgc actcaaattt ttagcttcta
67681 aagcattcaa gccccaaata acggctttat cacttaaatg ccctttgcct gttaaagata
67741 aagagccata aagaatgatt tgaatttttt caatttttc taattttta gcaatttttt

67801 tacaaaataa atttcctgca agcataggtc ctaaagtatg cgatgatgaa ggacctacac
67861 ctattttaaa aatatttaaa ttactcattc gctacacctt aaaaagtata aattacggtg
67921 ataattgtta aaacacctgt aacaaaaaca aaagcatcta aagctttatt ttggaatttt
67981 ttcatttttg aaaccgtata aattgcaata attggcatta aaaataagat tgctgcgata
68041 attggaccgc ctaaactttc aataaaaact aaaatactag gattgatata agcagtaatt
68101 agcataataa tatacataac taaagttgaa caaatagcaa ttttttttcaa atcaggattt
68161 tcactacctg ctaatttgca acattttctt actataccat aagcaccctc tctagcacca
68221 aaataatgtc caaaaaatga actagaaatc gctaaaaatg caatcaatgg accaccatag
68281 gaaataaaag gattatcaag cttatttgca aaataagaaa gcacaggtat attttgtgct
68341 ctagcttcat taagctctgc aggacttaaa gaaagtacgc aagatatcac aaaaaacata
68401 acgaaaaaga gtagcattgt agaagttcta aataaaattt gatcagcttt ttctacggca
68461 tgttctttgt attgtctttt tacacttaaa gaaaaagtag aaatagcggg cgagtgatta
68521 aaagaaaaca ccaaaaccgg caaagtcagc cacactatag taataaattc ttttacccca
68581 ggaatagcac taaagctttc aaacgaccat tgtgggataa gatataaaga aaatataaat
68641 aaaatagcac acaaaggata cacaagccat tcacacactt tagtaatcaa ttcttcacta
68701 aaaagcatga taagcataaa aatacttacc aaaataaaag ctaaaagcaa gcgataaaaa
68761 gggaataaac tcgctacaac ttttccttta tcatctgcgc ttacctcata catagcacga
68821 atgatatccg caaatacacc ctctgaattt aaaagaggta aaaattgatg atagataaaa
68881 ctttcaaaag tattggttat acccacacaa tacgctaaac aaattggaaa aatagcaaag
68941 aaataaagca cagaaataaa aatactcacc cctctaccaa aatattcctc agcagcatga
69001 gtaatgtctt tatcattacc atttgcttgg cttacaaaac ggcttaaagc tctatgactt
69061 agataaacca ttggaaaaat aatcacagcc attgcaataa ccggccaaaa accacccaca
69121 cctgccttaa taggcaaaaa taatatcccc gcacccacag cggttccaaa aagagataaa
69181 acccatctcg tgtcataacg agtccactta ggagtattca tacattttcc ttaaaatttg
69241 atatcgcaaa attaagctta ttttaacaca cttaccaagc aaaaaatcta aattcctagc
69301 atttttcttt aatttatgta acattttgta aaatattagt cttattaaaa ttatatttta
69361 agatttaatt caagtaagtt ttgaagatgg tttttatat aaaaaaccat attggattaa
69421 caaagagaaa tttttaaact catttaaaaa tttggaaaca taaagtctcc aaatttacat
69481 gcttttaaaa ttaataatca aatttttgtc ctaagatatt accctgtgca tcagtataaa
69541 tttttaattg attgcttaat tcaaatttat atccgttgat ttctttgctt acttctatga
69601 tttgagcatt tgacgaagca ctgatttttg gcataatatt tggtaaaatg ctatttggta
69661 gagctttgta ttttccatct acatctttcc actctccagt gataataaag tcaatttctg
69721 ttccatcatt taaagtcaca tcataagaat ctatatcttt ttttactaaa gcaatttgtg
69781 ctgttttaaa attcttagat ataaaatctt gtgcatttct tggaagttca ctcgcaggaa
69841 caattatatc tctagcaaaa agcaccgtag cacccaataa agccgctaaa cctaatttta
69901 atttcatttt ttctcctttt tgtttgtgtt ttggttgaga gaagtttaac ttatctttgt
69961 gaagtgaatg tgaaactaaa aaataatttt taaactctca ttttcaacgc ctatgccttg
70021 acttttctta aaagaaagta ttctaccatc ttcaaatttt aaagtatcat tatcttttaa
70081 agttaaatca taagttaaaa tatgcaaaat aatgtcatac aaagtgaaga aaagctcttt
70141 ttcttctata tttgcatcac aaacctcaag ctctaattta tcgaaatttc taagaccata
70201 ggtataggca ttattgccat tatcacctgg ataaacgctt acataaatcc aattataaac
70261 aggcaagcga ttatcttcta gcatttttgc attttctata taaaacttag gctctaaaac
70321 aactcctcct gtataaaatc ccaaagcatg cttatcttgc aaacacacag atgatatttg
70381 agtaaaaagt ttagcttgat ctaattttgt ttcactctga cttaaaacgg atataagaag
70441 atgtgcttta tgtttttggg tttgctctaa agcatctttc cacataaagt taaaattagc
70501 ataatattct gcttcgttgt tgggtatggg atttttaatc aaatttaaag taatgagcat
70561 atttttaaaa gaaaaaatca ctccatcttg agtaaaattt tcatcagcaa tccatatatc
70621 aaaatcatct tcgagtatct tttttaattt caaaaaatca aacttggctt catcaagaag
70681 aacaaaagaa ataaaattat gattttcttg catgcttttc cttaagggtt aaagtattta
70741 aaaaattata acctaaattt ttaagccaaa ggcttgtgaa attatgatta tttaaaaaat
70801 tatgataatt aatattaatt ataattttat ttttattgta gtataatcgc caaaatttaa
70861 tcaaattatg gagttaaaga tggaattttt aaaagattat attgatttaa ttatctttat
70921 aattttagga gttatggcat ttatagcttt ttggtgcgta attgaaagag tattattttt
70981 tagaaaaata gattttaaaa attatgaaaa tcaagagcaa tttgatgatg caattagcga

71041 gaatttaact accatttata tcatttattc taatgctcct tacataggac ttttaggaac
71101 tgttgtagga attatgatca ctttttacga aatgggaata gcaggaaata tagatgttaa
71161 atccatagtc gtaggacttt ctttagctct taaagccaca gctttaggac ttttagtcgc
71221 tatacctgcc ttaatggctt ataatgcctt gcttcgtaag gtttcacttt taagcaatgc
71281 ttataaggta agtaaaaatg cttaaactac ctaaaaatga agggttaaat atagttcctt
71341 tcatagatat aatgcttgtt ttacttgcta tcattcttag tatttcaact ttcattgctc
71401 atggcaaaat tgaaatcaat ctacccgtta gcgaaaatgc tctaagtctt aacaatgatg
71461 aaaaaaaatt atccattagc attgatgaaa acaatatttt ctatctagac gataagacta
71521 cagatttaaa aggcttaagt gatgccattt cttcgcttca taaaaaaacc atagtggaat
71581 taaaaagcga taaaaatgca aaatttgaaa gctttgttca aatcattgat ttattaaaag
71641 ctaaagagca tgagaatttc caaattataa cagagaaaaa tagatgaaaa atgctttgtc
71701 aaatcacaaa tcacaagctt tttatatatc ctttttattt ttcctacctc tagcttttt
71761 actttttaat tctgccaatt tttttaaaat agaatttaaa agtgaagata gctttacttt
71821 ggcgatgaag caattcactc aaaatcaacc aacccaaact ccacctatac cgacaaattc
71881 tgagcctata cagcatcata aacccaaacc tataaaacaa cctattaaaa aaactaaaaa
71941 ggcagccgaa aaccaagtaa aacctataca agcgatcaat cctatacaaa aaagtacacc
72001 attcatagct caaacccata caactcacaa tcaagcttta aaaaattccg atgcaaagcc
72061 tactcaactt acctatggta aagataatca ccctgcatta gaaaaaattc aaaaagctat
72121 catggaacaa gcaagaaaaa actatccaag acaagcaaga aaaatgcgta tgcaaggcgt
72181 tgtggaggtg gaatttttat ggaaagaaaa caaaagctta gcagaattaa aaatcattca
72241 aagttctggg cacaatttat tggataaaag tgctttagaa agtatacgca aggcttcggc
72301 tttttttcct tattatcaaa gcgatctaag gatagttttg cctatagtgt ataatttaaa
72361 agcgtgattt aaatcaattt agtaattctt aatatctaat atttttaaat ttttacactc
72421 ttttatccac aaatatggct tcattgttac ctatttgcac cattaaatct tttttttaaat
72481 tttgctctaa attccctaaa gacttatttt gaagttcttc aaaaatttgt ggattacttg
72541 ctttaagttt ggcttgctca tctgcactta gggcacttag tcctttttct ttggcgattt
72601 caagcaaatc tttaacgtct ttttcatctt ttttcttttt gatttttacc tcatcgtttt
72661 catcactttt ttgcggatct gtgattttgg tagcatccgc acttccgccc gatgcatttt
72721 ttatatctct taaaatatct gttaaacttt ctttttttac atcaaattca ttgatatttc
72781 catcaaaatt cacatctttt tctatggcaa aattaagtct agtttgcaaa tttgcactta
72841 agcttaattc atcttcacct ttaaaagcta aattttgcat actttcaaat aaggtatttt
72901 tatgaaattt cacattgata tgatttgact cttctttatt atttaatgaa tttggattaa
72961 aaaactcgct gcttattttt tcaaaccaat tagcaataaa ttcattagct tcgccattaa
73021 gccttatagt tccatcatct ctagcgatat aatttgtaaa agaaccaaaa tgactttgta
73081 aatttttata attttcatca ctgattttta aagccataag ttcaccttgt ttgttttcaa
73141 aaaccacttt aaaacctgaa atttgattat ctgaaatatc atttactttg actttttctt
73201 gctcttcttt ttttaattct aaaatttctt tacgatttgt aatattttga gaacttggta
73261 aagaaacatt aaaatcattt attgttaatt tatccataat aactccttag aaattttaaa
73321 attggacttg tttattttat ttacaagctg caaataagcc caaacgacac gcttgtcttt
73381 tatgttgggt aggcaaagaa ttgcattgtt tcaatcttat ttcataatca acgcaagaat
73441 ttctaacctg tgagttttta ttattattct cagttttgcc ataagcaaaa gaattaacaa
73501 aacaagcaac cagtgcaata aaaagtaatt ttctcatagc aaagctccaa aaaataaaaa
73561 tattttataa tcacaatctt aaaattattt gatacaatta caaatttgat atcgacaaaa
73621 aatcaaattt atttatacta aaaaaattaa atttaaaaaa ggaatacaaa tgaaagcatt
73681 agcccttttt agtggcggac ttgatagtat gcttgcgatg aaacttatca cttcacaagg
73741 cattgaagta aaggctctaa atattaatat aggctttgga agcacaagcg ataaaagtga
73801 aatcatgaaa aagcgtgccg caatggtagg tgcggatttt gaaatgatag atgtaagaaa
73861 ttcgtatttg caaaaagtgc tttttaatcc acaatacggc tatggaaagc attttaaccc
73921 ctgtatagac tgccacgctt ttatgtttaa aactgcactt tcaatgctaa aagaagaaaa
73981 tgcaagcttt atcataacag gtgaagtttt aggacaacgc cctatgagcc aaagaggtga
74041 tgcgatggct aaggttaaaa aactggcttt agatgaggaa gacttgatac ttcgtcctat
74101 gtgtgcaaaa aatttgcctt taaccaagcc tgaaagagaa ggttgggtgg atagagaaaa
74161 gcttgaaggt attagcggaa gaagtcgtaa aagacaactc gaacttgcag ctaaatttgg
74221 tttagaagac tttgaaagtc ctggtggagg atgcttgctc acacttgata gttttgctaa

74281 aaaaattcgc gactttatag aatttgataa agatatgcaa gtaaatgatg cacaactttt
74341 aaaatatgga cgccatttaa gactgcctaa tggagctaaa atgatcatag gtagaaatga
74401 attagaaaat gagctactaa gaaatttaaa aacacctaaa tatgaagtga tagaacttgg
74461 agatttaatc ggagcttatt ctttacttag cgcaaatgca gatgaaaaag atttagaact
74521 tgccttaaaa attgctctaa cttacaccaa gcatgaagca aacaaaagct atgagcttcg
74581 atttaaaaat caaaactata aaagcatagc ttttgaagac aaaaaagaaa tcaatccatt
74641 tttcatttct taagcttaga aaaatacaga agccctgaga taaataggaa tcgtttcgct
74701 tggcatatta gaagatttat gcagggcttt agaagctgtt aaatttaagg aaagatgatt
74761 taaattatat ctaaaaccaa aagcgacacc agctaaaaag tctttattgg gcaaaagata
74821 atccctacca taacccatat ccaatccaac aaaaggctct aatgctctta gatatttagg
74881 tgcatttaaa aattgcaaat aaatcgtatt gttactatat gctccataat caagagcagc
74941 ggagctttct ttaaatcccc taactgtgta ttcatcaccg attaaaaatt tatcagcata
75001 caataaggat tcattagaat aactcgcacc cgagctgttt tgatataaaa atgctaggcc
75061 atcatttgca taaaaacttt tttgatagct taaattaaaa ttgactttgt taaattcgat
75121 tttatataaa gaatctttag aatctttttt agaacccaaa aatggaattc ctttttcgta
75181 ttctaaattt aaaaacaaaa atccaccaaa tgccagcgtt gaatactcaa ccccgctagc
75241 aatacttgaa tacctgccac ttgagctttc taaacgaaaa ttatcaatct cattaacatc
75301 atctttaatg cctaaattag cataaattga aaatttatcc ttacttgtgc gatgtaaaat
75361 ctttttttaac ataattttat gtcttaagct tgtatctgta tttttttataa aagaattatc
75421 atatccctca atcatattta tattatcact atattgcata gaataagaca attgataact
75481 ctgtataggt aaggtataat ttgctacata aacattttct cgctcttgac tcatatcttt
75541 taacaatcct ttgataaaac caaagcgtat ggaatcgttt aggttgaaaa tattacttaa
75601 agaaagataa gcgctagcct tatattctcc tttagcttta tagccactat tatcaaagtc
75661 taatatcaaa tcaggtggct ttgattgaag atttataaat atatcagaat aaccataatt
75721 ttcactggct ttaacagcaa ttttaacatc tcttgcacca ttgtttaaat tttctatgcc
75781 tgtatctaaa tcgtaaatat tgaatttatc gccctttttt aaaggcatac caaaatctaa
75841 tcttgttgta ttattatccc cattaagata tacctcccct acaaaaccat attttaaatc
75901 aagaaaaagt atatcgcgtt cttgattaat cccatcaata gttacgctag aagttgaata
75961 accctttgaa acgataaaat ttgtaagctc tttaatcata ttgtagatat cactgacact
76021 taaaggctga ttaatgtatt tttttaaaat attattcgtt tgcatagtca atgtatcgtt
76081 tttcttaaat tgaatttgtt tgaaaatata aattttatct tcagaatctt gattttctaa
76141 atacacatcc ttttcttgct gttgcttttt ttccaattca tcaaattctt tttgtatatg
76201 taattgctgt tgtcttttat ctagtaaatt aagcgttttt gcagagtcat ctgcttgcga
76261 taagcagata aaactcataa gcaaaaaaat aatttttaaa aatctcacct atagccttgt
76321 agtttgtgtg ctttcattga tataaggccc atctgattta gaaggttggt ttgatggttg
76381 attagaagta gaattagatc cagtaacctt gcttatccca ttttttattt tagagattga
76441 agaattaaga tttatagtac ctcctgccca agaatgatct tgcgtatctg tggtattttg
76501 agtatctgta cttataccat gatttttgtt attgtttata ttgatattga ctgcagaatt
76561 cacaccgcct gtcttatcga tatgatcatc tatatcaacc actcccccaa agctttgatt
76621 tgttccaccg cttaatctta tcgtagcccc atcgcttttt tcatagacac ttactttaga
76681 atttgtaact tttccatcaa caagtaaatt tcctttttga ctatcagaat ttaaaattcc
76741 cccttgcaaa tttaaatttc cattaacatg gccattgatt tcatttgctg cactgatacc
76801 actttgagtt gtaacagatg ttttatctgt tttagcatat cctatgccca aagtgcctgc
76861 aacacttcct gtaacaagat gattgcttga taagcctcca gttcctgaca catttaataa
76921 agcactatat ttttgagaat taagactatc agctaaagaa tttacttcca aatttccacc
76981 cacatttaaa atagcatcat tagaagcttt aacattggct ccattgagtg caaaatcttt
77041 acctgtgttg atattgacat tatttgcatt taaattcgtg ttagtaaatt tcgtttcatt
77101 tgttttagaa tgtgatgcac ttacaaagcc ctcaatacct tcacccacag ttccaccatc
77161 aactgcatgg tatcctgcag tttgtttgtt tgttgctgca atttccaaag aagaactaga
77221 agaagaattt ttagattttg cagcatttaa gataacatta tcttttgcct cgatattaat
77281 gctattagcc ttagcttcca ctccatttaa aacaatactt ccttttgtgc tttctaaatt
77341 taaattaccg cctgcattga ttttagaaat attctcacta gaactttggc ttttatcttg
77401 attgaattta aaacctagag tttgattaga agtagtattt atgagattgt tgctaaatat
77461 attagcagtg ttggctaaag cttgagaagc tactattcca taatttacac ctaaattttc

77521 atttttagca gctgcatttt gagctgattg attgatagca gaagctatag gactagtagc
77581 ttcgattctt tctttaatat acaaaccaaa gcctgtagtg ctttgtgttt tagtatcttc
77641 atatttagtg ctagcaatct ctcctgctct taaattaata tccttactag cctcaaaatt
77701 agctccgcct atgtctaaag cattgtttgc attgatatta atatttgaac ctttaattgt
77761 atttgaaaca taagaaagct catcacttat tttttcagtt ttagaaaatt ccaaaccaac
77821 ctctgcagtt cctaaaagat cagatcttac tccaccttgt gtgatactat tagagctatt
77881 tgtggtattg gcatttgctt tatcttgcac aaaactataa tttacagcaa ccccatttcc
77941 ggttaattct aaattagcat tagcaaaaac tccaaaagct aatgaatcac tttctgtgtg
78001 gattttattt tgtgcggcta taaaatcaac cttatcagct tgaatatcaa tctcgcctcc
78061 attaagctca cttcctttaa tgcttacatt tttttgagca tttaaattca aattatcggt
78121 ttgaatttgc gatgaagata ctaaagaagc atcagtattt acagaacttt gcttatatcc
78181 agcaatggaa cttgttgaag aatgagaaga gcttgaaatt ttattttctc cattttctat
78241 tgtgatattt tctccattta aattagcaga tttatccgct tgtaaagttg agcctgtgat
78301 tgctatattg ccattggtat taatattaat atcacctttt gaatttaaag tactgcttat
78361 tgattttgaa gtttctaaat tagcactttc ataactgatc tctacgggtt ttaaatacac
78421 accatcggtt ttagtcttgc taagctcgtg tgcatcttta atattaacat tgttagcatt
78481 gatatctatt ttagaatcag cattgatatt gctagctata atatttacat cttgattgga
78541 aacaattcct acatttgctc ctttgatatt ggtgcttaaa accccatcta tactcgttgt
78601 agtattggat ttttgactat ctgtattaat ttgttcaaat gaagatgatt tagaataatc
78661 atttttgata tttacatcgc ccttttgaga tataagaaca attttagaat cattcgcact
78721 agtagaaata tcactatttt ttatgtttaa atcatttacc gaatcaatat tcacaacatt
78781 atttgcttta atttctcctt tttgaattga attataagag cctttttcat ctatacctaa
78841 aacagtgtct atattgacat tattggcact taaatttata ttttgcgcat ttaaattaga
78901 actttgcaaa ttaatctcat tgccagcatt gatattaatc tgaccctctg aagttatacc
78961 acttaataaa gaattaaatg attgtgtatt aatatctatg cctgtttttc ctgtaagatt
79021 tgattttatt tcattatttg caattgtctc tttagaaaga tacaacatag gggccatgat
79081 atttttaccc ttttgtttga ttgttacata taaaatcata tcttgatcaa cagcttgatt
79141 tttagccaaa ccttgtccta aatttaagcc attttcattg gcgtattttg ctccattttc
79201 aagtaatttt ttaatttctt cttcgcttaa tttagtattg gattgtagtg catttgcata
79261 cattgcatta ataagctgat tttcaaaaaa aggatctcct attactataa attcatcaaa
79321 attcttattt ttaaattgat ttaaaatgtt taaaaatcca aaatactcac tttcatctat
79381 gatggaagta tttgtagtgt attcataagt aatgttattg tttgttttat tttcaagagt
79441 aaacaaaccc gaattaataa gatcaggtat agaatttaaa attaaatcct gactatttgt
79501 agcttttttt aaatccgaat caactaaggc aaagttttta tccaaagaac tgactaaatc
79561 ttgattgtat tctttagcat taagatctaa tttaccattt gaagcaatag cagcctcgga
79621 ttttgcttta aaaacaatct ttgcatcaag attccactct ttttgatcag gaacaaatct
79681 ttttgaagca taatcactgc caaacaataa agaaaaatat tggtttaaag ctgaattttt
79741 agctgctgca tctttaagtg cattccacgc agattctctt tgatgatctt ttgcttgagt
79801 ggtagaaaaa tatttcaaag catcaagcaa actttcacga tctacaaaat aataagttga
79861 atttgtattc caagaaccaa gatattcttc ggcaaaaaat ccattaaccc taatttgctc
79921 taaaacttcc ctcacagaaa tttctttact taaagattta ttgtctatat ttccaaaaga
79981 tacaatattg ccttgggcaa gaatttttcc gctgttgttg atttgtctag cattatcttg
80041 tgattgagta ttgatattta tatctttgcc tgcttggata gaagcttgct tgctatctaa
80101 taggttatta tattttacaa tacctgtaac ccctacttcc atattccatc gtttaaaagc
80161 agtatcacca tatcctactt gaccaaaacc ttgagcatac tcagtcaaaa tctctaaatt
80221 gcctgttaaa tttgcattat tatttagaat atttgcttcg atatttagat ctttatctgc
80281 aaaaattaaa gcattattat gaatcgtatg ggcgtttaaa tacaagttat ttaagctatt
80341 aaaagtagaa ttatttgtaa gtgttgtagc ttgggctcta aaatcaccat ttgaaaataa
80401 ataagcatca tttacaagat tttcatccgc atgaagattt aattcttttc cgctagcgac
80461 aagattttta acataaatat ttttcgcatc aagatctatt gaagaagcat tttcgagttt
80521 tgaattgata tttaaatcat tcaagctttt tatatgaagt aaattttgtg cgtataaatt
80581 tccatgattt aaagtcaaat ctttgttagc ttgtatttga atatcttttt ggcttaaaat
80641 tttgccatta ttcacaaaag catcatcatt aagcgttaag cttatatctt tatttgctga
80701 aatttctcct tgattatcaa aagaatttgt atcaacttta atatcattta aagcattgat

80761 aaagccaaaa ttgctagttt tatctagggt atttaaagct aaattgtctg cataaatttt
80821 tgagttttct tcatttatat caaaaatatc ttttgtctct tggctaaagt ttacttgttt
80881 tgcattttca aaattattgg cattgatttg aatttgcttt cctttataaa gtccttcatt
80941 agtaactttt ccacttgctt ttaaatcaat attatccgca ttgattaaag aggttttaat
81001 gtctttagca tctattttaa cacttccatt tgaatttaca cctatagctt gaaattcagc
81061 cttatctttt acttttacta taacatcttc gattccgcgt attactccac tatgtcttac
81121 gccaacgccc tcttctgtgc taataagagt aactgtattt gcatacatag atcccaaata
81181 cttcccatca ataccatagt caattttttc atctttttgt tttgaagcaa caatttttgg
81241 tgcatttggg gtgcttaaat ctactttgtt aagtccggct ataaaattga tatcggatag
81301 gtttttacca ctttgatagt gagcaatgct acctgcaaga tcaatcccac ggctgattac
81361 attaaaataa tccccatcaa ccattacccc cttttctcca atagccaccc tgccattact
81421 cgttacttct gtaaaatctc cattaaattt acccgtacta agcgtaaccc ctttagtatt
81481 taaaaaagct gctccattta cactaaaacc attttcatta gcaaaaatta aattagcact
81541 ttttccaaat acctcaacag caccattgat actagaagct tgcttgcttg aaatttcatt
81601 gatgatggta ttagcatttt gttttaaatt tggatttttc cctacccatc caccaatttg
81661 agaatttcca tcttttaaac tattattaaa aattactcca tcttggacac tgaattcatc
81721 aaattgattg tgtgatactc cattttgatt tggatttttg atattaatca catctgtttt
81781 attgcttgat tgtgtaagat ctgctgttct tgaaacaatg acattaccat ttctttgacg
81841 caaatctgct gagaacaata cgcaaggaaa taaagcgcca agaagcgtta aattcaacga
81901 taaacgaaca attttctttt tattaaattc catcaatccc tccttgtaat atttttaatc
81961 attttaatat taaaatggta atttttctca atattttttaa ttaatactaa aaaaatccta
82021 aataaaaatt cctttttttac acatttaaaa taaaaataat attaatttgt tttttttaaaa
82081 ttaccaaaat atataaaaat aaatgttaag aaatttatag aagagacgat atggagttaa
82141 atcttttcaa aatattgcaa attaaagccc aaaaaagggc tttagtatta ttttactcca
82201 taagcatttt ttaaattgtt taaattagca cttgcatgaa gcaataaaca atcagccaaa
82261 accaaacgca ccatagcact cactactaca ctacctctaa cgcctacaca aggatcatgt
82321 ctgcctttga gttcaaattc taaatcattt ccgaatttat ccatactgct ttgctttaaa
82381 aaaatagaag gagtgggttt aaaataagtc tttaaaataa gatcttgtcc atttgaaata
82441 cctcctaaaa tccctccgct gtgattactt aaaaattcac cccctttttaa agcatcatta
82501 ttttgcgagc ctcgcatttt actcgcattt ataccctcgc ctatctcaac agcttttaca
82561 gcattaattc ccattaaggc atgagctaat ttagaatcta atttatcata aagcacttct
82621 ccaagtccca caagcattcc cttagcccta gtataaacag aagcacccac gctatctttt
82681 gaatttctag cacctaaaat ttcattttta aattcatctt ctaaattagg atctaagcaa
82741 aaaatctcac tttgttttgc aaattcaaaa tccaattctt cctcttttaa atttgaaaca
82801 aatcttccta ctccaaaaac cccactttga acacaaatat caaattcacg caaaagcata
82861 gcagcaacag cacccgcggc cactctagca acactttctc tagcactagc tcttccgcct
82921 cctctgtgat ctcttatgcc gtatttatga aaataagtaa aatcagcatg agcaggacga
82981 aataaatctt ttaaattatc ataatcctta gaatgtgcat tttcattaaa tacaaccata
83041 gcaataggat gacccgtagt atagccttca aataccccgc ttaaaacctg caccttatcg
83101 ccttcttttc ttggagttgc aaatttgctt ccgcccttac gcttatcaag ttcgctttgc
83161 aaaaaatcct catcaaattt aacacccgcg ggcattccat cgatcacgca acccaccgcc
83221 gctccgtgag attctccaaa acttgtaaat tttaatcttg taccaaaagt attcactata
83281 aagctcctaa tttttcaagt gtaatttttg ctgccatttg ctgtgcttct tttttatttc
83341 ctgcaatggc tcttgcaagc tcttgtccat taaggattaa agcgatttca aattgtttta
83401 aatgatcagg cccaaaagca cgcacggttt catattgtgg agtttgccct aattttgctt
83461 gtgtaatttc ttgcaatttt gttttataat ccttaatgag aatttttgca tcaatttgtg
83521 ggaagttttt ttctatgagt tttaaagcga tattttttgc aaaatcaaat cctgcttcta
83581 aatgtatagc tcctataatc gcttctaaag catcagaaag tatagatggc ttttctttac
83641 ctccattgtt ttcttcagcc gcactcataa aaataaaatc gcccaaattt aaactatttg
83701 caatctttgc aaaagatttt tcattaacta aagccgctct taattttgac aaatcccccct
83761 cagcgtcttt agcaaattta tgaaaaagat attcgcccac aaccaaatca agcaccgcat
83821 cgcctaagaa ttccaaacgc tcattattgt aaggtttttt aaaactttta tgagttagag
83881 catggattaa aagatcttta tctttgaaat tataggctaa tttttgttct aataattcaa
83941 tatttttcat cattttccct tgcaattttt aaagcagctt cgcgcgctaa agtatcacac

84001 ctttcatttt ccaaatgccc attatgtgct tttacccaat aagcttttat cttatgaggt
84061 tttgccatat tgatatattc tttccataaa tctacatttt ttttaccctt gaaatctttt
84121 tttatccaag cttctaacca ttcattaatg ctttgaacca tcaaattaga gtccgtaaaa
84181 agtgaaattt cacaaggttc ttttaaagct tttaaagctt cgataatcgc ttttagttcc
84241 attctattgt ttgtagtatt agcctctgct ccaaaacctt ctttttgatg ttgattatag
84301 cgtaagatat aagcccatcc tccaaaacct ggatttttaa gacaagaacc atctgtataa
84361 atttcaatat gtctcattat ttttcacttc ataaatgatt tgacaagtat taaattcata
84421 acaaacagga caatgataaa aaaataaagg cattacactt ttacactctg tacaacgata
84481 tgaaaattca agtcttgctt taaaggaatt gtcttttaaa atttttaaca ttttaaaatt
84541 ggaattttta aaaactatat ctttaggctt ttcatcttct atcaaaccca aagcataaaa
84601 aaattcaaaa tattctttat tttgcaaatt aaaagcttta ttttccttat aaagcaaatc
84661 acaaatactc gaaaaatctt gatttaaaaa aatatgatat ttttcaaaaa ccaagcgtcc
84721 tagcatagaa ttattgtcta tttgtagctt taaaatgtgt tctttttttt cttcatcact
84781 taaactagaa gcaagaaaat ccaaggcttt taaaaattct ttttcttctt ttacattttc
84841 tccaagttca aacaaacagc ccaaaagctc caaattttcc ttatgttttc ttaatttaag
84901 ataaacaatt tttaaaagct ttagagtttg tatatttctg ggtcgaattt ttaaagcttg
84961 caacaaaact tcctttgcac gctctaaaaa ccctgcttta aaatatactt ttgccaatgc
85021 gaaaaagata aattcacgct cttgcttgtc cttacttttt tcaagggcga taagataaat
85081 ttgcgtggcc ttttcaaatt caccactttt ggtaaaaatt tcagccaaaa aactcaaatt
85141 tccaaaactc aaaccttcat ttctcaacaa atctttatgg atattatcca tctcaaattt
85201 tttaataaat ttttcaagct tttgctcttc atctttactc gcaaatactt tccaaatata
85261 atttgccaca gctacaacga agattaaaac agttaaaatt ataagcccca ctaagggatc
85321 tctatattct acgaagaaaa aatccatcat cctgctctaa aaaattttat gccaaattat
85381 agctaaatct ttgtataatt ttatttaagg ttttaaaatg ataactaaag aaagtataga
85441 aaatttatcc caaagactta atattgttga tattattgaa aattatatcg aagtaaaaaa
85501 acaaggttcg agctttgttt gtgtatgccc ttttcacgca gataaaaacc cttctatgca
85561 tataaatcct acaaaaggat tttatcattg ttttgcatgt aaagcaggag gagatgcttt
85621 taaatttgta atggattatg aaaaattaag ttttgctgat gcagttgaaa aagtcgcaaa
85681 tctttgtaat tttaccctaa gctacaccaa agaaaaaaat gaaaataaaa aagaactaag
85741 aactatacta cccagcctca atgcttattt taaatccaat ctcaaacacc ataaagaagc
85801 tttggattat ctatataaaa gaatgctaaa tgatcaagat atagccaaat ttgaattagg
85861 ttttgcaggc tcaagcgagg atagtataag attgtttcat aatgaaaaaa tccctttaga
85921 agatgctatg agtgtaggtg ctttaaaaaa ggatgaaaat aatgaatttt atgcaagttt
85981 tatatggcgt atcacttttc caatttatga tcataaagac ttattagtag gttttggagg
86041 gcgtacttta aatcctaatg tcgcagcaaa atatgtgaat tctccccaaa atatattatt
86101 tgataaaagt agaatttttt atgcttttaa tatagccaaa gaaaatatag ccaaaaaaaa
86161 agaaatgata gtatgcgaag gctatatgga tgctatagct tttcataaag caggttttaa
86221 caatgccgta gcagtccttg ggacagcatt aactgaaaat catctgcctt tgatccgtcg
86281 ttatgaggct aaagtgattt tatgttttga taatgatgaa gcaggactaa atgcagccac
86341 tcgttcggct tttttactta gcactcataa aatagatggc aaagtagccc ttttagaagg
86401 tggaaaagat cctgccgaac ttgttgccaa caaccaaagc gcaaaacttc ataccctact
86461 tgaaaaaggg atggatttgg gggagtttta cattagacgc atactttcaa gcttttcttt
86521 aaattccgcc ctagataaac aaaaagccct tgaaaatata caaaaataca cctttagcct
86581 tgaacccttg gtagctagtt cctatgtaag tttggtttca aaactcttgg gtgtagatga
86641 aaaactcatc gttcttagct caaatcaaaa gtcaaataaa actatcgatt ttaacgcctc
86701 tttccagcaa acgcaaaatc ataatcctaa atcccacatc accgaacttg agctcttaaa
86761 ttatttaaaa aacaatccta acacacacga gctttttata aagatcggcg atattagatg
86821 ttttaaacat agggtattat tagaaaaaat tttagaacac aaaagctacg aagatagtga
86881 tataagagaa tttgaaagta aaaattttag taaagattta agccaaattg aattcttgtg
86941 ggggatttgc aaggtgaatc tagccttttt aaatcatatc aatatcacaa ctccttatct
87001 tgctttaaaa aaacaaatct ttactcttat agaaaaaaat tttactcttc ttagtaaaaa
87061 tttaaacgat tatgagctta tagggttttt gaaagagaat ttgtttttac taaaaaatga
87121 aaaagatgaa gagaaactag aagctttatt taaaaatttg catagatttt tttctgctaa
87181 aaatttggat attaaaattt taaaaaacaa ttctgaaatt gattacaatg aagaaccctt

87241 ttaagcttca tttagaaaaa taacgaaaaa actatccgat ttaccttttt ttaaacacaa
87301 tcacgctaaa gtatagaaaa atttattaag gatatattat gatgcctttt agcgatgaag
87361 agcttataaa tcctgtaaaa gcaagtttag ataaaagtat gcctatgtta gaacgcgatg
87421 gaggcggtct tgaattttta gggattaaaa atggtgtggt ttatgttcat ttaattgggg
87481 cttgcaaagg atgcgcttca agtggaacga cattaaaata cggacttgaa agacaactta
87541 aaatcgatat acatccagaa attactatag tcaatctaaa tggcggagct gaagattttg
87601 caaaattata aaaaacttgg aatcgagcat tttttacgca aagactttaa aacagcaaaa
87661 atatattttt ctttagctta tgaaaaacgc aaaaacaagc gtttgtttca ttttatcact
87721 ctttgtgatt tagctttaaa atctccaaaa gaagcatttt tactttttga attttattta
87781 gagcattatc aaattaaaaa tattgataaa gatttagaag aaattttaca caccgtagaa
87841 cttaaaaacg aaaaaaatgc cgatgaagca gaaaatgaaa ttcaagaagc ccatgcttta
87901 aactataaag attttctaaa aagtgaagag cagatcggct ttaaaaaaag ttttgaaaat
87961 attatacata gcacaaaatt agtcattgat aatcgtgatg atttcttaga ctttttagaa
88021 aaattgcttg ataatggtta taaagatata actttaaact atatagaaag cgttatgcca
88081 cacttttggg caaatgaacg ctttataaaa ctccaagaaa aacttatagg atttaaaagt
88141 gaaattaaag cttgatcact cttttatcac cgacaatacc ttagaatgcg aaaaagattg
88201 ctttttctta aaaactgcac aaaatatgaa tttttctagt aaagctctag aaaaaggtgc
88261 aaaaattatc gatgtagaag aatgcaaaaa actcttaaag atcgatgaaa atatcaaaat
88321 catagggatt acaggaacca atggcaaaac cactaccgca gcggctattt attcgatttt
88381 gcttgactta ggttttaagt gtggactttg tggcactcgt ggagccttta tcaatgatga
88441 acaaattgat gaaaaatctc taaccacttc acccatttta aaaactttag aatatttaca
88501 acttgccacg caaaaaaaat gtgatttttt catcatggaa gtcagctcac atgctttagt
88561 gcaaaatcgc atcgaaggct taaattttgc agccaaaatt tttaccaata tcactcaaga
88621 tcatttagat tttcatggca attttgaaaa ttacaaagca gcaaaagagc tcttttttac
88681 cgatgagagt ttaaaattta tcaacaaaga tgccttaatg attaaattta atgtgcgcaa
88741 tgcttttact tatggcatag aaaatccttc tttgtatcaa gttaaggctt attctttaga
88801 agatggcatc aatgctattg tggcattaaa agatcaaagt tttcatatcg actctccctt
88861 gctaggactt tttaatcttt acaatcttct agccgctagt gcttgtgtta atgaacttgt
88921 aaagccggat ttaaaagact tagaaaaagc tatcagtggc tttggtgggg tttgtggtag
88981 ggttgagcaa gtagccaatg gtgtaatcgt agattttgct cacactcctg atggtattga
89041 aaaagtactt gatacactca aaaataaaaa acttatagtg gtttttggag caggtggaga
89101 tagggataaa accaaacgac ctttaatggg aaaaatcgtt gaacattttg caaaaatagc
89161 cattatcaca agcgacaatc cacgcagtga agagcctaaa gatatcatga atgaaattct
89221 atcaggcttt caaaaccctg ataaagcttt gatgatagaa gatagaaaag aagctataaa
89281 taaagcttta aaacttaaag aaaaagacga tttagtagtt attttaggca agggtgatga
89341 aaatacccaa gaaatcaaag gggtaaaata cccttttagc gataaagcaa ttgtaaatga
89401 aattttaaaa aatcaaggat aataaatgtt tgaaaatatg gatttttcta agatgggaga
89461 acttttaaat caagttcaag aaaaggcaaa aaatatcgaa ttagaattgg ccaatcgaga
89521 atttagtgct aaaagcggag caggacttgt gaaagtcagt gctaatggca agggtgagat
89581 tatcgatgtt agcattgatg attctttact tgaagataaa gaatctttgc aaattttact
89641 catttctgct atcaatgatg ttttagctat ggtagcgcaa aatcgaacaa gcttagccag
89701 tgatgtttta ggaggatttg ggaaattatg aaatttttgt ttttttttagt agccttttta
89761 gtatctctaa gtgcaatcga gcgtcctaaa tttgaagatt ttgctgcagg ctatgaaaga
89821 aataaagcca gcatgctcaa ttatgaagga atgcccgctt ttgccctgag tgaaaattta
89881 cttgcggttt taaaacagcc taatgctaaa ctcaataaat atgtaaaata tgatccattt
89941 ttaaatcttt atcttgtaag aactgatttt agcttaatcc ctacacctat gggcgatgaa
90001 gaaaaactta cgcgtaacga ttggatagga atttgggatc ctaatagagc ctatataggt
90061 cacatcaaat accttgcaca aaatattgat gaaaaagatc agcttgattt ttctacaaaa
90121 gtaggacttt taggcactcc ttgctgcgaa atgatgggaa ttgcgctcaa taatagctct
90181 tttataggaa atcgctattt aaaacatttt atgaaatata atgatgtgta ttggggcgac
90241 ataggggttg attttgcagt gcgtgaaaat aaaatttatg tgaataatgt tcgaaaaagt
90301 gggcaatttc taatcaacga tgaaatcata agcgtagatg gaacaccttt taaagatata
90361 agaaagctaa atgaaaaaat tctctttgca gatcgcggaa gcacacttta ttttcaagta
90421 ttgcgagata atgtggattt aaatatttct actgaagttt ttgccaaaga tttaagcaaa

90481 tttaatcttc cgagtaataa accaaaacca aaaccaacaa gttttagaag caatataggt
90541 ctaagcgtca attcaagtct tgtagttaca agagttgatg caggctccaa agctgcttta
90601 gcaggattta tggtaggaga taaaattttg cgcgcaaaca atgtgatatt aaaagatttt
90661 aaagaacttc aaaccctact tggcacagga aatgatttta atattttaat cgaacgcaaa
90721 agtgataaac ttcctttatc aggattaaac aatgatttag gaggagttca ttcaggtgga
90781 gatggaaaat ttcaattttt cataaggctt gtgaagtgaa agaactcttt atcaaacatt
90841 tagaaaatca tctgccaaaa gttcaaagct tccatccttt tttcaatgaa gccttagcgg
90901 tgatgttaag ggcgggcggg aagcactttc gcgcccaact tttacttggc attgtagaag
90961 caaaaatgcc aagtcttata ccaaacgctc taaacgcggc tttggcatta gaatttatcc
91021 acacctactc actcatacac gatgatttgc ctgctatgga taatgcagat tttagaagag
91081 gaactcctac tttacacaaa acttatgatg aaaccacggc tattttagtg ggcgatgctt
91141 taaacactga agcttttttg cttttatcta atttaaattt aaaagaaagc attaaaatca
91201 atctgattag aactctagct tttaacgcag ggcttaatgg tatgatttta ggtcaagcaa
91261 ttgattgtta ttttgaagat aagaaattaa atttagaaga acttgagttt ttacacatac
91321 acaaaaccgc aaggcttatt gcagcggctt tgaaaatggg ttgtgaaatt tgcgagctag
91381 atgaaaaaga aagcgaaaaa atttatcaaa tcggcttaaa gcttggactt atttttcaaa
91441 tcaacgatga tatcatcgat gcgaccatga gcgaagaaga aagtggaaag ccaacaaagc
91501 acgatgagca taaaaattct tttgtgaatt tattaggact taaaaatgct attcaaagca
91561 aggaagattt gattaatgag tgtaaaaacg atcttaatga tcttgatgct aatttagcaa
91621 aaatgattaa aagcttaatg attaaatatt tataaaggtt acaaatggat ctatcaatac
91681 tacaagaaca agcaaatact ttaagatttt taagtgcaga tatggtgcaa aaggctaatt
91741 caggtcaccc aggtgcacct ttaggaatgg ctgatatttt aacggcttta agttatcatc
91801 tttctcataa tcccaaaaat ccaacttggc taaaccgcga tagattaata ttttcaggcg
91861 gacatgccag tgctttgctt tatagctttt tgcatttaag tggatatgat ttaagtttag
91921 aagatcttaa aaatttccgc caacttcatt caaaaactcc aggccatcct gaaatttcaa
91981 ctcaaggagt agaaattgct acagggccat taggacaagg tatagctaat gctgtaggct
92041 ttgctatggc agctaaaaaa gctcaaaact tactaggaaa agaattgatc aatcacaagg
92101 tttattgtct ttgtggagat ggggatttac aagaaggaat ttcttatgaa gcgtgttctt
92161 tagcagggct tcataagctt gataatttaa tcatcatcta tgatagcaat gaaatttcta
92221 tcgaaggaga tgtaagctta gcctttaatg aagatgtaaa aatgcgtttt gagtcgcaag
92281 gttttgaagt tttaagtatc aatggacacg attatgaaga aattgataaa gctctaaaac
92341 aagctaaaga agccaacaaa ccttgtctta tcatcgcaaa aacaaccata gcaaaaggtg
92401 caggagagct tgaaggaagc cataaaagcc acggcgcacc tttgggtgaa gaggttatta
92461 aaaaagccaa agaaaaagca ggatttgatc caaatttaag ttttcatata cctaaagcgg
92521 caaaaattcg ttttgaaagc gctgtagaac ttggagattt agaagaggct aaatggaaag
92581 ataaactcga aaaatcagag aaaaaagaac tcttagaaaa acttttaaat cctgatttta
92641 ccaagataaa ttatcctgat tttaaaggca aggatttagc tacaagagac agtaatggag
92701 aaattttaaa tgccttagct aaagatttag aaggattttt gggcggaagt gcggacttag
92761 ctccatctaa taaaaccgaa ctttatggaa tgggtgattt tgtagaagga aaaaatattc
92821 attttggtat cagagaacat gctatggctg cgatcaataa tgcctttgca agatatggaa
92881 ttttcttgcc tttttcagca acattttttа tctttagcga atatttaaaa cccgcagcta
92941 ggatagcagc tttaatgaaa atcaaacatt ttttcatttt cacacatgat agcataggcg
93001 ttggcgaaga tggaccaact catcaaccta tcgaacaact cagcaccttt agagctatgc
93061 cgaatttctt gacttttaga ccggctgatg gcgtggagaa tgttaaagct tggcaaatag
93121 ctttaaatgc agatattcca agcgcttttg tgctttcacg ccaaaagctt aaagcattaa
93181 acgagcctgt ttttggtgat gtaaaaaatg gggcgtattt acttaaagaa agtaaagatg
93241 ctaaatttac acttttagca agtggaagtg aagtttcgct ttgtttagag agtgcgaatg
93301 aattagaaaa acaaggtttt gcttgcaatg taatctctat gccttgcttt gaactctttg
93361 aaaaacaaga caaagcctat aaagatagac ttttacaagg tgaagttata ggggttgaag
93421 cagcgcattc taatgagctt tataaattct gtgatagggt ttatggcata gaaagctttg
93481 gagaaagtgg aaaagataaa gatgtattta aacattttgg ctttagcact ccgcaaattt
93541 taaatttcat tctcgataaa taaagatgga agcaaatttc aaatttcaaa aagaagaaaa
93601 taaagttttt atttttggaa tttgggataa aacgagtgta tctaaaatta aaatcaaaga
93661 tctattgagt caaatttctc aaacaaattg tatttttgat tttagtcaat taaatgaaat

93721 tgatatggct ggagtgagat tttttcttgc tctagagcat gatttgaaaa aaaataatat
93781 cttgatttta agacaaggat taaacacacg ctttaaaagt ctttttgaac tttgtgaaaa
93841 aaactaccaa agaatcgatg aaaaacaaga gaaaaaattc aattttacag acttttttat
93901 ttctttagga attttaagtt ttgatctttt aaaaatttttg caaaaattta tcagctttac
93961 aggtgccttt tttactagtc ttttttttatg tgtaaaaaat cccaaaaatt tccgctttat
94021 atcttttttta tatcatattg aaaattcagc ttttaaagcc ttgcctattg ttattttaac
94081 tgctctttta gtcggtgttg tacttgcata ccaagctgct tatcaacttg cacaatttgg
94141 agctaatatt ttcatagtag atttagtagg aatttcagcc actagagaac ttgctccttt
94201 gatagctgcg atagtcatag caggtagaag tgcaagctct tatacagcgc aaattggggt
94261 tatgaaaatc acagatgaga tcaatgctat gaatactatg ggctttcgct cttttgaatt
94321 tatcatcatt ccgcgagtta tggctttggt tattgctatg cctttaatcg ttgccttaag
94381 cgatgcaatc agcatattgg gaggaatggt aatagcaaaa ataaatcttg atatatcttt
94441 tggagaattt ttaagacgct ttagagaagc tgtggcaatg aaacatatta ttatagggct
94501 tgtaaaagct cctatttttg gtttttttaat aggactgatt gcttgttttc ggggatttga
94561 ggtcaaaaat accactcaaa gtataggaat ttatactaca aaaagtgttg taaatgctat
94621 attttgggta atagcatttg atgctttatt ttctgtggtt ttaaccataa tggaagttta
94681 aatgattata aaagcgcaaa atatcatcac ccgatttgga aataaagttg tccacgatgg
94741 agtaagtttc gaaatcaata aaaatgaaat ttttgggatt ttaggaggaa gtggaagtgg
94801 taaatccgta ctcttaaaac aaatgttaat gcttgaacat tttgatgagg gagaatatga
94861 aattttgggc tttaagctaa aaaatatcag tgaagaagat gccttagcac tgcgtaaaaa
94921 atggggtgta gtgtttcagt tttctgcact ttttagcttt tttagtgttt atgaaaatat
94981 agctatccct cttaaagaat atacaaattt agatcccata gctataaaag aactggtgtt
95041 gatgaaactt aaaatggtcg gtttaaatga aagtgtttta aagcagcttc caagtgagct
95101 aagcggtggt atgcaaaaaa gagttgccat cgctagggct ttggctcttg atagcaaatt
95161 gctttttttta gatgaaccaa cttctggact tgatccgcat agtagtcgtg aatttgatga
95221 tttgatacta gaactcaaac aaagctttga tcttaatatt gttttagtaa cacatgataa
95281 agaaaagtatg aaaaatctac tcgagcgttt tatcatccta gaagataaaa aagttgcctt
95341 ttgtggaaat tatgaggagt taaaggcgca aaatgaaaga ctttttaaaa gatttatgga
95401 gtaaaagtgg aaaataaagc gaattatttt tttgtagggc tttttgtttt tggagttttt
95461 tttgctagta ttggcttcat gctttggctt ggaggatact caaaagaaga aagttttgaa
95521 tactatgaaa ttcatacaca agaatccgtt gcaggccttg gaatcaaagc tcctgtaaga
95581 cttttgggag tagagatagg aaatgtagaa gatatcagta tttacgataa agaaaattta
95641 ggggtaaata ttttaatcaa aatcaaaaaa ggaaccccta taaaagaaga tacttttgca
95701 actttgcaac ttcaaggtat tacaggactt aaatttgtac aacttcaagg aggttctaaa
95761 aatagtccaa aacttcaagc aaaagataaa gaagaatttc cagtgattca gtttaaagaa
95821 agtttttttg ctgccataga tagacaaagt gagcatattt tttctttgat aaaaagagca
95881 gatgataaat caaaagaact ttttagtgat aaaaatttaa aaaatttaga aatactttta
95941 aataatttag cacaactaag cgctacttta aatgaaaatt ctaaaacatt aaatcacaat
96001 atcgcaaaca ctagcttaaa acttggagaa atggcggaga atgtaagctt gagtgcaaaa
96061 ggttttaacg cgagcttaaa ggatatccaa gaaggggctt tagcacttag ttcttttgca
96121 aaaaaagcca atgctaaact tgattcttat gatgacttgc gactttcctt agatgaaaat
96181 ttagaacttt tgaaaaaagt acttttagaa agtaacattt taatacaaaa tttacaaaaa
96241 agtccatcag atttaatctt taaagaaacc aaacccaaat taggaccagg agaaaaataa
96301 tgaaaaaatt tatactcata gccatcttag cccttatatt taatgcatgc tcgattccaa
96361 ctagcactct tgataaaacg caaagtttta tacttaaaaa tgatgggatt cctcttcagg
96421 ctaattttat taaaagcgat aaaattctta aaattcaaaa tgcgtccttg cctttatatc
96481 ttcactcaag atcaattatt tatatacaaa aaggtgtaag tggagcttat gcgtatcatt
96541 attgggcgga tttacccaac aatctttatc aatccttact cctatctaaa tttgaacaaa
96601 gccaaatctt taaaacccta ctttcccaag gaagttctat aggaagtgat tatatacttg
96661 aaagcagaat agattctttt gaacaagtga tcaatgaaaa tgaaaattat attagcattt
96721 ctattagtgt gaatttcatt gacgcaaaag aaagtaaaat tattgcccat cgcttgtttt
96781 cagtcaaaga agagcttgat aaaatagata tttttaatgt ctatcaatcc tttgaaaaag
96841 ctttaaattt tataggaaat gaaatagttt tgtggataaa tactacactt gatgaaaaat
96901 aggaacactt tttgcttttt aaacctttag caatattttt tgaaaggtgt aaaatgaaaa

96961 atgaattgga tgtttttaag aaacacttgg gcgaaataca aggcgtaagt gaatttaaag
97021 ctacacaaat ttgttcacaa ataaatgatg caaatgattt tatcggtgct ctacaaatac
97081 ttgatttaac gcttaaaaaa attgaaaaaa gtatcaccga aagacttgat gagcaaaacg
97141 atgatttaca aaaaagaacc ttagatgcaa cgagttctca acttatccaa aactgctctt
97201 ttatgggtac agcacttttt ggaaatattt tcaatgtata tgtaggtcaa aaactttttg
97261 aatttgaaat ttcaaatcct cttttagttt tagaaaaagc aggatatgag ggtgttttag
97321 cttatgttca agataaaaga gatgaaatta aagatatttt atgcgaatta tcgagtgcta
97381 ttactatggg aataggtatg gataatttga atttatctaa ttctactaca gactttaaaa
97441 atttattcag ataagggtta aaactcttat cgctttaaat gtattcgtta aaaaaataat
97501 cttaagaagt ttttatattt tagcttaaag agatattcta ctcttcttta aagctaggtt
97561 gaaaaaaagg gggagggaag caacctagcc tgtgagatct ttgaagatct ctttaaacta
97621 aaatatcaaa tcatgttttg gtttaattta taaatagtta ttttaaaaat gacattttaa
97681 tttaaattta cttaaatatt tataaaaaaa taatttaata atttaaatta ttaaattatt
97741 ggaaaatttt tgtaaaaatt ttccaatttt tacaaataaa aactcttata aagtagataa
97801 aatttcagca cgcccattaa taatagccac acaatgttca taatgtgcac tattaagctc
97861 atccacactt cctgcatccc attttccatt aaaatgcttt ggagttccat ctttttggca
97921 tatcataggt tctatacaaa ataccattcc atttttaatt tttggcccac tttttgcatt
97981 tgcacctttt tctaaataat ttaaaatttc aggttctcca tgaggtttgc gtcctatacc
98041 atgcccgcaa tatccacgaa gtggaacaaa acctcttgaa tctataaatt cccctaaagc
98101 tgcagaaagt tctttaaaac gcattccttc gcgaataata tctatagcat gatacaaagc
98161 gtccttagca caagaaatca aagcctcatc tgtggatgaa attttaccta tggctatggt
98221 acgcgccgca tcaccataat acccatcgat caagcttccc acatcaagtc ctaaaatgtc
98281 accctctttg atgatttttt catcaggaat tccatggatg caaacctgat ttaaagaagt
98341 gcaaattgca ccaggaaaac cataaagccc tttaaatgaa ggttttgctc caagacttaa
98401 gataaaatct tctgccattt tatcgatttg cttaagactc atacccgctt taatctcatt
98461 ttctaaaaaa tccaaagtct tagcgacaat ttgatttgct attcttaatt tttctatttc
98521 cgctggtttt tttaattcta tcattgtatt tccttataaa tttagccaaa ttttagcagt
98581 attttctaaa gacttgacat cgctcgaagc ataaaaatgt aattttgctt ttttttcttt
98641 taataaaagt ttttgccttt gttttaaaaa ctccactata gcatctcctg aatgaataag
98701 ctttgtcttt gaaccaaaat aagcactcaa ggaatttgcc agcaaaggaa aatgagtgca
98761 agcaagtatc aaagcattag gagtttcaat ccctttaaaa taatgtcgca tagcactttg
98821 caaaaattct ccttcaaaaa taccctcttc aaccataggt acaaaaagac ctgtagctaa
98881 agcatctaca ttagtaaaac cttgttcttg taagcgattt tgatattctt ttgaatgaat
98941 tgttgccttg gtggcaataa ctagaatttt tttattttta tccaataaag ctttttttagt
99001 agcctcaaca cccgcatcaa taaccccata aataggaaaa tcagctcttg atcttaaagc
99061 atctaaagca taagcactgg ctgtattaca cgcgataata agcatatcga tattaaattt
99121 tgaaaaaaaa tctaaagcct caaggcaaaa tttgactata gtatccttat ctttaacccc
99181 ataaggaacg cgagcagtat ctccataata aataatttcg tcaaaaagtc ttgcctcgta
99241 aagcgatttt aaaacgctaa gtccgcctac accactatca aaaacaccta ttttcatctt
99301 aatatcttct agcacctata tatcgatttt tccaataaga actatttaaa tccacttctc
99361 ttactccgcc ttttgtagaa agatggataa atttaccctt gctcatataa attcccacat
99421 gcataccatt tggacctctt cctgttttaa aaaaaacaag atctcctgtt tttaattttg
99481 atttagaaac ttttataccc gagcgtagtt gctttgttgt agtacgcggt atatgtgtat
99541 taaactgagc taaagtactt tgagcaaaac ccgaacaatc agctcctctt tttgtagatc
99601 cacccaaaac ataaggagtt tttttccatt cattagcaat ggctcttaat cttgtttctt
99661 tatcaacatt ataatttatt ttagtatgac ttgtattttg agataagcta catccaccta
99721 tacaaaaagc aaccataata acaaaaaaaa taaatctcat aaattttcct aagaacgcgc
99781 ggtaaaaaat ttaagataga taaaacccca aattccagaa caaaatgaag caatcaaaat
99841 agcaagatta tccgcatagt taaaaatatc actgacctca tatgctaaac catctataaa
99901 taaactcata gtaaagccaa tacctgttaa aatacaaacc ccataaagct gctttaaatt
99961 tgcattttga ggtaagcttg ctaatttaaa tttgatcgca aagtatgaaa acaaaaagac
100021 tcctaattgt tttcctataa acaaacccaa aaaaatacct atactaacgc cagaaaaaat
100081 agcatttaaa tcaatatttg ataaattaac ccccgcattt gcaaaagcaa ataaaggtaa
100141 aataacaaaa gctatccaaa atttcaaact ttcataaatt ccttctaaaa aatcttctcc

100201 atttttagtt tgcataggta taaaaaatgc tgtaataatt ccagctaaag ttgcatgcac
100261 cccacttttt aaaacgctta cccaaagaat gattgaacaa ataaaataaa aagatttgcg
100321 tgttatgcct aaaatattta aaatcaacat cataaaaata gcaattcctg cgactataaa
100381 agcaaatatt gaaagtttgg ttgtatagaa aattgcaata attaaaatcg cacccacatc
100441 atcaaaaata gccaaagaaa gtaaaaacaa cttaagacta gaggggatat gttttccaca
100501 catcatcaaa atagccaaag caaatgcagt atccgtggct gtggggatag cccatccttt
100561 taaagtgtaa gcatcgccta tattgactat agcaaatatt aaagcgggta taagtatacc
100621 gcctaatgcc cccataaaag gtaaaactat atttttaggg tttttaaagt ccccgtgtag
100681 aaattctttt ttaagctcaa ggcctatagc gaagaaaaat atcgatataa gaccatcgtt
100741 aatccaaagt aagaaaggct tgttaagctc aaactcaccc acactaactc caacttttaa
100801 attgagtagt tcgcgataat gctcacttaa agaaccattt tgcacaagta gagcaagtat
100861 agtacaaatt atgagtaaaa atccaccaaa ggcctcattt aatactaaag cttttaattt
100921 taataaaacc ttattcattt aacaatttta agatatataa atccaacaat agcacttaaa
100981 aaactcgcta ttaaaatagc tagtttatcc gcgtgctcaa aaatattgct gtttttgtat
101041 gccaagccat cgataaataa actcatagta aaaccaatac ctgttaaaat acaaattccg
101101 tagaattttt tatacttaat attttcaggc agttttgcaa gctttaattt tatagccaaa
101161 taacaaaacc caaaaactcc tagttgtttt cctaaaaata aacccagcat aattcccata
101221 cttacaggtg aaacaataga ctcaaaatgc atatctctta aatcaatacc cgcatttgca
101281 aaagcaaata aaggcaaaat aaaatacacc acccaagggt ttaaatcctt gtgcacttcg
101341 tgaagatagg gtttttttatt tttagtatca agaggaataa ataaagctat gattacaccc
101401 gctaaagtcg catgaacgcc actttttaac atagctatcc ataaaaccac tccaactaaa
101461 acataaagag gcaagtgagt aacatgaaaa taattcaaaa caaataaaat aaaaacacat
101521 aaaagacata caataattgc taaagtagat aattgatctg tataaaataa agcaatgata
101581 ataatagctc ccaagtcatc aaaaatagct aaagaaagca agaataactt aagactagaa
101641 ggaattttgc ttcccaaaag cattaaaata cctactgcaa atgctatatc cgtagctgta
101701 gggatagccc atcctttcat agcaaattca tgagaataat taatagcaga aaaaatcaaa
101761 gcagggacaa tcataccgcc taaagctcca aatataggca aagaaactga tcttatattt
101821 ttaagttgtc cgcgtaaaat ttcatatttt aattcaagtc ctatacaaag gaaaaaaata
101881 gcaatcaaac catcatttat ccataaatcc aaagatttag caatctgaaa atgatcaaat
101941 cctatagtaa aattagcatg aaaaaaatct gtataaataa cgcttaagga agaattttta
102001 aaaagtaatg ctaaaagagt aaaaaatatg agcaaaatgc ctgggaaagt ttcacttaaa
102061 attattttct ttacagcttg cattcacgcc ccttgattat aaattcaata tgctaattat
102121 tataaccaaa aaatattaaa gaaatgatta ttttaacaag atatatcctt atttagggtg
102181 taaaaacacc ctaaaattta agatacttaa gcataaatat ctaaaccact agagctagaa
102241 gcttgtggag cagaggctcc tgatgcactc tccatacctt tgataagctc attcatcaac
102301 acctcattcg tatccattga ttttttcaaa accgaagtat ttactgctgt caataaatta
102361 gcattactca tagttgcgtc tgaaatcata acaccctcct aagtataatt tatgtatgaa
102421 gacttaagta gtatcggaaa aaaatttaaa cacttaaccc tttcaataaa aaattatttt
102481 tatctttatt taatatgatt cgtattatac ttctatcaat attgataata aatattattt
102541 tttatatttt taaggaaatg aatgaaattt tttaaaatct tgtttgttct ctctatgctc
102601 tttctagcaa attctgcttt tgctagagtt gatgattata tagcagaagc taataccatt
102661 aaaaatatac tagaagagag tataaaaagc tataaaaaag gcgataatct aaatgctaaa
102721 aaactaagcg aagacgctta ttttcaacac tttgaaaata tggaaggttc tattggacga
102781 aatataggca aaaaagccat caccatggaa agaaaattta caaatttgcg tcgcttgtat
102841 aaagataaag cacccataga gcaaattgat gctttaattg aaagccttta ttatgattta
102901 gatgaagttg caccgatttt gcaaaatggc tatcgcttaa aagcagaagc aagtgataca
102961 aactacgata aagcagcagc tgaaaaatca agcatgcaag ccaatgccaa gcgcgaagca
103021 gaagcagaag ctttgattgc tcaaatgatg ggtattgata agaatgattt agcaaaaacc
103081 aatccctctt cttttgatac tcaaaacgaa actatagatg atagtgtaag tagcgatttg
103141 caagcggctg cagctatgga tgcaagattg caatttatat tagataatat atctactaaa
103201 ttctctcaag cagccaatgc ctttaaaaac aaagattatc aagcaagtaa agattttta
103261 aatgatgctt tatttaatga ttatcgcaat accaaagttg aagttttaat caacaaattc
103321 accaaagcag gtaatgatca aaaaatccaa caagccatac gaactctcat acgccaaatc
103381 aacaacacaa atattgatga aaaaaaccta agaaatgatt tagattcgat agaagaacag

103441 ctttttgata tctttttaca aattccaaat tcagagcttt caaatttaaa aatcgaagga
103501 tttaatgatg aaacaaaggg taaagattac actaaagttt ctgatgatat caaacttgcc
103561 ttagatgaaa ttttaaaaaa ttacgagggt tttagtgctt ctatagtaga tgatttacaa
103621 gcaatttatt tagatatttt tgaagccagt ggaatggaaa ataaaatcgg tgctgtagat
103681 agtgctttaa aacttaaaat cgaaagttta ttttccaaaa gcgtagctct tatcaaagca
103741 agtgcagata aaaaagagct tgaagccact tttaacgact tagaacaact gattgcaagt
103801 agcgtggata aaattcaaga ttcttcgcct tattcgcttt ttatatgggc tttgggtatt
103861 atcttgcgtg aaggtttgga ggctttgatt atagttgtgg ctatagtttc ttatcttgta
103921 caaagcggca ataaaaatcg ccttaatatc gcttatagtg ccttatttac aggtattata
103981 ctaagcttca tcactgcttt tgggctctct tggcttttta aagaaaatgc gggacaaagt
104041 agagagctta tagaaggcat taccatgctt atagctgtgt ttttactctt ttatgtaggc
104101 ttttggctac tttcaaatgc gcaaaataaa aaatgggtaa gttttatcaa gcaaggtgct
104161 attgatgcta tttcaaacaa tagtgcaaaa accctttggt ttacagtttt tttagcagtt
104221 tatagagagg gtgctgaaac cgtgcttttt tatcaagctt tattttttga tgctaaaacc
104281 agtactgatt taagtgctat ttttggcggg cttgggcttg gaattttagt gcttattgtc
104341 ttatattttc ttttaaaagc aggcgcgatt cgtatcccta taaaacaatt tttctatata
104401 tgctcttata ttattttta tatgattttt gtctttacag gcaaaggcat agccgaactc
104461 atagaaggaa aacttatcgt tcctagtctt atatctatga attttgaacc tattttatgg
104521 cttggaattt atccttatta tgagacttta atccctcaat ttatagtttt aattttgcta
104581 attattggca ttttattaac aaaacaaatt tcaaaaaaag gagtaaaatc atgataaaaa
104641 aatttttatc agtagttgct gcagctgcag ccatcagtac taatttgttt gcaggcgaag
104701 ttccaatcgg tgatcctaaa gagttaaacg gcatggaaat agctgctgtt tatcttcagc
104761 ctatcgaaat ggaaccaagg ggtattgatt tggctgcgtc cttagcagat attcacctag
104821 aagcagacat tcatgctctt aaaaacaatc caaacggttt tccagaaggc ttttggatgc
104881 cttatttaac catagcttat gagcttaaaa atacagacac aggcgctatt aaacgcggaa
104941 ctttaatgcc tatggtagca gatgatggcc ctcactatgg agcaaacatt gctatggaaa
105001 aggataaaaa aggcggattt ggtgtaggta attatgagct tactttttat atttcaaatc
105061 cagaaaaaca aggatttgga cgccatgtcg atgaagaaac aggtgtgggt aaatggtttg
105121 agccttttaa ggttgattat aaattcaaat acacaggcac gccaaaatga ttttacactc
105181 ggattttccg agtgtttaaa tatttttatt tttattttaa aatcaaaata acaatgttta
105241 aagagtaaat tatgagtata tattttgtgc attttctttc tagcatcttg cctttaagta
105301 ttttaatggc ttttatcaca cccaataaaa aatatatttt taaaagcttt ttagctgttt
105361 ttttgggctt tttgtttgga tattttgctt tttttattgc ggcacaattt ctaaaaactg
105421 agaatttgat atttaatttc aattttattt ttatagcctg tttgcttttg agttttgtgc
105481 tttatttttg gcaaaaaata gaaattttga gttttatttt actaggaatt ttgagttttt
105541 gtatagcttt gcattattat aatttaactc aagattttcc tatatttaac ggtgttttaa
105601 tcgatagtga agccatttct tctttgggtt ttattgcttt agctttatta atttgtattt
105661 taattttctt ctttttaaaa tggcaaaaga atatcaacaa aaaagcaagt tttgtacttt
105721 ttttaatcct tatttttata gaaggagata aggttttagc aaatatattg cttacactga
105781 tgagaaattc tttcatagaa actcatactt ttttagtaag ttttgttgga aaaagtaatt
105841 attttggtgt ttttggaatt tatgtttatt tatttttcat catttttta gcatttttaa
105901 gtttgcaaat tcgcaaaaaa aatattgtaa aaaagcaaat tttagatatt gcttatcgca
105961 aaaatgaagc aaaaaatact ctcataaatc gctatttttc tagtgttttt atctcttgta
106021 ttttgagttt ttgtatcatt ttatactttt ttatggtaag ttctaaacct ttaagtatag
106081 atgagcctac agagctttta cctgataaaa atggcaaatt tatttttgat gtagcccttt
106141 taagagataa caaacttcat cgttttgctt atataagtga gcaaggcaaa gtgataagat
106201 ttttcctcat caataaaaga gaagataggg attctcccgt ggctgttttt gatgcttgtg
106261 ctatttgtgg ggatatgggt tatattaaaa aagaagggga gttaatttgc atttcgtgca
106321 atgtgcgaat ttttctaccg agcgtaggaa aaacaggcgg ttgcaatcct atcccattaa
106381 aatatgatta tgatggtgaa aaaatcacca tagatgttaa agatgttgtg gcaggatcta
106441 attattttag tcaaatcaaa gatatacaag tgcaagatcc tgtttcaaaa gataagatca
106501 ttaacactca agcacctttt tcttacagct ataaaggcat aacttattat ttttcaaatg
106561 aaaaaaacta tgaggaattt aagaaagatc ctatgaaata tgtagaagat acagaagcct
106621 tatttttaat ccaaaggaga aataatgcta gttaaaatga taactcattc tatctttaga

106681 aataaaattc aaaaatttct agcttttctt acttgttttt tggctacgct tttgctatgt
106741 acaatgttaa atatcactct aagtataggc gatgaagtga ctaagcaact taaaagttat
106801 ggatctaata tactggtttt acctaaaggc tcaagcctta gcatagaaat aggtaatgag
106861 ctttacgaac ctttaaaaaa caaaaactat ttagaagaaa aaaatttata tatgttaaaa
106921 gatatttatt ggcggaacaa tatcacagcc tttgctcctt ttttagaagg aaaagtagaa
106981 ataaaaagca atgattcttc aaaaaaagca ctcctttatg gtacttattt tcaaaaagcg
107041 attgagataa aagatgatga cgattttatc acaggtatta aaagtcttta tccttattta
107101 aaagtacaag gagaatgggc caaagatgat agcaatgaaa tcatgctagg tgaagatttt
107161 gctaaaaata acgatcttaa aatcaatgat acgataaaac tcataggaga gaataaaatt
107221 tcaaaagaag taaaagtcgt aggaatttta ctccatgcca atcctaaaat gtcaaacaaa
107281 atcatcgctc ctttaaattt agctcaagaa ttattagaaa aaaaaggctt gtatgcaagt
107341 gctgaagtaa gagcttttac tatacctgaa tcagctcttt ctgaaaaggt acgccgaatg
107401 ggcgaggaaa aactcgatca aatagaatat gataaatggt attgctcagc ttatgtagga
107461 tcaattgcta gtcaaattag cgatggcttg ccaggagcgg atgcaaaagc actcaatgcc
107521 ataagtgatg cacaaagctt ggttgttaaa aaaattcagt ctttaatggc tattacttgt
107581 attatttgtc ttattgttgc aagtattgct atatcaagtt taatgagctc tgaaattcat
107641 cgtagaaaaa aagaaatagg acttttgaaa gtgcttggag caaatacatt tcaaatttat
107701 ttactgtttg caggtgaaaa tttgatcata gctttagttt ctgctgtatt tggatttatt
107761 tttggcatag gactttcaga gcttatcagc cttggaattt ttggatactt tatagacatt
107821 gcttttatag ccttgccttt aagtcttgta tttgcaggtt tgatagctat acttggatgc
107881 ttgcttccta taaaaaatat cgcaaatctt tctcccgcag gagttcttta tggtagataa
107941 gtttttttta aatgaacttt ttaaaagcat tgctttttct tatcaaaggc tttttattat
108001 tgtcttaagt gtttttatag gagcactaac ttgctcggct tttttaaata tttattttga
108061 tattgataca aagctttcta aagaattaaa agcctatggg gcaaatgtta tcatcgaacc
108121 caaaaatgat aaagaactta tcttaaatca agactatgaa gaagcaaaaa agaatttaaa
108181 agctagagcc ttaactcctt ttttgtatac atttttaaat ttgggtagta ctagtggagt
108241 ggttttgggt acagatttta gagccttaaa gataactaag ccttttatag aagttaaaga
108301 agggagtttt tctctaagtg attttgatga aaattcagct tttttgggta ttaatttatc
108361 caaacaacta ggacttaaag caggcaatga gcttcaaatt tacaacccag aaaatggcaa
108421 aactaccaag cttacgataa aaggcatact cttaagcaat gatgaattag acagcatagt
108481 tttagcgcct ttaagcgtag tacaaaattt aagcgacact tttggcattc agtatgctaa
108541 tgcggttgtt tatggaaatt ttgatgagat aaatacaaaa acaaaaaata ttagcaatga
108601 aagcattaat gcaaaaccta tttcatcagt atccttaagc gaaggcctag ttttagacaa
108661 aataaaagct ttaatgtttt taatcatact tgttgtctta atcatagtca caacaagtgt
108721 aaataccact ctaagctcga ttatattttc gcgtaaaaaa gaaattgcct tgcgtttggc
108781 cttgggcgct aaaaaaagtg aaattttcaa gctttttgct tgtgaatatt ttatcattag
108841 tctttttgct agcattatag gagctttttg tgggatattt ttagcaaata tatttggata
108901 tttgattttt aatgcaagta tagattttag atttaaggct gtatttatgg ctttaattat
108961 ttctttgttt tttgcttttt tagctgcctt ttttcctata aaaaaagcat taaaaatcaa
109021 tgtctgtgaa aatttgaaag gtgaataatg aaagaactta tacaaattaa aaatttaagc
109081 aaagaatttg gtaaagtaaa agcactagat aatataaatt taaatattta caaaggagaa
109141 tggcttgcca tcatgggtcc ttctggaagt ggaaaatcaa cgcttttaaa tatattatct
109201 cttatggata atccaagcag tggcaaatac attttagata atgaagattt agagcaaatc
109261 aatgaagagc aaaaaatcac tttgcgccga gaaaaaatcg gtcttatttt tcagcaattt
109321 catcttattc cttatcttag tgccttagaa aatgttatgc tttcgcaata ctatcattct
109381 agtgtagatg aagaggatgc aaaagctgta cttgaaaaag taggactttc acaccgcctt
109441 agccatttac caagccaact aagcggtgga gagcaacaaa gagtttgcat agcaagggcg
109501 ttgattaaca atcctgaaat tttacttgct gacgagccta cagggaattt ggatgaagcc
109561 aatgaaaaaa tcgtccttga aaccttgcaa aaattaaaaa atgagggtaa aaccatagtc
109621 ttgatcacac acaatcccga attagcaaaa tttgcagatc gcacccttat cttacagcat
109681 ggagttttaa aatgaaaaaa gtatttttat ttttcataag tcttttttg ctcaatgctt
109741 gctcttttga tacaaatcaa aaccaaggta aaattggcca aataggagct gaaatttcag
109801 ctaaagatac ttcgggaaag gctgttaaat tagctgatga taaaagcgat ataaaggttt
109861 tggttttctt tcaaaatggt tgtccttctt gtttgcaaga gctgccttct ttggataaat

109921 ttatgcaaga gcatcctaat aaaatcagcg tttatgctat aaattcggta gataaagctg
109981 aagtcgttca ggttttggcc gaacaattaa attttaaaaa tatcaaagtt ttaaccgatg
110041 atcttaaaat caccaatgat cgctacgctg tattttcaac cccgaccacc atcatactta
110101 aagatgggat tataaaagaa agaattttag gagaaaaacc atggcaaact ttcgaatcca
110161 aacttatctc tttgctttaa ttacagcttt attttttata gcttgcgata gtggagaaaa
110221 tttcaaagct ttaaatagtg ataaaattta taatttttct tataatggtt ttgaaaaaag
110281 tttaaaacta gacgagcaaa cacaaaattt tgcattagta tttttttacta aagattgtgg
110341 ggtttgtaaa gaacaaattc ctattttgca agatttagct aaaaattatg attttaatat
110401 ttttgtggtt ttaggtgatg ctaaagacgc aaaggacgct aaagcttggg cggatgaaaa
110461 gggtttatcg catctagctt tgttttatga aagaaaagca gctaaatatc tctcgggtgc
110521 agttggggaa atatatgggg tacctgtact tagcttttt aaagaaagta aaatggatga
110581 aaaattcata ggtttaacgc cttataatgt gcttgaaaat gagattaaaa aactaaaaat
110641 ttgattttat attaaaatat ttttaccgca aatacctaag actagctagg tatagcgata
110701 aataaatcaa attttaaaca aatatctaaa aaggtgatta tcttatcacc ttttttcagg
110761 cttttctctt aattttttaa tctcttctaa aagcataaat ttaagctctt taagtccagt
110821 tttttcaaga cttgatacct tgattaaaaa gctttgtgga ttatcaatac tttttaaata
110881 ttcttctaaa ttcgctatat tttgggtgat ctgctcggca aaatcttcac ccaaattcac
110941 actatcactt tttgaaatca ttatgccaaa ctcacgccca aaaagctcat ttgaaaattt
111001 ctcaagctca tttcttagca ctataaattg ctcttttaga ggcatttctc tcataggatc
111061 tagtacaaaa agtaaaaaac tcgtgcgttc aatatgtttt aaaaatgcaa gtcctaagcc
111121 tttgccccca cttgcacctt cgataatccc tggaatatca gccatcacaa aagaattata
111181 ctcatccaca tctacaagcc ctaattttgg tgtgagcgtg gtaaattcat aatttgcgat
111241 ttcaggctta gcatttgaaa ctacacttat aagggtggat tttcccacat ttggaaagcc
111301 tacaagccct acatcggcga taagtttaag ttcaagtctt actaaacgac tttcaccttt
111361 aatacctggc tgtgcgtaat cagggcgttg gttggtagcg tgcttaaagt gtgtattccc
111421 aagtcctcct ttgccacctt ttagaaaaag ctctctttga ccctcttttg taagatctag
111481 taaaacttca ttggtttgtg catcgataac ttgagttccc tcagggacaa taagctctaa
111541 attttcaccc ttttttccat ttttattgcg tcccattcca cctgcgccat tttgcgcacg
111601 aagctctctt ttgcctttga aatttacaag tgtatgtgta ttgttatcac aaacaaagat
111661 gatatcgcca ccatttcctc catcgccacc atcaggccca ccgagaggaa catgcttttc
111721 acgacgaaaa ctcaccgcac ccttgccacc atcgccagaa gctaaagtaa tcttaacact
111781 atcaataaac attataattg cctttgattt taaatttata aagctaaatt agaataaaaa
111841 gaagttaaat ttataaattt aaagtttcta aggcgtaaaa acgccctaaa aacttatgca
111901 ggatatacag aaactttttt tctgtctttg tcttttcttt caaatttcac aaaaccatca
111961 attaaagcaa agatagtgtg atcttttcct atgcctacat tatttcctgc atgagttgca
112021 gttcctcttt ggcgaatgat gatatttcct gctctaacga attcaccacc aaattttta
112081 acacctaagc gacgacctat agaatcgcgg ttattttgag ttgaaccctg acctttcttg
112141 tgtgccattc tttactcctt aagctttgat atcttttact acaacgcgag tgaattgtct
112201 tctaaaaccg cgtttaagtt tagaatcttt tctgcgtctt tttttataaa tcacaacttt
112261 tttatctttt ccgtgattaa tcacttccaa aacaactttt gcacctgcta cgaaaggcgc
112321 acctaccttt aattctttat cattgatagc aagaacttcg cttacttcaa tatttgcttt
112381 gctttcagct tcaaagtgat ctagctttaa ctcatcgcca acgctaactt tatactgctt
112441 tccgctgtgt ttaataatag catacattgc tttttcctta ataatttggt aagtaccaca
112501 aagcaccgaa attcggattt gaaagcttta atggctagaa aagttttaat tttacaaaaa
112561 ttttctttaa tctttgatga atttaagaaa gactttagag tttgaaacca aaccctaaag
112621 tggtttaaaa gccaagatta accccgccat tttcatgaag tttataagta ttttctttcc
112681 cttcttgttt gttatcttct tctaaagtat caaaaaatag tgatttttcc atttctcttt
112741 gcataaataa cgcaatgttt tcccaatttt tttgctcttt tttgctgtcg ccagataaag
112801 taaaagttgt attgaaggtt ttagaaaaaa caagctctct tgtaggataa aatataagct
112861 tataggctat atttatactt gcttttgtat tgttttgata gcttatcttg ctttgttttg
112921 cttgagtaat atctaaaatc tcaaattcca agatataatc agcgcccaaa acattaccca
112981 atttatagct ttcttcacta gagctatctt cactttttat aatcttattt tcatctttaa
113041 aatcatgctc tctttctaaa agagtaaaat tctcatcctg cgaaaaagca tcatataaag
113101 ctctttttaa atatgtttct tctttatttt tatcatttaa aattgctaat tttggtttat

113161 tatcgttttg atacaaagat ttaaaagccg aaatttcaac ttgccaaagc gttccatttt
113221 ggtcaatttt atctactttg taagtatcga tgcgaccctt gcttacattt tgaagaatgc
113281 ttttagattt ttgtgaaatt tgccctaagt gagagaaatt taaatcaaca ctttgaattt
113341 cttgtcttaa attgacacta gaaactcctt taaattgcga caaagcatcg attaaagcat
113401 tatttaaagc ctcttctttg ctttctcctg ttccattacc ttgcaccact ttttgaaatt
113461 tatttctttg gtgtggcaag gttttaagac tctgaacagg atctaaagaa cttaaattaa
113521 catcctctaa tggaacataa tcataaataa taacatcatc ttgagcccac ataaaactca
113581 agatggttaa gaataaaaag attttttca ttaccaagtt accgatttgc ttgaacctgt
113641 tttatcaatc gttctttcat cttcccaata agcaagtcct gaatttaaat ccgtaacgct
113701 tagataaaag aaatactctg cttgtatatc accattttta agcttgacat tgtcttgtcg
113761 gatcttacca ctgattgaaa aatcaggtgc aaccaaagtt cctttgctag aaatggtatt
113821 ttgattaaat tcatcattat ctcttaattc tcttactgct tcactcattt catctctagc
113881 tccacccgct gcaacagctg ttgttaaaac aaatttacct gattttctaa gcgctatggt
113941 gatcttacta atgagcttat ctgtatcaat gcgttgggga gtatcattga taattttgcc
114001 tatagcaata acctttcttt gacctggctg aatattagca aaagcaggat cgttcaacat
114061 gctatctatc ataatttcag cagttttttc aaaatcttct ctatcaagcc ccaaagttaa
114121 agcatcgcct tgttttttt gacttgcata accatctgta taaacaggcg ccccgcaagc
114181 tgcaaaaaat actgcaaaaa atgcactaaa gattataaaa atattttttt tcattatttt
114241 ctcctttttt tgtaattata ctcttttagt tttcaatttt gtaaactcta gtgggaaatt
114301 gagaagcaaa agaacgtact acaatcaata catttttgtc aatatccact tcactagaat
114361 aaattttttg atttttatta tcataaattt gcaccaatcc atcattttca atcatcataa
114421 tttgtatatt tttaggtaaa aaattccaaa atcttaaatc acttcttgtg gttacaatgc
114481 taaaaatatt tgtagcaaga cttaaaattc cactctcatc atttttagct acagccatat
114541 ttaaacttgt ttttaaagtg cttgagatta aagatttgat gataatcggg gtcaaatttg
114601 ctttaaattc acttgcaacg atttgatcaa aatcaaaagt attggaagtc gtaaaatttt
114661 gatgattgtt tgcaatattt aaataatcat aagaagcatt acgctttgtt aaaataggca
114721 aagcaatact aactgttgta atataatcat caaagataaa aggtaaagtt agactaaatt
114781 catccaaagc gggacttaat ccattttcat atattataaa aatatatttt tttaattttt
114841 ttggatttaa actgcttgaa tattgatcaa aaatcttaaa ttctttttct atttcttcat
114901 ttaaatttag cacataaact tctttaagta aatctgctgc tttttttataa tcataatcca
114961 taaagaaaaa tatagatgaa atataagtta cataaggatt agtatattct ttttgtgcat
115021 taaaatcttt aaaaagattt tcatattgtt ttgaaacaat ttttatatta ttatcgatat
115081 tttgccttaa tttttcttcc ttgctggcct cttgatatgc ttttttaatt tcggaagcaa
115141 aatactcttt tgccttttct tgtctcatca atgcgcggtt aaattccacc ctagcattaa
115201 caaaatctcc caaactcata aaatttaaac ccttgtaaac attaagcatg gttctttcat
115261 accaagcacc ttgataatca tttacaccct catttatcaa agtagatgca agtgttcttg
115321 tgcttttctt ggataaactt tgcaaatcca catcgtattt ataagaatct tcaaccttat
115381 caaaaataga attacttaag ttaaaatcct tacaatctct tgcaatatag cccgtattta
115441 aacctatgaa aatcacatct ttattttgat tgatcttttc ttgctgctct tgaaaaaatt
115501 gcttattaca agtttgagag aataaatttt gttgatagaa tacattttgt tcttgatgag
115561 tagagcatgc agaaaaaacc atagccaaaa tagaaaaaac aattgtttta tatctcatta
115621 taattccaaa aataaaaatt ataatttaat atatcataaa attgataata gcccaaaaaa
115681 gggctattac attaacatag gagcgactac aaagcctaaa gctacagcga ttgcgatagc
115741 taaaactcct ggtataaaga aagagtgatt aaagataaat tttccgattc tagtagtacc
115801 tgtatcatcc atttgcacag cacctaataa agtaggataa gtcggaagca caaaaagtgc
115861 tgaaactgct gcaaaacaag ctacaagcat ataagaatcc tgtggatttg ttgcgctaat
115921 tcctaaagca gtaacaatta caggtacaat agcctttgcg gttgccgctt gagaatataa
115981 aagcatactt gcaaagaaaa aagccaccgc aagcatagct gggctttgtt ttacccaatc
116041 acctgctact tctttaatag aagcttcatg ccctgcaaca aaagtatttc caagccatgc
116101 tacaccaaaa acacaaatgc aagctgtcat accactttta aagacgcttg tatcaagaat
116161 cttactaggt tcaaccttgc aaagccaagt tattaaagcc gcggcagcca ataaaaatcc
116221 cataatagct tgatctcttg atacttttac aggatcgata taagacctta ctatttcaaa
116281 aaaagctact ccttgaaccc tagaagaatt taaactagca agttttgaaa aatggtcttc
116341 tagtttgact ttgaaatttt gtatattaaa ccctgtctga ctcaactctt taaccaatac

116401 atcattgttt aaagtcgata aaattgattt tttactatca ccaatagctt gagtaaatac
116461 acttgtattt gcatcagaaa ctaatgtttt ttctatgatg gcaaattgca tattaatact
116521 ttttgaaaga ttatttctc ccaaattacc gagtaaatct tgtttggttt ccataatttg
116581 atcatatgca ggataaattt tagaattaac ataatttact ataggatttc ttacaacatc
116641 agaaattgtt gtggcataaa acacaaccgc taaaacagta ataagaaaaa tcgctactga
116701 aagtttagca cctggcttat cttcgctatg taagatagca cccgcatctt taacaagacc
116761 tgcttttagg cgttcttgat aaactggatc tttacttaaa tccataggag tgattaaact
116821 cactataaaa gcagttatca tacaagctat aaaagtagta cctatccaaa ttccaattaa
116881 agcaggataa ctccaaccaa atggttctaa aaccccactc atataaacca cagccgcact
116941 tacaggagat gcagtaattc caatctgtga ggatacgacc atcaacgaaa gcggggcact
117001 tggtttgata ttttgagatt ttgccacatc aactacaaca ggcattaaag aaaatacagc
117061 atttcccgtt cctgccaaga tagtaagcaa ccatccgcaa gctggggcaa gataattaat
117121 aaatctaggc ttagatctta aaattttttc tgtaacccta accatataat caagcccacc
117181 agcttgttgc atcgcagaaa tagctgcaat agctgcagct atgattaaaa ttacatccca
117241 aggaatattt cctggtttca ttcccaaaac aagccccaaa acaacaacac caagtcctcc
117301 tgcatagcct atggctatac ctcctaagcg aattcctata aaaattgctc caaggagaac
117361 gattatttgc aaaattatca ttattgtatc cattggctag cctttatatt attttttagc
117421 ttccatatgt ggatttaaca tatttgcagg agttaaaatt tcatcaattt gttccttact
117481 taaaagccct ctttcaagtg cgatatcagc tactttttta cctgtactca tagcctcttt
117541 agcaatgctg gctgaatttt cataacctat ataaggattt aaagcagtta cgattcctac
117601 tgagttataa acaaaatcag aacaaatttt ttcattagca ctaatgccat ctatacattt
117661 atctgctaaa gtatgcattg ctttttcaag cataacaata gaattaaaca agctataagc
117721 aattacaggt tcaaaaacat taagctgtaa ttgtccgcct tcacttgcaa aagtaacggt
117781 tacatctgct ccaattactg cgtaacaaac ttgatttacc acttcaggga taacaggatt
117841 tactttacct ggcatgatag agctacctgg ttgcattttt ggcaaattga tttcattaag
117901 accgcatttt ggaccactgc ttaaaagtct taagtcatta catactttag aaagttttgt
117961 ggcaacgcgt tttaaaacac ctgaaatttg gacataagct cctgtatctt gagttgcttc
118021 gataagatct tctgccacag tgtattcaaa gccggttact tctcttattt ttctttctac
118081 tacttttgga taattaggat gagaattaat acctgttcct atagctgttc cacctagatt
118141 gatttctaaa attagttttc tagcttctaa aactctttgt atatcttcac ccatcatcac
118201 agcaaaagtt ttaaactcgc gacccaaagt cataggtact gcatcttgaa gttgagttct
118261 acccattttt aatacatctt taaattcttc tgcttttctc tcataagcct ttttaagatg
118321 ctccatagct tttgctaaat cacttagata atcatgcaaa gcaagatgca aagcagtagg
118381 ataagcatca tttgtacttt ggcttaaatt tacatgatcg tttggatgaa ggtattgata
118441 ctcacctttt ttatggccca taagctcaag tgctatattg gctataactt cattggcatt
118501 catatttgta cttgttccag ctccaccttg tatcatatca actacaaatt gatcataata
118561 tccgccctct aaaattttat cgcaagcttt aatgatagca tcttggatat ctttatctaa
118621 aagacctaat tcataatttg ccatagcagc tgctttttta acccttgcta aagctcttac
118681 aaagcgtggg aaatccttta acctctcgtg tgaaatatta aaattttcca ccgctctaaa
118741 ggtttgcact ccataataaa cctcatcaga aatttctaat tccccgataa aatcgtgctc
118801 ttttcttgtt cccatttgaa acctttcttg tttaattttt tatacaagct ttgctgcttg
118861 tactcatatc agtataatca tattgtaatt ttggttaata attggttaat aatattaatt
118921 ctttaaaaat actataaaat agacttttag gacaatttaa ctctttttat atttaatttt
118981 tatttatcaa ttgattttat ttatttcagt aaaaattaat atattttttct tagcttattt
119041 gataaaatat acctttagtt tatttttgga agtttttatg aaagaaatta ctataaacat
119101 tgacgatatt aaaataaata acgattggaa agaattttta aaagaagaat ttagtaaaag
119161 ctattttttta gaaatcaaaa aacattatat acaagcttta aacaataaag ctatcatcta
119221 tcctccttca aatttgactt ttaatgcttt taatcttact cctttaaacg agcttaaaat
119281 tgtactttta ggacaagatc cttaccatca acccaatcaa gctatggggc ttagtttttc
119341 tgtgccttat ggtatcaaaa ttccgcccag tcttttaaat atttataaag aattaaaagc
119401 agatcttgat atagaaccct ctaaaagcgg agatttaagt tcttgggcaa agcaaggggt
119461 tttactttta aattccattc ttagtgttga agcaggaaaa cctgcaagcc atagttcatg
119521 gggttggcaa gaatttagcg atgcggtgat atcaaaacta agtcttgaaa aatcaggact
119581 tatttttatg ctttggggaa attatgcaaa atccaaaaaa gctttaatcg atacaaaaaa

119641 acattttatt ttagaagcag cacatcctag cccactagct agaacaggct ttttaggttg
119701 caaacatttt tcaaaagcta atgaaatttt aaaaaattta ggaaaaaaac ctataaattg
119761 gcagttggct tagaaaaatt atataaatat ttttttaaca atttaaaaat aaaacaatta
119821 atataaattt gtataattta tcgttaaaat ataaaaaatt taaggagata aaggtgaaaa
119881 cgataggtat tataggtgga atgagttttg aaagcaccat cacctactat aaaaccataa
119941 atgaagctat taataatcaa cttggcaatt taaattctgc aaaaatttta ctctatagtg
120001 tcaattttga agaaatagca aatttgcaaa aaaatggcga gtgggaaaaa gctgcacaaa
120061 tcttaggaga gtgtgctaaa aaactagaac ttggcggagc tgattttata cttattgcca
120121 ccaacaccat gcataaaatt tttcatcaaa ttcaaaaaaa tacaaaaatt cctctcgttc
120181 acattgctca aagtacagct aaaattctaa agcaacaaaa tatacatacc ataggcttgc
120241 taggcactca atatactatg atggaagatt tttataaaaa tgtattaaaa gaagaaaata
120301 taaaaacaat tactccaaat caaaacgata tgcaagaact aagtgatatt atctttaatg
120361 aactttgtag ggggaaaatt aaagaaaatt ctaaagaaaa atacctaaaa attatccaaa
120421 aactaaaaga cgaaggtgct caaggtatag ttttgggctg cacagagatt ggattgttga
120481 tttctcaaaa agatacaagc attccgattt ttgatacagc cctaatccac gcccttgatg
120541 ctgtaaattt ggctttaaaa gattaatctt tctttttaga gataggacaa actcccaaag
120601 gacaagcttg ctttcctgtc caaactctta taggacatac gccaaaaata cctgtaagca
120661 aaggaataaa tcctatcaaa gcccaccaag acgcataata aaccccctata ctaaagataa
120721 ccattgctag gattattctt attgttcttt ctaatttacc catttcttct cctttttgta
120781 taatttaatc aaagtataaa caaaaaaaat tttattgtca gtgataaaag tcactaaaaa
120841 taattaatct aaaaaagata caataaaaat taaattcaca aaggtagtct atcttggata
120901 aggaaaaaat tttaaaagaa tattttaaaa actataatct tgaaaacaaa gactttgaag
120961 ctatggtgga aaaatcatac tttaaagaat ttgacaaaaa tacaatttta gatgattgtt
121021 taggatttgt gatagtttta aaagggggat ttcgtgcatt tattttaggt caaaatgcta
121081 aagaaatcac tgtttttaaa ctgaaacaaa atgaagaatg tgtcatttgt tctcattgta
121141 tctttgaaac tatatcttat aatctcacct tagaaagttt tgacgatact caaattttgg
121201 ttgttcctgt aaaaatatat tctcaactaa aagataaata tcctttgata gcaaattaca
121261 ctcttaatct tattgccaaa cgctttaatt ctttgatcaa tattttagag caagcccttt
121321 ttactcctct tcatcacagg gttaaaatgt ttcttaaaga aaatgcaaaa gaaggaaaaa
121381 tcactttcac tcatgaagaa atagctttgc atttaggtag cactagagag gttatttcac
121441 gaattttaaa aacaatgcaa aaagaaggtt ttattcagca aaatcgtaaa gagattacac
121501 ttttaaaagc tttgtgataa atgcccatat aaaatatggg caaaattgca atattttgaa
121561 aagatttaat cctaaatcgg ttaaatcttt aagaacttta tagtttgtta tctttttcta
121621 attgattgat attaagcatg ataaatttat agcttacata aatcaccaaa ggccaaacca
121681 ataaaaacaa agcgtgtagc atttttctc ctcatttaat ataagtgttc gctgtctttc
121741 atttcttcgc gagtgatttt tactctatcc attgcattcc aaacataaac aatataagct
121801 aaaacaaaag gcacaagcaa agatacataa gccattacac taagggtgta ataactcgaa
121861 ctcgcattgt gtatggttaa agaactttgt aaatcactaa gcgaaggata aaaagcactt
121921 gatccaagcc ctatgcttaa aaataaagca aaaacggtca aaaccgatcc aaaacctaaa
121981 gtaaaaatcg ttttagaaca tccctttgat ccttgtatca tacctaaaag cacaaataca
122041 actccaatga taaataaaat agcaaaaaga atattatcta aaaaattgta taaatataca
122101 ttttcagcca tactcacaat gccctttca tctactccaa aaccttcttt tataaaaatc
122161 catgccaaaa atcctaagaa aaaaggtaaa aataaaatac tatttatact aagttttta
122221 acagcttttg ctttaatatt ttcatctttg atattattca taaaataagc agcacccaaa
122281 attctagata aaaacaccaa agcaataccc aaaagataat taaaaggatt aaacaaaagc
122341 tccaaaccgc gtaaaggatt ttgccaagaa acaaaattgt gttcatttaa tacaaaatgc
122401 gatccactaa aaaaactgct tattgcaacc ccgattaaaa acactcctaa ataaccattg
122461 attttaagaa aaacttcata ggttttagaa ccataaacat tgttttcttt tttacgatac
122521 tcataagcaa cagcttgcaa aataaagcaa aataaaatac aaagccaagc ccaataagct
122581 ccaccaaagc ttgtgctata aaaaagtgga aaagcagcaa aagcagcacc accaaaaagt
122641 acaagagtag taaaaccaag ctcccattta cggccaagtg aatttacaag cattgttttt
122701 tcaagttcat cagaacttaa ttcatcaatt aaggtttgac ctccttgaac aaaaaaacata
122761 aagaccaaaa gtccgcccaa taaacttaaa atcacccacc aataaatttg tagtcccca
122821 agttctaaac cgaaaaacat tatttttctc ctttacccat aagagtacta tgtcctgcat

122881 gcgcgtcaaa accttttttg atttgagtaa gcatgatctt aatctctgca atcaaaagtg
122941 cagtaaataa aactgcaaaa atccaaaatg aaatttgaac attgactttt ccaagctcgg
123001 ttgctgctac atgaacaggc attaaatctt gtatagccca aggctgtcta cccacttcag
123061 caactatcca tcctgcctct gctgcaatat atcccaaagg aatacacagc aaacaaagcc
123121 aaagaatttt tctaaatttc tcaatatcat ttgccatagt aagataaagc gttgcgataa
123181 aaagtaagaa aaataaactt ccaagagcta ccatgatatg aaaactataa aaagttaaag
123241 ccacaggcgg gatagcatct gttggttttt ctaaataacc ataaccaaaa tctttaaaat
123301 tcgcctctaa aatacttttg tgaattgcca tagccattgt atcgttatta tctttagcca
123361 atctataatc ttttaaggcc tgtatagcga tttttccttt atcgatgcgt tcttgcatag
123421 gttctatacc cctatcttcg tttccgtgta ttaaatcctc aatgcctggc acaaaagaat
123481 taggatcacg atttcctaat atagataaag cataaggtat agtaatatca aataaaaaca
123541 cactttcatt attgtctata gttttcttag gatttaaaat tccaaaagga acaagtcctg
123601 cacggtgctc accttgataa accccttcca tagcagcaag tttcataggt tgtgtttggg
123661 taacacgata agcactctca tctccgctaa aaaataagaa aaccgaacaa acaagcccaa
123721 aacttgcacc cacaactaaa cttttcttag cttcgataat atgacgccct tttaatataa
123781 accaagctga aattcccatc acaaataaag cagaaatcac ataaccgcta cctatggtat
123841 gtaaaaattt agagattgct acaggagaaa acgcgacttc aaaaaagctt tgcatttcat
123901 ttcttgctgt atcgggatta aattgcattc ccacagggta ttgcatccaa ccatttgcaa
123961 ccaaaatcca aaatgccgat aaattactgc ctatagccac acaccaagtc gaaacaagat
124021 ggaatttctt agaaacttta tcccaaccaa aaaacataac agcaaaaaag gtagcctcta
124081 agaaaaaggc cattataccт tcaactgcta aaggagcacc aaaaatatca cctacaaacc
124141 agctataatt tgcccagttg gttccaaatt caaactccat aataatgcca gtagctacgc
124201 caatagcaaa attgatagca aataaagaaa gccaaaattt agtaattttt ttccatcttt
124261 cattgcccgt tttgacataa atagtttcca taatagcaat gataaaagat aagcccaaag
124321 tcaaaggcac aaataaaaaa tgataaagag cggttaatgc aaactgcgct cttgaccaat
124381 ccacactgct aagttcactc attatttctc cttgataaga ttttctatga caaaattact
124441 tttttcatta tcatttttaa agatagaatt gaaatttaca tcaaaaacaa aaagtttaag
124501 tatgacaagc attataaaaa gttttataaa aatgattttc cataaagttt ttccaagagt
124561 aaggttttta aacccttctt tgtaaaagtt aaaaattcgc ataaaaaaga aataaaaaca
124621 ttttaaatac attaaattaa accttataaa ataatgaaaa tttaaattgt aaaaaagctc
124681 aaaattataa tgaaatttat gttattaaat acttaaaaaa atattattaa attttgatct
124741 tggaaaaaaa tattgtataa aaaacactac aatatatttt taaaaattca ctttttaatt
124801 gattaaatcc ttaaaatttt gcttttatca caaatgaaat ttttagtgct tgtgattata
124861 aattctaaat tttagtaatt aaccaacaaa aatattctat aaaaatcaca aaacaaaaag
124921 agattttact ctcattatag ttatattttt atattatatt tatatttttc tatactctat
124981 atgttaccaa attgcacaaa ataatacata agacgcttta taatttttct taaaatttaa
125041 tttcattata tatattttaa gaatttgtta gaatgaaaag aaactttaat gaaaataaaa
125101 caaaggagaa gacacattat gtggcaacaa atttatgatc cattcaatag catttggctc
125161 agcgccttat ttgctttttt acctattctt tgctttttag cttgtttggt atttttcaag
125221 ttaaaaggtt atcaagcggg ttttttaacc gtggttttgg caagcgttat agctttattt
125281 gtttatgata tgccttggaa tttagtaggt gctagttttg tacaaggctt tacaaatggt
125341 atgtggccta tagcttggat tattatcgcg gctatttttc tttacaaact ctctattaaa
125401 tcaggctctt ttgagataat taaaaaaagc gtgatgagca ttacccctga tcatcgtatt
125461 caagtgattt tgataggttt ttgttttggt tctttcttag aaggtgctat aggttttggc
125521 ggtccagtag ctatcacagc agctcttttg gtcggacttg gtttgcgccc tttacaagca
125581 gcagggcttt gtcttatagc aaataccgcg cctgttgcct ttggtgctgt ggggattcct
125641 atcatcgcta tggcaaattt agtaggagtt gaagctcatt ctgtatctgc tatggtgggt
125701 agaatgcttg ttccacttag ccttactata cctttttca tagtgttttt aatggatggt
125761 ttaaaaggag taaaagaaac ttttcctgct attttagttg ccgcattaag ctttacagcg
125821 acccaatttt ggagctcaaa tcacctagga gctgaacttc ctgatattat ttcagctatt
125881 gtttctttag cagttacaac tatcttttta aaattttgga aaccaaaaaa catttttaga
125941 ttagatggag aaactcattt tactgaagat aataccttaa attttggaga tgtattaaaa
126001 gcttggacac cttttatttt acttataatt tgtattatca tttggactca accttggttt
126061 aaagctatct ttgataagga tggtttttta agctatacaa gcattacttt acaatttagc

126121 aatcttacag gcggtatttt aagtccctct atcacaggta taggcgaagc taaacctctt
126181 tctttaggac ttggaatcga tcttatcaat ggtaaaactg tagcccaagc aggaactgcg
126241 attttgcttg cagctttttt aaccatagct gttttaagaa taaaagctga agatgctgct
126301 gaatgttttt gggccaccct taaagaaatg gctatccctt gtcttaccat aggtttggtt
126361 gtagcctttg cttttatttc taaaaatagt ggtatgagta ctactttagg acttgctttt
126421 gctcatacag gagatgcttt tgcattcttt tcacctatta taggttggat tggcgttttc
126481 ctaacaggat cggatacaag tgctaactta ctttttggaa ccctacaaca agtaagtgcg
126541 caaaaattag gcgttagcga agctttattc ttggcagcaa actccgtagg tggggtagta
126601 ggaaaaatga tcagccctca aagtatagct attgcttgcg cagctgttgg acttgtgggt
126661 aaagaatcag atcttttcaa attcacactc aaatactcta tagcttttat cattttaata
126721 ggaatttgga cttgcattat cgcattcttt ttaccaggaa tcattccaga agtagtagct
126781 aaataaaaat aaacttgcaa gacttattct tgcaagttta agcaaatttt aaatgaaaaa
126841 attttgaaaa tatttttatt attgatttaa tttttatttt atttttctaa agctagaata
126901 attgtttata ttaaatctct taaaatcaag gagaaaagat gaagaaggtg tatttttacg
126961 ccacatgctt aggaactgct gctatgcaag aaagcgttct taatggtatt aaacttttgc
127021 gccgagaagg agtggaagtt atttttaaaa aaaatcaaac ctgttgtgct caaccctctt
127081 ttaattcagg atattttaat gaaagtcgcg aaatagcctt atacaatgta gatttatttg
127141 ataaagacta tcctatcgtt gtacctagtg gatcttgcgc aggtatgatg agtcacgatt
127201 ataaggaact tttcaaggat agacctgaat tttctagagt gaaagatttt agctcccgtg
127261 ttatagagct ttcgcaatat cttgatgaag ttttaaatgt aaattatgaa gataaaggag
127321 agcctattaa agtgacttgg cattctaatt gccatgcttt aagagttcaa aaaagcatac
127381 aagcaagtaa aaacctcatt agaaaactta aaaatgttga acttgtagaa ttacaatacg
127441 aagaagaatg ttgcggtttt ggagggactt tttctgtaaa agagcctgaa atttcaaatg
127501 ctatggtaag aaataagatt aaagatatac aaaatactgg tgtaaaatac ctcatcagtg
127561 gagatggtgg ctgtctttta aacatagatg gaaccatgcg tagaatgggt ttggatgtta
127621 aaggcataca cttgtatgaa tttttattta agcgtttgga aggagagaga ttatgagtca
127681 aaaaattcca cacgaagaaa ttgttcatat taaattaaac gacattcaaa tgcgtgaaaa
127741 tttgatgact gctatgcata ctttgcaaaa aaatagactc ggtgtcatcg atgcaagatt
127801 tcaggattgg caaggattaa gagccaaagc aaaacaagct aaaaacaatg ctttaatgag
127861 cttaaaagat agacttttag aatttgaaaa aaacgctaca aaaaatggta tacaagtaca
127921 ttgggcaagt agcgatgaag atgcttgtga aatagtctat gagattatgc gtgaaaaaaa
127981 catcaccaag cttttaaaag gcaagtctat ggcgagtgag gaaataggct taaatcatta
128041 tttagaaaaa aaaggtttaa aagccataga aaccgatctt ggagagctta ttttacaact
128101 caacgaagaa cctcctttgc atatagttgt tcctgctatc catagaaatc gccatgaaat
128161 aggtcaaatt tttaaagata aactcggtgc aaaattagaa aacgacgagc ctgaaagctt
128221 aaatgccata gcaagagatc acttaagaaa agactttgag ggtttaaagt tagggcttag
128281 tggagtaaat tttgcaatgt caagagaagg ggctttttgg cttatagaaa atgagggaaa
128341 tggtagaatg tgtaccacgg cacctgatat tcacatcgca ctttgtggcg tagaaaaagt
128401 tatggaaagc tttgaagatg cagcgactat ggtttcttta cttactcctt cagccacagg
128461 acaatttata cctacttata ataacatcat cacaggcccg cgtaaaaatg gagatttaga
128521 tggcccaaaa gaagtgcatg tgattttatt tgatcacaat agaaccaaaa tgctagctca
128581 taaagattat tatgaagctt tgcgttgtat tcgatgtggg gcttgtatga atttttgccc
128641 tgtttatgat caaatcggcg gacacgcata ccaaaccacc tatccaggcc ctattggtga
128701 agtaatcagt ccaaatattt ttggtataga tcatacaggt gatattttaa atttctgttc
128761 gctttgcgga cgctgctcag aggtttgtcc ggtgcaaatt cctttggctg atctgattag
128821 aaaactaaga tgcgataaaa taggacaagg agaaaatcca cctatgggtg caaaaaatat
128881 ccatcataac gcaatggaag cctttgcatt caaacaattc aaaaaagcag caacaaaagg
128941 cgaaaggtgg cgttttgcac tttcaaaagc gcattatttt aattggtttg tgcaaaattt
129001 atcaggagct ttaccggtga taaaaaaatg gtatgctttt aaagaattgc ctcaaatcaa
129061 gaaagatcta tataaagaaa ttcaggctat agaaggggta atttatgagt aaaatcaatg
129121 aaatttctaa caaaagcaaa gaaaaaattt tatcagcact caatgaagct tatgaagcaa
129181 aagattttac ttatcaagcc agtatagatc ctgtagaaca tattcaaaca aaagaagatg
129241 ccttagctga aatgaagcaa aaaatgagtg ataataaata cattgttgaa aatgctacca
129301 aagaaacctt ggaagaaaaa atcaatgaaa ttatcacaaa atacaactac aaaaaaatga

129361 tatacggcac tgatttgggt ttggatttaa acaaaattca agcagaagaa aaaatttgct
129421 ttgataagga aattgaaaat ttacgatatg aagtttttca tagcgatttt tctatagtcc
129481 atgcaagggc tggagtaagc tctcatggcg ttgctttgat accttcaagt aaaggacagc
129541 ctagaatgct aagtcttgca cctaagcttt gtatagtgtt gttaaaaaaa gaagacatag
129601 taaaaagcct tagtgaagct ttgaatttgg taaaaacaga aaatgaaatt ttgcctacaa
129661 atattttatt tatcgcaggt ccttcaagaa cagcagatat agaattaatc actgtttttg
129721 gcgttcatgg gcctcaaatt gctcatattg taatttatta aaatttagct ctagtgttat
129781 ttgctagagc taaaattggc ttaagttaaa taaatattat acaagataga ttaaaaattc
129841 tatatcttaa atatagttaa ataagaatat ttatatccta tttaattata tatcattttt
129901 aatatttatt taatataaaa tacgataaca tacctcaata tcaaaatatg caaataattg
129961 aaatttattt ggagaaaact ttggataaat taattcctgc ttgggatgaa aaatacagca
130021 taaatgatga gaaaatagac gcacaacacc aaaaactttt tgaattagct gctaaaattg
130081 aaaatgctgt ttacaaattc gttcaacgcg atgaacttaa agaaattctt accgaactct
130141 ttaattatat gaaagaacac ttcagtaatg aagaggatta tatgcaagaa atttcttatc
130201 cttatttaaa tgaacataaa ataatgcata aaaacattat ccacgatatg tcacatctta
130261 tccaaaacat aaagactaca aatgatttaa aagaaaaact ttatactata atgtcagatt
130321 ggttattaga acatattttg catcatgatg ttatgatagg aaattggctt tcagagcaat
130381 tattggccaa acaagaagaa gaattagaaa aacttcagca agaagatctt caacaagaag
130441 aaaaacaata tgaaaataat ccaaatccaa ttcctcaagt caaaacagaa tatgatttta
130501 tctattcttg tccttgtagg acaaatcact ttctttcata tatagagcat cttgatatta
130561 tttgccataa aaaacaacta agatgtaaaa aatgtcaaaa aaatctattt tatgtaagaa
130621 gtgaaatcaa aactgaaaaa aggaataatg atgtttccaa agtgggatga taattatagc
130681 gttcataatg ccaagattga cgagcaacac aaaaaacttt ttgaactagc aggcaaggtt
130741 gaatttatgt ttgatcgacc cgtgtataaa gatgatatca aagctctatt ggctgatttt
130801 tttaactata tgaaagatca ttttaatgat gaagaaaaat acatgaagct aataggatat
130861 cctgatttgg aagaacataa aaagatacat aaagaaatca tccaatctat gatagacctc
130921 attaaaaaca taaagtcaac caacgattta aaagaaaaac tttatacagt ttctaaaaaa
130981 tggctcttag agcatatact ttatgaagat atgaaagtag aacaatacag acgctcttct
131041 ttatcctctg aagatgacag cgaagtcagt tttgaagaag taaaagaaga aaaagacgat
131101 gaaacggctt tgtattttta tacttgcgaa tgtgttggac aaattcacga tgtgcctttt
131161 ggaattcatc aaaaaataca actaaaaggt gctaaattta aatgcaaaaa atgcaaaatg
131221 gccatacaat tttataaaaa acattccgaa tcttttgaat aataaccatt ctatagtaga
131281 ataaaataaa aaaataattg gttctactat gaataaaaaa ataaaatatt atatctcaaa
131341 taaaacaaag tattcttatc ctattttaac caaggaagtt caatgtttta attgtaaaaa
131401 attttactcg atcgaatatg cttcgaattt gaaaaaaata gaaaaagaat gcccatcttg
131461 caaaacaaaa atggacatta agcttaaaga ttagtttttt gccttgctta caatattgat
131521 gatattttga gcaacttcat cagcaagttc taagtgtgag gtaaagccta cgcaagaaca
131581 atttatctca cctaaaacat acttatcgtt gccctttca tcattatcca aaataaaatc
131641 agccgtccaa ataagtggta aatcataatt gcctaatttt gcacgcactt taggaagctc
131701 tcctaaaaac atatctacca aattttgcca atcctcgggt ttatcatagc gatattgcgc
131761 cccactaaaa agtgtagcag aaaaagcatc agcatcttcg gctggttttt tatggactac
131821 atttacagga gtattataaa gcattaaaag tcttatctcc ccttctttga tacgtggtaa
131881 aaaagtcata tccacaagca taccattatc acctataatg tattgctcgc aaaaatccat
131941 aaattcaccc aattctctgt gctctacatg attgtctttt gcttcggtgc attttatttt
132001 agtatctaaa ggtaaaactt ctccttttgc ttcaccctct acactcacac gccaaattcc
132061 ctcacctgta gagccacgat tttgttttaa aactctttct cccttagcta aactttttgg
132121 aaaatttct ttaaaagtct taatatcata gtatgcataa gtatcacttg gcacaagatc
132181 tgtatctgca agttttgtta aagcatcttt tgccccataa cctatcatag catcaggatg
132241 aggcatacca acaagacctt cttggcaaag ttttcttaac atatcaaaat actcattttc
132301 ttctttaaga ttgccaggat tgatgcgcga aacatagcca tcaaaatttt ctttcacata
132361 attgtaaatt tcatcctttt ttcccacttc aaaaaaaatc acctcggcat tccaaccctt
132421 agcttttaaa gcatttacca taggcatagt atcttttctg tagccatctt cacccttatc
132481 gctaccgcct ctaacctcaa aaaagactat atttttttc attgcacact gctcctttaa
132541 gtttaaaata taaacaatta tttcataaaa atgttataaa agttaaacga ataagagcct

132601 ttttctccta taaaacacaa aaatttagaa ttattttatt ttaattctaa ggcattcgca
132661 ttcaatctaa gtataacatc gcccattact ttacttaaaa aattagaact ttggctttct
132721 aaacgattta aaaatctatc aattctaccc tcttctttcc aaataggatt taaaacttta
132781 gcaagttctt gaagttcttt ttttgcattt tcataattgc ttaactcatc aataagccct
132841 aaatctttcg cattttgtgc taaaaatacc cttgcgtttg cccaatcatt tcttttgtct
132901 aattccaaat tgcgctcttt agctacaaat tccgaaaaaa gatcataact ttcattgatt
132961 aaattttgca agaattctcg ctctttttca ctccaagccc ttgtcaaagt tcctgcgctt
133021 ttaaattccc ctgcttgtat ggtttgttct ttaattccta ttttcttagc caactcactc
133081 aaatcagcac cctgcataat cacacctata gatcctataa aactagcagg gtttgctaaa
133141 attttatttg ctcctactcc agccaaataa ctcccactcg ccatagttcc acttgcataa
133201 gctataacag gtttttttagc ttttaaatct ttaatcgcca aagctagctc catacttggc
133261 gcaaaagcac ctccaggaga atctatcaca aaaagcacgc ctttgatatt gtcattattt
133321 ttagcattga taattttttc taaaacttct gagctatcta gtatctcacc ttttaaatca
133381 atgcgttcta aattggcata agatgagttt gagtttgaac ttggtgtaaa aagccaaact
133441 acgatcaaca ataaaacaaa agttttaaaa taggtgttga taaattttat cacgctgcct
133501 aaacccttaa aaaatgattt taaaatttgc attcttttcc ttttataaat aatttttgta
133561 cttctttagc atttaaaata aattgcaatg gtgcttgctc cttgtcacat tcccctagct
133621 caaatacact aaaatcagct atttttccct gcttgatttc gcctaaattt aaatttaaag
133681 ctttggccgg ataaagcgtt gccatttgca aaagcttagg agctaatttt aacaaatcaa
133741 aattggtatg cacaagcaaa ttagcacgca tttcatctaa aatcgataaa gaaatattag
133801 aacttaagcc atctgtaccc aaatggatat ttaaaccgct ctttaaagcc tttttttaaat
133861 caaatgtttt ttgacttaaa agacgatttg aaaaagcaca atgggtaata gaatgtaaat
133921 ttttatcaag caaatccatt tcttttaaat acacacaatg agtaaaaagc gtcctaatgc
133981 ctttgaataa ttggataaat tcactaactt tataaagtgg cttaggattt ggtgtaaagt
134041 ttttaaacca ttctttaaac cctccttttg cctctttaag ccaagcattt tcagccttac
134101 tttctaaaaa atgcgtgctg attaataact tttgcttttt agcaagattg gttgcaaatt
134161 ttgcaagttc gggatgggtt gaataagcag aatgcacaga aatagcaggc ataaaaaact
134221 catctttaaa ttttaaagaa ttttcaaatc ttgttaaaaa ctcttgtttt ttatcttgaa
134281 tttgggcttc atttatgcct aaaatttcat taaaaaatat aacacgcatt ccattttgac
134341 ttgctttaac acaaggattt aagtcactgc caaagcttga aatttcacct atggtaccta
134401 taccgctttt ttgcattttt tgtatatttg tcaatataag ctcttcttta gcttgctcat
134461 ttaacataga acgagattta atcacacttt taagccaagt taaaaactct ccaaaatgca
134521 aagtagtcga atttgcactg aattctaaat gtgtatgtgg attgataaaa gcgggtaaaa
134581 taacacaatt aggacttgtt tttataagct tagcttttgg gtatcgtttt tttaattctt
134641 ctaattcgcc taattctaaa attttatcct caaaaacaaa ggctttattt tctaaaatac
134701 taaattgctc atcgcataaa aagatatatt ttgcactgat tatatacatt ctttttttcct
134761 tgaataaaat cacattgtag cgaaaattta ttttaaaatg ttataataaa aggttaaaaa
134821 taaaaatttt aaggtgaaat tatgaaaata atggtcatcc aaggcccaaa tatcaacatg
134881 ctaggcgtaa gagaagttgg aatttatggt gcaatgaaaa tggaagacat tcatacgcaa
134941 atgaaaatgg cagcttccca aaacaatgtt gaattagatt tttttcaaag taattttgaa
135001 ggtgaaattg tagataaaat tcaagaatgc cttggcacag tagatggtat catcatcaat
135061 gctgcaggct atacccatac ttcagtagcc atccgcgatg cgatttctgc agtagcttta
135121 cctactatag aagtgcatat tagcaatgtt tatcgcagag aagaatttcg tcaaaaaagc
135181 cttatagctc ctgtatgctc tggatctatt gtaggatttg gtccttttgg ttatcactta
135241 gctttaatgg gtgttattca aatttgcgag caagtaaaaa acttgcgtgc tgcacaagca
135301 cagcaaatgc aagctggggc tcaaaattaa aaatgctaaa aataaaagga gcaagacgac
135361 ttgaaacaag ccgttttttt ccttatttta gccaaaagaa aaaaggattt aaatatcttg
135421 ctctagtagg cttaggaagc aatatagagc cagaaaaaac aagatttaac aagctttttc
135481 gcgtgatgat ggaagataaa aggtttaaaa ttctagccac atctccattt ttaatcaatg
135541 aagcttttgg ctttaaagct caaaaagact tcaccaatgc aacaatgctt atgcaaacaa
135601 atctacacgc aagagctttt ttgaaagttt tgcttttttta tgaacttaaa ttcaagcgaa
135661 aaagaacttt caaaaacgct cctagaacac ttgatttaga tcttttatat ttttcacaaa
135721 aagtcaagcg tgatgagggg tgtatggtgc ctcatacagg ggccaatcaa agaataagtg
135781 taattttgcc cttgggctta acaaaaggat tataaatggg acaacttatc catacctttta

135841 ctgtagaaga tacagagcaa attatcccga aagtaaaaga ggattatggc gacaaagctt
135901 taattattac caataaacaa attcgtccca aaacacttaa ccgcgctgct ctttatgaag
135961 taatggttgc catagaagag agtgattatg aagagcattt gaaaaaaatg gggaaatcct
136021 tacctcctaa aaaaactatc gctaaaaccc tagaaaaaca aaatcttgaa gaaaaagtca
136081 atgcttcaat accacaaaat aatgaagatg aagatgttat ccttgatttt tcaaatacta
136141 gagcaaattt agcaaaaact aaaactaacg atactaatta ccaaagcttt ccacaaagta
136201 aaatcaatcc taatcccacc atgggcttta atgattttaa agaacgcttg agtgaagtaa
136261 gcaatgaaat ttcaaaagta accaataccc ctttagtgga taattacgcg cccaatccaa
136321 actataacaa aaaaatagag aattttgaaa aacaaatcaa taaactcaat gataaaatcg
136381 atttacttgc agatatgatg tgggatgaaa aagcagaagc tcgcaacaat cttatcattc
136441 ctcctgaatt tgcaaccatt tacaaaaaag ctaaagaaag cggtatgctt gaaaagcatt
136501 tagaagctat catgaaagcg actatagaaa atatgcctgc ggctatgaaa acaaacaaag
136561 acgctgtgca acgatatttt tattctcttt tgagaaatat tttaccttgc cgagtagaaa
136621 gtgaaatcaa aaaacaaaaa attatgatgc ttgtaggccc aacaggagtt ggaaaaacta
136681 caactctagc aaaactagct tttcgctatg cttacgggga taagcgttat aaaacaggta
136741 taatcacttt agacacttat agaatcggtg ccgtagagca actttttcaa tatgcaaaaa
136801 tgatgaaact tcctatcatt gatagcatag aaccaaaaga tttagacgag gcgattaaaa
136861 gtcttaacaa ctgcgaagta attttagtcg ataccatagg aaattcacaa tacgatcaaa
136921 acaaacttgc caaaacaaaa gaatttctca tgcattcaaa tgctgaaatt gatgttagcc
136981 tagtcgtttc tgctaatacc aaacacgaag atttgatgga aatttacaaa aatttttcat
137041 ttttaaatat cgacactctc atcatcacaa aatttgatga aaccaaagtt tttggaaata
137101 ttttctcttt aatttatgaa actaatatcc cgctaagctt tttctctaca ggacaagaag
137161 ttcctgatga tatagaagta gccaatagcg atttttttagt tcgctgtatc ttagaaggtt
137221 ttaacaaagg aaaagataat gaataaccaa gccaataaac tccgtagcct tatgaatcaa
137281 aatggaggaa aaaaatcaca aaacactcat tttatagcga ttacaagtgg aaaaggtgga
137341 gtgggtaaaa gcactatcag tgcaaatttg gcaaatgtgc tagctaataa tggatataaa
137401 gtaggacttt ttgatgcaga tattggccta gcaaatcttg atgttatttt aaatgttcgt
137461 attcaaaaaa atcttttaca tgttttgcgc ggggaatgct ctttagaaga tattttaatc
137521 gaagtaaagc caaatttatg gctcatccct ggtgaaagtg gtgatgaaat tttaaaatac
137581 aatgataaaa acatttatga aagattttta aatcaagcaa gcattttaga tgaacttgat
137641 tttcttatca ttgatacagg tgcaggaata ggaggaaata tactgaattt cttagaaatg
137701 tcagatgagg ttgtagtagt aaccgttcct gatcctgctg caattaccga tgcatatgca
137761 actattaaaa ccacttctaa gacaaaagaa aatttgctta tgctgtttaa tgttgtcaaa
137821 aatgaaaatg aggctttaaa ggtttttgaa aatattaaaa aagttgccga tgcaaatatt
137881 aaaaatcatc taaaattaga atttttagga catttaagcg cttcaaaaga tgtaagcggt
137941 agcattaaaa aacgcacctt gtttacagat gaaaattctg cttcaagtga tgagcttaaa
138001 gccctagctt ccaagctttt atataggttg gaacggaaag tgcttgataa tgtcacgagt
138061 cgaagttttt caaatttctt taggaaaatc atcgaacgat tttaagcaat aaagagaaga
138121 tattatgaga cctgaaaatt atgtagcatt ttttactgtt tgtggatttt ttataggatt
138181 ggcttttagt gtgattagca ttgaagaagc ttttgatatt ttgattttta cttgttttat
138241 tacctttatg ttttatgttt ttatacacat tgctattatg aattttatag atattaaaaa
138301 aatcagcgga cgaattttca ataaacatga ttatgaaaag actagcaata atatcataaa
138361 cgatttagtc attcgtgaaa agaaaatgga tataatctta gaaaagctta acgaagaaag
138421 agaagagctt aagaaaaacg aatcaaaaga aagacgcaaa aatgcaaaaa gagccgccta
138481 aagcttatgc tcaaatgata aaagatgaac aagatcagct agttttatcc tatatgcctg
138541 cacttcgtgc aatggctttt aggttaaagg agcgcttgcc ttctagcatt gatgtgaatg
138601 atcttatcag cattggtgtt gaagagatga tcaagctttc acgccgctac gataaagaac
138661 aaaatgatag cttttggggg tttgctagaa agcgtgttaa tggcgctatg ctcgattttt
138721 tacgctcttt ggatgtaatg agtcgcaata accgcaaaat tattaaagat atcgatattt
138781 taatcgatga atattttaga gaacacgaag aagagcctga tgatgaatat ctagctcaaa
138841 aacttggctt ggatatagaa aagatcaaag aagtacgcac cgcacatgct attagttata
138901 ctttatctat cgatgatcaa ttagaacttt ataatgaaga tgatacttta gaaaaattag
138961 aaaaagaaca acttttagaa aaaattcatg aagtattaga agatcttaaa gagcgtgatc
139021 aactcattat ccaactttat tattatgaag aactcagcct taaggagatc agtgaaattt

139081 tagagattag cgagagtaga atttcacaaa ttcataaaaa acttttaaaa aaacttagag
139141 aaaggctagt gtaatggcag aaatactctc ccaagaagaa attgacgctc tacttgaagt
139201 tgttgatgat aataccgata cgcctacagc ctccaattca aaagatggaa aagatgaaag
139261 aaatatagtc gtatatgact ttaagcgtcc aaatagggta tctaaagagc agcttagaac
139321 gatcaaaggt atacacgata aactagcaag aaatttagct tctcaaattt catctatgat
139381 gagaagtatt gttgaaacca agcttcactc ggtggatcaa atgacttatg gtgagttctt
139441 gatgtcccta ccaagcccta caagctttaa cgttttctct atcaagcctc ttgatggaaa
139501 ctgcgtttta gagattaacc caagcattgc tttccctatg atagatagac ttttaggagg
139561 tcaaggcgat agctatgagg cttcgcgaga gcttaccgat atagagctta atttgcttga
139621 ttcgattttg cgtattatta tgcaaagact taaagaaagc tgggctacag taactgaaat
139681 ttatcctagt atagaagcta aagaatcaag ccccaatgtt gtacaaatcg tatcgcaaaa
139741 cgaaatcgtt atcatggtgg taatggaaat catcatagga aattcaagcg gtatggtaaa
139801 tatctgctat cctgttgtcc atttagaaag tatcttgagt cgtttagcaa accgcgatat
139861 tatgatgggt gaaacttcgg ctaaaaaatc gcgtaacaaa gagcttaaaa ctctcatcgg
139921 tcgtgctgaa gtagtttatg aagcgatttt aggaaaaact ctcatcaatg ttcatgaatt
139981 tctagaactc aaacaaggcg atatcttaag gctagataga gaagctgatg ataaagccat
140041 agtaagcata gataaaaaag atgttttctt ggcacaaatt ggcttgcatc gctttagaaa
140101 atctattaaa attcttgaac ttatacgcac tgataaagat gaaattaaag aaattctaga
140161 aagatatgaa gaggagcgta aggcaaaagc tagcgtttat gatgagcctg aagaggagga
140221 agaagaaata tgatcaatga atttttaaaa ttatttacaa acgaatgcac cagcacaata
140281 gaaggactta cgggaaaaag tgcagaattt agcgaatata aagaatttga tgtttcatcg
140341 caagatacac ttaagccccc tcttgtttat gcggtttttta acatcaaaga aggtggtaaa
140401 ataggtatat tagcaggtgc tgtgcttatg agtgctattg gcgaatggat gatgggcgaa
140461 gaagaaattt ctaaaaacga tcaactaggg cctgatgaaa tggacgctgc taaagaagca
140521 attcaaaaca tcatttcggc tttttctacc actttaggcg cacaaaaaga catacccaaaa
140581 atggattttg aaatcagctc ttgcgaattt gtagcagaaa atgttgattt taaagatttt
140641 acaaaacttt atttctttga tgtcaaaata gaagacttag aagagcaaat ttctttgatt
140701 gtagatcaaa ctcttcacaa tattttaagc ggcaaagctg cagatgatgg ccatgcacac
140761 tctagctcaa attccgaaaa taaaggcttt gagataagtg aggagcttaa aaatatcaat
140821 ctcatcatgg atgtgcgttt gcctgtgcgt gtaagaattg gcaacaaaaa aatgctttta
140881 aaagatgttt taaccatgga tataggctct gtagtagagc ttaatcaact tgcaaatgat
140941 ccacttgaaa tattaatcgg tgataaaaga attgcctatg gcgaagtagt aatcgtcgat
141001 ggaaatttcg gcgttcaaat cacagaaatt ggctctaaaa aagaaagatt agaacaatta
141061 agataattta tactaaacca tctatcattt ttgccgattt atcttgtaaa tcttatttta
141121 caaggtagca aagatgatta atggtatcaa ttcttattca agctataatt acacaagcac
141181 tttcaataat actttcaata caaaatcatc taatatcata aatgataatt caaaccttgt
141241 aagcgataaa tctggggcag taagtaaaat actaggctat ggtgtagata aagatggctt
141301 ttttacaagc gattttaacg aagctgcagg cttaccaaaa gattataaga tttatgctaa
141361 agatatagaa aattttgtta atattcaaac aaaaagtcta ttatcaagtt atattaatat
141421 tgatatagca aaaagtatag gaaatgctta taaagtattt tcacaacttt taaatgatga
141481 gttaaacaat aaaatcaatt ttacaaaaga agacttagta aaaataccac aaggtttcga
141541 atttgatcaa aatactttgg agataaataa aaaatataat ttaaatgata aagaatataa
141601 agattttatg tctaattatc aaaattactc tccgacttcc cataatataa aaataatgac
141661 ttttttctca cttgatgata aaagtgatat ttttaaatat tctttaatag atcctactcc
141721 ttatagtaat aatgataaaa ctataacaaa aagtggtatt tttatgactt tctttactgg
141781 atttcaagca aaaaactatt ttatcgaagg acaaacaagt attttaggca aaatgcaagg
141841 atttgataaa actatgagtc aaagtgaaat tgataattta aacaaattca taagtgaaaa
141901 tagtatgaat tttaaaggaa atatttttaa gctttttgat ttattaaatt cagatatgag
141961 cattgatgat tttaaacaaa aaatactaga acaaaaagaa aatttaaaca atcctacaaa
142021 aagcacaaat aacaaagaaa cttcagaaga agacaaaaaa aagcctttta aacctataca
142081 agcgcaaagt tcaagcgaaa cctacaaaga tgatagtaaa atgaatgaac ttttaaaaaa
142141 gttactagaa actaaatttg gcacaagcga tgagctagaa attctttttg gtatgaaatt
142201 tagtgatgat gatacaggag aatttaataa aattctttct ttaaattctg caccaaaaag
142261 tatagatatt aaggcttaaa aatataaaaa tttaaattat aaaaaggatt aaaaatgata

142321 ggtatcaact caactttcaa taactccttt gtcaatctta atattaaaaa caataacaac
142381 tcttccttaa attcaaatac taatgtaaaa aatttacaag atacaaaaca atactctaaa
142441 acaagcaata aagttttgga ttatgaaata gataacgagg gttattttac aagtgatttt
142501 aacgagaaag caggaattcc aaaagatata aaaattcatt ctagtactat ggaaaatttt
142561 attaaggctc aaaataatgg aagtttaaaa agttatgcaa atatcgatat tgcaaaaacc
142621 gtgggaaatg cttataaaat agtttctcaa cttgtagaaa aaactcccca attaaaaagg
142681 aaaagtagct tttcaaaaga agatttagca aattattttc cacaaaacta cctaatagat
142741 aaaaacactt tagaagtaaa acaaactttt tcttacgaag aaatgaaaga cattatcgca
142801 aaaggtggat ttaaaaatat taacgagaat gaaaaattaa gtcctagttt ttttaatttt
142861 gacaatacag atgaaaaatc agaactttta cctttcaatc ctgatatttt acatagtgct
142921 aaaaacactg ctttatcttg ggatactagt gctgataaat atacaaataa tgatggctct
142981 attactatgg gtggtttatt aataggtttt ttatctaaca atgtgaatac tggagtaacc
143041 gcacaacctt taattgctgg cgaaacaacc gtatggggaa agttacaagg gcttgataat
143101 tctttaagcg aaaatgaaat taaagaactt aataataaac ttagcaatac ttatttttca
143161 aatttaagcg atgcttttgc attacttaat ggcacaagtg atattaaaga atttgaaaat
143221 aaattaaata gtttaaatca aaataataaa acaaattcaa gtaatttttt agaatgggtg
143281 caacaaatca ccaaggagca tcaaaaatct ttatttgaaa ttctaaaaca accagataat
143341 attaataaaa tatatttatt gataaatata gacataaggg cttaaggcat tcaaacttta
143401 tgattttata taaatttaaa tgagcttatt tgaaacctac ataaagaact tattataaat
143461 ttttagctag aatgaaattt attttatcta aaggttgcaa aatggatacg aaaataagcc
143521 aagatttaga aaaatttgct aatataccaa acctaaaaaa tgccataact ttttttggtt
143581 ctgcaagatt aaaagaagat agtttttatt atcaagaggc caaaaaatta gctaatttat
143641 gtgtagaaaa tggcttttgt gtcgttagcg gcggaggtgg aggcattatg cgtgcggcta
143701 atgctggcgc tgccaaatgt aaaaacgatg caaattttat aaaatcggta ggttttaata
143761 tacttttgcc ttttgagcaa aaattaaacg attttgtaga atataacata acttttgaga
143821 gcttagccat acgcaaaatg gctcttattg aaaagagtct agcttttgtg attttcccag
143881 gcggctttgg aacacttgat gaattgtgtg aagtacttac actaaaacaa cttggattta
143941 aaaacaatgt tcctattatc ttgtttggga gtgaattttg gcaaggcttt gatgagttcg
144001 tcagaaattc tttgctaaaa cttgaagtca tttccaaagg agacgagtta aagtatgaaa
144061 tcactgacga tttagattat ataatcaata ctttaaagga actctaatga aaattctagt
144121 cgctatgagt ggtggggtcg atagcacagt cactgcttat aaactcaaaa atgcagggca
144181 tgaagttata ggctgttata tgaaacttca tggcaaacct aattatcatg aagaaaatat
144241 caaaaaagta gaaaaagttg ccaatttctt acaaattcct tatcatgttt tagacttgca
144301 agaagacttt aaaaataaag tttatatgcc ttttgtagat acttacaaag agggtaaaac
144361 gcctaatcct tgtgctttat gcaatcgctt tattaaactt ggaaaactct tagaatttgc
144421 taaaagttta ggctgtgaaa agcttgctac gggacattat gcaagacttg aaaacaatct
144481 tatccgcaca gctgttgatg aaagcaagga tcaaagctat tttttagcca gtgcggacaa
144541 agaagcttta aaatacctca tctttccatt aggtgaaatg aaaaaagaag atgtgaaaaa
144601 atttgcttct actatagaag ttttaaaatc atttgcaact caaaaagaaa gttctgaaat
144661 ttgctttgtt gaagatactt atgtgcaagt tttagatcaa tttatggata ctaaaattcc
144721 aggagaagtg ctagatagta gcggtaaggt tgtaggaaaa cacgaaggct atatgcacta
144781 taccataggc aaaagacgcg gttttgaagt gcgcggggca cacgaaccgc attttgtttt
144841 aaagatcaat cctaaacaaa atcaaatcat cgttggcacc aaagaagaat taaaaatcaa
144901 tgaatttaaa ctcaaaaata tcaatctttt catcgatgca aaagaacttg attgcgaagt
144961 aaaaatccgc tatagatcaa aatctacccc atgtaaagtc attataagtg atgataaaag
145021 cgcgagcatt tctttaaaag agagtgttta tggcttagca agtgggcaaa tggcagtatt
145081 ttacgatgat gataaagtca ttgcgagtgg gtttatagaa taagacttat caaagcttta
145141 aaagctttga taagatttta ataaaatccc aaaaatttcc accatacaag tccaacaagt
145201 atcattacgg ttatatctat caccgaaata acaaatccta cggaccacca tttttttaaga
145261 cttacataac ctgttccaaa aataactgga gctgttcctg ttgcataatg cgttaaggcc
145321 atcataacat ttcctgatgc tatcataata aaagcataaa gaagtggtgg agcccctaaa
145381 gcaagtcctg cactataaaa tacaaaaaac atagctgaaa tatgcgctgt ggttgaagca
145441 aagaaataat gcgtatataa aaaggcaagg cttaagaaaa tcataataga aatttcacca
145501 agtcccatag cagaggcaaa ttcacccaaa gcttcagcca aaaactgcgt tacgccgagc

145561 tttccaagca aagttgccat cattacaagg gctgaaaacc atactaaagt attccaagca
145621 gctttttctg ctaacacctc accaaaagtc aaaactccac ttactaaaac caaagaaaga
145681 ccgagcaaag caacacttgt agcatcaaga gaaattccaa aaagaaagcc caaagcccct
145741 gcccataaaa gcaaaagcaa tacaaaaaca cctagcataa tcttttcact acccttcata
145801 gcgccaagtt cttctaactt accctttgca aaaatcttag catttggggt tgatttaatc
145861 tctggtggag aaagaaaata aatcaccaaa ggcattaaaa gcatagccgc tatgccaggt
145921 aaaaacatac ctaaagccca ttgtccccaa gttaaatgta cttctaaatt tgtagcttga
145981 gcgaccaaat caacaactaa tggatttgga gcagtggctg tgataaacat tgccgaagaa
146041 ataggattgg cttggaaatt caccaaagaa agataagtgc ctattttatt ttgtgttcca
146101 tcttcaggag tggatttaaa acttcttgcg atagcctgaa ctataggatt gattatcgcg
146161 cccgctcttg ctgtatttga aggagttaca ggagctaaga tagtttcaca aaatgcgatc
146221 gaatacgcaa tacctaaagt tttcttacca aagatactga taaaataata cgccaaacgc
146281 tctccaagtc ctgttttaat aactccacga gctacaatga tagaaactac tatgagccaa
146341 attaaagaat tagaaaatcc acttaaagca tccttaatac ctgtttttga ttttaaactt
146401 gatattgcag tattaacttt ttcatctata tctttttgag acataaattc aagagctaaa
146461 gagtttagtg ctgcgatctt gctctcatca acaagctttt gtgtagcaac ttttgaagtc
146521 gcttttgcat aaagtttgct taggctatct atttgatatt ctatgtttga attagatttt
146581 aaaatttcat ttactttagg ttctaaattt ttagccacat ttgctttgat gagattttcc
146641 aacacgctag cttgcgattt tttatccata gcaactttat agatattgga attatctttt
146701 aagacaatgg cttcaagagt ttttatatgc gtagcacgaa cagaactttg agtagtcgtg
146761 attccgctta gtgcgacaat agcaatagct atcatacaca cagctcctaa aggcatcact
146821 tgtaagatga ccgccataat caccgcgata aaaagtccaa gaaaatgcca agaattctcc
146881 tctaaaccta taggagtagg gattatccaa aatgcaagtg cgataaggat acacaatata
146941 agcatgggaa ttttcttttt ctccatttat cctcctttgt tgtattaaaa ttgttaaatt
147001 ttaattcaaa aattattaat taaatcttaa aaaaattttg ccaaagtctt gaaattctat
147061 accaagaata aaaatattta agagatataa aaaggaagtt taaatgaaat aaacccttttc
147121 ccagatggct gcggcacaca ctacaatcaa gtgctctgct gtgttcccac cctgaagcgg
147181 tgcttaaaaa tagcattgca caggtctaag aaaaggcgat gaaaattata tcatactagg
147241 cttaaaaagt cttgagttgt aaaaattaaa aatttgtata aatatgtaac aaatataagt
147301 taaatttaaa aaattaaaca ccatctttaa agacataaac taagttttta taaaaattta
147361 taaattcaat gaaagcttta atgcaaatca agcaagattt aacgcgctat ataaaaaaga
147421 tttatcgagt tgaaaattga tacaaaaata ataagcatta gcaaaagata attcctaaga
147481 attatctttt catattgata gctttttgta tcatttgatc acttgtagtg atgcttttag
147541 aacttgcatc aaaagatttt tgagttgcta taagattgga taattgaaca ctcaaatcaa
147601 cattagattg ttctaaaaat ccacccttaa attcagctgt attgatcact tgtccatctt
147661 tcatgataaa gctagcatct ccgctattgg ctgttgcagc aaagatatta tcacccatag
147721 cagccaatcc ttgctcattg ataaaattat acaaagcaag ttttcctact atggcggctt
147781 taccattgct aaattgcgcg actatgctac catcatctga tatgctgtat tgatcaaaaa
147841 acccttcagc cacaccatct tgctgtgtaa ctatattttt tgcaacccct tctttgatat
147901 aaattccact atagcccgaa cctggtttgt ttgcgtcata aggagtgcct aaatctatat
147961 tgatagtccc gccgtctgga tttgcaatac ttgtgatatt gttttgaagc aaggctccat
148021 ttttatcaaa aaccatactt ccttcagttg gattgcccac cgcttcgcca tcagaattaa
148081 aaatttgcgc gatagcttta tattgtatat tctcgccctc ttgaggcaaa attctctcaa
148141 gcgtaagcct aagaatgctt ttactgccat ctgcattata aatggaagat tctaaaatat
148201 ctttattggc cttttgctgt tctgttgaaa tttgtgctgt ttcaagtttt atactattag
148261 gatctaaacc ttttaaatca agctcattac ttgtaaaatt caaattttca tccaaagtag
148321 cttcaaaact tgtctttact ccattatcat cgataaaatt taaaagtatt ctatcgcctg
148381 ccttagcact aaatacatct tctttgctta cactaccgct aactatatac tttccatctt
148441 cagtcttttg gacattaaat ttcgcaggat caagatcgac tttaaccact tctgttgttg
148501 catttgtatt taaacttcca ctccatttta cattttgagt tacttgcgga ggcaaataca
148561 tattaacagg aactttaatc actccttgtg aagccgttgt ccctatatta aaaacatcat
148621 ttgaatttac agtaaaacca ctatttacag gagttcctgt gttaagataa tcccccataa
148681 gtcctgcaac tctatcacta taactcactc cagcaaaagc aggattcata gttcctaaaa
148741 caaaattccc ataagaatcc actaaattgc cagcagcatc tcttctaaag gctccatttc

148801 gagtataata agcctctcca tcagctccta aaacaccaaa aaacccttttt ccttgaagcg
148861 ctacatcaaa ttcgccctca cttgccacag gcgaaccttg ctcaaaaaca atctgagaag
148921 aagccgctcc cgttccatag ccccccttgag taggattggt agattgagat gtgatcgtgc
148981 tataaaaaat atccttaaat tgagtagtag aatacttaaa acccatagta ttgacatttg
149041 cgatattgtt agcagcaaca tctatatcaa aactattggt tttaactcct gaaagtccgt
149101 tataaaatga agtcatcatg gcttatgccc tctcttcgct ctcactatct tctgtagttc
149161 ctgatgagcc tccacttgaa tcacttcctg aactaccact tccggtgcta gaagaagagc
149221 ttaatttata atcagtaatt tctgttatag catcaaaagg aacttcttga cctgccatct
149281 ttgcataagc taaaccttct ttaaaaatga ttccttcgat agggtaagta ccataatttg
149341 cagtgatttt ttcgccattt ttattgttat aaaccatttt aacagtatac tcgccatcgc
149401 ctgcataaac gccatcatta tttcttcctg gccattccat tgtaaaaagt ccttgagaaa
149461 cagattgctc gccacttttt tcgctaaata ctagcttgtt attgctgtca tatacttcca
149521 aagtaactcc attttcatca gaatcttcag gcaaatacat tttaagcgcg ataatctcat
149581 ctgaacccgt aagcttgatt ttattatccg aaacagttgc catctttcct atggctccga
149641 tagctgaagt actcatactt gtgctaaaag aatttgaaag caattgcata gtttcaacca
149701 tttttttgcat agtggtattt gtattttgct gcatctcaag tgctgataat tgagaagttt
149761 gcgtaagcat tttatcacta tccataggat cagttggatc ttgatgctga agctcgatta
149821 aaagaagttt caaaaaggca tctttatcaa gagttgcatt tggattagaa gcaagacctg
149881 aactactagt tgaacttgta tctgaagtag tgtttgtgct agtagtagta gcggtagcgg
149941 tattggtatt tgtgttggta tttgtgtttg tcatagtcca atctgatgaa gttaacatta
150001 taaactcctt tttatattaa gtcttttttt aataaaagca ataagtgttc caatttagaa
150061 atacttagcc aaaacaagct ctagatttac cttttcattt tcggttttcc cttctaaagc
150121 atctttaaaa ccatatccac ttcgattttt accttgattt tgattttgct ctcttttttcc
150181 ttgatcgcta aaattcattt caagtcctgt aaatcccata tttacaagag aatttttaaa
150241 ttcagcttga ttttggataa ataaattcat cgcattggta ttggaattaa aattgatatg
150301 aagattattt cctctttgta ccaaagtcac ctcaacttct ccaaggttgt gaggatttag
150361 tgtgatacta agcttggtga ttggggcttt gtattgctcc attgctgctt ttaaatcctg
150421 cgaaaaatgt tgtaaggttt ccttaggagt gatattttta gtattgtttt gtgaaatttt
150481 atttaaatcc tttactaaag aatttaaatc atctaaagga ttttttttcgg tattctcact
150541 ctgtatttta ccttgctctt gtgtataatt ttctttatta gtgtagtgag tgaaatttgt
150601 attatcttta ttggatacaa attgagtttt gtttaaattt tcaaaactaa ctttttgctc
150661 tgtgaaattt tgcttgatat cttttaaggt attgtcttta acgctcgatt tattgtcttt
150721 attgagatta agatcctttg gatttaaatt taaactcaaa ttttgagaat cccttatatt
150781 ttctttattg gaagttttgt taaaatttgt attttcattc ttaacttgtt ctttaggatt
150841 ttcacttaga ttattatttt tatccaaggc ttgatttaaa tcttttgtat ttttaatatt
150901 gtttgtttct tgatttaaac cctctttgtt attaagaggc ttttcttgca ctgatgcctt
150961 attatcttgc aagattgtct tttcttgtgt cgatactttc tcttgtggca ttgtcttttc
151021 ttgtgtagat accttctctt gcattggcaa tttatcttgc gtggtattat ttttactagg
151081 tgtcaaatct tgcgcattag ctttatttgt attttttaat tctttatcat cttttttttaa
151141 aggttcattt aaaagtgtat ttttatcatt tttaagttct ttggtgtgct ctttgttttg
151201 cacctcttga tttggaagtt ttttatcatt attggttaaa ttttcttgat caagttcttt
151261 taaagactcg ctatggttta aattttaga agtattttttt aattctgttg tttcgccatc
151321 tgtttgaatt ttaacattat catttttgat ttcggctttg tcttttttttt catttttact
151381 tgaaatgata gcatctaaat tttttaaagt ttgagaaagc aaagaagtat catctttgga
151441 ttttatattt ttagaatttt gattaagatt agcgtgcgaa gtgtgattta aattttgtac
151501 attttgattt aaatttttac tgatatgggc aagtttattg tttataatct ctttaaaaac
151561 attatccact gcacccttaa agaaattagc cttatctaaa tttggaaaag tagccttaag
151621 atctagcaaa cgatctactt tgatattttt tacattcaaa cccaaatctt tagctatatt
151681 caaaagctcg ctaagattgc ttgcactttt tagggcatta aaattagctt ctgtttttac
151741 aagttgagaa agctgggtgg aaagattggc aagtttgata tcggctgtat ctgcttttaa
151801 cttatcaagc aaagataaaa tttgcataaa agaggcggat tcaaaaatac ttattttatc
151861 actctcgcta aaaaccccat tttgtaattt atccatagct tcattaacaa cttctttttg
151921 actgattttc atatgatcag gcaaaaactc atttgtctct tcgatagaat tgagtaaaga
151981 atttaaaaaa ctctctgtat catcttgtgt tgtattgtca gatttttttta aatcttgctc

152041 tttagtttta ctcgatgaac tatgggttgt atttgtctta cttggagtaa gatttaagat
152101 gtcattactt ggggtcaaat ttgacatgat tgttccttaa ttattttaaa aattgacttg
152161 acgctaaatg aggaaatttt tctttcaaat aagcattctc atatactttg atcatcttat
152221 cttcatttaa agcaactaaa atatccaaaa gctcttctaa attttgagca ttggtaattt
152281 tttcaagatt ttcttcattt tccaaaagct ctgtaagctc gcttgatatt cttgccattt
152341 tttcattttg attagatttt aaagtttcta aaagatctaa aacttgaagc aatacttcac
152401 tttttctgtt gattaattga gtatcaagct ctataaaaaa ctctgtattt aatggttttg
152461 acattttaac ttccttttta aaaatgatat ctaagcaaat tagaagcaag aagcgttcca
152521 acttttgctt tttgaaaaat taagcaaaag ttctatataa ttttggcttt gaatttaaat
152581 ttggagagta tcttggaaaa tattagaaat atcgcagtta tagcccacgt tgaccatggt
152641 aaaactacta tggtagatga actactgaaa caatcaggta cttttagtga aagagaacaa
152701 atttcagaac gcgttatgga tagtaacgat atagaaaaag aacgcggtat caccatactt
152761 tcaaaaaaca cagctatcaa ttataaaggc acaaaaatca acatcataga cactccaggc
152821 cacgccgatt ttggtggaga agtagagcgt gttttaaaaa tgatcgatgg ggttttactc
152881 ttagtcgatg cacaagaagg cgttatgcca caaactaaat tcgtagttaa aaaagcccta
152941 tctttaggac ttaaacctat agttgtaatc aacaaaatcg acaaaccagc tgctgatcct
153001 gaaagagtaa tcaacgaaat tttcgacctt tttgtagcct tagatgcaaa cgatgagcaa
153061 cttgactttg ctatcgttta tgctgcagca aaaaatggtt atgctaagct tgatctaaac
153121 gatgagagcg acaatatgga acctcttttt aaaaccatac tcgaacgcgt tccagcacca
153181 agcggtacaa atgaaaatcc tttacaactt caagtcttca ctttaggata tgataacttc
153241 gtaggtaaaa taggaattgc tagaattttc aacggcgttg tgaagaaaaa tcaaagcgta
153301 atgcttgcaa aagccgatgg cactaaagta aatggtagaa tttcaaaact catcggtttt
153361 atgggtttag aaaaaatgga tatagaagaa gcaggcagtg gcgatatcgt tgcgatcgca
153421 ggttttgaag ctttagatgt gggcgatagc gttgtagatc caaacaatcc tatgccactt
153481 gatcctttac atatcgaaga accaacctta agcatagtat tttcagtaaa tgatggtccc
153541 ttagcaggaa ccgaaggaaa gcatgtaact tcaaacaaaa tcgctgaacg cttagaagct
153601 gaaatgaaaa ccaatatcgc gatgaaatac gaaagcacag gcgaaggtaa atttaaagta
153661 agcggtaggg gcgaacttca aatcaccatt ttagctgaaa atatgcgtcg tgagggcttt
153721 gagttttgca tgggaagacc tgaagttatc gtaaaagttg aagatggggt taagacagaa
153781 ccatttgaac atttagttat cgatgtgcct gaagaattta gtggtgcagt gatagaaaag
153841 cttggaaaaa gaaaagctga aatgaaaact atggcaccta caggcgatgg acaaacaaga
153901 cttgaatttg aaattcctgc acgcggactt ataggcttta gatctcaatt tttaaccgat
153961 accaaaggcg aaggcgtaat gaaccatagc tttttagaat tccgcccttt cagtggtgca
154021 gttgaaaagc gtaacaatgg tgctttgatc tctatggaaa atggcgtggc tttggggtat
154081 tctttgttta accttcaaga acgcggcgtg cttttcatag aacctcaaac caaagtatat
154141 acagggatga ttataggtga gcactctcgt ccaaacgact tagatgttaa tcctatcaaa
154201 ggaaaaaatc ttaccaatgt aagagcaagt ggaagcgatg atgcgatcaa gcttgtaccg
154261 cctagaaaat taagccttga aagagcttta gagtggatag aagaagatga acttgtagaa
154321 gttacacctc aaaatgtgcg tgttcgcaaa cgctatcttg atccaactca aagaaaaaga
154381 atggaaaaag caaaatctta atggacttaa aaagcttaga aaatagaaga ctttatatac
154441 ttaaacgctt aggaatttta aaattcttaa gcattataga agctttgctt gtaggctttt
154501 tagcctttgt ttttacaaaa gatattttaa tagccatcat ccttgctgtg tttgtaggca
154561 ttttttttctt tcgttttaca gctaaaaaac tcaaacttgc aaaaaaagaa ttagaatttg
154621 atgctttgaa tttatttttg cgtcgttttg gagctaaatt tagaaaagaa agtttaagcc
154681 aaaaagattt tttaaaacta gaacttagcg acaatttaaa ggattttaaa agccaaaatt
154741 gctttgaatt taaagaattt aaaatttatg atatccattt catcgatgaa aacaaacgct
154801 tttttttgtgg aattttgctt gaaattttaa agcctagcaa aaatcctagt tttgaagatg
154861 aagagaaaat ttatgttaaa ttacaagata aaaatttcac tctaaatcat atcttttcta
154921 aggacaatca ctatcttatc gctactctaa aaaacccttt tttcatagac ttaaaagaaa
154981 gtctagaaaa aaatttcaaa aatttagaaa acaatctcaa gctaatagaa gaaaaaatca
155041 tcaaaattta atcctcatct ttagccaaat tctccaagct tttaagtcct tcttcgccta
155101 tcaaatttcc gccttgcaag tcgctaaata tcgcatttaa agaactcact atggcagtag
155161 aattaaagct taaattttct atggctcttt ctcttttttt ccatatattt tgcaaggctc
155221 ttttttctgc ttctagctct tctttcatat tttgataagt tttgagtatg aaggtgattt

155281 gagtgttaaa ttctttagaa tttaggtatt cgtataagat atgatttttc tcatctttgt
155341 tttgcaaagc atttttgagc ttgtaagcat tgactatgct ttctctaagc accgccaaaa
155401 cacctttaaa ttcagcaaaa gtgcagatta aaatcccctc tttaaaatgc gtttttttgct
155461 tttctttagg catggttttg gtgattaaaa ccgctatatc actcttagca gctatgctgt
155521 caagcttaag cttgtcgatc cattctttat taaactcctt agtgcgtttg ctctcataca
155581 aaatactacc gcaaatattg ccaaagctgt cttttacgat atgcaaacaa tccgccccat
155641 ttacaccctt tggcacttct tttacctcat cgccgatgta ttcgttttgg atgtattctt
155701 ctatgagtag ttctgctgct tctccttgga gttgctgtga gccttgctct atcctgcgtt
155761 gtgcgtcctc tagcttttgg gctacgcttt taaaatttgt ttctaattct ttgtatttta
155821 actcctgctc ttgagtgctt ttttgaaatt caagacgcat cttatctctt tcaaattcca
155881 aatttttact ttcttgctct ttaaattcct taaaagcttc ttctttggct tgaaatttca
155941 gtctttcttc gttttctttt tgctctcttt ttagtctttc attttcagct tttacactta
156001 agaattcggt gagttctttg ctcttttttt ctaattcttc ttgcatgatt ttcatctcat
156061 tttcatgctc tttgttaaat ttctcactga attctttttg gaaattttgc ctttgtatta
156121 gcaattcttg ctcgaatttt tgtttttcta aaagaagttt ttggcttact tgctcgttta
156181 tgagtttttc ttgctctagc ttttgggctt ttaagtcgtt taaggccttg atgtactcgg
156241 ctcttttggc atttacctca tcttcaaatt cttttttttg cttgagcatt tcttgcttgg
156301 ctttgtctaa aatttgagca tataaagcag tgctgatatc gataaaactt ccacaacttg
156361 ggcatttgat ttgctcgttt tgattaaaat ttggcataaa gacttccttg agaatttttc
156421 tataaattta acatagaaaa acaaagatta ttctaagatt gacttgttaa aatccttgtt
156481 ttaaaaataa ttaggattac aaatgcaaaa acacaccaaa attcacggct tttcaaagct
156541 caaactcatc atcatccttg ctttgatgtc aagcatagca ccactttcta cagatatgta
156601 tttgcctgcg cttagccatg tagaacaaag ctttcatacc tctaaagcac tgactcaact
156661 tagtatcgcg agctttttc ttgcttttgc cttagggcaa ctcatatatg ggcctttaag
156721 cgatatcttt gggcgtaaaa ttcccgcttt ggtaggaatt ttcttcttta tcgtttcaag
156781 cttgttttgt gtgatcatcg acaatatcta tgcctttatc gctttgagat tttttgaagc
156841 actaggaggc tgtgcaggag tagtgatcgc aagggctata gtaaatgact tgtttgagat
156901 caaagaggct gcggggatct ttgctttgat gatggtattt agctcccttg ctcctatgct
156961 ttcgccgact tttggaagct ttttgctgga gtatttttca tggcatagca tttttacgac
157021 tttgtttgct ttgggaattt tgcttttttt gatgatactt tttggcttaa aagaatccgc
157081 accgcacctt aaaaataaaa aattctccca cgaagaagcg atgaaaagct ataaatttgt
157141 tttaaaagat aaacgctttt taacctatgt cttttgtgct gctttggctt tggctgcgat
157201 gtttgcttat atcacgggtt cgagttttgt atttacacaa ttttttggct taagcgagca
157261 acaatttggc atacttttg gagtaaatgc cctaggcttt gtcatctgtg caaatatcaa
157321 tgcaaggcta gtgcgtaaat ttgaaagtga aaaaatactt tcaaaagcct tgataatcat
157381 gtttgtatca actttgatct tgcttatcaa tgcctttttg tatccgaatt tatttctttt
157441 tgaagcaagc atttttacaa gtatcgctat gcttggcttt atcgcaccca ataccacgac
157501 tttggctatg gcgagattta aagaccatag cggaaccgct tctgcggtgc ttggctttat
157561 acaatttgcc ctagcaggac tcatctcctc tgtagtcagt gctttagatg ccaacacacc
157621 gataatcctt gcttgcgtga tgtgtgcttg tgtcttgata gcaaatggag tgtatttcca
157681 agctaagcgt aaggcttagc ttggcactat ggaatttttt cgtgcggttt tttggagtga
157741 gaaaaggact aaagccaaaa gtatagtata aatcgtaatc aaaacatttt caaaaccatt
157801 ataactgtta tcgcctataa gatttacgat tccaaatatt attttcggct tataacatat
157861 cacccataaa accgcgatca agttaaaata cacaaaaaaa tcaaattttt ttatacaccc
157921 aaaccaatac aaacaaagca aattaaaaac accaactata cttaaacata ctccgtattg
157981 atttaaaact tttaacaaat tgatatcttg aatcacacgc gaagtaaaat acagtaaagc
158041 aaacgagcct atcaacacaa ccaaccatct taaatttaaa agtaagtctg tatgatgatc
158101 gcaaagcttg tgataaaagg aaagttgcca tttgtctatc ttgtccttaa gggttttatt
158161 ttttttgttt tttagctcta gctctttaca ataaagctcg tatttatgaa aatttgaagc
158221 gtctaagaga ttattatctt ttatcaaggt gcttttgaaa ttcctaaaag aatctctaaa
158281 atcatttgca attttatcta aaggtttttt ttctttagct ttattaaaat ctttgtattc
158341 ttgtttgatt ctctcttgta aatcatcaaa agtaaaattt aaatttgtat tgaccacatt
158401 tatgtttcct ttaaaaatag cttgggggaa attcggagtt ttatcaaatg ttactccata
158461 aaaacaagct gtcttttcaa attcacatgt ataaaaatta atataatccc taaaataaga

158521 actattaaaa aaagtgtttt cattaaaaac acccgaaaaa taaacagctt tataaaagtg
158581 gcaattttcc atagttaata gcttaaattt tttttgccat gtaaaaaaaa cttcttcaaa
158641 aaaattacaa tttacaaaag ctaaattttt atattctaaa tacataactt ctataaccgt
158701 tatatgtttt ggttcgcggt aatattcttc tcctatccta cacttaaaat cgcactgttt
158761 aaattgtaaa gaaaactttg taaataaccc tatatttact ttttttatag gcaatctttc
158821 ggacaattgt ctttttatag catctttcat atcctctcca acagaataat gtgcccgtgg
158881 tctaatatat attgtatcgc tatttacaaa aatatttttt tcttcaattt gcaattcttt
158941 tgataatatt tttctagata tcatctcata tgatattttc tttacctctg ttttcaataa
159001 gtcctcatta atatctttgc ttgcattttc ccaatatcct ataatacgct tataaatatt
159061 ttttttaagt cctttttttt cttcattctc taaaagtttt tccgataata cttcttttct
159121 gacttgtcga attaaggaaa ttctcacatc tttatttaaa gtttttttac acattaccta
159181 tcctctataa tttctatttc tttggttttt agtgttttgc tataaatttt catcacaata
159241 ccttaaattt ttcagcttgg cactatggaa ttttttcgtg cggttttttg gagtgagaac
159301 aacaccaata ccacaagtat aaaatacacc acagacaaac taaacaacaa aggataagta
159361 gtattagaat ctttatctaa aaagcttcca aatataggaa gcaataagct aggtttagcc
159421 aacaatataa aaatgcagac tgcataagaa atacttacaa aaagaaatct ttcaaattca
159481 tcatcaagaa ccaatataaa aacaaataaa catacaaatg acaatataat caataaatga
159541 ctaaatatcg acttaggtag tcctatataa aaaacaatta cctcaagtaa aagcaaaaat
159601 aaaaaccaca aacaattagc attagacaat atcacaactg caaataataa acccaaagta
159661 aaagtgaaac aaaatttaga tatatctaag tcctgaattt ctttctctat atttaaagat
159721 aagagattgt aagtagtttt attgtgttct ttaaaaatat aattttcatt agaatccact
159781 ttaacgatat tatattttat atcattttct atatgactgg gttgcttaaa tttgtcaaac
159841 acaaaagaaa acataccaaa aagcataatg actataagta agttatgaaa tgctttgagt
159901 aagtctgtat gatgatcgca aagcttacga taaaagaaaa gttgatatct gtctactata
159961 tctttaaaag ttgtagcttt tttattttt agttctagct ctttacaata aagttcacat
160021 ttatgaaaat ttgaagcatc taaaagatta ttatctttta tcaaagcatt tttaaaagtt
160081 ctgaaagaat ctctaaaatc atttgaaaat ttgtataaag gtttttttatc ttgttctttt
160141 ttatctttgt taaaatcttc atattcttga tcaatttgtt tttctaaatc atcaaataca
160201 aaatttgaag ttatatttaa agcattcaaa ttacctttta aaactacttg agaaaaattc
160261 ggagccttat aaaacttcac tccataaaaa caagctatat tatcaaattt acactcgtga
160321 aaatcagcat aattataaaa ttcagagttg ttaaaataaa cttcatcttc aaaatgagca
160381 tttgaaaaat ttaattttac ttgattatct ttatctttat atccaaattt agaatctaca
160441 aaataacacc tatttttaaa tttatttttc cacaaactta tattttttat ggtgcaattt
160501 tttataaaaa gatatccttt aaattcgtta tcttgcaaat ctaaggatat gattttttta
160561 tcattttgtt cattggagca atttataaaa accaaatgat ctaaaaattg gttttgatat
160621 atttctaagt cattataaaa cgcacactga ttaaaagtta atgtttttttc aaaaataata
160681 ttttctaatt ttattaacgc tttttcaaaa gtacaattgt aaaagtatag attggtttta
160741 acaatgcgat tattctctga ttgcttatca cttaagctaa tattacattc actaaaaata
160801 atattacact gggcaatagc attatactgg atattgttca tattatcaca taatgaagat
160861 aagtctagtg ttgccttgcg aatcaaatac catttttcat catcagaaaa taactcatct
160921 ccttgcctaa agtcttcttt tccttttatt tcttttattg tttcttttgt tttttcatca
160981 cttgggatag atatatcata ttcttttata ttatttggat tgttttttga taaaatatta
161041 aataactgtc ttctaatttc atactcactc ttaatttcat actcactctt atcattttga
161101 tgtgtttctt cacaacattt ctctagggta ttaagtattt tttcaaatgc tttcctcatc
161161 atttaatcct tcctattttt cagcttggca ctatggaatt ttttctaatt gtctttataa
161221 aagaaaatac aacaaaacca aaaacgattg tataaattcc acctattgtg ctaagcggat
161281 ctagcataga tcttttatct gtaaaaattc ccatagcagg gataagaatt ttcggtgaaa
161341 ccgctaggac tgaaagagtt accccataag atatgattat aaaaaattct cttatatatt
161401 ttagacataa agcaaatcca actaaaccca aataaccaaa aagcattgca aaatttactt
161461 ttaccacaaa caaataattg tcctgtatga tagaatttat atgatgatta taaaaatcca
161521 atgaaaaata ccaatgctcc aagataggct tatatccaag acgatagtta aaactagcaa
161581 ccatcacaac gcttgatatc acaaaaatcc ctatcaccaa aataagtgaa tttaaacttt
161641 gtaaaatatc agtatgatga tcgcaaagct tgcgataaaa ggaaagttgc catttgtcta
161701 tcctgtcttt aagggtttta tctttttat tttttagttc tagctcttta caataaagtt

161761 catatttatg aaaatttgaa gcgtctaaaa gattgttatc ttttatcaaa gtgcttttga
161821 aattcctaaa agaatctcta aaatcatttg caaatttatc taaggatttt tcttgatata
161881 aatttggtat aacacctact tctttagcta ttctttgact tttgtaagaa ccatattctt
161941 gtttgatttt ttcttctaaa ttatcaaaag taaaatttaa atttgtatta actacattta
162001 aatttccttt aaagatagct tgggaaaaat tcggagtttt atcaaatttt actccataaa
162061 aacaagctat tttttcaaat tcacattcat gaaaatcagc ataattataa aattcagagt
162121 tgttaaaata agcattattt tcaaagtgag catttgaaaa atttaattct gctttattca
162181 ctttatcctt atatccaaat ttagaatcta caaaataaca cctatcttta aatttatttt
162241 tccacaaact tatattttct atgatgcaat tttgcataaa aaaatacttt ttaaactcac
162301 ttaagtttat atccaaatat tttattttag tttcttcttc aaatttgata ctttctgaaa
162361 aaatactatt atttaaactt agctctttaa attcgcaagc tctaaatact attggttttt
162421 tttgttcact ctcgtatcta catccatcta tctcaaaatt tttaaaacaa atattttta
162481 attctatcac cttaaaatcg caattgttaa aatataaatt ttgtccaatt tttatttttt
162541 ctttagaaaa ggaatcagtg aactttattg tatagccttc aaatttaatt tgataatcca
162601 tatcagccaa actagacatt ttttttaaat ctatttctaa atctaaaaga ttttttttctt
162661 tattgcttat gataaataaa ttcttatgct ttttcttatc atgagcaaaa ccgaaattgt
162721 ttatatttac ttcatttatt acttctattt tatctttgca acgatagcct agaatacgac
162781 ataactcttt tttaatattt tcaatttcat cttgtgataa agttaaaatt tcagaatcca
162841 ttttatttcc ttgtcttttt tgagctttgt actatagaat ttaatttgac ttttttttcat
162901 cttcaaatga tttatacacg ctatacaatc gcggataatt gctcttatta gcttctttaa
162961 tgctaaagta tatatcacaa tatgaaaaaa tcatctcatt attcaaaaaa tctcttattt
163021 cttcttctga tgatttattt attttttcca aatgagcata aaaatcgcta taacttttta
163081 tatatttata tttatcttgg attttttgta tctttttgga aaaattattt tgttttattg
163141 ttatttcttt gatttctaag ccctcatagt attgtttcaa tgcttcatca ccattacata
163201 ttatattaat aaatttttca tcatatttac aatcttttgc taattctaag caatattttt
163261 tcatttttag atttctttca tttttatcaa aatgaaatac cctatcaagt gctataaaat
163321 ttataaaacc caactcgtat acaaaatcat ctaattgttt ttcatcaaca ctattaaaaa
163381 aaatttcaag tttatttta ttggtgccat ttggatactg aatgctatct tctaataact
163441 ttctcaatgc aaataaacca ctataaaacc tatattcttt caattctttt tcaaaatctt
163501 cctttttatc attaggtata gaataaatca aaccattgtt agtataatct ttaattatac
163561 tattgatagt ttttaaaaat atacgcggca aaggtatacc ttgttgcttt tcttcattta
163621 aattatatga atccatattt tcatattcta atattatttt ttttgatatt tctataaaga
163681 aagctttata ctctttgttt tgttccaatc tatctgcaag ctgcaattct ttattgaaaa
163741 attcaagagc ttttattatt ttatttaaat ttcttagatt ttcaatttga tatatagaat
163801 ctggaattat ttctttagat atttctggat ttttttcttt taaaatttgc gatacaactt
163861 ctgttttgga acgtatgtgt atatttaaat ctatacactt ttctttatat gtatcaaaaa
163921 tttctttatc tttttcttta ttatttgata attcatcttt atttgatatc aaaataactt
163981 tacatttttc ctctttaagc tgattaatca aacctaaaat atctttaata tcaagcttat
164041 ccgattttct ttctaaatca tcaaggcata caattatatt tttgaaattt tttgttataa
164101 atagttgcaa aaatgttccc acagataact tccaaaaagt tatatttcta ataaaatata
164161 aaatcttgtt atgtttatag gcttttaggg ttatttcttc taaaacttct ttataatgtt
164221 cttttccaaa taaagaaaca taaacaatat tttttacttg tttatttgga tttttaatat
164281 aattacaaag attaaaatac tctgtaatac ttataagggt aattccgaca cattgtttta
164341 tgatggaaaa tagtattatt aaatggttaa gaaaatttat attagactta aactttttcc
164401 acaaattttc cttatgtatt atttttggat taatttcatt ttcaatttct ttccataaat
164461 aagtttttcc aactccccaa gagccactta tcaataaact ataattttcc tcatcttcca
164521 aaaaattttt aatctgttgt ttatattcca ttatttactt tcaatgattt ttatctcatc
164581 ttcagtgaga ttgtagagtt tataaaccaa agaattgatt ttattttcat tatctttagc
164641 ttttagtgtt ttgctataaa ttttcatcac aataccttaa aattttcagc ttggcactat
164701 ggaatttttt ctaattgtct ttataaaaga aaatacaaca aaaccaaaaa cgattgtata
164761 aattccaccc attgtgctaa gcggatctag catagctctt ttatctgtaa aaaatcccat
164821 agcagggata agaattttcg gtgaaaccac taggactaaa agagttatcg cataagatat
164881 gattataaaa aattctctta tatattttag acataaagca aatccaacta aaatcaaata
164941 accaaaaagc attgcaaaat ttacttttac cacaaacaaa taattgtcct gtatgataga

```
   165001 atttatatga tgattataaa aatccaatga aaaataccaa tgctccaaga taggcttata
   165061 tccaagacga tagttaaaaa tagcaaccat cgcaacgctt gatagcacaa aaatccctat
   165121 caccaaaata agtgaattta aactttgtaa aatatcagta tgatgatcac aaagcttgtg
   165181 ataaaaggaa agttgccatc gttcgatttt ttctcttaag cttttctttt ctttatactt
   165241 tagctccaac tccctagcat aaagctcttg tgttcttagc tctgaagcgt cgattagatt
   165301 gtgatgagct atgcggttgc tttttaattt tctaaataaa tctcttaaat ttagcaaggc
   165361 acgaaattta tcatttttat cttgtatatt atttatctta ttttctatat ctttatcact
   165421 atatttgata tattgatgtt ctatgttaaa ttggttagaa aaagtgcagt ttataaattg
   165481 cggtttggta ttgagctttg tattgctaaa ggttacaaca ccattaaaca aaacattatc
   165541 aaatctcaat ctttcaaatt ccattccttc aaagcttaat gtatccttaa atattgcgtt
   165601 ttggaataca taagattctg cataagattc tacatttata tcatcattat gaataaaatt
   165661 tattttatta aattctacat tatgaaatat acaattattt ggatttatat taaaaaagag
   165721 tgcctcatct ttaaatatcg aaaacaaaaa gctaattctt gctttaagca caagactatc
   165781 aaaagccacc tttcccttaa agatagtttc cctaaattca ttttctattg tttcattatt
   165841 actttgctct gctagaaact gagtttctat aaaccttacc tcttctttaa actgtgaaag
   165901 tccaaaacta acccttgctt taaatgtagc gttagaaaaa aatgctcttc cttcaaaaat
   165961 aacttcttca aaaatatttt ctattgtttc attattactt tgctctgcta gaaactgagt
   166021 ttctataaac cttacctctt ctttaaactg tgaaagtcca aaactaaccc ttgctttaaa
   166081 tgtagcatga ttaaagattg tctttcctat aaaaacaact cctaaaaatt gattttcgat
   166141 tatatcactg tcgttttgat ttgcttgaaa tttggtgttt gtaaaaattg tttcattttt
   166201 aaactgtgaa agtgcaaaac taactcttgc tttaaatgta gcgttagaaa aagatgctct
   166261 tccttcaaaa ataacttctt caaaaatatt ttctattatt tcattattac tttgctctgc
   166321 tagaaactga gtttctataa accttatctc ttctttaaac tgtgaaattc taaaaccaac
   166381 ccttgcttta aatgtagcgt tagaaaaaga tgctcttcct tcaaaaataa cttccctaaa
   166441 atcattttct attattttgc tattattttg ctctgcttgg aactgagttt ctataaatct
   166501 tacctcttct ttaaactgtg aaagtccaaa actaaccctt gctttaaata tagcatgatt
   166561 aaagattgtc tttcctataa aaacaactcc taaaaattga ttttcgatta tatcactgtc
   166621 gttttgattt gcttgaaatt cagtttttat aaagtttgct tcacttttaa actgtgaaag
   166681 tccaaaacta acccttgctt taaatatagc atgattaaag attgtctttc ctataaaaac
   166741 aactcctaaa aattgatttt cgattatatc actgtcgttt tgatttgctt gaaatt
//
LOCUS       DMACINML_5            165586 bp    DNA     linear       16-DEC-2020
DEFINITION  Campylobacter species strain strain.
ACCESSION
VERSION
KEYWORDS    .
SOURCE      Campylobacter species
  ORGANISM  Campylobacter species
            Unclassified.
COMMENT     Annotated using prokka 1.13 from
            https://github.com/tseemann/prokka.
FEATURES             Location/Qualifiers
     source          1..165586
                     /organism="Campylobacter species"
                     /mol_type="genomic DNA"
                     /strain="strain"
     gene            complement(1487..1996)
                     /locus_tag="DMACINML_00905"
     CDS             complement(1487..1996)
                     /locus_tag="DMACINML_00905"
                     /inference="ab initio prediction:Prodigal:2.6"
                     /inference="similar to AA sequence:RefSeq:CAL34420.1"
                     /codon_start=1
                     /transl_table=11
```

```
                     /product="putative integral membrane protein"
                     /protein_id="Prokka:DMACINML_00905"
                     /translation="MQFTIFHILAFVILIFCFILICILIFLKVKQKEFALISYTIATI
                     FTALLIYSVFLTINQFTIQASLSKLTFSRDLRHESIIITGKVQNLTKFNIRKCYLMLS
                     ILNKKQVGGEIFDDKNVRNAKMQNTSVSYTIEIIDTLPGNTYKTFRAEAPFPPSFENP
                     EFYHTLKCI"
     gene            complement(1996..2526)
                     /locus_tag="DMACINML_00906"
     CDS             complement(1996..2526)
                     /locus_tag="DMACINML_00906"
                     /inference="ab initio prediction:Prodigal:2.6"
                     /inference="similar to AA sequence:RefSeq:CAL34421.1"
                     /codon_start=1
                     /transl_table=11
                     /product="putative integral membrane protein"
                     /protein_id="Prokka:DMACINML_00906"
                     /translation="MERLKEIVHFYTTHLYLVDYMLILFVFFLFTCVLLLCIFLRHKP
                     VAALLIIALNIIICFLVYTYGYKLIDSQARSRKTTIVEQKFIQSSGALIVDFNITNTS
                     KNDFKQCKIIAKIFKDKLPQDNLINTYKNKFIPFRQKSREIFNLKQNTTQFQRIAFDN
                     FNYDNNYTIRLNSECF"
     gene            complement(2537..3619)
                     /locus_tag="DMACINML_00907"
     CDS             complement(2537..3619)
                     /locus_tag="DMACINML_00907"
                     /inference="ab initio prediction:Prodigal:2.6"
                     /inference="similar to AA sequence:RefSeq:CAL34422.1"
                     /codon_start=1
                     /transl_table=11
                     /product="putative transmembrane protein"
                     /protein_id="Prokka:DMACINML_00907"
                     /translation="MPADLNDYFNKKNGNSNNNNNRQNFNFKAPEFNFKGFGKFSPFV
                     YGAIIIVLFLIIAKPFMVINSGEMGIKSTTGKYDPNPLEPGLHFFLPFIQKITIIDTR
                     VRQINYASIEGSNENLSSGSGVINKNSISVLDSRGLPVSIDVTVQYRLNPLQVPQTIA
                     TWSLNWENKIIDPVVRDVVRSVVGKYTAEELPTNRNAIATQIEEGIRKTIEAQPNEPV
                     ELRAVQLREIILPLKVKEQIERVQIAKQEAERTKYEVERANQEALKKAALAEGEANAT
                     IISAKGKAMAVKIEADAQAYSNKEIANSLNTPLLNLKQIETQKEFNEALKVNQDAKIF
                     LTPGGAVPNIWVDTKDAKKQSSANMN"
     gene            complement(3635..4549)
                     /gene="ilvE"
                     /locus_tag="DMACINML_00908"
     CDS             complement(3635..4549)
                     /gene="ilvE"
                     /locus_tag="DMACINML_00908"
                     /EC_number="2.6.1.42"
                     /inference="ab initio prediction:Prodigal:2.6"
                     /inference="similar to AA sequence:RefSeq:CAL34423.1"
                     /codon_start=1
                     /transl_table=11
                     /product="branched-chain amino acid aminotransferase"
                     /protein_id="Prokka:DMACINML_00908"
                     /translation="MISADKIWMDGKLVDFKDATLHFLTHSLHYGNAVFEGTRAYKTD
                     KGLAIFRLEDHTKRLLESAKITLLNCPFSQKELEKAQIELLKVNDFKNNVYIRPVIFL
                     GDGVMGLYHVKAPVRVGIAAWEWGAYLGEEGLEKGIKVKISSFVRNSVKSCMGKAKAS
                     ANYLNSQIAKFEAIEAGYEEALMLDEEGFIAEGTGECFFIVKDGVLITPPNDFSLKSI
```

```
                     TQDTVLKIAHDLGLTVLRQRISRDEVYTADEAFFTGTAAEITPINNVDARIIGSGIRG
                     PITKKLQDAYFDVVYGRNEKYASMLTYI"
     gene            4893..5099
                     /locus_tag="DMACINML_00909"
     CDS             4893..5099
                     /locus_tag="DMACINML_00909"
                     /EC_number="5.3.2.-"
                     /inference="ab initio prediction:Prodigal:2.6"
                     /inference="similar to AA sequence:RefSeq:CAL34424.1"
                     /codon_start=1
                     /transl_table=11
                     /product="putative tautomerase family protein"
                     /protein_id="Prokka:DMACINML_00909"
                     /translation="MPIVNIKLAKPALSKEQKAELIADITELLSTKYNKSKERVVVML
                     EDIESYDIGFGGESVEAIKAKASK"
     gene            5096..5554
                     /locus_tag="DMACINML_00910"
     CDS             5096..5554
                     /locus_tag="DMACINML_00910"
                     /inference="ab initio prediction:Prodigal:2.6"
                     /inference="similar to AA sequence:RefSeq:CAL34425.1"
                     /codon_start=1
                     /transl_table=11
                     /product="bacterioferritin comigratory protein"
                     /protein_id="Prokka:DMACINML_00910"
                     /translation="MSILKEGDEAPQFELSNQDGVKIALKDFIGKKVILYFYPKDNTP
                     GCTTEACDFSANYDKFCDKNAVIIGVSPDGVASHDKFIAKFNLKHILLSDSEKEVAKA
                     YGAWGLKKNYGKEYEGIIRSTFVIDEAGKIAQIYSSVRVKDHALKVLESL"
     gene            5554..6645
                     /locus_tag="DMACINML_00911"
     CDS             5554..6645
                     /locus_tag="DMACINML_00911"
                     /inference="ab initio prediction:Prodigal:2.6"
                     /inference="similar to AA sequence:RefSeq:CAL34426.1"
                     /note="conserved hypothetical protein Cj0272"
                     /codon_start=1
                     /transl_table=11
                     /product="hypothetical protein"
                     /protein_id="Prokka:DMACINML_00911"
                     /translation="MLVHICCSVDSHYFIEELRKEYPKEKIIGYFYDPNIHPLSEYEL
                     RFLDVKRSCDKLGIKLYKGEYEYEKWLKAVKGYEDEPEKGARCEICFDLRMGSSVEFA
                     AKIGEKKLTTTLLTSPKKDLEQLKNALQKECEPYGVEFLAPDFRKNGGTQRQFALAKK
                     EMLYHQNYCGCIYGLKKQKQDKNFIDELMSPINAQILPASVEARIALYKKVNLLEKKG
                     IKFEIIRQKFLNYRLLNALIKLDKKAVKSHILFYSHFKNHYTRFSLDEKILNENLKDG
                     IYKSMKDEISFIEFWRFNSYFKNKWKNFEDFLKYPLKIEEEIKWRNKHFGAYDLSPVI
                     VLEKILPTRYEIVAKSEIYFDIKEVIKRM"
     gene            6759..7199
                     /gene="fabZ"
                     /locus_tag="DMACINML_00912"
     CDS             6759..7199
                     /gene="fabZ"
                     /locus_tag="DMACINML_00912"
                     /EC_number="4.2.1.-"
                     /inference="ab initio prediction:Prodigal:2.6"
```

```
                     /inference="similar to AA sequence:RefSeq:CAL34427.1"
                     /codon_start=1
                     /transl_table=11
                     /product="(3R)-hydroxymyristoyl-[acyl carrier protein]
                     dehydratase"
                     /protein_id="Prokka:DMACINML_00912"
                     /translation="MIDVMQIQEILPHRYPFLLVDKITELKVGEVVRGYKNISISDHV
                     FMGHFPGHPIYPGVLILEGMAQTGGVLAFESMDAKVDPQSKVVYFTGIDGAKFRNPVR
                     PGDRLDYEMQVVKNRGNMWIFKGQAFVDGNLVAEAELKAMIVDK"
gene                 7210..8001
                     /gene="lpxA"
                     /locus_tag="DMACINML_00913"
CDS                  7210..8001
                     /gene="lpxA"
                     /locus_tag="DMACINML_00913"
                     /EC_number="2.3.1.129"
                     /inference="ab initio prediction:Prodigal:2.6"
                     /inference="similar to AA sequence:RefSeq:CAL34428.1"
                     /codon_start=1
                     /transl_table=11
                     /product="acyl-[acyl-carrier-protein]--UDP-N-
                     acetylglucosamine O-acyltransferase"
                     /protein_id="Prokka:DMACINML_00913"
                     /translation="MKKIHPSAVIEDGAILGDDVVIEAYAYVSKEAKIGNGVVIKQGA
                     RILSDTTIGDHSRVFSYAIVGDIPQDISYKDEQKSGVIIGQNSTIREFATINSGTAKG
                     DGFTRIGDNAFIMAYCHIAHDCLLGDNIILANNATLAGHVELGDFTVVGGLTPIHQFV
                     KVGEGCMIAGASALSQDIVPFCLAEGNRASIRSLNLVGIRRRFDKDEVDRLSKAFKFL
                     FRQGDLKENAQKLLENNESENINKMCKFILETKRGIPVYRGKNNA"
gene                 7994..9217
                     /gene="clpX"
                     /locus_tag="DMACINML_00914"
CDS                  7994..9217
                     /gene="clpX"
                     /locus_tag="DMACINML_00914"
                     /inference="ab initio prediction:Prodigal:2.6"
                     /inference="similar to AA sequence:RefSeq:CAL34429.1"
                     /codon_start=1
                     /transl_table=11
                     /product="ATP-dependent Clp protease ATP-binding subunit"
                     /protein_id="Prokka:DMACINML_00914"
                     /translation="MPRKCSFCNEVESPQRRILANENDDAFICEYCVEGAYSIIYGEE
                     KEFKEPKHEHGVDFKDITPKELKAYLDRYVIGQDRAKKVFSVGVYNHYKRLFKSELQD
                     DDVELFKSNILLIGPTGSGKTLLAQTLAKFLDVPIAICDATSLTEAGYVGEDVENILT
                     RLLQAADGDVERAQKGIVFIDEIDKIARMSENRSITRDVSGEGVQQALLKIIEGSLVN
                     IPPKGGRKHPNQDFIQIDTSNILFVCGGAFDGLETILKRKLGDKVVGFFDNEKENSKA
                     LLEKIEPDDLVHFGLIPELIGRLHVITSLNELNEDDMVRILTEPKNAIVKQYQKLFAI
                     DGVNLKFEDDALREIAKLALERKTGARGLRSIIEEIMVDLMFELPEYKNYDIVITKEV
                     VRDGAKALLIKKKIS"
gene                 9230..10270
                     /gene="mreB"
                     /locus_tag="DMACINML_00915"
CDS                  9230..10270
                     /gene="mreB"
                     /locus_tag="DMACINML_00915"
```

```
                     /inference="ab initio prediction:Prodigal:2.6"
                     /inference="similar to AA sequence:RefSeq:CAL34430.1"
                     /codon_start=1
                     /transl_table=11
                     /product="of E. coli rod shape-determining protein"
                     /protein_id="Prokka:DMACINML_00915"
                     /translation="MILDQLIGFFSSDMGIDLGTANTLVLVKDKGIVINEPSVVAVER
                     EKYGSRAKILAVGKEAKDMVGKTPGNIEAIRPMKDGVIADFDMTEKMIRYFIEKTHRR
                     KSFLRPRIIISVPYGLTQVERKAVRESALSAGAREVFLIEEPMAAAIGANLPIQEPKG
                     NLVVDIGGGTTEIGVISLGGLVISKSIRTAGDKLDTSIVNYVKEKYNLIIGERTGEEI
                     KIAIGSAIQLPKELSMVVKGRDQVSGLLSRIELTSEDVREAMREYLKEIADALKMVLE
                     MMPPDLASDIVENGVVLTGGGALIRGLDKYLSEIVRLPVYIADEPLLAVAKGTGKALE
                     EISLLQQLTNEE"
     gene            10260..11009
                     /gene="mreC"
                     /locus_tag="DMACINML_00916"
     CDS             10260..11009
                     /gene="mreC"
                     /locus_tag="DMACINML_00916"
                     /inference="ab initio prediction:Prodigal:2.6"
                     /inference="similar to AA sequence:RefSeq:CAL34431.1"
                     /codon_start=1
                     /transl_table=11
                     /product="of E. coli rod shape-determining protein"
                     /protein_id="Prokka:DMACINML_00916"
                     /translation="MKNKLIFVLVLFFLVFISFYYGGLVKKNILLVNDAVLEKFYGVK
                     DFIAESFSKHFNQVEQIEQLKARNQELEYAAVLTTSFANQLNRILEDKNSTRYLPQVS
                     LVRAISYVRLNDYKRLWLDFAKVSPNKNKGLIHQGYTIGIAINKDGRAMALLQGDEQC
                     VFSVYVGKNKIPGLIQGEDDKLKVKFIPKWAKINIGDEILTSGLDNIFFSDVPVGIVS
                     AIEDGDVYQSAEVKPYANINIPAYLYIVDSL"
     gene            complement(11004..11927)
                     /locus_tag="DMACINML_00917"
     CDS             complement(11004..11927)
                     /locus_tag="DMACINML_00917"
                     /inference="ab initio prediction:Prodigal:2.6"
                     /codon_start=1
                     /transl_table=11
                     /product="hypothetical protein"
                     /protein_id="Prokka:DMACINML_00917"
                     /translation="MLNSKDRTQVILEKSGLSLSKFATILGKDRRTLAKFIENDTVKE
                     LDTKSKEKICEFFRYPFKIWDSSENEFYTLLNQIENDEIRIIDEGYIGGLKYIFENEN
                     EGSLILHPAFPNPAYRDFIVPLVYQNSDSEEARIYRMRRGEKMRAHSFNASEWYSIKS
                     LLEFCFSPIGNFYTKEQKIQILELMINTFKDNLNKSLYFFDSYDKKIYGLDMFYLSIN
                     AKENTMFFKAPLEMLLVEIKNSTLVHKIHEHYTHAKKCPAHILAQDACSIMEILLQCL
                     KENLNIKESCEILDKKSPYGNLFKKSLSIDL"
     gene            12025..14082
                     /gene="acs_1"
                     /locus_tag="DMACINML_00918"
     CDS             12025..14082
                     /gene="acs_1"
                     /locus_tag="DMACINML_00918"
                     /EC_number="6.2.1.1"
                     /inference="ab initio prediction:Prodigal:2.6"
                     /inference="similar to AA sequence:RefSeq:CAL35637.1"
```

```
                     /codon_start=1
                     /transl_table=11
                     /product="acetyl-coenzyme A synthetase"
                     /protein_id="Prokka:DMACINML_00918"
                     /translation="MHTKIKILFKKYTKIIFLATKSHNQTKKLAFYFSNKNSIMQIQL
                     IKKGWIMGIVYEQTYRESLQNPEAFWAEAAKKVHWYNEWDRVLDDSDGHYRWFVGGCM
                     NTCYNALDLHVHNGRGDQLALIYDSPVTDTKKKYTYKQLRDRVAKVAGILANKGVVKG
                     DRVVIYMPMIPEALIAMLACARLGAIHSVVFGGFAAHELATRIEDAKPRMIISASCGI
                     EVSSIIEYKPILDEAIKKCTHKPTTCLIWQRPQYRANMLPWRDIDWELEEEKTRGVDP
                     VPVLATDPLYILYTSGTTGSPKGVIRSNGGHSVAMKWSMDNIYNAKAGDVFFTASDVG
                     WVVGHSYIVYAPLMNGCTTIVYEGKPVRTPNPSAFWRIIEEYKVNVLFSAPTAFRAIK
                     KEDPKGEWIKKFNLDSLRSIFVAGERCDSDTLKWIEKLTKKPVIDNWWQTETGWAIAA
                     NPLGLEAQVVKAGSPTKPMPGFNLKVLDEKGQELGAGKKGILCLKLPLPPACLMGIWE
                     NDERYRRGYLDQFPGYYLTGDTGYIDKDGYVYVLGRMDGIINVAGHRLSTGEMEEIIA
                     KHPDVAECAVIGVNDELKGEIPMGFIVLKEGIERDHRGIVEGVVALVRQEIGAVASFK
                     IATVVSALPKTRSGKILRKNLREIADGSTLNVPATIEDSNVLKACEKAINDLGYPKNK
                     GEK"
     gene            14082..14957
                     /gene="aepX"
                     /locus_tag="DMACINML_00919"
     CDS             14082..14957
                     /gene="aepX"
                     /locus_tag="DMACINML_00919"
                     /inference="ab initio prediction:Prodigal:2.6"
                     /inference="similar to AA sequence:RefSeq:ASN93130.1"
                     /codon_start=1
                     /transl_table=11
                     /product="phosphoenolpyruvate mutase"
                     /protein_id="Prokka:DMACINML_00919"
                     /translation="MSAGKKFKELVKTSKPLTIVGAVNAYSALQATKIGHKALYLSGS
                     GVASASYGLPDLGIIALEEVCIDVRRICSRVDTPLLVDADTGFGGAFNIARTIKELIR
                     AGAAATHIEDQVAQKRCGHRPNKELVSTEEMCDRIKAAVDAKIDPDFVIMARTDAHAI
                     EGQQKAIERALAYVEAGAEMVFAEAITSLEEYEEFVKNVKVPILANITEFGKTPYFTK
                     EELNRAGVSMVLYPLSANRAMNKAAVQVYESILKNGHQKDVLEIMQTREELYEMLDYY
                     SYEQKLDELFKRGAK"
     gene            14954..16081
                     /locus_tag="DMACINML_00920"
     CDS             14954..16081
                     /locus_tag="DMACINML_00920"
                     /inference="ab initio prediction:Prodigal:2.6"
                     /inference="similar to AA sequence:RefSeq:ADN91805.1"
                     /codon_start=1
                     /transl_table=11
                     /product="Citrate synthase"
                     /protein_id="Prokka:DMACINML_00920"
                     /translation="MSISEATKKTGGLAGVIAGQSAICTCGLGNGLNYYGYSIEELAQ
                     NAEFEEIAYLLQFGELPNAKELENYKEKIIAQRALSENLKSVLKAIPKEVHPMNLMQA
                     AVAALGALEGENEDFSDQDEKIIRLLGILPSVLCYWHHYVNFGKEIDFDSKQTSIAGY
                     FLEKLKLEAPKEDFIKAMQCSLILYAEHEFNASTFTARICASTKSDIFSAVAAAIGAL
                     RGPLHGGANEAAMHLIESFKSVEDAIKGVNEKLENKELLMGFGHRVYGLGGDPRNALI
                     KVWSKHLGGDDLLFKISEAIEALMKEKRPSLPPNADFYSASAYHFMGIPTEYFTPIFI
                     MSRVSGWCAHIKEQRANNKLIRPSSEYIGVEPRKFVKIQDR"
     gene            16091..17545
                     /gene="prpD"
```

```
                     /locus_tag="DMACINML_00921"
     CDS             16091..17545
                     /gene="prpD"
                     /locus_tag="DMACINML_00921"
                     /EC_number="4.2.1.79"
                     /inference="ab initio prediction:Prodigal:2.6"
                     /inference="similar to AA sequence:UniProtKB:P77243"
                     /codon_start=1
                     /transl_table=11
                     /product="2-methylcitrate dehydratase"
                     /protein_id="Prokka:DMACINML_00921"
                     /translation="MSDMGILEAKRPEFDELLTKIAKYADEFEITSDLALETARYCLM
                     DTIGCGLLALKYPACTKLLGPSVEGAEFRPLGAKVFGTSYQLEPIRGAFNVGAMVRWL
                     DFNDTWLAAEWGHPSDNLGAIWAVADYVSRKNISEGKEPLKVKCVLKAMIKAHEIQGV
                     LALENCFNKVGMDHVLLVRIASTAVAAKLLGCNFEEIRNAISHAFIDGGALRTYRHAP
                     NTGSRKSWAAGDASSRGVDLALKAKTGEMGYPSALSAKFWGYEDVKMKGQKLTIPQEF
                     GSYVMENVLFKISFPAEFHAQTAVEAALALHDEVKDRLDQIEKIIITTQESGHRIINK
                     VGELANPADRDHCIQYMVAVPLIFGRLNADDYEDSVASDERIDALRAKMQVEVDDRYT
                     KEYLESDKRSIANAVQVFFKDGTSTKKVEVEYPIGHKRRRNEGIPLLIAKFKANLATR
                     LSPKQCEKIMQICEDQKSLEQMNFNEFSDLFWLG"
     gene            17708..18601
                     /locus_tag="DMACINML_00922"
     CDS             17708..18601
                     /locus_tag="DMACINML_00922"
                     /inference="ab initio prediction:Prodigal:2.6"
                     /inference="similar to AA sequence:RefSeq:CAL34988.1"
                     /codon_start=1
                     /transl_table=11
                     /product="putative integral membrane protein"
                     /protein_id="Prokka:DMACINML_00922"
                     /translation="MLKNLKFQAFFFAFLAAIFNAFVGIFSVFLFKKGFMSSEVAFYK
                     CFVASLILFAILIYMRKLGALLVFIKQKIFTLLLLAFFGFFMLYHFESAAYTSMSVAN
                     VVFVLFGVGMIITFICEALDAKRFFHLNELLAMVFALLGLWMIFLAEGGTLENFTHLK
                     DLVNAILAGLGYALFLFFTRKLKLGFGLIPLCALLFIGSLYLSIPLFGANLNLNFSLE
                     SLVLLLALAFLPTIGGFYCTTRALSLAKSNSVQLIELSEPLFAMLFGFLFLAQSVSFL
                     QIVGGVFILFAIFVHEFKLKL"
     gene            complement(18585..19544)
                     /locus_tag="DMACINML_00923"
     CDS             complement(18585..19544)
                     /locus_tag="DMACINML_00923"
                     /inference="ab initio prediction:Prodigal:2.6"
                     /inference="similar to AA sequence:RefSeq:AON64954.1"
                     /codon_start=1
                     /transl_table=11
                     /product="TPR repeat protein, Sel1 subfamily"
                     /protein_id="Prokka:DMACINML_00923"
                     /translation="MKKIKILVFLIFFCQFSVLANNLPQNIKENLGINQFLDENLKEI
                     LNQPLKENFALTPKGEYFFESDLSLFQKQCLENNATACAYLGGEFAADTRLSLDLNKA
                     LLLLQKACKLGRLDSCARINNIKFKLLKKLDEKTYQSNLKYFLDQNSSSALLEHIATL
                     SKKDTKSAIILAKKSCEQGHDAICAGLSYMYACGQGVEKDLIKAKELSSKICEKDTKL
                     CFAQGWVELFNKNDILAQKLFYKSCQAGYYADCNMAVDKNSKDQAMQLYLIKITQKNS
                     QNQCQNSSNKQSFVCHKASALSKEILKIQLKKISKKLLFTILI"
     sig_peptide     complement(19482..19544)
                     /locus_tag="DMACINML_00923"
```

```
                     /inference="ab initio prediction:SignalP:4.1"
                     /note="predicted cleavage at residue 21"
     gene            19651..22920
                     /gene="carB"
                     /locus_tag="DMACINML_00924"
     CDS             19651..22920
                     /gene="carB"
                     /locus_tag="DMACINML_00924"
                     /EC_number="6.3.5.5"
                     /inference="ab initio prediction:Prodigal:2.6"
                     /inference="similar to AA sequence:RefSeq:CAL34432.1"
                     /codon_start=1
                     /transl_table=11
                     /product="carbamoyl-phosphate synthase large chain"
                     /protein_id="Prokka:DMACINML_00924"
                     /translation="MPKRTDIKNILLIGSGPIVIGQACEFDYSGTQAAKTLKEQGYRV
                     VLINSNPATIMTDPEFADATYIEPVTKESILSIIKKEKIDAILPTMGGQVALNVAMEV
                     YESGLLGDVKFLGANPEAIKKGEDRQVFKECMKKIGMDLPKSMYAYNYDEALKAVDEI
                     GFPLMIRASYTLGGAGSGVVYNMDEFKELANAALALSPIHEILIEESLLGWKEYEMEV
                     IRDKNDNCIIVCSIENLDPMGVHTGDSITVAPALTLTDKEYQVMRNASFAILREIGVD
                     TGGSNVQFAVNPTNGRMIVIEMNPRVSRSSALASKATGYPIAKVATLLAIGFSLDEIK
                     NDITGTPASFEPVIDYIVTKIPRFTFEKFPGANTTLGTAMKSVGEVMAIGRTFKESIQ
                     KALCSLERNLSGFDRVKFEDKNDLIFKIRNANEKRLLYVAQAFREGFDVKELYELCKI
                     DPWFLSQIKEIVDFEEQIDMDILHNKTLLRKAKTMGFSDKMIALLVNLKDNLELSQND
                     IYYARMKQKIIAEFSEVDTCAGEFEALTPYLYSSINVSELTQSKNEARDKKEKKVMII
                     GGGPNRIGQGIEFDYACVHASFALKDLGVKTIMYNCNPETVSTDYDTSDILYFEPIDF
                     EHLRGVIEREKPDGVIVHFGGQTPLKFAKRLSAFGAKIIGTSARVIDMAEDRKKFAEF
                     ITKLGINQPKNSTATSVEEAVLKASEIGYPVLVRPSYVLGGRAMRVVNDESELRLYMQ
                     EAVDVSDKSPVLIDQYLDNATEIDVDAISDGKDVYVAGIMEHIEEAGIHSGDSACSLP
                     PCNIDEKMQEQIIQKTANIALNLGVVGLLNIQFAIYNNELYMIEVNPRASRTVPFVSK
                     ATGIPLAKVATRVMWQGNLKEALKFYDTFDVVNFDNTILRPKLSKHISVKEAVFPFAK
                     LSGSDLELGPEMRSTGEVMGISKDFANSYAKSQISSFNHLPENGVVFISLKEKDKKYA
                     KKLASEYSKLGFKLMATSGTCKEIVENGFECELVHKISEGRPNVEDKLKNGEIQLVIN
                     TSDSHTFAGDTKKIRENIIRFKIPYFTNLRSALAGAKSIKAIQSQSYLEVKSLQEYLK
                     A"
     gene            22917..23339
                     /locus_tag="DMACINML_00925"
     CDS             22917..23339
                     /locus_tag="DMACINML_00925"
                     /inference="ab initio prediction:Prodigal:2.6"
                     /inference="similar to AA sequence:RefSeq:CAL34433.1"
                     /note="hypothetical protein Cj0280"
                     /codon_start=1
                     /transl_table=11
                     /product="hypothetical protein"
                     /protein_id="Prokka:DMACINML_00925"
                     /translation="MIYLAQTDTTAGFLSKNLEEINALKGRAKDQPCLITSAKFSELK
                     KIVRVPNQFKNLVRRAKKTTFIYPNNQAIRIVKECKHAQFLKEKGCFYSSSANKHGQK
                     FDELWARSVADVVVDEIFFENTPSKIIKLHRKKLRKIR"
     gene            complement(23336..24322)
                     /gene="tal"
                     /locus_tag="DMACINML_00926"
     CDS             complement(23336..24322)
                     /gene="tal"
```

```
                     /locus_tag="DMACINML_00926"
                     /EC_number="2.2.1.2"
                     /inference="ab initio prediction:Prodigal:2.6"
                     /inference="similar to AA sequence:RefSeq:CAL34434.1"
                     /codon_start=1
                     /transl_table=11
                     /product="putative transaldolase"
                     /protein_id="Prokka:DMACINML_00926"
                     /translation="MKKFSLWCDFIENSFLDDDFLNLLSRGINGATSNPAIFKNAILN
                     SPIYKEKIAKLKGKKAKEIYEELAVADIQKAADKLAPLYHTGNDGFISLEIDPRFYDN
                     TSLSLGEAKKLYSAIGKDNVMIKVPATNASYEVMYELMKNGISVNATLIFSLEQSQKC
                     FEALNAGLVEFRKNNIALQGKNTRLRTPQAVISIFVSRFDRLLNSKAKEQNRIGILNA
                     NLAYNNIISKNEPNIRALFASTGVKGDDLPKDYYIKELLFENSINTAPLDAINAFKTE
                     FEFKKPLMNFEIYTQLNAIISQNEREKACDELLKDGLEQFCIAFEEILQALN"
     gene            complement(24324..24947)
                     /gene="serB"
                     /locus_tag="DMACINML_00927"
     CDS             complement(24324..24947)
                     /gene="serB"
                     /locus_tag="DMACINML_00927"
                     /EC_number="3.1.3.3"
                     /inference="ab initio prediction:Prodigal:2.6"
                     /inference="similar to AA sequence:RefSeq:CAL34435.1"
                     /codon_start=1
                     /transl_table=11
                     /product="putative phosphoserine phosphatase"
                     /protein_id="Prokka:DMACINML_00927"
                     /translation="MIKLCVFDFDSTLMDGETIDILAKAYGKEKEVVDITHRAMAGEL
                     DFFESLQERVSFLKGMPYDLVLKIGQDLPLMNGAYELIEFLNSKNIFVVIFSGGFHEG
                     IDPAMKKLKVNLGFANYLHHKNNTLSGLVGGEMMFSNSKGLMLQRLKNFLNLQTHEVM
                     CVGDGANDIAMFNESGLKVAFCAKEILRSKADICIDNKDLKEIIKVI"
     gene            complement(24947..25468)
                     /locus_tag="DMACINML_00928"
     CDS             complement(24947..25468)
                     /locus_tag="DMACINML_00928"
                     /inference="ab initio prediction:Prodigal:2.6"
                     /inference="similar to AA sequence:RefSeq:ADT65674.1"
                     /codon_start=1
                     /transl_table=11
                     /product="chemotaxis protein"
                     /protein_id="Prokka:DMACINML_00928"
                     /translation="MSNEKLEQILQKQQTQIAGPVGDQKEDDIIQLVGFVVGDEEYAI
                     PILNIQEIIKPIEYTRVPSVPDYVLGVFNMRGNVMPLIDLAQRFNLGSSKMTPQTRYI
                     VLRGESNGSGVGGNAGFVIDRLTEAIKIHRNRIDPPPETLVKDKGMIYGIGKRDENIL
                     TILKVEALLKREF"
     gene            complement(25473..27770)
                     /gene="cheA"
                     /locus_tag="DMACINML_00929"
     CDS             complement(25473..27770)
                     /gene="cheA"
                     /locus_tag="DMACINML_00929"
                     /EC_number="2.7.3.-"
                     /inference="ab initio prediction:Prodigal:2.6"
                     /inference="similar to AA sequence:RefSeq:CAL34437.1"
```

```
                     /codon_start=1
                     /transl_table=11
                     /product="chemotaxis histidine kinase"
                     /protein_id="Prokka:DMACINML_00929"
                     /translation="MEDMQEILEDFLVEAFELVEQIDHDLVELETNPEDLELLNRIFR
                     VAHTVKGSSSFLNFDVLTKLTHHMEDVLNKARHGELKITPDIMDVVLESIDRMKTLLN
                     SIRDNGNDTAIEMDIGPICARLTAISEGESPSSAKLNETPVETPQKEEPAPAPEPEVD
                     VNQLSDSEVEAEIERLLKARKAEDQARRAQKKQNAASPTNSKPAASTPKAENTEKKVP
                     ASGGGGGGMDQTIRVEVKRLDHLMNLIGELVLGKNRLLKIYDDVEERYEGEKFLEELN
                     QVVSQLSIITTDVQLAVMKTRMQPIAKVFNKFPRVVRDLSRELGKHIELEITGEETEL
                     DKSIVEEIGDPIMHMIRNSCDHGIEDPATRAANGKPEKGIVQLKAYNEGNHIVVEITD
                     DGKGLDANGLKAKAIEKNLITEREADQMTDKEAFALIFKPGFSTATKVTNVSGRGVGM
                     DVVKTNIEKLNGVIEIDSELGKGSSFKLKIPLTLAIIQSLLVGTQEEYYAIPLASVLE
                     TVRVPIDDIYTIEGKNVLRLRDEVLSLVRLSDVFGVKQVLESGDQTYVVVIGVAESKL
                     GIIVDTLIGQEEIVIKSMGDYLQNIQGIAGATIRGDGRVTLIIDVGAMMDMAKEIKVD
                     IKAQLESSAKKPKEKPSDYKVLIVDDSKMDRTLMQKALEPLGVSLIEATNGVEALNII
                     KSGDHDIDAILIDIEMPRMDGYTLAGEIRKYSKYRNLPLIAVTSRTSKTDRLRGVEVG
                     MTEYITKPYSPEYLENVVRKNLKLG"
     gene            complement(27774..28730)
                     /gene="cheV"
                     /locus_tag="DMACINML_00930"
     CDS             complement(27774..28730)
                     /gene="cheV"
                     /locus_tag="DMACINML_00930"
                     /EC_number="2.7.3.-"
                     /inference="ab initio prediction:Prodigal:2.6"
                     /inference="similar to AA sequence:RefSeq:CAL34438.1"
                     /codon_start=1
                     /transl_table=11
                     /product="chemotaxis protein"
                     /protein_id="Prokka:DMACINML_00930"
                     /translation="MFDENIVKTGSNEMELVDFRIFKQGHDKVYEGIYGVNVSKVREI
                     IKIPSLTELPGVPDYIEGIFDLRGVVIPVVNLAKWMQITEPESTMLKPRVIITEFSNI
                     LIGFIVHEAKRIRRINWKDIEPATFSTGSGALDKGKITGVTRIENDEVLLILDLESVV
                     EDLGIYSPKTDIDFSKIEKFSGSALILDDSMTARKRVKEMMQQMGFQVIEVKDGVEGL
                     NKLEELSQIYGENLSSILKIIVSDVEMPQMDGFHFAARIKEDARFKDIPIVFNSSLSN
                     EFMNEKGVHEAGGESYLVKFNASDFFNEIAKVIKKHQSQEQG"
     gene            complement(28723..29358)
                     /locus_tag="DMACINML_00931"
     CDS             complement(28723..29358)
                     /locus_tag="DMACINML_00931"
                     /inference="ab initio prediction:Prodigal:2.6"
                     /inference="similar to AA sequence:RefSeq:AAW34924.1"
                     /note="conserved hypothetical protein"
                     /codon_start=1
                     /transl_table=11
                     /product="hypothetical protein"
                     /protein_id="Prokka:DMACINML_00931"
                     /translation="MGDIFDLLIGEISATHEFAKPYIKLLEELALKMEIIYLEGNHDF
                     NLARFFQRVKIFSLQEQPLKLNLHTSKGKNTDFKNSFITLAHGDIFLSPCLQFTLKSL
                     RNHYLLVFLNFLNQISSNSISNKILKNQNKKNLFYKIENFKNLAKERYKKYGNLGFWV
                     CEGHYHQNLILDEKDIKYFNLASFAYERSFFVVECYQEIKFREQKLRGQNV"
     gene            complement(29491..29976)
                     /gene="greA"
```

```
                     /locus_tag="DMACINML_00932"
     CDS             complement(29491..29976)
                     /gene="greA"
                     /locus_tag="DMACINML_00932"
                     /inference="ab initio prediction:Prodigal:2.6"
                     /inference="similar to AA sequence:RefSeq:CAL34440.1"
                     /codon_start=1
                     /transl_table=11
                     /product="transcription elongation factor"
                     /protein_id="Prokka:DMACINML_00932"
                     /translation="MKKEPMSQFGYDKLSAELKDLKDNQRPAVVIEIDTARSHGDLKE
                     NAEYHAAREKQALIESRIAELSDLIARAQIIDPSSYEHDSVKFGSSVVIMDLDTEKES
                     KYTLVGICEGDLDKGYISIASPIAKAMLGKKEGDDFKVRLPKGESEFEIISISYEPLK
                     F"
     gene            complement(29993..31084)
                     /locus_tag="DMACINML_00933"
     CDS             complement(29993..31084)
                     /locus_tag="DMACINML_00933"
                     /EC_number="2.4.1.182"
                     /inference="ab initio prediction:Prodigal:2.6"
                     /inference="similar to AA sequence:RefSeq:AGQ95761.1"
                     /codon_start=1
                     /transl_table=11
                     /product="ipid-A-disaccharide synthase"
                     /protein_id="Prokka:DMACINML_00933"
                     /translation="MKNLLVCALEPSANLHLKEVLKAYKEEFGEFKIYGIYDENLCKE
                     FALNSKPLYSSHEFSAMGFIEILPLIFKAKRAIKELVNLTLSQTMDAVLCIDSPAFNI
                     PFAKALKKANSKTKRIYYILPQVWAWKKGRIPVIENHFDVLASILPFDEQFFSKSIYV
                     GHPLLDEIKDFKNENDIKILLNKNESEKTIAFLPGSRRSEIKRLMPVFRELSRKFEGE
                     KILCVPPFNLERLEIYGDIKDFKIQSNTPQVLKKADFAFICSGTATLEAALVGTPFVL
                     AYKAKAIDIFIARLFVKLKHIGLANIFCDFAGKEALNPEFLQEQVNVLNLYEAYNKYD
                     YKAFFDKVHFLKEYLKFGSAKNLAKILNQ"
     gene            31230..32105
                     /gene="zupT"
                     /locus_tag="DMACINML_00934"
     CDS             31230..32105
                     /gene="zupT"
                     /locus_tag="DMACINML_00934"
                     /inference="ab initio prediction:Prodigal:2.6"
                     /inference="similar to AA sequence:RefSeq:CAL34417.1"
                     /codon_start=1
                     /transl_table=11
                     /product="zinc transporter"
                     /protein_id="Prokka:DMACINML_00934"
                     /translation="MEFTLDQILVAMALTILAGLSTAIGSVIAFFSRNDNLRILSFGL
                     GFSAGVMIYISFMEILPSAFVDFKKYYHSGYGELLALLCFFGGIFLSAIIDKFIPEDV
                     NPHDPKEDLSELKICPLPQKNEKAPAFHAGEPLKKINVKALKRTGIFTALAIAIHNFP
                     EGFATFISSLDNLSFGVAIAVAVAIHNIPEGMAVSLPIYHATKDKKKAFIYSALSGIA
                     EPMGAIVGAFLILPIMNELTLAITFAVVAGIMVFISFDELLPAAKTYDKAHDSLYGLV
                     LGMAVMAVSLILLNP"
     gene            complement(32229..32801)
                     /gene="cdtC"
                     /locus_tag="DMACINML_00935"
     CDS             complement(32229..32801)
```

```
                     /gene="cdtC"
                     /locus_tag="DMACINML_00935"
                     /inference="ab initio prediction:Prodigal:2.6"
                     /inference="similar to AA sequence:RefSeq:CAL34250.1"
                     /codon_start=1
                     /transl_table=11
                     /product="cytolethal distending toxin C"
                     /protein_id="Prokka:DMACINML_00935"
                     /translation="MKKIFILFFALLSFLKAGPSLDELEDFTPMFAIRSLETGISLSP
                     FRKTSKRLEDQNWFLKEIVPSEELKAKDMHAKDLPFGYVQFVSPKGDDICLAVLSEKS
                     FGTKSCKQDLQDKKMQTIFSIIPMTNGSIQIRSLINGGNQCMSTFPDSSIAIENRFGL
                     GECLLDRSIVTELSKLFFFSPAIIEASVIY"
     sig_peptide     complement(32733..32801)
                     /gene="cdtC"
                     /locus_tag="DMACINML_00935"
                     /inference="ab initio prediction:SignalP:4.1"
                     /note="predicted cleavage at residue 23"
     gene            complement(32811..33614)
                     /gene="cdtB"
                     /locus_tag="DMACINML_00936"
     CDS             complement(32811..33614)
                     /gene="cdtB"
                     /locus_tag="DMACINML_00936"
                     /inference="ab initio prediction:Prodigal:2.6"
                     /inference="similar to AA sequence:RefSeq:CAL34251.1"
                     /codon_start=1
                     /transl_table=11
                     /product="cytolethal distending toxin B"
                     /protein_id="Prokka:DMACINML_00936"
                     /translation="MKKIVFLILSFNVLFAALENYNTGTWNLQGSSAATESKWNVSIR
                     QLITGANPMDVLAVQEAGVLPSTAMMTPRQVQPVGVGIPIHEYIWNLGSVSRPSSVYI
                     YYSRVDVGANRVNLAIVSRVQADEVFVLPPPTVASRPIIGIRIGNDAFFNIHALASGG
                     NDAGAIVAAVDMFFRNRPEINWMILGDFNRESGALVTLLDPDLRARTRVVVPPSSTQT
                     GGRTIDYAITGNSNTAALYNPPPIVAILALAGLRTFLASDHFPVNFRRP"
     sig_peptide     complement(33564..33614)
                     /gene="cdtB"
                     /locus_tag="DMACINML_00936"
                     /inference="ab initio prediction:SignalP:4.1"
                     /note="predicted cleavage at residue 17"
     gene            complement(33639..34415)
                     /gene="cdtA"
                     /locus_tag="DMACINML_00937"
     CDS             complement(33639..34415)
                     /gene="cdtA"
                     /locus_tag="DMACINML_00937"
                     /inference="ab initio prediction:Prodigal:2.6"
                     /inference="similar to AA sequence:RefSeq:CAL34252.1"
                     /codon_start=1
                     /transl_table=11
                     /product="cytolethal distending toxin A"
                     /protein_id="Prokka:DMACINML_00937"
                     /translation="MQKIKLSFMFLIVTIIFSACSSKEQQINPLGRSYGKFSDNDPLR
                     LGSKPTPPVKQNTPSLVEGKKFPAIPLVPPVITPNTFKGDNAVKGPLPRLKSPNEFAS
                     NALYENTGMVSDFVTIMNPNGASLTIWALNPGNWIWGYSLFASRPFGDARAWQLIEFP
```

```
                     NNTVMIKNAKTFTCLNAYRNGIVHYPCDQTNFAQFWRLYPMTNGAYQIQNFATQQCIQ
                     TPVSNVMEEFNLSFYNIYLTDCLKEKEKNLDRQWYIGAPI"
     sig_peptide     complement(34350..34415)
                     /gene="cdtA"
                     /locus_tag="DMACINML_00937"
                     /inference="ab initio prediction:SignalP:4.1"
                     /note="predicted cleavage at residue 22"
     gene            34938..35714
                     /gene="surE"
                     /locus_tag="DMACINML_00938"
     CDS             34938..35714
                     /gene="surE"
                     /locus_tag="DMACINML_00938"
                     /EC_number="3.1.3.5"
                     /inference="ab initio prediction:Prodigal:2.6"
                     /inference="similar to AA sequence:RefSeq:CAL34444.1"
                     /codon_start=1
                     /transl_table=11
                     /product="multifunctional protein SurE"
                     /protein_id="Prokka:DMACINML_00938"
                     /translation="MKEILITNDDGFESEGLKKLIKMLKKEFKAKITIVAPATEKSAC
                     SHSITLTKPLRFIKVGKRFYKLDDGTPADCVYLALHALYKTRLPDLVISGINKGANVG
                     EDITYSGTCAGAMEAVLQGIPAIALSQFYKKSEKELDFKNALKVTKKIVQNIFDKGFP
                     LGKKEFLNINFPAKSNIKGIKICKAGKRVYNFEAHSNINPRGMEYYWLAAANLDFEDE
                     KDSDIALLKKGYVTITPIMLDLSAYDKMKKVKKWLKANNE"
     gene            35707..36363
                     /locus_tag="DMACINML_00939"
     CDS             35707..36363
                     /locus_tag="DMACINML_00939"
                     /inference="ab initio prediction:Prodigal:2.6"
                     /inference="similar to AA sequence:RefSeq:AMP64630.1"
                     /codon_start=1
                     /transl_table=11
                     /product="tRNA cyclic N6-threonylcarbamoyladenosine(37)
                     synthase TcdA"
                     /protein_id="Prokka:DMACINML_00939"
                     /translation="MNDRFTRIKWLVSEENFDKISQTKVLVCGLGGVGGMCVDALYRS
                     GFQNLTLIDADKFEITNQNRQIHSENLGEEKAKVFARIYNAKGIVSKIDNEFLANFDL
                     SEFDLIVDAIDDIPAKVALANLIDFKRQIFISSTGGARKLDPTRIKTTSIFKTHGDAL
                     AKKFRYELRKSGFKGNFDVVFSDEEAHCKDLGSFMGVTASFGLVLASLALRKVLDKKS
                     "
     gene            36347..36805
                     /locus_tag="DMACINML_00940"
     CDS             36347..36805
                     /locus_tag="DMACINML_00940"
                     /EC_number="2.3.1.-"
                     /inference="ab initio prediction:Prodigal:2.6"
                     /inference="similar to AA sequence:RefSeq:CAL34446.1"
                     /codon_start=1
                     /transl_table=11
                     /product="putative acetyltransferase"
                     /protein_id="Prokka:DMACINML_00940"
                     /translation="MIRKADIKDLNACLSLFEQSVITLCAKDYTKDQIRAWIKIDRRQ
                     WEEKFKYDEIFLYEKRGEIASFISVKKEQQLLDLLFTHPDFVRQGLAQNLLDFILKTY
```

```
                     LHNEIYTFASLSAKDFFLKNGFELIRENKVIKEGQNLKNFLMKKDVVYKN"
     gene            complement(36917..37297)
                     /gene="panD"
                     /locus_tag="DMACINML_00941"
     CDS             complement(36917..37297)
                     /gene="panD"
                     /locus_tag="DMACINML_00941"
                     /EC_number="4.1.1.11"
                     /inference="ab initio prediction:Prodigal:2.6"
                     /inference="similar to AA sequence:RefSeq:CAL34447.1"
                     /codon_start=1
                     /transl_table=11
                     /product="aspartate 1-decarboxylase precursor"
                     /protein_id="Prokka:DMACINML_00941"
                     /translation="MNITLLKSKIHRASVTEARLDYVGSISIDEKLLQASGILEYEKV
                     QVVNINNGARFETYTIATQEEGVVCLNGAAARLAEVGDKIIIMSYADFNEEEAKTFKP
                     KVVFVDENNTATKITNYEKHGAIF"
     gene            complement(37294..38142)
                     /gene="panC"
                     /locus_tag="DMACINML_00942"
     CDS             complement(37294..38142)
                     /gene="panC"
                     /locus_tag="DMACINML_00942"
                     /EC_number="6.3.2.1"
                     /inference="ab initio prediction:Prodigal:2.6"
                     /inference="similar to AA sequence:RefSeq:CAL34448.1"
                     /codon_start=1
                     /transl_table=11
                     /product="pantoate--beta-alanine ligase"
                     /protein_id="Prokka:DMACINML_00942"
                     /translation="MEVITSVKEAKQITKNWKSHNLSIGYVPTMGFLHDGHLSLIKNA
                     KTQDKVIVSIFVNPMQFGPNEDFSSYPRDLERDIKMCQDNGVDMVFIPDAAQMYLKNF
                     STYVDMNTITDKLCGAKRPGHFRGVCTVLAKFFNILNPDIVYMGQKDAQQCVVVRHMV
                     DDLNFDLKIQICPIIREEDGLAKSSRNVYLSEEERKASLAISQSIFLAEKLVQEGEKD
                     SSKIIQAMKDILEKEKLIKIDYIELVDFNTMENIENIADNVLGAVAAFVGKTRLIDNF
                     LVQGLK"
     gene            complement(38153..38977)
                     /gene="panB"
                     /locus_tag="DMACINML_00943"
     CDS             complement(38153..38977)
                     /gene="panB"
                     /locus_tag="DMACINML_00943"
                     /EC_number="2.1.2.11"
                     /inference="ab initio prediction:Prodigal:2.6"
                     /inference="similar to AA sequence:RefSeq:CAL34449.1"
                     /codon_start=1
                     /transl_table=11
                     /product="3-methyl-2-oxobutanoate
                     hydroxymethyltransferase"
                     /protein_id="Prokka:DMACINML_00943"
                     /translation="MRKSMISFLEKKAKNEKITMVSAYDYHSARILDNSDIDIILVGD
                     SLAMTVLGMQDTLSVTMDEMLIFTKAVSRGAKKSFVLADMPFMSYQSSDRDAILNASR
                     FIKESHANGVKVEGGVEIASKIKLISQSGIPVVAHLGLTPQAVNMLGGYRVQGKDLQS
                     AQKIIDDAKAVQDSGACMLVLECVPVKLAQKISSILEIPTIGIGSGKYCDGQVLVYHD
```

```
                     LLGLNKDFKAKFVKHFDKIDPQVGVEKYRDEVKSGIFPSQEHSFDYLDDELLDKLY"
     gene            complement(39332..39709)
                     /locus_tag="DMACINML_00944"
     CDS             complement(39332..39709)
                     /locus_tag="DMACINML_00944"
                     /inference="ab initio prediction:Prodigal:2.6"
                     /codon_start=1
                     /transl_table=11
                     /product="hypothetical protein"
                     /protein_id="Prokka:DMACINML_00944"
                     /translation="MKFYNVITMVIFILFFAGCSVSGMITADHNGKMYWVPADCPRYK
                     YFYNEPDKLICTDSNGIETGRILYPADEQQIANYRYEQQRQDEISQRNMEQLHQNTEN
                     LKEINRHFYENFMPKRHNVYIHY"
     gene            39799..40050
                     /locus_tag="DMACINML_00945"
     CDS             39799..40050
                     /locus_tag="DMACINML_00945"
                     /inference="ab initio prediction:Prodigal:2.6"
                     /codon_start=1
                     /transl_table=11
                     /product="hypothetical protein"
                     /protein_id="Prokka:DMACINML_00945"
                     /translation="MKDDFSNSSEEEILNFYKKVSSNIRKFREKKGISQLELALDIGI
                     KSVAFYSNCECNRYGKHFNLEHLYKISKSLEIPLKDLLE"
     gene            complement(40146..41030)
                     /gene="modC"
                     /locus_tag="DMACINML_00946"
     CDS             complement(40146..41030)
                     /gene="modC"
                     /locus_tag="DMACINML_00946"
                     /EC_number="3.6.3.29"
                     /inference="ab initio prediction:Prodigal:2.6"
                     /inference="similar to AA sequence:RefSeq:EAQ73357.1"
                     /codon_start=1
                     /transl_table=11
                     /product="molybdenum ABC transporter, ATP-binding protein"
                     /protein_id="Prokka:DMACINML_00946"
                     /translation="MIKIDIKLPINTAKGKKQLELNTCLKANEITAIFGESGAGKTTL
                     LKIIAGLIKPEFGRIKVGDELWLDTQKNINLAIQKRKIGFVFQDYALFPNMSVKENIS
                     YAATSKQKAEELLSLMNLENLAKIYPKNLSGGQAQRVALARALAREPQILLLDEPLSA
                     LDFKMRSFLQDELVKILQHFKITTLLVSHDLAEIYKLSHRILELSDGKIIKDARTNEF
                     FTSSNLSAKLRLSATLLEMKKSDILVIFTLLLNQDIVKITLSEEEFLRSYKDIKIGDT
                     LLLSIKAFNPIIVGKLDK"
     gene            complement(41039..41707)
                     /gene="modB"
                     /locus_tag="DMACINML_00947"
     CDS             complement(41039..41707)
                     /gene="modB"
                     /locus_tag="DMACINML_00947"
                     /inference="ab initio prediction:Prodigal:2.6"
                     /inference="similar to AA sequence:RefSeq:AAW34935.1"
                     /codon_start=1
                     /transl_table=11
                     /product="molybdenum ABC transporter, permease protein"
```

```
                     /protein_id="Prokka:DMACINML_00947"
                     /translation="MDNDFLQTLYLTFKLAFITTFILFFIGVFLAYLLSFVKFPFKSL
                     VQTFVALPLVLPPSVLGFYLLISFSQNSFLGQFLKEYFNLSLVFSFEGLVFASLIFSL
                     PFMVNPLQSAFSSINQNLLDASYTLGKGKIYTLFRVILPNSKVGIFSACAMSFAHTIG
                     EFGVVMMIGGHKKGETLVASIAIYDELEVLNYTLAHQYAFTLFAISFTVLLSLYFVNK
                     KIAL"
     gene            complement(41700..42101)
                     /locus_tag="DMACINML_00948"
     CDS             complement(41700..42101)
                     /locus_tag="DMACINML_00948"
                     /inference="ab initio prediction:Prodigal:2.6"
                     /inference="similar to AA sequence:RefSeq:AAW34936.1"
                     /codon_start=1
                     /transl_table=11
                     /product="TOBE domain protein"
                     /protein_id="Prokka:DMACINML_00948"
                     /translation="MNILNGKIIELLNEGEIVIVKISIKEQIFKVLMLDLHSLQDLKI
                     GTKIQVLFKEHELGFALPHSILSVENSFLARIKSIKKGKILYHIFFDFKGDEISSIIT
                     KEKALELKLEEGQEWLCFVKENDIILKAVYG"
     gene            complement(42098..42847)
                     /gene="modA"
                     /locus_tag="DMACINML_00949"
     CDS             complement(42098..42847)
                     /gene="modA"
                     /locus_tag="DMACINML_00949"
                     /inference="ab initio prediction:Prodigal:2.6"
                     /inference="similar to AA sequence:RefSeq:AAW34937.1"
                     /codon_start=1
                     /transl_table=11
                     /product="molybdenum ABC transporter, periplasmic
                     molybdenum-binding protein"
                     /protein_id="Prokka:DMACINML_00949"
                     /translation="MKKLVLCFIVLFLSLNLNAQNLSVFVASSASKAMEEVKNEFLKS
                     HPNDNIDLVFGASGKYYQLLKQGREFDLFFSADTKYAQAIYEDQNALDKPKVYVLGIL
                     ALYSLDESLLEGGIEKLKDKANKIHHLSLANPKVAPYGVAAKEVLENLNLDKLFEDKI
                     VLGENISVPVLHVDSNNADLGIVAYSLVSSINHPKGKAVLIDQKYFTPLKQSYVITKY
                     AKDKKLAFEFSDFISSQKAKEIFKKYGFDTP"
     sig_peptide     complement(42788..42847)
                     /gene="modA"
                     /locus_tag="DMACINML_00949"
                     /inference="ab initio prediction:SignalP:4.1"
                     /note="predicted cleavage at residue 20"
     gene            complement(42901..43587)
                     /locus_tag="DMACINML_00950"
     CDS             complement(42901..43587)
                     /locus_tag="DMACINML_00950"
                     /inference="ab initio prediction:Prodigal:2.6"
                     /inference="similar to AA sequence:RefSeq:AAW34938.1"
                     /codon_start=1
                     /transl_table=11
                     /product="biotin biosynthesis protein BioC"
                     /protein_id="Prokka:DMACINML_00950"
                     /translation="MNFLKAKDYQKHAKVQDLMGLKLCEILKNLEISHFKKVFEFGCG
                     RGEFSDKLSKIITFDEYLKNDILDYPDNLNVEIFDMNTLATQNLSKQKFDLITSNASL
```

```
                     QWLDLKQVLPTLANMLNEKGILLLSTFGEMNLKEIKQSTGLGLKYFSTKELEQIFKPY
                     FSDIKITEEIVNLEFQNALEVFRHLKLSGVNSLGFYRLNKQFLKEFEEKFQNKLTYHP
                     IFILCKKDTK"
     gene            complement(43584..44195)
                     /locus_tag="DMACINML_00951"
     CDS             complement(43584..44195)
                     /locus_tag="DMACINML_00951"
                     /inference="ab initio prediction:Prodigal:2.6"
                     /inference="similar to AA sequence:RefSeq:AAW34939.1"
                     /note="conserved hypothetical protein"
                     /codon_start=1
                     /transl_table=11
                     /product="hypothetical protein"
                     /protein_id="Prokka:DMACINML_00951"
                     /translation="MKISNLIQNENSQELILVFGGFASHPSHFAHLKSDKNVVLVYDY
                     ENLDFKFDLNSFSKITLIAFSMGVCVASRVLKDIEFSQKIAINGTPFGIDKLKGIHPA
                     IFARQIKKFDLTTFKKSLFKERENEAKDFIFKDEKDLKTELEKLFEFALKERNESFIW
                     DKIYSSNNDEIFPQNALKNTFSKLIFLNEPHFAFFHFKTWDEI"
     gene            complement(44192..45334)
                     /gene="bioF"
                     /locus_tag="DMACINML_00952"
     CDS             complement(44192..45334)
                     /gene="bioF"
                     /locus_tag="DMACINML_00952"
                     /EC_number="2.3.1.47"
                     /inference="ab initio prediction:Prodigal:2.6"
                     /inference="similar to AA sequence:RefSeq:AAW34940.1"
                     /codon_start=1
                     /transl_table=11
                     /product="8-amino-7-oxononanoate synthase"
                     /protein_id="Prokka:DMACINML_00952"
                     /translation="MSLENILETLEKNHNLRKLTSLKHEGNFVFKEGKKLLNLAGNDY
                     LNLSSCKSLKDEFLDNIKEQDLFFSSSSSRSLSGNFTIYEELESFLKSKFKEKEILHF
                     NSGYHLNISCIAALASIPNTLFLADKFIHASMIDGLKLGGAKFFRFHHNNMNHLENLI
                     QKHYDHYENIIILSEALFSMDGDFSDFNTLIAFKEKYPKIKLYIDEAHSVGCFDEEGL
                     GLVKALNLEEKVDFIVFTFGKALASMGACMICKNLYKSFFINKARAFIYSTALPPINI
                     AWTKFIFEKMSELKEKREKLQIIGNFFKNQLIEKNHQILGDAYIISLVLGENQKAIEL
                     AKKLETNGIFAPAIKEPTVPKNTARIRFSLHAGLSKEELKKVVELL"
     gene            45401..46684
                     /locus_tag="DMACINML_00953"
     CDS             45401..46684
                     /locus_tag="DMACINML_00953"
                     /inference="ab initio prediction:Prodigal:2.6"
                     /inference="similar to AA sequence:RefSeq:AMP64640.1"
                     /codon_start=1
                     /transl_table=11
                     /product="adenosylmethionine--8-amino-7-oxononanoate
                     aminotransferase BioA"
                     /protein_id="Prokka:DMACINML_00953"
                     /translation="MQNEILKKLDLEHIWHPCTQMKDHENLPLIPIKRAKGVWLYDFD
                     DKAYMDCVSSWWVNLFGHCNEKIANAIKKQVDELEHVILAGFTHEPIIKLSARLCEKV
                     GRNFNKCFYADNGSSAIEVALKMSFHYHLNKGVKKSKFLSLSNSYHGETLGALSVGDV
                     ALYKDTYKPLLLECLSTPVPQSKDYTQELEILKDILEKNASEICAFILEPLVQCAGNM
                     HMYEAGFIDEAIKLCHQFGVQVIFDEIAVGFGRTGSLFALHQCKESPDFICLSKGITG
```

```
                     GFMPLSVVLTRDEIYNAFYDTYESQKAFLHSHSYTGNTLACAAANAVLDIFENENILE
                     KNQILSEFIKKEFSRLEKFDFLGNFRTCGMISAFDILSTKYKRVGLFVFRKALEKGLL
                     LRPLANTIYFMPPYIITKEQISFVVDTLEQIFKDF"
     gene            complement(46658..47230)
                     /locus_tag="DMACINML_00954"
     CDS             complement(46658..47230)
                     /locus_tag="DMACINML_00954"
                     /inference="ab initio prediction:Prodigal:2.6"
                     /inference="similar to AA sequence:RefSeq:APA48630.1"
                     /codon_start=1
                     /transl_table=11
                     /product="dethiobiotin synthase"
                     /protein_id="Prokka:DMACINML_00954"
                     /translation="MKIYISGIDTDVGKTYLSARICKELGFDYFKLIQAGVPKDSDEV
                     AKFSPKTKIFKEGVCLQTPASPHKARILEKLNYKAFDIQIPQSDKLIIELAGGLFSPI
                     DDEKTMIDYMSEFKHPTLLVAKDYLGSINHTLLSIEALKQRGIEILALVLKSEDHFSK
                     DFISKYTQIPIVEFDDRVCENLSKILKNLL"
     gene            complement(47238..47552)
                     /locus_tag="DMACINML_00955"
     CDS             complement(47238..47552)
                     /locus_tag="DMACINML_00955"
                     /inference="ab initio prediction:Prodigal:2.6"
                     /inference="similar to AA sequence:RefSeq:CAL34460.1"
                     /codon_start=1
                     /transl_table=11
                     /product="putative efflux protein"
                     /protein_id="Prokka:DMACINML_00955"
                     /translation="MEWIFLFIAAIFEVLGVIVMKQLVLTKNKFYLLIFIICFALSFS
                     FLSLSMQNIAMTVAYSIWTGAGTAGGVIVGVLFYKESKSFLKLFLIALIIACTVGLKI
                     LS"
     gene            complement(47554..47883)
                     /locus_tag="DMACINML_00956"
     CDS             complement(47554..47883)
                     /locus_tag="DMACINML_00956"
                     /inference="ab initio prediction:Prodigal:2.6"
                     /inference="similar to AA sequence:RefSeq:ABV51886.1"
                     /codon_start=1
                     /transl_table=11
                     /product="multidrug resistance protein, SMR family"
                     /protein_id="Prokka:DMACINML_00956"
                     /translation="MNTNIAWFMVIFGGLIECFWVGGLKYSTELWQYILTIIGICISF
                     ICLFKACEKLEVSIAYSVFVGIGTVGIVLNEIFLFDIEVSVAKIVLIALILLSTIGLK
                     FTSKESK"
     gene            48017..48553
                     /gene="rplY"
                     /locus_tag="DMACINML_00957"
     CDS             48017..48553
                     /gene="rplY"
                     /locus_tag="DMACINML_00957"
                     /inference="ab initio prediction:Prodigal:2.6"
                     /inference="similar to AA sequence:RefSeq:CAL34462.1"
                     /codon_start=1
                     /transl_table=11
                     /product="putative 50S ribosomal protein L25 (general
```

```
                     stress protein Ctc)"
                     /protein_id="Prokka:DMACINML_00957"
                     /translation="MLEGIVRESIGRKAAKALKRDGYLIANIYGKGLENINAAFKVND
                     FIKEVRKKTTLAFDVKVGSQTLNVVVVDYQKDPVTAELKHVDLKVAQKGVISKYMVPV
                     KITGTAIGLKNKGVLIQSKRRLKVKCAAENLPNFFELDVSKLDVGDALLVRDVVVPEG
                     VTMIDADRVAVVGVEKAR"
gene                 48550..49092
                     /gene="pth"
                     /locus_tag="DMACINML_00958"
CDS                  48550..49092
                     /gene="pth"
                     /locus_tag="DMACINML_00958"
                     /EC_number="3.1.1.29"
                     /inference="ab initio prediction:Prodigal:2.6"
                     /inference="similar to AA sequence:RefSeq:CAL34463.1"
                     /codon_start=1
                     /transl_table=11
                     /product="peptidyl-tRNA hydrolase"
                     /protein_id="Prokka:DMACINML_00958"
                     /translation="MILVVGLGNIGKEYENTRHNVGFMLVDLLLKEGNFTNLTNSKFK
                     GELFKMGSSLFLKPHTYMNNSGLSVKAVNDFYKCERIIVVHDDIDINLGALRFKKGGS
                     SGGHNGLKSIDNLCGSDYERVRIGVGKGENVISHVLGKFRAEEELILSKVLEHSKKAL
                     LELIRNDDLSAISSKYSLKA"
gene                 49094..50152
                     /locus_tag="DMACINML_00959"
CDS                  49094..50152
                     /locus_tag="DMACINML_00959"
                     /inference="ab initio prediction:Prodigal:2.6"
                     /inference="similar to AA sequence:RefSeq:CAL34464.1"
                     /codon_start=1
                     /transl_table=11
                     /product="putative integral membrane protein"
                     /protein_id="Prokka:DMACINML_00959"
                     /translation="MWIFFRFISGIYLKNFFIIFLSLLGFYCGIDLLLNFNDLPDAAN
                     LSLLYVIFLAFSAITYVLPVSLIFALVLSLVSMIRANEFVSLYALGLSKNLVILFPFL
                     WALFFCFVYVGLNFTPFAYANDYKRNILKNGTMLKQSGEVFLKFNDEFVYISKVNNGQ
                     STAQNIKIFNIKDLNLSSFIEAKSGVFEKKNWTLKEGNLTLLPKEYELGDKGLNISEF
                     KELDTLEGFKPKIIEGVASNSDYSILDILESLELFKTQNISIEALKINLYKLVFMPFF
                     APFLMLIMYYFFPVIARFFNLAFVAFVAFVVTLLTWGMLFLLSRLSENGVILSELGIV
                     MPIVLLIFLGGFMFYKHR"
gene                 50194..51402
                     /gene="lysA"
                     /locus_tag="DMACINML_00960"
CDS                  50194..51402
                     /gene="lysA"
                     /locus_tag="DMACINML_00960"
                     /EC_number="4.1.1.20"
                     /inference="ab initio prediction:Prodigal:2.6"
                     /inference="similar to AA sequence:RefSeq:CAL34465.1"
                     /codon_start=1
                     /transl_table=11
                     /product="diaminopimelate decarboxylase"
                     /protein_id="Prokka:DMACINML_00960"
                     /translation="MDYKKLKDEFQTPFYIYDFDFIKERFLRLKNAFKARKSQIFYAV
```

KANSNLSLLQMLVKLDSGFDCVSIGEVKRALKAGAKSYKIIFSGVGKTQEELKLALEY
DILYINLESEAEMMLLESVAKEFNLKARISIRVNPNVNAKTHPYISTGLNENKFGVEI
DLARKMYLYAKNSPHLEPIGVHFHIGSQLLDISPIHEAATIVAKLVRELKALQIELKF
[truncated: 414,266 more chars]
