# Supplementary material for: Characterization of Campylobacter spp. Strains Isolated From Wild Birds in Turkey
Source: Front Microbiol. 2021 Aug 18;12:712106. doi: 10.3389/fmicb.2021.712106 (PMC8416542; doi:10.3389/fmicb.2021.712106)
Supplement: Supplementary file 7 [file Table_5.pdf]

```
>>BfR-CA-15822_min_cov_30 putative plasmid location

LOCUS       DODKMMJL_1             12696 bp    DNA     linear       12-APR-2021
DEFINITION  Campylobacter species strain strain.
ACCESSION
VERSION
KEYWORDS    .
SOURCE      Campylobacter species
  ORGANISM  Campylobacter species
            Unclassified.
COMMENT     Annotated using prokka 1.14.6 from
            https://github.com/tseemann/prokka.
FEATURES             Location/Qualifiers
     source          1..12696
                     /organism="Campylobacter species"
                     /mol_type="genomic DNA"
                     /strain="strain"
     gene            224..1510
                     /gene="trxB_1"
                     /locus_tag="DODKMMJL_00001"
     CDS             224..1510
                     /gene="trxB_1"
                     /locus_tag="DODKMMJL_00001"
                     /inference="ab initio prediction:Prodigal:002006"
                     /inference="similar to AA sequence:RefSeq:CKG86610.1"
                     /codon_start=1
                     /transl_table=11
                     /product="thioredoxin reductase"
                     /protein_id="Prokka:DODKMMJL_00001"
                     /translation="MSETYEIYTPNGLIMDVYKDANKIIFSGNAKPTGNYTEEYSKAL
                     FEGDRILRNSPYKDYKPIYLDPNFYTGQASTLLEFKEWQSIYLKDPIKGAIAPWTKAE
                     KAYYKSLKTKRERYKYLIIRSGIRSTVIDIPYDAYCNVDEKGYLINEEYAYIYDEVNN
                     NKETLKSSLFRQEWGIAAGILGKPEYFVRSKNHGFNARMIQCFILYIQLTGGGYEELG
                     IKRGIYNYADNLLEIGIGMAGIHKNPLRAKLVKDLAKTIQPDEFGMLPFIDEIMGVDW
                     VIDLNKYDFAYDEEGRIIWALYNDIEKGKLKDPRDVDSTPESRNEFDDAMDGYRNGMK
                     TNFDVDIRNERDERSAKLTMDTLVLSAKLAALTPPQGYPNAPYYFTPERLEWIYKRGY
                     LDKLLDPRIPAIYRYNFPRELRAKILKFGEENGIKD"
     gene            complement(1515..2414)
                     /locus_tag="DODKMMJL_00002"
     CDS             complement(1515..2414)
                     /locus_tag="DODKMMJL_00002"
                     /inference="ab initio prediction:Prodigal:002006"
                     /inference="similar to AA sequence:RefSeq:CKG87069.1"
                     /note="Uncharacterized conserved protein%2C contains FHA
                     domain"
                     /codon_start=1
                     /transl_table=11
                     /product="hypothetical protein"
                     /protein_id="Prokka:DODKMMJL_00002"
                     /translation="MIEKEELGIAIENYDECISGSKAFVFDTKGGTIGSGDDCTFRIQ
                     DKLEQIKNTHAIVSYEEGSFTIAAFEDSDIYYNKSFSRMPSGYEIIISIGDIFKIGNL
                     EFRFVDAKSIEESIKENKKYLEDIEKRNHFDEIEIEPRGKTSINFDKNEELKEILKNN
                     NYKFIEEEKADDSFLKEIIQRDPNSLGYENILKSLVKNLKDIKTKQQSSKLNNNYSPI
                     NIKDFESIINNIPLIKSTRLINILALSLIAKELYTPIFEEMEENMFIKYLEAAIQNNI
```

```
                     KEDKILFENLTLKALEKYIKDFE"
     gene            complement(2411..5938)
                     /locus_tag="DODKMMJL_00003"
     CDS             complement(2411..5938)
                     /locus_tag="DODKMMJL_00003"
                     /inference="ab initio prediction:Prodigal:002006"
                     /inference="similar to AA sequence:RefSeq:CKG87105.1"
                     /note="Uncharacterized protein conserved in bacteria"
                     /codon_start=1
                     /transl_table=11
                     /product="hypothetical protein"
                     /protein_id="Prokka:DODKMMJL_00003"
                     /translation="MFKKIFDFIKSRLFITAFLLCCIFLFSVLFWFWSPLIAFNDIYI
                     FSSSFLRFSIILIIWLIVFLFFLLKPIINFISSLKSEKRLKFKDLKKEADEFIYKSKR
                     NFFLSLKDAKETWKNDLKTKNLPLIIIIGNEGAGKSTFINYSDIEYPLSDSLESYKKF
                     HKSTRNFALYVSKKGALLDTEGNYFSQEEFFKPASSDEIPEDDIDKNRDFLIKKNIWK
                     KFLTFLNKNFFHSKLNGIILVVDTVLFLNNPKEYSKNLIRYLTKRVNECEKTLNLKLP
                     IYIVFSKLDLMEGMKEYFDIFDKKISDKILGLSFDKILSEEFLNNEFKELSNSLLCSL
                     MSKNSHIYNIENKNKSYLFLKQLDNLFALAKTFILEVQEENKLKNNSYLRGVYFVSAY
                     QENIPRNFLLDAICEKYNCKKVLSKSSIIYNKQSYFVKSLLEDLIFTDYALNTMKSYS
                     KKLSFLIIILIIGFGTYTISSYFISKNDKEFEKSQNTLRALQSLLEDQDYQNLNIKQK
                     ADFLIELRNILNTYPELWQDDNIFQYLNLNLSYKGFKEAKQLYYKLNEDVLKNTLLKE
                     MEYTLLTDTNKENLIKTLYLYRSLFEQKYFNKEMLKIWINENWNTLSKYLISKDDFLG
                     GVDELKQFNLKSFTEDENSINIGKRKLESISRTQRIYTLLNFLNSDKPKEKYLIKEDL
                     GFAANSVFSNNSQITSIDKIYTKVGMMDFLNDLNQQVDTAINIESWMLDNNFKENKNT
                     LTMGILKLYLSEYQNAWQNLLASLQPVRYNTKEAMLNELNILSKKENPLYSLLKIVSS
                     NTNLNDAVLLTQAYNLGLNAGEIRSNFIGVSNAFTQYHKLVNKNTLLSVGNIEVGKGT
                     DDEKILDILNTSITNMSNKIIDFSSNNNQSAEEKISYALGGNKDANDPFAVFQMSIKK
                     LPSDLERYYNQLSDHSWNFIENHGISLFNTAWINEVYNPFINDIAPFYPFNEESTANL
                     SMDSFKTFFGRNGVLNSFYKKYLNNVLVKRKNNYSINSQFASKLNFSKEFLDFITNAG
                     NLSSLILNANDNIRVNFTIQSLDLSADFSFVKLGYNNKIIQYDHTLDQTLQIVAEEFN
                     NGTSLNFTAYNYSNPNLNYTKSYKGEWAWYKFIKDNKNNSTYSIMFNNNKNLYFDFEI
                     INGASDLNNIVYILNNLKIVENITGVNKQ"
     gene            6073..6588
                     /locus_tag="DODKMMJL_00004"
     CDS             6073..6588
                     /locus_tag="DODKMMJL_00004"
                     /inference="ab initio prediction:Prodigal:002006"
                     /inference="similar to AA sequence:RefSeq:AJK71137.1"
                     /codon_start=1
                     /transl_table=11
                     /product="major exported protein"
                     /protein_id="Prokka:DODKMMJL_00004"
                     /translation="MAEPAFIRIEGSTQGLISSGASTEASIGNRYKSGHEDEIMAQEV
                     SHIVTVPVDQQSGQPSGQRVHKPFSFTCSLNKSVPLLYNALTKGERLPTVEVHWFRTA
                     TSGGSEHFFTTKLEDAIITNIELIMPNAQESSNHDKTELLKVSMSYRKVVWEHTAAGT
                     SGSDDWREGKA"
     gene            6846..7406
                     /locus_tag="DODKMMJL_00005"
     CDS             6846..7406
                     /locus_tag="DODKMMJL_00005"
                     /inference="ab initio prediction:Prodigal:002006"
                     /codon_start=1
                     /transl_table=11
```

```
                     /product="hypothetical protein"
                     /protein_id="Prokka:DODKMMJL_00005"
                     /translation="MTYILLLIIISTILSYLILKFIYKIIFKSTKSVSKFLVFLGSIG
                     LIIFYYTPYSYYLEPSYWQFRNMCKLNELPNNEEKYNKILGYFDKKLGDIDDFRHLKK
                     YGRIEMWLCIYYDDTYKTPSNFQEKIEMDNIALKTKNMQMSLHKIVDMHISPYWNTRR
                     YILEGNEGNMNFEFIEDELACGNLYQ"
     gene            7425..7646
                     /locus_tag="DODKMMJL_00006"
     CDS             7425..7646
                     /locus_tag="DODKMMJL_00006"
                     /inference="ab initio prediction:Prodigal:002006"
                     /inference="similar to AA sequence:RefSeq:CKG86895.1"
                     /codon_start=1
                     /transl_table=11
                     /product="methyltransferase small"
                     /protein_id="Prokka:DODKMMJL_00006"
                     /translation="MTNKEQINNLKDYTDSYFNFVYTEGQKALGFKISKTRFFKDKSE
                     LEKLDYFKALHDEYNGYLVIDDSLFFMRN"
     gene            8407..8601
                     /locus_tag="DODKMMJL_00007"
     CDS             8407..8601
                     /locus_tag="DODKMMJL_00007"
                     /inference="ab initio prediction:Prodigal:002006"
                     /codon_start=1
                     /transl_table=11
                     /product="hypothetical protein"
                     /protein_id="Prokka:DODKMMJL_00007"
                     /translation="MSKNIKTQEAKLDLITKFFDYANIADASYAMLDLIDENDENSTL
                     APLWLYADGVTNLKNHSKRQ"
     gene            8605..9171
                     /locus_tag="DODKMMJL_00008"
     CDS             8605..9171
                     /locus_tag="DODKMMJL_00008"
                     /inference="ab initio prediction:Prodigal:002006"
                     /codon_start=1
                     /transl_table=11
                     /product="hypothetical protein"
                     /protein_id="Prokka:DODKMMJL_00008"
                     /translation="MKYIILTFILGLIVFVSVKGCFYPTSYRAFIPKSLDSEYKEFER
                     LCKEEVGKVVYARPIKHGIKLEDLFNLPSTSARRVALSRHTYMLIENVTTREDEIFYL
                     HIAGFEYYTGWTRKSFSMHDDSKKYIRCEPLLYGKTWKENAKIMEQKAFDNKEYIGTE
                     EYLQYSPYHYEYNTLYRQIVNFKQKKEN"
     gene            9171..9350
                     /locus_tag="DODKMMJL_00009"
     CDS             9171..9350
                     /locus_tag="DODKMMJL_00009"
                     /inference="ab initio prediction:Prodigal:002006"
                     /codon_start=1
                     /transl_table=11
                     /product="hypothetical protein"
                     /protein_id="Prokka:DODKMMJL_00009"
                     /translation="MIYNYKYSLGIYVNDWGFMMGSEGNPHVFLGLTKGKSPDELDKI
                     DEELRREYLKNKNSN"
     gene            9410..9901
```

```
                     /locus_tag="DODKMMJL_00010"
     CDS             9410..9901
                     /locus_tag="DODKMMJL_00010"
                     /inference="ab initio prediction:Prodigal:002006"
                     /inference="similar to AA sequence:RefSeq:CKG86435.1"
                     /codon_start=1
                     /transl_table=11
                     /product="hypothetical protein"
                     /protein_id="Prokka:DODKMMJL_00010"
                     /translation="MQIPIYSKLKTKLESRGIDNITSGQIAWFVCSVIFTYLLFLLIV
                     IINTRTISFENLKFFTFEGFFKSLAFIFANHFFLFIFMWIVCIINFFNIKNLIPITLN
                     WIISYCYFVLEINIYFILILCITICVVFYFLQKRLLYTFLFYFVYMVLTYTLFYLGGG
                     FYK"
     gene            9898..10035
                     /locus_tag="DODKMMJL_00011"
     CDS             9898..10035
                     /locus_tag="DODKMMJL_00011"
                     /inference="ab initio prediction:Prodigal:002006"
                     /inference="similar to AA sequence:RefSeq:CKG86410.1"
                     /codon_start=1
                     /transl_table=11
                     /product="hypothetical protein"
                     /protein_id="Prokka:DODKMMJL_00011"
                     /translation="MKYYVTIYIDPLFDFEDATHAFLGLTKGKSPDELDKIDKELRQK
                     F"
     gene            10082..10507
                     /locus_tag="DODKMMJL_00012"
     CDS             10082..10507
                     /locus_tag="DODKMMJL_00012"
                     /inference="ab initio prediction:Prodigal:002006"
                     /inference="similar to AA sequence:RefSeq:CKG86435.1"
                     /codon_start=1
                     /transl_table=11
                     /product="hypothetical protein"
                     /protein_id="Prokka:DODKMMJL_00012"
                     /translation="MQIPIYSKLKAKLESKGIDNISSGQIAWFVCSVGIFIIPMMYLY
                     ISNNYNQFIILFFMIFIFILNMLYTKNFFALLLLVILIFIYMKYHEFIYNFKILFFIF
                     APIILIFLRAWRILITFLCGYIFLVAMVYIFELLGGFYK"
     gene            10504..10698
                     /locus_tag="DODKMMJL_00013"
     CDS             10504..10698
                     /locus_tag="DODKMMJL_00013"
                     /inference="ab initio prediction:Prodigal:002006"
                     /inference="similar to AA sequence:RefSeq:CKG86410.1"
                     /codon_start=1
                     /transl_table=11
                     /product="hypothetical protein"
                     /protein_id="Prokka:DODKMMJL_00013"
                     /translation="MKYYATIYIDPLVECFLDFKNQCYWQREDFTHAFLGLTKDKSPD
                     ELDKIDEELRREYLKNKNSN"
     gene            10758..11183
                     /locus_tag="DODKMMJL_00014"
     CDS             10758..11183
                     /locus_tag="DODKMMJL_00014"
```

```
                     /inference="ab initio prediction:Prodigal:002006"
                     /inference="similar to AA sequence:RefSeq:CKG86435.1"
                     /codon_start=1
                     /transl_table=11
                     /product="hypothetical protein"
                     /protein_id="Prokka:DODKMMJL_00014"
                     /translation="MQIPIYSKLKTKLESKGIDNISSGQIAWFVCSVGIFIIPMIYFY
                     ISNNYDQFTILFIMIFIFILNMFYTKNFFALLLLVILIFIYMKYHEFIYNFKILFFIF
                     APIILIFLRAWRILITFLCGYIFLVAMVYIFELLGGFYK"
     gene            11180..12457
                     /locus_tag="DODKMMJL_00015"
     CDS             11180..12457
                     /locus_tag="DODKMMJL_00015"
                     /inference="ab initio prediction:Prodigal:002006"
                     /inference="similar to AA sequence:RefSeq:CKG86410.1"
                     /codon_start=1
                     /transl_table=11
                     /product="hypothetical protein"
                     /protein_id="Prokka:DODKMMJL_00015"
                     /translation="MKYYATIYIDPLVECFLDFKNQCYWQREDFTHAFLGLTKDKSPD
                     ELDKIDKELRQEYLKNKEWDKIDQKWYEAKEPNEFNDGFWGFGTGIANVAESLIGSPG
                     KVFNNNQYVLDNNVDKNRYIIKKLRSPQTFKPSNRCTLEISEEQYETLLANIKADFEA
                     TKEITPNSEKPINEEFTYKLLENNCVTWVVKKLSSIGIEVVNPVVKDPSGLLMATKLI
                     QSIHSIFLKFQNIDDNLQSVKGAKAFKNWARMLINNSLMQEIYLDEYKKQYNSENLSP
                     EQEEVLYALWNITRVYKILGILLSRLITKSGTKNLKGTFELIYFDKNDKQAKLLKASN
                     HYEAIIIQELDLSKKYNNFSASKFYPFIFIPKDEMLSRTLYHKYDYGNISQEYQKDRN
                     EFYFNVLAGEKSDKYWSSSYHKMTKSLRKVYVS"
ORIGIN
        1 agactagaaa ttatttataa aagacataag cttgataaac tccttgatcc aagaatccct
       61 gctatttata gatataattt cccaagagaa cttagagcta agatactcaa atttgctgaa
      121 gaaaatgaca ttaaagatta aagtaatttc ataggtagac tggccactac tataatcata
      181 gaagatggtt tatacatggg ataaatttaa gaaaggataa ataatgagcg aaacttatga
      241 gatttataca cccaatgggt taataatgga tgtatataaa gatgctaata aaatcatttt
      301 ttcaggaaat gctaaaccaa caggcaatta taccgaagaa tattccaaag ctctcttcga
      361 aggtgaccgc atattaagaa actcccccta caaagactat aaaccaatat atctagatcc
      421 taacttttat acagggcaag cttctacttt acttgaattt aaagaatggc aaagcatata
      481 tttaaaagat cccattaaag gagctatagc tccttggact aaagcagaaa aagcttatta
      541 taaatcttta aaaaccaaaa gagaaagata taaatattta attataagaa gcggtataag
      601 aagtaccgtt atagatatac cctatgatgc ttattgtaat gtagatgaaa aaggatattt
      661 aatcaatgaa gaatacgcct atatttatga tgaagttaac aataataaag aaaccctaaa
      721 atcttctctt tttagacaag aatggggtat agcagcagga atactaggaa aacctgaata
      781 ttttgtgcgt tctaaaaatc atggctttaa tgctagaatg atacaatgtt ttattttata
      841 tattcaactc acaggtggag gatatgaaga attaggtata aaaagaggaa tttataatta
      901 tgctgataat cttttagaaa taggtatagg tatggcaggg atacataaaa atcctttaag
      961 ggctaaattg gtaaaagatt tagctaaaac tatacagcct gatgaatttg gtatgctacc
     1021 ttttatagat gagattatgg gagtagattg ggtgattgat ttaaataaat atgattttgc
     1081 ctatgatgaa gaaggtagaa tcatttgggc tttatataat gatattgaaa aaggcaaatt
     1141 aaaagatcca agagatgtgg attctactcc tgaaagtaga aatgaatttg atgatgctat
     1201 ggatggatat agaaatggga tgaaaactaa ttttgatgta gatattagaa acgaaagaga
     1261 tgagagatct gctaaactga ctatggatac cctagtatta agtgccaaac tcgcagccct
     1321 cactcctcct caaggctatc ctaatgcccc ttattatttt acacctgaaa gattggaatg
     1381 gatttataaa agaggatatt tagataaact ccttgatcca agaatccctg ctatttatag
     1441 atataatttc ccaagggaac ttagagctaa gatactaaaa tttggcgaag aaaatggcat
     1501 taaagattaa aaaatcactc aaaatcttta atatactttt ccaatgcttt tagggtaaga
```

```
1561 ttctcaaaaa gaatcttatc ttctttgata ttgttttgta tggcagcttc aagatattta
1621 ataaacatat tttcttccat ttcttcaaag atgggagtgt ataattcttt tgcgattagg
1681 ctaagggcta aaatattaat caatcttgtg gatttgatta aaggtatatt attaataata
1741 ctttcaaaat ctttaatgtt gataggagaa taattattat ttaatttaga gctttgttgt
1801 tttgttttaa tatcttttaa attttttacc aaacttttta gaatattttc ataccctaat
1861 gaatttggat ctctttgaat gatttctttt aaaaagctat catctgcctt ttcttcctcg
1921 ataaacttat aattattatt ttttaaaatc tctttcaact cttcgttttt gtcaaaattt
1981 atacttgttt ttcctctagg ttcaatttca atttcatcaa aatgattgcg tttttcaata
2041 tcttctaagt atttttttatt ttctttgata ctttcttcaa tgctttttgc atccacaaat
2101 ctaaattcta aattaccgat tttaaaaata tcgcctatgc tgattattat ttcatagcca
2161 cttggcattc tagaaaagga cttgttataa taaatgtcag aatcttcaaa agctgcaatt
2221 gtaaaagatc cttcttcata agatactatg gcatgggtat ttttgatttg ctctagttta
2281 tcttgaatac gaaaagtgca atcatcacca cttcctatgg ttcctccctt tgtatcaaat
2341 acaaatgctt tagaaccact aatgcattcg tcataattct caatagcaat tcctaattct
2401 tctttttcta tcattgttta tttactcctg tgatattttc tactatttta agattattta
2461 aaatataaac tatattattt aaatcactag caccgttaat aatttcaaag tcaaaatata
2521 agtttttatt attattaaac ataatgctgt atgtagaatt gtttttatta tcttttataa
2581 acttatacca tgcccattct cccttatatg attttgtata atttaaatta ggattggagt
2641 agttgtaggc tgtaaaattt aaacttgttc cattattgaa ttcttctgct acaatttgta
2701 gggtttgatc aagagtatga tcgtattgaa taattttatt gttgtagcca agctttacaa
2761 aggaaaaatc tgcacttaaa tcaaggcttt gaatagtaaa attaactctt atattatcat
2821 tagcattcag tatgaggctt gagagattac ctgcattggt gataaaatct aaaaattctt
2881 tagagaaatt taattttgaa gcaaattgag aattgataga atagttattt tttcttttta
2941 ctaaaacatt gttcaagtat tttttataaa aactatttaa tactccattt ctaccaaaaa
3001 atgttttaaa gctatccata ctaagattag ctgtgctttc ttcattaaaa ggataaaatg
3061 gcgcaatatc attgataaat ggattataca cttcattaat ccaagcagta ttaaataaag
3121 aaataccatg attttctata aaattccaag aatgatcaga aagctgatta taataccttt
3181 ctaaatcact aggtaatttt tttatactca tttgaaatac agcaaaagga tcattggcat
3241 ctttattgcc accaagagca tatgaaattt tttcttcagc gctttggtta ttattggagc
3301 taaagtctat aattttattg gacatatttg taatacttgt attaagaata tctaatattt
3361 tttcatcatc agtgcctttg cccacttcta tattaccaac actcaaaagt gtatttttgt
3421 ttactagctt atgatattgc gtaaaagcat tgcttacccc aataaaattt gatctaattt
3481 cccctgcatt taatcctaaa ttgtaggctt gagttagtaa aactgcatca tttaaatttg
3541 tattagaact aacaattttt aacaaagaat ataaagggtt ttcttttttt gataagatat
3601 taagctcatt aagcattgcc tctttggtat tatacctaac aggttgcaat gaagcgagta
3661 gattttgcca agcattttgg tattcactta agtataattt taaaataccc atagtcaaag
3721 tgtttttatt ttctttaaaa ttgttatcta gcatccaaga ttcaatattg attgcggtgt
3781 caacttgttg atttaaatca tttaaaaagt ccatcatacc gacttttgta tatattttat
3841 ctatagaagt tatttgagaa ttatttgaaa atacactatt tgcagcaaaa cctaaatcct
3901 cttttataag atatttttct ttaggtttat cgctatttaa aaaattaaga agagtgtaaa
3961 ttctttgagt tctacttata ctctcaagtt ttcttttttcc tatatttata ctgttttcat
4021 cttctgtaaa ggatttaaga ttaaattgtt taagttcatc cacacctcct agaaagtcat
4081 ctttagagat taaatactta cttagagtat tccaattctc gttaatccaa atttttagca
4141 tttctttatt aaaatatttt tgctcaaaca aagatctata taaatataag gttttgatta
4201 aattttcttt atttgtgtca gtaagtaaag tatactccat ttcttttaat aaagtatttt
4261 ttaacacatc ttcatttagt ttataataaa gttgtttagc ttctttaaaa cctttgtatg
4321 agagattaag gtttaaatat tgaaatatat tatcatcttg ccaaagttca ggataagtat
4381 ttaaaatatt tctcaattca attaaaaagt cggctttttg tttgatattt aaattttgat
4441 aatcttgatc ttcaagaagc gattgtaaag ctcttaaagt gttttggctt ttttcaaatt
4501 ctttatcgtt tttacttata aaataagaag atatagtata tgttccaaag cctatgatta
4561 aaattatgat taaaaaagat aattttttgg aataagattt catagtattt aaagcataat
4621 ctgtaaaaat taaatcttca agtagtgatt taacaaaata actttgttta ttatatataa
4681 tactagattt tgataatact tttttacagt tgtatttttc acatatggca tcgagcaaga
4741 aatttctagg aatattttct tgataagcac taacaaagta aactcctcta agataagaat
```

4801 tatttttaag cttgttttct tcttgcactt ccaaaataaa tgtttttgcc aatgcaaata
4861 aattatcaag ttgttttaaa aataagtagc ttttattttt attttctata ttgtaaatat
4921 gggaattttt actcattaaa gagcataata atgaattact taattcttta aattcattat
4981 ttaaaaattc ttcacttaag attttatcaa aagataatcc taaaatttta tccgaaattt
5041 ttttatcgaa aatatcaaaa tactctttca tgccttccat caagtcaagt tttgaaaaaa
5101 ctatatagat aggcagcttt aaattcaaag tttttcgca ttcgtttact ctttttgtta
5161 gatatctaat aagatttttg gaatactcct taggattatt taagaaaagg acagtatcaa
5221 caactaaaat aattccattt agtttgctat gaaaaaaatt tttattgagg aaagtaagga
5281 atttttttcca tatatttttt ttaattaaaa aatctctatt tttatctata tcatcttcgg
5341 gtatttcgtc gctacttgca ggtttaaaaa attcttcttg tgagaagtag tttccttctg
5401 tatctaacaa agcacctttt tttgatacat aaagcgcaaa atttctagta gacttatgga
5461 atttttttata agactctaaa ctatcgctaa gtggatattc gatgtcagaa taattaataa
5521 aagtgctttt tccagcccct tcatttccta tgataataat taagggtaaa tttttttgttt
5581 ttaaatcatt cttccatgtt tctttggcat ctttaagaga taaaaagaag tttcttttag
5641 atttgtagat aaactcatca gcttcttttt ttaggtcttt aaattttaat ctttttttcac
5701 tttttaaaga agatataaaa tttataatag gctttaataa aaaaaataaa aaaacaataa
5761 gccaaattat caagataata ctaaatctta aaaatgagct actaaaaata tatatgtcgt
5821 taaaagctat taaaggactc caaaaccaaa ataaaacact aaaaagaaaa atacaacaaa
5881 gtaaaaaagc agttataaat aatcttgatt ttataaaatc aaatattttt ttaaacatcg
5941 ttattcctta tttaaaagta aaaacaaata aatattaata tcaatataca aatattttac
6001 aaaaatttat atagattaag attaaaatga tacaattttg aatgttaaaa aaaattaaag
6061 gaggttcttt ttatggctga accagcgttt ataagaattg aaggttccac acaaggtttg
6121 atttcaagtg gtgcatctac tgaagcgagt attggcaata gatataaatc aggtcatgaa
6181 gatgaaatca tggcgcaaga ggtatctcat atcgtaactg tacccgttga tcagcaaagt
6241 gggcaacctt ctggacaaag agttcataag ccctttctt ttacttgctc attgaataag
6301 tccgtgcctt tgttatataa tgcacttacc aaaggagaaa gacttccaac agtcgaagtt
6361 cattggttta gaactgcaac aagtggcgga tctgaacatt tttttactac aaaattggaa
6421 gatgcaatta ttacaaatat tgaattaata atgccaaatg cacaagagtc aagtaatcat
6481 gataaaaccg agcttttaaa agtttcaatg agttatagaa aagtagtttg ggaacatact
6541 gctgctggaa ctagcggaag cgatgattgg agagaaggta aagcttaatt ataattatct
6601 taaaaaaccc cgactaaaat gcgtaatttt atagtcgggg ttatatctta aaaatatatt
6661 ttgttttctt ggttttaatt acaaaactac tttctaaatt atagtactat ttattcatac
6721 atgaaatcat ttatcttaat gagcaatttt cgtcttctat gtttggatac tagcgagtta
6781 atctttatca aaccacaaaa ttacatctta attttaataa aatagagagt ctaaggagat
6841 aacgtatgac atacatatta cttcttatta taatttctac tattctttct tatcttatat
6901 taaaatttat tttataaaatc atctttaaat ctacaaaaag cgtttcaaag tttttagtct
6961 ttcttggaag tataggctta attatctttt attatactcc ttattcttat tatttagagc
7021 ctagttattg gcaatttagg aatatgtgta agttaaatga acttcctaat aacgaagaga
7081 aatataataa gattctaggg tattttgata aaaagttagg tgacatagat gattttcgac
7141 atctaaaaaa atatggtagg atagaaatgt ggctttgtat atattacgat gatacttaca
7201 aaactccttc aaattttcaa gaaaagatag agatggataa tattgcacta aaaacaaaaa
7261 atatgcaaat gtctttacac aagattgttg atatgcatat aagtccatat tggaatacta
7321 gaaggtatat attagaaggc aatgagggta atatgaattt tgagtttata gaggatgagc
7381 ttgcttgtgg caatctttat caataataat ataaggaata aataatgacc aataaggaac
7441 aaatcaacaa tttaaaagat tatacagata gttattttaa ttttgtttat actgaaggac
7501 aaaaagcttt aggatttaaa atatctaaaa ctagattttt taaagataag agtgaattag
7561 aaaaattgga ctattttaaa gcattacatg atgaatataa tggatatttg gttatagatg
7621 attctttatt ttttatgcga aactaaatgg agatttagag aattacaagc tagaaatttt
7681 tctcaaagat atgagattaa atttcatcag tccaacacta caagtggctt tagcactgct
7741 ttgtcttata aaaagacaga gaaaattaag aggaattaat aaatactaga attctctatt
7801 aatcttttaa tatttattct tatatttttc tttttgttt ttatattttc tattatttta
7861 tattttatct ttaaaatttt ttttaaaata aaaaataaaa taaaaactat aaattttaaa
7921 aaaatattta aaaaacttac tttatttctt ttgcctgtat atcttttatt attcttaaca
7981 ggaggttgtt cttataaata tatggatccg cgatattatg agtttgagag cttgtgtaca

8041 gatatagata ataaagtaat aatgtacgat aaagcttatt gggaaatatt tagcaataga
8101 gaaaaaggag aattctttat ttaatcaaaa aataaataaa aaatatattt tgattttaaa
8161 aaatcaaaaa aacattaatg tattacaaaa aataaattta ttttaaaaga agttacattt
8221 gaagattatt atgataatat tcattattca acgcatttat cctatattta tgataattat
8281 ggtacattct taaaaggtga tgagggcgct ggattacatt ttagatatat ggaagtatta
8341 agttgtaaga atattgataa aaaatttaag aaagatgatt ggcaagttga aactttggaa
8401 taacaaatga gcaaaaacat taaaacacaa gaagctaaat tagacttaat cactaaattt
8461 tttgactatg ctaatatagc agatgcaagt tatgcaatgc ttgatttaat cgatgagaat
8521 gatgagaatt caacccttgc acctttatgg ctttatgcag atggggtaac taatctaaaa
8581 aatcatagta aaaggcaata aagtatgaaa tatattatat tgacttttat tctaggtttg
8641 attgtatttg tttcagtaaa aggttgtttt tatcctacaa gttatcgtgc ttttatacca
8701 aaaagcttag attccgagta taaagagttt gaaagattat gtaaagagga agtaggaaag
8761 gtagtatatg caagacctat aaaacatggt ataaagttgg aagatttgtt taacttgcct
8821 tctacgagtg ctagacgtgt tgctttaagc agacatacat atatgttgat agaaaatgtt
8881 acaacaagag aagacgaaat tttttactta catatagctg gttttgaata ttatacaggc
8941 tggacaagaa aaagtttttc tatgcatgat gattctaaaa aatatataag atgtgagcct
9001 ttgctttatg gtaaaacttg gaaagaaaat gctaagataa tggaacaaaa ggcatttgat
9061 aataaagaat atataggcac agaagaatat ttacaatatt caccttatca ttatgagtat
9121 aatacacttt atcgccaaat agttaatttc aaacaaaaga aagagaatta atgatatata
9181 attacaaata ctctcttgga atctatgtta atgattgggg ctttatgatg ggcagtgagg
9241 gaaaccctca tgtgttctta ggacttacca agggcaaaag tcctgatgag ttagataaaa
9301 tagatgaaga attaagacga gaatatctta aaaacaaaaa ttcaaattaa taaaaaattt
9361 taagcaaatt agtttataat aaaacaattt atctgtatta ggagtaaatt tgcaaatccc
9421 tatttattct aaattaaaaa caaagctaga aagtagagga atagacaata taacaagcgg
9481 acaaattgct tggtttgtgt gtagtgtaat ttttacctat ttattatttt tattaattgt
9541 aattataaat actagaacaa tatcttttga aaaccttaaa tttttttactt ttgaaggttt
9601 tttcaaatct cttgctttta tttttgcaaa tcattttttt ttatttattt ttatgtggat
9661 tgtttgtata ataaattttt ttaatataaa aaatttaatt ccaataacac taaattggat
9721 aataagctat tgttattttg ttcttgaaat taatatttat tttattttga ttttatgtat
9781 aaccatttgt gttgtatttt atttttttaca aaaaagatta ttatatactt ttttatttta
9841 ttttgtttat atggtattaa cttatacttt gttttatctt ggtggaggat tttataaatg
9901 aaatattatg ttacgattta tattgatccg ctatttgatt ttgaagatgc tacccacgcc
9961 tttttaggac ttaccaaagg caaaagtcct gatgagttag ataaaaataga taaagaatta
10021 agacaaaaat tttaagcaaa ttagtttata ataaaacaat ttatctatat taggagtaaa
10081 tttgcaaatt cctatttatt ctaaactcaa agcaaagcta gaaagtaaag gaatagacaa
10141 tatatcaagt gggcaaattg cttggtttgt gtgtagcgtg ggtattttta tcattcctat
10201 gatgtatctt tatattagca acaattacaa tcaatttatt attttgtttt ttatgatatt
10261 tatttttata ttaaatatgc tttatactaa aaattttttt gcattattat tgcttgtaat
10321 attaatattt atatatatga aatatcatga gtttatctat aattttaaaa ttttattttt
10381 tatttttgca cctataattc ttatcttttt aagggcttgg agaattttaa taacttttttt
10441 atgcggatat atttttttag tggctatggt atatatattt gagttattag gaggatttta
10501 taaatgaaat attatgcaac aatttatatt gatccattgg tagaatgttt tttggatttt
10561 aaaaatcagt gttattggca aagagaagat ttcacccacg ctttcttagg gcttactaaa
10621 gataaaagtc ctgatgagtt agataaaaata gatgaagaat taagacgaga atatcttaaa
10681 aacaaaaatt caaattaata aaaaatttta agtaaattag tttataataa aacaatttat
10741 ctgtattagg agtaaatttg caaatcccta tttattctaa attaaaaaca aagctagaaa
10801 gtaaaggaat agacaatata tcaagcggac aaattgcttg gtttgtgtgt agtgtaggta
10861 tttttatcat tcctatgata tatttttata tcagcaacaa ttatgatcaa ttcactattt
10921 tatttattat gatatttatt tttatattaa atatgtttta tactaaaaat ttttttgcat
10981 tattattact tgtaatatta atatttatat atatgaaata tcatgagttt atctataatt
11041 ttaaaatttt atttttatt tttgcaccta taattcttat ctttttaagg gcttggagaa
11101 ttttaataac tttttttatgc ggatatattt ttttagtggc tatggtatat atatttgagt
11161 tattaggagg attttataaa tgaaatatta tgcaacaatt tatattgatc cattggtaga
11221 atgtttttttg gattttaaaa atcagtgtta ttggcaaaga gaagatttca cccacgcttt

```
    11281 cttagggctt actaaagata aaagtcctga tgagttagat aagatagata aagaattaag
    11341 acaagaatat cttaaaaaca aagaatggga caagatagat caaaaatggt atgaagctaa
    11401 agagcctaat gagtttaatg atggtttttg gggttttgga actggaattg ctaatgttgc
    11461 agaatctctt ataggatctc ctggcaaagt atttaataat aaccaatatg tattagataa
    11521 caatgttgat aaaaatcgct atattattaa aaaactaaga agccctcaaa cttttaaacc
    11581 ctctaatcgt tgcactttag aaatttctga agaacaatat gaaaccctac ttgcaaatat
    11641 taaagctgat tttgaagcca caaaagaaat cacacctaat agcgaaaagc caatcaatga
    11701 agaatttacc tataaacttc ttgaaaataa ttgcgttact tgggttgtta aaaagctttc
    11761 tagcatagga atagaagttg taaatcccgt tgttaaagat cctagcggat tattaatggc
    11821 tacaaaatta atacaaagca ttcattctat ctttttaaaa tttcaaaaca ttgatgataa
    11881 tttacaaagc gtaaaaggtg caaaagcttt taaaaattgg gctagaatgc ttataaataa
    11941 ttcattaatg caagaaattt atcttgatga atataaaaaa caatacaata gtgaaaatct
    12001 tagtccagaa caagaagaag ttttatatgc tttatggaat attacaaggg tttataaaat
    12061 tttaggcatt cttttaagtc gtttaattac aaaatcgggc actaaaaacc taaaaggcac
    12121 ttttgaatta atttattttg ataaaaatga taagcaagca aagcttttaa aagcgagtaa
    12181 tcattatgag gcgattatta tacaagaact agatttaagt aaaaaataca ataattttag
    12241 tgcgagtaaa ttttatcctt ttatctttat acctaaagat gaaatgctct caagaacgct
    12301 ttatcataag tatgattatg gtaatatctc acaagaatat caaaaagata gaaatgaatt
    12361 ttattttaat gttttagcag gagaaaaaag cgataaatat tggagttcta gttatcataa
    12421 aatgactaaa agtttaagga aagtctatgt ctcttgaaaa aatcaaagaa acgatttttg
    12481 cttatgataa agaagtgatt gattgtgaag ttttaagggc taaaaatatt gatgtaattc
    12541 attctaaaat ttattttcaa ggtatactcc ttacaggctc caccgaactt cctaataatc
    12601 ctttttactt tggaaaatta gatcaagata atactataaa acaagatata cccagttatt
    12661 atttttctcc taaagatgaa agcagtggtt taggaa
//
LOCUS       DODKMMJL_2              5506 bp    DNA     linear       12-APR-2021
DEFINITION  Campylobacter species strain strain.
ACCESSION
VERSION
KEYWORDS    .
SOURCE      Campylobacter species
  ORGANISM  Campylobacter species
            Unclassified.
COMMENT     Annotated using prokka 1.14.6 from
            https://github.com/tseemann/prokka.
FEATURES             Location/Qualifiers
     source          1..5506
                     /organism="Campylobacter species"
                     /mol_type="genomic DNA"
                     /strain="strain"
     gene            complement(244..4155)
                     /locus_tag="DODKMMJL_00016"
     CDS             complement(244..4155)
                     /locus_tag="DODKMMJL_00016"
                     /inference="ab initio prediction:Prodigal:002006"
                     /inference="similar to AA sequence:RefSeq:AAW35464.1"
                     /note="conserved hypothetical protein"
                     /codon_start=1
                     /transl_table=11
                     /product="hypothetical protein"
                     /protein_id="Prokka:DODKMMJL_00016"
                     /translation="MFISSKTELIQTIKNLTINNKEVKQIDFIYPNFSILHAILKNID
                     FHQRKILFKNQILAMEILEILKQTRDNHNNIEVNIHTLNELDAGQNVNLKDLSSSEIC
                     TKVYLEDLEEEIQDKKQGKDFNLFYHTDKNNLCIEFINTLKELAKDSEDITTLDELIE
```

```
                     NIDPDDISSEKFDDFKKKVLEKIGNVIESDFFKNSISPALKNAYEKFIDELLEHIKKL
                     AEEKEYEKIKIILSAIAGVTIATVVAVLTGGAGLAILAAGLFSLMGSASEYYEFAQRD
                     YGTLLTPLAAFLATRCAMFIHYINSRLLSCVLIIDKKEIIDLSGFGLYLGSDESNIAS
                     NLAYKITLSEEIKSIENIFSEIFLNPEQKDNESKNTANDSKSSNLPAFISTQELSNLY
                     LKNNAILKNLQKDIKQFIYFSYIESPNFNSSKLAEFFAQKNQNTQIDLNTTKMSKNYL
                     IISNIPNKYNAGLNESLTQEIANYNIQDKYRTIKKNIDTSKKTYDSTRDYSEYSIEMP
                     QDENEPYILQLSPFVKISKEAYENSKKAYEDMQEIYKYLTNFVATPQKLPEFAKTAKE
                     KLAISENESMIEIVKKTENLRHCERINILAYFDYSKKINDKKWLPYFYIKKFFKQNED
                     KESIIEKEKKIAHQYLTFLKKELFEKSDKSSFGNYSLMECLFISFVLYQAKLIFPNIN
                     LDLKKYGGSNFVYIKYLEETYVLWTDEYPIYVNNEPTNITAYEVVSQLLKILDKKDYS
                     FVENDSSITIDDFFNELSKDLDVTNTLDKKIIKLKQMYHLDEQKCIKEFMNLDEEAQQ
                     AFFVWMDIEKQISDLSKLLEQMYGKEYLNKAKEQKTSEIFTYAMLKILLSASPFISFT
                     AQMLWDFNFLKTIEYVIQACIKINKEGFKEFFKEQLGIFTPLKKNSIYHIHIKSNLII
                     KARLQQIEKELTHTKIQMILAQEIKSNLIIQKNTSSLNLFITKIKEYFNEKQIKIYWS
                     NKYMKQLFIYSNQESNVSKIALMHSIDIQTQKYTIYTYAKLNKLDLASIVAGEFVGIF
                     LNYLFPTNYKALQEQYESIVYQLYTFKDDSPLAVIRDHFITYPFAINSKFISFDLKKI
                     FYGIDLCTGVFDTHFSFHYEIDQKLEENKEFLLKRLLSYLIIDEKRSALRIIKDENET
                     IQERDDYTFFNQSLNSIELIKSEIYILKNTTLEKYYKEVNDEINLTAIQAYNEALQIL
                     QDFANGLYIVKYEEGKCIEDKNKTRRLLEKLNIIGQNNLKAMYMGINGNEESLKNKDN
                     EEGNEEDIESVTKNKQSQKLPPNFIGRLATTIIIEDGLYMG"
     gene            complement(4422..4781)
                     /locus_tag="DODKMMJL_00017"
     CDS             complement(4422..4781)
                     /locus_tag="DODKMMJL_00017"
                     /inference="ab initio prediction:Prodigal:002006"
                     /inference="similar to AA sequence:RefSeq:CKG86986.1"
                     /codon_start=1
                     /transl_table=11
                     /product="hypothetical protein"
                     /protein_id="Prokka:DODKMMJL_00017"
                     /translation="MVIFKNILLFLFFSTYLLAQEDFTPLEQCTYENEKFWIKILNLC
                     PEGNITCDKIVYVGVNKNNGKYIVLNGKSISDVNMNFKGYVFKNGIYEYNIFNNFLYI
                     SKNKQIIQEYRLKLCEK"
     gene            complement(4775..5488)
                     /locus_tag="DODKMMJL_00018"
     CDS             complement(4775..5488)
                     /locus_tag="DODKMMJL_00018"
                     /inference="ab initio prediction:Prodigal:002006"
                     /inference="similar to AA sequence:RefSeq:APA47419.1"
                     /codon_start=1
                     /transl_table=11
                     /product="lysozyme"
                     /protein_id="Prokka:DODKMMJL_00018"
                     /translation="MKNLKLQIAQGLDKILEDEDRKQELYVCKFVVVNGVSENKIKAK
                     NKDVNIDNDTKNINTTKSNNISSSENNAQEKITHLSNDGQNLLKNIEKLRLKPYNDQN
                     GKEITSYVKGATIGYGHLIGQNEWDLYKNGITLQEADKLFKSDLLPFENAVKNSINSS
                     LAQNEFDALVILCFNIGIDNFKNSSVAKIINGEKTGYKTLKEAWMAWNKSQNKVMRGL
                     INRRNAEYKLYTQGGYEKW"
ORIGIN
        1 aagtggattt ttgccctgta taaaagttag gatctagata tattggttta tagtctttgt
       61 agggggagtt tcgtttaatg tcgtgtgctt caaacaaagc tttggaatat tcttcggtat
      121 aattgcctgt gggtttaata ttttctttaa atagtatttt attggtatct ttttccacat
      181 ctaatgctaa tccattgggt gtataaattt tataagtttc actcattttt atcctttctt
      241 aatttaaccc atatataaac catcttctat gattatagta gtggctagtc tgcctatgaa
```

301 gttaggcggt aatttttggg attgtttgtt tttagttaca ctttctatat cctcctcgtt
361 tccttcttca ttgtctttgt ttttcaagct ttcttcattt ccatttatcc ccatatacat
421 agctttaaga ttgttttgtc ctatgatatt gagcttttct agtaaccttc tggttttatt
481 tttatcttct atacattttc cttcttcata tttgacaata taaagtccat tagcaaaatc
541 ttgtaaaatt tgtaaagctt cattgtaagc ttgaatggct gttaaattga tttcatcatt
601 aacttcttta taatactttt ctaatgtagt gtttttttagg atataaattt cagatttaat
661 gagttctata ctatttaaac tttgattaaa aaaagtataa tcatctcttt cttgtatagt
721 ttcattttca tcttttatta tccttaaagc ggatcttttc tcatcaataa tcaaataaga
781 caaaagtctt tttaaaagaa attctttatt ttcttcaagc ttttgatcta tttcataatg
841 aaaggaaaaa tgagtgtcaa aaactcctgt gcaaagatct atgccataaa aaattttctt
901 taaatcaaag ctaatgaatt ttgaattgat tgcaaaagga taagttataa aatgatctct
961 tataaccgcc aaaggagaat catctttaaa ggtgtaaagc tgataaacaa tgctttcata
1021 ttgttcttgc agggctttat aatttgttgg aaagagataa tttaaaaaaa tacccacaaa
1081 ttctccagcc acaatagaag caaggtcaag tttatttagc ttagcataag tataaatagt
1141 atatttttga gtttgtatat ctatgctatg cataagagca attttagata cattgctttc
1201 ttgattggag tatataaata attgtttcat gtatttatta ctccaataaa tcttaatttg
1261 tttttcatta aagtattctt ttatctttgt gataaataga tttaaactgg atgtattttt
1321 ttgtattatt aaattggatt ttatctcttg tgctaaaatc atttggattt tagtatgagt
1381 taattctttt tctatttgtt gcaagcgcgc ttttattatt aaattagatt ttatgtgtat
1441 atgatatata ctattctttt ttagaggtgt aaaaatacca agctgttctt taaaaaattc
1501 tttaaatcct tctttgttta ttttaataca agcttgaata acatattcta ttgtttttaa
1561 aaaattaaaa tcccacaaca tttgagctgt aaagctgata aaggactag cgcttaataa
1621 aatttttaac attgcataag taaatatttc acttgttttt tgttcttttg ccttatttaa
1681 atattctttt ccatacatct gttccaaaag ctttgataaa tctgatattt gtttctctat
1741 atccatccat acaaaaaaag cttgttgcgc ttcttcgtct aaattcataa attctttaat
1801 gcatttttgt tcatctaaat gatacatttg cttcaatttg attatttttt tatctaatgt
1861 atttgttaca tctaaatctt ttgataattc attaaaaaaa tcatctatag tgatcgaact
1921 atcattttcc acaaaagaat aatctttttt gtcaagtatt tttaataatt gcgatacaac
1981 ttcatatgct gtaatatttg ttggctcatt atttacatat attggatatt catctgtcca
2041 taaaacataa gtttcctcta agtattttat atatacaaaa ttactgcctc catatttttt
2101 caaatccaaa ttgatatttg gaaaaataag cttagcttga tacaagacaa atgaaataaa
2161 taaacattcc attaaagaat agtttccaaa tgaagattta tccgattttt caaaaagctc
2221 tttttttcaaa aaagttaaat attgatgagc tattttttttt tctttttcta tgatggattc
2281 tttgtcttcg ttttgtttaa agaatttttt gatataaaaa taaggaagcc atttttttatc
2341 atttattttt ttactgtagt caaaataagc taaaatgttt attcgttcgc aatgtcgtaa
2401 attttccgtc tttttttacta tttctatcat tgactcattt tctgatattg ctaatttttc
2461 tttagctgtt tttgcgaatt ctggtagctt ctgcggtgta gcaacaaaat ttgttaaata
2521 tttataaatc tcttgcatat cttcataagc tttttttacta ttctcataag cttctttgga
2581 tattttcaca aaagggctta attgtaaaat ataaggttca ttttcatctt gtggcatttc
2641 tatgctatat tcgctataat ctcttgtgct gtcataagtt tttttttgaag tatcaatatt
2701 tttttttaatt gttctgtatt tatcttgtat attatagttg gcgatttctt gagttaagct
2761 ttcattgagt cctgcattgt atttattggg gatatttgaa ataatcaaat aatttttact
2821 cattttagtt gtatttaaat ctatttgtgt gttttgattt ttttgtgcga aaaattcagc
2881 tagtttactt gagttaaaat ttggactttc tatataagaa aaataaataa attgtttaat
2941 atctttttgc aaattcttta aaatagcatt gtttttttaaa tataaattag ataattcttg
3001 cgttgaaatg aaagcgggta aattgctcga ttttgaatca ttagctgtat ttttactctc
3061 attgtctttt tgttctgggt ttaaaaagat ttcactaaaa atattttcga tgctttttat
3121 ttcttcgctt aaagtgattt tataggctaa atttgaagcg atattgcttt catcacttcc
3181 aagatataat ccaaaaccac ttaaatcaat aatttctttt ttatcaataa ttaaaacaca
3241 agataacaag cgagaattaa tataatgaat aaacatagca catctagtgg ctaaaaatgc
3301 ggctaaaggc gttaaaagtg tgccataatc tctttgagca aattcataat actcgcttgc
3361 agaacccatt aaagaaaata aaccagcagc caaaatagct agtcctgccc cacctgtaag
3421 aacagctacc actgtggcta ttgtaacacc cgctatagca cttagtatga ttttaatttt
3481 ttcatattct ttttcttcgg caagtttttt aatgtgttct aacaactcat cgataaattt

```
     3541 ttcataagca ttttttaaag cgggacttat gctatttttg aaaaaatccg attctatgac
     3601 atttccaatc ttttctaaaa ctttttttctt aaaatcatca aatttctcac tgcttatatc
     3661 atcaggatcg atgttttcga ttaactcgtc caaggttgtt atatcttcag aatctttggc
     3721 taattctttt aaagtattga taaattcgat acaaagatta tttttatctg tatgataaaa
     3781 aagattaaaa tctttacctt gtttcttatc ttgaatttct tcttctaagt cttctaaata
     3841 aactttagtg caaatttcac tgcttgataa atcttttaaa tttacattct gtccagcatc
     3901 aagctcatta agggtatgga tatttacttc tatattattg tgattatcac gagtttgttt
     3961 taaaatttct agtatttcca tagccaatat ttgattttta aataggattt ttctttgatg
     4021 aaaatcaata ttttttaata tagcgtgtaa aatagaaaaa ttaggataaa taaaatcaat
     4081 ctgtttaact tctttattgt tgattgttaa atttttaata gtttgaatta actctgtttt
     4141 agaacttata aacataaaag attccttata taaagcatta ttatatcatt ttttatttgc
     4201 taatattttt tgtctttatt aatctctcct atccatctta taatattacc ctcatcatca
     4261 atctctactt ctaaatcttt gtatttgtta taaaatttag gagctaagat ttcattaatt
     4321 aggattttta taactttaaa aacttcttgt aattcttatt gcatcttttt aactttttac
     4381 atattctagg tatttgttgt tgcttgttgc ctttgttgtg tctatttttc acataatttc
     4441 agacgatatt cttgtataat ctgcttgttt ttagaaatat ataaaaaatt attaaaaata
     4501 ttgtattcat aaataccatt tttaaaaaca tatcctttaa aattcatatt tacatcacta
     4561 atagacttac catttaggac aatatatttt ccattatttt tattgacacc cacatataca
     4621 attttatcac aagttatatt accctctgga catagattta aaatttttat ccaaaacttt
     4681 tcattttcat aggtacattg ctctagtgga gtaaaatcct cttgcgctaa caaataagta
     4741 ctaaaaaata aaaacaataa tatattttta aaaattacca tttttcataa cctccttgag
     4801 tgtaaagctt atattcagca tttctcctat taattagccc ccgcattact ttattttgac
     4861 ttttgttcca cgccatccaa gcttccttta atgttttata acccgttttc tctccattga
     4921 tgattttagc aacagaagaa tttttaaaat tatcaatccc tatattaaaa cataatataa
     4981 ccaaggcatc aaattcgttc tgtgctaagg aagagtttat tgaatttttt acagcatttt
     5041 caaagggtaa caaatcgctt ttaaacaatt tatccgcttc ttgtaaagta ataccatttt
     5101 tatacaaatc ccattcattt tgccctataa gatgtccata tccgatagtt gcacctttaa
     5161 catatgaagt aatttctttt ccattttgat cattataagg tttgagtctt agcttctcta
     5221 tattttttaa aagattttga ccatcattgc ttagatgagt gatttttttct tgagcattgt
     5281 tttcactaga acttatatta ttgcttttttg tagtgtttat attctttgtg tcattatcta
     5341 tatttacatc tttatttttt gcttttattt tgttttcgct aaccccattt accaccacaa
     5401 atttacaaac ataaagctct tgttttctat cttcatcttc taagatttta tctaatcctt
     5461 gtgcgatttg aagttttaaa tttttcattt ttgcaggagc taaaaa
//
LOCUS       DODKMMJL_3               5277 bp    DNA     linear       12-APR-2021
DEFINITION  Campylobacter species strain strain.
ACCESSION
VERSION
KEYWORDS    .
SOURCE      Campylobacter species
  ORGANISM  Campylobacter species
            Unclassified.
COMMENT     Annotated using prokka 1.14.6 from
            https://github.com/tseemann/prokka.
FEATURES             Location/Qualifiers
     source          1..5277
                     /organism="Campylobacter species"
                     /mol_type="genomic DNA"
                     /strain="strain"
     gene            19..135
                     /locus_tag="DODKMMJL_00019"
     CDS             19..135
                     /locus_tag="DODKMMJL_00019"
                     /inference="ab initio prediction:Prodigal:002006"
```

```
                     /inference="similar to AA sequence:RefSeq:CKG86393.1"
                     /codon_start=1
                     /transl_table=11
                     /product="methyltransferase small"
                     /protein_id="Prokka:DODKMMJL_00019"
                     /translation="MKNLKLQIAQGLDKILEDEDRKQELYVCKFVVVNGVKV"
gene                 132..3647
                     /locus_tag="DODKMMJL_00020"
CDS                  132..3647
                     /locus_tag="DODKMMJL_00020"
                     /inference="ab initio prediction:Prodigal:002006"
                     /codon_start=1
                     /transl_table=11
                     /product="hypothetical protein"
                     /protein_id="Prokka:DODKMMJL_00020"
                     /translation="MKIKNFTLITSNHNLEIELKNLTKNANKIIFICSDFDGYKEAEN
                     SLYKTLHTIEDNFKIDNTKTFKIIHLNQTNKDEITKEQKFSSDKNLSSIKKIIYNNLK
                     LLNDTINQNENKTLNKIIDESNEKNIENFLKEIQKELNILLKNIKESNYFDQEIKPKI
                     QVTLDTLFNTLLKNIAQSNDDEYKQIIRAVAKFFINILDSITSLKKPISLIKKNPYVL
                     IVNTLFEAYNSYSEYQEYKEKKYYYDFAYPLLELITSKLYPIIALCNEEILSDVLIID
                     DKVLLDFSSYTNAPSISLYKNSFYKIILDEEFQGVFDNFLENNLKFENEKLDNTLLVN
                     SSLNNSDTTLKKAINLNISKLNHFFYAEYPDLNSSMLVEMILDKKNADQINTTKMGKN
                     YLFITNPPQLNNAGLCEDLYQKKLGAIIKNRPKALTKNEKLALKGDFPSKRDYGSYFI
                     ERDVKKEDDFVLQLCPFAKLDGQIYQKYKGYFNFYNQTYSWMKLYLLEYINERFEYER
                     FKQDFAYIDNFFKLPKELEKISEKEKEKVKEYVELFNDYLDKINFRYDQIQFFLDSEP
                     LMFKTYENVDFKKYSPLTVVLLTLTLYVLQKDLAFTQEYSLGVKNIAIPNPDYGDKEI
                     SLNYQRIKIPNYFNEYILFIPDDIYFYDNDKKIYIFLSKELNKNKKEDEVIYLDELAK
                     AMFENDLKEDDQTYVKFLNTLNENFDDEIEFEENIQTNQTLPTKKDLDKSIQEIMLDN
                     ELAKLSLDITFFNIIKQLFPFTEFFMSQPDFFKKMILFFINSSSDKKYYGNKLFSSFK
                     AHFFKELGIFYALNPKGRFIKIIPKNKKPFNKLFLYQIGTNYYYFSQSEYASELKKSK
                     SFSRLDKAKQIHLISKARKQLAIDFSIKVSKNLSADVLKNLAGSFIDTLLPTNYERLK
                     ILYEKLMYEKLTYNYDFPLAVKKEEYITYPLLINSRFMSFDLSEFIFGSLLCTGGLNY
                     YPSIACTTTSSEAREFALKRLLSYIILDEKRSAFMEDKINEGKYILNDKEFFEKNNIE
                     LRNCNIYKLEHFQRKEEYKTQNPMIKQHPILAYNDTIQILYEYINGIYNTIQDEKGNF
                     IEDRQKARRLMENLEAIGQHNLEVMYKGINEENDETEKIISQNKRHYKNIENLITNNK
                     SQDYSNKFIGRLATTIIMEDGLYMG"
gene                 3665..4957
                     /gene="trxB_2"
                     /locus_tag="DODKMMJL_00021"
CDS                  3665..4957
                     /gene="trxB_2"
                     /locus_tag="DODKMMJL_00021"
                     /inference="ab initio prediction:Prodigal:002006"
                     /inference="similar to AA sequence:RefSeq:CKG86288.1"
                     /codon_start=1
                     /transl_table=11
                     /product="thioredoxin reductase"
                     /protein_id="Prokka:DODKMMJL_00021"
                     /translation="MSETYEIYTPEGFILNVDKQSGKILFSESKSKKPTGKYTQEYSK
                     ALFEADRILRTSPYINYQPRYLDPEFHTGQKSTLLEFKDWQSIYLKDPIKGSIAPWTK
                     AEKAYYNSLKTKKERYKYLVIRSGIRSVVIDIPYEAIGAVDEKGNVDPKYEELYRTVD
                     DNKHNLRSSLFHNEWGMAAGILGDYKYLANDMSQNGFNARFIQATILYIQLSGGSSIL
                     DKPNLLGAIYGYADIAVGSGLVGVHKNPLREQQIKTLAKTLKPDEFGMLPFIDEIMGV
                     DWVIDLNVYQFNFDAWGKIYRALRSDVVEGKIKDPRDIDSTYESRREFDRHMDGYQNG
```

```
                     MTINYHVDINNYRSEESAKLFMDSLVLEAKLAALTPPQGYPNAPYYFTPERLEWDYKN
                     HQLDKLLDPRIPAIYRYNFPRELRAKILKFGEENGIKE"
ORIGIN
        1 tttttagctc ctgcaaaaat gaaaaattta aaacttcaaa tcgcacaagg attagataaa
       61 atcttagaag atgaagatag aaaacaagag ctttatgttt gtaaatttgt ggtggtaaat
      121 ggggttaaag tatgaaaata aaaaatttta ctttaattac atcaaatcat aatttagaaa
      181 tcgagttaaa aaatcttacc aaaaatgcta ataaaattat ttttatatgc agtgattttg
      241 atggatataa agaagctgag aattctctgt ataaaacatt acatactatt gaagataatt
      301 ttaaaattga caatacaaaa acttttaaaa tcatacattt aaatcaaacc aataaagacg
      361 aaatcacaaa agaacaaaaa tttagctctg ataaaaattt atctagcatt aaaaaaatta
      421 tctataataa tttaaaactt ttaaatgata caatcaatca aaatgaaaat aaaacactca
      481 ataaaattat tgatgaaagc aatgaaaaaa atatcgaaaa tttttaaaa gaaattcaaa
      541 aagaattaaa tatattacta aaaaatatca aagaatctaa ttattttgat caagaaatca
      601 agccaaagat acaagttact ttagatacgc tttttaacac acttttaaaa aatattgccc
      661 aatctaatga tgatgaatat aagcaaatta ttagagctgt agcgaaattt tttatcaata
      721 tattagattc tataaccagc cttaaaaagc ccatttcttt aattaaaaaa aatccttatg
      781 ttttgattgt aaatacatta tttgaagctt ataattctta ctctgaatat caagagtata
      841 aggaaaaaaa atattattat gattttgctt atccactttt agaacttatc actagcaaac
      901 tttatcctat tattgcttta tgcaatgaag agattttatc tgatgtgtta attatagatg
      961 ataaagtatt attagatttt tcatcttata ctaatgcacc ttctatcagt ctttataaaa
     1021 atagttttta taaaattatt ttagatgaag aatttcaagg tgtttttgat aattttttag
     1081 aaaataatct aaaatttgaa aatgaaaaat tagacaacac tcttttagtg aattctagtt
     1141 taaataattc tgacacaaca ttaaaaaagg cgataaattt aaatatctcc aaactcaatc
     1201 atttctttta tgcagaatac cctgatttaa attcttccat gctagtagaa atgatactgg
     1261 ataaaaaaaa tgctgatcaa atcaatacaa ccaaaatggg taaaaattat ttatttatta
     1321 ccaatccacc acagcttaat aatgcagggc tttgcgaaga tttgtatcaa aaaaaattag
     1381 gagcaataat aaaaaatcgc cccaaagctc ttacaaaaaa tgaaaaactt gcattaaaag
     1441 gcgattttcc tagcaaaaga gattatggtt cttattttat agaaagagat gttaaaaaag
     1501 aagatgattt tgtcttgcaa ctttgtcctt ttgcgaaact tgatggacag atttatcaaa
     1561 aatataaggg atattttaat ttttataatc aaacatattc ttggatgaaa ctttatttat
     1621 tagaatatat aaatgaacga tttgaatatg aaagatttaa acaagatttt gcttatattg
     1681 ataacttttt taaattacct aaagagcttg agaaaattag cgaaaaagaa aaagaaaaag
     1741 taaaagaata tgtcgagctt tttaatgatt atttggataa gatcaatttt cgttatgatc
     1801 aaatacaatt ctttcttgac tcagaaccat taatgttcaa aacatatgaa aatgtagatt
     1861 ttaagaaata ctcaccttta acggttgttt tattgacttt aactttgtat gttttacaaa
     1921 aagacttagc ttttacgcaa gaatattctc ttggtgttaa aaatattgca attccaaatc
     1981 cagattatgg cgataaagaa atttcactta attaccaaag gattaaaata ccaaattatt
     2041 ttaatgagta tatcttattt atccccgatg atatttattt ttatgataat gataaaaaaa
     2101 tttacatctt tttatccaaa gagttaaata aaaataaaaa agaagatgaa gttatctatt
     2161 tagatgaatt agctaaagct atgtttgaaa atgatcttaa agaagatgat caaacttatg
     2221 tgaaattttt aaacacctta aatgaaaact ttgatgatga aattgaattt gaagaaaata
     2281 tacaaacaaa tcaaacactt cctacaaaaa aggatttaga taaaagtatt caagaaatta
     2341 tgcttgataa tgagcttgca aaactatcct tagatatcac ttttttaat atcattaagc
     2401 aactttttcc ttttacagaa ttttttatga gtcagcctga tttttttaaa aaaatgattt
     2461 tattttttat aaattcaagc tctgataaaa agtattatgg caataaatta ttttctagtt
     2521 ttaaagcaca ttttttaaa gaactaggga tattttatgc acttaatcct aaagggcgtt
     2581 ttataaaaat catcccaaaa aataaaaagc cattcaataa gcttttttta tatcaaatag
     2641 gaacaaatta ttattatttt agtcaatcag aatatgcaag cgagttgaaa aaatcaaaga
     2701 gtttttcaag attggataaa gctaagcaaa ttcatttgat atcaaaagca agaaaacaat
     2761 tagctattga tttttctatt aaagtaagta aaaatttaag tgcggatgta ttaaaaaatt
     2821 tagcaggttc ttttattgat acacttttgc ctacaaatta tgaaagatta aaaattttat
     2881 atgaaaaact aatgtatgaa aaattgactt acaattatga ttttccttta gctgtaaaaa
     2941 aagaagagta cataacctat cctttattga taaattctag atttatgagt tttgatttat
     3001 ctgagtttat ttttggttct ttattgtgca caggtgggct taattattat cctagtattg
```

```
     3061 cttgcactac aacttcaagt gaagctagag aatttgcctt aaaaagactt ttatcttata
     3121 ttattttaga tgagaaaaga agtgctttta tggaagataa aatcaatgag ggcaaatata
     3181 tcttaaatga taaggaattt tttgaaaaaa acaatataga acttagaaat tgtaatatat
     3241 ataagctaga acattttcaa agaaaagaag agtataaaac acaaaatcct atgataaaac
     3301 aacaccctat cttagcttat aatgatacta tacaaatttt atatgaatac ataaatggaa
     3361 tttacaatac tatacaggat gaaaagggta atttcataga agataggcaa aaggcaagaa
     3421 gattgatgga aaatttagaa gccataggac aacataattt agaagtgatg tataagggga
     3481 taaatgaaga gaatgatgaa acagaaaaaa taatatcgca aaacaaaaga cattataaaa
     3541 atattgagaa tttaattacc aataataaat cccaagatta ttctaataag tttataggca
     3601 ggctagccac tactataatc atggaagatg gtttatatat gggataaatt aagaaaggat
     3661 aaaaatgagt gaaacttatg aaatttatac acccgaaggt tttatattaa atgtagataa
     3721 acaaagcgga aaaatactat tttcagagtc aaagtcaaaa aaacctacag gcaaatacac
     3781 ccaagaatat tccaaagctt tgtttgaagc cgatagaatt ttaagaacct ctccttatat
     3841 taattatcaa cctaggtatc tagatcctga atttcacaca gggcaaaaat ccactttgct
     3901 tgaatttaaa gattggcaaa gcatatattt aaaagatccc attaaaggaa gcatagctcc
     3961 ttggactaaa gcagaaaaag cttattataa ctctttaaaa actaaaaaag aaagatataa
     4021 atatttagtt ataagaagtg gtataagaag tgttgttata gatatacctt atgaagccat
     4081 aggagctgta gatgaaaaag gaaatgtaga tcctaagtat gaagaattat atagaacagt
     4141 agatgataat aaacataatc taagatcaag tttatttcat aatgaatggg gtatggcagc
     4201 aggaatacta ggtgattata aatatctagc taacgatatg tctcaaaatg gttttaatgc
     4261 aagatttatc caagcaacta tactttatat acaattaagc ggaggaagta gtatactaga
     4321 taaacctaat ttattgggtg ctatttatgg ttatgcagat attgcagtag gaagtggttt
     4381 agtaggagtg cataaaaatc ctttaagaga acaacaaatt aaaaccttag ctaagacttt
     4441 aaaacctgat gaatttggta tgcttccttt tatagatgag attatgggag tagattgggt
     4501 gattgattta aatgtttatc aatttaattt tgatgcatgg ggaaaaatat atagagcctt
     4561 aagaagtgat gtagttgagg gtaaaataaa agatccaaga gatatagatt ctacttatga
     4621 aagcagaagg gagtttgatc gccatatgga tggatatcaa aatggtatga caattaatta
     4681 ccatgtagat ataaataatt atagaagcga agaatcagct aaattattta tggattcttt
     4741 ggtattagag gccaaactcg cagccctcac tcctcctcaa ggctatccta atgcacctta
     4801 ttattttacc cctgaaagac tagaatggga ttataaaaat catcaattag acaaactcct
     4861 tgatccaaga atccctgcta tttatagata taatttccca agggaactta gagctaagat
     4921 actcaaattt ggtgaagaaa atggcattaa agaataaagt aacctcatag gcaggctagc
     4981 cactactata atcatggaag atggtttata tatgggataa attaagaaag gataaaaatg
     5041 agtgataata aagatattta cgagatctat acttctaatg gacttatatt agaagttgat
     5101 aaaaatacaa atcaaattat atttgataaa agaaaagatg ggcgtgaagt aggcaaatac
     5161 acccaagaat attccaaagc tttgtttgaa gccgatagaa ttttaagaac ctctccttat
     5221 attaattatc aacctaggta tctagatcct gaatttcaca cagggcaaaa atccact
//
LOCUS       DODKMMJL_4              4481 bp    DNA     linear       12-APR-2021
DEFINITION  Campylobacter species strain strain.
ACCESSION
VERSION
KEYWORDS    .
SOURCE      Campylobacter species
  ORGANISM  Campylobacter species
            Unclassified.
COMMENT     Annotated using prokka 1.14.6 from
            https://github.com/tseemann/prokka.
FEATURES             Location/Qualifiers
     source          1..4481
                     /organism="Campylobacter species"
                     /mol_type="genomic DNA"
                     /strain="strain"
     gene            complement(214..417)
```

```
                     /locus_tag="DODKMMJL_00022"
     CDS             complement(214..417)
                     /locus_tag="DODKMMJL_00022"
                     /inference="ab initio prediction:Prodigal:002006"
                     /codon_start=1
                     /transl_table=11
                     /product="hypothetical protein"
                     /protein_id="Prokka:DODKMMJL_00022"
                     /translation="MDNITIIEKTLSNIFLILVIKNNHRLFTIGGKIIHPITMRNSLN
                     LSGLCPIKGDIIQAKIKNIIQQI"
     gene            627..875
                     /locus_tag="DODKMMJL_00023"
     CDS             627..875
                     /locus_tag="DODKMMJL_00023"
                     /inference="ab initio prediction:Prodigal:002006"
                     /inference="similar to AA sequence:RefSeq:CKG86895.1"
                     /codon_start=1
                     /transl_table=11
                     /product="methyltransferase small"
                     /protein_id="Prokka:DODKMMJL_00023"
                     /translation="MSKDYKDYFIYDDSLAFYPKLNGEFREIQTQNFAKRYTIKFHQP
                     NTFSGFSATLFYDIQKDEYIASFRGTEGIVSMDAFVPI"
     gene            1121..1555
                     /locus_tag="DODKMMJL_00024"
     CDS             1121..1555
                     /locus_tag="DODKMMJL_00024"
                     /inference="ab initio prediction:Prodigal:002006"
                     /codon_start=1
                     /transl_table=11
                     /product="hypothetical protein"
                     /protein_id="Prokka:DODKMMJL_00024"
                     /translation="MGILKKVYIVWGVFWGIIFLFSSLYAYFSDIEFFGDMLKIAFFI
                     YSYPLLPYISYLTLQETIKSKNIFLYFSLFFFILLYFALFFFISGGFFIYVIILGIYT
                     ILFLIISIKFRKELSYYFSLLFCIFLYIFLYLFIFYIELKNE"
     gene            1659..1955
                     /locus_tag="DODKMMJL_00025"
     CDS             1659..1955
                     /locus_tag="DODKMMJL_00025"
                     /inference="ab initio prediction:Prodigal:002006"
                     /codon_start=1
                     /transl_table=11
                     /product="hypothetical protein"
                     /protein_id="Prokka:DODKMMJL_00025"
                     /translation="MKILKSLYIIWMIFWGFGAILGYYMGYDATFTKMFFFLVILYLF
                     PLLPYLHYLIFEKTKNKNIFISICYYRLLFYFNIFMWNKFFNIFYNDYFIYDYL"
     gene            1927..2151
                     /locus_tag="DODKMMJL_00026"
     CDS             1927..2151
                     /locus_tag="DODKMMJL_00026"
                     /inference="ab initio prediction:Prodigal:002006"
                     /codon_start=1
                     /transl_table=11
                     /product="hypothetical protein"
                     /protein_id="Prokka:DODKMMJL_00026"
```

```
                     /translation="MITLYMIICSAIILWKFKINLRLYFYIILCIFTYLYIVLHIFIA
                     CYEIEWTDILLDKDVRIYIYKEIINLLTEQ"
     gene            2311..2616
                     /locus_tag="DODKMMJL_00027"
     CDS             2311..2616
                     /locus_tag="DODKMMJL_00027"
                     /inference="ab initio prediction:Prodigal:002006"
                     /codon_start=1
                     /transl_table=11
                     /product="hypothetical protein"
                     /protein_id="Prokka:DODKMMJL_00027"
                     /translation="MGVFRKFYIVWVVFCISGFAISPAVGHNPNRVYEFFVMLGWIIF
                     PLILLMLYRFFSLCEIKFLYIALLLLLYYPIALILYYMFYYHNSFYVTLYIFLSLFK"
     gene            2626..4158
                     /locus_tag="DODKMMJL_00028"
     CDS             2626..4158
                     /locus_tag="DODKMMJL_00028"
                     /inference="ab initio prediction:Prodigal:002006"
                     /inference="similar to AA sequence:RefSeq:AAW35443.1"
                     /note="conserved domain protein"
                     /codon_start=1
                     /transl_table=11
                     /product="hypothetical protein"
                     /protein_id="Prokka:DODKMMJL_00028"
                     /translation="MKEKINKLRDYAELAWASYFYFDLKGYILQENETIVTLSELVSL
                     SYNGKIAGKKEKVRQEYSFIGKGELNGEFGELQARNFSQRYEIKFHQANTLSGFSATL
                     FYDKEKDKFVVGIRGTEVSFKDLKATWETALDVTQDLVLSLNGNLQSSPLLEFLEQVD
                     KIIKNENKSIIFVGHSLGGYLAQMALIYCDIKYKDKLSFSPNEVYTFNAPSVYGWNFP
                     NIAINPNTIKIMQDLLGKYTIDVSEKITHIYDNGGVEIIASAQYGASNALDIYTGKND
                     HSIKPLVNTLYFYSYLLELKENEDKIIKNNSLKESLENLNHFMKNIQIYTETFVLKNN
                     DINNKNFARKNGPLGLFRSSPEKINHFEYFLSLIATIMQETNDILEELGDNYYTNYKA
                     PTISQTKIIDFILKVQEKEKYILILDKNDFNKYRKDCSFINNQENLAHKIAIGEFRIF
                     IAVYEDMKCLENINNITKIYGYNSKVYRLKDQIWDERYLGGVCKISQALYFSGKAKIG
                     II"
ORIGIN
        1 aaaaagtcag acaagaatat agttttattg gcaaaggtga attaaatgga gaatttggag
       61 aattacaagc tagaaatttc tctcaaagat atgagattaa attccatcaa gccaacactt
      121 taagtggctt tagtgctact ttgttttatg ataaagaaaa agataaattt gttgtaggaa
      181 ttagagggac tgaatgcgga ttctaaaaat actttatatt tgctggatta tattttttat
      241 ctttgcttgg ataatatcac ctttaattgg gcataatcca gatagattta aggaatttct
      301 catagtgatt ggatggataa ttttgcctcc aatagtgaac aatctatggt tatttttaat
      361 aacaagaatt aaaaaaatat ttgataaggt tttttctatt attgttatat tgtccattgg
      421 caattttatt atatcttata tttgattaaa aggaaaaaaa tatgaagcaa caaatcaaga
      481 atcttagaga ctatgctgag ttagcttgag caagctattt caaccttatc tatattgatg
      541 gacaaaaaac taatgatttc acaataggaa cagaaagaat cttaaaagat ggagaaaata
      601 tagaaaaatt taaatattat aaagcattgt ctaaagacta taaagattat tttatttatg
      661 atgattcgtt agctttttat ccaaaactca atggtgaatt tagagaaatc caaacacaaa
      721 attttgcaaa acgctataca atcaaatttc accaacccaa tacttttagt ggtttttctg
      781 ctacactttt ttatgacatt caaaaagatg agtatattgc tagttttcga ggaacagaag
      841 gaattgtaag tatggatgca tttgtgccga tataggactt acttttggta aaggagattt
      901 tcaagctaat ggcttaaaac aatttttatt agatatagca cctataggac attctctggt
      961 gggtatttag ctcaaatggc tttgatatat tgtgattaaa tataaagatt tatactttta
     1021 atactcttag tatttacgtt tgaaatgttc ctagtgtggt tatattttga tttttaaaaa
     1081 agtaggataa ttttattaaa aatattaaaa aggaaaataa atgggtatat taaaaaaagt
```

1141 atacattgtt tggggggtct tttggggaat tattttttttg tttagttctt tatatgctta
1201 ttttagtgat atagaatttt ttggtgatat gttaaagatt gcattttttta tatattctta
1261 tccattgctg ccttacatat catatttaac actacaagaa actattaaat ccaaaaatat
1321 ttttctttat ttttctttat ttttctttat tttgctttat tttgctttat ttttctttat
1381 ctcgggagga ttttttattt atgtaataat attaggtatt tatacaatat tattttttaat
1441 aatatctatt aaatttagaa aagaactttc ttattatttt tctttattat tttgtatttt
1501 tttatacatt tttttgtatc tttttatttt ttatatagaa ttaaaaaaatg agtaataaag
1561 ctcaaattaa caatctcaaa aattacgcta aacttgtata gattggtttt ttttaatttt
1621 ttaataataa tttaaatttc aacaaaaaag gaagtagtgt gaaaatatta aaaagtttat
1681 atattatttg gatgatattt tggggatttg gggctatttt aggttattat atgggatatg
1741 atgcaacttt tactaaaatg ttcttttttc ttgttatatt atatcttttt ccattgttgc
1801 cttatttgca ttatttaatt tttgagaaaa caaagaacaa aaatatattc atttctattt
1861 gttattaccg cttattattt tactttaata tattcatgtg gaacaaattt tttaacattt
1921 tttataatga ttactttata tatgattatt tgtagtgcta taatcttatg gaaatttaaa
1981 ataaatttaa gattatattt ttacataatt ttatgtattt ttacttatct ctatatagtt
2041 ttacatattt ttatagcatg ttatgaaata gaatggacag atatactgct agataaagat
2101 gtaaggatat atatttacaa agaaataatt aatttgctta cggagcaata atttgtttta
2161 taataaatct tatttttata gggggaataa attaatgaaa acccaaatca acaatttaag
2221 agattatgta gaacttgcat gggctagtta tttttatttt aatttttaaa aaagtagaaa
2281 atttactaaa aatattaaaa aggaaaataa atgggtgtat ttcgtaagtt ttatattgtt
2341 tgggtagtat tttgtataag tggatttgct atttctcctg ctgtagggca taatccaaat
2401 agagtgtatg aattttttgt aatgctaggt tggattattt tccctttaat tctattaatg
2461 ctttatcgct ttttttctct ttgtgaaata aagtttttat atatagcatt gctattgtta
2521 ctttattatc ctatagcttt gatattgtat tacatgtttt attatcataa tagtttttat
2581 gttacattat atattttttt atcattattt aaataaagga taattttgaa agaaaaaatc
2641 aataagctaa gagattatgc agagcttgca tgggctagtt atttttattt tgatttaaaa
2701 ggttatatat tacaagaaaa tgaaactatc gttactttaa gtgagcttgt aagtttaagt
2761 tataacggaa aaatagctgg aaaaaaagaa aaagtcagac aagaatatag ttttattggc
2821 aaaggtgaat taaatggaga atttggagaa ttacaagcta gaaatttctc tcaaagatat
2881 gagattaaat tccatcaagc caacacttta agtggtttta gtgctacttt gttttatgat
2941 aaagaaaaag ataaatttgt tgtaggaatt agagggactg aagtttcttt taaagattta
3001 aaagcgacat gggaaactgc tttagatgta acccaagact tggttttatc cctcaatggc
3061 aatctccaat cttctcctct tttagaattt ttagaacaag tagataaaat catcaaaaat
3121 gaaaataaaa gtatcatatt tgtaggacat tcattgggtg ggtatttagc tcaaatggct
3181 ttgatatatt gcgatattaa atataaagat aaactatctt ttagccctaa tgaagtttat
3241 acttttaatg cccctagtgt ttatggttgg aattttccta atatagctat caaccctaat
3301 actataaaaa ttatgcaaga tcttttagga aaatatacta tagatgtttc agaaaaaatc
3361 actcatattt atgataatgg tggagttgaa atcattgctt cagctcaata tggtgcaagc
3421 aatgccttag atatatatac agggaaaaat gatcactcta taaaacctct tgttaatact
3481 ctctattttt attcttatct tttagagctt aaggaaaatg aagataaaat aataaaaaat
3541 aattctttaa aagaaagttt ggaaaattta aatcatttca tgaaaaatat tcaaatttat
3601 acagaaactt ttgtgttaaa aaataatgat attaataata aaaattttgc tcgtaaaaat
3661 ggacctcttg ggcttttttag aagctcccca gaaaaaatca atcattttga atactttctt
3721 tcattaatag ccaccataat gcaagaaacc aatgatattt tagaagagct tggagataat
3781 tattatacaa actacaaagc tcctaccatt agtcaaacaa aaatcataga ttttattttta
3841 aaggtgcaag aaaaagaaaa atatatttta attcttgata aaaatgattt taataaatac
3901 agaaaagatt gttcctttat taataatcaa gaaaatttag ctcataaaat tgccattggg
3961 gaatttagaa ttttcattgc agtttatgaa gatatgaaat gtcttgaaaa tattaacaat
4021 ataacaaaaa tatatggata taattcaaaa gtttataggc taaaagatca aatttgggat
4081 gagcggtatt taggtggagt ttgtaaaata tcacaagctt tatattttag tggaaaagcc
4141 aaaataggta ttatttagga aaattatgac aggtagagac tttttatatt ctattcataa
4201 agataaaaaa agtatttatt tattttgtga aaataaaagc atcattgatt gtcaaagcat
4261 ttatgaagaa ctttataaat tagaagcaac gacagatttt acttttgaag aactccaaag
4321 ctatcaagct tatatcttcc taaactccac cctacttaca ggctccaccg aacttcctaa

```
     4381 taatcctttt tactttggaa aattagatca agataatact ataaaacaag atatacccag
     4441 ttattatttt tctcctaaag atgaaagcag tggtttagga a
//
LOCUS       DODKMMJL_5              1234 bp    DNA     linear       12-APR-2021
DEFINITION  Campylobacter species strain strain.
ACCESSION
VERSION
KEYWORDS    .
SOURCE      Campylobacter species
  ORGANISM  Campylobacter species
            Unclassified.
COMMENT     Annotated using prokka 1.14.6 from
            https://github.com/tseemann/prokka.
FEATURES             Location/Qualifiers
     source          1..1234
                     /organism="Campylobacter species"
                     /mol_type="genomic DNA"
                     /strain="strain"
ORIGIN
        1 cttacaggct ccaccgaact tcctaataat cctttttact ttggaaaatt agatcaagat
       61 aatactataa aacaagatat acccagttat tatttttctc ctaaagatga aagcagtggt
      121 ttaggaaaac ttagcatctt ttataaaaat gatgagcttt gcttgcttaa ttattttata
      181 ttagaaaatt ctttaaatat caaattagaa tgcttaagca agcaaagctt agaatacaaa
      241 gatttaattt caaacacttt taaaactcaa aaaaccacac aagtagatac aaaacaaagc
      301 atagccaaac ttcacgctct tttagaaaat caaaacctag aatgtatcca tggaggcaaa
      361 gtcatactta aatcaaacaa aggaaaatcc tttaaaagtg atggtatccc tataatgcta
      421 gaaagtgatt tattaaactc tagtatagta gcttgtccta ataccatagc aaatgtaagt
      481 tatccttgta ctaaggtagt ggatattaaa ggctccttat ctcaaaagaa agtaaataat
      541 gaatttgtaa tcttacaaga actcatctca gcttgcacta cagataaagg ctttgcttta
      601 aaagtaagct ttactccaag taagtttaaa tttgatcaca gctttgatcc tgaggagggc
      661 ttaggagaac aaagtaaaaa tcaaatagag ttaaaagaac ctataataag acttcattat
      721 aaaagtgata gatttcaaaa agataattta cctatctata atcttttaat caataatgaa
      781 aaaaaagaac aagataaggc tttaaatgaa ttcaatatag aacaaaaaga tttaaaagat
      841 atagaggatg taaatatctt taatcaattt gaacaaggct ttagtaaaga ttatgaattt
      901 aaagaattaa atcttagctt tgatactaat ttaatcaagc tttatttcat tatccccaaa
      961 aacattgcca aagtttataa aagtgcttat aaagaatttg aaaacaaaga tctaggagca
     1021 gggtatttta cacagctaca tgagtatgat aaaatcatca aaaatgcttt agaagataat
     1081 aaagaattaa acgaatatca tttaactttt ttagctcctg caaaaatgaa aaatttaaaa
     1141 cttcaaatcg cacaaggatt agataaaatc ttagaagatg aagatagaaa acaagagctt
     1201 tatgtttgta aatttgtggt ggtaaatggg gtta
//
LOCUS       DODKMMJL_6               654 bp    DNA     linear       12-APR-2021
DEFINITION  Campylobacter species strain strain.
ACCESSION
VERSION
KEYWORDS    .
SOURCE      Campylobacter species
  ORGANISM  Campylobacter species
            Unclassified.
COMMENT     Annotated using prokka 1.14.6 from
            https://github.com/tseemann/prokka.
FEATURES             Location/Qualifiers
     source          1..654
                     /organism="Campylobacter species"
```

```
                     /mol_type="genomic DNA"
                     /strain="strain"
ORIGIN
        1 ttcaactcac aggtggagga tatgaagaat taggtataaa aagaggaatt tataattatg
       61 ctgataatct tttagaaata ggtataggta tggcagggat acataaaaat cctttaaggg
      121 ctaaattagt aaaagattta gctaaaacta tacagcctga tgaatttggt atgctacctt
      181 ttatagatga gattatggga gtagattggg tgattgattt aaatcgatat aaatttgctc
      241 ttgatgaaga aggtagaatc atttgggctt tatatgatga tattgaaaaa ggtaaattaa
      301 aagatccaag agatgtggat tctactcctg aaagcaggaa agaatttgat cattatatgg
      361 atggatataa aaatggaatg gaaactagat tcgatgctga tattccaaat gattggggtg
      421 atcgacaagc cactttattc aaagataccc tagtattaag tgccaaactc gcagccctca
      481 ctcctcctca aggttatcct aatgctccaa gatactatag ccctgaaaga ctagaaatta
      541 tttataaaag acataagctt gataaactcc ttgatccaag aatccctgct atttatagat
      601 ataatttccc aagagaactt agagctaaga tactcaaatt tgctgaagaa aatg
//
```

```
>>BfR-CA-15829_min_cov_30 putative plasmid location

LOCUS       FAFJHLOP_1              3230 bp    DNA     linear       12-APR-2021
DEFINITION  Campylobacter species strain strain.
ACCESSION
VERSION
KEYWORDS    .
SOURCE      Campylobacter species
  ORGANISM  Campylobacter species
            Unclassified.
COMMENT     Annotated using prokka 1.14.6 from
            https://github.com/tseemann/prokka.
FEATURES             Location/Qualifiers
     source          1..3230
                     /organism="Campylobacter species"
                     /mol_type="genomic DNA"
                     /strain="strain"
     gene            complement(443..1720)
                     /gene="trxB_1"
                     /locus_tag="FAFJHLOP_00001"
     CDS             complement(443..1720)
                     /gene="trxB_1"
                     /locus_tag="FAFJHLOP_00001"
                     /inference="ab initio prediction:Prodigal:002006"
                     /inference="similar to AA sequence:RefSeq:CKG86610.1"
                     /codon_start=1
                     /transl_table=11
                     /product="thioredoxin reductase"
                     /protein_id="Prokka:FAFJHLOP_00001"
                     /translation="MSDTYEIYTPHGSILDVNKKTNEIYFDQSSKPTGKYTKEYSKAL
                     FKAWEIMENSPYKDYEPRYLDPNFYTGQKSTLVEFKDWQSIYLKEPIKGSIAPWTKAE
                     KAYYKSLKTKRERYKYLVIRSGIRSTVIDIPYDAYANVDEKGNLINEDYKELYKEVEA
                     NRGLAHLSDGYLFMGEWELAAGILGDIKGFIGALQLSMTGFKARTQAVNFLLIQLGHE
                     QGFKSLYDSYAYRGLTDGIHKNPLKAQMLKDFSKNPPYDEFGMLPFLDELMGVDWVID
                     LTEYEFADDADGKAIRSLDDDVLKGKLKDPRDIDSTPESRREFNREMWAYRRGSVAFY
```

```
                     NTDIPNDWTNQEAKLYIDSLILESKLAVLTPPQGYPNAPYYWVPEHLEYVYKKHKLDK
                     LLDPRIPAIYRYNFPRELRAKILKFGEENGIKD"
     gene            complement(1792..3069)
                     /gene="trxB_2"
                     /locus_tag="FAFJHLOP_00002"
     CDS             complement(1792..3069)
                     /gene="trxB_2"
                     /locus_tag="FAFJHLOP_00002"
                     /inference="ab initio prediction:Prodigal:002006"
                     /inference="similar to AA sequence:RefSeq:CKG86610.1"
                     /codon_start=1
                     /transl_table=11
                     /product="thioredoxin reductase"
                     /protein_id="Prokka:FAFJHLOP_00002"
                     /translation="MSDTYMIYAPNGRGVEVDKKTKEIYFAQSVDPVGKYTKEYTKVF
                     FKAWDIMENSPYKDYQPIYLDPNFYTGQKSTLVEFKDWQSIYLKEPIKGSIAPWTKAE
                     KAYYKSLKTKRERYKYLIIRSGIRSTVIDIPYEAYCNVDEKGNLINKDYKELYKEVEA
                     NRGMANMHKGWLFMAEWELAAGILGDIKGFVGALQLSMTGFKARTQAINFLLIQLGHE
                     QGFKSLYDSYAYRDLTDGIHKNSLKAQMLKDFAKNPPYDEFGMLPFLDELIGVDWVID
                     VNKYQFAYDEKGRVNDALKDDVEKGILKDPRDIDSTPESRWEFDKEMDAYKNGMRTRF
                     DGDNPNHWSKEQVERFNDTLILCAKLAALTPPQGYPNAPYYYSPERLEFIYKKHKLDR
                     LEDPRIPAIYRYNFPRELRAKILKFGEENGIKD"
ORIGIN
        1 atatttatat ctttctcttt tagtttttaa agatttataa taagcttttt ctgctttagt
       61 ccaaggagct atacttcctt tgataggttc ttttaaatat atactttgcc aatctttaaa
      121 ttctactaga gtggattttt gccctgtata aaagttagga tctaaatatc ttggttcata
      181 atctttgtag gggagttttt ccatgatttc ccaagctttg aagagggctt tggaatgttg
      241 ctcaacatac ttatctacag gcttattaat tgtatcgcca attaatattt tatttgtgtc
      301 tttttctaca tttaatataa gtccatttgg tgtataaatc tcataagttt cactcatctt
      361 ttgtcctttt atctttaatt tatcctatat gtaaaccatt tttcatgatt atagtggtgg
      421 ctagtctgcc tatgaaatta ctttaatctt taatgccatt ttcttcgcca aattttagta
      481 tcttagctct aagttccctt gggaaattat atctataaat agcagggatt cttggatcaa
      541 ggagtttatc aagcttatgt tttttgtaaa catattctaa atgttctgga acccaataat
      601 aaggtgcatt aggatagcct tgtggaggag ttaagactgc tagtttagat tctaaaataa
      661 gagaatctat atatagtttc gcttcttgat tagtccaatc attgggaata tccgtattgt
      721 aaaatgcaac agaacctcta cgatatgccc acatttcacg attaaactct cttctactct
      781 ctggagtaga gtctatatct cttggatctt ttaatttacc ttttaaaaca tcatcatcta
      841 aagatctaat agctttacca tcagcatcat ctgcaaattc atattctgtt aaatcaatca
      901 cccaatccac ccccataagc tcatctaaga aaggtagcat tccaaattca tcataaggag
      961 gatttttaga aaagtctttt agcatttgag ctttaagagg gtttttatgt ataccatctg
     1021 ttaaacctct atatgcataa gaatcataaa gacttttaaa accttgttca tgtcctagtt
     1081 gtatgagtaa aaaatttact gcttgggttc ttgctttaaa tcctgtcata gaaagttgta
     1141 aggctcctat aaatcctttt atatctccta gtatccctgc tgctaattcc cattcaccca
     1201 taaataaata tccatcactt aaatgagcta agcctctatt agcttctact tctttgtaaa
     1261 gttctttata atcttcattg attaaatttc ctttttcatc tacattagca taagcatcat
     1321 agggtatatc tataacagtg cttcttatac cacttctaat cactagatat ttatatcttt
     1381 ctcttttagt ttttaaagat ttataataag ctttttctgc tttagtccaa ggagctatac
     1441 ttcctttgat aggttctttt aaatatatac tttgccaatc tttaaattct actagagtgg
     1501 atttttgccc tgtataaaag ttaggatcta aatatcttgg ttcataatct ttgtaggggg
     1561 agttttccat gatttcccaa gctttgaaga gggctttgga atattctttg gtatattttc
     1621 ctgtgggttt ggaactttga tcaaaatata tttcattagt ttttttatta acatctaata
     1681 tggaaccatg tggtgtataa atctcataag tatcactcat cttttatcct ttctttaatt
     1741 aacctatatg caaaccattt ttcataatga tagtagtggc ttgtaattac tttaatcttt
     1801 aatgccattt tcttcgccaa atttgagtat cttagctcta agttcccttg ggaaattata
```

```
     1861 tctataaata gcagggattc ttggatcttc taatctatca agcttgtgtt ttttgtaaat
     1921 aaattctaat ctttcaggag agtaataata aggggcatta ggatagcctt gaggggggagt
     1981 tagggctgcg agtttggcac ataaaatcaa agtatcatta aatctctcta cttgctcttt
     2041 actccaatga tttggattat ctccatcaaa tctagttctc attccattct tataagcatc
     2101 catctctttg tcaaactccc acctactctc tggagtagaa tctatatctc ttggatcttt
     2161 gagtattcct ttttcaacat catcttttag agcatcatta actcttcctt tttcatcata
     2221 ggcaaattga tatttattca catctatcac ccaatccact cctataagtt catctagaaa
     2281 aggtagcata ccaaattcat cataaggagg atttttagca aagtctttta gcatttgagc
     2341 tttaagagag tttttgtgta tgccatctgt taaatctcta tatgcataag aatcataaag
     2401 acttttaaaa ccttgttcat gtcctagttg tatgagtaaa aaatttattg cttgagttct
     2461 tgctttaaat cctgtcatgg aaagttgtaa ggctcctaca aatcctttta tatctcctag
     2521 tattcctgct gctaattccc attcagccat aaagagccaa cctttatgca tattagccat
     2581 acctctattg gcttctactt ctttatagag ttctttatag tctttattga ttaaatttcc
     2641 tttttcatct acattacaat atgcttcata aggtatatct ataacagtgc ttcttatacc
     2701 acttcttata attaaatatt tatatctttc tcttttagtt tttaaagatt tataataagc
     2761 tttttctgct ttagtccaag gagctatact tcctttgata ggttctttta aatatatact
     2821 ttgccaatct ttaaattcta ctagagtgga tttttgccct gtataaaagt taggatctag
     2881 atatattggt tgatagtctt tgtaggggga gttttccata atatcccaag ctttaaaaaa
     2941 aaccttagtg tattctttag tatatttacc tacaggatca acgctttgtg caaaatatat
     3001 ttcttttgtt tttttatcta cctctacccc tcttccattg ggagcgtata tcatataagt
     3061 atcactcatc ttttatcctt tcttaattta tcctatatgt aagccatttt tcatgattat
     3121 agtggtggct agtctgccta tgaggttact ttaatcttta atgctatttt cttcgccaaa
     3181 ttttagtatc ttagctctaa gttcccttgg gaaattatat ctataaatag
//
LOCUS       FAFJHLOP_2              1016 bp    DNA     linear       12-APR-2021
DEFINITION  Campylobacter species strain strain.
ACCESSION
VERSION
KEYWORDS    .
SOURCE      Campylobacter species
  ORGANISM  Campylobacter species
            Unclassified.
COMMENT     Annotated using prokka 1.14.6 from
            https://github.com/tseemann/prokka.
FEATURES             Location/Qualifiers
     source          1..1016
                     /organism="Campylobacter species"
                     /mol_type="genomic DNA"
                     /strain="strain"
     gene            complement(447..944)
                     /gene="trxB_3"
                     /locus_tag="FAFJHLOP_00003"
     CDS             complement(447..944)
                     /gene="trxB_3"
                     /locus_tag="FAFJHLOP_00003"
                     /inference="ab initio prediction:Prodigal:002006"
                     /inference="similar to AA sequence:RefSeq:CKG87041.1"
                     /codon_start=1
                     /transl_table=11
                     /product="thioredoxin reductase"
                     /protein_id="Prokka:FAFJHLOP_00003"
                     /translation="MLPFIDEIMGVDWVIDLNRYKFALDEEGRIIWALYDDIEKGKLK
                     DPRDVDSTPESRKEFDHYMDGYKNGMVTRFDVDIRNERDERSAKLTMDTLVLSAKLAA
                     LTPPQGYPNAPRYYSPERLEIIYKRHKLDKLLDPRIPAIYRYNFPRELRAKILKFAEE
```

```
                     NGIKD"
ORIGIN
        1 cacttcttat aattaaatat ttatatcttt ctcttttggt ttttaaagat ttataataag
       61 ctttttctgc tttagtccaa ggagctatgc ttcctttaat gggatctttt aaatatatgc
      121 tttgccattc tttaaattca agtaaagtag aagcttgccc tgtataaaag ttaggatcta
      181 gatatattgg tttatagtct ttgtaggggg agtttcttaa tatgcggtca ccttcgaaga
      241 gagctttgga atgttgtttg gtgtatgtac cattgctttt tgccgtaaat aatattttgt
      301 ttgtatcttt ttctacattt aatattgaac catctggtgt ataaatctca taagtttcgc
      361 tcattattta tcctttctta aatttatccc atgtataaac catcttctat gattatagta
      421 gtggctagtc tacctatgca attactttaa tctttaatgc cattttcttc agcaaatttt
      481 agtatcttag ctctaagttc ccttgggaaa ttatatctat aaatagcagg gattcttgga
      541 tcaaggagtt tatcaagctt atgtctttta taaataattt ctagtctttc agggctatag
      601 tatcttggag cattaggata gccttgtgga ggagtgaggg ctgcgagttt ggcacttaat
      661 actagggtat ccatagtcag tttagcagat ctctcatctc tttcgtttct aatatctaca
      721 tcaaatctag ttaccattcc atttttatat ccatccatat aatgatcaaa ttctttcctg
      781 ctttcaggag tagaatccac atctcttgga tcttttaatt taccttttc aatatcatca
      841 tataaagccc aaatgattct accttcttca tcaagagcaa atttatatcg atttaaatca
      901 atcacccaat ctactcccat aatctcatct ataaaaggta gcataccaaa ttcatcaggc
      961 tgtatagttt tagctaaatc ttttactaat ttagccctta aaggattttt atgtat
//
LOCUS       FAFJHLOP_3               958 bp    DNA     linear       12-APR-2021
DEFINITION  Campylobacter species strain strain.
ACCESSION
VERSION
KEYWORDS    .
SOURCE      Campylobacter species
  ORGANISM  Campylobacter species
            Unclassified.
COMMENT     Annotated using prokka 1.14.6 from
            https://github.com/tseemann/prokka.
FEATURES             Location/Qualifiers
     source          1..958
                     /organism="Campylobacter species"
                     /mol_type="genomic DNA"
                     /strain="strain"
     gene            complement(383..952)
                     /gene="trxB_4"
                     /locus_tag="FAFJHLOP_00004"
     CDS             complement(383..952)
                     /gene="trxB_4"
                     /locus_tag="FAFJHLOP_00004"
                     /inference="ab initio prediction:Prodigal:002006"
                     /inference="similar to AA sequence:RefSeq:CKG86288.1"
                     /codon_start=1
                     /transl_table=11
                     /product="thioredoxin reductase"
                     /protein_id="Prokka:FAFJHLOP_00004"
                     /translation="MHKNPLREQQIKTLAKTLKPDEFGMLPFIDEIMGVDWVIDLNKY
                     DIASDQFGSMYKALRSDVVEGKIKDPRDIDSTYESRREFDRHRGGYKNGMLQGYGTDT
                     PNDWSEERAQLFNDTLILHAKLAALTPPQGYPNAPYYFTPENLEWYYKRHKLDKLLDP
                     RIPAIYRYNFPRELRAKILKFGEENGIKE"
ORIGIN
        1 ctttagtcca aggagctatg cttcctttaa tgggatcttt taaatatatg ctttgccaat
       61 ctttaaattc aagcaaagtg gatttttgcc ctgtgtgaaa ttcaggatct agatacctag
```

```
      121 gttgatagtc tttgtagggg gagttttgtt tgatattgtg tgcttcaaaa agggctctag
      181 aatattcttg ggtgtatttg cctaccttag ctccccccatc acttaataaa attttattcg
      241 tttctttttc tacttctaat ataacaccat taggcatata aatttcataa gtttcactca
      301 tcttttatcc tttcttaatt tatcccatat ataaaccatc ttccatgatt atagtagtgg
      361 ctaatctgcc taggtaatta ctttattctt taatgccatt ttcttcacca aatttgagta
      421 tcttagctct aagttccctt gggaaattat atctataaat agcagggatt cttggatcaa
      481 ggagtttatc aagcttatgt cttttataat accattctag gttttcaggg gtaaaataat
      541 aaggtgcatt aggatagcct tgaggaggag tgagggctgc tagtttggca tgaagtatta
      601 aagtatcatt aaaaagttgc gccctttctt cgctccaatc attgggtgta tcggttccat
      661 agccttgcag catgccattt ttatatccac ctctatggcg atcaaattcc cttctgcttt
      721 cataagtaga atctatatcc cttggatctt ttatcttgcc ctcaaccaca tcacttctta
      781 aggctttata catacttcca aattgatcac tagctatatc atatttattt aaatcaatca
      841 cccaatctac tcccataatc tcatctataa aaggaagcat accaaattca tcaggtttta
      901 aagtcttagc taaggtttta atttgttgct ctcttaaagg atttttatgc actcctac
//
LOCUS       FAFJHLOP_4               874 bp    DNA     linear       12-APR-2021
DEFINITION  Campylobacter species strain strain.
ACCESSION
VERSION
KEYWORDS    .
SOURCE      Campylobacter species
  ORGANISM  Campylobacter species
            Unclassified.
COMMENT     Annotated using prokka 1.14.6 from
            https://github.com/tseemann/prokka.
FEATURES             Location/Qualifiers
     source          1..874
                     /organism="Campylobacter species"
                     /mol_type="genomic DNA"
                     /strain="strain"
     gene            7..570
                     /gene="trxB_5"
                     /locus_tag="FAFJHLOP_00005"
     CDS             7..570
                     /gene="trxB_5"
                     /locus_tag="FAFJHLOP_00005"
                     /inference="ab initio prediction:Prodigal:002006"
                     /inference="similar to AA sequence:RefSeq:CKG86288.1"
                     /codon_start=1
                     /transl_table=11
                     /product="thioredoxin reductase"
                     /protein_id="Prokka:FAFJHLOP_00005"
                     /translation="MHKNPLREQQIKTLAKTLKPDEFGMLPFIDEIMGVDWVIDYNEY
                     QISEDEFGDIYKALRSDVVEGKIKDPRDIDSTYESRREFDYYMDGYSNGMINAYGYDI
                     PNDWNDAQLKIDSMILTAKLAALTPPQGYPNAPYYFTPEKLEWYYKRHKLDKLLDPRI
                     PAIYRYNFPRELRAKILKFGEENGIKE"
ORIGIN
        1 gtaggagtgc ataaaaatcc tttaagagag caacaaatta aaaccttagc taagacttta
       61 aaacctgatg aatttggtat gcttcctttt atagatgaga ttatgggagt agattgggtg
      121 attgattata acgaatatca aatttctgaa gatgaatttg gggatatata taaagcctta
      181 agaagtgatg tagttgaggg caagataaaa gatccaaggg atatagattc tacttatgaa
      241 agtagaaggg agtttgatta ttatatggat ggttattcta atggcatgat taatgcctat
      301 ggttatgata ttccaaatga ttggaatgat gcgcaattaa aaatagattc tatgatatta
      361 actgccaaac tcgcagccct cactcctcca caaggttatc ctaatgcacc ttattatttt
```

```
      421 acccctgaaa aactagaatg gtattataaa agacataagc ttgataaact ccttgatcca
      481 agaatccctg ctatttatag atataatttc ccaagggaac ttagagctaa gatactcaaa
      541 tttggcgaag aaaatggcat taaagaataa agtaattaca taggcagatt agccactact
      601 ataatcatgg aagatggttt atatatggga taaattaaga aaggataaaa gatgagtgaa
      661 acttatgaaa tttatacacc taatggtatt attttagatg tagaaaaaga aacgaataaa
      721 attttattaa gtgatggggg agctaaggta ggcaaataca cccaagaata ttctaaagct
      781 ttgtttgaag ccgatagaat tttaagaacc tctccttata ttaattatca acctaggtat
      841 ctagatcctg aatttcacac agggcaaaaa tcca
//
LOCUS       FAFJHLOP_5               702 bp    DNA     linear       12-APR-2021
DEFINITION  Campylobacter species strain strain.
ACCESSION
VERSION
KEYWORDS    .
SOURCE      Campylobacter species
  ORGANISM  Campylobacter species
            Unclassified.
COMMENT     Annotated using prokka 1.14.6 from
            https://github.com/tseemann/prokka.
FEATURES             Location/Qualifiers
     source          1..702
                     /organism="Campylobacter species"
                     /mol_type="genomic DNA"
                     /strain="strain"
ORIGIN
        1 tataggcgta ttcttcattg attaaatatc ctttttcatc tacattacaa taagcatcat
       61 aaggtatatc tataacagtg cttcttatac cacttctaat cactagatat ttatatcttt
      121 ctcttttggt ttttaaagat ttataataag ctttttctgc tttagtccaa ggagctatag
      181 ctcctttgat aggatctttt aaataaatgt tttgccattc tttaaattct actagagtgg
      241 atttttgccc tgtataaaag ttaggatcta gatatattgg tttatagtct ttgtaggggg
      301 agtttttcat aatataataa gattcaaaaa ccgctttgga atattcttcg gtataattgc
      361 ctgtgggttt aatattttct ttaaatagta ttttattggt atcttttttcc acatctaatg
      421 ttaatccatt gggtgtataa atctcataag tttcgctcat tatttatcct ttcttaattt
      481 aacctatata taaaccatct tctatgatta tagtagtggc tagtctacct atgcaattac
      541 tttattcttt aatgccattt tcttcgccaa atttgagtat cttagctcta agttcccttg
      601 ggaaattata tctataaata gcagggattc ttggatcaag gagtttatct aaatatcctc
      661 ttttataaat ccattccaat ctttcaggtg taaaataata ag
//
LOCUS       FAFJHLOP_6               664 bp    DNA     linear       12-APR-2021
DEFINITION  Campylobacter species strain strain.
ACCESSION
VERSION
KEYWORDS    .
SOURCE      Campylobacter species
  ORGANISM  Campylobacter species
            Unclassified.
COMMENT     Annotated using prokka 1.14.6 from
            https://github.com/tseemann/prokka.
FEATURES             Location/Qualifiers
     source          1..664
                     /organism="Campylobacter species"
                     /mol_type="genomic DNA"
                     /strain="strain"
ORIGIN
```

```
        1 aatcaatcac ccaatctact cccataatct catctataaa aggaagcata ccaaattcat
       61 caggttttaa agtcttagct aaggttttaa tttgttgctc tcttaaagga tttttatgca
      121 ctcctactaa accacttcct actgcaatat ctgcataacc ataaatagca cccaataaat
      181 taggtttatc tagtatacta cttcctccac ttaattgtat ataaagtata gttgcttgga
      241 taaatcttgc attaaaacca ttttgagaca tatcgttagc tagatattta taatcaccca
      301 gtattcctgc tgccataccc cattcattat gaaataaact tgatcttaga ttatgtttat
      361 tatcatctac tgttctatat aattcttcat acttaggatc tacatttcct ttttcatcta
      421 cagctcctat ggcttcataa ggtatatcta taacaacact tcttatacca cttcttataa
      481 ctagatattt atatctttct cttttggttt ttaaagattt ataataagct ttttctgctt
      541 tagtccaagg agctatgctt cctttaatgg gatcttttaa atatatgctt tgccaatctt
      601 taaattcaag caaagtggat ttttgccctg tgtgaaattc aggatctaga tacctaggtt
      661 gata
//
LOCUS       FAFJHLOP_7               634 bp    DNA     linear       12-APR-2021
DEFINITION  Campylobacter species strain strain.
ACCESSION
VERSION
KEYWORDS    .
SOURCE      Campylobacter species
  ORGANISM  Campylobacter species
            Unclassified.
COMMENT     Annotated using prokka 1.14.6 from
            https://github.com/tseemann/prokka.
FEATURES             Location/Qualifiers
     source          1..634
                     /organism="Campylobacter species"
                     /mol_type="genomic DNA"
                     /strain="strain"
ORIGIN
        1 gaactttcta ataatccttt ttactttgga gaattagatc aagataatct tataaaacaa
       61 gatataccca gttattattt ttctcctaaa gatgaaagca gtggtttagg aagacttagt
      121 atcttttata aaaacgatga actttgcttg cttaattatt ctatattaga aaattcttta
      181 aatatcaaat tagaatgctt aagcaagcaa agcttagaat acaaagattt aatttcaaac
      241 actctaaaag aacaaaaaac cacacaagta gataaaaaac aaagcatagc taaacttcac
      301 gctcttttag aaaatcaaaa cctagaatgt atccatggag gtaaagtcat acttaaatca
      361 aacaaaggaa aatcctttaa aagtgatggt atccctataa tgctagaaag tgatttatta
      421 aactctagta tagtagcttg tcctcacacc atagcaaatg taagttatcc ttgtactaaa
      481 gtcgttaatg taaaaggctc cttatctcaa aagaaagtaa ataatgaatt tgtaatctta
      541 caagaactca tctcagcttg cactacagat aaaggctttg ctttaaaagt aagctttact
      601 ccaagtaagt ttaaatttga tcacagcttt gatc
//
LOCUS       FAFJHLOP_8               607 bp    DNA     linear       12-APR-2021
DEFINITION  Campylobacter species strain strain.
ACCESSION
VERSION
KEYWORDS    .
SOURCE      Campylobacter species
  ORGANISM  Campylobacter species
            Unclassified.
COMMENT     Annotated using prokka 1.14.6 from
            https://github.com/tseemann/prokka.
FEATURES             Location/Qualifiers
     source          1..607
                     /organism="Campylobacter species"
```

```
                     /mol_type="genomic DNA"
                     /strain="strain"
     gene            complement(209..385)
                     /locus_tag="FAFJHLOP_00006"
     CDS             complement(209..385)
                     /locus_tag="FAFJHLOP_00006"
                     /inference="ab initio prediction:Prodigal:002006"
                     /codon_start=1
                     /transl_table=11
                     /product="hypothetical protein"
                     /protein_id="Prokka:FAFJHLOP_00006"
                     /translation="MSKKFIA*VLALNPVMESCKAPINPFISPSIPAANSHSAIKSQP
                     LCILAIPLLASTSL"
ORIGIN
        1 tatatttaaa agatcctatc aaaggagcta tagctccttg gactaaagca gaaaaagctt
       61 attataaatc tttaaaaact aaaagagaaa gatataaata tttaattata agaagtggac
      121 ttagaagcac tgttatagat ataccttatg atgcttattg taatgtagat gaaaaaggaa
      181 atttaatcaa taaagactat aaagaacttt acaaagaagt agaagctaat agaggtatgg
      241 ctaatatgca taaaggttgg ctctttatgg ctgaatggga attagcagca ggaatactag
      301 gagatataaa aggatttata ggagccttac aactttccat gacaggattt aaagcaagaa
      361 ctcaagcaat aaattttttta ctcatacaac taggacatga acaaggtttt aaaagtcttt
      421 atgattctta tgcatataga ggtttaacag atggtataca caaaaactct cttaaagctc
      481 aaatgctaaa agactttgct aaaaatcctc cttatgatga atttggaatg ctacctttct
      541 tagatgagct tataggagtg gattgggtga ttgatttgaa tgaatatcaa tttgcttatg
      601 atggtga
//
LOCUS       FAFJHLOP_9               592 bp    DNA     linear       12-APR-2021
DEFINITION  Campylobacter species strain strain.
ACCESSION
VERSION
KEYWORDS    .
SOURCE      Campylobacter species
  ORGANISM  Campylobacter species
            Unclassified.
COMMENT     Annotated using prokka 1.14.6 from
            https://github.com/tseemann/prokka.
FEATURES             Location/Qualifiers
     source          1..592
                     /organism="Campylobacter species"
                     /mol_type="genomic DNA"
                     /strain="strain"
     gene            complement(48..590)
                     /gene="trxB_6"
                     /locus_tag="FAFJHLOP_00007"
     CDS             complement(48..590)
                     /gene="trxB_6"
                     /locus_tag="FAFJHLOP_00007"
                     /inference="ab initio prediction:Prodigal:002006"
                     /inference="similar to AA sequence:RefSeq:CKG86288.1"
                     /codon_start=1
                     /transl_table=11
                     /product="thioredoxin reductase"
                     /protein_id="Prokka:FAFJHLOP_00007"
                     /translation="MLKDFAKNPPYDEFGMLPFLDELIGVDWVIDLNEYQFAYDGDGG
```

```
                     SVKALDEKVRQGKLKDPRDVDSTPESRKEFDEEANAYTFGNRMNFSSDIRDSRSKESA
                     RLFDDLLILHAKLAALTPPQGYPNAPYYFSPEQLEDFYKRQVLDAKLDPRIPAIYRYN
                     FPRELRAKILKFGEENSIKD"
ORIGIN
        1 ccatttttca tgattatagt ggtggctagt ctgcctatga ggttacttta atctttaatg
       61 ctattttctt cgccaaattt tagtatctta gctctaagtt cccttgggaa attatatcta
      121 taaatagcag ggattcttgg atcaagttta gcgtctaaaa cttgtctttt gtaaaaatct
      181 tctaattgtt caggactaaa ataataaggt gcattaggat agccttgagg aggagtgagg
      241 gctgcgagtt tggcatgaag tatcaaaagg tcatcaaata atctagccga ttctttactt
      301 ctactatcgc gaatatcaga ggaaaaattc attctattac caaaagtata agcattagct
      361 tcttcatcga attctttcct gctctctgga gtagaatcta catctcttgg atcttttaat
      421 ttaccttgtc ttactttctc atctaaagcc ttaacactac cgccatcacc atcataagca
      481 aattgatatt cattcaaatc aatcacccaa tccactccta taagctcatc taagaaaggt
      541 agcattccaa attcatcata aggaggattt ttagcaaagt cttttagcat tt
//
LOCUS       FAFJHLOP_10              554 bp    DNA     linear       12-APR-2021
DEFINITION  Campylobacter species strain strain.
ACCESSION
VERSION
KEYWORDS    .
SOURCE      Campylobacter species
  ORGANISM  Campylobacter species
            Unclassified.
COMMENT     Annotated using prokka 1.14.6 from
            https://github.com/tseemann/prokka.
FEATURES             Location/Qualifiers
     source          1..554
                     /organism="Campylobacter species"
                     /mol_type="genomic DNA"
                     /strain="strain"
ORIGIN
        1 taaaaatcct ttaagagagc aacaaattaa aaccttagct aagactttaa aacctgatga
       61 atttggtatg cttcctttta tagatgagat tatgggagta gattgggtga ttgatttaaa
      121 taaatataga ttagctaatg atgaatttgg aaaaatatac aaagccttaa gaagtgatgt
      181 agttgagggc aagataaaag atccaaggga tatagattct acttatgaaa gcagaaggga
      241 gtttgatcgt tatagagttg gatatagaaa gggtatggcg caaggctatg gaaccgatac
      301 acccaatgat tggagcgaag aagaagcaca actttttaat gatactttaa tacttcatgc
      361 caaactcgca gccctcactc ctcctcaagg ctatcctaat gcaccttatt attttacccc
      421 tgaaaatcta gaatggtatt ataaaagaca taagcttgat aaactccttg atccaagaat
      481 ccctgctatt tatagatata atttcccaag ggaacttaga gctaagatac tcaaatttgg
      541 cgaagaaaat ggca
//
LOCUS       FAFJHLOP_11              535 bp    DNA     linear       12-APR-2021
DEFINITION  Campylobacter species strain strain.
ACCESSION
VERSION
KEYWORDS    .
SOURCE      Campylobacter species
  ORGANISM  Campylobacter species
            Unclassified.
COMMENT     Annotated using prokka 1.14.6 from
            https://github.com/tseemann/prokka.
FEATURES             Location/Qualifiers
     source          1..535
```

```
                     /organism="Campylobacter species"
                     /mol_type="genomic DNA"
                     /strain="strain"
ORIGIN
        1 atacataaaa atcctttaag ggctaaatta gtaaaagatt tagctaaaac tatacagcct
       61 gatgaatttg gtatgctacc ttttatagat gagattatgg gagtagattg ggtgattgat
      121 ttaaatcagt atcaatttgc gtatgacggt aaaggtgatt ctacttgggc tttatatgat
      181 gatattgaaa aaggtaaatt aaaagatcca agagatgtgg attctactcc tgaaagcagg
      241 aaagaatttg atgaagaaac tgacgtttat agatttggta acagaatgaa ttttgcctct
      301 gatattcgtg atagtagaag tgaagaatca actagattat ttgatgatac tttaatactt
      361 catgccaaac tcgcagccct cactcctcca caaggctatc ctaatgcccc ttattatttt
      421 acacctgaaa gattggaatg gatttataaa agaggatatt tagataaact ccttgatcca
      481 agaatccctg ctatttatag atataatttc ccaagggaac ttagagctaa gatac
//

>>BfR-CA-15831_min_cov_30 putative plasmid location
LOCUS       EDCPFOPP_1              2138 bp    DNA     linear       12-APR-2021
DEFINITION  Campylobacter species strain strain.
ACCESSION
VERSION
KEYWORDS    .
SOURCE      Campylobacter species
  ORGANISM  Campylobacter species
            Unclassified.
COMMENT     Annotated using prokka 1.14.6 from
            https://github.com/tseemann/prokka.
FEATURES             Location/Qualifiers
     source          1..2138
                     /organism="Campylobacter species"
                     /mol_type="genomic DNA"
                     /strain="strain"
     gene            345..647
                     /locus_tag="EDCPFOPP_00001"
     CDS             345..647
                     /locus_tag="EDCPFOPP_00001"
                     /inference="ab initio prediction:Prodigal:002006"
                     /codon_start=1
                     /transl_table=11
                     /product="hypothetical protein"
                     /protein_id="Prokka:EDCPFOPP_00001"
                     /translation="MILCIFSSNFSSKYFSLASCFFNSSSSSPKIKSLKKPATSVPTT
                     APTTVPTIGKLEPTAAPVAAPAAIRESMDTPALAAILDATLELAILVAALEATLLV"
     gene            714..932
                     /locus_tag="EDCPFOPP_00002"
     CDS             714..932
                     /locus_tag="EDCPFOPP_00002"
                     /inference="ab initio prediction:Prodigal:002006"
                     /codon_start=1
                     /transl_table=11
                     /product="hypothetical protein"
                     /protein_id="Prokka:EDCPFOPP_00002"
                     /translation="MIIVILTPTLVSVKALTLDFPSALARKKFNIPKFIIILECIILA
                     ICELSSMPNFTISFSKALVAKPSVMPKA"
     gene            complement(1026..1664)
```

```
                     /locus_tag="EDCPFOPP_00003"
     CDS             complement(1026..1664)
                     /locus_tag="EDCPFOPP_00003"
                     /inference="ab initio prediction:Prodigal:002006"
                     /codon_start=1
                     /transl_table=11
                     /product="hypothetical protein"
                     /protein_id="Prokka:EDCPFOPP_00003"
                     /translation="MSEINNNTEIKEQDTQDEIIWEGKKHIPSFLLFWITYIVLLCFA
                     LYLFPRLFNPSKEIDYKWFIVFFTFLLVIYALAQKIYRMANIKRIYITKEKLVIEYYI
                     KNDLVFPLGTFFVYYRRLSFVLTPGHIVIYTFGDKVKEYLEPGLFSDGDDPTKDCFER
                     INAIIKPHVMPYLLSLSDEEFEKIIFHVSGFSEIDTTFLKEAMELRKEKKDE"
     gene            complement(1715..2107)
                     /locus_tag="EDCPFOPP_00004"
     CDS             complement(1715..2107)
                     /locus_tag="EDCPFOPP_00004"
                     /inference="ab initio prediction:Prodigal:002006"
                     /codon_start=1
                     /transl_table=11
                     /product="hypothetical protein"
                     /protein_id="Prokka:EDCPFOPP_00004"
                     /translation="MANIKRIYITKEKLVIEYYIKNDLVFPLGTFFVYYRKISYSLSP
                     GHIVIYTFGDKVKEYLEPSLFSDGDDPTKGCFERINAIIKPHVMPYLLSLSDEEFEKI
                     FFHVNATSKIDTTFLKEAMELRKEKKDE"
ORIGIN
        1 attcatcctc atctgcttca tcataaggta tgccagtatc ttcttcaaaa gcaacttcat
       61 agttttctgg atctaaatta tcatacgcac cttccataaa atcatctata taattttta
      121 ctgccttatt agctatgggt tgtattctga ttatagttgg atttttttct tgagcttctt
      181 tataactttg ctcatcttta tctaaataat aaggaaaact tagcatcaaa agaatggttt
      241 taaaataagc actattaata tcacttattt ctttacaaag cttttctgct tcatcttcat
      301 ttaacactct taattctata tactgatgag tgatgagata attgatgatt ttatgcattt
      361 tttcatctaa tttttcctct aaatattttt ctttggcttc ttgttttttt aattcctcat
      421 cttcatcgcc aaaaattaag tcattaaaaa aacctgctac aagtgttccc accactgcac
      481 ccaccactgt cccaacaata ggaaagctcg agcctacagc tgctccagtt gctgcacctg
      541 cggcaatacg agaaagtatg gatacgccag ctctagctgc gatattagat gctactcttg
      601 agctagctat cttggttgcg gcattagaag caaccctgct ggtttgagtt aaggtttgtt
      661 tggcaacatg ttcgctacct ttttctattg ttgctccaat aggagtttca ttcatgatta
      721 tagtgatact aacaccaacg cttgtttcag tcaaagctct tacattggat tttccctctg
      781 ctttagccag aaaaaaattc aatattccaa aattcattat tattttagaa tgcatcattc
      841 tcgcaatatg cgaattatct tctatgccaa acttcactat ttcttttca aaagctttag
      901 tagcaaaacc ttctgtaatg cctaaggcat aaggagcaac atctccaaat actcctaaat
      961 cccttttatc actaggagtg ctttgagttt ctttagaata aaactctaaa cttccataaa
     1021 tattttcatt catctttttt ctcttttctg agttccattg cttctttaag gaaagtagta
     1081 tcaatttcgc taaaaccgct tacatggaaa atgatttttt caaattcttc atcacttaag
     1141 cttaataaat aaggcattac atgaggtttt ataatggcat taattctttc aaaacaatct
     1201 tttgttggat catcgccatc actgaataaa ccaggttcaa ggtattcttt gactttatct
     1261 ccgaaggtgt atatcactat atgtcctgga gtaagcacaa atgacaggcg acgataatac
     1321 acaaaaaaag ttcctaaagg aaaaaccaaa tcatttttga tataatactc aatcacaagt
     1381 ttttcttttg tgatataaat tcttttaata tttgccattc tatagatctt ttgtgcaagt
     1441 gcatatataa ctaaaagaaa tgtaaaaaaa actataaacc atttataatc tatttctttg
     1501 ctaggattaa ataatctagg aaacaaataa agcgcaaaac ataaaagcac aatatatgtt
     1561 atccaaaaaa gcaaaaacga aggtatatgc ttttttcctt cccaaatgat ttcatcttgg
     1621 gtgtcttgct ctttgatttc tgtgttattg tttatttcac tcatttttat cctttaaata
     1681 agttttaatc tcttgtatat aaaataaaat attttcattc atctttttc tcttttctga
```

```
     1741 gttccattgc ttctttaagg aaagtggtat caattttact agtagcgttt acatggaaaa
     1801 agattttttc aaattcttca tcacttaagc ttaataaata aggcattaca tgaggtttta
     1861 taatggcatt aattctttca aaacaacctt ttgttggatc atcgccatca ctgaataaac
     1921 taggttcaag gtattctttg actttatctc cgaaggtgta tatcactata tgtcctggag
     1981 aaagtgaata tgaaatttta cgataataca caaaaaaagt tcctaaagga aaaaccaaat
     2041 catttttgat ataatactca atcacaagtt tttcttttgt gatataaatt cttttaatat
     2101 ttgccatttt atagatcgct cgtgcaaatg catatatg
//
LOCUS       EDCPFOPP_2               863 bp    DNA     linear       12-APR-2021
DEFINITION  Campylobacter species strain strain.
ACCESSION   
VERSION
KEYWORDS    .
SOURCE      Campylobacter species
  ORGANISM  Campylobacter species
            Unclassified.
COMMENT     Annotated using prokka 1.14.6 from
            https://github.com/tseemann/prokka.
FEATURES             Location/Qualifiers
     source          1..863
                     /organism="Campylobacter species"
                     /mol_type="genomic DNA"
                     /strain="strain"
     gene            complement(125..670)
                     /gene="tssI1"
                     /locus_tag="EDCPFOPP_00005"
     CDS             complement(125..670)
                     /gene="tssI1"
                     /locus_tag="EDCPFOPP_00005"
                     /inference="ab initio prediction:Prodigal:002006"
                     /inference="similar to AA sequence:RefSeq:AOH52008.1"
                     /codon_start=1
                     /transl_table=11
                     /product="type VI secretion system, secreted protein"
                     /protein_id="Prokka:EDCPFOPP_00005"
                     /translation="MGGEYLTNVGLSKDTIVGLSNTLNVGVDNKVRIAKNSHEFVGEN
                     KDIEIGANQNTIIHKDEIRNVKGENKLLIEKSLTQTIEKEFFLNVHQNLSAHIQDNTS
                     LKSNSMQTKIEEQYSLESENSTFDFQTDCGVKAGNQILHQVGDTQIVTKKDCVIIKAG
                     GVEVIIDSNGLVVKGGEVKAE"
ORIGIN
        1 caatatatgt tatccaaaaa agcaaaaacg aaggtatatg ctttttttcct tcccaaatga
       61 tttcatcttg ggtgtcttgc tctttgattt ctgtgttatt gtttatttca ctcattttta
      121 tcctttactc tgccttaacc tctccacctt taaccacaag tccattagag tctataatca
      181 cttctactcc acctgcttta attataacac aatctttctt ggtaacaatt tgagtatcac
      241 ctacttgatg gaggatttga ttgcctgctt ttactccaca atctgtttga aagtcaaaag
      301 tagaattttc tgattctaaa gaatattgct cttctatctt tgtttgcatg gaattagact
      361 ttaaagatgt gttatcttgt atatgggcgg ataaattttg atgaacatta aggaaaaatt
      421 ctttttctat tgtttgggtt aaagattttt ctattaataa tttattttct cccttcacat
      481 tccttatctc atctttatgg ataatagtat tttgatttgc acctatttct atatctttgt
      541 tttctcctac aaactcatga gagtttttag ctattcttac tttattatct actcctacat
      601 ttaaagtatt gcttaatcct actatagtgt ctttagataa acctacatta gttaagtatt
      661 ctcctcccac attgacattt ttagctaagt ctatggtttg agtatgaacc tttttaatcc
      721 tttcattata aattccatct accctagaat ctttatcatt taaaattctt tgagtgaaat
      781 tatgctggac taattcatca taatcctttt gggcttttaa ataaatttgt tctttatctt
```

```
      841 ttatatttga gagggtaagt tca
//


>>BfR-CA-15837_min_cov_30 putative plasmid location

LOCUS       FDNNOFDK_1              3982 bp    DNA     linear       12-APR-2021
DEFINITION  Campylobacter species strain strain.
ACCESSION
VERSION
KEYWORDS    .
SOURCE      Campylobacter species
  ORGANISM  Campylobacter species
            Unclassified.
COMMENT     Annotated using prokka 1.14.6 from
            https://github.com/tseemann/prokka.
FEATURES             Location/Qualifiers
     source          1..3982
                     /organism="Campylobacter species"
                     /mol_type="genomic DNA"
                     /strain="strain"
     gene            49..1071
                     /locus_tag="FDNNOFDK_00001"
     CDS             49..1071
                     /locus_tag="FDNNOFDK_00001"
                     /inference="ab initio prediction:Prodigal:002006"
                     /inference="similar to AA sequence:RefSeq:APB38683.1"
                     /codon_start=1
                     /transl_table=11
                     /product="replication initiation protein"
                     /protein_id="Prokka:FDNNOFDK_00001"
                     /translation="MSDIVKYHNDFNKIQLPSFTEIEQDLLCGLMVKLKEEKGKVTFY
                     PWDLRNILKSSYDNNSLMEFASSLKRKFFKADFTIIEKTTRGEKEVEAHKTINLFTEF
                     AIYVYASSKDLESIEIQVNPQFEYILNQLTANFTAFELSEFIALSGKYTKTLYRLLKQ
                     YRTTGKARFEWEEFCRVMDIPQDYRQSAIDKWILKPAIKELSKERNLFDQVRVPFKNL
                     AYEKEKAKGSRGRGGKVSGITFTFKPENIEMQKLENESQKIMSDEQKYLKILNNMKLN
                     QVRFVYNDKLWQFNDFDFNEFKIIAVELQRDEYENLNFGNHMHFNAKNQEQFFKMIET
                     FRKGIR"
     gene            1101..1475
                     /locus_tag="FDNNOFDK_00002"
     CDS             1101..1475
                     /locus_tag="FDNNOFDK_00002"
                     /inference="ab initio prediction:Prodigal:002006"
                     /codon_start=1
                     /transl_table=11
                     /product="hypothetical protein"
                     /protein_id="Prokka:FDNNOFDK_00002"
                     /translation="MTMQIIQDQIPQELSGRMTKQSLERLANHLHIATQNEMGDIPLW
                     LLWDLAITCKQLDLKENYQKVLIEIQMNLETQHKANLAEQRADLAEQRAEKAEQRAEK
                     AEQLYQQIKNFRISMMKKYQTN"
     gene            1806..1970
                     /locus_tag="FDNNOFDK_00003"
     CDS             1806..1970
                     /locus_tag="FDNNOFDK_00003"
```

```
                     /inference="ab initio prediction:Prodigal:002006"
                     /codon_start=1
                     /transl_table=11
                     /product="hypothetical protein"
                     /protein_id="Prokka:FDNNOFDK_00003"
                     /translation="MDYEKYSKMNLRQLINAYTNAEKKEKKLIDDFQKKLDENKNLME
                     FLKTKIKESL"
     gene            1974..3302
                     /locus_tag="FDNNOFDK_00004"
     CDS             1974..3302
                     /locus_tag="FDNNOFDK_00004"
                     /inference="ab initio prediction:Prodigal:002006"
                     /inference="similar to AA sequence:RefSeq:APA51640.1"
                     /codon_start=1
                     /transl_table=11
                     /product="mobilization protein"
                     /protein_id="Prokka:FDNNOFDK_00004"
                     /translation="MSYDEIEKVKRSAYTKTASESTKRNYISSGNFKKSTSFQVFHNA
                     NVRPDYAIGGELVFDLGGYEALELKNKIVSEAKEAYEKNKSPKAPKFKAKSYEWSLVV
                     NIKPDTSMNDLKNLSKHFQEKYGFQCYQIAIHRDEGHFNEQGEKLINHHAHMEFITLD
                     KETGKNNFRREKISPKVLREIQTEIAEILGMERGEDKRISGAKRIEPRAYAALKEKEK
                     REKKRNINLEQEQEKEQFLNKSEYKKIIENFRKSQINQGLDKDFFKELSELKKNFQEI
                     KEDNLSELLNDILREHLERIEKLKKENSILKAENELLKQKEQKNDKSIDQEQKNASER
                     NFEPKNDNNTEDKKIFFEKLATYRLNEGLSKIYSKPLKRIVEPETEAKKKERIKILTE
                     TMEKTEAYLPETLKKWLENNKDIDLKEILVAYQNTLSKELKQSKSIYKGR"
ORIGIN
        1 aggggtagag aagcccttg ccttgattgt cttactttgg atatgcaaat gagcgatata
       61 gttaaatacc ataatgattt taataaaatt cagttgccta gttttacaga aatcgaacaa
      121 gatttattgt gtggtttaat ggtaaaactt aaagaagaaa aaggcaaagt aacattctat
      181 ccttgggact taagaaacat tctcaaaagt agttatgata ataatagtct aatggaattt
      241 gcgtcttctc taaaaaggaa atttttcaaa gctgacttta caataattga aaaaacaact
      301 aggggtgaaa aagaagttga agcacacaaa acaataaacc tttttacaga atttgcaatt
      361 tatgtttatg caagctcaaa agatttagag agtatagaaa tacaagttaa tccgcaattt
      421 gaatatattt taaatcaact aaccgctaat tttacagcct ttgaacttag cgaatttatt
      481 gcccttagcg gtaaatatac taaaactctt tatagacttt taaagcaata cagaactaca
      541 ggtaaagcac gatttgagtg ggaagaattt tgcagggtta tggatatacc gcaagattat
      601 agacaaagtg caatcgataa atggatttta aaacctgcta taaaagaact ttctaaagaa
      661 cgcaatcttt ttgatcaagt gcgagtgcct tttaaaaatc ttgcctatga aaaagaaaaa
      721 gcaaaaggaa gtaggggtag gggtggcaag gtttcaggca ttacctttac ttttaaacct
      781 gaaaatatcg aaatgcaaaa gctagaaaat gaaagtcaaa aaataatgag cgatgagcaa
      841 aaatatttaa aaattttaaa caatatgaaa cttaatcaag tccgcttcgt ttataatgac
      901 aagctttggc aatttaacga ttttgatttt aatgaattta aaattattgc agtagagctt
      961 caaagagatg agtatgagaa tttaaacttt ggaaatcata tgcactttaa tgctaaaaat
     1021 caagagcagt tttttaaaat gattgaaacc tttagaaaag gtatcagata agcgaaaaag
     1081 acaaaaacag gagaaagaaa atgacaatgc aaataataca agatcaaatt ccacaggaat
     1141 tgtcagggag aatgacaaaa cagagcctag agagattagc aaatcatctt cacatagcga
     1201 cccaaaacga aatgggggat attccactat ggctactttg ggacttagcg ataacttgca
     1261 aacaattaga tttaaaagag aattatcaga aagtcttaat agaaatacag atgaacctag
     1321 aaacacagca caaagcaaac ttagcagagc agagagcaga cttagcagag cagagagcag
     1381 agaaagcaga gcagagagca gagaaagcgg agcaattata tcagcaaata aagaatttca
     1441 gaatatcaat gatgaaaaaa tatcaaacaa attaaataaa gaagaaagca aggaatttaa
     1501 tatccccgct ctaagacggg gcaacctatc taaaagataa gctaattctt cgctaaactt
     1561 tcatttagga agtcaaataa aattctatca taaataattt tatttaatac ttttacatta
     1621 aatgagcttg cgaattaatg caaagccacg cggtgcaaat tattaatcat ttttttattt
```

```
     1681 ataaaatatg attggataat ttgcaaatta tccgaactaa aaaagttcta taatttgttt
     1741 ttaaaaaata tgctataatc tcacaagctc gattacattt atattttttc aaaaaaggga
     1801 aaaaaatgga ttatgaaaaa tattctaaaa tgaatttaag acaattgata aacgcttaca
     1861 caaacgcaga aaaaaaagaa aaaaaattaa tcgatgattt tcaaaaaaaa cttgatgaga
     1921 ataaaaattt aatggagttt ttaaaaacaa aaatcaaaga aagtttataa aaaatgtctt
     1981 acgatgagat tgaaaaggtt aaaagaagtg cttacacaaa aactgcaagt gaaagcacaa
     2041 aaagaaatta tatttcaagc ggaaatttta aaaaatcaac ttcttttcaa gtcttccata
     2101 atgccaatgt aagacctgat tacgcaatag gcggtgagct tgtttttgat ttaggcggtt
     2161 atgaagcttt agagcttaaa aataagatag tttcggaagc gaaagaagcc tatgaaaaaa
     2221 ataaaagccc caaagctcca aaatttaaag ctaagagcta tgaatggagc ttagttgtca
     2281 atatcaagcc tgatacctca atgaatgatt taaaaaacct ttctaaacat tttcaagaaa
     2341 aatacggctt tcagtgctat caaatcgcta tacaccgcga tgaagggcat tttaacgagc
     2401 aaggcgaaaa gcttataaac caccacgcac atatggaatt tatcactctt gacaaagaaa
     2461 caggtaaaaa caactttagg cgtgaaaaaa taagcccaaa agtcttaaga gaaattcaaa
     2521 ccgaaatcgc tgaaatctta ggaatggagc gtggcgaaga taagagaata agcggagcaa
     2581 aacgcataga gcctagagct tatgcagcct taaaagaaaa ggaaaaaagg gagaaaaagc
     2641 gaaacattaa tttagagcaa gagcaagaaa aagagcaatt tttaaataaa agcgagtata
     2701 aaaaaatcat tgaaaatttt agaaaaagtc aaattaatca agggcttgac aaagatttct
     2761 tcaaagagtt aagcgaacta aaaaagaact ttcaagagat taaagaagac aatttatcag
     2821 agcttttaaa cgatatttta agagagcatt tagaacgcat tgaaaagtta aaaaaagaga
     2881 atagcatttt aaaagccgaa aatgagcttt taaagcaaaa agagcaaaaa aacgataaaa
     2941 gtatcgacca agagcaaaaa aacgcttcag aacgcaattt cgagccgaaa aacgataata
     3001 atacagaaga taaaaaaata tttttcgaaa aattagccac ttacagactg aatgaaggat
     3061 taagtaagat ttattccaag ccccttaaaa gaattgtgga gcctgaaacc gaagccaaaa
     3121 agaaagaaag aattaaaatt ttaacagaaa caatggaaaa aacagaagct tacctacctg
     3181 aaactttaaa aaaatggctt gaaaataaca aagatattga tttaaaagaa atacttgtcg
     3241 cttatcaaaa taccccttagc aaagaattaa aacagagcaa gagtatttac aaaggcagat
     3301 aaacactttc tctccctttt tagcttagca aatcgcacct ttggtgctat gatttgcgat
     3361 aatcggggtg cgggggcgtg aagttagcct agagctaacg aacggacaag ccaccgccct
     3421 aatgtatatc atcaaaactt tttttgtttt ctctctcgcg cgcgtacgcg tacgcgattt
     3481 tttttatttt ttatatattt tttatatttt tagggggctt tctaagccct atataatggg
     3541 ggctagcgaa atattaaggg tagaaatcta aactattaag ggtagaaatc taaactatta
     3601 agggtagaaa tctaaactat taagggtaga aatctaaact attaagggga caaaattcta
     3661 ctattaaggg tagaaatata ttgacaacct atccttaata tgctacaata aaaaatccgc
     3721 cttgagtttt cactcttggc ggaaatttat caatcaaggc taactatgaa acgacatagt
     3781 aagcgttttg tgagattata cctaaatttt aggtttttta agcttaaagt tgaagtattt
     3841 ttctaacaaa accgcagggg tagagaagcc ccttgccttg attgtcttac tttggatatg
     3901 caaatgagcg atatagttaa ataccataat gattttaata aaattcagtt gcctagtttt
     3961 acagaaatcg aacaagattt at
//
LOCUS       FDNNOFDK_2              2761 bp    DNA     linear       12-APR-2021
DEFINITION  Campylobacter species strain strain.
ACCESSION
VERSION
KEYWORDS    .
SOURCE      Campylobacter species
  ORGANISM  Campylobacter species
            Unclassified.
COMMENT     Annotated using prokka 1.14.6 from
            https://github.com/tseemann/prokka.
FEATURES             Location/Qualifiers
     source          1..2761
                     /organism="Campylobacter species"
                     /mol_type="genomic DNA"
```

```
                     /strain="strain"
     gene            23..1219
                     /locus_tag="FDNNOFDK_00005"
     CDS             23..1219
                     /locus_tag="FDNNOFDK_00005"
                     /inference="ab initio prediction:Prodigal:002006"
                     /inference="similar to AA sequence:RefSeq:EAQ72546.1"
                     /codon_start=1
                     /transl_table=11
                     /product="putative sugar transferase"
                     /protein_id="Prokka:FDNNOFDK_00005"
                     /translation="MLDPKKLNNDTEKINYKNITAELAWEMNLPLPDKYDFIFISSHG
                     TGEKAFQEMLARCDGVKVLQKNKWYDNGKDRYRAFYKDLVIDCTAKKCIPILECNFLY
                     YGKYLHMFGKKVPLICLVRDPIKMVASVYNYKVIKDKQKLHFSNKNNNEVMFDNYFYT
                     TTKSKSIIDLNELKKRIIHQRTITSNMRFTYIAKTHIQNLKNMDKVYYIDSSEISASK
                     AYLKLKEIGDIVGFSVPEDFNLINKISFTHYLRFIFDIDRPIIYTHQINPDLNLQIFI
                     FHMYQQTERQDITHLFNLQQYDSSMHNIFICTDTQDYDIIIHDEKILYEIRDNILNFF
                     NKINNHIAILRKEAISEEDILKIYKEDKQTSDAFKKLIEDEFKDVLEKESMLKKWNHY
                     LKFIKL"
     gene            1243..2631
                     /locus_tag="FDNNOFDK_00006"
     CDS             1243..2631
                     /locus_tag="FDNNOFDK_00006"
                     /inference="ab initio prediction:Prodigal:002006"
                     /inference="similar to AA sequence:RefSeq:AHY40622.1"
                     /codon_start=1
                     /transl_table=11
                     /product="putative sugar transferase"
                     /protein_id="Prokka:FDNNOFDK_00006"
                     /translation="MYNIVLASDNNYIKYVGVVLSSIIKNTKENDNFANEKYIFHVLT
                     SDISLNNQQKLQTLVDYLSNIYPCELKIHIIDDSQFHEFPKAWHVNHATYYRFEIANI
                     LGDEVSKCLYIDADVLACDDIREIFHIDLSGKIVGVVTDSCSQLWTKLKSRHTDNLNI
                     NFDPIYYFNAGVIMIDLNKWRQCDIKQKCIQAFLDYDHGGLADQSYLNIALKDSIYKL
                     PLGWNLIAPEYVLLQGYERHYVQKCLDEGKDYNIAYTRKEFSQALNEKKIVHFCAAKP
                     WWNLYYKNERVDFNERKIWWDIAINMEGFKEDFRYLRLNLELEYLNRKINSIASNGIK
                     HVTKIIQVNHNAKSRIQNQLSYKLGQIALINSKSILGLLCMPVYIISTIIVHVQDKKI
                     YHNAIKKDPSLKLLPLEQYPDYNEAIKLKQHFSYKLGSVIMHSLKYWYLGGILALPFR
                     VFKLYKNHKKKI"
ORIGIN
        1 ggaaaaccat ccttatcctt ctttgcttga tcctaaaaag ttaaataatg atactgaaaa
       61 aattaattat aaaaatatta cagcggaact tgcctgggaa atgaatttac ctcttccaga
      121 taagtatgat tttatattta taagtagtca tggaactggc gaaaaagctt ttcaagaaat
      181 gctagcacgt tgtgatggtg taaaagtctt gcaaaaaaat aaatggtatg ataatggaaa
      241 agatagatat agggcttttt ataaagatct tgtcattgat tgtactgcta aaaaatgtat
      301 tccaatttta gaatgtaatt ttttatacta tggaaagtat ttacatatgt ttggtaaaaa
      361 agttccatta atatgcttag taagagatcc aattaaaatg gttgctagtg tatataatta
      421 caaagtgata aaagataaac aaaaattaca tttcagtaat aagaataata atgaagttat
      481 gtttgataat tatttttaca ccacaacaaa atctaaatca attattgatt taaatgaatt
      541 aaagaaaaga attattcatc aaagaactat tacaagtaat atgagattta catatattgc
      601 aaaaactcat attcaaaatt taaaaaatat ggataaggtg tattatatcg attcttctga
      661 aataagtgca tctaaggcat atttaaaatt aaaagagatt ggggatattg tagggtttag
      721 tgttcctgaa gattttaatt tgattaataa gataagtttt acacattatt taagattcat
      781 atttgatatt gataggccaa ttatatatac acatcaaatt aatcctgacc ttaatttgca
      841 aatttttata tttcatatgt atcaacaaac tgagagacag gatatcacac atctttttaa
```

```
      901 tcttcaacag tatgatagtt ctatgcataa tatttttatt tgtacagata cacaagatta
      961 tgatataata atacatgatg aaaaaattct gtatgaaatt cgtgataata tattgaattt
     1021 ttttaataaa ataaataatc atatagccat attaagaaag gaagcaataa gtgaagagga
     1081 tattttgaaa atttataaag aagataagca aacatctgat gcttttaaaa agcttattga
     1141 agatgaattt aaggatgttt tagaaaagga aagtatgttg aaaaaatgga accattattt
     1201 aaaatttata aaactgtaaa aaaaagaggg ggagtttaaa agatgtacaa tattgtacta
     1261 gcttcagata ataattatat taaatatgtt ggagttgttt tatcaagtat tattaaaaat
     1321 acaaaagaaa atgataattt tgcaaatgaa aaatatattt ttcatgtttt gacaagcgat
     1381 ataagtttga ataatcaaca aaaactacaa acccttgttg attatttatc aaatatatat
     1441 ccatgtgaat taaaaattca tattattgat gatagccagt ttcatgaatt tccaaaagca
     1501 tggcatgtaa atcatgcaac ttattataga tttgaaattg ctaatatttt aggagatgaa
     1561 gtatccaaat gtctatatat tgatgcggat gttttagcat gtgatgatat tagagaaatt
     1621 tttcatattg atttaagtgg taaaattgtt ggagttgtaa cagattcatg ttcgcaatta
     1681 tggacaaagt taaaatcaag acatactgac aatttaaata ttaattttga tccgatatat
     1741 tattttaatg ctggcgttat tatgattgat ttaaataaat ggagacagtg tgatattaag
     1801 caaaaatgta tccaagcttt tttggattat gatcacggcg gtctagctga tcaaagctat
     1861 cttaatatag cactaaaaga ctctatatat aaattacctt taggttggaa tctaatagca
     1921 cctgaatatg ttttgctaca aggttatgag agacattatg ttcaaaaatg tctagatgaa
     1981 ggaaaagatt acaatatagc atatacaaga aaagaattta gtcaagcgct aaatgaaaaa
     2041 aaaatagttc atttttgtgc tgcaaaacct tggtggaatt tatattataa aaatgagaga
     2101 gtagatttta acgaaaggaa aatttggtgg gatatcgcaa taaatatgga aggttttaag
     2161 gaagatttta gatatttaag attaaatcta gaattagaat acctaaatag aaagataaat
     2221 tcaatagcta gcaatgggat taaacatgta actaaaatta ttcaagttaa tcataatgcc
     2281 aaatcccgta tccaaaatca attatcttat aaactaggtc aaatagcctt aattaattct
     2341 aagagtattt taggtttgtt atgtatgcct gtgtatataa tatctaccat cattgtgcat
     2401 gttcaagata aaaaaatata tcataatgca ataaaaaaag atcctagtct aaaattattg
     2461 cccttagaac aataccctga ttacaatgag gcaattaaac ttaaacagca tttttcatat
     2521 aaattaggaa gtgtaattat gcattcattg aaatattggt atttaggtgg aattttagct
     2581 ctacctttta gagtttttaa gctttataaa aatcataaaa aaaagatatg acaaatgaaa
     2641 accgtaggta tagtaatccc tatctataat gtggaaaaat acttaagaga atgtttggat
     2701 agtgttatca atcaaaccta tgctaactta gaaatcatac tagtaaatga tggtagcata
     2761 g
//
```

>>BfR-CA-15838_min_cov_30 putative plasmid location

```
LOCUS       OIDGCHHC_1             37091 bp    DNA     linear       12-APR-2021
DEFINITION  Campylobacter species strain strain.
ACCESSION
VERSION
KEYWORDS    .
SOURCE      Campylobacter species
  ORGANISM  Campylobacter species
            Unclassified.
COMMENT     Annotated using prokka 1.14.6 from
            https://github.com/tseemann/prokka.
FEATURES             Location/Qualifiers
     source          1..37091
                     /organism="Campylobacter species"
                     /mol_type="genomic DNA"
                     /strain="strain"
     gene            complement(376..2841)
                     /locus_tag="OIDGCHHC_00001"
```

```
CDS             complement(376..2841)
                /locus_tag="OIDGCHHC_00001"
                /inference="ab initio prediction:Prodigal:002006"
                /inference="similar to AA sequence:RefSeq:ASI88309.1"
                /codon_start=1
                /transl_table=11
                /product="IncP-type conjugative transfer protein TrbE"
                /protein_id="Prokka:OIDGCHHC_00001"
                /translation="MLALKEFRSKAKAFPDILNYASFIDEGILLNKDGSLTAGFYYRA
                GDISSMTINERNTLSARINSVLKTLGNGWAVHIDCSRIKTENYIDGDTFYKNNIAQIL
                ENERKHYFDNVEHFENLFTMIFTYLPPHKSINKITDLMIIDETKNKNQGDKILEYFKN
                TLQKLEDSLSNYIKIERMLPRIAIDEYNNEYILDDMLEYINFCICGERQKIILPNAPM
                FLDCLLGGRHFETGMHPRIDNQYIGIIAIEGFPSESYPNILNALTELNFDYRFNTRFI
                FLDDFEAQNSLNKYRKKWQQKTKGWLDQLLDRPARKQDDHAVLMVQELDSALSESKSG
                MIGYGYYTANIVVFDRDYESLEYKLKDIRKVLENLGFLSRTETINSVEAYLGTLPGFV
                YPNLRRPILNTLNLTHLIPLASIWAGDKYNSSERFPPNSPPLMQVVTSGNTPFRLNLH
                VSDLGHTLIFGPPGSGKSTLLANIFLAWQKYPNAKIYAFDKGQSLLAPTLATGGIHYD
                VAGDHSSLAFAPLANIKTDAEIAWAEFWIETCLKLQNVNITPKQKKLIHEALISHINT
                NSTSLTEFVSALQDSDLRDALNYYTISGTGGFLFDSEEDKLSLSSITTFEIEELMNLG
                DQYIIPALLYIFNRIEKGLDGSPTLLIIDEAWIALKHPAFKDKIVNWLKVLRKANVAV
                IMATQSLSDSAKSGILDILQEYCPTKIFLPNPEAYKKGSENVLAPYDFYKIFGLNDVQ
                ISIINNAIQKREYYYTSPLGTRLFNLALGKTNLAFTTISDKVSVKRVRQLYEKHKENW
                AYKWLEEKEINYKRFDNSQKEEA"
gene            complement(2845..3132)
                /locus_tag="OIDGCHHC_00002"
CDS             complement(2845..3132)
                /locus_tag="OIDGCHHC_00002"
                /inference="ab initio prediction:Prodigal:002006"
                /inference="similar to AA sequence:RefSeq:ASI88308.1"
                /codon_start=1
                /transl_table=11
                /product="IncP-type conjugative transfer protein TrbD"
                /protein_id="Prokka:OIDGCHHC_00002"
                /translation="MEELQSIDIYSALNKPNLIFGADRELILMVGVISFALIFTGATL
                LTSIIGIFLFFFCNMLLRLMAKSDPLMRQIFLRQIKYKKFYYAQSTPFSKD"
gene            complement(3132..3428)
                /locus_tag="OIDGCHHC_00003"
CDS             complement(3132..3428)
                /locus_tag="OIDGCHHC_00003"
                /inference="ab initio prediction:Prodigal:002006"
                /inference="similar to AA sequence:RefSeq:ASI88307.1"
                /codon_start=1
                /transl_table=11
                /product="Conjugative transfer protein TrbC"
                /protein_id="Prokka:OIDGCHHC_00003"
                /translation="MKKFLILFLAFSLEYAFASTTGAGLPWEGPLEQIKASLSGPVAF
                VISILAIIGAGAGLIWGGELSGFIKTLFYIVLVIALIVGANSFMSLFSTSGALI"
gene            complement(3438..4376)
                /locus_tag="OIDGCHHC_00004"
CDS             complement(3438..4376)
                /locus_tag="OIDGCHHC_00004"
                /inference="ab initio prediction:Prodigal:002006"
                /inference="similar to AA sequence:RefSeq:ASI88306.1"
                /codon_start=1
```

```
                     /transl_table=11
                     /product="Conjugative transfer protein TrbB"
                     /protein_id="Prokka:OIDGCHHC_00004"
                     /translation="MSNLEFQARINEKIKREFGDILTHLEDKETIELMLNSDGKLWLE
                     KLGQDMICLGEFSEARAKSIITAISTLLDTTTNADNPILECELPLDGSRFEALLPPIV
                     AKPTFTIRKKAVKIFTLGDYVDSNIITPNQKEVLINAIIERQNILVVGGTGSGKTTFS
                     NAIIDGISKITPDHRIVIIEDTAELQCASENKVILRATDKVDMLRLLKATMRLRPDRI
                     IVGETRGKEALDLLKAWNTGHPGGIATIHANSANGGLTRMEQLISEATSAKMSKLIAE
                     AVNLIVFISKSKEGRKVKEIIKVIDYENDKYITQTI"
gene                 complement(4916..5656)
                     /locus_tag="OIDGCHHC_00005"
CDS                  complement(4916..5656)
                     /locus_tag="OIDGCHHC_00005"
                     /inference="ab initio prediction:Prodigal:002006"
                     /codon_start=1
                     /transl_table=11
                     /product="hypothetical protein"
                     /protein_id="Prokka:OIDGCHHC_00005"
                     /translation="MLFNYEIINCQKPNNTMNIKRTYKVIRCIFKNKTDEASKYYQQA
                     WDLAKQVNPLKANDSTSQRDKERLILDALGGILAEYGWFYYIKRNFGDIVNFTEFKSS
                     IGQIDLLLNNGKTLEVRSSFPRNGINFAICNAKYNFKNICKYDNLYKPNEMDKDFFAC
                     VLFETTKNQMVKADEIIFYLIGGSTRKMMLDNTISYTDNLTAEDDLIQQKTNYKVIRL
                     YNALDIEGFEKYMTEELGYPKILNDQTF"
gene                 complement(5646..6563)
                     /locus_tag="OIDGCHHC_00006"
CDS                  complement(5646..6563)
                     /locus_tag="OIDGCHHC_00006"
                     /inference="ab initio prediction:Prodigal:002006"
                     /codon_start=1
                     /transl_table=11
                     /product="hypothetical protein"
                     /protein_id="Prokka:OIDGCHHC_00006"
                     /translation="MNKNSSSKLNNAKNIKNDEFYTQYEDINKELNFYKNAFKDRIVY
                     CNCDDPQKSNFTKFFKLNFDRLKLKKLISTSFNSNGKGKIFIIEKNKPKYQGLLENNG
                     DFRSDETIKLLKESNIIVTNPPFSLFREFIDILISNQKDFLIIGNANAISYKNCFNYM
                     MKNKLWLGQNCVRWFINTKGELVEGARSFWFSNINNQKRNKKISLNRKYNNQDYITYD
                     NYNAIEISKSKDIPSDYTGIMGVPITFLDKYNPQQFEIIGADYQVNSGELIYIKRNDW
                     NGKTDRAYINGKRLYSRIFIKHRGVNNAI"
gene                 complement(6586..6999)
                     /locus_tag="OIDGCHHC_00007"
CDS                  complement(6586..6999)
                     /locus_tag="OIDGCHHC_00007"
                     /inference="ab initio prediction:Prodigal:002006"
                     /inference="similar to AA sequence:RefSeq:ABS43377.1"
                     /note="conserved hypothetical protein"
                     /codon_start=1
                     /transl_table=11
                     /product="hypothetical protein"
                     /protein_id="Prokka:OIDGCHHC_00007"
                     /translation="MKKVYLDTNIFLDYFNTERTYHNEAKQLMYYLLTNNIQIVFSED
                     MISTIAYLIKKENLNNFTHFLKDISLEKNFNITSFGVSVINMACDCYEKYRGDFEDYL
                     QYFCAEKEGCSAIYTMDKKFPNLKIPIKRYGEIDI"
gene                 complement(6996..7910)
                     /locus_tag="OIDGCHHC_00008"
```

```
     CDS             complement(6996..7910)
                     /locus_tag="OIDGCHHC_00008"
                     /inference="ab initio prediction:Prodigal:002006"
                     /inference="similar to AA sequence:RefSeq:CKG86570.1"
                     /codon_start=1
                     /transl_table=11
                     /product="hypothetical protein"
                     /protein_id="Prokka:OIDGCHHC_00008"
                     /translation="MSNEIEIKDLNEINKNNKRQVVYRKDNLSTDYITGEVVERNQII
                     VKKVQTKESFIKLFTENIDYIINLESQEKTLFFTLLSSVDYKNVVRFDKSMKQNIVLD
                     KVLSRAGMYRALNGLIEKKVIFKINREFVKSYAGVGSDDCYLFNPNLVGRGSWNDLKN
                     LRHTITREWDFENLEVRENLEIEEEYLGFDEIRNNQKQYQVKEINQEHNETTKVKNTE
                     IVIEEKNNSINNQYSFDKKEEPSINKKVLISNDTQPSLFDENQKEQEARLFIEKYAGI
                     LTGAYDSTKSAKEMKRELLDKDLEEGRI"
     gene            complement(8181..8534)
                     /locus_tag="OIDGCHHC_00009"
     CDS             complement(8181..8534)
                     /locus_tag="OIDGCHHC_00009"
                     /inference="ab initio prediction:Prodigal:002006"
                     /codon_start=1
                     /transl_table=11
                     /product="hypothetical protein"
                     /protein_id="Prokka:OIDGCHHC_00009"
                     /translation="MGCGVCSRLERQTNLTPPYCCKKSKRRLVSSNLQMSVSEICCKK
                     FFVFKSRLRRKRLKKNAFKYRYNSALIRFLIFPPVGKKLLVVGKLFLEKFIFPNKLGK
                     NYFFCLNMYHFLGKS"
     gene            8971..9414
                     /locus_tag="OIDGCHHC_00010"
     CDS             8971..9414
                     /locus_tag="OIDGCHHC_00010"
                     /inference="ab initio prediction:Prodigal:002006"
                     /inference="similar to AA sequence:RefSeq:ADN91794.1"
                     /codon_start=1
                     /transl_table=11
                     /product="hypothetical protein"
                     /protein_id="Prokka:OIDGCHHC_00010"
                     /translation="MKKLALVLAALVALNVSAFAGVITIHNSNLVKVSGSDDIYAKAA
                     LEDAKEDMRSQFNTDNLEIYAIESIKKDDPHEYDVLQRGDLKIERENMAKGKTVYYNI
                     FLVVDRKTNWASTWKIECDHRDRPIKLSNGNKCKAGIYINRYNFK"
     gene            complement(9443..9691)
                     /locus_tag="OIDGCHHC_00011"
     CDS             complement(9443..9691)
                     /locus_tag="OIDGCHHC_00011"
                     /inference="ab initio prediction:Prodigal:002006"
                     /codon_start=1
                     /transl_table=11
                     /product="hypothetical protein"
                     /protein_id="Prokka:OIDGCHHC_00011"
                     /translation="MFSAEQFGGLKAAGSEPMAWMLIELAKGLLKVFAYIASAYVMWK
                     TILGGPDWTFRLMGISNSASDVITEDVGRRMENKTFMV"
     gene            complement(9914..11008)
                     /locus_tag="OIDGCHHC_00012"
     CDS             complement(9914..11008)
                     /locus_tag="OIDGCHHC_00012"
```

```
                     /inference="ab initio prediction:Prodigal:002006"
                     /codon_start=1
                     /transl_table=11
                     /product="hypothetical protein"
                     /protein_id="Prokka:OIDGCHHC_00012"
                     /translation="MKDIMDFRKLVEKYKKGNEAINDTIAKEILHYEILESLFVSTEV
                     ANKLVFQGGTALRICYDNNRYSEDLDFTLVDVEDFDSMKLDDFKKHFSQKIKDKYDLN
                     VEIIEPKKASQFVKKWEAKIALNFLNRKSKINIKIACIPSYDNQNMPIKENYKEIGAN
                     MILKVETLREILADKLVALLCRDYIKYRDLWDIKYLKDRNTISDYELVRKKLIDYNHA
                     DENTIKIMFENKEKQLNNKELENEFYNEMIRFLDEKIFDYCKNNNYFSDVLKIVKDEF
                     ENLKKKILSNQDLSLEDELSQLSKDLKINIIELARLFERYKNLNNKDDDMFWIGYNDV
                     KDILKKCFDKYCNSYKCENHTENKTKRKFH"
     gene            complement(10986..11537)
                     /locus_tag="OIDGCHHC_00013"
     CDS             complement(10986..11537)
                     /locus_tag="OIDGCHHC_00013"
                     /inference="ab initio prediction:Prodigal:002006"
                     /codon_start=1
                     /transl_table=11
                     /product="hypothetical protein"
                     /protein_id="Prokka:OIDGCHHC_00013"
                     /translation="MTHLELMDFLDYWDNKDKWLFTLSNFAVCFHKESLQNLKISLSR
                     LSKKGYIVHVSKGLYANPRTRCSMLFREYEVANHLRNKGYSYLSLETLLSQEGLISQI
                     PNRLTFVSLKFSHTYHTPYGIIEYVQKKENPERFFDDCYYDEKCQVWVANQKKAIDDI
                     YRFNRSIDLYEEQKMKDERYYGF"
     gene            complement(11627..12340)
                     /locus_tag="OIDGCHHC_00014"
     CDS             complement(11627..12340)
                     /locus_tag="OIDGCHHC_00014"
                     /inference="ab initio prediction:Prodigal:002006"
                     /codon_start=1
                     /transl_table=11
                     /product="hypothetical protein"
                     /protein_id="Prokka:OIDGCHHC_00014"
                     /translation="MTLEENIFLAKRNIIDNIYKSARLEGIAVTFPQTEAIYNGVNVN
                     NLKVNDIVAINNLKHAWNFLFETMSYPEVDFAYICEINKTIGANLINKAGFIRTYEVS
                     IGGTTWKPKILHKEEILEELNKIKEIDNATQKAIALMLYLMRKQIFSDGNKRTAVLCA
                     NRVLIENGAGLVNIPVELISEFKEKLIKYYETNDMKDIMDFIYKNCIEGFVSVEPSEE
                     EILESKKNQEMFARYRKKK"
     gene            complement(12375..12938)
                     /locus_tag="OIDGCHHC_00015"
     CDS             complement(12375..12938)
                     /locus_tag="OIDGCHHC_00015"
                     /inference="ab initio prediction:Prodigal:002006"
                     /codon_start=1
                     /transl_table=11
                     /product="hypothetical protein"
                     /protein_id="Prokka:OIDGCHHC_00015"
                     /translation="MEYEEYQEYKKNKNINYENILIGIGLNQVDNLKPSEYFYKAVEN
                     SSSYEELEEKIIEYYKNADNKEIARTKECDLVSQRIAKIIDDEGFSFSPITLKSIHRK
                     IFTDIFPQTMEKYVGNFRKVNIFKEEANLNGKSVNYANYDDILDLLEYDFEKEKKNDY
                     SKMNNDELIKKISNFISNIWQVHPFTE"
     gene            complement(12958..13173)
                     /locus_tag="OIDGCHHC_00016"
```

```
     CDS             complement(12958..13173)
                     /locus_tag="OIDGCHHC_00016"
                     /inference="ab initio prediction:Prodigal:002006"
                     /codon_start=1
                     /transl_table=11
                     /product="hypothetical protein"
                     /protein_id="Prokka:OIDGCHHC_00016"
                     /translation="MAKTINRDILNSISKSAEMQENKTIEFEGNKNFTISFKVSQYNS
                     LKEHCEKNHTSMGKLIKDLLKEKKLIN"
     gene            complement(13200..13403)
                     /locus_tag="OIDGCHHC_00017"
     CDS             complement(13200..13403)
                     /locus_tag="OIDGCHHC_00017"
                     /inference="ab initio prediction:Prodigal:002006"
                     /codon_start=1
                     /transl_table=11
                     /product="hypothetical protein"
                     /protein_id="Prokka:OIDGCHHC_00017"
                     /translation="MLLIIDNITSQIDNCKHFSIAKNIIRDRIIFKNSLAEGKGILEV
                     DSKGASDLKAKEELNSLFKEFNL"
     gene            13914..14108
                     /locus_tag="OIDGCHHC_00018"
     CDS             13914..14108
                     /locus_tag="OIDGCHHC_00018"
                     /inference="ab initio prediction:Prodigal:002006"
                     /codon_start=1
                     /transl_table=11
                     /product="hypothetical protein"
                     /protein_id="Prokka:OIDGCHHC_00018"
                     /translation="MNKDKKYLNFSEYYEVRDFLELYPEKDRENIENILMTIGENKKS
                     KNLSFEFLYDFFKELGFEKS"
     gene            complement(14122..14451)
                     /locus_tag="OIDGCHHC_00019"
     CDS             complement(14122..14451)
                     /locus_tag="OIDGCHHC_00019"
                     /inference="ab initio prediction:Prodigal:002006"
                     /codon_start=1
                     /transl_table=11
                     /product="hypothetical protein"
                     /protein_id="Prokka:OIDGCHHC_00019"
                     /translation="MQRGEAKIEFYSNINYLKKEFENGCVVSKFLYDKALKEKNFKMT
                     YKQFNKYFNDEFKNKIKIKETIQEDNQLQSLVLKNKEPIKLKINTKSKKVFDAKFGKD
                     IKEDDLL"
     gene            complement(14465..14914)
                     /locus_tag="OIDGCHHC_00020"
     CDS             complement(14465..14914)
                     /locus_tag="OIDGCHHC_00020"
                     /inference="ab initio prediction:Prodigal:002006"
                     /codon_start=1
                     /transl_table=11
                     /product="hypothetical protein"
                     /protein_id="Prokka:OIDGCHHC_00020"
                     /translation="MDIKIATFEDIEFIKYCFLLGFKEKHFYNVRFIEKLEDIIKNNG
                     VSGGIFIIKDGTNEKAGFAFCGKSKGSNIYEINMIYILEKFRNKKLATNFIKYYEKLI
```

```
                     PNNKIIARCDINYSQQAIKIFKHLGYQELSKKEKTNQYEFMILEKSI"
     gene            15124..15507
                     /locus_tag="OIDGCHHC_00021"
     CDS             15124..15507
                     /locus_tag="OIDGCHHC_00021"
                     /inference="ab initio prediction:Prodigal:002006"
                     /codon_start=1
                     /transl_table=11
                     /product="hypothetical protein"
                     /protein_id="Prokka:OIDGCHHC_00021"
                     /translation="MRKDKRIAIRLSEDEYKHIMQKAQNHGLTISRYLRDLSMNYPIT
                     CIVDQKVAHNVLNIAGDIGRLGGLFKHWLVRNEDNKVNFSNKRTYNDIDEIVDQILDL
                     QILLKEQAKRIIQNDNQKDSKQKAE"
     gene            15470..17626
                     /locus_tag="OIDGCHHC_00022"
     CDS             15470..17626
                     /locus_tag="OIDGCHHC_00022"
                     /inference="ab initio prediction:Prodigal:002006"
                     /inference="similar to AA sequence:RefSeq:ASI88299.1"
                     /codon_start=1
                     /transl_table=11
                     /product="IncP-type DNA relaxase TraI"
                     /protein_id="Prokka:OIDGCHHC_00022"
                     /translation="MIIKKIPSKKQNKSSFKNLSNYILDKDNNNAKVLVDYMLDKNNE
                     MDKVEGYHFTNCSFDNDEDNINEIINTQKLNTTTKQDKTLHLVVSFQEDENPTLEILQ
                     AIEEEIAKSLGMEDHQRLSVVHSNTNNLHIHIAINKVNPHTLKVINPYNDVRILQETA
                     MKLEKKYNLKLDNHISQKDKQSNKYNTHTMNCNFETWVKEKLSKQVDLMLKDEKTTFK
                     DIKQFLAEYDLEFRERRKGFVIASKSEKLFCKASSIHRGLSKQALEKRFKELDLKQEK
                     ENTKNIKEEKHEIKEQYQRPNKETSKTLWEKYLRIENEKKAELDKELRMLKLRRNEFK
                     TSIPSMKFNKNTFAHVKNQRMIFKNKQKELYQKYKRVSYRDFLISESLSGNEEATRAL
                     RRTKTKINENENTLSSTQEKPKIFENVDYITKEGYAVYKSGFNKAIDKGEMLKVSLIN
                     DKDDKEFLLNSLLIAIDRFGNHLNITGDDKFKRNILEVANDYNLNVSFSDQRMQKIQE
                     ENNNKRKEMKTRKVLKNIIELKIKLTEQDKDIDENIKEKELKALKFSLKKISNSTPSI
                     FKGELKKLGFSYKEIDSMDTESVCIEIDGFIVNSANKEGLIAMNEEIKQTLIDDEEIK
                     RFEKFEHLFLNTNKITDIAKGFYTNRKIDADEYIKRFSMQESKINKVANAISMLSDKN
                     IDIMEKYAKKLEKDLKYQYLKKVEVIESNDINLNNF"
     gene            complement(17860..18411)
                     /locus_tag="OIDGCHHC_00023"
     CDS             complement(17860..18411)
                     /locus_tag="OIDGCHHC_00023"
                     /inference="ab initio prediction:Prodigal:002006"
                     /codon_start=1
                     /transl_table=11
                     /product="hypothetical protein"
                     /protein_id="Prokka:OIDGCHHC_00023"
                     /translation="MIKQIFNKITKHFNLKKSSIKLNIAEHKDIDFIMWCIDDGIKHG
                     HFINISKENIEKVITTFSLSKIFIINFNTVPIGIIIENHLLHYYDHYIINHSNNQKLY
                     GLEINVLYITKEFRNKGIGSAIITYYEKETDYNKILARCEQNNSKKSIIFFEKLGYKK
                     IGFNKQPKHNMTHIILEKNLQKE"
     gene            complement(18404..18799)
                     /locus_tag="OIDGCHHC_00024"
     CDS             complement(18404..18799)
                     /locus_tag="OIDGCHHC_00024"
                     /inference="ab initio prediction:Prodigal:002006"
```

```
                     /codon_start=1
                     /transl_table=11
                     /product="hypothetical protein"
                     /protein_id="Prokka:OIDGCHHC_00024"
                     /translation="MNNKQKDQNSETEEKIIEIIKNQKDQNSETEKNTNDEENLDSDL
                     NKKQAEQYLKIAEDKHKLAINYFKYTYWIIIGYISIITSIVICIGFESLIYDNYVINI
                     FLGTTSINIIGLGYIAMRWLFPNSYKKYD"
     gene            complement(18796..19266)
                     /locus_tag="OIDGCHHC_00025"
     CDS             complement(18796..19266)
                     /locus_tag="OIDGCHHC_00025"
                     /inference="ab initio prediction:Prodigal:002006"
                     /inference="similar to AA sequence:RefSeq:AVS36970.1"
                     /note="DUF4065 domain-containing protein"
                     /codon_start=1
                     /transl_table=11
                     /product="hypothetical protein"
                     /protein_id="Prokka:OIDGCHHC_00025"
                     /translation="MEKNSKLYSSKAIANEFIRLAQKDNKTLTHMQLQKLIYFTHAVT
                     LVIFKRPLVKDGFQAWPFGPVNLELYLELQGSGKNGIEKYIDLDQYHNDIKDDRETME
                     IIESIYKKLGDKDGWELSKITHQKGHPWSSVYEKGKSNLIEDDAIIYFYSKDLK"
     gene            19362..19490
                     /locus_tag="OIDGCHHC_00026"
     CDS             19362..19490
                     /locus_tag="OIDGCHHC_00026"
                     /inference="ab initio prediction:Prodigal:002006"
                     /codon_start=1
                     /transl_table=11
                     /product="hypothetical protein"
                     /protein_id="Prokka:OIDGCHHC_00026"
                     /translation="MGLKPGPKRTAKSTGELDQRQRDNKNTPGNTSKLKPSKSSKK"
     gene            complement(19596..19820)
                     /locus_tag="OIDGCHHC_00027"
     CDS             complement(19596..19820)
                     /locus_tag="OIDGCHHC_00027"
                     /inference="ab initio prediction:Prodigal:002006"
                     /codon_start=1
                     /transl_table=11
                     /product="hypothetical protein"
                     /protein_id="Prokka:OIDGCHHC_00027"
                     /translation="MGFKKLSSEAPKNENDFVNSARGETGNAKTGNAKRNKKLMINFT
                     EEEYQILNQEAHSMGLSLNSYVRFKIFRKD"
     gene            complement(19823..20509)
                     /locus_tag="OIDGCHHC_00028"
     CDS             complement(19823..20509)
                     /locus_tag="OIDGCHHC_00028"
                     /inference="ab initio prediction:Prodigal:002006"
                     /inference="similar to AA sequence:RefSeq:ASI88294.1"
                     /codon_start=1
                     /transl_table=11
                     /product="Plasmid partitioning protein ParA"
                     /protein_id="Prokka:OIDGCHHC_00028"
                     /translation="MIISVVNEKGGSGKTTLAVNLSARLAEDGDNVLLIDADPQKSTE
                     VFSDMRSQSNLEPLFSNVSKTGVSLGDEIKRMKNAFDSIIVDTGGRDSKEMRKAILSS
```

```
                     NIIIIPTIPSQYDVNVLDHMLEIYNEVIEINPNLLALVLVNRVSPNPFLAKELENLKE
                     YINEAKKEKGLDRVIMLESVIYERQAYRKAVIEGKSMKEFCDKNDKALNDFENFYQEL
                     LKIANEKLGV"
     gene            complement(20601..20996)
                     /locus_tag="OIDGCHHC_00029"
     CDS             complement(20601..20996)
                     /locus_tag="OIDGCHHC_00029"
                     /inference="ab initio prediction:Prodigal:002006"
                     /codon_start=1
                     /transl_table=11
                     /product="hypothetical protein"
                     /protein_id="Prokka:OIDGCHHC_00029"
                     /translation="MNKKELIEEIAKKHHIILDENDPILAVVSANEIIFDDFLEKIDL
                     LFTKYKTDLESYKFNIFNEIREYSKSNQEALKDIITQVQANQNTIKPSQEKTEDTKEK
                     KNTNINFWIIAIASQIIFLLVGLIIGIII"
     gene            complement(21062..21529)
                     /locus_tag="OIDGCHHC_00030"
     CDS             complement(21062..21529)
                     /locus_tag="OIDGCHHC_00030"
                     /inference="ab initio prediction:Prodigal:002006"
                     /codon_start=1
                     /transl_table=11
                     /product="hypothetical protein"
                     /protein_id="Prokka:OIDGCHHC_00030"
                     /translation="MRKTIAIFAFSFAFISILSYLIFYFGGFHFNYTRSMPLGLYKEI
                     NSSTFNKNDIVLLKIPQKKEILLKKIIAVSGDFVEVNTQGVFINESLLPNSKIFSFDS
                     EGNLLEFKPFKRKLKENELFVMGENIKSYDSRYFGVVNILQNEVKKVKLMISF"
     gene            complement(21534..22196)
                     /locus_tag="OIDGCHHC_00031"
     CDS             complement(21534..22196)
                     /locus_tag="OIDGCHHC_00031"
                     /inference="ab initio prediction:Prodigal:002006"
                     /inference="similar to AA sequence:RefSeq:ASI88290.1"
                     /codon_start=1
                     /transl_table=11
                     /product="Cell filamentation protein"
                     /protein_id="Prokka:OIDGCHHC_00031"
                     /translation="MWDSQENLSEFDKFLIKTNKIGAKSLDELFRKERDITNAKALEL
                     SKNPIKGNFDYQHLKNIHKYIFQDVFHWAGKDRMEVGLHGNFGKYAPNGTITNFVPGK
                     DLNATAQQIFTWLKEDNYLKNSKDLNDFAKNLAEFARNLNALHPFREGNGRTQRIFLN
                     ELAKNAGYKLDLNLIPKDKTIMASVEASQLKLGKLEAIIKTNLKSFRQNLDLEQNKGI
                     SL"
     gene            complement(22196..22435)
                     /locus_tag="OIDGCHHC_00032"
     CDS             complement(22196..22435)
                     /locus_tag="OIDGCHHC_00032"
                     /inference="ab initio prediction:Prodigal:002006"
                     /codon_start=1
                     /transl_table=11
                     /product="hypothetical protein"
                     /protein_id="Prokka:OIDGCHHC_00032"
                     /translation="MQKQYPEVHSLEESLAILQKYKDDLTKEQYEAIRSNIGNFAIED
                     MFLNERNIIDNIKIIKGEATANEIITEYKKEWGIS"
     gene            complement(22526..23245)
```

```
                     /locus_tag="OIDGCHHC_00033"
     CDS             complement(22526..23245)
                     /locus_tag="OIDGCHHC_00033"
                     /inference="ab initio prediction:Prodigal:002006"
                     /inference="similar to AA sequence:RefSeq:ASI88288.1"
                     /codon_start=1
                     /transl_table=11
                     /product="IncP-type DNA transfer protein TraL"
                     /protein_id="Prokka:OIDGCHHC_00033"
                     /translation="MAKIHFVLNGKGGIGKSFISSLLCQYFLDKGESIVAIDTDPNNT
                     TLLNVKALKAKFLQLIDDDGKFDVRAFDKIVELAFEKKAENYIIDSGATTFIPLIDYL
                     KENEVLDFLTNNNLEVYMHVPIVGGQARDDTILGLTQLTEAFNCSFVVWINEYHGSII
                     KDNLEFEETKEYQAIKEKIKAIVYLNALSKDTFGKDLLELTSKNLTFGEAMQDKSFSL
                     MSKQRLKIFKDKAFSQIGNIL"
     gene            complement(23256..25079)
                     /locus_tag="OIDGCHHC_00034"
     CDS             complement(23256..25079)
                     /locus_tag="OIDGCHHC_00034"
                     /inference="ab initio prediction:Prodigal:002006"
                     /inference="similar to AA sequence:RefSeq:ASI88287.1"
                     /codon_start=1
                     /transl_table=11
                     /product="IncP-type DNA transfer coupling protein TraG"
                     /protein_id="Prokka:OIDGCHHC_00034"
                     /translation="MKKQTNQALVYILGLIGIFLTFTIISQIATQYLAKSFNYSPSLG
                     ETLLYGFYNPFKWISWSYAYYSFYPDFFKKFFMVMLAGIAFCFIVFILVKLAFLRKAK
                     AIENLHGSAHWATLEEVKESGVFDKDKGVYIGGFEHKKTLHYLRHDGPEHVMLFAPTR
                     SGKGVSLLLPTLLSWEESALIFDTKGELWALTAGWRQKYAKNKVLKLDPTCLDDSAVK
                     FNILEEIRLETMHEIKDTQNIAINLIFKGETPPTNPTQGSTSYFKSEAGNFLVSIILY
                     ALHLKKYRGENTPNLTDIYKFINDPNQSIDELLEEMVECDISTMDKNTQEIIQSISRS
                     MKNKATQELSGVVGTASEALNLYIDPILAKNTAKSDFKIKDLMNHESPISLYLIIPPG
                     QKDRLRPFFNLLINQIFRTLTDDSLNFKNGENVKRYKHKMLVLADELTMFGKLGVLEE
                     NLAYMAGYGMKFYGSIQDIQQLYSIYGEKETIISNCHIRIAFAPNKIETAKLLSEMGG
                     ITTIVKKSITSSGKRTAVMLGNVSETIQEVQRPLITADECMRLPSAKKDKDDKIIAPG
                     DMLIFIAGQAPIYGKQILYFKDPAFLARSKVTLDKQVSDIL"
     gene            complement(25076..27289)
                     /locus_tag="OIDGCHHC_00035"
     CDS             complement(25076..27289)
                     /locus_tag="OIDGCHHC_00035"
                     /inference="ab initio prediction:Prodigal:002006"
                     /inference="similar to AA sequence:RefSeq:ASI88286.1"
                     /codon_start=1
                     /transl_table=11
                     /product="IncP-type DNA transfer primase TraC"
                     /protein_id="Prokka:OIDGCHHC_00035"
                     /translation="MKNKDFIQETADKIIESLKNGTAPWIKPWKGIDLANNMPYNPIT
                     NKPYNGINSINLMLQNYNDPRWLTYKQAQSINAQVRKGEKSTLIQYWQFSEMVDKLDE
                     EGNVITNENGEVEQIEVKLENPKVFFAYVFNAEQIENMPKLEQKQEIDNFQIIKEAQK
                     ILDDSKAIIHHKGNRAFYNAANDTITLPPKENFLSEGAYYTTALHELVHWSGHESRLN
                     RDLKNPFGSKEYAKEELRAEIASFLFNGKIGLDYDPGQHLSYIDSWVQILEDKPNEIF
                     KATSDATKIVNFLESLSLEQKQKIELENSNTQNQALKQDKELNLATRKTYLYVPFEEK
                     EFAKKAGAKWDKEAKMWYAPKGADLKNFTQWLNQSPLTQSYNNTHDEFKAFLEKYELN
                     IEGQPIMDGKLHRVSVIGDKGREKSGAYVGFLNGHPAGFVQNYKTGIKENWKSANSLE
                     NTKNKEINLQNALEHNKALKEQRDKELNQAYENTAVKLENEYNNARWANSEHPYLKEK
```

GFDKNFYLKQDKNGNLLIPLRDIEGKYWATQRIFANGDKMIGATRTAEEKEQGIEYPA
KKQGNFFLLGAKNLNNVNEVYICEGFATSASVYEATKSPTIMGVDAGNLEIIVTNIKE
KYPKMNIIIAGDNDVKKELNGNKNVGKETALGIQKKYPEVKVVLPNFTNQEAQKGLSD
FNDLMKSRGLEEIKKQLKEQIAKQLMVEKATTKALNKNKGINKEITARKDIGISL"

```
gene            complement(27294..27434)
                /locus_tag="OIDGCHHC_00036"
CDS             complement(27294..27434)
                /locus_tag="OIDGCHHC_00036"
                /inference="ab initio prediction:Prodigal:002006"
                /codon_start=1
                /transl_table=11
                /product="hypothetical protein"
                /protein_id="Prokka:OIDGCHHC_00036"
                /translation="MKDFKELETIPNFSYEVDPLTAEELGAFKENALSEDDAKEGTED
                QG"
gene            complement(27421..27783)
                /locus_tag="OIDGCHHC_00037"
CDS             complement(27421..27783)
                /locus_tag="OIDGCHHC_00037"
                /inference="ab initio prediction:Prodigal:002006"
                /inference="similar to AA sequence:RefSeq:ASI88284.1"
                /codon_start=1
                /transl_table=11
                /product="Single-stranded DNA-binding protein"
                /protein_id="Prokka:OIDGCHHC_00037"
                /translation="MINNITIGGRLTADAELRHCNQGSIICTFTLANNRKYKEIENST
                YIEVSLFGSFANAMHPYLKKGISVDVVGELTQDTWEHEGKIYSKHRIKAKEIDFRTPK
                KEKQTIPNIEEGENYEGF"
gene            complement(27776..27877)
                /locus_tag="OIDGCHHC_00038"
CDS             complement(27776..27877)
                /locus_tag="OIDGCHHC_00038"
                /inference="ab initio prediction:Prodigal:002006"
                /codon_start=1
                /transl_table=11
                /product="hypothetical protein"
                /protein_id="Prokka:OIDGCHHC_00038"
                /translation="MENENLEENKPTQFDDNEPSLFDNLEDEEQDND"
gene            complement(27880..28425)
                /locus_tag="OIDGCHHC_00039"
CDS             complement(27880..28425)
                /locus_tag="OIDGCHHC_00039"
                /inference="ab initio prediction:Prodigal:002006"
                /inference="similar to AA sequence:RefSeq:ASI88282.1"
                /codon_start=1
                /transl_table=11
                /product="T-DNA transferring Peptidoglycan hydrolase
                VirB1"
                /protein_id="Prokka:OIDGCHHC_00039"
                /translation="MIDIALIEQCKNPNVETQIIQKIIQVESNNQQFAININKVGSFI
                LKNKKEAENLANNFIKGGYSVDIGLMQFNSNNLKSSTFSHYSVEDLLDTCKNIKAGSD
                IFYLAYEMTNKNLAKEDRINQALSIYNTGDLKKGFSNGYVAKYDSTAKTIDDLEEKAR
                KSDTRIKITYNLFNLQTQKKE"
gene            complement(28418..29659)
```

```
                     /locus_tag="OIDGCHHC_00040"
     CDS             complement(28418..29659)
                     /locus_tag="OIDGCHHC_00040"
                     /inference="ab initio prediction:Prodigal:002006"
                     /inference="similar to AA sequence:RefSeq:ASI88281.1"
                     /codon_start=1
                     /transl_table=11
                     /product="Conjugative transfer protein TrbI"
                     /protein_id="Prokka:OIDGCHHC_00040"
                     /translation="MLEVNMSPNKFLPKNNKRLSKVPIIIIFAIIAIVLFSIFYVALS
                     RKQNLERQTAQTTPKEETQIATNHNNDLNLYIDELQVQKAQENNIISPPMPNATQQPI
                     QETNSQTTQQPPNNTPVLVQNNAIDEKEMKIKKDMQLKALLSNTKLDIKTEATDSKKN
                     QAINFNPPNNNEDINLGAINPQNTGLGLESGTNKDVQGQQFLSQKKDNGYLKYVKQKP
                     LSTYEIKAGWNIPAILITGVNSDLPGQILAQVTQNVYDSATGKYLLIPQGTKVVGAYS
                     SNIIYGQSRLLVAWNKLIFPNGDTLNLDGMQGTSQDGYTGFEDQVDNHYFRIFGSAFL
                     LSSISAGIALSDNSDTNSEKETASDKAIAQAIQQMGQVASEMIRKNMNISPTLKIRPG
                     YKFNIFVTKDIILEPLELNND"
     gene            complement(29663..30106)
                     /locus_tag="OIDGCHHC_00041"
     CDS             complement(29663..30106)
                     /locus_tag="OIDGCHHC_00041"
                     /inference="ab initio prediction:Prodigal:002006"
                     /codon_start=1
                     /transl_table=11
                     /product="hypothetical protein"
                     /protein_id="Prokka:OIDGCHHC_00041"
                     /translation="MKILFLALIAFFITSCAKMNSDYFNSSFAVEETKINYNKIADDF
                     SQFIISYYAPNKTTFFINEDKNNQDFSNYFINTLRKKGYAISNDNSKKDLTFLSYQIS
                     QIDNENIVAIFNINESKINVIYKIIENKLVKPNITSFNFTPKTKG"
     gene            complement(30103..31257)
                     /locus_tag="OIDGCHHC_00042"
     CDS             complement(30103..31257)
                     /locus_tag="OIDGCHHC_00042"
                     /inference="ab initio prediction:Prodigal:002006"
                     /codon_start=1
                     /transl_table=11
                     /product="hypothetical protein"
                     /protein_id="Prokka:OIDGCHHC_00042"
                     /translation="MKKFISNTNTATREIHHTPTSGARLISLDCNYTKEYNYNYDEFS
                     GIHLSSFRGLDFRHKGANLVANTPKNYTQKLKKLSLIASLLLFIGSSAYANNLNERDF
                     SNLTPKEKKDLAIAQKWINSKTTTIQGNNGEVIFLFGESMPSIVTAPLRLTDIALEPG
                     EVIKDVQIGDSIRWAVSLSVSGEEPYLISHIIIKPTDKNLQTTMNIMTNKRVYRLNLI
                     SEAKKFMPAVSFNYPNSIIKTLEDYKNQMKAKSESKNFYKTKDDEIPSNIENLDFGYS
                     VEGNAPFKPLRIYNDGIKTYIQMPKNLKFYEAPALMILDSSNEKQIVNYRLKYDTFIV
                     DRLFNKAILLSNVGSKQEKIKITKHSNKANQDIVNNVLYDLSLQNKKENK"
     gene            complement(31270..31965)
                     /locus_tag="OIDGCHHC_00043"
     CDS             complement(31270..31965)
                     /locus_tag="OIDGCHHC_00043"
                     /inference="ab initio prediction:Prodigal:002006"
                     /codon_start=1
                     /transl_table=11
                     /product="hypothetical protein"
                     /protein_id="Prokka:OIDGCHHC_00043"
```

```
                     /translation="MNIFRQKQKIEKNEFDTSSNPYLNAKTEWLERYGDYITRAKNWQ
                     MVAIISLAMCFVCVLFIGYIGSQNKLIPYVIEVDKLGNTAKVGMVQNIDLKNPNVIKY
                     SLNTFIYSWRSVWGNAETQRKFIFDAYAYVEPRSKAFNYLNSEFQKNNPFTRATKENV
                     RVKIKNIVPQNIDTWQVEWEETTTNLNEDILSKETYRGLIRIKQIIPNTEEQILKNPL
                     GIFITDINFAKIL"
gene                 complement(31984..32313)
                     /locus_tag="OIDGCHHC_00044"
CDS                  complement(31984..32313)
                     /locus_tag="OIDGCHHC_00044"
                     /inference="ab initio prediction:Prodigal:002006"
                     /codon_start=1
                     /transl_table=11
                     /product="hypothetical protein"
                     /protein_id="Prokka:OIDGCHHC_00044"
                     /translation="MLWNNKTNARVVSPYISVSQTAIIYFLDLKKIFLISSLKSVITT
                     AITAMKRLKSSIMDSYLFGFHPHTQPNSQKKSSISHKRTELKPNKQVVKPLELQSIYQ
                     MDKLIIS"
gene                 complement(32300..33508)
                     /locus_tag="OIDGCHHC_00045"
CDS                  complement(32300..33508)
                     /locus_tag="OIDGCHHC_00045"
                     /inference="ab initio prediction:Prodigal:002006"
                     /inference="similar to AA sequence:RefSeq:ASI88318.1"
                     /codon_start=1
                     /transl_table=11
                     /product="IncP-type conjugative transfer protein TrbL"
                     /protein_id="Prokka:OIDGCHHC_00045"
                     /translation="MKITTKTLLNGNKLAIIFLIFLAPNLAFGVESAEGVLSIIRNGL
                     LSWTPLIKTACLWVFWTLVAIDLIWTFGLKALSGFEFGEFLATLIKKIMYVGIFLFLF
                     NIDQWLQIIFNSFSQLATNVNGGLEISPSNIIQNAFKIVMIILSSMSVTSPGESFFKV
                     ICGVIILIAFVLMAVDLLIVYLKFFLMNVIVFFALALSGLNHFKQIGLNPIMTAIKVG
                     VELFMIQGLMALSITIIEKIYNEMDQKITYEITLGILVTALIFCMITKMVPGIIEAVF
                     NGSIGESAGTAAGFRAVATMAAGAAAGAAAGAVGITRAMNAAKALHLAEGGAGGMDLV
                     KGVAKNLAGAGGEHLRDNLTKGRMPNQMANRLQEKLKDIQGKTSEGGISSGSPKQENY
                     QSGINPDAME"
gene                 complement(33471..33749)
                     /locus_tag="OIDGCHHC_00046"
CDS                  complement(33471..33749)
                     /locus_tag="OIDGCHHC_00046"
                     /inference="ab initio prediction:Prodigal:002006"
                     /codon_start=1
                     /transl_table=11
                     /product="hypothetical protein"
                     /protein_id="Prokka:OIDGCHHC_00046"
                     /translation="MKHFIFSGLIGLGALAFLAGCGDEAKTSDYYKAHLEEAKARVAE
                     CKKMEKMNETQQRDCSNAKWAVEMQTDNRVPYGIGKNENYNKDALKWK"
gene                 complement(33799..34602)
                     /locus_tag="OIDGCHHC_00047"
CDS                  complement(33799..34602)
                     /locus_tag="OIDGCHHC_00047"
                     /inference="ab initio prediction:Prodigal:002006"
                     /codon_start=1
                     /transl_table=11
                     /product="hypothetical protein"
```

```
                     /protein_id="Prokka:OIDGCHHC_00047"
                     /translation="MKKSLFGVKKATLAIATTLLIGTNNAFAGGIPVIDVSAIANQVK
                     DYAMQLQQYEQMYSQLQQQIQMVQMQKQNLERLTKEDWDNLGTVLYQTRNVMNKVNGI
                     SYDIGNVSRKFEDTYKDFEGYSNDLENATNESERNKIYSDRYKQIAETNQNTFNGTLQ
                     QLELRYQDLESEDALIAKLKQNSQNSNGNLQAVQATNDLLAYQIDEIRKLRSVIMDQS
                     NMLTNYLASQNNKNVLEQAKINKQLESDNRVPYGIGKNENYNKDALKWK"
gene                 complement(34887..35471)
                     /locus_tag="OIDGCHHC_00048"
CDS                  complement(34887..35471)
                     /locus_tag="OIDGCHHC_00048"
                     /inference="ab initio prediction:Prodigal:002006"
                     /codon_start=1
                     /transl_table=11
                     /product="hypothetical protein"
                     /protein_id="Prokka:OIDGCHHC_00048"
                     /translation="MSNTSIININNQEVIFENKDEQVFCTSLDVAKVFGKQHKNVLRD
                     IENILNDLREIGTSNYLLNFELSFKVHKIQGFKGRERKYPYYQLTKDGFFLLAMGFTG
                     KKALQFKIAFINAFNEMERIIKNQYTPKLENYNKYKIPNTPYKENIIKAIMQTEQNRN
                     CEFEELIAYEIKEIYKDNKSHIEQKINFKVKGLI"
gene                 complement(35483..36349)
                     /locus_tag="OIDGCHHC_00049"
CDS                  complement(35483..36349)
                     /locus_tag="OIDGCHHC_00049"
                     /inference="ab initio prediction:Prodigal:002006"
                     /inference="similar to AA sequence:RefSeq:ASI88311.1"
                     /codon_start=1
                     /transl_table=11
                     /product="IncQ plasmid conjugative transfer protein TraQ"
                     /protein_id="Prokka:OIDGCHHC_00049"
                     /translation="MKNFIFGVNKTIGKSHPHKNEARLKSLDYNYTKEYNYNYEICVT
                     QFNHLFSEVKFSHRVAALWLTPKNYTQKLKKLSLIASLLLFIGSNAFADDILTGDTKL
                     ACEAILCLSSGTRPSECSSSLARYFSIKFKKPWETINARRAFLNLCPIQNDANIEDLV
                     LNNLVDDVLPVSDPRQCTPNYLNTQVETKRSYSTFGIMSYRINPNMPNFCHALINHAY
                     TDYKTPKYKCTGEFYNSLEWKLSAKLQLITQQAYESLSDDQRYMISRTCGDRNCYDYY
                     QKIPFTKECWTY"
gene                 complement(36346..36900)
                     /locus_tag="OIDGCHHC_00050"
CDS                  complement(36346..36900)
                     /locus_tag="OIDGCHHC_00050"
                     /inference="ab initio prediction:Prodigal:002006"
                     /inference="similar to AA sequence:RefSeq:ASI88310.1"
                     /codon_start=1
                     /transl_table=11
                     /product="Phage antirepressor protein"
                     /protein_id="Prokka:OIDGCHHC_00050"
                     /translation="MSKVILFENKELGQVRTIRDKNNEPLFCLNDVCKILNIKKTNSV
                     RDSINKEFDKGGVFEWHPFKTLGGIQKFIVINEPELYFVLMRSDKPKAKPFRQWVIKE
                     VLPSIRKQGYYSFDNTPKLENYNKYKIPNTPYKENIIKAIMQTEQNRNCEFEELIAYE
                     ITEIYKDNKSHIEQKINFKVKGLI"
gene                 complement(36897..37052)
                     /locus_tag="OIDGCHHC_00051"
CDS                  complement(36897..37052)
                     /locus_tag="OIDGCHHC_00051"
                     /inference="ab initio prediction:Prodigal:002006"
```

```
                     /inference="similar to AA sequence:RefSeq:ABS43435.1"
                     /codon_start=1
                     /transl_table=11
                     /product="Cpp32"
                     /protein_id="Prokka:OIDGCHHC_00051"
                     /translation="MGFTGKKALQFKIAFINAFNEMERIIKNQYTPKLENYNKYKIPN
                     TFYKEIA"
ORIGIN
        1 attgattttt aataattctt tccatttcat taaaagcatt aatgaaagcg attttgaatt
       61 gtaaagcttt tttgcctgta aagcccattg ctaaaagaaa gaatccatct tttgtaagtt
      121 gatagtatgg gtattttctt tctctacctt taaagccttg tattttatgg actttgaatg
      181 aaagctcaaa attgagcaga taattggaag tccctatttc tcgcaaatcg ttcaaaatga
      241 gtttaatatc tgctaataca ttatcatgtc ttttcccaaa aaccttagcc acatctaaac
      301 ttgtgcaaaa aacttgttcg tctttgtttt caaaaataac ttcttgatcg tttatattta
      361 taacagttag gttactcatg cttcctcctt ttgtgaatta tcaaatcttt tgtagtttat
      421 ttctttttct tctagccatt tataagccca attttcttta tgtttctcat aaagttgtct
      481 tactctttta acgcttactt tgtcgcttat tgttgtgaag gctaaattag tttttccgag
      541 tgccaaatta aaaagtctag tgccgagtgg agaggtgtaa taatattctc tttttttgaat
      601 ggcattattg ataatgctaa tttgcacatc gttaagtcca aaaattttat aaaagtcgta
      661 aggagctaaa acattttcac ttccttttttt ataagcttcg ggattaggta aaaatatttt
      721 agtagggcaa tattcttgta aaatatctaa aattccactt tttgcactat cgcttaagct
      781 ttgtgtagcc attataacgg ctacatttgc ttttcttaaa actttaagcc aattaactat
      841 tttatcttta aacgcagggt gctttaatgc aatccaagct tcatcaatga tgagtaatgt
      901 aggacttcca tctaagcctt tttctatacg attgaaaatg tagagtaagg cagggattat
      961 gtattgatct cctaaattca tcaattcttc tatttcaaat gttgtgatac tgcttaagct
     1021 taatttatct tcctcgctgt caaataaaaa accacctgtt ccagaaattg tataataatt
     1081 taaagcatct cttaaatcac tatcttgcaa agctgataca aattcagtca aagaagtaga
     1141 atttgtattg atatgagaaa ttaaggcttc atgtattaat ttttttgtt ttggtgtaat
     1201 atttacattt tgtaatttta agcatgtttc aatccaaaat tcagcccatg cgatttcggc
     1261 atctgttttg atatttgcca aaggagcaaa agccaaagat gaatgatctc ctgccacatc
     1321 ataatgaatt ccacctgttg ccagtgtcgg agctagcaag ctttgtcctt tatcaaatgc
     1381 gtaaatttta gcatttggat atttttgcca agcaagaaaa atatttgcca aaagagtaga
     1441 tttaccactc ccaggaggac cgaaaattaa agtgtgtcct aaatcgctaa catgtaaatt
     1501 taacctgaaa ggcgtattgc cagaagttac aacttgcatt aatgggggag aatttggagg
     1561 aaatctttca cttgaattat atttatcccc cgcccaaata gacgccaaag gaattaaatg
     1621 tgttaagtta agtgtattta atattggacg gcgtaaattt ggatatacga atccaggtaa
     1681 agttcctaaa tacgcttcta ctgaattgat tgtttcggtt cttgatagaa agcctaaatt
     1741 ttctaaaact tttcttatat cttttaattt atattctaag ctctcataat ctctatcaaa
     1801 aacgacaata tttgcggtat aatagccata tcctatcata ccgctttttg attctgataa
     1861 tgcagaatct agttcttgca ccattaacac agcatgatca tcttgttttc ttgcagggcg
     1921 atctagtaac tgatcaagcc aacctttttgt tttttgttgc catttttttac gatatttatt
     1981 aagtgaattt tgagcttcaa agtcgtctaa aaatataaat ctagtgttga atctgtaatc
     2041 gaaattaagc tctgtgagtg catttaagat atttggataa ctttcgcttg gaaagccctc
     2101 aatagcaata attcctatat attgattatc aattcttggg tgcattcctg tttcgaaatg
     2161 ccttccaccc aataaacaat caagaaacat aggggcattt ggtagtatta tttttttgtct
     2221 ctcaccacaa atacaaaaat taatatattc aagcatatca tctaaaatat actcattatt
     2281 atattcgtca atagctattc ttggtagcat tctttcgatt tttatataat tgcttaaact
     2341 atcttctaat ttttgaaggg tgtttttaaa atactctaat attttatcac cctgattttt
     2401 gttttttgtt tcgtcaatta tcatcaaatc ggtgatttta tttatgcttt tgtgcggtgg
     2461 aaggtaagtg aaaatcattg taaaaagatt ttcaaaatgt tctacattgt caaaataatg
     2521 ttttctttca ttttcaagaa tttgtgcaat attgtttttta taaaaagtat cgccgtcaat
     2581 gtagttttct gtttttatcc tagagcaatc aatatgcacc gcccagccat tacctagagt
     2641 ttttaaaaca ctattaatcc tagcacttaa agtatttctt tcatttattg tcatggaact
     2701 gatatcccct gcacgataat aaaaaccagc tgttaaactt ccgtctttgt tgagcaaaat
```

2761 gccttcatcg ataaaacttg catagtttag aatatcggga aatgcttttg ctttgcttct
2821 gaattcttta agtgctagca tattttaatc ctttgaaaat ggtgtgcttt gcgcataata
2881 aaatttttta tatttaattt ggcgcaaaaa tatttgtctc ataagcggat cgctttttgc
2941 cattaatctt aaaagcatgt tgcagaaaaa aaataagaat atacctataa tgcttgtaag
3001 gagcgttgca cctgtaaaga ttagtgcaaa tgaaatcact ccaaccataa gaataagctc
3061 tcgatctgcg ccaaatatta aatttggttt gttgagggca ctataaatat ctatgctttg
3121 caattcttcc attagattaa tgctccactt gttgaaaata gcgacataaa gctgtttgcg
3181 cctactatta atgctatgac tagcacgata taaaagaggg ttttaataaa accgcttaat
3241 tcgccgcccc aaattaaacc tgctccagct ccaatgattg ctagaattga aataacaaag
3301 gctacaggac cacttaagct cgctttaatt tgttctaatg gaccttccca aggcaaacct
3361 gctccagttg ttgaggcaaa agcatattcc aagctaaacg ccaaaaataa aattaaaaat
3421 tttttcattt aaactcctta aattgtttgt gtgatgtatt tatcattttc ataatcaatt
3481 actttaatga tttcctttac ttttctaccc tccttactct ttgagataaa aacaattaaa
3541 ttcacagctt cagcaattaa tttgctcatc tttgcacttg ttgcttcaga gattaattgt
3601 tccattcttg tgagtcctcc atttgcggaa ttagcgtgga ttgtagctat tcctccaggg
3661 tgtcctgtat tccaagcttt tagcaagtct aaagcttctt tgcctcttgt ttcacccact
3721 ataattctat cggggcgaag tcgcattgtg gctttcaaaa gcctaagcat atccacttta
3781 tccgtagccc ttaaaattac tttgttttca ctcgcacatt gaagttctgc tgtgtcttca
3841 ataattacta ttctgtgatc gggagtgatt ttagaaatgc cgtcaataat ggcatttgaa
3901 aaagtggttt taccactacc agttccgcct acaactaaaa tattttggcg ttcgataatg
3961 gcattaatca aaacttcttt ttgatttggt gtaataatat tagaatctac ataatcaccg
4021 agagtaaaga ttttgacagc tttttttctg attgtaaagg ttggttttgc gacgattggt
4081 ggcaataaag cttcaaacct tgatccatca agtggcaatt cacattctaa tataggatta
4141 tctgcatttg tagttgtatc aaggagggta gagattgctg taatgataga ttttgctcta
4201 gcttctgaaa attcgccaag acatatcata tcttgaccta gcttttcaag ccaaagtttg
4261 ccgtctgaat ttagcataag ctctattgtt tctttatctt ctaaatgagt taaaatatcg
4321 ccaaattctc tttttatttt ttcattgatc cttgcttgaa actcaagatt actcattgaa
4381 gagtcctttt gattcaattg caagagttaa aaatttgtca gaattttttt tgtatagatt
4441 gtggagttgt aaaagtttaa aaattaataa aagtttaaga ttattctgct ctctctctct
4501 ctctctctag ctattaagca aaaattaagt ttattttttc tcaaaaaatt ctccaaaaat
4561 taaaaactaa tatcgttatt atattatttt aaatttaatt gtcaagattt tttttctaaa
4621 aaaatgatac atttttaata tttgaatgtt tatttttttc attttttggg aaaaaataaa
4681 catttttat aaaaaatttt ctattttgtt tcattttttt tgagacaaaa tatagatttt
4741 ttgcttttgt cccaaaatat gatacatttt taacatttga atgtttattt ttttcatttt
4801 tttgagacaa aatacaattt ttaatttttg tgtcataaaa ttatcaaaaa tgaaatcaat
4861 tgaaataaaa cgtactaaaa ttatgaaaca aaatagaatt ttaaatattc ttattttaaa
4921 aagtttgatc gtttaatatt tttggataac ctaattcttc cgtcatatac ttttcaaatc
4981 cttcaatatc taaagcatta tataatctaa taaccttgta attagttttt tgttgaatta
5041 gatcatcttc tgctgttaaa ttatctgtat aactaatagt attatctaac atcatttttc
5101 ttgtagaacc tcctattaga taaaatataa tttcatctgc ctttaccatt tgatttttcg
5161 tggtttcaaa caatacacaa gcaaagaagt ctttatccat ttcattaggt ttgtaaagat
5221 tatcgtattt gcatatgttc ttaaaattat attttgcatt gcatattgca aaattaatgc
5281 catttcttgg aaaagaggaa cgaacctcta atgttttacc attgtttaac aataaatcta
5341 tttgccctat cgatgattta aattctgtaa aattaacaat atctccaaaa tttcttttta
5401 tatagtaaaa ccaaccatat tcggccaaaa ttccccctaa agcatctaaa atcagccttt
5461 ctttatctct ttgtgatgtg ctatcgtttg ctttaagtgg atttacttgt tttgctaagt
5521 cccaagcttg ttgataatat tttgaagctt catcagtttt atttttaaaa atacatctaa
5581 ttactttgta tgttcttttt atattcattg tattgtttgg tttttgacag tttattattt
5641 cataattaaa tagcattatt aacaccccta tgttttataa aaattctaga gtatagtctt
5701 tttccattta tatacgctct atcggttttt ccattccagt catttcgttt aatataaata
5761 agctctccac tgtttacttg ataatctgcc ccaataattt caaattgttg tggattgtat
5821 ttatctaaaa atgtaatagg aactcccatt attccagtat aatctgaagg tatatctttg
5881 gactttgaaa tttcaatagc attgtaatta tcatatgtaa tataatcttg attattatat
5941 tttctattta aagatatttt tttatttcgt ttttggttat taatatttga aaaccaaaaa

6001 gatcttgctc cctctaccaa ttctcctttt gtgtttataa accatctaac gcaattttgt
6061 cctaaccata atttattttt catcatatag ttgaaacaat ttttataaga aattgcatta
6121 gcatttccta taatcaaaaa atctttttga ttagatataa gtatatctat aaattcccta
6181 aatagtgaaa atggtggatt tgttacgatt atattagatt cttttagcaa tttaattgtt
6241 tcatcgcttc taaaatcacc gttattttct aacaaacctt gatattttgg cttatttttt
6301 tctataataa aaattttacc cttaccatta ctattaaatg aagtactaat taattttttt
6361 agttttaatc tatcaaaatt taatttaaaa aattttgtaa aattactttt ttgtggatca
6421 tcgcaattac aataaactat cctatcttta aacgcatttt tataaaaatt taattcttta
6481 tttatatctt catattgtgt ataaaattca tcgtttttta tatttttgc attatttaat
6541 ttagaactac tgtttttatt catttatact taaccttcta tatatttaaa tatcaatctc
6601 gccatatctt tttataggta ttttcaaatt tggaaatttt ttatccatgg tataaatagc
6661 actgcaacct tctttttcgg cacaaaaata ttgcaaatag tcttcaaagt ctcctcgata
6721 tttctcataa caatcacaag ccatattaat gacacttaca ccaaaagaag tgatattaaa
6781 atttttttct aatgaaatat cttttaagaa atgagtgaaa ttatttaaat tttcttttt
6841 gataagataa gcaatggtag aaatcatatc ttcagagaaa acaatttgaa tattgtttgt
6901 gagtaaataa tacattaatt gtttagcttc attatgatag gttctttcag tattgaaata
6961 atctagaaaa atatttgtat ctaaataaac tttttcata ttctaccttc ttctaaatct
7021 ttatccaata actctctttt catttctttt gctgattttg tagaatcgta tgcaccagta
7081 agtatcccag catatttttc tataaatagt ctagcttctt gttcttttg attttcatca
7141 aataaagaag gttgtgtatc attagagatt aagactttt tatttatgct cggctcttct
7201 tttttatcga agctatattg attgtttata gagttatttt tttcttcaat aacaatttct
7261 gtattttta ctttagtcgt ttcattatgc tcttgattta tttcttttac ctgatattgc
7321 ttttgattgt ttcttatttc atcaaagcct agatattctt cttctatttc taaattttct
7381 ctaacttcta aattttcaaa atcccattct ctcgtaatgg tatgtcttaa attttttaaa
7441 tcattccaag atccgcgtcc tactaaattt ggattaaata aataacaatc atcgctacca
7501 acaccagcat atgattttac aaattctcta tttattttaa aaatcacttt cttttctatc
7561 aacccattta aagctctata cattccagcc ctgcttaaaa ctttatctaa aacgatattt
7621 tgcttcatag atttatcaaa cctcacaaca tttttataat ctactgaaga aagcaaagta
7681 aaaaataaag tttttcttg agattctaaa ttgataatat aatcaatatt ttcagtaaaa
7741 agtttaataa aactttcttt tgtttgaact tttttaacaa taatttgatt tctttctaca
7801 acttcaccag taatataatc agtagataga ttatcttttc tataaactac ttgccgtttg
7861 ttatttttat taatctcatt taaatcttta atttctattt cattgctcat attttatcct
7921 tactgaattt ataaaatatt gtatcataat ttgagacaaa aagcaataaa tattttataa
7981 aatataaaat attgtatcat aatttgagaa aaagtaacca taatctgtct cacaggtgag
8041 ctaacttgtc tcacaggtga gactctttgt ctcactgatg agacagatta aaagtgcttt
8101 gaagcactat aaaataagca tttcaaagca tttttaatta agccatctct tctatatcta
8161 ttcttacaaa ttcaaactct tcaactttt cccaaaaaat gatacatatt taagcaaaaa
8221 aagtaatttt ttcccaattt atttggaaat ataaattttt ctaaaaataa ttttcccacc
8281 acgagcagtt tttttcccac tggtgggaaa attaaaaacc ttatcaaagc actattatac
8341 ctatatttga aagcattttt tttaagtctt ttccttctta atctactctt gaaaacaaaa
8401 aatttttac agcaaatttc gctaacgctc atttgcaaat ttgaagagac taagcgtctc
8461 ttactcttct tacaacaata gggcggggtc aagttcgttt gtcgctctag gcgactgcac
8521 accccgcacc ccatatctac gcaaattata gcacctactt aggtgcaatt tgctatatac
8581 accaaaaata aaatatttag tttgaaaatt ctacttttca aattaagttt aattgttttg
8641 aatatcgata atataggctt tttataaata aaaatatttc tttttaagtt tagagtattt
8701 ttctttgaaa cttaaactaa tggaaaaata ggattttta ggtttaagat ttttaaaaac
8761 ttaaactaaa atattcttaa aaaattctcg tgtcgtctct ttctatttta ataaattttt
8821 aatttttcta aaacctcttt gtgtcttaga tagataaaga gtttgagcta ctcaccaaaa
8881 ctataaaaat tactgatagg gctatggcgg cagccatata gccctatttt tttgtataat
8941 aacaaataaa ttttcaatca aggagaacaa atgaaaaaat tagctttagt tttagcagct
9001 ttagttgctt tgaatgtatc ggcatttgca ggtgttatta ctatacataa ctccaatttg
9061 gttaaagtat caggaagtga tgatatctat gcaaaagcag cactagaaga tgctaaagaa
9121 gatatgagaa gtcaattcaa tactgataat ttagagattt acgctataga aagtatcaaa
9181 aaagatgatc cgcatgaata tgatgtatta caaaggggag atttaaaaat agaaagagaa

9241 aatatggcaa aaggaaagac tgtctattat aatatatttt tagttgtaga tagaaaaaca
9301 aattgggctt ctacttggaa gatcgaatgc gatcatcgtg atagacctat aaaattaagt
9361 aatggtaaca aatgcaaagc tggtatttat atcaatcgtt ataatttcaa ataatacttg
9421 aaagctctaa aatttagagc tttcaaacca tgaaggtttt attttccatt cttctaccta
9481 catcttcagt gataacatca gaagctgagt tactaattcc cattaatcta aatgtccaat
9541 ctggtcctcc taaaatagtc ttccacataa cataagctga agcaatatag gcaaaaactt
9601 ttaataaacc ttttgctaat tcgattaaca tccaagccat aggctcactt ccagctgctt
9661 ttagaccacc aaattgttcc gcagaaaaca ctaagaacac atctttaata aaggaatgaa
9721 agaataaagc taaatagata aataaaacaa tcaatattaa taaaaggaat ttaagaagtt
9781 gcttagaaaa ttttgcctta aagttgattt tctgactcca tttatccctg taaccaaaag
9841 aatagctata cctaatacgc tttaattgta atattttcaa atggctctgc taaatgaata
9901 aggtttattt ttattaatga aactttctct ttgttttgtt ttctgtgtgg ttttcgcact
9961 tatatgaatt acaatacttg tcgaagcatt ttttcaaaat atctttgaca tcattatagc
10021 ctatccagaa catatcatca tctttattat ttagattttt atatctttca aacaatctag
10081 ctaattcgat tatatttatt tttaaatctt ttgacaattg gcttagctca tcttccaaag
10141 aaagatcttg atttgatagt attttctttt ttaaattttc aaattcatct tttacaatct
10201 ttaaaacatc ggaaaaataa ttattatttt tacaataatc aaagattttt tcatctaaaa
10261 atctaatcat ttcattataa aattcatttt ctaattcttt attgttgagt tgtttttctt
10321 tgttttcaaa cattattttt attgtatttt catctgcgtg attataatca ataagctttt
10381 ttctaactaa ttcataatcg cttattgtgt ttctatcttt taagtatttg atgtcccata
10441 aatctctata ttttatataa tctctgcaaa gtaaggctac taatttatct gctaagattt
10501 cccttagagt ttcaactttt aagatcatat tagcccctat ctctttgtaa ttttctttaa
10561 ttggcatatt ttgattatca tagcttggaa tgcaagcaat tttgatatta attttgcttt
10621 ttctgtttaa aaaattaaga gcaatttttg cttcccattt tttaacaaat tgtgaggctt
10681 tttttggttc tataatttca acatttaaat catatttgtc tttgattttt tgactaaaat
10741 gtttttttaaa atcatctaat ttcatgctat caaaatcttc tacatccaca agtgtaaaat
10801 ctaaatcttc agaatatcta ttattatcat aacatattct caaagccgtt ccgccttgaa
10861 aaacaagttt gtttgcaact tcggtagaaa caaataaact ttctagaatt tcataatgaa
10921 gtatttcttt agcaatagta tcattaattg cctcgttgcc tttttgtat ttttctacaa
10981 gttttctaaa atccataata tctttcatct ttcatttttt gttcctcata taaatctata
11041 ctacgattaa atctataaat atcatcaatt gctttttttt gattggctac ccaaacttga
11101 catttttcat cataataaca atcatcaaaa aatctttctg gattttcctt tttttgaaca
11161 tactcaataa taccgtaggg agtatggtaa gtatgagaaa atttcaaaga tacaaaagtt
11221 agcctgtttg gaatttgtga aatcaaacct tcttgcgaaa gcaaggtttc taaactaaga
11281 taagaataac ctttgtttct caaatgatta gcaacttcat attctcgaaa taacatactg
11341 catctcgttc taggattagc atataagcct ttgctcacat gaactatata tcctttttttg
11401 cttaatcggc tcaggcttat ttttaaattt tgtaaacttt ctttatgaaa acatacagca
11461 aaattactta atgtaaatag ccatttatct ttattatccc aataatcaag aaaatccatc
11521 agttctaaat gtgtcactta ttgtcctaaa taaaatatat atatttgtat attttattta
11581 atttaaataa aaataaattt aattataatc taatcctagc tcgttttcat ttcttttttc
11641 tatagcgagc aaacatttct tgattttttt tgctctcaag tatttcttct tcgctaggct
11701 ctacgctaac aaatccttct atgcaatttt tataaataaa atccataata tctttcatat
11761 catttgtttc atagtattta attagctttt ctttaaactc gctgattagc tctactggta
11821 tattgaccaa acctgcacca ttttcaatta aaactctatt agcacataaa acagctgttc
11881 ttttgttgcc atcgctaaag atttgttttc tcattaaata aagcattagg gcgatcgctt
11941 tttgagtagc attatctatc tcttttatct tgtttaattc ttctaagatt tcttctttgt
12001 gtagtatttt aggtttccaa gttgttccac caatagaaac ttcatatgtc cttataaaac
12061 ctgctttatt tatcaaattt gcaccgatag ttttattaat ttcgcaaata taagcaaaat
12121 caacttcagg ataactcata gtttcaaaaa gaaaattcca agcatgcttg agattattaa
12181 tggctacaat atcatttact tttaaattgt ttacatttac accattgtaa atagcttctg
12241 tttgcggaaa agtcacggct atgccttcaa gcctagctga tttatagata ttatctataa
12301 tgtttctttt tgctaaaaat atattttctt ctaatgtcat aatttgcttt ctagtgtatt
12361 accatgcaac gaatttattc tgtaaaaggg tgtacttgcc atatatttga aataaaattt
12421 gatatttttt taattaactc atcattgttc attttagagt aatcattctt tttttctttt

12481 tcaaaatcat attctaaaag gtctaaaata tcatcatagt tagcataatt tacagattta
12541 ccattcaaat tagcttcttc tttaaaaata tttacttttc taaaatttcc tacatatttc
12601 tccatggtct gtggaaaaat atcagtaaat atttttctat gaatactttt aagcgtaatg
12661 ggtgaaaaac taaaaccttc atcatcgata attttagcaa ttctttgaga tactaaatca
12721 cattctttag ttcttgcaat ctctttattg tctgcatttt tataatactc tatgattttt
12781 tcttctaatt cttcatagct tgatgaattt tctactgctt tataaaaata ctcagaaggc
12841 ttaagattat ctacttgatt aagtcctatg ccaattaaaa tattttcata attaatgttt
12901 ttattctttt tatattcttg gtattcttca tattccattc tcatacctca tttttttctta
12961 atttattaat ttcttctctt ttagcaaatc tttaattaat ttccccatgc ttgtatgatt
13021 tttctcgcaa tgttctttta aagaattgta ttggctaact ttgaacgata tagtaaagtt
13081 tttatttcct tcaaattcta tagttttgtt ttcttgcatt tctgcgctct tactaattga
13141 attcaaaata tctctattaa ttgtttttgc catttatatt ccttttatat tgtattgtat
13201 tataaattaa attctttgaa taaagaattt aattcttctt tagctttcaa atcagaagca
13261 cctttgctat cgacttccaa aattcctttt ccttctgcaa gagaattttt gaaaataatt
13321 ctatctctta tgatattttt agcaatagaa aaatgtttgc aattgtctat ttgagaggtt
13381 atattgtcta ttatcaataa caattgtttt ttgttgattt gtttctaaaa aatcaattaa
13441 agaattttca tttttgaaag aaataatttc aaaaggcgat aacccactta attttctaat
13501 tttgtttaga tttgttatgc tattttgtcc atctagatca atacaaacaa aatctttttt
13561 tttgtaattc caaatagact aaaaaattaa agcataaagt tgttttaccc acaccacctt
13621 ttggatgaga gaatgaaata ataggcattt actatcctta tattataata caatatatta
13681 caatacaata tattatattg tattgtaata tagttaatga aaaaaatatt tctttttaag
13741 tttagagtat ttttctttga aacttaaagt aatggtaaaa taggaattta attatttaag
13801 tttttgattt tcttaaaaaa ttaatttgta tagatttaga taaggtaaaa ttatatttta
13861 ttggaattag tgtataatat tgttaataat aaaaattttt aaaaggtaaa aaaatgaata
13921 aagataaaaa atatttaaat ttttctgaat attatgaggt tagggatttt ttagagcttt
13981 accctgaaaa agatagagaa aatatagaga atattttaat gacgataggc gaaaataaaa
14041 agtcaaaaaa tttatctttt gaatttttat acgatttttt taaagaatta ggttttgaaa
14101 aaagttgata tagatgattt gctataacaa atcatcttcc ttgatatctt ttccaaattt
14161 agcatcaaat actttttttg attttgtgtt aatctttagc ttgataggtt ctttattttt
14221 cagaactaaa gattgaagtt gattatcttc ttgtattgtt tcttttatct tgattttgtt
14281 tttaaattca tcattgaaat atttattgaa ttgtttataa gtcattttaa agtttttttc
14341 ttttaaagcc ttatcataaa gaaatttaga tactacacag ccattttcaa attcttttttt
14401 taaatagttt atatttgaat aaaattcgat tttagcttca cctctttgca ttatatatcc
14461 tttcttaaat agatttttct aatatcataa attcatattg gtttgttttt tctttttttag
14521 ataattcttg gtagcctaaa tgtttaaaaa ttttaatagc ttgctgggaa tagtttatat
14581 cacatctagc aattatttta ttgttgggaa taagtttttc ataatattta ataaaatttg
14641 tagctaactt tttgtttcta aatttttcca agatatagat catatttatt tcatatatat
14701 ttgatccttt gctttttcca caaaatgcga aacctgcttt ttcatttgta ccatctttaa
14761 taataaaaat tcctcctgat acaccattat tttttattat atcttctaat ttttcaataa
14821 atcttacatt gtaaaaatgt ttttctttga atcctagcaa aaaacaatat tttataaatt
14881 ctatatcttc aaatgttgca atttttatat ccatgattgt tctcctatat tctatttgta
14941 ttttttcatt ataggatatt ttaggttaat ttgagtttat ttaggttatt ttaggtaaag
15001 ttagttttat tacaatagaa ttacctaaat ttagttataa ttacttggag taataagaca
15061 ggatatgcaa atgtcgaaca tttgcaaagt tttatcgcta aagctctaaa ataaggacgc
15121 caaatgagaa aagataaaag aatagcaata agattatctg aagatgagta taaacatata
15181 atgcaaaaag cacaaaatca cggcttaacg atttcaagat atttgcgtga tttatcgatg
15241 aattatccta ttacttgcat agtagatcaa aaagtcgcac ataatgtttt aaacattgct
15301 ggagatatag gaagattagg cggacttttt aaacattggt tggttagaaa cgaagataat
15361 aaagtcaatt tttcaaacaa aagaacttat aatgatattg atgagattgt agatcaaatt
15421 ttagatttac aaatactttt aaaagaacaa gccaaaagga taatacaaaa tgataatcaa
15481 aaagattcca agcaaaaagc agaataaatc aagttttaaa aatttatcaa actatatttt
15541 agacaaagac aataataatg caaaagtatt ggttgattat atgcttgata aaaacaatga
15601 aatggataaa gtggaaggtt atcattttac aaattgttct tttgataacg atgaagataa
15661 tattaatgag attatcaata ctcaaaagct taataccaca acaaaacaag ataaaacctt

15721 gcatttagta gtttcttttc aagaagatga aaaccctact ttagaaattt tacaagcaat
15781 tgaagaagaa atcgcaaagt ctttaggaat ggaagatcat caaagattaa gcgtggttca
15841 ttccaacaca aataatcttc acatacatat tgcaattaat aaagttaatc cgcatacact
15901 aaaagttatt aatccttaca atgatgttag aattttacaa gaaacagcga tgaaacttga
15961 aaagaaatat aatttgaagc tagacaatca tatttcacaa aaagataagc aaagtaataa
16021 atataatacc cacactatga attgtaattt tgaaacctgg gtaaaagaaa agctttctaa
16081 acaagtagat cttatgttaa aagatgaaaa aactactttt aaagatatta aacaattttt
16141 agctgaatat gatcttgaat ttagagaaag aagaaaaggt tttgttattg cttctaaaag
16201 tgaaaaaatta ttttgtaagg caagtagtat tcacaggggg ttgtctaaac aagctttaga
16261 gaaaagattt aaagaattag atttaaaaca agaaaaagaa aatacgaaaa atattaaaga
16321 agaaaaacac gaaataaaag aacaatatca gcgaccaaat aaagaaactt ctaaaacatt
16381 atgggaaaag tatttaagaa ttgaaaatga gaaaaaagca gagcttgaca aagaacttag
16441 aatgttaaag cttaggcgaa atgaatttaa aacttcaata ccatcaatga aatttaataa
16501 aaataccttt gcacatgtta aaaaccaaag aatgattttt aaaaataaac aaaaagagct
16561 ttatcaaaaa tataaaagag tgtcttatag agattttta attagtgaat ccttatcagg
16621 aaacgaagag gctacaaggg ctttgcgaag aactaaaacg aaaatcaatg aaaatgaaaa
16681 tactctatca tcaacccagg aaaaacctaa aatatttgaa aatgtagatt atataacaaa
16741 agaaggttat gctgtatata aaagcggttt taataaagcc atagataaag gagaaatgct
16801 aaaagtatct cttatcaatg ataaagatga taaagaattt cttttaaatt ctttgcttat
16861 agcaattgat agattcggaa atcatcttaa tattacagga gatgataaat ttaaaagaaa
16921 tattttagaa gtcgcaaatg attataattt aaatgtaagt ttttcagatc agagaatgca
16981 aaagatacaa gaagaaaaca ataacaagcg taaagaaatg aaaactagaa aggttttaaa
17041 aaatattatt gaattaaaaa tcaaactcac agaacaggat aaagatatag atgaaaatat
17101 taaagaaaaa gaattgaaag ctttaaaatt ttcacttaaa aaaatatcaa actctacacc
17161 ttcaatattt aaaggcgagc tgaaaaaatt aggctttagc tataaagaaa tagattccat
17221 ggatactgaa agtgtttgta ttgaaattga cggctttatt gtaaattctg ccaataaaga
17281 aggacttatc gcaatgaatg aagaaattaa acaaaccttg atcgatgatg aagagataaa
17341 aaggtttgaa aaatttgaac atttattttt aaatactaat aaaataacag atattgccaa
17401 gggattttat acaaatagaa aaattgatgc tgatgagtat ataaaaagat tttcaatgca
17461 agaaaagcaaa attaataagg tggcaaacgc tatttctatg ttaagcgata agaatattga
17521 cataatggaa aagtatgcaa aaaaacttga aaaagattta aaatatcaat acttgaaaaa
17581 ggtggaagta attgaaagta atgatattaa tttgaataat ttttaatatc ataatattat
17641 tattatgata ttaaaataat gttaattaaa tatatatttc atgtatgcta aataaataaa
17701 atattctaca attaaaaaac cacaactata tgttaaaaaa atttaatatc aaaaagttgc
17761 caacttgttt taagaattgc aatgtgagaa tacaatgtga atattgttaa taaattaatt
17821 attataaaaa taaaaatata acatataata tttataattt tactcctttt gcaaattttt
17881 ttctaaaatt atatgtgtca tattatgttt aggttgttta ttgaaaccta tttttttata
17941 tccaagtttt tcaaaaaaaa taatactttt ctttgaatta ttttgttcgc atctagctaa
18001 tattttattg taatctgttt ctttttcata ataagtaatt atagcagaac caataccttt
18061 atttctaaat tcttttgtta tataaagaac attaatttcc aatccataga gtttttgatt
18121 gtttgaatgg tttataatat aatgatcata gtagtgcaat aaatgatttt ctatgattat
18181 tcctattgga acagtattaa aattaataat aaagatttta cttaagctaa aggtagttat
18241 tacttttttct atattttctt ttgaaatatt tataaagtgt ccatgtttga ttccatcatc
18301 aatgcaccac attatgaaat ctatatcttt atgttcagca atattgagtt ttatagaaga
18361 tttttttaag ttaaaatgct tagttatttt gttgaatatt tgcttaatca tatttcttat
18421 atgaatttgg aaatagccaa cgcatagcaa tatatcctaa tccaataata ttaatacttg
18481 ttgttccaag aaaaatatta ataacataat tatcatatat caaagattcg aatcctatac
18541 atataactat tgaagtaata attgagatgt aaccaattat aatccaatat gtatatttaa
18601 aataattaat agcaagttta tgtttatctt ccgctatttt taaatattgt tcagcttgtt
18661 tcttatttaa atcactatct aaattttcct catcatttgt attttttca gtttcactat
18721 tttgatcttt ttgattcttt ataatttcaa taattttttc ttcagtttca ctattttgat
18781 ctttttgctt attgttcatt ttaaatcttt actataaaaa tatataattg catcgtcttc
18841 aattaaatta gatttccctt tttcgtaaac actactccaa ggatgacctt tttgatgggt
18901 tatttttgat aattcccagc catctttatc accaagtttt ttatatatgc tttcgattat

18961 ttccattgtt tctctatcat cttttatatc attatgatat tgatcgaggt cgatatattt
19021 ttcaatccca ttttttccag aaccttgaag ttctaaatat aactcaagat ttactggccc
19081 aaatggccat gcttgaaatc catcttttac taatgggcgt ttaaaaatga ctaatgtaac
19141 tgcatgtgta aaatatatta atttttgtaa ttgcatatga gttagcgttt tattatcttt
19201 ttgtgccaaa cgaataaatt catttgcaat tgcttttgat gaatataatt tactattttt
19261 ttccatgata aactaccaat ttattaatgt ttatactata atactataat ttagtatata
19321 ttgtactaaa ttatagaaat ttttatagaa aaggatttaa aatgggattg aaaccaggtc
19381 caaaacgcac cgcaaaatct actggagaac ttgatcaaag acaaagagac aataaaaata
19441 ctccaggaaa tacatcaaaa ttaaaaccat ctaaaagttc taagaaataa catctatgtt
19501 ttaaactagg aatggagtta ttcctagttt aatatgataa aagatttgta aaagtattaa
19561 acaagtatta aaataaacta attttgtata gaatatcaat cttttctaaa aatcttaaat
19621 ctcacataag aatttaagct taagcccatt gaatgagcct cttgatttaa aatttggtat
19681 tcttcttctg tgaaatttat cataagcttt ttatttcttt ttgcatttcc tgtttttgca
19741 tttcctgttt ctccacgagc agaattaaca aaatcatttt catttttggg tgcttctgaa
19801 cttagttttt taaatcccat tattatactc ctaatttttc atttgctatt tttaaaagtt
19861 cttggtaaaa gttttcaaag tcatttaaag ctttgtcgtt tttatcgcaa aattctttca
19921 tgcttttacc ttctattact gctttgcgat aagcttgtct ttcatagatt acgctttcaa
19981 gcattattac tctatctaag ccctttttctt ttttagcttc gttaatgtat tcttttaaat
20041 tttctaattc ttttgctaaa aaaggattag gtgaaaccct attgactaaa accaaagcca
20101 ataaattagg attaatttca atcacttcat tataaatttc tagcatatga tctagcacat
20161 taacatcgta ttgacttgga attgtaggaa tgataatgat atttgatgaa agtatagctt
20221 ttcgcatttc tttactatcc ctaccacctg tatcaactat aatgctatca aaagcatttt
20281 tcattctttt aatctcatcg cctaaactaa caccagtttt tgaaacattt gaaaataaag
20341 gctctaaatt tgattgactt ctcatatccg aaaaaacttc agtcgatttt tgcggatctg
20401 catcaataag taaaacattg tctccatctt cggctaatct tgcggaaaga tttactgcta
20461 atgtagtttt accactcccg ccttttttcat ttactacgga tataatcata ataaaacctt
20521 gtttttagtt taaattaaaa taatatcata ataatatcat aataatatta atacaatatt
20581 aaaagaataa taaaataata ttatattatg atacctatga ttaaaccaac aagaagaaaa
20641 ataatttggc tagcaatagc gataatccaa aaattaatat ttgtattttt cttttctttg
20701 gtgtcttctg ttttttcttg tgatggctta attgtatttt gatttgcttg tacttgtgtt
20761 ataatatctt ttaatgcttc ttggttgctt ttactatatt ctcttatttc gttaaatata
20821 ttaaatttat aactttctaa atccgttttg tattttgtaa aaagtagatc tattttttct
20881 aaaaaatcat caaagataat ttcatttgca ctaacgactg caagtatagg atcattttca
20941 tctaagatta tatggtgctt ttttgcaatt tcttcaataa gttcttttttt attcatttta
21001 tttttccttt tatgatatta ttttgatatt aaataattaa tattttaata ttatactaat
21061 attaaaatga tatcatcaat ttaacttttt ttacttcatt ttggagtata ttaacaactc
21121 caaaatacct actatcataa ctttttatat tttcgcccat tacaaaaagt tcattttctt
21181 ttaatttgcg tttaaaaggt ttaaattcta gtaaattacc ttcgctatca aatgaaaaaa
21241 ttttagaatt aggcaaaaga ctttcattaa taaaaacacc ttgcgtatta acttctacaa
21301 aatcgccact tactgcgata atctttttta ataaaatttc tttcttttgt ggtattttta
21361 aaagcacaat atcgtttttg ttaaaagtgg aagagttaat ctctttgtat aatcctaaag
21421 gcatcgatct tgtataatta aaatgaaaac cgccaaaata aaaaataaga tatgagagta
21481 tagaaataaa agcaaaagaa aaggcaaata ttgcaatagt ttttctcata gcattacaat
21541 gatattcctt tattttgctc taaatctaag ttttgtctaa agctttttag attggtttta
21601 attatggctt caagttttcc taatttcaat tgtgaagctt ctacagatgc cattatcgtt
21661 ttatcttttg gtattaaatt taaatcaagt ttataacctg catttttagc caactcattt
21721 aaaaatattc tttgagttct gccgttacct tctctaaagg ggtgcaaagc atttagattt
21781 ctagcaaatt cggctaagtt ctttgcgaaa tcgttaagat cttttgaatt ttttaagtaa
21841 ttgtcttctt taagccaagt aaaaatttgt tgagcggttg catttaaatc ttttccagga
21901 acaaaattag taattgttcc atttggtgca tattttccaa aatttccatg taaacctact
21961 tccattctat cttttccagc ccagtgaaat acatcttgaa aaatatattt atgtatattt
22021 ttcaaatgct gataatcaaa attgccttta atgggatttt tgcttaattc caaggctttt
22081 gcgttggtta tatctctttc tttgcgaaat aattcatcta atgattttgc acctatttta
22141 ttagtcttga ttaaaaattt atcaaactct gataaatttt cttgagaatc ccacattaag

22201 atatccccca ttcttttta tattcagtga tgatttcatt agctgttgct tcgcctttta
22261 taattttaat attatcaatg atatttcttt catttaaaaa catatcttct attgcaaaat
22321 tacctatatt tgatcttata gcttcgtatt gctctttagt taaatcatct ttatattttt
22381 gaagtatcgc aaggctttct tctaaactat gcacttcggg atattgtttt tgcattttta
22441 ctcctttata aaaaatataa ttatattata ataatattaa aatagaatta aattaatatc
22501 ataatgatat cataatgata ttaatttata atatattacc tatttgacta aacgctttat
22561 ctttgaaaat tttaagcctt tgttttgaca ttagagaaaa gcttttatct tgcatagctt
22621 caccaaaagt taaattcttt gaagtgagtt ctaaaagatc tttgccgaaa gtatctttac
22681 ttaaagcatt aagatagaca atagctttga tttttcttt gatagcttga tactctttag
22741 tttcttcaaa ttctaaatta tctttgataa ttgatccgtg atattcatta atccacacca
22801 caaaagaaca attaaaagct tctgttagtt gtgttaatcc caaaatggta tcatctcttg
22861 cttgaccacc tacaattgga acatgcatat aaacttctaa attattgttt gttaaaaagt
22921 ctaaaacctc attttcttta agataatcaa ttaaaggtat aaaagtagtc gcaccgctat
22981 caataatata gttttcagcc ttttttcaa aagctaattc tacaatttta tcaaaagcac
23041 ggacatcaaa tttgccatca tcatcaataa gttgcaagaa tttagctttt aaggctttta
23101 cattaagtaa agttgtattg ttaggatcag tatcaatagc aacgatactt tctcctttat
23161 ctaaaaaata ttgacatagt aaagagctta taaagctttt tcctatacca cctttaccat
23221 ttaaaacaaa atgtattttt gccattttta ctcctttata aaatatcgct gacttgttta
23281 tctagtgtta cttttgatct tgctaaaaat gcgggatctt taaagtataa aatttgtttt
23341 ccataaatag gtgcttgacc tgctataaaa attaacatat caccaggagc gattatcttg
23401 tcatctttat cttttttagc tgaaggcaat cgcatacact catcagcagt aattaaaggg
23461 cgttgcactt cttgtatagt ttcactaaca ttgccaagca ttacagcggt tctttttcca
23521 gagcttgtga tagattttt aacaatagtt gtgattccgc ccatttcaga taagagtttt
23581 gcagtttcga ttttatttgg tgcaaatgct attcttatgt ggcagttcga aataattgtt
23641 tctttttcgc cataaataga ataaagctgt tgtatatctt ggatcgaccc gtaaaacttc
23701 attccatatc ctgccatata agcaagattt tcttcaagca ctcctaattt tccaaacata
23761 gttagctcat cagctaagac tagcatttta tgtttatacc ttttcacatt ttctccattt
23821 ttaaaattta gagaatcatc agttaaagtt ctaaaaattt gattaatgag taaattaaaa
23881 aatggacgca acctatcttt ttgccctggc ggaatgatta aataaagact gatagggctt
23941 tcatgattca ttaaatcttt gattttaaaa tcgcttttg ctgtattttt tgctaagata
24001 ggatcaatat aaagatttaa agcttccgaa gctgttccta ctacaccgct taattcttgt
24061 gtggctttat ttttcattga tcttgaaatg ctttgaataa tttcttgtgt atttttatcc
24121 attgtagaaa tatcacattc aaccatttct tctaatagct catcgatact ttgatttgga
24181 tcattgataa atttataaat atcggttaga tttggggtat tttcgcctcg atattttttt
24241 aagtgtaaag cataaagtat aattgaaact aagaaatttc ccgcctcact tttaaaataa
24301 gatgtgctac cttgagtggg attggtaggg ggcgtttcgc ctttgaaaat gagattaatt
24361 gcaatatttt gagtgtcttt aatctcgtgc attgtttcta gtcttatttc ttctaaaata
24421 ttaaatttta cggcactatc atctaaacaa gtcggatcta attttaatac tttatttttg
24481 gcgtattttt gtcgccaacc cgcagttaaa gcccatagct caccttttgt atcaaaaata
24541 agtgcagact cttcccagga taaaagtgta ggtaaaagta agcttacgcc tttaccgctt
24601 cttgtaggtg caaaaagcat tacatgttca ggaccatcat gtcgtaaata atgaagtgtt
24661 tttttgtgtt caaaaccacc gatatacacg cctttatctt tatcaaatac gccactttct
24721 ttaacttctt ctaaagtagc ccaatgtgct gatccatgaa gattttctat tgcttttgcc
24781 ttacgcaaaa aggcaagttt caccaaaata aaaacaataa aacaaaatgc aatacctgca
24841 agcattacca tgaagaattt tttgaaaaaa tcaggataaa aagaataata agcataactc
24901 caagaaatcc acttaaaagg attataaaat ccataaagta aagtttcacc taaactagga
24961 gagtaattaa aagattttgc caaatattga gttgcaattt ggctaataat agtaaatgtt
25021 aggaaaattc ctataagtcc taatatataa actaaagctt gattagtttg tttttcata
25081 aagaaatccc tatatctttt ctagctgtta tctctttatt gatgccttta tttttattta
25141 gtgctttagt agtggctttt tcaaccatta gttgctttgc aatttgttct tttaattgct
25201 ttttaatttc ttccaatcct ctgcttttca ttaaatcgtt aaaatcactt aaacctttct
25261 gcgcttcttg atttgtaaaa tttggtaata caacttttac ttcgggatat tttttttgta
25321 tgcctaaggc tgtttctttg cctacattct tattgccgtt taattctttt tttacatcat
25381 tatcccctgc aataatgata ttcattttag ggtatttttc ttttatattt gttacgataa

25441 tttctaaatt tccagcatct actcccatta tagtaggaga ttttgtagct tcatagacac
25501 tagcacttgt cgcaaatcct tcacaaatat aaacttcatt gacattattt aaattctttg
25561 caccaagtaa gaaaaaatta ccttgctttt tcgcagggta ttctatgccc tgctcttttt
25621 cttctgctgt gcgtgtagct cctatcattt tatcaccatt agcgaagatt ctttgcgtag
25681 cccaatattt gccttcaata tctcttaaag gaatcaataa gttgccgttt ttgtcttgtt
25741 tgagataaaa atttttatca aagccttttt cttttaaata cgggtgttct gaattagccc
25801 atcttgcatt gttgtattca ttttctaatt tcacagcggt attttcatac gcttgattta
25861 attctttatc tctttgctct tttaaggctt tattatgttc taaagcgttt tgcagattta
25921 tttctttatt tttggtgttt tccaaagaat tagcactttt ccaattttct ttaatgccag
25981 ttttgtaatt ttgcacaaat cctgcagggt gtccatttaa aaaacctaca taagctccgc
26041 ttttttctct ccctttatcc cctataacgc taactctatg aagtttgccg tccattatag
26101 gctgaccttc aatattaagc tcatattttt ctaaaaaagc tttaaattca tcatgagtat
26161 tgttgtaact ttgtgttaat ggtgattgat ttagccattg agtgaaattt tttaaatccg
26221 caccccttagg agcataccac attttagctt ctttatccca ttttgcacct gctttttttag
26281 caaattcttt ttcctcgaaa ggaacatata agtaagtttt tctagttgcc aagtttaact
26341 ccttatcttg tttaagcgct tgattttgag tatttgaatt ttcaagctct atttttttgtt
26401 tttgctccaa agaaagactt tcaagaaaat taacaatttt ggtagcgtct gaagtggctt
26461 taaaaatttc atttggctta tcttctaaaa tttgcaccca actatcaata tatgataagt
26521 gttgtcctgg atcataatct aagcctattt taccattaaa tagaaaactt gcaatttcgg
26581 ctcttaattc ttctttggcg tattctttag aaccaaaagg gtttttttaaa tctctattta
26641 atcgactttc atgaccgctc caatgtacta attcatgcaa agcagttgta tagtaagcgc
26701 cttcacttaa aaaattttct ttcgggggta gtgtaatagt atcatttgcg gcattataaa
26761 aagctctatt tcctttgtga tgaataattg cttttgaatc atctaaaatt ttttgcgctt
26821 ctttaattat ttgaaaatta tcaatctctt gcttttgctc caattttggc atattttcga
26881 tttgctcggc attaaaaaca taagcgaaaa atactttagg attctctaat tttacttcga
26941 tttgttctac ttctccgttt tcatttgtaa taacattacc ttcctcatct aatttatcaa
27001 ccatttcgct aaattgccaa tattgaatca aagtcgattt ttcacctttt ctaacttgtg
27061 cattaatact ttgtgcttgt ttataagtaa gccacctagg atcattataa ttttgtagca
27121 ttaaattaat gctatttatg ccgttataag gcttatttgt aatcgggtta taaggcatat
27181 tgttagctag atcaatacct ttccaaggtt taatccatgg tgcagttcca tttttttaagc
27241 tttctatgat tttatcggct gtttcttgta tgaaatcctt atttttcatg aaattatcct
27301 tgatcttctg tgccttcttt ggcatcatct tcgcttaagg cattttcttt aaatgcacct
27361 aattcttcag cagttaatgg atcaacctca tagctgaaat ttggtattgt ttctagttct
27421 ttaaaatcct tcataatttt ctccttcttc aatattaggt attgtttgtt tttcttttttt
27481 tggtgttcta aaatctatct cttttgcttt tattcggtgt tttgagtaaa tttttccttc
27541 atgctcccaa gtgtcttgag ttagttctcc aaccacatca actgaaatgc cttttttttaa
27601 atatgggtgc atggcattag caaaagaacc aaaaagagat acttcgatat aagttgaatt
27661 ttctatctct ttgtattttc tgttgtttgc taaggtaaag gtgcatatga tactaccttg
27721 attacaatgt cttaattcag catcagctgt taatcttccg cctattgtaa tattattaat
27781 cattatcttg ctcctcatct tctaagttat caaataagct tggctcatta tcatcaaatt
27841 gtgtaggttt attttcttcc aaattttcat tttccatttt tactcctttt tttgtgtttg
27901 caaattgaaa agattgtaag taatcttaat tcttgtgtct gattttcttg ctttttcttc
27961 caagtcatct atcgtttttg ctgttgaatc gtatttagct acatagccat tagaaaatcc
28021 tttttttaaa tccccagtat tataaatact taaagcctga tttatacgat cttctttcgc
28081 tagattttta tttgtcattt catacgccaa ataaaaaata tcactacccg ctttaatatt
28141 tttacaagta tctaataaat cttctacgct gtaatgtgag aatgttgaag atttaagatt
28201 gttagaatta aattgcatta gtccaatatc tacactatat ccccctttaa tgaaattgtt
28261 cgctaaattt tcagcttctt ttttattttt taatataaaa gagccaacct tattaatgtt
28321 aattgcaaat tgttgattgt tgctttctac ttgaataatc ttttgtatga tttgtgtttc
28381 tacatttgga tttttgcatt gttcaattag agcaatatca atcattattt aactccaaag
28441 gctctaaaat aatatcctta gttacaaaga tattaaattt atatccagga cgaattttta
28501 aagtagggct gatattcata ttttttctta tcatctcgct tgcgacttga cccatttgtt
28561 gaatagcttg tgcgattgct ttatccgaag cagtttcttt ttcgctgttt gtgtctgaat
28621 tatcagacaa cgcaatacct gcgctgatcg atgaaagtaa aaaagcagag ccgaaaatcc

28681 taaaataatg attatccact tgatcttcaa agcctgtata tccatcttgt gaagttcctt
28741 gcatgccatc aagatttaag gtatcaccat tcggaaaaat aagcttattc catgctacta
28801 agagtcttga ttgaccataa ataatattac ttgaataagc tcctacaact ttagttcctt
28861 gtggaattaa aaggtatttt ccagttgcgc tatcataaac attttgtgtt acttgtgcca
28921 atatttgacc aggtaaatcc gaattaacac ctgtaattaa aatggcaggt atattccaac
28981 ccgctttaat ttcataagtg cttaatggtt tttgttttac atattttaaa tagccattgt
29041 cttttttttg agataaaaat tgttgccctt gcacatcttt gtttgtgcca ctttctaagc
29101 ccagtcctgt attttgtgga ttaattgctc caagattaat atcttcatta ttattaggtg
29161 gattgaaatt aattgcttgg ttttttttgc tatccgttgc ttctgttttg atatcaagct
29221 tggtattgct taataaagct tttaactgca tatctttttt aatcttcatt tctttttcat
29281 ctatagcatt gttttggaca aggacaggag tattgttagg tggctgttgt gtagtttgag
29341 aatttgtttc ttgaataggt tgttgtgtgg catttggcat aggtggtgaa attatattat
29401 tttcttgtgc tttttgaact tgtaattcgt caatatataa attaagatca ttgttatgat
29461 ttgtagctat ttgtgtttcc tcttttggtg tggtttgcgc agtttgtctt tctaaatttt
29521 gctttcttga taaggctaca taaaatatgc taaataatac tattgcgatg attgcaaata
29581 ttattataat aggaactttc gaaagccttt tgttattttt tggtaaaaat ttgttaggag
29641 acatattcac ttcaagcata atttaacctt tagtttttgg agtaaaatta aaacttgtta
29701 tatttggttt tacaagttta ttttcgatga ttttataaat cacattgatt ttactttcat
29761 tgatattaaa tatcgcaact atattttcat tatcaatttg tgaaatttga tagcttaaga
29821 aggttaaatc tttttttagaa ttgtcattgc ttattgcata acctttttttt cttaaggtat
29881 tgataaaata gttagaaaaa tcttgattat ttttatcttc attgataaaa aatgtagttt
29941 tgttaggtgc gtaatatgaa ataatgaact gcgaaaaatc gtctgcaatt ttattgtagt
30001 ttatttttgt ttcttcaact gcaaagctag agttaaaata atccgaattc attttagcac
30061 acgaggttat aaagaaagcg attaacgcca aaaataaaat tttcatttgt tttccttttt
30121 attttggagt gataagtcat aaagcacatt attaactata tcttgattag ctttgtttga
30181 atgctttgtg attttaattt tttcttgttt tgagcctaca tttgaaagta aaatcgcttt
30241 gttaaaaagc ctatctacaa taaaagtatc atatttcaag cgataattta cgatttgttt
30301 ttcattacta gaatctaaaa tcataagcgc aggtgcttca tagaatttta aattttttgg
30361 catttgaata taagttttta tgccatcatt gtaaattctt aaaggtttaa aaggtgcatt
30421 accttctaca ctatagccaa agtccaaatt ttcaatattg ctcggaattt catcatcttt
30481 tgttttatag aaatttttag attctgattt tgctttcatt tgatttttat aatcttcaag
30541 agtttttatg atactatttg gataattaaa tgaaaccgca ggcataaatt tcttagcttc
30601 tgatattaaa tttaagcgat aaactcgctt atttgtcatg atattcatag ttgtttgtaa
30661 gtttttatcg gtaggcttaa tgatgatatg agagattaaa taaggctctt caccacttac
30721 gcttaatgaa acagcccatc ttatactatc gcctatttgc acatctttaa tcacttcacc
30781 aggttctaaa gcaatatcag taagtcttaa gggtgcggtt acaatacttg gcattgattc
30841 accaaaaaga aaaattactt ctccattatt tccttgtata gtagtagttt ttgaatttat
30901 ccatttttgt gctattgcta gatctttttt ttcttttggt gtaagatttg aaaaatctct
30961 ttcgtttaga ttgttagcgt atgcactact accaataaat aaaagtaaag acgcaattaa
31021 agaaagtttt ttgagttttt gagtataatt ttttggggtg tttgccacaa ggttcgcacc
31081 cttgtggcga aaatctaagc ccctaaagga gctaagatga ataccactaa attcatcata
31141 gttgtaatta tactcctttg tatagttaca gtcaaggctt attagccttg ccccacttgt
31201 tggggtgtgg tggatttccc ttgtggctgt atttgtatta gaaataaact ttttcatata
31261 tttaactcct tataaaattt ttgcaaaatt aatatcagtg atgaaaatcc ctaaaggatt
31321 ttttaaaatt tgttcttctg tatttggaat gatttgttta attcggatta atcctcgata
31381 agtttcttta gaaagaatat cttcatttaa atttgtagtc gtctcttccc attcaacttg
31441 ccaagtgtca atattttgtg gaacgatatt tttaattttc actcgcacat tttcctttgt
31501 agctcgtgtg aaaggattgt ttttttgaaa ttctgaattt aaataattaa atgcttttga
31561 tcttggctct acataagcat aggcatcaaa tatgaatttt ctttgagttt cggcatttcc
31621 ccaaaccgaa cgccaagaat aaataaaagt atttaggcta tactttatga catttggatt
31681 ttttaagtcg atattttgca ccattcctac tttagctgta ttgcctaact tatcaacttc
31741 aataacataa gggataagct tgttttgaga gccgatatag cctataaaga gaacacatac
31801 aaagcacatg gctaaactaa ttatcgctac catttgccag tttttagcac gagtgatata
31861 atcaccgtat ctttctagcc attcggtttt tgcattaaga taaggattgc ttgaagtgtc

31921 aaactcattc ttttctatct tttgcttttg cctaaaaata ttcattattt tttttactc
31981 cttttatgat ataattaatt tatccatttg gtagatggac tgtaactcta aaggtttcac
32041 aacctgttta tttggtttaa gctcggttct cttatgagaa atcgagcttt ttttttggct
32101 atttggctgg gtgtgcgggt ggaaaccaaa taagtaggag tccataatgg aactttttaa
32161 tcttttcatt gcggtaattg cagtagtaat tacacttttt agagaagaaa taagaaaaat
32221 ctttttttaag tctaaaaagt aaattatcgc cgtttgtgaa acgctaatgt aaggacttac
32281 caccccttgca ttggttttat tattccatag catcgggatt tattcctgat tgatagtttt
32341 cttgttttgg tgatcctgat gatatgccgc cttcacttgt ttttccttgt atatctttaa
32401 gtttttcttg taagcgatta gccatttggt ttggcattct accttttgta agattatccc
32461 ttaaatgttc gccccctgca cctgctagat tttttgccac acctttaacc aagtccattc
32521 caccggctcc accttctgct aaatgcaagg ctttagctgc attcatagca cgagtaatgc
32581 cgacagctcc tgcagctgca cctgccgcag cacctgctgc catggtagca accgccctaa
32641 agcctgccgc agtgcctgca ctttctccga tactaccatt aaatacggct tcaataatac
32701 caggaaccat ttttgtaatc atgcagaaga ttaaagcggt aacaagaatt cctaatgtta
32761 tttcgtaagt tattttttga tccatttcat tatagatttt ttcaataatt gttatagata
32821 gtgccattaa tccttgtatc ataaagagtt ctacacccac tttaatggct gtcattatgg
32881 gatttaagcc tatttgttta aaatgattta aaccgcttaa cgccaaagca aaaaatacaa
32941 ttacattcat taagaaaaat ttgagataga caataagtaa atccacagcc attaacacaa
33001 aagcaattag tataataacg ccacagataa ctttaaaaaa gctttctcct ggacttgtta
33061 cactcataga actaagaata atcataacta ttttaaaagc attttgaata atattactag
33121 gtgaaatttc caatccacca tttacatttg tagctaattg tgaaaaacta ttaaaaataa
33181 tttgtaacca ttgatcgata ttaaataaaa ataggaatat gcctacatac attatttttt
33241 taataagagt ggctaaaaac tctccaaatt caaaaccgct caaagctttt aaaccaaaag
33301 tccaaatcaa atcaatagca actaaagtcc aaaacaccca taaacaagcg gttttaataa
33361 gaggcgtcca agataaaagc ccattgcgta ttatgcttaa aacgccttct gcactttcaa
33421 ccccaaaagc taaatttgga gcaagaaaaa ttaaaaaaat aatcgctagt ttatttccat
33481 ttaagagcgt ctttgttgta attttcattt tttccaattc cgtaaggaac acgattgtcg
33541 gtttgcattt caactgccca ttttgcatta gagcaatccc tttgttgagt ttcattcatt
33601 ttctccattt ttttacattc tgcaactcta gcctttgctt cctctaagtg cgctttataa
33661 taatcgcttg tttttgcctc atcgccacaa cctgctaaaa aagccaaagc acctaaaccg
33721 attaatccgc taaaaataaa atgtttcatt gtttttctcc ttgttttaaa tatagaaaca
33781 aaatatttta acatcatttt atttccattt aagagcgtct ttgttgtaat tttcattttt
33841 tccaattccg taaggaacac gattgtcgga ttctaattgt ttattgattt ttgcttgttc
33901 caaaacattt ttgttatttt gcgacgctaa ataattagta agcatattgc tttgatccat
33961 gataaccgat cttagttttc ttatctcatc aatctgataa gctaaaagat cattggtggc
34021 ttgcacagcc tgcaaatttc cattagaatt ttgtgaattt tgcttaagtt ttgcgataag
34081 cgcatcttca ctttctaagt cttgatatcg aagctcgagt tgttgcaatg ttccgttaaa
34141 tgtattttgg tttgtttccg caatttgctt gtatctatca gaataaattt tatttctctc
34201 gctttcattt gttgcgtttt caagatcatt actataacct tcaaaatctt tgtaagtatc
34261 ttcaaatttg cgcgaaacat tacctatatc ataagaaatt ccatttactt tgttcattac
34321 atttcgggtt tgataaagaa ccgttcctaa gttatcccaa tcttctttag ttaatctttc
34381 taaattctgt ttttgcattt gcaccatttg tatttgctgt tgcaattgag aatacatttg
34441 ctcgtattgc tgaagttgca ttgcataatc ttttacttga ttggcaatag cgctaacatc
34501 aataacagga ataccacctg caaaggcatt attggttcct atgagtaaag ttgtagctat
34561 tgctagagta gcttttttta cgccaaataa tgattttttc attttgtata tttttgaagt
34621 ttaagtaggt tttagttaga atacccatta agggttagcc gtaggtctga ccccctatgg
34681 ctaaatttta cccgtgaaag tgggtgattt tatgtccatg attgaccgcg taatcattgt
34741 tattatacta ttatgtataa taatagtcaa ggcttattag ccttgcccta cttgttgggg
34801 ttatgcggta acctacctct taaacttcta cttctccttt cttttaaatt tttaatgttt
34861 attttgtatc gccaaaaatt aaaatttcat attaatcctt ttactttaaa attaattttt
34921 tgttcgatat gagatttatt gtctttatat atttctttaa tttcatatgc gattagctct
34981 tcaaattcac aattacgatt ttgttcggtt tgcataatcg ctttaataat attttcttta
35041 taaggtgtat ttggaatctt atatttatta taattttcta gttttggtgt gtattgattt
35101 ttaataattc tttccatttc attaaaagca ttaatgaaag cgattttgaa ttgtaaagct

```
    35161 tttttgcctg taaagcccat tgctaaaaga aagaatccat cttttgtaag ttgatagtaa
    35221 gggtattttc tttctctacc tttaaagcct tgtattttat ggactttgaa tgaaagctca
    35281 aaattgagca gataattgga agtccctatt tctcgcaaat catttaaaat gttttcgata
    35341 tctcttagaa catttttatg ttgtttccca aaaaccttag ctacatctaa acttgtgcaa
    35401 aaaacttgtt cgtctttgtt ttcaaaaata acttcttgat tgtttatgtt gattatgctt
    35461 gtattgctca tatctactcc ttttagtaag tccagcattc tttagtgaat ggtatttttt
    35521 gataatagtc ataacaattc ctatctccgc aagttcgaga aatcatatat ctttgatcgt
    35581 cagaaaggct ttcataagct tgttgagtta taagctgtaa ttttgcactt agtttccatt
    35641 ctaaggaatt gtaaaattcc cctgtgcatt tatattttgg agttttataa tcggtataag
    35701 catggttgat taatgcgtgg caaaagttag gcatatttgg attgattcta tagctcataa
    35761 ttccaaaagt agaataactt cttttagttt ctacttgcgt gtttaaataa tttggtgtgc
    35821 attgcctagg atcacttaca ggtaatacat catcgactaa gttatttaac actaaatctt
    35881 ctatgtttgc gtcattttgt atagggcata aatttaaaaa tgctcttctt gcattaatcg
    35941 tttcccaagg ttttttaaat ttaatagaaa aataccttgc taaagaagat gaacactcgc
    36001 tcgggcgtgt gccactactt aaacataaaa tcgcctcgca tgctaatttt gtatcgcctg
    36061 ttaaaatatc atcagcaaaa gcgttactac caataaataa aagtaaagac gcaattaaag
    36121 aaagtttttt gagtttttga gtataatttt ttggggttag ccatagggct gcaaccctgt
    36181 ggctaaattt aacctcagaa aagaggtggt taaattgagt tacacaaatc tcatagttat
    36241 aattatactc ctttgtataa ttatagtcaa ggctttttag ccttgcctca tttttatggg
    36301 ggtgagattt ccctatggtt ttattaacac caaaaataaa atttttcata ttaatccttt
    36361 tactttaaaa ttaatttttt gttcgatatg agatttattg tctttatata tttctgtaat
    36421 ttcatatgcg attagctctt caaattcaca attacgattt tgttcggttt gcataatcgc
    36481 tttaataata ttttctttat aaggtgtatt tggaatctta tatttattat aattttctag
    36541 ttttggtgtg ttatcaaatg aataataacc ttgctttcta atacttggta agacttcttt
    36601 aataacccat tgtctaaaag gttttgcttt tggtttatcg cttctcatta ggacaaaata
    36661 aagttcaggt tcatttatta caataaattt ttgtattcca ccaagagttt taaaggggtg
    36721 ccactcaaaa acgccccctt tatcaaactc cttatttata gagtctctaa cgctatttgt
    36781 tttttaata tttaatattt tacaaacatc atttagacaa aataatggct cattattttt
    36841 atctctaatt gttcttactt gtcccaattc tttattttca aataaaataa ctttgctcat
    36901 gctatctcct tgtagaatgt atttggaatc ttatatttat tataattttc tagttttggt
    36961 gtgtattgat ttttaataat tctttccatt tcattaaaag cattaatgaa agcgattttg
    37021 aattgtaaag ctttttttgcc tgtaaagccc attgctaaaa gaaagaatcc atcttttgta
    37081 agttgatagt a
//
LOCUS       OIDGCHHC_2              7365 bp    DNA     linear       12-APR-2021
DEFINITION  Campylobacter species strain strain.
ACCESSION
VERSION
KEYWORDS    .
SOURCE      Campylobacter species
  ORGANISM  Campylobacter species
            Unclassified.
COMMENT     Annotated using prokka 1.14.6 from
            https://github.com/tseemann/prokka.
FEATURES             Location/Qualifiers
     source          1..7365
                     /organism="Campylobacter species"
                     /mol_type="genomic DNA"
                     /strain="strain"
     gene            complement(388..1674)
                     /gene="trxB_1"
                     /locus_tag="OIDGCHHC_00052"
     CDS             complement(388..1674)
                     /gene="trxB_1"
```

```
                     /locus_tag="OIDGCHHC_00052"
                     /inference="ab initio prediction:Prodigal:002006"
                     /inference="similar to AA sequence:RefSeq:CKG86288.1"
                     /codon_start=1
                     /transl_table=11
                     /product="thioredoxin reductase"
                     /protein_id="Prokka:OIDGCHHC_00052"
                     /translation="MSETYEIYTPNGLILDVEKETNKILLSDGGAKVGKYTQEYSKAL
                     FEAHNIKQNSPYKDYQPRYLDPEFHTGQKSTLLEFKDWQSIYLKDPIKGSIAPWTKAE
                     KAYYNSLKTKKERYKYLVIRSGIRSVVIDIPYEAIGAVDEKGNVDPKYEELYRTVDDN
                     KHNLRSSLFHNEWGMAAGILGDYKYLANDMSQNGFNARFIQATILYIQLSGGSSILDK
                     PNLLGAIYGYADIAVGSGLVGVHKNPLREQQIKTLAKTLKPDEFGMLPFIDEIMGVDW
                     VIDYNKYRIARDEFGSMYKALRSDVVEGKIKDPRDVDSTYESRREFDRHRGGYYNGMV
                     NGYGTDTPNDWSEERAQLFNDTLILHAKLAALTPPQGYPNAPYYFTPENLEWYYKRHK
                     LDKLLDPRIPAIYRYNFPRELRAKILKFGEENGIKE"
     gene            complement(1756..3051)
                     /gene="trxB_2"
                     /locus_tag="OIDGCHHC_00053"
     CDS             complement(1756..3051)
                     /gene="trxB_2"
                     /locus_tag="OIDGCHHC_00053"
                     /inference="ab initio prediction:Prodigal:002006"
                     /inference="similar to AA sequence:RefSeq:CKG86288.1"
                     /codon_start=1
                     /transl_table=11
                     /product="thioredoxin reductase"
                     /protein_id="Prokka:OIDGCHHC_00053"
                     /translation="MSDNKDIYEIYTSNGLILEVDKNTNQIIFDKRKDGREVGKYTQE
                     YSKALFEADRILRTSPYINYQPRYLDPEFHTGQKSTLLEFKDWQSIYLKDPIKGSIAP
                     WTKAEKAYYNSLKTKKERYKYLVIRSGIRSVVIDIPYEAIGAVDEKGNVDPKYEELYR
                     TVDDNKHNLRSSLFHNEWGMAAGILGDYKYLANDMSQNGFNARFIQATILYIQLSGGS
                     SILDKPNLLGAIYGYADIAVGSGLVGVHKNPLREQQIKTLAKTLKPDEFGMLPFIDEI
                     MGVDWVIDYNEYQISEDEFGDIYKALRSDVVEGKIKDPRDIDSTYESRREFDYYMDGY
                     SNGMINAYGYDIPNDWNDAQLKIDSMILTAKLAALTPPQGYPNAPYYFTPEKLEWYYK
                     RHKLDKLLDPRIPAIYRYNFPRELRAKILKFGEENGIKE"
     gene            complement(3069..6566)
                     /locus_tag="OIDGCHHC_00054"
     CDS             complement(3069..6566)
                     /locus_tag="OIDGCHHC_00054"
                     /inference="ab initio prediction:Prodigal:002006"
                     /codon_start=1
                     /transl_table=11
                     /product="hypothetical protein"
                     /protein_id="Prokka:OIDGCHHC_00054"
                     /translation="MKIKNFTLITSNHNLEIELKNLTKNANKIIFICSDFDGYKEAEN
                     SLYKTLHTIEDNFKIDNTKTFKIIHLNQTNKDEITKEQKFSSDKNLSSIKKIIYNNLK
                     LLNDTINQNENKTLNKIIDESNEKNIENFLKEIQKELNILLKNIKESNYFDQEIKPKI
                     QVTLDTLFNTLLKNIAQSNDDEYKQIIRAVAKFFINILDSITSLKKPISLIKKNPYVL
                     IVNTLFEAYNSYSEYQEYKEKKYYYDFAYPLLELITSKLYPIIALCNEEILSDVLIID
                     DKVLLDFSSYTNAPSISLYKNSFYKIILDEEFQGVFDNFLENNLKFENEKLDNTLLVN
                     SSLNNSDTTLKKAINLNISKLNHFFYAEYPDLNSSMLVEMILDKKNADQINTTKMGKN
                     YLFITNPPQLNNAGLCEDLYQKKLGAIIKNRPKALTKNEKLALKGDFPSKRDYGSYFI
                     ERDVKKEDDFVLQLCPFAKLDGQIYQKYKGYFNFYNQTYSWMKLYLLEYINERFEYER
                     FKQDFAYIDNFFKLPKELEKISEKEKEKMEEYVELFNDYLDKINFRYDQIQFFLDSEP
```

```
                     LMFKTYENVDFKKYSPLTVVLLTLTLYVLQKDLAFTQEYSLGVKNIVIPNPDYGDKEI
                     SLNYQRIKIPNYFNEYILFIPDDIYFYDNDKKIYIFLSKELNKNKKEDEVIYLDELAK
                     AMFENNLKEDDQTYVKFLNTLNENFDDEIEFEENIQTNQTLPTKKDLDKSIQEIMLDN
                     ELAKLSLDITFFNIIKQLFPFTEFFMSQPDFFKKMILFFINSSSDKKYYGNKLFSSFK
                     AHFFKELGIFYALNPKGRFIKIIPKNKKPFNKLFLYQIGTNYYYFSQSEYASELKKSK
                     SFSRLDKAKQIHLISKARKQLAIDFSIKVSKNLSVDVLKNLAGSFIDTLLPTNYERLK
                     ILYEKLMYEKLTYNYDFPLAVKKEEYITYPLLINSRFMSFDLSEFIFGSLLCTGGLNY
                     YPSIACTTTSSEAREFALKRLLSYIILDEKRSAFMEDKINEGKYILNDKEFFEKNNIE
                     LRNCNIYKLEHFQRKEEYKTQNPMIKQHPILAYNDTIQILYEYINGIYNTIQDEKGNF
                     IEDRQKARRLMENLEAIGQHNLEVMYKGINNKDVDNERNRRKDKESFNDIGKIQDYSN
                     KFIGRLATTIIMEDGLYMG"
     gene            complement(6563..7306)
                     /locus_tag="OIDGCHHC_00055"
     CDS             complement(6563..7306)
                     /locus_tag="OIDGCHHC_00055"
                     /inference="ab initio prediction:Prodigal:002006"
                     /inference="similar to AA sequence:RefSeq:CKG86671.1"
                     /codon_start=1
                     /transl_table=11
                     /product="methyltransferase small"
                     /protein_id="Prokka:OIDGCHHC_00055"
                     /translation="MDIKGSLSQKKVNNEFVILQELISACTTDKGFALKVSFTPSKFK
                     FDHSFDPKEGLGEQSKNQIELKEPIIRLHYKSDRFQKDNLPIYNLLINNEKKEQDKAL
                     NEFNIEQKDLKDIEDVNIFNQFKQDFSEDYEFKELNLSFDTNLIKLYFIIPKNIAKVY
                     KSAYKEFENKDLGAGYFTQLHEYDKIIKNSLEDKKELNEYHLTFLTPAKMQNLKLQIA
                     QGLDKILEDEDRKQELYVCKFVVVNGVKI"
ORIGIN
        1 ctttagtcca aggagctatg cttcctttga taggatcttt taaatatatg ctttgccaat
       61 ctttaaattc aagcaaagtg gatttttgcc ctgtgtgaaa ttcaggatct agatacctag
      121 gttgataatt aatataagga gaggttctta aaattctatc agcttcaaac aaagctttag
      181 aatattcttg ggtgtatttg cctgtaggtt tttttgactt tgactctgaa aatagtattt
      241 ttccgctttg tttatctaca tttaatataa aaccttcggg tgtataaatt tcataagttt
      301 cactcatttt tatcctttct taatttatcc catatataaa ccatcttcca tgattatagt
      361 tgtagctaat ctgcctatgt aattacttta ttctttaatg ccattttctt cgccaaattt
      421 gagtatctta gctctaagtt cccttgggaa attatatcta taaatagcag ggattcttgg
      481 atcaaggagt ttatcaagct tatgtctttt ataataccat tctaggtttt caggggtaaa
      541 ataataaggt gcattaggat agccttgtgg aggagtgagg gctgcgagtt tggcatgaag
      601 tattaaagta tcattaaaaa gttgtgccct ttcttcactc caatcattag gtgtatctgt
      661 tccatagcca tttaccatgc cattataata ccctccccta tggcgatcaa attcccttct
      721 gctttcataa gtagaatcta catcccttgg atcttttatc ttgccctcaa ctacatcact
      781 tcttaaggct ttatacatac ttccaaattc atcgcgagca attctatatt tattataatc
      841 aatcacccaa tctactccca taatctcatc tataaaagga agcataccaa attcatcagg
      901 ttttaaagtc ttagctaagg ttttaatttg ttgctctctt aaaggatttt tatgcactcc
      961 tactaaacca cttcctactg caatatctgc ataaccataa atagcaccca ataaattagg
     1021 tttatctagt atactacttc ctccgcttaa ttgtatataa agtatagttg cttggataaa
     1081 tcttgcatta aaaccatttt gagacatatc gttagctaga tatttataat cacccagtat
     1141 tcctgctgcc ataccccatt cattatgaaa taaacttgat cttagattat gtttattatc
     1201 atctactgtt ctatataatt cttcatactt aggatctaca tttccttttt catctacagc
     1261 tcctatagct tcataaggta tatctataac aacacttctt ataccacttc ttataactaa
     1321 atatttatat ctttcttttt tagtttttag tgagttataa taagcttttt ctgctttagt
     1381 ccaaggagct atgcttcctt tgataggatc ttttaaatat atgctttgcc aatctttaaa
     1441 ttcaagcaaa gtggattttt gccctgtgtg aaattcagga tctagatacc taggttgata
     1501 gtctttgtag ggggagtttt gtttgatatt gtgtgcttca aaaagggctt tagaatattc
     1561 ttgggtgtat ttgcctacct tagctccccc atcacttaat aaaatttttat tcgtttcttt
```

1621 ttctacatct aatataagtc cattaggtgt ataaatttca taagtttcac tcatctttta
1681 tcctttctta atttatccca tatataaacc atcttccatg attatagttg tagctagtct
1741 gcctatgtaa ttactttatt ctttaatacc attttcttcg ccaaatttta gtatcttagc
1801 tctaagttcc cttgggaaat tatatctata aatagcaggg attcttggat caaggagttt
1861 atcaagctta tgtcttttat aataccattc tagtttttca ggggtaaaat aataaggtgc
1921 attaggataa ccttgtggag gagtgagggc tgcgagtttg gcagttaata tcatagaatc
1981 tatttttaat tgcgcatcat tccaatcatt tggaatatca taaccatagg cattaatcat
2041 gccattagaa taaccatcca tataataatc aaactccctt ctactttcat aagtagaatc
2101 tatatccctt ggatctttta tcttgccctc aactacatca cttcttaagg ctttatatat
2161 atccccaaat tcatcttcag aaatttgata ttcgttataa tcaatcaccc aatctactcc
2221 cataatctca tctataaaag gaagcatacc aaattcatca ggttttaaag tcttagctaa
2281 ggttttaatt tgttgctctc ttaaaggatt tttatgcact cctactaaac cacttcctac
2341 tgcaatatct gcataaccat aaatagcacc caataaatta ggtttatcta gtatactact
2401 tcctccgctt aattgtatat aaagtatagt tgcttggata aatcttgcat taaaaccatt
2461 ttgagacata tcgttagcta gatatttata atcacccagt attcctgctg ccatacccca
2521 ttcattatga aataaacttg atcttagatt atgtttatta tcatctactg ttctatataa
2581 ttcttcatac ttaggatcta catttccttt ttcatctaca gctcctatag cttcataagg
2641 tatatctata acaacacttc ttataccact tcttataact aaatatttat atctttcttt
2701 tttagttttt agtgagttat aataagcttt ttctgcttta gtccaaggag ctatgcttcc
2761 tttgatagga tcttttaaat atatgctttg ccaatcttta aattcaagca aagtggattt
2821 ttgccctgtg tgaaattcag gatctagata cctaggttga taattaatat aaggagaggt
2881 tcttaaaatt ctatcagctt caaacaaagc tttagaatat tcttgggtgt atttgcctac
2941 ttcacgccca tcttttcttt tatcaaatat aatttgattt gtatttttat caacttctaa
3001 tataagtcca ttagaagtat agatctcgta aatatcttta ttatcactca tttttatcct
3061 ttcttaattt atcccatata taaaccatct tccatgatta tagttgtagc tagtctgcct
3121 ataaacttat tagaataatc ttgtatttta ccaatatcat taaaactttc tttatctttt
3181 cttctgtttc tttcattatc aacatcttta ttgtttatcc ccttatacat cacttctaaa
3241 ttatgttgtc ctatggcttc taaattttcc atcaatcttc ttgccttttg cctatcttct
3301 atgaaattac ccttttcatc ctgtatagta ttgtaaattc catttatgta ttcatataaa
3361 atttgtatag tatcattata agctaagata gggtgttgtt ttatcatagg attttgtgtt
3421 ttatactctt cttttctttg aaaatgttct agcttatata tattacaatt tctaagttct
3481 atattgtttt tttcaaaaaa ttccttatca tttaagatat atttgccctc attgatttta
3541 tcttccataa aagcacttct tttctcatct aaaataatat aagataaaag tctttttaag
3601 gcaaattctc tagcttcact tgaagttgta gtgcaagcaa tactaggata gtaattaagc
3661 ccacctgtgc acaataaaga accaaaaata aactcagata aatcaaaact cataaatcta
3721 gaatttatca ataaaggata ggttatgtac tcttcttttt ttacagctaa aggaaaatca
3781 taattgtaag tcaatttttc atacattagt ttttcatata aaatttttaa tctttcataa
3841 tttgtaggca aaagtgtatc aataaaagaa cctgctaaat tttttaatac atccacactt
3901 aaatttttac ttactttaat agaaaaatca atagctaatt gttttcttgc ttttgatatc
3961 aaatgaattt gcttagcttt atccaatctt gaaaaactct ttgacttttt taactcgctt
4021 gcatattctg attgactaaa ataataataa tttgttccta tttgatataa aaaaagctta
4081 ttgaatggct ttttattttt tgggatgatt tttataaaac gccctttagg attaagtgca
4141 taaaatatcc ctagttcttt aaaaaaatgt gctttaaaac tagaaaataa tttattgcca
4201 taatactttt tatcagagct tgaatttata aaaaataaaa tcattttttt aaaaaaatca
4261 ggctgactca taaaaaattc tgtaaaagga aaaagttgct taatgatatt aaaaaaagtg
4321 atatctaagg atagttttgc aagctcatta tcaagcataa tttcttgaat acttttatct
4381 aaatcctttt ttgtaggaag tgtttgattt gtttgtatat tttcttcaaa ttcaatttca
4441 tcatcaaagt tttcatttaa ggtgtttaaa aatttcacat aagtttgatc atcttcttta
4501 agattatttt caaacatagc tttagctaat tcatctaaat agatgacttc atcttctttt
4561 ttatttttat ttaactcttt ggataaaaag atgtaaattt ttttatcatt atcataaaaa
4621 taaatatcat cggggataaa taagatatac tcattaaaat aatttggtat tttaatcctt
4681 tggtaattaa gtgaaatttc tttatcgcca taatctggat ttggaattac aatattttta
4741 acaccaagag aatattcttg cgtaaaagct aagtcttttt gtaaaacata caaagttaaa
4801 gtcaataaaa caaccgttaa aggtgagtat ttcttaaaat ctacattttc atatgttttg

```
     4861 aacattaatg gttctgagtc aagaaagaat tgtatttgat catagcgaaa attgatctta
     4921 tccaaataat cattaaaaag ttcgacatat tcttccatct tttcttttc tttttcgcta
     4981 attttctcaa gctctttagg taatttaaaa aagttatcaa tataagcaaa atcttgttta
     5041 aatctttcat attcaaatcg ttcatttata tattctaata aataaagttt catccaagaa
     5101 tatgtttgat tataaaaatt aaaatatccc ttatattttt gataaatctg tccatcaagt
     5161 ttcgcaaaag gacaaagttg caagacaaaa tcatcttctt ttttaacatc tctttctata
     5221 aaataagaac cataatctct tttgctagga aaatcgcctt ttaatgcaag tttttcattt
     5281 tttgtaagag ctttggggcg attttttatt attgctccta atttttttg atacaaatct
     5341 tcgcaaagcc ctgcattatt aagctgtggt ggattggtaa taaataaata atttttaccc
     5401 attttggttg tattgatttg atcagcattt tttttatcta gtatcatttc tactagcatg
     5461 gaagaattta aatcagggta ttctgcataa aagaaatgat tgagtttgga gatatttaaa
     5521 tttatcgcct tttttaatgt tgtgtcagaa ttatttaaac tagaattcac taaaagagtg
     5581 ttgtctaatt tttcattttc aaattttaga ttattttcta aaaaattatc aaaaacacct
     5641 tgaaattctt catctaaaat aattttataa aaactatttt tataaagact gatagaaggt
     5701 gcattagtat aagatgaaaa atctaataat actttatcat ctataattaa cacatcagat
     5761 aaaatctctt cattgcataa agcaataata ggataaagtt tgctagtgat aagctctaaa
     5821 agtggataag caaaatcata ataatatttt ttttccttat actcttgata ttcagagtaa
     5881 gaattataag cttcaaataa tgtatttaca atcaaaacat aaggattttt tttaattaaa
     5941 gaaatgggct ttttaaggct ggttatagaa tctaatatat tgataaaaaa tttcgctaca
     6001 gctctaataa tttgcttata ttcatcatca ttagattggg caatattttt taaaagtgtg
     6061 ttaaaaagcg tatctaaagt aacttgtatc tttggcttga tttcttgatc aaaataatta
     6121 gattctttga tattttttag taatatattt aattcttttt gaatttcttt taaaaaattt
     6181 tcgatatttt tttcattgct ttcatcaata attttattga gtgttttatt ttcattttga
     6241 ttgattgtat catttaaaag ttttaaatta ttatagataa tttttttaat gctagataaa
     6301 tttttatcag agctaaattt ttgttctttt gtgatttcgt ctttattggt ttgatttaaa
     6361 tgtatgattt taaaagtttt tgtattgtca attttaaaat tatcttcaat agtatgtaat
     6421 gttttataca gagaattctc agcttcttta tatccatcaa aatcactgca tataaaaata
     6481 attttattag catttttggt aagatttttt aactcgattt ctaaattatg atttgatgta
     6541 attaaagtaa aattttttat tttcatattt taacccccatt taccaccaca aatttacaaa
     6601 cataaagctc ttgttttcta tcttcatctt ctaagatttt atctagtcct tgtgcgattt
     6661 gaagttttaa attttgcatt tttgcaggag ttaaaaaagt taaatgatat tcgtttaatt
     6721 ctttcttatc ttctaaagaa tttttaatga ttttatcata ttcatgtagc tgtgtaaaat
     6781 accctgctcc tagatctttg ttttcaaatt ccttataagc acttttataa actttagcaa
     6841 tgtttttggg gataatgaaa taaagcttga ttaaattagt atcaaagcta agatttaatt
     6901 ctttaaattc ataatcttca ctaaagtctt gtttgaattg attaaagata tttacatcct
     6961 ctatatcttt taaatctttt tgttctatat tgaattcatt taaagcctta tcttgttctt
     7021 ttttttcatt attgattaaa agattataga taggcaaatt atctttttga aatctatcac
     7081 ttttataatg aagtcttatt ataggctctt ttaactctat ttgattttta ctttgttctc
     7141 ctaagccctc tttaggatca aagctgtgat caaatttaaa cttacttgga gtaaagctta
     7201 cttttaaagc aaagccttta tctgtagtgc aagctgatat gagttcttgt aagattacaa
     7261 attcattatt tactttcttt tgagataagg agcctttaat atccactacc ttagtacaag
     7321 gataacttac atttgctatg gtattaggac aagctactat actag
//
LOCUS       OIDGCHHC_3              6566 bp    DNA     linear       12-APR-2021
DEFINITION  Campylobacter species strain strain.
ACCESSION   
VERSION     
KEYWORDS    .
SOURCE      Campylobacter species
  ORGANISM  Campylobacter species
            Unclassified.
COMMENT     Annotated using prokka 1.14.6 from
            https://github.com/tseemann/prokka.
FEATURES             Location/Qualifiers
```

```
source          1..6566
                /organism="Campylobacter species"
                /mol_type="genomic DNA"
                /strain="strain"
gene            complement(303..1304)
                /locus_tag="OIDGCHHC_00056"
CDS             complement(303..1304)
                /locus_tag="OIDGCHHC_00056"
                /inference="ab initio prediction:Prodigal:002006"
                /inference="similar to AA sequence:RefSeq:AAW35443.1"
                /note="conserved domain protein"
                /codon_start=1
                /transl_table=11
                /product="hypothetical protein"
                /protein_id="Prokka:OIDGCHHC_00056"
                /translation="MGGYLAQMALIYCDIKYKDKLSFSPNEVYTFNAPSVYGWNFPNI
                AINPNTIKIMQDLLGKYTIDVSEKITHIYDNGGVEIIASAQYGASNALDIYTGKNDHS
                IKPLVNTLYFYSYLLELDANYNKVKDKSLSECIEYLNHFMKNIQIYTETFVLKNNATN
                NKNFARKNGPLGFFRSSPEKINHFEYFLSLIATIMQETNDILEELGDSYYTSYKTPII
                SQTEIIDFILKAQEKEKYILILDKNDFNKYRKDCSFINNQENLAHKIAIGEFRIFIAV
                YKDMKCLENINNITKIYGYNSKSYKIKDQIWDERYLGGVCKISQALYFSGKAKIGII"
gene            complement(1313..1501)
                /locus_tag="OIDGCHHC_00057"
CDS             complement(1313..1501)
                /locus_tag="OIDGCHHC_00057"
                /inference="ab initio prediction:Prodigal:002006"
                /inference="similar to AA sequence:RefSeq:AAW35443.1"
                /note="conserved domain protein"
                /codon_start=1
                /transl_table=11
                /product="hypothetical protein"
                /protein_id="Prokka:OIDGCHHC_00057"
                /translation="MFYDKEKDKFVVGFRGTEGFWNMDTMQDITLSLNGNLQSSPLLE
                FLEQVDKIIKNENKISYL"
gene            complement(1597..1803)
                /locus_tag="OIDGCHHC_00058"
CDS             complement(1597..1803)
                /locus_tag="OIDGCHHC_00058"
                /inference="ab initio prediction:Prodigal:002006"
                /inference="similar to AA sequence:RefSeq:AAW35443.1"
                /note="conserved domain protein"
                /codon_start=1
                /transl_table=11
                /product="hypothetical protein"
                /protein_id="Prokka:OIDGCHHC_00058"
                /translation="MKEKINKLRDYAELAQASYFYFDLKGYILQENETIVILNELVSL
                SYNGKIAGKKKRSDKNIVLLAKVN"
gene            complement(1813..2118)
                /locus_tag="OIDGCHHC_00059"
CDS             complement(1813..2118)
                /locus_tag="OIDGCHHC_00059"
                /inference="ab initio prediction:Prodigal:002006"
                /codon_start=1
                /transl_table=11
```

```
                     /product="hypothetical protein"
                     /protein_id="Prokka:OIDGCHHC_00059"
                     /translation="MGVFRKFYIVWVVFCISGFAISPAVGHNPNRVYEFFVMLGWIIF
                     PLILLMLYRFFSLCEIKFLYIALLLLLYYPIALILYYMFYYHNSFYVTLYIFLSLFK"
     gene            complement(2246..2572)
                     /locus_tag="OIDGCHHC_00060"
     CDS             complement(2246..2572)
                     /locus_tag="OIDGCHHC_00060"
                     /inference="ab initio prediction:Prodigal:002006"
                     /codon_start=1
                     /transl_table=11
                     /product="hypothetical protein"
                     /protein_id="Prokka:OIDGCHHC_00060"
                     /translation="MNTLKKIYIIWGMFWTIGLGIFVPTAHGFDSMDFVDFFILALQF
                     IAIIWLPPLLIFNIVYFAIKKERKYIINTLWILLYYPIALILYYMFYYHNSFYVTLYI
                     FLSLFK"
     gene            complement(2693..3220)
                     /locus_tag="OIDGCHHC_00061"
     CDS             complement(2693..3220)
                     /locus_tag="OIDGCHHC_00061"
                     /inference="ab initio prediction:Prodigal:002006"
                     /codon_start=1
                     /transl_table=11
                     /product="hypothetical protein"
                     /protein_id="Prokka:OIDGCHHC_00061"
                     /translation="MIRLKKILKISSFILLPLIIIWSMTFGWGWFVPYSLQPSYHEFK
                     EMCKLDPEIYQANGGKIDKEYYNKLLAYFNTNFEEVLQGNKGLSREGNSFNEKINIYR
                     FRGEIKNNRQEIMLSLNIEKNKTTNEILPLKKVFFYVEWHNRRKYLTIKSVASYEFEW
                     RENYENCAYFKKEIK"
     gene            complement(3221..3796)
                     /locus_tag="OIDGCHHC_00062"
     CDS             complement(3221..3796)
                     /locus_tag="OIDGCHHC_00062"
                     /inference="ab initio prediction:Prodigal:002006"
                     /inference="similar to AA sequence:RefSeq:AVS37480.1"
                     /note="DUF2974 domain-containing protein"
                     /codon_start=1
                     /transl_table=11
                     /product="hypothetical protein"
                     /protein_id="Prokka:OIDGCHHC_00062"
                     /translation="MFVADASLAIGSIPKAQYDDMINFYETCIKDYPQIKEKDSLAIT
                     GHSLGGCLAQLFALSICDDKNRNNIKALYTYNAPGARKIIPPYDYIVKLFIFHSKEQQ
                     ERFIKEEIENIANRARDLGKDNIFLESKIREILHKIIQEKQSQYYGIIMSISTHTTMM
                     TLDINAIPILADIAPYYRQLAYNNIESKESA"
     gene            complement(3800..4312)
                     /locus_tag="OIDGCHHC_00063"
     CDS             complement(3800..4312)
                     /locus_tag="OIDGCHHC_00063"
                     /inference="ab initio prediction:Prodigal:002006"
                     /inference="similar to AA sequence:RefSeq:AVS37480.1"
                     /note="DUF2974 domain-containing protein"
                     /codon_start=1
                     /transl_table=11
                     /product="hypothetical protein"
```

```
                     /protein_id="Prokka:OIDGCHHC_00063"
                     /translation="MKNKAVINKLKDNSELAWASYGYFHLANPRYDFNKDNIDKERLE
                     YFRKLYKNNKLESSKTDEQLQSIYPTPADILNIECKYFKDNNGKPQDSWYHKHFLGGD
                     FSPLQAKQFFSRYDLLKHCPNTNSGFSATLFGEKRKQIDSKTKEESYTSEYGYINYIL
                     AIRGTEMSSF"
     gene            complement(4316..4888)
                     /locus_tag="OIDGCHHC_00064"
     CDS             complement(4316..4888)
                     /locus_tag="OIDGCHHC_00064"
                     /inference="ab initio prediction:Prodigal:002006"
                     /codon_start=1
                     /transl_table=11
                     /product="hypothetical protein"
                     /protein_id="Prokka:OIDGCHHC_00064"
                     /translation="MTYILLLIIISTILSYLILKFIYKIIFKSKKSVSKFLVFLGSIG
                     LIIFYYTPYSYYLEPSFYKFKEICKLDPEIYQANGGKLDEEYYNKVLRYFDTDLDNMS
                     KSYKKELGVTNDKKYSRYWFETWIDGRTEISFEIWFKSNIAIKDNIDFSIKPFFYANW
                     DKMRPLPAGNEGTGFFLGSVPISCIYFKKD"
     gene            complement(5073..5576)
                     /locus_tag="OIDGCHHC_00065"
     CDS             complement(5073..5576)
                     /locus_tag="OIDGCHHC_00065"
                     /inference="ab initio prediction:Prodigal:002006"
                     /codon_start=1
                     /transl_table=11
                     /product="hypothetical protein"
                     /protein_id="Prokka:OIDGCHHC_00065"
                     /translation="MELEKKREEFRSKYLGSEIKVGNETHFVNFSYLKKKFPQVLSNG
                     CKYYRNDYNYDVKDPDYDFNFDDKPEFAYYEDQFKAYMGEENYKKLRPYLGMTTYYVC
                     EGRKYPVVFATMIDYKVKNYGLFGDEGRGFSFSSISRKSAGGGSFHYFTNGKFIKSDE
                     KYTGQSY"
     gene            complement(5938..6423)
                     /locus_tag="OIDGCHHC_00066"
     CDS             complement(5938..6423)
                     /locus_tag="OIDGCHHC_00066"
                     /inference="ab initio prediction:Prodigal:002006"
                     /codon_start=1
                     /transl_table=11
                     /product="hypothetical protein"
                     /protein_id="Prokka:OIDGCHHC_00066"
                     /translation="MFLLPVYLLLFLVGGCSYKYMDPQYYEFRSLCKDIDNKVIIYNK
                     AYWEIFSNREKGNTMHDEKGEFFFNQKINKKIYFDFKKSESINVLQKNKFTLTEVTFE
                     DYYDGIHYSTHLSYIYDNYGMFLKGDEGAGLYFRYMEVLSCKNIDKKFKKDDWQVETL
                     E"
ORIGIN
        1 ctttaggaga aaaataataa ctgggtatat cttgttttat agtattatct tgatctaatt
       61 ctccaaaata aaaaggatta ttagaaagtt catttgagcc tgtaaggagg gtggagttta
      121 ggaagatata agcttgatag ctttgcaact cttcaaaagt aaaatctgtc gttgcttcta
      181 atttataaag ttcttcataa atgctttggc aatcaatgat gcttttattt tcgcaaaata
      241 aataaatact ttttttatct ttatgaatag aatataaaaa gtctctacct atcataattt
      301 tcctaaataa tacctatttt ggcttttcca ctaaaatata aagcttgcga tattttacaa
      361 actccaccta aataccgctc atcccaaatt tgatctttta ttttatagct ttttgaatta
      421 tatccatata tttttgttat attattaata ttttcaagac atttcatatc tttataaact
      481 gcaatgaaaa ttctaaattc cccaatggca attttatggg ctaaattttc ttgattatta
```

541 ataaaggaac aatcttttct gtatttatta aaatcatttt tatcaagaat taaaatatat
601 ttttcttttt cttgcgcctt taaaataaaa tctataattt ctgtttgact aataatagga
661 gttttataac ttgtataata actatctcca agctcttcta aaatatcatt ggtttcttgc
721 attatagtgg ctattaatga aagaaagtat tcaaaatgat tgattttttc tggggagctt
781 ctaaaaaacc caagaggtcc atttttacga gcaaaatttt tattattagt agcattattt
841 tttaacacaa aagtttctgt ataaatttga atatttttca tgaaatgatt aagatactct
901 atacactcac taaggctttt atctttgact ttgttatagt tagcatctaa ttctaaaaga
961 taagaataaa aatagagagt attaacaaga ggttttatag agtgatcatt tttccctgta
1021 tatatatcta aggcattgct tgcaccatat tgagctgaag caatgatttc aactccacca
1081 ttatcataaa tatgagtgat tttttctgaa acatctatag tatattttcc taaaagatct
1141 tgcataattt ttatagtatt agggttgata gctatattag gaaaattcca accataaaca
1201 ctaggggcat taaaagtata aacttcatta gggctaaaag atagtttatc tttatattta
1261 atatcgcaat atatcaaagc catttgagct aaatacccac ccaatgaatg tcctacaaat
1321 atgatatttt attttcattt ttgatgattt tatctacttg ttctaaaaat tctaaaagag
1381 gagaagattg gagattgcca ttaagagata gagttatgtc ttgcatcgta tccatattcc
1441 aaaaaccctc agtccctcta aatcctacaa caaatttatc tttttcttta tcataaaaca
1501 aagtagcact aaaaccactt aaagtgttgg cttgatggaa tttaatctca tatctttgag
1561 agaaatttct agcttgtaat tctccaaatt ctccatttaa tttacctttg ccaataaaac
1621 tatattcttg tccgaccttt tcttttttcc agctattttt ccgttataac ttaaacttac
1681 aagctcattt aaaataacga tagtttcatt ttcttgtaat atataacctt ttaaatcaaa
1741 ataaaaataa ctagcttgtg caagctcggc ataatctctt agcttattga tttttttcttt
1801 caaaattatc ctttatttaa ataatgataa aaaaatatat aatgtaacat aaaaactatt
1861 atgataataa aacatgtaat acaatatcaa agctatagga taataaagta acaatagcaa
1921 tgctatatac aaaaacttta tttcacaaag agaaaaaaag cgataaagca ttaatagaat
1981 taaagggaaa ataatccaac ctagcattac aaaaaattca tacactctat ttggattatg
2041 ccctacagca ggagaaatag caaatccact tatacaaaat actacccaaa caatataaaa
2101 cttacgaaat acacccattt attttccttt ttaatatttt tagtaaaatt cttctacttt
2161 tttaaaaatt aaaataaaaa taactagctt gtgcaagctc ggcataatct cttagcttat
2221 cgattctttc tttcaaaatt atcctttatt taaataatga taaaaaaata tataatgtaa
2281 cataaaaact attatgataa taaaacatgt aatacaatat caaagctata ggataataca
2341 ataatatcca taatgtattt ataatatatt ttctttcttt ttttatcgca aaatatacaa
2401 tattaaaaat gagtaatgga ggcaaccaaa ttatagctat aaattgtaaa gcaagtatga
2461 aaaaatccac aaaatccata ctatcaaatc catgtgctgt aggcacaaaa attccaagtc
2521 caatagtcca aaacatcccc caaatgatat aaattttctt taatgtattc atttattttc
2581 ctttttaata tttttagtaa aattcttcta ctttttttaaa aattaaaata aaaataacta
2641 gcccaagcta gttctgcatt gtctctaagc tttttaatct gttctttatt gttcatttta
2701 tttccttttt aaaataagca caattttcat aattttcacg ccattcaaat tcataagaag
2761 caacactctt tattgtaaga tacttccttc tattatgcca ttctacatag aaaaaaactt
2821 tttttaatgg taaaatttca ttagtagttt tattttttttc tatgtttagt gataacatta
2881 tttcttgtct attatttttt atctcacctc taaaacgata gatattgatt ttttcattaa
2941 aactatttcc ttctcgagat aaacctttat ttccctgtaa aacttcttca aaatttgtgt
3001 taaaataagc taataattta ttatagtatt ccttgtctat cttaccacca ttagcttgat
3061 aaatctctgg gtctagctta cacatctctt taaattcgtg ataactcggt tgtaggctat
3121 aaggcacaaa ccaccccccaa ccaaatgtca tactccatat aattatcaaa ggaagtagaa
3181 taaaacttga tatttttaga atctttttaa gccttatcat ttatgcactc tctttagatt
3241 ctatattatt ataagcaagt tgtctataat atggtgcaat atctgctaag ataggaatgg
3301 cattaatatc aagtgtcatc attgttgtat gagtagaaat actcattatt atcccataat
3361 attgactttg tttttcttgg attatcttgt gtaatatctc tcgtatctta ctttctaaga
3421 aaatattatc tttgcctaaa tctcttgctc tatttgctat attttctatt tcttctttta
3481 taaatctttc ttgttgttct ttactgtgaa atataaagag tttgactata taatcataag
3541 gaggtattat ctttctagct ccaggtgcat tataagtata taaagctttg atattgtttc
3601 tattcttatc atcacagata cttaaagcaa agagttgtgc tagacagcct cccaatgagt
3661 gtcctgtaat ggcaagagaa tctttttctt tgatttgtgg ataatcttta atacaagttt
3721 cataaaagtt tatcatatca tcatattggg ctttgggtat agagcctatg gctaaagaag

```
     3781 catcagctac aaacaaatct taaaaagaac tcatttctgt tcctctaata gctaaaatat
     3841 aattgatata gccatattca ctggtatagc tttcttcttt agttttacta tcaatttgtt
     3901 ttcttttttc tccaaataaa gtagcagaaa aacctgaatt tgtattagga caatgtttta
     3961 ataaatcata gcgagaaaag aattgtttag cttggagtgg ggagaagtcg ccacctaaga
     4021 aatgtttatg ataccaagaa tcttgaggct taccattatt gtctttaaaa tatttgcatt
     4081 ctatattaag aatgtctgca ggggtagggt aaatggattg taattgttca tcggttttac
     4141 tagattctaa tttattgttt ttatataatt ttctaaaata ctctaatctt tctttgtcta
     4201 tgttatcttt attgaaatca taccttggat tagccagatg aaaatatcca taactagccc
     4261 aagctagttc tgaattatct tttagcttat taatcactgc tttatttttc attgtttagt
     4321 ccttttttgaa atatatgcaa cttattggga cacttcctaa aaaaaatcct gtcccctcat
     4381 tacctgctgg gaggggtctc attttatccc aattagcata aaaaaaaggt tttatactaa
     4441 agtcaatatt gtcttttatt gcaatattac ttttaaacca tatttcaaag cttatttcgg
     4501 ttcttccatc tatccatgtt tcaaaccagt atctactata ttttttatca ttagttaccc
     4561 ctagctcttt tttatatgac tttgacatat tatccaaatc cgtatcaaaa taccttagca
     4621 ctttattata atattcctca tctaatttac caccattggc ttggtaaatc tctgggtcta
     4681 gcttgcatat ttctttaaac ttataaaagc ttggctctaa ataataagaa taaggagtat
     4741 aataaaagat aattaagcct atacttccaa gaaagactaa aaactttgaa acactttttt
     4801 tagatttaaa gatgatttta taaataaatt ttaatataag ataagaaaga atagtagaaa
     4861 ttataataag aagtaatatg tatgtcatta taagttattt cctttagatt atctatttta
     4921 ttaaaattaa gatgtaattt tgtggtttag tgttagcagt ataacttttt ctttatcatt
     4981 aaaataatag agcttgtttt ttcatatatt tttatccttt atagtttata tcttctagtc
     5041 tttgatttga gagataagtt ctttagggtt catcagtaac tttgtcctgt atatttttca
     5101 tcacttttta taaatttacc attggtaaaa taatgaaaag atcctccgcc tgcactttta
     5161 cgacttatgc tagaaaagct aaatcctctt ccttcatctc caaataatcc ataatttttt
     5221 actttataat ctatcatggt agcaaaaaca acaggatatt ttctaccctc acacacataa
     5281 taagttgtca tacctaaata aggtcttaat tttttatagt tttcttcacc catatatgct
     5341 ttaaattgat cttcatagta tgcaaattcg ggtttatcgt cgaaattaaa atcataatct
     5401 gggtctttta catcataatt ataatcatta cgataatatt tacaaccatt agaaaggact
     5461 tgggggaatt ttttcttcaa ataagaaaaa ttcacaaaat gagtttcatt tcctacttta
     5521 atttcactac ccaaatactt actacgaaat tcttctcttt ttttctctaa ttccaatatc
     5581 tcttctctta tttttttatc aaagatataa attccaccat aattacttgc aagagtttct
     5641 atttcacttg cttttttttct ttaaaagcac atgcacttaa aaatactaaa gaaaataagc
     5701 ttagaattat cattgtgatt ttcatttaat actccttttt aaattgtatt atttcaaata
     5761 ccttatataa ataaaattta gtttttatat ttaatatatc gcaaaaaacc tttttcatca
     5821 ttctcatcga ttaaatcaag cattgcataa ctagcatctg ctatattagc atagtcaaaa
     5881 aaatttagtg attaagtcta atttagcttc ttgtgtttta atgtttttac tcatttgtta
     5941 ttccaaagtt tcaacttgcc aatcatcttt cttaaatttt ttatcaatat tcttacaact
     6001 taatacttcc atatatctaa aatataatcc agcaccctca tcacctttta agaacatacc
     6061 ataattatca taaatatagg ataaatgcgt tgaataatga ataccatcat aataatcttc
     6121 aaatgtaact tctgttaaag taaatttatt tttttgcaac acattaatgc tttctgattt
     6181 tttaaaatca aaatatattt ttttatttat tttttgatta aaaaagaatt ctccttttttc
     6241 atcatgcatt gtatttcctt tttctctatt gctgaatatt tcccaataag ctttattgta
     6301 aattattact ttattatcta tatctttaca caagctccta aactcataat attgcggatc
     6361 catatattta taagaacatc ctcctactaa gaataataaa agatatacag gtaaaagaaa
     6421 caaagtaaat tttttaaata ttgttttaaa atttatagtt tttgttttat tttttatttt
     6481 aaaaaagact ttaaagataa aatataaaat aatagaaaat ataaaaacaa aaaagaaaaa
     6541 tataagaata aatattaaaa gattaa
//
LOCUS       OIDGCHHC_4              5820 bp    DNA     linear      12-APR-2021
DEFINITION  Campylobacter species strain strain.
ACCESSION
VERSION
KEYWORDS    .
SOURCE      Campylobacter species
```

```
  ORGANISM  Campylobacter species
            Unclassified.
COMMENT     Annotated using prokka 1.14.6 from
            https://github.com/tseemann/prokka.
FEATURES             Location/Qualifiers
     source          1..5820
                     /organism="Campylobacter species"
                     /mol_type="genomic DNA"
                     /strain="strain"
     gene            22..600
                     /locus_tag="OIDGCHHC_00067"
     CDS             22..600
                     /locus_tag="OIDGCHHC_00067"
                     /inference="ab initio prediction:Prodigal:002006"
                     /inference="similar to AA sequence:RefSeq:APA51619.1"
                     /codon_start=1
                     /transl_table=11
                     /product="lysozyme"
                     /protein_id="Prokka:OIDGCHHC_00067"
                     /translation="MQNLKLQIAQGLDKILEDEDRKQELYVCKFVVVNGASENKIKAK
                     NKDVNIDNDTKNINTTKSNNISSSENNAQEIIPDNVIRIQTQDGSGEFIEIVFDELKE
                     WEKSSSGAIKPLFTPLEGIVEDISNSLENVSIIGDTLAMLGAVKNPKNAKNIIKKKSL
                     TKDEVLQIVDKLPKNVRDKCRRIVENKEELDK"
     gene            686..817
                     /locus_tag="OIDGCHHC_00068"
     CDS             686..817
                     /locus_tag="OIDGCHHC_00068"
                     /inference="ab initio prediction:Prodigal:002006"
                     /inference="similar to AA sequence:RefSeq:CKG86671.1"
                     /codon_start=1
                     /transl_table=11
                     /product="methyltransferase small"
                     /protein_id="Prokka:OIDGCHHC_00068"
                     /translation="MIKLLYNIKKLLKVGGETIEINSNKPRENLRAIHIENGVKNEV"
     gene            807..1544
                     /locus_tag="OIDGCHHC_00069"
     CDS             807..1544
                     /locus_tag="OIDGCHHC_00069"
                     /inference="ab initio prediction:Prodigal:002006"
                     /inference="similar to AA sequence:RefSeq:CKG86653.1"
                     /codon_start=1
                     /transl_table=11
                     /product="hypothetical protein"
                     /protein_id="Prokka:OIDGCHHC_00069"
                     /translation="MKYKYIKWNKEEKCAIFFPDIDYSLWKKTQLKENVYNNPSLIIS
                     SCLITKMDILELFEYYILFICGKSMGTSRWECNNLILLDTYNIDNLSNPFSLNTPKPT
                     YTSEYINILGQLFLAGYIDFGSYCDEEDRNKIDYPSNLSYYKEDKYQAWIYFRDNFFY
                     QDKFIRDLDDPETLDEDGYSLILGDVSWDTPQYWSQYNIWVATTPKGTKYFNEILAPK
                     FYNKYKDLEVEIDDKSNVLRWIGEINR"
     gene            1554..2324
                     /locus_tag="OIDGCHHC_00070"
     CDS             1554..2324
                     /locus_tag="OIDGCHHC_00070"
                     /inference="ab initio prediction:Prodigal:002006"
```

```
                     /codon_start=1
                     /transl_table=11
                     /product="hypothetical protein"
                     /protein_id="Prokka:OIDGCHHC_00070"
                     /translation="MAIGKVIHKYGSFFPDFDEIFYNLGYDGICFDEKSDIKLFLQIP
                     NLKKTDILEYFEDHFLGSIFEDACEFNDLDCPLFYTEDENLKNSIKPNYNSEYVNIIG
                     QLFLAGYIDFGITPRSEDKYTRTDYPTNLSYYKEDKYQAWIYFRDNFFYTNAFLKGYY
                     DDILIYKGKEYTANTLPKLKKGETIHSTMHSAASWDMPRYWSGYNIWVTRTQKGTKYL
                     REILEPRVYNKYKDLEVEIDDKGNVLRWIGEINRDKKY"
ORIGIN
        1 acttttttaa ctcctgcaaa aatgcaaaat ttaaaacttc aaatcgcaca aggactagat
       61 aaaatcttag aagatgaaga tagaaaacaa gagctttatg tttgtaaatt tgtggtggta
      121 aatggggcta gcgaaaacaa aataaaagca aaaaataaag atgtaaatat agataatgac
      181 acaaagaata taaacactac aaaaagcaat aatataagtt ctagtgaaaa caatgctcaa
      241 gaaatcatcc ctgataatgt tataagaata caaacacaag atggtagtgg agagtttatt
      301 gagatagtat ttgatgaact taaagaatgg gaaaaatcat caagcggagc cataaagcct
      361 ttatttaccc cattagaagg tatagtggaa gatatttcaa attcacttga aaatgtcagc
      421 attatcggtg ataccttagc aatgcttggt gcagtgaaaa atcctaaaaa tgcaaaaaat
      481 attattaaga aaaaatcatt gacaaaggat gaagtgttgc agatagtgga taaactgcca
      541 aaaaatgtcc gagataaatg cagaagaata gttgaaaaca aagaagaatt ggacaaatga
      601 tggaaaaagt taactaaaaa ttctaaagag ttagaaaata aagtagataa aaaatacgga
      661 caatcaactt acatgagaaa attagatgat caaactatta tacaatataa aaaagcttct
      721 aaaagttgga ggcgagacca tagagataaa ttctaacaaa ccaagagaaa atcttagagc
      781 aatacatata gaaaatgggg tgaaaaatga agtataaata tatcaaatgg aataaagaag
      841 aaaaatgtgc tatatttttt cctgatatag attatagctt atggaagaaa acacaattaa
      901 aagaaaatgt ctacaataat ccctctttaa ttatatcttc ttgcctgata acaaaaatgg
      961 atattttaga gttatttgag tattatattt tatttatttg cggaaaaagc atgggcacat
     1021 caagatggga gtgtaataat ttaattttat tggatactta taacattgac aatcttagca
     1081 accctttctc tttaaataca cccaagccta cctatacaag tgaatatata aacatcttag
     1141 gacaactatt cttagcagga tatatagact ttggttctta ttgcgatgaa gaagatagaa
     1201 ataaaataga ctatccctca aacttatctt attataaaga agataaatac caagcttgga
     1261 tatattttag agataatttc ttttatcaag acaaatttat tagagatttg gacgatcctg
     1321 aaacattgga cgaggatgga tatagtctta ttttaggtga tgtttcttgg gatacgccac
     1381 aatattggtc acaatacaat atctgggtag caacaacccc caaaggcaca aaatatttta
     1441 atgaaatctt agctcctaaa ttttataata aatacaaaga tttagaagta gagattgatg
     1501 ataagagtaa tgtgcttagg tggattggag agattaatag atgaaagata aaaatggcga
     1561 ttggtaaagt aattcataaa tacggttcgt tctttcctga ttttgatgag atattttaca
     1621 atttaggtta tgatggtatt tgttttgatg aaaaatcaga tataaagctt tttctacaaa
     1681 ttccaaacct taagaaaaca gatattttag aatattttga agatcatttt ttaggatcaa
     1741 tttttgagga tgcatgcgaa tttaatgact tggattgtcc tttgttttac acagaagatg
     1801 aaaacttaaa aaattccata aaaccaaatt acaatagtga atatgtaaat attataggac
     1861 aactattttt agcaggttat atagactttg ggattactcc aagatcggaa gataaatata
     1921 caagaacaga ctatcctact aatttatctt attataaaga agataaatat caagcttgga
     1981 tatattttag agataatttc ttttatacta atgcattttt aaaaggatat tatgacgata
     2041 ttttaatcta taaaggcaaa gaatatacag caaatactct acccaaactt aaaaaaggag
     2101 aaacgatcca tagtacaatg catagtgctg cttcttggga tatgccaaga tattggagtg
     2161 ggtataatat atgggtaaca agaactcaaa aaggcacaaa atatctccgt gagattttag
     2221 aacctagagt ttataacaaa tacaaagatt tagaagtaga gattgatgat aagggtaatg
     2281 tgcttagatg gataggagag attaatagag acaaaaaata ttagcaaata aaaaatgata
     2341 taataatgct ttatataagg aatcttttat gtttataagt tctaaaacag agttaattca
     2401 aactattaaa aatttaacaa tcaacaataa agaagttaaa cagattgatt ttatttatcc
     2461 taatttttct attttacacg ctatattaga aaatattgat tttcatcaaa gaaaaatcct
     2521 atttaaaaat caaatattgg ctatggaaat attagaaatt ttaaaacaaa ctcgtgataa
     2581 tcacaataat atagaagtaa atatccatac ccttaatgag cttgatgctg gacaaaatgt
```

2641 aaatttaaaa gatttatcaa gcagtgaaat ttgcactaaa gtttatttag aagacttaga
2701 agaagaaatt caagataaga aacaaggtaa agattttaat ctttttttatc atacagataa
2761 aaataatctt tgtatcgaat ttattaatac tttaaaagaa ttagccaaag attctgaaga
2821 tataacaacc ttggacgagt taatccacaa cattaatcct gatgatataa gcagtgagaa
2881 atttgatgat tttaagaaaa aagttttaga aaagattgta aatgtcatag aatcggattt
2941 tttcaaaaat agcataagtc ccactttaaa aaatacttat gaaaaattta tcgatgagtt
3001 gttagaacac attaaaaaac ttgccgaaga aaaagaatat gaaaaaatta aaatcatact
3061 aagtgctata gcgggtgtta caatagccac agtggtagct gttcttacag gtggggcagg
3121 actagctatt ttggctgctg gtttattttc tttaatgggt tctgcaagcg agtattatga
3181 atttgctcaa agagattatg gcacactttt aacgccttta gccgcatttt tagccactag
3241 atgtgctatg tttattcatt atattaattc tcgcttgtta tcttgtgttt taattattga
3301 taaaaaagaa attattgatt taagtggttt tggattatat cttggaagtg atgaaagcaa
3361 tatcgcttca aatttagcct ataaaataac cttaagcgaa gaaataaaaa gcatcgaaaa
3421 tattttttagt gaaatctttt taaacccaga acaaaaagac aatgagagta aaaatacagc
3481 taatgattca aaatcgagca atttacccgc tttcatttca acgcaaaaat tatctaattt
3541 gtatttaaaa aacaatgcta ttttaaagaa tttacaagaa gatattaaac aatttattta
3601 tttttcttat atagaaagtc caaattttaa ctcaagtaaa ctagctgaat ttttcgcaca
3661 aaaaaatcaa aacacacaaa tagatttaaa tacaactaaa atgagtaaaa attatttgat
3721 tatttcaaat atccccaata aatacaatgc aggacttagt gaaagtttaa ttcaagaaat
3781 cgccaactat aatatacaag ataaatacag aacaattaaa aaaaatattg atacttcaaa
3841 aaaaacttat gacagcacaa gagattatag cgaatatagc atagaaatgc cacaagatga
3901 aaatgaacct tatattttac aattaagccc ttttgtgaaa atatccaaag aagcttatga
3961 gaatagtaaa aaagcttatg aagatatgca agagatttat aaatatttaa caaattttgt
4021 tgctacaccg cagaagctac cagaattcgc aaaaacagct aaagaaaaat tagcaatatc
4081 agaaaatgag tcagtaaaaa agacggaaaa tttacgacat tgcgaacgaa taaacatttt
4141 agcttatatt gactacaata aaaaaataaa tgataaaaaa tggcttcctt atttttatat
4201 caaaaaaattc tttaaacaaa acgaagacaa agaatccatc atagaaaaag aaaaaaaaat
4261 agctcatcaa tatttaactt ttttgaaaaa agagcttttt gaaaaatcgg ataaatcttc
4321 atttggaaac tattctttat cggataaatc ttcatttgga aactattctt taatggaatg
4381 tttatttatt tcatttgtct tgtatcaagc taagcttatt tttccaaata tcaatttgga
4441 tttgaaaaaa tatggaggca gtaattttgt atatataaaa tacttagagg aaacttatgt
4501 tttatggaca gatgaatatc caatatatgt aaataatgag ccaacaaata ttacagcata
4561 tgaagttgta tcgcaattat taaaaatact tgacaaaaaa gattattctt ttgtggaaaa
4621 tgatagttcg atcactatag atgatttttt taatgaatta tcaaaagatt tagatgcaac
4681 aaatacatta gataaaaaaa taatcaaatt gaagcaaatg tatcatttag atgaacaaaa
4741 atgcattaaa gaatttatga atttagacga agaagcgcaa caagcttttt ttgtatggat
4801 ggatatagag aaacaaatat cagatttatc aaagcttttg gaacagatgt atggaaaaga
4861 atatttaaat aaggcaaaag aacaaaaaac aagtgaaata tttacttatg caatgttaaa
4921 aattttatta agtgctagtc cttttatcag ctttacagct caaatgttgt gggattttaa
4981 ttttttaaaa acaatagaat atgttattca agcttgtatt aaaataaaca aagaaggatt
5041 taaagaattt tttaaagaac agcttggtat ttttacacct ctaaaatatc atatacacat
5101 aaaatctaat ttaataataa aagcgcgctt gcaacaaata gaaaaagaat taactcatac
5161 taaaatccaa atgattttag cacaagagat aaaatccaat ttaataatac aaaaaaatac
5221 atccagttta aatctattta tcacaaagat aaaagaatac tttaatgaaa aacaaattaa
5281 gatttattgg agtgataaat atatgaaaca attatttata tactccaatc aagaaagcaa
5341 tatatctaaa attgctctta tgcatagcat agatatacaa actcaaaaat atactattta
5401 tacttatgct aagctaaata aacttgacct tgcttctatt gtggctggag aatttgtggg
5461 tattttttta aattatctct ttccaacaaa ttttaaagcc ttacaagaac aatatgaaag
5521 cattgtttat cagctttaca cctttaaaga tgattctcct ttggcggtta taagagatca
5581 ttttataact tatccttttg caatcaattc aaaattcatt agctttgatt taaagaaaat
5641 tttttatggc atagatcttt gcacaggagt ttttgacact catttttcct ttcattatga
5701 aatagatcaa aagcttgaag aaaataaaga atttctttta aaaagacttt tgtcttattt
5761 gattattgat gagaaaagaa gtgcaactgc tttagaggat ataaaagaac aaaaatatat
//

```
LOCUS       OIDGCHHC_5              4447 bp    DNA     linear       12-APR-2021
DEFINITION  Campylobacter species strain strain.
ACCESSION
VERSION
KEYWORDS    .
SOURCE      Campylobacter species
  ORGANISM  Campylobacter species
            Unclassified.
COMMENT     Annotated using prokka 1.14.6 from
            https://github.com/tseemann/prokka.
FEATURES             Location/Qualifiers
     source          1..4447
                     /organism="Campylobacter species"
                     /mol_type="genomic DNA"
                     /strain="strain"
     gene            32..754
                     /locus_tag="OIDGCHHC_00071"
     CDS             32..754
                     /locus_tag="OIDGCHHC_00071"
                     /inference="ab initio prediction:Prodigal:002006"
                     /codon_start=1
                     /transl_table=11
                     /product="hypothetical protein"
                     /protein_id="Prokka:OIDGCHHC_00071"
                     /translation="MYHYPKVQLEFKNFEKMTLNPSPKDMIKIVDDHQPKLEDFKDLN
                     VKMQKAIFDFKVAKLFGFEDRYYGVILVAYSNIFTISTTKEHIYFNYLNFISNLNSNE
                     KQKYLSLRASTKDLEKQIFEEKLKFIKHYDDFYDYLERIGYLDKGAWYKGMANIYKII
                     IYYFTYDMPKNLKKFYSLEDKKLALEKMKRSYEIFNNLDLNSTSKIPSIANKNWRNAF
                     KDFSNASYNWINEIQKALDECK"
     gene            741..842
                     /locus_tag="OIDGCHHC_00072"
     CDS             741..842
                     /locus_tag="OIDGCHHC_00072"
                     /inference="ab initio prediction:Prodigal:002006"
                     /codon_start=1
                     /transl_table=11
                     /product="hypothetical protein"
                     /protein_id="Prokka:OIDGCHHC_00072"
                     /translation="MNANENNANNENNTINIEESIKEALRGARNCRK"
     gene            874..2343
                     /locus_tag="OIDGCHHC_00073"
     CDS             874..2343
                     /locus_tag="OIDGCHHC_00073"
                     /inference="ab initio prediction:Prodigal:002006"
                     /codon_start=1
                     /transl_table=11
                     /product="hypothetical protein"
                     /protein_id="Prokka:OIDGCHHC_00073"
                     /translation="MQVSDGATNLFPNSPTKQTIQPYIQKIQVITDKVDNRLKFVEYT
                     LEYINNGDTTRVIIKLGKEVLEDKILTKGLEISKDIALIAMKSVPKGKTPLGIAIFAG
                     GSIISYFFADEIEDLAKELFFNKNNIFYRTNKAVEQFFTPPIKCPKSSEWPTGECKSI
                     DVIFNEMQIILGKSLFTPHPYPSYSKEYKFALARKKELITKTSIASNSYNFMDEEYLK
                     NIQDLQEQDQIINQEEQKYQLALKFYKEDNKIKNEIFNPLFKTLNENLSFQLKQERNT
                     FINPNQSLSIISTKKYSLKTLNLQDINNIRINSEFAYLRALFLCESFLVLNNNDEVLL
```

```
                     NEKITKEILECNEDEYILFILHKDKLNDTYLQARKELYKSIDEFKRLGFKDLENAYQT
                     YINSLIINKEINSKDENKEINKNLNQDENSNQENNIKENTSSIIKNTNSKNNPSLILK
                     QKDISINQEINNTKEQDFNLLFDKIIIQNNIIFKGKKIA"
     gene            2336..3205
                     /locus_tag="OIDGCHHC_00074"
     CDS             2336..3205
                     /locus_tag="OIDGCHHC_00074"
                     /inference="ab initio prediction:Prodigal:002006"
                     /codon_start=1
                     /transl_table=11
                     /product="hypothetical protein"
                     /protein_id="Prokka:OIDGCHHC_00074"
                     /translation="MLKISKRISTIVFIVLVFIAVVSSVYEFIHEALKFKEDNESKAR
                     ENLSALIKWSENEGKEELEYAKKLNKETYNQEKVTQMIIKNLKMIQTGIEDIRILSSY
                     YPVEEDVELMRQAGHVISDSNTDIILYLLDNEKTFIGHQTYFLFDKERFKIFEDFLFF
                     LSTRLEEDFLKKDIDKFDSFDVIRIGMYINALIGYNRASTDMYLSEFSQDYICDLNTP
                     KTMTILNGMSQINTAIDRILLFFNKELEKYTDDYSKMQLEKMIYNFKKLKLGQKQINE
                     LKSIQSKLKECKQ"
ORIGIN
        1 ttattctcct ttatatacga tttattgggg aatgtatcat tatcctaaag ttcaactaga
       61 atttaaaaac tttgaaaaaa tgactttaaa tccaagccct aaggatatga ttaaaattgt
      121 agatgatcat cagccaaaat tagaagattt taaagattta aatgtaaaaa tgcaaaaggc
      181 tatttttgat tttaaggtgg ctaaattatt tggatttgag gataggtatt atggggttat
      241 ccttgtagcc tacagcaata tatttacaat atcaacaacc aaggaacata tttatttcaa
      301 ttacctaaat ttcataagca atttaaactc aaatgaaaaa caaaaatacc taagcttaag
      361 agcatctact aaagatttag aaaaacaaat ctttgaagaa aaacttaaat ttataaaaca
      421 ttatgatgat ttttatgatt atttagagcg tataggttat ttggataagg gagcttggta
      481 taagggaatg gcaaacatat ataaaattat aatatattat tttacctacg atatgccaaa
      541 aaatcttaaa aaattttact ccctagaaga taaaaaatta gcgcttgaaa aaatgaaaag
      601 aagctatgaa atattcaata atttagattt aaattcgact tctaaaattc ctagcatagc
      661 taataagaat tggagaaatg cttttaaaga tttttctaat gcaagttata attggataaa
      721 tgaaatccaa aaggctttag atgaatgcaa atgaaaacaa tgctaataat gagaataata
      781 ctattaatat tgaagaaagt ataaaagaag ctttaagagg ggcaagaaat tgtagaaagt
      841 agtagagaag agatagggaa agcaaatacc tcattgcaag tcagcgatgg tgctactaac
      901 cttttcccca actcccccac caagcaaact atacaacctt atatccaaaa aattcaagtt
      961 attacagata aggtagacaa tagattaaaa tttgttgaat atacactaga atatattaac
     1021 aatggcgata caactagagt tattattaaa ttaggaaaag aagtcttaga agataaaatt
     1081 cttacaaaag gtttagaaat ttccaaagat atagcactta ttgcaatgaa atctgtaccc
     1141 aaaggcaaaa ctcctttagg catagctatt tttgctggag gaagtattat atcttatttt
     1201 tttgctgatg aaattgaaga tttagcaaaa gaattatttt ttaataaaaa taacattttt
     1261 tatcgtacca ataaagctgt tgagcaattt ttcacacctc ctatcaaatg tccaaaaagt
     1321 tcagaatggc caactggaga gtgtaaaagc atagatgtaa tcttcaatga aatgcaaata
     1381 atccttggaa aatccctctt taccccacac ccctacccta gctattccaa agaatacaaa
     1441 tttgccctag caagaaaaaa agaacttata actaaaacaa gcatagcttc taattcctat
     1501 aattttatgg atgaagagta tttaaaaaac atacaagatt tacaagaaca agatcaaatc
     1561 atcaatcaag aagaacaaaa atatcaactt gctttaaaat tttacaaaga agataataaa
     1621 atcaaaaatg agatttttaa tcctttattt aaaaccttaa atgaaaatct aagctttcaa
     1681 ttaaaacaag aaagaaatac ttttataaat ccaaaccaaa gtttaagcat tatctcgact
     1741 aaaaaatact cattaaagac tttaaattta caagatatta ataatatccg catcaattca
     1801 gaatttgctt atttaagggc tttgttttta tgtgaaagtt ttttagtttt aaataataat
     1861 gatgaagttt tattaaatga gaaaataact aaagaaattt tagaatgtaa tgaagatgaa
     1921 tacattcttt ttattctcca caaagacaaa ttaaacgata cttatttaca agcaagaaaa
     1981 gagctttata agagtattga tgaatttaaa agacttggtt ttaaagattt agaaaatgct
     2041 tatcaaactt atattaattc tttgattata aataaagaaa taaatagtaa agatgaaaac
```

```
     2101 aaagaaataa ataaaaattt aaatcaagat gaaaattcaa atcaagaaaa caatataaaa
     2161 gaaaatacaa gcagtataat aaaaaataca aattctaaaa ataatccttc tttaatttta
     2221 aaacaaaaag atataagtat aaatcaagag ataaacaaca caaaagaaca agactttaat
     2281 cttttatttg ataagattat aatacaaaac aatattattt ttaaaggtaa aaaaattgct
     2341 taaaatatca aaaagaattt ctactatagt ttttattgta ttggttttta ttgcagttgt
     2401 tagtagtgtt tatgagttta tccatgaagc tctaaagttt aaagaagata atgaaagcaa
     2461 agcaagagaa aatcttagtg ctttaatcaa gtggagtgaa aatgaaggca aagaagaatt
     2521 agagtatgct aaaaaattaa acaaagaaac ttataatcaa gaaaaagtta cgcaaatgat
     2581 tattaaaaat cttaagatga tacaaactgg tattgaagat ataagaattt taagttctta
     2641 ctatcccgta gaagaagatg ttgagcttat gagacaggca ggtcatgtta tttcagactc
     2701 aaatactgac attatacttt atcttttaga taatgaaaaa acttttatag gacatcaaac
     2761 ttattttta tttgataagg aaagatttaa aatttttgaa gattttttat ttttttaag
     2821 cactcgttta gaagaggatt ttttgaaaaa agatattgat aagtttgata gctttgatgt
     2881 aattagaatt ggaatgtata ttaatgctct aattggctat aatcgcgcat ctactgatat
     2941 gtatttgagt gaattttctc aagattatat ttgtgattta aacaccccta aaactatgac
     3001 aattttaaac ggtatgagcc aaattaatac tgctattgat agaattttat tattttcaa
     3061 taaagaatta gaaaaatata ctgatgatta ctcaaaaatg caattagaaa aaatgatcta
     3121 taattttaaa aaactcaaac tcggtcaaaa acaaatcaat gaacttaaat ccatacaatc
     3181 taaactaaaa gagtgtaaac aatgaatgaa gaattgcaaa aagaagaatt aaatgaaaca
     3241 gaaaaagatt tgcaacttcc tttaaaagat gcaggtgtag atgaaatagg agaaaaacta
     3301 cttaaagaaa tagaagatgc agataaaaca gaaaatttta tgcaccaaaa tgaagttcaa
     3361 gcctggaaaa gtgctactgg atttataagc aatacaaacg atacaatatc tagctttaac
     3421 aaaaacctcg aaccctacct actccctttt aaaatcatca taaaaagagc agataatact
     3481 cttagtgtaa ttgatgcttt aatagaatat caaaaaacaa aagatggctt aaaggttatt
     3541 gtaaaatttg gttctgaaat tatagcttca caactttttg caataggaac aagaatggta
     3601 gtccaagcag caatttctgc ttttgtagcg gtggctattt gcacaggcag tataatattt
     3661 ggaattttag caggtgttgc tattgttgct gctggaatag cagctctttg gtggattaat
     3721 tctaaaatag aagactggtt aaaagaaagc ggtatttcct ttattgattt catgagagaa
     3781 tttgaaactc atttaagttc tgaagaaaaa tattattata atttagcaca ttgtcataaa
     3841 acacaaatta gaaatatgca aagaattcaa ttatattaca atggcaaaat ttcagaagat
     3901 ttaaaaaatt attattatcc tttttatgac tttaaaaaag ctcctatact tgatggcaaa
     3961 gatattatta aaaaaatcaa tgagttgcaa aataattaca aaatattaag cccaaaagat
     4021 acaatagaca ctattcatat aagttcaaaa ccttatttat taaaagcttt atatttttgt
     4081 gaaagtttta ctgaattaaa acaagatgat aaagttctat gggatgaaaa aagtataaaa
     4141 gagtcatttc cttacgattc tagtttttat attatattct tactacacaa ggataaaatc
     4201 acagatactt atttaaaagc aagaaaagag ctttataaga gtattatagc atttaatcaa
     4261 attcaaaata agaatatatt agaacaaaat aaagaagatt ttcaagaata caacccaatc
     4321 tctcactcta aaaacctaaa ccatttcatc ttagaaaaca aagataaaaa taaaagaatt
     4381 atctttttaa ataatgcttc atcgatgtca aatttaatcc atatttatga taatcattgt
     4441 gatgtat
//
LOCUS       OIDGCHHC_6              4184 bp    DNA     linear       12-APR-2021
DEFINITION  Campylobacter species strain strain.
ACCESSION
VERSION
KEYWORDS    .
SOURCE      Campylobacter species
  ORGANISM  Campylobacter species
            Unclassified.
COMMENT     Annotated using prokka 1.14.6 from
            https://github.com/tseemann/prokka.
FEATURES             Location/Qualifiers
     source          1..4184
                     /organism="Campylobacter species"
```

```
                     /mol_type="genomic DNA"
                     /strain="strain"
     gene            97..228
                     /locus_tag="OIDGCHHC_00075"
     CDS             97..228
                     /locus_tag="OIDGCHHC_00075"
                     /inference="ab initio prediction:Prodigal:002006"
                     /codon_start=1
                     /transl_table=11
                     /product="hypothetical protein"
                     /protein_id="Prokka:OIDGCHHC_00075"
                     /translation="MPYLLSLSDEEFEKIIFHVSGFSEIDTTFLKEAMELRKEKKDE"
     gene            221..2314
                     /locus_tag="OIDGCHHC_00076"
     CDS             221..2314
                     /locus_tag="OIDGCHHC_00076"
                     /inference="ab initio prediction:Prodigal:002006"
                     /codon_start=1
                     /transl_table=11
                     /product="hypothetical protein"
                     /protein_id="Prokka:OIDGCHHC_00076"
                     /translation="MNENIYGSLEFYSKETQSTPSDKRDLGVFGDVAPYALGITEGFA
                     TKAFEKEIVKFGIKHNSHIATMMHSKIIMNFGILNFFLAKAEGKSNVRALGETGVGIM
                     IGIGIELTMDNTSIGAKIKQGGEYVAKQTLTQTSRVASNAATKIASSRVASNIAARAG
                     VSILSRIAAGAATGAAVGSSFPIVGTVVGAVVGTLVAGFFNDLIFGDEDEELKKQEEA
                     KEKYLEEKLDEKMHKIINYLITHQYIELRVLNEDEAEKLCKEISDINSAYFKTILLML
                     SFPYYLDKDEQSYKEAQEKNPTIIRIQPIANALNIKIEINECFLAKNGEALKNKEIYV
                     YNHRFDRVVTKAMSDDEGKIVFENVYVGKESTIDKISFIIDRENFNEDNFYESVLKYA
                     PMFNVQKKHKQEGQAFIGKMFFSFTYAQEIMQDNEVLKLEALRNNFHIVFDYEVRKQE
                     ESYKNYIILSYLVFDVKEDIEEYIRHITIENRAFRGLELLGRGWKNQYSIKDEWRDKG
                     VVFFAYFNSQKFTPYKKMAFIDKPIVILDIEKFDKENILEDIKFHFKTLAKAYKIFVI
                     DLDANTQIQEKKSIVNNIKKNTQNLELLYLQLKLFDDKDTNKCKVQYFHNENKYANQE
                     MKWIEYCKKQFNALNNKDNPIYKNKNSFDMEVPFVSISFGSLIYDKERLAKKGVRQIF
                     GVGLAESCRRYFYEK"
ORIGIN
        1 gtcaaagaat accttgaacc tggtttattc agtgatggcg atgatccaac aaaagattgt
       61 tttgaaagaa ttaatgccat tataaaacct catgtaatgc cttatttatt aagcttaagt
      121 gatgaagaat ttgaaaaaat cattttccat gtaagcggtt ttagcgaaat tgatactact
      181 ttccttaaag aagcaatgga actcagaaaa gagaaaaaag atgaatgaaa atatttatgg
      241 aagtttagag ttttattcca aagaaactca aagcactcct agtgataaaa gagatttagg
      301 agtatttgga gatgttgctc cttatgcctt aggcattaca gaaggttttg ctactaaagc
      361 ttttgaaaaa gaaatagtga agtttggcat aaaacataat tcgcatattg cgacaatgat
      421 gcattctaaa ataataatga attttggaat attgaatttt tttctggcta aagcagaggg
      481 aaaatccaat gtaagagctt tgggtgaaac aggtgttggt attatgattg gaattggtat
      541 tgaattgacc atggataata cttctattgg ggcaaaaata aaacaaggtg gtgaatatgt
      601 tgccaagcaa accttaactc aaactagcag ggttgcttct aatgccgcaa ccaagatagc
      661 tagctcaaga gtagcatcta atatcgcagc tagagctggc gtatccatac tttctcgtat
      721 tgccgcaggt gcagcaactg gagcagctgt aggctcgagc tttcctattg ttgggacagt
      781 ggtgggtgca gtagtgggaa cacttgtagc aggttttttt aatgacttaa tttttggcga
      841 tgaagatgag gaattaaaaa aacaagaaga agccaaagag aaatacttag aggaaaaatt
      901 agatgaaaaa atgcataaaa tcatcaatta tctcatcact catcagtata tagaattaag
      961 agtgttaaat gaagatgaag cagaaaagct ttgtaaagaa ataagtgata ttaatagtgc
     1021 ttattttaaa accattcttt tgatgctaag ttttccttat tatttagata aagatgagca
     1081 aagttataaa gaagctcaag aaaaaaatcc aactataatc agaatacaac ccatagctaa
```

```
     1141 tgccttaaat ataaaaatag aaatcaatga atgctttctt gctaagaatg gagaagcttt
     1201 aaaaaataaa gaaatttatg tttataatca tcgctttgat agagtagtaa caaaagctat
     1261 gagtgatgat gagggtaaaa tagtatttga aaatgtttat gtgggtaaag agagcactat
     1321 agataaaata agctttatta tagacagaga aaatttcaat gaagacaatt tttatgaaag
     1381 tgttttaaaa tacgctccaa tgtttaatgt ccaaaaaaag cataagcaag aaggacaagc
     1441 ttttattggt aaaatgtttt ttagcttcac ttatgcacaa gaaataatgc aagataatga
     1501 ggtgttaaaa ctagaggctt taagaaataa ttttcatatc gtttttgatt atgaagtaag
     1561 aaaacaagaa gaatcttata aaaattatat cattttatct tatttggttt ttgatgttaa
     1621 agaagatata gaagaatata tacgccatat tactatagaa aatagagctt ttcgtggatt
     1681 agaattgtta ggtcgtggat ggaaaaatca atacagcatc aaagacgaat ggagagataa
     1741 gggtgtggtt ttttttgctt attttaattc tcaaaaattt actccttata aaaaaatggc
     1801 ttttattgat aagcctatag ttattttaga tatagaaaaa tttgataaag aaaatatact
     1861 agaagatata aaattccatt ttaaaactct tgctaaagcc tataaaattt ttgtaataga
     1921 tttagatgct aatactcaaa tacaagaaaa aaaatccatt gtcaataata taaaaaagaa
     1981 tactcaaaac ttagaactgc tttatctgca acttaaactt tttgatgata aagatactaa
     2041 caaatgcaag gtgcaatact ttcacaatga aaacaaatat gctaatcaag aaatgaaatg
     2101 gatagaatat tgcaaaaagc aatttaacgc acttaataat aaagataatc ctatttataa
     2161 aaataaaaac tcgtttgata tggaagtgcc ttttgtaagc atttcttttg ggtctttgat
     2221 ttatgataaa gaaagactgg ctaaaaaagg agttaggcaa atttttggag tgggtttagc
     2281 agaatcttgc aggcgatatt tttatgagaa atgatttgat tttagataat taaagaatat
     2341 actacaaact ttattttacc caccttatgc taaaataaat cacataagct ataggctatt
     2401 aatcatcaac aaacccaaaa ggatcttcaa tgtcccttaa ttcttatatg agtcttgata
     2461 tattaaatca tacttcaaat cccctaccct ttaaaatcac taaagccctc atcaaagaaa
     2521 gtcttgaaga gctttttttct atagaatgtg aaggcttttt tgaaagttta gaacaagatt
     2581 tattttcttt aaatacttta accaatgctt catctcatct aagcacccct acagctttta
     2641 attttcatcc taatgtttta atcgataaag aagctatttt aagtatccat aatccttatg
     2701 agaacaatac cttaaacttt gatcataatg aagtaaaaaa ctataaagga atcatcactt
     2761 atataaaata tctaggtatt aatcatgaga gtgctttaaa tatcaatgat agcacttcaa
     2821 acactaagca aataaaatac aagcattttt tttcttttaa gcttcaatca gtcttgataa
     2881 gactttcttt aaataaagcc aatcgtattt atactcatac tcatatcata gaagtgatta
     2941 aacaaaccct tggtttttat caagatatct tacataaaga aattgattat tctcatatac
     3001 attttaatta tgaagaacaa gaacttatct ctcaatacaa tgaaagcgat ttagaattta
     3061 tcacaagatt agctcacaat aatggaatct tttttatga agatgaaaac actatttatt
     3121 tttgtgatgt gtataaaaat gtaaaaaata aagaaattga gtacaatcct aatgtgaata
     3181 atattttaaa tcaagcttgt atttcttcta tctataaaga gcaaagttta agaactaatg
     3241 cttttacaca ttctagtatc aatgctaata ctcctttaaa tcttttatct ttacactcta
     3301 gtaaagttcc ttatgaacaa gaacttaaca ataaagctta ttataatgaa catttttatg
     3361 agagtgaata ttcttttaca agaagtattg atttaaagac taagccttct cttaaagaaa
     3421 aaagagcctt gatcttaaat gaaagcttac tagcaaaaag taatatttat catcttagtt
     3481 tgggagattt tatcacttta aattataagg aatttaattt gagtggattg agtgatgatg
     3541 aagatactaa aagtcaagat gaagaaaaac aagataaagc ttctttgctt aaagacttta
     3601 ttattattgc caatactcaa atcttaatag atgatgcttt actagctaat tctatcaata
     3661 ctaatgatca tttaaattta aaagatttaa atttaagtaa gtcttattct aatactttaa
     3721 ctttattaaa aaagaatatt atctttactc caagttttaa agctaagcct aaagcccctc
     3781 ataatactca aggtattgtt ataggagaga gtaaggatat agaaagtgag agaaatacta
     3841 tttatactga tgagtatgga agggttaagg taaggattaa tctttatgct aatcaagagg
     3901 aattagataa taatctttta agtgcaaaca taaaaaccaa tactcatgag aatacaaaca
     3961 atactgattc taaagataat acaaacttca caaactcttc taatcattta tcaagcaatg
     4021 cttataaatc ttatcatcat actccttttt taagagtagc aacaagtata gcaagcaatc
     4081 attcaggttt ttttcatact ccaagagtgg gagatgaagt gattgtttct tttttagatg
     4141 atgatataga taaaccctat gtgagctcta gtttgtataa tggg
//
LOCUS       OIDGCHHC_7              3545 bp    DNA     linear      12-APR-2021
DEFINITION  Campylobacter species strain strain.
```

```
ACCESSION
VERSION
KEYWORDS    .
SOURCE      Campylobacter species
  ORGANISM  Campylobacter species
            Unclassified.
COMMENT     Annotated using prokka 1.14.6 from
            https://github.com/tseemann/prokka.
FEATURES             Location/Qualifiers
     source          1..3545
                     /organism="Campylobacter species"
                     /mol_type="genomic DNA"
                     /strain="strain"
     gene            62..955
                     /gene="tssI1"
                     /locus_tag="OIDGCHHC_00077"
     CDS             62..955
                     /gene="tssI1"
                     /locus_tag="OIDGCHHC_00077"
                     /inference="ab initio prediction:Prodigal:002006"
                     /inference="similar to AA sequence:RefSeq:AOH52008.1"
                     /codon_start=1
                     /transl_table=11
                     /product="type VI secretion system, secreted protein"
                     /protein_id="Prokka:OIDGCHHC_00077"
                     /translation="MIVSFLDDDIDKPYVSSSLYNGTNPSLVNLPFNDHQTSLSSKTI
                     GVNEEGYNELTLSNIKDKEQIYLKAQKDYDELVQHNFTQRILNDKDSKVDGIYNERIK
                     KVHTQTIDLAKNVNVGGEYLTNVGLSKDTIVGLSNTLNVGVDNKVRIAKNSHEFVGEN
                     KDIEIGANQNTIIHKDEIRNVKGENKLLIEKSLTQTIEKEFFLNVHQNLSAHIQDNTS
                     LKSNSMQTKIEEQYSLESENSTFDFQTDCEVKAGNQILHQVGDTQIVTKKDCVIIKAG
                     GVEVIIDSNGLVVKGGELKAE"
     gene            966..1781
                     /locus_tag="OIDGCHHC_00078"
     CDS             966..1781
                     /locus_tag="OIDGCHHC_00078"
                     /inference="ab initio prediction:Prodigal:002006"
                     /codon_start=1
                     /transl_table=11
                     /product="hypothetical protein"
                     /protein_id="Prokka:OIDGCHHC_00078"
                     /translation="MKKPFYKLKRFYIPCIIIIIIFAVLAKLLYSPLYTIYWGMYHYP
                     KVQLEFKNFEKMTLNPSPKDMIKIVDDYQPKLEDFKDLNVKMQKAIFDFKVAKLFGFE
                     DRYFEAFIKSCAGNFFVLHGKEQIYFNYLNFISGINSTSNEKQKYLNLRASTRDLERQ
                     IFEEKLKFIKHYEEFYDYLEGVGYLDKGTEYIGTAISFKTLIQNVFLSNNAQLCSFED
                     RNLMFKNMKENYEIFKNLDVKNIDDLKRNIYKKILIDMENLLNETQKALDECK"
     gene            1768..1896
                     /locus_tag="OIDGCHHC_00079"
     CDS             1768..1896
                     /locus_tag="OIDGCHHC_00079"
                     /inference="ab initio prediction:Prodigal:002006"
                     /codon_start=1
                     /transl_table=11
                     /product="hypothetical protein"
                     /protein_id="Prokka:OIDGCHHC_00079"
```

```
                     /translation="MNANENNTSVNNENNTTINFEESIKEALRKGQEIVESGKGIR"
     gene            1897..2244
                     /locus_tag="OIDGCHHC_00080"
     CDS             1897..2244
                     /locus_tag="OIDGCHHC_00080"
                     /inference="ab initio prediction:Prodigal:002006"
                     /codon_start=1
                     /transl_table=11
                     /product="hypothetical protein"
                     /protein_id="Prokka:OIDGCHHC_00080"
                     /translation="MSIYKECEVCKTKINKLQNIWNIFSGKLEIKCKNCKSKYGVSSK
                     IIDWIYFLFFENLLLFPPIVILNILYLGRYFSKYGLFEMIFYLSIAILLSRKSVNIIL
                     LLLCKLKRLENDK"
ORIGIN
        1 agcaacaagt atagcaagca atcattcagg tttttttcat actccaagag tgggagatga
       61 agtgattgtt tctttttag atgatgatat agataaaccc tatgtgagct ctagtttgta
      121 taatgggact aatccaagct tagtaaatct tccttttaat gatcatcaaa cctccctttc
      181 ttctaaaacc ataggtgtta atgaagaagg ttataatgaa cttaccctct caaatataaa
      241 agataaagaa caaatttatt taaaagccca aaaggattat gatgaattag tgcaacataa
      301 tttcactcaa agaattttaa atgataaaga ttctaaggta gatggaattt ataatgaaag
      361 gattaaaaag gttcatactc aaaccataga tttggctaaa aatgtcaatg taggaggaga
      421 atacttaact aatgtaggct tatctaaaga taccatagta ggattaagca atactttaaa
      481 tgtaggagta gataataaag taagaatagc taaaaactct catgagtttg taggagaaaa
      541 caaagacata gaaataggtg caaatcaaaa tactattatc cataaagatg agataaggaa
      601 tgtgaaggga gaaaataaat tattaataga aaaatcttta acccaaacaa tagaaaaaga
      661 atttttcctt aatgtccatc aaaatttatc cgcccatata caagataaca catctttaaa
      721 gtctaattcc atgcaaacaa agatagaaga gcaatattct ttagaatcag aaaattctac
      781 ttttgacttt caaacagatt gtgaagtaaa agcaggcaat caaatcctcc atcaagtagg
      841 tgatactcaa attgttacca agaaagattg tgttataatt aaagcaggtg gagtagaagt
      901 gattatagac tctaatggac ttgtggttaa aggtggagag cttaaggcag agtaaaggat
      961 aagcaatgaa aaaacctttt tacaaactta aaagatttta tataccttgt ataataatta
     1021 ttataatatt tgccgtgctt gcaaaattac tttattctcc tttatatacg atttattggg
     1081 gaatgtatca ttatcctaaa gttcaactag aatttaaaaa ctttgaaaaa atgactttaa
     1141 atccaagccc taaggatatg attaaaattg tagatgatta ccaacctaag ctagaagatt
     1201 ttaaagattt aaatgtaaaa atgcaaaagg ctatttttga ttttaaggtg gctaaattat
     1261 ttggatttga ggataggtat tttgaagctt ttattaaaag ctgtgctggt aacttctttg
     1321 ttttacatgg taaagaacaa atttatttta attatcttaa tttcataagc ggtataaatt
     1381 caacttccaa tgaaaagcaa aaatatctaa acttaagagc atcaactaga gatttagaaa
     1441 gacaaatttt tgaagaaaaa cttaaattta taaaacatta tgaagaattt tatgattact
     1501 tagagggtgt aggttatttg gataagggaa cagaatatat aggtacggca atttctttta
     1561 aaacattgat tcaaaatgta tttttgtcta ataatgctca attatgctct tttgaagata
     1621 gaaacttaat gtttaaaaac atgaaagaaa attatgaaat atttaaaaat ttagatgtaa
     1681 aaaatataga tgatttaaag agaaatattt ataaaaaaat attaattgat atggaaaatc
     1741 tcctaaatga aacccaaaag gctttagatg aatgcaaatg aaaataatac cagtgttaat
     1801 aatgaaaata acaccactat caactttgaa gaaagcataa aagaagcttt aagaaaaggg
     1861 caagaaattg tagaaagtgg caaaggaata agataaatgt ctatttataa agaatgtgaa
     1921 gtttgtaaaa caaaaatcaa taaattgcaa aatatttgga atatattctc aggcaaatta
     1981 gaaataaaat gtaaaaattg caaaagtaaa tatggtgttt ctagtaaaat tatagattgg
     2041 atatattttc tttttttga aaatttatta ctctttccgc caattgtaat tttaaatatc
     2101 ctatatctag gtagatattt ttcaaaatat ggattatttg aaatgatatt ttatttatct
     2161 atagccattc ttcttagtag aaaatcagtc aatattattt tacttcttct ttgcaaatta
     2221 aaaaggctag aaaatgacaa ataaaggatt aatagaacat cttaaggatg caaaagatag
     2281 tgctgtagat ttttatatag ggctttatga ttttggagac ttcttagcta gaatttcagg
     2341 ttttaaagat tggcaagaaa aaagtgttgt tttcgatgat ttttataaaa aacaagcatt
```

```
     2401 tgaagaattg ggctttttag aagatgttct taaaaatgaa acttctagaa attttataat
     2461 agaacattta acaaaagata ccaatgaaag acctttatat tatttagcac cattaggagc
     2521 tctaacaata attagtcaaa caaccaaagt aaaaaaacta ttcataataa gcttaacctc
     2581 cttatctggg aaagccactt ccactgccca ctctctctac ccctactaca aaaacataca
     2641 aaattccttt accccttat gttctataga taataaaaat agtaatttgt taaaagaatt
     2701 taattttgat agtttttctt gtaattttta taataataat attttcaatc aaagtcttaa
     2761 gcatttaaat aatatacaaa atgaaaaaga atatatcaat gagtataaaa tattttttaga
     2821 aaatcttaat tattttaatc aagaatataa taattataag cttgtatcta tacaagtaat
     2881 caataattta aaaatagcct atatacaaaa gctatcttta caagatatta ataatattaa
     2941 tatcaattca gaactttatt tgctaaaagc tttatatttt tgtgaagatt atgttttatt
     3001 tgataaagat aagcaagctt tatttaataa aaacacaata aaagaattag aatttgattc
     3061 tagtttttat actatctttg tctctcacaa agccaaacta aacgatactt atttaaaagc
     3121 aagaaaagag ctttataaaa gcataaatga atttaaaaga cttggtttta aagatttaga
     3181 aaatgcttat caaacttata tccattcttt gattgtaaat aaagaaataa atagtaaaga
     3241 taaaacaata aataaaaatg aaaataaaga aataaataaa aatgaaaata aagaaataaa
     3301 taaaaatgaa aataaagaaa taaataaaaa tttaaatcaa gatgaaaatt caaatcagaa
     3361 aagcaatata aaagaaaatg caagtaacac aaaaaaagaa gattttaatc ttttatcttc
     3421 tcactctaaa aacctaaacc atttcatctt agaaaacaaa gataaaaata aaagaattat
     3481 ctttttaaat aatgcttcat cgatgtcaaa tttaatccat atttatgata atcattgtga
     3541 tgtat
//
LOCUS       OIDGCHHC_8              3158 bp    DNA     linear       12-APR-2021
DEFINITION  Campylobacter species strain strain.
ACCESSION
VERSION
KEYWORDS    .
SOURCE      Campylobacter species
  ORGANISM  Campylobacter species
            Unclassified.
COMMENT     Annotated using prokka 1.14.6 from
            https://github.com/tseemann/prokka.
FEATURES             Location/Qualifiers
     source          1..3158
                     /organism="Campylobacter species"
                     /mol_type="genomic DNA"
                     /strain="strain"
     gene            22..519
                     /locus_tag="OIDGCHHC_00081"
     CDS             22..519
                     /locus_tag="OIDGCHHC_00081"
                     /inference="ab initio prediction:Prodigal:002006"
                     /codon_start=1
                     /transl_table=11
                     /product="hypothetical protein"
                     /protein_id="Prokka:OIDGCHHC_00081"
                     /translation="MFKKLALFLLPVYLLLFLAGGCSYKYMDPQYYEFKKLCKDNSNK
                     MIVFNKDYLDLKKQFVQAMTNNSNIRIKNNGIKYFYNEKLNLEIQPSNWKEIETKSRE
                     IKLGIGKVKEKEIEAWYIDDKNNIKYQEFKRYLYYNYSIFLEGDEGAGFHFEYEEILD
                     CGDVG"
ORIGIN
        1 aaaactataa attttaaaac aatgtttaaa aaacttgctt tatttctttt acctgtatat
       61 cttttattat tcttagcagg aggatgttct tataaatata tggatccgca gtattatgag
      121 tttaagaagt tgtgtaaaga taatagtaat aaaatgatag tgtttaataa agattatttg
      181 gatttaaaaa aacaatttgt tcaagctatg acgaataatt ctaatattag aattaaaaat
```

```
      241 aatggtataa aatattttta taatgaaaaa ttgaatttgg aaattcaacc ttcaaattgg
      301 aaagaaatag aaactaaatc acgcgaaatt aaacttggaa taggaaaagt aaaagaaaaa
      361 gaaatagaag cttggtatat tgatgataaa aataacatta agtatcaaga atttaaacga
      421 tatttgtatt ataattatag tatattttta gaaggcgatg agggtgctgg gtttcatttt
      481 gaatatgagg aaatattaga ttgtggagat gttggataaa aaatttaaaa gcataatgag
      541 cttaagatag aaatataaaa tgagcaaaaa cattaaaaca caagaagcta aattagactt
      601 aattgctaaa ttcttagact atgctaatgt agctgatgct agttatgcga tgttgcattg
      661 gattgattat gtaaaaaaag aaaatgacaa aaaacatgaa aatggtgata cccaaaaact
      721 aggcgataaa cacaataatc aaaattctac ttatgcaaga gctatacaag ctcgttttga
      781 acaaaataaa atagttaaaa tagaacctaa atattgtatt tctcttataa atacttgttt
      841 tgatagtaaa gaaataacat tggataatga tattagtagg gtaggattaa atgatgcact
      901 tagtaaaaga actattgatt ttgtaaatag atttaaactt ttaaaacatc agcccaacac
      961 tacaagtggc tttagtgcta ctttgtttga agatactaaa gataataatc aaaagattat
     1021 agtcatacga ggaacagagc ctacaagtaa ttttagtgta gatattttgg atgctgatgt
     1081 tgatttagct ttaggcaaag ttccctataa tcaatatctt gatatgatta aattttattc
     1141 tgagtgtgtt aaagaatttc caaatattat aaaagacaaa ggtttggtaa ttgtaggtca
     1201 ttctttaggc ggagctttgg cacaactact tactctttca ttagcgagtg ttaattctag
     1261 tgctaatgtt aaagaaatct acactttcaa ttcgcctggt gcaaaagaac taaaagcttt
     1321 aaatttaaat caaatttaca gaatagatgg aaaaattatc aattctgata ataaagagca
     1381 ggcattgttt tatcaaataa gatcttataa atatcaaaaa agtatagaaa taaacttaat
     1441 aggatatgat tctaatttat ttcaaaatat aaaatcatac tttcatgata aaacaaactt
     1501 aaaagaacat tatatcttta taaaaacaaa tataatttat agtaaatcaa cgaattattt
     1561 ttatgatatt tttgaaattg atgaagtatt tataaattgt gttaatcagc tccttgctaa
     1621 tcttaatatc aaaaatattt tagctacaag tgataatact tatcatattg aaacagatac
     1681 tgattccgat gcttctaata caaaaggggt gatacaagat ttaggagtag atatagacgg
     1741 gaagcattat attgttaatt taggggatcg attttgggat tctcattttt tagagcctac
     1801 tatcatagaa ttaaattata ttttaaatct tatgaaaaat aaagaaattg ataacctttt
     1861 agagtataat ataaataagg atgaagagct atggactttt atcaaatacc ataataatgt
     1921 tttggctacc gcaagaactc gttatgatga tcgtattttg tcatatttta gcattcctaa
     1981 actatctaat gatttttcaa attctttgga atatttgcaa attcattatt taccaaaaga
     2041 acccaaaaaa cctttatttg cagcaaatat tcaaggtttc tcatctacgc aattgcaagc
     2101 tttggaggaa tataaaataa aaaaagctaa atatgatgag gagttaaaat ggtatgaact
     2161 ttataaagta aaatatgaaa gagaacaaca aatttttaat cagaaattta aagagtattt
     2221 ttcagaaata atttttatc aaaatagact ctattctcat aaaaaaacac aagagaaaat
     2281 tatattaaat gctataatac aacaaattaa tcaaaaagat ataacatatt ttaatttgta
     2341 ttctgtgata gaatttttaa aagaaaacaa ggcttattat aaatatatag atttaaatga
     2401 aatccaagat ttaattttaa atgatttaga taataaattg ggttattttt attgccttta
     2461 tgtatgtata aatttaatag tattaagaga agataaaaat acttatttca cccaagctac
     2521 cgctataaac aatctaggct ataattctac tttcacaaag atcttcatcc tagataaaga
     2581 aaaccttaat aatacctatc ttgatgctag aaaaaggctt tataaatcta tcaatacttt
     2641 aaaagaaaaa agaaatgaaa aaataaaaga tatccaagcc tatttagata aagaaatttc
     2701 cttacaagat aatctaaata atcaaagctt taatactaag gaaaatgatg ttaatttaat
     2761 tttaaacgaa gtatttaaca tagagcaatt ttatgataaa aataccaata ccatagtatt
     2821 aaaaacaaat aatataaaaa tcatattctt agataaagta aatttacaag atttgcaaga
     2881 taattctaac aatacttctt tcatctctat gtcaaattta atccatattt atgataatca
     2941 ttgtgatatt ttcacaaaag atagaagtgt tttggatata aagaacatag aagaaaaata
     3001 tcaaatagat tttaaaagtt tagatacaaa aatcttccta aactccaccc tccttacagg
     3061 ctcaaatgaa ctttctaata atccttttta ttttggagaa ttagatcaag ataatactat
     3121 aaaacaagat atacccagtt attatttttc tcctaaag
//
LOCUS       OIDGCHHC_9              2845 bp    DNA     linear       12-APR-2021
DEFINITION  Campylobacter species strain strain.
ACCESSION
VERSION
```

```
KEYWORDS    .
SOURCE      Campylobacter species
  ORGANISM  Campylobacter species
            Unclassified.
COMMENT     Annotated using prokka 1.14.6 from
            https://github.com/tseemann/prokka.
FEATURES             Location/Qualifiers
     source          1..2845
                     /organism="Campylobacter species"
                     /mol_type="genomic DNA"
                     /strain="strain"
     gene            complement(77..217)
                     /locus_tag="OIDGCHHC_00082"
     CDS             complement(77..217)
                     /locus_tag="OIDGCHHC_00082"
                     /inference="ab initio prediction:Prodigal:002006"
                     /inference="similar to AA sequence:RefSeq:CKG86746.1"
                     /codon_start=1
                     /transl_table=11
                     /product="hypothetical protein"
                     /protein_id="Prokka:OIDGCHHC_00082"
                     /translation="MRCIKIFKDYQKFTPYGLDFKLYSKDFITKLDFKDKAYNVLYNT
                     GV"
     gene            complement(204..698)
                     /locus_tag="OIDGCHHC_00083"
     CDS             complement(204..698)
                     /locus_tag="OIDGCHHC_00083"
                     /inference="ab initio prediction:Prodigal:002006"
                     /inference="similar to AA sequence:RefSeq:CKG86821.1"
                     /codon_start=1
                     /transl_table=11
                     /product="hypothetical protein"
                     /protein_id="Prokka:OIDGCHHC_00083"
                     /translation="MGDSIMLSTSLGLVGFIFAFTNIKFYLTTLSVLIVFSILNYWIK
                     INIKEFVKSYFSIYFIIFFTAIVIIFYLTYGIETSKDLALLSSLEAKELSPQEYKARN
                     YFGFLTCMTMFFLVITFIILPCLLQAYLFKCYSTEEKRKKLSWFIGFVCNIFLLIASV
                     YAMY"
     gene            complement(699..2234)
                     /locus_tag="OIDGCHHC_00084"
     CDS             complement(699..2234)
                     /locus_tag="OIDGCHHC_00084"
                     /inference="ab initio prediction:Prodigal:002006"
                     /inference="similar to AA sequence:RefSeq:CKG86849.1"
                     /codon_start=1
                     /transl_table=11
                     /product="hypothetical protein"
                     /protein_id="Prokka:OIDGCHHC_00084"
                     /translation="MDEGLFWENLVSTEYIGAMQEKISNESFSNNISFRSLEYDATVL
                     PAELECCYNEFKYHQKSNNEIAALGWAVSYQLQALWQNSPKIDTIQNYHLPIKQNKPN
                     NPQAEQRLKEIQNFLQSKGIEPLILHYTPYPKNTQTQEMKRLRQNSERFSRHVDNKTN
                     EVREEKRSIKFYTTLDWDKFYSHFPIIKRLSKKFHTKEDIYTQNFTKQIEIILQKTHN
                     ILTLKNLSQGEYKFDFAIDIKENRSDKNEESYIIIKENDEMPKDYKRVLACKPYELDT
                     LNCQYIGAGDDEFFRQIRQNLEVNIARQFDIEFSHSWLIKTYLRFNPGTRKHMLTYDT
                     FEIGKQTYDELMQDLSLYACSGKFSFNFVPSKLSIKKEIQGDREKVLYRVDEMLVYVY
```

```
                     DSFDFLDQGHEFDDEGNFIKLGQPVGAWDFNEKSFSIWGSTRQMETYKPKEYLFFIPQ
                     ITFADILQSPKTKQYYLYNQDYQDYQKFTPYGLDFKLYSKDFITKLDFKDKAYNVLYD
                     TGV"
     gene            complement(2436..2717)
                     /locus_tag="OIDGCHHC_00085"
     CDS             complement(2436..2717)
                     /locus_tag="OIDGCHHC_00085"
                     /inference="ab initio prediction:Prodigal:002006"
                     /codon_start=1
                     /transl_table=11
                     /product="hypothetical protein"
                     /protein_id="Prokka:OIDGCHHC_00085"
                     /translation="MLLWFGYFFIPLEWQSSFKEFESLCENLKNREVIYNQEIYDRVK
                     GKNNAIRVKFVENNIGLRIQKVEYQYYKIVTNILYKNYYTYGWLYMYVC"
ORIGIN
        1 aaaattttat attagtaaaa gcaaaaataa atccaaataa accccaaaaa atagaaaatg
       61 taatatcttc tcccatttaa actcctgtat tataaagcac attataagct ttatctttaa
      121 aatcaagctt ggtgataaaa tctttactat aaagtttaaa atccaaacca taaggagtga
      181 atttttggta gtctttaaag attttaatac atcgcataca cactcgcaat taataaaaag
      241 atattgcaca caaaccctat aaaccaactt aattttttgc gtttttcttc tgtgctataa
      301 catttaaata aataagcttg caataaacaa gggagtataa taaatgtgat tactaaaaaa
      361 aacatagtca tacaagtcaa aaatccaaaa taattacgtg ctttatattc ttgtggtgat
      421 aattctttag cttctagaga gctaagcaat gcaagatcct tagaagtttc aataccatag
      481 gtaagataaa atatgataac aatagctgta aaaaagataa taaaatatat tgaaaaataa
      541 gattttacaa attcttttat attaatctta atccaataat ttaaaataga aaagacaata
      601 agaacactta aagtggtaag ataaaatttt atattagtaa aagcaaaaat aaatcctacc
      661 aaacctaaag aagtagaaag cataatacta tctcccattt aaactcctgt atcataaagc
      721 acattataag ctttatcttt aaaatcaagt ttagtgataa aatctttact ataaagttta
      781 aaatccaaac cataaggagt gaatttttgg tagtcttggt aatcttgatt gtaaagatag
      841 tattgtttgg tttttgggga ttgtagaata tcagcaaaag ttatttgtgg tataaaaaat
      901 aaatattcct ttggtttata agtttccatc tgtcttgtag aaccccaaat gctaaaactc
      961 ttttcattaa aatcccaagc cccaacaggt tgtcctaatt tgataaaatt tccctcatca
     1021 tcaaattcat gtccttgatc taaaaaatca aagctatcat aaacataaac aagcatttca
     1081 tcaacgcggt ataatacctt ttccctatct ccttgaattt ctttttaat gcttaatttt
     1141 gaaggaacaa aattaaaaga aaatttacca ctacaagcat aaagagatag atcttgcata
     1201 agttcatcat aagtttgttt cccaatctca aatgtatcat aagttaacat atgttttctt
     1261 gttcctggat tgaatcttaa atatgtttta ataagccaag aatgagaaaa ttctatatca
     1321 aattgccttg caatatttac ttctaaattt tgccgaattt gtctgaagaa ctcatcatca
     1381 cccgcaccta tatattgaca atttaaagta tcaagctcat aaggtttgca agctaaaacg
     1441 cgtttataat cttttggcat ttcatcattt tctttaatga tgatataaga ttcttcattt
     1501 ttatcgcttc gattttcttt tatatcgatt gcaaaatcaa atttgtattc tccttgggat
     1561 aaatttttta atgtcaaaat attatgagtt ttttgtaaga ttatttctat ttgctttgta
     1621 aaattttgag tgtatatgtc ttcttttgta tgaaattttt tacttagtct ttttattata
     1681 ggaaaatgag aataaaactt atcccaatct agcgtagtgt aaaatttaat agaacgcttt
     1741 tcttctctta cttcatttgt tttattatct acatggcgcg aaaaacgctc tgaattttgc
     1801 cttagtcttt tcatctcttg tgtttgtgtg tttttggat aaggggtata gtgcaaaata
     1861 aggggttcta tacctttact ttgtaagaaa ttttgtattt cttttagtct ttgttctgct
     1921 tggggattat taggcttatt ttgttttata gggagatgat aattttgtat tgtatctatc
     1981 ttagggctat tttgccacaa tgcttgaagt tgataactaa cagcccaacc caatgctgct
     2041 atttcattat tgcttttttg atggtactta aattcattgt agcaacactc taattccgca
     2101 ggcaaaactg ttgcatcata ttctagagat ctaaaactta tattatttga aaatgattca
     2161 tttgatattt tctcttgcat tgctcctata tattctgttg aaacaagatt ttcccaaaaa
     2221 agtccttcat ccatcatttt atcctattgt ttttatattt tttagaaatt tttcaagtat
     2281 gttttcattt aaatttacaa ataactaatc cagcatattc taacatattt ataaagtttc
```

```
     2341 atatatttgg cttgtttttt atgcattagt agtctctttc ttttattttt ttcttatatt
     2401 catcaatgcc atcacaacct aatattcttc catttttagc aaacatacat atatagccaa
     2461 ccatatgtat agtaattttt atataaaata tttgttacaa ttttataata ttgatattct
     2521 actttttgta tccttaatcc tatattgttc tcaacaaatt ttactcttat agcattattt
     2581 ttgcctttaa ctctatcata aatctcttga ttataaatca cttctctatt ttttaaattc
     2641 tcacacaaac tctcaaattc tttaaaacta ctttgccatt caagaggtat aaaaaagtat
     2701 ccgaaccaaa gaagcataaa ggtcttaatt ttttatagtt ttcttcaccc atatatgctt
     2761 taaattgatc ttcatagtat gcaaattcgg gtttatcgtc gaaattaaaa tcataatctg
     2821 ggtcttttac atcataatta taatc
//
LOCUS       OIDGCHHC_10             2521 bp    DNA     linear       12-APR-2021
DEFINITION  Campylobacter species strain strain.
ACCESSION
VERSION
KEYWORDS    .
SOURCE      Campylobacter species
  ORGANISM  Campylobacter species
            Unclassified.
COMMENT     Annotated using prokka 1.14.6 from
            https://github.com/tseemann/prokka.
FEATURES             Location/Qualifiers
     source          1..2521
                     /organism="Campylobacter species"
                     /mol_type="genomic DNA"
                     /strain="strain"
     gene            74..571
                     /gene="trxB_3"
                     /locus_tag="OIDGCHHC_00086"
     CDS             74..571
                     /gene="trxB_3"
                     /locus_tag="OIDGCHHC_00086"
                     /inference="ab initio prediction:Prodigal:002006"
                     /inference="similar to AA sequence:RefSeq:CKG87041.1"
                     /codon_start=1
                     /transl_table=11
                     /product="thioredoxin reductase"
                     /protein_id="Prokka:OIDGCHHC_00086"
                     /translation="MLPFIDEIMGVDWVIDLNRYKFALDEEGRIIWALYDDIEKGKLK
                     DPRDVDSTSESRKEFDHYMDGYKNGMVTRFDVDIRNERDERSAKLTMDTLVLSAKLAA
                     LTPPQGYPNAPRYYSPERLEIIYKRHKLDKLLDPRIPAIYRYNFPRELRAKILKFGEE
                     NGIKD"
     gene            complement(576..1475)
                     /locus_tag="OIDGCHHC_00087"
     CDS             complement(576..1475)
                     /locus_tag="OIDGCHHC_00087"
                     /inference="ab initio prediction:Prodigal:002006"
                     /inference="similar to AA sequence:RefSeq:CKG87069.1"
                     /note="Uncharacterized conserved protein%2C contains FHA
                     domain"
                     /codon_start=1
                     /transl_table=11
                     /product="hypothetical protein"
                     /protein_id="Prokka:OIDGCHHC_00087"
                     /translation="MIEKEELGITIENYDECISGSKAFVFDTKGGTIGSGDDCTFRIQ
```

```
                     DKLEQIKNTHAIVSYEEGSFTIAAFEDSDIYYNKSFSRMPSGYEIIISIGDIFKIGNL
                     EFRFVDAKSIEESIKENKKYLEDIEKRNHFDEIEIEPRGKTSINFDKNEELKEILKNN
                     NYKFIEEEKADDSFLKEIIQRDPNSLGYENILKSLVKNLKDIKTKQQSSKLNNDYSPI
                     SIKDFESIINNIPLIKSTRLINILALSLIAKELYTPIFEEMEENMFIKYLEAAIQNNI
                     KEDKILFENLTLKALEKYIKDFE"
     gene            complement(1472..2518)
                     /locus_tag="OIDGCHHC_00088"
     CDS             complement(1472..2518)
                     /locus_tag="OIDGCHHC_00088"
                     /inference="ab initio prediction:Prodigal:002006"
                     /inference="similar to AA sequence:RefSeq:CKG87105.1"
                     /note="Uncharacterized protein conserved in bacteria"
                     /codon_start=1
                     /transl_table=11
                     /product="hypothetical protein"
                     /protein_id="Prokka:OIDGCHHC_00088"
                     /translation="MDLYAYQQSCVKTFAYFLFLVQYYPSLSFLENLKEFCKYHRITN
                     VSNKIIDFSSNNNQSAEEKIAYALDGNKDANDPFTVFQMSIKKLPSDLERYYNQLSDH
                     SWNLIENYGISLFNTAWINEVYNPFINDIAPFYPFNEESTANLSMDSFKTFFGRNGVL
                     NSFYKKYLNNVLVKRKNNYSINSQFASKLNFSKEFLDFITNAGNLSSLMLNANDNIRV
                     NFTIQSLDLSADFSFVKLGYNNKNIQYDHTLDQTLQIVAEEFNNGTSLNFTAYNYSNP
                     NLNYTKSYKGEWAWYKFIKDNKNNSTYSIMFNNNKNLYFDFEIINGASDLNNIVYILN
                     NLKIVENITGVNKQ"
ORIGIN
        1 gatacataaa aatcctttaa gggctaaatt agtaaaagat ttagctaaaa ctatacagcc
       61 tgatgaattt ggtatgctac cttttataga tgagattatg ggagtagatt gggtgattga
      121 tttaaatcga tataaatttg ctcttgatga agaaggtaga atcatttggg ctttatatga
      181 tgatattgaa aaaggtaaat taaaagatcc aagagatgtg gattctactt ctgaaagcag
      241 gaaagaattt gatcattata tggatggata taaaaatgga atggtaacta gatttgatgt
      301 agatattaga aacgaaagag atgagagatc tgctaaactg actatggata ccctagtatt
      361 aagtgccaaa ctcgcagccc tcactcctcc tcaaggttat cctaatgctc caagatacta
      421 tagccctgaa agactagaaa ttatttataa aagacataag cttgataaac tccttgatcc
      481 aagaatccct gctatttata gatataattt cccaagagaa cttagagcta agatactcaa
      541 atttggcgaa gaaaatggca ttaaagatta aaaaatcact caaaatcttt aatatacttt
      601 tccaatgctt ttagggtaag attctcaaaa agaatcttat cttctttgat attgttttgt
      661 atggcagctt caagatattt aataaacata ttttcttcca tttcttcaaa gatgggagtg
      721 tataattctt ttgcgattaa actaagggct aaaatattaa tcaatcttgt ggatttgatt
      781 aaaggtatat tattaataat actttcaaaa tctttaatac tgataggaga ataatcatta
      841 tttaatttag agctttgttg ttttgtttta atatctttta aattttttac caaacttttt
      901 agaatatttt catacсctaa tgaatttgga tctctttgaa tgatttcttt taaaaagcta
      961 tcatctgcct tttcttcctc gataaactta taattattat tttttaaaat ctctttcaac
     1021 tcttcatttt tgtcaaaatt tatacttgtt tttcccctag gttcaatttc aatttcatca
     1081 aaatgattgc gtttttcaat atcttctaag tattttttat tttctttgat actttcttca
     1141 atgctttttg catccacaaa tctaaattct aaattaccga ttttaaaaat atcgcctatg
     1201 ctaattatta tttcatagcc acttggcatt ctagaaaagg acttgttata ataaatgtca
     1261 gaatcttcaa aagctgcaat tgtaaaagat ccttcttcat aagatactat ggcatgggta
     1321 tttttgattt gctctagttt atcttgaata cgaaaagtac aatcatcacc acttcctata
     1381 gttccaccct ttgtatcaaa tacaaatgct ttagaaccac taatgcattc gtcataattc
     1441 tcaatagtaa ttcctaattc ttctttttct atcattgttt atttacccct gtgatatttt
     1501 ctactatttt aagattattt aaaatataaa ctatattatt taaatcacta gcaccgttaa
     1561 taatttcaaa gtcaaaatat aagtttttat tattattaaa cataatgctg tatgtagaat
     1621 tgtttttatt atcttttata aacttatacc atgcccattc tcccttatat gattttgtat
     1681 aatttaaatt aggattggag tagttgtagg ctgtaaaatt taaacttgtt ccattattga
     1741 attcttctgc tacaatttgt agggtttgat caagagtatg atcgtattga atatttttat
```

```
     1801 tgttgtagcc aagctttaca aaggaaaaat ctgcacttaa atcaaggctt tgaatggtaa
     1861 aattaactct tatattatca ttagcattca gcatgagact tgagagattg cctgcattgg
     1921 tgataaaatc taaaaattct ttagagaaat ttaattttga agcaaattga gaattaatag
     1981 aatagttatt ttttcttttt actaaaacat tattcaagta ttttttataa aaactattta
     2041 atactccatt tctgccaaaa aatgttttaa agctatccat actcagatta gctgtgcttt
     2101 cttcattaaa aggataaaat ggtgcaatat cattgataaa tggattatac acttcgttaa
     2161 tccaagcagt attaaataat gaaataccat agttctcaat caaattccaa gaatgatcag
     2221 aaagctgatt gtaatacctt tctaaatcac taggtaattt ttttatactc atttgaaata
     2281 cagtaaaagg atcattggca tctttattgc catcaagagc atatgcgatt ttttcttcgg
     2341 cgctttggtt attgttggag ctaaagtcta taattttatt ggacacattt gtaatacgat
     2401 gatacttaca aaactccttt aaattttcaa gaaaagatag agatggataa tattgcacta
     2461 aaaacaaaaa atatgcaaat gtctttacac aagattgttg atatgcatat aagtccatat
     2521 t
//
LOCUS       OIDGCHHC_11             2464 bp    DNA     linear       12-APR-2021
DEFINITION  Campylobacter species strain strain.
ACCESSION
VERSION
KEYWORDS    .
SOURCE      Campylobacter species
  ORGANISM  Campylobacter species
            Unclassified.
COMMENT     Annotated using prokka 1.14.6 from
            https://github.com/tseemann/prokka.
FEATURES             Location/Qualifiers
     source          1..2464
                     /organism="Campylobacter species"
                     /mol_type="genomic DNA"
                     /strain="strain"
     gene            complement(483..1760)
                     /gene="trxB_4"
                     /locus_tag="OIDGCHHC_00089"
     CDS             complement(483..1760)
                     /gene="trxB_4"
                     /locus_tag="OIDGCHHC_00089"
                     /inference="ab initio prediction:Prodigal:002006"
                     /inference="similar to AA sequence:RefSeq:CKG86610.1"
                     /codon_start=1
                     /transl_table=11
                     /product="thioredoxin reductase"
                     /protein_id="Prokka:OIDGCHHC_00089"
                     /translation="MSDTYMIYAPNGLGVEVDKKTKEIYFAQSVDPVGKYTKEYTRVF
                     FKAWEIKQNSPYKDYQPIYLDPNFYTGQKSTLVEFKEWQSIYLKEPIKGSIAPWTKAE
                     KAYYKSLKTKRERYKYLIIRSGIRSTVIDIPYDAYCNVDEKGNLINKDYKELYKEVEA
                     NRGMANMHKGWLFMAEWELAAGILGDIKGFIGALQLSMTGFKARTQAINFLLIQLGHE
                     QGFKSLYDSYAYRDLTDGIHKNPLKAQMLKDFSKNPPYDELGMLPFLDELIGVDWVID
                     VNKYQFAYDEKGRVNDALKDDVEKGILKDPRDIDSTPESRWGFDKEMDAYKNGMRTRF
                     DGDNPNHWSKEQVERFNDTLILCAKLAALTPPQGYPNAPYYYSPERLEFIYKKHKLDK
                     LLDPRIPAIYRYNFPRELRAKILKFGEENGIKD"
     gene            complement(1842..2387)
                     /gene="trxB_5"
                     /locus_tag="OIDGCHHC_00090"
     CDS             complement(1842..2387)
                     /gene="trxB_5"
```

```
                     /locus_tag="OIDGCHHC_00090"
                     /inference="ab initio prediction:Prodigal:002006"
                     /inference="similar to AA sequence:RefSeq:CKG86610.1"
                     /codon_start=1
                     /transl_table=11
                     /product="thioredoxin reductase"
                     /protein_id="Prokka:OIDGCHHC_00090"
                     /translation="MLKDFSKNPPYDELGMLPFLDELIGVDWVIDPNDYYFVYDGAKG
                     RVNDALKDDVEKGTLKDPRDVDSTPESREEFIDNFERYGNGMRERYTLEISNDTPETS
                     ANIWLETMLLEAKIKALTPPQGYPSAPYYYSPERLEWIYKNHNLDRLLDPRIPAIYRY
                     NFSRELRAKILKFGEENGIKD"
ORIGIN
        1 agggtatatc tataaccaca gatcttaatc cacttcttat tgccaaatac ttacatcttt
       61 ctcttttagt ttttaaagat ttataataag ctttttctgc tttagtccaa ggagctatgc
      121 ttcctttaac aggatctttt aaatatatgc tttgccattc tttaaattca agcaaagtag
      181 atttttgccc tgtataaaag ttaggatcta gatatattgg tttatagtct ttgtaggggg
      241 agtttttcat aatataataa gattcaaaaa ccgctttgga atattcttga gtataattgc
      301 ctgtgggttt aatattttct ttaaatagta ttttattggt atctttttcc acatctaatg
      361 ttaatccatt gggtgtataa atctcataag tttcgctcac tatttatcct ttcttaaatt
      421 tatcccatgt ataaaccatc ttctatgatt atagtagtgg ctagtctgcc tatgaggtta
      481 ctttaatctt taatgccatt ttcttcgcca aatttgagta tcttagctct aagttctctt
      541 gggaaattat atctataaat agcagggatt cttggatcaa ggagtttatc aagcttgtgt
      601 tttttgtaaa taaattctaa tctttcagga gagtaataat aaggtgcatt aggatagcct
      661 tgaggaggag tgagggctgc gagtttggca cataaaatca aagtatcatt aaatctctct
      721 acttgctctt tactccaatg atttggatta tctccatcaa atctagttct cattccattc
      781 ttataagcat ccatctcttt gtcaaacccc catctactct ctggagtaga atctatatct
      841 cttggatctt tgagtattcc tttttcaaca tcatctttta gagcatcatt aactcttcct
      901 ttttcatcat aggcaaattg atatttattc acatctatca cccaatccac tcctataagt
      961 tcatctagaa aaggtagcat acccaattca tcataaggag gatttttaga aaagtctttt
     1021 agcatttgag ctttaagagg gtttttgtgt atgccatctg ttaaatctct atatgcataa
     1081 gaatcataaa gacttttaaa accttgttca tgtcctagtt gtatgagtaa aaaatttatt
     1141 gcttgagttc ttgctttaaa tcctgtcata gaaagttgta aggctcctat aaatcctttt
     1201 atatctccta aaattcctgc tgctaattcc cattcagcca taaagagcca acctttatgc
     1261 atattagcca tacctctatt ggcttctact tctttgtaaa gttctttata gtctttattg
     1321 attaaatttc ctttttcatc tacattacaa taagcatcat aaggtatatc tataacagta
     1381 cttcttatac cacttcttat aattaaatat ttatatcttt ctcttttagt ttttaaagat
     1441 ttataataag ctttttctgc tttagtccaa ggagctatac ttcctttgat aggttctttt
     1501 aaatatatgc tttgccattc tttaaattct actagagtgg atttttgccc tgtataaaag
     1561 ttaggatcta gatatattgg ttgatagtct ttgtaggggg agttttgttt aatttcccaa
     1621 gctttaaaaa aaactctagt atattcttta gtatatttac ctacaggatc aacgctttgt
     1681 gcaaaatata tttcttttgt ttttttatct acctctactc ctaatccatt aggagcatat
     1741 atcatataag tatcactcat cttttatcct ttcttaattt aacccatata taaaccatct
     1801 tctatgatta tagtagtggc tagtctgcct atgcaattac tttaatcttt aatgccattt
     1861 tcttcaccaa attttagtat cttagctcta agttcccttg agaaattata tctataaatt
     1921 gcagggattc taggatcaag aagcctatct aaattatgat ttttataaat ccattctaat
     1981 ctttcaggag agtaataata aggtgcacta ggatagcctt gagggggagt tagggcttta
     2041 attttagctt ctaaaagcat tgtttctagc caaatattag cagatgtttc aggagtatca
     2101 ttgcttattt ccaaagtata gcgctccctc attccattcc cataacgctc gaagttatca
     2161 atgaattctt ccctactctc tggagtggaa tctacatctc ttggatcttt gagtgttcct
     2221 ttttcaacat catcttttag ggcatcatta actcttcctt ttgctccatc ataaacaaaa
     2281 taataatcat ttggatctat cacccaatcc actcctataa gttcatctag aaaaggtagc
     2341 atacccaatt catcataagg aggattttta gaaaagtctt ttagcatttg agctttaaga
     2401 gggtttttgt gtatgccatc tgttaaatct ctatatgcat aagaatcata aagactttta
     2461 aaac
```

```
//
LOCUS       OIDGCHHC_12             2262 bp    DNA     linear       12-APR-2021
DEFINITION  Campylobacter species strain strain.
ACCESSION
VERSION
KEYWORDS    .
SOURCE      Campylobacter species
  ORGANISM  Campylobacter species
            Unclassified.
COMMENT     Annotated using prokka 1.14.6 from
            https://github.com/tseemann/prokka.
FEATURES             Location/Qualifiers
     source          1..2262
                     /organism="Campylobacter species"
                     /mol_type="genomic DNA"
                     /strain="strain"
     gene            370..510
                     /locus_tag="OIDGCHHC_00091"
     CDS             370..510
                     /locus_tag="OIDGCHHC_00091"
                     /inference="ab initio prediction:Prodigal:002006"
                     /inference="similar to AA sequence:RefSeq:CKG86746.1"
                     /codon_start=1
                     /transl_table=11
                     /product="hypothetical protein"
                     /protein_id="Prokka:OIDGCHHC_00091"
                     /translation="MRCIKIFKDYQKFTPYGLDFKLYSKDFITKLDFKDKAYNVLYDT
                     GV"
     gene            511..1020
                     /locus_tag="OIDGCHHC_00092"
     CDS             511..1020
                     /locus_tag="OIDGCHHC_00092"
                     /inference="ab initio prediction:Prodigal:002006"
                     /inference="similar to AA sequence:RefSeq:CKG86768.1"
                     /codon_start=1
                     /transl_table=11
                     /product="hypothetical protein"
                     /protein_id="Prokka:OIDGCHHC_00092"
                     /translation="MGEDITLSIFWGLVGFIFRLVTIDTILYITALSVLIVFSIINYW
                     IKINIKEFVKSYFLVYFIIFFTAIIIIFYLSYDLETSRGYVMLGPPEVEELSPQEYIV
                     YKCSKFLIFINFIFFTITFIIFPCLLQAYLFKRYSTEEKRKKLSWFIGFLCNIIILIA
                     SAYLMYIFI"
     gene            1139..1645
                     /locus_tag="OIDGCHHC_00093"
     CDS             1139..1645
                     /locus_tag="OIDGCHHC_00093"
                     /inference="ab initio prediction:Prodigal:002006"
                     /inference="similar to AA sequence:RefSeq:CKG86719.1"
                     /codon_start=1
                     /transl_table=11
                     /product="hypothetical protein"
                     /protein_id="Prokka:OIDGCHHC_00093"
                     /translation="MGDSITLSIIMGMLFSILYFVIFIKNYIIIALIVLIVFSILNYW
                     IKINIKEFVKSYFLISFIIFFTAIVIVFYLTYDLETSRGLVLLGPPEIEELSPQEYKV
```

```
                     HGYFGFLFFTSIGFCTITFFILPCLLQAYLFKCYSTGEKRKKLSWFIGFVCNPILITS
                     MYVIYENL"
     gene            1632..1763
                     /locus_tag="OIDGCHHC_00094"
     CDS             1632..1763
                     /locus_tag="OIDGCHHC_00094"
                     /inference="ab initio prediction:Prodigal:002006"
                     /inference="similar to AA sequence:RefSeq:CKG86746.1"
                     /codon_start=1
                     /transl_table=11
                     /product="hypothetical protein"
                     /protein_id="Prokka:OIDGCHHC_00094"
                     /translation="MKIFKDYQKFTPYGLDFKLYSKDFITKLDFKDKAYNVLYNTGV"
ORIGIN
        1 ttttaaatta ttggatcaag attaatataa aagaatttgt aaaatcttat tttttaatat
       61 cttttattat ctttttttaca gctattgtta tcgtatttta tcttacttat gatcttgaaa
      121 cttctagagg gcttgtatta cttggtcctc cagaagttga aaaattatca ccacaagaat
      181 ataaagcaca taattatttt ggatttttaa gtggtataac tattattttt ttagcaatca
      241 catttttttat actcccttgt ttattacaag cttatttttt taaatgctat agcacaggag
      301 aaaaacgcaa aaaattaagt tggtttatag gatttgtgtg caatatcttt ttattaacgg
      361 cgagtgtgta tgcgatgtat taaaatcttt aaagactacc aaaaattcac tccttatggt
      421 ttggatttta aactttatag taaagatttt atcactaaac ttgattttaa agataaagct
      481 tataatgtgc tttatgatac aggagtttaa atgggagaag atattacact ttctattttt
      541 tggggtttgg taggatttat ttttaggctt gttactattg atacaatact ttatattaca
      601 gccttaagcg ttcttattgt cttttctatt ataaattatt ggattaaaat taatataaaa
      661 gaatttgtaa agtcttattt tttagtatat tttattatct tttttacagc tattattatc
      721 atattttatc ttagctatga tcttgaaact tctagaggat atgtaatgct cggtcctcca
      781 gaagttgaag aattatcacc acaagaatat atagtatata agtgttctaa atttttaatc
      841 tttataaatt ttattttttt tacaatcaca tttattatat ttccttgttt attgcaagct
      901 tatttattta aacgctatag cacagaagaa aaacgcaaaa aattaagttg gtttataggg
      961 tttttatgca atattattat attaattgca agtgcatatt tgatgtatat atttatttaa
     1021 agactaccaa aaattcactc cttatggttt ggattttaaa ctttatagta aagattttat
     1081 cactaaactt gattttagag ataaagctta taatgtgctt tataatacag gagtttaaat
     1141 gggagatagt attacattgt caattattat gggaatgtta ttttctattc tttattttgt
     1201 aatttttata aaaaactata ttataatagc tttgattgtc cttattgtct tttctatttt
     1261 aaattattgg atcaagatta atataaaaga atttgtaaaa tcttattttt taatatcttt
     1321 tattatcttt tttacagcta ttgttatcgt attttatctt acttatgatc ttgaaacttc
     1381 tagagggctt gtattacttg gtcctccaga aattgaagaa ttatcaccac aagagtataa
     1441 agtacatggt tatttcggat ttttattttt tacaagtatt ggcttttgta caatcacatt
     1501 ttttatactt ccttgtttat tacaagctta tttatttaaa tgttatagca caggagaaaa
     1561 acgcaaaaaa ttaagttggt ttatagggtt tgtgtgtaat cctatattaa ttacaagtat
     1621 gtatgtgata tatgaaaatc tttaaagact atcaaaaatt cactccttat ggtttggatt
     1681 ttaaacttta tagtaaagat tttatcacca agcttgattt taaagataaa gcttataatg
     1741 tgctttataa tacaggagtt taaatgggag atagtattac gctttctatt tttgtaggtt
     1801 cattgctatc cattgctgtt atcacaaaat tttatcttat agctttatct gtgcttattg
     1861 tcttttctat tttaaattat tggattaaga ttaatataaa agaatttgta aaatcttatt
     1921 ttttaatatc ttttattatc ttttttacag ctattgttat cgtattttat cttacttatg
     1981 atcttgaaac ttctagggga cttgtattac ttggtcctcc agaagttgaa aaattatcac
     2041 cacaagaata taaagcacat aattatttta aatttttaac ctttacaact atgttttttt
     2101 tggcaatcac tttttttata ctcccttgtt tattacaagc ttatttattt aaatgttata
     2161 gcacaggaga aaaacgcaaa aaattaagtt ggtttatagg gtttgcgtgt aatatctttt
     2221 tattaattgt gagtttttat aagttatatg aaaatcttca aa
//
LOCUS       OIDGCHHC_13              2057 bp    DNA     linear       12-APR-2021
```

```
DEFINITION  Campylobacter species strain strain.
ACCESSION
VERSION
KEYWORDS    .
SOURCE      Campylobacter species
  ORGANISM  Campylobacter species
            Unclassified.
COMMENT     Annotated using prokka 1.14.6 from
            https://github.com/tseemann/prokka.
FEATURES             Location/Qualifiers
     source          1..2057
                     /organism="Campylobacter species"
                     /mol_type="genomic DNA"
                     /strain="strain"
     gene            130..768
                     /locus_tag="OIDGCHHC_00095"
     CDS             130..768
                     /locus_tag="OIDGCHHC_00095"
                     /inference="ab initio prediction:Prodigal:002006"
                     /codon_start=1
                     /transl_table=11
                     /product="hypothetical protein"
                     /protein_id="Prokka:OIDGCHHC_00095"
                     /translation="MSEINNNAEIKEQDTQDEIIWEGKKHIPSFLLFWITYIVLLCLF
                     LYLFPRLFNPSKEIDYKWFIVFFTFLLGIYAFARAIYKMANIKRIYITKEKLVIEYYI
                     KNDLVFPLGTFFIYYRQISYSPSPGHIVIHTFGDKAKEYLEPGLFSDGDDPTKGCFEK
                     INAIIKPHVMPYLLSLSDEEFEKIFFHVNTTSEIDTTFLKEAMELRKEKKDE"
     gene            761..913
                     /locus_tag="OIDGCHHC_00096"
     CDS             761..913
                     /locus_tag="OIDGCHHC_00096"
                     /inference="ab initio prediction:Prodigal:002006"
                     /codon_start=1
                     /transl_table=11
                     /product="hypothetical protein"
                     /protein_id="Prokka:OIDGCHHC_00096"
                     /translation="MNENIYGSLELYSKETQSTPSDKRDLGVFGDLAPYALQKVLLLR
                     LLRGKQ"
     gene            910..1527
                     /locus_tag="OIDGCHHC_00097"
     CDS             910..1527
                     /locus_tag="OIDGCHHC_00097"
                     /inference="ab initio prediction:Prodigal:002006"
                     /codon_start=1
                     /transl_table=11
                     /product="hypothetical protein"
                     /protein_id="Prokka:OIDGCHHC_00097"
                     /translation="MNNSTLLWKSKRKIIPFSLFFCSLYFILAFFLCWFCWFVLLKES
                     LWYSLLAIFLTFFYISELYETLNFKSIILTEQGLTLKMRWFDTEIFYPYGSFTIKSIT
                     IQFIKFDETISFLSNTTIKNFKLLNGFESFGSFENNHTFKELCTKYTQKALETMNLEE
                     KVNLYKLYQANIEIFFNQEIDHNVFIIDFSPYIQEIKTYLKDKNE"
ORIGIN
        1 gtaggtgata ctcaaattgt taccaagaaa gattgtgtta taattaaagc aggtggagta
       61 gaagtgatta tagactctaa tggacttgtg gttaaaggtg gagagcttaa ggcagagtaa
```

```
      121 aggataaaaa tgagtgaaat aaacaataac gcagaaatca aagagcaaga cactcaagat
      181 gaaatcattt gggaaggaaa aaagcatata ccttcgtttt tgcttttttg gataacatat
      241 attgtgcttt tatgtctttt tctttattta tttcctagat tatttaatcc tagcaaagaa
      301 atagattata aatggtttat agtttttttt acatttcttt taggcatata tgcatttgca
      361 cgagcgatct ataaaatggc aaatattaaa agaatttata tcacaaaaga aaaacttgtg
      421 attgagtatt atatcaaaaa tgatttggtt tttcctttag gaactttttt tatatattat
      481 cgtcaaattt catattcacc ttctccagga catatagtga tacatacctt tggagataag
      541 gccaaagaat accttgaacc tggtttattc agtgatggcg atgatccaac aaaaggttgt
      601 tttgaaaaaa ttaatgccat tataaaacct catgtaatgc cttatttatt aagcttaagc
      661 gatgaagaat ttgaaaaaat ctttttccat gtaaacacta ctagtgaaat tgatactact
      721 ttccttaaag aagcaatgga actcagaaaa gagaaaaaag atgaatgaaa atatttatgg
      781 aagtttagag ctttattcta aagaaactca aagcactcct agtgataaaa gagatttagg
      841 agtatttgga gatcttgctc cttatgcctt acagaaagtt ttactactaa ggcttttaag
      901 gggtaagcaa tgaataattc tactttactt tggaaaagta agagaaaaat tattcctttt
      961 agtttgtttt tttgctctct ttattttata ttagcttttt ttctgtgttg gttttgttgg
     1021 tttgttttac ttaaagaatc tttgtggtat agtttattag ctatatttct tacatttttt
     1081 tatatttctg aattgtatga aacattaaat tttaaatcta taattttaac agagcaagga
     1141 ttaacattaa aaatgcggtg gtttgatacg gaaattttct atccttatgg aagttttact
     1201 ataaaatcta taaccataca atttattaaa tttgatgaaa ctataagttt tttatcaaat
     1261 actactatta aaaattttaa attacttaat ggttttgaat cttttggtag ttttgaaaac
     1321 aatcacacat ttaaagagtt atgcaccaaa tatacacaaa aagctttaga aactatgaat
     1381 ttagaagaaa aagtaaatct ttataagctt tatcaagcaa atatagaaat atttttttaat
     1441 caagaaatag atcacaatgt ttttattatt gatttctccc cttatataca agagattaaa
     1501 acttatttaa aggataaaaa tgagtgaaat aaacaataac acagaaatca aagagcaaga
     1561 cacccaagat gaaatcattt gggaaggaaa aaagcatata ccttcgtttt tgcttttttg
     1621 gataacatat attgtgcttt tatgttttgc gctttatttg tttcctagat tatttaatcc
     1681 tagcaaagaa atagattata aatggtttat agtttttttt acatttcttt tagttatata
     1741 tgcacttgca caaaagatct atagaatggc aaatattaaa agaatttata tcacaaaaga
     1801 aaaacttgtg attgagtatt atatcaaaaa tgatttggtt tttcctttag gaactttttt
     1861 tgtgtattat cgtcgcctgt catttgtgct tactccagga catatagtga tatacacctt
     1921 cggagataaa gtcaaagaat accttgaacc tggtttattc agtgatggcg atgatccaac
     1981 aaaagattgt tttgaaagaa ttaatgccat tataaaacct catgtaatgc cttatttatt
     2041 aagcttaagt gatgaag
//
LOCUS       OIDGCHHC_14             1994 bp    DNA     linear       12-APR-2021
DEFINITION  Campylobacter species strain strain.
ACCESSION
VERSION
KEYWORDS    .
SOURCE      Campylobacter species
  ORGANISM  Campylobacter species
            Unclassified.
COMMENT     Annotated using prokka 1.14.6 from
            https://github.com/tseemann/prokka.
FEATURES             Location/Qualifiers
     source          1..1994
                     /organism="Campylobacter species"
                     /mol_type="genomic DNA"
                     /strain="strain"
     gene            96..239
                     /locus_tag="OIDGCHHC_00098"
     CDS             96..239
                     /locus_tag="OIDGCHHC_00098"
                     /inference="ab initio prediction:Prodigal:002006"
```

```
                     /codon_start=1
                     /transl_table=11
                     /product="hypothetical protein"
                     /protein_id="Prokka:OIDGCHHC_00098"
                     /translation="MAQAQLLEDLSKQVKFIRSEKENIISLKVAYPEKIDNKIIFVGK
                     VYE"
     gene            232..1485
                     /locus_tag="OIDGCHHC_00099"
     CDS             232..1485
                     /locus_tag="OIDGCHHC_00099"
                     /inference="ab initio prediction:Prodigal:002006"
                     /inference="similar to AA sequence:RefSeq:CKG88360.1"
                     /codon_start=1
                     /transl_table=11
                     /product="hypothetical protein"
                     /protein_id="Prokka:OIDGCHHC_00099"
                     /translation="MNKTLIFHENSHIDLNASFAPGTYIELQLEKESDKTLNWSYVEC
                     NSEKKMRYLLDTDCHSIEAKDLKFIDSFKGNICGFHLPISRDNEPTKDIKDYKYYIIV
                     FAYDEKKAIPSLEDFHIVIDMSFRVGVGKDESVEESKPRNDFNSDIIQTNCIFSVFEA
                     VEFLKACQSFKEKNEIDNYVFFKNYPQFAGKIAYIYYRFDLANEEFIKSVSDGNKYLK
                     EREKFYKVASDVYIKNPIYKMGFEKKLQNQVVDINIAKDFLKDFAKRLGIDEKVLPVI
                     GYNPQRGDSGTYDYTVNTLDLNPKASYVDFIDTIIHEFRHFYIYCIKNNSKNSIEKLL
                     YLDMVQFYIPWNFYKIFNSYDKKCLIYDNEDETKKCLKNRTYYGSWKFLNPTLDLLPS
                     PLYYLQPSELDSRIMAYYFRKEIKL"
ORIGIN
        1 aaaaagataa aaaagagcaa agcataataa aagataatct atatctttat agcatcaatg
       61 aaaagggaga gtttggcagt agtggtagca taggtatggc acaagcacag ttattagagg
      121 atttaagtaa acaggttaaa tttatacgaa gcgaaaagga aaatataatc tcactcaaag
      181 tggcttaccc tgaaaaaata gataataaaa ttatatttgt aggaaaagtt tatgaataaa
      241 acactaatat ttcatgaaaa ttcccatatt gatttaaacg cttcttttgc accagggact
      301 tatatagaat tgcaattaga aaaagaaagc gataaaacgc taaattggag ttatgtagag
      361 tgcaatagtg agaaaaaaat gcgatatttg ttggatactg attgtcattc tattgaggct
      421 aaagatctta aatttataga tagttttaaa ggtaatattt gtggatttca tttacctata
      481 agtagagata atgaaccaac aaaagatata aaagattata aatattatat tatagttttt
      541 gcttatgatg aaaaaaaggc tatacctagt ttagaagatt ttcatatagt tatagatatg
      601 agctttagag taggagttgg aaaagatgag agtgtagaag aaagtaagcc aaggaatgat
      661 tttaatagtg atataattca aacaaattgt atttttagtg tttttgaggc tgtagagttt
      721 ttaaaagctt gccaaagttt taaagaaaaa aatgaaatag ataattatgt ttttttcaaa
      781 aactatcctc aatttgcagg aaaaattgct tatatttatt atagatttga tttagctaat
      841 gaagaattta ttaaaagtgt aagtgatgga aataagtatt taaaagaaag ggagaaattt
      901 tataaagttg cttcagatgt ttatattaaa aaccctattt ataaaatggg ctttgagaaa
      961 aaacttcaaa atcaagttgt tgatataaac attgcaaaag attttttaaa ggactttgct
     1021 aaaagactag ggatagacga aaaagttttg cctgtaattg gatataatcc tcaacgaggg
     1081 gattctggaa catatgacta tacagtaaat actttagatt tgaatccaaa agcttcttat
     1141 gttgatttta ttgataccat tattcatgaa tttaggcatt tttatattta ttgcataaaa
     1201 aataattcta aaaatagtat agaaaaattg ctttatcttg atatggtaca attttatata
     1261 ccgtggaatt tttataaaat tttcaatagt tatgataaaa aatgtttgat ttatgataat
     1321 gaagatgaaa cgaagaagtg tttaaaaaat cgcacatatt atggaagttg gaaatttta
     1381 aatcccacat tagatctctt gccaagtcct ttatactatc ttcagccaag tgagttagac
     1441 tctaggatta tggcatatta ttttagaaag gaaattaaat tataatgact ccaccatctt
     1501 taccagatgt agaaaaacat gaagatttct tacaaaccag aaaagaacct tatgctattt
     1561 atttagccat aaacacaaac ataaaaagtt ataataatat ttgtcctagc gagaaatatt
     1621 tttggaaatt taataatatg aatgaacttg ataaatggta taacccaaaa tttggcatat
     1681 accttggcaa aattgttttt gataaaaaag acaacaaact catcccaaaa tatatagccg
```

```
     1741 ctaaatttga aaatttagaa gaagaagtta aaaagataaa aaatccttta tggcttgcta
     1801 ataaaaatcc taattatatt gaacctaaat tttatgatgg tatgagtgga ggttattatt
     1861 ttgaaagtcc aaataattta gagtatcaat gcaaaataga aaaagacact caagttttaa
     1921 gccaagaaca aatcatttct tatgtgaaag aactttatag taaaaacaca ataataataa
     1981 aaaattatat tgat
//
LOCUS       OIDGCHHC_15             1445 bp    DNA     linear       12-APR-2021
DEFINITION  Campylobacter species strain strain.
ACCESSION
VERSION
KEYWORDS    .
SOURCE      Campylobacter species
  ORGANISM  Campylobacter species
            Unclassified.
COMMENT     Annotated using prokka 1.14.6 from
            https://github.com/tseemann/prokka.
FEATURES             Location/Qualifiers
     source          1..1445
                     /organism="Campylobacter species"
                     /mol_type="genomic DNA"
                     /strain="strain"
     gene            76..855
                     /locus_tag="OIDGCHHC_00100"
     CDS             76..855
                     /locus_tag="OIDGCHHC_00100"
                     /inference="ab initio prediction:Prodigal:002006"
                     /inference="similar to AA sequence:RefSeq:CKG88360.1"
                     /codon_start=1
                     /transl_table=11
                     /product="hypothetical protein"
                     /protein_id="Prokka:OIDGCHHC_00100"
                     /translation="MSFRVGVGKDESVEESKLSSGLKSDTDFIEEWKKLNLIADFYEA
                     YNFILIMIFIYKNDWDNFIVNPMNYPQLAGKIAYIYYRFDFRKIYHQIKLKYDMQMKY
                     KNYFQDLKYYEETIKMVVKKYNYKSSQTYPVQWWERLIKEICNEFKINTKIEFIPMKV
                     KQYGLYNNSSYTISINVNNIKVTQEILKTIVHEIRHAYMQQKKLKNDALQRYMYFTYK
                     KVYFNAEQIYLDENETKDVYYFQPSETEARAIENNIIKEIQ"
ORIGIN
        1 tattatatta tagtttttgc ttatgatgaa aaaaaggcta tacctagttt agaagatttt
       61 catatagtta tagatatgag ctttagagta ggagttggaa aagatgagag tgtagaagaa
      121 agtaagctat ctagtggttt aaaatctgat accgatttta tagaggaatg gaaaaagtta
      181 aatttaatag ctgattttta tgaagcttac aactttatac tcataatgat ctttatatat
      241 aaaaacgatt gggataattt tatagtaaat ccaatgaatt atccccaact tgcaggaaaa
      301 attgcttata tttattatag atttgatttt agaaaaatat atcatcaaat taaattaaaa
      361 tatgatatgc aaatgaagta taaaaattat tttcaagact taaaatacta cgaagaaaca
      421 attaagatgg tggttaaaaa atataattac aaatcaagtc aaacttatcc tgtgcaatgg
      481 tgggaaagat taattaaaga aatttgcaat gaatttaaaa taaatacaaa aattgaattt
      541 attccaatga aagttaaaca atacggttta tataataatt caagttacac aataagtata
      601 aatgtaaata atataaaagt tacacaagaa atattaaaga caatcgttca tgaaatacgc
      661 cacgcttata tgcaacaaaa aaaacttaaa aacgatgctt tgcaaagata tatgtatttt
      721 acttacaaaa aagtttattt taatgcagaa caaatatatt tggatgaaaa tgagactaaa
      781 gatgtgtatt attttcagcc aagtgaaaca gaggctagag caatagaaaa caatataatt
      841 aaggaaatac aatgagattt atcgagccag acttagaaaa acataagtat tttttacaaa
      901 ccagaaaaga gccttttgct atttatttag ccatcaatac taatattaaa agctttaata
      961 atatttgtcc tagcgaggaa aattcgtggt attttcgcag caaagaaatt ctagaaaatt
```

```
     1021 ctcttaataa tttttataac ccaaaatttg gcatatatct aggtaaaatt atatttgata
     1081 aaaaaggcaa taaacttatt ccaaaataca tacccactaa atttgaaaat ttagaagaag
     1141 aacttaaaaa gataaaaaat cctttatggc ttgctaataa aaaccctaat tatattaagc
     1201 ctaaaccatt gaaacctatg aaacctaaaa caaaagaaag ttctttggaa aatataagtc
     1261 aattggagtt agcaatacaa gatgtagtgg taagtcgtgg tgattataaa ataaccactc
     1321 caaataattt agagtatcaa tgcaaaatag aaaaagacac tcaagtttta agccaagaac
     1381 aaatcatttc ttatgtgaaa gaactttata gtaaaaacac aataataata aaaaattata
     1441 ttgat
//
LOCUS       OIDGCHHC_16             1355 bp    DNA     linear       12-APR-2021
DEFINITION  Campylobacter species strain strain.
ACCESSION
VERSION
KEYWORDS    .
SOURCE      Campylobacter species
  ORGANISM  Campylobacter species
            Unclassified.
COMMENT     Annotated using prokka 1.14.6 from
            https://github.com/tseemann/prokka.
FEATURES             Location/Qualifiers
     source          1..1355
                     /organism="Campylobacter species"
                     /mol_type="genomic DNA"
                     /strain="strain"
ORIGIN
        1 tctcactcta aaaacctaaa ccatttcatc ttagaaaaca aagataaaaa taaaagaatt
       61 atctttttaa ataatgcttc atcgatgtca aatttaatcc atatttatga taatcattgt
      121 gatgtattta ttaaaaatga ttctttgctt gatattaaaa gaatagaaaa agaatatgat
      181 attaaattta aagaatttaa tacaagagtg ttttttttatg aaaaacttct tttaggtgca
      241 aaagaaaata atgaattttt atttgaagaa aaagaagaaa atttacttta tcattttaaa
      301 tacgataaag aggatttaag ttctttaaaa gatttaagta taaaatataa tatttataat
      361 gcaaggatta agaattattc tttactagaa gaaagtttaa atattaaatt agaacccaaa
      421 gagcttaaaa aagaaattgc ctacaacaat gcaagtttta ataaactttc aaaagaacct
      481 ttaaacaaat cctcatctaa cgctaaagaa ccctgcttta tcactttata tctaaaagat
      541 gaacacaata aacccattgc taatgctaaa atcatcataa aaggttttca acataatgaa
      601 gccttaagag tagttaattt aaaaagacgc agcgatgaaa aaggcaagat aagatttgat
      661 aaagaaaagt attttagtga ttgttatagc tttaaagtaa agcttgatga aagcaaagct
      721 tattttccta ctcctttaca aaatgctaaa agaatattta ataattatac ccatcacaaa
      781 gttggactta tacttaaatt taaagctaaa gatcatttag tttatgatgg atttaatgtc
      841 tatcattaca aaggaaatga attaattaat tcttatatgg caagaagtgg cactgctaag
      901 gcagacaatc aaaaaagaag taaaaaacaa attgcacatt atttttacca agatgaaaaa
      961 gataaaagca aaagcggaaa ggcttatttt tactatgatg atgaaagcat tcaagataga
     1021 tttggcactt tacctgaagg ggagtattat ttaaaaatca atgaaatagc taaagatact
     1081 aagcctaact ttttaaaaga ttatcccttt gaaataggaa aaacctgggg aagatactgc
     1141 gtaagattat atactgataa agaatgctct aaaactttta aagaaataga aataaaagaa
     1201 agtaatgaaa aagaaaacaa aacaagcaaa aaagataaaa aagagcaaag cataataaaa
     1261 gataatctat atctttatag catcaatgaa aagggagagt ttggcagtag tggtagcata
     1321 ggtatggcac aagcacagtt attagaggat ttaag
//
LOCUS       OIDGCHHC_17             1314 bp    DNA     linear       12-APR-2021
DEFINITION  Campylobacter species strain strain.
ACCESSION
VERSION
KEYWORDS    .
```

```
SOURCE      Campylobacter species
  ORGANISM  Campylobacter species
            Unclassified.
COMMENT     Annotated using prokka 1.14.6 from
            https://github.com/tseemann/prokka.
FEATURES             Location/Qualifiers
     source          1..1314
                     /organism="Campylobacter species"
                     /mol_type="genomic DNA"
                     /strain="strain"
ORIGIN
        1 aaaaagataa aaaagagcaa agcataataa aagataatct atatctttat agcatcaatg
       61 aaaagggaga gtttggcagt agtggtagca taggtatggc acaagcacag ttattagagg
      121 atttaagcaa gcaagctaag tttatacaag atgagaagga aggaattgtt tctttaagag
      181 tagagtatcc taagcaagag aaatctaaag taaatttcat aaaagaaata gaatgggaac
      241 tcgatgaaga tgaagatgtg gtaagtgctg aaaaaagtgt aaggattatt atcgatgaaa
      301 aggcaaattt tgcaccaggg acttatgtag aatgcgaagt tgatggttta gaagatgatg
      361 taaatatcgt ttgggcttta gctcaaattc aaaatgaagc aagacttcaa agaattttag
      421 aaagtaaaga gcatttagaa tttaaagata taaagttttt aaaagacgaa agtaaatatt
      481 ttcatgaatg ctgggaaatg gatgatgaag aaaaagatga agaatttgaa aaaataggct
      541 ttagtttacc tctaaaaagg gctaatgaag aagaatttga taaatattat gctttagttt
      601 ttgcttatga agataaaaat aaatttaaaa gacttcctaa tttaaatgat gcttataaaa
      661 tcatagatat gagctttaaa gttggctctg gtagagatga tagggtgaaa gaattattta
      721 caagagagga gctaaaatcc cttaatgtat ataatcttaa ccgacaaaaa tcaatcgata
      781 gtgttgaaga ggcaaattgg tttttaaggg caaatttaaa aaatcaagaa aaaagagact
      841 tttttaccta tcttagaacc tatcctcaac ttgccggaaa aattgcttat atatactata
      901 gatttgattt aatcaataag gaatttttaa caagtgtatc agaaaaagat aaaggaaatt
      961 atcttcaaga tagagacggc aaatatatta gcaccataat taataaaatt ttacctttgg
     1021 aatataagaa tggtaaaagt tttaaacaat tccacacaga gcttttaaat aacaaaaaca
     1081 gagatcttaa agaatgtaaa gatttttaa aagctttaac tcaaactctt tgtaaagaat
     1141 ttaatttacc tttagaaaat gtagtctttt atagccaaaa gcctagcgga tctttatcat
     1201 ttggagctta tgaagaaaat aaggtaagat taaatcaata cattttaaaa gcttatttta
     1261 aagaatttgc aaaaacggtt tttcacgaat gtaggcattt ttatatagaa aaat
//
LOCUS       OIDGCHHC_18             1132 bp    DNA     linear       12-APR-2021
DEFINITION  Campylobacter species strain strain.
ACCESSION
VERSION
KEYWORDS    .
SOURCE      Campylobacter species
  ORGANISM  Campylobacter species
            Unclassified.
COMMENT     Annotated using prokka 1.14.6 from
            https://github.com/tseemann/prokka.
FEATURES             Location/Qualifiers
     source          1..1132
                     /organism="Campylobacter species"
                     /mol_type="genomic DNA"
                     /strain="strain"
     gene            40..393
                     /gene="trxB_6"
                     /locus_tag="OIDGCHHC_00101"
     CDS             40..393
                     /gene="trxB_6"
```

```
                     /locus_tag="OIDGCHHC_00101"
                     /inference="ab initio prediction:Prodigal:002006"
                     /inference="similar to AA sequence:RefSeq:CKG86610.1"
                     /codon_start=1
                     /transl_table=11
                     /product="thioredoxin reductase"
                     /protein_id="Prokka:OIDGCHHC_00101"
                     /translation="MDSTPESRNEFDDAMDRYENGMVTRFDVDTPNDWGEQQAALDRD
                     TLVLSAKLAALTPPQGYPNAPYYFTPERLEWIYKRGYLDKLLDPRIPAIYRYNFPREL
                     RAKILKFGEENGIKD"
ORIGIN
        1 aatgatattg aaaaaggtaa attaaaagat ccaagagatg tggattctac tcctgaaagt
       61 agaaatgaat ttgatgatgc tatggataga tacgaaaatg gaatggtaac tagatttgat
      121 gtagatactc caaatgattg gggtgaacaa caagctgctt tagatagaga tactttagta
      181 ttaagtgcca aactcgcagc cctcactcct ccacaaggct atcctaatgc tccttattat
      241 tttacacctg aaagattaga atggatttat aaaagaggat atttagataa actccttgat
      301 ccaagaatcc ctgctattta tagatataat ttcccaaggg aacttagagc taagatactc
      361 aaatttggcg aagaaaatgg cattaaagat taaagtaatt gcataggcag actagccact
      421 actataatca tagaagatgg tttatatata ggttaaatta agaaaggata aaaatgagtg
      481 aaacttatga gatttataca cccaatgggt taataatgga tgtatataaa gatactaata
      541 aaatcatttt ttcaggaaat gctaaaccca caggcaatta taccgaagaa tattccaaag
      601 ctttgtttga agcacacgac attaaacgaa actcccccta caaagactat aaaccaatat
      661 atctagatcc taacttttat acagggcaag cttctacttt acttgaattt aaagaatggc
      721 aaagcatata tttaaaagat cctatcaaag gagctcaaaa tatcacaaat atttgtatta
      781 attatgtaaa aaaaggtttt aaagaagttt taatcgaaca acttggcatt atatatttat
      841 taacacatcc acaaatcgaa gttgaagtta agaaatatgg aaaaaaatat tttaaggcta
      901 taaacaagga aattgctgca aaaagatttt ctacaattaa agaagaatta tttttcaata
      961 aaacaaaaat atcctttatg gcagaacaag taaaaaacgg tattgattat gaaatagatc
     1021 aaaagcttga agaaaataaa gaatttcttt taaaaagact tttgtcttat ttgattattg
     1081 atgagaaaag aagtgcaact gctttagagg atataaaaga acaaaaatat at
//
LOCUS       OIDGCHHC_19             1037 bp    DNA     linear       12-APR-2021
DEFINITION  Campylobacter species strain strain.
ACCESSION
VERSION
KEYWORDS    .
SOURCE      Campylobacter species
  ORGANISM  Campylobacter species
            Unclassified.
COMMENT     Annotated using prokka 1.14.6 from
            https://github.com/tseemann/prokka.
FEATURES             Location/Qualifiers
     source          1..1037
                     /organism="Campylobacter species"
                     /mol_type="genomic DNA"
                     /strain="strain"
     gene            1..954
                     /locus_tag="OIDGCHHC_00102"
     CDS             1..954
                     /locus_tag="OIDGCHHC_00102"
                     /inference="ab initio prediction:Prodigal:002006"
                     /inference="similar to AA sequence:RefSeq:AVS37497.1"
                     /codon_start=1
                     /transl_table=11
```

```
                     /product="type VI secretion protein VgrG"
                     /protein_id="Prokka:OIDGCHHC_00102"
                     /translation="MPQYNYPRYKREIATNALSYLSVAPAMIDKGLDTLKLQENTDYE
                     LSSINNHYLTLANSTIGVDNADARARNEITLKNDKDKEEIYILAQKDYKEEIGNNYEQ
                     TIKNNKTSEVGALYTEFITLGHMQNIIGFKNVNVGAEYLENTLLSKDTNVGLNNTLNV
                     GISNEVNIGQNHEEKIGNDKRVIINNNLEQDIKNDFIQRIGHNKNETIKGSYVLQTNQ
                     SIKFYSKQDLSIETNDCFKAEADTFVSFKAKKNCSFTADEVYTMANKESFLTAQKQIV
                     SRVGNTTITQTKDKIILQVGTTQVIIDSKGLRVQGGDLRAD"
ORIGIN
        1 atgccccaat ataattatcc aagatacaaa agagaaatcg ctacaaatgc attgagctat
       61 ttaagcgtag ctcctgctat gattgataaa ggtttagata cattaaaact tcaagaaaat
      121 actgattatg aattaagctc tataaacaat cattatctta ctttagcaaa ttctacaata
      181 ggagtagata atgccgatgc aagagcaagg aatgaaatta ccctaaaaaa cgacaaagac
      241 aaagaagaaa tttatatcct agcacaaaaa gattataaag aagaaatagg taataattac
      301 gagcaaacca taaaaaataa taaaacttca gaagtgggag ccttatatac cgaatttatc
      361 actttaggac atatgcaaaa tattataggt tttaaaaatg tcaatgtagg agctgaatat
      421 ttagaaaata ccttgctttc taaagatact aatgtgggtt taaataatac tttgaatgtg
      481 ggaatcagca atgaagtcaa tatcggacaa aaccacgaag aaaagatagg aaacgataaa
      541 agagtaataa tcaataacaa tctagaacaa gatatcaaaa atgattttat ccaaagaata
      601 ggacataata aaaatgaaac cataaaaggt tcttatgtgc ttcaaaccaa tcaaagtata
      661 aaattttatt ccaaacaaga tctaagcata gaaacaaacg attgctttaa agctgaagct
      721 gatacttttg ttagtttcaa agccaagaag aattgctctt ttacagccga tgaagtgtat
      781 actatggcaa ataaagaaag ttttttaaca gcacaaaaac aaattgtttc tagggtagga
      841 aatacaacta tcacacaaac aaaagataag attatactcc aagtaggaac aacacaagtg
      901 attatagata gtaaaggttt aagagtgcaa ggaggtgatt taagagcaga ttaaaagcaa
      961 ttttcttaaa attaaaacca aaatcataaa taaacctaat ttacaataaa aggaaaaaca
     1021 atgaaaacat tagaaga
//
LOCUS       OIDGCHHC_20             1028 bp    DNA     linear       12-APR-2021
DEFINITION  Campylobacter species strain strain.
ACCESSION
VERSION
KEYWORDS    .
SOURCE      Campylobacter species
  ORGANISM  Campylobacter species
            Unclassified.
COMMENT     Annotated using prokka 1.14.6 from
            https://github.com/tseemann/prokka.
FEATURES             Location/Qualifiers
     source          1..1028
                     /organism="Campylobacter species"
                     /mol_type="genomic DNA"
                     /strain="strain"
     gene            complement(554..910)
                     /locus_tag="OIDGCHHC_00103"
     CDS             complement(554..910)
                     /locus_tag="OIDGCHHC_00103"
                     /inference="ab initio prediction:Prodigal:002006"
                     /inference="similar to AA sequence:RefSeq:APB39663.1"
                     /codon_start=1
                     /transl_table=11
                     /product="VgrG protein"
                     /protein_id="Prokka:OIDGCHHC_00103"
                     /translation="MKGNKKEVVEGKLELHVNKGINYFTEEHFSMQTNNYIDIYTEQN
```

```
                     LSTQTKKQHTELAESKYSDFQTDSEVKAGNQILHQVGDTQIVTKKDCVIIKAGGVEVI
                     IDSNGLVVKGGELKAE"
ORIGIN
        1 cttcatcact taagcttaat aaataaggca ttacatgagg ttttataatg gcattaattc
       61 tttcaaaaca atcttttgtt ggatcatcgc catcactgaa taaaccaggt tcaaggtatt
      121 ctttgacctt atctccaaag gtgtatatca ccatatctcc tggatttgat atggatggaa
      181 gtgacgaact acgataatat acaaaaaaag ttcctaaagg aaaaaccaaa tcatttttaa
      241 tataatactc aatcacaagt ttttcttttg tgatataaaa tcttttaata tttgccattt
      301 gatagatatt ttttacaagt atatataacc caaaaagaaa tataacaaaa actataaacc
      361 atttataatc tatttctttg ttaggattaa ataatctagg aaacaaataa agaataaaac
      421 ataaaaatac aatatttgct atccaataaa gcaaaaacga aggtatatgc ttttttcctt
      481 cccaaatgat ttcatcttgg gtgtcttgct ctttgatttc tgtgttattg tttatttcac
      541 tcatttttat cctttactct gccttaagct ctccaccttt aaccacaagt ccattagagt
      601 ctataatcac ttctactcca cctgctttaa tgataacaca atctttctta gtaacaattt
      661 gagtatcacc tacttgatgg aggatttgat tgcctgcttt tacttcagaa tctgtttgaa
      721 agtcactata tttagattct gcaagttctg tatgttgttt tttggtttgc gtgcttaaat
      781 tttgttctgt gtatatatca atgtaattgt tagtttgcat actaaaatgt tcttccgtaa
      841 agtaatttat tcctttatta acatgaagct caagttttcc ctcaaccacc tcctttttat
      901 ttcccttcac attccttatc tcatctttat ggataatagt attttgattt gcacctattt
      961 ctatgtcttt gttttctcct acaaactcat gagagttttt agctattctt actttattat
     1021 ctactcct
//
LOCUS       OIDGCHHC_21             1005 bp    DNA     linear       12-APR-2021
DEFINITION  Campylobacter species strain strain.
ACCESSION
VERSION
KEYWORDS    .
SOURCE      Campylobacter species
  ORGANISM  Campylobacter species
            Unclassified.
COMMENT     Annotated using prokka 1.14.6 from
            https://github.com/tseemann/prokka.
FEATURES             Location/Qualifiers
     source          1..1005
                     /organism="Campylobacter species"
                     /mol_type="genomic DNA"
                     /strain="strain"
ORIGIN
        1 ccttatcctt tcttatggct tcatttaata aatcacagta ttcattacga tcttcataat
       61 ccttataaaa gatattaaat ttcttttcaa gttgatcgga agtcattgca cctgcagctt
      121 ttaaaagctt tctatttcta gctccttcgg cttcatctaa aggtgtcgct aaatagggaa
      181 caaaattaac attagctcca agctctatta gaagttgcac ggttctgtaa tttcctggct
      241 ctctcactgc tctaaataag ggtgtattct gatatttata gattgattca aactcatatt
      301 cttccatatc cacattagcc cctcctttga tggcaagctc tagaaattta taatcattat
      361 tactagccca aaaatgcata ggagaccagc ccattttgtc gcgatatact tcaaatttag
      421 ctccatgatt tgctaggtat tggataattt gggggtttgg attatcagca gttaggatat
      481 acttatagat taaagcttct ttgtaaatgc taggattatc ttccatgtat cttactacaa
      541 catatttatc attagcttct ttgatatatt tcatatctgt aaagcgaaaa ttatatttag
      601 gatcttttag acttaagcca tactcatcaa ataaataatc taaaatagaa aaatcattat
      661 tattttcttc acaagcaaat gcagctctca ttatcaaata ataaggatca aataaaggat
      721 attcttcgtt atcattttta aaatgaagct cataacaaga aatttttaca ggataatctt
      781 ttaaaatctc ttttacttta gctaaatcat tatcatctat agcctcaaga actaaatctt
      841 ctaacttatc cttttcctca taactcatag ccttaatatc ttctaatgtt ttcattgttt
      901 ttccttttat tgtaaattag gtttatttat gattttggtt ttaattttaa gaaaattgct
```

```
      961 tttaatctgc tcttaaatca cctccttgca ctcttaaacc tttac
//
LOCUS       OIDGCHHC_22              961 bp    DNA     linear       12-APR-2021
DEFINITION  Campylobacter species strain strain.
ACCESSION
VERSION
KEYWORDS    .
SOURCE      Campylobacter species
  ORGANISM  Campylobacter species
            Unclassified.
COMMENT     Annotated using prokka 1.14.6 from
            https://github.com/tseemann/prokka.
FEATURES             Location/Qualifiers
     source          1..961
                     /organism="Campylobacter species"
                     /mol_type="genomic DNA"
                     /strain="strain"
     gene            120..620
                     /locus_tag="OIDGCHHC_00104"
     CDS             120..620
                     /locus_tag="OIDGCHHC_00104"
                     /inference="ab initio prediction:Prodigal:002006"
                     /inference="similar to AA sequence:RefSeq:CKG86719.1"
                     /codon_start=1
                     /transl_table=11
                     /product="hypothetical protein"
                     /protein_id="Prokka:OIDGCHHC_00104"
                     /translation="MGDSITLYIFLGLLGFISVFTFARTYFITLAVFIIFSIINYWMR
                     INIKEFVKSYFLIFFVILFADIIGVFYFGYNIENPAKFTSLFLEVKQLPSEEYLVFSC
                     LAFLFIITMEFFIITFFILPCLLQAYFFKCYSTGEKRKKLSWFIGFACNIFLLIVSFY
                     KLYENL"
     gene            607..738
                     /locus_tag="OIDGCHHC_00105"
     CDS             607..738
                     /locus_tag="OIDGCHHC_00105"
                     /inference="ab initio prediction:Prodigal:002006"
                     /inference="similar to AA sequence:RefSeq:CKG86746.1"
                     /codon_start=1
                     /transl_table=11
                     /product="hypothetical protein"
                     /protein_id="Prokka:OIDGCHHC_00105"
                     /translation="MKIFKDYQKFTPYGLDFKLYSKDFITKLDFRDKAYNVLYDIGV"
ORIGIN
        1 aagactatca aaaattcact ccttatggtt tggattttaa actttatagt aaagatttta
       61 tcactaaact tgattttaaa gataaagctt ataatgtgct ttataataca ggagtttaaa
      121 tgggagatag cattacactt tatattttttt tgggcttgtt gggatttatt agtgtcttta
      181 cttttgcaag aacttatttt ataactttag ctgtgtttat tatcttttct attataaatt
      241 attggatgag aattaacata aaagaatttg taaaatctta ttttttgata ttttttgtta
      301 ttttatttgc agatataatt ggagtatttt atttcggcta taatattgaa aatcctgcga
      361 aattcacttc tcttttttta gaggttaagc aattaccatc tgaagaatac ctagtattta
      421 gctgtcttgc attcttattt atcataacta tggaattttt tataatcaca ttctttatac
      481 ttccttgttt attacaagct tatttttttta aatgctatag cacaggagaa aaacgcaaaa
      541 aattaagttg gtttataggg tttgcgtgta atatcttttt attaattgtg agtttttata
      601 agttatatga aaatctttaa agactatcaa aaattcactc cttatggttt ggattttaaa
```

```
      661 ctttatagta aagattttat cactaagctt gattttagag ataaagctta taatgtgctt
      721 tatgatatag gagtttaaat gggagatagc attacacttt ctatttttg gggtttgatt
      781 ttgtttattg ctagttttac tgttgcaaga atttatctta tagctctaag cgttcttatt
      841 gtcttttcta ttttaaatta ttggatcaag attaatataa aagaatttgt aaaatcttat
      901 tttttaatat cttttattat cttttttaca gctattgtta tcgtatttta tcttacttat
      961 g
//
LOCUS       OIDGCHHC_23              932 bp    DNA     linear       12-APR-2021
DEFINITION  Campylobacter species strain strain.
ACCESSION
VERSION
KEYWORDS    .
SOURCE      Campylobacter species
  ORGANISM  Campylobacter species
            Unclassified.
COMMENT     Annotated using prokka 1.14.6 from
            https://github.com/tseemann/prokka.
FEATURES             Location/Qualifiers
     source          1..932
                     /organism="Campylobacter species"
                     /mol_type="genomic DNA"
                     /strain="strain"
     gene            126..539
                     /locus_tag="OIDGCHHC_00106"
     CDS             126..539
                     /locus_tag="OIDGCHHC_00106"
                     /inference="ab initio prediction:Prodigal:002006"
                     /codon_start=1
                     /transl_table=11
                     /product="hypothetical protein"
                     /protein_id="Prokka:OIDGCHHC_00106"
                     /translation="MFKKLALFLLPVYLLLFLAGGCSYKYMDPQYYEFKSLCYFNLGK
                     VLIEDESIKNKTNIIVTPKSKQIKDRIVETRFERKNENQVVYYRINTYFYDNYGIFLK
                     GDEGAGLYFRYMEVLSCKNIDKKFKKDDWQVETLE"
     gene            543..683
                     /locus_tag="OIDGCHHC_00107"
     CDS             543..683
                     /locus_tag="OIDGCHHC_00107"
                     /inference="ab initio prediction:Prodigal:002006"
                     /codon_start=1
                     /transl_table=11
                     /product="hypothetical protein"
                     /protein_id="Prokka:OIDGCHHC_00107"
                     /translation="MNKNIKTQEAKLDLITKFLDYANCADASYAMLHLIDENDEKGFL
                     KG"
ORIGIN
        1 ttaatctttt aatatttatt cttatatttt tctttttgt ttttatattt tctattattt
       61 tatattttat ctttaaagtc ttttttaaaa taaaaaataa aacaaaaact ataaatttta
      121 aaacaatgtt taaaaaactt gctttatttc ttttacctgt atatctttta ttattcttag
      181 caggaggctg ttcttataaa tatatggatc cgcagtatta tgagtttaag agcttgtgtt
      241 attttaattt gggaaaagtg ttgattgagg atgaatctat aaagaataaa acgaatataa
      301 tagtgacacc taaaagtaag caaataaaag ataggatagt ggaaactaga tttgaaagaa
      361 aaaatgaaaa ccaagtagtg tattatcgta ttaatacata tttttatgat aattatggta
      421 tattcttgaa aggcgatgag ggtgctggat tatattttag atatatggaa gtattaagtt
```

```
      481 gtaagaatat tgacaaaaaa tttaagaaag atgactggca agttgaaact ttggaataac
      541 aaatgaacaa aaacattaaa acgcaagaag ctaaattaga cttaatcact aaatttttag
      601 actatgctaa ttgtgcagat gctagttatg cgatgttgca tttaatcgat gagaatgatg
      661 aaaaaggttt tttaaaagga taataatgct agaattcttt gttaatcttt taatatttat
      721 tcttatattt ttcttttttg tttttatatt ttctattatt ttatatttta tctttaaagt
      781 ctttttttaaa ataaaaaata aaacaaaaac tataaatttt aaaacaatgt ttaaaaaact
      841 tgctttattt cttttacctg tatatctttt attattctta gcaggaggat gttcttataa
      901 atatatggat ccgcagtatt atgagtttaa ga
//
LOCUS       OIDGCHHC_24              747 bp    DNA     linear       12-APR-2021
DEFINITION  Campylobacter species strain strain.
ACCESSION
VERSION
KEYWORDS    .
SOURCE      Campylobacter species
  ORGANISM  Campylobacter species
            Unclassified.
COMMENT     Annotated using prokka 1.14.6 from
            https://github.com/tseemann/prokka.
FEATURES             Location/Qualifiers
     source          1..747
                     /organism="Campylobacter species"
                     /mol_type="genomic DNA"
                     /strain="strain"
ORIGIN
        1 acaggcaatt ataccgaaga atattccaaa gctttgtttg aagccgatcg catattaaga
       61 aactcccccct acaaagacta taaaccaata tatctagatc ctaactttta tacagggcaa
      121 aaatccactt tgcttgaatt taaagaatgg caaagcatat atttaaaaga tcctatcaaa
      181 ggagctatag ctccttggac taaagcagaa aaagcttatt ataaatcttt aaaaaccaaa
      241 agagaaagat ataaatatct aattataaga agtggtataa gaagcactgt tatagatata
      301 ccctatgatg cttattgtaa tgtagatgaa aaaggatatt taatcaatga agaatacgcc
      361 tatatttatg atgaagttaa caataataaa gaaaccttaa aatcttctct ttttagacaa
      421 gaatggggta tagcagcagg aatactagga aaacctgaat attttgtgcg ttctaaaaat
      481 catggcttta atgctagaat gatacaatgt tttattttat atattcaact cacaggtgga
      541 ggatatgaag aattaggtat aaaaagagga atttataatt atgctgataa tcttttagaa
      601 ataggtatag gtatggcagg gatacataaa aatcctttaa gggctaaatt agtaaaagat
      661 ttagctaaaa ctatacagcc tgatgaattt ggtatgctac cttttataga tgagattatg
      721 ggagtagatt gggtgattga tttaaat
//
LOCUS       OIDGCHHC_25              738 bp    DNA     linear       12-APR-2021
DEFINITION  Campylobacter species strain strain.
ACCESSION
VERSION
KEYWORDS    .
SOURCE      Campylobacter species
  ORGANISM  Campylobacter species
            Unclassified.
COMMENT     Annotated using prokka 1.14.6 from
            https://github.com/tseemann/prokka.
FEATURES             Location/Qualifiers
     source          1..738
                     /organism="Campylobacter species"
                     /mol_type="genomic DNA"
                     /strain="strain"
```

```
     gene            49..222
                     /locus_tag="OIDGCHHC_00108"
     CDS             49..222
                     /locus_tag="OIDGCHHC_00108"
                     /inference="ab initio prediction:Prodigal:002006"
                     /codon_start=1
                     /transl_table=11
                     /product="hypothetical protein"
                     /protein_id="Prokka:OIDGCHHC_00108"
                     /translation="MDNIALKTKNMQMSLHKIVDMHISPYWNTRRYVLEGNEGNMNFE
                     FIEDELACGNLYQ"
     gene            394..609
                     /locus_tag="OIDGCHHC_00109"
     CDS             394..609
                     /locus_tag="OIDGCHHC_00109"
                     /inference="ab initio prediction:Prodigal:002006"
                     /inference="similar to AA sequence:RefSeq:CKG86895.1"
                     /codon_start=1
                     /transl_table=11
                     /product="methyltransferase small"
                     /protein_id="Prokka:OIDGCHHC_00109"
                     /translation="MDYFKALHDEYNGYLVIDDSLFFYAKLNGDFRELQARNFSQRYE
                     IKFHQSNTTSGFSTALSYEKTEKIKRS"
ORIGIN
        1 tacgatgata cttacaaaac tcctttaaat tttcaagaaa agatagagat ggataatatt
       61 gcactaaaaa caaaaaatat gcaaatgtct ttacacaaga ttgttgatat gcatataagt
      121 ccatattgga atactagaag gtatgtgtta gaaggcaatg agggtaatat gaattttgag
      181 tttatagagg atgagctcgc ttgtggcaat ctttatcaat aataatataa ggaataaata
      241 atgaccaata aagaacaaat caacaattta aaagattata cagaatttgt gtaagctagt
      301 tattttaatt ttgtttatac tgaaggacaa aaagctttag gatttaaaat atctaaaact
      361 agatttttta aagataagag tgaattagaa aaattggact attttaaagc attacatgat
      421 gaatataatg gatatttggt tatagatgat tctttatttt tttatgcgaa actaaatgga
      481 gattttagag aattacaagc tagaaatttt tctcaaagat atgagattaa atttcatcag
      541 tccaacacta caagtggttt tagcactgct ttgtcttatg aaaagacaga gaaaattaag
      601 aggagttaat aaatgctaga attctttgtt aatcttttaa tatttattct tatatttttc
      661 ttttttgttt ttatattttc tattatttta tattttatct ttaaagtctt ttttaaaata
      721 aaaaataaaa caaaaact
//
LOCUS       OIDGCHHC_26              730 bp    DNA     linear       12-APR-2021
DEFINITION  Campylobacter species strain strain.
ACCESSION
VERSION
KEYWORDS    .
SOURCE      Campylobacter species
  ORGANISM  Campylobacter species
            Unclassified.
COMMENT     Annotated using prokka 1.14.6 from
            https://github.com/tseemann/prokka.
FEATURES             Location/Qualifiers
     source          1..730
                     /organism="Campylobacter species"
                     /mol_type="genomic DNA"
                     /strain="strain"
ORIGIN
```

```
        1 tatctacatc aaatctagtt accattccat tttcgtatct atccatagca tcatcaaatt
       61 catttctact ttcaggagta gaatccacat ctcttggatc ttttaattta cctttttcaa
      121 tatcattata taaagcccaa atgattctac cttcttcatc ataggcaaaa tcatatttat
      181 ttaaatcaat cgtccaatct gctcctataa tctcatctaa aaaaggtagc ataccaaatt
      241 catctaacaa actctcataa tgatgatcgt gtaaatcgta aatcattttt gctctaagct
      301 tattccaatg ttgccctgta agaaaagaat tagaacacaa tactgctaaa cctaaactct
      361 taaaagcatt tttatctcca aattgagctt gtaaaaataa agcttgttgt tgtcttgctt
      421 taaatcctac agcggctgta ggactagctc tcatatctcc tagaagtaaa gcagctaatt
      481 cccattcatt aaagaaagaa taagatttta atgttccacg atgagaactt acttcatcat
      541 aaatataggc gtattcttca ttgattaaat atcctttttc atctacatta caataagcat
      601 catagggtat atctataacc acagatctta atccacttct tattgccaaa tacttacatc
      661 tttctctttt agtttttaaa gatttataat aagctttttc tgctttagtc caaggagcta
      721 tgcttccttt
//
LOCUS       OIDGCHHC_27              671 bp    DNA     linear       12-APR-2021
DEFINITION  Campylobacter species strain strain.
ACCESSION
VERSION
KEYWORDS    .
SOURCE      Campylobacter species
  ORGANISM  Campylobacter species
            Unclassified.
COMMENT     Annotated using prokka 1.14.6 from
            https://github.com/tseemann/prokka.
FEATURES             Location/Qualifiers
     source          1..671
                     /organism="Campylobacter species"
                     /mol_type="genomic DNA"
                     /strain="strain"
     gene            complement(55..615)
                     /locus_tag="OIDGCHHC_00110"
     CDS             complement(55..615)
                     /locus_tag="OIDGCHHC_00110"
                     /inference="ab initio prediction:Prodigal:002006"
                     /inference="similar to AA sequence:RefSeq:AAW35464.1"
                     /note="conserved hypothetical protein"
                     /codon_start=1
                     /transl_table=11
                     /product="hypothetical protein"
                     /protein_id="Prokka:OIDGCHHC_00110"
                     /translation="MSYLIIDEKRSATALEDIKEQKYIIDDYTFYFKELKSLELKKCT
                     IYQLPCNHPLFKEFRNKEKQISAFKKDEDSPIHAYNEALQILQDFANGIYNTSKDKTG
                     KCIEDKNKTRRLLEKLNIIGQNNLKAMYMGINGNKESLKAIGKRENNTSKANINADIA
                     SETKAPPNFIGRLATTIIIEDGLYMG"
ORIGIN
        1 ccattgggtg tataaatctc ataagtttcg ctcactattt atcctttctt aaatttatcc
       61 catgtataaa ccatcttcta tgattatagt agtggctagt ctacctatga agttaggcgg
      121 agcttttgtt tccgatgcaa tatcagcatt tatatttgct ttgctagtgt tattttctct
      181 ttttcctatg gctttcaagc tttctttatt tccatttatc cccatataca tagctttaag
      241 attgttttgt cctatgatat tgagtttttc tagtaatctt ctggttttat ttttatcttc
      301 tatacatttt cctgttttat ctttgcttgt attgtaaatt ccattagcaa aatcttgtaa
      361 aatttgtaaa gcttcattat aagcatggat gggactatct tcatcctttt taaaagcact
      421 aatttgcttt tctttatttc taaactcttt aaacaaagga tgattacaag ggagttgata
      481 tatagtgcat tttttaagct ctaaagattt taactctttg aaataaaaag tataatcatc
```

```
      541 tataatatat ttttgttctt ttatatcctc taaagcagtt gcacttcttt tctcatcaat
      601 aatcaaataa gacaaaagtc tttttaaaag aaattcttta ttttcttcaa gcttttgatc
      661 tatttcataa t
//
LOCUS       OIDGCHHC_28              639 bp    DNA     linear       12-APR-2021
DEFINITION  Campylobacter species strain strain.
ACCESSION
VERSION
KEYWORDS    .
SOURCE      Campylobacter species
  ORGANISM  Campylobacter species
            Unclassified.
COMMENT     Annotated using prokka 1.14.6 from
            https://github.com/tseemann/prokka.
FEATURES             Location/Qualifiers
     source          1..639
                     /organism="Campylobacter species"
                     /mol_type="genomic DNA"
                     /strain="strain"
     gene            complement(20..562)
                     /gene="trxB_7"
                     /locus_tag="OIDGCHHC_00111"
     CDS             complement(20..562)
                     /gene="trxB_7"
                     /locus_tag="OIDGCHHC_00111"
                     /inference="ab initio prediction:Prodigal:002006"
                     /inference="similar to AA sequence:RefSeq:CKG87041.1"
                     /codon_start=1
                     /transl_table=11
                     /product="thioredoxin reductase"
                     /protein_id="Prokka:OIDGCHHC_00111"
                     /translation="MLKDFSKNPPYDELGMLPFLDELIGVDWVIDPNRYRFAEDEKGR
                     VNDALKDDVEKGILKDPRDIDSTSESRLEFEYKLDAYRNGMKTRFDGDNPNHWSKEQV
                     ERFNDTLILCAKLAALTPPQGYTNAPYYYSPERLEFIYKNHNLDKLLDPRIPAIYRYN
                     FPRELRAKILKFGEENGIKD"
ORIGIN
        1 gtctgcctat gaggttactt taatctttaa tgccattttc ttcgccaaat ttgagtatct
       61 tagctctaag ttctcttggg aaattatatc tataaatagc agggattctt ggatcaagga
      121 gtttatctaa attatgattt ttgtaaataa actctaatct ttcaggagag taataataag
      181 gtgcattagt atagccttga ggaggagtga gggctgcgag tttggcacat aaaatcaaag
      241 tatcattaaa tctttctact tgctctttac tccaatgatt tggattatct ccatcaaatc
      301 tagttttcat tccatttcta taagcatcca atttatattc aaattccaat ctactctctg
      361 aagtagaatc tatatctctt ggatctttga gtattccttt ttcaacatca tcttttagag
      421 catcattaac tcttcctttt tcgtcttctg caaatcgata cctatttgga tctatcaccc
      481 aatccactcc tataagttca tctaagaaag gaagcatacc caattcatca taaggaggat
      541 ttttagaaaa gtcttttagc atttgagctt taagagggtt tttgtgtatg ccatctgtta
      601 aatctctata tgcataagaa tcataaagac ttttaaaac
//
LOCUS       OIDGCHHC_29              629 bp    DNA     linear       12-APR-2021
DEFINITION  Campylobacter species strain strain.
ACCESSION
VERSION
KEYWORDS    .
SOURCE      Campylobacter species
```

```
  ORGANISM  Campylobacter species
            Unclassified.
COMMENT     Annotated using prokka 1.14.6 from
            https://github.com/tseemann/prokka.
FEATURES             Location/Qualifiers
     source          1..629
                     /organism="Campylobacter species"
                     /mol_type="genomic DNA"
                     /strain="strain"
     gene            complement(50..616)
                     /locus_tag="OIDGCHHC_00112"
     CDS             complement(50..616)
                     /locus_tag="OIDGCHHC_00112"
                     /inference="ab initio prediction:Prodigal:002006"
                     /inference="similar to AA sequence:RefSeq:CKG88165.1"
                     /codon_start=1
                     /transl_table=11
                     /product="ribosomal protein L7/L12"
                     /protein_id="Prokka:OIDGCHHC_00112"
                     /translation="MAKNVNVGGEYLTNVGLSKDTIVGLSNTLNVGVDNKVRVAKNSH
                     EFVGENKDIEIGANQNTIIHKDEIRNVKGNKKEMVEGHYDINISDKMQVLSEKEMDYK
                     SKDNILFTSNESIGFESDKNTSMVADNITTYAKTIHELKADSEATIQVGETIINAKPD
                     CVIIKAGGVEVIIDSNGLVVKGGELKAE"
ORIGIN
        1 tatataaaat cttttaagtt tgtaaaaagg ttttttcatt gcttatcctt tactctgcct
       61 taagctctcc acctttaacc acaagtccat tagagtctat aatcacttct actccacctg
      121 ctttaatgat aacacaatca ggttttgcat taataattgt ttctcctacc tgtatagtcg
      181 cttcgctatc ggcttttaac tcatgaatag tttttgcgta agttgtgata ttatcagcta
      241 ccatgcttgt atttttatca ctttcaaatc ctatggattc attacttgta aaaagaatat
      301 tgtctttgct tttataatcc atctctttct cgcttaatac ttgcatttta tcgctgatat
      361 taatatcata atgcccctca accatttcct ttttatttcc cttcacattc cttatctcat
      421 ctttatggat aatagtattt tgattagcac ctatttctat atctttgttt tctcctacaa
      481 actcatgaga gtttttagcc actcttactt tattatctac tcctacattt aaagtattgc
      541 ttaatcctac tatggtatct ttagataagc ctacattagt taagtattct cctcctacat
      601 tgacattttt agccaaatct atggtttga
//
LOCUS       OIDGCHHC_30              587 bp    DNA     linear       12-APR-2021
DEFINITION  Campylobacter species strain strain.
ACCESSION
VERSION
KEYWORDS    .
SOURCE      Campylobacter species
  ORGANISM  Campylobacter species
            Unclassified.
COMMENT     Annotated using prokka 1.14.6 from
            https://github.com/tseemann/prokka.
FEATURES             Location/Qualifiers
     source          1..587
                     /organism="Campylobacter species"
                     /mol_type="genomic DNA"
                     /strain="strain"
ORIGIN
        1 atgagttctt gtaagattac aaattcatta tttactttct tttgagataa ggagcctta
       61 atatccacta ccttagtaca aggataactt acatttgcta tggtattagg acaagctact
```

```
      121 atactagagt ttaataaatc actttctagc attataggga taccatcact tttaaaggat
      181 tttcctttgt ttgatttaag tatgacttta cctccatgga tacattctag gttttgattt
      241 tctaaaagag cgtgaagttt ggctatgctt tgttttttat ctacttgtgt ggttttttgt
      301 tcttttagag tgtttgaaat taaatctttg tattctaagc tttgtttgct taagcattct
      361 aatttgatat ttaaagattt ttctaatata gaataattaa gcaagcaaag ttcatcattt
      421 ttataaaaga tactaagtct tcctaaacca ctgctttcat ctttaggaga aaaataataa
      481 ctgggtatat cttgttttat agtattatct tgatctaatt ctccaaaata aaaaggatta
      541 ttagaaagtt catttgagcc tgtaaggagg gtggagttta ggaagat
//
LOCUS       OIDGCHHC_31              584 bp    DNA     linear       12-APR-2021
DEFINITION  Campylobacter species strain strain.
ACCESSION
VERSION
KEYWORDS    .
SOURCE      Campylobacter species
  ORGANISM  Campylobacter species
            Unclassified.
COMMENT     Annotated using prokka 1.14.6 from
            https://github.com/tseemann/prokka.
FEATURES             Location/Qualifiers
     source          1..584
                     /organism="Campylobacter species"
                     /mol_type="genomic DNA"
                     /strain="strain"
     gene            218..316
                     /locus_tag="OIDGCHHC_00113"
     CDS             218..316
                     /locus_tag="OIDGCHHC_00113"
                     /inference="ab initio prediction:Prodigal:002006"
                     /codon_start=1
                     /transl_table=11
                     /product="hypothetical protein"
                     /protein_id="Prokka:OIDGCHHC_00113"
                     /translation="MSKNIKTQEAKLDLITKFLDYANIADASYAML"
ORIGIN
        1 aaaataaatt tactttaaca gaagttacat ttgaagatta ttatgatggt attcattatt
       61 caacgcattt atcctatatt tatgataatt atggtatgtt cttaaaaggt gatgagggtg
      121 ctggattcta ttttagatat cataaaagac tgtattgtga agatgttaga taaaaaatta
      181 gaaagataat ttgagattta gatttttgga ataacaaatg agcaaaaaca ttaaaacaca
      241 agaagctaaa ttagacttaa tcactaaatt tttagactat gctaatatag cagatgctag
      301 ttatgctatg ttgtaatatg tttgggaaaa tatagagcaa gatgaaaaga ataatattta
      361 tacttttggt gataaattaa agcaagatat tgttatgaaa aatagtaaag gagaagatat
      421 tgtaaaacct aaaaatacaa acacagctta tgcttgtgct atacaagctc gttttgaaca
      481 aaataaaata gttaaaatag aacctaaata ttgtatttct cttataaata cttgttttga
      541 tagtaaagaa ataacattgg ataatgatat tagtagggta ggat
//
LOCUS       OIDGCHHC_32              517 bp    DNA     linear       12-APR-2021
DEFINITION  Campylobacter species strain strain.
ACCESSION
VERSION
KEYWORDS    .
SOURCE      Campylobacter species
  ORGANISM  Campylobacter species
            Unclassified.
```

```
COMMENT     Annotated using prokka 1.14.6 from
            https://github.com/tseemann/prokka.
FEATURES             Location/Qualifiers
     source          1..517
                     /organism="Campylobacter species"
                     /mol_type="genomic DNA"
                     /strain="strain"
     gene            complement(365..496)
                     /locus_tag="OIDGCHHC_00114"
     CDS             complement(365..496)
                     /locus_tag="OIDGCHHC_00114"
                     /inference="ab initio prediction:Prodigal:002006"
                     /codon_start=1
                     /transl_table=11
                     /product="hypothetical protein"
                     /protein_id="Prokka:OIDGCHHC_00114"
                     /translation="MTSDQLEKKFNIFYKDYEDRNEYCDLLNEAIRKDKEKENLQKN"
ORIGIN
        1 atatttatca ttagcttctt tgatatattt catatctgca aagcgaaaat tatatttagg
       61 atcttttaga cttaagccat actcatcaaa taaataatct aaaatagaaa aatcattatt
      121 atttgctcca caagtgcgag ctgctctcat tatcaaataa taaggatcaa ataaaggata
      181 ttcttcgtta tcatttttaa aatgaagctc ataacaagaa atttttacag gataatcttt
      241 taaaatctct tttactttag ctaaattatt atctcttata gcctcaagaa ctaaatcttc
      301 taacttatcc ttttcctcat aactcatagc cttaatatct tctaatgttt tcattgttgc
      361 tcctttaatt tttttgtagg ttttcttttt ccttatcctt tcttatggct tcatttaata
      421 aatcacagta ttcattacga tcttcataat ccttataaaa gatattaaat ttcttttcaa
      481 gttgatcgga agtcattgca cctgcagctt ttaaaag
//
LOCUS       OIDGCHHC_33              504 bp    DNA     linear       12-APR-2021
DEFINITION  Campylobacter species strain strain.
ACCESSION
VERSION
KEYWORDS    .
SOURCE      Campylobacter species
  ORGANISM  Campylobacter species
            Unclassified.
COMMENT     Annotated using prokka 1.14.6 from
            https://github.com/tseemann/prokka.
FEATURES             Location/Qualifiers
     source          1..504
                     /organism="Campylobacter species"
                     /mol_type="genomic DNA"
                     /strain="strain"
ORIGIN
        1 cattattact agcccaaaaa tgcataggag accagcccat tttgtcgcga tatacttcaa
       61 atttagctcc atgatttgct aggtattgga taatttgggg gtttggatta tcagcagtta
      121 ggatataagc atagattaaa gcttctttgt aaatgctagg attatcttcc atgtatctta
      181 ctacaatata tttatcatta gcttctttga tatatttcat atctgtaaag caaaaattat
      241 atttaagatc ttttagactt aagccatact catcaaataa ataatctaaa atagaaaaat
      301 cattattatt ttcttcacaa gcatatgcag ctctcattat caaataataa ggatcaaata
      361 aaggatattc ttcattttca tttttaaaat gaagctcata acaagaaatt tttacaggat
      421 aatcttttaa aatctctttt actttagcta aattattatc tcttatagcc tcaagaacta
      481 aatcttctaa cttatccttt tcct
//
```

```
>>BfR-CA-15839_min_cov_30 putative plasmid location

LOCUS       BKOFBKEE_1              7180 bp    DNA     linear       12-APR-2021
DEFINITION  Campylobacter species strain strain.
ACCESSION
VERSION
KEYWORDS    .
SOURCE      Campylobacter species
  ORGANISM  Campylobacter species
            Unclassified.
COMMENT     Annotated using prokka 1.14.6 from
            https://github.com/tseemann/prokka.
FEATURES             Location/Qualifiers
     source          1..7180
                     /organism="Campylobacter species"
                     /mol_type="genomic DNA"
                     /strain="strain"
     gene            348..851
                     /locus_tag="BKOFBKEE_00001"
     CDS             348..851
                     /locus_tag="BKOFBKEE_00001"
                     /inference="ab initio prediction:Prodigal:002006"
                     /codon_start=1
                     /transl_table=11
                     /product="hypothetical protein"
                     /protein_id="Prokka:BKOFBKEE_00001"
                     /translation="MKILEKDFDLICKIYIQWKELNNNIKKFSSRGVNFPDCISEPIV
                     CYAFNYNWNNNNETGDATSDSGELIEIKATSNFDSDLSSFSPNTKFDKLIFVRLDQER
                     DLAYIYDLELNFEQFQILEVNKKETVLDQQKQNRRPRLSLIQYIEKEKIEPLYYVDFI
                     KKIVTKN"
     gene            921..1844
                     /locus_tag="BKOFBKEE_00002"
     CDS             921..1844
                     /locus_tag="BKOFBKEE_00002"
                     /inference="ab initio prediction:Prodigal:002006"
                     /inference="similar to AA sequence:RefSeq:ASE91810.1"
                     /codon_start=1
                     /transl_table=11
                     /product="DNA (cytosine-5-)-methyltransferase"
                     /protein_id="Prokka:BKOFBKEE_00002"
                     /translation="MKVASFFAGVGGIDLAFSNHDIVYVNEIDKNATKTYKFNFDVEI
                     DCRDIQTVNEKELPDFDILLGGFPCQAFSVAGYRKGFEDSRGNLFFDIIRIAKEKKPK
                     IIFLENVKNLVSHDCGKTFNIILKSLESLGYYVKYDVLNAMHYGNTPQNRERIYIVCF
                     KNKKQYKDFNFPNPIFLNNKIDNIVDFNLKVDDKFYYTEKNFKHYEMLLQNITKKNVI
                     YQWRRKYVRENKSNVCPTLTANMGTGGHNVPLVLTKHGIRKLTPKECFMFQGFPNSYK
                     LPDIANSHLYKQAGNSVCVKVIQRIADRISN"
     gene            complement(2128..2328)
                     /locus_tag="BKOFBKEE_00003"
     CDS             complement(2128..2328)
                     /locus_tag="BKOFBKEE_00003"
                     /inference="ab initio prediction:Prodigal:002006"
                     /codon_start=1
```

```
                     /transl_table=11
                     /product="hypothetical protein"
                     /protein_id="Prokka:BKOFBKEE_00003"
                     /translation="MGFKNINTEKASINFINNARGDKINTQKRDKKILVSFTQDEYDR
                     VKESALQIGMGVNQYIRYKLFI"
     gene            complement(2329..3009)
                     /locus_tag="BKOFBKEE_00004"
     CDS             complement(2329..3009)
                     /locus_tag="BKOFBKEE_00004"
                     /inference="ab initio prediction:Prodigal:002006"
                     /inference="similar to AA sequence:RefSeq:ASI88294.1"
                     /codon_start=1
                     /transl_table=11
                     /product="Plasmid partitioning protein ParA"
                     /protein_id="Prokka:BKOFBKEE_00004"
                     /translation="MIISICNEKGGSGKSTLAVNLSSRLTEDGDSILLFDSDPQRSTE
                     VFINIRNNSNLKQNFKHSFDINLLNSGFKNELNNYDAIIIDTGGRDSREMRKAMLLSN
                     VIIIPTIASQYDVSVLDHMLNIYKEVKLINKNIILLILINRVSPNPFLIKELNNLIDY
                     IHEKKEVNNLNDVFLLESVLYERQAYKRAVIEGKSISEFCDKNDKALKDFEKFYQEIF
                     QILKNKDK"
     gene            complement(3589..4917)
                     /locus_tag="BKOFBKEE_00005"
     CDS             complement(3589..4917)
                     /locus_tag="BKOFBKEE_00005"
                     /inference="ab initio prediction:Prodigal:002006"
                     /inference="similar to AA sequence:RefSeq:APA51640.1"
                     /codon_start=1
                     /transl_table=11
                     /product="mobilization protein"
                     /protein_id="Prokka:BKOFBKEE_00005"
                     /translation="MSYDEIEKVKRSAYTKTASESTKRNYISSGNFKKSTSFQVFHNA
                     NVRPDYAIGGELVFDLGGYEALELKNKIVSEAKEAYEKNKSPKAPKFKAKSYEWSLVV
                     NIKPDTSMNDLKNLSKHFQEKYGFQCYQIAIHRDEGHFNEQGEKLINHHAHMEFITLD
                     KETGKNNFRREKISPKVLREIQTEIAEILGMERGEDKRISGAKRIEPRAYAALKEKEK
                     REKKRNINLEQEQEKEQFLNKSEYKKIIENFRKSQINQGLDKDFFKELSELKKNFQEI
                     KEDNLSELLNDILREHLERIEKLKKENSILKAENELLKQKEPKNDNNIDQEQKNASER
                     NFEPKNDNNTEDKKIFFEKLATYRLNEGLSKIYSKPLKRIVEPETEAKKKERIKILTE
                     TMEKTEAYLPETLKKWLENNKDIDLKEILVAYQNTLSKELKQSKSIYKGR"
     gene            complement(4921..5085)
                     /locus_tag="BKOFBKEE_00006"
     CDS             complement(4921..5085)
                     /locus_tag="BKOFBKEE_00006"
                     /inference="ab initio prediction:Prodigal:002006"
                     /codon_start=1
                     /transl_table=11
                     /product="hypothetical protein"
                     /protein_id="Prokka:BKOFBKEE_00006"
                     /translation="MDYEKYSKMNLRQLINAYTNAEKKEKKLIDDFQKKLDENKNLME
                     FLKTKIKESL"
     gene            complement(5488..5823)
                     /locus_tag="BKOFBKEE_00007"
     CDS             complement(5488..5823)
                     /locus_tag="BKOFBKEE_00007"
                     /inference="ab initio prediction:Prodigal:002006"
```

```
                     /codon_start=1
                     /transl_table=11
                     /product="hypothetical protein"
                     /protein_id="Prokka:BKOFBKEE_00007"
                     /translation="MSEKTKLQIAEHILKNCYWGNTTMTPSFIVEHIQDKDFAKRIFS
                     AIFQNSLTMFEDLKIIDNEEWIKEFIISQNQRLGYHKRFYYEERLDELIAHYGIKNAG
                     KRREVHFIA"
     gene            complement(5820..6857)
                     /locus_tag="BKOFBKEE_00008"
     CDS             complement(5820..6857)
                     /locus_tag="BKOFBKEE_00008"
                     /inference="ab initio prediction:Prodigal:002006"
                     /inference="similar to AA sequence:RefSeq:ALW35761.1"
                     /codon_start=1
                     /transl_table=11
                     /product="replication initiation protein"
                     /protein_id="Prokka:BKOFBKEE_00008"
                     /translation="MSDIVKYHNDFNKIQLPNFSEQEQNLLFLIFAKIKEKGIDNVIN
                     IYANDFKINQKLSNSREYLTENINSLKYKFFKANFKQIIETKTEVIHKYVNLFETMEI
                     HYVKRNPDDGYDGSTLFNRITLKINPDFAYLVNQLTTNFTAFELEEFISLSGRYAKTL
                     YRLLKQFRTTGKARFEWEEFCRIMDIPQDYRQSEIDKRIIKPAIKELSKERNLFDQMR
                     VPFKNLAYEKEKAKGRGQGGKVSGITFTFKPENIEMQKLENESQKIMSDEQKYLKILN
                     NMKLNQVRFNYNDKLWQFNDFDFNEFKIIAVELIRDEYENLNFGNHMYFNAKNQEQFF
                     KMIEIFRKGIR"
ORIGIN
        1 catgttaagg gtatattgtc aatatttgtc cccttaatac ctacaatttg tccccttaat
       61 acctacaatt tgtcccctta atacctacaa tttgtcccct taatacctac aatttgtccc
      121 cttaatacct acaatttgtc cccttaatat tccatatagc tcccatcata tagtgcttag
      181 aaagatgcct aaaaataata aaaataataa aaaaatagaa aaatattacc atagtttcta
      241 aatctactaa atatattatg atttattgta ttcttacaat gaaatagtag taaatcgaga
      301 tttaaaaata ttttgtatat aatatttaaa gtttagacgc aagttgtatg aaaatacttg
      361 aaaaagattt tgacttaatt tgtaaaattt atattcaatg gaaagaatta aataataaca
      421 taaaaaaatt tagttctaga ggtgtaaatt ttcctgattg tatttccgaa cctatagtat
      481 gctatgcttt caactataat tggaataaca ataacgaaac aggagatgct actagtgatt
      541 caggtgaatt aatagaaata aaagcgactt cgaatttcga tagcgattta agttcttttt
      601 cgccaaatac taaatttgat aagctaattt ttgttagatt agatcaagaa agggatttag
      661 cttatattta tgatttggaa ttaaattttg aacaatttca aattctagaa gtgaataaaa
      721 aggaaactgt tttagatcaa caaaaacaaa ataggcgtcc taggttaagt ttaattcaat
      781 atattgaaaa ggaaaagata gaaccgcttt attatgttga ttttataaaa aaaatagtta
      841 ccaaaaatta aagaaaggag tggcgtctaa actcctttct ttaaaaaagc agcatgctgc
      901 aattataatg gagataaaat atgaaagtgg cttctttttt tgccggtgta ggtggtattg
      961 atttggcctt tagtaatcat gatatcgttt atgttaatga aatagataaa aatgctacaa
     1021 aaacatataa atttaatttt gatgtcgaaa tagattgcag ggatatacaa actgttaatg
     1081 aaaaagaatt gcccgatttt gatattttat taggagggtt tccatgccaa gcattcagtg
     1141 ttgctggtta tagaaaagga tttgaagatt ctagaggaaa tctttttttc gatattatac
     1201 ggattgcaaa agaaaaaaaa ccaaaaataa tatttctaga aaatgtaaaa aatttggtat
     1261 cgcatgattg tggaaaaact tttaatatta tattaaaatc attggaaagt ttaggttatt
     1321 atgttaaata tgatgtttta aatgcaatgc attatggaaa tacaccacaa aatagagaaa
     1381 gaatttatat tgtttgtttt aaaaataaga aacaatataa agattttaat tttccaaacc
     1441 caattttttt gaacaataaa atagacaata ttgtagattt taatttaaaa gtagatgata
     1501 aattttatta tactgaaaaa aattttaaac attatgaaat gttattgcaa aatataacaa
     1561 aaaaaaatgt tatttatcaa tggagaagaa aatatgtaag agaaaataaa agcaatgtat
     1621 gtcctacttt aacagcaaat atgggtactg gtggacacaa tgtaccgtta gtcttaacaa
     1681 aacatggaat aagaaaacta acccctaaag aatgctttat gtttcaaggt tttccaaata
```

1741 gttataaatt acctgatata gcaaatagcc atttatataa acaagcagga aatagtgtat
1801 gcgtcaaagt gatacagcgt atagcagatc gtatttcaaa ttagaaaaat ttttatattt
1861 tatttggtaa acaaccttta tatgattttt aaaattaaaa aaattctgat ttaaaatata
1921 gtttaagttt ttaaaaaact taaagcaata aattcccatt tttccataac tttaagtttt
1981 taacttttta cctcgaaact taaatataaa acaaaatgta ttaaaacaat ggagtagcca
2041 tatttttatc aaaaaatata aagaatgaaa attgcacttt tcaatacaaa atatatttta
2101 catatttata taaaatatat atatttatta tataaataat ttatatctta tatactgatt
2161 aactcccata cctatttgca aagcactttc tttcacacgg tcatactcat cctgtgtgaa
2221 actaaccaat atttttttat ctcttttttg tgtattaatt ttatctccac gagcattatt
2281 aataaaatta atacttgctt tttctgtgtt gatattttta aaccccattt atttatcctt
2341 atttttttaat atttgaaaaa tttcttgata aaatttttca aaatctttca atgctttatc
2401 atttttatca caaaactcac taatactctt tccttctatt acggctcttt tgtaggcttg
2461 cctttcatat aaaacgcttt ctagcaaaaa gacatcattt aaattattga cttctttttt
2521 ctcatgaata taatcaatta gattatttaa ttctttaatt aaaaatggat taggagagac
2581 tctattgatt aatattaata atataatatt tttatttatt aatttaactt ctttatatat
2641 attcaacata tgatcaagaa cacttacatc atattggcta gctatggtag gtataattat
2701 tacatttgat aaaagcatag cttttctcat ttctctacta tctctaccac ctgtatcaat
2761 aataatagca tcgtaattgt ttaattcatt tttaaaacca ctatttagca aattaatatc
2821 aaaactgtgt ttaaaatttt gttttaagtt agagttgttt ctgatattga taaaaacttc
2881 agttgatctt tgcggatcac tgtcaaacag caaaatgctg tcaccatctt cagttaatct
2941 tgaagataaa ttaacagcta aagtgctttt accactaccg ccttttttcat tgcatataga
3001 tataatcatt atatactctt tatatattta ttatatttat attatatact atttttttgag
3061 taattgatca aaaacttgat ttttgcttta ttaaaaagtt tatatttata ccatttgccc
3121 ttttaatatt tcgctatctc ctatcatata gtgcttagaa agatatctaa aaatatataa
3181 aaataaaaaa gaagttttat aaataagcca caattgcagt atttctgtag gttatatcat
3241 agatataacc tacataaaat atcaaaaaaa cgctttacaa atgcttaaaa tttgaaattt
3301 taaaattttt atggtataat atcattcaga ataacattga tttatagtgt tattgattaa
3361 ttttataagg aggataaatg actctactag aattagtagc aatttgctac tatttattaa
3421 caaattgctg taatgtattt taacagaaat ttcattatag caaatttttt ttaattataa
3481 attagacggg ggcttttat tagctttaag ttaacttcgc acccctgttt ccaaaatcat
3541 agcacctata aggtgcgatt tgctaagcta aaaagggaga gaaagtgttt atctgccttt
3601 gtaaatactc ttgctctgtt ttaattcttt gctaagggta ttttgataag cgacaagtat
3661 ttcttttaaa tcaatatctt tgttattttc aagccatttt tttaaagttt caggtaggta
3721 agcttctgtt ttttccattg tttctgttaa aattttaatt ctttctttct ttttggcttc
3781 ggtttcaggc tccacaattc ttttaagggg cttggaataa atcttactta atccttcatt
3841 cagtctgtaa gtggctaatt tttcgaaaaa tattttttta tcttctgtat tattatcgtt
3901 tttcggctcg aaattgcgtt ctgaagcgtt tttttgctct tggtcgatat tattatcgtt
3961 tttcggctct ttttgcttta aaagctcatt ttcggctttt aaaatgctat tctctttttt
4021 taacttttca atgcgttcta aatgctctct taaaatatcg tttaaaagct ctgataaatt
4081 gtcttcttta atctcttgga agttcttttt tagttcgctt aactctttga agaaatcttt
4141 gtcaagccct tgattaattt gactttttct aaaattttca atgatttttt tatactcgct
4201 tttatttaaa aattgctctt tttcttgctc ttgctctaaa ttaatgtttc gctttttctc
4261 ccttttttcc ttttctttta aggctgcata agctctaggc tctatgcgtt ttgctccgct
4321 tattctctta tcttcgccac gctccattcc taagatttca gcgatttcgg tttgaatttc
4381 tcttaagact tttgggctta ttttttcacg cctaaagttg tttttacctg tttctttgtc
4441 aagagtgata aattccatat gtgcgtggtg gtttataagc ttttcgcctt gctcgttaaa
4501 atgcccttca tcgcggtgta tagcgatttg atagcactga aagccgtatt tttcttgaaa
4561 atgtttagaa aggtttttta aatcattcat tgaggtatca ggcttgatat tgacaactaa
4621 gctccattca tagctcttag ctttaaattt tggagctttg gggcttttat tttttttcata
4681 ggcttctttc gcttccgaaa ctatcttatt tttaagctct aaggcttcat aaccgcctaa
4741 atcaaaaaca agctcaccgc ctattgcgta atcaggtctt acattggcat tatggaagac
4801 ttgaaaagaa gttgattttt taaaatttcc gcttgaaata taatttcttt ttgtgctttc
4861 acttgcagtt tttgtgtaag cacttctttt aacttttttca atctcatcgt aagacatttt
4921 ttataaactt tctttgattt ttgtttttaa aaactccatt aaatttttat tctcatcaag

```
     4981 ttttttttga aaatcatcga ttaatttttt ttctttttttt tctgcgtttg tgtaagcgtt
     5041 tatcaattgt cttaaattca ttttagaata tttttcataa tccatttttt ttccctttttt
     5101 tgaaaaaata taaatgtaat cgagcttgtg agattatagc atatttttta aaaacaaatt
     5161 atagaacttt tttagttcgg ataatttgca aattatccaa tcatatttta taaataaaaa
     5221 aatgattaat aatttgcacc gcgtggcttt gcattaattc gcaagctcat ttaatgtaaa
     5281 agtttaaata aaattattta tgatagaatt ttattcgact tcctaaatga aagtttagcg
     5341 aagaattagc ttatctttta gataggttgc cccgtcttag agcggggata ttaaattcct
     5401 tgcttattgt atttaggaca ttcaaatatt ctatctttta aatttaaatc atctttgata
     5461 tatccacaac aactacaagt tttagagcta ggcaatgaaa tgcacttctc ttctttttcc
     5521 tgcatttttt atcccataat gtgcgataag ctcgtcaagc ctttcttcat aataaaatct
     5581 tttgtgatag cctaatcttt ggttttgaga aatgataaat tcttttatcc attcttcatt
     5641 atcgataatc tttaaatctt caaacattgt gagtgaattt tgaaaaatgg cactaaaaat
     5701 tctttttgca aagtctttat cttgtatatg ttctactata aaactagggg tcatagtagt
     5761 atttccccaa tagcaatttt ttaaaatatg ttctgcgatt tgaagttttg tttttttcgct
     5821 catctgatac cttttctaaa gatttcaatc attttaaaaa actgctcttg atttttagca
     5881 ttaaagtaca tatgatttcc aaagtttaaa ttctcgtatt catctcttat aagctctact
     5941 gcaataattt taaattcatt aaaatcaaaa tcgttaaatt gccaaagctt gtcattataa
     6001 ttaaatctaa cttgattaag tttcatattg tttaaaatct ttaaatattt ttgctcatcg
     6061 ctcattattt tttgactttc attttctagc ttttgcattt cgatattttc aggtttaaaa
     6121 gtaaaggtaa tacctgaaac cttgccacct tgcccccgcc cttttgcttt ttctttttca
     6181 taggcaagat ttttaaaagg cactcgcatt tgatcaaaaa gattgcgttc tttagaaagt
     6241 tcttttatgg caggtttaat aattctttta tcgatttcgc tttgtctata atcttgcggt
     6301 atatccataa tcctacaaaa ctcttcccat tcaaatcgtg ctttacctgt agttctaaat
     6361 tgctttaaaa ggcggtaaag tgttttagca tatctaccgc taagagatat aaattcttca
     6421 agctcaaaag ctgtaaaatt agtagttaat tgattaacta aataagcaaa atcagggttt
     6481 atttttaggg ttattctgtt aaataaagtg cttccatcat atccatcatc aggatttctt
     6541 ttaacatagt gtatttccat tgtttcaaac aaatttacat atttgtgtat aacttctgtc
     6601 tttgtttcta tgatttgttt aaaatttgcc ttgaagaatt tatattttag gctattgata
     6661 ttctctgtta aatattctct tgaattagaa agtttttgat taattttaaa atcattggca
     6721 tagatattaa ttacattgtc tataccсttt tctttaatct ttgcaaagat taaaaataaa
     6781 aggttttgct cttgctctga aaagttaggc aactgaattt tattaaaatc attatggtat
     6841 ttaactatat cactcataga ttttccttat aagccctaag gcaaggggct tttctgcccc
     6901 tgcggtttcg ctagaaaaat acttcaactt taagtttaaa aaacctaaaa tttaggtata
     6961 atctcacaaa acgcttacta tgtcgtttca tagtcaagcc tttagggtta tttccgccaa
     7021 gagtgaaaac tcaaggcgga tttttttattg tagcatgtta agggtatatt gtcaatattt
     7081 gtccccttaa tacctacaat ttgtcccctt aatacctaca atttgtcccc ttaataccta
     7141 caatttgtcc ccttaatacc tacaatttgt ccccttaata
//


>>BfR-CA-15840_min_cov_30 putative plasmid location

LOCUS       HKBNLMBC_1              4687 bp    DNA     linear       12-APR-2021
DEFINITION  Campylobacter species strain strain.
ACCESSION
VERSION
KEYWORDS    .
SOURCE      Campylobacter species
  ORGANISM  Campylobacter species
            Unclassified.
COMMENT     Annotated using prokka 1.14.6 from
            https://github.com/tseemann/prokka.
FEATURES             Location/Qualifiers
     source          1..4687
```

```
                     /organism="Campylobacter species"
                     /mol_type="genomic DNA"
                     /strain="strain"
     gene            complement(117..386)
                     /locus_tag="HKBNLMBC_00001"
     CDS             complement(117..386)
                     /locus_tag="HKBNLMBC_00001"
                     /inference="ab initio prediction:Prodigal:002006"
                     /inference="similar to AA sequence:RefSeq:AMK28635.1"
                     /codon_start=1
                     /transl_table=11
                     /product="addiction module toxin RelE"
                     /protein_id="Prokka:HKBNLMBC_00001"
                     /translation="MKYNIKYSSKFKKHYKKLNTKEQQETKTIIERLANDGPLEPKYK
                     DHALKGDFLGFRECHIKPDLLLIYQKQDDKIILYCFDIGSHSELF"
     gene            complement(386..700)
                     /locus_tag="HKBNLMBC_00002"
     CDS             complement(386..700)
                     /locus_tag="HKBNLMBC_00002"
                     /inference="ab initio prediction:Prodigal:002006"
                     /codon_start=1
                     /transl_table=11
                     /product="hypothetical protein"
                     /protein_id="Prokka:HKBNLMBC_00002"
                     /translation="MLAQEKQDNSDYIYIEKLKTKSERQLKVMLDTLEKETIKIQAEK
                     AKLESKLKDKIKKGHFVKDALMKKWRTPSAELLEAMQELKNGGGTLCRSMEEFDRQMA
                     ED"
     gene            complement(871..1971)
                     /locus_tag="HKBNLMBC_00003"
     CDS             complement(871..1971)
                     /locus_tag="HKBNLMBC_00003"
                     /inference="ab initio prediction:Prodigal:002006"
                     /codon_start=1
                     /transl_table=11
                     /product="hypothetical protein"
                     /protein_id="Prokka:HKBNLMBC_00003"
                     /translation="MAKNTDIVELKEGFTRNQSNVSNAIANARTKMTALELKTFYQVS
                     TLIQMDDTEFKEYEISVSDFLKALNISDSNREQVVKLCRRLIRQVFEINQENGDYIAY
                     TIFSRMHYKHKEQKISMKFNEEFRPFLLELKQFTKIQQVKYIKSFDSKYSIRFYALLK
                     DYRKMSQRDFNLENIFNIFELPKTYNYTRFYQYVLKPAIDEINAKSDLWVSEPEIIGK
                     KGKKITDIRLYFGNKNEKMSDDFTKELIKQYNKFKSFNVFANCRYLADGFNTINDLMK
                     ITRISTEKNTYFRAYHSNSFGEESCIIGTPNKDDFLNSLANGIYRAINLIYEKEKKEQ
                     LPTMQWQDDKDKLKRFKSILKEWQENPEAKIY"
     gene            2315..2965
                     /locus_tag="HKBNLMBC_00004"
     CDS             2315..2965
                     /locus_tag="HKBNLMBC_00004"
                     /inference="ab initio prediction:Prodigal:002006"
                     /codon_start=1
                     /transl_table=11
                     /product="hypothetical protein"
                     /protein_id="Prokka:HKBNLMBC_00004"
                     /translation="MNNIDDIRIKEKDYDITIKYIEWCNELKNKNIELQDKIVMTIST
                     ALFGIIIVNLDKISLLYTHPWTKILFITLIILNSVTIIFLLISLKMANKSIENDIESV
```

```
                     YNNTIKRKYYFIAKWTQYIYLLSFAFVTIIFCIILCIVSYKELTMDKKQENNIIHMCN
                     ESYLGTSKPILFSDYLSKQNNQENNNKINSNNTDNSSTLNLNNIQSSTLENEKEKD"
     gene            complement(2962..4677)
                     /locus_tag="HKBNLMBC_00005"
     CDS             complement(2962..4677)
                     /locus_tag="HKBNLMBC_00005"
                     /inference="ab initio prediction:Prodigal:002006"
                     /codon_start=1
                     /transl_table=11
                     /product="hypothetical protein"
                     /protein_id="Prokka:HKBNLMBC_00005"
                     /translation="MGAISSINFKKSNAIQTRHNDRDLPPNYLIGGDCECNRTHKEAL
                     TLKNQIIKNGIKKYTEITGQKFQAKSYEWSSVVNIKPDTTMQDLERLALFLNKKYGFQ
                     CYQIAIHRDEGHINEQGQKEINHHAHLEFITLNKENGRNMWRRELITPKVLRQMQSEV
                     AEILGMQRGQDKRITKRQRIEPRKYAQMKEAEKKDLKQELLSAKEIKERLEAERKAWI
                     LEKNHTAEEYKALKELKNKIYTDKTELEKEINTLKERLKSLGDENTLLREENRNLKQE
                     MGLNEVNILAYFPPFSDFKTKLISEVYQNIKHDFSNYYFDKEKKEFSNRKLDFKVQDN
                     GNSIALNSSNNQNMSEKVSLMLKMAMVKGWDLDNLNIKGSDKFKAECYKQIAEIQKNK
                     IKELESNLKTKEQKNDEIHQKENKNDLKPISDTQNTKDKTELEEINEQIEENNKKIEK
                     LLNNNRNLYFVKELDKNNLSDFDKNEIIKISEDFINNYGESIWLDRDRIKDKDYKCIV
                     TYLKDELPRRFPNQEEYEAEYNKLTSISLELRQKKRKLENPFKNKLQDFTNQQTRKND
                     QGFSR"
ORIGIN
        1 cgtgcgatta cattcacaat cgccaccaat taagtaatta ggcggtaaat ctctgtcatt
       61 atgccttgtt tgaatagcat tgcttttttt aaaattaatg ctagagatag cacccattaa
      121 aataactcgc tatgtgagcc tatatcaaaa caataaagaa ttattttatc atcttgtttt
      181 tggtagatta ataacaaatc aggcttaatg tggcattctc taaaacctaa aaaatcacct
      241 tttaaagcgt gatctttgta ttttggttcc aaaggcccat cattagctaa acgctcaata
      301 attgtctttg tttcttgttg ttctttggtg tttagcttct tgtaatgctt tttaaattta
      361 gaagaatatt tgatattgta tttcattaat cttccgccat ttgtctgtca aattcttcca
      421 ttgatctaca aagcgttcca ccgccatttt ttaactcttg catagcttct aaaagttctg
      481 cactaggtgt tcgccatttt ttcattaaag catcttttac aaagtgccct tttttaattt
      541 tgtcttttag tttgctttct agctttgctt tttctgcttg aatttttatt gtttcttttt
      601 ctaaagtgtc aagcattact tttagctgtc tctcgctttt tgtttttaat ttttcaatgt
      661 agatataatc agaattatct tgtttttctt gtgctaacat tgcttcgctc cttttaaaaa
      721 atatataatt gaaattatga aattataaca tattttttaa aacaaattat tatcttattt
      781 taataaggtg gataatttgc aaattatcca cctttacaag caaagtgtta atttgaactg
      841 cgtgacttta tatattaatt cataagttta ttaatatatt tttgcttctg gattttcttg
      901 ccattctttg agtatgcttt taaatctttt taatttatct ttatcatctt gccattgcat
      961 tgtaggtaat tgttcttttt tctctttttc atagattaga ttaattgctc tataaattcc
     1021 attggctaaa gaatttaaaa aatcatcttt atttggagtt ccaataatac aactttcttc
     1081 accaaaacta ttagaatggt aagctctaaa gtatgtattt ttttcggtgc ttattcttgt
     1141 tattttcatt aaatcgttga tagtgttaaa accatctgca agataacgac aattagcaaa
     1201 tacattaaag cttttaaatt tgttgtattg tttgataagt tcttttgtaa aatcatcgct
     1261 catttttttcg tttttattcc caaaatatag gcgtatatca gttatctttt taccttttttt
     1321 tcctatgatt tcaggctcac ttacccacaa atcactttta gcgttgattt catcaatagc
     1381 gggttttagg acatattgat aaaaccttgt gtagttgtat gtttttggta actcaaatat
     1441 attaaaaata ttttctaggt taaaatctct ttggctcatt ttacgataat cttttaaaag
     1501 ggcataaaag cgtatagaat acttactatc aaagctttta atgtatttta cttgttgtat
     1561 ttttgtaaat tgttttagtt ctaataaaaa ggggcgaaac tcttcattga atttcattga
     1621 aatcttttgc tctttatgtt tatagtgcat tcttgaaaag attgtgtaag caatataatc
     1681 gccattttct tggttgattt caaatacttg tcttatcaaa cgcctacaaa gctttactac
     1741 ttgctctctg ttgctatcgc taatatttaa agcttttaaa aaatcgctca cgctgatttc
     1801 atactcttta aactctgtat cgtccatttg tataagcgta gaaacttgat aaaaggtttt
```

```
     1861 aagctctaaa gctgtcattt tagttcttgc attagcgatt gcattgctaa cattgctttg
     1921 atttctggta aaaccctctt taagctccac aatatcggta tttttagcca ttttaagcct
     1981 ttgtattttt cacattaaaa atcttacttt tatgtattat aatatatatt agtaagtttt
     2041 attattaatc ttacttttat attcaaattc acattaaaaa tcttactttt atattcaaat
     2101 tcacattaaa aatcttactt ttcacattaa aaatcttact tttcgttctt tctagctccc
     2161 taaaatacgt acttttatat aatcgacata ttcaatacat atctatttat attacatatt
     2221 agaaaaatct ggaaaatatt ttgatttttt gttaaatttt gctttgaaac ttaaataaga
     2281 taaaatttca aataaaatat tataaggaat aactttgaat aatattgatg atattcgcat
     2341 taaagaaaag gattacgata taacaataaa atatatagaa tggtgcaatg aattaaaaaa
     2401 caagaacata gaattacaag ataaaattgt tatgacaatt tcaacagcat tatttgggat
     2461 tatcatagtt aaccttgata aaatttcact tttatataca catccttgga caaaaatact
     2521 ttttattaca ctaataatat taaattctgt tacaataatt ttcctattaa tttcattaaa
     2581 aatggccaat aaatctatag aaaatgatat agaaagtgtt tacaataata caataaaaag
     2641 aaaatattac tttatagcta aatggacaca atatatatat ttactctcat ttgcttttgt
     2701 tactattatt ttttgtataa ttctatgtat agtttcttat aaggaattaa caatggataa
     2761 aaaacaagaa aacaatataa tacatatgtg caatgaaagc tatcttggaa ccagtaagcc
     2821 tatcctattt agtgattatc tgtctaagca aaacaaccaa gaaaacaaca acaaaataaa
     2881 ttcaaacaat acagataatt catctacttt aaatttaaac aatattcaaa gctcaacgct
     2941 agaaaatgaa aaagaaaaag attaacgact aaaaccctga tcgttttttc ttgtttgctg
     3001 atttgtaaaa tcttgtaatt tgtttttaaa ggggttttct aattttcttt tcttttgacg
     3061 aagttctaag ctaatagaag ttaatttgtt gtattctgct tcgtattctt cttgattagg
     3121 aaatcttctt ggtagttcat cttttaaata agtcactatg cacttataat ctttgtcttt
     3181 aattctatca cgatcaagcc aaatactttc accataatta ttgataaaat cttcgcttat
     3241 tttaataatc tcatttttat caaaatcgct tagattattt ttgtctaatt cttttacaaa
     3301 atataaattt ctgttgttat taagcaattt ctctattttt ttattatttt cttcaatttg
     3361 ttcatttatt tcttctagtt ctgttttatc tttagtattt tgcgtgtcag aaataggctt
     3421 taaatcattt ttattttctt tttggtgtat ttcatcgttt ttttgctctt ttgtttttaa
     3481 attgctttct agctctttta tcttgttttt ttgaatttct gctatttgtt tataacattc
     3541 tgctttgaat ttatcacttc ctttgatatt taaattatct aaatcccacc ccttaaccat
     3601 tgccatttta agcattaaac taactttttc gctcatattt tgattattgc ttgaatttaa
     3661 agcaatagaa tttccattat cttgcacttt aaaatctagt ttacgattag aaaattcttt
     3721 tttctctttg tcaaagtaat aattgctaaa atcgtgttta atgttctgat atacttcact
     3781 aataagcttt gttttaaaat cggaaaaagg ggggaaataa gcaagaatat ttacttcatt
     3841 taagcccatt tcttgcttta aatttcggtt ttcttctctt agtaaagtat tttcatcacc
     3901 taaactcttt aaacgctctt ttagggtgtt tatttctttt tctagttctg ttttatctgt
     3961 gtaaatttta ttttttaatt cttttaaagc tttgtattct tcggcggtgt ggttttttttc
     4021 taaaatccac gctttacgct cggcttctag tctctcttta atttctttag cacttaaaag
     4081 ctcttgcttt agatcttttt tttctgcttc tttcatttgt gcgtatttgc gtggttctat
     4141 tctctgtctt tttgtaatac gcttatcttg tccccttttgc attcctagaa tttcggcgac
     4201 ttcgctttgc atttgtctta aaacttttgg cgtaataagt tctctacgcc acatattacg
     4261 cccatttttct ttatttagcg taataaattc taaatgggcg tgatgattga tttcttttttg
     4321 tccttgctcg ttgatgtgtc cttcatctct gtggattgct atttgatagc attgaaagcc
     4381 atattttttta tttaaaaaca atgcaagtct ttcaagatct tgcattgttg tatctggttt
     4441 tatattgaca accgaactcc attcataact ttttgcttga aatttttgtc ctgtaatttc
     4501 tgtatatttc ttaatgccgt tttttataat ttgatttttt agcgttaatg cttctttgtg
     4561 cgtgcgatta cattcacaat cgccaccaat taagtaatta ggcggtaaat ctctgtcatt
     4621 atgccttgtt tgaatagcat tgcttttttt aaaattaatg ctagagatag cacccattaa
     4681 aataact
//
LOCUS       HKBNLMBC_2              4059 bp    DNA     linear       12-APR-2021
DEFINITION  Campylobacter species strain strain.
ACCESSION
VERSION
KEYWORDS    .
```

```
SOURCE      Campylobacter species
  ORGANISM  Campylobacter species
            Unclassified.
COMMENT     Annotated using prokka 1.14.6 from
            https://github.com/tseemann/prokka.
FEATURES             Location/Qualifiers
     source          1..4059
                     /organism="Campylobacter species"
                     /mol_type="genomic DNA"
                     /strain="strain"
     gene            complement(1057..2445)
                     /locus_tag="HKBNLMBC_00006"
     CDS             complement(1057..2445)
                     /locus_tag="HKBNLMBC_00006"
                     /inference="ab initio prediction:Prodigal:002006"
                     /codon_start=1
                     /transl_table=11
                     /product="hypothetical protein"
                     /protein_id="Prokka:HKBNLMBC_00006"
                     /translation="MSYDEIEKVKRSAYTKTASENTKRNYISSGNFKKSTSFQVFHNA
                     NVRPDYAIGGELVFDLGGYEALELKNKIISEAKEAYEKNKSPKAPKFKAKSYEWSLVV
                     NIKPDTSMNDLKNLSKHFQEKYGFQCYQIAIHRDEGHFNEQGEKLINHHAHMEFITLD
                     KETGKNNFRREKISPKVLREIQTEIAEILGMERGEDKRISGAKRIEPRAYAALKEKEK
                     REKKRNINLEQEQEKEQFLNKSEYKKIIENFRKSQINQGLDKDFFKELSELKKNFQEA
                     KEDDLSELLNDILREHLERIEKLKKENSILKAENELLKQKEQKNNINIDQEQENASKR
                     EIEPKNDKISKLEDDFNFVKNINPNDMSEENYFKLKKLYTENYNNLVEDKNFPNYDYL
                     VDFFKKAPQNINDLIVQKASIMKDVELQENIQFYLKKTLEKHSKDHTQDLVKTTKNYR
                     QNYLKKGLGFER"
     gene            complement(2449..2613)
                     /locus_tag="HKBNLMBC_00007"
     CDS             complement(2449..2613)
                     /locus_tag="HKBNLMBC_00007"
                     /inference="ab initio prediction:Prodigal:002006"
                     /codon_start=1
                     /transl_table=11
                     /product="hypothetical protein"
                     /protein_id="Prokka:HKBNLMBC_00007"
                     /translation="MDYEKYSKMNLRQLINAYTNAEKKEKKLIDDFQKKLDENKNLME
                     FLKTKIKESL"
     gene            complement(2844..3881)
                     /locus_tag="HKBNLMBC_00008"
     CDS             complement(2844..3881)
                     /locus_tag="HKBNLMBC_00008"
                     /inference="ab initio prediction:Prodigal:002006"
                     /inference="similar to AA sequence:RefSeq:ALW35761.1"
                     /codon_start=1
                     /transl_table=11
                     /product="replication initiation protein"
                     /protein_id="Prokka:HKBNLMBC_00008"
                     /translation="MSDIVKYHNDFNKIKLPSFTEQEQNLLFLIFSKIKEKGIDNVIN
                     LYANDFKINEKLSNSREYLTENINSLKYKFFKANFKQIIETKTEVIHRYVNLFETMEI
                     HYIKRNPDDGYDESTLFNRITLKINPDFAYLVNQLTTNFTAFELEEFISLSGKYTKTL
                     YRLLKQYRTTGKAYFKWEEFCRVMDISEKLAMRDIDKSILKPVIKELSKERNLFDQMR
                     VPFKNLAYEKEKTAGRGRGGKVSGIVFTFKPENIEMQKLENESQKIMSDEQKYLKILN
```

```
                 NMKLNQVRFNYNDKLWQFNDFDFNEFKIIAVELQRDEYENLNFANHMHFNAKNQEQFF
                 KMIDTFRNGIR"
ORIGIN
        1 cggttttgct agaaaaatac ttcaacttta agtttaaaaa acctaaaatt tagatataat
       61 ctcacaaaac gcttactatg ttgtttcata gttaagcctt taaagggtta tttccgccaa
      121 gagtgaagac tcaaggcgga tttttttattg taacatatta agtaggtatt gtcaaataat
      181 ataccactta atacctacaa tttacccact taatacctac aatttaccca cttaatacct
      241 acaatttacc cacttaatac ctacaattta cccacttaat atttcgctag accccatcat
      301 atagggctta gaaagccacc taaaaatata aaaaatatat aaaaaataaa aaaattcgcg
      361 tatgcgtacg cgcgcttgat aataaatata aattaatatt atatttttat aagcgatttt
      421 catttttgt ttaaaaaacg aatttttgag aaaaactcgg taattctcgt ttacacatta
      481 gggcggtggc ttgttcgttc gttagctcta ggctaacttc acgcccccgc accccatttc
      541 gcaaatcata acaccttata ggtgtgattt gctaagctaa aaagggaaaa atgtgtgatt
      601 tgctaagcta aaaagggaaa aatgtgtgat ttgcttcaac tttatttatt aaagtcatta
      661 tttacatagt tttaaaatta ttttttatta aattttaagc ataaataaac tataattgcg
      721 aggtttctct ttcatattaa agataaacgt gcaaaatatc tttaaagaaa ggaggtttat
      781 aatgctttat cttatcatag atagcgaatt gttagatata tttcatttga caattttaat
      841 tatcaaatca ttgcctattt aataggtaat gattatcttg ttaaccttat ctattaattt
      901 atctttaagc tccagtgtgt gtattatttt tttattcatg acgtcgaaat tatatcataa
      961 aaatatataa ataaccttaa aaaactatta atatttatta aaaattactt taaatatact
     1021 tataaatata tttataagta tatttaaatt tttaaattat ctttcaaatc ccaaaccttt
     1081 ttttagatag ttttgacgat aatttttagt tgttttaact aaatcttgcg tatgatcttt
     1141 gctatgcttt tcaagtgttt tcttaagata gaattgtata ttttcttgca attcaacatc
     1201 tttcataatt gaagcttttt gcactattaa atcattaata ttttgtggtg ctttcttaaa
     1261 gaaatctact aaataatcat agtttggaaa gttcttatct tctactaaat tattgtaatt
     1321 ttcagtgtaa agtttttttaa gtttgaaata gttttcttca ctcatatcat ttggattaat
     1381 atttttttaca aaattgaaat catcttctag ctttgaaatt ttatcgtttt tcggctcaat
     1441 ttcgcgtttt gacgcgtttt cttgttcttg gtcgatattt atattgtttt tttgctcttt
     1501 ttgctttaaa agctcatttt cagcttttaa aatgctattc tcttttttta atttttcaat
     1561 gcgttctaaa tgctctctta aaatatcgtt taaaagttct gataaatcat cttcttttgc
     1621 ttcttggaag ttcttttttaa gttcgcttaa ctctttgaag aaatctttgt caagcccttg
     1681 attaatttga ctttttctaa aattttcaat gattttttta tactcgcttt tatttaaaaa
     1741 ttgctctttt tcttgctctt gctctaaatt aatgtttcgc tttttctccc ttttttcctt
     1801 ttcttttaag gctgcataag ctctaggctc tatgcgtttt gctccgctta ttctcttatc
     1861 ttcgccacgc tccattccta agatttcagc gatttcggtt tgaatttctc ttaagacttt
     1921 tgggcttatt ttttcgcgcc taaagttgtt tttacctgtt tctttgtcaa gagtgataaa
     1981 ttccatatgt gcgtggtggt ttataagctt ttcgccttgt tcgttaaaat gcccttcatc
     2041 gcggtgtata gcgatttgat agcactgaaa gccgtatttt tcttgaaaat gtttagaaag
     2101 gtttttttaaa tcattcattg aggtatcagg cttgatattg acaactaagc tccattcata
     2161 gctcttagct ttaaattttg gagctttggg gcttttattt ttttcatagg cttctttcgc
     2221 ttccgaaatt atcttatttt taagctctaa ggcttcataa ccgcctaaat caaaaacaag
     2281 ctcaccgcct attgcgtaat caggtcttac attggcatta tggaagacct gaaaagaagt
     2341 tgattttttta aaatttccgc ttgaaatata atttcttttt gtgttttcac ttgcagtttt
     2401 tgtgtaagca cttcttttaa ctttttcaat ctcatcgtaa gacatttttt ataaactttc
     2461 tttgattttt gtttttaaaa actccattaa atttttattc tcatcaagtt tttttttgaaa
     2521 atcatcgatt aatttttttt cttttttttc tgcgtttgtg taagcgttta tcaattgtct
     2581 taaattcatt ttagaatatt tttcataatc cattttttttc ccttttttga aaaaatataa
     2641 atgtaatcga gcttatgaga ttataccata ttttttaaaa acaaattata gaactttttt
     2701 agttcggata atttgcaaat tatccaatca tattttataa ataaaaaaat gattaataat
     2761 ttgcaccgcg tggctttgca ttaattcgca agctcattta atgaaatgtt agattattca
     2821 gcaatgcact tttgtttttt cgcttatcta atcccatttc taaaagtatc aatcatttta
     2881 aaaaactgct cttgattttt tgcgttaaag tgcatatgat ttgcaaagtt taaattctca
     2941 tactcatctc tttgaagctc tactgcaata attttaaatt cattaaaatc aaaatcgtta
     3001 aattgccaaa gcttgtcatt ataattaaat ctaacttgat taagtttcat attgtttaaa
```

```
     3061 atttttaaat atttttgctc atcgctcatt attttttgac tttcattttc tagcttttgc
     3121 atttcgatat tttcaggttt aaaagtaaaa actatacctg aaaccttgcc gccacgccca
     3181 cgtcctgcgg ttttttcttt ttcataggca agatttttaa aaggcactcg catctgatca
     3241 aaaagattgc gttctttaga aagttctttg ataacaggtt taagaatgct tttgtcaata
     3301 tctctcattg caagtttttc agatatatcc ataactctac aaaattcttc ccatttaaaa
     3361 taagccttgc ccgtagttct atattgcttt aataaacgat aaagtgtttt tgtatattta
     3421 ccactaagag atataaattc ttcaagttca aaagctgtaa aattagttgt caattgattg
     3481 actaaataag caaaatcagg attaattttt agtgttattc tgttaaataa agtgctttca
     3541 tcatatccat catcaggatt tcttttaata tagtgtattt ccattgtttc aaacaaattt
     3601 acatatctgt gtataacttc tgtctttgtt tctatgattt gtttaaaatt tgccttgaaa
     3661 aatttatatt ttaaactatt gatattctct gttaaatatt ctctagagtt agaaagtttt
     3721 tcattgattt taaaatcatt ggcataaaga ttaattacat tatctatacc cttttcttta
     3781 atctttgaaa agattaaaaa taaaaggttt tgctcttgct ctgtaaagct aggcaacttt
     3841 attttattaa aatcattgtg gtatttaaca atatcgctca tattttacct tttgaagtat
     3901 aaccctaaag gcaaggggct ttttcgcccc tgcggttttg ctagaaaaat acttcaactt
     3961 taagtttaaa aaacctaaaa tttagatata atctcacaaa acgcttacta tgttgtttca
     4021 tagttaagcc tttaaagggt tatttccgcc aagagtgaa
//


>>BfR-CA-15842_min_cov_30 putative plasmid location

LOCUS       BDFFJJMB_1             44520 bp    DNA     linear       12-APR-2021
DEFINITION  Campylobacter species strain strain.
ACCESSION
VERSION
KEYWORDS    .
SOURCE      Campylobacter species
  ORGANISM  Campylobacter species
            Unclassified.
COMMENT     Annotated using prokka 1.14.6 from
            https://github.com/tseemann/prokka.
FEATURES             Location/Qualifiers
     source          1..44520
                     /organism="Campylobacter species"
                     /mol_type="genomic DNA"
                     /strain="strain"
     gene            970..1212
                     /locus_tag="BDFFJJMB_00001"
     CDS             970..1212
                     /locus_tag="BDFFJJMB_00001"
                     /inference="ab initio prediction:Prodigal:002006"
                     /codon_start=1
                     /transl_table=11
                     /product="hypothetical protein"
                     /protein_id="Prokka:BDFFJJMB_00001"
                     /translation="MRSEVFVLTSSPLPINFQITFKLFAKAPPAKVTRLSSYLSIFSG
                     FALISKLSFLIEIGNFPLDSPATAFKSVFGVQELDI"
     gene            complement(3203..3601)
                     /locus_tag="BDFFJJMB_00002"
     CDS             complement(3203..3601)
                     /locus_tag="BDFFJJMB_00002"
                     /inference="ab initio prediction:Prodigal:002006"
                     /inference="similar to AA sequence:RefSeq:EAQ71728.1"
```

```
                     /codon_start=1
                     /transl_table=11
                     /product="Ssb"
                     /protein_id="Prokka:BDFFJJMB_00002"
                     /translation="MNEVVLCGFLGKDWEISQGKKDLIAKNSLAITTVTKKLNEEIKH
                     TDWIPLVVFGSNRVNALANFLKKGDKFLCKGRIFTSVYEDENQKTRTGFQVIVDSFEF
                     VHQREKQDMQKESEKNISAEDFEENKEEVF"
     gene            complement(3612..5654)
                     /locus_tag="BDFFJJMB_00003"
     CDS             complement(3612..5654)
                     /locus_tag="BDFFJJMB_00003"
                     /EC_number="5.6.2.1"
                     /inference="ab initio prediction:Prodigal:002006"
                     /inference="similar to AA sequence:RefSeq:APA82025.1"
                     /codon_start=1
                     /transl_table=11
                     /product="DNA topoisomerase III"
                     /protein_id="Prokka:BDFFJJMB_00003"
                     /translation="MKLFIAEKPELGRAIAEGLDGDINKYEGYIQKGDNLITWAFGHI
                     LKLATPEIYNEKWKLWNLNDLPLDIKEFKYVPIENSKKQLSIICKLIHNDQVDSIVHC
                     GDADDEGQILIDEIIQYSKTQKPIFRVLINDLTPKSVRSEISKMKPNSQFKGMSERGF
                     ARSQADWIVGINLTRAFTSVAQKSGFKEVLTLGRVQTPILGLVTTRDYEYENFKSIDY
                     YSLLGKFELNGKVFNARLKTEEKIIDENTAKNIKEININKTGLLNTSKEKKKEYPPLP
                     YSLLVLQAECAKVYGYAPDKTLEVTQILREKYKAITYNRSDCQYLPETMFEQAPNIVN
                     AIKSNIKNSEKDLNLLIENSDLSIKGKAFNDKNISAHYGIIPTQNNFDYGLLKEEEKI
                     IYNLIAKRFMIQFFKPREYEVTNLEIQTYDKNIFFTSQSKNISDGFRKFFNHEEKDNH
                     EEKDSEDANNHLDISTMENNSICKIKDIEIEKKQTKPRPYYTMDSLLRDLNSVAKYVK
                     DEKIKKLLIEKDKDKKGENGGIGTPATRSTHIKNLIEQGYVEVSKDKKQVVKSTKKGK
                     LLISLAPETLKYPDMTALWFEQQKMIEQGTIKKEDFLKSIAKEVNNEILKIKENDDMK
                     QFLQLSKEKQYSCPECKNGYLIRRKNAKGTYWWGCSEYKNGCKFICYDEKKKPKLK"
     gene            complement(5665..6306)
                     /locus_tag="BDFFJJMB_00004"
     CDS             complement(5665..6306)
                     /locus_tag="BDFFJJMB_00004"
                     /inference="ab initio prediction:Prodigal:002006"
                     /inference="similar to AA sequence:RefSeq:ADT73517.1"
                     /codon_start=1
                     /transl_table=11
                     /product="Hypothetical Protein"
                     /protein_id="Prokka:BDFFJJMB_00004"
                     /translation="MKANYIELEKKEGQISDIPYTIIKVEYPNLKKLQDNDLLMANDK
                     GQWLCGFIGLKENQKVDFKELDNGYIVPQYLYLNSKDQKEIDLFSNNLEITKVLQFDL
                     NHSWNKTEKNTQLDEEECLKIIKNTIEYINNFNKNISKQEFEELSKEENIHKTNKLIE
                     ELITQEEGVSGLIEKDLKEIKKDKSYFFNKMSSFVKNQTIEKSRTKEDYSINN"
     gene            complement(6390..7070)
                     /locus_tag="BDFFJJMB_00005"
     CDS             complement(6390..7070)
                     /locus_tag="BDFFJJMB_00005"
                     /inference="ab initio prediction:Prodigal:002006"
                     /inference="similar to AA sequence:RefSeq:EAQ71790.1"
                     /codon_start=1
                     /transl_table=11
                     /product="cpp47"
                     /protein_id="Prokka:BDFFJJMB_00005"
```

```
                     /translation="MAFIKLSNGSNPRLMVDVNNALNYKDAQTGEIKQRQVATALVDV
                     IEEAGKVAGMDQGAVTASFKINNEWKNYFVNRDKDSHNIVLMPTDAKERSNRDNHVFI
                     NNHWNKESKRFYHAINDKREAGKALIEGIGISEFQNQDGSKSFYLDTHIKLSNNEIKE
                     ALEKTKLEKGDGYLAIVRNGGFEIKNEVEMKEQKSKQQDGFSKEETIGQTQEIKEPEK
                     TQNQEFGR"
     gene            complement(7146..8027)
                     /locus_tag="BDFFJJMB_00006"
     CDS             complement(7146..8027)
                     /locus_tag="BDFFJJMB_00006"
                     /inference="ab initio prediction:Prodigal:002006"
                     /inference="similar to AA sequence:RefSeq:EAQ71821.1"
                     /codon_start=1
                     /transl_table=11
                     /product="cpp46"
                     /protein_id="Prokka:BDFFJJMB_00006"
                     /translation="MMAEENQNLFDDEQINQENESNSLFDEVEIKICNYQEIIDEQIK
                     ILEILNNIINIGLNNTLDENTKEGIFKDLEQNFFKLKTNIKTLISYFKNPPSQEELEI
                     INKTLLMKKELETLNSSFLNLKIEDFAQQVTQLRALLDNFLVNFDNTLLESLNSALKT
                     GDDNLKDFKEKIELRLSSSLQTYDQFLKLFLEKEKKAILDFSQIFSRITKNIKRALIT
                     SLIFFTFFGISFGVFSYIVYLKYEDYKKITHNAQKLSSIVIKENKDKSLTLSFPKNKT
                     ILNKDENGIHITLQGGE"
     gene            complement(8027..8785)
                     /locus_tag="BDFFJJMB_00007"
     CDS             complement(8027..8785)
                     /locus_tag="BDFFJJMB_00007"
                     /inference="ab initio prediction:Prodigal:002006"
                     /inference="similar to AA sequence:RefSeq:ADT73520.1"
                     /codon_start=1
                     /transl_table=11
                     /product="YggA-like protein"
                     /protein_id="Prokka:BDFFJJMB_00007"
                     /translation="MKKITFATILFFGVVSSSNAIKIDVLTGDTKLACEAMLCLASPV
                     KPPECGAALARYFSIDARKWADVIAKRRSFLNLCPVDASSDPEMYKYKNDILVHLDGE
                     CTIPVLNKRIESIVLRTETIRNCSHGDGCTTEIIKHYGYRINPQLTNSCRLLASSKYT
                     DYHLKYTCNTKFYEKKDWERGYELKEVSQEIYNALPISQKEQGEKLVPVSYAEFVRLP
                     VNKRKQIGFGKYYRIDIAFYQKIIIKKDCWINEK"
     gene            complement(8782..9213)
                     /locus_tag="BDFFJJMB_00008"
     CDS             complement(8782..9213)
                     /locus_tag="BDFFJJMB_00008"
                     /inference="ab initio prediction:Prodigal:002006"
                     /inference="similar to AA sequence:RefSeq:EAQ71817.1"
                     /codon_start=1
                     /transl_table=11
                     /product="cpp44"
                     /protein_id="Prokka:BDFFJJMB_00008"
                     /translation="MKKTLSFLLLTSIVFTGCAVSAPKPKKLEGGSKLSLNNSLLEER
                     YKFVPKDSRLSNMDWIYQLELAKNKEGLLSNEQVVKTFLLAHNAKRIILIGEKNVIQE
                     YNEYFKKNGVQAEILLQPLNIIKGNKNTVNMLFFHAKGENK"
     gene            complement(9197..10993)
                     /locus_tag="BDFFJJMB_00009"
     CDS             complement(9197..10993)
                     /locus_tag="BDFFJJMB_00009"
                     /inference="ab initio prediction:Prodigal:002006"
```

/inference="similar to AA sequence:RefSeq:EAQ71814.1"
/codon_start=1
/transl_table=11
/product="cmgD4"
/protein_id="Prokka:BDFFJJMB_00009"
/translation="MKDKIIMFIFISIFSLLIAYFLSPFVMFYLNDVSLKRIFEFYNL
KFTFEALINSYPKAYVSFFAMFGFSAIVLIFLALIAKSKKTQFGEARFANLAEIKKMK
LFSDNGIIIGKYKGKFLRFKSPQFVALGAPTRSGKGVGIVIPNLMDWKESAVVQDIKQ
ECFDFTSKYRKEILGQDVFLFNPFSKRTHRYNPLTYIDMSDSDNCDSQLMDLANIIYP
KEGDSTTQFFNGLAQNLFIGLCYLWKDLNLREDGKLFLETFNLKTEFSFFGILQLSKG
FTFEHEGKKINGFDNTYKYLSNCDDLLSDITKRRLETYFSISSDNTKSGVMSSFNEPL
TPFEAEVLKLSTQASDFDLRDLRKKKMTIYIGITPDQLTNAKFILNIFWSQLILLNTK
ELPEQNPQIKYTCLMLMDEFTAPRKIPIYLSAVSFMAGYWLRSLMIYQSKSQLKAPAP
DGYGDDGAKTLLTNHACQIFYTPREQEDAEEISRILGNTTFKTRSRSVGASGGSTSYS
EASRALMLPQELREMSFEDELITIDNGKPILCKKAFYYNDPYFIDKFKQVSPTLAKIK
GIPEENILKGAFQRGECRIQIPTTGENNEKNA"

gene complement(10990..12003)
/gene="virB11"
/locus_tag="BDFFJJMB_00010"

CDS complement(10990..12003)
/gene="virB11"
/locus_tag="BDFFJJMB_00010"
/inference="ab initio prediction:Prodigal:002006"
/inference="similar to AA sequence:RefSeq:ATG66343.1"
/codon_start=1
/transl_table=11
/product="P-type DNA transfer ATPase VirB11"
/protein_id="Prokka:BDFFJJMB_00010"
/translation="MSSSRTLEKLANDFFGKHLTNPSVTEICYNGKENIFIQTTDGKW
QEETSKLDYEASKSFATSLASFKDDKIDESRPILSCILHRGERVQVVIPPATKKNHIS
ITIRKPSEKRFLIQDHVKSGLFEEIKTDHLVTNPLDEELKKLYQNKNFKDFICKAVEY
EKNIIIAGGTGSGKTTFMKTLIDFIPKEQRIITIEDVEEIKFYEHNNFVQLFYPSEAK
SEDFLNSATLLKSCLRMKPDRILLAELRGAETYDFINVLSSGHGGSITSCHAGSTHET
FERLALMTLQNSQGQKIPFEKIQDMLKNLIDVVVHIRAHNGKRHISQIYFKEMNNDKR
SEK"

gene complement(12004..13170)
/locus_tag="BDFFJJMB_00011"

CDS complement(12004..13170)
/locus_tag="BDFFJJMB_00011"
/inference="ab initio prediction:Prodigal:002006"
/inference="similar to AA sequence:RefSeq:EAQ71812.1"
/codon_start=1
/transl_table=11
/product="cmgB10"
/protein_id="Prokka:BDFFJJMB_00011"
/translation="MSNNEKQELKDMDLQSELQTQSVNVKKIQIYVFFIACVFILLLF
GYIFAKSFISSSDEAANSNIKETNKDIASSVKLRDFILPKEEPIQEETKSFEDLLKSR
EQEESVKVAPKENPFEVISKPLQPRIVKGVGMVVVSNSNSENSSYEQENSSQEGYSNN
IFNSESNQLSNGGNSNEFVGDVFTPTSASVSQFDPNLLLSKGTYIGCSLKTRLVSTIK
GGIACIVSNNVYSSNGNVLLIEKGSTITGSFSSGQLNDGMDRLFVIWQEIRTPNNIII
PVYSGATDELGASGIQGWVDHHYMKRFGSAILLSIIDDSLNILANQVINKKENSVDYT
ENSRETSREIANTALEKMINIQPTLYKNHGDLVGVYVNRDIDFSKVYQLKRRGF"

gene complement(13163..14056)
/gene="virB9"

```
                     /locus_tag="BDFFJJMB_00012"
     CDS             complement(13163..14056)
                     /gene="virB9"
                     /locus_tag="BDFFJJMB_00012"
                     /inference="ab initio prediction:Prodigal:002006"
                     /inference="similar to AA sequence:RefSeq:ATG66341.1"
                     /codon_start=1
                     /transl_table=11
                     /product="P-type conjugative transfer protein VirB9"
                     /protein_id="Prokka:BDFFJJMB_00012"
                     /translation="MKNITLISLSFFFLASTAFALNEPQKSEYDQRIAHAEYNSKDVF
                     KMIARNGFVSVIEFGDDEIITNTATGFAEGWDLIEKKNLLFIKPKAYKTTLFVENDPY
                     NQSQQKEMIVDPNPRDWKTNLIVTTNLKTYIFDLLLTNDSKQATYKLSFNYSDKILKQ
                     ESEIKKELEKKIIKEELDKNIVPRNWDFLMKANKGSEDIAPNYAYDDGVFTYLGFDNT
                     KTFPSVFLYENGKEMILNTHIRKDGKYDVLVVQKIAKQILLRSGDKVVGIFNNGFGKN
                     PLDRTRETSNENIQRVVNNHE"
     gene            complement(14053..14721)
                     /locus_tag="BDFFJJMB_00013"
     CDS             complement(14053..14721)
                     /locus_tag="BDFFJJMB_00013"
                     /inference="ab initio prediction:Prodigal:002006"
                     /inference="similar to AA sequence:RefSeq:ADT73526.1"
                     /codon_start=1
                     /transl_table=11
                     /product="type IV secretion system protein VirB8,
                     putative"
                     /protein_id="Prokka:BDFFJJMB_00013"
                     /translation="MKNQNEINSNALNFEASVRYLIEKSNQRAWLVAFISMAISIISI
                     VAVVFLTPLKTTEPYVIRVDNTTGMVDIITSLQEEQISSIEALDKYFISQYIKAREGY
                     YYKFLNQDYLLTQLMSDENVANDYRAIYAGANALDQIYKDNVEVSVKILSIVLGNSNG
                     VKTSTIRALINKKDSVTKSTQQSTKVITLSYDFLQAKTSEENRILNPLGFKVTNYRID
                     EEIR"
     gene            complement(14744..15190)
                     /locus_tag="BDFFJJMB_00014"
     CDS             complement(14744..15190)
                     /locus_tag="BDFFJJMB_00014"
                     /inference="ab initio prediction:Prodigal:002006"
                     /codon_start=1
                     /transl_table=11
                     /product="hypothetical protein"
                     /protein_id="Prokka:BDFFJJMB_00014"
                     /translation="MKHKLVLLALFTIIFSACSTKNNTNYNYLFDDKNGWVAINNIKE
                     QEKFLKIENNTPVFEKKETLIKENKNTKKKKIIVLNHPTMSYVILRKNVNDSNSFSEN
                     KIIKASEFDYSISYYTEQGDVFVKVLKQNLFFKEKDIQIIETEVLE"
     gene            complement(15180..16190)
                     /locus_tag="BDFFJJMB_00015"
     CDS             complement(15180..16190)
                     /locus_tag="BDFFJJMB_00015"
                     /inference="ab initio prediction:Prodigal:002006"
                     /inference="similar to AA sequence:RefSeq:ALW65300.1"
                     /codon_start=1
                     /transl_table=11
                     /product="conjugal transfer protein TrbL"
                     /protein_id="Prokka:BDFFJJMB_00015"
```

```
                     /translation="MQGNFFTELGNATHAILEVVRNAALSEKMTQLAVLFSITLVVTI
                     MYKGYEILFGRSQSPTKEVIWDLVAKAMIITFALNIGGWLTLVINAMDGFHEWAGGSL
                     SMYKQIDIIFNEVRNLADNIWAKASGFDTVIAGITVIIVYIAFGIAVLPALVVINATA
                     FTQTILVIAAPLMFYCLLYKSTKNVFTQWLALLISNTLLVLFMSLFFKVFAKEIYRFA
                     RYAQDQYMNADADAFFIGISMIFSSVLLVAMIFVAKVFAEKVGSVALDSAMQSVFSRV
                     SNPISSVAGKTLGNLFGHTQKGGSKMAEGVYSAMKSGASVAGSNTMSLGKNLLQRMRN
                     EA"
     gene            complement(16222..16776)
                     /locus_tag="BDFFJJMB_00016"
     CDS             complement(16222..16776)
                     /locus_tag="BDFFJJMB_00016"
                     /inference="ab initio prediction:Prodigal:002006"
                     /inference="similar to AA sequence:RefSeq:ASI88310.1"
                     /codon_start=1
                     /transl_table=11
                     /product="Phage antirepressor protein"
                     /protein_id="Prokka:BDFFJJMB_00016"
                     /translation="MSSVILFENKELGQVRTIRDKYNEPLFCLNDICKILNIKKTNSV
                     RDSINKEFDKGGGFEWYPFKTSGGTQKFIVINEPELYFVLMRSDKPKAKPFRQWVIKE
                     VLPSIRKQGYYASNNAPKLENYNNKYDLPDTPYKEKIVKAINEIEQKQNSKFIELIEY
                     EIVEKFKNGKTQVLQKLNFKTKLI"
     gene            complement(16790..17707)
                     /locus_tag="BDFFJJMB_00017"
     CDS             complement(16790..17707)
                     /locus_tag="BDFFJJMB_00017"
                     /inference="ab initio prediction:Prodigal:002006"
                     /inference="similar to AA sequence:RefSeq:ADT66946.1"
                     /codon_start=1
                     /transl_table=11
                     /product="cmgB5"
                     /protein_id="Prokka:BDFFJJMB_00017"
                     /translation="MFDDIKVFCKKSKIKSSVIATKIKCVNFILIKSHGYCSVLNPKT
                     KKQNKFLFSIISSLCIVNLAFSAGIPVVDATANSQMATQNAKEIAEWTKQATRWTDTV
                     KHYQQQIQAYKDELLSKTGIRDSVSFMNDTKQIYLDFAEAGQNIQSFYNDVLQDPQSF
                     LSDKSKETFEKFTLFDRCNASYLSEERKRICRMDMITYAAQVEHYNQASIQVNTVAKS
                     LEKLHQKLIESKDIKETNDINNAINVEKTKLEIIKTNLEISNMHYENQRRIRQDQAAQ
                     LFAESMGKEPLDYGELRKKYDKEVDALFD"
     gene            complement(17752..18096)
                     /locus_tag="BDFFJJMB_00018"
     CDS             complement(17752..18096)
                     /locus_tag="BDFFJJMB_00018"
                     /inference="ab initio prediction:Prodigal:002006"
                     /codon_start=1
                     /transl_table=11
                     /product="hypothetical protein"
                     /protein_id="Prokka:BDFFJJMB_00018"
                     /translation="MRLSLSKNRENFRSPHEKNCVLFYYVRLKKSFISSLKKIVIVQA
                     TDTMVLIKSSIVFPLCVEIPFHAPTAKIRQKKTRLLKKAIGYFNTVTQFLSNDKDTTT
                     ELDPNIVVKIVA"
     gene            complement(18093..20867)
                     /locus_tag="BDFFJJMB_00019"
     CDS             complement(18093..20867)
                     /locus_tag="BDFFJJMB_00019"
                     /inference="ab initio prediction:Prodigal:002006"
```

```
                     /inference="similar to AA sequence:RefSeq:EAQ71805.1"
                     /codon_start=1
                     /transl_table=11
                     /product="cmgB3/4"
                     /protein_id="Prokka:BDFFJJMB_00019"
                     /translation="MNNYFDPIFKGLTRPALLFGVPLMPFLINILGFILISVYTQQFF
                     LLIFGVISHFIMKAMTKKDEQMFRLFFLKIKFISNFLSKKYHKAKTFNSSTYKRLPTN
                     NDFPKLSIFPIHTEPSLEKLIPYSSLITDSIVITKEHMLISTFFIEGIEFECESDENL
                     TFKKNLLNMMIMSFSNEPVSFYFHNVRFKLHEFLDSYFENPFLKEIDEKYHKSFDKKS
                     FQQNSLYLTLIYKPLKSNIDLKTFNKLNYKSKAKEISNFVIKFQEYLGKFEANIKDFK
                     PKILSVYKKNNIRYSEQLEFYNYLISGKFNPIAVCDSPIYQYLTGSLKNIQFNHDMIQ
                     LNYNDDTKRFARCIEIKDYSNQTFSGILDSLMYLNTDYIITQTFVPLSKFDSKSALNK
                     QINQLISSEDDGVSQIEQLNLALDDLTSNVISFGNYHFNICVFGDSIDECKQSTNKII
                     TSLNSLGFTSTLANIALPASYFSQFPSNFAIRPRVHMISSLNFSSLVALHNFLMGKKE
                     KNCWGNAVTILKTPNKQPYFLNFHKSNGRNKDEFGDLNLANTLILGQSGGGKTVFMSF
                     IIDQMIKYANKDSFPANTPEDKKKFTAVFLDKDKGALGNILCAGGRYISINNGKPTGF
                     NPFMVENNEENLRFLKQLIKLCVTRNGEILNTKEEKQINEAVNTLMFQFDINERKYPI
                     SLLLENITDDFNDNNSLKSRLSLFQRGKQFGWVFDNEYDNLDFPDDINLFGIDGTEFL
                     DEKDVSALISYFILWRVMNLADGRRLCIDIDEAWKWLENDIVAEEVKNKFKTIRKQNG
                     FLRLATQSIEDFLKLKIANTLVEQSATKIFLENPLAKEDEYLKLGLSKDEFLIVKNFA
                     PHKRQFLVKRQDEAVVCSLDLSSLGKENLKILSTGTSYIEKIESIFNQENKTLEEKIN
                     ELKTFYKEEQ"
     gene            complement(20871..21131)
                     /locus_tag="BDFFJJMB_00020"
     CDS             complement(20871..21131)
                     /locus_tag="BDFFJJMB_00020"
                     /inference="ab initio prediction:Prodigal:002006"
                     /inference="similar to AA sequence:RefSeq:ADT73535.1"
                     /codon_start=1
                     /transl_table=11
                     /product="TraC protein"
                     /protein_id="Prokka:BDFFJJMB_00020"
                     /translation="MFKQIFLMALAVNLAFAVGGIDKVNTLAQDIEAALFGVSVIAIT
                     IAFGIAGYRMAILKQQWTEVLPVVIGGIVIGAASSLATLLVG"
     gene            21417..21896
                     /locus_tag="BDFFJJMB_00021"
     CDS             21417..21896
                     /locus_tag="BDFFJJMB_00021"
                     /inference="ab initio prediction:Prodigal:002006"
                     /inference="similar to AA sequence:RefSeq:EAQ71736.1"
                     /note="conserved hypothetical protein"
                     /codon_start=1
                     /transl_table=11
                     /product="hypothetical protein"
                     /protein_id="Prokka:BDFFJJMB_00021"
                     /translation="MLDYEKFQTMSKEEYFKKYNVGIRFLFGCDINQKDEIEMISLRV
                     VLPKKYFQEYKNIDIFKAMDLFKETLLFKELTEQSIKIDFEKREFVMPDFFIKHDIEI
                     IPYFTQGGEKEEELSKEKFFELLKQNKMKELNYLCFLFFGLFCEEEYKYFCKVKELK"
     gene            21893..22339
                     /locus_tag="BDFFJJMB_00022"
     CDS             21893..22339
                     /locus_tag="BDFFJJMB_00022"
                     /inference="ab initio prediction:Prodigal:002006"
                     /inference="similar to AA sequence:RefSeq:ADC29285.1"
```

```
                     /note="conserved hypothetical phage protein"
                     /codon_start=1
                     /transl_table=11
                     /product="hypothetical protein"
                     /protein_id="Prokka:BDFFJJMB_00022"
                     /translation="MKLSEFDFRVWDKDEKCFLDPYTKSRAVVKNLPNEKLLNLEIEL
                     FTGIKDKNGKKIFEGDILLYKKNKKKYIFKIFDGLSHFKEFLFLKYSFDKESQTIIQS
                     PYSQIELSHKLDCDCNFHNKANEIYFEVIGNIHENEEILKNTELLK"
     gene            22336..22773
                     /locus_tag="BDFFJJMB_00023"
     CDS             22336..22773
                     /locus_tag="BDFFJJMB_00023"
                     /inference="ab initio prediction:Prodigal:002006"
                     /codon_start=1
                     /transl_table=11
                     /product="hypothetical protein"
                     /protein_id="Prokka:BDFFJJMB_00023"
                     /translation="MKDEIINIIKEAMGENYKKVLWIFQEHIKNLINCYPSNENGLVF
                     EYKSRISSNDVISIIMFEKTGQKINISLFFENTRKKEKILELNPEEKNSEELILKALF
                     IKNKKVLLSDLFILDKVSMYYTRNKMFKEFQKLMIDNKEKGIV"
     gene            22782..22940
                     /locus_tag="BDFFJJMB_00024"
     CDS             22782..22940
                     /locus_tag="BDFFJJMB_00024"
                     /inference="ab initio prediction:Prodigal:002006"
                     /inference="similar to AA sequence:RefSeq:EAQ71797.1"
                     /note="conserved domain protein"
                     /codon_start=1
                     /transl_table=11
                     /product="hypothetical protein"
                     /protein_id="Prokka:BDFFJJMB_00024"
                     /translation="MYPYLFNDKIKNHSMILCTEIECKKCGEKIYVEFQANEKFIFKC
                     PNCSSEKK"
     gene            22952..23260
                     /locus_tag="BDFFJJMB_00025"
     CDS             22952..23260
                     /locus_tag="BDFFJJMB_00025"
                     /inference="ab initio prediction:Prodigal:002006"
                     /inference="similar to AA sequence:RefSeq:ADT73510.1"
                     /codon_start=1
                     /transl_table=11
                     /product="Hypothetical Protein"
                     /protein_id="Prokka:BDFFJJMB_00025"
                     /translation="MRSSLEKVIKNYSENFSNKRDNDFAQGLIKYLYENAIIGKNDKF
                     FLEKNCQYIRIINEKEKRLINVYFCEKPIIDFFQNAEYEKELKYYGINEKDFIELAKE
                     "
     gene            23264..23638
                     /locus_tag="BDFFJJMB_00026"
     CDS             23264..23638
                     /locus_tag="BDFFJJMB_00026"
                     /inference="ab initio prediction:Prodigal:002006"
                     /inference="similar to AA sequence:RefSeq:EAQ71782.1"
                     /codon_start=1
                     /transl_table=11
```

```
                     /product="cpp8"
                     /protein_id="Prokka:BDFFJJMB_00026"
                     /translation="MIEKNLTKKEMFSYAIFIAIIALICIFKNEIWEFVKFIFSNLDF
                     IVGEDNIVFFPITTLLIVFTIAICAVWGLSNVLALSLFIMPIFVAVFYIFNINISLEN
                     LYGSYFIFLIIINGILWSVNQR"
     gene            23635..24147
                     /locus_tag="BDFFJJMB_00027"
     CDS             23635..24147
                     /locus_tag="BDFFJJMB_00027"
                     /inference="ab initio prediction:Prodigal:002006"
                     /inference="similar to AA sequence:RefSeq:ADT73509.1"
                     /codon_start=1
                     /transl_table=11
                     /product="cpp9"
                     /protein_id="Prokka:BDFFJJMB_00027"
                     /translation="MKKITIVAYAIYFLSGLWFLFSAFKEHFDILSFILGIVFIYFGV
                     INTKRILNDSNENKTELETLFATANIIFVSISSIFLLMLITFFAFQNEINIKFFSDQG
                     LVLKDDKKISIYFKEKEIKSFDENKYGFVMAVYGDKHIEVVLQNKENDTFFELRCNEE
                     GCKEVLKENK"
     gene            24144..24701
                     /locus_tag="BDFFJJMB_00028"
     CDS             24144..24701
                     /locus_tag="BDFFJJMB_00028"
                     /inference="ab initio prediction:Prodigal:002006"
                     /inference="similar to AA sequence:RefSeq:EAQ71826.1"
                     /codon_start=1
                     /transl_table=11
                     /product="cpp10"
                     /protein_id="Prokka:BDFFJJMB_00028"
                     /translation="MRVVNLSKKFVDKILFLRINHVVNIFMLILALFSTIAIAVFLYH
                     FIEEENYYAYFVSSFFVVNGFLLTIHLIGEKKCAKEVYYDDNFISKLNNSLRKELEDD
                     LLKLQEYYDGINVLEYKTFALLKCSSYYEKGRIDEELIDKIKKYYINLFKKKIAKELE
                     EMQKEKERSNILKTNQELILKKIGE"
     gene            25217..25888
                     /locus_tag="BDFFJJMB_00029"
     CDS             25217..25888
                     /locus_tag="BDFFJJMB_00029"
                     /inference="ab initio prediction:Prodigal:002006"
                     /inference="similar to AA sequence:RefSeq:EAQ71730.1"
                     /codon_start=1
                     /transl_table=11
                     /product="para protein"
                     /protein_id="Prokka:BDFFJJMB_00029"
                     /translation="MIISICNEKGGSGKSTLAVNLAIKAAAENKKVLLCDSDPQKSVM
                     TFANIRLEEGINRTFSIVSKVGESLKEFIQKEKKNNEYDLIIIDTGGRDSKEMRIALG
                     FSDVVIIPTIPSQFDVSVLDKMFTIIKMAKELNKELQTFVLINKSSPNPFLNKKIEDL
                     RNYIKEQENEDIKLLDNIICEREKYKTCIQMGLGISETETNSNKALEEFNTFYNELKN
                     KIKGL"
     gene            25890..26111
                     /locus_tag="BDFFJJMB_00030"
     CDS             25890..26111
                     /locus_tag="BDFFJJMB_00030"
                     /inference="ab initio prediction:Prodigal:002006"
                     /codon_start=1
```

```
                     /transl_table=11
                     /product="hypothetical protein"
                     /protein_id="Prokka:BDFFJJMB_00030"
                     /translation="MSFKKNTNVDLNSFIDGATGETSKQRVSGRTNKRDKKLLVGFTN
                     EEYERLSSMAEQMGMDKGAFIRFKIFNNY"
     gene            26219..26821
                     /locus_tag="BDFFJJMB_00031"
     CDS             26219..26821
                     /locus_tag="BDFFJJMB_00031"
                     /inference="ab initio prediction:Prodigal:002006"
                     /inference="similar to AA sequence:RefSeq:EAQ71825.1"
                     /codon_start=1
                     /transl_table=11
                     /product="cpp27"
                     /protein_id="Prokka:BDFFJJMB_00031"
                     /translation="MVIAYIRISTAKQDSEVQKLQIYEYCIKNKINIDEVMEVEMSSR
                     KSLEKRRMNELKDKLNEGDLLICTELSRLGRSMLEVITLILELSEKGIQFAFLRQPEL
                     SSFNNPSQKLLLSFYAYVSETERDFISQRTKAGLANARKNGKNIGRPVNSFNSMYDKD
                     TDLIKELISKKITLRNIWKFLGEKGTYNNFHYFCKSRKLA"
     gene            27023..32803
                     /locus_tag="BDFFJJMB_00032"
     CDS             27023..32803
                     /locus_tag="BDFFJJMB_00032"
                     /inference="ab initio prediction:Prodigal:002006"
                     /inference="similar to AA sequence:RefSeq:APB40411.1"
                     /codon_start=1
                     /transl_table=11
                     /product="helicase"
                     /protein_id="Prokka:BDFFJJMB_00032"
                     /translation="MAKKKKLNDDQPSLFYFIEEELIITSERNGNEQQNGKLSEYGNI
                     TKEFKQTSISSNTPSVDRQENSTIQLDNTREMGRVLSEHNQESRNVRSNDTSIATNTN
                     GISNLGEQRSRQEERTGVNRYGDTSRRANKSQSIHSDRTPNLFSFISRNDDERQGTDA
                     ARSYNQQSVLETAARNDGYERRSLKSGQKLDFVSDDEIYLGSLKDRFQKNLQAIKLSK
                     TIEQENRYATKQEQEILNKFSGWGGIPQAFDHQNKEWEKEFKELISTLDYAEYEKAKT
                     STLDAFYTPKIIIDTIYQGLNQLGFNNDDHTKEIFEPSAGIGSFLSYAKNYSDKYHFT
                     CVELDTISANILKHLHPNQTIYNKAFQHHLFDKPYDAFIGNPPFGQKKILDLNDPTLN
                     KTSVHNYFIGNAIKNLKEDGIAAFVVSSYFLDSKNSTIRNFIAEQATFLGAVRLPNNA
                     FKKRANTEVTTDIIFFKKGKDLNIDKSWLESVEYYDDRFDEAEKRDMHPDVFNDFRIN
                     EYFKNNPQNILGKMNIKSSQYGYSLECLDDGRDLKIALENFTKTLPKNIYKYQETKIK
                     LSYYRIDRESPEYEKYSKTLSKLKDGNYFEYNNEIYMKIRSDDELVFSKPVLNEHDTR
                     RIKKLIALRDQFNTLIEYEKNESSDLLVENQRKYLNKLYDEFVAKEGYLNRDANKKAF
                     REDVEANKILALEKNYSSGISKSVALKEGIDPIAPSATKADIFFKRTINAQKEIKIST
                     PKEALIASINEYGRIDLKFLETNLNQPLEETLKSLEQDRLIFKDHKNPANYVLAEKYL
                     SGNVKAKHKEVKKLVEDGFNEFANNLESLEQVLPKDLKAVDISISLGTTWIPIKYYKQ
                     FIEETLQITPKDYDLFLMERTGEWSLKGFGYSLSSYIRSEYATERIGCFDLLEHGLLR
                     KPIRIYDKKLDPSGKEIRELNPKETAIANSKLENLKNEFIEWIYKDYDRRTDLENIYN
                     ERFNTNIEPRYNGEHLELPNFNANIKLKKHQKNAIYRAIQENSIIFDHQVGAGKTLVA
                     ICSVMEQKRMGLLNKPLIVVPNHLTRQWNDEFYHAYTNANILVATNDDLKKDNREKFF
                     SKIATNNFDAIIMTHSQFKLIPAPYEVMKKQYEEDINILEQTLEKKQNDDNVMRYSVK
                     DLEKRIENFKVKLSDLQTTHKKSKAIDFSELGIDALIVDEAHEFKNLLITTSMGDISG
                     LGNLKGSQKAYDLYSKTQYIHEQNKKLYFLTGTPISNSITELFTLQRYIQPNVLKEKG
                     IQAFDSWASTFGEVTNAWELDSSGINYKIVARFNKFKNVPELSTMYKSVADIVTNNDI
                     MKYQKDFVPKLYNDKPINIVAPRSEEIAEFIGIQDENGRWNEGSIVWRMEHQQDDITR
                     NNLLACTTDARKAGLDFRLINPHCDDYANSKINKLIENVFNEYNAWNDDKGTQLIFCD
```

```
                     LSTPKQHSQKVSLNSNIATPNLNKEEFVNINEAIEQTEEENSKSLDELLAEQAKFDVY
                     TDILKKLVAKGIPQNEIRFIHDAKTDLQKGQLFADMNSGKARILIGSTQKMGAGTNVQ
                     KRIVALHHLDCPWRPSDLEQRSGRVIRQGNELFMRDPQNFRVKEFRYATERTYDARMW
                     QVIESKARSIEQFRKAGADVRTLEDVTSGAADAAEMKAEATGNPLILLQVQLNSDLRQ
                     EEMLYSGYKRQIHNNEESLITNLSKIELLKKDIAKMEILNSIIKENNSEHFQGKYYIN
                     DDEKDFIVFKNDTSDLNKSRQAELERNFEKIVKTIADDYQKEYKVLEFKGLLISGERT
                     DLNTLNFYVSTTDGKTIMEPENLIYKRDEKTFFKLESYVNFGGFVKRLNNFYNDLEKF
                     TQLNKNKIENLEKEIVELKSITGDNTPKYKRKDYLEALREDNRIIMDEIEKMSKQKNY
                     KSDFVPKSAKILEDLNKHNEIEVLSR"
     gene            complement(32864..33715)
                     /locus_tag="BDFFJJMB_00033"
     CDS             complement(32864..33715)
                     /locus_tag="BDFFJJMB_00033"
                     /inference="ab initio prediction:Prodigal:002006"
                     /codon_start=1
                     /transl_table=11
                     /product="hypothetical protein"
                     /protein_id="Prokka:BDFFJJMB_00033"
                     /translation="MEMLEKITYQDKENNLLILNNILDLLEDSLYKKKNTINDKFDLI
                     FKQPKQFNDEYIKSHYLSIYRAIESVNINRYFVFWEIASGMPQKIITQTIIYDMARFY
                     NTIKHKEKLIDESLQYIVKEINNIIGSIQGLGDYIAPQESFCNSFGFSQLYTPFHTSL
                     IGKAFLIRMALEKKIKWMIFGDLQEHKINLHEVLNKLKENHRQYFDLPSNVDFDKIML
                     VKNWCNRIIHNGFFPYIWVVWDAIEIIEPLFCISDKKGRIVLEGFNYRKDITQDISKF
                     FLEDEKR"
     gene            complement(33785..35098)
                     /locus_tag="BDFFJJMB_00034"
     CDS             complement(33785..35098)
                     /locus_tag="BDFFJJMB_00034"
                     /inference="ab initio prediction:Prodigal:002006"
                     /inference="similar to AA sequence:RefSeq:EAQ71823.1"
                     /codon_start=1
                     /transl_table=11
                     /product="cpp17"
                     /protein_id="Prokka:BDFFJJMB_00034"
                     /translation="MSRLKIFDDEIIAKRVYTKIEIGSKSTYNFKNRSSNKTNRILKK
                     NTEVVVKITSGSKNLQAVCKHLDYISREGTVDVITSDFDTLKGKDELKFLKKIYENEG
                     APIPRFGQEKKEKRHTINMVFSMQDYTNAPPEKMKKAVLMTLKKMYPDNFFAIAFHND
                     TDNPHCHVCLKVADKNGKRINPKKTDLMNLRLSFAKALNELGVEAKATIRSKNNEVSY
                     KLEKNKEQTKMHYYQVLDYGDAKYKFSLDKNAEDSFYVKYRTKKGIATIWSKDLARVI
                     KENKILKGEYAKFKIVGQETYQIKKKVKVNGKIVEVTKMAKKSVWDASVLGREKELKL
                     LQSDEANNPKYTIKENNERDYTKQYRQRILQELSNKSTIRRDAAQERDSMRILSEQSM
                     VHDQKGTQMPMHINALNQLQQGKRGRTDSSLRWTSARNNEIERDG"
     gene            complement(35098..35628)
                     /locus_tag="BDFFJJMB_00035"
     CDS             complement(35098..35628)
                     /locus_tag="BDFFJJMB_00035"
                     /inference="ab initio prediction:Prodigal:002006"
                     /inference="similar to AA sequence:RefSeq:ASI88230.1"
                     /codon_start=1
                     /transl_table=11
                     /product="copG family domain Ribbon-helix-helix protein"
                     /protein_id="Prokka:BDFFJJMB_00035"
                     /translation="MKKTVIYFDEKNYSFLVGLKKEMKTSFSKIINTILKNISNDEKE
                     NINNFDKINHKKNEPTKEIRFKLTEEEYKIFSDFAKSNGNGTATKQIRILTLNHIYND
```

```
                     KFFDKKYINEFILTRTEINTIGRNIYQLLKILRSGKSVRINENNLEKSFKDISLNIEK
                     LSKELEKIIIKNNERI"
     gene            35832..36113
                     /locus_tag="BDFFJJMB_00036"
     CDS             35832..36113
                     /locus_tag="BDFFJJMB_00036"
                     /inference="ab initio prediction:Prodigal:002006"
                     /codon_start=1
                     /transl_table=11
                     /product="hypothetical protein"
                     /protein_id="Prokka:BDFFJJMB_00036"
                     /translation="MKKESKEIELLKQEYKEKIQKAKQKEKDKKEKLCKKLGSEIIYL
                     LDNDDFRNLFFDYIQKNNLEKIKSLCSDIDDFFNINYIKNKEDENDQDK"
     gene            36174..36875
                     /locus_tag="BDFFJJMB_00037"
     CDS             36174..36875
                     /locus_tag="BDFFJJMB_00037"
                     /inference="ab initio prediction:Prodigal:002006"
                     /inference="similar to AA sequence:RefSeq:EAQ71803.1"
                     /codon_start=1
                     /transl_table=11
                     /product="cpp20"
                     /protein_id="Prokka:BDFFJJMB_00037"
                     /translation="MENKEMQENITFDNIKVRITDNSKLCKEYQECVFNGTYDNTSNP
                     KEIESLFNTLKTFDSCEKLKQIENLSFSKQEIDFEIITDDFKIPLKVRLGEGHFEKGL
                     DELIKLGSKNIDTNINKKINPDIKEKFDKILSPLKPENFQNILNGTFEVIKEAGSLTN
                     TNEISSFIQEFLKAYKKTQKLSENNKTIDNPKEDKKKDKNYFLKKVDEFFQKKLDSRE
                     REHVRTNEKSFSKSI"
     gene            36893..37957
                     /locus_tag="BDFFJJMB_00038"
     CDS             36893..37957
                     /locus_tag="BDFFJJMB_00038"
                     /inference="ab initio prediction:Prodigal:002006"
                     /inference="similar to AA sequence:RefSeq:EAQ71815.1"
                     /codon_start=1
                     /transl_table=11
                     /product="cpp22"
                     /protein_id="Prokka:BDFFJJMB_00038"
                     /translation="MYSLNIPSAIQSFKQALEEEGLIIDGSPIMDGKIHRVKTTNDKG
                     RELSGAYIGYLNNNFPAGFVQNFKTGIKKNWKMQIEKNQSNNIKSPKELHEQIKKDQE
                     LREKEILALQEKTALKLEDEYNNARWANSNHAYLKKKGFDENFYLKQDNKGSLLIPLK
                     DENGKLWSVQRIFPNGDKIIGVIKTQEEKDQGVEYLAKKQGCFHIIGAKTLHNLKEFY
                     ICEGFATGATLYKALNKPIIMAVDAGNLENVVKKLSTLYSDRNITIFADNDLKKELKG
                     LENTGKKTALEIKEKYSNVSVILPNFTQEQSSQGMSDFNDLMSLKGLEFVAKEIKMQM
                     FREKVVCLKKENSDKELTRG"
     gene            complement(38025..39230)
                     /locus_tag="BDFFJJMB_00039"
     CDS             complement(38025..39230)
                     /locus_tag="BDFFJJMB_00039"
                     /inference="ab initio prediction:Prodigal:002006"
                     /codon_start=1
                     /transl_table=11
                     /product="hypothetical protein"
                     /protein_id="Prokka:BDFFJJMB_00039"
```

```
                     /translation="MSKDKNEKLEELQEEINKVKELYEQLKSDYSINKAFIDSSSDDS
                     LKSLINNEINSYQEVEREIQERIYKLNEAYNYLLNNDQSGKSRLTRIQEVTSDNIIND
                     IKQKINNINNSYAEIFLSDKGNIPGDLLNKYEQIKNIYNDLIVVKNSENLNKVEDLNR
                     KIDDFLSKYSEIIGNEENSKYKTFISNYTDFEKKKRELDDFYKIVFGSDDGNIQSLKK
                     ELEERKEQLKKVEVEAKKVIDLSSDAGLAAGFFKQVEQANDNKNKNLYFFAGAILCMA
                     IFNFFTIDWKNLNNIDLVSAIVRLMINTPFIWAATVANINLNKYIKLEQEYAHKESLA
                     KSFERYKDQIKDLSANYETDEKQVELMTKLLDTNIEAFKKNPSDNIEKIRSNNIFTEM
                     LARKNKNDG"
gene                 complement(39243..39446)
                     /locus_tag="BDFFJJMB_00040"
CDS                  complement(39243..39446)
                     /locus_tag="BDFFJJMB_00040"
                     /inference="ab initio prediction:Prodigal:002006"
                     /codon_start=1
                     /transl_table=11
                     /product="hypothetical protein"
                     /protein_id="Prokka:BDFFJJMB_00040"
                     /translation="MLELKSILKYCKENRDSMLKDKTSKEYVGCENAFKADMSRSMWK
                     ELLDYGELMKQAEEKAKASGLFD"
gene                 39982..40854
                     /locus_tag="BDFFJJMB_00041"
CDS                  39982..40854
                     /locus_tag="BDFFJJMB_00041"
                     /inference="ab initio prediction:Prodigal:002006"
                     /inference="similar to AA sequence:RefSeq:CKG86570.1"
                     /codon_start=1
                     /transl_table=11
                     /product="hypothetical protein"
                     /protein_id="Prokka:BDFFJJMB_00041"
                     /translation="MAKIVNLENRDTKKLYSSVQEKIDATTGEVTTVISSFLQREKTK
                     DDFIKLFVENISFLTDNLSNPALRVVLMMVKNLNYQNVFNYNSDFVHYFESKKILGKS
                     SVYRAIKELEDKQVIFKVTEEQRKEYDIIGSNSYIMNPQIIGKGSFKDLKKLRQTVVK
                     TFDFDNLEFKQEISVESEYEGFEQVKKNPDNYVIEEVKQIASNKNTIENQVIISEKDN
                     DQDYPKEEIIDVKVDDNKEEQPALFVENKNEQNAKAFINEFAGMFNGAFHPSKTVKEM
                     KKESLENDLKEGNI"
gene                 40851..41264
                     /locus_tag="BDFFJJMB_00042"
CDS                  40851..41264
                     /locus_tag="BDFFJJMB_00042"
                     /inference="ab initio prediction:Prodigal:002006"
                     /inference="similar to AA sequence:RefSeq:CKG86535.1"
                     /codon_start=1
                     /transl_table=11
                     /product="PIN domain protein"
                     /protein_id="Prokka:BDFFJJMB_00042"
                     /translation="MKKVFLDTNIFIDFFDEARVNNEQAKQLIYFLISNEIKIVFSED
                     MISTIAYLIDKNKLPALMSFFKSTITNPYFEIVSFGSSVINMACDFHLQNKGDFEDYL
                     QYFCAEKENCCAIYTMDKNFPNLKIPIKRYGDTNI"
gene                 41289..42497
                     /locus_tag="BDFFJJMB_00043"
CDS                  41289..42497
                     /locus_tag="BDFFJJMB_00043"
                     /inference="ab initio prediction:Prodigal:002006"
                     /inference="similar to AA sequence:RefSeq:EAQ71815.1"
```

```
                     /codon_start=1
                     /transl_table=11
                     /product="cpp22"
                     /protein_id="Prokka:BDFFJJMB_00043"
                     /translation="MFLFVPYQDIHEAKKLGAMWDNKQKQWFAPKSLDINIFKKWLDN
                     NKSNSYSINIPSAIQSFKQALEEEGLIIDGSPIMDGKIHRVKTTNDKGRELSGAYIGY
                     LNNNFPAGFVQNFKTGIKKNWKMQIEKNQSNNIKSPKELHEQIKKDQELREKEILALQ
                     EKTALKLEDEYNNARWANSNHAYLKKKGFDENFYLKQDNKGSLLIPLKDENGKLWSVQ
                     RIFPNGDKIIGVIKTQEEKDQGVEYLAKKQGCFHIIGAKTLHNLKEFYICEGFATGAT
                     LYKALNKPIIMAVDAGNLENVVKKLSTLYSDRNITIFADNDLKKELKGLENTGKKTAL
                     EIKEKYSNVSVILPNFTQEQSSQGMSDFNDLMSLKGLEFVAKEIKMQMFREKVVCLKK
                     ENSDKELTRG"
     gene            complement(42563..42865)
                     /locus_tag="BDFFJJMB_00044"
     CDS             complement(42563..42865)
                     /locus_tag="BDFFJJMB_00044"
                     /inference="ab initio prediction:Prodigal:002006"
                     /codon_start=1
                     /transl_table=11
                     /product="hypothetical protein"
                     /protein_id="Prokka:BDFFJJMB_00044"
                     /translation="MKKITLGLASFSLIVFLGACSSEPKSMEYFKEAKNEKELKDTLK
                     YCKENKDSIFKDTTSKEYQECKNAFRADMSKSMGKEPLDYGELRKKYDKEVDALFD"
     gene            complement(42965..43606)
                     /locus_tag="BDFFJJMB_00045"
     CDS             complement(42965..43606)
                     /locus_tag="BDFFJJMB_00045"
                     /inference="ab initio prediction:Prodigal:002006"
                     /codon_start=1
                     /transl_table=11
                     /product="hypothetical protein"
                     /protein_id="Prokka:BDFFJJMB_00045"
                     /translation="MNIKLNIPYSEKDKLKEYGIKWNQEHKTWYLQDYKNLNYITNYL
                     ENENFTFYVTETLNIVQGFINCWKCSKIIPVYALGSSKFIYKEKGEWFFQPKFKIFRN
                     ITAYSEDFTSILKRMNIKSLEKSFSNTVGSSYLMNKCQYCQSKQGEFFLFEELNSIFS
                     PNSIKEAQQLKIYDFPLLFDLGFTGEIIEILTSECSDINALIWFNAERIKIIK"
     gene            complement(43816..44457)
                     /gene="pinR"
                     /locus_tag="BDFFJJMB_00046"
     CDS             complement(43816..44457)
                     /gene="pinR"
                     /locus_tag="BDFFJJMB_00046"
                     /EC_number="3.1.22.-"
                     /inference="ab initio prediction:Prodigal:002006"
                     /inference="similar to AA sequence:UniProtKB:P0ADI0"
                     /codon_start=1
                     /transl_table=11
                     /product="Serine recombinase PinR"
                     /protein_id="Prokka:BDFFJJMB_00046"
                     /db_xref="COG:COG1961"
                     /translation="MIFAYIRVSTDKQNTENQRFEILQWSKRNEIKINYFVQEVISGK
                     IAIDHRNLGRLFKKLKNGDSLVVTELSRLGRSLLDIMEKLNWCIKNNITIYSIKENFI
                     LNDNINTKVIAFAFSLSAEIERQLISQRTKEALARKKMEGVVLGRPKGKKNRIEVNPC
                     YPALKQILKWDKQGYSHSKIAKRIGVHRDTLRKFLITMGYAHLLKRYKPQFQN"
```

```
ORIGIN
        1 ggtgattttc cttgtccatt taagtaagcc tttccttcgt tgtcaaaagt tgcacttttt
       61 gaaactattg ttccgtcccc acttaaagtc cattccacac ttgcatttgg ctttccacca
      121 cgagcagtca ctttaaaagt ttcatcacca acaagctcag ttttatcgct ttgaatcgat
      181 aatgtatatt gatgaagctt aacaatgtct ttaacttcta ttttatttgc cacactttga
      241 ttatataaag ccgagatctc taaattttta atattataat cattaatcgc ttcaaaagtt
      301 acttgacttt ccccttttc atttgaggtg ctttgctgac tgctgttaat tttaattttc
      361 tttccgctat ctgcgtcttt tgctgtccac tctactgtag tatttggcat taagcctgta
      421 agtttaacca caaattcgct ctcataatca attgttccat tttcaaatgg agcatttgta
      481 atcggatgat tgtgcgttgg caaaatcatt gtggctttat aatttttaac ttctaattct
      541 ttactttttt cgctcacatg atcatcattt acatttttag caaaaatttt aactttgccg
      601 tcgttttcta aacctataat tttaacttga ctttcccctt tttcatcagc gataagctgt
      661 aattcattta cagtagagct attaccattt tcatcttcta aaaggaataa tgccttatct
      721 tctccttcta atctccattt aacattagtt ttgtttgggc gtaaattttt ataagttgcg
      781 atcgcaattt ctttatttcc tattacttcc ttgtcaatac tcaaatcgtt atcatcatta
      841 ccaaatatca aaacttgcac aggtgctttg aactctactc cattaacact agcaataatt
      901 tcaacttctt ccttatcttt catgcttgga tctgcttgat atctaattgt agattcgccg
      961 ttttcatcag tgagatccga agtatttgtt ttaacaagct cccctttgcc tatcaatttc
     1021 caaattactt ttaaattatt cgctaaagct ccacctgcta aagtaacaag gctttcttca
     1081 tatctttcta ttttttcagg atttgcatta atttcaaaac taagcttttt gattgaaata
     1141 gggaattttc cgcttgattc tcctgctacg gcatttaaat cagtgtttgg agtgcaagaa
     1201 ctagatattt gaatataaac ctttccgcta ttgtctgtga tataatcaaa accattttca
     1261 aacaatacgc aagaatgtgt cggagttaca cgcataggtg tatttttaac aacttcatca
     1321 aatccatttt ttaacaatat gcaatgttta tcgttgccta aatctccaca ataagaaata
     1381 tgatatttgt ttggagttgc tctatattca agcaccatag aatagtccct gtcaataaat
     1441 tgagttctac ttccttcaat tgtattttcg attcctgcta attttggttg aaatgctaac
     1501 ttcattgctt tttcatcaag tggaatatta atattcgcat tgattgaagt attctttttt
     1561 gaactttcag cactcctgct atgacttaaa gtgaaagtta gtgcaggaaa aggggaataa
     1621 gaaatccccc cttcataaat gaaaggattt ctttctaaat cttcgacatt gcttgatcca
     1681 aaaacaccta ctttttctcc ataccattgt tgcatttttg caaagactga aacatttaca
     1741 agcgaaggaa aagcatattt tatctttgca tcccaaccac tagcaggacg ctcttgaaca
     1801 taatctttat caaaatcata actatctatc caattgctta gtctatgata aacatttgca
     1861 ttaaaggcaa gcgtatctat cattgcttct gcaccaactg acattctttt atgctctcta
     1921 ctaaaatcgt gatcgtagac tagatttaat ccgaacatca tatttcctga atcatttgtt
     1981 gtcactgcat ttggataata tctcataccg cctccaaagt gtcctatagt tctatcatta
     2041 gaaaattccc ctataccgct ttgaataaaa taagatactt taggatcact ttggtttaag
     2101 ataggaaaaa gtgctttagc atttccgcta aatccgcttc tttcattata attaaatgaa
     2161 aaatctgcat taacagaagt gtttaaagag tcaatatagt ttctaactaa ctcatttgct
     2221 ttagaattta aataagagct tgccatattt tttataattt cttttctact ctcactatcc
     2281 tcgctactat aaattgctcc tgcggtttga agtgttttca ttatgttatt tgaaagctct
     2341 tgatcttcct tgcttaaatt attatcatta tttttacttt tttttatcgt ttcttgtgga
     2401 cttaaatttt tattatcgta atttgcactt atgaaattat ttgaataggt attatctaaa
     2461 ggcttttcgg ttattttgc ttctttaatt gtattttctt ctttaatttc ttttactaca
     2521 tcttgctttt gttcttgctc ttgcaaagct atttgttgat ttgcattttc ttcttcatct
     2581 tttgcttcat catcaaactc aagccatttt gcagatatat attcattttt atttgttaat
     2641 ttagcccatt tcgtgcctgt atcatttgtt ataatgtctt caatatataa aattctattt
     2701 tctctataaa tttctacttg cttactttta acattcggtt cttctcttga aattatacga
     2761 tatgctttta cttttgcttt taaaattgtt tgttgatttg tattttttaa ttccttacca
     2821 aaattatttt tttcatactc aatttgttga taatcacgct ttctttcaac tgattgttct
     2881 tttttaaaa ttaaatcatt tttcagttct gatttatcca catttattcg cttgttagtt
     2941 tgtgtttgct gcggtttaat agcaatttct tgattatttg agggcaaatt tgaaacatca
     3001 ggtaaagaaa taattttttg acttaaatta tcgatctcac tgtctatatt ctcaaaaata
     3061 acactagagt gtgtttggtt tactaaagat gaacttagca aaaaagacaa taagattttt
     3121 cttaaaattt ttatttttgg acgctttgtt tttaaagctt ttctatataa tattgatttc
```

3181 attttaccct ctctaacttt tcttaaaata cttcttcttt attctcttca aaatcttcag
3241 cagaaatatt tttttcactt tctttttgca tatcttgctt ttctctttga tgaacaaatt
3301 caaaactatc aactattact tgaaaacctg ttcttgtttt ttggttctca tcctcataaa
3361 cagaagtaaa aattcttcct ttgcacaaaa atttatctcc ttttttttaaa aaattcgcca
3421 aagcatttac acgatttgat ccaaaaacaa cgagaggaat ccaatctgta tgttttattt
3481 cttcatttaa tttcttagtg acagttgtaa tcgctaagct attttttgca attaaatctt
3541 tttttccctg cgatatttcc caatccttac caagaaaacc acataacaca acttcattca
3601 tatttatctc cttattttaa tttcggtttt ttcttttcat cataacaaat aaacttgcag
3661 ccatttttgt attcactgca cccccaccaa taagttcctt ttgcattttt tcttcttatt
3721 aaataaccat ttttacattc agggcagcta tattgcttct ctttagataa ctgcaaaaat
3781 tgcttcatgt catcattttc ttttattttt aaaatttcat tatttacctc ctttgctatg
3841 ctttttaaaa aatcttcttt ttttattgtg ccttgctcaa tcattttttg ctgttcaaac
3901 cataaagcag tcatatcagg atatttaaga gtttcaggtg ctaaagatat taaaagtttt
3961 cctttttttgg tagacttaac aacttgcttt ttatctttgc taacttcgac ataccccttgt
4021 tcaattaaat ttttaatatg agttgatcta gttgcaggtg ttccaatgcc tccattctcg
4081 ccttttttat ctttatcttt ttcaattaat aattttttta ttttttcatc tttcacatat
4141 ttagcaacac tatttaaatc ccttaacaac gaatccattg tataataagg acgcggtttt
4201 gtttgctttt tctctatttc aatatcttta atcttgcaaa tactgttatt ttccattgtt
4261 gaaatatcta aatgattatt agcgtcttcg ctatcttttt cttcatgatt atctttttct
4321 tcatgattga aaaattttct aaatccatca ctgatatttt tactttgaga agtaaagaat
4381 atattcttat cgtaagtttg tatttcaaga tttgtaactt cgtattctct tggtttaaaa
4441 aattgaatca taaacctttt tgctattaaa ttatagatga ttttttcttc ttcttttaaa
4501 agtccataat cgaaattatt ttgagttggt attatcccgt aatgagcaga aatattttta
4561 tcattaaaag ctttaccttt aatacttaaa tcgctatttt cgattaataa atttaaatct
4621 ttttcgctgt tttttatatt tgatttaatg gcatttacaa tatttggagc ttgttcaaac
4681 attgtttcag gtaaatattg acaatctgat ctattataag ttatagcctt atatttttct
4741 cttaaaattt gagtgacctc taatgtttta tcaggagcgt aaccatatac tttagcacat
4801 tctgcttgta aaactaaaag actataaggt aaaggcggat actctttctt tttttcttta
4861 gaagtattaa gtaaacctgt cttattgata ttaatttctt ttatgttttt agcagtattt
4921 tcatcaataa tcttttcttc tgtttttaat ctcgcattaa aaacttttcc atttagttca
4981 aattttccta ataacgaata ataatctata cttttaaaat tttcatattc ataatctctt
5041 gttgttacaa gtcctaaaat aggagtttgc actctaccta aggtaagaac ctctttgaag
5101 ccacttttct gtgctacgct tgtaaaagct cttgtaagat taattcccac aatccaatct
5161 gcttggcttc tcgcaaaacc tctttcactc attcctttaa attgagaatt tggtttcatt
5221 ttagaaattt cgcttcttac agacttagga gttaaatcgt taattaaaac tctaaaaatg
5281 ggtttttgtg tttttgaata ctgaataatt tcatcaataa gaatctgacc ctcatcatct
5341 gcatcaccgc aatgaacaat actatcgact tgatcattat gaatcaattt gcaaataatg
5401 cttaattgtt ttttgctatt ttctatagga acatatttaa attctttaat atctaaaggc
5461 aaatcattca aattccataa cttccatttt tcattataaa tttcaggtgt tgcaagcttt
5521 aaaatatgac caaaagccca agtaattaaa ttatcgcctt tttgaatata tccctcatat
5581 ttattaatat caccatctaa accttctgct atagctctgc caagttcagg tttttctgct
5641 ataaataatt tcattttcac ctttctagtt attaatgcta taatcttctt ttgtcctcga
5701 tttttctata gtttgatttt ttacaaaact actcatcttg ttaaaaaaat aacttttatc
5761 tttttttatt tcctttaaat ctttttctat taatccactt actccttctt cttgcgttat
5821 aagttcttca attaatttat tagttttatg aatattttct tctttgctta gctcttcaaa
5881 ttcttgtttg ctgatatttt tattgaaatt attaatatat tctatggtgt ttttaatgat
5941 ttttaagcat tcttcttcat ctaattgagt attttttttcg gttttattcc aagaatgatt
6001 taaatcaaat tgtaaaactt ttgtaatttc tagattattc gaaaataaat caatttcttt
6061 ttgatctttt gagtttaaat ataaatattg aggaactata tatccattat ctaattcttt
6121 aaaatctact ttttgatttt cttttaaacc tataaaaccg caaagccatt gtcctttatc
6181 atttgccatt aaaagatcat tgtcttgtaa tttttttaga ttcggatatt caacttttat
6241 tatagtatac ggaatatcgc ttatttgccc ttcttttttt tcaagctcta tataatttgc
6301 tttcattttt attcctttta attttgttac ttataattac tattaataaa taataactat
6361 aaatatgcac ccttataagg tgcatatttt tatcttccaa actcttggtt ttgagttttt

6421 tcaggctctt tgatttcttg agtttgccct attgtttctt ccttagaaaa accatcttgt
6481 tgttttgatt tttgctcttt catttcaact tcattttta tttcgaaccc accatttcgg
6541 acaatagcta aataaccatc acctttttca agcttagttt tttccagagc ttctttaatc
6601 tcattattag aaagctttat gtgcgtgtca agataaaaac ttttactacc atcttgattt
6661 tgaaattcgc taattccaat tccttcaatt aaagcttttc cagcttctct tttatcattg
6721 attgcatgat agaatctttt agactcttta ttccaatgat tattgatgaa aacatgatta
6781 tctctattgc ttctttcttt tgcatctgtt ggcattagca caatattatg agaatcctta
6841 tctctattca caaaataatt tttccattca ttatttattt taaaagacgc agtaactgca
6901 ccttgatcca tgccagcaac ttttccagcc tcttctatca catcaactaa agcagtagca
6961 acttgtcttt gcttaatctc acctgtttga gcgtctttat aatttaatgc attatttaca
7021 tcaaccataa gtcttggatt gcttccattt gataatttta tgaatgccat tttttctcct
7081 tttaaaaatt ttttgttttt tggttttgtt ttgggaattt aaaaaaaata tatataatta
7141 catgttcatt cacctccttg tagtgtgata tgaatgccat tctcatcttt attaagaatg
7201 gttttgtttt ttgggaagct caaagttaag cttttatctt tattttcttt tataacgata
7261 gagcttaatt tttgggcgtt atgagttatt tttttataat cttcgtattt taaatatacg
7321 atataagaaa aaacgccaaa agaaatccca aagaaagtaa aaaatattaa tgaagtaatt
7381 aatgctcgtt taatattttt agttattctg gaaaatattt gagaaaaatc taaaattgct
7441 tttttttcct tttctaaaaa caatttcaaa aactgatcat atgtttgtaa actagaactt
7501 agcctaagtt caattttttc tttaaaatct tttagattgt catcgcctgt ttttaaagca
7561 ctatttaaag attcaagaag cgtattatca aaattaacca aaaaattgtc aagcaaagct
7621 cttaattgag ttacttgctg agcaaaatct tctattttta ggtttaaaaa agatgaattt
7681 aatgtttcaa gttctttttt cattaaaagt gttttattaa taatttccaa ttcttcttga
7741 ctcgggggat ttttaaaata tgaaatcaaa gttttaatat ttgtcttaag tttaaagaaa
7801 ttttgttcta aatctttaaa aattccctct ttagtatttt catcaagagt gttatttaat
7861 cctatgttga tgatattgtt aagaatctct aaaattttta tttgctcatc aataatttct
7921 tgataattac aaatttttat ctctacttca tcaaacaaag aattactttc attctcttga
7981 tttatttgtt catcatcaaa taaattttga ttttcttcag ccatcattat ttctcattta
8041 tccaacaatc tttttaata attatttttt ggtaaaaagc aatatcaatt ctgtaatatt
8101 taccaaatcc tatttgtttc cttttattga ctggcaatct aacaaactct gcataactta
8161 caggaactaa cttctctcct tgttcttttt gagagatagg taaggcatta taaatttctt
8221 gcgatacttc ttttaactca taacctcttt cccagtcttt tttttcataa aatttagtat
8281 tacaagtata ttttagatga taatctgtgt atttcgatga agctaaaagc ctgcaagaat
8341 ttgtaagttg tggattaatt ctatatccat aatgctttat aatttcagtt gtacaaccat
8401 ctccatgaga gcagtttcgt atagtttcag ttcttaatac gatactttca attcgtttat
8461 ttaatacagg tatagtacat tccccatcca aatgaaccaa aatatcattt ttatatttat
8521 acatttcagg atcacttgaa gcatcgacag ggcataaatt taaaaaactt ctgcgtttag
8581 cgattacatc tgcccatttt cgtgcatcaa tgctaaaata tcttgccaaa gctgcaccgc
8641 attcaggtgg tttaacagga cttgcaagac aaagcatcgc ttcacaagct aatttagtat
8701 ctcctgttaa aacatctatt tttattgcgt ttgatgaaga tacaactcca aaaaataaaa
8761 ttgttgcgaa agtaattttt ttcatttatt ttctcctttt gcatggaaaa aaagcatatt
8821 gacagtattt ttattaccct tgataatatt taatggttga agaaggattt ctgcttgaac
8881 gccattcttt ttaaaatatt cattatattc ttgaatgaca tttttttcgc ctattaaaat
8941 aatccttttt gcgttatgag ccaaaagaaa agttttaacc acttgctcat tgctaagtaa
9001 accttctttg tttttgcta attctaattg ataaatccaa tccatattgc tcaatcttga
9061 atctttagga acaaatttat atctttcttc caataaagaa ttatttaaag aaagttttga
9121 accaccttcc aatttttag gttttggtgc agaaacagca catcctgtaa aaactatact
9181 tgttagtaaa agaaaactaa gcgttttttt cattattctc tcctgttgtt ggtatttgta
9241 ttcgacattc tcctctttga aaagcccctt ttaaaatatt ttcttcaggt atgcctttaa
9301 tttttgccaa agtgggagaa acttgtttga atttatcgat aaaataagga tcattgtaat
9361 aaaaagcttt tttgcataaa ataggctttc cattatctat agtaatgagt tcatcctcaa
9421 aagacatttc tcgaagttct tgtggaagca ttaatgctct gctcgcttcg ctataacttg
9481 tagatccacc actcgctcct acacttcttg atcttgtttt aaatgtagta tttccaagta
9541 ttctactaat ttcttctgca tcttcttgtt ctctgggtgt ataaaaaatt tgacaagcgt
9601 gattagtcaa aagagttttt gctccatcat ctccatatcc atcaggagca ggtgctttaa

9661 gttgagattt tgattgataa atcatcaaag agcgtaacca atatccagcc ataaaactta
9721 ctgccgaaag ataaatagga atttttctag gtgctgtaaa ttcatccata agcattaagc
9781 aagtatattt aatttgtggg ttttgttctg gaagttcttt tgtattcaaa agaataagtt
9841 gtgaccaaaa aatatttagt ataaactttg cattagttaa ttgatcaggg gttataccta
9901 tgtaaatagt cattttcttt ttgcgtaaat cccttaaatc aaaatcgcta gcctgtgtag
9961 aaagttttaa tacttctgct tcaaaaggag ttaaaggctc gttaaaagaa gacataaccc
10021 ctgattttgt attatcactc gaaatgctaa aataagtttc tagccttctt tttgtaatat
10081 ctgaaagcag atcatcacaa tttgataaat acttataagt attgtcaaat ccatttattt
10141 ttttgccctc atgctcaaaa gtaaaacctt ttgagagttg caatattcca aaaaaagaaa
10201 actctgtttt cagattaaaa gtttctaaaa aaagtttccc atcttctctt aagttcaaat
10261 ctttccataa atagcaaagc cctataaata aattttgcgc aagcccatta aaaaactgag
10321 ttgtagaatc tccttctttt ggataaatga tatttgccaa atccatcagt tgactatcac
10381 aattatcact atcactcata tcaatgtaag tcaagggatt ataacgatga gttctttttg
10441 aaaatggatt aaaaagaaaa acatcttgac ctaaaatctc ttttctatat ttgcttgtaa
10501 aatcaaagca ttcctgctta atatcttgca caacagcact ttctttccaa tccattaaat
10561 ttggtattac aataccaacc cctttaccgc ttcttgtagg agcacctaag gctacaaact
10621 gaggactttt aaatcttaaa aattttcctt tgtattttcc gatgattatt ccattatctg
10681 aaaataattt catttttttg atttctgcta aatttgcaaa tcttgcttca ccaaattgag
10741 tttttttact tttggctatt aacgctaaaa atattaaaac aatggcacta aagccaaaca
10801 tagcaaaaaa agaaacataa gcttttgggt aagaatttat taatgcttca aaagtaaatt
10861 tgagattata aaattcaaaa attcttttta aactcacatc attgagataa aacattacaa
10921 atgggcttaa aaaataagct attaagagtg aaaaaatact tataaaaata aacattataa
10981 ttttatcttt catttttcgc tccttttatc attattcatt tccttaaaat atatttgact
11041 aatatgtctt tttccattat gagctctaat gtgaacaact acatcaataa gatttttaag
11101 catatcttgt atttttttcaa aaggaatttt ttgaccttgt gaattttgta aagtcattaa
11161 agctaaacgc tcaaatgttt cgtgagtact tcctgcatga caacttgtaa tacttccgcc
11221 atgtccacta gagagaacat ttataaaatc ataagtttcc gcacctctaa gctctgcaag
11281 taaaattctg tcaggcttca ttcttagaca agactttaaa agcgttgcag aatttaaaaa
11341 atcttcactt ttagcttcgc taggataaaa aagttgaaca aaattattat gctcataaaa
11401 tttaatttct tcaacatctt ctatagttat aattctttgt tctttgggaa taaaatcaat
11461 aagagttttc ataaaagtag tttttccact gcctgtacct cctgctataa tgatattttt
11521 ttcatactct actgctttac aaataaaatc tttaaaattt ttatttttggt aaagcttctt
11581 taattcctca tccaaaggat ttgttaccaa atgatcggtt tttatttctt caaaaagccc
11641 acttttaaca tgatcttgta ttaagaatct tttctcactt ggttttctaa ttgtaattga
11701 aatatgattt tttttagtcg ctggaggtat tactacttga accctttcgc ctcgatgtaa
11761 aatacaagaa agaatagggc gtgattcatc tattttatca tctttaaaag aagccaaaga
11821 agtagcaaac gatttagagg cttcataatc tagtttactt gtttcttctt gccattttcc
11881 atcagtggtt tgtataaaga tattttcttt gccgttgtaa catatttcag ttacactagg
11941 attagttaaa tgttttccaa aaaaatcatt agctaatttt tctaaagttc ttgaactact
12001 catttaaaaa cctcttcttt ttaattgata aaccttagaa aaatcaatat ctctatttac
12061 ataaacacca actaaatcac catgattttt ataaagagtt ggttgaatat tgatcatttt
12121 ttctaaagca gtattagcta tttcacgaga agtttctctt gaattttctg tataatcaac
12181 gctattttct tttttattaa taacttgatt ggctaaaata tttaaagaat catcgatgat
12241 tgagagtaaa attgcggaac caaaacgctt catataatga tgatctaccc atccttgtat
12301 accacttgca ccaagctcat cggttgcacc agaataaaca gggatgataa tattgttcgg
12361 cgttctaatt tcttgccaaa tcacaaaaag tctatccatg ccatcattta actgtccaga
12421 actaaaactt cctgtaatag tgcttccttt ttcaattaaa agaacattac catttgatga
12481 atagacattg tttgaaacaa tacaagcaat accaccttta atagtgctga ctaaacgagt
12541 ttttaatgaa catccaatat aagtgccttt tgataacaat aaatttggat caaactgact
12601 aacactggca ctagtcggag taaaaacatc tccgacaaat tcattagaat tgccaccatt
12661 ggataattga ttagattcgg aattaaaaat attatttgaa taaccctctt gtgaactatt
12721 ttcctgctcg taagaagaat tttcactgtt actattactt actacaacca ttcctactcc
12781 cttaactatt cttggctgta atggttttga gataacctca aaaggatttt cttttggagc
12841 tactttaaca gattcttcct gctctcttga ttttaataaa tcttcaaaac ttttcgtttc

12901 ttcttggata ggctcttctt ttggtagaat aaaatctctt agttttacac tacttgcaat
12961 atctttatta gtttctttaa tgttcgagtt tgctgcctca tcactactgc ttatgaaaga
13021 ttttgcaaag atatatccga aaagcaataa tataaataca caagcaataa aaaacacata
13081 gatttgaatt tttttaacat ttacactttg agtttgtaac tcactttgca aatccatatc
13141 ttttaattct tgtttttcat tgttactcat gattatttac cactctttgt atattttcat
13201 tggaagtttc tctagttcta tccaaaggat ttttaccaaa accattattg aaaataccaa
13261 caactttatc accacttctt aacagaattt gttttgcaat tttttgaacc actaaaacat
13321 catattttcc atcttttcga atatgagtat ttaaaatcat ttctttgcca ttttcatata
13381 agaaaacact cggaaatgtt ttggtattgt caaatcccaa ataagtaaaa acaccatcat
13441 cataagcata atttggtgct atatcttcac ttcctttatt agctttcatt aaaaagtccc
13501 agtttctagg aactatattt ttatcaagct cttcttttat gattttcttt tctaattctt
13561 tttttatttc actttcttgc tttaaaattt tgtcagaata attaaaagac aatttataag
13621 ttgcttgttt gctatcatta gttaacaaca aatcaaaaat ataagtcttc aaattcgtag
13681 taacaattaa atttgttttc caatctcttg gatttggatc aacaatcatt tccttttgtt
13741 ggctttggtt atacggatca ttttctacaa aaagtgttgt tttataggct ttcggtttaa
13801 taaataaaag atttttcttt tctattaaat cccaaccttc tgcaaaacct gtggcagtgt
13861 ttgtaataat ttcatcatcg ccaaattcaa tcacgctcac aaaaccattt ctagctatca
13921 ttttaaatac atctttagag ttgtattctg catgtgctat gcgttgatca tattctgact
13981 tttgtggctc atttaacgca aaagcggtag acgccaaaaa gaaaaaagaa agggaaatca
14041 aagtaatatt tttcatctta tctcctcatc aattctgtaa ttagttactt taaaaccaag
14101 agggtttaaa attctatttt cttcacttgt ttttgcttgt aaaaagtcat atgataaagt
14161 tatgacttta gtagattgct gtgtgctttt ggttacggaa tctttttat taattaatgc
14221 tcttatggtt gaagttttaa caccattact atttccaaga acgatactta aaattttaac
14281 acttacttca acattatctt tatagatttg atctaaagcg ttagcaccag cataaatagc
14341 tctataatca ttcgcaacat tctcatcgct cattaattga gtgagtaaat aatcttgatt
14401 taagaattta taataatatc cctctcgagc ttttatatat tgagaaataa aatatttatc
14461 cagagcttca atagagctaa tttgctcttc ttgtaagcta gtgataatat ctaccatgcc
14521 agtagtatta tctaccctaa taacatatgg ttctgtggtt tttaatggtg ttagaaaaac
14581 tacagccact atagaaataa ttgatattgc catgcttata aatgcaacta gccaagctct
14641 ttgatttgat ttttcaatta aatatcttac acttgcttca aaatttaaag cattagaatt
14701 gatttcattt tggtttttca attttttctc ctttctttaa aatttattcc aaaacttcag
14761 tttcaatgat ttgaatatct ttttctttaa aaaaaagatt ttgttttaaa acttttacaa
14821 aaacatctcc ttgctctgta tagtaggaaa tagaataatc aaactcacta gctttaatta
14881 ttttattttc agaaaaagag ttagagtcat ttacattttt tcttaaaatc acataagaca
14941 tagtcggatg atttaaaaca attatttttt tctttttggt gtttttgttt tcctttatca
15001 aagtttcttt tttttcaaat actggcgtat tattttctat ttttaaaaac ttctcttgtt
15061 ctttgatatt attaattgca acccaaccat ttttatcatc aaataaatag ttatagttag
15121 tattattttt agtagaacaa gcactaaaaa taattgtaaa taatgctaat aaaaccaact
15181 tatgcttcat ttctcatcct ttgtaataaa tttttgccca aagacatagt attgctccct
15241 gctacactag cacctgattt catcgcagaa taaacacctt ctgccatttt agaacctcct
15301 ttttgagtgt gtccaaataa atttccaaga gtttttccag caacagaact tataggatta
15361 ctcactcttg aaaacacact ttgcatagca gaatctaaag caacacttcc tactttttca
15421 gcaaaaacct ttgctacaaa aatcatagcg actaataaaa cagaggaaaa aatcattgat
15481 attcctataa aaaaagcgtc tgcatctgca ttcatatatt gatcttgagc atatctagca
15541 aaacgatata tttcttttgc aaaaacctta aaaaacaaac tcataaaaag cactaataag
15601 gtattagaaa taagcaatgc aagccattga gtaaaaacat ttttagtgct tttatataaa
15661 agacaataaa acattaaagg tgcggcgatc accaatatag tttgagtaaa ggcagttgca
15721 ttaatcacta ccaaagcagg taacacagct ataccaaatg ctatataaac tataattaca
15781 gtaattccag ctattactgt atcaaatcca ctagccttag cccatatatt atcagctaaa
15841 tttctaactt cattaaaaat aatatcaatt tgcttataca tactaaggct gccgcccgcc
15901 cactcatgaa agccatccat agcattaatg actaaagtaa gccatccacc gatatttaaa
15961 gcaaaagtga tgatcatagc cttagcaact aaatcccata tcacttcttt agtagggctt
16021 tgagaacgac cgaataaaat ttcataacct ttatacataa tagtaaccac aagcgtaatg
16081 gaaaaaagta cagcaagttg agtcattttt tcactcaatg ccgcatttct aacaacctcc

16141 aaaattgcat gtgtagcatt accgagctct gtaaagaaat taccttgcat ggcagctcct
16201 ttctttaaaa tttgttttta tttaaattaa tttggtttta aaattaagtt tttgtaaaac
16261 ttgtgttttt ccatttttaa atttttctac tatttcatac tctattagtt ctataaattt
16321 agaattttgt ttttgttcta tttcatttat agctttgact atcttttcct tataaggtgt
16381 atcgggaaga tcatatttgt tgttataatt ttctaatttt ggtgcattgt tagacgcata
16441 ataaccttgc tttctaatac ttggcaagac ttctttaata acccattgtc taaaaggctt
16501 tgcttttggc ttatcacttc tcattaaaac aaagtaaagt tcaggttcat taattacaat
16561 aaatttttgc gttcctccag aagttttaaa ggggtaccac tcaaaaccgc cccctttatc
16621 aaattcctta tttatagagt ctctaacact atttgtcttt tttatattta atattttgca
16681 tatatcattt aggcaaaaca aaggttcatt gtatttatct cttattgttc ttacttgtcc
16741 caattcttta ttttcaaata aaataacact actcattttt cactcctatt taatcaaaca
16801 aagcatcaac ttctttatca tattttttc tgagctcgcc ataatccaaa ggctctttcc
16861 ccattgattc tgcaaatagt tgtgccgctt gatcttgtct tattcttctt tgattttcat
16921 aatgcatatt agaaatttca agattagttt taataatttc tagtttggtt ttttctacat
16981 taattgcatt attaatgtca ttagtttctt ttatgtcctt agattctata agcttttggt
17041 ggagtttttc caaagacttt gctacggtat taacttggat agaagcttga ttataatgct
17101 ccacttgagc agcataggta atcatatcca ttctacatat tctttttctc tcttcactca
17161 aataactagc attgcaacga tcaaaaagag taaatttttc aaaagtttct ttacttttat
17221 cgcttaaaaa actttgtgga tcttgtaaaa catcattata aaaagattga atattttgcc
17281 cagcctctgc aaaatctaaa taaatttgtt ttgtatcatt cataaatgat accgaatccc
17341 ttattcctgt tttcgataag agctcatctt tgtatgcttg tatttgttgt tgatagtgtt
17401 taaccgtatc tgtccatctt gtagcttgtt tagtccattc tgcaatttct tttgcatttt
17461 gggttgccat ttgtgaattg gcagtcgcat caactacagg aatgcctgca ctaaaagcta
17521 aatttacaat acataacgat gagattatgg agaataaaaa tttattttgt tttttggttt
17581 tgggatttag cacagagcaa taaccatgtg acttaattaa gatgaagttc acacacttta
17641 tcttagtagc aattacacta ctttttattt ttgatttttt acagaaaact ttaatatcat
17701 caaacatcac ttctttactc cttgttttta ttaaaactac ttttattttt tttatgctac
17761 aatttttaca acgatattag ggtctaattc agtagttgta tctttatcgt tagaaagaaa
17821 ttgtgtaact gtgttgaaat acccgattgc ttttttaagt agtcgggttt ttttttgcct
17881 tattttggcg gtaggggcgt ggaagggaat ttcaacacat aaaggaaaaa caatggaact
17941 tttaataagc accattgtgt ctgttgcttg cactatgaca attttcttta aagaagagat
18001 aaaactcttt tttaaacgaa catagtaaaa caacacacaa tttttttcat ggggagaacg
18061 aaagttctct ctattcttac ttaaacttaa tctcattgtt cttccttata aaatgtttta
18121 agctcattaa ttttttcttc taaagtttta ttttcttggt taaaaatact ttctattttt
18181 tctatatagc ttgtgcctgt agataaaatt tttaggtttt ctttgcctaa agatgaaaga
18241 tccaaagaac aaacaactgc ttcatcttgt cttttaacaa ggaattgtct tttgtgcggg
18301 gcaaagtttt taactatcaa aaattcatct ttactaagac ctagttttaa atactcatct
18361 tcttttgcta atgggttttc taaaaatatt tttgtagcag attgctcaac taaagtattt
18421 gctatcttta gtttcaaaaa gtcctctata ctttgtgtag caagtcttaa gaatccattt
18481 tgttttctta tggttttaaa tttattcttg acctcctctg cgacaatatc attttctaac
18541 catttccaag cttcatcaat atctatacaa agtcttcttc catctgctaa attcataaca
18601 cgccaaagaa taaaataaga aattagtgcc gaaacatctt tttcgtctaa aaattcggtt
18661 ccgtcaatac caaataaatt aatatcatca ggaaaatcca aattgtcata ttcgttgtcg
18721 aatacccagc caaactgctt ccctctttga aaaagagata atctactttt taaagaatta
18781 ttgtcattaa aatcatctgt gatattttca agaagcaatg aaataggata ttttctttca
18841 ttaatatcaa attgaaacat taaggtattt acagcttcat tgatttgttt ttcttctttt
18901 gtattaagaa tttcgccatt gcgtgttaca caaagtttta ttagctgttt tagaaacctt
18961 aaattttctt cattattttc aaccataaag ggattgaaac ctgtcggctt gccattgttt
19021 atagaaatat atctacctcc agcacaaagt atgttgccta acgcaccttt atctttatct
19081 aaaaatacag cagtaaattt ctttttatct tcaggagtat ttgcgggaaa actatcttta
19141 ttagcatatt ttatcatttg atcaatgatg aaactcataa ataccgtttt accaccgccg
19201 ctttgtccta aaattaaggt attagctaaa ttcaagtcgc caaactcatc tttatttcta
19261 ccattgcttt tgtgaaaatt taaaaaatat ggttgtttgt ttggtgtttt taaaatagtg
19321 acagcattac cccagcaatt tttttctttt ttacccatta agaaattatg taatgcaacc

19381 aaagatgaaa aatttaaaga tgaaatcata tgaactcttg ggcgtattgc gaaattgctt
19441 ggaaattgtg aaaagtaaga agcaggcaat gcaatatttg ccaatgttga agtaaaccct
19501 agactattta aagaagtaat tatcttgttc gtactttgct tacattcatc aatcgaatca
19561 ccaaaaacac aaatattgaa atgataattt ccaaaactta tgacattact agttaaatca
19621 tctaatgcta aatttaactg ctctatttga cttacgccat catcctcact tgaaattaac
19681 tgatttattt gtttgtttaa tgcagatttt gaatcaaatt ttgacaaagg gacaaatgtt
19741 tgcgtgatga tataatctgt attaagatac attaaagaat ctaaaatacc agaaaaagtt
19801 tgattagaat aatctttaat ttcaatacat ctagcaaatc ttttcgtatc atcattatag
19861 ttcaattgaa tcatatcatg attgaattga atattttta aggaacctgt taaatattga
19921 tatatcggac tatcacaaac cgcaatagga ttaaattttc cgcttattaa ataattataa
19981 aattccaact gttctgaata tctaatatta ttctttttat atacgcttaa tattttggt
20041 ttaaaatctt taatattagc ctcaaatttt cctaagtatt cttgaaattt aataacaaaa
20101 ttcgaaattt cttttgcttt tgatttataa tttagtttat taaaggtttt taaatcaata
20161 ttagatttca aaggcttgta aatcagagtt aaataaagcg agttttgctg aaaacttttt
20221 ttatcaaaac ttttatgata tttttcatca atctctttta aaaaaggatt ttcaaaataa
20281 gaatctaaaa attcatgcaa tttgaatctt acattgtgaa aataaaagct tacaggctcg
20341 ttagaaaacg acattatcat catattcaaa agatttttct taaaagttaa attctcatct
20401 gactcacatt caaattctat accctcaatg aaaaatgtgc taattaacat atgttctttt
20461 gtaatgacaa tgctatcagt aattaaagaa ctataaggaa ttaacttttc taaacttggc
20521 tcagtatgaa tcggaaatat ggataatttc ggaaaatcat tgttagtcgg caatcgttta
20581 taagtcgaac tattaaaggt tttagcttta tgatattttt tacttaaaaa attgctgata
20641 aatttaattt ttaaaaaaaa caatctaaac atttgctcat ctttcttagt cattgctttc
20701 ataataaaat gtgaaatgac tccaaaaatt aaaagaaaaa actgttgagt ataaacagat
20761 attaaaatga agcctaaaat atttatcaaa aaaggcatta aaggcactcc aaaaaggagt
20821 gcagggcgag ttaatccttt aaaaatagga tcaaaataat tattcatatt ttatcctact
20881 aaaagtgtcg ccaaagaaga agctgctccg ataacaatac caccaatgac tactggtaga
20941 acttcagtcc attgttgttt caagattgcc attcgataac ctgcaatacc aaaagctatt
21001 gtaatagcaa tcactgaaac accaaataaa gctgcttcta tatcttgtgc taaggtatta
21061 actttgtcaa taccaccaac agcaaaagct aaatttacag ctaatgccat taaaaaaatt
21121 tgcttaaaca tttaagaaat cctttcatta aaaaataacg aacgataaat ttagttttta
21181 tattaaatat aaatgtatta tttgattttt atttttgtt aaaattaaat atataataga
21241 tatttttttt gattttggat aaaaaggagt aaatttggta accaaaaaat caaatctgta
21301 tataaaaatt ttaaaagcaa gaataaatat taaaccaagc ttaaaaaata gattaagagt
21361 gtatttaaat ttaaaatctg aaaaagttaa aaagaatata gcaggcgaga acaataatgc
21421 ttgattatga aaaatttcaa acaatgagta aagaagaata ttttaaaaaa tataatgtcg
21481 gaattaggtt tttgtttggg tgcgatatca atcaaaaaga tgaaattgaa atgataagcc
21541 ttagagttgt tttgccaaag aagtattttc aagaatacaa aaacattgac atttttaaag
21601 caatggactt gtttaaagaa acgctattat ttaaagaatt aacagaacaa agcattaaga
21661 ttgattttga aaaaagagaa tttgtaatgc ctgatttttt tattaagcac gatatagaaa
21721 tcattcctta ttttacgcaa ggtggcgaaa aagaagaaga attaagcaaa gagaaatttt
21781 ttgaactttt aaaacaaaac aagatgaaag aattaaacta tttatgcttt ttgtttttg
21841 gcttgttttg tgaagaagag tataaatatt tttgcaaagt aaaggagtta aaatgaaact
21901 aagtgaattt gatttcaggg tttgggacaa agatgaaaaa tgctttttag atccttacac
21961 aaaaagcaga gcagttgtaa aaaatttacc aaatgaaaaa ttattaaatt tagaaataga
22021 actttttaca ggaataaaag acaaaaatgg caaaaaaatc tttgaaggag atattctttt
22081 atataaaaaa aataagaaaa aatacatttt taaaatattt gatggtttaa gtcattttaa
22141 agaattttta ttcttaaaat atagctttga taaagaaagt caaacgatca tacaatctcc
22201 ctattctcaa atagaattat cacacaaatt agattgtgat tgcaattttc ataacaaagc
22261 aaatgaaatt tattttgaag ttatcggcaa tatccacgag aatgaagaaa tcttaaaaaa
22321 tacggagttg ctaaagtgaa agatgaaata attaatatca taaaagaagc tatgggagaa
22381 aactataaaa aggttttatg gatatttcaa gaacatatta aaaatttaat caattgttat
22441 cctagcaatg aaaacggact tgtttttgaa tataaatcaa gaatatcttc aaatgatgtt
22501 atatcgataa taatgtttga aaaaacaggg caaaaaatta atataagttt atttttgaa
22561 aatacaagaa aaaaagaaaa aattttagaa ttaaatccag aagaaaaaaa ttcagaagag

22621 ttaatattaa aagctttatt tatcaaaaat aaaaaagttt tattgtcaga tttatttatc
22681 ttagacaaag taagtatgta ttatacccga aacaaaatgt ttaaagaatt tcaaaaactg
22741 atgatagata ataaagaaaa aggaatagtt tgaaaaatac tatgtatcca tatttattta
22801 atgataaaat caaaaatcat tctatgattt tatgcacaga gattgaatgt aaaaaatgcg
22861 gagaaaagat ttatgttgaa tttcaggcaa atgaaaaatt tatcttcaaa tgtcctaatt
22921 gctcgagtga aaaaaaatag aaaggttttt tatgcgttct agcttagaaa aagtgataaa
22981 aaactatagt gaaaattttt caaataaaag agataatgat tttgcacagg gtttaataaa
23041 gtatctttat gaaaatgcta ttataggcaa aaatgataaa tttttttag aaaaaaattg
23101 tcaatatatt aggataatca atgaaaaaga aaaaagattg atcaatgtgt atttttgtga
23161 aaaaccaata attgattttt ttcaaaatgc agaatatgaa aaagaattga aatactatgg
23221 aataaatgaa aaagatttta tagaattagc aaaggagtaa aaaatgattg aaaaaaactt
23281 aactaaaaaa gaaatgtttt cttatgcaat ttttatagca attatcgctt taatttgcat
23341 ttttaaaaat gaaatttggg aatttgtaaa atttattttt tctaatttag atttcattgt
23401 aggcgaagat aatatagtat tttttccaat tacaactctt ttaatagtat ttactatagc
23461 tatttgtgct gtttggggat tatctaatgt tttagctctc tcattattta taatgcctat
23521 atttgtcgca gttttttata tttttaatat aaatataagt ttagaaaacc tttatggctc
23581 ttattttata tttttaatca ttattaatgg tattttgtgg agtgtaaatc aaagatgaaa
23641 aaaattacaa tagtagcata cgcaatatat tttctatcag gcttatggtt tttgtttagt
23701 gcttttaaag agcattttga cattttatct tttattcttg gaattgtatt catttatttt
23761 ggtgttatta atacaaaaag aattttgaat gattctaacg agaataaaac cgagcttgaa
23821 actctatttg ctacagcaaa tattattttt gtatcgataa gttctatttt tcttttaatg
23881 ctcattacct tttttgcatt tcaaaatgaa ataaatatta aattcttttc agatcaaggg
23941 cttgtattaa aagatgataa aaagatttct atttatttta aagaaaaaga gattaaaagc
24001 tttgatgaaa acaaatatgg atttgtaatg gctgtttatg gcgataaaca cattgaagtt
24061 gttttgcaaa acaaagaaaa cgataccttt tttgagttaa gatgtaatga ggaaggttgc
24121 aaagaagtat taaaggagaa taaatgagag tagtaaattt gtcaaaaaaa tttgtggata
24181 aaattttgtt tttgagaata aatcatgttg ttaatatctt tatgttaatt ttggctttgt
24241 tttcaactat tgctattgca gtatttttat accattttat agaagaagaa aattactatg
24301 cttattttgt atctagtttt tttgtggtga acgggttttt gctaactatt catttaattg
24361 gtgaaaaaaa gtgtgcaaaa gaagtgtatt atgatgataa ttttatcagt aaacttaaca
24421 actctttgag aaaagaatta gaagatgatc ttttaaagct tcaggaatac tatgatggaa
24481 ttaatgtttt agaatacaaa acttttgctt tgttaaaatg tagttcttat tatgaaaaag
24541 gcagaattga tgaggaactc atagataaaa tcaaaaaata ttacattaat ctttttaaaa
24601 agaaaaatagc aaaagaatta gaagaaatgc agaaagagaa agagagaagc aatattttaa
24661 aaacaaatca agagttaatc ttgaaaaaga taggagaata aatgagaata aattatgaaa
24721 aattaaccat taagccaaaa gttttcggaa caatttttga gaagaattaa aaaaaatatt
24781 ctccgatgct attagctatg ataatatagg cttgcatgat tcaagaattg ctgacattac
24841 aaaaattcct ttagcaacat taaacaattg gaaaaaagat gagtcaagct atagaaagaa
24901 aatttattat tttttaaaag gctctgatga aagcatattg aggaaaattt ttatttaaac
24961 tttttgata gatattttt ccatacaagc ttcgttttcc atatactcaa aaagtgaatc
25021 actttttttt ggctttcttc tgtatacatg aatataactt ttgcttctga aataaaaaac
25081 aacaataaat tttttatcat tgacacataa tatatttgat aaatattttt aacatttttt
25141 atgtatatct tcttaatata tattttatat ttttatgata taattatgat atataataaa
25201 tatcataagg ataagtatga ttatatccat ttgtaacgaa aaaggcggaa gtggcaaaag
25261 tacactagct gtaaatttag caattaaagc agcagctgag aataaaaaag ttttattgtg
25321 tgatagcgat ccgcaaaaat ctgttatgac ttttgcaaat attagattag aagaaggtat
25381 taatagaaca ttttctattg tgtctaaagt tggtgaatct ttaaaagagt ttatacaaaa
25441 agaaaagaag aataatgagt atgatttgat tattattgat actggtggca gggattcaaa
25501 agaaatgaga attgcactag gcttttcaga tgttgtgatt attcctacaa ttccaagcca
25561 atttgatgtt agtgttttgg ataaaatgtt tacaattatc aaaatggcaa aagagctaaa
25621 taaagagcta caaacatttg tcttaatcaa caaaagttcc ccaaatcctt tcttaaataa
25681 aaaaattgaa gatttaagaa actatatcaa agaacaagaa aatgaagata ttaaactttt
25741 agataatatt atctgtgaaa gagaaaaata taaaacttgt attcaaatgg gtttagggat
25801 tagtgaaaca gaaactaact ccaataaagc tttagaagaa tttaatactt tttacaacga

25861 gttaaaaaat aaaattaaag gattataaaa tgtcatttaa aaaaaataca aatgtagatt
25921 taaatagttt tattgatggt gctactggag aaacctcaaa gcaaagagta tctggtagaa
25981 ctaataaaag agataaaaaa ctgctcgttg gttttactaa cgaagaatat gaaagattat
26041 catctatggc agaacaaatg ggcatggata aaggtgcttt tataagattt aaaattttca
26101 ataactatta attaaatatt ataaagatat gttttagata tgtatataat atataaaata
26161 ttatatacac acttttaatt aaaacagcac ttcaagtaat tttagatata attatacaat
26221 ggtaatagca tatataagaa ttagcacagc aaaacaagat tcagaagttc aaaaactcca
26281 aatatacgaa tattgtatta aaaataaaat caatattgat gaagttatgg aagttgaaat
26341 gagttcaaga aaaagtctgg aaaaaagacg catgaatgaa ttaaaagata aacttaacga
26401 gggtgattta ttaatttgca cagaactttc aagacttgga agaagtatgc tagaagtcat
26461 tacactaata ctagaacttt ctgaaaaagg tattcaattt gctttttttaa ggcaacctga
26521 gttaagctca tttaacaatc cttctcaaaa actacttctt tccttttatg cctatgtttc
26581 agaaacagaa agagatttta tttctcaaag aacaaaagcg ggacttgcta acgctagaaa
26641 aaatggaaaa aatataggtc gtcctgtaaa tagttttaat agtatgtatg ataaagatac
26701 agatttaata aaagagctta tatctaaaaa aataacttta cgcaatatct ggaaattttt
26761 aggagaaaaa ggaacttata acaatttcca ttatttttgc aaaagtagaa aattagcata
26821 atatattata ctaatttaca aataatatta tttacatatt taaataatat atataatata
26881 tgtatagaat atatatgtta tatatattta atatttattt tattaaataa tgctatactt
26941 ttaaaattca atttttaatt ttctttgtat aaaatgttat ttccaaaaaa caaaacattt
27001 tcttaaaaaa ggatattctt gcatggcaaa gaaaaaaaaa ttgaacgacg atcaacctag
27061 tctattttat tttatagaag aggaattaat tattacaagc gaaaggaatg ggaatgagca
27121 acagaatgga aagttatccg aatatggaaa tattacaaaa gagtttaaac aaactagcat
27181 ttcatcaaat acaccaagcg tggatagaca agaaaattcc acaatacagc ttgataatac
27241 tagagagatg ggcagagttt tatccgaaca caatcaagaa tctaggaatg tcagatctaa
27301 tgacactagc attgccacaa acacaaatgg aattagcaat cttggagagc aaagaagcag
27361 acaagaagag agaacagggg ttaacagata tggagatact agcagaagag caaataaatc
27421 tcaatcaata catagcgata gaaccccaaa tttattctcc tttatttcaa gaaatgatga
27481 tgaaagacaa ggaacagatg ctgcaagaag ctacaatcaa caatcagtat tggaaactgc
27541 agcaagaaat gatggatatg aaagaagaag cctcaaatct gggcaaaaat tagattttgt
27601 aagcgatgat gaaatctatc ttggctcttt aaaagataga tttcaaaaaa acctccaagc
27661 cattaaactt tcaaaaacta ttgaacaaga aaatcgttac gctaccaaac aagaacaaga
27721 aattctaaat aaattttcag gatggggcgg tattcctcaa gcttttgatc atcagaataa
27781 agaatgggaa aaagaattta aagaacttat tagcacactt gattatgcag aatatgaaaa
27841 ggcaaaaact tcaactttag atgcttttta cactccaaaa attattatcg atacaattta
27901 tcaagggctt aatcagctag gatttaataa tgatgatcac accaaagaaa tatttgagcc
27961 aagtgcaggt ataggctctt ttttgtctta tgcaaaaaat tatagcgata aatatcattt
28021 tacttgcgta gagcttgata ctataagtgc aaatatatta aagcatttgc acccaaatca
28081 aacaatttat aataaagcat ttcagcacca tttatttgat aagccatacg atgcttttat
28141 aggtaatccg ccttttggtc aaaaaaagat cttggattta aatgatccga cacttaataa
28201 aacgagtgtg cataattatt ttattggtaa tgcaattaaa aatttaaaag aagatggaat
28261 tgctgctttt gtagtatcaa gttatttttt agattctaaa aatagcacaa taagaaattt
28321 tatagccgaa caagcaacat ttttaggtgc tgtaagattg ccaaataatg cttttaaaaa
28381 acgagctaat acagaagtta ctaccgatat tatctttttt aaaaaaggta aagatttaaa
28441 tatagataaa tcttggctag aaagcgtaga atactatgac gatcgttttg atgaagctga
28501 aaaaagagat atgcaccctg atgtttttaa tgactttaga ataaatgagt attttaaaaa
28561 taatccacaa aatatcttag gaaaaatgaa tattaaaagc tctcaatatg gttatagttt
28621 agaatgctta gatgatggaa gagatttaaa aatcgcttta gaaaatttta caaaaacctt
28681 acctaaaaac atttataaat atcaagaaac aaaaatcaag ctatcttatt acaggattga
28741 tagagaatcg cctgaatacg aaaaatatag caaaactcta agtaagttaa aagatggaaa
28801 ttattttgaa tacaataatg aaatttacat gaaaatcaga agcgatgatg agttagtctt
28861 ttcaaaacct gttttaaatg aacacgatac aagaaggatt aaaaaactca ttgctcttag
28921 agatcaattt aacactctta ttgaatatga aaaaaatgaa tctagcgatc ttttagtaga
28981 aaatcaaaga aaatatctaa acaagcttta tgatgaattt gtagccaaag aaggatattt
29041 aaatagagat gcaaataaaa aagcctttag agaagatgta gaagcaaaca aaattttagc

29101 actagaaaaa aattatagca gtggtatttc aaaaagtgta gctttaaaag aaggtataga
29161 tcctattgca ccaagtgcga caaaagcaga tattttttc aaaagaacta taaatgcaca
29221 aaaagaaatt aaaatctcga ctccaaaaga agcattaatt gctagtatca atgaatatgg
29281 gagaattgat ttaaaatttt tagaaacgaa tttaaatcag cctttagaag aaacccttaa
29341 atctttagag caagatagat taatctttaa agatcacaaa aatcctgcaa attatgtttt
29401 ggctgaaaaa tatctttctg gcaatgttaa agcaaagcat aaagaagtaa aaaaattagt
29461 agaagatgga tttaatgaat ttgctaataa tttagaaagc ttagaacaag tcttgcctaa
29521 agatttaaaa gcagttgata tttcaataag cttaggaaca acatggatac ctataaaata
29581 ttacaaacaa tttatagaag aaaccttaca aattactcct aaagattatg atttattttt
29641 aatggaaaga acaggcgaat ggagtcttaa aggttttggc tattctttaa gttcttatat
29701 tcgttcagaa tatgccacag aaagaatagg ttgctttgat ttgttggagc atggattatt
29761 aagaaagcct attcgcattt atgataaaaa actagatcca tcaggaaaag aaatcagaga
29821 attaaatcct aaagaaactg caatcgctaa ttctaaatta gaaaatttaa aaaatgaatt
29881 tatagaatgg atttataaag actatgatag acgcacagat ttggaaaata tctataatga
29941 aaggtttaat acaaatatag aaccacgata caatggagag catttagaac ttccaaattt
30001 caatgccaat atcaaactca aaaaacacca aaaaaatgct atatataggg ctattcaaga
30061 aaatagtatt atttttgatc atcaagtagg tgctggaaaa actttagtgg caatttgctc
30121 tgtgatggag caaaaaagaa tggggctttt aaataaaccc ctgattgtag tccctaatca
30181 tttaacaaga caatggaatg atgagtttta tcacgcctat acaaatgcaa acattttagt
30241 tgccacaaat gatgatttaa aaaaagataa tagagaaaag tttttttcaa agattgcaac
30301 aaataatttt gatgccatca ttatgacaca ctcgcaattt aaattaatcc ctgcacctta
30361 tgaagtcatg aaaaaacaat atgaagaaga tattaatatc ttagaacaaa cattagaaaa
30421 aaaacaaaac gatgataatg ttatgcgata ttctgtaaaa gacttagaaa aacgcattga
30481 aaattttaag gtgaaattga gtgatttgca aactacacat aaaaaatcta aagcaataga
30541 tttttcagaa ttaggaattg atgctttaat agttgatgaa gcacacgaat ttaaaaattt
30601 actcattaca acttcaatgg gtgatataag tggtcttggc aatttaaaag gctcacaaaa
30661 agcctatgat ctttattcta aaacacaata tattcacgag caaaataaaa aactctattt
30721 tttaacagga acgcctataa gcaattcaat tacagagctt tttaccttgc aaagatatat
30781 acaaccaaat gtattaaaag aaaaagggat acaagctttt gactcttggg cttccacttt
30841 cggtgaagtt acaaacgctt gggagcttga tagttctgga ataaattata aaatcgttgc
30901 aagatttaac aaattcaaaa atgttcctga actttctacc atgtataaaa gtgtggctga
30961 tattgtaaca aataatgata ttatgaaata tcaaaaagat ttcgtgccaa agctttacaa
31021 tgacaaacct attaatatcg ttgctcctag aagtgaagaa atagcagaat ttataggcat
31081 tcaagatgaa aatggaagat ggaatgaagg ctctattgtg tggagaatgg agcatcaaca
31141 agatgatatt actagaaata acttacttgc ttgtaccaca gatgctagaa aagctggact
31201 tgattttaga cttattaatc cacattgtga tgattatgca aattctaaaa ttaataagct
31261 tattgaaaat gtttttaatg agtataatgc ttggaatgat gataaaggca cacaattaat
31321 attttgtgat ttatccactc caaaacaaca ctcacaaaaa gtttctctaa atagcaatat
31381 tgctacacca aatttaaata aagaagaatt tgtcaatatt aatgaagcaa tagaacaaac
31441 agaagaagag aatagcaaaa gccttgatga actcttagca gaacaagcaa aatttgatgt
31501 atatactgat atcttaaaaa aacttgtcgc aaaaggtatt ccacaaaatg aaattcgttt
31561 cattcatgat gcaaaaaccg atttacaaaa agggcaactt tttgcggata tgaattcagg
31621 aaaagctaga attttaattg gatcaacgca aaaaatgggt gctggaacaa atgtgcaaaa
31681 aagaattgta gccttacacc atttagattg cccttggcgt ccgagcgatt tagaacaaag
31741 aagcggtagg gttataagac aaggcaatga actctttatg agagatccgc aaaactttag
31801 agtaaaagag tttagatatg caactgaaag aacctatgat gctagaatgt ggcaagtcat
31861 tgaaagcaaa gcaagatcca tagaacaatt tagaaaagct ggtgctgatg taagaacctt
31921 agaagatgtt acaagtggtg cagctgatgc tgcagaaatg aaagcagaag caacaggcaa
31981 tccactcatt ttactacagg tgcaattaaa tagtgatcta agacaagaag aaatgcttta
32041 tagtggttat aaaagacaaa tacacaataa tgaagaaagt cttataacca atctttcaaa
32101 gattgaacta ctaaaaaaag atatagccaa aatggaaatt ttaaattcaa tcattaaaga
32161 aaataatagc gagcattttc aaggaaagta ttacattaat gatgatgaaa aagatttcat
32221 tgtctttaaa aatgatacga gtgatctaaa caaaagcaga caagcagaac ttgaaagaaa
32281 ttttgaaaag attgttaaaa ccatagccga tgactatcaa aaagaatata aggttttgga

32341 gttcaaagga ctgcttataa gtggagaaag aacagattta aacactctta atttttatgt
32401 gagtactact gatggtaaaa caataatgga gcctgaaaat ttgatttata aaagagatga
32461 aaaaaccttt tttaaattgg aaagttatgt aaattttggt ggttttgtaa agagattaaa
32521 caatttttat aatgacttag aaaaatttac tcaactcaat aaaaataaga tagaaaatct
32581 tgaaaaagaa attgtggagc taaaaagcat tacaggggat aacactccaa aatacaaaag
32641 aaaggattat ttagaggcat taagagaaga taatcgcatt atcatggatg aaatagaaaa
32701 aatgagtaaa caaaaaaact acaaaagtga ttttgtgcca aaatctgcaa agattttaga
32761 agatttaaat aaacataatg aaattgaagt cttgtcaaga tagagtgtaa tcaaaaaatg
32821 ccctaaaatt agcttattga tcttagggca gtattatttc tttttatctt ttttcatctt
32881 ctaaaaaaaa tttgctaata tcttgtgtta tatcttttcg gtaattaaaa ccctcaagaa
32941 caattcgtcc tttttatca gaaatacaaa aaagtggctc tatgatctcg atagcatccc
33001 atacaaccca aatataagga aagaaaccat tgtgtataat tctattgcac caatttttaa
33061 ctagcataat cttatcaaaa tccacatttg aaggtaaatc aaaatattgt ctatgatttt
33121 cttttagctt attaagaact tcatgtaaat ttattttatg ctcttgtaaa tcaccaaata
33181 tcatccactt tatttttttc tctagtgcca tacggattaa aaaagccttt ccaatgagag
33241 aggtgtgaaa tggtgtatac aattgcgaga atccaaatga attgcaaaat gattcttgtg
33301 gtgcaatata atcacccaaa ccttgtatgc tcccaattat attattgatt tccttaacga
33361 tatactgcaa agactcatca attaattttt ctttgtgctt tatcgtatta taaaatcttg
33421 ccatatcata aataatagtc tgcgttatta tcttttgtgg cataccacta gcaatttccc
33481 aaaatacaaa atacctattt atatttaccg attcaatggc tctataaatt gataaataat
33541 ggcttttaat atattcatca ttaaattgct ttggttgctt aaaaatcaaa tcaaacttat
33601 catttattgt attttttttc ttgtataaac tatcttctaa taaatcaagt atattattca
33661 aaattaataa attgttttct ttatcttgat atgttatttt ttcaagcatt tccattataa
33721 ttctccttaa aaacctatat tataaaatat ataatgcgat atttatacta tgattctcta
33781 taaatcaccc atctctttca atctcattat tcctagcact tgtccatcgc aagctactat
33841 cggttcgtcc tcgttttcct tgttgtaact gattgagtgc attgatgtgc ataggcattt
33901 gagttccttt ttggtcatga accatagact gttcggacaa tattcgcata gagtctcttt
33961 cttgggcagc atctcttcgg atagttgatt tattactgag ctcttgtaag attctctgtc
34021 tatattgttt tgtataatct ctctcattat tttcctttat agtatatttt ggattattcg
34081 cttcatctga ttgcaataac ttcaattctt tttctctacc taaaacagaa gcgtcccaaa
34141 cagatttttt tgccatcttg gttacttcta caatcttacc gtttactttg actttttct
34201 taatttgata agtttcttgt cctacaattt taaattttgc atattctcct tttaaaattt
34261 tattttcttt aataactctt gctaaatctt tactccatat tgtagcaatt ccttttttgg
34321 ttctatattt cacataaaaa ctatcttcag catttttatc taaactgaat ttatattttg
34381 catctccata atctaaaact tgataataat gcattttagt ttgttcttta tttttctcaa
34441 gtttataaga tacttcatta ttttttgaac gaatagttgc ttttgcctcc acgcctaatt
34501 cattcaatgc ttttgcaaaa gataatctta aattcattaa atctgttttt tttggattaa
34561 ttcgtttgcc attttatcc gcaactttca agcaaacatg gcaatgagga ttatcagtgt
34621 cattatgaaa agcaattgca aaaaaattat ctggatacat ttttttaat gtcattaaaa
34681 ctgctttttt cattttttct ggtggagcat ttgtataatc ttgcatagaa aaaaccatat
34741 taatggtatg tcttttttct tttttttctt gtccgaatct tgggatcgga gctccctcat
34801 tttcataaat ctttttttaaa aattttaact catctttgcc ttttaatgta tcaaaatcac
34861 ttgtaattac atctactgtt ccttctcttg aaatataatc taaatgtttg caaactgctt
34921 gcaaattctt tgaacctgat gtaattttaa caacaacctc agtatttttt tttaaaattc
34981 tattcgtttt atttgatgat ctatttttaa aattatatgt agattttgag cctatttcta
35041 ttttagtata aactcttttt gctatgattt catcatcaaa tattttaagt cttgacatta
35101 aattctttca ttattttttaa taattatttt ttctagctct ttagataatt tttcaatatt
35161 taatgaaata tctttgaaag atttttctaa attgttttca tttattctta cacttttacc
35221 acttcttaga atttttaaaa gctgataaat atttctacct attgtattta tttctgttct
35281 tgtcagtatg aattcattta tatatttttt atcaaaaaac ttatcattat atatatgatt
35341 taaagttaaa attcttattt gtttagttgc tgtgccattc ccatttgatt ttgcaaaatc
35401 tgaaaatatt ttatattctt cttctgtaag tttaaatcta atctcttttg taggttcatt
35461 tttttatgg tttattttat caaaattatt tatattttct ttttcatcat tgctgatgtt
35521 ttttaagata gtatttatta tcttactaaa agaagttttc atttcttttt ttaatccaac

35581 aagaaaagaa tagttttttt catcaaaata aatgacagtt tttttcatat aaattataac
35641 accttttatt gagcctcgca gagaatattt cgctatttac ttaaaaaatg atgtcatttt
35701 ttggagtaaa tagctatcct gctattatta cattttctaa acctatattt tagttaaatt
35761 atctttatat aaaaattaat attttcaata tttatgatat aattaatata caaaaattta
35821 taaggagtaa aatgaaaaaa gagagcaaag aaatagaatt gcttaagcaa gaatataaag
35881 aaaaaatcca aaaggcaaaa caaaaagaaa aagataaaaa agaaaaactt tgcaagaagt
35941 taggatctga aattatatat ttattagata atgatgattt tagaaattta ttttttgatt
36001 atatacaaaa aaataattta gaaaagataa aatcattatg tagtgatatt gatgatttct
36061 ttaatattaa ttacattaaa aataaggagg atgaaaacga tcaagataaa taatattttt
36121 aaaaaataat accccaaaac aaaaccaaaa aacaaaatta ttaaggatga aaaatggaaa
36181 ataaagaaat gcaagaaaat ataacatttg ataatatcaa agtaagaatt actgataatt
36241 ctaagctatg taaagaatat caagaatgtg tttttaatgg aacatatgat aatacttcaa
36301 atccaaaaga gatagaatct ctttttaata ctctaaagac ttttgattct tgtgagaaat
36361 taaagcaaat agaaaattta tctttttcaa aacaagaaat tgattttgaa atcataacag
36421 atgacttcaa aatacctcta aaagtaagac tgggagaagg acattttgaa aaagggttag
36481 atgagttaat taaattaggt agtaaaaata tcgacactaa cattaataaa aaaattaatc
36541 ctgatataaa agagaaattt gacaaaatat tatctccact caaacctgaa aattttcaaa
36601 acattttaaa tggaaccttt gaagtaatta aagaagctgg atctttgaca aatacgaatg
36661 aaatctctag ttttattcaa gaatttttaa aagcttataa aaaaactcaa aaattatctg
36721 aaaataataa aactatagat aatccaaaag aagataagaa aaaagacaaa aactattttt
36781 taaaaaaggt tgatgaattt tttcaaaaaa agcttgattc tcgcgaaagg gagcatgttc
36841 ggactaacga aaaatctttt tctaaatcaa tataaaatta aaaggattat tgatgtatag
36901 tttaaacata ccctcagcca ttcaatcttt taaacaagct ttggaagaag aaggattaat
36961 tattgatggt tctcctatca tggatggcaa aattcataga gtaaaaacaa ctaatgataa
37021 aggtagagaa ttaagtggtg cttatattgg atatttaaac aataattttc ctgcaggatt
37081 tgtgcaaaat tttaaaacag gcataaagaa aaattggaaa atgcaaatag aaaaaaatca
37141 aagcaataat ataaaaagtc cgaaagaatt gcatgagcaa attaaaaaag atcaagagct
37201 tagagaaaaa gaaattttag ctttgcaaga aaaaaccgct ttaaaacttg aagatgagta
37261 taataatgca agatgggcta attctaatca cgcttattta aagaaaaaag gctttgatga
37321 aaactttat cttaaacaag acaacaaagg ttctttgctt attcctttaa aagacgagaa
37381 tggaaaatta tggtctgtcc aaagaatatt tccaaatgga gataagatta taggagtcat
37441 taaaactcaa gaagaaaaag atcaaggcgt agaatatctt gcaaaaaaac aaggttgttt
37501 tcatataata ggagctaaaa cactgcataa tttaaaagaa ttttatattt gcgagggttt
37561 tgctactggt gctacattat ataaagcctt aaataaacct attattatgg cagtagatgc
37621 aggaaattta gaaaatgtag taaaaaaatt aagcaccctt tattctgata gaaatattac
37681 aatatttgcc gacaatgatc tcaaaaaaga attaaaaggc ttagaaaata caggtaaaaa
37741 aacagcttta gagattaaag aaaaatattc taatgtaagt gtaattttac caaattttac
37801 acaagagcaa agctctcaag gtatgagtga ttttaatgat ttaatgagtt taaaaggatt
37861 ggaatttgta gcaaaagaaa ttaaaatgca gatgtttaga gaaaaagtag tgtgcttaaa
37921 aaaagaaaat tctgacaagg aattaactag aggctaaaat ctctagttaa tatttgtttt
37981 tagatattta tgttatatgt aattgatatt aggtagatat tttattatcc atcattttta
38041 ttttttcttg ccaacatctc cgtaaaaata ttatttgaac gaatttttc aatattatca
38101 gaaggatttt tcttaaaagc ctcaatattc gtatctagca attttgtcat taactcaact
38161 tgcttctcat ctgtttcata atttgctgac aaatctttga tctgatcttt atatctttca
38221 aatgattttg ctaaagattc tttatgtgca tattcttgtt caagtttgat atatttattt
38281 aaattaatat ttgcaacagt agcagcccat ataaaagggg tattaatcat taatctaaca
38341 atagctgaaa ctaaatcaat attatttaaa tttttccaat caatagtgaa aaaattaaat
38401 attgccatac ataatattgc tccagcaaaa aaatataaat ttttattttt attatcatta
38461 gcttgttcta cttgtttaaa aaatccagct gcaagtcctg catcactaga taaatctata
38521 acttttttg cttccacttc tactttttt aattgctctt ttctctcttc taattctttc
38581 tttaaagatt gaatattgcc atcatctgaa ccaaatacaa ttttataaaa atcatctaat
38641 tctcttttt tcttttcaaa atcagtataa tttgaaataa atgtcttata ttttgaattt
38701 tcttcattac ctattatttc agaatatttg ctcaaaaaat catctatttt tctgtttaaa
38761 tcttctacct tatttaagtt ttcactattt ttcacaacaa ttaaatcatt ataaatattt

38821 ttaatttgtt catatttgtt caataaatca ccaggtatat ttcccttatc acttaaaaat
38881 atttctgcat acgaattatt aatattattt attttttgct taatatcatt tattatatta
38941 tcagatgtaa cttcttgaat tcgtgttagt ctacttttac cactctgatc attattcaac
39001 aaataattat aagcttcatt taatttgtat attctttctt gtatctctct ttctacttct
39061 tgatagctat taatctcgtt attaataagt gatttcaaag aatcatcact actactatca
39121 ataaatgctt tatttataga gtaatcgctc ttaagttgct cataaagctc ttttacctta
39181 ttaatttctt cttgtaattc ctctaatttt tcatttttat ctttagacat ttttactcct
39241 atttagtcaa ataagccact agcttttgct ttttcttcag cttgcttcat aagctcgcca
39301 taatccaaaa gctctttcca cattgatcta ctcatatctg ctttaaaagc attttcacat
39361 cctacatatt cttttgatgt tttatctttt agcatagaat ctctattttc tttacaatat
39421 tttaaaatac tttttaattc gagcatcctt ataatatatt ttaaggatat tgaaatgata
39481 tctaataaat attaattaga tataaaaata acaacatttt acttaaaact taaatttttt
39541 tatattttca gaattatttt aacataaaat ttaatttata catttcattc tttggatttt
39601 gattataata aaagttcaaa agtaatttta ttacttttga tttacattat ttgtaaatga
39661 gcgttagcga aataaaaata attgaattta tatttttgtt ttcaagagta gattaagaat
39721 aaaagattaa atagaaaatg cttctaaata cctataaaat agttatttgt aaaggttttt
39781 gattttccca cctgtgggag aaattttccc gcctgtggga gaaaaactgc tcgtggtggg
39841 aaaaaattaa gaagtaattt ttcccaaaat aattaataaa tttcatcata tgaattataa
39901 tttgtgtttt tatttgggaa attatcattt tttgggaaaa atgtgttata atgtatttta
39961 tttttttggga aaaggttaat aatggcaaaa atagttaatt tagagaacag agatactaaa
40021 aaattatata gtagtgttca ggaaaaaatt gatgctacaa ctggagaagt tactaccgta
40081 ataagttctt ttttacaaag agaaaaaact aaagatgatt ttattaaatt atttgttgaa
40141 aatattagtt ttcttactga taatctttca aatcctgctt taagggttgt tttgatgatg
40201 gttaaaaact taaattatca aaatgtcttt aactataata gcgattttgt tcattatttc
40261 gaaagtaaaa agattttagg gaagtcgagt gtttatcgtg caataaaaga acttgaagac
40321 aagcaagtta tttttaaagt aacagaagaa caaagaaaag aatatgatat catagttca
40381 aattcatata ttatgaatcc gcagatcata ggcaaaggtt cttttaaaga tcttaaaaaa
40441 ttaagacaaa ctgttgttaa aacttttgat tttgataatt tagaatttaa acaagaaata
40501 tcagttgaga gtgaatatga aggatttgaa caggttaaga aaaatccaga taattatgtt
40561 atcgaggaag tcaaacaaat agcctcaaat aagaatacaa tagaaaatca agttattatt
40621 tctgaaaaag acaatgatca agattatcca aaagaagaaa taattgatgt taaagtggat
40681 gataacaaag aagagcaacc tgctcttttt gttgaaaata aaaatgaaca aaacgcaaaa
40741 gcttttatta atgaatttgc aggtatgttt aatggagctt ttcatccgag caaaacagtt
40801 aaagaaatga aaaaagaatc tttagagaat gatttaaaag agggtaatat atgaaaaaag
40861 tatttcttga tacaaatatt tttattgatt tttttgatga agcaagggta aataatgagc
40921 aagctaagca attaatatat tttttaatat caaatgaaat aaaaattgta ttttctgaag
40981 atatgatttc aaccattgcc tatttaatag acaaaaacaa gttaccagct cttatgtctt
41041 tttttaaaag tacaataaca aacccatatt ttgaaatagt atcgttcggt tcaagtgtta
41101 ttaatatggc ttgtgatttt catttgcaaa ataaaggtga ttttgaagat tatttgcaat
41161 acttttgtgc agaaaaagaa aattgctgcg caatttatac tatggataaa aattttccaa
41221 atttaaaaat acctataaaa agatatggtg atacaaatat atagggggaat atcttgccaa
41281 aaaaaagaat gtttttgttt gttccatatc aagatattca tgaagctaaa aaactcggtg
41341 caatgtggga taacaagcaa aaacaatggt ttgcaccaaa atcattggat attaatatat
41401 tcaaaaaatg gctagataac aataaaagta attcttattc aatcaacata ccctcagcca
41461 ttcaatcttt taaacaagct ttggaagaag aaggattaat tattgatggt tctcctatca
41521 tggatggcaa aattcataga gtaaaaacaa ctaatgataa aggtagagaa ttaagtggtg
41581 cttatattgg atatttaaac aataattttc ctgcaggatt tgtgcaaaat tttaaaacag
41641 gcataaagaa aaattggaaa atgcaaatag aaaaaaatca aagcaataat ataaaaagtc
41701 cgaaagaatt gcatgagcaa attaaaaaag atcaagagct tagagaaaaa gaaattttag
41761 ctttgcaaga aaaaaccgct ttaaaacttg aagatgagta taataatgca agatgggcta
41821 attctaatca cgcttattta aagaaaaaag gctttgatga aaacttttat cttaaacaag
41881 acaacaaagg ttctttgctt attcctttaa aagacgagaa tggaaaatta tggtctgtcc
41941 aaagaatatt tccaaatgga gataagatta taggagtcat taaaactcaa gaagaaaaag
42001 atcaaggcgt agaatatctt gcaaaaaaac aaggttgttt tcatataata ggagctaaaa

```
    42061 cactgcataa tttaaaagaa ttttatattt gcgagggttt tgctactggt gctacattat
    42121 ataaagcctt aaataaacct attattatgg cagtagatgc aggaaattta gaaaatgtag
    42181 taaaaaaatt aagcaccctt tattctgata gaaatattac aatatttgcc gacaatgatc
    42241 tcaaaaaaga attaaaaggc ttagaaaata caggtaaaaa aacagcttta gagattaaag
    42301 aaaaatattc taatgtaagt gtaattttac caaattttac acaagagcaa agctctcaag
    42361 gtatgagtga ttttaatgat ttaatgagtt taaaaggatt ggaatttgta gcaaaagaaa
    42421 ttaaaatgca gatgtttaga gaaaaagtag tgtgcttaaa aaaagaaaat tctgacaagg
    42481 aattaactag aggctaaaat ctctagttaa tatttgtttt tagatattta tgttatatgt
    42541 aattgatatt aggtagatat ttttaatcaa acaaagcatc aacttcttta tcatattttt
    42601 ttctgagctc gccataatcc aaaggctctt tccccattga tttactcata tcagctctaa
    42661 aagcattttt acattcttga tattcttttg aagtagtatc cttgaatata gagtctttat
    42721 tttctttaca atattttaaa gtatctttta attctttttc atttttggct tctttaaaat
    42781 attccataga cttaggctct gatgagcaag cacctaaaaa gacaataaga ctaaaagaag
    42841 ctaaacctaa agtaattttt ttcattcgag catccttata atatatttta aatatgttcg
    42901 ttatatttta atgatattga aattatatct aataaatatt aattagatat aaaaatataa
    42961 aaaattattt gatgatttta atgcgttcgg cgttaaacca aattaaagca ttaatatccg
    43021 agcattcgct tgtaagtatc tcaataattt ctccagtaaa acctaaatca aataataaag
    43081 gaaaatcgta aatctttaat tgttgtgctt ctttgatgct gttagggcta aaaatagaat
    43141 ttaattcttc aaacaagaaa aactcacctt gttttgattg acaatattgg catttattca
    43201 ttaaataaga acttccaact gtatttgaaa atgatttttc gagagatttt atattcatac
    43261 gctttaagat gctagtaaaa tcttcagagt atgcagttat gtttctaaat attttaaatt
    43321 taggttgaaa gaaccattca ccttttttctt tgtatataaa tttagagctt cctaaagcat
    43381 atactggtat aattttggag catttccaac aattaataaa tccctgaaca atatttaaag
    43441 tttcagttac ataaaatgta aaattttcat tctctaaata attagtaata tagtttaaat
    43501 ttttataatc ttgtaaatac caagttttat gttcttgatt ccattttatt ccatattctt
    43561 tcaatttatc tttttctgaa taaggaatat taagttttat attcataaat tgcccttaga
    43621 taataaaaac acttaataat taattacata tataaataat taatataaat tttataaata
    43681 ttctgttaaa atttatcgtt cgttattttt taatttttta ttctatcaaa aaaatactat
    43741 gttttatctt aaaataatat tgaatttatt acttgattta gtaatattta tcgacattct
    43801 tggatattta aagaattaat tttgaaattg aggtttgtat cttttcaaca agtgagcata
    43861 acccattgta atgagaaatt ttcttaatgt atctctatgc acacctattc tttttgctat
    43921 ttttgaatgt gagtagcctt gcttatccca ttttaaaatt tgttttaatg ctggatagca
    43981 tggatttact tcaattctat tttttttgcc tttgggacgc cctaagacaa caccttccat
    44041 tttttttctt gctaaagcct ctttggttct ttgtgaaatc aactgccttt caatttcagc
    44101 cgagagagag aaggcaaaag caattacttt tgtattaata ttatcattaa gaataaaatt
    44161 ttctttaatt gaatatatag tgatattatt tttaatacac caatttaatt tttccataat
    44221 atccaaaaga cttcttccaa gtcttgaaag ttctgtgaca accaaagaat ctccattttt
    44281 tagtttttta aataatcttc ctaaatttct atgatcaatc gctattttttc cactaattac
    44341 ttcttgaaca aagtaattga ttttaatttc atttctttta ctccattgaa gtatttcaaa
    44401 tctttgattt tcagtgtttt gtttgtcggt gctaactctg atataggcaa aaatcattaa
    44461 tattccttac aataaaaaat gaagaatgtt ttataccaaa aaagataaaa aaattccacc
//
LOCUS       BDFFJJMB_2              1175 bp    DNA     linear       12-APR-2021
DEFINITION  Campylobacter species strain strain.
ACCESSION
VERSION
KEYWORDS    .
SOURCE      Campylobacter species
  ORGANISM  Campylobacter species
            Unclassified.
COMMENT     Annotated using prokka 1.14.6 from
            https://github.com/tseemann/prokka.
FEATURES             Location/Qualifiers
     source          1..1175
```

```
                     /organism="Campylobacter species"
                     /mol_type="genomic DNA"
                     /strain="strain"
     gene            57..581
                     /locus_tag="BDFFJJMB_00047"
     CDS             57..581
                     /locus_tag="BDFFJJMB_00047"
                     /inference="ab initio prediction:Prodigal:002006"
                     /codon_start=1
                     /transl_table=11
                     /product="hypothetical protein"
                     /protein_id="Prokka:BDFFJJMB_00047"
                     /translation="MDTLIQISNLYFTIGETAQYGFDICPYHICSIGEFGNNGYGGTG
                     YYKNNYREIHVTKAYNNYPTMELYIFGFDTQQDAIDSINKINFTYQPTYGGNTKYDMK
                     CLANSVGFASIGDGKSPQFPSNKWGVWLVDFGGLGHGNQCLAVTKKHNGIYTFKTVYK
                     GKTYTSILHLKLSE"
     gene            complement(640..1152)
                     /locus_tag="BDFFJJMB_00048"
     CDS             complement(640..1152)
                     /locus_tag="BDFFJJMB_00048"
                     /inference="ab initio prediction:Prodigal:002006"
                     /codon_start=1
                     /transl_table=11
                     /product="hypothetical protein"
                     /protein_id="Prokka:BDFFJJMB_00048"
                     /translation="MEAKAFNQTLKASAKISVFQTRGILAHPTGNLNGGNGRTTLLGT
                     NNGEDGQAFGSYSQGYSIFTLTNHNGNFDLGIYGYQNTNDVLNANIKVTYTPAYGGNQ
                     KWNLNYYINSKNYYLGNNFNPFDMKHYTYLLFSNEEALIPTKQNNGIYKFEDLNNKYI
                     KPTYLHLKLP"
ORIGIN
        1 caccgtttaa taacgacatt aatactaata tcaaaagctt agggcaaagc ctagctttgg
       61 atactttaat acaaatttct aatttgtatt ttactatagg agaaacggca caatatggtt
      121 tcgatatttg tccatatcat atttgctcaa ttggagaatt tggcaataat ggttatggtg
      181 gaacagggta ttataaaaac aattacagag aaatacatgt cactaaggct tataataatt
      241 atcctacaat ggagctatat atttttggtt ttgatactca acaagatgct atagattcta
      301 taaataaaat aaattttact tatcagccta cctatggagg taatactaag tatgatatga
      361 aatgtcttgc taatagtgta ggatttgcat cgataggtga tggaaaaagc ccacaatttc
      421 cttcaaataa atgggggtt tggttagtcg acttcggagg tttaggacat ggcaatcagt
      481 gccttgctgt tactaaaaaa cacaacggta tttatacttt taaaacagtt tataaaggta
      541 aaacttatac aagtatttta catttaaaat taagtgaata atcctttagc ctataaaaaa
      601 tcaaatttta tataggctat tttaaattcc attagatttt taaggtaact tcaaatgtaa
      661 ataagttggt tttatatatt tattatttaa gtcttcaaac ttatatatac cgttgttttg
      721 cttggttgga attaaagctt cttcattact aaataacaaa taagtataat gcttcatatc
      781 aaaagggttg aaattgtttc ctaaataata attttttgaa ttgatataat aatttaaatt
      841 ccatttttga ttaccccat aagcaggagt ataagttact tttatatttg catttaatac
      901 atcatttgtg ttttgataac cgtaaattcc taaatcaaaa ttaccattat gatttgttag
      961 cgtaaaaata gaataacctt gagagtaact gccaaaagcc tgtccatcct ctccattatt
     1021 agttcctagc aaagtagttc ttccattacc tccgtttaaa ttccctgtag gatgtgctaa
     1081 tattcccctt gtttgaaata cactaatctt agcactagct ttcaatgttt gattaaaagc
     1141 tttagcctcc acattaatgc tgttattaaa tggtg
//
```

>>BfR-CA-15843_min_cov_30 putative plasmid location

```
LOCUS       IKNDEOKD_1             19540 bp    DNA     linear       12-APR-2021
DEFINITION  Campylobacter species strain strain.
ACCESSION
VERSION
KEYWORDS    .
SOURCE      Campylobacter species
  ORGANISM  Campylobacter species
            Unclassified.
COMMENT     Annotated using prokka 1.14.6 from
            https://github.com/tseemann/prokka.
FEATURES             Location/Qualifiers
     source          1..19540
                     /organism="Campylobacter species"
                     /mol_type="genomic DNA"
                     /strain="strain"
     gene            51..251
                     /locus_tag="IKNDEOKD_00001"
     CDS             51..251
                     /locus_tag="IKNDEOKD_00001"
                     /inference="ab initio prediction:Prodigal:002006"
                     /codon_start=1
                     /transl_table=11
                     /product="hypothetical protein"
                     /protein_id="Prokka:IKNDEOKD_00001"
                     /translation="MTFGWGWFVPYSLQPSYHEFKEMCKLNELPNDEHKYNKILSYFG
                     LSLDTLDWEELNERSLKLIKGF"
     gene            522..872
                     /locus_tag="IKNDEOKD_00002"
     CDS             522..872
                     /locus_tag="IKNDEOKD_00002"
                     /inference="ab initio prediction:Prodigal:002006"
                     /inference="similar to AA sequence:RefSeq:AVS37480.1"
                     /note="DUF2974 domain-containing protein"
                     /codon_start=1
                     /transl_table=11
                     /product="hypothetical protein"
                     /protein_id="Prokka:IKNDEOKD_00002"
                     /translation="MNNKEQIKKLKDNSELAWASYGYFDFIDKQYSFDEKDKDKFKTL
                     YAKINDLKKSDEKVQNAKATYTDILNTECNSLFSGDFSPLQAKQFFSRYDLLKHCPNT
                     NSGFSATLFGEKRK"
     gene            969..1529
                     /locus_tag="IKNDEOKD_00003"
     CDS             969..1529
                     /locus_tag="IKNDEOKD_00003"
                     /inference="ab initio prediction:Prodigal:002006"
                     /codon_start=1
                     /transl_table=11
                     /product="hypothetical protein"
                     /protein_id="Prokka:IKNDEOKD_00003"
                     /translation="MTYILLLIIISTILSYLILKFIYKIIFKSTKSVSKFLVFLGSIG
                     LIIFYYTPYSYYLEPSYWQFRNMCKLNELPNNEEKYNKILGYFDKKLGDIDDFRHVKK
                     YGSIEMWLYIYYDDTYKTPSNFQEKIEMDNIALKTKNMQMSLHKIVDMHISPYWNTRR
                     YVLEGNEGNMNFEFIEDELACGNLYQ"
```

```
gene            1548..1853
                /locus_tag="IKNDEOKD_00004"
CDS             1548..1853
                /locus_tag="IKNDEOKD_00004"
                /inference="ab initio prediction:Prodigal:002006"
                /inference="similar to AA sequence:RefSeq:AVS37480.1"
                /note="DUF2974 domain-containing protein"
                /codon_start=1
                /transl_table=11
                /product="hypothetical protein"
                /protein_id="Prokka:IKNDEOKD_00004"
                /translation="MTNKQLINKLRDMAELAQASYGYFDLVTHDKNDFFIKIKDEATE
                SAFFNKHITQANVLDISYKNYKAYQYDSSLKKDIKIGTLNGDFSPLKPNNSFLAMIY"
gene            2265..2699
                /locus_tag="IKNDEOKD_00005"
CDS             2265..2699
                /locus_tag="IKNDEOKD_00005"
                /inference="ab initio prediction:Prodigal:002006"
                /codon_start=1
                /transl_table=11
                /product="hypothetical protein"
                /protein_id="Prokka:IKNDEOKD_00005"
                /translation="MGILKKVYIAWGVFWGIIFLFSSLYAYFSDIEFFGDMLKIAFFI
                YSYPLLPYISYLTLQETIKSKNIFLYFSLFCFIFLYFALFFFISGGFFIYVIILGIYT
                ILFLIISIKFRKELSYYFSLLFCIFLYIFLYLFIFYIELKNE"
gene            2802..3020
                /locus_tag="IKNDEOKD_00006"
CDS             2802..3020
                /locus_tag="IKNDEOKD_00006"
                /inference="ab initio prediction:Prodigal:002006"
                /codon_start=1
                /transl_table=11
                /product="hypothetical protein"
                /protein_id="Prokka:IKNDEOKD_00006"
                /translation="MKILKSLYIIWMIFWGFGAILGYYMGYDATFTKMFFFLVILYLF
                PLLPYLHYLIYKKARNTKNIFVAICCYY"
gene            3235..3561
                /locus_tag="IKNDEOKD_00007"
CDS             3235..3561
                /locus_tag="IKNDEOKD_00007"
                /inference="ab initio prediction:Prodigal:002006"
                /codon_start=1
                /transl_table=11
                /product="hypothetical protein"
                /protein_id="Prokka:IKNDEOKD_00007"
                /translation="MNTLKKIYIIWGMFWTIGLGIFVPTAHGFDSMDFVDFFIFALQF
                IAIIWLPPLLIFNIVYFVIKKERKYIINTLWILLYYPIALILYYMFYYHNSFYVTLYI
                FLSLFK"
gene            3571..4017
                /locus_tag="IKNDEOKD_00008"
CDS             3571..4017
                /locus_tag="IKNDEOKD_00008"
                /inference="ab initio prediction:Prodigal:002006"
                /inference="similar to AA sequence:RefSeq:AAW35443.1"
```

```
                     /note="conserved domain protein"
                     /codon_start=1
                     /transl_table=11
                     /product="hypothetical protein"
                     /protein_id="Prokka:IKNDEOKD_00008"
                     /translation="MKEKIDKLRDYAELAQASYFYFDLKGYILQENETIVILNELVSL
                     SYNGKIAGKKEKVGQEYSFIGKGELNGEFGELQARNFSQRYEIKFHQSNTLSGFSATL
                     FYDKQKDEFVVGFRGTEGFWNMDTMQDITLSLNGNLQSSPLLEFLE"
     gene            4470..5183
                     /locus_tag="IKNDEOKD_00009"
     CDS             4470..5183
                     /locus_tag="IKNDEOKD_00009"
                     /inference="ab initio prediction:Prodigal:002006"
                     /inference="similar to AA sequence:RefSeq:APA47419.1"
                     /codon_start=1
                     /transl_table=11
                     /product="lysozyme"
                     /protein_id="Prokka:IKNDEOKD_00009"
                     /translation="MQNLKLQIAQGLDKILEDEDRKQELYVCKFVVVNGVSENKIKAK
                     NKDVNIDNDTKNINTTKSNNISSSENNAQEKITHLSNDGQNLLKNIEKLRLKPYNDQN
                     GKEITSYVKGATIGYGHLIGQNEWDLYKNGITLQEADKLFKSDLLPFENAVKNSINSS
                     LAQNEFDALVILCFNIGIDNFKNSSVAKIINGEKTGYKTLKEAWMAWNKSQNKVMQGL
                     INRRNAEYKLYTQGGYEKW"
     gene            5177..5536
                     /locus_tag="IKNDEOKD_00010"
     CDS             5177..5536
                     /locus_tag="IKNDEOKD_00010"
                     /inference="ab initio prediction:Prodigal:002006"
                     /inference="similar to AA sequence:RefSeq:CKG86986.1"
                     /codon_start=1
                     /transl_table=11
                     /product="hypothetical protein"
                     /protein_id="Prokka:IKNDEOKD_00010"
                     /translation="MVIFKNILLFLFFSTYLLAQEDFTPLEQCTYENEKFWIKILNLC
                     PEGNITCDKIVYVGVNKNNGKYIVLNGKSISDVNMNFKGYVFKNDIYEYNIFNNFLYI
                     SKNKQIIQEYRLKLCEK"
     gene            5803..5904
                     /locus_tag="IKNDEOKD_00011"
     CDS             5803..5904
                     /locus_tag="IKNDEOKD_00011"
                     /inference="ab initio prediction:Prodigal:002006"
                     /inference="similar to AA sequence:RefSeq:AAW35464.1"
                     /note="conserved hypothetical protein"
                     /codon_start=1
                     /transl_table=11
                     /product="hypothetical protein"
                     /protein_id="Prokka:IKNDEOKD_00011"
                     /translation="MFISSKTELIQTIKNSTINNKEVKQIDFIYPNF"
     gene            7084..8214
                     /locus_tag="IKNDEOKD_00012"
     CDS             7084..8214
                     /locus_tag="IKNDEOKD_00012"
                     /inference="ab initio prediction:Prodigal:002006"
                     /inference="similar to AA sequence:RefSeq:ALW56279.1"
```

```
                     /codon_start=1
                     /transl_table=11
                     /product="integrase"
                     /protein_id="Prokka:IKNDEOKD_00012"
                     /translation="MKNLVKLKEYKGIYFIDKDKKLEDIKEISLVELQRRLTIPYELA
                     IRYYEGKKSSYEIFPILNNTQTLKKNCELIALQRINGKFAKKFKIKEPKLNEVFEEWI
                     SMRKNTISKKHYEVTIFSYNAHIKDVIGNIKIKQVKLKDMQNIVNKMIDQGKSPRTTK
                     TIKDILSPVFEFAIKNDYIEKNIAREIEIQKFDNKRYFTIDDEDRNALYKAIINYENL
                     MIRAMFIFLLHGRRKSEVLTLKWDNIDFANKIYFLPSNQNKSRKNLQFPLSSLQIEAL
                     KNIGVKNKGYIFIKEDGNPYKDIRWHWDKINQKLKTPIRLHDLRHLIGYIGINMGISL
                     EAIGETLGHSSIGVTKRYANIKNETINNTLNAIFSNFKTEKH"
     gene            complement(8260..8538)
                     /locus_tag="IKNDEOKD_00013"
     CDS             complement(8260..8538)
                     /locus_tag="IKNDEOKD_00013"
                     /inference="ab initio prediction:Prodigal:002006"
                     /codon_start=1
                     /transl_table=11
                     /product="hypothetical protein"
                     /protein_id="Prokka:IKNDEOKD_00013"
                     /translation="MNMDNIIICKASDLEDIFKKILKENELIKNKTDENLNVQQASNY
                     LKISISKLYKDCNLGKIPFYKIGKKLVFKKNDLDEYLKFNSSKNSLKF"
     gene            8908..9597
                     /locus_tag="IKNDEOKD_00014"
     CDS             8908..9597
                     /locus_tag="IKNDEOKD_00014"
                     /inference="ab initio prediction:Prodigal:002006"
                     /codon_start=1
                     /transl_table=11
                     /product="hypothetical protein"
                     /protein_id="Prokka:IKNDEOKD_00014"
                     /translation="MFGRNLKKIRKASGLTQLELAKILNTTQRTISLWETGSFEPPLN
                     TILLLCNIFKVTPNELLLEGCFDDDNEYLAHHIVRLTKELNCKTYTKRLFNHVLYRLE
                     LKKLTSHLQKFKGDSFAKKLAHSWTGKGERMLLVFYYFIEYLEKYNINENNIDKNSFL
                     KLLNDFNISKDNTNTIHCFILKEKDKRNLIEWVEKEMQEFEIKVLVAMLKDEIKTLVK
                     NEINMLNKLLV"
     gene            9879..9983
                     /locus_tag="IKNDEOKD_00015"
     CDS             9879..9983
                     /locus_tag="IKNDEOKD_00015"
                     /inference="ab initio prediction:Prodigal:002006"
                     /inference="similar to AA sequence:RefSeq:APA79438.1"
                     /codon_start=1
                     /transl_table=11
                     /product="TraG-like protein"
                     /protein_id="Prokka:IKNDEOKD_00015"
                     /translation="MGVNPSSYIMQDASKSGNELAKEAQGLFDAMRKK"
     gene            complement(10004..10324)
                     /locus_tag="IKNDEOKD_00016"
     CDS             complement(10004..10324)
                     /locus_tag="IKNDEOKD_00016"
                     /inference="ab initio prediction:Prodigal:002006"
                     /codon_start=1
                     /transl_table=11
```

```
                     /product="hypothetical protein"
                     /protein_id="Prokka:IKNDEOKD_00016"
                     /translation="MDKAEVKLKKSKMTTTEKLILALWLSCGVAVIVLLLVYLKIITS
                     LIGILGLLSVSSLVIAFVLFLAKNIKIVPYDEKKHRRKSKIAGYIESDSRKYDYIFPS
                     IRDN"
     gene            complement(10446..11117)
                     /locus_tag="IKNDEOKD_00017"
     CDS             complement(10446..11117)
                     /locus_tag="IKNDEOKD_00017"
                     /inference="ab initio prediction:Prodigal:002006"
                     /codon_start=1
                     /transl_table=11
                     /product="hypothetical protein"
                     /protein_id="Prokka:IKNDEOKD_00017"
                     /translation="MQEKEEKYIQLYKTQDKVLDLVAKEKLDFYLTGGTALQRFHYNQ
                     FRFSDDLDFFLINNGIKNHTPKEFNDFASMLQDNNIDFEIKVSNQYFTQLYIKENNLK
                     IDLVNDYVFHEKNDYVKLENGLLIDSIQNIFVNKLETSISRDEPRDLFDIYTILRNSK
                     IDIEKSFDILDKKTNIEPKDIINKLKNIKLEQIQYNKINTKSDFIIKDFLQNFHKSID
                     ENFNS"
     gene            complement(11080..11412)
                     /locus_tag="IKNDEOKD_00018"
     CDS             complement(11080..11412)
                     /locus_tag="IKNDEOKD_00018"
                     /inference="ab initio prediction:Prodigal:002006"
                     /codon_start=1
                     /transl_table=11
                     /product="hypothetical protein"
                     /protein_id="Prokka:IKNDEOKD_00018"
                     /translation="MKKDKMQIAEHILQNCYWGNTTMTPSFIVEHINDKDFARTIFSA
                     IFQNSLTMFEDLKIIDNEEWIKEFIISQNQRLGYHKRFYYEERLDELIAHYGIKDAGK
                     RREVYPIV"
     gene            11879..12379
                     /locus_tag="IKNDEOKD_00019"
     CDS             11879..12379
                     /locus_tag="IKNDEOKD_00019"
                     /inference="ab initio prediction:Prodigal:002006"
                     /codon_start=1
                     /transl_table=11
                     /product="hypothetical protein"
                     /protein_id="Prokka:IKNDEOKD_00019"
                     /translation="MQSYYSPSAASTIHKGNYGTVDSSAVGFGNNYEADKKTMGNGHL
                     DFAGQNTAWGQGNSQGTVQQEVQGQVANATNQFSKDAPKRTVDDGVNVAEMAVNGAKG
                     SFKNTVNAVGTITQTAINNPSHLLDSNFWIGARDANGNLIQPSNSGGNNSAPSSIPPA
                     PKIPSN"
     gene            complement(12538..12981)
                     /locus_tag="IKNDEOKD_00020"
     CDS             complement(12538..12981)
                     /locus_tag="IKNDEOKD_00020"
                     /inference="ab initio prediction:Prodigal:002006"
                     /codon_start=1
                     /transl_table=11
                     /product="hypothetical protein"
                     /protein_id="Prokka:IKNDEOKD_00020"
                     /translation="MDKAVNIKLKIIFAVLFVLILTNIGILFISKNEIFTKTLVGLIA
```

IAFILYYALAWIKTIKSIKKDSSKSGVKNLFIGILKLPYELVKVVLKIIKKTFSFFFG
LIDACVDDTVKSRNNKHMYDPSFINAEGIGELYTQNKKGEKVFFN"

```
gene            complement(13574..14446)
                /locus_tag="IKNDEOKD_00021"
CDS             complement(13574..14446)
                /locus_tag="IKNDEOKD_00021"
                /inference="ab initio prediction:Prodigal:002006"
                /inference="similar to AA sequence:RefSeq:CKG86076.1"
                /note="Domain of uncharacterised function (DUF3825)"
                /codon_start=1
                /transl_table=11
                /product="hypothetical protein"
                /protein_id="Prokka:IKNDEOKD_00021"
                /translation="MTESGFNLEKDENEPSLVYVKKATTRNKTLNREQIQNKVEEKPL
                LFQFAYFPNFLNTLQELSNIAQKEFWGSGNNILFSYLFKYFEFIHENKNYPDIITYNK
                DKTKACFNTGLYSTGVFPIFAYFEKQENGGYIFRKFCSNGDRVLDDLEIPKSLNDFDS
                FKNELIFDSKLDFRINHLHLFERKERLPEIIKKLNDRFIGHIINGELKIIKDNYNHQK
                MIIPAAYKQKVVLYIPLKLQEESVDTIVVVEKEEVKNEQYYAVRTILNPHDNIYKTAR
                VLSIVESEWVKNTI"
gene            complement(14582..15013)
                /locus_tag="IKNDEOKD_00022"
CDS             complement(14582..15013)
                /locus_tag="IKNDEOKD_00022"
                /inference="ab initio prediction:Prodigal:002006"
                /inference="similar to AA sequence:RefSeq:ABS43376.1"
                /codon_start=1
                /transl_table=11
                /product="putative thioredoxin"
                /protein_id="Prokka:IKNDEOKD_00022"
                /translation="MILKKIKILFLAIFLAFSPMTLEAKIYDNVYAAQKQALKEAKLM
                VFFVLSNTCNYCHKLMSDALNNRDLMEYLDENFIVAISDLNADGLIPRDLLFNGVTPT
                TYILTPTGKVIGTPIEGAVDSNMLFGLLKGLEDYKKGQLGF"
gene            complement(15026..15808)
                /locus_tag="IKNDEOKD_00023"
CDS             complement(15026..15808)
                /locus_tag="IKNDEOKD_00023"
                /inference="ab initio prediction:Prodigal:002006"
                /inference="similar to AA sequence:RefSeq:SQE23558.1"
                /codon_start=1
                /transl_table=11
                /product="TraT complement resistance protein"
                /protein_id="Prokka:IKNDEOKD_00023"
                /translation="MKINKSILKMGLSSILAASLLTGCLTTTLQTNATMSQSIFLDPV
                AKEKRVVFLNIKNTSGHEVNLEPKLRSALETKGYKIIDDPDKATYILSTNVLYCDKKQ
                ENNVAGGAVAAGATGAAISGYNGGGAGGMVAAGAAGALVGGLLGKLTEDTIWQMQVDI
                NIKQKADGKVLTSSGNVSGQASVRDQRASGFLNSFGGSVRSDKVGQLNSNQINSTQQT
                YEGKYIEKNSIIFAEAVKTGLKLPEATPILENKIATQIAGLF"
gene            complement(16202..16330)
                /locus_tag="IKNDEOKD_00024"
CDS             complement(16202..16330)
                /locus_tag="IKNDEOKD_00024"
                /inference="ab initio prediction:Prodigal:002006"
                /codon_start=1
                /transl_table=11
```

```
                     /product="hypothetical protein"
                     /protein_id="Prokka:IKNDEOKD_00024"
                     /translation="MKKQIIGSILLCSSLFAYKSTKDLDKNDVLEKLIQNINDSLY"
     gene            complement(16351..17010)
                     /locus_tag="IKNDEOKD_00025"
     CDS             complement(16351..17010)
                     /locus_tag="IKNDEOKD_00025"
                     /inference="ab initio prediction:Prodigal:002006"
                     /inference="similar to AA sequence:RefSeq:ARJ53355.1"
                     /codon_start=1
                     /transl_table=11
                     /product="transcriptional regulator"
                     /protein_id="Prokka:IKNDEOKD_00025"
                     /translation="MYIRDFRKRLHMTQSEFAQALGTKQTNISMYEKHRVKPTCELIV
                     KLIIRFNANPNFLFFGKEPHLNTDTFKNEISQELSQLISELSLYENEENIIVELENKA
                     LGKILSLVPNKEIWERLFFLFRINRKLHFVTSFICRVAKRLKQRDDEAKAYLISIINS
                     FNEGDFTELNEYIKLDLIALFDEKITEEEAKTIIEDCLVVFKHIENTAPIHKMVELGK
                     D"
     gene            complement(17024..17200)
                     /locus_tag="IKNDEOKD_00026"
     CDS             complement(17024..17200)
                     /locus_tag="IKNDEOKD_00026"
                     /inference="ab initio prediction:Prodigal:002006"
                     /inference="similar to AA sequence:RefSeq:ARJ53354.1"
                     /codon_start=1
                     /transl_table=11
                     /product="conjugal transfer protein TraU"
                     /protein_id="Prokka:IKNDEOKD_00026"
                     /translation="MPIWRKSSYRLQILAPVPHPMGMGIGQSGILWSYAKNYPVAGDN
                     FTFMLFKKKECCAF"
     gene            complement(17221..17415)
                     /locus_tag="IKNDEOKD_00027"
     CDS             complement(17221..17415)
                     /locus_tag="IKNDEOKD_00027"
                     /inference="ab initio prediction:Prodigal:002006"
                     /inference="similar to AA sequence:RefSeq:ARJ53354.1"
                     /codon_start=1
                     /transl_table=11
                     /product="conjugal transfer protein TraU"
                     /protein_id="Prokka:IKNDEOKD_00027"
                     /translation="MIATLIDAACMNITGGVDIAYMTEVDPLWQDDELTALINPEALL
                     FGNPILNLACMADSASAQGN"
     gene            17808..18128
                     /locus_tag="IKNDEOKD_00028"
     CDS             17808..18128
                     /locus_tag="IKNDEOKD_00028"
                     /inference="ab initio prediction:Prodigal:002006"
                     /codon_start=1
                     /transl_table=11
                     /product="hypothetical protein"
                     /protein_id="Prokka:IKNDEOKD_00028"
                     /translation="MDKAEVKLKKSKMTITEKLILTLWLSCGVAAIVLLLVYLKIITS
                     FIGILGLLSVSSLVIAFVLFLAKNIKIVPYDEKKHRRKSKFAEYMSSGRCKYYHIFGA
                     IRDN"
```

```
     gene            18363..18923
                     /locus_tag="IKNDEOKD_00029"
     CDS             18363..18923
                     /locus_tag="IKNDEOKD_00029"
                     /inference="ab initio prediction:Prodigal:002006"
                     /inference="similar to AA sequence:RefSeq:ABS43512.1"
                     /codon_start=1
                     /transl_table=11
                     /product="helix-turn-helix domain protein"
                     /protein_id="Prokka:IKNDEOKD_00029"
                     /translation="MKRLSVKGNKGKNKKTYDQIMEILSIQVKENLPSKVVASRYDIT
                     LQTVYKWRKKYTKYLENYEKVKQEAEEKTLKESDLENEHIHISDCAKRLRTLRIALNL
                     SQQRIANILGISQALYVRFEKGYGQLNSFMIKKLYKTLGINPIYLICGEGDCFLIKDI
                     DLFERINNEQKKYNKNQLEQENILNY"
     gene            19205..19360
                     /locus_tag="IKNDEOKD_00030"
     CDS             19205..19360
                     /locus_tag="IKNDEOKD_00030"
                     /inference="ab initio prediction:Prodigal:002006"
                     /inference="similar to AA sequence:RefSeq:ABS43172.1"
                     /codon_start=1
                     /transl_table=11
                     /product="single-strand binding protein family protein"
                     /protein_id="Prokka:IKNDEOKD_00030"
                     /translation="MKFLDSKPADNNQSGNPYGNVQATPEPVNQSVQKQVPIHQFEEQ
                     QGEDLPF"
ORIGIN
        1 ttctaaaaat atcaagtttt attctacttc ctttgataat tatatggaat atgacatttg
       61 gttgggggtg gtttgtgcct tatagcctac aaccgagtta tcacgaattt aaagagatgt
      121 gtaagcttaa tgaacttcca aatgatgaac acaaatataa taagattcta agttattttg
      181 gattaagttt ggatacattg gattgggagg agttgaatga gagatcgtta aagttgatta
      241 aaggttttta atgcagatta tgttaaaagc aagttagaat acagagcaaa ggtcacagca
      301 attcgaaaaa aacgctataa tatttcagtt gatttgtttg ccaataacaa aggattatca
      361 aaggagaata taacttacat tcaaatatac ggtacttgga acacaaatcg ctattttta
      421 gagcaaaaaa gtatggcaag ttataatctt gtatggcgtg aaaaagaatt aagttgtact
      481 gatattgcac ccttgaaata tgactatgca gaaaggataa aatgaacaat aaagaacaga
      541 ttaaaaagct taaagacaat tcagaacttg cttgggctag ttatggttat tttgacttta
      601 tagacaagca atatagcttt gatgaaaagg ataaagataa gtttaaaact ttatatgcaa
      661 aaataaatga tttaaagaaa agcgatgaaa aagtacaaaa tgccaaagct acctacaccg
      721 acattctcaa cacagaatgc aactctttat ttagtggcga cttctcccca ctccaagcta
      781 aacaattctt ttctcgctat gatttattaa aacattgtcc taatacaaat tcaggttttt
      841 ctgctacttt gtttggagaa aaaagaaaat aaatttatag taaaataaaa gaaaaagtta
      901 tactgctgaa tataacttat aaactatatt ttaattttaa taaaatagag agtctaagga
      961 gataacccat gacatacata ttacttctta ttataatttc tactattctt tcttatctta
     1021 tattaaaatt tatttataaa atcatcttta aatctacaaa aagcgtttca aagtttttag
     1081 tctttcttgg aagcataggt ttaattatct tttattatac tccttattct tattatttag
     1141 agcctagtta ttggcaattt aggaatatgt gtaagttaaa tgaacttcct aataacgaag
     1201 agaaatataa taagattcta gggtattttg ataaaaagtt aggtgacata gatgattttc
     1261 gacatgtaaa aaaatatggt agtatagaaa tgtggcttta tatatattac gatgatactt
     1321 acaaaactcc ttcaaatttt caagaaaaga tagagatgga taatattgca ctaaaaacaa
     1381 aaaatatgca aatgtcttta cacaagattg ttgatatgca tataagtcca tattggaata
     1441 ctagaaggta tgtgttagaa ggcaatgagg gtaatatgaa ttttgagttt atagaggatg
     1501 agctcgcttg tggcaatctt tatcaataat aatataagga ataaataatg accaataaac
     1561 aacttattaa taaactaagg gatatggcgg aacttgcaca ggcaagttat ggttattttg
```

1621 atttggtaac acatgacaag aatgattttt tcattaaaat taaagatgaa gctacagaat
1681 ctgctttctt taataagcat attactcaag ctaatgtctt agatattagt tataaaaatt
1741 ataaggcata tcaatatgac tcatctttaa agaaagatat taaaattggc actctcaatg
1801 gcgacttctc cccactcaag ccaaacaatt cttttctcgc tatgatttat taaaacattg
1861 tcctaacaca aattcaggtt tttctgctac actttttat gacattcaaa aagatgagta
1921 tattgctagt tttcgaggaa cagaaggaat tgtaagtatg gatgcatttg tgccgatata
1981 ggacttactt ttggtaaagg agattttcaa gctaatgcct taaaacaatt tttattagat
2041 atagcaccta ttttaaataa tatagattct agtaaaatta cctttgtagg acattctctg
2101 gtggatattt agctcaaatg gctttgatat atattgtgat taaatataaa gatttatact
2161 tttaatactc ttagtattta cgtttgaaat gttcctagtg tggttatatt ttgattttta
2221 aaaaagtagg ataattttat taaaaatatt aaaaaggaaa ataaatgggt atattaaaaa
2281 aagtatacat tgcttggggg gtcttttggg gaattatttt tttgtttagt tctttatatg
2341 cttattttag tgatatagaa tttttggtg atatgttaaa gattgcattt tttatatatt
2401 cttatccatt gttgccttac atatcatatt taacactaca agaaactatt aaatccaaaa
2461 atatttttct ttatttttct ttattttgct ttatttttct ttattttgct ttatttttct
2521 ttatctcggg aggatttttt atttatgtaa taatattagg tatttataca atattatttt
2581 taataatatc tattaaattt agaaaagaac tttcttatta tttttcttta ttattttgta
2641 ttttttata catttttttg tatcttttta tttttatat agaattaaaa aatgagtaat
2701 aaagctcaaa ttaacaatct caaaaattac gctaaacttg tatagattgg tttttttaat
2761 tttttaataa taatttaaat ttcaacaaaa aaggaagtag tgtgaaaata ttaaaaagtt
2821 tatatattat ttggatgata ttttggggat ttggggctat tttaggttat tatatgggat
2881 atgatgcaac ttttactaaa atgttctttt ttcttgttat attatatctt tttccattgt
2941 tgccttattt gcattatttg atttataaaa aagcaagaaa tacaaaaaat atatttgttg
3001 ctatttgttg ctactattga ttaatagaaa taatagtaat tttgagctgt acttaattat
3061 tattgtgata catattataa tatttgcaat aatttattta attaaaagaa ataaaaatga
3121 aaactcaaat caataattta agagattatg tagaactagc ttgggctagt tatttttatt
3181 ttaattttta aaaaagtaga agaattttac taaaaatatt aaaaaggaaa ataaatgaat
3241 acattaaaga aaatttatat catttggggg atgttttgga ctattggact tggaattttt
3301 gtgcctacag cacatggatt tgatagtatg gattttgtgg attttttcat atttgcttta
3361 caatttatag ctataatttg gttgcctcca ttactcattt ttaatattgt atattttgtg
3421 ataaaaaaag aaagaaaata tattataaat acattatgga tattattgta ttatcctata
3481 gctttgatat tgtattacat gttttattat cataatagtt tttatgttac attatatatt
3541 tttttatcat tatttaaata aaggataatt ttgaaagaaa aaatcgataa gctaagagat
3601 tatgccgagc ttgcacaagc tagttatttt tattttgatt taaagggtta tatattacaa
3661 gaaaatgaaa ctatcgttat tttaaatgag cttgtaagtt taagttataa cggaaaaata
3721 gctggaaaaa aagaaaaggt cggacaagaa tatagtttta ttggcaaagg tgaattaaat
3781 ggagaatttg gagaattaca agctagaaat ttctctcaaa gatatgagat taaatttcat
3841 caatccaaca ctttaagtgg tttttctgct actttatttt atgataaaca aaaagatgaa
3901 ttcgtggtgg gatttagagg aactgagggt ttttggaata tggatacgat gcaagacata
3961 actctatctc ttaatggcaa tctccaatct tctcctcttt tagaattttt agaataagta
4021 gataaaatca tcaaaaatga aaataaaagt atcatatttg tagggcattc attgggtgag
4081 atttgggaga tgcaatgaaa atattaaaaa tactttatat ttgctggatt atattttta
4141 tctttgcttg gataatatca cctttaattg ggcataatcc agatagattt aaggaatttc
4201 tcatagtgat tggatggata attttgcctc caatagtgaa caatctatgg ttatttttaa
4261 taacaagaat taaaaaatat ttgataaggt tttttctatt attgttatat tgtccattgg
4321 caattttatt atatcttata tttgattaaa aggaaaaaat atgaagctat tttacacagc
4381 tacatgaata tgataaaatc attaaaaatt ctttagaaga taagaaagaa ttaaacgaat
4441 atcatttaac ttttttaact cctgcaaaaa tgcaaaattt aaaacttcaa atcgcacaag
4501 gattagataa aatcttagaa gatgaagata gaaaacaaga gctttatgtt tgtaaatttg
4561 tggtggtaaa tggggttagc gaaaacaaaa taaaagcaaa aaataaagat gtaaatatag
4621 ataatgacac aaagaatata aacactacaa aaagcaataa tataagttct agtgaaaaca
4681 atgctcaaga aaaaatcact catctaagca atgatggtca aaatctttta aaaaatatag
4741 agaagctaag actcaaacct tataatgatc aaaatggaaa agaaattact tcatatgtta
4801 aaggtgcaac tatcggatat ggacatctta tagggcaaaa tgaatgggat ttgtataaaa

4861 atggtattac tttacaagaa gcggataaat tgtttaaaag cgatttgtta ccttttgaaa
4921 atgctgtaaa aaattcaata aactcttcct tagcacagaa cgaatttgat gccttggtta
4981 tattatgttt taatataggg attgataatt ttaaaaattc ttctgttgct aaaatcatca
5041 atggagagaa aacgggttac aaaacattaa aggaagcttg gatggcgtgg aacaaaagtc
5101 aaaataaagt aatgcagggg ctaattaata ggagaaatgc tgaatataag ctttacactc
5161 aaggaggtta tgaaaaatgg taatttttaa aaatatatta ttgtttttat tttttagtac
5221 ttatttgtta gcgcaagagg attttactcc actagagcaa tgtacctatg aaaatgaaaa
5281 gttttggata aaaattttaa atctatgtcc agagggtaat ataacttgtg ataaaattgt
5341 atatgtgggt gtcaataaaa ataatggaaa atatattgtc ctaaatggta agtctattag
5401 tgatgtaaat atgaatttta aaggatatgt ttttaaaaat gatatttatg aatacaatat
5461 ttttaataat tttttatata tttctaaaaa caagcagatt atacaagaat atcgtctaaa
5521 attatgtgaa aaatagacac aacaaaggca acaagcaaca acaaatacct agaatatgta
5581 aaaagttaaa aagatgcaat aagaattaca agaagttttt aaagttataa aaatcctaat
5641 taatgaaatc ttagctccta aattttataa caaatacaaa gatttagaag tagagattga
5701 tgataagggt aatattataa gatggatagg agagattaat aaagacaaaa aatattagca
5761 aataaaaaat gatataataa tgctttatat aaggaatctt ttatgtttat aagttctaaa
5821 acagagttaa ttcaaactat taaaaattca acaatcaaca ataaagaagt taaacagatt
5881 gattttattt atcctaattt ttaaagaatg atgaaaatct taatacttct tttactaatc
5941 ttaatgcttt aaaattagct ttagaagaaa agaattttta tcttaatttt atagacttaa
6001 acagcaaaga aagtctttta agtttttata atcaaataat aagtaataat catcttagaa
6061 attattttta ttttaaggat aatcaatttt ttattaaaaa agatataaaa aataaaattt
6121 tatttcatag tattcactct tcttctattg taagtaatta tcttagcttg ttaaatgatt
6181 ttaatacttt aaacaagata gaactcataa aaatattaga aagtttaagt ttgtataacc
6241 tttcaacttt aatgcaaact atgcaatcaa aaaatgaata tgactcaaaa ggctttgata
6301 aagatggtat ctttttatct aatatgatta tgaatgtgag attgaaataa gtaaatgata
6361 gatttagcag gtgtgatttg ttaatgaaaa tatacatctc caattgattt gaaaaaatag
6421 caaggtttta aaaaagcaat acaagagaga aaatttaaag aatttgggta atgcaattga
6481 atgcaactag attttagtag atataaattt aaataaaatt tatcaagaag tttttttctat
6541 aaagactatg catttttttaa aaaaaagtat ttttaaacca aagtttttac tatgataata
6601 aaacctatta tttaagaaat taaacttaaa aatgaaaatt tgattatcaa agttcctagt
6661 gatttttcaa agctttagaa gatattataa aactcacata gtataatcag gaagctattt
6721 ttgaaaatat aaaaactctt ttttaagatt ttaatataaa ttgccaaaaa ataacacttg
6781 agagtaaaaa aaacaaatgg tgcctgaggt cggactcgaa ccgacacaag gttgccctta
6841 ccagattttg agtctaatgc gttttgttat catttttttat caaagtattg aaaaataggt
6901 attttattgt ttaagtttat cttaaaaata gcattatttt ataccatttt gagaaaaaaa
6961 ccgctaccaa aaccgctacc actaaaatcc atttgagata ctatttttag tatattttta
7021 attttatatt atgtaaaaaa taataaaatt ttctgcgtaa attaaaagca aaaaaggaat
7081 atcttgaaaa atctagtaaa actaaaagaa tataagggta tatattttat agacaaagat
7141 aagaaattgg aggatataaa agaaattagc ttagtagaat tacaaagaag attaactata
7201 ccatacgaat tagctataag atattatgaa ggaaaaaaat caagttatga gatatttcct
7261 attttaaata acactcagac ccttaaaaag aattgtgagt taattgcctt gcaaagaatt
7321 aatggaaaat ttgcaaagaa atttaaaatc aaagaaccta aactaaatga agtgtttgaa
7381 gaatggatta gtatgcgaaa aaatactatt tctaaaaaac attatgaggt aaccatattt
7441 tcatataatg cacatataaa agatgttata ggaaacatca aaataaaaca agtcaaatta
7501 aaagatatgc aaaatattgt caataaaatg attgatcaag gcaaatctcc aagaacaaca
7561 aaaacaataa aagatatact ttctccagta tttgaatttg ctattaaaaa tgattatata
7621 gaaaaaaata tagcaagaga aatagagata caaaaatttg ataacaaaag atattttact
7681 attgatgatg aggatagaaa tgctctttat aaagctatta taaattatga aaatttaatg
7741 ataagagcta tgtttatatt tcttttacat ggaagaagaa aaagtgaagt tcttacatta
7801 aaatgggaca atattgactt tgctaataaa atttattttt taccttcaaa ccaaaacaaa
7861 tcaaggaaaa acttacaatt cccgttaagt tctttacaaa tagaagcttt aaaaaacatc
7921 ggagtaaaaa ataaaggata tatttttatc aaagaagatg gaaaccctta taaagatata
7981 agatggcatt gggataaaat aaatcaaaaa ttaaaaactc ctattagatt acatgactta
8041 agacatttaa taggatatat aggtattaat atgggtatat ctttagaagc tattggagaa

8101 acattaggac attcaagcat aggagttaca aaaagatatg caaatattaa aaatgaaact
8161 attaataata ctctaaatgc tatttttagt aattttaaaa ctgaaaaaca ctaatttaca
8221 atggtttttt aggtttttcg gttttttagt taagtgtgat taaaatttta aactattttt
8281 agaactgtta aattttaaat attcatcaag atcatttttt ttaaaaacaa gtttttttgcc
8341 aattttataa aaaggaattt ttcctaaatt acaatccttg taaagttttg aaatgcttat
8401 ttttaaataa tttgaagcct gttgtacatt taaattttca tcagttttat ttttgattaa
8461 ttcattttct tttaaaatct ttttaaagat atcttcaaga tcactagctt tgcaaattat
8521 tatattatcc atattcatta tttttcctca ttttttaagt aataataaat cgaattgtta
8581 tcaaaacgat ctttgagcat gtaccgtttt ccttattaat atagaatact ttggactgat
8641 tgttttttta aaatcatttt gcaataaaca taaagaaata attgtttttt tcttgctata
8701 gtagaattta acaaccgccc atcctcattt gagctaaaat cacttttctg tataaactat
8761 tgtatttatt agcatcgcgc tctttcataa tttttatgga tatattataa cataaaaaat
8821 cattaaattt caattaaata aaaaaaagat aatttatgct atattaagca atattatttt
8881 ataattacaa aaaaatggag ttgtaaaatg tttggaagaa atctaaaaaa aataagaaaa
8941 gcaagtggtt taactcagtt agagctagca aaaatactaa atacaacaca aagaacaatt
9001 agtctttggg aaacaggaag ctttgagcca ccgcttaata caattttgct tttatgcaat
9061 atttttaaag ttactccaaa tgaactattg ttagagggat gttttgatga tgataatgaa
9121 tatcttgcgc atcatattgt aagattaaca aaagaactga attgtaaaac ttatacaaaa
9181 agattattca atcatgtttt atatagacta gagttaaaaa aacttacatc gcatctgcag
9241 aaatttaaag gtgattcctt tgctaaaaaa ttagctcatt cttggactgg aaaaggtgaa
9301 agaatgcttt tagtttttta ttattttatt gaatatttag aaaaatacaa tattaatgaa
9361 aataatattg ataaaaattc ttttttgaag ctcctaaatg attttaatat atctaaagat
9421 aatacaaata ctatacattg ttttatatta aaagaaaaag ataagagaaa tcttatagaa
9481 tgggtggaaa aagaaatgca agaatttgaa ataaaagttt tggtagctat gttaaaagat
9541 gaaattaaaa cactggttaa aaatgaaata aatatgctga ataaattact ggtgtagtat
9601 atattatagc tttgattaca agttgatatg taattttatc tctattatag tccttcaagc
9661 gtaggggctc acaatgctca taataatgat gcggtagcta gaactaatca aaataattta
9721 gacaaattag gaacagaagg ggtgacaata taagaaaaga atttaataat gataataaag
9781 atgctatggc taattttaga aatcaagagg ttaaaactca taatgtagtc cacaacagtg
9841 ctaaagatgc catagcagaa caataagcta acaggagaat gggagtaaat cctagctctt
9901 atataatgca agatgcaagc aaatcaggca atgagcttgc aaaagaagct caaggcttgt
9961 ttgatgctat gagaaaaaaa taagtctagc tttttggcta ggcttagtta tctcgtatac
10021 tagggaatat ataatcatat ttacgagaat cgctttctat atatcctgcg attttacttt
10081 ttcttctatg ctttttctca tcataaggaa ctatttttat atttttagca aggaataata
10141 caaaagcaat tacaaggcta gatacagata aaagccctaa aatacctata aggctagtga
10201 taatttttaa atataccaaa agcaaaacaa taactgcaac tccgcaagaa agccaaagag
10261 caagtatgag tttttcggtt gtagtcattt ttgatttttt aagttttact tctgctttat
10321 ccattgttat tcctttcata ttaacttatt tatataacat aattatatta tattttaata
10381 aatttttgta agtatttttg ctattaaata cctattttat aggattttaa gcgaatttct
10441 tttatttaag aattaaaatt ttcatcaatg gacttgtgaa aattttgtaa aaaatctttt
10501 ataataaaat cactctttgt attaatttta ttatattgaa tttgttctaa tttaatattt
10561 tttaatttat taataatatc ttttggttcg atattggttt ttttatctaa aatatcaaaa
10621 ctttttttcaa tatctatttt actgtttctt aaaatcgtgt aaatatcaaa caaatctctt
10681 ggttcatctc tagaaattga agtttctaac ttattaacaa agatattttg tatgctgtct
10741 ataagcaaac cattttcaag ttttacataa tcgttttttt cgtgaaatac ataatcattg
10801 actaaatcta ttttgagatt attttctttg atataaagtt gagtaaaata ttgattggaa
10861 acttttattt caaaatctat attattatct tgtaacatac ttgcaaaatc attaaattct
10921 tttggagtat ggtttttaat tccattgtta atcaagaaaa aatccaaatc atcagaaaat
10981 ctaaattgat tatagtgaaa tctttgcaaa gctgttccac ctgtaagata aaaatctaat
11041 ttttcttttg caactagatc aagaactttg tcttgtgttt tatacaattg gatatacttc
11101 tcttcttttt cctgcatctt ttatcccata atgtgcgata agctcgtcaa gcctttcttc
11161 ataataaaat cttttgtgat agcctaatct ttggttttga gaaatgatga attcttttat
11221 ccattcttca ttatcgataa tctttaaatc ttcaaacatt gtaagcgaat tttgaaaaat
11281 agcactaaaa attgttcttg caaagtcttt atcgttaata tgttctacta taaaactagg

11341 tgtcatagta gtatttcccc aatagcaatt ttgtaaaatg tgttctgcta tttgcatttt
11401 atcttttttc attagaattt tccttttttt gcaattttac taaaattatt ttttgaaaaa
11461 taagtggata atttattttt gcaatgcttt cttaaaatat ttgcagttat caaaaaccct
11521 acgatctaca ggattagtta aagccactac ggtttcttct gaagaaacaa aaccttcttg
11581 atgaattcaa gtattgcttt ttggagaaag acttactacg ctttgtacag ctttatctat
11641 attatttaca gttaacatca taagtagctc ctttctcaat taatataaca taattatatc
11701 atattttaat aaatttcacg agtaatatta gtattaaata ctattttatg gggctttaaa
11761 tgaaattctt tttttatctg actacactta actttaaatt gattacaagt taatatgcaa
11821 taaatacttt aatgcaggag attttaatag tccgcaaaat actataggtt ccgtaggtat
11881 gcaaagctat tatagtcctt ctgcagcttc tacaatccat aaaggtaatt atggcacagt
11941 agattcatcg gctgtaggat ttggaaataa ttatgaagct gataagaaaa ctatgggaaa
12001 tggacactta gatttcgcag gacaaaatac agcttgggga caaggcaatt cacaaggaac
12061 agtgcaacaa gaagttcaag gacaggttgc taatgcgaca aatcaattta gcaaagatgc
12121 ccctaaaaga acagttgatg atggtgttaa tgttgcagaa atggctgtta atggagcaaa
12181 aggttcattt aagaataccg taaatgctgt aggaactata actcaaacag ccataaacaa
12241 tccttcgcac cttcttgatt ctaatttttg gataggtgcg agagatgcaa atggaaattt
12301 aatacaacct tcaaatagtg gaggcaataa ctctgcacca agttctattc ctcctgctcc
12361 taaaatacct tcaaattaat aatataagca agctaaattt ggcttgctta taaatattgt
12421 taaaatatat ttttgctttt tatttgtgga aatgttaagc atgtaataaa aatttgaaaa
12481 aatgtttgca agaatgataa ttagaataag ctagaaattt ttctagcttt gtaaaagtta
12541 attaaaaaaa accttttcac cttttttatt ttgtgtatat aattctccta taccttcagc
12601 attaataaaa gaaggatcgt acatatgttt attatttctc gacttaactg tatcatcaac
12661 acaagcatct attaaaccaa agaaaaaaga aaatgttttt ttgatgatct ttaaaaccac
12721 tttcactaat tcataaggca attttaaaat acctatgaaa agatttttaa ctccactttt
12781 agaactatct tttttaatac tttttatagt ttttatccaa gctaaagcat aatataaaat
12841 gaaagcaatc gctattaagc ctacaagtgt ttttgtgaat atttcattct tgcttataaa
12901 taaaatgcct atatttgtta aaatcaatac aaataatacg gcaaaaatga tttttaattt
12961 tatatttact gctttatcca ttgttattcc tttcatatta acttatttat ataacataat
13021 tatattatat tttaataaat tttttgtaag tatttttgct attaaacgac tatttttatg
13081 gggtttaaaa tggatttctt tttttatctg actacaactt aacttcaagt tgattacaag
13141 ttaatatgta attttaatct ctattatagt ccttcaagcg tagggattaa caatgcccat
13201 aattcgatta agtgtttttt tggtaaattg tcttaagctt aggccaatcg tggtttattt
13261 ataattattt cggttttttt aagtgagata atcaacgagc ttgattatct cgttaattga
13321 ttctaagaca agaactaaaa actcaatgat cttactcatt attcctcctt cttataatcg
13381 aaagagtagt catacctaat acactttaat tatagtattt ttgaatgaat gttttagcct
13441 cttgttaaat gtttgtatga taaaattcca aaaacaaaag ttccaaaatg cgtaagagtt
13501 ttagcctctt gttaaatgtt tgtatgataa aatagactta ttttaaaagc ctattttatc
13561 aatattttta ttttcaaatg gtgtttttaa cccattcaga ttcaactatc gaaagaactc
13621 ttgcagtttt gtagatatta tcatgcggat ttagtatagt tcttacagcg taatactgct
13681 catttttttac ttcctctttt tcaactacaa ctattgtgtc aactgattct tcttgaagtt
13741 tcaaaggaat atataaaact accttttgct tataagctgc aggaataatc atcttttgat
13801 gattataatt atccttgatg attttcaact caccattgat aatgtgtcct atgaacctat
13861 catttagttt ttttattatt tcgggcaatc gttcttttct ctcgaagaga tgtaaatggt
13921 taattctaaa atctaacttg ctatcaaaga tgagttcatt tttaaacgaa tcaaaatcgt
13981 tcaaagattt gggtatttcc aaatcatcta aaactctatc cccattagag caaaattttc
14041 taaaaatata tcctccattt tcctgctttt caaagtatgc aaatattgga aaaacacctg
14101 tgctatacaa acctgtattg aaacacgcct ttgttttgtc tttgttataa gtgataatat
14161 caggatagtt tttattttca tgaataaatt caaaatattt aaatagatag gagaataaaa
14221 tattatttcc gctcccccaa aattcttttt gagctatatt agaaagctct tgaagtgtat
14281 ttagaaaatt tggaaaatag gcaaattgga ataataaagg cttctcttca actttgtttt
14341 gaatttgctc acgatttaaa gttttgtttc ttgtggtagc tttttttaca taaactaaag
14401 aaggttcgtt ctcatctttt tcaagattaa atcctgattc tgtcaacgcc ttactacaat
14461 taattttcaa atcatatttt ttttcaaata ttgactgata tgtcctgtag taacatgttc
14521 aaatttagtt ttgctatctt cataggcttc taaaattaat ttttctatgt ttttttccac

14581 gctaaaaacc taattgtcct ttcttataat cttctaaacc ttttaataat ccaaaaagca
14641 tattagagtc aaccgctcct tcaatagggg tgcctattac ttttcctgtt ggagttaaaa
14701 tataagttgt tggagtcact ccattaaata ataagtctct aggaattagt ccatctgcat
14761 tcaaatcaga gatagcaacg ataaaatttt catcaagata ttccatcaaa tctctattgt
14821 ttaacgcatc actcatgagt ttgtggcaat aattgcaagt gtttgaaaga acaaagaaaa
14881 ccattaactt tgcttctttt aatgcttgtt tttgagcagc atatacatta tcatagattt
14941 tagcctctag cgtcatgggg ctaaaggcta aaaagatagc taaaaataaa atttttattt
15001 tttttagtat cataagatat cctttttaga acaatcctgc aatttgagtt gcgattttgt
15061 tttccaaaat aggggttgct tcaggaagtt ttagtcctgt ttttacagct tctgcaaaaa
15121 taattgaatt tttttctata tatttaccct cataggtttg ttgcgtacta ttaatttggt
15181 ttgaattaag ctgtcctact ttatcgctac gaacacttcc gccaaagcta tttaaaaatc
15241 ctgatgctct ttgatctcta acgcttgctt gtccacttac atttccgcta gaagttaaaa
15301 ctttaccatc ggctttttgc ttgatattaa tgtcaacttg catttgccaa atagtgtctt
15361 cggtaagttt tcctaaaagt cctcctacta aagcacctgc tgcacctgct gcaaccatac
15421 cgcctgcacc gcctccatta tatccgctaa ttgctgcacc tgtagcacct gctgctacag
15481 caccgcctgc aacattattt tcttgttttt tatcgcaata taaaacatta gtacttaaaa
15541 tataagttgc tttatcagga tcatctataa ttttatatcc cttggtttct aatgcgcttc
15601 ttagtttagg ttctaagtta acttcgtgtc cgcttgtatt tttaatattt aagaatacaa
15661 ctcttttttc ttttgccaca ggatctaaaa agatgctttg actcatagtt gcattagttt
15721 gtaaagttgt tgttagacaa cccgtaagca agcttgctgc caaaatactg cttaatccca
15781 tttttaaaat tgatttattt attttcatcg ttttcctttt tagtgaatat tttttcaaaa
15841 atttctttaa cctgcttttt aaattttagc acgactttct tccgtttcgc ccgttagctc
15901 atccaagctt ttttctatag cattgaaaat tttttaaaat aatttttttc ttgctctttg
15961 gcatgatgtt ttgaaaattc cttagaaatt ttagattcta aataagttct agagaaatct
16021 gttttattta aaatcacttc taagtataca gtagctttcg cttctatgtt atatttatca
16081 ttgacaaagc tatcaaccaa aattcaacta cattcctgtc cttatttgtt tttattctcc
16141 tttataaaaa tatatgttat agtgtttaat tttctatatt taagtcctgt tcctccttta
16201 tttagtataa actatcattt atattttgaa ttaatttttc caatacatcg tttttatcta
16261 aatctttggt agatttatat gcaaataacg aagagcataa taaaatagaa cctataattt
16321 gtttttttcat tttttaacc tttcttatta ttaatctttt ccaagttcta ccattttgtg
16381 gataggagct gtattttcaa tatgcttaaa tacaaccaag cagtcttcga taatagtttt
16441 tgcttcttct tcggtgattt tttcatcaaa tagtgcaatt aaatctaatt tgatatattc
16501 attaagttcg gtgaaatcac cttcattaaa actattaata atagaaatta gatatgcttt
16561 ggcttcatca tctctttgtt tgagtctttt agctactcta caaataaatg aagtaacaaa
16621 atgtaatttt ctgtttattc taaataagaa aaacaatctt tcccaaatct ctttgtttgg
16681 cacaagcgaa agaatcttcc ctaaggcttt attttctagt tccactatga tattttcttc
16741 attttcatat agagataatt cactgattaa ttgactcaat tcttgtgaaa tttcattttt
16801 gaatgtatct gtattcaaat gaggttcttt gccaaagaaa agaaagtttg gattagcatt
16861 gaatcttata attaatttta caataagctc gcaggttggc tttactctgt gtttttcata
16921 cattgagata tttgtctgtt tagtgcctaa tgcttgtgca aattctgatt gtgtcatgtg
16981 caatcttttt ctaaaatctc ttatatacat taaacctcac ttattaaaat gcacagcatt
17041 ctttttttctt aaaaagcata aaggtgaaat tatctcctgc tacaggataa ttcttagcat
17101 aactccacaa tatgccactt tgtcctattc ccattcccat aggatgagga acaggtgcta
17161 aaatttgcaa tctataggat gattttctcc aaataggcat aggatattct ccacaaagtc
17221 ctaattgccc tgtgctgatg cactatctgc catacaagca agatttaaaa tgggattacc
17281 aaatagcaaa gcctcaggat tgattaaagc tgttaattca tcatcttgcc acaaaggatc
17341 tacttctgtc atataggcta tatccacacc gcctgtaata ttcatacatg ctgcatcaat
17401 taaagttgct atcatgccta gaactaggat gatattttta atataaaaaa ctttaattag
17461 aaatttcttg ataattatat cattttttag aaaaaaatat atgtttatta tatatattta
17521 taaatttttt ttataaatat atataataaa catataataa tttttatatt gaagatattt
17581 tagatataaa aattcaattg aaaaaagtca aaaacaaatg ctttttaagt aatgaattct
17641 ttagcatgaa ttgaaagttt acccaattgc agatagctat catcaatttg taaaaattca
17701 cttttaatcc tataaaataa gcatttaata gcaaaaatac ttacaaaaat ttattaaaat
17761 ataatataat tatgttatat aaataagtta atatgaaagg aataacaatg gataaagcag

```
     17821 aagtaaaact taaaaaatca aaaatgacta taaccgaaaa actcatactt actctttggc
     17881 tttcttgcgg agttgcagct attgttttgc ttttggtata tttaaaaatt atcactagct
     17941 ttataggtat tttagggctt ttatctgtat ctagccttgt aattgctttt gtattattcc
     18001 ttgctaaaaa tataaaaata gttccctatg atgagaaaaa gcatagaaga aaaagtaaat
     18061 ttgcagaata tatgagtagc ggacgttgta aatattatca tatattcggt gctatacgag
     18121 ataactaagt ctagccaaaa agctaggatt tactcccatt ttcctattag catcttgttc
     18181 tattttacat ataattttac atataatttt acatatataa tttaaaaata tataaattaa
     18241 atataaaaat tactaaatat atttaataat tatgtaaaac attattctat tttttcaaat
     18301 atatttttag atacaatcag ggctaatatt aacacacaaa acaaggcaaa aaagaatatt
     18361 caatgaaaag attatctgta aaaggcaata agggaaaaaa caaaaaaact tatgatcaaa
     18421 taatggaaat tttatcaata caagtaaaag aaaatctacc atcaaaggta gtagcttcta
     18481 ggtatgatat aaccttgcaa acagtatata agtggagaaa gaaatataca aaatatttag
     18541 aaaattacga aaaagtaaaa caagaagctg aagaaaaaac tttaaaagaa tccgatttag
     18601 aaaatgaaca tattcatata tcagattgtg caaaaagact aagaacttta agaatagcac
     18661 tcaatctaag tcaacagcgt atagcaaata tacttggaat atcccaagct ctttatgtta
     18721 gatttgagaa aggatatgga caactcaaca gctttatgat taaaaaactt tacaaaactc
     18781 taggaatcaa tcctatttat ctaatttgtg gagaaggcga ttgctttcta atcaaagaca
     18841 ttgatttatt tgaaagaata aataacgaac aaaagaaata caacaaaaat caacttgaac
     18901 aagaaaacat cttaaactat tagataaatt tatctatatt aaattaataa tataaattaa
     18961 ttctcttttt taaaataaac cgatctttta aataatttct taaagagcgt tattataaca
     19021 gttccatttt ttacctaagt aattgaaaaa atgggtgcag tgaggtttta agacgaatac
     19081 agcgaaattt tacttcatta aaacaaaagg ctcaaaaatc tttttgcaag gaagattggt
     19141 ttaagaaaat tatacagata aggaagaaaa aaatagaaca agctataaga ttgctgttga
     19201 aactatgaag tttttagata gcaagcctgc tgataataat caatcgggta atccttatgg
     19261 taatgttcaa gcaactcctg aacctgtgaa tcagtctgtg caaaaacaag tacctattca
     19321 tcagtttgag gaacaacaag gagaagatct tcctttttaa ctctaagcac ttaacccata
     19381 gttgggatat ttttatccca ttatgggata ttaatacccc ttgactttta gcattttttt
     19441 gttataagat aaggagaaga aatatgaaag attttttcaa aaaactatcg atatttacaa
     19501 aaatacccac aaaagcaatc ttaagattca tggaatctat
//
LOCUS       IKNDEOKD_2             12446 bp    DNA     linear       12-APR-2021
DEFINITION  Campylobacter species strain strain.
ACCESSION
VERSION
KEYWORDS    .
SOURCE      Campylobacter species
  ORGANISM  Campylobacter species
            Unclassified.
COMMENT     Annotated using prokka 1.14.6 from
            https://github.com/tseemann/prokka.
FEATURES             Location/Qualifiers
     source          1..12446
                     /organism="Campylobacter species"
                     /mol_type="genomic DNA"
                     /strain="strain"
     gene            77..592
                     /locus_tag="IKNDEOKD_00031"
     CDS             77..592
                     /locus_tag="IKNDEOKD_00031"
                     /inference="ab initio prediction:Prodigal:002006"
                     /inference="similar to AA sequence:RefSeq:AOH52017.1"
                     /codon_start=1
                     /transl_table=11
                     /product="ankyrin domain protein"
```

```
                     /protein_id="Prokka:IKNDEOKD_00031"
                     /translation="MIEYFFKKGCTFETYNEESGSTPLHEWILCGEEVKYLEEALKLG
                     ANPNMRSIKTENKFSFSGAGETLLHRAAESDEKPIGACVEVLIKYGADVNAANCRISD
                     IEDGKISYYKPATPLDRANRARNVSKNIKILKKAGAKTWEELVEEYNIDTSLERIEQI
                     KMYEERRKDKK"
     gene            629..2560
                     /locus_tag="IKNDEOKD_00032"
     CDS             629..2560
                     /locus_tag="IKNDEOKD_00032"
                     /inference="ab initio prediction:Prodigal:002006"
                     /codon_start=1
                     /transl_table=11
                     /product="hypothetical protein"
                     /protein_id="Prokka:IKNDEOKD_00032"
                     /translation="MEFNDINFKIKEFESVDIISDYSLVFYLPKEEHMIGEKDQDGKI
                     RINKSNSLKYIEKINEEKEKEIEKLKLQSNEKINEEFDKLKDDALPTKEYKRKNYEYD
                     VFYDVYTSKDNINKCIKKIPIKNGVGTLKSNFLRRFYENSVKGEYNCKWCLIPRLIRN
                     KGESSFDPVPILNSSFVISELSNIKQKTLTFQRTIANVEHPNHFALQSPKEFKRMLEN
                     EQSFASYGGKEKQIEELLKTAQHKLETIEKAVGISEETKEDLEKKQRKEVEKCKKLEH
                     SGKKISPKLIGDTGEYVASLLFGKRSTRYLSNQRKLDANFNIYSFNHTIPDFLVSING
                     IPSLVEVKNVQVQSLTEQISFELKLAREYELDYIFVCNHYTHLTENVAGSALTREEHE
                     ELARDDGDKVGEFNKETKRLKKAKIAEVLNEKDDLANEEFIKSVSDGNKYLKEREKFY
                     KVASDVYIKNPIYKMGFEKKLQNQVVDINIVKDFLKDFAKRLGIDEKVLPVIGYNPQR
                     GDSGTYDYTVNTLDLNPKASYVDFIDTIIHEFRHFYIYCIKNNSENSIEKLLYLDMVQ
                     FYIPWNFYKIFNSYDKKCLIYDNEDETKKCLKNRTYYGSWKFLNPTLDLLPSPLYYLQ
                     PSELDSRIMAYYFRKEIKL"
     gene            2560..3825
                     /locus_tag="IKNDEOKD_00033"
     CDS             2560..3825
                     /locus_tag="IKNDEOKD_00033"
                     /inference="ab initio prediction:Prodigal:002006"
                     /inference="similar to AA sequence:RefSeq:CKG88389.1"
                     /codon_start=1
                     /transl_table=11
                     /product="hypothetical protein"
                     /protein_id="Prokka:IKNDEOKD_00033"
                     /translation="MTPPSLPDVEKHEDFLQTRKEPYAIYLAINTNIKSYNNICPSEK
                     YFWKFDNMNELDKWYNPKFGIYLGKIVFDKKDNKLIPKYIAAKFENLEEEVKKIKNPL
                     WLANKNPNYIEPKFYDGMSGGYYFESPNNLEYQCKIEKDTQVLSQEQIISYVKELYSK
                     NTIIIKNYIDAINKDYGIKPFVFSDEIYDELGEVGILTKEQANNFKDKSYIKKDPILL
                     AMLDYLAKQNKKDEDYLITFDDEYFYTYLVWSLKDFLLELSYGLFQDETELLFNPAAY
                     MDDTKIYYQNLNEEINKRYEKILLNMGFEGENGYFKDYYDYGFGNNGIFEFSIYDYFA
                     YDEIGVQPIQQSPYVPPKSPFDSPNFVYSDGNYHGDAKLVPSALGKYYFELLYQKEIY
                     IELLRPYYPSIKDLPEGWDNKMLEKANLK"
     gene            4517..4723
                     /locus_tag="IKNDEOKD_00034"
     CDS             4517..4723
                     /locus_tag="IKNDEOKD_00034"
                     /inference="ab initio prediction:Prodigal:002006"
                     /codon_start=1
                     /transl_table=11
                     /product="hypothetical protein"
                     /protein_id="Prokka:IKNDEOKD_00034"
                     /translation="MDALGRYVFYSEKAYINEKIIFDKLSRICDPSENYLVGCNIGAI
```

```
                     QDAYEIQPNERDPRYVETLIGRSL"
     gene            4736..4918
                     /locus_tag="IKNDEOKD_00035"
     CDS             4736..4918
                     /locus_tag="IKNDEOKD_00035"
                     /inference="ab initio prediction:Prodigal:002006"
                     /codon_start=1
                     /transl_table=11
                     /product="hypothetical protein"
                     /protein_id="Prokka:IKNDEOKD_00035"
                     /translation="MKTIKIPLKDYISNIREDKTIYLGLQSNMSIKGLDEEFLKKNII
                     LREISNLKQAFIWVKL"
     gene            4903..5151
                     /locus_tag="IKNDEOKD_00036"
     CDS             4903..5151
                     /locus_tag="IKNDEOKD_00036"
                     /inference="ab initio prediction:Prodigal:002006"
                     /codon_start=1
                     /transl_table=11
                     /product="hypothetical protein"
                     /protein_id="Prokka:IKNDEOKD_00036"
                     /translation="MGKIIIKEKNIIFKAFNIDENCFEGLKELFFKNNPYTIIDNLEL
                     KKYFLKLYKDNIEFLTSYINEINQVHGIKLYYILHLML"
     gene            5879..6154
                     /locus_tag="IKNDEOKD_00037"
     CDS             5879..6154
                     /locus_tag="IKNDEOKD_00037"
                     /EC_number="3.6.1.-"
                     /inference="ab initio prediction:Prodigal:002006"
                     /inference="similar to AA sequence:RefSeq:ABS44566.1"
                     /codon_start=1
                     /transl_table=11
                     /product="F-box only protein 18"
                     /protein_id="Prokka:IKNDEOKD_00037"
                     /translation="MDEAQDINACVIDIVLNQKQAKKVFIGDTFQSIYKFRGAVNSLE
                     ILASMPKSHTLYLTQSFRCPQNIAFITNSYLKILNAPRDFKGTKKIK"
     gene            6114..6752
                     /locus_tag="IKNDEOKD_00038"
     CDS             6114..6752
                     /locus_tag="IKNDEOKD_00038"
                     /EC_number="3.6.1.-"
                     /inference="ab initio prediction:Prodigal:002006"
                     /inference="similar to AA sequence:RefSeq:ABS44566.1"
                     /codon_start=1
                     /transl_table=11
                     /product="F-box only protein 18"
                     /protein_id="Prokka:IKNDEOKD_00038"
                     /translation="MLREISKEQKKSSNENVKNEAKAIVCRTNAKLFDIAVENLDKKL
                     FFVGGINSYSFDELLDIQNLLFKKHEYIKNQFIVKFADLKELLEYINETKEVDLKQKI
                     FVLFKYVHGDIIKLIKAIEKSSVKKQEQADLILSTGHKSKGLEWNNVEIIDDFLNIKQ
                     ELEERVRITIAKEELNLLYVAITRAKNSLSISKDYLFEKEFIQENLERIIIE"
     gene            6749..6961
                     /locus_tag="IKNDEOKD_00039"
     CDS             6749..6961
```

/locus_tag="IKNDEOKD_00039"
/inference="ab initio prediction:Prodigal:002006"
/inference="similar to AA sequence:RefSeq:AVL47291.1"
/codon_start=1
/transl_table=11
/product="helix-turn-helix domain-containing protein"
/protein_id="Prokka:IKNDEOKD_00039"
/translation="MKIIKKLQKDGSFKEQVYYSVHEVSQMHSVTHVTVRLWISRGIL
KGMKLGKGFYMSKEAIIEFQKNDGLL"
gene 7729..8649
/locus_tag="IKNDEOKD_00040"
CDS 7729..8649
/locus_tag="IKNDEOKD_00040"
/inference="ab initio prediction:Prodigal:002006"
/inference="similar to AA sequence:RefSeq:ADC28003.1"
/note="conserved hypothetical protein"
/codon_start=1
/transl_table=11
/product="hypothetical protein"
/protein_id="Prokka:IKNDEOKD_00040"
/translation="MEAKGIAIFPTNVAFSKKTNAFFTELEDQFLKEGVGNKSAIKLD
FEYYILYCQFLQIDKESLFFGRNYELVFCFLLYYRALLNKNNTQKIVAKIINVIRCQD
RLPEKVHYREKIICMDNQHRDLSQTLSSCIAEFYNSIDLYNTFRKNIGFENRKKIAIE
MLNRASEHYGFFGLSRGGITYKEEKISCVPIQALDEFHHGMSHLCAVFLKEDGRYTNL
ERAIPHFKRGMMEVYKTIIRDFMILNKDALGVKQEALKIRELEYQTIGKPKTVTKNPT
TNLTNKKEEKVIPGYKELCEKIVSQIKNAK"
gene complement(8749..9777)
/gene="flgS"
/locus_tag="IKNDEOKD_00041"
CDS complement(8749..9777)
/gene="flgS"
/locus_tag="IKNDEOKD_00041"
/inference="ab initio prediction:Prodigal:002006"
/inference="similar to AA sequence:RefSeq:CAL34921.1"
/codon_start=1
/transl_table=11
/product="signal transduction histidine kinase"
/protein_id="Prokka:IKNDEOKD_00041"
/translation="MNENILKSLDSSEKETLQKGLQSLIEQTYIVENEYKILNENYNS
LRAMVDEIIEVLPSALWILDKEKNIILQNQEALKKPKLLDIISLEKNRDELEFENRFY
AVKIIAHNEKTIVSATDISDEKRNERLASMGSVAAHLAHEIRNPIGSISLLASTLFSR
SELKNKHIVLEIQKAIARVERIVNSTLLFTKGVHINASEFNLLELTEECESAINSYNY
SSQIDFEINFLDMKIYGDKALLSLVLQNLIYNAIDAIEESECENPKIKIQASCDEKNL
YIRVYDNGCEIKDENLVFEAFKTTKLKGNGLGLSLSKEIINAHKGELKFEIWPKNFYF
VLPLHYNS"
gene complement(9774..10595)
/locus_tag="IKNDEOKD_00042"
CDS complement(9774..10595)
/locus_tag="IKNDEOKD_00042"
/inference="ab initio prediction:Prodigal:002006"
/inference="similar to AA sequence:RefSeq:CAL34920.1"
/note="conserved hypothetical protein Cj0792"
/codon_start=1
/transl_table=11

```
                     /product="hypothetical protein"
                     /protein_id="Prokka:IKNDEOKD_00042"
                     /translation="MAKIFDFYSVFEGFESLDSLNFEEDVFHNIEYLLLKNYLDIKDC
                     FKLDETASYALSLLAKNNRKRFSINRKIQHFKALSTLKYLLRVGIIKLEHSKEAKRVR
                     DKRQKLKKELRSYVIQDKIIFANQFTRFFFYFLKPNEKLILQNRYEEVLTLIKEKFEL
                     YQSFCFEQLSRELLEKKFQVSGVQSYWDKNLELDLYYKDDEISFIGEVKFKNKKICRN
                     ILNLLELKAKSLKLEPNNYIIISKNGFSKEFYKICKQDLLLLDLNDFKILLEENK"
gene                 10699..11967
                     /locus_tag="IKNDEOKD_00043"
CDS                  10699..11967
                     /locus_tag="IKNDEOKD_00043"
                     /inference="ab initio prediction:Prodigal:002006"
                     /inference="similar to AA sequence:RefSeq:CAL34919.1"
                     /codon_start=1
                     /transl_table=11
                     /product="putative aminotransferase"
                     /protein_id="Prokka:IKNDEOKD_00043"
                     /translation="MQISDLKKEIILKKGTLHFDFTASALALKCVEKEIAKILPSYAN
                     THSDSSLNSFKTQQIYEQARKDIKTSLNLSDEFALIACGSGSSSAIKKFQELLGIYIP
                     PLVKEKYFEKVDKSTLPLVIVGPYEHHSNELSFREGLCECIRIPLDKKGNIDFEILEK
                     VLKENQHRKIIASFSLASNVTGILSDYKRISKLVRKFKGIVAFDASSFIPYKNIACKY
                     YDALFISSHKLIGGIGGSGLLAIKKELCGNKPSFAAGGTVGYVSRTSQCYLCNEEALE
                     EGGTPGILQLIRASLAFKIKDEIGIEEIEKKEEILKEYFFEKLKTIPHLILYANNIKA
                     RLPIFAFNIKNISPFDLAYELSKKYHIETRAGCACAGPYGHDLLGLKDNEELKTKPGW
                     LRISLHYTHEKEDIDYFFEALQKSIIKLKF"
gene                 complement(11971..12047)
                     /locus_tag="IKNDEOKD_00044"
tRNA                 complement(11971..12047)
                     /locus_tag="IKNDEOKD_00044"
                     /product="tRNA-Asp"
                     /inference="COORDINATES:profile:Aragorn:001002"
                     /note="tRNA-Asp(gtc)"
gene                 complement(12069..12144)
                     /locus_tag="IKNDEOKD_00045"
tRNA                 complement(12069..12144)
                     /locus_tag="IKNDEOKD_00045"
                     /product="tRNA-Val"
                     /inference="COORDINATES:profile:Aragorn:001002"
                     /note="tRNA-Val(tac)"
gene                 complement(12153..12228)
                     /locus_tag="IKNDEOKD_00046"
tRNA                 complement(12153..12228)
                     /locus_tag="IKNDEOKD_00046"
                     /product="tRNA-Glu"
                     /inference="COORDINATES:profile:Aragorn:001002"
                     /note="tRNA-Glu(ctc)"
gene                 complement(12242..12317)
                     /locus_tag="IKNDEOKD_00047"
tRNA                 complement(12242..12317)
                     /locus_tag="IKNDEOKD_00047"
                     /product="tRNA-Lys"
                     /inference="COORDINATES:profile:Aragorn:001002"
                     /note="tRNA-Lys(ttt)"
gene                 complement(12321..12397)
```

```
                     /locus_tag="IKNDEOKD_00048"
     tRNA            complement(12321..12397)
                     /locus_tag="IKNDEOKD_00048"
                     /product="tRNA-Asp"
                     /inference="COORDINATES:profile:Aragorn:001002"
                     /note="tRNA-Asp(gtc)"
ORIGIN
        1 acttaaagct gatgaagtac actttggata caatgcactc agtagatata tcgatagaga
       61 tggaaaaaat aaagaagtga tagagtattt ttttaaaaaa ggatgcacat ttgaaactta
      121 taatgaagaa agtggtagca ctcctttgca tgaatggata ttatgtggcg aagaagtgaa
      181 gtatttagaa gaagctttaa aactaggagc aaatcctaat atgagaagta ttaaaacaga
      241 aaataaattt agctttagtg gtgcaggaga aacccttttg catagagctg cagaatctga
      301 tgagaaacct ataggagctt gcgtggaagt gctaatcaaa tatggagctg atgtaaatgc
      361 ggctaattgt cgcataagtg atattgaaga tggaaaaatc agctattata aacctgcaac
      421 cccattggat agagccaaca gagctcgcaa tgtgagcaaa aatataaaaa tcttaaaaaa
      481 agcaggagct aaaacttggg aggaattggt tgaagaatac aatatcgata caagcttaga
      541 aagaatagaa caaattaaaa tgtatgaaga aagaagaaag gataaaaaat aagattaata
      601 aggcaaagaa caaaaaaagg atttttcatt ggaatttaat gatataaatt ttaaaatcaa
      661 agaatttgaa agtgtggata tcatcagtga ttattctctt gtcttttatt tgcctaaaga
      721 agagcatatg ataggagaaa aagatcaaga tggtaaaatt cgcatcaata aaagtaattc
      781 tttaaaatat atagaaaaaa tcaatgaaga aaaagaaaaa gaaattgaaa aacttaaact
      841 tcaaagcaat gaaaaaatca atgaagaatt tgataaatta aaagatgatg ctttacctac
      901 taaagaatat aaaagaaaaa attatgaata cgatgtattt tatgatgttt atacaagtaa
      961 ggataatata aacaagtgca taaaaaaaat cccgataaaa aatggtgtag gaacgctaaa
     1021 atcaaatttc ttacgaagat tttatgaaaa ttctgtcaag ggtgagtata attgtaaatg
     1081 gtgtcttata cctagactaa tccgcaataa aggagaatct agttttgatc ctgtgcctat
     1141 tttaaatagt agctttgtca tttcagaatt aagcaatatc aaacaaaaaa cccttacttt
     1201 tcaaagaaca atagccaatg tggaacatcc aaatcatttt gctttacaaa gtcctaagga
     1261 atttaaacgt atgcttgaaa atgagcaaag ctttgcttct tatggtggca aagaaaaaca
     1321 aatagaagag cttttaaaaa ccgcacaaca taaacttgaa accattgaaa aagcagttgg
     1381 aatttctgaa gaaaccaagg aagatttaga aaaaaagcaa agaaaagaag ttgaaaaatg
     1441 caaaaaatta gaacatagcg gtaaaaaaat ttcacccaaa ctcataggag atacgggaga
     1501 atatgtcgct tctttacttt ttgggaaaag atctacacgc tatttaagca atcaaagaaa
     1561 attggatgct aattttaata tctatagttt taatcatacc atacctgatt ttttagtaag
     1621 tattaatggt attcctagtt tagtagaagt taaaaatgtt caagttcaaa gcctaacaga
     1681 acaaataagc tttgagttaa aactggctag agaatatgaa cttgattaca tctttgtgtg
     1741 taatcattat acacatttaa cagaaaatgt agcaggaagt gcattgactc gtgaagaaca
     1801 cgaagagcta gcacgagatg atggagacaa agtgggtgag tttaataaag aaacaaaaag
     1861 attgaaaaaa gcaaaaatag cagaagtctt aaatgaaaag gatgatttag ctaatgaaga
     1921 atttattaaa agtgtaagtg atggaaataa gtatttaaaa gaaagggaga aattttataa
     1981 agttgcttca gatgtttata ttaaaaaccc tatttataaa atgggctttg agaaaaaact
     2041 tcaaaatcaa gttgttgata taaacattgt aaaagatttt ttaaaggact ttgctaaaag
     2101 actagggata gacgaaaaag ttttgcctgt aattggatat aatcctcaac gaggggattc
     2161 tggaacatat gactatacag taaatacttt agatttgaat ccaaaagctt cttatgttga
     2221 ttttattgat accattattc atgaatttag gcatttttat atttattgca taaaaaataa
     2281 ttctgaaaat agtatagaaa aattgcttta tcttgatatg gtacaatttt atataccgtg
     2341 gaatttttat aaaattttca atagttatga taaaaaatgc ttaatttatg ataatgaaga
     2401 tgaaacgaag aagtgtttaa aaaatcgcac atattatgga agttggaaat ttttaaatcc
     2461 cacattagat ctcttgccaa gtcctttata ctatcttcag ccaagtgagt tagactctag
     2521 gattatggca tattatttta gaaaggaaat taaattataa tgactccacc atctttacca
     2581 gatgtagaaa aacatgaaga tttcttacaa accagaaaag aaccttatgc tatttattta
     2641 gccataaaca caaacataaa aagttataat aatatttgtc ctagcgagaa atatttttgg
     2701 aaatttgata atatgaatga acttgataaa tggtataacc caaaatttgg catatacctt
     2761 ggcaaaattg tttttgataa aaaagacaac aaactcatcc caaaatatat agccgctaaa
```

2821 tttgaaaatt tagaagaaga agttaaaaag ataaaaaatc ctttatggct tgctaataaa
2881 aatcctaatt atattgaacc taaattttat gatggtatga gtgggggtta ttattttgaa
2941 agtccaaata atttagagta tcaatgcaaa atagaaaaag acactcaagt tttaagccaa
3001 gaacaaatca tttcttatgt gaaagaactt tatagtaaaa acacaataat aataaaaaat
3061 tatattgatg caatcaataa agactatggc ataaaacctt tcgtatttag tgatgaaatt
3121 tatgatgaat taggagaagt tggcattctt acaaaagaac aagctaataa ttttaaagat
3181 aaaagttata ttaaaaaaga tccgatattg cttgctatgc ttgattattt agcaaaacaa
3241 aataaaaaag atgaagatta tttaatcact tttgatgatg agtattttta tacttatttg
3301 gtttggagtt taaaagattt cttattagag ctttcttatg gtttgtttca agatgaaacc
3361 gagcttcttt ttaatcctgc agcttatatg gatgacacaa aaatatatta tcaaaattta
3421 aacgaagaaa taaataagcg ttatgaaaaa atattgctta atatgggatt tgaaggagaa
3481 aatggttatt ttaaagatta ttatgattat ggctttggca ataatggtat ttttgaattt
3541 agcatatatg attattttgc ttatgatgaa ataggagtac aaccaattca acaaagtcca
3601 tatgttccac ctaaaagccc ttttgatagc cctaattttg tctattctga tggaaattat
3661 cacggagatg caaagcttgt cccttctgct ttaggaaagt attattttga gcttttatat
3721 caaaaagaaa tttacataga acttttacgc ccttattatc caagcataaa agatttgcca
3781 gaaggttggg acaataaaat gctagaaaaa gctaatttaa aataaaatta tagatatgag
3841 ctttagagta ggagttggaa gggatgagag tgtaaaagaa agcaagctat gaaataattt
3901 taacaacgct gtaaaaaata attagacgcc taaagacatt atctaaacaa attgtatttt
3961 tagtgttttt gaagctgtag agtttttaaa agcttgtcaa agttttaaag aaaaaaaatg
4021 aaacagataa ttatattttt tcaaaaccta tcctcaattt gcaggaaaaa ttgcttatat
4081 atactataga tttgatttaa tcaacaagga atttttaaca agcgtatcag aaaaagataa
4141 aggaaattat cttcaagtta gagatggcaa atatattagc accataatta ataaaatttt
4201 acctttagag tataagaatg gtaaaagttt taaacaattc cacacagagc ttttaaataa
4261 caaaaacaga gatcttaaag aatgtaaaga ttttctaaaa gctttaactc aaactctttg
4321 taaagaattt aatttacctt tagaaaatat agtcttttat agccaaaagc ctagtggatc
4381 tttatcattt ggagcttatg aagaaaataa ggtcagatta aatcaataca ttttaaaagc
4441 ttattttaaa gaatttgcaa aaacggtttt tcacgaatgt aggcattttt atatagaaaa
4501 atattataat cacaatatgg atgctctagg cagatatgtt ttttattctg aaaaagctta
4561 tatcaatgag aaaattattt ttgataaatt gagtagaatt tgcgatccta gtgagaatta
4621 tttagtaggt tgcaatatag gtgctataca agatgcttat gaaattcagc ccaatgaaag
4681 agatccaaga tatgtagaaa ccctcatagg tagaagttta taatcaagga aaataatgaa
4741 aactataaaa ataccgctta aagattatat ctccaatata agagaagata aaactattta
4801 cttgggcttg caaagtaaca tgagtataaa aggacttgat gaagaattct taaaaaagaa
4861 tataatctta agagaaattt caaatttgaa acaagcattt atttgggtaa aattataatc
4921 aaagaaaaaa atatcatctt taaagctttt aatattgatg agaattgctt tgaagggctt
4981 aaagaacttt tcttcaaaaa taacccctat acaattatag ataatttaga attaaaaaaa
5041 tatttcttaa aactttataa agataatatt gaatttttaa cttcttatat taatgaaatt
5101 aatcaagttc atggtattaa actttactat attttacatt taatgcttta aaattctaag
5161 tcctccataa agcatgagtt taattaacat agcattctta aaactgctat aatcatcttt
5221 tttttgcttt ttccttctaa aacattattt agccttatag tttcattctc gctaagataa
5281 tttaactttt cttctgcttt ttcagtttta aacctaattt tttcatatca aagctaaaaa
5341 ggaataagtc atcattatta tcacttataa aaaagctagt taatactctc aaataagtaa
5401 ttttgttctt tttgataatt tattgctatt ttttaaatta ttttccaaat aattaagaaa
5461 attaatcaaa aaagttgttt ttatatcatt tatttgcatt tcatcctgat aatccagtaa
5521 atattccaca agctctaaaa atacacctat aaaacaataa agtgttcttt aatataatat
5581 tatttatata aataaaattt catataatcc acatacaaca tcagaaattg ataataaata
5641 tttatcttga tttatcttat tgatgttttt aagttttatt ccaacaattt cataaaactt
5701 tggttttatt gtatttaatt tatcttcagt gtaaaaaatt tatattttgc aattagtcct
5761 tcaaacaatt ttataaatgc aaaatctata ttttcaagtt ctttgctata caaataatta
5821 tttataactt cttttaattc aacttacaaa accaaagcta aagtatgatt ttattttagt
5881 ggatgaagca caggatatca acgcttgtgt tatcgacatt gttcttaatc aaaaacaagc
5941 taaaaaagtt tttatagggg atacctttca gagtatttat aaatttagag gagcggtaaa
6001 ttctttagaa atattagcat ctatgccaaa atctcacact ttatacctaa cacaaagttt

6061 taggtgtccg caaaatatag cttttattac caactcttat ttgaaaattt taaatgctcc
6121 gcgagatttc aaaggaacaa aaaaaatcaa gtaatgaaaa tgtcaaaaac gaagcaaaag
6181 ccatagtgtg tagaactaat gctaagttat ttgatatagc cgtagagaat ttagataaaa
6241 aattattttt tgtaggagga ataaatagtt atagttttga tgagttacta gatatacaaa
6301 atcttctttt taaaaaacac gaatatatca aaaaccaatt tatagtaaag tttgctgatt
6361 taaaggagct tcttgaatat attaatgaaa ctaaagaagt tgatttaaag caaaaaatct
6421 ttgttctttt caaatatgtg catggcgata ttataaagct tattaaagcc attgaaaaaa
6481 gtagcgtaaa aaaacaagag caagctgatt taatcttaag cacaggacat aagagcaaag
6541 gcttggaatg gaacaatgtt gaaattattg atgatttttt aaacatcaag caagaacttg
6601 aagaaagggt tcgaatcacc atagcaaaag aagaattaaa ccttttatat gtggcaatca
6661 caagagccaa aaattctcta agtattagca aagattattt gtttgaaaaa gaatttatac
6721 aagaaaattt agaaaggata ataatagaat gaaaataatt aaaaaattac aaaaagatgg
6781 aagcttcaaa gagcaagttt attattctgt tcatgaagtt tcacaaatgc actcagtaac
6841 gcatgtaaca gttcgcctat ggatttcaag aggaattcta aaaggtatga aattaggcaa
6901 aggattttat atgtcaaaag aagccattat tgaatttcag aaaaatgatg gtttattata
6961 attaaggaga gaaaaaatga cttttagtag aaaagaatgt gctaaaattc ttgggatatc
7021 tttgcaaact ttagatagat tgacaaaaga taaaaaataa aatcaatgaa tataactcgt
7081 tttgtattat tctccattga agaaatagct aaaattctct caggggctaa gtcaacaaaa
7141 tgaatagaaa agatagggtt gcactagctg cttatcataa aaataaaaag ctcacttgct
7201 ttggtaagtg agtaatttaa aagtatatgt taaagtatat gtttttttaga tatttaattt
7261 ttaaagtatg gatatttaga tatttgatta ataaatcaca cgccgacctc aggcaccatt
7321 gcttcttaaa atcataaaat tccaaaaaaa ttttattgtt tatatttgct cgaaaaaatg
7381 cttctaaaat agctgaaata tttatttata gatattaaaa atatttttat ataaataaaa
7441 tatgctttaa attatgttaa atatttaatt ataaattatt taaaaataat aagctcgctc
7501 ccttaaaata agataaaaat agccctatat tcttaaataa gactaaaaat gtatgattaa
7561 acttaaatta aactaaaatt tgatatagtt cgcgcgatca aaaaagttta aataaaaaat
7621 taaaaagtca tagttattta aactgaaaca tttgtgttat ttttcataga aaatcataat
7681 acaaataaag atctatatac cgaataacta aaaaaaaagg gggggggtat ggaagcaaaa
7741 ggaatagcga tatttcctac aaatgttgca tttagtaaaa aaacaaatgc tttttttcact
7801 gaattagaag atcaattctt aaaagaaggg gtcggtaata aaagtgccat aaaactagat
7861 tttgaatatt atattttata ttgtcaattt ttacaaatag acaaagaatc cctatttttt
7921 ggaagaaact atgaacttgt gttttgtttt ttactttatt atagagcttt attaaataaa
7981 aacaatactc aaaaaatcgt agcaaaaata ataaatgtta tacgttgcca agatagatta
8041 ccagagaaag tacactatag agaaaaaata atttgtatgg ataatcaaca cagagatctt
8101 tctcaaacac taagttcatg tattgcagaa ttttataatt caattgatct atacaatacc
8161 tttagaaaaa atataggatt tgaaaataga aagaaaatag cgatagaaat gttaaatcgc
8221 gcatcagaac actatggttt ttttggctta agcaggggag gaataacata taaagaagaa
8281 aaaatctctt gcgtccctat acaagcatta gatgagtttc atcatggtat gtcgcatcta
8341 tgtgcagttt tcttaaaaga agatggacga tataccaatt tagaaagagc gatccctcac
8401 ttcaaacgtg gaatgatgga agtatacaaa acaataatca gagattttat gatattaaat
8461 aaagatgctt taggagtcaa acaagaagcc ttaaaaataa gagaattaga atatcaaacc
8521 ataggaaaac ccaaaacagt aacaaaaaac cctaccacaa acctaaccaa taaaaaagaa
8581 gagaaagtta tcccaggata taaagaactg tgcgaaaaaa ttgtcagtca aatcaaaaat
8641 gcaaagtaaa atcggtatga attgagcttg agtatgcaat ttagcttatt tgctatttgc
8701 atacttagag ccattctttg aagaaattaa aacaaatcct atcataaatt aactattata
8761 atgtaaaggc aaaacaaaat aaaaattttt aggccaaatt tcaaatttta attctccttt
8821 atgagcatta atgatttctt tagaaagcga taggcccaat ccatttccct taagtttagt
8881 agtcttaaaa gcttcaaaaa ccaaattttc atctttgatt tcacaaccat tatcataaac
8941 cctaatgtat aaatttttct catcacagct tgcttgaatt ttgattttag gattttcgca
9001 ctcactttct tcgatcgcat ctatggcatt ataaatcaaa ttttgcaaaa ccaaagataa
9061 aagtgcctta tctccataaa ttttcatatc taaaaaatta atttcaaaat caatttgaga
9121 tgagtaatta taagaattta tagcactttc acactcttct gttaattcta aaagattaaa
9181 ctcgcttgca ttgatatgca caccccttggt aaaaagcaag gtagaattta caatacgctc
9241 taccctagca atagcctttt gaatttcaag cacaatatgt ttgtttttaa gctcactacg

9301 agaaaaaagc gtggaggcaa gtaaggatat agagcctata ggatttctta tctcatgcgc
9361 taaatgcgcc gcaacacttc ccatactagc aagtctttca ttgcgttttt catcgctaat
9421 atccgtagct gaaactatgg ttttttcatt atgagcaata attttaaccg cataaaatct
9481 attctcaaat tcaagttcat cacgattttt ttcaagactg ataatatcaa gcaatttagg
9541 tttttttcaaa gcttcttgat tttgcaaaat aatatttttt tctttatcta aaatccaaag
9601 cgcactaggc aaaacctcga taatctcatc taccatagca cgcaaagaat tataattttc
9661 gtttaaaatt ttatattcgt tttctacaat ataagtttgc tctataaggc tttgtaagcc
9721 tttttgtaag gtttcttttt cacttgagtc tagacttttt aaaatatttt cattcattta
9781 ttttcctcta ataaaatttt aaaatcattt aaatccaata aaagcaaatc ttgtttacaa
9841 attttataaa attctttact aaaaccattt tttgaaataa taatataatt atttggctct
9901 agttttaaag attttgcttt taattctaaa agatttaaaa tatttctaca aattttttta
9961 tttttaaatt taacttcacc gataaaactt atctcatcat ccttataata aagatcaagt
10021 tctaaatttt tatcccaata actttgcacc ccgctcactt gaaatttttt ttcaagtaat
10081 tctctggaaa gttgctcaaa acaaaaactt tgataaagtt caaatttctc tttaatcaaa
10141 gttaaaactt cttcatagcg attttgcaaa atcaactttt cattgggttt tagaaaataa
10201 aaaaagaaac gagtaaattg attggcaaag ataattttat cttgtatcac ataagatctt
10261 aattcttttt taagtttttg tcgtttatct cttacccttt tcgcctcttt gctatgctca
10321 agttttataa tcccaactct taaaagatat tttaaagttg ataaagcctt aaaatgctga
10381 atttttctat tgatagaaaa acgcttacga ttgtttttag ctagcaagct aagagcataa
10441 gaagcggtct catcgagctt gaaacaatcc ttgatatcta aatagttttt gagcaaaaga
10501 tattctatat tgtgaaatac atcttcttca aaatttaaac tatccaagct ctcaaaaccc
10561 tcaaaaacac tataaaaatc aaaaatttta gccaaagctg aagtgttgca aatttttgca
10621 aaatgattaa aatttatatt tttccttttt aaaagtattt ttatagtata attttagcaa
10681 atttttaaag gattgtgctt gcaaatttca gatttaaaaa aagaaataat tttaaaaaaa
10741 ggcaccttgc attttgactt taccgctagt gctttggcat taaaatgtgt agaaaaagaa
10801 attgcaaaaa tactgccaag ttatgcaaat actcattctg acagctcctt aaactctttt
10861 aaaactcaac aaatttacga acaagcacgc aaagatatca aaacaagtct taatctaagt
10921 gatgaatttg cacttattgc ttgtggatca ggctctagca gtgctattaa aaaatttcaa
10981 gaattacttg gaatttatat cccaccactt gttaaagaaa agtattttga aaaggtagat
11041 aaaagcaccc taccottagt tatcgtaggg ccttatgaac atcattctaa cgaactttcc
11101 ttccgtgaag gtttgtgcga atgtataaga attcccctag ataaaaaagg aaacatagac
11161 tttgaaattt tagaaaaagt tttaaaagaa aatcaacaca gaaaaattat cgctagtttt
11221 tccctagctt caaatgttac aggtatatta agtgattata aacgcatttc taaattagtg
11281 cgtaaattta aaggcatagt tgcttttgat gcatcaagct ttatccctta taaaaacata
11341 gcttgcaagt attatgatgc tttatttata agctctcata aattaatcgg gggtatagga
11401 ggctctggtc ttttagcaat caaaaaagaa ctttgcggaa acaaaccaag ctttgccgca
11461 ggaggaactg tgggttatgt ttcgcgcacc tctcaatgtt atctttgcaa tgaagaggct
11521 ttagaagaag gagggactcc tggaatttta caactaattc gtgcgagctt ggcttttaaa
11581 ataaaagatg aaataggcat agaagaaata gaaaaaaaag aagaaatttt aaaagaatac
11641 ttctttgaaa aactaaaaac tatacctcat ttaatcctat atgctaataa tataaaagct
11701 cgcttaccca tctttgcttt taatattaaa aacatttctc cttttgactt agcctatgag
11761 ctaagtaaaa aataccacat agaaacacgc gcgggctgtg cttgtgctgg tccttatggg
11821 catgatttac taggcttaaa agataatgaa gaattaaaaa ctaaaccagg ttggctaaga
11881 attagtttac attatactca cgaaaaagaa gatattgact acttctttga agcactgcaa
11941 aaaagtataa taaaattgaa attttaatga tggcgcagcg gacggggctc gaacccgcga
12001 cctccgccgt gacagggcgg cattctaacc agctgaacta ccgctgcacc caagaagtaa
12061 taatttaatg gtggtcacta taagactcga acttatgaca tccaccttgt aagggtggcg
12121 ctctaccaac tgagctaagc gaccttaatt tttggcgacc cctagaggat ttgaacctct
12181 gtttccacac agagagagtg ttgtcctggg ccactagacg aaagggtcta aacccgataa
12241 gtggtgtccc gtgttggatt cgaaccaacg gccccctcct taaaagggag atgctctacc
12301 ggctgagcta acgagacata tggcgcagcg gacggggctc gaacccgcga cctccgccgt
12361 gacagggcgg cattctaacc agctgaacta ccgctgcacc caagaagtaa taatttaatg
12421 gtggtcacta taagactcga acttat
//

```
LOCUS       IKNDEOKD_3              4643 bp    DNA     linear       12-APR-2021
DEFINITION  Campylobacter species strain strain.
ACCESSION
VERSION
KEYWORDS    .
SOURCE      Campylobacter species
  ORGANISM  Campylobacter species
            Unclassified.
COMMENT     Annotated using prokka 1.14.6 from
            https://github.com/tseemann/prokka.
FEATURES             Location/Qualifiers
     source          1..4643
                     /organism="Campylobacter species"
                     /mol_type="genomic DNA"
                     /strain="strain"
     gene            complement(3..755)
                     /locus_tag="IKNDEOKD_00049"
     CDS             complement(3..755)
                     /locus_tag="IKNDEOKD_00049"
                     /inference="ab initio prediction:Prodigal:002006"
                     /inference="similar to AA sequence:RefSeq:CKG86653.1"
                     /codon_start=1
                     /transl_table=11
                     /product="hypothetical protein"
                     /protein_id="Prokka:IKNDEOKD_00049"
                     /translation="MIRVIYLDGLERLIDESKNFRIKNKNSQFERFKMKYKVLNKEHM
                     NFYFYDIPYELGKKISKYYEMHLFLQIPNLKKTDILELFEMTILTSFDNEAEMDETIF
                     ADYLFDFSNLYKLANHYADDYVAIVGQLFLDDCIDFWRCAYHKMDNANTKTPNLSQYK
                     KENKYETWKYFRDNYIYSYNDNLVNHATKGDFWGNLDNWDMYNINCFRTQKGTKYLNE
                     ILAPKFYNKYKDLEVEIDDKGNIIRWIGKINR"
     gene            complement(709..1467)
                     /locus_tag="IKNDEOKD_00050"
     CDS             complement(709..1467)
                     /locus_tag="IKNDEOKD_00050"
                     /inference="ab initio prediction:Prodigal:002006"
                     /codon_start=1
                     /transl_table=11
                     /product="hypothetical protein"
                     /protein_id="Prokka:IKNDEOKD_00050"
                     /translation="MAIGKVIHKYGSFFPDFDEIFYNLGYDGICFDEKSDIKLFLQIP
                     NLKKTDILEYFEDHFLGSIFEDACEFNDLDCPLFYTEDENLKNSIKPNYNSEYLSIIG
                     QLFLAGYIDFGITPRSEDKYTRTDYPTNLSYYKEDKYQAWIYFRDNFFYTNAFLKGYY
                     DDILIYKGKEYTANTLPKLKKGETIYSTMHSAASWDMPRYWSGYNIWVTRTQKGTKYL
                     REILEPRVYNKYKDLEVEIDDKGNILRWIGEINR"
     gene            complement(1477..2214)
                     /locus_tag="IKNDEOKD_00051"
     CDS             complement(1477..2214)
                     /locus_tag="IKNDEOKD_00051"
                     /inference="ab initio prediction:Prodigal:002006"
                     /inference="similar to AA sequence:RefSeq:CKG86653.1"
                     /codon_start=1
                     /transl_table=11
                     /product="hypothetical protein"
                     /protein_id="Prokka:IKNDEOKD_00051"
```

```
                     /translation="MKYKYIKWNKEEKCAIFFPDIDYSLWKKTQLKENVYNNPSLIIS
                     SCLITKMDILELFEYYILFICGKSMGTSRWECNNLILLDTYNIDNLSNPFSLNTPKPT
                     YISEYINILGQLFLAGYIDFGSYCDEEDRNKIDYPSNLSYYKEDKYQAWIYFRDNFFY
                     QDKFIRDLDNPETLDEDGYSLILGDISWDTPQYWSQYNIWVATTPKGTKYFNEILAPK
                     FYNKYKDLEVEIDDKGNVLRWIGEINR"
     gene            complement(2204..2971)
                     /locus_tag="IKNDEOKD_00052"
     CDS             complement(2204..2971)
                     /locus_tag="IKNDEOKD_00052"
                     /inference="ab initio prediction:Prodigal:002006"
                     /inference="similar to AA sequence:RefSeq:APA51619.1"
                     /codon_start=1
                     /transl_table=11
                     /product="lysozyme"
                     /protein_id="Prokka:IKNDEOKD_00052"
                     /translation="MQKIDFEIAKGLDERLDNENVCCFNVYTKDYFNANDKDMLISDN
                     IKDDTNTTQAHINENYNNAQELIPDNVIRIQTQDGSGEFIEIVFDEFKEWEKSSSGAI
                     KPLFTPLEGIVEDISNSLENVSIIGDTLAMLGAVKNPKNAKNIIKKKSLTRDEVLQIV
                     DKLPKNVRDKCRRIVENKEELDKLWKKLTKNSKELENKVDKKYGQSIYMRKLDDQTII
                     QYKKASKSGGETIEINSNKPRGNLRTIHIENGVKNEV"
     gene            complement(3271..3741)
                     /locus_tag="IKNDEOKD_00053"
     CDS             complement(3271..3741)
                     /locus_tag="IKNDEOKD_00053"
                     /inference="ab initio prediction:Prodigal:002006"
                     /codon_start=1
                     /transl_table=11
                     /product="hypothetical protein"
                     /protein_id="Prokka:IKNDEOKD_00053"
                     /translation="MQIPIYSKLKAKLESKGIDNISSGQIAWFVCSLILALVTYIILT
                     FHYIYISYGKYDKNLFLLIFDRNAMELFIIIYSCIMFNLLYIYNAIPLVLSAVFIFLA
                     FYFSSLSLFIVGCCGMGVFFLISIFKRKLIPSVLLFAMGYICSYLLLHAIGTYH"
     gene            complement(3951..4100)
                     /locus_tag="IKNDEOKD_00054"
     CDS             complement(3951..4100)
                     /locus_tag="IKNDEOKD_00054"
                     /inference="ab initio prediction:Prodigal:002006"
                     /inference="similar to AA sequence:RefSeq:AAW35443.1"
                     /note="conserved domain protein"
                     /codon_start=1
                     /transl_table=11
                     /product="hypothetical protein"
                     /protein_id="Prokka:IKNDEOKD_00054"
                     /translation="MKEKIDKLRDYAELAQASYFYFDLKGYILQENETIVILNELVSL
                     SYNGN"
     gene            complement(4110..4415)
                     /locus_tag="IKNDEOKD_00055"
     CDS             complement(4110..4415)
                     /locus_tag="IKNDEOKD_00055"
                     /inference="ab initio prediction:Prodigal:002006"
                     /codon_start=1
                     /transl_table=11
                     /product="hypothetical protein"
                     /protein_id="Prokka:IKNDEOKD_00055"
```

```
                     /translation="MGVFRKFYIVWVVFCISGFAISPAVGHNPNRVYEFFVMLGWIIF
                     PLILLMLYRFFSLCEIKFLYIALLLLLYYPIALILYYMFYYHNSFYVTLYIFLSLFK"
ORIGIN
        1 actcatctat taatctttcc tatccatctt ataatattac ccttatcatc aatttctact
       61 tctaaatctt tgtatttgtt ataaaattta ggagctaaga tttcattgag atattttgtg
      121 ccttttttgag tgcgaaagca attgatatta tacatatccc aattatccaa attcccccaa
      181 aaatcgcctt ttgttgcatg atttactaaa ttatcattat aagaataaat ataattatct
      241 ctaaaatatt tccaagtttc atatttattt tcttttttgt attgcgataa attaggagtt
      301 tttgtatttg cattgtccat tttatggtat gcacaacgcc aaaaatcaat acaatcatct
      361 aaaaaaagtt gtcctacaat agctacataa tcatccgcat aatgatttgc taatttatat
      421 aaatttgaaa aatcaaataa ataatcagca aaaattgttt catccatttc tgcttcgtta
      481 tcaaaacttg tcaaaattgt catttcaaac aattctaaaa tatctgtttt tttaaggttt
      541 ggaatttgta gaaaaaggtg catttcatag tatttactta tttttttacc taactcataa
      601 gggatatcgt aaaaataaaa attcatatgc tctttattta atactttata tttcatctta
      661 aatctctcaa attgtgaatt tttgtttttg attctaaaat ttttagattc atctattaat
      721 ctctccaatc catctaagta tattaccctt atcatcaatc tctacttcta aatctttgta
      781 tttgttataa actctaggtt ctaaaatctc acggagatat tttgtgcctt tttgagttct
      841 tgttacccat atattatacc cactccaata tcttggcata tcccaagaag cagcactatg
      901 cattgtacta taaatcgttt ctcctttttt aagtttgggt agagtatttg ctgtatattc
      961 tttgccttta tagattaaaa tatcgtcata atatcctttt aaaaatgcat tagtataaaa
     1021 gaaattatct ctaaaatata tccaagcttg atatttatct tctttataat aagataaatt
     1081 agtaggatag tctgttcttg tatatttatc ttccgatctt ggagtaatcc caaagtctat
     1141 atatcctgct aagaatagtt gccctataat acttagatac tcgctattgt aatttggttt
     1201 tatggaattt tttaagtttt catcttctgt gtaaaacaaa ggacaatcca agtcattaaa
     1261 ttcgcatgca tcctcaaaaa ttgatcctaa aaaatgatct tcaaaatatt ctaaaatatc
     1321 tgttttctta aggtttggaa tttgtagaaa aagctttata tctgattttt catcaaaaca
     1381 aataccatca taacctaaat tgtaaaatat ctcatcaaaa tcagggaaaa acgaaccgta
     1441 tttatgaatt actttaccaa tcgccatttt tatctttcat ctattaatct ctccaatcca
     1501 cctaagcaca ttacccttat catcaatctc tacttctaaa tctttgtatt tattataaaa
     1561 tttaggagct aagatttcat taaaatattt tgtgcctttg ggggttgttg ctacccagat
     1621 attgtattgt gaccaatatt gtggtgtatc ccaagaaata tcacctaaaa taagactata
     1681 tccatcctca tctaatgttt caggattgtc caaatctcta ataaatttgt cttgataaaa
     1741 gaaattatct ctaaaatata tccaagcttg gtatttatct tctttataat aagataagtt
     1801 tgagggatag tctattttat ttctatcttc ttcatcgcaa taagaaccaa agtctatata
     1861 tcctgctaag aatagttgtc ctaagatgtt tatatattca cttatatagg taggcttggg
     1921 cgtatttaaa gagaaagggt tgctaagatt gtcaatgtta taagtatcca ataaaattaa
     1981 attattacat tcccatcttg atgtgcccat gctttttccg caaataaata aaatataata
     2041 ctcaaataac tctaaaatat ccatttttgt tatcaggcaa gaagatataa ttaaagaggg
     2101 attattgtag acattttctt ttaattgtgt tttcttccat aagctataat ctatatcagg
     2161 aaaaaatata gcacattttt cttctttatt ccatttgata tatttatact tcatttttca
     2221 ccccattttc tatatgtatt gttctaagat ttcctcttgg tttgttagaa tttatctcta
     2281 tggtctcgcc tccactttta gaagcttttt tatattgtat aatagtttga tcatctaatt
     2341 ttctcatgta aattgattgt ccgtattttt tatctacttt atttctaac tctttagaat
     2401 ttttagttaa ctttttccat aatttgtcca attcttcttt gttttcaact attcttctgc
     2461 atttatctcg gacatttttt ggcagtttat ccactatctg caacacttca tcccttgtca
     2521 atgacttttt cttaataata ttttttgcat ttttaggatt tttcactgca ccaagcattg
     2581 ctaaggtatc accgataatg ctgacatttt caagtgaatt tgaaatatct tccactatac
     2641 cttctaatgg ggtaaataaa ggctttatag ctccgcttga tgatttttcc cattctttaa
     2701 attcatcaaa tactatctca ataaactctc cactgccatc ttgtgtttgt attcttataa
     2761 cattatcagg gatgagttct tgagcattgt tatagttttc attgatgtga gcttgtgtgg
     2821 tatttgtatc atcttttata ttatcactta taagcatatc tttatcattt gcattaaaat
     2881 aatctttagt ataaacatta aagcaacaca cattttcatt atcaagccgc tcatctaaac
     2941 cttttgcaat ttcaaaatca atcttttgca ttttagcagg agttaaaaaa actccagttc
     3001 caaaacccca aaaaccatca ttaaactcat taggctcttt agcttcatac catttttgat
```

```
     3061 ctatcttgtc ccattctttg tttttaaaat attcttgtct taattcttta tctattttat
     3121 ctaactcatc aggacttttg tctttggtaa gtcctaaaaa ggcgtgggta aaatcttctc
     3181 tttaccaata acactgattt ttaaaatcca aaaaaacatt ctaccaatgg atcaatataa
     3241 attgttacat aatacttcat ttataaaatc ctaatgataa gttccaattg catgaagtaa
     3301 gagatatgag caaatataac ccatagcaaa caacaaaacg ctaggtataa gttttctttt
     3361 gaaaatggat attaagaaaa atactcccat gccacaacaa ccaacaataa acaagcttaa
     3421 actagaaaaa taaaatgcca aaaatataaa tactgcagat aaaaccaaag gaatggcatt
     3481 gtaaatatat aatagattaa acattataca agaatatata ataataaata attccattgc
     3541 atttctatca aatattaata aaaataaatt cttatcatat ttaccataac taatgtaaat
     3601 gtagtgaaaa gttagaataa tataagtcac caaagcaaga atcaaactac acacaaacca
     3661 agcaatttgc ccactcgata tattgtctat tcctttactt tctagttttg ctttgagttt
     3721 agaataaata ggaatttgca aatttactcc taatataaca agttattcta ttataaaata
     3781 atttatttaa aaatttgatt gtttcagtaa gaaaaaattt cttgaaatca ttattaaatt
     3841 cacaaggctc tttagcttca tttattttat ttacttattc taaaagagga aaaaattgga
     3901 taatgtcatt aaaagatagg gctatgtctt gaaccgagtc aataaaatta tcagtttccg
     3961 ttataactta aacttacaag ctcatttaaa ataacgatag tttcattttc ttgtaatata
     4021 taacccttta aatcaaaata aaaataacta gcttgtgcaa gctcggcata atctcttagc
     4081 ttatcgattt tttctttcaa aattatcctt tatttaaata atgataaaaa aatatataat
     4141 gtaacataaa aactattatg ataataaaac atgtaataca atatcaaagc tataggataa
     4201 taaagtaaca atagcaatgc tatatacaaa aactttattt cacaaagaga aaaaaagcga
     4261 taaagcatta atagaattaa agggaaaata atccaaccta gcattacaaa aaattcatac
     4321 actctatttg gattatgccc tacagcagga gaaatagcaa atccacttat acaaaatact
     4381 acccaaacaa tataaaactt acgaaataca cccatttatt ttccttttta atatttttag
     4441 taaattttct actttttaa aaattaaaat aaaaataact agcccatgca agctcggcat
     4501 aatctcttag cttattgatt ttttctttca aaattatcct ttatttaaat aatgataaaa
     4561 aaatatataa tgtaacataa aaactattat gataataaaa catgtaatac aatatcaaag
     4621 ctataggata atacaataat atc
//
LOCUS       IKNDEOKD_4              1042 bp    DNA     linear       12-APR-2021
DEFINITION  Campylobacter species strain strain.
ACCESSION
VERSION
KEYWORDS    .
SOURCE      Campylobacter species
  ORGANISM  Campylobacter species
            Unclassified.
COMMENT     Annotated using prokka 1.14.6 from
            https://github.com/tseemann/prokka.
FEATURES             Location/Qualifiers
     source          1..1042
                     /organism="Campylobacter species"
                     /mol_type="genomic DNA"
                     /strain="strain"
     gene            77..589
                     /locus_tag="IKNDEOKD_00056"
     CDS             77..589
                     /locus_tag="IKNDEOKD_00056"
                     /inference="ab initio prediction:Prodigal:002006"
                     /inference="similar to AA sequence:RefSeq:AOH52017.1"
                     /codon_start=1
                     /transl_table=11
                     /product="ankyrin domain protein"
                     /protein_id="Prokka:IKNDEOKD_00056"
                     /translation="MIEYFFKKGCTFETYNEKHGSTPLHEWILCGEEVKYLEEALKLG
```

```
                     ANPNMRRIKTENKFSFSGAGETLLHRAAGSKEKPIGACVEVLIKYGADVNAVNCGICD
                     IEDGKIEYYDPATPLDEANTYDISKNIKILKKAGAKTWEELVEEYNIDTSLERIEQIK
                     MYEERRKDKK"
     gene            601..894
                     /locus_tag="IKNDEOKD_00057"
     CDS             601..894
                     /locus_tag="IKNDEOKD_00057"
                     /inference="ab initio prediction:Prodigal:002006"
                     /codon_start=1
                     /transl_table=11
                     /product="hypothetical protein"
                     /protein_id="Prokka:IKNDEOKD_00057"
                     /translation="MDFNTDILESLDNFKAFLDTKPSKELLKAVKNYIDDFMEGAYDN
                     LDPENYEVAFEEDTGIPYDEADEDEFEDWFIKNVLYHDDLSEIYKILKSLVKD"
ORIGIN
        1 acttaaagct gatgaagtac actttggata caatgcactc agtagatata tcgatagaga
       61 tggaaaaaat aaagaagtga tagagtattt ttttaaaaaa ggatgcacat ttgaaactta
      121 taatgaaaaa catggcagca ctcctttgca tgaatggata ctatgtggcg aagaagtgaa
      181 gtatttagaa gaagctttaa aactaggagc aaatcctaat atgagaagaa ttaaaacaga
      241 aaataaattt agctttagtg gtgcaggaga aacccttttg catagagctg caggaagtaa
      301 agaaaagcct ataggagctt gcgtggaagt gctaatcaaa tatggagctg atgtaaatgc
      361 agttaattgt ggtatatgtg atattgaaga tgggaaaatt gaatattatg atcctgctac
      421 cccattagat gaggccaaca cctatgatat aagcaaaaat ataaaaatct taaaaaaagc
      481 aggagctaag acttgggaag aattggttga agaatacaat atcgatacaa gcttagaaag
      541 aatagaacaa attaaaatgt atgaagaaag aagaaaagat aaaaaataaa aggaaaaatt
      601 atggatttta acacagatat tttagaaagc ttagataatt tcaaagcatt tttagacaca
      661 aaacctagca aagaactttt aaaggcagta aaaaattata tagatgattt tatggaaggt
      721 gcgtatgata atttagatcc agaaaactat gaagttgctt ttgaagaaga tactggcata
      781 ccttatgatg aagcagatga ggatgaattt gaggattggt ttattaaaaa tgtgctttat
      841 catgatgatt taagtgagat ttataaaata ctaaaatcac ttgttaaaga ttaaaaacta
      901 aatttataaa gaaagaaagg aaaaacaatg aaaacattag aagaattaat agctttagaa
      961 gaaaaatatc taaaagaatt atgtttagaa ttttatgatc ttgtagaggc taacaaactt
     1021 gaagaagtta aagaattttt aa
//


>>BfR-CA-15848_min_cov_30 putative plasmid location

LOCUS       IHOPMAOI_1             21417 bp    DNA     linear       12-APR-2021
DEFINITION  Campylobacter species strain strain.
ACCESSION
VERSION
KEYWORDS    .
SOURCE      Campylobacter species
  ORGANISM  Campylobacter species
            Unclassified.
COMMENT     Annotated using prokka 1.14.6 from
            https://github.com/tseemann/prokka.
FEATURES             Location/Qualifiers
     source          1..21417
                     /organism="Campylobacter species"
                     /mol_type="genomic DNA"
                     /strain="strain"
     gene            complement(124..432)
```

```
                     /locus_tag="IHOPMAOI_00001"
     CDS             complement(124..432)
                     /locus_tag="IHOPMAOI_00001"
                     /inference="ab initio prediction:Prodigal:002006"
                     /codon_start=1
                     /transl_table=11
                     /product="hypothetical protein"
                     /protein_id="Prokka:IHOPMAOI_00001"
                     /translation="MKNSKGEDIVKPKNTNTAYACAIQARFEQNKIVKIEPKYCISLI
                     NTCFDSKEITLDNDISRVGLNDALSKRTIDFVNRFKLLKHQPNTTNCLMKKTDKIKRN
                     "
     gene            complement(526..624)
                     /locus_tag="IHOPMAOI_00002"
     CDS             complement(526..624)
                     /locus_tag="IHOPMAOI_00002"
                     /inference="ab initio prediction:Prodigal:002006"
                     /codon_start=1
                     /transl_table=11
                     /product="hypothetical protein"
                     /protein_id="Prokka:IHOPMAOI_00002"
                     /translation="MSKNIKTQQAKLDLITKFLDYANIADASYAML"
     gene            complement(669..1271)
                     /locus_tag="IHOPMAOI_00003"
     CDS             complement(669..1271)
                     /locus_tag="IHOPMAOI_00003"
                     /inference="ab initio prediction:Prodigal:002006"
                     /codon_start=1
                     /transl_table=11
                     /product="hypothetical protein"
                     /protein_id="Prokka:IHOPMAOI_00003"
                     /translation="MLEFFVNLLIFILIFFFFVFIFSIILYFIFKIFYKLKNKTKTIN
                     FKTIFKKFTLFLLPVYLLLFLVGGCSYKYMDPQYYEFRSLCKDIDNKVIIYNKAYWEI
                     FSNREKGNTMHDEKGEFFFNQKINKKIYFDFKKSESINVLQKNKFTLTEVTFEDYYDG
                     IHYSTHLSYIYNDYGIFLGGDEGAGFYFRYHKRLYCEDVR"
     gene            complement(1291..1608)
                     /locus_tag="IHOPMAOI_00004"
     CDS             complement(1291..1608)
                     /locus_tag="IHOPMAOI_00004"
                     /inference="ab initio prediction:Prodigal:002006"
                     /inference="similar to AA sequence:RefSeq:CKG86955.1"
                     /codon_start=1
                     /transl_table=11
                     /product="methyltransferase small"
                     /protein_id="Prokka:IHOPMAOI_00004"
                     /translation="MDNKQQINKLRDMAELAQASYGYFHYVDNKFDIKDEDKIVTFED
                     VLDITYKNSKIIDEREFKIGTLKGDFSPLQAKQFFSRYDLLKHCPNTNSGFSATLSYE
                     KNR"
     gene            complement(1601..2029)
                     /locus_tag="IHOPMAOI_00005"
     CDS             complement(1601..2029)
                     /locus_tag="IHOPMAOI_00005"
                     /inference="ab initio prediction:Prodigal:002006"
                     /codon_start=1
                     /transl_table=11
```

```
                     /product="hypothetical protein"
                     /protein_id="Prokka:IHOPMAOI_00005"
                     /translation="MKKYKKILVLLSPIILIILFYTPFIYPSYWEFRGLCKLNNYPKS
                     EEKYNTILAYFDKSLDGSIGKNGYAKIGYSNRIDLGVYIYYKNPNNKTLIFKNINKIY
                     FRPIWKNYAPNIYGNEGNMDFRLKFDGEIDCRSLLGELDG"
     gene            complement(2026..2130)
                     /locus_tag="IHOPMAOI_00006"
     CDS             complement(2026..2130)
                     /locus_tag="IHOPMAOI_00006"
                     /inference="ab initio prediction:Prodigal:002006"
                     /codon_start=1
                     /transl_table=11
                     /product="hypothetical protein"
                     /protein_id="Prokka:IHOPMAOI_00006"
                     /translation="MQNIKKQIQTLKDNSELAWAAYGYFHLATSKAIE"
     gene            complement(2117..2737)
                     /locus_tag="IHOPMAOI_00007"
     CDS             complement(2117..2737)
                     /locus_tag="IHOPMAOI_00007"
                     /inference="ab initio prediction:Prodigal:002006"
                     /codon_start=1
                     /transl_table=11
                     /product="hypothetical protein"
                     /protein_id="Prokka:IHOPMAOI_00007"
                     /translation="MTYILLLIIISTILSYLIFKCIYRIIFKSKKSVSKFLVFLGSIG
                     LIIFYYTPYSYYLEPSFYKFKEICKLDPEIYQANSGKLDEEYYNKVLRYFDTDLDNMS
                     KSYKKELGVTNDKKYSRYWFETWIDGRTEISFEIWFKSNIAIKDNIDFSIKPFFYASW
                     RDLRPFPSGNEGTGVYLSGDRLSCFEILSKSEYKKFKEWKENNAKY"
     gene            complement(2958..3731)
                     /locus_tag="IHOPMAOI_00008"
     CDS             complement(2958..3731)
                     /locus_tag="IHOPMAOI_00008"
                     /inference="ab initio prediction:Prodigal:002006"
                     /inference="similar to AA sequence:RefSeq:AJK71138.1"
                     /codon_start=1
                     /transl_table=11
                     /product="glutamyl-tRNA amidotransferase"
                     /protein_id="Prokka:IHOPMAOI_00008"
                     /translation="MKEINEEKKALSLLLDSNFDGLKNNKIIDYSLELLLLSYRLSKI
                     SSMDTSNINQLRETLINKILDITAKLSMCKEYDEKEIIKFKYCLCVFIDESLMKNELF
                     INFWAHNTLTVRLFDETLGGNNFYDIASSWINNPFKFKDFLEFIYACLILGYKGKYNE
                     AKDRDEKIIHFCNNIATSLRPVYKTEEELAFNKAYKIGLEENIWQKFIRLYFKKLIII
                     VPVLIVLGVLSFAIFNLETNNLKVDNNISVLIKNLTHIE"
     gene            complement(3728..4309)
                     /locus_tag="IHOPMAOI_00009"
     CDS             complement(3728..4309)
                     /locus_tag="IHOPMAOI_00009"
                     /inference="ab initio prediction:Prodigal:002006"
                     /inference="similar to AA sequence:RefSeq:AJK71139.1"
                     /codon_start=1
                     /transl_table=11
                     /product="type VI secretion protein"
                     /protein_id="Prokka:IHOPMAOI_00009"
                     /translation="MSMSIKIHPEFLYEKFLEFQGELAAFSNEESFLDFIPYKHDNLY
```

```
                     NTFLNMMNNLRLLFSKITSPKYFIAKTSINGNGFYDFIFDNSGILEDAEIYFAIRADV
                     TTEYLLNNFKIQSKIHTQSKIKNIVATQLEGLNVDQVPNIPSSIPYLNGYVYYKIDKR
                     DQLFKDFKNESIISMYLTNNIKNPDIIMWAVFQ"
     gene            4319..6517
                     /locus_tag="IHOPMAOI_00010"
     CDS             4319..6517
                     /locus_tag="IHOPMAOI_00010"
                     /inference="ab initio prediction:Prodigal:002006"
                     /inference="similar to AA sequence:RefSeq:AVS37497.1"
                     /codon_start=1
                     /transl_table=11
                     /product="type VI secretion protein VgrG"
                     /protein_id="Prokka:IHOPMAOI_00010"
                     /translation="MIKQTLGFYQDILHKEIDYSHIHFNYEEQELISQYNESDLEFIT
                     RLAHNNGIFFYEDENTIYFCDVYKNVKNKEIEYNPNVNNILNQACISSIYKEQSLRTN
                     AFTHSSINANTPLNLLSLHSSKVPYEQELNNKAYYNEHFYESEYSFTRSIDLKTKPTL
                     KEKRALVLNESLLAKSNIYHLSLGDFITLNYKEFNLSGLSDDEDTKSQDKASLLKDFI
                     IIANTQILIDDALLANSINTNDHLNLKDLNLSKSYSNTLTLLKKNIIFTPSFKAKPKA
                     PHNTQGIVIGESKDIESERNTIYTDEYGRVKVRINLYANQEELDNNLLSANIKTNTHE
                     NTNNTDSKDNTNFTNSSNHLPSNAYKSYHHTPFLRVATSIASNHSGFFHTPRVGDEVI
                     VSFLDDDIDKPYVSSSLYNGTTTMPQYNYPRYKREIATNALSYLSVAPAMIDKGLDTL
                     KLQENTDYELSSINNHYLTLANSTIGVDNADARARNEITLKNDKDKEEIYILAQKDYK
                     EEIGNNYEQTIKNNKTSEVGALYTEFITLGHMQNIIGFKNVNVGAEYLENTLLSKDTN
                     VGLNNTLNVGISNEVNIGQNHEEKIGNDKRVIINNNLEQDIKNDFIQRIGHNKNETIK
                     GSYVLQTNQSIKFYSKQDLSIETNDCFKAEADTFVSFKAKKNCSFTADEVYTMANKES
                     FLTAQKQIVSRVGNTTITQTKDKIILQVGTTQVIIDSKGLRVQGGDLRAD"
     gene            6584..7465
                     /locus_tag="IHOPMAOI_00011"
     CDS             6584..7465
                     /locus_tag="IHOPMAOI_00011"
                     /inference="ab initio prediction:Prodigal:002006"
                     /inference="similar to AA sequence:RefSeq:AOH52011.1"
                     /codon_start=1
                     /transl_table=11
                     /product="ankyrin domain protein"
                     /protein_id="Prokka:IHOPMAOI_00011"
                     /translation="MKTLEELINFEENSSEEYLEELCLEFYDLVEANKLEEVKEFLKD
                     YPVPEIFFEKCYTPYWDRENKRAIIDTAITLACAGLAYDKSKSFEMMEYFESLGLKAD
                     EVHFGYNALSRYIDRDGKNKEVIEYFFKKGCTFETYNEESGDTPLHVWILLNHANYLE
                     EALKLGANPNMRSIKTENEFSFSGAGETLLHQAAESDEKPIGACVEVLIKYGADVNAA
                     NCRICDIEDGKIEYYDPATPLDRANRARNVSKNIKILKKAGAKTWEELVEEYNIDTSL
                     EEPEQIKMYEERRKDKK"
     gene            7477..8346
                     /locus_tag="IHOPMAOI_00012"
     CDS             7477..8346
                     /locus_tag="IHOPMAOI_00012"
                     /inference="ab initio prediction:Prodigal:002006"
                     /inference="similar to AA sequence:RefSeq:AOH52011.1"
                     /codon_start=1
                     /transl_table=11
                     /product="ankyrin domain protein"
                     /protein_id="Prokka:IHOPMAOI_00012"
                     /translation="MKTLEELIALEEKYLKELCLEFYDLVEANKLEEVKEFLKDYPVP
                     EIFFEKCYKAYYDYKERAIIDTAITLACAGLAYDKSKSFEMMEYFESLGLKADEVHFG
```

YNALSRYIDRDGKNKEVIEYFFKKGCTFETYNEESGDTPLHEWILCGEEVKYLEEALK
LGANPNMRSIKTENKFSFSGAGETLLHRAAESDEKPIGACVEVLIKYGADVNAANCRI
SDIEDGKISYYKPATPLDRANRTRNVSKNIKILKKAGAKTWEELVEEYNIDTSLERIE
QIKMYEERRKDKK"

gene 8358..8651
/locus_tag="IHOPMAOI_00013"
CDS 8358..8651
/locus_tag="IHOPMAOI_00013"
/inference="ab initio prediction:Prodigal:002006"
/codon_start=1
/transl_table=11
/product="hypothetical protein"
/protein_id="Prokka:IHOPMAOI_00013"
/translation="MDFNTDILESLDNFKAFLDTKPSKELLKAVKNYIDDFMEGAYDN
LDPENYEVAFEEDTGIPYDEADEDEFEDWFIKNVLYHDDLSEIYKILKSLVKD"

gene 8681..9559
/locus_tag="IHOPMAOI_00014"
CDS 8681..9559
/locus_tag="IHOPMAOI_00014"
/inference="ab initio prediction:Prodigal:002006"
/inference="similar to AA sequence:RefSeq:AOH52017.1"
/codon_start=1
/transl_table=11
/product="ankyrin domain protein"
/protein_id="Prokka:IHOPMAOI_00014"
/translation="MKTLEELINFEENSSEEYLEELCLEFYDLVEANKLEEVKEFLKD
YPVPEIFFEKCYTPYWDRENKRAIIDTAITLACAGLAYDKSKSFEMMEYFESLGLKAD
EVHFGYNALSRYIDRDGKNKEVIEYFFKKGCTFETYNEESGSTPLHVWILLNHANYLE
EALKLGANPNMRRIKTENEFSFSGAGETLLHRAAGSKEKPIGACVEVLIKYGADVNAV
NCGICDIEDGKIEYYDPATPLDEANTYDINKNIKILKKAGAKTWEELVEEYNIDTSLE
EPEQIKMYEERRKDKK"

gene 9596..11527
/locus_tag="IHOPMAOI_00015"
CDS 9596..11527
/locus_tag="IHOPMAOI_00015"
/inference="ab initio prediction:Prodigal:002006"
/codon_start=1
/transl_table=11
/product="hypothetical protein"
/protein_id="Prokka:IHOPMAOI_00015"
/translation="MEFNDINFKIKEFESVDIISDYSLAFYLPKEEHMIGEKDQDGKI
RINKSNSLKYIEKINEEKEKEIEKLKLQSNEKINEEFDKLKDGALPTKEYKRKNYEYD
VFYDVYTSKDNINKCIKKIPIKNGVGTLKSNFLRRFYENSVKGKYNCKWCLIPRLIRN
KGESSFDPVPILNSSFVISELSNIKQKTLTFQRTIANVEHPSHFALQSPKEFKSMLEN
EQSFASYGGKEKQIEDLLKTAQHKLETIKKANGISKKTKEDLQEKQRKEIKKCKELKN
SKKKISPKLIGDTGEYVASLLFGKRSTRYLSNQRKLDANFNIYSFNHTIPDFLVSING
IPSLVEVKNVQVQSLTEQISFELKLAREYELDYIFVCNHYTHLTENVAGSALTREEHE
ELARDDKGKVGEFNKETKRLKKAKVAEVLNEKDDLANEEFIKSVSDGNKYLKEREKFY
KVASDVYIKNPIYKMGFEKKLQNQVVDINIVKDFLKDFAKRLGIDEKVLPVIGYNPQR
GDSGTYDYTVNTLDLNPKASYVDFIDTIIHEFRHFYIYCIKNNSENSIEKLLYLDMVQ
FYISWNFYKIFNSYDKKCLIYDNEDETKKCLKNRTYYGSWKFLNPTLDLLPSPLYYLQ
SSELDSRIMAYYFRKEIKL"

gene 11527..12792
/locus_tag="IHOPMAOI_00016"

```
CDS             11527..12792
                /locus_tag="IHOPMAOI_00016"
                /inference="ab initio prediction:Prodigal:002006"
                /inference="similar to AA sequence:RefSeq:CKG88389.1"
                /codon_start=1
                /transl_table=11
                /product="hypothetical protein"
                /protein_id="Prokka:IHOPMAOI_00016"
                /translation="MTPPSLPDVEKHEDFLQTRKEPYAIYLAINTNIKSYNNICPSEK
                YFWKFDNMNELDKWYNPKFGIYLGKIVFDKKDNKLIPKYIAAKFENLEEEVKKIKNPL
                WLANKNPNYIEPKFYDGMSGGYYFESPNNLEYQCKIEKDTQVLSQEQIISYVKELYSK
                NTIIIKNYIDAINKDYGIKPFVFSDEIYDELGEVGILTKEQANNFKDKSYIKKDPILL
                AMLDYLAKQNKKDEDYLITFDDEYFYTYLVWSLKDFLLELSYGLFQDETELLFNPAAY
                MDDTKIYYQNLNEEINKRYEKILLNMGFEGENGYFKDYYDYGFGNNGIFEFSIYDYFA
                YDEIGVQPIQQSPYVPPKSPFDSPNFVYSDGNYHGDAKLVPSALGKYYFELLYQKEIY
                IELLRPYYPSIKDLPEGWDNKMLEKANLK"
gene            13002..13694
                /locus_tag="IHOPMAOI_00017"
CDS             13002..13694
                /locus_tag="IHOPMAOI_00017"
                /inference="ab initio prediction:Prodigal:002006"
                /inference="similar to AA sequence:RefSeq:CKG88360.1"
                /codon_start=1
                /transl_table=11
                /product="hypothetical protein"
                /protein_id="Prokka:IHOPMAOI_00017"
                /translation="MFFFKNYPQFAGKIAYIYYRFDLINKEFLTSVSEKDKEFYLQDR
                DGKYISTIINKILPLEYKNGKSFKQFHTELLNNKNRDLKECKDFLKALTQTLCKEFNL
                PLENVVFYSQKPSGSLSFGAYEENKVRLNQYILKAYFKEFAKTVFHECRHFYIEKYYN
                HNMDALGRYVFYSEKAYINEKIIFDKLSRICDPGENYLVGCNIGAIQDAYEIQPNERD
                PRYVETLIGRSL"
gene            13707..14051
                /locus_tag="IHOPMAOI_00018"
CDS             13707..14051
                /locus_tag="IHOPMAOI_00018"
                /inference="ab initio prediction:Prodigal:002006"
                /codon_start=1
                /transl_table=11
                /product="hypothetical protein"
                /protein_id="Prokka:IHOPMAOI_00018"
                /translation="MKTIKIPLKDYISNIREDKTIYLGLQSDMSIKGLDEEFLKKEYN
                LKRNFKFETSIYLGKIIIKEKNIIFKAFNIDENCFEGLKELFFKNSPYKIVDNLELKK
                YFLKLYKDNIEF"
gene            14850..15125
                /locus_tag="IHOPMAOI_00019"
CDS             14850..15125
                /locus_tag="IHOPMAOI_00019"
                /EC_number="3.6.1.-"
                /inference="ab initio prediction:Prodigal:002006"
                /inference="similar to AA sequence:RefSeq:ABS44566.1"
                /codon_start=1
                /transl_table=11
                /product="F-box only protein 18"
                /protein_id="Prokka:IHOPMAOI_00019"
```

```
                     /translation="MDEAQDINACVIDIVLNQKQAKKVFIGDTFQSIYKFRGAVNSLE
                     ILASMPKSHTLYLTQSFRCPQNIAFITNSYLKILNAPRDFKGTKKIK"
     gene            15085..15723
                     /locus_tag="IHOPMAOI_00020"
     CDS             15085..15723
                     /locus_tag="IHOPMAOI_00020"
                     /EC_number="3.6.1.-"
                     /inference="ab initio prediction:Prodigal:002006"
                     /inference="similar to AA sequence:RefSeq:ABS44566.1"
                     /codon_start=1
                     /transl_table=11
                     /product="F-box only protein 18"
                     /protein_id="Prokka:IHOPMAOI_00020"
                     /translation="MLREISKEQKKSSNENVKNEAKAIVCRTNAKLFDIAVENLDKKL
                     FFVGGINSYSFDELLDIQNLLFKKHEYIKNQFIVKFADLKELLEYINETKEVDLKQKI
                     FVLFKYVHGDIIKLIKAIEKSSVKKQEQADLILSTGHKSKGLEWNNVEIIDDFLNIKQ
                     ELEERDQITIAKEELNLLYVAITRAKNSLSISKDYLLEKEFIQENLERIIIE"
     gene            15720..15932
                     /locus_tag="IHOPMAOI_00021"
     CDS             15720..15932
                     /locus_tag="IHOPMAOI_00021"
                     /inference="ab initio prediction:Prodigal:002006"
                     /inference="similar to AA sequence:RefSeq:AVL47291.1"
                     /codon_start=1
                     /transl_table=11
                     /product="helix-turn-helix domain-containing protein"
                     /protein_id="Prokka:IHOPMAOI_00021"
                     /translation="MKIIKKLQKDGSLKKQVYYSVQEVSQMHSVTHVTVRLWISRGIL
                     KGMKLGKGFYMSKETILDFQKTDGLS"
     gene            16701..17621
                     /locus_tag="IHOPMAOI_00022"
     CDS             16701..17621
                     /locus_tag="IHOPMAOI_00022"
                     /inference="ab initio prediction:Prodigal:002006"
                     /inference="similar to AA sequence:RefSeq:ADC28003.1"
                     /note="conserved hypothetical protein"
                     /codon_start=1
                     /transl_table=11
                     /product="hypothetical protein"
                     /protein_id="Prokka:IHOPMAOI_00022"
                     /translation="MEAKGIAIFPTNVAFSKKTNAFFTELEDQFLKEGVGNKSAIKLD
                     FEYYILYCQFLQIDKESLFFGRNYELVFCFLLYYRALLNKNNTQKIVAKIINVIRCQD
                     RLPEKVHYREKIICMDNQHRDLSQTLSSCIAEFYNSIDLYNTFRKNIGFENRKKIAIE
                     MLNRASEHYGFFGLSRGGITYKEEKISCVPIQALDEFHHGMSHLCAVFLKEDGRYTNL
                     ERAIPHFKRGMMEVYKTIIRDFMILNKDALGVKQEALKIRELEYQTIGKPKTVTKNPT
                     TNLTNKKEEKVIPGYKELCEKIVSQIKNAK"
     gene            complement(17721..18749)
                     /gene="flgS"
                     /locus_tag="IHOPMAOI_00023"
     CDS             complement(17721..18749)
                     /gene="flgS"
                     /locus_tag="IHOPMAOI_00023"
                     /inference="ab initio prediction:Prodigal:002006"
                     /inference="similar to AA sequence:RefSeq:CAL34921.1"
```

```
                     /codon_start=1
                     /transl_table=11
                     /product="signal transduction histidine kinase"
                     /protein_id="Prokka:IHOPMAOI_00023"
                     /translation="MNENILKSLDSSEKETLQKGLQSLIEQTYIVENEYKILNENYNS
                     LRAMVDEIIEVLPSALWILDKEKNIILQNQEALKKPKLLDIISLEKNRDELEFENRFY
                     AVKIIAHNEKTIVSATDISDEKRNERLASMGSVAAHLAHEIRNPIGSISLLASTLFSR
                     SELKNKHIVLEIQKAIARVERIVNSTLLFTKGVHINASEFNLLELTEECESAINSYNY
                     SSQIDFEINFLDMKIYGDKALLSLVLQNLIYNAIDAIEESECENPKIKIQASCDEKNL
                     YIRVYDNGCEIKDENLVFEAFKTTKLKGNGLGLSLSKEIINAHKGELKFEIWPKNFYF
                     VLPLHYNS"
gene                 complement(18746..19567)
                     /locus_tag="IHOPMAOI_00024"
CDS                  complement(18746..19567)
                     /locus_tag="IHOPMAOI_00024"
                     /inference="ab initio prediction:Prodigal:002006"
                     /inference="similar to AA sequence:RefSeq:CAL34920.1"
                     /note="conserved hypothetical protein Cj0792"
                     /codon_start=1
                     /transl_table=11
                     /product="hypothetical protein"
                     /protein_id="Prokka:IHOPMAOI_00024"
                     /translation="MAKIFDFYSVFEGFESLDSLNFEEDVFHNIEYLLLKNYLDIKDC
                     FKLDETASYALSLLAKNNRKRFSINRKIQHFKALSTLKYLLRAGIIKLEYSKEAKRVR
                     DKRQKLKKELRSYVIQDKIIFANQFTRFFFYFLKPNEKLILQNRYEEVLTLIKEKFEL
                     YQSFCFEQLSRELLEKKFQVSGVQSYWDKNLELDLYYKDDKISFIGEVKFKNKKICRN
                     ILNLLELKAKSLKLEPNNYIIISKNGFSKEFYKICKQDLLLLDLNDFKILLEENK"
gene                 19671..20939
                     /locus_tag="IHOPMAOI_00025"
CDS                  19671..20939
                     /locus_tag="IHOPMAOI_00025"
                     /inference="ab initio prediction:Prodigal:002006"
                     /inference="similar to AA sequence:RefSeq:CAL34919.1"
                     /codon_start=1
                     /transl_table=11
                     /product="putative aminotransferase"
                     /protein_id="Prokka:IHOPMAOI_00025"
                     /translation="MQISDLKKEIILKKGTLHFDFTASALALKCVEKEIAKILPSYAN
                     THSDSSLNSFKTQQIYEQARKDIKTSLNLSDEFALIACGSGSSSAIKKFQELLGIYIP
                     PLVKEKYFEKVDKSTLPLVIVGPYEHHSNELSFREGLCECIRIPLDKKGNIDFEILEK
                     VLKENQHRKIIASFSLASNVTGILSDYKRISKLVRKFKGIVAFDASSFIPYKNIACKY
                     YDALFISSHKLIGGIGGSGLLAIKKELCGNKPSFAAGGTVGYVSRTSQCYLCNEEALE
                     EGGTPGILQLIRASLAFKIKDEIGIEEIEKKEEILKEYFFEKLKTIPHLILYANNIKT
                     RLPIFAFNIKNISPFDLAYELSKKYHIETRAGCACAGPYGHDLLGLKDNEELKTKPGW
                     LRISLHYTHEKEDIDYFFEALQKSIIKLKF"
gene                 complement(20943..21019)
                     /locus_tag="IHOPMAOI_00026"
tRNA                 complement(20943..21019)
                     /locus_tag="IHOPMAOI_00026"
                     /product="tRNA-Asp"
                     /inference="COORDINATES:profile:Aragorn:001002"
                     /note="tRNA-Asp(gtc)"
gene                 complement(21041..21116)
                     /locus_tag="IHOPMAOI_00027"
```

```
     tRNA            complement(21041..21116)
                     /locus_tag="IHOPMAOI_00027"
                     /product="tRNA-Val"
                     /inference="COORDINATES:profile:Aragorn:001002"
                     /note="tRNA-Val(tac)"
     gene            complement(21125..21200)
                     /locus_tag="IHOPMAOI_00028"
     tRNA            complement(21125..21200)
                     /locus_tag="IHOPMAOI_00028"
                     /product="tRNA-Glu"
                     /inference="COORDINATES:profile:Aragorn:001002"
                     /note="tRNA-Glu(ctc)"
     gene            complement(21214..21289)
                     /locus_tag="IHOPMAOI_00029"
     tRNA            complement(21214..21289)
                     /locus_tag="IHOPMAOI_00029"
                     /product="tRNA-Lys"
                     /inference="COORDINATES:profile:Aragorn:001002"
                     /note="tRNA-Lys(ttt)"
     gene            complement(21293..21369)
                     /locus_tag="IHOPMAOI_00030"
     tRNA            complement(21293..21369)
                     /locus_tag="IHOPMAOI_00030"
                     /product="tRNA-Asp"
                     /inference="COORDINATES:profile:Aragorn:001002"
                     /note="tRNA-Asp(gtc)"
ORIGIN
        1 tgttttattt tttaatttat aaaaaatttt aaagataaaa tataaaataa tagaaaatat
       61 aaaaacaaaa aagaaaaata taagaataaa tattaaaaga ttaacaaaga attctagcat
      121 ttattaattc ctcttaattt tatctgtttt tttcataaga caatttgtag tgttgggctg
      181 atgttttaaa agtttaaatc tatttacaaa atcaatagtt cttttactca gtgcatcatt
      241 caatcctacc ctactaatat cattatccaa tgttatttct ttactatcaa aacaagtatt
      301 tataagagaa atacaatatt taggttctat tttaactatt ttattttgtt caaaacgagc
      361 ttgtatagca caagcataag ctgtgtttgt atttttaggt tttacaatat cttctccttt
      421 actatttttc ataacaatat cttgctttaa tttatcacca aaagtaagtt tatctgcttt
      481 ataaatatta ttctttttat cttgctctat attttcccaa acatattaca acatcgcata
      541 actagcatct gctatattag catagtctaa aaatttagtg attaagtcta atttagcttg
      601 ttgtgtttta atgtttttgc tcatttgtta ttccaaaaat ctaaatctca aattatcttt
      661 ctaatttttt atctaacatc ttcacaatac agtcttttat gatatctaaa atagaatcca
      721 gcaccctcat cacctcccaa aaatatacca taatcattat aaatataaga taagtgtgtt
      781 gaataatgaa taccatcata ataatcttca aatgtaactt ctgttaaagt aaatttattt
      841 ttttgcaaca cattaatgct ttctgatttt ttaaaatcaa aatatatttt tttatttatt
      901 ttttgattaa aaaagaattc tcctttttca tcatgcattg tatttccttt ttctctattg
      961 ctgaatattt cccaataagc tttattgtaa attattactt tattatctat atctttacac
     1021 aagctcctaa actcataata ttgcggatcc atatatttat aagaacatcc tcctactaag
     1081 aataataaaa gatatacagg taaaagaaac aaagtaaatt ttttaaatat tgttttaaaa
     1141 tttatagttt ttgttttatt ttttaattta taaaaaattt taaagataaa atataaaata
     1201 atagaaaata taaaaacaaa aaagaaaaat ataagaataa atattaaaag attaacaaag
     1261 aattctagca tttattaact cctcttaatt ttatctgttt ttttcataag acaaagtagc
     1321 agaaaaacct gaatttgtat taggacaatg ttttaataaa tcatagcgag aaaagaattg
     1381 tttagcttgg agtggggaga agtcgccttt gagagtgccg attttaaatt ctctttcatc
     1441 aataatttta gagtttttat aggtaatatc taaaacatct tcaaaggtaa caattttatc
     1501 ttcatctttt atatcaaatt tattatcaac gtaatgaaaa tatccataac ttgcctgtgc
     1561 aagttctgcc atatccctta gtttattgat ttgttgttta ttatccatct aactctccta
```

1621 ataagcttct gcaatctatt tcaccgtcaa actttaatct aaaatccata ttaccctcat
1681 taccataaat atttggtgca tagtttttcc atattggtct aaaatatatt ttattaatat
1741 ttttaaaaat taatgttttg ttatttggat tcttataata aatatataca cctaaatcta
1801 ttctattact ataacctatt tttgcataac catttttttcc tatactacca tctaaactct
1861 tatcaaaata agctaagatt gtattatatt tttcctcgct ttttgggtaa ttatttaatt
1921 tacataaccc tctaaattcc caataactag gatatataaa aggggtataa aataatataa
1981 ttaaaattat tggtgataat agcactaaaa tctttttata tttttttcatt ctatcgcctt
2041 agatgttgct aagtgaaaat atccataggc tgcccaagca agttctgaat tgtcttttaa
2101 tgtttgtatt tgttttttaa tattttgcat tattttcttt ccattctttg aattttttat
2161 attcactctt gcttaatatt tcaaagcaac tcaatctatc tcctgaaaga tagactcctg
2221 tcccctcatt acctgatgga aatgggcgta aatcacgcca agaagcataa aaaaaaggtt
2281 ttatactaaa gtcaatattg tcttttattg caatattact tttaaaccat atttcaaagc
2341 ttatttcggt tcttccatct atccatgttt caaaccagta tctactatat tttttatcat
2401 tagttacccc tagctctttt ttatatgact ttgacatatt atccaaatcc gtatcaaaat
2461 accttagcac tttattataa tattcctcat ctaatttacc actattggct tgataaatct
2521 ctgggtctag cttacatatt tctttaaact tataaaagct tggctctaaa taataagaat
2581 atggagtata ataaaagata attaaaccta tgcttccaag aaagactaaa aactttgaaa
2641 cactttttttt agatttaaag atgattctat atatacactt aaaaattaga taagaaagaa
2701 tagtagaaat tataataaga agtaatatat atgtcatggg ttatctcctt agactctcta
2761 ttttattaaa attaagatgt aattttgtgg tttagtgtta gcagtataac tttttcttta
2821 tcattaaaat aatagagctt gtttttttcat atatttttat cctttagatt tgcatttgat
2881 ttttattcta gctgtaaaat aatttttatt taattttact cataacaaat aaattgatat
2941 ttaaaaattt aatatcttta ttctatatgt gttaaatttt ttatcaatac ggatatatta
3001 ttatccactt taagattatt ggtttctaaa ttaaaaattg caaagcttaa tactcctaat
3061 acaatcaata ctggcacaat gataataagt tttttaaaat acaatcttat aaatttttgc
3121 caaatatttt cttctaaacc aattttgtat gctttattaa aagctaactc ttcttctgtc
3181 ttataaactg gtcttaaaga tgtggcaata ttattgcaaa aatggattat tttttcatct
3241 ctatccttag cctcattata ttttccttta taaccaagaa tcaaacaagc ataaataaat
3301 tccaaaaaat ctttaaactt aaaaggatta ttaatccaag atgatgcaat atcataaaaa
3361 ttatttccac ctaaggtttc atcaaataat cttacagtta gagtattgtg agcccaaaaa
3421 ttgataaaaa gttcattttt catcaagctt tcatctataa agacacaaag acaatatttg
3481 aatttaataa tttctttttc atcatattct ttacacatgc ttagtttagc ggttatatct
3541 aaaattttat taatcaatgt ttctctaagt tgattaatat tacttgtatc catagagctt
3601 atttttgaaa gtctgtaaga aagcaaaagt aactccaaag aataatcaat aattttattg
3661 ttttttaaac catcaaaatt tgaatcaaga agtaagctta atgccttttt ttcttcgttt
3721 atttctttca ttgaaatacc gcccacatta taatatcagg attttttata ttgttggtca
3781 agtacatgct aataatactt tcatttttaa aatctttaaa cagctgatct cttttatcta
3841 ttttataata aacataacca tttaaataag gaatactaga tggaatatta ggcacttgat
3901 ctacatttaa gccttcaagc tgtgttgcta ctatattttt aattttactt tgcgtatgta
3961 tcttgctttg aattttaaaa ttattaagta gatattctgt agtaacatct gcacgaattg
4021 caaaataaat ttcagcatcc tctaaaattc ccgaattatc aaaaataaaa tcataaaaac
4081 cattgccatt aatactggtc ttagctataa aatatttagg acttgtgatt ttggaaaata
4141 acaatcttaa attattcatc atatttaaaa aagtattata aagattatca tgtttataag
4201 gaataaaatc taaaaaagat tcttcattac taaaagctgc aagctctcct tgaaattcta
4261 aaaatttttc gtataaaaat tcaggatgta tttttatact catactcata tcatagaagt
4321 gattaaacaa acccttggtt tttatcaaga tatcttacat aaagaaatag attattctca
4381 tatacatttt aattatgaag aacaagaact catctctcaa tacaatgaaa gtgatttaga
4441 atttatcaca agattagctc acaataatgg aatctttttt tatgaagatg aaaacactat
4501 ttatttttgt gatgtgtata aaaatgtaaa aaataaagaa attgagtaca atcctaatgt
4561 gaataatatt ttaaatcaag cttgtatttc ttctatctat aaagagcaaa gcttaagaac
4621 taatgctttt acacattcta gtatcaatgc taatactcct ttaaatcttt tatctttaca
4681 ctctagtaaa gttccttatg agcaagaact taacaacaaa gcttattata atgaacattt
4741 ttatgagagt gaatattctt ttacaagaag tattgattta aagactaaac ctactcttaa
4801 agaaaaaaga gccttggtat taaatgaaag cttactagca aaaagtaata tttatcatct

4861 tagtttggga gattttatca ctttaaatta taaggaattt aatttgagtg gattgagtga
4921 tgatgaagat actaaaagtc aagataaagc ttctttgctt aaagacttta ttattattgc
4981 caatactcaa atcttaatag atgatgcttt actagctaat tctatcaata ctaatgatca
5041 tttaaattta aaagatttaa atttaagtaa gtcttattct aatactttaa ctttattaaa
5101 aaagaatatt atctttactc caagttttaa agctaagcct aaagcccctc ataatactca
5161 aggtattgtt ataggagaga gtaaagatat agaaagtgag agaaatacta tttatactga
5221 tgagtatgga agggttaagg taaggattaa tctttatgct aatcaagagg aattagataa
5281 taatctttta agtgcaaaca taaaaaccaa tactcatgaa aatacaaaca atactgattc
5341 taaagataat acaaacttca caaactcttc taatcattta ccaagcaatg cttataaatc
5401 ttatcatcat actccttttt taagagtagc aacaagtata gcaagcaatc attcaggctt
5461 ttttcatact ccaagagtgg gagatgaagt gattgtttct tttttagatg atgatataga
5521 taaaccctat gtgagctcta gtttgtataa tgggactaca acaatgcccc aatataatta
5581 tccaagatac aaaagagaaa tcgctacaaa tgcattgagc tatttaagcg tagctcctgc
5641 tatgattgat aaaggtttag atacattaaa acttcaagaa aatactgatt atgaattaag
5701 ctctataaac aatcattatc ttactttagc aaattctaca ataggagtag ataatgccga
5761 tgcaagagca aggaatgaaa ttaccctaaa aaacgacaaa gacaaagaag aaatttatat
5821 cctagcacaa aaagattata aagaagaaat aggtaataat tacgagcaaa ccataaaaaa
5881 taataaaact tcagaagtgg gagccttata taccgaattt atcactttag gacatatgca
5941 aaatatcata ggttttaaaa atgtcaatgt aggagctgaa tatctagaaa ataccttgct
6001 ttctaaagat actaatgtgg gtttaaataa tactttgaat gtgggaatca gcaatgaagt
6061 caatatcgga caaaaccacg aagaaaagat aggaaacgat aaaagagtaa taatcaataa
6121 caatctagaa caagatatca aaaatgattt tatccaaaga ataggacata ataaaaatga
6181 aaccataaaa ggttcttatg tgcttcaaac caatcaaagt ataaaatttt attccaaaca
6241 agatctaagc atagaaacaa acgattgctt taaagctgaa gctgatactt ttgttagttt
6301 caaagccaag aagaattgct cttttacagc cgatgaagtg tatactatgg caaataaaga
6361 aagtttttta acagcacaaa aacaaattgt ttctagggta ggaaatacaa ctatcacaca
6421 aacaaaagat aagattatac tccaagtagg aacaacacaa gtgattatag atagtaaagg
6481 tttaagagtg caaggaggtg atttaagagc agattaaaag caatttttctt aaaattaaaa
6541 ccaaaatcat aaataaacct aatttacaat aaaaggaaaa acaatgaaaa cattagaaga
6601 attaataaat tttgaagaaa attcttcaga agaatattta gaagaattat gtttagaatt
6661 ttatgatctt gtagaggcta acaaacttga agaagttaaa gaatttttaa aagactatcc
6721 tgtacctgaa atattctttg aaaaatgcta tacaccttat tgggatagag aaaacaaaag
6781 agctattatc gatactgcta tcactttagc ttgtgcagga cttgcttatg ataaaagtaa
6841 aagttttgaa atgatggagt attttgaaag cctaggactt aaagccgatg aagtacactt
6901 tggatacaat gcactcagta gatatatcga tagagatgga aaaaataaag aagtaataga
6961 gtattttttt aaaaaaggat gcacatttga aacttataat gaagaaagtg gcgatactcc
7021 tttgcatgtt tggatattgc taaatcatgc aaattattta gaagaagctt taaaactagg
7081 agcaaatcct aatatgagaa gtattaaaac agaaaatgaa tttagcttta gtggtgcagg
7141 agaaaccctt ttacatcaag ctgcagaatc tgatgagaaa cctataggag cttgtgtgga
7201 agtgctaatc aaatatggag ctgatgtaaa tgcggctaat tgtcgcatat gtgatattga
7261 agatgggaaa attgaatatt atgatcctgc aaccccattg gatagagcca acagagctcg
7321 caatgtgagc aaaaatataa aaatcttaaa aaaagcagga gctaagactt gggaagaatt
7381 ggttgaagaa tacaatatcg atacaagctt agaagaacca gaacaaatta aaatgtatga
7441 agaaagaaga aaagataaaa aataaaagga aaaacaatga aaacattaga agaattaata
7501 gctttagaag aaaaatatct aaaagaatta tgtttagaat tttatgatct tgtagaggct
7561 aacaaacttg aagaagttaa agaattttta aaagactatc ctgtacctga aatattcttt
7621 gaaaaatgct ataaagcata ttatgattat aaagaaagag ctattatcga tactgctatc
7681 actttagctt gtgcaggact tgcttatgat aaaagtaaaa gttttgaaat gatggagtat
7741 tttgaaagcc taggacttaa agccgatgaa gtacactttg gatacaatgc actcagtaga
7801 tatatcgata gagatggaaa aaataaagaa gtaatagagt atttttttaa aaaaggatgc
7861 acatttgaaa cttataatga agaaagtggc gatactcctt tgcatgaatg gatactatgt
7921 ggcgaagaag tgaagtattt agaagaagct ttaaaactag gagcaaatcc taatatgaga
7981 agtattaaaa cagaaaataa atttagcttt agtggtgcag gagaaaccct tttgcataga
8041 gctgcagaat ctgatgagaa acctatagga gcttgcgtgg aagtgctaat caaatatgga

8101 gctgatgtaa atgcggctaa ttgtcgcata agtgatattg aagatggaaa aatcagctat
8161 tataaacctg caaccccatt ggatagagcc aacagaactc gcaatgtgag caaaaatata
8221 aaaatcttaa aaaaagcagg agctaaaact tgggaggaat tggttgaaga atacaatatc
8281 gatacaagct tagaaagaat agaacaaatt aaaatgtatg aagaaagaag aaaagataaa
8341 aaataaaagg aaaaattatg gattttaaca cagatatttt agaaagctta gataatttca
8401 aagcattttt agacacaaaa cctagcaaag aacttttaaa ggcagtaaaa aattatatag
8461 atgattttat ggaaggtgcg tatgataatt tagatccaga aaactatgaa gttgcttttg
8521 aagaagatac tggcatacct tatgatgaag cagatgagga tgaatttgag gattggttta
8581 ttaaaaatgt gctttatcat gatgatttaa gtgagattta taaaatacta aaatcacttg
8641 ttaaagatta aaaactaaat ttacaataaa aggaaaaaca atgaaaacat tagaagaatt
8701 aataaatttt gaagaaaatt cttcagaaga atatttagaa gaattatgtt tagaatttta
8761 tgatcttgta gaggctaaca aacttgaaga agttaaagaa tttttaaaag actatcctgt
8821 acctgaaata ttctttgaaa aatgctatac accttattgg gatagagaaa acaaaagagc
8881 tattatcgat actgctatca ctttagcttg tgcaggactt gcttatgata aaagtaaaag
8941 ttttgaaatg atggagtatt ttgaaagcct aggacttaaa gctgatgaag tacactttgg
9001 atacaatgca ctcagtagat atatcgatag agatggaaaa aataaagaag taatagagta
9061 tttttttaaa aaaggatgca catttgaaac ttataatgaa gaaagtggca gcactccttt
9121 gcatgtttgg atattgctaa atcatgcaaa ttatttagaa gaagctttaa aactaggagc
9181 aaatcctaat atgagaagaa ttaaaacaga aaatgaattt agctttagtg gtgcaggaga
9241 aacccttttg catagagctg caggaagtaa agaaaagcct ataggagctt gcgtggaagt
9301 gctaatcaaa tatggagctg atgtgaatgc agttaattgt ggtatatgtg atattgaaga
9361 tgggaaaatt gaatattatg atcctgctac tccattggat gaagctaata cttacgatat
9421 aaataaaaat ataaaaatct taaaaaaagc aggagctaag acttgggaag aattggttga
9481 agaatacaat atcgatacaa gcttagaaga accagaacaa attaaaatgt atgaagaaag
9541 aagaaaggat aaaaaataag attaataagg caaagaacaa aaaaaggatt tttcattgga
9601 atttaatgat ataaatttta aaatcaaaga atttgaaagt gtggatatca tcagtgatta
9661 ttctcttgcc ttttatttgc ctaaagaaga gcatatgata ggagaaaaag atcaagatgg
9721 taaaattcgc atcaataaaa gtaattcttt aaaatatata gaaaaaatca atgaagaaaa
9781 agaaaaagaa attgaaaaac ttaaacttca aagcaatgaa aaaatcaatg aagaatttga
9841 taaattaaaa gatggtgctt tacctactaa agaatataaa agaaaaaatt atgaatacga
9901 tgtattttat gatgtttata caagtaagga taatataaac aagtgcataa aaaaaatccc
9961 gataaaaaat ggtgtaggaa cgctaaaatc aaatttctta cgaagatttt atgaaaattc
10021 tgtcaagggt aagtataatt gtaaatggtg tcttatacct agactaatcc gcaataaagg
10081 agaatctagt tttgatcctg tgcctatttt aaatagtagc tttgtcattt cagaattaag
10141 caatatcaaa caaaaaaccc ttacttttca aagaacaata gccaatgtgg aacatccaag
10201 tcattttgct ttacaaagtc ctaaggaatt taaaagtatg cttgaaaatg agcaaagctt
10261 tgcttcttat ggtggcaaag aaaaacaaat agaagatctt ttaaaaaccg cacaacataa
10321 acttgaaacc attaaaaaag caaatggaat ttctaaaaaa accaaggaag atttacaaga
10381 aaagcaaaga aaagaaatta aaaaatgcaa agaattaaaa aatagcaaga aaaaaatttc
10441 acccaaactc ataggagata cgggagaata tgtcgcttct ttacttttg ggaaaagatc
10501 tacacgctat ttaagcaatc aaagaaaact ggatgctaat tttaatatct atagttttaa
10561 tcataccata cctgattttt tagtaagtat taatggtatt cctagtttag tcgaagttaa
10621 aaatgttcaa gttcaaagcc taacagaaca aataagcttt gagctaaaac tggctagaga
10681 atatgaactt gattacatct ttgtgtgtaa tcattataca catttaacag aaaatgtagc
10741 aggaagcgca ttgactcgtg aagaacacga agagctagca cgagatgata aaggcaaagt
10801 gggtgagttt aataaagaaa caaaaagatt gaaaaaagca aaggtggcag aagtcttaaa
10861 tgaaaaggat gatttagcta atgaagaatt tattaaaagt gtaagtgatg gaaataagta
10921 tttaaaagaa agggagaaat tttataaagt tgcttcagat gtttatatta aaaaccctat
10981 ttataaaatg ggctttgaga aaaaacttca aaatcaagtt gttgatataa acattgtaaa
11041 agattttta aaggactttg ctaaaagact agggatagac gaaaaagttt tgcctgtaat
11101 tggatataat cctcaacgag gggattctgg aacatatgac tatacagtaa atactttaga
11161 tttgaatcca aaagcttctt atgttgattt tattgatacc attattcatg aatttaggca
11221 tttttatatt tattgcataa aaaataattc tgaaaatagt atagaaaaat tgctttatct
11281 tgatatggta caattttata tatcgtggaa tttttataaa attttcaata gttatgataa

11341 aaaatgcttg atttatgata atgaagatga aacgaagaag tgtttaaaaa atcgcacata
11401 ttatggaagt tggaaatttt taaatcccac attagatctc ttgccaagtc ctttatacta
11461 tcttcagtca agtgagttag actctaggat tatggcatat tattttagaa aggaaattaa
11521 attataatga ctccaccatc tttaccagat gtagaaaaac atgaagattt cttacaaacc
11581 agaaaagaac cttatgctat ttatttagcc ataaacacaa acataaaaag ttataataat
11641 atctgtccta gcgagaaata tttttggaaa tttgataata tgaatgaact tgataaatgg
11701 tataacccaa aatttggcat ataccttggc aaaattgttt ttgataaaaa agacaacaaa
11761 ctcatcccaa aatatatagc cgctaaattt gaaaatttag aagaagaagt taaaaagata
11821 aaaaatcctt tatggcttgc taataaaaat cctaattata ttgaacctaa attttatgat
11881 ggtatgagtg ggggttatta ttttgaaagt ccaaataatt tagagtatca atgcaaaata
11941 gaaaaagaca ctcaagtttt aagccaagaa caaatcattt cttatgtgaa agaactttat
12001 agtaaaaaca caataataat aaaaaattat attgatgcaa tcaataaaga ctatggcata
12061 aaacctttcg tatttagtga tgaaatttat gatgaattag gagaagttgg cattcttaca
12121 aaagaacaag ctaataattt taaagataaa agttatatta aaaaagatcc gatattgctt
12181 gctatgcttg attatttagc aaaacaaaat aaaaaagatg aagattattt aatcactttt
12241 gatgatgagt atttttatac ttatttggtt tggagtttaa aagatttctt attagagctt
12301 tcttatggtt tgtttcaaga tgaaaccgag cttcttttta atcctgcagc ttatatggat
12361 gacacaaaaa tatattatca aaatttaaac gaagaaataa ataagcgtta tgaaaaaata
12421 ttgcttaata tgggatttga aggagaaaat ggttatttta aagattatta tgattatggc
12481 tttggcaata atggtatttt tgaatttagc atatatgatt attttgctta tgatgaaata
12541 ggagtacaac caattcaaca aagtccatat gttccaccta aaagcccttt tgatagccct
12601 aatttgtct attctgatgg aaattatcac ggagatgcaa agcttgtccc ttctgcttta
12661 ggaaagtatt attttgagct tttatatcaa aaagaaattt acatagaact tttacgccct
12721 tattatccaa gcataaaaga tttgccagaa ggttgggaca ataaaatgct agaaaaagct
12781 aatttaaaat aaaattatag atatgagctt tagagtagga gttggaaaag atgagagtgt
12841 aaaagaaagc aagctatgaa ataattttaa caacaatgta aaaaataatt agacgcctga
12901 agacattatc taaacaaatt gtatttttag tgtttttgaa gctgtagagt ttttaaaagc
12961 ttgtcaaagt tttaaagaag aaaaaaatga aatagataat tatgtttttt ttcaaaaact
13021 atccccaatt tgccggaaaa atcgcttata tatactatag atttgattta atcaataagg
13081 aatttttaac aagtgtatca gaaaaagata aggaatttta tcttcaagat agagacggca
13141 aatatattag caccataatt aataaaattt tacctttgga atataagaat ggtaaaagtt
13201 ttaaacaatt ccacacagag cttttaaata acaaaaacag agatcttaaa gaatgtaaag
13261 attttttaaa agctttaact caaactcttt gcaaagaatt taatttacct ttagaaaatg
13321 tagtctttta tagccaaaag cctagtggat ctttatcatt tggagcttat gaagaaaata
13381 aggtaagatt aaatcaatac attttaaaag cttattttaa agaatttgca aaaacggttt
13441 ttcacgaatg taggcatttt tatatagaaa aatattataa tcacaatatg gatgctctag
13501 gcagatatgt tttttattct gaaaaagctt atatcaatga gaaaattatt tttgataaat
13561 tgagtagaat ttgcgatcct ggtgagaatt atttagtagg ttgcaatata ggtgctatac
13621 aagatgctta tgaaattcag cccaatgaaa gagatccaag atatgtagaa accctcatag
13681 gtagaagttt ataatcaagg aaaataatga aaactataaa aataccgctt aaagattata
13741 tctccaatat aagagaagat aaaactattt acttgggctt gcaaagtgat atgagtataa
13801 aaggacttga tgaagaattc ttaaaaaaag aatataatct taagagaaat ttcaaatttg
13861 aaacaagcat ttatttgggt aaaattataa tcaaagaaaa aaatatcatc tttaaagctt
13921 ttaatattga tgagaattgc tttgaagggc ttaaagaact tttcttcaaa aatagcccct
13981 ataaaattgt agataattta gaattaaaaa aatatttctt aaaactttat aaagataata
14041 ttgaatttta acttcttata ttaatgaaat taatcaagtt catggtatta aactttacta
14101 tattttacat ttaatgcttt aaaattccaa gtcctccata aagcatgagt ttaattaaca
14161 tagcattctt aaaactgcta taatcatctt ttttttgctt tttccttcta aaacattatt
14221 tagccttata gtttcattct cgctaagata atttaacttt tcttctgctt tttcagtttt
14281 aaacctaatt ttttcatatc aaagctaaaa aggaataagt catcattatt atcacttata
14341 aaaaagctag ttaatactct caaataagta attttgttct ttttgataat ttattgctat
14401 tttttaaatt attttccaaa taattaagaa aattaatcaa aaaagttgtt tttatatcat
14461 ttatttgcat ttcatcctga taatctagta aatattccgc aagctctaaa aatacaccta
14521 taaaacaata aagtgttctt taatataata ttatctatat aaataaaatt tcatataatc

14581 cacatacaac atcagaaatt gataataaat atttatcttg atttatctta ttgatgtttt
14641 taagttttat tccaacaatt tcataaaact ttggttttat tgtatttaat ttatcttcag
14701 tgtaaaaaat ttatattttg caattagtcc ttcaaacaat tttataaatg caaaatctat
14761 attttcaagt tctttgctat acaaataatt atttataact tcttttaatt caacttacaa
14821 aaccaaagct aaagtatgat tttattttag tggatgaagc acaggatatc aacgcttgtg
14881 ttatcgacat tgttcttaat caaaaacaag ctaaaaaagt ttttataggg gatacctttc
14941 agagtattta taaatttaga ggagcggtaa attctttaga aatattagca tctatgccaa
15001 aatctcacac tttataccta acacaaagtt ttaggtgtcc gcaaaatata gcttttatta
15061 ccaactctta tttgaaaatt ttaaatgctc cgcgagattt caaaggaaca aaaaaaatca
15121 agtaatgaaa atgtcaaaaa cgaagcaaaa gccatagtgt gtagaactaa tgctaagtta
15181 tttgatatag ccgtagagaa tttagataaa aaattatttt ttgtaggagg gataaatagt
15241 tatagtttcg atgagttact agatatacaa aatcttcttt ttaaaaaaca cgaatatatc
15301 aaaaaccaat ttatagtaaa gtttgctgat ttaaaggagc ttcttgaata tattaatgaa
15361 actaaagaag ttgatttaaa gcaaaaaatc tttgttcttt tcaaatatgt gcatggcgat
15421 attataaagc ttattaaagc cattgaaaaa agtagcgtaa aaaaacaaga gcaagctgat
15481 ttaatcttaa gcacaggaca taagagcaaa ggcttggaat ggaacaatgt tgaaattatt
15541 gatgattttt taaacatcaa gcaagaactt gaagaaagag atcaaatcac catagcaaaa
15601 gaagaattaa acctttttata tgtggcaatc acaagagcca aaaattctct aagtattagc
15661 aaagattact tgcttgaaaa agaatttata caagaaaatt tagaaaggat aataatagaa
15721 tgaaaataat taaaaaatta caaaaagatg gaagccttaa aaagcaagtt tattattctg
15781 ttcaggaggt ttcacaaatg cactcagtaa cgcatgtaac agttcgccta tggatttcaa
15841 gaggaattct aaaaggtatg aaattaggca aaggatttta tatgtcaaaa gaaaccattc
15901 ttgattttca gaaaactgat ggtttatcat aattaaggag agaaaaaatg acttttagta
15961 gaaaagaatg tgctaaaatt cttgggatat ctttgcaaac tttagataga ttgacaaaag
16021 ataaaaaata aaatcaatga atataactcg ttttgtatta ttctccattg aagaaatagc
16081 taaaattctc tcaggggcta agtcaacaaa atgaatagaa aagataggt tgcactagct
16141 gcttatcata aaaataaaaa gctcacttgc tttggtaagt gagtaattta aaagtatatg
16201 ttaaagtata tgttttttag atatttaatt tttaaagtat ggatatttag atatttgatt
16261 aataaatcac acgccgacct caggcaccat tgcttcttaa aatcataaaa ttccaaaaaa
16321 attttattgt ttatatttgc tcgaaaaaat gcttctaaaa tagctgaaat atttatttat
16381 agatattaaa aatattttta tataaataaa atatgcttta aattatgtta aatatttaat
16441 tataaattat ttaaaaataa taagctcgct cccttaaaat aagataaaaa tagccctata
16501 ttcttaaata agactaaaaa tgtatgatta aacttaaatt aaactaaaat ttgatatagt
16561 tcgcgcgatc aaaaaagttt aaataaaaaa ttaaaaagtc atagttattt aaactgaaac
16621 atttgtgtta tttttcatag aaaatcataa tacaaataaa gatctatata ccgaataact
16681 aaaaaaaaag gggggggggt atggaagcaa aaggaatagc gatatttcct acaaatgttg
16741 catttagtaa aaaaacaaat gctttttttca ctgaattaga agatcaattc ttaaaagaag
16801 gggtcggtaa taaaagtgcc ataaaactag attttgaata ttatatttta tattgtcaat
16861 ttttacaaat agacaaagaa tccctatttt ttggaagaaa ctatgaactt gtgttttgtt
16921 ttttacttta ttatagagct ttattaaata aaaacaatac tcaaaaaatc gtagcaaaaa
16981 taataaatgt tatacgttgc caagatagat taccagagaa agtacactat agagaaaaaa
17041 taatttgtat ggataatcaa cacagagatc tttctcaaac actaagttca tgtattgcag
17101 aattttataa ttcaattgat ctatacaata cctttagaaa aaatatagga tttgaaaata
17161 gaaagaaaat agcgatagaa atgttaaatc gcgcatcaga acactatggt ttttttggct
17221 taagcagggg aggaataaca tataaagaag aaaaaatctc ttgcgtccct atacaagcat
17281 tggatgagtt tcatcatggt atgtcgcatc tatgtgcagt tttcttaaaa gaagatggac
17341 gatataccaa tctagaaaga gcgatccctc acttcaaacg tggaatgatg gaagtataca
17401 aaacaataat cagagatttt atgatattaa ataaagatgc tttaggagtc aaacaagaag
17461 ccttaaaaat aagagaatta gaatatcaaa ccataggaaa acccaaaaca gtaacaaaaa
17521 accctaccac aaacctaacc aataaaaaag aagagaaggt tatcccagga tataaagaac
17581 tgtgcgaaaa aattgtcagt caaatcaaaa atgcaaagta aaatcggtat gaattgagct
17641 tgagtatgca atttagctta tttgctattt gcatacttag agccattctt tgaagaaatt
17701 aaaacaaatc ctatcataaa ttaactatta taatgtaaag gcaaaacaaa ataaaaattt
17761 ttaggccaaa tttcaaattt taattctcct ttatgagcat taatgatttc tttagaaagc

17821 gataggccca atccatttcc cttaagttta gtagtcttaa aagcttcaaa aactaaattt
17881 tcatctttga tttcacaacc attatcataa accctaatgt ataaattttt ctcatcacag
17941 cttgcttgaa ttttgatttt aggattttcg cactcacttt cttcgatcgc atctatggca
18001 ttataaatca aattttgcaa aaccaaagat aaaagtgctt tatctccata aattttcata
18061 tctaaaaaat taatttcaaa atcaatttga gatgagtaat tataagaatt tatagcactt
18121 tcacactctt ctgttaattc taaaagatta aactcgcttg cattgatatg tacaccctta
18181 gtaaaaagta aggtagaatt tacaatacgc tctaccctag caatagcctt ttgaatttca
18241 agcacaatat gtttgttttt aagctcacta cgagaaaaaa gcgtggaggc aagtaaggat
18301 atagagccta tgggatttct tatctcatgc gctaaatgtg ccgcaacact tcccatacta
18361 gcaagtcttt cattgcgttt ttcatcgcta atatccgtag ctgaaactat ggtttttttca
18421 ttatgagcaa taattttaac cgcataaaat ctattctcaa attcaagttc atcacgattt
18481 ttttcaagac tgataatatc aagcaattta ggtttttca aagcttcttg attttgcaaa
18541 ataatatttt tttctttatc taaaatccaa agcgcactag gcaaaacctc gataatctca
18601 tctaccatag cacgcaaaga attataattt tcgtttaaaa ttttatattc gttttctaca
18661 atataagttt gctctataag gctttgtaag ccttttgta gggtttcttt ttcacttgag
18721 tctagacttt ttaaaatatt ttcgttcatt tattttcctc taataaaatt ttaaaatcat
18781 ttaaatccaa taaaagcaaa tcttgtttac aaattttata aaattcttta ctaaaaccat
18841 tttttgaaat aataatataa ttatttggct ctagttttaa agattttgct tttaattcta
18901 aaaggtttaa aatatttcta caaatttttt tatttttaaa tttaacttca ccgataaaac
18961 ttatcttgtc atccttataa taaagatcaa gttctaaatt tttatcccaa taactttgca
19021 ccccactcac ttgaaatttt ttttcaagta attctctgga aagttgctca aaacaaaaac
19081 tttgataaag ttcaaatttc tctttaatca aagttaaaac ttcttcatag cgattttgca
19141 aaatcaactt ttcattgggt tttagaaaat aaaaaaagaa acgagtaaat tgattagcaa
19201 agataatttt atcttgtatc acataagatc ttaattcttt tttaagtttt tgtcgtttat
19261 ctcttaccct tttcgcctct ttgctatact caagttttat aatccccgct cttaaaagat
19321 attttaaagt tgataaagcc ttaaaatgct gaatttttct attgatagaa aaacgcttac
19381 gattgttttt agctagcaag ctaagagcat aagaagcggt ctcatcgagc ttgaaacaat
19441 ccttgatatc taaatagttt ttgagcaaaa gatattctat attgtgaaat acatcttctt
19501 caaaatttaa actatccaag ctctcaaaac cctcaaaaac actataaaaa tcaaaaattt
19561 tagccaaagc tgaagtgttg caaatttttg caaaatgatt aaaatttata tttttccttt
19621 ttaaaagtat ttttatagta taattttagc aaatttttaa aggattgtgc ttgcaaattt
19681 cagatttaaa aaaagaaata attttaaaaa aaggcacctt gcattttgac tttaccgcta
19741 gtgctttggc attaaaatgc gtagaaaaag aaattgcaaa aatactgcca agttatgcaa
19801 atactcattc tgacagctcc ttaaactctt ttaaaactca acaaatttac gaacaagcac
19861 gcaaagatat caaaacaagt cttaatctaa gtgatgaatt tgcacttatt gcttgtggat
19921 caggctctag cagtgctatt aaaaaatttc aagaattact tggaatttat atcccgccac
19981 ttgtcaaaga aaagtatttt gaaaaggtag ataaaagcac cctaccctta gttatcgtag
20041 ggccttatga acatcattct aacgaacttt ccttccgtga aggtttgtgc gaatgtataa
20101 gaattcccct agataaaaaa ggaaacatag actttgaaat tttagaaaaa gttttaaaag
20161 aaaatcaaca cagaaaaatt atcgctagtt tttccctagc ttcaaatgtt acaggtatat
20221 taagcgatta taaacgcatt tctaaattag tgcgtaaatt taaaggcata gtcgcttttg
20281 atgcatcaag ctttatccct tataaaaaca tagcttgcaa gtattatgat gctttattta
20341 taagctctca taaattaatc gggggtatag gaggctctgg tcttttagca atcaaaaaag
20401 aactttgcgg aaacaaacca agctttgccg caggaggaac tgtgggttat gtttcgcgca
20461 cctctcaatg ttatctttgc aatgaagagg ctttagaaga aggagggact cctggaattt
20521 tacaactaat tcgcgcgagc ttggctttta aaataaaaga tgaaataggc atagaagaaa
20581 tagaaaaaaa agaagaaatt ttaaaagaat acttctttga aaaactaaaa actatacctc
20641 atttaatcct atatgctaat aatataaaaa ctcgcttacc catctttgct tttaatatta
20701 aaaacatttc tccttttgac ttagcctatg agctgagtaa aaaataccac atagaaacac
20761 gcgcgggctg tgcttgcgct ggtccttatg ggcatgattt actaggctta aaagataatg
20821 aagaattaaa aactaaacca ggttggctaa gaattagttt gcattatact cacgaaaaag
20881 aagatattga ctatttcttt gaagcactgc aaaaaagtat aataaaattg aaattttaat
20941 gatggcgcag cggacggggc tcgaacccgc gacctccgcc gtgacagggc ggcattctaa
21001 ccagctgaac taccgctgca cccaagaagt aataatttaa tggtggtcac tataagactc

```
    21061 gaacttatga catccacctt gtaagggtgg cgctctacca actgagctaa gcgaccttaa
    21121 tttttggcga cccctagagg atttgaacct ctgtttccac acagagagag tgttgtcctg
    21181 ggccactaga cgaaagggtc taaacccgat aagtggtgtc ccgtgttgga ttcgaaccaa
    21241 cggccccctc cttaaaaggg agatgctcta ccggctgagc taacgagaca gatggcgcag
    21301 cggacggggc tcgaacccgc gacctccgcc gtgacagggc ggcattctaa ccagctgaac
    21361 taccgctgca cccaagaagt aataatttaa tggtggtcac tataagactc gaactta
//
LOCUS       IHOPMAOI_2              7392 bp    DNA     linear       12-APR-2021
DEFINITION  Campylobacter species strain strain.
ACCESSION
VERSION
KEYWORDS    .
SOURCE      Campylobacter species
  ORGANISM  Campylobacter species
            Unclassified.
COMMENT     Annotated using prokka 1.14.6 from
            https://github.com/tseemann/prokka.
FEATURES             Location/Qualifiers
     source          1..7392
                     /organism="Campylobacter species"
                     /mol_type="genomic DNA"
                     /strain="strain"
     gene            309..503
                     /locus_tag="IHOPMAOI_00031"
     CDS             309..503
                     /locus_tag="IHOPMAOI_00031"
                     /inference="ab initio prediction:Prodigal:002006"
                     /inference="similar to AA sequence:RefSeq:ARJ53354.1"
                     /codon_start=1
                     /transl_table=11
                     /product="conjugal transfer protein TraU"
                     /protein_id="Prokka:IHOPMAOI_00031"
                     /translation="MIATLIDAACMNITGGVDIAYMTEVDPLWQDDELTALINPEALL
                     FGNPILNLACMADSASAQGN"
     gene            524..700
                     /locus_tag="IHOPMAOI_00032"
     CDS             524..700
                     /locus_tag="IHOPMAOI_00032"
                     /inference="ab initio prediction:Prodigal:002006"
                     /inference="similar to AA sequence:RefSeq:ARJ53354.1"
                     /codon_start=1
                     /transl_table=11
                     /product="conjugal transfer protein TraU"
                     /protein_id="Prokka:IHOPMAOI_00032"
                     /translation="MPIWRKSSYRLQILAPVPHPMGMGIGQSGILWSYAKNYPVAGDN
                     FTFMLFKKKECCAF"
     gene            714..1373
                     /locus_tag="IHOPMAOI_00033"
     CDS             714..1373
                     /locus_tag="IHOPMAOI_00033"
                     /inference="ab initio prediction:Prodigal:002006"
                     /inference="similar to AA sequence:RefSeq:ARJ53355.1"
                     /codon_start=1
                     /transl_table=11
```

```
                     /product="transcriptional regulator"
                     /protein_id="Prokka:IHOPMAOI_00033"
                     /translation="MYIRDFRKRLHMTQSEFAQALGTKQTNISMYEKHRVKPTCELIV
                     KLIIRFNANPNFLFFGKEPHLNTDTFKNEISQELSQLISELSLYENEENIIVELENKA
                     LGKILSLVPNKEIWERLFFLFRINRKLHFVTSFICRVAKRLKQRDDEAKAYLISIINS
                     FNEGDFTELNEYIKLDLIALFDEKITEEEAKTIIEDCLVVFKHIENTAPIHKMVELGK
                     D"
gene                 1394..1522
                     /locus_tag="IHOPMAOI_00034"
CDS                  1394..1522
                     /locus_tag="IHOPMAOI_00034"
                     /inference="ab initio prediction:Prodigal:002006"
                     /codon_start=1
                     /transl_table=11
                     /product="hypothetical protein"
                     /protein_id="Prokka:IHOPMAOI_00034"
                     /translation="MKKQIIGSILLCSSLFAYKSTKDLDKNDVLEKLIQNINDSLY"
gene                 1916..2698
                     /locus_tag="IHOPMAOI_00035"
CDS                  1916..2698
                     /locus_tag="IHOPMAOI_00035"
                     /inference="ab initio prediction:Prodigal:002006"
                     /inference="similar to AA sequence:RefSeq:SQE23558.1"
                     /codon_start=1
                     /transl_table=11
                     /product="TraT complement resistance protein"
                     /protein_id="Prokka:IHOPMAOI_00035"
                     /translation="MKINKSILKMGLSSILAASLLTGCLTTTLQTNATMSQSIFLDPV
                     AKEKRVVFLNIKNTSGHEVNLEPKLRSALETKGYKIIDDPDKATYILSTNVLYCDKKQ
                     ENNVAGGAAAAGATGVAISGYNGGGAGGMVAAGAAGALVGGLLGKLTEDTIWQMQVDI
                     NIKQKADGKVLTSSGNVSGQASIRDQRASGFLNSFGGSVRSDKVGQLNSNQINSTQQT
                     YEGKYIEKNSIIFAEAVKTGLKLPEATPILENKIATQIAGLF"
gene                 2711..3142
                     /locus_tag="IHOPMAOI_00036"
CDS                  2711..3142
                     /locus_tag="IHOPMAOI_00036"
                     /inference="ab initio prediction:Prodigal:002006"
                     /inference="similar to AA sequence:RefSeq:ABS43376.1"
                     /codon_start=1
                     /transl_table=11
                     /product="putative thioredoxin"
                     /protein_id="Prokka:IHOPMAOI_00036"
                     /translation="MILKKIKILFLAIFLAFSPMTLEAKIYDNVYIAQKRALKEAKLM
                     VFFVLSNTCNYCHKLMSDALNNRDLMEYLDENFIVTISDLNADGLIPRDLLFNGVTPT
                     TYILTPTGKVIGTPIEGAVDSNMLFGLLKGLEDYKKGQLGF"
gene                 3278..4150
                     /locus_tag="IHOPMAOI_00037"
CDS                  3278..4150
                     /locus_tag="IHOPMAOI_00037"
                     /inference="ab initio prediction:Prodigal:002006"
                     /inference="similar to AA sequence:RefSeq:CKG86076.1"
                     /note="Domain of uncharacterised function (DUF3825)"
                     /codon_start=1
                     /transl_table=11
```

```
                     /product="hypothetical protein"
                     /protein_id="Prokka:IHOPMAOI_00037"
                     /translation="MTESGFNLEKDENEPSLVYVKKATTRNKTLNREQIQNKVEEKPL
                     LFQFAYFPNFLNTLQELSNIAQKEFWGSGNNILFSYLFKYFEFIHENKNYPDIITYNK
                     DKTKACFNTGLYSTGVFPIFAYFEKQENGGYIFRKFCSNGDRVLDDLEIPKSLNDFDS
                     FKNELIFDSKLDFRINHLHLFERKERLPEIIKKLNDRFIGHIINGELKIIKDNYNHQK
                     MIIPAAYKQKVVLYIPLKLQEESVDTIVVVEKEEVKNEQYYAVRTILNPHDNIYKTAR
                     VLSIVESEWVKNTI"
     repeat_region   4191..4351
                     /note="CRISPR with 3 repeat units"
                     /rpt_family="CRISPR"
                     /rpt_type=direct
                     /rpt_unit_seq="attttatcatacaaacatttaacaagagg"
     gene            4808..5416
                     /locus_tag="IHOPMAOI_00038"
     CDS             4808..5416
                     /locus_tag="IHOPMAOI_00038"
                     /inference="ab initio prediction:Prodigal:002006"
                     /codon_start=1
                     /transl_table=11
                     /product="hypothetical protein"
                     /protein_id="Prokka:IHOPMAOI_00038"
                     /translation="MDKAVNIKLKIIFAVLFVLILTNIGILFISKNEIFTKTLVGLIA
                     IAFILYYALAWIKTIKNIKKDSSKSGVKNLFIGILKLPYELVKILFKLPKIIFMAILT
                     EFDYFLGYFSDKFKNKKQNNYDNFDDIDYIINNAKAIGGNAITKIGNTTFVGGDMVTK
                     IGNTTFIGGNMVTKIGNTTFVGGDMVTKIGNNTFVGGNLVLW"
     gene            complement(5444..5923)
                     /locus_tag="IHOPMAOI_00039"
     CDS             complement(5444..5923)
                     /locus_tag="IHOPMAOI_00039"
                     /inference="ab initio prediction:Prodigal:002006"
                     /codon_start=1
                     /transl_table=11
                     /product="hypothetical protein"
                     /protein_id="Prokka:IHOPMAOI_00039"
                     /translation="MQSYYSPSAASTIHKGNYGTVDSSAVGFGNNYEADKKTMGNGHL
                     DFAGQNTAWGQGNSQGTVQQEVQGQVANATNQFNKPTEQRMGGGHFVDATVGEGSLLN
                     RAGSGMIKWGESVGETAKMFGWGSGIPSTPPPQNNSTTSTPPNYAPPAPKISPEFLK"
     gene            6390..6722
                     /locus_tag="IHOPMAOI_00040"
     CDS             6390..6722
                     /locus_tag="IHOPMAOI_00040"
                     /inference="ab initio prediction:Prodigal:002006"
                     /codon_start=1
                     /transl_table=11
                     /product="hypothetical protein"
                     /protein_id="Prokka:IHOPMAOI_00040"
                     /translation="MKKDKMQIAEHILQNCYWGNTTMTPSFIVEHINDKDFARTIFSA
                     IFQNSLTMFEDLKIIDNEEWIKEFIISQNQRLGYHKRFYYEERLDELIAHYGIKDAGK
                     RREVYPIV"
     gene            6685..7356
                     /locus_tag="IHOPMAOI_00041"
     CDS             6685..7356
                     /locus_tag="IHOPMAOI_00041"
```

```
                     /inference="ab initio prediction:Prodigal:002006"
                     /codon_start=1
                     /transl_table=11
                     /product="hypothetical protein"
                     /protein_id="Prokka:IHOPMAOI_00041"
                     /translation="MQEKEEKYIQLYKTQDKVLDLVAKEKLDFYLTGGTALQRFHYNQ
                     FRFSDDLDFFLINNGIKNHTPKEFNDFASMLQDNNIDFEIKVSNQYFTQLYIKENNLK
                     IDLVNDYVFHEKNDYVKLENGLLIDSIQNIFVNKLETSISRDEPRDLFDIYTILRNSK
                     IDIEKSFDILDKKTNIEPKDIINKLKNIKLEQIQYNKINTKSDFIIKDFLQNFHKSID
                     ENFNS"
ORIGIN
        1 gcttatttta taggattaaa agtgaatttt tacaaattga tgatagctat ctgcaattgg
       61 gtaaactttc aattcatgct aaagaattca ttacttaaaa agcatttgtt tttgactttt
      121 ttcaattgaa tttttatatc taaaatatct tcaatataaa aaattattat atgtttatta
      181 tatatattta taaaaaaaat ttataaatat atataataaa catatatttt tttctaaaaa
      241 atgatataat tatcaagaaa tttctaatta aagtttttta tattaaaaat atcatcctag
      301 ttctaggcat gatagcaact ttaattgatg cagcatgtat gaatattaca ggcggtgtgg
      361 atatagccta tatgacagaa gtagatcctt tgtggcaaga tgatgaatta acagctttaa
      421 tcaatcctga ggctttgcta tttggtaatc ccattttaaa tcttgcttgt atggcagata
      481 gtgcatcagc acagggcaat taggactttg tggagaatat cctatgccta tttggagaaa
      541 atcatcctat agattgcaaa ttttagcacc tgttcctcat cctatgggaa tgggaatagg
      601 acaaagtggc atattgtgga gttatgctaa gaattatcct gtagcaggag ataatttcac
      661 ctttatgctt tttaagaaaa aagaatgctg tgcattttaa taagtgaggt ttaatgtata
      721 taagagattt tagaaaaaga ttgcacatga cacaatcaga atttgcacaa gcattaggca
      781 ctaaacagac aaatatctca atgtatgaaa aacacagagt aaagccaacc tgcgagctta
      841 ttgtaaaatt aattataaga ttcaatgcta atccaaactt tcttttcttt ggcaaagaac
      901 ctcatttgaa tacagataca ttcaaaaatg aaatttcaca agaattgagt caattaatca
      961 gtgaattatc tctatatgaa aatgaagaaa atatcatagt ggaactagaa aataaagcct
     1021 tagggaagat tctttcgctt gtgccaaaca aagagatttg ggaaagattg tttttcttat
     1081 ttagaataaa cagaaaatta cattttgtta cttcatttat ttgtagagta gctaaaagac
     1141 tcaaacaaag agatgatgaa gccaaagcat atctaatttc tattattaat agttttaatg
     1201 aaggtgattt caccgaactt aatgaatata tcaaattaga tttaattgca ctatttgatg
     1261 aaaaaatcac cgaagaagaa gcaaaaacta ttatcgaaga ctgcttggtt gtatttaagc
     1321 atattgaaaa tacagctcct atccacaaaa tggtagaact tggaaaagat taataataag
     1381 aaaggttaaa aaaatgaaaa aacaaattat aggttctatt ttattatgct cttcgttatt
     1441 tgcatataaa tctaccaaag atttagataa aaacgatgta ttggaaaaat taattcaaaa
     1501 tataaatgat agtttatact aaataaagga ggaacaggac ttaaatatag aaaattaaac
     1561 actataacat atatttttat aaaggagaat aaaaacaaat aaggacagga atgtagttga
     1621 attttggttg atagctttgt caatgataaa tataacatag aagcgaaagc tactgtatac
     1681 ttagaagtga ttttaaataa aacagatttc tctagaactt atttagaatc taaaatttct
     1741 aaggaatttt caaaacatca tgccaaagag caagaaaaaa attattttaa aaaatttca
     1801 atgctataga aaaaagcttg gatgagctaa cgggcgaaac ggaagaaagt cgtgctaaaa
     1861 tttaaaaagc aggttaaaga aatttttgaa aaaatattca ctaaaaagga aaacgatgaa
     1921 aataaataaa tcaattttaa aaatgggatt aagcagtatt ttggcagcaa gcttgcttac
     1981 gggttgtcta acaacaactt tacaaactaa tgcaactatg agtcaaagca tctttttaga
     2041 tcctgtggca aaagaaaaaa gagttgtatt cttaaatatt aaaaatacaa gcggacacga
     2101 agttaactta gaacctaaac taagaagcgc attagaaacc aagggatata aaattataga
     2161 tgatcctgat aaagcaactt atattttaag tactaatgtt ttatattgcg ataaaaaaca
     2221 agaaaataat gttgcaggcg gtgctgcagc cgcaggtgct acaggcgtag caattagtgg
     2281 atataatgga ggcggtgcag gcggtatggt tgcagcaggt gcagcaggtg ctttagtagg
     2341 aggactttta ggaaaactta ccgaagacac tatttggcaa atgcaagttg acattaatat
     2401 caagcaaaaa gccgatggta aagttttaac ttctagcgga aatgtaagtg gacaagcaag
     2461 cattagagat caaagagcat caggattttt aaatagcttt ggcggaagtg ttcgtagcga
     2521 taaagtagga cagcttaatt caaaccaaat taatagtacg caacaaacct atgagggtaa
```

2581 atatatagaa aaaaattcaa ttatttttgc agaagctgta aaaacaggac taaaacttcc
2641 tgaagcaacc cctattttgg aaaacaaaat cgcaactcaa attgcaggat tgttctaaaa
2701 aggatatctt atgatactaa aaaaaataaa aattttattt ttagctatct ttttagcctt
2761 tagccctatg acgctagagg ctaaaatcta tgataatgta tatattgctc aaaaacgagc
2821 attaaaagaa gcaaagttaa tggttttctt tgttctttca aacacttgca attattgcca
2881 caaactcatg agtgatgcgt taaataatag agatttgatg gaatatcttg atgaaaattt
2941 tatcgttact atctctgatt tgaatgcaga tggactaatt cctagagact tattatttaa
3001 tggagtgact ccaacaactt atattttaac tccaacagga aaagtaatag gcactcctat
3061 tgaaggagcg gttgactcta atatgctttt tggattatta aaaggtttag aagattataa
3121 gaaaggacaa ttaggttttt agcgtggaaa aaaacataga aaaattaatt ttagaagcct
3181 atgaagatag caaaactaaa tttgaacatg ttactacagg acatatcagt caatatttga
3241 aaaaaaatat gatttgaaaa ttaattgtag taaggcgttg acagaatcag gatttaatct
3301 tgaaaaagat gagaacgaac cttctttagt ttatgtaaaa aaagctacca caagaaacaa
3361 aactttaaat cgtgagcaaa ttcaaaacaa agttgaagag aagcctttat tattccaatt
3421 tgcctatttt ccaaattttc taaatacact tcaagagctt tctaatatag ctcaaaaaga
3481 attttggggg agcggaaata atattttatt ctcctatcta tttaaatatt ttgaatttat
3541 tcatgaaaat aaaaactatc ctgatattat cacttataac aaagacaaaa caaaggcgtg
3601 tttcaataca ggtttgtata gcacaggtgt ttttccaata tttgcatact ttgaaaagca
3661 ggaaaatgga ggatatattt ttagaaaatt ttgctctaat ggggatagag ttttagatga
3721 tttggaaata cccaaatctt tgaacgattt tgattcgttt aaaaatgaac tcatctttga
3781 tagcaagtta gattttagaa ttaaccattt acatctcttc gagagaaaag aacgattgcc
3841 cgaaataata aaaaaactaa atgataggtt cataggacac attatcaatg gtgagttgaa
3901 aatcatcaag gataattata atcatcaaaa gatgattatt cctgcagctt ataagcaaaa
3961 ggtagtttta tatattcctt tgaaacttca agaagaatca gttgacacaa tagttgtagt
4021 tgaaaaagag gaagtaaaaa atgagcagta ttacgctgta agaactatac taaatccgca
4081 tgataatatc tacaaaactg caagagttct ttcgatagtt gaatctgaat gggttaaaaa
4141 caccatttga aaataaaaat attgataaaa taggctttta aaataagtct attttatcat
4201 acaaacattt aacaagaggc taaaaccgaa ttaagcggtt ttattaaaac cctcttattt
4261 tatcatacaa acatttaaca agaggctaaa acttgctatt gatgattgct attcatgatt
4321 gcattttatc atacaaacat ttaacaagag gttaaaacat tcattcaaaa atactataat
4381 taaagtgtat taggtatgac tactctttcg attataagaa ggaggaataa tgagtaagat
4441 cattgagttt ttagttcttg tcttagaatc aattaacgag ataatcaagc tcgttgatta
4501 tctcacttaa aaaaaccgaa ataattataa ataaaccacg attagcctaa gcttaagaca
4561 atttaccaaa aaaacactta atcgaattat gggcattgtt aatccctacg cttgaaggac
4621 tataatagag attaaaatta catattaact tgtaatcaac ttgaagttaa gttgtagtca
4681 gataaaaaaa gaaatccatt ttaaacccca taaaaatagt cgtttaatag caaaaatact
4741 tacaaaaaat ttattaaaat ataatataat tatgttatat aaataagtta atatgaaagg
4801 aataacaatg gataaagcag taaatataaa attaaaaatc atttttgccg tattatttgt
4861 attgatttta acaaatatag gcattttatt tataagcaag aatgaaatat tcacaaaaac
4921 acttgtaggc ttaatagcga ttgctttcat tttatattat gctttagctt ggataaaaac
4981 tataaaaaac attaagaaag atagctctaa aagtggagtt aaaaatcttt ttataggtat
5041 tttaaaatta ccttatgagt tagtgaaaat attatttaaa ttgcctaaaa ttatatttat
5101 ggctatttta acagagtttg attatttttt gggctatttt tcagataaat ttaaaaacaa
5161 aaaacaaaac aactatgata attttgatga tatagattat attataaaca atgcaaaagc
5221 tataggcgga aatgctatta ctaaaatagg taatacaaca tttgtaggcg gtgatatggt
5281 taccaaaata ggtaatacta catttatagg cggtaatatg gttactaaaa taggtaatac
5341 aacatttgta ggcggtgata tggttaccaa aataggaaac aatacatttg tgggggggaaa
5401 tttagtttta tggtaattta taagcaagcc taaattagct tgcttatttt aaaaattcag
5461 gagatatttt aggcgcagga ggagcataat ttggaggcgt agaagttgta gaattatttt
5521 gcggaggcgg agtgctaggt attccacttc cccaaccaaa cattttggca gtttctccta
5581 cactttcacc ccattttatc attccacttc ctgctctatt taataatgag ccttctccta
5641 cagttgcatc tacaaaatgc cccccccca ttctttgttc agtaggctta ttaaattgat
5701 ttgtcgcatt agcaacctgt ccttgaactt cttgttgcac tgttccttgt gaattgcctt
5761 gtccccaagc tgtattttgt cctgcgaaat ctaagtgtcc atttcccata gtttcttat

```
     5821 cagcttcata attatttcca aatcctacag ccgatgaatc tactgtgcca taattacctt
     5881 tatggattgt agaagctgca gaaggactat aatagctttg catacctacg gaacctatag
     5941 tattttgcgg actattaaaa tctcctgcat taaagtattt attgcatatt aacttgtaat
     6001 caatttaaag ttaagtgtag tcagataaaa aaagaaattc atttaaagcc ccataaaata
     6061 gtatttaata ctaatattac tcgtgaaatt tattaaaata tgatataatt atgttatatt
     6121 aattgagaaa ggagctactt atgatgttaa ctgtaaataa tatagataaa gctgtacaaa
     6181 gcgtagtaag tctttctcca aaaagcaata cttgaattca tcaagaaggt tttgtttctt
     6241 cagaagaaac cgtagtggct ttaactaatc ctgtagatcg tagggttttt gataactgca
     6301 aatattttaa gaaagcattg caaaaataaa ttatccactt atttttcaaa aaataatttt
     6361 agtaaaattg caaaaaaagg aaaattctaa tgaaaaaaga taaaatgcaa atagcagaac
     6421 acattttaca aaattgctat tggggaaata ctactatgac acctagtttt atagtagaac
     6481 atattaacga taaagacttt gcaagaacaa tttttagtgc tatttttcaa aattcgctta
     6541 caatgtttga agatttaaag attatcgata atgaagaatg gataaaagaa ttcatcattt
     6601 ctcaaaacca aagattaggc tatcacaaaa gattttatta tgaagaaagg cttgacgagc
     6661 ttatcgcaca ttatgggata aaagatgcag gaaaaagaag agaagtatat ccaattgtat
     6721 aaaacacaag acaaagttct tgatctagtt gcaaaagaaa aattagattt ttatcttaca
     6781 ggtggaacag ctttgcaaag atttcactat aatcaattta gattttctga tgatttggat
     6841 tttttcttga ttaacaatgg aattaaaaac catactccaa aagaatttaa tgattttgca
     6901 agtatgttac aagataataa tatagatttt gaaataaaag tttccaatca atattttact
     6961 caactttata tcaaagaaaa taatctcaaa atagatttag tcaatgatta tgtatttcac
     7021 gaaaaaaacg attatgtaaa acttgaaaat ggtttgctta tagacagcat acaaaatatc
     7081 tttgttaata agttagaaac ttcaatttct agagatgaac caagagattt gtttgatatt
     7141 tacacgattt taagaaacag taaaatagat attgaaaaaa gttttgatat tttagataaa
     7201 aaaaccaata tcgaaccaaa agatattatt aataaattaa aaaatattaa attagaacaa
     7261 attcaatata ataaaattaa tacaaagagt gattttatta taaaagattt tttacaaaat
     7321 tttcacaagt ccattgatga aaattttaat tcttaaataa aagaaattcg cttaaaatcc
     7381 tataaaatag gt
//
LOCUS       IHOPMAOI_3              1938 bp    DNA     linear       12-APR-2021
DEFINITION  Campylobacter species strain strain.
ACCESSION
VERSION
KEYWORDS    .
SOURCE      Campylobacter species
  ORGANISM  Campylobacter species
            Unclassified.
COMMENT     Annotated using prokka 1.14.6 from
            https://github.com/tseemann/prokka.
FEATURES             Location/Qualifiers
     source          1..1938
                     /organism="Campylobacter species"
                     /mol_type="genomic DNA"
                     /strain="strain"
     gene            150..512
                     /locus_tag="IHOPMAOI_00042"
     CDS             150..512
                     /locus_tag="IHOPMAOI_00042"
                     /inference="ab initio prediction:Prodigal:002006"
                     /codon_start=1
                     /transl_table=11
                     /product="hypothetical protein"
                     /protein_id="Prokka:IHOPMAOI_00042"
                     /translation="MCKLNELPNNEEKYNKILGYFDKKLGDIDDFRHVKKYGSIEMWL
                     YIYYDDTYKTPSNFQEKIEMDNIALKTKNMQMSLHKIVDMHISPYWNTRRYVLEGNEG
```

```
                     NMNFEFIEDELACGNLYQ"
     gene            531..1262
                     /locus_tag="IHOPMAOI_00043"
     CDS             531..1262
                     /locus_tag="IHOPMAOI_00043"
                     /inference="ab initio prediction:Prodigal:002006"
                     /inference="similar to AA sequence:RefSeq:AVS37480.1"
                     /note="DUF2974 domain-containing protein"
                     /codon_start=1
                     /transl_table=11
                     /product="hypothetical protein"
                     /protein_id="Prokka:IHOPMAOI_00043"
                     /translation="MTNKQLINKLRDMAELAQASYGYFDLVTHDKNDFFIKIKDEATE
                     SAFFNKHITQANVLDISYKNYKAYQYDSSLKKDIKIGTLNGDFSPLQAKQFFSRYDLL
                     KHCPNTNSGFSATLFGEKRKQINSKTKEKSYTSEYGYINYILAIRGTEMSSFKDLFVA
                     DASLAIGSIPKAQYDDMINFYETCIKDYPQIKEKDSLTITGHSLGGCLAQLFALSICD
                     DKSEANINEICNIESKPLKELQCYL"
     gene            1250..1816
                     /locus_tag="IHOPMAOI_00044"
     CDS             1250..1816
                     /locus_tag="IHOPMAOI_00044"
                     /inference="ab initio prediction:Prodigal:002006"
                     /codon_start=1
                     /transl_table=11
                     /product="hypothetical protein"
                     /protein_id="Prokka:IHOPMAOI_00044"
                     /translation="MLFVIICFLLIVFTLSYFIWWLIYRKAFKSKKKISKFLVFIGGI
                     GLIVFYYTPYSYYLEPSFYKFKEICKLDPEIYQANGSKLDEEYYNKVLRYFDTDLDNM
                     SKIKTLRISDDKKHFSYWFEKWIGDRISFDFVIWFKNENATKDNIKKVSVYVWWRDLR
                     PLPAGNEGAGFYLSGDRENCAYFKKEIK"
ORIGIN
        1 taatttttaa gtgtatatat agaatcatct ttaaatctaa aaaaagtgtt tcaaagtttt
       61 tagtctttct tggaagcata ggtttaatta tcttttatta tactccttat tcttattatt
      121 tagagcctag ttattggcaa tttaggaata tgtgtaagtt aaatgaactt cctaataacg
      181 aagagaaata taataagatt ctagggtatt ttgataaaaa gttaggtgac atagatgatt
      241 ttcgacatgt aaaaaaatat ggtagtatag aaatgtggct ttatatatat tacgatgata
      301 cttacaaaac tccttcaaat tttcaagaaa agatagagat ggataatatt gcactaaaaa
      361 caaaaaatat gcaaatgtct ttacacaaga ttgttgatat gcatataagt ccatattgga
      421 atactagaag gtatgtgtta gaaggcaatg agggtaatat gaattttgag tttatagagg
      481 atgagctcgc ttgtggcaat ctttatcaat aataatataa ggaataaata atgaccaata
      541 aacaacttat taataaacta agggatatgg cggaacttgc acaggcaagt tatggttatt
      601 ttgatttggt aacacatgac aagaatgatt ttttcattaa aattaaagat gaagctacag
      661 aatctgcttt ctttaataag cacattactc aagctaatgt cttagatatt agttataaaa
      721 attataaggc atatcaatat gactcatctt taaagaaaga tattaaaatt ggcactctca
      781 atggcgactt ctctccactc caagctaaac aattcttttc tcgctatgat ttattaaaac
      841 attgccctaa tacaaattca ggtttttctg ctactttatt tggagaaaaa agaaaacaaa
      901 ttaatagtaa aacaaaagaa aaaagctata ccagtgaata tggctatatc aattatattt
      961 tagctattag aggaacagaa atgagttctt ttaaagattt gtttgtagct gatgcttctt
     1021 tagccatagg ctctatacct aaagcccaat atgatgatat gataaacttt tatgaaactt
     1081 gtattaaaga ttatccacaa atcaaagaaa aagactctct taccattaca ggacactctc
     1141 ttggaggctg tcttgcacaa ctctttgctt taagtatctg tgatgataaa agcgaagcaa
     1201 atatcaatga aatttgtaat atagaatcta aacccctaaa ggaattacaa tgctatttgt
     1261 aatcatatgt ttttctcctta ttgtctttac tctctcatat tttatatggt ggcttatcta
     1321 tagaaaagca tttaaatcaa agaaaaaaat ctctaagttt ctagtattta ttggtgggat
```

```
     1381 aggattgata gtattttatt atactcctta ttcttattat ttagagccaa gcttttataa
     1441 atttaaagaa atatgtaagc tagacccaga gatttaccaa gccaatggta gtaaattaga
     1501 tgaggaatat tataataaag tgctaaggta ttttgatacg gatttggata atatgtcaaa
     1561 gataaaaaca ttaagaataa gcgacgataa aaagcacttt tcatattggt ttgaaaaatg
     1621 gattggagat agaatctctt ttgattttgt catttggttt aaaaatgaaa acgctacaaa
     1681 agacaatatc aagaaagtta gcgtttatgt ttggtggcgt gatttacgac cattgccagc
     1741 aggtaatgag ggggcgggat tctatctttc aggagatagg gaaaattgtg cttattttaa
     1801 aaaggaaata aaatgaacaa taaagaacag attaaaaagc tcaaagataa tgccgaatta
     1861 gcaatggcaa gttatgggta ttttcattta gcagataaat cctataatca taatgataaa
     1921 tatgaaaagc gattaaag
//
LOCUS       IHOPMAOI_4               830 bp    DNA     linear       12-APR-2021
DEFINITION  Campylobacter species strain strain.
ACCESSION
VERSION
KEYWORDS    .
SOURCE      Campylobacter species
  ORGANISM  Campylobacter species
            Unclassified.
COMMENT     Annotated using prokka 1.14.6 from
            https://github.com/tseemann/prokka.
FEATURES             Location/Qualifiers
     source          1..830
                     /organism="Campylobacter species"
                     /mol_type="genomic DNA"
                     /strain="strain"
     gene            complement(130..702)
                     /locus_tag="IHOPMAOI_00045"
     CDS             complement(130..702)
                     /locus_tag="IHOPMAOI_00045"
                     /inference="ab initio prediction:Prodigal:002006"
                     /codon_start=1
                     /transl_table=11
                     /product="hypothetical protein"
                     /protein_id="Prokka:IHOPMAOI_00045"
                     /translation="MTYILLLVIISTILSYLILKFIYKIIFKSTKSVSKFLVFLGSIG
                     LIIFYYTPYSYYLEPSFYKFKEICKLDPEIYQANGGKLDEEYYNKVLRYFDTDLDNMS
                     KSYKKELGVTNDKKYSRYWFETWIDGRTEISFEIWFKSNIAIKDNIDFSIKPFFYANW
                     DKMRPLPAGNEGTGFFLGSVPISCIYFKKD"
ORIGIN
        1 ctttaatcgc ttttcatatt tatcattatg attataggat ttatctgcta aatgaaaata
       61 cccataactt gccattgcta attcggcatt atctttgagc tttttaatct gttctttatt
      121 gttcattgtt tagtcctttt tgaaatatat gcaacttatt gggacacttc ctaaaaaaaa
      181 tcctgtcccc tcattacctg ctgggagggg tctcatttta tcccaattag cataaaaaaa
      241 aggttttata ctaaagtcaa tattgtcttt tattgcaata ttacttttaa accatatttc
      301 aaagcttatt tcggttcttc catctatcca tgtttcaaac caatatctac tatattttt
      361 atcattagtt acccctagct ctttttata tgactttgac atattatcca aatccgtatc
      421 aaaatacctt agcactttat tataatactc ctcatctaat ttaccaccat tagcttggta
      481 aatctctggg tctagcttac atatttcttt aaacttataa aagcttggct ctaaataata
      541 agaataagga gtataataaa agataattaa gcctatactt ccaagaaaga ctaaaaactt
      601 tgaaacgctt tttgtagatt taaagatgat tttataaata aattttaata taagataaga
      661 aagaatagta gaaattataa caagaagtaa tatatatgtc ataggttatc tccttagact
      721 ctctatttta ttaaaattaa gatgtaattt tgtggtttag tgttagcagt ataacttttt
      781 ctttatcatt aaaataatag agcttgtttt ttcatatatt tttatccttt
```

```
//

>>BfR-CA-15849_min_cov_30 putative plasmid location

LOCUS       BGIOAKBI_1              3741 bp    DNA     linear       12-APR-2021
DEFINITION  Campylobacter species strain strain.
ACCESSION
VERSION
KEYWORDS    .
SOURCE      Campylobacter species
  ORGANISM  Campylobacter species
            Unclassified.
COMMENT     Annotated using prokka 1.14.6 from
            https://github.com/tseemann/prokka.
FEATURES             Location/Qualifiers
     source          1..3741
                     /organism="Campylobacter species"
                     /mol_type="genomic DNA"
                     /strain="strain"
     gene            complement(66..242)
                     /locus_tag="BGIOAKBI_00001"
     CDS             complement(66..242)
                     /locus_tag="BGIOAKBI_00001"
                     /inference="ab initio prediction:Prodigal:002006"
                     /inference="similar to AA sequence:RefSeq:CKG86410.1"
                     /codon_start=1
                     /transl_table=11
                     /product="hypothetical protein"
                     /protein_id="Prokka:BGIOAKBI_00001"
                     /translation="MKYYATIYIDPLFDFEDATHAFLGLTKDKSPDELDKIDKELRQE
                     YLKNKEFKLIKSIK"
     gene            complement(239..697)
                     /locus_tag="BGIOAKBI_00002"
     CDS             complement(239..697)
                     /locus_tag="BGIOAKBI_00002"
                     /inference="ab initio prediction:Prodigal:002006"
                     /inference="similar to AA sequence:RefSeq:CKG86435.1"
                     /codon_start=1
                     /transl_table=11
                     /product="hypothetical protein"
                     /protein_id="Prokka:BGIOAKBI_00002"
                     /translation="MQIPIYSKLKAKLESKGIDNISSGQIAWFVCSVGIVYLLSIFLY
                     LHGVNKEGISFNRIFIAFYINHFFCFIFMWFILLLNLLYKENTISLILAILTTIVYFY
                     NPYFAYILIFIICIIMRIIKKQRWLYVYFIGLIYIYATYSIFNLRGGFYK"
     gene            complement(744..917)
                     /locus_tag="BGIOAKBI_00003"
     CDS             complement(744..917)
                     /locus_tag="BGIOAKBI_00003"
                     /inference="ab initio prediction:Prodigal:002006"
                     /inference="similar to AA sequence:RefSeq:CKG86410.1"
                     /codon_start=1
                     /transl_table=11
                     /product="hypothetical protein"
                     /protein_id="Prokka:BGIOAKBI_00003"
```

```
                     /translation="MKYCATIYIDPLVECFLDCENKNFINAEDFTHAFLGLTKGKSLD
                     ELDKIDKELRQKF"
     gene            complement(914..1339)
                     /locus_tag="BGIOAKBI_00004"
     CDS             complement(914..1339)
                     /locus_tag="BGIOAKBI_00004"
                     /inference="ab initio prediction:Prodigal:002006"
                     /inference="similar to AA sequence:RefSeq:CKG86435.1"
                     /codon_start=1
                     /transl_table=11
                     /product="hypothetical protein"
                     /protein_id="Prokka:BGIOAKBI_00004"
                     /translation="MQIPIYSKLKAKLESKGIDNITSGQIAWFVCSVGIFIIPMIYFY
                     ISNNYDQFTILFIMIFIFILNMFYTKNFFALLLLVILIFIYMKYHEFIYNFKILFFIF
                     APIILIFLRAWRILITFLCGYIFLVAMVYIFELLGGFYK"
     gene            complement(1609..2076)
                     /locus_tag="BGIOAKBI_00005"
     CDS             complement(1609..2076)
                     /locus_tag="BGIOAKBI_00005"
                     /inference="ab initio prediction:Prodigal:002006"
                     /codon_start=1
                     /transl_table=11
                     /product="hypothetical protein"
                     /protein_id="Prokka:BGIOAKBI_00005"
                     /translation="MQIPIYSKLKAKLESKGIDNISSGQIAWFVCSLILALVTYIILT
                     FHYIYISYGKYDKNLFLLIFDRNAMELFIIIYSCIMFNLLYIYNAIPLVLSAVFIFLA
                     FYFSSLSLFIVGCCGIGVFFLISIFKRKFMFSVLLFALGYTCTLFFFYGLATY"
     gene            complement(2286..2435)
                     /locus_tag="BGIOAKBI_00006"
     CDS             complement(2286..2435)
                     /locus_tag="BGIOAKBI_00006"
                     /inference="ab initio prediction:Prodigal:002006"
                     /inference="similar to AA sequence:RefSeq:AAW35443.1"
                     /note="conserved domain protein"
                     /codon_start=1
                     /transl_table=11
                     /product="hypothetical protein"
                     /protein_id="Prokka:BGIOAKBI_00006"
                     /translation="MKEKINKLRDYAELAWASYFYFDLKGYILQENETIVTLSELVSL
                     SYNGN"
     gene            complement(2445..2750)
                     /locus_tag="BGIOAKBI_00007"
     CDS             complement(2445..2750)
                     /locus_tag="BGIOAKBI_00007"
                     /inference="ab initio prediction:Prodigal:002006"
                     /codon_start=1
                     /transl_table=11
                     /product="hypothetical protein"
                     /protein_id="Prokka:BGIOAKBI_00007"
                     /translation="MGVFRKFYIVWVVFCISGFAISPAVGHNPNRVYEFFVMLGWIIF
                     PLILLMLYRFFSLCEIKFLYIALLLLLYYPIALILYYMFYYHNSFYVTLYIFLSLFK"
     gene            complement(2909..3421)
                     /locus_tag="BGIOAKBI_00008"
     CDS             complement(2909..3421)
```

```
                     /locus_tag="BGIOAKBI_00008"
                     /inference="ab initio prediction:Prodigal:002006"
                     /codon_start=1
                     /transl_table=11
                     /product="hypothetical protein"
                     /protein_id="Prokka:BGIOAKBI_00008"
                     /translation="MKILKSLYIIWMIFWGFGAILGYYMGYDATFTKMFFFLVILYLF
                     PLLPYLHYLIFEKTKNKNIFISICYYLLLFVITAYYFTLIYSCGTNFLTFFIMITLYM
                     IICSAIILWKFKINLRLYFYIILCIFTYLYIVLHIFIACYEIEWTDILLDKDVRIYIY
                     KEIINLLTEQ"
ORIGIN
        1 tttagaataa ataggaattt gcaaatttac tcctaatata gataaattgt tttattataa
       61 ccaatctact taatactttt aattaattta aattctttgt ttttaagata ttcttgtctt
      121 aattctttat ctattttatc taactcatca ggacttttat ctttggtaag tcctaaaaag
      181 gcgtgggtag catcttcaaa atcaaatagt ggatcaatat aaattgttgc ataatacttc
      241 atttataaaa tcctcctctt aagttaaata tactataggt ggcataaata taaattaaac
      301 caataaaata tacataaagc catctttgtt tctttattat acgcataatg atgcaaatga
      361 taaatatcaa aatataagca aaatatggat tataaaaata gacaatagtt gttaatattg
      421 ctaatattaa tgaaatagta ttttctttat agagtaaatt taacaataaa ataaaccaca
      481 taaatataaa acaaaagaaa tgatttatat aaaatgcaat aaatattcta ttaaacgaaa
      541 ttccctcttt attaacgcca tgcaaatata aaaaaataga aagcaaatat actatcccta
      601 cactacacac aaaccaagca atttgtccac ttgatatatt gtctattcct ttactttcta
      661 gttttgcttt gagtttagaa taaataggaa tttgcaaatt tgctcctaat atagataaat
      721 tgttttatta taaactaatt tgcttaaaat ttttgtctta attctttatc tattttatct
      781 aactcatcaa gacttttgcc tttggtaagt cctaaaaagg cgtgggtaaa atcttccgca
      841 ttaataaaat ttttattttc gcaatctaaa aaacattcta ccaatggatc aatataaatt
      901 gttgcacaat acttcattta taaaatcctc ctaataactc aaatatatat accatagcca
      961 ctaaaaaaat atatccgcat aaaaaagtta ttagaattct ccaagccctt aaaaagataa
     1021 gaattatagg tgcaaaaata aaaaataaaa ttttaaaatt atagataaac tcatgatatt
     1081 tcatatatat aaatattaat attacaagta ataataatgc aaaaaaattt ttagtataaa
     1141 acatatttaa tataaaaata aatatcataa taaataaaat agtgaattga tcataattgt
     1201 tgctgatata aaaatatatc ataggaatga taaaaatacc tacactacac acaaaccaag
     1261 caatttgtcc gcttgttata ttgtctattc ctttactttc tagttttgct ttgagtttag
     1321 aataaatagg aatttgcaaa tttgctccta atatagataa attgttttat tataaactaa
     1381 tttgcttaaa atttttttatt aatttgaatt tttgttttta agatattctc gtcttaattc
     1441 ttcatctatt ttatctaact catcaggact tttgcctttg gtaagtccta aaaaggcgtg
     1501 ggtaaaatct tctatttgcc aataacactg atttttaaaa tccaaaaaaa cattctacca
     1561 atggatcaat ataaattgtt gcataatatt tcatttataa aatcctttct aatatgttgc
     1621 cagtccataa aaaaagaaca atgtgcaagt ataacccaag gcaaatagta aaacactaaa
     1681 cataaacttt cttttaaaaa tggatattaa gaaaaatact cctataccac aacaaccaac
     1741 aataaacaag cttaaactag aaaaataaaa tgctaaaaat ataaatactg cagataaaac
     1801 caaaggaatg gcattgtaaa tatataatag attaaacatt atacaagaat atataataat
     1861 aaataattcc attgcatttc tatcaaatat taataaaaat aaattcttat catatttacc
     1921 ataactaatg taaatgtagt gaaaagttag aataatataa gtcaccaaag caagaatcaa
     1981 gctacataca aaccaagcaa tttgcccact tgatatattg tctattcctt tactttctag
     2041 ctttgctttg agtttagaat aaataggaat ttgcaaattt actcctaata taacaagtta
     2101 ttctattata agacaattta tttaaaaatt tgattgtttc agtaagaaaa aatttcttga
     2161 aatcatcatt aaattcacaa ggctctttag cttcatttat tttatctact tattctaaaa
     2221 gaggaaaaaa ttggataatg tcattaaaag atagggctat gtcttgaacc gagtcaataa
     2281 aattatcagt ttccgttata acttaaactt acaagctcac ttaaagtaac gatagtttca
     2341 ttttcttgta atatataacc ttttaaatca aaataaaaat aactagccca tgcaagctct
     2401 gcataatctc ttagcttatt gattttttct ttcaaaatta tcctttattt aaataatgat
     2461 aaaaaaatat ataatgtaac ataaaaacta ttatgataat aaaacatgta atacaatatc
     2521 aaagctatag gataataaag taacaatagc aatgctatgt acaaaaactt tatttcacaa
```

```
     2581 agagaaaaaa agcgataaag cattaataga attaaaggga aaataatcca acctagcatt
     2641 acaaaaaatt catacactct atttggatta tgccctacag caggagaaat agcaaatcca
     2701 cttatacaaa atactaccca aacaatataa aacttacgaa atacacccat ttattttcct
     2761 ttttaatatt tttagtaaat tttctacttt tttaaaaatt aaaataaaaa taactagccc
     2821 aagctagttc tacataatct cttaaattgt tgatttgggt tttcattaat ttattcccct
     2881 ataaaaataa gatttattat aaaacaaatt attgctccgt aagcaaatta attatttctt
     2941 tgtaaatata tatccttaca tctttatcta gcagtatatc tgtccattct atttcataac
     3001 atgctataaa aatatgtaaa actatataga gataagtaaa aatacataaa attatgtaaa
     3061 aatataatct taaatttatt ttaaatttcc ataagattat agcactacaa ataatcatat
     3121 ataaagtaat cattataaaa aatgttaaaa aatttgttcc acatgaatat attaaagtaa
     3181 aataataagc ggtaataaca aataatagta gataataaca aatagaaatg aatatatttt
     3241 tgttctttgt tttctcaaaa attaaataat gcaaataagg caacaatgga aaaagatata
     3301 atataacaag aaaaaagaac attttagtaa aagttgcatc atatcccata taataaccta
     3361 aaatagcccc aaatccccaa aatatcatcc aaataatata taaacttttt aatattttca
     3421 cactacttcc ttttttgttg aaatttaaat tattattaaa aaattaaaaa aaccaatcta
     3481 tacaagttta gcgtaatttt tgagattgtt aatttgagct ttattactca ttttttaatt
     3541 ctatataaaa aataaaaaga tacaaaaaaa tgtataaaaa aatacaaaat aataaagaaa
     3601 aataataaga aagttctttt ctaaatttaa tagatattat taaaaataat attgtataaa
     3661 tacctaatat tattacataa ataaaaaatc ctcccgagat aaagcaaaat aaagcaaaat
     3721 aaagcaaaat aaagaaaaat a
//
LOCUS       BGIOAKBI_2              3223 bp    DNA     linear       12-APR-2021
DEFINITION  Campylobacter species strain strain.
ACCESSION
VERSION
KEYWORDS    .
SOURCE      Campylobacter species
  ORGANISM  Campylobacter species
            Unclassified.
COMMENT     Annotated using prokka 1.14.6 from
            https://github.com/tseemann/prokka.
FEATURES             Location/Qualifiers
     source          1..3223
                     /organism="Campylobacter species"
                     /mol_type="genomic DNA"
                     /strain="strain"
     gene            432..764
                     /locus_tag="BGIOAKBI_00009"
     CDS             432..764
                     /locus_tag="BGIOAKBI_00009"
                     /inference="ab initio prediction:Prodigal:002006"
                     /codon_start=1
                     /transl_table=11
                     /product="hypothetical protein"
                     /protein_id="Prokka:BGIOAKBI_00009"
                     /translation="MNTLKKIYIIWGMFWTIGLGIFVPTAHGFDSMDFVDFFILALQF
                     IAIIWLPPLLIFNIVYFVIKKERKYIINTLWILLYYPIALILYYMFYYHNSFYVTLYI
                     FYHYLNKG"
     gene            767..1213
                     /locus_tag="BGIOAKBI_00010"
     CDS             767..1213
                     /locus_tag="BGIOAKBI_00010"
                     /inference="ab initio prediction:Prodigal:002006"
                     /inference="similar to AA sequence:RefSeq:AAW35443.1"
```

```
                     /note="conserved domain protein"
                     /codon_start=1
                     /transl_table=11
                     /product="hypothetical protein"
                     /protein_id="Prokka:BGIOAKBI_00010"
                     /translation="MKEKIDKLRDYAELAQASYFYFDLKGYILQENETIVILNELVSL
                     SYNGKIAGKKEKVGQEYSFIGKGELNGEFGELQARNFSQRYEIKFHQSNTLSGFSATL
                     FYDKQKDEFVVGFRGTEGFWNMDTMQDITLSLNGNLQSSPLLEFLE"
     gene            1666..2379
                     /locus_tag="BGIOAKBI_00011"
     CDS             1666..2379
                     /locus_tag="BGIOAKBI_00011"
                     /inference="ab initio prediction:Prodigal:002006"
                     /inference="similar to AA sequence:RefSeq:APA47419.1"
                     /codon_start=1
                     /transl_table=11
                     /product="lysozyme"
                     /protein_id="Prokka:BGIOAKBI_00011"
                     /translation="MQNLKLQIAQGLDKILEDEDRKQELYVCKFVVVNGASENKIKAK
                     NKDVNIDNDTKNINTTKSNNISSSENNAQEKITHLSNDGQNLLKNIEKLRLKPYNDQN
                     GKEITSYVKGATIGYGHLIGQNEWDLYKNGITLQEADKLFKSDLLPFENAVKNSINSS
                     LAQNEFDALVILCFNIGIDNFKNSSVAKIINGEKTGYKTLKEAWMAWNKSQNKVMQRL
                     INRRNAEYKLYTQGGYEKW"
     gene            2373..2732
                     /locus_tag="BGIOAKBI_00012"
     CDS             2373..2732
                     /locus_tag="BGIOAKBI_00012"
                     /inference="ab initio prediction:Prodigal:002006"
                     /inference="similar to AA sequence:RefSeq:CKG86986.1"
                     /codon_start=1
                     /transl_table=11
                     /product="hypothetical protein"
                     /protein_id="Prokka:BGIOAKBI_00012"
                     /translation="MVIFKNILLFLFFSTYLLAQEDFTPLEQCTYENEKFWIKILNLC
                     PEGNITCDKIVYVGVNKNNGKYIVLNGKSISDVNMNFKGYVFKNGIYEYNIFNNFLYI
                     SKNKQIIQEYRLKLCEK"
     gene            2999..3100
                     /locus_tag="BGIOAKBI_00013"
     CDS             2999..3100
                     /locus_tag="BGIOAKBI_00013"
                     /inference="ab initio prediction:Prodigal:002006"
                     /inference="similar to AA sequence:RefSeq:AAW35464.1"
                     /note="conserved hypothetical protein"
                     /codon_start=1
                     /transl_table=11
                     /product="hypothetical protein"
                     /protein_id="Prokka:BGIOAKBI_00013"
                     /translation="MFISSKTELIQTIKNLTINNKEVKQIDFIYPNF"
ORIGIN
        1 atattatttg gatgatattt tggggatttg gggctatttt aggttattat atgggatatg
       61 atgcaacttt tactaaaatg ttcttttttc ttgttatatt atatcttttt ccattgttgc
      121 cttattttta ttatttgatt tataaaaaag caagaaatac aaaaaatata tttattgcta
      181 tttattgcta tttattgtta tttgttgcta ctattgatta atagaaataa tagtaatttt
      241 gagctgtact taattattat tgtgatacat attataatat ttgcaataat ttatttaatt
```

```
      301 aaaagaaata aaaatgaaaa ctcaaatcaa taatttaaaa gattatgcag aacttgcatg
      361 ggctagttat gtttatttta atttttaaaa aagtagaaga attttactaa aaatattaaa
      421 aaggaaaata aatgaataca ttaaagaaaa tttatatcat ttgggggatg ttttggacta
      481 ttggacttgg aatttttgtg cctacagcac atggatttga tagtatggat tttgtggatt
      541 ttttcatact tgctttacaa tttatagcta taatttggtt gcctccatta ctcattttta
      601 atattgtata ttttgtgata aaaaaagaaa gaaaatatat tataaataca ttatggatat
      661 tattgtatta tcctatagct ttgatattgt attacatgtt ttattatcat aatagttttt
      721 atgttacatt atatattttt tatcattatt taaataaagg ataattttga aagaaaaaat
      781 cgataagcta agagattatg ccgagcttgc acaagctagt tatttttatt ttgatttaaa
      841 gggttatata ttacaagaaa atgaaactat cgttatttta aatgagcttg taagtttaag
      901 ttataacgga aaaatagctg gaaaaaaaga aaaggtcgga caagaatata gttttattgg
      961 caaaggtgaa ttaaatggag aatttggaga attacaagct agaaatttct ctcaaagata
     1021 tgagattaaa tttcatcaat ccaacacttt aagtggtttt tctgctactt tattttatga
     1081 taaacaaaaa gatgaattcg tggtgggatt tagaggaact gagggttttt ggaatatgga
     1141 tacgatgcaa gacataactc tatctcttaa tggcaatctc caatcttctc ctcttttaga
     1201 atttttagaa taagtagata aaatcatcaa aaatgaaaat aaaagtatca tatttgtagg
     1261 gcattcattg ggtgagattt gggggatgca atgaaaatat taaaaatact ttatatttgc
     1321 tggattatat tttttatctt tgcttggata atatcacctt taattgggca taatccagat
     1381 agatttaagg aatttctcat agtgattgga tggataattt tgcctccaat agtgaacaat
     1441 ctatggttat ttttaataac aagaattaaa aaatatttga taaggttttt tctattattg
     1501 ttatattgtc cattggcaat tttattatat cttatatttg attaaaagga aaaaatatga
     1561 agctatttta cacagctaca tgagtatgat aaaatcatca aaaattcttt agaagataag
     1621 aaagaattaa acgaatatca tttaactttt ttaactcctg caaaaatgca aaatttaaaa
     1681 cttcaaatcg cacaaggact agataaaatc ttagaagatg aagatagaaa acaagagctt
     1741 tatgtttgta aatttgtggt ggtaaatggg gctagcgaaa acaaaataaa agcaaaaaat
     1801 aaagatgtaa atatagataa tgacacaaag aatataaaca ctacaaaaag caataatata
     1861 agttctagtg aaaacaatgc tcaagaaaaa atcactcatc taagcaatga tggtcaaaat
     1921 cttttaaaaa atatagagaa gctaagactc aaaccttata atgatcaaaa tggaaaagaa
     1981 attacttcat atgttaaagg tgcaactatc ggatatggac atcttatagg gcaaaatgaa
     2041 tgggatttgt ataaaaatgg tattacttta caagaagcgg ataaattgtt taaaagcgat
     2101 ttgttacctt ttgaaaatgc tgtaaaaaat tcaataaact cttccttagc acagaacgaa
     2161 tttgatgcct tggttatatt atgttttaat atagggattg ataattttaa aaattcttct
     2221 gttgctaaaa tcatcaatgg agagaaaacg ggttacaaaa cattaaagga agcttggatg
     2281 gcgtggaaca aaagtcaaaa taaagtaatg cagaggctaa ttaataggag aaatgctgaa
     2341 tataagcttt acactcaagg aggttatgaa aaatggtaat ttttaaaaat atattattgt
     2401 ttttattttt tagtacttat ttgttagcgc aagaggattt tactccacta gagcaatgta
     2461 cctatgaaaa tgaaaagttt tggataaaaa ttttaaatct atgtccagag ggtaatataa
     2521 cttgtgataa aattgtatat gtaggtgtca ataaaaataa tggaaaatat attgtcctaa
     2581 atggtaagtc tattagtgat gtaaatatga attttaaagg atatgttttt aaaaatggta
     2641 tttatgaata caatattttt aataattttt tatatatttc taaaaacaag cagattatac
     2701 aagaatatcg tctgaaatta tgtgaaaaat agacacagca aaggcaacaa gcaacaacaa
     2761 atacctagaa tatgtaaaaa gttaaaaaga tgcaataaga attacaagaa gtttttaaag
     2821 ttataaaaat cctaattaat gaaatcttag ctcctaaatt ttataacaaa tacaaagatt
     2881 tagaagtaga gattgatgat aagggtaata ttataagatg gataggagag attaataaag
     2941 acaaaaaata ttagcaaata aaaaatgata taataatgct ttatataagg aatcttttat
     3001 gtttataagt tctaaaacag agttaattca aactattaaa aatttaacaa tcaacaataa
     3061 agaagttaaa cagattgatt ttatttatcc taatttttaa agaatgatga aaatcttaat
     3121 acttctttta ctaatcttaa tgctttaaaa ttcgctttag aagaaaagaa tttttatctt
     3181 aattttatag acttaaacag caaagaaagt cttttaagtt ttt
//
LOCUS       BGIOAKBI_3               929 bp    DNA     linear       12-APR-2021
DEFINITION  Campylobacter species strain strain.
ACCESSION
VERSION
```

```
KEYWORDS    .
SOURCE      Campylobacter species
  ORGANISM  Campylobacter species
            Unclassified.
COMMENT     Annotated using prokka 1.14.6 from
            https://github.com/tseemann/prokka.
FEATURES             Location/Qualifiers
     source          1..929
                     /organism="Campylobacter species"
                     /mol_type="genomic DNA"
                     /strain="strain"
     gene            complement(73..378)
                     /locus_tag="BGIOAKBI_00014"
     CDS             complement(73..378)
                     /locus_tag="BGIOAKBI_00014"
                     /inference="ab initio prediction:Prodigal:002006"
                     /inference="similar to AA sequence:RefSeq:AVS37480.1"
                     /note="DUF2974 domain-containing protein"
                     /codon_start=1
                     /transl_table=11
                     /product="hypothetical protein"
                     /protein_id="Prokka:BGIOAKBI_00014"
                     /translation="MTNKQLINKLRDMAELAQASYGYFDLVTHDKNDFFIKIKDEATE
                     SAFFNKHITQANVLDISYKNYKAYQYDSSLKKDIKIDTLNGDFSPLKLNNSFLAMIY"
     gene            complement(397..759)
                     /locus_tag="BGIOAKBI_00015"
     CDS             complement(397..759)
                     /locus_tag="BGIOAKBI_00015"
                     /inference="ab initio prediction:Prodigal:002006"
                     /codon_start=1
                     /transl_table=11
                     /product="hypothetical protein"
                     /protein_id="Prokka:BGIOAKBI_00015"
                     /translation="MCKLNELPNNEEKYNKILGYFDKKLGDIDDFRHVKKYGSIEMWL
                     YIYYDDTYKTPSNFQEKIEMDNIALKTKNMQMSLHKIVDMHISPYWNTRRYVLEGNEG
                     NMNFEFIEDELACGNLYQ"
ORIGIN
        1 tttgagctaa atacccacca gagaatgtcc tacaaatatg atacttttat tttcattttt
       61 taggacaatg ttttaataaa tcatagcgag aaaagaattg tttagcttga gtggggagaa
      121 gtcgccattg agagtgtcaa ttttaatatc tttctttaaa gatgagtcat attgatatgc
      181 cttataattt ttataactaa tatctaagac attagcttga gtaatgtgct tattaaagaa
      241 agcagattct gtagcttcat ctttaatttt aatgaaaaaa tcattcttgt catgtgttac
      301 caaatcaaaa taaccataac ttgcctgtgc aagttccgcc atatccctta gtttattaat
      361 aagttgttta ttggtcatta tttattcctt atattattat tgataaagat taccacaagc
      421 aagctcatcc tctataaact caaaattcat attaccctca ttgccttcta acacatacct
      481 tctagtattc caatatggac ttatatgcat atcaacaatc ttgtgtaaag acatttgcat
      541 attttttgtt tttagtgcaa tattatccat ctctatcttt tcttgaaaat ttgaaggagt
      601 tttgtaagta tcatcgtaat atatataaag ccacatttct atactaccat attttttac
      661 atgtcgaaaa tcatctatgt cacctaactt tttatcaaaa taccctagaa tcttattata
      721 tttctcttcg ttattaggaa gttcatttaa cttacacata ttcctaaatt gccaataact
      781 tggctctaaa taataagaat atggagtata ataaaagata attaaaccta tgcttccaag
      841 aaagactaaa aactttgaaa cgctttttgt agatttaaag atgattttat aaataaattt
      901 taatataaga taagaaagaa tagtagaaa
//
```

```
>>BfR-CA-15851_min_cov_30 putative plasmid location

LOCUS       FHGGBLIP_1             13234 bp    DNA     linear       12-APR-2021
DEFINITION  Campylobacter species strain strain.
ACCESSION
VERSION
KEYWORDS    .
SOURCE      Campylobacter species
  ORGANISM  Campylobacter species
            Unclassified.
COMMENT     Annotated using prokka 1.14.6 from
            https://github.com/tseemann/prokka.
FEATURES             Location/Qualifiers
     source          1..13234
                     /organism="Campylobacter species"
                     /mol_type="genomic DNA"
                     /strain="strain"
     gene            complement(1570..2478)
                     /locus_tag="FHGGBLIP_00001"
     CDS             complement(1570..2478)
                     /locus_tag="FHGGBLIP_00001"
                     /inference="ab initio prediction:Prodigal:002006"
                     /inference="similar to AA sequence:RefSeq:AJK71146.1"
                     /codon_start=1
                     /transl_table=11
                     /product="type VI secretion protein"
                     /protein_id="Prokka:FHGGBLIP_00001"
                     /translation="MNDLASYSFFKLIKKLEKDYGRKNIFLRTNKSLKHPNKDIEKII
                     FSEHEQSVVELFINFMGLHGVSSQLPSFMLDKLSRNEDAEQGWTLFFDFFNHYLLWIF
                     FDVVSLKNYPRSFNENFKDSISQILFNILGIKEHDIAKKYLPFAPLLLSLRRPKTHIE
                     RVLQVNFKLKDKLSIIENLPHQILISDSQKNKLGIKNHTLGNNFILGDKFISYQNKIA
                     IYIKDISYQEAVKFMPNGTKHDDLKNSIMFLTNNEFCVDLYLKINYSSEMKFVLGDEN
                     TAKLGWAKILGNAQKKYTIVYMKLCE"
     gene            complement(2475..4196)
                     /locus_tag="FHGGBLIP_00002"
     CDS             complement(2475..4196)
                     /locus_tag="FHGGBLIP_00002"
                     /inference="ab initio prediction:Prodigal:002006"
                     /inference="similar to AA sequence:RefSeq:AJK71145.1"
                     /codon_start=1
                     /transl_table=11
                     /product="type VI secretion protein"
                     /protein_id="Prokka:FHGGBLIP_00002"
                     /translation="MKDNIFYYQKELEYLYEKREHFIKNYPKLTPFLAYDSKDPDIER
                     IIENLAILSSKIHQELDENIPHIAESLINIISPNYTNPLPSLCMQEFKFEQNSKENNL
                     IIPKGTLIKSKPIDKCVCEFKTVYDVYLYPISINEVFISSKNQDYTFNLTLQVNKAET
                     KICDLGLEKINLYLGNDTYMSSTLLLYMHSYLKELKIQSLDTDEEFFLNTYNIEKIGL
                     NPDESSLSYNDLGFEAFSLLREYFFMPHKFNFLRINGLDILNNCQGKTINIEFKFSKP
                     FPANCIFRKELLSLGMTPIINIFAKSAEPLINNHKKDSYRIFVDRSQPKAYEIIQTLQ
                     VKAHNSEGGKRLLKNYKSFERFEFLKDNQKDFYSVNTKKNSKGEVFSEISFFSSYLID
                     ETISIDLLCSNGDLPSKLKIGDINTCNLKGVDTKNVEIPSETRRCSVDGNLLWKLVSV
                     LSFSYQTILSKQAFFGVLESYSFLDNQSNWKIYKLLQEAIIDIQSKSTYLIDENITKK
```

```
                     GTVAIFSIKDSKFYTLGEVYLLGLIISKFLASFASINSFCELKIKCLDSKEILHYPAS
                     FGKKALI"
     gene            complement(4193..4585)
                     /locus_tag="FHGGBLIP_00003"
     CDS             complement(4193..4585)
                     /locus_tag="FHGGBLIP_00003"
                     /inference="ab initio prediction:Prodigal:002006"
                     /inference="similar to AA sequence:RefSeq:CKG87312.1"
                     /codon_start=1
                     /transl_table=11
                     /product="type VI secretion system lysozyme-like protein"
                     /protein_id="Prokka:FHGGBLIP_00003"
                     /translation="MSLLDKLIHNLDEQNIHIPFYQNDFEDVKNNIKVLLNAKINDCY
                     AVKNLGMPNMADINLNSNELCVSMAKEIRKLIDNYEKRICVVSITYDNSLSPWQLSFI
                     VKCFFQDDRFKEFNIEIIFKNNRYCEVK"
     gene            complement(4588..6042)
                     /locus_tag="FHGGBLIP_00004"
     CDS             complement(4588..6042)
                     /locus_tag="FHGGBLIP_00004"
                     /inference="ab initio prediction:Prodigal:002006"
                     /inference="similar to AA sequence:RefSeq:AJK71143.1"
                     /codon_start=1
                     /transl_table=11
                     /product="type VI secretion protein"
                     /protein_id="Prokka:FHGGBLIP_00004"
                     /translation="MANTKVIDTPIIEQIMEKSKYSKTDESYSIAKRGVAEFISEIVK
                     SDNAEEKINKFALDEMIAHIDHLLSKQMDEVLHNEEFQKLESTWRGLRFLVERTDFNE
                     NIKIDLFDITKEEALEDFENNPDITQSIVYKNIYSSEYGQFGGEPVGAIIGDYQLGSA
                     SPDMTFLNKMASIAAMSHSPFLTSFGPKFFGLDDYSELASIQDLQGLLEGPQYTRWRT
                     FRENEDSKYTGLLVTRFLARSPYDPEENPIKSFNYKENVHTSHNHLLWANSSYTFCTR
                     LTESFAKYRWCGNIIGPKSGGTVRDLPTYLYENFGTIQSKIPTEVLITDRREYELAEA
                     GFITLTLRRDSNNAAFFSANSPLKPKIFQNTPEGKEAETNYRLGTQLPYIFLISRLAH
                     YLKVLQREEIGSWKERSDIENGLNEWIRQYISDQENPPSEVRSRRPFRAAQVKVDDIP
                     GEPGWYKIGLSVRPHFKYMGGNFELSLVGKLDKE"
     gene            complement(6044..6529)
                     /locus_tag="FHGGBLIP_00005"
     CDS             complement(6044..6529)
                     /locus_tag="FHGGBLIP_00005"
                     /inference="ab initio prediction:Prodigal:002006"
                     /inference="similar to AA sequence:RefSeq:AJK71142.1"
                     /codon_start=1
                     /transl_table=11
                     /product="type VI secretion protein"
                     /protein_id="Prokka:FHGGBLIP_00005"
                     /translation="MSDGSHAPKERINITYKAKTNGQNEDVELPLKLMVMANLKGKNE
                     TPLEEREILQINKINFDQVMRKLDITTSFSVKNTLGTGAEELDVKLNIASMKDFSPDS
                     LAKQIPELNKLLQLREALMALKGPMGNIPDFRKAVLEALKNEKTKEKLLLEIKQEEQG
                     N"
     gene            complement(6598..7845)
                     /locus_tag="FHGGBLIP_00006"
     CDS             complement(6598..7845)
                     /locus_tag="FHGGBLIP_00006"
                     /inference="ab initio prediction:Prodigal:002006"
                     /inference="similar to AA sequence:RefSeq:AJK71141.1"
```

```
                     /codon_start=1
                     /transl_table=11
                     /product="nucleobase:cation symporter"
                     /protein_id="Prokka:FHGGBLIP_00006"
                     /translation="MSFCDQLKNNLDELQEFHLLEDEMSKYKTLNHENISWDKVYQYS
                     QFILLHHSLDFKVCNYFLLSCFNLNNEECFEKLLLLFQHLKKLMDENNTYILAQKRKI
                     QIFIQNFIQEYNKTKVPVSSEIINQFIALFSEFENLLSCNFAKIEIAQVVPQLPKSPV
                     TQVSINIRSDNINSLNEREYKNFYQKLAFELLEEDENNLNIYALFTQAMWGKIKCLPE
                     CNNEKITKIRKPDTDIIRILLSDKKNDIEYIKCFMHELILNPFWIEGVQLFCNFLEKK
                     KKNKQLDILIILTSDFISKFDTIELLRFQNGDFICKEEVYKYFVKPKENKKSFFSSKK
                     TDKEHTLQDFEQMLMNIDKENFNNSIMNNINSLLDMVKIFESKGMKKNSKILNIYLVE
                     LMEKTLLKDYLAEEYENAKNKIK"
     gene            7969..8415
                     /locus_tag="FHGGBLIP_00007"
     CDS             7969..8415
                     /locus_tag="FHGGBLIP_00007"
                     /inference="ab initio prediction:Prodigal:002006"
                     /inference="similar to AA sequence:RefSeq:AJK71140.1"
                     /codon_start=1
                     /transl_table=11
                     /product="type VI secretion protein"
                     /protein_id="Prokka:FHGGBLIP_00007"
                     /translation="MFCKKSLFFLLPLFFCACSSVVSVKINNIENSNLNNRHDDVPVT
                     AIIYQLKDIKKFEEASDIDLATREDGVLGKDKLDSIKTQIAPKDNIIALKVDEGEVPY
                     VGVLVLFANNTKKVTKIWAKTEDANGFGKNKYLKFEISKEGIKRIK"
     gene            8425..9822
                     /locus_tag="FHGGBLIP_00008"
     CDS             8425..9822
                     /locus_tag="FHGGBLIP_00008"
                     /inference="ab initio prediction:Prodigal:002006"
                     /inference="similar to AA sequence:RefSeq:AJK71139.1"
                     /codon_start=1
                     /transl_table=11
                     /product="type VI secretion protein"
                     /protein_id="Prokka:FHGGBLIP_00008"
                     /translation="MANKLKVAWFDGLNIGQTHFEQQERFFNRNIDLKTINIYSNLYG
                     IIDLEFSQEMLLQGKIALSKISGIAQDGSIFNAPEQDLLPEPIEINYESLIDSVVVLK
                     IPIGVTDIADLSLRNTFPNSKYICLRNSIALRNYDDSKSNAMDKIEDEYELENLTFTQ
                     EKKDLLLASLRLKLGILGNSTPDELELPIAKIKNIDINKKIELESDFIPTCLNISKIS
                     TIRSFLEEIIFSINQHKKVLSNVFKGIDQTKNTLDFSTFLSLNLLKKWYLIFSHLSKK
                     DKIHPEFLYEKFLEFQGELAAFSNEESFLDFIPYKHDNLYNTFLNMMNNLRLLFSKIT
                     SPKYFIAKTSISGNGFYDFIFDNSGILEDAEIYFAIRADVATEYLLNNFKIQSKIHTQ
                     SKIKNIVATQLEGLNVDQVPNVPSSIPYLNGYVYYKIDKRDQLFKDFKNESIISMYLT
                     NNIKNPDIIMWAVFQ"
     gene            9819..10592
                     /locus_tag="FHGGBLIP_00009"
     CDS             9819..10592
                     /locus_tag="FHGGBLIP_00009"
                     /inference="ab initio prediction:Prodigal:002006"
                     /inference="similar to AA sequence:RefSeq:AJK71138.1"
                     /codon_start=1
                     /transl_table=11
                     /product="glutamyl-tRNA amidotransferase"
                     /protein_id="Prokka:FHGGBLIP_00009"
```

```
                     /translation="MKEINEEKKALSLLLDSNFDGLKNNKIIDYSLELLLLSYRLSKI
                     SSMDTSNINQLRETLINKILDITAKLSMCKEYDEKEIIKFKYCLCVFIDESLMKNELF
                     INFWAHNTLTVRLFDETLGGNNFYDIASSWINNPFKFKDFLEFIYACLILGYKGKYNE
                     AKDRDEKIIHFCNNIATSLRPVYKTEEDLAFNKAYKIGLKEDIWQKFIRLYFKKLIII
                     VPVLIVLGVLSFAIFNLETNNLKVDNNISVLIKNLTHIK"
gene                 10818..11390
                     /locus_tag="FHGGBLIP_00010"
CDS                  10818..11390
                     /locus_tag="FHGGBLIP_00010"
                     /inference="ab initio prediction:Prodigal:002006"
                     /codon_start=1
                     /transl_table=11
                     /product="hypothetical protein"
                     /protein_id="Prokka:FHGGBLIP_00010"
                     /translation="MTYILLLIIISTILSYLILKFIYKIIFKSTKSVSKFLVFLGSIG
                     LIIFYYTPYSYYLEPSFYKFKEICKLDPEIYQANGSKLDEEYYNKVLRYFDTDLDNMS
                     KSYKQELGVTNDKKYSRYWFETWIDGRTEISFEIWFKSNIAIKDNIDFSIKPFFYANW
                     DKMRPLPAGNEGIGFFLGSVPISCIYFKKD"
gene                 11394..11495
                     /locus_tag="FHGGBLIP_00011"
CDS                  11394..11495
                     /locus_tag="FHGGBLIP_00011"
                     /inference="ab initio prediction:Prodigal:002006"
                     /codon_start=1
                     /transl_table=11
                     /product="hypothetical protein"
                     /protein_id="Prokka:FHGGBLIP_00011"
                     /translation="MKNKAVINKLKDNSELAWASYGYFHLATSKAIE"
gene                 11492..11920
                     /locus_tag="FHGGBLIP_00012"
CDS                  11492..11920
                     /locus_tag="FHGGBLIP_00012"
                     /inference="ab initio prediction:Prodigal:002006"
                     /codon_start=1
                     /transl_table=11
                     /product="hypothetical protein"
                     /protein_id="Prokka:FHGGBLIP_00012"
                     /translation="MKKYKKILVLLSPIILIILFYTPFIYPSYWEFRGLCKLNNYPKS
                     EEKYNTILAYFDKSLDGSIGKNGYAKIGYSNRIDLGVYIYYKNPNNKTLTFKNINKIY
                     FRPIWKNYAPNIYGNEGNMDFRLKLDGEIDCRSLLGELGG"
gene                 11913..12146
                     /locus_tag="FHGGBLIP_00013"
CDS                  11913..12146
                     /locus_tag="FHGGBLIP_00013"
                     /inference="ab initio prediction:Prodigal:002006"
                     /inference="similar to AA sequence:RefSeq:AVS37480.1"
                     /note="DUF2974 domain-containing protein"
                     /codon_start=1
                     /transl_table=11
                     /product="hypothetical protein"
                     /protein_id="Prokka:FHGGBLIP_00013"
                     /translation="MDNKQQINKLRDMAELAWAAYGYFHYVDNKFDIKDEDKIVTFED
                     VLDITYKNSKIIDERGFKIGTLNGDFSPLQAKQ"
gene                 12227..12865
```

```
                     /locus_tag="FHGGBLIP_00014"
     CDS             12227..12865
                     /locus_tag="FHGGBLIP_00014"
                     /inference="ab initio prediction:Prodigal:002006"
                     /codon_start=1
                     /transl_table=11
                     /product="hypothetical protein"
                     /protein_id="Prokka:FHGGBLIP_00014"
                     /translation="MLEFFVNLLIFILIFFFFVFIFSIILYFIFKIFYKLKNKTKTIN
                     FKTMFKKLALFLLPVYLLLFLAGGCSYKYMDPQYYEFKKLCKDNSNKMIVFNKDYLDL
                     KKQFVQAMTNNSNIRIKNNGIKYFYNEKLNLEIQPSNWKEIETKSREIKLGIGKVKEK
                     EIEAWYIDDKNNIKYQEFKRYLYYNYSIFLEGDEGAGFHFEYEEILDCGDVR"
     gene            12906..13034
                     /locus_tag="FHGGBLIP_00015"
     CDS             12906..13034
                     /locus_tag="FHGGBLIP_00015"
                     /inference="ab initio prediction:Prodigal:002006"
                     /codon_start=1
                     /transl_table=11
                     /product="hypothetical protein"
                     /protein_id="Prokka:FHGGBLIP_00015"
                     /translation="MSKNIKTQQAKLDLIAKFLDYANVADASYAMLHWIDYVKKRK"
ORIGIN
        1 catcagtata aatagtattt ctctcacttt ctatatcctt actctctcct ataacaatac
       61 cttgagtatt atgaggggct ttaggcttag ctttaaaact tggagtaaag ataatattct
      121 tttttaataa agttaaagta ttagaataag acttacttaa atttaaatct tttaaattta
      181 aatgatcatt agtattgata gaattagcta gtaaagcatc atctattaag atttgagtat
      241 tggcaataat aataaagtct ttaagcaaag aagctttatc ttgactttta gtatcttcat
      301 catcactcaa tccactcaaa ttaaattcct tataatttaa agtgataaaa tctcccaagc
      361 taagatgata aatattactt tttgctagta agctttcatt taagatcaag gctctttttt
      421 ctttaagaga aggcttagtc tttaaatcaa tacttcttgt aaaagaatat tcactctcat
      481 aaaaatgttc attataataa gctttgttat taagttcttg ttcataagga actttactag
      541 agtgtaaaga taaaagatgt aaaggagtat tagcattgat actagaatgt gtaaaagcat
      601 tagttcttaa actttgctct ttatagatag aagaaataca agcttgattt aaaatattat
      661 tcacattagg attgtactca atttctttat tttttacatt tttatacaca tcacaaaaat
      721 aaatagtgtt ttcatcttca taaaaaaaga ttccattatt gtgagctaat cttgtgataa
      781 attctaaatc gctttcattg tattgagaga tgagttcttg ttcttcataa ttaaaatgta
      841 tatgagaata atcaatttct ttatgtaaga tatcttgata aaaaccaagg gtttgtttaa
      901 tcacttctat gatatgagta tgagtataaa tacgattggc tttatttaaa gaaagtctta
      961 tcaagactga ttgaagctta aaagaaaaaa aatgcttgta ttttatttgc ttagtgtttg
     1021 aagtgctatc attgatattt aaagcactct catgattgat acctagatat tttatataag
     1081 tgatgattcc tttatagttt tttacttcat tatgatcaaa gtttaaggta ttgttctcat
     1141 aaggattatg gatacttaaa atagcttctt tatcgattaa aacattagga tgaaaattaa
     1201 aagttgtagg ggtgcttgga tgagatgaag cattggttaa agtatttaaa gaaaataaat
     1261 cttgttctaa actttcaaaa aagccttcac attctataga aaaaagctct tcaagacttt
     1321 ctttgatgag ggccttagtg attttaaagg gtaggggatt tgaagtatga tttaatatat
     1381 caagactcat ataagaatta agggacattg atgatccttt tgggtttgtt gatgattaat
     1441 agcctatagc ttatgtgatt tattttagca taaggtgggt aaaataaagt ttattttagg
     1501 gtatggttta tgggtaaaga gagattgtgg ttttttgaaa taaaatttta aatatatttt
     1561 tgtgttaggc tattcgcata gtttcatata tactattgta tatttttttt gtgcattgcc
     1621 taaaattttt gcccaaccaa gtttggcagt attttcatct cctaacacaa acttcatttc
     1681 tgaagaatag ttgattttaa gatataaatc aacacaaaat tcattatttg ttaaaaacat
     1741 tatactattt ttcaaatcat catgtttggt tccattgggc ataaatttaa cggcttcttg
     1801 atatgaaata tcttttatat aaattgctat tttgttttga taagagataa atttatcgcc
```

1861 caatataaag ttatttccta gggtatgatt ttttatccct agtttatttt tttgagaatc
1921 tgaaattaaa atttgatggg gtaaattttc aataattgat aatttatcct taagtttaaa
1981 attgacttgt aaaactcttt caatgtgtgt tttaggtcgt cttaaactta gtagtaaagg
2041 tgcaaatggt aaatattttt ttgcgatatc atgttcttta attcctaata tattaaacaa
2101 aatttgcgaa atcgaatctt tgaaattttc attaaaagat ctagggtaat tttttaaact
2161 gactacatca aaaaatatcc aaagcaaata atgattaaaa aaatcaaaaa ataaagtcca
2221 accttgctct gcatcttcat ttcttgataa tttatctaac ataaatgaag gaagttgcga
2281 agagactcca tgtaacccca tgaaatttat aaacaattct acaactgatt gttcatgttc
2341 tgaaaaaatg atttttctta tatctttatt tggatgtttt aaacttttat ttgttcttaa
2401 aaatatattc tttcttccat agtctttttc tagttttttt attaatttaa aaaaactata
2461 tgaagcaaga tcattcatat taaagctttt tttccaaagg aggcaggata atgtaaaatt
2521 tcttttgagt caaggcattt tatctttaat tcacaaaagg agttaatact agcaaaagat
2581 gctaaaaatt tagatataat caatcctaat aaatatacct cgcctaaagt ataaaatttc
2641 gaatctttaa tactaaaaat agctacggtt ccttttttttg tgatattttc atctattaaa
2701 taagtacttt tgctttgaat atcaattata gcttcttgta aaagcttata aatcttccaa
2761 tttgattggt tatctaaaaa gctatagctt tccaacacac caaaaaaagc ttgtttgctc
2821 aatatggttt gatatgaaaa agataataca gaaacaagct tccataataa gttaccatca
2881 acactacatc ttcttgtttc gctcggaatt tccacatttt tggtgtcgac accttttagg
2941 ttgcaggtat tgatatcacc tatttttaat tttgaaggca aatctccatt agagcataaa
3001 agatcaatac ttatagtttc atctatcaaa taagaggaaa aaaaggaaat ttcgctaaat
3061 acctctccct tagaattctt tttggtattt acactataaa aatctttttg attatctttt
3121 aaaaattcaa agcgttcaaa acttttatag ttttttaata gccttttacc gccttcacta
3181 ttatgcgctt taacttgtag tgtctgtatg atttcataag ctttaggttg acttctatcc
3241 acaaaaattc tataactgtc ttttttatga ttatttataa gaggttctgc actttttgca
3301 aagatattga taataggcgt catgcctaaa gaaagcaatt cttttctaaa aatacaattt
3361 gccggaaatg gtttggagaa tttaaattca atattaattg tctttccttg gcaattattt
3421 aaaatatcta aaccattgat tcttaaaaaa ttaaacttat gtggcataaa aaaatattct
3481 cttaacaaag aaaaagcttc aaaacctaaa tcattataag ataagctact ttcatctgga
3541 tttaatccta ttttttcaat attataggta ttaagaaaaa attcttcatc ggtatctaaa
3601 ctttgaattt ttaattcttt taaataagaa tgcatgtata acaataaagt gctagacata
3661 taagtatcat tacctagata taaattaatt ttttctaaac ccaaatcaca tatttttgtt
3721 tctgctttat taacttgtaa tgttaaattg aaagtataat cttgattttt actagatata
3781 aaaacttcat tgatggagat aggataaaga tagacatcat aaactgtttt aaattcgcaa
3841 acacatttat ctatcggttt tgatttaata agagttcctt tgggaatgat taaattgttt
3901 tctttactat tttgttcaaa tttaaattct tgcatacata aagaaggtaa aggatttgta
3961 tagtttgggg aaatgatatt gattagggac tctgctatat gtggtatatt ttcatcaagc
4021 tcttgatgaa ttttagaact gagaatggct aaattttcaa taatcctttc tatatcagga
4081 tctttactat cgtatgctaa aaatggagtt agttttggat agttttttat aaaatgctct
4141 ctttttttcat atagatattc aagttctttt tggtaataaa aaatattatc cttcatttaa
4201 cctcgcaata tctattattt ttaaagatga tttctatatt gaattcttta aaacgatcat
4261 cctgaaagaa acattttaca ataaaagaaa gttgccaagg acttaaactg ttatcgtaag
4321 tgatagaaac tacacatatt ctttttttcat agttatcaat cagttttctt atttccttag
4381 ccattgatac gcataattca ttagagttaa gattaatatc agccatatta ggcattccta
4441 agttttttac agcgtaacaa tcattaattt ttgcattgag caaaaccttt atattgtttt
4501 ttacatcttc aaaatcattt tggtaaaatg gaatatgaat gttttgctcg tctaaattat
4561 gaatcagttt atctagcagg gacattatta ttctttatct aattttccta ctaaagaaag
4621 ttcaaaatta ccacccatat atttaaaatg aggtcggacg cttagtccta ttttatacca
4681 tcctggctct cctggtatat catcaacttt cacttgtgct gctctgaatg gacgacgact
4741 tcttacttca gaaggtggat tttcttgatc ggaaatatat tgtctaatcc attcgttaag
4801 accattttcg atatcgcttc tttctttcca gctccctatt tcttctcgtt gtaaaacctt
4861 taaatagtgc gccaatcttg aaattaaaaa gatatatgga agctgagttc ctagtctata
4921 atttgtttca gcttctttac cttctggggt attttgaaat atctttggtt ttaatggaga
4981 atttgctgaa aaaaaagctg cattattact atctcttctt agtgttaaag ttataaaacc
5041 tgcttcagca agttcatact ctctcctatc tgtaatcaaa acttctgtag gtatttttga

5101 ttgtatagtt ccaaaatttt cataaagata agtaggtaga tccctaacag tccctccgct
5161 ttttggtcct ataatattgc cgcaccatct atacttagca aagctttccg ttaatcttgt
5221 gcaaaaagta tatgaagaat tagcccacaa taaatgatta tgtgaagtat gaacattttc
5281 tttatagtta aagcttttaa tagggttttc ttcagggtca taagggcttc ttgctaaaaa
5341 tcttgttact aacaagcctg tatatttaga gtcttcattt tctctaaaag ttctccatct
5401 tgtatattga ggtccttcta gcaaaccttg taaatcttga atacttgcaa gctcagaata
5461 atcatcaagc ccaaaaaact ttggtccaaa agatgtcaaa aagggagaat gactcatagc
5521 tgcaatacta gccattttat ttaaaaaagt catatcaggg cttgctgatc caagttgata
5581 atctccgatg attgctccta cgggttctcc tccaaattgt ccatattctg aagaataaat
5641 atttttataa acaatacttt gtgtgatgtc tggattattt tcaaaatcct ctaaggcttc
5701 ttcttttgtt atatcaaata ggtcgatttt tatattttca ttaaaatcag ttctttcaac
5761 cagaaatcta agtccgcgcc aagtagattc aagtttttga aattcttcat tatgtaaaac
5821 ttcatccatt tgttttgaga gtaaatggtc aatatgggct atcatttcat caagtgcaaa
5881 tttattgatt ttttcttcgg cattatcgct ttttacaatt tctgaaataa attcagcaac
5941 acctcttttg gcaatactat aactttcatc agttttagag tatttgcttt tctccataat
6001 ttgttcaata attggcgtat caattacttt ggtatttgcc attttaattt ccttgctctt
6061 cttgtttaat ttctaaaagt aacttttctt tagttttttc attttttaac gcttctaaaa
6121 cagcttttct aaaatcagga atattaccca taggaccttt taaagccatt aaagcttctc
6181 ttagctgcaa taatttgttt aactcaggta tttgtttagc taagctatct ggagaaaaat
6241 ccttcatact agcaatattg agcttaacat caagctcttc agctcctgtt ccaagagtat
6301 tttttacgct aaaactcgtt gttatatcga gttttctcat aacttgatca aaattaatct
6361 tgttaatttg caaaatttct ctttcctcta aaggggtttc gtttttttcct tttaaatttg
6421 ccattaccat taattttaac ggcagctcca catcctcatt ttgtccatta gttttagctt
6481 tatatgtaat attaattcgt tcctttggag catgagatcc atctgacatt ttaaatcctt
6541 aaaaataaaa attttagctt aattattata ttaaaatttg ctaaatttaa tcttgcatca
6601 tttaatttta ttttttgcgt tttcatattc ttcggctaga taatctttta gtagagtttt
6661 ttccattaat tctactaaat atatatttaa aattttagag ttctttttca ttccttttga
6721 ttcaaaaatt tttaccatat ctagcaagga attaatatta ttcataatag aattattaaa
6781 attttcctta tcaatattca tcagcatttg ctcaaaatct tgtaatgtat gttctttgtc
6841 agttttttta cttgaaaaaa aacttttttt attttctttg ggtttaacga aatatttata
6901 aacttcttct ttgcagataa agtcaccatt ttggaaacgg agaagttcaa tagtatcaaa
6961 tttagaaata aaatcacttg ttaatattat aagaatgtct aattgtttat tttttttctt
7021 tttctctaaa aaattacaaa agagttgcac accctctatc caaaaaggat ttaaaataag
7081 ttcatgcata aaacatttaa tatattctat atcatttttt ttatcagaaa gcaatatacg
7141 aattatatcc gtatcaggtt ttcttatttt ggtaattttt tcattattac attcgggcaa
7201 acacttgatt tttccccaca ttgcttgggt gaataaagcg tagatattga gattgttttc
7261 atcctcttcc aaaagttcaa atgcaagttt ttgataaaaa tttttatatt ctctctcgtt
7321 taaagagtta atattatcac ttctgatgtt tatagatact tgggttacag gagattttgg
7381 aagttgtggt acaacttgag caatttcaat ttttgcaaaa ttacaagata gtaaattttc
7441 aaattcggaa aataaagcaa taaattgatt aataatctca gatgatacag gtacttttgt
7501 tttattatat tcttgaataa aattttgaat aaaaatttga atttttcgct tttgagctaa
7561 tatataagta ttattttcat ccataagttt ttttaaatgt tgaaaaagaa gcaaaagttt
7621 ttcaaagcat tcttcattgt ttaaattgaa gcacgataaa agaaaatagt tacaaacttt
7681 aaaatctaaa gaatgatgca ataatataaa ttgagaatat tgataaactt tatcccaaga
7741 aatattttca tgatttaaag tcttatattt tgacatctca tcttcaagta agtgaaattc
7801 ttgtagctca tctaaattat tttttaattg gtcacaaaaa gacatttaat tcctccaaaa
7861 atttgcctca ttataattaa attttaaaaa atattatgtt aattttttaa aaaaaattac
7921 ccaaattaat cacaaattat attattatag aaaggatacc ttagttttat gttttgtaaa
7981 aaaagcttat ttttttttatt gccgttgttc ttttgtgctt gctcaagtgt tgttagtgtt
8041 aagataaata atatagaaaa ttccaatctt aataacagac atgatgatgt tcctgttaca
8101 gctattatat atcaacttaa ggatattaaa aaatttgaag aagcaagtga tatagatttg
8161 gccactagag aagatggagt attgggcaag gataagcttg attctattaa aacgcaaatt
8221 gcacctaaag acaatattat cgcacttaaa gtagatgaag gagaggttcc ttatgtaggt
8281 gttcttgtat tgtttgcaaa taatacaaaa aaagtaacaa aaatatgggc aaagaccgaa

8341 gatgcaaatg ggtttggaaa gaataagtat ttaaaatttg aaatatccaa agaaggtatt
8401 aaaagaatta aataggaatg atatatggca aataaattaa aagttgcttg gtttgatggg
8461 cttaatattg gacaaaccca ttttgagcag caagaaagat tttttaatag aaatatagat
8521 cttaaaacta taaatatata tagtaattta tatggaataa tagacttgga attttctcaa
8581 gagatgttat tgcaaggaaa aattgcactt tctaaaattt caggtattgc gcaagatgga
8641 agtattttta atgcacctga gcaagatttg ttacccgaac ctatcgaaat caattatgaa
8701 tctttaattg attcagttgt ggtattaaaa attcctattg gagttactga tattgctgat
8761 ttgtctttaa gaaacacttt tccaaattca aagtatatat gtcttcgaaa tagtattgct
8821 ttaagaaatt atgatgattc aaaatccaac gcaatggaca aaattgaaga tgagtacgaa
8881 cttgaaaatc tgacttttac gcaagagaaa aaagatctac ttcttgctag tttaagatta
8941 aaactcggca ttttaggcaa ttcaactcct gatgaacttg aattgccaat tgcaaaaatt
9001 aaaaatatcg atatcaataa aaaaatcgaa ttagaaagtg attttatacc cacttgttta
9061 aatatcagta aaatatctac cataagatcc tttttagaag aaatcatttt ttcaatcaat
9121 caacataaaa aagttctatc taatgttttc aaaggaattg accaaacaaa aaatacattg
9181 gattttagta ctttttatc tcttaatctt ttaaaaaaat ggtatttaat attttcacat
9241 ctatcaaaaa aagataaaat acatcctgaa tttttatacg aaaaattttt agaatttcaa
9301 ggagagcttg cagcttttag taatgaagaa tcttttttag attttattcc ttataaacat
9361 gataatcttt ataatacttt tttaaatatg atgaataatc taagattgtt attttccaaa
9421 atcacaagtc ctaaatattt tatagctaag accagtatta gtggcaatgg tttttatgat
9481 tttattttg ataactcggg aattttagag gatgctgaaa tttattttgc aattcgtgca
9541 gatgttgcta cagaatatct acttaataat tttaaaattc aaagcaagat acatacgcaa
9601 agtaaaatta aaaatatagt agcaacacag cttgaaggct taaatgtaga tcaagtacct
9661 aatgttccat ctagtattcc ttatttaaat ggttatgttt attataaaat agataaaaga
9721 gatcagctgt ttaaagattt taaaaatgaa agtattatta gcatgtactt gaccaacaat
9781 ataaaaaatc ctgatattat aatgtgggcg gtatttcaat gaaagaaata aacgaagaaa
9841 aaaaggcatt aagcttactt cttgattcaa attttgatgg tttaaaaaac aataaaatta
9901 ttgattattc tttggagtta cttttgcttt cttacaggct ttcaaaaata agctctatgg
9961 atacaagtaa tattaatcaa cttagagaaa cattgattaa taaaatttta gatataaccg
10021 ctaaactaag tatgtgtaaa gaatatgatg aaaaagaaat tattaaattc aaatattgtc
10081 tttgtgtctt tatagatgaa agcttgatga aaaatgaact ttttatcaat ttttgggctc
10141 acaatactct aactgtaaga ttatttgatg aaaccttagg tggaaataat ttttatgata
10201 ttgcatcatc ttggattaat aatcctttta agtttaaaga ttttttggaa tttatttatg
10261 cttgtttgat tcttggttat aaaggaaaat ataatgaggc taaggataga gatgaaaaaa
10321 taatccattt ttgcaataat attgccacat ctttaagacc agtttataag acagaagaag
10381 atctagcttt taacaaagca tacaaaattg gtttaaagga agatatttgg caaaaattta
10441 taagattgta ttttaaaaaa cttattatca ttgtgccagt attgattgta ttaggagtat
10501 taagctttgc aatttttaat ttagaaacca ataatcttaa agtggataac aatatatctg
10561 tattgataaa aaatttaaca catataaaat aaagatatta aattttttaa atatcaattt
10621 atttgttatg agtaaaatta aataaaaatt attttacagt tagaataaaa atcaaatgca
10681 aatttaaagg ataaaaatat atgaaaaaac aagctctatt attttaatga taaagaaaaa
10741 gttatactgc taacactaaa ccacaaaatt acatcttaat tttaataaaa tagataatct
10801 aaaggaaata acttataatg acatacatat tacttcttat tataatttct actattcttt
10861 cttatcttat attaaaattt atttataaaa tcatctttaa atctacaaaa agcgtttcaa
10921 agtttttagt ctttcttgga agtataggct taattatctt ttattatact ccttattctt
10981 attatttaga gccaagcttt tataagttta aagaaatatg caagctagac ccagagattt
11041 accaagccaa tggtagtaaa ttagatgagg agtattataa taaagtgcta aggtattttg
11101 atacggattt ggataatatg tcaaagtcat ataaacaaga gctaggggta actaatgata
11161 aaaaatatag tagatactgg tttgaaacat ggatagatgg aagaaccgaa ataagctttg
11221 aaatatggtt taaaagtaat attgcaataa aagacaatat tgactttagt ataaaacctt
11281 tttttatgc taattgggat aaaatgagac ccctcccagc aggtaatgag gggataggat
11341 tttttttagg aagtgtccca ataagttgta tatatttcaa aaaggactaa acaatgaaaa
11401 ataaagcagt gattaataag ctaaaagata attcagaact tgcttgggct agttatggat
11461 attttcacct agcaacatct aaggcgatag aatgaaaaaa tataaaaaga ttttagtgct
11521 attatcacca ataattttaa ttatattatt ttatacccct tttatatatc ctagttattg

```
    11581 ggaatttaga gggttatgta aattaaataa ttacccaaaa agcgaggaaa aatataatac
    11641 aatcttagct tattttgata agagtttaga tggtagtata ggaaaaaatg gttatgcaaa
    11701 aataggttat agtaatagaa tagatttagg tgtatatatt tattataaga atccaaataa
    11761 caaaacatta acttttaaaa atattaataa aatatatttt agaccaatat ggaaaaacta
    11821 tgcaccaaat atttatggta atgaaggtaa tatggatttt agattaaagc ttgacggtga
    11881 aatagattgc agaagcttat taggagagtt aggtggataa taaacaacaa atcaataaac
    11941 taagggacat ggcagaactt gcatgggcag cttatggata ttttcattac gttgataata
    12001 aatttgatat aaaagatgaa gataaaattg ttacctttga agatgtttta gatattacct
    12061 ataaaaactc taaaattatt gatgaaagag gatttaaaat cggcactctc aatggcgact
    12121 tctccccact ccaagctaaa caataaaaca ttgtcctaat acaaatttag gttttagcac
    12181 tgctttgtct tatgaaaaga cagagaaaat taagaggagt taataaatgc tagaattctt
    12241 tgttaatctt ttaatattta ttcttatatt tttctttttt gtttttatat tttctattat
    12301 cttatatttt atctttaaaa ttttttataa attaaaaaat aaaacaaaaa ctataaattt
    12361 taaaacaatg tttaaaaaac ttgctttatt tcttttacct gtatatcttt tattattctt
    12421 agcaggagga tgttcttata aatatatgga tccgcaatat tatgaattta agaagttgtg
    12481 taaagataat agtaataaaa tgatagtgtt taataaagat tatttggatt taaaaaaaca
    12541 atttgttcaa gctatgacga ataattctaa tattagaatt aaaaataatg gtataaaata
    12601 tttttataat gaaaaattga atttggaaat tcaaccttca aattggaaag aaatagaaac
    12661 taaatcacgc gaaattaaac ttggaatagg aaaagtaaaa gaaaaagaaa tagaagcttg
    12721 gtatattgat gataaaaata acattaagta tcaagaattt aaacgatatt tgtattataa
    12781 ttatagtata tttttagaag gcgatgaggg tgctgggttt cattttgaat atgaggaaat
    12841 attagattgt ggagatgtta gataaaaaat ttaaaagcat aatgagctta agatagaaat
    12901 ataaaatgag caaaaacatt aaaacacaac aagctaaatt agacttaatt gctaaattct
    12961 tagactatgc taatgtagct gatgctagtt atgcgatgtt gcattggatt gattatgtaa
    13021 aaaaaagaaa atgacaaaaa acatgaaaat ggtgataccc aaaaactagg cgataaacac
    13081 aataatcaaa attctactta tgcaagagct atacaagctc gttttgaaca aaataaaata
    13141 gttaaaatag aacctaaata ttgtatttct cttataaata cttgttttga tagtaaagaa
    13201 ataacattgg ataatgatat tagtagggta ggat
//
LOCUS       FHGGBLIP_2              4242 bp    DNA     linear       12-APR-2021
DEFINITION  Campylobacter species strain strain.
ACCESSION
VERSION
KEYWORDS    .
SOURCE      Campylobacter species
  ORGANISM  Campylobacter species
            Unclassified.
COMMENT     Annotated using prokka 1.14.6 from
            https://github.com/tseemann/prokka.
FEATURES             Location/Qualifiers
     source          1..4242
                     /organism="Campylobacter species"
                     /mol_type="genomic DNA"
                     /strain="strain"
     gene            complement(95..1375)
                     /gene="trxB_1"
                     /locus_tag="FHGGBLIP_00016"
     CDS             complement(95..1375)
                     /gene="trxB_1"
                     /locus_tag="FHGGBLIP_00016"
                     /inference="ab initio prediction:Prodigal:002006"
                     /inference="similar to AA sequence:RefSeq:CKG86610.1"
                     /codon_start=1
                     /transl_table=11
```

```
                     /product="thioredoxin reductase"
                     /protein_id="Prokka:FHGGBLIP_00016"
                     /translation="MSETYEIYTPDGSILNVEKDTNKILFTAKSNGTYTKQHSQALFE
                     GDRILRNSPYKDYQPKYLDPNFYTGEKSTLLEFKEWQSIYLKDPIKGSIAPWTKEEKA
                     YYKSLKTKRERYKYLIIRSGIRSTVIDIPYDAYCNVDEKGYLINEEYAYIYDEVNNNK
                     ETLKSSLFRQEWGIAAGILGKPEYFVRSKNHGFNARMIQCFILYIQLTGGGYEELGIK
                     RGIYNYADNLLEIGIGMAGIHKNPLRAKLVKDLAKTIQPDEFGMLPFIDEIMGVDWVI
                     DLNQYQFAYDGKGDSTWALYDDIEKGKLKDPRDVDSTPESRKEFDEETDVYRFGNRMN
                     FASDIRDSRSEESTRLFDDTLILHAKLAALTPPQGYPNAPYYFTPERLEWIYKRGYLD
                     KLLDPRIPAIYRYNFPRELRAKILKFAEENGIKD"
     gene            complement(1394..4150)
                     /locus_tag="FHGGBLIP_00017"
     CDS             complement(1394..4150)
                     /locus_tag="FHGGBLIP_00017"
                     /inference="ab initio prediction:Prodigal:002006"
                     /inference="similar to AA sequence:RefSeq:AAW35464.1"
                     /note="conserved hypothetical protein"
                     /codon_start=1
                     /transl_table=11
                     /product="hypothetical protein"
                     /protein_id="Prokka:FHGGBLIP_00017"
                     /translation="MYLKNNAILKNLQEDIKQFIYFSYIESPNFNSSKLAEFFAQKNQ
                     NAQIDLNTTKMSKNYLIISNIPNKYNAGLSESLTQEIANYNIQDKYRTIKKNIDTSKK
                     TYDSTRDYSEYSIEMPQDENEPYILQLSPFVKISKEAYENSKKAYEDMQEIYKYLTNF
                     VATPQKLPEFAKTAKEKLAISENESMIEIVKKTENLRHCERINILAYIDYNKKINDKK
                     WLPYFYIKKFFKQNEDKESIIEKEKKIAHQYLTFLKKELFEKSDKSSFGNYSLSDKSS
                     FGNYSLMECLFISFVLYQAKLIFPNINLDLKKYGGSNFVYIKYLEETYVLWTDEYPIY
                     VNNEPTNITAYEVVSQLLKILDKKDYSFVENDSSITIDDFFNELSKDLDVTNTLDKKI
                     IKLKQMYHLDEQKCIKEFMNLDEEAQQAFFVWMDIEKQISDLSKLLEQMYGKEYLNKA
                     KEQKTSEIFTYAMLKILLSASPFISFTAQMLWDFNFLKTIEYVIQACIKINKEGFKEF
                     FKEQLGIFTPLKYHIHIKSNLIIKARLQQIEKELTHTKIQMILAQEIKSNLIIQKNTS
                     SLNLFITKIKEYFNEKQIKIYWSDKYMKQLFIYSNQESNISKIALMHSIDIQTQKYTI
                     YTYAKLNKLDLASIVAGEFVGIFLNYLFPTNYKALQEQYESIVYQLYTFKDDSPLAVI
                     RDHFITYPFAINSKFISFDLKKIFYGIDLCTGVFDTHFSFHYEIKPNLEENKEFLLKR
                     LLSYLIIDEKRSALKIIKDENETILERDDYTFFNQSLNSIELIKSEIYILKNTTLEKY
                     YKEVNDEINLTAIQAYNEALQILQDFANGIYNTSKDKTGKYIEDKNKTRRLLEKLNII
                     GQNNLKAMYMGIEVKEIQNQNQMRENNFYQVIIGKNKDKKSPPNFIGRLATTIITEDG
                     LYMG"
ORIGIN
        1 gccatacaaa acaatatcaa agaagataag attctttttg agaatcttac cctaaaagca
       61 ttggaaaagt atattaaaga ttttgagtga ttttttaatc tttaatgcca ttttcttcag
      121 caaattttag tatcttagct ctaagttccc ttgggaaatt atatctataa atagcaggga
      181 ttcttggatc aaggagttta tctaaatatc ctcttttata aatccattcc aatctttcag
      241 gtgtaaaata ataaggagca ttaggatagc cttgaggagg agtgagggct gcgagtttag
      301 catgaagtat taaagtatca tcaaataatc tagttgattc ttcacttcta ctatcacgaa
      361 tatcagaggc aaaattcatt ctgttaccaa atctataaac gtcagtttct tcatcaaatt
      421 ctttcctgct ttcaggagta gaatccacat ctcttggatc ttttaatttg cctttttcaa
      481 tatcatcata taaagcccaa gtagaatcac ctttaccgtc atacgcaaat tgatactgat
      541 ttaaatcaat cacccaatct actcccataa tctcatctat aaaaggtagc atgccaaatt
      601 catcaggctg tatagtttta gctaaatctt ttaccaattt agcccttaaa ggatttttat
      661 gtatccctgc catacctata cctatttcta aaagattatc agcataatta taaattcctc
      721 tttttatacc taattcttca tatcctccac ctgtgagttg aatatataaa ataaaacatt
      781 gtatcattct agcattaaag ccatgatttt tagaacgcac aaaatattca ggttttccta
      841 gtattcctgc tgctataccc cattcttgtc taaaaagaga agatttaag gtttctttat
```

901 tattgttaac ttcatcataa atataggcgt attcttcatt gattaaatat cctttttcat
961 ctacattaca ataagcatca tagggtatat ctataacagt gcttcttata ccacttctta
1021 taattaaata tttatatctt tctcttttgg tttttaaaga tttataataa gctttttctt
1081 ctttagtcca aggagctatg cttcctttga taggatcttt taaatatatg ctttgccatt
1141 ctttaaattc aagtaaagtg gatttctctc ctgtataaaa gttaggatct agatacttgg
1201 gttgatagtc tttgtagggg gagtttctta atatgcggtc accctcgaag agagcttgcg
1261 aatgttgttt agtgtatgta ccattgcttt ttgccgtaaa taatattttg tttgtatctt
1321 tttctacatt taatattgaa ccatctggtg tataaatctc ataagtttcg ctcattattt
1381 atcctttcct aatttaaccc atatataaac catcttctgt gattatagta gtggctagtc
1441 tacctatgaa gttaggcggg gatttttgt ctttattttt acctattatc acttgataaa
1501 agttattttc tctcatttgg ttttgatttt gtatctcttt tacttcaatc cccatataca
1561 tagctttaag attgttttgt cctatgatat tgagtttttc tagtaatctt ctggttttat
1621 ttttatcttc tatatatttt cctgttttat ctttacttgt attataaatt ccattagcaa
1681 aatcttgtaa aatttgtaaa gcttcattgt aagcttgaat ggctgttaaa ttgatttcat
1741 cattaacttc tttataatac ttttctaatg tagtgttttt taggatataa atttcagatt
1801 taatgagttc tatactattt aaactttgat taaaaaaagt ataatcatct ctttctagta
1861 tggtttcgtt ttcatctttt attatcttta aagccgatct tttctcatca ataatcaaat
1921 aagacaaaag tctttttaaa agaaattctt tatttcttc aagatttggt tttatttcat
1981 aatgaaagga aaaatgagtg tcaaaaactc ctgtgcaaag atctatgcca taaaaaattt
2041 tctttaaatc aaagctaatg aattttgaat tgattgcaaa aggataagtt ataaaatgat
2101 ctcttataac cgccaaagga gaatcatctt taaaggtgta aagctgataa acaatgcttt
2161 catattgttc ttgcagggct ttataatttg ttggaaagag ataatttaaa aaaataccca
2221 caaattctcc agccacaata gaagcaaggt caagtttatt tagcttagca taagtataaa
2281 tagtatattt ttgagtttgt atatctatgc tatgcataag agcaatttta gatatattgc
2341 tttcttgatt agagtatata aataattgtt tcatatattt atcactccaa taaatcttaa
2401 tttgtttttc attaaagtat tcttttatct ttgtgataaa tagatttaaa ctggatgtat
2461 tttttgtat tattaaattg gattttatct cttgtgctaa aatcatttgg attttagtat
2521 gagttaattc tttttctatt tgttgcaagc gcgcttttat tattaaatta gattttatgt
2581 gtatatgata ttttagaggt gtaaaaatac caagctgttc tttaaaaaat tctttaaatc
2641 cttctttgtt tattttaata caagcttgaa taacatattc tattgttttt aaaaaattaa
2701 aatcccacaa catttgagct gtaaagctga taaaaggact agcacttaat aaaattttta
2761 acattgcata agtaaatatt tcacttgttt tttgttcttt tgccttattt aaatattctt
2821 ttccatacat ctgttccaaa agctttgata aatctgatat ttgtttttct atatccatcc
2881 atacaaaaaa agcttgttgc gcttcttcgt ctaaattcat aaattcttta atgcattttt
2941 gttcatctaa atgatacatt tgcttcaatt tgattatttt tttatctaat gtatttgtta
3001 catctaaatc ttttgataat tcattaaaaa aatcatctat agtgatcgaa ctatcatttt
3061 ccacaaaaga ataatctttt ttgtcaagta tttttaataa ttgcgataca acttcatatg
3121 ctgtaatatt tgttggctca ttatttacat atattggata ttcatctgtc cataaaacat
3181 aagtttcctc taagtatttt atatatacaa aattactgcc tccatatttt ttcaaatcca
3241 aattgatatt tggaaaaata agcttagctt gatacaagac aaatgaaata aataaacatt
3301 ccattaaaga atagtttcca aatgaagatt tatccgataa agaatagttt ccaaatgaag
3361 atttatccga tttttcaaaa agctcttttt tcaaaaaagt taaatattga tgagctattt
3421 tttttctttt ttctatgatg gattctttgt cttcgttttg tttaaagaat tttttgatat
3481 aaaaataagg aagccatttt ttatcattta ttttttttatt gtagtcaata taagctaaaa
3541 tgtttattcg ttcgcaatgt cgtaaatttt ccgtcttttt tactatttct atcattgact
3601 catttctga tattgctaat ttttctttag ctgtttttgc gaattctggt agcttctgcg
3661 gtgtagcaac aaaatttgtt aaatatttat aaatctcttg catatcttca taagcttttt
3721 tactattctc ataagcttct ttggatattt tcacaaaagg gcttaattgt aaaatataag
3781 gttcattttc atcttgtggc atttctatgc tatattcgct ataatctctt gtgctgtcat
3841 aagttttttt tgaagtatca atattttttt taattgttct gtatttatct tgtatattat
3901 agttggcgat ttcttgagtt aaactttcac taagtcctgc attgtattta ttggggatat
3961 ttgaaataat caaataattt ttactcattt tagttgtatt taaatctatt tgtgcatttt
4021 gatttttttg tgcgaaaaat tcagctagtt tacttgagtt aaaatttgga ctttctatat
4081 aagaaaaata aataaattgt ttaatatctt cttgcaaatt ctttaaaata gcattgtttt

```
     4141 ttaaatacaa attagataat ttttgcgttg aaatgaaagc gggtaaattg ctcgattttg
     4201 aatcattagc tgtattttta ctctcattgt ctttttgttc tg
//
LOCUS       FHGGBLIP_3              3467 bp    DNA     linear       12-APR-2021
DEFINITION  Campylobacter species strain strain.
ACCESSION
VERSION
KEYWORDS    .
SOURCE      Campylobacter species
  ORGANISM  Campylobacter species
            Unclassified.
COMMENT     Annotated using prokka 1.14.6 from
            https://github.com/tseemann/prokka.
FEATURES             Location/Qualifiers
     source          1..3467
                     /organism="Campylobacter species"
                     /mol_type="genomic DNA"
                     /strain="strain"
     gene            179..595
                     /locus_tag="FHGGBLIP_00018"
     CDS             179..595
                     /locus_tag="FHGGBLIP_00018"
                     /inference="ab initio prediction:Prodigal:002006"
                     /codon_start=1
                     /transl_table=11
                     /product="hypothetical protein"
                     /protein_id="Prokka:FHGGBLIP_00018"
                     /translation="MVQKNDYEVVDFSDDLFYSYLVWSLRKFIIAYDIGLYQDKNNLF
                     FNLATYKIHKNHSFYIEANNNLNFNLIELFFSNISPLEYAVGYLQKEEIIYNGALEYS
                     LGTLQKTLNTTTQFHNKFYFKLKERPFEYAQYFKQR"
     gene            complement(1152..1400)
                     /locus_tag="FHGGBLIP_00019"
     CDS             complement(1152..1400)
                     /locus_tag="FHGGBLIP_00019"
                     /inference="ab initio prediction:Prodigal:002006"
                     /inference="similar to AA sequence:RefSeq:AVL47240.1"
                     /codon_start=1
                     /transl_table=11
                     /product="recombinase XerC"
                     /protein_id="Prokka:FHGGBLIP_00019"
                     /translation="MEYLLDYQDEMQINDIKTTFLINFLNYLENNLKNSNKLSKRTKI
                     TYLRVLTSFFISDNNDDLFLFSFDMKKLGLKLKKQKKS"
     gene            1751..2140
                     /locus_tag="FHGGBLIP_00020"
     CDS             1751..2140
                     /locus_tag="FHGGBLIP_00020"
                     /EC_number="3.6.1.-"
                     /inference="ab initio prediction:Prodigal:002006"
                     /inference="similar to AA sequence:RefSeq:ABS44566.1"
                     /codon_start=1
                     /transl_table=11
                     /product="F-box only protein 18"
                     /protein_id="Prokka:FHGGBLIP_00020"
                     /translation="MDEVQDINACVIDIMLNQKQAKKVFIGDTFQSIYKFRGAVISLE
```

```
                     ILASMPKSHTLCLTQSFRCPQNIAFIANSYLKILNAPRNFKGTKKSSNENVKNEAKAI
                     VCRTNAKLFDIAVENLDKKLFFCERDK"
     gene            2124..2327
                     /locus_tag="FHGGBLIP_00021"
     CDS             2124..2327
                     /locus_tag="FHGGBLIP_00021"
                     /EC_number="3.6.1.-"
                     /inference="ab initio prediction:Prodigal:002006"
                     /inference="similar to AA sequence:RefSeq:ABS44566.1"
                     /codon_start=1
                     /transl_table=11
                     /product="F-box only protein 18"
                     /protein_id="Prokka:FHGGBLIP_00021"
                     /translation="MREINSYSFDELLDIQNLLFKKHEYIKNQFIAKFADLKEFLEYI
                     NETKEVDLKQKIFVLLNMCIAIL"
     gene            2309..2623
                     /locus_tag="FHGGBLIP_00022"
     CDS             2309..2623
                     /locus_tag="FHGGBLIP_00022"
                     /EC_number="3.6.1.-"
                     /inference="ab initio prediction:Prodigal:002006"
                     /inference="similar to AA sequence:RefSeq:ABS44566.1"
                     /codon_start=1
                     /transl_table=11
                     /product="F-box only protein 18"
                     /protein_id="Prokka:FHGGBLIP_00022"
                     /translation="MHSDIIKLIKAIEKSSVKKQEQTDLILSTGHKNKGLEWNNIEII
                     DDFLNIKQELEERDQITIAKEELNLLYMAITRAKNSLSISKDYLLEKEFIQENLERII
                     IE"
     gene            2620..2832
                     /locus_tag="FHGGBLIP_00023"
     CDS             2620..2832
                     /locus_tag="FHGGBLIP_00023"
                     /inference="ab initio prediction:Prodigal:002006"
                     /inference="similar to AA sequence:RefSeq:AVL47291.1"
                     /codon_start=1
                     /transl_table=11
                     /product="helix-turn-helix domain-containing protein"
                     /protein_id="Prokka:FHGGBLIP_00023"
                     /translation="MKIIKKLQKDGSLKKQVYYSVHEVAQMHSVTHTTVRLWISRGIL
                     KGVKLGKGFYMSKETILDFQKTDGLS"
     gene            2848..2997
                     /locus_tag="FHGGBLIP_00024"
     CDS             2848..2997
                     /locus_tag="FHGGBLIP_00024"
                     /inference="ab initio prediction:Prodigal:002006"
                     /inference="similar to AA sequence:RefSeq:ARJ53387.1"
                     /codon_start=1
                     /transl_table=11
                     /product="DNA-binding protein"
                     /protein_id="Prokka:FHGGBLIP_00024"
                     /translation="MSSIAKDSHVEMRDFLFNKYNKITFSRKECAEILGISLQTLDRL
                     TKDKK"
ORIGIN
```

1 cccctataca attatagata atttagaatt aaaaaaatat ttcttaaaac tttataaaga
61 taatattgaa tttttaactt cttatattaa tgaaattaat caagttcatg gtattaaact
121 ttaccatatc gaaaataaaa atttaaataa atttgaacta atttcaatac tagatgagat
181 ggtgcaaaaa aatgactatg aagtagtaga ctttagcgat gatttatttt attcttatct
241 tgtatggagt ttgagaaaat ttatcattgc ttatgatata ggtttatatc aagataaaaa
301 taatttattt tttaatttag ctacctataa aatacataag aatcattctt tttatataga
361 agcgaataat aatttaaatt ttaatcttat tgaattattt ttctcaaata tctctccttt
421 ggaatatgct gtgggttatt tacaaaaaga agagataata tataatggag ctttggaata
481 ttctttagga actcttcaaa aaactttaaa tactacaacc caatttcata ataaatttta
541 ttttaaactg aaagaaagac cctttgaata tgcgcaatat tttaaacaaa gatagactta
601 gctgaaataa attaaatttg ctcactttaa tgcaatcaat caggatgaaa aatcggcgtg
661 aagactgact tattttagaa agtttattta gaaaagatga atattaaaaa gtaaaagaaa
721 tatattttat aggatttttg acataaaaat aattataatc agcttttgca tatagttgtt
781 gtatttagat ttgcatgctt taattttttt gtataactgc aagatttgtt cctttggcga
841 ttaatttcat agctaaaagt atgctttaat aagcgcagtc ctttcttgac tattgaagct
901 ttggcataaa tattatttac aatcataaaa gcattacttc tatttaaatg cttccctgtc
961 ctagtttaca tcattatact tgaaacactc aaacccgtca tcaatttctg ctttttttata
1021 aatacagttt attctttact acccttgcct attatagaga tttttaatat ttcatcatta
1081 acctctttaa aatcacaaag ttttacattt aacattattt agccttatag tttcattctc
1141 gctaagataa tttaactttt cttctgcttt ttcagtttta aacctaattt tttcatatca
1201 aagctaaaaa ggaataagtc atcattatta tcacttataa aaaagctagt taatactctc
1261 aaataagtaa tttttgttct ttttgataat ttattgctat tttttaaatt attttccaaa
1321 taattaagaa aattaatcaa aaaagttgtt tttatatcat ttatttgcat ttcatcctga
1381 taatctagta aatattccac aagctctaaa aatacatcta taaaacaata aagtgttctt
1441 taaatataat attatctata taaataaaat ttcatataat ccacatacaa catcagaaat
1501 tgataataaa tatttatctt gatttatctt attgatgttt ttaagtttta ttccaacaat
1561 ttcataaaac tttggtttta ttgtgtttaa tttatcttca gtataaaaaa tttatatttt
1621 gcaattagtc ctttaaacaa ttttataaat gtaaaatcta tattttcaag ttctttgcta
1681 tacaaataat tatttataac ttcttttaat tcaacttaca aaaccaaagc tagaatatga
1741 ttttatttta gtggatgaag ttcaggatat caatgcttgt gttattgaca ttatgcttaa
1801 ccaaaagcaa gctaaaaaag tttttatagg agataccttt cagagtattt ataaatttag
1861 aggagcggta atttctttag aaatattagc atctatgcca aaatctcaca ctttatgcct
1921 aacacaaagt tttaggtgtc cgcaaaatat agcttttatc gccaactctt atttgaaaat
1981 tttaaatgct ccgcgaaatt tcaaaggaac aaaaaaatca agtaatgaaa atgtcaaaaa
2041 cgaagcaaaa gccatagtgt gtagaactaa tgctaagtta tttgatatag ccgtagagaa
2101 tttagataaa aaattatttt tttgtgagag agataaatag ttatagtttt gatgagttat
2161 tagatataca aaatcttctt tttaaaaaac acgaatatat taaaaaccaa tttatagcaa
2221 agtttgctga tttaaaggag tttcttgaat atattaatga aaccaaagaa gttgatttaa
2281 agcaaaaaat ctttgttctt ttaaatatgt gcatagcgat attataaagc ttattaaagc
2341 cattgaaaaa agtagcgtaa aaaaacaaga gcaaacggac ttaattttaa gtacaggaca
2401 caagaacaaa ggcttggaat ggaacaatat tgaaattatt gatgattttt taaacatcaa
2461 gcaagaactt gaagaaagag atcaaatcac catagcaaaa gaagaattaa acctttttata
2521 tatggcaatc acaagagcca aaaattctct aagtattagc aaagattact tacttgaaaa
2581 agaatttata caagaaaatt tagaaaggat aataatagaa tgaaaataat taaaaaatta
2641 caaaaagatg gaagccttaa aaagcaagtt tattattctg ttcatgaagt tgcgcaaatg
2701 cactcagtaa cgcatacaac agttcgccta tggatttcaa gaggaattct aaaaggcgtg
2761 aaattaggca aaggatttta tatgtcaaaa gaaaccattc ttgattttca gaaaactgat
2821 ggtttatcat aattaaggag agaaaaaatg agtagtatag caaaagatag tcatgttgaa
2881 atgagagatt ttttatttaa taaatataac aaaataactt ttagcagaaa agaatgtgcc
2941 gaaattcttg ggatatcttt gcaaacttta gatagattga caaaagataa aaaataaaat
3001 caatgaatat aactcgtttt gtattattct ccattgaaga aatggctaaa attctctcag
3061 gggctaagtt aacaaaatga atagaaaaga tagggttgcg ctagcttctt atcataaaaa
3121 taaaaagctc acttgctttg gtaagtgggc aattcaaaag tataagttaa agtatatgtt
3181 ttttagatat ttaattttta aagtatggat atttaggtat ttgattaata aatcacacgc

```
     3241 cgacctcagg caccatttgt ttttttactc tcaagtgtta ttttttggca atttatatta
     3301 aaatcttaaa aaagagtttt atattttcaa aaatagcttg ctgattatac tgtgtgagtt
     3361 ttataatatc ttctaaagct ttgaaaaatc actaggaact ttgataatca aattttcatt
     3421 tttaagttta atttcttaaa taataggttt tattatcata gtaaaaa
//
LOCUS       FHGGBLIP_4              2982 bp    DNA     linear       12-APR-2021
DEFINITION  Campylobacter species strain strain.
ACCESSION   
VERSION
KEYWORDS    .
SOURCE      Campylobacter species
  ORGANISM  Campylobacter species
            Unclassified.
COMMENT     Annotated using prokka 1.14.6 from
            https://github.com/tseemann/prokka.
FEATURES             Location/Qualifiers
     source          1..2982
                     /organism="Campylobacter species"
                     /mol_type="genomic DNA"
                     /strain="strain"
     gene            complement(415..1014)
                     /locus_tag="FHGGBLIP_00025"
     CDS             complement(415..1014)
                     /locus_tag="FHGGBLIP_00025"
                     /inference="ab initio prediction:Prodigal:002006"
                     /codon_start=1
                     /transl_table=11
                     /product="hypothetical protein"
                     /protein_id="Prokka:FHGGBLIP_00025"
                     /translation="MANLSELRESLTYVIPLQELLENEDILGEAVNRYIKKSLSHLDL
                     VTNFIESEEVYHKTKNFRKAVVHFTSNLVSGPIIAVGIVGGIRTGQVEVAVITIIHAN
                     DIPVAIENATNKAFDIYDNVLDAILEFIFNGTASRIITNFLNSIYERFLHQEILSKFA
                     IVQKNVKYYNRIIFKILQMVYPSSPHTPTLAIPKNTNLP"
     gene            complement(1020..1694)
                     /locus_tag="FHGGBLIP_00026"
     CDS             complement(1020..1694)
                     /locus_tag="FHGGBLIP_00026"
                     /inference="ab initio prediction:Prodigal:002006"
                     /inference="similar to AA sequence:RefSeq:CKG88211.1"
                     /codon_start=1
                     /transl_table=11
                     /product="hypothetical protein"
                     /protein_id="Prokka:FHGGBLIP_00026"
                     /translation="MNLFYKIYFFILYHPIKAFFILVLIVSSFSFVDKFILPKKEKEL
                     SPIIQIVIKDSESIPDYISNTKSKEVFLSQNPSLKDLFEKMATEYPLKSGLNFGKPEY
                     IAGVATIEKDWGEYMEKILKEDSEELLKKANSCKIIDVKSKMQCFIEKINYVFKIIDS
                     QSENCTLNLINNKNFYNNLYEVIYFAFYDDEYDKNLKKEEYFFSKAYYAARVHFLANK
                     QCFRME"
     gene            complement(1735..2508)
                     /locus_tag="FHGGBLIP_00027"
     CDS             complement(1735..2508)
                     /locus_tag="FHGGBLIP_00027"
                     /inference="ab initio prediction:Prodigal:002006"
                     /inference="similar to AA sequence:RefSeq:CKG88190.1"
```

```
                     /codon_start=1
                     /transl_table=11
                     /product="hypothetical protein"
                     /protein_id="Prokka:FHGGBLIP_00027"
                     /translation="MNKPFYKLKRFYIPCGVLVVLIIFISLTYHFLQRPLELIFWNKY
                     YHEKEYQNAKDVSKLLWNNKEEFKKVFKEQNLNQELKTNQKELLNYMHYFKKDFKFMQ
                     ILGLDNAYLVALKNKDVLFGLQMQNNLDYFYFISNNTDLEEINNYLNVVDELLIFMNE
                     IEKLPPKYNLGKIMFEINFMNYNTLFFGFTLDKNFICSIPQKEQLLENMINSYEKMNL
                     FHDVDLKFQDQELYEAIYVTKELNYFINIAKGRLNACGK"
     gene            complement(2519..2971)
                     /locus_tag="FHGGBLIP_00028"
     CDS             complement(2519..2971)
                     /locus_tag="FHGGBLIP_00028"
                     /inference="ab initio prediction:Prodigal:002006"
                     /inference="similar to AA sequence:RefSeq:CKG88165.1"
                     /codon_start=1
                     /transl_table=11
                     /product="ribosomal protein L7/L12"
                     /protein_id="Prokka:FHGGBLIP_00028"
                     /translation="MAKNSHEFVGENKDIEIGANQNTIIHKDEIRNVKGNKKEMVEGH
                     YDINISDKMQVLSEKEMDYKSKDNILFTSNESIGFESDKNTSMVADNITTYAKTIHEL
                     KADSEATIQVGETIINAKPDCVIIKAGGVEVIIDSNGLVVKGGELKAE"
ORIGIN
        1 catcattatt atctaaaact aaaaaacttt cacataaaaa caaagctctg aaataagcaa
       61 tttctgaatt gatattaata ttatcaatat cttgtaaatt taaagtcttt aatgagtatt
      121 ttttagtcgc gataatactt aaactttggt ttggatttat aaaagtattt ctttcttgtt
      181 ttaattgaaa gcttagattt tcatttaagg atttaaataa agaattaaaa acctcatttt
      241 tgattttatt atcttctttg taaaatttta acgcaagttg atatttttgt tcttcttgat
      301 caatgatttg atcttgttct tttaaatctt gtatgttttt tagatactct ttattcataa
      361 aattatagga attagaagct atacttgttt tagctataag ttcttttttt cttactaggg
      421 caaatttgta ttctttggaa tagctagggt aggggtgtgg ggtgaagagg gataaaccat
      481 ttgtaagatt ttgaaaatta tcctgttgta atatttgaca ttcttctgga ctattgcaaa
      541 tttgcttaaa atttcttggt gcaaaaatct ttcatatata gaattaagaa aatttgttat
      601 tatgcgagaa gctgtaccat taaaaataaa ttctaatatt gcatctaaaa cattatcata
      661 gatatcgaaa gctttattgg ttgcattctc gattgcgaca ggaatatcat ttgcgtgaat
      721 aattgtaatt acagcaactt ctacttgacc agttcttatg ccacctacaa tacctactgc
      781 aataattgga ccgctaacta aattacttgt aaaatgaact acagcttttc taaaattctt
      841 agtcttatga tatacttctt ctgattctat aaaatttgtt actaaatcca gatggctaag
      901 tgatttttta atatatctat taacagcttc tcctagaata tcttcgtttt ctaacaattc
      961 ttgtagtggt atgacataag tcaatgattc tcttaattcg cttaagttag ccatttgttt
     1021 tactccattc tgaagcattg cttatttgct aaaaaatgga ctcttgctgc atagtaagct
     1081 tttgaaaaga aatattcttc tttttttcaaa tttttatcgt attcatcatc ataaaaagca
     1141 aagtaaatta cttcataaag attattataa aaattttttat tattaataag attaagagtg
     1201 caattttcgc tttgagagtc tataatttta aaaacataat ttatcttttc tataaaacat
     1261 tgcattttac tttttacatc tataatttta caagagttag ctttttttcaa gagctcttcg
     1321 ctatcttctt ttaaaatttt ttccatatat tctccccaat ccttttctat ggttgcaact
     1381 cctgcaatgt attcaggttt tccaaaatta agtcctgatt ttaaaggata ttctgtggcc
     1441 attttttcaa ataaatcttt taagcttggg ttttgggaaa gaaaaacttc tttgcttttg
     1501 gtattgctta tataatcagg gatgctttca gaatctttta ttacaatttg tataattgga
     1561 gatagctcct tttctttttt aggtagaata aatttatcaa caaaagaaaa agaactcaca
     1621 ataaggacaa gaataaaaaa agcttttata gggtggtata aaataaaaaa ataaattttg
     1681 taaaataaat tcataattac tccttaattt ataatattat ctttaaaact taaattattt
     1741 tccgcatgca tttaatcttc ctttggcaat atttatgaaa taattaagtt cttttgttac
     1801 atatatcgct tcgtataatt cttgatcttg aaattttaaa tccacatcat gaaataaatt
```

```
     1861 catttttca taagaattaa tcatattctc taataattgt tctttttgag gtatagagca
     1921 aataaaattt ttatctaaag taaatccaaa aaacaaagta ttataattca taaaattaat
     1981 ttcaaacatt attttaccta aattatattt gggcggtaat ttttcaattt cattcataaa
     2041 gataagcaac tcatccacga catttaaata attattaatt tcctctaaat cggtattatt
     2101 ggaaataaaa taaaaataat caagattatt ttgcatttgt aagccaaata atacatcctt
     2161 atttttaaga gccactaaat aagcattatc caagcctaaa atttgcataa atttaaaatc
     2221 ttttttgaaa tagtgcatat agtttaacaa ttctttttga tttgttttta attcttgatt
     2281 taagttttgt tctttaaaaa ctttttaaa ttcttctttg ttgttccaaa gtaatttgga
     2341 tacatcttta gcattttgat attctttttc atgataatac ttattccaaa aaataagctc
     2401 taaagggcgt tgtaaaaaat gataagttaa gcttatgaaa ataatcaaaa ctacaagcac
     2461 accacaaggt atataaaatc ttttaagttt gtaaaaaggt ttattcattg cttatccttt
     2521 actctgcttt aagctctcca cctttaacca caagtccatt agagtctata attacttcta
     2581 ctccacctgc tttaatgata acacaatcag gttttgcatt aataattgtt tctcctacct
     2641 gtatagtcgc ttcgctatcg gcttttaact catgaatagt ttttgcgtaa gttgtgatat
     2701 tatcagctac catgcttgta tttttatcac tttcaaatcc tatggattca ttacttgtaa
     2761 aaagaatatt gtctttgctt ttataatcca tctctttctc gcttaatact tgcattttat
     2821 cgctgatatt aatatcataa tgcccctcaa ccatttcctt tttatttccc ttcacatttc
     2881 ttatctcatc tttatggata atagtatttt gatttgcacc tatttctatg tctttgtttt
     2941 ctcctacaaa ctcatgagag tttttagcca ctcttacttt at
//
LOCUS       FHGGBLIP_5              2702 bp    DNA     linear       12-APR-2021
DEFINITION  Campylobacter species strain strain.
ACCESSION
VERSION
KEYWORDS    .
SOURCE      Campylobacter species
  ORGANISM  Campylobacter species
            Unclassified.
COMMENT     Annotated using prokka 1.14.6 from
            https://github.com/tseemann/prokka.
FEATURES             Location/Qualifiers
     source          1..2702
                     /organism="Campylobacter species"
                     /mol_type="genomic DNA"
                     /strain="strain"
ORIGIN
        1 tccatggata cattctaggt tttgattttc taaaagagcg tgaagtttgg ctatgctttg
       61 ttttttatct acttgtgtgg ttttttgagt tttaaaagtg tttgaaatta aatctttgta
      121 ttctaagttt tgtttgctta agcattctaa tttgatattt aaagaatttt ctataataga
      181 ataattaagc aaacaaagtt catcattttt ataaaagata ctaagttttc ctaaaccact
      241 gctttcatct ttaggagaaa aataataact gggtatatct tgttttataa gattatcttg
      301 atctaattct ccaaagtaaa aaggattatt agaaagttca tttgagcctg taaggagggt
      361 ggagtttagg aagatataag cttggtagct ttggagttct tcaaaagtaa cacctacttt
      421 actagccact ctttcataaa gctcatcaca atcaatcaaa tccttacaat tagcaaaaat
      481 aaaaacttct tcttttgata aattaacaaa tatagtcgaa cttgaagaat taaattttac
      541 ttttgtttta atgggttggt ttttattaag ataataactt gtgcaattat tttcataaat
      601 caaattaatc atttgctttt ttctagcttg aaaaaaatct aaatcatttc ttaaactaag
      661 acatttaaag cagttggaat tataatttaa gacttcccct atattatctt ctgtaagtat
      721 tttttgatta tttttatctt taacaataaa aggcaaccac ttataaactg cttcagcatg
      781 atctaaatct tgcttgaagt cattattaga tagtttttct aatatagatt tatcaagtat
      841 ttttatataa atttgcatag cttccaaaac caaaaaagaa tcaggaataa caaatttatt
      901 gacattttta tattcactta cataaaacaa ctcttgcata tctttataaa taagattaaa
      961 ttctttcttg ttatatatgc tatttttaca caagctatca aaaattttat ccaatactat
     1021 actaatataa ttttgattct catcttggat atctacttta tcttctgata aaagcttatt
```

```
     1081 tttctcactt aagtcgatta aattttgtgt ataagaattt agataatcca tagcacaagc
     1141 aacatcccct ttaaattctt tgatattttc attatttttt accaaatgct cataaaaata
     1201 actcgcccaa actatattat acataaaatg actagttagc aaatagcttt tttcaagctc
     1261 tcctatatga attaaatgca acttgtctgt atttttataa tcaaacttat ctttaatatc
     1321 taatccatat ttataaccca aatcagctat cgcagatctt gaggactcta gtgcttgtct
     1381 atattctcgt gaaaaatctt ctttattagg gataggttta gctatagttt ctatatgatg
     1441 tatttctata cttgttttac ctttacttgc ttgttttata tttaaagcac attcatcaag
     1501 tctttttga tctatttgtc cttgtgaatt tttacattct tcgatcaagc catcggttcc
     1561 attttctata tttaaaactt tagagcaatt atctaaagtt tttctagtca taccaaagcc
     1621 aaaaagtttt ttaattcctt taatgagtaa tttaaaaaca accctaacag ctctaataaa
     1681 aatatttata aaacttgcta cagcacctcc tataccagct ccattataag tataaagttt
     1741 ttgaatatat tttttttgcc caaagataca aaaacattga gccaaatgtc cccctaaaga
     1801 atgccccaca acatctattt taaaatcatc gctcccatgc ttttctctaa tgatttttaa
     1861 aacttcatca ctataattaa ccaaagacac cacttgtgga attccaagtc ccaaagctat
     1921 acaaccatct gtaaaaaatg catcttttaa tatctgtgtt ccacctgttt cagtgccacg
     1981 aaaagccaag atataagtat tatcattttt acttgtatct ttaaaaacag tatgagaata
     2041 accactaatg ctagtatttt ttacatgatc tataacttca taacgagata caaagttttt
     2101 agtgcgtggg ctaagatgat gtcttaaatt tctaggattt tcattgtgct tagcaaaatt
     2161 caaagccagt agattttttt gatcttttgc ttctttgtct tctttgtttc tataaataaa
     2221 gctttgagct ttatttccaa taacttgatc ctcatctaaa gcttcattgt cttttctagg
     2281 ttttttaaag ataagatcag cagaaaatct agcctctata atggtagcat aagccgtagg
     2341 atctcctatt tcgcgtttat tttttaaaat taagcttttt gaagattcat tttgaatatg
     2401 tttatcttct atctcatacc ctaactttat tccatctgca taaagccaca aaggaggagt
     2461 aaaagcacta tcaaaaaaac ctttttcatc attctcatcg attaaatcaa gcattgcata
     2521 actagcatct gctatattag catagtcttt gatttgagag ataagttctt taggattcat
     2581 cagtaacttt gtcctgtata tttttcatca ctttttataa atttaccatt ggtaaaataa
     2641 tgaaaagatc ctccgcctgc acttttacga ctaatgcttg aaaagctaaa tcctcttcct
     2701 tc
//
LOCUS       FHGGBLIP_6              2651 bp    DNA     linear       12-APR-2021
DEFINITION  Campylobacter species strain strain.
ACCESSION
VERSION
KEYWORDS    .
SOURCE      Campylobacter species
  ORGANISM  Campylobacter species
            Unclassified.
COMMENT     Annotated using prokka 1.14.6 from
            https://github.com/tseemann/prokka.
FEATURES             Location/Qualifiers
     source          1..2651
                     /organism="Campylobacter species"
                     /mol_type="genomic DNA"
                     /strain="strain"
     gene            complement(294..668)
                     /locus_tag="FHGGBLIP_00029"
     CDS             complement(294..668)
                     /locus_tag="FHGGBLIP_00029"
                     /inference="ab initio prediction:Prodigal:002006"
                     /inference="similar to AA sequence:RefSeq:CKG86895.1"
                     /codon_start=1
                     /transl_table=11
                     /product="methyltransferase small"
                     /protein_id="Prokka:FHGGBLIP_00029"
```

```
                     /translation="MTNMEQINNLKDYTEFVQASYFNFVYTEGQKALGFKISKTRFFK
                     DKSELEKLDYFKALHDEYNGYLVIDDSFFYAKLNRDLENYKLEIFLKDMRLNFISPTL
                     QVALALLCLMKRQRKLRGINKY"
     gene            complement(687..1049)
                     /locus_tag="FHGGBLIP_00030"
     CDS             complement(687..1049)
                     /locus_tag="FHGGBLIP_00030"
                     /inference="ab initio prediction:Prodigal:002006"
                     /codon_start=1
                     /transl_table=11
                     /product="hypothetical protein"
                     /protein_id="Prokka:FHGGBLIP_00030"
                     /translation="MCKLNELPNNEEKYNKILGYFDKKLGDIDDFRHLKKYSRIEMWL
                     CIYYDDTYKTPSNFQEKIEMDNIALKTKNMQMSLHKIVDMHISPYWNTRRYILEGNEG
                     NMNFEFIEDELACGNLYQ"
     gene            complement(1441..1641)
                     /locus_tag="FHGGBLIP_00031"
     CDS             complement(1441..1641)
                     /locus_tag="FHGGBLIP_00031"
                     /inference="ab initio prediction:Prodigal:002006"
                     /codon_start=1
                     /transl_table=11
                     /product="hypothetical protein"
                     /protein_id="Prokka:FHGGBLIP_00031"
                     /translation="MQTKLYFICNYLSIIYFLNYLVTLSFVRNYPPKLKAIDSYILFL
                     YKKLLKYDYIVFYKDSYITKKT"
     gene            complement(1687..2055)
                     /locus_tag="FHGGBLIP_00032"
     CDS             complement(1687..2055)
                     /locus_tag="FHGGBLIP_00032"
                     /inference="ab initio prediction:Prodigal:002006"
                     /inference="similar to AA sequence:RefSeq:ASI87259.1"
                     /codon_start=1
                     /transl_table=11
                     /product="Hypothetical protein"
                     /protein_id="Prokka:FHGGBLIP_00032"
                     /translation="MIKAYFENNAINVKAFARAHNDVFDKYITKILKSLTKQGSNIDY
                     VKAICISSKKQEFTGSLTGDKYSINPMTWKEIISTRKKDLENHIKDVKSNLSLSKYKD
                     IHNFKEKEIKKERVGNLKKI"
ORIGIN
        1 aataagcttt attgtaaatt attactttat tatctatatc tttacacaag ctcctaaact
       61 cataatatcg cggatccata tatttataag aacatcctcc tgctaagaat aataaaagat
      121 atacaggtaa aagaaacaaa gtaaattttt taaatattgt tttaaaattt atagtttttg
      181 ttttattttt tattttaaaa aagactttaa agataaaata taagataata gaaaatataa
      241 aaacaaaaaa aagaaaaata taagaataaa tattaaaaga ttaataaaga attctagtat
      301 ttattaattc ctcttaattt tctctgtctt ttcataagac aaagcagtgc taaagccact
      361 tgtagtgttg gactgatgaa atttaatctc atatctttga gaaaaatttc tagcttgtaa
      421 ttctctaaat ctctatttag tttcgcataa aaaaaagaat catctataac caaatatcca
      481 ttatattcat catgtaatgc tttaaaatag tccaattttt ctaattcact cttatcttta
      541 aaaaatctag ttttagatat tttaaatcct aaagcttttt gtccttcagt ataaacaaaa
      601 ttaaaataac tagcttgcac aaattctgta taatctttta aattgttgat ttgttccata
      661 ttagtcatta tttattcctt atattattat tgataaagat tgccacaagc aagctcatcc
      721 tctataaact caaaattcat attaccctca ttgccttcta atatatacct tctagtattc
      781 caatatgggc ttatatgcat atcaacaatc ttgtgtaaag acatttgcat atttttttgtt
```

```
      841 tttagtgcaa tattatccat ctctatcttt tcttgaaaat ttgaaggagt tttgtaagta
      901 tcatcgtaat atatacaaag ccacatttct atcctactat attttttag atgtcgaaaa
      961 tcatctatgt cacctaactt tttatcaaaa taccctagaa tcttattata tttctcttcg
     1021 ttattaggaa gttcatttaa cttacacata ttcctaaatt gccaataatt aggctctaaa
     1081 taataagaat aaggagtata ataaaagata attaagccta tacttccaag aaagactaaa
     1141 aactttgaaa cgcttttttt agatttgtaa ggggtatttt aaaaagaata tgtaactatc
     1201 cctaaataat tttagcaaaa ctcccaaata ataggatttt aaagcctgca aaaatgactt
     1261 tttatctttc ctaatttaaa gtgtaaaaaa tcccaaatat cccaaaatgt ctgttttatc
     1321 cctaaaaagt aattaaatat tgtttaaaac gcaattaaaa gcgatatttg attttaaatt
     1381 tttatatccc aaaaatgttt ttgatcctat aagatagtta cattttttact taaagtattc
     1441 ttatgttttc ttagttatgt aactatcctt ataaaacact atataatcat attttaacaa
     1501 ctttttatac aaaaataata tgtaactatc tatagcttta agttttggag gatagttacg
     1561 cacaaagctt aatgttacta aataatttaa aaaataaata atagacaggt agttgcaaat
     1621 aaaatatagt tttgtttgca taattatcgt attggaaatt ttttattata caaaatattc
     1681 aaatttttat attttttttta aattgccaac cctttccttt tttatttctt tttctttaaa
     1741 attgtgaata tctttatatt ttgatagaga taaattactt ttaacatctt ttatgtggtt
     1801 ttctaagtct ttttttcttg tactaattat ttctttccaa gtcattggat ttatgctata
     1861 tttatctcca gttaagcttc ctgtaaattc ttgctttttt gatgaaatgc atatagcctt
     1921 cacataatca atattacttc cttgctttgt taatgattta agaattttag tgatatattt
     1981 atcaaacaca tcgttgtgag ccctagcaaa agcttttaca ttaatagcat tgttttcaaa
     2041 atatgcttta atcatttttt ctcctttaat acttcaattt taattgaagt ttatctttaa
     2101 ttgtgataat atttcaatta aaattgaagt attatactat taaaagttag tataagtcaa
     2161 gtaaaaaact aatttttttat agtatttttg ttaaggttac gattatatgg aagtcttaga
     2221 tatattgcaa aaaatgtttg aagctgtagg ggtaaaaaat aaaaatcagt tggcaaaata
     2281 tttaaaaata gagaatagcc ttatagctgg ttgggagaaa agaggaaaaa ttcccgaaaa
     2341 gtatgtgtat aaaatttctc aagatagtaa tttgccatat gaatatttta tcaacaatac
     2401 taataaaaat gagtataata ataacaataa taagcgagat gttaattttt attcaatacc
     2461 aaaacttaat gtttcggttt ctgctggtag tggcaatgag ttaattgggc ttgaagaata
     2521 tgaaacagga gaaatgcttg aacttagcaa agcgtttttt aaaacaacac ctaaaaatgt
     2581 aaaagcgatt aaagttgatg gttattctat ggttccaatg cttttgcctg atagttgggt
     2641 tgtctttgag g
//
LOCUS       FHGGBLIP_7              2243 bp    DNA     linear       12-APR-2021
DEFINITION  Campylobacter species strain strain.
ACCESSION
VERSION
KEYWORDS    .
SOURCE      Campylobacter species
  ORGANISM  Campylobacter species
            Unclassified.
COMMENT     Annotated using prokka 1.14.6 from
            https://github.com/tseemann/prokka.
FEATURES             Location/Qualifiers
     source          1..2243
                     /organism="Campylobacter species"
                     /mol_type="genomic DNA"
                     /strain="strain"
     gene            21..785
                     /gene="trxB_2"
                     /locus_tag="FHGGBLIP_00033"
     CDS             21..785
                     /gene="trxB_2"
                     /locus_tag="FHGGBLIP_00033"
                     /inference="ab initio prediction:Prodigal:002006"
```

```
                     /inference="similar to AA sequence:RefSeq:CKG86288.1"
                     /codon_start=1
                     /transl_table=11
                     /product="thioredoxin reductase"
                     /protein_id="Prokka:FHGGBLIP_00033"
                     /translation="MAAGILGDYKYLANDMSQNGFNARFIQATILYIQLSGGSSILDK
                     PNLLGAIYGYADIAVGSGLVGVHKNPLREQQIKTLAKTLKPDEFGMLPFIDEIMGVDW
                     VIDLNKYDIASDQFGSMYKALRSDVVEGKIKDPRDIDSTYESRREFDRHRGGYKNGML
                     QGYGTDTPNDWSEERAQLFNDTLILHAKLAALTPPQGYPNAPYYFTPENLEWYYKRHK
                     LDKLLDPRIPAIYRYNFPRELRAKILKFGEENGIKE"
     gene            866..2158
                     /gene="trxB_3"
                     /locus_tag="FHGGBLIP_00034"
     CDS             866..2158
                     /gene="trxB_3"
                     /locus_tag="FHGGBLIP_00034"
                     /inference="ab initio prediction:Prodigal:002006"
                     /inference="similar to AA sequence:RefSeq:CKG86288.1"
                     /codon_start=1
                     /transl_table=11
                     /product="thioredoxin reductase"
                     /protein_id="Prokka:FHGGBLIP_00034"
                     /translation="MSETYEIYTPEGFILNVDKQSGKILFSESKSKKPTGKYTQEYSK
                     ALFEADRILRTSPYINYQPRYLDPEFHTGQKSTLLEFKDWQSIYLKDPVKGSIAPWTK
                     AEKAYYKSLKTKKERYKYLVIRSGIRSVVIDIPYEAIGAVDEKGNVDPKYEELYRTVD
                     DNKHNLRSSLFHNEWGMAAGILGDYKYLANDMSQNGFNARFIQATILYIQLSGGSSIL
                     DKPNLLGAIYGYADIAVGSGLVGVHKNPLREQQIKTLAKTLKPDEFGMLPFIDEIMGV
                     DWVIDLNKYRLANDQFGNIYKALRSDVVEGKIKDPRDIDSTYESRREFDRYRVGYRKG
                     MAQGYGTDTPNDWSEEEAQLFNDTLILHAKLAALTPPQGYPNAPYYFTPENLEWYYKR
                     HKLDKLLDPRIPAIYRYNFPRELRAKILAYAKEHNIKD"
ORIGIN
        1 tatttcataa tgaatggggt atggcagcag gaatactggg tgattataaa tatctagcta
       61 acgatatgtc tcaaaatggt tttaatgcaa gatttatcca agcaactata ctttatatac
      121 aattaagtgg aggaagtagt atactagata aacctaattt attgggtgct atttatggtt
      181 atgcagatat tgcagtagga agtggtttag taggagtgca taaaaatcct ttaagagaac
      241 aacaaattaa aaccttagct aagactttaa aacctgatga atttggtatg cttccttta
      301 tagatgagat tatgggagta gattgggtga ttgatttaaa taaatatgat atagctagtg
      361 atcaatttgg aagtatgtat aaagccttaa gaagtgatgt ggttgagggt aagataaaag
      421 atccaaggga tatagattct acttatgaaa gcagaaggga atttgatcgc catagaggtg
      481 gatataaaaa tggcatgctg caaggctatg gaaccgatac acctaatgat tggagtgaag
      541 aaagggcaca actttttaat gatactttaa tacttcatgc caaactcgca gccctcactc
      601 ctcctcaagg ctatcctaat gcaccttatt attttacccc tgaaaaccta gaatggtatt
      661 ataaaagaca taagcttgat aaactccttg atccaagaat ccctgctatt tatagatata
      721 atttcccaag ggaacttaga gctaagatac tcaaatttgg cgaagaaaat ggcattaaag
      781 aataaagtaa ttacctaggc agattagcca ctactataat catggaagat ggtttatata
      841 tgggataaat taagaaagga taaaaatgag tgaaacttat gaaatttata cacccgaagg
      901 ttttatatta aatgtagata aacaaagcgg aaaaatacta ttttcagagt caaagtcaaa
      961 aaaacctaca ggcaaataca cccaagaata ttctaaagct ttgtttgaag ctgatagaat
     1021 tttaagaacc tctccttata ttaattatca acctaggtat ctagatcctg aatttcacac
     1081 agggcaaaaa tccactttgc ttgaatttaa agattggcaa agtatatatt taaaagatcc
     1141 tgttaaagga agcatagctc cttggactaa agcagaaaaa gcttattata aatctttaaa
     1201 aactaaaaaa gaaagatata aatatttagt tataagaagt ggtataagaa gtgttgttat
     1261 agatatacct tatgaagcca taggagctgt agatgaaaaa ggaaatgtag atcctaagta
     1321 tgaagaatta tatagaacag tagatgataa taaacataat ctaagatcaa gtttatttca
```

```
     1381 taatgaatgg ggtatggcag caggaatact gggtgattat aaatatctag ctaacgatat
     1441 gtctcaaaat ggttttaatg caagatttat ccaagcaact atactttata tacaattaag
     1501 cggaggaagt agtatactag ataaacctaa tttattgggt gctatttatg gttatgcaga
     1561 tattgcagta ggaagtggtt tagtaggagt gcataaaaat cctttaagag agcaacaaat
     1621 taaaacctta gctaagactt taaaacctga tgaatttggt atgcttcctt ttatagatga
     1681 gattatggga gtagattggg tgattgattt aaataaatat agattggcta atgatcaatt
     1741 tggaaatata tataaagcct taagaagtga tgtagttgag ggtaagataa aagatccaag
     1801 ggatatagat tctacttatg aaagcagaag ggagtttgat cgttatagag ttggatatag
     1861 aaagggtatg gcgcaaggct atggaaccga tacacccaat gattggagcg aagaagaagc
     1921 acaacttttt aatgatactt taatacttca tgccaaactc gcagccctca ctcctccaca
     1981 aggctatcct aatgcacctt attattttac ccctgaaaac ctagaatggt attataaaag
     2041 acataagctt gataaactcc ttgatccaag aatccctgct atttatagat ataatttccc
     2101 aagggaactt agagctaaga tactagctta tgctaaagaa cataatatca aagattaaac
     2161 cacaaaagaa aggaaaaaca aatgcaaaaa acatttcaaa tcgatttaga aaaaagaaaa
     2221 gctttagaga attttaaaaa tct
//
LOCUS       FHGGBLIP_8              2165 bp    DNA     linear       12-APR-2021
DEFINITION  Campylobacter species strain strain.
ACCESSION
VERSION
KEYWORDS    .
SOURCE      Campylobacter species
  ORGANISM  Campylobacter species
            Unclassified.
COMMENT     Annotated using prokka 1.14.6 from
            https://github.com/tseemann/prokka.
FEATURES             Location/Qualifiers
     source          1..2165
                     /organism="Campylobacter species"
                     /mol_type="genomic DNA"
                     /strain="strain"
     gene            117..566
                     /locus_tag="FHGGBLIP_00035"
     CDS             117..566
                     /locus_tag="FHGGBLIP_00035"
                     /inference="ab initio prediction:Prodigal:002006"
                     /codon_start=1
                     /transl_table=11
                     /product="hypothetical protein"
                     /protein_id="Prokka:FHGGBLIP_00035"
                     /translation="MVGGGWFVPYSLQPSYHEFKEMCKLNELPNDEHKYNKILSYFGL
                     NLDTLDWENIKKNSTLLTQGYLEYNSQNDRYMYRYENKSDPRIRKVFFLFFNDSIKLS
                     NLYKIDFEVFWNDKRDYIHTKVTSYDTVIEENFRTCGYFSGYLKGIK"
     gene            566..931
                     /locus_tag="FHGGBLIP_00036"
     CDS             566..931
                     /locus_tag="FHGGBLIP_00036"
                     /inference="ab initio prediction:Prodigal:002006"
                     /inference="similar to AA sequence:RefSeq:AVS37480.1"
                     /note="DUF2974 domain-containing protein"
                     /codon_start=1
                     /transl_table=11
                     /product="hypothetical protein"
                     /protein_id="Prokka:FHGGBLIP_00036"
```

```
                     /translation="MHHKQQIQNFRDNAELAWASYGYFHFIGKNYNLRNKDKEKLDSF
                     IKDNNRMPTYTDILNMEYNSLFDGEFSPLQAKQFFERYDLLKHCPNTNSGFSATLFQN
                     KETKEYTLAIRGTEPKEVK"
     gene            931..1419
                     /locus_tag="FHGGBLIP_00037"
     CDS             931..1419
                     /locus_tag="FHGGBLIP_00037"
                     /inference="ab initio prediction:Prodigal:002006"
                     /codon_start=1
                     /transl_table=11
                     /product="hypothetical protein"
                     /protein_id="Prokka:FHGGBLIP_00037"
                     /translation="MKKFLTPKKIILAFIALFGIYLLGIPLYYTPYELQPSYWKFKKI
                     CEATFQLHNQHIEHTQRIKPLMQIFNVDNQKIKKYAKLENNTYISRIPYPHFLMRIEN
                     AQLNIYSDLNWNIIKIDTGDLVWYNWRPMILGNEGNMDLRVKWNNFLMCGNLDKRIEY
                     EQ"
ORIGIN
        1 acttgcttat aataatatag aatctaaaga gagtgcataa atgataaggc ttaaaaagat
       61 tctaaaaata tcaagtttta ttctacttcc tttgataatt atatggagta tgacatttgg
      121 ttgggggggg gtggtttgtg ccttatagcc tacaaccgag ttatcacgaa tttaaagaga
      181 tgtgtaagct taatgaactt ccaaatgatg aacacaaata taataagatt ctaagttatt
      241 ttggattaaa tttggataca ttggattggg aaaatattaa aaaaaattca acattattaa
      301 cacaaggtta tttagaatat aattcacaaa atgatagata tatgtatcga tatgaaaata
      361 aaagtgatcc aagaataaga aaagtttttt tcttattttt taatgacagt ataaagctaa
      421 gcaatcttta taaaatagat tttgaagtat tttggaatga taaaagagat tatattcata
      481 caaaagtaac ttcttatgat actgtaatag aagagaactt tagaacttgt ggatatttta
      541 gtggatacct gaaaggaatc aaataatgca ccataaacag caaatacaaa attttagaga
      601 taacgcagaa ttagcttggg caagttatgg ttattttcat tttataggta aaaactataa
      661 tttacgaaat aaagacaagg aaaaattaga tagttttatt aaagataaca atagaatgcc
      721 tacctatacc gacattctca atatggaata taattcctta tttgatggcg aattctcccc
      781 actccaagcc aaacaattct ttgaaagata tgatttatta aaacattgtc ctaatacaaa
      841 ttcaggtttt tctgctactc tctttcaaaa caaagaaact aaggaatata ctttagctat
      901 taggggaaca gagcctaagg aagtcaaata atgaaaaagt ttctgacacc taaaaagatt
      961 atcttagctt ttattgcatt atttggaatt tatctacttg gaatcccact ttattatact
     1021 ccttatgaat tacaaccaag ttattggaag tttaagaaaa tatgcgaggc tacatttcaa
     1081 cttcataatc aacatataga gcatacacaa agaataaaac ccttaatgca aatttttaat
     1141 gtagataacc aaaaaattaa aaagtatgct aagttggaga ataataccta tatctctcgt
     1201 atcccatatc cacatttttt gatgcgaata gaaaatgctc agttaaatat ttatagtgat
     1261 ttaaattgga atattatcaa aattgatacg ggggatttag tatggtataa ttggagaccc
     1321 atgatattag gtaatgaggg caatatggat ttaagggtta aatggaataa tttttttaatg
     1381 tgtggaaatt tggataaaag gatagagtat gagcaataaa gagcagatta gaaagcttcg
     1441 tgataattca gaactagctt gggcatcgta tggatatttc cactattttt tagaacagca
     1501 aaaaaagtct tacgctgttt ggctacaaga tgaatcaggc aaaaatagac tagatgaaaa
     1561 taaaaaagca atcacaaaat ccattgattt aaacgatgta ttagatattg aatataaagg
     1621 gcttgaagtt attaaactag atatatttgg aaacccagaa aaaattgaca ctctcaaagg
     1681 cgacttctcc ccactccaaa ctaaacaatt cttttctcgc tatgatttat taaaacattg
     1741 tcctaataca aattcaggtt tttctgctac tttatttgga gaaaaaagaa aacaaattaa
     1801 tagtaaaaca aaagaagaaa gctataccag tgaatatggc tatatcaatt atattttagc
     1861 tattagagga acagaaatga gttcttttaa agatttgctt gtagctgatg cttctttagc
     1921 cataggctct atacctaaag cccaatatga tgatatgata aacttttatg aaacttgtat
     1981 taaagattat ccacaaatca aagaaaaaga ctctcttacc attacaggac actcattggg
     2041 aggctgtctt gcacaactct ttgctttaag tatctgtgat gataagaata gaaataatat
     2101 caaagcttta tatacttata atgcacctgg agctagaaag ataatacctc cttatgatta
     2161 tatag
```

```
//
LOCUS       FHGGBLIP_9              1815 bp    DNA     linear       12-APR-2021
DEFINITION  Campylobacter species strain strain.
ACCESSION
VERSION
KEYWORDS    .
SOURCE      Campylobacter species
  ORGANISM  Campylobacter species
            Unclassified.
COMMENT     Annotated using prokka 1.14.6 from
            https://github.com/tseemann/prokka.
FEATURES             Location/Qualifiers
     source          1..1815
                     /organism="Campylobacter species"
                     /mol_type="genomic DNA"
                     /strain="strain"
     gene            82..882
                     /locus_tag="FHGGBLIP_00038"
     CDS             82..882
                     /locus_tag="FHGGBLIP_00038"
                     /inference="ab initio prediction:Prodigal:002006"
                     /inference="similar to AA sequence:RefSeq:CKG87105.1"
                     /note="Uncharacterized protein conserved in bacteria"
                     /codon_start=1
                     /transl_table=11
                     /product="hypothetical protein"
                     /protein_id="Prokka:FHGGBLIP_00038"
                     /translation="MSIKKLPSDLERYYNQLSDHSWNFIENHGISLFNTAWINEVYNP
                     FINDIAPFYPFNEESTANLSMDSFKTFFGRNGVLNSFYKKYLNNVLVKRKNNYSINSQ
                     FASKLNFSKEFLDFITNAGNLSSLILNANDNIRVNFTIQSLDLSADFSFVKLGYNNKI
                     IQYDHTLDQTLQIVAEEFNNGTSLNFTAYNYSNPNLNYTKSYKGEWAWYKFIKDNKNN
                     STYSIMFNNNKNLYFDFEIINGASDLNNIVYILNNLKIVENITGVNKQ"
     gene            879..1778
                     /locus_tag="FHGGBLIP_00039"
     CDS             879..1778
                     /locus_tag="FHGGBLIP_00039"
                     /inference="ab initio prediction:Prodigal:002006"
                     /inference="similar to AA sequence:RefSeq:CKG87069.1"
                     /note="Uncharacterized conserved protein%2C contains FHA
                     domain"
                     /codon_start=1
                     /transl_table=11
                     /product="hypothetical protein"
                     /protein_id="Prokka:FHGGBLIP_00039"
                     /translation="MIEKEELGITIENYDECISGSKAFVFDTKGGTIGSGDDCTFRIQ
                     DKLEQIKNTHAIVSYEEGSFTIAAFEDSDIYYNKSFSRMPSGYEIIISIGDIFKIGNL
                     EFRFVDAKSIEESIKENKKYLEDIEKRNHFDEIEIEPRGKTSINFDKNEELKEILKNN
                     NYKFIEEEKADDSFLKEIIQRDPNSLGYENILKSLVKNLKDIKTKQQSSKLNNNYSPI
                     SIKDFESIINNIPLIKSTRLINILALSLIAKELYTPIFEEMEENMFIKYLEAAIQNNI
                     KEDKILFENLTLKALEKYIKDFE"
ORIGIN
        1 aataaccaaa gcgccgaaga aaaaatttca tatgctcttg gtggcaataa agatgccaat
       61 gatccttttg ctgtatttca aatgagtata aaaaaattac ctagtgattt agaaaggtat
      121 tataatcagc tttctgatca ttcttggaat tttatagaaa atcatggtat ttctttattt
```

```
      181 aatactgctt ggattaatga agtgtataat ccatttatca atgatattgc gccattttat
      241 ccttttaatg aagaaagcac agctaatctg agtatggata gctttaaaac atttttttggt
      301 agaaatggag tattaaatag tttttataaa aaatacttga acaatgtttt agtaaaaaga
      361 aaaaataact attctatcaa ttctcaattt gcttcaaaat taaatttctc taaagaattt
      421 ttagatttta tcaccaatgc aggtaatctc tcaagcctca tactgaatgc taatgataat
      481 ataagagtta attttactat tcaaagcctt gatttaagtg cagattttttc ctttgtaaag
      541 cttggctaca acaataaaat tattcaatac gatcatactc ttgatcaaac cctacaaatt
      601 gtagcagaag aatttaataa tggaacaagt ttaaatttta cagcctacaa ctactccaat
      661 cctaatttaa attatacaaa atcatataag ggagaatggg catggtataa gtttataaaa
      721 gataataaaa acaattctac atacagcatt atgtttaata ataataaaaa cttatatttt
      781 gactttgaaa ttattaacgg tgctagtgat ttaaataata tagtttatat tttaaataat
      841 cttaaaatag tagaaaatat cacaggagta aataaacaat gatagaaaaa gaagaattag
      901 gaattactat tgagaattat gacgaatgca ttagtggttc taaagcattt gtatttgata
      961 caaagggtgg aactatagga agtggtgatg attgcacttt tcgtattcaa gataaactag
     1021 agcaaatcaa aaatacccat gccatagtat cttatgaaga aggatctttt acaattgcag
     1081 cttttgaaga ttctgacatt tattataaca agtccttttc tagaatgcca agtggctatg
     1141 aaataataat cagcataggc gatattttta aaatcggtaa tttagaattt agatttgtgg
     1201 atgcaaaaag cattgaagaa agtatcaaag aaaataaaaa atacttagaa gatattgaaa
     1261 aacgcaatca ttttgatgaa attgaaattg aacctagagg aaaaacaagt ataaattttg
     1321 acaaaaacga agagttgaaa gagattttaa aaaataataa ttataagttt attgaggaag
     1381 aaaaggcaga tgatagcttt ttaaaagaaa tcattcaaag agatccaaat tcattagggt
     1441 atgaaaatat tctaaaaagt ttggtaaaaa atttaaaaga tattaaaaca aaacaacaaa
     1501 gctctaaatt aaataataat tattctccta tcagtattaa agattttgaa agtattatta
     1561 ataatatacc tttaatcaaa tccacaagat tgattaatat tttagccctt agcctaatcg
     1621 caaaagaatt atacactccc atctttgaag aaatggaaga aaatatgttt attaaatatc
     1681 ttgaagctgc catacaaaac aatatcaaag aagataagat tctttttgag aatcttaccc
     1741 taaaagcatt ggaaaagtat attaaagatt ttgagtgatt ttttaatctt taatgccatt
     1801 ttcttcagca aattt
//
LOCUS       FHGGBLIP_10             1455 bp    DNA     linear       12-APR-2021
DEFINITION  Campylobacter species strain strain.
ACCESSION   
VERSION     
KEYWORDS    .
SOURCE      Campylobacter species
  ORGANISM  Campylobacter species
            Unclassified.
COMMENT     Annotated using prokka 1.14.6 from
            https://github.com/tseemann/prokka.
FEATURES             Location/Qualifiers
     source          1..1455
                     /organism="Campylobacter species"
                     /mol_type="genomic DNA"
                     /strain="strain"
     gene            144..695
                     /gene="trxB_4"
                     /locus_tag="FHGGBLIP_00040"
     CDS             144..695
                     /gene="trxB_4"
                     /locus_tag="FHGGBLIP_00040"
                     /inference="ab initio prediction:Prodigal:002006"
                     /inference="similar to AA sequence:RefSeq:CKG87041.1"
                     /codon_start=1
                     /transl_table=11
```

```
                     /product="thioredoxin reductase"
                     /protein_id="Prokka:FHGGBLIP_00040"
                     /translation="MIYDLHDHHYESLLDEFGMLPFLDEIIGADWTIDLNRYKFALDE
                     EGRIIWALYDDIEKGKLKDPRDVDSTPESRKEFDHYMDGYKNGMVTRFDVDIRNERDE
                     RSAKLTMDTLVLSAKLAALTPPQGYPNAPRYYSPERLEIIYKRHKLDKLLDPRIPAIY
                     RYNFPRELRAKILKFGEENSIKD"
ORIGIN
        1 taggatttaa agcaagacaa caacaagctt tatttttaca agctcaattt ggagataaaa
       61 atgcttttaa gagtttaggt ttagcagtat tgtgttctaa ttcttttctt acagggcaac
      121 attggaacaa gcttagagca aaaatgattt acgatttaca cgatcatcat tatgagagtt
      181 tgttagatga atttggtatg ctaccttttt tagatgagat tataggagca gattggacga
      241 ttgatttaaa tcgatataaa tttgctcttg atgaagaagg tagaatcatt tgggctttat
      301 atgatgatat tgaaaaaggt aaattaaaag atccaagaga tgtggattct actcctgaaa
      361 gcaggaaaga atttgatcat tatatggatg gatataaaaa tggaatggta actagatttg
      421 atgtagatat tagaaacgaa agagatgaga gatctgctaa actgactatg gataccctag
      481 tattaagtgc caaactcgca gccctcactc ctcctcaagg ttatcctaat gctccaagat
      541 actatagccc tgaaagacta gaaattattt ataaaagaca taagcttgat aaactccttg
      601 atccaagaat ccctgctatt tatagatata atttcccaag ggaacttaga gctaagatac
      661 tcaaatttgg cgaagaaaat agcattaaag attaaagtaa cttcataggc agactagcca
      721 ctactataat catagaagat ggtttataca tgggataaat ttaagaaagg ataaaaatga
      781 gtgaaactta taaaatttat acacccaatg gattagcatt agatgtggaa aaagatacca
      841 ataaaatact atttaaagaa aatattaaac ccacaggcaa ttataccgaa gaatattcca
      901 aagctttgtt tgaagcacac gacattaaac gaaactcccc ctacaaagac tataaaccaa
      961 tatatctaga tcctaacttt tatacagggc aaaaatccac tctagtagaa tttaaagaat
     1021 ggcaaagcat atatttaaaa gatcctatca aagtagctca aaatatcaca aatatttgta
     1081 ttaattatgt aaaaaaaggt tttaaagaag ttttaatcga acaacttggc attatatatt
     1141 tattaacaca tccacaaatc gaagttgaag ttaagaaata tggaaaaaaa tattttaagg
     1201 ctataaacaa ggaaattgct gcaaaaagat tttctacaat taaagaagaa ttatttttca
     1261 ataaaacaaa aatatccttt atggcagaac aagtaaaaaa cggtattgat tatgaaatag
     1321 atcaaaagct tgaagaaaat aaagaatttc ttttaaaaag acttttgtct tatttgatta
     1381 ttgatgagaa aagaagtgca actgctttag aggatataaa agaacaaaaa tatattatag
     1441 atgattatac ttttt
//
LOCUS       FHGGBLIP_11             1304 bp    DNA     linear       12-APR-2021
DEFINITION  Campylobacter species strain strain.
ACCESSION
VERSION
KEYWORDS    .
SOURCE      Campylobacter species
  ORGANISM  Campylobacter species
            Unclassified.
COMMENT     Annotated using prokka 1.14.6 from
            https://github.com/tseemann/prokka.
FEATURES             Location/Qualifiers
     source          1..1304
                     /organism="Campylobacter species"
                     /mol_type="genomic DNA"
                     /strain="strain"
     gene            119..250
                     /locus_tag="FHGGBLIP_00041"
     CDS             119..250
                     /locus_tag="FHGGBLIP_00041"
                     /inference="ab initio prediction:Prodigal:002006"
                     /codon_start=1
```

```
                     /transl_table=11
                     /product="hypothetical protein"
                     /protein_id="Prokka:FHGGBLIP_00041"
                     /translation="MNPQELISQIKDYANIADASYAMLDLIDENDEKGFLRYIKYKN"
     gene            303..947
                     /locus_tag="FHGGBLIP_00042"
     CDS             303..947
                     /locus_tag="FHGGBLIP_00042"
                     /inference="ab initio prediction:Prodigal:002006"
                     /codon_start=1
                     /transl_table=11
                     /product="hypothetical protein"
                     /protein_id="Prokka:FHGGBLIP_00042"
                     /translation="MKIAMIILSLFSLVFLSACAFKEKKASEIETLASNYGGIYIFDK
                     KIREEILELEKKREEFRSKYLGSEIKVGNETHFVNFSYLKKKFPQILSNGCNYYRSDY
                     RYKGKAHFGFKDKPEFAYYEDQFKAYMGEENYKKLRPYLGMTTYYVCEGKKYPVVFAT
                     MIDYKVKNYGLFGDEGRGFSFSNISRKSAGGGSFHYFTNGKFIKSDEKYTGQSY"
ORIGIN
        1 gaagaggatt tagcttttca agcattagtc gtaaaagtgc aggcggagga tcttttcatt
       61 attttaccaa tggtaaattt ataaaaagtg atgaaaaata tacaggacaa agttactgat
      121 gaatcctcaa gaacttatct ctcaaatcaa agactatgct aatatagcag atgctagtta
      181 tgcaatgctt gatttaatcg atgagaatga tgaaaaaggt tttttgcgat atattaaata
      241 taaaaactaa atttgattta tataaggtat ttgaaataat acaatttaaa aaggagtatt
      301 aaatgaaaat cgcaatgata attctaagct tatttttcttt agtattttta agtgcatgtg
      361 cttttaaaga aaaaaaagca agtgaaatag aaactcttgc aagtaattat ggtggaattt
      421 atatctttga taaaaaaata agagaagaga tattggaatt agagaaaaaa agagaagaat
      481 ttcgtagtaa gtatttgggt agtgaaatta aagtaggaaa tgaaactcat tttgtgaatt
      541 tttcttattt gaagaaaaaa ttcccccaaa tcctttctaa tggttgtaac tattatcgta
      601 gtgattatag atataaaggc aaggctcatt ttggtttcaa agataaaccc gaatttgcat
      661 actatgaaga tcaatttaaa gcatatatgg gtgaagaaaa ctataaaaaa ttaagacctt
      721 atttaggtat gacaacttat tatgtgtgtg agggtaaaaa atatcctgtt gtttttgcta
      781 ccatgataga ttataaagta aaaaattatg gattatttgg agatgaagga agaggattta
      841 gcttttcaaa cataagtcgt aaaagtgcag gcggaggatc ttttcattat tttactaatg
      901 gtaaatttat aaaaagtgat gaaaaatata caggacaaag ttactgatga accctaaaga
      961 acttatctct caaatcaaag actatgctaa tatagcagat gctagttatg ctatgttgca
     1021 atatgtttgg gaaaatatag agcaagatga aaagaataat atttataaag cagataaact
     1081 tacttttggt gataaattaa agcaagatat tgttatgaaa aatagtaaag gggaaaatat
     1141 tgtaaaacct aaaaatacaa acacagctta tacttgtgct atacaagctc gttttgaaca
     1201 aaataaaata gttaaaatag aacctaaata ttgtatttct cttataaata cttgttttga
     1261 tagtaaagaa ataacattgg ataatgatat tagtagggta ggat
//
LOCUS       FHGGBLIP_12             1284 bp    DNA     linear       12-APR-2021
DEFINITION  Campylobacter species strain strain.
ACCESSION
VERSION
KEYWORDS    .
SOURCE      Campylobacter species
  ORGANISM  Campylobacter species
            Unclassified.
COMMENT     Annotated using prokka 1.14.6 from
            https://github.com/tseemann/prokka.
FEATURES             Location/Qualifiers
     source          1..1284
                     /organism="Campylobacter species"
```

```
                     /mol_type="genomic DNA"
                     /strain="strain"
     gene            complement(584..1273)
                     /gene="trxB_5"
                     /locus_tag="FHGGBLIP_00043"
     CDS             complement(584..1273)
                     /gene="trxB_5"
                     /locus_tag="FHGGBLIP_00043"
                     /inference="ab initio prediction:Prodigal:002006"
                     /inference="similar to AA sequence:RefSeq:CKG86610.1"
                     /codon_start=1
                     /transl_table=11
                     /product="thioredoxin reductase"
                     /protein_id="Prokka:FHGGBLIP_00043"
                     /translation="MTGFKARTQAVNFLLIQLGHEQGFTSLYDSYAYRGLTDGIHKNP
                     LKAQMLKDFAKNPPYDELGMLPFLDELIGVDWVIDANNYDIAYDGAKGNIIWALDDDV
                     LKGKLKDPRDIDSTPESRDEFDNYLDGYKNGMTEHYTLEISHDTPEKSANLWLETMLL
                     EAKIKALTPPQGYPNAPYYFTPERLEFIYKNHDLDKLLDPRIPAIYRYNFPRDLRAKI
                     LKFGEENGIKD"
ORIGIN
        1 catcacttaa atgagctaag cctctattgg cttctacttc tttgtaaagt tctttatagt
       61 ctttattgat taaatttcct ttttcatcta cattacaata agcatcataa ggtatatcta
      121 taacagtact tctaagtcca cttcttataa ttaaatattt atatctttct cttttggttt
      181 ttaaagattt ataataagct ttttctgctt tagtccaagg agctatgctt cctttgatag
      241 gatcttttaa atatatgctt tgccattctt taaattcaag caaagtggat ttttgccctg
      301 tataaaagtt aggatctaga tatattggtt tatagtcttt gtaggggggag tttcttaata
      361 tgcgatcacc ctcgaagaga gcttgagaat gttgtttagt gtatgtacca ttgctttttg
      421 ccgtaaataa tattttattt gtatcttttt ctacatttaa tattgagcca tctggtgtat
      481 aaatctcata agtatcactc atcttttatc ctttctttaa ttaacctata tgcaaaccat
      541 ttttcataat gatagtagtg gctagcctgc ctatgtaatt actttaatcc ttaatgccat
      601 tttcttcgcc aaatttgagt atcttagctc ttaaatccct tgggaaatta tatctataaa
      661 tagcagggat tcttggatca aggagtttgt ctaaatcatg atttttgtaa ataaattcta
      721 atctttcagg tgtaaaataa taaggtgcat taggatagcc ttgaggaggg gttagggctt
      781 taattttggc ttctaacagc attgtttcta accaaagatt tgcggatttt tcgggtgtgt
      841 catggcttat ttctagagta taatgctctg tcattccatt tttgtaacca tctaaatagt
      901 tgtcaaattc atctctactc tccggagtag aatctatatc tcttggatct tttaatttac
      961 cttttaacac atcatcatct aaagcccaaa taatattacc ctttgcccca tcataagcga
     1021 tatcataatt attcgcatca atcacccaat ccacccctat aagttcatct aagaaaggaa
     1081 gcattcccaa ttcatcataa ggaggatttt tagcaaagtc ttttagcatt tgagctttaa
     1141 gagggttttt gtgtatgcca tctgttaaac ctctatatgc ataagaatca taaagacttg
     1201 taaagccttg ttcatgtcct agttgtatga gtaaaaaatt tactgcttgg gttcttgctt
     1261 taaatcctgt catagaaagt tgta
//
LOCUS       FHGGBLIP_13             1228 bp    DNA     linear       12-APR-2021
DEFINITION  Campylobacter species strain strain.
ACCESSION
VERSION
KEYWORDS    .
SOURCE      Campylobacter species
  ORGANISM  Campylobacter species
            Unclassified.
COMMENT     Annotated using prokka 1.14.6 from
            https://github.com/tseemann/prokka.
FEATURES             Location/Qualifiers
```

```
     source          1..1228
                     /organism="Campylobacter species"
                     /mol_type="genomic DNA"
                     /strain="strain"
     gene            66..869
                     /locus_tag="FHGGBLIP_00044"
     CDS             66..869
                     /locus_tag="FHGGBLIP_00044"
                     /inference="ab initio prediction:Prodigal:002006"
                     /inference="similar to AA sequence:RefSeq:CKG86263.1"
                     /codon_start=1
                     /transl_table=11
                     /product="hypothetical protein"
                     /protein_id="Prokka:FHGGBLIP_00044"
                     /translation="MQKTFQIDLEKRKALENFKNLALLGLGGTMLLNLKEQPLDFNQE
                     LTYINTYDKYLNLKLDSFYIPSTLLSLLQLNQKSKEAFYINVLINSDTYYKNPLLQYA
                     ITQKQEQENVVYALKNDENLNTSFTNLNALKLALEEKNFYLNFIDLNSKESLLSFYNQ
                     IISNNHLRNYFYFKDNQFFIKKDIKNKILFHSIHSSSIISNYLNLLNGFNTLSKIELI
                     KILESLSLYNLSTLMQTMQTIQSKNEYGSKGFDKDGIFLSNMIMNVRLK"
ORIGIN
        1 aagatactag cttatgctaa agaacataat atcaaagatt aaaccacaaa agaaaggaaa
       61 aacaaatgca aaaaacattt caaatcgatt tagaaaaaag aaaagcttta gagaatttta
      121 aaaatctagc tttattagga ttaggcggaa caatgctttt aaatttaaaa gaacagcctt
      181 tagattttaa tcaagaatta acttatataa atacttatga taagtattta aacttaaagc
      241 ttgattcttt ttatatccct tctacacttt tatctttact tcaattaaat caaaaaagca
      301 aagaagcttt ttatatcaat gttttaatca atagtgatac atactataaa aaccctttat
      361 tacaatatgc tatcactcaa aaacaagaac aagaaaatgt cgtttatgct ttaaagaatg
      421 atgaaaatct taatacttct tttactaatc ttaatgcttt aaaattagct ttagaagaaa
      481 agaattttta tcttaatttt atagacttaa acagcaaaga aagtctttta agtttttata
      541 atcaaataat aagtaataat catcttagaa attattttta ttttaaagat aatcaatttt
      601 ttattaaaaa agatataaaa aataaaattt tatttcatag tattcactct tcttctatta
      661 taagtaatta tcttaactta ttaaatggtt ttaatacttt aagcaagata gaactcataa
      721 aaatattaga aagtttaagt ttgtataacc tttcaacttt aatgcaaact atgcaaacta
      781 tacaatcaaa aaatgaatat ggctcaaaag gctttgataa agatggtatc tttttatcta
      841 atatgattat gaatgtgaga ttgaaataag taaatgatag atttagcagg tgtgatttgt
      901 taatgaaaat atacatctcc aattgatttg aaaaaatagc aaggttttaa aaaagcaata
      961 caagagagaa aatttaaaga atttgggtaa tgcatttgaa tgcaactgga ttttagtaga
     1021 tataaattta aataaaattt atcaagaagt tttttctata aagactatgc atttttttaaa
     1081 aaaagtattt ttaaaccaaa gtttttacta tgataataaa acctattatt taagaaatta
     1141 aacttaaaaa tgaaaatttg attatcaaag ttcctagtga tttttcaaag ctttagaaga
     1201 tattataaaa ctcacacagt ataatcag
//
LOCUS       FHGGBLIP_14             1209 bp    DNA     linear       12-APR-2021
DEFINITION  Campylobacter species strain strain.
ACCESSION
VERSION
KEYWORDS    .
SOURCE      Campylobacter species
  ORGANISM  Campylobacter species
            Unclassified.
COMMENT     Annotated using prokka 1.14.6 from
            https://github.com/tseemann/prokka.
FEATURES             Location/Qualifiers
     source          1..1209
```

```
                     /organism="Campylobacter species"
                     /mol_type="genomic DNA"
                     /strain="strain"
     gene            complement(590..1132)
                     /gene="trxB_6"
                     /locus_tag="FHGGBLIP_00045"
     CDS             complement(590..1132)
                     /gene="trxB_6"
                     /locus_tag="FHGGBLIP_00045"
                     /inference="ab initio prediction:Prodigal:002006"
                     /inference="similar to AA sequence:RefSeq:CKG86288.1"
                     /codon_start=1
                     /transl_table=11
                     /product="thioredoxin reductase"
                     /protein_id="Prokka:FHGGBLIP_00045"
                     /translation="MLKDFAKNPPYDEFGMLPFLDELIGVDWVIDLNEYQFAYDGDGG
                     SVKALDEKVRQGKLKDPRDIDSTPESRKEFDEEANAYTFGNRMNFSSDIRDSRSKESA
                     RLFDDLLILHAKLAALTPPQGYPNAPYYFSPEQLEDFYKRQVLDAKLDPRIPAIYRYN
                     FPRELRAKILKFGEENGIKD"
ORIGIN
        1 catcacttaa atgagctaag cctctattgg cttctacttc tttgtaaagt tctttatagt
       61 ctttattgat taaatttcct ttttcatcta cattacaata agcatcataa ggtatatcta
      121 taacagtgct tcttatacca cttcttataa ttaaatattt atatctttct cttttagttt
      181 ttaaagattt ataataagct ttttctgctt tagtccaagg agctatgctt cctttgatag
      241 gatcttttaa atatatactt tgccattctt taaattctac tagagtggat ttttgccctg
      301 tataaaagtt aggatctaga tactggggtt gatagtcttt gtggggggag ttttccatga
      361 tttcccaagc tttgaagagg gctttggaat attctttggt atattttcct gtgggtttgg
      421 aacttttatc aaaatatatt tcattagttt ttttattaac atctaatatg gaaccatgtg
      481 gtgtataaat ctcataagta tcactcatct tttatccttt ctttatttaa cctatatgca
      541 aaccattttt cataatgata gtagtggcta gcctgcctat gcaattactt taatctttaa
      601 tgccattttc ttcgccaaat ttgagtatct tagctctaag ttcccttggg aaattatatc
      661 tataaatagc agggattctt ggatcaagtt tagcgtctaa aacttgtctt ttgtaaaaat
      721 cttctaattg ttcaggacta aaataataag gtgcattagg ataaccttga ggaggagtga
      781 gggctgcgag tttggcatga agtatcaaaa ggtcatcaaa taatctagcc gattctttac
      841 ttctactatc gcgaatatca gaggaaaaat tcattctatt accaaaagta taagcattag
      901 cttcttcatc gaattctttc ctgctctctg gagtagaatc tatatctctt ggatctttta
      961 atttaccttg tcttactttc tcatctaaag ccttaacact accgccatca ccatcataag
     1021 caaattgata ttcattcaaa tcaatcaccc aatccactcc tataagttca tctaagaaag
     1081 gaagcattcc aaattcatca taaggaggat ttttagcaaa gtcttttagc attttctctc
     1141 ttaaaggatt tttatgtcct ccacctgcta aggctacaga agatgagtaa gtagtgagtt
     1201 ggtgttcta
//
LOCUS       FHGGBLIP_15              994 bp    DNA     linear       12-APR-2021
DEFINITION  Campylobacter species strain strain.
ACCESSION
VERSION
KEYWORDS    .
SOURCE      Campylobacter species
  ORGANISM  Campylobacter species
            Unclassified.
COMMENT     Annotated using prokka 1.14.6 from
            https://github.com/tseemann/prokka.
FEATURES             Location/Qualifiers
     source          1..994
```

```
                     /organism="Campylobacter species"
                     /mol_type="genomic DNA"
                     /strain="strain"
     gene            complement(366..908)
                     /gene="trxB_7"
                     /locus_tag="FHGGBLIP_00046"
     CDS             complement(366..908)
                     /gene="trxB_7"
                     /locus_tag="FHGGBLIP_00046"
                     /inference="ab initio prediction:Prodigal:002006"
                     /inference="similar to AA sequence:RefSeq:CKG86610.1"
                     /codon_start=1
                     /transl_table=11
                     /product="thioredoxin reductase"
                     /protein_id="Prokka:FHGGBLIP_00046"
                     /translation="MLKDFAKNPPYDELGMLPFLDELIGVDWVIDVNKYQFAYDEKGR
                     VNDALKDDVEKGILKDPRDIDSTPESRWEFDKEMDAYKNGMRTRFDGDNPNHWSKEQV
                     ERFNDTLILCAKLAALTPPQGYPNAPYYYSPERLEFIYKKHKLDRLEDPRIPAIYRYN
                     FPRELRAKILKFGEENGIKD"
ORIGIN
        1 gcttcctttg ataggatctt ttaaatatat gttttgccat tctttaaatt ctactagagt
       61 ggatttttgc cctgtataaa agttaggatc tagatactgg ggttgatagt ctttgtaggg
      121 ggagtttctt aggattttat cagctttaaa aaccgctttg gaatattctt gagtgtattt
      181 gcctgtaggc ttggagttct tattgaaata tattttattt gtaagtttat ttacctctaa
      241 ttctgctgta ttgggaacta gcaccttata aatatcttta ttattaccac tcattgtttg
      301 tcctttcttt aattaaccta tatgcaaacc atttttcata atgatagtag tggcttgtaa
      361 ttactttaat ctttaatgcc attttcttcg ccaaatttga gtatcttagc tctaagttcc
      421 cttgggaaat tatatctata aatagcaggg attcttggat cttctaatct atcaagcttg
      481 tgttttttgt aaataaattc taatctttca ggagagtaat aataaggggc attaggatag
      541 ccttgagggg gagttagggc tgcgagtttg gcacataaaa tcaaagtatc attaaatctc
      601 tctacttgct ctttactcca atgatttgga ttatctccat caaatctagt tctcattcca
      661 ttcttataag catccatctc tttgtcaaac tcccacctac tctctggagt agaatctata
      721 tctcttggat ctttgagtat tcctttttca acatcatctt ttagagcatc attaactctt
      781 ccttttttcat cataggcaaa ttgatattta ttcacatcta tcacccaatc cactcctata
      841 agttcatcta gaaaaggtag catacccaat tcatcataag gaggattttt agcaaagtct
      901 tttagcattt gagctttaag agggtttttg tgtatgccat ctgttaaatc tctatatgca
      961 taagaatcat aaagactttt aaagccttgt tcat
//
LOCUS       FHGGBLIP_16              861 bp    DNA     linear       12-APR-2021
DEFINITION  Campylobacter species strain strain.
ACCESSION
VERSION
KEYWORDS    .
SOURCE      Campylobacter species
  ORGANISM  Campylobacter species
            Unclassified.
COMMENT     Annotated using prokka 1.14.6 from
            https://github.com/tseemann/prokka.
FEATURES             Location/Qualifiers
     source          1..861
                     /organism="Campylobacter species"
                     /mol_type="genomic DNA"
                     /strain="strain"
     gene            complement(97..609)
```

```
                     /locus_tag="FHGGBLIP_00047"
     CDS             complement(97..609)
                     /locus_tag="FHGGBLIP_00047"
                     /inference="ab initio prediction:Prodigal:002006"
                     /inference="similar to AA sequence:RefSeq:AVS37480.1"
                     /note="DUF2974 domain-containing protein"
                     /codon_start=1
                     /transl_table=11
                     /product="hypothetical protein"
                     /protein_id="Prokka:FHGGBLIP_00047"
                     /translation="MNNYNPFNPTYADILNITYKYYLDDNGKPKDGFFDNGFLGGEFS
                     PLQAKQFFSRYDLLKHCPNTNSGFSATLFGEKRKQINSKTKEKSYTSEYGYINYILAT
                     RGTEMSSFKDLLVADASLAIGSIPKAQYDDMINFYETCIKDYPQITKKKTLLPLQDIL
                     LEAVLHNSLL"
     gene            complement(587..790)
                     /locus_tag="FHGGBLIP_00048"
     CDS             complement(587..790)
                     /locus_tag="FHGGBLIP_00048"
                     /inference="ab initio prediction:Prodigal:002006"
                     /codon_start=1
                     /transl_table=11
                     /product="hypothetical protein"
                     /protein_id="Prokka:FHGGBLIP_00048"
                     /translation="MKNKAVINKLKDNSELAWAAYGYFHLANESYKPEENSKDWKRLK
                     YFRKLYKNNNLESSKTDEQLQSI"
ORIGIN
        1 ctatataatc ataaggaggt attatctttc tagctccagg tgcattataa gtatataaag
       61 ctttgatatt atttctattc ttatcatcac agatacttaa agcaaagagt tgtgcaagac
      121 agcctccaag agaatgtcct gtaatggtaa gagagtcttt ttctttgtga tttgtggata
      181 atctttaata caagtttcat aaaagtttat catatcatca tattgggctt tgggtataga
      241 gcctatggct aaagaagcat cggctacaag caaatcttta aaagaactca tttctgttcc
      301 tctagtggct aaaatataat tgatatagcc atattcactg gtatagcttt tttctttagt
      361 tttactatta atttgttttc ttttttctcc aaataaagta gcagaaaaac ctgaatttgt
      421 attagggcaa tgttttaata aatcatagcg agaaaagaat tgtttagctt ggagtgggga
      481 gaattcacca cctaagaagc cattatcaaa gaagccatct ttgggtttgc cattatcatc
      541 aagatagtat ttataggtaa tgttgagtat atctgcgtag gtggggttaa atggattgta
      601 attgttcatc ggttttacta gattctaaat tattgttttt atataatttt ctaaaatatt
      661 ttaatctttt ccaatcctta ctattttcct ctggcttata actttcatta gctaaatgaa
      721 aatatccata ggctgcccaa gcaagttctg aattatcttt tagcttatta atcactgctt
      781 tatttttcat tgtttagtcc tttttgaaat atatacaact tattgggaca cttcctaaaa
      841 aaaatcctat cccctcatta c
//
LOCUS       FHGGBLIP_17              804 bp    DNA     linear       12-APR-2021
DEFINITION  Campylobacter species strain strain.
ACCESSION
VERSION
KEYWORDS    .
SOURCE      Campylobacter species
  ORGANISM  Campylobacter species
            Unclassified.
COMMENT     Annotated using prokka 1.14.6 from
            https://github.com/tseemann/prokka.
FEATURES             Location/Qualifiers
     source          1..804
```

```
                     /organism="Campylobacter species"
                     /mol_type="genomic DNA"
                     /strain="strain"
     gene            complement(86..793)
                     /gene="trxB_8"
                     /locus_tag="FHGGBLIP_00049"
     CDS             complement(86..793)
                     /gene="trxB_8"
                     /locus_tag="FHGGBLIP_00049"
                     /inference="ab initio prediction:Prodigal:002006"
                     /inference="similar to AA sequence:RefSeq:CKG87041.1"
                     /codon_start=1
                     /transl_table=11
                     /product="thioredoxin reductase"
                     /protein_id="Prokka:FHGGBLIP_00049"
                     /translation="MTGFKARTQAVNFLLIQLGHEQGFTSLYDSYAYRGLTDGIHKNP
                     LKAQMLKDFAKNPPYDEFGMLPFLDELIGVDWVIDANKTIATNENNIVIDSQGYIIRA
                     LEDRVKKGKIKDPRDIDSTPESRDEFDNYLDGYKNGMTEHHYTLEISHDTPEKSANLW
                     LETMLLEAKIKALTPPQGYPNAPRYYSPERLEFIYNKHKLDSKLDPRIPAIYRANFPE
                     ELRAKILAYAKEHNIKD"
ORIGIN
        1 agatttttaa aattctctaa agcttttctt ttttctaaat cgatttgaaa tgttttttgc
       61 atttgttttt cctttctttt gtggtttaat ctttgatatt atgttcttta gcataagcta
      121 gtatcttggc tctaagctct tcaggaaagt tagctctata aatagcaggg attctaggat
      181 caagtttaga atcaagtttg tgtttgttgt aaataaattc taatctttca ggactatagt
      241 atcttggtgc attaggataa ccttgaggag gggttagggc tttaattttg gcttctaaca
      301 gcattgtttc taaccaaaga tttgcggatt tttcgggtgt gtcatggctt atttctagag
      361 tataatgatg ctctgtcatt ccatttttgt aaccatctaa atagttgtca aattcatctc
      421 tactctccgg agtagaatct atatctcttg gatcttttat ttttaccttt ttaactctat
      481 cctctaaagc acgaataata taaccttgac tatcgataac tatattattt tcatttgtgg
      541 ctatagtttt attcgcatca atcacccaat ctactcctat aagttcatct aagaaaggta
      601 gcattccaaa ttcatcataa ggaggatttt tagcaaagtc ttttagcatt tgagctttaa
      661 gagggttttt gtgtatacca tctgttaaac ctctatatgc ataagaatca taaagacttg
      721 taaagccttg ttcatgtcct agttgtatga gtaaaaaatt tactgcttgg gttcttgctt
      781 taaatcctgt catagaaagt tgta
//
LOCUS       FHGGBLIP_18              789 bp    DNA     linear       12-APR-2021
DEFINITION  Campylobacter species strain strain.
ACCESSION
VERSION
KEYWORDS    .
SOURCE      Campylobacter species
  ORGANISM  Campylobacter species
            Unclassified.
COMMENT     Annotated using prokka 1.14.6 from
            https://github.com/tseemann/prokka.
FEATURES             Location/Qualifiers
     source          1..789
                     /organism="Campylobacter species"
                     /mol_type="genomic DNA"
                     /strain="strain"
     gene            144..695
                     /gene="trxB_9"
                     /locus_tag="FHGGBLIP_00050"
```

```
     CDS             144..695
                     /gene="trxB_9"
                     /locus_tag="FHGGBLIP_00050"
                     /inference="ab initio prediction:Prodigal:002006"
                     /inference="similar to AA sequence:RefSeq:CKG86610.1"
                     /codon_start=1
                     /transl_table=11
                     /product="thioredoxin reductase"
                     /protein_id="Prokka:FHGGBLIP_00050"
                     /translation="MIYDLHDHHYESLLDEFGMLPFLDEIIGADWTIDLNKYDFAYDE
                     EGRIIWALYNDIEKGKLKDPRDVDSTPESRNEFDDAMDGYRNGMKTNFDVDTPNEWSE
                     QQAALDRDTLVLSAKLAALTPPQGYPNAPYYFTPERLEWIYKRGYLDKLLDPRIPAIY
                     RYNFPRELRAKILKFAEENGIKD"
ORIGIN
        1 taggatttaa agcaagacaa caacaagctt tatttttaca agctcaattt ggagataaaa
       61 atgcttttaa gagtttaggt ttagcagtat tgtgttctaa ttcttttctt acagggcaac
      121 attggaataa gcttagagca aaaatgattt acgatttaca cgatcatcat tatgagagtt
      181 tgttagatga atttggtatg ctaccttttt tagatgagat tataggagca gattggacga
      241 ttgatttaaa taaatatgat tttgcctatg atgaagaagg tagaatcatt tgggctttat
      301 ataatgatat tgaaaaaggc aaattaaaag atccaagaga tgtggattct actcctgaaa
      361 gtagaaatga atttgatgat gctatggatg gatatagaaa tgggatgaaa actaactttg
      421 atgtagatac tccaaatgaa tggagtgaac aacaagctgc tttagataga gatactttag
      481 tattaagtgc caaactcgca gccctcactc ctccacaagg ctatcctaat gccccttatt
      541 attttacacc tgaaagatta gaatggattt ataaaagagg atatttagat aaactccttg
      601 atccaagaat ccctgctatt tatagatata atttcccaag ggaacttaga gctaagatac
      661 tcaaatttgc tgaagaaaat ggcattaaag attaaaaaat cactcaaaat ctttaatata
      721 cttttccaat gcttttaggg taagattctc aaaaagaatc ttatcttctt tgatattgtt
      781 ttgtatggc
//
LOCUS       FHGGBLIP_19              760 bp    DNA     linear       12-APR-2021
DEFINITION  Campylobacter species strain strain.
ACCESSION
VERSION
KEYWORDS    .
SOURCE      Campylobacter species
  ORGANISM  Campylobacter species
            Unclassified.
COMMENT     Annotated using prokka 1.14.6 from
            https://github.com/tseemann/prokka.
FEATURES             Location/Qualifiers
     source          1..760
                     /organism="Campylobacter species"
                     /mol_type="genomic DNA"
                     /strain="strain"
     gene            complement(420..599)
                     /locus_tag="FHGGBLIP_00051"
     CDS             complement(420..599)
                     /locus_tag="FHGGBLIP_00051"
                     /inference="ab initio prediction:Prodigal:002006"
                     /inference="similar to AA sequence:RefSeq:AAW35464.1"
                     /note="conserved hypothetical protein"
                     /codon_start=1
                     /transl_table=11
                     /product="hypothetical protein"
```

```
                     /protein_id="Prokka:FHGGBLIP_00051"
                     /translation="MYMGINGNKDKTSNTSRSKEDMASKIDANADIASETKTPPNFIG
                     RLATTIIIEDGLYMG"
ORIGIN
        1 agggtatatc tataaccaca gatcttaatc cacttcttat tgccaaatac ttacatcttt
       61 ctcttttagt ttttaaagat ttataataag ctttttctgc tttagtccaa ggagctatgc
      121 ttccttttat aggatctttt aaataaatgt tttgccattc tttaaattca agtaaagtgg
      181 atttctctcc tgtataaaag ttaggatcta gatatattgg tttatagtct ttgtaggggg
      241 agtttcttaa tatgcgatcg gcttcaaaca aagctttgga atattcttcg gtataattgc
      301 ctgtgggttt agcatttcct gaaaaaatga ttttattagt atctttatat acatccatta
      361 tcaacccatt gggtgtataa atctcataag tttcgctcac tatttatcct ttcttaaatt
      421 tatcccatgt ataaaccatc ttctatgatt atagtagtag ctagtctacc tatgaagtta
      481 ggcggagttt ttgtttctga tgcaatatca gcatttgcat ctatcttact agccatatct
      541 tccttgcttc tacttgtatt agaagtttta tctttatttc catttatccc catatacata
      601 gctttaagat tgttttgtcc tatgatattg agtttttcta gtaatcttct ggttttattt
      661 ttatcttcta tacattttcc tgttttatct ttgcttgtat tgtaaattcc attagcaaaa
      721 tcttgtaaaa tttgtaaagc ttcattataa gcatggatgg
//
LOCUS       FHGGBLIP_20              738 bp    DNA     linear       12-APR-2021
DEFINITION  Campylobacter species strain strain.
ACCESSION
VERSION
KEYWORDS    .
SOURCE      Campylobacter species
  ORGANISM  Campylobacter species
            Unclassified.
COMMENT     Annotated using prokka 1.14.6 from
            https://github.com/tseemann/prokka.
FEATURES             Location/Qualifiers
     source          1..738
                     /organism="Campylobacter species"
                     /mol_type="genomic DNA"
                     /strain="strain"
     gene            complement(353..706)
                     /gene="tssI1"
                     /locus_tag="FHGGBLIP_00052"
     CDS             complement(353..706)
                     /gene="tssI1"
                     /locus_tag="FHGGBLIP_00052"
                     /inference="ab initio prediction:Prodigal:002006"
                     /inference="similar to AA sequence:RefSeq:AOH52008.1"
                     /codon_start=1
                     /transl_table=11
                     /product="type VI secretion system, secreted protein"
                     /protein_id="Prokka:FHGGBLIP_00052"
                     /translation="MKGENKLLIEKSLTQTIEKEFFLNVHQNLSAHIQDNTSLKSNSM
                     QTKIEEQYSLESDNSTFDFQTDCEVKAGNQILHQVGDTQIVTRKDCVIIKAGGVEVVI
                     DSNGLVVRGGEVKAE"
ORIGIN
        1 caaaaaaagt tcctaaagga aaaaccaaat catttttgat ataatactca atcacaagtt
       61 tttcttttgt gatataaatt cttttaatat ttgccattct atagatcttt tgtgcaagtg
      121 catatatgcc taaaagaaat gtaaaaaaaa ctataaacca tttataatct atttctttgc
      181 taggattaaa taatctagga aataaataaa gaaaaagaca taaaagcaca atatatgtta
      241 tccaaaaaag caaaaacgaa ggtatatgct tttttccttc ccaaatgatt tcatcttggg
```

```
      301 tgtcttgctc tttgatttct gtgttattgt ttatttcact catttttatc ctttactctg
      361 cttttacttc tccacctcta accacaagtc cattagagtc tatcactact tcaactccac
      421 ctgctttaat gataacacaa tctttcctag taacaatttg agtatcacct acttgatgga
      481 ggatttgatt gcctgctttt acttcacaat ctgtttgaaa gtcaaaagtt gaattatctg
      541 attctaaaga atattgctct tctatctttg tttgcatgga attagacttt aaagatgtat
      601 tatcttgtat gtgggcggat aaattttgat ggacattaag gaaaaattct ttttctattg
      661 tttgggttaa agatttttct attaataatt tattttctcc cttcacattc cttatctcat
      721 ctttatggat aatagtat
//
LOCUS       FHGGBLIP_21              699 bp    DNA     linear       12-APR-2021
DEFINITION  Campylobacter species strain strain.
ACCESSION
VERSION
KEYWORDS    .
SOURCE      Campylobacter species
  ORGANISM  Campylobacter species
            Unclassified.
COMMENT     Annotated using prokka 1.14.6 from
            https://github.com/tseemann/prokka.
FEATURES             Location/Qualifiers
     source          1..699
                     /organism="Campylobacter species"
                     /mol_type="genomic DNA"
                     /strain="strain"
     gene            complement(288..467)
                     /locus_tag="FHGGBLIP_00053"
     CDS             complement(288..467)
                     /locus_tag="FHGGBLIP_00053"
                     /inference="ab initio prediction:Prodigal:002006"
                     /inference="similar to AA sequence:RefSeq:AAW35464.1"
                     /note="conserved hypothetical protein"
                     /codon_start=1
                     /transl_table=11
                     /product="hypothetical protein"
                     /protein_id="Prokka:FHGGBLIP_00053"
                     /translation="MYMGINGNEDKTSNTSRSKEDMASKIDANADITSETKTPPNCIG
                     RLATTIIIEDGLYMG"
ORIGIN
        1 gatcttttaa atatatgctt tgccattctt taaattcaag caaagtggat ttttgccctg
       61 tataaaagtt aggatctaga tatattggtt tatagtcttt gtagggggag tttcttaata
      121 tgcgatcggc ttcaaacaaa gctttggaat attcttcggt ataattgcct gttggtttag
      181 catttcctga aaaaatgatt ttattagtat ctttatatac atccattatc aacccattgg
      241 gtgtataaat ctcataagta tcactcatta tttatccttt cttaaattta tcccatgtat
      301 aaaccatctt ctatgattat agtagtggct agtctgccta tgcaattagg cggagttttt
      361 gtttctgaag taatatcagc atttgcatct atcttactag ccatatcttc cttgcttcta
      421 cttgtattag aagttttatc ttcatttcca tttatcccca tatacatagc tttaagattg
      481 ttttgtccta tgatattgag cttttctagt aatcttctgg ttttattttt atcttctata
      541 cattttcctt cttcatattt gacaatataa agtccattag caaaatcttg taaaatttgt
      601 aaagcttcat tgtaagcttg aatggctgtt aaattgattt catcattaac ttctttataa
      661 tacttttcta atgtagtgtt ttttaggata taaatttca
//
```

>>BfR-CA-15855_min_cov_30 putative plasmid location

```
LOCUS       JOBBOHMI_1             28513 bp    DNA     linear       12-APR-2021
DEFINITION  Campylobacter species strain strain.
ACCESSION
VERSION
KEYWORDS    .
SOURCE      Campylobacter species
  ORGANISM  Campylobacter species
            Unclassified.
COMMENT     Annotated using prokka 1.14.6 from
            https://github.com/tseemann/prokka.
FEATURES             Location/Qualifiers
     source          1..28513
                     /organism="Campylobacter species"
                     /mol_type="genomic DNA"
                     /strain="strain"
     gene            complement(198..1001)
                     /locus_tag="JOBBOHMI_00001"
     CDS             complement(198..1001)
                     /locus_tag="JOBBOHMI_00001"
                     /inference="ab initio prediction:Prodigal:002006"
                     /codon_start=1
                     /transl_table=11
                     /product="hypothetical protein"
                     /protein_id="Prokka:JOBBOHMI_00001"
                     /translation="MKHFIFGVNQKSKKLSLALISSLIIGNNAFAGGIPVIDVSAIAN
                     QVKDYAMQLQQYEQMYSQLQQQIQMVQMQKQNLERLTKEDWDNLGTVLYQTRNVMNKV
                     NGISYDIGNVSRKFEDTYKDFEGYSNDLENATNESEKNKIYSDRYKQIAETNQNTFNG
                     TLQQLELRYQDLESEDALIAKLKQNSQNSNGNLQAVQATNDLLAYQIDEIRKLRSVIM
                     DQSNMLTNYLASQNNQRIMQQAKIDKFIEDTGYIEKTWGKSNPDALKWK"
     gene            complement(998..1603)
                     /locus_tag="JOBBOHMI_00002"
     CDS             complement(998..1603)
                     /locus_tag="JOBBOHMI_00002"
                     /inference="ab initio prediction:Prodigal:002006"
                     /codon_start=1
                     /transl_table=11
                     /product="hypothetical protein"
                     /protein_id="Prokka:JOBBOHMI_00002"
                     /translation="MLDLLKGVDMSNTSIININNQEVIFENKDEQVFCTSLDVAKVFG
                     KQHKHILELIGEKFNNNEIKNFCEPNFRLSFKTRKIEGFRGRERKYPYYQLTKDGFSF
                     IAMGLTGRKADKFKIAFINAFNEMERIIKNQYTPKLKNYNKYKIPNTPYKENIIKAIM
                     QTEQNRNCEFEELIAYEITETYKDNKSHIEQKINFKVKGLI"
     gene            complement(1588..2259)
                     /locus_tag="JOBBOHMI_00003"
     CDS             complement(1588..2259)
                     /locus_tag="JOBBOHMI_00003"
                     /inference="ab initio prediction:Prodigal:002006"
                     /inference="similar to AA sequence:RefSeq:ASI88311.1"
                     /codon_start=1
                     /transl_table=11
                     /product="IncQ plasmid conjugative transfer protein TraQ"
                     /protein_id="Prokka:JOBBOHMI_00003"
                     /translation="MKNFIFGVKKITLALSATLLISSNAFADDILTGDTKLACEAILC
                     LSSGTRPSECSSSLARYFSIKFKKPWETINARRAFLNLCPIQNDANIEDLVLNNLVDD
```

```
                     VLPVSDPRQCTPNYLNTQVETKRSYSTFGIMSYRINPNMPNFCHALINHAYTDYKTPK
                     YKCTGEFYNSLEWKLSAKLQLITQQAYESLSDDQRYMISRTCGDRNCYDYYQKIPFTK
                     ECWTY"
     gene            complement(2256..4721)
                     /locus_tag="JOBBOHMI_00004"
     CDS             complement(2256..4721)
                     /locus_tag="JOBBOHMI_00004"
                     /inference="ab initio prediction:Prodigal:002006"
                     /inference="similar to AA sequence:RefSeq:ASI88309.1"
                     /codon_start=1
                     /transl_table=11
                     /product="IncP-type conjugative transfer protein TrbE"
                     /protein_id="Prokka:JOBBOHMI_00004"
                     /translation="MLALKEFRSKAKAFPDILNYASFIDEGILLNKDGSLTAGFYYRA
                     GDISSMTINERNTLSARINSVLKTLGNGWAVHIDCSRIKTENYIDGDTFYKNNIAQIL
                     ENERKHYFDNVEHFENLFTMIFTYLPPHKSINKITDLMIIDETKNKNQGDKILEYFKN
                     TLQKLEDSLSNYIKIERMLPRIAIDEYNNEYILDDMLEYINFCICGERQKIILPNAPM
                     FLDCLLGGRHFETGMHPRIDNQCIGIIAIEGFPSESYPNILNALTELNFDYRFNTRFI
                     FLDDFEAQNSLNKYRKKWQQKTKGWLDQLLDRPARKQDDHAVLMVQELDSALSESKSG
                     MIGYGYYTANIVVFDRDYESLEYKLKDVRKVLENLGFLSRTETINSVEAYLGTLPGFV
                     YPNLRRPILNTLNLTHLIPLASIWAGDKYNSSERFPPNSPPLMQVVTSGNTPFRLNLH
                     VSDLGHTLIFGPPGSGKSTLLANIFLAWQKYPNAKIYAFDKGQSLLAPTLATGGIHYD
                     VAGDHSSLAFAPLANIKTDAEIAWAEFWIETCLKLQNVNITPKQKKLIHEALISHINT
                     NSTSLTEFVSALQDSDLRDALNYYTISGTGGFLFDSEEDKLSLSSITTFEIEELMNLG
                     DQYIIPALLYIFNRIEKGLDGSPTLLIIDEAWIALKHPAFKDKIVNWLKVLRKANVAV
                     IMATQSLSDSAKSGILDILQEYCPTKIFLPNPEAYKKGSENVLAPYDFYKIFGLNDVQ
                     ISIINNAIQKREYYYTSPLGTRLFNLALGKTNLAFTTISDKVSVKRVRQLYEKHKENW
                     AYKWLEEKEINYKRFDNSQKEQI"
     gene            complement(4725..5012)
                     /locus_tag="JOBBOHMI_00005"
     CDS             complement(4725..5012)
                     /locus_tag="JOBBOHMI_00005"
                     /inference="ab initio prediction:Prodigal:002006"
                     /inference="similar to AA sequence:RefSeq:ASI88308.1"
                     /codon_start=1
                     /transl_table=11
                     /product="IncP-type conjugative transfer protein TrbD"
                     /protein_id="Prokka:JOBBOHMI_00005"
                     /translation="MEELQRIDIYSALNKPNLIFGADRELILMVGVISFALIFTGATL
                     LTSIIGIFLFFFCNMLLRLMAKSDPLMRQIFLRQIKYKKFYYAQSTPFSKD"
     gene            complement(5012..5308)
                     /locus_tag="JOBBOHMI_00006"
     CDS             complement(5012..5308)
                     /locus_tag="JOBBOHMI_00006"
                     /inference="ab initio prediction:Prodigal:002006"
                     /inference="similar to AA sequence:RefSeq:ASI88307.1"
                     /codon_start=1
                     /transl_table=11
                     /product="Conjugative transfer protein TrbC"
                     /protein_id="Prokka:JOBBOHMI_00006"
                     /translation="MKKFLILFLALGLGYALASTTGAGLPWEGPLEQIKASLSGPVAF
                     VISILAIIGAGAGLIWGNEFSGFIKTLFYIVIVIALIVGANSFMSLFSTSGALI"
     gene            complement(5318..6256)
                     /locus_tag="JOBBOHMI_00007"
```

```
     CDS             complement(5318..6256)
                     /locus_tag="JOBBOHMI_00007"
                     /inference="ab initio prediction:Prodigal:002006"
                     /inference="similar to AA sequence:RefSeq:ASI88306.1"
                     /codon_start=1
                     /transl_table=11
                     /product="Conjugative transfer protein TrbB"
                     /protein_id="Prokka:JOBBOHMI_00007"
                     /translation="MSNLEFQARINEKIKREFGDILTHLEDKETIELMLNSDGKLWLE
                     KLGQDMICLGEFSEARAKSIITAISTLLDTTTNADNPILECELPLDGSRFEALLPPIV
                     AKPTFTIRKKAVKIFTLGDYVDSNIITPNQKEVLINAIIERQNILVVGGTGSGKTTFS
                     NAIIDGISKITPNHRIVIIEDTAELQCASENKVILRATDKVDMLRLLKATMRLRPDRI
                     IVGETRGKEALDLLKAWNTGHPGGIATIHANSANGGLTRMEQLISEATNAKMSKLIAE
                     AVNLIVFISKSKEGRKVKEIIKVIGYENDKYITQAI"
     gene            complement(6797..7537)
                     /locus_tag="JOBBOHMI_00008"
     CDS             complement(6797..7537)
                     /locus_tag="JOBBOHMI_00008"
                     /inference="ab initio prediction:Prodigal:002006"
                     /codon_start=1
                     /transl_table=11
                     /product="hypothetical protein"
                     /protein_id="Prokka:JOBBOHMI_00008"
                     /translation="MLFNYEIINCQKPNNTMNIKRTYKVIRCIFKNKTDEASKYYQQA
                     WDLAKQVNPLKANDSTSQRDKERLILDALGGILAEYGWFYYIKRNFGDIVNFTEFKSS
                     IGQIDLLLNNGKTLEVRSSFPRNGINFAICNAKYNFKNICKYDNLYKPNEMDKDFFAC
                     VLFETTKNQMVKADEIIFYLIGGSTRKMMLDNTISYTDNLTAEDDLIQQKTNYKVIRL
                     YNALDIEGFEKYMTEELGYPKILNDQTF"
     gene            complement(7527..8444)
                     /locus_tag="JOBBOHMI_00009"
     CDS             complement(7527..8444)
                     /locus_tag="JOBBOHMI_00009"
                     /inference="ab initio prediction:Prodigal:002006"
                     /codon_start=1
                     /transl_table=11
                     /product="hypothetical protein"
                     /protein_id="Prokka:JOBBOHMI_00009"
                     /translation="MNKNSSSKLNNAKNIKNDEFYTQYEDINKELNFYKNAFKDRIVY
                     CNCDDPQKSNFTKFFKLNFDRLKLKKLISTSFNSNGKGKIFIIEKNKPKYQGLLENNG
                     DFRSDETIKLLKESNIIVTNPPFSLFREFIDMLISNQKDFLIIGNANAISYKNCFNYM
                     MKNKLWLGQNCVRWFINTKGELVEGARSFWFSNINNQKRNKKISLNRKYNNQDYITYD
                     NYNAIEISKSKDIPSDYTGIMGVPITFLDKYNPQQFEIIGADYQVNSGELIYIKRNDW
                     NGKTDRAYINGKRLYSRIFIKHRGVNNAI"
     gene            complement(8467..8880)
                     /locus_tag="JOBBOHMI_00010"
     CDS             complement(8467..8880)
                     /locus_tag="JOBBOHMI_00010"
                     /inference="ab initio prediction:Prodigal:002006"
                     /inference="similar to AA sequence:RefSeq:ABS43377.1"
                     /note="conserved hypothetical protein"
                     /codon_start=1
                     /transl_table=11
                     /product="hypothetical protein"
                     /protein_id="Prokka:JOBBOHMI_00010"
```

```
                     /translation="MKKVYLDTNIFLDYFNTERTYHNEAKQLMYYLLTNNIQIVFSED
                     MISTIAYLIKKENLNNFTHFLKDISLEKNFNITSFGVSVINMACDYYDKYRGDFEDYL
                     QYFCAEKEGCSAIYTMDKNFPNLKIPIKRYGEIDI"
     gene            complement(8877..9791)
                     /locus_tag="JOBBOHMI_00011"
     CDS             complement(8877..9791)
                     /locus_tag="JOBBOHMI_00011"
                     /inference="ab initio prediction:Prodigal:002006"
                     /inference="similar to AA sequence:RefSeq:CKG86570.1"
                     /codon_start=1
                     /transl_table=11
                     /product="hypothetical protein"
                     /protein_id="Prokka:JOBBOHMI_00011"
                     /translation="MSNEIEIKDLNEINKNNKRQVVYRKDNLSTDYITGEVVERNQII
                     VKKVQTKESFIKLFTENIDYIINLESQEKTLFFTLLSSVDYKNVVRFDKSMKQNIVLD
                     KVLSRAGMYRALNGLIEKKVIFKINREFVKSYAGVGSDDCYLFNPNLVGRGSWNDLKN
                     LRHTITREWDFENLEVRENLEIEEEYLGFDEIRNNQKQYQVKEINQEHDETTKVKNTE
                     IVIEEKNNSINNQYSFDKKEEPSINKKVLISNDTQPSLFDENQKEQEARLFIEKYAGI
                     LTGAYDSTKSAKEMKRELLDKDLEEGRI"
     gene            10523..10723
                     /locus_tag="JOBBOHMI_00012"
     CDS             10523..10723
                     /locus_tag="JOBBOHMI_00012"
                     /inference="ab initio prediction:Prodigal:002006"
                     /codon_start=1
                     /transl_table=11
                     /product="hypothetical protein"
                     /protein_id="Prokka:JOBBOHMI_00012"
                     /translation="MNKDKKYLNFSEYYEVRDFLELYPEKDRENIENILITIGENKKS
                     KNLSFEFLYDFFKKLGFEKKLI"
     gene            complement(10732..11061)
                     /locus_tag="JOBBOHMI_00013"
     CDS             complement(10732..11061)
                     /locus_tag="JOBBOHMI_00013"
                     /inference="ab initio prediction:Prodigal:002006"
                     /codon_start=1
                     /transl_table=11
                     /product="hypothetical protein"
                     /protein_id="Prokka:JOBBOHMI_00013"
                     /translation="MQRGEAKIEFYSNINYLKKEFENGCVVSKFLYDKALKEKNFKMT
                     YKQFNKYFNDEFKNKIKIKETMQEDNQLQSLVLKNKEPIKLKINTKPKKVFDAKFGKD
                     IKEDDLL"
     gene            complement(11075..11524)
                     /locus_tag="JOBBOHMI_00014"
     CDS             complement(11075..11524)
                     /locus_tag="JOBBOHMI_00014"
                     /inference="ab initio prediction:Prodigal:002006"
                     /codon_start=1
                     /transl_table=11
                     /product="hypothetical protein"
                     /protein_id="Prokka:JOBBOHMI_00014"
                     /translation="MDIKIATFEDIEFIKYCFLLGFKEKHFYNVRFIEKLEDIIKNNG
                     VSGGIFIIKDGTNEKAGFAFCGKSKGSNIYEINMIYILEKFRNKKLATNFIKYYEKLI
                     PNNKIIARCDINYSQQAIKIFKHLGYQELSKKEKTNQYEFMILEKSI"
```

```
     gene            11734..12117
                     /locus_tag="JOBBOHMI_00015"
     CDS             11734..12117
                     /locus_tag="JOBBOHMI_00015"
                     /inference="ab initio prediction:Prodigal:002006"
                     /codon_start=1
                     /transl_table=11
                     /product="hypothetical protein"
                     /protein_id="Prokka:JOBBOHMI_00015"
                     /translation="MRKDKRIAIRLSEDEYKHIMQKAQNHGLTISRYLRDLSMNYPIT
                     CIVDQKVAHNVLNIAGDIGRLGGLFKHWLVRNEDNKVNFSNKRTYNDIDEIVDQILDL
                     QILLKEQAKRIIQNDNQKDSKQKAE"
     gene            12080..14236
                     /locus_tag="JOBBOHMI_00016"
     CDS             12080..14236
                     /locus_tag="JOBBOHMI_00016"
                     /inference="ab initio prediction:Prodigal:002006"
                     /inference="similar to AA sequence:RefSeq:ASI88299.1"
                     /codon_start=1
                     /transl_table=11
                     /product="IncP-type DNA relaxase TraI"
                     /protein_id="Prokka:JOBBOHMI_00016"
                     /translation="MIIKKIPSKKQNKSSFKNLSNYILDKDNNNAKVLVDYMLDKNNE
                     MDKVEGYHFTNCSFDNDEDNINEIINTQKLNTTTKQDKTLHLVVSFQEDENPTLEILQ
                     AIEEEIAKSLGMEDHQRLSVVHSNTNNLHIHIAINKVNPHTLKVINPYNDVRILQETA
                     MKLEKKYNLKLDNHISQKDKQSNKYNTHTMNCNFETWVKEKLSKQVDLMLKDEKTTFK
                     DIKQFLAEYDLEFRERRKGFVIASKSEKLFCKASSIHRGLSKQALEKRFKELDLKQEK
                     ENTKNIKEEKHEIKEQYQRPNKETSKTLWEKYLRIENEKKAELDKELRMLKLRRNEFK
                     TSIPSMKFNKNTFAHVKNQRMIFKNKQKELYQKYKRVSYRDFLISESLSGNEEATRAL
                     RRTKTKINENENTLSSTQEKPKIFENVDYITKEGYAVYKSGFNKAIDKGEMLKVSLIN
                     NKDDKEFLLNSLLIAIDRFGNHLNITGDDKFKRNILEVANDYNLNVSFSDQRMQKIQE
                     ENNNKRKEMKTRKVLKNIIELKIKLTEQDKDIDENIKEKELKALKFSLKKISNSTPSI
                     FKGELKKLGFSYKEIDSMDTESVCIEIDGFIVNSANKEGLVAMNEEIKQTLIDDEEIK
                     RFEKFEHLFLNTNKITDIAKGFYTNRKIDADEYIKRFSMQESKINKVANAISMLSDKN
                     IDIMEKYAKKLEKDLKYQYLKKVEVFASNDIDLNNF"
     gene            complement(14233..14697)
                     /locus_tag="JOBBOHMI_00017"
     CDS             complement(14233..14697)
                     /locus_tag="JOBBOHMI_00017"
                     /inference="ab initio prediction:Prodigal:002006"
                     /codon_start=1
                     /transl_table=11
                     /product="hypothetical protein"
                     /protein_id="Prokka:JOBBOHMI_00017"
                     /translation="MQYLIDNADYINIIIKLIQGLASIGVVIGLIIACLQLRNSSKNN
                     RINILKTEFDAFEKISNAEKNFVDYIEHLDLEKNQLDEKNNNLLNKKREEYYKTLNLI
                     CLYVLEKCFTKKHFLNEYKNKLLKIYKILKNDNMLDKYINIKDVVEKYNLKP"
     gene            complement(14701..14895)
                     /locus_tag="JOBBOHMI_00018"
     CDS             complement(14701..14895)
                     /locus_tag="JOBBOHMI_00018"
                     /inference="ab initio prediction:Prodigal:002006"
                     /codon_start=1
                     /transl_table=11
```

```
                     /product="hypothetical protein"
                     /protein_id="Prokka:JOBBOHMI_00018"
                     /translation="MKNQEYNVENLKICNESYDDQRVFAALDLSYKMQTAYNQNQTNS
                     NQNQTNSNQNQTNSNQSDKK"
     gene            complement(14947..15171)
                     /locus_tag="JOBBOHMI_00019"
     CDS             complement(14947..15171)
                     /locus_tag="JOBBOHMI_00019"
                     /inference="ab initio prediction:Prodigal:002006"
                     /codon_start=1
                     /transl_table=11
                     /product="hypothetical protein"
                     /protein_id="Prokka:JOBBOHMI_00019"
                     /translation="MGFKKLSSEAPKNENNFVNSARGETGNAKTGNAKRNKKLMINFT
                     EEEYQILNQEAHSMGLSLNSYVRFKIFRKD"
     gene            complement(15174..15860)
                     /locus_tag="JOBBOHMI_00020"
     CDS             complement(15174..15860)
                     /locus_tag="JOBBOHMI_00020"
                     /inference="ab initio prediction:Prodigal:002006"
                     /inference="similar to AA sequence:RefSeq:ASI88294.1"
                     /codon_start=1
                     /transl_table=11
                     /product="Plasmid partitioning protein ParA"
                     /protein_id="Prokka:JOBBOHMI_00020"
                     /translation="MIISIVNEKGGSGKTTLAVNLSARLAEDGDNVLLIDADPQKSTE
                     VFSDMRSQSNLEPLFSNVSKTGISLGDEIKRMKNAFDSIIVDTGGRDSKEMRKAILSS
                     NIIIIPTIPSQYDVNVLDHMLEIYNEVIEINPNLLALVLVNRVSPNPFLAKELENLKE
                     YINEAKKEKGLDRVIMLESVIYERQAYRKAVIEGKSMKEFCDKNDKALNDFENFYQEL
                     LKIANEKLGV"
     gene            complement(15952..16347)
                     /locus_tag="JOBBOHMI_00021"
     CDS             complement(15952..16347)
                     /locus_tag="JOBBOHMI_00021"
                     /inference="ab initio prediction:Prodigal:002006"
                     /codon_start=1
                     /transl_table=11
                     /product="hypothetical protein"
                     /protein_id="Prokka:JOBBOHMI_00021"
                     /translation="MNKKELIEEIAKKHHIILDENDPILAVVSANEIIFDDFLEKIDL
                     LFIKHKTDLESYKFNILNEIREYSKNNQEALKDILIQAQTNQNTIKASQEKTEDTKEK
                     KNTNINFWIIAIASQIIFLLVGLIIGIII"
     gene            complement(16413..16880)
                     /locus_tag="JOBBOHMI_00022"
     CDS             complement(16413..16880)
                     /locus_tag="JOBBOHMI_00022"
                     /inference="ab initio prediction:Prodigal:002006"
                     /codon_start=1
                     /transl_table=11
                     /product="hypothetical protein"
                     /protein_id="Prokka:JOBBOHMI_00022"
                     /translation="MRKTIAIFAFSFAFLSILSYLIFYFGGFHFNYTRSMPLGLYKEI
                     NSSTFNKNDIVLLKIPQKKEILLKKIIAVNGDFVEVNTQGVFINESLLPNSKIFSFDS
                     EGNLLEFKPFKRKLKENELFVMGENIKSYDSRYFGVVNILQNEVKKVKLMISF"
```

```
     gene            complement(16885..17538)
                     /locus_tag="JOBBOHMI_00023"
     CDS             complement(16885..17538)
                     /locus_tag="JOBBOHMI_00023"
                     /inference="ab initio prediction:Prodigal:002006"
                     /inference="similar to AA sequence:RefSeq:ASI88290.1"
                     /codon_start=1
                     /transl_table=11
                     /product="Cell filamentation protein"
                     /protein_id="Prokka:JOBBOHMI_00023"
                     /translation="MKESNEELFKRLITDNKLGIKDEKLFDKESRKYTSQRIDELITS
                     PIKGNFDYQHLKNIHKHIFQDVFHWAGKDRMEVGLHGNFGKYAPNGTITNFVPGKDLN
                     ATAQQIFTWLKEDNYLKNSKDLNDFAKNLAEFARNLNALHPFREGNGRTQRIFLNELA
                     KNAGYKLDLNLIPKDKTIMASVEASQLKLGKLEAIIKTNLKSFRQNLDLEQNKGISL"
     gene            complement(17522..17776)
                     /locus_tag="JOBBOHMI_00024"
     CDS             complement(17522..17776)
                     /locus_tag="JOBBOHMI_00024"
                     /inference="ab initio prediction:Prodigal:002006"
                     /codon_start=1
                     /transl_table=11
                     /product="hypothetical protein"
                     /protein_id="Prokka:JOBBOHMI_00024"
                     /translation="MQKQYPEVHSLEESLAILKKYKDDVTKEQYENIKSNIGTHAIES
                     IYLNELDIIMLVKKNVYGLSADEIIAEYQEKGFVEYERKQ"
     gene            complement(17867..18586)
                     /locus_tag="JOBBOHMI_00025"
     CDS             complement(17867..18586)
                     /locus_tag="JOBBOHMI_00025"
                     /inference="ab initio prediction:Prodigal:002006"
                     /inference="similar to AA sequence:RefSeq:ASI88288.1"
                     /codon_start=1
                     /transl_table=11
                     /product="IncP-type DNA transfer protein TraL"
                     /protein_id="Prokka:JOBBOHMI_00025"
                     /translation="MAKIHFVLNGKGGIGKSFISSLLCQYFLDKGESIVAIDTDPNNT
                     TLLNVKALKAKFLQLIDDDGKFDVRAFDKIVELAFEKKAENYIIDSGATTFIPLIDYL
                     KENEVLDFLTNNNLEVYMHVPIVGGQARDDTILGLTQLTEAFNCSFVVWINEYHGSII
                     KDNLEFEETKEYQAIKEKIKAIVYLNALSKDTFGKDLLELTSKNLTFGEAMQDKSFSL
                     MSKQRLKIFKDKAFSQIGNIL"
     gene            complement(18597..20420)
                     /locus_tag="JOBBOHMI_00026"
     CDS             complement(18597..20420)
                     /locus_tag="JOBBOHMI_00026"
                     /inference="ab initio prediction:Prodigal:002006"
                     /inference="similar to AA sequence:RefSeq:ASI88287.1"
                     /codon_start=1
                     /transl_table=11
                     /product="IncP-type DNA transfer coupling protein TraG"
                     /protein_id="Prokka:JOBBOHMI_00026"
                     /translation="MKKQTNQTLVYILAITGILLAFITISQIATQYLAKSFNYSPSLG
                     ETLLYGFYNPFKWISWSYAYYSFYPDFFKKFFMVMFAGIAFCFIVFILVKLAFLRKAK
                     AIENLHGSAHWATLEEVKESGVFDKDKGVYIGGFEHKKTLHYLRHDGPEHVMLFAPTR
                     SGKGVSLLLPTLLSWEESALIFDIKGELWALTAGWRQKYAKNKVLKLDPTCLDDSAVK
```

```
                     FNILEEIRLETMHEIKDTQNIAINLIFKGETPPTNPTQGSTSYFKSEAGNFLVSIILY
                     ALHLKKYRGENTPNLTDIYKFINDPNQSIDELLEEMVECDISTMDKNTQEIIQSISRS
                     MKNKATQELSGVVGTASEALNLYIDPILAKNTAKSDFKIKDLMNHESPISLYLIIPPG
                     QKDRLRPFFNLLINQIFRTLTDDSLNFKNGENVKRYKHKMLVLADELTMFGKLGVLEE
                     NLAYMAGYGMKFYGSIQDIQQLYSIYGEKETIISNCHIRIAFAPNKIETAKLLSEMGG
                     ITTIVKKSITSSGKRTAVMLGNVSETIQEVQRPLITADECMRLPSAKKDKDDKIIAPG
                     DMLIFIAGQAPIYGKQILYFKDPAFLARSKVTLDKQVSDIL"
     gene            complement(20417..22630)
                     /locus_tag="JOBBOHMI_00027"
     CDS             complement(20417..22630)
                     /locus_tag="JOBBOHMI_00027"
                     /inference="ab initio prediction:Prodigal:002006"
                     /inference="similar to AA sequence:RefSeq:ASI88286.1"
                     /codon_start=1
                     /transl_table=11
                     /product="IncP-type DNA transfer primase TraC"
                     /protein_id="Prokka:JOBBOHMI_00027"
                     /translation="MKNKDFIQETADKIIESLKNGTAPWIKPWKGIDLANNMPYNPIT
                     NKPYNGINSINLMLQNYNDPRWLTYKQAQSINAQVRKGEKSTLIQYWQFSEMVDKLDE
                     EGNVITNENGEVEQIEVKLENPKVFFAYVFNAEQIENMPKLEQKQEIDNFQIIKEAQK
                     ILDDSKAIIHHKGNRAFYNAANDTITLPLKENFLSEGAYYTTALHELGHWSGHESRLN
                     RDLKNPFGSKEYAKEELRAEIASFLFNGKIGLDYDPGQHLSYIDSWVQILEDKPNEIF
                     KATSDATKIVNFLESLSLEQKQKIELENSNTQNQALKQDKELNLATRKTYLYVPFEEK
                     EFAKKAGAKWDKEAKMWYAPKGADLKNFTQWLNQSQLTQSYNNTHDEFKAFLEKYELN
                     IEGQPIMDGKLHRVSVIGDKGREKSGAYVGFLNGHPAGFVQNYKTGIKENWKSANSLE
                     NTKNKEINLQNALEHNKALKEQRDKELNQAYENTAVKLENEYNNARWANSEHPYLKEK
                     GFDKNFYLKQDKNGNLLIPLRDIEGKYWATQRIFANGDKMIGATRTAEEKEQGIEYPA
                     KKQGNFFLLGAKNLNNVNEVYICEGFATSASVYEATKSPTIMGVDAGNLEIIVTNIKE
                     KYPKMNIIIAGDNDVKKELNGNKNVGKETALGIQKKYPEVKVVLPNFTNQEAQKGLSD
                     FNDLMKSRGLEEIKKQLKEQIAKQLMVEKATTKALDKNKGINKEITAKKDIGISL"
     gene            complement(22635..22775)
                     /locus_tag="JOBBOHMI_00028"
     CDS             complement(22635..22775)
                     /locus_tag="JOBBOHMI_00028"
                     /inference="ab initio prediction:Prodigal:002006"
                     /codon_start=1
                     /transl_table=11
                     /product="hypothetical protein"
                     /protein_id="Prokka:JOBBOHMI_00028"
                     /translation="MKDFKELETIPNFSYEVDPLTAEELGAFKENALSEDDAKEGTED
                     QG"
     gene            complement(22762..23124)
                     /locus_tag="JOBBOHMI_00029"
     CDS             complement(22762..23124)
                     /locus_tag="JOBBOHMI_00029"
                     /inference="ab initio prediction:Prodigal:002006"
                     /inference="similar to AA sequence:RefSeq:ASI88284.1"
                     /codon_start=1
                     /transl_table=11
                     /product="Single-stranded DNA-binding protein"
                     /protein_id="Prokka:JOBBOHMI_00029"
                     /translation="MINNITIGGRLTADAELRHCNQGSIICTFTLANNRKYKEIENST
                     YIEVSLFGSFANAMHPYLKKGISVDVVGELTQDTWEHEGKIYSKHRIKAKEIDFRTPK
                     KEKQTIPNIEEGENYEGF"
```

```
     gene            complement(23117..23218)
                     /locus_tag="JOBBOHMI_00030"
     CDS             complement(23117..23218)
                     /locus_tag="JOBBOHMI_00030"
                     /inference="ab initio prediction:Prodigal:002006"
                     /codon_start=1
                     /transl_table=11
                     /product="hypothetical protein"
                     /protein_id="Prokka:JOBBOHMI_00030"
                     /translation="MENENLEENKPTQFDDNEPSLFDNLEDEEQDND"
     gene            complement(23221..23766)
                     /locus_tag="JOBBOHMI_00031"
     CDS             complement(23221..23766)
                     /locus_tag="JOBBOHMI_00031"
                     /inference="ab initio prediction:Prodigal:002006"
                     /inference="similar to AA sequence:RefSeq:ASI88282.1"
                     /codon_start=1
                     /transl_table=11
                     /product="T-DNA transferring Peptidoglycan hydrolase
                     VirB1"
                     /protein_id="Prokka:JOBBOHMI_00031"
                     /translation="MIDIALIEQCKNPNVETQIIQKIIQVESNNQQFAININKVGSFI
                     LKNKKEAENLANNFIKGGYSVDIGLMQFNSNNLKSSTFSHYSVEDLLDTCKNIKAGSD
                     IFYLAYEMTNKNLAKEDRINQALSIYNTGDLKKGFSNGYVAKYDSTAKTIDDLEEKAR
                     KSDTRIKITYNLFNLQTQKKE"
     gene            complement(23759..25000)
                     /locus_tag="JOBBOHMI_00032"
     CDS             complement(23759..25000)
                     /locus_tag="JOBBOHMI_00032"
                     /inference="ab initio prediction:Prodigal:002006"
                     /inference="similar to AA sequence:RefSeq:ASI88281.1"
                     /codon_start=1
                     /transl_table=11
                     /product="Conjugative transfer protein TrbI"
                     /protein_id="Prokka:JOBBOHMI_00032"
                     /translation="MLEVNMSPNKFLPKNNKRLSKVPIIIIFAIIAIVLFSIFYVALS
                     RKQNLERQTAQTTPKEETQIATNHNNDLNLYIDELQVQKAQENNIISPPMPNATQQPI
                     QETNSQTTQQPPNNTPVLVQNNAIDEKEMKIKKDMQLKALLSNTKLDIKTEATDSKKN
                     QAINFNPPNNNEDINLGAINPQNTGLGLESGTNKDVQGQQFLSQKKDNGYLKYVKQKP
                     LSTYEIKAGWNIPAILITGVNSDLPGQILAQVTQNVYDSATGKYLLIPQGTKVVGAYS
                     SNIIYGQSRLLVAWNKLIFPNGDTLNLDGMQGTSQDGYTGFEDQVDNHYFRIFGSAFL
                     LSSISAGIALSDNSDTNSEKETASDKAIAQAIQQMGQVASEMIRKNMNISPTLKIRPG
                     YKFNIFVTKDIILEPLELNND"
     gene            complement(25004..25447)
                     /locus_tag="JOBBOHMI_00033"
     CDS             complement(25004..25447)
                     /locus_tag="JOBBOHMI_00033"
                     /inference="ab initio prediction:Prodigal:002006"
                     /codon_start=1
                     /transl_table=11
                     /product="hypothetical protein"
                     /protein_id="Prokka:JOBBOHMI_00033"
                     /translation="MKILFLALIAFFITSCAKMNSDYFNSSFAVEETKINYNKIADDF
                     SQFIISYYAPNKTTFFINEDKNNQDFSNYFINTLRKKGYAISNDNSKKDLTFLSYQIS
```

```
                     QIDNENIVAIFNINESKINVIYKIIENKLVKPNITSFNFTPKTKG"
     gene            complement(25444..26367)
                     /locus_tag="JOBBOHMI_00034"
     CDS             complement(25444..26367)
                     /locus_tag="JOBBOHMI_00034"
                     /inference="ab initio prediction:Prodigal:002006"
                     /codon_start=1
                     /transl_table=11
                     /product="hypothetical protein"
                     /protein_id="Prokka:JOBBOHMI_00034"
                     /translation="MKKIILLSSLIASSLIANNLNERDFSNLTPKEKKDLAIAQKWIN
                     SKTTTIQGNNGEVIFLFGESMPSIVTAPLRLTDIALEPGEVIKDVQIGDSIRWAVSLS
                     VSGEEPYLISHIIIKPTDKNLQTTMNIMTNKRVYRLNLISEAKKFMPAVSFNYPNSII
                     KTLEDYKNQMKAKSESKNFYKTKDDEIPSNIENLDFGYSVEGNAPFKPLRIYNDGIKT
                     YIQMPKNLKFYEAPALMILDSSNEKQIVNYRLKYDTFIVDRLFNKAILLSNVGSKQEK
                     IKITKHSNKANQDIVNNVLYDLSLQNKKENK"
     gene            complement(26380..27075)
                     /locus_tag="JOBBOHMI_00035"
     CDS             complement(26380..27075)
                     /locus_tag="JOBBOHMI_00035"
                     /inference="ab initio prediction:Prodigal:002006"
                     /codon_start=1
                     /transl_table=11
                     /product="hypothetical protein"
                     /protein_id="Prokka:JOBBOHMI_00035"
                     /translation="MNIFRQKQKIEKNEFDTSSNPYLNAKTEWLERYGDYITRAKNWQ
                     IVAMISLAICFVCVLFIGYIGSQNKLIPYVIEVDKLGNTAKVGMVQNIDLKNPNVIKY
                     SLNTFIYSWRSVWGNAETQRKFIFDAYAYVEPRSKAFNYLNSEFQKNNPFARSTKENV
                     RVKIKNIVPQNIDTWQVEWEETTTNLNEDILSKETYRGLIQIKQIIPNTEEQILKNPL
                     GIFITDINFAKIL"
     gene            complement(27095..28381)
                     /locus_tag="JOBBOHMI_00036"
     CDS             complement(27095..28381)
                     /locus_tag="JOBBOHMI_00036"
                     /inference="ab initio prediction:Prodigal:002006"
                     /inference="similar to AA sequence:RefSeq:ASI88318.1"
                     /codon_start=1
                     /transl_table=11
                     /product="IncP-type conjugative transfer protein TrbL"
                     /protein_id="Prokka:JOBBOHMI_00036"
                     /translation="MKLNKGIVLMQNGQLKQKIQVILKKLGVNQTQTLLNGNKLAIIF
                     LIFLAPNLAFGAENAEGVLNIIRNGLLSWTPLIKTACLWVFWTLVIIDLVWTFGLKAL
                     SGFEFGEFLATLIKKIMYVGIFLFLFNIDQWLQIIFNSFSQLATNVNNGVSVSPNNII
                     AQALSIVIKIIRSMDFWSPGDSILKVIAGVIILIAFVLMAIDLLIVYLKFFLMNVIVF
                     FALALGGLNHFKQIGLNPIMTAIKVGVELFMIQGLMALCINMIDTINNEITQNSSAEV
                     ILTILVTSVTFCLITKMVPGIIEAVFNGSIGESAGAAAGFRAVATMAAGAAAGAAVGA
                     VGVTRAMNAAKALHLAEGGAGGMDLVKGVAKNLASAGGEHLRDNLTKGRMPNQMANRL
                     QEKLKDIQGKTSEGGISSGSPKQENYQSGINPDAME"
ORIGIN
        1 ttctccattt ttttacattc tgcaactcta gcttttgctt cctctaagtg cgctttataa
       61 taatcgcttg tttttgcctc atcgccacaa cctgctaaaa aagccaaagc acctaaacca
      121 attaatccgc taaaaataaa atgtttcatt gtttttctcc ttgatttaat atagaaacaa
      181 aatattttaa catcatttta tttccattta agagcgtctg ggtttgattt accccaagtt
      241 ttttcaatat aacctgtatc ttctataaat ttatctattt ttgcctgttg cattattctt
```

301 tgattatttt gcgatgctaa ataattagta agcatattgc tttgatccat aatgaccgat
361 cttagttttc ttatctcatc aatctgataa gctaaaagat cattggtggc ttgcacagcc
421 tgcaaatttc cattagaatt ttgtgaattt tgcttaagtt ttgcgataag cgcatcttca
481 ctttctaagt cttgatatcg aagctcgagt tgttgcaatg ttccgttaaa tgtattttgg
541 tttgtttccg caatttgctt gtatctatca gaataaattt tatttttctc gctttcattt
601 gttgcgtttt caagatcatt actataacct tcaaaatctt tgtaagtatc ttcaaatttg
661 cgcgaaacat tacctatatc ataagaaatt ccatttactt tgttcattac atttcgggtt
721 tgataaagaa ccgttcctaa gttatcccaa tcttctttag ttaatctttc taaattctgt
781 ttttgcattt gcaccatttg tatttgctgt tgcaattgag aatacatttg ctcgtattgt
841 tgaagttgca ttgcataatc ttttacttga ttggcaatag cactaacatc aataacagga
901 ataccacctg caaaggcatt atttccgatg attaatgatg atattaaagc taatgatagc
961 tttttacttt tttgattaac tccaaaaata aaatgtttca tattagccct ttcactttaa
1021 aattaatttt ttgttcgata tgagatttat tatctttata tgtttctgta atttcatatg
1081 caattaattc ttcaaattca caattacgat tttgttcggt ttgcataatc gctttaataa
1141 tatttctttt ataaggtgtg ttaggaatct tatatttatt ataatttttt agttttggtg
1201 tgtattgatt tttaataatt ctttccattt cattaaaagc attaatgaaa gcgattttga
1261 atttatccgc ttttctaccc gttaatccca tagctataaa tgagaaacca tcttttgtaa
1321 gttgatagta tggatatttt ctttctctac ctctaaaacc ctctatttta cgggttttaa
1381 atgaaagccg aaaattcggc tcacaaaaat ttttaatctc attattgtta aatttttcac
1441 caatcaattc taaaatatgc ttatgttgtt taccaaaaac cttagccaca tctaaacttg
1501 tgcaaaaaac ttgttcgtct ttgttttcaa aaataacttc ttgattgttt atgttgatta
1561 tgcttgtatt gctcatatct actcctttta gtaagtccag cattctttag tgaatggtat
1621 tttttgataa tagtcataac aattcctatc tccgcaagtt cgagaaatca tatatctttg
1681 atcgtcagaa aggctttcat aagcttgttg agttataagc tgtaattttg cacttagttt
1741 ccattctaag gaattgtaaa attcccctgt gcatttatat tttggagttt tataatcggt
1801 ataagcatgg ttgattaatg cgtggcaaaa gttaggcata tttggattga ttctatagct
1861 cataattcca aaagtagaat aacttctttt agtttctact tgcgtgttta aataatttgg
1921 tgtgcattgc ctaggatcgc ttacaggtaa tacatcatcg actaagttat ttagcactaa
1981 atcttctatg tttgcgtcat tttgtatagg gcataaattt aaaaatgctc ttcttgcatt
2041 aatcgtttcc caaggttttt taaatttaat agaaaaatac cttgctaaag aagatgaaca
2101 ctcgctcggg cgtgtgccac tacttaaaca taaaattgcc tcgcatgcta attttgtatc
2161 gcctgttaaa atatcatcag caaaagcgtt actgctaatt aataaagttg cacttaaagc
2221 taatgtaatt ttttttacgc caaaaataaa atttttcata tttgctcctt ttgtgaatta
2281 tcaaatcttt tgtagtttat ttcttttttct tctagccatt tataagccca attttcttta
2341 tgtttctcat aaagttgtct tactctttta acgcttactt tgtcgcttat tgttgtgaag
2401 gctaaattag tttttccgag tgccaaatta aaaagtctag tgccgagtgg agaggtgtaa
2461 taatattctc ttttttgaat ggcattattg ataatgctaa tttgcacatc gttaagtcca
2521 aaaattttat aaaagtcgta aggagctaaa acattttcac ttccttttttt ataagcttcg
2581 ggattaggta aaaatatttt agtagggcaa tattcttgta aaatatctaa aattccactt
2641 tttgcactat cgcttaagct ttgtgtagcc attataacgg ctacatttgc ttttcttaaa
2701 actttaagcc aattaactat tttatcttta aacgcagggt gctttaatgc aatccaagct
2761 tcatcaatga tgagtaatgt aggacttcca tctaagcctt tttctatgcg attgaaaatg
2821 tagagtaagg cagggattat gtattgatct cctaaattca tcaattcttc tatttcaaat
2881 gttgtgatac tgcttaaact taatttatct tcctcgctgt caaataaaaa accacctgtt
2941 ccagaaattg tataataatt taaagcatct cttaaatcac tatcttgcaa agctgataca
3001 aattcagtca aagaggtaga atttgtattg atatgagaaa ttaaggcttc atgtattaat
3061 tttttttgtt ttggtgtaat atttacattt tgtaatttta agcatgtttc aatccaaaat
3121 tcagcccatg cgatttcggc atctgttttg atatttgcca aaggagcaaa agccaaagat
3181 gaatgatctc ctgccacatc ataatgaatt ccacctgttg ccagtgtcgg agctagcaag
3241 ctttgtcctt tatcaaatgc gtaaatttta gcatttggat atttttgcca agcaagaaaa
3301 atatttgcca aaagagtaga tttaccactc ccaggaggac cgaaaattaa agtgtgtcct
3361 aaatcgctaa catgtaaatt taacctgaaa ggcgtattgc cagaagttac aacttgcatt
3421 aatgggggag aatttggagg aaatctttca cttgaattat atttatcccc cgcccaaata
3481 gacgccaaag gaattaaatg tgttaagtta agtgtattta atattggacg gcgtaaattt

3541 ggatatacga atccaggtaa agttcctaaa tacgcttcta ctgaattgat tgtttcggtt
3601 cttgatagaa agcctaaatt ttctaaaact tttcttacat cttttaattt atattctaag
3661 ctctcataat ctctatcaaa aacgacaata tttgcggtat aatagccata tcctatcata
3721 ccgctttttg attctgataa tgcagaatct agttcttgca ccattaacac agcatgatca
3781 tcttgttttc ttgcagggcg atctagtaac tgatcaagcc aacctttgt tttttgttgc
3841 catttttac gatatttatt aagtgaattt tgagcttcaa agtcgtctaa aaatataaat
3901 ctagtgttga atctgtaatc gaaattaagc tctgtgagtg catttaagat atttggataa
3961 ctttcgcttg gaaagccctc aatagcaata attcctatac attgattatc aattcttggg
4021 tgcattcctg tttcgaaatg ccttccaccc aataaacaat caagaaacat aggggcattt
4081 ggtagtatta ttttttgtct ctcaccacaa atacaaaaat taatatattc aagcatatca
4141 tctaaaatat actcattatt atattcgtca atagctattc ttggtagcat tctttcgatt
4201 tttatataat tgcttaaact atcttctaat ttttgaaggg tgtttttaaa atactctaat
4261 attttatcac cctgattttt gtttttgtt tcgtcaatta tcatcaaatc ggtgatttta
4321 tttatgcttt tgtgcggtgg aaggtaagtg aaaatcattg taaaaagatt ttcaaaatgt
4381 tctacattgt caaaataatg ttttctttca ttttcaagaa tttgtgcaat attgttttta
4441 taaaaagtat cgccgtcaat gtagttttct gtttttatcc tagagcaatc aatatgcacc
4501 gcccagccat tacctagagt ttttaaaaca ctattaatcc tagcacttaa agtatttctt
4561 tcatttattg tcatggaact gatatcccct gcacgataat aaaaaccagc tgttaaactt
4621 ccgtctttgt tgagcaaaat gccttcatcg ataaaacttg catagtttag aatatcggga
4681 aatgcttttg ctttgcttct gaattcttta agtgctagca tattttaatc ctttgaaaat
4741 ggtgtgcttt gcgcataata aaatttttta tatttaattt ggcgcaaaaa tatttgtctc
4801 ataagcggat cgctttttgc cattaatctt aaaagcatgt tgcagaaaaa aaataagaat
4861 atacctataa tgcttgtaag gagcgttgca cctgtaaaga ttagtgcaaa tgaaatcact
4921 ccaaccataa gaataagctc tcgatctgcg ccaaatatta aatttggttt gttgagggcg
4981 ctataaatat ctattctttg caattcttcc attagattaa tgctccactt gttgaaaaaa
5041 gcgacataaa gctgtttgcg cctactatta atgctattac aattacgata taaaagaggg
5101 ttttaataaa accgctaaat tcattgcccc aaattaaacc tgctccagct ccaatgattg
5161 ctagaattga aataacaaag gctacaggac cacttaagct cgctttaatt tgttctaatg
5221 gaccttccca aggcaaaccc gctccagttg ttgaggctaa agcatatcct aatcccaacg
5281 ccaaaaataa aattaaaaat tttttcattt aaactcctta aattgcttgt gtaatgtatt
5341 tgtcgttctc ataaccaatt acttttatga tttcctttac ttttctaccc tccttgcttt
5401 ttgagataaa aacaattaaa ttcacagctt cagcaattaa tttgctcatc tttgcatttg
5461 ttgcttcaga gattaattgc tccattcttg tgagtcctcc atttgcggaa ttagcgtgga
5521 ttgtagctat tcctccaggg tgtcctgtat tccaagcttt tagcaagtct aaagcttctt
5581 tgcctcttgt ttcgcccact ataattctat cggggcgaag tcgcattgtg gctttcaaaa
5641 gcctaagcat atccacttta tccgtagccc ttaaaattac tttgttttca ctcgcacatt
5701 gaagttctgc tgtgtcttca atgattacta ttctgtgatt gggagtgatt ttagaaatgc
5761 cgtcaataat ggcatttgaa aaagtggttt taccactacc agttccgcct acaactaaaa
5821 tattttggcg ttcgataatg gcattaatca aaacttcttt ttgatttggt gtaataatat
5881 tagaatctac ataatcacca agagtaaaga ttttgacagc ttttttttctg attgtaaagg
5941 ttggttttgc gacgattggt ggcaataaag cttcaaacct tgatccatca agtggcaatt
6001 cacattctaa tataggatta tctgcatttg tagttgtatc aaggagggta gagattgctg
6061 taatgataga ttttgctcta gcttctgaaa attcgccaag acatatcata tcttgaccta
6121 gcttttcaag ccaaagtttg ccgtctgaat ttagcataag ctctattgtt tctttatctt
6181 ctaaatgagt taaaatatcg ccaaattctc tttttatttt ttcattgatc cgtgcttgaa
6241 actcaagatt actcattgaa gagtcctttt gattcaattg caagagttaa aaatttgtca
6301 ggattttttt tgtatagatt gtggagttgt aaaagtttaa aaattaataa aagtttaaga
6361 ttattctgct ctctctctct ctctctctct agctattaag caaaaattaa gtttattttt
6421 tctcaaaaaa ttctccaaaa attaaaaact aatatcgtta ttatattatt ttaaatttaa
6481 ttgtcaagat ttttttctaa aaaaatgata catttttaat atttgaatgt ttattttttt
6541 catttttttgg gaaaaaataa acatttttta taaaaaattt tctattttgt ttcatttttt
6601 ttgagacaaa atatagattt tttgcttttg tcccaaaata tgatacattt ttaacatttg
6661 aatgtttatt tttttcattt ttttgagaca aaatacaatt tttaattttt gtgtcataaa
6721 attatcaaaa atgaaatcaa ttgaaataaa acggactaaa attatgaaac aaaatagaat

6781 tttaaatatt cttattttaa aaagtttgat cgtttaatat ttttggataa cctaattctt
6841 ccgtcatata cttttcaaat ccttcaatat ctaaagcatt atataatcta ataaccttgt
6901 aattggtttt ttgttgaatt agatcatctt ctgctgttaa attatctgta taactaatag
6961 tattatctaa catcattttt cttgtagaac ctcctattag ataaaatata atttcatctg
7021 cctttaccat ttgatttttc gtggtttcaa acaatacaca agcaaagaag tctttatcca
7081 tttcattagg tttgtaaaga ttatcgtatt tgcatatgtt cttaaaatta tattttgcat
7141 tgcatattgc aaaattaatg ccatttcttg gaaaagagga acgaacctct aatgttttac
7201 cattgtttaa caataaatct atttgcccta tcgatgattt aaattctgta aaattaacaa
7261 tatctccaaa atttcttttt atatagtaaa accaaccata ttcggccaaa attcccccta
7321 aagcatctaa aatcagcctt tctttatctc tttgtgatgt gctatcgttt gctttaagtg
7381 gatttacttg ttttgctaag tcccaagctt gttgataata ttttgaagct tcatcagttt
7441 tatttttaaa aatacatcta attactttgt atgttctttt tatattcatt gtattgtttg
7501 gtttttgaca gtttattatt tcataattaa atagcattat taacacccct atgttttata
7561 aaaattctag agtatagtct ttttccattt atatacgctc tatcggtttt tccattccag
7621 tcatttcgtt taatataaat aagctctcca ctgtttactt gataatctgc cccaataatt
7681 tcaaattgtt gtggattgta tttatctaaa aatgtaatag gaactcccat tattccagta
7741 taatctgaag gtatatcttt ggactttgaa atttcaatag cattgtaatt atcatatgta
7801 atataatctt gattattata ttttctattt aaagatattt ttttatttcg tttttggtta
7861 ttaatatttg aaaaccaaaa agatcttgct ccctctacca attctccttt tgtgtttata
7921 aaccatctaa cgcaattttg tcctaaccat aatttatttt tcatcatata gttgaaacaa
7981 tttttataag aaattgcatt agcatttcct ataatcaaaa aatctttttg attagatata
8041 agcatatcta taaattccct aaatagtgaa aatggtggat ttgttacgat tatattagat
8101 tcttttagca atttaattgt ttcatcgctt ctaaaatcac cgttattttc taacaaacct
8161 tgatattttg gcttattttt ttctataata aaaattttac ccttaccatt actattaaat
8221 gaagtactaa ttaatttttt tagttttaat ctatcaaaat ttaatttaaa aaattttgta
8281 aaattacttt tttgtggatc atcgcaatta caataaacta tcctatcttt aaacgcattt
8341 ttataaaaat ttaattcttt atttatatct tcatattgtg tataaaattc atcgtttttt
8401 atatttttttg cattatttaa tttagaacta ctgtttttat tcatttatac ttaaccttct
8461 atatatttaa atatcaatct cgccatatct ttttataggt attttcaaat ttggaaaatt
8521 tttatccatg gtataaatag cactgcaacc ttctttttcg gcacaaaaat attgcaaata
8581 gtcttcaaag tctcctcgat atttatcata ataatcacaa gccatattaa tgacacttac
8641 accaaaagaa gtgatattaa aattttttttc taatgaaata tcttttaaga aatgagtgaa
8701 attatttaaa ttttcttttt tgataagata agcaatggta gaaatcatat cttcagagaa
8761 aacaatttga atattgtttg tgagtaaata atacattaat tgtttagctt cattatgata
8821 ggttctttca gtattgaaat aatctagaaa aatatttgta tctaaataaa ctttttttcat
8881 attctacctt cttctaaatc tttatccaat aactctcttt tcatttcttt tgctgatttt
8941 gtagaatcgt atgcaccagt aagtatccca gcatattttt ctataaatag tctagcttct
9001 tgttcttttt gattttcatc aaataaagaa ggttgtgtat cattagagat taagactttt
9061 ttatttatgc tcggctcttc tttttatcg aagctatatt gattgtttat agagttattt
9121 ttttcttcaa taacaatttc tgtatttttt actttagtcg tttcatcatg ctcttgattt
9181 atttctttta cttgatattg cttttgattg tttcttattt catcaaagcc tagatattct
9241 tcttctattt ctaaattttc tctaacttct aaattttcaa aatcccattc tctcgtaatg
9301 gtatgtctta aattttttaa atcattccaa gatccgcgtc ctactaaatt tggattaaat
9361 aaataacaat catcgctacc aacaccagca tatgatttta caaattctct atttatttta
9421 aaaatcactt tcttttctat caacccattt aaagctctat acattccagc cctgcttaaa
9481 actttatcta aaacgatatt ttgcttcata gatttatcaa acctcacaac attttttataa
9541 tctactgaag aaagcaaagt aaaaaataaa gtttttttctt gagattctaa attgataata
9601 taatcaatat tttcagtaaa aagtttaata aaactttctt ttgtttgaac ttttttaaca
9661 ataatttgat ttctttctac aacttcacca gtaatataat cagtagatag attatctttt
9721 ctataaacta cttgccgttt gttattttta ttaatctcat ttaaatcttt aatttctatt
9781 tcattgctca tattttatcc ttactgaatt tataaaatat tgtatcataa tttgagacaa
9841 aaagcaataa atattttata aaatataaaa tattgtacca taatttgaga caaagtaact
9901 ttaatctgtc tcactgatga gctaacttgt ctcacaggtg agacgctttg tctcactgat
9961 gagacagatt aaaagtgctt caaagcacta taaaatagac atttgaaagc atttttaatt

10021 aaactatctc ttctatatct attcttacaa attcaaactc taattttttta cagcaaattt
10081 cgctaacgct catttgcaaa tttgaagaga ctatgcgtct cttactcttc ttacaacaat
10141 agggcggggt caagttcgtt tgtcgctcta ggcgactgca caccccgcac cccatatcta
10201 cgcaaattat agcacctact taggtgcaat ttgctatata caccaaaaat aaaatattta
10261 gtttgaaaat tctacttttc aaattaagtt taattgtttt gaatatcgat aatataggct
10321 ttttataaat aaaaatattt ctttttaagt ttagagtatt tttctttgaa acttaaagta
10381 atggtaaaat aggaatttaa ttatttaagt ttttgatttt cttaaaaaat taatttgtat
10441 agatttagat aaggtaaagt tatattttat tggaattagt gtataatatt gttaataata
10501 aaaattttta aaaggtaaaa aaatgaataa agataaaaaa tatttaaatt tttctgaata
10561 ttatgaggtt agggattttt tagagcttta ccctgaaaaa gatagagaaa atatagagaa
10621 tattttaata acgataggcg aaaataaaaa gtcaaaaaat ttatcttttg aatttttata
10681 cgattttttt aaaaaattag gttttgaaaa aaagttgata tagatgattt gttataacaa
10741 atcatcttcc ttgatatctt ttccaaattt agcgtcaaat actttttttg gttttgtgtt
10801 aatctttagc ttgataggtt ctttattttt cagaactaaa gattgaagtt gattatcttc
10861 ttgcattgtt tcttttatct tgattttgtt tttaaattca tcattgaaat atttattgaa
10921 ttgtttataa gtcattttaa agtttttttc ttttaaagcc ttatcataaa gaaatttaga
10981 tactacacag ccattttcaa attctttttt taaatagttt atatttgaat aaaattcgat
11041 tttagcttca cctctttgca ttatatatcc tttcttaaat agatttttct aatatcataa
11101 attcatattg gtttgttttt tctttttttag ataattcttg gtagcctaaa tgtttaaaaa
11161 ttttaatagc ttgctgggaa tagtttatat cacatctagc aattatttta ttgttgggaa
11221 taagtttttc ataatattta ataaaatttg tagctaactt tttgtttcta aatttttcca
11281 agatatagat catatttatt tcatatatat ttgatccttt gctttttcca caaaatgcga
11341 aacctgcttt ttcatttgta ccatctttaa taataaaaat tcctcctgat acaccattat
11401 tttttattat atcttctaat ttttcaataa atcttacatt gtaaaaatgt ttttctttga
11461 atcctagcaa aaaacaatat tttataaatt ctatatcttc aaatgttgca atttttatat
11521 ccatgattgt tctcctatat tctatttgta tttttttcatt ataggatatt ttaggttaat
11581 ttgagtttat ttaggttatt ttaggtaaag ttaattttat tacaatagaa ttacctaaat
11641 ttagttataa ttacttggag taataagaca ggatatgcaa atgtcgaaca tttgcaaagt
11701 tttatcgcta aagctctaaa ataaggacgc caaatgagaa aagataaaag aatagcaata
11761 agattatctg aagatgagta taaacatata atgcaaaaag cgcaaaatca cggcttaacg
11821 atttcaagat atttgcgtga tttatcgatg aattatccta ttacttgcat agtagatcaa
11881 aaagtcgcac ataatgtttt aaacattgct ggagatatag gaagattagg cggacttttt
11941 aaacattggt tggttagaaa cgaagataat aaagtcaatt tttcaaacaa aagaacttat
12001 aatgatattg atgagattgt agatcaaatt ttagatttac aaatactttt aaaagaacaa
12061 gccaaaagga taatacaaaa tgataatcaa aaagattcca agcaaaaagc agaataaatc
12121 aagttttaaa aatttatcaa actatatttt agacaaagac aataataatg caaaagtatt
12181 ggttgattat atgcttgata aaaacaatga aatggataaa gtggaaggtt atcattttac
12241 aaattgttct tttgataacg atgaagataa tattaatgag attatcaata ctcaaaagct
12301 taataccaca acaaaacaag ataaaacctt gcatttagta gtttcttttc aagaagatga
12361 aaaccctact ttagaaattt tacaagcaat tgaagaagaa atcgcaaagt ctttaggaat
12421 ggaagatcat caaagattaa gcgtggttca ttccaacaca aataatcttc acatacatat
12481 tgcaattaat aaagttaatc cgcatacact aaaagttatt aatccttaca atgatgttag
12541 aattttacaa gaaacagcga tgaaacttga aaagaaatat aatttgaagc tagacaatca
12601 tatttcacaa aaagataagc aaagtaataa atataatacc cacactatga attgtaattt
12661 tgaaacctgg gtaaaagaaa agctttctaa acaagtagat cttatgttaa aagatgaaaa
12721 aactactttt aaagatatta aacaattttt agctgaatat gatcttgaat ttagagaaag
12781 aagaaaaggt tttgttattg cttctaaaag tgaaaaatta ttttgtaagg caagtagtat
12841 tcacaggggg ttgtctaaac aagctttaga gaaaagattt aaagaattag atttaaaaca
12901 agaaaaagaa aatacgaaaa atattaaaga agaaaaacac gaaataaaag aacaatatca
12961 gcgaccaaat aaagaaactt ctaaaacatt atgggaaaag tatttaagaa ttgaaaatga
13021 gaaaaaagca gagcttgaca aagaacttag aatgttaaag cttaggcgaa atgaatttaa
13081 aacttcaata ccatcaatga aatttaataa aaatacctttt gcacatgtta aaaaccaaag
13141 aatgattttt aaaaataaac aaaaagagct ttatcaaaaa tataaaagag tgtcttatag
13201 agatttttta attagtgaat ccttatcagg aaacgaagag gctacaaggg ctttgcgaag

13261 aactaaaacg aaaatcaatg aaaatgaaaa tactctatca tcaacccagg aaaaacctaa
13321 aatatttgaa aatgtagatt atataacaaa agaaggttat gctgtatata aaagcggttt
13381 taataaagcc atagataaag gagaaatgct aaaagtatct cttattaata ataaagacga
13441 taaagaattt cttttaaatt ctttgcttat agcaattgat agattcggaa atcatcttaa
13501 tattacagga gatgataaat ttaaaagaaa tattttagaa gtcgcaaatg attataattt
13561 aaatgtaagt ttttcagatc agagaatgca aaagatacaa gaagaaaaca acaacaagcg
13621 taaagaaatg aaaactagaa aggttttaaa aaatattatt gaattaaaaa tcaaactcac
13681 agaacaggat aaagatatag atgaaaatat taaagaaaaa gaattgaaag ctttaaaatt
13741 ttcacttaaa aaaatatcaa actctacacc ttcaatattt aaaggcgagc tgaaaaaatt
13801 aggctttagc tataaagaaa tagattccat ggatactgaa agtgtttgta ttgaaattga
13861 cggctttatt gtaaattctg ccaataaaga aggacttgtc gcaatgaatg aagaaattaa
13921 acaaaccttg atcgatgatg aagagataaa aaggtttgaa aaatttgaac atttattttt
13981 aaatactaat aaaataacag atattgccaa gggattttat acaaatagaa aaattgatgc
14041 tgatgagtat ataaaaagat tttcaatgca agaaagcaaa attaataagg tggcaaacgc
14101 tatttctatg ttaagcgata agaatattga cataatggaa aagtatgcaa aaaaacttga
14161 aaaagattta aaatatcaat acttgaaaaa ggtggaagtc tttgcaagta atgatattga
14221 tttgaataat ttttaaggtt tgagattata tttctcaacc acatctttaa tattgatata
14281 tttgtctaac atattatcat tttttagtat tttataaatc tttaatagtt tatttttgta
14341 ttcatttaaa aaatgttttt ttgtgaaaca tttttctaat acatataagc agattaaatt
14401 tagtgttttg taatattctt ctcttttttt attcagcaaa ttgttatttt tttcatctaa
14461 ttggtttttt tctaaatcta agtgttctat gtaatcaaca aaattttttt ccgcgttaga
14521 aatttttttca aaagcatcaa attcagtttt taaaatatta attctattat ttttagagct
14581 atttcttaat tgcaaacaag caattattag tccaatgact acaccaatgg aagccaatcc
14641 ttgtattaat ttgatgataa tattaatata atcggcattg tctattaaat attgcatgta
14701 ttacttttttg tcactctgat ttgaattagt ttgattctga tttgaattag tttgattctg
14761 atttgaatta gtttgattct gattatatgc agtttgcatt ttataggata aatctaaggc
14821 agcaaaaact ctttgatcat cataactttc gttgcaaatt tttaaatttt caacattata
14881 ttcttgattt ttcataaaaa atactcctag tatcaaaatt aatatatttt gtgattttaa
14941 ttatacctaa tcttttctaa aaattttaaa tcttacataa gaatttaagc ttaagcccat
15001 tgaatgagcc tcttgattta aaatttggta ttcttcttct gtaaaattta tcataagctt
15061 tttatttctt tttgcatttc ctgtttttgc atttcctgtt tctccacgag cagaattaac
15121 aaaattattt tcattttttg gtgcttctga acttagtttt ttaaatccca ttattatact
15181 cctaattttt catttgctat ttttaaaagt tcttggtaaa agttttcaaa gtcatttaaa
15241 gctttgtcgt ttttatcgca aaattctttc atgcttttac cttctattac tgctttgcga
15301 taagcttgtc tttcatagat tacgctttca agcattatta ctctatctaa gcccttttct
15361 tttttagctt cgttaatgta ttcttttaaa ttttctaatt cttttgctaa aaaaggatta
15421 ggtgaaaccc tattgactaa aaccaaagcc aataaattag gattaatttc aatcacttca
15481 ttataaattt ctagcatatg atctagcaca ttaacatcgt attgacttgg aattgtagga
15541 atgataatga tatttgatga aagtatagct tttcgcattt ctttactatc cctaccgcct
15601 gtatcaacta taatgctatc aaaagcattt ttcattcttt taatctcatc gcctaaacta
15661 atgcctgttt tcgaaacatt tgaaaataaa ggctctaaat ttgattgact tctcatatcc
15721 gaaaaaactt cagtcgattt ttgcggatct gcgtcaataa gtaaaacatt gtctccatct
15781 tcggctaatc ttgcggaaag atttactgct aatgtagttt taccactccc gcctttttca
15841 tttactatgg atataatcat aataaaacct tgtttttagt ttaaattaaa ataatatcat
15901 aataatatca taataatatt aatacaatat taaaagaata ataaaataat attatattat
15961 gatacctatg attaaaccaa caagaagaaa aataatttgg ctagcaatag cgataatcca
16021 aaaattaata tttgtatttt tcttttcttt ggtgtcttct gttttttctt gtgatgcctt
16081 aattgtgttt tgatttgtct gtgcttgtat taaaatatct tttaaagctt cttgattgtt
16141 tttgctatat tctcttattt cattcaatat attgaattta tagctttcta aatctgtttt
16201 gtgttttata aagagtagat ctattttttc taaaaaatca tcaaagataa tttcatttgc
16261 actaacgact gcaagtatag gatcattttc atctaagatt atatggtgct tttttgcaat
16321 ttcttcaata agttcttttt tattcatttt atttttcctt ttatgatatt attttgatat
16381 taaataatta atattttaat attatattaa tattaaaacg atatcatcaa tttaactttt
16441 tttacttcat tttggagtat attaacaact ccaaaatacc tactatcata actttttata

16501 ttttcaccca ttacaaaaag ttcattttct tttaatttgc gtttaaaagg tttaaattct
16561 agtaaattac cttcgctatc aaatgaaaaa attttagaat taggcaaaag actttcatta
16621 ataaaaacac cttgcgtatt aacttctaca aaatcgccat ttactgcgat aatctttttt
16681 aataaaattt ctttcttttg tggtattttt aaaagcacaa tatcgttttt gttaaaagtg
16741 gaagagttaa tctctttgta taatcctaaa ggcatcgatc ttgtataatt aaaatgaaaa
16801 ccgccaaaat aaaaaataag atatgagagt atagaaagaa aagcaaaaga aaaggcaaat
16861 attgcaatag tttttctcat agcattacaa tgatattcct ttattttgct ctaaatctaa
16921 gttttgtcta aagcttttta gattggtttt aattatggct tcaagttttc ctaatttcaa
16981 ttgtgaagct tctacagatg ccattatcgt tttatctttt ggtattaaat ttaaatcaag
17041 tttataacct gcatttttag ccaactcatt taaaaatatt ctttgagttc tgccgttacc
17101 ttctctaaag ggtgcaaag catttagatt tctagcaaat tcggctaagt tctttgcgaa
17161 atcgttaaga tcttttgaat tttttaagta attgtcttct ttaagccaag taaaaatttg
17221 ttgagcggtt gcatttaaat cttttccagg aacaaaatta gtaattgttc catttggtgc
17281 atattttcca aaatttccat gcaagcctac ttccattcta tcttttccag cccagtgaaa
17341 tacatcttga aaaatatgtt tatgtatatt ttttaagtgc tgataatcga aattgccttt
17401 aatgggactt gtaatcaatt cgtcaattct ttgtgaagta tattttctcg attctttatc
17461 aaataatttt tcatctttta taccgagttt attatctgta attaatcttt taaaaagttc
17521 ttcattgctt tctttcatat tctacaaatc ctttttcttg atattcagcg ataatttcat
17581 cagcgcttaa accatataca ttttttttaa ctaacataat aatatctaac tcgttaaggt
17641 aaatgctttc aatagcgtga gttcctatgt ttgatttaat attctcgtat tgctctttgg
17701 ttacatcatc tttatatttt ttaagtattg caaggctttc ttctaagcta tgcacttcgg
17761 gatattgttt ttgcattttt actcctttat aaaaaatata attatattat aataatatta
17821 aaatagaatt aaattaatat cataatgata tcataatgat attaatttat aatatattac
17881 ctatttgact aaacgcttta tctttgaaaa ttttaagcct ttgttttgac attagagaaa
17941 agcttttatc ttgcatagct tcaccaaaag ttaaattctt tgaagtgagt tctaaaagat
18001 ctttgccgaa agtatcttta cttaaagcat taagatagac aatagctttg atttttttctt
18061 tgatagcttg atactcttta gtttcttcaa attctaaatt atctttgata attgatccgt
18121 gatattcatt aatccacacc acaaaagaac aattaaaagc ttctgttagt tgtgttaatc
18181 ccaaaatggt atcatctctt gcttgaccac ctacaattgg aacatgcata taaacttcta
18241 aattattgtt tgttaaaaag tctaaaacct cattttcttt aagataatca attaaaggta
18301 taaaagtagt cgcaccgcta tcaataatat agttttcagc cttttttttca aaagctaatt
18361 ctacaatttt atcaaaagca cggacatcaa atttgccatc atcatcaata agttgcaaga
18421 atttagcttt taaggctttt acattaagta aagttgtatt attaggatcg gtatcaatag
18481 cgactatact ttctccttta tctaaaaaat attgacatag taaagagctt ataaagcttt
18541 ttcctatacc acctttacca tttaaaacaa aatgtatttt tgccattttt actcctttat
18601 aaaatatcgc tgacttgttt atctagtgtt actttgatc ttgctaaaaa tgcgggatct
18661 ttaaagtata aaatttgttt tccataaata ggtgcttgac ctgctataaa aattaacata
18721 tcaccaggag cgattatctt gtcatcttta tcttttttag ctgaaggcaa tcgcatacac
18781 tcatcagcag taattaaagg gcgttgcact tcttgtatag tttcactaac attgccaagc
18841 attacagcgg ttctttttcc agagcttgtg atagattttt taacaatagt tgtgattccg
18901 cccatttcag ataagagttt tgcagtttcg attttatttg gtgcaaatgc tattcttatg
18961 tggcagttcg aaataattgt ttctttttcg ccataaatag aataaagctg ttgtatatct
19021 tggatcgacc cgtaaaactt cattccatat cctgccatat aagcaagatt ttcttcaagc
19081 actcctaatt ttccaaacat agttagctca tcagctaaga ctagcatttt atgtttatac
19141 cttttcacat tttctccatt tttaaaattt agagaatcat cagttaaagt tctaaaaatt
19201 tgattaatga gtaaattaaa aaatggacgc aacctatctt tttgccctgg cggaatgatt
19261 aaataaagac tgatagggct ttcatgattc attaaatctt tgattttaaa atcgcttttt
19321 gctgtatttt ttgctaagat aggatcaata taaagattta aagcttccga agctgttcct
19381 actacaccgc ttaattcttg tgtggcttta tttttcattg atcttgaaat gctttgaata
19441 atttcttgtg tatttttatc cattgtagaa atatcacatt caaccatttc ttctaatagc
19501 tcatcgatac tttgatttgg atcgttaata aatttataaa tatcggttag atttggggta
19561 ttttcgcctc gatatttttt taagtgtaaa gcataaagta taattgaaac taagaaattt
19621 cccgcctcac ttttaaaata agatgtgcta ccttgagtgg gattggtagg gggcgtttcg
19681 cctttgaaaa tgagattaat tgcaatattt tgagtgtctt taatctcgtg cattgtttct

19741 agtcttattt cttctaaaat attaaatttt acggcactat catctaaaca agtcggatct
19801 aattttaata ctttattttt ggcgtatttt tgtcgccaac ccgcagttaa agcccatagc
19861 tcacctttta tatcaaaaat aagtgcagac tcttcccagg ataaaagtgt aggtaaaagt
19921 aagcttacgc ctttaccgct tcttgtaggt gcaaaaagca ttacatgttc aggaccatca
19981 tgtcgtaaat aatgaagtgt ttttttgtgt tcaaaaccac cgatatacac gcctttatct
20041 ttatcaaata cgccactttc tttaacttct tctaaagtag cccaatgtgc tgatccatga
20101 agattttcta ttgcttttgc cttacgcaaa aaggcaagtt tcaccaaaat aaaaacaata
20161 aaacaaaatg caatacctgc aaacattacc atgaagaatt ttttgaaaaa atcaggataa
20221 aaagaataat aagcataact ccaagaaatc cacttaaaag gattataaaa tccataaagt
20281 aaagtttcgc ctaaactagg agagtaatta aaagattttg ccaaatattg tgttgcaatt
20341 tggcttattg tgataaacgc tagtaagata ccagttattg ccaatatata aactaaagtt
20401 tgattggttt gtttttcat aaagaaatcc ctatatcttt tttggctgtt atctctttat
20461 tgataccttt atttttatct agcgctttag tagtggcttt ttcaaccatt agttgctttg
20521 caatttgttc ttttaattgc tttttaattt cttccaatcc tctgcttttc attaaatcat
20581 taaaatcact taaacctttt tgcgcttctt gatttgtaaa atttggcaat acaactttta
20641 cttcgggata ttttttttgt atgcctaagg ctgtttcttt gcctacattc ttattgccgt
20701 ttaattcttt ttttacatca ttatcccctg caataatgat attcatttta gggtattttt
20761 cttttatatt tgttacgata atttctaaat ttccagcatc tactcccatt atagtaggag
20821 attttgtagc ttcatagaca ctagcacttg tcgcaaatcc ttcacaaata taaacttcat
20881 tgacattatt taaattcttt gcaccaagta agaaaaaatt accttgcttt ttcgcagggt
20941 attctatgcc ttgctctttt tcttctgctg tgcgtgtagc tcctatcatt ttatcaccat
21001 tagcgaagat tctttgcgta gcccaatatt tgccttcaat atctcttaaa ggaatcaata
21061 agttgccgtt tttgtcttgt ttgagataaa aatttttatc aaagcctttt tcttttaaat
21121 acgggtgttc tgaattagcc catcttgcat tgttgtattc attttctaat ttcacagcgg
21181 tattttcata cgcttgattt aattctttat ctctttgctc ttttaaggct ttattatgtt
21241 ctaaagcgtt ttgcagattt atttctttat ttttggtgtt ttccaaagaa ttagcacttt
21301 tccaattttc tttaatgcca gttttgtaat tttgcacaaa tcctgcaggg tgtccattta
21361 aaaaacctac ataagctccg ctttttttctc tccctttatc ccctataacg ctaactctat
21421 gaagtttgcc gtccattata ggctgacctt caatattaag ctcatatttt tctaaaaaag
21481 ctttaaattc atcatgagta ttgttgtaac tttgtgttaa ttgtgattga tttagccatt
21541 gagtgaaatt ttttaaatcc gcacccttag gagcatacca cattttagct tctttatccc
21601 attttgcacc tgctttttta gcaaattctt tttcctcgaa aggaacatat aagtaagttt
21661 ttctagttgc caagtttaac tccttatctt gtttaagcgc ttgattttga gtatttgaat
21721 tttcaagctc tatttttttgt ttttgctcca aagaaagact ttcaagaaaa ttaacaattt
21781 tggtagcgtc tgaagtggct ttaaaaattt catttggctt atcttctaaa atttgcaccc
21841 aactatcaat atatgataag tgttgtcctg gatcataatc taagcctatt ttaccattaa
21901 atagaaaact tgcaatttcg gctcttaatt cttctttggc gtattcttta gaaccaaaag
21961 ggtttttttaa atctctattt aatcgacttt catgaccgct ccaatgtcct aattcatgta
22021 aagcagttgt atagtaagcg ccttcactta aaaaatttc tttcaggggt agtgtaatag
22081 tatcatttgc ggcattataa aaagctctat ttcctttgtg atgaataatt gcttttgaat
22141 catctaaaat tttttgcgct tctttaatta tttgaaaatt atcaatctct tgcttttgct
22201 ccaattttgg catattttcg atttgctcgg cattaaaaac ataagcgaaa aatactttag
22261 gattctctaa ttttacttcg atttgttcta cttctccgtt ttcatttgta ataacattac
22321 cttcctcatc taatttatca accatttcgc taaattgcca atattgaatc aaagtcgatt
22381 tttcaccttt tctaacttgt gcattaatac tttgtgcttg tttataagta agccacctag
22441 gatcattata attttgtagc attaaattaa tgctatttat gccgttataa ggcttatttg
22501 taatcgggtt ataaggcata ttgttagcta gatcaatacc tttccaaggt ttaatccatg
22561 gtgcagttcc atttttaag ctttctatga ttttatcggc tgtttcttgt atgaaatcct
22621 tattttttcat gaaattatcc ttgatcttct gtgccttctt tggcatcatc ttcgcttaag
22681 gcattttctt taaatgcacc taattcttca gcagttaatg gatcaacctc atagctgaaa
22741 tttggtattg tttctagttc tttaaaatcc ttcataattt tctccttctt caatattagg
22801 tattgtttgt ttttcttttt ttggtgttct aaaatctatc tcttttgctt ttattcggtg
22861 ttttgagtaa atttttcctt catgctccca agtgtcttga gttagttctc caaccacatc
22921 aactgaaatg cctttttttta aatatgggtg catggcatta gcaaaagaac caaaaagaga

22981 tacttcgata taagttgaat tttctatctc tttgtatttt ctgttgtttg ctaaggtaaa
23041 ggtgcatatg atactacctt gattacaatg tcttaattca gcatcagctg ttaatcttcc
23101 gcctattgta atattattaa tcattatctt gctcctcatc ttctaagtta tcaaataagc
23161 ttggctcatt atcatcaaat tgtgtaggtt tattttcttc caaattttca ttttccattt
23221 ttactccttt ttttgtgttt gcaaattgaa aagattgtaa gtaatcttaa ttcttgtgtc
23281 tgattttctt gctttttctt ccaagtcatc tatcgttttt gctgttgaat cgtatttagc
23341 tacatagcca ttagaaaatc ctttttttaa atccccagta ttataaatac ttaaagcctg
23401 atttatacga tcttctttcg ctagattttt atttgtcatt tcatacgcca aataaaaaat
23461 atcactaccc gctttaatat ttttacaagt atctaataaa tcttctacgc tgtaatgtga
23521 gaatgttgaa gatttaagat tgttagaatt aaattgcatt agtccaatat ctacactata
23581 tccccccttta atgaaattgt tcgctaaatt ttcagcttct tttttatttt ttaatataaa
23641 agagccaacc ttattaatgt taattgcaaa ttgttgattg ttgctttcta cttgaataat
23701 cttttgtatg atttgtgttt ctacatttgg atttttgcat tgttcaatta gagcaatatc
23761 aatcattatt taactccaaa ggctctaaaa taatatcctt agttacaaag atattaaatt
23821 tatatccagg acgaattttt aaagtagggc tgatattcat attttttctt atcatctcgc
23881 ttgcgacttg acccatttgt tgaatagctt gtgcgattgc tttatccgaa gcagtttctt
23941 tttcgctgtt tgtgtctgaa ttatcagaca acgcaatacc tgcgctgatc gatgaaagta
24001 aaaaagcaga gccgaaaatc ctaaaataat gattatccac ttgatcttca aagcctgtat
24061 atccatcttg tgaagttcct tgcatgccat caagatttaa ggtatcacca ttcggaaaaa
24121 taagcttatt ccatgctact aagagtcttg attgaccata aataatatta cttgaataag
24181 ctcctacaac tttagttcct tgtggaatta aaaggtattt tccagttgcg ctatcataaa
24241 cattttgtgt tacttgtgcc aatatttgac caggtaaatc cgaattaaca cctgtaatta
24301 aaatggcagg tatattccaa cccgctttaa tttcataagt gcttaatggt ttttgtttta
24361 catattttaa atagccattg tctttttttt gagataaaaa ttgttgccct tgcacatctt
24421 tgtttgtgcc actttctaag cccagtcctg tattttgtgg attgattgct ccaagattaa
24481 tatcttcatt attattaggt ggattgaaat taattgcttg gttttttttg ctatccgttg
24541 cttctgtttt gatatcaagc ttggtattgc ttaataaagc ttttaactgc atatcttttt
24601 taatcttcat ttctttttca tctatagcat tgttttggac aaggacagga gtattgttag
24661 gtggctgttg tgtagtttga gaatttgttt cttgaatagg ttgttgtgtg gcatttggca
24721 taggtggtga aattatatta ttttcttgtg ctttttgaac ttgtaattcg tcaatatata
24781 aattaagatc attgttatga tttgtagcta tttgtgtttc ctcttttggt gtggtttgcg
24841 cagtttgtct ttctaaattt tgctttcttg ataaggctac ataaaatatg ctaaataata
24901 ctattgcgat gattgcaaat attattataa taggaacttt cgaaagcctt ttgttatttt
24961 ttggtaaaaa tttgttagga gacatattca cttcaagcat aatttaacct ttagtttttg
25021 gagtaaaatt aaaacttgtt atatttggtt ttacaagttt attttcgatg attttataaa
25081 tcacattgat tttactttca ttgatattaa atatcgcaac tatattttca ttatcaattt
25141 gtgaaatttg atagcttaag aaggttaaat ctttttttaga attgtcattg cttattgcat
25201 aaccttttttt tcttaaggta ttgataaaat agttagaaaa atcttgatta tttttatctt
25261 cattgataaa aaatgtagtt ttgttaggtg cgtaatatga aataatgaac tgcgaaaaat
25321 cgtctgcaat tttattgtag tttattttttg tttcttcaac tgcaaagcta gagttaaaat
25381 aatccgaatt cattttagca cacgaggtta taaagaaagc gattaacgcc aaaaataaaa
25441 ttttcatttg ttttcctttt tattttggag tgataaatca taaagcacat tattaactat
25501 atcttgatta gctttgtttg aatgctttgt gattttaatt ttttcttgtt ttgagcctac
25561 atttgaaagt aaaatcgctt tgttaaaaag cctatctaca ataaaagtat catatttcaa
25621 gcgataattt acgatttgtt tttcattact agaatctaaa atcataagcg caggtgcttc
25681 atagaatttt aaattttttg gcatttgaat ataagttttt atgccgtcat tgtaaattct
25741 taaaggttta aaaggtgcat taccttctac actatagcca aagtccaaat tttcaatatt
25801 actcggaatt tcatcatctt ttgttttata gaaattttta gattctgatt ttgctttcat
25861 ttgattttta taatcttcaa gagtttttat gatactattt ggataattaa atgaaaccgc
25921 aggcataaat ttcttagctt ctgatattaa atttaagcga taaactcgct tatttgtcat
25981 gatattcata gttgtttgta agtttttatc ggtaggctta atgatgatat gagagattaa
26041 ataaggctct tcaccactta cgcttaatga aacagcccat cttatactat cgcctatttg
26101 cacatcttta atcacttcac caggttctaa agcaatatca gtaagtctta agggtgcggt
26161 tacaatactt ggcattgatt caccaaaaag aaaaattact tctccattat ttccttgtat

```
    26221 agtagtagtt tttgaattta tccatttttg tgctattgct agatcttttt tttcttttgg
    26281 tgtaagattt gaaaaatctc tttcatttag attgttagca attaaagaac ttgcaattaa
    26341 agaacttagt aatattattt ttttcatata ttcaactcct tataaaattt ttgcaaaatt
    26401 aatatcagtg atgaaaatcc ctaaaggatt ttttaaaatt tgttcttctg tatttggaat
    26461 gatttgttta atttggatta atcctcgata agtttcttta gaaagaatat cttcatttaa
    26521 atttgtagtc gtctcttccc attcaacttg ccaagtgtca atattttgtg gaacgatatt
    26581 tttaattttc actcgcacat tttcctttgt agatcgtgcg aaaggattgt ttttttgaaa
    26641 ttctgaattt aaataattaa atgcttttga tcttggctcc acataagcat aggcatcaaa
    26701 tatgaatttt ctttgagttt cggcatttcc ccaaaccgaa cgccaagaat aaataaaagt
    26761 atttagacta tactttatta catttgggtt ttttaagtcg atattttgca ccattcctac
    26821 tttagctgta ttgcctaact tatcaacttc aataacataa gggataagct tgttttgaga
    26881 gccgatatag cctataaaga gaacacatac aaagcatata gctaagctga tcatcgctac
    26941 aatttgccaa tttttagcac gagtgatata atctccgtat ctttctagcc attcggtttt
    27001 tgcattaaga taaggattac ttgaagtgtc aaactcattc ttttctatct tttgtttttg
    27061 tctgaaaata ttcattcttt cttactcctt gtttttattc catagcatcg ggatttattc
    27121 ctgattgata gttttcttgt tttggtgatc ctgatgatat accgccttca cttgtttttc
    27181 cttgtatatc tttaagtttt tcttgtaagc gattagccat ttggtttggc attctacctt
    27241 ttgtaagatt gtctcttaag tgttcgccac ccgcgcttgc tagatttttt gccacacctt
    27301 taaccaagtc cattccaccg gctccacctt ctgctaaatg caaggcttta gctgcattca
    27361 tagcacgagt aacgccgaca gctcctacag ctgcacctgc cgcagcgcct gctgccatgg
    27421 tagcaaccgc cctaaagcct gccgcagcgc ctgcgctttc tccaatacta ccattaaata
    27481 cggcttcaat aataccagga accatttttg taattaggca gaatgtaacg gaagtgacta
    27541 gaattgtcaa aattacttca gcgctcgaat tttgtgttat ttcattattt attgtatcta
    27601 tcatgttaat acaaagagcc attaagcctt gtatcataaa gagttctaca cctactttaa
    27661 tggctgtcat tataggattt aagcctattt gtttaaaatg atttaatcca cctaacgcca
    27721 aagcaaaaaa tacaattaca ttcattaaga aaaattttaa atagacaatc agtaaatcaa
    27781 ttgccattaa aacaaaagct attaaaataa taacacctgc tataactttt aatatcgaat
    27841 caccaggact ccaaaagtcc atagaacgaa taatttttat tacaatagat aatgcttgag
    27901 ctataatatt attaggagaa accgatacac cattatttac atttgtggct aattgcgaaa
    27961 agctattaaa aataatttgt aaccattgat cgatattaaa taaaaatagg aatatgccta
    28021 catacattat ttttttaata agagtggcta aaaactctcc aaattcaaaa ccgctcaaag
    28081 cttttaagcc aaaagtccaa actaaatcaa taataactaa agtccaaaat acccataaac
    28141 aagcggtttt aataagaggc gtccaagata agagtccatt tcgtattata tttaaaacgc
    28201 cttcagcatt ttcagctcca aaagctaaat ttggagcaag aaaaattaaa aaaataatcg
    28261 ctagtttatt tccatttaag agcgtctggg tttgatttac cccaagtttt ttcaatataa
    28321 cctgtatctt ctgtttcaac tgcccatttt gcattagaac aatccctttg ttgagtttca
    28381 ttcattttct ccattttttt acattctgca actctagctt ttgcttcctc taagtgcgct
    28441 ttataataat cgcttgtttt tgcctcatcg ccacaacctg ctaaaaaagc caaagcacct
    28501 aaaccaatta atc
//
```

>>BfR-CA-15857_min_cov_30 putative plasmid location

```
LOCUS       LBFOAKKB_1             17297 bp    DNA     linear       12-APR-2021
DEFINITION  Campylobacter species strain strain.
ACCESSION
VERSION
KEYWORDS    .
SOURCE      Campylobacter species
  ORGANISM  Campylobacter species
            Unclassified.
COMMENT     Annotated using prokka 1.14.6 from
            https://github.com/tseemann/prokka.
```

```
FEATURES             Location/Qualifiers
     source          1..17297
                     /organism="Campylobacter species"
                     /mol_type="genomic DNA"
                     /strain="strain"
     gene            complement(64..303)
                     /locus_tag="LBFOAKKB_00001"
     CDS             complement(64..303)
                     /locus_tag="LBFOAKKB_00001"
                     /inference="ab initio prediction:Prodigal:002006"
                     /codon_start=1
                     /transl_table=11
                     /product="hypothetical protein"
                     /protein_id="Prokka:LBFOAKKB_00001"
                     /translation="MLGTKIFWVQYLKKRQIVINRVETTMKWSFSTLAWVQKCPCLNL
                     DFLMLNLKLKSLKMGILYFSLGTKMPMLKLGLFNA"
     gene            803..1234
                     /locus_tag="LBFOAKKB_00002"
     CDS             803..1234
                     /locus_tag="LBFOAKKB_00002"
                     /inference="ab initio prediction:Prodigal:002006"
                     /inference="similar to AA sequence:RefSeq:CKG86054.1"
                     /codon_start=1
                     /transl_table=11
                     /product="hypothetical protein"
                     /protein_id="Prokka:LBFOAKKB_00002"
                     /translation="MPQTDTKPNAKNRIHKAIETWFTKIYLNKIIHNAKDTSIFINKS
                     SCLAFILSIYGKTEENKNKMTPAVITHINTTKNNFATKLKRVKNHENIVELQAKYPKL
                     DIISAYQFLTLKDKFKITKSEIQDFETLIDILSKNAQKSKK"
     gene            2561..2905
                     /locus_tag="LBFOAKKB_00003"
     CDS             2561..2905
                     /locus_tag="LBFOAKKB_00003"
                     /inference="ab initio prediction:Prodigal:002006"
                     /codon_start=1
                     /transl_table=11
                     /product="hypothetical protein"
                     /protein_id="Prokka:LBFOAKKB_00003"
                     /translation="MDKAVNMKSPKMKLINKIAIGLGIAVMFALILLSCLLINIYNIG
                     IKHIILSFIISLVCLFLVLLLINTANKPFKKTYPKRGNCYDTHFKQSGISKYSTLSLS
                     NPFYNNLYSNED"
     gene            3070..3420
                     /locus_tag="LBFOAKKB_00004"
     CDS             3070..3420
                     /locus_tag="LBFOAKKB_00004"
                     /inference="ab initio prediction:Prodigal:002006"
                     /codon_start=1
                     /transl_table=11
                     /product="hypothetical protein"
                     /protein_id="Prokka:LBFOAKKB_00004"
                     /translation="MENQEQKGLFELYNFYNENKRNTTCYIFDKEDYQSITSENYFIK
                     NYLKKRKISYLLEDLFDLNSSNNLYQNCKIYKECVDILNKFTNQNIGILYKKPTYFSR
                     RVIYNILQRLANQA"
     gene            complement(3675..4316)
```

```
                     /locus_tag="LBFOAKKB_00005"
     CDS             complement(3675..4316)
                     /locus_tag="LBFOAKKB_00005"
                     /inference="ab initio prediction:Prodigal:002006"
                     /codon_start=1
                     /transl_table=11
                     /product="hypothetical protein"
                     /protein_id="Prokka:LBFOAKKB_00005"
                     /translation="MFVGFSKNLGNGFRIGIGYNLNNNKHSKRDNDREEFVSKVINEV
                     RNLFQEMCNKLNVSRETFEFAVEQDCQIKITDILTEDNSKKVSEISDIFSTISELIEK
                     VSFSKSLSTKTKETITDLIFKSKKIIFTLESENDLEQRLISLLSEKLQKEEKKIRKMI
                     KKMNGKKKKIGCLTYIVLFFIFMLIISFITHEDKTNNLEHSNVEHSNIDKKTK"
     gene            4702..5037
                     /locus_tag="LBFOAKKB_00006"
     CDS             4702..5037
                     /locus_tag="LBFOAKKB_00006"
                     /inference="ab initio prediction:Prodigal:002006"
                     /inference="similar to AA sequence:RefSeq:SQE24169.1"
                     /codon_start=1
                     /transl_table=11
                     /product="hypothetical protein"
                     /protein_id="Prokka:LBFOAKKB_00006"
                     /translation="MKEITMEKAHLITYLISLAFIVSGLIVCCILYLFKDKINKITPD
                     NIDNINSNVKDFIEKNICGGSSTIDHSFTTNFGDCSSISGNTSSSTNYFIDPTYSYMP
                     ENIYHNNQD"
     gene            6041..7183
                     /locus_tag="LBFOAKKB_00007"
     CDS             6041..7183
                     /locus_tag="LBFOAKKB_00007"
                     /inference="ab initio prediction:Prodigal:002006"
                     /inference="similar to AA sequence:RefSeq:ALW56296.1"
                     /codon_start=1
                     /transl_table=11
                     /product="recombinase RecR"
                     /protein_id="Prokka:LBFOAKKB_00007"
                     /translation="MLRKIEISGLYSFGIETQIVDFTAKPKSKLKNTKYEYNFNLSQA
                     GKPMKSAVFFGKNTSGKTNFFIGVKILLNIIKYGFSHALREHLVDNAFNKNSNSIKLS
                     IELSNNNKDIFTYSIEFDKKYILKESFLKNNKIIYKFENDKAIFNIYSIEDNKLLESF
                     FSRNLSENIFYLLKDFEIEQKEIFINLINNIEVYMNSNYSKKYNFEFEENKKKYFLEH
                     KESILEIFQLLDKTIKDFEFKKYKKDEKNIYEIFFVRNGKKFNYQIESEGVKKIVYFA
                     DKITQIIKEGKIVFIDELDSSISTLALILIFNNLINTEENHNGQLIVSSHNVLLFDVS
                     FLNSQQIFLIQKDQHLESKIRTYYDYDIRSEKKKAYIDYLKGLYDE"
     gene            7173..7775
                     /locus_tag="LBFOAKKB_00008"
     CDS             7173..7775
                     /locus_tag="LBFOAKKB_00008"
                     /inference="ab initio prediction:Prodigal:002006"
                     /codon_start=1
                     /transl_table=11
                     /product="hypothetical protein"
                     /protein_id="Prokka:LBFOAKKB_00008"
                     /translation="MMNKKTKPSIQIWCEGKTEEDYFNSLFKYLNYDNVKIDVKNLKN
                     NNSYKNILYKIEKAPYVDKLIIVMDLDRAKSDTNELKILEKLIKHIKNSKGKHLFLTL
                     GDFEDWLRFHFVDHSRNSKTIFYKKLGYSDSKNFKSSTNDLYSQILNKGGHIENAEKY
```

```
                     FCNIKVFFNEQCEIDKNSLNNLTQSNLYYFRKLLEQYNGA"
     gene            complement(8469..8564)
                     /locus_tag="LBFOAKKB_00009"
     CDS             complement(8469..8564)
                     /locus_tag="LBFOAKKB_00009"
                     /inference="ab initio prediction:Prodigal:002006"
                     /codon_start=1
                     /transl_table=11
                     /product="hypothetical protein"
                     /protein_id="Prokka:LBFOAKKB_00009"
                     /translation="MIINEDKKIEDLIKEAFAEKIWLPEFQRPFA"
     gene            8743..9201
                     /locus_tag="LBFOAKKB_00010"
     CDS             8743..9201
                     /locus_tag="LBFOAKKB_00010"
                     /inference="ab initio prediction:Prodigal:002006"
                     /inference="similar to AA sequence:RefSeq:AVS36970.1"
                     /note="DUF4065 domain-containing protein"
                     /codon_start=1
                     /transl_table=11
                     /product="hypothetical protein"
                     /protein_id="Prokka:LBFOAKKB_00010"
                     /translation="MEQNNAYCGKKIANEFIRLNCNKNNILTHMHIQKLVYFAHAMSL
                     ATYNKPLVKETFQAWPYGPVNVEIYQELKGSGKDNIISMLKTEDDSAELDENSKKIIH
                     AVYDKFGGKNAWKLSEISHNNSPWKKVFEKGKSNPISNEDIINYYKSSGE"
     gene            9207..9761
                     /locus_tag="LBFOAKKB_00011"
     CDS             9207..9761
                     /locus_tag="LBFOAKKB_00011"
                     /inference="ab initio prediction:Prodigal:002006"
                     /codon_start=1
                     /transl_table=11
                     /product="hypothetical protein"
                     /protein_id="Prokka:LBFOAKKB_00011"
                     /translation="MDFLEKNINKEEIRENQENQENQENQENQESKESKEITCSLYYA
                     FYKLEENYNTIEERIKNESNKFEERIKNESNKFEERIKETQKQYITILGIFASIVLTF
                     VSSFAFSSSILSHMHQVSIYRLIFVILCIALFVVNILYGLYSFLIKIHNGNIKSNNKP
                     ITNFNMIILVLIVINYFCYLARYY"
     gene            complement(9904..10593)
                     /locus_tag="LBFOAKKB_00012"
     CDS             complement(9904..10593)
                     /locus_tag="LBFOAKKB_00012"
                     /inference="ab initio prediction:Prodigal:002006"
                     /codon_start=1
                     /transl_table=11
                     /product="hypothetical protein"
                     /protein_id="Prokka:LBFOAKKB_00012"
                     /translation="MFGRNLKKIRKASGLTQLELAKILNTTQRTISLWETGNFEPPLN
                     TILLLCDIFKVTPNDLLLEGCYDDDNEYLAHHIVRLTKELNCRTYTKRLFNHILRRLE
                     LEKLTSHLQKFKGDSFARKLAHSWTGKGERMLLVFYYFIEYLEKYNINENNIDKNSFL
                     KLLNDFNLSKDNTNTIHCFILKEKDKINLIEWVEKEMQEFEIKVLVAMLKDEIKTLIK
                     KEINTLNKLLV"
     gene            10964..11242
                     /locus_tag="LBFOAKKB_00013"
```

```
CDS             10964..11242
                /locus_tag="LBFOAKKB_00013"
                /inference="ab initio prediction:Prodigal:002006"
                /codon_start=1
                /transl_table=11
                /product="hypothetical protein"
                /protein_id="Prokka:LBFOAKKB_00013"
                /translation="MNMDNIIICKASDLEDIFKKILKENELIKNKTDENLNVQQASNY
                LKISTSKLYKDCNLGKIPFCKIGKKLVFKKNDLDEYLKFNSSKNSLKF"
gene            complement(11288..12418)
                /locus_tag="LBFOAKKB_00014"
CDS             complement(11288..12418)
                /locus_tag="LBFOAKKB_00014"
                /inference="ab initio prediction:Prodigal:002006"
                /inference="similar to AA sequence:RefSeq:ALW56279.1"
                /codon_start=1
                /transl_table=11
                /product="integrase"
                /protein_id="Prokka:LBFOAKKB_00014"
                /translation="MKNLVKIKEYKGIYFIDKDKKLEDIKEISLVELQRRLTIPYELA
                IRYYEGKKSSYEIFPILNNTQTLKKNCELIALQRINGKFAKKFKIKEPKLNEVFEEWI
                SMRKNTISKKHYEVTIFSYNAHIKDVIGNIKIKQVKLKDMQNIVNKMIDQGKSPRTTK
                TIKDILSPVFEFAIKNDYIEKNIAREIEIQKFDNKRYFTIDDEDRNALYKAIINYENL
                MIRAMFIFLLHGRRKSEVLTLKWDNIDFANKIYFLPSNQNKSRKNLQFPLSSLQIEAL
                KSIGVKNKGYIFIKEDGNPYKDIRWHWDKINQKLKTPIRLHDLRHLIGYIGINMGISL
                EAIGETLGHSSIGVTKRYANIKNETINNTLNAIFSNFKTEKH"
gene            complement(13627..14628)
                /gene="flgS"
                /locus_tag="LBFOAKKB_00015"
CDS             complement(13627..14628)
                /gene="flgS"
                /locus_tag="LBFOAKKB_00015"
                /inference="ab initio prediction:Prodigal:002006"
                /inference="similar to AA sequence:RefSeq:CAL34921.1"
                /codon_start=1
                /transl_table=11
                /product="signal transduction histidine kinase"
                /protein_id="Prokka:LBFOAKKB_00015"
                /translation="MNEDILKSLDSSEKETFQKGLLSLIEQTYIVENEYKNLRAMLDG
                IIEVLPTALWVLDKEKNIILQNQEALKKPRLLDIISLEKNRDELEFENRFYTVKIISD
                NEKTIVSATDISDEKRNERLASMGSVAAHLAHEIRNPIGSISLLASTLFSRSELKNKH
                IVLEIQKAIARVERIVNSTLLFTKGVHINASEFNLLELTEECESAINSYNYSSQIDFE
                IKFLDMKIYGDKALLSLVLQNLIYNAIDAIEESECENPKIKIQASCDEQNLYIRVYDN
                GCEIKDENLVFEAFRTTKLKGNGLGLSLSKEIINAHKGELKFEIQPKNFYFILPCKL"
gene            complement(14625..15446)
                /locus_tag="LBFOAKKB_00016"
CDS             complement(14625..15446)
                /locus_tag="LBFOAKKB_00016"
                /inference="ab initio prediction:Prodigal:002006"
                /inference="similar to AA sequence:RefSeq:CAL34920.1"
                /note="conserved hypothetical protein Cj0792"
                /codon_start=1
                /transl_table=11
                /product="hypothetical protein"
```

```
                     /protein_id="Prokka:LBFOAKKB_00016"
                     /translation="MTKIFDFYSVFEGFESLDSLNFEKDIFENIECLLLKNYLHIKEH
                     FELDETASYALSLLAKNNRKRFSINRKIQHFKALSTLKYLLRAGIIRLEYSKEAKRIK
                     DKRQKLKKELRSYVIQDKIIFANHFTRFFFYFLKPNEKLILQNQYEEVLGLIKEKFEL
                     YQSFCFEQLSRELLEKKFQVSGVQSYWDKNLELDLYYKDDEISFIGEVKFKNKKICRN
                     ILNLLESKAKSLKLEPNNYIIISKNGFSKEFYKICKQNLLLMDLNDFKILLEEDR"
     gene            15550..16818
                     /locus_tag="LBFOAKKB_00017"
     CDS             15550..16818
                     /locus_tag="LBFOAKKB_00017"
                     /inference="ab initio prediction:Prodigal:002006"
                     /inference="similar to AA sequence:RefSeq:CAL34919.1"
                     /codon_start=1
                     /transl_table=11
                     /product="putative aminotransferase"
                     /protein_id="Prokka:LBFOAKKB_00017"
                     /translation="MQISDLKKEIILKKGTLHFDFTASALALKCVEKEIAKILPSYAN
                     THSDSSLNSFKTQQIYEQARKDIKTSLELNDDFALIACGSGSSSAIKKFQELLGIYIP
                     PLIKEKYFDKVDKSTLPLVIVGPYEHHSNELSFREGLCECIRIPLDKKGGIDFEILEK
                     VLKENQHRKIIASFSLASNVTGILSDYKRISKLVRKFKGIVAFDASSFIPYKNIACKY
                     YDALFISSHKLIGGIGGSGLLAIKKELCGSKPSFAAGGTVGYVSRTSQCYLCNEEALE
                     EGGTPGILQLIRASLAFKIKDKIGIEEIEKKEETLKEYFFEKLKTIPHLILYANNIKA
                     RLPIFAFNIENISPFDLAYELSKKYHIETRAGCACAGPYGHDLLGLKDNEELKTKPGW
                     LRISLHYTHEKEDIDYFFEALQKSITKLKF"
     gene            complement(16822..16898)
                     /locus_tag="LBFOAKKB_00018"
     tRNA            complement(16822..16898)
                     /locus_tag="LBFOAKKB_00018"
                     /product="tRNA-Asp"
                     /inference="COORDINATES:profile:Aragorn:001002"
                     /note="tRNA-Asp(gtc)"
     gene            complement(16920..16995)
                     /locus_tag="LBFOAKKB_00019"
     tRNA            complement(16920..16995)
                     /locus_tag="LBFOAKKB_00019"
                     /product="tRNA-Val"
                     /inference="COORDINATES:profile:Aragorn:001002"
                     /note="tRNA-Val(tac)"
     gene            complement(17004..17079)
                     /locus_tag="LBFOAKKB_00020"
     tRNA            complement(17004..17079)
                     /locus_tag="LBFOAKKB_00020"
                     /product="tRNA-Glu"
                     /inference="COORDINATES:profile:Aragorn:001002"
                     /note="tRNA-Glu(ctc)"
     gene            complement(17093..17168)
                     /locus_tag="LBFOAKKB_00021"
     tRNA            complement(17093..17168)
                     /locus_tag="LBFOAKKB_00021"
                     /product="tRNA-Lys"
                     /inference="COORDINATES:profile:Aragorn:001002"
                     /note="tRNA-Lys(ttt)"
     gene            complement(17172..17248)
                     /locus_tag="LBFOAKKB_00022"
```

```
     tRNA            complement(17172..17248)
                     /locus_tag="LBFOAKKB_00022"
                     /product="tRNA-Asp"
                     /inference="COORDINATES:profile:Aragorn:001002"
                     /note="tRNA-Asp(gtc)"
ORIGIN
        1 atgggcattt ttgtacccaa gctaaagtag agaatgccca tttttaggga ctttaatttt
       61 aaattaagca ttaaaaagtc caagtttaag catgggcatt tttgtaccca agctaaagta
      121 gagaatgccc atttttaggg actttaattt taaattaagc attaaaaagt ccaagtttaa
      181 gcatgggcat ttttgtaccc aagctaaagt agagaatgac catttcatag tcgtttcaac
      241 gcgatttatt acaatttgtc ttttttttaa atattgcacc caaaaaattt ttgtacctaa
      301 cattcattaa cttcgttaat aatattgcca tttttgtacc taacccaaag catttttagc
      361 ccctgaaata gaaaagtctt cttctcgccc tttatcacct catttatccc cctcttcgaa
      421 ttagacacat ttttcttgaa agtttccctc atttatcacc tcatttatca cctcatttat
      481 cccctcgatt ctcatcacac tttccttagc cccataagca aaatccatct tctcatctcg
      541 atcatatacc tacaagcttc ttacaatagc cctttccctc atttattcac tcatttattc
      601 actcatttat cccctcattc tttacctcat tcttcccctc atttattcct tttctcgcct
      661 ctatttcgat cctaaatgcc tgtttatttt tagccacttt tgatataaac tataccccac
      721 atataaaaac actcatttag tacccaagtt taataaaaat acggtttaac tccacaaaac
      781 caccaaatcc ataggagcat ttatgcctca aacagataca aaaccaaacg caaaaaaccg
      841 aatacacaaa gccatagaaa cttggtttac taaaatctac ttgaacaaaa taattcacaa
      901 cgccaaagac actagcatct ttatcaacaa aagcagttgc ctagcattca tactctcaat
      961 ctacggcaaa accgaagaaa acaaaaacaa aatgacaccc gctgttatca cccacataaa
     1021 cactaccaaa aacaatttcg ctaccaagct aaaaagagtt aaaaaccacg aaaacattgt
     1081 cgagctacaa gccaaatatc caaagcttga tataatatca gcctatcaat tcctaaccct
     1141 caaagataaa ttcaaaatta ccaaatcaga aattcaagat ttcgaaacat taatcgatat
     1201 tctttcaaaa aacgcccaaa aatcaaaaaa gtaaccaaga ccattaaaaa aacccttta
     1261 gccttttaag ggctattttt gtgattttaa acgctatatc cactcaagat attcacataa
     1321 aatcaaaaaa acccgcaaaa attaacccaa aacaaaaacc acagcacatt tcaaaattgc
     1381 aacgcagttt tgaaaattcg ctagaacttc taaaatcagc cctagctctc atattatcgc
     1441 tataaatcaa gcgcaatcaa aaacacacgc catgaagaaa attttgcagt ttttggctct
     1501 aaagtctatt tttgccaaaa tattacatga taacttgata atcaataatg gcaatcacct
     1561 aaaaatagat attttaaaag ccttttacag atcgcaaaat gtcggtaaaa cattgatgat
     1621 tacttgcccg caataataag ctttaaatgc ttatattttg gctatactct tgcattatgc
     1681 ccattggctc aactttttac attttttca aaaaaagcaa aagaacctaa aaataagggt
     1741 tttaacatag ataagccctg aatattgata ttttattttt ttattacact ttttaaagat
     1801 gattttcatc tttaaaaata atattaacac aaagaaatcc cataaaatag cattttagac
     1861 aattttttaa gtgtaataaa cttgcaataa acctgcaata aacttgtaat caacttgtag
     1921 ttaatacgca ataaaattgt atttggagtg ctgataacta cgctttaata tcaacttgag
     1981 ttcaaaaatt atacattttt gtagtaaaag tgtaatcaac ttgaaataaa aatcacacaa
     2041 acataaacta aacttgtagt taagttgtat ttcacgataa aaattttatc gttaagttgt
     2101 atttaatgcg tatcataatt gaaatgaaat tgtatattac ttgaaaacaa cttgtaatgt
     2161 atgtaaataa cattttgtat agttgacttg tagttgactt gtagttaatt tacaataact
     2221 tgtatctatc ttgcaatatt attgaaatta aattgtattt atcttgtact tattttgctt
     2281 ttagtttttt atattgttgt gatacattgt ataaaataat tgcacagcac ctttcatact
     2341 catgctcaaa tgaaacagat tcttctagga ggaaatcttt agtaataggt tgatgtttca
     2401 atccaatatt gtattttta aaataattgc caccaattga caaagaaatc catttaaaac
     2461 cctataaaat aggcatttga tagcaaaaat acttacaaaa atttattaaa atatggtata
     2521 attatgttat ataaataact taattatgaa aggaataata atggataaag cagtaaatat
     2581 gaaatcacca aaaatgaaat taataaataa aatagcaata ggattgggaa tagcagtaat
     2641 gtttgctttg atattattaa gctgtttatt gataaatatt tataatatag gcattaaaca
     2701 tataatttta agttttataa ttagtttagt gtgtttattt ttagtattac ttttaataaa
     2761 cactgcaaat aaaccattca aaaaaactta tcctaaaaga ggcaactgtt acgatacgca
     2821 tttcaaacaa agcggaatat caaaatattc aactctttcg ttgtctaatc ctttttataa
```

2881 caatttatat tccaatgaag actaattgag aaattaatct caattaatct tagtgtatag
2941 cgggatcccc tgtttttaca tttattagat gattagcttt tttaattttt tctccgactt
3001 cattgtttaa attagatttg actgctacac ccttaacttc ttgactatct gggcataggc
3061 gattttatta tggaaaatca agagcaaaag ggattgtttg aattgtataa tttttataat
3121 gaaaataagc gtaataccac ttgttatatc ttcgataagg aagattatca aagcataacc
3181 tcagaaaact attttataaa aaactattta aaaaaaagga aaatatctta cctgctagaa
3241 gatctatttg atttaaattc aagtaacaat ctttatcaaa attgtaaaat ctataaggaa
3301 tgtgtagata ttttaaataa atttacaaat caaaatatag gcattttata taaaaagccc
3361 acttacttta gcaggcgggt aatttacaat atattacaga ggctagctaa tcaagcatag
3421 agcgttacaa tcacccgacc gtgaaagctc aaaaactttg cgatagaata attcaaactc
3481 actcaaatga aggtgacacc attttcattc cttttgtagg aagcataagc gagataattt
3541 cggtcataaa aaataataga aaagcctttg gttgcgaaat atacaattta gcaaaagata
3601 taataaatac cttgttaaaa gtagatagat actattaaaa aattatcaat ttgtaaaaat
3661 ttataataaa taatttactt tgttttctta tctatattgg aatgttctac attggaatgt
3721 tcaagattgt ttgtcttgtc ttcatgggtt ataaaagaaa tgataagcat aaaaataaaa
3781 aataaaacaa tatatgttaa acatcctatt tttttctttt taccgttcat ttttttgatc
3841 attttcctta tcttcttttc ttctttttga agtttttctg aaagtaatga gatcaatctt
3901 tgctctaagt cgttttcaga ttctagtgta aaaattattt tttttgattt aaatataaga
3961 tctgtaattg tttctttcgt tttagttgat aaactttttg aaaaactgac tttttctatc
4021 aattcagata ttgttgaaaa aatatccgat atctcagaga ctttttttgga gttatcttct
4081 gttaatatat ctgtaatttt tatttgacaa tcttgctcta cagcaaattc aaaagtttcc
4141 cgagatacat ttagtttatt gcacatttct tgaaataaat ttctaacttc atttataact
4201 tttgaaacaa attcttctct atcattatct ctttttgaat gtttgttatt attaaggtta
4261 taacctatac cgattctaaa tccatttcct aaatttttac taaaaccaac aaacataaat
4321 tttcctaaca aaattttata taaataacaa aagacttaat aatataaaaa acttttaatt
4381 cgttataccg ccttgagttt tcactcttgg cggaaataac cctaaaggct taactatgaa
4441 agaacatagt aagcgttttg tgagattata tcaaaatttt aagttttttt aaacttaaag
4501 ttgaagtatt tttctaacaa aaccgcagga gcagaaaagt cccttgcctt tattagtgta
4561 tgaaaccaca aattcgcatt tattttttca agttaaataa aaaagttcgt tccaagcccc
4621 ataaaataag tatttaatag caaaaatact tacaaaaatt tattaaaata taatataatt
4681 atgctatata aataagttaa tatgaaagaa ataacaatgg aaaaagcaca tttaatcact
4741 tatttaataa gtcttgcatt tattgtaagc ggtttaatag tttgttgtat tttatactta
4801 ttcaaagata agataaataa aataacacct gataatatag ataatataaa ttcaaatgtt
4861 aaagatttta ttgaaaaaaa tatttgtggt ggttcatcaa caatagatca ctcatttacg
4921 acaaattttg gcgactgctc atctatatca ggcaatacta gctcatcaac aaattatttt
4981 atagatccaa catatagcta tatgccagaa aatatatacc ataataatca agactaagcc
5041 tagctaaaaa gctagactcg attttacata tttctatttt ggctaccaaa atctatgact
5101 ttgctaatgt cagcttttcc atctttaaca tctttttgta cgctagtgta atccaatctt
5161 attatttctt tcctcaatag cagttcctgt tttatattca gcaacaccta ctactttaaa
5221 ctacttttta tatcaaaagc ccagatggct attttctcca aaaaattgtt taatacaata
5281 ttctattaat tttataaaac aacattattt atttttggat tttgtttaat atcgtcttta
5341 cgaagtaaat taggtaatga gctttaaaat aagcttggag ttttaatact atgagcaaaa
5401 actaagtcca gctttttagc tgtactcaat tatctcgtat agggctaaat atataattca
5461 atttaatata aaaattgcaa ttgttttcta tatatcctgt gattatagat ctagaagtaa
5521 atctccttat ttgacttaaa atcgatttgg aaatattaaa caatacttcc aaatctttta
5581 ttgcgataat tattttaagc aaacattatt tgttagtgat gagctaaagg atagacatat
5641 ttttactttt tcatcctctt gatgcttttt atatagtagg aattttaaat ttatgtcatt
5701 taactgatta gaattatcta cactatcagc aggagtgtta gctttgttta tatcacgcct
5761 attccatctg ctgatattta aatcattaag aacaaatata tttttgtaga tttagttttt
5821 tatatgctta tgaaagtaaa atattgaaat ttgctaagtt attcattttt taccttgatc
5881 aagaatgtat ttttattaat tcatttttat agctttaatt ctttagtgaa tattataaaa
5941 ttatatcaaa acaggcttta aaattatgta ttttaaatat aaacatattt tatgtttata
6001 ttaacattaa tgctttataa taacacaaaa ggaaaatact atgttaagaa aaatagaaat
6061 atctggtttg tattcttttg gaatagaaac acaaattgtt gattttacag caaaaccaaa

6121 aagtaagttg aaaaatacaa aatatgagta taattttaat ttatcacaag ctggaaaacc
6181 tatgaaatca gcagtttttt ttggaaagaa tactagcgga aaaactaact tttttatagg
6241 cgtaaaaatt ttactaaata taattaaata tggcttttct catgccctaa gagagcattt
6301 agtagacaat gcttttaata aaaattcaaa ttctataaaa ttaagtattg agttatctaa
6361 taacaataaa gatattttta catattcgat agagtttgat aaaaaataca tcctaaaaga
6421 atcttttttg aaaaataata aaattatata taaatttgag aatgataagg ccatttttaa
6481 tatttacagt atagaagata ataagctatt agaatcattt ttttcaagaa atcttagtga
6541 aaatatattt tacttgttaa aagattttga aatagagcaa aaagaaatat ttataaatct
6601 tataaacaat attgaagttt atatgaatag taactattca aaaaaatata attttgaatt
6661 tgaagaaaat aaaaagaaat attttttaga acacaaagaa agtattttgg aaattttttca
6721 attactggat aaaactataa aagattttga gtttaaaaag tataaaaaag acgaaaagaa
6781 tatatatgaa atcttttttg ttcgcaacgg aaagaaattt aactatcaaa tagaatcaga
6841 aggtgtcaaa aaaatagtat attttgcaga taaaataacc caaattatca aggaaggaaa
6901 aattgttttt attgatgaat tagactcttc aataagcact ttggcattaa tacttatatt
6961 caacaatctt attaatacag aggaaaatca taatggacaa ttaattgttt catctcataa
7021 tgttttattg tttgatgtaa gtttttttaaa ttctcaacaa atatttttaa ttcaaaaaga
7081 tcaacaccta gaaagtaaga taagaactta ctacgattat gatattagaa gcgaaaagaa
7141 aaaagcatat attgattatt taaaagggct ttatgatgaa taaaaaaaca aagcccagta
7201 tacagatttg gtgcgaaggt aaaacagaag aagattattt taattccctt tttaaatatc
7261 taaattatga taatgtcaaa atagatgtaa aaaatttaaa aaataataat tcatataaaa
7321 acattcttta taaaatagaa aaagcacctt atgttgataa gcttattatt gtaatggatc
7381 tagatagagc aaaaagtgat actaacgagt tgaaaatttt agaaaaactt ataaaacata
7441 taaaaaattc aaaaggcaag catttatttc ttactcttgg tgattttgaa gattggctta
7501 gatttcattt tgtagatcat agtagaaata gcaaaacaat tttttataaa aaattaggat
7561 atagcgatag taagaatttt aaatcatcta ctaacgattt atactctcaa atattaaata
7621 aaggtggaca tatagaaaat gctgaaaagt atttttgcaa tatcaaagtt tttttcaatg
7681 agcagtgtga gattgataaa aattcactaa ataatctaac tcaaagtaat ttatattatt
7741 ttagaaaatt attagaacaa tataatggtg cttaaaatat attataaaga aattctactt
7801 cttttttata ttgcaattat atacagaaat tggtttttttc cttagtcaag ataatttaaa
7861 attatgagca ggtttatttt gtcttatttt tacaattttt agcttaattt atataattga
7921 tcttcctcaa agctatcatt caatcctact tcagcaagct ctctaactgt tctttgttaa
7981 tttcttcatc atctttaaac gcttcaaaac tttttatcca taaggctttt ctctatttta
8041 tgtattcttc gtttttaaac atatctatcc ttttgattta ttatttaatt tttacttgtt
8101 ttaatttctc taagatgatt tcaaaagtag tttcgtttat acccataaaa atccttttat
8161 taaataacaa agtataataa aaatagactt tattgcttat aaataatttt tttctgtaaa
8221 catattattt aaactattct taatagaaat ttgtcaattt tataaataac aaatatatca
8281 acaaagcaac catgaatata agtatgttat ttcatcgttt tcattttgcc tgtcaggaat
8341 tttaaaatct tttatacttc caccaactct tcttcttgca agttcatttc ctcctctcca
8401 ggataaaata tactaattgt gtagttatga aacaaactat caatgagtaa ccttatttgg
8461 ttgttatctc atgcaaatgg tctttgaaat tctggtagcc aaattttttc agcaaatgct
8521 tctttgatca agtcttcaat tttttttatct tcatttatta tcatgatttt aatccttgta
8581 aaataaccgt ataagttatt atagaaataa aaataatatt acatgcaaat ccgaaaaatt
8641 atcatttaaa tatgtaaatt taagtatctt tatcattaac ggtttttatt tatgatattt
8701 atgatataat ttatagctaa aacatgaagt aggatgtaga atatggaaca aaacaatgct
8761 tattgtggaa aaaaaattgc aaatgaattt atcagattaa attgtaataa aaataacata
8821 ctcacacata tgcatataca aaaacttgtt tattttgctc atgctatgag tttagctaca
8881 tataataagc ctttggtgaa agaaactttt caagcatggc catatggacc tgttaatgtg
8941 gaaatttatc aagaattaaa agggtcggga aaagacaata ttataagcat gcttaagaca
9001 gaagatgatt ctgctgaatt ggatgaaaat tctaaaaaaa ttattcatgc tgtatatgat
9061 aaatttgggg ggaaaaatgc ttggaagttg tctgaaattt ctcataataa ttctccttgg
9121 aaaaaagtat ttgagaaagg taaatcaaat ccaatttcaa atgaagatat aattaattat
9181 tacaagtcta gtggagaata aagcaggtgg atttttttaga aaaaaatata aataaagagg
9241 aaataagaga aaatcaagaa aatcaagaaa atcaagaaaa tcaagaaaat caagaaagta
9301 aagaaagtaa agaaatcaca tgctctttat attatgcatt ttacaagttg gaggaaaatt

9361 ataatacaat cgaagaaaga ataaagaatg aaagtaataa atttgaagaa agaataaaga
9421 atgaaagtaa taaatttgaa gaaagaataa aagaaactca aaaacaatat attaccattc
9481 taggtatttt tgcttcaatt gtattaacat ttgttagttc ttttgcattt tctagttcaa
9541 tcttatctca tatgcatcaa gttagtattt acagattaat ttttgttatt ctttgcattg
9601 ctctttttgt tgtaaatatt ctttatggac tttatagttt tttaatcaaa attcataatg
9661 gtaatataaa atctaataat aagccaataa caaattttaa tatgataata ttagtactta
9721 tagtaattaa ctatttctgt tatcttgcgc gatactattg aggcattttt agaatttgct
9781 tttaattata ataataaatt ccaaattaaa aactcaaagt caaagatatc gttagccgac
9841 atccatttta agaagaataa tttgaaattg aagcaagatg tttaatattg gctataagat
9901 atactacact agtaatttat tcagcgtatt tatttctttt ttaatcaatg tcttaatttc
9961 atcttttaac atagccacca aaacttttat ttcgaattct tgcatttctt tttcaaccca
10021 ttctataaga tttatcttat ctttttcttt taaaataaaa caatggatgg tatttgtatt
10081 atctttagat agattaaaat catttaagag tttcaaaaaa gaatttttat caatattatt
10141 ttcattaata ttgtattttt ctaaatattc aataaaataa taaaaaacta aaagcattct
10201 ttcacctttt ccagtccaag aatgagctaa ttttctagca aaagaatcac ctttaaattt
10261 ttgcagatgt gatgtaagtt tttctaactc tagtctacgt aaaatgtgat taaataatct
10321 ttttgtataa gttctacaat taagttcttt tgttaatctt acaatatgat gtgcaagata
10381 ttcattatca tcatcatagc acccctctag caatagatca tttggggtaa ctttaaaaat
10441 atcacataaa agcaaaattg tattaagcgg tggctcaaaa tttcctgttt cccaaagact
10501 aatcgttctt tgtgttgtat ttagtatttt tgctagctct aactgagtta aaccacttgc
10561 ttttcttatt ttttttagat ttcttccaaa cattttacaa ctccattttt ttgtaattat
10621 aaaatagtat tgcttaatat atcataaatt atcttttttt tatttaattg aaatttaatg
10681 atttttttatg ttataatata tcataaaaaa ttatgaaaga gcgcgatgct aataaataca
10741 atagtttata cagaaaagtg attttagctc aaatgaggat gggcggttgt taaattctac
10801 tatagcaaga aaaaaacaat tatttcttta tgtttattgc aaaatgattt taaaaaaaac
10861 aatcagtcca aagtattcta tattaataag gaaaacggta catgctcaaa gatcgttttg
10921 ataacaattc gatttattat tacttaaaaa atgaggaaaa ataatgaata tggataatat
10981 aataatttgc aaagctagtg atcttgaaga tatctttaaa aagattttaa aagaaaatga
11041 attaatcaaa aataaaactg atgaaaattt aaatgtacaa caggcttcaa attatttaaa
11101 aataagcact tcgaaactct acaaggattg taatttagga aaaattcctt tttgtaaaat
11161 tggaaaaaaa cttgttttta aaaaaaatga tcttgatgaa tatttaaaat ttaacagttc
11221 taaaaatagt ttaaaatttt aatcacactt aactaaaaac ccgaaaaacc taaaaaacca
11281 ttgtaaatta gtgtttttca gttttaaaat tactaaaaat agcatttaga gtattattaa
11341 tagtttcatt tttaatattt gcatatcttt ttgtaactcc tatgcttgaa tgtcctaatg
11401 tttctccaat agcttctaaa gatataccca tattaatacc tatatatcct attaaatgtc
11461 ttaagtcatg taatctaata ggagttttta acttttgatt tattttatcc caatgccatc
11521 ttatatcttt ataagggttt ccatcttctt tgataaaaat atatccttta ttttttactc
11581 caatgctttt taaagcttct atttgtaaag aacttaacgg aaattgtaag tttttccttg
11641 atttattttg gtttgaaggt aaaaaataaa ttttattagc aaagtcaata ttgtcccatt
11701 ttaatgtaag aacttcactt tttcttcttc catgtaaaag aaatataaac atagctctta
11761 tcattaaatt ttcataattt ataatagctt tataaagagc atttctatcc tcatcatcaa
11821 tagtaaaata tcttttgtta tcaaattttt gtatctctat ttctcttgct atattttttt
11881 ctatataatc atttttaata gcaaattcaa atactggaga aagtatatct tttattgttt
11941 ttgttgttct tggagatttg ccttgatcaa tcattttatt gacaatattt tgcatatctt
12001 ttaatttgac ttgttttatt ttgatgtttc ctataacatc ttttatatgt gcattatatg
12061 aaaatatagt tacctcataa tgtttttttag aaatagtatt ttttcgcata ctaatccatt
12121 cttcaaacac ttcatttaat ttaggttctt taattttaaa tttctttgca aattttccat
12181 taattctttg taaggcaatc aactcacaat tctttttaag ggtctgggtg ttatttaaaa
12241 taggaaatat ctcataactt gatttttttc cttcataata tcttatagct agttcatatg
12301 gtatagttaa tcttctttgt aattctacta agctaatttc ttttatatcc tccaatttct
12361 tatctttgtc tataaaatat ataccccttgt attcttttat ttttactaga tttttcaaga
12421 tattcctttt ttgcttttaa tttacgcata aaattttatt atttttttaca taatataaaa
12481 ttaaaaatat actaaaaatg gtatctcaaa tggattttag tggtagcggt tttggtagcg
12541 gtttttttct caaaatggta taaaataatg ctatttttaa gataaactta aacaataaaa

12601 cacctatttt tcaatacttt gataaaaaat gataacaaaa cgcattagac tcaaaatctg
12661 gtaagggcaa ccttgtgtcg gttcgagtcc gacctcaggc accatttgtt tttttactct
12721 caagtgttat tttttagcaa tttatattaa aatcttaaaa aagagtttta tattttcaaa
12781 aatagcttgc tgattatact atgtgagttt tataatatct tctaaagctt tgaaaaatca
12841 ctaggaactt tgataatcaa tttttcattt ttaagtttaa tttcttaaat aataggtttt
12901 attatcatag taaaaacttt ggtttaaaaa tacttttttt aaaaaatgca tagtctttat
12961 agaaaaaact tcttgataaa ttttatttaa atttatatct actaaaattc agttgcattc
13021 aaatgtatta cccaaattct ttaaattttc tctcttgtat tgctttttta aaaccttgct
13081 atttttttcaa atcaattgga gatgtatatt ttcattaaca aatcacacct gctaaatcta
13141 tcatttactt atttcaatct cacattcata atcatattag ataaaaagat accatcttta
13201 tcaaagcctt ttgagtcata ttcatttttt gattgtatag tttgcatagt ttgcattaaa
13261 gttgaaaggt tatacaaact taaactttct aatattttta tgagttctat cttgcttaaa
13321 gtattaaaac catttaataa gctaagataa ttacttacaa tagaagaaga gtgaatacta
13381 tgaaataaaa ttttattttt tatatctttt ttaataaaaa attgattatc tttaaaataa
13441 aaataatttc taagatgatt attacttatt atttgattat aaaaaactta aaagactttc
13501 tttgctgttt aagtctataa aattaagata aaaattcttt tcttctaaag ctaattttaa
13561 agcattaaga ttagtaaaag aagtattaag attttcatca ttcctcaggc gccattgctt
13621 ctcaaactac aacttacaag gcaaaataaa ataaaaattt ttaggctgaa tttcaaattt
13681 taactcccct ttgtgagcgt taatgatttc tttagaaagc gataagccca gcccatttcc
13741 tttgagttta gtagttctaa aagcttcaaa gaccaaattt tcatctttta tttcacaacc
13801 gttatcataa actctaatgt ataaattttg ctcatcgcag cttgcttgaa ttttgatttt
13861 aggattttca cactcacttt cttcgattgc atctatggca ttataaatta aattttgcaa
13921 aaccaaagat aaaagcgctt tatctccata aattttcata tctaaaaatt tgatttcaaa
13981 atcaatttga gatgagtaat tgtaagaatt tatagcactt tcacattctt ctgttagctc
14041 caaaagatta aactcacttg cattgatatg cacaccctta gtaaaaagta aggtggaatt
14101 tacgatacgc tctaccctag caatagcctt ttgaatttca agtacaatat gtttgttttt
14161 aagctcacta cgagaaaaaa gcgtagaggc gagcaaggat atggagccta taggatttct
14221 tatctcatgc gccaaatgtg ccgcaacact tcccatgcta gcaagtcttt cattacgctt
14281 ttcatcgcta atatccgtag ccgaaactat agttttttca ttatctgaga tgattttaac
14341 cgtgtagaat ctattctcaa attcaagttc atcacgattt ttttcaagac taataatatc
14401 aagcaatcta ggctttttta aagcttcttg attttgcaaa ataatatttt tttccttatc
14461 taaaacccaa agtgcggtcg gtaaaacctc gatgatccca tccaacatag cgcgcaaatt
14521 tttatattcg ttttctacga tgtaagtttg ctctataaga cttagcaagc ctttttgaaa
14581 ggtttccttt tcgcttgaat ctaggctttt taaaatatct tcattcatct atcttcctct
14641 aataaaattt taaaatcatt taaatccatc aaaagtaaat tctgtttgca aattttataa
14701 aattctttac taaaaccatt ttttgaaatg ataatataat tatttggttc taactttaaa
14761 gattttgctt ttgattctaa aagatttaaa atatttctac aaattttttt atttttaaat
14821 ttgacttcac cgataaaact tatctcatca tccttataat aaagatcaag ttctaaattc
14881 ttatcccaat aactctgcac tccactcacc tgaaatttct tttcaagcaa ttctctagaa
14941 agttgctcaa aacaaaaact ctgataaagc tcaaatttct ctttgatcag tcctaaaacc
15001 tcttcatatt gattttgcaa aatcaacttt tcattaggtt ttaaaaagta aaaaaagaaa
15061 cgagtaaaat ggttagcaaa aatgatttta tcttgaatca cataagatct taattctttt
15121 ttaagttttt gtcgtttgtc ttttatcctt tttgcttctt tgctatactc aagccttata
15181 atacccgctc ttaaaagata ttttaaagtt gataaagcct taaaatgctg aatttttcta
15241 ttgatagaaa agcgtttgcg attattttta gctagcaagc taagagcata agaagcggtc
15301 tcatcaagct caaaatgttc cttgatatgt aaatagtttt taagtaaaag acattctata
15361 ttttcaaata tatctttttc aaaatttaag ctgtctaaac tctcaaaacc ctcaaaaaca
15421 ctataaaaat caaaaatttt agtcaaagct gaagtattgc aaatttttgc aaaatgatta
15481 aaatttatat ttttcctttt taaaagtatt tttatagtat aattttagca aatttttaaa
15541 ggattatgct tgcaaatttc agatttaaaa aaagaaataa ttttaaaaaa aggcaccttg
15601 cattttgatt ttactgccag cgccttggca ttaaaatgcg tggaaaaaga aattgcaaaa
15661 atactgccaa gttatgctaa tactcactct gacagttctt taaactcttt taaaactcaa
15721 caaatttacg aacaagcacg caaagatatc aaaacaagtc tcgaactaaa cgatgatttt
15781 gcgcttattg cttgcggttc aggctctagc agtgctatta aaaaatttca agaattactt

```
    15841 ggaatttaca tcccgccact tatcaaagaa aagtattttg ataaagtgga taaaagcacc
    15901 ctacccttag ttatcgtagg accttatgaa catcattcta acgagctttc tttccgtgaa
    15961 ggtttatgcg aatgtataag aattccccta gacaaaaaag ggggtataga ctttgaaatt
    16021 ttagaaaaag ttttaaaaga aaatcaacac agaaaaatta tcgccagttt ttccctagct
    16081 tcaaatgtta cgggtatatt aagcgattat aaacgcattt ctaaattagt gcgtaaattt
    16141 aaaggtatag tggcttttga cgcatcaagt tttatccctt ataaaaacat agcttgcaag
    16201 tattatgatg ctttatttat aagctctcat aaattaatcg gtggcatagg aggttctggc
    16261 cttttggcaa ttaaaaaaga actttgtgga agcaaaccca gttttgctgc aggagggact
    16321 gtgggctatg tttcgcgtac ttctcaatgc tatctttgca atgaagaagc tttagaagaa
    16381 gggggaactc ctggaatttt acaattaatt cgtgcgagtc tagccttcaa aataaaagat
    16441 aaaataggta tagaagaaat agaaaaaaaa gaagaaacct taaaagaata cttctttgaa
    16501 aaactaaaaa ccatacctca tttaatccta tatgctaata atataaaagc tcgcttgcct
    16561 atctttgctt ttaatatcga aaatatctct cctttcgact tagcctatga acttagcaaa
    16621 aaataccaca tagaaacacg cgcaggatgt gcttgtgctg gtccttatgg gcatgattta
    16681 ttgggcttaa aagacaatga agaattaaaa accaaaccag gttggctaag aatcagttta
    16741 cattacaccc atgaaaaaga agatattgat tatttctttg aagcactgca aaaaagtatc
    16801 acgaaattga aattttaaat atggcgcagc ggacggggct cgaacccgcg acctccgccg
    16861 tgacagggcg gcattctaac cagctgaact accgctgcac ctaagaagta ataatttaat
    16921 ggtggtcact ataagactcg aacttatgac atccaccttg taagggtggc gctctaccaa
    16981 ctgagctaag cgaccttaat ttttggcgac ccctagagga tttgaacctc tgtttccaca
    17041 cagagagagt gttgtcctgg gccactagac gaaagggtct aaacccgata agtggtgtcc
    17101 cgtgttggat tcgaaccaac ggccccctcc ttaaaaggga gatgctctac cggctgagct
    17161 aacgagacag atggcgcagc ggacggggct cgaacccgcg acctccgccg tgacagggcg
    17221 gcattctaac cagctgaact accgctgcac ctaagaagta ataatttaat ggtggtcact
    17281 ataagactcg aacttat
//
```

>>BfR-CA-15860_min_cov_30 putative plasmid location

```
LOCUS       PIMPEBOA_1             17665 bp    DNA     linear       12-APR-2021
DEFINITION  Campylobacter species strain strain.
ACCESSION
VERSION
KEYWORDS    .
SOURCE      Campylobacter species
  ORGANISM  Campylobacter species
            Unclassified.
COMMENT     Annotated using prokka 1.14.6 from
            https://github.com/tseemann/prokka.
FEATURES             Location/Qualifiers
     source          1..17665
                     /organism="Campylobacter species"
                     /mol_type="genomic DNA"
                     /strain="strain"
     gene            complement(370..1278)
                     /locus_tag="PIMPEBOA_00001"
     CDS             complement(370..1278)
                     /locus_tag="PIMPEBOA_00001"
                     /inference="ab initio prediction:Prodigal:002006"
                     /inference="similar to AA sequence:RefSeq:AJK71146.1"
                     /codon_start=1
                     /transl_table=11
                     /product="type VI secretion protein"
```

```
                     /protein_id="Prokka:PIMPEBOA_00001"
                     /translation="MNDLASYSFFKLIKKLEKDYGRKNIFLRTNKSLKHPNKDIEKII
                     FSEHEQSVVELFINFMGLHGVSSQLPSFMLDKLSRNEDGEQGWTLFFDFFNHYLLWIF
                     FDVVSLKNYPRSFNENFKDSISQILFNILGIKEHDIAKKYLPFAPLLLSLRRPKTHIE
                     RVLQVNFKLKDKLSIIENLPHQILISDSQKNKLGIKNHTLGNNFILGDKFISYQNKIA
                     IYIKDISYQEAVKFMPNGTKHDDLKNSIMFLTNNEFCVDLYLKINYSSEMKFVLGDEN
                     TAKLGWAKILGNAQKKYTIVYMKLCE"
     gene            complement(1275..2996)
                     /locus_tag="PIMPEBOA_00002"
     CDS             complement(1275..2996)
                     /locus_tag="PIMPEBOA_00002"
                     /inference="ab initio prediction:Prodigal:002006"
                     /inference="similar to AA sequence:RefSeq:AJK71145.1"
                     /codon_start=1
                     /transl_table=11
                     /product="type VI secretion protein"
                     /protein_id="Prokka:PIMPEBOA_00002"
                     /translation="MKDNIFYYQKELEYLYEKREHFIKNYPKLTPFLAHDSKDPDIER
                     IIENLAILSSKIHQELDENIPHIAESLINIISPNYTNPLPSLCMQEFKFEQNSKENNL
                     IIPKGTLIKSKPIDKCVCEFKTVYDVYLYPISINEVFISSKNQDYTFNLTLQVNKAET
                     KICDLGLEKINLYLGNDTYMSSTLLLYMHSYLKELKIQSLDTDEEFFLNTYNIEKIGL
                     NPDESSLSYNDLGFEAFSLLREYFFMPHKFNFLRINGLDILNNCQGKTINIEFKFSKP
                     FPANCIFRKELLSLGMTPIINIFAKSAEPLINNHKKDSYRIFVDRSQPKAYEIIQTLQ
                     VKAHNSEGGKRLLKNYKSFERFEFLKDNQKDFYSVNTKKNSKGEVFSEISFFSSCLID
                     ETISIDLLCSNGDLPSKLKIGDINTCNLKGVDTKNVEIPSETRRCSVDGNLLWKLVSV
                     LSFSYQTILSKKAFFGVLESYSFLDNQSNWKIYKLLQEAIIDIQSKSTYLIDENITKK
                     GTVAIFSIKDSKFYTLGEVYLLGLIISKFLASFASINSFCELKIKCLDSKEILHYPAS
                     FGKKALI"
     gene            complement(2993..3385)
                     /locus_tag="PIMPEBOA_00003"
     CDS             complement(2993..3385)
                     /locus_tag="PIMPEBOA_00003"
                     /inference="ab initio prediction:Prodigal:002006"
                     /inference="similar to AA sequence:RefSeq:CKG87312.1"
                     /codon_start=1
                     /transl_table=11
                     /product="type VI secretion system lysozyme-like protein"
                     /protein_id="Prokka:PIMPEBOA_00003"
                     /translation="MSLLDKLIHNLDEQNIHIPFYQNDFEDVKNNIKVLLNAKINDCY
                     AVRNLGMPNMADINLNSNELCVSMAKEIRKLIDNYEKRICVVSITYDNSLSPWQLSFI
                     VKCFFQDDRFKEFNIEIIFKNNRYCEVK"
     gene            complement(3388..4842)
                     /locus_tag="PIMPEBOA_00004"
     CDS             complement(3388..4842)
                     /locus_tag="PIMPEBOA_00004"
                     /inference="ab initio prediction:Prodigal:002006"
                     /inference="similar to AA sequence:RefSeq:AJK71143.1"
                     /codon_start=1
                     /transl_table=11
                     /product="type VI secretion protein"
                     /protein_id="Prokka:PIMPEBOA_00004"
                     /translation="MANTKVIDTPIIEQIMEKSKYSKTDESYSIAKRGVAEFISEIVK
                     SDNAEEKINKFALDEMIAHIDHLLSKQMDEVLHNEEFQKLESTWRGLRFLVERTDFNE
                     NIKIDLFDITKEEALEDFENNPDITQSIVYKNIYSSEYGQFGGEPVGAIIGDYQLGSA
```

SPDMTFLNKMASIAAMSHSPFLTSFGPKFFGLDDYSELANIQDLQGLLEGPQYTRWRT
FRENEDSKYTGLLVTRFLARSPYDPEENPIKSFNYKENVHASHNHLLWANSSYTFCTR
LTESFAKYRWCGNIIGPKSGGTVKDLPTYLYENFGTIQSKIPTEVLITDRREYELAEA
GFITLTLRRDSNNAAFFSANSPLKPKIFQNTPEGKEAETNYRLGTQLPYIFLISRLAH
YLKVLQREEIGSWKERSDIENGLNEWIRQYISDQENPPSEVRSRRPFRAAQVKVDDIP
GEPGWYKIGLSVRPHFKYMGGNFELSLVGKLDKE"

```
gene            complement(4844..5329)
                /locus_tag="PIMPEBOA_00005"
CDS             complement(4844..5329)
                /locus_tag="PIMPEBOA_00005"
                /inference="ab initio prediction:Prodigal:002006"
                /inference="similar to AA sequence:RefSeq:AJK71142.1"
                /codon_start=1
                /transl_table=11
                /product="type VI secretion protein"
                /protein_id="Prokka:PIMPEBOA_00005"
                /translation="MSDGSHAPKERINITYKAKTNGQNEDVELPLKLMVMANLKGKNE
                TPLEEREILQINKINFDQVMRKLDITTSFSVKNTLGTGAEELDVKLNIASMKDFSPDS
                LAKQIPELNKLLQLREALMALKGPMGNIPDFRKAVLEALKNEKTKEKLLLEIKQEEQG
                N"
gene            complement(5399..6646)
                /locus_tag="PIMPEBOA_00006"
CDS             complement(5399..6646)
                /locus_tag="PIMPEBOA_00006"
                /inference="ab initio prediction:Prodigal:002006"
                /inference="similar to AA sequence:RefSeq:AJK71141.1"
                /codon_start=1
                /transl_table=11
                /product="nucleobase:cation symporter"
                /protein_id="Prokka:PIMPEBOA_00006"
                /translation="MSFCNQLKNNLDELQEFHLLEDEMSKYKTLNHENISWDKVYQYS
                QFILLHHSLDFKVCNYFLLSCFNLNNEECFEKLLLLFQHLKKLMDENNTYISAQKRKI
                QIFIQNFIQEYNKTKVLVSSEIIDQFIALFSEFENLLSCNFAKIEIAQVAPQLPKSPV
                AQVSVNIRSDNINSLNEREYKNFYQKLAFELLEEDENNLNIYALFTQAMWGKIKCLPE
                CNNEKITKIRKPDADIIRILLSDKENDIEHIKCFMHELILNPFWIEGVQLFCDFLEKK
                KKNKQLDILIMLTSDFISKFDAIELLRFQNGDFICKEEVYKYFVKPKENKKSFFSSKK
                TDKEHTLQDFEQMLMNIDKENFNNSIMNNINSLLDMVKIFESKGMKKNSKILNIYLVE
                LMEKTLLKDYLAEEYENAKNKIK"
gene            6769..7215
                /locus_tag="PIMPEBOA_00007"
CDS             6769..7215
                /locus_tag="PIMPEBOA_00007"
                /inference="ab initio prediction:Prodigal:002006"
                /inference="similar to AA sequence:RefSeq:AJK71140.1"
                /codon_start=1
                /transl_table=11
                /product="type VI secretion protein"
                /protein_id="Prokka:PIMPEBOA_00007"
                /translation="MFRKKSLFFLLPLFFCACSSVVSVKINNIENSNLNNRYDDVPVT
                AIVYQLKDIKKFEEASDIDLATREDGVLGKDKLDSIKTQIAPKDNIIAVKVDEGEVPY
                VGVLVLFANNTKKVTKIWAKTEDANGFGKNKYLKFEISKEGIKRIK"
gene            7225..8622
                /locus_tag="PIMPEBOA_00008"
CDS             7225..8622
```

```
                     /locus_tag="PIMPEBOA_00008"
                     /inference="ab initio prediction:Prodigal:002006"
                     /inference="similar to AA sequence:RefSeq:AJK71139.1"
                     /codon_start=1
                     /transl_table=11
                     /product="type VI secretion protein"
                     /protein_id="Prokka:PIMPEBOA_00008"
                     /translation="MANKLKVAWFDGLNIGQTHFEQQERFFNRNIDLKTINIYSNLYG
                     IIDLEFSQEMLLQGKIALSKISGIAQDGSIFNAPEQDLLPEPIEINYESLIDSVVVLK
                     IPIGVTDIADLSLRNTFPNSKYICLRNSIALRNYDDSKSNAMDKIEDEYELENLTFTQ
                     EKKDLLLASLRLKLGILGNSTPDELELPIAKIKNIDINKKIELESDFIPTCLNISKIS
                     TIRSFLEEIIFSINQHKKVLSNVFKGIDQTKNTLDFSTFLSLNLLKKWYLIFSHLSKK
                     DKIHPEFLYEKFLEFQGELAAFSNEESFLDFIPYKHDNLYNTFLNMMNNLRLLFSKIT
                     SPKYFIAKTSINGNGFYDFIFDNSGILEDAEIYFAIRADVTTEYLLNNFKIQSKIHTQ
                     SKIKNIVATQLEGLNVDQVPNIPSSIPYLNGYVYYKIDKRDHLFKDFKNESIISMYLT
                     NNIKNPDIIMWAVFQ"
     gene            8619..9392
                     /locus_tag="PIMPEBOA_00009"
     CDS             8619..9392
                     /locus_tag="PIMPEBOA_00009"
                     /inference="ab initio prediction:Prodigal:002006"
                     /inference="similar to AA sequence:RefSeq:AJK71138.1"
                     /codon_start=1
                     /transl_table=11
                     /product="glutamyl-tRNA amidotransferase"
                     /protein_id="Prokka:PIMPEBOA_00009"
                     /translation="MKEINEEKKALSLLLDSNFDGLKNNKIIDYSLELLLLSYRLSKI
                     SSMDTSNINQLRETLINKILDITAKLSMCKEYDEKEIIKFKYCLCVFIDESLMKNELF
                     INFWAHNTLTVRLFDETLGGNNFYDIASSWINNPFKFKDFLEFIYACLILGYKGKYNE
                     AKDRDEKIIHFCNNIATSLRPVYKTEEELAFNKAYKIGLEENIWQKFIRLYFKKLIII
                     VPVLIVLGVLSFAIFNLETNNLKVDNNISVLIKNLTHIE"
     gene            9615..10175
                     /locus_tag="PIMPEBOA_00010"
     CDS             9615..10175
                     /locus_tag="PIMPEBOA_00010"
                     /inference="ab initio prediction:Prodigal:002006"
                     /codon_start=1
                     /transl_table=11
                     /product="hypothetical protein"
                     /protein_id="Prokka:PIMPEBOA_00010"
                     /translation="MTYILLLIIISTILSYLILKFIYKIIFKSTKSVSKFLVFLGSIG
                     LIIFYYTPYSYYLEPSYWQFRNMCKLNELPNNEEKYNKILGYFDKKLGDIDDFRHVKK
                     YGSIEMWLYIYYDDTYKTPSNFQEKIEMDNIALKTKNMQMSLHKIVDMHISPYWNTRR
                     YVLEGNEGNMNFEFIEDELACGNLYQ"
     gene            10194..10520
                     /locus_tag="PIMPEBOA_00011"
     CDS             10194..10520
                     /locus_tag="PIMPEBOA_00011"
                     /inference="ab initio prediction:Prodigal:002006"
                     /inference="similar to AA sequence:RefSeq:CKG86955.1"
                     /codon_start=1
                     /transl_table=11
                     /product="methyltransferase small"
                     /protein_id="Prokka:PIMPEBOA_00011"
```

```
                     /translation="MTNKQLINKLRDMAELAQASYGYFDLVTHDKNDFFIKIKDEATE
                     SAFFNKHITQANVLDISYKNYKAYQYDSSLKKDIKIGTLKGDFSPLQAKRFFSRYDLL
                     KHCPKK"
     gene            10719..10820
                     /locus_tag="PIMPEBOA_00012"
     CDS             10719..10820
                     /locus_tag="PIMPEBOA_00012"
                     /inference="ab initio prediction:Prodigal:002006"
                     /codon_start=1
                     /transl_table=11
                     /product="hypothetical protein"
                     /protein_id="Prokka:PIMPEBOA_00012"
                     /translation="MGILKKVYIVFFGGGYFGELFFCLVLYMLILVI"
     gene            11244..11756
                     /locus_tag="PIMPEBOA_00013"
     CDS             11244..11756
                     /locus_tag="PIMPEBOA_00013"
                     /inference="ab initio prediction:Prodigal:002006"
                     /codon_start=1
                     /transl_table=11
                     /product="hypothetical protein"
                     /protein_id="Prokka:PIMPEBOA_00013"
                     /translation="MKILKSLYIIWMIFWGFGAILGYYMGYDATFTKTFFFLVILYLF
                     PLLPYLHYLIFEKTKNKNIFISICYYLLLFVITAYYFTLIYSCGTNFLTFFIMITLYM
                     IICSAIILWKFKINLRLYFYIILCIFTYLYIVLHIFIACYEIEWTDILLDKDVRIYIY
                     KEIINLLTEQ"
     gene            11799..11948
                     /locus_tag="PIMPEBOA_00014"
     CDS             11799..11948
                     /locus_tag="PIMPEBOA_00014"
                     /inference="ab initio prediction:Prodigal:002006"
                     /inference="similar to AA sequence:RefSeq:AAW35443.1"
                     /note="conserved domain protein"
                     /codon_start=1
                     /transl_table=11
                     /product="hypothetical protein"
                     /protein_id="Prokka:PIMPEBOA_00014"
                     /translation="MKTQINNLRDYTELAWASYFYFDLKGYILQENETIVILNELVSL
                     SYNGN"
     gene            12158..12628
                     /locus_tag="PIMPEBOA_00015"
     CDS             12158..12628
                     /locus_tag="PIMPEBOA_00015"
                     /inference="ab initio prediction:Prodigal:002006"
                     /codon_start=1
                     /transl_table=11
                     /product="hypothetical protein"
                     /protein_id="Prokka:PIMPEBOA_00015"
                     /translation="MQIPIYSKLKAKLESKGIDNISSGQIAWFVCSLILALVTYIILT
                     FHYIYISYGKYDKNLFLLIFDRNAMELFIIIYSCIMFNLLYIYNAIPLVLSAVFIFLA
                     FYFSSLSLFIVGCCGIGVFFLISIFKRKLIPSVLLFTMGYICSYLLLHAIGTYH"
     gene            12639..12791
                     /locus_tag="PIMPEBOA_00016"
     CDS             12639..12791
```

```
                     /locus_tag="PIMPEBOA_00016"
                     /inference="ab initio prediction:Prodigal:002006"
                     /inference="similar to AA sequence:RefSeq:CKG86410.1"
                     /codon_start=1
                     /transl_table=11
                     /product="hypothetical protein"
                     /protein_id="Prokka:PIMPEBOA_00016"
                     /translation="MKYCVTIYIDPLVECFLDFKNQCYWQREDFTHAFLGLTKDKSPD
                     ELDKYR"
     gene            12894..13319
                     /locus_tag="PIMPEBOA_00017"
     CDS             12894..13319
                     /locus_tag="PIMPEBOA_00017"
                     /inference="ab initio prediction:Prodigal:002006"
                     /inference="similar to AA sequence:RefSeq:CKG86435.1"
                     /codon_start=1
                     /transl_table=11
                     /product="hypothetical protein"
                     /protein_id="Prokka:PIMPEBOA_00017"
                     /translation="MQIPIYSKLKAKLESKGIDNITSGQIAWFVCSVGIFIIPMMYLY
                     ISNNYNQFIILFFMIFIFILNMLYTKNFFALLLLVILIFIYMKYHEFIYNFKILFFIF
                     APIILIFLRAWRILITFLCGYIFLVAMVYIFELLGGFYK"
     gene            13316..13528
                     /locus_tag="PIMPEBOA_00018"
     CDS             13316..13528
                     /locus_tag="PIMPEBOA_00018"
                     /inference="ab initio prediction:Prodigal:002006"
                     /inference="similar to AA sequence:RefSeq:CKG86410.1"
                     /codon_start=1
                     /transl_table=11
                     /product="hypothetical protein"
                     /protein_id="Prokka:PIMPEBOA_00018"
                     /translation="MKYYATIYIDPLVECFLDFKNQCYWQIEDFTHAFLGLTKGKSLD
                     ELDKIDKELKQEYLKNKEFKLIKSIK"
     gene            13570..14028
                     /locus_tag="PIMPEBOA_00019"
     CDS             13570..14028
                     /locus_tag="PIMPEBOA_00019"
                     /inference="ab initio prediction:Prodigal:002006"
                     /inference="similar to AA sequence:RefSeq:CKG86435.1"
                     /codon_start=1
                     /transl_table=11
                     /product="hypothetical protein"
                     /protein_id="Prokka:PIMPEBOA_00019"
                     /translation="MQIPIYSKLKAKLESKGIDNISSGQIAWFVCSVGIVYLLSIFLY
                     LHGVNKEGISFNRIFIAFYINHFFCFMLMWFILLLNLLYKENTISLILTLLTTIAYFY
                     NPYFAYILIFIICIIMRIINKQRWLYIYFIGLIYIYATYSIFNLRGGFYK"
     gene            14025..14126
                     /locus_tag="PIMPEBOA_00020"
     CDS             14025..14126
                     /locus_tag="PIMPEBOA_00020"
                     /inference="ab initio prediction:Prodigal:002006"
                     /codon_start=1
                     /transl_table=11
```

```
                     /product="hypothetical protein"
                     /protein_id="Prokka:PIMPEBOA_00020"
                     /translation="MKYYATIYIDPLFDFKDATHAFLGLPKAKVLMS"
     gene            14208..14687
                     /locus_tag="PIMPEBOA_00021"
     CDS             14208..14687
                     /locus_tag="PIMPEBOA_00021"
                     /inference="ab initio prediction:Prodigal:002006"
                     /codon_start=1
                     /transl_table=11
                     /product="hypothetical protein"
                     /protein_id="Prokka:PIMPEBOA_00021"
                     /translation="MQIPIYSKLKAKLESKGIDNITSGQIAWCLGSGYIALICAVFVV
                     VYKLKNLEFELYIVFFFPILLFYIAIFWFIFLSFYKNFFFIHLFCYSFMYIFNIIFND
                     FYLNLVVMNILLITIWLFFVKLHLKFRFLFLMMYFCFEFAIFIGIIPYGLLEGTISK"
     gene            14684..14899
                     /locus_tag="PIMPEBOA_00022"
     CDS             14684..14899
                     /locus_tag="PIMPEBOA_00022"
                     /inference="ab initio prediction:Prodigal:002006"
                     /inference="similar to AA sequence:RefSeq:CKG86410.1"
                     /codon_start=1
                     /transl_table=11
                     /product="hypothetical protein"
                     /protein_id="Prokka:PIMPEBOA_00022"
                     /translation="MKEIIKYYVTIYIDELLENEMLKHVDFSLLFKAKFSQISKQIDF
                     THAFLGLTKDKSSDELDKIDEELRQKF"
     gene            14946..15221
                     /locus_tag="PIMPEBOA_00023"
     CDS             14946..15221
                     /locus_tag="PIMPEBOA_00023"
                     /inference="ab initio prediction:Prodigal:002006"
                     /codon_start=1
                     /transl_table=11
                     /product="hypothetical protein"
                     /protein_id="Prokka:PIMPEBOA_00023"
                     /translation="MQIPIYSKLKTKLESKGIDNISSGQIAWFVCSVIFTYLLFLLIV
                     IINTRTISFENLKFFTFEGFFKSLAFIFANHFFLFIFMWIVCIINFF"
     gene            15468..15647
                     /locus_tag="PIMPEBOA_00024"
     CDS             15468..15647
                     /locus_tag="PIMPEBOA_00024"
                     /inference="ab initio prediction:Prodigal:002006"
                     /inference="similar to AA sequence:RefSeq:CKG86410.1"
                     /codon_start=1
                     /transl_table=11
                     /product="hypothetical protein"
                     /protein_id="Prokka:PIMPEBOA_00024"
                     /translation="MVECFLDFKNQCYWQREDFTHAFLGLTKGKSPDELDKIDEELRQ
                     EYLKNKEFKLIKSIK"
     gene            15689..16147
                     /locus_tag="PIMPEBOA_00025"
     CDS             15689..16147
                     /locus_tag="PIMPEBOA_00025"
```

```
                     /inference="ab initio prediction:Prodigal:002006"
                     /codon_start=1
                     /transl_table=11
                     /product="hypothetical protein"
                     /protein_id="Prokka:PIMPEBOA_00025"
                     /translation="MQIPIYSKLKAKLESKGIDNISSGQIAWFVCSVVLALVTFLIIY
                     FTVIIEDKNLFLAIFELFQWQLFIFIIYFCIPFNLLYIHNAIPLVLSAIFMFLTLYFS
                     SLNLFIIGCCGIGAFFLISIFKRKLMFSVLLFAMGYICIYFFLYAITRYH"
     gene            16203..17429
                     /locus_tag="PIMPEBOA_00026"
     CDS             16203..17429
                     /locus_tag="PIMPEBOA_00026"
                     /inference="ab initio prediction:Prodigal:002006"
                     /inference="similar to AA sequence:RefSeq:CKG86410.1"
                     /codon_start=1
                     /transl_table=11
                     /product="hypothetical protein"
                     /protein_id="Prokka:PIMPEBOA_00026"
                     /translation="MIKDLLNMKGLDFTHAFLGLTKDKSPDELDKIDKELRQEYLKNK
                     EWDKIDQKWYEAKEPNEFNDGFWGFGTGIANVAESLIGSPGKVFNNNQYVLDNNVDKN
                     RYIIKKLRSPQTFKPSNRCTLEISKEQYETLLANIKADFEATKEITPNSEKPINEEFT
                     YKLLENNCVTWVVKKLSSIGIEVVNPVVKDPSGLLMATKLIQSIHSIFLKFQNIDDNL
                     QSVKGAKAFKNWARMLINNSLMQEIYLDEYKKQYNSENLSPEQEEVLYALWNITRVYK
                     ILGILLSRLITKSGTKNLKGTFELIYFDKNDKQAKLLKASNHYEAIIIQELDLSKKYN
                     NFSASKFYPFIFIPKDEMLSRTLYHKYDYGNISQEYQKDRNEFYFNVLAGEKSDKYWS
                     SSYHKMTKNLRKVYVS"
ORIGIN
        1 gttgtagggg tgcttagatg agatgaagca ttggttaaag tatttaaaga aaataaatct
       61 tgttctaaac tttcaaaaaa gccttcacat tctatagaaa aaagctcttc aagactttct
      121 ttgatgaggg ctttagtgat tttaaagggt aggggatttg aagtatgatt taatatatca
      181 agactcatat aagaattaag ggacattgat gatccttttg ggtttgttga tgattaatag
      241 ccataagctt atgtgattta ttttagcata aggtgggtaa aataaagttt attttagggt
      301 gtgggtttat gggtaaagag agattgtggt tttttgaaat aaaattttta aatatatttt
      361 tgtgttaggc tattcgcata gtttcatata tactattgta tatttttttt gtgcattgcc
      421 taaaattttt gcccaaccaa gtttggcagt attttcatct cctagcacaa acttcatttc
      481 tgaagaatag ttgattttaa gatataaatc aacacaaaat tcattatttg ttaaaaacat
      541 tatactattt ttcaaatcat catgtttggt tccattgggc ataaatttaa cggcttcttg
      601 atatgaaata tcttttatat aaattgctat tttgttttga taagagataa atttatcgcc
      661 caatataaag ttatttccta gggtatgatt ttttatccct agtttatttt tttgagaatc
      721 tgaaattaaa atttgatggg gtaaattttc aataattgat aatttatcct taagtttaaa
      781 attgacttgt aaaactcttt caatgtgtgt tttaggtcgt cttaaactta gtagtaaagg
      841 tgcaaatggt aaatattttt ttgcgatatc atgttcttta attcctaata tattaaacaa
      901 aatttgcgaa atcgaatctt tgaaattttc attaaaagat cgagggtaat tttttaaact
      961 gactacatca aaaaatatcc aaagcaaata atgattaaaa aaatcaaaaa ataaagtcca
     1021 accttgctct ccatcttcat ttcttgataa tttatctagc ataaatgaag gaagttgcga
     1081 agagactcca tgtagcccca tgaaatttat aaacaattct acaactgatt gttcatgttc
     1141 tgaaaaaaatg atttttttcta tatctttatt tggatgtttt aagcttttat ttgttcttaa
     1201 aaatatattc tttcttccat agtctttttc tagttttttt attaatttaa aaaaactata
     1261 tgaagcaaga tcattcatat taaagctttt tttccaaagg aggcaggata atgtaaaatt
     1321 tcttttgagt caaggcattt tatctttaat tcacaaaagg agttgatact agcaaaagat
     1381 gctaaaaatt tagatataat caatcctaat aaatatacct cgcctaaagt ataaaatttc
     1441 gaatctttaa tactaaaaat agctacggtt ccttttttttg tgatattttc atctattaaa
     1501 taagtacttt tgctttgaat atcaattata gcttcttgta aaagcttata aatcttccaa
     1561 tttgattggt tatctaaaaa gctatagctt tccaacacac caaaaaaagc ttttttgctc
```

1621 aatatggttt gatatgaaaa agataataca gaaacaagct tccataataa gttaccatca
1681 acactacatc ttcttgtttc gctcggaatt tccacatttt tggtatcgac accttttagg
1741 ttgcaggtat tgatatcacc tatttttaat tttgaaggca aatctccatt agagcataaa
1801 agatcaatac ttatagtttc atctattaaa caagaggaaa aaaaggaaat ttcgctaaat
1861 acctctccct tagaattctt tttggtattt acactataaa aatctttttg attatctttt
1921 aaaaattcaa agcgttcaaa acttttatag tttttaata gccttttacc gccttcacta
1981 ttatgtgctt taacttgtag tgtctgtatg atttcataag ctttaggttg acttctatcc
2041 acaaaaattc tataactgtc ttttttatga ttatttataa gaggttctgc actttttgca
2101 aagatattga taataggcgt catacctaaa gaaagtaatt cttttctaaa aatacaattt
2161 gccggaaatg gtttggagaa tttaaattca atattaattg tctttccttg gcaattgttt
2221 aaaatatcta aaccattgat tcttaaaaaa ttaaacttat gtggcataaa aaaatattct
2281 cttaacaaag aaaaagcttc aaaacctaaa tcattataag ataagctact ttcatctgga
2341 tttaatccta ttttttcaat attataggta ttaagaaaaa attcttcatc ggtatctaaa
2401 ctttgaattt ttaattcttt taaataagaa tgcatgtata ataataaagt gctagacata
2461 taagtatcat tgcctagata taaattaatt ttttctaaac ctaaatcaca tatttttgtt
2521 tctgctttat taacttgtaa tgttaaattg aaagtataat cttgattttt gctagatata
2581 aaaacttcat tgatggagat aggataaaga tagacatcat aaactgtttt aaattcacaa
2641 acacatttgt ctatcggttt tgatttaata agagttcctt taggaatgat taaattgttt
2701 tctttactat tttgttcaaa ttttaaattct tgcatacata aagaaggtaa aggatttgta
2761 tagtttgggg aaatgatatt gattagagac tctgctatat gtggtatatt ttcatcaagc
2821 tcttgatgaa ttttagaact gagaatggct aaattttcaa taatcctttc tatatcagga
2881 tctttactat cgtgtgctaa aaatggagtt agctttggat agtttttat aaaatgctct
2941 ctttttcat atagatattc aagttctttt tggtaataaa aaatattatc cttcatttaa
3001 cctcgcaata tctattgttt ttaaagatga tttctatatt gaattcttta aaacgatcat
3061 cctgaaagaa acattttaca ataaaagaaa gttgccaagg acttaaactg ttgtcgtaag
3121 tgatagaaac tacacatatt ctttttcat agttgtcaat cagttttctt atttccttag
3181 ccattgatac gcataattca ttagagttaa gattaatatc agccatatta ggcattccta
3241 agtttcttac agcgtaacaa tcattaattt ttgcattgag caaaaccttt atattgtttt
3301 ttacatcttc aaaatcattt tggtaaaatg gaatatgaat gttttgctcg tctaaattat
3361 ggatcagttt atctagcagg gacattatta ttctttatct aattttccta ctaaagaaag
3421 ttcaaaatta ccacccatat atttaaaatg aggtcggacg cttagtccta ttttatacca
3481 tcctggctct cctggtatat catcaacttt cacttgtgct gccctgaatg gacgacgact
3541 tcttacttca gaaggtggat tttcttgatc ggaaatatat tgtctaatcc attcattaag
3601 accattttcg atatcgcttc tttctttcca actccctatt tcttctcgtt gtaaaacctt
3661 taaatagtgc gccaatcttg aaattaaaaa gatatatgga agctgagttc ctagtctata
3721 atttgtttca gcttctttac cttctggggt attttgaaat atctttggtt ttaatggaga
3781 atttgctgaa aaaaatgccg cattattact atctcttctt agtgttaaag ttataaaacc
3841 tgcttcagca agttcatact ctctcctatc tgtaatcaaa acttctgtag gtatttttga
3901 ttgtatagtt ccaaaatttt cataaagata agtaggcaga tccttaacag tccctccgct
3961 ttttggtcct ataatatttc cacaccatct atatttagca aagctttctg ttaatcttgt
4021 acaaaaagta tatgaagaat tagcccacaa taaatgatta tgtgaagcat gaacattttc
4081 tttataatta aagcttttaa tagggttttc ttcaggatca taagggcttc ttgctaaaaa
4141 tcttgtcact agcaagcctg tatatttaga gtcttcattt tctctaaaag ttctccatct
4201 tgtatattga ggcccttcta gcaagccttg taaatcttga atatttgcaa gttcagaata
4261 atcatcaagt ccaaaaaact ttggtccaaa agatgttaaa aagggagaat gactcatagc
4321 tgcaatacta gccattttat ttaaaaaagt catatcagga cttgctgatc caagttgata
4381 atctccgatg attgctccta cgggttctcc tccaaattgt ccatattctg aagaatatat
4441 atttttataa acaatacttt gtgtgatgtc tggattattt tcaaaatcct ctaaggcttc
4501 ttcttttgtt atatcaaata ggtcaatttt tatattttca ttaaaatcag ttctttcaac
4561 tagaaatcta agtccacgcc aagtagattc aagtttttga aattcttcat tatgtaaaac
4621 ttcatccatt tgttttgaga gcaaatggtc aatatgggct atcatttcat caagtgcaaa
4681 tttattgatt ttttcttcag cattatcact ttttacaatt tctgaaataa attcagcaac
4741 acctcttttg gcaatactat aactttcatc agttttagag tatttgcttt tttccataat
4801 ttgttcaata attggcgtat caattacttt agtatttgcc attttaattt ccttgctctt

4861 cttgtttgat ttctaaaagt aacttttctt tagtttttc attttttaac gcttctaaaa
4921 cagcttttct aaaatcagga atattgccca taggaccttt taaagccatt aatgcttctc
4981 ttagctgcaa taatttattt agctcaggta tttgtttagc taagctatct ggagaaaaat
5041 ccttcatact agcaatattg agcttaacat caagctcttc agctcctgtt ccaagagtat
5101 tttttacgct aaaactcgtt gttatatcga gttttctcat aacttgatca aaattgatct
5161 tattaatttg caaaatttct ctttcctcta agggagtttc gtttttttcct tttaaatttg
5221 ccattaccat taattttaat ggcagctcca catcctcatt ttgtccatta gttttagctt
5281 tatatgtaat attaattcgt tcctttggag catgagatcc atctgacatt ttaaatcctt
5341 aaaaataaaa atttttagtt caattattat attaaaattt gctaaattta atcttgcatc
5401 atttaatttt attttttgcg ttttcatatt cttcagctag ataatctttt agtagagttt
5461 tttccattaa ctctactaag tatatattta aaattttaga gttctttttc attccttttg
5521 attcaaaaat ttttaccata tctagcaagg aattaatatt attcataata gaattattaa
5581 aattttcttt atcaatattc atcagcattt gctcaaaatc ttgtaatgta tgttctttgt
5641 cagtttttttt acttgaaaaa aaactttttt tattttcttt aggtttaacg aaatatttat
5701 aaacttcttc tttgcagata aagtcaccat tttgaaaacg gagaagttca atagcatcaa
5761 atttagaaat aaaatcactt gttagcatta tgagaatgtc taattgttta tttttttttct
5821 ttttctctaa aaaatcacaa aagagttgca caccctctat ccaaaaagga tttaaaatga
5881 gttcatgcat aaaacattta atatgttcta tatcattttc tttatcagaa agtaatatac
5941 gaattatatc cgcatcaggt tttcttattt tagtaatttt ttcattatta cattcaggca
6001 aacatttgat ttttccccac attgcttggg tgaataaagc gtagatattg agattgtttt
6061 catcctcttc taaaagctca aatgcaagtt tttgataaaa atttttatat tctctctcgt
6121 ttaaggagtt aatattatcg cttctaatgt ttacagatac ttgagctaca ggagattttg
6181 gaagctgtgg tgcaacttga gcaatttcaa tttttgcaaa attgcaagat agtaaatttt
6241 caaattcgga aaacaaagcg ataaattgat caataatctc agatgataca agcacttttg
6301 ttttgttata ttcttgaata aaattttgaa taaaaatttg aatttttcgc ttttgggctg
6361 atatataagt attattttca tccataagtt tttttaagtg ttgaaaaaga agcaaaagtt
6421 tttcaaagca ttcttcattg tttaaattga agcacgataa aagaaaatag ttacaaactt
6481 taaaatccaa agaatgatgc aataatataa attgagaata ttgataaact ttatcccaag
6541 aaatattttc atgatttaaa gtcttatatt ttgacatctc atcttcaagt aagtgaaatt
6601 cttgcagctc atctaaatta tttttcaatt ggttacaaaa agacatttaa ttcctccaaa
6661 aatttgcctc attataatta aattttaaaa aatattatgt taattttttta aaaaaattac
6721 ccaattaatc acaaattata ttatttatag aaaggatacc ttagttttat gtttcgtaaa
6781 aaaagcttat ttttttttatt gccgttgttc ttttgtgctt gctcaagtgt tgttagtgtt
6841 aagataaata atatagaaaa ttccaatctt aataacagat atgatgatgt tcctgttaca
6901 gctattgtat atcaacttaa ggatattaaa aaatttgaag aagcaagtga tatagatttg
6961 gccactagag aagatggggt attaggcaag gataagcttg attctattaa aacgcaaatt
7021 gcacctaaag acaatattat cgcggttaaa gtagatgaag gagaggttcc ttatgtaggt
7081 gttcttgtat tgtttgcaaa taatacaaaa aaagtaacaa aaatatgggc aaagaccgaa
7141 gatgcaaatg ggtttggaaa gaataagtat ttaaaatttg aaatatccaa agaaggcatt
7201 aaaagaatta aataggaatg atatatggca aataaattaa aagttgcttg gtttgatggg
7261 cttaatattg gacaaaccca ttttgagcag caagaaagat tttttaatag aaatatagat
7321 cttaaaacta taaatatata tagtaattta tatggaataa tagacttgga attttctcaa
7381 gagatgttat tgcaaggaaa aattgcactt tctaaaattt caggcatcgc gcaagatgga
7441 agtattttta atgcacctga gcaagattta ttacccgaac ctatcgaaat caattatgaa
7501 tctttaattg attcagttgt ggtattaaaa attcctattg gggttactga tattgctgat
7561 ttgtctttaa gaaacacttt tccaaattca aagtatatat gtcttcgaaa tagtattgct
7621 ttaagaaatt atgatgattc aaaatccaac gcaatggaca aaattgaaga tgagtacgaa
7681 cttgaaaatc tgacttttac gcaagagaaa aaagatctac ttcttgctag tttaagatta
7741 aaacttggca ttttaggtaa ttcaactcct gatgaacttg aattgccaat tgcaaaaatt
7801 aaaaatatcg atatcaataa aaaaatcgaa ttagaaagtg attttatacc tacttgttta
7861 aatatcagta aaatatctac cataagatct tttttggaag aaatcatttt ttcaatcaat
7921 caacataaaa aagttctatc taatgttttc aaaggaattg accaaacaaa aaatacattg
7981 gattttagta ctttttttatc tcttaatctt ttaaaaaaat ggtatttaat attttcacat
8041 ctatcaaaaa aagataaaat acatcctgaa tttttatacg aaaaattttt agaatttcaa

8101 ggagagcttg cagcttttag caacgaagaa tcttttttag attttattcc ttataaacat
8161 gataatcttt ataatacttt tttaaatatg atgaataatt taagattgtt attttccaaa
8221 atcacaagtc ctaaatattt tatagctaag accagtatta atggcaatgg tttttatgat
8281 tttatttttg ataattcggg aattttagag gatgctgaaa tttattttgc aattcgtgca
8341 gatgttacta cagaatatct acttaataat tttaaaattc aaagcaagat acatacgcaa
8401 agtaaaatta aaaatatagt agcaacacag cttgaaggct taaatgtaga tcaagtacct
8461 aatattccat ctagtattcc ttatttaaat ggttacgttt attataaaat agataaaaga
8521 gatcatctgt ttaaagattt taaaaatgaa agtatcatta gtatgtactt gaccaacaat
8581 ataaaaaatc ctgatattat aatgtgggcg gtatttcaat gaaagaaata aacgaagaaa
8641 aaaaggcatt aagcttactt cttgattcaa attttgatgg tttaaaaaac aataaaatta
8701 ttgattattc tttggagtta cttttgcttt cttacagact ttcaaaaata agctctatgg
8761 atacaagtaa tattaatcaa cttagagaaa cattgattaa taaaatttta gatataaccg
8821 ctaaactaag catgtgtaaa gaatatgatg aaaaagaaat tattaaattc aaatattgtc
8881 tttgtgtctt tatagatgaa agcttgatga aaaatgaact ttttatcaat ttttgggctc
8941 acaatactct aactgtaaga ttatttgatg aaaccttagg tggaaataat ttttatgata
9001 ttgcatcatc ttggattaat aatcctttta agtttaaaga ttttttggaa tttatttatg
9061 cttgtttgat tcttggttat aaaggaaaat ataatgaggc taaggataga gatgaaaaaa
9121 taatccattt ttgcaataat attgccacat ctttaagacc agtttataag acagaagaag
9181 agttagcttt taataaagca tacaaaattg gtttagaaga aaatatttgg caaaaattta
9241 taagattgta ttttaaaaaa cttattatca ttgtgccagt attgattgta ttaggagtat
9301 taagctttgc aatttttaat ttagaaacca ataatcttaa agtggataat aatatatccg
9361 tattgataaa aaatttaaca catatagaat aaagatatta aatttttta aatatcaatt
9421 tatttgtgat gagtaaaatt aaataaaaat tattttacag ctagaataaa aatcaaatgc
9481 aaatctaaag gataaaaata tacgaaaaaa caagctctat tattttaatg ataaagaaaa
9541 agttatactg ctaacactaa actacaaaat tacatcttaa ttttaataaa atagagagtc
9601 taaggagata acctatgaca tatatattac ttcttattat aatttctact attctttctt
9661 atcttatatt aaaatttatt tataaaatca tctttaaatc tacaaaaagc gtttcaaagt
9721 ttttagtctt tcttggaagc ataggtttaa ttatctttta ttatactcct tattcttatt
9781 atttagagcc tagttattgg caatttagga atatgtgtaa gttaaatgaa cttcctaata
9841 acgaagagaa atataataag attctagggt attttgataa aaagttaggt gacatagatg
9901 attttcgaca tgtaaaaaaa tatggtagta tagaaatgtg gctttatata tattacgatg
9961 atacttacaa aactccttca aattttcaag aaaagataga gatggataat attgcactaa
10021 aaacaaaaaa tatgcaaatg tctttacaca agattgttga tatgcatata agtccatatt
10081 ggaatactag aaggtatgtg ttagaaggca atgagggtaa tatgaatttt gagtttatag
10141 aggatgagct cgcttgtggc aatctttatc aataataata taaggaataa ataatgacca
10201 ataaacaact tattaataaa ctaagggata tggcggaact tgcacaggca agttatggtt
10261 attttgattt ggtaacacat gacaagaatg atttttttcat taaaattaaa gatgaagcta
10321 cagaatctgc tttctttaat aagcacatta ctcaagctaa tgtcttagat attagttata
10381 aaaattataa ggcatatcaa tatgactcat ctttaaagaa agatattaaa attggcactc
10441 tcaaaggcga cttctcccca ctccaagcca aacgattctt ttctcgctat gatttattaa
10501 aacattgtcc taaaaaatga aaataaaagt atcatatttg taggacattc tctggtgggt
10561 atttagctca aatggctttg atatattgtg attaaatata aagatttata cttttaatac
10621 tcttagtatt tacgtttgaa atgttcctag tgtggttata ttttgatttt taaaaaaaag
10681 taggataatt ttattaaaaa tattaaaaag gaaaataaat gggtatatta aaaaaagtat
10741 acattgtttt ttttgggggg gggtattttg gggaattatt tttttgttta gttctttata
10801 tgcttatttt agtgatatag aatttttttgg tgatatgtta aagattgcat tttttatata
10861 ttcttatcca ttgttgcctt acatatcata tttaacacta caagaaagta ttaaatccaa
10921 aaatattttt ctttattttt ctttattttg ctttattttt ctttatctcg ggaggatttt
10981 ttatttatgc aataatatta ggtatttata caatattatt tttaataata tctattaaat
11041 ttagaaaaga actttcttat tatttttctt tattgttttg tattttttta tacatttttt
11101 tgtatctttt tattttttat atagaattaa aaaatgagta ataaagctca aattaacaat
11161 ctcaaaaatt acgctaaact tgtatagatt ggtttttttta atttttaat aataatttaa
11221 atttcaacaa aaaaggaagt agtgtgaaaa tattaaaaag tttatatatt atttggatga
11281 tattttgggg atttggggct attttaggtt attatatggg atatgatgca acttttacta

11341 aaacgttctt ttttcttgtt atattatatc tttttccatt gttgccttat ttgcattatt
11401 taatttttga gaaaacaaag aacaaaaata tattcatttc tatttgttat tatctactat
11461 tatttgttat taccgcttat tattttactt taatatattc atgtggaaca aatttttttaa
11521 catttttttat aatgattact ttatatatga ttatttgtag tgctataatc ttatggaaat
11581 ttaaaataaa tttaagatta tatttttaca taattttatg tatttttact tatctctata
11641 tagttttaca tatttttata gcatgttatg aaatagaatg gacagatata ctgctagata
11701 aagatgtaag gatatatatt tacaaagaaa taattaattt gcttacagag caataatttg
11761 ttttataata aatcttattt ttatagggga ataaattaat gaaaacccaa atcaacaatt
11821 taagagatta tacagagctt gcatgggcta gttattttta ttttgattta aaaggttata
11881 tattacaaga aaatgaaact atcgttattt taaatgagct tgtaagttta agttataacg
11941 gaaactgata attttattga ctcggttcaa gacatagccc tatcttttaa tgacattatc
12001 caatttttttc ctcttttaga ataagtagat aaaataaatg aagctaaaga gccttgtgaa
12061 tttaatgatg atttcaagaa attttttctt actgaaacaa tcaaattttt aaataaattg
12121 tcttataata gaataactta ttatattagg agcaaatttg caaattccta tttattctaa
12181 actcaaagca aaactagaaa gcaaaggaat agacaatata tcaagcggac aaattgcttg
12241 gtttgtgtgt agtttgattc ttgctttggt gacttatatt attctaactt ttcactacat
12301 ttacattagt tatggtaaat atgataagaa tttattttta ttaatatttg atagaaatgc
12361 aatggaatta tttattatta tatattcttg tataatgttt aatctattat atatttacaa
12421 tgccattcct ttggttttat ctgcagtatt tatatttttg gcattttatt tttctagttt
12481 aagcttgttt attgttggtt gttgtggcat aggagtattt ttcttaatat ccattttcaa
12541 aagaaaactt atacctagcg ttttgttgtt tactatgggt tatatttgct catatctctt
12601 acttcatgca attggaactt atcattagga ttttataaat gaagtattgt gtaacaattt
12661 atattgatcc attggtagaa tgttttttgg attttaaaaa tcagtgttat tggcaaagag
12721 aagatttcac ccacgccttt ttaggactta ccaaagataa aagtcccgat gagttagata
12781 aatatagatg aagaattaaa acaagaatat cttaaaaaca aagaatttaa attaattaaa
12841 agtattaagt agattggtta taataaaaca atttatctat attaggagta aatttgcaaa
12901 ttcctattta ttctaaactc aaagcaaaac tagaaagcaa aggaatagac aatataacaa
12961 gcggacaaat tgcttggttt gtgtgtagcg taggtatttt tatcattcct atgatgtatc
13021 tttatatcag caacaattac aatcaattta ttattttgtt ttttatgata tttatttttta
13081 tattaaatat gctttatact aaaaattttt ttgcattatt attacttgta atattgatat
13141 ttatatatat gaaatatcat gagtttatct ataattttaa aattttattt tttatttttg
13201 cacctataat tcttatcttt ttaagggctt ggagaatttt aataactttt ttatgcggat
13261 atattttttt agtggctatg gtatatatat ttgagttatt aggaggattt tataaatgaa
13321 atattatgca acgatttata ttgatccatt ggtagaatgc tttttggatt ttaaaaatca
13381 gtgttattgg caaatagaag attttaccca cgctttctta ggacttacca aaggcaaaag
13441 tcttgatgaa cttgataaaa tagataaaga attaaaacaa gaatatctta aaaacaaaga
13501 atttaaatta attaaaagta ttaagtagat tggttataat aaaacaattt atctatatta
13561 ggagtaaatt tgcaaattcc tatttattct aaactcaaag caaaactaga aagcaaagga
13621 atagacaata tatcaagtgg acaaattgct tggtttgtgt gtagtgtagg gatagtatat
13681 ttgctttcta tttttttata tttgcatggc gttaataaag agggaatttc gtttaataga
13741 atatttattg cattttatat aaatcatttc ttttgtttta tgttgatgtg gtttatttta
13801 ttgttaaatt tactttataa agaaaatact atttcattaa tattaacatt attaacaacc
13861 attgcctatt tttataatcc atattttgct tatattttga tatttatcat ttgcatcatt
13921 atgcgtataa taaataaaca aagatggctt tatatatatt ttattggttt aatttatatt
13981 tatgccacct atagtatatt taatttaaga ggaggatttt ataaatgaaa tattatgcaa
14041 caatttatat tgatccgcta tttgatttta aagatgctac ccatgccttt ttgggcttac
14101 caaaggcaaa agtcctgatg agttagataa aatagataaa gaattaagac aaaaatttta
14161 agcaaattag tttataataa aacaatttat ctatattagg agtaaatttg caaattccta
14221 tttattctaa actcaaagca aaactagaaa gtaaaggaat agacaatata acaagcggac
14281 aaattgcttg gtgtttaggg agtggctata ttgccttaat atgtgccgtg tttgttgttg
14341 tatataaact taagaatttg gaatttgagc tttatattgt ttttttcttt cctatcttgc
14401 tattttatat tgctatattt tggtttattt tcttaagttt ttataaaaac ttcttttttta
14461 ttcatctttt ttgctattct tttatgtata tctttaatat aatttttaat gatttttatt
14521 taaatcttgt ggtaatgaat attttattaa taacaatatg gctctttttt gttaaattac

```
    14581 atttaaaatt tagattttta tttttaatga tgtatttttg ttttgaattt gcgatattta
    14641 taggaatcat accttatgga cttttagaag ggactatttc aaaatgaaag aaataataaa
    14701 atattatgta acaatttata tagatgagtt attagaaaat gaaatgttaa agcatgttga
    14761 tttttcttta ctatttaaag caaaattttc tcaaatttct aagcaaatcg acttcaccca
    14821 cgccttttta ggacttacca aagataaaag ttctgatgag ttagataaaa tagatgaaga
    14881 attaagacaa aaattttaag caaattagtt tataataaaa taatttatct atattaggag
    14941 taaatttgca aatccctatt tattctaaat taaaaacaaa gctagaaagt aaaggaatag
    15001 acaatatatc aagcggacaa attgcttggt ttgtgtgtag tgtaattttt acctatttat
    15061 tatttttatt aattgtaatt ataaatacta gaacaatatc ttttgaaaac cttaaatttt
    15121 ttacttttga aggtttttc aaatctcttg cttttatttt tgcaaatcat ttttttttat
    15181 ttatttttat gtggattgtt tgtataataa atttttttta atataaaaaa tttaattcca
    15241 ataacactaa attggataat aagctattgt tattttgttc ttgaaattaa tatttatttt
    15301 attttgattt tatgtataac cctttgtgtt gtattttatt ttttacaaaa aagattatta
    15361 tatacttttt tattttattt tgtttatatg gtattaactt atactttgtt ttatcttggt
    15421 ggaggatttt ataaataaaa tattatgtaa caatttatat tgatccattg gtagaatgtt
    15481 ttttggattt taaaaatcag tgttattggc aaagagaaga tttcacccac gcctttttag
    15541 gacttaccaa aggtaaaagt cctgatgagt tagataaaat agatgaagaa ttaagacaag
    15601 aatatcttaa aaacaaagaa tttaaattaa ttaaaagtat taagtagatt ggttataata
    15661 aaacaattta tctatattag gagtaaattt gcaaattcct atttattcta aactcaaagc
    15721 aaaactagaa agtaaaggaa tagacaacat atcaagtgga caaattgctt ggtttgtgtg
    15781 tagtgtagtt cttgctttgg tgacttttct tattatctat tttacagtga ttattgaaga
    15841 taaaaattta tttttagcaa tatttgaatt gtttcaatgg caactattta tttttatcat
    15901 atacttttgt atccctttta atctattata tattcataat gctattcctt tagtgctgtc
    15961 tgcaatattt atgtttttaa cattgtattt ttctagctta aatttattta ttatcggctg
    16021 ttgtggtata ggagcatttt tcttaatatc catttttaaa agaaagctta tgtttagtgt
    16081 tttgttgttt gctatgggtt atatttgtat ctattttttt ctttacgcaa taactcgcta
    16141 tcattaagga acaaaatgaa gtattatgta acgatttata ttgatgagtt attagaattc
    16201 aaatgataaa agatttgctg aatatgaaag gtttagactt cacccacgct ttcttagggc
    16261 ttactaaaga taaaagtcct gatgagttag ataagataga taaagaatta agacaagaat
    16321 atcttaaaaa caaagaatgg gacaagatag atcaaaaatg gtacgaagct aaagagccta
    16381 atgagtttaa tgatggtttt tggggttttg gaactggaat tgctaatgtt gcagaatctc
    16441 ttataggatc tcctggcaaa gtatttaata ataaccaata tgtattagat aacaatgttg
    16501 ataaaaatcg ctatattatt aaaaaactaa gaagccctca aacttttaaa ccctctaatc
    16561 gttgcacttt agaaatttct aaggaacaat atgaaaccct acttgcaaat attaaagctg
    16621 attttgaagc cacaaaagaa atcacaccta atagcgaaaa gccaatcaat gaagaattta
    16681 cctataaact tcttgaaaat aattgcgtta cttgggttgt taaaaagctt tctagcatag
    16741 gaatagaagt tgtaaatccc gttgttaaag atcctagcgg attattaatg gctacaaaat
    16801 taatacaaag cattcattct atctttttaa aatttcaaaa cattgatgat aatttacaaa
    16861 gcgtaaaagg tgcaaaagct tttaaaaatt gggctagaat gcttataaat aattcattaa
    16921 tgcaagaaat ttatcttgat gaatataaaa aacaatacaa tagtgaaaat cttagtccag
    16981 aacaagaaga agttttatat gctttatgga atattacaag ggtttataaa attttaggca
    17041 ttcttttaag tcgtttaatt acaaaatcgg gcactaaaaa cctaaaaggc acttttgaat
    17101 taatttattt tgataaaaat gataagcaag caaagctttt aaaagcgagt aatcattatg
    17161 aggcgattat tatacaagaa ctagatttaa gtaaaaaata caataatttt agtgcgagta
    17221 aattttatcc ttttatcttt atacctaaag atgaaatgct ctcaagaacg ctttatcata
    17281 agtatgatta tggtaatatc tcacaagaat atcaaaaaga tagaaatgaa ttttatttta
    17341 atgttttagc aggagaaaaa agcgataaat attggagttc tagctatcat aaaatgacta
    17401 aaaatttaag gaaagtctat gtctcttgaa aaaatcaaag aaacgatttt tgcttatgat
    17461 aaagaagtga ttgattgtga agttttaagg gctaaaaata ttgatgtaat tcattctaaa
    17521 atttattttc aaggtatact ccttacaggc tccaccgaac tccctaataa tcctttttac
    17581 tttggagaat tagatcaaga taatactata aaacaagata tacccagtta ttatttttct
    17641 cctaaagatg aaagcagtgg tttag
//
LOCUS       PIMPEBOA_2             7372 bp    DNA     linear       12-APR-2021
```

```
DEFINITION  Campylobacter species strain strain.
ACCESSION
VERSION
KEYWORDS    .
SOURCE      Campylobacter species
  ORGANISM  Campylobacter species
            Unclassified.
COMMENT     Annotated using prokka 1.14.6 from
            https://github.com/tseemann/prokka.
FEATURES             Location/Qualifiers
     source          1..7372
                     /organism="Campylobacter species"
                     /mol_type="genomic DNA"
                     /strain="strain"
     gene            19..489
                     /locus_tag="PIMPEBOA_00027"
     CDS             19..489
                     /locus_tag="PIMPEBOA_00027"
                     /inference="ab initio prediction:Prodigal:002006"
                     /codon_start=1
                     /transl_table=11
                     /product="hypothetical protein"
                     /protein_id="Prokka:PIMPEBOA_00027"
                     /translation="MQNLKLQIAQGLDKILEDEDRKQELYVCKFVVVNGVSENKIKAK
                     NKDVNIDNDTKNINTTKSNNISSSESNAQEKITHLSNDGQNLLKNIEKLRLKPYNDQN
                     GKEITSYVKGATIGYGHLIGQNEWDLYKNGITLQEADKLFKSDLLPFENAVKIQ"
     gene            725..1084
                     /locus_tag="PIMPEBOA_00028"
     CDS             725..1084
                     /locus_tag="PIMPEBOA_00028"
                     /inference="ab initio prediction:Prodigal:002006"
                     /inference="similar to AA sequence:RefSeq:CKG86986.1"
                     /codon_start=1
                     /transl_table=11
                     /product="hypothetical protein"
                     /protein_id="Prokka:PIMPEBOA_00028"
                     /translation="MVIFKNILLFLFFSTYLLAQEDFTPLEQCTYENEKFWIKILNLC
                     LEGNITCDKIVYVGVNKNNGKYIVLNGKSISDVNMNFKGYVFKNGIYEYNIFNNFLYI
                     SKNKQIIQEYRLKLCEK"
     gene            1349..5344
                     /locus_tag="PIMPEBOA_00029"
     CDS             1349..5344
                     /locus_tag="PIMPEBOA_00029"
                     /inference="ab initio prediction:Prodigal:002006"
                     /inference="similar to AA sequence:RefSeq:AAW35464.1"
                     /note="conserved hypothetical protein"
                     /codon_start=1
                     /transl_table=11
                     /product="hypothetical protein"
                     /protein_id="Prokka:PIMPEBOA_00029"
                     /translation="MFISSKTELIQTIKNSTINNKEVKQIDFIYPNFSILHAILKNID
                     FHQRKILFKNQILAMEILEILKQTRDNHNNIEVNIHTLNELDAGQNVNLKDLSSSEIC
                     TKVYLEDLEEEIQDKKQGKDFNLFYHTDKNNLCIEFINTLKELAKDSEDITTLDELIE
                     NIDPDDISSEKFDDFKKKVLEKIGNVIESDFFKNSISPTLKNTYEKFIDELLEHIKKL
```

```
                     AEEKEYEKIKIILSAIAGVAIATVVAVLTGGAGLAILAAGLFSLMGSASEYYEFAQRD
                     YGTLLTPLAAFLATRCAMFIHYINSRLLSCVLIIDKKEIIDLSGFGLYLGSDESNIAS
                     NLAYKITLSEEIKSIENIFSEIFLNPEQKDNESKNTANDSKSSNLLAFISTQKLSNLY
                     LKNNAILKNLQEDIKQFIYFSYIESPNFNSSKLAEFFAQKNQNTQIDLNTTKMSKNYL
                     IISNIPNKYNAGLSESLTQEIANYNIQDKYRTIKKNIDTSKKTYDSTRDYSEYSIEIP
                     QDENEPYILQLSPFVKISKEAYENSKKAYEDMQEIYKYLTNFVATPQKLPEFAKTAKE
                     KLAISENESMIEIVKKTENLRHCERINILAYIDYNKKINDKKWLPYFYIKKFFKQNED
                     KESIIEKEKKIAHQYLTFLKKELFEKSDKSSFGNYSLSDKSSFGNYSLSDKSSFGNYS
                     LMECLFISFVLYQAKLIFPNINLDLKKYGGSNFVYIKYLEETYVLWTDEYPIYVNNEP
                     TNITAYEVVSQLLKILDKKDYSFVENDSSITIDDFFNELSKDLDVTNTLDKKIIKLKQ
                     MYHLDEQKCIKEFMNLDEEAQRAFFVWMDIEKQISDLSKLLEQMYGKEYLNKAKEQKT
                     SEIFTYAMLKILLSASPFISFTAQMLWDFNFLKTIEYVIQACIKINKEGFKEFFKEQL
                     GIFTPLKKNSIYHIHIKSNLIIKACLQQIEKELTHTKIQMILAQEIKSNLIIQKNTSD
                     LNLFITKIKEYFNEKQIKIYWSDKYMKQLFIYSNQESNISKIALMHSIDIQTQKYTIY
                     TYAKLNKLDLASIVAGEFVGIFLNYLFPTNYKALQEQYESIVYQLYTFKDDSPLAVIR
                     DHFITYPFAINSKFISFDLKKIFYGIDLCTGVFDTHFSFHYEIDQKLEENKEFLLKRL
                     LSYLIIDEKRSATALEDIKEQKYIIDDYTFYFKELKSLELKKCTIYQLPCNHSLFKEF
                     RNKEKQISAFKKDEDSPIHAYNEALQILQDFANGLYIVKYEEGKCIEDKNKTRRLLEK
                     LNIIGQNNLKAMYMGINGNKESLKAIGKRENNTSKANINADIVSETKAPPNFIGRLAT
                     TIIIEDGLYIG"
     gene            5363..6631
                     /gene="trxB_1"
                     /locus_tag="PIMPEBOA_00030"
     CDS             5363..6631
                     /gene="trxB_1"
                     /locus_tag="PIMPEBOA_00030"
                     /inference="ab initio prediction:Prodigal:002006"
                     /inference="similar to AA sequence:RefSeq:CKG87041.1"
                     /codon_start=1
                     /transl_table=11
                     /product="thioredoxin reductase"
                     /protein_id="Prokka:PIMPEBOA_00030"
                     /translation="MSETYEIYTPNGLTLDVEKDTNKILFKENIKPTGNYTEEYSKAV
                     FESYYIMKNSPYKDYKPIYLDPNFYTGEKSTLLEFKEWQNIYLKDPIKGSIAPWTKAE
                     KAYYKSLKTKRERCKYLAIRSGLRSVVIDIPYDAYCNVDEKGYLINEEYAYIYDEVSS
                     HRGTLKSYSFFNEWELAALLLGDMRASPTAAVGFKARQQQALFLQAQFGDKNAFKSLG
                     LAVLCSNSFLTGQHWNKLRAKMIYDLHDHHYESLLDEFGMLPFLDEIIGADWTIDLNK
                     YDFAYDEEGRIIWALYNDIEKGKLKDPRDVDSTPESRNEFDDAMDRYENGMVTRFDVD
                     TPNEWSEQQAALDRDTLVLSAKLAALTPPQGYPNAPYYFTPERLEWIYKRGYLDKLLD
                     PRIPAIYRYNFPRELRAKILKFAEENGIKE"
ORIGIN
        1 tttttaactc ctgcaaaaat gcaaaattta aaacttcaaa tcgcacaagg attagataaa
       61 atcttagaag atgaagatag aaaacaagag ctttatgttt gtaaatttgt ggtggtaaat
      121 ggggttagcg aaaacaaaat aaaagcaaaa aataaagatg taaatataga taatgacaca
      181 aagaatataa acactacaaa aagcaataat ataagttcta gtgaaagcaa tgctcaagaa
      241 aaaatcactc atctaagcaa tgatggtcaa aatcttttaa aaaatataga gaagctaaga
      301 ctcaaacctt ataatgatca aaatggaaaa gagattactt catatgttaa aggtgcaact
      361 atcggatatg gacatcttat agggcaaaat gaatgggatt tgtataaaaa tggtattact
      421 ttacaagaag cggataaatt gtttaaaagc gatttgttac cctttgaaaa tgctgtaaaa
      481 attcaataaa ctcttcctta gcacagaacg aatttgatgc cttggttata ttatgtttta
      541 atatagggat tgataatttt aaaaattctt ctgttgctaa aatcatcaat ggagagaaaa
      601 cgggttacaa aacattaaag gaagcttgga tggcgtggaa caaaagtcaa aataaagtaa
      661 tgcagaggct aattaatagg agaaatgctg aatataagct ttacactcaa ggaggttatg
      721 aaaaatggta atttttaaaa atatattatt gtttttattt tttagtactt atttgttagc
```

781 gcaagaggat tttactccac tagagcaatg tacctatgaa aatgaaaagt tttggataaa
841 aattttaaat ctatgtctag agggtaatat aacttgtgat aaaattgtat atgtaggtgt
901 caataaaaat aatggaaaat atattgtcct aaatggtaag tctattagtg atgtaaatat
961 gaattttaaa ggatatgttt ttaaaaatgg tatttatgaa tacaatattt ttaataattt
1021 tttatatatt tctaaaaaca agcagattat acaagaatat cgtctaaaat tatgtgaaaa
1081 atagacacaa caaaggcaac aagtaacaac aaatacctag aatgtaaaaa gttaaaaaga
1141 tgcaataaga attacaagaa gtttttaaag ttataaaaat cctaattaat gaaatcttag
1201 ctcctaaatt ttataacaaa tacaaagatt tagaagtaga gattgatgat aggggtaata
1261 ttataagatg gataggagag attaataaag acaaaaaata ttagcaaata aaaaatgata
1321 taataatgct ttatataagg aatcttttat gtttataagt tctaaaacag agttaattca
1381 aactattaaa aattcaacaa tcaacaataa agaagttaaa cagattgatt ttatttatcc
1441 taatttttct attttacacg ctatattaaa aaatattgat tttcatcaaa gaaaaatcct
1501 atttaaaaat caaatattgg ctatggaaat actagaaatt ttaaaacaaa ctcgtgataa
1561 tcacaataat atagaagtaa atatccatac ccttaatgag cttgatgctg gacaaaatgt
1621 aaatttaaaa gatttatcaa gcagtgaaat ttgcactaaa gtttatttag aagacttaga
1681 agaagaaatt caagataaga aacaaggtaa agattttaat ctttttttatc atacagataa
1741 aaataatctt tgtatcgaat ttattaatac tttaaaagaa ttagccaaag attctgaaga
1801 tataacaacc ttggacgagt taatcgaaaa catcgatcct gatgatataa gcagtgagaa
1861 atttgatgat tttaagaaaa aagttttaga aaagattgga aatgtcatag aatcggattt
1921 tttcaaaaat agcataagtc ccactttaaa aaatacttat gaaaaattta tcgatgagtt
1981 gttagaacac attaaaaaac ttgccgaaga aaaagaatat gaaaaaatta aaatcatact
2041 aagtgctata gcgggtgttg caatagccac agtggtagct gttcttacag gtggggcagg
2101 actagctatt ttggctgctg gtttattttc tttaatgggt tctgcaagtg agtattatga
2161 atttgctcaa agagattatg gcacactttt aacgccttta gccgcatttt tagccactag
2221 atgtgctatg tttattcatt atattaattc tcgcttgtta tcttgtgttt taattattga
2281 taaaaaagaa attattgatt taagtggttt tggattatat cttggaagtg atgaaagcaa
2341 tatcgcttca aatttagcct ataaaataac cttaagcgaa gaaataaaaa gcatcgaaaa
2401 tatttttagt gaaatctttt taaacccaga acaaaaagac aatgagagta aaaatacagc
2461 taatgattca aaatcgagca atttactcgc tttcatttca acgcaaaaat tatctaattt
2521 gtatttaaaa aacaatgcta ttttaaagaa tttgcaagaa gatattaaac aatttattta
2581 tttttcttat atagaaagtc caaattttaa ctcaagtaaa ctagctgaat tttcgcaca
2641 aaaaaatcaa aacacacaaa tagatttaaa tacaactaaa atgagtaaaa attatttgat
2701 tatttcaaat atccccaata aatacaatgc aggacttagt gaaagtttaa ctcaagaaat
2761 cgccaactat aatatacaag ataaatacag aacaattaaa aaaaatattg atacttcaaa
2821 aaaaacttat gacagcacaa gagattatag cgaatatagc atagaaatac cacaagatga
2881 aaatgaacct tatattttac aattaagccc ttttgtgaaa atatccaaag aagcttatga
2941 gaatagtaaa aaagcttatg aagatatgca agagatttat aaatatttaa caaattttgt
3001 tgctacaccg cagaagctac cagaattcgc aaaaacagct aaagaaaaat tagcaatatc
3061 agaaaatgag tcaatgatag aaatagtaaa aaagacggaa aatttacgac attgcgaacg
3121 aataaacatt ttagcttata ttgactacaa taaaaaaata aatgataaaa aatggcttcc
3181 ttatttttat atcaaaaaat tctttaaaca aaacgaagac aaagaatcca tcatagaaaa
3241 agaaaaaaaa atagctcatc aatatttaac tttttgaaa aaagagcttt ttgaaaaatc
3301 ggataaatct tcatttggaa actattcttt atcggataaa tcttcatttg gaaactattc
3361 tttatcggat aaatcttcat ttggaaacta ttctttaatg gaatgtttat ttatttcatt
3421 tgtcttgtat caagctaagc ttatttttcc aaatatcaat ttggatttga aaaaatatgg
3481 aggcagtaat tttgtatata taaaatactt agaggaaact tatgttttat ggacagatga
3541 atatccaata tatgtaaata atgagccaac aaatattaca gcatatgaag ttgtatcgca
3601 attattaaaa atacttgaca aaaaagatta ttcttttgtg gaaaatgata gttcgatcac
3661 tatagatgat ttttttaatg aattatcaaa agatttagat gtaacaaata cattagataa
3721 aaaaataatc aaattgaagc aaatgtatca tttagatgaa caaaaatgca ttaaagaatt
3781 tatgaattta gacgaagaag cgcaacgagc ttttttttgtg tggatggata tagagaaaca
3841 aatatcagat ttatcaaagc ttttggaaca gatgtatgga aaagaatatt taaataaggc
3901 aaaagaacaa aaaacaagtg aaatatttac ttatgcaatg ttaaaaattt tattaagcgc
3961 tagtcctttt atcagcttta cagctcaaat gttgtgggat tttaattttt taaaaacaat

4021 agaatatgtt attcaagctt gtattaaaat aaacaaagaa ggatttaaag aatttttaa
4081 agaacagctt ggtattttta cacctctaaa aagaatagt atatatcata tacacataaa
4141 atctaattta ataataaaag cgtgcttgca acaaatagaa aaagaattaa ctcatactaa
4201 aatccaaatg attttagcac aagagataaa atccaattta ataatacaaa aaaatacatc
4261 cgatttaaat ctatttatca caaagataaa agaatacttt aatgaaaaac aaattaagat
4321 ttattggagt gataaatata tgaaacaatt atttatatac tccaatcaag aaagcaatat
4381 atctaaaatt gctcttatgc atagcataga tatacaaact caaaaatata ctatttatac
4441 ttatgctaag ctaaataaac ttgaccttgc ttctattgtg gctggagaat ttgtgggtat
4501 ttttttaaat tatctctttc caacaaatta taaagccctg caagaacaat atgaaagcat
4561 tgtttatcag ctttacacct ttaaagatga ttctcctttg gcggttataa gagatcattt
4621 tataacttat ccttttgcaa tcaattcaaa attcattagc tttgatttaa agaaaatttt
4681 ttatggcata gatctttgca caggagtttt tgacactcat ttttcctttc attatgaaat
4741 agatcaaaag cttgaagaaa ataaagaatt tcttttaaaa agacttttgt cttatttgat
4801 tattgatgag aaaagaagtg caactgcttt agaggatata aaagaacaaa aatatattat
4861 agatgattat actttttatt tcaaagagtt aaaatcttta gagcttaaaa aatgcactat
4921 atatcaactc ccttgtaatc attctttgtt taaagagttt agaaataaag aaaagcaaat
4981 tagtgctttt aaaaaggatg aagatagtcc catccatgct tataatgaag ctttacaaat
5041 tttacaagat tttgctaatg gactttatat tgtcaaatat gaagaaggaa aatgtataga
5101 agataaaaat aaaaccagaa gattactaga aaaactcaat atcataggac aaaacaatct
5161 taaagctatg tatatgggga taaatggaaa taaagaaagc ttgaaagcca taggaaaaag
5221 agaaaataac actagcaaag caaatataaa tgctgatatt gtatcagaaa caaaagctcc
5281 gcctaacttc ataggtagac tagccactac tataatcata gaagatggtt tatatatagg
5341 ttaaattaag aaaggataaa tagtgagcga aacttatgag atttatacac ccaatggatt
5401 aacattagat gtggaaaaag ataccaataa aatactattt aaagaaaata ttaaacccac
5461 aggcaattat accgaagaat attccaaagc ggtttttgaa tcttattata ttatgaaaaa
5521 ctccccctac aaagactata aaccaatata tctagatcct aacttttata caggagagaa
5581 atccacttta cttgaattta aagaatggca aaacatttat ttaaaagatc ctataaaagg
5641 aagcatagct ccttggacta aagcagaaaa agcttattat aaatctttaa aaactaaaag
5701 agaaagatgt aagtatttgg caataagaag tggattaaga tctgtggtta tagatatacc
5761 ctatgatgct tattgtaatg tagatgaaaa aggatattta atcaatgaag aatacgccta
5821 tatttatgat gaagtaagtt ctcatcgtgg aacattaaaa tcttattctt tctttaatga
5881 atgggaatta gctgctttac ttctaggaga tatgagagct agtcctacag ccgctgtagg
5941 atttaaagca agacaacaac aagctttatt tttacaagct caatttggag ataaaaatgc
6001 ttttaagagt ttaggtttag cagtattgtg ttctaattct tttcttacag ggcaacattg
6061 gaataagctt agagcaaaaa tgatttacga tttacacgat catcattatg agagtttgtt
6121 agatgaattt ggtatgctac ctttttaga tgagattata ggagcagatt ggacgattga
6181 tttaaataaa tatgattttg cctatgatga agaaggtaga atcatttggg ctttatataa
6241 tgatattgaa aaaggcaaat taaaagatcc aagagatgtg gattctactc ctgaaagtag
6301 aaatgaattt gatgatgcta tggatagata cgaaaatgga atggtaacta gatttgatgt
6361 agatactcca aatgaatgga gtgaacaaca agctgcttta gatagagata ctttagtatt
6421 aagtgccaaa ctcgcagccc tcactcctcc acaaggctat cctaatgccc cttattattt
6481 tacacctgaa agattagaat ggatttataa aagaggatat ttagataaac tccttgatcc
6541 aagaattcct gctatttata gatataattt cccaagggaa cttagagcta agatactcaa
6601 atttgctgaa gaaaatggca ttaaagaata aagtaattgc ataggcagac tagccactac
6661 tataatcata gaagatggtt tatacatggg ataaatttaa gaaaggataa ataatgagcg
6721 aaacttatga gatttataca cccaatggat taacattaga tgtggaaaaa gataccaata
6781 aaatactatt taaagaaaat attaaaccca caggcaatta tactcaagaa tattccaaag
6841 cggtttttaa atcttattat attatgaaaa actcccccta caaagactat aaaccaatat
6901 atctagatcc taacttttat acagggcaaa aatccactct agtagaattt aaagaatggc
6961 aaagcatata tttaaaagat cctatcaaag tagctcaaaa tatcacaaat atttgtatta
7021 attatgtaaa aaaaggtttt aaagaagttt taatcgaaca acttggcatt atatatttat
7081 taacacatcc acaaatcgaa gttgaagtta agaaatatgg aaaaaaatat tttaagacta
7141 taaacaagga aattgctgca aaaagatttt ctacaattaa agaagaatta tttttcaata
7201 aaacaaaaat atcctttatg gcagaacaag taaaaaacgg tattgattat gaaatagatc

```
     7261 aaaagcttga agaaaataaa gaatttcttt taaaaagact tttgtcttat ttgattattg
     7321 atgagaaaag aagtgcaact gctttagagg atataaaaga acaaaaatat at
//
LOCUS       PIMPEBOA_3              4049 bp    DNA     linear       12-APR-2021
DEFINITION  Campylobacter species strain strain.
ACCESSION
VERSION
KEYWORDS    .
SOURCE      Campylobacter species
  ORGANISM  Campylobacter species
            Unclassified.
COMMENT     Annotated using prokka 1.14.6 from
            https://github.com/tseemann/prokka.
FEATURES             Location/Qualifiers
     source          1..4049
                     /organism="Campylobacter species"
                     /mol_type="genomic DNA"
                     /strain="strain"
     gene            complement(403..3918)
                     /locus_tag="PIMPEBOA_00031"
     CDS             complement(403..3918)
                     /locus_tag="PIMPEBOA_00031"
                     /inference="ab initio prediction:Prodigal:002006"
                     /codon_start=1
                     /transl_table=11
                     /product="hypothetical protein"
                     /protein_id="Prokka:PIMPEBOA_00031"
                     /translation="MKIKNFTLITSNHNLKIELKNLTKNANKIIFICSDFDGYKEAEN
                     SLYKTLHTIEDNFKIDNTKTFKIIHLNQTNKDEITKEQKFSSDKNLSSIKKIIYNNLK
                     LLNDTINQNENKTLNKIIDESNEKNIENFLKEIQKELNILLKNIKESNYFDQEIKPKI
                     QVTLDTLFNTLLKNIAQSNDDEYKQIIRAVAKFFINILDSITSLKKPISLIKKNPYVL
                     IVNTLFEAYNSYSEYQEYKEKKYYYDFAYPLLELITSKLYPIIALCNEEILSDVLIID
                     DKVLLDFSSYTNAPSISLYKNSFYKIILDEEFQGVFDNFLENNLKFENEKLDNTLLVN
                     SSLNNSDTTLKKAINLNISKLNHFFYAEYPDLNSSMLVEMILDKKNADQINTTKMGKN
                     YLFITNPPQLNNAGLCEDLYQKKLGAIIKNRPKALTKNEKLALKGDFPSKRDYGSYFI
                     ERDVKKEDDFVLQLCPFAKLDGQIYQKYKGYFNFYNQTYSWMKLYLLEYINERFEYER
                     FKQDFAYIDNFFKLPKELEKISEKEKEKVKEYVELFNDYLDKINFRYDQIQFFLGSEP
                     LMFKTYENVDFKKYSPLTVVLLTLTLYVLQKDLAFTQEYSLGVKNIAIPNPDYGDKEI
                     SLNYQRIKIPNYFNEYILFIPDDIYFYDNDKKIYIFLSKELNKNKKEDEVIYLDELAK
                     AMFENDLKEDDQTYVKFLNTLNENFDDEIEFEENIQTNQTLPTKKDLDKSIQEIMLDN
                     ELAKLSLDITFFNIIKQLFPFTEFFMSQPDFFKKMILFFINSSSDKKYYGNKLFSSFK
                     AHFFKELGIFYALNPKGRFIKIIPKNKKPFNKLFLYQIGTNYYYFSQSEYASELKKSK
                     SFSRLDKAKQIHLISKARKQLAIDFSIKVSKNLSADVLKNLAGSFIDTLLPTNYERLK
                     ILYEKLMYEKLTYNYDFPLAVKKEEYITYPLLINSRFMSFDLSEFIFGSLLCTGGLNY
                     YPSIACTTTSSEAREFALKRLLSYIILDEKRSAFMEDKINEGKYILNDKEFFEKNNIE
                     LRNCNIYKLEHFQRKEEYKTQNPMIKQHPILAYNDTIQILYEYINGIYNTIQDEKGNF
                     IEDRQKARRLMENLETIGQHNLEVMYKGINEENDETEKIISQNKRHYKNIENLITNNK
                     SQDYSNKFIGRLATTIIMEDGLYMG"
     gene            complement(3915..4031)
                     /locus_tag="PIMPEBOA_00032"
     CDS             complement(3915..4031)
                     /locus_tag="PIMPEBOA_00032"
                     /inference="ab initio prediction:Prodigal:002006"
                     /inference="similar to AA sequence:RefSeq:CKG86393.1"
```

```
                     /codon_start=1
                     /transl_table=11
                     /product="methyltransferase small"
                     /protein_id="Prokka:PIMPEBOA_00032"
                     /translation="MQNLKLQIAQGLDKILEDEDRKQELYVCKFVVVNGVKV"
ORIGIN
        1 ttcttatacc acttcttata actaaatatt tatatctttc ttttttagtt tttagtgagt
       61 tataataagc tttttctgct ttagtccaag gagctatgct tcctttaatg ggatctttta
      121 aatatatact ttgccaatct ttaaattcaa gcaaagtgga tttttgccct gtgtgaaatt
      181 caggatctag atacctaggt tgataattaa tataaggaga ggttcttaaa attctatcgg
      241 cttcaaacaa agctttagaa tattcttggg tgtatttgcc tgtaggtttt tttgactttg
      301 actctgaaaa tagtattttt ccgctttgtt tatctacatt taatataaaa ccttcgggtg
      361 tataaatttc ataagtttca ctcattttta tcctttctta atttatccca tatataaacc
      421 atcttccatg attatagtag tggctagtct gcctataaac ttattagaat aatcttggga
      481 tttattattg gtaattaaat tctcaatatt tttataatgt cttttgtttt gcgatattat
      541 tttttctgtt tcatcattct cttcatttat ccccttatac atcacttcta aattatgttg
      601 tcctatggtt tctaaatttt ccatcaatct tcttgccttt tgcctatctt ctatgaaatt
      661 acccttttca tcctgtatag tattgtaaat tccatttatg tattcatata aaatttgtat
      721 agtatcatta taagctaaga tagggtgttg ttttatcata ggattttgtg ttttatactc
      781 ttcttttctt tgaaaatgtt ctagcttata tatattacaa tttctaagtt ctatattgtt
      841 tttttcaaaa aattccttat catttaagat atatttgccc tcattgattt tatcttccat
      901 aaaagcactt cttttctcat ctaaaataat ataagataaa agtcttttta aggcaaattc
      961 tctagcttca cttgaagttg tagtgcaagc aatactagga taataattaa gcccacctgt
     1021 gcacaataaa gaaccaaaaa taaactcaga taaatcaaaa ctcataaatc tagaatttat
     1081 caataaagga taggttatgt actcttcttt ttttacagct aaaggaaaat cataattgta
     1141 agtcaatttt tcatacatta gtttttcata taaaattttt aatctttcat aatttgtagg
     1201 caaaagtgta tcaataaaag aacctgctaa atttttaat acatccgcac ttaaattttt
     1261 acttacttta atagaaaaat caatagctaa ttgttttctt gcttttgata tcaaatgaat
     1321 ttgcttagct ttatccaatc ttgaaaaact ctttgatttt ttcaactcgc ttgcatattc
     1381 tgattgacta aaataataat aatttgttcc tatttgatat aaaaaaagct tattgaatgg
     1441 ctttttattt tttgggatga tttttataaa acgcccttta ggattaagtg cataaaatat
     1501 ccctagttct ttaaaaaaat gtgctttaaa actagaaaat aatttattgc cataatactt
     1561 tttatcagag cttgaattta taaaaaataa aatcattttt ttaaaaaaat caggctgact
     1621 cataaaaaat tctgtaaaag gaaaaagttg cttaatgata ttaaaaaaag tgatatctaa
     1681 ggatagtttt gcaagctcat tatcaagcat aatttcttga atacttttat ctaaatcctt
     1741 ttttgtagga agtgtttgat ttgtttgtat attttcttca aattcaattt catcatcaaa
     1801 gttttcattt aaggtgttta aaaatttcac ataagtttga tcatcttctt taagatcatt
     1861 ttcaaacata gctttagcta attcatctaa atagatgact tcatcttctt ttttattttt
     1921 atttaactct ttggataaaa agatgtaaat ttttttatca ttatcataaa aataaatatc
     1981 atcgggggata aataagatat actcattaaa ataatttggt attttaatcc tttggtaatt
     2041 aagtgaaatt tctttatcgc cataatctgg atttggaatt gcaatatttt taacaccaag
     2101 agaatattct tgcgtaaaag ctaagtcttt ttgtaaaaca tacaaagtta aagtcaataa
     2161 aacaaccgtt aaaggtgagt atttcttaaa atctacattt tcatatgttt tgaacattaa
     2221 tggttctgag ccaagaaaga attgtatttg atcataacga aaattgatct tatccaaata
     2281 atcattaaaa agctcgacat attcttttac tttttctttt tctttttcgc taattttctc
     2341 aagctcttta ggtaatttaa aaaagttatc aatataagca aaatcttgtt taaatctttc
     2401 atattcaaat cgttcattta tatattctaa taaataaagt ttcatccaag aatatgtttg
     2461 attataaaaa ttaaaatatc ccttatattt ttgataaatc tgtccatcaa gtttcgcaaa
     2521 aggacaaagt tgcaagacaa aatcatcttc ttttttaaca tctctttcta taaaataaga
     2581 accataatct cttttgctag gaaaatcgcc ttttaatgca agtttttcat tttttgtaag
     2641 agctttgggg cgattttttta ttattgctcc taattttttt tgatacaaat cttcgcaaag
     2701 ccctgcatta ttaagctgtg gtggattggt aataaataaa taatttttac ccattttggt
     2761 tgtattgatt tgatcagcat tttttttatc cagtatcatt tctactagca tggaagaatt
     2821 taaatcaggg tattctgcat aaaagaaatg attgagtttg gagatattta aatttatcgc
```

```
     2881 cttttttaat gttgtgtcag aattatttaa actagaattc actaaaagag tgttgtctaa
     2941 tttttcattt tcaaatttta gattattttc taaaaaatta tcaaaaacac cttgaaattc
     3001 ttcatctaaa ataattttat aaaaactatt tttataaaga ctgatagaag gtgcattagt
     3061 ataagatgaa aaatctaata atactttatc atctataatt aacacatcag ataaaatctc
     3121 ttcattgcat aaagcaataa taggataaag tttgctagtg ataagttcta aaagtggata
     3181 agcaaaatca taataatatt tttttccttt atactcttga tattcagagt aagaattata
     3241 agcttcaaat aatgtattta caatcaaaac ataaggattt tttttaatta aagaaatggg
     3301 ctttttaagg ctggttatag aatctaatat attgataaaa aatttcgcta cagctctaat
     3361 aatttgctta tattcatcat cattagattg ggcaatattt tttaaaagtg tgttaaaaag
     3421 cgtatctaaa gtaacttgta tctttggctt gatttcttga tcaaaataat tagattcttt
     3481 gatatttttt agtaatatat ttaattcttt ttgaatttct tttaaaaaat tttcgatatt
     3541 tttttcattg ctttcatcaa taattttatt gagtgtttta ttttcatttt gattgattgt
     3601 atcatttaaa agttttaaat tattatagat aattttttta atgctagata aatttttatc
     3661 agagctaaat ttttgttctt ttgtgatttc gtctttattg gtttgattta aatgtatgat
     3721 tttaaaagtt tttgtattgt caattttaaa attatcttca atagtatgta atgttttata
     3781 cagagaattc tcagcttctt tatatccatc aaaatcactg catataaaaa taattttatt
     3841 agcatttttg gtaagatttt ttaactcaat ttttaaatta tgatttgatg taattaaagt
     3901 aaaatttttt attttcatac tttaacccca tttaccacca caaatttaca aacataaagc
     3961 tcttgttttc tatcttcatc ttctaagatt ttatctaatc cttgtgcgat ttgaagtttt
     4021 aaattttgca tttttgcagg agttaaaaa
//
LOCUS       PIMPEBOA_4              3161 bp    DNA     linear       12-APR-2021
DEFINITION  Campylobacter species strain strain.
ACCESSION
VERSION
KEYWORDS    .
SOURCE      Campylobacter species
  ORGANISM  Campylobacter species
            Unclassified.
COMMENT     Annotated using prokka 1.14.6 from
            https://github.com/tseemann/prokka.
FEATURES             Location/Qualifiers
     source          1..3161
                     /organism="Campylobacter species"
                     /mol_type="genomic DNA"
                     /strain="strain"
     gene            368..550
                     /locus_tag="PIMPEBOA_00033"
     CDS             368..550
                     /locus_tag="PIMPEBOA_00033"
                     /inference="ab initio prediction:Prodigal:002006"
                     /inference="similar to AA sequence:RefSeq:CKG87535.1"
                     /codon_start=1
                     /transl_table=11
                     /product="methyltransferase small"
                     /protein_id="Prokka:PIMPEBOA_00033"
                     /translation="MPHRGRHPNEYHEYILEKMNKIDKIARGDKNKFLKEFEKLKEEI
                     KNNPAILRKEYYKERK"
     gene            550..1116
                     /locus_tag="PIMPEBOA_00034"
     CDS             550..1116
                     /locus_tag="PIMPEBOA_00034"
                     /inference="ab initio prediction:Prodigal:002006"
                     /inference="similar to AA sequence:RefSeq:CKG87555.1"
```

```
                     /codon_start=1
                     /transl_table=11
                     /product="hypothetical protein"
                     /protein_id="Prokka:PIMPEBOA_00034"
                     /translation="MRYYKMMYNGQHNDVDNWINCVKPDIKNNDKYALLESKPITNWQ
                     TPTFEIDKDDGKILTDLISNVYNWRIVSPKFINLMQDLIKDCVQYLDVEIRSQEINYY
                     DCKIMHVTKSLEALDYEHSVYTYMGDNDEYLSITKAVLKKSKLDGSHIFRIKDDEIPV
                     FVSNEFRKRVRENNLLGFSFSEVMVYEN"
     gene            1199..1750
                     /locus_tag="PIMPEBOA_00035"
     CDS             1199..1750
                     /locus_tag="PIMPEBOA_00035"
                     /inference="ab initio prediction:Prodigal:002006"
                     /inference="similar to AA sequence:RefSeq:CKG87579.1"
                     /codon_start=1
                     /transl_table=11
                     /product="hypothetical protein"
                     /protein_id="Prokka:PIMPEBOA_00035"
                     /translation="MTREQAELIIKEEKLIDTDWYPSYKHSGEYHLTMWFDSDNNKYE
                     AVYIGERGSVEFEYSFDSEKEAIDKMLQMNYEQKKYDGKDIKGYLTKEVALRIIKEEN
                     LEVIWYDEALKPLCAGIKHDKQRDKYISFITNAKAEIIEYRSIEFDGDEEFEGKIYND
                     EDIALYALINRARMIKQNKISFV"
     gene            1821..2171
                     /locus_tag="PIMPEBOA_00036"
     CDS             1821..2171
                     /locus_tag="PIMPEBOA_00036"
                     /inference="ab initio prediction:Prodigal:002006"
                     /inference="similar to AA sequence:RefSeq:CKG87641.1"
                     /codon_start=1
                     /transl_table=11
                     /product="hypothetical protein"
                     /protein_id="Prokka:PIMPEBOA_00036"
                     /translation="MEDLKLLQRRWEEAYEAMPKLYETPDELIINFTLSEDTDTILFK
                     EPWENFELDDEDKEVKWRLSFFSISKDEPLGYLEYKEALEKLQDFSLIQSEERILIKA
                     MSLEELESLELKGW"
     gene            2171..2803
                     /locus_tag="PIMPEBOA_00037"
     CDS             2171..2803
                     /locus_tag="PIMPEBOA_00037"
                     /inference="ab initio prediction:Prodigal:002006"
                     /inference="similar to AA sequence:RefSeq:CKG87668.1"
                     /codon_start=1
                     /transl_table=11
                     /product="hypothetical protein"
                     /protein_id="Prokka:PIMPEBOA_00037"
                     /translation="MLPQPIFLNKLIELLSDENLDIVNMKKGVDKIFSKKDFYAILEL
                     NIKIMNIAKLIFKSDEKLNILLPQISDHKNINYFLQELELKKEMDVNLNDLKNYDNKK
                     IIEIIKQKYQNNPVKELDINSQKEKIKILFIVNEVPKFLKSIYKECKFKEYGLANYLE
                     FDEIWDFYENNTRYFAILTPMGANKIKLRLENTQNSEKELNIIEVNLDKK"
ORIGIN
        1 cttgaaggtg cagcaattgt aaaactaatg aatttagata aagaagaatt taaacaatac
       61 aaatactttc cttatgattt aatttaatct tagtgggttt aaaaacccac aaggcttttc
      121 atggctagaa tttgctatac ctaaaaagaa taagattcaa tttgctttag ctaaaataca
      181 agctagtcct cttgctaaga ttgagtatga taggataaga tgggaaggga taggaggtaa
```

```
      241 aagaaatcaa ttaccaatcc aaacccatca cattgctact gataagagta aaaaatatac
      301 tccaaaattt caagagatac ttaatttata tgatttaaaa ctaaatggag attggaataa
      361 agttaaaatg ccacatcgcg gaagacatcc caatgaatat catgaataca ttcttgaaaa
      421 aatgaataaa attgataaaa tagctagagg ggataaaaat aaattcttaa aagaatttga
      481 aaaactaaaa gaagaaatca aaaataatcc tgctatactt cgtaaagaat actataagga
      541 gagaaaataa tgagatatta taaaatgatg tataatggac aacataatga tgtcgataat
      601 tggataaatt gtgttaaacc agatataaaa aataacgata aatatgcact tttagaatct
      661 aaacctatta caaattggca aacacctaca tttgaaatcg ataaagatga tggtaaaata
      721 ctgactgatt taatatctaa tgtatataat tggcgtatag tctctccaaa atttataaat
      781 ttaatgcaag atttaatcaa agattgtgtg caatatttag atgtagaaat tagaagtcag
      841 gaaataaatt attatgattg taaaattatg catgtaacaa aatcacttga ggctttagat
      901 tatgaacatt ctgtatatac ttatatgggt gataatgatg aatatttaag cattactaag
      961 gctgttttaa aaaaatcaaa acttgatgga agtcatatat ttagaattaa agatgatgaa
     1021 atacctgttt ttgtatcaaa tgaatttaga aaaagagtga gagaaaacaa tttattaggt
     1081 tttagtttta gtgaagttat ggtatatgaa aattaataaa catataaaaa tattataatc
     1141 ataaattata taaacttatt aggttttatc aaacaaatac cataataaag gatagaaaat
     1201 gacaagagaa caagcagaat taattataaa agaagaaaag ttgattgata cagattggta
     1261 tccttcttat aaacattcag gagaatacca tcttactatg tggtttgatt ctgataataa
     1321 taaatacgaa gctgtttata ttggagaaag aggcagtgtt gaatttgagt attcttttga
     1381 tagtgaaaaa gaagctattg ataaaatgtt gcagatgaat tatgaacaaa aaaaatacga
     1441 tggaaaagac attaaaggat atctaacaaa agaagtagca ctaagaatca tcaaagaaga
     1501 aaatctagaa gtgatttggt atgatgaagc acttaaacct ttatgtgcag gtattaaaca
     1561 cgataaacaa agagataaat acatttcttt tatcactaat gctaaagcag agattataga
     1621 atacagaagt attgagtttg atggggatga agaatttgaa ggaaaaatat ataatgatga
     1681 agatatagcc ctttatgctc ttatcaatcg tgccagaatg attaagcaaa ataagattag
     1741 ctttgtgtaa atactttcct tatgatttaa tttaaaaaga tttttagcta taatttcatt
     1801 ttatatttaa ggaaaaattt atggaagatt taaaactttt acaaaggcgt tgggaagaag
     1861 cttatgaagc tatgcctaaa ctttatgaaa caccggatga gttaataata aatttcaccc
     1921 taagtgaaga tacagatact attcttttta aagaaccttg ggaaaatttt gaattagatg
     1981 atgaagataa agaagtcaaa tggagactaa gtttttttag cattagtaaa gatgagcctt
     2041 tggggtattt agagtataaa gaagctttag agaaattgca agatttttct ttaattcaat
     2101 cagaagaaag aattttaatt aaagcgatga gtttagaaga gcttgaaagt ttagagctta
     2161 aagggtggta atgcttcctc aacctatatt tttaaataaa cttatagaat tgttatctga
     2221 tgagaattta gatattgtaa atatgaaaaa aggcgtagat aaaatttttt ctaaaaaaga
     2281 tttttacgct atactagaat taaatatcaa aattatgaat atagcaaaat taattttaa
     2341 aagtgatgaa aaattaaata tattattacc acaaatttct gatcataaga atataaatta
     2401 ttttcttcaa gaattagagt tgaaaaaaga aatggatgtt aatcttaatg atttaaaaaa
     2461 ttacgataat aaaaaaataa tagaaataat taaacaaaaa tatcaaaaca atccggttaa
     2521 agaattggat attaattctc aaaaagaaaa aatcaaaata ctttttattg ttaatgaagt
     2581 accaaaattt ttaaaatcaa tctataaaga atgcaaattt aaagaatatg gattagcaaa
     2641 ctatttggaa tttgatgaaa tttgggattt ttatgaaaat aatactagat attttgctat
     2701 tttaactcct atgggtgcaa ataaaataaa attgcgttta gaaaatacac aaaacagcga
     2761 aaaagaactc aatatcatag aagtaaattt agataaaaaa taaattttaa taacaaaaat
     2821 aagcaatttc ttttttctac tcaaatttag actatctgtg ttaaaatatt tcttaataga
     2881 tataaaaaca attataataa tagaaaactt aatgagttta cactaaaaac tacaacaaac
     2941 ccaaaaggat catcaatgtc ccttaattct tatatgagtc ttgatatatt aaatcatact
     3001 tcaaatcccc taccctttaa aatcactaaa gccttcatca aagaaagtct tgaagagctt
     3061 ttttctatag aatgtgaagg ctttttgaa agtttagaac aagatttatt ttctttaaat
     3121 actttaacca atgcttcatc tcatctaagc acccctacaa c
//
LOCUS       PIMPEBOA_5              2630 bp    DNA     linear       12-APR-2021
DEFINITION  Campylobacter species strain strain.
ACCESSION
VERSION
```

```
KEYWORDS    .
SOURCE      Campylobacter species
  ORGANISM  Campylobacter species
            Unclassified.
COMMENT     Annotated using prokka 1.14.6 from
            https://github.com/tseemann/prokka.
FEATURES             Location/Qualifiers
     source          1..2630
                     /organism="Campylobacter species"
                     /mol_type="genomic DNA"
                     /strain="strain"
     gene            28..552
                     /locus_tag="PIMPEBOA_00038"
     CDS             28..552
                     /locus_tag="PIMPEBOA_00038"
                     /inference="ab initio prediction:Prodigal:002006"
                     /codon_start=1
                     /transl_table=11
                     /product="hypothetical protein"
                     /protein_id="Prokka:PIMPEBOA_00038"
                     /translation="MLLCFALYLFPRLFNPSKEIDYKWFIVFFTFLLGIYAFARAIYK
                     MANIKRIYITKEKLVIEYYIKNDLVFPLGTFFVYYRKISYSLSPGHIVIYTFGDKVKE
                     YLEPGLFSDGDDPTKGCFERINAIIKPHVMPYLLSLSDEEFEKIFFHVNATSKIDTTF
                     LKEAMELRKEKKDE"
     gene            603..1241
                     /locus_tag="PIMPEBOA_00039"
     CDS             603..1241
                     /locus_tag="PIMPEBOA_00039"
                     /inference="ab initio prediction:Prodigal:002006"
                     /codon_start=1
                     /transl_table=11
                     /product="hypothetical protein"
                     /protein_id="Prokka:PIMPEBOA_00039"
                     /translation="MSEINNNTEIKEQDTQDEIIWEGKKHIPSFLLFWITYIVLLCFA
                     LYLFPRLFNPSKEIDYKWFIVFFTFLLGIYAFARAIYKMANIKRIYITKEKLVIEYYI
                     KNDLVFPLGTFFIYYRQISYSPSPGHIVIHTFGDKAKEYLEPGLFSDGDDPTKGCCEK
                     INAIIKPHVMPYLLSLSDEEFEKIFFHVNATSEIDTTFLKEAMELRKEKKDE"
     gene            1234..1386
                     /locus_tag="PIMPEBOA_00040"
     CDS             1234..1386
                     /locus_tag="PIMPEBOA_00040"
                     /inference="ab initio prediction:Prodigal:002006"
                     /codon_start=1
                     /transl_table=11
                     /product="hypothetical protein"
                     /protein_id="Prokka:PIMPEBOA_00040"
                     /translation="MNENIYGSLELYSKETQSTPSDKRDLGVFGDVAPYALGITEGFA
                     TKAFKG"
     gene            1390..2007
                     /locus_tag="PIMPEBOA_00041"
     CDS             1390..2007
                     /locus_tag="PIMPEBOA_00041"
                     /inference="ab initio prediction:Prodigal:002006"
                     /codon_start=1
```

```
                     /transl_table=11
                     /product="hypothetical protein"
                     /protein_id="Prokka:PIMPEBOA_00041"
                     /translation="MNNSTLPWKSKRKFYFLAILQYIIYLALGLFVCWFCWFVLLKES
                     LWYGFFAFGSLYYVFELHRALNFKSIILTEQGLILKMRWFDTEIFYPYGSFTMSYDIA
                     YGIRFFERIEIQNIKHIRKEFIFPCGVDSFGSFENNHTFKELCTKYTQKALETMNLEE
                     KVNLYKLYRANIKIFFNQEIDHNVFIIDFSPYIQEIKLNLKDKNE"
     gene            complement(2347..2496)
                     /locus_tag="PIMPEBOA_00042"
     CDS             complement(2347..2496)
                     /locus_tag="PIMPEBOA_00042"
                     /inference="ab initio prediction:Prodigal:002006"
                     /codon_start=1
                     /transl_table=11
                     /product="hypothetical protein"
                     /protein_id="Prokka:PIMPEBOA_00042"
                     /translation="MALILSKQPFVGSSPSLNKPGSRYSLTLSPKVYITISPGFDMDG
                     SDELR"
ORIGIN
        1 tttttgcttt tttggataac atatattgtg cttttatgtt ttgcgcttta tttgtttcct
       61 agattattta atcctagcaa agaaatagat tataaatggt ttatagtttt ttttacattt
      121 cttttaggca tatatgcatt tgcacgagcg atctataaaa tggcaaatat taaaagaatt
      181 tatatcacaa aagaaaaact tgtgattgag tattatatca aaaatgattt ggttttttcct
      241 ttaggaactt tttttgtgta ttatcgtaaa atttcatatt cactttctcc aggacatata
      301 gtgatataca ccttcggaga taaagtcaaa gaataccttg aacctggttt attcagtgat
      361 ggcgatgatc caacaaaagg ttgttttgaa agaattaatg ccattataaa acctcatgta
      421 atgccttatt tattaagctt aagtgatgaa gaatttgaaa aaatcttttt ccatgtaaac
      481 gctactagta aaattgatac cactttcctt aaagaagcaa tggaactcag aaaagagaaa
      541 aaagatgaat gaaaatattt tattttatat acaagagatt aaaacttatt taaaggataa
      601 aaatgagtga aataaacaat aacacagaaa tcaaagagca agacacccaa gatgaaatca
      661 tttgggaagg aaaaaagcat ataccttcgt ttttgctttt ttggataaca tatattgtgc
      721 ttttatgttt tgcgctttat ttgtttccta gattatttaa tcctagcaaa gaaatagatt
      781 ataaatggtt tatagttttt tttacatttc ttttaggcat atatgcattt gcacgagcga
      841 tctataaaat ggcaaatatt aaaagaattt atatcacaaa agaaaaactt gtgattgagt
      901 attatatcaa aaatgatttg gtttttcctt taggaacttt ttttatatat tatcgtcaaa
      961 tttcatattc accttctcca ggacatatag tgatacatac ctttggagat aaggccaaag
     1021 aataccttga acctggttta ttcagtgatg gcgatgatcc aacaaaaggt tgttgtgaaa
     1081 aaattaatgc cattataaaa cctcatgtaa tgccttattt attaagctta agtgatgaag
     1141 aatttgaaaa aatctttttc catgtaaacg ctactagtga aattgatact actttcctta
     1201 aagaagcaat ggaactcaga aaagagaaaa aagatgaatg aaaatattta tggaagttta
     1261 gagctttatt ctaaagaaac tcaaagcact cctagtgata aaagggattt aggagtattt
     1321 ggagatgttg ctccttatgc cttaggcatt acagaaggtt ttgctactaa agcttttaag
     1381 gggtaagcaa tgaataattc tactttacct tggaaaagca agagaaaatt ttacttcctt
     1441 gctattttgc aatatattat ttatcttgca ttaggtcttt ttgtgtgttg gttttgttgg
     1501 tttgttttgc ttaaagaatc tttgtggtat ggttttttttg cctttgggag tctttattat
     1561 gtttttgaat tacatagggc attgaatttt aaatccataa ttttaacaga gcagggatta
     1621 atattaaaaa tgcggtggtt tgatacagaa attttctatc cttatggaag ttttacaatg
     1681 agttatgaca ttgcctatgg gataagattt tttgaaagaa tagaaataca aaatataaaa
     1741 cacataagaa aagaatttat ttttccttgc ggtgtggatt cttttggtag ttttgaaaac
     1801 aatcatacat ttaaagaatt atgcaccaaa tatacacaaa aagctttaga gactatgaat
     1861 ttagaagaaa aagtaaatct ttataagctt tatagggcaa atataaaaat atttttttaat
     1921 caagaaatag atcacaatgt ttttattatt gatttctccc cttatataca agagattaaa
     1981 cttaatttaa aggataaaaa tgagtgaaat aaacaataac acagaaatca aagagcaaga
     2041 cacccaagat gaaatcattt gggaaggaaa aaaggatata ccttcgtttt tgctttattg
```

```
     2101 gatagcaaat attgtatttt tatgttttat tctttatttg tttcctagat tatttaatcc
     2161 tagcaaagaa atagattata aatggtttat agtttttgtt atatttcttt ttgggttata
     2221 tatacttgta aaaaatatct atcaaatggc aaatattaaa agaatttata tcacaaaaga
     2281 aaaacttgtg attgagtatt atattaaaaa tgatttggtt tttcctttag gaacttttttt
     2341 tgtatattat cgtagttcgt cacttccatc catatcaaat ccaggagata tagtgatata
     2401 cacctttgga gataaggtca aagaatacct tgaacctggt ttattcagtg atggcgatga
     2461 tccaacaaaa ggttgttttg aaagaattaa tgccattata aaacctcatg taatgcctta
     2521 tttattaagc ttaagtgatg aagaatttga aaaaatcatt tcacatgtaa gcggttataa
     2581 cgaaattggt accactttcc ttaaagaagc aatggaactc agaaaagaga
//
LOCUS       PIMPEBOA_6              2407 bp    DNA     linear       12-APR-2021
DEFINITION  Campylobacter species strain strain.
ACCESSION
VERSION
KEYWORDS    .
SOURCE      Campylobacter species
  ORGANISM  Campylobacter species
            Unclassified.
COMMENT     Annotated using prokka 1.14.6 from
            https://github.com/tseemann/prokka.
FEATURES             Location/Qualifiers
     source          1..2407
                     /organism="Campylobacter species"
                     /mol_type="genomic DNA"
                     /strain="strain"
     gene            40..510
                     /locus_tag="PIMPEBOA_00043"
     CDS             40..510
                     /locus_tag="PIMPEBOA_00043"
                     /inference="ab initio prediction:Prodigal:002006"
                     /codon_start=1
                     /transl_table=11
                     /product="hypothetical protein"
                     /protein_id="Prokka:PIMPEBOA_00043"
                     /translation="MPAFTSQSV*KSKVEFSDSKEYCSSIFVCMELDFKDVLSCIWAD
                     KF*WTLRKNSFSIVWVKDFSINNLFSPFTFLISSLWIIVF*FAPISISLFSPTNS*EF
                     LATLTLLSTPTFKVLLNPTMVSLDKPTLIKYSAPTLTFLAKSMV*V*TFLILSL"
ORIGIN
        1 gtaacaattt gagtatcacc tacttgatgg aggatttgat tgcctgcttt tacttcacaa
       61 tctgtttgaa agtcaaaagt agaattttct gattctaaag aatattgctc ttctatcttt
      121 gtttgcatgg aattagactt taaagatgtg ttatcttgta tatgggcgga taaattttga
      181 tggacattaa ggaaaaattc tttttctatt gtttgggtta aagatttttc tattaataat
      241 ttattttctc ccttcacatt ccttatctca tctttatgga taatagtatt ttgatttgca
      301 cctatttcta tatctttgtt ttctcctaca aactcatgag agtttttagc cactcttact
      361 ttattatcta ctcctacatt taaagtattg cttaatccta ctatggtatc tttagataaa
      421 cctacattga ttaaatattc agcccctaca ttgacatttt tagccaaatc tatggtttga
      481 gtatgaacct ttttaatcct ttcattataa attccatcta ctatagaatc tttatcattt
      541 aaaattcttt gagtgaaatt atgttgcact aattcatcat aatccttttg ggcttttaaa
      601 taaatttgtt ctttgtcttt tatatttgag agggtaagtt cattataacc ttcttcattt
      661 gtgcctatgg ttttagaaga aagagaagtt tgatgatcat taaaaggaag atttactaag
      721 cttggattag ccccattata caaactagag ctcacatagg gtttatctat atcatcatct
      781 aaaaaagaaa caatcacttc atctcccact cttggagtat gaaaaaagcc tgaatgattg
      841 cttgctatac ttgttgctac tcttaaaaaa ggagtatgat gataagattt ataagcattg
      901 cttgataaat gattagaaga gtttgtgaag tttgtattat ctttagaatc agtattgttt
```

```
      961 gtattttcat gagtattggt ttttatgttt gcacttaaaa gattattatc taattcctct
     1021 tgattagcat aaagattaat ccttacctta acccttccat actcatcagt ataaatagta
     1081 tttctctcac tttctatatc cttactctct cctataacaa taccttgagt attatgaggg
     1141 gctttaggct tagctttaaa acttggagta aagataatat tcttttttaa taaagttaaa
     1201 gtattagaat aagacttact taaatttaaa tcttttaaat ttaaatgatc attagtattg
     1261 atagaattag ctagtaaagc atcatctatt aagatttgag tattggcaat aataataaag
     1321 tctttaagca aagaagcttt atcttgtttt tcttcatctt gacttttagt atcttcatca
     1381 tcactcaatc cactcaaatt aaattcctta taatttaaag tgataaaatc tcccaaacta
     1441 agatgataaa tattactttt tgctagcaag ctttcattta agatcaaagc tctttttct
     1501 ttaagagaag gcttagtctt taaatcaata cttcttgtaa aagaatattc actctcataa
     1561 aaatgttcat tataataagc tttgttatta agttcttgct cataaggaac tttactagaa
     1621 tgtaaagata aaagatttaa aggagtatta gcattgatac tagaatgtgt aaaagcatta
     1681 gttcttaagc tttgctcttt atagatagaa gaaatacaag cttgatttaa aatattattc
     1741 acattaggat tgtactcaat ttctttattt tttacatttt tatacacatc acaaaaataa
     1801 atagtgtttt catcttcata aaaaaagatt ccattattgt gagctaatct tgtgataaat
     1861 tctaaatcgc tttcattgta ttgagagatg agttcttgtt cttcataatt aaaatgtata
     1921 tgagaataat caatttcttt atgtaagata tcttgataaa aaccaagggt ttgtttaatc
     1981 acttctatga tatgagtatg agtataaata cgattggctt tatttaaaga aagtcttatc
     2041 aagactgatt gaagcttaaa agaaaaaaaa tgcttgtatt ttatttgctt agtgtttgaa
     2101 gtgctatcat tgatatttaa agcactctca tgattgatac ctagatattt tatataagtg
     2161 atgattcctt tatagttttt tacttcatta tgatcaaagt ttaaggtatt gttctcataa
     2221 ggattatgga tacttaaaat agcttcttga tcgattaaaa cattaggatg aaaattaaaa
     2281 gttgtagggg tgcttagatg agatgaagca ttggttaaag tatttaaaga aaataaatct
     2341 tgttctaaac tttcaaaaaa gccttcacat tctatagaaa aaagctcttc aagactttct
     2401 ttgatga
//
LOCUS       PIMPEBOA_7              2139 bp    DNA     linear       12-APR-2021
DEFINITION  Campylobacter species strain strain.
ACCESSION
VERSION
KEYWORDS    .
SOURCE      Campylobacter species
  ORGANISM  Campylobacter species
            Unclassified.
COMMENT     Annotated using prokka 1.14.6 from
            https://github.com/tseemann/prokka.
FEATURES             Location/Qualifiers
     source          1..2139
                     /organism="Campylobacter species"
                     /mol_type="genomic DNA"
                     /strain="strain"
     gene            complement(23..667)
                     /locus_tag="PIMPEBOA_00044"
     CDS             complement(23..667)
                     /locus_tag="PIMPEBOA_00044"
                     /inference="ab initio prediction:Prodigal:002006"
                     /inference="similar to AA sequence:RefSeq:CKG87616.1"
                     /note="Domain of uncharacterised function (DUF1910)"
                     /codon_start=1
                     /transl_table=11
                     /product="hypothetical protein"
                     /protein_id="Prokka:PIMPEBOA_00044"
                     /translation="MVRDTSKDEAYFTKHIIECEEKIKKSEKILLELPFGKRETCLFC
                     ITDRKKCIAMDKYSRGDDINPVKKDLEAYVLLKQKNRLEFAIDIGYYRGNALELCIRV
```

```
                     LLDMDSACVLELIEEDERKRRDILNRDWFLHFMGSKGKNLNLERKCNCKEHELIKEFV
                     ATKDIEFLHQYMKKHTRLRDPLDTWDLEGAAIVKLMNLDKEEFKQYKYFPYDLI"
     gene            complement(826..1515)
                     /locus_tag="PIMPEBOA_00045"
     CDS             complement(826..1515)
                     /locus_tag="PIMPEBOA_00045"
                     /inference="ab initio prediction:Prodigal:002006"
                     /codon_start=1
                     /transl_table=11
                     /product="hypothetical protein"
                     /protein_id="Prokka:PIMPEBOA_00045"
                     /translation="MIQQENLDDIVLKFCKKLDKFSAQYGLKTEEFITYGVFDDLLFT
                     FEHYLEEEDDCISFSVSFRVKSMKSDEILCKIIGLDQPNLDFKILNQHGVRSFPFSFK
                     SMEFTNLNDLKQSGLDDMPSDSYHSFLKAYKESIDTLIEAFKTDTTNTIKGLYDFIYN
                     YEENEDTIWQDRTLMQVLLLIELNQKEQALKILKNNQKAIEERDFTIEYDDGSIEVFD
                     VLKHSIENDLC"
     gene            complement(1516..2133)
                     /locus_tag="PIMPEBOA_00046"
     CDS             complement(1516..2133)
                     /locus_tag="PIMPEBOA_00046"
                     /inference="ab initio prediction:Prodigal:002006"
                     /codon_start=1
                     /transl_table=11
                     /product="hypothetical protein"
                     /protein_id="Prokka:PIMPEBOA_00046"
                     /translation="MSIKGYPIFAIPKKNKIQFALAKIQASPLAKIEYDRIRWEGIGG
                     KRSKKNHSCKILFSSTQFNDKDKKRKQLKENQINGKRFEQKDFKIFKKECPNAQSQVT
                     LRVYDKNKPIVTTRVDAIAHCKGKIIIREAKSSKDAPLTQNQIKAFNLISQKNNDITI
                     ELRGNKVLKYDNKMLKKGDKILSKNVTIKISRPSGITTYKSTKGI"
ORIGIN
        1 tgggttttta aacccactaa gattaaatta aatcataagg aaagtatttg tattgtttaa
       61 attcttcttt atctaaattc attagtttta caattgctgc accttcaaga tcccaagtat
      121 ctagaggatc tcttaatctt gtatgttttt tcatatattg atgtaaaaac tcaatatcct
      181 tagtagctac aaattcttta ataagctcgt gttctttaca attacatttg cgttctaaat
      241 ttaaattctt acccttagaa cccataaaat gtaaaaacca atctctattg agaatatctc
      301 ttctttttct ctcatcttct tctattaatt ctaatacaca agcactatcc atatctaata
      361 atactctaat acaaagttca agagcatttc ctctataata accaatatca attgcaaatt
      421 ctaaacgatt tttttgtttt aataatacgt atgcttctaa atcttttttta actggattta
      481 tatcatcgcc acgagagtat ttatccatag caatacattt ttttctatct gttatacaaa
      541 aaagacaagt ttctcttttt ccaaaaggaa gctctaaaag tattttttcg gatttttttaa
      601 tttttttcttc acactctatt atatgtttcg taaaatacgc ttcatcttta ctagtatccc
      661 ttaccatttt ctatcctttt tattatggta tttgtttgat aaaacctaat aagtttatat
      721 aatttatgat tataatattt tatatattaa tcaatcttca catactaaaa cttcactaaa
      781 actaaaacct aataaattgt tttctcttac acattttaaa aaatcctaac aaagatcatt
      841 ttctatagaa tgttttaaaa catcaaatac ttctatactt ccatcatcat attctatagt
      901 aaagtctctt tcttcaatgg ctttttgatt attttttaga atttttaatg cttgttcttt
      961 ttgattaagt tctataagta gtaaaacttg cattaaggtt ctatcttgcc aaatagtatc
     1021 ttcattttct tcataattgt aaataaaatc ataaagccct tttattgtgt tagtagtatc
     1081 agttttaaaa gcttcaataa gagtatctat actttcttta taagctttta aaaaactatg
     1141 ataagaatca cttggcatat catctaatcc agattgtttt aaatcattca aattggtaaa
     1201 ttccatactt ttaaaagaaa atggaaaaga tctaacgcca tgttgattta aaattttaaa
     1261 atctaaatta ggctgatcaa gtcctattat cttacataaa atttcatcac ttttcataga
     1321 ttttactcta aaagaaacac taaaacttat gcaatcatct tcttcttcta aataatgttc
     1381 aaaagtaaaa agtaagtcgt caaaaacacc ataagtgata aattcttctg tttttaaacc
```

```
     1441 atattgtgct gaaaatttgt ccagtttttt gcaaaatttt agaacaatat catctaagtt
     1501 ttcttgttgt atcatttaaa ttccttttgt tgatttgtat gttgttattc cgctagggcg
     1561 tgatattttt attgtaacat tttttgataa tattttatct ccttttttta gcattttatt
     1621 gtcatatttc aaaactttat tacctcgtag ttctattgtt atgtcattgt ttttttgact
     1681 tattaaatta aatgctttta tttgattttg tgttagggga gcatctttag atgattttgc
     1741 ctccctgata ataattttac ctttacaatg agctattgca tccactctgg ttgtaacaat
     1801 gggtttattt ttatcataaa ctctgagagt tacttgggat tgtgcattag ggcattcttt
     1861 tttaaatatc ttaaaatcct tttgctcaaa tcgtttgccg ttaatttgat tttccttaag
     1921 ttgtttgcgt tttttatctt tatcattaaa ttgagtgcta gaaaaaagta ttttacatga
     1981 atgatttttc ttacttcttt tacctcctat cccttcccat cttatcctat catactcaat
     2041 cttagcaaga ggactagctt gtattttagc taaagcaaat tgaatcttat tctttttagg
     2101 tatagcaaat ataggatagc cttttatact cactcctat
//
LOCUS       PIMPEBOA_8              1875 bp    DNA     linear       12-APR-2021
DEFINITION  Campylobacter species strain strain.
ACCESSION
VERSION
KEYWORDS    .
SOURCE      Campylobacter species
  ORGANISM  Campylobacter species
            Unclassified.
COMMENT     Annotated using prokka 1.14.6 from
            https://github.com/tseemann/prokka.
FEATURES             Location/Qualifiers
     source          1..1875
                     /organism="Campylobacter species"
                     /mol_type="genomic DNA"
                     /strain="strain"
     gene            7..630
                     /locus_tag="PIMPEBOA_00047"
     CDS             7..630
                     /locus_tag="PIMPEBOA_00047"
                     /inference="ab initio prediction:Prodigal:002006"
                     /codon_start=1
                     /transl_table=11
                     /product="hypothetical protein"
                     /protein_id="Prokka:PIMPEBOA_00047"
                     /translation="MSIKGYPIFAIPKKNKIQFALAKIQASPLAKIEYDRIRWEEIGG
                     KLKAAQRRRREKSKEKAKMLLYLENENKKDKVSDQEVHLYKHNGIWPKITPKPRSSDY
                     ILEDGEIILNIKQRAMEIKNTLNGGYNSISIKTKDKLTRYDLDGKPHYEKTSKKIIDT
                     PHKIEYTKHINPQDTTKYRMSQGAVEPISHKDLDIVENYLKRQNNEI"
     gene            620..1006
                     /locus_tag="PIMPEBOA_00048"
     CDS             620..1006
                     /locus_tag="PIMPEBOA_00048"
                     /inference="ab initio prediction:Prodigal:002006"
                     /codon_start=1
                     /transl_table=11
                     /product="hypothetical protein"
                     /protein_id="Prokka:PIMPEBOA_00048"
                     /translation="MKFENLKHGSLFKREFYLKDVKIDLRNDFYVYSITMNKNTLEIF
                     FKSEKKDIQDLQIIFYDYKLLHPKSYCKNFNQLEANFIDLIRNNWDDITGYIEKEEEM
                     LISIYFDNDLEYITLTCKYFNIDFID"
     gene            1021..1578
```

```
                     /locus_tag="PIMPEBOA_00049"
     CDS             1021..1578
                     /locus_tag="PIMPEBOA_00049"
                     /inference="ab initio prediction:Prodigal:002006"
                     /inference="similar to AA sequence:RefSeq:CKG87579.1"
                     /codon_start=1
                     /transl_table=11
                     /product="hypothetical protein"
                     /protein_id="Prokka:PIMPEBOA_00049"
                     /translation="MTREQAELIIKEEKLIDTDWYPSYKHSGEFHLTMWFDSDNNKYE
                     TFYVGERGSVELEYSFDSEKEAIDKMLQMNYEQKKYDGKDIKEYLTKEVALRIIKEEN
                     LEVIWYDEALKPMRAGIKHDKQRDKYISFITNAKAEIIEYRSIEFDGDRYSETFKEML
                     NDENIALYALINRARMIKQNRISFT"
ORIGIN
        1 ataggagtga gtataaaagg ctatcctata tttgctatac ctaaaaagaa taagattcaa
       61 tttgctttag ctaaaataca agctagtcct cttgctaaga ttgagtatga taggataaga
      121 tgggaagaga taggaggtaa attaaaagct gcacaaagaa ggagaagaga aaaaagtaaa
      181 gagaaagcaa aaatgctttt atatttggaa aatgaaaata aaaaagataa agtaagtgat
      241 caagaagttc atctttataa acataatgga atatggccta aaattactcc aaaacctaga
      301 agttctgatt atattcttga agatggagag attattttaa acatcaaaca aagagccatg
      361 gaaataaaaa atactttaaa tggagggtat aatagtattt ctataaagac aaaagataaa
      421 cttaccagat atgatttaga tggtaaacca cattatgaga agacatcaaa gaaaataatt
      481 gatacacctc ataaaataga atatactaaa catataaatc ctcaagatac aacaaagtat
      541 agaatgtcac agggagcagt agagccaata tctcataaag atttagatat tgtagaaaat
      601 tatttaaaaa ggcaaaataa tgaaatttga gaatttaaag catggaagtt tattcaaaag
      661 agaattttat cttaaagatg taaaaataga tttaagaaat gacttttatg tttattctat
      721 cacaatgaat aaaaacactt tggaaatatt ttttaaatca gagaaaaaag atatacaaga
      781 tttgcaaatt atttttatg attataagtt gctacatcca aaatcatatt gtaaaaattt
      841 taatcaatta gaagctaatt ttatagattt gataagaaat aattgggatg atataacagg
      901 ctatattgaa aaagaagaag agatgttgat aagcatatat tttgataatg atttagaata
      961 tattacttta acttgtaagt attttaatat agattttata gactgaataa aggatagaaa
     1021 atgacaagag aacaagcaga attaatcata aaagaagaaa agttgattga tacagattgg
     1081 tatccttctt ataaacattc aggagaattt catcttacta tgtggtttga ttctgataat
     1141 aataaatatg aaactttcta tgttggagaa aggggcagtg ttgaacttga gtattctttt
     1201 gatagtgaaa aagaagccat tgataaaatg ttgcagatga attatgaaca aaaaaaatac
     1261 gatggaaaag acattaaaga atatctaaca aaagaagtag cactaagaat catcaaagaa
     1321 gaaaatctag aagtgatttg gtatgatgaa gcacttaaac caatgcgagc aggtatcaaa
     1381 cacgataaac aaagagataa atatatttct tttatcacta atgctaaagc agagattata
     1441 gaatacagaa gtattgagtt tgatggggat agatacagtg aaacttttaa ggaaatgctt
     1501 aacgatgaaa atatagccct ttatgctctt atcaatcgtg ccagaatgat taagcaaaat
     1561 aggattagct ttacgtaaat actttcctta taatttaatt taatcttagt gggtttaaaa
     1621 acccacaagg cttttcatgg ctagaatttg ctatacccaa aaagaataag attaaatttg
     1681 ctttagctaa aatacaagct agtcctcttg ctaagattga gtatgatagg ataagatggg
     1741 aagggatagg aggtaaaaga agtaagaaaa ataaaaatga tagagcaaag caaatacaag
     1801 cattaaaaaa taatcagcaa aacaacaaac aaaattcatt tttacaagaa gttgaacaac
     1861 aaaaagaaga ggctg
//
LOCUS       PIMPEBOA_9             1821 bp    DNA     linear       12-APR-2021
DEFINITION  Campylobacter species strain strain.
ACCESSION
VERSION
KEYWORDS    .
SOURCE      Campylobacter species
  ORGANISM  Campylobacter species
```

```
            Unclassified.
COMMENT     Annotated using prokka 1.14.6 from
            https://github.com/tseemann/prokka.
FEATURES             Location/Qualifiers
     source          1..1821
                     /organism="Campylobacter species"
                     /mol_type="genomic DNA"
                     /strain="strain"
     gene            67..321
                     /locus_tag="PIMPEBOA_00050"
     CDS             67..321
                     /locus_tag="PIMPEBOA_00050"
                     /inference="ab initio prediction:Prodigal:002006"
                     /inference="similar to AA sequence:RefSeq:CKG86821.1"
                     /codon_start=1
                     /transl_table=11
                     /product="hypothetical protein"
                     /protein_id="Prokka:PIMPEBOA_00050"
                     /translation="MLDPLEIEKLSPQEYRVRNYFKFLTFATMFFLVITFIILPCLLQ
                     AYLFKCYSTGEKRKKLSWFIGFVCNAPILIASVYVVYENL"
     gene            440..931
                     /locus_tag="PIMPEBOA_00051"
     CDS             440..931
                     /locus_tag="PIMPEBOA_00051"
                     /inference="ab initio prediction:Prodigal:002006"
                     /inference="similar to AA sequence:RefSeq:CKG86719.1"
                     /codon_start=1
                     /transl_table=11
                     /product="hypothetical protein"
                     /protein_id="Prokka:PIMPEBOA_00051"
                     /translation="MGEDITLSIFWGLIGFILACSVIEFYLIALSVLIVFSILNYWIK
                     INIKEFVKSYFLISFIIFFMAIVIVFYLTYDLETSRGLVLLGPPEIEKLSPQEYKARN
                     YFDFLGGITFIFLVITFFILPCLLQAYLFKCYSTGEKRKKLSWFIGFVCNPILMAGVY
                     VMY"
ORIGIN
        1 tttttttacag ctattattat catattttat cttagctatg atcttgaatt ttataaatcg
       61 cttgtattgc ttgatcctct agaaattgaa aaattatcac cgcaagaata tagagtgcgt
      121 aattatttta aatttttaac ctttgcaact atgttttttt tagtaatcac atttattata
      181 cttccttgtt tattgcaagc ttatttattt aaatgttata gcacaggaga aaaacgcaaa
      241 aaattaagtt ggtttatagg gtttgtgtgt aatgctccta tattaattgc gagtgtgtat
      301 gtggtatatg aaaatcttta aagactatca aaaattcact ccttatggtt tggattttaa
      361 actttatagt aaagatttta tcactaagct tgattttaaa gataaagctt ataatgtgct
      421 ttataataca ggagtttaaa tgggagaaga tattacactt tctatttttt ggggtttgat
      481 aggatttatt cttgcttgta gtgttataga attttatctt atagctctaa gcgttcttat
      541 tgtcttttct attttaaatt attggatcaa gattaatata aaagaatttg taaaatctta
      601 tttttaata tcttttatta tcttttttat ggctattgtt atcgtatttt atcttactta
      661 tgatcttgaa acttctaggg gacttgtatt acttggtcct ccagaaattg aaaaattatc
      721 accacaagaa tataaagcac gtaattattt tgatttttta ggcggtataa cttttatttt
      781 tttagtaatc acatttttta tacttccttg tttattacaa gcttatttat ttaaatgcta
      841 tagcacagga gaaaaacgca aaaaattaag ttggtttata gggtttgtgt gtaatcctat
      901 attaatggcg ggtgtgtatg tgatgtatta aaatctttaa agacttttaa aaaatttaat
      961 atatttgata taatatttat ttatcttaaa tatttagaga aaaaatgaat agagaattat
     1021 ttataacaag acttgcattt ataaacaatg atgaaataaa taaaataaat aaaaattcat
     1081 ctattgcata tttaaaagcc ttatatcaat gtgaaaattc catagctgta aattatgatg
```

```
     1141 aagaagagct tttcacccaa accactgcta taaacaatct aggatatggc aatgatttca
     1201 caaaaatctt catcctagat aaagaaaacc tcaatgacac ctatcttgat gctagaaaaa
     1261 agctttataa atctatcaat actttaaaag aagaaaaaaa tgaaaaaata aaaaatatcc
     1321 aagcttatgt aaataaagaa aattccttgc aagacaatct aaataatcaa agctttaata
     1381 ctgaggaaaa tgatgttaat ttaattttaa acgaagtatt taacatagag caattttatg
     1441 ataaaaatac caataccata gtattaaaga caaataatat aaaaatcata ttcttagata
     1501 aagtaaattt acaagatttg caagataatt ctaacaatac ttctttcatc tctatgtcaa
     1561 atttaatcca tatttatgag aatcattgtg atatttttac gaaagataga agtattttag
     1621 atataaaatt tatagaagaa aaatatcaaa tagattttaa aagtttagat acaaaaatct
     1681 tcctaaactc caccctcctt acaggctcca ccgaactccc taataatcct ttttactttg
     1741 gagaattaga tcaagataat actataaaac aagatatacc cagttattat ttttctccta
     1801 aagatgaaag cagtggttta g
//
LOCUS       PIMPEBOA_10             1275 bp    DNA     linear       12-APR-2021
DEFINITION  Campylobacter species strain strain.
ACCESSION
VERSION
KEYWORDS    .
SOURCE      Campylobacter species
  ORGANISM  Campylobacter species
            Unclassified.
COMMENT     Annotated using prokka 1.14.6 from
            https://github.com/tseemann/prokka.
FEATURES             Location/Qualifiers
     source          1..1275
                     /organism="Campylobacter species"
                     /mol_type="genomic DNA"
                     /strain="strain"
     gene            112..609
                     /locus_tag="PIMPEBOA_00052"
     CDS             112..609
                     /locus_tag="PIMPEBOA_00052"
                     /inference="ab initio prediction:Prodigal:002006"
                     /inference="similar to AA sequence:RefSeq:CKG86821.1"
                     /codon_start=1
                     /transl_table=11
                     /product="hypothetical protein"
                     /protein_id="Prokka:PIMPEBOA_00052"
                     /translation="MGDSITLSIFVGSLLSIAVITKFYLIALSVLIVFSILNYWIKIN
                     IKEFVKSYFLVYFTIFFTAIVIIFYLTYGIETSKDLALLSSLEAKELSPQEYRVRNYF
                     KFLTFATMFFLVITFIILPCLLQAYLFKCYSAGEKRKKLSWFIGFVCNIIILIASAYL
                     MYIFI"
     gene            728..1231
                     /locus_tag="PIMPEBOA_00053"
     CDS             728..1231
                     /locus_tag="PIMPEBOA_00053"
                     /inference="ab initio prediction:Prodigal:002006"
                     /inference="similar to AA sequence:RefSeq:CKG86821.1"
                     /codon_start=1
                     /transl_table=11
                     /product="hypothetical protein"
                     /protein_id="Prokka:PIMPEBOA_00053"
                     /translation="MGEDITFSIFWGLFGFILAFTNIKFYLTTLSVLIVFSILNYWIK
                     INIKEFVKSYFSIYFIIFFTAIVIIFYLTYGIETSKDLALLSSLEAKELSPQEYKARN
```

```
                     YFGFLTCMTMFFLVITFIILPCLLQAYLFKRYSTEEKRKKLSWFIGFLCNIIILIASA
                     YLMYIFI"
ORIGIN
        1 caaaaattca ctccttatgg tttggatttt agactttata gtaaagattt tatcactaaa
       61 cttgatttta aagataaagc ttataatgtg ctttataata caggagttta aatgggagat
      121 agtattacgc tttctatttt tgtaggttca ttgctatcca ttgctgttat cacaaaattt
      181 tatcttatag ctttatctgt tcttattgtc ttttctattt taaattattg gattaagatt
      241 aatataaaag aatttgtaaa atcttatttt ttagtatatt ttactatctt ttttacagct
      301 attgttatca tattttatct tacctatggt attgaaactt ctaaggatct tgcattgctt
      361 agctctctag aagctaaaga attatcaccg caagaatata gagtgcgtaa ttattttaaa
      421 tttttaacct ttgcaactat gtttttttta gtaatcacat ttattatact cccttgttta
      481 ttgcaagctt atttatttaa atgttatagc gcaggagaaa aacgcaaaaa attaagttgg
      541 tttatagggt ttgtgtgcaa tattattata ttaattgcaa gtgcatattt gatgtatata
      601 tttatttaaa gactatcaaa aattcactcc ttatggtttg gattttagac tttgtagtaa
      661 agattttatc actaagcttg attttaaaga taaagcttat aatgtgcttt atgatacagg
      721 agtttaaatg ggagaagata ttacattttc tattttttgg ggtttatttg gatttattct
      781 tgcttttact aatataaaat tttatcttac cactttaagt gttcttattg tcttttctat
      841 tttaaattat tggattaaaa ttaatataaa agaatttgta aaatcttatt tttcaatata
      901 ttttattatc tttttacag ctattgttat catattttat cttacctatg gtattgaaac
      961 ttctaaggat cttgcattgc ttagctctct agaagctaaa gaattatcac cacaagaata
     1021 taaagcacgt aattattttg gatttttgac ttgtatgact atgttttttt tagtaatcac
     1081 atttattata cttccttgtt tattgcaagc ttatttattt aaacgctata gcacagaaga
     1141 aaaacgcaaa aaattaagtt ggtttatagg gtttttatgc aatattatta tattaattgc
     1201 aagtgcatat ttgatgtata tatttattta aagactatca aaaattcact ccttatggtt
     1261 tggattttag acttt
//
LOCUS       PIMPEBOA_11             1237 bp    DNA     linear       12-APR-2021
DEFINITION  Campylobacter species strain strain.
ACCESSION
VERSION
KEYWORDS    .
SOURCE      Campylobacter species
  ORGANISM  Campylobacter species
            Unclassified.
COMMENT     Annotated using prokka 1.14.6 from
            https://github.com/tseemann/prokka.
FEATURES             Location/Qualifiers
     source          1..1237
                     /organism="Campylobacter species"
                     /mol_type="genomic DNA"
                     /strain="strain"
ORIGIN
        1 ctccttacag gctccaccga actccctaat aatccttttt actttggaga attagatcaa
       61 gataatacta taaaacaaga tatacccagt tattattttt ctcctaaaga tgaaagcagt
      121 ggtttaggaa gacttagcat cttttataaa aatgatgaac tttgcttgct taattattct
      181 atattagaca attctttaaa tatcaaatta gaatgcttaa gcaaacaaag cttagaatac
      241 aaagatttaa tttcaaacac tctaaaagaa caaaaaacca cacaagtaga taaaaaacaa
      301 agcatagcca aacttcacgc tcttttagaa aatcaaaacc tagaatgtat ccatggaggt
      361 aaagtcatac ttaaatcaaa caaaggaaaa tcctttaaaa gtgatggtat ccctataatg
      421 ctagaaagtg atttattaaa ctctagtata gtagcttgtc ctcacaccat agcaaatgta
      481 agttatcctt gtactaaggt agtggatatt aaaggctcct tatctcaaaa gaaagtaaat
      541 aatgaatttg taatcttaca agaactcatc tcagcttgca ctacagataa aggctttgct
      601 ttaaaagtaa gctttactcc aagtaagttt aaatttgatc acagctttga tcctgaggag
      661 ggcttaggag aacaaagtaa aaatcaaata gagttaaaag agcctataat aagacttcat
```

```
      721 tataaaagtg atagatttca aaaagataat ttgcctattt ataatctttt aatcaataat
      781 gaaaaaaaag aacaagataa ggctttaaat gaattcaata tagaacaaaa agatttaaaa
      841 gatatagagg atataaatat ctttaatcaa tttaaacaag actttagtaa agatcatgaa
      901 tttaaagaat taaatcttag cttcgatact aatttaatca agctttattt cattatcccc
      961 aaaaacattg ctaaagttta taaaagtgct tataaagaat ttaaaaacaa agatctagga
     1021 gcagggtatt ttacacagct acatgaatat gataaaatca ttaaaaattc tttagaagat
     1081 aagaaagaat taaacgaata tcatttaact tttttaactc ctgcaaaaat gcaaaattta
     1141 aaacttcaaa tcgcacaagg attagataaa atcttagaag atgaagatag aaaacaagag
     1201 ctttatgttt gtaaatttgt ggtggtaaat ggggtta
//
LOCUS       PIMPEBOA_12             1123 bp    DNA     linear       12-APR-2021
DEFINITION  Campylobacter species strain strain.
ACCESSION
VERSION
KEYWORDS    .
SOURCE      Campylobacter species
  ORGANISM  Campylobacter species
            Unclassified.
COMMENT     Annotated using prokka 1.14.6 from
            https://github.com/tseemann/prokka.
FEATURES             Location/Qualifiers
     source          1..1123
                     /organism="Campylobacter species"
                     /mol_type="genomic DNA"
                     /strain="strain"
     gene            complement(113..265)
                     /locus_tag="PIMPEBOA_00054"
     CDS             complement(113..265)
                     /locus_tag="PIMPEBOA_00054"
                     /inference="ab initio prediction:Prodigal:002006"
                     /codon_start=1
                     /transl_table=11
                     /product="hypothetical protein"
                     /protein_id="Prokka:PIMPEBOA_00054"
                     /translation="MNENIYGSLEFYSKETQSTPSDKRDLGVFGDVAPYALGITESFT
                     TKAFKG"
     gene            complement(258..890)
                     /locus_tag="PIMPEBOA_00055"
     CDS             complement(258..890)
                     /locus_tag="PIMPEBOA_00055"
                     /inference="ab initio prediction:Prodigal:002006"
                     /codon_start=1
                     /transl_table=11
                     /product="hypothetical protein"
                     /protein_id="Prokka:PIMPEBOA_00055"
                     /translation="MSEINNNTEIKEQDTQDEIIWEGKKEIPSFLLYWITYIVLLCFM
                     LYLLPRTFDNTKNFKWFVFFIMFLLGIYIFAREIYKMANIKRIYVTKEKLVVEYYIKN
                     DLVFPLGTFFVYYRSLPFGLTPGDMVIYTFGDKVKEYLEPGLFSAGDDPTKGCCEKIN
                     AIIKPHVMPYLLSLSDEEFEKIISHVSGFSEIDTTFLKEAMELRKEKKDE"
ORIGIN
        1 aacaaaacca acacagaaaa aaagctaata taaaataaag agagcaaaaa aacaaactaa
       61 aaggaataat ttttctctta cttttccaaa gtaaagtaga attattcatt gcttacccct
      121 taaaagcctt agtagtaaaa ctttctgtaa tgcctaaagc ataaggagca acatctccaa
      181 atactcctaa atctctttta tcactaggag tgctttgagt ttctttagaa taaaactcta
```

```
      241 aacttccata aatattttca ttcatctttt ttctcttttc tgagttccat tgcttcttta
      301 aggaaagtag tatcaatttc gctaaaaccg cttacatgtg aaatgatttt ttcaaattct
      361 tcatcactta agcttaataa ataaggcatt acatgaggtt ttataatggc attaattttt
      421 tcacaacaac cttttgttgg atcatcgcca gcactgaata aaccaggttc aaggtattct
      481 ttgaccttat ctccaaaggt gtatatcacc atatctcctg gagtaagccc aaatggcaaa
      541 ctacgataat atacaaaaaa agttcctaaa ggaaaaacca aatcattttt aatataatac
      601 tcaaccacaa gtttttcttt tgtgacataa attcttttaa tatttgccat tttatagatc
      661 tctcgtgcaa atatatatat gcctaaaaga aacataataa aaaacacaaa ccatttaaaa
      721 ttcttagtat tgtcaaatgt cctaggaagc aaataaagca taaaacataa aagcacaata
      781 tatgttatcc aataaagcaa aaacgaaggt atttcctttt ttccttccca aatgatttca
      841 tcttgagtgt cttgttcttt gatttctgtg ttattgttta tttcactcat ttttatcctt
      901 tactctgctt ttacttctcc acctttaacc acaagtccat tagagtctat aatcacttct
      961 actccacctg ctttaatgat aacacaatct ttcttggtaa caatttgagt atcacctact
     1021 tgatggagga tttgattgcc tgcttttact tcacaatctg tttgaaagtc aaaagtagaa
     1081 ttttctgatt ctaaagaata ttgctcttct atctttgttt gca
//
LOCUS       PIMPEBOA_13             1121 bp    DNA     linear       12-APR-2021
DEFINITION  Campylobacter species strain strain.
ACCESSION
VERSION
KEYWORDS    .
SOURCE      Campylobacter species
  ORGANISM  Campylobacter species
            Unclassified.
COMMENT     Annotated using prokka 1.14.6 from
            https://github.com/tseemann/prokka.
FEATURES             Location/Qualifiers
     source          1..1121
                     /organism="Campylobacter species"
                     /mol_type="genomic DNA"
                     /strain="strain"
     gene            161..541
                     /locus_tag="PIMPEBOA_00056"
     CDS             161..541
                     /locus_tag="PIMPEBOA_00056"
                     /inference="ab initio prediction:Prodigal:002006"
                     /inference="similar to AA sequence:RefSeq:CKG87598.1"
                     /codon_start=1
                     /transl_table=11
                     /product="methyltransferase small"
                     /protein_id="Prokka:PIMPEBOA_00056"
                     /translation="MQMDKYYKAKGYKRISLNKVKGLDDKIHHEMDGIYYNPDKNPKY
                     IIAEAKFGTSKLDRKTKQMSDEWLIKNKLKQLKDNVGEKQAKEIKKLLNSNEVRKDLF
                     HVDEGGKETIDYLDKDANKIKPKG"
ORIGIN
        1 cctctagata cttgggatct tgaaggtgca gcaattgtaa aactaatgaa tttagataaa
       61 gaagaattta aacaatacaa atactttcct tatgatttaa tttaatctta gtgggtttaa
      121 aaacccaaaa aataatcagc aaaaaggtaa ttatgctgaa atgcaaatgg ataaatatta
      181 caaagctaaa ggctataaaa gaattagttt aaataaagtc aaagggcttg atgataaaat
      241 tcatcatgaa atggatggaa tttattataa tccagataaa aacccaaaat atatcatcgc
      301 agaagctaag tttggaacaa gcaaattaga tagaaaaact aagcaaatga gtgatgaatg
      361 gttaataaag aataaactaa aacagcttaa agataatgta ggagaaaaac aggccaaaga
      421 aataaaaaaa cttcttaata gcaatgaagt cagaaaagat ttatttcatg ttgatgaagg
      481 aggtaaagaa actattgatt atttagataa agatgcaaat aaaataaaac ccaaaggata
```

```
      541 gaaaatggta agggatacta gtaaagatga agcgtatttt actaaagaga taatagaaag
      601 tgaagaagat atcaaagaat ccgaaaaaat acttttagag cttcctttg gacaaagaga
      661 aacttgtctt ttttgtatag aagatagaaa atttcatctt gctatggata aatactctcg
      721 tggtgatgat atagacatag ttaaaaaaga tttagaagca tacatactag aaaaagaaaa
      781 aaatcgttta gaaaaaggat tggatattag ctggtttgat ggaaatgctc ttgaactttg
      841 tgttagagta ttactagata tggatagtgc ttgtgtatta gaattaatag aagaagatga
      901 gagaaaaaga agagatattc tcaatagaga ttggttttta cattttatag gatctaaggg
      961 taagaattta aatttagaac gcaaatgtat tcgcaaagaa cacgagctta ttaaagaatt
     1021 tgtagctact aaggatattg agtttttaca tcaatatatg aaaaaacata caagattaag
     1081 agatcctcta gatacttggg atcttgaagg tgcagcaatt g
//
LOCUS       PIMPEBOA_14             1024 bp    DNA     linear       12-APR-2021
DEFINITION  Campylobacter species strain strain.
ACCESSION
VERSION
KEYWORDS    .
SOURCE      Campylobacter species
  ORGANISM  Campylobacter species
            Unclassified.
COMMENT     Annotated using prokka 1.14.6 from
            https://github.com/tseemann/prokka.
FEATURES             Location/Qualifiers
     source          1..1024
                     /organism="Campylobacter species"
                     /mol_type="genomic DNA"
                     /strain="strain"
     gene            complement(475..972)
                     /gene="trxB_2"
                     /locus_tag="PIMPEBOA_00057"
     CDS             complement(475..972)
                     /gene="trxB_2"
                     /locus_tag="PIMPEBOA_00057"
                     /inference="ab initio prediction:Prodigal:002006"
                     /inference="similar to AA sequence:RefSeq:CKG86288.1"
                     /codon_start=1
                     /transl_table=11
                     /product="thioredoxin reductase"
                     /protein_id="Prokka:PIMPEBOA_00057"
                     /translation="MLPFIDEIMGVDWVIDLNKYDIASDESGKIYKALRSDVVEGKIK
                     DPRDIDSTYESRREFDRHRGGYKNGMREGYETDTPNDWSEEEAQLFNDTLILHAKLAA
                     LTPPQGYPNAPYYFTPENLEWYYKRHKLDKLLDPRIPAIYRYNFPRELRAKILKFGEE
                     NGIKE"
ORIGIN
        1 ttcttatacc acttcttata actaaatatt tatatctttc ttttttagtt tttagtgagt
       61 tataataagc tttttctgct ttagtccaag gagctatgct tcctttaatg ggatctttta
      121 aatatatgct ttgccaatct ttaaattcaa gcaaagtgga tttttgccct gtgtgaaatt
      181 caggatctag atacctaggt tgatagtctt tgtagggaga gttttgtttg atattgtgtg
      241 cttcaaaaag agctctagaa tattcttggg tgtatttgcc tacttcacgc ccatcttttc
      301 ttttatcaaa tataatttga tttgtatttt tatcaacttc taatataagt ccattagaag
      361 tatagatctc gtaaatatct ttattatcac tcatttttat cctttcttaa tttatcccat
      421 atataaacca tcttccatga ttatagtagt ggctaatctg cctatgtaat tactttattc
      481 tttaatacca ttttcttcgc caaatttgag tatcttagct ctaagttccc ttgggaaatt
      541 atatctataa atagcaggga ttcttggatc aaggagttta tcaagcttat gtcttttata
      601 ataccattct agattttcag ggtaaaata ataaggtgca ttaggatagc cttgaggagg
```

```
      661 agtgagggct gctagtttgg catgaagtat taaagtatca ttaaaaagtt gtgcttcttc
      721 ttcgctccaa tcattgggtg tatcggtttc atagccttcc ctcatgccat ttttataccc
      781 acctctatgg cgatcaaatt cccttctgct ttcataagta gaatctatat ctcttggatc
      841 ttttatcttg ccctcaacta catcacttct taaggctttg tatatttttc cagattcatc
      901 actagctata tcatatttat ttaaatcaat cacccaatct actcccataa tctcatctat
      961 aaaaggaagc ataccaaatt catcaggttt taaagtctta gctaaggttt taatttgttg
     1021 ttct
//
LOCUS       PIMPEBOA_15              909 bp    DNA     linear       12-APR-2021
DEFINITION  Campylobacter species strain strain.
ACCESSION
VERSION
KEYWORDS    .
SOURCE      Campylobacter species
  ORGANISM  Campylobacter species
            Unclassified.
COMMENT     Annotated using prokka 1.14.6 from
            https://github.com/tseemann/prokka.
FEATURES             Location/Qualifiers
     source          1..909
                     /organism="Campylobacter species"
                     /mol_type="genomic DNA"
                     /strain="strain"
     gene            29..160
                     /locus_tag="PIMPEBOA_00058"
     CDS             29..160
                     /locus_tag="PIMPEBOA_00058"
                     /inference="ab initio prediction:Prodigal:002006"
                     /inference="similar to AA sequence:RefSeq:CKG87616.1"
                     /note="Domain of uncharacterised function (DUF1910)"
                     /codon_start=1
                     /transl_table=11
                     /product="hypothetical protein"
                     /protein_id="Prokka:PIMPEBOA_00058"
                     /translation="MKKHTRLRDPLDTWDLEGAAIVKLMNLDKEEFKSYKYFPYDLI"
ORIGIN
        1 taaggatatt gagtttttac atcaatatat gaaaaaacat acaagattaa gagatcctct
       61 agatacttgg gatcttgaag gtgcagcaat tgtaaaacta atgaatttag ataaagaaga
      121 atttaaaagc tataaatatt ttccttatga tttaatttaa tcttatttgg ataacagaaa
      181 agagtagaga tagacttggc attgtagata aaaaacatag ggaaaaaata gaagaacttc
      241 ttgaaaaaaa tagcaatgaa gtcagaaaag atttatttca tgttgatgaa ggaggtaaag
      301 aaactattga ttatttagat aaagatgcaa ataaaataaa acccaaagga taaaaaatgc
      361 taagagatac taagagagat gaagcgtatt ttacgaaaca tataatagag tgtgaagaag
      421 aaattaaaga agatgaaaaa atacttttag agcttccttt tggaaaaaga gaaacttgtc
      481 ttttttgtat aacagataga aaaaaatgta ttgctatgga taaatactct cgtggtgatg
      541 atataaatct agttaaaaaa gatttagaag cacttatgct attaagacaa aaaaatcgtt
      601 tagaattagg tggatttgat actagcgatt atagaggaaa tgctattgaa ctttgtgtta
      661 gagtattatt agatatggat agtgcttgtg tattagaatt aatagaagaa gatgagagaa
      721 agaaaaaaga tatttttaat agagattggt ttttacattt tatgggttct aagggtaaaa
      781 acttaaattt agaacgcaaa tgtaattgta aagaacacga gcttattaaa gaatttgtag
      841 ctactaagga tattgagttt ttacatcaat atatgaaaaa acatacaaga ttaagagatc
      901 ctctagata
//
LOCUS       PIMPEBOA_16              895 bp    DNA     linear       12-APR-2021
```

```
DEFINITION  Campylobacter species strain strain.
ACCESSION
VERSION
KEYWORDS    .
SOURCE      Campylobacter species
  ORGANISM  Campylobacter species
            Unclassified.
COMMENT     Annotated using prokka 1.14.6 from
            https://github.com/tseemann/prokka.
FEATURES             Location/Qualifiers
     source          1..895
                     /organism="Campylobacter species"
                     /mol_type="genomic DNA"
                     /strain="strain"
     gene            complement(320..889)
                     /gene="trxB_3"
                     /locus_tag="PIMPEBOA_00059"
     CDS             complement(320..889)
                     /gene="trxB_3"
                     /locus_tag="PIMPEBOA_00059"
                     /inference="ab initio prediction:Prodigal:002006"
                     /inference="similar to AA sequence:RefSeq:CKG86288.1"
                     /codon_start=1
                     /transl_table=11
                     /product="thioredoxin reductase"
                     /protein_id="Prokka:PIMPEBOA_00059"
                     /translation="MHKNPLREQQIKTLAKTLKPDEFGMLPFIDEIMGVDWVIDLNKY
                     DIASDQFGSMYKALRSDVVEGKIKDPRDIDSTYESRREFDRHRGGYKNGMLQGYGTDT
                     PNDWSEERAQLFNDTLILHAKLAALTPPQGYPNAPYYFTPENLEWYYKRHKLDKLLDP
                     RIPAIYRYNFPRELRAKILKFGEENGIKE"
ORIGIN
        1 tggatttttg ccctgtgtga aattcaggat ctagatacct aggttgataa ttaatataag
       61 gagaggttct taaaattcta tcggcttcaa acaaagcttt agaatattct tgggtgtatt
      121 tgcctacttc acgcccatct tctcttttat caaatataat ttgatttgta tttttatcaa
      181 cttctaatat aagtccatta gaagtataga tctcgtaaat atctttatta tcactcatct
      241 tttatccttt cttaatttat cccatatata aaccatcttc catgattata gtagtggcta
      301 atctgcctag gtaattactt tattctttaa tgccattttc ttcgccaaat ttgagtatct
      361 tagctctaag ttcccttggg aaattatatc tataaatggc agggattctt ggatcaagga
      421 gtttatcaag cttatgtctt ttataatacc attctaggtt ttcaggggta aaataataag
      481 gtgcattagg atagccttga ggaggagtga gggctgcgag tttggcatga agtattaaag
      541 tatcattaaa aagttgtgcc ctttcttcac tccaatcatt aggtgtatcg gttccatagc
      601 cttgcagcat gccattttta tatccacctc tatggcgatc aaattccctt ctgctttcat
      661 aagtagaatc tatatccctt ggatctttta tcttaccctc aaccacatca cttcttaagg
      721 ctttatacat acttccaaat tgatcactag ctatatcata tttatttaaa tcaatcaccc
      781 aatctactcc cataatctca tctataaaag gaagcatacc aaattcatca ggttttaaag
      841 tcttagctaa ggttttaatt tgttgttctc ttaaaggatt tttatgcact cctac
//
LOCUS       PIMPEBOA_17              877 bp    DNA     linear       12-APR-2021
DEFINITION  Campylobacter species strain strain.
ACCESSION
VERSION
KEYWORDS    .
SOURCE      Campylobacter species
  ORGANISM  Campylobacter species
```

```
            Unclassified.
COMMENT     Annotated using prokka 1.14.6 from
            https://github.com/tseemann/prokka.
FEATURES             Location/Qualifiers
     source          1..877
                     /organism="Campylobacter species"
                     /mol_type="genomic DNA"
                     /strain="strain"
     gene            2..571
                     /gene="trxB_4"
                     /locus_tag="PIMPEBOA_00060"
     CDS             2..571
                     /gene="trxB_4"
                     /locus_tag="PIMPEBOA_00060"
                     /inference="ab initio prediction:Prodigal:002006"
                     /inference="similar to AA sequence:RefSeq:CKG86288.1"
                     /codon_start=1
                     /transl_table=11
                     /product="thioredoxin reductase"
                     /protein_id="Prokka:PIMPEBOA_00060"
                     /translation="MHKNPLREQQIKTLAKTLKPDEFGMLPFIDEIMGVDWVIDLNVY
                     QFNFDAWGKIYRALRSDVVEGKIKDPRDVDSTYESRREFDRYMDGYQNGMTINYHVDI
                     NNYRSEESAKLFMDSLVLEAKLAALTPPQGYPNAPYYFTPERLEWDYKNYQLDKLLDP
                     RIPAIYRYNFPRELRAKILKFGEENGIKE"
ORIGIN
        1 agtgcataaa aatcctttaa gagaacaaca aattaaaacc ttagctaaga ctttaaaacc
       61 tgatgaattt ggtatgcttc cttttataga tgagattatg ggagtagatt gggtgattga
      121 tttaaatgtt tatcaattta attttgatgc atggggaaaa atatatagag ccttaagaag
      181 tgatgtagtt gagggcaaga taaaagatcc aagggatgta gattctactt atgaaagcag
      241 aagggagttt gatcgttata tggatggata tcaaaatggt atgacaatta attaccatgt
      301 agatataaat aattatagaa gcgaagaatc agctaaatta tttatggatt ctttggtatt
      361 agaggccaaa ctcgcagccc tcactcctcc acaaggctat cctaatgcac cttattattt
      421 tacccctgaa agactagaat gggattataa aaattatcaa ttggataaac tccttgatcc
      481 aagaatccct gctatttata gatataattt cccaagggaa cttagagcta agatactaaa
      541 atttggtgaa gaaaatggca ttaaagaata aagtaattac ataggcagat tagccactac
      601 tataatcatg gaagatggtt tatatatggg ataaattaag aaaggataaa agatgagtga
      661 aacttatgaa atttatacac ctaatggtgg tatattagat gtggaaaaag aaacgaataa
      721 aattttactc tatgatgggg gagctaaggt aggcaaatac acccaagaat attctaaagc
      781 tttgtttgaa gccgatagaa ttttaagaac ctctccttat attaattatc aacctaggta
      841 tctagatcct gaatttcaca cagggcaaaa atccact
//
LOCUS       PIMPEBOA_18              835 bp    DNA     linear       12-APR-2021
DEFINITION  Campylobacter species strain strain.
ACCESSION
VERSION
KEYWORDS    .
SOURCE      Campylobacter species
  ORGANISM  Campylobacter species
            Unclassified.
COMMENT     Annotated using prokka 1.14.6 from
            https://github.com/tseemann/prokka.
FEATURES             Location/Qualifiers
     source          1..835
                     /organism="Campylobacter species"
```

```
                     /mol_type="genomic DNA"
                     /strain="strain"
     gene            145..639
                     /locus_tag="PIMPEBOA_00061"
     CDS             145..639
                     /locus_tag="PIMPEBOA_00061"
                     /inference="ab initio prediction:Prodigal:002006"
                     /inference="similar to AA sequence:RefSeq:CKG87598.1"
                     /codon_start=1
                     /transl_table=11
                     /product="methyltransferase small"
                     /protein_id="Prokka:PIMPEBOA_00061"
                     /translation="MKKKIQELKSINNLSTKQKGNYGEAKMDLYYLKQGYKRISLNKV
                     KGLDDKIHHGIDGIYHNPDKNPKYIIAEAKFGKGDLKKTGKYKQMSDEWITEESRNRL
                     GVVDKKHRKKIEELLKENNNEVRKDLFHVDKKGNVSIKELDKNAKTKNLIERIENGKG
                     YEER"
ORIGIN
        1 ggaggtaaaa gaagtaagaa aaataaaaat gatagagcaa agcaaataca agcattaaaa
       61 aataatcagc aaaacaacaa acaaaattca tttttacaag aagttgaaca acaaaaagaa
      121 gaggctgaaa aagttttagc agatatgaaa aaaaagatac aagaattaaa aagtattaat
      181 aacttaagca caaaacaaaa aggtaattat ggcgaagcta aaatggattt gtattattta
      241 aaacaaggct ataaaagaat tagtttaaat aaagtcaaag ggcttgatga taaaattcat
      301 catggaattg atggaattta tcataatcca gataaaaacc caaaatatat catcgcagaa
      361 gctaagtttg gaaaaggtga tttaaaaaaa actggaaaat acaaacaaat gagtgatgaa
      421 tggataacag aagagagtag aaatagactt ggtgttgtag ataaaaaaca taggaaaaaa
      481 atagaagaac ttcttaagga aaataacaat gaagtcagaa aagatttatt tcatgttgat
      541 aaaaaaggca atgtgagtat taaagaatta gataaaaatg caaaaaccaa aaacttaata
      601 gaaaggatag aaaatggtaa gggatacgaa gagagatgaa gcgtatttta cgaaacatat
      661 aatagagtgt gaaaaaaaaa tcaaagaaga tgaaaaaaga ctcttagaac ttccactagg
      721 agatgcaaga agggaaaatt atttttttc tataacagat aaaaaaaaat gtattgctat
      781 ggataaatac tctcgtggcg atgatataaa tccagttaaa aaagatttag aagca
//
LOCUS       PIMPEBOA_19              736 bp    DNA     linear       12-APR-2021
DEFINITION  Campylobacter species strain strain.
ACCESSION
VERSION
KEYWORDS    .
SOURCE      Campylobacter species
  ORGANISM  Campylobacter species
            Unclassified.
COMMENT     Annotated using prokka 1.14.6 from
            https://github.com/tseemann/prokka.
FEATURES             Location/Qualifiers
     source          1..736
                     /organism="Campylobacter species"
                     /mol_type="genomic DNA"
                     /strain="strain"
     gene            52..183
                     /locus_tag="PIMPEBOA_00062"
     CDS             52..183
                     /locus_tag="PIMPEBOA_00062"
                     /inference="ab initio prediction:Prodigal:002006"
                     /codon_start=1
                     /transl_table=11
```

```
                     /product="hypothetical protein"
                     /protein_id="Prokka:PIMPEBOA_00062"
                     /translation="MPYLLSLSDEEFEKIIFHVSGFSEIDTTFLKEAMELRKEKKDE"
ORIGIN
        1 ccaacaaaag attgttttga aagaattaat gccattataa aacctcatgt aatgccttat
       61 ttattaagct taagtgatga agaatttgaa aaaatcattt tccatgtaag cggttttagc
      121 gaaattgata ctactttcct taaagaagca atggaactca gaaaagagaa aaaagatgaa
      181 tgaaaatatt ttattttata tacaagagat taaaacttgt ttaaaggata aaaatgagcc
      241 aaacaaacaa taacacagaa atcaaagagc aagacaccca agatgaaatc atttgggaag
      301 gaaaaaagca tataccttcg tttttgcttt attggataac atatattgtg cttttatgtt
      361 ttatgcttta tttgcttcct agggcatttg acaatactaa gaattttaaa tggtttgtgg
      421 tttttattat gtttcttgta acggtatatg cacttgcaca agagatctat aaagcggcaa
      481 atattaaaag aatttatatc acaaaagaaa aacttgtgat tgagtattat atcaaaaatg
      541 atttggtttt tcctttagga actttttttg tgtattatcg tcacgtgtca tttgtgctta
      601 ctccaggaga tatagtgata tacaccttcg gagataaagt caaagaatac cttgaacctg
      661 gtttattcag tgatggcgat gatccaacaa aagattgttt tgaaagaatt aatgccatta
      721 taaaacctca tgtaat
//
LOCUS       PIMPEBOA_20              708 bp    DNA     linear       12-APR-2021
DEFINITION  Campylobacter species strain strain.
ACCESSION
VERSION
KEYWORDS    .
SOURCE      Campylobacter species
  ORGANISM  Campylobacter species
            Unclassified.
COMMENT     Annotated using prokka 1.14.6 from
            https://github.com/tseemann/prokka.
FEATURES             Location/Qualifiers
     source          1..708
                     /organism="Campylobacter species"
                     /mol_type="genomic DNA"
                     /strain="strain"
     gene            28..558
                     /locus_tag="PIMPEBOA_00063"
     CDS             28..558
                     /locus_tag="PIMPEBOA_00063"
                     /inference="ab initio prediction:Prodigal:002006"
                     /codon_start=1
                     /transl_table=11
                     /product="hypothetical protein"
                     /protein_id="Prokka:PIMPEBOA_00063"
                     /translation="MLLCFALYLFPRLFNPSKEIDYKWFIVFFTFLLVIYALAQKIYK
                     MANIKRIYVTKEKLVVEYYIKNDLVFPLGTFFVYYRSSSLPSISNPGDIVIYTFGDKV
                     KEYLEPGLFSDGDDPTKDCFERINAIIKPHVMPYLLSLSDEEFEKIISHVSGYNEIGT
                     TFLKEAMELRKEKKDE"
     gene            551..703
                     /locus_tag="PIMPEBOA_00064"
     CDS             551..703
                     /locus_tag="PIMPEBOA_00064"
                     /inference="ab initio prediction:Prodigal:002006"
                     /codon_start=1
                     /transl_table=11
                     /product="hypothetical protein"
```

```
                     /protein_id="Prokka:PIMPEBOA_00064"
                     /translation="MNENIYGSLEFYSKETQSTPSDKRDLGVFGDVAPYALGITEGFA
                     TKAFKG"
ORIGIN
        1 tttttgcttt tttggataac atatattgtg cttttatgtt ttgcgcttta tttgtttcct
       61 agattattta atcctagcaa agaaatagat tataaatggt ttatagtttt ttttacattt
      121 cttttagtta tatatgcact tgcacaaaag atctataaaa tggcaaatat taaaagaatt
      181 tatgtcacaa aagaaaaact tgtggttgag tattatatca aaaatgattt ggtttttcct
      241 ttaggaactt tttttgtata ttatcgtagt tcgtcacttc catccatatc aaatccagga
      301 gatatagtga tatacacctt cggagataaa gtcaaagaat accttgaacc tggtttattc
      361 agtgatggcg atgatccaac aaaagattgt tttgaaagaa ttaatgccat tataaagcct
      421 catgtaatgc cttatttatt aagcttaagt gatgaagaat ttgaaaaaat catttcacat
      481 gtaagcggtt ataacgaaat tggtaccact ttccttaaag aagcaatgga actcagaaaa
      541 gagaaaaaag atgaatgaaa atatttatgg aagtttagag ttttattcta aagaaactca
      601 aagcactcct agtgataaaa gggatttagg agtatttgga gatgttgctc cttatgcctt
      661 aggcattaca gaaggttttg ctactaaagc ttttaagggg taagcaat
//
LOCUS       PIMPEBOA_21              586 bp    DNA     linear       12-APR-2021
DEFINITION  Campylobacter species strain strain.
ACCESSION
VERSION
KEYWORDS    .
SOURCE      Campylobacter species
  ORGANISM  Campylobacter species
            Unclassified.
COMMENT     Annotated using prokka 1.14.6 from
            https://github.com/tseemann/prokka.
FEATURES             Location/Qualifiers
     source          1..586
                     /organism="Campylobacter species"
                     /mol_type="genomic DNA"
                     /strain="strain"
ORIGIN
        1 aatcaatcac ccaatctact cccataatct catctataaa aggaagcata ccaaattcat
       61 caggttttaa agtcttagct aaggttttaa tttgttgttc tcttaaagga tttttatgca
      121 ctcctactaa accacttcct actgcaatat ctgcataacc ataaatagca cccaataaat
      181 taggtttatc tagtatacta cttcctccgc ttaattgtat ataaagtata gttgcttgga
      241 taaatcttgc attaaaacca ttttgagaca tatcgttagc tagatattta taatcaccca
      301 gtattcctgc tgccataccc cattcattat gaaataaact tgatcttaga ttatgtttat
      361 tatcatctac tgttctatat aattcttcat acttaggatc tacatttcct ttttcatcta
      421 cagctcctat agcttcataa ggtatatcta taacaacact tcttatacca cttcttataa
      481 ctaaatattt atatctttct tttttagttt ttagtgagtt ataataagct ttttctgctt
      541 tagtccaagg agctatgctt cctttaatgg gatcttttaa atatat
//
LOCUS       PIMPEBOA_22              554 bp    DNA     linear       12-APR-2021
DEFINITION  Campylobacter species strain strain.
ACCESSION
VERSION
KEYWORDS    .
SOURCE      Campylobacter species
  ORGANISM  Campylobacter species
            Unclassified.
COMMENT     Annotated using prokka 1.14.6 from
            https://github.com/tseemann/prokka.
```

```
FEATURES             Location/Qualifiers
     source          1..554
                     /organism="Campylobacter species"
                     /mol_type="genomic DNA"
                     /strain="strain"
ORIGIN
        1 tgccattttc ttcgccaaat ttgagtatct tagctctaag ttcccttggg aaattatatc
       61 tataaatagc agggattctt ggatcaagga gtttatcaag cttatgtctt ttataatacc
      121 attctagttt ttcaggggta aaataataag gtgcattagg ataaccttgt ggaggagtga
      181 gggctgcgag tttggcagtt aatatcatag aatctatttt taattgcgca tcattccaat
      241 catttggaat atcataacca taggcattaa tcatgccatt agaataacca tccatataat
      301 aatcaaactc ccttctgctt tcataagtag aatctatatc ccttggatct tttatcttgc
      361 cctcaactac atcacttctt aaggctttat atatatcccc aaattcatct tcagaaattt
      421 gatattcgtt ataatcaatc acccaatcta ctcccataat ctcatctata aaaggaagca
      481 taccaaattc atcaggtttt aaagtcttag ctaaggtttt aatttgttgt tctcttaaag
      541 gatttttatg cact
//
LOCUS       PIMPEBOA_23              518 bp    DNA     linear       12-APR-2021
DEFINITION  Campylobacter species strain strain.
ACCESSION
VERSION
KEYWORDS    .
SOURCE      Campylobacter species
  ORGANISM  Campylobacter species
            Unclassified.
COMMENT     Annotated using prokka 1.14.6 from
            https://github.com/tseemann/prokka.
FEATURES             Location/Qualifiers
     source          1..518
                     /organism="Campylobacter species"
                     /mol_type="genomic DNA"
                     /strain="strain"
ORIGIN
        1 cccttgggaa attatatcta taaatagcag ggattcttgg atcaaggagt ttatcaagct
       61 tatgtctttt ataataccat tctagatttt caggggtaaa ataataaggt gcattaggat
      121 agccttgtgg aggagtgagg gctgcgagtt tggcagttaa tatcatagaa tctattctta
      181 attgtgcact ttcttcactc cgatcatttg gaatatcata accataagca tttaccatgc
      241 cgttataata tcctccccta tggcgatcaa attcccttct gctttcataa gtagaatcta
      301 tatctcttgg atcttttatc ttgccctcaa ctacatcact tcttaaggct ttatacatac
      361 ttccaaattc atcgcgagca attctatacc tgttataatc aatcacccaa tctactccca
      421 taatctcatc tataaaagga agcataccaa attcatcagg ttttaaagtc ttagctaagg
      481 ttttaatttg ttgttctctt aaaggatttt tatgcact
//
LOCUS       PIMPEBOA_24              518 bp    DNA     linear       12-APR-2021
DEFINITION  Campylobacter species strain strain.
ACCESSION
VERSION
KEYWORDS    .
SOURCE      Campylobacter species
  ORGANISM  Campylobacter species
            Unclassified.
COMMENT     Annotated using prokka 1.14.6 from
            https://github.com/tseemann/prokka.
FEATURES             Location/Qualifiers
```

```
     source          1..518
                     /organism="Campylobacter species"
                     /mol_type="genomic DNA"
                     /strain="strain"
ORIGIN
        1 taagttccct tgggaaatta tatctataaa tagcagggat tcttggatca aggagtttat
       61 caagcttatg tcttttataa taccattcta gtttttcagg ggtaaaataa taaggtgcat
      121 taggatagcc ttgaggagga gtgagggctg cgagtttggc atgaagtatt aaagtatcat
      181 taaaaagttg tgccctttct tcactccaat cattgggtgt atcggtttca taaccttgcc
      241 tcataccctt tctatatcca actctataac gatcaaattc ccttctgctt tcataagtag
      301 aatctatatc tcttggatct tttatcttgc cctcaactac atcacttctt aaggctttat
      361 atatatcccc aaattcatca ttagctaatc tatatttatt taaatcaatc acccaatcta
      421 ctcccataat ctcatctata aaaggaagca taccaaattc atcaggtttt aaagtcttag
      481 ctaaggtttt aatttgttgt tctcttaaag gattttta
//
LOCUS       PIMPEBOA_25              507 bp    DNA     linear       12-APR-2021
DEFINITION  Campylobacter species strain strain.
ACCESSION
VERSION
KEYWORDS    .
SOURCE      Campylobacter species
  ORGANISM  Campylobacter species
            Unclassified.
COMMENT     Annotated using prokka 1.14.6 from
            https://github.com/tseemann/prokka.
FEATURES             Location/Qualifiers
     source          1..507
                     /organism="Campylobacter species"
                     /mol_type="genomic DNA"
                     /strain="strain"
     gene            67..207
                     /locus_tag="PIMPEBOA_00065"
     CDS             67..207
                     /locus_tag="PIMPEBOA_00065"
                     /inference="ab initio prediction:Prodigal:002006"
                     /inference="similar to AA sequence:RefSeq:CKG86746.1"
                     /codon_start=1
                     /transl_table=11
                     /product="hypothetical protein"
                     /protein_id="Prokka:PIMPEBOA_00065"
                     /translation="MWYMKIFKDYQKFTPYGLDFRLYSKDFIAKLDFKDKAYNVLYNT
                     GV"
ORIGIN
        1 aacgcaaaaa attaagttgg tttatagggt ttgtgtgtaa tgctcctata ttaattgcga
       61 gtgtgtatgt ggtatatgaa aatctttaaa gactatcaaa aattcactcc ttatggtttg
      121 gattttagac tttatagtaa agattttatt gccaaacttg attttaaaga taaagcttat
      181 aatgtgcttt ataatacagg agtttaaatg ggagaagata ttacattttc tattttttgg
      241 ggtttatttg gatttattct tgcttttact aatataaaat tttatcttac cactttaagc
      301 gttcttattg tcttttctat tttaaattat tggatcaaga ttaatataaa agaatttgta
      361 aaatcttatt ttttaatatc ttttattatc ttttttacag ctattgttat catattttat
      421 cttacctatg gtattgaaac ttctaaggat cttgcattgc ttagctctct agaagctaaa
      481 gaattgtcac cacaagaata taaagcg
//
```

```
>>BfR-CA-15861_min_cov_30 putative plasmid location

LOCUS       ECFEEEMN_1             17665 bp    DNA     linear       12-APR-2021
DEFINITION  Campylobacter species strain strain.
ACCESSION
VERSION
KEYWORDS    .
SOURCE      Campylobacter species
  ORGANISM  Campylobacter species
            Unclassified.
COMMENT     Annotated using prokka 1.14.6 from
            https://github.com/tseemann/prokka.
FEATURES             Location/Qualifiers
     source          1..17665
                     /organism="Campylobacter species"
                     /mol_type="genomic DNA"
                     /strain="strain"
     gene            complement(370..1278)
                     /locus_tag="ECFEEEMN_00001"
     CDS             complement(370..1278)
                     /locus_tag="ECFEEEMN_00001"
                     /inference="ab initio prediction:Prodigal:002006"
                     /inference="similar to AA sequence:RefSeq:AJK71146.1"
                     /codon_start=1
                     /transl_table=11
                     /product="type VI secretion protein"
                     /protein_id="Prokka:ECFEEEMN_00001"
                     /translation="MNDLASYSFFKLIKKLEKDYGRKNIFLRTNKSLKHPNKDIEKII
                     FSEHEQSVVELFINFMGLHGVSSQLPSFMLDKLSRNEDGEQGWTLFFDFFNHYLLWIF
                     FDVVSLKNYPRSFNENFKDSISQILFNILGIKEHDIAKKYLPFAPLLLSLRRPKTHIE
                     RVLQVNFKLKDKLSIIENLPHQILISDSQKNKLGIKNHTLGNNFILGDKFISYQNKIA
                     IYIKDISYQEAVKFMPNGTKHDDLKNSIMFLTNNEFCVDLYLKINYSSEMKFVLGDEN
                     TAKLGWAKILGNAQKKYTIVYMKLCE"
     gene            complement(1275..2996)
                     /locus_tag="ECFEEEMN_00002"
     CDS             complement(1275..2996)
                     /locus_tag="ECFEEEMN_00002"
                     /inference="ab initio prediction:Prodigal:002006"
                     /inference="similar to AA sequence:RefSeq:AJK71145.1"
                     /codon_start=1
                     /transl_table=11
                     /product="type VI secretion protein"
                     /protein_id="Prokka:ECFEEEMN_00002"
                     /translation="MKDNIFYYQKELEYLYEKREHFIKNYPKLTPFLAHDSKDPDIER
                     IIENLAILSSKIHQELDENIPHIAESLINIISPNYTNPLPSLCMQEFKFEQNSKENNL
                     IIPKGTLIKSKPIDKCVCEFKTVYDVYLYPISINEVFISSKNQDYTFNLTLQVNKAET
                     KICDLGLEKINLYLGNDTYMSSTLLLYMHSYLKELKIQSLDTDEEFFLNTYNIEKIGL
                     NPDESSLSYNDLGFEAFSLLREYFFMPHKFNFLRINGLDILNNCQGKTINIEFKFSKP
                     FPANCIFRKELLSLGMTPIINIFAKSAEPLINNHKKDSYRIFVDRSQPKAYEIIQTLQ
                     VKAHNSEGGKRLLKNYKSFERFEFLKDNQKDFYSVNTKKNSKGEVFSEISFFSSCLID
                     ETISIDLLCSNGDLPSKLKIGDINTCNLKGVDTKNVEIPSETRRCSVDGNLLWKLVSV
                     LSFSYQTILSKKAFFGVLESYSFLDNQSNWKIYKLLQEAIIDIQSKSTYLIDENITKK
                     GTVAIFSIKDSKFYTLGEVYLLGLIISKFLASFASINSFCELKIKCLDSKEILHYPAS
```

```
                     FGKKALI"
     gene            complement(2993..3385)
                     /locus_tag="ECFEEEMN_00003"
     CDS             complement(2993..3385)
                     /locus_tag="ECFEEEMN_00003"
                     /inference="ab initio prediction:Prodigal:002006"
                     /inference="similar to AA sequence:RefSeq:CKG87312.1"
                     /codon_start=1
                     /transl_table=11
                     /product="type VI secretion system lysozyme-like protein"
                     /protein_id="Prokka:ECFEEEMN_00003"
                     /translation="MSLLDKLIHNLDEQNIHIPFYQNDFEDVKNNIKVLLNAKINDCY
                     AVRNLGMPNMADINLNSNELCVSMAKEIRKLIDNYEKRICVVSITYDNSLSPWQLSFI
                     VKCFFQDDRFKEFNIEIIFKNNRYCEVK"
     gene            complement(3388..4842)
                     /locus_tag="ECFEEEMN_00004"
     CDS             complement(3388..4842)
                     /locus_tag="ECFEEEMN_00004"
                     /inference="ab initio prediction:Prodigal:002006"
                     /inference="similar to AA sequence:RefSeq:AJK71143.1"
                     /codon_start=1
                     /transl_table=11
                     /product="type VI secretion protein"
                     /protein_id="Prokka:ECFEEEMN_00004"
                     /translation="MANTKVIDTPIIEQIMEKSKYSKTDESYSIAKRGVAEFISEIVK
                     SDNAEEKINKFALDEMIAHIDHLLSKQMDEVLHNEEFQKLESTWRGLRFLVERTDFNE
                     NIKIDLFDITKEEALEDFENNPDITQSIVYKNIYSSEYGQFGGEPVGAIIGDYQLGSA
                     SPDMTFLNKMASIAAMSHSPFLTSFGPKFFGLDDYSELANIQDLQGLLEGPQYTRWRT
                     FRENEDSKYTGLLVTRFLARSPYDPEENPIKSFNYKENVHASHNHLLWANSSYTFCTR
                     LTESFAKYRWCGNIIGPKSGGTVKDLPTYLYENFGTIQSKIPTEVLITDRREYELAEA
                     GFITLTLRRDSNNAAFFSANSPLKPKIFQNTPEGKEAETNYRLGTQLPYIFLISRLAH
                     YLKVLQREEIGSWKERSDIENGLNEWIRQYISDQENPPSEVRSRRPFRAAQVKVDDIP
                     GEPGWYKIGLSVRPHFKYMGGNFELSLVGKLDKE"
     gene            complement(4844..5329)
                     /locus_tag="ECFEEEMN_00005"
     CDS             complement(4844..5329)
                     /locus_tag="ECFEEEMN_00005"
                     /inference="ab initio prediction:Prodigal:002006"
                     /inference="similar to AA sequence:RefSeq:AJK71142.1"
                     /codon_start=1
                     /transl_table=11
                     /product="type VI secretion protein"
                     /protein_id="Prokka:ECFEEEMN_00005"
                     /translation="MSDGSHAPKERINITYKAKTNGQNEDVELPLKLMVMANLKGKNE
                     TPLEEREILQINKINFDQVMRKLDITTSFSVKNTLGTGAEELDVKLNIASMKDFSPDS
                     LAKQIPELNKLLQLREALMALKGPMGNIPDFRKAVLEALKNEKTKEKLLLEIKQEEQG
                     N"
     gene            complement(5399..6646)
                     /locus_tag="ECFEEEMN_00006"
     CDS             complement(5399..6646)
                     /locus_tag="ECFEEEMN_00006"
                     /inference="ab initio prediction:Prodigal:002006"
                     /inference="similar to AA sequence:RefSeq:AJK71141.1"
                     /codon_start=1
```

```
                     /transl_table=11
                     /product="nucleobase:cation symporter"
                     /protein_id="Prokka:ECFEEEMN_00006"
                     /translation="MSFCNQLKNNLDELQEFHLLEDEMSKYKTLNHENISWDKVYQYS
                     QFILLHHSLDFKVCNYFLLSCFNLNNEECFEKLLLLFQHLKKLMDENNTYISAQKRKI
                     QIFIQNFIQEYNKTKVLVSSEIIDQFIALFSEFENLLSCNFAKIEIAQVAPQLPKSPV
                     AQVSVNIRSDNINSLNEREYKNFYQKLAFELLEEDENNLNIYALFTQAMWGKIKCLPE
                     CNNEKITKIRKPDADIIRILLSDKENDIEHIKCFMHELILNPFWIEGVQLFCDFLEKK
                     KKNKQLDILIMLTSDFISKFDAIELLRFQNGDFICKEEVYKYFVKPKENKKSFFSSKK
                     TDKEHTLQDFEQMLMNIDKENFNNSIMNNINSLLDMVKIFESKGMKKNSKILNIYLVE
                     LMEKTLLKDYLAEEYENAKNKIK"
gene                 6769..7215
                     /locus_tag="ECFEEEMN_00007"
CDS                  6769..7215
                     /locus_tag="ECFEEEMN_00007"
                     /inference="ab initio prediction:Prodigal:002006"
                     /inference="similar to AA sequence:RefSeq:AJK71140.1"
                     /codon_start=1
                     /transl_table=11
                     /product="type VI secretion protein"
                     /protein_id="Prokka:ECFEEEMN_00007"
                     /translation="MFRKKSLFFLLPLFFCACSSVVSVKINNIENSNLNNRYDDVPVT
                     AIVYQLKDIKKFEEASDIDLATREDGVLGKDKLDSIKTQIAPKDNIIAVKVDEGEVPY
                     VGVLVLFANNTKKVTKIWAKTEDANGFGKNKYLKFEISKEGIKRIK"
gene                 7225..8622
                     /locus_tag="ECFEEEMN_00008"
CDS                  7225..8622
                     /locus_tag="ECFEEEMN_00008"
                     /inference="ab initio prediction:Prodigal:002006"
                     /inference="similar to AA sequence:RefSeq:AJK71139.1"
                     /codon_start=1
                     /transl_table=11
                     /product="type VI secretion protein"
                     /protein_id="Prokka:ECFEEEMN_00008"
                     /translation="MANKLKVAWFDGLNIGQTHFEQQERFFNRNIDLKTINIYSNLYG
                     IIDLEFSQEMLLQGKIALSKISGIAQDGSIFNAPEQDLLPEPIEINYESLIDSVVVLK
                     IPIGVTDIADLSLRNTFPNSKYICLRNSIALRNYDDSKSNAMDKIEDEYELENLTFTQ
                     EKKDLLLASLRLKLGILGNSTPDELELPIAKIKNIDINKKIELESDFIPTCLNISKIS
                     TIRSFLEEIIFSINQHKKVLSNVFKGIDQTKNTLDFSTFLSLNLLKKWYLIFSHLSKK
                     DKIHPEFLYEKFLEFQGELAAFSNEESFLDFIPYKHDNLYNTFLNMMNNLRLLFSKIT
                     SPKYFIAKTSINGNGFYDFIFDNSGILEDAEIYFAIRADVTTEYLLNNFKIQSKIHTQ
                     SKIKNIVATQLEGLNVDQVPNIPSSIPYLNGYVYYKIDKRDHLFKDFKNESIISMYLT
                     NNIKNPDIIMWAVFQ"
gene                 8619..9392
                     /locus_tag="ECFEEEMN_00009"
CDS                  8619..9392
                     /locus_tag="ECFEEEMN_00009"
                     /inference="ab initio prediction:Prodigal:002006"
                     /inference="similar to AA sequence:RefSeq:AJK71138.1"
                     /codon_start=1
                     /transl_table=11
                     /product="glutamyl-tRNA amidotransferase"
                     /protein_id="Prokka:ECFEEEMN_00009"
                     /translation="MKEINEEKKALSLLLDSNFDGLKNNKIIDYSLELLLLSYRLSKI
```

SSMDTSNINQLRETLINKILDITAKLSMCKEYDEKEIIKFKYCLCVFIDESLMKNELF
INFWAHNTLTVRLFDETLGGNNFYDIASSWINNPFKFKDFLEFIYACLILGYKGKYNE
AKDRDEKIIHFCNNIATSLRPVYKTEEELAFNKAYKIGLEENIWQKFIRLYFKKLIII
VPVLIVLGVLSFAIFNLETNNLKVDNNISVLIKNLTHIE"

```
gene            9615..10175
                /locus_tag="ECFEEEMN_00010"
CDS             9615..10175
                /locus_tag="ECFEEEMN_00010"
                /inference="ab initio prediction:Prodigal:002006"
                /codon_start=1
                /transl_table=11
                /product="hypothetical protein"
                /protein_id="Prokka:ECFEEEMN_00010"
                /translation="MTYILLLIIISTILSYLILKFIYKIIFKSTKSVSKFLVFLGSIG
                LIIFYYTPYSYYLEPSYWQFRNMCKLNELPNNEEKYNKILGYFDKKLGDIDDFRHVKK
                YGSIEMWLYIYYDDTYKTPSNFQEKIEMDNIALKTKNMQMSLHKIVDMHISPYWNTRR
                YVLEGNEGNMNFEFIEDELACGNLYQ"
gene            10194..10520
                /locus_tag="ECFEEEMN_00011"
CDS             10194..10520
                /locus_tag="ECFEEEMN_00011"
                /inference="ab initio prediction:Prodigal:002006"
                /inference="similar to AA sequence:RefSeq:CKG86955.1"
                /codon_start=1
                /transl_table=11
                /product="methyltransferase small"
                /protein_id="Prokka:ECFEEEMN_00011"
                /translation="MTNKQLINKLRDMAELAQASYGYFDLVTHDKNDFFIKIKDEATE
                SAFFNKHITQANVLDISYKNYKAYQYDSSLKKDIKIGTLKGDFSPLQAKRFFSRYDLL
                KHCPKK"
gene            10719..10820
                /locus_tag="ECFEEEMN_00012"
CDS             10719..10820
                /locus_tag="ECFEEEMN_00012"
                /inference="ab initio prediction:Prodigal:002006"
                /codon_start=1
                /transl_table=11
                /product="hypothetical protein"
                /protein_id="Prokka:ECFEEEMN_00012"
                /translation="MGILKKVYIVFFGGGYFGELFFCLVLYMLILVI"
gene            11244..11756
                /locus_tag="ECFEEEMN_00013"
CDS             11244..11756
                /locus_tag="ECFEEEMN_00013"
                /inference="ab initio prediction:Prodigal:002006"
                /codon_start=1
                /transl_table=11
                /product="hypothetical protein"
                /protein_id="Prokka:ECFEEEMN_00013"
                /translation="MKILKSLYIIWMIFWGFGAILGYYMGYDATFTKTFFFLVILYLF
                PLLPYLHYLIFEKTKNKNIFISICYYLLLFVITAYYFTLIYSCGTNFLTFFIMITLYM
                IICSAIILWKFKINLRLYFYIILCIFTYLYIVLHIFIACYEIEWTDILLDKDVRIYIY
                KEIINLLTEQ"
gene            11799..11948
```

```
                     /locus_tag="ECFEEEMN_00014"
     CDS             11799..11948
                     /locus_tag="ECFEEEMN_00014"
                     /inference="ab initio prediction:Prodigal:002006"
                     /inference="similar to AA sequence:RefSeq:AAW35443.1"
                     /note="conserved domain protein"
                     /codon_start=1
                     /transl_table=11
                     /product="hypothetical protein"
                     /protein_id="Prokka:ECFEEEMN_00014"
                     /translation="MKTQINNLRDYTELAWASYFYFDLKGYILQENETIVILNELVSL
                     SYNGN"
     gene            12158..12628
                     /locus_tag="ECFEEEMN_00015"
     CDS             12158..12628
                     /locus_tag="ECFEEEMN_00015"
                     /inference="ab initio prediction:Prodigal:002006"
                     /codon_start=1
                     /transl_table=11
                     /product="hypothetical protein"
                     /protein_id="Prokka:ECFEEEMN_00015"
                     /translation="MQIPIYSKLKAKLESKGIDNISSGQIAWFVCSLILALVTYIILT
                     FHYIYISYGKYDKNLFLLIFDRNAMELFIIIYSCIMFNLLYIYNAIPLVLSAVFIFLA
                     FYFSSLSLFIVGCCGIGVFFLISIFKRKLIPSVLLFTMGYICSYLLLHAIGTYH"
     gene            12639..12791
                     /locus_tag="ECFEEEMN_00016"
     CDS             12639..12791
                     /locus_tag="ECFEEEMN_00016"
                     /inference="ab initio prediction:Prodigal:002006"
                     /inference="similar to AA sequence:RefSeq:CKG86410.1"
                     /codon_start=1
                     /transl_table=11
                     /product="hypothetical protein"
                     /protein_id="Prokka:ECFEEEMN_00016"
                     /translation="MKYCVTIYIDPLVECFLDFKNQCYWQREDFTHAFLGLTKDKSPD
                     ELDKYR"
     gene            12894..13319
                     /locus_tag="ECFEEEMN_00017"
     CDS             12894..13319
                     /locus_tag="ECFEEEMN_00017"
                     /inference="ab initio prediction:Prodigal:002006"
                     /inference="similar to AA sequence:RefSeq:CKG86435.1"
                     /codon_start=1
                     /transl_table=11
                     /product="hypothetical protein"
                     /protein_id="Prokka:ECFEEEMN_00017"
                     /translation="MQIPIYSKLKAKLESKGIDNITSGQIAWFVCSVGIFIIPMMYLY
                     ISNNYNQFIILFFMIFIFILNMLYTKNFFALLLLVILIFIYMKYHEFIYNFKILFFIF
                     APIILIFLRAWRILITFLCGYIFLVAMVYIFELLGGFYK"
     gene            13316..13528
                     /locus_tag="ECFEEEMN_00018"
     CDS             13316..13528
                     /locus_tag="ECFEEEMN_00018"
                     /inference="ab initio prediction:Prodigal:002006"
```

```
                     /inference="similar to AA sequence:RefSeq:CKG86410.1"
                     /codon_start=1
                     /transl_table=11
                     /product="hypothetical protein"
                     /protein_id="Prokka:ECFEEEMN_00018"
                     /translation="MKYYATIYIDPLVECFLDFKNQCYWQIEDFTHAFLGLTKGKSLD
                     ELDKIDKELKQEYLKNKEFKLIKSIK"
     gene            13570..14028
                     /locus_tag="ECFEEEMN_00019"
     CDS             13570..14028
                     /locus_tag="ECFEEEMN_00019"
                     /inference="ab initio prediction:Prodigal:002006"
                     /inference="similar to AA sequence:RefSeq:CKG86435.1"
                     /codon_start=1
                     /transl_table=11
                     /product="hypothetical protein"
                     /protein_id="Prokka:ECFEEEMN_00019"
                     /translation="MQIPIYSKLKAKLESKGIDNISSGQIAWFVCSVGIVYLLSIFLY
                     LHGVNKEGISFNRIFIAFYINHFFCFMLMWFILLLNLLYKENTISLILTLLTTIAYFY
                     NPYFAYILIFIICIIMRIINKQRWLYIYFIGLIYIYATYSIFNLRGGFYK"
     gene            14025..14126
                     /locus_tag="ECFEEEMN_00020"
     CDS             14025..14126
                     /locus_tag="ECFEEEMN_00020"
                     /inference="ab initio prediction:Prodigal:002006"
                     /codon_start=1
                     /transl_table=11
                     /product="hypothetical protein"
                     /protein_id="Prokka:ECFEEEMN_00020"
                     /translation="MKYYATIYIDPLFDFKDATHAFLGLPKAKVLMS"
     gene            14208..14687
                     /locus_tag="ECFEEEMN_00021"
     CDS             14208..14687
                     /locus_tag="ECFEEEMN_00021"
                     /inference="ab initio prediction:Prodigal:002006"
                     /codon_start=1
                     /transl_table=11
                     /product="hypothetical protein"
                     /protein_id="Prokka:ECFEEEMN_00021"
                     /translation="MQIPIYSKLKAKLESKGIDNITSGQIAWCLGSGYIALICAVFVV
                     VYKLKNLEFELYIVFFFPILLFYIAIFWFIFLSFYKNFFFIHLFCYSFMYIFNIIFND
                     FYLNLVVMNILLITIWLFFVKLHLKFRFLFLMMYFCFEFAIFIGIIPYGLLEGTISK"
     gene            14684..14899
                     /locus_tag="ECFEEEMN_00022"
     CDS             14684..14899
                     /locus_tag="ECFEEEMN_00022"
                     /inference="ab initio prediction:Prodigal:002006"
                     /inference="similar to AA sequence:RefSeq:CKG86410.1"
                     /codon_start=1
                     /transl_table=11
                     /product="hypothetical protein"
                     /protein_id="Prokka:ECFEEEMN_00022"
                     /translation="MKEIIKYYVTIYIDELLENEMLKHVDFSLLFKAKFSQISKQIDF
                     THAFLGLTKDKSSDELDKIDEELRQKF"
```

```
     gene            14946..15221
                     /locus_tag="ECFEEEMN_00023"
     CDS             14946..15221
                     /locus_tag="ECFEEEMN_00023"
                     /inference="ab initio prediction:Prodigal:002006"
                     /codon_start=1
                     /transl_table=11
                     /product="hypothetical protein"
                     /protein_id="Prokka:ECFEEEMN_00023"
                     /translation="MQIPIYSKLKTKLESKGIDNISSGQIAWFVCSVIFTYLLFLLIV
                     IINTRTISFENLKFFTFEGFFKSLAFIFANHFFLFIFMWIVCIINFF"
     gene            15468..15647
                     /locus_tag="ECFEEEMN_00024"
     CDS             15468..15647
                     /locus_tag="ECFEEEMN_00024"
                     /inference="ab initio prediction:Prodigal:002006"
                     /inference="similar to AA sequence:RefSeq:CKG86410.1"
                     /codon_start=1
                     /transl_table=11
                     /product="hypothetical protein"
                     /protein_id="Prokka:ECFEEEMN_00024"
                     /translation="MVECFLDFKNQCYWQREDFTHAFLGLTKGKSPDELDKIDEELRQ
                     EYLKNKEFKLIKSIK"
     gene            15689..16147
                     /locus_tag="ECFEEEMN_00025"
     CDS             15689..16147
                     /locus_tag="ECFEEEMN_00025"
                     /inference="ab initio prediction:Prodigal:002006"
                     /codon_start=1
                     /transl_table=11
                     /product="hypothetical protein"
                     /protein_id="Prokka:ECFEEEMN_00025"
                     /translation="MQIPIYSKLKAKLESKGIDNISSGQIAWFVCSVVLALVTFLIIY
                     FTVIIEDKNLFLAIFELFQWQLFIFIIYFCIPFNLLYIHNAIPLVLSAIFMFLTLYFS
                     SLNLFIIGCCGIGAFFLISIFKRKLMFSVLLFAMGYICIYFFLYAITRYH"
     gene            16203..17429
                     /locus_tag="ECFEEEMN_00026"
     CDS             16203..17429
                     /locus_tag="ECFEEEMN_00026"
                     /inference="ab initio prediction:Prodigal:002006"
                     /inference="similar to AA sequence:RefSeq:CKG86410.1"
                     /codon_start=1
                     /transl_table=11
                     /product="hypothetical protein"
                     /protein_id="Prokka:ECFEEEMN_00026"
                     /translation="MIKDLLNMKGLDFTHAFLGLTKDKSPDELDKIDKELRQEYLKNK
                     EWDKIDQKWYEAKEPNEFNDGFWGFGTGIANVAESLIGSPGKVFNNNQYVLDNNVDKN
                     RYIIKKLRSPQTFKPSNRCTLEISKEQYETLLANIKADFEATKEITPNSEKPINEEFT
                     YKLLENNCVTWVVKKLSSIGIEVVNPVVKDPSGLLMATKLIQSIHSIFLKFQNIDDNL
                     QSVKGAKAFKNWARMLINNSLMQEIYLDEYKKQYNSENLSPEQEEVLYALWNITRVYK
                     ILGILLSRLITKSGTKNLKGTFELIYFDKNDKQAKLLKASNHYEAIIIQELDLSKKYN
                     NFSASKFYPFIFIPKDEMLSRTLYHKYDYGNISQEYQKDRNEFYFNVLAGEKSDKYWS
                     SSYHKMTKNLRKVYVS"
ORIGIN
```

1 gttgtagggg tgcttagatg agatgaagca ttggttaaag tatttaaaga aaataaatct
61 tgttctaaac tttcaaaaaa gccttcacat tctatagaaa aaagctcttc aagactttct
121 ttgatgaggg ctttagtgat tttaaagggt aggggatttg aagtatgatt taatatatca
181 agactcatat aagaattaag ggacattgat gatccttttg ggtttgttga tgattaatag
241 ccataagctt atgtgattta ttttagcata aggtgggtaa aataaagttt attttagggt
301 gtgggtttat gggtaaagag agattgtggt tttttgaaat aaaattttta aatatatttt
361 tgtgttaggc tattcgcata gtttcatata tactattgta tatttttttt gtgcattgcc
421 taaaattttt gcccaaccaa gtttggcagt attttcatct cctagcacaa acttcatttc
481 tgaagaatag ttgattttaa gatataaatc aacacaaaat tcattatttg ttaaaaacat
541 tatactattt ttcaaatcat catgtttggt tccattgggc ataaatttaa cggcttcttg
601 atatgaaata tcttttatat aaattgctat tttgttttga taagagataa atttatcgcc
661 caatataaag ttatttccta gggtatgatt ttttatccct agtttatttt tttgagaatc
721 tgaaattaaa atttgatggg gtaaattttc aataattgat aatttatcct taagtttaaa
781 attgacttgt aaaactcttt caatgtgtgt tttaggtcgt cttaaactta gtagtaaagg
841 tgcaaatggt aaatattttt ttgcgatatc atgttcttta attcctaata tattaaacaa
901 aatttgcgaa atcgaatctt tgaaattttc attaaaagat cgagggtaat tttttaaact
961 gactacatca aaaaatatcc aaagcaaata atgattaaaa aaatcaaaaa ataaagtcca
1021 accttgctct ccatcttcat ttcttgataa tttatctagc ataaatgaag gaagttgcga
1081 agagactcca tgtagcccca tgaaatttat aaacaattct acaactgatt gttcatgttc
1141 tgaaaaaatg atttttctta tatctttatt tggatgtttt aagcttttat ttgttcttaa
1201 aaatatattc tttcttccat agtcttttc tagttttttt attaatttaa aaaaactata
1261 tgaagcaaga tcattcatat taaagctttt tttccaaagg aggcaggata atgtaaaatt
1321 tcttttgagt caaggcattt tatctttaat tcacaaaagg agttgatact agcaaaagat
1381 gctaaaaatt tagatataat caatcctaat aaatatacct cgcctaaagt ataaaatttc
1441 gaatctttaa tactaaaaat agctacggtt cctttttttg tgatattttc atctattaaa
1501 taagtacttt tgctttgaat atcaattata gcttcttgta aaagcttata aatcttccaa
1561 tttgattggt tatctaaaaa gctatagctt tccaacacac caaaaaaagc tttttttgctc
1621 aatatggttt gatatgaaaa agataataca gaaacaagct tccataataa gttaccatca
1681 acactacatc ttcttgtttc gctcggaatt tccacatttt tggtatcgac accttttagg
1741 ttgcaggtat tgatatcacc tatttttaat tttgaaggca aatctccatt agagcataaa
1801 agatcaatac ttatagtttc atctattaaa caagaggaaa aaaaggaaat ttcgctaaat
1861 acctctccct tagaattctt tttggtattt acactataaa aatctttttg attatctttt
1921 aaaaattcaa agcgttcaaa acttttatag tttttttaata gccttttacc gccttcacta
1981 ttatgtgctt taacttgtag tgtctgtatg atttcataag ctttaggttg acttctatcc
2041 acaaaaattc tataactgtc ttttttatga ttatttataa gaggttctgc actttttgca
2101 aagatattga taataggcgt catacctaaa gaaagtaatt cttttctaaa aatacaattt
2161 gccggaaatg gtttggagaa tttaaattca atattaattg tctttccttg gcaattgttt
2221 aaaatatcta aaccattgat tcttaaaaaa ttaaacttat gtggcataaa aaaatattct
2281 cttaacaaag aaaaagcttc aaaacctaaa tcattataag ataagctact ttcatctgga
2341 tttaatccta ttttttcaat attataggta ttaagaaaaa attcttcatc ggtatctaaa
2401 ctttgaattt ttaattcttt taaataagaa tgcatgtata ataataaagt gctagacata
2461 taagtatcat tgcctagata taaattaatt ttttctaaac ctaaatcaca tatttttgtt
2521 tctgctttat taacttgtaa tgttaaattg aaagtataat cttgattttt gctagatata
2581 aaaacttcat tgatggagat aggataaaga tagacatcat aaactgtttt aaattcacaa
2641 acacatttgt ctatcggttt tgatttaata agagttcctt taggaatgat taaattgttt
2701 tctttactat tttgttcaaa tttaaattct tgcatacata aagaaggtaa aggatttgta
2761 tagtttgggg aaatgatatt gattagagac tctgctatat gtggtatatt ttcatcaagc
2821 tcttgatgaa ttttagaact gagaatggct aaattttcaa taatcctttc tatatcagga
2881 tctttactat cgtgtgctaa aaatggagtt agctttggat agttttttat aaaatgctct
2941 ctttttttcat atagatattc aagttctttt tggtaataaa aaatattatc cttcatttaa
3001 cctcgcaata tctattgttt ttaaagatga tttctatatt gaattcttta aaacgatcat
3061 cctgaaagaa acattttaca ataaaagaaa gttgccaagg acttaaactg ttgtcgtaag
3121 tgatagaaac tacacatatt cttttttcat agttgtcaat cagttttctt atttccttag
3181 ccattgatac gcataattca ttagagttaa gattaatatc agccatatta ggcattccta

3241 agtttcttac agcgtaacaa tcattaattt ttgcattgag caaaaccttt atattgtttt
3301 ttacatcttc aaaatcattt tggtaaaatg gaatatgaat gttttgctcg tctaaattat
3361 ggatcagttt atctagcagg gacattatta ttctttatct aattttccta ctaaagaaag
3421 ttcaaaatta ccacccatat atttaaaatg aggtcggacg cttagtccta ttttatacca
3481 tcctggctct cctggtatat catcaacttt cacttgtgct gccctgaatg gacgacgact
3541 tcttacttca gaaggtggat tttcttgatc ggaaatatat tgtctaatcc attcattaag
3601 accattttcg atatcgcttc tttctttcca actccctatt tcttctcgtt gtaaaacctt
3661 taaatagtgc gccaatcttg aaattaaaaa gatatatgga agctgagttc ctagtctata
3721 atttgtttca gcttctttac cttctggggt attttgaaat atctttggtt ttaatggaga
3781 atttgctgaa aaaaatgccg cattattact atctcttctt agtgttaaag ttataaaacc
3841 tgcttcagca agttcatact ctctcctatc tgtaatcaaa acttctgtag gtatttttga
3901 ttgtatagtt ccaaaatttt cataaagata agtaggcaga tccttaacag tccctccgct
3961 ttttggtcct ataatatttc cacaccatct atatttagca aagctttctg ttaatcttgt
4021 acaaaaagta tatgaagaat tagcccacaa taaatgatta tgtgaagcat gaacattttc
4081 tttataatta aagcttttaa tagggttttc ttcaggatca taagggcttc ttgctaaaaa
4141 tcttgtcact agcaagcctg tatatttaga gtcttcattt tctctaaaag ttctccatct
4201 tgtatattga ggcccttcta gcaagccttg taaatcttga atatttgcaa gttcagaata
4261 atcatcaagt ccaaaaaact ttggtccaaa agatgttaaa aagggagaat gactcatagc
4321 tgcaatacta gccattttat ttaaaaaagt catatcagga cttgctgatc caagttgata
4381 atctccgatg attgctccta cgggttctcc tccaaattgt ccatattctg aagaatatat
4441 atttttataa acaatacttt gtgtgatgtc tggattattt tcaaaatcct ctaaggcttc
4501 ttcttttgtt atatcaaata ggtcaatttt tatattttca ttaaaatcag ttctttcaac
4561 tagaaatcta agtccacgcc aagtagattc aagtttttga aattcttcat tatgtaaaac
4621 ttcatccatt tgttttgaga gcaaatggtc aatatgggct atcatttcat caagtgcaaa
4681 tttattgatt ttttcttcag cattatcact ttttacaatt tctgaaataa attcagcaac
4741 acctcttttg gcaatactat aactttcatc agttttagag tatttgcttt tttccataat
4801 ttgttcaata attggcgtat caattacttt agtatttgcc attttaattt ccttgctctt
4861 cttgtttgat ttctaaaagt aacttttctt tagtttttttc atttttaac gcttctaaaa
4921 cagcttttct aaaatcagga atattgccca taggaccttt taaagccatt aatgcttctc
4981 ttagctgcaa taatttattt agctcaggta tttgtttagc taagctatct ggagaaaaat
5041 ccttcatact agcaatattg agcttaacat caagctcttc agctcctgtt ccaagagtat
5101 tttttacgct aaaactcgtt gttatatcga gttttctcat aacttgatca aaattgatct
5161 tattaatttg caaaatttct ctttcctcta agggagtttc gtttttttcct tttaaatttg
5221 ccattaccat taattttaat ggcagctcca catcctcatt ttgtccatta gttttagctt
5281 tatatgtaat attaattcgt tcctttggag catgagatcc atctgacatt ttaaatcctt
5341 aaaaataaaa atttttagtt caattattat attaaaattt gctaaattta atcttgcatc
5401 atttaatttt attttttgcg ttttcatatt cttcagctag ataatctttt agtagagttt
5461 tttccattaa ctctactaag tatatattta aaattttaga gttcttttttc attccttttg
5521 attcaaaaat ttttaccata tctagcaagg aattaatatt attcataata gaattattaa
5581 aattttcttt atcaatattc atcagcattt gctcaaaatc ttgtaatgta tgttctttgt
5641 cagtttttttt acttgaaaaa aaactttttt tattttcttt aggtttaacg aaatatttat
5701 aaacttcttc tttgcagata aagtcaccat tttgaaaacg gagaagttca atagcatcaa
5761 atttagaaat aaaatcactt gttagcatta tgagaatgtc taattgttta ttttttttct
5821 ttttctctaa aaaatcacaa aagagttgca caccctctat ccaaaaagga tttaaaatga
5881 gttcatgcat aaaacattta atatgttcta tatcattttc tttatcagaa agtaatatac
5941 gaattatatc cgcatcaggt tttcttattt tagtaatttt ttcattatta cattcaggca
6001 aacatttgat ttttccccac attgcttggg tgaataaagc gtagatattg agattgtttt
6061 catcctcttc taaaagctca aatgcaagtt tttgataaaa atttttatat tctctctcgt
6121 ttaaggagtt aatattatcg cttctaatgt ttacagatac ttgagctaca ggagattttg
6181 gaagctgtgg tgcaacttga gcaatttcaa tttttgcaaa attgcaagat agtaaatttt
6241 caaattcgga aaacaaagcg ataaattgat caataatctc agatgataca agcacttttg
6301 ttttgttata ttcttgaata aaattttgaa taaaaatttg aatttttcgc ttttgggctg
6361 atatataagt attattttca tccataagtt tttttaagtg ttgaaaaaga agcaaaagtt
6421 tttcaaagca ttcttcattg tttaaattga agcacgataa aagaaaatag ttacaaactt

6481 taaaatccaa agaatgatgc aataatataa attgagaata ttgataaact ttatcccaag
6541 aaatattttc atgatttaaa gtcttatatt ttgacatctc atcttcaagt aagtgaaatt
6601 cttgcagctc atctaaatta tttttcaatt ggttacaaaa agacatttaa ttcctccaaa
6661 aatttgcctc attataatta aattttaaaa aatattatgt taattttttta aaaaaattac
6721 ccaattaatc acaaattata ttatttatag aaaggatacc ttagttttat gtttcgtaaa
6781 aaaagcttat tttttttatt gccgttgttc ttttgtgctt gctcaagtgt tgttagtgtt
6841 aagataaata atatagaaaa ttccaatctt aataacagat atgatgatgt tcctgttaca
6901 gctattgtat atcaacttaa ggatattaaa aaatttgaag aagcaagtga tatagatttg
6961 gccactagag aagatggggt attaggcaag gataagcttg attctattaa aacgcaaatt
7021 gcacctaaag acaatattat cgcggttaaa gtagatgaag gagaggttcc ttatgtaggt
7081 gttcttgtat tgtttgcaaa taatacaaaa aaagtaacaa aaatatgggc aaagaccgaa
7141 gatgcaaatg ggtttggaaa gaataagtat ttaaaatttg aaatatccaa agaaggcatt
7201 aaaagaatta aataggaatg atatatggca aataaattaa aagttgcttg gtttgatggg
7261 cttaatattg gacaaaccca ttttgagcag caagaaagat tttttaatag aaatatagat
7321 cttaaaacta taaatatata tagtaattta tatggaataa tagacttgga attttctcaa
7381 gagatgttat tgcaaggaaa aattgcactt tctaaaattt caggcatcgc gcaagatgga
7441 agtattttta atgcacctga gcaagattta ttacccgaac ctatcgaaat caattatgaa
7501 tctttaattg attcagttgt ggtattaaaa attcctattg ggttactga tattgctgat
7561 ttgtctttaa gaaacacttt tccaaattca aagtatatat gtcttcgaaa tagtattgct
7621 ttaagaaatt atgatgattc aaaatccaac gcaatggaca aaattgaaga tgagtacgaa
7681 cttgaaaatc tgacttttac gcaagagaaa aaagatctac ttcttgctag tttaagatta
7741 aaacttggca ttttaggtaa ttcaactcct gatgaacttg aattgccaat tgcaaaaatt
7801 aaaaatatcg atatcaataa aaaaatcgaa ttagaaagtg attttatacc tacttgttta
7861 aatatcagta aaatatctac cataagatct tttttggaag aaatcatttt ttcaatcaat
7921 caacataaaa aagttctatc taatgttttc aaaggaattg accaaacaaa aaatacattg
7981 gatttagta ctttttttatc tcttaatctt ttaaaaaaat ggtatttaat attttcacat
8041 ctatcaaaaa aagataaaat acatcctgaa tttttatacg aaaaattttt agaatttcaa
8101 ggagagcttg cagcttttag caacgaagaa tcttttttag attttattcc ttataaacat
8161 gataatcttt ataatacttt tttaaatatg atgaataatt taagattgtt attttccaaa
8221 atcacaagtc ctaaatattt tatagctaag accagtatta atggcaatgg tttttatgat
8281 tttatttttg ataattcggg aattttagag gatgctgaaa tttattttgc aattcgtgca
8341 gatgttacta cagaatatct acttaataat tttaaaattc aaagcaagat acatacgcaa
8401 agtaaaatta aaaatatagt agcaacacag cttgaaggct taaatgtaga tcaagtacct
8461 aatattccat ctagtattcc ttatttaaat ggttacgttt attataaaat agataaaaga
8521 gatcatctgt ttaaagattt taaaaatgaa agtatcatta gtatgtactt gaccaacaat
8581 ataaaaaaatc ctgatattat aatgtgggcg gtatttcaat gaaagaaata aacgaagaaa
8641 aaaaggcatt aagcttactt cttgattcaa attttgatgg tttaaaaaac aataaaatta
8701 ttgattattc tttggagtta cttttgcttt cttacagact ttcaaaaata agctctatgg
8761 atacaagtaa tattaatcaa cttagagaaa cattgattaa taaaatttta gatataaccg
8821 ctaaactaag catgtgtaaa gaatatgatg aaaaagaaat tattaaattc aaatattgtc
8881 tttgtgtctt tatagatgaa agcttgatga aaaatgaact ttttatcaat ttttgggctc
8941 acaatactct aactgtaaga ttatttgatg aaaccttagg tggaaataat ttttatgata
9001 ttgcatcatc ttggattaat aatcctttta agtttaaaga ttttttggaa tttatttatg
9061 cttgtttgat tcttggttat aaaggaaaat ataatgaggc taaggataga gatgaaaaaa
9121 taatccattt ttgcaataat attgccacat ctttaagacc agtttataag acagaagaag
9181 agttagcttt taataaagca tacaaaattg gtttagaaga aaatatttgg caaaaattta
9241 taagattgta ttttaaaaaa cttattatca ttgtgccagt attgattgta ttaggagtat
9301 taagctttgc aatttttaat ttagaaacca ataatcttaa agtggataat aatatatccg
9361 tattgataaa aaatttaaca catatagaat aaagatatta aattttttta aatatcaatt
9421 tatttgtgat gagtaaaatt aaataaaaat tattttacag ctagaataaa aatcaaatgc
9481 aaatctaaag gataaaaata tacgaaaaaa caagctctat tattttaatg ataaagaaaa
9541 agttatactg ctaacactaa actacaaaat tacatcttaa ttttaataaa atagagagtc
9601 taaggagata acctatgaca tatatattac ttcttattat aatttctact attctttctt
9661 atcttatatt aaaatttatt tataaaatca tctttaaatc tacaaaaagc gtttcaaagt

9721 ttttagtctt tcttggaagc ataggtttaa ttatctttta ttatactcct tattcttatt
9781 atttagagcc tagttattgg caatttagga atatgtgtaa gttaaatgaa cttcctaata
9841 acgaagagaa atataataag attctagggt attttgataa aaagttaggt gacatagatg
9901 attttcgaca tgtaaaaaaa tatggtagta tagaaatgtg gctttatata tattacgatg
9961 atacttacaa aactccttca aattttcaag aaaagataga gatggataat attgcactaa
10021 aaacaaaaaa tatgcaaatg tctttacaca agattgttga tatgcatata agtccatatt
10081 ggaatactag aaggtatgtg ttagaaggca atgagggtaa tatgaatttt gagtttatag
10141 aggatgagct cgcttgtggc aatctttatc aataataata taaggaataa ataatgacca
10201 ataaacaact tattaataaa ctaagggata tggcggaact tgcacaggca agttatggtt
10261 attttgattt ggtaacacat gacaagaatg atttttttcat taaaattaaa gatgaagcta
10321 cagaatctgc tttctttaat aagcacatta ctcaagctaa tgtcttagat attagttata
10381 aaaattataa ggcatatcaa tatgactcat ctttaaagaa agatattaaa attggcactc
10441 tcaaaggcga cttctcccca ctccaagcca aacgattctt ttctcgctat gatttattaa
10501 aacattgtcc taaaaaatga aaataaaagt atcatatttg taggacattc tctggtgggt
10561 atttagctca aatggctttg atatattgtg attaaatata aagatttata cttttaatac
10621 tcttagtatt tacgtttgaa atgttcctag tgtggttata ttttgatttt taaaaaaaag
10681 taggataatt ttattaaaaa tattaaaaag gaaaataaat gggtatatta aaaaaagtat
10741 acattgtttt ttttgggggg gggtattttg gggaattatt tttttgttta gttctttata
10801 tgcttatttt agtgatatag aattttttgg tgatatgtta aagattgcat tttttatata
10861 ttcttatcca ttgttgcctt acatatcata tttaacacta caagaaagta ttaaatccaa
10921 aaatattttt ctttattttt ctttattttg ctttattttt ctttatctcg ggaggatttt
10981 ttatttatgc aataatatta ggtatttata caatattatt tttaataata tctattaaat
11041 ttagaaaaga actttcttat tatttttctt tattgttttg tattttttta tacatttttt
11101 tgtatctttt tatttttat atagaattaa aaaatgagta ataaagctca aattaacaat
11161 ctcaaaaatt acgctaaact tgtatagatt ggttttttta attttttaat aataatttaa
11221 atttcaacaa aaaaggaagt agtgtgaaaa tattaaaaag tttatatatt atttggatga
11281 tattttgggg atttggggct attttaggtt attatatggg atatgatgca acttttacta
11341 aaacgttctt ttttcttgtt atattatatc tttttccatt gttgccttat ttgcattatt
11401 taatttttga gaaaacaaag aacaaaaata tattcatttc tatttgttat tatctactat
11461 tatttgttat taccgcttat tattttactt taatatattc atgtggaaca aattttttaa
11521 cattttttat aatgattact ttatatatga ttatttgtag tgctataatc ttatggaaat
11581 ttaaaataaa tttaagatta tatttttaca taattttatg tatttttact tatctctata
11641 tagttttaca tatttttata gcatgttatg aaatagaatg gacagatata ctgctagata
11701 aagatgtaag gatatatatt tacaaagaaa taattaattt gcttacagag caataatttg
11761 ttttataata aatcttattt ttatagggga ataaattaat gaaaacccaa atcaacaatt
11821 taagagatta tacagagctt gcatgggcta gttattttta ttttgattta aaaggttata
11881 tattacaaga aaatgaaact atcgttattt taaatgagct tgtaagttta agttataacg
11941 gaaactgata attttattga ctcggttcaa gacatagccc tatcttttaa tgacattatc
12001 caattttttc ctcttttaga ataagtagat aaaataaatg aagctaaaga gccttgtgaa
12061 tttaatgatg atttcaagaa attttttctt actgaaacaa tcaaattttt aaataaattg
12121 tcttataata gaataactta ttatattagg agcaaatttg caaattccta tttattctaa
12181 actcaaagca aaactagaaa gcaaaggaat agacaatata tcaagcggac aaattgcttg
12241 gtttgtgtgt agtttgattc ttgctttggt gacttatatt attctaactt ttcactacat
12301 ttacattagt tatggtaaat atgataagaa tttattttta ttaatatttg atagaaatgc
12361 aatggaatta tttattatta tatattcttg tataatgttt aatctattat atatttacaa
12421 tgccattcct ttggttttat ctgcagtatt tatatttttg gcattttatt tttctagttt
12481 aagcttgttt attgttggtt gttgtggcat aggagtattt ttcttaatat ccattttcaa
12541 aagaaaactt atacctagcg ttttgttgtt tactatgggt tatatttgct catatctctt
12601 acttcatgca attggaactt atcattagga ttttataaat gaagtattgt gtaacaattt
12661 atattgatcc attggtagaa tgttttttgg attttaaaaa tcagtgttat tggcaaagag
12721 aagatttcac ccacgccttt ttaggactta ccaaagataa aagtcccgat gagttagata
12781 aatatagatg aagaattaaa acaagaatat cttaaaaaca aagaatttaa attaattaaa
12841 agtattaagt agattggtta taataaaaca atttatctat attaggagta aatttgcaaa
12901 ttcctattta ttctaaactc aaagcaaaac tagaaagcaa aggaatagac aatataacaa

12961 gcggacaaat tgcttggttt gtgtgtagcg taggtatttt tatcattcct atgatgtatc
13021 tttatatcag caacaattac aatcaattta ttattttgtt ttttatgata tttattttta
13081 tattaaatat gctttatact aaaaattttt ttgcattatt attacttgta atattgatat
13141 ttatatatat gaaatatcat gagtttatct ataattttaa aattttattt tttatttttg
13201 cacctataat tcttatcttt ttaagggctt ggagaatttt aataactttt ttatgcggat
13261 atattttttt agtggctatg gtatatatat ttgagttatt aggaggattt tataaatgaa
13321 atattatgca acgatttata ttgatccatt ggtagaatgc tttttggatt ttaaaaatca
13381 gtgttattgg caaatagaag attttaccca cgctttctta ggacttacca aaggcaaaag
13441 tcttgatgaa cttgataaaa tagataaaga attaaaacaa gaatatctta aaaacaaaga
13501 atttaaatta attaaaagta ttaagtagat tggttataat aaaacaattt atctatatta
13561 ggagtaaatt tgcaaattcc tatttattct aaactcaaag caaaactaga aagcaaagga
13621 atagacaata tatcaagtgg acaaattgct tggtttgtgt gtagtgtagg gatagtatat
13681 ttgctttcta tttttttata tttgcatggc gttaataaag agggaatttc gtttaataga
13741 atatttattg cattttatat aaatcatttc ttttgtttta tgttgatgtg gtttatttta
13801 ttgttaaatt tactttataa agaaaatact atttcattaa tattaacatt attaacaacc
13861 attgcctatt tttataatcc atattttgct tatattttga tatttatcat ttgcatcatt
13921 atgcgtataa taaataaaca aagatggctt tatatatatt ttattggttt aatttatatt
13981 tatgccacct atagtatatt taatttaaga ggaggatttt ataaatgaaa tattatgcaa
14041 caatttatat tgatccgcta tttgatttta aagatgctac ccatgccttt ttgggcttac
14101 caaaggcaaa agtcctgatg agttagataa aatagataaa gaattaagac aaaaatttta
14161 agcaaattag tttataataa aacaatttat ctatattagg agtaaatttg caaattccta
14221 tttattctaa actcaaagca aaactagaaa gtaaaggaat agacaatata acaagcggac
14281 aaattgcttg gtgtttaggg agtggctata ttgccttaat atgtgccgtg tttgttgttg
14341 tatataaact taagaatttg gaatttgagc tttatattgt ttttttcttt cctatcttgc
14401 tattttatat tgctatattt tggtttattt tcttaagttt ttataaaaac ttctttttta
14461 ttcatctttt ttgctattct tttatgtata tctttaatat aatttttaat gatttttatt
14521 taaatcttgt ggtaatgaat attttattaa taacaatatg gctctttttt gttaaattac
14581 atttaaaatt tagattttta tttttaatga tgtatttttg ttttgaattt gcgatattta
14641 taggaatcat accttatgga cttttagaag ggactatttc aaaatgaaag aaataataaa
14701 atattatgta acaatttata tagatgagtt attagaaaat gaaatgttaa agcatgttga
14761 tttttcttta ctatttaaag caaaattttc tcaaatttct aagcaaatcg acttcaccca
14821 cgccttttta ggacttacca aagataaaag ttctgatgag ttagataaaa tagatgaaga
14881 attaagacaa aaattttaag caaattagtt tataataaaa taatttatct atattaggag
14941 taaatttgca aatccctatt tattctaaat taaaaacaaa gctagaaagt aaaggaatag
15001 acaatatatc aagcggacaa attgcttggt ttgtgtgtag tgtaattttt acctatttat
15061 tatttttatt aattgtaatt ataaatacta gaacaatatc ttttgaaaac cttaaatttt
15121 ttacttttga aggtttttc aaatctcttg cttttatttt tgcaaatcat tttttttat
15181 ttatttttat gtggattgtt tgtataataa attttttta atataaaaaa tttaattcca
15241 ataacactaa attggataat aagctattgt tattttgttc ttgaaattaa tatttatttt
15301 attttgattt tatgtataac cctttgtgtt gtattttatt ttttacaaaa aagattatta
15361 tatacttttt tattttattt tgtttatatg gtattaactt atactttgtt ttatcttggt
15421 ggaggatttt ataaataaaa tattatgtaa caatttatat tgatccattg gtagaatgtt
15481 ttttggattt taaaaatcag tgttattggc aaagagaaga tttcacccac gcctttttag
15541 gacttaccaa aggtaaaagt cctgatgagt tagataaaat agatgaagaa ttaagacaag
15601 aatatcttaa aaacaaagaa tttaaattaa ttaaaagtat taagtagatt ggttataata
15661 aaacaattta tctatattag gagtaaattt gcaaattcct atttattcta aactcaaagc
15721 aaaactagaa agtaaaggaa tagacaacat atcaagtgga caaattgctt ggtttgtgtg
15781 tagtgtagtt cttgctttgg tgacttttct tattatctat tttacagtga ttattgaaga
15841 taaaaattta tttttagcaa tatttgaatt gtttcaatgg caactattta tttttatcat
15901 atacttttgt atccctttta atctattata tattcataat gctattcctt tagtgctgtc
15961 tgcaatattt atgtttttaa cattgtattt ttctagctta aatttattta ttatcggctg
16021 ttgtggtata ggagcatttt tcttaatatc catttttaaa agaaagctta tgtttagtgt
16081 tttgttgttt gctatgggtt atatttgtat ctattttttt ctttacgcaa taactcgcta
16141 tcattaagga acaaaatgaa gtattatgta acgatttata ttgatgagtt attagaattc

```
    16201 aaatgataaa agatttgctg aatatgaaag gtttagactt cacccacgct ttcttagggc
    16261 ttactaaaga taaaagtcct gatgagttag ataagataga taaagaatta agacaagaat
    16321 atcttaaaaa caaagaatgg gacaagatag atcaaaaatg gtacgaagct aaagagccta
    16381 atgagtttaa tgatggtttt tggggttttg gaactggaat tgctaatgtt gcagaatctc
    16441 ttataggatc tcctggcaaa gtatttaata ataaccaata tgtattagat aacaatgttg
    16501 ataaaaatcg ctatattatt aaaaaactaa gaagccctca aacttttaaa ccctctaatc
    16561 gttgcacttt agaaatttct aaggaacaat atgaaaccct acttgcaaat attaaagctg
    16621 attttgaagc cacaaaagaa atcacaccta atagcgaaaa gccaatcaat gaagaattta
    16681 cctataaact tcttgaaaat aattgcgtta cttgggttgt taaaaagctt tctagcatag
    16741 gaatagaagt tgtaaatccc gttgttaaag atcctagcgg attattaatg gctacaaaat
    16801 taatacaaag cattcattct atctttttaa aatttcaaaa cattgatgat aatttacaaa
    16861 gcgtaaaagg tgcaaaagct tttaaaaatt gggctagaat gcttataaat aattcattaa
    16921 tgcaagaaat ttatcttgat gaatataaaa aacaatacaa tagtgaaaat cttagtccag
    16981 aacaagaaga agttttatat gctttatgga atattacaag ggtttataaa attttaggca
    17041 ttcttttaag tcgtttaatt acaaaatcgg gcactaaaaa cctaaaaggc acttttgaat
    17101 taatttattt tgataaaaat gataagcaag caaagctttt aaaagcgagt aatcattatg
    17161 aggcgattat tatacaagaa ctagatttaa gtaaaaaata caataatttt agtgcgagta
    17221 aattttatcc ttttatcttt atacctaaag atgaaatgct ctcaagaacg ctttatcata
    17281 agtatgatta tggtaatatc tcacaagaat atcaaaaaga tagaaatgaa ttttatttta
    17341 atgttttagc aggagaaaaa agcgataaat attggagttc tagctatcat aaaatgacta
    17401 aaaatttaag gaaagtctat gtctcttgaa aaaatcaaag aaacgatttt tgcttatgat
    17461 aaagaagtga ttgattgtga agttttaagg gctaaaaata ttgatgtaat tcattctaaa
    17521 atttattttc aaggtatact ccttacaggc tccaccgaac tccctaataa tcctttttac
    17581 tttggagaat tagatcaaga taatactata aaacaagata tacccagtta ttatttttct
    17641 cctaaagatg aaagcagtgg tttag
//
LOCUS       ECFEEEMN_2             17527 bp    DNA     linear       12-APR-2021
DEFINITION  Campylobacter species strain strain.
ACCESSION
VERSION
KEYWORDS    .
SOURCE      Campylobacter species
  ORGANISM  Campylobacter species
            Unclassified.
COMMENT     Annotated using prokka 1.14.6 from
            https://github.com/tseemann/prokka.
FEATURES             Location/Qualifiers
     source          1..17527
                     /organism="Campylobacter species"
                     /mol_type="genomic DNA"
                     /strain="strain"
     gene            177..1025
                     /locus_tag="ECFEEEMN_00027"
     CDS             177..1025
                     /locus_tag="ECFEEEMN_00027"
                     /inference="ab initio prediction:Prodigal:002006"
                     /codon_start=1
                     /transl_table=11
                     /product="hypothetical protein"
                     /protein_id="Prokka:ECFEEEMN_00027"
                     /translation="MPIYNLLINNEKKEQDKALNEFNIEQKDLKDIEDINIFNQFKQD
                     FSKDHEFKELNLSFDTNLIKLYFIIPKNIAKVYKSAYKEFKNKDLGAGYFTQLHEYDK
                     IIKNSLEDKKELNEYHLTFLTPAKMQNLKLQIAQGLDKILEDEDRKQELYVCKFVVVN
                     GVSENKIKAKNKDVNIDNDTKNINTTKSNNISSSESNAQEKITHLSNDGQNLLKNIEK
```

```
                     LRLKPYNDQNGKEITSYVKGATIGYGHLIGQNEWDLYKNGITLQEADKLFKSDLLPFE
                     NAVKIQ"
     gene            1261..1620
                     /locus_tag="ECFEEEMN_00028"
     CDS             1261..1620
                     /locus_tag="ECFEEEMN_00028"
                     /inference="ab initio prediction:Prodigal:002006"
                     /inference="similar to AA sequence:RefSeq:CKG86986.1"
                     /codon_start=1
                     /transl_table=11
                     /product="hypothetical protein"
                     /protein_id="Prokka:ECFEEEMN_00028"
                     /translation="MVIFKNILLFLFFSTYLLAQEDFTPLEQCTYENEKFWIKILNLC
                     LEGNITCDKIVYVGVNKNNGKYIVLNGKSISDVNMNFKGYVFKNGIYEYNIFNNFLYI
                     SKNKQIIQEYRLKLCEK"
     gene            1885..5880
                     /locus_tag="ECFEEEMN_00029"
     CDS             1885..5880
                     /locus_tag="ECFEEEMN_00029"
                     /inference="ab initio prediction:Prodigal:002006"
                     /inference="similar to AA sequence:RefSeq:AAW35464.1"
                     /note="conserved hypothetical protein"
                     /codon_start=1
                     /transl_table=11
                     /product="hypothetical protein"
                     /protein_id="Prokka:ECFEEEMN_00029"
                     /translation="MFISSKTELIQTIKNSTINNKEVKQIDFIYPNFSILHAILKNID
                     FHQRKILFKNQILAMEILEILKQTRDNHNNIEVNIHTLNELDAGQNVNLKDLSSSEIC
                     TKVYLEDLEEEIQDKKQGKDFNLFYHTDKNNLCIEFINTLKELAKDSEDITTLDELIE
                     NIDPDDISSEKFDDFKKKVLEKIGNVIESDFFKNSISPTLKNTYEKFIDELLEHIKKL
                     AEEKEYEKIKIILSAIAGVAIATVVAVLTGGAGLAILAAGLFSLMGSASEYYEFAQRD
                     YGTLLTPLAAFLATRCAMFIHYINSRLLSCVLIIDKKEIIDLSGFGLYLGSDESNIAS
                     NLAYKITLSEEIKSIENIFSEIFLNPEQKDNESKNTANDSKSSNLLAFISTQKLSNLY
                     LKNNAILKNLQEDIKQFIYFSYIESPNFNSSKLAEFFAQKNQNTQIDLNTTKMSKNYL
                     IISNIPNKYNAGLSESLTQEIANYNIQDKYRTIKKNIDTSKKTYDSTRDYSEYSIEIP
                     QDENEPYILQLSPFVKISKEAYENSKKAYEDMQEIYKYLTNFVATPQKLPEFAKTAKE
                     KLAISENESMIEIVKKTENLRHCERINILAYIDYNKKINDKKWLPYFYIKKFFKQNED
                     KESIIEKEKKIAHQYLTFLKKELFEKSDKSSFGNYSLSDKSSFGNYSLSDKSSFGNYS
                     LMECLFISFVLYQAKLIFPNINLDLKKYGGSNFVYIKYLEETYVLWTDEYPIYVNNEP
                     TNITAYEVVSQLLKILDKKDYSFVENDSSITIDDFFNELSKDLDVTNTLDKKIIKLKQ
                     MYHLDEQKCIKEFMNLDEEAQRAFFVWMDIEKQISDLSKLLEQMYGKEYLNKAKEQKT
                     SEIFTYAMLKILLSASPFISFTAQMLWDFNFLKTIEYVIQACIKINKEGFKEFFKEQL
                     GIFTPLKKNSIYHIHIKSNLIIKACLQQIEKELTHTKIQMILAQEIKSNLIIQKNTSD
                     LNLFITKIKEYFNEKQIKIYWSDKYMKQLFIYSNQESNISKIALMHSIDIQTQKYTIY
                     TYAKLNKLDLASIVAGEFVGIFLNYLFPTNYKALQEQYESIVYQLYTFKDDSPLAVIR
                     DHFITYPFAINSKFISFDLKKIFYGIDLCTGVFDTHFSFHYEIDQKLEENKEFLLKRL
                     LSYLIIDEKRSATALEDIKEQKYIIDDYTFYFKELKSLELKKCTIYQLPCNHSLFKEF
                     RNKEKQISAFKKDEDSPIHAYNEALQILQDFANGLYIVKYEEGKCIEDKNKTRRLLEK
                     LNIIGQNNLKAMYMGINGNKESLKAIGKRENNTSKANINADIVSETKAPPNFIGRLAT
                     TIIIEDGLYIG"
     gene            5899..7167
                     /gene="trxB_1"
                     /locus_tag="ECFEEEMN_00030"
     CDS             5899..7167
```

```
                     /gene="trxB_1"
                     /locus_tag="ECFEEEMN_00030"
                     /inference="ab initio prediction:Prodigal:002006"
                     /inference="similar to AA sequence:RefSeq:CKG87041.1"
                     /codon_start=1
                     /transl_table=11
                     /product="thioredoxin reductase"
                     /protein_id="Prokka:ECFEEEMN_00030"
                     /translation="MSETYEIYTPNGLTLDVEKDTNKILFKENIKPTGNYTEEYSKAV
                     FKSYYIMKNSPYKDYKPIYLDPNFYTGEKSTLLEFKEWQNIYLKDPIKGSIAPWTKAE
                     KAYYKSLKTKRERCKYLAIRSGLRSVVIDIPYDAYCNVDEKGYLINEEYAYIYDEVSS
                     HRGTLKSYSFFNEWELAALLLGDMRASPTAAVGFKARQQQALFLQAQFGDKNAFKSLG
                     LAVLCSNSFLTGQHWNKLRAKMIYDLHDHHYESLLDEFGMLPFLDEIIGADWTIDLNK
                     YDFAYDEEGRIIWALYNDIEKGKLKDPRDVDSTPESRNEFDDAMDRYENGMVTRFDVD
                     TPNEWSEQQAALDRDTLVLSAKLAALTPPQGYPNAPYYFTPERLEWIYKRGYLDKLLD
                     PRIPAIYRYNFPRELRAKILKFAEENGIKE"
     gene            7250..8395
                     /locus_tag="ECFEEEMN_00031"
     CDS             7250..8395
                     /locus_tag="ECFEEEMN_00031"
                     /inference="ab initio prediction:Prodigal:002006"
                     /codon_start=1
                     /transl_table=11
                     /product="hypothetical protein"
                     /protein_id="Prokka:ECFEEEMN_00031"
                     /translation="MSETYEIYTPNGLTLDVEKDTNKILFKENIKPTGNYTQEYSKAV
                     FKSYYIMKNSPYKDYKPIYLDPNFYTGQKSTLVEFKEWQSIYLKDPIKVAQNITNICI
                     NYVKKGFKEVLIEQLGIIYLLTHPQIEVEVKKYGKKYFKTINKEIAAKRFSTIKEELF
                     FNKTKISFMAEQVKNGIDYEIDQKLEENKEFLLKRLLSYLIIDEKRSATALEDIKEQK
                     YIIDDYTFYFKELKSLELKKCTIYQLPCNHSLFKEFRNKEKQISAFKKDEDSPIHAYN
                     EALQILQDFANGLYIVKYEEGKCIEDKNKTRRLLEKLNIIGQNNLKAMYMGINGNKES
                     LKAIGKRENNTSKANINADIVSETKAPPNFIGRLATTIIIEDGLYIG"
     gene            8414..9682
                     /gene="trxB_2"
                     /locus_tag="ECFEEEMN_00032"
     CDS             8414..9682
                     /gene="trxB_2"
                     /locus_tag="ECFEEEMN_00032"
                     /inference="ab initio prediction:Prodigal:002006"
                     /inference="similar to AA sequence:RefSeq:CKG87041.1"
                     /codon_start=1
                     /transl_table=11
                     /product="thioredoxin reductase"
                     /protein_id="Prokka:ECFEEEMN_00032"
                     /translation="MSETYEIYTPNGLTLDVEKDTNKILFKENIKPTGNYTEEYSKAV
                     FKSYYIMKNSPYKDYKPIYLDPNFYTGEKSTLLEFKEWQNIYLKDPIKGSIAPWTKAE
                     KAYYKSLKTKRERCKYLAIRSGLRSVVIDIPYDAYCNVDEKGYLINEEYAYIYDEVSS
                     HRGTLKSYSFFNEWELAALLLGDMRASPTAAVGFKARQQQALFLQAQFGDKNAFKSLG
                     LAVLCSNSFLTGQHWNKLRAKMIYDLHDHHYESLLDEFGMLPFLDEIIGADWTIDLNK
                     YDFAYDEEGRIIWALYNDIEKGKLKDPRDVDSTPESRNEFDDAMDRYENGMVTRFDVD
                     TPNEWSEQQAALDRDTLVLSAKLAALTPPQGYPNAPYYFTPERLEWIYKRGYLDKLLD
                     PRIPAIYRYNFPRELRAKILKFAEENGIKD"
     gene            complement(9687..10586)
                     /locus_tag="ECFEEEMN_00033"
```

```
CDS             complement(9687..10586)
                /locus_tag="ECFEEEMN_00033"
                /inference="ab initio prediction:Prodigal:002006"
                /inference="similar to AA sequence:RefSeq:CKG87069.1"
                /note="Uncharacterized conserved protein%2C contains FHA
                domain"
                /codon_start=1
                /transl_table=11
                /product="hypothetical protein"
                /protein_id="Prokka:ECFEEEMN_00033"
                /translation="MIEKEKLGIAIENYDECISGSKAFVFDTKGGTIGSGDDCTFRIQ
                DKLEQIKNTHAIVSYEEGSFTIAAFEDSDIYYNKSFSRMPSGYEIIISIGDIFKIGNL
                EFRFVDAKSIEESIKENKKYLEDIEKRNHFDEIEIEPRGKTSINFDKNEELKEILKNN
                NYKFIEEEKADDSFLKEIIQRDPNSLGYENILKSLVKNLKDIKTKQQSSKLNNDYSPI
                NIKDFESIINNIPLIKSTRLINILALSLIAKELYTPIFEEMEENMFIKYLEAAIQNNI
                KEDKILFENLTLKALEKYIKDFE"
gene            complement(10583..14110)
                /locus_tag="ECFEEEMN_00034"
CDS             complement(10583..14110)
                /locus_tag="ECFEEEMN_00034"
                /inference="ab initio prediction:Prodigal:002006"
                /inference="similar to AA sequence:RefSeq:CKG87105.1"
                /note="Uncharacterized protein conserved in bacteria"
                /codon_start=1
                /transl_table=11
                /product="hypothetical protein"
                /protein_id="Prokka:ECFEEEMN_00034"
                /translation="MFKKIFDFIKSRLFITAFLLCCIFLFSVLFWFWGPLVAFNDIYI
                FSSSFLRFSIISIIWLIVFLFFLLKPIINFISSLKSEKRLKFKDLKKEADEFIYKSKR
                NFFLSLKDAKETWKNDLKTKNLPLIIIIGNEGAGKSTFINYSDIEYPLSDSLESYKKF
                HKSTRNFALYVSKKGALLDTEGNYFSQEEFFKPASSDEIPEDDIDKNRDFLIKKNTWK
                KFLTFLNKNFFHSKLNGIILVVDTVLFLNNPKEYSKNLIRYLTKRVNECEKTLNLKLP
                IYIVFSKLDLMEGMKEYFDIFDKKISDKILGLSFDKILSEEFLNNEFKELSDSLLCSL
                MSKNSHIYNIENKNKSYLFLKQLDNLFALAKTFILEVQEENKLKNNSYLRGIYFVSAY
                QENIPRNFLLDAICEKYNCKKVLSKSSIIYNKQSYFVKSLLEDLIFTDYALSTMKSYS
                KKLSFLIMILIIGFGTYTISSYFISKNDKEFEKSQNTLRALQSLLENQDYQNLNIKQK
                ADFLIELRNILNTYPELWQDDNIFQYLNLNLSYKGFKEAKQLYYKLNEDVLKNTLLKE
                MEYTLLTDTNKENLIKTLYLYRSLFEQKYFNKEMLKIWINENWNTLSKYLISKDDFLG
                GVDELKQFNLKSFTEDENSINIGKRKLESISRTQRIYTLLNFLNSDKPKEKYLIKEDL
                GFAANSVFSNNSQITSIDKIYTKVGMMSFLKDLNQQVDTAINIESWMLNSNFKENKNT
                LTMGILKLYLNEYQNAWQNLLASLQPVKYNTKEAMLNELDILSKRENPLYSLLKIVSS
                NTNLNDAVLLTQAYNLGLNAGEIKSNFIGVSNAFTQYHKLVNKNTLLSVGNIEVGKGT
                DDEKILDILNTNITNVSNKIIDFSSNNNQSAEEKIAYALGGNKDANDPFTAFQMSIKK
                LPSDLERYYNQLSDHSWNLIENYGISLFNTAWINEVYNPFINDIAPFYPFNEESTANL
                SMDSFKTFFGRNGVLNSFYKKYLNNVLVKRKNNYSINSQFASKLNFSKEFLDFITNAG
                NLSSLILNANDNIRVNFTIQSLDLSADFSFVKLGYNNKNIQYDHTLDQTLQIVAEEFN
                NGTSLNFTAYNYSNPNLNYTKSYKGEWAWYKFIKDNKNNSTYSIMFNNNKNLYFDFEI
                INGAGDLNNIVYILNNLKIVENITGVNKQ"
gene            14243..14758
                /locus_tag="ECFEEEMN_00035"
CDS             14243..14758
                /locus_tag="ECFEEEMN_00035"
                /inference="ab initio prediction:Prodigal:002006"
                /inference="similar to AA sequence:RefSeq:AJK71137.1"
```

```
                     /codon_start=1
                     /transl_table=11
                     /product="major exported protein"
                     /protein_id="Prokka:ECFEEEMN_00035"
                     /translation="MAEPAFIRIEGSTQGLISSGASTEASIGNRYKSGHEDEIMAQEV
                     SHIVTVPVDQQSGQPSGQRVHKPFSFTCSLNKSVPLLYNALTKGERLPIVEVHWFRTA
                     TSGGSEHFFTTKLEDAIITNIELIMPNAQESSNHDKTELLKVSMSYRKVVWEHTAAGT
                     SGSDDWREGKA"
     gene            complement(15310..17304)
                     /locus_tag="ECFEEEMN_00036"
     CDS             complement(15310..17304)
                     /locus_tag="ECFEEEMN_00036"
                     /inference="ab initio prediction:Prodigal:002006"
                     /inference="similar to AA sequence:RefSeq:CKG88019.1"
                     /codon_start=1
                     /transl_table=11
                     /product="hypothetical protein"
                     /protein_id="Prokka:ECFEEEMN_00036"
                     /translation="MEALNQKNSLNIRLLSSNNILLHNQEFKLYEIAQGNKSEIKTIF
                     TTNRIGEAHLNASEIFSKEAQSFELILNQSSVYKNKPIYNHRFLLRSYEYGHWCEIKF
                     ERKADKGSINSIRLKSKEFTLENNEKIVRNFYTFGDIVEFEAIYEDTKIKEEQIKWGY
                     VFIQDKNTLDKLNKNQLTNDKKESSLLDEEILKDCFYIEKEEDKALLSSSIIYRHLEN
                     NKAYCGKHLSLQLPNPKDTQEQKALLVFAYKEIPNYNASYLIRINDYPQITIDCTLAE
                     TLRAHTNKDEISRLGWGEVSYICQRLWHDNPSDAKELKDLTFRSSTSQDFIDTIPNET
                     MKTIVKEILPRVEMQQLKTDNTKSRDKARFYAELDWDNFYMKFPIMQELKGEYMNLKK
                     LFGEESDFQKQFEDFLNDTLLDIKNIKNTQEYTMALNIQAMKENKTKNAEVFKLYKKE
                     ETLPETHKAIKDLQKPSDIIDNSLYFQRFDIKNDVFLPHLGLSKFDAFSLQNSIQHEK
                     EVLDKFHSNPKYKQNFALYSIAGAFNILYIPSLIQITRVTRFYNYHSLYAACKKVRAF
                     IIDTVNFNNNGSDQPIGAWDYEKVGFDLYNSIMQYRNTKFHFKYTSPKSFLFPLYNSD
                     FLKTKQYFNLGLDFFLYSSTFLDMDFSHMQMQDTAFIVGK"
ORIGIN
        1 cttgcactac agataaaggc tttgctttaa aagtaagctt tactccaagt aagtttaaat
       61 ttgatcacag ctttgatcct gaggagggct taggagaaca aagtaaaaat caaatagagt
      121 taaaagagcc tataataaga cttcattata aaagtgatag atttcaaaaa gataatttgc
      181 ctatttataa tcttttaatc aataatgaaa aaaaagaaca agataaggct ttaaatgaat
      241 tcaatataga acaaaaagat ttaaaagata tagaggatat aaatatcttt aatcaattta
      301 aacaagactt tagtaaagat catgaattta aagaattaaa tcttagcttc gatactaatt
      361 taatcaagct ttatttcatt atccccaaaa acattgctaa agtttataaa agtgcttata
      421 aagaatttaa aaacaaagat ctaggagcag ggtattttac acagctacat gaatatgata
      481 aaatcattaa aaattcttta gaagataaga aagaattaaa cgaatatcat ttaacttttt
      541 taactcctgc aaaaatgcaa aatttaaaac ttcaaatcgc acaaggatta gataaaatct
      601 tagaagatga agatagaaaa caagagcttt atgtttgtaa atttgtggtg gtaaatgggg
      661 ttagcgaaaa caaaataaaa gcaaaaaata aagatgtaaa tatagataat gacacaaaga
      721 atataaacac tacaaaaagc aataatataa gttctagtga aagcaatgct caagaaaaaa
      781 tcactcatct aagcaatgat ggtcaaaatc ttttaaaaaa tatagagaag ctaagactca
      841 aaccttataa tgatcaaaat ggaaaagaga ttacttcata tgttaaaggt gcaactatcg
      901 gatatggaca tcttataggg caaaatgaat gggatttgta taaaaatggt attactttac
      961 aagaagcgga taaattgttt aaaagcgatt tgttaccctt tgaaaatgct gtaaaaattc
     1021 aataaactct tccttagcac agaacgaatt tgatgccttg gttatattat gttttaatat
     1081 agggattgat aattttaaaa attcttctgt tgctaaaatc atcaatggag agaaaacggg
     1141 ttacaaaaca ttaaaggaag cttggatggc gtggaacaaa agtcaaaata aagtaatgca
     1201 gaggctaatt aataggagaa atgctgaata taagctttac actcaaggag gttatgaaaa
     1261 atggtaattt ttaaaaatat attattgttt ttattttta gtacttattt gttagcgcaa
     1321 gaggatttta ctccactaga gcaatgtacc tatgaaaatg aaaagttttg gataaaaatt
```

1381 ttaaatctat gtctagaggg taatataact tgtgataaaa ttgtatatgt aggtgtcaat
1441 aaaaataatg gaaaatatat tgtcctaaat ggtaagtcta ttagtgatgt aaatatgaat
1501 tttaaaggat atgtttttaa aaatggtatt tatgaataca atatttttaa taattttta
1561 tatatttcta aaaacaagca gattatacaa gaatatcgtc taaaattatg tgaaaaatag
1621 acacaacaaa ggcaacaagt aacaacaaat acctagaatg taaaaagtta aaaagatgca
1681 ataagaatta caagaagttt ttaaagttat aaaaatccta attaatgaaa tcttagctcc
1741 taaattttat aacaaataca aagatttaga agtagagatt gatgataggg gtaatattat
1801 aagatggata ggagagatta ataaagacaa aaaatattag caaataaaaa atgatataat
1861 aatgctttat ataaggaatc ttttatgttt ataagttcta aaacagagtt aattcaaact
1921 attaaaaatt caacaatcaa caataaagaa gttaaacaga ttgattttat ttatcctaat
1981 ttttctattt tacacgctat attaaaaaat attgattttc atcaaagaaa aatcctattt
2041 aaaaatcaaa tattggctat ggaaatacta gaaattttaa aacaaactcg tgataatcac
2101 aataatatag aagtaaatat ccataccctt aatgagcttg atgctggaca aaatgtaaat
2161 ttaaaagatt tatcaagcag tgaaatttgc actaaagttt atttagaaga cttagaagaa
2221 gaaattcaag ataagaaaca aggtaaagat tttaatcttt tttatcatac agataaaaat
2281 aatctttgta tcgaatttat taatacttta aaagaattag ccaaagattc tgaagatata
2341 acaaccttgg acgagttaat cgaaaacatc gatcctgatg atataagcag tgagaaattt
2401 gatgatttta agaaaaaagt tttagaaaag attggaaatg tcatagaatc ggattttttc
2461 aaaaatagca taagtcccac tttaaaaaat acttatgaaa aatttatcga tgagttgtta
2521 gaacacatta aaaaacttgc cgaagaaaaa gaatatgaaa aaattaaaat catactaagt
2581 gctatagcgg gtgttgcaat agccacagtg gtagctgttc ttacaggtgg ggcaggacta
2641 gctattttgg ctgctggttt attttcttta atgggttctg caagtgagta ttatgaattt
2701 gctcaaagag attatggcac acttttaacg cctttagccg catttttagc cactagatgt
2761 gctatgttta ttcattatat taattctcgc ttgttatctt gtgttttaat tattgataaa
2821 aaagaaatta ttgatttaag tggttttgga ttatatcttg gaagtgatga aagcaatatc
2881 gcttcaaatt tagcctataa aataacctta agcgaagaaa taaaaagcat cgaaaatatt
2941 tttagtgaaa tctttttaaa cccagaacaa aaagacaatg agagtaaaaa tacagctaat
3001 gattcaaaat cgagcaattt actcgctttc atttcaacgc aaaaattatc taatttgtat
3061 ttaaaaaaca atgctatttt aaagaatttg caagaagata ttaaacaatt tatttatttt
3121 tcttatatag aaagtccaaa ttttaactca agtaaactag ctgaattttt cgcacaaaaa
3181 aatcaaaaca cacaaataga tttaaataca actaaaatga gtaaaaatta tttgattatt
3241 tcaaatatcc ccaataaata caatgcagga cttagtgaaa gtttaactca agaaatcgcc
3301 aactataata tacaagataa atacagaaca attaaaaaaa atattgatac ttcaaaaaaa
3361 acttatgaca gcacaagaga ttatagcgaa tatagcatag aaataccaca agatgaaaat
3421 gaaccttata ttttacaatt aagccctttt gtgaaaatat ccaaagaagc ttatgagaat
3481 agtaaaaaag cttatgaaga tatgcaagag atttataaat atttaacaaa ttttgttgct
3541 acaccgcaga agctaccaga attcgcaaaa acagctaaag aaaaattagc aatatcagaa
3601 aatgagtcaa tgatagaaat agtaaaaaag acggaaaatt tacgacattg cgaacgaata
3661 aacattttag cttatattga ctacaataaa aaaataaatg ataaaaaatg gcttccttat
3721 ttttatatca aaaaattctt taaacaaaac gaagacaaag aatccatcat agaaaaagaa
3781 aaaaaaatag ctcatcaata tttaactttt ttgaaaaaag agctttttga aaaatcggat
3841 aaatcttcat ttggaaacta ttctttatcg gataaatctt catttggaaa ctattcttta
3901 tcggataaat cttcatttgg aaactattct ttaatggaat gtttatttat ttcatttgtc
3961 ttgtatcaag ctaagcttat ttttccaaat atcaatttgg atttgaaaaa atatggaggc
4021 agtaattttg tatatataaa atacttagag gaaacttatg ttttatggac agatgaatat
4081 ccaatatatg taaataatga gccaacaaat attacagcat atgaagttgt atcgcaatta
4141 ttaaaaaatac ttgacaaaaa agattattct tttgtggaaa atgatagttc gatcactata
4201 gatgattttt ttaatgaatt atcaaaagat ttagatgtaa caaatacatt agataaaaaa
4261 ataatcaaat tgaagcaaat gtatcattta gatgaacaaa aatgcattaa agaatttatg
4321 aatttagacg aagaagcgca acgagctttt tttgtgtgga tggatataga gaaacaaata
4381 tcagatttat caaagctttt ggaacagatg tatggaaaag aatatttaaa taaggcaaaa
4441 gaacaaaaaa caagtgaaat atttacttat gcaatgttaa aaattttatt aagcgctagt
4501 ccttttatca gctttacagc tcaaatgttg tgggatttta attttttaaa aacaatagaa
4561 tatgttattc aagcttgtat taaaataaac aaagaaggat ttaaagaatt ttttaaagaa

4621 cagcttggta tttttacacc tctaaaaaag aatagtatat atcatataca cataaaatct
4681 aatttaataa taaaagcgtg cttgcaacaa atagaaaaag aattaactca tactaaaatc
4741 caaatgattt tagcacaaga gataaaatcc aatttaataa tacaaaaaaa tacatccgat
4801 ttaaatctat ttatcacaaa gataaaagaa tactttaatg aaaaacaaat taagatttat
4861 tggagtgata aatatatgaa acaattattt atatactcca atcaagaaag caatatatct
4921 aaaattgctc ttatgcatag catagatata caaactcaaa aatatactat ttatacttat
4981 gctaagctaa ataaacttga ccttgcttct attgtggctg gagaatttgt gggtattttt
5041 ttaaattatc tctttccaac aaattataaa gccctgcaag aacaatatga aagcattgtt
5101 tatcagcttt acacctttaa agatgattct cctttggcgg ttataagaga tcattttata
5161 acttatcctt ttgcaatcaa ttcaaaattc attagctttg atttaaagaa aattttttat
5221 ggcatagatc tttgcacagg agtttttgac actcattttt cctttcatta tgaaatagat
5281 caaaagcttg aagaaaataa agaatttctt ttaaaaagac ttttgtctta tttgattatt
5341 gatgagaaaa gaagtgcaac tgctttagag gatataaaag aacaaaaata tattatagat
5401 gattatactt tttatttcaa agagttaaaa tctttagagc ttaaaaaatg cactatatat
5461 caactccctt gtaatcattc tttgtttaaa gagtttagaa ataaagaaaa gcaaattagt
5521 gcttttaaaa aggatgaaga tagtcccatc catgcttata atgaagcttt acaaatttta
5581 caagattttg ctaatggact ttatattgtc aaatatgaag aaggaaaatg tatagaagat
5641 aaaaataaaa ccagaagatt actagaaaaa ctcaatatca taggacaaaa caatcttaaa
5701 gctatgtata tggggataaa tggaaataaa gaaagcttga aagccatagg aaaaagagaa
5761 aataacacta gcaaagcaaa tataaatgct gatattgtat cagaaacaaa agctccgcct
5821 aacttcatag gtagactagc cactactata atcatagaag atggtttata tataggttaa
5881 attaagaaag gataaatagt gagcgaaact tatgagattt atacacccaa tggattaaca
5941 ttagatgtgg aaaaagatac caataaaata ctatttaaag aaaatattaa acccacaggc
6001 aattataccg aagaatattc caaagcggtt tttaaatctt attatattat gaaaaactcc
6061 ccctacaaag actataaacc aatatatcta gatcctaact tttatacagg agagaaatcc
6121 actttacttg aatttaaaga atggcaaaac atttatttaa aagatcctat aaaaggaagc
6181 atagctcctt ggactaaagc agaaaaagct tattataaat ctttaaaaac taaaagagaa
6241 agatgtaagt atttggcaat aagaagtgga ttaagatctg tggttataga tataccctat
6301 gatgcttatt gtaatgtaga tgaaaaagga tatttaatca atgaagaata cgcctatatt
6361 tatgatgaag taagttctca tcgtggaaca ttaaaatctt attctttctt taatgaatgg
6421 gaattagctg ctttacttct aggagatatg agagctagtc ctacagccgc tgtaggattt
6481 aaagcaagac aacaacaagc tttattttta caagctcaat ttggagataa aaatgctttt
6541 aagagtttag gtttagcagt attgtgttct aattcttttc ttacagggca acattggaat
6601 aagcttagag caaaaatgat ttacgattta cacgatcatc attatgagag tttgttagat
6661 gaatttggta tgctaccttt tttagatgag attataggag cagattggac gattgattta
6721 aataaatatg attttgccta tgatgaagaa ggtagaatca tttgggcttt atataatgat
6781 attgaaaaag gcaaattaaa agatccaaga gatgtggatt ctactcctga aagtagaaat
6841 gaatttgatg atgctatgga tagatacgaa aatggaatgg taactagatt tgatgtagat
6901 actccaaatg aatggagtga acaacaagct gctttagata gagatacttt agtattaagt
6961 gccaaactcg cagccctcac tcctccacaa ggctatccta atgcccctta ttattttaca
7021 cctgaaagat tagaatggat ttataaaaga ggatatttag ataaactcct tgatccaaga
7081 attcctgcta tttatagata taatttccca agggaactta gagctaagat actcaaattt
7141 gctgaagaaa atggcattaa agaataaagt aattgcatag gcagactagc cactactata
7201 atcatagaag atggtttata catgggataa atttaagaaa ggataaataa tgagcgaaac
7261 ttatgagatt tatacaccca atggattaac attagatgtg gaaaaagata ccaataaaat
7321 actatttaaa gaaaatatta aacccacagg caattatact caagaatatt ccaaagcggt
7381 ttttaaatct tattatatta tgaaaaactc cccctacaaa gactataaac caatatatct
7441 agatcctaac ttttatacag ggcaaaaatc cactctagta gaatttaaag aatggcaaag
7501 catatattta aaagatccta tcaaagtagc tcaaaatatc acaaatattt gtattaatta
7561 tgtaaaaaaa ggttttaaag aagttttaat cgaacaactt ggcattatat atttattaac
7621 acatccacaa atcgaagttg aagttaagaa atatggaaaa aaatatttta agactataaa
7681 caaggaaatt gctgcaaaaa gattttctac aattaaagaa gaattatttt tcaataaaac
7741 aaaaatatcc tttatggcag aacaagtaaa aaacggtatt gattatgaaa tagatcaaaa
7801 gcttgaagaa aataaagaat ttcttttaaa aagacttttg tcttatttga ttattgatga

7861 gaaaagaagt gcaactgctt tagaggatat aaaagaacaa aaatatatta tagatgatta
7921 tacttttat ttcaaagagt taaaatcttt agagcttaaa aaatgcacta tatatcaact
7981 cccttgtaat cattctttgt ttaaagagtt tagaaataaa gaaaagcaaa ttagtgcttt
8041 taaaaaggat gaagatagtc ccatccatgc ttataatgaa gctttacaaa ttttacaaga
8101 ttttgctaat ggactttata ttgtcaaata tgaagaagga aaatgtatag aagataaaaa
8161 taaaaccaga agattactag aaaaactcaa tatcatagga caaaacaatc ttaaagctat
8221 gtatatgggg ataaatggaa ataaagaaag cttgaaagcc ataggaaaaa gagaaaataa
8281 cactagcaaa gcaaatataa atgctgatat tgtatcagaa acaaaagctc cgcctaactt
8341 cataggtaga ctagccacta ctataatcat agaagatggt ttatatatag gttaaattaa
8401 gaaaggataa atagtgagcg aaacttatga gatttataca cccaatggat taacattaga
8461 tgtggaaaaa gataccaata aaatactatt taaagaaaat attaaaccca caggcaatta
8521 taccgaagaa tattccaaag cggtttttaa atcttattat attatgaaaa actcccccta
8581 caaagactat aaaccaatat atctagatcc taacttttat acaggagaga aatccacttt
8641 acttgaattt aaagaatggc aaaacattta tttaaaagat cctataaaag gaagcatagc
8701 tccttggact aaagcagaaa aagcttatta taaatcttta aaaactaaaa gagaaagatg
8761 taagtatttg gcaataagaa gtggattaag atctgtggtt atagatatac cctatgatgc
8821 ttattgtaat gtagatgaaa aaggatattt aatcaatgaa gaatacgcct atatttatga
8881 tgaagtaagt tctcatcgtg gaacattaaa atcttattct ttctttaatg aatgggaatt
8941 agctgcttta cttctaggag atatgagagc tagtcctaca gccgctgtag gatttaaagc
9001 aagacaacaa caagctttat ttttacaagc tcaatttgga gataaaaatg cttttaagag
9061 tttaggttta gcagtattgt gttctaattc ttttcttaca gggcaacatt ggaataagct
9121 tagagcaaaa atgatttacg atttacacga tcatcattat gagagtttgt tagatgaatt
9181 tggtatgcta cctttttag atgagattat aggagcagat tggacgattg atttaaataa
9241 atatgatttt gcctatgatg aagaaggtag aatcatttgg gctttatata atgatattga
9301 aaaaggcaaa ttaaaagatc caagagatgt ggattctact cctgaaagta gaaatgaatt
9361 tgatgatgct atggatagat acgaaaatgg aatggtaact agatttgatg tagatactcc
9421 aaatgaatgg agtgaacaac aagctgcttt agatagagat actttagtat taagtgccaa
9481 actcgcagcc ctcactcctc cacaaggcta tcctaatgcc ccttattatt ttacacctga
9541 aagattagaa tggatttata aaagaggata tttagataaa ctccttgatc caagaattcc
9601 tgctatttat agatataatt tcccaaggga acttagagct aagatactca aatttgctga
9661 agaaaatggc attaaagatt aaagaatcac tcaaaatctt taatatactt ttccaatgct
9721 tttagggtaa gattctcaaa aagaatctta tcttctttga tattgttttg tatggcagct
9781 tcaagatatt taataaacat attttcttcc atttcttcaa agatgggagt gtataattct
9841 tttgcgatta ggctaagggc taaaatattg atcaatcttg tggatttgat taaaggtata
9901 ttattaataa tactttcaaa atctttaatg ttgataggag aataatcatt atttaattta
9961 gagctttgtt gttttgtttt aatatctttt aaatttttta ccaaactttt tagaatattt
10021 tcatacccta atgaatttgg atctctttga atgatttctt ttaaaaagct atcatctgcc
10081 ttttcttcct cgataaactt ataattatta tttttaaaa tctctttcaa ctcttcattt
10141 ttgtcaaaat ttatacttgt ttttcctcta ggttcaattt caatttcatc aaaatgattg
10201 cgtttttcaa tatcttctaa gtatttttta ttttctttga tactttcttc aatgcttttt
10261 gcatccacaa atctaaattc taaattaccg attttaaaaa tatcgcctat gctaattatt
10321 atttcatagc cacttggcat tctagaaaag gacttgttat aataaatgtc agaatcttca
10381 aaagctgcaa ttgtaaaaga tccttcttca taagatacta tggcatgggt atttttgatt
10441 tgttctagtt tatcttgaat acgaaaagtg caatcatcac cacttcctat agttccaccc
10501 tttgtatcaa atacaaatgc tttagaacca ctaatgcatt cgtcataatt ctcaatagca
10561 attcctaatt tttcttttc tatcattgtt tatttactcc tgtgatattt tctactattt
10621 taagattatt taaaatataa actatattat ttaaatcgcc agcaccgtta ataatttcaa
10681 agtcaaaata taagttttta ttattattaa acataatgct gtatgtagaa ttgtttttat
10741 tatcttttat aaacttatac catgcccatt ctcccttata tgattttgta taatttaaat
10801 taggattgga gtagttgtag gctgtaaaat ttaaacttgt tccattattg aattcttctg
10861 ctacaatttg tagggtttga tcaagagtat gatcgtattg aatattttta ttgttgtagc
10921 caagctttac aaaggaaaaa tctgcactta aatcaaggct ttgaatagta aaattaactc
10981 ttatattatc attagcattc agtatgagac ttgagagatt gcctgcattg gtgataaaat
11041 ctaaaaattc tttagagaaa tttaattttg aagcaaattg ggaattaata gaatagttat

11101 tttttctttt tactaaaaca ttattcaagt attttttata aaaactattt aatactccat
11161 ttctgccaaa aaatgtttta aagctatcca tactcagatt agctgtgctt tcttcattaa
11221 aaggataaaa tggcgcaata tcattgataa atggattgta tacttcgtta atccaagcag
11281 tattaaataa tgaaatacca tagttctcaa tcaaattcca agaatgatca gaaagctgat
11341 tgtaatacct ttctaaatca ctaggtaatt tttttatact catttgaaat gcagtaaaag
11401 gatcattggc atctttattg ccaccaagag catatgcgat tttttcttcg gcgctttggt
11461 tattattgga gctaaaatct ataattttat tggacacatt tgtaatattt gtattaagaa
11521 tatctaatat tttttcatca tcagtgcctt tgcctacttc tatattgcca acacttaaaa
11581 gtgtattttt gtttacaagc ttatgatatt gcgtaaaagc attacttact ccaataaagt
11641 ttgatttaat ttctccagca tttaatccta aattgtaggc ttgagtcagt aaaaccgcat
11701 catttaaatt tgtattagaa ctaacaattt ttaataaaga atataaaggg ttttctcttt
11761 ttgataagat atcaagctca ttaagcattg cctctttggt attatactta acaggctgca
11821 atgaagcgag tagattttgc caagcatttt ggtattcatt taaatatagt tttaaaatac
11881 ccatagtcaa agtattttta ttttctttaa aattgctatt taacatccaa gattcaatat
11941 tgattgcggt gtcaacttgt tgatttaagt cttttaaaaa gctcatcata ccaacttttg
12001 tatatatttt atctatagaa gttatttgag aattatttga aaatacacta tttgcagcaa
12061 agcctaaatc ctctttgata agatattttt ctttaggttt atcactattt aaaaaattaa
12121 gaagagtgta aattctttga gttctactta tgctctcaag ttttcttttt cctatattta
12181 tactgttttc atcttctgta aaggatttaa gattaaattg tttaagttca tccacacctc
12241 ctagaaagtc atctttagag attaaatact tgcttaaagt attccaattc tcgttaatcc
12301 aaatttttag catttctttg ttaaagtatt tttgctcaaa caaagatcta tataaatata
12361 aggttttgat taaattttct ttatttgtgt cggtaagtaa agtatactcc atttctttta
12421 ataaagtatt ttttaacaca tcttcattca gtttataata aagttgttta gcttctttaa
12481 atcctttgta tgagagatta aggtttaaat attgaaatat attatcatct tgccaaagtt
12541 caggataagt atttaaaata tttctcaatt caattaaaaa gtcggctttt tgtttgatat
12601 ttaaattttg ataatcttga ttttcaagaa gtgattgtaa agctcttaaa gtgttttggc
12661 tttttttcaaa ttctttatcg tttttactta taaaatagga agatatagta tatgttccaa
12721 agcctatgat taaaatcata attaaaaaag ataatttttt ggaataagat ttcatagtac
12781 ttaaagcata atctgtaaaa attaaatctt caagtagtga tttaacaaaa taactttgtt
12841 tattatatat aatactagat tttgataata ctttttttaca gttgtatttt tcacatatgg
12901 catcgagcaa gaaatttcta ggaatatttt cttgataagc actaacaaaa taaattcccc
12961 taagataaga attattttta agcttgtttt cttcttgcac ttccaaaata aatgtttttg
13021 ccaatgcaaa taaattatca agttgtttta aaaataagta gcttttattt ttattttcta
13081 tattgtaaat atgggaattt ttactcatta aagagcataa taatgaatca cttaattctt
13141 taaattcatt atttaaaaat tcttcactta agattttatc aaaagataat cctaaaattt
13201 tatccgaaat ttttttatcg aaaatatcaa aatactcttt catgccttcc atcaagtcaa
13261 gttttgaaaa aactatatag ataggcaact ttaaattcaa agttttttca cattcgttta
13321 ctctttttgt tagatatcta ataagatttt tggaatactc cttaggatta tttaagaaaa
13381 ggacagtatc aacaactaaa ataattccat ttagtttgct atgaaaaaaa tttttattga
13441 ggaaagtaag gaattttttc catgtatttt ttttaattaa aaaatctcta tttttatcta
13501 tatcatcttc gggtatttcg tcgctacttg caggtttaaa aaattcttct tgtgagaaat
13561 agtttccttc tgtatctagc aaagcacctt tttttgatac ataaagcgca aaatttctag
13621 tagacttatg gaatttttta taagactcta aactatcgct aagcggatat tcgatgtcag
13681 aataattaat aaaagtgctt tttccagccc cttcgtttcc tatgatgata attaagggta
13741 aattttttgt ttttaaatca ttcttccatg tttctttggc atctttaaga gataaaaaga
13801 agtttctttt agatttgtaa atgaactcat cagcttcttt ttttaggtct ttaaatttta
13861 atcttttttc gctttttaaa gaagatataa aatttataat aggctttaat aaaaaaaaca
13921 aaaaaacaat aagccaaatt atcgagataa tgctaaatct taaaaatgaa ctactaaaaa
13981 tatatatatc attaaaagct actaaaggac cccaaaacca aaataaaaca ctaaaaagaa
14041 aaatacaaca aagtaaaaaa gcagttataa ataatcttga tttaataaaa tcaaatattt
14101 ttttaaacat cattattcct taattaaagt aaaaacaaat aaatattaat atcaatatac
14161 aaatatttta taaaaattta atagattaag attaaaatga tacaattttg aatgttaaaa
14221 aaaattaaag gaggttcttt ttatggctga accagcgttt ataagaattg aaggttccac
14281 acaaggtttg atttcaagcg gtgcatctac tgaagcgagt attggcaata gatataaatc

14341 aggtcatgaa gatgaaatca tggctcaaga ggtatctcat atcgtaactg tacccgttga
14401 tcagcaaagt ggacaacctt ctggacaaag agttcataag ccttttcttt ttacttgctc
14461 attaaataag tccgtacctt tgctatataa tgcacttacc aaaggagaaa gacttccaat
14521 agtcgaggtt cattggttta gaactgcaac aagtggcgga tctgaacatt tttttactac
14581 aaaattggaa gatgcaatta ttacaaatat tgaattaata atgccaaatg cacaagagtc
14641 aagtaatcat gataaaactg agcttttaaa agtttcaatg agctatagaa aagtagtttg
14701 ggaacatact gctgctggaa ctagcggaag tgatgattgg agagagggta aagcttaatt
14761 ataatttatc ttaaataatc ccgactaaaa tacataatta tagtcggggt tgtatcttaa
14821 aaatatattt tgtttttctt ggtttgaatt acaaaattac tttctaaatt ataatactat
14881 tattcatata taaaatcatt tgtcttaacg agcaattttt gtcttctatg cctagatact
14941 atgtttttta tctgtgtttt ctaagccctt taactctttt ttaagatcat tatcggcaaa
15001 tattgtaata tttctatcag gatagagggt atttaacttt tttactacac tttctaaatt
15061 accagcatct actgctataa taataggttt atttaaggct ttatataatg tagcaccagt
15121 agcaaaaccc tcgcaaatat aaaattcttt taaattatgc agtgttttag ctcctattat
15181 atgaaagcaa ccttttgagg cttatattta tacaataaaa gcgtcagaca ataaaggggt
15241 agaaaatagt atgcaataag actaaaccca tatataaaag gctcataatc accaccagca
15301 aaaaacatct tacttcccca ctataaaagc agtatcttgc atttgcatat gactaaaatc
15361 catatccaaa aatgtagagc tatataaaaa gaaatctaag cctagattaa aatactgttt
15421 tgtttttaga aagtctgaat tatataaggg aaataagaat gattttggtg aggtatattt
15481 aaaatgaaat tttgtatttc ggtattgcat tatcgaatta tataaatcaa atcctacctt
15541 ctcataatcc catgccccaa taggctgatc gctaccatta ttattaaaat tcacagtatc
15601 aatgataaaa gcacgcacct ttttgcacgc agcatataaa gaatggtaat tatagaatct
15661 tgtaactctc gtgatttgta taaggcttgg tatatataag atattaaaag cccctgcgat
15721 agagtataga gcaaaattct gcttgtattt tgggttagag tggaatttat ctaaaacttc
15781 tttttcatgt tgtatggagt tttgcaatga aaatgcatca aatttactta agcctaaatg
15841 cggtaaaaac acatcatttt ttatatcaaa cctttgaaaa tataaactat tatcgataat
15901 atctgatggt ttttgtaagt cttttatagc tttatgagtt tctgggagtg tttcctcttt
15961 tttatatagc ttaaagactt ctgcattctt cgtcttattc tctttcattg cttggatatt
16021 gagtgccatt gtatattctt gtgtattttt aatgtttttt atatctagta aagtatcatt
16081 aagaaaatct tcgaattgtt tttgaaaatc agattcttct ccaaaaagct ttttgagatt
16141 catatactcg ccctttagct cttgcattat ggggaacttc atgtaaaaat tatcccaatc
16201 taattccgca taaaatcttg ctttatctct tgattttgtg ttgtcagtct ttaattgttg
16261 catctctact ctaggcaata tctcctttac tattgttttc attgtttcat tagggatagt
16321 atctataaaa tcttgcgaag tagagcttct aaaggttaaa tcttttaatt ctttagcatc
16381 acttggatta tcatgccata atctttgaca gatatagctt acctcccccc aaccaagcct
16441 tgagatttca tctttgtttg tatgtgccct tagtgtttct gctagagtgc aatcaatggt
16501 aatttgtgga taatcattaa ttcttataag ataagaggca ttataattgg gtatttcttt
16561 ataagcaaag actaatagcg ctttttgctc ttgtgtgtct ttagggtttg gtagttgcaa
16621 actaagatgt tttccacaat aagctttgtt gttttctaaa tgtctataaa tgatagaaga
16681 agataatagg gctttatctt cttctttttc aatataaaaa caatctttta aaatttcttc
16741 atcaagcaaa gaactttcct ttttgtcatt agttaattgg tttttgttta acttatctaa
16801 ggtgttttta tcctgaataa aaacataacc ccatttaatt tgctcttctt tgatttttgt
16861 atcctcataa atagcttcaa attctactat atcaccaaaa gtatagaaat tacgcactat
16921 tttttcatta ttttctaagg taaattcttt ggattttagc ctaatagagt ttatagaacc
16981 tttatctgcc tttctttcaa attttatttc acaccaatgc ccatactcat aacttcttaa
17041 taaaaatcta tgattataga taggtttgtt tttataaaca ctagattggt ttaaaattaa
17101 ttcaaagctt tgtgcttctt tactaaaaat ttcactagca tttaaatgag cttctcctat
17161 tctattagtt gtaaaaatag tcttgatttc acttttattg ccctgtgcta tttcataaag
17221 cttaaattct tgattatgca gaagaatatt attagagctt aaaagcctaa tatttaaact
17281 atttttttga ttgagtgctt ccattactct gccttaagct ctccaccttt aaccacaagt
17341 ccattagagt ctataatcac ttctactcca cctgctttaa tgataacaca atctttctta
17401 gtaacaattt gagtatcacc tacttgatgg aggatttgat tgcctgcttt tacttcacaa
17461 tctgtttgaa agtcaaaagt agaattttct gattctaaag aatattgctc ttctatcttt
17521 gtttgca

```
//
LOCUS       ECFEEEMN_3              4060 bp    DNA     linear       12-APR-2021
DEFINITION  Campylobacter species strain strain.
ACCESSION
VERSION
KEYWORDS    .
SOURCE      Campylobacter species
  ORGANISM  Campylobacter species
            Unclassified.
COMMENT     Annotated using prokka 1.14.6 from
            https://github.com/tseemann/prokka.
FEATURES             Location/Qualifiers
     source          1..4060
                     /organism="Campylobacter species"
                     /mol_type="genomic DNA"
                     /strain="strain"
     gene            complement(619..771)
                     /locus_tag="ECFEEEMN_00037"
     CDS             complement(619..771)
                     /locus_tag="ECFEEEMN_00037"
                     /inference="ab initio prediction:Prodigal:002006"
                     /codon_start=1
                     /transl_table=11
                     /product="hypothetical protein"
                     /protein_id="Prokka:ECFEEEMN_00037"
                     /translation="MNENIYGSLEFYSKETQSTPSDKRDLGVFGDVAPYALGITEGFA
                     TKAFKG"
     gene            complement(764..1408)
                     /locus_tag="ECFEEEMN_00038"
     CDS             complement(764..1408)
                     /locus_tag="ECFEEEMN_00038"
                     /inference="ab initio prediction:Prodigal:002006"
                     /codon_start=1
                     /transl_table=11
                     /product="hypothetical protein"
                     /protein_id="Prokka:ECFEEEMN_00038"
                     /translation="MSEINNNTEIKEQDTQDEIIWEGKKDIPSFLLYWIANIVFLCFI
                     LYLFPRLFNPSKEIDYKWFIVFVIFLFGLYILVKNIYQMANIKRIYITKEKLVIEYYI
                     KNDLVFPLGTFFVYYRSSSLPSISNPGDIVIYTFGDKVKEYLEPGLFSDGDDPTKGCF
                     ERINAIIKPHVMPYLLSLSDEEFEKIISHVSGYNEIGTTFLKEAMELRKEKKDE"
     gene            complement(1401..2018)
                     /locus_tag="ECFEEEMN_00039"
     CDS             complement(1401..2018)
                     /locus_tag="ECFEEEMN_00039"
                     /inference="ab initio prediction:Prodigal:002006"
                     /codon_start=1
                     /transl_table=11
                     /product="hypothetical protein"
                     /protein_id="Prokka:ECFEEEMN_00039"
                     /translation="MNNSTLPWKSKRKFYFLAILQYIIYLALGLFVCWFCWFVLLKES
                     LWYGFFAFGSLYYVFELHRALNFKSIILTEQGLILKMRWFDTEIFYPYGSFTMSYDIA
                     YGIRFFERIEIQNIKHIRKEFIFPCGVDSFGSFENNHTFKELCTKYTQKALETMNLEE
                     KVNLYKLYRANIKIFFNQEIDHNVFIIDFSPYIQEIKLNLKDKNE"
     gene            complement(2022..2174)
```

```
                     /locus_tag="ECFEEEMN_00040"
     CDS             complement(2022..2174)
                     /locus_tag="ECFEEEMN_00040"
                     /inference="ab initio prediction:Prodigal:002006"
                     /codon_start=1
                     /transl_table=11
                     /product="hypothetical protein"
                     /protein_id="Prokka:ECFEEEMN_00040"
                     /translation="MNENIYGSLELYSKETQSTPSDKRDLGVFGDVAPYALGITEGFA
                     TKAFKG"
     gene            complement(2167..2805)
                     /locus_tag="ECFEEEMN_00041"
     CDS             complement(2167..2805)
                     /locus_tag="ECFEEEMN_00041"
                     /inference="ab initio prediction:Prodigal:002006"
                     /codon_start=1
                     /transl_table=11
                     /product="hypothetical protein"
                     /protein_id="Prokka:ECFEEEMN_00041"
                     /translation="MSEINNNTEIKEQDTQDEIIWEGKKHIPSFLLFWITYIVLLCFA
                     LYLFPRLFNPSKEIDYKWFIVFFTFLLGIYAFARAIYKMANIKRIYITKEKLVIEYYI
                     KNDLVFPLGTFFIYYRQISYSPSPGHIVIHTFGDKAKEYLEPGLFSDGDDPTKGCCEK
                     INAIIKPHVMPYLLSLSDEEFEKIFFHVNATSEIDTTFLKEAMELRKEKKDE"
     gene            complement(2856..3488)
                     /locus_tag="ECFEEEMN_00042"
     CDS             complement(2856..3488)
                     /locus_tag="ECFEEEMN_00042"
                     /inference="ab initio prediction:Prodigal:002006"
                     /codon_start=1
                     /transl_table=11
                     /product="hypothetical protein"
                     /protein_id="Prokka:ECFEEEMN_00042"
                     /translation="MSQTNNNTEIKEQDTQDEIIWEGKKHIPSFLLYWITYIVLLCFM
                     LYLLPRAFDNTKNFKWFVVFIMFLVTVYALAQEIYKAANIKRIYITKEKLVIEYYIKN
                     DLVFPLGTFFVYYRHVSFVLTPGDIVIYTFGDKVKEYLEPGLFSDGDDPTKDCFERIN
                     AIIKPHVMPYLLSLSDEEFEKIISHVSGFSEIGTTFLKEAMELRKEKKDE"
     gene            complement(3539..4039)
                     /locus_tag="ECFEEEMN_00043"
     CDS             complement(3539..4039)
                     /locus_tag="ECFEEEMN_00043"
                     /inference="ab initio prediction:Prodigal:002006"
                     /codon_start=1
                     /transl_table=11
                     /product="hypothetical protein"
                     /protein_id="Prokka:ECFEEEMN_00043"
                     /translation="MFPRLFNPSKEIDYKWFIVFFTFLLVIYALAQKIYRMANIKRIY
                     ITKEKLVIEYYIKNDLVFPLGTFFVYYRRLSFVLTPGHIVIYTFGDKVKEYLEPGLFS
                     DGDDPTKDCFERINAIIKPHVMPYLLSLSDEEFEKIIFHVSGFSEIDTTFLKEAMELR
                     KEKKDE"
ORIGIN
        1 ctcattttta tcctttaaat aagttttaat ctcttgtata taaggggaga aatcaataat
       61 aaaaacattg tgatctattt cttgattaaa aaatatttct atatttgctt gataaagctt
      121 ataaagattt actttttctt ctaaattcat agtttctaaa gctttttgtg tatatttggt
      181 gcataattct ttaaatgtgt gattgttttc aaaactacca aaagattcaa aaccattaag
```

241 taatttaaaa tttttaatat tagtatttga taaaaaactt atagtttcat caaatttaat
301 aaattgtatg gttatagatt ttatagtaaa acttccataa ggatagaaaa tttccgtatc
361 aaaccaccgc atttttaatg ttaatccttg ctctgttaaa attatagatt taaaatttaa
421 tgtttcatac aattcagaaa tataaaaaaa tgtaagaaat atagctaata aactatacca
481 caaagattct ttaagtaaaa caaaccaaca aaaccaacac agaaaaaaag ctaatataaa
541 ataaagagag caaaaaaaca aactaaaagg aataattttt ctcttacttt tccaaagtaa
601 agtagaatta ttcattgctt accccttaaa agctttagta gcaaaacctt ctgtaatgcc
661 taaggcataa ggagcaacat ctccaaatac tcctaaatcc cttttatcac taggagtgct
721 ttgagtttct ttagaataaa actctaaact tccataaata ttttcattca tctttttct
781 cttttctgag ttccattgct tctttaagga aagtggtacc aatttcgtta taaccgctta
841 catgtgaaat gatttttca aattcttcat cacttaagct taataaataa ggcattacat
901 gaggttttat aatggcatta attctttcaa aacaaccttt tgttggatca tcgccatcac
961 tgaataaacc aggttcaagg tattctttga ccttatctcc aaaggtgtat atcactatat
1021 ctcctggatt tgatatggat ggaagtgacg aactacgata atatacaaaa aaagttccta
1081 aaggaaaaac caaatcattt ttaatataat actcaatcac aagtttttct tttgtgatat
1141 aaattctttt aatatttgcc atttgataga tattttttac aagtatatat aacccaaaaa
1201 gaaatataac aaaaactata aaccatttat aatctatttc tttgctagga ttaaataatc
1261 taggaaacaa ataaagaata aaacataaaa atacaatatt tgctatccaa taaagcaaaa
1321 acgaaggtat atcctttttt ccttcccaaa tgatttcatc ttgggtgtct tgctctttga
1381 tttctgtgtt attgtttatt tcactcattt ttatccttta aattaagttt aatctcttgt
1441 atataagggg agaaatcaat aataaaaaca ttgtgatcta tttcttgatt aaaaaatatt
1501 tttatatttg ccctataaag cttataaaga tttacttttt cttctaaatt catagtctct
1561 aaagcttttt gtgtatattt ggtgcataat tctttaaatg tatgattgtt ttcaaaacta
1621 ccaaaagaat ccacaccgca aggaaaaata aattcttttc ttatgtgttt tatattttgt
1681 atttctattc tttcaaaaaa tcttatccca taggcaatgt cataactcat tgtaaaactt
1741 ccataaggat agaaaatttc tgtatcaaac caccgcattt ttaatattaa tccctgctct
1801 gttaaaatta tggatttaaa attcaatgcc ctatgtaatt caaaaacata ataaagactc
1861 ccaaaggcaa aaaaaccata ccacaaagat tctttaagca aaacaaacca acaaaaccaa
1921 cacacaaaaa gacctaatgc aagataaata atatattgca aaatagcaag gaagtaaaat
1981 tttctcttgc ttttccaagg taaagtagaa ttattcattg cttacccctt aaaagcttta
2041 gtagcaaaac cttctgtaat gcctaaggca taaggagcaa catctccaaa tactcctaaa
2101 tcccttttat cactaggagt gctttgagtt tctttagaat aaagctctaa acttccataa
2161 atattttcat tcatcttttt tctcttttct gagttccatt gcttctttaa ggaaagtagt
2221 atcaatttca ctagtagcgt ttacatggaa aaagattttt tcaaattctt catcacttaa
2281 gcttaataaa taaggcatta catgaggttt tataatggca ttaatttttt cacaacaacc
2341 ttttgttgga tcatcgccat cactgaataa accaggttca aggtattctt tggccttatc
2401 tccaaaggta tgtatcacta tatgtcctgg agaaggtgaa tatgaaattt gacgataata
2461 tataaaaaaa gttcctaaag gaaaaaccaa atcatttttg atataatact caatcacaag
2521 tttttctttt gtgatataaa ttcttttaat atttgccatt ttatagatcg ctcgtgcaaa
2581 tgcatatatg cctaaaagaa atgtaaaaaa aactataaac catttataat ctatttcttt
2641 gctaggatta aataatctag gaaacaaata aagcgcaaaa cataaaagca caatatatgt
2701 tatccaaaaa agcaaaaacg aaggtatatg ctttttttcct tcccaaatga tttcatcttg
2761 ggtgtcttgc tctttgattt ctgtgttatt gtttatttca ctcattttta tcctttaaat
2821 aagttttaat ctcttgtata taaaataaaa tattttcatt catcttttt ctcttttctg
2881 agttccattg cttctttaag gaaagtggta ccaatttcgc taaaaccgct tacatgcgaa
2941 atgatttttt caaattcttc atcacttaag cttaataaat aaggcattac atgaggtttt
3001 ataatggcat taattctttc aaaacaatct tttgttggat catcgccatc actgaataaa
3061 ccaggttcaa ggtattcttt gactttatct ccgaaggtgt atatcactat atctcctgga
3121 gtaagcacaa atgacacgtg acgataatac acaaaaaaag ttcctaaagg aaaaaccaaa
3181 tcatttttga tataatactc aatcacaagt tttctttttg tgatataaat tcttttaata
3241 tttgccgctt tatagatctc ttgtgcaagt gcatataccg ttacaagaaa cataataaaa
3301 accacaaacc atttaaaatt cttagtattg tcaaatgccc taggaagcaa ataaagcata
3361 aaacataaaa gcacaatata tgttatccaa taaagcaaaa acgaaggtat atgctttttt
3421 ccttcccaaa tgatttcatc ttgggtgtct tgctctttga tttctgtgtt attgtttgtt

```
     3481 tggctcattt ttatccttta aacaagtttt aatctcttgt atataaaata aaatattttc
     3541 attcatcttt tttctctttt ctgagttcca ttgcttcttt aaggaaagta gtatcaattt
     3601 cgctaaaacc gcttacatgg aaaatgattt tttcaaattc ttcatcactt aagcttaata
     3661 aataaggcat tacatgaggt tttataatgg cattaattct ttcaaaacaa tcttttgttg
     3721 gatcatcgcc atcactgaat aaaccaggtt caaggtattc tttgacttta tctccgaagg
     3781 tgtatatcac tatatgtcct ggagtaagca caaatgacag gcgacgataa tacacaaaaa
     3841 aagttcctaa aggaaaaacc aaatcatttt tgatataata ctcaatcaca agtttttctt
     3901 ttgtgatata aattctttta atatttgcca ttctatagat cttttgtgca agtgcatata
     3961 taactaaaag aaatgtaaaa aaaactataa accatttata atctatttct ttgctaggat
     4021 taaataatct aggaaacaaa taaagcgcaa aacataaaag
//
LOCUS       ECFEEEMN_4              4049 bp    DNA     linear       12-APR-2021
DEFINITION  Campylobacter species strain strain.
ACCESSION
VERSION
KEYWORDS    .
SOURCE      Campylobacter species
  ORGANISM  Campylobacter species
            Unclassified.
COMMENT     Annotated using prokka 1.14.6 from
            https://github.com/tseemann/prokka.
FEATURES             Location/Qualifiers
     source          1..4049
                     /organism="Campylobacter species"
                     /mol_type="genomic DNA"
                     /strain="strain"
     gene            complement(403..3918)
                     /locus_tag="ECFEEEMN_00044"
     CDS             complement(403..3918)
                     /locus_tag="ECFEEEMN_00044"
                     /inference="ab initio prediction:Prodigal:002006"
                     /codon_start=1
                     /transl_table=11
                     /product="hypothetical protein"
                     /protein_id="Prokka:ECFEEEMN_00044"
                     /translation="MKIKNFTLITSNHNLKIELKNLTKNANKIIFICSDFDGYKEAEN
                     SLYKTLHTIEDNFKIDNTKTFKIIHLNQTNKDEITKEQKFSSDKNLSSIKKIIYNNLK
                     LLNDTINQNENKTLNKIIDESNEKNIENFLKEIQKELNILLKNIKESNYFDQEIKPKI
                     QVTLDTLFNTLLKNIAQSNDDEYKQIIRAVAKFFINILDSITSLKKPISLIKKNPYVL
                     IVNTLFEAYNSYSEYQEYKEKKYYYDFAYPLLELITSKLYPIIALCNEEILSDVLIID
                     DKVLLDFSSYTNAPSISLYKNSFYKIILDEEFQGVFDNFLENNLKFENEKLDNTLLVN
                     SSLNNSDTTLKKAINLNISKLNHFFYAEYPDLNSSMLVEMILDKKNADQINTTKMGKN
                     YLFITNPPQLNNAGLCEDLYQKKLGAIIKNRPKALTKNEKLALKGDFPSKRDYGSYFI
                     ERDVKKEDDFVLQLCPFAKLDGQIYQKYKGYFNFYNQTYSWMKLYLLEYINERFEYER
                     FKQDFAYIDNFFKLPKELEKISEKEKEKVKEYVELFNDYLDKINFRYDQIQFFLGSEP
                     LMFKTYENVDFKKYSPLTVVLLTLTLYVLQKDLAFTQEYSLGVKNIAIPNPDYGDKEI
                     SLNYQRIKIPNYFNEYILFIPDDIYFYDNDKKIYIFLSKELNKNKKEDEVIYLDELAK
                     AMFENDLKEDDQTYVKFLNTLNENFDDEIEFEENIQTNQTLPTKKDLDKSIQEIMLDN
                     ELAKLSLDITFFNIIKQLFPFTEFFMSQPDFFKKMILFFINSSSDKKYYGNKLFSSFK
                     AHFFKELGIFYALNPKGRFIKIIPKNKKPFNKLFLYQIGTNYYYFSQSEYASELKKSK
                     SFSRLDKAKQIHLISKARKQLAIDFSIKVSKNLSADVLKNLAGSFIDTLLPTNYERLK
                     ILYEKLMYEKLTYNYDFPLAVKKEEYITYPLLINSRFMSFDLSEFIFGSLLCTGGLNY
                     YPSIACTTTSSEAREFALKRLLSYIILDEKRSAFMEDKINEGKYILNDKEFFEKNNIE
                     LRNCNIYKLEHFQRKEEYKTQNPMIKQHPILAYNDTIQILYEYINGIYNTIQDEKGNF
```

```
                     IEDRQKARRLMENLETIGQHNLEVMYKGINEENDETEKIISQNKRHYKNIENLITNNK
                     SQDYSNKFIGRLATTIIMEDGLYMG"
     gene            complement(3915..4031)
                     /locus_tag="ECFEEEMN_00045"
     CDS             complement(3915..4031)
                     /locus_tag="ECFEEEMN_00045"
                     /inference="ab initio prediction:Prodigal:002006"
                     /inference="similar to AA sequence:RefSeq:CKG86393.1"
                     /codon_start=1
                     /transl_table=11
                     /product="methyltransferase small"
                     /protein_id="Prokka:ECFEEEMN_00045"
                     /translation="MQNLKLQIAQGLDKILEDEDRKQELYVCKFVVVNGVKV"
ORIGIN
        1 ttcttatacc acttcttata actaaatatt tatatctttc ttttttagtt tttagtgagt
       61 tataataagc tttttctgct ttagtccaag gagctatgct tcctttaatg ggatctttta
      121 aatatatact ttgccaatct ttaaattcaa gcaaagtgga tttttgccct gtgtgaaatt
      181 caggatctag atacctaggt tgataattaa tataaggaga ggttcttaaa attctatcgg
      241 cttcaaacaa agctttagaa tattcttggg tgtatttgcc tgtaggtttt tttgactttg
      301 actctgaaaa tagtattttt ccgctttgtt tatctacatt taatataaaa ccttcgggtg
      361 tataaatttc ataagtttca ctcattttta tcctttctta atttatccca tatataaacc
      421 atcttccatg attatagtag tggctagtct gcctataaac ttattagaat aatcttggga
      481 tttattattg gtaattaaat tctcaatatt tttataatgt cttttgtttt gcgatattat
      541 tttttctgtt tcatcattct cttcatttat ccccttatac atcacttcta aattatgttg
      601 tcctatggtt tctaaatttt ccatcaatct tcttgccttt tgcctatctt ctatgaaatt
      661 accctttca tcctgtatag tattgtaaat tccatttatg tattcatata aaatttgtat
      721 agtatcatta taagctaaga tagggtgttg ttttatcata ggattttgtg ttttatactc
      781 ttcttttctt tgaaaatgtt ctagcttata tatattacaa tttctaagtt ctatattgtt
      841 tttttcaaaa aattccttat catttaagat atatttgccc tcattgattt tatcttccat
      901 aaaagcactt cttttctcat ctaaaataat ataagataaa agtcttttta aggcaaattc
      961 tctagcttca cttgaagttg tagtgcaagc aatactagga taataattaa gcccacctgt
     1021 gcacaataaa gaaccaaaaa taaactcaga taaatcaaaa ctcataaatc tagaatttat
     1081 caataaagga taggttatgt actcttcttt ttttacagct aaaggaaaat cataattgta
     1141 agtcaatttt tcatacatta gtttttcata taaaattttt aatctttcat aatttgtagg
     1201 caaaagtgta tcaataaaag aacctgctaa attttttaat acatccgcac ttaaattttt
     1261 acttacttta atagaaaaat caatagctaa ttgttttctt gcttttgata tcaaatgaat
     1321 ttgcttagct ttatccaatc ttgaaaaact ctttgatttt ttcaactcgc ttgcatattc
     1381 tgattgacta aaataataat aatttgttcc tatttgatat aaaaaaagct tattgaatgg
     1441 ctttttattt tttgggatga tttttataaa acgcccttta ggattaagtg cataaaatat
     1501 ccctagttct ttaaaaaaat gtgctttaaa actagaaaat aatttattgc cataatactt
     1561 tttatcagag cttgaattta taaaaaataa aatcattttt ttaaaaaaat caggctgact
     1621 cataaaaaat tctgtaaaag gaaaaagttg cttaatgata ttaaaaaaag tgatatctaa
     1681 ggatagtttt gcaagctcat tatcaagcat aatttcttga atacttttat ctaaatcctt
     1741 ttttgtagga agtgtttgat ttgtttgtat attttcttca aattcaattt catcatcaaa
     1801 gttttcattt aaggtgttta aaaatttcac ataagtttga tcatcttctt taagatcatt
     1861 ttcaaacata gctttagcta attcatctaa atagatgact tcatcttctt ttttattttt
     1921 atttaactct ttggataaaa agatgtaaat ttttttatca ttatcataaa aataaatatc
     1981 atcggggata aataagatat actcattaaa ataatttggt attttaatcc tttggtaatt
     2041 aagtgaaatt tctttatcgc cataatctgg atttggaatt gcaatatttt taacaccaag
     2101 agaatattct tgcgtaaaag ctaagtcttt ttgtaaaaca tacaaagtta aagtcaataa
     2161 aacaaccgtt aaaggtgagt atttcttaaa atctacattt tcatatgttt tgaacattaa
     2221 tggttctgag ccaagaaaga attgtatttg atcataacga aaattgatct tatccaaata
     2281 atcattaaaa agctcgacat attcttttac tttttctttt tctttttcgc taattttctc
     2341 aagctcttta ggtaatttaa aaaagttatc aatataagca aaatcttgtt taaatctttc
```

```
     2401 atattcaaat cgttcattta tatattctaa taaataaagt ttcatccaag aatatgtttg
     2461 attataaaaa ttaaaatatc ccttatattt ttgataaatc tgtccatcaa gtttcgcaaa
     2521 aggacaaagt tgcaagacaa aatcatcttc ttttttaaca tctctttcta taaaataaga
     2581 accataatct cttttgctag gaaaatcgcc ttttaatgca agtttttcat tttttgtaag
     2641 agctttgggg cgattttttta ttattgctcc taattttttt tgatacaaat cttcgcaaag
     2701 ccctgcatta ttaagctgtg gtggattggt aataaataaa taatttttac ccattttggt
     2761 tgtattgatt tgatcagcat ttttttatc cagtatcatt tctactagca tggaagaatt
     2821 taaatcaggg tattctgcat aaaagaaatg attgagtttg gagatattta aatttatcgc
     2881 ctttttaat gttgtgtcag aattatttaa actagaattc actaaaagag tgttgtctaa
     2941 tttttcattt tcaaatttta gattattttc taaaaaatta tcaaaaacac cttgaaattc
     3001 ttcatctaaa ataattttat aaaaactatt tttataaaga ctgatagaag gtgcattagt
     3061 ataagatgaa aaatctaata atactttatc atctataatt aacacatcag ataaaatctc
     3121 ttcattgcat aaagcaataa taggataaag tttgctagtg ataagttcta aaagtggata
     3181 agcaaaatca taataatatt ttttttcctt atactcttga tattcagagt aagaattata
     3241 agcttcaaat aatgtattta caatcaaaac ataaggattt tttttaatta aagaaatggg
     3301 ctttttaagg ctggttatag aatctaatat attgataaaa aatttcgcta cagctctaat
     3361 aatttgctta tattcatcat cattagattg ggcaatattt tttaaaagtg tgttaaaaag
     3421 cgtatctaaa gtaacttgta tctttggctt gatttcttga tcaaaataat tagattcttt
     3481 gatattttt agtaatatat ttaattcttt ttgaatttct tttaaaaaat tttcgatatt
     3541 tttttcattg ctttcatcaa taattttatt gagtgtttta ttttcatttt gattgattgt
     3601 atcatttaaa agttttaaat tattatagat aattttttta atgctagata aatttttatc
     3661 agagctaaat ttttgttctt ttgtgatttc gtctttattg gtttgattta aatgtatgat
     3721 tttaaaagtt tttgtattgt caattttaaa attatcttca atagtatgta atgttttata
     3781 cagagaattc tcagcttctt tatatccatc aaaatcactg catataaaaa taattttatt
     3841 agcatttttg gtaagatttt ttaactcaat ttttaaatta tgatttgatg taattaaagt
     3901 aaaatttttt attttcatac tttaacccca tttaccacca caaatttaca aacataaagc
     3961 tcttgttttc tatcttcatc ttctaagatt ttatctaatc cttgtgcgat ttgaagtttt
     4021 aaattttgca tttttgcagg agttaaaaa
//
LOCUS       ECFEEEMN_5              3161 bp    DNA     linear       12-APR-2021
DEFINITION  Campylobacter species strain strain.
ACCESSION
VERSION
KEYWORDS    .
SOURCE      Campylobacter species
  ORGANISM  Campylobacter species
            Unclassified.
COMMENT     Annotated using prokka 1.14.6 from
            https://github.com/tseemann/prokka.
FEATURES             Location/Qualifiers
     source          1..3161
                     /organism="Campylobacter species"
                     /mol_type="genomic DNA"
                     /strain="strain"
     gene            368..550
                     /locus_tag="ECFEEEMN_00046"
     CDS             368..550
                     /locus_tag="ECFEEEMN_00046"
                     /inference="ab initio prediction:Prodigal:002006"
                     /inference="similar to AA sequence:RefSeq:CKG87535.1"
                     /codon_start=1
                     /transl_table=11
                     /product="methyltransferase small"
                     /protein_id="Prokka:ECFEEEMN_00046"
```

```
                     /translation="MPHRGRHPNEYHEYILEKMNKIDKIARGDKNKFLKEFEKLKEEI
                     KNNPAILRKEYYKERK"
     gene            550..1116
                     /locus_tag="ECFEEEMN_00047"
     CDS             550..1116
                     /locus_tag="ECFEEEMN_00047"
                     /inference="ab initio prediction:Prodigal:002006"
                     /inference="similar to AA sequence:RefSeq:CKG87555.1"
                     /codon_start=1
                     /transl_table=11
                     /product="hypothetical protein"
                     /protein_id="Prokka:ECFEEEMN_00047"
                     /translation="MRYYKMMYNGQHNDVDNWINCVKPDIKNNDKYALLESKPITNWQ
                     TPTFEIDKDDGKILTDLISNVYNWRIVSPKFINLMQDLIKDCVQYLDVEIRSQEINYY
                     DCKIMHVTKSLEALDYEHSVYTYMGDNDEYLSITKAVLKKSKLDGSHIFRIKDDEIPV
                     FVSNEFRKRVRENNLLGFSFSEVMVYEN"
     gene            1199..1750
                     /locus_tag="ECFEEEMN_00048"
     CDS             1199..1750
                     /locus_tag="ECFEEEMN_00048"
                     /inference="ab initio prediction:Prodigal:002006"
                     /inference="similar to AA sequence:RefSeq:CKG87579.1"
                     /codon_start=1
                     /transl_table=11
                     /product="hypothetical protein"
                     /protein_id="Prokka:ECFEEEMN_00048"
                     /translation="MTREQAELIIKEEKLIDTDWYPSYKHSGEYHLTMWFDSDNNKYE
                     AVYIGERGSVEFEYSFDSEKEAIDKMLQMNYEQKKYDGKDIKGYLTKEVALRIIKEEN
                     LEVIWYDEALKPLCAGIKHDKQRDKYISFITNAKAEIIEYRSIEFDGDEEFEGKIYND
                     EDIALYALINRARMIKQNKISFV"
     gene            1821..2171
                     /locus_tag="ECFEEEMN_00049"
     CDS             1821..2171
                     /locus_tag="ECFEEEMN_00049"
                     /inference="ab initio prediction:Prodigal:002006"
                     /inference="similar to AA sequence:RefSeq:CKG87641.1"
                     /codon_start=1
                     /transl_table=11
                     /product="hypothetical protein"
                     /protein_id="Prokka:ECFEEEMN_00049"
                     /translation="MEDLKLLQRRWEEAYEAMPKLYETPDELIINFTLSEDTDTILFK
                     EPWENFELDDEDKEVKWRLSFFSISKDEPLGYLEYKEALEKLQDFSLIQSEERILIKA
                     MSLEELESLELKGW"
     gene            2171..2803
                     /locus_tag="ECFEEEMN_00050"
     CDS             2171..2803
                     /locus_tag="ECFEEEMN_00050"
                     /inference="ab initio prediction:Prodigal:002006"
                     /inference="similar to AA sequence:RefSeq:CKG87668.1"
                     /codon_start=1
                     /transl_table=11
                     /product="hypothetical protein"
                     /protein_id="Prokka:ECFEEEMN_00050"
                     /translation="MLPQPIFLNKLIELLSDENLDIVNMKKGVDKIFSKKDFYAILEL
```

NIKIMNIAKLIFKSDEKLNILLPQISDHKNINYFLQELELKKEMDVNLNDLKNYDNKK
IIEIIKQKYQNNPVKELDINSQKEKIKILFIVNEVPKFLKSIYKECKFKEYGLANYLE
FDEIWDFYENNTRYFAILTPMGANKIKLRLENTQNSEKELNIIEVNLDKK"

ORIGIN

```
        1 cttgaaggtg cagcaattgt aaaactaatg aatttagata aagaagaatt taaacaatac
       61 aaatactttc cttatgattt aatttaatct tagtgggttt aaaaacccac aaggcttttc
      121 atggctagaa tttgctatac ctaaaaagaa taagattcaa tttgctttag ctaaaataca
      181 agctagtcct cttgctaaga ttgagtatga taggataaga tgggaaggga taggaggtaa
      241 aagaaatcaa ttaccaatcc aaacccatca cattgctact gataagagta aaaaatatac
      301 tccaaaattt caagagatac ttaatttata tgatttaaaa ctaaatggag attggaataa
      361 agttaaaatg ccacatcgcg gaagacatcc caatgaatat catgaataca ttcttgaaaa
      421 aatgaataaa attgataaaa tagctagagg ggataaaaat aaattcttaa aagaatttga
      481 aaaactaaaa gaagaaatca aaaataatcc tgctatactt cgtaaagaat actataagga
      541 gagaaaataa tgagatatta taaaatgatg tataatggac aacataatga tgtcgataat
      601 tggataaatt gtgttaaacc agatataaaa aataacgata aatatgcact tttagaatct
      661 aaacctatta caaattggca aacacctaca tttgaaatcg ataaagatga tggtaaaata
      721 ctgactgatt taatatctaa tgtatataat tggcgtatag tctctccaaa atttataaat
      781 ttaatgcaag atttaatcaa agattgtgtg caatatttag atgtagaaat tagaagtcag
      841 gaaataaatt attatgattg taaaattatg catgtaacaa aatcacttga ggctttagat
      901 tatgaacatt ctgtatatac ttatatgggt gataatgatg aatatttaag cattactaag
      961 gctgttttaa aaaaatcaaa acttgatgga agtcatatat ttagaattaa agatgatgaa
     1021 atacctgttt ttgtatcaaa tgaatttaga aaaagagtga gagaaaacaa tttattaggt
     1081 tttagtttta gtgaagttat ggtatatgaa aattaataaa catataaaaa tattataatc
     1141 ataaattata taaacttatt aggttttatc aaacaaatac cataataaag gatagaaaat
     1201 gacaagagaa caagcagaat taattataaa agaagaaaag ttgattgata cagattggta
     1261 tccttcttat aaacattcag gagaatacca tcttactatg tggtttgatt ctgataataa
     1321 taaatacgaa gctgtttata ttggagaaag aggcagtgtt gaatttgagt attcttttga
     1381 tagtgaaaaa gaagctattg ataaaatgtt gcagatgaat tatgaacaaa aaaaatacga
     1441 tggaaaagac attaaaggat atctaacaaa agaagtagca ctaagaatca tcaaagaaga
     1501 aaatctagaa gtgatttggt atgatgaagc acttaaacct ttatgtgcag gtattaaaca
     1561 cgataaacaa agagataaat acatttcttt tatcactaat gctaaagcag agattataga
     1621 atacagaagt attgagtttg atggggatga agaatttgaa ggaaaaatat ataatgatga
     1681 agatatagcc ctttatgctc ttatcaatcg tgccagaatg attaagcaaa ataagattag
     1741 ctttgtgtaa atactttcct tatgatttaa tttaaaaaga tttttagcta taatttcatt
     1801 ttatatttaa ggaaaaattt atggaagatt taaaactttt acaaaggcgt tgggaagaag
     1861 cttatgaagc tatgcctaaa ctttatgaaa caccggatga gttaataata aatttcaccc
     1921 taagtgaaga tacagatact attcttttta aagaaccttg ggaaaatttt gaattagatg
     1981 atgaagataa agaagtcaaa tggagactaa gtttttttag cattagtaaa gatgagcctt
     2041 tggggtattt agagtataaa gaagctttag agaaattgca agatttttct ttaattcaat
     2101 cagaagaaag aattttaatt aaagcgatga gtttagaaga gcttgaaagt ttagagctta
     2161 aagggtggta atgcttcctc aacctatatt tttaaataaa cttatagaat tgttatctga
     2221 tgagaattta gatattgtaa atatgaaaaa aggcgtagat aaaatttttt ctaaaaaaga
     2281 tttttacgct atactagaat taaatatcaa aattatgaat atagcaaaat taattttttaa
     2341 aagtgatgaa aaattaaata tattattacc acaaatttct gatcataaga atataaatta
     2401 ttttcttcaa gaattagagt tgaaaaaaga aatggatgtt aatcttaatg atttaaaaaa
     2461 ttacgataat aaaaaaataa tagaaataat taaacaaaaa tatcaaaaca atccggttaa
     2521 agaattggat attaattctc aaaaagaaaa aatcaaaata ctttttattg ttaatgaagt
     2581 accaaaattt ttaaaatcaa tctataaaga atgcaaattt aaagaatatg gattagcaaa
     2641 ctatttggaa tttgatgaaa tttgggattt ttatgaaaat aatactagat attttgctat
     2701 tttaactcct atgggtgcaa ataaaataaa attgcgttta gaaaatacac aaaacagcga
     2761 aaaagaactc aatatcatag aagtaaattt agataaaaaa taaattttaa taacaaaaat
     2821 aagcaatttc ttttttctac tcaaatttag actatctgtg ttaaaatatt tcttaataga
     2881 tataaaaaca attataataa tagaaaactt aatgagttta cactaaaaac tacaacaaac
     2941 ccaaaaggat catcaatgtc ccttaattct tatatgagtc ttgatatatt aaatcatact
```

```
     3001 tcaaatcccc taccctttaa aatcactaaa gccttcatca aagaaagtct tgaagagctt
     3061 ttttctatag aatgtgaagg ctttttttgaa agtttagaac aagatttatt ttctttaaat
     3121 actttaacca atgcttcatc tcatctaagc acccctacaa c
//
LOCUS       ECFEEEMN_6              2527 bp    DNA     linear       12-APR-2021
DEFINITION  Campylobacter species strain strain.
ACCESSION
VERSION
KEYWORDS    .
SOURCE      Campylobacter species
  ORGANISM  Campylobacter species
            Unclassified.
COMMENT     Annotated using prokka 1.14.6 from
            https://github.com/tseemann/prokka.
FEATURES             Location/Qualifiers
     source          1..2527
                     /organism="Campylobacter species"
                     /mol_type="genomic DNA"
                     /strain="strain"
     gene            complement(192..1442)
                     /locus_tag="ECFEEEMN_00051"
     CDS             complement(192..1442)
                     /locus_tag="ECFEEEMN_00051"
                     /inference="ab initio prediction:Prodigal:002006"
                     /inference="similar to AA sequence:RefSeq:CKG87785.1"
                     /codon_start=1
                     /transl_table=11
                     /product="hypothetical protein"
                     /protein_id="Prokka:ECFEEEMN_00051"
                     /translation="MKQILSANRVSFHLLSTKTFSDEKYLNAWSLKGERLNAKDWFEF
                     KQANKDKFYNKHLENTLKAMQEFKAYFSVDKFEGINFGGNDEWLIFLPKNYTQIYNRL
                     CRDKSKYILHRYYQKNDLAEIGALKNLAPNFPFWDEKGENFSQSQIDTFIEKDNLAPY
                     FNKYDIYFNSSLELSFNFSDESLEYLINNYNHRIDHTFTPKSQDEIQKELNLSKEEKI
                     ILELENMFLNLCKGISADYAFMDFSYFFVGYDHTLDSKNNTLKSYPINQDKLIHKRSF
                     KEYIEEYANTYFLNYYSKEFLNELDDQELNKNRLNYYLINYNKIPKNTEEENTQGILS
                     KQKDININFVNFIDYKDYVYNNKDDFWEFSAEENSILWEKFLSKNTLKPFPLNFDCSL
                     EEKLLYAKDKQLENILKTHKAFHG"
     gene            complement(1439..1900)
                     /locus_tag="ECFEEEMN_00052"
     CDS             complement(1439..1900)
                     /locus_tag="ECFEEEMN_00052"
                     /inference="ab initio prediction:Prodigal:002006"
                     /inference="similar to AA sequence:RefSeq:CKG87488.1"
                     /codon_start=1
                     /transl_table=11
                     /product="hypothetical protein"
                     /protein_id="Prokka:ECFEEEMN_00052"
                     /translation="MKNKNLLDKDTQEVFKYYLKNDSYLKGALDGLKKYERRAFISSI
                     LSYEYKNLVENGNLKVDEKYKAILPPNLIAILEYDKSEFNGANLRNFINDLEFELEHI
                     EMELYDDPADFEGGDYYNEDSECDEDYTVEDWVEDQVSDIRDFLYALEDFK"
     gene            complement(1946..2374)
                     /locus_tag="ECFEEEMN_00053"
     CDS             complement(1946..2374)
                     /locus_tag="ECFEEEMN_00053"
```

```
                     /inference="ab initio prediction:Prodigal:002006"
                     /inference="similar to AA sequence:RefSeq:AOH52017.1"
                     /codon_start=1
                     /transl_table=11
                     /product="ankyrin domain protein"
                     /protein_id="Prokka:ECFEEEMN_00053"
                     /translation="MHFWVMQNNYELLELAIKGGANVDMQTRLIQESEYNETLLFEAV
                     KEAETYRVTQLLIELGANVNFATPSTPLDIAKGSRNKKLLKDAGAMTSEQIRKKFNLP
                     AYDSSHCEIDGKTDFDLLGKYHDEYSKLLNDAIKKAKESE"
ORIGIN
        1 ctccttgtat ttcatcatta agcataatag gaacattgtc tgattttatt cttttagctt
       61 tttttgcttt taacttaact ctacctccat gtaagcatac taaagtatta tcttctaata
      121 taggatgcaa acaagaagta tcaggactta cattaatatc ttttattcta ggaatagtga
      181 ttattctttt tttagccatg aaaagccttg tgggttttaa gtatgttttc tagttgttta
      241 tctttggcat aaagaagctt ttcttctaaa gagcaatcaa aatttaaagg aaagggtttt
      301 agggtgtttt tacttagaaa tttttcccaa agtatagaat tttcttctgc tgaaaactcc
      361 caaaaatcat ctttattgtt ataaacatag tctttataat ctataaaatt tacaaaatta
      421 atatttatat ctttttgttt agaaagtatg ccttgtgtat tttcttcttc tgtgtttttt
      481 ggaattttgt tgtaattaat aagataataa ttaagtctat ttttatttaa ttcttgatcg
      541 tcaagttcat ttaaaaactc tttagaataa taatttaaaa aataagtatt tgcgtattct
      601 tctatatatt ctttaaaact tcttttatga attaatttat cttgattaat gggataagat
      661 tttaaagtat tatttttaga atctaaagta tgatcatatc ctacaaaaaa ataagaaaaa
      721 tccataaaag cataatcagc actgatgcct ttgcaaagat ttaaaaacat attttcgagt
      781 tctaaaatga ttttttcttc tttgcttaaa tttaattctt tttgtatctc atcttgactt
      841 tttggagtaa aagtatgatc gattctgtga ttataattgt taattaaata ttctaagctt
      901 tcatcactaa aattaaagct taactcaagc gaagaattaa aataaatatc atatttatta
      961 aagtagggtg ctaaattatc tttttctata aaagtatcaa tttgactttg agaaaaattt
     1021 tctccttttt catcccaaaa aggaaaatta ggagcaagat ttttaagtgc accaatctct
     1081 gccaaatcat ttttttgata atatcgatgt aagatatatt tgcttttatc cctgcataaa
     1141 cgattataaa tttgagtata atttttttggt aaaaaaatta accattcatc attgccaccg
     1201 aagttaattc cttcaaattt atcaacacta aaataagctt taaactcttg catagctttt
     1261 aaggtatttt ctaaatgttt gttataaaat ttatctttat tagcttgttt aaattcaaac
     1321 caatctttgg catttaaacg ctcgcctttt aaactccaag cattaagata tttttcatca
     1381 ctaaaagttt tagtagataa aagatgaaag cttactcggt tagcgcttaa tatttgtttc
     1441 atttaaaatc ttccaaggca tataaaaagt ctctaatatc gcttacttga tcttctaccc
     1501 aatcctccac tgtataatcc tcatcacatt cgctatcttc gttataataa tctccccctt
     1561 caaagtcagc aggatcatca tagagttcca tttctatatg ttctaattca aattccaaat
     1621 catttataaa atttcgaaga tttgcaccat taaattcact tttatcatat tctaaaatgg
     1681 cgattaagtt aggtggtaaa atagctttat atttttcatc gacttttaaa ttgccatttt
     1741 ccactagatt tttatattca tagcttaaga tgctacttat aaaagctctt cgctcgtatt
     1801 tttttaaacc atctaaagca ccttttaaat aactatcatt tttaagatag tacttaaata
     1861 cttcttgcgt atctttatcc aataaatttt tatttttcat tatctttcct tatagtttat
     1921 tttagcaaag tgaatttcta ttccctcatt cactttcttt agcttttttt atagcatcgt
     1981 ttaaaagctt ggaatattca tcatgatatt tacccaataa gtcaaaatca gtttttccat
     2041 caatttcaca atgagaagaa tcatatgctg gtaaattaaa tttctttctg atttgttctg
     2101 aagtcattgc tcctgcatct tttaaaagct ttttatttct agatcctttt gcaatatcta
     2161 aaggggtgct tggggtagca aaatttacat tggctccaag ttcaattaaa agctgtgtaa
     2221 ccctataggt ttctgcttct tttacagctt caaataaaag ggtttcatta tattcactct
     2281 cttgaataag tcgggtttgc atatctacat tagctcctcc tttaatggct agttctaata
     2341 attcataatt attttgcatt acccaaaaat gcataggagt cctaccggaa tatccttcag
     2401 catgcacttc aaatttagcc ccacgattga ctagatactt aatgatttga gagtttggat
     2461 ttttagcgcc taatatataa gcataaatta aagcttttcg acaaataatg ttgctttctt
     2521 ttgcttt
//
```

```
LOCUS       ECFEEEMN_7              2407 bp    DNA     linear       12-APR-2021
DEFINITION  Campylobacter species strain strain.
ACCESSION
VERSION
KEYWORDS    .
SOURCE      Campylobacter species
  ORGANISM  Campylobacter species
            Unclassified.
COMMENT     Annotated using prokka 1.14.6 from
            https://github.com/tseemann/prokka.
FEATURES             Location/Qualifiers
     source          1..2407
                     /organism="Campylobacter species"
                     /mol_type="genomic DNA"
                     /strain="strain"
     gene            40..510
                     /locus_tag="ECFEEEMN_00054"
     CDS             40..510
                     /locus_tag="ECFEEEMN_00054"
                     /inference="ab initio prediction:Prodigal:002006"
                     /codon_start=1
                     /transl_table=11
                     /product="hypothetical protein"
                     /protein_id="Prokka:ECFEEEMN_00054"
                     /translation="MPAFTSQSV*KSKVEFSDSKEYCSSIFVCMELDFKDVLSCIWAD
                     KF*WTLRKNSFSIVWVKDFSINNLFSPFTFLISSLWIIVF*FAPISISLFSPTNS*EF
                     LATLTLLSTPTFKVLLNPTMVSLDKPTLIKYSAPTLTFLAKSMV*V*TFLILSL"
ORIGIN
        1 gtaacaattt gagtatcacc tacttgatgg aggatttgat tgcctgcttt tacttcacaa
       61 tctgtttgaa agtcaaaagt agaattttct gattctaaag aatattgctc ttctatcttt
      121 gtttgcatgg aattagactt taaagatgtg ttatcttgta tatgggcgga taaattttga
      181 tggacattaa ggaaaaattc tttttctatt gtttgggtta aagatttttc tattaataat
      241 ttattttctc ccttcacatt ccttatctca tctttatgga taatagtatt ttgatttgca
      301 cctatttcta tatctttgtt ttctcctaca aactcatgag agtttttagc cactcttact
      361 ttattatcta ctcctacatt taaagtattg cttaatccta ctatggtatc tttagataaa
      421 cctacattga ttaaatattc agcccctaca ttgacatttt tagccaaatc tatggtttga
      481 gtatgaacct ttttaatcct ttcattataa attccatcta ctatagaatc tttatcattt
      541 aaaattcttt gagtgaaatt atgttgcact aattcatcat aatccttttg ggcttttaaa
      601 taaatttgtt ctttgtcttt tatatttgag agggtaagtt cattataacc ttcttcattt
      661 gtgcctatgg ttttagaaga aagagaagtt tgatgatcat taaaaggaag atttactaag
      721 cttggattag ccccattata caaactagag ctcacatagg gtttatctat atcatcatct
      781 aaaaaagaaa caatcacttc atctcccact cttggagtat gaaaaaagcc tgaatgattg
      841 cttgctatac ttgttgctac tcttaaaaaa ggagtatgat gataagattt ataagcattg
      901 cttgataaat gattagaaga gtttgtgaag tttgtattat ctttagaatc agtattgttt
      961 gtattttcat gagtattggt ttttatgttt gcacttaaaa gattattatc taattcctct
     1021 tgattagcat aaagattaat ccttacctta acccttccat actcatcagt ataaatagta
     1081 tttctctcac tttctatatc cttactctct cctataacaa taccttgagt attatgaggg
     1141 gctttaggct tagctttaaa acttggagta aagataatat tcttttttaa taaagttaaa
     1201 gtattagaat aagacttact taaatttaaa tcttttaaat ttaaatgatc attagtattg
     1261 atagaattag ctagtaaagc atcatctatt aagatttgag tattggcaat aataataaag
     1321 tctttaagca aagaagcttt atcttgtttt tcttcatctt gacttttagt atcttcatca
     1381 tcactcaatc cactcaaatt aaattcctta taatttaaag tgataaaatc tcccaaacta
     1441 agatgataaa tattactttt tgctagcaag ctttcattta agatcaaagc tcttttttct
     1501 ttaagagaag gcttagtctt taaatcaata cttcttgtaa aagaatattc actctcataa
```

```
     1561 aaatgttcat tataataagc tttgttatta agttcttgct cataaggaac tttactagaa
     1621 tgtaaagata aaagatttaa aggagtatta gcattgatac tagaatgtgt aaaagcatta
     1681 gttcttaagc tttgctcttt atagatagaa gaaatacaag cttgatttaa aatattattc
     1741 acattaggat tgtactcaat ttctttattt tttacatttt tatacacatc acaaaaataa
     1801 atagtgtttt catcttcata aaaaaagatt ccattattgt gagctaatct tgtgataaat
     1861 tctaaatcgc tttcattgta ttgagagatg agttcttgtt cttcataatt aaaatgtata
     1921 tgagaataat caatttcttt atgtaagata tcttgataaa aaccaagggt ttgtttaatc
     1981 acttctatga tatgagtatg agtataaata cgattggctt tatttaaaga aagtcttatc
     2041 aagactgatt gaagcttaaa agaaaaaaaa tgcttgtatt ttatttgctt agtgtttgaa
     2101 gtgctatcat tgatatttaa agcactctca tgattgatac ctagatattt tatataagtg
     2161 atgattcctt tatagttttt tacttcatta tgatcaaagt ttaaggtatt gttctcataa
     2221 ggattatgga tacttaaaat agcttcttga tcgattaaaa cattaggatg aaaattaaaa
     2281 gttgtagggg tgcttagatg agatgaagca ttggttaaag tatttaaaga aaataaatct
     2341 tgttctaaac tttcaaaaaa gccttcacat tctatagaaa aaagctcttc aagactttct
     2401 ttgatga
//
LOCUS       ECFEEEMN_8              2139 bp    DNA     linear       12-APR-2021
DEFINITION  Campylobacter species strain strain.
ACCESSION
VERSION
KEYWORDS    .
SOURCE      Campylobacter species
  ORGANISM  Campylobacter species
            Unclassified.
COMMENT     Annotated using prokka 1.14.6 from
            https://github.com/tseemann/prokka.
FEATURES             Location/Qualifiers
     source          1..2139
                     /organism="Campylobacter species"
                     /mol_type="genomic DNA"
                     /strain="strain"
     gene            complement(23..667)
                     /locus_tag="ECFEEEMN_00055"
     CDS             complement(23..667)
                     /locus_tag="ECFEEEMN_00055"
                     /inference="ab initio prediction:Prodigal:002006"
                     /inference="similar to AA sequence:RefSeq:CKG87616.1"
                     /note="Domain of uncharacterised function (DUF1910)"
                     /codon_start=1
                     /transl_table=11
                     /product="hypothetical protein"
                     /protein_id="Prokka:ECFEEEMN_00055"
                     /translation="MVRDTSKDEAYFTKHIIECEEKIKKSEKILLELPFGKRETCLFC
                     ITDRKKCIAMDKYSRGDDINPVKKDLEAYVLLKQKNRLEFAIDIGYYRGNALELCIRV
                     LLDMDSACVLELIEEDERKRRDILNRDWFLHFMGSKGKNLNLERKCNCKEHELIKEFV
                     ATKDIEFLHQYMKKHTRLRDPLDTWDLEGAAIVKLMNLDKEEFKQYKYFPYDLI"
     gene            complement(826..1515)
                     /locus_tag="ECFEEEMN_00056"
     CDS             complement(826..1515)
                     /locus_tag="ECFEEEMN_00056"
                     /inference="ab initio prediction:Prodigal:002006"
                     /codon_start=1
                     /transl_table=11
                     /product="hypothetical protein"
```

```
                     /protein_id="Prokka:ECFEEEMN_00056"
                     /translation="MIQQENLDDIVLKFCKKLDKFSAQYGLKTEEFITYGVFDDLLFT
                     FEHYLEEEDDCISFSVSFRVKSMKSDEILCKIIGLDQPNLDFKILNQHGVRSFPFSFK
                     SMEFTNLNDLKQSGLDDMPSDSYHSFLKAYKESIDTLIEAFKTDTTNTIKGLYDFIYN
                     YEENEDTIWQDRTLMQVLLLIELNQKEQALKILKNNQKAIEERDFTIEYDDGSIEVFD
                     VLKHSIENDLC"
     gene            complement(1516..2133)
                     /locus_tag="ECFEEEMN_00057"
     CDS             complement(1516..2133)
                     /locus_tag="ECFEEEMN_00057"
                     /inference="ab initio prediction:Prodigal:002006"
                     /codon_start=1
                     /transl_table=11
                     /product="hypothetical protein"
                     /protein_id="Prokka:ECFEEEMN_00057"
                     /translation="MSIKGYPIFAIPKKNKIQFALAKIQASPLAKIEYDRIRWEGIGG
                     KRSKKNHSCKILFSSTQFNDKDKKRKQLKENQINGKRFEQKDFKIFKKECPNAQSQVT
                     LRVYDKNKPIVTTRVDAIAHCKGKIIIREAKSSKDAPLTQNQIKAFNLISQKNNDITI
                     ELRGNKVLKYDNKMLKKGDKILSKNVTIKISRPSGITTYKSTKGI"
ORIGIN
        1 tgggttttta aacccactaa gattaaatta aatcataagg aaagtatttg tattgtttaa
       61 attcttcttt atctaaattc attagtttta caattgctgc accttcaaga tcccaagtat
      121 ctagaggatc tcttaatctt gtatgttttt tcatatattg atgtaaaaac tcaatatcct
      181 tagtagctac aaattcttta ataagctcgt gttctttaca attacatttg cgttctaaat
      241 ttaaattctt acccttagaa cccataaaat gtaaaaacca atctctattg agaatatctc
      301 ttctttttct ctcatcttct tctattaatt ctaatacaca agcactatcc atatctaata
      361 atactctaat acaaagttca agagcatttc ctctataata accaatatca attgcaaatt
      421 ctaaacgatt tttttgtttt aataatacgt atgcttctaa atcttttttta actggattta
      481 tatcatcgcc acgagagtat ttatccatag caatacattt ttttctatct gttatacaaa
      541 aaagacaagt ttctcttttt ccaaaaggaa gctctaaaag tattttttcg gatttttttaa
      601 ttttttcttc acactctatt atatgtttcg taaaatacgc ttcatcttta ctagtatccc
      661 ttaccatttt ctatcctttt tattatggta tttgtttgat aaaacctaat aagtttatat
      721 aatttatgat tataatattt tatatattaa tcaatcttca catactaaaa cttcactaaa
      781 actaaaacct aataaattgt tttctcttac acattttaaa aaatcctaac aaagatcatt
      841 ttctatagaa tgttttaaaa catcaaatac ttctatactt ccatcatcat attctatagt
      901 aaagtctctt tcttcaatgg ctttttgatt atttttttaga atttttaatg cttgttcttt
      961 ttgattaagt tctataagta gtaaaacttg cattaaggtt ctatcttgcc aaatagtatc
     1021 ttcattttct tcataattgt aaataaaatc ataaagccct tttattgtgt tagtagtatc
     1081 agttttaaaa gcttcaataa gagtatctat actttcttta taagctttta aaaaactatg
     1141 ataagaatca cttggcatat catctaatcc agattgtttt aaatcattca aattggtaaa
     1201 ttccatactt ttaaaagaaa atggaaaaga tctaacgcca tgttgattta aaattttaaa
     1261 atctaaatta ggctgatcaa gtcctattat cttacataaa atttcatcac ttttcataga
     1321 ttttactcta aaagaaacac taaaacttat gcaatcatct tcttcttcta aataatgttc
     1381 aaaagtaaaa agtaagtcgt caaaaacacc ataagtgata aattcttctg tttttaaacc
     1441 atattgtgct gaaaatttgt ccagtttttt gcaaaatttt agaacaatat catctaagtt
     1501 ttcttgttgt atcatttaaa ttccttttgt tgatttgtat gttgttattc cgctagggcg
     1561 tgatattttt attgtaacat tttttgataa tattttatct cctttttttta gcattttatt
     1621 gtcatatttc aaaactttat tacctcgtag ttctattgtt atgtcattgt tttttttgact
     1681 tattaaatta aatgctttta tttgattttg tgttagggga gcatctttag atgattttgc
     1741 ctccctgata ataattttac ctttacaatg agctattgca tccactctgg ttgtaacaat
     1801 gggtttattt ttatcataaa ctctgagagt tacttgggat tgtgcattag ggcattcttt
     1861 tttaaatatc ttaaaatcct tttgctcaaa tcgtttgccg ttaatttgat tttccttaag
     1921 ttgtttgcgt tttttatctt tatcattaaa ttgagtgcta gaaaaaagta ttttacatga
     1981 atgatttttc ttacttcttt tacctcctat cccttcccat cttatcctat catactcaat
```

```
     2041 cttagcaaga ggactagctt gtattttagc taaagcaaat tgaatcttat tctttttagg
     2101 tatagcaaat ataggatagc cttttatact cactcctat
//
LOCUS       ECFEEEMN_9              2022 bp    DNA     linear       12-APR-2021
DEFINITION  Campylobacter species strain strain.
ACCESSION
VERSION
KEYWORDS    .
SOURCE      Campylobacter species
  ORGANISM  Campylobacter species
            Unclassified.
COMMENT     Annotated using prokka 1.14.6 from
            https://github.com/tseemann/prokka.
FEATURES             Location/Qualifiers
     source          1..2022
                     /organism="Campylobacter species"
                     /mol_type="genomic DNA"
                     /strain="strain"
     gene            68..229
                     /locus_tag="ECFEEEMN_00058"
     CDS             68..229
                     /locus_tag="ECFEEEMN_00058"
                     /inference="ab initio prediction:Prodigal:002006"
                     /inference="similar to AA sequence:RefSeq:AOH52015.1"
                     /codon_start=1
                     /transl_table=11
                     /product="ankyrin domain protein"
                     /protein_id="Prokka:ECFEEEMN_00058"
                     /translation="MTSNEIRKKFNLPAYDSSHCEIDGKTDFDLLGKYHDECSKLLND
                     AIKKAKENE"
     gene            222..1889
                     /locus_tag="ECFEEEMN_00059"
     CDS             222..1889
                     /locus_tag="ECFEEEMN_00059"
                     /inference="ab initio prediction:Prodigal:002006"
                     /inference="similar to AA sequence:RefSeq:AOH52014.1"
                     /codon_start=1
                     /transl_table=11
                     /product="restriction endonuclease fold toxin 7 domain
                     protein"
                     /protein_id="Prokka:ECFEEEMN_00059"
                     /translation="MSRVIKINFANVNFHIKEFQKVYILENCKLCFYKPFHLQAKVKN
                     NGKKFKDKNKKIHQEQEIKKINSQDQSDEKEDINEEQAKAYAEKIDEEISNFDKNNFT
                     SNIQTQTIEKRQDKFKDYDIFYDVYTSKDNKDKSLNKELGCGDGFEVNNGIAEVGAEF
                     FDKCFKKGIYVLIPRLISLDKNEKLHFDPVPLEDKGFVVTDFRKGKKEEKNIDLTFQR
                     SIAKIKNPNLALQSPEDFLKALNGNENRDFTEKDKIQQAMGIKKACGKKVDEITKILL
                     KDDEKLKDIIRGEEKLKKEIGRLRDKQNKSPKELSLLGRLEFLQRNQDKVEKHKDIID
                     NLQNSQQAGDNKQNNSAKGGAKGGLKNKQKQTTTSNQEEQKNKNINSKDIGDAGEYAA
                     SMLFTKRSTRYLSNRRKLDANFNTKGFNHTIPDFLVTLNDYPTLVEVKNIQDQALTEQ
                     ISFELKLAREYELDYLFICNHYTKLVDNITNLKVFNKDNEKHKGSRSGFIYHRHFHRS
                     IKTIFKEMYLHHIKGVAPNKIMIKRLNLNNPANIEEELNKNKQIQKN"
ORIGIN
        1 cccaagaacc cctttagatg atgcaaaagg atctagaaat aaaaagcttt taaaagatgc
       61 aggagcaatg acttcaaatg aaattagaaa gaaatttaat ttaccagcat atgattcttc
```

```
      121 tcattgtgaa attgatggaa aaactgattt tgacttattg ggtaaatatc atgatgaatg
      181 ttccaaactt ttaaacgacg ctataaaaaa agccaaagag aatgagtaga gtgataaaaa
      241 ttaactttgc taacgtcaat tttcatatca aagaatttca aaaggtttat atattagaaa
      301 attgtaaatt gtgtttttat aagccttttc atttgcaagc aaaagttaaa aacaatggaa
      361 aaaaatttaa agataaaaat aaaaaaattc atcaagaaca agaaatcaaa aaaataaatt
      421 ctcaagatca atcagatgaa aaagaagata tcaatgaaga acaagcaaaa gcttatgctg
      481 aaaaaatcga tgaagagata agtaattttg ataaaaataa ctttacttca aacatacaaa
      541 cacaaaccat agaaaagcga caagataaat ttaaagacta tgatatcttt tacgatgttt
      601 atacaagtaa agacaataaa gataaaagtt taaataaaga attaggatgc ggtgatggct
      661 ttgaagtaaa taatggcata gctgaagttg gagcagaatt ttttgataaa tgctttaaaa
      721 aaggaattta tgttttaata ccaagattaa tatctttaga taaaaacgaa aaattacact
      781 ttgatcctgt gcctttagaa gataagggct ttgtggttac tgattttaga aaaggaaaaa
      841 aggaagaaaa aaatatagac ttaacctttc aaagaagcat agctaaaata aaaaaaccta
      901 atttagcttt gcaaagtcct gaagattttt taaaagcttt gaatgggaat gaaaatagag
      961 attttacaga aaaagataaa atacaacaag cgatgggtat taaaaaagct tgtggaaaaa
     1021 aagtagatga aataaccaaa atattactta aagatgatga aaaattaaaa gatattatta
     1081 ggggtgaaga gaaattaaag aaagaaattg gtagattacg agataaacaa aacaaatcac
     1141 ctaaagaact aagcctacta ggtagacttg aatttttaca aagaaatcaa gataaagttg
     1201 aaaaacataa agatattatt gacaatcttc aaaactctca acaagctggt gataacaaac
     1261 aaaacaatag cgcaaaaggt ggagcaaagg gtggattaaa aaacaaacaa aaacaaacca
     1321 ccacctcaaa ccaagaagaa caaaaaaaca aaaatatcaa ttcaaaagat ataggagatg
     1381 caggagaata tgctgcttct atgcttttta ctaaaagatc aactaggtat ttatctaata
     1441 gaagaaaact agatgccaat tttaatacta aaggttttaa tcatactata cctgattttt
     1501 tagtaacctt aaatgattac cctactttag tagaggttaa aaatatccaa gatcaagctt
     1561 taacagaaca aataagcttt gaactaaaac tggctagaga atatgaactt gattatttat
     1621 ttatatgcaa tcactataca aaattggtag ataatataac caatctaaaa gtatttaata
     1681 aagacaatga aaaacacaaa ggatcaagaa gtggctttat ctaccacaga catttccaca
     1741 gaagcataaa aacaatattt aaagaaatgt atttacacca tatcaaagga gtagcaccta
     1801 ataaaatcat gataaaaaga ttaaatttaa ataatcctgc taatatagaa gaagaattaa
     1861 acaaaaacaa acaaatacaa aaaaactaaa ggagcaacaa tgaaaacatt agaagatatc
     1921 aaaaaattaa attttaaaat aaaaatggaa attcaaaaac aattatttga ttttattagt
     1981 aataatgact tagaaaatgt taaaaatctt ttaaaagatt at
//
LOCUS       ECFEEEMN_10             1875 bp    DNA     linear       12-APR-2021
DEFINITION  Campylobacter species strain strain.
ACCESSION
VERSION
KEYWORDS    .
SOURCE      Campylobacter species
  ORGANISM  Campylobacter species
            Unclassified.
COMMENT     Annotated using prokka 1.14.6 from
            https://github.com/tseemann/prokka.
FEATURES             Location/Qualifiers
     source          1..1875
                     /organism="Campylobacter species"
                     /mol_type="genomic DNA"
                     /strain="strain"
     gene            7..630
                     /locus_tag="ECFEEEMN_00060"
     CDS             7..630
                     /locus_tag="ECFEEEMN_00060"
                     /inference="ab initio prediction:Prodigal:002006"
                     /codon_start=1
```

```
                     /transl_table=11
                     /product="hypothetical protein"
                     /protein_id="Prokka:ECFEEEMN_00060"
                     /translation="MSIKGYPIFAIPKKNKIQFALAKIQASPLAKIEYDRIRWEEIGG
                     KLKAAQRRRREKSKEKAKMLLYLENENKKDKVSDQEVHLYKHNGIWPKITPKPRSSDY
                     ILEDGEIILNIKQRAMEIKNTLNGGYNSISIKTKDKLTRYDLDGKPHYEKTSKKIIDT
                     PHKIEYTKHINPQDTTKYRMSQGAVEPISHKDLDIVENYLKRQNNEI"
     gene            620..1006
                     /locus_tag="ECFEEEMN_00061"
     CDS             620..1006
                     /locus_tag="ECFEEEMN_00061"
                     /inference="ab initio prediction:Prodigal:002006"
                     /codon_start=1
                     /transl_table=11
                     /product="hypothetical protein"
                     /protein_id="Prokka:ECFEEEMN_00061"
                     /translation="MKFENLKHGSLFKREFYLKDVKIDLRNDFYVYSITMNKNTLEIF
                     FKSEKKDIQDLQIIFYDYKLLHPKSYCKNFNQLEANFIDLIRNNWDDITGYIEKEEEM
                     LISIYFDNDLEYITLTCKYFNIDFID"
     gene            1021..1578
                     /locus_tag="ECFEEEMN_00062"
     CDS             1021..1578
                     /locus_tag="ECFEEEMN_00062"
                     /inference="ab initio prediction:Prodigal:002006"
                     /inference="similar to AA sequence:RefSeq:CKG87579.1"
                     /codon_start=1
                     /transl_table=11
                     /product="hypothetical protein"
                     /protein_id="Prokka:ECFEEEMN_00062"
                     /translation="MTREQAELIIKEEKLIDTDWYPSYKHSGEFHLTMWFDSDNNKYE
                     TFYVGERGSVELEYSFDSEKEAIDKMLQMNYEQKKYDGKDIKEYLTKEVALRIIKEEN
                     LEVIWYDEALKPMRAGIKHDKQRDKYISFITNAKAEIIEYRSIEFDGDRYSETFKEML
                     NDENIALYALINRARMIKQNRISFT"
ORIGIN
        1 ataggagtga gtataaaagg ctatcctata tttgctatac ctaaaaagaa taagattcaa
       61 tttgctttag ctaaaataca agctagtcct cttgctaaga ttgagtatga taggataaga
      121 tgggaagaga taggaggtaa attaaaagct gcacaaagaa ggagaagaga aaaaagtaaa
      181 gagaaagcaa aaatgctttt atatttggaa aatgaaaata aaaaagataa agtaagtgat
      241 caagaagttc atctttataa acataatgga atatggccta aaattactcc aaaacctaga
      301 agttctgatt atattcttga agatggagag attattttaa acatcaaaca aagagccatg
      361 gaaataaaaa atactttaaa tggagggtat aatagtattt ctataaagac aaaagataaa
      421 cttaccagat atgatttaga tggtaaacca cattatgaga agacatcaaa gaaaataatt
      481 gatacacctc ataaaataga atatactaaa catataaatc ctcaagatac aacaaagtat
      541 agaatgtcac agggagcagt agagccaata tctcataaag atttagatat tgtagaaaat
      601 tatttaaaaa ggcaaaataa tgaaatttga gaatttaaag catggaagtt tattcaaaag
      661 agaattttat cttaaagatg taaaaataga tttaagaaat gacttttatg tttattctat
      721 cacaatgaat aaaaacactt tggaaatatt ttttaaatca gagaaaaaag atatacaaga
      781 tttgcaaatt atttttatg attataagtt gctacatcca aaatcatatt gtaaaaattt
      841 taatcaatta gaagctaatt ttatagattt gataagaaat aattgggatg atataacagg
      901 ctatattgaa aaagaagaag agatgttgat aagcatatat tttgataatg atttagaata
      961 tattacttta acttgtaagt attttaatat agattttata gactgaataa aggatagaaa
     1021 atgacaagag aacaagcaga attaatcata aaagaagaaa agttgattga tacagattgg
     1081 tatccttctt ataaacattc aggagaattt catcttacta tgtggtttga ttctgataat
     1141 aataaatatg aaactttcta tgttggagaa aggggcagtg ttgaacttga gtattctttt
```

```
     1201 gatagtgaaa aagaagccat tgataaaatg ttgcagatga attatgaaca aaaaaaatac
     1261 gatggaaaag acattaaaga atatctaaca aaagaagtag cactaagaat catcaaagaa
     1321 gaaaatctag aagtgatttg gtatgatgaa gcacttaaac caatgcgagc aggtatcaaa
     1381 cacgataaac aaagagataa atatatttct tttatcacta atgctaaagc agagattata
     1441 gaatacagaa gtattgagtt tgatggggat agatacagtg aaacttttaa ggaaatgctt
     1501 aacgatgaaa atatagccct ttatgctctt atcaatcgtg ccagaatgat taagcaaaat
     1561 aggattagct ttacgtaaat actttcctta taatttaatt taatcttagt gggtttaaaa
     1621 acccacaagg cttttcatgg ctagaatttg ctatacccaa aaagaataag attaaatttg
     1681 ctttagctaa aatacaagct agtcctcttg ctaagattga gtatgatagg ataagatggg
     1741 aagggatagg aggtaaaaga agtaagaaaa ataaaaatga tagagcaaag caaatacaag
     1801 cattaaaaaa taatcagcaa aacaacaaac aaaattcatt tttacaagaa gttgaacaac
     1861 aaaaagaaga ggctg
//
LOCUS       ECFEEEMN_11             1821 bp    DNA     linear       12-APR-2021
DEFINITION  Campylobacter species strain strain.
ACCESSION
VERSION
KEYWORDS    .
SOURCE      Campylobacter species
  ORGANISM  Campylobacter species
            Unclassified.
COMMENT     Annotated using prokka 1.14.6 from
            https://github.com/tseemann/prokka.
FEATURES             Location/Qualifiers
     source          1..1821
                     /organism="Campylobacter species"
                     /mol_type="genomic DNA"
                     /strain="strain"
     gene            67..321
                     /locus_tag="ECFEEEMN_00063"
     CDS             67..321
                     /locus_tag="ECFEEEMN_00063"
                     /inference="ab initio prediction:Prodigal:002006"
                     /inference="similar to AA sequence:RefSeq:CKG86821.1"
                     /codon_start=1
                     /transl_table=11
                     /product="hypothetical protein"
                     /protein_id="Prokka:ECFEEEMN_00063"
                     /translation="MLDPLEIEKLSPQEYRVRNYFKFLTFATMFFLVITFIILPCLLQ
                     AYLFKCYSTGEKRKKLSWFIGFVCNAPILIASVYVVYENL"
     gene            440..931
                     /locus_tag="ECFEEEMN_00064"
     CDS             440..931
                     /locus_tag="ECFEEEMN_00064"
                     /inference="ab initio prediction:Prodigal:002006"
                     /inference="similar to AA sequence:RefSeq:CKG86719.1"
                     /codon_start=1
                     /transl_table=11
                     /product="hypothetical protein"
                     /protein_id="Prokka:ECFEEEMN_00064"
                     /translation="MGEDITLSIFWGLIGFILACSVIEFYLIALSVLIVFSILNYWIK
                     INIKEFVKSYFLISFIIFFMAIVIVFYLTYDLETSRGLVLLGPPEIEKLSPQEYKARN
                     YFDFLGGITFIFLVITFFILPCLLQAYLFKCYSTGEKRKKLSWFIGFVCNPILMAGVY
                     VMY"
```

```
ORIGIN
        1 ttttttacag ctattattat catattttat cttagctatg atcttgaatt ttataaatcg
       61 cttgtattgc ttgatcctct agaaattgaa aaattatcac cgcaagaata tagagtgcgt
      121 aattatttta aatttttaac ctttgcaact atgttttttt tagtaatcac atttattata
      181 cttccttgtt tattgcaagc ttatttattt aaatgttata gcacaggaga aaaacgcaaa
      241 aaattaagtt ggtttatagg gtttgtgtgt aatgctccta tattaattgc gagtgtgtat
      301 gtggtatatg aaaatcttta aagactatca aaaattcact ccttatggtt tggattttaa
      361 actttatagt aaagatttta tcactaagct tgattttaaa gataaagctt ataatgtgct
      421 ttataataca ggagtttaaa tgggagaaga tattacactt tctatttttt ggggtttgat
      481 aggatttatt cttgcttgta gtgttataga attttatctt atagctctaa gcgttcttat
      541 tgtcttttct attttaaatt attggatcaa gattaatata aaagaatttg taaaatctta
      601 ttttttaata tcttttatta tctttttat ggctattgtt atcgtatttt atcttactta
      661 tgatcttgaa acttctaggg gacttgtatt acttggtcct ccagaaattg aaaaattatc
      721 accacaagaa tataaagcac gtaattattt tgattttta ggcggtataa cttttatttt
      781 tttagtaatc acatttttta tacttccttg tttattacaa gcttatttat ttaaatgcta
      841 tagcacagga gaaaaacgca aaaaattaag ttggtttata gggtttgtgt gtaatcctat
      901 attaatggcg ggtgtgtatg tgatgtatta aaatctttaa agacttttaa aaaatttaat
      961 atatttgata taatatttat ttatcttaaa tatttagaga aaaaatgaat agagaattat
     1021 ttataacaag acttgcattt ataaacaatg atgaaataaa taaaataaat aaaaattcat
     1081 ctattgcata tttaaaagcc ttatatcaat gtgaaaattc catagctgta aattatgatg
     1141 aagaagagct tttcacccaa accactgcta taaacaatct aggatatggc aatgatttca
     1201 caaaaatctt catcctagat aaagaaaacc tcaatgacac ctatcttgat gctagaaaaa
     1261 agctttataa atctatcaat actttaaaag aagaaaaaaa tgaaaaaata aaaaatatcc
     1321 aagcttatgt aaataaagaa aattccttgc aagacaatct aaataatcaa agctttaata
     1381 ctgaggaaaa tgatgttaat ttaattttaa acgaagtatt taacatagag caattttatg
     1441 ataaaaatac caataccata gtattaaaga caaataatat aaaaatcata ttcttagata
     1501 aagtaaattt acaagatttg caagataatt ctaacaatac ttctttcatc tctatgtcaa
     1561 atttaatcca tatttatgag aatcattgtg atatttttac gaaagataga agtattttag
     1621 atataaaatt tatagaagaa aaatatcaaa tagattttaa aagtttagat acaaaaatct
     1681 tcctaaactc caccctcctt acaggctcca ccgaactccc taataatcct ttttactttg
     1741 gagaattaga tcaagataat actataaaac aagatatacc cagttattat ttttctccta
     1801 aagatgaaag cagtggttta g
//
LOCUS       ECFEEEMN_12             1275 bp    DNA     linear       12-APR-2021
DEFINITION  Campylobacter species strain strain.
ACCESSION
VERSION
KEYWORDS    .
SOURCE      Campylobacter species
  ORGANISM  Campylobacter species
            Unclassified.
COMMENT     Annotated using prokka 1.14.6 from
            https://github.com/tseemann/prokka.
FEATURES             Location/Qualifiers
     source          1..1275
                     /organism="Campylobacter species"
                     /mol_type="genomic DNA"
                     /strain="strain"
     gene            112..609
                     /locus_tag="ECFEEEMN_00065"
     CDS             112..609
                     /locus_tag="ECFEEEMN_00065"
                     /inference="ab initio prediction:Prodigal:002006"
                     /inference="similar to AA sequence:RefSeq:CKG86821.1"
```

```
                     /codon_start=1
                     /transl_table=11
                     /product="hypothetical protein"
                     /protein_id="Prokka:ECFEEEMN_00065"
                     /translation="MGDSITLSIFVGSLLSIAVITKFYLIALSVLIVFSILNYWIKIN
                     IKEFVKSYFLVYFTIFFTAIVIIFYLTYGIETSKDLALLSSLEAKELSPQEYRVRNYF
                     KFLTFATMFFLVITFIILPCLLQAYLFKCYSAGEKRKKLSWFIGFVCNIIIILIASAYL
                     MYIFI"
     gene            728..1231
                     /locus_tag="ECFEEEMN_00066"
     CDS             728..1231
                     /locus_tag="ECFEEEMN_00066"
                     /inference="ab initio prediction:Prodigal:002006"
                     /inference="similar to AA sequence:RefSeq:CKG86821.1"
                     /codon_start=1
                     /transl_table=11
                     /product="hypothetical protein"
                     /protein_id="Prokka:ECFEEEMN_00066"
                     /translation="MGEDITFSIFWGLFGFILAFTNIKFYLTTLSVLIVFSILNYWIK
                     INIKEFVKSYFSIYFIIFFTAIVIIFYLTYGIETSKDLALLSSLEAKELSPQEYKARN
                     YFGFLTCMTMFFLVITFIILPCLLQAYLFKRYSTEEKRKKLSWFIGFLCNIIIILIASA
                     YLMYIFI"
ORIGIN
        1 caaaaattca ctccttatgg tttggatttt agactttata gtaaagattt tatcactaaa
       61 cttgatttta aagataaagc ttataatgtg ctttataata caggagttta aatgggagat
      121 agtattacgc tttctatttt tgtaggttca ttgctatcca ttgctgttat cacaaaattt
      181 tatcttatag ctttatctgt tcttattgtc ttttctattt taaattattg gattaagatt
      241 aatataaaag aatttgtaaa atcttatttt ttagtatatt ttactatctt ttttacagct
      301 attgttatca tattttatct tacctatggt attgaaactt ctaaggatct tgcattgctt
      361 agctctctag aagctaaaga attatcaccg caagaatata gagtgcgtaa ttattttaaa
      421 tttttaacct ttgcaactat gttttttta gtaatcacat ttattatact cccttgttta
      481 ttgcaagctt atttatttaa atgttatagc gcaggagaaa aacgcaaaaa attaagttgg
      541 tttatagggt ttgtgtgcaa tattattata ttaattgcaa gtgcatattt gatgtatata
      601 tttatttaaa gactatcaaa aattcactcc ttatggtttg gattttagac tttgtagtaa
      661 agattttatc actaagcttg attttaaaga taaagcttat aatgtgcttt atgatacagg
      721 agtttaaatg ggagaagata ttacattttc tattttttgg ggtttatttg gatttattct
      781 tgcttttact aatataaaat tttatcttac cactttaagt gttcttattg tcttttctat
      841 tttaaattat tggattaaaa ttaatataaa agaatttgta aaatcttatt tttcaatata
      901 ttttattatc ttttttacag ctattgttat catattttat cttacctatg gtattgaaac
      961 ttctaaggat cttgcattgc ttagctctct agaagctaaa gaattatcac cacaagaata
     1021 taaagcacgt aattattttg gatttttgac ttgtatgact atgttttttt tagtaatcac
     1081 atttattata cttccttgtt tattgcaagc ttatttattt aaacgctata gcacagaaga
     1141 aaaacgcaaa aaattaagtt ggtttatagg gtttttatgc aatattatta tattaattgc
     1201 aagtgcatat ttgatgtata tatttattta aagactatca aaaattcact ccttatggtt
     1261 tggattttag acttt
//
LOCUS       ECFEEEMN_13             1215 bp    DNA     linear       12-APR-2021
DEFINITION  Campylobacter species strain strain.
ACCESSION
VERSION
KEYWORDS    .
SOURCE      Campylobacter species
  ORGANISM  Campylobacter species
            Unclassified.
```

```
COMMENT     Annotated using prokka 1.14.6 from
            https://github.com/tseemann/prokka.
FEATURES             Location/Qualifiers
     source          1..1215
                     /organism="Campylobacter species"
                     /mol_type="genomic DNA"
                     /strain="strain"
     gene            complement(176..940)
                     /gene="trxB_3"
                     /locus_tag="ECFEEEMN_00067"
     CDS             complement(176..940)
                     /gene="trxB_3"
                     /locus_tag="ECFEEEMN_00067"
                     /inference="ab initio prediction:Prodigal:002006"
                     /inference="similar to AA sequence:RefSeq:CKG86288.1"
                     /codon_start=1
                     /transl_table=11
                     /product="thioredoxin reductase"
                     /protein_id="Prokka:ECFEEEMN_00067"
                     /translation="MGEWELAAGILGDKLGFAKGGGGLWKTGFTTRAYQALFLAAQLG
                     HQPSLEHQLTTYSSSVALAGGGHKNPLREKMLKDFAKNPPYDEFGMLPFLDELIGVDW
                     VIDLNEYQFAYDGDGGSVKALDEKVRQGKLKDPRDIDSTPESRKEFDEEANAYTFGNR
                     MNFSSDIRDSRSKESARLFDDLLILHAKLAALTPPQGYPNAPYYFSPEQLEDFYKRQV
                     LDAKLDPRIPAIYRYNFPRELRAKILKFGEENGIKD"
ORIGIN
        1 cattgctttt tgccgtaaat aatattttat ttgtatcttt ttctacattt aatattgagc
       61 catctggtgt ataaatctca taagtatcac tcatctttta tcctttcttt atttaaccta
      121 tatgcaaacc atttttcata atgatagtag tggctagcct gcctatgcaa ttactttaat
      181 ctttaatgcc attttcttcg ccaaatttga gtatcttagc tctaagttcc cttgggaaat
      241 tatatctata aatagcaggg attcttggat caagtttagc gtctaaaact tgtcttttgt
      301 aaaaatcttc taattgttca ggactaaaat aataaggtgc attaggataa ccttgaggag
      361 gagtgagggc tgcgagtttg gcatgaagta tcaaaaggtc atcaaataat ctagccgatt
      421 ctttacttct actatcgcga atatcagagg aaaaattcat tctattacca aaagtataag
      481 cattagcttc ttcatcgaat tctttcctgc tctctggagt agagtctata tctcttggat
      541 cttttaattt accttgtctt actttctcat ctaaagcctt aacactaccg ccatcaccat
      601 cataagcaaa ttgatattca ttcaaatcaa tcacccaatc cactcctata agttcatcta
      661 agaaaggtag cattccaaat tcatcataag gaggattttt agcaaagtct tttagcattt
      721 tctctcttaa aggattttta tgtcctccac ctgctaaggc tacagaagat gagtaagtag
      781 tgagttggtg ttctaaagaa ggttgatgtc ctagttgtgc tgctaaaaat aatgcttgat
      841 aagccctagt agtaaatcct gttttccata atcctcctcc accttttgca aagcctaatt
      901 tatctcctaa aattcctgct gctaattccc attcacccat aaataaatat ccatcactta
      961 aatgagctaa gcctctattg gcttctactt ctttgtaaag ttctttatag tctttattga
     1021 ttaaatttcc tttttcatct acattacaat aagcatcata aggtatatct ataacagtgc
     1081 ttcttatacc acttcttata attaaatatt tatatctttc tcttttagtt tttaaagatt
     1141 tataataagc tttttctgct ttagtccaag gagctatagc tcctttgata ggatctttta
     1201 aatatatgct ttgcc
//
LOCUS       ECFEEEMN_14             1183 bp    DNA     linear       12-APR-2021
DEFINITION  Campylobacter species strain strain.
ACCESSION
VERSION
KEYWORDS    .
SOURCE      Campylobacter species
  ORGANISM  Campylobacter species
```

```
            Unclassified.
COMMENT     Annotated using prokka 1.14.6 from
            https://github.com/tseemann/prokka.
FEATURES             Location/Qualifiers
     source          1..1183
                     /organism="Campylobacter species"
                     /mol_type="genomic DNA"
                     /strain="strain"
     gene            complement(159..572)
                     /locus_tag="ECFEEEMN_00068"
     CDS             complement(159..572)
                     /locus_tag="ECFEEEMN_00068"
                     /inference="ab initio prediction:Prodigal:002006"
                     /inference="similar to AA sequence:RefSeq:AOH52023.1"
                     /codon_start=1
                     /transl_table=11
                     /product="immunity protein 70"
                     /protein_id="Prokka:ECFEEEMN_00068"
                     /translation="MVGFSIDNLWYEIGSGDFLHAFFSNVAYHLEKGNWGSRFPILMN
                     EVYQGKLNFSKASLALNELENIQKEFKNFTPKQIIWDIENLSLTPPWGDNISSDITDL
                     SNYFVTSDGNDFIELFKKVLQLADNEKANLTIKSF"
     gene            complement(587..1183)
                     /locus_tag="ECFEEEMN_00069"
     CDS             complement(587..1183)
                     /locus_tag="ECFEEEMN_00069"
                     /inference="ab initio prediction:Prodigal:002006"
                     /codon_start=1
                     /transl_table=11
                     /product="hypothetical protein"
                     /protein_id="Prokka:ECFEEEMN_00069"
                     /translation="MSIKGYPIFAIPKKNKIQFALAKIQASPLAKIEYDRIRWEGIES
                     KEEAKMLLYLENENKNDSKIKQISISNIPKKPHWRESEEDISKLYYDYEKQKSFLNGN
                     EVPYGTKHSVRPDLYKNGSSIEIKNYNLDKTSSANNLINIITKQYQQRLQHLPPKTEQ
                     IFIIDSRGQNISKEIQEKIKQKIRIKLNCDILIQFKTK"
ORIGIN
        1 tcttttttta gccatgaaaa gccttgtggg ttttaagtat gttttctagt tgtttatctt
       61 tggcataaag aagcttttct tctaaagagc aatcaaaatt taaaggaaag ggttttaggg
      121 tgtttttgct tagaaatttt tcccaaagta taattatttt aaaaactttt aatggtaagg
      181 ttagcttttt cattgtcagc tagttgtaag acctttttaa agagttctat aaaatcatta
      241 ccatcgcttg ttacaaaata atttgatagg tctgtaatat cactactgat attatcaccc
      301 caaggtggcg taagagataa gttttcaata tcccaaatta tttgttttgg ggtaaaattt
      361 ttaaattctt tttgtatatt ctctagttca tttaacgcta aagaagcttt tgaaaaattt
      421 aatttacctt gatatacttc attcattaat atagggaatc tacttcccca gtttcctttt
      481 tcaagatgat aagccacatt gctaaaaaat gcatgtaaaa aatcaccact gcctatttca
      541 taccaaagat tatctatact aaaacccacc attattcttc ctaaatttac ttagttttaa
      601 attgaataag tatatcacaa tttaatttaa tgcgaatttt ttgttttatt ttttcttgta
      661 tttctttaga tatattttgt cctctagagt ctataatgaa gatttgttca gtttttggtg
      721 gcaaatgttg caatctttgt tgatattgtt tggtaataat attaattaaa ttgtttgcac
      781 tggatgtttt atcaagatta tagtttttaa tttcaatgct agaaccattt ttgtataagt
      841 ctggacgaac agaatgtttt gttccgtatg gaacttcatt gccattaaga aaagatttct
      901 gtttttcata atcatagtat aactttgata tatcttcttc agattctcgc caatgaggtt
      961 ttttaggtat gtttgatata gatatttgtt ttattttgct atcgttttta ttttcatttt
     1021 ccaaatataa aagcattttt gcttcttctt tgctttctat cccttcccat cttatcctat
     1081 catactcaat cttagcaaga ggactagctt gtattttagc taaagcaaat tgaatcttat
```

```
     1141 tctttttagg tatagcaaat ataggatagc cttttatact cac
//
LOCUS       ECFEEEMN_15             1123 bp    DNA     linear       12-APR-2021
DEFINITION  Campylobacter species strain strain.
ACCESSION
VERSION
KEYWORDS    .
SOURCE      Campylobacter species
  ORGANISM  Campylobacter species
            Unclassified.
COMMENT     Annotated using prokka 1.14.6 from
            https://github.com/tseemann/prokka.
FEATURES             Location/Qualifiers
     source          1..1123
                     /organism="Campylobacter species"
                     /mol_type="genomic DNA"
                     /strain="strain"
     gene            234..866
                     /locus_tag="ECFEEEMN_00070"
     CDS             234..866
                     /locus_tag="ECFEEEMN_00070"
                     /inference="ab initio prediction:Prodigal:002006"
                     /codon_start=1
                     /transl_table=11
                     /product="hypothetical protein"
                     /protein_id="Prokka:ECFEEEMN_00070"
                     /translation="MSEINNNTEIKEQDTQDEIIWEGKKEIPSFLLYWITYIVLLCFM
                     LYLLPRTFDNTKNFKWFVFFIMFLLGIYIFAREIYKMANIKRIYVTKEKLVVEYYIKN
                     DLVFPLGTFFVYYRSLPFGLTPGDMVIYTFGDKVKEYLEPGLFSAGDDPTKGCCEKIN
                     AIIKPHVMPYLLSLSDEEFEKIISHVSGFSEIDTTFLKEAMELRKEKKDE"
     gene            859..1011
                     /locus_tag="ECFEEEMN_00071"
     CDS             859..1011
                     /locus_tag="ECFEEEMN_00071"
                     /inference="ab initio prediction:Prodigal:002006"
                     /codon_start=1
                     /transl_table=11
                     /product="hypothetical protein"
                     /protein_id="Prokka:ECFEEEMN_00071"
                     /translation="MNENIYGSLEFYSKETQSTPSDKRDLGVFGDVAPYALGITESFT
                     TKAFKG"
ORIGIN
        1 tgcaaacaaa gatagaagag caatattctt tagaatcaga aaattctact tttgactttc
       61 aaacagattg tgaagtaaaa gcaggcaatc aaatcctcca tcaagtaggt gatactcaaa
      121 ttgttaccaa gaaagattgt gttatcatta aagcaggtgg agtagaagtg attatagact
      181 ctaatggact tgtggttaaa ggtggagaag taaaagcaga gtaaaggata aaaatgagtg
      241 aaataaacaa taacacagaa atcaaagaac aagacactca agatgaaatc atttgggaag
      301 gaaaaaagga aataccttcg tttttgcttt attggataac atatattgtg cttttatgtt
      361 ttatgcttta tttgcttcct aggacatttg acaatactaa gaattttaaa tggtttgtgt
      421 tttttattat gtttctttta ggcatatata tatttgcacg agagatctat aaaatggcaa
      481 atattaaaag aatttatgtc acaaaagaaa aacttgtggt tgagtattat attaaaaatg
      541 atttggtttt tcctttagga actttttttg tatattatcg tagtttgcca tttgggctta
      601 ctccaggaga tatggtgata tacacctttg gagataaggt caaagaatac cttgaacctg
      661 gtttattcag tgctggcgat gatccaacaa aaggttgttg tgaaaaaatt aatgccatta
```

```
       721 taaaacctca tgtaatgcct tatttattaa gcttaagtga tgaagaattt gaaaaaatca
       781 tttcacatgt aagcggtttt agcgaaattg atactacttt ccttaaagaa gcaatggaac
       841 tcagaaaaga gaaaaaagat gaatgaaaat atttatggaa gtttagagtt ttattctaaa
       901 gaaactcaaa gcactcctag tgataaaaga gatttaggag tatttggaga tgttgctcct
       961 tatgctttag gcattacaga aagttttact actaaggctt ttaaggggta agcaatgaat
      1021 aattctactt tactttggaa aagtaagaga aaaattattc cttttagttt gtttttttgc
      1081 tctctttatt ttatattagc tttttttctg tgttggtttt gtt
//
LOCUS       ECFEEEMN_16             1091 bp    DNA     linear       12-APR-2021
DEFINITION  Campylobacter species strain strain.
ACCESSION   
VERSION     
KEYWORDS    .
SOURCE      Campylobacter species
  ORGANISM  Campylobacter species
            Unclassified.
COMMENT     Annotated using prokka 1.14.6 from
            https://github.com/tseemann/prokka.
FEATURES             Location/Qualifiers
     source          1..1091
                     /organism="Campylobacter species"
                     /mol_type="genomic DNA"
                     /strain="strain"
     gene            complement(551..931)
                     /locus_tag="ECFEEEMN_00072"
     CDS             complement(551..931)
                     /locus_tag="ECFEEEMN_00072"
                     /inference="ab initio prediction:Prodigal:002006"
                     /inference="similar to AA sequence:RefSeq:CKG87598.1"
                     /codon_start=1
                     /transl_table=11
                     /product="methyltransferase small"
                     /protein_id="Prokka:ECFEEEMN_00072"
                     /translation="MQMDKYYKAKGYKRISLNKVKGLDDKIHHEMDGIYYNPDKNPKY
                     IIAEAKFGTSKLDRKTKQMSDEWLIKNKLKQLKDNVGEKQAKEIKKLLNSNEVRKDLF
                     HVDEGGKETIDYLDKDANKIKPKG"
ORIGIN
         1 ctagaggatc tcttaatctt gtatgttttt tcatatattg atgtaaaaac tcaatatcct
        61 tagtagctac aaattcttta ataagctcgt gttctttgcg aatacatttg cgttctaaat
       121 ttaaattctt acccttagat cctataaaat gtaaaaacca atctctattg agaatatctc
       181 ttctttttct ctcatcttct tctattaatt ctaatacaca agcactatcc atatctagta
       241 atactctaac acaaagttca agagcatttc catcaaacca gctaatatcc aatccttttt
       301 ctaaacgatt tttttctttt tctagtatgt atgcttctaa atcttttta actatgtcta
       361 tatcatcacc acgagagtat ttatccatag caagatgaaa ttttctatct tctatacaaa
       421 aaagacaagt ttctctttgt ccaaaaggaa gctctaaaag tattttttcg gattctttga
       481 tatcttcttc actttctatt atctctttag taaaatacgc ttcatcttta ctagtatccc
       541 ttaccatttt ctatcctttg ggttttattt tatttgcatc tttatctaaa taatcaatag
       601 tttctttacc tccttcatca acatgaaata aatcttttct gacttcattg ctattaagaa
       661 gtttttttat ttctttggcc tgtttttctc ctacattatc tttaagctgt tttagtttat
       721 tctttattaa ccattcatca ctcatttgct tagtttttct atctaatttg cttgttccaa
       781 acttagcttc tgcgatgata tattttgggt ttttatctgg attataataa attccatcca
       841 tttcatgatg aattttatca tcaagccctt tgactttatt taaactaatt cttttatagc
       901 ctttagcttt gtaatattta tccatttgca tttcagcata attacctttt tgctgattat
       961 tttttgggtt tttaaaccca ctaagattaa attaaatcat aaggaaagta tttgtattgt
```

```
     1021 ttaaattctt ctttatctaa attcattagt tttacaattg ctgcaccttc aagatcccaa
     1081 gtatctagag g
//
LOCUS       ECFEEEMN_17              980 bp    DNA     linear       12-APR-2021
DEFINITION  Campylobacter species strain strain.
ACCESSION
VERSION
KEYWORDS    .
SOURCE      Campylobacter species
  ORGANISM  Campylobacter species
            Unclassified.
COMMENT     Annotated using prokka 1.14.6 from
            https://github.com/tseemann/prokka.
FEATURES             Location/Qualifiers
     source          1..980
                     /organism="Campylobacter species"
                     /mol_type="genomic DNA"
                     /strain="strain"
     gene            complement(12..641)
                     /locus_tag="ECFEEEMN_00073"
     CDS             complement(12..641)
                     /locus_tag="ECFEEEMN_00073"
                     /inference="ab initio prediction:Prodigal:002006"
                     /codon_start=1
                     /transl_table=11
                     /product="hypothetical protein"
                     /protein_id="Prokka:ECFEEEMN_00073"
                     /translation="MSFELIFLFFCSSWFEVVVLSAAIISKVFALSVPSFSLSLAC*G
                     F*RLSIISLCFSTLS*FLCKNSSLPSRLSFSFVLGFFRNSSIFFFNFSSSVITSFKLS
                     SSLSKILVISSAFFPQAFLIPIACCILSSSVNFSFPFKAFKKSSGLCKAKLGFFILAM
                     LL*KVKSIFFSPFFPFLKSVTTKPLSSKGTGSKCNFSFLSKDINLGIKT"
ORIGIN
        1 aaaaaaggaa tttatgtttt aataccaaga ttaatatctt tagataaaaa cgaaaaatta
       61 cactttgatc ctgtgccttt agaagataag ggctttgtgg ttactgattt tagaaaagga
      121 aaaaagggag aaaaaaatat agatttaacc tttcaaagaa gcatagctaa aataaaaaac
      181 cctaatttag ctttgcaaag tcctgaagat tttttaaaag ctttgaatgg gaatgaaaaa
      241 tttacagaag atgataaaat acaacaagcg atgggtatta aaaaagcttg tggaaaaaaa
      301 gcagatgaaa tgactaaaat cttgcttaaa gatgatgaaa gtttaaaaga tgttattaca
      361 gatgaagaaa aattaaagaa aaaaatcgaa gaattacgaa aaaaacccaa aacaaatgaa
      421 aaactaagcc tactaggcag acttgaattt ttacaaagaa atcaagataa agttgaaaaa
      481 cataaagata ttattgacaa tcttcaaaac cctcaacaag ctagtgataa tgaaaaagat
      541 ggtactgata atgcaaatac tttagatata atggcagcag ataacaccac cacctcaaac
      601 caagaagaac aaaaaaacaa aaatatcaat tcaaaagaca taggagatgc aggagaatat
      661 gctgtctcta tgctttttac taaaagatca actaggtatt tatccaatag aagaaaacta
      721 gatgctaatt ttaatactaa aggttttaat catactatac ctgatttttt agtaacctta
      781 aatgattacc ctactttagt agaggttaaa aatatccaag atcaagcttt aacagaacaa
      841 ataagctttg aattaaaact ggctagagaa tatgaacttg attatttatt tatatgcaat
      901 cactatacaa aattggtaga taatataacc aatctaaaag tatttaataa agacaatgaa
      961 aaacacaaag gatcaagaag
//
LOCUS       ECFEEEMN_18              965 bp    DNA     linear       12-APR-2021
DEFINITION  Campylobacter species strain strain.
ACCESSION
VERSION
```

```
KEYWORDS    .
SOURCE      Campylobacter species
  ORGANISM  Campylobacter species
            Unclassified.
COMMENT     Annotated using prokka 1.14.6 from
            https://github.com/tseemann/prokka.
FEATURES             Location/Qualifiers
     source          1..965
                     /organism="Campylobacter species"
                     /mol_type="genomic DNA"
                     /strain="strain"
     gene            complement(637..849)
                     /locus_tag="ECFEEEMN_00074"
     CDS             complement(637..849)
                     /locus_tag="ECFEEEMN_00074"
                     /inference="ab initio prediction:Prodigal:002006"
                     /codon_start=1
                     /transl_table=11
                     /product="hypothetical protein"
                     /protein_id="Prokka:ECFEEEMN_00074"
                     /translation="MTSEQIRKKFNIYWDDKECKKDKNYIEKHSKLLNDAIKKAKESE
                     *GNRNLLY*CQFSYQRISKGLYTGKL"
ORIGIN
        1 tctatatttt tttctccctt ttttcctttt ctaaaatcag taaccacaaa gcccttatct
       61 tctaaaggca caggatcaaa gtgtaatttt tcgtttttat ctaaagatat taatcttggt
      121 attaaaatat aaattccttc ttgaaagcat ttatcaaaaa aatctgctcc aactttggct
      181 atgccattat ttactttaaa gccatcaccg aatcctaatt ctttatttaa acttttatct
      241 ttattgtctt tacttgtata aacatcgtaa aagatatcat agtctttaaa atcatctgta
      301 tttcttgata aaggtttagc ttctctttct tcataggctt catttagcgt attaatatta
      361 ttctcatcaa aataagcatt gattgcatca tttccatgtt catcattgct aaataataca
      421 tttttacttt tttcctcttt ttctctttct ttttctcttt cttttatttt gttttgcaca
      481 gaagttttat taatgcctac agcttcttcc tctattttgt tttttaattc attttctttt
      541 tcaactataa aagaaaattt atttttattt tgttctatcc tgattacttt ttttccattt
      601 tgcgtttgta aatgaaaagg actgtaaaaa cataatttac aattttccag tatataaacc
      661 ttttgaaatt ctttgatatg aaaattgaca tcagtaaagt aaatttctat tccctcattc
      721 actttcttta gccttttttta tagcgtcgtt taaaagtttg gagtgttttt ctatatagtt
      781 tttatctttt ttacactctt tgtcatccca atatatatta aacttctttc tgatttgctc
      841 tgaagtcatt gctcctgcat cttttaaaag ctttttattt ctagatcctt ttgcaatatc
      901 taaaggggtg cttggggtag caaaatttac attggctcca agttcaatta aaagctgtgt
      961 aaccc
//
LOCUS       ECFEEEMN_19              909 bp    DNA     linear       12-APR-2021
DEFINITION  Campylobacter species strain strain.
ACCESSION
VERSION
KEYWORDS    .
SOURCE      Campylobacter species
  ORGANISM  Campylobacter species
            Unclassified.
COMMENT     Annotated using prokka 1.14.6 from
            https://github.com/tseemann/prokka.
FEATURES             Location/Qualifiers
     source          1..909
                     /organism="Campylobacter species"
```

```
                     /mol_type="genomic DNA"
                     /strain="strain"
     gene            29..160
                     /locus_tag="ECFEEEMN_00075"
     CDS             29..160
                     /locus_tag="ECFEEEMN_00075"
                     /inference="ab initio prediction:Prodigal:002006"
                     /inference="similar to AA sequence:RefSeq:CKG87616.1"
                     /note="Domain of uncharacterised function (DUF1910)"
                     /codon_start=1
                     /transl_table=11
                     /product="hypothetical protein"
                     /protein_id="Prokka:ECFEEEMN_00075"
                     /translation="MKKHTRLRDPLDTWDLEGAAIVKLMNLDKEEFKSYKYFPYDLI"
ORIGIN
        1 taaggatatt gagtttttac atcaatatat gaaaaaacat acaagattaa gagatcctct
       61 agatacttgg gatcttgaag gtgcagcaat tgtaaaacta atgaatttag ataaagaaga
      121 atttaaaagc tataaatatt ttccttatga tttaatttaa tcttatttgg ataacagaaa
      181 agagtagaga tagacttggc attgtagata aaaaacatag ggaaaaaata gaagaacttc
      241 ttgaaaaaaa tagcaatgaa gtcagaaaag atttatttca tgttgatgaa ggaggtaaag
      301 aaactattga ttatttagat aaagatgcaa ataaaataaa acccaaagga taaaaaatgc
      361 taagagatac taagagagat gaagcgtatt ttacgaaaca tataatagag tgtgaagaag
      421 aaattaaaga agatgaaaaa atacttttag agcttccttt tggaaaaaga gaaacttgtc
      481 ttttttgtat aacagataga aaaaaatgta ttgctatgga taaatactct cgtggtgatg
      541 atataaatct agttaaaaaa gatttagaag cacttatgct attaagacaa aaaaatcgtt
      601 tagaattagg tggatttgat actagcgatt atagaggaaa tgctattgaa ctttgtgtta
      661 gagtattatt agatatggat agtgcttgtg tattagaatt aatagaagaa gatgagagaa
      721 agaaaaaaga tatttttaat agagattggt ttttacattt tatgggttct aagggtaaaa
      781 acttaaattt agaacgcaaa tgtaattgta aagaacacga gcttattaaa gaatttgtag
      841 ctactaagga tattgagttt ttacatcaat atatgaaaaa acatacaaga ttaagagatc
      901 ctctagata
//
LOCUS       ECFEEEMN_20              897 bp    DNA     linear       12-APR-2021
DEFINITION  Campylobacter species strain strain.
ACCESSION
VERSION
KEYWORDS    .
SOURCE      Campylobacter species
  ORGANISM  Campylobacter species
            Unclassified.
COMMENT     Annotated using prokka 1.14.6 from
            https://github.com/tseemann/prokka.
FEATURES             Location/Qualifiers
     source          1..897
                     /organism="Campylobacter species"
                     /mol_type="genomic DNA"
                     /strain="strain"
     gene            7..576
                     /gene="trxB_4"
                     /locus_tag="ECFEEEMN_00076"
     CDS             7..576
                     /gene="trxB_4"
                     /locus_tag="ECFEEEMN_00076"
                     /inference="ab initio prediction:Prodigal:002006"
```

```
                     /inference="similar to AA sequence:RefSeq:CKG86288.1"
                     /codon_start=1
                     /transl_table=11
                     /product="thioredoxin reductase"
                     /protein_id="Prokka:ECFEEEMN_00076"
                     /translation="MHKNPLREQQIKTLAKTLKPDEFGMLPFIDEIMGVDWVIDLNKY
                     DIASDQFGSMYKALRSDVVEGKIKDPRDIDSTYESRREFDRHRGGYKNGMLQGYGTDT
                     PNDWSEERAQLFNDTLILHAKLAALTPPQGYPNAPYYFTPENLEWYYKRHKLDKLLDP
                     RIPAIYRYNFPRELRAKILKFGEENGIKE"
ORIGIN
        1 gtaggagtgc ataaaaatcc tttaagagaa caacaaatta aaaccttagc taagacttta
       61 aaacctgatg aatttggtat gcttcctttt atagatgaga ttatgggagt agattgggtg
      121 attgatttaa ataaatatga tatagctagt gatcaatttg gaagtatgta taaagcctta
      181 agaagtgatg tggttgaggg taagataaaa gatccaaggg atatagattc tacttatgaa
      241 agcagaaggg aatttgatcg ccatagaggt ggatataaaa atggcatgct gcaaggctat
      301 ggaaccgata cacctaatga ttggagtgaa gaaagggcac aactttttaa tgatacttta
      361 atacttcatg ccaaactcgc agccctcact cctcctcaag gctatcctaa tgcaccttat
      421 tattttaccc ctgaaaacct agaatggtat tataaaagac ataagcttga taaactcctt
      481 gatccaagaa tccctgccat ttatagatat aatttcccaa gggaacttag agctaagata
      541 ctcaaatttg gcgaagaaaa tggcattaaa gaataaagta attacctagg cagattagcc
      601 actactataa tcatggaaga tggtttatat atgggataaa ttaagaaagg ataaaagatg
      661 agtgataata aagatattta cgagatctat acttctaatg gacttatatt agaagttgat
      721 aaaaatacaa atcaaattat atttgataaa agagaagatg ggcgtgaagt aggcaaatac
      781 acccaagaat attctaaagc tttgtttgaa gccgatagaa ttttaagaac ctctccttat
      841 attaattatc aacctaggta tctagatcct gaatttcaca cagggcaaaa atccact
//
LOCUS       ECFEEEMN_21              869 bp    DNA     linear       12-APR-2021
DEFINITION  Campylobacter species strain strain.
ACCESSION
VERSION
KEYWORDS    .
SOURCE      Campylobacter species
  ORGANISM  Campylobacter species
            Unclassified.
COMMENT     Annotated using prokka 1.14.6 from
            https://github.com/tseemann/prokka.
FEATURES             Location/Qualifiers
     source          1..869
                     /organism="Campylobacter species"
                     /mol_type="genomic DNA"
                     /strain="strain"
     gene            68..565
                     /gene="trxB_5"
                     /locus_tag="ECFEEEMN_00077"
     CDS             68..565
                     /gene="trxB_5"
                     /locus_tag="ECFEEEMN_00077"
                     /inference="ab initio prediction:Prodigal:002006"
                     /inference="similar to AA sequence:RefSeq:CKG86288.1"
                     /codon_start=1
                     /transl_table=11
                     /product="thioredoxin reductase"
                     /protein_id="Prokka:ECFEEEMN_00077"
                     /translation="MLPFIDEIMGVDWVIDLNKYRLANDEFGDIYKALRSDVVEGKIK
```

```
                     DPRDIDSTYESRREFDRYRVGYRKGMRQGYETDTPNDWSEERAQLFNDTLILHAKLAA
                     LTPPQGYPNAPYYFTPEKLEWYYKRHKLDKLLDPRIPAIYRYNFPRELRAKILKFGEE
                     NGIKE"
ORIGIN
        1 taaaaatcct ttaagagaac aacaaattaa aaccttagct aagactttaa aacctgatga
       61 atttggtatg cttcctttta tagatgagat tatgggagta gattgggtga ttgatttaaa
      121 taaatataga ttagctaatg atgaatttgg ggatatatat aaagccttaa gaagtgatgt
      181 agttgagggc aagataaaag atccaagaga tatagattct acttatgaaa gcagaaggga
      241 atttgatcgt tatagagttg gatatagaaa gggtatgagg caaggttatg aaaccgatac
      301 acccaatgat tggagtgaag aaagggcaca actttttaat gatactttaa tacttcatgc
      361 caaactcgca gccctcactc ctcctcaagg ctatcctaat gcaccttatt attttacccc
      421 tgaaaaacta gaatggtatt ataaaagaca taagcttgat aaactccttg atccaagaat
      481 ccctgctatt tatagatata atttcccaag ggaacttaga gctaagatac tcaaatttgg
      541 cgaagaaaat ggcattaaag aataaagtaa ttacataggc agattagcta caactataat
      601 catggaagat ggtttatata tgggataaat taagaaagga taaaagatga gtgaaactta
      661 tgaaatttat acacctaatg gtattatttt agatgtagaa aaagaaacga ataaaatttt
      721 attaagtgat gggggagcta aggtaggcaa atacacccaa gaatattcta aagctttgtt
      781 tgaagccgat agaattttaa gaacctctcc ttatattaat tatcaaccta ggtatctaga
      841 tcctgaattt cacacagggc aaaaatcca
//
LOCUS       ECFEEEMN_22              859 bp    DNA     linear       12-APR-2021
DEFINITION  Campylobacter species strain strain.
ACCESSION
VERSION
KEYWORDS    .
SOURCE      Campylobacter species
  ORGANISM  Campylobacter species
            Unclassified.
COMMENT     Annotated using prokka 1.14.6 from
            https://github.com/tseemann/prokka.
FEATURES             Location/Qualifiers
     source          1..859
                     /organism="Campylobacter species"
                     /mol_type="genomic DNA"
                     /strain="strain"
     gene            2..571
                     /gene="trxB_6"
                     /locus_tag="ECFEEEMN_00078"
     CDS             2..571
                     /gene="trxB_6"
                     /locus_tag="ECFEEEMN_00078"
                     /inference="ab initio prediction:Prodigal:002006"
                     /inference="similar to AA sequence:RefSeq:CKG86288.1"
                     /codon_start=1
                     /transl_table=11
                     /product="thioredoxin reductase"
                     /protein_id="Prokka:ECFEEEMN_00078"
                     /translation="MHKNPLREQQIKTLAKTLKPDEFGMLPFIDEIMGVDWVIDLNVY
                     QFNFDAWGKIYRALRSDVVEGKIKDPRDVDSTYESRREFDRYMDGYQNGMTINYHVDI
                     NNYRSEESAKLFMDSLVLEAKLAALTPPQGYPNAPYYFTPERLEWDYKNYQLDKLLDP
                     RIPAIYRYNFPRELRAKILKFGEENGIKE"
ORIGIN
        1 agtgcataaa aatcctttaa gagaacaaca aattaaaacc ttagctaaga ctttaaaacc
       61 tgatgaattt ggtatgcttc cttttataga tgagattatg ggagtagatt gggtgattga
```

```
      121 tttaaatgtt tatcaattta attttgatgc atggggaaaa atatatagag ccttaagaag
      181 tgatgtagtt gagggcaaga taaaagatcc aagggatgta gattctactt atgaaagcag
      241 aagggagttt gatcgttata tggatggata tcaaaatggt atgacaatta attaccatgt
      301 agatataaat aattatagaa gcgaagaatc agctaaatta tttatggatt ctttggtatt
      361 agaggccaaa ctcgcagccc tcactcctcc acaaggctat cctaatgcac cttattattt
      421 tacccctgaa agactagaat gggattataa aaattatcaa ttggataaac tccttgatcc
      481 aagaatccct gctatttata gatataattt cccaagggaa cttagagcta agatactaaa
      541 atttggtgaa gaaaatggca ttaaagaata aagtaattac ataggcagat tagccactac
      601 tataatcatg gaagatggtt tatatatggg ataaattaag aaaggataaa agatgagtga
      661 aacttatgaa atttatacac ctaatggtgg tatattagat gtggaaaaag aaacgaataa
      721 aattttactc tatgatgggg gagctaaggt aggcaaatac acccaagaat attctaaagc
      781 tttgtttgaa gccgatagaa ttttaagaac ctctccttat attaattatc aacctaggta
      841 tctagatcct gaatttcac
//
LOCUS       ECFEEEMN_23              835 bp    DNA     linear       12-APR-2021
DEFINITION  Campylobacter species strain strain.
ACCESSION
VERSION
KEYWORDS    .
SOURCE      Campylobacter species
  ORGANISM  Campylobacter species
            Unclassified.
COMMENT     Annotated using prokka 1.14.6 from
            https://github.com/tseemann/prokka.
FEATURES             Location/Qualifiers
     source          1..835
                     /organism="Campylobacter species"
                     /mol_type="genomic DNA"
                     /strain="strain"
     gene            145..639
                     /locus_tag="ECFEEEMN_00079"
     CDS             145..639
                     /locus_tag="ECFEEEMN_00079"
                     /inference="ab initio prediction:Prodigal:002006"
                     /inference="similar to AA sequence:RefSeq:CKG87598.1"
                     /codon_start=1
                     /transl_table=11
                     /product="methyltransferase small"
                     /protein_id="Prokka:ECFEEEMN_00079"
                     /translation="MKKKIQELKSINNLSTKQKGNYGEAKMDLYYLKQGYKRISLNKV
                     KGLDDKIHHGIDGIYHNPDKNPKYIIAEAKFGKGDLKKTGKYKQMSDEWITEESRNRL
                     GVVDKKHRKKIEELLKENNNEVRKDLFHVDKKGNVSIKELDKNAKTKNLIERIENGKG
                     YEER"
ORIGIN
        1 ggaggtaaaa gaagtaagaa aaataaaaat gatagagcaa agcaaataca agcattaaaa
       61 aataatcagc aaaacaacaa acaaaattca tttttacaag aagttgaaca acaaaaagaa
      121 gaggctgaaa aagttttagc agatatgaaa aaaaagatac aagaattaaa aagtattaat
      181 aacttaagca caaaacaaaa aggtaattat ggcgaagcta aaatggattt gtattattta
      241 aaacaaggct ataaaagaat tagtttaaat aaagtcaaag ggcttgatga taaaattcat
      301 catggaattg atggaattta tcataatcca gataaaaacc caaaatatat catcgcagaa
      361 gctaagtttg gaaaaggtga tttaaaaaaa actggaaaat acaaacaaat gagtgatgaa
      421 tggataacag aagagagtag aaatagactt ggtgttgtag ataaaaaaca taggaaaaaa
      481 atagaagaac ttcttaagga aaataacaat gaagtcagaa aagatttatt tcatgttgat
      541 aaaaaaggca atgtgagtat taaagaatta gataaaaatg caaaaaccaa aaacttaata
```

```
      601 gaaaggatag aaaatggtaa gggatacgaa gagagatgaa gcgtatttta cgaaacatat
      661 aatagagtgt gaaaaaaaaa tcaaagaaga tgaaaaaaga ctcttagaac ttccactagg
      721 agatgcaaga agggaaaatt atttttttc tataacagat aaaaaaaaat gtattgctat
      781 ggataaatac tctcgtggcg atgatataaa tccagttaaa aaagatttag aagca
//
LOCUS       ECFEEEMN_24              730 bp    DNA     linear       12-APR-2021
DEFINITION  Campylobacter species strain strain.
ACCESSION
VERSION
KEYWORDS    .
SOURCE      Campylobacter species
  ORGANISM  Campylobacter species
            Unclassified.
COMMENT     Annotated using prokka 1.14.6 from
            https://github.com/tseemann/prokka.
FEATURES             Location/Qualifiers
     source          1..730
                     /organism="Campylobacter species"
                     /mol_type="genomic DNA"
                     /strain="strain"
ORIGIN
        1 aatcaagttc atattctcta gccagtttta attcaaagct tatttgttct gttaaagctt
       61 gatcttggat atttttaacc tctactaaag tagggtaatc atttaaggtt actaaaaaat
      121 caggtatggt atgattaaaa cctttagtat taaaattagc atcaagtttt cttctattgg
      181 ataaatacct agttgatctt ttagtaaaaa gcatagagac ggcatattct cctgcatctc
      241 ctatatcttt tgaattgatg gttttatttt tttgttcttc ttggtttgag gtggtgttat
      301 tcgttttgtt atctgaaaag tcatcatcag caccatcttt ttcattatca ctagcttgtt
      361 gagggttttg aagattgtca ataatatctt tatgttcttt aaccttttt tgatttcttt
      421 gtaaaaattc aagtctacct agtaggctta gtttttatt tgttttgggt tctcctcgca
      481 attcttcgat tttttctct aattttttt catctctaat aatatctttt aaattttcat
      541 catctctaat aatatctttt aatttttctt catctctaag taatattttg gttatttcat
      601 ctacttttt tccacaagct tttttaatac ccatcgcttg ttgtatttta tcatcttctg
      661 taaatttttc attcccattc aaagctttta aaaaatcttc aggactttgc aaagctaaat
      721 tagggttttt
//
LOCUS       ECFEEEMN_25              701 bp    DNA     linear       12-APR-2021
DEFINITION  Campylobacter species strain strain.
ACCESSION
VERSION
KEYWORDS    .
SOURCE      Campylobacter species
  ORGANISM  Campylobacter species
            Unclassified.
COMMENT     Annotated using prokka 1.14.6 from
            https://github.com/tseemann/prokka.
FEATURES             Location/Qualifiers
     source          1..701
                     /organism="Campylobacter species"
                     /mol_type="genomic DNA"
                     /strain="strain"
ORIGIN
        1 ctccttacag gctccaccga actccctaat aatccttttt actttggaga attagatcaa
       61 gataatacta taaaacaaga tatacccagt tattattttt ctcctaaaga tgaaagcagt
      121 ggtttaggaa gacttagcat cttttataaa aatgatgaac tttgcttgct taattattct
```

```
      181 atattagaca attctttaaa tatcaaatta gaatgcttaa gcaaacaaag cttagaatac
      241 aaagatttaa tttcaaacac tctaaaagaa caaaaaacca cacaagtaga taaaaaacaa
      301 agcatagcca aacttcacgc tcttttagaa aatcaaaacc tagaatgtat ccatggaggt
      361 aaagtcatac ttaaatcaaa caaaggaaaa tcctttaaaa gtgatggtat ccctataatg
      421 ctagaaagtg atttattaaa ctctagtata gtagcttgtc ctcacaccat agcaaatgta
      481 agttatcctt gtactaaggt agtggatatt aaaggctcct tatctcaaaa gaaagtaaat
      541 aatgaatttg taatcttaca agaactcatc tcagcttgca ctacagataa aggctttgct
      601 ttaaaagtaa gctttactcc aagtaagttt aaatttgatc acagctttga tcctgaggag
      661 ggcttaggag aacaaagtaa aaatcaaata gagttaaaag a
//
LOCUS       ECFEEEMN_26              684 bp    DNA     linear       12-APR-2021
DEFINITION  Campylobacter species strain strain.
ACCESSION
VERSION
KEYWORDS    .
SOURCE      Campylobacter species
  ORGANISM  Campylobacter species
            Unclassified.
COMMENT     Annotated using prokka 1.14.6 from
            https://github.com/tseemann/prokka.
FEATURES             Location/Qualifiers
     source          1..684
                     /organism="Campylobacter species"
                     /mol_type="genomic DNA"
                     /strain="strain"
     gene            complement(218..394)
                     /locus_tag="ECFEEEMN_00080"
     CDS             complement(218..394)
                     /locus_tag="ECFEEEMN_00080"
                     /inference="ab initio prediction:Prodigal:002006"
                     /codon_start=1
                     /transl_table=11
                     /product="hypothetical protein"
                     /protein_id="Prokka:ECFEEEMN_00080"
                     /translation="MSKKFIA*VLALNPVMESCKAPINPFISPSIPAANSHSAIKSQP
                     LCILAIPLLASTSL"
ORIGIN
        1 ggcaaagcat atatttaaaa gatcctatca aaggagctat agctccttgg actaaagcag
       61 aaaaagctta ttataaatct ttaaaaacta aaagagaaag atataaatat ttaattataa
      121 gaagtggact tagaagtact gttatagata taccttatga tgcttattgt aatgtagatg
      181 aaaaaggaaa tttaatcaat aaagactata aagaactcta taaagaagta gaagctaata
      241 gaggtatggc taatatgcat aaaggttggc tctttatggc tgaatgggaa ttagcagcag
      301 gaatactagg agatataaaa ggatttatag gagccttaca actttccatg acaggattta
      361 aagcaagaac tcaagcaata aattttttac tcatacaact aggacatgaa caaggtttta
      421 aaagtcttta tgattcttat gcatatagag gtttaacaga tggtatacac aaaaactctc
      481 ttaaagctca aatgctaaaa gactttgcta aaaatcctcc ttatgatgaa tttggaatgc
      541 tacctttctt agatgagctt ataggagtgg attgggtgat tgatttgaat gaatatcaat
      601 ttgcttatga tggtgatggc ggtagtgtta aggctttaga tgagaaagta agacaaggta
      661 aattaaaaga tccaagagat atag
//
LOCUS       ECFEEEMN_27              614 bp    DNA     linear       12-APR-2021
DEFINITION  Campylobacter species strain strain.
ACCESSION
VERSION
```

```
KEYWORDS    .
SOURCE      Campylobacter species
  ORGANISM  Campylobacter species
            Unclassified.
COMMENT     Annotated using prokka 1.14.6 from
            https://github.com/tseemann/prokka.
FEATURES             Location/Qualifiers
     source          1..614
                     /organism="Campylobacter species"
                     /mol_type="genomic DNA"
                     /strain="strain"
ORIGIN
        1 atctagaatg gtattataaa agacataagc ttgataaact ccttgatcca agaatccctg
       61 ctatttatag atataatttc ccaagggaac ttagagctaa gatactcaaa tttggcgaag
      121 aaaatggtat taaagaataa agtaattaca taggcagatt agccactact ataatcatgg
      181 aagatggttt atatatggga taaattaaga aaggataaaa atgagtgata ataaagatat
      241 ttacgagatc tatacttcta atggacttat attagaagtt gataaaaata caaatcaaat
      301 tatatttgat aaaagaaaag atgggcgtga agtaggcaaa tacacccaag aatattctag
      361 agctcttttt gaagcacaca atatcaaaca aaactctccc tacaaagact atcaacctag
      421 gtatctagat cctgaatttc acacagggca aaaatccact ttgcttgaat ttaaagattg
      481 gcaaagcata tatttaaaag atcccattaa aggaagcata gctccttgga ctaaagcaga
      541 aaaagcttat tataactcac taaaaactaa aaaagaaaga tataaatatt tagttataag
      601 aagtggtata agaa
//
LOCUS       ECFEEEMN_28              586 bp    DNA     linear       12-APR-2021
DEFINITION  Campylobacter species strain strain.
ACCESSION
VERSION
KEYWORDS    .
SOURCE      Campylobacter species
  ORGANISM  Campylobacter species
            Unclassified.
COMMENT     Annotated using prokka 1.14.6 from
            https://github.com/tseemann/prokka.
FEATURES             Location/Qualifiers
     source          1..586
                     /organism="Campylobacter species"
                     /mol_type="genomic DNA"
                     /strain="strain"
ORIGIN
        1 aatcaatcac ccaatctact cccataatct catctataaa aggaagcata ccaaattcat
       61 caggttttaa agtcttagct aaggttttaa tttgttgttc tcttaaagga tttttatgca
      121 ctcctactaa accacttcct actgcaatat ctgcataacc ataaatagca cccaataaat
      181 taggtttatc tagtatacta cttcctccgc ttaattgtat ataaagtata gttgcttgga
      241 taaatcttgc attaaaacca ttttgagaca tatcgttagc tagatattta taatcaccca
      301 gtattcctgc tgccataccc cattcattat gaaataaact tgatcttaga ttatgtttat
      361 tatcatctac tgttctatat aattcttcat acttaggatc tacatttcct ttttcatcta
      421 cagctcctat agcttcataa ggtatatcta taacaacact tcttatacca cttcttataa
      481 ctaaatattt atatctttct tttttagttt ttagtgagtt ataataagct ttttctgctt
      541 tagtccaagg agctatgctt cctttaatgg gatcttttaa atatat
//
LOCUS       ECFEEEMN_29              537 bp    DNA     linear       12-APR-2021
DEFINITION  Campylobacter species strain strain.
ACCESSION
```

```
VERSION
KEYWORDS    .
SOURCE      Campylobacter species
  ORGANISM  Campylobacter species
            Unclassified.
COMMENT     Annotated using prokka 1.14.6 from
            https://github.com/tseemann/prokka.
FEATURES             Location/Qualifiers
     source          1..537
                     /organism="Campylobacter species"
                     /mol_type="genomic DNA"
                     /strain="strain"
ORIGIN
        1 agaacaacaa attaaaacct tagctaagac tttaaaacct gatgaatttg gtatgcttcc
       61 ttttatagat gagattatgg gagtagattg ggtgattgat ttaaataaat atgatatagc
      121 tagtgatgaa tctggaaaaa tatacaaagc cttaagaagt gatgtagttg agggcaagat
      181 aaaagatcca agagatatag attctactta tgaaagcaga agggaatttg atcgccatag
      241 aggtgggtat aaaaatggca tgagggaagg ctatgaaacc gatacaccca atgattggag
      301 cgaagaagaa gcacaacttt ttaatgatac tttaatactt catgccaaac tagcagccct
      361 cactcctcct caaggctatc ctaatgcacc ttattatttt acccctgaaa atctagaatg
      421 gtattataaa agacataagc ttgataaact ccttgatcca agaatccctg ctatttatag
      481 atataatttc ccaagggaac ttagagctaa gatactcaaa tttggcgaag aaaatgg
//
LOCUS       ECFEEEMN_30              518 bp    DNA     linear       12-APR-2021
DEFINITION  Campylobacter species strain strain.
ACCESSION
VERSION
KEYWORDS    .
SOURCE      Campylobacter species
  ORGANISM  Campylobacter species
            Unclassified.
COMMENT     Annotated using prokka 1.14.6 from
            https://github.com/tseemann/prokka.
FEATURES             Location/Qualifiers
     source          1..518
                     /organism="Campylobacter species"
                     /mol_type="genomic DNA"
                     /strain="strain"
ORIGIN
        1 cccttgggaa attatatcta taaatagcag ggattcttgg atcaaggagt ttatcaagct
       61 tatgtctttt ataataccat tctagatttt caggggtaaa ataataaggt gcattaggat
      121 agccttgtgg aggagtgagg gctgcgagtt tggcagttaa tatcatagaa tctattctta
      181 attgtgcact ttcttcactc cgatcatttg gaatatcata accataagca tttaccatgc
      241 cgttataata tcctccccta tggcgatcaa attcccttct gctttcataa gtagaatcta
      301 tatctcttgg atcttttatc ttgccctcaa ctacatcact tcttaaggct ttatacatac
      361 ttccaaattc atcgcgagca attctatacc tgttataatc aatcacccaa tctactccca
      421 taatctcatc tataaaagga agcataccaa attcatcagg ttttaaagtc ttagctaagg
      481 ttttaatttg ttgttctctt aaaggatttt tatgcact
//
LOCUS       ECFEEEMN_31              518 bp    DNA     linear       12-APR-2021
DEFINITION  Campylobacter species strain strain.
ACCESSION
VERSION
KEYWORDS    .
```

```
SOURCE      Campylobacter species
  ORGANISM  Campylobacter species
            Unclassified.
COMMENT     Annotated using prokka 1.14.6 from
            https://github.com/tseemann/prokka.
FEATURES             Location/Qualifiers
     source          1..518
                     /organism="Campylobacter species"
                     /mol_type="genomic DNA"
                     /strain="strain"
ORIGIN
        1 taagttccct tgggaaatta tatctataaa tagcagggat tcttggatca aggagtttat
       61 caagcttatg tctttttataa taccattcta gtttttcagg ggtaaaataa taaggtgcat
      121 taggataacc ttgtggagga gtgagggctg cgagtttggc agttaatatc atagaatcta
      181 tttttaattg cgcatcattc caatcatttg gaatatcata accataggca ttaatcatgc
      241 cattagaata accatccata taataatcaa actcccttct gctttcataa gtagaatcta
      301 tatcccttgg atcttttatc ttgccctcaa ctacatcact tcttaaggct ttatatatat
      361 ccccaaattc atcttcagaa atttgatatt cgttataatc aatcacccaa tctactccca
      421 taatctcatc tataaaagga agcataccaa attcatcagg ttttaaagtc ttagctaagg
      481 ttttaatttg ttgttctctt aaaggatttt tatgcact
//
LOCUS       ECFEEEMN_32              514 bp    DNA     linear       12-APR-2021
DEFINITION  Campylobacter species strain strain.
ACCESSION
VERSION
KEYWORDS    .
SOURCE      Campylobacter species
  ORGANISM  Campylobacter species
            Unclassified.
COMMENT     Annotated using prokka 1.14.6 from
            https://github.com/tseemann/prokka.
FEATURES             Location/Qualifiers
     source          1..514
                     /organism="Campylobacter species"
                     /mol_type="genomic DNA"
                     /strain="strain"
     gene            complement(231..380)
                     /locus_tag="ECFEEEMN_00081"
     CDS             complement(231..380)
                     /locus_tag="ECFEEEMN_00081"
                     /inference="ab initio prediction:Prodigal:002006"
                     /codon_start=1
                     /transl_table=11
                     /product="hypothetical protein"
                     /protein_id="Prokka:ECFEEEMN_00081"
                     /translation="MALILSKQSFVGSSPSLNKPGSRYSLTLSPKVYITISPGFDMDG
                     SDELR"
ORIGIN
        1 cttttatgtt ttgcgcttta tttgtttcct agattattta atcctagcaa agaaatagat
       61 tataaatggt ttatagtttt ttttacattt cttttagtta tatatgcact tgcacaaaag
      121 atctataaaa tggcaaatat taaaagaatt tatgtcacaa aagaaaaact tgtggttgag
      181 tattatatca aaaatgattt ggtttttcct ttaggaactt tttttgtata ttatcgtagt
      241 tcgtcacttc catccatatc aaatccagga gatatagtga tatacacctt cggagataaa
      301 gtcaaagaat accttgaacc tggtttattc agtgatggcg atgatccaac aaaagattgt
```

```
      361 tttgaaagaa ttaatgccat tataaagcct catgtaatgc cttatttatt aagcttaagt
      421 gatgaagaat ttgaaaaaat catttcacat gtaagcggtt ataacgaaat tggtaccact
      481 ttccttaaag aagcaatgga actcagaaaa gaga
//
LOCUS       ECFEEEMN_33              507 bp    DNA     linear       12-APR-2021
DEFINITION  Campylobacter species strain strain.
ACCESSION
VERSION
KEYWORDS    .
SOURCE      Campylobacter species
  ORGANISM  Campylobacter species
            Unclassified.
COMMENT     Annotated using prokka 1.14.6 from
            https://github.com/tseemann/prokka.
FEATURES             Location/Qualifiers
     source          1..507
                     /organism="Campylobacter species"
                     /mol_type="genomic DNA"
                     /strain="strain"
     gene            complement(301..441)
                     /locus_tag="ECFEEEMN_00082"
     CDS             complement(301..441)
                     /locus_tag="ECFEEEMN_00082"
                     /inference="ab initio prediction:Prodigal:002006"
                     /inference="similar to AA sequence:RefSeq:CKG86746.1"
                     /codon_start=1
                     /transl_table=11
                     /product="hypothetical protein"
                     /protein_id="Prokka:ECFEEEMN_00082"
                     /translation="MWYMKIFKDYQKFTPYGLDFRLYSKDFIAKLDFKDKAYNVLYNT
                     GV"
ORIGIN
        1 cgctttatat tcttgtggtg acaattcttt agcttctaga gagctaagca atgcaagatc
       61 cttagaagtt tcaataccat aggtaagata aaatatgata acaatagctg taaaaaagat
      121 aataaaagat attaaaaaat aagattttac aaattctttt atattaatct tgatccaata
      181 atttaaaata gaaaagacaa taagaacgct taaagtggta agataaaatt ttatattagt
      241 aaaagcaaga ataaatccaa ataaacccca aaaaatagaa aatgtaatat cttctcccat
      301 ttaaactcct gtattataaa gcacattata agctttatct ttaaaatcaa gtttggcaat
      361 aaaatcttta ctataaagtc taaaatccaa accataagga gtgaattttt gatagtcttt
      421 aaagattttc atataccaca tacacactcg caattaatat aggagcatta cacacaaacc
      481 ctataaacca acttaatttt ttgcgtt
//

>>BfR-CA-15863_min_cov_30 putative plasmid location

LOCUS       CIKGKLNE_1              5012 bp    DNA     linear       12-APR-2021
DEFINITION  Campylobacter species strain strain.
ACCESSION
VERSION
KEYWORDS    .
SOURCE      Campylobacter species
  ORGANISM  Campylobacter species
            Unclassified.
COMMENT     Annotated using prokka 1.14.6 from
```

```
                     https://github.com/tseemann/prokka.
FEATURES             Location/Qualifiers
     source          1..5012
                     /organism="Campylobacter species"
                     /mol_type="genomic DNA"
                     /strain="strain"
     gene            complement(474..1034)
                     /locus_tag="CIKGKLNE_00001"
     CDS             complement(474..1034)
                     /locus_tag="CIKGKLNE_00001"
                     /inference="ab initio prediction:Prodigal:002006"
                     /codon_start=1
                     /transl_table=11
                     /product="hypothetical protein"
                     /protein_id="Prokka:CIKGKLNE_00001"
                     /translation="MTYILLLIIISTILSYLILKFIYKIIFKSTKSVSKFLVFLGSIG
                     LIIFYYTPYSYYLEPSYWQFRNMCKLNELPNNEEKYNKILGYFDKKLGDIDDFRHVKK
                     YGSIEMWLYIYYDDTYKTPSNFQEKIEMDNIALKTKNMQMSLHKIVDMHISPYWNTRR
                     YVLEGNEGNMNFEFIEDELACGNLYQ"
     gene            complement(1261..2034)
                     /locus_tag="CIKGKLNE_00002"
     CDS             complement(1261..2034)
                     /locus_tag="CIKGKLNE_00002"
                     /inference="ab initio prediction:Prodigal:002006"
                     /inference="similar to AA sequence:RefSeq:AJK71138.1"
                     /codon_start=1
                     /transl_table=11
                     /product="glutamyl-tRNA amidotransferase"
                     /protein_id="Prokka:CIKGKLNE_00002"
                     /translation="MKEINEEKKALSLLLDSNFDGLKNNKIIDYSLELLLLSYRLSKI
                     SSMDTSNINQLRETLINKILDITAKLSMCKEYDEKEIIKFKYCLCVFIDESLMKNELF
                     INFWAHNTLTVRLFDETLGGNNFYDIASSWINNPFKFKDFLEFIYACLILGYKGKYNE
                     AKDRDEKIIHFCNNIATSLRPVYKTEEELAFNKAYKIGLEENIWQKFIRLYFKKLIII
                     VPVLIVLGVLSFAIFNLETNNLKVDNNISVLIKNLTHIE"
     gene            complement(2031..2612)
                     /locus_tag="CIKGKLNE_00003"
     CDS             complement(2031..2612)
                     /locus_tag="CIKGKLNE_00003"
                     /inference="ab initio prediction:Prodigal:002006"
                     /inference="similar to AA sequence:RefSeq:AJK71139.1"
                     /codon_start=1
                     /transl_table=11
                     /product="type VI secretion protein"
                     /protein_id="Prokka:CIKGKLNE_00003"
                     /translation="MSMSIKIHPEFLYEKFLEFQGELAAFSNEESFLDFIPYKHDNLY
                     NTFLNMMNNLRLLFSKITSPKYFIAKISINGNGFYDFIFDNSGILEDAEIYFAIRADV
                     TTEYLLNNFKIQSKIHTQSKIKNIVATQLEGLNVDQVPNVPSSIPYLNGYVVYYKIDKR
                     DQLFKDFKNESIISMYLTNNIKNPDIIMWAVFQ"
     gene            2622..4835
                     /locus_tag="CIKGKLNE_00004"
     CDS             2622..4835
                     /locus_tag="CIKGKLNE_00004"
                     /inference="ab initio prediction:Prodigal:002006"
                     /inference="similar to AA sequence:RefSeq:AVS37497.1"
```

```
                     /codon_start=1
                     /transl_table=11
                     /product="type VI secretion protein VgrG"
                     /protein_id="Prokka:CIKGKLNE_00004"
                     /translation="MIKQTLGFYQDILHKEIDYSHIHFNYEEQELISQYNESDLEFIT
                     RLAHNNGIFFYEDENTIYFCDVYKNVKNKEIEYNPNVNNILNQACISSIYKEQSLRTN
                     AFTHSSINANTPLNLLSLHSSKVPYEQELNNKAYYNEHFYESEYSFTRSIDLKTKPSL
                     KEKRALILNESLLAKSNIYHLSLGDFITLNYKEFNLSGLSDDEDTKSQDEEKQDKASL
                     LKDFIIIANTQILIDDALLANSINTNDHLNLKDLNLSKSYSNTLTLLKKNIIFTPSFK
                     AKPKAPHNTQGIVIGESKDIESERNTIYTDEYGRVKVRINLYANQEELDNNLLSANIK
                     TNTHENTNNTDSKDNTNFTNSSNHLSSNAYKSYHHTPFLRVATSIASNHSGFFHTPRV
                     GDEVIVSFLDDDIDKPYVSSSLYNGATTMPQYNYPRYKREIATNALSYLSVAPAMIDK
                     GLDTLKLQENTDYELSSINNHYLTLANSTIGVDNADARARNEITLKNDKDKEEIYILA
                     QKDYKEEIGNNYEQTIKNNKTSEVGALYTEFITLGHMQNIIGFKNVNVGAEYLENTLL
                     SKDTNVGLNNTLNVGISNEVNIGQNHEEKIGNDKRVIINNNLEQDIKNDFIQRIGHNK
                     NETIKGSYVLQTNQSIKFYSKQDLSIETNDCFKAEADTFVSFKAKKNCSFTADEVYTM
                     ANKESFLTAQKQIVSRVGNTTITQTKDKIILQVGTTQVIIDSKGLRVQGGDLRAD"
ORIGIN
        1 aaatataaaa taatagaaaa tataaaaaca aaaaagaaaa atataagaat aaatattaaa
       61 agattaacaa agaattctag catttattaa ctcctcttaa ttttctctgt cttttcataa
      121 gacaaagcag tgctaaaacc acttgtagtg ttggactgat gaaatttaat ctcatatctt
      181 tgagaaaaat ttctagcttg taattctcta aaatctccat ttagtttcgc ataaaaaaat
      241 aaagaatcat ctataaccaa atatccatta tattcatcat gtaatgcttt aaaatagtcc
      301 aatttttcta attcactctt atctttaaaa aatctagttt tagatatttt aaatcctaaa
      361 gctttttgtc cttcagtata aacaaaatta aaataactag cttacacaaa ttctgtataa
      421 tcttttaaat tgttgatttg ttctttattg gtcattattt attccttata ttattattga
      481 taaagattgc cacaagcgag ctcatcctct ataaactcaa aattcatatt accctcattg
      541 ccttctaaca catacccttct agtattccaa tatggactta tatgcatatc aacaatcttg
      601 tgtaaagaca tttgcatatt ttttgttttt agtgcaatat tatccatctc tatcttttct
      661 tgaaaatttg aaggagtttt gtaagtatca tcgtaatata tataaagcca catttctata
      721 ctaccatatt tttttacatg tcgaaaatca tctatgtcac ctaacttttt atcaaaatac
      781 cctagaatct tattatattt ctcttcgtta ttaggaagtt catttaactt acacatattc
      841 ctaaattgcc aataacttgg ctctaaataa taagaatatg gagtataata aaagataatt
      901 aaacctatac ttccaagaaa gactaaaaac tttgaaacgc tttttgtaga tttaaagatg
      961 attttataaa taaattttaa tataagataa gaaagaatag tagaaattat aataagaagt
     1021 aatatgtatg tcattataag ttatttcctt tagattatct attttattaa aattaagatg
     1081 taattttgtg gtttagtgtt agcagtataa ctttttcttt atcattaaaa taatagagct
     1141 tgttttttca tatattttta tcctttagat ttgtatttga tttttattct agctgtaaaa
     1201 taatttttat ttaattttac tcataacaaa taaattgata tttaaaaaaa tttaatatct
     1261 ttattctata tgtgttaaat tttttatcaa tacagatata ttgttatcca ctttaagatt
     1321 attggtttct aaattaaaaa ttgcaaagct taatactcct aatacaatca atactggcac
     1381 aatgataata agttttttaa aatacaatct tataaatttt tgccaaatat tttcttctaa
     1441 accaattttg tatgctttat taaaagctaa ctcttcttct gtcttataaa ctggtcttaa
     1501 agatgtggca atattattgc aaaaatggat tatttttttca tctctatcct tagcctcatt
     1561 atattttcct ttataaccaa gaatcaaaca agcataaata aattccaaaa aatctttaaa
     1621 cttaaaagga ttattaatcc aagatgatgc aatatcataa aaattatttc cacctaaggt
     1681 ttcatcaaat aatcttacag ttagagtatt gtgagcccaa aaattgataa aaagttcatt
     1741 tttcatcaag ctttcatcta taaagacaca aagacaatat ttgaatttaa taatttcttt
     1801 ttcatcatat tctttacaca tgcttagttt agcggttata tctaaaattt tattaatcaa
     1861 tgtttctcta agttgattaa tattacttgt atccatagag cttattttttg aaagtctgta
     1921 agaaagcaaa agtaactcca aagaataatc aataatttta ttgtttttta aaccatcaaa
     1981 atttgaatca agaagtaagc ttaatgcctt tttttcttcg tttatttctt tcattgaaat
     2041 accgcccaca ttataaatatc aggatttttt atattgttgg tcaagtacat actaatgata
     2101 ctttcatttt taaaatcttt aaacagctga tctcttttat ctattttata ataaacgtaa
```

```
     2161 ccatttaaat aaggaatact agatggaaca ttaggtactt gatctacatt taagccttca
     2221 agctgtgttg ctactatatt tttaatttta ctttgcgtat gtatcttgct ttgaatttta
     2281 aaattattaa gtagatattc tgtagtaaca tctgcacgaa ttgcaaaata aatttcagca
     2341 tcctctaaaa ttcccgagtt atcaaaaata aaatcataaa aaccattgcc attaatactg
     2401 atcttagcta taaaatattt aggacttgtg attttggaaa ataacaatct taaattattc
     2461 atcatattta aaaaagtatt ataaagatta tcatgtttat aaggaataaa atctaaaaaa
     2521 gattcttcat tactaaaagc tgcaagctct ccttgaaatt ctaaaaattt ttcgtataaa
     2581 aattcaggat gtatttttat actcatactc atatcataga agtgattaaa caaacccttg
     2641 gtttttatca agatatctta cataaagaaa ttgattattc tcatatacat tttaattatg
     2701 aagaacaaga acttatctct caatacaatg aaagcgattt agaatttatc acaagattag
     2761 ctcacaataa tggaatcttt ttttatgaag atgaaaacac tatttatttt tgtgatgtgt
     2821 ataaaaatgt aaaaaataaa gaaattgagt acaatcctaa tgtgaataat attttaaatc
     2881 aagcttgtat ttcttctatc tataaagagc aaagtttaag aactaatgct tttacacatt
     2941 ctagtatcaa tgctaatact cctttaaatc ttttatcttt acactctagt aaagttcctt
     3001 atgagcaaga acttaacaat aaagcttatt ataatgaaca tttttatgag agtgaatatt
     3061 cttttacaag aagtattgat ttaaagacta agccttctct taaagaaaaa agagccttga
     3121 tcttaaatga aagcttacta gcaaaaagta atatttatca tcttagcttg ggagatttta
     3181 tcactttaaa ttataaggaa tttaatttga gtggattgag tgatgatgaa gatactaaaa
     3241 gtcaagatga agaaaaacaa gataaagctt ctttgcttaa agactttatt attattgcca
     3301 atactcaaat cttaatagat gatgctttac tagctaattc tatcaatact aatgatcatt
     3361 taaatttaaa agatttaaat ttaagtaagt cttattctaa tactttaact ttattaaaaa
     3421 agaatattat ctttactcca agttttaaag ctaagcctaa agcccctcat aatactcaag
     3481 gtattgttat aggagagagt aaggatatag aaagtgagag aaatactatt tatactgatg
     3541 agtatggaag ggttaaggta aggattaatc tttatgctaa tcaagaggaa ttagataata
     3601 atcttttaag tgcaaacata aaaaccaata ctcatgaaaa tacaaacaat actgattcta
     3661 aagataatac aaacttcaca aactcttcta atcatttatc aagcaatgct tataaatctt
     3721 atcatcatac tccttttttta agagtagcaa caagtatagc aagcaatcat tcaggctttt
     3781 ttcatactcc aagagtggga gatgaagtga ttgtttcttt tttagatgat gatatagata
     3841 aaccctatgt gagctctagt ttgtataatg gggctacaac catgccccaa tataattatc
     3901 caagatacaa aagagaaatc gctacaaatg cattgagcta tttaagcgta gctcctgcta
     3961 tgattgataa aggtttagat acattaaaac ttcaagaaaa tactgattat gaattaagct
     4021 ctataaacaa tcattatctt actttagcaa attctacaat aggagtagat aatgccgatg
     4081 caagagcaag gaatgaaatt accctaaaaa acgacaaaga caaagaagaa atttatatcc
     4141 tagcacaaaa agattataaa gaagaaatag gtaataatta cgagcaaacc ataaaaaata
     4201 ataaaacttc agaagtggga gccttatata ccgaatttat cactttagga catatgcaaa
     4261 atattatagg ttttaaaaat gtcaatgtag gagctgaata tctagaaaat accttgcttt
     4321 ctaaagatac taatgtgggt ttaaataata ctttgaatgt gggaatcagc aatgaagtca
     4381 atatcggaca aaaccacgaa gaaaagatag gaaacgataa aagagtaata atcaataaca
     4441 atctagaaca agatatcaaa aatgatttta tccaaagaat aggacataat aaaaatgaaa
     4501 ccataaaagg ttcttatgtg cttcaaacca atcaaagtat aaaattttat tccaaacaag
     4561 atctaagcat agaaacaaac gattgcttta aagctgaagc tgatactttt gttagtttca
     4621 aagccaagaa gaattgctct tttacagccg atgaagtgta tactatggca aataaagaaa
     4681 gtttttttaac agcacaaaaa caaattgttt ctagggtagg aaatacaact atcacacaaa
     4741 caaaagataa gattatactc caagtaggaa caacacaagt gattatagat agtaaaggtt
     4801 taagagtgca aggaggtgat ttaagagcag attaaaagca attttcttaa aattaaaacc
     4861 aaaatcataa ataaacctaa tttacaataa aaggaaaaac aatgaaaaca ttagaagaat
     4921 taatagcttt agaagaaaaa tatctaaaag aattatgttt agaattttat gatcttgtag
     4981 aggctaacaa acttgaagaa gttaaagaat tt
//
LOCUS       CIKGKLNE_2             3592 bp    DNA     linear       12-APR-2021
DEFINITION  Campylobacter species strain strain.
ACCESSION
VERSION
KEYWORDS    .
```

```
SOURCE      Campylobacter species
  ORGANISM  Campylobacter species
            Unclassified.
COMMENT     Annotated using prokka 1.14.6 from
            https://github.com/tseemann/prokka.
FEATURES             Location/Qualifiers
     source          1..3592
                     /organism="Campylobacter species"
                     /mol_type="genomic DNA"
                     /strain="strain"
     gene            480..761
                     /locus_tag="CIKGKLNE_00005"
     CDS             480..761
                     /locus_tag="CIKGKLNE_00005"
                     /inference="ab initio prediction:Prodigal:002006"
                     /codon_start=1
                     /transl_table=11
                     /product="hypothetical protein"
                     /protein_id="Prokka:CIKGKLNE_00005"
                     /translation="MLLWFGYFFIPLEWQSSFKEFESLCENLKNREVIYNQEIYDRVK
                     GKNNAIRVKFVENNIGLRIQKVEYQYYKIVTNILYKNYYTYGWLYMYVC"
     gene            733..831
                     /locus_tag="CIKGKLNE_00006"
     CDS             733..831
                     /locus_tag="CIKGKLNE_00006"
                     /inference="ab initio prediction:Prodigal:002006"
                     /codon_start=1
                     /transl_table=11
                     /product="hypothetical protein"
                     /protein_id="Prokka:CIKGKLNE_00006"
                     /translation="MVGYICMFAKNGRILGCDGIDEYKKKIKERDY"
     gene            963..2465
                     /locus_tag="CIKGKLNE_00007"
     CDS             963..2465
                     /locus_tag="CIKGKLNE_00007"
                     /inference="ab initio prediction:Prodigal:002006"
                     /inference="similar to AA sequence:RefSeq:CKG86849.1"
                     /codon_start=1
                     /transl_table=11
                     /product="hypothetical protein"
                     /protein_id="Prokka:CIKGKLNE_00007"
                     /translation="MDEGLFWENLVSTEYIGAMQEKISNESFSNNISFRSLEYDATVL
                     PAELECCYNEFKYHQKSNNEIATLGWAVSYQLQALWQNSPKIDTIQNYHLPIKQNKPN
                     NPQAEQRLKEIQNFLQSKGIEPLILHYTPYPKNTQTQEMKRLRQNSERFSRHVDNKTN
                     EVREEKRSIKFYTTLDWDKFYSHFPIIKRLSKKFHTKEDIYTQNFTKQIEIILQKTHN
                     ILILKNLPQGEYKFDFAIDIKENRSDKNEESYIIIKENDEISKDYKRILDCKPYELDT
                     LNCQYIGAGDDEFLQQALQIIDINIKQQFDLDFATKTLIKNSTIPNKIYFTYKDYYHA
                     MQDLSLYACSGKFSFNFVPSKLSIKKEIQGDREKVLYRVDEMLVYVYDSFDFLDQGHE
                     FDDEGNFIKLGQPVGAWDFNEKSFSIWGSTRQMETYKPKEYLFFIPQITFADILQSPK
                     TKQYYLYNQDYQDYQKFTPYGLDFKLYSKDFITKLDFKDKAYNVLYDTGV"
     gene            2466..2969
                     /locus_tag="CIKGKLNE_00008"
     CDS             2466..2969
                     /locus_tag="CIKGKLNE_00008"
```

```
                     /inference="ab initio prediction:Prodigal:002006"
                     /inference="similar to AA sequence:RefSeq:CKG86821.1"
                     /codon_start=1
                     /transl_table=11
                     /product="hypothetical protein"
                     /protein_id="Prokka:CIKGKLNE_00008"
                     /translation="MGDSIMLSTSLGLVGFIFAFTNIKFYLTTLSVLIVFSILNYWIK
                     INIKEFVKSYFSIYFIIFFTAIVIIFYLTYGIKTSKDLALLSSLEAKELSPQEYKARN
                     YFGFLTCMTMFFLVITFIILPCLLQAYLFKCYSTGEKRKKLSWFIGFVCNAPILIASV
                     YVVYENL"
     gene            2947..3087
                     /locus_tag="CIKGKLNE_00009"
     CDS             2947..3087
                     /locus_tag="CIKGKLNE_00009"
                     /inference="ab initio prediction:Prodigal:002006"
                     /inference="similar to AA sequence:RefSeq:CKG86746.1"
                     /codon_start=1
                     /transl_table=11
                     /product="hypothetical protein"
                     /protein_id="Prokka:CIKGKLNE_00009"
                     /translation="MWYMKIFKDYQKFTPYGLDFRLYSKDFITKLDFKDKAYNVLYNI
                     GV"
     gene            3088..3588
                     /locus_tag="CIKGKLNE_00010"
     CDS             3088..3588
                     /locus_tag="CIKGKLNE_00010"
                     /inference="ab initio prediction:Prodigal:002006"
                     /inference="similar to AA sequence:RefSeq:CKG86719.1"
                     /codon_start=1
                     /transl_table=11
                     /product="hypothetical protein"
                     /protein_id="Prokka:CIKGKLNE_00010"
                     /translation="MGDSIALSIFWGLIGFILTCSVIEFYLIALSVLIVFSILNYWIK
                     INIKEFVKSYFLISFIIFFMAIVIVFYLTYDLETSRGLVLLGPPEIEKLSPQEYKARN
                     YFDFLGGITFIFLVITFFILPCLLQAYLFKCYSTGEKRKKLSWFIGFVCNPILITSMY
                     VIYENL"
ORIGIN
        1 aattttattt atataaggta tttgaaataa tacaatttaa aaaggagtat taaatgaaaa
       61 tcacaatgat aattctaagc ttattttctt tagtattttt aagtgcatgt gcttttaaag
      121 aaaaaaagca agtgaaatag aaactcttgc aagtaattat ggtggaattt atatctttga
      181 taaaaaaata agagaagaga tattggaatt agagaaaaaa gagaagaatt tcgtagtaag
      241 tatttgggta gtgaaattaa agtaggaaat gaaactcatt ttgtgaattt ttcttatttg
      301 aagaaaaaat tccccccaagt cctttctaat ggttgtaaat attatcgtaa tgattataat
      361 tatgatgtaa aagacccaga ttatgatttt aatttcgacg ataaacccga atttgcatac
      421 tatgaagatc aatttaaagc atatatgggt gaagaaaact ataaaaaatt aagacccttta
      481 tgcttctttg gttcggatac tttttttatac ctcttgaatg gcaaagtagt tttaaagaat
      541 ttgagagttt gtgtgagaat ttaaaaaata gagaagtgat ttataatcaa gagatttatg
      601 atagagttaa aggcaaaaat aatgctataa gagtaaaatt tgttgagaac aatataggat
      661 taaggataca aaaagtagaa tatcaatatt ataaaattgt aacaaatatt ttatataaaa
      721 attactatac atatggttgg ctatatatgt atgtttgcta aaaatggaag aatattaggt
      781 tgtgatggca ttgatgaata taagaaaaaa ataaaagaaa gagactacta atgcataaaa
      841 aacaagccaa atatatgaaa ctttataaat atgttagaat atgctggatt agttatttgt
      901 aaatttaaat gaaaacatac ttgaaaaatt tctaaaaaat ataaaaacaa taggataaaa
      961 tgatggatga aggacttttt tgggaaaatc ttgtttcaac agaatatata ggagcaatgc
```

```
     1021 aagagaaaat atcaaatgaa tcattttcaa ataatataag ttttagatct ctagaatatg
     1081 atgcaacagt tttgcctgcg gaattagagt gttgctacaa tgaatttaag taccatcaaa
     1141 aaagcaataa tgaaatagca acactaggtt gggctgttag ttatcaactt caagcattgt
     1201 ggcaaaatag ccctaagata gatacaatac aaaattatca tctccctata aaacaaaata
     1261 agcctaataa tccccaagca gaacaaagac taaaagaaat acaaaatttc ttacaaagta
     1321 aaggtataga acccctta tt ttgcactata ccccttatcc aaaaaacaca caaacacaag
     1381 agatgaaaag actaaggcaa aattcagagc gtttttctcg ccatgtagat aataaaacaa
     1441 atgaagtaag agaagaaaag cgttctatta aattttacac tacgctagat tgggataagt
     1501 tttattctca ttttcctata atcaaaagac taagtaaaaa atttcataca aaagaagaca
     1561 tatacactca aaattttaca aagcaaatag aaataatctt acaaaaaact cataatattt
     1621 tgatattaaa aaatttgcct caaggagaat ataaatttga ttttgctatc gatataaaag
     1681 aaaatcgaag cgataaaaat gaagaatctt atatcatcat taaagaaaat gatgaaattt
     1741 caaaagatta taaacgcatt ttagactgta agccctatga gcttgatact ttaaattgtc
     1801 aatatatagg tgcgggtgat gatgagtttt tacagcaagc tttacaaatt atagatatta
     1861 atattaaaca acaatttgat ttagattttg caacaaaaac tttaattaaa aattcaacaa
     1921 ttccaaataa aatttatttt acatataaag attattatca tgcaatgcaa gatctatctc
     1981 tttatgcttg tagtggtaaa ttttctttta attttgttcc ttcaaaatta agcattaaaa
     2041 aagaaattca aggagatagg gaaaaggtat tataccgcgt tgatgaaatg cttgtttatg
     2101 tttatgatag ctttgatttt ttagatcaag gacatgaatt tgatgatgag ggaaatttta
     2161 tcaaattagg acaacctgtt ggggcttggg attttaatga aaagagtttt agcatttggg
     2221 gttctacaag acagatggaa acttataaac caaaggaata tttatttttt ataccacaaa
     2281 taacttttgc tgatattcta caatccccaa aaccaaaaca atactatctt tacaatcaag
     2341 attaccaaga ctaccaaaaa ttcactcctt atggtttgga ttttaaactt tatagtaaag
     2401 attttatcac taaacttgat tttaaagata aagcttataa tgtgctttat gatacaggag
     2461 tttaaatggg agatagtatt atgctttcta cttctttagg tttggtagga tttatttttg
     2521 cttttactaa tataaaattt tatcttacca ctttaagtgt tcttattgtc ttttctattt
     2581 taaattattg gatcaagatt aatataaaag aatttgtaaa atcttatttt tcaatatatt
     2641 ttattatctt ttttacagct attgttatca tattttatct tacctatggt attaaaactt
     2701 ctaaggatct tgcattgctt agctctctag aagctaaaga attatcacca caagaatata
     2761 aagcacgtaa ttatttttgga tttttgactt gtatgactat gtttttttta gtaatcacat
     2821 ttatcatact tccttgttta ttgcaagctt atttatttaa atgttatagc acaggagaaa
     2881 aacgcaaaaa attaagttgg tttatagggt ttgtgtgtaa tgctcctata ttaattgcga
     2941 gtgtgtatgt ggtatatgaa aatctttaaa gactatcaaa aattcactcc ttatggtttg
     3001 gattttagac tttatagtaa agattttatc actaagcttg attttaaaga taaagcttat
     3061 aatgtgcttt ataatatagg agtttaaatg ggagatagta ttgcactttc tatttttttgg
     3121 ggtttgatag gatttattct tacttgtagt gttatagaat tttatcttat agctctaagc
     3181 gttcttattg tcttttctat tttaaattat tggatcaaga ttaatataaa agaatttgta
     3241 aaatcttatt ttttaatatc ttttattatc tttttatgg ctattgttat cgtattttat
     3301 cttacttatg atcttgaaac ttctagggga cttgtattac ttggtcctcc agaaattgaa
     3361 aaattatcac cacaagaata taaagcacgt aattattttg atttttagg cggtataact
     3421 tttattttttt tagtaatcac atttttata ctcccttgtt tattgcaagc ttatttattt
     3481 aaatgttata gcacaggaga aaaacgcaaa aaattaagtt ggtttatagg gtttgtgtgt
     3541 aatcctatat taattacaag tatgtatgtg atatatgaaa atctttaaag ac
//
LOCUS       CIKGKLNE_3              3244 bp    DNA     linear       12-APR-2021
DEFINITION  Campylobacter species strain strain.
ACCESSION
VERSION
KEYWORDS    .
SOURCE      Campylobacter species
  ORGANISM  Campylobacter species
            Unclassified.
COMMENT     Annotated using prokka 1.14.6 from
            https://github.com/tseemann/prokka.
```

```
FEATURES             Location/Qualifiers
     source          1..3244
                     /organism="Campylobacter species"
                     /mol_type="genomic DNA"
                     /strain="strain"
     gene            110..445
                     /locus_tag="CIKGKLNE_00011"
     CDS             110..445
                     /locus_tag="CIKGKLNE_00011"
                     /inference="ab initio prediction:Prodigal:002006"
                     /codon_start=1
                     /transl_table=11
                     /product="hypothetical protein"
                     /protein_id="Prokka:CIKGKLNE_00011"
                     /translation="MDPQYYEFKSLCYFNLGKVLIEDESIKNKTNIIVTPKSKQIKDR
                     IVETRFERKNENQVVYYRINTYFYDNYGIFLKGDEGAGLYFRYMEVLSCKNIDKKFKK
                     DDWQVETLE"
     gene            449..589
                     /locus_tag="CIKGKLNE_00012"
     CDS             449..589
                     /locus_tag="CIKGKLNE_00012"
                     /inference="ab initio prediction:Prodigal:002006"
                     /codon_start=1
                     /transl_table=11
                     /product="hypothetical protein"
                     /protein_id="Prokka:CIKGKLNE_00012"
                     /translation="MSKNIKTQEAKLDLITKFLDYANCADASYAMLHLIDENDEKGFL
                     KG"
     gene            592..1230
                     /locus_tag="CIKGKLNE_00013"
     CDS             592..1230
                     /locus_tag="CIKGKLNE_00013"
                     /inference="ab initio prediction:Prodigal:002006"
                     /codon_start=1
                     /transl_table=11
                     /product="hypothetical protein"
                     /protein_id="Prokka:CIKGKLNE_00013"
                     /translation="MLEFFVNLLIFILIFFFFVFIFSIILYFIFKVFFKIKNKTKTIN
                     FKTMFKKLALFLLPVYLLLFLAGGCSYKYMDPQYYEFKKLCKDNSNKMIVFNKDYLDL
                     KKQFVQAMTNNSNIRIKNNGIKYFYNEKLNLEIQPSNWKEIETKSREIKLGIGKVKEK
                     EIEAWYIDDKNNIKYQEFKRYLYYNYSIFLGGDEGAGFHFEYEEILDCGDVR"
     gene            1362..1466
                     /locus_tag="CIKGKLNE_00014"
     CDS             1362..1466
                     /locus_tag="CIKGKLNE_00014"
                     /inference="ab initio prediction:Prodigal:002006"
                     /codon_start=1
                     /transl_table=11
                     /product="hypothetical protein"
                     /protein_id="Prokka:CIKGKLNE_00014"
                     /translation="MLDLIDENDENSTLAPLWLYADGVTNLKNHSKRQ"
     gene            1470..2036
                     /locus_tag="CIKGKLNE_00015"
     CDS             1470..2036
```

```
                     /locus_tag="CIKGKLNE_00015"
                     /inference="ab initio prediction:Prodigal:002006"
                     /codon_start=1
                     /transl_table=11
                     /product="hypothetical protein"
                     /protein_id="Prokka:CIKGKLNE_00015"
                     /translation="MKYIILTFILGLIVFVSVKGCFYPTSYRAFIPKSLDSEYKEFER
                     LCKEEVGKVVYARPIKHGIKLEDLFNLPSTSARRVALSRHMYMLIENVTTREDEIFYL
                     HIAGFEYYIGWTRKSFSMHDDSKKYIRCEPLLYGKTWKENAKIMEQKAFDNKEYIGTE
                     EYLQYSPYHYEYNTLYRQIVNFKQKKEN"
     gene            2036..2188
                     /locus_tag="CIKGKLNE_00016"
     CDS             2036..2188
                     /locus_tag="CIKGKLNE_00016"
                     /inference="ab initio prediction:Prodigal:002006"
                     /codon_start=1
                     /transl_table=11
                     /product="hypothetical protein"
                     /protein_id="Prokka:CIKGKLNE_00016"
                     /translation="MIYNYKYSLGIYVNDWGFMMGSEGNPHVFLGLKREQVHCGDMQI
                     KNTIYK"
     gene            2241..2777
                     /locus_tag="CIKGKLNE_00017"
     CDS             2241..2777
                     /locus_tag="CIKGKLNE_00017"
                     /inference="ab initio prediction:Prodigal:002006"
                     /inference="similar to AA sequence:RefSeq:CKG86719.1"
                     /codon_start=1
                     /transl_table=11
                     /product="hypothetical protein"
                     /protein_id="Prokka:CIKGKLNE_00017"
                     /translation="MGDSITLSIFVGLLLSIAIITKFYLIALSVLIVFSILNYWIKIN
                     IKEFVKSYFLISFIIFFTAIVIVFYLTYDLETSRGLVLLGPPEVEKLSPQEYKAHNYF
                     KFLTFTTMFFLAITFFILPCLLQAYLFKCYSTGEKRKKLSWFIGFACNIFLLIVSFYK
                     LYENLQRLSKIHSLWFGF"
     gene            2725..2856
                     /locus_tag="CIKGKLNE_00018"
     CDS             2725..2856
                     /locus_tag="CIKGKLNE_00018"
                     /inference="ab initio prediction:Prodigal:002006"
                     /inference="similar to AA sequence:RefSeq:CKG86746.1"
                     /codon_start=1
                     /transl_table=11
                     /product="hypothetical protein"
                     /protein_id="Prokka:CIKGKLNE_00018"
                     /translation="MKIFKDYQKFTPYGLDFKLYSKDFITKLDFKDKAYNVLYDTGV"
ORIGIN
        1 aaataaaaca aaaactataa attttaaaac aatatttaaa aaatttactt tgtttctttt
       61 acctgtatat cttttattat tcttagtagg aggatgttct tataaatata tggatccgca
      121 gtattatgag tttaagagct tgtgttattt taatttggga aaagtgttga ttgaggatga
      181 atctataaag aataaaacga atataatagt gacacctaaa agtaagcaaa taaaagatag
      241 gatagtggaa actagatttg aaagaaaaaa tgaaaaccaa gtagtgtatt atcgtattaa
      301 tacatatttt tatgataatt atggtatatt cttgaaaggc gatgagggtg ctggattata
      361 ttttagatat atggaagtat taagttgtaa gaatattgac aaaaaattta agaaagatga
```

```
      421 ctggcaagtt gaaactttgg aataacaaat gagcaaaaac attaaaacac aagaagctaa
      481 attagactta atcactaaat ttttagacta tgctaattgt gcagatgcta gttatgcgat
      541 gttgcattta atcgatgaga atgatgaaaa aggtttttta aaaggataat aatgctagaa
      601 ttctttgtta atcttttaat atttattctt atatttttct tttttgtttt tatattttct
      661 attattttat attttatctt taaagtcttt tttaaaataa aaaataaaac aaaaactata
      721 aattttaaaa caatgtttaa aaaacttgct ttatttcttt tacctgtata tcttttatta
      781 ttcttagcag gaggatgttc ttataaatat atggatccgc aatattatga atttaagaag
      841 ttgtgtaaag ataatagtaa taaaatgata gtgtttaata aagattattt ggatttaaaa
      901 aaacaatttg ttcaagctat gacgaataat tctaatatta gaattaaaaa taatggtata
      961 aaatattttt ataatgaaaa attgaatttg gaaattcaac cttcaaattg gaaagaaata
     1021 gaaactaaat cacgcgaaat taaacttgga ataggaaaag taaaagaaaa agaaatagaa
     1081 gcttggtata ttgatgataa aaataacatt aagtatcaag aatttaaacg atatttgtat
     1141 tataattata gtatattttt aggaggcgat gagggcgctg ggtttcattt tgaatatgag
     1201 gaaatattag attgtggaga tgttagataa aaaatttaaa agcataatga gcttaagata
     1261 ggaatataaa atgagcaaaa acattaaaac acaagaagct aaattagact taatcactaa
     1321 atttttttga ctatgctaat atagcagatg ctagttatgc aatgcttgat ttaatcgatg
     1381 agaatgatga gaattcaacc cttgcacctt tatggcttta tgcagatggg gtaactaatc
     1441 taaaaaatca tagtaaaagg caataaagta tgaaatatat tatattgact tttattctag
     1501 gtttgattgt atttgtttca gtaaaaggtt gtttttatcc tacaagttat cgtgctttta
     1561 taccaaaaag cttagattcc gagtataaag agtttgaaag attatgtaaa gaggaagtag
     1621 gaaaggtagt atatgcaaga cctataaaac atggtataaa gttggaagat ttgtttaact
     1681 tgccttctac gagtgctagg cgtgttgctt taagcagaca tatgtatatg ttgatagaaa
     1741 atgttacaac aagagaagac gaaatttttt acttacatat agctggtttt gaatattata
     1801 taggctggac aagaaaaagt ttttctatgc atgatgattc taaaaaatat ataagatgtg
     1861 agcctttgct ttatggtaaa acttggaaag aaaatgctaa gataatggaa caaaaggcat
     1921 ttgataataa agaatatata ggcacagaag aatatttaca atattcacct tatcattatg
     1981 agtataatac actttatcgc caaatagtta atttcaaaca aaagaaagag aattaatgat
     2041 atataattac aaatactctc ttggaatcta tgttaatgat tggggcttta tgatgggcag
     2101 tgagggaaac cctcatgtgt tcttaggact aaaaagagag caagtgcatt gtggcgatat
     2161 gcaaataaaa aacacaatat ataaataaac ttgattttag agataaagct tataatgtgc
     2221 tttataatac aggagtttaa atgggagata gtattacgct ttctattttt gtaggtttat
     2281 tgctatccat tgctattatc acaaaatttt atcttatagc tttatctgtg cttattgtct
     2341 tttctatttt aaattattgg attaagatta atataaaaga atttgtaaaa tcttattttt
     2401 taatatcttt tattatcttt tttacagcta ttgttatcgt attttatctt acttatgatc
     2461 ttgaaacttc taggggactt gtattacttg gtcctccaga agttgaaaaa ttatcaccac
     2521 aagaatataa agcacataat tattttaaat ttttaacctt tacaactatg ttttttttgg
     2581 caatcacttt ttttatactc ccttgtttat tacaagctta tttatttaaa tgttatagca
     2641 caggagaaaa acgcaaaaaa ttaagttggt ttatagggtt tgcgtgtaat atctttttat
     2701 taattgtgag tttttataag ttatatgaaa atcttcaaag actatcaaaa attcactcct
     2761 tatggtttgg attttaaact ttatagtaaa gattttatca ctaagcttga ttttaaagat
     2821 aaagcttata atgtgcttta tgatacagga gtttaaatgg gagatagtat tacattgtca
     2881 attattatgg gaatgttatt ttctattctt tattttgtaa tttttataaa aaactatatt
     2941 ataatagctt tgattgtcct tgttgtcttt tctattataa attattggat taagattaac
     3001 ataaaagaat ttgtaaaatc ttattttta atatctttta ttatcttttt tacagctatt
     3061 gttatcgtat tttatcttac ttatgatctt gaaacttcta gagggcttgt attacttggt
     3121 cctccagaaa ttgaagaatt atcaccacaa gagtataaag tacatggtta tttcggattt
     3181 ttatttttta caagtattgg cttttgtaca atcacatttt ttatacttcc ttgtttatta
     3241 caag
//
LOCUS       CIKGKLNE_4              1602 bp    DNA     linear       12-APR-2021
DEFINITION  Campylobacter species strain strain.
ACCESSION   
VERSION     
KEYWORDS    .
```

```
SOURCE      Campylobacter species
  ORGANISM  Campylobacter species
            Unclassified.
COMMENT     Annotated using prokka 1.14.6 from
            https://github.com/tseemann/prokka.
FEATURES             Location/Qualifiers
     source          1..1602
                     /organism="Campylobacter species"
                     /mol_type="genomic DNA"
                     /strain="strain"
     gene            218..316
                     /locus_tag="CIKGKLNE_00019"
     CDS             218..316
                     /locus_tag="CIKGKLNE_00019"
                     /inference="ab initio prediction:Prodigal:002006"
                     /codon_start=1
                     /transl_table=11
                     /product="hypothetical protein"
                     /protein_id="Prokka:CIKGKLNE_00019"
                     /translation="MSKNIKTQEAKLDLITKFLDYANCADASYAML"
     gene            395..712
                     /locus_tag="CIKGKLNE_00020"
     CDS             395..712
                     /locus_tag="CIKGKLNE_00020"
                     /inference="ab initio prediction:Prodigal:002006"
                     /codon_start=1
                     /transl_table=11
                     /product="hypothetical protein"
                     /protein_id="Prokka:CIKGKLNE_00020"
                     /translation="MKNSKGEDIVKPKNTNTAYACAIQARFEQNKIVKIEPKYCISLI
                     NTCFDSKEITLDNDISRVGLNDALSKRTIDFVNRFKLLKHQPNTTNCLMKKQRKLRGV
                     NKC"
     gene            706..1332
                     /locus_tag="CIKGKLNE_00021"
     CDS             706..1332
                     /locus_tag="CIKGKLNE_00021"
                     /inference="ab initio prediction:Prodigal:002006"
                     /codon_start=1
                     /transl_table=11
                     /product="hypothetical protein"
                     /protein_id="Prokka:CIKGKLNE_00021"
                     /translation="MLEFFINLLIFILIFFFFVFIFSIILYFIFKIFYKLKNKTKTIN
                     FKTIFKKFTLFLLPVYLLLFLVGGCSYKYMDPQYYKFKSLCKDIDSKVIIYDKAYWEL
                     YSDFTKKKPSIEKRVKDDGYEYFYYEKLNETFAYYDIKDMIKSKKRNGNIITIVYDKK
                     YKKMPKPFATYIRYKYENDGVFLRGDEGSGLYFRYEEVFTCSYFDNFK"
     gene            1341..1496
                     /locus_tag="CIKGKLNE_00022"
     CDS             1341..1496
                     /locus_tag="CIKGKLNE_00022"
                     /inference="ab initio prediction:Prodigal:002006"
                     /codon_start=1
                     /transl_table=11
                     /product="hypothetical protein"
                     /protein_id="Prokka:CIKGKLNE_00022"
```

```
                     /translation="MSKNIKTQEAKLDLITKFLDYANIADASYAMLDLIDENDEKGFL
                     WYIKYKN"
ORIGIN
        1 aaaataaatt tactttaaca gaagttacat ttgaagatta ttatgatggt attcattatt
       61 caacgcattt atcctatatt tatgataatt atggtatgtt cttaaaaggt gatgagggtg
      121 ctggattcta ttttagatat cataaaagac tgtattgtga agatgttaga taaaaaatta
      181 gaaagataat ttgagattta gatttttgga ataacaaatg agcaaaaaca ttaaaacaca
      241 agaagctaaa ttagacttaa tcactaaatt tttagactat gctaattgtg cagatgctag
      301 ttatgcaatg ttgtaatatg tttgggaaaa tatagagcaa gatgaaaaga ataatattta
      361 tacttttggt gataaattaa agcaagatat tgttatgaaa aatagtaaag gagaagatat
      421 tgtaaaacct aaaaatacaa acacagctta tgcttgtgct atacaagctc gttttgaaca
      481 aaataaaata gttaaaatag aacctaaata ttgtatttct cttataaata cttgttttga
      541 tagtaaagaa ataacattgg ataatgatat tagtagggta ggattgaatg atgcactgag
      601 taaaagaact attgattttg taaatagatt taaactttta aaacatcagc ccaacactac
      661 aaattgtctt atgaaaaaac agagaaaatt aagaggagtt aataaatgct agaattcttt
      721 attaatcttt taatatttat tcttatattt ttcttttttg tttttatatt ttctattatt
      781 ttatatttta tctttaaaat tttttataaa ttaaaaaata aaacaaaaac tataaatttt
      841 aaaacaatat ttaaaaaatt tactttgttt cttttacctg tatatctttt attattctta
      901 gtaggaggat gttcttataa atatatggat ccgcagtatt ataagtttaa gagcttgtgt
      961 aaagatatag atagtaaagt aatcatttat gataaagctt attgggaatt gtatagcgat
     1021 tttacaaaaa aaaagccttc tatagaaaaa cgagttaagg atgatggtta tgaatatttt
     1081 tattatgaaa aacttaatga aacatttgct tattatgata taaaagatat gatcaaatct
     1141 aagaaacgaa atggtaatat tataaccata gtatatgata aaaaatataa aaaaatgccc
     1201 aagccttttg ctacatatat aagatataaa tatgaaaatg atggtgtatt cttaagaggt
     1261 gatgagggct ctggattata ttttagatat gaagaagtat tcacttgtag ctattttgat
     1321 aattttaagt aaggatataa atgagcaaaa acattaaaac acaagaagct aaattagact
     1381 taatcactaa atttttagac tatgctaata tagcagatgc tagttatgca atgcttgatt
     1441 taatcgatga gaatgatgaa aaaggttttt tgtggtatat taaatataaa aactaaattt
     1501 tatttatata aggtatttga aataatacaa tttaaaaagg agtattaaat gaaaatcaca
     1561 atgataattc taagcttatt ttctttagta tttttaagtg ca
//
LOCUS       CIKGKLNE_5              1595 bp    DNA     linear       12-APR-2021
DEFINITION  Campylobacter species strain strain.
ACCESSION
VERSION
KEYWORDS    .
SOURCE      Campylobacter species
  ORGANISM  Campylobacter species
            Unclassified.
COMMENT     Annotated using prokka 1.14.6 from
            https://github.com/tseemann/prokka.
FEATURES             Location/Qualifiers
     source          1..1595
                     /organism="Campylobacter species"
                     /mol_type="genomic DNA"
                     /strain="strain"
     gene            242..778
                     /locus_tag="CIKGKLNE_00023"
     CDS             242..778
                     /locus_tag="CIKGKLNE_00023"
                     /inference="ab initio prediction:Prodigal:002006"
                     /inference="similar to AA sequence:RefSeq:CKG86719.1"
                     /codon_start=1
                     /transl_table=11
```

```
                     /product="hypothetical protein"
                     /protein_id="Prokka:CIKGKLNE_00023"
                     /translation="MGDSITLSIFVGSLLSIAVITKFYLIALSVLIVFSILNYWIKIN
                     IKEFVKSYFLISFIIFFTAIVIVFYLTYGIETSRGLVLLGPPEVEKLSPQEYKAHNYF
                     KFLTFTTMFFLAITFFILPCLLQAYLFKCYSTGEKRKKLSWFIGFACNIFLLIVSFYK
                     LYENLQRLSKIHSLWFGF"
     gene            857..1360
                     /locus_tag="CIKGKLNE_00024"
     CDS             857..1360
                     /locus_tag="CIKGKLNE_00024"
                     /inference="ab initio prediction:Prodigal:002006"
                     /inference="similar to AA sequence:RefSeq:CKG86821.1"
                     /codon_start=1
                     /transl_table=11
                     /product="hypothetical protein"
                     /protein_id="Prokka:CIKGKLNE_00024"
                     /translation="MGDGITLSTSLGLVGFIFAFTNIKFYLTTLSVLIVFSILNYWIK
                     INIKEFVKSYFSIYFIIFFTAIVIIFYLTYGIETSRDLALLSSLEAKELSPQEYKAYN
                     YFGFLTCMTMFFLVITFIILPCLLQAYLFKCYSTEEKRKKLSWFIGFLCNIIILIASV
                     YLMYIFI"
ORIGIN
        1 caagcttatt tatttaaatg ttatagcaca ggagaaaaac gcaaaaaatt aagttggttt
       61 atagggtttg tgtgtaatcc tatattaatt acaagtatgt atgtgatata tgaaaatctt
      121 taaagactac caaaaattca ctccttatgg tttggatttt aaactttata gtaaagattt
      181 tatcaccaag cttgatttta aagataaagc ttataatgtg ctttataata caggagttta
      241 aatgggagat agtattacgc tttctatttt tgtaggttca ttgctatcca ttgctgttat
      301 cacaaaattt tatcttatag ctttatctgt gcttattgtc ttttctattt taaattattg
      361 gatcaagatt aatataaaag aatttgtaaa atcttatttt ttaatatctt ttattatctt
      421 ttttacagct attgttatcg tattttatct tacctatggt attgaaactt ctaggggact
      481 tgtattactt ggtcctccag aagttgaaaa attatcacca caagaatata aagcacataa
      541 ttattttaaa tttttaacct ttacaactat gtttttttg gcaatcactt tttttatact
      601 cccttgttta ttacaagctt atttatttaa atgttatagc acaggagaaa aacgcaaaaa
      661 attaagttgg tttatagggt ttgcgtgtaa tatcttttta ttaattgtga gtttttataa
      721 gttatatgaa aatcttcaaa gactatcaaa aattcactcc ttatggtttg gattttaaac
      781 tttatagtaa agatttttg ctaaacttga ttttaaagat aaagcttata atgtgcttta
      841 taatacagga gtttaaatgg gagatggtat tacgctttct acttctttag gtttggtagg
      901 atttattttt gcttttacta atataaaatt ttatcttacc actttaagtg ttcttattgt
      961 cttttctatt ttaaattatt ggatcaagat taatataaaa gaatttgtaa aatcttattt
     1021 ttcaatatat ttattatct tttttacagc tattgttatc atattttatc ttacctatgg
     1081 tattgaaact tctagggatc ttgcattgct tagctctcta gaagctaaag aattgtcacc
     1141 acaagaatat aaagcgtata attattttgg atttttgact tgtatgacta tgttttttt
     1201 agtaatcaca tttattatac tcccttgttt attgcaagct tatttattta aatgttatag
     1261 cacagaagaa aaacgcaaaa aattaagttg gtttataggg tttttatgca atattattat
     1321 attaattgca agtgtgtatt tgatgtatat atttatttaa agactaccaa aaattcactc
     1381 cttatggttt ggattttaaa ctttatagta aagattttat cactaagctt gattttaaag
     1441 ataaagctta taatgtgctt tataatacag gagtttaaat gggagaagat attacatttt
     1501 ctattttttg ggtttattt ggatttattt ttgcttttac taatataaaa ttttatctta
     1561 ccactttaag tgttcttatt gtcttttcta tttta
//
LOCUS       CIKGKLNE_6               718 bp    DNA     linear       12-APR-2021
DEFINITION  Campylobacter species strain strain.
ACCESSION
VERSION
KEYWORDS    .
```

```
SOURCE      Campylobacter species
  ORGANISM  Campylobacter species
            Unclassified.
COMMENT     Annotated using prokka 1.14.6 from
            https://github.com/tseemann/prokka.
FEATURES             Location/Qualifiers
     source          1..718
                     /organism="Campylobacter species"
                     /mol_type="genomic DNA"
                     /strain="strain"
     gene            208..714
                     /locus_tag="CIKGKLNE_00025"
     CDS             208..714
                     /locus_tag="CIKGKLNE_00025"
                     /inference="ab initio prediction:Prodigal:002006"
                     /inference="similar to AA sequence:RefSeq:CKG86719.1"
                     /codon_start=1
                     /transl_table=11
                     /product="hypothetical protein"
                     /protein_id="Prokka:CIKGKLNE_00025"
                     /translation="MGEDITLSIFWGLVGFIFRLVTIDTILYITALSVLIVFSIINYW
                     IKINIKEFVKSYFLVYFIIFFTAIIIIFYLSYDLETSRGYVMLGPPEIEELSPQEYKV
                     HGYFGFLFFTSIGFCTITFFILPCLLQAYLFKCYSTGEKRKKLSWFIGFVCNPILITS
                     MYVIYENL"
ORIGIN
        1 aacgcaaaaa attaagttgg tttatagggt ttttatgcaa tattattata ttaattgcaa
       61 gtgcatattt gatgtatata tttatttaaa gactatcaaa aattcactcc ttatggtttg
      121 gattttagac tttatagtaa agattttatc actaaacttg attttaaaga taaagcttat
      181 aatgtgcttt atgatacagg agtttaaatg ggagaagata ttacactttc tatttttggg
      241 ggtttggtag gatttatttt taggcttgtt actattgata caatacttta tattacagcc
      301 ttaagcgttc ttattgtctt ttctattata aattattgga ttaaaattaa tataaaagaa
      361 tttgtaaagt cttatttttt agtatatttt attatctttt ttacagctat tattatcata
      421 ttttatctta gctatgatct tgaaacttct agaggatatg taatgctcgg tcctccagaa
      481 attgaagaat tatcaccaca agagtataaa gtacatggtt atttcggatt tttatttttt
      541 acaagtattg gcttttgtac aatcacattt tttatacttc cttgtttatt acaagcttat
      601 ttatttaaat gttatagcac aggagaaaaa cgcaaaaaat taagttggtt tatagggttt
      661 gtgtgtaatc ctatattaat tacaagtatg tatgtgatat atgaaaatct ttaaagac
//
LOCUS       CIKGKLNE_7                649 bp    DNA     linear       12-APR-2021
DEFINITION  Campylobacter species strain strain.
ACCESSION
VERSION
KEYWORDS    .
SOURCE      Campylobacter species
  ORGANISM  Campylobacter species
            Unclassified.
COMMENT     Annotated using prokka 1.14.6 from
            https://github.com/tseemann/prokka.
FEATURES             Location/Qualifiers
     source          1..649
                     /organism="Campylobacter species"
                     /mol_type="genomic DNA"
                     /strain="strain"
     gene            253..384
```

```
                     /locus_tag="CIKGKLNE_00026"
     CDS             253..384
                     /locus_tag="CIKGKLNE_00026"
                     /inference="ab initio prediction:Prodigal:002006"
                     /inference="similar to AA sequence:RefSeq:CKG86746.1"
                     /codon_start=1
                     /transl_table=11
                     /product="hypothetical protein"
                     /protein_id="Prokka:CIKGKLNE_00026"
                     /translation="MKIFKDYQKFTPYGLDFKLYSKDFITKLDFKDKAYNVLYNTGV"
ORIGIN
        1 ctgcgaaatt cacttctctt tttttagagg ttaagcaatt accatctgaa gaatacctag
       61 tatttagctg tcttgcattc ttatttatca taactatgga attttttata atcacattct
      121 ttatacttcc ttgtttatta caagcttatt tttttaaatg ctatagcaca ggagaaaaac
      181 gcaaaaaatt aagttggttt atagggtttg cgtgtaatat ctttttatta attgtgagtt
      241 tttataagtt atatgaaaat ctttaaagac tatcaaaaat tcactcctta tggtttggat
      301 tttaaacttt atagtaaaga ttttatcacc aagcttgatt ttaaagataa agcttataat
      361 gtgctttata atacaggagt ttaaatggga gatagcatta cactttctat tttttggggt
      421 ttgattttgt ttattgctag ttttactgtt gcaagaattt atcttatagc tctaagcgtt
      481 cttattgtct tttctatttt aaattattgg atcaagatta atataaaaga atttgtaaaa
      541 tcttattttt taatatcttt tattatcttt tttacagcta ttgttatcgt attttatctt
      601 acttatgatc ttgaaacttc tagagggctt gtattacttg gtcctccag
//
LOCUS       CIKGKLNE_8               598 bp    DNA     linear       12-APR-2021
DEFINITION  Campylobacter species strain strain.
ACCESSION
VERSION
KEYWORDS    .
SOURCE      Campylobacter species
  ORGANISM  Campylobacter species
            Unclassified.
COMMENT     Annotated using prokka 1.14.6 from
            https://github.com/tseemann/prokka.
FEATURES             Location/Qualifiers
     source          1..598
                     /organism="Campylobacter species"
                     /mol_type="genomic DNA"
                     /strain="strain"
     gene            complement(10..453)
                     /locus_tag="CIKGKLNE_00027"
     CDS             complement(10..453)
                     /locus_tag="CIKGKLNE_00027"
                     /inference="ab initio prediction:Prodigal:002006"
                     /codon_start=1
                     /transl_table=11
                     /product="hypothetical protein"
                     /protein_id="Prokka:CIKGKLNE_00027"
                     /translation="MHEWILCGEEVKYLEEALKLGANPNMRRIKTENKFSFSGAGETL
                     LHRAARSKEKPIGACVEVLIKYGADVNAVNCGICDIEDGKIEYYDPATPLDEANTYDI
                     NKNIKILKKAGAKTWEELVEEYNIDTSLEEPEQIKMYEERRKDKK"
ORIGIN
        1 tttttccttt tattttttat cttttcttct ttcttcatac attttaattt gttctggttc
       61 ttctaagctt gtatcgatat tgtattcttc aaccaattct tcccaagtct tagctcctgc
      121 tttttttaag atttttatat ttttatttat atcgtaagta ttagcttcat ccaatggagt
```

```
      181 agcaggatca taatattcaa ttttcccatc ttcaatatca catataccac aattaactgc
      241 atttacatca gctccatatt tgattagcac ttccacgcaa gctcctatag gcttttcttt
      301 acttcttgca gctctatgca aaagggtttc tcctgcacca ctaaagctaa atttattttc
      361 tgttttaatt cttctcatat taggatttgc tcctaatttt aaagcttctt ctaaatactt
      421 cacttcttcg ccacatagta tccattcatg caaaggagtg ctgccacttt tttcattata
      481 agtttcaaat gtgcatcctt ttttaaaaaa atactctatt acttctttat tttttccatc
      541 tctatcgata tatctactga gtgcattgta tccaaagtgt acttcatcag ctttaagt
//

>>BfR-CA-15866_min_cov_30 putative plasmid location

LOCUS       HHHABCBJ_1              1844 bp    DNA     linear       12-APR-2021
DEFINITION  Campylobacter species strain strain.
ACCESSION
VERSION
KEYWORDS    .
SOURCE      Campylobacter species
  ORGANISM  Campylobacter species
            Unclassified.
COMMENT     Annotated using prokka 1.14.6 from
            https://github.com/tseemann/prokka.
FEATURES             Location/Qualifiers
     source          1..1844
                     /organism="Campylobacter species"
                     /mol_type="genomic DNA"
                     /strain="strain"
     gene            1..468
                     /gene="trxB_1"
                     /locus_tag="HHHABCBJ_00001"
     CDS             1..468
                     /gene="trxB_1"
                     /locus_tag="HHHABCBJ_00001"
                     /inference="ab initio prediction:Prodigal:002006"
                     /inference="similar to AA sequence:RefSeq:CKG87041.1"
                     /codon_start=1
                     /transl_table=11
                     /product="thioredoxin reductase"
                     /protein_id="Prokka:HHHABCBJ_00001"
                     /translation="MDWVIDLTEYEFADDADGKAIRSLDDDVLKGKLKDPRDIDSTPE
                     SRREFNREMWAYRRGSVAFYNTDIPNDWTNQEAKLYIDSLILESKLAVLTPPQGYPNA
                     PYYWVPEHLEYVYKKHKLDKLLDPRIPAIYRYNFPRELRAKILKFAEENGIKD"
     gene            550..1827
                     /gene="trxB_2"
                     /locus_tag="HHHABCBJ_00002"
     CDS             550..1827
                     /gene="trxB_2"
                     /locus_tag="HHHABCBJ_00002"
                     /inference="ab initio prediction:Prodigal:002006"
                     /inference="similar to AA sequence:RefSeq:CKG86610.1"
                     /codon_start=1
                     /transl_table=11
                     /product="thioredoxin reductase"
                     /protein_id="Prokka:HHHABCBJ_00002"
                     /translation="MSDTYEIYTPHGSILDVNKKTNEIYFDQSSKPTGKYTKEYSKAL
```

```
                     FKAWEIMENSPYKDYEPRYLDPNFYTGQKSTLVEFKEWQSIYLKDPIKGSIAPWTKAE
                     KAYYKSLKTKRERYKYLVIRSGIRSTVIDIPYDAYANVDEKGNLINEDYKELYKEVEA
                     NRGLAHLSDGYLFMGEWELAAGILGDIKGFIGALQLSMTGFKARTQAVNFLLIQLGHE
                     QGFKSLYDSYAYRGLTDGIHKNPLKAQMLKDFSKNPPYDEFGMLPFLDELMGVDWVID
                     LTEYEFADDADGKAIRSLDDDVLKGKLKDPRDIDSTPESRREFNREMWAYRRGSVAFY
                     NTDIPNDWTNQEAKLYIDSLILESKLAVLTPPQGYPNAPYYWVPEHLEYVYKKHKLDK
                     LLDPRIPAIYRYNFPRELRAKILKFAEENGIKD"
ORIGIN
        1 gtggattggg tgattgattt aacagaatat gaatttgcag atgatgctga tggtaaagct
       61 attagatctt tagatgatga tgttttaaaa ggtaaattaa aagatccaag agatatagac
      121 tctactccag agagtagaag agagtttaat cgtgaaatgt gggcatatcg tagaggttct
      181 gttgcatttt acaatacgga tattcccaat gattggacta atcaagaagc gaaactatat
      241 atagattctc ttattttaga atctaaacta gcagtcttaa ctcctccaca aggctatcct
      301 aatgcacctt attattgggt tccagaacat ttagaatatg tttacaaaaa acataagctt
      361 gataaactcc ttgatccaag aatccctgct atttatagat ataatttccc aagagaactt
      421 agagctaaga tactcaaatt tgctgaagaa aatggcatta aagattaaag taattgcata
      481 ggcagactag ccactactat cattatgaaa aatggtttgc atataggtta aataaagaaa
      541 ggataaaaga tgagtgatac ttatgagatt tatacaccac atggttccat attagatgtt
      601 aataaaaaaa ctaatgaaat atattttgat caaagttcca aacccacagg aaaatatacc
      661 aaagaatatt ccaaagccct cttcaaagct tgggaaatca tggaaaactc cccctacaaa
      721 gattatgaac caagatattt agatcctaac ttttatacag ggcaaaaatc cactctagta
      781 gaatttaaag aatggcaaag catatattta aaagatccta tcaaaggaag catagctcct
      841 tggactaaag cagaaaaagc ttattataaa tctttaaaaa ctaaaagaga aagatataaa
      901 tatctagtga ttagaagtgg tataagaagc actgttatag atatacccta tgatgcttat
      961 gctaatgtag atgaaaaagg aaatttaatc aatgaagatt ataaagaact ttacaaagaa
     1021 gtagaagcta atagaggctt agctcattta agtgatggat atttatttat gggtgaatgg
     1081 gaattagcag cagggatact aggagatata aaaggattta taggagcctt acaactttct
     1141 atgacaggat ttaaagcaag aacccaagca gtaaattttt tactcataca actaggacat
     1201 gaacaaggtt ttaaaagtct ttatgattct tatgcatata gaggtttaac agatggtata
     1261 cataaaaacc ctcttaaagc tcaaatgcta aaagactttt ctaaaaatcc tccttatgat
     1321 gaatttggaa tgctaccttt cttagatgag cttatggggg tggattgggt gattgattta
     1381 acagaatatg aatttgcaga tgatgctgat ggtaaagcta ttagatcttt agatgatgat
     1441 gttttaaaag gtaaattaaa agatccaaga gatatagact ctactccaga gagtagaaga
     1501 gagtttaatc gtgaaatgtg ggcatatcgt agaggttctg ttgcatttta caatacggat
     1561 attcccaatg attggactaa tcaagaagcg aaactatata tagattctct tattttagaa
     1621 tctaaactag cagtcttaac tcctccacaa ggctatccta atgcacctta ttattgggtt
     1681 ccagaacatt tagaatatgt ttacaaaaaa cataagcttg ataaactcct tgatccaaga
     1741 atccctgcta tttatagata taatttccca agagaactta gagctaagat actcaaattt
     1801 gctgaagaaa atggcattaa agattaaagt aattgcatag gcag
//
LOCUS       HHHABCBJ_2              1739 bp    DNA     linear       12-APR-2021
DEFINITION  Campylobacter species strain strain.
ACCESSION
VERSION
KEYWORDS    .
SOURCE      Campylobacter species
  ORGANISM  Campylobacter species
            Unclassified.
COMMENT     Annotated using prokka 1.14.6 from
            https://github.com/tseemann/prokka.
FEATURES             Location/Qualifiers
     source          1..1739
                     /organism="Campylobacter species"
                     /mol_type="genomic DNA"
```

```
                     /strain="strain"
     gene            complement(703..1482)
                     /gene="trxB_3"
                     /locus_tag="HHHABCBJ_00003"
     CDS             complement(703..1482)
                     /gene="trxB_3"
                     /locus_tag="HHHABCBJ_00003"
                     /inference="ab initio prediction:Prodigal:002006"
                     /inference="similar to AA sequence:RefSeq:CKG86610.1"
                     /codon_start=1
                     /transl_table=11
                     /product="thioredoxin reductase"
                     /protein_id="Prokka:HHHABCBJ_00003"
                     /translation="MHKGWLFMAEWELAAGILGDIKGFIGALQLSMTGFKARTQAVNF
                     LLIQLGHEQGFKSLYDSYAYRGLTDGIHKNSLKAQMLKDFSKNPPYDEFGMLPFLDEL
                     IGVDWVIDLNEYDFAYDEAGSIIRALDDDVLKGKLKDPRDIDSTPESRLEFEYKRDAY
                     KNGMLNGYGDNIPSDWTQKEVKLWLDTLVLQAKLAVLTPPQGYPNAPYYFSPERLEWD
                     YKNHQLDKLLDPRIPAIYRYNFPRELRAKILKFGEENGIKD"
ORIGIN
        1 tgcttgagtt cttgctttaa atcctgtcat ggaaagttgt aaggctccta taaatccttt
       61 tatatctcct agtattcctg ctgctaattc ccattcaccc ataaataaat atccatcact
      121 taaatgagct aagcctctat tagcttctac ttctttgtaa agttctttat agtctttatt
      181 gattaaattt ccttttttcat ctacattaca ataagcatca taaggtatat ctataaccac
      241 agatcttaat ccacttctta taattaaata tttatatctt tctcttttag tttttaaaga
      301 tttataataa gctttttctg ctttagtcca aggagctata gctcctttga taggatcttt
      361 taaatatatg ctttgccatt ctttaaattc aagcaaagtg gatttttgcc ctgtataaaa
      421 gttaggatct agatatattg gttgatagtc tttgtatggg gagttttgtt taatttccca
      481 agccttaaaa aaaactctag tatattcttt agtatattta cctacaggat caacgctttg
      541 tgcaaaatat atttcttttg ttttttatc tacctctact cctaatccat taggagcata
      601 tatcatataa gtatcactca tcttttatcc tttcttaatt taacctatat ataaaccatc
      661 ttctatgatt atagtagtgg ctagtctgcc tatgcaatta ctttaatctt taatgccatt
      721 ttcttcacca aatttgagta tcttagctct aagttccctt gggaaattat atctataaat
      781 agcagggatt cttggatcaa ggagtttgtc taattgatga tttttataat cccattccag
      841 cctttctggg ctaaaataat aaggtgcatt aggatagcct tgtggaggag ttaagaccgc
      901 tagtttggct tgtaaaacta aagtatctag ccataatttt acttcttttt gcgtccaatc
      961 actaggaata ttatcaccat agccatttaa catcccattt ttataagcat ctcttttgta
     1021 ttcaaattcc aatctactct ctggagtaga gtctatatct cttggatctt ttaatttacc
     1081 ttttaaaaca tcatcatcta aagccctaat aatgctacct gcttcatcat aagcaaaatc
     1141 atattcattc aaatcaatca cccaatccac tcctataagc tcatctaaga aaggtagcat
     1201 tccaaattca tcataaggag gatttttaga aaagtctttt agcatttgag ctttaagaga
     1261 gtttttgtgt atgccatctg ttaaacctct atatgcataa gaatcataaa gacttttaaa
     1321 gccttgttca tgtcctagtt gtatgagtaa aaaatttact gcttgagttc ttgctttaaa
     1381 tcctgtcata gaaagttgta aggctcctat aaatcctttt atatctccta gtattcctgc
     1441 tgctaattcc cattcagcca taaagagcca acctttatgc atattagcca tacctctatt
     1501 agcttctact tctttataga gttctttata gtctttattg attaaatttc ctttttcatc
     1561 tacattacaa taagcatcat aaggtatatc tataacagta cttctaagtc cacttcttat
     1621 aattaaatat ttatatcttt ctcttttggt ttttaaagat ttataataag ctttttctgc
     1681 tttagtccaa ggagctatag ctcctttgat aggatctttt aaatatatgc tttgccatt
//
LOCUS       HHHABCBJ_3              1177 bp    DNA     linear       12-APR-2021
DEFINITION  Campylobacter species strain strain.
ACCESSION
VERSION
KEYWORDS    .
```

```
SOURCE      Campylobacter species
  ORGANISM  Campylobacter species
            Unclassified.
COMMENT     Annotated using prokka 1.14.6 from
            https://github.com/tseemann/prokka.
FEATURES             Location/Qualifiers
     source          1..1177
                     /organism="Campylobacter species"
                     /mol_type="genomic DNA"
                     /strain="strain"
     gene            74..571
                     /gene="trxB_4"
                     /locus_tag="HHHABCBJ_00004"
     CDS             74..571
                     /gene="trxB_4"
                     /locus_tag="HHHABCBJ_00004"
                     /inference="ab initio prediction:Prodigal:002006"
                     /inference="similar to AA sequence:RefSeq:CKG86610.1"
                     /codon_start=1
                     /transl_table=11
                     /product="thioredoxin reductase"
                     /protein_id="Prokka:HHHABCBJ_00004"
                     /translation="MLPFIDEIMGVDWVIDLNKYDFAYDEEGRIIWALYNDIKKGKLK
                     DPRDVDSTPESRNEFDDAMDGYENGMVTRFDVDTPNEWSEQQAALDRDTLVLSAKLAA
                     LTPPQGYPNAPYYFTPERLEWIYKRGYLDKLLDPRIPAIYRYNFPRELRAKILKFAEE
                     NGIKD"
ORIGIN
        1 gatacataaa aatcctttaa gggctaaatt agtaaaagat ttagctaaaa ctatacagcc
       61 tgatgaattt ggtatgctac cttttataga tgagattatg ggagtagatt gggtgattga
      121 tttaaataaa tatgattttg cctatgatga agaaggtaga atcatttggg ctttatataa
      181 tgatattaaa aaaggtaaat taaaagatcc aagagatgtg gattctactc ctgaaagtag
      241 aaatgaattt gatgatgcta tggatggata cgaaaatgga atggtaacta gatttgatgt
      301 agatactcca aatgaatgga gtgaacaaca agctgcttta gatagagata ccctagtatt
      361 aagtgccaaa ctcgcagccc tcactcctcc acaaggctat cctaatgccc cttattattt
      421 tacacctgaa agattggaat ggatttataa aagaggatat ttagataaac tccttgatcc
      481 aagaattcct gctatttata gatataattt cccaagggaa cttagagcta agatactaaa
      541 atttgctgaa gaaaatggca ttaaagatta aagtaatttc ataggtagac tagccactac
      601 tataatcata gaagatggtt tatatatggg ttaaattaag aaaggataaa aatgagtgaa
      661 acttataaaa tttatacacc caatggatta gcattagatg tggaaaaaga taccaataaa
      721 atactattta aagaaaatat taaacccaca ggcaattata ccgaagaata ttccaaagct
      781 ttgtttgaag cacacgacat taaacgaaac tccccctaca aagactataa accaatatat
      841 ctagatccta acttttatac agggcaaaaa tccactctag tagaatttaa agaatggcaa
      901 agcatatatt taaaagatcc cattaaggga agcatagctc cttggactaa agcagaaaaa
      961 gcttattata aatctttaaa aactaaaaga gaaagatata aatatctaat tataagaagt
     1021 ggtataagaa gcactgttat agatatacct tatgatgctt attgtaatgt agatgaaaaa
     1081 ggatatttaa tcaatgaaga atacgcctat atttatgatg aagttaacaa taataaagaa
     1141 accctaaaat cttctctttt tagacaagaa tggggta
//
LOCUS       HHHABCBJ_4              1010 bp    DNA     linear       12-APR-2021
DEFINITION  Campylobacter species strain strain.
ACCESSION
VERSION
KEYWORDS    .
SOURCE      Campylobacter species
```

```
  ORGANISM  Campylobacter species
            Unclassified.
COMMENT     Annotated using prokka 1.14.6 from
            https://github.com/tseemann/prokka.
FEATURES             Location/Qualifiers
     source          1..1010
                     /organism="Campylobacter species"
                     /mol_type="genomic DNA"
                     /strain="strain"
     gene            complement(440..937)
                     /gene="trxB_5"
                     /locus_tag="HHHABCBJ_00005"
     CDS             complement(440..937)
                     /gene="trxB_5"
                     /locus_tag="HHHABCBJ_00005"
                     /inference="ab initio prediction:Prodigal:002006"
                     /inference="similar to AA sequence:RefSeq:CKG86610.1"
                     /codon_start=1
                     /transl_table=11
                     /product="thioredoxin reductase"
                     /protein_id="Prokka:HHHABCBJ_00005"
                     /translation="MLPFIDEIMGVDWVIDLNQYQFAYDGKGDSTWALYDDIEKGKLK
                     DPRDVDSTPESRKEFDEETDVYRFGNRMNFASDIRDSRSEESTRLFDDTLILHAKLAA
                     LTPPQGYPNAPYYFTPERLEWIYKRGYLDKLLDPRIPAIYRYNFPRELRAKILKFAEE
                     NGIKD"
ORIGIN
        1 ctagatattt atatctttct cttttggttt ttaaagattt ataataagct ttttctgctt
       61 tagtccaagg agctatgctt cctttaatgg gatcttttaa atatatgctt tgccattctt
      121 taaattctac tagagtggat ttttgccctg tataaaagtt aggatctaga tatattggtt
      181 tatagtcttt gtagggggag tttttcataa tataataaga ttcaaaaacc gctttggaat
      241 attcttcggt ataattgcct gtgggtttaa tattttcttt aaatagtatt ttattggtat
      301 ctttttccac atctaatgtt aacccattgg gtgtataaat ctcataagtt tcgctcatta
      361 tttatccttt cttaatttaa cccatatata aaccatcttc tatgattata gtagtggcta
      421 gtctacctat gcaattactt taatctttaa tgccattttc ttcagcaaat ttgagtatct
      481 tagctctaag ttcccttggg aaattatatc tataaatagc agggattctt ggatcaagga
      541 gtttatctaa atatcctctt ttataaatcc attccaatct ttcaggtgta aaataataag
      601 gggcattagg atagccttgt ggaggagtga gggctgcgag tttggcatga agtattaaag
      661 tatcatcaaa taatctagtt gattcttcac ttctactatc acgaatatca gaggcaaaat
      721 tcattctgtt accaaatcta taaacgtcag tttcttcatc aaattctttc ctgctttcag
      781 gagtagaatc cacatctctt ggatctttta atttaccttt ttcaatatca tcatataaag
      841 cccaagtaga atcaccttta ccgtcatacg caaattgata ctgatttaaa tcaatcaccc
      901 aatctactcc cataatctca tctataaaag gtagcatacc aaattcatca ggctgtatag
      961 ttttagctaa atcttttact aatttagccc ttaaaggatt tttatgtatc
//
LOCUS       HHHABCBJ_5                997 bp    DNA     linear       12-APR-2021
DEFINITION  Campylobacter species strain strain.
ACCESSION
VERSION
KEYWORDS    .
SOURCE      Campylobacter species
  ORGANISM  Campylobacter species
            Unclassified.
COMMENT     Annotated using prokka 1.14.6 from
            https://github.com/tseemann/prokka.
```

```
FEATURES             Location/Qualifiers
     source          1..997
                     /organism="Campylobacter species"
                     /mol_type="genomic DNA"
                     /strain="strain"
     gene            36..818
                     /gene="trxB_6"
                     /locus_tag="HHHABCBJ_00006"
     CDS             36..818
                     /gene="trxB_6"
                     /locus_tag="HHHABCBJ_00006"
                     /inference="ab initio prediction:Prodigal:002006"
                     /inference="similar to AA sequence:RefSeq:CKG86610.1"
                     /codon_start=1
                     /transl_table=11
                     /product="thioredoxin reductase"
                     /protein_id="Prokka:HHHABCBJ_00006"
                     /translation="MHKGWLFMAEWELAAGILGDIKGFIGALQLSMTGFKARTQAVNF
                     LLIQLGHEQGFKSLYDSYAYRDLTDGIHKNPLKAQMLKDFAKNPPYDEFGMLPFLDEL
                     IGVDWVIDPNDYYFVYDGAKGRVNDALKDDVEKGTLKDPRDVDSTPESREEFIDNFER
                     YGNGMRERYTLEISNDTPETSANIWLETMLLEAKIKALTPPQGYPSAPYYYSPERLEW
                     IYKNHNLDRLLDPRIPAIYRYNFPESLREKIQAYAKEHNIKE"
ORIGIN
        1 ataaagaagt agaagctaat agaggtatgg ctaatatgca taaaggttgg ctctttatgg
       61 ctgaatggga attagcagca ggaatactag gagatataaa aggatttata ggagccttac
      121 aactttccat gacaggattt aaagcaagaa ctcaagcagt aaatttttta ctcatacaac
      181 taggacatga acaaggtttt aaaagtcttt atgattctta tgcatataga gatttaacag
      241 atggcataca caaaaaccct cttaaagctc aaatgctaaa agactttgct aaaaatcctc
      301 cttatgatga atttggaatg ctaccttttc tagatgaact tataggagtg gattgggtga
      361 tagatccaaa tgattattat tttgtttatg atggagcaaa aggaagagtt aatgatgccc
      421 taaaagatga tgttgaaaaa ggaacactca aagatccaag agatgtagat tccactccag
      481 agagtaggga agaattcatt gataacttcg agcgttatgg gaatggaatg agggagcgct
      541 atactttgga aataagcaat gatactcctg aaacatctgc taatatttgg ctagaaacaa
      601 tgcttttaga agctaaaatt aaagccctaa ctccccctca aggctatcct agtgcacctt
      661 attattactc tcctgaaaga ttagaatgga tttataaaaa tcataattta gataggcttc
      721 ttgatcctag aatccctgca atttatcgtt ataactttcc agaaagccta agagagaaga
      781 tacaagctta tgctaaagag cataatatca aagaataaac caccaaaaat gataggcaga
      841 ctagctacta ctatcattat gaaaaatggc ttgcatatag gttaaataaa gaaaggataa
      901 aagatgagtg atacttatga gatttataca ccaaatggac ttatattaaa tgtagaaaaa
      961 gacacaaata aaatattaat tggcgataca attaata
//
LOCUS       HHHABCBJ_6               892 bp    DNA     linear       12-APR-2021
DEFINITION  Campylobacter species strain strain.
ACCESSION
VERSION
KEYWORDS    .
SOURCE      Campylobacter species
  ORGANISM  Campylobacter species
            Unclassified.
COMMENT     Annotated using prokka 1.14.6 from
            https://github.com/tseemann/prokka.
FEATURES             Location/Qualifiers
     source          1..892
                     /organism="Campylobacter species"
```

```
                     /mol_type="genomic DNA"
                     /strain="strain"
     gene            complement(393..890)
                     /gene="trxB_7"
                     /locus_tag="HHHABCBJ_00007"
     CDS             complement(393..890)
                     /gene="trxB_7"
                     /locus_tag="HHHABCBJ_00007"
                     /inference="ab initio prediction:Prodigal:002006"
                     /inference="similar to AA sequence:RefSeq:CKG86288.1"
                     /codon_start=1
                     /transl_table=11
                     /product="thioredoxin reductase"
                     /protein_id="Prokka:HHHABCBJ_00007"
                     /translation="MLPFLDELIGVDWVIDLNEYQFAYDGDGGSVKALDEKVRQGKLK
                     DPRDIDSTPESRKEFDEEANAYTFGNRMNFSSDIRDSRSKESARLFDDLLILHAKLAA
                     LTPPQGYPNAPYYFSPEQLEDFYKRQVLDAKLDPRIPAIYRYNFPRELRAKILKFGEE
                     NGIKD"
ORIGIN
        1 taataagctt tttctgcttt agtccaagga gctatacttc ctttgatagg ttcttttaaa
       61 tatatacttt gccaatcttt aaattctact agagtggatt tttgccctgt ataaaagtta
      121 ggatctagat atattggttg atagtctttg tatggggagt ttcttaatat gcgatcaccc
      181 tcgaagagag cttgagaatg ttgtttagtg tatgtaccat tgctttttgc cgtaaataat
      241 attttatttg tatctttttc tacatttaat attgagccat ctggtgtata aatctcataa
      301 gtatcactca ttttttatcc tttctttatt taacctatat gcaaaccatt tttcataatg
      361 atagtagtag ctagtctgcc tatgaaatta ctttaatctt taatgccatt ttcttcgcca
      421 aattttagta tcttagctct aagttccctt gggaaattat atctataaat agcagggatt
      481 cttggatcaa gtttagcgtc taaaacttgt cttttgtaaa aatcttctaa ttgttcagga
      541 ctaaaataat aaggtgcatt aggataacct tgaggaggag tgagggctgc gagtttggca
      601 tgaagtatca aaaggtcatc aaataatcta gccgattctt tacttctact atcgcgaata
      661 tcagaggaaa aattcattct attaccaaaa gtataagcat tagcttcttc atcgaattct
      721 ttcctgctct ctggagtaga gtctatatct cttggatctt ttaatttacc ttgtcttact
      781 ttctcatcta aagccttaac actaccgcca tcaccatcat aagcaaattg atattcattc
      841 aaatcaatca cccaatccac tcctataagt tcatctagaa aaggtagcat ac
//
LOCUS       HHHABCBJ_7               849 bp    DNA     linear       12-APR-2021
DEFINITION  Campylobacter species strain strain.
ACCESSION
VERSION
KEYWORDS    .
SOURCE      Campylobacter species
  ORGANISM  Campylobacter species
            Unclassified.
COMMENT     Annotated using prokka 1.14.6 from
            https://github.com/tseemann/prokka.
FEATURES             Location/Qualifiers
     source          1..849
                     /organism="Campylobacter species"
                     /mol_type="genomic DNA"
                     /strain="strain"
     gene            114..656
                     /gene="trxB_8"
                     /locus_tag="HHHABCBJ_00008"
     CDS             114..656
```

```
                     /gene="trxB_8"
                     /locus_tag="HHHABCBJ_00008"
                     /inference="ab initio prediction:Prodigal:002006"
                     /inference="similar to AA sequence:RefSeq:CKG87041.1"
                     /codon_start=1
                     /transl_table=11
                     /product="thioredoxin reductase"
                     /protein_id="Prokka:HHHABCBJ_00008"
                     /translation="MLKDFAKNPPYDEFGMLPFLDELIGVDWVIDPNRYRFAEDEKGR
                     VNDALKDDVEKGILKDPRDIDSTSESRLEFEYKLDAYRNGMKTRFDGDNPNHWSKEQV
                     ERFNDTLILCAKLAALTPPQGYPNAPYYYSPERLEFIYKKHKLDKLKDPRIPAIYRYN
                     FPRELRAKILKFGEENGIKD"
ORIGIN
        1 taaatttttt actcatacaa ctaggacatg aacaaggctt taaaagtctt tatgattctt
       61 atgcatatag agatttaaca gatggcatac acaaaaactc tcttaaagct caaatgctaa
      121 aagactttgc taaaaatcct ccttatgatg aatttggtat gctacctttt ctagatgaac
      181 ttataggagt ggattgggtg atagatccaa ataggtatcg atttgcagaa gacgaaaaag
      241 gaagagttaa tgatgctcta aaagatgatg ttgaaaaagg aatactcaaa gatccaagag
      301 atatagattc tacttcagag agtagattgg aatttgaata taaattggat gcttatagaa
      361 atggaatgaa aactagattt gatggagata atccaaatca ttggagtaaa gagcaagtag
      421 aaagatttaa tgatactttg attttatgtg ccaaactcgc agccctcact cctcctcaag
      481 gctatcctaa tgcaccttat tattactctc ctgaaagatt agaatttatt tacaaaaaac
      541 acaaacttga taaattaaaa gatccaagaa tccctgctat ttatagatat aatttcccaa
      601 gggaacttag agctaagata ctcaaatttg gcgaagaaaa tggcattaaa gattaaagta
      661 attgcatagg cagactagct accactataa tcatgaaaaa tggtttatat ataggttaaa
      721 ttaagaaagg ataaaagatg agtgatactt atgagattta tacaccacat ggttccatat
      781 tagatgttaa taaaaaaact aatgaaatat attttgatca aagttccaaa cccacaggaa
      841 aatatacca
//


>>BfR-CA-15867_min_cov_30 putative plasmid location

LOCUS       NLMJPNJI_1             24409 bp    DNA     linear       12-APR-2021
DEFINITION  Campylobacter species strain strain.
ACCESSION
VERSION
KEYWORDS    .
SOURCE      Campylobacter species
  ORGANISM  Campylobacter species
            Unclassified.
COMMENT     Annotated using prokka 1.14.6 from
            https://github.com/tseemann/prokka.
FEATURES             Location/Qualifiers
     source          1..24409
                     /organism="Campylobacter species"
                     /mol_type="genomic DNA"
                     /strain="strain"
     gene            159..1055
                     /locus_tag="NLMJPNJI_00001"
     CDS             159..1055
                     /locus_tag="NLMJPNJI_00001"
                     /inference="ab initio prediction:Prodigal:002006"
                     /inference="similar to AA sequence:RefSeq:AAW35468.1"
```

```
                     /note="conserved domain protein"
                     /codon_start=1
                     /transl_table=11
                     /product="hypothetical protein"
                     /protein_id="Prokka:NLMJPNJI_00001"
                     /translation="MIVSFLDDDIDKPYVSSSLYNGTNPSLVNLPFNDHQTSLSSKTI
                     GVNEEGYNELTLSNIKDKEQIYLKAQKDYDELVQHNFTQRILNDKDSKVDGIYNERIK
                     KVHTQTIDLAKNVNVGAEYLINVGLSKDTIVGLSNTLNVGVDNKVRIAKNSHEFVGEN
                     KDIEIGANQNTIIHKDEIRNVKGNKKEVVEGHYDINIKETLKIQTEKETSIRSKNNLL
                     ITTNASMGFETDKNNTFVSDNSLSQTKTDYEVKAGNQILHQVGDTQIVTKKDCVIIKA
                     GGVEVIIDSNGLVVKGGELKAE"
     gene            1055..3046
                     /locus_tag="NLMJPNJI_00002"
     CDS             1055..3046
                     /locus_tag="NLMJPNJI_00002"
                     /inference="ab initio prediction:Prodigal:002006"
                     /inference="similar to AA sequence:RefSeq:CKG88019.1"
                     /codon_start=1
                     /transl_table=11
                     /product="hypothetical protein"
                     /protein_id="Prokka:NLMJPNJI_00002"
                     /translation="MEALNQKNSLNIRLLSSNNILLHNQEFKLYEIAQGNKNEIKTIF
                     TTNRIGEAHLNASEIFSKEAQSFELILNQSSVYKNKPIYNHRFLLRNYEYGHWCEIKF
                     ERKADKGSINSIRLKSKEFTLENNEKIVRNFYTFGDIVEFEAIYEDTKIKEEQIKWGY
                     VFIQDKNTLDKLNKNQLTNDKKESSLLDEEILKDCFYIEKEEDKALLSSSIIYRHLEN
                     NKAYCGKHLSLQLPNPKDTQEQKALLVFAYKEIPNYNASYLIRINDYPQITIDCTLAE
                     TLRAHTNKDEISRLGWGVSYICQRLWHDNPSDAKELKDLTFRSSTSQDFIDTIPNETM
                     KTIVKEILPRVEMQQLKTDNTKSRDKARFYAELDWDNFYMKFSIMQELKGEYMNLKKL
                     FGEESDFQKQFEDFLNDTLLDIKNIKNTQEYTMALNIQAMKENKTKNAEVFKLYKKEE
                     TLPETHKAIKDLQKPSDIIDNSLYFQRFDIKNDVFLPHLGLSKFDAFSLQNSIQHEKE
                     VLDKFHSNPKYKQNFALYSIAGAFNILYIPSLIQITRVTRFYNYHSLYAACKKVRAFI
                     IDTVNFNNNGSDQPIGAWDYEKVGFDLYNSIMQYRNTKFHFKYTSPKSFLFPLYNSDF
                     LKTKQYFNLGLDFFLYSSTFLDMDFSHMQMQDTAFIVGK"
     gene            3048..3659
                     /locus_tag="NLMJPNJI_00003"
     CDS             3048..3659
                     /locus_tag="NLMJPNJI_00003"
                     /inference="ab initio prediction:Prodigal:002006"
                     /inference="similar to AA sequence:RefSeq:CKG88032.1"
                     /codon_start=1
                     /transl_table=11
                     /product="hypothetical protein"
                     /protein_id="Prokka:NLMJPNJI_00003"
                     /translation="MFFAGGDYEPFIYGLSLIAYYFLPLYCLTLLLYKYKPQYVNLAL
                     HIGASVLLYLAMWVIFVDTTIISLFEIFQMFLIPIILQIIHLTFILYAIIYTYFDMQK
                     ISFTKRLGRYIVSFFIGIGLLILSFVMMYAIGYYISSWCDRYYLKIDERNEAIRTITF
                     YVLVVVFPSIIALLLSYILYKNHKSFFTKILYRILSMIGKDKP"
     gene            3659..3820
                     /locus_tag="NLMJPNJI_00004"
     CDS             3659..3820
                     /locus_tag="NLMJPNJI_00004"
                     /inference="ab initio prediction:Prodigal:002006"
                     /codon_start=1
                     /transl_table=11
```

/product="hypothetical protein"
/protein_id="Prokka:NLMJPNJI_00004"
/translation="MQKLIYQNHDYQQIQIQQTQKHFNLGCDFRILTSTFKDIEVCSF
SMPISIKVD"
gene 4062..4301
/locus_tag="NLMJPNJI_00005"
CDS 4062..4301
/locus_tag="NLMJPNJI_00005"
/inference="ab initio prediction:Prodigal:002006"
/codon_start=1
/transl_table=11
/product="hypothetical protein"
/protein_id="Prokka:NLMJPNJI_00005"
/translation="MDSVSIVFLGAMIFNIYSTIYLLLFKFLSNNNQNIFPSKKEKYC
LFGLNLLFSLLFYAIWFAAFEYDLADKVVKFFNGV"
gene 4304..4561
/locus_tag="NLMJPNJI_00006"
CDS 4304..4561
/locus_tag="NLMJPNJI_00006"
/inference="ab initio prediction:Prodigal:002006"
/codon_start=1
/transl_table=11
/product="hypothetical protein"
/protein_id="Prokka:NLMJPNJI_00006"
/translation="MNGAKLLDNIKPSDLKRSQMQFYGIANKEFGIEYKLSSKERSNQ
VIALYALSGKFQIYYVLDSFEVERMNEKEIVIYPTQIESWY"
gene 4710..5153
/locus_tag="NLMJPNJI_00007"
CDS 4710..5153
/locus_tag="NLMJPNJI_00007"
/inference="ab initio prediction:Prodigal:002006"
/inference="similar to AA sequence:RefSeq:CKG88076.1"
/codon_start=1
/transl_table=11
/product="hypothetical protein"
/protein_id="Prokka:NLMJPNJI_00007"
/translation="MTDGFGFLAYVFTLPVLILGGISCVCIVFLIRFQSFCGFVCSKS
FWIAWLFPYYLLFLIICITCEWELLTMYICGISLPLVYFLRKFFMQSHLNLAPNEFSL
WIIPMSIFSLCIHSSLCVAIAWVLIFFQTNYDTQALQFWLRFLFI"
gene 5237..5707
/locus_tag="NLMJPNJI_00008"
CDS 5237..5707
/locus_tag="NLMJPNJI_00008"
/inference="ab initio prediction:Prodigal:002006"
/codon_start=1
/transl_table=11
/product="hypothetical protein"
/protein_id="Prokka:NLMJPNJI_00008"
/translation="MQKILIVLGLCLMLAISANAETNNKANSTHATHLNKWLGTYTLQ
ENLYMGSFTIKKCDKFNICEARYEAILNKSEFNDMHECDITLKLQIKSPQEAIALNDD
KDFANCKIYIRKNQQGIVFIRDEKVFNSPDYFCSTMCGNQTIFEWGDIYKKEMQ"
gene 5707..6111
/locus_tag="NLMJPNJI_00009"
CDS 5707..6111

```
                     /locus_tag="NLMJPNJI_00009"
                     /inference="ab initio prediction:Prodigal:002006"
                     /codon_start=1
                     /transl_table=11
                     /product="hypothetical protein"
                     /protein_id="Prokka:NLMJPNJI_00009"
                     /translation="MLYDVKKLNENDYKKECAKLLIAFENPNYKQENAIHITGNVTIG
                     YGLDLKTGNNPKLLLEGYLPDDSKKLKIGDEELTFYEIIEKYRANKAGFTDPKIVKGY
                     LQNKVFNFSLTQEQAQEILERTFDSYKKYISQ"
     gene            6433..7029
                     /gene="tssI1"
                     /locus_tag="NLMJPNJI_00010"
     CDS             6433..7029
                     /gene="tssI1"
                     /locus_tag="NLMJPNJI_00010"
                     /inference="ab initio prediction:Prodigal:002006"
                     /inference="similar to AA sequence:RefSeq:AOH52008.1"
                     /codon_start=1
                     /transl_table=11
                     /product="type VI secretion system, secreted protein"
                     /protein_id="Prokka:NLMJPNJI_00010"
                     /translation="MLLASDREKLNLANEKYPFYRLGGIGISAFEKLVGLSNTLNVGV
                     DNKVRIAKNSHEFVGENKDIEIGANQNTIIHKDEIRNVKGENKLLIEKSLTQTIEKEF
                     FLNVHQNLSAHIQDNTSLKSNSMQTKIEEQYSLESENSTFDFQTDSEVKAGNQILHQV
                     GDTQIVTKKDCVIIKAGGVEVIIDSNGLVVRGGEIKAE"
     gene            7046..7684
                     /locus_tag="NLMJPNJI_00011"
     CDS             7046..7684
                     /locus_tag="NLMJPNJI_00011"
                     /inference="ab initio prediction:Prodigal:002006"
                     /codon_start=1
                     /transl_table=11
                     /product="hypothetical protein"
                     /protein_id="Prokka:NLMJPNJI_00011"
                     /translation="MSEINNNTEIKEQDTQDEIIWEGKKHIPSFLFFWITYIVLLCLF
                     LYLFPRLFNPSKEIDYKWFIVFFTFLLGIYAFARAIYKMANIKRIYITKEKLVIEYYI
                     KNDLVFPLGTFFVYYRKISYSLSPGHIVIHTFGDKAKEYLEHGLFSDGDDPTKDCFER
                     INAIIKPHVMPYLLSLSDEEFEKIFFHVNATSEINTTFLKEAMELRKEKKDE"
     gene            7677..9263
                     /locus_tag="NLMJPNJI_00012"
     CDS             7677..9263
                     /locus_tag="NLMJPNJI_00012"
                     /inference="ab initio prediction:Prodigal:002006"
                     /codon_start=1
                     /transl_table=11
                     /product="hypothetical protein"
                     /protein_id="Prokka:NLMJPNJI_00012"
                     /translation="MNENIYGSLEFYSKETQSTPSDKRDLGVFGDVAPYALGITEGFA
                     TKAFEKEIVKFGIKHNSRIATMMHSKIIMNFGILNFFLAKAEGKSNVRALTETSVGVS
                     ITIIMNETPIGATIEKGSEHVAKQTLTQTSRVASNAATKIAGSRVASNIAARAGVSIL
                     SRIAAGTATGAAVGSSFPIVGTVVGAVVGTLVAGFFNDLIFGDEDEELKKQEEAKEKY
                     LEEKLDEKMHKIINYLITHQYIELRVLNEDEAEKLCKEISDINSAYFKTILLMLSFPY
                     YLDKDEQSYKEAQEKNPTIIRIQPIANALNIKIEINECFLAKNGEALKNKEIYVYNHR
                     FDRVVTKAMSDDEGKIVFENVYVGKESTIDKISFIIDRENFNEDNFYESVLKYAPMFN
```

```
                     VQKKHKQEGQAFIGKMFFSFTYAQEIMQDNEVLKLEALRNNFHIVFDYEVRKQEESYK
                     NYIILSYLVFDVKEDIEEYIRHTTIENRAFRGLELLGRGWKNQYSIKDEWRDKGVVFL
                     LILILKNLLLIKKWLLLTSL"
     gene            9239..9460
                     /locus_tag="NLMJPNJI_00013"
     CDS             9239..9460
                     /locus_tag="NLMJPNJI_00013"
                     /inference="ab initio prediction:Prodigal:002006"
                     /codon_start=1
                     /transl_table=11
                     /product="hypothetical protein"
                     /protein_id="Prokka:NLMJPNJI_00013"
                     /translation="MAFIDKPIVILDIEKFDKENILEDIKFHFKTLAKAYKIFVIDLD
                     ANTQIKAGGVEVIIDSNGLVVKGGELKAE"
     gene            9471..10286
                     /locus_tag="NLMJPNJI_00014"
     CDS             9471..10286
                     /locus_tag="NLMJPNJI_00014"
                     /inference="ab initio prediction:Prodigal:002006"
                     /codon_start=1
                     /transl_table=11
                     /product="hypothetical protein"
                     /protein_id="Prokka:NLMJPNJI_00014"
                     /translation="MKKPFYKLKRFYIPCIIIIIIFAVLAKLLYSPLYTIYWGMYHYP
                     KVQLEFKNFEKMTLNPSPKDMIKIVDDYQPKLEDFKDLNTKMQKAIFDFKVAKLFGFE
                     DRYFEAFIKSCAGNFFVLHGKEQIYFNYLNFISGINSTSNEKQKYLNLRASTRDLERQ
                     IFEEKLKFIKHYEEFYDYLEGVGYLDKGTEYIGTAISFKTLIQNVFLSNNAQLCSFED
                     RNLMFKNMKENYEIFKNLDVKNIDDLKRNIYKKILIDMENLLNETQKALDECK"
     gene            10273..10401
                     /locus_tag="NLMJPNJI_00015"
     CDS             10273..10401
                     /locus_tag="NLMJPNJI_00015"
                     /inference="ab initio prediction:Prodigal:002006"
                     /codon_start=1
                     /transl_table=11
                     /product="hypothetical protein"
                     /protein_id="Prokka:NLMJPNJI_00015"
                     /translation="MNANENNTSVNNENNTTINFEESIKEALREGQEIVESGKGIR"
     gene            10402..10749
                     /locus_tag="NLMJPNJI_00016"
     CDS             10402..10749
                     /locus_tag="NLMJPNJI_00016"
                     /inference="ab initio prediction:Prodigal:002006"
                     /codon_start=1
                     /transl_table=11
                     /product="hypothetical protein"
                     /protein_id="Prokka:NLMJPNJI_00016"
                     /translation="MSIYKECEVCKTKINKLQNIWNIFSGKLEIKCKNCKSKYGVSSK
                     IIDWIYFLFFENLLLFPPIVILNILYLGRYFSKYGLFEMIFYLSIAILLSRKSVNIIL
                     LLLCKLKRLENDK"
     gene            10739..13342
                     /locus_tag="NLMJPNJI_00017"
     CDS             10739..13342
                     /locus_tag="NLMJPNJI_00017"
```

```
                     /inference="ab initio prediction:Prodigal:002006"
                     /codon_start=1
                     /transl_table=11
                     /product="hypothetical protein"
                     /protein_id="Prokka:NLMJPNJI_00017"
                     /translation="MTNKGLIEHLKDAKDSAVDFYIGLYDFGDFLARISGFKDWQEKS
                     VVFDDFYKKQAFEELGFLEDVLKNETSRNIILEYLIKDVNERPLYYLAPSSILKIISK
                     TTKAKTIPTIGLISLSGKATSTAHSLYPYYKNIQNSFTPLCSIDNKNSNLLKEFNFDS
                     FSCNFYNNNIFNQSLKHLNNIQNEKEYINEYKIFLENLNYFNQEYNNYKLVSIQVINN
                     LKIAYIQKLSLQDINNININSELYLLKALYFCEDYVLFDKDKQALFNKNTIKELEFDS
                     SFYTIFVSHKAKLNDTYLQARKELYKSINEFKRLGFKDLENAYQTYIHSLIVNKEINS
                     KDKTINKNENKEINKNLNQDENSNQESNIKENASNTKKEDFNLLSSHSKNLNHFILEN
                     KDKNKRIIFLNNASSMSNLIHIYDNNCDVFIKNDSLLDIKKIEKEYDIKFKEFNTRVF
                     FYEKLLLGAKENNEFLFEEKEENLLYHFKYDKEDLSSLKDLSIKYNIYNARIKNYSLL
                     EESLNIKLEPKELKKEIAYNNASFNKLSKEPLNEFSSSAKEPCFITLYLKDEHNKPIA
                     NAKIIIKGFQHNEALRVVNLKRRSDEKGKIRFDKEKYFSDCYSFKVKLDESKAYFPTP
                     LQNAKRIFNNYTHHKVGLILKFKAKDHLVYDGFNVYHYKGNELINSYMARSGTAKADN
                     EKRSKKQIAHYFYQDEKDKSKSGKAYFYYDDESIQDRFGTLPEGEYYLKINEIAKDTK
                     PNFLKDYPFEIGKTWGRYCVRLYTDKECSKTFKEIEIKESNEKENKTSKKDKKEQSII
                     KDNLYLYSINEKGEFGSSGSIGIAQAQLLEDLSKQVKFIRSEKENIISLKVAYPEKID
                     NKIIFVGKVYE"
     gene            13335..14090
                     /locus_tag="NLMJPNJI_00018"
     CDS             13335..14090
                     /locus_tag="NLMJPNJI_00018"
                     /inference="ab initio prediction:Prodigal:002006"
                     /inference="similar to AA sequence:RefSeq:CKG88360.1"
                     /codon_start=1
                     /transl_table=11
                     /product="hypothetical protein"
                     /protein_id="Prokka:NLMJPNJI_00018"
                     /translation="MNKTLIFHENSHIDLNASFAPGTYIELQLEKESDKTLNWSYVEC
                     NSEKKMRYLLDTDCHSIEAKDLKFIDSFKGNICGFHLPISRDNEPTKDIKDYKYYIIV
                     FAYDEKKAIPSLEDFHIVIDMSFRAGVGKDESVEESKLRNDFNSDIIQTNCIFSVFEA
                     VEFLKACQSLKEEKNEIDNYIFFKNYPQLAGKIAYIYYRFDLANEEFIKSVSDGNKYL
                     KEREKFYKVASDVYIKNPIYKMGFEKKTSKSSC"
     gene            14059..14592
                     /locus_tag="NLMJPNJI_00019"
     CDS             14059..14592
                     /locus_tag="NLMJPNJI_00019"
                     /inference="ab initio prediction:Prodigal:002006"
                     /inference="similar to AA sequence:RefSeq:CKG88360.1"
                     /codon_start=1
                     /transl_table=11
                     /product="hypothetical protein"
                     /protein_id="Prokka:NLMJPNJI_00019"
                     /translation="MRKKLQNQVVDINIVKDFLKDFAKRLGIDEKVLPVIGYNPQRGD
                     SGTYDYTVNTLDLNPKASYVDFIDTIIHEFRHFYIYCIKNNSKNSIEKLLYLDMVQFY
                     IPWNFYKIFNSYDKKCLIYDNEDETKKCLKNRTYYGSWKFLNPTLDLLPSPLYYLQPS
                     ELDSRIMAYYFRKEIKL"
     gene            14592..15857
                     /locus_tag="NLMJPNJI_00020"
     CDS             14592..15857
                     /locus_tag="NLMJPNJI_00020"
```

```
                     /inference="ab initio prediction:Prodigal:002006"
                     /inference="similar to AA sequence:RefSeq:CKG88389.1"
                     /codon_start=1
                     /transl_table=11
                     /product="hypothetical protein"
                     /protein_id="Prokka:NLMJPNJI_00020"
                     /translation="MTPPSLPDVEKHEDFLQTRKEPYAIYLAINTNIKSYNNICPSEK
                     YFWKFDNMNELDKWYNPKFGIYLGKIVFDKKDNKLIPKYIAAKFENLEEEVKKIKNPL
                     WLANKNPNYIEPKFYDGMSGGYYFESPNNLEYQCKIEKDTQVLSQEQIISYVKELYSK
                     NTIIIKNYIDAINKDYGIKPFVFSDEIYDELGEVGILTKEQANNFKDKSYIKKDPILL
                     AMLDYLAKQNKKDEDYLITFDDEYFYTYLVWSLKDFLLELSYGLFQDETELLFNPAAY
                     MDDTKIYYQNLNEEINKRYEKILLNMGFEGENGYFKDYYDYGFGNNGIFEFSIYDYFA
                     YDEIGVQPIQQSPYVPPKSPFDSPNFVYSDGNYHGDAKLVPSALGKYYFELLYQKEIY
                     IELLRPYYPSIKDLPEGWDNKMLEKANLK"
     gene            16413..16619
                     /locus_tag="NLMJPNJI_00021"
     CDS             16413..16619
                     /locus_tag="NLMJPNJI_00021"
                     /inference="ab initio prediction:Prodigal:002006"
                     /codon_start=1
                     /transl_table=11
                     /product="hypothetical protein"
                     /protein_id="Prokka:NLMJPNJI_00021"
                     /translation="MNALGRYVFYSEKAYINEKIIFDKLSRICDPSENYLVGCNIGAI
                     QDTYEIQPNERDPRYVETLIGRSL"
     gene            16632..16802
                     /locus_tag="NLMJPNJI_00022"
     CDS             16632..16802
                     /locus_tag="NLMJPNJI_00022"
                     /inference="ab initio prediction:Prodigal:002006"
                     /codon_start=1
                     /transl_table=11
                     /product="hypothetical protein"
                     /protein_id="Prokka:NLMJPNJI_00022"
                     /translation="MKTIKIPLKDYISNIREDKTIYLGLQSDMSIKGLDKEFLKKNII
                     LREISNLKQAFI"
     gene            complement(17176..17394)
                     /locus_tag="NLMJPNJI_00023"
     CDS             complement(17176..17394)
                     /locus_tag="NLMJPNJI_00023"
                     /inference="ab initio prediction:Prodigal:002006"
                     /inference="similar to AA sequence:RefSeq:AVL47240.1"
                     /codon_start=1
                     /transl_table=11
                     /product="recombinase XerC"
                     /protein_id="Prokka:NLMJPNJI_00023"
                     /translation="MQINDIKTTFLINFLNYLENNLKNSNKLSKRTKITYLRVLTSFF
                     ISDNNDDLFLFSFDMKKLGLKLKKQKKS"
     gene            17775..18647
                     /locus_tag="NLMJPNJI_00024"
     CDS             17775..18647
                     /locus_tag="NLMJPNJI_00024"
                     /EC_number="3.6.1.-"
                     /inference="ab initio prediction:Prodigal:002006"
```

```
                     /inference="similar to AA sequence:RefSeq:ABS44566.1"
                     /codon_start=1
                     /transl_table=11
                     /product="F-box only protein 18"
                     /protein_id="Prokka:NLMJPNJI_00024"
                     /translation="MDEAQDINACVIDIVLNQKQAKKVFIGDTFQSIYKFRGAVNSLE
                     ILASMPKSHTLYLTQSFRCPQNIAFIANSYLKILNAPQDFKGTKKSSNENVKNEAKAI
                     VCRTNAKLFDIAVENLDKKLFFVGGINSYSFDELLDIQNLLFKKHEYIKNQFIVKFTD
                     LKELLEYINETKEVDLKRKIFVLFKYAHGDIIKLIKAIEKSSVKKQEQADLILSTGHK
                     SKGLEWNNVEIIDDFLNIKQELEERDQITIAKEELNLLYVAITRAKNSLSISKDYLLE
                     KEFIQENLERIIIE"
gene                 18644..18856
                     /locus_tag="NLMJPNJI_00025"
CDS                  18644..18856
                     /locus_tag="NLMJPNJI_00025"
                     /inference="ab initio prediction:Prodigal:002006"
                     /inference="similar to AA sequence:RefSeq:AVL47291.1"
                     /codon_start=1
                     /transl_table=11
                     /product="helix-turn-helix domain-containing protein"
                     /protein_id="Prokka:NLMJPNJI_00025"
                     /translation="MKIIKKLQKDGSLKKQVYYSVHEVAQMHSVTHVTVRLWISRGIL
                     KGVKLGKGFYMSKEAIIEFQKNDGLS"
gene                 18874..19023
                     /locus_tag="NLMJPNJI_00026"
CDS                  18874..19023
                     /locus_tag="NLMJPNJI_00026"
                     /inference="ab initio prediction:Prodigal:002006"
                     /inference="similar to AA sequence:RefSeq:ARJ53387.1"
                     /codon_start=1
                     /transl_table=11
                     /product="DNA-binding protein"
                     /protein_id="Prokka:NLMJPNJI_00026"
                     /translation="MSSIAKDSHVEMRDFLFNKYNKITFSSKECAKILGISLQTLDRL
                     TKDKK"
gene                 19693..20613
                     /locus_tag="NLMJPNJI_00027"
CDS                  19693..20613
                     /locus_tag="NLMJPNJI_00027"
                     /inference="ab initio prediction:Prodigal:002006"
                     /inference="similar to AA sequence:RefSeq:ADC28003.1"
                     /note="conserved hypothetical protein"
                     /codon_start=1
                     /transl_table=11
                     /product="hypothetical protein"
                     /protein_id="Prokka:NLMJPNJI_00027"
                     /translation="MEAKGIAIFPTNVAFSKKTNAFFTELEDQFLKEGVGNKSAIKLD
                     FEYYILYCQFLQIDKESLFFGRNYELVFCFLLYYRALLNKNNTQKIVAKIINVIRCQD
                     RLPEKVHYREKIICMDNQHRDLSQTLSSCIAEFYNSIDLYNTFRKNIGFENRKKIAIE
                     MLNRASEHYGFFGLSRGGITYKEEKISCVPIQALDEFHHGMSHLCAVFLKEDGRYTNL
                     ERAIPHFKRGMMEVYKTIIRDFMILNKDALGVKQEALKIRELEYQTIGKPKTVTKNPT
                     TNLTNKKEEKVIPGYKELCEKIVSQIKNAK"
gene                 complement(20713..21741)
                     /gene="flgS"
```

```
                     /locus_tag="NLMJPNJI_00028"
     CDS             complement(20713..21741)
                     /gene="flgS"
                     /locus_tag="NLMJPNJI_00028"
                     /inference="ab initio prediction:Prodigal:002006"
                     /inference="similar to AA sequence:RefSeq:CAL34921.1"
                     /codon_start=1
                     /transl_table=11
                     /product="signal transduction histidine kinase"
                     /protein_id="Prokka:NLMJPNJI_00028"
                     /translation="MNENILKSLDSSEKETLQKGLQSLIEQTYIVENEYKILNENYNS
                     LRAMVDEIIEVLPSALWILDKEKNIILQNQEALKKPKLLDIISLEKNRDELEFENRFY
                     AVKIIAHNEKTIVSATDISDEKRNERLASMGSVAAHLAHEIRNPIGSISLLASTLFSR
                     SELKNKHIVLEIQKAIARVERIVNSTLLFTKGVHINASKFNLLELTEECESAINSYNY
                     SSQIDFEINFLDMKIYGDKALLSLVLQNLIYNAIDAIEESECENPKIKIQASCDEQNL
                     YIRVYDNGCEIKDENLVFEAFKTTKLKGNGLGLSLSKEIINAHKGELKFEIWPKNFYF
                     VLPLHYNS"
     gene            complement(21738..22559)
                     /locus_tag="NLMJPNJI_00029"
     CDS             complement(21738..22559)
                     /locus_tag="NLMJPNJI_00029"
                     /inference="ab initio prediction:Prodigal:002006"
                     /inference="similar to AA sequence:RefSeq:CAL34920.1"
                     /note="conserved hypothetical protein Cj0792"
                     /codon_start=1
                     /transl_table=11
                     /product="hypothetical protein"
                     /protein_id="Prokka:NLMJPNJI_00029"
                     /translation="MAKIFDFYSVFEGFESLDSLNFEEDVFHNIEYLLLKNYLDIKDC
                     FKLDETASYALSLLAKNNRKRFSINRKIQHFKALSTLKYLLRAGIIKLEHSKEAKRVR
                     DKRQKLKKELRSYVIQDKIIFANQFTRFFFYFLKPNEKLILQNRYEEVLTLIKEKFEL
                     YQSFCFEQLSRELLEKKFQVSGVQSYWDKNLELDLYYKDDKISFIGEVKFKNKKICRN
                     ILNLLELKAKSLKLEPNNYIIISKNGFSKEFYKICKQDLLLLDLNDFKILLEENK"
     gene            22663..23931
                     /locus_tag="NLMJPNJI_00030"
     CDS             22663..23931
                     /locus_tag="NLMJPNJI_00030"
                     /inference="ab initio prediction:Prodigal:002006"
                     /inference="similar to AA sequence:RefSeq:CAL34919.1"
                     /codon_start=1
                     /transl_table=11
                     /product="putative aminotransferase"
                     /protein_id="Prokka:NLMJPNJI_00030"
                     /translation="MQISDLKKEIILKKGTLHFDFTASALALKCVEKEIAKILPSYAN
                     THSDSSLNSFKTQQIYEQARKDIKTSLNLSDEFALIACGSGSSSAIKKFQELLGIYIP
                     PLVKEKYFEKIDKSTLPLVIVGPYEHHSNELSFREGLCECIRIPLDKKGNIDFEILEK
                     VLKENQHRKIIASFSLASNVTGILSDYKRISKLVRKFKGIVAFDASSFIPYKNIACKY
                     YDALFISSHKLIGGIGGSGLLAIKKELCGNKPSFAAGGTVDYVSRTSQCYLCNEEALE
                     EGGTPGILQLIRASLAFKIKDEIGIEEIEKKEEILKEYFFEKLKTIPHLILYANNIKT
                     RLPIFAFNIKNISPFDLAYELSKKYHIETRAGCACAGPYGHDLLGLKDNEELKTKPGW
                     LRISLHYTHEKEDIDYFFEALQKSIIKLKF"
     gene            complement(23935..24011)
                     /locus_tag="NLMJPNJI_00031"
     tRNA            complement(23935..24011)
```

```
                     /locus_tag="NLMJPNJI_00031"
                     /product="tRNA-Asp"
                     /inference="COORDINATES:profile:Aragorn:001002"
                     /note="tRNA-Asp(gtc)"
     gene            complement(24033..24108)
                     /locus_tag="NLMJPNJI_00032"
     tRNA            complement(24033..24108)
                     /locus_tag="NLMJPNJI_00032"
                     /product="tRNA-Val"
                     /inference="COORDINATES:profile:Aragorn:001002"
                     /note="tRNA-Val(tac)"
     gene            complement(24117..24192)
                     /locus_tag="NLMJPNJI_00033"
     tRNA            complement(24117..24192)
                     /locus_tag="NLMJPNJI_00033"
                     /product="tRNA-Glu"
                     /inference="COORDINATES:profile:Aragorn:001002"
                     /note="tRNA-Glu(ctc)"
     gene            complement(24206..24281)
                     /locus_tag="NLMJPNJI_00034"
     tRNA            complement(24206..24281)
                     /locus_tag="NLMJPNJI_00034"
                     /product="tRNA-Lys"
                     /inference="COORDINATES:profile:Aragorn:001002"
                     /note="tRNA-Lys(ttt)"
     gene            complement(24285..24361)
                     /locus_tag="NLMJPNJI_00035"
     tRNA            complement(24285..24361)
                     /locus_tag="NLMJPNJI_00035"
                     /product="tRNA-Asp"
                     /inference="COORDINATES:profile:Aragorn:001002"
                     /note="tRNA-Asp(gtc)"
ORIGIN
        1 atactgattc taaagataat acaaacttca caaactcttc taatcattta ccaagcaatg
       61 cttataaatc ttatcatcat actccttttt taagagtagc aacaagtata gcaagcaatc
      121 attcaggctt ttttcatact ccaagagttg gagatgaagt gattgtttct tttttagatg
      181 atgatataga taaaccctat gtgagctcta gtttgtataa tgggaccaat ccaagcttag
      241 taaatcttcc ttttaatgat catcaaacct ccctttcttc taaaaccata ggtgttaatg
      301 aagaaggtta taatgaactt accctctcaa atataaaaga taaagaacaa atttatttaa
      361 aagcccaaaa ggattatgat gaattagtcc agcataattt cactcaaaga attttaaatg
      421 ataaagattc taaggtagat ggaatttata atgaaaggat taaaaaggtt catactcaaa
      481 ccatagattt ggctaaaaat gtcaatgtag gggctgaata tttaatcaat gtaggcttat
      541 ctaaagatac catagtagga ttaagcaata ctttaaatgt aggagtagat aataaagtaa
      601 gaatagctaa aaactctcat gagtttgtag gagaaaacaa agatatagaa ataggtgcaa
      661 atcaaaatac tattatccat aaagatgaga taaggaatgt gaaggggaat aaaaaggagg
      721 tggttgaggg gcattatgat attaatataa aagaaacatt aaaaatccaa acagaaaaag
      781 agacctctat aagaagcaaa aataatctac taatcaccac caatgcttca atgggctttg
      841 aaacagataa aaataatacc tttgtatctg ataattcatt atcgcaaaca aaaacagatt
      901 atgaagtaaa agcaggcaat caaatcctcc atcaagtagg cgatactcaa attgttacta
      961 agaaagattg tgttataatt aaagcaggtg gagtagaagt gattatagac tctaatggac
     1021 ttgtggttaa aggtggagag cttaaggcag agtaatggaa gcactcaatc aaaaaaatag
     1081 tttaaatatt aggcttttaa gttctaataa tattcttctg cataatcaag aatttaagct
     1141 ttatgaaata gcacagggca ataaaaatga aatcaagact atttttacaa ctaatagaat
     1201 aggagaagct catttaaatg ctagtgaaat ttttagtaaa gaagcacaaa gctttgaatt
```

1261 aattttaaac caatctagtg tttataaaaa caaacctatc tataatcata gatttttatt
1321 aagaaattat gagtatgggc attggtgtga aataaaattt gaaagaaagg cagataaagg
1381 ttctataaac tctattaggc taaaatccaa agaatttacc ttagaaaata atgaaaaaat
1441 agtgcgtaat ttctatactt ttggtgatat agtagaattt gaagctattt atgaggatac
1501 aaaaatcaaa gaagagcaaa ttaaatgggg ttatgttttt attcaggata aaaacacctt
1561 agataagtta aacaaaaacc aattaactaa tgacaaaaaa gaaagttctt tgcttgatga
1621 agaaatttta aaagattgtt tttatattga aaaagaagaa gataaagccc tattatcttc
1681 ttctatcatt tatagacatt tagaaaacaa caaagcttat tgtggaaaac atcttagttt
1741 gcaactacca aaccctaaag acacacaaga gcaaaaagcg ctattagtct ttgcttataa
1801 agaaataccc aattataatg cctcttatct tataagaatt aatgattatc cacaaattac
1861 cattgattgc actctagcag aaacactaag ggcacataca aacaaagatg aaatctcaag
1921 gcttggttgg ggggtaagct atatctgtca aagattatgg catgataatc caagtgatgc
1981 taaagaatta aaagatttaa cctttagaag ctctacttcg caagatttta tagatactat
2041 ccctaatgaa acaatgaaaa caatagtaaa ggagatattg cctagagtag agatgcaaca
2101 attaaagact gacaacacaa aatcaagaga taaagcaaga ttttatgcgg aattagattg
2161 ggataatttt tacatgaagt tctccataat gcaagagcta aagggcgagt atatgaatct
2221 caaaaagctt tttggagaag aatctgattt tcaaaaacaa ttcgaagatt ttcttaatga
2281 tactttacta gatataaaaa acattaaaaa tacacaagaa tatacaatgg cactcaatat
2341 ccaagcaatg aaagagaata agacgaagaa tgcagaagtc tttaagctat ataaaaaaga
2401 ggaaacactc ccagaaactc ataaagctat aaaagactta caaaaaccat cagatattat
2461 cgataatagt ttatattttc aaaggtttga tataaaaaat gatgtgtttt taccgcattt
2521 aggcttaagt aaatttgatg cattttcatt gcaaaactcc atacaacatg aaaaagaagt
2581 tttagataaa ttccactcta acccaaaata caagcagaat tttgctctat actctatcgc
2641 aggggctttt aatatcttat atataccaag ccttatacaa atcacgagag ttacaagatt
2701 ctataattac cattctttat atgctgcgtg caaaaaggtg cgtgctttta tcattgatac
2761 tgtgaatttt aataataatg gtagcgatca gcctattggg gcatgggatt atgagaaggt
2821 aggatttgat ttatataatt cgataatgca ataccgaaat acaaaaatttc attttaaata
2881 tacctcacca aaatcattct tatttccctt atataattca gactttctaa aaacaaaaca
2941 gtattttaat ctaggcttag atttcttttt atatagctct acatttttgg atatggattt
3001 tagtcatatg caaatgcaag atactgcttt tatagtgggg aagtaagatg tttttttgctg
3061 gtggtgatta tgagcctttt atatatgggc ttagtcttat tgcatactat tttctacccc
3121 tttattgtct gacgctttta ttgtataaat ataagcctca atatgtgaat cttgctctgc
3181 atattggagc ttcagtgttg ttatacttag ctatgtgggt aatatttgtc gatacaacta
3241 ttatcagcct ttttgaaatt tttcaaatgt ttttaatccc cattatcttg caaattatac
3301 atcttacctt tatactctat gctattatct atacttattt tgatatgcaa aagatttcat
3361 ttacaaagag actaggcaga tatatcgttt catttttttat tgggattgga ctacttatcc
3421 tttcttttgt aatgatgtat gctattggat attatatcag ctcatggtgt gatagatatt
3481 atcttaaaat cgatgagaga aatgaggcga ttcgcactat aactttttat gttcttgttg
3541 tggtttttcc cagtataatt gcactactcc ttagctatat cctctataaa aatcataagt
3601 catttttttac taagattcta tatagaatct tatctatgat aggcaaggat aaaccataat
3661 gcaaaaactt atatatcaaa accacgacta ccaacaaata caaatccaac aaacacagaa
3721 acatttcaat cttggttgtg attttagaat ccttacttct acatttaagg atatagaagt
3781 ttgctctttt tcaatgccta taagtataaa ggtggattga taaatattac agatttattg
3841 ataattctac caaaagcaat tttgatagca tttctttata gtcttgctat gttatggctt
3901 gtgattccag caatattacc atttattctc acgacattta tccctaaaat aaacaaaata
3961 ttgcttaaaa atagaagtat tgtttattgg attattggca gttttatttc ttatattatt
4021 tttattatgg ctcatattat atcgttcttt ttaaatttaa tatggattct gtatctatag
4081 tgtttcttgg agcaatgatc tttaatattt attccacaat ataccttttta ttgtttaaat
4141 ttttatccaa caataatcaa aatattttttc caagtaaaaa agaaaaatat tgtctctttg
4201 gcttaaatct tttattttct ttgctatttt atgcaatatg gtttgcagca tttgaatatg
4261 atttagctga caaagtagtt aaattcttta atggagtgta acaatgaatg gggctaaact
4321 ccttgataat ataaaaccta gtgatttaaa aagaagccaa atgcaatttt atggaattgc
4381 taataaggaa tttgggatag aatataaact aagctctaaa gaaagatcaa atcaagttat
4441 agctctttat gccctctctg gtaaatttca aatctattat gtgttagata gctttgaagt

4501 agaaagaatg aatgaaaaag aaatagttat ctatcctaca caaattgaat cgtggtacta
4561 ggtattatcg actaccaaaa tacgcaatct cactttaatt tgggtttgga ttttataatt
4621 acaagcccaa tatttaaaaa tattaagatt ccaaataatt tggatacaaa gaatctattg
4681 aggattatag taaaaagtga ggcgagataa tgactgatgg atttgggttt ttagcatatg
4741 tctttacatt gcctgtattg attcttggtg gtatatcttg tgtatgtata gtatttttga
4801 taagatttca aagcttttgt gggtttgtat gttcaaaaag tttttggatt gcttggcttt
4861 ttccatatta tttattattt ttgattatct gcattacttg tgaatgggag ttattaacaa
4921 tgtatatctg tggtatttct ttgcctttgg tatatttttt aagaaaattt tttatgcaat
4981 cacatttaaa tctagctcca aacgaatttt cattatggat tatcccaatg agtattttct
5041 ctctttgtat tcatagtagc ctgtgtgttg ctattgcgtg ggtgttaata ttttttcaaa
5101 caaattatga tacccaagca cttcaatttt ggcttagatt tctttttata tagctctata
5161 tttttagata ttgtgctaga ggagtagtta ttatgtttga agcgacaaaa atacggagta
5221 aattaaagga gttcttatgc aaaagatttt aatagtttta ggattgtgtt taatgttagc
5281 cattagtgct aatgcagaaa ctaacaataa ggctaactcc actcatgcaa cacatttaaa
5341 caaatggctt gggacttata cattacaaga aaatctttat atggggagct ttacaataaa
5401 aaagtgtgat aagtttaata tttgtgaagc tagatatgag gctatcctaa ataaaagtga
5461 atttaatgat atgcacgaat gcgatataac tttgaaatta cagataaaat ctccgcaaga
5521 agctatagct ttaaatgatg ataaggattt tgcaaattgt aaaatttata ttaggaaaaa
5581 tcaacagggt attgtattta taagagatga gaaagtattt aattcacccg attatttttg
5641 tagcacaatg tgtggcaacc aaacgatatt tgagtgggga gatatctata aaaaggaaat
5701 gcaataatgc tttatgatgt taaaaaatta aatgaaaatg attataaaaa agaatgtgca
5761 aagcttctta ttgcttttga aaatccaaat tacaaacaag aaaatgctat acatattaca
5821 ggcaatgtaa ctattggata tggacttgat ttaaagacag gaaataatcc aaagttactt
5881 ttagaagggt atttaccaga tgattctaaa aaacttaaaa taggagatga agaattaact
5941 ttttatgaaa ttatagagaa atatagagcc aataaggcag gttttacaga cccaaagata
6001 gtaaaaggtt atttacaaaa taaagtgttt aatttttcgc ttacgcaaga acaagctcaa
6061 gaaattttgg aaagaacctt tgattcatat aaaaagtata tatcacaata acttgttgga
6121 atatagtcaa gaaaaagctt ccttggtttc tttacattat cacggtagat ttaataatat
6181 caagataaag cctcaaaaat gctaaaaatc tatttaaaac tagaaagata tagtagcgaa
6241 gagattttat cacatagagg ggaaattttg aattaacact taataatctt aagattttag
6301 ataaaaatgg gaataatcat gaaaccataa ctaaaatatt tcttcataat tgtgagaaca
6361 gaagaattta tgaaagttta aagctagagt atcaagaagg catttataag agtagtttta
6421 aataatacct ctatgctttt agcaagtgat agggaaaagc ttaatcttgc aaatgaaaaa
6481 tatccatttt atcggttagg tggtatagga atttccgctt ttgaaaaatt agtaggatta
6541 agcaatactt taaatgtagg agtagataat aaagtaagaa tagctaaaaa ctctcatgag
6601 tttgtaggag aaaacaaaga tatagaaata ggtgctaatc aaaatactat tatccataaa
6661 gatgagataa ggaatgtaaa gggagaaaat aaattattaa tagaaaaatc tttaacccaa
6721 acaatagaaa aagaattttt ccttaatgtc catcaaaatt tatccgccca tatacaagat
6781 aacacatctt taaagtctaa ttccatgcaa acaaagatag aagagcaata ttctttagaa
6841 tcagaaaatt ctacttttga ctttcaaaca gattctgaag taaaagcagg caatcaaatc
6901 ctccatcaag taggtgatac tcaaattgtt actaagaaag attgtgttat cattaaagca
6961 ggtggagtag aagtgattat agactctaat ggacttgtgg ttagaggtgg agagattaaa
7021 gcagaatgat aactcggaga taaaaatgag tgaaataaac aataacacag aaatcaaaga
7081 gcaagacacc caagatgaaa tcatttggga aggaaaaaag catatacctt cgtttttgtt
7141 tttttggata acatatattg tgcttttatg tctttttctt tatttatttc ctagattatt
7201 taatcctagc aaagaaatag attataaatg gtttatagtt ttttttacat ttcttttagg
7261 catatatgca tttgcacgag cgatctataa aatggcaaat attaaaagaa tttatatcac
7321 aaaagaaaaa cttgtgattg agtattatat caaaaatgat ttggtttttc ctttaggaac
7381 ttttttttgtg tattatcgta aaatttcata ttcactttct ccaggacata tagtgataca
7441 tacctttgga gataaggcca aagaatacct tgaacatggt ttattcagtg atggcgatga
7501 tccaacaaaa gattgttttg aaagaattaa tgccattata aaacctcatg taatgcctta
7561 tttattaagc ttaagtgatg aagaatttga aaaaatcttt ttccatgtaa acgctactag
7621 tgaaattaat accactttcc ttaaagaagc aatggaactc agaaaagaga aaaaagatga
7681 atgaaaacat ttatggaagt ttagagtttt attctaaaga aactcaaagc actcctagtg

7741 ataaaagaga tttaggagta tttggagatg ttgctcctta tgccttaggc attacagaag
7801 gttttgctac taaagctttt gaaaaagaaa tagtgaagtt tggcataaaa cataattcgc
7861 gtattgcgac aatgatgcat tctaaaataa taatgaattt tggaatattg aattttttc
7921 tggctaaagc agagggaaaa tccaatgtaa gagctttgac tgaaacaagc gttggtgtta
7981 gtatcactat aatcatgaat gaaactccta ttggagcaac aatagaaaaa ggtagcgaac
8041 atgttgccaa gcaaacctta actcaaacta gcagggttgc ttctaatgcc gcaaccaaga
8101 tagctggctc aagagtagca tctaatatcg cagctagagc tggcgtatcc atactttctc
8161 gtattgccgc aggtacagca actggagcag ctgtaggctc gagctttcct attgttggga
8221 cagtggtggg tgcagtggtg ggaacacttg tagcaggttt ttttaatgac ttaattttttg
8281 gcgatgaaga tgaggaatta aaaaaacaag aagaagccaa agagaaatac ttagaggaaa
8341 aattagatga aaaaatgcac aaaatcatca attatctcat cactcatcag tatatagaat
8401 taagagtgtt aaatgaagat gaagcagaaa agctttgtaa agaaataagt gatattaata
8461 gtgcttattt taaaaccatt cttttgatgc taagttttcc ttattattta gataaagatg
8521 agcaaagtta taaagaagct caagaaaaaa atccaactat aatcagaata caacccatag
8581 ctaatgcctt aaatataaaa atagaaatca atgaatgctt tcttgctaag aatggagaag
8641 ctttaaaaaa taaggaaatt tatgtttata atcatcgctt tgatagagta gtaacaaaag
8701 ctatgagtga tgatgagggt aaaatagtat ttgaaaatgt ttatgtgggt aaagagagca
8761 ctatagataa aataagcttt attatagaca gagaaaattt caatgaagac aatttttatg
8821 aaagtgtttt aaaatacgct ccaatgttta atgtccaaaa aaagcataag caagaaggac
8881 aagcttttat tggtaaaatg ttttttagct tcacttatgc acaagaaata atgcaagata
8941 atgaggtgtt aaaactagag gctttaagaa ataattttca tatcgttttt gattatgaag
9001 taagaaaaca agaagaatcc tataaaaatt atatcatttt atcttatttg gtttttgatg
9061 ttaaagaaga tatagaagaa tatatacgcc atactactat agaaaataga gcttttcgtg
9121 gattagaatt gttaggtcgt ggatggaaaa atcaatacag catcaaagac gaatggagag
9181 ataagggtgt ggtttttttg cttattttaa ttctcaaaaa tttactcctt ataaaaaaat
9241 ggcttttatt gacaagccta tagttatttt agatatagaa aatttgata aagaaaatat
9301 actagaagat ataaaattcc attttaaaac tcttgctaaa gcctataaaa tttttgtaat
9361 agatttagat gctaatactc aaattaaagc aggtggagta gaagtgatta tagactctaa
9421 tggacttgtg gttaaaggtg gagagcttaa ggcagagtaa aggataagca atgaaaaaac
9481 ctttttacaa acttaaaaga ttttatatac cttgtataat aattattata atatttgccg
9541 tgcttgcaaa attactttat tctcctttat atacgattta ttggggaatg tatcattatc
9601 ctaaagttca actagaattt aaaaactttg aaaaaatgac tttaaatcca agccctaagg
9661 atatgattaa aattgtagat gattaccaac ctaagctaga agattttaaa gatttaaaca
9721 caaaaatgca aaaggctatt tttgatttta aggtggctaa attatttgga tttgaggata
9781 ggtattttga agcttttatt aaaagctgtg ctggtaactt ctttgtttta catggtaaag
9841 aacaaattta ttttaattat cttaatttca taagcggtat aaattcaact tccaatgaaa
9901 agcaaaaata tctaaactta agagcatcaa ctagagattt agaaagacaa atttttgaag
9961 aaaaacttaa atttataaaa cattatgaag aattttatga ttacttagag ggtgtaggtt
10021 atttggataa gggaacagaa tatataggta cggcaatttc ttttaaaaca ttgattcaaa
10081 atgtattttt gtctaataat gctcaattat gctcttttga agatagaaac ttaatgttta
10141 aaaacatgaa agaaaattat gaaatattta aaaatttaga tgtaaaaaat atagatgatt
10201 taaagagaaa tatctataaa aaaatattaa ttgatatgga aaatctccta aatgaaaccc
10261 aaaaggcttt agatgaatgc aaatgaaaat aataccagtg ttaataatga aaataacacc
10321 actatcaact ttgaagaaag cataaaagaa gctttaagag aggggcaaga aattgtagaa
10381 agtggcaaag gaataagata aatgtctatt tataaagaat gtgaagtttg taaaacaaaa
10441 atcaataaat tgcaaaatat ttggaatata ttctcaggca aattagaaat aaaatgtaaa
10501 aattgcaaaa gtaaatatgg tgtttctagt aaaattatag attggatata ttttcttttt
10561 tttgaaaatt tattactctt tccgccaatt gtaattttaa atatcctata tctaggtaga
10621 tatttttcaa aatatggatt atttgaaatg atattttatt tatcaatagc cattcttctt
10681 agtagaaaat cagtcaatat tattttactt cttctttgca aattaaaaag gctagaaaat
10741 gacaaataaa ggattaatag aacatcttaa ggatgcaaaa gatagtgctg tagattttta
10801 tataggactt tatgattttg gagatttttt agctagaatt tcaggtttta aagattggca
10861 agaaaaaagt gttgttttcg atgattttta taaaaaacaa gcatttgaag aattgggctt
10921 tttagaagat gttcttaaaa atgaaacttc tagaaatatt atactagaat atttaataaa

10981 agatgtcaat gaaagacctt tatattattt agcaccatca tcaattttaa aaataattag
11041 taaaacaact aaagcaaaaa caatacctac aataggctta atctccttat ctggaaaagc
11101 aacatccacc gctcactctc tctaccccta ctacaaaaac atacaaaatt cctttacccc
11161 tttatgttct atagataata aaaatagtaa tttgttaaaa gaatttaatt ttgatagttt
11221 ttcttgtaat ttttataata ataatatttt caatcaaagt cttaagcatt taaataatat
11281 acaaaatgaa aaagaatata tcaatgagta taaaatattt ttagaaaatc ttaattattt
11341 taatcaagaa tataataatt ataagcttgt atctatacaa gtaatcaata atttaaaaat
11401 agcctatata caaaagctat ctttacaaga tattaataat attaatatca attcagaact
11461 ttatttgcta aaagctttat atttttgtga agattatgtt ttatttgata aagataagca
11521 agctttattt aataaaaaca caataaaaga attagaattt gattctagtt tttatactat
11581 ctttgtctct cacaaagcca aattaaacga tacttattta caagcaagaa aagaacttta
11641 taaaagcata aatgaattta aaagacttgg ctttaaagat ttagaaaatg cttatcaaac
11701 ttatatccat tctttgattg taaataaaga aataaatagt aaagataaaa caataaataa
11761 aaatgaaaat aaagaaataa ataaaaattt aaatcaagat gaaaattcaa atcaagaaag
11821 caatataaaa gaaaatgcaa gtaacacaaa aaaagaagat tttaatcttt tatcttctca
11881 ttctaaaaac ctaaaccatt tcatcttaga aaacaaagat aaaaataaaa gaattatctt
11941 tttaaataat gcttcatcga tgtcaaattt aatccatatt tatgataata attgtgatgt
12001 gtttattaaa aatgattctt tgcttgatat taaaaaaata gaaaaagaat atgatattaa
12061 atttaaagaa tttaatacaa gggtattttt ttacgaaaaa cttcttttag gtgcaaaaga
12121 aaataatgaa tttttatttg aagaaaaaga agaaaattta ctttatcatt ttaaatacga
12181 taaagaggat ttaagttctt taaaagattt aagtataaaa tataatattt ataatgcaag
12241 gattaagaat tattctttac tagaagaaag tttaaatatt aaattagaac ccaaagagct
12301 taaaaaagaa attgcctaca acaatgcaag ttttaataaa ctttcaaaag aacctttaaa
12361 tgaattctcg tccagtgcta aagaaccctg ctttatcact ttatatctaa aagatgaaca
12421 caataaaccc attgctaatg ctaaaatcat cataaaaggt tttcaacata atgaagcctt
12481 aagagtagtt aatctaaaaa gacgcagtga tgaaaaaggc aagataagat ttgataaaga
12541 aaagtatttt agtgattgtt atagctttaa agtaaagctt gatgaaagca aagcttattt
12601 tcctactcct ttacaaaatg ctaaaagaat atttaataat tatacccatc acaaagttgg
12661 acttatactt aaatttaaag ctaaagatca tttagtttat gatggattta atgtctatca
12721 ttacaaagga aatgaattaa ttaattctta tatggcaaga agtggcactg ctaaggcaga
12781 caatgaaaaa agaagtaaaa aacaaattgc acattatttt taccaagatg aaaaagataa
12841 aagcaaaagt ggaaaggctt atttttacta tgatgatgaa agcattcaag atagatttgg
12901 cactttacct gaagggggagt attatttaaa aatcaatgaa atagctaaag atactaagcc
12961 taacttttta aaagattatc cctttgaaat aggaaaaacc tggggaagat actgcgtaag
13021 attatatact gataaagaat gctctaaaac ttttaaagaa atagaaataa aagaaagtaa
13081 tgaaaaagaa aacaaaacaa gcaaaaaaga taaaaaagag caaagcataa taaaagataa
13141 tctatatctt tatagcatca atgaaaaggg agagtttggc agtagtggta gcataggtat
13201 agcacaagca cagttattag aggatttaag caaacaggtt aaatttatac gaagcgaaaa
13261 ggaaaatata atctcactca aagtggctta ccctgaaaaa atagataata aaattatatt
13321 tgtaggaaaa gtttatgaat aaaacactaa tatttcatga aaattcccat attgatttaa
13381 acgcttcttt tgcaccaggg acttatatag aattgcaatt agaaaaagaa agcgataaaa
13441 cgctaaattg gagttatgta gagtgcaata gtgagaaaaa aatgcgatat ttgttggata
13501 ctgattgtca ttctattgag gctaaagatc ttaaatttat agatagtttt aaaggtaata
13561 tttgtggatt tcatttacct ataagtagag ataatgaacc aacaaaagat ataaaagatt
13621 ataaatatta tattatagtt tttgcttatg atgaaaaaaa ggctataccт agtttagaag
13681 attttcatat agttatagat atgagcttta gagcaggagt tggaaaagat gagagtgtag
13741 aagaaagtaa gctaaggaat gattttaata gtgatataat tcaaacaaat tgtatttttta
13801 gtgtttttga ggctgtagag tttttaaaag cttgccaaag tttaaaagaa gaaaaaaatg
13861 aaatagataa ttatattttt ttcaaaaact atccccaact tgcaggaaaa attgcttata
13921 tatactatag atttgattta gctaatgaag aatttattaa aagtgtaagt gatggaaata
13981 agtatttaaa agaaagggag aaattttata aagttgcttc agatgtttat attaaaaacc
14041 ctatttataa aatgggcttt gagaaaaaaa cttcaaaatc aagttgttga tataaacatt
14101 gtaaaagatt ttttaaagga ctttgctaaa agactaggga tagacgaaaa agttttgcct
14161 gtaattggat ataatcctca acgaggggat tctggaacat atgactatac agtaaatact

14221 ttagatttga atccaaaagc ttcttatgtt gattttattg ataccattat tcatgaattt
14281 aggcattttt atatttattg cataaaaaat aattctaaaa atagtataga aaaattgctt
14341 tatcttgata tggtacaatt ttatataccg tggaattttt ataaaatttt caatagttat
14401 gataaaaaat gcttaattta tgataatgaa gatgaaacga agaagtgttt aaaaaatcgc
14461 acatattatg gaagttggaa atttttaaat cccacattag atctcttgcc aagtccttta
14521 tactatcttc agccaagtga gttagactct aggattatgg catattattt tagaaaggaa
14581 attaaattat aatgactcca ccatctttac cagatgtaga aaaacatgaa gatttcttac
14641 aaaccagaaa agaaccttat gctatttatt tagccataaa cacaaacata aaaagttata
14701 ataatatttg tcctagcgag aaatattttt ggaaatttga taatatgaat gaacttgata
14761 aatggtataa cccaaaattt ggcatatacc ttggcaaaat tgtttttgat aaaaaagaca
14821 acaaactcat cccaaaatat atagccgcta aatttgaaaa tttagaagaa gaagttaaaa
14881 agataaaaaa tcctttatgg cttgctaata aaaatcctaa ttatattgaa cctaaatttt
14941 atgatggtat gagtggaggt tattattttg aaagtccaaa taatttagag tatcaatgca
15001 aaatagaaaa agacactcaa gttttaagcc aagaacaaat catttcttat gtgaaagaac
15061 tttatagtaa aaacacaata ataataaaaa attatattga tgcaatcaat aaagactatg
15121 gcataaaacc tttcgtattt agtgatgaaa tttatgatga attaggagaa gttggcattc
15181 ttacaaaaga acaagctaat aattttaaag ataaaagtta tattaaaaaa gatccgatat
15241 tgcttgctat gcttgattat ttagcaaaac aaaataaaaa agatgaagat tatttaatca
15301 cttttgatga tgagtatttt tatacttatt tggtttggag tttaaaagat ttcttattag
15361 agctttctta tggtttgttt caagatgaaa ccgagcttct ttttaatcct gcagcttata
15421 tggatgacac aaaaatatat tatcaaaatt taaacgaaga aataaataag cgttatgaaa
15481 aaatattgct taatatggga tttgaaggag aaaatggtta ttttaaagat tattatgatt
15541 atggctttgg caataatggt atttttgaat ttagcatata tgattatttt gcttatgatg
15601 aaataggagt acaaccaatt caacaaagtc catatgttcc acctaaaagc cctttttgata
15661 gccctaattt tgtctattct gatggaaatt atcacggaga tgcaaagctt gtcccttctg
15721 ctttaggaaa gtattatttt gagcttttat atcaaaaaga aatttacata gaacttttac
15781 gcccttatta tccaagcata aaagatttgc cagaaggttg ggacaataaa atgctagaaa
15841 aagctaattt aaaataaaat tatagatatg agctttagag taggagttgg aagggatgag
15901 agtgtaaaag aaagcaagct atgaaataat tttaacaacg ctgtaaaaaa taattagacg
15961 cctaaagaca ttatctaaac aaattgtatt tttagtgttt ttgaagctgt agagttttta
16021 aaagcttgtc aaagttttaa agaaaaaaat gaaacagata attatatttt ttcaaaacct
16081 atcctcaatt tgcaggaaaa attgcttata tatactatag atttgattta atcaataagg
16141 aatttttaac aagcgtatca gaaaaagata aaggaaatta tcttcaagtt agagatggca
16201 aatatattag caccataatt aataaaattt tacctttaga aaatgtagtc ttttatagcc
16261 aaaagcctag tggatcttta tcatttggag cttatgaaga aaataaggtc agattaaatc
16321 aatacatttt aaaagcttat tttaaagaat ttacaaaaac ggtttttcac gaatgtaggc
16381 atttttatat agaaaaatat tataatcaca atatgaatgc tctaggcaga tatgtttttt
16441 attctgaaaa agcttatatc aatgagaaaa ttattttttga taaattgagt agaatttgcg
16501 atcctagtga gaattattta gtaggttgca atataggtgc tatacaagat acttatgaaa
16561 ttcagcccaa tgaaagagat ccaagatatg tagaaaccct cataggtaga agtttataat
16621 caaggaaaat aatgaaaact ataaaaatac cgcttaaaga ttatatctcc aatataagag
16681 aagataaaac tatttacttg ggcttgcaaa gtgatatgag tataaaagga cttgataaag
16741 aattcttaaa aaagaatata atcttaagag aaatttcaaa tttgaaacaa gcatttattt
16801 aggtaaaatt ataatcaaag aaaaaaatat catttttaaa gcttttaata ttgatgagaa
16861 ttgctttgaa gggcttaaag aacttttctt caaaaataac ccctatacaa ttatagataa
16921 tttagaatta aaaaaatatt tcttaaaact ttataaagat aatattgaat ttttaacttc
16981 ttatattaat gaaattaatc aagttcatgg tattaaactt taccatgttt tacatttaat
17041 gctttaaaat tctaagtctt ccataaagca tgagtttaat taacatagca ttcttaaaac
17101 tgctataatc atcttttttg ctttttcctt ctaaaacatt atttagcctt atagtttcat
17161 tctcgctaag ataatttaac ttttcttctg ctttttcagt tttaaaccta attttttcat
17221 atcaaagcta aaaaggaata agtcatcatt attatcactt ataaaaaagc tagttaatac
17281 tctcaaataa gtaatttttg ttctttttga taatttattg ctatttttta aattattttc
17341 caaataatta agaaaattaa tcaaaaaagt tgtttttata tcatttattt gcatttcatc
17401 ctgataatct agtaaatatt ccgcaagctc taaaaataca cctataaaac aataaagtgt

17461 tctttaaata taatattatt tatataaata aaatttcata taatccacat acaacatcag
17521 aaattgataa taaatattta tcttgattta tcttattgat gtttttaagt tttattccaa
17581 caatttcata aaactttggt tttgttgtat ttaatttatc ttcagtgtaa aaaatttata
17641 ttttgcaatt agtccttcaa acaattttat aaatgtaaaa tctatatttt caagttcttt
17701 gctatacaaa taattattta taacttcttt taattcaact tacaaaacca aagctagaat
17761 atgattttat tttagtggat gaagcacagg atatcaacgc ttgtgttatc gacattgttc
17821 ttaatcaaaa acaagctaaa aaagttttta taggagatac ctttcagagt atttataaat
17881 ttagaggagc ggtaaattct ttagaaatat tagcatctat gccaaaatct cacactttat
17941 acctaacaca aagttttagg tgtccgcaaa atatagcttt tattgccaac tcttatttga
18001 aaattttaaa tgctccgcaa gatttcaaag gaacaaaaaa atcaagtaat gaaaatgtca
18061 aaaacgaagc aaaagccata gtgtgtagaa ctaatgctaa gttatttgat atagccgtag
18121 agaatttaga taaaaaatta ttttttgtag gagggataaa tagttatagt tttgatgagt
18181 tattagacat acaaaatctt ctttttaaaa aacacgaata tatcaaaaac caatttatag
18241 taaagtttac tgatttaaag gagcttcttg aatatattaa tgaaaccaaa gaagttgatt
18301 taaaacgaaa aatctttgtt cttttcaaat atgcgcatgg cgatattata aagcttatta
18361 aagccattga aaaaagtagc gtaaaaaaac aagagcaagc tgatttaatc ttaagcacag
18421 gacataagag caaaggcttg gaatggaaca atgttgaaat tattgatgat tttttaaaca
18481 tcaagcaaga acttgaagaa agagatcaaa tcaccatagc aaaagaagaa ttaaaccttt
18541 tatatgtggc aatcacaaga gccaaaaatt ctctaagtat tagcaaagat tacttgcttg
18601 aaaaagaatt tatacaagaa aatttagaaa ggataataat agaatgaaaa taattaaaaa
18661 attacaaaaa gatggaagcc ttaaaaagca agtttattat tctgttcatg aagttgcgca
18721 aatgcactca gtaacgcatg taacagttcg cctatggatt tcaagaggaa ttctaaaagg
18781 cgtgaaatta ggcaaaggat tttatatgtc aaaagaagcc attattgaat ttcagaaaaa
18841 tgatggttta tcataattaa ggagagagaa aaaatgagca gtatagcaaa agatagtcat
18901 gttgaaatga gagatttttt atttaataaa tataacaaaa taacttttag cagcaaagaa
18961 tgtgctaaaa ttcttgggat atctttgcaa actttagata gattgacaaa agataaaaaa
19021 taaaatcaat gaatataact cgttttgtat tattctccat tgaagaaata gctaaaattc
19081 tctcaggggc taagtcaaca aaatgaatag aaaagatagg gttgcgctag ctgcttatca
19141 taaaaacaaa aagctcgctt gctttgataa gtgagtaatt taaaagtata tgttaaagta
19201 tatgtttttt agatatttaa tttttaaagt atggatattt agatatttga ttaataaatc
19261 acaccccgac ctcaggcacc attacttctt aaaatcataa aattccaaaa aaattttatt
19321 gtttatattt gctcgaaaaa atacttccaa aatagctgaa atatttattt atagatatta
19381 agaatatttt tatataaata aaatatgctt taaattatgt taaatattta attataaatt
19441 atttaaaaac aataagctcg ctctcttaaa ataagataaa aatagcccta tattcttaaa
19501 taagactaaa aatgtatgat taaacttaaa ttaaactaaa atttgatata gttcgcgcga
19561 tcaaaaaagt ttaaataaaa aattaaaaag tcatagttat ttaaactgaa acatttgtgt
19621 tatttttcat agaaaatcat aatacaaata aagatctata taccgaataa ctaaaaaaaa
19681 aagggggggg gtatggaagc aaaaggaata gcgatatttc ctacaaatgt tgcatttagt
19741 aaaaaaacaa atgctttttt cactgaatta gaagatcaat tcttaaaaga aggggtcggt
19801 aacaaaagtg ccataaaact agattttgaa tattatattt tatattgtca atttttacaa
19861 atagacaaag aatccctatt ttttggaaga aactatgaac ttgtgttttg ttttttactt
19921 tattataggg ctttattaaa taaaaacaat actcaaaaaa tcgtagcaaa aataataaat
19981 gttatacgtt gccaagatag attaccagag aaagtacact atagagaaaa aataatttgt
20041 atggataatc aacacagaga tctttctcaa acactaagtt catgtattgc agaattttat
20101 aattcaattg atctatacaa tacctttaga aaaaatatag gatttgaaaa tagaaagaaa
20161 atagcgatag aaatgttaaa tcgcgcatca gaacactatg gtttttttgg cttaagcagg
20221 ggaggaataa catataagga agaaaaaatc tcttgcgtcc ctatacaagc attggatgag
20281 tttcatcatg gtatgtcgca tctatgtgca gttttcttaa aagaagatgg acgatatacc
20341 aatctagaaa gagcgatccc tcacttcaaa cgtggaatga tggaagtata caaaacaata
20401 atcagagatt ttatgatatt aaataaagat gctttaggag tcaaacaaga agccttaaaa
20461 ataagagaat tagaatatca aaccatagga aaacccaaaa cagtaacaaa aaaccctacc
20521 acaaacctaa ccaataaaaa agaagagaag gttatcccag gatataaaga actgtgcgaa
20581 aaaattgtca gtcaaatcaa aaatgcaaag taaaatcggt atgaattgag cttgagtatg
20641 caatttagct tatttgctat ttgcatactt agagccattc tttgaagaaa ttaaaacaaa

20701 tcctatcata aattaactat tataatgtaa aggcaaaaca aaataaaaat ttttaggcca
20761 aatttcaaat tttaattctc ctttatgagc attaatgatt tctttagaaa gcgataggcc
20821 caatccattt cccttaagtt tagtagtctt aaaagcttca aaaaccaaat tttcatcttt
20881 gatttcacaa ccattatcat aaaccctaat gtataaattt tgttcatcac agcttgcttg
20941 aattttgatt ttaggatttt cgcactcact ttcttcgatc gcatctatgg cattataaat
21001 caaattttgc aaaaccaaag ataaaagtgc cttatctcca taaattttca tatctaaaaa
21061 attaatttca aaatcaattt gagatgagta attgtaagaa tttatagcac tttcacactc
21121 ttctgttaat tctaaaagat taaacttgct tgcattgata tgtacaccct tagtaaaaag
21181 caaggtagaa tttacaatac gctctaccct agcaatagcc ttttgaattt caagcacaat
21241 atgtttgttt ttaagctcac tacgagaaaa aagcgtggag gcaagtaagg atatagagcc
21301 tataggattt cttatctcat gtgctaaatg cgccgcaaca cttcccatac tagcaagtct
21361 ttcattgcgt ttttcatcgc taatatccgt agctgaaact atggtttttt cattatgagc
21421 aataatttta accgcataaa atctattctc aaattcaagt tcatcacgat ttttttcaag
21481 actgataata tcaagcaatt taggtttttt caaagcttct tgattttgca aaataatatt
21541 tttttcttta tctaaaatcc aaagcgcact aggcaaaacc tcgataatct catctaccat
21601 agcacgcaaa gaattataat tttcgtttaa aattttatat tcgttttcta caatataagt
21661 ttgctctata aggctttgta agcctttttg tagggtttct ttttcacttg agtctagact
21721 ttttaaaata ttttcattca tttattttcc tctaataaaa ttttaaaatc atttaaatcc
21781 aataaaagca aatcttgttt acaaatttta taaaattctt tactaaaacc atttttttgaa
21841 ataataatat aattatttgg ctctagtttt aaagattttg cttttaattc taaaaggttt
21901 aaaatatttc tacaaatttt tttattttta aatttaactt cgccgataaa acttatcttg
21961 tcatccttat aataaagatc aagttctaaa tttttatccc aataactttg caccccactc
22021 acttgaaatt ttttttcaag taattctctg gaaagttgct caaaacaaaa actttgataa
22081 agttcaaatt tctctttaat caaagttaaa acttcttcat agcgattttg caaaatcaac
22141 ttttcattgg gttttagaaa ataaaaaaag aaacgagtaa attgattggc aaagataatt
22201 ttatcttgta tcacataaga tcttaattct tttttaagtt tttgtcgttt atctcttacc
22261 cttttcgcct ctttgctatg ctcaagtttt ataatccccg ctcttaaaag atattttaaa
22321 gttgataaag ccttaaaatg ctgaattttt ctattgatag aaaaacgctt acgattgttt
22381 ttagctagca agctaagagc ataagaagcg gtctcatcga gcttgaaaca atccttgata
22441 tctaaatagt ttttgagcaa aagatattct atattgtgaa atacatcttc ttcaaaattt
22501 aaactatcca agctctcaaa accctcaaaa acactataaa aatcaaaaat tttagccaaa
22561 gctgaagtgt tgcaaatttt tgcaaaatga ttaaaattta tatttttcct ttttaaaagt
22621 atttttatag tataatttta gcaaattttt aaaggattgt gcttgcaaat ttcagattta
22681 aaaaaagaaa taattttaaa aaaaggcacc ttgcattttg actttaccgc tagtgctttg
22741 gcattaaaat gcgtagaaaa agaaattgca aaaatactgc caagttatgc aaatactcat
22801 tctgacagct ccttaaactc ttttaaaact caacaaattt acgaacaagc acgcaaagat
22861 atcaaaacaa gtcttaatct aagtgatgaa tttgcactta ttgcttgtgg atcaggctct
22921 agcagtgcta ttaaaaaatt tcaagaatta cttggaattt atatcccgcc acttgtcaaa
22981 gaaaagtatt ttgaaaagat agataaaagc accctaccct tagttatcgt agggccttat
23041 gaacatcatt ctaacgaact ttccttccgt gaaggtttgt gcgaatgtat aagaattccc
23101 ctagataaaa aaggaaacat agactttgaa attttagaaa aagttttaaa agaaaatcaa
23161 cacagaaaaa ttatcgctag tttttcccta gcttcaaatg ttacaggtat attaagcgat
23221 tataaacgca tttctaaatt agtgcgtaaa tttaaaggca tagtcgcttt tgatgcatca
23281 agctttatcc cttataaaaa catagcttgc aagtattatg atgctttatt tataagttct
23341 cataaattaa tcgggggcat aggaggttct ggtcttttag caatcaaaaa agaactttgc
23401 ggaaacaaac caagctttgc cgcaggagga actgtggatt atgtttcgcg cacctctcaa
23461 tgttatcttt gcaatgaaga ggctttagaa gaagggggga ctcctggaat tttacaacta
23521 attcgtgcga gcttggcttt taaaataaaa gatgaaatag gcatagaaga aatagaaaaa
23581 aaagaagaaa ttttaaaaga atacttcttt gaaaaactaa aaactatacc tcatttaatc
23641 ctatatgcta ataatataaa aactcgctta cctatctttg cttttaatat caaaaacatt
23701 tctccttttg acttagccta tgagctaagt aaaaaatacc acatagaaac acgcgcgggc
23761 tgtgcttgcg ctggtcctta tgggcatgat ttactaggct taaaagataa tgaagaatta
23821 aaaactaaac caggttggct aagaattagt ttacattata ctcacgaaaa agaagatatt
23881 gactatttct ttgaagcact gcaaaaaagt ataataaaat tgaaatttta atgatggcgc

```
    23941 agcggacggg gctcgaaccc gcgacctccg ccgtgacagg gcggcattct aaccagctga
    24001 actaccgctg cacccaagaa gtaataattt aatggtggtc actataagac tcgaacttat
    24061 gacatccacc ttgtaagggt ggcgctctac caactgagct aagcgacctt aatttttggc
    24121 gacccctaga ggatttgaac ctctgtttcc acacagagag agtgttgtcc tgggccacta
    24181 gacgaaaggg tctaaacccg ataagtggtg tcccgtgttg gattcgaacc aacggccccc
    24241 tccttaaaag ggagatgctc taccggctga gctaacgaga cagatggcgc agcggacggg
    24301 gctcgaaccc gcgacctccg ccgtgacagg gcggcattct aaccagctga actaccgctg
    24361 cacccaagaa gtaataattt aatggtggtc actataagac tcgaactta
//
LOCUS       NLMJPNJI_2              7512 bp    DNA     linear       12-APR-2021
DEFINITION  Campylobacter species strain strain.
ACCESSION
VERSION
KEYWORDS    .
SOURCE      Campylobacter species
  ORGANISM  Campylobacter species
            Unclassified.
COMMENT     Annotated using prokka 1.14.6 from
            https://github.com/tseemann/prokka.
FEATURES             Location/Qualifiers
     source          1..7512
                     /organism="Campylobacter species"
                     /mol_type="genomic DNA"
                     /strain="strain"
     gene            complement(400..1680)
                     /gene="trxB_1"
                     /locus_tag="NLMJPNJI_00036"
     CDS             complement(400..1680)
                     /gene="trxB_1"
                     /locus_tag="NLMJPNJI_00036"
                     /inference="ab initio prediction:Prodigal:002006"
                     /inference="similar to AA sequence:RefSeq:CKG87041.1"
                     /codon_start=1
                     /transl_table=11
                     /product="thioredoxin reductase"
                     /protein_id="Prokka:NLMJPNJI_00036"
                     /translation="MSDTYMIYAPNGLGVEVDKKTKEIYFAQSVDPVGKYTKEYTRVF
                     FKAWEIKQNSPYKDYKPIYLDPNFYTGQASTLLEFKDWQSIYLKDPIKGAIAPWTKAE
                     KAYYKSLKTKRERYKYLIIRSGLRSVVIDIPYEAYCNVDEKGNLINKDYKELYKEVEA
                     NRGMANMHKGWLFMAEWELAAGILGDIKGFVGALQLSMTGFKARTQAINFLLIQLGHE
                     QGFKSLYDSYAYRDLTDGIHKNPLKAQMLKDFSKNPPYDELGMLPFLDELIGVDWVID
                     PNDYYFVVYDGAKGRVNDALKDDVEKGILKDPRDIDSTSESRLEFEYKLDAYRNGMKTR
                     FDGDNPNHWSKEQVERFNDTLILCAKLAALTPPQGYTNAPYYYSPERLEFIYKNHNLD
                     KLLDPRIPAIYRYNFPRELRAKILKFGEENGIKE"
     gene            complement(1701..5414)
                     /locus_tag="NLMJPNJI_00037"
     CDS             complement(1701..5414)
                     /locus_tag="NLMJPNJI_00037"
                     /inference="ab initio prediction:Prodigal:002006"
                     /codon_start=1
                     /transl_table=11
                     /product="hypothetical protein"
                     /protein_id="Prokka:NLMJPNJI_00037"
                     /translation="MKNKEFTCQYIYNQNELKNLIDKYCKQAKNIVLYCNNFSRKNVL
```

```
                     NLAISDYEIAKDCYYSLDKYLYNTLKISAYKGKKVQIYDYSYKQFTDSNKTDNNSSNF
                     ITTSLSCDMASIFDDFIKALEKSEDETTSEAMTQISKKENKGIDFLITCINEKLKTPI
                     LQKLKSFDEYQKLNEYKLNEQIELIFGELIQNIKDIYAEGYEFAYKTIINAFLNIVNI
                     FKLKSFLTKTHPLLIASDIAFESWRVFQDISEWSENKYSYVFYNAIIKLLISDIGYIF
                     TVFENKIHHYTLIIDDKILFDFSGFGAVNFQSSVYHKQDLAICLKSNEEIFKDFWSAN
                     SSNFFTENPKEEFILYKQYSIKEALKRQIPLDQNRLSYIESPYFNSIALAQIIKDLKD
                     KKSESYHAKNFLIITNAPQKYNAGLHQKLTEEIFENTIIYPDTIKDEKQNDILVKAPK
                     DYDVMIDYSKYKINIIDNETNAFVLSLSPFVKLNYQTYQSFEDVVKEKEKLLNSITDD
                     NPENVFQYLNFIKNPSNADKIHNYHYLQSVFYQANNKKTFLEKQKQYFQEGIDCLRAY
                     INSIIQTKQQNIGTYTGVTYSEKFVDKKNITITEVLLLALSYYVIKHFYSSIKTDYQA
                     WWKLFTNYESIQIEEEESVDMLPKDVFLNINEEKIPLCFSKQQKAKLAKKEIFCIEEF
                     VDYLEQLKDYEISDEDDRQFLFSLEEPQDENNQIQDTAVECNLKQLNALIENEKKSNR
                     KIDDELDELIKDIEKNNINKTPYNPQKTIVAMMDAFIDKFFPFADFLRGDDFKFAKQL
                     SKTVLNFLTLEAQKALYENIGASYLLFLMTKSNIHMKKVTIHNQKEFDKRFKKLSKMA
                     LLELCYMQKNKNKHEAIMLVDGMTFKELKNRKGFKDLTNISKKQIFRYKFNFTLKTIR
                     NIAKESFISLPQVIITDLVNQLWITHYEKSKKEYERLKFFNFSDKYNPPYVLMKKPTN
                     TQEGFISYPMFINNTFISFNFTSMFTGGKLQTGGLGEKESLFVYHKDLSGTKKVRNYL
                     LNKLFAYLCLDELRSKTGDLKIIEDIDFFNAYSYLHDLPIKPRLLKLKHEEDFLISNH
                     LSKTQKENIQKYNQKLQAINEDDIKKALYKEYKNREAKGLAAIDAYHKCMDYLEDMDN
                     DIFNTKATNNASDPSKHREFLRALDTIGQNNIKALYVGINGDKNISQKKESEENKEYM
                     IKVVNNKEEKEKCPKFVGRLATTIIMKNGLHIG"
     gene            complement(5476..6063)
                     /locus_tag="NLMJPNJI_00038"
     CDS             complement(5476..6063)
                     /locus_tag="NLMJPNJI_00038"
                     /inference="ab initio prediction:Prodigal:002006"
                     /codon_start=1
                     /transl_table=11
                     /product="hypothetical protein"
                     /protein_id="Prokka:NLMJPNJI_00038"
                     /translation="MKFKVLETINAELFPQWDVLLDLHINSFLDIFSTHKQVNKNDIT
                     EIIEYSFLCDFLECSDYAEFFMIFNLYTKDYQGEFVKVFTKLFLHDLIDFYINDEKQN
                     TLSAYTKDKDKNWKYFLDNYINKECFYIDDFCIASWDIPSSWNKYNINAIITPKGTKY
                     FNEILAPKFYNKYKDLEVEIDDKGNVLRWIGEINR"
     gene            complement(6063..6542)
                     /locus_tag="NLMJPNJI_00039"
     CDS             complement(6063..6542)
                     /locus_tag="NLMJPNJI_00039"
                     /inference="ab initio prediction:Prodigal:002006"
                     /codon_start=1
                     /transl_table=11
                     /product="hypothetical protein"
                     /protein_id="Prokka:NLMJPNJI_00039"
                     /translation="MEEWRNKSSRVIKPLFTPLEGIVGDISNSLENVSIIGDALAMLG
                     AVKNPKNAKNIIKKKSLTKDEILNEIKKLKKSHNRIYLVDDINELMVLWDKLTKNYQK
                     EIVETNSKRIRIIRILEDDTRLMLRNYSSNKSDNMPTIDTKINNKDYKIHINNLGKP"
     gene            complement(6621..7508)
                     /locus_tag="NLMJPNJI_00040"
     CDS             complement(6621..7508)
                     /locus_tag="NLMJPNJI_00040"
                     /inference="ab initio prediction:Prodigal:002006"
                     /inference="similar to AA sequence:RefSeq:CKG86671.1"
                     /codon_start=1
                     /transl_table=11
```

```
                     /product="methyltransferase small"
                     /protein_id="Prokka:NLMJPNJI_00040"
                     /translation="MLESDLLNSSIVACPNTIANVSYPCTKVVDIKGSLSQKKVNGEF
                     VILQELISACTTDKGFALKVSFTPSKFKFDHSFDPKEGLGEQSKNQIELKEARLRMYY
                     KEHKYQKDNLLITNLYLNDTLYQNEQGLDKLELSKNDLFNLNDINVLNTLKQDFSKDY
                     EFKECKIKLGVHLLKLIFIIPKNIAKIYKSAYKEFENKDLGAGYFTQLHEYDKDYREN
                     ENIIHHRVFLTPSKMQKIDFEIAKGLDEWLDNENVCCFNVYTKDYFNANDKDMLISDN
                     IKDDTNTTQAHTNENYNNT"
ORIGIN
        1 taataagctt tttctgcttt agtccaagga gctatagctc cttttatagg atcttttaaa
       61 tatatgcttt gccaatcttt aaattcaagt aaagtagaag cttgtcctgt ataaaagtta
      121 ggatctaaat atattggttt atagtctttg tagggggagt tttcttctat ttcaacagcc
      181 ttgagcaaaa cttcagcatg ctttttgtta tatctacctc cttttttgt tcctttataa
      241 agaataacct catttgtttc ttgattaaat tttgctcctt ttccattagg ggcatatata
      301 taagtttcac tcatcttttg tccttttatc tttaatttat cctatatgta aaccattttt
      361 catgattata gtggtagcta gtctgcctat gaggttactt tattctttaa tgccattttc
      421 ttcaccaaat ttgagtatct tagctctaag ttcccttggg aaattatatc tataaatagc
      481 agggattctt ggatcaagga gtttatctaa attatgattt ttgtaaataa actctaatct
      541 ttcaggagag taataataag gtgcattagt atagccttga ggaggagtga gggctgcgag
      601 tttggcacat aaaatcaaag tatcattaaa tctttctact tgctctttac tccaatgatt
      661 tggattatct ccatcaaatc tagttttcat tccatttcta taagcatcca atttatattc
      721 aaattccaat ctactctctg aagtagaatc tatatctctt ggatctttga gtattccttt
      781 ttcaacatca tcttttaggg catcattaac tcttcctttt gctccatcat aaacaaaata
      841 ataatcattt ggatctatca cccaatccac tcctataagt tcatctagaa aaggtagcat
      901 acccaattca tcataaggag gatttttaga aaagtctttt agcatttgag ctttaagagg
      961 gtttttgtgt atgccatctg ttaaatctct atatgcataa gaatcataaa gacttttaaa
     1021 gccttgttca tgtcctagtt gtatgagtaa aaaatttatt gcttgagttc ttgctttaaa
     1081 tcctgtcatg gaaagttgta aggctcctac aaatcctttt atatctccta aaattcctgc
     1141 tgctaattcc cattcagcca taaagagcca acctttatgc atattagcca tacctctatt
     1201 ggcttctact tctttataga gttctttata gtctttattg attaaatttc ctttttcatc
     1261 tacattacaa tatgcttcat aaggtatatc tataaccaca gatcttaatc cacttcttat
     1321 aattaaatat ttatatcttt ctcttttagt ttttaaagat ttataataag ctttttctgc
     1381 tttagtccaa ggagctatag ctccttttat aggatctttt aaatatatgc tttgccaatc
     1441 tttaaattca agtaaagtag aagcttgtcc tgtataaaag ttaggatcta gatatattgg
     1501 tttatagtct ttgtaggggg agttttgttt aatttcccaa gccttaaaaa aaactctagt
     1561 atattcttta gtatatttac ctacaggatc aacgctttgt gcaaaatata tttcttttgt
     1621 ttttttatct acctctactc ctaatccatt aggagcatat atcatataag tatcactcat
     1681 cttttatcct ttctttaaat ttatcctata tgtaagccat ttttcatgat tatagtggtg
     1741 gctagtctgc ctacaaattt agggcatttt tctttttcct ctttattgtt gacaacctta
     1801 atcatatatt ctttgttttc ctcactttct tttttttgtg agatattttt atcaccattt
     1861 atccccacat acaaagcttt aatattattt tgccctatag tgtctaaggc tcttaagaat
     1921 tctctgtgtt ttgagggatc agaagcgtta tttgtcgctt tagtattaaa aatatcatta
     1981 tccatatctt ctaaataatc catgcattta tgataagcat ctatggctgc taaacctttt
     2041 gcttctctat ttttatactc tttatataaa gcttttttta tatcatcttc attaatagct
     2101 tgtaattttt gattgtattt ttgtatgttt tctttttggg ttttagaaag atgattagag
     2161 attaaaaaat cttcttcatg ttttagtttt aaaagtcttg gttttatagg taaatcatgt
     2221 aaataagaat aagcattaaa aaagtctata tcttctatga tttttaaatc accggttttt
     2281 gatcttaact catctaaaca caaatatgca aaaagtttgt ttaaaagata atttctcacc
     2341 tttttttgtgc cgcttagatc tttatgatag acaaataatg attctttttc tcctaaacct
     2401 cctgtttgta gttttccacc tgtaaacata gaagtgaaat taaaagaaat aaaagtattg
     2461 ttgataaaca taggataaga gataaatcct tcttgtgtat ttgtgggttt tttcattaac
     2521 acatagggag gattgtattt atcactaaaa ttgaaaaatt tcaatctttc gtattctttt
     2581 ttgctttttt catagtgggt tatccataat tgattaacta aatctgtgat gatgacttga
     2641 ggcaaggata tgaaagattc tttggcaata ttgcgaatgg tttttaaagt aaaattaaat
```

2701 ttatatctga aaatttgttt tttactaata tttgtcaagt ctttaaaacc tttgcgattt
2761 tttaattctt taaaagtcat accatctact aacatgatag cttcgtgttt gtttttattt
2821 ttttgcatat agcaaagctc taatagggcc attttgctta gctttttaaa tcttttgtca
2881 aattcttttt ggttgtgtat tgtaactttt ttcatatgaa tatttgattt agtcatcaaa
2941 aacaacaaat aactcgcacc tatattttca taaagggctt tttgtgcttc aagggttaaa
3001 aaattaagga cagttttgct aagttgcttg gcaaatttaa aatcatctcc tcttaaaaaa
3061 tccgcaaaag gaaagaattt gtctataaaa gcatccatca tagctactat agtttttttgt
3121 ggattataag gcgttttatt tatattgttt ttttctatat cttttatcaa ttcatcaagc
3181 tcatcatcga ttttcctgtt tgattttttc tcattttcaa tcaaagcatt taattgcttt
3241 agattacact ccactgctgt atcttggatt tgattatttt catcttgagg ctcttctaga
3301 gaaaataaaa actgcctatc atcctcatca ctaatttcat aatcttttaa ctgctctaaa
3361 taatccacaa attcttctat acagaaaatt tctttttttg cgagtttggc tttttgttgt
3421 tttgaaaagc ataggggaat tttttcctca tttatattta aaaagacatc tttaggaagc
3481 atatcaacac tttcttcctc ttctatttgt atactttcat aatttgtaaa caatttccac
3541 caagcttgat aatcggtttt aatagacgaa taaaaatgct ttattacata ataacttaag
3601 gcaagtaaaa gaacttctgt gatggtaata tttttcttat cgacaaattt ttccgaataa
3661 gttacacctg tgtaagtacc aatgttttgt tgtttggttt gaataatgct atttatataa
3721 gctcttaagc aatcaatgcc ttcttgaaag tattgttttt gtttttctaa aaaagttttt
3781 ttattatttg cttgataaaa aacagattgt aaataatgat aattgtgtat tttatcagca
3841 ttactaggat tttttataaa atttagatat tgaaatacat tttcaggatt gtcatctgtg
3901 atactattta ataacttttc tttttctttt accacatcct caaagctttg ataagtttga
3961 taatttaatt taacaaaagg acttaaagaa agcacaaaag cattagtttc attatcgata
4021 atatttattt tatatttact ataatctatc attacatcat agtcttttgg agctttgact
4081 aaaatatcat tttgtttttc atcttttatg gtatctggat aaatgatggt attttcaaaa
4141 atttcttctg taagtttttg atgaagtcca gcattatatt tttgcggagc attagtgatg
4201 atgagaaaat tttttgcatg gtaagattcg cttttttttat cctttaaatc ctttatgatt
4261 tgagctagcg caatagagtt aaaatacgga ctttctatat aacttagtct attttgatct
4321 aaaggtattt gccttttaag tgcctcttta atggagtatt gtttataaag aataaattct
4381 tctttgggat tttcagtgaa aaaatttgaa gaatttgcac tccaaaaatc tttaaaaatt
4441 tcttcatttg atttaagaca tattgctaaa tcttgtttat gatatactga tgattgaaaa
4501 tttaccgctc caaagcctga aaaatcaaaa agaattttat catctataat caaagtatag
4561 tgatgaattt tgttttcaaa aacagtaaaa atatagccaa tatcacttat aagtagtttt
4621 ataatagcat tataaaagac ataagagtat ttattctcac tccactctga aatatcttga
4681 aaaaccctcc atgattcaaa agcaatatca ctagcaatta ataatggatg cgtttttgtc
4741 aaaaagcttt ttagtttaaa gatattaaca atattaagaa aagcattaat tattgttttg
4801 tatgcaaact cataaccttc tgcgtaaata tctttaatat tttgtatcaa ttcaccaaat
4861 atcagttcta tttgttcatt tagtttatat tcgtttaatt tttgatattc atcaaatgat
4921 tttagttttt gaagtatagg agtttttaat ttctcattaa tacaagtaat taaaaaatca
4981 attcctttgt tttctttttt tgaaatttgc gtcatagctt ctgaagtagt ttcatcttcg
5041 gattttteta aggcttttat aaaatcatca aaaatgcttg ccatatcaca agataaagaa
5101 gttgtgataa agtttgagga attattatca gttttattag aatcagtaaa ttgtttataa
5161 gaataatcat aaatttgaac ttttttacct ttataggcag atattttaag agtgttatat
5221 aagtatttat ctaaactata ataacagtcc ttggcaattt cgtaatctga tattgctaga
5281 ttaagtacat tttttcttga aaaattatta caatataaaa ctatattttt agcttgtttg
5341 caatatttgt ctatcaagtt tttaagctcg ttttggttat aaatatattg acaagtaaat
5401 tctttatttt tcattattta cctttaagta aatattgtct tagtattttt ttatttaaat
5461 aaaatttaag caaaatcatc tattaatctc tccaatccac ctaagcacat taccccttatc
5521 atcaatctct acttctaaat ctttgtattt attataaaat ttaggagcta agatttcatt
5581 aaaatatttt gtgcctttgg gggtaataat tgcattaata ttatatttat tccaactact
5641 tggaatatcc caagaagcaa tacaaaaatc atctatataa aaacactctt tattgatata
5701 attatctaaa aaatatttcc aatttttatc tttatcttta gtataagctg ataatgtatt
5761 ttgtttttca tcgtttatat aaaaatcaat taaatcatgt agaaataatt ttgtaaatac
5821 ttttacaaat tcaccttgat aatcttttgt ataaaggtta aaaatcataa aaaattctgc
5881 ataatcacta cattctaaga aatcgcataa aaaactatac tctataatct ctgttatatc

```
     5941 atttttattt acttgtttat gtgtggaaaa tatatcaaga aatgaattta tatgcaaatc
     6001 aagcaagaca tcccattgtg gaaataattc tgcatttata gtttctaaaa ctttaaattt
     6061 cattaaggtt ttcctaggtt attaatatgt attttataat ctttattatt aattttagta
     6121 tctatcgtgg gcatattgtc acttttgttg cttgaataat ttctcaacat taatcttgtg
     6181 tcatcttcta aaattcttat aattctaatt cttttactat ttgtttccac tatttctttt
     6241 tgataatttt ttgttaactt gtcccataaa accatcaatt catttatatc atctactaaa
     6301 taaattctat tgtgagattt ttttaatttt ttaatttcat ttaaaatttc atcctttgtc
     6361 aatgattttt tcttaataat attttttgca tttttaggat ttttcactgc accaagcatt
     6421 gctaaggcat caccgataat gctgacattt tcaagtgaat ttgaaatatc tcccactata
     6481 ccttctaatg gggtaaataa aggctttatg actctgcttg atttatttct ccattcctcc
     6541 aaatcatcaa atattatctc aataaactct ccactaccat cttgtgtttg tattcttata
     6601 acattatcag ggataatttc ttaagtattg ttatagtttt cattggtgtg agcttgtgta
     6661 gtatttgtat catcttttat attatcactt ataagcatat ctttatcatt tgcgttaaaa
     6721 taatccttag tataaacatt aaagcaacac acattttcat tatcaagcca ctcatctaaa
     6781 ccttttgcaa tttcaaaatc aatcttttgc attttagaag gagttaaaaa aactctatgg
     6841 tggatgatat tttcattttc cctataatct ttatcatatt catgtagctg tgtaaaatac
     6901 cctgctccta gatctttgtt ttcaaattct ttataagcac ttttataaat cttggcaatg
     6961 tttttgggga taataaagat taattttagc aaatgcacac caagtttaat tttgcactct
     7021 ttaaattcat aatctttact aaagtcttgt ttaagagtat ttagtacatt aatatcattt
     7081 aaattgaata aatcattttt agataattct aatttatcta aaccttgttc gttttgatat
     7141 agggtatcgt ttagatataa attagtgatt aaaagattgt ctttttgata tttgtgttct
     7201 ttataataca ttcttaatct agcttctttt aactctattt gatttttact ttgttctcct
     7261 aagccctcct taggatcaaa gctgtgatca aatttaaact tacttggagt aaagcttact
     7321 tttaaagcaa agcctttatc tgtagtgcaa gctgatatga gttcttgtaa gattacaaat
     7381 tctccattta ctttcttttg agataaggag cctttaatat ccactacctt agtacaagga
     7441 taacttacat ttgctatggt attaggacaa gctactatac tagagtttaa taaatcactt
     7501 tctagcatta ta
//
LOCUS       NLMJPNJI_3              7385 bp    DNA     linear       12-APR-2021
DEFINITION  Campylobacter species strain strain.
ACCESSION
VERSION
KEYWORDS    .
SOURCE      Campylobacter species
  ORGANISM  Campylobacter species
            Unclassified.
COMMENT     Annotated using prokka 1.14.6 from
            https://github.com/tseemann/prokka.
FEATURES             Location/Qualifiers
     source          1..7385
                     /organism="Campylobacter species"
                     /mol_type="genomic DNA"
                     /strain="strain"
     gene            629..892
                     /locus_tag="NLMJPNJI_00041"
     CDS             629..892
                     /locus_tag="NLMJPNJI_00041"
                     /inference="ab initio prediction:Prodigal:002006"
                     /codon_start=1
                     /transl_table=11
                     /product="hypothetical protein"
                     /protein_id="Prokka:NLMJPNJI_00041"
                     /translation="MSKIKTLRISDDKKHFSYWFEKWIGDRISFDFVIWFKNENATKD
                     NIKKVSVYVWWRDLRPLPAGNEGAGFYLSGDRENCAYFKKEIK"
```

```
gene            889..1008
                /locus_tag="NLMJPNJI_00042"
CDS             889..1008
                /locus_tag="NLMJPNJI_00042"
                /inference="ab initio prediction:Prodigal:002006"
                /inference="similar to AA sequence:RefSeq:CKG86955.1"
                /codon_start=1
                /transl_table=11
                /product="methyltransferase small"
                /protein_id="Prokka:NLMJPNJI_00042"
                /translation="MNNKEQIKKLKDNAELAMASYGYFHLADKSYNHNDKYEK"
gene            1366..2307
                /locus_tag="NLMJPNJI_00043"
CDS             1366..2307
                /locus_tag="NLMJPNJI_00043"
                /inference="ab initio prediction:Prodigal:002006"
                /codon_start=1
                /transl_table=11
                /product="hypothetical protein"
                /protein_id="Prokka:NLMJPNJI_00043"
                /translation="MSSFKDLLVADASLAIGSIPKAQYDDMINFYETCIKDYPQIKEK
                DSLTITGHSLGGCLAQLFALSICDDKNRNNIKALYTYNAPGARKIIPPYDYIVKLFIF
                HSKEQQERFIKEEIENIACPHTIANVSYPCTKVVDIKGSLSQKKVNNEFVILQELISA
                CTTDKGFALKVSFTPSKFKFDHSFDPEEGLGEQSKNQTELKEPIIRLHYKSDRFQKDN
                LPIYNLLINNEKKEQDKALNEFNIEQKDLKDIEDINIFNQFKQDFSKDHEFKELNLSF
                DTNLIKLYFIIPKNIAKVYKSAYKEFKNKDLGAGYFT"
gene            2398..2514
                /locus_tag="NLMJPNJI_00044"
CDS             2398..2514
                /locus_tag="NLMJPNJI_00044"
                /inference="ab initio prediction:Prodigal:002006"
                /inference="similar to AA sequence:RefSeq:CKG86393.1"
                /codon_start=1
                /transl_table=11
                /product="methyltransferase small"
                /protein_id="Prokka:NLMJPNJI_00044"
                /translation="MQNLKLQIAQGLDKILEDEDRKQELYVCKFVVVNGVKV"
gene            2511..5918
                /locus_tag="NLMJPNJI_00045"
CDS             2511..5918
                /locus_tag="NLMJPNJI_00045"
                /inference="ab initio prediction:Prodigal:002006"
                /codon_start=1
                /transl_table=11
                /product="hypothetical protein"
                /protein_id="Prokka:NLMJPNJI_00045"
                /translation="MKIKNFTLITSNHNLEIELKNLTKNANKIIFICSDFDGYKEAEN
                SLYKTLHTIEDNFKIDNTKTFKIIHLNQTNKDEITKEQKFSSDKNLSSIKKIIYNNLK
                LLNDTINQNENKTLNKIIDESNEKNIENFLKEIQKELNILLKNIKESNYFDQEIKPKI
                QVTLDTLFNTLLKNIAQSNDDEYKQIIRAVAKFFINILDSITSLKKPISLIKKNPYVL
                IVNTLFEAYNSYSEYQEYKEKKYYYDFAYPLLELITSKLYPIIALCNEEILSDVLIID
                DKVLLDFSSYTNAPSISLYKNSFYKIILDEEFQGVFDNFLENNLKFENEKLDNTLLVN
                SSLNNSDTTLKKAINLNISKLNHFFYAEYPDLNSSMLVEMILDKKNADQINTTKMGKN
                YLFITNPPQLNNAGLCEDLYQKKLGAIIKNRPKALTKNEKLALKGDFPSKRDYGSYFI
```

```
                     ERDVKKEDDFVLQLCPFAKLDGQIYQKYKGYFNFYNQTYSWMKLYLLEYINERFEYEK
                     FKQDFAYIDNFFKLPKELEKISEKEKEKVEEYVELFNDYLDKMNKSLAPYSVQNLYEY
                     SVLHLFLATCALYVFQKEFEKHKYEIYADRGLYYYQALNKLKVIFSKSYTMFDDKKRY
                     IFINKKLDIQADEKNRINEVIYLDELAKAMFENDLEEDDQTYVKFLNTLNENFDDEIE
                     FEENIQTNQTLPTKKDLDKSIQEIMLDNELAKLSLDITFFNIIKQLFPFTEFFMSQPD
                     FFKKMILFFIDSSLDKKYYGNKLFSSFKAHFFKELGIFYALNPKGRFIKIIPKSKKPF
                     NKLFLYQIGTNYYYFSQSEYASELKKSKSFSRLDKAKQIHLISKARKQLAIDFSIKVS
                     KNLSADVLKNLAGSFIDTLLPTNYERLKILYEKLMYEKLTYNYDFPLAVKKEEYITYP
                     LLINSRFMSFDLSEFIFGSLLCTGGLNYYPSIACTTTSSEAREFALKRLLSYIILDEK
                     RSAFMEDKINEGKYILNDKEFFEKNNIELRNCNIYKLEHFQRKEEYKTQNPMIKQHPI
                     LAYNDTIQILYEYINGIYNTIQDEKGNFIEDRQKARRLMENLEAIGQHNLEVMYKGMG
                     DKKIKDERKNENDDDESDDINKIQDYSNKFIGRLATTIIMEDGLYMG"
     gene            5937..7223
                     /gene="trxB_2"
                     /locus_tag="NLMJPNJI_00046"
     CDS             5937..7223
                     /gene="trxB_2"
                     /locus_tag="NLMJPNJI_00046"
                     /inference="ab initio prediction:Prodigal:002006"
                     /inference="similar to AA sequence:RefSeq:CKG86288.1"
                     /codon_start=1
                     /transl_table=11
                     /product="thioredoxin reductase"
                     /protein_id="Prokka:NLMJPNJI_00046"
                     /translation="MSETYEIYMPNGVILEVEKETNKILLSDGGAKVGKYTQEYSKAL
                     FEAHNIKQNSPYKDYQPRYLDPEFHTGQKSTLLEFKDWQSIYLKDPIKGSIAPWTKAE
                     KAYYNSLKTKKERYKYLVIRSGIRSVVIDIPYEAIGAVDEKGNVDPKYEELYRTVDDN
                     KHNLRSSLFHNEWGMAAGILGDYKYLANDMSQNGFNARFIQATILYIQLSGGSSILDK
                     PNLLGAIYGYADIAVGSGLVGVHKNPLREQQIKTLAKTLKPDEFGMLPFIDEIMGVDW
                     VIDLNKYRLANDEFGDIYKALRSDVVEGKIKDPRDIDSTYESRREFDRYRVGYRKGMR
                     QGYETDTPNDWSEERAQLFNDTLILHAKLAALTPPQGYPNAPYYFTPENLEWYYKRHK
                     LDKLLDPRIPAIYRYNFPRELRAKILAYAKEYNIKD"
ORIGIN
        1 attacgttga taataaattt gatataaaag atgaagataa aattgttacc tttgaagatg
       61 ttttagatat tacctataaa aactctaaaa ttattgatga aagaggattt aaaatcggca
      121 ctctcaatgg cgacttctct ccactccaag ctaaacaatt cttttctcgc tatgatttat
      181 taaaacattg tcctaataca aattcaggtt tttctgctac tttgtttgga gaaaaaagaa
      241 aataaattta tagtaaaata aaagaaaaag ttatactgct gaatataact tataaactat
      301 attttaattt taataaaata gagagtctaa ggagataacc catgacatac atattacttc
      361 ttattataat ttctactatt ctttcttatc ttatattaaa atttatttat aaaatcatct
      421 ttaaatctac aaaaagcgtt tcaaagtttt tagtctaggc ttaattatct tttattatac
      481 tccttattct tattatttag agccaagctt ttataagttt aaagaaatat gcaagctaga
      541 cccagagatt taccaagcca atggtagtaa attagatgag gagtattata ataaagtgct
      601 aaggtatttt gatacggatt tggataatat gtcaaagata aaaacattaa gaataagcga
      661 cgataaaaag cacttttcat attggtttga aaaatggatt ggagatagaa tctcttttga
      721 ttttgtcatt tggtttaaaa atgaaaacgc tacaaaagac aatatcaaga aagttagcgt
      781 ttatgtttgg tggcgtgatt tacgaccatt gccagcaggt aatgaggggg cgggattcta
      841 tctttcagga gatagggaaa attgtgctta ttttaaaaag gaaataaaat gaacaataaa
      901 gaacagatta aaaagctcaa agataatgcc gaattagcaa tggcaagtta tgggtatttt
      961 catttagcag ataaatccta taatcataat gataaatatg aaaagtgatt aaaggaattt
     1021 agagaactta aagcaaaagc attaggaaaa gaaattgatg aaagtattta ccccacctac
     1081 gcagatatac tcgacattac ctataaatac tatcttgatg ataatggcaa acccaaagat
     1141 ggcttatttg atgatgagtt atttagtggc gacttctctc cactccaagc taaacaattc
     1201 ttttctcgct atgatttatt aaaacattgt cctaatacaa attcaggttt ttctgctact
```

1261 ttatttggag aaaaaagaaa acaaaagaat acagaatcta aagagattag ctatacaagt
1321 gaatatggct atatcaatta tattttagct attagaggaa cagaaatgag ttcttttaaa
1381 gatttgcttg tagctgatgc ttctttagcc ataggctcta tacccaaagc ccaatatgat
1441 gatatgataa acttttatga aacttgtatt aaagattatc cacaaatcaa agaaaaagat
1501 tctcttacta ttacaggaca ctctcttgga ggctgtcttg cgcaactctt tgctttgagt
1561 atctgtgatg ataagaatag aaataatatc aaagctttat atacttataa tgcacctgga
1621 gctagaaaga taatacctcc ttatgattat atagtcaaac tctttatatt tcacagtaaa
1681 gaacaacaag aaagatttat aaaagaagaa atagaaaata tagcttgtcc tcacaccata
1741 gcaaatgtaa gttatccttg tactaaggta gtggatatta aaggctcctt atctcaaaag
1801 aaagtaaata atgaatttgt aatcttacaa gaactcatat cagcttgcac tacagataaa
1861 ggctttgctt taaaagtaag ctttactcca agtaagttta aatttgatca cagctttgat
1921 cctgaggagg gcttaggaga acaaagtaaa aatcaaacag agttaaaaga gcctataata
1981 agacttcatt ataaaagtga tagatttcaa aaagataatt tgcctattta taatctttta
2041 atcaataatg aaaaaaaaga acaagacaag gctttaaatg aattcaatat agaacaaaaa
2101 gatttaaaag atatagagga tataaatatc tttaatcaat ttaaacaaga ctttagtaaa
2161 gaccatgaat ttaaagaatt aaatcttagc ttcgatacta atttaatcaa gctttatttc
2221 attatcccca aaaacattgc caaagtttat aaaagtgctt ataaagaatt taaaaacaaa
2281 gatctaggag cagggtattt tacatagcta catgaatatg ataaaatcat taaaaattct
2341 ttagaagata agaaagaatt aaacgaatat catttaactt ttttaactcc tgcaaaaatg
2401 caaaatttaa aacttcaaat cgcacaagga ttagataaaa tcttagaaga tgaagataga
2461 aaacaagagc tttatgtttg taaatttgtg gtggtaaatg ggggttaaagt atgaaaataa
2521 aaaattttac tttaattaca tcaaatcata atttagaaat cgagttaaaa aatcttacca
2581 aaaatgctaa taaaattatt tttatatgca gtgattttga tggatataaa gaagctgaga
2641 attctctgta taaaacatta catactattg aagataattt taaaattgac aatacaaaaa
2701 cttttaaaat catacattta aatcaaacca ataaagacga aatcacaaaa gaacaaaaat
2761 ttagctctga taaaaattta tctagcatta aaaaaattat ctataataat ttaaaacttt
2821 taaatgatac aatcaatcaa aatgaaaata aaacactcaa taaaattatt gatgaaagca
2881 atgaaaaaaa tatcgaaaat tttttaaaag aaattcaaaa agaattaaat atattactaa
2941 aaaatatcaa agaatctaat tattttgatc aagaaatcaa gccaaagata caagttactt
3001 tagatacgct ttttaacaca cttttaaaaa atattgccca atctaatgat gatgaatata
3061 agcaaattat tagagctgta gcgaaatttt ttatcaatat attagattct ataaccagcc
3121 ttaaaaagcc catttcttta attaaaaaaa atccttatgt tttgattgta aatacattat
3181 ttgaagctta taattcttac tctgaatatc aagagtataa ggaaaaaaaa tattattatg
3241 attttgctta tccactttta gaacttatca ctagcaaact ttatcctatt attgctttat
3301 gcaatgaaga gattttatct gatgtgttaa ttatagatga taaagtatta ttagattttt
3361 catcttatac taatgcacct tctatcagtc tttataaaaa tagtttttat aaaattattt
3421 tagatgaaga atttcaaggt gtttttgata attttttaga aaataatcta aaatttgaaa
3481 atgaaaaatt agacaacact cttttagtga attctagttt aaataattct gacacaacat
3541 taaaaaaggc gataaattta aatatctcca aactcaatca tttcttttat gcagaatacc
3601 ctgatttaaa ttcttccatg ctagtagaaa tgatactgga taaaaaaaat gctgatcaaa
3661 tcaatacaac caaaatgggt aaaaattatt tatttattac caatccacca cagcttaata
3721 atgcagggct ttgcgaagat ttgtatcaaa aaaaattagg agcaataata aaaaatcgcc
3781 ccaaagctct tacaaaaaat gaaaaacttg cattaaaagg cgattttcct agcaaaagag
3841 attatggttc ttattttata gaaagagatg ttaaaaaaga agatgatttt gtcttgcaac
3901 tttgtccttt tgcgaaactt gatggacaga tttatcaaaa atataaggga tattttaatt
3961 tttataatca aacatattct tggatgaaac tttatttatt agaatatata aatgaacgat
4021 ttgaatatga aaaatttaaa caagattttg cttatattga taacttttt aaattaccta
4081 aagagcttga gaaaattagc gaaaaagaaa aagaaaaggt ggaagaatat gtcgaacttt
4141 ttaatgatta tttggataag atgaataaaa gtttggctcc ttattctgtt caaaatttat
4201 atgaatattc tgttttacat ttgtttttag cgacttgcgc tttatatgtt tttcaaaaag
4261 aatttgaaaa acataaatat gaaatatatg ccgatagagg attgtattat tatcaggcgc
4321 ttaataagct aaaagtcatt ttttcaaaat cttataccat gtttgacgat aaaaaaagat
4381 atattttcat aaataaaaaa ttggatatac aagcagatga aaaaaataga ataaatgaag
4441 tcatctattt agatgaatta gctaaagcta tgtttgaaaa tgatcttgaa gaagatgatc

```
     4501 aaacttatgt gaaattttta aacaccttaa atgaaaactt tgatgatgaa attgaatttg
     4561 aagaaaatat acaaacaaat caaacacttc ctacaaaaaa ggatttagat aaaagtattc
     4621 aagaaattat gcttgataat gagcttgcaa aactatcctt agatatcact ttttttaata
     4681 tcattaagca actttttcct tttacagaat tttttatgag tcagcctgat ttttttaaaa
     4741 aaatgatttt atttttttata gattcaagct tagataaaaa gtattatggt aataaattat
     4801 tttctagttt taaagcacat ttttttaaag aactagggat attttatgcg cttaatccta
     4861 aagggcgttt tataaaaatc atcccaaaaa gtaaaaagcc attcaataag cttttttttat
     4921 atcaaatagg aacaaattat tattatttta gtcaatcaga atatgcaagc gagttgaaaa
     4981 aatcaaagag tttttcaaga ttggataaag ctaagcaaat tcatttgata tcaaaagcaa
     5041 gaaaacaatt agctattgat ttttctatta aagtaagtaa aaatttaagt gcggatgtat
     5101 taaaaaaattt agcaggttct tttattgata cacttttgcc tacaaattat gaaagattaa
     5161 aaattttata tgaaaaacta atgtatgaaa aattgactta caattatgat tttcctttag
     5221 ctgtaaaaaa agaagagtac ataacctatc ctttattgat aaattctaga tttatgagtt
     5281 ttgatttatc tgagtttatt tttggttctt tattgtgcac aggtgggctt aattattatc
     5341 ctagtattgc ttgcactaca acttcaagtg aagctagaga atttgcctta aaaagacttt
     5401 tatcttatat tattttagat gagaaaagaa gtgcttttat ggaagataaa atcaatgagg
     5461 gcaaatatat cttaaatgat aaggaatttt ttgaaaaaaa caatatagaa cttagaaatt
     5521 gtaatatata taagctagaa cattttcaaa gaaaagaaga gtataaaaca caaaatccta
     5581 tgataaaaca acaccctatc ttagcttata atgatactat acaaatttta tatgaataca
     5641 taaatggaat ttacaatact atacaggatg aaaagggtaa ttttatagaa gataggcaaa
     5701 aggcaagaag attgatggaa aatttagaag ccataggaca acataattta gaagtgatgt
     5761 ataaggggat gggagataag aagataaaag atgaaagaaa aaatgaaaat gatgatgatg
     5821 aaagtgatga tattaataaa atacaagatt attctaataa gtttataggc aggctagcca
     5881 ctactataat catggaagat ggtttatata tgggataaat taagaaagga taaaagatga
     5941 gtgaaactta tgaaatttat atgcctaatg gtgttatatt agaagtagaa aaagaaacga
     6001 ataaaatttt attaagtgat gggggagcta aggtaggcaa atacacccaa gaatattcta
     6061 aagccctttt tgaagcacac aatatcaaac aaaactctcc ctacaaagac tatcaaccta
     6121 ggtatctaga tcctgaattt cacacagggc aaaaatccac tttgcttgaa tttaaagatt
     6181 ggcaaagtat atatttaaaa gatcctatca aaggaagcat agctccttgg actaaagcag
     6241 aaaaagctta ttataactct ttaaaaacta aaaaagaaag atataaatat ttagttataa
     6301 gaagtggtat aagaagtgtt gttatagata taccttatga agctatagga gctgtagatg
     6361 aaaaaggaaa tgtagatcct aagtatgaag aattatatag aacagtagat gataataaac
     6421 ataatctaag atcaagttta tttcataatg aatggggtat ggcagcagga atactgggtg
     6481 attataaata tctagctaac gatatgtctc aaaatggttt taatgcaaga tttatccaag
     6541 caactatact ttatatacaa ttaagtggag gaagtagtat actagataaa cctaatttat
     6601 tgggtgctat ttatggttat gcagatattg cagtaggaag tggtttagta ggagtgcata
     6661 aaaatccttt aagagagcaa caaattaaaa ccttagctaa gactttaaaa cctgatgaat
     6721 ttggtatgct tccttttata gatgagatta tgggagtaga ttgggtgatt gatttaaata
     6781 aatatagatt agctaatgat gaatttgggg atatatataa agccttaaga agtgatgtag
     6841 ttgagggtaa gataaaagat ccaagggata tagattctac ttatgaaagc agaagggagt
     6901 ttgatcgtta tagagttgga tatagaaagg gtatgaggca aggctatgaa accgatacac
     6961 ccaatgattg gagtgaagaa agggcgcaac tttttaatga tactttaata cttcatgcca
     7021 aactcgcagc cctcactcct cctcaaggct atcctaatgc accttattat tttacccctg
     7081 aaaacctaga atggtattat aaaagacata agcttgataa actccttgat ccaagaatcc
     7141 ctgctattta tagatataat ttcccaaggg aacttagagc taagatacta gcttatgcta
     7201 aagagtataa tatcaaagat taaaccacaa aagaaaggaa aaacaatgca aaaaacactt
     7261 caaatcgatt tagaaaaaag aaaagcttta gagaatttta aaaatctagc tttattagga
     7321 ttaggcggaa caatgctttt aaatttaaaa gaacagcctt tagattttaa tcaagaatta
     7381 actta
//
LOCUS       NLMJPNJI_4              6436 bp    DNA     linear       12-APR-2021
DEFINITION  Campylobacter species strain strain.
ACCESSION
VERSION
```

```
KEYWORDS    .
SOURCE      Campylobacter species
  ORGANISM  Campylobacter species
            Unclassified.
COMMENT     Annotated using prokka 1.14.6 from
            https://github.com/tseemann/prokka.
FEATURES             Location/Qualifiers
     source          1..6436
                     /organism="Campylobacter species"
                     /mol_type="genomic DNA"
                     /strain="strain"
     gene            complement(305..1516)
                     /locus_tag="NLMJPNJI_00047"
     CDS             complement(305..1516)
                     /locus_tag="NLMJPNJI_00047"
                     /inference="ab initio prediction:Prodigal:002006"
                     /inference="similar to AA sequence:RefSeq:CKG86410.1"
                     /codon_start=1
                     /transl_table=11
                     /product="hypothetical protein"
                     /protein_id="Prokka:NLMJPNJI_00047"
                     /translation="MKYYVTIYIDELLEFQMIKDLLNMKGLDFTHAFLGLTKDKSPDE
                     LDKIDQEWYKAKETNKFNDGFWGFGTGTDSVYCTAGKVFNNNQYVLDNNVDKNRYIIK
                     KLRSPQTFKPSNRCTLEISKEQYETLLANIKADFEATKEITPNSEKPINEEFTYKLLE
                     NNCVTWVVKKLSSIGIEVVNPVVKDPSGLLMATKLIQSIHSIFLKFQNIDDNLQSVKG
                     AKAFKNWARMLINNSLMQEIYLDEYKKQYNSENLSPEQEEVLYALWNITRVYKILDIL
                     LSRLITKSGTKNLKGDFELIYFDRKDKQIKLLKADNDYEAIIIQDLDLSKKYNNFSAS
                     KFYPFIFIPKDEMLSRTLYHKYDYGNISQEYQKDRNEFYFNVLAGEKSDKYWSSSYHK
                     MTKNLRKVYVS"
     gene            complement(1525..1983)
                     /locus_tag="NLMJPNJI_00048"
     CDS             complement(1525..1983)
                     /locus_tag="NLMJPNJI_00048"
                     /inference="ab initio prediction:Prodigal:002006"
                     /codon_start=1
                     /transl_table=11
                     /product="hypothetical protein"
                     /protein_id="Prokka:NLMJPNJI_00048"
                     /translation="MQIPIYSKLKAKLESKGIDNISSGQIAWFVCSVVLALVTFLIIY
                     FTVIIEDKNLFLAIFELFQWQLFIFIIYFCIPFNLLYIHNAIPLVLSAIFMFLTLYFS
                     SLNLFIIGCCGIGAFFLISIFKRKLMFSVLLFAMGYICIYFFLYAITRYH"
     gene            complement(2025..2204)
                     /locus_tag="NLMJPNJI_00049"
     CDS             complement(2025..2204)
                     /locus_tag="NLMJPNJI_00049"
                     /inference="ab initio prediction:Prodigal:002006"
                     /inference="similar to AA sequence:RefSeq:CKG86410.1"
                     /codon_start=1
                     /transl_table=11
                     /product="hypothetical protein"
                     /protein_id="Prokka:NLMJPNJI_00049"
                     /translation="MVECFLDFKNQCYWQIEDFTHAFLGLTKGKSPDELDKIDEELRQ
                     EYLKNKEFKLIKSIK"
     gene            complement(2234..2482)
```

```
                     /locus_tag="NLMJPNJI_00050"
     CDS             complement(2234..2482)
                     /locus_tag="NLMJPNJI_00050"
                     /inference="ab initio prediction:Prodigal:002006"
                     /codon_start=1
                     /transl_table=11
                     /product="hypothetical protein"
                     /protein_id="Prokka:NLMJPNJI_00050"
                     /translation="MWIVCIINFFNIKNLIPITLNWIISYCYFVLEINIYFILILCIT
                     LCVVFYFLQKRLLYTFLFYFVYMVLTYTLFYLGGGFYK"
     gene            complement(2464..2724)
                     /locus_tag="NLMJPNJI_00051"
     CDS             complement(2464..2724)
                     /locus_tag="NLMJPNJI_00051"
                     /inference="ab initio prediction:Prodigal:002006"
                     /codon_start=1
                     /transl_table=11
                     /product="hypothetical protein"
                     /protein_id="Prokka:NLMJPNJI_00051"
                     /translation="MQIPIYSKLKTKLESKGIDNITSGQIAWFVCSVIFTYLLFLLIV
                     IINTRTISFENLKFFTFEGFFKSLAFIFANHFFYLFLCGLFV"
     gene            complement(2784..3020)
                     /locus_tag="NLMJPNJI_00052"
     CDS             complement(2784..3020)
                     /locus_tag="NLMJPNJI_00052"
                     /inference="ab initio prediction:Prodigal:002006"
                     /inference="similar to AA sequence:RefSeq:CKG86410.1"
                     /codon_start=1
                     /transl_table=11
                     /product="hypothetical protein"
                     /protein_id="Prokka:NLMJPNJI_00052"
                     /translation="MKEIIKYYVTIYIDELLENEMLKHVDFSLLFKAKFSQISKQIDF
                     THAFLGLTKDKSPDELDKIDEELRREYLKNKNSN"
     gene            complement(3017..3496)
                     /locus_tag="NLMJPNJI_00053"
     CDS             complement(3017..3496)
                     /locus_tag="NLMJPNJI_00053"
                     /inference="ab initio prediction:Prodigal:002006"
                     /codon_start=1
                     /transl_table=11
                     /product="hypothetical protein"
                     /protein_id="Prokka:NLMJPNJI_00053"
                     /translation="MQIPIYSKLKAKLESKGIDNISSGQIAWCLGSGYIALICAVFVV
                     VYKLKNLEFELYIVFFFPILLFYIAIFWFIFLSFYKNFFFVHLFCYSFMYIFNIIFND
                     FYLNLVVMNILLITIWLFFVKLHLKFRFLFLMMYFCFEFAIFIGIIPYGLLEGTISK"
     gene            complement(3543..3716)
                     /locus_tag="NLMJPNJI_00054"
     CDS             complement(3543..3716)
                     /locus_tag="NLMJPNJI_00054"
                     /inference="ab initio prediction:Prodigal:002006"
                     /inference="similar to AA sequence:RefSeq:CKG86410.1"
                     /codon_start=1
                     /transl_table=11
                     /product="hypothetical protein"
```

```
                     /protein_id="Prokka:NLMJPNJI_00054"
                     /translation="MKYCATIYIDPLVECFLDCENKNFINAEDFTHAFLGLTKGKSLD
                     ELDKIDKELRQKF"
     gene            complement(3713..4138)
                     /locus_tag="NLMJPNJI_00055"
     CDS             complement(3713..4138)
                     /locus_tag="NLMJPNJI_00055"
                     /inference="ab initio prediction:Prodigal:002006"
                     /inference="similar to AA sequence:RefSeq:CKG86435.1"
                     /codon_start=1
                     /transl_table=11
                     /product="hypothetical protein"
                     /protein_id="Prokka:NLMJPNJI_00055"
                     /translation="MQIPIYSKLKAKLESKGIDNITSGQIAWFVCSVGIFIIPMMYFY
                     ISNNYDQFTILFIMIFIFILNMFYTKNFFALLLLVILIFIYMKYHEFIYNFKILFFIF
                     APIILIFLRAWRILITFLCGYIFLVAMVYIFELLGGFYK"
     gene            complement(4180..4341)
                     /locus_tag="NLMJPNJI_00056"
     CDS             complement(4180..4341)
                     /locus_tag="NLMJPNJI_00056"
                     /inference="ab initio prediction:Prodigal:002006"
                     /codon_start=1
                     /transl_table=11
                     /product="hypothetical protein"
                     /protein_id="Prokka:NLMJPNJI_00056"
                     /translation="MLNWGFMMGSEGNPHVFLGLTKGKSPDELDKIDEELRQEYLKNK
                     EFKLIKSIK"
     gene            complement(4375..4974)
                     /locus_tag="NLMJPNJI_00057"
     CDS             complement(4375..4974)
                     /locus_tag="NLMJPNJI_00057"
                     /inference="ab initio prediction:Prodigal:002006"
                     /codon_start=1
                     /transl_table=11
                     /product="hypothetical protein"
                     /protein_id="Prokka:NLMJPNJI_00057"
                     /translation="MKYIILTFILGLIVFVSVKGCFYPTSYRAFIPKSLDSEYKEFER
                     FSQQELGRVLYARPLTEIEKNCLNKNDFYIKTNRYGIKSKDMDMFVGIEFIYSAERLV
                     YVYIKELGYYGGWNKWKFNLFILPNITQRNYNSIESITINNSQIMQNKEIIGNLKIGD
                     KLDRSYTYDYLNILVLENLLDKPSYLNHFEENYDYFEDL"
     gene            complement(4978..5082)
                     /locus_tag="NLMJPNJI_00058"
     CDS             complement(4978..5082)
                     /locus_tag="NLMJPNJI_00058"
                     /inference="ab initio prediction:Prodigal:002006"
                     /codon_start=1
                     /transl_table=11
                     /product="hypothetical protein"
                     /protein_id="Prokka:NLMJPNJI_00058"
                     /translation="MLDLMNENDENSTLAPLWLYADGVTNLKNHSKRQ"
     gene            complement(5326..5574)
                     /locus_tag="NLMJPNJI_00059"
     CDS             complement(5326..5574)
                     /locus_tag="NLMJPNJI_00059"
```

```
                     /inference="ab initio prediction:Prodigal:002006"
                     /codon_start=1
                     /transl_table=11
                     /product="hypothetical protein"
                     /protein_id="Prokka:NLMJPNJI_00059"
                     /translation="MFLLPVYLLLFLAGGCSYQYMDPQYYEFGSLCYFNLEKVLIEDE
                     SIKNKTNIIVTPKSKQIKDRIVETRFERKNEKPSSVLS"
     gene            complement(5726..6094)
                     /locus_tag="NLMJPNJI_00060"
     CDS             complement(5726..6094)
                     /locus_tag="NLMJPNJI_00060"
                     /inference="ab initio prediction:Prodigal:002006"
                     /inference="similar to AA sequence:RefSeq:CKG86895.1"
                     /codon_start=1
                     /transl_table=11
                     /product="methyltransferase small"
                     /protein_id="Prokka:NLMJPNJI_00060"
                     /translation="MTNKEQINNLKDYTEFMQASYFNFVYTEGQKALGFKISKTRFFK
                     DKSELEKLDYFKALHDEYNGYLVIDDSLFFYAKLNGDFRELQARNFSQRYEIKFHQSN
                     TTSGFSATLSYEKIERIKRN"
     gene            complement(6113..6352)
                     /locus_tag="NLMJPNJI_00061"
     CDS             complement(6113..6352)
                     /locus_tag="NLMJPNJI_00061"
                     /inference="ab initio prediction:Prodigal:002006"
                     /codon_start=1
                     /transl_table=11
                     /product="hypothetical protein"
                     /protein_id="Prokka:NLMJPNJI_00061"
                     /translation="MWLYIYYDDTYKTPSNFQEKIEMDNIALKTKNMQMSLHKIVDMY
                     ISPYWNTRRYVLEGNEGNMNFEFIEDELACGNLYQ"
ORIGIN
        1 agaattctct aatatagaat aattaagcaa gcaaagttca tcatttttat aaaagatact
       61 aagtcttcct aaaccactgc tttcatcttt aggagaaaaa taataactgg gtatatcttg
      121 ttttatagta ttatcttgat ctaattctcc aaagtaaaaa ggattattag gaagttcggt
      181 ggagcctgta aggagtatac cttgaaaata aattttagaa tgaattaaat caacattttt
      241 agcccttaaa acttcacaat caatcacttc tttatcataa gcaaaaatcg tttcttttat
      301 tttttcaaga gacatagact ttccttaaat ttttagtcat tttatgatag ctagaactcc
      361 aatatttatc gctttttttct cctgctaaaa cattaaaata aaattcattt ctatcttttt
      421 gatattcttg tgagatatta ccataatcat acttatgata aagcgttctt gagagcattt
      481 catctttagg tataaagata aaaggataaa atttactcgc actaaaatta ttatattttt
      541 tacttaaatc caaatcttgt ataatgatag cttcataatc attatctgct tttaaaagtt
      601 tgatttgttt atcttttcta tcaaaataaa ttaattcaaa atcgcctttt aggtttttag
      661 tgcccgattt tgtaattaaa cgacttaaaa gaatgtctaa aattttataa acccttgtaa
      721 tattccataa agcatataaa acttcttctt gttctggact aagattttca ctattgtatt
      781 gtttttata ttcatcaaga taaatttctt gcattaatga attatttata agcattctag
      841 cccaattttt aaaagctttt gcacctttta cgctttgtaa attatcatca atgttttgaa
      901 attttaaaaa gatagaatga atgctttgta ttaattttgt agccattaat aatccgctag
      961 gatctttaac aacgggattt acaacttcta ttcctatgct agaaagcttt ttaacaaccc
     1021 aagtaacgca attattttca agaagtttat aggtaaattc ttcattgatt ggcttttcgc
     1081 tattaggtgt gatttctttt gtggcttcaa aatcagcttt aatatttgca agtagggttt
     1141 catattgttc cttagaaatt tctaaagtgc aacgattaga gggtttaaaa gtttgagggc
     1201 ttcttagttt tttaataata tagcgatttt tatcaacatt attatctaat acatattggt
     1261 tattattaaa tactttgcca gctgtacaat agacagaatc tgttccagtt ccaaaacccc
```

1321 aaaaaccatc attgaattta ttggtttctt tagctttata ccattcttga tctatcttat
1381 ccaactcatc aggactttta tctttggtaa gtcctaaaaa ggcgtgggtg aagtctaaac
1441 ctttcatatt cagcaaatct tttatcattt gaaattctaa taactcatca atataaatcg
1501 ttacataata cttcattttg ttccttaatg atagcgagtt attgcgtaaa gaaaaaaata
1561 gatacaaata taacccatag caaacaacaa aacactaaac ataagctttc ttttaaaaat
1621 ggatattaag aaaaatgctc ctataccaca acagccgata ataaataaat ttaagctaga
1681 aaaatacaat gttaaaaaca taaatattgc agacagcact aaaggaatag cattatgaat
1741 atataataga ttaaaaggga tacaaaagta tatgataaaa ataaatagtt gccattgaaa
1801 caattcaaat attgctaaaa ataaattttt atcttcaata atcactgtaa aatagataat
1861 aagaaaagtc accaaagcaa gaactacact acacacaaac caagcaattt gtccacttga
1921 tatgttgtct attcctttac tttctagttt tgctttgagt ttagaataaa taggaatttg
1981 caaatttact cctaatatag ataaattgtt ttattataac caatctactt aatactttta
2041 attaatttaa attctttgtt tttaagatat tcttgtctta attcttcatc tattttatct
2101 aactcatcag gacttttacc tttggtaagt cctaaaaagg cgtgggtaaa atcttctatt
2161 tgccaataac actgattttt aaaatccaaa aaacattcta ccaatggatc aatataaatt
2221 gttacataat attttattta taaaatcctc caccaagata aaacaaagta taagttaata
2281 ccatataaac aaaataaaat aaaaaagtat ataataatct tttttgtaaa aaataaaata
2341 caacacaaag ggttatacat aaaatcaaaa taaaataaat attaatttca agaacaaaat
2401 aacaatagct tattatccaa tttagtgtta ttggaattaa atttttata ttaaaaaaat
2461 ttattataca aacaatccac ataaaaataa ataaaaaaaa tgatttgcaa aaataaaagc
2521 aagagatttg aaaaaacctt caaaagtaaa aaatttaagg ttttcaaaag atattgttct
2581 agtatttata attacaatta ataaaaataa taaataggta aaaattacac tacacacaaa
2641 ccaagcaatt tgtccgcttg ttatattgtc tattccttta ctttctagct ttgtttttaa
2701 tttagaataa atagggattt gcaaatttac tcctaataca gataaattgt tttattataa
2761 actaatttgc ttaaaatttt ttattaattt gaatttttgt ttttaagata ttctcgtctt
2821 aattcttcat ctattttatc taactcatca ggacttttat ctttggtaag tcctaaaaag
2881 gcgtgggtga agtcgatttg cttagaaatt tgagaaaatt ttgctttaaa tagtaaagaa
2941 aaatcaacat gctttaacat ttcattttct aataactcat ctatataaat tgttacataa
3001 tattttatta tttctttcat tttgaaatag tcccttctaa aagtccataa ggtatgattc
3061 ctataaatat cgcaaattca aaacaaaaat acatcattaa aaataaaaat ctaaatttta
3121 aatgtaattt aacaaaaaag agccatattg ttattaataa aatattcatt accacaagat
3181 ttaaataaaa atcattaaaa attatattaa agatatacat aaaagaatag caaaaaagat
3241 gaacaaaaaa gaagttttta taaaaactta agaaaataaa ccaaaatata gcaatataaa
3301 atagcaagat aggaaagaaa aaaacaatat aaagctcaaa ttccaaattc ttaagtttat
3361 atacaacaac aaacacggca catattaagg caatatagcc actccctaaa caccaagcaa
3421 tttgtccgct tgatatattg tctattcctt tgctttctag ttttgctttg agtttagaat
3481 aaataggaat ttgcaaattt gctcctaata tagataaatt gttttattat aaactaattt
3541 gcttaaaatt tttgtcttaa ttctttatct attttatcta actcatcaag acttttgcct
3601 ttggtaagtc ctaaaaaggc gtgggtaaaa tcttccgcat taataaaatt tttattttcg
3661 caatctaaaa aacattctac caatggatca atataaattg ttgcacaata cttcatttat
3721 aaaatcctcc taataactca aatatatata ccatagccac taaaaaaata tatccgcata
3781 aaaaagttat tagaattctc caagccctta aaaagataag aattataggt gcaaaaataa
3841 aaaataaaat tttaaaatta tagataaact catgatattt catatatata aatatcaata
3901 ttacaagtaa taataatgca aaaaaatttt tagtataaaa catatttaat ataaaaataa
3961 atatcataat aaataaaata gtgaattgat cataattgtt gctgatataa aaatacatca
4021 taggaatgat aaaaatacct acgctacaca caaaccaagc aatttgtccg cttgttatat
4081 tgtctattcc tttgctttct agttttgctt tgagtttaga ataaatagga atttgcaaat
4141 ttactcctaa tatagataaa ttgttttatt ataaccaacc tacttaatac ttttaattaa
4201 tttaaattct ttgtttttaa gatattcttg tcttaattct tcatctattt tatctaactc
4261 atcgggactt ttgcctttgg taagccctaa gaacacatga gggtttccct cactgcccat
4321 cataaagccc caatttaaca tagattccaa gagagtattt gtaattaaaa gtcattataa
4381 gtcctcaaaa taatcatagt tttcttcgaa atgattaaga tagcttggtt tgtctaataa
4441 attttctaac acgagaatat tcagataatc ataagtataa gatctatcta atttatcacc
4501 tatttttaga ttgcctatta tctctttgtt ttgcattatt tgagagttgt ttattgtaat

```
     4561 actctctatt gaattgtaat ttctttgtgt tatatttgga agaataaata aattaaattt
     4621 ccacttattc caaccaccat aatagcccaa ttcttttata taaacatata caagtctttc
     4681 tgctgaatat ataaattcaa tacctacaaa catatccata tctttgctct taatgccata
     4741 tcgatttgtt ttgatataaa aatcattttt attgagacaa ttttttcta tttctgttaa
     4801 aggtctagca taaagtactc tgcccaattc ttgttgtgaa aatctttcaa actctttata
     4861 ctcggaatct aagctttttg gtataaaagc acgataactt gtaggataaa aacaaccttt
     4921 tactgaaaca aatacaatca aacctagaat aaaagtcaat ataatatatt tcatacttta
     4981 ttgcctttta ctatgatttt ttagattagt taccccatct gcataaagcc ataaaggtgc
     5041 aagggttgaa ttctcatcat tctcattcat taaatcaagc attgtataac tagcatctgc
     5101 tatattagca tagtcaaaaa aatttagtga ttaagtctaa tttagcttct tgtgttttaa
     5161 tgtttttgct catttgttat tccaaagctt caacttgcca atcatctttc ttaaattttt
     5221 tgtcaatatt cttacaactt aatacttcca tatatctaaa atataatcca gcgccctcat
     5281 cacctttaa gaatatacca taattatcat aaaaatatgt attaattacg ataatacact
     5341 acttggtttt tcattttttc tttcaaatct agtttccact atcctatctt ttatttgctt
     5401 acttttaggg gttactatta tattcgtttt attctttata gattcatcct caatcaacac
     5461 tttttccaaa ttaaaataac acaagctccc aaactcataa tactgtggat ccatatattg
     5521 ataagaacag cctcctgcta agaataacaa aagatataca ggcaaaagaa acaaagtaag
     5581 tttttaaat atttttttta aaatttatag tttttgtttt atttttatt ttaaaaaaga
     5641 ttttaaagat aaaatataaa ataatagaaa atataaaaac gaaaaaagaa aaatataaaa
     5701 gattaataaa gaattctagt gtctattaat tcctcttaat tctctctatt ttttcataag
     5761 acaaagtagc actaaagcca cttgtagtgt tggactgatg aaatttaatc tcatatcttt
     5821 gagaaaaatt tctagcttgt aattctctaa aatctccatt tagtttcgca taaaaaaata
     5881 aagaatcatc tataaccaaa tatccattat attcatcatg taatgcttta aaatagtcca
     5941 atttttctaa ttcactctta tctttaaaaa atctagtttt agatatttta aatcctaaag
     6001 ctttttgtcc ttcagtataa acaaaattaa aataactagc ttgcataaat tctgtataat
     6061 cttttaaatt gttgatttgt tctttattgg tcattattta ttccttatat tattattgat
     6121 aaagattgcc acaagcgagc tcatcctcta taaactcaaa attcatatta ccctcattgc
     6181 cttctaacac ataccttcta gtattccaat atggacttat atacatatca acaatcttgt
     6241 gtaaagacat ttgcatattt tttgttttta gtgcaatatt atccatctct atcttttctt
     6301 gaaaatttga aggagttttg taagtatcat cgtaatatat ataaagccac atttctatac
     6361 taccatattt ttttacatgt cgaaaatcat ctatgtcacc taacttttta tcaaaatacc
     6421 ctagaatctt attata
//
LOCUS       NLMJPNJI_5              2833 bp    DNA     linear       12-APR-2021
DEFINITION  Campylobacter species strain strain.
ACCESSION
VERSION
KEYWORDS    .
SOURCE      Campylobacter species
  ORGANISM  Campylobacter species
            Unclassified.
COMMENT     Annotated using prokka 1.14.6 from
            https://github.com/tseemann/prokka.
FEATURES             Location/Qualifiers
     source          1..2833
                     /organism="Campylobacter species"
                     /mol_type="genomic DNA"
                     /strain="strain"
     gene            9..560
                     /locus_tag="NLMJPNJI_00062"
     CDS             9..560
                     /locus_tag="NLMJPNJI_00062"
                     /inference="ab initio prediction:Prodigal:002006"
                     /codon_start=1
```

```
                     /transl_table=11
                     /product="hypothetical protein"
                     /protein_id="Prokka:NLMJPNJI_00062"
                     /translation="MTFILGLIVFVSVKGCFYPTSYRAFIPKSLDSEYKEFERLCKEE
                     VGKVVYARPIKHGIKLEDLFNLPSTSVRRVALSRHMYMLIENVTTREDEIFYLHIAGF
                     EYYTGWTRKSFSMHDDSKKYIRCEPLLYGKTWKENAKIMEQKAFDNKEYIGTEEYLRY
                     SPYHYEYNTLYRQIVNFKQKKEN"
     gene            560..712
                     /locus_tag="NLMJPNJI_00063"
     CDS             560..712
                     /locus_tag="NLMJPNJI_00063"
                     /inference="ab initio prediction:Prodigal:002006"
                     /codon_start=1
                     /transl_table=11
                     /product="hypothetical protein"
                     /protein_id="Prokka:NLMJPNJI_00063"
                     /translation="MIYNYKYSLGIYVNDWGFMMGSEGNPHVFLGLKREQVHCGDMQI
                     KNTIYK"
     gene            765..1262
                     /locus_tag="NLMJPNJI_00064"
     CDS             765..1262
                     /locus_tag="NLMJPNJI_00064"
                     /inference="ab initio prediction:Prodigal:002006"
                     /inference="similar to AA sequence:RefSeq:CKG86719.1"
                     /codon_start=1
                     /transl_table=11
                     /product="hypothetical protein"
                     /protein_id="Prokka:NLMJPNJI_00064"
                     /translation="MGDSITLSIFVGSLLSIAVITKFYLIALSVLIVFSILNYWIKIN
                     IKEFVKSYFLISFIIFFTAIVIVFYLTYDLETSRGLVLLGPPEVEKLSPQEYKAHNYF
                     KFLTFTTMFFLAITFFILPCLLQAYLFKCYSTEEKRKKLSWFIGFACNIFLLIVSFYK
                     LYENL"
     gene            1249..1380
                     /locus_tag="NLMJPNJI_00065"
     CDS             1249..1380
                     /locus_tag="NLMJPNJI_00065"
                     /inference="ab initio prediction:Prodigal:002006"
                     /inference="similar to AA sequence:RefSeq:CKG86746.1"
                     /codon_start=1
                     /transl_table=11
                     /product="hypothetical protein"
                     /protein_id="Prokka:NLMJPNJI_00065"
                     /translation="MKIFKDYQKFTPYGLDFKLYSKDFIAKLDFKDKAYNVLYDTGV"
     gene            1381..1875
                     /locus_tag="NLMJPNJI_00066"
     CDS             1381..1875
                     /locus_tag="NLMJPNJI_00066"
                     /inference="ab initio prediction:Prodigal:002006"
                     /inference="similar to AA sequence:RefSeq:CKG86719.1"
                     /codon_start=1
                     /transl_table=11
                     /product="hypothetical protein"
                     /protein_id="Prokka:NLMJPNJI_00066"
                     /translation="MGDSIALSIFWGLIGFILTCSVIEFYLIALSVLIVFSILNYWIK
```

```
                     INIKEFVKSYFLISFIIFFTAIVIIFYLTYDLETSRGLVLLGPPEVEKLSPQEYKAHN
                     YFGFLSGITIIFLVITFIILPCLLQAYLFKCYSTGEKRKKLSWFIGFVCNIFLLTASV
                     YAMY"
ORIGIN
        1 atattatatt gacttttatt ctaggtttga ttgtatttgt ttcagtaaaa ggttgttttt
       61 atcctacaag ttatcgtgct tttataccaa aaagcttaga ttccgagtat aaagagtttg
      121 aaagattatg taaagaggaa gtaggaaagg tagtatatgc aagacctata aaacatggta
      181 taaagttgga agatttgttt aacttgcctt ctacgagtgt tagacgtgtt gctttaagca
      241 gacatatgta tatgttgata gaaaatgtta caacaagaga agacgaaatt ttttacttac
      301 atatagctgg ttttgaatat tatacaggct ggacaagaaa aagtttttct atgcatgatg
      361 attctaaaaa atatataaga tgtgagcctt tgctttatgg taaaacttgg aaagaaaatg
      421 ctaagataat ggaacaaaag gcatttgata ataaagaata tataggcaca gaagaatatt
      481 tacgatattc accttatcat tatgagtata atacacttta tcgccaaata gttaatttca
      541 aacaaaagaa agagaattaa tgatatataa ttacaaatac tctcttggaa tctatgttaa
      601 tgattggggc tttatgatgg gcagtgaggg aaaccctcat gtgttcttag gactaaaaag
      661 agagcaagtg cattgtggcg atatgcaaat aaaaaacaca atatataaat aaacttgatt
      721 ttaaagataa agcttataat gtgctttata atataggagt ttaaatggga gatagtatta
      781 cgctttctat ttttgtaggt tcattgctat ccattgctgt tatcacaaaa ttttatctta
      841 tagctttatc tgtgcttatt gtcttttcta ttttaaatta ttggattaag attaatataa
      901 aagaatttgt aaaatcttat tttttaatat cttttattat cttttttaca gctattgtta
      961 tcgtatttta tcttacttat gatcttgaaa cttctagggg gcttgtatta cttggtcctc
     1021 cagaagttga aaaattatca ccacaagaat ataaagcaca taattatttt aaatttttaa
     1081 cctttacaac tatgtttttt ttggcaatca cttttttat actcccttgt ttattacaag
     1141 cttatttatt taaatgttat agcacagaag aaaaacgcaa aaaattaagt tggtttatag
     1201 ggtttgcgtg taatatcttt ttattaattg tgagttttta taagttatat gaaaatcttt
     1261 aaagactatc aaaaattcac tccttatggt ttggatttta aactttatag taaagatttt
     1321 attgccaaac ttgattttaa agataaagct tataatgtgc tttatgatac aggagtttaa
     1381 atgggagata gtattgcact ttctattttt tggggtttga taggatttat tcttacttgt
     1441 agtgttatag aattttatct tatagctcta agcgttctta ttgtcttttc tattttaaat
     1501 tattggatca agattaatat aaaagaattt gtaaaatctt atttttaat atcttttatt
     1561 atcttttta cagctattgt tatcatattt tatcttactt atgatcttga aacttctaga
     1621 gggcttgtat tacttggtcc tccagaagtt gaaaaattat caccacaaga atataaagca
     1681 cataattatt ttggattttt aagtggtata actattattt ttttagtaat cacatttatt
     1741 atactccctt gtttattaca agcttattta tttaaatgct atagcacagg agaaaaacgc
     1801 aaaaaattaa gttggtttat aggatttgtg tgcaatatct ttttattaac ggcgagtgtg
     1861 tatgcgatgt attaaaatct ttaaagactt ttaaaaaatt taatatattt gatataatat
     1921 ttatttatct taaatattta gagaaaaaat gaatagagaa ttatttataa caagacttgc
     1981 atttataaac aatgatgaaa taaataaaat aaataaaaat tcatctattg catatttaaa
     2041 agccttatat caatgtgaaa attccatagc tgtaaattat gatgaagaag agcttttcac
     2101 ccaaaccact gctataaaca atctaggata tggcaatgat ttcacaaaaa tcttcatcct
     2161 agataaagaa aacctcaatg acacctatct tgatgctaga aaaaagcttt ataaatctat
     2221 caatacttta aaagaagaaa aaaatgaaaa aataaaaaat atccaagctt atgtaaataa
     2281 agaaaattcc ttgcaagaca atctaaataa tcaaagcttt aatactgagg aaaatgatct
     2341 taatttagct ttaaacgaag tatttaatat agaacaattt tatgataaaa ataccaatac
     2401 catagtatta aaaacaaata atataaaaat catattctta gataaagtaa atttacaaga
     2461 tttacaatct aatactagta ataactcttt tatctctatg tcaaatttaa tccatattta
     2521 tgagaatcat tgtgatattt ttacaaaaga tagaagtatc ttagatataa aatttataga
     2581 agaaaaatat caaatagatt ttaaaagttt agatacaaaa atcttcctaa actccaccct
     2641 ccttacaggc tccaccgaac tttctaataa tccttttac tttggagaat tagatcaaga
     2701 taatcttata aaacaagata tacccagtta ttatttttct cctaaagatg aaagcagtgg
     2761 tttaggaaga cttagtatct tttataaaaa tgatgaactt tgcttgctta attattctat
     2821 attagagaat tct
//
LOCUS       NLMJPNJI_6             1956 bp    DNA     linear       12-APR-2021
```

```
DEFINITION  Campylobacter species strain strain.
ACCESSION
VERSION
KEYWORDS    .
SOURCE      Campylobacter species
  ORGANISM  Campylobacter species
            Unclassified.
COMMENT     Annotated using prokka 1.14.6 from
            https://github.com/tseemann/prokka.
FEATURES             Location/Qualifiers
     source          1..1956
                     /organism="Campylobacter species"
                     /mol_type="genomic DNA"
                     /strain="strain"
     gene            113..334
                     /locus_tag="NLMJPNJI_00067"
     CDS             113..334
                     /locus_tag="NLMJPNJI_00067"
                     /inference="ab initio prediction:Prodigal:002006"
                     /inference="similar to AA sequence:RefSeq:CKG87598.1"
                     /codon_start=1
                     /transl_table=11
                     /product="methyltransferase small"
                     /protein_id="Prokka:NLMJPNJI_00067"
                     /translation="MSDEWITEESRNRLGIVDKKDREKIANLLKKNSNEVRKDLFHVD
                     KKGNVSIKELDKNAKTKNLIERIENGKGY"
     gene            318..965
                     /locus_tag="NLMJPNJI_00068"
     CDS             318..965
                     /locus_tag="NLMJPNJI_00068"
                     /inference="ab initio prediction:Prodigal:002006"
                     /inference="similar to AA sequence:RefSeq:CKG87616.1"
                     /note="Domain of uncharacterised function (DUF1910)"
                     /codon_start=1
                     /transl_table=11
                     /product="hypothetical protein"
                     /protein_id="Prokka:NLMJPNJI_00068"
                     /translation="MVRDTSKDEAYFTKHIIECEEDIKESEKILLELPFGKRETCLFC
                     ITDRKKCIAMDKYSRGDDINLVKKDLEALMLLRQKNRLELGGFDTSDYRGNAIELCVR
                     VLLDMDSACVLELIEEDERKKKDIFNRDWFLHFMGSKGKNLNLERKCNCKEHELIKEF
                     VATKDIEFLHQYMKKHTRLRDPLDTWDLEGAAIVKLMNLDKEEFKSYKYFPYDLI"
     gene            1162..1815
                     /locus_tag="NLMJPNJI_00069"
     CDS             1162..1815
                     /locus_tag="NLMJPNJI_00069"
                     /inference="ab initio prediction:Prodigal:002006"
                     /inference="similar to AA sequence:RefSeq:CKG87616.1"
                     /note="Domain of uncharacterised function (DUF1910)"
                     /codon_start=1
                     /transl_table=11
                     /product="hypothetical protein"
                     /protein_id="Prokka:NLMJPNJI_00069"
                     /translation="MLRDTKRDEAYFTKHIIECEEKIKEDEKRLLELPLGDARRENYF
                     FSITDKKKCIAMDKYSRGDDINLVKKDLEALMLLRQKNRLELGGFDTSDYRGNAIELC
```

```
                     VRVLLDMDSACVLELIEEDERKKKDIFNRDWFLHFMGSKGKNLNLERKCNCKEHELIK
                     EFVATKDIEFLHQYMKKHTRLRDPLDTWDLEGAAIVKLMNLDKEEFKQYKYFPYDLI"
ORIGIN
        1 taaaattcat catggaattg atggaattta tcacaatcca aataaaaacc caaaatatat
       61 catcgcagaa gctaagtttg gaacaagcaa attagataga aaaactaagc aaatgagtga
      121 tgaatggata acagaagaga gtagaaatag acttggcatt gtagataaaa aagatagaga
      181 aaaaatagca aatcttctta aaaaaaatag caatgaagtc agaaaagatt tatttcatgt
      241 tgataaaaaa ggcaatgtga gtattaaaga attagataaa aatgcaaaaa ccaaaaactt
      301 aatagaaagg atagaaaatg gtaagggata ctagtaaaga tgaagcgtat tttacgaaac
      361 atataataga gtgtgaagaa gatatcaaag aatccgaaaa aatactttta gagcttcctt
      421 ttggaaaaag agaaacttgt ctttttgta taacagatag aaaaaaatgt attgctatgg
      481 ataaatactc tcgtggtgat gatataaatc tagttaaaaa agatttagaa gcacttatgc
      541 tattaagaca aaaaaatcgt ttagaattag gtggatttga tactagcgat tatagaggaa
      601 atgctattga actttgtgtt agagtattat tagatatgga tagtgcttgt gtattagaat
      661 taatagaaga agatgagaga aagaaaaaag atattttaa tagagattgg tttttacatt
      721 ttatgggttc taagggtaaa aatttaaatt tagaacgcaa atgtaattgt aaagaacacg
      781 agcttattaa agaatttgta gctactaagg atattgagtt tttacatcaa tatatgaaaa
      841 aacatacaag attaagagat cctctagata cttgggatct tgaaggtgca gcaattgtaa
      901 agctaatgaa tttagataaa gaagaattta aaagctataa atattttcct tatgatttaa
      961 tttaatctta tttggttaac agaagagagt agaaatagac ttggcattgt agataaaaaa
     1021 catagagaaa aaatagcaaa tcttcttgaa aaaaatagca atgaagtcag aaaagattta
     1081 tttcatgttg atgaaggagg taaagaaact attgattatt tagataaaaa tgcaaataaa
     1141 ataaaaccca aaggataaaa aatgctaaga gatactaaga gagatgaagc gtattttacg
     1201 aaacatataa tagagtgtga agaaaaaatc aaagaagatg aaaaaagact cttagaactt
     1261 ccactaggag atgcaagaag ggaaaattat ttttttcta taacagataa aaaaaaatgt
     1321 attgctatgg ataaatactc tcgtggtgat gatataaatc tagttaaaaa agatttagaa
     1381 gcacttatgc tattaagaca aaaaaatcgt ttagaattag gtggatttga tactagcgat
     1441 tatagaggaa atgctattga actttgtgtt agagtattat tagatatgga tagtgcttgt
     1501 gtattagaat taatagaaga agatgagaga aagaaaaaag atattttaa tagagattgg
     1561 tttttacatt ttatgggttc taagggtaaa aatttaaatt tagaacgcaa atgtaattgt
     1621 aaagaacacg agcttattaa agaatttgta gctactaagg atattgagtt tttacatcaa
     1681 tatatgaaaa aacatacaag attaagagat cctctagata cttgggatct tgaaggtgca
     1741 gcaattgtaa aactaatgaa tttagataaa gaagaattta aacaatacaa atactttcct
     1801 tatgatttaa tttaatctta gtgggtttaa aaacccacaa ggcttttcat ggctaaaaaa
     1861 agaacaatcc ttattcctag aataaaagat attaatgtaa gtcctgatac ttcttgtatg
     1921 catcctatat tagaagataa tactttagta tgctta
//
LOCUS       NLMJPNJI_7              1591 bp    DNA     linear       12-APR-2021
DEFINITION  Campylobacter species strain strain.
ACCESSION
VERSION
KEYWORDS    .
SOURCE      Campylobacter species
  ORGANISM  Campylobacter species
            Unclassified.
COMMENT     Annotated using prokka 1.14.6 from
            https://github.com/tseemann/prokka.
FEATURES             Location/Qualifiers
     source          1..1591
                     /organism="Campylobacter species"
                     /mol_type="genomic DNA"
                     /strain="strain"
     gene            complement(267..824)
                     /locus_tag="NLMJPNJI_00070"
```

```
     CDS             complement(267..824)
                     /locus_tag="NLMJPNJI_00070"
                     /inference="ab initio prediction:Prodigal:002006"
                     /inference="similar to AA sequence:RefSeq:CKG87579.1"
                     /codon_start=1
                     /transl_table=11
                     /product="hypothetical protein"
                     /protein_id="Prokka:NLMJPNJI_00070"
                     /translation="MTREQAELIIKEEKLIDTDWYPSYKHSGEFHLTMWFDSDNNKYE
                     AVYIGERGSVELEYSFDSEKEAIDKMLQMNYEQKKYDGKDIKEYLTKEVALRIIKEEN
                     LEVIWYDEALKPMRAGIKHDKQRDKYISFITNAKAEIIEWKSIEFDGDRYSETFKEML
                     NDENIALYALINRARMIKQNRISFT"
     gene            complement(838..1413)
                     /locus_tag="NLMJPNJI_00071"
     CDS             complement(838..1413)
                     /locus_tag="NLMJPNJI_00071"
                     /inference="ab initio prediction:Prodigal:002006"
                     /inference="similar to AA sequence:RefSeq:AOH52020.1"
                     /note="putative protein (DUF4237 domain)"
                     /codon_start=1
                     /transl_table=11
                     /product="hypothetical protein"
                     /protein_id="Prokka:NLMJPNJI_00071"
                     /translation="MLLYLENENKKGKVSDKEVHLYKHNGIWPKNTPKPRSPDYIRKN
                     GRIKYPDDDGYKIPPKPREITLEKGTKLDRYGDNSGSFVCPFEEKKGAISYEKRSLPY
                     EDNEAMQKTYKRYEVIEDINMENIERKIEMSGNRELKGKIDKLKEKNKFHSPKEGKIS
                     PYFDQEGGGTQIKLPISIENLMQLGFIKQIP"
ORIGIN
        1 ttgcatttca gcataattac ctttttgctg attatttttt aatgcttgta tttgctttgc
       61 tctatcattt ttattttttct tacttctttt atctcctatc ccttcccatc ttatcctatc
      121 atactcagtc ttagcaagag gactagcttg tattttagct aaagcaaatt gaatcttatt
      181 ctttttaggt atagcaaatt ctagccatga aaagccttgt gggtttttaa acccactaag
      241 attaaattaa attataagga aagtatttac gtaaagctaa tcctattttg cttaatcatt
      301 ctggcacgat tgataagagc ataaagggct atattttcat cgttaagcat ttccttaaaa
      361 gtttcactgt atctatcccc atcaaattca atacttttcc attctataat ctctgcttta
      421 gcattagtga taaaagaaat atatttatct ctttgtttat cgtgtttgat acctgctcgc
      481 attggtttaa gtgcttcatc ataccaaatc acttctagat tttcttcttt gatgattctt
      541 agtgctactt cttttgttag atattcttta atgtcttttc catcgtattt tttttgttca
      601 taattcatct gcaacatttt atcaatggct tctttttcac tatcaaaaga atactcaagt
      661 tcaacactgc ccctttctcc aatataaaca gcttcgtatt tattattatc agaatcaaac
      721 cacatagtaa gatgaaattc tcctgaatgt ttataagaag gataccaatc tgtatcaatc
      781 aacttttctt cttttatgat taattctgct tgttctcttg tcattttttta tcctttatta
      841 tggtatttgt ttgataaagc ctaattgcat taagttttct atggaaatag gaagtttaat
      901 ctgtgttcct ccaccttctt gatcaaagta tggagatatt ttaccttctt ttggggaatg
      961 gaatttgttt ttttctttta atttgtcaat tttacctttt agttctctat ttccactcat
     1021 ttcaatcttt ctctcaatat tttccatatt aatatcttct attacttcat atcttttata
     1081 ggttttttgc atggcttcat tatcttcata aggcaatgat ctttttttcat aagatattgc
     1141 tccttttttc tcttcaaaag gacaaacaaa gctacctgaa ttatctccat atctgtctag
     1201 ctttgttcct ttttctagag tgatttctct tggtttaggt gggattttat agccatcatc
     1261 atcaggatat ttaatcctac catttttacg aatataatca ggacttctag gttttggagt
     1321 gtttttaggc catattccat tatgtttata aagatgaact tctttatcac ttaccttacc
     1381 ttttttattt tcattttcca aatataaaag catttttgct ttctctttac ttttttctct
     1441 tctccttctt tgtgctgctc ctaatttacc tcctatccct tcccatctta tcctatcata
     1501 ctcaatctta gcaagaggac tagcttgtat tttagctaaa gcaaatttaa tcttattctt
```

```
     1561 tttaggtata gcaaatatag gatagccttt t
//
LOCUS       NLMJPNJI_8              1343 bp    DNA     linear       12-APR-2021
DEFINITION  Campylobacter species strain strain.
ACCESSION
VERSION
KEYWORDS    .
SOURCE      Campylobacter species
  ORGANISM  Campylobacter species
            Unclassified.
COMMENT     Annotated using prokka 1.14.6 from
            https://github.com/tseemann/prokka.
FEATURES             Location/Qualifiers
     source          1..1343
                     /organism="Campylobacter species"
                     /mol_type="genomic DNA"
                     /strain="strain"
     gene            14..664
                     /locus_tag="NLMJPNJI_00072"
     CDS             14..664
                     /locus_tag="NLMJPNJI_00072"
                     /inference="ab initio prediction:Prodigal:002006"
                     /codon_start=1
                     /transl_table=11
                     /product="hypothetical protein"
                     /protein_id="Prokka:NLMJPNJI_00072"
                     /translation="MGLIGVSIKGYPIFAIPKKNKIQFALAKIQASPLAKIEHDRIRW
                     EGIGGKLKAAQRRRREKSKEKAKMLLYLENENKNDSKIKQISISNIPKKPHWRESEED
                     ISKLYYDYEKQKSFLNGNEVPYGTKHSVRPDLYKNGSSIEIKNYNLDKTSSANNLINI
                     ITKQYQQRLQHLPPKTEQIFIIDSRGQNISKEIQEKIKQKIRIKLNCDILIQFKTK"
     gene            679..1092
                     /locus_tag="NLMJPNJI_00073"
     CDS             679..1092
                     /locus_tag="NLMJPNJI_00073"
                     /inference="ab initio prediction:Prodigal:002006"
                     /inference="similar to AA sequence:RefSeq:AOH52023.1"
                     /codon_start=1
                     /transl_table=11
                     /product="immunity protein 70"
                     /protein_id="Prokka:NLMJPNJI_00073"
                     /translation="MVGFSIDNLWYEIGSGDFLHAFFSNVAYHLEKGNWGSRFPILMN
                     EVYQGKLNFSKASLALNELENIQKEFKNFTPKQIIWDIENLSLTPPWGDNISSDITDL
                     SNYFVTSDGNDFIELFKKVLQLADNEKANLTIKSF"
ORIGIN
        1 ttctgtttta caaatgggtt tgataggagt gagtataaaa ggctatccta tatttgctat
       61 acctaaaaag aataagattc aatttgcttt agctaaaata caagctagtc ctcttgctaa
      121 gattgagcat gataggataa gatgggaagg gataggaggt aaattaaaag ctgcacaaag
      181 aaggagaaga gaaaaaagta aagagaaagc aaaaatgctt ttatatttgg aaaatgaaaa
      241 taaaaacgat agcaaaataa aacaaatatc tatatcaaac atacctaaaa aacctcattg
      301 gcgagaatct gaagaagata tatcaaagtt atactatgat tatgaaaaac agaaatcttt
      361 tcttaatggc aatgaagttc catacggaac aaaacattct gttcgtccag acttatacaa
      421 aaatggttct agcattgaaa ttaaaaacta taatcttgat aaaacatcca gtgcaaacaa
      481 tttaattaat attattacca aacaatatca acaaagattg caacatttgc caccaaaaac
      541 tgaacaaatc ttcattatag actctagagg acaaaatata tctaaagaaa tacaagaaaa
```

```
      601 aataaaacaa aaaattcgca ttaaattaaa ttgtgatata cttattcaat ttaaaactaa
      661 gtaaatttaa gaagaataat ggtgggtttt agtatagata atctttggta tgaaataggc
      721 agtggtgatt ttttacatgc attttttagc aatgtggctt atcatcttga aaaaggaaac
      781 tggggaagta gattccctat attaatgaat gaagtatatc aaggtaaatt aaatttttca
      841 aaagcttctt tagcgttaaa tgaactagag aatatacaaa aagaatttaa aaattttacc
      901 ccaaaacaaa taatttggga tattgaaaac ttatctctta caccaccttg gggtgataat
      961 atcagtagtg atattacaga cctatcaaat tattttgtaa caagcgatgg taatgatttt
     1021 atagaactct ttaaaaaggt cttacaacta gctgacaatg aaaaagctaa ccttaccatt
     1081 aaaagttttt aaaataatta tactttggga aaaatttcta agtaaaaaca ccctaaaacc
     1141 ctttccttta aattttgatt gctctttaga agaaaagctt ctttatacca aagataaaca
     1201 actagaaaac atacttaaaa cccacaaggc ttttcatggc taaaaaaaga acaatcctta
     1261 ttcctagaat aaaagatatt aatgtaagtc ctgatacttc ttgtatgcat cctatattag
     1321 aagataatac tttagtatgc tta
//
LOCUS       NLMJPNJI_9               519 bp    DNA     linear       12-APR-2021
DEFINITION  Campylobacter species strain strain.
ACCESSION
VERSION
KEYWORDS    .
SOURCE      Campylobacter species
  ORGANISM  Campylobacter species
            Unclassified.
COMMENT     Annotated using prokka 1.14.6 from
            https://github.com/tseemann/prokka.
FEATURES             Location/Qualifiers
     source          1..519
                     /organism="Campylobacter species"
                     /mol_type="genomic DNA"
                     /strain="strain"
     gene            113..277
                     /locus_tag="NLMJPNJI_00074"
     CDS             113..277
                     /locus_tag="NLMJPNJI_00074"
                     /inference="ab initio prediction:Prodigal:002006"
                     /codon_start=1
                     /transl_table=11
                     /product="hypothetical protein"
                     /protein_id="Prokka:NLMJPNJI_00074"
                     /translation="MSDEWLIKNKLKRLKNDVGEVEAEKIANLLKKIAMKSEKIYFML
                     IKKAM*VLKN"
ORIGIN
        1 taaaattcat catggaattg atggaattta tcacaatcca aataaaaacc caaaatatat
       61 catcgcagaa gctaagtttg gaacaagcaa attagataga aaaactaagc aaatgagtga
      121 tgaatggtta ataaagaata aactaaaacg gcttaaaaat gatgtaggag aagtagaagc
      181 tgaaaaaata gcaaatcttc ttaaaaaaat agcaatgaag tcagaaaaga tttatttcat
      241 gttgataaaa aaggcaatgt gagtattaaa gaattagata aaaatgcaaa aaccaaaaac
      301 ttaatagaaa ggataaaaaa tgctaagaga tacgaagaga gatgaagcgt attttacgaa
      361 acatataata gagtgtgaag aaaaaattaa aaaatccgaa aaaatacttt tagagcttcc
      421 ttttggaaaa agagaaactt gtcttttttg tataacagat agaaaaaaat gtattgctat
      481 ggataaatac tctcgtggtg atgatataaa tctagttaa
//

>>BfR-CA-15875_min_cov_30 putative plasmid location
```

```
LOCUS       JIMAIPOP_1              7184 bp    DNA     linear       12-APR-2021
DEFINITION  Campylobacter species strain strain.
ACCESSION
VERSION
KEYWORDS    .
SOURCE      Campylobacter species
  ORGANISM  Campylobacter species
            Unclassified.
COMMENT     Annotated using prokka 1.14.6 from
            https://github.com/tseemann/prokka.
FEATURES             Location/Qualifiers
     source          1..7184
                     /organism="Campylobacter species"
                     /mol_type="genomic DNA"
                     /strain="strain"
     gene            348..851
                     /locus_tag="JIMAIPOP_00001"
     CDS             348..851
                     /locus_tag="JIMAIPOP_00001"
                     /inference="ab initio prediction:Prodigal:002006"
                     /codon_start=1
                     /transl_table=11
                     /product="hypothetical protein"
                     /protein_id="Prokka:JIMAIPOP_00001"
                     /translation="MKILEKDFDLICKIYIQWKELNNNIKKFSSRGVNFPDCISEPIV
                     CYAFNYNWNNNNETGDATSDSGELIEIKATSNFNSDLSSFSPNTKFDKLIFVRLDQER
                     DLAYIYDLELNFEQFQILEVNKKETVLDQQKQNRRPRLSLIQYIEKEKIEPLYYVDFI
                     KKIVTKN"
     gene            921..1844
                     /locus_tag="JIMAIPOP_00002"
     CDS             921..1844
                     /locus_tag="JIMAIPOP_00002"
                     /inference="ab initio prediction:Prodigal:002006"
                     /inference="similar to AA sequence:RefSeq:ASE91810.1"
                     /codon_start=1
                     /transl_table=11
                     /product="DNA (cytosine-5-)-methyltransferase"
                     /protein_id="Prokka:JIMAIPOP_00002"
                     /translation="MKVASFFAGVGGIDLAFSNHDIVYVNEIDKNATKTYKFNFDVEI
                     DCRDIQTVNEKELPDFDILLGGFPCQAFSVAGYRKGFEDSRGNLFFDIIRIAKEKKPK
                     IIFLENVKNLVSHDCGKTFNIILKSLESLGYYVKYDVLNAMHYGNTPQNRERIYIVCF
                     KNKKQYKDFNFPNPIFLNNKIDNIVDFNLKVDDKFYYTEKNFKHYEMLLQNITKKNVI
                     YQWRRKYVRENKSNVCPTLTANMGTGGHNVPLVLTKHGIRKLTPKECFMFQGFPNSYK
                     LPDIANSHLYKQAGNSVCVKVIQRIADRISN"
     gene            complement(2128..2328)
                     /locus_tag="JIMAIPOP_00003"
     CDS             complement(2128..2328)
                     /locus_tag="JIMAIPOP_00003"
                     /inference="ab initio prediction:Prodigal:002006"
                     /codon_start=1
                     /transl_table=11
                     /product="hypothetical protein"
                     /protein_id="Prokka:JIMAIPOP_00003"
                     /translation="MGFKNINTEKASINFINNARGDKINTQKRDKKILVSFTQDEYDR
```

```
                     VKESALQIGMGVNQYIRYKLFI"
gene                 complement(2329..3009)
                     /locus_tag="JIMAIPOP_00004"
CDS                  complement(2329..3009)
                     /locus_tag="JIMAIPOP_00004"
                     /inference="ab initio prediction:Prodigal:002006"
                     /inference="similar to AA sequence:RefSeq:ASI88294.1"
                     /codon_start=1
                     /transl_table=11
                     /product="Plasmid partitioning protein ParA"
                     /protein_id="Prokka:JIMAIPOP_00004"
                     /translation="MIISICNEKGGSGKSTLAVNLSSRLTEDGDSILLFDSDPQRSTE
                     VFINIRNNSNLKQNFKHSFDINLLNSGFKNELNNYDAIIIDTGGRDSREMRKAMLLSN
                     VIIIPTIASQYDVSVLDHMLNIYKEVKLINKNIILLILINRVSPNPFLIKELNNLIDY
                     IHEKKEVNNLNDVFLLESVLYERQAYKRAVIEGKSISEFCDKNDKALKDFEKFYQEIF
                     QILKNKDK"
gene                 complement(3542..4945)
                     /locus_tag="JIMAIPOP_00005"
CDS                  complement(3542..4945)
                     /locus_tag="JIMAIPOP_00005"
                     /inference="ab initio prediction:Prodigal:002006"
                     /codon_start=1
                     /transl_table=11
                     /product="hypothetical protein"
                     /protein_id="Prokka:JIMAIPOP_00005"
                     /translation="MSYDEIEKVKRSAYTKTASESTKRNYISSGNFKKSTSFQVFHNA
                     NVRPDYAIGGELVFDLGGYEALELKNKIVSEAKEAYEKNKSPKAPKFKAKSYEWSLVV
                     NIKPDTSMNDLKNLSKHFQEKYGFQCYQIAIHRDEGHFNEQGEKLINHHAHMEFITLD
                     KETGKNNFRREKISPKVLREIQTEIAEILGMERGEDKRISGAKRIEPRAYAALKEKEK
                     REKKRNINLEQEKGQFLNKSEYKKIIENFRKSQINQGLDKDFFKELSELKKNFQEAKE
                     DDLSELLNDILREHLERIEKLKKENSILKAENELLKQKEQKNDKSIDQEQKNASERNF
                     EPKNDKISKLEDDFNFVKNINPNDMSEENYFKLKKLYTENYDNLAEDKSFPNYDYLVN
                     FFKKAPQNINDLIVQKASIMKDVELQENIQFYLKKTLEKHSKDHTQNLVKTTDKTIKT
                     TKNYHQNYLKKGLGFER"
gene                 complement(4949..5113)
                     /locus_tag="JIMAIPOP_00006"
CDS                  complement(4949..5113)
                     /locus_tag="JIMAIPOP_00006"
                     /inference="ab initio prediction:Prodigal:002006"
                     /codon_start=1
                     /transl_table=11
                     /product="hypothetical protein"
                     /protein_id="Prokka:JIMAIPOP_00006"
                     /translation="MDYEKYSKMNLRQLINAYTNAEKKEKKLIDDFQKKLDENKNLME
                     FLKTKIKESL"
gene                 complement(5515..5850)
                     /locus_tag="JIMAIPOP_00007"
CDS                  complement(5515..5850)
                     /locus_tag="JIMAIPOP_00007"
                     /inference="ab initio prediction:Prodigal:002006"
                     /codon_start=1
                     /transl_table=11
                     /product="hypothetical protein"
                     /protein_id="Prokka:JIMAIPOP_00007"
```

```
                     /translation="MSEKTKLQIAEHILKNCYWGNTTMTPSFIVEHIQDKDFAKRIFS
                     AIFQNSLTMFEDLKIIDNEEWIKEFIISQNQRLGYHKRFYYEERLDELIAHYGIKNAG
                     KRREVHFIA"
     gene            complement(5847..6884)
                     /locus_tag="JIMAIPOP_00008"
     CDS             complement(5847..6884)
                     /locus_tag="JIMAIPOP_00008"
                     /inference="ab initio prediction:Prodigal:002006"
                     /inference="similar to AA sequence:RefSeq:ALW35761.1"
                     /codon_start=1
                     /transl_table=11
                     /product="replication initiation protein"
                     /protein_id="Prokka:JIMAIPOP_00008"
                     /translation="MSDIVKYHNDFNKIQLPNFSEQEQNLLFLIFAKIKEKGIDNVIN
                     IYANDFKINQKLSNSREYLTENINSLKYKFFKANFKQIIETKTEVIHKYVNLFETMEI
                     HYVKRNPDDGYDGSTLFNRITLKINPDFAYLVNQLTTNFTAFELEEFISLSGRYAKTL
                     YRLLKQFRTTGKARFEWEEFCRIMDIPQDYRQSEIDKRIIKPAIKELSKERNLFDQMR
                     VPFKNLAYEKEKAKGRGQGGKVSGITFTFKPENIEMQKLENESQKIMSDEQKYLKILN
                     NMKLNQVRFNYNDKLWQFNDFDFNEFKIIAVELIRDEYENLNFGNHMYFNAKNQEQFF
                     KMIEIFRKGIR"
ORIGIN
        1 tcaaggcgga tttttattgt agcatgttaa gggtatattg tcaatatttg tccccttaat
       61 acctacaatt tgtcccctta atacctacaa tttgtcccct taatacctac aatttgtccc
      121 cttaatacct acaatttgtc cccttaatat tccatatagc tcccatcata tagtgcttag
      181 aaagatgcct aaaaataata aaaataataa aaaaatagaa aaatattacc atagtttcta
      241 aatctactaa atatatttat gatttattgt attcttacaa tgaaatagta gtaaatcgag
      301 atttaaaaat attttgtata taatattaaa gtttagacgc aagttgtatg aaaatacttg
      361 aaaaagattt tgacttaatt tgtaaaattt atattcaatg gaaagaatta aataataaca
      421 taaaaaaatt tagttctaga ggtgtaaatt ttcctgattg tatttccgaa cctatagtat
      481 gctatgcttt caactataat tggaataaca ataacgaaac aggagatgct actagtgatt
      541 caggtgaatt aatagaaata aaagcgactt caaatttcaa tagcgattta agttcttttt
      601 cgccaaatac taaatttgat aagctaattt ttgttagatt agatcaagaa agggatttag
      661 cttatattta tgatttggaa ttaaattttg aacaatttca aattctagaa gtgaataaaa
      721 aggaaactgt tttagatcaa caaaaacaaa ataggcgtcc taggttaagt ttaattcaat
      781 atattgaaaa ggaaaagata gaaccgcttt attatgttga ttttataaaa aaaatagtta
      841 ccaaaaatta aagaaaggag tggcgtctaa actcctttct ttaaaaaagc agcatgctgc
      901 aattataatg gagataaaat atgaaagtgg cttctttttt tgccggtgta ggtggtattg
      961 atttggcctt tagtaatcat gatatcgttt atgttaatga aatagataaa aatgctacaa
     1021 aaacatataa atttaatttt gatgtcgaaa tagattgcag ggatatacaa actgttaatg
     1081 aaaaagaatt gcccgatttt gatattttat taggagggtt tccatgccaa gcattcagtg
     1141 ttgctggtta tagaaaagga tttgaagatt ctagaggaaa tctttttttc gatattatac
     1201 ggattgcaaa agaaaaaaaa ccaaaaaataa tatttctaga aaatgtaaaa aatttggtat
     1261 cgcatgattg tggaaaaact tttaatatta tattaaaatc attggaaagt ttaggttatt
     1321 atgttaaata tgatgtttta aatgcaatgc attatggaaa tacaccacaa aatagagaaa
     1381 gaatttatat tgtttgtttt aaaaataaga aacaatataa agattttaat tttcccaacc
     1441 caattttttt gaacaataaa atagacaata ttgtagattt taatttaaaa gtagatgata
     1501 aattttatta tactgaaaaa aattttaaac attatgaaat gttattgcaa aatataacaa
     1561 aaaaaaatgt tatttatcaa tggagaagaa aatatgtaag agaaaataaa agcaatgtat
     1621 gtcctacttt aacagcaaat atgggtactg gtggacacaa tgtaccgtta gtcttaacaa
     1681 aacatggaat aagaaaacta acccctaaag aatgctttat gtttcaaggt ttttccaaata
     1741 gttataaatt acctgatata gcaaatagcc atttatataa acaagcagga aatagtgtat
     1801 gcgtcaaagt gatacagcgt atagcagatc gtatttcaaa ttagaaaaat ttttatattt
     1861 tatttggtaa acaaccttta tatgattttt aaaattaaaa aaattctgat ttaaaatata
     1921 gtttaagttt ttaaaaaact taaagcaata aattcccatt tttccataac tttaagtttt
```

1981 taacttttta cctcgaaact taaatataaa acaaaatgca ttaaaacaat ggagtagcca
2041 tatttttatc aaaaaatata aagaatgaaa attgcacttt tcaatacaaa atatatttta
2101 catatttata taaaatatat atatttatta tataaataat ttatatctta tatactgatt
2161 aactcccata cctatttgca aagcactttc tttcacacgg tcatactcat cctgtgtgaa
2221 actaaccaat atttttttat ctcttttttg tgtattaatt ttatctccac gagcattatt
2281 aataaaatta atacttgctt tttctgtgtt gatattttta aaccccattt atttatcctt
2341 atttttttaat atttgaaaaa tttcttgata aaatttttca aaatctttca atgctttatc
2401 atttttatca caaaactcac taatactctt tccttctatt acggctcttt tgtaggcttg
2461 cctttcatat aaaacgcttt ctagcaaaaa gacatcattt aaattattga cttctttttt
2521 ctcgtgaata taatcaatta gattatttaa ttctttaatt aaaaatggat taggagagac
2581 tctattgatt aatattaata atataatatt tttatttatt aatttaactt ctttatatat
2641 atttaacata tgatcaagaa cacttacatc atattggcta gctatggtag gtataattat
2701 tacatttgat aaaagcatag cttttctcat ttctctacta tctctaccac ctgtatcaat
2761 aataatagca tcgtaattgt ttaattcatt tttaaaacca ctatttagca aattaatatc
2821 aaaactgtgt ttaaaatttt gttttaagtt agagttgttt ctgatattga taaaaacttc
2881 agtcgatctt tgcggatcac tgtcaaacag taaaatgctg tcaccatctt cagttaatct
2941 tgaagataaa ttaacagcta aagtgctttt accactaccg ccttttttcat tgcatataga
3001 tataatcatt atatactctt tatatattta ttatatttat attatatact atttttttgag
3061 taattgatca aaaaattgat ttttgcttta ttaaaaagtt tacatttata ccatttgccc
3121 ttttaatatt tcgctatact ccatcatata gtgcttagaa agccccttaa aaatataaaa
3181 aatatataaa aaataaaaaa atacacaacc cgcacatttt taaaaagaaa aaagaaaaag
3241 aagcaaaaag aaaaaagaaa aagatatttc aagataagaa taaatttaaa aaataaagtc
3301 aattagatac tttggactat aaaaatcaaa aatataaata tatacgatac aaccattaaa
3361 aagttgtatg aaaaaaataa ggagtttata tgaataaaat atataatttt atacataaac
3421 ttttatacga tcttttgtat gatcttttgt atgattatat acaatacaac atatacatat
3481 atatttaaaa atcacactaa atatatatga taatttatta aactaaaaaa aggagggagt
3541 gtcatctttc aaatcccaaa cctttttttta gatagttttg gtgataattt ttagttgttt
3601 ttatagtctt gtcagttgtt ttaactaagt tttgcgtatg atctttgcta tgcttttcaa
3661 gtgttttctt aagatagaat tgtatatttt cttgcaattc aacatctttc ataattgaag
3721 ctttttgcac tattaaatca ttaatatttt gtggtgcttt cttaaagaaa tttactaagt
3781 aatcatagtt tggaaagctc ttatcttctg ctaaattatc gtaattttca gtgtaaagtt
3841 ttttaagttt gaaatagttt tcttcactca tatcatttgg attaatattt tttacaaaat
3901 tgaaatcatc ttctagtttt gaaattttat cgtttttcgg ctcgaaattg cgttctgaag
3961 cgtttttttg ctcttggtcg atacttttat cgtttttttg ctctttttgc tttaaaagct
4021 cattttcggc ttttaaaatg ctattctctt tttttaactt ttcaatgcgt tctaaatgct
4081 ctcttaaaat atcgtttaaa agttctgata aatcatcttc ttttgcttct tggaagttct
4141 ttttaagttc gcttaactct ttgaagaaat ctttgtcaag cccttgatta atttgacttt
4201 ttctaaaatt ttcaatgatt tttttatact cgcttttatt taaaaattgc ccttttttctt
4261 gctctaaatt aatgtttcgc tttttctccc tttttttcctt ttcttttaag gctgcataag
4321 ctctaggctc tatgcgtttt gctccgctta ttctcttatc ttcgccacgc tccattccta
4381 agatttcagc gatttcggtt tgaatttctc ttaagacttt tgggcttatt ttttcacgcc
4441 taaagttgtt tttacctgtt tctttgtcaa gagtgataaa ttccatatgt gcgtggtggt
4501 ttataagctt ttcgccttgc tcgttaaaat gcccttcatc gcggtgtata gcgatttgat
4561 agcactgaaa gccgtatttt tcttgaaaat gtttagaaag gtttttttaaa tcattcattg
4621 aggtatcagg cttgatattg acaactaagc tccattcata gctcttagct ttaaattttg
4681 gagctttggg gcttttattt ttttcatagg cttctttcgc ttccgaaact atcttatttt
4741 taagctctaa ggcttcataa ccgcctaaat caaaaacaag ctcaccgcct attgcgtaat
4801 caggtcttac attggcatta tggaagactt gaaaagaagt tgattttttta aaatttccgc
4861 ttgaaatata atttcttttt gtgctttcac ttgcagtttt tgtgtaagca cttcttttaa
4921 ctttttcaat ctcatcgtaa gacatttttt ataaactttc tttgattttt gtttttaaaa
4981 actccattaa atttttattc tcatcaagtt ttttttgaaa atcatcgatt aatttttttt
5041 ctttttttttc tgcgtttgtg taagcgttta tcaattgtct taaattcatt ttagaatatt
5101 tttcataatc catttttttc ccttttttga aaaaatataa atgtaatcga gcttgtgaga
5161 ttatagcata tttttttaaaa acaaattata gaactttttt agttcggata atttgcaaat

```
5221 tatccaatca tatttataaa taaaaaaatg attaataatt tgcaccgcgt ggctttgcat
5281 taattcgcaa gctcatttaa tgtaaaagta ttaaataaaa ttatttatga tagaatttta
5341 tttgacttcc taaatgaaag tttagcgaag aattagctta tcttttagat aggttgcccc
5401 gtcttagagc ggggatatta aattccttgc ttattgtatt taggacattc aaatattcta
5461 tcttttaaat ttaaatcatc tttgatatat ccataacaac tacaagtttt agagctaggc
5521 aatgaaatgc acttctcttc tttttcctgc attttttatc ccataatgtg cgataagctc
5581 gtcaagcctt tcttcataat aaaatctttt gtgatagcct aatctttggt tttgagaaat
5641 gataaattct tttatccatt cttcattatc gataatcttt aaatcttcaa acattgtgag
5701 tgaattttga aaaatggcac taaaaattct ttttgcaaag tctttatctt gtatatgttc
5761 tactataaaa ctaggggtca tagtagtatt tccccaatag caattttttta aaatatgttc
5821 tgcgatttga agttttgttt tttcgctcat ctgatacctt ttctaaagat ttcaatcatt
5881 ttaaaaaact gctcttgatt tttagcatta aagtacatat gatttccaaa gtttaaattc
5941 tcgtattcat ctcttataag ctctactgca ataattttaa attcattaaa atcaaaatcg
6001 ttaaattgcc aaagcttgtc attataatta aatctaactt gattaagttt catattgttt
6061 aaaatcttta aatattttttg ctcatcgctc attatttttt gactttcatt ttctagcttt
6121 tgcatttcga tattttcagg tttaaaagta aaggtaatac ctgaaacctt gccaccttgc
6181 ccccgccctt ttgctttttc tttttcatag gcaagatttt taaaaggcac tcgcatttga
6241 tcaaaaagat tgcgttcttt agaaagttct tttatggcag gtttaataat tcttttatcg
6301 atttcgcttt gtctataatc ttgcggtata tccataatcc tacaaaactc ttcccattca
6361 aatcgtgctt tacctgtagt tctaaattgc tttaaaaggc ggtaaagtgt tttagcatat
6421 ctaccgctaa gagatataaa ttcttcaagc tcaaaagctg taaaattagt agttaattga
6481 ttaactaaat aagcaaaatc agggtttatt tttagggtta ttctgttaaa taaagtgctt
6541 ccatcatatc catcatcagg atttctttta acatagtgta tttccattgt ttcaaacaaa
6601 tttacatatt tgtgtataac ttctgtcttt gtttctatga tttgtttaaa atttgccttg
6661 aagaatttat attttaggct attgatattc tctgttaaat attctcttga attagaaagt
6721 ttttgattaa ttttaaaatc attggcatag atattaatta cattgtctat acccttttct
6781 ttaatctttg caaagattaa aaataaaagg ttttgctctt gctctgaaaa gttaggcaac
6841 tgaattttat taaaatcatt atggtattta actatatcac tcatagattt tccttataag
6901 ccctaaggca aggggctttt ctgcccctgc ggtttcgcta gaaaaatact tcaactttaa
6961 gtttaaaaaa cctaaaattc aggtataatc tcacaaaacg cttactatgt cgtttcatag
7021 ttaagccttt agggttattt ccgccaagag tgaaaactca aggcggattt ttattgtagc
7081 atgttaaggg tatattgtca atatttgtcc ccttaatacc tacaatttgt ccccttaata
7141 cctacaattt gtccccttaa tacctacaat ttgtcccctt aata
//
```
