# Supplementary material for: Characterization of Campylobacter spp. Strains Isolated From Wild Birds in Turkey
Source: Front Microbiol. 2021 Aug 18;12:712106. doi: 10.3389/fmicb.2021.712106 (PMC8416542; doi:10.3389/fmicb.2021.712106)
Supplement: Supplementary file 2 [file Image_2.pdf]

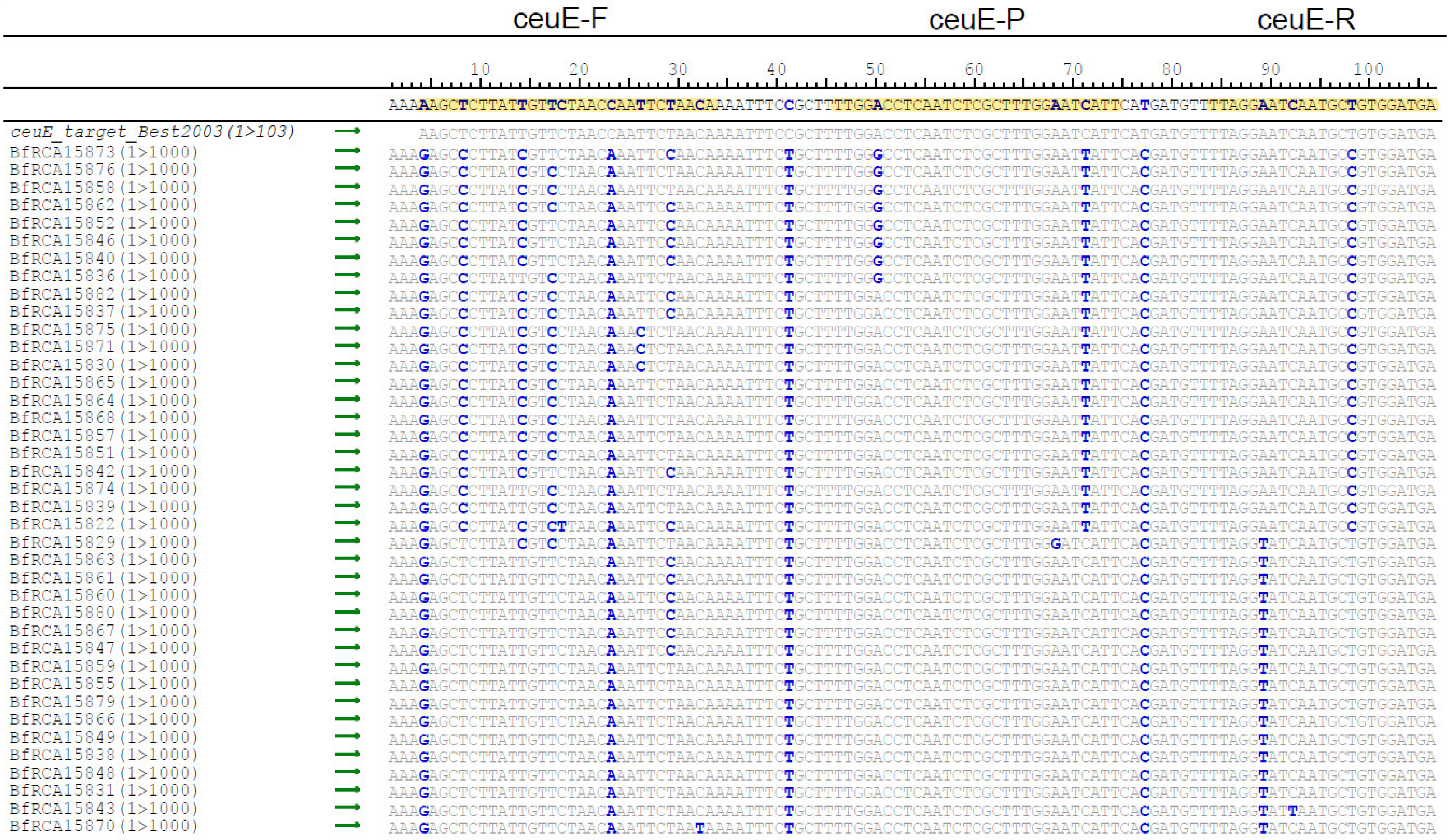

Image 2. Alignment of the 103 bp target fragment of *ceuE*. Oligo and probe annealing sites are highlighted in yellow, with mismatches in blue font.
